# Ubiquitous Micro-Modular Homologies among Genomes from Viruses to Bacteria to Human Mitochondrial DNA: Platforms for Recombination during Evolution?

**DOI:** 10.3390/v14050885

**Published:** 2022-04-24

**Authors:** Stefanie Weber, Christina M. Ramirez, Walter Doerfler

**Affiliations:** 1 Institute for Clinical and Molecular Virology, University Hospital, Friedrich-Alexander University Erlangen-Nürnberg, Schlossgarten 4, 91054 Erlangen, Germany; siweber@gmx.de; 2 Department of Biostatistics, UCLA School of Public Health, Los Angeles, CA 90095-1772, USA; cr@ucla.edu; 3 Institute of Genetics, University of Cologne, 50674 Cologne, Germany

**Keywords:** micro-modular sequence identities, *SARS-CoV-2* RNA compared to genomes of many viruses, protobacteria, *Escherichia coli*, patch type sequence similarities, the 4,641,652 nt DNA sequence of *Escherichia coli K12*, micro-modular identities in 1000 randomized *SARS-CoV-2* genomes, four letter genetic encoding system, signals for evolution, annealing and integrative recombination

## Abstract

The emerging *Severe Acute Respiratory Syndrome Coronavirus-2 (SARS-CoV-2)* and its variants have raised tantalizing questions about evolutionary mechanisms that continue to shape biology today. We have compared the nucleotide sequence of *SARS-CoV-2* RNA to that of genomes of many different viruses, of endosymbiotic proteobacterial and bacterial DNAs, and of human mitochondrial DNA. The entire 4,641,652 nt DNA sequence of *Escherichia coli K12* has been computer-matched to *SARS-CoV-2* RNA. Numerous, very similar micro-modular clusters of 3 to 13 nucleotides lengths were detected with sequence identities of 40 to >50% in specific genome segments between *SARS-CoV-2* and the investigated genomes. These clusters were part of patch-type homologies. Control sequence comparisons between 1000 randomly computer-composed sequences of 29.9 kb and with the A, C, G, T base composition of *SARS-CoV-2* genome versus the reference Wuhan *SARS-CoV-2* sequence showed similar patterns of sequence homologies. The universal A, C, G, T genetic coding mode might have succeeded in evolution due in part to its built-in capacity to select for a substantial reservoir of micro-modular domains and employ them as platforms for integrative recombination. Their role in *SARS-CoV-2* interspecies transition and the generation of variants appears likely, but their actual involvement will require detailed investigations.

## 1. Introduction

Like many researchers [1,2,3], we have followed the active replication and worldwide spread of *SARS-CoV-2*, the time course of rapidly emerging new mutations and its more complex variants [4,5]. These analyses led to the finding that more than 50% of the new mutations were C to T transitions, but there was a compensatory “repair” of T and A base pairs to C and G’s (Table 13 in [5]) to counteract the otherwise deleterious depletion of cytosines in the sequence. The possibility exists that the cellular APOBEC cytosine deaminase might be involved in the high incidence of C to U (T) transitions [6].

In search of an independent approach to the study of basic nucleotide sequence design, vestiges of evolution as it were, different nucleotide sequences were juxtaposed to investigate fundamental nucleotide sequence commonalities across species barriers. Here we report on marked nucleotide sequence homologies between *SARS-CoV-2* RNA and the genomes of many classes of viruses, the genomes of proteobacteria, of bacteria, and of human mitochondrial DNA. The entire 4,641,652 nt DNA sequence of *E. coli K12* has been computer-matched to *SARS-CoV-2* RNA, and similar patchy sequence identity patterns have been observed. These similarities manifest themselves as micro-modules of two to maximally 13 nucleotide clusters. Could these sequence coincidences be explained statistically, when taking into account the fundamental biochemical design of genetic information that is limited to only four nucleosides? Initially five, subsequently expanded to 1000, randomized sequences were created in silico with the only premise of comparable sequence lengths (29.9 kb) and base composition to that of *SARS-CoV-2* RNA. And indeed, very similar patches of sequence identities among up to 1000 randomized DNA sequences and of each of these to the *SARS-CoV-2* sequence were noted at comparable frequencies, possibly with shorter homologies. So, has it all been statistically predictable? Certainly in the affirmative. However, in the information storage system of DNA or RNA that has successfully sustained evolution to this day, nucleotide sequence patches have facilitated base-pairing among genomes of very different derivations. Hence, the actual existence of micro-modular connectedness among vastly different genomes—though statistically predictable—still facilitates segmental sequence exchanges between genomes across the genetic universe. This genetic repertoire might have played a decisive role during evolution and still function as the molecular engine for exchanges among nucleotide sequence patches by whatever recombination-based mechanisms. In biology, the statistically predicted still holds highly significant functional alternatives.

The mechanisms operative during the emergence of *SARS-CoV-2* from a poorly identified source to the worldwide pandemic pathogen with the ability to generate an abundance of highly replication-efficient mutants and variants have left us with a major enigma. Could principles of structure design in genomes in general offer a possible clue for further investigations? We have set out to compare the nucleotide sequences of the major groups of viruses, of proteobacteria, of *Escherichia coli K12*, and of the presumably bacterial plasmid-derived mitochondrial DNA of *Homo sapiens* to the nucleotide sequence of *SARS-CoV-2*. We found complex patterns of nucleotide sequence homologies at very similar levels among all genomes investigated. We have quantitated these extensive homologies and recorded the number of 3- to 9-mers, in some cases of up to 13-mers, of continuous sequence identities between the *SARS-CoV-2* genome and the genomes selected for study. Aside from numerical documentation, complex patchy patterns of sequence identities have been found that might carry structural signals, e.g., for integrative recombination and nucleic acid-protein interactions. In control experiments, 1000 randomized sequences of the size and base composition of the *SARS-CoV-2* genome were similarly quantitated and revealed very similar micro-modular patch homologies. We pursue the notion that the four-letter system of encoding genetic information has proved successful during evolution partly due to its inherent capability of providing identity platforms for integrative recombination and specific DNA-protein interactions.

## 2. Materials and Methods

All DNA sequences on which this study is based were taken from the NCBI database, and table accession numbers were summarized in Table S1. For sequence comparisons, VectorNTI Advance 11 was employed. The default settings of the align tool of the software were used (pairwise alignment: K-tuple size: 2; number of best diagonals: 4; window size: 4; gap penalty: 5; gap opening penalty: 15; gap extension penalty: 6.66; multiple alignment: gap opening penalty: 15; gap extension penalty: 6.66; gap separation penalty range: 8; % identity for alignment delay: 40; transition weighting: 0). We have performed sequence comparisons with these settings for years.

**Figure 1:**
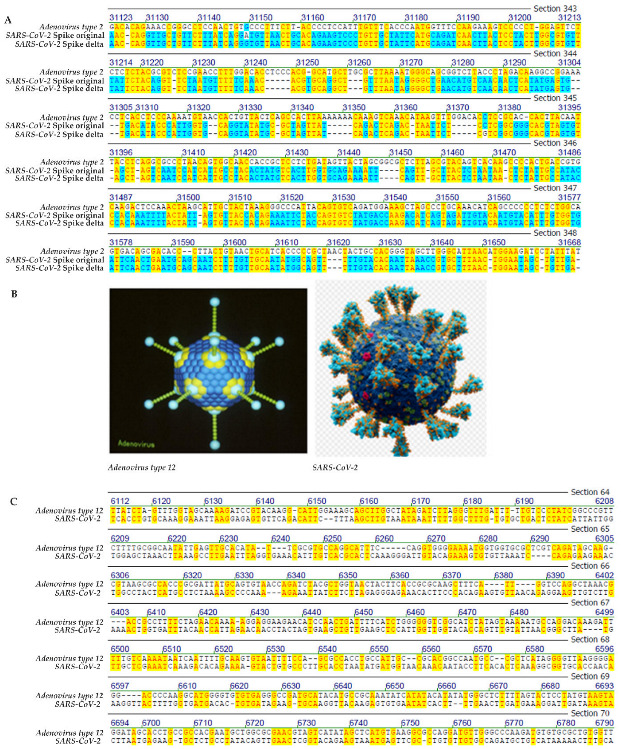
(**A**) *SARS-CoV-2* original Wuhan and delta spike sequences versus fiber protein of *human adenovirus type 2 (Ad2)*. Segmental homology 45.1%. (**B**) Viral models of *human adenovirus type 2* and *SARS-CoV-2*. (**C**) Sequence alignment between Wuhan *SARS-CoV-2* and *adenovirus type 12 (Ad12)*. The yellow highlight designates sequence identities between the genomes of *SARS-CoV-2* and *Ad2* (**A**) or *Ad12* (**C**). The blue highlight stands for sequence identities between the nucleotide sequences of the SARS-CoV-2 spike in the WUHAN isolate and its delta variant.

Many sequence alignments have been included under Supplemental Materials, e.g., as Figure S1C from which Figure 1C was taken as a screen shot. Similar selections of complete sequence alignments are Figures S1–S4. These alignments will be available from the authors upon request.

The random and shuffle sequences were created using the Sequence Manipulation Suite (https://www.bioinformatics.org/sms2/about.html, accessed on 9 September 2021). Further technical details have been published previously [4,5]. We then used the PRSS software to shuffle the random sequences 1000 times and then estimated the statistical significance from the shuffled alignment scores using E values. The E values ranged from 0.2–1 with a median value of 0.53. The sequences were aligned as described previously. The number of matches in the alignment were counted using the stringer package in R Studio (1.2.5033).

## 3. Results

### 3.1. Problems Studied

We have set out to investigate overall similarities in basic genome architecture among genomes from very different organisms. How closely related are their sequences? Our study has focused on comparisons between different viral, proteobacterial, and bacterial genomes to *SARS-CoV-2* RNA. Since genetic information storage is based on DNA or RNA molecules, in which the sequence of only four nucleotides carries the entire genetic message, sequence similarities can be expected solely on statistical grounds. It will be much harder, however, to search beyond statistical considerations and explain the frequently observed micro-modular clusters that resemble one another among seemingly unrelated genomes. Of course, all genomes are interconnected through their common evolutionary history.

**Figure 2:**
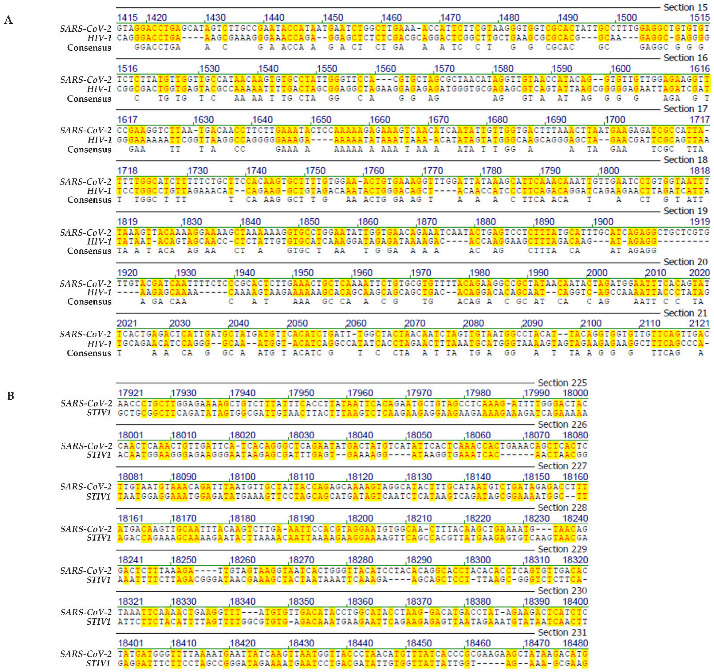
(**A**) Sequence alignment between Wuhan *SARS-CoV-2* and *HIV-1*. (**B**) Sequence alignment between Wuhan *SARS-CoV-2* and *Sulfolobus turreted icosahedral virus 1* (*STIV1*). The yellow highlight designates sequence identities between the genomes of *SARS-CoV-2* and *HIV* (**A**) and *Sulfolobus turreted icosahedral virus* (*STIV1*) (**B**), respectively.

**Figure 3:**
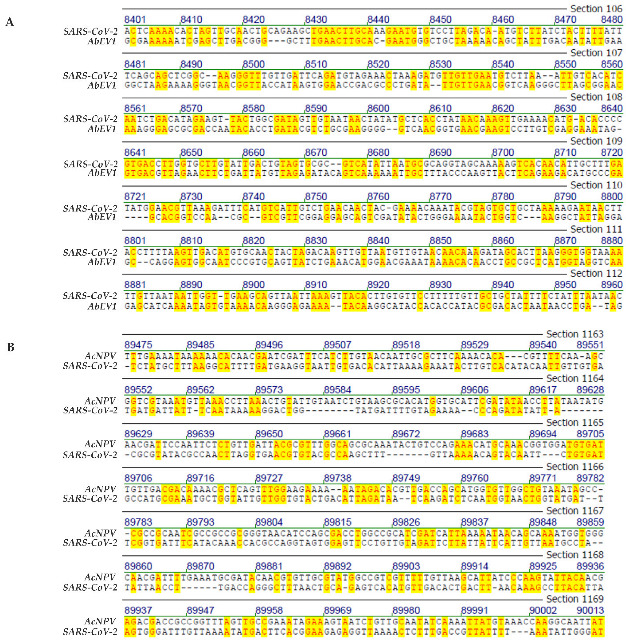
(**A**) Sequence alignment between the genomes of Wuhan *SARS-CoV-2* and of *Alternaria brassicicola endornavirus (AbEV1)*. (**B**) Sequence alignment between the genomes of Wuhan *SARS-CoV-2* and of *Autographa californica nuclear Polyhedrosis Virus (AcNPV)*. The yellow highlight designates sequence identities between the genome of *Alternaria brassicola endornavirus* (*AbEV1*) (**A**), or *Autographa californica Nuclear Polyhedrosis Virus (AcNPV)* (**B**) with the genome of *SARS-CoV-2*.

Table 1 lists the viral, proteobacterial, bacterial, and human mitochondrial genomes studied in this analysis. Levels of mutual overall sequence homologies can vary, but sequence homologies in selected segments of different genomes range between 44 and 47%, and in some cases reach > 50% (see Figures 1–4 in [6]). In comparisons between sequences in selected segments of a given genome and that of *SARS-CoV-2* RNA, clusters between 3 and up to 13 nucleotides of identical sequences were registered (Figure 1, Figure 2, Figure 3 and Figure 4 in [6]). Similarly, extensive sequence homologies, at on average 44.5%, between *SARS-CoV-2* and randomized DNA sequences have also been documented (Table 2).

### 3.2. Comparisons of Selected Segments of SARS-CoV-2 RNA with Segments of High Sequence Homologies in the Genomes of Diverse Viral and Non-Viral Families

**Figure 4:**
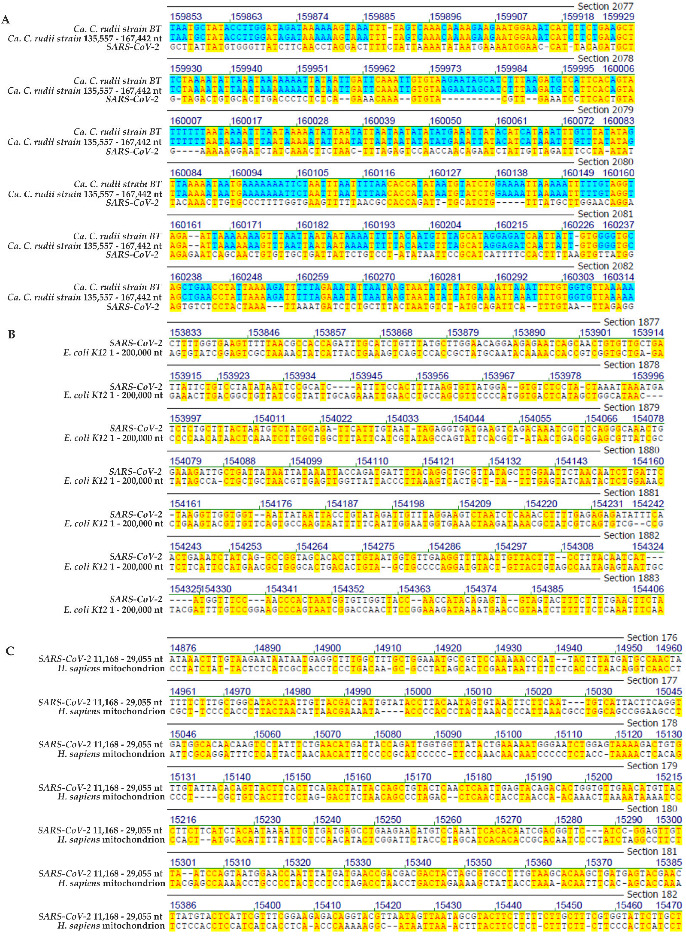
(**A**) Sequence alignment between Wuhan *SARS-CoV-2* and *Candidatus Carsonella ruddii*. (**B**) Sequence alignment between Wuhan *SARS-CoV-2* and *E. coli K12*. (**C**) Sequence alignment between Wuhan *SARS-CoV-2* and human mitochondrial DNA. The yellow highlight designates sequence identities between the genomes of *SARS-CoV-2* and two segments of the proteobacterium *Candidatus Carsonella ruddii* (**A**), a segment of the *Escherichia coli K12* genome (**B**) or mitochondrial DNA of *Homo sapiens* (**C**). The blue highlight stands for sequence identities between the nucleotide sequences of two different segments of the *Candidatus Carsonella ruddii genome*.

In Figure 1, Figure 2, Figure 3 and Figure 4, selected frame shots from total sequence alignments between genomes of many different viruses, proteobacteria, bacteria, plus human mitochondrial DNA and *SARS-CoV-2* RNA were reproduced. In Figure 1, Figure 2, Figure 3 and Figure 4, sequence identities in specific genome parts were highlighted in yellow. Data compilations in Table 1 present overall sequence similarities across the entire genomes of the listed species and the *SARS-CoV-2* Wuhan reference RNA sequence. Table 1 also contains sequence homologies in selected frame shots with high values of sequence identities. Finally, data from a control series that aligned the *SARS-CoV-2* RNA sequence to computer-randomized nucleotide sequences of 29.9 kbp length, have been presented as comparisons to the five computer-generated sequence artifacts (Table 2).

1. Comparisons of the Wuhan *SARS-CoV-2* nucleotide sequences to sequences from diverse virus families
(i) Genomes of *SARS-CoV-2* and *human adenovirus types 2* and *12*

In Figure 1A, the sequence of *SARS-CoV-2* RNA from the coding part of the viral spike protein between nucleotides 1863 and 2365, both from the Wuhan reference sequence and that of the delta variant, were aligned with *human adenovirus type 2* (*Ad2*) sequence between nucleotides 31,081 and 31,668, a segment coding for part of the *Ad2* fiber protein. In the analyzed genome segment, the Wuhan and delta *SARS-CoV-2* sequences differed in only one nucleotide in position 31,149. Segmental homology/sequence identity in the depicted section between the *SARS-CoV-2* and *Ad2* sequences was 43.2%. The lengths of identical micro-modular elements between the two biologically unrelated sequences ranged from n = 2 [frequent] to n = 7 [to n = 10 with one interruption, see bottom array].

We also compared the amino acid sequences of the *Ad2* fiber and the *SARS-CoV-2* spike proteins (https://pubmed.ncbi.nlm.nih.gov/; accessed on 9 September 2021). There were no common sequences, not even at the level of oligopeptides. The four-letter system of DNA/RNA encoding represents an amazingly simple framework at the statistical level but facilitates a high level of micro-modular homologies and, above all, has generated a recombinatorial platform for the enormous complexity of life.

This overall theme—occurrence of micro-modular sequence identities among unrelated viral nucleotide sequences and those from bacteria or human mitochondrial DNA—will be documented by a series of relevant examples.

The alignment of the adenovirus type 12 sequence between nucleotides 5989 and 6640 to the 6112–6790 region in *SARS-CoV-2* RNA revealed further micro-modular sequence homologies between n = 2 and n = 6 nucleotides with several closely-spaced alternating spots of homology and non-homologies that are thought to favor recombinatorial integration events (Figure 1C).

(ii) Genome comparison: *SARS-CoV-2* and *human immunodeficiency virus-1* (*HIV-1*)

Figure 2A depicts the sequence alignment between the designated regions of the *SARS-CoV-2* and *HIV-1* genomes with an overall homology of 45.8%. Apart from the extent of this homology, this arbitrarily selected frame shot demonstrates the staccato-like alternations between short modules with and those without sequence homologies. In a different field of our earlier research, i.e., the characterization of sequence elements at the sites of integrative recombination between *Ad2* or *Ad12* DNA and (hamster) cellular DNA in *Ad2*-transformed hamster cells or in *Ad12*-induced hamster tumor cells, that carry multiple copies of integrated *Ad2* or *Ad12* DNA, very similar patchy sequence homologies have been described (see Section 5).

(iii) Genome comparison: *SARS-CoV-2* and of an archaea phage

*Archaea* are extremophiles that constitute the third branch of living organisms [7]. They have been categorized together with bacteria as prokaryotes and are distinguished from eukaryotes by the absence of a distinct nucleus. There are a considerable number of archaea phages whose nucleotide sequences have also been compared to that of *SARS-CoV-2* RNA (Table 1). Overall sequence homologies range from 30 to 45, and in selected genome segments can reach 47%.

As an example, Figure 2B presents a screen shot of the genome segment of Wuhan reference *SARS-CoV-2* RNA between nucleotides 17,921 and 18,480 that encodes part of ORF1b. It likely codes for the *SARS-CoV-2* exoribonuclease (nsp14) and/or other nsp (non-structural) proteins involved in *SARS-CoV-2* replication [8]. This *SARS-CoV-2* genome segment has been aligned to the genome segment between nucleotides 13,472 and 14,001 of the archaea phage *Sulfolobus turreted icosahedral virus.* In this particular genome segment, there is 45.5% sequence homology between the two viral genomes with micro-modular sequence patches of between n = 2 and n = 5 (6) nucleotides, the latter encompassing one mismatch.

(iv) Genome comparison: *SARS-CoV-2* and plant *Alternaria brassicola endornavirus*

In selected segments, there is also up to 44.5% nucleotide sequence homology between the genomes of *SARS-CoV-2* and the plant virus *Alternaria brassicola endornavirus* (*AbEV1*) (Table 1). In the screen shot segment shown in Figure 3A, sequence homology between the two genomes amounts to 44.5%. There are distinct patterns of micro-modular arrays with sequence tracks of 2 to 10 nucleotides identity. It might be the staccato-like alternations of short sequence identities and non-homologies that can serve as target sites for recombinatorial mechanisms.

(v) Genome comparison: *SARS-CoV-2* and insect *Autographa californica nucleo- polyhedrovirus* (*AcNPV*)

As shown in Figure 3B and described in its legend, patchy genome homologies between 2 and 6 nucleotides also exist between these taxonomically unrelated viruses. Again, several sites of alternating homologous and non-homologous regions generate recognizable patterns with potentially functional significance.

2. Homologies between *SARS-CoV-2* RNA and proteobacterial and bacterial genomes
(i) Genome comparison: *SARS-CoV-2* and *Candidatus Carsonella ruddii strain BT*

Psyllids are primitive plant-specific and plant-feeding insects that harbor obligate endosymbionts like *Candidatus Carsonella ruddii*, a proteobacterium with one of the smallest known genomes, a circular DNA molecule of 174,004 nt [9]. We have aligned this genome with that of *SARS-CoV-2* and registered 8.5% overall and 46% homologies in specific segments of the viral and proteobacterial genomes (Table 1).

In the *SARS-CoV-2* genome segment between nucleotides 22,352 and 22,773 located in its *spike* protein coding region that was compared to sequence positions 159,853 to 160,314 in the *Candidatus Carsonella ruddii* strain BT genome, sequence homology reached 44.8% (Figure 4A).

(ii) Genome comparison: *SARS-CoV-2* and *Escherichia coli strain K12*

Significant sequence homologies between *SARS-CoV-2* RNA and segments of the *Escherichia coli K12* genome were also documented (Table 1). The graph in Figure 4B visualizes one prominent example with the nucleotide 22,572 to 23,116 *spike*-coding region in the *SARS-CoV-2* genome exhibiting 47.3% homology to the *E. coli K12* genome region between nucleotides 153,328 and 153,888. Comparable findings were documented in the adjacent genome segments (data not shown). (i) 48% homology to nucleotide segment 4317 to 4845 in *SARS-CoV*-*2 PL proteinase* (ORF1a) to an *E. coli K12* genome region of nucleotides 255,598 to 256,156. (ii) 46.9% homology to nucleotide segment 22,300 to 22,813 in *SARS-CoV-2 spike* protein to an *E. coli K12* genome region of nucleotides 270,286 to 270,851. In both comparisons the lengths of micro-modular homologies vary between n = 2 and n = 7. More extensive comparisons between the entire *E. coli* nucleotide sequence and the *SARS-CoV-2* genome will be described in Section 4.

3. Genome comparison: *SARS-CoV-2* RNA to human mitochondrial DNA.

Current concepts pursue the notion that during evolution, human mitochondrial DNA might have been derived from a prokaryotic endosymbiont, possibly related to bacterial plasmid DNA. The data in Figure 4C reveal patchy nucleotide sequence homologies of 45.3% in the SARS-CoV-2 region 11,168–29,055 nt compared to the entire mitochondrial DNA sequence. The latter falls in the mitochondrial DNA region coding for the ND5 [*NADH dehydrogenase*] gene. The lengths of the patchy sequence homologies in this segment range between two and seven nucleotides. There are multiple segments that exhibit closely spaced short sequence identities interrupted by one or two non-congruent nucleotides. As suggested in the description of micro-modular sequence arrays with several viral genomes and *SARS-CoV-2* RNA, similar patchy sequence homology arrangements have been observed at junction sites between adenoviral or *SV40* DNA [10] and cellular DNA at sites of adenoviral DNA integration due to recombinatorial integration events [11,12,13,14,15,16].

4. Genome comparison: *SARS-CoV-2* RNA to the entire *Escherichia coli K12* chromosome of 4,641,652 nucleotide pairs

**Figure 5:**
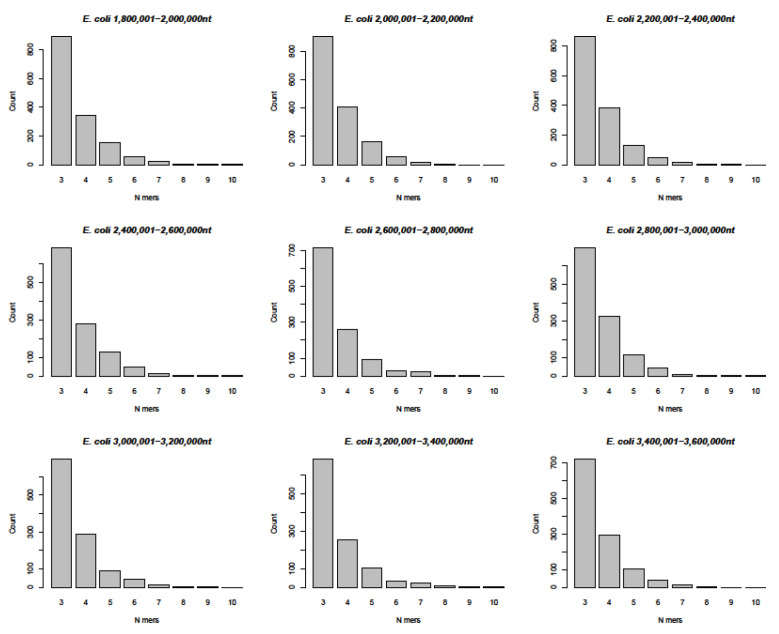
Histograms of nucleotide sequence identities between the *SARS-CoV-2* genome and different segments of the entire *Escherichia (E) coli K12* DNA as indicated in individual panels. For details of the programmed analyses of the entire *E. coli K12* genome see Materials and Methods. Very similar data obtained for the *E. coli* DNA segments 1 to 1.8 million and 3,400,001 to 4,641,652 have been deposited under Supplemental Materials (Figure S5A,B).

The comparison between the 29,903 nucleotide sequence of *SARS-CoV-2* and the entire *Escherichia coli K12* nucleotide sequence of 4,641,652 nucleotide pairs produced a statistically very rigorous insight into nucleotide sequence relationships. The data in Figure 5 and Figure S5A,B (Supplemental Materials) confirm the occurrence of n = 3-mers to n = 10-mers, as described above, for many different sets of sequences but fail to reflect the actual complexities of the micro-modular arrays (as apparent from the sequence alignments in Figure 1, Figure 2, Figure 3 and Figure 4).

5. Statistical analysis (bar graphs) of inter-genomic micro-modular homology clusters of 3 to 9 and up to rarely occurring 13 nucleotides.

**Figure 6:**
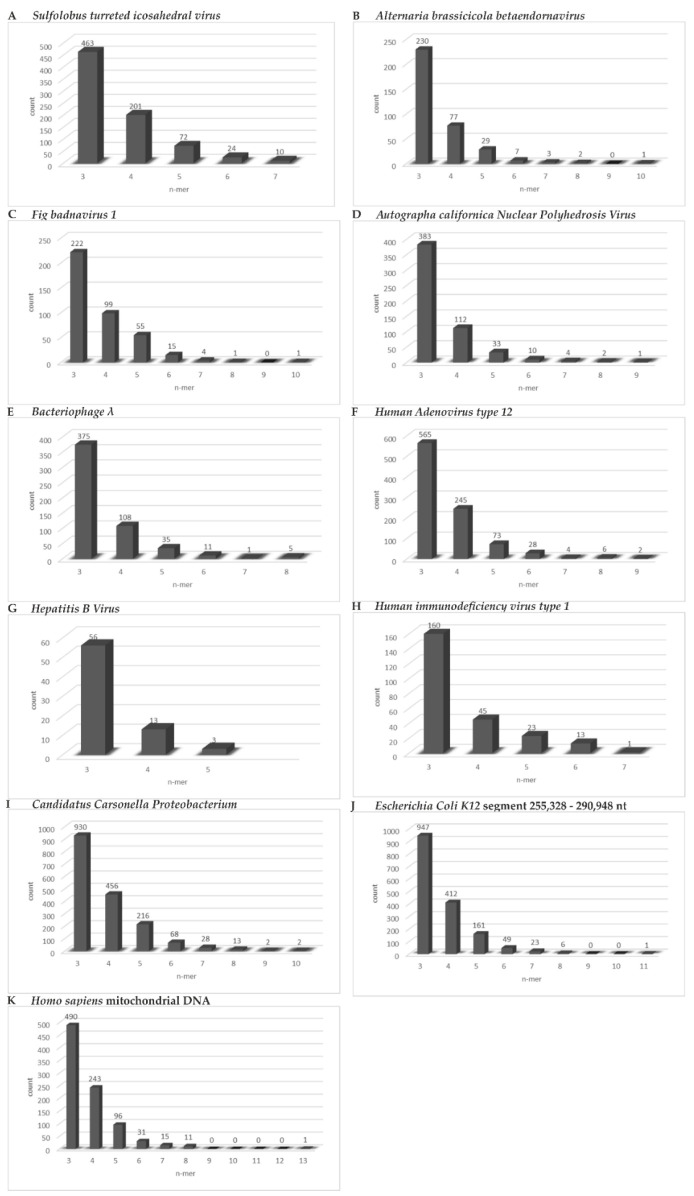
Table S2. Bar graphs. As described under Methods, the individual nucleotide sequences as indicated in the Table/Figures were aligned to the DNA-converted RNA sequence of *SARS-CoV-2*. We analyzed these complex patterns of sequence identities by counting the total number of 3 to up to maximally 13-mers in each of the sequence alignments chosen for representative organisms. As apparent from the nucleotide sequence alignments [see Supplementary Material Figures S1–S4], sequence identities were highlighted in yellow. Individual identities of n-mers were quantitated and expressed as percent of total 3- to n-mers, as indicated. (**A**–**K**) summarize the results in the form of bar graphs, in Table S1 the actual numbers of 3- to 13-mers have been compiled. The bar graphs did not, however, reflect the complexity of actual patterns of sequence identities in individual classes that wee, however, apparent in the sequence alignments in Figure 1, Figure 2, Figure 3 and Figure 4. The nucleotide sequences of the following organisms were aligned with the genome sequence of *SARS-CoV-2*: (**A**) Circular double-stranded DNA with 17,663 nucleotides of the Archaea phage *Sulfolobus turreted icosahedral virus*. (**B**) RNA with 10,290 nucleotides of the plant virus *Alternaria brassicicola betaendornavirus*. (**C**) DNA with 7140 nucleotides of the plant virus *Fig badna virus 1*. (**D**) Double-stranded DNA with 133,894 base pairs of the hymenopteran insect virus *Autographa californica Nuclear Polyhedrosis Virus.* (**E**) Double-stranded DNA with 48,502 nucleotide pairs of *Escherichia coli bacteriophage lambda*. (**F**) Double-stranded DNA with 34,125 nucleotide pairs of *human adenovirus type 12*. (**G**) Partly double-stranded DNA with 3182 nucleotide pairs of human *Hepatitis B Virus (HBV)*. (**H**) Single-stranded RNA with 9719 bp of *Human Immunodeficiency Virus-1 (HIV-1)*. (**I**) Circular double-stranded DNA of the proteobacterium *Candidatus Carsonella* with 174,004 nucleotide pairs. (**J**) Sequence sub-segment comprising nucleotides 255,328 to 290,948 (arbitrary selection) from the 4,608,319 bp double-stranded circular genome of *Escherichia coli K12*. (**K**) Circular double-stranded DNA of *Homo sapiens* mitochondrial DNA with 16,269 base pairs.

The actual patterns of homologies between the different analyzed genomes are characterized by high complexity. There are constantly shifting runs of sequence homologies and non-homologies that change from segment to segment. A realistic approach to their evaluation can be reached by scrolling through the entire sequence alignments of individual genomes with the nucleotide sequence of *SARS-CoV-2* (Supplemental Materials Figures S1–S4). As a manageable method of analyses, the number of 3- to 9-mers, plus the very rare 10- to 13-mers of homologies were counted and subsequently in part analyzed by a computer program. The results of this quantitation for the following eleven organisms (Table 1 and Figure 1, Figure 2, Figure 3 and Figure 4), selected as examples, were converted to the bar graphs in (Figure 6): *Sulfolobus turreted icosahedral virus*, an Archaea virus (Figure 6A), *Alternaria brassicicola betaendorna virus*, a plant virus (Figure 6B), *Fig badna virus 1*, another plant virus (Figure 6C), *Autographa californica Nuclear Polyhedrosis Virus, AcNPV*, a baculovirus of hymenoptera (Figure 6D), *Bacteriophage lambda* of *Escherichia coli K12* (Figure 6E), *Human Adenovirus type 12* (Figure 6F), Human *Hepatitis B virus*, *HBV* (Figure 6G), *Human Immunodeficiency Virus type 1*, *HIV* (Figure 6H), the Proteobacterium *Candidatus Carsonella* (Figure 6I), *Escherichia coli K12* (human endo-bacterium), sub-segment comprising nucleotides 255,328–290,948 (Figure 6J), and *Homo sapiens*, mitochondrial DNA (Figure 6K). The numerical data for the composition of these graphs has been submitted as Table S2 under Supplemental Materials. The graphs drawn from a control computer-aided evaluation of n-mers of homology between the entire 4,641,652 nucleotide sequence of *Escherichia coli* DNA and *SARS-CoV-2* RNA, were presented in Figure 5 and Figure S5A,B. The graphs are very similar to the one presented in Figure 6.

Among the micro-modular clusters of sequence identities, 3-mers (54 to 78), 4-mers (18 to 27), and 5-mers (6 to 14) were found in all inspected sequence alignments. Very long stretches of identities (10-, 11-, 13-mers) were solitary and found only in some of the alignments (Figure 6A–C,I–K). Aside from statistical considerations, the occurrence of all homologies, particularly the longer ones, facilitates sequence recognition in DNA-protein interactions and provides platforms for integrative recombination. The frequencies of distinct micro-modular clusters do not, however, sufficiently reflect the actual mosaic of modular patterns and their unknown recognition functions (See alignments in Figures S1–S4). In these patterns, homologies alternate with non-homologies leading to patchy sequence arrangements that might have signal potential.

6. Control analyses with randomly computer-synthesized 29.9 kb DNA

**Figure 7:**
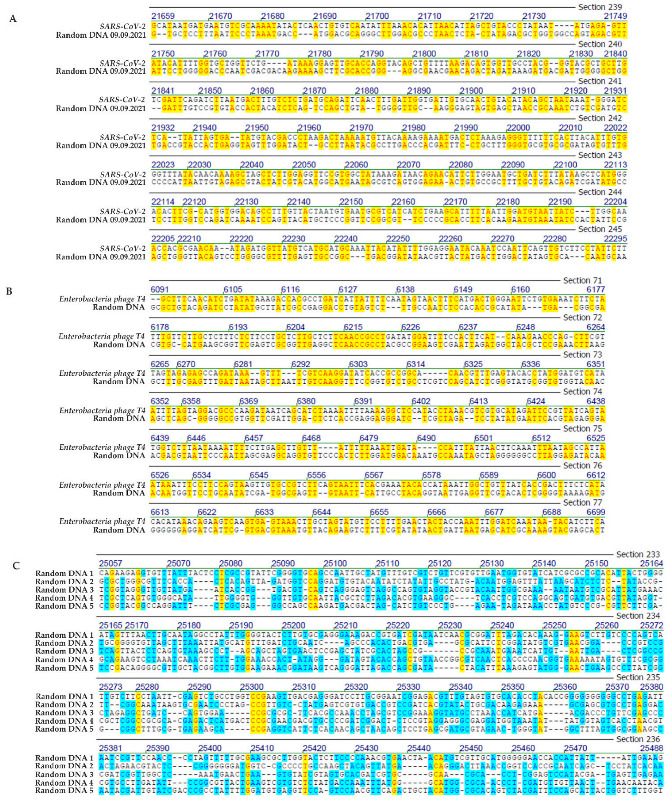
(**A**) Sequence alignment between Wuhan *SARS-CoV-2* and a randomly computer-generated DNA. (**B**) Sequence alignment between *bacteriophage T4* and a randomly computer-generated DNA. (**C**) Sequence alignments between five randomly computer-generated DNAs. The different highlights have been explained in the legends of Figure 1, Figure 2, Figure 3 and Figure 4. The blue highlights in part (**C**) indicate sequence identities among different randomized sequences.

The data presented above suggest significant patch type micro-modular homologies between the 29.9 kb Wuhan sequence of *SARS-CoV-2* that was chosen as a highly relevant reference genome to conduct sequence comparisons in a current biomedical context and a selection of very different genomes. What controls could be performed to reflect on the biological significance of the 44.2 to 47% sequence homologies between *SARS-CoV-2* and a spectrum of viral, proteobacterial, bacterial, and human mitochondrial DNA sequences? Obviously, there is no planet B harboring life with a completely different evolutionary history. Instead, five computer-generated, random nucleotide sequences of 30 kb lengths (Figure 7C) were chosen for comparisons among these artefactual sequences and of some of the randomized sequences to the Wuhan *SARS-CoV-2* sequence (Figure 7A) or to the sequence of *bacteriophage T4* (Figure 7B).

A selected frame in Figure 7A shows the sequence alignment between *SARS-CoV-2* sequence and one of the randomized nucleotide sequences and demonstrates the occurrence of very similar patchy sequence homologies at about comparable frequencies as discussed in Figure 1, Figure 2, Figure 3 and Figure 4. In another example, a 30 kb randomized sequence was compared to the DNA sequence of *bacteriophage T4* (Figure 7B). The nucleotide sequence of *bacteriophage T4* DNA (nucleotides 5858 to 6442) was aligned to the sequence of a 30 kb computer-generated randomized sequence (nucleotides 5803 to 6390). Sequence homologies of micro-modular patches of 2 to 10 nucleotides lengths amounted to 49%. Numbers represent lengths of homologous matches between the two sequences, dots stand for non-homologies. In Figure 7C five randomized 30 kb nucleotide sequences have been aligned to each other and again exhibit micro-domains of homologies among each other.

**Figure 8:**
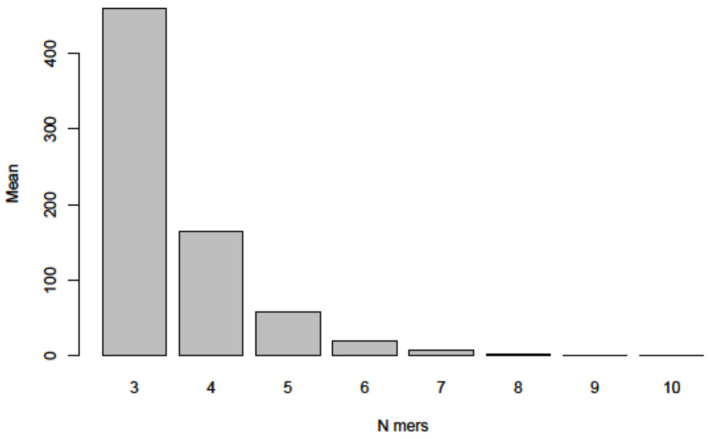
By using programs described under Methods, 1000 randomly generated nucleotide sequences were computer-screened for sequence identities among these randomized sequences of 3- to 9-mers (up to 10-mers in rare instances).

This series of random sequence controls was expanded in two ways. First, the randomization was now based on the 29.903 nucleotide length and additionally on the specific nucleoside composition of the *SARS-CoV-2* RNA molecule. Second, a total of 1000 such randomized sequences were analyzed by the program described in Section 2. Figure 8 documents that the frequency distributions of 3- to 9- (10-)-mers of sequence identities among 1000 randomized A, C, G, T sequences followed the same patterns very closely as shown in Figure 5A–K and Figure 6 for comparisons between the *SARS-CoV-2* and the analyzed nucleotide sequences in this report. Hence, the data further support the notion that platforms of annealable nucleotide sequences can be attributed to the inherent characteristics of the four letter code that generated all of biology.

## 4. Discussion and Outlook

(i) Synopsis and interpretation of data

**Figure 9:**
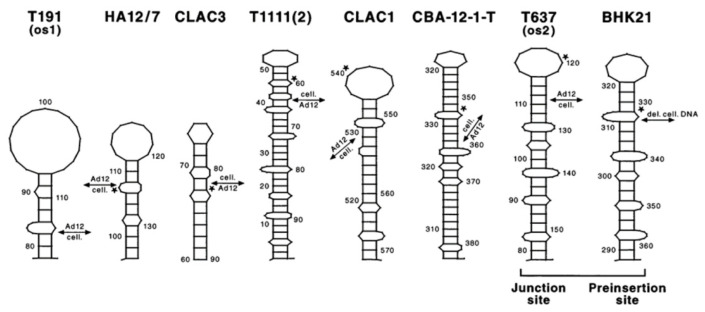
Stem-loop structures at the sites of linkage between *Ad12* DNA and cellular DNA from several *Ad12*-transformed hamster cell lines and *Ad12*-induced hamster or mouse tumor cell lines. A stem-loop structure at the preinsertion site in *BHK21* cells was also shown. Details are described in the text. Double-headed arrows, *Ad12* DNA-cell DNA junction sites; asterisks, *topoisomerase I* recognition sites (from [15]).

A comparison of the nucleotide sequences of the *SARS-CoV-2* genome to a number of viral, proteobacterial, and bacterial genomes as well as to human mitochondrial DNA has revealed common patch-like homologies with micro-domains of up to 40 and 50%, with the highest values in selected regions of these genomes. These homologies comprise between 2 to maximally 13 nucleotides, in rare instances, and are further characterized by longer stretches of sequences with alternating patch homologies interrupted, often by just one nucleotide pair of non-homology. Similar patch-like homologies, sometimes permitting the formation of stem-loop structures (Figure 9), have been previously reported from this laboratory at sites of integrative recombination between *adenovirus* DNA and cellular DNA in *Adenovirus type 2-, Ad12*-transformed rodent cells, and *Ad12*-induced hamster tumor cells [11,12,13,14,15,16]. Similar patch homologies at junction sites of integrated viral DNA and the recipient host DNA have also been described for *SV40* [10], *Hepatitis B Virus* [17,18]. Patch-type micro-domains have served as signals for heterologous integrative recombination.

Control studies on homologies between the *SARS-CoV-2* genome and five artificially computer-generated randomized nucleotide sequences of 29.9 kb have revealed similar patch homologies, possibly of slightly shorter lengths. Control studies on computer-generated randomized nucleotide sequences have been extended to 1000 sequences. Their comparisons to the sequence of *SARS-CoV-2* RNA yielded patterns very similar to the ones described for all comparisons described (Figure 8). In light of the results of these control studies, one might question the biological significance of the sequence comparisons reported here. In fact, however, these ubiquitously occurring homologies do exist and are inherent in the reality of a four-letter coding system on which all life is based. We submit that it would be a serious neglect to discount the possible biological significance of such wide-spread sequence homologies and their possible role in integrative recombinatorial events between very different genomes or their fragments. The recombinatorial repertoire available through these ubiquitous nucleotide sequence homologies might have played an important role during evolution. In the current *SARS-CoV-2* pandemic, it is worth considering similar mechanisms that have been at work during the continuing evolution of *SARS-CoV-2* from undefined animal to human hosts and the generation of ever more efficient variants during the current unabated COVID-19 pandemic. Of interest to note, *SARS-CoV-2*-superinfected *HIV*-positive, often immunocompromised individuals (compare our Figure 2A) have been purported as possible driving forces in the emergence of the *omicron variant* of *SARS-CoV-2* [19].

(ii) Patch homologies and stem-loop structures between DNA molecules of very diverse origins

In this article, nucleotide sequence alignments and comparisons in a study on genomes of very different organisms—the entire spectrum of viruses, proteobacteria, bacteria (*Escherichia coli K12*), and human mitochondria, presumably of endo-symbiotic origin—have unequivocally documented that a plethora of patch-type or micro-modular homologies exist between these heterogeneous populations of genomes and the *SARS-CoV-2* genome. In addition, randomized, computer-synthesized 29.9 kbp genomes exhibit similar micro-modular sequence homologies among each other and to the *SARS-CoV-2* genome.

In a research program that was pursued over two decades, i.e., an extensive analysis of sites of integrative recombination between DNA molecules that were unlikely to meet in nature, like the DNA of *Ad12* and mammalian (hamster) cellular DNA, almost identical patch structures have been observed (Figure 9, reproduced from [15,16]). Special sequence patterns, including patchy homologies, were also reported for a number of non-homologous recombination events, e.g., the insertion site of unique and repetitive DNA fragments into the *aprt* locus in the hamster genome [20] or a hotspot for novel amplification joints in *Alu*-like repeats [21] as well as for virus-cell DNA integration sites [22,23]. These structural similarities of DNA patterns in several entirely different biological systems cannot necessarily prove that the reaction products would be the same. However, the observation calls for more detailed investigations of the biological meaning of ubiquitous sequence homologies.

(iii) The four-letter coding system and its selection during evolution—a hypothesis

The history of genetic research in the 1930s [24], reminds us that vivid discussions were then dominated by speculations about the chemical nature of the genetic principle or as Oswald Avery and colleagues, who succeeded in identifying it as DNA called it—the *transforming principle* [25]. Today, we have long since internalized that a genetic alphabet of four chemical compounds, often referred to by their initials A, C, G and T, suffices to store all information for the structure and activities for all of biology. Another important aspect in evolution has been the exchange and scavenging of genetic information among different organisms with serendipitous encounters of their genomes. Under favorable conditions, mobile genetic elements, or viral genomes, probably any form of foreign DNA, can find entry via integrative recombination into established genomes that is often mediated by short sequence homologies. In analyses of junction sites between cellular DNA and different integrating viral genomes, it has been documented that patchy sequence homologies between the recombination partners—viral and receiving mammalian genomes—do occur and can direct the localization in integrative recombination with genomes of foreign derivation. In detail, alternations of base pairs with and without homologies in these patches provide distinct recognition signals that aid targeting possible sites for this type of heterologous recombination. A well-documented example of base pairing of short stretches of nucleotides providing enough stability to ascertain essential biological function is the 12 nucleotide homology in the cohesive termini in DNA of *Escherichia virus lambda* (*λ*) [26] that facilitate genome circularization as the first essential step in *lambda* DNA replication or in site-specific integration into the bacterial genome [5′ GGGCGGCGACCT 3′ and 5′ AGGTCGCCGCCC 3′]. Hydrogen bond stability, of course, is strengthened in this short sequence by the predominance of C-G base pairs [27]. We intend to pursue the role that micro-module-based sequence platforms might have played and continue to play in the emergence of *SARS-CoV-2* and the selection of variants of concern. While this research was initiated with a focus on *SARS-CoV-2* genetics, the data presented now bear on a much wider scope and intend to shed light on important mechanisms in past and on-going evolution.

## 5. Conclusions

- Genome structures of several organisms across the evolutionary spectrum were aligned with the *SARS-CoV-2* genome sequence. Micro-modular homologies of n = 3 to n = 9, and up to n = 13 (rare case) were recorded to gain insights into generalities in the design of existing and evolving (*SARS-CoV-2*) genomes.
- We found abundant micro-modular identities across the spectrum of genomes and investigated and documented these data in the bar graphs depicted in Figure 6A–K.
- In a computer-aided sequence comparison, the sequence of the *SARS-CoV-2* genome was matched to the entire 4,641,652 nt DNA sequence of *Escherichia coli K12.* This analysis yielded very similar results (Figure 6 and Figure S6A,B) with respect both to the numerical n = 3 to n = 9, and up to n > 10-mer arrays and to the complex patch pattern sequence homologies, reminiscent of those described in integrative recombination reactions [11,12,13,14,15,16].
- In an extensive control project, we subjected 1000 randomly generated nucleotide sequences of the length and base composition of the *SARS-CoV-2* genome to the same type of analyses for micro-modular sequence identities and found very similar patterns of micro-modular homologies, now presented in Figure 8.
- The four letter A, C, G, T genetic encoding strategy has proven exceptionally successful during 4 billion years of evolution. We submit that the system-inherent capability to generate a vast abundance of sequence and pattern identities alternating with non-identities to the tune of “patchy homologies” has provided opportunities for biochemically meaningful platforms for DNA-protein interactions and for (integrative) recombination reactions. In the course of evolutionary time periods, this system has facilitated the annealing of nucleotide sequences required for repeated extensions of initially smaller nucleotide sequences to the real genomes of enormous lengths that exist today. Moreover, the actually existing patterns (see alignments in Figures S1–S4) offer unidentified recognition signals for recombination events between otherwise unrelated genomes.
- It will take systematic investigations to explore the possibility of whether these mechanisms might have served also in the generation and selection of variants of newly emerging pathogenic viruses like *SARS-CoV-2* and others.

## Figures and Tables

**Table 1:** Nucleotide sequences that have been compared to the *SARS-CoV-2* sequence: percentages of sequence homologies to *SARS-CoV-2*. Nucleotide sequences from different viruses, to proteobacteria, bacteria, and to human mitochondrial DNA have been analyzed for sequence homologies, overall or in selected segments, to the *SARS-CoV-2* genome.

Genomes	Overall Homology	In Selected Segments
***Archaea* Viruses**		
*Acidianus rod-shaped virus 1*	41.4%	46.2%
*Nitrosopumilus spindle-shaped virus*	45.8%	47%
*Sulfolobus turreted icosahedral virus*	30.3%	45.5%
**Bacteriophages**		
*Escherichia virus lambda* (*λ*)	30.6%	45.3%
*Escherichia virus T4*	9%	46.4%
*Escherichia virus mu*	39.7%	44.2%
**Plant Viruses**		
*Alternaria brassicicola endornavirus*	13.1%	44.5%
*Carrot mottle mimic umbravirus* (ds-RNA)	5.9%	45%
*Fig badnavirus 1*	11.8%	45.5%
**Insect Virus**		
*Autographa californica Nuclear Polyhedrosis Virus*	11.2%	45.6%
**Human Viruses**		
*Adenovirus type 2*	38.4%	43.2%
*Adenovirus type 5*	40%	43.4%
*Adenovirus type 12*	43.2%	44.9%
*Hepatitis B Virus*	5.4%	45.7%
*Human Immunodeficiency Virus 1*	16.6%	45.8%
**Bacterial Genomes (Segments)**		
*Escherichia coli K12* (129,062–161,212 nt)		45.2%
*Candidatus Carsonella ruddii* strain BT	8.5%	46%
**Mitochondrial DNA—*homo sapiens***	27.3%	45.3%

**Table 2:** Randomized computer-generated nucleotide sequences of 30 kbp and their sites of sequence coincidences with the sequence of the *SARS-CoV-2* genome.

Random DNA	Homology	
Random DNA 1 versus *SARS-CoV-2*	44.7%	Average: 44.5%
Random DNA 2 versus *SARS-CoV-2*	44.8%	
Random DNA 3 versus *SARS-CoV-2*	44.4%	
Random DNA 4 versus *SARS-CoV-2*	44.5%	
Random DNA 5 versus *SARS-CoV-2*	44.3%	

## Data Availability

All data presented in this article will be freely available from the authors on request.

## Supplementary Materials

The following supporting information can be downloaded at: https://www.mdpi.com/article/10.3390/v14050885/s1, Figures S1–S4—total sequence alignments; Figure S5A,B—parts of the analysis of the total *E. coli* genome versus *SARS-CoV-2*; Table S1—accession numbers for analyzed sequences; Table S2—actual numbers the bar graphs in Figure 6 were derived from.
