# Supplementary material for: Ubiquitous Micro-Modular Homologies among Genomes from Viruses to Bacteria to Human Mitochondrial DNA: Platforms for Recombination during Evolution?
Source: Viruses. 2022 Apr 24;14(5):885. doi: 10.3390/v14050885 (PMC9147251; doi:10.3390/v14050885)
Supplement: Supplementary file 1 [file viruses-14-00885-s001.zip › Fig. S1C, SARS-CoV-2 & Ad12 DNA Alignment.pdf]

Section 1

```
                         (1)  1       10        20        30        40        50        60        70        80              97
Ad 12 X73487             (1)  CCTATCTAATAATATACCTTATACTGGACTAGTGCCAATATTAAAATGAAGTGGGCGTAGTGTGTAATTTGATTGGGTGGAGGTGTGGCTTTGGCGT
SARS-CoV-2 NC_045512.2   (1)  -------------------------------------------------------------------------------------------------
```

Section 2

```
                        (98)  98         110       120       130       140       150       160       170       180           194
Ad 12 X73487            (98)  GCTTGTAAGTTTGGGCGGATGAGGAAGTGGGGCGCGGCGTGGGAGCCGGGCGCGCCGGATGTGACGTTTTAGACGCCATTTTACACGGAAATGATGT
SARS-CoV-2 NC_045512.2   (1)  -------------------------------------------------------------------------------------------------
```

Section 3

```
                       (195)  195  200       210       220       230       240       250       260       270       280       291
Ad 12 X73487           (195)  TTTTTGGGCGTTGTTTGTGCAAATTTTGTGTTTTAGGCGCGAAAACTGAAATGCGGAAGTGAAAATTGATGACGGCAATTTTATTATAGGCGCGGAA
SARS-CoV-2 NC_045512.2   (1)  -------------------------------------------------------------------------------------------------
```

Section 4

```
                       (292)  292     300       310       320       330       340       350       360       370             388
Ad 12 X73487           (292)  TATTTACCGAGGGCAGAGTGAACTCTGAGCCTCTACGTGTGGGTTTCGATACGTGAGCGACGGGGAAACTCCACGTTGGCGCTCAAAGGGCGCGTTT
SARS-CoV-2 NC_045512.2   (1)  -------------------------------------------------------------------------------------------------
```

Section 5

```
                       (389)  389        400       410       420       430       440       450       460       470            485
Ad 12 X73487           (389)  ATTGTTCTGTCAGCTGATCGTTTGGGTATTTAATGCCGCCGTGTTCGTCAAGAGGCCACTCTTGAGTGCCAGCGAGAAGAGTTTTCTCTGCCAGCTC
SARS-CoV-2 NC_045512.2   (1)  -------------------------------------------------------------------------------------------------
```

Section 6

```
                       (486)  486           500       510       520       530       540       550       560       570         582
Ad 12 X73487           (486)  ATTTTCACGGCGCCATTATGAGAACTGAAATGACTCCCTTGGTCCTGTCGTATCAGGAAGCTGACGACATATTGGAGCATTTGGTGGACAACTTTTT
SARS-CoV-2 NC_045512.2   (1)  -------------------------------------------------------------------------------------------------
```

Section 7

```
                       (583)  583    590       600       610       620       630       640       650       660                679
Ad 12 X73487           (583)  TAACGAGGTACCCAGTGATGATGATCTTTATGTTCCGTCTCTTTACGAACTGTATGATCTTGATGTGGAGTCTGCCGGTGAAGATAATAATGAACAG
SARS-CoV-2 NC_045512.2   (1)  -------------------------------------------------------------------------------------------------
```

```
                                                                                                           Section 8
                             (680) 680       690       700       710       720       730       740       750       760            776
              Ad 12 X73487   (680) GCGGTGAATGAGTTTTTTCCCGAATCGCTTATTTTAGCTGCCAGTGAGGGGTTGTTTTTACCGGAGCCTCCTGTACTTTCTCCTGTCTGTGAGCCTA
      SARS-CoV-2 NC_045512.2   (1) -------------------------------------------------------------------------------------------------
                                                                                                           Section 9
                             (777) 777          790       800       810       820       830       840       850       860            873
              Ad 12 X73487   (777) TTGGGGGCGAATGTATGCCACAACTGCACCCTGAAGATATGGATTTATTGTGCTACGAGATGGGCTTTCCCTGTAGCGATTCGGAAGACGAGCAAGA
      SARS-CoV-2 NC_045512.2   (1) -------------------------------------------------------------------------------------------------
                                                                                                           Section 10
                             (874) 874   880       890       900       910       920       930       940       950       960       970
              Ad 12 X73487   (874) CGAGAACGGAATGGCGCATGTTTCTGCATCCGCAGCTGCTGCTGCCGCTGATAGGGAACGTGAGGAGTTTCAGTTAGACCATCCAGAGTTGCCCGGA
      SARS-CoV-2 NC_045512.2   (1) -------------------------------------------------------------------------------------------------
                                                                                                           Section 11
                             (971) 971      980       990       1000      1010      1020      1030      1040      1050           1067
              Ad 12 X73487   (971) CACAATTGTAAGTCCTGTGAGCACCACCGGAATAGTACTGGAAATACTGACTTAATGTGCTCTTTGTGCTATCTGCGAGCCTACAACATGTTCATTT
      SARS-CoV-2 NC_045512.2   (1) -------------------------------------------------------------------------------------------------
                                                                                                           Section 12
                            (1068) 1068        1080      1090      1100      1110      1120      1130      1140      1150           1164
              Ad 12 X73487  (1068) ACAGTAAGTGTGCTATGGGAGGTGGGAGGTGATTTTTTTTTCTTAAGCAGTGAAAAATAATATTTTGTTGTTTTTAGGTCCTGTTTCCGATAATGAG
      SARS-CoV-2 NC_045512.2   (1) -------------------------------------------------------------------------------------------------
                                                                                                           Section 13
                            (1165) 1165 1170      1180      1190      1200      1210      1220      1230      1240      1250      1261
              Ad 12 X73487  (1165) CCTGAACCTAATAGCACTTTGGATGGCGATGAGCGACCCTCACCCCCGAAACTAGGAAGTGCGGTTCCAGAAGGAGTAATAAAACCTGTGCCTCAGC
      SARS-CoV-2 NC_045512.2   (1) -------------------------------------------------------------------------------------------------
                                                                                                           Section 14
                            (1262) 1262   1270      1280      1290      1300      1310      1320      1330      1340           1358
              Ad 12 X73487  (1262) GGGTGACTGGGAGGCGTAGATGTGCTGTGGAAAGCATTTTGGATTTGATTCAAGAGGAAGAAAGAGAACAAACAGTGCCTGTTGATCTGTCAGTGAA
      SARS-CoV-2 NC_045512.2   (1) -------------------------------------------------------------------------------------------------
```

Section 15

```
                              (1359) 1359      1370      1380      1390      1400      1410      1420      1430      1440      1455
          Ad 12 X73487        (1359) ACGCCCTAGATGTAATTAATGGACTTTGAGCACCTGGGCAATAAAATAGGGGTAATGTGGTTTTTGTGAGTCATGTATAATAAAACTGGTTTCGGTT
SARS-CoV-2 NC_045512.2           (1) -------------------------------------------------------------------------------------------------
```

Section 16

```
                              (1456) 1456          1470      1480      1490      1500      1510      1520      1530      1540      1552
          Ad 12 X73487        (1456) GAAGTGTCTTGTTAATGTTTGTTTGGGCGTGGTTAAACAGGGATATAAAGCTGGGTTGGTGTTGCTTTGAATAGTTCATCTTAGTAATGGAGTTGGA
SARS-CoV-2 NC_045512.2           (1) -------------------------------------------------------------------------------------------------
```

Section 17

```
                              (1553) 1553   1560      1570      1580      1590      1600      1610      1620      1630           1649
          Ad 12 X73487        (1553) AACTGTGCTGCAAAGTTTTCAGAGCGTTCGCCAGCTCTTGCAGTATACCTCTAAAAACACTTCAGGTTTTTGGAGGTATCTGTTTGGCTCTACCTTA
SARS-CoV-2 NC_045512.2           (1) -------------------------------------------------------------------------------------------------
```

Section 18

```
                              (1650) 1650      1660      1670      1680      1690      1700      1710      1720      1730           1746
          Ad 12 X73487        (1650) AGCAAGGTGGTAAATAGGGTGAAAGAAGACTATAGAGAGGAATTTGAAAACATATTGGCCGACTGTCCAGGGCTTTTGGCTTCACTAGACCTTTGTT
SARS-CoV-2 NC_045512.2           (1) -------------------------------------------------------------------------------------------------
```

Section 19

```
                              (1747) 1747          1760      1770      1780      1790      1800      1810      1820      1830      1843
          Ad 12 X73487        (1747) ACCACTTGGTGTTTCAGGAAAAAGTGGTCAGATCCTTAGATTTTTCATCTGTGGGACGAACGGTTGCTTCTATTGCTTTTTTGGCAACCATATTGGA
SARS-CoV-2 NC_045512.2           (1) -------------------------------------------------------------------------------------------------
```

Section 20

```
                              (1844) 1844  1850      1860      1870      1880      1890      1900      1910      1920      1930      1940
          Ad 12 X73487        (1844) TAAATGGAGCGAGAAATCCCACCTGAGTTGGGATTACATGCTGGATTACATGTCAATGCAGCTGTGGAGGGCATGGCTGAAGAGGAGGGTTTGCATT
SARS-CoV-2 NC_045512.2           (1) -------------------------------------------------------------------------------------------------
```

Section 21

```
                              (1941) 1941     1950      1960      1970      1980      1990      2000      2010      2020           2037
          Ad 12 X73487        (1941) TACTCGCTGGCGCGGCCTTTGACCATGCCGCCGCTGCCGACGTTGCAAGAGGAGAAGGAGGAGGAGCGGAACCCTGCGGTGGTGGAGAAGTAAACAT
SARS-CoV-2 NC_045512.2           (1) -------------------------------------------------------------------------------------------------
```

```
                                                                                                         Section 22
                           (2038) 2038        2050      2060      2070      2080      2090      2100      2110      2120          2134
          Ad 12 X73487     (2038) GGAACAACAGGTGCAAGAAGGCCATGTACTTGACTCTGGCGAAGGGCCTAGTTGCGCAGATGATAGAGATAAGCAGGAAAAAAAAGAAAGTTTAAAG
 SARS-CoV-2 NC_045512.2       (1) -------------------------------------------------------------------------------------------------
                                                                                                         Section 23
                           (2135) 2135 2140      2150      2160      2170      2180      2190      2200      2210      2220         2231
          Ad 12 X73487     (2135) GAAGCTGCTGTTCTTAGTAGGCTAACTGTTAATCTGATGTCCCGCCCGCGTTTGGAAACTGTATATTGGCAGGAGTTGCAGGATGAATTTCAGCGGG
 SARS-CoV-2 NC_045512.2       (1) -------------------------------------------------------------------------------------------------
                                                                                                         Section 24
                           (2232) 2232    2240      2250      2260      2270      2280      2290      2300      2310                2328
          Ad 12 X73487     (2232) GTGATATGCATTTACAGTACAAATACAGTTTTGAACAATTAAAAACCCACTGGTTAGAGCCATGGGAGGATATGGAGTGTGCTATTAAAGCTTTTGC
 SARS-CoV-2 NC_045512.2       (1) -------------------------------------------------------------------------------------------------
                                                                                                         Section 25
                           (2329) 2329       2340      2350      2360      2370      2380      2390      2400      2410             2425
          Ad 12 X73487     (2329) TAAATTGGCCTTACGTCCTGATTGTAGCTACAGAATTACTAAAACAGTAACCATTACTTCATGCGCCTATATTATAGGTAACGGGGCAATAGTTGAG
 SARS-CoV-2 NC_045512.2       (1) -------------------------------------------------------------------------------------------------
                                                                                                         Section 26
                           (2426) 2426           2440      2450      2460      2470      2480      2490      2500      2510       2522
          Ad 12 X73487     (2426) GTAGATACAAGCGACAGAGTTGCTTTTAGATGTCGAATGCAGGGTATGGGCCCAGGGGTGGTGGGTTTGGATGGAATTACATTTATAAATGTTAGGT
 SARS-CoV-2 NC_045512.2       (1) -------------------------------------------------------------------------------------------------
                                                                                                         Section 27
                           (2523) 2523   2530      2540      2550      2560      2570      2580      2590      2600                 2619
          Ad 12 X73487     (2523) TTGCTGGAGATAAGTTTAAAGGCATTATGTTCGAAGCTAATACCTGTCTTGTCTTGCATGGTGTTTACTTTCTTAACTTTAGTAACATTTGTGTAGA
 SARS-CoV-2 NC_045512.2       (1) -------------------------------------------------------------------------------------------------
                                                                                                         Section 28
                           (2620) 2620      2630      2640      2650      2660      2670      2680      2690      2700            2716
          Ad 12 X73487     (2620) GTCTTGGAATAAGGTTTCTGCTAGGGGCTGTACTTTTTATGGATGTTGGAAGGGTTTGGTGGGTAGACCAAAAAGTAAACTGTCTGTAAAAAAGTGT
 SARS-CoV-2 NC_045512.2       (1) -------------------------------------------------------------------------------------------------
```

```
                                                                                                    Section 29
                        (2717) 2717       2730     2740     2750     2760     2770     2780     2790     2800      2813
          Ad 12 X73487  (2717) TTGTTTGAAAAATGTGTACTTGCTTTAATTGTAGAGGGGGATGCACATATTAGGCATAATGCAGCTTCAGAAAATGCCTGTTTTGTATTATTGAAGG
SARS-CoV-2 NC_045512.2     (1) -------------------------------------------------------------------------------------------------
                                                                                                    Section 30
                        (2814) 2814 2820     2830     2840     2850     2860     2870     2880     2890     2900  2910
          Ad 12 X73487  (2814) GAATGGCTATTTTAAAGCATAATATGGTTTGTGGGGTGTCTGATCAAACTATGCGACGTTTTGTTACCTGTGCTGATGGAAATTGTCATACCTTAAA
SARS-CoV-2 NC_045512.2     (1) -------------------------------------------------------------------------------------------------
                                                                                                    Section 31
                        (2911) 2911     2920     2930     2940     2950     2960     2970     2980     2990        3007
          Ad 12 X73487  (2911) AACTGTTCATATTGTGAGCCACAGTAGACATTGTTGGCCTGTATGTGATCATAACATGTTTATGCGCTGTACCATACATTTAGGCTTAAGGCGGGGT
SARS-CoV-2 NC_045512.2     (1) -------------------------------------------------------------------------------------------------
                                                                                                    Section 32
                        (3008) 3008        3020     3030     3040     3050     3060     3070     3080     3090      3104
          Ad 12 X73487  (3008) ATGTTTAGACCTTCCCAATGTAACTTCAGCCACTCAAACATTATGCTGGAACCTGAAGTGTTTTCTAGAGTGTGTTTAAATGGGGTATTTGATTTAT
SARS-CoV-2 NC_045512.2     (1) -------------------------------------------------------------------------------------------------
                                                                                                    Section 33
                        (3105) 3105 3110     3120     3130     3140     3150     3160     3170     3180     3190  3201
          Ad 12 X73487  (3105) CTGTGGAATTATGTAAGGTTATAAGATATAATGATGATACTCGACATCGTTGCCGACAGTGTGAGTGTGGTAGCAGTCATCTAGAACTTCGTCCCAT
SARS-CoV-2 NC_045512.2     (1) -------------------------------------------------------------------------------------------------
                                                                                                    Section 34
                        (3202) 3202 3210     3220     3230     3240     3250     3260     3270     3280            3298
          Ad 12 X73487  (3202) TGTGCTAAATGTAACTGAGGAGCTGAGAAGTGACCACCTTACCCTGTCTTGCCTGCGGACTGACTATGAGTCAAGTGATGAAGACGACAACTGAGGT
SARS-CoV-2 NC_045512.2     (1) -------------------------------------------------------------------------------------------------
                                                                                                    Section 35
                        (3299) 3299       3310     3320     3330     3340     3350     3360     3370     3380      3395
          Ad 12 X73487  (3299) AAGTGGGTGGAGCTAGGTGGGATTATAAAAGGCTGGAAGTCAACTAAAAATTGTTTTTGTTCTTTTAACAGCACGATGAACGGAACTACTCAGAACA
SARS-CoV-2 NC_045512.2     (1) -------------------------------------------------------------------------------------------------
```

Section 36

		3396 3410 3420 3430 3440 3450 3460 3470 3480 3492
	(3396)	
Ad 12 X73487	(3396)	ACGCTGCGCTTTTTGATGGAGGGGTTTTTAGCCCTTATTTGACTTCCAGGTTACCATATTGGGCCGGAGTACGTCAGAATGTGGTAGGATCTACAGT
SARS-CoV-2 NC_045512.2	(1)	---

Section 37

		3493 3500 3510 3520 3530 3540 3550 3560 3570 3589
	(3493)	
Ad 12 X73487	(3493)	GGACGGTCGACCTGTGGCACCTGCAAATTCATCAACATTAACCTATGCAACTATTGGACCCTCGCCTTTGGATACCGCCGCCGCCGCTGCAGCTTCC
SARS-CoV-2 NC_045512.2	(1)	---

Section 38

		3590 3600 3610 3620 3630 3640 3650 3660 3670 3686
	(3590)	
Ad 12 X73487	(3590)	GCGGCCGCTTCTACGGCTCGCAGTATGGCAGCTGATTTCAGCTTCTACAATCACTTGGCTTCGAATGCTGTGACACGCACCGCAGTTCGAGAGGACA
SARS-CoV-2 NC_045512.2	(1)	---

Section 39

		3687 3700 3710 3720 3730 3740 3750 3760 3770 3783
	(3687)	
Ad 12 X73487	(3687)	TTCTGACTGTTATGCTTGCCAAGCTTGAAACTCTAACTGCTCAGCTGGAAGAGCTATCGCAAAAGGTTGAGGAATTAG--CTG-ATGCTACTACCCA
SARS-CoV-2 NC_045512.2	(1)	--ATTAAAGGTTTATACCTTCCCAGGTAACAAACCAACCAACTTTCGATCTCTTG

Section 40

		3784 3790 3800 3810 3820 3830 3840 3850 3860 3870 3880
	(3784)	
Ad 12 X73487	(3781)	TAC--------CCCAGCCCAACCTGTAACCCAATAAAGAAAAAACTTAAATTGAGATGGTGTT--ATGAATC---TTTATTGATA-CTT--------
SARS-CoV-2 NC_045512.2	(54)	TAGATCTGTTCTCTAAACGAACTTTAAAATCTGTGTGGCTGTCACTCGGCTGCATGCTTAGTGCACTCACGCAGTATAATTAATAACTAATTACTGT

Section 41

		3881 3890 3900 3910 3920 3930 3940 3950 3960 3977
	(3881)	
Ad 12 X73487	(3856)	GTTTTTTCTGACATGGTAAGCTCTTGACCACCGTTCCCTATCAT--TAAGAACACGGTGAATGTGTTCCAGTATT--TTGTAAAGATGAGCCTGTAT
SARS-CoV-2 NC_045512.2	(151)	CGTTGACAGGACACG--AGTAACTCGTCTAT-CTTCTGCAGGCTGCTTACGGTTTCGTCCGTGT-TGCAGCCGATCATCAGCACATCTAGG---TTT

Section 42

		3978 3990 4000 4010 4020 4030 4040 4050 4060 4074
	(3978)	
Ad 12 X73487	(3949)	-ATTAAGGTACATTGGCATTAGGCCATCTTTGGGA----TGAAGGTAGGACCATTGAAGGGCTTCATGTTCCGGGTTAGTGT-----TGTAGATAA-
SARS-CoV-2 NC_045512.2	(241)	CGTCCGGGT--GTGACCGAAAGGTAAGATGGAG-AGCCTTGTCCCTGGTTTCAACG-AGAAAACACACGTCCAACTCAGTTTGCCTGTTTTACAGGT

```
                                                                                                              Section 43
                             (4075) 4075 4080          4090           4100          4110          4120          4130          4140          4150          4160          4171
             Ad 12 X73487    (4035) TCCAGTCATAGCA--ACAACGCTGGGCATGG-TGATTAAATATATCTT-TTAACAACAAGCTAATTG-CTAATGGAAG-ACCTTTAGTATAGGTATT
 SARS-CoV-2 NC_045512.2      (334)  TCGCGACGTGCTCGTACGTGGCTTTGGAGACTCCGTGGAGGAGGTCTTATCAGAGGCACGT-CAACATCTTAAAGATGGCACTTGTGGCTTAGTAGA
                                                                                                              Section 44
                             (4172) 4172     4180          4190          4200          4210          4220          4230          4240          4250                4268
             Ad 12 X73487    (4126) GATA-AAACGGTTAAGCTGGGTGGGATGCATCCGAGGTGACATGATAT--GAAGTTTTGATTGTATTTTGAGATTGGCAA-TGTTACCTGCCAAATC
 SARS-CoV-2 NC_045512.2      (430)  AGTTGAAAAAGGCGTTTTGCCTCAACTTGAACAGCCCTATGTGTTCATCAAACGTT-----CGGATGCTCGAACTGCACCTCATGGTCATGTTATGG
                                                                                                              Section 45
                             (4269) 4269        4280          4290          4300          4310          4320          4330          4340          4350          4365
             Ad 12 X73487    (4219) TCTTCTTGGATTCATAT-TGTGGAGAACCACGAAAACGGTGT--AGCCAGTACACTTGGGAA-ATTTGTCATGGAGTTTAGAAGGAAAGGCATGGAA
 SARS-CoV-2 NC_045512.2      (522)  TTGAGCTGGTAGCAGAACT-CGAAGGCATTCAGTACGGTCGTAGTGGTGAGACACTTGGTGTCCTTGTCCCTCATGT-------------GGGCGAA
                                                                                                              Section 46
                             (4366) 4366           4380          4390          4400          4410          4420          4430          4440          4450          4462
             Ad 12 X73487    (4312) AAACTTGGAAACGCCTTTGTGACTTCCCAAATTTTCCATACACTCATCCATTATTATGGCA-ATTGGACCGCGAGCAGCGGC-TTGAGCAAAAATGT
 SARS-CoV-2 NC_045512.2      (605)  ATACCAGTG---GC--TTACCGCAAGGTTCTTCTTCGTAAGAACGGTAATAAA-----GGAGCTGGTGGCCATAGTTACGGCGCCGATCTAA-A-GT
                                                                                                              Section 47
                             (4463) 4463   4470          4480          4490          4500          4510          4520          4530          4540                4559
             Ad 12 X73487    (4407) TTTCTGGATCAGAA-ACATCAT-AGTTGTGGTCTAGAGTTAGGTCATCGT-AGGACAACTTAACAAATTTAGGACACAGCGTTCCAGATTGTGGAAT
 SARS-CoV-2 NC_045512.2      (690)  CATTTGACTTAGGCGACGAGCTTGGCACTGATCCTTATGAAGATTTTCAAGAAAACTGGAACACTAAACATAGCAGTGGTGTTAC---CCGTGAACT
                                                                                                              Section 48
                             (4560) 4560       4570          4580          4590          4600          4610          4620          4630          4640                4656
             Ad 12 X73487    (4501) AATAGTTCCCTCTGGTCCTGGGACA--TAATTTCCCTCACAAATTTGCATTTCCCAAGATTTAATTTCAGA-----TGGGGGAATC----ATGTCCA
 SARS-CoV-2 NC_045512.2      (784)  CATGCGTGAGCTTAACGGAGGGGCATACACTCGCTATG-TCGATAACAACTTCTGTGGCCCTGATGGCTACCCTCTTGAGTGCATTAAAGACCTTCT
                                                                                                              Section 49
                             (4657) 4657         4670          4680          4690          4700          4710          4720          4730          4740          4753
             Ad 12 X73487    (4587) --CTTGCGGA-ACAATAAAAAAAACAGTTTC--TGGAGCAGGTGTAACCAGCTGGGCAGAAAGCAAATTACGCAACAACTGAGACTTCCCACAGCCA
 SARS-CoV-2 NC_045512.2      (880)  AGCACGTGCTGGTAAAGCTTCATGCACTTTGTCCGAACAACTGGACTTTATTGACACTAAGAGGGGTGTATACTGCTGCCGTGAACATGAGCATGAA
```

```
                                                                                           Section 50
                        (4754) 4754  4760      4770      4780      4790      4800      4810      4820      4830      4840     4850
          Ad 12 X73487  (4679) GTGGGTCCATAAATTACCCAATTACAGGTTGCAAGTGATAGTTTA-ACGAGGTGCAGCTGCCGTCTTCGTGGAGAAGCGGAGCCACTTCATTCATC
SARS-CoV-2 NC_045512.2   (977) ATTGCTTGGTACACGGAACGTTCTGAAAAGAG--CTATGAATTGCAGACACCTTT----TGAAATTAAATTGGCAAAGAAATTTGACACCTTC---A
                                                                                           Section 51
                        (4851) 4851     4860      4870      4880      4890      4900      4910      4920      4930           4947
          Ad 12 X73487  (4775) ATTTGTCGGACGCGGATGTTTTGCTTGGCCAGTTCCCCTAACAGACGCTCTCCGCCTAAGGAAAGTAACTCTTGTAAAGATTTGAAATTTTTAAGTG
SARS-CoV-2 NC_045512.2  (1065) ATGGG--GAATGTCCAAATTTTGTAT------TTCCCTTAAATTCCATAATCAAGACTATTCAACCAAGGGTTGAAAAG----AAAAAGCTTGATGG
                                                                                           Section 52
                        (4948) 4948        4960      4970      4980      4990      5000      5010      5020      5030      5044
          Ad 12 X73487  (4872) GCTTTAGGCCATCGGCCATAGGCATGTGGTCCAGGGTTTGCTT-CAGCAGTTGCAAGCGATCCCATAGCTCAGTTATATTTTCTATGCCATCTCGAT
SARS-CoV-2 NC_045512.2  (1150) CTTTATGGGTAGAATTC-GATCTGTCTATCCAGTTGCGT-CACCAAATGAATGCAACCAAATGTGCCTTTCAACT-CTCATGAAGTGTGATCATTGT
                                                                                           Section 53
                        (5045) 5045 5050      5060      5070      5080      5090      5100      5110      5120      5130     5141
          Ad 12 X73487  (4968) CCAGCAAACTTCCTCGTTGCGGGGTTTGGCTGGCTGTTGCTGTAAG-----GAACGAGGCGGTGAGCATCCAAATGGACGAGGGTTTTGTCCTTCC
SARS-CoV-2 NC_045512.2  (1244) GGTG-AAACTTCATGGCAGACGGGC--------GATTTTGTTAAAGCCACTTGCGAATTTTGTGGCACTGAGAATTTGACTAAAGAAGGTGCCACTA
                                                                                           Section 54
                        (5142) 5142    5150      5160      5170      5180      5190      5200      5210      5220           5238
          Ad 12 X73487  (5060) AGGGACGTAATGTGCGCGTCAGGGTTGTTTCGGTCACGGTGAATGGATGCGCTCCTGGTTGAGCGCTGGCCAGTGTGCGCTTTAAACTGAGGCGGCT
SARS-CoV-2 NC_045512.2  (1332) CTTGTGGTTACTTA--CCCCAAAATGCTGTTGTTAAAATTTA----T--T-GTCCAGCATG-----TCACAATTCAGAAGTAGGACCTGAGCATAGT
                                                                                           Section 55
                        (5239) 5239       5250      5260      5270      5280      5290      5300      5310      5320          5335
          Ad 12 X73487  (5157) GGTGCTGAAGCGCGTGTCTTCTCCCTGTGCTTCGGCAAGGTAGCATTTTAACATAAGATCATAAGACAAAGCCTCTGTAGCG--TGGCCTTTAGCCC
SARS-CoV-2 NC_045512.2  (1415) CTTGCCGAATACCAT----AATGAATCTGGCTTGAAAACCATTCTTCGTAAG-GGTGGTCGCACTATT--GCCTTTGGAGGCTGTGTGTTCTCTTAT
                                                                                           Section 56
                        (5336) 5336          5350      5360      5370      5380      5390      5400      5410      5420     5432
          Ad 12 X73487  (5252) GTATTTTTCCTTTGGAGGTGCTCCCGCAGTGAGGACACT----GAAGGCATTTAAGGGCGTACAGTTTTGGAGCCAAAAAAACAGATTCTGGAGAAT
SARS-CoV-2 NC_045512.2  (1505) GTTGGTTGCCATAACAAGTGTGCCTATTGGGTTCCACGTGCTAGCGCTAACATAGGTTGTAACCATACAGGTGTTGTTGGAGAAGGTTCCGAAGGTC
```

```
                                                                                                    Section 57
                         (5433) 5433   5440                5450                5460                5470                5480                5490                5500                5510                    5529
Ad 12 X73487             (5345) AAGCATCTG--C-GCCACAATAACTACAAACAGTTTCACATTCAACTGACCAGGTCAGCTCAGGACATGATGGATCAAAAACAAGTTTCCCTCCGTA
SARS-CoV-2 NC_045512.2   (1602) TTAATGACAACCTTCTTGAAATACTCCAAAAAG--AGAAAGTCAAC--ATCAATATTGTTGGTGACTTTAAACTTAATGAAGA-GAT---CGCCATT

                                                                                                    Section 58
                         (5530) 5530       5540                5550                5560                5570                5580                5590                5600                5610            5626
Ad 12 X73487             (5439) CTTTTTGATGCGTTTCTTAC-----CTT-GCGACTCCATAAGGCGGCGTCCTTTCTCTGTGACAA-AAAGACTGTCAG-TGTCTCCGTATACAGATT
SARS-CoV-2 NC_045512.2   (1691) ATTTTGGCATCTTTTTCTGCTTCCACAAGTGCTTTTGTGGAAACTGTGAAAGGT---TTGGATTATAAAG-CATTCAACAAATTGTTGAATCCTGT

                                                                                                    Section 59
                         (5627) 5627         5640                5650                5660                5670                5680                5690                5700                5710          5723
Ad 12 X73487             (5528) TAAGGGGTCTATCCTTC-AGTGGTA---TTCCGCGGTCCTCCTCGTACAGGAATTCTGACCACTCTGACACAAAAGCTCTAGTCCAAGCAAGTACAA
SARS-CoV-2 NC_045512.2   (1784) GGTAATTTTAAAGTTACAAAAGGAAAAGCTAAAAAAGGTGCCTGGAATATTGGTGAACAGAAATCAATACTGAGTCCTCTTTATGCATTTGCATCAG

                                                                                                    Section 60
                         (5724) 5724  5730                5740                5750                5760                5770                5780                5790                5800                5810    5820
Ad 12 X73487             (5621) AGGAAGCCACATGGGAAGGGTACCGATCGTTGTTAATTAAAGGGTTAGAACTTTCTAAGGTGTGTAAACA-----CATGTCTCCTTCTTCAGCGTCC
SARS-CoV-2 NC_045512.2   (1881) AGGCTGCTCGTGTTGTACGATCAATTTTCTCCCGCACT-CTTGAAACTGCTCAA-AATTCTGTGCGTGTTTTACAGAAGGCCGCTATAACAATACTA

                                                                                                    Section 61
                         (5821) 5821     5830                5840                5850                5860                5870                5880                5890                5900            5917
Ad 12 X73487             (5713) --ATGAATGTGATTGGTTTG--TAGGTGTAAGTCACGTGTTCACAA-TTTTCTGGTGGTGGGCTATAAAAAGGGGCGGGTCCTT-GGTCTTCA-TCG
SARS-CoV-2 NC_045512.2   (1976) GATGGAATTTCACAGTATTCACTGAGACTCATTGA--TGCTATGATGTTCACATCTGATTTGGCTACTAACAATCTAGTTGTAATGGCCTACATTAC

                                                                                                    Section 62
                         (5918) 5918        5930                5940                5950                5960                5970                5980                5990                6000          6014
Ad 12 X73487             (5803) CTTTCTTCTGCTTCGCTG-TTTACGAGCGCCAACTGGTTGGGTGAGTACACGCGCTCAAAGGCAGGCATTACCTCTGTACTCAACGTGTCAGTTTCT
SARS-CoV-2 NC_045512.2   (2071) AGGTGGTGTTGTTCAGTTGACTTCG-CAGTGGCTAACTAACAT-CTTTGGCACTGTTTATGAAAAAC-TCAAACCCGTCCTTGATTGGCTTGAAG--

                                                                                                    Section 63
                         (6015) 6015 6020                6030                6040                6050                6060                6070                6080                6090                6100       6111
Ad 12 X73487             (5899) ATAAAC--GATGAGGATTTGATGTTTAATCGCCCCGCTGCAA--TTTCTTTCATTAGGCT-TTC--TTCCATTTGATCAGAAAAAACTATTTTTTTG
SARS-CoV-2 NC_045512.2   (2163) AGAAGTTTAAGGAAGGTGTAGAGTTTCTTAGAGACGGTTGGGAAATTGTTAAATTTATCTCAACCTGTGCTTGTGAAATTGTCGGTGGACAAATT-G
```

```
                                                                                                    Section 64
                           (6112) 6112    6120      6130      6140      6150      6160      6170      6180      6190           6208
Ad 12 X73487               (5989) TTATCTA-GTTTGGTAGCAAAAGATCCGTACAAGG-CATTGGAAAGCAGCTTGGCTATAGATCTTAGGGTTTGATTT-TTGTCCCTATCGGCCCGTT
SARS-CoV-2 NC_045512.2     (2259) TCACCTGTGCAAAGGAAATTAAGGAGAGTGTTCAGACATTC--TTTAAGCTTGTAAATAAATTTTTGGCTTTG-TGTGCTGACTCTATCATTATTGG
                                                                                                    Section 65
                           (6209) 6209       6220      6230      6240      6250      6260      6270      6280      6290      6305
Ad 12 X73487               (6083) CTTTTGCGGCAATATTGAGTTGCACATA--T---TCGCGTGCCAGGCATTTC-----CAGGTGGGGAAAATGGTGGTGCGCTCGTCAGATAGCAAG-
SARS-CoV-2 NC_045512.2     (2353) TGGAGCTAAACTTAAAGCCTTGAATTTAGGTGAAACATTTGTCACGCACTCAAAGGGATTGTACAGAAAGTGTGTTAAATC----CAGAGAAGAAAC
                                                                                                    Section 66
                           (6306) 6306          6320      6330      6340      6350      6360      6370      6380      6390      6402
Ad 12 X73487               (6169) CGTAAGCGCCACCCGCGATTATGCAGTGTAACCAGATCTACGCTGGTAACTACTTCACCGCGCAAGCTTTCA----TT----GGTCCAGGCTAAACG
SARS-CoV-2 NC_045512.2     (2446) TGGCCTACTCATGCCTCTAAAAGCCCCAAA---AGAAATTATCTTCTTAGAGGGAGAAACACTTCCCACAGAAGTGTTAACAGAGGAAGTTGTCTTG
                                                                                                    Section 67
                           (6403) 6403   6410      6420      6430      6440      6450      6460      6470      6480           6499
Ad 12 X73487               (6258) ---ACCGCCTTTTCTAGAACAAAA-AGGAGGAAGAACATCCAACTGATTTTCATCTGGGGGGTCGGCATCTATAGTAAAAATGCCAGGACAAAGATT
SARS-CoV-2 NC_045512.2     (2540) AAAACTGGTGATTTACAACCATTAGAACAACCTACTAGTGAAGCTGTTGAAGCTCCATTGGTTGGTACACCAGTTTGTATTAACGGGCTTA----TG
                                                                                                    Section 68
                           (6500) 6500      6510      6520      6530      6540      6550      6560      6570      6580           6596
Ad 12 X73487               (6351) TTTGTCAAAATAATCAATTTTGCAAGTGTAATTTTCCA--GCGCCACCTGCCATTGC--CGCACGGCCAATGCC--CGCTCATAGGGGTTAAGGGGA
SARS-CoV-2 NC_045512.2     (2633) TTGCTCGAAATCAAAGACACAGAAAA-GTACTGTGCCCTTGCACCTAATATGATGGTAACAAACAATACCTTCACACTCAAAGGCGGTGCACCAACA
                                                                                                    Section 69
                           (6597) 6597         6610      6620      6630      6640      6650      6660      6670      6680      6693
Ad 12 X73487               (6442) GG----ACCCCAAGGCATGGGGTGTGTGAGGGCCGATGCATACATGCCGCAAATATCATATACATATATGGGCTCTTTTAGTACTCCTATGTAAGTA
SARS-CoV-2 NC_045512.2     (2729) AAGGTTACTTTTGGTGATGACAC-TGTGATAGAAG-TGCAAGGTTACAAGAGTGTGAATATCACTT--TTGAACTTGATGAAAGGATTGATAAAGTA
                                                                                                    Section 70
                           (6694) 6694  6700      6710      6720      6730      6740      6750      6760      6770      6780    6790
Ad 12 X73487               (6535) GGATAGCACCTGCCGCCACGAATGCTGGCGCGAACGTAGTCATATAGCTCATGTGAAGGCGCCAGGATGTTGGGCCCAAGATGTGTGCGCTGTGGTT
SARS-CoV-2 NC_045512.2     (2822) CTTAATGAGAAG-TGCTCTGCCTATACAGTTGAACTCGGTACAGAAGTAAATGAGTTCGC-CTGTGTTGTGGCAGATGCTGTCATAAAAACTTTGCA
```

Section 71

```
                         (6791) 6791     6800        6810        6820        6830        6840        6850        6860        6870              6887
Ad 12 X73487             (6632) TTTCGGCGCGGTACAAAATTTGTCTGAAAATTGCATGAGAGTTAGAGGAAATGGTAGGACGCTGAAACACATTAAAATGTGCCGCGTCAAGACCCAC
SARS-CoV-2 NC_045512.2   (2917) ACCAGTATCTGAATTACT----TACACCACTGGCATTGATTTAGATGAGTGG-AGTATGGCTACATACTACTTATT--TGATGAG------TCTGG
```

Section 72

```
                         (6888) 6888           6900        6910        6920        6930        6940        6950        6960        6970        6984
Ad 12 X73487             (6729) TGCGTCAGTA-ACAAACTGGGCGTATGAGCTACGCAGTTTTTCTACCAATGAGGCAGTCACAAGTACATCCAGGGCACAATAGTTTAATGTTTCCCC
SARS-CoV-2 NC_045512.2   (3001) TGAGTTTAAATTGGCTTCA--CATATGTATTGTTCTTTCTACCCTCCAGATGAGGATGAAGAAGAAGGTGATTGTGAAGAAGAA---GAGTTTGAGC
```

Section 73

```
                         (6985) 6985 6990        7000        7010        7020        7030        7040        7050        7060        7070     7081
Ad 12 X73487             (6825) GATAAG---ATTGT-AATTTTTTCTCCTTTTTTTTTCCATAGTTCTTGATTTAGGAGGTATTCCTCCTTATCCTTCCAGT-ACTCCTCCAGGGGAA
SARS-CoV-2 NC_045512.2   (3093) CATCAACTCAATATGAGTATGGTACTGAAGATGATTACCAAGGTAA--ACCTTTGGAATTTGGTGCCA-----CTTCTGCTG-CTCTTCAACCTGAA
```

Section 74

```
                         (7082) 7082    7090        7100        7110        7120        7130        7140        7150        7160              7178
Ad 12 X73487             (6917) ACCCATTTGCATCTGCACGGTA-AGAACCA-AGCATATAAAACTGATTTACCGCCTTGTACGGACAACATCCTTTTTCTACAGGCAGGGCATACGCT
SARS-CoV-2 NC_045512.2   (3182) ---GAAGAGCAAGAAGAAGATTGGTTAGATGATGATAGTCAACAAACTGTTGGTCAACAA--GACGGCAG---TG-AGGACAATCAGACAA--CTAC
```

Section 75

```
                         (7179) 7179       7190        7200        7210        7220        7230        7240        7250        7260              7275
Ad 12 X73487             (7012) TGTGCAGCCTTTCTTAAAGATGTATGAGTAAGAGCAAAGGTATCTCTGACCATTACTTTTAAATACTGGTATTTAAAATCTTGGTCGTCACACCCTC
SARS-CoV-2 NC_045512.2   (3268) TATTCAAACAATTGTTGAGGTTCAACCTCAATTAGAGATGGAACTTACACCAGTTGTTCAGACTATTGAAGTGAATAGTTTTAGTGGTTATTTAAAA
```

Section 76

```
                         (7276) 7276          7290        7300        7310        7320        7330        7340        7350        7360        7372
Ad 12 X73487             (7109) CGTGTTCCCACAGTAGGAAG--TTAGTTCGCTTTTTGTAGTGGGATTGGGAAGGGCAAAAGTAATATCATTAAATAATATTTTGCCAG---CTCTT
SARS-CoV-2 NC_045512.2   (3365) CTTACTGACAATGTATACATTAAAAATGCAGACATTGT-GGAAGAAGCTAAAAAGGTAAAACCAACAGTGGTTGTTAATGC--AGCCAATGTTTACC
```

Section 77

```
                         (7373) 7373   7380        7390        7400        7410        7420        7430        7440        7450              7469
Ad 12 X73487             (7201) GGAA-----TAAAATTTCTAGAAATTTTAAAGGGTCCAGGGACGTCCAAG--CGGTTATTGATTACCTGAGCGGCAAGAACAATTTCATCAAATCCA
SARS-CoV-2 NC_045512.2   (3459) TTAAACATGGAGGAGGTGTTGCAGGAGCCTTAAATAAGGCTACTAACAATGCCATGCAAGTTGAATCTGATGATTACATAGCTACTAATGGACCACT
```

```
                                                                                          Section 78
                               (7470) 7470      7480      7490      7500      7510      7520      7530      7540      7550           7566
Ad 12 X73487                   (7291) TTAATATTGTGTCCTACTATATACAACTCTACAAATCTTGGCTCACCCTTAATTGCAGGGGCTCTTTTAAGATCTTCGTAG---GAAAGATCTTCAA
SARS-CoV-2 NC_045512.2         (3556) TAAAGTGGGTGGTAGTTGTGTTTTAAGCGGACACAATCTTGCTAAACACTGTCTTCATGTTGTCGGCCCAAATGTTAACAAAGGTGAAGACATTCAA
                                                                                          Section 79
                               (7567) 7567         7580      7590      7600      7610      7620      7630      7640      7650        7663
Ad 12 X73487                   (7385) GC----GCGACTAGTCCGTTTTCTTCTTGAGCCCATTGAGACAAGTGTGGATTTTTTTGTAAAAAAGT-CATCCAAAGATCAGTAGCTAAGGAGGTT
SARS-CoV-2 NC_045512.2         (3653) CTTCTTAAGAGTGCTTATGAAAATTTTAATCAGCACGAAGTTCTACTTGCACCATTATTATCAGCTGGTATTTTTG-GTGCTGACCCTATACAT-TC
                                                                                          Section 80
                               (7664) 7664  7670      7680      7690      7700      7710      7720      7730      7740      7750    7760
Ad 12 X73487                   (7477) TGTAAGCGGTTTCTATAGGTACGAAACTGTTGACCGACCTTCATTTTTTCTGGGGTTAAGCAGTAGAAAGTAGTAGAGTCTTTTTCCCATTGGTCCC
SARS-CoV-2 NC_045512.2         (3748) TTTAAGAGTTTG-TGTAGATACTGTTCGCACAAATGTCTACTTAGCTGTCTTTGATAAAAATCTCTATGACAAACTTGTTTCAAGCTTTTTGGA--A
                                                                                          Section 81
                               (7761) 7761     7770      7780      7790      7800      7810      7820      7830      7840           7857
Ad 12 X73487                   (7574) ATCCAAGTTCTAATGCAAGTTGTAAGGCATGTTTGACAAGATTGTCATCCCCAGACAGTTTCATCACCAGCATAAATGGGACAAGTTGCTTTC---C
SARS-CoV-2 NC_045512.2         (3842) ATGAAGAGTGAAAAGCAAGTTGAACA-AAAGATCGCTGAGATTCCTAAA---GAGGAAGTTAAGC-CATTTATAACTGAAAGTAAAC-CTTCAGTTG
                                                                                          Section 82
                               (7858) 7858        7870      7880      7890      7900      7910      7920      7930      7940        7954
Ad 12 X73487                   (7668) AAATGCCCCCATCCAGGTGTAGGTTTCTACATCATAGGTAATAAAAAGGCGCTCAGTGCGAGGATGCGAACCGATTGGGAAAAAGTGGATCTCCTGC
SARS-CoV-2 NC_045512.2         (3933) AACAGAGAAAACAAGATGATAAGAAAAT-CAAAGCTTGTGTTGAAGA-------AGTT--ACA--AC--AACTCTGGAAGAAACTAAGTTCCTCACA
                                                                                          Section 83
                               (7955) 7955 7960      7970      7980      7990      8000      8010      8020      8030      8040     8051
Ad 12 X73487                   (7765) CACCAGTTGGAAGAATGGCTGTTGATGTGATGAAAGTAGAAATCTCGTCGGCGGACAGAGCATTCATGCTGATGTTTGTAAAAGCGTGCGCAGTGTT
SARS-CoV-2 NC_045512.2         (4016) GAAAACTTGTTACTTTATATTGACATTAATGGCAA-T------CTTCATCCAGATTCTGCCACTCTTGTTAGTGACATTGA---CAT-CACTTTCTT
                                                                                          Section 84
                               (8052) 8052    8060      8070      8080      8090      8100      8110      8120      8130           8148
Ad 12 X73487                   (7862) CGCATCGTTGCACGGGCTGTATCTGTTGAATGAGGTGTACCTGGCGGCCTCGCACCAGAAAGCAGA-TGGGAAAATCAATACCACTTGGCAGCTGCC
SARS-CoV-2 NC_045512.2         (4102) AAA---GAAAGATGCTCCATATATAGTGGGTGATGTTGTTCAAGAGGGTGTTT----------TAACT-GCTGTGGTTATACCTAC-TAAAAAGGCT
```

Section 85

```
                            (8149) 8149       8160      8170      8180      8190      8200      8210      8220      8230      8245
Ad 12 X73487                (7958) GTTCGTCCTCTTCCTCTTCTGCTGCATTGCCACTACCGTTTGGATCCTCGAAAGCGAGAACGGAGA---GGGTGACGGTGCCCCTCGACCTGCATGT
SARS-CoV-2 NC_045512.2      (4184) GGTGGCACTACTGAAATGCTAGCGAAAGCTTTGAGAAAAGTG-CCAACAGACAATTATATAACCACTTACCCGGGTCAGGGTTTAAATGGTTACACT
```

Section 86

```
                            (8246) 8246          8260      8270      8280      8290      8300      8310      8320      8330      8342
Ad 12 X73487                (8052) CCAGATTTCAGCACGAGAGGGCGGAAACGGGAAATCAGGGCGTACAGCCTGGAGCTGTCCATGGTATC-AGTCAGAGAGAAAAGCATGTCCGCGGG
SARS-CoV-2 NC_045512.2      (4280) GTAGAGGAGGCAAAGACAGTGCTTAAAAAGTGTAAAAGTGCCTTTTACATTCTACCA-TCTATTATCTCTAATGAGAAGCAAGAAATTCTTGGAACT
```

Section 87

```
                            (8343) 8343   8350      8360      8370      8380      8390      8400      8410      8420          8439
Ad 12 X73487                (8148) GACAGCGCGCAAGTTGACTTCGCACAGGCGGGTAAGAGCAGGCTGGAGGTGCAGGTAAT---ACTTAATTTCTAGAGGCGTGCCGTTGGCAGAGT--
SARS-CoV-2 NC_045512.2      (4376) --------GTTTCTTGGAATTTGCGAGAAATGCTTGCACATGCAGAAGAAACACGCAAATTAATGCCTGTCTGTGTGGAA-ACTAAAGCCATAGTTT
```

Section 88

```
                            (8440) 8440      8450      8460      8470      8480      8490      8500      8510      8520          8536
Ad 12 X73487                (8240) CTAT--TGCGTGAAGTATTCCATGAGCCCGGGGACTAACCACGGT----TCCACGGTGCACT------TTTCCAATGCGCCTGCTT--AAAATCGGC
SARS-CoV-2 NC_045512.2      (4464) CAACTATAC-AGCGTAAATATAAGGGTATTAAAATACAAGAGGGTGTGGTTGATTATGGTGCTAGATTTTACTTTTACACCAG--TAAAACAACTGT
```

Section 89

```
                            (8537) 8537         8550      8560      8570      8580      8590      8600      8610      8620      8633
Ad 12 X73487                (8323) GGCGCGGACGAGCTCCCGGAGGAAGCGGCGGTTCGGGTCC-TGCGGGAAGCGGGGGAAGCGGTATGTCGGCCTGACGCTCTGG--CAGGGGAAGGTG
SARS-CoV-2 NC_045512.2      (4558) AGCGTCACTTATCAACACACTTAACGATCTAAATGAAACTC-TTGTTACAATGCCACTTGGCTATGTA-ACACATGGCTTAAATTTGGAAGAAGCTG
```

Section 90

```
                            (8634) 8634  8640      8650      8660      8670      8680      8690      8700      8710      8720    8730
Ad 12 X73487                (8417) TTGAGCCCGAAGTTGACTGGCATGG-GCGACTACCCGGCGATT---GATATCTTGAATCTGTCGGCGTTGTGTAAACACTACCGGCCCTGTTGTTTT
SARS-CoV-2 NC_045512.2      (4653) CTC-GGTATATGAGATCTCTCAAAGTGCCAGCTACAGTTTCTGTTTCTTCACCTGATGCTGTTACAGC-GTATAATGGTTATCTTACTTCTTCTTCT
```

Section 91

```
                            (8731) 8731     8740      8750      8760      8770      8780      8790      8800      8810          8827
Ad 12 X73487                (8510) GAA-CCTGA-AAGAAAGTTCAACAGAATCAATCTCAGTGTCATTTACTGCAGCCTGTCTTAAAATCTCCTGAACGTCGCCTGAGTT---ATCTTGGT
SARS-CoV-2 NC_045512.2      (4748) AAAACACCTGAAGAACATTTTATTGAAACCATCTCAC------TTGCTGGTTCCTATAAAGATTGGTCCT-ATTCTGGACAATCTACACAACTAGGT
```

```
                                                                      Section 92
                              (8828) 8828       8840     8850     8860     8870     8880     8890     8900     8910      8924
              Ad 12 X73487    (8602) AGGCAATTTCTGCCATTAATTGATCAATTTCTTCCTCCTGG-AGGTCTCCATGTCCCGC--ACGTTCAAT-AGTGGCTGCAAGGTCATTAGA-TATC
SARS-CoV-2 NC_045512.2        (4838) ATAGAATTTCTTAAGAGAGGTGATAAAAGTGTATATTACACTAG---TAATCCTACCACATTCCACCTAGATGGTGAAGTTATCACCTTTGACAATC
                                                                      Section 93
                              (8925) 8925 8930      8940     8950     8960     8970     8980     8990     9000     9010     9021
              Ad 12 X73487    (8694) C---GACTCATA-AGCTGTGAAAATGC----GTTTAGTCCAATTTCGTTCCAGACTCGGCTGTATACT-ACCCCTCCTTCGCTGTCCCGAGCGCGCA
SARS-CoV-2 NC_045512.2        (4932) TTAAGACACTTCTTTCTTGAGAGAAGTGAGGACTATTA-AGGTGTTTACAACAGTAGACAACATTAACCTCCAC-ACGCAAGTTGTGGACATGTCA
                                                                      Section 94
                              (9022) 9022   9030     9040     9050     9060     9070     9080     9090     9100          9118
              Ad 12 X73487    (8782) TAACCACTTGCGCCAAGTTGAGTTCC-ACGAGCCGTGC-GAACACGCCGTAGTTGCGCAAGC-GCTGAAACAGGTAGTTTAAGGTGGT-GGCAACGT
SARS-CoV-2 NC_045512.2        (5027) ATGACATATGGACAACAGTTTGGTCCAACTTATTTGGATGGAGCTGATGTTACTAAAATAAAACCTCA------TAATTCACA-TGAAGGTAAAACA
                                                                      Section 95
                              (9119) 9119       9130     9140     9150     9160     9170     9180     9190     9200      9215
              Ad 12 X73487    (8875) GT-TCTGAGACGAAGAA-ATACAGAATCCACCGACGAAGCGTCAGCTCGTTGATGTCACCTAAGGCTTC-AAGACGT-TCCATGGCTTCGTA-----
SARS-CoV-2 NC_045512.2        (5117) TTTTATGTTTTACCTAATGATGACACTCTACGT--GTTGAGGCTTTTGAGTACTACCACACAACTGATCCTAGTTTTCTGGGTAGGTACATGTCAGC
                                                                      Section 96
                              (9216) 9216          9230     9240     9250     9260     9270     9280     9290     9300     9312
              Ad 12 X73487    (8963) ---AAAGTCTACTGCAAAATTGAAAAACTGGGAGTTGCGAGCTGCCACCGTCAATTCTTCTTCCAACAGACGAATAAGCTCGGCCACCGTCTCGCGC
SARS-CoV-2 NC_045512.2        (5212) ATTAAATCACACTAAAAAGTGGAAATACCCACAAGTTAATGGTTTAACTT----CTATTAAATGGGCAGA----TAACAACTGTTATCTTGCCACTG
                                                                      Section 97
                              (9313) 9313  9320      9330     9340     9350     9360     9370     9380     9390           9409
              Ad 12 X73487    (9057) ACTTCTTGCTGAAATGCGCCCGGAACTATTTCTTGTTCTTCCTCTTCTA-----CCTCCATTATTTCTTCCTCGACCACAGGTGGTGGGGTTGT--
SARS-CoV-2 NC_045512.2        (5301) CATTGTT------AACACTCCAACAAATAGAGTTGAAGT--TTAATCCACCTGCTCTACAAGATG-CTTATTACAGAGCAAGGGCTGGTGAAGCTGC
                                                                      Section 98
                              (9410) 9410      9420     9430     9440     9450     9460     9470     9480     9490           9506
              Ad 12 X73487    (9147) CTTCTTCGACGCCGGCGAACGGGCAGC-CTGTCTACA---AATCTTTCAATCATTTCGCCGCGACGGCGGCGCATAGTTTCGGTTACTGCTCGACCG
SARS-CoV-2 NC_045512.2        (5389) TAACTTTTGTGCACTTAT-CTTAGCCTACTGTAATAAGACAGTAGGTGAGTTAGGTGAT-GTTAGAGAAACAATGAGTTACTTGT-TTCAACATGCC
```

```
                                                                                                      Section 99
                          (9507) 9507         9520      9530      9540      9550      9560      9570      9580      9590      9603
          Ad 12 X73487    (9240) TTTTCACGTGGTCGTAACTCAAAAACTCCACCTCTAAGTTCTGTTTCATGTAAAATGGGAAATGAGGCGTTGCGAGGGGCGTTAGGTAGGGATACAG
SARS-CoV-2 NC_045512.2    (5483) AATTTAGATTCTTGCAA-------AAGAGTC--TTGAACGTGGTGTGTAAAACTTGTGGACAACAGCAGA---CAACCCTTAAGGGTGTAGAAGCTG
                                                                                                      Section 100
                          (9604) 9604  9610      9620      9630      9640      9650      9660      9670      9680      9690   9700
          Ad 12 X73487    (9337) CGCTGATTATGCATTT-TATTATTTGCTGCGTAGGAACTCCGC-GC-AAGGAGCTAAGCGTCTGCATATCCACCGGGTCGGAGA------ACCTT-T
SARS-CoV-2 NC_045512.2    (5568) TT---A-TGTACATGGGCACACTTTCTTATGAACAA--TTTAAGAAAGGTGTTCAGATACCTTGTACGTGTGGTAAACAAGCTACAAAATATCTAGT
                                                                                                      Section 101
                          (9701) 9701     9710      9720      9730      9740      9750      9760      9770      9780            9797
          Ad 12 X73487    (9424) CAAGAAAGGCATCTAGC---CAGTCACAGTCACAAGGTAGGCTAAGT-TTTGTTTCTTCTAAAGTACCAGGAAGCTGAGCAA-TGCTACTAATAATG
SARS-CoV-2 NC_045512.2    (5659) ACAACAGGAGTCACCTTTTGTTATGATGTCAGCACCACCTGCTCAGTATGAACTTAAG--CATGGTACATTTACTTGTGCTAGTGAGTACACTGGT-
                                                                                                      Section 102
                          (9798) 9798        9810      9820      9830      9840      9850      9860      9870      9880      9894
          Ad 12 X73487    (9516) TAATTGAAG-TAAGCTGTTTTAAGCCCACGAATGGTTTTAAG--AAGCACCACATCTTTGGGTCCGGCTTGTTGAATTCGCAGGCGGTCTGCCATTC
SARS-CoV-2 NC_045512.2    (5753) -AATTACCAGTGTGGTCACT-ATAAACATATAACTTCTAAAGAAACTTTGTATTGCATAGACGGTGCTTTACTTACAAAGTCCTCAGAATACAAAGG
                                                                                                      Section 103
                          (9895) 9895 9900      9910      9920      9930      9940      9950      9960      9970      9980      9991
          Ad 12 X73487    (9610) CCCACACGTCACTTTGACATCGTCCAAGATCTTTGTAGTAGTCT-TGCATT-AACCTTTCCACCTCTACCTC---GCGG----TTT---CCGCGATC
SARS-CoV-2 NC_045512.2    (5848) TCCTATT--ACGGATGTTTTCTACAAAGAAAACAGTTACACAACAACCATAAAACCAGT-TACTTATAAATTGGATGGTGTTGTTTGTACAGAAATT
                                                                                                      Section 104
                          (9992) 9992    10000     10010     10020     10030     10040     10050     10060     10070          10088
          Ad 12 X73487    (9695) AGCCATGTGCGTGCTTCCGTAGCCTTGCAGCGGTTGTAATAAAGCTAAATCTGCC-A-CTACCCGTTCCGCAAGCACTGCCTGTTGAATTTGGGTAA
SARS-CoV-2 NC_045512.2    (5942) GACCCTA----AGTTGGA--C-AATTATTATAAGAAAGACAATTCTTATTTCACAGAGCAACCAATTGATCTTGTACCAAACCAACCATATCC-AAA
                                                                                                      Section 105
                         (10089) 10089     10100     10110     10120     10130     10140     10150     10160     10170          10185
          Ad 12 X73487    (9790) GGGTGGTTGCAAAGTCATCC-ACATCTACAAAGCGGTGATAA-GCTCCT-GCATTAATGGTG--TAGCTGCAGTTTGTCATTACTGACCAATTAACA
SARS-CoV-2 NC_045512.2    (6031) CGCAAGCTTCGA--TAATTTTAAGTTTGT-ATGTGATAATATCAAATTTGCTGATGATTTAAACCAGTTAAC--TGGTTATAAGA----AACCTGCT
```

```
                                                                                                                      Section 106
                          (10186) 10186         10200     10210     10220     10230     10240     10250     10260     10270      10282
            Ad 12 X73487   (9882) GTTTGCGTGCCTGGCTGTACAGTTTCTGTGTAT-CGCAAGCGTGAGTAAGCCCGAGAGTCAAA-AACATAGTCATTGCAGGTGCGCACTAGGTATTG
SARS-CoV-2 NC_045512.2     (6119) TCAAGAGAGC-TTAAAGTTACATTTTTCCCTGACTTAAATGGTGATGTGG-TGGCTATTGATTATAAACACT-AC--------ACACCCTCTTTTAAG
                                                                                                                      Section 107
                          (10283) 10283  10290     10300     10310     10320     10330     10340     10350     10360           10379
            Ad 12 X73487   (9977) ATAGCCCACAAGGAAATGAGGAGGAGGTTCGCGATACAACGGCCAGCCAAGCGTAGCCG--CAGCACCTGGAGCGAGATCTTCCAACATGAGGCGGT
SARS-CoV-2 NC_045512.2     (6206) AAAGGAGCTAAATTGTTACATAAACC--TATTGTTGGCATGTTAACAATGC--AACTAAT-AAAGCCACGT-----ATAAACCAAAT--ACCTGGT
                                                                                                                      Section 108
                          (10380) 10380     10390     10400     10410     10420     10430     10440     10450     10460          10476
            Ad 12 X73487  (10072) GGTATTCATATA---TGTATCTGGACATCCATGTGATGCCGGCAGCGGTAGTTGTTGCTCGCATAAATTCGCGGCTCGGTTCCAAATATTGCGCAG
SARS-CoV-2 NC_045512.2     (6291) G-------TATACGTTGTCTTTGGAGCA-CAAAACCAGTTGAA------ACATCAAATTCGTTTGA-TGTACTGAA-----GTCAGAGGACGCGCAG
                                                                                                                      Section 109
                          (10477) 10477         10490     10500     10510     10520     10530     10540     10550     10560      10573
            Ad 12 X73487  (10166) GGG-TAAAAAGCGTTCAATAGTTGCCACGCTTTGACCGGTCAGGCGTGCGCAGTCTTGAATGCTCTGGACATGGAAAAAATGAAAGTTGGTAAGCGA
SARS-CoV-2 NC_045512.2     (6368) GGAATGGATAATCTTGCCTGCGAAG--ATCTAAAACCAGTCTCTGAAGAAGTAG-TGGAAAA-TCCTACCA--TACAGAAAGACGTTCTT---GAGT
                                                                                                                      Section 110
                          (10574) 10574 10580     10590     10600     10610     10620     10630     10640     10650     10660 10670
            Ad 12 X73487  (10262) -CTCCCTTCCGTGGTTTGGTGGA-AAAGTCACA--AGGGTACCAT-AGCGAGGAACCCCGGTTCGA-------AACCGGCAG----GATCC--GCTA
SARS-CoV-2 NC_045512.2     (6456) GTAATGTGAAAACTACCGAAGTTGTAGGAGACATTATACTTAAACCAGCAAATAA----TAGTTTAAAAATTACAGAAGAGGTTGG--CCACACAGA
                                                                                                                      Section 111
                          (10671) 10671    10680     10690     10700     10710     10720     10730     10740     10750          10767
            Ad 12 X73487  (10341) T---------------GAGCACAAGTGAG-----------GCGCTTGC----------GCGT----TGAACCCGGCCAAGG-------ACCCCC---
SARS-CoV-2 NC_045512.2     (6547) TCTAATGGCTGCTTATGTAGACAATTCTAGTCTTACTATT-AAGAAACCTAATGAATTATCTAGAGTATTAGGTTTGAAAACCCTTGCTACTCATGG
                                                                                                                      Section 112
                          (10768) 10768       10780     10790     10800     10810     10820     10830     10840     10850        10864
            Ad 12 X73487  (10388) ---AGACACGGAGAGGA----------GTCTTT--------TTTTA----------TTTATT--TTTTCTTAGATGCA-TCCTGTCCTGCGACAAATG
SARS-CoV-2 NC_045512.2     (6643) TTTAGCTGCTGTTAATAGTGTCCCTTGGGATACTATAGCTAATTATGCTAAGCCT----TTTCTTAACAAAGTTGTTA----GTACAACTACTAA--
```

```
                                                                                                              Section 113
                              (10865) 10865 10870          10880          10890          10900          10910          10920          10930          10940          10950     10961
              Ad 12 X73487 (10452) CGACC-TCAGCC------------------CAGGGCAACC-----ACGGCCTCAGCAGCGGTG---GCGCTTT---CGGGCTCTGGCGAACAGGAA-
    SARS-CoV-2 NC_045512.2  (6730) -CATAGTTACACGGTGTTTAAACCGTGTTTGTACTAATTATATGC-CTTATTTCTTTACTTTATTGCTACAATTGTGTACTTT-TACTAGAAGTACA
                                                                                                              Section 114
                              (10962) 10962     10970          10980          10990          11000          11010          11020          11030          11040               11058
              Ad 12 X73487 (10518) GAGCCTCAATGTCCT-ACATT----GGAGTTGGAAG--AAGGAGAAGGCATAGCCCGATTGGGCGCCCACTCTCCTGAGCGTCACCCAAGGGTGCAG
    SARS-CoV-2 NC_045512.2  (6824) AATTCTAGAATTAAAGCATCTATGCCGACTACTATAGCAAAGAATACTGTTAAGAGTGTCGGTA---AATTTTGTCTAG----AGGCTTCATTTAAT
                                                                                                              Section 115
                              (11059) 11059          11070          11080          11090          11100          11110          11120          11130          11140          11155
              Ad 12 X73487 (10608) CTCGCCCGGGACAGTCGCGTGGCATTTGTGCCTCGTCAGAACATGTT-TCGCGACAACAGCGGGGAGGA--AGCTGAGGAAAT--GCGAGACTGCAG
    SARS-CoV-2 NC_045512.2  (6914) TATTTGAAGTCACCTAATTTTTCTAAACTGATAAATATTATAATTTGGTTTTTACTATTAAGTGTTTGCCTAGGTTCTTTAATCTACTCAACCGCTG
                                                                                                              Section 116
                              (11156) 11156               11170          11180          11190          11200          11210          11220          11230          11240     11252
              Ad 12 X73487 (10700) GTTTA---GGGCCGGTCGCG-AGCTGCGCCGCGGATTTAATCGCGAGCGACTGCTGCGTGAGGAGGACTTTGAGCCAG-ATGAACATTCG--GGGAT
    SARS-CoV-2 NC_045512.2  (7011) CTTTAGGTGTTTTAATGTCTAATTTAGGCATGC---CTTCTTACTGT--ACTGGTTACAGAGAAGGCTATTTGAACTCTACTAATGTCACTATTGCA
                                                                                                              Section 117
                              (11253) 11253  11260          11270          11280          11290          11300          11310          11320          11330               11349
              Ad 12 X73487 (10790) TAGTTCTGCACGGGC------CCATGTATC---AGCAGC--CAACTTAGTAACAGCATATGAACAAACGGTTACAGAGGAACGTAACTTT-CAAAA-
    SARS-CoV-2 NC_045512.2  (7103) ACCTACTGTACTGGTTCTATACCTTGTAGTGTTTGTCTTAGTGGTTTAGATTCTTTAGACACCTATCCTTCTTTAGAAACTATACAAATTACCATTT
                                                                                                              Section 118
                              (11350) 11350     11360          11370          11380          11390          11400          11410          11420          11430               11446
              Ad 12 X73487 (10874) AAGCTTTAATAACCATGTGCGCACACTAATAGCGCGAGAAGA-AGTAGCCATTGGTTTA-ATGCATCTTTGGGACTTTGTAGAAGCTTATGTACATA
    SARS-CoV-2 NC_045512.2  (7200) CATCTTTT-AAATGGGATTTAACTGCTTTTGGCTTAGTTGCAGAGTGGTTTTTGGCATATATTCTTTTCACTAGGTTTTTCTATGTACTTGGATTGG
                                                                                                              Section 119
                              (11447) 11447          11460          11470          11480          11490          11500          11510          11520          11530          11543
              Ad 12 X73487 (10969) ATCCAGCAAGTAAACCCCTAACTGCCCAGCTGTTCTTAATAGTTCAACATAGTAGAGACAATGAAACTTTTAG--GGATGCAATG-CTTAACATAGC
    SARS-CoV-2 NC_045512.2  (7296) CTGCA----ATCATGCAATTGTTTTTCAGCTATTTTGC--AGTACATTTTATTAGT---AATTCTTGGCTTATGTGGTT--AATAA-TTAATCTTGT
```

Section 120

```
(11544)                          11544 11550     11560     11570     11580     11590     11600     11610     11620     11630     11640
Ad 12 X73487 (11063)             TG-AACCCCAGGGTCGGTGGTTACTCGATTTAATTAACATTCTGCAGAGCATTGTGGTTCA-GGAACGCAGTCTTAGTTTGGCAGACAAGGTGGCCG
SARS-CoV-2 NC_045512.2 (7381)    ACAAATGGCCCCGATTTCAGCT-ATGGTTAGAATGTACAT----C------TTCT-TTGCAT------C-ATTTTATTATGTATGGAAAAGTTATGT
```

Section 121

```
(11641)                          11641    11650     11660     11670     11680     11690     11700     11710     11720           11737
Ad 12 X73487 (11158)             CCATTAATTACTCCATGTTAA--GTTTGGGAAAGTTTTATGCTCGTAAAATCTACAAAAGTCCGTATG-TTCCCATTGACAAGGAA-----GTGAAG
SARS-CoV-2 NC_045512.2 (7459)    GCATGTTGTAGACGGTTGTAATTCATCAA-CTTGTATGATGTGT-TACAAACGTAATAGAGCAACAAGAGTCGAATGTACAACTATTGTTAATGGTG
```

Section 122

```
(11738)                          11738       11750     11760     11770     11780     11790     11800     11810     11820        11834
Ad 12 X73487 (11247)             ATAGA--CAGCTTTTA----TATGCGCATGGCTTTAA----AGGTACTAACATTAAGCGACGATCTTGGAGTGTACCGCAATG-ACCGAATCCACAA
SARS-CoV-2 NC_045512.2 (7554)    TTAGAAGGTCCTTTTATGTCTATGCTAATGGAGGTAAAGGCTTTTGCAAAC--TACACAATTGGAATTGTGTTAATTGTGATACATTCTGTGCTGGT
```

Section 123

```
(11835)                          11835 11840     11850     11860     11870     11880     11890     11900     11910     11920     11931
Ad 12 X73487 (11333)             AGCAGTAAGCGCCAGTCGCCGCA-GAGAGCTAAGCGACAAAGAGCTTATGCATAGCTTACAAAGGGCGCTGACGGGAGCAG-GA-ACAGAGGACGAG
SARS-CoV-2 NC_045512.2 (7649)    AGTACATTTATTAGTGATGAAGTTGCGAGAGACTTGTCACTA--CAGTT---TAAAAGACCAATAAATCCTACTGA-CCAGTCTTCT------TACA
```

Section 124

```
(11932)                          11932   11940     11950     11960     11970     11980     11990     12000     12010          12028
Ad 12 X73487 (11427)             TCGTTCTTTGATATGGGCGCAGACCTACGGTGGCAGCCAAGCGCTCGCGCTTTGGAGGCAGCTGGAGTGGCGTCTGCTGACG-TCACTGGCGATGAC
SARS-CoV-2 NC_045512.2 (7734)    TCGTTGAT-AGT-GTTACAGTGAAGAATGGT-----TCCATCCATCTTTACTTTGATAAAGCTGGTCAAAAGACTTATGAAAGACATTCTCTCTCTC
```

Section 125

```
(12029)                          12029      12040     12050     12060     12070     12080     12090     12100     12110      12125
Ad 12 X73487 (11523)             GATGACGAAGACCAGTACGAGGACTGATCGGCCGT--ACCTTTTGTTAGATGCAGCGACCGGCGATCATC--GCGGAGAGGGCTCCTAACCT-----
SARS-CoV-2 NC_045512.2 (7824)    ATTTTGTTA-AC--TTAGACAACCTGAGAGCTAATAACACTAAAGGTTCATT--TGCCTATTAATGTTATAGTTTTTGATGGTA---AATCAAAATGT
```

Section 126

```
(12126)                          12126         12140     12150     12160     12170     12180     12190     12200     12210    12222
Ad 12 X73487 (11611)             ------------GGATCCCGCGGTTTTGGCGGCCATGCAAAGCCAGCCTTCTGGCGTTACAGCTTCAGATGACTGGACAGCGGCCATGGATCGTATT
SARS-CoV-2 NC_045512.2 (7913)    GAAGAATCATCTGCAAAATCAGCGTCTGTTTAC--TACAGT--CAGC-TTATGTGTCAACCTATACTG-TTACTAGATCA-GGCATTAGT--GTCTG
```

		Section 127
(12223)		12223 12230 12240 12250 12260 12270 12280 12290 12300 12319
Ad 12 X73487	(11696)	ATGGCTTTAACGGCGC-GCAGTCCTGATGCT-TTTCGCCAGCAGCCCCAAGCTAACCGCTTTTCGGCCATTT-TGGAAGCAGTAGTGCCGTCTCGTA
SARS-CoV-2 NC_045512.2	(8001)	ATGTTGGTGATAGTGCGGAAGTT--GCAGTTAAAATGTTTGATGCTTACGTTAATACGTTT-T----CATCAA--CTTTTA--ACGTACCAATGGAA
		Section 128
(12320)		12320 12330 12340 12350 12360 12370 12380 12390 12400 12416
Ad 12 X73487	(11790)	CTAACCCTAC-TCACG-----AGAAAGTGTTAACCATTGTAAATGCTTTGTTGGATAGCAAAGCCATCCGCAAAGATGAGGCTGGTTTAATATACAA
SARS-CoV-2 NC_045512.2	(8087)	AAACTCAAAACACTAGTTGCAACTGCAGAAGCTGAACTTGCAAAGAATGTGTCCTTAGACAATGTCTTATCT-ACTTTTATTTCAGCAGCTCGGCAA
		Section 129
(12417)		12417 12430 12440 12450 12460 12470 12480 12490 12500 12513
Ad 12 X73487	(11881)	CGCTTTGCTTGAGCGCG----TGGCACGCTATAACAGTACCAATGTGC-AGGCTAACTTAG-ACCGGATGGGTACAGATGTAAAGGAGGCGCTGGCT
SARS-CoV-2 NC_045512.2	(8183)	GGGTTTGTTGATTCAGATGTA-GAAACTAAAGATGTTGTTGAATGTCTTAAATTGTCACATCAATCTGACATAGAAG-TTACTGGCGATAGTTGTAA
		Section 130
(12514)		12514 12520 12530 12540 12550 12560 12570 12580 12590 12600 12610
Ad 12 X73487	(11972)	CAACGAGAG-CGCTTTCATCGCG--ATGGTAATCTTGGTTCGCTAGTAGCATTAAACGCT-----TTTTG-AGTACTCAGCCGGCTAATGTTCCGC
SARS-CoV-2 NC_045512.2	(8278)	TAACTATATGCTCACCTATAACAAAGTTGAAAACATGACAC-CCCGTGAC-CTTGGTGCTTGTATTGACTGTAGTGCGCGTCATATTAATGCGCAGG
		Section 131
(12611)		12611 12620 12630 12640 12650 12660 12670 12680 12690 12707
Ad 12 X73487	(12060)	GTGGTCAGGAAGATTATACAAACTTCATCAGCGCCTTGCGACTA--ATGGTTACTGAAGTGCCTCAAAGTGAAGTGTATCAGTCTGGACCCGATTAC
SARS-CoV-2 NC_045512.2	(8373)	TAGCAAAAAGTCACAACATTGCTTTGATATGGAACGTTAAAGATTTCATGTCATTGTCTGAACAACTACGAAAACAAATACGTAGTGCTGCTAAAAA
		Section 132
(12708)		12708 12720 12730 12740 12750 12760 12770 12780 12790 12804
Ad 12 X73487	(12155)	TTTTTTCAAACGTC---CAGGCAGGGTTTGCAA-ACCGTAAACTTAACTCAGGCT-TTTAAAAATTTGCAAGGTTTGTGGGGGTTCGTGCTCCAGT
SARS-CoV-2 NC_045512.2	(8470)	GAATAACTTACCTTTTA-AGTTGACATGTGCAAC----TA----CTAGACAAGTTGTTAATGTTGTAACAACAAAGATAGCACTTAAGGGTGGTA-A
		Section 133
(12805)		12805 12810 12820 12830 12840 12850 12860 12870 12880 12890 12901
Ad 12 X73487	(12247)	AGGCGATCGT--TCAACTTTGTCCAGTTT-ACTAACACCAAACTCGCGCCTATTACTGTTGCT-AATTGCCCCCTTTACCAACACCA--ACA-----
SARS-CoV-2 NC_045512.2	(8557)	AATTGTTAATAATTGG--TTGAAGCAGTTAATTAAAGTTACACTTGTGTTCCTTTTTGTTGCTGCTATTTTCTATTTAATAACACCTGTTCATGTCA

```
                                                                                                   Section 134
                               (12902) 12902   12910       12920       12930       12940       12950       12960       12970       12980      12998
             Ad 12 X73487 (12333) -GTTTAAGTCGAGATTCATACCTGGGTCACTTAGTTACTTTGTACCGC-GAAGCCATTGGTCAAGCGCAGGTAGACGAA-CAA-ACTTATC-AAGAA
SARS-CoV-2 NC_045512.2    (8652) TGTCTAAACATACTGACTTTTCAAGTGAAATCA--TAGGATACAAGGCTATTGATGGTGGTGTCACTCGTGACATAGCATCTACAGATACTTGTTTT
                                                                                                   Section 135
                               (12999) 12999         13010       13020       13030       13040       13050       13060       13070       13080      13095
             Ad 12 X73487 (12425) ATAACCAGTGTTAGTCGCGCA--CTGGGCCAGGAGGACACTGGCAGTTTAGAGGCCACACTTAACTTTTTACTAACTAACCGTCGCCAGCAAGTGCC
SARS-CoV-2 NC_045512.2    (8747) GCTAACA----AACATGCTGATTTTGACACATGGTTTAGCCAG------CGTGGTGGTAGTTA--TACTAA-TGACAAAGCTTGCCCATT--GA--T
                                                                                                   Section 136
                               (13096) 13096             13110       13120       13130       13140       13150       13160       13170       13180      13192
             Ad 12 X73487 (12520) TCCTCAGTACACTTTAAATGCGGAAGAAG-AACGCATATTGCGCTATGTACAGCAATCTGTAAGTTTGTATCTTATGCGTGAGGGTGCCACCCCCAG
SARS-CoV-2 NC_045512.2    (8827) TGCT--GCAGTCATAACAAG---A-GAAGTGGGTTTTGTCGTGCCTGGTTTGCCTGGCACGATATTACGCACA-ACTAATG--GTGACTTTTTGCAT
                                                                                                   Section 137
                               (13193) 13193   13200       13210       13220       13230       13240       13250       13260       13270            13289
             Ad 12 X73487 (12616) TGCCGCCTTAGACATGACAGCGCGCAATATGGA---GCCGTCCTTCTACG-CTTCCAATCGAGCTTCATTAATCGCTTGATGGATTACCTTCACCG
SARS-CoV-2 NC_045512.2    (8915) TTCTTACCTAGAGTTTTTAG--TGCAGTTGGTAACATCTGTTACACACCATCAAAACTTATAGAGTACACTGACT--TTGCAACATCAGCTT----G
                                                                                                   Section 138
                               (13290) 13290       13300       13310       13320       13330       13340       13350       13360       13370           13386
             Ad 12 X73487 (12709) CGCTGCGGCCATGAACGGGGAATACTT--TACAAATGCAATTCTAAATCCGCATTGGTTGCCCCCTCCTGGATTTTACACTGGTGAATTTGATTTGC
SARS-CoV-2 NC_045512.2    (9004) TGTTTTGGC--TGCTGAATGTACAATTTTTAAAGATGCTTCTGGTAAGC--C--AG--TACCATATTGT-TATG--ATACCAATGTACTAGAAGGTT
                                                                                                   Section 139
                               (13387) 13387             13400       13410       13420       13430       13440       13450       13460       13470      13483
             Ad 12 X73487 (12804) CGGAAGGAAATGATGGCTTTTTGTGG-GATGATGTTACGGACAGTCTGTTTAGTCCTGCAGTTATTGGACACCATGGTAA-AAAGGAA---GCAGGT
SARS-CoV-2 NC_045512.2    (9090) CTGTTGCTTATGAAAGTTTACGCCCTGACACACGTTATGTGCTCA---TGGATGGCT-CTATTATT---CAATTTCCTAAC-ACCTACCTTGAAGGT
                                                                                                   Section 140
                               (13484) 13484  13490       13500       13510       13520       13530       13540       13550       13560       13570  13580
             Ad 12 X73487 (12896) G--ATGAAGGTC--CCTTGCTTGACTCTCGGGCGAGTTCT----CCATTCCCCAGTTTAACTAGTTTACCCGCCAGTGTTAACAGCGGTCGTACCAC
SARS-CoV-2 NC_045512.2    (9179) TCTGTTAGAGTGGTAACAACTTTTGATTCT---GAGTACTGTAGGCACGGCACTTGTGAAAGATCAGAAGCTGGTGTTGTGTATCTACTA--GTGG
```

```
                                                                                     Section 141
                           (13581) 13581    13590           13600           13610           13620           13630           13640           13650           13660         13677
            Ad 12 X73487 (12985) CAGACCCCGACT-AACAGGTGAAAGTGAATACTTAAATGACCCCATCTTGTTTCCAGTGCGCGACAAAAATTTTCCCAACAATGGCATAGAAAGTTT
SARS-CoV-2 NC_045512.2  (9271) TAGATGGGTACTTAACA----ATGATTATTACAGATCTTTACCAGGA--GTTT--TCTG--TGGTGTAGATGCTGTAAATTTACTTACTAATATGTT
                                                                                     Section 142
                           (13678) 13678            13690           13700           13710           13720           13730           13740           13750           13760         13774
            Ad 12 X73487 (13081) GGTAGATAAAATGTCTCGCTGGAAAACATATGCACAAGAGCGGCGAGAATGGGAGGAAAGACAGCCAAGACCAGTTCGCCCTCCTAGGCAACGTTGG
SARS-CoV-2 NC_045512.2  (9358) TA-------CACCACTAAT---TCAACCTA--TT--GGTGC----------TTTGGACA----TATCAG--CA----TCTATAGTAGCTGGTGGT--
                                                                                     Section 143
                           (13775) 13775 13780          13790           13800           13810           13820           13830           13840           13850           13860       13871
            Ad 12 X73487 (13178) CAGCGACGCAAAAAAGGGGCACATGCGGGGGATGAAGGAAGCGATGACTCAGCTGACGACAGTAGTGTATTAGATTTAGGAGGGTCAGGAAACCCAT
SARS-CoV-2 NC_045512.2  (9419) -ATTGTAGCTATCGTAGTA-ACATGCCTTGCCTACT-----------ATTTTATGAGGTT--TAG---AAGAGCTTTTGGTGAATACAGTCATGTAG
                                                                                     Section 144
                           (13872) 13872       13880           13890           13900           13910           13920           13930           13940           13950                 13968
            Ad 12 X73487 (13275) TTGCTCATTTGCGCCCACAGGGTTGCATAGGGTCATTGTATTAAAT-TGAATAAAAGCATACTTACCAAAGCCATGGCGACCAGTGTTCGTCTTATT
SARS-CoV-2 NC_045512.2  (9498) TTGCCTTTAATA-CTTT-ACTATTC-CTTATGTCATTCACTGTACTCTGTTTAACACC--AGTT---TACTCATTCTTACCTGGTGTTTATTCTGTT
                                                                                     Section 145
                           (13969) 13969        13980           13990           14000           14010           14020           14030           14040           14050           14065
            Ad 12 X73487 (13371) TTCCTTCTTCCGTTAGCTGTGAAATGAGGCGCGCGGTGGAACTGCAGACAGTGGCTTTTCCTGAGACACCACCTCC--CTCTTACGAA-ACCGT--G
SARS-CoV-2 NC_045512.2  (9587) AT----------TTACTTGTACT-TG--ACATT--TT-ATCTTAC-TAATGATGTTTCTTTTTAGCACATATTCAGTGGATGGTTATGTTCACACC
                                                                                     Section 146
                           (14066) 14066              14080           14090           14100           14110           14120           14130           14140           14150       14162
            Ad 12 X73487 (13463) ATGGCAGC--GGCGCCACCCTACGTGCCTCCCCGCTATTTGGGTCCTACGGAGGGAAGAAACAGTATCCGTTACTCGGAATTGTCACCGTTGTACGA
SARS-CoV-2 NC_045512.2  (9667) TTTAGTACCTTTCTGGATAACAATTGCT--TATATCATTTGTATTTCCA----CAAAGCATTTCTATTGGTT-C-TTTAGTAATTACCTAAAG---A
                                                                                     Section 147
                           (14163) 14163  14170           14180           14190           14200           14210           14220           14230           14240                 14259
            Ad 12 X73487 (13558) TACCACTCGAGTGTACTTGGTGGACAACAAGTCTTCTGACAT----TGCTTCATTGAATTACCAGAATGATCACAGCAACTTTTTAACCACTGTAGT
SARS-CoV-2 NC_045512.2  (9753) GACGTGTAGTCTTTAATGGTGTTTCCTTTAGTACTTTTGAAGAAGCTGCGCTGTGCACCTTTTGTTAAATAAAGAAATGTATCTAAAGT-TGCGTA
```

```
                                                                                                        Section 148
                       (14260) 14260           14270           14280           14290           14300           14310           14320           14330           14340           14356
       Ad 12 X73487 (13651) GCAAAATAATGACTATTCCCCTATAGAGGCTGGCACGCAAA-CTATTAACTTTGATGAAAGGTCTAGATGGGGTGGAGATTTAAAAACCATCTTACA
SARS-CoV-2 NC_045512.2  (9849) GTGATGTGCTATTACCTCTTACGCAATATAATAGATACTTAGCTCTTTATAATAAGTACAAGTATTTTAGTGG-AGCAATGGATACAACTAGCTACA
```

```
                                                                                                        Section 149
                       (14357) 14357        14370           14380           14390           14400           14410           14420           14430           14440           14453
       Ad 12 X73487 (13747) TACCAACATGCCAAACGTGAAC-----GATTTTATGTTTACCACCAAATTTAAGGC-CAGGGTAATGGTGGCTAGGAAA-ACAAACAACGA--AGGC
SARS-CoV-2 NC_045512.2  (9945) GAGA-AGCTGCTTGTTGTCATCTCGCAAAGGCTCTCAATGACTTCAGTAACTCAGGTTCTGATGTTCTTTACCAACCACCACAAACCTCTATCACCT
```

```
                                                                                                        Section 150
                       (14454) 14454 14460           14470           14480           14490           14500           14510           14520           14530           14540 14550
       Ad 12 X73487 (13835) CAAACCATTTTAGAATATGAGTGGGCAGAATTTGTGCTACCCGAGGGT-AACTATTCGGAAACCATGACTATTGACTTAATGAACAATGCTATTATT
SARS-CoV-2 NC_045512.2 (10041) CAGCTGT-TTTGCAGAGTGGTTTAGAAAAATGGCATTCCCATCTGGTAAAGTTGAGGGTTGTATGGTACAAGTAACTTGTGG----TACAACTACA
```

```
                                                                                                        Section 151
                       (14551) 14551    14560           14570           14580           14590           14600           14610           14620           14630                14647
       Ad 12 X73487 (13931) GAGCATTATTTGCGAGTAGGAAGACAGCATGGAGTGCTGGAAAGTGACATTGGAGTTAAGTTTGACACCAGAAACTTTCGTCTGGGTTGGGACCCCG
SARS-CoV-2 NC_045512.2 (10133) C----TTAACGGTCTTTGGCTTGATGACGTAGTTTACTGTCCAAGACATGTGATCTGCACCTCT-GAAGACATGC-TTAACCCTAATTATGAAG---
```

```
                                                                                                        Section 152
                       (14648) 14648       14660           14670           14680           14690           14700           14710           14720           14730           14744
       Ad 12 X73487 (14028) AAACCCAATTAGTAACTCCGGGAGTGTAC-ACTAATG----AGGCTTTTCATCCAGATATAGTACTGCTTCCAGGTTGCGGGGTTGATTTTACAGAG
SARS-CoV-2 NC_045512.2 (10221) --ATTTACTCATTCGTAAGTCTAATCATAATTTCTTGGTACAGGCTGGTAATGTTCAACTCAGGGTT-------ATTGGACATTCTATGCAAAATTG
```

```
                                                                                                        Section 153
                       (14745) 14745 14750           14760           14770           14780           14790           14800           14810           14820           14830 14841
       Ad 12 X73487 (14120) AGCAGAT--TAAGCAACATACTAGGTATAAGAAAGAGGCAGCCGTTTCAGGAAGGATTTGTG--ATTATGTATGAACACTTAGAGGGAGGCAATATTT
SARS-CoV-2 NC_045512.2 (10309) TGTACTTAAGCTTAAGGTTGATACAGCCAATCCTAAGACA-CC--TAAGTATAAGTTTGTTCGCATTCAACCAGGACAGACTTTTTCAGTG-----T
```

```
                                                                                                        Section 154
                       (14842) 14842   14850           14860           14870           14880           14890           14900           14910           14920                14938
       Ad 12 X73487 (14213) CCAGCTCTTTTGGATGTAAAA--------------AAATA--CGAAAA----------CAGTCTGCAGGATCAAAAC---ACTGTAA----------
SARS-CoV-2 NC_045512.2 (10398) TAGCTTGTTACAATGGTTCACCATCTGGTGTTTACCAATGTGCTATGAGGCCCAATTTCACTATTAAGGGTTCATTCCTTAATGGTTCATGTGGTAG
```

```
                                                                                               Section 155
(14939)                      14939 14950 14960 14970 14980 14990 15000 15010 15020 15035
Ad 12 X73487 (14271)         ----------------GAGGA-----------------------GACAACTTT---ATTGCCTTAA-ATAAG----GCTG-----------------
SARS-CoV-2 NC_045512.2 (10495) TGTTGGTTTTAACATAGATTATGACTGTGTCTCTTTTTGTTACATGCACCATATGGAATTACCAACTGGAGTTCATGCTGGCACAGACTTAGAAGGT
                                                                                               Section 156
(15036)                      15036 15050 15060 15070 15080 15090 15100 15110 15120 15132
Ad 12 X73487 (14304)         -------------------CTAGGATTGAACCGGTTGAAACA-----------GACC-CCAAAGGACGCA--GTTACAACTTGCTTC-CA--GACAA
SARS-CoV-2 NC_045512.2 (10592) AACTTTTATGGACCTTTTGTTGACAGGCAAACAGCACAAGCAGCTGGTACGGACACAACTATTACAGTTAATGTTTTAGCTTGGTTGTACGCTGCTG
                                                                                               Section 157
(15133)                      15133 15140 15150 15160 15170 15180 15190 15200 15210 15229
Ad 12 X73487 (14365)         AAAAAATACTAAATATCGCAGCTGGTATTTGGCATACAACTACGGAGACCCAGAAAAAGGAGTTC--GGTCATGGACTCTACTAACAACTCCAGATG
SARS-CoV-2 NC_045512.2 (10689) TTATAAATGGAGACAGGTGGTTTCTCAATCGATTTACCACAACTCTTAATGACTTT-A--ACCTTGTGGCTATGAA--GTACAATTATGAACCTCTA
                                                                                               Section 158
(15230)                      15230 15240 15250 15260 15270 15280 15290 15300 15310 15326
Ad 12 X73487 (14460)         TAACAGG-CGGCTCCGAACAGGTGTACTGGTCCCTACCCGATATGA-TGCAAGATCCGGTGACTTTTCGCTCCTCGCGTCAAGTTAGCAACTATCCT
SARS-CoV-2 NC_045512.2 (10781) ACACAAGA-----CCATGTTGACATACTAGGACCTCTTTCTGCTCAA-ACTGGAATTGCCGTTTTAGATATGTGTGCTTCATTAAAAGAATTA--CT
                                                                                               Section 159
(15327)                      15327 15340 15350 15360 15370 15380 15390 15400 15410 15423
Ad 12 X73487 (14555)         G-TAGTTGCAGCAGAATTACTG-CCAGTTCATGCTAAAAGCTTCTACAACGAGCAAGCCGTCTACTCACAGCTT---ATTCGCCAGTCAACCGCGCT
SARS-CoV-2 NC_045512.2 (10870) GCAAAATGGTATGAATGGACGTACCA--TATTGGGTAGTGCT-TTATTAGAAGATGAATTTACACCTTTTGATGTTGTTAGACAATGCTCAGGTGTT
                                                                                               Section 160
(15424)                      15424 15430 15440 15450 15460 15470 15480 15490 15500 15510 15520
Ad 12 X73487 (14647)         TACG-CGCGTGTTTAATCGCTTTCCCGAGAACCAGATAC-TGGTGCGTCCACCAGCCGCTACC--ATCACTACCGTCAG-TGAAAACGT-----TCC
SARS-CoV-2 NC_045512.2 (10964) ACTTTCCAAAGTGCAGTGAAAAGAACAATCAAGGGTACACACCACTGGTTGTTACTCACAATTTTGACTTCACTTTTAGTTTTAGTCCAGAGTACTC
                                                                                               Section 161
(15521)                      15521 15530 15540 15550 15560 15570 15580 15590 15600 15617
Ad 12 X73487 (14734)         C--GCCCTTACAGATCACGGGACCCTGCCGCTGCGTAGC----AGTATCAGTG-GAGTT--CAGCGAGTCACCATCAC---TGACGCCCGCC-GCCG
SARS-CoV-2 NC_045512.2 (11061) AATGGTCTTTGTTCTTTTTTTTGTATGAAAATGCCTTTTTACCTTTTGCTATGGGTATTATTGCTATGTCTGCTTTTGCAATGATGTTTGTCAAACA
```

```
                                                                                              Section 162
                       (15618) 15618       15630     15640     15650     15660     15670     15680     15690     15700     15714
         Ad 12 X73487 (14818) GAC--------CTGTCCCTACGTTTACAAAGCACTGGGCATAGTTTCTCCACGAGTGCTTTCTAGTCGCACTTTTTAAAAAAGTGTGGTAACATGTC
SARS-CoV-2 NC_045512.2 (11158) TAAGCATGCATTTCTCTGTTTGTTTTTGTTACCTTCTCTTGCCACTGTAGCTTATTTTAATATGGTCTA--TATGCCTGCTAGTTGGGTGATGCGTA
                                                                                              Section 163
                       (15715) 15715 15720     15730     15740     15750     15760     15770     15780     15790     15800     15811
         Ad 12 X73487 (14907) CA---TTTTGGTTTCGCCAAGTAACAACACGGGCTGGGGACTGGGTGCCGCCCGCA------------TGTATGGAGGAGCTAAAACAAGGTCTAGC
SARS-CoV-2 NC_045512.2 (11253) TTATGACATGGTTGGATATGGTTGATAC--T---AGTTTGTCTGGTTTTAAGCTAAAAGACTGTGTTATGTATGCATCAGCTGTA-----GTGTTAC
                                                                                              Section 164
                       (15812) 15812    15820     15830     15840     15850     15860     15870     15880     15890     15908
         Ad 12 X73487 (14989) CAA-CATCCAGTGCGCGTACGCGGACATTACCGAGCTCCATGGGG-CG-CGCATACCCGA-GGACGCACTGG--TCGCACCACTGTAGACGATGTTA
SARS-CoV-2 NC_045512.2 (11340) TAATCCTTATGACAGCAAGAACTGTGTATGATGATGGTGCTAGGAGAGTGTGGACACTTAT--G---AATGTCTTGACACTCGTTTATAA-AGTTTA
                                                                                              Section 165
                       (15909) 15909      15920     15930     15940     15950     15960     15970     15980     15990     16005
         Ad 12 X73487 (15080) TTGA-CTCGGTAGTGGCCGATGCTCGCAAGTACCGCGCGCCCGCTGAAACAGCAGGGTCTACTGTTGATGCAGTAATTGATGAG------GTAGTGG
SARS-CoV-2 NC_045512.2 (11431) TTATGGT-AATGCTTTAG--ATCAAGCCATTTCCATGTGGGCTCTTATAA--TCTCTGTTACTTCTAACTACTCAGGTGTAGTTACAACTGTCATGT
                                                                                              Section 166
                       (16006) 16006         16020     16030     16040     16050     16060     16070     16080     16090     16102
         Ad 12 X73487 (15170) CAAACGCGCGGGCTTATGCAA-----GGCGCCGCAGACGGCTG---CGTCGCC---GGCGTAGACCAACCAC--CGCCATGCGCGCGGCCAGAG--C
SARS-CoV-2 NC_045512.2 (11523) TTTTGGCCAGAGGTATTGTTTTTATGTGTGTTGAGTATTGCCCTATTTTCTTCATAACTGGTAATACACTTCAGTGTATAATGCTAGTTTATTGTTT
                                                                                              Section 167
                       (16103) 16103  16110     16120     16130     16140     16150     16160     16170     16180     16199
         Ad 12 X73487 (15252) GTTGGTTCGACGGGCCAGGCG---CATTGGGCGG--CGAGCTA-TGATGCGGGCAGCC-------AGG-CGGGCTG-C----AACGCC----TGCC-
SARS-CoV-2 NC_045512.2 (11620) CTTAGGCTATTTTTGTACTTGTTACTTTGGCCTCTTTTGTTTACTCAACCG--CTACTTTAGACTGACTCTTGGTGTTTATGATTACTTAGTTTCTA
                                                                                              Section 168
                       (16200) 16200     16210     16220     16230     16240     16250     16260     16270     16280     16296
         Ad 12 X73487 (15325) ----GGTCGAGC-------GCGGAGAC-GGGCCGCAGCTGCGGCCGCAACAGCTA--TTG----CAAACCTAGCTGCTCCGCGACGAGGAAATGTAT
SARS-CoV-2 NC_045512.2 (11715) CACAGGAGTTTAGATATATGAATTCACAGGGACTACTCCCACCCAAGAATAGCATAGATGCCTTCAAAC-TCAACATTAAATTGTTGGGTGTTGGTG
```

Section 169

```
                             (16297) 16297        16310      16320      16330      16340      16350      16360      16370      16380      16393
              Ad 12 X73487 (15404) ACTGGGTGCGCGACTCAGTGACCGG-GACGCGTGT-----GCCAGTTCGTACGCGTCCACCTC-ACCCTTAGA-----AGACAAAG--AGT--GACT
SARS-CoV-2 NC_045512.2 (11811) GCAAACCTTGTATCAAAGTAGCCACTGTACAGTCTAAAATGTCAGATGTAAAGTGCACATCAGTAGTCTTACTCTCAGTTTTGCAACAACTCAGAGT
```

Section 170

```
                             (16394) 16394 16400      16410      16420      16430      16440      16450      16460      16470      16480 16490
              Ad 12 X73487 (15485) CAATGTCTGTTATGTAT--------------GCCCAGC------ATGACCAAACGCAAGTTCAAAGAAGAGCTGCTGCAGGCCTTAGCGCCTGAAAT
SARS-CoV-2 NC_045512.2 (11908) AGAATCATCATCTAAATTGTGGGCTCAATGTGTCCAGTTACACAATGACATTCTCTTAGCT---AAAGATACTACTGAAG-CCTTTGAAAAAATGGT
```

Section 171

```
                             (16491) 16491    16500      16510      16520      16530      16540      16550      16560      16570         16587
              Ad 12 X73487 (15562) ATATGGCCCATCGGATAACCTTACCAAGCGCGATATCAAGCATGTTAAAAAACGGG---AAAAAAAAGAGGAAGAAGTCGCCGCGGCGTCAGCAGAC
SARS-CoV-2 NC_045512.2 (12001) TTCACTACTTTCTGTTTTGCTTTCCATGCAGGGTGCTGTAGACATAAACAAGCTTTGTGAAGAAATGCTGGA------CAACAGGGCAACCTTACAA
```

Section 172

```
                             (16588) 16588        16600      16610      16620      16630      16640      16650      16660      16670      16684
              Ad 12 X73487 (15656) GGCGTCGAGTTTGTGCGCTCATTTGCG--CCCAGACGTAGGGTACAGTGGAAGGGACGGCAAGTAAA---ACGCATT-TTGCGACCGGGCACCACAG
SARS-CoV-2 NC_045512.2 (12092) GCTAT--AGCCTCAGAGTTTAGTTCCCTTCCATCATATGCAGCTTTTGCTACTGCTCAAGAAGCTTATGAGCAGGCTGTTGCTAAT--GGTG-ATTC
```

Section 173

```
                             (16685) 16685 16690      16700      16710      16720      16730      16740      16750      16760      16770 16781
              Ad 12 X73487 (15747) TGGTTTTTTCTCCCGGAGAGCGAACGATTATGCGTCCCCTAAAGCGCGAGTACGACGAAGTGTACGCAGACGATGACATTTTGGAGCAAGCGGCACA
SARS-CoV-2 NC_045512.2 (12184) TGAAGTTGTTCTTAAAAAGTTGAAGAAG---TCTT---TG--AATGTGGCTA-----------AATCTGAATTTGACCGT---GATGCAGCCATGCA
```

Section 174

```
                             (16782) 16782   16790      16800      16810      16820      16830      16840      16850      16860          16878
              Ad 12 X73487 (15844) ACAGACT-GGGGAATTTGCATATGGAA-AAAAAGGGCGTTACGGAGACAAAATTGCTATTCCTTTGGACGAGGGAAATCCAAC--ACCCAGTTTAAA
SARS-CoV-2 NC_045512.2 (12259) ACGTAAGTTGGAA--AAGATGGCTGATCAAGCTATGACCCAAATGTATAAACAGGCTAGATCTGAGGACAAGAGG--GCAAAAGTTACTAGTGCTAT
```

Section 175

```
                             (16879) 16879      16890      16900      16910      16920      16930      16940      16950      16960         16975
              Ad 12 X73487 (15937) GGCTGTCA--CTTTGCAACAAGTGTTGCCCGTCCTTGGGCCTTCGGAAGAAAAGCGTGGAATTAAAAGGGAAGCCATGGATGAATTGCAGCCTACAA
SARS-CoV-2 NC_045512.2 (12352) GCAGACAATGCTTTTCACTATGCTTAG---AAAGTTGGATAATGATGCACTCAACAA--CATTATCAACAATGCAAGAGATGGTTGTGTTCCC-TTG
```

Section 176

```
                              (16976) 16976         16990     17000     17010     17020     17030     17040     17050     17060     17072
                   Ad 12 X73487 (16032) TGCA----ACTGATGGTGCCTAAGCGGCAAAAGTTAGAGGACGTACTAGAGC-ACATGAAGGTGG--AT-CCTAGCGTACAGCCAGATGTAAAAGTA
SARS-CoV-2 NC_045512.2 (12443) AACATAATACCTCTTACAACAGCAGCCAAACTAATGGTTGTCATACCAGACTATAACACATATAAAAATACGTGTGATGGTACAACATTTACTTATG
```

Section 177

```
                              (17073) 17073  17080     17090     17100     17110     17120     17130     17140     17150             17169
                   Ad 12 X73487 (16121) CGTCCGATAAAAAAGGTAGCTCCAGG--ATTGGGAGTTCAAACAGTGGAC--ATTCAAATTCCT-------GTGCAAACTGCATTGGGT-------G
SARS-CoV-2 NC_045512.2 (12540) CATCAGCAT--TGTGGGAAATCCAACAGGTTGTAGATGCAGATAGTAAAATTGTTCAACTTAGTGAAATTAGTATGGACAATTCACCTAATTTAGC-
```

Section 178

```
                              (17170) 17170     17180     17190     17200     17210     17220     17230     17240     17250         17266
                   Ad 12 X73487 (16200) AAACTATGGAAATCCAAAC--TTCGCCAATAAAAACAACGGTGA----ACGCAAGCGTGCAAACAGACCCTTGGTACCCGCCAGTGCTTTCAACA--
SARS-CoV-2 NC_045512.2 (12634) ATGGCCTCTTATTGTAACAGCTTTAAGGGCCAATTCTGCTGTCAAATTACAGAATAATGAGCTTAGT-CCTGTTGCACTACGACAGATGTCTTGTGC
```

Section 179

```
                              (17267) 17267        17280     17290     17300     17310     17320     17330     17340     17350     17363
                   Ad 12 X73487 (16289) -----AAAAAAAAAGCGTC----------------------------------ACTACAGACAAACAAGTTCGCTTTTGCCAGACTACGTTTTACAT
SARS-CoV-2 NC_045512.2 (12730) TGCCGGTACTACACAAACTGCTTGCACTGATGACAATGCGTTAGCTTACTACAACACAACAAAGGGAGGTAGGTTTGTACTTG-CACTGTTATCCGA
```

Section 180

```
                              (17364) 17364 17370     17380     17390     17400     17410     17420     17430     17440     17450 17460
                   Ad 12 X73487 (16346) CCTTCCATTGTGCCCACGCCTGGGTACCGTGGGACAACTTTTCAGCGCCGAGCCACAGCCCCTAGCCGTAGACGAGGTCCATCACGCCGTAGACGTC
SARS-CoV-2 NC_045512.2 (12826) TTTACAGGA---------TTTGAAATGGGCTAGATTCCCTAAGAGTGATGGAACTGGT--ACTATCTATACAGAACTGGAACCACCTTGTAGGTTTG
```

Section 181

```
                              (17461) 17461    17470     17480     17490     17500     17510     17520     17530     17540         17557
                   Ad 12 X73487 (16443) GACGCAAAGCCACTTTAGCCCCAGCGG--CAGTACGTCGCGTTGTACAAAGGGGGC-GCACACTAATACTTCCATCCGTGCGTTACCACCCTAGCAT
SARS-CoV-2 NC_045512.2 (12912) TTACAGACACACCTAAAGGTCCTAAAGTGAAGTATTTATACTTTATTA----AAGGATTAAACAACC--TAAATAGAGGTATGGTACTTGGTAGTTT
```

Section 182

```
                              (17558) 17558       17570     17580     17590     17600     17610     17620     17630     17640         17654
                   Ad 12 X73487 (16537) T-CTCTAACA--AGCTG-CGCTGCCGTTTTTTCAGAT--GGCTCTTACTTGCCGAATGCGCATACCCATTCCAGGATACAGAGGACGAC--CCCGCC
SARS-CoV-2 NC_045512.2 (13003) AGCTGCCACAGTACGTCTACAAGCTGGTAATGCAACAGAAGTGCCTGCCAATTCAA-CTGTATTATCTTTCTGTGCTTTTGCTGTAGATGCTGCTAA
```

```
                                                                                                                    Section 183
                          (17655) 17655 17660              17670               17680               17690               17700               17710               17720               17730               17740          17751
           Ad 12 X73487 (16626) GGAGGAAAGGGCTGACC-GGGAACGGTC-GATTT-----------------------------CG-GCGGCGTAGTATGCGCAGACGCATGAAGGGT
SARS-CoV-2 NC_045512.2 (13099) AGCTTACAAAGATTATCTAGCTAGTGGGGGACAACCAATCACTAATTGTGTTAAGATGTTGTGTACACACACTGGTACTGGTCAGGCAAT-AACAGT
                                                                                                                    Section 184
                          (17752) 17752 17760 17770 17780 17790 17800 17810 17820 17830 17848
           Ad 12 X73487 (16691) GGGGT--GCTGCCCTTCCTAAT-TCCACT--TATTGCTGCGGCCATTGGAGCCGTTCCCGGAATTGCCTCAGTAGCCTTGCAGGCTTCTCGAAAAA-
SARS-CoV-2 NC_045512.2 (13195) TACACCGGAAGCCAATATGGATCAAGAATCCTTTGGTGGTGCATCGTGTTGTCTGTACTGCCGTTGCCAC-ATAGATCATCCAAATCCTAAAGGATT
                                                                                                                    Section 185
                          (17849) 17849 17860 17870 17880 17890 17900 17910 17920 17930 17945
           Ad 12 X73487 (16782) ATTAAAATAAAATA-----AAACTTCCAACTTATTACTGGTACTATGACTGT---TTTATGCAG--ACTA----AATGGAAGACATCAATTTTTCGT
SARS-CoV-2 NC_045512.2 (13291) TTGTGACTTAAAAGGTAAGTATGTACAAATACCTA--------CAACTTGTGCTAATGACCCTGTGGGTTTTACACTTAAAAACA-CAGTCTGTACC
                                                                                                                    Section 186
                          (17946) 17946 17960 17970 17980 17990 18000 18010 18020 18030 18042
           Ad 12 X73487 (16865) CGCTGGCCCCGCGACACGGCACGCGGCCGTACATGGGCACCTGGAACGAGATCG---GCACGAGCCAGCTGAACGGGGGCGC---CTTCAATTGGAA
SARS-CoV-2 NC_045512.2 (13379) GTCTGCGGTATGTGGAAAGGTTATGGCTGTAGTTGTG-ATCAACTCCGCGAACCCATGCTTCAGTCAGCTG-ATGCACAATCGTTTTTAAACGGGTT
                                                                                                                    Section 187
                          (18043) 18043 18050 18060 18070 18080 18090 18100 18110 18120 18139
           Ad 12 X73487 (16956) CAGTATCTG-GAGCGGTCTT---AAAAATTTTGGTTCCACGATTAAGA-CATATGGCA----CCAAGGCGTGGAACAGCCAACCGGCCAGATGCTA
SARS-CoV-2 NC_045512.2 (13474) TGCGGTGTAAGTGCAGCCCGTCTTACACCGTGCGGCACAGGCACTAGTACTGATGTCGTATACAGGGCTTTTGACATCTACAATGATAAAGTAGCT-
                                                                                                                    Section 188
                          (18140) 18140 18150 18160 18170 18180 18190 18200 18210 18220 18236
           Ad 12 X73487 (17044) AGGGACAAGTTAAAAGACCAAAATTTTCAACAGAAAGTTGTAG-ATGGTCTGGCTTCGGGAATTAATGGAGTTGTAGACATAGCCAATCAGGCTGTA
SARS-CoV-2 NC_045512.2 (13570) --GGTTTTGCTAAATTCCTAAAAAC-T----A-ATTGTTGTCGCTTCCAAGAAAAGGACGAAG--ATGACAATTTAATTGATTCTTACTTTGTAGTT
                                                                                                                    Section 189
                          (18237) 18237 18250 18260 18270 18280 18290 18300 18310 18320 18333
           Ad 12 X73487 (17140) CAGAAAAAAATT---------GCCAACCGTTTAGAGCCGCGGCCCGACGAGGTAATGGTAGAGGAAAAGCTGCCACCTCTAGAAACTG-TGCCCGGA
SARS-CoV-2 NC_045512.2 (13657) AAGAGACACACTTTCTCTAACTACCAACATGAAGAAACAATTTATAATTTACTTAAG-GATTGTCCAG-CTGTTGCTAAACATGACTTCTTTAAGTT
```

Section 190

```
                               (18334) 18334 18340     18350     18360     18370     18380     18390     18400     18410     18420  18430
                  Ad 12 X73487 (17227) TCCGTTCCAACCAAAGGAGAAAGCGGCCACGGCCGGATGCAGAGGAAACCTTAGTAACGCACACAACAG-AACCGCCGTCCTATGAGGAAGCAATA
SARS-CoV-2 NC_045512.2 (13752) TAGAATAGAC-----GGTGACATGGTACCAC--ATATATCACGTCAACGTCTTACTAAAT-ACACAATGGCAGACCTCGTCTATGCTTTAAGGCATT
```

Section 191

```
                               (18431) 18431    18440     18450     18460     18470     18480     18490     18500     18510          18527
                  Ad 12 X73487 (17323) AAACAAGGAGCCGCTCTGTCACCTACCACCTATCCCATGACCAAGCCTATTTTACCCATGGCTACTAGAGTGTATGGAAAAACGAAAATGTGCCTA
SARS-CoV-2 NC_045512.2 (13841) TTGATGAAGGTAATTGTGACACATTAAAAGAAATACTTGTCACATACAATTGTTGTGATGATGATTATT-TCAATAAAAAGGACTGG--------TA
```

Section 192

```
                               (18528) 18528       18540     18550     18560     18570     18580     18590     18600     18610         18624
                  Ad 12 X73487 (17420) TGACCCTTGAGCTGCCTCCTTTGCCAGAACCCACTATCGCGGATCCCGTAGGTTCCGTTCCTGTTG----CATCTGTTCCAGTTGCATCGACAGTGA
SARS-CoV-2 NC_045512.2 (13929) TGATTTTGTAGAAAA-CCCAGATATATTA--------CGCGTATACGCCAACTTAGGTGAACGTGTACGCCAAGCTTT---GTTAAAAACAGTACAA
```

Section 193

```
                               (18625) 18625 18630     18640     18650     18660     18670     18680     18690     18700     18710  18721
                  Ad 12 X73487 (17513) G-CCGTCCAGCAGTGCGGCCTGT------TGCC-GTGGCTAG-CTTGCGAAACCCACGATC-CAGT---AATTGGCAA-----------AGTACC---
SARS-CoV-2 NC_045512.2 (14014) TTCTGTGATGCCATGCGAAATGCTGGTATTGTTGGTGTACTGACATTAGATAATCAAGATCTCAATGGTAACTGGTATGATTTCGGTGATTTCATAC
```

Section 194

```
                               (18722) 18722    18730     18740     18750     18760     18770     18780     18790     18800          18818
                  Ad 12 X73487 (17584) ---CTAAACAGTATTGTGGGACTGGGAGTAAAGTCTCTCAAACGCCGACGCTGCTACTAACATTAAAAGACGAGTGTTAATTCCCATCTGTGTATAC
SARS-CoV-2 NC_045512.2 (14111) AAACCACGCCAGGTAGTGGAGTTCCTGTTGTAGATTCTTATTATTCATTGTTAATGCCTA--TATTAACCT-----TGACCAGGGCTTTAACTGCA-
```

Section 195

```
                               (18819) 18819      18830     18840     18850     18860     18870     18880     18890     18900        18915
                  Ad 12 X73487 (17678) GCCTCCTATGTTAGCGCCAGAGGACCAACGCGTGAATCGCAGTCA-CCACCAGCGCTTTC-AAG--ATGGCCAC--TCCCTCGATGATGCCGCAGTG
SARS-CoV-2 NC_045512.2 (14200) GAGTCACATGTTGACACTGACTTAACAAAGCCTTACATTAAGTGGGATTTGTTAAAATATGACTTCACGGAAGAGAGGTTAAAACTCTTTGACCGTT
```

Section 196

```
                               (18916) 18916         18930     18940     18950     18960     18970     18980     18990     19000     19012
                  Ad 12 X73487 (17769) GTCTTACATGCACATCGCCGGTCAGGATGCCTCGGAGTACCTGAG-TCCCGGTCTGGTGCAATT-CGCCCGCGCCACGGACACCTACTTCACCC--T
SARS-CoV-2 NC_045512.2 (14297) ATTTT------AAATATTGGGATCAGACATACCACCCAAATTGTGTTAACTGTTTGGATGACAGATGCATTCTGCATTGTGCAAACTTTAATGTTTT
```

```
                                                                                                    Section 197
                     (19013) 19013  19020          19030          19040          19050          19060          19070          19080          19090               19109
           Ad 12 X73487 (17862) GGGAAACAAGTTTAGAAACCCC---ACCGTGGCTCCCACCCATGATGTTACCACCGATCGCTCGCAGCGTCTGACGCTGCGT--TTTGTGCCCGTGG
SARS-CoV-2 NC_045512.2 (14388) ATTCTCTACA-GTGTTCCCACCTACAAGTTTTGGACCACTAGTGAGAAAAATAT---TTGTTGATGGTGTTCCA---TTTGTAGTTTCAACTGGATA
                                                                                                    Section 198
                     (19110) 19110          19120          19130          19140          19150          19160          19170          19180          19190          19206
           Ad 12 X73487 (17954) ATCGGGAAGA----TACTACCTACTCCTAC-AAGGCTCGCTTTACGCTGGCTGTGGGTGACAACCGCGTGTTAGACATGGCTAGTTCTTACTTTGAC
SARS-CoV-2 NC_045512.2 (14478) CCACTTCAGAGAGCTAGGTGTTGTACATAATCAGGATGTAAACTTAC--ATAGCTCTAGACT-TAGT-TTTAAGGAATTACTTGTGTATGCTGCT-G
                                                                                                    Section 199
                     (19207) 19207               19220          19230          19240          19250          19260          19270          19280          19290          19303
           Ad 12 X73487 (18046) ATTCGAGGGGTACTGGATCGT----GGTCCCAGTTTTAAGCCCTA----TTCCGGAACCGCCTACAATTCTTTGGCACCAAAAGGCGCTCCT----A
SARS-CoV-2 NC_045512.2 (14570) ACCCT-GCTATGCACGCTGCTTCTGGTAATCTATT--ACTAGATAAACGCACTACGTGCTTTTCAGTAGCTGCACTTACTAACAATGTTGCTTTTCA
                                                                                                    Section 200
                     (19304) 19304  19310          19320          19330          19340          19350          19360          19370          19380          19390   19400
           Ad 12 X73487 (18131) ATGCTTCACAATGG-----TCAGAT--AACGCTA--AGCTTAATACCTTTGCTC-AGGCGCCGTAT--CTTAGCGACACTA---TCACCGCCGCCGA
SARS-CoV-2 NC_045512.2 (14664) AACTGTCAAACCCGGTAATTTTAACAAAGACTTCTATGACT--TTGCTGTGTCTAAGGGTTTCTTTAA-GGAAGGAAGTTCTGTTGAAT-TAAAACA
                                                                                                    Section 201
                     (19401) 19401          19410          19420          19430          19440          19450          19460          19470          19480               19497
           Ad 12 X73487 (18213) -------TGGTATTAAAGTTGGAACAGACACCGCCCAGGCAGGCG-CGGCGGTG--------------------TATGCCAACAAAA-----CTTAT
SARS-CoV-2 NC_045512.2 (14757) CTTCTTC-TTTGCTCAGGATGGTAATGCTGCTATCAGCGATTATGACTACTATCGTTATAATCTACCAACAATGTGTGATATCAGACAACTACTATT
                                                                                                    Section 202
                     (19498) 19498               19510          19520          19530          19540          19550          19560          19570          19580          19594
           Ad 12 X73487 (18277) CAGCCAGAGCCGCAAGTAGGACCAAGTGAATGGAACACCAGCATTGAAAACGTTAAAGCTGGCGGGAGGCATTAAAGCAAACCACTGCAATGCAGC
SARS-CoV-2 NC_045512.2 (14853) TGTAGTTGAAGTTGTTGATAAGTACTTTGATTGTTACGATG--GTGGCTGTATTAATGCTAA--CCAAGTCA-T--CGTCAACAACCT------AGA
                                                                                                    Section 203
                     (19595) 19595 19600          19610          19620          19630          19640          19650          19660          19670          19680   19691
           Ad 12 X73487 (18374) CGTGCTATGGCTCCTACGCTCGTCCAACCAACGAACACGGAGGACAATCCAAGGATG--------ACAACATTGAAC---TTAAGTTC--TTTGATT
SARS-CoV-2 NC_045512.2 (14937) CAAATCAG--CTGGT-TTTCCATTTAATAAATGGGTAAG--GCTA--GACTTTATTATGATTCAATGAG-TTATGAGGATCAAGATGCACTTTTCG
```

```
                                                                                                   Section 204
                      (19692) 19692   19700     19710     19720     19730     19740     19750     19760     19770          19788
        Ad 12 X73487 (18458) CAGCTAA------------CAATGCAGCAAACACTGCTCAAGTT----GTGTTCTATACCGAAGACGTAAACCTTGAAATGCCAGACACGCATCTTG
SARS-CoV-2 NC_045512.2 (15026) CATATACAAAACGTAATGTCATCCCTACTATA---ACTCAAATGAATC-TTAAGTATGCCATTAGTGCAAAGAATAGAGCTCGCACCGTAGCTGGTG
                                                                                                   Section 205
                      (19789) 19789      19800     19810     19820     19830     19840     19850     19860     19870       19885
        Ad 12 X73487 (18539) TGTTTAAGCC---TACTGTTACCAATGGAACAATTGCTTCTGAGTCGCTGTTGGGACAGCAAGCAGCGCCAAATAGAGCAAACTACATTGCATTCAG
SARS-CoV-2 NC_045512.2 (15119) TCTCTATCTGTAGTACTATGACCAATAGACAGTTTCATCAAAAATTATT-----GAAATCAATAGCCGCC-ACTAGAG-GAGCTAC--TGTA-----
                                                                                                   Section 206
                      (19886) 19886         19900     19910     19920     19930     19940     19950     19960     19970   19982
        Ad 12 X73487 (18633) AGATAATTTT---ATTGGCCTGATGTA----TTACAACAGTACAG-GCAACATGGGTGTATTGGCCGGGCAAGCTTCCCAACTTAACGCAGTAGTAG
SARS-CoV-2 NC_045512.2 (15202) --GTAATTGGAACAAGCAAATTCTATGGTGGTTGGCACAACATGTTAAAAACTGTTTATAGTGATGTAGAAAACCCTC-ACCTTATGGGT----TGG
                                                                                                   Section 207
                      (19983) 19983  19990     20000     20010     20020     20030     20040     20050     20060           20079
        Ad 12 X73487 (18722) -ACCTGCAAGACAGAAATACAGAGCTGTCATACC-AGTTAATGCTGGATGCTTTGGGAGAC------AG--AACACGGTACTTTTCCT----TGTGG
SARS-CoV-2 NC_045512.2 (15292) GATTATCCTAAATGTGATAGAGCCATGCCTAACAT-GCTTAGAATTATGGCCTCACTTGTTCTTGCTCGCAAACATACAACGTGTTGTAGCTTGTC-
                                                                                                   Section 208
                      (20080) 20080     20090     20100     20110     20120     20130     20140     20150     20160        20176
        Ad 12 X73487 (18805) AATTCCGCAGTGGACAGTTACGACCCTGACGT-----TCGCGTTATTGAGAATCACGGGGTAGAGGATGAACTACCAAATTATTGCTT-TCCTCTTA
SARS-CoV-2 NC_045512.2 (15387) -ACACCGTTTCTATAGATTAGCTAATGAGTGTGCTCAAGTATTGAGTGAAATGGTCATGTGTGGCGGTTCACTATATGTTAAACCAGGTGGAACCTC
                                                                                                   Section 209
                      (20177) 20177        20190     20200     20210     20220     20230     20240     20250     20260      20273
        Ad 12 X73487 (18896) GCGCAGTAGGTGAAATAAAAAAT--TACAAAGGCAT---TAAGCCAGATAACGGAGGAGGAGGTGGCTGGACTGCCGACAACACTGTCAGTGAAGC-
SARS-CoV-2 NC_045512.2 (15483) ATCAGGAGATGCCACAACTGCTTATGCTAATAGTGTTTTTAACATTTGTCAAGCTG--TCACG--GCCAATGTTAATGCACTTTTATCTACTGATGG
                                                                                                   Section 210
                      (20274) 20274 20280     20290     20300     20310     20320     20330     20340     20350     20360 20370
        Ad 12 X73487 (18987) AAACCACATAGGCATTGGGAATATAG-CCGCC---ATGGAAATTAATTTGCAGGCTA-ATTTGTGGAGAAGCTTCTTGTAC-----TCAAATGTGGG
SARS-CoV-2 NC_045512.2 (15576) TAACAAAATTGCCGATAAGTATGTCCGCAATTTACAACACAGACTTTATGAGTGTCTCTATAGAAATAGAGATGTTGACACAGACTTTGTGAATGAG
```

```
                                                                                                   Section 211
                          (20371) 20371    20380     20390     20400     20410     20420     20430     20440     20450          20467
                Ad 12 X73487 (19074) CTTAT----ACCTACCAGAC---GACTTAAATACACTCCAGGAAACATAAAA------CTACCT-GATA--ACAAGAACACCTACGAGTACATGAA
SARS-CoV-2 NC_045512.2 (15673) TTTTACGCATATTTGCGTAAACATTTCTCAATGATGATACTCTCTGACGATGCTGTTGTGTGTTTCAATAGCACTTATGCATCTCAAGGTCTAGTGG
                                                                                                   Section 212
                          (20468) 20468       20480     20490     20500     20510     20520     20530     20540     20550          20564
                Ad 12 X73487 (19155) CG--GGCGTGTGACTGCCCCGGGGTTGGTGGATACCTATGTCAATATCGGCGCTCGCTGGTCCCCAGATGTGATGGATAATGTAAACCCTTTTAACC
SARS-CoV-2 NC_045512.2 (15770) CTAGCATAAAGAACTTTAAGTCAGTTCTTTATTATCAAAACAATGTTTTTATGTCTGAAGCA-AA--ATGTTGGACTGAGACTGACCTTACTAAAGG
                                                                                                   Section 213
                          (20565) 20565 20570     20580     20590     20600     20610     20620     20630     20640     20650     20661
                Ad 12 X73487 (19250) ACCACCGAAACGCAGGGTTGCG-CTACAGATCCA-TGTTGCTAGGCAATGGG-AGATTTGTTCCTTTTCACATTCAGGTGCCGC-AAAAAT-TTTTT
SARS-CoV-2 NC_045512.2 (15864) ACCTCATGAATTTTGCTCTCAACATACAATGCTAGTTAAACAGGGTGATGATT--ATGTGTACCTT-CCTTACCCAGATCCATCAAGAATCCTAGGG
                                                                                                   Section 214
                          (20662) 20662   20670     20680     20690     20700     20710     20720     20730     20740          20758
                Ad 12 X73487 (19342) GCCATCAG------AAATTTGTTGCTGTTGCCCGGTTCCTACACTTACGAATGGA--ACTTTAGAAAGGATGTAAACATGATTCTTCAGAGCACACT
SARS-CoV-2 NC_045512.2 (15958) GCCGGCTGTTTTGTAGAT-GATATCGTAAAAACAGATGGTACACTTATGATTGAACGGTTCGTGTCTTTAGCTATAGATGCTTACCCACTTA---CT
                                                                                                   Section 215
                          (20759) 20759     20770     20780     20790     20800     20810     20820     20830     20840          20855
                Ad 12 X73487 (19431) G--GGAAATGATCTTCGGGTGGACGGAGCCAGCGTTCGCTTTGACAAC--ATTGCCCTGTATGCTAACTT-TTTTCCCATGGCACATAACACAGC--
SARS-CoV-2 NC_045512.2 (16051) AAAC--A-TCCTAATCAGGAGTATGCTGATGTCTTTCATTTGTACTTACAATACATAAGAAAGCTACATGATGAGTTAACAGGACACATGTTAGACA
                                                                                                   Section 216
                          (20856) 20856          20870     20880     20890     20900     20910     20920     20930     20940     20952
                Ad 12 X73487 (19521) TTCTACTTTAGAAGCCATGTTAAGAAATGACACCAACGACCAGTCTTTTAACGATTA--TTTGTG-TGCTGCAAACATGCTGTATCCCATCCCA---
SARS-CoV-2 NC_045512.2 (16145) TGTATTCTGTTATGCTTACTAATGATAACACTTCAAGG-----TATTGGGAACCTGAGTTTTATGAGGCTATGTACACACCGCATACAGTCTTACAG
                                                                                                   Section 217
                          (20953) 20953  20960     20970     20980     20990     21000     21010     21020     21030          21049
                Ad 12 X73487 (19612) GCTAACGCCAC---CAGCGTGCCCATTTCAATACCTTCGCGAAATTGGGCGGCATTTAGAGGCTGGAGCTTTACTC-GCCTAAAAACTAAAGAAACT
SARS-CoV-2 NC_045512.2 (16237) GCTGTTGGGGCTTGTGTTCTTTGCAATTCACAGACTTCATTAAGATGTGGTGCTTGCATACGTAG-ACCATTCTTATGTTGTAAATGCTGTTACGAC
```

```
Section 218
                                (21050) 21050     21060     21070     21080     21090     21100     21110     21120     21130          21146
Ad 12 X73487                    (19705) CCTTCCCTGGGTTCAGGGTTTGACCCCTACTTTGTATA-----CTCTGGAACCATTCCCTATTTA-GACGGCACCTTTTAC--CTAAACCACACTTT
SARS-CoV-2 NC_045512.2          (16333) CATG--------TC---ATATCAACATCAC---ATAAATTAGTCTTGTCTGTTAATCCGTATGTTT----GCAA----TGCTCCAGGTTGTGATGTC

Section 219
                                (21147) 21147        21160     21170     21180     21190     21200     21210     21220     21230     21243
Ad 12 X73487                    (19794) TAAGAAGGTGTCAATCATGTTTGACTCCTCCGTGAGTTGGCCTGGAAATGACCGTTTGCTAACCCCAAATGAATTTGAAATAAAGCGTTCTGTG---
SARS-CoV-2 NC_045512.2          (16408) ACAGATGTGACTCAACTTTACTTAGG-AGGTATGAGCTATTATTGTAA--ATCACATA--AACCACCCATTAGTTTTCCATT--GTGTGCTAATGGA

Section 220
                                (21244) 21244 21250     21260     21270     21280     21290     21300     21310     21320     21330  21340
Ad 12 X73487                    (19888) GATGGGGAGGGATACAATGTGGCCCAATGCAATATGACTAAGGATTGGTTCCTAATACAAATGCTTAGTCATTACAACATTGGATACCAAGGTTTTT
SARS-CoV-2 NC_045512.2          (16498) CAAGTTTTTGGTTTATATAAAAATACATGTGTTGGTAGCGATAA-TGTTACTGACTTTAA----TGCAATTG--CAACATGTGACTGGACAAATGCT

Section 221
                                (21341) 21341    21350     21360     21370     21380     21390     21400     21410     21420          21437
Ad 12 X73487                    (19985) ACATTCCAGAGAGCTACAAGGACCGCATGTATTCTTTCTTTAGAAACTTTCAGCCCATGAGTAGGCAAGTTGTG-GATACCACAGAATATAAGAACT
SARS-CoV-2 NC_045512.2          (16588) GGTGATTACATTTTAGCTAACACCTG-TACTGAAAGACTCAAGCTTTTTGCAGCAGAAACGCTCAAAGCTACTGAGGAGACATTTAAACTGTCTTAT

Section 222
                                (21438) 21438       21450     21460     21470     21480     21490     21500     21510     21520        21534
Ad 12 X73487                    (20081) ACAAAAAAGTAACCGTAGAGTTTCAACATAACAACTCAGGATTC---GTGGGATACCTGGGCCCCACTATGCGGGAGGGACAAGCTTACCCCGCCAA
SARS-CoV-2 NC_045512.2          (16684) GGTATT--GCTACTGTACGTGAAGTGCTGTCTGACAGAGAATTACATCTTTCATGGGAAGTTGGTAAACCTAGAC-----CACCACTTAACCGA-AA

Section 223
                                (21535) 21535 21540     21550     21560     21570     21580     21590     21600     21610     21620  21631
Ad 12 X73487                    (20175) CTATCCCTACCCTCTTATAGGCCAAACAGCTGT-GGAAAGCATCACACAGAAAAAGTTTCTATGCGATCGTGTTATGTGGCGCATCCCATTTTC-TA
SARS-CoV-2 NC_045512.2          (16773) TTATGTCTTTACTGGTTATCGTGTAACTAAAAACAGTAAAGTACAAATAGGAGA-G--TACA--CCTTTGAAAAAGGTGACTATGGTGATGCTGTTG

Section 224
                                (21632) 21632   21640     21650     21660     21670     21680     21690     21700     21710          21728
Ad 12 X73487                    (20270) GTAACTT----CATGTCTATGGGGGCGCTAACGGATCTTGGGCAAAATATGCTGTACGCAAACTCAGCCCATGCTCTAGACATGACATTTGAGGTGG
SARS-CoV-2 NC_045512.2          (16865) TTTACCGAGGT-ACAACAACTTACAAATTAAATGTTGGTGATTATTTTGTGCTG-ACATCACATACAGTAATGC-----------CATTA--AGTGC
```

```
                                                                                    Section 225
                     (21729) 21729      21740     21750     21760     21770     21780     21790     21800     21810       21825
           Ad 12 X73487 (20363) ATCCAATGGATGAGCCTACCCTTCTTTATGTTTTATTTGAAGTTTTCGACGTGGTACGCATTCACCAGCCACACCGCGGCGTCATTGAAGCGGTCTA
SARS-CoV-2 NC_045512.2 (16947) ACCTACACTA-GTGCCACAAGAGCACTATGTTAGAATT-----------ACTGGCTTATACCCAACACTCAATATCTCA--G--ATG--AGTTTTCTA
                                                                                    Section 226
                     (21826) 21826         21840     21850     21860     21870     21880     21890     21900     21910   21922
           Ad 12 X73487 (20460) CCTGCGCACGCCCTTCTCGGCGGGTAACGCTACCACCTAAGAAG---GCACCCTC--CCAGA-CTGCTG-TAATGGGTTCAAGCGAACAGGAGCTGA
SARS-CoV-2 NC_045512.2 (17027) GCAATGTTGCAAATTATCAA-----AAGGTTGGTATGCAA-AAGTATTCTACACTCCAGGGACCACCTGGTACTGGTAAGAGTCATTTTGCTATTGG
                                                                                    Section 227
                     (21923) 21923  21930     21940     21950     21960     21970     21980     21990     22000          22019
           Ad 12 X73487 (20550) CGGCCATTGTTCGAGATCTAGGCTGTGGACCCTATTTT-TTGGGAACCTTTGACAAACGTTTTCCGGGTTT-TGTGTCTCGCGACCGCTTATCATGT
SARS-CoV-2 NC_045512.2 (17118) CCTAGCTCTCTACTACCCTTCTGCTCGCATAGTGTATACAGCTTGCTCTCATGCCGCTGTTGATGCACTATGTGAGAAGGCATT-AAAATAT--TTG
                                                                                    Section 228
                     (22020) 22020     22030     22040     22050     22060     22070     22080     22090     22100        22116
           Ad 12 X73487 (20645) GCTATTGTTAA---CACTGCCGGT-CGCGAAACTGGGGGCGTACACTGGCTGGCTTTTGGATGGAACCCCAAATCGCACACTTGCTATTTATTCGAT
SARS-CoV-2 NC_045512.2 (17212) CCTATAGATAAATGTAGTAGAATTATACCTGCACGTGCTCGTGTAGAGTGTTTTGATAAATTCAAGTGAATTCAACATTAG-A-ACAGTAT----G
                                                                                    Section 229
                     (22117) 22117        22130     22140     22150     22160     22170     22180     22190     22200    22213
           Ad 12 X73487 (20738) CCATTTGGATTTTCTGAT-CAACGACTAAAACAAATCTATCAGTTTGAGTACGAAAGTCTGTTGCGCCGTAGTGC--GCTAGCGGCCACTAAAGACC
SARS-CoV-2 NC_045512.2 (17303) TCTTTTGTACTGTAA-ATGCATTGCCTGAGAC---GACAGCA---GATATAGTTGTCTTTGATGAA--ATTTCAATGGCCACAAATTATGAT--TTG
                                                                                    Section 230
                     (22214) 22214 22220     22230     22240     22250     22260     22270     22280     22290     22300 22310
           Ad 12 X73487 (20832) GATGCGTTACCCTAGAAAAGTCAACCCAAACTGTACAAGGACCGTTTTCTGCAGCGTGCGGCCTGTTTTGTTGTATGTTCTTACACGCTTTTACTCA
SARS-CoV-2 NC_045512.2 (17389) AGTGTTGTCAATGCCAGATTACGTGCTAAGCACTATGTGTACA-TTGGCGAC--CCTGCTCAATTACCTGCACCACGC----ACATTGC-TAACTAA
                                                                                    Section 231
                     (22311) 22311    22320     22330     22340     22350     22360     22370     22380     22390          22407
           Ad 12 X73487 (20929) CTGGCCTGACCATCCAATGGATAAAAATCCCACTATGGACCTACTTACTGGGGTGCCTAATTGTATGCTACAAAGTCCTCAGGTAGTGGGCACATTG
SARS-CoV-2 NC_045512.2 (17478) GGGCACACTAGAACCAGAATATTTCAATTC-AGTGTGTAG--ACTTA------TG---AAAACTATAGGTCCAGACATGTTCCTCGGAACTTGTCGG
```

```
                                                                                                          Section 232
                           (22408) 22408       22420      22430      22440      22450      22460      22470      22480      22490      22504
            Ad 12 X73487 (21026) CAACGCAATCAGAATGAATTGTATAAATTCTTAAACAATC-TGTCCCCTTACTTT---CGTCACAACCGCGAGCGCATAGAAAAAGCTACATCTTTT
SARS-CoV-2 NC_045512.2 (17563) CGTTGT--CCTGCTGAAATTGTTGACACTGTGA---GTGCT--TTGGTTTATGATAATAAGCTTAAAGCACATAAAGACAAATCAGCTCAATGCTTT
                                                                                                          Section 233
                           (22505) 22505 22510     22520      22530      22540      22550      22560      22570      22580      22590   22601
            Ad 12 X73487 (21119) ACTAAAATGCAAAATGGACTCAAA---TAAACGTGTACACAATGCATTAATAATAAAACCA-----TTTTATTAGC----TCATTGGAGTACAAGCT
SARS-CoV-2 NC_045512.2 (17653) AAAATGTTTTATAAGGGTGT-TATCACGCATGATGTTTCATCTGCAATTAACAGGCCACAAATAGGCGTGGTAAGAGAATTCCTTACACGT-AACCC
                                                                                                          Section 234
                           (22602) 22602   22610      22620      22630      22640      22650      22660      22670      22680           22698
            Ad 12 X73487 (21204) TGACTGTTTTATTAAAAATCAAATG-GCTCTTCGCGACAGTCGCCG--TGGTTGGTGGGCAGGGATATGTTTCTGTACT-GCAAACGCTGATGCCAC
SARS-CoV-2 NC_045512.2 (17748) TGCTTGGA---GAAAAGCTGTCTTTATTTCACCTTATAATTCACAGAATGCTGTAGCCTCAAAGATTTT----GGGACTACCAACTCAAACTGTTGA
                                                                                                          Section 235
                           (22699) 22699      22710      22720      22730      22740      22750      22760      22770      22780       22795
            Ad 12 X73487 (21297) TTGAATTCTGG---AATAACAAGCCTAGGGGGGGAGC---CGTCAAAATTTTC---TCCCCACAGCT-GGCGCACAAG----TTGCAGGGCGCCCA-
SARS-CoV-2 NC_045512.2 (17838) TTCATCACAGGGCTCAGAATATGACTA--T-GTCATATTCACTCAAACCACTGAAACAGCTCACTCTTGTAATGTAAACAGATTTAATGTT-GCTAT
                                                                                                          Section 236
                           (22796) 22796         22810      22820      22830      22840      22850      22860      22870      22880     22892
            Ad 12 X73487 (21379) -----TAACATCAGGAGCAGAAATCTTGAAGTCGCAA-TTAGGGCCA--GCATTGC-CGCGCGCATTGCGA-TAAACTGGATTTGCGCACTGAAAAA
SARS-CoV-2 NC_045512.2 (17931) TACCAGAGCAAAA-GTAGGCATACTTTGCAT----AATGTCTGATAGAGACCTTTATGACAAG--TTGCAATTTACAAGTCTTGA--AATTCCACGT
                                                                                                          Section 237
                           (22893) 22893  22900      22910      22920      22930      22940      22950      22960      22970           22989
            Ad 12 X73487 (21466) CCAACAAACACGGATACTTAATACTGGCTAACGCTCCAGGGTCGGTTACTTCGTTGATATCAATGTTATCCACATTGCT--GAGGTTAAAGGAGTG
SARS-CoV-2 NC_045512.2 (18019) --AGG-AATGTGGCAACTTTACAAGCTGAAAATGTAACAGGACTCTTTAAAGATTGTAGT--AAGGTAATCACTGGGTTACATCCTACACAGGC-AC
                                                                                                          Section 238
                           (22990) 22990     23000      23010      23020      23030      23040      23050      23060      23070           23086
            Ad 12 X73487 (21561) ATTTTACACA-------GTTGACGCCCCATCCGTGGCAGGCCATCTTGCTTGTTTAAACATTCGCAGCGCACTGGCAT----AAGGAGACGTTTTTG
SARS-CoV-2 NC_045512.2 (18110) ---CTACACACCTCAGTGTTGACACTAAATTCAAAACT------GAAGGTTTATGT---GTTGACAT--ACCTGGCATACCTAAGGACATGACCTAT
```

```
                                                                                                                        Section 239
                           (23087) 23087        23100      23110      23120      23130      23140      23150      23160      23170    23183
         Ad 12 X73487 (21647) CCCATGTCGCATGTGAGGGTAGTCGGCCAGCATAAAAGC--TTCAATTTGCCTAAAAGCTATTTGAGCCTTCATTCCTTCAGAATAAAACAAGCCGC
SARS-CoV-2 NC_045512.2 (18193) AGAAGACT-CATCTCTATGA--TGGGTTTTAAAATGAATTA-TCAAGTTAATGGTTACCCTA---A--CAT-GTT-TATCACCCGCGAAGAAGCTAT
                                                                                                                        Section 240
                           (23184) 23184 23190      23200      23210      23220      23230      23240      23250      23260      23270 23280
         Ad 12 X73487 (21742) AGGACTTTCCGGAGAAAGAATTATTCCCGCAGCCAACATCATGAAAACAGCAGCGGGCATCGTCGTTTTTAATTTGAACTACATTACGCCCCCAGCG
SARS-CoV-2 NC_045512.2 (18279) AAGACATGTACGTGCATGGATTGGCTTCGATGTCGAGGGGTGTCATGCTACTAGAGAAGCTGTTGGTACCAATTT-----ACCTTTACAGCTAGGTT
                                                                                                                        Section 241
                           (23281) 23281    23290      23300      23310      23320      23330      23340      23350      23360        23377
         Ad 12 X73487 (21839) GTTTTGCGCCACCTTGGCTTTCGAGGGGTTC-TCTTTCAACGCTCGTTGCCCACTTTCGCTGGTTACATCCATTTCCACCAAATGCTCTTTGCGCAC
SARS-CoV-2 NC_045512.2 (18371) TTTCTACAGG-TGTTAACCTAGTTGCTGTACCTACAGGTTATGTTGATACACCTAATAATA---CAGATTTTTCCAGAGTTAG---TGCTAAACCAC
                                                                                                                        Section 242
                           (23378) 23378        23390      23400      23410      23420      23430      23440      23450      23460      23474
         Ad 12 X73487 (21935) CATCTCCATTCCATGCAGGCATCTAAGCTCCCCTTCGC------GCTCGGTACACTTATGCTCCC--ACACGCAGCAACCGGTGGGTTCCCAGGAAT
SARS-CoV-2 NC_045512.2 (18461) CGCCTGGAGATCA-------ATTTAAACACCTCATACCACTTATGTACAAAGGACTTCCTTGGAATGTAGTGCGTATAA-AGATTGTAC----AAAT
                                                                                                                        Section 243
                           (23475) 23475 23480     23490      23500      23510      23520      23530      23540      23550      23560    23571
         Ad 12 X73487 (22024) TCTGTTGGACACCGGCA-TAAGCTTGCATATATCCTTGCAAAAAGCGTCCCATGAGCTCCTGAAAGGTTTTTTGGGATGAAAAAGTC-AGCTGCAAA
SARS-CoV-2 NC_045512.2 (18546) GTTAAGTGACACACTTAAAAATCTCTCTGACAGAGTCGTATTTGT---CTTATGGGCACATGGCTTTGA-GTTGACATCTATGAAGTATTTTGTGAA
                                                                                                                        Section 244
                           (23572) 23572    23580      23590      23600      23610      23620      23630      23640      23650        23668
         Ad 12 X73487 (22119) CCGCGCTTTTCTTCGTTGAGCCATG--TTGTGCATATTTTCTTGTACACGCTGCCCTGATCCGGCAAAAAACGAAAGGTGGCGCGCTCGTCGTGATC
SARS-CoV-2 NC_045512.2 (18639) AATAGGAC--CTGAGCGCACCTGTTGTCTATGTGATAGACGTGCCACATGCTTTTCCACTGCTTCAGACACTTATGCC---TGTTGGCATCATTCTA
                                                                                                                        Section 245
                           (23669) 23669       23680      23690      23700      23710      23720      23730      23740      23750      23765
         Ad 12 X73487 (22214) CACATGGTACTTTT--CCATTAGCA--TAGCCAT-GGCTTCC---ATGCCTTTTTCCCAAGCTGAAACTAGGGGCTGGCTTGCCGGATTGCGAACAA
SARS-CoV-2 NC_045512.2 (18731) TTGGATTTGATTACGTCTATAATCCGTTTATGATTGATGTTCAACAATGGGGTTTTACAGG--TAACCTACAAAGCAACCATGATCTGTATTGTCAA
```

Section 246

(23766) 23766 23780 23790 23800 23810 23820 23830 23840 23850 23862

Ad 12 X73487 (22303) CAACAACATTCTTTTCATTTTCGTCGCTGTTTTGAGCGGAAGCC----TTCAAA-ACGTGTACCTGCCTGGTTT-CCATTTTTTGAAAAGACTG---

SARS-CoV-2 NC_045512.2 (18826) GTCCATGGTAATGCACATGTAGCTAGTTGTGATGCAATCATGACTAGGTGTCTAGCTGTCCACGAGTGCTTTGTTAAGCGTGTTGACTGGACTATTG

Section 247

(23863) 23863 23870 23880 23890 23900 23910 23920 23930 23940 23959

Ad 12 X73487 (22391) -AGA----ACCGTCTGC-ATGATGCATA-ATGCGGACGGGCGGCAT---GCTGAAACCCATTACTCCTAAAACTGCTCTTGGTGGTTCTGCCTCTTC

SARS-CoV-2 NC_045512.2 (18923) AATATCCTATAATTGGTGATGAACTGAAGATTAATGCGGCTTGTAGAAAGGTTCAACACATGGTTGTTAAAGCTGCATTATTAGCAGACAAATTCCC

Section 248

(23960) 23960 23970 23980 23990 24000 24010 24020 24030 24040 24056

Ad 12 X73487 (22478) TTCTTCTGCACTCTCTGGGGAAAG---AGGTATCGCAGCCAT------AGATT-TCTTGACTTTT--TTCTTTGG-------------AGGTAAA--

SARS-CoV-2 NC_045512.2 (19020) AGTTCTTCACGACATTGGTAACCCTAAAGCTATTAAGTGTGTACCTCAAGCTGATGTAGAATGGAAGTTCTATGATGCACAGCCTTGTAGTGACAAA

Section 249

(24057) 24057 24070 24080 24090 24100 24110 24120 24130 24140 24153

Ad 12 X73487 (22548) -----------GGCACA---GCTTCCAGT------TCTTCTTCGCTTTCGGAATCCAGAAA--GTATCTGCCCATTTTT--GGCGGCGGCGGCTGAG

SARS-CoV-2 NC_045512.2 (19117) GCTTATAAAATAGAAGAATTATTCTA--TTCTTATGCCACACATTCTGACAAATTCACAGATGGTGTATGCCTATTTTGGAATTGCAATGTCGATAG

Section 250

(24154) 24154 24160 24170 24180 24190 24200 24210 24220 24230 24240 24250

Ad 12 X73487 (22621) -------------------------------CGCTGC-GGTCTGGGG--TGCGCTCCCTCTGTGAGTGCTGATTGCTGGCCATTATTTAATCCTAGG

SARS-CoV-2 NC_045512.2 (19212) ATATCCTGCTAATTCCATTGTTTGTAGATTTGACACTA-GAGTGCTATCTAACCTTAACTTGCCTGGTTGTGATGGTGGCAGTTTGTATGTAAATAA

Section 251

(24251) 24251 24260 24270 24280 24290 24300 24310 24320 24330 24347

Ad 12 X73487 (22684) CAAAGAAACACATGATGGA-TCTG--GAGCCACAGGAAAGCTTAACCGCCC-CCACCGC--TCCCGCCATTGGCGCTACGGCTGTCATGGA-GAAGG

SARS-CoV-2 NC_045512.2 (19308) ACATGCATTCCACACACCAGCTTTTGATAAAAGTGCTTTTGTTAATTTAAAACAATTACCATTTTTCTATTACTCTGACAGT--CCATGTGAGTCTC

Section 252

(24348) 24348 24360 24370 24380 24390 24400 24410 24420 24430 24444

Ad 12 X73487 (22774) ACAAAAGTCTACTCAT-------ACCCC----AAGACGCACCGGTT---GAGCAGAAC-------TTG---------GGCTACGAGACTCCCCCCGA

SARS-CoV-2 NC_045512.2 (19403) ATGGAAAACAAGTAGTGTCAGATATAGATTATGTACCACTAAAGTCTGCTACGTGTATAACACGTTGCAATTTAGGTGGTGCTGTCTGTAGACATCA

Section 253

(24445) 24445 24450 24460 24470 24480 24490 24500 24510 24520 24530 24541
Ad 12 X73487 (22841) GGAATTTGAAGGCTTTCTTCAAATCCA----AAAGCAAC---CAAATGAGCAAA---ACGCTGGGCTCGAGGAC------CATG--ACTACCTAAAC
SARS-CoV-2 NC_045512.2 (19500) TGCTAATGAGTACAGATTGTATCTCGATGCTTATAACATGATGATCTCAGCTGGCTTTAGCTTG-TGGGTTTACAAACAATTTGAT-ACTTATAACC

Section 254

(24542) 24542 24550 24560 24570 24580 24590 24600 24610 24620 24638
Ad 12 X73487 (22920) GAGGGAGATGTCCTGTTTAAACATCTACAGCGACAAAGCACTATCGTTCGCGACGCCATATCTGATCGC-----TCTTCAATACCAGTTTCAAT-TG
SARS-CoV-2 NC_045512.2 (19595) TCTGGA-ACACT-TTTACAAGACTTCAGAGTTTAGAA--AATGTGGCTTTTAATGTTGTAAATAAGGGACACTTTGATGGACAACAGGGTGAAGTAC

Section 255

(24639) 24639 24650 24660 24670 24680 24690 24700 24710 24720 24735
Ad 12 X73487 (23011) CAGAACTAT--CTTGCATCTACGAACGCAACCTGTTCTCCCCACGTGTGCCCCCTAAACG-GCAAGCCAACGGCACATGCGAGCC---AAATCCTCG
SARS-CoV-2 NC_045512.2 (19688) CAGTT-TCTATCATTA--ATAACACTGTTTACACAAAAGTTGATGGTGTTGATGTAGAATTGTTTGAAAATAAAACAACATTACCTGTTAATGTAGC

Section 256

(24736) 24736 24750 24760 24770 24780 24790 24800 24810 24820 24832
Ad 12 X73487 (23102) CCTTAACT-TCTACCCAGTTTTTGCAGTG--CCAGAAGCACTGG-CAACATACCATATTTTCTTTAAAAATCACAAAATACCCCTATCCTGTCGAGC
SARS-CoV-2 NC_045512.2 (19782) ATTTGAGCTTTGGGCTAAGCGCAACATTAAACCAGTACCAGAGGTGAAAATACTCAATAATTTGGGTGTGGACATTGCTGCTAATACTGTGATCTGG

Section 257

(24833) 24833 24840 24850 24860 24870 24880 24890 24900 24910 24929
Ad 12 X73487 (23195) TAACCGCAGCCGCGCAGATGAGCTTCTTGCTTTAAGGGCTGGC---GCTTCCATACCTGGGATTGTGTCCTTGGAAGAGGTGCCTAAAATTTTTGAA
SARS-CoV-2 NC_045512.2 (19879) GACTACAAAAGAGATGCTCCAGCACATATATCTA-CTATTGGTGTTTGTTCTATGACTGACAT--AGCCAAGAAACCAACTGAAACGATTTGTGCAC

Section 258

(24930) 24930 24940 24950 24960 24970 24980 24990 25000 25010 25026
Ad 12 X73487 (23289) GGTTTAGGTCGGGATGAAAAACGAGCAGCAAATGCCCTGCAAAAAGAA-----AATGAACAAAATCACCATGGGAATAGTGCTCTAATAGAACTGGA
SARS-CoV-2 NC_045512.2 (19973) CACTCACTGTCTTTTTTGATGGTAGAGTTGATGGTCAAGTAGACTTATTTAGAAATGCCCGTAATGGTGTTCTTATTACAGA---AGGTAGTGTTAA

Section 259

(25027) 25027 25040 25050 25060 25070 25080 25090 25100 25110 25123
Ad 12 X73487 (23381) AGGT-GACAATGCCCGCCTGGCAGTTTTAAAGCGCAATATTG----AGGTTACTCACTTTG-CCTACCCGGCAGTAAATCTTCCGCCAAAGGTAATG
SARS-CoV-2 NC_045512.2 (20067) AGGTTTACAA---CCATCTGTAGGTCCCAAA---CAAGCTAGTCTTAATGGAGTCACATTAATTGGAGAAGCCGTAAAAACACAGTTCAATTATTAT

```
                                                                              Section 260
                          (25124) 25124 25130     25140     25150     25160     25170     25180     25190     25200     25210 25220
          Ad 12 X73487 (23472) AGCGCAGTGATGAATCAGCTACTAATTAAGCGAGCCCAACCCATTGACAA--AGATGCAAACTTGCAAGACCCGGAGGCAACAGATGATGGAAAGCC
SARS-CoV-2 NC_045512.2 (20158) AAGAAAGT--TGATGGTGTTGTCCAACAATTACCTGAAACTTACTTTACTCAGAGTAGAAATTTACAAGAATTT-A---AACCCAGGAGTCAAATGG
                                                                              Section 261
                          (25221) 25221   25230     25240     25250     25260     25270     25280     25290     25300         25317
          Ad 12 X73487 (23567) G-GTTGTAAGCGACGAGCAATTAACTAAGTGGTTGGGAACAGACAATTCCAACGAACTACAACAGCGGCGTAAAC-TCATGATGGCCGCCGTAC-TT
SARS-CoV-2 NC_045512.2 (20249) AAATTG---ATTTCTTAGAATTAGCTATG-GATGA-----ATTCATTGAA-CGG---TATAAATTAGAAGGCTATGCCTTCGAACATATCGTTTATG
                                                                              Section 262
                          (25318) 25318       25330     25340     25350     25360     25370     25380     25390     25400     25414
          Ad 12 X73487 (23661) GTAACTGTGGAACTCGAGTGCATGCAT-CGTTTTTTCTCCGACATCACCACATTGCGCAAAATTG-AGG----AATGTCTTCACTACACTTTCCGCC
SARS-CoV-2 NC_045512.2 (20333) GAGATTTTAGTCATAGTCAGT-TAGGTG---GTTTACATCTACTGATTGGACTAGCTAAACGTTTTAAGGAATCACCTTTTGAAT-TAGAAGATTTT
                                                                              Section 263
                          (25415) 25415 25420     25430     25440     25450     25460     25470     25480     25490     25500 25511
          Ad 12 X73487 (23752) ATGGCTACGTGCGCCAAGCCTGTAAAATTTCTAATGTGGAGCTGAGCAATCTAGTTTCTTACATGGGCATCTTG--CATGAAAACCGATTGGGACAG
SARS-CoV-2 NC_045512.2 (20425) ATTCCTATGGACAGTACAGTTAAAAACTATTTCATA-ACAGATGCGCAAACAGGTTCATCTAAGTGTGTGTGTTCTGTTATTGATTTATTACTTGAT
                                                                              Section 264
                          (25512) 25512   25520     25530     25540     25550     25560     25570     25580     25590         25608
          Ad 12 X73487 (23847) AACGTGCTACACTCAACACTACGCGATGAAGCA-CGCAGAGATTACGTGCGA-GACTGCATTTACCTTTTCCTGTTACATACCTGGCAAACTGGGAT
SARS-CoV-2 NC_045512.2 (20521) GATTTTGTTGAAATAATAAAATCCCAAGATTTATCTG------TAGTTTCTAAGGTTG----------TCAAAGTGAC---TATTGACTATACAGAA
                                                                              Section 265
                          (25609) 25609      25620     25630     25640     25650     25660     25670     25680     25690      25705
          Ad 12 X73487 (23942) GGGTGTTTGGCAGCAATGCTTGGAAGAAAAAACCTTCGAGAACTAAACAAAC-TGTTAGACAGAGCACTAAAATCCCTATGGACCGGTTTTGACGA
SARS-CoV-2 NC_045512.2 (20599) ATTTCATT-----TATGCTTTGGTGTA--AAGATGGCCATGT-----AGAAACAT--TTTACCCAAAATTACAAT--CTAGTCA---AGCGTGGCAA
                                                                              Section 266
                          (25706) 25706         25720     25730     25740     25750     25760     25770     25780     25790 25802
          Ad 12 X73487 (24038) ACGGACAGTAGCTGCAGAGCTAGCTGACATAATTTTCCCAGAAAGGTTAATGATAACCTTGCAAAACGGCTTGCCTGACTTTATGAGTCAAAGTATG
SARS-CoV-2 NC_045512.2 (20677) CCGGGT----GTTGCT----ATGCCTA-AT--CTTTACAAAATGC---AAAGAATGCTATTAGAAAAGTGTGACCTTC----------AAAATTATG
```

```
                                                                                     Section 267
                          (25803) 25803  25810           25820           25830           25840           25850           25860           25870           25880                     25899
              Ad 12 X73487 (24135) CTGCACAATTATCGCTCTTTTATATTAGAGCGTTCTGGGATGCTTCCTAGCATGTGTTGTGCACTTCCTTCAGATTTTGTGCCTATATATTTTAGAG
    SARS-CoV-2 NC_045512.2 (20750) GTGATAGTGCAACA-----TTACCTAAAGGC--ATAATGATGAAT----GTCGC--AA-AATATACTCAACTGTGTCAATATTTAAACACATTAACA
                                                                                     Section 268
                          (25900) 25900           25910           25920           25930           25940           25950           25960           25970           25980                25996
              Ad 12 X73487 (24232) AGTGCCCCCCTCCCCTGT----GGAGCCACTGCTACTTACTACGACTTGCT-AACTACCT--AGCTTACCACTCAGACCTTATGACAGATTCAAGCG
    SARS-CoV-2 NC_045512.2 (20833) TTAGCTGTACCCTATAATATGAGAGTTATACATTTTGGTGCTGGTTCTGATAAAGGAGTTGCACCAGGTACAGCTG---T-TTTAAGACAGTGGTTG
                                                                                     Section 269
                          (25997) 25997        26010           26020           26030           26040           26050           26060           26070           26080           26093
              Ad 12 X73487 (24322) GCGAAGGCCTAATGGAGTGTCACTGCCGCTGCAATCTTTGC-ACCCCCCACCGTTCTTTGGTTTGCAATACTGAACTATTAAGTGAAAGTCAAGTCA
    SARS-CoV-2 NC_045512.2 (20926) CCTACGGGTACGCTGCTTGTCGATTCAGATCTTAATGACTTTGTCTCTGATGCAGATTCAACTTTGATTGGTGATTGTGCAACTGTACATACAGCTA
                                                                                     Section 270
                          (26094) 26094 26100           26110           26120           26130           26140           26150           26160           26170           26180  26190
              Ad 12 X73487 (24418) TTGGTA-------CCTTCGAA-----ATGCAGGGACCGCAGTCTGACAGCAATTTCACGAC---GAACCTAAGACTTACCCCTGG--GCTTTGGACT
    SARS-CoV-2 NC_045512.2 (21023) ATAAATGGGATCTCATTATTAGTGATATGTACGACCCTAAGACTAAAAATG-TTACAAAAGAAAATGACTCTAAAGAGGGTTTTTTCACTTACATTT
                                                                                     Section 271
                          (26191) 26191    26200           26210           26220           26230           26240           26250           26260           26270                26287
              Ad 12 X73487 (24498) TCTGCCTACCTGCGCAA--ATTTGAACCCCAAGATTACCACGCCCACAGTATCAATTTTTACGAAGACCAATCCAAACCCCCAAAAGCGCCACTAAC
    SARS-CoV-2 NC_045512.2 (21119) GTGGGTTTATACAACAAAAGCTAGCTCTTGGAGGTTCCGTGGCTATAAAGATAACAGAACATTCTTG-GAATGCTGATCTTTATAAGCTCATGGGAC
                                                                                     Section 272
                          (26288) 26288        26300           26310           26320           26330           26340           26350           26360           26370           26384
              Ad 12 X73487 (24593) GGCTTGCGTCATTACGCAGGGAAAAATTCTAGCCCAATTGCATGCTATTAAGCAAGCGCGCGAAGAGTTTTTACTTAAAAAAGGACACGGAGTGTAC
    SARS-CoV-2 NC_045512.2 (21215) ACTTCGCAT-----GGTGGACAGCCTTTGTTACTAATGTGAA------TGCGTCATCATCTGAAGCATTTTTAATTGG---ATGTAATTATCTTGGC
                                                                                     Section 273
                          (26385) 26385 26390           26400           26410           26420           26430           26440           26450           26460           26470      26481
              Ad 12 X73487 (24690) CTTGATCCCCAAACCGGCGAGGAACTAAACCTTCCATCACCTTTGTGTGCTACTGCGTCTCCCCATTCGCAGCATGTCCCCGAAAGCCGCAAAACAG
    SARS-CoV-2 NC_045512.2 (21298) AAACCACGCGAACAAATAGATGGTT---ATGT-CATGCATGC-------AAATTACATAT-----TTTGGAGGAA----TACAAATCCAATTCAGTT
```

Section 274

```
(26482)                        26482   26490        26500        26510        26520        26530        26540        26550        26560        26578
Ad 12 X73487 (24787)           GCTATTGCGCAGCAACGCTCAAAGAAA-CAGCAGCAACGGCAGGAAATCTGGAGGAAGAATCTTGGGAGAGTCAGGCAGAGGACGAGGTCGAGGAC
SARS-CoV-2 NC_045512.2 (21375) G-TCTTCCT---ATTCTTTATTTGACATGAGTAAATTTCCCCTTAAATTAAGGGGTACTGCTGTTATGTCTTTAAA--AGAAGGTCAAATCAATGAT
```

Section 275

```
(26579)                        26579   26590        26600        26610        26620        26630        26640        26650        26660        26675
Ad 12 X73487 (24883)           TTGGAAGAATGGGAGGAGGAGGAGGCGGACAGCCTAGACGAGGATCCAGAGGAGGAGGAGGAAGGTTCCAAGGACGGAGCG--ACC----GCCGCCA
SARS-CoV-2 NC_045512.2 (21466) ATG--------------ATTTTATCTCTTCTTAGTAAAGGTAGA-----CTTATAATTAGAGA-----AAACAACAGAGTTGTTATTTCTAGTGATG
```

Section 276

```
(26676)                        26676   26690        26700        26710        26720        26730        26740        26750        26760        26772
Ad 12 X73487 (24974)           AACCGTCGCTTTCAACCAAGCCCTCTCCAA-TGAAACC---CGCTGTGAGCAAATC----TCAGAAAGCCAACCGTAGATGGGACACCATTGAAACC
SARS-CoV-2 NC_045512.2 (21539) TTCTTGTTAACAACT--AAACGAACAATGTTTGTTTTTCTTGTTTTATTGCCACTAGTCTCTAGTCAGTGT---GTTAATCTTACA--ACCAGAACT
```

Section 277

```
(26773)                        26773   26780        26790        26800        26810        26820        26830        26840        26850        26869
Ad 12 X73487 (25063)           -AGCGCCGCAAACTTGGGTAAGA--ATCGCAAGCAGGCGCGTCGGGGCTACTGCTCATGGCG-GGCTCACCAAAGTAATATTGTAGCCTGCTTTCAG
SARS-CoV-2 NC_045512.2 (21629) CAATTACCCC----CTGCATACACTAATTCTTTCACACGT------------GGTGTTTATTACCCTGACAAAGTTTTCAG-ATCCT---CAGTTTT
```

Section 278

```
(26870)                        26870   26880        26890        26900        26910        26920        26930        26940        26950        26966
Ad 12 X73487 (25156)           CACTGCGGGGGGAATATCTCATTTGCAAGGCGGTATTTGCTATACCATGATGGAGTGGCGATTCCAA-GGAATGTCCTCCATTACTACCGTCATCTC
SARS-CoV-2 NC_045512.2 (21706) ACATTC---------AACTCA--GGACTTGTTCT------TA-----------CCTTTCTTTTCCAATGTTACTTGGTTCCATGCTATACATGTCTC
```

Section 279

```
(26967)                        26967   26980        26990        27000        27010        27020        27030        27040        27050        27063
Ad 12 X73487 (25252)           TACAGCCCCTT-TGAAGAGCTCGACAAGGAACCGACCTGCAACAG-CCAAGCG------------GC--CCACTAGAATCGGCAACAGCAGCAACAA
SARS-CoV-2 NC_045512.2 (21775) TGGGACCAATGGTACTAAGAG-GTTTGATAACCCTGTCCTACCATTTAATGATGGTGTTTATTTTGCTTCCACTGAGAAGTCTAACATAAT-AAGAG
```

Section 280

```
(27064)                        27064   27070        27080        27090        27100        27110        27120        27130        27140        27150   27160
Ad 12 X73487 (25333)           GGAAAGTCCTGAG----GCGCGCGAGTTAAGAAAACGCATTTTTCCCACTTTATATGCTATTTTTC-AGCAGAGTCGAGGTCAAGAACA-CGAACTG
SARS-CoV-2 NC_045512.2 (21870) GCTGGATTTTGGTACTACTT--TAGATTCGAAGACCCAGT---CCCTACTTATTGTTAATAACGCTACTAATGTTGTTATTAAAGTCTGTGAATTT
```

Section 281

```
(27161)                        27161    27170     27180     27190     27200     27210     27220     27230     27240            27257
Ad 12 X73487 (25424)           AAAATAAAAAACCGTTCCCTGCGTTCACTTACCCGCAGCTGTCTCTACCTCAAAAGCGAAGATCAGTTGCAACGCACCTTGCAGGACGCAGAAGCTC
SARS-CoV-2 NC_045512.2 (21962) CAATTTT-GTAATGATCCAT---------TTTTG-G--GTGTTTATTACCACAAAAACAACAAAAGTTGGA----TGGAAAGTGAGTTCAGAGTTTA
```

Section 282

```
(27258)                        27258       27270     27280     27290     27300     27310     27320     27330     27340      27354
Ad 12 X73487 (25521)           TGTTCAATAAATACTGCTCCCTCTCGCTTAAAGAGTAAA-------------AAAAGCCCGCGCGCGGACTT--TCA-------ACAGGCGGGAAAA
SARS-CoV-2 NC_045512.2 (22042) T-TCTAGTGCGAATAATT-GCACTTT-TGAATATGTCTCTCAGCCTTTTCTTATGGACCTTGAAGGAAAACAGGGTAATTTCAAAAATCTTAGGGAA
```

Section 283

```
(27355)                        27355 27360     27370     27380     27390     27400     27410     27420     27430     27440  27451
Ad 12 X73487 (25596)           G---TGACGTCA-CAACAAGATGAGTA----AAGATATTCCCAC--GCCTTACATGTGGAGCTTTCAACCCCAAATGGGACTGGCGGCCGGCGCGGC
SARS-CoV-2 NC_045512.2 (22136) TTTGTGTT-TAAGAATATTGATGGTTATTTTAAAATATATTCTAAGCACACGCCTATTAA--TT-TAGTGCGTGATC-TCCCTCAGGGTTTTTCGGC
```

Section 284

```
(27452)                        27452   27460     27470     27480     27490     27500     27510     27520     27530          27548
Ad 12 X73487 (25683)           TCAAGAC--TATTCTAGCAA--AATGAATTGGTTAAGCGCCGGACCCCACATGATTTCCAGGGTGAATGGGGTACGAGCCCGGCGTAACCAAATACT
SARS-CoV-2 NC_045512.2 (22228) TTTAGAACCATTGGTAGATTTGCCAATAGGTATTAACATCACTAGGTTTCAAACTTTACTTGCTTTACATAGAAGTTATTTGACTCCTGGTGATTCT
```

Section 285

```
(27549)                        27549      27560     27570     27580     27590     27600     27610     27620     27630      27645
Ad 12 X73487 (25776)           GCTAGAACAAGCCGCTCTCACCGCTACACCACGTAATCAACTTAACCCTCCCTCTTGGCCAGCTGCCCTGATATATCAGGAAAATCCCCCTCCTACC
SARS-CoV-2 NC_045512.2 (22325) TCTTCAGGTTGGACAGCTGG-TGCTGCAGCTTATTATGTGGGTTATCTTCAACCTAGGACTTTTCTATTAAAATAT---A---ATGAAAATGGAACC
```

Section 286

```
(27646)                        27646          27660     27670     27680     27690     27700     27710     27720     27730    27742
Ad 12 X73487 (25873)           ACTGTACTTTTGCCTCGCGACGCCCAGGCCGAAGTCCATATGACTAACGCT--GGGGCACAGCTTGCGGGCGG----TGCACGTCACAGTTTCAGGT
SARS-CoV-2 NC_045512.2 (22415) ATTACAGATGCT-GT-AGACTGTGC------------ACTTGACCCTCTCTCAGAAACAAAGTGTACGTTGAAATCCTTCACTGTAGAA---AAGG
```

Section 287

```
(27743)                        27743  27750     27760     27770     27780     27790     27800     27810     27820           27839
Ad 12 X73487 (25964)           ATAAAGGTCGCACTGAGCCCTATCCGTCTCCAGCTATAAAAAGAGTACTCATCAG-AGGGAAAGGTATTCAGCTGAACGACGAA-GTCACATCGCCA
SARS-CoV-2 NC_045512.2 (22495) A---ATCTATCAAACT-------T-----CTAACTTTAGAG-TCCAACCAA-CAGAATCTATTGTTA-------GATTTCCTAATATTACAAACT-T
```

```
                                                                                                   Section 288
                              27840     27850     27860     27870     27880     27890     27900     27910     27920        27936
(27840)
Ad 12 X73487 (26059)          TTGGAGTCAG-ACCCGACGGAGTGTTTCA-GCTCGGAGGGTCCGGACGTTCCTCCTTTACC--GCTCGTCAAGCCTACCTG--ACACTACAGA-GC
SARS-CoV-2 NC_045512.2 (22567) GTGCCCTTTTGG-------TGAAGTTTTTAACGCCACCAGATTTGCAT-CTGTT--TATGCTTGGAACAGGAAGAGAATCAGCAACTGTGTTGCTGA
                                                                                                   Section 289
                              27937        27950     27960     27970     27980     27990     28000     28010     28020     28033
(27937)
Ad 12 X73487 (26149)          TCATCCTCAGCTCCGAGAT--CTGGTGGTATTGGAACTCTCCAATTTGTGGAGGAATTTACTCCATCTGTTTACTTCAATCCTTTTTCGGGCTCGCC
SARS-CoV-2 NC_045512.2 (22654) TTATTCTGTCCTATATAATTCCGCATCATTTTCCACTTTTA--AGT-GTTATGGAGTGTCTCCTACTAAATTAAATGAT------CTCTGCTTTACT
                                                                                                   Section 290
                              28034 28040     28050     28060     28070     28080     28090     28100     28110     28120 28130
(28034)
Ad 12 X73487 (26244)          TGGACACTATCCTGACGCCTTCATACCCAACTTTGACGCAGTGAGT-GAATCTGTGGATGGCTATGATTAATGTCTAATGGAGCGGCTGACAGAGCG
SARS-CoV-2 NC_045512.2 (22742) AATGTC-TATGCAGATTCATTTGTAATTAGAGGTGATGAAGTCAGACAAATCGCTCCAGGGCAAACTGGAAAGATTGCTGATTATAATTATAAA--T
                                                                                                   Section 291
                              28131    28140     28150     28160     28170     28180     28190     28200     28210        28227
(28131)
Ad 12 X73487 (26340)          CGGCTGCGACATTTAGACCACTGTCGCCAACCTCACTGCTTTGCTCGAGAC--ATCTGTGTCTTTACCTACTTT-GAGCTTCCAGAGGAGCACCCCC
SARS-CoV-2 NC_045512.2 (22836) TACCAGATGATTTTA--CAGGCTGCG----TTATA--GCTTGGAATTCTAACAATCTTG---ATTCTAAGGTTGGTGGTAATTATA--ATTACCTGT
                                                                                                   Section 292
                              28228       28240     28250     28260     28270     28280     28290     28300     28310     28324
(28228)
Ad 12 X73487 (26434)          AGGGGCCAGCTCACGGTGTCAGAATAACAGTTGAAAAAGGAATTGATACACACCTCATTAAATTTTTCACCAAACGCCCGCTATTGGTGGAAAAAGA
SARS-CoV-2 NC_045512.2 (22920) ATA----GATTGT----TT-AGGAAGTCTAATCTCAAACCTTTTGAGAGAGAT--------ATTTCAACTGAAATCTATCAGGCCGGTAGCACACCT
                                                                                                   Section 293
                              28325 28330     28340     28350     28360     28370     28380     28390     28400     28410 28421
(28325)
Ad 12 X73487 (26531)          TCAAGGAAATACTATATTAACTTTATATTGCATTTGTCCTG----TTCCCGGATTACATGAA--GATTTCTGCTGTCATT----TGTGTGCTGAATT
SARS-CoV-2 NC_045512.2 (23000) TGTA--ATG--GTGTTGAAGGTTTTAATTGTTACTTTCCTTTACAATCATATGGTTTCCAACCCACTAATGGTGTTGGTTACCAACCATACAGAGTA
                                                                                                   Section 294
                              28422    28430     28440     28450     28460     28470     28480     28490     28500        28518
(28422)
Ad 12 X73487 (26618)          TAATCATC------------TGTAGTGGCGCTGTACCGCCTGAAGAAGAACCTAACTGTCATC-CGCATTTAAGCAACATTAAAATCAACCTTTCGA
SARS-CoV-2 NC_045512.2 (23093) GTAGTACTTTCTTTTGAACTTCTACATGCACCA-GCAACTGTTTGTGGACCTAAAAAGTC--TACTAATTTGG------TTAAAAACA--AATGTG-
```

Section 295

```
                              28519      28530     28540     28550     28560     28570     28580     28590     28600          28615
                     (28519)
Ad 12 X73487 (26702)          TCCCTCATATCACTCTTCGCTGCAGTTTTTTTTCCACACATCTCACCTGGACCTTTAACGGAAAACACGTTACCAATACAGATATAAAGTTTAAACT
SARS-CoV-2 NC_045512.2 (23178) ----TCAATTTCAACTTCAATGG--TTTAACAGGCACAGGTGTTCTTACTGAGTCTAACAAAAAGTT--TCTGCCTTTC--CAACAATTTG-GCAGA
```

Section 296

```
                              28616         28630     28640     28650     28660     28670     28680     28690     28700    28712
                     (28616)
Ad 12 X73487 (26799)          ACACAAAGAAAACATCACTCTATTT-CAACCTATTAACCTGGGATA-------CTACCGCTGCTCAGCTCCACCCTGTACGCAAGCATTTTTTGTTG
SARS-CoV-2 NC_045512.2 (23264) GA-CATTGCTGACACTACTGATGCTGTCCGTGATCCACAGACACTTGAGATTCTTGACATTAC--ACCATGTTCTTTTGGTGGTGTCAGTGTTATAA
```

Section 297

```
                              28713  28720     28730     28740     28750     28760     28770     28780     28790          28809
                     (28713)
Ad 12 X73487 (26888)          CTCCAGTTATTGACAAACGCCCTGCTCCGACAACAGCTGCTGTCACTGAGCACATCACCGAGGCAGTTTCTCCTTCTAAAGGTACAGAGGAAATTGT
SARS-CoV-2 NC_045512.2 (23358) CACCAGGAAC---AAATACTTCTA--AC--CAG--GTTGCTGTTC-TTT----ATC-AGGATGTTAACTGCAC----AGAAGTCCCTGTT----GCT
```

Section 298

```
                              28810     28820     28830     28840     28850     28860     28870     28880     28890          28906
                     (28810)
Ad 12 X73487 (26985)          GTACTTTTCAAACTTTACA-AACCACTTAG-TTTTAAATTGT-----TCCTGTTCTAACTCCTTAATTTCATGGTTTGCTAACAGCTCTCT--GTGC
SARS-CoV-2 NC_045512.2 (23432) ATTCATGCAGATCAACTTACTCCTACTTGGCGTGTTTATTCTACAGGTTCTAATGTTTTTCAAACACGTGCAGGCTGTTTAA------TAGGGGCTG
```

Section 299

```
                              28907        28920     28930     28940     28950     28960     28970     28980     28990     29003
                     (28907)
Ad 12 X73487 (27073)          AAAACTTTCTACCAAGGAAAACTTTTGTATTCTGCTAAACTCACATTGTGTAACCAGAGCACCCCTTCCCACCTTACTCTATTGCCACCTTTT-GTT
SARS-CoV-2 NC_045512.2 (23523) AACA-TGTCAAC-------AACTCATATGA-GTGTGACATACCCATTGGTG--CAGGTATATGCGCT---A-GTTATCAGACTCAGACTAATTCTCC
```

Section 300

```
                              29004 29010     29020     29030     29040     29050     29060     29070     29080     29090 29100
                     (29004)
Ad 12 X73487 (27169)          GCC----GGTC-----GTTACTTTTGCATAGGAGCTGCACGTACTAGCCCCTGTCAACAGCATTGGAATTTAACTTACTG-----TCCCCACCAGT
SARS-CoV-2 NC_045512.2 (23605) TCGGCGGGCACGTAGTGTAGCTAGTCAATCCATC----ATTGCCTACACTATGTCACTTGGTGCAG-----AAAATTCAGTTGCTTACTCTAATAAC
```

Section 301

```
                              29101    29110     29120     29130     29140     29150     29160     29170     29180          29197
                     (29101)
Ad 12 X73487 (27252)          GTCGCCCTTTGTG--ATCAATACTGAATATTTA-GACTATAATCCCTTGCTTGCTTACGGCGGTCTCGCAGCTCTTATTTTATTC----CTGATTTC
SARS-CoV-2 NC_045512.2 (23693) TCTATTGCCATACCC-ACAAATTTTACTATTAGTGTTACCACAGAAATTCT-ACCAGTGTCTATGACCAAGACATCAGTAGATTGTACAATGTACAT
```

Section 302

(29198)		29198 29210 29220 29230 29240 29250 29260 29270 29280 29294
Ad 12 X73487	(27342)	TAACTTGTT-TCTAGTGCAACATTTGTATTCAT-ACTAACAATGCTTTCCATTTTTC----TTTTATTTCTCTTTTCTTTACCTTCTGGCTTGTATG
SARS-CoV-2 NC_045512.2	(23788)	TTGTGGTGATTCAACT--GAATGCAGCAATCTTTTGTTGCAAT--ATGGCAGTTTTTGTAC-ACAATTAAACCGTGCTTTA---ACTGGAATAGCTG

Section 303

(29295)		29295 29300 29310 29320 29330 29340 29350 29360 29370 29380 29391
Ad 12 X73487	(27433)	CTCAAACAGCCGAAAGACCACTAAAAGTCGTGGTGGAAGCTGG-CCATAATGTAACCCT---TCCCCACCTTTCTGGTTCAC-ACCAAACTGGCCAT
SARS-CoV-2 NC_045512.2	(23877)	TTGAACAAGACAAAAACACCCAAGAAGTTTTTGCACAAGTCAAA-CAAATTTACAAAACACCACCAATTAAAGATTTTGGTGGTTTTAATTTTTCAC

Section 304

(29392)		29392 29400 29410 29420 29430 29440 29450 29460 29470 29488
Ad 12 X73487	(27525)	GTTACTTGGCTAGTAGAGACATCAGATTATGGTTCAGCTTCTCCAGACAACTTCATTTTCAGTG----GACAAAAACTATGCCAGTTTACTGACAGA
SARS-CoV-2 NC_045512.2	(23973)	AAATATTACC----AGATCCATCA--AAACCAAGCAAGAGGTCAT-------TTATTGAAGATCTACTTTTCAACAAAGTGACACTTGCA-------

Section 305

(29489)		29489 29500 29510 29520 29530 29540 29550 29560 29570 29585
Ad 12 X73487	(27618)	ACCATGGTGTGGCCTTATTACAATTTACATTTTAACTGTGAA--AATTA-TGACCTTAATCT---GTTTTGGCTTA------AGGTGGAAAATTCGG
SARS-CoV-2 NC_045512.2	(24050)	G--ATGCTGGCTTCATCAAACAATATGGTG-ATTGCCTTGGTGATATTGCTGCTAGAGACCTCAT-TTGTGCACAAAAGTTTAACGGCCTTA----C

Section 306

(29586)		29586 29600 29610 29620 29630 29640 29650 29660 29670 29682
Ad 12 X73487	(27703)	CTATTTACAACGTTAAAAA-TACAGTCAATGCTT-CTGAAACAAATATTTACTATGATTTAAGAGTAGTACAAATTTTTCCACCTAAATGCATCATT
SARS-CoV-2 NC_045512.2	(24139)	TGTTTTGCCACCTTTGCTCACAGATGAAATGATTGCTCAATACACT-T-CTGCACTGTT--AGC-GGGTACAATCACTTCTGGTTGGACCTTTGGTG

Section 307

(29683)		29683 29690 29700 29710 29720 29730 29740 29750 29760 29779
Ad 12 X73487	(27798)	ACTTCAAAGTACCTTA--CAAACGATTAT-TGT-CACATTACAATTAACTGCACTAACTCTGATTACCCCAATAAGGTTGTG----TTTA------A
SARS-CoV-2 NC_045512.2	(24231)	CAG--GTGCTGCATTACAAATACCATTTGCTATGCAAATGGCTTATAGGTTTAATGGTATTGGAGTTACACAGAATGTTCTCTATGAGAACCAAAAA

Section 308

(29780)		29780 29790 29800 29810 29820 29830 29840 29850 29860 29876
Ad 12 X73487	(27881)	T--AATGTCAGTCGATGGTACTACGGATACGGTA-AGGGCAGCC----CAACCCTTCCCAACT-ACTTT---ATAACTAACTTTAATGTTTCAGGTA
SARS-CoV-2 NC_045512.2	(24326)	TTGATTGCCAACCAATTTAATAGTGCTATTGGCAAAATTCAAGACTCACTTTC--TTCCACAGCAAGTGCACTTGGAAAACTTCAAGATGT--GGTC

Section 309

```
(29877)                         29877 29890 29900 29910 29920 29930 29940 29950 29960 29973
Ad 12 X73487 (27967)            TTACTAAAAG------CTTTAATCACACTTACCCTTTTAATGAGCTCTGTGATTATCCCACATCCCAATCTCAACACAGTTTAACACATACAG-TAA
SARS-CoV-2 NC_045512.2 (24419)  AACCAAAATGCACAAGCTTTAAACACGCTTGTTAAACAACTTAGCTCCAATTTTGGTGCAATTTCA-AGTGTTTTAAATGATATCCTTTCACGTCTT
```

Section 310

```
(29974)                         29974 29980 29990 30000 30010 30020 30030 30040 30050 30060 30070
Ad 12 X73487 (28057)            GCACAGTAATCTTTTTAGGAATAATTGGCT--TCAGCATTTTGA-TTATTATAGCAGCCTTT--ATTTATCTGTGCTGGCATAGAAAATCTTTGTGT
SARS-CoV-2 NC_045512.2 (24515)  GACAAAGTTGAGGCTGAAGTGCAAATTGATAGGTTG-ATCACAGGCAGACTTCAAAGTTTGCAGACATATGTGACTCAACAA-----TTAATTAGAG
```

Section 311

```
(30071)                         30071 30080 30090 30100 30110 30120 30130 30140 30150 30167
Ad 12 X73487 (28149)            GTTTCTAAAACAGAACCTCT--TATGCCGATTCCTTACTAGTTTTCTTTTTTCTTACAGTATGGT-------GACGGTTCTTCTCATCTTTTTATGC
SARS-CoV-2 NC_045512.2 (24606)  CTGCAGAAATCAGAGCTTCTGCTAATCTTGCTGCTACTAAAATGTCAGAGTGTGTACTTGGACAATCAAAAAGAG-------TTGATTTTTGTGGAA
```

Section 312

```
(30168)                         30168 30180 30190 30200 30210 30220 30230 30240 30250 30264
Ad 12 X73487 (28237)            CTGCCAGTCATTT---TTTC-TTCTTCG------ACTTTTGCCGCAGTCAGTGACCTTGATCCCGAGTGTTTAGCCCCCTTTGCGGTGTACCTGATT
SARS-CoV-2 NC_045512.2 (24696)  AGGGCTATCATCTTATGTCCTTCCCTCAGTCAGCACCTCA--TGGTGTAGTCTTCTTGCATGT-GACTTATGTCCCTGCACAAGAAAAGAACTTCAC
```

Section 313

```
(30265)                         30265 30270 30280 30290 30300 30310 30320 30330 30340 30350 30361
Ad 12 X73487 (28324)            TTCACATTTGTGACTGCTACCTGCGTCTGC-AGTATTATTACTCTGCTAATCAC-CTC-GCTCCAATTTTTTGATTACTACTACGTGAGAATTGTTT
SARS-CoV-2 NC_045512.2 (24790)  AACT-GCTCCTGCCATTTGTCATGATGGAAAAGCACACTTTCCTCGTGAAGGTGTCTTTGTTTCAAATGGCACA---C-ACTG-GTTTGTAACACAA
```

Section 314

```
(30362)                         30362 30370 30380 30390 30400 30410 30420 30430 30440 30458
Ad 12 X73487 (28418)            ACCGCAGACACCACCCCCGTTACCAAAACC-----CTCAAATTGCGGCTCTTTTGCAGCTCCAACCATGAAAACAGCATTAGTTCTTTTCTTTATGT
SARS-CoV-2 NC_045512.2 (24881)  AGGAATTTTTATGAACCACAAATCATTACTACAGACAACACATTTGTGTCTGGTA--ACTGTGATGTTGTAA-TAGGAATTGTCAACAACAC--AGT
```

Section 315

```
(30459)                         30459 30470 30480 30490 30500 30510 30520 30530 30540 30555
Ad 12 X73487 (28510)            TAATCCCAGTTTGGGCTAGTTCTTG---TCAACTACATAAACCATG-------------GAATTTTTTAGATTGTTATAC-TAAAGAAA-CAAACTA
SARS-CoV-2 NC_045512.2 (24973)  TTATGATCCTTTGCAACCTGAATTAGACTCATTCAAGGAGG---AGTTAGATAAATATTTTAAGAATCATACATCACCAGATGTTGATTTAGGTGAC
```

Section 316

```
                            (30556) 30556        30570      30580      30590      30600      30610      30620      30630      30640       30652
                  Ad 12 X73487 (28589) --CATAGGCTGGGTTTATGGAATTATGTCTGGCTTAGTATTTGTC-TCCTCTGTAGTTTCTTTACAACTGTATGCGCGCCTTAATTTTAGTTGGAAT
  SARS-CoV-2 NC_045512.2 (25067) ATCTCTGGCATTAATGCTTCAGTTGTAAACATTCAAAAGAAATTGACCGCCTCAATGAGGTTGCCAA-GAAT--TTAAATGAATCTCTCATCGATC
```

Section 317

```
                            (30653) 30653  30660      30670      30680      30690      30700      30710      30720      30730          30749
                  Ad 12 X73487 (28683) AAGTATAC--TG--ATGATCTTCCGAATATCCAAACCCCCAGGATGATTT-AC-----CCCTAAATATTGTA-TTT---CCAG-----AGCC----
  SARS-CoV-2 NC_045512.2 (25161) TCCAAGAACTTGGAAAGTATGAGCAGTATATAAAA-TGGCCATGGTACATTTGGCTAGGTTTTATAGCTGGCTTGATTGCCATAGTAATGGTGACAA
```

Section 318

```
                            (30750) 30750     30760      30770      30780      30790      30800      30810      30820      30830       30846
                  Ad 12 X73487 (28757) C-CCGCGTCCTCCTTCTGTTGTTAGCTATTTTAA-GTTCACCGG--TGAAGATGATT--GAA-CCTGATCTAGAAATTGATGGAAGAATCACCGAAC
  SARS-CoV-2 NC_045512.2 (25257) TTATGCTTT---GCTGTATGACCAGTTGCTGTAGTTGTCTCAAGGGCTGTTGTTCTTGTGGATCCT--GCTGCAAATTTGATGAAGA-CGACT---C
```

Section 319

```
                            (30847) 30847         30860      30870      30880      30890      30900      30910      30920      30930       30943
                  Ad 12 X73487 (28847) AGAGGCTCCTCACTGATCGCGCTAGGCGACGCC-AACAGGATCAAAAAAATAAAGAGTTAATTGATTTACAA-ACCGTGCATCAGTGTAAAAAAGGA
  SARS-CoV-2 NC_045512.2 (25345) TGAG---CCAGT-----GCTCAAAGGAGTCAAATTACATTACACATAAACGAACTTATGGATTTGTTTATGAGAATCTTCAC--------AATTGGA
```

Section 320

```
                            (30944) 30944 30950      30960      30970      30980      30990      31000      31010      31020      31030 31040
                  Ad 12 X73487 (28942) CTTTTTTGCCTGGTAAAACAAGCTACCCTTCGCT-ATGAATCTTTACCAGGCAAAGAACATCAACTGTGCTACACGCTGCCCACTCAGC--GACAAA
  SARS-CoV-2 NC_045512.2 (25426) ACTGTAACTTTGAAGCAAG--GTGAAATCAAGGATGCTACTCCTTCAGAT-TTTGTTCGCGCTACTGCAACGATACCGATACAAGCCTCACTCCCTT
```

Section 321

```
                            (31041) 31041    31050      31060      31070      31080      31090      31100      31110      31120          31137
                  Ad 12 X73487 (29036) CCTTTACTGCAATGGTGGGCTCGGTACCTATTAAAGTGTCCCAACAAGCAG-GAGAACAAGAAGGCTCTATTCGGTGCCTATGTGATAACCCTGAAT
  SARS-CoV-2 NC_045512.2 (25520) TCGGATGGCTTATTGTTGGCGTTGCACTTCTTGCTGTTTTTCAGAGCGCTTCCAAAATCATAACCCTCAAAAGAGATGGCAA--CTAGCACTCTCC
```

Section 322

```
                            (31138) 31138       31150      31160      31170      31180      31190      31200      31210      31220       31234
                  Ad 12 X73487 (29132) GTTT--GTACACTTTAATAAAAAC--ACTGTGCGGTTTAAGAAATCTTTTACC-AATGAATTAAATAAATTA----CTTACCGGAAATCTGAAAATA
  SARS-CoV-2 NC_045512.2 (25615) AAGGGTGTTCACTTTGTTTGCAACTTGCTGTTGTTGTTTGTAACAGTTTACTCA---CACCTTTTGCTCGTTGCTGCTGGCCTTGAAGCCCCTTTTC
```

```
                                                                                                     Section 323
                                  31235 31240     31250     31260     31270     31280     31290     31300     31310     31320      31331
                         (31235)
             Ad 12 X73487 (29220) CATCATGGTCTCCGTGTACTCTTATAAAA----TTTCCCTCTTCCCAACTGTCAAACCTGACAGACTTGC-AAACAGCAAA---CTTTCTCCAAATC
SARS-CoV-2 NC_045512.2 (25709) TCTATC-TTTATGCTTTAGTCTACTTCTTGCAGAGTATAAACTTTGTAAGAATAATAATGAGGCTTTGGCTTTGCTGGAAATGCCGTTCCAAAAACC
                                                                                                     Section 324
                                  31332   31340     31350     31360     31370     31380     31390     31400     31410          31428
                         (31332)
             Ad 12 X73487 (29309) TTA-AATGGAAGG-TCAGATTCTTC---TTCC---CAATCCCTACCCA----CCATCTT-CATCTT-TTCTAGATGAAGCGCA-GCAGAACCCAGTA
SARS-CoV-2 NC_045512.2 (25805) CATTACTTTATGATGCCAACTATTTTCTTTGCTGGCATACTAATTGTTACGACTATTGTATACCTTACAATAG-TGTAACTTCTTCAATTGTC-ATT
                                                                                                     Section 325
                                  31429      31440     31450     31460     31470     31480     31490     31500     31510       31525
                         (31429)
             Ad 12 X73487 (29391) TGCTGAAGAAACAGAAGAAAATGATG-ACTTCAAC---CCCGTTTACCCTTTTGACCC---ATTTGACACATCAGACGTACCCTTTGTTACACCCCC
SARS-CoV-2 NC_045512.2 (25900) ACTTCAGGTGATGGCACAACAAGTCCTATTTCTGAACATGACTACCAGATTGGTGGTTATACTGAAAAATGGGAATC-TGGAGTAAAAGACTGTGTT
                                                                                                     Section 326
                                  31526         31540     31550     31560     31570     31580     31590     31600     31610    31622
                         (31526)
             Ad 12 X73487 (29481) TTTTACTTCTTCCAATGGTCTTCAAGAAAAAACCACCAGGTGTATTAGCACTTAATTACAAAGACCCCATTGTAACTGAAAATGGAACCCT-TACAC-
SARS-CoV-2 NC_045512.2 (25996) GTATTACACAGT-TACTTCACTTCAGACTATT-ACCAGCTGTACTCAACTCA-ATTGAGTACAGACACTGGTG-TTGAACATGTTACCTTCTTCATC
                                                                                                     Section 327
                                  31623  31630     31640     31650     31660     31670     31680     31690     31700           31719
                         (31623)
             Ad 12 X73487 (29576) -TCAAGCTAGGGGACGGAATAAAACTTAATGCCCAA--GGTCAACTTACAGCTAGTAATAATATCAATG--TTTTGGA-GCCCCTTACCAACACCTC
SARS-CoV-2 NC_045512.2 (26089) TACAATAAAATTGTTGATGAGCCTGAAGAACATGTCCAAATTCACACAATCGACGGTTCATCCGGAGTTGTTAATCCAGTAATGGAACCAATTTATG
                                                                                                     Section 328
                                  31720     31730     31740     31750     31760     31770     31780     31790     31800          31816
                         (31720)
             Ad 12 X73487 (29667) A--CAAGGTCTTAAACTTTCTTGGAGCGCCCCCCTAGCAGTAAAGGCTAGTGCCCTCACACTTAACACAAGAGCGCCCTTAACCACAACGGATGAAA
SARS-CoV-2 NC_045512.2 (26186) ATGAACCGACGACGACTACTAGCGTGC----CTTTGTAAGCACAAGCTGATG-------AGTACGAACTTATGTACTCATTCGTTTCGGAAGAGACA
                                                                                                     Section 329
                                  31817        31830     31840     31850     31860     31870     31880     31890     31900       31913
                         (31817)
             Ad 12 X73487 (29762) GCTTAGCCTT--AATAACCGCCCCTCCCATTACAGTAGAGTCTTCGC-GTTTGGGCTTGGCCACCAT--AGCCCCTCTAAGCTTAGATG----GAGG
SARS-CoV-2 NC_045512.2 (26272) GGTACGTTAATAGTTAATAGCGTACTTCTTTTTCTTGCTTTCGTGGTATTCTTGCTAGTTACACTAGCCATCCTTACTGCGCTTCGATTGTGTGCGT
```

Section 330

(31914) 31914 31920 31930 31940 31950 31960 31970 31980 31990 32000 32010
Ad 12 X73487 (29850) TGGAAACCTAGGTTTAAATCTTTCTGCTCCCCTGG---ACGTTAGTAACAACAATTTGCATCTCACCACTG-AAACTCCCTTAGTTG-TAAATTCTA
SARS-CoV-2 NC_045512.2 (26369) ACTGCTGCAATATTGTTAACGTGAGTCTTGTAAAACCTTCTTTTTACGTTTACTCTCGTGTTAAAAATCTGAATTCTTCTAGAGTTCCTGATCTTCT

Section 331

(32011) 32011 32020 32030 32040 32050 32060 32070 32080 32090 32107
Ad 12 X73487 (29942) GCGGTGCCCTATCTGTTGCTACTGCAG-ACCCCATAA-GTGTTCGCAACAACGCTCTTACCCTACCTACGGCAGATCCGTTAATGGTGAGCTCCGAT
SARS-CoV-2 NC_045512.2 (26466) GGTCTAAAC--GAACTAAATATTATATT---AGTTTTTCTGTTTGGAACTTTAATTTTAG-----CCATGGCAGATTCCAACGGTACTATTACCGTT

Section 332

(32108) 32108 32120 32130 32140 32150 32160 32170 32180 32190 32204
Ad 12 X73487 (30037) GGGTTGGGAATAAGTGTCACT-AGTCCCATT-ACAGTAA-TAAACGGTTCCTTAGCCTTGT-CTACAACTGCTCCCCTCAACAG-CACAGGATCCA-
SARS-CoV-2 NC_045512.2 (26553) GAAGAGCTTAAAAA-GCTCCTT-GAACAATGGAACCTAGTAATAGGTTTCCTATTCCTTACATGGATTTGTCTTCTACAATTTGCCTATGCCAACAG

Section 333

(32205) 32205 32210 32220 32230 32240 32250 32260 32270 32280 32290 32301
Ad 12 X73487 (30128) CTTTAAGTCTG---TCTGTTGCCAAT-CCTCTGACTATTTCACA-AGACACATTGACTGTTTC-CACT-------GGTAAC-GGTCTTCAAGTGTCG
SARS-CoV-2 NC_045512.2 (26648) GAATAGGTTTTTGTATATAATTAAGTTAATTTTCCTCTGGCTGTTATGGCCAGTAACTTTAGCTTGTTTTGTGCTTGCTGCT-GTTTACAGAATAAA

Section 334

(32302) 32302 32310 32320 32330 32340 32350 32360 32370 32380 32398
Ad 12 X73487 (30211) G--GGTCTCAATTAGTAACAAGAATAGGGGATGGTTTAACATTCG-----ATAATG-GGGTCATGAAAGTAAACGTTGC---CGGGGGAATGAGAAC
SARS-CoV-2 NC_045512.2 (26744) TTGGAT---CACCGGTGGAATTGCTATCGCAATGGCTTGTCTTGTAGGCT-TGATGTGGCTCAGCTACTTCA---TTGCTTCTTTCAGACTG-----

Section 335

(32399) 32399 32410 32420 32430 32440 32450 32460 32470 32480 32495
Ad 12 X73487 (30297) TTCTGGCGGTAGAATAATTTTAGATGTTAA-TTATCCCTTTGATGCGAGCAATAACCTGTCCTTAAGACGGGGATTGGGACTAATTTATAACCAATC
SARS-CoV-2 NC_045512.2 (26829) TTTGCGCGTACGCG--TTCCATGTGGTCATTCAATCCA---GAAACTAACATTCTTCTC----AACGTGCCACTCCATGGCACTATTCTGACCA-GA

Section 336

(32496) 32496 32510 32520 32530 32540 32550 32560 32570 32580 32592
Ad 12 X73487 (30393) TACAAAC-TGGAACTTAACAACTGATATTAGTA-----CCGAA----AAAGGTT-TAATGTTTAGTGGCAATCAAATAGCTCTTAAT--GCA-----
SARS-CoV-2 NC_045512.2 (26916) CCGCTTCTAGAAAGTGAACTCGTAATCGGAGCTGTGATCCTTCGTGGACATCTTCGTATTGCTGGACACCATCTAGGACGCTGTGACATCAAGGACC

```
                                                                                                  Section 337
                              32593 32600          32610          32620          32630          32640          32650          32660          32670              32689
(32593)
Ad 12 X73487 (30472)          --GGTCAGGGCTTACATTTAATAATGG-CCAAC--TTAGGGTT---AAGTTGGGAGCTGGACTTA-TTTTTGA-TTCAAACAATAACATTGCCTTA
SARS-CoV-2 NC_045512.2 (27013) TGCCTAAAGAAATCACTGTTGCTACATCACGAACGCTTTCTTATTACAAATTGGGAGCTTCGCAGCGTGTAGCAGGT--GACTCAGGTTTTGCTGCA
```

```
                                                                                                  Section 338
                              32690       32700          32710          32720          32730          32740          32750          32760          32770          32786
(32690)
Ad 12 X73487 (30559)          GGCAGCAGCAGCAACACTCCATACGACCCTC--TGACACTGTGGACAACTCCTGACCCACCACCAAACTGCAGCCTCATACAAGAGCTAGATGCAAA
SARS-CoV-2 NC_045512.2 (27108) TACAGTCGCTACAGGATTGGCAACT---ATAAATTAAAC-ACAGACCATTCC--AG---TAGC---AGTGACAAT--ATTGCTTTGCTTGTACAGTA
```

```
                                                                                                  Section 339
                              32787         32800          32810          32820          32830          32840          32850          32860          32870          32883
(32787)
Ad 12 X73487 (30654)          ACTCAC--CCTGTGCTTAACAAAAAACGGA---TC----TATTGT--TAATGGCAT-TGTAAGTTTAGTGGG---TGTTAAGGGTAATCTCCTAAAT
SARS-CoV-2 NC_045512.2 (27191) AGTGACAACAGATGTTTCATCTC--GTTGACTTTCAGGTTACTATAGCAGAGATATTACTAATTATTATGAGGACTTTTAAAGTTTCCATTTGGAAT
```

```
                                                                                                  Section 340
                              32884 32890          32900          32910          32920          32930          32940          32950          32960          32970 32980
(32884)
Ad 12 X73487 (30736)          ATCCAA-AGTACTACTACCACTGTAGGAGTGCATTTAGTGTTTGATGAACAGGGAAGATTAA-TCACATCAACCCCTACTGCCCTGGTTCCCCAAGC
SARS-CoV-2 NC_045512.2 (27286) CTTGATTACATCATAAACCTCATAATTAAAAATTTATCTAAGTCACTAACTGAGAA---TAAATATTCTCAATTAGATGA--AGAGCAACCAATGGA
```

```
                                                                                                  Section 341
                              32981    32990          33000          33010          33020          33030          33040          33050          33060              33077
(32981)
Ad 12 X73487 (30831)          TTCGTGGGGATATAGACAAGGCCAATCAGTGTCTACCAATACTGTT-ACCAATGGTCTAGGTTTTATGCCTAATGTGAGTGCTTACCCTAGACCAAA
SARS-CoV-2 NC_045512.2 (27378) GATT-GATTAAACGAACATGAAAATTATTCTTTTCTTGGCACTGATAACACTCGCTACTTGTGAGCTTTATCACTACCAAGAGTGTGTTAGAGGTAC
```

```
                                                                                                  Section 342
                              33078            33090          33100          33110          33120          33130          33140          33150          33160          33174
(33078)
Ad 12 X73487 (30927)          TGCCAGTGAGGCTAAAAGCCAAATGGTAAGTCTCACGTACTTAC-AGGGAGAT---ACATCTAAACC-------TATAA-CAATGAAAGTTG-CATT
SARS-CoV-2 NC_045512.2 (27474) AA-CAGTACTTTTAAAAGAACCTTGCTC---TTCTGGAACATACGAGGGCAATTCACCATTTCATCCTCTAGCTGATAACAAATTTGCACTGACTTG
```

```
                                                                                                  Section 343
                              33175 33180          33190          33200          33210          33220          33230          33240          33250          33260 33271
(33175)
Ad 12 X73487 (31011)          TAATGGCATT-----------------ACGTCGCTAAATGGATACTCTTTAACATT--CATGT-GGTCAGGTCTATCAA--ACTAT--ATAAATC--
SARS-CoV-2 NC_045512.2 (27567) CTTTAGCACTCAATTTGCTTTTGCTTGTCCTGACGGCGTAAAACACGTCTATCAGTTACGTGCCAGATCAGTTTCACCTAAACTGTTCATCAGACAA
```

```
                                                                                                        Section 344
                              (33272) 33272   33280     33290     33300     33310     33320     33330     33340     33350              33368
              Ad 12 X73487 (31082) --AGCCTTTCTCTA-CACCATCCTGCTCCTTTTCTTACA-TTACC-CAAGAATAAAAACACACA------------CAA--------------AA--
   SARS-CoV-2 NC_045512.2 (27664) GAGGAAGTTCAAGAACTTTACTCTCCAATTTTTCTTATTGTTGCGGCAATAGTGTTTATAACACTTTGCTTCACACTCAAAAGAAAGACAGAATGAT
                                                                                                        Section 345
                              (33369) 33369      33380     33390     33400     33410     33420     33430     33440     33450        33465
              Ad 12 X73487 (31146) CACAAATTGCGTACTTATTGTTTATTTTTTTTTTT-------TTTTACACTATACGCGTGGTTAAACTGCCTCCTTCCCATTT--TACCTTGTATAC
   SARS-CoV-2 NC_045512.2 (27761) TGAACTTTCATTAATTGACTTCTATTTGTGCTTTTTAGCCTTTCTGCTA----TTCC-TTGTTTTAATTA-TGCTTATTATCTTTTGGTTCTCACTT
                                                                                                        Section 346
                              (33466) 33466         33480     33490     33500     33510     33520     33530     33540     33550   33562
              Ad 12 X73487 (31234) CTCCCTTTCCCCC-TTTGTAGCTGAAAACAACTGCACTTGAATATTTCGACTTAGGTTTTTGGCGTTAGCGTCCACACAGTTTC-TTTACGGGCAA
   SARS-CoV-2 NC_045512.2 (27852) GAACTGCAAGATCATAATGAAACTTGTCACGCCTAAACGAA--CATGAAATTTCTTGTTTTCTTAGGAATCATCACAACTGTAGCTGCATTTCACCA
                                                                                                        Section 347
                              (33563) 33563  33570     33580     33590     33600     33610     33620     33630     33640          33659
              Ad 12 X73487 (31329) AGCGAGGGTCGGTGATGGAAACGAA-TCCCTCGCCCG-CACAGTCAC--TCAAGCGGCATTCCCCATCCAAAACCAGGTCCATGATTTTATCCTACA
   SARS-CoV-2 NC_045512.2 (27947) AGAATG----TAGTTTACAGTCATGTACTCAACATCAACCATATGTAGTTGATGACCCGTGTCCTATTCACTTCTATTCTAAATGGTATATTAGAGT
                                                                                                        Section 348
                              (33660) 33660     33670     33680     33690     33700     33710     33720     33730     33740        33756
              Ad 12 X73487 (31422) AAAAGTAACAACAGTCAGTGTCCATCAGCCGCCCAAGGATTCTCTCG-TTGATT-ATAATCTCCAAATAAAATTGCTCGATGATGCATAATTAAACC
   SARS-CoV-2 NC_045512.2 (28040) AGGAGCTAG-AAAATCAGCACCTTTAATTGAATTGTGCGTGGATGAGGCTGGTTCTAAATCACC-CATTCAGTACATCGATATCGGT-AATTATAC-
                                                                                                        Section 349
                              (33757) 33757        33770     33780     33790     33800     33810     33820     33830     33840     33853
              Ad 12 X73487 (31517) CTTTAGCAGTTGCTGAC-GATAACGTTCATGCCGACTATGTTTTAGAGGGCGAACAGTGTTTTCAGCAATTACTTGAACAACTTTTAACATTAGCAG
   SARS-CoV-2 NC_045512.2 (28133) -------AGTTT--C-CTGTTTACCTTT-----TACAA--TTAATTGCCAGGAAC-CTAAATTGGGTAGT--CTTGTAGTGCGTTGTTC----G---
                                                                                                        Section 350
                              (33854) 33854 33860     33870     33880     33890     33900     33910     33920     33930     33940  33950
              Ad 12 X73487 (31613) TCTGGTACGACGAGCGCAACAGCGCATGCGTATCTCACTTAAGTCTTTACAATAATCACAAC-ACAGCACTAACATGTTA-TTTAAAATTCCATAAT
   SARS-CoV-2 NC_045512.2 (28203) --TTCTATGAAG-ACTTTTTAGAGTATCATGACGTTCGTGTTGTTTTAGATTTCATCTAAACGAACAAACTAAAATGTCTG---ATAATGGACCCCA
```

```
                                                                                                    Section 351
                       (33951) 33951    33960      33970      33980      33990      34000      34010      34020      34030         34047
            Ad 12 X73487 (31708) TAA--AG-GCGCTCCATCCAAAACTAACTTTTTCTAACGC--TAACCAGGCATGGCCATCATACATAATTTTAAAGT-AAATTAAAT--GGCGACCT
SARS-CoV-2 NC_045512.2 (28294) AAATCAGCGAAATGCACCCCGCATTACGTTTGGTGGACCCTCAGATTCAAC-TGGCA--GTAACCAGA-----ATGGAGAACGCAGTGGGGCG--CG
                                                                                                    Section 352
                       (34048) 34048       34060      34070      34080      34090      34100      34110      34120      34130      34144
            Ad 12 X73487 (31797) CTAACAAAGGTGCTTCCCACATACATCACCTCTTTAGGCATTAAAT--GGTTA---ACAACCTCCCGATACCAAAAACACCTTTT---GTTAATTAA
SARS-CoV-2 NC_045512.2 (28381) ATCAAAAACAACGTCGGCCCCAAGGTTTACCCAATAATACTGCGTCTTGGTTCACCGCTCTCACTCAACATGGCAAGGAAGACCTTAAATTCCCTCGA
                                                                                                    Section 353
                       (34145) 34145 34150     34160      34170      34180      34190      34200      34210      34220      34230   34241
            Ad 12 X73487 (31886) GGCGCCATATACGGCCATTTTGAACCAGCGTCCCAAAAG-CATCCCAGCTGACATACACTGTAGTGAACCCGGACGCTGGCAATGACAATGAATAAG
SARS-CoV-2 NC_045512.2 (28478) GGA--CAAGGCGTTCCAATTAACACCAATAGCAGTCCAGATGACCAAATTGGCTACTACCGAAGAGCTACCAGACGAATTCG-TGGTGGTGA--CGG
                                                                                                    Section 354
                       (34242) 34242    34250      34260      34270      34280      34290      34300      34310      34320         34338
            Ad 12 X73487 (31982) CCACCGCTCATGACCATGTAATA-ATTGAGTAACTTCAACATTT--ATAGTGGCACAACACATACATACACTCATGTATTTTTTCAAAAT----AAA
SARS-CoV-2 NC_045512.2 (28570) TAAAATGAAAGATCTCAGTCCAAGATGGTATTTCTACTACCTAGGAACTGGGCCAGAAGCTGGACTTCCC--TATGGTGCTAACAAAGACGGCATCA
                                                                                                    Section 355
                       (34339) 34339    34350      34360      34370      34380      34390      34400      34410      34420         34435
            Ad 12 X73487 (32072) CATCTCATAATCAGTTAGAATCATATCCCACGGTATTGGCCATTCCTGCAGCACTGTAAAACCTACACATGAAGGA---ATGCCTCTTA------CC
SARS-CoV-2 NC_045512.2 (28665) TATGGGTTGC-AACTGAGGGAGCCTTGA-ATACACCAAAAGATCACATTGGCACCCGCAATCCTGCTAAC-AATGCTGCAATCGTGCTACAACTTCC
                                                                                                    Section 356
                       (34436) 34436          34450      34460      34470      34480      34490      34500      34510      34520   34532
            Ad 12 X73487 (32160) TCA-CTTACATTATGTAAAGTCAGACTATTACACTCAGGCCATAAAGAATTTTCCGAAGTACTCAACGTAG-CTTTGACTGTTCCTCACAGGGCGG
SARS-CoV-2 NC_045512.2 (28759) TCAAGGAACAACATTGCCA---AAAGGCTTCTACGCAG--AAGGGAGCAGAGGCGGC---------AGTCAAGCCTCTTCTCGTTCCTCATCACGTAG
                                                                                                    Section 357
                       (34533) 34533  34540      34550      34560      34570      34580      34590      34600      34610         34629
            Ad 12 X73487 (32255) TAGTTGGT-ACTTGTTG--TATGGTGC-CAATCTGTAGCGATACCGTCTGTCGCGCTGCATCGTAA-ACAACAGACTTGCGAGCGTCTTCGTACTTA
SARS-CoV-2 NC_045512.2 (28843) TCGC--AACAGTTCAAGAA------ATTCAACT--C---CAGGCAG-CAGTAGGGAACTTCTCCTG---CTAGAATGGCTGGCAATGGCGGTGATG
```

```
                                                                                                   Section 358
                          (34630) 34630     34640     34650     34660     34670     34680     34690     34700     34710          34726
        Ad 12 X73487 (32347) AAAAAACAAAACCACGTACGACCAC---TGGTTATCGCACCTCGTCCTTTT--TGTTTGCAGCGTTGGCGTTCCGTCAAAAAAGCAAAGTACAACCA
SARS-CoV-2 NC_045512.2 (28923) CTGCT-CTTGCTTTGCTGCTGCTT-GACAGATTGAACCAGCTTGAGAGCAAAATGTCTGGTAAAGGCC------AACAACAACAAGG-------CCA
                                                                                                   Section 359
                          (34727) 34727        34740     34750     34760     34770     34780     34790     34800     34810          34823
        Ad 12 X73487 (32439) CTCTCGCAGGCTTGCTAAAATGTAT--TCAGCTTC-AGGTGTTATC-TTCAAATCATGATGTTTAATAAAGC---GCAGAGTATCCACACAGGATGC
SARS-CoV-2 NC_045512.2 (29005) AACTGTCA--CTAAGAAATCTGCTGCTGAGGCTTCTAAGAAGCCTCGGCAAAAACGTACTGC-CACTAAAGCATACAATGTAACACAAGCTTTCGGC
                                                                                                   Section 360
                          (34824) 34824 34830     34840     34850     34860     34870     34880     34890     34900     34910 34920
        Ad 12 X73487 (32529) ATGG-----GCTAAACCAAGCCATGCTATGCAGGCAGCCGTGTCCCGACTTACAGGAGGAGGAGGA-ATACAAGGTAGAGGCATAAAAACTT----A
SARS-CoV-2 NC_045512.2 (29099) AGACGTGGTCCAGAACAAACCCAAGGAAATTTTGGGGACCAGGAACTAATCAGACAAGGAACTGATTACAAACATTGGCCGCAAATTGCACAATTTG
                                                                                                   Section 361
                          (34921) 34921    34930     34940     34950     34960     34970     34980     34990     35000          35017
        Ad 12 X73487 (32616) ATCAAGACG-GTCAGCAAGGATTTGAATGCGTAAATCTCGCAGGTGGCAGCGATCGCCTCCGCTGTGCTG-GTGAAAGATCACA-GCCAGATCAAAT
SARS-CoV-2 NC_045512.2 (29196) CCCCCAGCGCTTCAGCGTTCTTCGGAATGTCGCGCATTGGCATG--GAAGTCACACCTTCGG--GAACGTGGTTGACCTACACAGGTGCCATCAAAT
                                                                                                   Section 362
                          (35018) 35018        35030     35040     35050     35060     35070     35080     35090     35100          35114
        Ad 12 X73487 (32710) TGTAAGCGATTTTCCAAATGTTCAACAACAGCTTCTAAAAGAGCCACAGCTCTGATTTCGATAAACAAA--AGCAAAGC--AAATGCATTATCATGA
SARS-CoV-2 NC_045512.2 (29289) TGGATGACAAAGATCCAAATTTCAAAGATCAAGTCATTTTGCTGAATAAGCATATTGACGCATACAAAACATTCCCACCAACAGAGCCT----AAAA
                                                                                                   Section 363
                          (35115) 35115 35120     35130     35140     35150     35160     35170     35180     35190     35200 35211
        Ad 12 X73487 (32803) AACTCTTCTATCATCA--AACTGCCTGACTGAACC--ATTCCCAGGTAATTTTCATTCTTCCACTGTT-GTATTATTTGAACACACTGATTTTGCAG
SARS-CoV-2 NC_045512.2 (29382) AGGACAAAAAGAAGAAGGCTGATGAAACTCAAGCCTTACCGCAGAGACAGAAGAAACAGCAAACTGTGA-CTCTTCTTCCTGCTGCAGATTTGGATG
                                                                                                   Section 364
                          (35212) 35212  35220     35230     35240     35250     35260     35270     35280     35290          35308
        Ad 12 X73487 (32895) GTTTAAACCGTGAATAT-TAAAAAGCTCTGTAAGGGCGCCCTCCACCGCCATCCGCAGGCAGTACTTCATATTTGCTGAAAAAA--GTCTGGATCTT
SARS-CoV-2 NC_045512.2 (29478) -ATTTCTCCAAACAATTGCAACAATC--CATGA-GCAGTGCTGACTCAAC----TCAGGCCTAAACTCAT-GCAGACCACACAAGGCAGATGGGCTA
```

```
                                                                  Section 365
                       (35309) 35309 35320 35330 35340 35350 35360 35370 35380 35390 35405
          Ad 12 X73487 (32989) CAAACACCTGCAGTAAATTCAGTAGATTTACATTAGGCTCCACACCTTGGTCTCGCAGCTGACATCTTAATGCCAGTTGTATAAAATCATACAAATC
SARS-CoV-2 NC_045512.2 (29566) TATAAACGT--TT---------TCGCTTTTCCGTTTACGAT--ATATAGTCTACTCTTGTGCAGAATGAATTCTCGTAACTACATAGCACAAGTAGA
                                                                  Section 366
                       (35406) 35406 35420 35430 35440 35450 35460 35470 35480 35490 35502
          Ad 12 X73487 (33086) AGAAGCCAGCAGCAAAGAAAGTTCACCTCCAGGTACAAGTTCCGGAGTTCCCACAGAACATACAACTTGCACAAATGGACC-CATATTAGTAAGCGT
SARS-CoV-2 NC_045512.2 (29650) TGTAG---TTAACTTTAATCTCACATAGCAATCTT--TAATCAGTGTGTAACATT--AGGGAGGACTTG-AAAGAGCCACCA-CATTTT--CACCGA
                                                                  Section 367
                       (35503) 35503 35510 35520 35530 35540 35550 35560 35570 35580 35599
          Ad 12 X73487 (33182) GGCGCCAACGTAGACATCGCGCATAGGAGGAGTTAAATAATG-CATTACCAGCAGCCAA------AA----CTCA--GGTAGCA---CGTCTTTAAG
SARS-CoV-2 NC_045512.2 (29736) GGCCAC---GCGGAGTACGATCG-AGTGTACAGTGAACAATGCTAGGGAGAGCTGCCTATATGGAAGAGCCCTAATGTGTAAAATTAATTTTAGTAG
                                                                  Section 368
                       (35600) 35600 35610 35620 35630 35640 35650 35660 35670 35680 35696
          Ad 12 X73487 (33263) AAACGTCACCACCTCAAAATCTAAGCCATGCAAATAGTTCCGTAAAGACTCCG-GAAACAACACGGAGTAATGAACAAGCGACCTCTGAAACATGCT
SARS-CoV-2 NC_045512.2 (29829) TGCTATCCCCA--TGTGATTTTAATAGCTTCTTAGGAGAATGACAAAAAAAAAAAAAAAAAAAAAAAAAAAAAAAAA--------------------
                                                                  Section 369
                       (35697) 35697 35710 35720 35730 35740 35750 35760 35770 35780 35793
          Ad 12 X73487 (33359) TTAGGTTAGCCTGAAAAATAAAAATATGTTAAATTAAAGATGCCTGGCAAACGGGTGGAAAAACAACTCTACTTAAAAGCAAGCGCGCGACTGGCTG
SARS-CoV-2 NC_045512.2 (29904) -------------------------------------------------------------------------------------------------
                                                                  Section 370
                       (35794) 35794 35800 35810 35820 35830 35840 35850 35860 35870 35880 35890
          Ad 12 X73487 (33456) CTTTGCGCGAACATCGCAAAACACGTCGGAATGATTAAACAACAAAACACTGAGCTCCATTCTTGAGCCTGGATAAAGCGTTTCAGCGCCAACAAAA
SARS-CoV-2 NC_045512.2 (29904) -------------------------------------------------------------------------------------------------
                                                                  Section 371
                       (35891) 35891 35900 35910 35920 35930 35940 35950 35960 35970 35987
          Ad 12 X73487 (33553) ACCCCTCTGGCGTTCATGTCGCATAATGAAAACAATGTTCCCAAATATCCAGGAGGAATATCAACTGCTATGTGCAAATATAAAAGCACAACTCCAT
SARS-CoV-2 NC_045512.2 (29904) -------------------------------------------------------------------------------------------------
```

```
                                                                                                                        Section 372
                          (35988) 35988          36000       36010       36020       36030       36040       36050       36060       36070        36084
              Ad 12 X73487 (33650) GTGGAGGTATAACAAAATTCGCAGGAGAAAATAACACATAAGCATTAGAGTCGCCCTCTTGTTTAGGCAACATAGCCCCAGGTCCCGTAAAATACAC
SARS-CoV-2 NC_045512.2 (29904) -------------------------------------------------------------------------------------------------
                                                                                                                        Section 373
                          (36085) 36085 36090       36100       36110       36120       36130       36140       36150       36160       36170    36181
              Ad 12 X73487 (33747) ATAAAGAGTCTCAAAAGCAGCCATAATGCCTTACCAGAAAAACAGTACAAAGCCAGGCACAGCAGACACAATCTGCCGCAAGTGCGCACCTTTAATA
SARS-CoV-2 NC_045512.2 (29904) -------------------------------------------------------------------------------------------------
                                                                                                                        Section 374
                          (36182) 36182   36190       36200       36210       36220       36230       36240       36250       36260           36278
              Ad 12 X73487 (33844) CTGAAAAATAGTGACGTAAATGGCCAAAGTTCGCCTACACAACACAAAAAAAACCCCAAAAGCCCGCGAAAAAAATCACTTCCGCATATGACTCGGC
SARS-CoV-2 NC_045512.2 (29904) -------------------------------------------------------------------------------------------------
                                                                                                                        Section 375
                          (36279) 36279        36290       36300       36310       36320       36330       36340       36350       36360        36375
              Ad 12 X73487 (33941) ATAATACGGTGTTCTCACGACACGTCACATCCGGCGCGCCCGGCTCCCACGCCGCGCCCCACTTCCTCATCCGCCCAAACTTACAAGCACGCCAAAG
SARS-CoV-2 NC_045512.2 (29904) -------------------------------------------------------------------------------------------------
                                                                                                                        Section 376
                          (36376) 36376          36390       36400       36410       36420       36430       36440       36450       36463
              Ad 12 X73487 (34038) CCACACCTCCACCCAATCAAATTACACACTACGCCCACTTCATTTTAATATTGGCACTAGTCCAGTATAAGGTATATTATTAGATAGG
SARS-CoV-2 NC_045512.2 (29904) ----------------------------------------------------------------------------------------
```
