# Supplementary material for: Ubiquitous Micro-Modular Homologies among Genomes from Viruses to Bacteria to Human Mitochondrial DNA: Platforms for Recombination during Evolution?
Source: Viruses. 2022 Apr 24;14(5):885. doi: 10.3390/v14050885 (PMC9147251; doi:10.3390/v14050885)
Supplement: Supplementary file 1 [file viruses-14-00885-s001.zip › Fig. S2A, SARS-CoV-2 & HIV-1 DNA Alignment.pdf]

```
                                          Section 1
                          (1) 1       10        20        30        40        50        60        70        80               97
                HIV-1 HXB2 (1) -------------------------------------------------------------------------------------------------
SARS-CoV-2 NC_045512.2     (1) ATTAAAGGTTTATACCTTCCCAGGTAACAAACCAACCAACTTTCGATCTCTTGTAGATCTGTTCTCTAAACGAACTTTAAAATCTGTGTGGCTGTCA
                                          Section 2
                         (98) 98          110       120       130       140       150       160       170       180            194
                HIV-1 HXB2 (1) -------------------------------------------------------------------------------------------------
SARS-CoV-2 NC_045512.2    (98) CTCGGCTGCATGCTTAGTGCACTCACGCAGTATAATTAATAACTAATTACTGTCGTTGACAGGACACGAGTAACTCGTCTATCTTCTGCAGGCTGCT
                                          Section 3
                        (195) 195  200       210       220       230       240       250       260       270       280        291
                HIV-1 HXB2 (1) -------------------------------------------------------------------------------------------------
SARS-CoV-2 NC_045512.2   (195) TACGGTTTCGTCCGTGTTGCAGCCGATCATCAGCACATCTAGGTTTCGTCCGGGTGTGACCGAAAGGTAAGATGGAGAGCCTTGTCCCTGGTTTCAA
                                          Section 4
                        (292) 292     300       310       320       330       340       350       360       370              388
                HIV-1 HXB2 (1) -------------------------------------------------------------------------------------------------
SARS-CoV-2 NC_045512.2   (292) CGAGAAAACACACGTCCAACTCAGTTTGCCTGTTTTACAGGTTCGCGACGTGCTCGTACGTGGCTTTGGAGACTCCGTGGAGGAGGTCTTATCAGAG
                                          Section 5
                        (389) 389         400       410       420       430       440       450       460       470          485
                HIV-1 HXB2 (1) -------------------------------------------------------------------------------------------------
SARS-CoV-2 NC_045512.2   (389) GCACGTCAACATCTTAAAGATGGCACTTGTGGCTTAGTAGAAGTTGAAAAAGGCGTTTTGCCTCAACTTGAACAGCCCTATGTGTTCATCAAACGTT
                                          Section 6
                        (486) 486           500       510       520       530       540       550       560       570        582
                HIV-1 HXB2 (1) -------------------------------------------------------------------------------------------------
SARS-CoV-2 NC_045512.2   (486) CGGATGCTCGAACTGCACCTCATGGTCATGTTATGGTTGAGCTGGTAGCAGAACTCGAAGGCATTCAGTACGGTCGTAGTGGTGAGACACTTGGTGT
                                          Section 7
                        (583) 583    590       600       610       620       630       640       650       660               679
                HIV-1 HXB2 (1) -------------------------------------------------------------------------------------------------
SARS-CoV-2 NC_045512.2   (583) CCTTGTCCCTCATGTGGGCGAAATACCAGTGGCTTACCGCAAGGTTCTTCTTCGTAAGAACGGTAATAAAGGAGCTGGTGGCCATAGTTACGGCGCC
```

```
                                                                                                                    Section 8
                              (680)  680       690       700       710       720       730       740       750       760                 776
HIV-1 HXB2                      (1)  -------------------------------------------------------------------------------------------------
SARS-CoV-2 NC_045512.2        (680)  GATCTAAAGTCATTTGACTTAGGCGACGAGCTTGGCACTGATCCTTATGAAGATTTTCAAGAAAACTGGAACACTAAACATAGCAGTGGTGTTACCC
                                                                                                                    Section 9
                              (777)  777          790       800       810       820       830       840       850       860             873
HIV-1 HXB2                      (1)  -------------------------------------------------------------------------------------------------
SARS-CoV-2 NC_045512.2        (777)  GTGAACTCATGCGTGAGCTTAACGGAGGGGCATACACTCGCTATGTCGATAACAACTTCTGTGGCCCTGATGGCTACCCTCTTGAGTGCATTAAAGA
                                                                                                                    Section 10
                              (874)  874   880       890       900       910       920       930       940       950       960       970
HIV-1 HXB2                      (1)  -------------------------------------------------------------------------------------------------
SARS-CoV-2 NC_045512.2        (874)  CCTTCTAGCACGTGCTGGTAAAGCTTCATGCACTTTGTCCGAACAACTGGACTTTATTGACACTAAGAGGGGTGTATACTGCTGCCGTGAACATGAG
                                                                                                                    Section 11
                              (971)  971      980       990       1000      1010      1020      1030      1040      1050              1067
HIV-1 HXB2                      (1)  -------------------------------------------------------------------------------------------------
SARS-CoV-2 NC_045512.2        (971)  CATGAAATTGCTTGGTACACGGAACGTTCTGAAAAGAGCTATGAATTGCAGACACCTTTTGAAATTAAATTGGCAAAGAAATTTGACACCTTCAATG
                                                                                                                    Section 12
                             (1068)  1068        1080      1090      1100      1110      1120      1130      1140      1150          1164
HIV-1 HXB2                      (1)  -------------------------------------------------------------------------------------------------
SARS-CoV-2 NC_045512.2       (1068)  GGGAATGTCCAAATTTTGTATTTCCCTTAAATTCCATAATCAAGACTATTCAACCAAGGGTTGAAAAGAAAAAGCTTGATGGCTTTATGGGTAGAAT
                                                                                                                    Section 13
                             (1165)  1165 1170      1180      1190      1200      1210      1220      1230      1240      1250      1261
HIV-1 HXB2                      (1)  -------------------------------------------------------------------------------------------------
SARS-CoV-2 NC_045512.2       (1165)  TCGATCTGTCTATCCAGTTGCGTCACCAAATGAATGCAACCAAATGTGCCTTTCAACTCTCATGAAGTGTGATCATTGTGGTGAAACTTCATGGCAG
                                                                                                                    Section 14
                             (1262)  1262    1270      1280      1290      1300      1310      1320      1330      1340              1358
HIV-1 HXB2                      (1)  -------------------------------------------------------------------------------------------------
SARS-CoV-2 NC_045512.2       (1262)  ACGGGCGATTTTGTTAAAGCCACTTGCGAATTTTGTGGCACTGAGAATTTGACTAAAGAAGGTGCCACTACTTGTGGTTACTTACCCCAAAATGCTG
```

Section 15

```
                          (1359) 1359       1370      1380      1390      1400      1410      1420      1430      1440           1455
              HIV-1 HXB2     (1) -------------------------------------------------------------------------------------------------
SARS-CoV-2 NC_045512.2  (1359) TTGTTAAAATTTATTGTCCAGCATGTCACAATTCAGAAGTAGGACCTGAGCATAGTCTTGCCGAATACCATAATGAATCTGGCTTGAAAACCATTCT
```

Section 16

```
                          (1456) 1456          1470      1480      1490      1500      1510      1520      1530      1540        1552
              HIV-1 HXB2     (1) -------------------------------------------------------------------------------------------------
SARS-CoV-2 NC_045512.2  (1456) TCGTAAGGGTGGTCGCACTATTGCCTTTGGAGGCTGTGTGTTCTCTTATGTTGGTTGCCATAACAAGTGTGCCTATTGGGTTCCACGTGCTAGCGCT
```

Section 17

```
                          (1553) 1553   1560      1570      1580      1590      1600      1610      1620      1630               1649
              HIV-1 HXB2     (1) -------------------------------------------------------------------------------------------------
SARS-CoV-2 NC_045512.2  (1553) AACATAGGTTGTAACCATACAGGTGTTGTTGGAGAAGGTTCCGAAGGTCTTAATGACAACCTTCTTGAAATACTCCAAAAAGAGAAAGTCAACATCA
```

Section 18

```
                          (1650) 1650      1660      1670      1680      1690      1700      1710      1720      1730            1746
              HIV-1 HXB2     (1) -------------------------------------------------------------------------------------------------
SARS-CoV-2 NC_045512.2  (1650) ATATTGTTGGTGACTTTAAACTTAATGAAGAGATCGCCATTATTTTGGCATCTTTTTCTGCTTCCACAAGTGCTTTTGTGGAAACTGTGAAAGGTTT
```

Section 19

```
                          (1747) 1747         1760      1770      1780      1790      1800      1810      1820      1830         1843
              HIV-1 HXB2     (1) -------------------------------------------------------------------------------------------------
SARS-CoV-2 NC_045512.2  (1747) GGATTATAAAGCATTCAAACAAATTGTTGAATCCTGTGGTAATTTTAAAGTTACAAAAGGAAAAGCTAAAAAAGGTGCCTGGAATATTGGTGAACAG
```

Section 20

```
                          (1844) 1844  1850      1860      1870      1880      1890      1900      1910      1920      1930      1940
              HIV-1 HXB2     (1) -------------------------------------------------------------------------------------------------
SARS-CoV-2 NC_045512.2  (1844) AAATCAATACTGAGTCCTCTTTATGCATTTGCATCAGAGGCTGCTCGTGTTGTACGATCAATTTTCTCCCGCACTCTTGAAACTGCTCAAAATTCTG
```

Section 21

```
                          (1941) 1941     1950      1960      1970      1980      1990      2000      2010      2020             2037
              HIV-1 HXB2     (1) -------------------------------------------------------------------------------------------------
SARS-CoV-2 NC_045512.2  (1941) TGCGTGTTTTACAGAAGGCCGCTATAACAATACTAGATGGAATTTCACAGTATTCACTGAGACTCATTGATGCTATGATGTTCACATCTGATTTGGC
```

Section 22

```
                              2038        2050      2060      2070      2080      2090      2100      2110      2120          2134
                    (2038)
HIV-1 HXB2             (1)    -------------------------------------------------------------------------------------------------
SARS-CoV-2 NC_045512.2 (2038) TACTAACAATCTAGTTGTAATGGCCTACATTACAGGTGGTGTTGTTCAGTTGACTTCGCAGTGGCTAACTAACATCTTTGGCACTGTTTATGAAAAA
```

Section 23

```
                              2135 2140      2150      2160      2170      2180      2190      2200      2210      2220        2231
                    (2135)
HIV-1 HXB2             (1)    -------------------------------------------------------------------------------------------------
SARS-CoV-2 NC_045512.2 (2135) CTCAAACCCGTCCTTGATTGGCTTGAAGAGAAGTTTAAGGAAGGTGTAGAGTTTCTTAGAGACGGTTGGGAAATTGTTAAATTTATCTCAACCTGTG
```

Section 24

```
                              2232    2240      2250      2260      2270      2280      2290      2300      2310              2328
                    (2232)
HIV-1 HXB2             (1)    -------------------------------------------------------------------------------------------------
SARS-CoV-2 NC_045512.2 (2232) CTTGTGAAATTGTCGGTGGACAAATTGTCACCTGTGCAAAGGAAATTAAGGAGAGTGTTCAGACATTCTTTAAGCTTGTAAATAAATTTTTGGCTTT
```

Section 25

```
                              2329       2340      2350      2360      2370      2380      2390      2400      2410           2425
                    (2329)
HIV-1 HXB2             (1)    -------------------------------------------------------------------------------------------------
SARS-CoV-2 NC_045512.2 (2329) GTGTGCTGACTCTATCATTATTGGTGGAGCTAAACTTAAAGCCTTGAATTTAGGTGAAACATTTGTCACGCACTCAAAGGGATTGTACAGAAAGTGT
```

Section 26

```
                              2426          2440      2450      2460      2470      2480      2490      2500      2510        2522
                    (2426)
HIV-1 HXB2             (1)    -------------------------------------------------------------------------------------------------
SARS-CoV-2 NC_045512.2 (2426) GTTAAATCCAGAGAAGAAACTGGCCTACTCATGCCTCTAAAAGCCCCAAAAGAAATTATCTTCTTAGAGGGAGAAACACTTCCCACAGAAGTGTTAA
```

Section 27

```
                              2523   2530      2540      2550      2560      2570      2580      2590      2600               2619
                    (2523)
HIV-1 HXB2             (1)    -------------------------------------------------------------------------------------------------
SARS-CoV-2 NC_045512.2 (2523) CAGAGGAAGTTGTCTTGAAAACTGGTGATTTACAACCATTAGAACAACCTACTAGTGAAGCTGTTGAAGCTCCATTGGTTGGTACACCAGTTTGTAT
```

Section 28

```
                              2620      2630      2640      2650      2660      2670      2680      2690      2700            2716
                    (2620)
HIV-1 HXB2             (1)    -------------------------------------------------------------------------------------------------
SARS-CoV-2 NC_045512.2 (2620) TAACGGGCTTATGTTGCTCGAAATCAAAGACACAGAAAAGTACTGTGCCCTTGCACCTAATATGATGGTAACAAACAATACCTTCACACTCAAAGGC
```

```
Section 29
                    (2717) 2717         2730      2740      2750      2760      2770      2780      2790      2800         2813
HIV-1 HXB2             (1) -------------------------------------------------------------------------------------------------
SARS-CoV-2 NC_045512.2 (2717) GGTGCACCAACAAAGGTTACTTTTGGTGATGACACTGTGATAGAAGTGCAAGGTTACAAGAGTGTGAATATCACTTTTGAACTTGATGAAAGGATTG

Section 30
                    (2814) 2814  2820      2830      2840      2850      2860      2870      2880      2890      2900      2910
HIV-1 HXB2             (1) -------------------------------------------------------------------------------------------------
SARS-CoV-2 NC_045512.2 (2814) ATAAAGTACTTAATGAGAAGTGCTCTGCCTATACAGTTGAACTCGGTACAGAAGTAAATGAGTTCGCCTGTGTTGTGGCAGATGCTGTCATAAAAAC

Section 31
                    (2911) 2911     2920      2930      2940      2950      2960      2970      2980      2990            3007
HIV-1 HXB2             (1) -------------------------------------------------------------------------------------------------
SARS-CoV-2 NC_045512.2 (2911) TTTGCAACCAGTATCTGAATTACTTACACCACTGGGCATTGATTTAGATGAGTGGAGTATGGCTACATACTACTTATTTGATGAGTCTGGTGAGTTT

Section 32
                    (3008) 3008        3020      3030      3040      3050      3060      3070      3080      3090          3104
HIV-1 HXB2             (1) -------------------------------------------------------------------------------------------------
SARS-CoV-2 NC_045512.2 (3008) AAATTGGCTTCACATATGTATTGTTCTTTCTACCCTCCAGATGAGGATGAAGAAGAAGGTGATTGTGAAGAAGAAGAGTTTGAGCCATCAACTCAAT

Section 33
                    (3105) 3105 3110      3120      3130      3140      3150      3160      3170      3180      3190       3201
HIV-1 HXB2             (1) -------------------------------------------------------------------------------------------------
SARS-CoV-2 NC_045512.2 (3105) ATGAGTATGGTACTGAAGATGATTACCAAGGTAAACCTTTGGAATTTGGTGCCACTTCTGCTGCTCTTCAACCTGAAGAAGAGCAAGAAGAAGATTG

Section 34
                    (3202) 3202   3210      3220      3230      3240      3250      3260      3270      3280             3298
HIV-1 HXB2             (1) -------------------------------------------------------------------------------------------------
SARS-CoV-2 NC_045512.2 (3202) GTTAGATGATGATAGTCAACAAACTGTTGGTCAACAAGACGGCAGTGAGGACAATCAGACAACTACTATTCAAACAATTGTTGAGGTTCAACCTCAA

Section 35
                    (3299) 3299       3310      3320      3330      3340      3350      3360      3370      3380           3395
HIV-1 HXB2             (1) -------------------------------------------------------------------------------------------------
SARS-CoV-2 NC_045512.2 (3299) TTAGAGATGGAACTTACACCAGTTGTTCAGACTATTGAAGTGAATAGTTTTAGTGGTTATTTAAAACTTACTGACAATGTATACATTAAAAATGCAG
```

```
                                                                                                    Section 36
                   (3396) 3396          3410      3420      3430      3440      3450      3460      3470      3480            3492
HIV-1 HXB2            (1) -------------------------------------------------------------------------------------------------
SARS-CoV-2 NC_045512.2 (3396) ACATTGTGGAAGAAGCTAAAAAGGTAAAACCAACAGTGGTTGTTAATGCAGCCAATGTTTACCTTAAACATGGAGGAGGTGTTGCAGGAGCCTTAAA
                                                                                                    Section 37
                   (3493) 3493   3500      3510      3520      3530      3540      3550      3560      3570                  3589
HIV-1 HXB2            (1) -------------------------------------------------------------------------------------------------
SARS-CoV-2 NC_045512.2 (3493) TAAGGCTACTAACAATGCCATGCAAGTTGAATCTGATGATTACATAGCTACTAATGGACCACTTAAAGTGGGTGGTAGTTGTGTTTTAAGCGGACAC
                                                                                                    Section 38
                   (3590) 3590      3600      3610      3620      3630      3640      3650      3660      3670                 3686
HIV-1 HXB2            (1) -------------------------------------------------------------------------------------------------
SARS-CoV-2 NC_045512.2 (3590) AATCTTGCTAAACACTGTCTTCATGTTGTCGGCCCAAATGTTAACAAAGGTGAAGACATTCAACTTCTTAAGAGTGCTTATGAAAATTTTAATCAGC
                                                                                                    Section 39
                   (3687) 3687         3700      3710      3720      3730      3740      3750      3760      3770            3783
HIV-1 HXB2            (1) -------------------------------------------------------------------------------------------------
SARS-CoV-2 NC_045512.2 (3687) ACGAAGTTCTACTTGCACCATTATTATCAGCTGGTATTTTTGGTGCTGACCCTATACATTCTTTAAGAGTTTGTGTAGATACTGTTCGCACAAATGT
                                                                                                    Section 40
                   (3784) 3784  3790      3800      3810      3820      3830      3840      3850      3860      3870      3880
HIV-1 HXB2            (1) -------------------------------------------------------------------------------------------------
SARS-CoV-2 NC_045512.2 (3784) CTACTTAGCTGTCTTTGATAAAAATCTCTATGACAAACTTGTTTCAAGCTTTTTGGAAATGAAGAGTGAAAAGCAAGTTGAACAAAAGATCGCTGAG
                                                                                                    Section 41
                   (3881) 3881     3890      3900      3910      3920      3930      3940      3950      3960                 3977
HIV-1 HXB2            (1) -------------------------------------------------------------------------------------------------
SARS-CoV-2 NC_045512.2 (3881) ATTCCTAAAGAGGAAGTTAAGCCATTTATAACTGAAAGTAAACCTTCAGTTGAACAGAGAAAACAAGATGATAAGAAAATCAAAGCTTGTGTTGAAG
                                                                                                    Section 42
                   (3978) 3978         3990      4000      4010      4020      4030      4040      4050      4060            4074
HIV-1 HXB2            (1) -------------------------------------------------------------------------------------------------
SARS-CoV-2 NC_045512.2 (3978) AAGTTACAACAACTCTGGAAGAAACTAAGTTCCTCACAGAAAACTTGTTACTTTATATTGACATTAATGGCAATCTTCATCCAGATTCTGCCACTCT
```

```
                                                                                                          Section 43
                      (4075) 4075 4080            4090            4100            4110            4120            4130            4140            4150            4160     4171
           HIV-1 HXB2    (1) -------------------------------------------------------------------------------------------------
SARS-CoV-2 NC_045512.2 (4075) TGTTAGTGACATTGACATCACTTTCTTAAAGAAAGATGCTCCATATATAGTGGGTGATGTTGTTCAAGAGGGTGTTTTAACTGCTGTGGTTATACCT
                                                                                                          Section 44
                      (4172) 4172   4180            4190            4200            4210            4220            4230            4240            4250                   4268
           HIV-1 HXB2    (1) -------------------------------------------------------------------------------------------------
SARS-CoV-2 NC_045512.2 (4172) ACTAAAAAGGCTGGTGGCACTACTGAAATGCTAGCGAAAGCTTTGAGAAAAGTGCCAACAGACAATTATATAACCACTTACCCGGGTCAGGGTTTAA
                                                                                                          Section 45
                      (4269) 4269       4280            4290            4300            4310            4320            4330            4340            4350               4365
           HIV-1 HXB2    (1) -------------------------------------------------------------------------------------------------
SARS-CoV-2 NC_045512.2 (4269) ATGGTTACACTGTAGAGGAGGCAAAGACAGTGCTTAAAAAGTGTAAAAGTGCCTTTTACATTCTACCATCTATTATCTCTAATGAGAAGCAAGAAAT
                                                                                                          Section 46
                      (4366) 4366          4380            4390            4400            4410            4420            4430            4440            4450            4462
           HIV-1 HXB2    (1) -------------------------------------------------------------------------------------------------
SARS-CoV-2 NC_045512.2 (4366) TCTTGGAACTGTTTCTTGGAATTTGCGAGAAATGCTTGCACATGCAGAAGAAACACGCAAATTAATGCCTGTCTGTGTGGAAACTAAAGCCATAGTT
                                                                                                          Section 47
                      (4463) 4463  4470            4480            4490            4500            4510            4520            4530            4540                     4559
           HIV-1 HXB2    (1) -------------------------------------------------------------------------------------------------
SARS-CoV-2 NC_045512.2 (4463) TCAACTATACAGCGTAAATATAAGGGTATTAAAATACAAGAGGGTGTGGTTGATTATGGTGCTAGATTTTACTTTTACACCAGTAAAACAACTGTAG
                                                                                                          Section 48
                      (4560) 4560      4570            4580            4590            4600            4610            4620            4630            4640                 4656
           HIV-1 HXB2    (1) -------------------------------------------------------------------------------------------------
SARS-CoV-2 NC_045512.2 (4560) CGTCACTTATCAACACACTTAACGATCTAAATGAAACTCTTGTTACAATGCCACTTGGCTATGTAACACATGGCTTAAATTTGGAAGAAGCTGCTCG
                                                                                                          Section 49
                      (4657) 4657         4670            4680            4690            4700            4710            4720            4730            4740            4753
           HIV-1 HXB2    (1) -------------------------------------------------------------------------------------------------
SARS-CoV-2 NC_045512.2 (4657) GTATATGAGATCTCTCAAAGTGCCAGCTACAGTTTCTGTTTCTTCACCTGATGCTGTTACAGCGTATAATGGTTATCTTACTTCTTCTTCTAAAACA
```

```
                                                                                                                              Section 50
                   (4754) 4754 4760      4770      4780      4790      4800      4810      4820      4830      4840      4850
           HIV-1 HXB2    (1) -------------------------------------------------------------------------------------------------
SARS-CoV-2 NC_045512.2 (4754) CCTGAAGAACATTTTATTGAAACCATCTCACTTGCTGGTTCCTATAAAGATTGGTCCTATTCTGGACAATCTACACAACTAGGTATAGAATTTCTTA
                                                                                                                              Section 51
                   (4851) 4851     4860      4870      4880      4890      4900      4910      4920      4930              4947
           HIV-1 HXB2    (1) -------------------------------------------------------------------------------------------------
SARS-CoV-2 NC_045512.2 (4851) AGAGAGGTGATAAAAGTGTATATTACACTAGTAATCCTACCACATTCCACCTAGATGGTGAAGTTATCACCTTTGACAATCTTAAGACACTTCTTTC
                                                                                                                              Section 52
                   (4948) 4948         4960      4970      4980      4990      5000      5010      5020      5030          5044
           HIV-1 HXB2    (1) -------------------------------------------------------------------------------------------------
SARS-CoV-2 NC_045512.2 (4948) TTTGAGAGAAGTGAGGACTATTAAGGTGTTTACAACAGTAGACAACATTAACCTCCACACGCAAGTTGTGGACATGTCAATGACATATGGACAACAG
                                                                                                                              Section 53
                   (5045) 5045 5050      5060      5070      5080      5090      5100      5110      5120      5130      5141
           HIV-1 HXB2    (1) -------------------------------------------------------------------------------------------------
SARS-CoV-2 NC_045512.2 (5045) TTTGGTCCAACTTATTTGGATGGAGCTGATGTTACTAAAATAAAACCTCATAATTCACATGAAGGTAAAACATTTTATGTTTTACCTAATGATGACA
                                                                                                                              Section 54
                   (5142) 5142    5150      5160      5170      5180      5190      5200      5210      5220               5238
           HIV-1 HXB2    (1) -------------------------------------------------------------------------------------------------
SARS-CoV-2 NC_045512.2 (5142) CTCTACGTGTTGAGGCTTTTGAGTACTACCACACAACTGATCCTAGTTTTCTGGGTAGGTACATGTCAGCATTAAATCACACTAAAAAGTGGAAATA
                                                                                                                              Section 55
                   (5239) 5239       5250      5260      5270      5280      5290      5300      5310      5320            5335
           HIV-1 HXB2    (1) -------------------------------------------------------------------------------------------------
SARS-CoV-2 NC_045512.2 (5239) CCCACAAGTTAATGGTTTAACTTCTATTAAATGGGCAGATAACAACTGTTATCTTGCCACTGCATTGTTAACACTCCAACAAATAGAGTTGAAGTTT
                                                                                                                              Section 56
                   (5336) 5336           5350      5360      5370      5380      5390      5400      5410      5420      5432
           HIV-1 HXB2    (1) -------------------------------------------------------------------------------------------------
SARS-CoV-2 NC_045512.2 (5336) AATCCACCTGCTCTACAAGATGCTTATTACAGAGCAAGGGCTGGTGAAGCTGCTAACTTTTGTGCACTTATCTTAGCCTACTGTAATAAGACAGTAG
```

```
                                                                                    Section 57
                           (5433) 5433   5440      5450      5460      5470      5480      5490      5500      5510                5529
HIV-1 HXB2                    (1) -------------------------------------------------------------------------------------------------
SARS-CoV-2 NC_045512.2     (5433) GTGAGTTAGGTGATGTTAGAGAAACAATGAGTTACTTGTTTCAACATGCCAATTTAGATTCTTGCAAAAGAGTCTTGAACGTGGTGTGTAAAACTTG
                                                                                    Section 58
                           (5530) 5530      5540      5550      5560      5570      5580      5590      5600      5610             5626
HIV-1 HXB2                    (1) -------------------------------------------------------------------------------------------------
SARS-CoV-2 NC_045512.2     (5530) TGGACAACAGCAGACAACCCTTAAGGGTGTAGAAGCTGTTATGTACATGGGCACACTTTCTTATGAACAATTTAAGAAAGGTGTTCAGATACCTTGT
                                                                                    Section 59
                           (5627) 5627         5640      5650      5660      5670      5680      5690      5700      5710          5723
HIV-1 HXB2                    (1) -------------------------------------------------------------------------------------------------
SARS-CoV-2 NC_045512.2     (5627) ACGTGTGGTAAACAAGCTACAAAATATCTAGTACAACAGGAGTCACCTTTTGTTATGATGTCAGCACCACCTGCTCAGTATGAACTTAAGCATGGTA
                                                                                    Section 60
                           (5724) 5724  5730      5740      5750      5760      5770      5780      5790      5800      5810       5820
HIV-1 HXB2                    (1) -------------------------------------------------------------------------------------------------
SARS-CoV-2 NC_045512.2     (5724) CATTTACTTGTGCTAGTGAGTACACTGGTAATTACCAGTGTGGTCACTATAAACATATAACTTCTAAAGAAACTTTGTATTGCATAGACGGTGCTTT
                                                                                    Section 61
                           (5821) 5821     5830      5840      5850      5860      5870      5880      5890      5900              5917
HIV-1 HXB2                    (1) -------------------------------------------------------------------------------------------------
SARS-CoV-2 NC_045512.2     (5821) ACTTACAAAGTCCTCAGAATACAAAGGTCCTATTACGGATGTTTTCTACAAAGAAAACAGTTACACAACAACCATAAAACCAGTTACTTATAAATTG
                                                                                    Section 62
                           (5918) 5918        5930      5940      5950      5960      5970      5980      5990      6000           6014
HIV-1 HXB2                    (1) -------------------------------------------------------------------------------------------------
SARS-CoV-2 NC_045512.2     (5918) GATGGTGTTGTTTGTACAGAAATTGACCCTAAGTTGGACAATTATTATAAGAAAGACAATTCTTATTTCACAGAGCAACCAATTGATCTTGTACCAA
                                                                                    Section 63
                           (6015) 6015 6020      6030      6040      6050      6060      6070      6080      6090      6100        6111
HIV-1 HXB2                    (1) -------------------------------------------------------------------------------------------------
SARS-CoV-2 NC_045512.2     (6015) ACCAACCATATCCAAACGCAAGCTTCGATAATTTTAAGTTTGTATGTGATAATATCAAATTTGCTGATGATTTAAACCAGTTAACTGGTTATAAGAA
```

```
Section 64
                          (6112) 6112    6120      6130      6140      6150      6160      6170      6180      6190            6208
HIV-1 HXB2                   (1) -------------------------------------------------------------------------------------------------
SARS-CoV-2 NC_045512.2    (6112) ACCTGCTTCAAGAGAGCTTAAAGTTACATTTTTCCCTGACTTAAATGGTGATGTGGTGGCTATTGATTATAAACACTACACACCCTCTTTTAAGAAA

Section 65
                          (6209) 6209       6220      6230      6240      6250      6260      6270      6280      6290           6305
HIV-1 HXB2                   (1) -------------------------------------------------------------------------------------------------
SARS-CoV-2 NC_045512.2    (6209) GGAGCTAAATTGTTACATAAACCTATTGTTTGGCATGTTAACAATGCAACTAATAAAGCCACGTATAAACCAAATACCTGGTGTATACGTTGTCTTT

Section 66
                          (6306) 6306          6320      6330      6340      6350      6360      6370      6380      6390      6402
HIV-1 HXB2                   (1) -------------------------------------------------------------------------------------------------
SARS-CoV-2 NC_045512.2    (6306) GGAGCACAAAACCAGTTGAAACATCAAATTCGTTTGATGTACTGAAGTCAGAGGACGCGCAGGGAATGGATAATCTTGCCTGCGAAGATCTAAAACC

Section 67
                          (6403) 6403   6410      6420      6430      6440      6450      6460      6470      6480            6499
HIV-1 HXB2                   (1) -------------------------------------------------------------------------------------------------
SARS-CoV-2 NC_045512.2    (6403) AGTCTCTGAAGAAGTAGTGGAAAATCCTACCATACAGAAAGACGTTCTTGAGTGTAATGTGAAAACTACCGAAGTTGTAGGAGACATTATACTTAAA

Section 68
                          (6500) 6500      6510      6520      6530      6540      6550      6560      6570      6580            6596
HIV-1 HXB2                   (1) -------------------------------------------------------------------------------------------------
SARS-CoV-2 NC_045512.2    (6500) CCAGCAAATAATAGTTTAAAAATTACAGAAGAGGTTGGCCACACAGATCTAATGGCTGCTTATGTAGACAATTCTAGTCTTACTATTAAGAAACCTA

Section 69
                          (6597) 6597         6610      6620      6630      6640      6650      6660      6670      6680       6693
HIV-1 HXB2                   (1) -------------------------------------------------------------------------------------------------
SARS-CoV-2 NC_045512.2    (6597) ATGAATTATCTAGAGTATTAGGTTTGAAAACCCTTGCTACTCATGGTTTAGCTGCTGTTAATAGTGTCCCTTGGGATACTATAGCTAATTATGCTAA

Section 70
                          (6694) 6694  6700      6710      6720      6730      6740      6750      6760      6770      6780  6790
HIV-1 HXB2                   (1) -------------------------------------------------------------------------------------------------
SARS-CoV-2 NC_045512.2    (6694) GCCTTTTCTTAACAAAGTTGTTAGTACAACTACTAACATAGTTACACGGTGTTTAAACCGTGTTTGTACTAATTATATGCCTTATTTCTTTACTTTA
```

```
                                                                                                        Section 71
                          (6791) 6791     6800      6810      6820      6830      6840      6850      6860      6870                         6887
HIV-1 HXB2                   (1) -------------------------------------------------------------------------------------------------
SARS-CoV-2 NC_045512.2    (6791) TTGCTACAATTGTGTACTTTTACTAGAAGTACAAATTCTAGAATTAAAGCATCTATGCCGACTACTATAGCAAAGAATACTGTTAAGAGTGTCGGTA
                                                                                                        Section 72
                          (6888) 6888        6900      6910      6920      6930      6940      6950      6960      6970                   6984
HIV-1 HXB2                   (1) -------------------------------------------------------------------------------------------------
SARS-CoV-2 NC_045512.2    (6888) AATTTTGTCTAGAGGCTTCATTTAATTATTTGAAGTCACCTAATTTTTCTAAACTGATAAATATTATAATTTGGTTTTTACTATTAAGTGTTTGCCT
                                                                                                        Section 73
                          (6985) 6985 6990      7000      7010      7020      7030      7040      7050      7060      7070                  7081
HIV-1 HXB2                   (1) -------------------------------------------------------------------------------------------------
SARS-CoV-2 NC_045512.2    (6985) AGGTTCTTTAATCTACTCAACCGCTGCTTTAGGTGTTTTAATGTCTAATTTAGGCATGCCTTCTTACTGTACTGGTTACAGAGAAGGCTATTTGAAC
                                                                                                        Section 74
                          (7082) 7082   7090      7100      7110      7120      7130      7140      7150      7160                         7178
HIV-1 HXB2                   (1) -------------------------------------------------------------------------------------------------
SARS-CoV-2 NC_045512.2    (7082) TCTACTAATGTCACTATTGCAACCTACTGTACTGGTTCTATACCTTGTAGTGTTTGTCTTAGTGGTTTAGATTCTTTAGACACCTATCCTTCTTTAG
                                                                                                        Section 75
                          (7179) 7179       7190      7200      7210      7220      7230      7240      7250      7260                       7275
HIV-1 HXB2                   (1) -------------------------------------------------------------------------------------------------
SARS-CoV-2 NC_045512.2    (7179) AAACTATACAAATTACCATTTCATCTTTTAAATGGGATTTAACTGCTTTTGGCTTAGTTGCAGAGTGGTTTTTGGCATATATTCTTTTCACTAGGTT
                                                                                                        Section 76
                          (7276) 7276          7290      7300      7310      7320      7330      7340      7350      7360                    7372
HIV-1 HXB2                   (1) -------------------------------------------------------------------------------------------------
SARS-CoV-2 NC_045512.2    (7276) TTTCTATGTACTTGGATTGGCTGCAATCATGCAATTGTTTTTCAGCTATTTTGCAGTACATTTTATTAGTAATTCTTGGCTTATGTGGTTAATAATT
                                                                                                        Section 77
                          (7373) 7373  7380      7390      7400      7410      7420      7430      7440      7450                              7469
HIV-1 HXB2                   (1) -------------------------------------------------------------------------------------------------
SARS-CoV-2 NC_045512.2    (7373) AATCTTGTACAAATGGCCCCGATTTCAGCTATGGTTAGAATGTACATCTTCTTTGCATCATTTTATTATGTATGGAAAAGTTATGTGCATGTTGTAG
```

```
                                                                                                        Section 78
                        (7470) 7470      7480      7490      7500      7510      7520      7530      7540      7550          7566
           HIV-1 HXB2      (1) -------------------------------------------------------------------------------------------------
SARS-CoV-2 NC_045512.2  (7470) ACGGTTGTAATTCATCAACTTGTATGATGTGTTACAAACGTAATAGAGCAACAAGAGTCGAATGTACAACTATTGTTAATGGTGTTAGAAGGTCCTT
                                                                                                        Section 79
                        (7567) 7567         7580      7590      7600      7610      7620      7630      7640      7650      7663
           HIV-1 HXB2      (1) -------------------------------------------------------------------------------------------------
SARS-CoV-2 NC_045512.2  (7567) TTATGTCTATGCTAATGGAGGTAAAGGCTTTTGCAAACTACACAATTGGAATTGTGTTAATTGTGATACATTCTGTGCTGGTAGTACATTTATTAGT
                                                                                                        Section 80
                        (7664) 7664  7670      7680      7690      7700      7710      7720      7730      7740      7750    7760
           HIV-1 HXB2      (1) -------------------------------------------------------------------------------------------------
SARS-CoV-2 NC_045512.2  (7664) GATGAAGTTGCGAGAGACTTGTCACTACAGTTTAAAAGACCAATAAATCCTACTGACCAGTCTTCTTACATCGTTGATAGTGTTACAGTGAAGAATG
                                                                                                        Section 81
                        (7761) 7761     7770      7780      7790      7800      7810      7820      7830      7840            7857
           HIV-1 HXB2      (1) -------------------------------------------------------------------------------------------------
SARS-CoV-2 NC_045512.2  (7761) GTTCCATCCATCTTTACTTTGATAAAGCTGGTCAAAAGACTTATGAAAGACATTCTCTCTCTCATTTTGTTAACTTAGACAACCTGAGAGCTAATAA
                                                                                                        Section 82
                        (7858) 7858        7870      7880      7890      7900      7910      7920      7930      7940          7954
           HIV-1 HXB2      (1) -------------------------------------------------------------------------------------------------
SARS-CoV-2 NC_045512.2  (7858) CACTAAAGGTTCATTGCCTATTAATGTTATAGTTTTTGATGGTAAATCAAAATGTGAAGAATCATCTGCAAAATCAGCGTCTGTTTACTACAGTCAG
                                                                                                        Section 83
                        (7955) 7955 7960      7970      7980      7990      8000      8010      8020      8030      8040     8051
           HIV-1 HXB2      (1) -------------------------------------------------------------------------------------------------
SARS-CoV-2 NC_045512.2  (7955) CTTATGTGTCAACCTATACTGTTACTAGATCAGGCATTAGTGTCTGATGTTGGTGATAGTGCGGAAGTTGCAGTTAAAATGTTTGATGCTTACGTTA
                                                                                                        Section 84
                        (8052) 8052    8060      8070      8080      8090      8100      8110      8120      8130            8148
           HIV-1 HXB2      (1) -------------------------------------------------------------------------------------------------
SARS-CoV-2 NC_045512.2  (8052) ATACGTTTTCATCAACTTTTAACGTACCAATGGAAAAACTCAAAACACTAGTTGCAACTGCAGAAGCTGAACTTGCAAAGAATGTGTCCTTAGACAA
```

```
                                                                                                     Section 85
                          (8149) 8149       8160      8170      8180      8190      8200      8210      8220      8230           8245
HIV-1 HXB2                   (1) -------------------------------------------------------------------------------------------------
SARS-CoV-2 NC_045512.2    (8149) TGTCTTATCTACTTTTATTTCAGCAGCTCGGCAAGGGTTTGTTGATTCAGATGTAGAAACTAAAGATGTTGTTGAATGTCTTAAATTGTCACATCAA
                                                                                                     Section 86
                          (8246) 8246          8260      8270      8280      8290      8300      8310      8320      8330        8342
HIV-1 HXB2                   (1) -------------------------------------------------------------------------------------------------
SARS-CoV-2 NC_045512.2    (8246) TCTGACATAGAAGTTACTGGCGATAGTTGTAATAACTATATGCTCACCTATAACAAAGTTGAAAACATGACACCCCGTGACCTTGGTGCTTGTATTG
                                                                                                     Section 87
                          (8343) 8343   8350      8360      8370      8380      8390      8400      8410      8420               8439
HIV-1 HXB2                   (1) -------------------------------------------------------------------------------------------------
SARS-CoV-2 NC_045512.2    (8343) ACTGTAGTGCGCGTCATATTAATGCGCAGGTAGCAAAAAGTCACAACATTGCTTTGATATGGAACGTTAAAGATTTCATGTCATTGTCTGAACAACT
                                                                                                     Section 88
                          (8440) 8440      8450      8460      8470      8480      8490      8500      8510      8520              8536
HIV-1 HXB2                   (1) -------------------------------------------------------------------------------------------------
SARS-CoV-2 NC_045512.2    (8440) ACGAAAACAAATACGTAGTGCTGCTAAAAAGAATAACTTACCTTTTAAGTTGACATGTGCAACTACTAGACAAGTTGTTAATGTTGTAACAACAAAG
                                                                                                     Section 89
                          (8537) 8537         8550      8560      8570      8580      8590      8600      8610      8620         8633
HIV-1 HXB2                   (1) -------------------------------------------------------------------------------------------------
SARS-CoV-2 NC_045512.2    (8537) ATAGCACTTAAGGGTGGTAAAATTGTTAATAATTGGTTGAAGCAGTTAATTAAAGTTACACTTGTGTTCCTTTTTGTTGCTGCTATTTTCTATTTAA
                                                                                                     Section 90
                          (8634) 8634  8640      8650      8660      8670      8680      8690      8700      8710      8720      8730
HIV-1 HXB2                   (1) -------------------------------------------------------------------------------------------------
SARS-CoV-2 NC_045512.2    (8634) TAACACCTGTTCATGTCATGTCTAAACATACTGACTTTTCAAGTGAAATCATAGGATACAAGGCTATTGATGGTGGTGTCACTCGTGACATAGCATC
                                                                                                     Section 91
                          (8731) 8731     8740      8750      8760      8770      8780      8790      8800      8810               8827
HIV-1 HXB2                   (1) -------------------------------------------------------------------------------------------------
SARS-CoV-2 NC_045512.2    (8731) TACAGATACTTGTTTTGCTAACAAACATGCTGATTTTGACACATGGTTTAGCCAGCGTGGTGGTAGTTATACTAATGACAAAGCTTGCCCATTGATT
```

Section 92

	(8828)	8828 8840 8850 8860 8870 8880 8890 8900 8910 8924
HIV-1 HXB2	(1)	---
SARS-CoV-2 NC_045512.2	(8828)	GCTGCAGTCATAACAAGAGAAGTGGGTTTTGTCGTGCCTGGTTTGCCTGGCACGATATTACGCACAACTAATGGTGACTTTTTGCATTTCTTACCTA

Section 93

	(8925)	8925 8930 8940 8950 8960 8970 8980 8990 9000 9010 9021
HIV-1 HXB2	(1)	---
SARS-CoV-2 NC_045512.2	(8925)	GAGTTTTTAGTGCAGTTGGTAACATCTGTTACACACCATCAAAACTTATAGAGTACACTGACTTTGCAACATCAGCTTGTGTTTTGGCTGCTGAATG

Section 94

	(9022)	9022 9030 9040 9050 9060 9070 9080 9090 9100 9118
HIV-1 HXB2	(1)	---
SARS-CoV-2 NC_045512.2	(9022)	TACAATTTTTAAAGATGCTTCTGGTAAGCCAGTACCATATTGTTATGATACCAATGTACTAGAAGGTTCTGTTGCTTATGAAAGTTTACGCCCTGAC

Section 95

	(9119)	9119 9130 9140 9150 9160 9170 9180 9190 9200 9215
HIV-1 HXB2	(1)	---
SARS-CoV-2 NC_045512.2	(9119)	ACACGTTATGTGCTCATGGATGGCTCTATTATTCAATTTCCTAACACCTACCTTGAAGGTTCTGTTAGAGTGGTAACAACTTTTGATTCTGAGTACT

Section 96

	(9216)	9216 9230 9240 9250 9260 9270 9280 9290 9300 9312
HIV-1 HXB2	(1)	---
SARS-CoV-2 NC_045512.2	(9216)	GTAGGCACGGCACTTGTGAAAGATCAGAAGCTGGTGTTTGTGTATCTACTAGTGGTAGATGGGTACTTAACAATGATTATTACAGATCTTTACCAGG

Section 97

	(9313)	9313 9320 9330 9340 9350 9360 9370 9380 9390 9409
HIV-1 HXB2	(1)	---
SARS-CoV-2 NC_045512.2	(9313)	AGTTTTCTGTGGTGTAGATGCTGTAAATTTACTTACTAATATGTTTACACCACTAATTCAACCTATTGGTGCTTTGGACATATCAGCATCTATAGTA

Section 98

	(9410)	9410 9420 9430 9440 9450 9460 9470 9480 9490 9506
HIV-1 HXB2	(1)	---
SARS-CoV-2 NC_045512.2	(9410)	GCTGGTGGTATTGTAGCTATCGTAGTAACATGCCTTGCCTACTATTTTATGAGGTTTAGAAGAGCTTTTGGTGAATACAGTCATGTAGTTGCCTTTA

Section 99

```
                              (9507) 9507         9520      9530      9540      9550      9560      9570      9580      9590         9603
HIV-1 HXB2                       (1) -------------------------------------------------------------------------------------------------
SARS-CoV-2 NC_045512.2        (9507) ATACTTTACTATTCCTTATGTCATTCACTGTACTCTGTTTAACACCAGTTTACTCATTCTTACCTGGTGTTTATTCTGTTATTTACTTGTACTTGAC
```

Section 100

```
                              (9604) 9604  9610      9620      9630      9640      9650      9660      9670      9680      9690    9700
HIV-1 HXB2                       (1) -------------------------------------------------------------------------------------------------
SARS-CoV-2 NC_045512.2        (9604) ATTTTATCTTACTAATGATGTTTCTTTTTAGCACATATTCAGTGGATGGTTATGTTCACACCTTTAGTACCTTTCTGGATAACAATTGCTTATATC
```

Section 101

```
                              (9701) 9701     9710      9720      9730      9740      9750      9760      9770      9780            9797
HIV-1 HXB2                       (1) -------------------------------------------------------------------------------------------------
SARS-CoV-2 NC_045512.2        (9701) ATTTGTATTTCCACAAAGCATTTCTATTGGTTCTTTAGTAATTACCTAAAGAGACGTGTAGTCTTTAATGGTGTTTCCTTTAGTACTTTTGAAGAAG
```

Section 102

```
                              (9798) 9798        9810      9820      9830      9840      9850      9860      9870      9880          9894
HIV-1 HXB2                       (1) -------------------------------------------------------------------------------------------------
SARS-CoV-2 NC_045512.2        (9798) CTGCGCTGTGCACCTTTTTGTTAAATAAAGAAATGTATCTAAAGTTGCGTAGTGATGTGCTATTACCTCTTACGCAATATAATAGATACTTAGCTCT
```

Section 103

```
                              (9895) 9895 9900      9910      9920      9930      9940      9950      9960      9970      9980      9991
HIV-1 HXB2                       (1) -------------------------------------------------------------------------------------------------
SARS-CoV-2 NC_045512.2        (9895) TTATAATAAGTACAAGTATTTTAGTGGAGCAATGGATACAACTAGCTACAGAGAAGCTGCTTGTTGTCATCTCGCAAAGGCTCTCAATGACTTCAGT
```

Section 104

```
                              (9992) 9992    10000     10010     10020     10030     10040     10050     10060     10070          10088
HIV-1 HXB2                       (1) -------------------------------------------------------------------------------------------------
SARS-CoV-2 NC_045512.2        (9992) AACTCAGGTTCTGATGTTCTTTACCAACCACCACAAACCTCTATCACCTCAGCTGTTTTGCAGAGTGGTTTTAGAAAAATGGCATTCCCATCTGGTA
```

Section 105

```
                             (10089) 10089      10100     10110     10120     10130     10140     10150     10160     10170         10185
HIV-1 HXB2                       (1) -------------------------------------------------------------------------------------------------
SARS-CoV-2 NC_045512.2       (10089) AAGTTGAGGGTTGTATGGTACAAGTAACTTGTGGTACAACTACACTTAACGGTCTTTGGCTTGATGACGTAGTTTACTGTCCAAGACATGTGATCTG
```

```
                                                                                                      Section 106
                          (10186) 10186         10200     10210     10220     10230     10240     10250     10260     10270    10282
                HIV-1 HXB2    (1) -------------------------------------------------------------------------------------------------
SARS-CoV-2 NC_045512.2 (10186) CACCTCTGAAGACATGCTTAACCCTAATTATGAAGATTTACTCATTCGTAAGTCTAATCATAATTTCTTGGTACAGGCTGGTAATGTTCAACTCAGG
                                                                                                      Section 107
                          (10283) 10283  10290     10300     10310     10320     10330     10340     10350     10360            10379
                HIV-1 HXB2    (1) -------------------------------------------------------------------------------------------------
SARS-CoV-2 NC_045512.2 (10283) GTTATTGGACATTCTATGCAAAATTGTGTACTTAAGCTTAAGGTTGATACAGCCAATCCTAAGACACCTAAGTATAAGTTTGTTCGCATTCAACCAG
                                                                                                      Section 108
                          (10380) 10380     10390     10400     10410     10420     10430     10440     10450     10460            10476
                HIV-1 HXB2    (1) -------------------------------------------------------------------------------------------------
SARS-CoV-2 NC_045512.2 (10380) GACAGACTTTTTCAGTGTTAGCTTGTTACAATGGTTCACCATCTGGTGTTTACCAATGTGCTATGAGGCCCAATTTCACTATTAAGGGTTCATTCCT
                                                                                                      Section 109
                          (10477) 10477        10490     10500     10510     10520     10530     10540     10550     10560         10573
                HIV-1 HXB2    (1) -------------------------------------------------------------------------------------------------
SARS-CoV-2 NC_045512.2 (10477) TAATGGTTCATGTGGTAGTGTTGGTTTTAACATAGATTATGACTGTGTCTCTTTTTGTTACATGCACCATATGGAATTACCAACTGGAGTTCATGCT
                                                                                                      Section 110
                          (10574) 10574 10580     10590     10600     10610     10620     10630     10640     10650     10660   10670
                HIV-1 HXB2    (1) -------------------------------------------------------------------------------------------------
SARS-CoV-2 NC_045512.2 (10574) GGCACAGACTTAGAAGGTAACTTTTATGGACCTTTTGTTGACAGGCAAACAGCACAAGCAGCTGGTACGGACACAACTATTACAGTTAATGTTTTAG
                                                                                                      Section 111
                          (10671) 10671    10680     10690     10700     10710     10720     10730     10740     10750            10767
                HIV-1 HXB2    (1) -------------------------------------------------------------------------------------------------
SARS-CoV-2 NC_045512.2 (10671) CTTGGTTGTACGCTGCTGTTATAAATGGAGACAGGTGGTTTCTCAATCGATTTACCACAACTCTTAATGACTTTAACCTTGTGGCTATGAAGTACAA
                                                                                                      Section 112
                          (10768) 10768       10780     10790     10800     10810     10820     10830     10840     10850         10864
                HIV-1 HXB2    (1) -------------------------------------------------------------------------------------------------
SARS-CoV-2 NC_045512.2 (10768) TTATGAACCTCTAACACAAGACCATGTTGACATACTAGGACCTCTTTCTGCTCAAACTGGAATTGCCGTTTTAGATATGTGTGCTTCATTAAAAGAA
```

```
                                                                                                   Section 113
                          (10865) 10865 10870     10880     10890     10900     10910     10920     10930     10940     10950    10961
HIV-1 HXB2                    (1) -------------------------------------------------------------------------------------------------
SARS-CoV-2 NC_045512.2    (10865) TTACTGCAAAATGGTATGAATGGACGTACCATATTGGGTAGTGCTTTATTAGAAGATGAATTTACACCTTTTGATGTTGTTAGACAATGCTCAGGTG
                                                                                                   Section 114
                          (10962) 10962   10970     10980     10990     11000     11010     11020     11030     11040            11058
HIV-1 HXB2                    (1) -------------------------------------------------------------------------------------------------
SARS-CoV-2 NC_045512.2    (10962) TTACTTTCCAAAGTGCAGTGAAAAGAACAATCAAGGGTACACACCACTGGTTGTTACTCACAATTTTGACTTCACTTTTAGTTTTAGTCCAGAGTAC
                                                                                                   Section 115
                          (11059) 11059      11070     11080     11090     11100     11110     11120     11130     11140       11155
HIV-1 HXB2                    (1) -------------------------------------------------------------------------------------------------
SARS-CoV-2 NC_045512.2    (11059) TCAATGGTCTTTGTTCTTTTTTTTGTATGAAAATGCCTTTTTACCTTTTGCTATGGGTATTATTGCTATGTCTGCTTTTGCAATGATGTTTGTCAAA
                                                                                                   Section 116
                          (11156) 11156         11170     11180     11190     11200     11210     11220     11230     11240    11252
HIV-1 HXB2                    (1) -------------------------------------------------------------------------------------------------
SARS-CoV-2 NC_045512.2    (11156) CATAAGCATGCATTTCTCTGTTTGTTTTTGTTACCTTCTCTTGCCACTGTAGCTTATTTTAATATGGTCTATATGCCTGCTAGTTGGGTGATGCGTA
                                                                                                   Section 117
                          (11253) 11253  11260     11270     11280     11290     11300     11310     11320     11330           11349
HIV-1 HXB2                    (1) -------------------------------------------------------------------------------------------------
SARS-CoV-2 NC_045512.2    (11253) TTATGACATGGTTGGATATGGTTGATACTAGTTTGTCTGGTTTTAAGCTAAAAGACTGTGTTATGTATGCATCAGCTGTAGTGTTACTAATCCTTAT
                                                                                                   Section 118
                          (11350) 11350     11360     11370     11380     11390     11400     11410     11420     11430        11446
HIV-1 HXB2                    (1) -------------------------------------------------------------------------------------------------
SARS-CoV-2 NC_045512.2    (11350) GACAGCAAGAACTGTGTATGATGATGGTGCTAGGAGAGTGTGGACACTTATGAATGTCTTGACACTCGTTTATAAAGTTTATTATGGTAATGCTTTA
                                                                                                   Section 119
                          (11447) 11447        11460     11470     11480     11490     11500     11510     11520     11530     11543
HIV-1 HXB2                    (1) -------------------------------------------------------------------------------------------------
SARS-CoV-2 NC_045512.2    (11447) GATCAAGCCATTTCCATGTGGGCTCTTATAATCTCTGTTACTTCTAACTACTCAGGTGTAGTTACAACTGTCATGTTTTTGGCCAGAGGTATTGTTT
```

```
                                                                                                            Section 120
                   (11544) 11544 11550     11560     11570     11580     11590     11600     11610     11620     11630 11640
HIV-1 HXB2             (1) -------------------------------------------------------------------------------------------------
SARS-CoV-2 NC_045512.2 (11544) TTATGTGTGTTGAGTATTGCCCTATTTTCTTCATAACTGGTAATACACTTCAGTGTATAATGCTAGTTTATTGTTTCTTAGGCTATTTTTGTACTTG
                                                                                                            Section 121
                   (11641) 11641    11650     11660     11670     11680     11690     11700     11710     11720         11737
HIV-1 HXB2             (1) -------------------------------------------------------------------------------------------------
SARS-CoV-2 NC_045512.2 (11641) TTACTTTGGCCTCTTTTGTTTACTCAACCGCTACTTTAGACTGACTCTTGGTGTTTATGATTACTTAGTTTCTACACAGGAGTTTAGATATATGAAT
                                                                                                            Section 122
                   (11738) 11738        11750     11760     11770     11780     11790     11800     11810     11820     11834
HIV-1 HXB2             (1) -------------------------------------------------------------------------------------------------
SARS-CoV-2 NC_045512.2 (11738) TCACAGGGACTACTCCCACCCAAGAATAGCATAGATGCCTTCAAACTCAACATTAAATTGTTGGGTGTTGGTGGCAAACCTTGTATCAAAGTAGCCA
                                                                                                            Section 123
                   (11835) 11835 11840     11850     11860     11870     11880     11890     11900     11910     11920 11931
HIV-1 HXB2             (1) -------------------------------------------------------------------------------------------------
SARS-CoV-2 NC_045512.2 (11835) CTGTACAGTCTAAAATGTCAGATGTAAAGTGCACATCAGTAGTCTTACTCTCAGTTTTGCAACAACTCAGAGTAGAATCATCATCTAAATTGTGGGC
                                                                                                            Section 124
                   (11932) 11932   11940     11950     11960     11970     11980     11990     12000     12010         12028
HIV-1 HXB2             (1) -------------------------------------------------------------------------------------------------
SARS-CoV-2 NC_045512.2 (11932) TCAATGTGTCCAGTTACACAATGACATTCTCTTAGCTAAAGATACTACTGAAGCCTTTGAAAAAATGGTTTCACTACTTTCTGTTTTGCTTTCCATG
                                                                                                            Section 125
                   (12029) 12029      12040     12050     12060     12070     12080     12090     12100     12110       12125
HIV-1 HXB2             (1) -------------------------------------------------------------------------------------------------
SARS-CoV-2 NC_045512.2 (12029) CAGGGTGCTGTAGACATAAACAAGCTTTGTGAAGAAATGCTGGACAACAGGGCAACCTTACAAGCTATAGCCTCAGAGTTTAGTTCCCTTCCATCAT
                                                                                                            Section 126
                   (12126) 12126          12140     12150     12160     12170     12180     12190     12200     12210 12222
HIV-1 HXB2             (1) -------------------------------------------------------------------------------------------------
SARS-CoV-2 NC_045512.2 (12126) ATGCAGCTTTTGCTACTGCTCAAGAAGCTTATGAGCAGGCTGTTGCTAATGGTGATTCTGAAGTTGTTCTTAAAAAGTTGAAGAAGTCTTTGAATGT
```

```
                                                                                                       Section 127
                      (12223) 12223 12230     12240     12250     12260     12270     12280     12290     12300            12319
HIV-1 HXB2                (1) -------------------------------------------------------------------------------------------------
SARS-CoV-2 NC_045512.2 (12223) GGCTAAATCTGAATTTGACCGTGATGCAGCCATGCAACGTAAGTTGGAAAAGATGGCTGATCAAGCTATGACCCAAATGTATAAACAGGCTAGATCT
                                                                                                       Section 128
                      (12320) 12320     12330     12340     12350     12360     12370     12380     12390     12400            12416
HIV-1 HXB2                (1) -------------------------------------------------------------------------------------------------
SARS-CoV-2 NC_045512.2 (12320) GAGGACAAGAGGGCAAAAGTTACTAGTGCTATGCAGACAATGCTTTTCACTATGCTTAGAAAGTTGGATAATGATGCACTCAACAACATTATCAACA
                                                                                                       Section 129
                      (12417) 12417        12430     12440     12450     12460     12470     12480     12490     12500       12513
HIV-1 HXB2                (1) -------------------------------------------------------------------------------------------------
SARS-CoV-2 NC_045512.2 (12417) ATGCAAGAGATGGTTGTGTTCCCTTGAACATAATACCTCTTACAACAGCAGCCAAACTAATGGTTGTCATACCAGACTATAACACATATAAAAATAC
                                                                                                       Section 130
                      (12514) 12514 12520     12530     12540     12550     12560     12570     12580     12590     12600  12610
HIV-1 HXB2                (1) -------------------------------------------------------------------------------------------------
SARS-CoV-2 NC_045512.2 (12514) GTGTGATGGTACAACATTTACTTATGCATCAGCATTGTGGGAAATCCAACAGGTTGTAGATGCAGATAGTAAAATTGTTCAACTTAGTGAAATTAGT
                                                                                                       Section 131
                      (12611) 12611    12620     12630     12640     12650     12660     12670     12680     12690         12707
HIV-1 HXB2                (1) -------------------------------------------------------------------------------------------------
SARS-CoV-2 NC_045512.2 (12611) ATGGACAATTCACCTAATTTAGCATGGCCTCTTATTGTAACAGCTTTAAGGGCCAATTCTGCTGTCAAATTACAGAATAATGAGCTTAGTCCTGTTG
                                                                                                       Section 132
                      (12708) 12708       12720     12730     12740     12750     12760     12770     12780     12790      12804
HIV-1 HXB2                (1) -------------------------------------------------------------------------------------------------
SARS-CoV-2 NC_045512.2 (12708) CACTACGACAGATGTCTTGTGCTGCCGGTACTACACAAACTGCTTGCACTGATGACAATGCGTTAGCTTACTACAACACAACAAAGGGAGGTAGGTT
                                                                                                       Section 133
                      (12805) 12805 12810     12820     12830     12840     12850     12860     12870     12880     12890  12901
HIV-1 HXB2                (1) -------------------------------------------------------------------------------------------------
SARS-CoV-2 NC_045512.2 (12805) TGTACTTGCACTGTTATCCGATTTACAGGATTTGAAATGGGCTAGATTCCCTAAGAGTGATGGAACTGGTACTATCTATACAGAACTGGAACCACCT
```

```
                                                                                                    Section 134
                  (12902) 12902   12910      12920      12930      12940      12950      12960      12970      12980         12998
       HIV-1 HXB2     (1) -----------TGGAAGGGCTAATTCACTCCCAACGAA--GACAAGATATCCTTGATCTGTGGATCTACCACACACAAGGCTACTTCCCTGATTAGC
SARS-CoV-2 NC_045512.2 (12902) TGTAGGTTTGTTACAGACACACCTAAAGGTCCTA-AAGTGAAGTATTTATACTTTATTA------AAGGA----TTAAACAACC-----TAAATAGA
                                                                                                    Section 135
                  (12999) 12999       13010      13020      13030      13040      13050      13060      13070      13080      13095
       HIV-1 HXB2    (85) AGAACTACACACCAGGGCCAG---GGATCAG-ATATCCACTGACCTTTGGATGGTGCTACAAGCTAGTACCAGTTGAGCCAGAGAAGTTAGAAGAAG
SARS-CoV-2 NC_045512.2 (12983) GGTATGGTACTTGGTAGTTTAGCTGCCACAGTACGTCTACAAGCTGG--TAATGCAACAGAAGTGCCTGCCAATTCAA---CTGTATTATCTTTCTG
                                                                                                    Section 136
                  (13096) 13096          13110      13120      13130      13140      13150      13160      13170      13180  13192
       HIV-1 HXB2   (178) CCAACAAAGGAGAGAACACCAGCTTGTTACACCCTGTGAGCCTGCATGGAATGGATGACCCGGAGAGAGAAGTGTTAGAGTGGAGGTTTGACAGCCG
SARS-CoV-2 NC_045512.2 (13075) TGCTTTTGCTGTAGATGCTGCTAAAGCTTACAAAGATTATCTAGCTAGTGGGGGACAACCAATCACTAATTGTGTTAAGATGTTGTGTACACACACT
                                                                                                    Section 137
                  (13193) 13193  13200      13210      13220      13230      13240      13250      13260      13270         13289
       HIV-1 HXB2   (275) CCTAGCATTTCATCACATGGCCCGAGAGCTGCATCCGGAGTACTTCAAGAACTGCT------GACATCGAGCTTGCTAC----AAGGGACTTTCCGC
SARS-CoV-2 NC_045512.2 (13172) GGTA-CTGGTCAGGCAAT-AACAGTTACACCGGAAGCCAATATGG-ATC-AAGAATCCTTTGGTGGTGCATCGTGTTGTCTGTACTGCCGTTGCCAC
                                                                                                    Section 138
                  (13290) 13290     13300      13310      13320      13330      13340      13350      13360      13370      13386
       HIV-1 HXB2   (362) TGGGGACTTTCCAG-----GGAGGCGTGGCCTGGGCGGGACTGGGGAGTGGCGAGCCCTCAGATCCTGCATATAAGCAGCTGCTTTTTGCCTGT---
SARS-CoV-2 NC_045512.2 (13265) -ATAGATCATCCAAATCCTAAA---GGATTTTGTGACTTAAAAGG---TAAGTATGTACAAATACCTA----CAACTTGTGCTAATGACCCTGTGGG
                                                                                                    Section 139
                  (13387) 13387        13400      13410      13420      13430      13440      13450      13460      13470   13483
       HIV-1 HXB2   (451) ------ACTGGGTCTCTCTGGTTAGACCAGATCTGAGCCTGGGA---GCTCTCTGGCTAACTAGGGAACCCACTGCTTAAGCCTCAATAAAGCTTGC
SARS-CoV-2 NC_045512.2 (13351) TTTTACACTTAAAAACA-CAGTCTGTACCGTCTGCGGTATGTGGAAAGGTTATGGCTGTAGTTGTG-ATCAACTCCGCGAACCCATGCTTCAGTCAG
                                                                                                    Section 140
                  (13484) 13484 13490      13500      13510      13520      13530      13540      13550      13560      13570 13580
       HIV-1 HXB2   (539) CTTGAGTGCTTCAAGTAGTGTGTGCCCGTCTGTTGTGTGACTCTGGTAACTAGAGATCCCTCAGACCCTTTTAGTCAGTG-----TGGAA------A
SARS-CoV-2 NC_045512.2 (13446) CTG--ATGCACAATCGTTTTTAAACGGGTTTGCGGTGTAAGTGCAGCCCGTCTTACACCGTGCGGCACAGGCACT-AGT-ACTGATGTCGTATACAG
```

```
                                                                                              Section 141
                         (13581) 13581    13590     13600     13610     13620     13630     13640     13650     13660      13677
HIV-1 HXB2                 (625) ATCTCTAGCAGTGGCGCCCGAACAGGGACCTGAAAGCGAAAGGGAAACCAGAGGAGC--TCTCTCGACGCAGGACTCGGCTTGCTGAAG--CGCGCA
SARS-CoV-2 NC_045512.2   (13539) GGCTTTTGACATCTACAATGATAAAGTAGCT---GGTTTTGCTAAATTCCTAAAAACTAATTGTTGTCGCTTCCAAGAAAAGGACGAAGATGACAAT
                                                                                              Section 142
                         (13678) 13678        13690     13700     13710     13720     13730     13740     13750     13760      13774
HIV-1 HXB2                 (718) CGGCAAGAGGCGAGGGGCGGCGA-CTGGTGAGTACGC-------CAAAAATTTTGACTAGCGGAGGCTAGAA---GGAGAGAGATGGGTGCG-AGAG
SARS-CoV-2 NC_045512.2   (13633) TTAATTGATTCTTACTTTGTAGTTAAGAGACACACTTTCTCTAACTACCAACATGAAGAAACAATTTATAATTTACTTAAG-GATTGTCCAGCTGTT
                                                                                              Section 143
                         (13775) 13775 13780    13790     13800     13810     13820     13830     13840     13850     13860  13871
HIV-1 HXB2                 (803) CGTCAGTATTAAGCGGGGGAGAATTAGATCGA-TGGGAAAAAATTCGGTTAAGGCCAGGGGGAAAGAAAAAATATAAATTAAAACATATAGTATGGG
SARS-CoV-2 NC_045512.2   (13729) GCTAAACATGACTTCTTTAAGTTTAGAATAGAC--GGTGACATG-GTACCAC--ATATATCACGT-CAACGTCTTACTAAAT-ACACAATGGCAGAC
                                                                                              Section 144
                         (13872) 13872   13880     13890     13900     13910     13920     13930     13940     13950         13968
HIV-1 HXB2                 (899) CAAGCAGGGAGCTAGAACGATTCGCAGT-----TAATCCTGGCCTGTTAGAAACATCAGAAGGCT--------------GTAGACAAATACTGGGAC
SARS-CoV-2 NC_045512.2   (13819) CTCG-TCTATGCTTTAAGGCATTTTGATGAAGGTAATTGTGACACATTAAAAGAAATACTTGTCACATACAATTGTTGTGATGATGATTATT-TCAA
                                                                                              Section 145
                         (13969) 13969   13980     13990     14000     14010     14020     14030     14040     14050         14065
HIV-1 HXB2                 (977) -AGCTACAACCATCCCTTCAGACAGGATCAGAAGAACTTAGATCATTATATAATACAGTAGCAACCCTCTATTGTGTGCAT--------CAAAGGAT
SARS-CoV-2 NC_045512.2   (13914) TAAAAAGGACTGG--TATGATTTTGTAGAAAA--CCCAGATATATT-A---------CGCGTATACGCCAACTTAGGTGAACGTGTACGCCAAGCTTT
                                                                                              Section 146
                         (14066) 14066         14080     14090     14100     14110     14120     14130     14140     14150  14162
HIV-1 HXB2                (1065) AGAGATAAAAGACACCAAGGAAGCTTTAGACAAG-ATAGAGGAA----G-------AGCAAAACAAAAGTAAGAAAAAAAGCACAGCA--AGCAGCAG
SARS-CoV-2 NC_045512.2   (13998) ---GTTAAAAACAGTACAATT-----CTGTGATGCCATGCGAAATGCTGGTATTGTTGGTGTACTGACATTAGATAA--TCAAGATCTCAATGGTAA
                                                                                              Section 147
                         (14163) 14163 14170     14180     14190     14200     14210     14220     14230     14240          14259
HIV-1 HXB2                (1148) CTGACAC----------AGGACACAG----CAATCAGGTCAGCCAAAATTACCCTATAG-TGCAGAACATCCAGGGGCAAATGGTACATCAGGCCAT
SARS-CoV-2 NC_045512.2   (14085) CTGGTATGATTTCGGTGATTTCATACAAACCACGCCAGGTAGTGGAGTTCCTGTTGTAGATTCTTATTATTCATTGTTAATGCCTA--TATTAACCT
```

Section 148

```
                          (14260) 14260     14270     14280     14290     14300     14310     14320     14330     14340      14356
HIV-1 HXB2                 (1230) ATCACCTAGAACTTTAAATGCATGGGTAAAAGTAGTAGAAGAGAAGGCTTTCAGCCCAGAAGTGATACCCATGTTTTCAGCATTATCAG--AAGGAG
SARS-CoV-2 NC_045512.2    (14180) --TGACCAGGGCTTTAACTGCA-GAGTCACA-TGTTGACACTGACTT-AACAAAGCCTTACATTAAGTGGGATTTGTTAAAATATGACTTCACGGAA
```

Section 149

```
                          (14357) 14357        14370     14380     14390     14400     14410     14420     14430     14440    14453
HIV-1 HXB2                 (1325) CC--ACCCCACAAGATTTAAACACCATGCTAAACACAGTGGGGGGACATCAAGCAGCCATGCAAATGTTAAAAGAGACCATCAATG-AGGAAGCTGC
SARS-CoV-2 NC_045512.2    (14272) GAGAGGTTAAAACTCTTTGACCGTTATTTT--AAATATTGGGATCAGACATACCACCC-AAATTGTGTTAACTGTTTGGATGACAGATGCATTCT--
```

Section 150

```
                          (14454) 14454 14460     14470     14480     14490     14500     14510     14520     14530     14540 14550
HIV-1 HXB2                 (1419) AGAATGGGATAGAGTGCATCC------------AGTGCATGCAGGGC--CTATTGCACCAGGCCAGATGAGAGAACCAAGGGGAAGTGACATAGCAGG
SARS-CoV-2 NC_045512.2    (14364) GCATTGTGCAAACTTTAATGTTTTATTCTCTACA-GTGTTCCCACCTACAAGTTTTGGACCACTAGTGAGAAAAATAT----TTGTTGATGGTGTTC
```

Section 151

```
                          (14551) 14551    14560     14570     14580     14590     14600     14610     14620     14630        14647
HIV-1 HXB2                 (1503) AACTACTAGTACCCTTCAGGAACAAATAGGATGGATGACAAATAATCCACCTATCCCAGTAGGAGAAATTTATAAAAGATGGATAATCCTGGGATTA
SARS-CoV-2 NC_045512.2    (14456) CA---TTTGTAG--TTTCAACTGGATACCACTTCA-GAGAGCTAGGTGTTGTACATAATCAGGATGTAAACTTAC--ATAGCTCTAGACT--TAGT-
```

Section 152

```
                          (14648) 14648       14660     14670     14680     14690     14700     14710     14720     14730      14744
HIV-1 HXB2                 (1600) AATAAAATAGTAAGAATGTATAGCCCTACCAGCATTCTGGACATAAGACAAGGACCAAAGGAACCCTTTAGAGACTATGTAGACCGGT----TCTAT
SARS-CoV-2 NC_045512.2    (14542) TTTAAGGAATTA--CTTGTGTAT-GCTGCT--GACCCT-GCTATGCACGCTGCTTCTG-GTAATCTATTACTAGATAAACGCACTACGTGCTTTTCA
```

Section 153

```
                          (14745) 14745 14750     14760     14770     14780     14790     14800     14810     14820     14830 14841
HIV-1 HXB2                 (1693) AAAACTCTAAGAGCCGAGCAAGCTTCA-CAGGAGGTAAAAAATTGGATGACAGAAACCTTGTTGGTCCAAAATGCGAACCCAGATTGTAAGACTATT
SARS-CoV-2 NC_045512.2    (14632) GTAGCTGCACTTACTAACAATGTTGCTTTTCAAACTGTCAAACCCGGTAA----------TTTTAACAAAGACTTCTATGACT--TTGCTGTGTCT--
```

Section 154

```
                          (14842) 14842   14850     14860     14870     14880     14890     14900     14910     14920          14938
HIV-1 HXB2                 (1789) TTAAAAGCATTGGGACCAGCGGCTACACTAGAAGAAATGATGACAGCATGTCAGGGAGTAGGAGGACCCGGCCATAAGGCAAGAGTTTTGGCTGAAG
SARS-CoV-2 NC_045512.2    (14716) -AAGGGTTTCTTTAA----------GGAAGGAAGTTCTGTTGAAT-----TAAAACACTTCTTC--TTTGCTCAGGATGGTAATGCTGCTATCAGCG
```

```
                                                                                                        Section 155
                               14939            14950          14960          14970          14980          14990          15000          15010          15020          15035
                       (14939)
HIV-1 HXB2              (1886) CAATGAGCCAAGTAACAAATTCAGCTACCATAAT--GATGCAGAGAGGCAA--TTTTAGGAACCAAAGAAAG--ATTGTTAAGTGTTTCAATTGTGG
SARS-CoV-2 NC_045512.2 (14795) ATTA-TGACTACTATCGTTATAATCTACCAACAATGTGTGATATCAGACAACTACTATTTGTAGTT--GAAGTTGTTGATAAGTACTTTGATTGTTA
                                                                                                        Section 156
                               15036                 15050          15060          15070          15080          15090          15100          15110          15120          15132
                       (15036)
HIV-1 HXB2              (1977) CAAAG--AAGGGCACACAGCCAGAAATTGCAGGGCCCCTAGGAAAAAGGGCTGTTGGAAATGTGGAAAGGAAGG-ACACCAAATGAAAGATTGTACT
SARS-CoV-2 NC_045512.2 (14889) CGATGGTGGCTGTATTAATGCTAA----CCAAGTCA-T--CGTCAACAACCT------AGACAAATCAG-CTGGT-TTTCCATTTAATAAATGGGGT
                                                                                                        Section 157
                               15133     15140          15150          15160          15170          15180          15190          15200          15210                    15229
                       (15133)
HIV-1 HXB2              (2071) GAGAGACAGGCTAATTTTTTAGGG----AAGATCTGGCCTTCCTACAAGGGAAGGCC--AGGGAATTTTCTTCAGAGCAGAC---------CAGAGC
SARS-CoV-2 NC_045512.2 (14971) AAG--GCTA--GACTTTATTATGATTCAATGAG-TTATGAGG------ATCAAGATGCA------CTTTTCGCATATACAAAACGTAATGTCATCCC
                                                                                                        Section 158
                               15230          15240          15250          15260          15270          15280          15290          15300          15310          15326
                       (15230)
HIV-1 HXB2              (2153) CAACAGCCC-CACCAGAAGAGAGCTTCAGGTCTGGGGTAGAGACAACAACTCCCCCTCAGAAGCAGGAGCCGATAGACAAGGAACTGTA-TCCTTTA
SARS-CoV-2 NC_045512.2 (15051) TACTATA--ACTCAAATGAATC---TTAAGTATGCCATTAGTGCAAAGAATAGAGCTCGCACCGTAGCT---GGTGTCTC-TATCTGTAGTACTATG
                                                                                                        Section 159
                               15327            15340          15350          15360          15370          15380          15390          15400          15410          15423
                       (15327)
HIV-1 HXB2              (2248) ACTTCCCTCAGG-TCACTCTTTGGCAACGACCCCTCGTCACAATAAAGATAGGGGGGCAACTAAAGGAAGCTCTATTAGATACAGGAGCAGATGATA
SARS-CoV-2 NC_045512.2 (15139) ACCAATAGACAGTTTCAT--------CAAAA-TTATT-----GAAATCAATAGCCGCC-ACTAGAG-GAGCTAC--TGTA-------GTAATTGGAA
                                                                                                        Section 160
                               15424 15430          15440          15450          15460          15470          15480          15490          15500          15510 15520
                       (15424)
HIV-1 HXB2              (2344) CAGTATTAGAAGAAATGAGTTTGC-CAGGAAGATGGAAACCAAAAATGATAGGGGGAATTGGAGGTTTTATCAAAGTAAGACAGTATGATCAGAT--
SARS-CoV-2 NC_045512.2 (15212) CAAGCAAATTCTATGGTGGTTGGCACAACATGTTAAAAACTGTT-----TATAGTGATGTAGAAAACCCTC-ACCTTATGGGT-TGGGATTATCCTA
                                                                                                        Section 161
                               15521     15530          15540          15550          15560          15570          15580          15590          15600                    15617
                       (15521)
HIV-1 HXB2              (2438) ACTCATAGAAATCTGTGGACATAA-AGCTATAGGTACAGTATTAGTAGGACCTACACCTGTCAACATAATTGGAAGAAATCTGTTGACTCAGATTGG
SARS-CoV-2 NC_045512.2 (15302) AATGTGATAGAGCCATGCCTAACAT-GCTTAGAATTATGGCCT-CACTTGTTCTTGCTCGCAAACATACAACGTGTTGT----A--GCTTGTC---A
```

```
                                                                                    Section 162
                             (15618) 15618      15630        15640        15650        15660        15670        15680        15690        15700       15714
HIV-1 HXB2                    (2534) TTGCACTTTAAA--TTTTCCATTAGCCCTATTG-AGACTGTACCAGTAAAATTAAAGCCAGGAATGGATGGCCCAAAAGTTAAACAATGGCCATTG
SARS-CoV-2 NC_045512.2       (15388) CACCGTTTCTATAGATTAGCTAATGAGTGTGCTCAAGTATTGAGTGAAATGGTCATGTGTGGCG---GTTCACTATATGTTAAACCAGGTGGAACCT
```

```
                                                                                    Section 163
                             (15715) 15715 15720        15730        15740        15750        15760        15770        15780        15790        15800  15811
HIV-1 HXB2                    (2628) ACAGAAGAAAAATAAAAGCATTA---GTAGAAATTT-GTACAGAGATGGAAAAGGAAGGGAAAATTTCAAAAATTGGGCCTGAAAATCCATACAAT
SARS-CoV-2 NC_045512.2       (15482) CATCAGGAGATGCCACAACTGCTTATGCTAATAGTGTTTT-TAACATTTGTCAAGCTG-TCACG--GCCAATGTT-AATGCACTTTTATCTACTGAT
```

```
                                                                                    Section 164
                             (15812) 15812      15820        15830        15840        15850        15860        15870        15880        15890           15908
HIV-1 HXB2                    (2721) ACTCCAGTATTTGCCATAAAGAAAAAAGACAGTACTAAATGGAGAAAATTAGTAGATTTC-AGAGAACTTAATAAGAGAACTCAAGACTTCTGGGAA
SARS-CoV-2 NC_045512.2       (15574) GGTAACAAAATTGCCGATAAGTATGTCCGCAATTTACAACACAGACTTTATGAGTGTCTCTATAGAAATAGAGATGTTGACACAGACTTTGTGAATG
```

```
                                                                                    Section 165
                             (15909) 15909        15920        15930        15940        15950        15960        15970        15980        15990       16005
HIV-1 HXB2                    (2817) GTTCAATTAGGAATACCACATC-----CCGCAGGGTTAAAAAAGAAAAAATCAGTAAC--AGTACTG-GATGTGGGTGA------TGCATATTTTTC
SARS-CoV-2 NC_045512.2       (15671) AGTTTTACGCATATTTGCGTAAACATTTCTCAATGATGATACTCTCTGACGATGCTGTTGTGTGTTTCAATAGCACTTATGCATCTCAAGGTCTAGT
```

```
                                                                                    Section 166
                             (16006) 16006          16020        16030        16040        16050        16060        16070        16080        16090   16102
HIV-1 HXB2                    (2900) AGTTCCCTTAGATGAAGACTTCAGGAAGTATACTGCATT-TACCATACCTAGTATAAACAATGAGACACCAGGGATTAGATATCAGTACAATGTGCT
SARS-CoV-2 NC_045512.2       (15768) GGCTAGCATAAAGAA--CTTTAAGTCAGT-TCTTTATTATCAAAACAATGTTTTTATGTCTGAAGCA-AA--ATGTTGGACTGAGACTGACCTTACT
```

```
                                                                                    Section 167
                             (16103) 16103 16110        16120        16130        16140        16150        16160        16170        16180           16199
HIV-1 HXB2                    (2996) TCCACAGGGATGGAAAG--GATCACCA-GCAATATTCCAAAGTAGCATGACAAAAAT-CTTAGAGCCTTTTAGAAAACAAAATCCAGACATAGTTAT
SARS-CoV-2 NC_045512.2       (15859) AAAGGACCTCATGAATTTTGCTCTCAACATACAATGCTAGTTAAACAGGGTGATGATTATGTGTACCTT--CCTTACCCAGATCCATCAAGAATCC-
```

```
                                                                                    Section 168
                             (16200) 16200     16210        16220        16230        16240        16250        16260        16270        16280           16296
HIV-1 HXB2                    (3089) CTATCAATACA---TG----GATGATTTGTATG--TAGGATCTGACTTAGAAATAGGCAGCATAGAACAAAAATAGAGGAGCTGAGACAACATCTG
SARS-CoV-2 NC_045512.2       (15953) ---TAGGGGCCGGCTGTTTTGTAGAT-GATATCGTAAAACAGATGGTACACTTATGATTGAACGGTTCGTGTCTTTAGCTATAGATGCTTACCCAC
```

```
                                     Section 169
                        (16297) 16297 16310 16320 16330 16340 16350 16360 16370 16380 16393
HIV-1 HXB2               (3177) TTGAGGTG-GGGACTTACCACACCAGACAAAAACATCAGAAAGAAC-CTCCATTCCTTTGGATGGG-TTATGAACTCCATC--CTGATAAATGGAC
SARS-CoV-2 NC_045512.2  (16046) TTA--CTAAAC-------A-TCCTAATCAGGAGTATGCTGATGTCTTTCATTTGTACTTACAATACATAAGAAAGCTACATGATGAGTTAACAGGAC

                                     Section 170
                        (16394) 16394 16400 16410 16420 16430 16440 16450 16460 16470 16480 16490
HIV-1 HXB2               (3269) AGTACAGCCTATAGTGCTGCCAGAAAAAGACAGCTGGACTGTCAATGACATACAGAAGTTAGTGGGGAAATTG---AATTG-GGCAAGTCAGATTTA
SARS-CoV-2 NC_045512.2  (16133) ACATGTTAGAC---ATGTATTCTGTTATGCTTACTAAT---GATAACACTTCAGG---TATTGGGAACCTGAGTTTTATGAGGCTATGTACACACC

                                     Section 171
                        (16491) 16491 16500 16510 16520 16530 16540 16550 16560 16570 16587
HIV-1 HXB2               (3362) CCCAGGGATTAAA----GTAAGGCAATTATGTAAACTCCTTAGAGGAACCAAAGCACTAACAGAAGTAATACCACTAACAGAAGAAGCAGAGCTAGA
SARS-CoV-2 NC_045512.2  (16221) GCATACAGTCTTACAGGCTGTTGGGGCTTG-TGTTCTTTGCAATTCACAGACTTCATT--AAGAT---GTGGTGCTTGCATACGTAG---ACCATTC

                                     Section 172
                        (16588) 16588 16600 16610 16620 16630 16640 16650 16660 16670 16684
HIV-1 HXB2               (3455) ACTGGCAGAAAACAGAGAGATTCTAAAAGAACCAGTACATGGAGTGTATTATGACCCATCAAAAGACTTAATAGCAGAAATACAGAAGCA-GGGGCA
SARS-CoV-2 NC_045512.2  (16309) TTATGTTGTAAATGCTGTTACGACCATG--------TC----ATATCAACATCAC---ATAAATTAGTCTTGTCTGTTAATCCGTATGTTT---GCA

                                     Section 173
                        (16685) 16685 16690 16700 16710 16720 16730 16740 16750 16760 16770 16781
HIV-1 HXB2               (3551) AGG-----CCAATGGACATATCAAATTTAT--CAAGAGCCATTTAAAAATCTGAAAACAGGAAAATATGCAAGAATGAGGGGTGCCCACACTAATGA
SARS-CoV-2 NC_045512.2  (16388) A--TGCTCCAGGTTGTGATGTCACAGATGTGACTCAACTTTACTTAG--G-AGGTATGAGCTATTATTGTAA--ATCACATAAACCACCCATTAGTT

                                     Section 174
                        (16782) 16782 16790 16800 16810 16820 16830 16840 16850 16860 16878
HIV-1 HXB2               (3641) TGTAAAACAATTAACAGAG---GCAGTGCAAAAAATAACCACAGAAAGCATAGTAATATGGGGAAAGA---CTCCTAAATTTAAACTGCCCATAC--
SARS-CoV-2 NC_045512.2  (16478) TTCCATT--GTGTGCTAATGGACAAGTTTTTGGT--TTATATAAAAATACATGTGTTGGTAGCGATAATGTTACTGAC-TTTAA--TGCAATTG-CA

                                     Section 175
                        (16879) 16879 16890 16900 16910 16920 16930 16940 16950 16960 16975
HIV-1 HXB2               (3730) -AAAAGGAAACATGGGAAACATGGTGGACAGAGTATTGGCAAGCCACC---TGGATTCCT-GAGTGGGAGTTTGTTAATA-CCCCTCCCTTAGTG--
SARS-CoV-2 NC_045512.2  (16567) ACATGTGACTGGACAAAT-GCTGGTGATTACATTTAGCTAACACCTGTACTGAAAGACTCAAGCTTTTTGCAGCAGAAACGCTCAAAGCTACTGAG
```

Section 176

```
                            16976         16990     17000     17010     17020     17030     17040     17050     17060    17072
                  (16976)
HIV-1 HXB2         (3819)   AAATTATGGTACCAGTTAGAGAAAGAACCCATAGTAGGAGCAGAAACCTTCTATG--TAGATGG---GGCAGCTAACAGGGAGACTAAATTAGGAAA
SARS-CoV-2 NC_045512.2 (16663) GAGACATTTAAACTGTCTTATGGTATTGCTACTGTACG-TGAAGTGCTGTCTGACAGAGAATTACATCTTTCATGGGAAGTTGGTAAACCTAGAC--
```

Section 177

```
                            17073  17080     17090     17100     17110     17120     17130     17140     17150           17169
                  (17073)
HIV-1 HXB2         (3911)   AGCAGGATA----TGTTACTAATAGAG--GAAGACAAAAAGTTGTCACCCTAAC--TGACACAACAAATCAGAAGACTGAGTTACAAGCAATTTATC
SARS-CoV-2 NC_045512.2 (16757) --CACCACTTAACCGA---AATTATGTCTTTACTGGTTATCGTGTAACTAAAAACAGTAAAGTACAAATAGGA--GA--G--TACA--CCT-TTGAA
```

Section 178

```
                            17170     17180     17190     17200     17210     17220     17230     17240     17250          17266
                  (17170)
HIV-1 HXB2         (4000)   TAGCTTTGCAGGATTCGGGATTAGAAGTAAACATAGTAACAGACTCACAATATGCATTAGGAATCATTCAAGCACAACCAGATCAAAGTGAATCAGA
SARS-CoV-2 NC_045512.2 (16840) AAAGGTGAC-TATGGTGATGCTGTTGTTTACCGAGGT---ACA-ACAACTTACAAATTAAATGTTGGT-GATTATTTTGTGCTG-ACATCACATACA
```

Section 179

```
                            17267       17280     17290     17300     17310     17320     17330     17340     17350      17363
                  (17267)
HIV-1 HXB2         (4097)   GTTAGTCAATCAAATAATAGAGCAG-TTAATAAAAAAGGAAAAGGTCTATCTGGCATGGGTACCAGCACACAAAGGAATTGGAGGAAATGAACAAGT
SARS-CoV-2 NC_045512.2 (16930) GTAATGC----CATTA--AGTGCACCTACACTA-GTGCCACAAGAGCACTATGTTAGAATT----------ACTGGC-TTATACCCAACACTCAATA
```

Section 180

```
                            17364 17370     17380     17390     17400     17410     17420     17430     17440     17450 17460
                  (17364)
HIV-1 HXB2         (4193)   AGATAAATTAGTCAGTGCTGGAATCAGGAAAGTACTATTTTTAGATGGAATAGATAAGGCCCAAGATG-----AACATGAGAAATATCACAGTAATT
SARS-CoV-2 NC_045512.2 (17009) TCTCA--G--ATGAGTTTTCTAGCAATGT--TGCAAATTATCAA-----AAGGTTGGTATGCAA-AAGTATTCTACACTCC--AGGGACCACCTGGT
```

Section 181

```
                            17461    17470     17480     17490     17500     17510     17520     17530     17540          17557
                  (17461)
HIV-1 HXB2         (4285)   GGAGAGCAATGGCTAG-TGATTTTAACCTGCCACCTGTAG----TAGCAAAAGAAATAGTA--------GCCAGCTGTGATAAA---TGTCAGCTAA
SARS-CoV-2 NC_045512.2 (17092) ACTGGTAAGAGTCATTTTGCTATTGGCCTAGCTCTCTACTACCCTTCTGCTCG-CATAGTGTATACAGCTTGCTCTCATGCCGCTGTTGATGCACTA
```

Section 182

```
                            17558       17570     17580     17590     17600     17610     17620     17630     17640      17654
                  (17558)
HIV-1 HXB2         (4366)   AAGGAGAAGCCATGCATGGACAAGTAGACTGTAGTCCA--GGAATATGGCAACT--AGATTGTACACATTTAGAAGGAAAAGTTATCCTGGTAGCAG
SARS-CoV-2 NC_045512.2 (17188) TGTGAGAAGGCATT-AAAATAT--TTGCCTATAGATAAATGTAGTAGAATTATACCTGCACGTGCTCGTGTAGAGTGTTTTGATAAATTCAAAGTGA
```

```
                                                                                         Section 183
                  (17655) 17655 17660     17670     17680     17690     17700     17710     17720     17730     17740     17751
              HIV-1 HXB2 (4459) TTCATGTAGCCAGTGG-ATATATAGAAGCAGAAGTTATTCCAGCAGAAACAGGGCAGGAA-ACAGCATATTTTCTTTTAAAATTAGCAGGAAGATGG
SARS-CoV-2 NC_045512.2 (17282) ATTCAACATTAG--A-ACAGTAT---GTCTTTTGTACTGTAA-ATGCATTGCCTGAGAC-GACAGCA---GATATAGTTGTCTTTGATGAA--ATTT
                                                                                         Section 184
                  (17752) 17752   17760     17770     17780     17790     17800     17810     17820     17830          17848
              HIV-1 HXB2 (4554) CCAGTAAAAACAATACATACTGACAATGGCAGCAATTT-----CACCGGTGCTACGGTTAGGGCCGCCTGTTGGTGG-GCGGGAATCAAGCAGGAAT
SARS-CoV-2 NC_045512.2 (17366) CAATGGCCACAAATTATGAT--TTGAGTGTTGTCAATGCCAGATTACGTGCTAAGCACTATGTGTACA-TTGGCGACCCTGCTCAATTACCTGCACC
                                                                                         Section 185
                  (17849) 17849      17860     17870     17880     17890     17900     17910     17920     17930      17945
              HIV-1 HXB2 (4645) TTGGAATTCCCTACAATCCCCAAAGTCAAGGAGTAGTAGAATCTATGAATAAAGAATTAAAGAAAATTATAGGACAGGTAAGAGATCAGGCTGAACA
SARS-CoV-2 NC_045512.2 (17460) ACGC---ACATTGC-TAACTAAGGGCACACTAGAACCAGAATATTTCAATTC-AGTGTGTAG--ACTTATG---AAAACTATAGGTCCAGACATGTT
                                                                                         Section 186
                  (17946) 17946         17960     17970     17980     17990     18000     18010     18020     18030    18042
              HIV-1 HXB2 (4742) TCTTAAGACAGCAGTACAAATG---GCAGTATTCATCCACAATTTTAAAAGA---AAAGGGGGGATTGG----GGGGTA---CAGTGCAGGGGAAAG
SARS-CoV-2 NC_045512.2 (17547) CCTCGGAACTTGTCGGCGTTGTCCTGCTGAAATTGTTGACACTGTGA---GTGCT--TTGGTTTATGATAATAAGCTTAAAGCACATAAAG-ACAAA
                                                                                         Section 187
                  (18043) 18043  18050     18060     18070     18080     18090     18100     18110     18120          18139
              HIV-1 HXB2 (4826) AATAGTA------G-ACATAATAGCAACAGACATACAAA--CTAAAGAATTACAAAAACAAATTACAAAAATTCAAA--------ATTTTCGGGTTT
SARS-CoV-2 NC_045512.2 (17638) TCAGCTCAATGCTTTAAAATGTTTTATAAGGGTGT-TATCACGCATGATGTTTCATCTGCAATTAACAGGCCACAAATAGGCGTGGTAAGAGAATTC
                                                                                         Section 188
                  (18140) 18140     18150     18160     18170     18180     18190     18200     18210     18220          18236
              HIV-1 HXB2 (4906) ATTACAGGGACAGCAGAAATCCACTTTGGA--AAGGACCAGCAAAGCTCCTCTGGAAAGGTGAAGGGGCAGTAGTAATACAAGATAATAGTGAC---
SARS-CoV-2 NC_045512.2 (17734) CTTA------CACGT--AACCCTGCTTGGAGAAAAGCTGTCTTTATTTCACCTTATAATTCACAGAATGCTGTAGCCTCAAAGATTTT-GGGACTAC
                                                                                         Section 189
                  (18237) 18237        18250     18260     18270     18280     18290     18300     18310     18320      18333
              HIV-1 HXB2 (4998) --A-TAAAAGTAGTGCCAAGAAGAAAAGCAAAGATCATTAG---GGATTAT---GGAAAACAGATG--GCAGGTGATGATTGTGTGGCAAGTA---G
SARS-CoV-2 NC_045512.2 (17822) CAACTCAAACTGTTGATTCATCACAGGGCTCAGAATATGACTATGTCATATTCACTCAAACCACTGAAACAGCTCACTCTTGTAATGTAAACAGATT
```

Section 190

```
                              (18334) 18334 18340 18350 18360 18370 18380 18390 18400 18410 18420 18430
HIV-1 HXB2                     (5081) ACAGGATGAGGATTA------GAACAT--GGAAAAGTTTAGTAAACACCATATGTATGTTTCAGGGAAAGCTAGGGGATGGTTTATAGACATCAC
SARS-CoV-2 NC_045512.2        (17919) TAATGTT----GCTATTACCAGAGCAAAGTAGGCATACTTTGCAT-----AATGTCTGATAGAGACCTTTATGACAAG--TTGCAATTTACAAGTC
```

Section 191

```
                              (18431) 18431 18440 18450 18460 18470 18480 18490 18500 18510 18527
HIV-1 HXB2                     (5170) TATGAAAGCCCTCATCCAAGAA----T-AAGTTCAGAAGTACACATCCCACTAGGGG--ATGCTAGATTGGTAATAACAACAT--ATTGGGGTCTGC
SARS-CoV-2 NC_045512.2        (18005) TTGA-AATTCCACGT--AGGAATGTGGCAACTTTACAAGCTGAAAATGTAACAGGACTCTTTAAAGATTGTAGT--AAGGTAATCACTGGGTT----
```

Section 192

```
                              (18528) 18528 18540 18550 18560 18570 18580 18590 18600 18610 18624
HIV-1 HXB2                     (5258) ATACAGGAGAAAGAGACTGGCATTTGGGTCAG--------GGAGTCTCCATAGAATGGAGGAAAAAGAGATATAGCACACAAGTAGACCCTGAACTA
SARS-CoV-2 NC_045512.2        (18093) --ACATCCTACACAGGC--AC---CTACACACCTCAGTGTTGACACTAAATTCAAAACT------GAAGGTTTATGT-----GTTGACAT--ACCTG
```

Section 193

```
                              (18625) 18625 18630 18640 18650 18660 18670 18680 18690 18700 18710 18721
HIV-1 HXB2                     (5347) GCAGACC-AACTAATTCATCTGTATTACTTTGACTGTTTTTCAGACTCTGCTATAAGAAAGGCCTTATTAGGACACATAGTTAGCCCTAGGTGTGAA
SARS-CoV-2 NC_045512.2        (18170) GCATACCTAAGGACATGACCTATAGAAGACT-CATCTCTATGA--TGGGTTTTAAAATGAATTA----TCAAGTTAATGGTTACCCTA---A---CA
```

Section 194

```
                              (18722) 18722 18730 18740 18750 18760 18770 18780 18790 18800 18818
HIV-1 HXB2                     (5443) TA--TCAAGCAGGACATAACAAG--GTAGGATCT---CTACAATACTTGGCACTAGCAGCATTAATAACACCAAAAAGATAAAGCCACCTTTGCCT
SARS-CoV-2 NC_045512.2        (18254) T-GTT-TATCACCCGCGAAGAAGCTATAAGACATGTACGTGCATGGATTGGCTTCGATGTCGAG--GGGTGTCATGCTACTAGAGAAGCTGTTGGTA
```

Section 195

```
                              (18819) 18819 18830 18840 18850 18860 18870 18880 18890 18900 18915
HIV-1 HXB2                     (5533) AGTGTTACGAAACTGACAGAGGATAGATGGAACAAGCCCCAGAAGACCAAGGGCCA-CAGAGGGAGCCAC-ACAATGAATGGACACTAGAGCTTTTA
SARS-CoV-2 NC_045512.2        (18347) CCAATTT-----------ACCTTTACAGCTAGGTTTTTCTACAGG-TGTTAACC--TAGTTGCTGT--ACCTACAGGTTATGTTGATACACCTAATA
```

Section 196

```
                              (18916) 18916 18930 18940 18950 18960 18970 18980 18990 19000 19012
HIV-1 HXB2                     (5628) GAGGAGCTTAAGAATGAAGCTGTTAGAC--ATTTTCCTAGGATTTGGCTCCATGGCTTAGGGCAACATATCTATGAAACTTATGGGGATACTTGGGC
SARS-CoV-2 NC_045512.2        (18428) ATA---CAGATTTTTCCAGAGTTAG---TGCTAAACCACCGCCTGGAGATCA---ATTTAAACACCTCATACCACTTAT-GTACAAAGGACTTCCTT
```

```
                                                                                                   Section 197
                             19013  19020       19030       19040       19050       19060       19070       19080       19090            19109
                   (19013)
HIV-1 HXB2          (5723)   AGGAGTGGAAGCCATAATAAGAATTCTGCAACAACTGCTGTTTATCCATTTTCAGAATTGGGTGTCGACATAGCAGAATAGGCGTTACTCGACAGAG
SARS-CoV-2 NC_045512.2 (18515) GGAAT-GTAGTGCGTATAAAGATTGTAC----AAATGTTAAGTGACACAC-TTAA-------------AAATCTCTCTGACAGAGTCGTATTTGT--
                                                                                                   Section 198
                             19110       19120       19130       19140       19150       19160       19170       19180       19190       19206
                   (19110)
HIV-1 HXB2          (5820)   GAGAGCAAGAAATGGAGCCAGTAGATCCTAGACTAGAGCCCTGGAAGCATCCAGGAAGTCAGCCTAAAACTGCTTGTACCAATTGCTATTGTAAAAA
SARS-CoV-2 NC_045512.2 (18591) ---CTTATGG-----G-CACATGGCTTTGA-GTTGACATCTATGAAGTATTTTGTGAAAA-TAGGAC--CTGAGCGCACCTGTTGTCTATGTGATAG
                                                                                                   Section 199
                             19207          19220       19230       19240       19250       19260       19270       19280       19290    19303
                   (19207)
HIV-1 HXB2          (5917)   GTGTTGCTTTCATTGCCAAGTTTGTTTCATAACAAAAGCCTTAGGCATCTCCTATGGCAGGAAGAAGCGGAGACAGCGACGAAGAGCTCATCAGAAC
SARS-CoV-2 NC_045512.2 (18675) ACGTGCCACATGCTTTTCCACTGCTTCAGACACTTATGCC-----TGTTGGC-AT-CATTCTATTGGATTTGATTACGTCTATAATCCGTTTATGAT
                                                                                                   Section 200
                             19304  19310       19320       19330       19340       19350       19360       19370       19380       19390  19400
                   (19304)
HIV-1 HXB2          (6014)   AGTCAGACTCATCAAGCTTCTCTATCA---AAGCAGTAAGTAGTACATGTAACGCAACCTATACCAATAGTAGCAATAGTAGCATTAGTAGTAGCAA
SARS-CoV-2 NC_045512.2 (18765) T--GATGTTCAACAATGGGGTTTTACAGGTAACCTA--CAAAGCAACCATGATCTGTATTGTCAAGTCCATGGTAATG---CACATGTAGCTAGTTG
                                                                                                   Section 201
                             19401      19410       19420       19430       19440       19450       19460       19470       19480          19497
                   (19401)
HIV-1 HXB2          (6108)   TAATAATAGCAATAGTTGTGTGGTCCATAGTAATCATAGAATATAGGAAAATATTAAGACAAAGAAAAA-T--AGACAGGTTAATTGATAGACTAAT
SARS-CoV-2 NC_045512.2 (18855) TGATGCAATCATGACTAGG----TGTCTAGCTGTCCACGAGTGCTTTGTTAAGCGTGTTGACTGGACTATTGAATATCCTATAATTGGTGATGAACT
                                                                                                   Section 202
                             19498         19510       19520       19530       19540       19550       19560       19570       19580       19594
                   (19498)
HIV-1 HXB2          (6202)   AGAAAGAGCAGAAGACAGTGGCAATG-AGAGTGAAGGAGAAATATCAGCACTTGTGGAGATGGGGGTGGAGATGGGGCACCATGCTCCTTGGGATGT
SARS-CoV-2 NC_045512.2 (18948) GAAGATTAATGCGGCTTGTAGAAAGGTTCAACACATGGTTGTTAAAGCTGCATTATTAGCAGACAAATTCCCAGTTCTT-CACGACATTGGTAACCC
                                                                                                   Section 203
                             19595 19600       19610       19620       19630       19640       19650       19660       19670       19680    19691
                   (19595)
HIV-1 HXB2          (6298)   TGATGATCTGTAGTGCTACAGAAAAATTGTGGGTCACAGTCTATTATGGGGTAC-CTGTGTGGAAGGAAGCAACCACCACT--CTATTTTGTGCATC
SARS-CoV-2 NC_045512.2 (19044) TAAAGCTATTAAGTGTGTACCTCAAGCTGATGTAGAATGGAAGTTCTATGATGCACAGCCTTG--T------AGTGACAAAGC-----TTATAAAAT
```

```
                                                                                              Section 204
                             (19692) 19692   19700      19710      19720      19730      19740      19750      19760      19770       19788
                     HIV-1 HXB2 (6392) AGATGCTAAAGCATATGATACAGAGGTACATAATGTTTGGGCCACACATGCCTGTGTACCCACAGACCCCAACCCACA-AGAAGTAGTATTGGTAAA
SARS-CoV-2 NC_045512.2 (19128) AGAAGAATTATTCTA--TTCTTATGCCACACA--TTCTGACAAATTCACAGATG-GTGTATGCCTATTTTG-------GAATTGCAATGTCGATAGA
                                                                                              Section 205
                             (19789) 19789      19800      19810      19820      19830      19840      19850      19860      19870      19885
                     HIV-1 HXB2 (6488) TGTGACAGAAATTTTAACATGTGGAAAAATGACATGGTAGA--ACAGATG-----CATGAGGATATAATCAGTTTATGGGATCAAAGCCTAAAGCC
SARS-CoV-2 NC_045512.2 (19213) TATCCTGCTAA---TTCCATTGTTTGTAGATT---TGACACTAGAGTGCTATCTAACCTTAACTTGCCTGGTTGTGAT-GGTGGCAGTTTGTATGTA
                                                                                              Section 206
                             (19886) 19886         19900      19910      19920      19930      19940      19950      19960      19970    19982
                     HIV-1 HXB2 (6578) ATGTGTAAAATTAACCCCACTCTGTGTTAGT---TTAAAGTGCACTGATTTGAAGAATGATACTAATACCAATAGTAGTAGCGGGAGAATGATAATG
SARS-CoV-2 NC_045512.2 (19303) A--ATAAACATGCATTCCACACACCAGCTTTTGATAAAAGTGCTTTTGTTAATTTAAAACAATTACCATT-TTTCTATTACTCTGACAGT--CCATG
                                                                                              Section 207
                             (19983) 19983  19990      20000      20010      20020      20030      20040      20050      20060        20079
                     HIV-1 HXB2 (6672) GAGAAAGGAGAGATAAAAAACTGCT----------C------TTTCAATATCAGC----ACAAGCATAA---------------GAGGTAAGGTGCA
SARS-CoV-2 NC_045512.2 (19395) TGA-GTCTCATGGAAAACAAGTAGTGTCAGATATAGATTATGTACCACTAAAGTCTGCTACGTGTATAACACGTTGCAATTTAGGTGGTGCTGTCTG
                                                                                              Section 208
                             (20080) 20080      20090      20100      20110      20120      20130      20140      20150      20160      20176
                     HIV-1 HXB2 (6734) GAAAGAATATGCATTTTTTTATAAACTTGATATAATA--CCAATAGATAATGATACTACCAGCTATAAGTTGACAAGTTGTAACACCTCAGTCATTA
SARS-CoV-2 NC_045512.2 (19491) TAGACATCATGCTAATGAGTACAGATTGTATCTCGATGCTTATAACATGATGATCTCAGCTGGCTTTAGCTTG-TGGGTTTACAAACAATTTGAT-A
                                                                                              Section 209
                             (20177) 20177         20190      20200      20210      20220      20230      20240      20250      20260      20273
                     HIV-1 HXB2 (6829) CACAGGCCTGTCCAAAGGTATCCTTTGAGCCAAT-TCCCATACATTATTGTGCCCCGGCTGGTTTTGCGATTCTAAAATGTAATAATAAGACGTTCA
SARS-CoV-2 NC_045512.2 (19586) CTTATAACCT-CTGGA-ACACT---TTTACAAGACTTCAGAGTTTAGAA--AATGTGGCTTTTAATGTTGTAAATAAGGGACACTTTGATGGACAAC
                                                                                              Section 210
                             (20274) 20274  20280      20290      20300      20310      20320      20330      20340      20350      20360   20370
                     HIV-1 HXB2 (6925) ATGGAACAGGACCATGTACAAATGTCAGCACAGTACAATGTACACATGGAATTAGGCCAGTAGTATCAACTCAACTGCTGTTAAATGGCAGTCTAGC
SARS-CoV-2 NC_045512.2 (19676) AGGGTGAAGTACCAGTTTCTATCATTA--ATAACACTGTTTACACAAAAGTTGATGGTGTTGATGTAGAATTGTTTGAA-AATAAAAC-AACATTAC
```

```
                                                                                          Section 211
                           20371    20380     20390     20400     20410     20420     20430     20440     20450         20467
                  (20371)
HIV-1 HXB2         (7022)  AGAAGAAGAGGTAGTAATTAGATCTGTCAATTTCACGGACAATGCTAAAACCATAATAGTACAGCTGAACACATCTGTAGAAATTAATTGTACAAGA
SARS-CoV-2 NC_045512.2 (19769)  CTGTTAATGTAG--CATTTGA-GCTTTGGGCTAAGCG----CAACATTAAA-CCAGTACCAGAGG-T-GAAAATACTCAATAA-TT-TGGGTGTGGA
                                                                                          Section 212
                           20468       20480     20490     20500     20510     20520     20530     20540     20550        20564
                  (20468)
HIV-1 HXB2         (7119)  CCCAACAACAA-TACAAGAAAAAGAATCCGTATCCAGAGAGGACCAGGGAGAGCATTTGTTACAATAGGAAAAATAGGAAATATGAGACAAGCACAT
SARS-CoV-2 NC_045512.2 (19854)  CATTGCTGCTAATACTGTGATCTGGGACTACAAAAGAGATGCTCCAGCACATATATCTA---CTATTGGTGTT-TGTTCTATGACTGACAT---AGC
                                                                                          Section 213
                           20565 20570     20580     20590     20600     20610     20620     20630     20640     20650  20661
                  (20565)
HIV-1 HXB2         (7215)  TGTAACATTAGTAGAGCAAAATGGAATAACACTTTAAAACAGATAGCTAGCAAATTAAGAGAACAATTTGGAAATAATAA--AACAATAATCTTTAA
SARS-CoV-2 NC_045512.2 (19944)  CAAGAAACCAACTGAAACGATTTGTGCACCACTCACTGTCTTTT-TTGAT--GGTAGAGTTGATGGTCAAGTAGACTTATTTAGAAATGCCCGTAAT
                                                                                          Section 214
                           20662   20670     20680     20690     20700     20710     20720     20730     20740          20758
                  (20662)
HIV-1 HXB2         (7310)  GCAATCCTCAGGAGGGGAC-CCAGAAATTGTAACGC--ACAGTTTTAATTGTGGAGGGGAATTTTTCTACTGTAATTCAACACAACTGTTTAATAGT
SARS-CoV-2 NC_045512.2 (20038)  GGTGTTCTTATTACAGA--AGGTAGTGTTA-AAGGTTTACAA---CCATC-TGTAGGTCCCAAA---CAAGCTAGTCTT----AATGGAGTCACATT
                                                                                          Section 215
                           20759      20770     20780     20790     20800     20810     20820     20830     20840       20855
                  (20759)
HIV-1 HXB2         (7404)  ACTTGGTTTAATAGTACTTGGAGTACTGAAGGGTCAAATAACACT-GAAGGAAGTGACACAATCACCCTCCCATGCAGAATAAAACAAATTATAAAC
SARS-CoV-2 NC_045512.2 (20121)  AATTGGAGAAGCCGTAAAAACACAGTTCAATTATTATAAGAAAGTTGATGGTGTT-GTCCAACAATTACCTGAAACTTACTTTACTCAGAGTAGAAA
                                                                                          Section 216
                           20856         20870     20880     20890     20900     20910     20920     20930     20940    20952
                  (20856)
HIV-1 HXB2         (7500)  ATGTGGCAGAAAGTAGGAAAAGCAATGTATGCCCCTCCCATCAGTGGACAAATTAGATGTTCATCAAATATTACAGGGCTGCTATTAACAAGAGATG
SARS-CoV-2 NC_045512.2 (20217)  TTTA-CAAGAATTT-A---AACCCAGGAGTCAAATGGAAATTG----ATTTCTTAGAATTAGCTATG-GATGA-------ATTCATTGAA-CGG---
                                                                                          Section 217
                           20953  20960     20970     20980     20990     21000     21010     21020     21030           21049
                  (20953)
HIV-1 HXB2         (7597)  GTGGTAATAGCAACAATGAGTCCGAGATCTTCAGACC-TGGAGGAGGAGATATGAGGGACAATTGGAGAAGTGAATTATATAA---ATATAAAGTAG
SARS-CoV-2 NC_045512.2 (20293)  TATAAATTAGAAGGCTATGCCTTCGAACATATCGTTTATGGAGATTTTAGTCATAGTCAGT-TAGGTG----GTTTACATCTACTGATTGGACTAGC
```

Section 218

```
                           21050     21060     21070     21080     21090     21100     21110     21120     21130    21146
                 (21050)
HIV-1 HXB2        (7690)   TAAAAATTG--------AACCATTAGGAGTAGCACCCACCAAGGCAAAGAGAAG--AGTGGTGCAGAGAGAAAAAAGAGCAGTGGGAATAGGAGCTT
SARS-CoV-2 NC_045512.2 (20385) TAAACGTTTTAAGGAATCACCTTTTGAATTAGAAGATTTTATTCCTATGGACAGTACAGT-TAAAAACTATTTCATA-ACAGATGCGCAAACAGGTT
```

Section 219

```
                           21147        21160     21170     21180     21190     21200     21210     21220     21230    21243
                 (21147)
HIV-1 HXB2        (7777)   TGTTCCTTGGGTTCTTGGGAGCAGCAGGAAGCACTATGGGCGCAGCCTCAATGACGCTGACGGTACAGGCCAGACAATTATTGTCTGGTATA-GTGC
SARS-CoV-2 NC_045512.2 (20480) CATCTAAGTGTGTGTGTTCTGTTATTG--ATTTATTACTTGATGATTTTGTTGAAATA-ATAAAATCCC---AAGATTTATCTG------TAGTTTC
```

Section 220

```
                           21244 21250     21260     21270     21280     21290     21300     21310     21320     21330 21340
                 (21244)
HIV-1 HXB2        (7873)   AGCAGCAGAACAATTTGCTGAGGGCTATTGAGGCGCAACAGCATCTGTTGCAACTCACAGTCTGGGGCATCAAGCAGCTCCAGGCAAGAATCCTGGC
SARS-CoV-2 NC_045512.2 (20565) TAAGGTTG----------TCAAAGTGAC---TATTGACTATACAGAAATTTCATT-------TATGCTTTGGTGTA--AAGATGGCCATGT-----A
```

Section 221

```
                           21341    21350     21360     21370     21380     21390     21400     21410     21420        21437
                 (21341)
HIV-1 HXB2        (7970)   TGTGGAAAGATACCTAAAGGATCAACAG-CTCCTGGGGATTTGGGGTTGCTCTGGAAAACTCATTTGCACCACTGCTGTGCCTTGGAATGCTAGTTG
SARS-CoV-2 NC_045512.2 (20635) ----GAAACAT--TTTACCCAAAATTACAAT-CTAGTCA---AGCGTGGCAACCGGGT----GTTGCT----ATGCCTA--AT--CTTTACAAAATG
```

Section 222

```
                           21438       21450     21460     21470     21480     21490     21500     21510     21520    21534
                 (21438)
HIV-1 HXB2        (8066)   GAGTAATAAATCTCTGGAACAGATTTGGAATCAC-ACGACCTGGATGGAGTGGGACAGAGAAATTAACAATTACACAAGCTTAATACACTCCTTAAT
SARS-CoV-2 NC_045512.2 (20710) C--AAAGAATGCTATTAGAAAAGTGTGACCTTC-AAAATTATGGTGATAGTGCAACA-----TTACCTAAAGGC---ATAATGATGAATGTCGC--A
```

Section 223

```
                           21535 21540     21550     21560     21570     21580     21590     21600     21610     21620 21631
                 (21535)
HIV-1 HXB2        (8162)   TGAAGAATCGCAAAACCAGCAAGAAA-AGAATGAACAAGAATTATTGGAATTAGATAAATGGGCAAGTTTGTGGAATTGGTTTAACATAACAAATTG
SARS-CoV-2 NC_045512.2 (20794) A-AATATACTCAACTGTGTCAATATTTAAACACATTAACATTAGCTGTACCCTATAATATGAGAGTTATACATTTTGGTGCTGGTTCTGATAAAGGA
```

Section 224

```
                           21632   21640     21650     21660     21670     21680     21690     21700     21710        21728
                 (21632)
HIV-1 HXB2        (8258)   GCTGTGGTATATAAAATTATTCATAATGATAGTAGGAGGCTTGGTAGGTTTAAGAATAGTTTTGCTGTACTTTCTATAGTGAATAGAGTTAGGCAG
SARS-CoV-2 NC_045512.2 (20890) GTTGCACCAGGTACAGCTG-T-TTTAAGACAGTGGTTGCCTACG--GGTACGCTGCTTGTCGATTCAGATCTTAATGACTT------TGTCTCTGAT
```

```
                                                                                                              Section 225
                            21729      21740      21750      21760      21770      21780      21790      21800      21810      21825
                  (21729)
HIV-1 HXB2         (8355) GGATATTCACCATT-ATCGTTTCAGACCCACCTCCCAACCCCGAGGGGACCCGACAGGCCCGAAGGAAT-AGAAGAAGAAGGTGGAGAGAGAGACAG
SARS-CoV-2 NC_045512.2 (20977) GCAGATTCAACTTTGATTGGTGATTGTGCAACTGTACATACAGCTAATAAATGGGATCTCATTATTAGTGATATGTACGACCCTAAGACTAAAAATG
                                                                                                              Section 226
                            21826             21840      21850      21860      21870      21880      21890      21900      21910      21922
                  (21826)
HIV-1 HXB2         (8450) AGACAGATCCA-TTCGATTAGTGAACGGATCCTTGGCACTTATCTGGGACGATCTGCGGAGCCT----GTGCCTCTTCAGCTACCACCGCTTGAGAG
SARS-CoV-2 NC_045512.2 (21074) TTACAAAAGAAAATGACTCTA--AAGAGGGTTTTTTCACTTACATTTGTGGGTTTATACAACAAAAGCTAGCTCTTGGAGGTTCCGTGGCTATAAAG
                                                                                                              Section 227
                            21923   21930      21940      21950      21960      21970      21980      21990      22000                22019
                  (21923)
HIV-1 HXB2         (8542) ACTTACT--CTTGATTGTAACGAGGAT-TGTGGAA-CTTCTGGGACGCAGGGGGTGGGAAGCCCTCAAATATTGGTGGAATCTCCTACAGTATTGGA
SARS-CoV-2 NC_045512.2 (21169) ATAACAGAACATTCTTGGAATGCTGATCTTTATAAGCTCATGGGACACTTCGCAT----GGTGGACAGCCTTTGTTACTAATGTGAA-------TGC
                                                                                                              Section 228
                            22020      22030      22040      22050      22060      22070      22080      22090      22100      22116
                  (22020)
HIV-1 HXB2         (8635) GTCAGGAACTAAAGAATAGTGCTGTTAGCTTGCTCAATGCCACAGCCATAGCAGTAGCTGAGGGGACAGATAGGGTTATAGAAGTAGTACAAGGAGC
SARS-CoV-2 NC_045512.2 (21255) GTCATCATCTGAAGCATTTTTA-ATTGG--------ATGTAATTATCTTGGCAAACCACGCGAACAAATAGATGGTTATGT----CATGCATGCAAA
                                                                                                              Section 229
                            22117         22130      22140      22150      22160      22170      22180      22190      22200      22213
                  (22117)
HIV-1 HXB2         (8732) TTGTAGAGCTATTCGCCACATACCTAGAAGAATAAGACAGGGCTTGGAAAGG-ATTTTGCTATAAGATGGGTGGCAAGTGGTCAAAAAGTAGTGTGA
SARS-CoV-2 NC_045512.2 (21339) TTACATAT---TTTGGAGGAA-----TACAAATCCAATTCAGTTG-TCTTCCTATTCT-TTATTTGACATGAGTAAATTTCCCCTTAAATTAAGGG-
                                                                                                              Section 230
                            22214   22220      22230      22240      22250      22260      22270      22280      22290      22300   22310
                  (22214)
HIV-1 HXB2         (8828) TTGGATGGC-CTACTGTAAGGGAAAGAATGAGACGAG-CTGAGCCAGCAGCAGATAGGGTGGGAGCAGCATCTCGAGACCTGGAAAAACATGGAGCA
SARS-CoV-2 NC_045512.2 (21425) --GTACTGCTGTTATGTCTTTAAAAGAAGGTCAAATCAATGATATG------------ATTTTATCTCTTCTTAGTAAAGGTAGA-----CTTATAA
                                                                                                              Section 231
                            22311     22320      22330      22340      22350      22360      22370      22380      22390                22407
                  (22311)
HIV-1 HXB2         (8923) ATCACAAGTAG-CAATACAGCAGCTACC---AATGCTG--CTTGTGCCTGGCTAGAAGCACAAGAGGAGGAGGAGGTGGGTTTTCCAGTCACACCTC
SARS-CoV-2 NC_045512.2 (21503) TTAGAGA--AAACAACAGAGTTGTTATTTCTAGTGATGTTCTTGTTAACAACT--AAACGAACAATGT-----T---TGTTTTTCTT----------
```

Section 232

```
                             22408       22420          22430          22440          22450          22460          22470          22480          22490        22504
                    (22408)
HIV-1 HXB2           (9014)  AGGTACCTTTAAGACCAATG-A--CTTACAAGGCAGCTGTAGATCTTAGCCACTTTTTAAAAGAAAAGGGGGGACTGGAAGGGCTAATTCACTCCCA
SARS-CoV-2 NC_045512.2 (21578)  ---GTTTTATTGC--CACTAGTCTCTAGTCAGTGT---GTTAATCTTACA-----ACCAGAACTCAATTACCCCCTGCATACAC----TAATTCTTT
```

Section 233

```
                             22505 22510          22520          22530          22540          22550          22560          22570          22580          22590  22601
                    (22505)
HIV-1 HXB2           (9108)  AAGAAGACAAGATATCCTTGATCTGTGGATCTACCACACACAAGGCTACTTCCCTGATTAGCAGAACTACACACCAGGGCCAGGGGTCAGATATCCA
SARS-CoV-2 NC_045512.2 (21658)  -----CACACGT-----------GGTGTTTATTACCCTGACAAAGTTTTCAG-ATCCT---CAGTTTTACATTC---------AACTCA----GGAC
```

Section 234

```
                             22602   22610          22620          22630          22640          22650          22660          22670          22680        22698
                    (22602)
HIV-1 HXB2           (9205)  CTGACCTTTGGATGGTGCTACAAGCTAGTA-CCAGTTGAGCCAGATAAGATAGAAGAGGCCAATAAAGGAGAGAACACCAGCTTGTTACACCCTGTG
SARS-CoV-2 NC_045512.2 (21722)  TTGTTCT------TA-----------CCTTTCTTTTCCAATGTTACTTGGTTCCATGCTATACATGTC-TCTGGGACCAATGGTACTAAGAG-----
```

Section 235

```
                             22699      22710          22720          22730          22740          22750          22760          22770          22780      22795
                    (22699)
HIV-1 HXB2           (9301)  AGCCTGCATGGGATGGATGACCCGGAGAGAGAAGTGTTAGAGTGGAGGTTTGACAGCCGCCTAGCATTTCATCACGTGGCCCGAGAGCTGCATCCGG
SARS-CoV-2 NC_045512.2 (21796)  -GTTT----GATAACCC-TGTCCTACCATTTAATGAT---------GGTGTTTATTTTGC-TTCCACTGAG-----AAGTCTAACATAATAAGAGGC
```

Section 236

```
                             22796             22810          22820          22830          22840          22850          22860          22870          22880   22892
                    (22796)
HIV-1 HXB2           (9398)  AGTACTTCAAGAACTGCTGACATCGAGCTTGCTACAAGGGACTTTCCGCTGGGGACTTTCCAGGGAGGCGTGGCCTGGGCGGGACTGGGGAGTGGCG
SARS-CoV-2 NC_045512.2 (21872)  TGGATTTTTGGTACTACTT-----TAGATTCGAAGACC-CAGT---CCCTACTTATTGTTAATAA--CGCT-ACTAATGTTGTTATTAAAGTCTGTG
```

Section 237

```
                             22893  22900          22910          22920          22930          22940          22950          22960          22970              22989
                    (22893)
HIV-1 HXB2           (9495)  AGCCCTCAGATCCTGCATATAAGCAGCTGCTTTTTGCCTGTACTGGGTCTCTCTGGTTAGACCAGATCTGAGCCTGGGAGCTCTCTGGCTAACTAGG
SARS-CoV-2 NC_045512.2 (21957)  AATTTCAATTTT-GTAATGATCCAT-------TTTTG--G--------GTGTTTAT-TACCACAAAACAACAA---AAGTTGGA----TGGAAAGT
```

Section 238

```
                             22990     23000          23010          23020          23030          23040          23050          23060          23070        23086
                    (22990)
HIV-1 HXB2           (9592)  GAACCCACTGCTTAAGCCTCAATAAAGCTTGCCTTGAGTGCTTCAAGTAGTGTGTGCCCGTCTGTTGT-GTGACTCTGGTAACTAGAGATCCCTCAG
SARS-CoV-2 NC_045512.2 (22028)  GAGTTCAGAGTTTAT-TCT-AGTGCGAATAATT-GCACTTT-TGAATATGTCTCTCA--GCCTTTTCTTATGGACCTTGAAGGAAAACAGGGTAATT
```

```
                                                                                                        Section 239
                            (23087) 23087        23100     23110     23120     23130     23140     23150     23160     23170     23183
                 HIV-1 HXB2  (9688) ----ACCCTTTTAGTCAG---TGTGGAAAATCTCTAGCA----------------------------------------------------------
SARS-CoV-2 NC_045512.2 (22119) TCAAAAATCTTAGGGAATTTGTGTT-TAAGAATATTGATGGTTATTTTAAAATATATTCTAAGCACACGCCTATTAATTTAGTGCGTGATCTCCCTC
                                                                                                        Section 240
                            (23184) 23184 23190     23200     23210     23220     23230     23240     23250     23260     23270 23280
                 HIV-1 HXB2  (9720) -------------------------------------------------------------------------------------------------
SARS-CoV-2 NC_045512.2 (22215) AGGGTTTTTCGGCTTTAGAACCATTGGTAGATTTGCCAATAGGTATTAACATCACTAGGTTTCAAACTTTACTTGCTTTACATAGAAGTTATTTGAC
                                                                                                        Section 241
                            (23281) 23281    23290     23300     23310     23320     23330     23340     23350     23360         23377
                 HIV-1 HXB2  (9720) -------------------------------------------------------------------------------------------------
SARS-CoV-2 NC_045512.2 (22312) TCCTGGTGATTCTTCTTCAGGTTGGACAGCTGGTGCTGCAGCTTATTATGTGGGTTATCTTCAACCTAGGACTTTTCTATTAAAATATAATGAAAAT
                                                                                                        Section 242
                            (23378) 23378       23390     23400     23410     23420     23430     23440     23450     23460     23474
                 HIV-1 HXB2  (9720) -------------------------------------------------------------------------------------------------
SARS-CoV-2 NC_045512.2 (22409) GGAACCATTACAGATGCTGTAGACTGTGCACTTGACCCTCTCTCAGAAACAAAGTGTACGTTGAAATCCTTCACTGTAGAAAAAGGAATCTATCAAA
                                                                                                        Section 243
                            (23475) 23475 23480     23490     23500     23510     23520     23530     23540     23550     23560 23571
                 HIV-1 HXB2  (9720) -------------------------------------------------------------------------------------------------
SARS-CoV-2 NC_045512.2 (22506) CTTCTAACTTTAGAGTCCAACCAACAGAATCTATTGTTAGATTTCCTAATATTACAAACTTGTGCCCTTTTGGTGAAGTTTTTAACGCCACCAGATT
                                                                                                        Section 244
                            (23572) 23572   23580     23590     23600     23610     23620     23630     23640     23650         23668
                 HIV-1 HXB2  (9720) -------------------------------------------------------------------------------------------------
SARS-CoV-2 NC_045512.2 (22603) TGCATCTGTTTATGCTTGGAACAGGAAGAGAATCAGCAACTGTGTTGCTGATTATTCTGTCCTATATAATTCCGCATCATTTTCCACTTTTAAGTGT
                                                                                                        Section 245
                            (23669) 23669      23680     23690     23700     23710     23720     23730     23740     23750         23765
                 HIV-1 HXB2  (9720) -------------------------------------------------------------------------------------------------
SARS-CoV-2 NC_045512.2 (22700) TATGGAGTGTCTCCTACTAAATTAAATGATCTCTGCTTTACTAATGTCTATGCAGATTCATTTGTAATTAGAGGTGATGAAGTCAGACAAATCGCTC
```

```
                                                                                                    Section 246
                    (23766) 23766         23780     23790     23800     23810     23820     23830     23840     23850     23862
           HIV-1 HXB2  (9720) -------------------------------------------------------------------------------------------------
SARS-CoV-2 NC_045512.2 (22797) CAGGGCAAACTGGAAAGATTGCTGATTATAATTATAAATTACCAGATGATTTTACAGGCTGCGTTATAGCTTGGAATTCTAACAATCTTGATTCTAA
                                                                                                    Section 247
                    (23863) 23863  23870     23880     23890     23900     23910     23920     23930     23940               23959
           HIV-1 HXB2  (9720) -------------------------------------------------------------------------------------------------
SARS-CoV-2 NC_045512.2 (22894) GGTTGGTGGTAATTATAATTACCTGTATAGATTGTTTAGGAAGTCTAATCTCAAACCTTTTGAGAGAGATATTTCAACTGAAATCTATCAGGCCGGT
                                                                                                    Section 248
                    (23960) 23960     23970     23980     23990     24000     24010     24020     24030     24040           24056
           HIV-1 HXB2  (9720) -------------------------------------------------------------------------------------------------
SARS-CoV-2 NC_045512.2 (22991) AGCACACCTTGTAATGGTGTTGAAGGTTTTAATTGTTACTTTCCTTTACAATCATATGGTTTCCAACCCACTAATGGTGTTGGTTACCAACCATACA
                                                                                                    Section 249
                    (24057) 24057        24070     24080     24090     24100     24110     24120     24130     24140     24153
           HIV-1 HXB2  (9720) -------------------------------------------------------------------------------------------------
SARS-CoV-2 NC_045512.2 (23088) GAGTAGTAGTACTTTCTTTTGAACTTCTACATGCACCAGCAACTGTTTGTGGACCTAAAAAGTCTACTAATTTGGTTAAAAACAAATGTGTCAATTT
                                                                                                    Section 250
                    (24154) 24154 24160     24170     24180     24190     24200     24210     24220     24230     24240 24250
           HIV-1 HXB2  (9720) -------------------------------------------------------------------------------------------------
SARS-CoV-2 NC_045512.2 (23185) CAACTTCAATGGTTTAACAGGCACAGGTGTTCTTACTGAGTCTAACAAAAAGTTTCTGCCTTTCCAACAATTTGGCAGAGACATTGCTGACACTACT
                                                                                                    Section 251
                    (24251) 24251    24260     24270     24280     24290     24300     24310     24320     24330           24347
           HIV-1 HXB2  (9720) -------------------------------------------------------------------------------------------------
SARS-CoV-2 NC_045512.2 (23282) GATGCTGTCCGTGATCCACAGACACTTGAGATTCTTGACATTACACCATGTTCTTTTGGTGGTGTCAGTGTTATAACACCAGGAACAAATACTTCTA
                                                                                                    Section 252
                    (24348) 24348       24360     24370     24380     24390     24400     24410     24420     24430     24444
           HIV-1 HXB2  (9720) -------------------------------------------------------------------------------------------------
SARS-CoV-2 NC_045512.2 (23379) ACCAGGTTGCTGTTCTTTATCAGGATGTTAACTGCACAGAAGTCCCTGTTGCTATTCATGCAGATCAACTTACTCCTACTTGGCGTGTTTATTCTAC
```

```
                                                                                                              Section 253
                           (24445) 24445 24450     24460     24470     24480     24490     24500     24510     24520     24530     24541
                    HIV-1 HXB2 (9720) -------------------------------------------------------------------------------------------------
SARS-CoV-2 NC_045512.2 (23476) AGGTTCTAATGTTTTTCAAACACGTGCAGGCTGTTTAATAGGGGCTGAACATGTCAACAACTCATATGAGTGTGACATACCCATTGGTGCAGGTATA
                                                                                                              Section 254
                           (24542) 24542   24550     24560     24570     24580     24590     24600     24610     24620     24638
                    HIV-1 HXB2 (9720) -------------------------------------------------------------------------------------------------
SARS-CoV-2 NC_045512.2 (23573) TGCGCTAGTTATCAGACTCAGACTAATTCTCCTCGGCGGGCACGTAGTGTAGCTAGTCAATCCATCATTGCCTACACTATGTCACTTGGTGCAGAAA
                                                                                                              Section 255
                           (24639) 24639      24650     24660     24670     24680     24690     24700     24710     24720     24735
                    HIV-1 HXB2 (9720) -------------------------------------------------------------------------------------------------
SARS-CoV-2 NC_045512.2 (23670) ATTCAGTTGCTTACTCTAATAACTCTATTGCCATACCCACAAATTTTACTATTAGTGTTACCACAGAAATTCTACCAGTGTCTATGACCAAGACATC
                                                                                                              Section 256
                           (24736) 24736         24750     24760     24770     24780     24790     24800     24810     24820     24832
                    HIV-1 HXB2 (9720) -------------------------------------------------------------------------------------------------
SARS-CoV-2 NC_045512.2 (23767) AGTAGATTGTACAATGTACATTTGTGGTGATTCAACTGAATGCAGCAATCTTTTGTTGCAATATGGCAGTTTTTGTACACAATTAAACCGTGCTTTA
                                                                                                              Section 257
                           (24833) 24833  24840     24850     24860     24870     24880     24890     24900     24910     24929
                    HIV-1 HXB2 (9720) -------------------------------------------------------------------------------------------------
SARS-CoV-2 NC_045512.2 (23864) ACTGGAATAGCTGTTGAACAAGACAAAAACACCCAAGAAGTTTTTGCACAAGTCAAACAAATTTACAAAACACCACCAATTAAAGATTTTGGTGGTT
                                                                                                              Section 258
                           (24930) 24930     24940     24950     24960     24970     24980     24990     25000     25010     25026
                    HIV-1 HXB2 (9720) -------------------------------------------------------------------------------------------------
SARS-CoV-2 NC_045512.2 (23961) TTAATTTTTCACAAATATTACCAGATCCATCAAAACCAAGCAAGAGGTCATTTATTGAAGATCTACTTTTCAACAAAGTGACACTTGCAGATGCTGG
                                                                                                              Section 259
                           (25027) 25027        25040     25050     25060     25070     25080     25090     25100     25110     25123
                    HIV-1 HXB2 (9720) -------------------------------------------------------------------------------------------------
SARS-CoV-2 NC_045512.2 (24058) CTTCATCAAACAATATGGTGATTGCCTTGGTGATATTGCTGCTAGAGACCTCATTTGTGCACAAAAGTTTAACGGCCTTACTGTTTTGCCACCTTTG
```

```
                                                                    Section 260
                           (25124) 25124 25130 25140 25150 25160 25170 25180 25190 25200 25210 25220
HIV-1 HXB2                  (9720) -------------------------------------------------------------------------------------------------
SARS-CoV-2 NC_045512.2     (24155) CTCACAGATGAAATGATTGCTCAATACACTTCTGCACTGTTAGCGGGTACAATCACTTCTGGTTGGACCTTTGGTGCAGGTGCTGCATTACAAATAC
                                                                    Section 261
                           (25221) 25221 25230 25240 25250 25260 25270 25280 25290 25300 25317
HIV-1 HXB2                  (9720) -------------------------------------------------------------------------------------------------
SARS-CoV-2 NC_045512.2     (24252) CATTTGCTATGCAAATGGCTTATAGGTTTAATGGTATTGGAGTTACACAGAATGTTCTCTATGAGAACCAAAAATTGATTGCCAACCAATTTAATAG
                                                                    Section 262
                           (25318) 25318 25330 25340 25350 25360 25370 25380 25390 25400 25414
HIV-1 HXB2                  (9720) -------------------------------------------------------------------------------------------------
SARS-CoV-2 NC_045512.2     (24349) TGCTATTGGCAAAATTCAAGACTCACTTTCTTCCACAGCAAGTGCACTTGGAAAACTTCAAGATGTGGTCAACCAAAATGCACAAGCTTTAAACACG
                                                                    Section 263
                           (25415) 25415 25420 25430 25440 25450 25460 25470 25480 25490 25500 25511
HIV-1 HXB2                  (9720) -------------------------------------------------------------------------------------------------
SARS-CoV-2 NC_045512.2     (24446) CTTGTTAAACAACTTAGCTCCAATTTTGGTGCAATTTCAAGTGTTTTAAATGATATCCTTTCACGTCTTGACAAAGTTGAGGCTGAAGTGCAAATTG
                                                                    Section 264
                           (25512) 25512 25520 25530 25540 25550 25560 25570 25580 25590 25608
HIV-1 HXB2                  (9720) -------------------------------------------------------------------------------------------------
SARS-CoV-2 NC_045512.2     (24543) ATAGGTTGATCACAGGCAGACTTCAAAGTTTGCAGACATATGTGACTCAACAATTAATTAGAGCTGCAGAAATCAGAGCTTCTGCTAATCTTGCTGC
                                                                    Section 265
                           (25609) 25609 25620 25630 25640 25650 25660 25670 25680 25690 25705
HIV-1 HXB2                  (9720) -------------------------------------------------------------------------------------------------
SARS-CoV-2 NC_045512.2     (24640) TACTAAAATGTCAGAGTGTGTACTTGGACAATCAAAAAGAGTTGATTTTTGTGGAAAGGGCTATCATCTTATGTCCTTCCCTCAGTCAGCACCTCAT
                                                                    Section 266
                           (25706) 25706 25720 25730 25740 25750 25760 25770 25780 25790 25802
HIV-1 HXB2                  (9720) -------------------------------------------------------------------------------------------------
SARS-CoV-2 NC_045512.2     (24737) GGTGTAGTCTTCTTGCATGTGACTTATGTCCCTGCACAAGAAAAGAACTTCACAACTGCTCCTGCCATTTGTCATGATGGAAAAGCACACTTTCCTC
```

```
                                                                                                     Section 267
                           (25803) 25803  25810      25820      25830      25840      25850      25860      25870      25880       25899
                      HIV-1 HXB2 (9720) -------------------------------------------------------------------------------------------------
SARS-CoV-2 NC_045512.2 (24834) GTGAAGGTGTCTTTGTTTCAAATGGCACACACTGGTTTGTAACACAAAGGAATTTTTATGAACCACAAATCATTACTACAGACAACACATTTGTGTC
                                                                                                     Section 268
                           (25900) 25900      25910      25920      25930      25940      25950      25960      25970      25980     25996
                      HIV-1 HXB2 (9720) -------------------------------------------------------------------------------------------------
SARS-CoV-2 NC_045512.2 (24931) TGGTAACTGTGATGTTGTAATAGGAATTGTCAACAACACAGTTTATGATCCTTTGCAACCTGAATTAGACTCATTCAAGGAGGAGTTAGATAAATAT
                                                                                                     Section 269
                           (25997) 25997        26010      26020      26030      26040      26050      26060      26070      26080  26093
                      HIV-1 HXB2 (9720) -------------------------------------------------------------------------------------------------
SARS-CoV-2 NC_045512.2 (25028) TTTAAGAATCATACATCACCAGATGTTGATTTAGGTGACATCTCTGGCATTAATGCTTCAGTTGTAAACATTCAAAAAGAAATTGACCGCCTCAATG
                                                                                                     Section 270
                           (26094) 26094 26100      26110      26120      26130      26140      26150      26160      26170      26180 26190
                      HIV-1 HXB2 (9720) -------------------------------------------------------------------------------------------------
SARS-CoV-2 NC_045512.2 (25125) AGGTTGCCAAGAATTTAAATGAATCTCTCATCGATCTCCAAGAACTTGGAAAGTATGAGCAGTATATAAAATGGCCATGGTACATTTGGCTAGGTTT
                                                                                                     Section 271
                           (26191) 26191    26200      26210      26220      26230      26240      26250      26260      26270       26287
                      HIV-1 HXB2 (9720) -------------------------------------------------------------------------------------------------
SARS-CoV-2 NC_045512.2 (25222) TATAGCTGGCTTGATTGCCATAGTAATGGTGACAATTATGCTTTGCTGTATGACCAGTTGCTGTAGTTGTCTCAAGGGCTGTTGTTCTTGTGGATCC
                                                                                                     Section 272
                           (26288) 26288       26300      26310      26320      26330      26340      26350      26360      26370    26384
                      HIV-1 HXB2 (9720) -------------------------------------------------------------------------------------------------
SARS-CoV-2 NC_045512.2 (25319) TGCTGCAAATTTGATGAAGACGACTCTGAGCCAGTGCTCAAAGGAGTCAAATTACATTACACATAAACGAACTTATGGATTTGTTTATGAGAATCTT
                                                                                                     Section 273
                           (26385) 26385 26390      26400      26410      26420      26430      26440      26450      26460      26470 26481
                      HIV-1 HXB2 (9720) -------------------------------------------------------------------------------------------------
SARS-CoV-2 NC_045512.2 (25416) CACAATTGGAACTGTAACTTTGAAGCAAGGTGAAATCAAGGATGCTACTCCTTCAGATTTTGTTCGCGCTACTGCAACGATACCGATACAAGCCTCA
```

Section 274

```
                          (26482) 26482   26490     26500     26510     26520     26530     26540     26550     26560           26578
                   HIV-1 HXB2 (9720) -------------------------------------------------------------------------------------------------
SARS-CoV-2 NC_045512.2 (25513) CTCCCTTTCGGATGGCTTATTGTTGGCGTTGCACTTCTTGCTGTTTTTCAGAGCGCTTCCAAAATCATAACCCTCAAAAAGAGATGGCAACTAGCAC
```

Section 275

```
                          (26579) 26579      26590     26600     26610     26620     26630     26640     26650     26660          26675
                   HIV-1 HXB2 (9720) -------------------------------------------------------------------------------------------------
SARS-CoV-2 NC_045512.2 (25610) TCTCCAAGGGTGTTCACTTTGTTTGCAACTTGCTGTTGTTGTTTGTAACAGTTTACTCACACCTTTTGCTCGTTGCTGCTGGCCTTGAAGCCCCTTT
```

Section 276

```
                          (26676) 26676         26690     26700     26710     26720     26730     26740     26750     26760       26772
                   HIV-1 HXB2 (9720) -------------------------------------------------------------------------------------------------
SARS-CoV-2 NC_045512.2 (25707) TCTCTATCTTTATGCTTTAGTCTACTTCTTGCAGAGTATAAACTTTGTAAGAATAATAATGAGGCTTTGGCTTTGCTGGAAATGCCGTTCCAAAAAC
```

Section 277

```
                          (26773) 26773  26780     26790     26800     26810     26820     26830     26840     26850               26869
                   HIV-1 HXB2 (9720) -------------------------------------------------------------------------------------------------
SARS-CoV-2 NC_045512.2 (25804) CCATTACTTTATGATGCCAACTATTTTCTTTGCTGGCATACTAATTGTTACGACTATTGTATACCTTACAATAGTGTAACTTCTTCAATTGTCATTA
```

Section 278

```
                          (26870) 26870     26880     26890     26900     26910     26920     26930     26940     26950            26966
                   HIV-1 HXB2 (9720) -------------------------------------------------------------------------------------------------
SARS-CoV-2 NC_045512.2 (25901) CTTCAGGTGATGGCACAACAAGTCCTATTTCTGAACATGACTACCAGATTGGTGGTTATACTGAAAAATGGGAATCTGGAGTAAAAGACTGTGTTGT
```

Section 279

```
                          (26967) 26967        26980     26990     27000     27010     27020     27030     27040     27050         27063
                   HIV-1 HXB2 (9720) -------------------------------------------------------------------------------------------------
SARS-CoV-2 NC_045512.2 (25998) ATTACACAGTTACTTCACTTCAGACTATTACCAGCTGTACTCAACTCAATTGAGTACAGACACTGGTGTTGAACATGTTACCTTCTTCATCTACAAT
```

Section 280

```
                          (27064) 27064 27070     27080     27090     27100     27110     27120     27130     27140     27150    27160
                   HIV-1 HXB2 (9720) -------------------------------------------------------------------------------------------------
SARS-CoV-2 NC_045512.2 (26095) AAAATTGTTGATGAGCCTGAAGAACATGTCCAAATTCACACAATCGACGGTTCATCCGGAGTTGTTAATCCAGTAATGGAACCAATTTATGATGAAC
```

```
                                                                                              Section 281
                          (27161) 27161    27170    27180    27190    27200    27210    27220    27230    27240    27257
                HIV-1 HXB2 (9720) -------------------------------------------------------------------------------------------------
SARS-CoV-2 NC_045512.2 (26192) CGACGACGACTACTAGCGTGCCTTTGTAAGCACAAGCTGATGAGTACGAACTTATGTACTCATTCGTTTCGGAAGAGACAGGTACGTTAATAGTTAA
                                                                                              Section 282
                          (27258) 27258    27270    27280    27290    27300    27310    27320    27330    27340    27354
                HIV-1 HXB2 (9720) -------------------------------------------------------------------------------------------------
SARS-CoV-2 NC_045512.2 (26289) TAGCGTACTTCTTTTTCTTGCTTTCGTGGTATTCTTGCTAGTTACACTAGCCATCCTTACTGCGCTTCGATTGTGTGCGTACTGCTGCAATATTGTT
                                                                                              Section 283
                          (27355) 27355 27360    27370    27380    27390    27400    27410    27420    27430    27440    27451
                HIV-1 HXB2 (9720) -------------------------------------------------------------------------------------------------
SARS-CoV-2 NC_045512.2 (26386) AACGTGAGTCTTGTAAAACCTTCTTTTTACGTTTACTCTCGTGTTAAAAATCTGAATTCTTCTAGAGTTCCTGATCTTCTGGTCTAAACGAACTAAA
                                                                                              Section 284
                          (27452) 27452    27460    27470    27480    27490    27500    27510    27520    27530    27548
                HIV-1 HXB2 (9720) -------------------------------------------------------------------------------------------------
SARS-CoV-2 NC_045512.2 (26483) TATTATATTAGTTTTTCTGTTTGGAACTTTAATTTTAGCCATGGCAGATTCCAACGGTACTATTACCGTTGAAGAGCTTAAAAAGCTCCTTGAACAA
                                                                                              Section 285
                          (27549) 27549    27560    27570    27580    27590    27600    27610    27620    27630    27645
                HIV-1 HXB2 (9720) -------------------------------------------------------------------------------------------------
SARS-CoV-2 NC_045512.2 (26580) TGGAACCTAGTAATAGGTTTCCTATTCCTTACATGGATTTGTCTTCTACAATTTGCCTATGCCAACAGGAATAGGTTTTTGTATATAATTAAGTTAA
                                                                                              Section 286
                          (27646) 27646    27660    27670    27680    27690    27700    27710    27720    27730    27742
                HIV-1 HXB2 (9720) -------------------------------------------------------------------------------------------------
SARS-CoV-2 NC_045512.2 (26677) TTTTCCTCTGGCTGTTATGGCCAGTAACTTTAGCTTGTTTTGTGCTTGCTGCTGTTTACAGAATAAATTGGATCACCGGTGGAATTGCTATCGCAAT
                                                                                              Section 287
                          (27743) 27743 27750    27760    27770    27780    27790    27800    27810    27820    27839
                HIV-1 HXB2 (9720) -------------------------------------------------------------------------------------------------
SARS-CoV-2 NC_045512.2 (26774) GGCTTGTCTTGTAGGCTTGATGTGGCTCAGCTACTTCATTGCTTCTTTCAGACTGTTTGCGCGTACGCGTTCCATGTGGTCATTCAATCCAGAAACT
```

Section 288

```
                           (27840) 27840     27850     27860     27870     27880     27890     27900     27910     27920     27936
                HIV-1 HXB2  (9720) -------------------------------------------------------------------------------------------------
SARS-CoV-2 NC_045512.2 (26871) AACATTCTTCTCAACGTGCCACTCCATGGCACTATTCTGACCAGACCGCTTCTAGAAAGTGAACTCGTAATCGGAGCTGTGATCCTTCGTGGACATC
```

Section 289

```
                           (27937) 27937        27950     27960     27970     27980     27990     28000     28010     28020     28033
                HIV-1 HXB2  (9720) -------------------------------------------------------------------------------------------------
SARS-CoV-2 NC_045512.2 (26968) TTCGTATTGCTGGACACCATCTAGGACGCTGTGACATCAAGGACCTGCCTAAAGAAATCACTGTTGCTACATCACGAACGCTTTCTTATTACAAATT
```

Section 290

```
                           (28034) 28034 28040     28050     28060     28070     28080     28090     28100     28110     28120 28130
                HIV-1 HXB2  (9720) -------------------------------------------------------------------------------------------------
SARS-CoV-2 NC_045512.2 (27065) GGGAGCTTCGCAGCGTGTAGCAGGTGACTCAGGTTTTGCTGCATACAGTCGCTACAGGATTGGCAACTATAAATTAAACACAGACCATTCCAGTAGC
```

Section 291

```
                           (28131) 28131    28140     28150     28160     28170     28180     28190     28200     28210     28227
                HIV-1 HXB2  (9720) -------------------------------------------------------------------------------------------------
SARS-CoV-2 NC_045512.2 (27162) AGTGACAATATTGCTTTGCTTGTACAGTAAGTGACAACAGATGTTTCATCTCGTTGACTTTCAGGTTACTATAGCAGAGATATTACTAATTATTATG
```

Section 292

```
                           (28228) 28228       28240     28250     28260     28270     28280     28290     28300     28310     28324
                HIV-1 HXB2  (9720) -------------------------------------------------------------------------------------------------
SARS-CoV-2 NC_045512.2 (27259) AGGACTTTTAAAGTTTCCATTTGGAATCTTGATTACATCATAAACCTCATAATTAAAAATTTATCTAAGTCACTAACTGAGAATAAATATTCTCAAT
```

Section 293

```
                           (28325) 28325 28330     28340     28350     28360     28370     28380     28390     28400     28410 28421
                HIV-1 HXB2  (9720) -------------------------------------------------------------------------------------------------
SARS-CoV-2 NC_045512.2 (27356) TAGATGAAGAGCAACCAATGGAGATTGATTAAACGAACATGAAAATTATTCTTTTCTTGGCACTGATAACACTCGCTACTTGTGAGCTTTATCACTA
```

Section 294

```
                           (28422) 28422   28430     28440     28450     28460     28470     28480     28490     28500     28518
                HIV-1 HXB2  (9720) -------------------------------------------------------------------------------------------------
SARS-CoV-2 NC_045512.2 (27453) CCAAGAGTGTGTTAGAGGTACAACAGTACTTTTAAAAGAACCTTGCTCTTCTGGAACATACGAGGGCAATTCACCATTTCATCCTCTAGCTGATAAC
```

```
                                                                                                  Section 295
                      (28519) 28519      28530      28540      28550      28560      28570      28580      28590      28600      28615
         HIV-1 HXB2    (9720) -------------------------------------------------------------------------------------------------
SARS-CoV-2 NC_045512.2 (27550) AAATTTGCACTGACTTGCTTTAGCACTCAATTTGCTTTTGCTTGTCCTGACGGCGTAAAACACGTCTATCAGTTACGTGCCAGATCAGTTTCACCTA
                                                                                                  Section 296
                      (28616) 28616         28630      28640      28650      28660      28670      28680      28690      28700    28712
         HIV-1 HXB2    (9720) -------------------------------------------------------------------------------------------------
SARS-CoV-2 NC_045512.2 (27647) AACTGTTCATCAGACAAGAGGAAGTTCAAGAACTTTACTCTCCAATTTTTCTTATTGTTGCGGCAATAGTGTTTATAACACTTTGCTTCACACTCAA
                                                                                                  Section 297
                      (28713) 28713  28720      28730      28740      28750      28760      28770      28780      28790          28809
         HIV-1 HXB2    (9720) -------------------------------------------------------------------------------------------------
SARS-CoV-2 NC_045512.2 (27744) AAGAAAGACAGAATGATTGAACTTTCATTAATTGACTTCTATTTGTGCTTTTTAGCCTTTCTGCTATTCCTTGTTTTAATTATGCTTATTATCTTTT
                                                                                                  Section 298
                      (28810) 28810      28820      28830      28840      28850      28860      28870      28880      28890          28906
         HIV-1 HXB2    (9720) -------------------------------------------------------------------------------------------------
SARS-CoV-2 NC_045512.2 (27841) GGTTCTCACTTGAACTGCAAGATCATAATGAAACTTGTCACGCCTAAACGAACATGAAATTTCTTGTTTTCTTAGGAATCATCACAACTGTAGCTGC
                                                                                                  Section 299
                      (28907) 28907         28920      28930      28940      28950      28960      28970      28980      28990    29003
         HIV-1 HXB2    (9720) -------------------------------------------------------------------------------------------------
SARS-CoV-2 NC_045512.2 (27938) ATTTCACCAAGAATGTAGTTTACAGTCATGTACTCAACATCAACCATATGTAGTTGATGACCCGTGTCCTATTCACTTCTATTCTAAATGGTATATT
                                                                                                  Section 300
                      (29004) 29004 29010      29020      29030      29040      29050      29060      29070      29080      29090 29100
         HIV-1 HXB2    (9720) -------------------------------------------------------------------------------------------------
SARS-CoV-2 NC_045512.2 (28035) AGAGTAGGAGCTAGAAAATCAGCACCTTTAATTGAATTGTGCGTGGATGAGGCTGGTTCTAAATCACCCATTCAGTACATCGATATCGGTAATTATA
                                                                                                  Section 301
                      (29101) 29101    29110      29120      29130      29140      29150      29160      29170      29180          29197
         HIV-1 HXB2    (9720) -------------------------------------------------------------------------------------------------
SARS-CoV-2 NC_045512.2 (28132) CAGTTTCCTGTTTACCTTTTACAATTAATTGCCAGGAACCTAAATTGGGTAGTCTTGTAGTGCGTTGTTCGTTCTATGAAGACTTTTTAGAGTATCA
```

```
                                                                                                  Section 302
                   (29198) 29198      29210     29220     29230     29240     29250     29260     29270     29280    29294
              HIV-1 HXB2    (9720) -------------------------------------------------------------------------------------------------
SARS-CoV-2 NC_045512.2 (28229) TGACGTTCGTGTTGTTTTAGATTTCATCTAAACGAACAAACTAAAATGTCTGATAATGGACCCCAAAATCAGCGAAATGCACCCCGCATTACGTTTG
                                                                                                  Section 303
                   (29295) 29295 29300     29310     29320     29330     29340     29350     29360     29370     29380    29391
              HIV-1 HXB2    (9720) -------------------------------------------------------------------------------------------------
SARS-CoV-2 NC_045512.2 (28326) GTGGACCCTCAGATTCAACTGGCAGTAACCAGAATGGAGAACGCAGTGGGGCGCGATCAAAACAACGTCGGCCCCAAGGTTTACCCAATAATACTGC
                                                                                                  Section 304
                   (29392) 29392   29400     29410     29420     29430     29440     29450     29460     29470           29488
              HIV-1 HXB2    (9720) -------------------------------------------------------------------------------------------------
SARS-CoV-2 NC_045512.2 (28423) GTCTTGGTTCACCGCTCTCACTCAACATGGCAAGGAAGACCTTAAATTCCCTCGAGGACAAGGCGTTCCAATTAACACCAATAGCAGTCCAGATGAC
                                                                                                  Section 305
                   (29489) 29489      29500     29510     29520     29530     29540     29550     29560     29570          29585
              HIV-1 HXB2    (9720) -------------------------------------------------------------------------------------------------
SARS-CoV-2 NC_045512.2 (28520) CAAATTGGCTACTACCGAAGAGCTACCAGACGAATTCGTGGTGGTGACGGTAAAATGAAAGATCTCAGTCCAAGATGGTATTTCTACTACCTAGGAA
                                                                                                  Section 306
                   (29586) 29586         29600     29610     29620     29630     29640     29650     29660     29670    29682
              HIV-1 HXB2    (9720) -------------------------------------------------------------------------------------------------
SARS-CoV-2 NC_045512.2 (28617) CTGGGCCAGAAGCTGGACTTCCCTATGGTGCTAACAAAGACGGCATCATATGGGTTGCAACTGAGGGAGCCTTGAATACACCAAAAGATCACATTGG
                                                                                                  Section 307
                   (29683) 29683  29690     29700     29710     29720     29730     29740     29750     29760           29779
              HIV-1 HXB2    (9720) -------------------------------------------------------------------------------------------------
SARS-CoV-2 NC_045512.2 (28714) CACCCGCAATCCTGCTAACAATGCTGCAATCGTGCTACAACTTCCTCAAGGAACAACATTGCCAAAAGGCTTCTACGCAGAAGGGAGCAGAGGCGGC
                                                                                                  Section 308
                   (29780) 29780     29790     29800     29810     29820     29830     29840     29850     29860          29876
              HIV-1 HXB2    (9720) -------------------------------------------------------------------------------------------------
SARS-CoV-2 NC_045512.2 (28811) AGTCAAGCCTCTTCTCGTTCCTCATCACGTAGTCGCAACAGTTCAAGAAATTCAACTCCAGGCAGCAGTAGGGGAACTTCTCCTGCTAGAATGGCTG
```

```
                                                                                                  Section 309
                    (29877) 29877        29890      29900      29910      29920      29930      29940      29950      29960     29973
        HIV-1 HXB2   (9720) -------------------------------------------------------------------------------------------------
SARS-CoV-2 NC_045512.2 (28908) GCAATGGCGGTGATGCTGCTCTTGCTTTGCTGCTGCTTGACAGATTGAACCAGCTTGAGAGCAAAATGTCTGGTAAAGGCCAACAACAACAAGGCCA
                                                                                                  Section 310
                    (29974) 29974  29980      29990      30000      30010      30020      30030      30040      30050      30060  30070
        HIV-1 HXB2   (9720) -------------------------------------------------------------------------------------------------
SARS-CoV-2 NC_045512.2 (29005) AACTGTCACTAAGAAATCTGCTGCTGAGGCTTCTAAGAAGCCTCGGCAAAAACGTACTGCCACTAAAGCATACAATGTAACACAAGCTTTCGGCAGA
                                                                                                  Section 311
                    (30071) 30071     30080      30090      30100      30110      30120      30130      30140      30150         30167
        HIV-1 HXB2   (9720) -------------------------------------------------------------------------------------------------
SARS-CoV-2 NC_045512.2 (29102) CGTGGTCCAGAACAAACCCAAGGAAATTTTGGGGACCAGGAACTAATCAGACAAGGAACTGATTACAAACATTGGCCGCAAATTGCACAATTTGCCC
                                                                                                  Section 312
                    (30168) 30168       30180      30190      30200      30210      30220      30230      30240      30250      30264
        HIV-1 HXB2   (9720) -------------------------------------------------------------------------------------------------
SARS-CoV-2 NC_045512.2 (29199) CCAGCGCTTCAGCGTTCTTCGGAATGTCGCGCATTGGCATGGAAGTCACACCTTCGGGAACGTGGTTGACCTACACAGGTGCCATCAAATTGGATGA
                                                                                                  Section 313
                    (30265) 30265 30270      30280      30290      30300      30310      30320      30330      30340      30350  30361
        HIV-1 HXB2   (9720) -------------------------------------------------------------------------------------------------
SARS-CoV-2 NC_045512.2 (29296) CAAAGATCCAAATTTCAAAGATCAAGTCATTTTGCTGAATAAGCATATTGACGCATACAAAACATTCCCACCAACAGAGCCTAAAAAGGACAAAAAG
                                                                                                  Section 314
                    (30362) 30362   30370      30380      30390      30400      30410      30420      30430      30440          30458
        HIV-1 HXB2   (9720) -------------------------------------------------------------------------------------------------
SARS-CoV-2 NC_045512.2 (29393) AAGAAGGCTGATGAAACTCAAGCCTTACCGCAGAGACAGAAGAAACAGCAAACTGTGACTCTTCTTCCTGCTGCAGATTTGGATGATTTCTCCAAAC
                                                                                                  Section 315
                    (30459) 30459      30470      30480      30490      30500      30510      30520      30530      30540         30555
        HIV-1 HXB2   (9720) -------------------------------------------------------------------------------------------------
SARS-CoV-2 NC_045512.2 (29490) AATTGCAACAATCCATGAGCAGTGCTGACTCAACTCAGGCCTAAACTCATGCAGACCACACAAGGCAGATGGGCTATATAAACGTTTTCGCTTTTCC
```

```
                                                                                                     Section 316
                            (30556) 30556         30570     30580     30590     30600     30610     30620     30630     30640     30652
HIV-1 HXB2                   (9720) -------------------------------------------------------------------------------------------------
SARS-CoV-2 NC_045512.2      (29587) GTTTACGATATATAGTCTACTCTTGTGCAGAATGAATTCTCGTAACTACATAGCACAAGTAGATGTAGTTAACTTTAATCTCACATAGCAATCTTTA
                                                                                                     Section 317
                            (30653) 30653  30660     30670     30680     30690     30700     30710     30720     30730               30749
HIV-1 HXB2                   (9720) -------------------------------------------------------------------------------------------------
SARS-CoV-2 NC_045512.2      (29684) ATCAGTGTGTAACATTAGGGAGGACTTGAAAGAGCCACCACATTTTCACCGAGGCCACGCGGAGTACGATCGAGTGTACAGTGAACAATGCTAGGGA
                                                                                                     Section 318
                            (30750) 30750     30760     30770     30780     30790     30800     30810     30820     30830            30846
HIV-1 HXB2                   (9720) -------------------------------------------------------------------------------------------------
SARS-CoV-2 NC_045512.2      (29781) GAGCTGCCTATATGGAAGAGCCCTAATGTGTAAAATTAATTTTAGTAGTGCTATCCCCATGTGATTTTAATAGCTTCTTAGGAGAATGACAAAAAAA
                                                                                                     Section 319
                            (30847) 30847        30860      30872
HIV-1 HXB2                   (9720) --------------------------
SARS-CoV-2 NC_045512.2      (29878) AAAAAAAAAAAAAAAAAAAAAAAAAA
```
