# Supplementary material for: Ubiquitous Micro-Modular Homologies among Genomes from Viruses to Bacteria to Human Mitochondrial DNA: Platforms for Recombination during Evolution?
Source: Viruses. 2022 Apr 24;14(5):885. doi: 10.3390/v14050885 (PMC9147251; doi:10.3390/v14050885)
Supplement: Supplementary file 1 [file viruses-14-00885-s001.zip › Fig. S2B, SARS-CoV-2 & Sulfolobus turreted icosahedral virus.pdf]

```
                                                                    Section 1
                                              (1) 1        10        20        30        40        50        60        70        80
SARS-CoV-2 Reference Genome NC_045512.2       (1) ATTAAAGGTTTATACCTTCCCAGGTAACAAACCAACCAACTTTCGATCTCTTGTAGATCTGTTCTCTAAACGAACTTTAA
Sulfolobus turreted icosahedral virus NC_005892 (1) --------------------------------------------------------------------------------
                                                                    Section 2
                                             (81) 81       90        100       110       120       130       140       150       160
SARS-CoV-2 Reference Genome NC_045512.2      (81) AATCTGTGTGGCTGTCACTCGGCTGCATGCTTAGTGCACTCACGCAGTATAATTAATAACTAATTACTGTCGTTGACAGG
Sulfolobus turreted icosahedral virus NC_005892 (1) --------------------------------------------------------------------------------
                                                                    Section 3
                                            (161) 161      170       180       190       200       210       220       230       240
SARS-CoV-2 Reference Genome NC_045512.2     (161) ACACGAGTAACTCGTCTATCTTCTGCAGGCTGCTTACGGTTTCGTCCGTGTTGCAGCCGATCATCAGCACATCTAGGTTT
Sulfolobus turreted icosahedral virus NC_005892 (1) --------------------------------------------------------------------------------
                                                                    Section 4
                                            (241) 241      250       260       270       280       290       300       310       320
SARS-CoV-2 Reference Genome NC_045512.2     (241) CGTCCGGGTGTGACCGAAAGGTAAGATGGAGAGCCTTGTCCCTGGTTTCAACGAGAAAACACACGTCCAACTCAGTTTGC
Sulfolobus turreted icosahedral virus NC_005892 (1) --------------------------------------------------------------------------------
                                                                    Section 5
                                            (321) 321      330       340       350       360       370       380       390       400
SARS-CoV-2 Reference Genome NC_045512.2     (321) CTGTTTTACAGGTTCGCGACGTGCTCGTACGTGGCTTTGGAGACTCCGTGGAGGAGGTCTTATCAGAGGCACGTCAACAT
Sulfolobus turreted icosahedral virus NC_005892 (1) --------------------------------------------------------------------------------
                                                                    Section 6
                                            (401) 401      410       420       430       440       450       460       470       480
SARS-CoV-2 Reference Genome NC_045512.2     (401) CTTAAAGATGGCACTTGTGGCTTAGTAGAAGTTGAAAAAGGCGTTTTGCCTCAACTTGAACAGCCCTATGTGTTCATCAA
Sulfolobus turreted icosahedral virus NC_005892 (1) --------------------------------------------------------------------------------
                                                                    Section 7
                                            (481) 481      490       500       510       520       530       540       550       560
SARS-CoV-2 Reference Genome NC_045512.2     (481) ACGTTCGGATGCTCGAACTGCACCTCATGGTCATGTTATGGTTGAGCTGGTAGCAGAACTCGAAGGCATTCAGTACGGTC
Sulfolobus turreted icosahedral virus NC_005892 (1) --------------------------------------------------------------------------------
```

Section 8

```
                                              (561) 561      570       580       590       600       610       620       630      640
SARS-CoV-2 Reference Genome NC_045512.2       (561) GTAGTGGTGAGACACTTGGTGTCCTTGTCCCTCATGTGGGCGAAATACCAGTGGCTTACCGCAAGGTTCTTCTTCGTAAG
Sulfolobus turreted icosahedral virus NC_005892 (1) --------------------------------------------------------------------------------
```

Section 9

```
                                              (641) 641      650       660       670       680       690       700       710      720
SARS-CoV-2 Reference Genome NC_045512.2       (641) AACGGTAATAAAGGAGCTGGTGGCCATAGTTACGGCGCCGATCTAAAGTCATTTGACTTAGGCGACGAGCTTGGCACTGA
Sulfolobus turreted icosahedral virus NC_005892 (1) --------------------------------------------------------------------------------
```

Section 10

```
                                              (721) 721      730       740       750       760       770       780       790      800
SARS-CoV-2 Reference Genome NC_045512.2       (721) TCCTTATGAAGATTTTCAAGAAAACTGGAACACTAAACATAGCAGTGGTGTTACCCGTGAACTCATGCGTGAGCTTAACG
Sulfolobus turreted icosahedral virus NC_005892 (1) --------------------------------------------------------------------------------
```

Section 11

```
                                              (801) 801      810       820       830       840       850       860       870      880
SARS-CoV-2 Reference Genome NC_045512.2       (801) GAGGGGCATACACTCGCTATGTCGATAACAACTTCTGTGGCCCTGATGGCTACCCTCTTGAGTGCATTAAAGACCTTCTA
Sulfolobus turreted icosahedral virus NC_005892 (1) --------------------------------------------------------------------------------
```

Section 12

```
                                              (881) 881      890       900       910       920       930       940       950      960
SARS-CoV-2 Reference Genome NC_045512.2       (881) GCACGTGCTGGTAAAGCTTCATGCACTTTGTCCGAACAACTGGACTTTATTGACACTAAGAGGGGTGTATACTGCTGCCG
Sulfolobus turreted icosahedral virus NC_005892 (1) --------------------------------------------------------------------------------
```

Section 13

```
                                              (961) 961      970       980       990       1000      1010      1020      1030    1040
SARS-CoV-2 Reference Genome NC_045512.2       (961) TGAACATGAGCATGAAATTGCTTGGTACACGGAACGTTCTGAAAAGAGCTATGAATTGCAGACACCTTTTGAAATTAAAT
Sulfolobus turreted icosahedral virus NC_005892 (1) --------------------------------------------------------------------------------
```

Section 14

```
                                             (1041) 1041     1050      1060      1070      1080      1090      1100      1110    1120
SARS-CoV-2 Reference Genome NC_045512.2      (1041) TGGCAAAGAAATTTGACACCTTCAATGGGGAATGTCCAAATTTTGTATTTCCCTTAAATTCCATAATCAAGACTATTCAA
Sulfolobus turreted icosahedral virus NC_005892 (1) --------------------------------------------------------------------------------
```

```
                                                           Section 15
                                            (1121) 1121     1130      1140      1150      1160      1170      1180      1190      1200
SARS-CoV-2 Reference Genome NC_045512.2     (1121) CCAAGGGTTGAAAAGAAAAAGCTTGATGGCTTTATGGGTAGAATTCGATCTGTCTATCCAGTTGCGTCACCAAATGAATG
Sulfolobus turreted icosahedral virus  NC_005892 (1) --------------------------------------------------------------------------------
                                                           Section 16
                                            (1201) 1201     1210      1220      1230      1240      1250      1260      1270      1280
SARS-CoV-2 Reference Genome NC_045512.2     (1201) CAACCAAATGTGCCTTTCAACTCTCATGAAGTGTGATCATTGTGGTGAAACTTCATGGCAGACGGGCGATTTTGTTAAAG
Sulfolobus turreted icosahedral virus  NC_005892 (1) --------------------------------------------------------------------------------
                                                           Section 17
                                            (1281) 1281     1290      1300      1310      1320      1330      1340      1350      1360
SARS-CoV-2 Reference Genome NC_045512.2     (1281) CCACTTGCGAATTTTGTGGCACTGAGAATTTGACTAAAGAAGGTGCCACTACTTGTGGTTACTTACCCCAAAATGCTGTT
Sulfolobus turreted icosahedral virus  NC_005892 (1) --------------------------------------------------------------------------------
                                                           Section 18
                                            (1361) 1361     1370      1380      1390      1400      1410      1420      1430      1440
SARS-CoV-2 Reference Genome NC_045512.2     (1361) GTTAAAATTTATTGTCCAGCATGTCACAATTCAGAAGTAGGACCTGAGCATAGTCTTGCCGAATACCATAATGAATCTGG
Sulfolobus turreted icosahedral virus  NC_005892 (1) --------------------------------------------------------------------------------
                                                           Section 19
                                            (1441) 1441     1450      1460      1470      1480      1490      1500      1510      1520
SARS-CoV-2 Reference Genome NC_045512.2     (1441) CTTGAAAACCATTCTTCGTAAGGGTGGTCGCACTATTGCCTTTGGAGGCTGTGTGTTCTCTTATGTTGGTTGCCATAACA
Sulfolobus turreted icosahedral virus  NC_005892 (1) --------------------------------------------------------------------------------
                                                           Section 20
                                            (1521) 1521     1530      1540      1550      1560      1570      1580      1590      1600
SARS-CoV-2 Reference Genome NC_045512.2     (1521) AGTGTGCCTATTGGGTTCCACGTGCTAGCGCTAACATAGGTTGTAACCATACAGGTGTTGTTGGAGAAGGTTCCGAAGGT
Sulfolobus turreted icosahedral virus  NC_005892 (1) --------------------------------------------------------------------------------
                                                           Section 21
                                            (1601) 1601     1610      1620      1630      1640      1650      1660      1670      1680
SARS-CoV-2 Reference Genome NC_045512.2     (1601) CTTAATGACAACCTTCTTGAAATACTCCAAAAAGAGAAAGTCAACATCAATATTGTTGGTGACTTTAAACTTAATGAAGA
Sulfolobus turreted icosahedral virus  NC_005892 (1) --------------------------------------------------------------------------------
```

```
                                                                                          Section 22
                                          (1681) 1681     1690      1700      1710      1720      1730      1740      1750    1760
SARS-CoV-2 Reference Genome NC_045512.2   (1681) GATCGCCATTATTTTGGCATCTTTTTCTGCTTCCACAAGTGCTTTTGTGGAAACTGTGAAAGGTTTGGATTATAAAGCAT
Sulfolobus turreted icosahedral virus  NC_005892 (1) --------------------------------------------------------------------------------
                                                                                          Section 23
                                          (1761) 1761     1770      1780      1790      1800      1810      1820      1830    1840
SARS-CoV-2 Reference Genome NC_045512.2   (1761) TCAAACAAATTGTTGAATCCTGTGGTAATTTTAAAGTTACAAAAGGAAAAGCTAAAAAAGGTGCCTGGAATATTGGTGAA
Sulfolobus turreted icosahedral virus  NC_005892 (1) --------------------------------------------------------------------------------
                                                                                          Section 24
                                          (1841) 1841     1850      1860      1870      1880      1890      1900      1910    1920
SARS-CoV-2 Reference Genome NC_045512.2   (1841) CAGAAATCAATACTGAGTCCTCTTTATGCATTTGCATCAGAGGCTGCTCGTGTTGTACGATCAATTTTCTCCCGCACTCT
Sulfolobus turreted icosahedral virus  NC_005892 (1) --------------------------------------------------------------------------------
                                                                                          Section 25
                                          (1921) 1921     1930      1940      1950      1960      1970      1980      1990    2000
SARS-CoV-2 Reference Genome NC_045512.2   (1921) TGAAACTGCTCAAAATTCTGTGCGTGTTTTACAGAAGGCCGCTATAACAATACTAGATGGAATTTCACAGTATTCACTGA
Sulfolobus turreted icosahedral virus  NC_005892 (1) --------------------------------------------------------------------------------
                                                                                          Section 26
                                          (2001) 2001     2010      2020      2030      2040      2050      2060      2070    2080
SARS-CoV-2 Reference Genome NC_045512.2   (2001) GACTCATTGATGCTATGATGTTCACATCTGATTTGGCTACTAACAATCTAGTTGTAATGGCCTACATTACAGGTGGTGTT
Sulfolobus turreted icosahedral virus  NC_005892 (1) --------------------------------------------------------------------------------
                                                                                          Section 27
                                          (2081) 2081     2090      2100      2110      2120      2130      2140      2150    2160
SARS-CoV-2 Reference Genome NC_045512.2   (2081) GTTCAGTTGACTTCGCAGTGGCTAACTAACATCTTTGGCACTGTTTATGAAAAACTCAAACCCGTCCTTGATTGGCTTGA
Sulfolobus turreted icosahedral virus  NC_005892 (1) --------------------------------------------------------------------------------
                                                                                          Section 28
                                          (2161) 2161     2170      2180      2190      2200      2210      2220      2230    2240
SARS-CoV-2 Reference Genome NC_045512.2   (2161) AGAGAAGTTTAAGGAAGGTGTAGAGTTTCTTAGAGACGGTTGGGAAATTGTTAAATTTATCTCAACCTGTGCTTGTGAAA
Sulfolobus turreted icosahedral virus  NC_005892 (1) --------------------------------------------------------------------------------
```

```
                                                                                                                          Section 29
                                                  (2241) 2241     2250      2260      2270      2280      2290      2300      2310     2320
    SARS-CoV-2 Reference Genome NC_045512.2       (2241) TTGTCGGTGGACAAATTGTCACCTGTGCAAAGGAAATTAAGGAGAGTGTTCAGACATTCTTTAAGCTTGTAAATAAATTT
    Sulfolobus turreted icosahedral virus  NC_005892 (1) --------------------------------------------------------------------------------
                                                                                                                          Section 30
                                                  (2321) 2321     2330      2340      2350      2360      2370      2380      2390     2400
    SARS-CoV-2 Reference Genome NC_045512.2       (2321) TTGGCTTTGTGTGCTGACTCTATCATTATTGGTGGAGCTAAACTTAAAGCCTTGAATTTAGGTGAAACATTTGTCACGCA
    Sulfolobus turreted icosahedral virus  NC_005892 (1) --------------------------------------------------------------------------------
                                                                                                                          Section 31
                                                  (2401) 2401     2410      2420      2430      2440      2450      2460      2470     2480
    SARS-CoV-2 Reference Genome NC_045512.2       (2401) CTCAAAGGGATTGTACAGAAAGTGTGTTAAATCCAGAGAAGAAACTGGCCTACTCATGCCTCTAAAAGCCCCAAAAGAAA
    Sulfolobus turreted icosahedral virus  NC_005892 (1) --------------------------------------------------------------------------------
                                                                                                                          Section 32
                                                  (2481) 2481     2490      2500      2510      2520      2530      2540      2550     2560
    SARS-CoV-2 Reference Genome NC_045512.2       (2481) TTATCTTCTTAGAGGGAGAAACACTTCCCACAGAAGTGTTAACAGAGGAAGTTGTCTTGAAAACTGGTGATTTACAACCA
    Sulfolobus turreted icosahedral virus  NC_005892 (1) --------------------------------------------------------------------------------
                                                                                                                          Section 33
                                                  (2561) 2561     2570      2580      2590      2600      2610      2620      2630     2640
    SARS-CoV-2 Reference Genome NC_045512.2       (2561) TTAGAACAACCTACTAGTGAAGCTGTTGAAGCTCCATTGGTTGGTACACCAGTTTGTATTAACGGGCTTATGTTGCTCGA
    Sulfolobus turreted icosahedral virus  NC_005892 (1) --------------------------------------------------------------------------------
                                                                                                                          Section 34
                                                  (2641) 2641     2650      2660      2670      2680      2690      2700      2710     2720
    SARS-CoV-2 Reference Genome NC_045512.2       (2641) AATCAAAGACACAGAAAAGTACTGTGCCCTTGCACCTAATATGATGGTAACAAACAATACCTTCACACTCAAAGGCGGTG
    Sulfolobus turreted icosahedral virus  NC_005892 (1) --------------------------------------------------------------------------------
                                                                                                                          Section 35
                                                  (2721) 2721     2730      2740      2750      2760      2770      2780      2790     2800
    SARS-CoV-2 Reference Genome NC_045512.2       (2721) CACCAACAAAGGTTACTTTTGGTGATGACACTGTGATAGAAGTGCAAGGTTACAAGAGTGTGAATATCACTTTTGAACTT
    Sulfolobus turreted icosahedral virus  NC_005892 (1) --------------------------------------------------------------------------------
```

```
                                                          Section 36
                                             (2801) 2801     2810      2820      2830      2840      2850      2860      2870     2880
SARS-CoV-2 Reference Genome NC_045512.2      (2801) GATGAAAGGATTGATAAAGTACTTAATGAGAAGTGCTCTGCCTATACAGTTGAACTCGGTACAGAAGTAAATGAGTTCGC
Sulfolobus turreted icosahedral virus  NC_005892 (1) --------------------------------------------------------------------------------
                                                          Section 37
                                             (2881) 2881     2890      2900      2910      2920      2930      2940      2950     2960
SARS-CoV-2 Reference Genome NC_045512.2      (2881) CTGTGTTGTGGCAGATGCTGTCATAAAAACTTTGCAACCAGTATCTGAATTACTTACACCACTGGGCATTGATTTAGATG
Sulfolobus turreted icosahedral virus  NC_005892 (1) --------------------------------------------------------------------------------
                                                          Section 38
                                             (2961) 2961     2970      2980      2990      3000      3010      3020      3030     3040
SARS-CoV-2 Reference Genome NC_045512.2      (2961) AGTGGAGTATGGCTACATACTACTTATTTGATGAGTCTGGTGAGTTTAAATTGGCTTCACATATGTATTGTTCTTTCTAC
Sulfolobus turreted icosahedral virus  NC_005892 (1) --------------------------------------------------------------------------------
                                                          Section 39
                                             (3041) 3041     3050      3060      3070      3080      3090      3100      3110     3120
SARS-CoV-2 Reference Genome NC_045512.2      (3041) CCTCCAGATGAGGATGAAGAAGAAGGTGATTGTGAAGAAGAAGAGTTTGAGCCATCAACTCAATATGAGTATGGTACTGA
Sulfolobus turreted icosahedral virus  NC_005892 (1) --------------------------------------------------------------------------------
                                                          Section 40
                                             (3121) 3121     3130      3140      3150      3160      3170      3180      3190     3200
SARS-CoV-2 Reference Genome NC_045512.2      (3121) AGATGATTACCAAGGTAAACCTTTGGAATTTGGTGCCACTTCTGCTGCTCTTCAACCTGAAGAAGAGCAAGAAGAAGATT
Sulfolobus turreted icosahedral virus  NC_005892 (1) --------------------------------------------------------------------------------
                                                          Section 41
                                             (3201) 3201     3210      3220      3230      3240      3250      3260      3270     3280
SARS-CoV-2 Reference Genome NC_045512.2      (3201) GGTTAGATGATGATAGTCAACAAACTGTTGGTCAACAAGACGGCAGTGAGGACAATCAGACAACTACTATTCAAACAATT
Sulfolobus turreted icosahedral virus  NC_005892 (1) --------------------------------------------------------------------------------
                                                          Section 42
                                             (3281) 3281     3290      3300      3310      3320      3330      3340      3350     3360
SARS-CoV-2 Reference Genome NC_045512.2      (3281) GTTGAGGTTCAACCTCAATTAGAGATGGAACTTACACCAGTTGTTCAGACTATTGAAGTGAATAGTTTTAGTGGTTATTT
Sulfolobus turreted icosahedral virus  NC_005892 (1) --------------------------------------------------------------------------------
```

Section 43

	(3361)	3361 3370 3380 3390 3400 3410 3420 3430 3440
SARS-CoV-2 Reference Genome NC_045512.2	(3361)	AAAACTTACTGACAATGTATACATTAAAAATGCAGACATTGTGGAAGAAGCTAAAAAGGTAAAACCAACAGTGGTTGTTA
Sulfolobus turreted icosahedral virus NC_005892	(1)	--------TAAAATGGGTTTAGGTTCAGAATTTGGAGAT-GTATTAGATGCTTTAAGGAAAAACATAGCAGATAGTTTAG

Section 44

	(3441)	3441 3450 3460 3470 3480 3490 3500 3510 3520
SARS-CoV-2 Reference Genome NC_045512.2	(3441)	ATGCAGCCAATGTTTACCTTAAACATGGAGGAGGTGTTGCAGGAGCCTTAAATAAGGCTACTAACAATGCCATGCAAGTT
Sulfolobus turreted icosahedral virus NC_005892	(72)	CAGAACTAGAAGCGTTAGGAAGAGGCGGAGGAGATGTAGCAGCAGCTAGAGCAGCGGAAGAATTGGCACCCGAAGAAATA

Section 45

	(3521)	3521 3530 3540 3550 3560 3570 3580 3590 3600
SARS-CoV-2 Reference Genome NC_045512.2	(3521)	GAATCTGATGATTACATAGCTACTAATGGACCACTTAAAGTGGGTGGTAGTTGTGTTTTAAGCGGACACAATCTTGCTAA
Sulfolobus turreted icosahedral virus NC_005892	(152)	GCAGCTAGACAATTAGACCTAGCAGATTTCTTACGAAGAATTAGAGGAAAGAGAGCTTTAGCCGAAGAAATAGCACCTAT

Section 46

	(3601)	3601 3610 3620 3630 3640 3650 3660 3670 3680
SARS-CoV-2 Reference Genome NC_045512.2	(3601)	ACACTGTCTTCATGTTGTCGGCCCAAATGTTAACAAAGGTGAAGACATTCAACTTCTTAAGAGTGCTTATGAAAATTTTA
Sulfolobus turreted icosahedral virus NC_005892	(232)	AGCAGATGAAGAAAGAGCATTAAGAGAAGCAGAAATAGCAGATGAAGAAAGAGCGCT-AAGAGAAAGAGTAGGAAGATTG

Section 47

	(3681)	3681 3690 3700 3710 3720 3730 3740 3750 3760
SARS-CoV-2 Reference Genome NC_045512.2	(3681)	ATCAGCACGAAGTTCTACTTGCACCATTATTATCAGCTGGTATTTTTGGTGCTGACCCTATACATTCTTTAAGAGTTTGT
Sulfolobus turreted icosahedral virus NC_005892	(311)	AGTGGCTTGAAAAAGCAGCGTTAGCAGGTTTAGGAAATTGGAGGCGCCGCATTAGGTGGATT-AGCATTATACAGGTTAC

Section 48

	(3761)	3761 3770 3780 3790 3800 3810 3820 3830 3840
SARS-CoV-2 Reference Genome NC_045512.2	(3761)	GTAGATACTGTTCGCACAAATGTCTACTTAGCTGTCTTTGATAAAAATCTCTATGACAAACTTGTTTCAAGCTTTTTGGA
Sulfolobus turreted icosahedral virus NC_005892	(390)	TTGAATAGAAATCAGCCCGGT-TCTCCCTACAATTTCCCAAATCCAAGCTCTCCAACTAACTTACCCCCTCCTTCCAGCC

Section 49

	(3841)	3841 3850 3860 3870 3880 3890 3900 3910 3920
SARS-CoV-2 Reference Genome NC_045512.2	(3841)	AATGAAGAGTGAAA--AGCAAGTTGAACAAAAGATCGCTGAGAT--TCCTAAAGAGGAAGTTAAGCCATTTATAACTGAA
Sulfolobus turreted icosahedral virus NC_005892	(469)	CCTACAACCTTCCTCCAACTAACTTACCAAACTCTCCCGGCGCTCTTGGCGCTGTCCCCGGAATCCCCTCTAGTCCTAGT

```
                                                    Section 50
                                               (3921) 3921     3930      3940      3950      3960      3970      3980      3990     4000
SARS-CoV-2 Reference Genome NC_045512.2        (3917) AGTAAACCTTCAGTTGAACAGAGAAAACAAGATGATAAGAAAATCAAAGCTTGTGTTGAAGAAGTTACAACAACTCTGGA
Sulfolobus turreted icosahedral virus NC_005892 (549) AGCCCAAGCTCTCCAGCGGGCGTAGTAGCAGGCAGCCCAATGGCTATGGCAGGCGTAGTACCAAGCCCAAGCAGCCCT-A

                                                    Section 51
                                               (4001) 4001     4010      4020      4030      4040      4050      4060      4070     4080
SARS-CoV-2 Reference Genome NC_045512.2        (3997) AGAAACTAAGTTCCTCACAGAAAACTTGTTA-------CTTTATATTGACATTAATGGCAATCTTCATCCAGATTC--TG
Sulfolobus turreted icosahedral virus NC_005892 (628) GCTCACCAAGCTCACCAAGTAGTCCATCTTCTGGAAGTCTTGAGGCTGACCTAAACAAAAAGGTATTTGGTGTACCAGTG

                                                    Section 52
                                               (4081) 4081     4090      4100      4110      4120      4130      4140      4150     4160
SARS-CoV-2 Reference Genome NC_045512.2        (4068) CCACTCTTGTTAGTGACATTGACATCACTTTCTTAAAGAAAGATGCTCCATATATAGTGGGTGATGTTGTTCAAG--AGG
Sulfolobus turreted icosahedral virus NC_005892 (708) TGGGTTTGGATAGTTATAGCGCTAGTGATCATAGCTTTAGTCATATATTATATCTATCATAAGAAGAAGCACAAGCACGG

                                                    Section 53
                                               (4161) 4161     4170      4180      4190      4200      4210      4220      4230     4240
SARS-CoV-2 Reference Genome NC_045512.2        (4146) GTGTTTTAACTGCTGTGGTTATACCTACTAAAAAGGCTGGTGGCACTACTGAAATGCTAGCGAAAGCTTTGAGAAAAGTG
Sulfolobus turreted icosahedral virus NC_005892 (788) GTGAGATAGATGGCATTAGGAGATGCATTAGGAGACATTGCTGATGAGATACGAAGTTATTTATCCGATTTATATGGAGG

                                                    Section 54
                                               (4241) 4241     4250      4260      4270      4280      4290      4300      4310     4320
SARS-CoV-2 Reference Genome NC_045512.2        (4226) CCAACAGACAATTATATAACCACTTACCCGGGTCAGGGTTTAAATGGTTACACTGTAGAGGAGGCAAAGACAGTGCTTAA
Sulfolobus turreted icosahedral virus NC_005892 (868) ATTA-AGAAGAGTATATAGC-ACTACGCCGGGTAAAATTCTAGCAATAGGAGGAGCTGCAGGGTTAGGCGCTG-GATTGC

                                                    Section 55
                                               (4321) 4321     4330      4340      4350      4360      4370      4380      4390     4400
SARS-CoV-2 Reference Genome NC_045512.2        (4306) AAAGTGTAAAAGTGCCTTTTACATTCTACCATCTATTATCTCTAATGAGAAGCAAGAAATTCTTGGAACTGTTTCTTGGA
Sulfolobus turreted icosahedral virus NC_005892 (945) TAGGTGCAGGAATTGGCGCCGGAATACACGGTTTCG-AGATAGGAGAATATGGAGGGAATCCGTTAAATCCTTTTGAGTA

                                                    Section 56
                                               (4401) 4401     4410      4420      4430      4440      4450      4460      4470     4480
SARS-CoV-2 Reference Genome NC_045512.2        (4386) ATTTGCGAGAAATGCTTGCACATGCAGAAGAAACACGCAATTAATGCCTGTCTGTGTGGAAACTAAAGCCATAGTTTCA
Sulfolobus turreted icosahedral virus NC_005892 (1024) CTTTTCGT-ACGTGCCATATTCTCCTTACGCCCCAC-CGAATGAAAGAGAACCCCCTCAGAAATTTATAGAAACTACGAG
```

Section 57

	(4481)	4481 4490 4500 4510 4520 4530 4540 4550 4560
SARS-CoV-2 Reference Genome NC_045512.2	(4466)	ACTATACAGCGTAAATATAAGGGTATTAAAATACAAGAGGGTGTGGTTGATTATGGTGCTAGATTTTACTTTTACACCAG
Sulfolobus turreted icosahedral virus NC_005892	(1102)	TCTTGGCGCTTTCTTTAATCCGTTAGTAATTACGATAATCATCATCTTGATCATAGTGATAGTGTTTCTAATAGCTACGC

Section 58

	(4561)	4561 4570 4580 4590 4600 4610 4620 4630 4640
SARS-CoV-2 Reference Genome NC_045512.2	(4546)	TAAAACAACTGTAGCGTCACTTATCAACACACTTAACGATCTAAATGAAACTCTTGTTACAATGCCACTTGGCTATGTAA
Sulfolobus turreted icosahedral virus NC_005892	(1182)	GGAAGTAGGGGGGATGTAAATGAACATTGAAGAAATTCAGTATGAGGTAGGACAGGAGGATCAGATACCTCCC-GCTCAA

Section 59

	(4641)	4641 4650 4660 4670 4680 4690 4700 4710 4720
SARS-CoV-2 Reference Genome NC_045512.2	(4626)	CACATGGCTTAAATT-TGGAAGAAGCTGCTCGGTATATGAGATCTCTCAAAGTGCCAGCTACAGTTTCTGTTTCTTCACC
Sulfolobus turreted icosahedral virus NC_005892	(1261)	ACTAAAACAGTAGTAGTTCAGCAGAATCCCAAGTT-ATTCGGGATTTCA--GTGTG-GATATGGATTATCATTATATTAG

Section 60

	(4721)	4721 4730 4740 4750 4760 4770 4780 4790 4800
SARS-CoV-2 Reference Genome NC_045512.2	(4705)	TGATGCTGTTACAGCGTATAATGGTTATCTTACTTCTTCTTCTAAAACACCT-GAAGAACATTTTATTGAAA-CCATCTC
Sulfolobus turreted icosahedral virus NC_005892	(1337)	TGCTAATTCTGTTAGTTTTGATAATAAAATGAGGTGAAACG-GTGAGAAGATCAAAGAGGTGGATTCAGAAGGCGATTAA

Section 61

	(4801)	4801 4810 4820 4830 4840 4850 4860 4870 4880
SARS-CoV-2 Reference Genome NC_045512.2	(4783)	ACTTGCTGGTTCCTATAAAGATTGGTCCTATTCTGGACAATCTACACAACT--------AGGTATAGAATTTCTTAAGAG
Sulfolobus turreted icosahedral virus NC_005892	(1416)	ACATTACGGAGCTTTGAAAAAATGGTTGAAGAAAGAACATCCTGAATTGTTGAAAGGAAACGGCGAAATAGCGTTTACAA

Section 62

	(4881)	4881 4890 4900 4910 4920 4930 4940 4950 4960
SARS-CoV-2 Reference Genome NC_045512.2	(4855)	AGGTGATAAAAGTGTATATTACACTA---GTAATCCTACCACATTCCACCTAGATGGTGAAGTTA--TCACCTTTGACAA
Sulfolobus turreted icosahedral virus NC_005892	(1496)	AACTTAGAGAATTTTATGAGAAACATAAGGATGAACTTACAGAT-CACCGTA-AGCGTCAAATTAACTTAGCATTTACAT

Section 63

	(4961)	4961 4970 4980 4990 5000 5010 5020 5030 5040
SARS-CoV-2 Reference Genome NC_045512.2	(4930)	TCTTAAGACACTTCTTTCTTTGAGAGAAGTGAGGACTATTAAGGTGTTTACAACAGTAGACAACATTAACCTCCACACGC
Sulfolobus turreted icosahedral virus NC_005892	(1574)	TA-CATAAAATGGCAATGAGAAGGAGGAAGAAGAAAAGTTAAGCGGTGGT--ATAGTGCAATTCTTAGACCCTGACTTGA

Section 64

		5041 5050 5060 5070 5080 5090 5100 5110 5120
	(5041)	
SARS-CoV-2 Reference Genome NC_045512.2	(5010)	AAGTTGTGGACATGTCAATGACATATGGACAACAGTTTGGTCCAACTTATTTGGATGGAGCTGATGTTACTAAAA-TAAA
Sulfolobus turreted icosahedral virus NC_005892	(1651)	ACACTGTCCTTATCACAATCGTAACTGGAATTGTTCTCTACGAATATCATTTTATTCGAAAATATGAGGAAAAGCTAAAC

Section 65

		5121 5130 5140 5150 5160 5170 5180 5190 5200
	(5121)	
SARS-CoV-2 Reference Genome NC_045512.2	(5089)	ACCTCATAATTCACATGAAGGTAAAACATTTTATGTTTTACCTAATGATGACACTCTACGTGTTGAGGCTTTTGAGTACT
Sulfolobus turreted icosahedral virus NC_005892	(1731)	AGGTTAGAGGAGAGAACAAGATGGATGGAATACATAATAAAAGAGAAAGAATTAAAGAAATGATAAGGCATTGGCTCA--

Section 66

		5201 5210 5220 5230 5240 5250 5260 5270 5280
	(5201)	
SARS-CoV-2 Reference Genome NC_045512.2	(5169)	ACCACACAACTGATCCTAGTTTTCTGGGTAGGTACATGTCAGCATTAAATCACACTAAAAAGTGGAAATACCCACAAGTT
Sulfolobus turreted icosahedral virus NC_005892	(1809)	ACTTT--CTTTTTTTCCCATTTGCT--GTCGGG-GATTTAGTCTTTGACTTCCTCAAAT---TCGCCGAAGA-TAAAATT

Section 67

		5281 5290 5300 5310 5320 5330 5340 5350 5360
	(5281)	
SARS-CoV-2 Reference Genome NC_045512.2	(5249)	AATGGTTTAACTTCTATTAAATGGGCAGATAACAACTGTTATCTTGCCACTGCATTGTTAACACTCCAACAAATAGAGTT
Sulfolobus turreted icosahedral virus NC_005892	(1880)	AGACGTAAGAG-----TGCAAGATGATACAATTATTTTTTATATTTCTTCTCC----TTCTCTTTATTGCAAATTAATAA

Section 68

		5361 5370 5380 5390 5400 5410 5420 5430 5440
	(5361)	
SARS-CoV-2 Reference Genome NC_045512.2	(5329)	GAAGTTTAATCCACCTGCTC-TACAAGATGC-TTATTACAGAGCAAGGGCTGGTGAAGCTGCTAACTTTTGTGCACTTAT
Sulfolobus turreted icosahedral virus NC_005892	(1951)	-TAGCAGAAGATAACGATACTATTTATATTCTCGATTCTGAGATGAATACTT-TGAATGAATTGAATGTGGAAGATTTAG

Section 69

		5441 5450 5460 5470 5480 5490 5500 5510 5520
	(5441)	
SARS-CoV-2 Reference Genome NC_045512.2	(5407)	CTTAGCCTACTGTAA-TAAGACAGTAGGTGA-GTTAGGTGATGTTAGAGAAACAATGAGTTACTTGTTTCAACATGCCAA
Sulfolobus turreted icosahedral virus NC_005892	(2029)	GAAAACTTACTCTTTCTGATTTTGTTTATAATAATTGTTTATATTCTCTTTTTAGAGAATT-----TCTCAAACTCTTAG

Section 70

		5521 5530 5540 5550 5560 5570 5580 5590 5600
	(5521)	
SARS-CoV-2 Reference Genome NC_045512.2	(5485)	T-TTAGATTCTTGCA-AAAGAGTCTTGAACGTGGTGTAAAACTTGTGGACAACAGCAGACAACCCTTAAGGGTGTAGA
Sulfolobus turreted icosahedral virus NC_005892	(2104)	TTAGAGTCTAATGCAGAAAAATTTTTAAACTCTTAGTTAGAGTCTATTTTTGAGGAAAAAATATGCCCGGGCATGTAGT

Section 71

```
                                                (5601) 5601      5610           5620           5630           5640           5650           5660           5670      5680
SARS-CoV-2 Reference Genome NC_045512.2         (5563) AGCTGTTATGTACATGGGCACACTTTCTTATGAACAATTTAAGAAAGGTGTTCAGATACCTTGTACGTGTGGTAAACAAG
Sulfolobus turreted icosahedral virus NC_005892 (2184) AGATTTGAGTCAAATTCCAAAATGGCAAAAGGAATTAGTTTTTAATACACTTAA-----GTTGAATCAGTTAAATAAGAC
```

Section 72

```
                                                (5681) 5681      5690           5700           5710           5720           5730           5740           5750      5760
SARS-CoV-2 Reference Genome NC_045512.2         (5643) CTACAAAATATCTAGTACAACAGGAGTCACCTTTTGTTATGATGTCAGCACCACCTGCTCAGTATGAACTTAAGCATGGT
Sulfolobus turreted icosahedral virus NC_005892 (2259) GGGTAGAAGAGGTAG---------ACCTAGCTTCTTCAAATTCGA-AGAGGGATATGTTGTTTTGTACATGATGAAGTAT
```

Section 73

```
                                                (5761) 5761      5770           5780           5790           5800           5810           5820           5830      5840
SARS-CoV-2 Reference Genome NC_045512.2         (5723) ACATTTACTTGTGCTAGTGAGTACACTGGTA-ATTACCAGTGTGGTCACTATAAACATATAACTTCTAAAGAAACTTTGT
Sulfolobus turreted icosahedral virus NC_005892 (2329) ATAT-GGGGTATGAGCGTAAGCAAGATACACGAATACTTGAACGAACA---AGCTAAAGAA--TT----AGGATTTAGTA
```

Section 74

```
                                                (5841) 5841      5850           5860           5870           5880           5890           5900           5910      5920
SARS-CoV-2 Reference Genome NC_045512.2         (5802) ATTGCATAGACGGTGCTTTACTTACAAAGTCCTCAGAATACAAAGGTCCTATTACGGATGTTTTCTACAAAGAAAA-CAG
Sulfolobus turreted icosahedral virus NC_005892 (2399) AGGAGGGGGAAGA----------ATATGTTTCACGTGATTCAA---TTGAAAGAA-GAT-TTGAAGAATTAGAAAAGCAG
```

Section 75

```
                                                (5921) 5921      5930           5940           5950           5960           5970           5980           5990      6000
SARS-CoV-2 Reference Genome NC_045512.2         (5881) TTACACAACAACCATAAAACCAGTTACTTATAAATTGGATGGTGTTGTTTGTACAGAAATTGACCCTAAGTTGGACAATT
Sulfolobus turreted icosahedral virus NC_005892 (2464) ATAGACAGTATAAGGGAAAAATG-GGAGCAGATTGCAAAGCAGCATGAAGAAGATGAAACTAGGGCTAGGCTGG----TT
```

Section 76

```
                                                (6001) 6001      6010           6020           6030           6040           6050           6060           6070      6080
SARS-CoV-2 Reference Genome NC_045512.2         (5961) ATTATA-AGAAAGACAATTCTTATTTCACAGAGCAACCAATTGATCTTGTACCAAACCAACCATATCCAAACGCAAGCTT
Sulfolobus turreted icosahedral virus NC_005892 (2539) GTAA-ATAGATCGGTGTGG--AATGA---------GTTATATGAATATATGTCAAACGTGAAACAGGGTAAAGAGAAGTT
```

Section 77

```
                                                (6081) 6081      6090           6100           6110           6120           6130           6140           6150      6160
SARS-CoV-2 Reference Genome NC_045512.2         (6040) CGATAATTTTAAGTTTGTATGTGATAATATCAAATTTGCTGATGATTTAAACCAGTTAACTGGTTATAAGAAACCTGCTT
Sulfolobus turreted icosahedral virus NC_005892 (2607) TGATACGGAAAAAGTTG-AGAAGTTAAT----------CGAAAGGATTAGCGAATTTAATGGAATA-AAGAGGCAGTTCA
```

```
                                                                                                                Section 78
                                              (6161) 6161     6170      6180      6190      6200      6210      6220      6230     6240
   SARS-CoV-2 Reference Genome NC_045512.2    (6120) CAAGAGAGCTTAAAGTTACATTTTTCCCTGACTTAAATGGTGATGTGGTGGCTATTGATTATAAACACTACACACCCTCT
Sulfolobus turreted icosahedral virus  NC_005892 (2675) GAAGATGGGTTAAA---ACGGTCATTG-GGGCTGCA--GGAAAAGGTGAGTCAATAAGCACATCTCAGTT-ACAAATTAG
                                                                                                                Section 79
                                              (6241) 6241     6250      6260      6270      6280      6290      6300      6310     6320
   SARS-CoV-2 Reference Genome NC_045512.2    (6200) TTTAAGAAAGGAGCTAAATTGTTACATAAACCTATTGTTTGGCATGTTAACAATGCA-ACTAATAAAGCCACGTATAAAC
Sulfolobus turreted icosahedral virus  NC_005892 (2748) AATAAAGAACTGGG-AGACTCTT-TATGAATTTATGTTAAAACAGAGGAAGAAGGCAAACCCG--GAGCAATGGAGTAAA
                                                                                                                Section 80
                                              (6321) 6321     6330      6340      6350      6360      6370      6380      6390     6400
   SARS-CoV-2 Reference Genome NC_045512.2    (6279) CAAATACCTGGTGTATACGTTGTCTTTGGAGCACAAAACCAGTTGAAACATCAAATTCGTTTGATGTACTGAAGTCAGAG
Sulfolobus turreted icosahedral virus  NC_005892 (2824) GAAG--ACT--TTGACGCGTTAA-TATTGTACATTAAAACAGTATTGGGCTATGATGAATCTA--CATAT--CGTCATTT
                                                                                                                Section 81
                                              (6401) 6401     6410      6420      6430      6440      6450      6460      6470     6480
   SARS-CoV-2 Reference Genome NC_045512.2    (6359) GACGCGCAGGGAATGGATAATCTTGCCTGCGAAGATCTAAAACCAGTCTCTGAAGAAGTAGTGGAAAATCCTACCATACA
Sulfolobus turreted icosahedral virus  NC_005892 (2895) --CGCTACTCACATTAAAGATTTTCCGAT---AAATCAAGATCTGGTAAACGAA---TTAAAAGGATTCCATGCAGGGAA
                                                                                                                Section 82
                                              (6481) 6481     6490      6500      6510      6520      6530      6540      6550     6560
   SARS-CoV-2 Reference Genome NC_045512.2    (6439) GAAAGACGTTCTTGAGTGTAATGTGAAAACTACCGAAGTTGTAGGAGACATTATACTTAAACCAGCAATAATAGTTTAA
Sulfolobus turreted icosahedral virus  NC_005892 (2967) GAAGCATCC-CGGCAGAAAAAAGACAAATCTGCATTATTTATATCCCGAAGAATATATAAAAG-TCAGCGAGTTCTGTAA
                                                                                                                Section 83
                                              (6561) 6561     6570      6580      6590      6600      6610      6620      6630     6640
   SARS-CoV-2 Reference Genome NC_045512.2    (6519) AAATTACAGAAGAGGTTGGCCACACAGATCTAATGGCTGCTTATGTAGAC-AATTCTAGTCTTACTATTAAGAAAC---C
Sulfolobus turreted icosahedral virus  NC_005892 (3045) TAGATTCAAAGGAGGAAAAAAAGAGAGATGTAAACGTTTCGAATTAACTCTACAGCTGCATTTAACGACAATGAGCAGGG
                                                                                                                Section 84
                                              (6641) 6641     6650      6660      6670      6680      6690      6700      6710     6720
   SARS-CoV-2 Reference Genome NC_045512.2    (6595) TAATGAATTATCTAGAGTATTAGGTTTGAAAACCCTTGCTACTCATGGTTTAGCTGCTGTTAATAGTGTCCCTTGGATA
Sulfolobus turreted icosahedral virus  NC_005892 (3125) AAGGGAATCATAAAGACA-------TTGAAAAG------TACGGTGTAGATAG-TAGTCTTTTCGGTCTGAAATGGCAAA
```

```
                                                                                                  Section 85
                                               (6721) 6721     6730      6740      6750      6760      6770      6780      6790      6800
    SARS-CoV-2 Reference Genome NC_045512.2    (6675) CTATAGCTAATTATGCTAAGCCTTTTCTTAACAAAGTTGTTAGTACAACTACTAACATAGTTACACGGTGTTTAAACCGT
Sulfolobus turreted icosahedral virus NC_005892 (3191) ACATTGATGT---TGTAAAGAGAACGATTGATGTCTATGAGTCAAAGACGGGAAAGACA-TGGTACGGCAT---AAACTT
                                                                                                  Section 86
                                               (6801) 6801     6810      6820      6830      6840      6850      6860      6870      6880
    SARS-CoV-2 Reference Genome NC_045512.2    (6755) GTTTGTACTAATTATATGCCTTATTTCTTTACTTTATTGCTACAATTGTGTACTTTTACTAGAAGTACAAATTCTAGAAT
Sulfolobus turreted icosahedral virus NC_005892 (3264) AGATCTTCTATTCCC-CGACTTATTGGA-CAATCTATT---ACGATTAAGGAAGGATAGC-GATATATACATTGTGAAAG
                                                                                                  Section 87
                                               (6881) 6881     6890      6900      6910      6920      6930      6940      6950      6960
    SARS-CoV-2 Reference Genome NC_045512.2    (6835) TAAAGC--ATCTATGCCGACTACTATAGCAAAGAATACTGTTAAGAGTGTCGGTAAATTTTGTCTAGAGGCTTCAT-TTA
Sulfolobus turreted icosahedral virus NC_005892 (3338) ATACGCTAGGACTCACATATGAGAGTTACAAAGCATGGTTAAAACAGT-TCAGCAAATTCT---TAGGGAAAGTAGATGA
                                                                                                  Section 88
                                               (6961) 6961     6970      6980      6990      7000      7010      7020      7030      7040
    SARS-CoV-2 Reference Genome NC_045512.2    (6912) ATTATTTGAAGTCACCTAATTTTTCTAAACTGATAAATATTATAATTTGGTTTTTACTATTAAGTGTTT-GCCTAGGTTC
Sulfolobus turreted icosahedral virus NC_005892 (3414) AAGAGGGAGAGGAACATTAACCCCCCATGATATTCGTAGATCTGCCGCTTACTGGCGTATAAATTATCTCGGGTTGCCTC
                                                                                                  Section 89
                                               (7041) 7041     7050      7060      7070      7080      7090      7100      7110      7120
    SARS-CoV-2 Reference Genome NC_045512.2    (6991) TTTAATCTACTCAACCGCTGCTTTAGGTGTTTTAATGTCTAATTTAGGCATGCCTTCTTACTGTACTGGTTACAGAGAAG
Sulfolobus turreted icosahedral virus NC_005892 (3494) TTGAATCGATAA----GCGGATTCGCCGGAGGTGAAAGATATTATAG--------TCCCTTTGGAGTAGCAT--GGGAAG
                                                                                                  Section 90
                                               (7121) 7121     7130      7140      7150      7160      7170      7180      7190      7200
    SARS-CoV-2 Reference Genome NC_045512.2    (7071) GCTATTTGAACTCTACTAATGTCACTATTGCAACCTACT-GTACTGGTTCTATACCTTGTAGTGTTTGTCTTAGTGGTTT
Sulfolobus turreted icosahedral virus NC_005892 (3560) ACCCG----AACACACTCATTTCT-TATTACGCGTCACTCGAAATGAGGCTACAAAAGCTCTTAGTTC---AATTCA---
                                                                                                  Section 91
                                               (7201) 7201     7210      7220      7230      7240      7250      7260      7270      7280
    SARS-CoV-2 Reference Genome NC_045512.2    (7150) AGATTCTTTAGACACCTATCCTTCTTTAGAAACTATACAAATTACCATTTCATCTTTTAAATGGGATTTAACTGCTTTTG
Sulfolobus turreted icosahedral virus NC_005892 (3629) AG--TCTTTAG-CTCAAGAATTAGTTCAGAACCCCGAAAAGGTCAAGCAAAAAGCGTTAAACGC--TCTAAGTTAGAGTT
```

Section 92

	(7281)	7281 7290 7300 7310 7320 7330 7340 7350 7360
SARS-CoV-2 Reference Genome NC_045512.2	(7230)	GCTTAGTTGCAGAGTGGTTTTTGGCATATATTCTTTTCAC--TAGGTTTTTCTATGTACTTGGATTGGCTGCAATCATGC
Sulfolobus turreted icosahedral virus NC_005892	(3704)	TTTCTCTTCAAAAATTTTTATACTTCTATTTTCTATTTAAGTTATGGACGCAGTAATAGCGGCATTGGC---AATGGCGC

Section 93

	(7361)	7361 7370 7380 7390 7400 7410 7420 7430 7440
SARS-CoV-2 Reference Genome NC_045512.2	(7308)	AATTGTTTTTCAGCTATTTTGCAGTACATTTTATTAGTAATTCTTGGCTTATGTGGTTAATAATTAATCTTGTACAAATG
Sulfolobus turreted icosahedral virus NC_005892	(3781)	TTTTTCCTATCGCCTATCTGATTATATCTTCTAAAAATAAGAAG-AGGAGGAGAAGGTAATATCTATTATAG---AAAT-

Section 94

	(7441)	7441 7450 7460 7470 7480 7490 7500 7510 7520
SARS-CoV-2 Reference Genome NC_045512.2	(7388)	GCCCCGATTTCAGCTATGGTTAGAATGTACATCTTCTTTGCATCATTTTATTATGTATGGAAAAGTTATGTGCATGTTGT
Sulfolobus turreted icosahedral virus NC_005892	(3856)	----CTTTTTTAGACTCTAAGACAAAGTATTTTTTGATGCTAGCAGA--AATAGCATTAG-AGGGATTTTTAGCAGCTAT

Section 95

	(7521)	7521 7530 7540 7550 7560 7570 7580 7590 7600
SARS-CoV-2 Reference Genome NC_045512.2	(7468)	AGACGGTTGTAATTCATCAACTTGTATGATGTGTTACAAACGTAATAGAGCAACAAGAGTCGAATGTA---CAACTATTG
Sulfolobus turreted icosahedral virus NC_005892	(3929)	AG---GCGCTATCTCAGCAGTTTTCATTATCGCCGAAGCAGCTCATC-TCTATAACGAGAAGATCAGAAATCAGTCATTC

Section 96

	(7601)	7601 7610 7620 7630 7640 7650 7660 7670 7680
SARS-CoV-2 Reference Genome NC_045512.2	(7545)	TTAATGGTGTTAGAAGGTCCTTTTATGTCTATGCTAATGGAGGTAAAGGCTTTTGCAAACTACACAATTGGAATTGTGTT
Sulfolobus turreted icosahedral virus NC_005892	(4005)	CAAAATGCTATAGACGCGATGGCGAAATCAAC---AGTTGTGGCTACAGAAAGTATTAAGGACACGACAGTTACTGGGAT

Section 97

	(7681)	7681 7690 7700 7710 7720 7730 7740 7750 7760
SARS-CoV-2 Reference Genome NC_045512.2	(7625)	AATTGTGATACATTCTGTGCTGGTAGTACATTTATTAGTGATGAAGTTGCGAGAGACTTGTCACTACAGTTTAAAAGACC
Sulfolobus turreted icosahedral virus NC_005892	(4082)	TAATGCATTA--GTGAAT-ATGGACACTCTCAGAGACGTAAACGATTTGGCAAAGTCG----AAATCTCAAAATCAGCCC

Section 98

	(7761)	7761 7770 7780 7790 7800 7810 7820 7830 7840
SARS-CoV-2 Reference Genome NC_045512.2	(7705)	AATAAATCCTACTGACCAGTCTTCTTACATCGTTGATAGTGTTACAGTGAAGAATGGTTCCATCCATCTTTACTTTGATA
Sulfolobus turreted icosahedral virus NC_005892	(4155)	GCTCA---AAAGTAATATCTTTTTTTTCTTGGCTCGAAGTAATATCTT-----CTTTTTTGGT---ATTTCTCTTTTATA

Section 99

```
                                                     (7841) 7841     7850      7860      7870      7880      7890      7900      7910      7920
    SARS-CoV-2 Reference Genome NC_045512.2          (7785) AAGCTGG--TCAAAAGACTTATGAAAGACATTCTCTCTCTCATTTTGTTAACTTAGACAACCTGAGAGCTAATAACACTA
Sulfolobus turreted icosahedral virus  NC_005892     (4224) TACTCTTTCTCTAACTCTAAGTTAGAGGTAATATGAAAATAAAAATA----CTTAAATCAGATTCGGGATATGGATAGAA
```

Section 100

```
                                                     (7921) 7921     7930      7940      7950      7960      7970      7980      7990      8000
    SARS-CoV-2 Reference Genome NC_045512.2          (7863) AAGGTTCATTGCCTATTAATGTTATAGTTTTTGATGGTAAATCAAAATGTGAAGAATCATCTGCAAAATCAGCGTCTGTT
Sulfolobus turreted icosahedral virus  NC_005892     (4300) ATGTCAGATG----AGGAACTTTTAAGTTTGTTCAATAAATTCTTAGAAAGTGTAA--ATAAGTATAGTGAAGAGGAAGC
```

Section 101

```
                                                     (8001) 8001     8010      8020      8030      8040      8050      8060      8070      8080
    SARS-CoV-2 Reference Genome NC_045512.2          (7943) TACTACAGTCAGCTTATGTGT-CAACCTATACTGTTACTAGATCAGGCATTAGTGTCTGATGTTGGTGATAGTGCGGAAG
Sulfolobus turreted icosahedral virus  NC_005892     (4374) GAAAAAAATGAACGAAAATATGCAAGATATTTTAGGACAAATTCTAGGGTTGGGATAA-ATGACAATGAGAATAATTACA
```

Section 102

```
                                                     (8081) 8081     8090      8100      8110      8120      8130      8140      8150      8160
    SARS-CoV-2 Reference Genome NC_045512.2          (8022) TTGCAGTTAAAATGTTTGATGCTTACGTTAATACGTTTTCATCAACTTTTAACGTACCAATGGAAAAACTCAAAACACTA
Sulfolobus turreted icosahedral virus  NC_005892     (4453) TTTAAGATAGAA----GAAGAACTA--TTAGAACTATTAGA---AAGATATGCGTTACGAT--ATAAATTAAATAGATCA
```

Section 103

```
                                                     (8161) 8161     8170      8180      8190      8200      8210      8220      8230      8240
    SARS-CoV-2 Reference Genome NC_045512.2          (8102) GTTGCAACT-GCAGAAGCTGAACTTGCAAAGAATGTGTCCTTAGACAATGTCTTATCTACTTTTATTTCAGCAGCTCGGC
Sulfolobus turreted icosahedral virus  NC_005892     (4522) GAGGCAATTAGGA-AAGCA-ATCG---AGAT-GTTAGT-AAAAGAAGAATTAGAAAAAGAGACTGTTCCGGTAGCT----
```

Section 104

```
                                                     (8241) 8241     8250      8260      8270      8280      8290      8300      8310      8320
    SARS-CoV-2 Reference Genome NC_045512.2          (8181) AAGGGTTTGTTGATTCAGATGTAGAAACTAAAGATGTTGTTGAATGTCTTAAATTGTCACATCAATCTGACATAGAAGTT
Sulfolobus turreted icosahedral virus  NC_005892     (4591) AAAGTAGAAAAGATAATGAGATTGTGAGCTAAAATGA----GAACGCTTTTTTTATACCTTCGATGGGGAGTGTGAGGC
```

Section 105

```
                                                     (8321) 8321     8330      8340      8350      8360      8370      8380      8390      8400
    SARS-CoV-2 Reference Genome NC_045512.2          (8261) ACTGGCGATAGTTGTAATAACTATATGCTCACCTATAACAAAGTTGAAAACATGACACCCCGTGACCTTGGTGCTTGTAT
Sulfolobus turreted icosahedral virus  NC_005892     (4667) TCCCCC-----TTAT--TGATTTTCTTGTCAAAAATGATATAGAATACGTGATTCTGTCACGTAGAAATCATGTAGCAGT
```

Section 106

	(8401)	8401 8410 8420 8430 8440 8450 8460 8470 8480
SARS-CoV-2 Reference Genome NC_045512.2	(8341)	TGACTGTAGTGCGCGTCATATTAATGCGCAGGTAGCAAAAAGTCACAACATTGCTTTGATATGGAACGTTAAAGATTTCA
Sulfolobus turreted icosahedral virus NC_005892	(4740)	TCA-------GAGGGAAATAGCGTTAGACATGT---------TCTTAGAAATGAAAGATTATGACACGTTAGCATTTTTA

Section 107

	(8481)	8481 8490 8500 8510 8520 8530 8540 8550 8560
SARS-CoV-2 Reference Genome NC_045512.2	(8421)	TGTCATTGTCTGAACAACTACGAAAACAAATACGTAGTGCTGCTAAAAAGAATAACTTACCTTTTAAGTTGACATGTGCA
Sulfolobus turreted icosahedral virus NC_005892	(4804)	GATGAG-----GATGTAGTACCGATAGAGATA------GATTTTCAAAAAGTAGAGGCTAAATTTAATGAGGGGTATGAT

Section 108

	(8561)	8561 8570 8580 8590 8600 8610 8620 8630 8640
SARS-CoV-2 Reference Genome NC_045512.2	(8501)	ACTACTAGACAAGTTGTTAATGTTGTAACAACAAAGATAGCACTTAAGGGTGGTAAAATTGTTAATAATTGGTTGAAGCA
Sulfolobus turreted icosahedral virus NC_005892	(4873)	GTTGTTTGTG--GCTATTATTATT-TAAAGACGCT--TAG-AGGCTACTCTGTTTATAGG---AAGGATTGGGAGAAGGA

Section 109

	(8641)	8641 8650 8660 8670 8680 8690 8700 8710 8720
SARS-CoV-2 Reference Genome NC_045512.2	(8581)	GTTAATTAAAGTTACACTTGTGTTCCTTTTTGTTGCTGCTATTTTCTATTTAATAACACCTGTTCATGTCATGTCTAAAC
Sulfolobus turreted icosahedral virus NC_005892	(4944)	AATATTTGATGGGGAAGTAAACGG--ATGTGGTCTAGGT-TTCACATTTATTAAAAGAGAATTTCTTGAGAAGA-TAAAG

Section 110

	(8721)	8721 8730 8740 8750 8760 8770 8780 8790 8800
SARS-CoV-2 Reference Genome NC_045512.2	(8661)	ATACTGACTTTTCAAGTGAAATCATAGGATACAAGGCTATTGATGGTGGTGTCACTCGTGACATAGCATCTACAGATACT
Sulfolobus turreted icosahedral virus NC_005892	(5020)	AGACCAGCATTCC-----------TAGCGTTCAAGCCTATAGAA----TCCCCGCATTGGATAG-GCGAAGATGTCTACT

Section 111

	(8801)	8801 8810 8820 8830 8840 8850 8860 8870 8880
SARS-CoV-2 Reference Genome NC_045512.2	(8741)	TGTTTTGCTAACAAACATGCTGATTTTGACACATGGTTTAGCCAGCGTGGTGGTAGTTATACTAATGACAAAGCTTGCCC
Sulfolobus turreted icosahedral virus NC_005892	(5084)	T-TTTCTCAACGCACAAGCCGCGGACTTACGCTTTAAGCAGTCTGAAAGCATATCATTTTATTGATGAGAGAT-TAGCAC

Section 112

	(8881)	8881 8890 8900 8910 8920 8930 8940 8950 8960
SARS-CoV-2 Reference Genome NC_045512.2	(8821)	ATTGATTGCTGCAGTCATAACAAGAGAAGTGGGTTTTGTCGTGCCTGGTTTGCCTGGCACGATATTACGCACAACTAATG
Sulfolobus turreted icosahedral virus NC_005892	(5162)	-----TTTCTCCAGATAGAA-------AATTAATTTTGCAAAAC---GATCACGTAGC-TAGAATAAAGTAATACTCATG

```
                                                   Section 113
                                        (8961) 8961     8970      8980      8990      9000      9010      9020      9030      9040
SARS-CoV-2 Reference Genome NC_045512.2 (8901) GTGACTTTTTGCATTTCTTACCTAGAGTTTTTAGTGCAGTTGGTAACATCTGTTACACACCATCAAAACTTATAGAG-TA
Sulfolobus turreted icosahedral virus NC_005892 (5226) ATAATTTTTTTACTCTAAGTTCGA--GTTATTATTGATGGGAGAAGTTTTTAAAGAAGTAAAAGAGAAGTTTGAGAGATA

                                                   Section 114
                                        (9041) 9041     9050      9060      9070      9080      9090      9100      9110      9120
SARS-CoV-2 Reference Genome NC_045512.2 (8980) CA---CTGACTTTGCAACATCAGCTTGTGTTTTGGCTGCTGAATGTACAATTTTTAAAGATGCTTCTGGTAAGCCAGTAC
Sulfolobus turreted icosahedral virus NC_005892 (5304) CAAGTTTGACGTAGTTTATGTAGATAGAGAATATCCAGTTTCTTCTAATAATTTAAACGTTTTTTTGAAATAGGAGAAA

                                                   Section 115
                                        (9121) 9121     9130      9140      9150      9160      9170      9180      9190      9200
SARS-CoV-2 Reference Genome NC_045512.2 (9057) CATATTGTTATGATACCAATGTACTAGAAGGTTCTGTTGCTTA-TGAAAG-TTTACGCCCTGACACACGTTATGTGCTCA
Sulfolobus turreted icosahedral virus NC_005892 (5384) GAAATTCGTTT--TCAGGATTGCTTATAAACGAAGGACAAGCAGTGATAGATGT----ACTTTTGCTAAAGAAAAGC-CA

                                                   Section 116
                                        (9201) 9201     9210      9220      9230      9240      9250      9260      9270      9280
SARS-CoV-2 Reference Genome NC_045512.2 (9135) TGGATGGCTCTATTATTCAATTTCCTAACACCTACCTTGAAGGTTCTGTTAGAGTGGTAACAACTTTTGATTCTGAGTAC
Sulfolobus turreted icosahedral virus NC_005892 (5457) CGAAGGACTAAG---TCCAATACCGGGCGAAGGAACGGGAA--TACAATTAAGCGCCGGACAGATTTTGAAGTTCTATAA

                                                   Section 117
                                        (9281) 9281     9290      9300      9310      9320      9330      9340      9350      9360
SARS-CoV-2 Reference Genome NC_045512.2 (9215) TGTAGGCACGGCACTTGTGAAAGATCAGAAGCTGGTGTTTGTGTATCTACTAGTGGTAGATGGGTACTTAACAATGATTA
Sulfolobus turreted icosahedral virus NC_005892 (5532) CGTACCAATTGCTGAGATTATTG-TC-GAATATGACCCTTCT--AACGTTTC-------AGGAGTAAGTAGCAATGTAAA

                                                   Section 118
                                        (9361) 9361     9370      9380      9390      9400      9410      9420      9430      9440
SARS-CoV-2 Reference Genome NC_045512.2 (9295) --TTACAGATCTTTACCAGGAGTTTTCTGTGGTGTAGATGCTGTAAATTTACTTACTAATATGTTTACACCACTAATTCA
Sulfolobus turreted icosahedral virus NC_005892 (5601) GCTGAAAGGGACAATACATCCCTTATTCGAGGTAC-----CATCTCAAATCAGTATCGAAAACTTTCAGCCT--ACTGAA

                                                   Section 119
                                        (9441) 9441     9450      9460      9470      9480      9490      9500      9510      9520
SARS-CoV-2 Reference Genome NC_045512.2 (9373) ACCTATTGGTGCTTTGGACATATCAGCATCTATAGTAGCTGGTGGTATTGTAGCTATCGTAGTAACATGCCTTGCCTACT
Sulfolobus turreted icosahedral virus NC_005892 (5674) AACTATC--TGATTTA-------CAGCGGTTTTGGAACTTCATTACCACAAACATATACAATT--CCAGCCAATGGCTAT
```

```
                                                                                                    Section 120
                                                (9521)  9521     9530      9540      9550      9560      9570      9580      9590     9600
SARS-CoV-2 Reference Genome NC_045512.2         (9453)  ATTTTATGAGGTTTAGAAGAGCTTTTGGTGAATACAGTCATGTAGTTGCCTTTAATACTTTACTATTCCTTATGTCATTC
Sulfolobus turreted icosahedral virus NC_005892 (5743)  TTAATAATAAGCATA-ACGAACACATC---AACAG-GCAATATAGGTCAAATAACACTGACAATCGGCAGTACAACAATG
                                                                                                    Section 121
                                                (9601)  9601     9610      9620      9630      9640      9650      9660      9670     9680
SARS-CoV-2 Reference Genome NC_045512.2         (9533)  ACTGTACTCTGTTTAACACCAGTTTACTCATTCTTACCTGGTGTTTATTCTGTTATTTACTTGTACTTGACATTTTATCT
Sulfolobus turreted icosahedral virus NC_005892 (5818)  ACTTTTAACCTACAGACAGGAGAGAATAAAATTCCAGTT-------ATCGCGGGA---ACACAAATAACG-AATCTGACT
                                                                                                    Section 122
                                                (9681)  9681     9690      9700      9710      9720      9730      9740      9750     9760
SARS-CoV-2 Reference Genome NC_045512.2         (9613)  TACTAATGATGTTTCTTTTTTAGCACATATTCAGTGGATGGTTATGTTCACACCTTTAGTACCTTTCTGGATAACAATTG
Sulfolobus turreted icosahedral virus NC_005892 (5887)  TTAACAAGCTCGTCAGCAATATTAATATATGAAGAG----GTGATATAATGAGTGTTACTACATTG--GGACAAAGTTTT
                                                                                                    Section 123
                                                (9761)  9761     9770      9780      9790      9800      9810      9820      9830     9840
SARS-CoV-2 Reference Genome NC_045512.2         (9693)  C------TTATATCATTTGTATTTCCACAAAGCATTTCTATTGGTTCTTTAGTAATTACCTAAAGAGACGTGTAGTCTTT
Sulfolobus turreted icosahedral virus NC_005892 (5961)  CCAGCTAATGCAAAAGTGAAATATTACTACAAACTTTCAGAG--------AAGCAAGATTTAGACGCGTTTGTAAAC---
                                                                                                    Section 124
                                                (9841)  9841     9850      9860      9870      9880      9890      9900      9910     9920
SARS-CoV-2 Reference Genome NC_045512.2         (9767)  AATGGTGTTTCCTTTAGTACTTTTGAAGAAGCTGCGCTGTGCACCTTTTTGTTAAATAAAGAAATGTATCTAAAGTTGCG
Sulfolobus turreted icosahedral virus NC_005892 (6030)  AGTATATTTGTAGGGAGCTATAAATTA-AAACAAATATCGTATTTATTGTATGGGAACACAAAAATAGTCTCAGCTCCAG
                                                                                                    Section 125
                                                (9921)  9921     9930      9940      9950      9960      9970      9980      9990    10000
SARS-CoV-2 Reference Genome NC_045512.2         (9847)  TAGTGATGTGCTATTACCTCTTACGCAATATAATAGATACTTAGCTCTTTATAATAAGTACAAGTATTTTAGTGGAGCAA
Sulfolobus turreted icosahedral virus NC_005892 (6109)  TCGTGCCTTTGGGGCCAAACGCATCAATTATTATAGACGACGAGCT---TCAAGAAGGACTA--TATTTAATTCGT----
                                                                                                    Section 126
                                               (10001)  10001    10010     10020     10030     10040     10050     10060     10070   10080
SARS-CoV-2 Reference Genome NC_045512.2         (9927)  TGGATACAACTAGCTACAGAGAAGCTGCTTGTTGTCATCTCGCAAAGGCTCTCAATGACTTCAGTAACTCAGGTTCTGAT
Sulfolobus turreted icosahedral virus NC_005892 (6180)  ---ATAAAAGTATATAATACAAATTCATTTTCTGTCACAGTAACACCATTTTTCAATA----ATAATAATACAATGACAT
```

```
                                                                                                   Section 127
                                                (10081) 10081    10090     10100     10110     10120     10130     10140     10150   10160
SARS-CoV-2 Reference Genome NC_045512.2         (10007) GTTCTTTACCAACCACCACAAACCTCTATCACCTCAGCTGTTTTGCAGAGTGGTTTTAGAAAAATGGCATTCCCATCTGG
Sulfobus turreted icosahedral virus NC_005892  (6253) ATTCTATTGGAGCAAATTCAGAGTTCGAG-ATTTATGATATATTTACAAAAGAACAAGGAAATATC-TATTACATTCAGC
                                                                                                   Section 128
                                                (10161) 10161    10170     10180     10190     10200     10210     10220     10230   10240
SARS-CoV-2 Reference Genome NC_045512.2         (10087) TAAAGTTGAGGGTTGTATGGTACAAGTAACTTGTGGTACAACTACACTTAACGGTCTTTGGCTTGATGACGTAG--TTTA
Sulfolobus turreted icosahedral virus NC_005892  (6331) TTCCTCCCGGTTTAGCAA--TACTAG-----------AATTCTCGTTAGAGAGAGTTTTCGAAAAAGGAAATAGAATT-A
                                                                                                   Section 129
                                                (10241) 10241    10250     10260     10270     10280     10290     10300     10310   10320
SARS-CoV-2 Reference Genome NC_045512.2         (10165) CTGTCCAAGACATGTGATCTGCACCTCTGAAGACATGCTTAACCCTAATTATGAAGATTTACTCATTCGTAAGTCTAATC
Sulfolobus turreted icosahedral virus NC_005892  (6397) ATATTCCAAAAATTATTCATACAAGCGGAAATGGATATATAAGTTTTCGATTGAGGAAGGGAACTT------ATGCAATA
                                                                                                   Section 130
                                                (10321) 10321    10330     10340     10350     10360     10370     10380     10390   10400
SARS-CoV-2 Reference Genome NC_045512.2         (10245) ATAATTTCTTGGTACAGGCTGGTAATGTTCAACTCAGGGTTATTGGACATTCTATGCAAAATTGTGTACTTAAGCTTAAG
Sulfolobus turreted icosahedral virus NC_005892  (6471) AAAATGCCTT--ACAGTTATAATA-ATACTACTTCAACTA-CTTTTACAA---ATTTTCAATTCGGGACT--ATATCAAC
                                                                                                   Section 131
                                                (10401) 10401    10410     10420     10430     10440     10450     10460     10470   10480
SARS-CoV-2 Reference Genome NC_045512.2         (10325) GT-TGATACAGCCAATCCTAAGACACCTAAGTATAAGTTTGTTCGCATTCAACCAGGACAGACTTTTTCAGTGTTAGCTT
Sulfolobus turreted icosahedral virus NC_005892  (6542) TTCTGTAGCTA-CCATCCCATTGGTAATAAGTTCGATACCAG-CTAATGGAAGCGGTTCAGGAACTTTCTTAGTT---TA
                                                                                                   Section 132
                                                (10481) 10481    10490     10500     10510     10520     10530     10540     10550   10560
SARS-CoV-2 Reference Genome NC_045512.2         (10404) GTTACAATGGTTCACCATCTGGTGTTTACCAATGTGCTATGAGGCCCAATTTCACTATTAAGGGTTCATTCCTTAATGGT
Sulfolobus turreted icosahedral virus NC_005892  (6617) TTTAAAGATTACGGGCGATTATGAAGATGTAAAGTTTAGTGTGACATACGGTGGTGGTTTAGGCGT--TCCTTTTACATT
                                                                                                   Section 133
                                                (10561) 10561    10570     10580     10590     10600     10610     10620     10630   10640
SARS-CoV-2 Reference Genome NC_045512.2         (10484) TCATGTGGTAGTGTTGGTTTTAACATAGATTATGACTGTGTCTCTTTTTGTTACATGCACCATATGGAATTACCAACTGG
Sulfolobus turreted icosahedral virus NC_005892  (6695) TGGACTGGAAGTAGAAGAAATAAATGAGTTAGTG-----GAAAATACTAATTT--CGTAACTCAAAGCGTAACTCTCAGT
```

Section 134

(10641) 10641 10650 10660 10670 10680 10690 10700 10710 10720
SARS-CoV-2 Reference Genome NC_045512.2 (10564) AGTTCATGC-TGGCACAGA-CTTAGAAGGTAACTTTTATGGACCTTTTGTTGACAGGCAAACAGCACAAGCAGCTGGTAC
Sulfolobus turreted icosahedral virus NC_005892 (6768) GGTTCTCAAGTAACTCAAAGCATACTA---AACGTTCAGGGTTCTGGATCACATCTGAGATTGAAATATGCTAGCGTGAG

Section 135

(10721) 10721 10730 10740 10750 10760 10770 10780 10790 10800
SARS-CoV-2 Reference Genome NC_045512.2 (10642) GGACACAACTATTACAGTTAATGTTTTAGCTTGGTTGTACGCTGCTGTTATAAATGGAGACAGGTGGTTTCTCAATCGAT
Sulfolobus turreted icosahedral virus NC_005892 (6845) CGGGCTAACCACTGCCGTAA--CTCAGTGTCAGCTTCAA-GCTACAAATCTAAATAGAAGCA--------CAACATA---

Section 136

(10801) 10801 10810 10820 10830 10840 10850 10860 10870 10880
SARS-CoV-2 Reference Genome NC_045512.2 (10722) TTACCACAACTCTTAATGACTTTAACCTTGTGGCTATGAAGTACAATTATGAACCTCTAACACAAGACCATGTTGACATA
Sulfolobus turreted icosahedral virus NC_005892 (6911) --TTCAACAGTA--TGGGACTTCATAGCAGGGGG-AAGTAGTACGCCGCCTAGT-TGGGACATAAGAGAAA-TAAATAGT

Section 137

(10881) 10881 10890 10900 10910 10920 10930 10940 10950 10960
SARS-CoV-2 Reference Genome NC_045512.2 (10802) CTAGGACCTCTTTCTGCTCAAACTGGAATTGCCGTTTTAGATATGTGTGCTTCATTAAAAGAATTACTGCAAAATGGTAT
Sulfolobus turreted icosahedral virus NC_005892 (6984) ATTCAGCTGGTAGCAAATGGTGGTTCATCAACATCAAGTGTTACAATTACATTAATATTAGTTTACGAACAAATAGCGGG

Section 138

(10961) 10961 10970 10980 10990 11000 11010 11020 11030 11040
SARS-CoV-2 Reference Genome NC_045512.2 (10882) GAATGGACGTACCATATTGGGTAGTGCTTTATTAGAAGATGAATTTACACCTTTTGATGTTGTTAGACAATGC-TCAGGT
Sulfolobus turreted icosahedral virus NC_005892 (7064) AGAACTAAGCTA-ATTTCCCCCCGTACCCCCCGGCGGG--GAATCTTTTTCTTTTATCTTTCTT---TAATTAGTAAATT

Section 139

(11041) 11041 11050 11060 11070 11080 11090 11100 11110 11120
SARS-CoV-2 Reference Genome NC_045512.2 (10961) GTTACTTTCCAAAGTGCAGTGAAAAGAACAATCAAGGGTACACACCACTGGTTGTTACTCACAATTTTGACTTCACTTTT
Sulfolobus turreted icosahedral virus NC_005892 (7138) GATTATTGC-----GGATTTAAAAAATACTGTCTTCTTTTGTTTTCTCGATTTTTGCCTAAAACTAT---CTCACCTAGG

Section 140

(11121) 11121 11130 11140 11150 11160 11170 11180 11190 11200
SARS-CoV-2 Reference Genome NC_045512.2 (11041) AGTTTTAGTCCAGAGTACTC-AATGGTCTTTGTT-CTTTTTTTTGTATG--AAAATGCCTTTTTACCTTTTGCTATGGGT
Sulfolobus turreted icosahedral virus NC_005892 (7210) TGAATTAGGAATACGTAATTTAAGGGATTTTTGCCATAAATTTTTAGGGCAAAAAGGGGGCAAAAATCTGGATTCGAG

Section 141

(11201) 11201 11210 11220 11230 11240 11250 11260 11270 11280
SARS-CoV-2 Reference Genome NC_045512.2 (11117) ATTATTGCTATGTCTGCTTTTGCAATGATGTTTGTCAAACATAAGCATGCATTTCTCTGTTTGTTTTTGTTACCTTCTCT
Sulfolobus turreted icosahedral virus NC_005892 (7290) TCTAAGTTAAAACGTGGAAGGTTTCTGAAGTTT--CATTCTT--CCATGAAATCTTTCAATTATTGTTGGATTGCGGTGA

Section 142

(11281) 11281 11290 11300 11310 11320 11330 11340 11350 11360
SARS-CoV-2 Reference Genome NC_045512.2 (11197) TGCCACTGTAGCTTATTTTAATATGGTCTATATGCCTGCTAGTTGGGTGATGCGTATTA---T---GACATGGTTGGA-T
Sulfolobus turreted icosahedral virus NC_005892 (7366) AAACACTGAAATATATTCAAAATGGGGTATAT--CAGCCAGCCGAAACCAGCCGTCTAGTATTGGAAAATAGTTTCAAT

Section 143

(11361) 11361 11370 11380 11390 11400 11410 11420 11430 11440
SARS-CoV-2 Reference Genome NC_045512.2 (11270) ATGGTTGATACTAGTTTGTCTGGTTTTAAGCTAAAAGACTGTGTTATGTATGCATCAGCTGTAGTGTTACTAATCCTTAT
Sulfolobus turreted icosahedral virus NC_005892 (7444) TTATCTTGTGATTGGGGGAGGGGTT--GACTGAAAGTATTCACGTTT--ATTTTACAA--TTCAGC--AGGAGATAGAAA

Section 144

(11441) 11441 11450 11460 11470 11480 11490 11500 11510 11520
SARS-CoV-2 Reference Genome NC_045512.2 (11350) GACAGCAAGAACTGTGTATGATGA--TGGTGC-TAGGAGAGTGTGGACACTTATGAATGTCTTGACAC-TCGTTTATAAA
Sulfolobus turreted icosahedral virus NC_005892 (7516) AATTCAAAAA-GTATATGGAAAGGGCTGAGGCTTTTTCTAGGATGTAACACAATAAAT----TGAAAAGTCACGTA-AAA

Section 145

(11521) 11521 11530 11540 11550 11560 11570 11580 11590 11600
SARS-CoV-2 Reference Genome NC_045512.2 (11426) GTTTATTATGGTAATGCTTTAGATCAAGCCATTTCCATGTGGGCTCTTATAATCTCTGTTACTTCTAACTACTCAGGTGT
Sulfolobus turreted icosahedral virus NC_005892 (7590) GCACGTCATTTACGTGTTACAAATCGATTTTTTGCCA---AGGGTCTTGT-------GTTACATTCATGT-TACACG---

Section 146

(11601) 11601 11610 11620 11630 11640 11650 11660 11670 11680
SARS-CoV-2 Reference Genome NC_045512.2 (11506) AGTTACAACTGTCATGTTTTTGGCCAGAGGTATTGTTTTTATGTGTGTTGAGTATTGCCCTATTTTCTTCATAACTGGTA
Sulfolobus turreted icosahedral virus NC_005892 (7656) -GTAAAAATTGTAAA-CGACCACAAAAATGTAACACAATACAGCTACAAAAATGTGGCATAACATTTTTAACATCAGACT

Section 147

(11681) 11681 11690 11700 11710 11720 11730 11740 11750 11760
SARS-CoV-2 Reference Genome NC_045512.2 (11586) ATACACTTCAGTGTATAATGCTAGTTTATTGTTTCTTAGGCTATTTTTGTACTTGTTACTTTGGCCTCTTTTGTTTACTC
Sulfolobus turreted icosahedral virus NC_005892 (7734) CTA-AGTTA-GAGTATA-TGAGGATAAATGTATATCTAAATAAGAAA-GAAGAAGAGAAATTAAACAAGATTTGC-AAAA

```
                                                                              Section 148
                                                  (11761) 11761    11770     11780     11790     11800     11810     11820     11830   11840
  SARS-CoV-2 Reference Genome NC_045512.2         (11666) AACCGCTACTTTAGACTGACT-CTTGGTGTTTATGATTACTTAGTTTCTACACAGGAGTTTAGATATATGAATTCACAGG
  Sulfolobus turreted icosahedral virus NC_005892  (7809) ATTTTAAATCAAAAAGCGAATGCATAAAGAAGATAATTGAAGAATAT-GAGAATTGTGTAACACTTGATGAATTAATACT
                                                                              Section 149
                                                  (11841) 11841    11850     11860     11870     11880     11890     11900     11910   11920
  SARS-CoV-2 Reference Genome NC_045512.2         (11745) GACTACTCCCACCCAAGAATAGCATAGATGCCTTCAAACTCAACATTAAATTGTTGGGTGTTGGTGGCAAACCTTGTATC
  Sulfolobus turreted icosahedral virus NC_005892  (7888) AACACTTTCTTCATTAGAA--GAAAAGAT-AGACTATATTAAGCAAAGAATAGACGGGCAA-------AAACATTTAAAT
                                                                              Section 150
                                                  (11921) 11921    11930     11940     11950     11960     11970     11980     11990   12000
  SARS-CoV-2 Reference Genome NC_045512.2         (11825) AAAGTAGCCACTGTACAGTCTAAAATGTCAGATGTAAAGTGCACATCAGTAGTCTTACTCTCAGTTTTGCAACAACTCAG
  Sulfolobus turreted icosahedral virus NC_005892  (7958) AGAGT--CTAAGTTAGAGTTTTAAATAGAGGGGATAAAATGGGTAAG----GTATTCCTCAC------GAACGCATTCTC
                                                                              Section 151
                                                  (12001) 12001    12010     12020     12030     12040     12050     12060     12070   12080
  SARS-CoV-2 Reference Genome NC_045512.2         (11905) AGTAGAATCATCATCTAAATTGTGGGCTCAATGTGTCCAGTTACACAA-TGACATTCTCTTAGCTAAAGATACTACTGAA
  Sulfolobus turreted icosahedral virus NC_005892  (8026) CATAAATATGTTAAAAGAATTC---CCTACGACAATTACGATAGATAAGTTAGATGA----AGAAGAC--TTCTGTTTAA
                                                                              Section 152
                                                  (12081) 12081    12090     12100     12110     12120     12130     12140     12150   12160
  SARS-CoV-2 Reference Genome NC_045512.2         (11984) GCCTTTGAAAAAATGGTTTCACTACTTTCTGTTTTGCTTTCCATGCAGGGTGCTGTAGACATAAACAAGCTTTGTGAAGA
  Sulfolobus turreted icosahedral virus NC_005892  (8097) AGCTA-GAGTTAAGG--CTCG-AAGATGGTACATTAATTAACGCG-ATAG---GGCATGATTCAACAATCAATCT-AGTA
                                                                              Section 153
                                                  (12161) 12161    12170     12180     12190     12200     12210     12220     12230   12240
  SARS-CoV-2 Reference Genome NC_045512.2         (12064) AATGCTGGACAACAGGGCAACCTTACAAGCTATAGCCTCAGAGTTTAGTTCCCTTC-CATCATATGCAGCTTTTGCTACT
  Sulfolobus turreted icosahedral virus NC_005892  (8168) AATACGCTA-TGCGGAACTCAACTACAA-----AAGAACAGAGTTGAGGTTAAGATGAATGAAGGAGATGAAGCGTTAAT
                                                                              Section 154
                                                  (12241) 12241    12250     12260     12270     12280     12290     12300     12310   12320
  SARS-CoV-2 Reference Genome NC_045512.2         (12143) GCTCAAGAAGCTTATGAGCA-GGCTGTTGCTAATGGTGATTCTGAAGTTGTTCTTAAAAAGTTGAAGAAGTCTTTGAATG
  Sulfolobus turreted icosahedral virus NC_005892  (8242) AATAATGATAAGTCAGAGACTGGAAGAGGGGAAAGTGCTTTCTGACAAAGAGATTAAGGATATGTATAGACAGGGTAA--
```

```
                                                          Section 155
                                      (12321) 12321    12330     12340     12350     12360     12370     12380     12390   12400
SARS-CoV-2 Reference Genome NC_045512.2 (12222) TGGCTAAATCTGAATTTGACCGTGATGCAGCCATGCAACGTAAGTTGGAA-AAGATGGCTGATCAAGCTATGACCCAAAT
Sulfolobus turreted icosahedral virus NC_005892 (8320) ------AATCTCA-TTTTATG-AGGTGTGGTG----ATG--AAAGTGGAAGAAGATTGGGAAGAAGTAGATGAAATTGAG
                                                          Section 156
                                      (12401) 12401    12410     12420     12430     12440     12450     12460     12470   12480
SARS-CoV-2 Reference Genome NC_045512.2 (12301) GTATAAACAGGCTAGATCTGAGGACAAGAGGGCAAAAGTTACTAGTGCTATGCAGACAATGCTT--TTCACTATGCTTAG
Sulfolobus turreted icosahedral virus NC_005892 (8386) GAAGAAGTAGATGAGATTTGCAGTAGAGTAACGTGCGAAGATTGCGTAGCTGAA--ATCTGCGGAGATCTTTGCG---AA
                                                          Section 157
                                      (12481) 12481    12490     12500     12510     12520     12530     12540     12550   12560
SARS-CoV-2 Reference Genome NC_045512.2 (12379) AAAGTTGGATAATGATGCACTCAACAACATT-ATCAACAATGCAAGAGATG-GTTGTGTTCCCTTGAACATAATACCTCT
Sulfolobus turreted icosahedral virus NC_005892 (8461) ATCCTTTGTGAAGAAG-AACAGGACTTAATGGAGGAGGTGGAATAAGAATGAGTGAAGCTGAAGTTCTCAGAGTTCTACA
                                                          Section 158
                                      (12561) 12561    12570     12580     12590     12600     12610     12620     12630   12640
SARS-CoV-2 Reference Genome NC_045512.2 (12457) TACAACAGCAGCCAAACTAATGGTTGTCATACCAGACTATAACACATATAAAAATACGTGTGATGGTACAACATTTACTT
Sulfolobus turreted icosahedral virus NC_005892 (8540) ATCAATGAAAGATAAAGAACTAGCAGATCTGATA----CGATCGCATATAAGTCAGCCTACTGAGCTGAAACTTCTCATG
                                                          Section 159
                                      (12641) 12641    12650     12660     12670     12680     12690     12700     12710   12720
SARS-CoV-2 Reference Genome NC_045512.2 (12537) ATGCATCAGCATTGTGGGAAATCCAACAGGTTGTAGATGCAGATAGTAAAATTGTTCAACTTAGTGAAATTAGTATGGAC
Sulfolobus turreted icosahedral virus NC_005892 (8616) ATAATGGCAAAGCTAGGGATGACTATAGG--TGAGGGAAAGTTAACGCAATAATTCATGTTAGTACTAATTATGAGGA-
                                                          Section 160
                                      (12721) 12721    12730     12740     12750     12760     12770     12780     12790   12800
SARS-CoV-2 Reference Genome NC_045512.2 (12617) AATTCACCTAATTTAGCATGGCCTCTTATTGTAACAGCTTTAAGGCCAAT-TCTGCTGTCAAATTACAGAATAATGAGC
Sulfolobus turreted icosahedral virus NC_005892 (8693) AAGAGGGTTAGTGTTTCAAAACATCGGAGGGATAATAATTGACGGAGTATTATATGTTTTACAACTAGTATATAATCC--
                                                          Section 161
                                      (12801) 12801    12810     12820     12830     12840     12850     12860     12870   12880
SARS-CoV-2 Reference Genome NC_045512.2 (12696) TTAGTCCTGTTGCACTACGACAGATGTCTTGTGCTGCCGGTACTACACAAACTGCTTGCACTGATGACAATGCGTTAGCT
Sulfolobus turreted icosahedral virus NC_005892 (8771) TAATTTGAATGAAATAAGAATAGAA--CTAGCTCAGC----AAGATATGATAGTATTACA---ATATCAATATTTAGGTG
```

```
                                                        Section 162
                                           (12881) 12881    12890     12900     12910     12920     12930     12940     12950   12960
SARS-CoV-2 Reference Genome NC_045512.2    (12776) TACTACAACACAACAAAGGGAGGTAGGTTTGTA--CTTGCACTGTTATCCGATTTAC-AGGATTTGAAA---TGGGCTAG
Sulfolobus turreted icosahedral virus NC_005892 (8842) AACAAAATGAGAAAAAAGATTAATTTGTATAAATGCTATTGCGGATATCAAACTTACGACTATTTGCAATTATTGAATCA
```

```
                                                        Section 163
                                           (12961) 12961    12970     12980     12990     13000     13010     13020     13030   13040
SARS-CoV-2 Reference Genome NC_045512.2    (12850) ATTCCCTAAGAGTGATGGAACTGGTACTATCTATACAGAACTGGAACCACCTTGTAGGTTTGTTACAGACACACCTAAAG
Sulfolobus turreted icosahedral virus NC_005892 (8922) TTACACGAAGCAT-ATAGAAGAGGAGTT---TGAGGAGATAAAGAAGGAAATAGAAGAAGATAGAAAGAAAGGACTCATT
```

```
                                                        Section 164
                                           (13041) 13041    13050     13060     13070     13080     13090     13100     13110   13120
SARS-CoV-2 Reference Genome NC_045512.2    (12930) GTCCTAAAGTGAAGTATTTATACTTTATTAAAGGATTAAACAACCTAAATAGAGGTATGGTACTTGGTAGTTTAGCTGCC
Sulfolobus turreted icosahedral virus NC_005892 (8998) GAGAGAAAGAGTGTAATTGATGTTAATAAGGAGGCGTTAGGA-CTTGCCTAGCGATATCAT------CAGATTAGGAGAT
```

```
                                                        Section 165
                                           (13121) 13121    13130     13140     13150     13160     13170     13180     13190   13200
SARS-CoV-2 Reference Genome NC_045512.2    (13010) ACAGTACGTCTACAAGCTGGTAATGCAACAGAAGTGCCTGCCAATTCAACTGTATTATCTTTCTGTGCTTTTGCTGTAGA
Sulfolobus turreted icosahedral virus NC_005892 (9071) C-AGTTAAATAAGAAGCAAGCTATA-AA----ATTGATCGATAAGATAAAAGACAAAAAATTAAAAGAATATTTAACAAC
```

```
                                                        Section 166
                                           (13201) 13201    13210     13220     13230     13240     13250     13260     13270   13280
SARS-CoV-2 Reference Genome NC_045512.2    (13090) TGCTGCTAAAGCTTACAAAGATTATCTAGCTAGTGGGGGACAACCAATCACTAATTGTGTTAAGATGTTGTGTACACACA
Sulfolobus turreted icosahedral virus NC_005892 (9145) TTATGTTCAATATAAATATCCGTCTG-ATCTATTAAAGGT-------------ATTGGATTATTTCAACATAAAGACTGA
```

```
                                                        Section 167
                                           (13281) 13281    13290     13300     13310     13320     13330     13340     13350   13360
SARS-CoV-2 Reference Genome NC_045512.2    (13170) CTGGTACTGGTCAGGCAATAACAGTTACACCGGAAGC-CAATATGGATCAAGAATCCTTTGGTGGTGCATCGTGTTGTCT
Sulfolobus turreted icosahedral virus NC_005892 (9211) TGTGGAAT---CGATTGATTACGTAATTACAGAAATCACAATATATAATGAAGATA-TATGGTTAGGGGCAATCATTCAA
```

```
                                                        Section 168
                                           (13361) 13361    13370     13380     13390     13400     13410     13420     13430   13440
SARS-CoV-2 Reference Genome NC_045512.2    (13249) GTACTGCCGTTGCCACATAGATCATCCAAATCCTAAAGGATTTTGTGACTTAAAAGGTAAGTATGTACAAATACCTACAA
Sulfolobus turreted icosahedral virus NC_005892 (9287) AATGGATTGATAT-ACTCA-ATTAGACTGAGATTAAATGATGAT------TATAGTGTGT-----TAGAAATGTACA-AA
```

Section 169

	(13441)	13441 13450 13460 13470 13480 13490 13500 13510 13520
SARS-CoV-2 Reference Genome NC_045512.2	(13329)	CTTGTGCTAATGACCCTGTGGGTTTTACACTTAAAAACACAGTCTGTACCGTCTGCG-GTATGTGGAAAGGTTATGGCTG
Sulfolobus turreted icosahedral virus NC_005892	(9353)	ATTTATTTGATAGCTCCAAT--TTGAAGGGGGAGAGAAGAAAATGGTTAAAATTGAAAAAATATACAATATATATAAGTG

Section 170

	(13521)	13521 13530 13540 13550 13560 13570 13580 13590 13600
SARS-CoV-2 Reference Genome NC_045512.2	(13408)	TAGTTGTGATCAACTCCGCGAACCCATGCTTCAGTCAGCTGATGCACAATCGTTTTTAAACGGGTTTGCGGTGTAAGTGC
Sulfolobus turreted icosahedral virus NC_005892	(9431)	CAATTGTG-------------------GATTTAA----------GACAAATGATTTTATAC---AATTCTTTGAACATTT

Section 171

	(13601)	13601 13610 13620 13630 13640 13650 13660 13670 13680
SARS-CoV-2 Reference Genome NC_045512.2	(13488)	AGCCCGTCTTACACCGTGCGGCACAGGCACTAGTACTGATGTCGTATACAGGGCTTTTGACATCTACAATGATAAAGTAG
Sulfolobus turreted icosahedral virus NC_005892	(9479)	CTTAAATCATAAGGAAATCGAGACAC---GATATGATCAAT---TAAAGAAGGAATT----AG---AAAAAATTAAGGAG

Section 172

	(13681)	13681 13690 13700 13710 13720 13730 13740 13750 13760
SARS-CoV-2 Reference Genome NC_045512.2	(13568)	CTGGTTTTGCTAAATTCCTAAAAACTAATTGTTGTCGCTTCCAAGAAAAGGACGAAGATGACAATTTAATTGATTCTTAC
Sulfolobus turreted icosahedral virus NC_005892	(9546)	GAGG----GAAGACTTGCCCAATG---ACGTTATTCGTTTGGGAGAACGTCTTCAAAAAGA-ACTTTCAGAAATT--GCA

Section 173

	(13761)	13761 13770 13780 13790 13800 13810 13820 13830 13840
SARS-CoV-2 Reference Genome NC_045512.2	(13648)	TTTGTAGTTAAGAGACACACTTTCTCTAACTACCAACATGAAGAAACAATTTATAATTTACTTAAGGATTGTCC-AGCTG
Sulfolobus turreted icosahedral virus NC_005892	(9616)	TTTACAAATAATATAT-----TACAAGAAGGAG-AACCAGATC-------TTAAGAGTACCATAACATTTCTCATAGAGA

Section 174

	(13841)	13841 13850 13860 13870 13880 13890 13900 13910 13920
SARS-CoV-2 Reference Genome NC_045512.2	(13727)	TTGCTAAACATGACTTCTTTAAGTTTAGAATAGACGGTGACATGGTACCACATATATCACGTCAACGTCTTACTAAATAC
Sulfolobus turreted icosahedral virus NC_005892	(9683)	GATATAGGCA--AGCTCAGAAAGAAGC-AGTAAA--GAAAGAAGCGATCACATTAAAGAAG---ATATATAACAAAATAG

Section 175

	(13921)	13921 13930 13940 13950 13960 13970 13980 13990 14000
SARS-CoV-2 Reference Genome NC_045512.2	(13807)	ACAATGGCAGACCTCGTCTATGCTTTAAGGCATTTTGATGAAGGTAATTGTGACACATTAAAAGAAATACTTGTCACATA
Sulfolobus turreted icosahedral virus NC_005892	(9755)	AAGGAGCTAAGTGCATTCAATGTAATAAAACGATACGATTAGGAGAATTATGTTACTTCGATCCAGAAAAT-----CATA

Section 176

```
                                                         14001    14010     14020     14030     14040     14050     14060     14070   14080
                                                (14001)
    SARS-CoV-2 Reference Genome NC_045512.2 (13887) CAATTGTTGTGATGATGATTATTTCAATAAAAAGGACTGGTATGATTTTGTAGAAAACCCAGATATATTACGCGTA--TA
Sulfolobus turreted icosahedral virus  NC_005892  (9830) AAGTTCTATG--TGCTAAATGTTTCGTAAGAAATAACAGTCAAGAGATCATGACAGAAGAAGCGATAAAAGCAGAACTTA
```

Section 177

```
                                                         14081    14090     14100     14110     14120     14130     14140     14150   14160
                                                (14081)
    SARS-CoV-2 Reference Genome NC_045512.2 (13965) CGCCAACTTAGGTGAACGTGTACGCCAAGCTTTGTTAAAAACAGTACAATTCTGTGATGCCATGCGAAATGCTGGTATTG
Sulfolobus turreted icosahedral virus  NC_005892  (9908) AGTTAGCTAGGCTGAAGGAAGAGATCAGAGCGTTAGAATCAGAGAAGAAAAAAATAATGAA---TGAAATGAAGACTGCA
```

Section 178

```
                                                         14161    14170     14180     14190     14200     14210     14220     14230   14240
                                                (14161)
    SARS-CoV-2 Reference Genome NC_045512.2 (14045) TTGGTGTACTGACATTAGATAATCAAGATCTCAATGGTAACTGGTATGATTTCGGTGATTTCATACAAACCACGCCAGGT
Sulfolobus turreted icosahedral virus  NC_005892  (9985) CAAATATACGAAGAGATGAATGAAAAAGCTCAGCTCTTTACTCAAAAAATAGAC---GA---AATGAAAAAATTCT--TT
```

Section 179

```
                                                         14241    14250     14260     14270     14280     14290     14300     14310   14320
                                                (14241)
    SARS-CoV-2 Reference Genome NC_045512.2 (14125) AGTGGAGTTCCTGTTGTAGATTCTTATTATTCATTGTTAATGCCTATATTAACCTTGACCAGGGCTTTAACTGCAGAGTC
Sulfolobus turreted icosahedral virus  NC_005892 (10057) ATGGAATAC----GTAAAAATAGTAAACCCA-AACGAAGACGAGAAAAATCTACTGAAACAAAAATTAGA-TGAAATGGT
```

Section 180

```
                                                         14321    14330     14340     14350     14360     14370     14380     14390   14400
                                                (14321)
    SARS-CoV-2 Reference Genome NC_045512.2 (14205) ACATGTTGACACTGACTTAACAAAGCCTTACATTAAGTGGGAT------TTGTTAAAATATGACTTCACGGAAGAGAGGT
Sulfolobus turreted icosahedral virus  NC_005892 (10131) AGAATTAGAGAAAGAGATCGCAGAGAGTTTTAAACTACTTGCTAAAGCATTGTTGAAGAAGGGGTAAGAAATGGAAAGAG
```

Section 181

```
                                                         14401    14410     14420     14430     14440     14450     14460     14470   14480
                                                (14401)
    SARS-CoV-2 Reference Genome NC_045512.2 (14279) TAAAACTCTTTGACCGTTATTTTAAATATTGGG-ATCAGACATACCACCCAAATTGTGTTAACTGTTTGGA--TGACAGA
Sulfolobus turreted icosahedral virus  NC_005892 (10211) TAATACTTTTATACTCTAACTCAGAGTCTAACATGGAGGGTAAAGAAATGAAAACGCATCTCCTCTTCAAAAATGTAAGA
```

Section 182

```
                                                         14481    14490     14500     14510     14520     14530     14540     14550   14560
                                                (14481)
    SARS-CoV-2 Reference Genome NC_045512.2 (14356) TGCA-TTCTGCATT---GTGCAAACTTTAATGTTTTATTCTCTACAGTGTTCCCACCTACAAGTTTTGGACCACTAGTGA
Sulfolobus turreted icosahedral virus  NC_005892 (10291) AAAGTATCTCAAACAGAGTTTGA-AATAGAATTAGACTGGACTGTAACAGTCTCGTTTAAGATAAAAAACGAAATACT--
```

Section 183

	(14561)	14561 14570 14580 14590 14600 14610 14620 14630 14640
SARS-CoV-2 Reference Genome NC_045512.2	(14432)	GAAAAATATTTGTTGATGGTGTTCCATTTGTA-GTTTCAACTGGATACCACTTCAGAGAGCTAGGTGTTGTACATAATCA
Sulfolobus turreted icosahedral virus NC_005892	(10368)	-AAAGATCATAGAGGAGATAGCT-AAGAAGCAAGGAAAAAATAAATCTGAAGTGATA-AGAGAGGCATTAAAAGAAGAAA

Section 184

	(14641)	14641 14650 14660 14670 14680 14690 14700 14710 14720
SARS-CoV-2 Reference Genome NC_045512.2	(14511)	GGATGTAAACTTACATAGCTCTAGACTTAGTTTTA-AGGAATTACTTGTGTATGCTGCTGACCCTGCTATGCACGCTGCT
Sulfolobus turreted icosahedral virus NC_005892	(10445)	TTGATGAAA--TAAAGAATTTAGGAAGTGGAAGAGTAGTAAGTTTTCGAGCAAAAT----ATAATGAAATTGA-G--ATG

Section 185

	(14721)	14721 14730 14740 14750 14760 14770 14780 14790 14800
SARS-CoV-2 Reference Genome NC_045512.2	(14590)	TCTGGTAATCTATTACTAGATAAACGCACTACGTGCTTTTCAGTAGCTGCACTTACTAACAATGTTGCTTTTCAAACTGT
Sulfolobus turreted icosahedral virus NC_005892	(10516)	ATAGATAATATAGCTAAACA-AAAAGGAGTAAGTAGAACTGATATAATTCATTCTAAAATAGCTAAATATCTTGAAAAGG

Section 186

	(14801)	14801 14810 14820 14830 14840 14850 14860 14870 14880
SARS-CoV-2 Reference Genome NC_045512.2	(14670)	CAAACCCGGTAATTT--TAACAAAGACTTCTATGACTTTGCTGTGTCTAAGGGTTTCTTTAAGGAAGGAAGTTCTGTT-G
Sulfolobus turreted icosahedral virus NC_005892	(10595)	AGGGGATAATGATTGGGTAAGAAAGTCTTCATGCCAGCAGAAGTGATAGAAGAGATTTACAGAAGAAAACGAAGTATTGG

Section 187

	(14881)	14881 14890 14900 14910 14920 14930 14940 14950 14960
SARS-CoV-2 Reference Genome NC_045512.2	(14747)	AATTAAAACACTTCTTCTTTGCTCAGGATGGTAATGCTGCTATCAGCGA--TTATGACTACTATC--GTTATAATCTACC
Sulfolobus turreted icosahedral virus NC_005892	(10675)	AAATAAAAGAGATGCTCTGCATTTGGCAATTTCATCTTTGTTAGAT-GAGGTTGAGAAGGCGAACGAAATAGAACTTATT

Section 188

	(14961)	14961 14970 14980 14990 15000 15010 15020 15030 15040
SARS-CoV-2 Reference Genome NC_045512.2	(14823)	AACAATGTGTGATATCAGAC-AACTACTATTTGTAGTTGAAGTTG-TTGATAAGTACTTTGATTGTTACGATGGTGGCTG
Sulfolobus turreted icosahedral virus NC_005892	(10754)	TGGAATA-AGAAGTTTTGGCTAGGAAAAGTGTATGTCAGAGATAGTAACTTAGAGAGGTTAAGGGTT-TTGGCGCAAAAG

Section 189

	(15041)	15041 15050 15060 15070 15080 15090 15100 15110 15120
SARS-CoV-2 Reference Genome NC_045512.2	(14901)	TATTAATGCTAACCAAGTCATCGTCAACAACCTAGACAAATCAGCTGGTTTTCCATTTAATAAATGGGGTAAGGCTAGAC
Sulfolobus turreted icosahedral virus NC_005892	(10832)	TATAACTGC------AGTTATGGTAG---AGTTTTAC---TAAGCTATATTATCCAAAAGAAAAAGAAAGAAGGA-ATAT

```
                                                                                              Section 190
                                                   (15121) 15121    15130     15140     15150     15160     15170     15180     15190   15200
           SARS-CoV-2 Reference Genome NC_045512.2 (14981) TTTATTATGATTC-AATGAGTTATGAGGATCAAGATGCACTTTTCGCATATACAAAACGTAATGTCATCCCTACTATAAC
Sulfolobus turreted icosahedral virus  NC_005892 (10899) TGGCATGGGGTGGGAAGTAATGGCAAAAGGTAAGAAGGTTTATAAAACTTTTCCTT---TAGATGAAGAAACATTTCAAC
                                                                                              Section 191
                                                   (15201) 15201    15210     15220     15230     15240     15250     15260     15270   15280
           SARS-CoV-2 Reference Genome NC_045512.2 (15060) TCAAATGAATCTTAAGTATGCCATTAGTGCAAAGAATAGAGCTC-GCACCGTAGCTGGTGTCTCTATCTGTAGTACTATG
Sulfolobus turreted icosahedral virus  NC_005892 (10976) T---ATTAGTTAAA-GCACAAGAGAAGGCGAAAGAAAAG---TATGGAATAGA---CACAAAGCTTGGAACAATAAAATT
                                                                                              Section 192
                                                   (15281) 15281    15290     15300     15310     15320     15330     15340     15350   15360
           SARS-CoV-2 Reference Genome NC_045512.2 (15139) ACCAATAGACAGTTTCATCAAAAATTATTGAAATCAATAGCCGCCACTAGAGGAGCTACTGTAGTAATTGGAACAAGCAA
Sulfolobus turreted icosahedral virus  NC_005892 (11046) AGCTTTAAAGAGGCTAATCGAAGATGA---GAATTGATA-TAGA-AGGAGAGAAGTTAGTAGCTGAGATTGAACGGA---
                                                                                              Section 193
                                                   (15361) 15361    15370     15380     15390     15400     15410     15420     15430   15440
           SARS-CoV-2 Reference Genome NC_045512.2 (15219) ATTCTATGGTGGTTGGCACAACATGTTAAAAACTGTTTATAGTGATGTAGAAAACCCTCACCTTATGGGTTGGGATTATC
Sulfolobus turreted icosahedral virus  NC_005892 (11118) ----TACGTTCAATAACAGGAC-TGTCTCAAAAGCAAGTTA-----CTGAACTTCTTTTAAATTATGCGTTAAGA--ATT
                                                                                              Section 194
                                                   (15441) 15441    15450     15460     15470     15480     15490     15500     15510   15520
           SARS-CoV-2 Reference Genome NC_045512.2 (15299) CTAAATGTGATAGAGCCATGCCTAACATGCTTAGAATTATGGCCTCACTTGTTCTTGCTCGCAAACATACAACGTGTTGT
Sulfolobus turreted icosahedral virus  NC_005892 (11186) CC-AGTGTGCAAATATTATGTAGAAAAC----AGATGTATTATTTTTAATG------------AACA-AAAACCTTTGTG
                                                                                              Section 195
                                                   (15521) 15521    15530     15540     15550     15560     15570     15580     15590   15600
           SARS-CoV-2 Reference Genome NC_045512.2 (15379) AGCTTGTCACACCGTTTCTATAGATTAGCTAATGAGTGTGCTCAAGTATTGAGTGAAATGGTCATG-TGTGGCGGTTCAC
Sulfolobus turreted icosahedral virus  NC_005892 (11248) TCTTTTTTGCAAACTTCGT------TAGCATACTATTTTTTTGAAAAATTCATGTAACTCTTCAAACTCTTTCATGCCCT
                                                                                              Section 196
                                                   (15601) 15601    15610     15620     15630     15640     15650     15660     15670   15680
           SARS-CoV-2 Reference Genome NC_045512.2 (15458) TATATGTTAAACCAGGTGGAACCTCATCAGGAGATGCCACAACTGCTTATGCTAATAGTGTTTTTAACATTTGTCAAGCT
Sulfolobus turreted icosahedral virus  NC_005892 (11322) TCTCTGT------AAGAAATAAAACCTTTCCATCCGGCGCTTCTTCTTCCCTTAGTATT-TTATCATCAATT--------
```

```
                                                         Section 197
                                          (15681) 15681    15690     15700     15710     15720     15730     15740     15750   15760
SARS-CoV-2 Reference Genome NC_045512.2   (15538) GTCACGGCCAATGTTAATGCACTTTTATCTACTGATGGTAACAAAATTGCCGATAAGTATGTCCGCAATTTACAACACAG
Sulfolobus turreted icosahedral virus  NC_005892 (11387) ---AATGAATCTATTAAAGGATATAATACACCATCAGAAATGTCTAT-----ATCAA-ATCTCTTCAATA--TCTCGCTT
                                                         Section 198
                                          (15761) 15761    15770     15780     15790     15800     15810     15820     15830   15840
SARS-CoV-2 Reference Genome NC_045512.2   (15618) ACTTTATGAGTGTCTCTATAG--AAATAGAGATGTTGACACAGACTTTGTGAATGAGTTTTACGCATATTTGCGTAAACA
Sulfolobus turreted icosahedral virus  NC_005892 (11456) CTTAGTCGTTTCTTC-TCAAGTCTACTTCCGTTTATTACCAATACTTTCAGAATAATGTAGTAGTTTAT--CCTCATATA
                                                         Section 199
                                          (15841) 15841    15850     15860     15870     15880     15890     15900     15910   15920
SARS-CoV-2 Reference Genome NC_045512.2   (15696) TTTCTCAATGATGATACTCTCTGACGATGCTGTTGTGTGTTTCAATAGCACT-TA---TGCATCTCAAGGTCTA-GTGGC
Sulfolobus turreted icosahedral virus  NC_005892 (11533) CTTTCTGATTTTCATACTTTCAGTTAACTCTGT-GTAGTTTTAAAGTCTACGCTAGACTCTATCTTATAATCCAAGTGGG
                                                         Section 200
                                          (15921) 15921    15930     15940     15950     15960     15970     15980     15990   16000
SARS-CoV-2 Reference Genome NC_045512.2   (15771) TAGCATAAAGAACTTTAAGTCAGTTCTTTATTATCAAAACAATGTTTTTATGTCTGAAG--CAAAATGTTGGACTGAGAC
Sulfolobus turreted icosahedral virus  NC_005892 (11612) AAACAT---GAACGATAATAC--TTATATACTTTCT-AACACAGAGTCTAAGATAGAGGGTAAAAATATGAAAGCGAAAG
                                                         Section 201
                                          (16001) 16001    16010     16020     16030     16040     16050     16060     16070   16080
SARS-CoV-2 Reference Genome NC_045512.2   (15849) TGACCTTACTAAAGGACCT-CATGAATTTTGCTCTCAACATACAATGCTAGTTAAACAGGGTGATGATTATGTGTACCTT
Sulfolobus turreted icosahedral virus  NC_005892 (11686) AAGCAAGAGAAAAGTATCTTCCAAAACTTTATTCTATACAAAGAAATTTAGGAGAAGC-GCTCAC-----AGAGGAGCAG
                                                         Section 202
                                          (16081) 16081    16090     16100     16110     16120     16130     16140     16150   16160
SARS-CoV-2 Reference Genome NC_045512.2   (15928) CCTTACCCAGATCCATCAAGAATCCTAGGGGCCGGCTGTTTTGTAGATGATATCGTAAAAACAGATGGTACACTTATGAT
Sulfolobus turreted icosahedral virus  NC_005892 (11760) CGAAAAGTAGAGCAGGGCGGGAG---AAAGTCAATAG---ATGCATATTATTATA-CTGTAAGGAT--TAGGGCTATTTT
                                                         Section 203
                                          (16161) 16161    16170     16180     16190     16200     16210     16220     16230   16240
SARS-CoV-2 Reference Genome NC_045512.2   (16008) TGAACGGTTCGTGTCTTTAGCTATAGATGCTTACCCACTTACTAAACATCCTAATCAGGAGTATGCTGATGTCTTTCATT
Sulfolobus turreted icosahedral virus  NC_005892 (11831) GAATGAGGTAA---CGGAAGTTATAGAAGCTTTAAAACAATACGATGATGATTTTGACGTTTATGAGGATGAAATCGAAA
```

Section 204
```
                                                       (16241) 16241    16250     16260     16270     16280     16290     16300     16310   16320
               SARS-CoV-2 Reference Genome NC_045512.2 (16088) TGTACTTACAATACATAAGAAAGCTACATGATGAGTTAACAGGACACATGTTAGAC-ATGTATTCTGTTATGCTTACTAA
Sulfolobus turreted icosahedral virus NC_005892 (11908) TGAAACTTAGAGTGATAAGAGAAGAAATAGCAGAACTACCATTGGCGATTTTATATGAGCAAGATAACGATTAATATTAA
```

Section 205
```
                                                       (16321) 16321    16330     16340     16350     16360     16370     16380     16390   16400
               SARS-CoV-2 Reference Genome NC_045512.2 (16167) TGATAACACTTCAAGGTATTGGGAACCTGAG---TTTTATGAGGCTATGTACA-CACCGCATACAGTCTTACAGGCTGTT
Sulfolobus turreted icosahedral virus NC_005892 (11988) AGATAATACA--ATAGAATACGGACACAAAGAATTTGTGCTAAGTAATTTACAAGAAGATATAAAAAATTTGGCAGAAAT
```

Section 206
```
                                                       (16401) 16401    16410     16420     16430     16440     16450     16460     16470   16480
               SARS-CoV-2 Reference Genome NC_045512.2 (16243) GGGGCTTGTGTTCTTTGC---AATTCACAGACTTCATTAAGATGTGGTGCTTGCATACGTAGACCATTCTTATGTTGTAA
Sulfolobus turreted icosahedral virus NC_005892 (12066) AGTTTATCAGTTAGCTAAGTTAATAGAAAAGCTTAGTCAATACGAAGAAGAAG--TAGATACAGAATTATA------TAA
```

Section 207
```
                                                       (16481) 16481    16490     16500     16510     16520     16530     16540     16550   16560
               SARS-CoV-2 Reference Genome NC_045512.2 (16320) ATGCTGTTACGACCATGTCATATCAACATCACATAAATTAGTCTTGTCTGTTAAT-CCGTATGTTTGCAATGCTCCAGGT
Sulfolobus turreted icosahedral virus NC_005892 (12138) TTTATTACACGAGTATGCAATATATCTAGCTGGAGCCACATCTCTCTTTATTGATTCTGAAAATAAGTAATACTCATGAT
```

Section 208
```
                                                       (16561) 16561    16570     16580     16590     16600     16610     16620     16630   16640
               SARS-CoV-2 Reference Genome NC_045512.2 (16399) TGTGATGTCACAGATGTGACTCAACTT-TACTTAGGAGGTATGAGCTATTATTGTAAATCACATAAACCACCCATTAGTT
Sulfolobus turreted icosahedral virus NC_005892 (12218) TTTTTTTAAACTCTTTTTTCGAGTCTAATATTGATGAGTC--AAGCTTCT---GAAAAGAAAGAAGAACAACAGCAAA--
```

Section 209
```
                                                       (16641) 16641    16650     16660     16670     16680     16690     16700     16710   16720
               SARS-CoV-2 Reference Genome NC_045512.2 (16478) TTCCATTGTGTGCTAATGGACAAGTTTTTGGTTTATATAAAAATACATGTGTTGGTAGCGATAATGTTACTGACTTTAAT
Sulfolobus turreted icosahedral virus NC_005892 (12291) -AACAAGAAGAGAAGAAAGAAGAACAAAAGACAGAGAAAAAGGACGAAAAAGTAGAAGAGAAAAAGGAA-GGAGAACAAA
```

Section 210
```
                                                       (16721) 16721    16730     16740     16750     16760     16770     16780     16790   16800
               SARS-CoV-2 Reference Genome NC_045512.2 (16558) GCAATTGCAACATGTGACTGGACAAATGCTGGTGATTACATTTTAGCTAACACCTGTACTGAAAGACTCAAGCTTTTTGC
Sulfolobus turreted icosahedral virus NC_005892 (12369) AGAAGG--AACAGGCTGAAAAACAAAAAGAGCCAAAGAAAGAAGAGAAAAAAGAGGA---GAAAGAAAAGAAA-----GT
```

Section 211

```
                                                        (16801) 16801    16810    16820    16830    16840    16850    16860    16870    16880
SARS-CoV-2 Reference Genome NC_045512.2                 (16638) AGCAGAAACGCTCAAAGCTACTGAGGAGACATTTAAACTGTCTTATGGTATTGCTACTGTACGTGAAGTGCTGTCTGACA
Sulfolobus turreted icosahedral virus  NC_005892        (12439) AGCAGAGAAAGAAGAGAAGAAAAAGGA--ACAAAAGAAGGAAGAGAAGAAAGAACAAAAGAAGAAAAAAGTGATAAAGCA
```

Section 212

```
                                                        (16881) 16881    16890    16900    16910    16920    16930    16940    16950    16960
SARS-CoV-2 Reference Genome NC_045512.2                 (16718) GAGAATTACATCTTTCATGGGAAGTTGGTAAACCTAGACCACCACTTAACCGAAATTATGTCTTTACTGGTTATCGTG-T
Sulfolobus turreted icosahedral virus  NC_005892        (12517) GAGAAAGAGAGAGGAAGTTAGTTACGGTT-TAATTGGATTAG-GTTTGGCGATTCTTATAGCGTCTTTAATCATGCTGAT
```

Section 213

```
                                                        (16961) 16961    16970    16980    16990    17000    17010    17020    17030    17040
SARS-CoV-2 Reference Genome NC_045512.2                 (16797) AACTAAAAACAGTAAAGTACAAATAGGAGAGTACACCTTTGAAAAAGGTGACTATGGTGATGCTGTTGTTTACCGAGGTA
Sulfolobus turreted icosahedral virus  NC_005892        (12595) TATTTCCATTTGG----AAAAGGTGGAAGAAATGA-----GGA-AAGAGGCAATTGAGGATTTAGAA-GAAGGCATGGTA
```

Section 214

```
                                                        (17041) 17041    17050    17060    17070    17080    17090    17100    17110    17120
SARS-CoV-2 Reference Genome NC_045512.2                 (16877) CAACAACT-TACAAATTAAATGTTGGTGATTATTTTGTGCTGACATCACATACAGTAATGCCATTAAGTGCACCTACACT
Sulfolobus turreted icosahedral virus  NC_005892        (12664) GTAGAGATATTTACAGGAAATATAG---ATTTC---GTAGGGACAGT---TACGAGAATCACAGA---TTCGTATATAGA
```

Section 215

```
                                                        (17121) 17121    17130    17140    17150    17160    17170    17180    17190    17200
SARS-CoV-2 Reference Genome NC_045512.2                 (16956) AGTGCCACAAGAGCACTATGTTAGAATTACTGGCTTATACCCAACACTCAATATCTCAGATGAGTTTTCTAGCAATGTTG
Sulfolobus turreted icosahedral virus  NC_005892        (12732) ATTG-------------ATCATACAAATAC---CTCTTA-ATGATAGAAATCAAGTAAA-AGAGGTAATTGCAAGAATAG
```

Section 216

```
                                                        (17201) 17201    17210    17220    17230    17240    17250    17260    17270    17280
SARS-CoV-2 Reference Genome NC_045512.2                 (17036) CAAATTATCAAAAGGTTGGTATGCAAAAGTATTCTACACTCCAGGGACCACCTGGTACTGGTAAGAGTCATTTTGCTATT
Sulfolobus turreted icosahedral virus  NC_005892        (12794) A----TCTCGTGAGTATTG-ACGCTATCATAATCCATAGTGGCG---CAAAGGTGAAGGAAAATGAGTGAGATATATGAC
```

Section 217

```
                                                        (17281) 17281    17290    17300    17310    17320    17330    17340    17350    17360
SARS-CoV-2 Reference Genome NC_045512.2                 (17116) GGCCTAGCTCTCTACTACCCTTCTGCTCGCATAGTGTATACAGCTTGCTCTCATGCCGCTGTTGATGCACT-ATGTGAGA
Sulfolobus turreted icosahedral virus  NC_005892        (12866) --CCTCAACAGGACTTAACTAAGATCTGTCAATCTG-AGTTCTTTGCGCCAGAACCAGAGAAAATTACGATCAACGGAAA
```

```
                                                                                                              Section 218
                                              (17361) 17361    17370         17380         17390         17400         17410         17420         17430    17440
          SARS-CoV-2 Reference Genome NC_045512.2 (17195) AGGCATTAAAATATTTGCCTATAGATAAATGTAGTAGAATTATACCTGCACGTGCTCGTGTAGAGTGTTTTGATAAATTC
Sulfolobus turreted icosahedral virus  NC_005892 (12943) AGAGATTATAATAAAAGGACGCAAAAGGATGGACATTATTAAGGATTTGTTGGACGCGGGTATAG----ATGAAGAATGT
                                                                                                              Section 219
                                              (17441) 17441    17450         17460         17470         17480         17490         17500         17510    17520
          SARS-CoV-2 Reference Genome NC_045512.2 (17275) AAAGTGAATTCAACATTAGAACAGTATGTCTTTTGTACTGTAAATGCATTGCCTGAGACGACAGCAGATATAGTTGTCTT
Sulfolobus turreted icosahedral virus  NC_005892 (13019) ATAGTGA--AGGGGATAGAATCATGGTGGAATAATAAGCATCCGAGAGAATATTTAAGGGCAATCAAATCAAA--GCTTA
                                                                                                              Section 220
                                              (17521) 17521    17530         17540         17550         17560         17570         17580         17590    17600
          SARS-CoV-2 Reference Genome NC_045512.2 (17355) TGATGAAATTTCAATGGCCACAAATTATGATTTGAGTGTTGTCAATGCCAGATTACGTGCTAAGCACTATGTGTACATTG
Sulfolobus turreted icosahedral virus  NC_005892 (13095) AACTCAAAG---AAGCAGCACAAA--ATGAGCAAAAAGTAGATGAGTCGTTAAAACTA---AACGTAGAAGAGGAAAAGA
                                                                                                              Section 221
                                              (17601) 17601    17610         17620         17630         17640         17650         17660         17670    17680
          SARS-CoV-2 Reference Genome NC_045512.2 (17435) GCGACCCTGCTCAATTACCTGCACCACGCACATTGCTAACTAAGGGCACACTAGAACCAGAATATTTCAATTCAGTGTGT
Sulfolobus turreted icosahedral virus  NC_005892 (13167) AGGAAGAAGGAGAGACAGAAGGAGAAGAAGCAC----AAGT-AGG----AGGAGAACTTCCGGAGCTAACTGCAGAAGGG
                                                                                                              Section 222
                                              (17681) 17681    17690         17700         17710         17720         17730         17740         17750    17760
          SARS-CoV-2 Reference Genome NC_045512.2 (17515) AGACTTATGAAAACTATAGGTCCAGACATGTTCCTCGGAACTTGTCGGCGTTGTCCTGCTGAAATTGTTGAC---ACTGT
Sulfolobus turreted icosahedral virus  NC_005892 (13238) CAAAAGCCTATAGAGATCACTGAGGAAGTTGTAGCCCTAGC-CTACGGGGCGCTCTTG--GAGCTTGTAGTCAGAATTCT
                                                                                                              Section 223
                                              (17761) 17761    17770         17780         17790         17800         17810         17820         17830    17840
          SARS-CoV-2 Reference Genome NC_045512.2 (17592) GAGTGCTTTGGTTTATGATAATAAGC--TTAAAGCACATAAA----GACAAATCAGCTCAATGCTTTA--AAATGTT-TT
Sulfolobus turreted icosahedral virus  NC_005892 (13315) GTCGGCTAGATACAAGAAAGACGTGGAACTAAACGACATAATTCCCGACGAAAGAATTAAACAACACGGGAAATATTACT
                                                                                                              Section 224
                                              (17841) 17841    17850         17860         17870         17880         17890         17900         17910    17920
          SARS-CoV-2 Reference Genome NC_045512.2 (17663) ATAAGGGTGTTATCACGCATGATGTTTCATCTGCAATTAACAGGCCACAAATAGGCGTGGTAAGAGAATTCCTTACACGT
Sulfolobus turreted icosahedral virus  NC_005892 (13395) ATCAACTCTTAGACGCTCTTGGTCTTTTAAACGAAA--GATATGTTC-AGTTATTCGTTCTAGGAATCGGAAGCGCCGGC
```

```
                                                                  Section 225
                                                  (17921) 17921    17930     17940     17950     17960     17970     17980     17990    18000
          SARS-CoV-2 Reference Genome NC_045512.2 (17743) AACCCTGCTTGGAGAAAAGCTGTCTTTATTTCACCTTATAATTCACAGAATGCTGTAGCCTCAAAG-ATTTTGGGACTAC
Sulfolobus turreted icosahedral virus  NC_005892 (13472) GCTGCGGCTTCAGATATAGTGGCGATTGTAACTTACTTTAAGTCTCAAGAAGAGGAAGAAGAAAAGAAAGATCAGAAAAA
                                                                  Section 226
                                                  (18001) 18001    18010     18020     18030     18040     18050     18060     18070    18080
          SARS-CoV-2 Reference Genome NC_045512.2 (17822) CAACTCAAACTGTTGATTCA-TCACAGGGCTCAGAATATGACTATGTCATATTCACTCAAACCACTGAAACAGCTCACTC
Sulfolobus turreted icosahedral virus  NC_005892 (13552) ACAATGGAAGGGAGAAGGGAATAAGAGCGATTTGAGT--GAAAAGG----ATAAGGTGAAATCAC------AACTAACGG
                                                                  Section 227
                                                  (18081) 18081    18090     18100     18110     18120     18130     18140     18150    18160
          SARS-CoV-2 Reference Genome NC_045512.2 (17901) TTGTAATGTAAACAGATTTAATGTTGCTATTACCAGAGCAAAAGTAGGCATACTTTGCATAATGTCTGATAGAGACCTTT
Sulfolobus turreted icosahedral virus  NC_005892 (13620) TAATGGAGGAAATGGAGATATGAAAGTTCCTAGCAGCATGATAGTCAATCTCATAAGTCAGATAGCGGAAAATGGC--TT
                                                                  Section 228
                                                  (18161) 18161    18170     18180     18190     18200     18210     18220     18230    18240
          SARS-CoV-2 Reference Genome NC_045512.2 (17981) ATGACAAGTTGCAATTTACAAGTCTTGA-AATTCCACGTAGGAATGTGGCAA-CTTTACAAGCTGAAAATG---TAACAG
Sulfolobus turreted icosahedral virus  NC_005892 (13698) AGACCAGAAAGCAAAAGAATACTTAAAACAATTAAAAGAAGGAAAAGTTCAGCCACGTTATGAAGAGTGTCAAGTAACGA
                                                                  Section 229
                                                  (18241) 18241    18250     18260     18270     18280     18290     18300     18310    18320
          SARS-CoV-2 Reference Genome NC_045512.2 (18056) GACTCTTTAAAGA----TTGTAGTAAGGTAATCACTGGGTTACATCCTACACAGGCACCTACACACCTCAGTGTTGACAC
Sulfolobus turreted icosahedral virus  NC_005892 (13778) AAATTTTCTTAGACGGGATAACGAAAGCTACTAATAAATTCAAAGA----AGCAGCTCCT-TTAAGC-GGGTCTCTTCA-
                                                                  Section 230
                                                  (18321) 18321    18330     18340     18350     18360     18370     18380     18390    18400
          SARS-CoV-2 Reference Genome NC_045512.2 (18132) TAAATTCAAAACTGAAGGTTT---ATGTGTTGACATACCTGGCATACCTAAG-GACATGACCTAT-AGAAGACTCATCTC
Sulfolobus turreted icosahedral virus  NC_005892 (13851) ATTCTTCTACATTTTAGTTTTGGCGTGTG-AGACAAATGAAGAATTCAGAAGAGAGTTAATAGAAATGTATAATCAACTT
                                                                  Section 231
                                                  (18401) 18401    18410     18420     18430     18440     18450     18460     18470    18480
          SARS-CoV-2 Reference Genome NC_045512.2 (18207) TATGATGGGTTTTAAAATGAATTATCAAGTTAATGGTTACCCTAACATGTTTATCACCCGCGAAGAAGCTATAAGACATG
Sulfolobus turreted icosahedral virus  NC_005892 (13930) GAGGATTTCTTCCTAGCCGGGATAGAAAATGAATCCTGACGATATTGTGGTTATTATTGGT-----AG--AAA-GCGAAG
```

```
                                                                    Section 232
                                           (18481) 18481    18490    18500    18510    18520    18530    18540    18550    18560
SARS-CoV-2 Reference Genome NC_045512.2    (18287) TACGTGCATGGATTGGCTTCGATGTCGAGGGGTGTCATGCTACTAGAGAAGCTGTTGGTACCAATTTACCTTTACAGCTA
Sulfolobus turreted icosahedral virus  NC_005892 (14002) CG-GTAAGA-GCTAC-CTT--ATTAAGCATTACTTTATTCCAGTCCTTAAG-----GCTCACAAAATCTCTT-ACATAAT
                                                                    Section 233
                                           (18561) 18561    18570    18580    18590    18600    18610    18620    18630    18640
SARS-CoV-2 Reference Genome NC_045512.2    (18367) GGTTTTTCTACAGGTGTTAACCTAGTTGCTGTACCTACAGGTTATGTTGATACACCTAATAATACAGATTTTTCCAGAGT
Sulfolobus turreted icosahedral virus  NC_005892 (14071) AGATGACCATAATCTTCTAAGA-AGTGGTTCAGAATACTCAAAATTCGGGTATAA-TGTTGTTACACTTT----CAGACA
                                                                    Section 234
                                           (18641) 18641    18650    18660    18670    18680    18690    18700    18710    18720
SARS-CoV-2 Reference Genome NC_045512.2    (18447) TAGTG-CTAAACCACCGCCTGGAGATCAATTTAAACACCTCATACCACTTATGTACAAAGGACTTCCTTGGAATGTAGTG
Sulfolobus turreted icosahedral virus  NC_005892 (14145) TAGTTTCCAAACAATA-TGTTGTCGTGTATGATAGAGAAAAAAATGATGTATTTTTTGAAAAATT--ATGGAATGGAGCT
                                                                    Section 235
                                           (18721) 18721    18730    18740    18750    18760    18770    18780    18790    18800
SARS-CoV-2 Reference Genome NC_045512.2    (18526) CGTATAAAGATTGTACAAATGTTAAGTGACACACTTAAAAATCTCTCTGACAGAGTCGTATTTGTCTTATGGGCACATGG
Sulfolobus turreted icosahedral virus  NC_005892 (14222) AAATTACACGCCAAGAAATGGGGAACT-AGTGTCCTCATTATA-----GATGAAGCT-TACTATCACTTTAAGTATA-GG
                                                                    Section 236
                                           (18801) 18801    18810    18820    18830    18840    18850    18860    18870    18880
SARS-CoV-2 Reference Genome NC_045512.2    (18606) CTTTGAGTTGACATCTATGAAGTATTTTGTGAAAATAGGACCTGAGCGCACCTGTTGTCTATGTGATAGACGTGCC-ACA
Sulfolobus turreted icosahedral virus  NC_005892 (14294) CAGAAAGTA-ACGCCGGCAATCGATGAGGC---------A-CTACATGCGAATCGACACGCAGGGATAGGGCTTATATTA
                                                                    Section 237
                                           (18881) 18881    18890    18900    18910    18920    18930    18940    18950    18960
SARS-CoV-2 Reference Genome NC_045512.2    (18685) TGCTTTTCCACTGCTTCAGACACTTATGCCTGTTGGCATCATTCTATTGGATTTGATTACGTCTATAATCCGTTTATGAT
Sulfolobus turreted icosahedral virus  NC_005892 (14363) AGTACACAGAGAGTCT-ATGATCTAATGCCGATTGTCTACAAACAAGCAGATCTCATTATAATGTTTATACCAGAGAGCC
                                                                    Section 238
                                           (18961) 18961    18970    18980    18990    19000    19010    19020    19030    19040
SARS-CoV-2 Reference Genome NC_045512.2    (18765) TGATGTTCAACAATGGGGTTTTACAGGTAACCTACAAAGCAACCATGATCTGTATTGTCAAGTCCATGGTAATGCACATG
Sulfolobus turreted icosahedral virus  NC_005892 (14442) TAATGA--ACTAAAGTGGATCTCAAAGTACATTAGCACGGAAGCAGCAGAGAAAGTGAAAACTC-----TTAAGCAGTAT
```

Section 239

```
                                                      19041     19050     19060     19070     19080     19090     19100     19110   19120
                                             (19041)
SARS-CoV-2 Reference Genome NC_045512.2      (18845) TAGCTAGTTGTGATGCAATCATGACTAGGTGTCTAGCTGTCCACGAGTGCTTTGTTAAGCG-TGTTGACTGGACTATTGA
Sulfolobus turreted icosahedral virus NC_005892 (14515) TACTT---TTTGAT----TTATGAC---GTGAATAGTCAAATGATAAAAATACATAAGCCGAT-TTGA--GAAAAATTTT
```

Section 240

```
                                                      19121     19130     19140     19150     19160     19170     19180     19190   19200
                                             (19121)
SARS-CoV-2 Reference Genome NC_045512.2      (18924) ATATCCTATAATTGGTGATGAACTGAAGATTAATGCGGCTTGTAGAAAGGTTCAACACATGGTTGTTAAAGCTGCATTAT
Sulfolobus turreted icosahedral virus NC_005892 (14582) TAAAGATAGA----GAGAG-AATTATCCATGGTTGTATCCCCTCTCCTTTTTTTTGACTTCTGATTGAACCGCTTAGGCT
```

Section 241

```
                                                      19201     19210     19220     19230     19240     19250     19260     19270   19280
                                             (19201)
SARS-CoV-2 Reference Genome NC_045512.2      (19004) TAGCAGACAAATTCCCAGTTCTTCACGACATTGGTAACCCTAAAGCTATTAAGTGTGTACCTC--AAGCTGATGTAGAAT
Sulfolobus turreted icosahedral virus NC_005892 (14657) GAGTTCATAAATTTTCTGAAACTTCTCATATTGAAAAC--TTTGTTTTCATAGCAGAATCCACGTTTTTTGTCCTCAAAT
```

Section 242

```
                                                      19281     19290     19300     19310     19320     19330     19340     19350   19360
                                             (19281)
SARS-CoV-2 Reference Genome NC_045512.2      (19082) GGAAGTTCTATGATGCACAGCCTTGTAGTGACAAAGCTTATAAAATAGAAGAATTATTCTATTCTTATGCCACACATTCT
Sulfolobus turreted icosahedral virus NC_005892 (14735) GTAAA--AAAACATAGAAATTTTAGAAAATTTGTT--TTCTTCATATGGATAAGGTTTATAGCGAAAAAACTAACAT---
```

Section 243

```
                                                      19361     19370     19380     19390     19400     19410     19420     19430   19440
                                             (19361)
SARS-CoV-2 Reference Genome NC_045512.2      (19162) GACAAATTCACAGATGGTGTATGCCTATTTTGGAATTGCAATGTCGATAGATATCCTGCTAATTCCATTGTTTGTAGATT
Sulfolobus turreted icosahedral virus NC_005892 (14808) --AGAGTTTTTTTGAGGAGAAAGAAATGAAAGAAGACGCTATGCATTATGTAATATTGTTAGTAG------TTGTAGCTA
```

Section 244

```
                                                      19441     19450     19460     19470     19480     19490     19500     19510   19520
                                             (19441)
SARS-CoV-2 Reference Genome NC_045512.2      (19242) TGACACTAGAGTGCTATCTAACCTTAACTTGCCTGGTTGTGATGGTGGCAGTTTGTATGTAAATAAACA-TGCATTCCAC
Sulfolobus turreted icosahedral virus NC_005892 (14880) TCGGTGTATGGGCAGGACTAACTCTA---TATAAATTCGTAAAACTATGAAAAGGTGGTGAAAAAATGGGAGAAATCTAT
```

Section 245

```
                                                      19521     19530     19540     19550     19560     19570     19580     19590   19600
                                             (19521)
SARS-CoV-2 Reference Genome NC_045512.2      (19321) ACACCAGCTTTTGATAAAAGTGCTTTTGTTAATTTAAAACAATTACCATT-TTTCTATTACTCTGACAGTCCATGTGAGT
Sulfolobus turreted icosahedral virus NC_005892 (14957) ACAGAGACATT--ACAACAGA-CTTA--TGCGTGGACTGCAGGGACAAATATACCTATTAAAATACCCCGCAATA-ATTT
```

Section 246

```
(19601)                                              19601     19610     19620     19630     19640     19650     19660     19670     19680
SARS-CoV-2 Reference Genome NC_045512.2 (19400)      CTCATGGAAAACAAGTAGTGTCAGATATAGATTATGTACCACTAAAGTCTGCTACGTGTATAACACGTTGCAATTTAGGT
Sulfolobus turreted icosahedral virus NC_005892 (15031) --TATCAGAAAGATTAGGGTTCAGCTAATAGG-ATCTATTTCTAATAGCGGTACTGCCGCTGTAACTCTTCCATCAGCGC
```

Section 247

```
(19681)                                              19681     19690     19700     19710     19720     19730     19740     19750     19760
SARS-CoV-2 Reference Genome NC_045512.2 (19480)      GGTGCTGTCTGTAGACATCA-TGCTAATGAGTACAGATTG-TATCTCGATGCTTATAACATGATGATCTCAGCTGGCTTT
Sulfolobus turreted icosahedral virus NC_005892 (15108) CT---TTCCCATACAATTTAGTGCAGACGTTTAACTTGAGCTATGAAGG-CTCAAAGACTCTTTATTCAGTCTCGGGTAC
```

Section 248

```
(19761)                                              19761     19770     19780     19790     19800     19810     19820     19830     19840
SARS-CoV-2 Reference Genome NC_045512.2 (19558)      AGCTTGTGGGTTTACAA---ACAAT--TTGATACTTATAACCTCTGGAACACTTTTACAAGACTTCAGAGTTTAGAAAAT
Sulfolobus turreted icosahedral virus NC_005892 (15184) AGGTCTTGGCATTTTAATGTACTATACTACAAAGGGACAGAATCCAGCATACCCCGCGCCCGGAACATCTGTACCTGCAT
```

Section 249

```
(19841)                                              19841     19850     19860     19870     19880     19890     19900     19910     19920
SARS-CoV-2 Reference Genome NC_045512.2 (19633)      GTGGCTTTTAATGTTGTAAATAAGGGACACTTTGATGGACAACAGGGTGAAGTACCAGTTTCTATCATTAATAACACTGT
Sulfolobus turreted icosahedral virus NC_005892 (15264) CTGG--TTCAGTGAAT--CTTAACGTAATGTGGGAATTCGATCTAGCTAGATTCCCTGCTACAATGGT-TCAAA-ATATT
```

Section 250

```
(19921)                                              19921     19930     19940     19950     19960     19970     19980     19990     20000
SARS-CoV-2 Reference Genome NC_045512.2 (19713)      TTACACAAAAGTT--G-ATGGTGTTGATGTAGAATTGTTTGAAAATAAAACAACAT-TACCTGTTAATGTAGCATTTGAG
Sulfolobus turreted icosahedral virus NC_005892 (15338) ATACTCAGCATTTTGACAGGACAGGCGCCTAGCGGTGTCTCCATAAACGCTAGTTTCTACATAACAATTACATATGAAAG
```

Section 251

```
(20001)                                              20001     20010     20020     20030     20040     20050     20060     20070     20080
SARS-CoV-2 Reference Genome NC_045512.2 (19789)      CTTTGGGCTAAGCGCAACATTAAACCAGTACCAGAGGTGAAAATACTCAATAATTTGGGTGTGGACATTGCTGCTAATAC
Sulfolobus turreted icosahedral virus NC_005892 (15418) AGTAACAGCACAGGAAATATTAAGTGAGGGAGGGTTAGGCGCAGACGGTGAAATGCC--TCTCGCTACGGTCTTACCGAA
```

Section 252

```
(20081)                                              20081     20090     20100     20110     20120     20130     20140     20150     20160
SARS-CoV-2 Reference Genome NC_045512.2 (19869)      TGTGATCTGGGACTACAAAAGAGATGCTCCAGCACATAT-ATCTACTATTGGTGTTTGTTCTATGACTGACATAGCCAAG
Sulfolobus turreted icosahedral virus NC_005892 (15496) GGTTATCGAG-ATTCCTACATTTAACGTACCGGCTAGCTCAGCGCCAATACATGTA-GCATACTTGCAGCCCGGACAGAT
```

Section 253

(20161) 20161 20170 20180 20190 20200 20210 20220 20230 20240
SARS-CoV-2 Reference Genome NC_045512.2 (19948) AAACCAACTGAAACGA-TTTGTGCACCACTCACTGTCTTTTTTGATGGTAGAGTTGATGGTCAAGTAGACTTATTTAGAA
Sulfolobus turreted icosahedral virus NC_005892 (15574) ATATAAGAGGCAGTTAGTTTATGTGATAAACAGTACCTCAGGAATCAATAACACAGATCCAACCGAATATGAGCTAAAAA

Section 254

(20241) 20241 20250 20260 20270 20280 20290 20300 20310 20320
SARS-CoV-2 Reference Genome NC_045512.2 (20027) ATGCCCGTAATGGTGTTCTTA-TTACAGAAGGTAGTGTTAAAGGTTTACA--ACCATCTGTAGGTCCCAAACAAGCTAGT
Sulfolobus turreted icosahedral virus NC_005892 (15654) TTGT---TAGAGGTGTACCAACTGACAAAATCAAGGTAAGTTGGGCTGCTCTTCAGGCAGA------GAACCAAGCTGAG

Section 255

(20321) 20321 20330 20340 20350 20360 20370 20380 20390 20400
SARS-CoV-2 Reference Genome NC_045512.2 (20104) CTTAATGGAGTCAC-ATTAATTGGAGAAGCCGTAAAAACACAGTTCAATTATTATAAGAAAGTTGATGGTGTTGTCCAAC
Sulfolobus turreted icosahedral virus NC_005892 (15725) TATCA-AGTAGCACCATATAGTGGAGCTTCCGCCATCATTGACTTTAGGAAATATTTTAATGGCGATTTAGATCTTACAC

Section 256

(20401) 20401 20410 20420 20430 20440 20450 20460 20470 20480
SARS-CoV-2 Reference Genome NC_045512.2 (20183) AATTACCTGAAACTTACTTTACTCAGAGTAGAAATTTACAAGAATTTAAACCCAGGAG--TC---AAATGGAAATTGATT
Sulfolobus turreted icosahedral virus NC_005892 (15804) ACGCTCCGTCAGATAGCATAGAATACGATCTAGCTTTACA-GAATCAAGACAATGTATACTCTCTATATGTTTCCTACGT

Section 257

(20481) 20481 20490 20500 20510 20520 20530 20540 20550 20560
SARS-CoV-2 Reference Genome NC_045512.2 (20258) TCTTAGAATTAGCTATGGATGAATTCATTGAACGGTATAAATTAGAAGGCTATGCCTTCGAACATATCGTTTATGGAGAT
Sulfolobus turreted icosahedral virus NC_005892 (15883) GCTTCCATACTACGATCAACTCGCAGCGTTACCTGCTCAAGTAGCAGCAATAGTACAAC-AATATGTTGCACGTCAAAAA

Section 258

(20561) 20561 20570 20580 20590 20600 20610 20620 20630 20640
SARS-CoV-2 Reference Genome NC_045512.2 (20338) TTTAGTCATAGTCAGTTAGGTGGTTTACATCTACTGATTGGACTAGCTAAACGTTTTAAGGAATCACCTTTTGAATTAGA
Sulfolobus turreted icosahedral virus NC_005892 (15962) AGAAGGATAAAG-AGATAGATGAGGTA-AGAT---GTCTTTTTTGGAGGATTGTTTAATGACGTAGAGAACGCAGTCAG

Section 259

(20641) 20641 20650 20660 20670 20680 20690 20700 20710 20720
SARS-CoV-2 Reference Genome NC_045512.2 (20418) AGATTTTATTCCTATGGACAGTACAGTTAAAAACTATTTCATAACAGATGCGCAAACAGGTTCATCTAAGTGTGTGTGTT
Sulfolobus turreted icosahedral virus NC_005892 (16037) TAATTTTACT-AAACAAGCTAAAAAATCAATTTCTGACGTAGAAAAA-GTAATC--CAGAAAGATATAACTCA-TCCACC

Section 260

```
(20721)                                              20721    20730     20740     20750     20760     20770     20780     20790     20800
SARS-CoV-2 Reference Genome NC_045512.2 (20498)      CTGTTATTGATTTATTACTTGATGATTTTGTTGAAATAATAAAATCCCAAGATTTATCTGTAGTTTCTAAGGTTGTCAAA
Sulfolobus turreted icosahedral virus NC_005892 (16112) GAAAT-TTTACCTACCACCTGT-CCATCTACCAA--ATATA---CATCAAATCGAAGCAGGAATAGGTCACGTAGAAAAA
```

Section 261

```
(20801)                                              20801    20810     20820     20830     20840     20850     20860     20870     20880
SARS-CoV-2 Reference Genome NC_045512.2 (20578)      GTGACTATTGACTATACAGAAATTTCATTTATGCTTTGGTGTAAAGATGGCCATGTAGAAACATTTTACCCAAAATTACA
Sulfolobus turreted icosahedral virus NC_005892 (16185) GTAATCCAGAAAGATATA-ACTCATCCACCGAAATTTTACCTA-------CCACCTGTCCATCT----ACCAAATATACA
```

Section 262

```
(20881)                                              20881    20890     20900     20910     20920     20930     20940     20950     20960
SARS-CoV-2 Reference Genome NC_045512.2 (20658)      ATCTAGTCAAGCGTGGCAACCGGGTGTTGCTATGCCTAATCTTTACAAAATGCAAAGAATGCTATTAGAAAAGTGTGACC
Sulfolobus turreted icosahedral virus NC_005892 (16253) TCAAATCGAAGCAGGAATAGGTC-ACGTAGAAAAAGTAAT-----CCAGAAAGATATAACTCATCCACCGAAATTTTACC
```

Section 263

```
(20961)                                              20961    20970     20980     20990     21000     21010     21020     21030     21040
SARS-CoV-2 Reference Genome NC_045512.2 (20738)      TTCAAAATTATGGTGATAGTGCAACATTACCTAAAGGCATAATGATGAATGTCGCAAAATATACTCAACTGTGTCAATAT
Sulfolobus turreted icosahedral virus NC_005892 (16327) TACCACCT---GTCCATCTACCAAATATACACCCGATCGAAATGGCTAT------AAGTCACGCTGGA--G-GTGAAGCT
```

Section 264

```
(21041)                                              21041    21050     21060     21070     21080     21090     21100     21110     21120
SARS-CoV-2 Reference Genome NC_045512.2 (20818)      TTAAACACATTAACATTAGCTGTACCCTATAATATGAGAGTTATACATTTTGGTGCTGGTTCTGATAAAGGAGTTGCACC
Sulfolobus turreted icosahedral virus NC_005892 (16395) TTAAACGATGT-GCAAAAGGCTCTCGCACAAGCATATCACTTA-AC----TAGAGAAGGATTCACTAATTTATTCGCT--
```

Section 265

```
(21121)                                              21121    21130     21140     21150     21160     21170     21180     21190     21200
SARS-CoV-2 Reference Genome NC_045512.2 (20898)      AGGTACAGCTGTTTTAAGACAGTGGTTGCCTACGGGTACGCTGCTTGTCGATTCAGATCTTAATGACTTTGTCTCTGATG
Sulfolobus turreted icosahedral virus NC_005892 (16467) -GGTGCAGC-------AGCGCTAGGAAGAGAAACGGGAG--AGTTAATAAGGTATCATCACTTCATCCCTTTTAACGAGG
```

Section 266

```
(21201)                                              21201    21210     21220     21230     21240     21250     21260     21270     21280
SARS-CoV-2 Reference Genome NC_045512.2 (20978)      CAGATTCAACTTTGATTGGTGATTGTGCAACTGTACATACAGCTAATAAATGGGATCTCATTATTAGTGATATGTAC-GA
Sulfolobus turreted icosahedral virus NC_005892 (16537) CAGTAGAACAAGTAGAGAATCAAAGCGTAGCAAATACGCCAATTTTCAGCGGTCTAACTAG-ATTCGGCATAAAAACAGG
```

Section 267

```
                                             (21281) 21281     21290          21300          21310          21320          21330          21340          21350    21360
    SARS-CoV-2 Reference Genome NC_045512.2 (21057) CCCTAAGACTA--AAAATGTTACAAAAGAAAATGACTCTAAAGAGGGTTTTTTCACTTACATTTGTGGGTTTATACAACA
Sulfolobus turreted icosahedral virus NC_005892 (16616) GTCTCA-ACTATTTAATGCCGAA--TCGCAATTAGCTGCGAACA---TCATACCCGTGACGG--GGACTTTTGGACACTT
```

Section 268

```
                                             (21361) 21361     21370          21380          21390          21400          21410          21420          21430    21440
    SARS-CoV-2 Reference Genome NC_045512.2 (21135) AAAGCTAGCTCTTGGAGGTTCCGTGGCTATAAAGATAACAGAACATTCTTGGAATGC-TGATCTTTATAAGCTCATGGGA
Sulfolobus turreted icosahedral virus NC_005892 (16688) GGCAGCGCATCCGGGAGAATCATTGGGGAGTAAGATT----AGTGATATAGCATTTGGTATAGCTGACCTGCTTCCGGGG
```

Section 269

```
                                             (21441) 21441     21450          21460          21470          21480          21490          21500          21510    21520
    SARS-CoV-2 Reference Genome NC_045512.2 (21214) CACTTCGCATGGTGGACAGCCTTTGTTACTAATGTGAATGCGTCATCATCTGAAGCATTTTTAATTGGATGTAATTATCT
Sulfolobus turreted icosahedral virus NC_005892 (16764) T--TAGATATAGCGTCTT--CTCTTCTACGCCCC-GCAGACGT-AGGAGCTGAAGCCGCGTTAGCCGGAGCTAGAGCTTT
```

Section 270

```
                                             (21521) 21521     21530          21540          21550          21560          21570          21580          21590    21600
    SARS-CoV-2 Reference Genome NC_045512.2 (21294) TGGCAAACCACGCGAACAAATAGATGGTTATGTCATGCATGCAAATTACATATTTTGGAGGAATACAAATCCAATTCAGT
Sulfolobus turreted icosahedral virus NC_005892 (16838) AG---AAACAGGAGGAGAAACAGCTACAGATCTATTAAATCCTCTTGCGAAAGATTT-AGGAAGTGAAA--CTACTGATT
```

Section 271

```
                                             (21601) 21601     21610          21620          21630          21640          21650          21660          21670    21680
    SARS-CoV-2 Reference Genome NC_045512.2 (21374) TGTCTTCCTATTCTTTATTTGACATGAGTAAATTTCCCCTTAAATTAAGGGGTACTGCTGTTATGTCTTTAAAAGAAGGT
Sulfolobus turreted icosahedral virus NC_005892 (16912) TGACTA---ATCCGGTAGAGAGGGATATTTTAGATGAATTAGACTTAGAGAAGAATGCAGAGAAAGATTTAGGAGAAGAA
```

Section 272

```
                                             (21681) 21681     21690          21700          21710          21720          21730          21740          21750    21760
    SARS-CoV-2 Reference Genome NC_045512.2 (21454) CAAATCAATGATATGATTTTATCTCTTCTTAGTAAAGGTAGACTTATAATTAGAGAAAACAACAGAGTTGTTATTTCTAG
Sulfolobus turreted icosahedral virus NC_005892 (16989) GGAAATGTAGAGAAAGATCTAGAAGACCTCACATCA-AACGATTTAGAAAAAG-GAGCTAGACTAAGGAATACGTTATTG
```

Section 273

```
                                             (21761) 21761     21770          21780          21790          21800          21810          21820          21830    21840
    SARS-CoV-2 Reference Genome NC_045512.2 (21534) TGATGTTCTTGTTAACAACTAAACGAACAATGTTTGTTTTTCTTGTTTT-ATTGCCACTAGTCTCTAGTCAGTGTGTTAA
Sulfolobus turreted icosahedral virus NC_005892 (17067) AAAA--------TAAGCAAATATGGAACATTGGGATTAGTAGGCGTAGGAATAGGAATTC--CATTAGGACTTTCATTAT
```

Section 274

(21841) 21841 21850 21860 21870 21880 21890 21900 21910 21920
SARS-CoV-2 Reference Genome NC_045512.2 (21613) TCTTACAACCAGAACTC---AATTACCCCCTGCATACACTAAT-TCTTTCACACGTGGTGTTTATTACCCTGACAAAGTT
Sulfolobus turreted icosahedral virus NC_005892 (17137) TCGGAGGCGGAAAAGGAGGAAATAATCAAACACAGACACAAACTACGCAAAACTATCCTAT-TCCTTCTTCTCCAAG--T

Section 275

(21921) 21921 21930 21940 21950 21960 21970 21980 21990 22000
SARS-CoV-2 Reference Genome NC_045512.2 (21689) TTCAGATCCTCAGTTTTACATTCAACTCAGGACTTGTTCTTACCTTTCTTTTCCAATGTTACTTGGTTCCATGCTATACA
Sulfolobus turreted icosahedral virus NC_005892 (17214) TTTCCATCATCTCCAAGCAGTC-----CTAGCATACAACTA--CCGCCAAAGATAACAATACCCAA-TCCAAGCTCTCCA

Section 276

(22001) 22001 22010 22020 22030 22040 22050 22060 22070 22080
SARS-CoV-2 Reference Genome NC_045512.2 (21769) TGTCTCTGGGACCAATGGTACTAAGAGGTTTGATAACCCTGTCCTACCATTTAATGATGGTGTTTATTTTGCTTCCACTG
Sulfolobus turreted icosahedral virus NC_005892 (17286) ACTAACTTACCCTCTAGCCCCTACAACTT-----ACCCCCCTCTCCCGGCGTAAC--AGGACTATATAATCCTTTTACT-

Section 277

(22081) 22081 22090 22100 22110 22120 22130 22140 22150 22160
SARS-CoV-2 Reference Genome NC_045512.2 (21849) AGAAGTCTAACATAATAAGAGGCTGGATTTTTGGTACTACTTTAGATTCGAAGACCCAGTCCCTACTTATTGTTAATAAC
Sulfolobus turreted icosahedral virus NC_005892 (17358) AATGGAGAACAGGCACAAGAAGCCGGGAC---AGCATTACAAAATATTC-AAGGTACTGCAGCTTCTCCAAGCTCTCAAA

Section 278

(22161) 22161 22170 22180 22190 22200 22210 22220 22230 22240
SARS-CoV-2 Reference Genome NC_045512.2 (21929) GCTACTAATGTTGTTATTAAAGTCTGTGAATTTCAATTTTGTAATGATCCATTTTTGGGTGTTTAT-TACCACAAAAACA
Sulfolobus turreted icosahedral virus NC_005892 (17434) CCAACTTACCC-GGAATTCCATCATCTCCA-AGCGCTCCCG----GTGCTGTCCCCGGAACCCCCTCTAGTCCTAGCAGC

Section 279

(22241) 22241 22250 22260 22270 22280 22290 22300 22310 22320
SARS-CoV-2 Reference Genome NC_045512.2 (22008) ACAAAAGTTGGATGGAAAG--TGAGTTCAGAGTTTATTCTAGTGCGAATAATTGCACTTTTGAATATGTCTCTCAGCCTT
Sulfolobus turreted icosahedral virus NC_005892 (17508) GCTATAGCGGGCGTAGTACCAAGCAGTCCAAGTAGCCCAAGCGGCCAAAGCGGAAGCTCTGGAGGATTTCTATCAAATTT

Section 280

(22321) 22321 22330 22340 22350 22360 22370 22380 22390 22400
SARS-CoV-2 Reference Genome NC_045512.2 (22086) TTCTTATGG-ACCTTGAAGGAAAACAGGGTAATTTCAAAAAATCTTAGGGAATTTGTGTTTAAGAATATTGATGGTTATTT
Sulfolobus turreted icosahedral virus NC_005892 (17588) ATTTCATAATAAATTCTTTTTAATAGGAATAATTGTAATAATATTAATAATAATAGGAATAATAGTCATGAGGTGA----

```
                                                                                                    Section 281
                                                   (22401) 22401    22410     22420     22430     22440     22450     22460     22470   22480
SARS-CoV-2 Reference Genome NC_045512.2 (22165) TAAAATATATTCTAAGCACACGCCTATTAATTTAGTGCGTGATCTCCCTCAGGGTTTTTCGGCTTTAGAACCATTGGTAG
Sulfolobus turreted icosahedral virus  NC_005892 (17664) --------------------------------------------------------------------------------
                                                                                                    Section 282
                                                   (22481) 22481    22490     22500     22510     22520     22530     22540     22550   22560
SARS-CoV-2 Reference Genome NC_045512.2 (22245) ATTTGCCAATAGGTATTAACATCACTAGGTTTCAAACTTTACTTGCTTTACATAGAAGTTATTTGACTCCTGGTGATTCT
Sulfolobus turreted icosahedral virus  NC_005892 (17664) --------------------------------------------------------------------------------
                                                                                                    Section 283
                                                   (22561) 22561    22570     22580     22590     22600     22610     22620     22630   22640
SARS-CoV-2 Reference Genome NC_045512.2 (22325) TCTTCAGGTTGGACAGCTGGTGCTGCAGCTTATTATGTGGGTTATCTTCAACCTAGGACTTTTCTATTAAAATATAATGA
Sulfolobus turreted icosahedral virus  NC_005892 (17664) --------------------------------------------------------------------------------
                                                                                                    Section 284
                                                   (22641) 22641    22650     22660     22670     22680     22690     22700     22710   22720
SARS-CoV-2 Reference Genome NC_045512.2 (22405) AAATGGAACCATTACAGATGCTGTAGACTGTGCACTTGACCCTCTCTCAGAAACAAAGTGTACGTTGAAATCCTTCACTG
Sulfolobus turreted icosahedral virus  NC_005892 (17664) --------------------------------------------------------------------------------
                                                                                                    Section 285
                                                   (22721) 22721    22730     22740     22750     22760     22770     22780     22790   22800
SARS-CoV-2 Reference Genome NC_045512.2 (22485) TAGAAAAAGGAATCTATCAAACTTCTAACTTTAGAGTCCAACCAACAGAATCTATTGTTAGATTTCCTAATATTACAAAC
Sulfolobus turreted icosahedral virus  NC_005892 (17664) --------------------------------------------------------------------------------
                                                                                                    Section 286
                                                   (22801) 22801    22810     22820     22830     22840     22850     22860     22870   22880
SARS-CoV-2 Reference Genome NC_045512.2 (22565) TTGTGCCCTTTTGGTGAAGTTTTTAACGCCACCAGATTTGCATCTGTTTATGCTTGGAACAGGAAGAGAATCAGCAACTG
Sulfolobus turreted icosahedral virus  NC_005892 (17664) --------------------------------------------------------------------------------
                                                                                                    Section 287
                                                   (22881) 22881    22890     22900     22910     22920     22930     22940     22950   22960
SARS-CoV-2 Reference Genome NC_045512.2 (22645) TGTTGCTGATTATTCTGTCCTATATAATTCCGCATCATTTTCCACTTTTAAGTGTTATGGAGTGTCTCCTACTAAATTAA
Sulfolobus turreted icosahedral virus  NC_005892 (17664) --------------------------------------------------------------------------------
```

Section 288

	(22961)	22961 22970 22980 22990 23000 23010 23020 23030 23040
SARS-CoV-2 Reference Genome NC_045512.2	(22725)	ATGATCTCTGCTTTACTAATGTCTATGCAGATTCATTTGTAATTAGAGGTGATGAAGTCAGACAAATCGCTCCAGGGCAA
Sulfolobus turreted icosahedral virus NC_005892	(17664)	--

Section 289

	(23041)	23041 23050 23060 23070 23080 23090 23100 23110 23120
SARS-CoV-2 Reference Genome NC_045512.2	(22805)	ACTGGAAAGATTGCTGATTATAATTATAAATTACCAGATGATTTTACAGGCTGCGTTATAGCTTGGAATTCTAACAATCT
Sulfolobus turreted icosahedral virus NC_005892	(17664)	--

Section 290

	(23121)	23121 23130 23140 23150 23160 23170 23180 23190 23200
SARS-CoV-2 Reference Genome NC_045512.2	(22885)	TGATTCTAAGGTTGGTGGTAATTATAATTACCTGTATAGATTGTTTAGGAAGTCTAATCTCAAACCTTTTGAGAGAGATA
Sulfolobus turreted icosahedral virus NC_005892	(17664)	--

Section 291

	(23201)	23201 23210 23220 23230 23240 23250 23260 23270 23280
SARS-CoV-2 Reference Genome NC_045512.2	(22965)	TTTCAACTGAAATCTATCAGGCCGGTAGCACACCTTGTAATGGTGTTGAAGGTTTTAATTGTTACTTTCCTTTACAATCA
Sulfolobus turreted icosahedral virus NC_005892	(17664)	--

Section 292

	(23281)	23281 23290 23300 23310 23320 23330 23340 23350 23360
SARS-CoV-2 Reference Genome NC_045512.2	(23045)	TATGGTTTCCAACCCACTAATGGTGTTGGTTACCAACCATACAGAGTAGTAGTACTTTCTTTTGAACTTCTACATGCACC
Sulfolobus turreted icosahedral virus NC_005892	(17664)	--

Section 293

	(23361)	23361 23370 23380 23390 23400 23410 23420 23430 23440
SARS-CoV-2 Reference Genome NC_045512.2	(23125)	AGCAACTGTTTGTGGACCTAAAAAGTCTACTAATTTGGTTAAAAACAAATGTGTCAATTTCAACTTCAATGGTTTAACAG
Sulfolobus turreted icosahedral virus NC_005892	(17664)	--

Section 294

	(23441)	23441 23450 23460 23470 23480 23490 23500 23510 23520
SARS-CoV-2 Reference Genome NC_045512.2	(23205)	GCACAGGTGTTCTTACTGAGTCTAACAAAAAGTTTCTGCCTTTCCAACAATTTGGCAGAGACATTGCTGACACTACTGAT
Sulfolobus turreted icosahedral virus NC_005892	(17664)	--

```
                                                                                      Section 295
                                           (23521) 23521    23530     23540     23550     23560     23570     23580     23590   23600
SARS-CoV-2 Reference Genome NC_045512.2 (23285) GCTGTCCGTGATCCACAGACACTTGAGATTCTTGACATTACACCATGTTCTTTTGGTGGTGTCAGTGTTATAACACCAGG
Sulfolobus turreted icosahedral virus  NC_005892 (17664) --------------------------------------------------------------------------------
                                                                                      Section 296
                                           (23601) 23601    23610     23620     23630     23640     23650     23660     23670   23680
SARS-CoV-2 Reference Genome NC_045512.2 (23365) AACAAATACTTCTAACCAGGTTGCTGTTCTTTATCAGGATGTTAACTGCACAGAAGTCCCTGTTGCTATTCATGCAGATC
Sulfolobus turreted icosahedral virus  NC_005892 (17664) --------------------------------------------------------------------------------
                                                                                      Section 297
                                           (23681) 23681    23690     23700     23710     23720     23730     23740     23750   23760
SARS-CoV-2 Reference Genome NC_045512.2 (23445) AACTTACTCCTACTTGGCGTGTTTATTCTACAGGTTCTAATGTTTTTCAAACACGTGCAGGCTGTTTAATAGGGGCTGAA
Sulfolobus turreted icosahedral virus  NC_005892 (17664) --------------------------------------------------------------------------------
                                                                                      Section 298
                                           (23761) 23761    23770     23780     23790     23800     23810     23820     23830   23840
SARS-CoV-2 Reference Genome NC_045512.2 (23525) CATGTCAACAACTCATATGAGTGTGACATACCCATTGGTGCAGGTATATGCGCTAGTTATCAGACTCAGACTAATTCTCC
Sulfolobus turreted icosahedral virus  NC_005892 (17664) --------------------------------------------------------------------------------
                                                                                      Section 299
                                           (23841) 23841    23850     23860     23870     23880     23890     23900     23910   23920
SARS-CoV-2 Reference Genome NC_045512.2 (23605) TCGGCGGGCACGTAGTGTAGCTAGTCAATCCATCATTGCCTACACTATGTCACTTGGTGCAGAAAATTCAGTTGCTTACT
Sulfolobus turreted icosahedral virus  NC_005892 (17664) --------------------------------------------------------------------------------
                                                                                      Section 300
                                           (23921) 23921    23930     23940     23950     23960     23970     23980     23990   24000
SARS-CoV-2 Reference Genome NC_045512.2 (23685) CTAATAACTCTATTGCCATACCCACAAATTTTACTATTAGTGTTACCACAGAAATTCTACCAGTGTCTATGACCAAGACA
Sulfolobus turreted icosahedral virus  NC_005892 (17664) --------------------------------------------------------------------------------
                                                                                      Section 301
                                           (24001) 24001    24010     24020     24030     24040     24050     24060     24070   24080
SARS-CoV-2 Reference Genome NC_045512.2 (23765) TCAGTAGATTGTACAATGTACATTTGTGGTGATTCAACTGAATGCAGCAATCTTTTGTTGCAATATGGCAGTTTTTGTAC
Sulfolobus turreted icosahedral virus  NC_005892 (17664) --------------------------------------------------------------------------------
```

```
                                                                                            Section 302
                                                      (24081) 24081    24090     24100     24110     24120     24130     24140     24150   24160
     SARS-CoV-2 Reference Genome NC_045512.2 (23845) ACAATTAAACCGTGCTTTAACTGGAATAGCTGTTGAACAAGACAAAAACACCCAAGAAGTTTTTGCACAAGTCAAACAAA
Sulfolobus turreted icosahedral virus  NC_005892 (17664) --------------------------------------------------------------------------------
                                                                                            Section 303
                                                      (24161) 24161    24170     24180     24190     24200     24210     24220     24230   24240
     SARS-CoV-2 Reference Genome NC_045512.2 (23925) TTTACAAAACACCACCAATTAAAGATTTTGGTGGTTTTAATTTTTCACAAATATTACCAGATCCATCAAAACCAAGCAAG
Sulfolobus turreted icosahedral virus  NC_005892 (17664) --------------------------------------------------------------------------------
                                                                                            Section 304
                                                      (24241) 24241    24250     24260     24270     24280     24290     24300     24310   24320
     SARS-CoV-2 Reference Genome NC_045512.2 (24005) AGGTCATTTATTGAAGATCTACTTTTCAACAAAGTGACACTTGCAGATGCTGGCTTCATCAAACAATATGGTGATTGCCT
Sulfolobus turreted icosahedral virus  NC_005892 (17664) --------------------------------------------------------------------------------
                                                                                            Section 305
                                                      (24321) 24321    24330     24340     24350     24360     24370     24380     24390   24400
     SARS-CoV-2 Reference Genome NC_045512.2 (24085) TGGTGATATTGCTGCTAGAGACCTCATTTGTGCACAAAAGTTTAACGGCCTTACTGTTTTGCCACCTTTGCTCACAGATG
Sulfolobus turreted icosahedral virus  NC_005892 (17664) --------------------------------------------------------------------------------
                                                                                            Section 306
                                                      (24401) 24401    24410     24420     24430     24440     24450     24460     24470   24480
     SARS-CoV-2 Reference Genome NC_045512.2 (24165) AAATGATTGCTCAATACACTTCTGCACTGTTAGCGGGTACAATCACTTCTGGTTGGACCTTTGGTGCAGGTGCTGCATTA
Sulfolobus turreted icosahedral virus  NC_005892 (17664) --------------------------------------------------------------------------------
                                                                                            Section 307
                                                      (24481) 24481    24490     24500     24510     24520     24530     24540     24550   24560
     SARS-CoV-2 Reference Genome NC_045512.2 (24245) CAAATACCATTTGCTATGCAAATGGCTTATAGGTTTAATGGTATTGGAGTTACACAGAATGTTCTCTATGAGAACCAAAA
Sulfolobus turreted icosahedral virus  NC_005892 (17664) --------------------------------------------------------------------------------
                                                                                            Section 308
                                                      (24561) 24561    24570     24580     24590     24600     24610     24620     24630   24640
     SARS-CoV-2 Reference Genome NC_045512.2 (24325) ATTGATTGCCAACCAATTTAATAGTGCTATTGGCAAAATTCAAGACTCACTTTCTTCCACAGCAAGTGCACTTGGAAAAC
Sulfolobus turreted icosahedral virus  NC_005892 (17664) --------------------------------------------------------------------------------
```

```
                                                                                                   Section 309
                                            (24641) 24641    24650     24660     24670     24680     24690     24700     24710 24720
SARS-CoV-2 Reference Genome NC_045512.2 (24405) TTCAAGATGTGGTCAACCAAAATGCACAAGCTTTAAACACGCTTGTTAAACAACTTAGCTCCAATTTTGGTGCAATTTCA
Sulfolobus turreted icosahedral virus  NC_005892 (17664) --------------------------------------------------------------------------------
                                                                                                   Section 310
                                            (24721) 24721    24730     24740     24750     24760     24770     24780     24790 24800
SARS-CoV-2 Reference Genome NC_045512.2 (24485) AGTGTTTTAAATGATATCCTTTCACGTCTTGACAAAGTTGAGGCTGAAGTGCAAATTGATAGGTTGATCACAGGCAGACT
Sulfolobus turreted icosahedral virus  NC_005892 (17664) --------------------------------------------------------------------------------
                                                                                                   Section 311
                                            (24801) 24801    24810     24820     24830     24840     24850     24860     24870 24880
SARS-CoV-2 Reference Genome NC_045512.2 (24565) TCAAAGTTTGCAGACATATGTGACTCAACAATTAATTAGAGCTGCAGAAATCAGAGCTTCTGCTAATCTTGCTGCTACTA
Sulfolobus turreted icosahedral virus  NC_005892 (17664) --------------------------------------------------------------------------------
                                                                                                   Section 312
                                            (24881) 24881    24890     24900     24910     24920     24930     24940     24950 24960
SARS-CoV-2 Reference Genome NC_045512.2 (24645) AAATGTCAGAGTGTGTACTTGGACAATCAAAAAGAGTTGATTTTTGTGGAAAGGGCTATCATCTTATGTCCTTCCCTCAG
Sulfolobus turreted icosahedral virus  NC_005892 (17664) --------------------------------------------------------------------------------
                                                                                                   Section 313
                                            (24961) 24961    24970     24980     24990     25000     25010     25020     25030 25040
SARS-CoV-2 Reference Genome NC_045512.2 (24725) TCAGCACCTCATGGTGTAGTCTTCTTGCATGTGACTTATGTCCCTGCACAAGAAAAGAACTTCACAACTGCTCCTGCCAT
Sulfolobus turreted icosahedral virus  NC_005892 (17664) --------------------------------------------------------------------------------
                                                                                                   Section 314
                                            (25041) 25041    25050     25060     25070     25080     25090     25100     25110 25120
SARS-CoV-2 Reference Genome NC_045512.2 (24805) TTGTCATGATGGAAAAGCACACTTTCCTCGTGAAGGTGTCTTTGTTTCAAATGGCACACACTGGTTTGTAACACAAAGGA
Sulfolobus turreted icosahedral virus  NC_005892 (17664) --------------------------------------------------------------------------------
                                                                                                   Section 315
                                            (25121) 25121    25130     25140     25150     25160     25170     25180     25190 25200
SARS-CoV-2 Reference Genome NC_045512.2 (24885) ATTTTTATGAACCACAAATCATTACTACAGACAACACATTTGTGTCTGGTAACTGTGATGTTGTAATAGGAATTGTCAAC
Sulfolobus turreted icosahedral virus  NC_005892 (17664) --------------------------------------------------------------------------------
```

```
                                                                                                                    Section 316
                                                    (25201) 25201    25210     25220     25230     25240     25250     25260     25270   25280
    SARS-CoV-2 Reference Genome NC_045512.2 (24965) AACACAGTTTATGATCCTTTGCAACCTGAATTAGACTCATTCAAGGAGGAGTTAGATAAATATTTTAAGAATCATACATC
Sulfolobus turreted icosahedral virus  NC_005892 (17664) --------------------------------------------------------------------------------
                                                                                                                    Section 317
                                                    (25281) 25281    25290     25300     25310     25320     25330     25340     25350   25360
    SARS-CoV-2 Reference Genome NC_045512.2 (25045) ACCAGATGTTGATTTAGGTGACATCTCTGGCATTAATGCTTCAGTTGTAAACATTCAAAAAGAAATTGACCGCCTCAATG
Sulfolobus turreted icosahedral virus  NC_005892 (17664) --------------------------------------------------------------------------------
                                                                                                                    Section 318
                                                    (25361) 25361    25370     25380     25390     25400     25410     25420     25430   25440
    SARS-CoV-2 Reference Genome NC_045512.2 (25125) AGGTTGCCAAGAATTTAAATGAATCTCTCATCGATCTCCAAGAACTTGGAAAGTATGAGCAGTATATAAAATGGCCATGG
Sulfolobus turreted icosahedral virus  NC_005892 (17664) --------------------------------------------------------------------------------
                                                                                                                    Section 319
                                                    (25441) 25441    25450     25460     25470     25480     25490     25500     25510   25520
    SARS-CoV-2 Reference Genome NC_045512.2 (25205) TACATTTGGCTAGGTTTTATAGCTGGCTTGATTGCCATAGTAATGGTGACAATTATGCTTTGCTGTATGACCAGTTGCTG
Sulfolobus turreted icosahedral virus  NC_005892 (17664) --------------------------------------------------------------------------------
                                                                                                                    Section 320
                                                    (25521) 25521    25530     25540     25550     25560     25570     25580     25590   25600
    SARS-CoV-2 Reference Genome NC_045512.2 (25285) TAGTTGTCTCAAGGGCTGTTGTTCTTGTGGATCCTGCTGCAAATTTGATGAAGACGACTCTGAGCCAGTGCTCAAAGGAG
Sulfolobus turreted icosahedral virus  NC_005892 (17664) --------------------------------------------------------------------------------
                                                                                                                    Section 321
                                                    (25601) 25601    25610     25620     25630     25640     25650     25660     25670   25680
    SARS-CoV-2 Reference Genome NC_045512.2 (25365) TCAAATTACATTACACATAAACGAACTTATGGATTTGTTTATGAGAATCTTCACAATTGGAACTGTAACTTTGAAGCAAG
Sulfolobus turreted icosahedral virus  NC_005892 (17664) --------------------------------------------------------------------------------
                                                                                                                    Section 322
                                                    (25681) 25681    25690     25700     25710     25720     25730     25740     25750   25760
    SARS-CoV-2 Reference Genome NC_045512.2 (25445) GTGAAATCAAGGATGCTACTCCTTCAGATTTTGTTCGCGCTACTGCAACGATACCGATACAAGCCTCACTCCCTTTCGGA
Sulfolobus turreted icosahedral virus  NC_005892 (17664) --------------------------------------------------------------------------------
```

```
                                                                                      Section 323
                                          (25761) 25761    25770     25780     25790     25800     25810     25820     25830   25840
SARS-CoV-2 Reference Genome NC_045512.2 (25525) TGGCTTATTGTTGGCGTTGCACTTCTTGCTGTTTTTCAGAGCGCTTCCAAAATCATAACCCTCAAAAAGAGATGGCAACT
Sulfolobus turreted icosahedral virus  NC_005892 (17664) --------------------------------------------------------------------------------
                                                                                      Section 324
                                          (25841) 25841    25850     25860     25870     25880     25890     25900     25910   25920
SARS-CoV-2 Reference Genome NC_045512.2 (25605) AGCACTCTCCAAGGGTGTTCACTTTGTTTGCAACTTGCTGTTGTTGTTTGTAACAGTTTACTCACACCTTTTGCTCGTTG
Sulfolobus turreted icosahedral virus  NC_005892 (17664) --------------------------------------------------------------------------------
                                                                                      Section 325
                                          (25921) 25921    25930     25940     25950     25960     25970     25980     25990   26000
SARS-CoV-2 Reference Genome NC_045512.2 (25685) CTGCTGGCCTTGAAGCCCCTTTTCTCTATCTTTATGCTTTAGTCTACTTCTTGCAGAGTATAAACTTTGTAAGAATAATA
Sulfolobus turreted icosahedral virus  NC_005892 (17664) --------------------------------------------------------------------------------
                                                                                      Section 326
                                          (26001) 26001    26010     26020     26030     26040     26050     26060     26070   26080
SARS-CoV-2 Reference Genome NC_045512.2 (25765) ATGAGGCTTTGGCTTTGCTGGAAATGCCGTTCCAAAAACCCATTACTTTATGATGCCAACTATTTTCTTTGCTGGCATAC
Sulfolobus turreted icosahedral virus  NC_005892 (17664) --------------------------------------------------------------------------------
                                                                                      Section 327
                                          (26081) 26081    26090     26100     26110     26120     26130     26140     26150   26160
SARS-CoV-2 Reference Genome NC_045512.2 (25845) TAATTGTTACGACTATTGTATACCTTACAATAGTGTAACTTCTTCAATTGTCATTACTTCAGGTGATGGCACAACAAGTC
Sulfolobus turreted icosahedral virus  NC_005892 (17664) --------------------------------------------------------------------------------
                                                                                      Section 328
                                          (26161) 26161    26170     26180     26190     26200     26210     26220     26230   26240
SARS-CoV-2 Reference Genome NC_045512.2 (25925) CTATTTCTGAACATGACTACCAGATTGGTGGTTATACTGAAAAATGGGAATCTGGAGTAAAAGACTGTGTTGTATTACAC
Sulfolobus turreted icosahedral virus  NC_005892 (17664) --------------------------------------------------------------------------------
                                                                                      Section 329
                                          (26241) 26241    26250     26260     26270     26280     26290     26300     26310   26320
SARS-CoV-2 Reference Genome NC_045512.2 (26005) AGTTACTTCACTTCAGACTATTACCAGCTGTACTCAACTCAATTGAGTACAGACACTGGTGTTGAACATGTTACCTTCTT
Sulfolobus turreted icosahedral virus  NC_005892 (17664) --------------------------------------------------------------------------------
```

```
                                                                                                                 Section 330
                                                   (26321) 26321    26330     26340     26350     26360     26370     26380     26390  26400
SARS-CoV-2 Reference Genome NC_045512.2 (26085) CATCTACAATAAAATTGTTGATGAGCCTGAAGAACATGTCCAAATTCACACAATCGACGGTTCATCCGGAGTTGTTAATC
Sulfolobus turreted icosahedral virus  NC_005892 (17664) --------------------------------------------------------------------------------
                                                                                                                 Section 331
                                                   (26401) 26401    26410     26420     26430     26440     26450     26460     26470  26480
SARS-CoV-2 Reference Genome NC_045512.2 (26165) CAGTAATGGAACCAATTTATGATGAACCGACGACGACTACTAGCGTGCCTTTGTAAGCACAAGCTGATGAGTACGAACTT
Sulfolobus turreted icosahedral virus  NC_005892 (17664) --------------------------------------------------------------------------------
                                                                                                                 Section 332
                                                   (26481) 26481    26490     26500     26510     26520     26530     26540     26550  26560
SARS-CoV-2 Reference Genome NC_045512.2 (26245) ATGTACTCATTCGTTTCGGAAGAGACAGGTACGTTAATAGTTAATAGCGTACTTCTTTTTCTTGCTTTCGTGGTATTCTT
Sulfolobus turreted icosahedral virus  NC_005892 (17664) --------------------------------------------------------------------------------
                                                                                                                 Section 333
                                                   (26561) 26561    26570     26580     26590     26600     26610     26620     26630  26640
SARS-CoV-2 Reference Genome NC_045512.2 (26325) GCTAGTTACACTAGCCATCCTTACTGCGCTTCGATTGTGTGCGTACTGCTGCAATATTGTTAACGTGAGTCTTGTAAAAC
Sulfolobus turreted icosahedral virus  NC_005892 (17664) --------------------------------------------------------------------------------
                                                                                                                 Section 334
                                                   (26641) 26641    26650     26660     26670     26680     26690     26700     26710  26720
SARS-CoV-2 Reference Genome NC_045512.2 (26405) CTTCTTTTTACGTTTACTCTCGTGTTAAAAATCTGAATTCTTCTAGAGTTCCTGATCTTCTGGTCTAAACGAACTAAATA
Sulfolobus turreted icosahedral virus  NC_005892 (17664) --------------------------------------------------------------------------------
                                                                                                                 Section 335
                                                   (26721) 26721    26730     26740     26750     26760     26770     26780     26790  26800
SARS-CoV-2 Reference Genome NC_045512.2 (26485) TTATATTAGTTTTTCTGTTTGGAACTTTAATTTTAGCCATGGCAGATTCCAACGGTACTATTACCGTTGAAGAGCTTAAA
Sulfolobus turreted icosahedral virus  NC_005892 (17664) --------------------------------------------------------------------------------
                                                                                                                 Section 336
                                                   (26801) 26801    26810     26820     26830     26840     26850     26860     26870  26880
SARS-CoV-2 Reference Genome NC_045512.2 (26565) AAGCTCCTTGAACAATGGAACCTAGTAATAGGTTTCCTATTCCTTACATGGATTTGTCTTCTACAATTTGCCTATGCCAA
Sulfolobus turreted icosahedral virus  NC_005892 (17664) --------------------------------------------------------------------------------
```

Section 337

```
                                              (26881) 26881    26890     26900     26910     26920     26930     26940     26950    26960
         SARS-CoV-2 Reference Genome NC_045512.2 (26645) CAGGAATAGGTTTTTGTATATAATTAAGTTAATTTTCCTCTGGCTGTTATGGCCAGTAACTTTAGCTTGTTTTGTGCTTG
Sulfolobus turreted icosahedral virus  NC_005892 (17664) --------------------------------------------------------------------------------
```

Section 338

```
                                              (26961) 26961    26970     26980     26990     27000     27010     27020     27030    27040
         SARS-CoV-2 Reference Genome NC_045512.2 (26725) CTGCTGTTTACAGAATAAATTGGATCACCGGTGGAATTGCTATCGCAATGGCTTGTCTTGTAGGCTTGATGTGGCTCAGC
Sulfolobus turreted icosahedral virus  NC_005892 (17664) --------------------------------------------------------------------------------
```

Section 339

```
                                              (27041) 27041    27050     27060     27070     27080     27090     27100     27110    27120
         SARS-CoV-2 Reference Genome NC_045512.2 (26805) TACTTCATTGCTTCTTTCAGACTGTTTGCGCGTACGCGTTCCATGTGGTCATTCAATCCAGAAACTAACATTCTTCTCAA
Sulfolobus turreted icosahedral virus  NC_005892 (17664) --------------------------------------------------------------------------------
```

Section 340

```
                                              (27121) 27121    27130     27140     27150     27160     27170     27180     27190    27200
         SARS-CoV-2 Reference Genome NC_045512.2 (26885) CGTGCCACTCCATGGCACTATTCTGACCAGACCGCTTCTAGAAAGTGAACTCGTAATCGGAGCTGTGATCCTTCGTGGAC
Sulfolobus turreted icosahedral virus  NC_005892 (17664) --------------------------------------------------------------------------------
```

Section 341

```
                                              (27201) 27201    27210     27220     27230     27240     27250     27260     27270    27280
         SARS-CoV-2 Reference Genome NC_045512.2 (26965) ATCTTCGTATTGCTGGACACCATCTAGGACGCTGTGACATCAAGGACCTGCCTAAAGAAATCACTGTTGCTACATCACGA
Sulfolobus turreted icosahedral virus  NC_005892 (17664) --------------------------------------------------------------------------------
```

Section 342

```
                                              (27281) 27281    27290     27300     27310     27320     27330     27340     27350    27360
         SARS-CoV-2 Reference Genome NC_045512.2 (27045) ACGCTTTCTTATTACAAATTGGGAGCTTCGCAGCGTGTAGCAGGTGACTCAGGTTTTGCTGCATACAGTCGCTACAGGAT
Sulfolobus turreted icosahedral virus  NC_005892 (17664) --------------------------------------------------------------------------------
```

Section 343

```
                                              (27361) 27361    27370     27380     27390     27400     27410     27420     27430    27440
         SARS-CoV-2 Reference Genome NC_045512.2 (27125) TGGCAACTATAAATTAAACACAGACCATTCCAGTAGCAGTGACAATATTGCTTTGCTTGTACAGTAAGTGACAACAGATG
Sulfolobus turreted icosahedral virus  NC_005892 (17664) --------------------------------------------------------------------------------
```

```
                                                                                                                   Section 344
                                              (27441) 27441    27450     27460     27470     27480     27490     27500     27510    27520
SARS-CoV-2 Reference Genome NC_045512.2       (27205) TTTCATCTCGTTGACTTTCAGGTTACTATAGCAGAGATATTACTAATTATTATGAGGACTTTTAAAGTTTCCATTTGGAA
Sulfolobus turreted icosahedral virus NC_005892 (17664) --------------------------------------------------------------------------------
                                                                                                                   Section 345
                                              (27521) 27521    27530     27540     27550     27560     27570     27580     27590    27600
SARS-CoV-2 Reference Genome NC_045512.2       (27285) TCTTGATTACATCATAAACCTCATAATTAAAAATTTATCTAAGTCACTAACTGAGAATAAATATTCTCAATTAGATGAAG
Sulfolobus turreted icosahedral virus NC_005892 (17664) --------------------------------------------------------------------------------
                                                                                                                   Section 346
                                              (27601) 27601    27610     27620     27630     27640     27650     27660     27670    27680
SARS-CoV-2 Reference Genome NC_045512.2       (27365) AGCAACCAATGGAGATTGATTAAACGAACATGAAAATTATTCTTTTCTTGGCACTGATAACACTCGCTACTTGTGAGCTT
Sulfolobus turreted icosahedral virus NC_005892 (17664) --------------------------------------------------------------------------------
                                                                                                                   Section 347
                                              (27681) 27681    27690     27700     27710     27720     27730     27740     27750    27760
SARS-CoV-2 Reference Genome NC_045512.2       (27445) TATCACTACCAAGAGTGTGTTAGAGGTACAACAGTACTTTTAAAAGAACCTTGCTCTTCTGGAACATACGAGGGCAATTC
Sulfolobus turreted icosahedral virus NC_005892 (17664) --------------------------------------------------------------------------------
                                                                                                                   Section 348
                                              (27761) 27761    27770     27780     27790     27800     27810     27820     27830    27840
SARS-CoV-2 Reference Genome NC_045512.2       (27525) ACCATTTCATCCTCTAGCTGATAACAAATTTGCACTGACTTGCTTTAGCACTCAATTTGCTTTTGCTTGTCCTGACGGCG
Sulfolobus turreted icosahedral virus NC_005892 (17664) --------------------------------------------------------------------------------
                                                                                                                   Section 349
                                              (27841) 27841    27850     27860     27870     27880     27890     27900     27910    27920
SARS-CoV-2 Reference Genome NC_045512.2       (27605) TAAAACACGTCTATCAGTTACGTGCCAGATCAGTTTCACCTAAACTGTTCATCAGACAAGAGGAAGTTCAAGAACTTTAC
Sulfolobus turreted icosahedral virus NC_005892 (17664) --------------------------------------------------------------------------------
                                                                                                                   Section 350
                                              (27921) 27921    27930     27940     27950     27960     27970     27980     27990    28000
SARS-CoV-2 Reference Genome NC_045512.2       (27685) TCTCCAATTTTTCTTATTGTTGCGGCAATAGTGTTTATAACACTTTGCTTCACACTCAAAAGAAAGACAGAATGATTGAA
Sulfolobus turreted icosahedral virus NC_005892 (17664) --------------------------------------------------------------------------------
```

Section 351

(28001) 28001 28010 28020 28030 28040 28050 28060 28070 28080
SARS-CoV-2 Reference Genome NC_045512.2 (27765) CTTTCATTAATTGACTTCTATTTGTGCTTTTTAGCCTTTCTGCTATTCCTTGTTTTAATTATGCTTATTATCTTTTGGTT
Sulfolobus turreted icosahedral virus NC_005892 (17664) --

Section 352

(28081) 28081 28090 28100 28110 28120 28130 28140 28150 28160
SARS-CoV-2 Reference Genome NC_045512.2 (27845) CTCACTTGAACTGCAAGATCATAATGAAACTTGTCACGCCTAAACGAACATGAAATTTCTTGTTTTCTTAGGAATCATCA
Sulfolobus turreted icosahedral virus NC_005892 (17664) --

Section 353

(28161) 28161 28170 28180 28190 28200 28210 28220 28230 28240
SARS-CoV-2 Reference Genome NC_045512.2 (27925) CAACTGTAGCTGCATTTCACCAAGAATGTAGTTTACAGTCATGTACTCAACATCAACCATATGTAGTTGATGACCCGTGT
Sulfolobus turreted icosahedral virus NC_005892 (17664) --

Section 354

(28241) 28241 28250 28260 28270 28280 28290 28300 28310 28320
SARS-CoV-2 Reference Genome NC_045512.2 (28005) CCTATTCACTTCTATTCTAAATGGTATATTAGAGTAGGAGCTAGAAAATCAGCACCTTTAATTGAATTGTGCGTGGATGA
Sulfolobus turreted icosahedral virus NC_005892 (17664) --

Section 355

(28321) 28321 28330 28340 28350 28360 28370 28380 28390 28400
SARS-CoV-2 Reference Genome NC_045512.2 (28085) GGCTGGTTCTAAATCACCCATTCAGTACATCGATATCGGTAATTATACAGTTTCCTGTTTACCTTTTACAATTAATTGCC
Sulfolobus turreted icosahedral virus NC_005892 (17664) --

Section 356

(28401) 28401 28410 28420 28430 28440 28450 28460 28470 28480
SARS-CoV-2 Reference Genome NC_045512.2 (28165) AGGAACCTAAATTGGGTAGTCTTGTAGTGCGTTGTTCGTTCTATGAAGACTTTTTAGAGTATCATGACGTTCGTGTTGTT
Sulfolobus turreted icosahedral virus NC_005892 (17664) --

Section 357

(28481) 28481 28490 28500 28510 28520 28530 28540 28550 28560
SARS-CoV-2 Reference Genome NC_045512.2 (28245) TTAGATTTCATCTAAACGAACAAACTAAAATGTCTGATAATGGACCCCAAAATCAGCGAAATGCACCCCGCATTACGTTT
Sulfolobus turreted icosahedral virus NC_005892 (17664) --

```
                                                                                                   Section 358
                                                    (28561) 28561    28570     28580     28590     28600     28610     28620     28630   28640
       SARS-CoV-2 Reference Genome NC_045512.2 (28325) GGTGGACCCTCAGATTCAACTGGCAGTAACCAGAATGGAGAACGCAGTGGGGCGCGATCAAAACAACGTCGGCCCCAAGG
Sulfolobus turreted icosahedral virus  NC_005892 (17664) --------------------------------------------------------------------------------
                                                                                                   Section 359
                                                    (28641) 28641    28650     28660     28670     28680     28690     28700     28710   28720
       SARS-CoV-2 Reference Genome NC_045512.2 (28405) TTTACCCAATAATACTGCGTCTTGGTTCACCGCTCTCACTCAACATGGCAAGGAAGACCTTAAATTCCCTCGAGGACAAG
Sulfolobus turreted icosahedral virus  NC_005892 (17664) --------------------------------------------------------------------------------
                                                                                                   Section 360
                                                    (28721) 28721    28730     28740     28750     28760     28770     28780     28790   28800
       SARS-CoV-2 Reference Genome NC_045512.2 (28485) GCGTTCCAATTAACACCAATAGCAGTCCAGATGACCAAATTGGCTACTACCGAAGAGCTACCAGACGAATTCGTGGTGGT
Sulfolobus turreted icosahedral virus  NC_005892 (17664) --------------------------------------------------------------------------------
                                                                                                   Section 361
                                                    (28801) 28801    28810     28820     28830     28840     28850     28860     28870   28880
       SARS-CoV-2 Reference Genome NC_045512.2 (28565) GACGGTAAAATGAAAGATCTCAGTCCAAGATGGTATTTCTACTACCTAGGAACTGGGCCAGAAGCTGGACTTCCCTATGG
Sulfolobus turreted icosahedral virus  NC_005892 (17664) --------------------------------------------------------------------------------
                                                                                                   Section 362
                                                    (28881) 28881    28890     28900     28910     28920     28930     28940     28950   28960
       SARS-CoV-2 Reference Genome NC_045512.2 (28645) TGCTAACAAAGACGGCATCATATGGGTTGCAACTGAGGGAGCCTTGAATACACCAAAAGATCACATTGGCACCCGCAATC
Sulfolobus turreted icosahedral virus  NC_005892 (17664) --------------------------------------------------------------------------------
                                                                                                   Section 363
                                                    (28961) 28961    28970     28980     28990     29000     29010     29020     29030   29040
       SARS-CoV-2 Reference Genome NC_045512.2 (28725) CTGCTAACAATGCTGCAATCGTGCTACAACTTCCTCAAGGAACAACATTGCCAAAAGGCTTCTACGCAGAAGGGAGCAGA
Sulfolobus turreted icosahedral virus  NC_005892 (17664) --------------------------------------------------------------------------------
                                                                                                   Section 364
                                                    (29041) 29041    29050     29060     29070     29080     29090     29100     29110   29120
       SARS-CoV-2 Reference Genome NC_045512.2 (28805) GGCGGCAGTCAAGCCTCTTCTCGTTCCTCATCACGTAGTCGCAACAGTTCAAGAAATTCAACTCCAGGCAGCAGTAGGGG
Sulfolobus turreted icosahedral virus  NC_005892 (17664) --------------------------------------------------------------------------------
```

```
                                                                                                Section 365
                                          (29121) 29121    29130     29140     29150     29160     29170     29180     29190  29200
SARS-CoV-2 Reference Genome NC_045512.2 (28885) AACTTCTCCTGCTAGAATGGCTGGCAATGGCGGTGATGCTGCTCTTGCTTTGCTGCTGCTTGACAGATTGAACCAGCTTG
 Sulfolobus turreted icosahedral virus NC_005892 (17664) --------------------------------------------------------------------------------
                                                                                                Section 366
                                          (29201) 29201    29210     29220     29230     29240     29250     29260     29270  29280
SARS-CoV-2 Reference Genome NC_045512.2 (28965) AGAGCAAAATGTCTGGTAAAGGCCAACAACAACAAGGCCAAACTGTCACTAAGAAATCTGCTGCTGAGGCTTCTAAGAAG
 Sulfolobus turreted icosahedral virus NC_005892 (17664) --------------------------------------------------------------------------------
                                                                                                Section 367
                                          (29281) 29281    29290     29300     29310     29320     29330     29340     29350  29360
SARS-CoV-2 Reference Genome NC_045512.2 (29045) CCTCGGCAAAAACGTACTGCCACTAAAGCATACAATGTAACACAAGCTTTCGGCAGACGTGGTCCAGAACAAACCCAAGG
 Sulfolobus turreted icosahedral virus NC_005892 (17664) --------------------------------------------------------------------------------
                                                                                                Section 368
                                          (29361) 29361    29370     29380     29390     29400     29410     29420     29430  29440
SARS-CoV-2 Reference Genome NC_045512.2 (29125) AAATTTTGGGGACCAGGAACTAATCAGACAAGGAACTGATTACAAACATTGGCCGCAAATTGCACAATTTGCCCCCAGCG
 Sulfolobus turreted icosahedral virus NC_005892 (17664) --------------------------------------------------------------------------------
                                                                                                Section 369
                                          (29441) 29441    29450     29460     29470     29480     29490     29500     29510  29520
SARS-CoV-2 Reference Genome NC_045512.2 (29205) CTTCAGCGTTCTTCGGAATGTCGCGCATTGGCATGGAAGTCACACCTTCGGGAACGTGGTTGACCTACACAGGTGCCATC
 Sulfolobus turreted icosahedral virus NC_005892 (17664) --------------------------------------------------------------------------------
                                                                                                Section 370
                                          (29521) 29521    29530     29540     29550     29560     29570     29580     29590  29600
SARS-CoV-2 Reference Genome NC_045512.2 (29285) AAATTGGATGACAAAGATCCAAATTTCAAAGATCAAGTCATTTTGCTGAATAAGCATATTGACGCATACAAAACATTCCC
 Sulfolobus turreted icosahedral virus NC_005892 (17664) --------------------------------------------------------------------------------
                                                                                                Section 371
                                          (29601) 29601    29610     29620     29630     29640     29650     29660     29670  29680
SARS-CoV-2 Reference Genome NC_045512.2 (29365) ACCAACAGAGCCTAAAAAGGACAAAAAGAAGAAGGCTGATGAAACTCAAGCCTTACCGCAGAGACAGAAGAAACAGCAAA
 Sulfolobus turreted icosahedral virus NC_005892 (17664) --------------------------------------------------------------------------------
```

```
                                                            Section 372
                                          (29681) 29681    29690     29700     29710     29720     29730     29740     29750   29760
  SARS-CoV-2 Reference Genome NC_045512.2 (29445) CTGTGACTCTTCTTCCTGCTGCAGATTTGGATGATTTCTCCAAACAATTGCAACAATCCATGAGCAGTGCTGACTCAACT
Sulfolobus turreted icosahedral virus  NC_005892 (17664) --------------------------------------------------------------------------------
                                                            Section 373
                                          (29761) 29761    29770     29780     29790     29800     29810     29820     29830   29840
  SARS-CoV-2 Reference Genome NC_045512.2 (29525) CAGGCCTAAACTCATGCAGACCACACAAGGCAGATGGGCTATATAAACGTTTTCGCTTTTCCGTTTACGATATATAGTCT
Sulfolobus turreted icosahedral virus  NC_005892 (17664) --------------------------------------------------------------------------------
                                                            Section 374
                                          (29841) 29841    29850     29860     29870     29880     29890     29900     29910   29920
  SARS-CoV-2 Reference Genome NC_045512.2 (29605) ACTCTTGTGCAGAATGAATTCTCGTAACTACATAGCACAAGTAGATGTAGTTAACTTTAATCTCACATAGCAATCTTTAA
Sulfolobus turreted icosahedral virus  NC_005892 (17664) --------------------------------------------------------------------------------
                                                            Section 375
                                          (29921) 29921    29930     29940     29950     29960     29970     29980     29990   30000
  SARS-CoV-2 Reference Genome NC_045512.2 (29685) TCAGTGTGTAACATTAGGGAGGACTTGAAAGAGCCACCACATTTTCACCGAGGCCACGCGGAGTACGATCGAGTGTACAG
Sulfolobus turreted icosahedral virus  NC_005892 (17664) --------------------------------------------------------------------------------
                                                            Section 376
                                          (30001) 30001    30010     30020     30030     30040     30050     30060     30070   30080
  SARS-CoV-2 Reference Genome NC_045512.2 (29765) TGAACAATGCTAGGGAGAGCTGCCTATATGGAAGAGCCCTAATGTGTAAAATTAATTTTAGTAGTGCTATCCCCATGTGA
Sulfolobus turreted icosahedral virus  NC_005892 (17664) --------------------------------------------------------------------------------
                                                            Section 377
                                          (30081) 30081    30090     30100     30110     30120                30139
  SARS-CoV-2 Reference Genome NC_045512.2 (29845) TTTTAATAGCTTCTTAGGAGAATGACAAAAAAAAAAAAAAAAAAAAAAAAAAAAAAAAA
Sulfolobus turreted icosahedral virus  NC_005892 (17664) -----------------------------------------------------------
```
