# Supplementary material for: Ubiquitous Micro-Modular Homologies among Genomes from Viruses to Bacteria to Human Mitochondrial DNA: Platforms for Recombination during Evolution?
Source: Viruses. 2022 Apr 24;14(5):885. doi: 10.3390/v14050885 (PMC9147251; doi:10.3390/v14050885)
Supplement: Supplementary file 1 [file viruses-14-00885-s001.zip › Fig. S3A, SARS-CoV-2 & Alternaria brassicicola endornavirus.pdf]

Sequence	Start	Alignment
**Section 1**	(1)	1 … 80
SARS-CoV-2 Reference Genome NC 045512.2	(1)	ATTAAAGGTTTATACCTTCCCAGGTAACAAACCAACCAACTTTCGATCTCTTGTAGATCTGTTCTCTAAACGAACTTTAA
Alternaria brassicicola endornavirus isolate 1 NC...	(1)	--
**Section 2**	(81)	81 … 160
SARS-CoV-2 Reference Genome NC 045512.2	(81)	AATCTGTGTGGCTGTCACTCGGCTGCATGCTTAGTGCACTCACGCAGTATAATTAATAACTAATTACTGTCGTTGACAGG
Alternaria brassicicola endornavirus isolate 1 NC...	(1)	--
**Section 3**	(161)	161 … 240
SARS-CoV-2 Reference Genome NC 045512.2	(161)	ACACGAGTAACTCGTCTATCTTCTGCAGGCTGCTTACGGTTTCGTCCGTGTTGCAGCCGATCATCAGCACATCTAGGTTT
Alternaria brassicicola endornavirus isolate 1 NC...	(1)	--
**Section 4**	(241)	241 … 320
SARS-CoV-2 Reference Genome NC 045512.2	(241)	CGTCCGGGTGTGACCGAAAGGTAAGATGGAGAGCCTTGTCCCTGGTTTCAACGAGAAAACACACGTCCAACTCAGTTTGC
Alternaria brassicicola endornavirus isolate 1 NC...	(1)	--
**Section 5**	(321)	321 … 400
SARS-CoV-2 Reference Genome NC 045512.2	(321)	CTGTTTTACAGGTTCGCGACGTGCTCGTACGTGGCTTTGGAGACTCCGTGGAGGAGGTCTTATCAGAGGCACGTCAACAT
Alternaria brassicicola endornavirus isolate 1 NC...	(1)	--------------------------------GAATTATAAAACGAATCAAATACTATCAATTTTAAAAAACAACACAAA
**Section 6**	(401)	401 … 480
SARS-CoV-2 Reference Genome NC 045512.2	(401)	CTTAAAGATGGCACTTGTGGCTTAGTAGAAGTTGAAAAAGGCGTTT-TGCCTCAACTTGAACAGCCCTATGTGTTCATCA
Alternaria brassicicola endornavirus isolate 1 NC...	(49)	CAAAATGGAGGGCCAACGAATTAACATCAACAACATAGAATCGGGTGTGAGAGCACCCGATTCTGAGTTTCCCCGAACTC
**Section 7**	(481)	481 … 560
SARS-CoV-2 Reference Genome NC 045512.2	(480)	AACGTTCGGATGCTCGAACTGCACCTCATGGTCATGTTATGGTTGAGCTGGTAGCAGAACTCGAAGGCATTCAGTACGGT
Alternaria brassicicola endornavirus isolate 1 NC...	(129)	ACAACAACAACGGGGAAAACAAAAATGGGGGACAATTTGTGCCCCATAGATCTACCGCTATCGA-GATAGAGAGGAGATC

Section 8

```
                                                          (561) 561      570       580       590       600       610       620       630       640
SARS-CoV-2 Reference Genome NC_045512.2                   (560) CGTAGTGGTGAGACACTTGGTGTCCTTGTCCCTCATGTGGGCGAAATACCAGTGGCTTACCGCAAGGTTCTTCTTCGTAA
Alternaria brassicicola endornavirus isolate 1 NC...      (208) AGCAGATGGGCCAGCTTTGGGCTACTGGGCCCCTTCATTTACACGATCCACTGGGCCGAGCCCGTCGGAGATCATTAGCC
```

Section 9

```
                                                          (641) 641      650       660       670       680       690       700       710       720
SARS-CoV-2 Reference Genome NC_045512.2                   (640) GAACGGT----AATAAAGGAGCTGGTGGCCATAGTTACGGCGCCGATCTAAAGTCATTTGACTTAGGCGACGAGCTTGGC
Alternaria brassicicola endornavirus isolate 1 NC...      (288) CGGTGGTGCCCATGATCGACAACTCTTTCGAGAGTAATCGACGCATGCGGGACCTAGATGAGTTGCTACAGCCGAATTGG
```

Section 10

```
                                                          (721) 721      730       740       750       760       770       780       790       800
SARS-CoV-2 Reference Genome NC_045512.2                   (716) ACTGATCCTTATGAAGATTTTCAAGAAAACTGGAACACTAAACATAGCAGTGGTGTTACCCGTGAACTCATGCGTGAGCT
Alternaria brassicicola endornavirus isolate 1 NC...      (368) AACAATGCTGAGGGCCTGTTCCTTGTCGTTGAAAGCACCTCAAGCAGGC-TGTTGCTTATGGAGAGCGGCGGGCGATTG
```

Section 11

```
                                                          (801) 801      810       820       830       840       850       860       870       880
SARS-CoV-2 Reference Genome NC_045512.2                   (796) TAACGGAGGGG-CATACACTCGCTATGTCGATAACAACTTCTGTGGCCCTGATGGCTACCCTCTTGAGTGCATTAAAGAC
Alternaria brassicicola endornavirus isolate 1 NC...      (447) AACGTCTGAGCTCACAAGCTCGTACTAGAAGCATAACAATCGGCCGTGCCGACGTCGAATTATCGCCAAGGCGACAGAGG
```

Section 12

```
                                                          (881) 881      890       900       910       920       930       940       950       960
SARS-CoV-2 Reference Genome NC_045512.2                   (875) CTTCTAGCACGTGCTGGTAA-AGCTTCATGCACTTTGTCCGAACAACTGGACTTTAT-TGACACTAAGAGGGGTGTATAC
Alternaria brassicicola endornavirus isolate 1 NC...      (527) TTGCTAGCCGCACATGTCATCAGCCCCATGGCTTTTGACTTTAGTAGTCTTATGGCTATGAGCCGCAGCCCTGTCAACAC
```

Section 13

```
                                                          (961) 961      970       980       990       1000      1010      1020      1030      1040
SARS-CoV-2 Reference Genome NC_045512.2                   (953) TGCTGCCGTGAACATGAGCATGAAATTGCTTGGTACACGGAACGTTCTGAAAGAGCTATGAATTGCAGACACCTTTTGA
Alternaria brassicicola endornavirus isolate 1 NC...      (607) AGCCGA----AACAACGCCAGTCACATGCTCGCCGGCCGCTGTGCGAAGCAA-GAGCTTTGTAAAGCTTCAAGAGGATGC
```

Section 14

```
                                                          (1041) 1041    1050      1060      1070      1080      1090      1100      1110      1120
SARS-CoV-2 Reference Genome NC_045512.2                   (1033) AATTAAATTGGCAAAGAAATTTGACACCTTCAATGGGAATGTCCAAATTTTGTATTTCCCTTAAATTCCATAATCAAGA
Alternaria brassicicola endornavirus isolate 1 NC...      (682) CAAAGCATTTGCAGGGATGCTTAAGGACATAACTGTC-AATCGCAAGCATCTGAAGACGCTATCAAGTCAGGCTGGAATT
```

```
                                                                                                   Section 15
                                                          1121      1130      1140      1150      1160      1170      1180      1190      1200
                                                  (1121)
SARS-CoV-2 Reference Genome NC 045512.2           (1113)  CTATTCAACCAAGGGTTGAAAAGAAAAAGCTTGATGGCTTTATGGGTAGAATTCGATCTGTCTATCCAGTTGCGTCACCA
Alternaria brassicicola endornavirus isolate 1 NC... (761)  GACTTGACGTCGTCTCGCAATCGCATGACGTATATGGCATGGATGGAACAGA-CGACCGGCTCGTTTGCACATATAACGA
                                                                                                   Section 16
                                                          1201      1210      1220      1230      1240      1250      1260      1270      1280
                                                  (1201)
SARS-CoV-2 Reference Genome NC 045512.2           (1193)  AATGAATGCAACCAAATGTGCCTTTCAACTCTCATGAAGTGTGATCATTGTGGTGAAACTTCATGGCAGACGGGCGATTT
Alternaria brassicicola endornavirus isolate 1 NC... (840)  ACGGCATGAAACACATTGGTGGCGAGAACATCGACCACTTGGTTCGTTCATTCCTTTGCGGCTTCGTGCAACCACTATCA
                                                                                                   Section 17
                                                          1281      1290      1300      1310      1320      1330      1340      1350      1360
                                                  (1281)
SARS-CoV-2 Reference Genome NC 045512.2           (1273)  TGTTAAAGCCACTTGCGAATTTTGTGGCACTGAGAATTTGACTAAAGAAGGTGCCACTACTTGTG-GTTACTTACCCCAA
Alternaria brassicicola endornavirus isolate 1 NC... (920)  ACGTTTAGCACAGCATGGGTTGTGCGTGCTTGCTACGACAAACTAGACCCGAGAATGGGCTTCAACGTTAAACCCCTAAC
                                                                                                   Section 18
                                                          1361      1370      1380      1390      1400      1410      1420      1430      1440
                                                  (1361)
SARS-CoV-2 Reference Genome NC 045512.2           (1352)  AATGCTGTTGTTAAAATTTATTGTCCAGCATGTCACAATTCAGAAGTAGGACCTGAGCATAGTCTTGCCGAATACCATAA
Alternaria brassicicola endornavirus isolate 1 NC... (1000) CAAGGCGGAGATGCTGCTCGTCGCTCAAAGAGAAACTGGGACAACAAAGTTCAGGTGCA-AGATCAGCCTATTCCAACAC
                                                                                                   Section 19
                                                          1441      1450      1460      1470      1480      1490      1500      1510      1520
                                                  (1441)
SARS-CoV-2 Reference Genome NC 045512.2           (1432)  TGAATCTGGCTTGAAAACCATTCTTCGTAAGGGTGGTCGCACTATTGCCTTTGGAGGCTGTGTGTTCTCTTATGTTGGTT
Alternaria brassicicola endornavirus isolate 1 NC... (1079) TGGATC-------A--ATGGGACAATGTACGTGGGGCTCA-TGTTGAGGAAGGCGATGATGAGGTCGATTTAATCGACT
                                                                                                   Section 20
                                                          1521      1530      1540      1550      1560      1570      1580      1590      1600
                                                  (1521)
SARS-CoV-2 Reference Genome NC 045512.2           (1512)  GCCATAACAAGTGTGCCTATTGGGTTCCACGTGCTAGCGCTAACATAGGTTGTAACCATACA---GGTGTTGTTGGAGAA
Alternaria brassicicola endornavirus isolate 1 NC... (1149) TCCACTTCGATTTCATATTTGGAAAGTCGAACGACATTGAGAAAGTGTTGAAGAACCACATAATAGGTGACGTTGCCACA
                                                                                                   Section 21
                                                          1601      1610      1620      1630      1640      1650      1660      1670      1680
                                                  (1601)
SARS-CoV-2 Reference Genome NC 045512.2           (1589)  GGTTCCGAAGGTCTTAATGACAACCTTCTTGAAATACTCCAAAAAGAGAAAGTCAACATCAATATTGTTGGTGACTTTAA
Alternaria brassicicola endornavirus isolate 1 NC... (1229) GCGATCAAGACGCAGCAGGTGCGCGGCAACTTAGATTTACACAACAGCAAAGTTATCAACCAGCTCGTCAAGGCGAACGC
```

```
                                                                      Section 22
                                              (1681) 1681     1690      1700      1710      1720      1730      1740      1750      1760
           SARS-CoV-2 Reference Genome NC_045512.2 (1669) ACTTAATGAAGAGATCGCCATTATTTTGGCATCTTTTTCTGCTTCCACAAGTGCTTTTGTGGAAACTGTGAAAGGTTTGG
Alternaria brassicicola endornavirus isolate 1 NC... (1309) TATGCGTGATTACCAAAT-ACCATCCAACACCAGTGATGCCATAGTAGACGCACTGTCGCTGGCGTGGCCCAACCGAACA
                                                                      Section 23
                                              (1761) 1761     1770      1780      1790      1800      1810      1820      1830      1840
           SARS-CoV-2 Reference Genome NC_045512.2 (1749) ATTATAAAGCATTCAAACAAATTGTTGAATCCTGTGGTAATTTTAAAGTTACAAAAGGAAAAGCTAAAAAAGGTGCCTGG
Alternaria brassicicola endornavirus isolate 1 NC... (1388) TTTATCAAAAGTAGTAACAAAGCGGACAATGGGCACGCTTATCACGCGGCGAACAGGGACTTGGTAAGAGAGAACCTAGT
                                                                      Section 24
                                              (1841) 1841     1850      1860      1870      1880      1890      1900      1910      1920
           SARS-CoV-2 Reference Genome NC_045512.2 (1829) AATATTGGTGAACAGAAATCAATACTGAGTCCTCTTTATGCATTTGCATCAGAGGCTGCTCGTGTTGTACGATCAATTTT
Alternaria brassicicola endornavirus isolate 1 NC... (1468) CATGA-GGTTCCCGAAGAGCGGCCTAGTTGTGGACATAGGTGGTGCATATAGCAGCCACATTAGAAGTGGCTTCTGGAAC
                                                                      Section 25
                                              (1921) 1921     1930      1940      1950      1960      1970      1980      1990      2000
           SARS-CoV-2 Reference Genome NC_045512.2 (1909) CTCCCGCACTCTTGAAACTGCTCAAAATTCTGTGCGTGTTTTACAGAAGGCCGCTATAACAATACTAGATGGAATTTCAC
Alternaria brassicicola endornavirus isolate 1 NC... (1547) GTGCATAGCTGCTTC--CCGGTTTTAGATGAGAATGATGCCAAGAGGAAAATGGAAAACGACACAGGAATCAAAAATTAC
                                                                      Section 26
                                              (2001) 2001     2010      2020      2030      2040      2050      2060      2070      2080
           SARS-CoV-2 Reference Genome NC_045512.2 (1989) AG-TATTCACTGAGACTCATTGATGCTATGATGTTCACATCTGATTTGGCTACTAACAATCTAGTTGTAATGGCCTACAT
Alternaria brassicicola endornavirus isolate 1 NC... (1625) ATACA-TGACACAGAAACTATGATGCAATCAAGC----AAAAGCGTTGTCAGCGCAGACAGGAGGGAGGCCGTTGCCAGA
                                                                      Section 27
                                              (2081) 2081     2090      2100      2110      2120      2130      2140      2150      2160
           SARS-CoV-2 Reference Genome NC_045512.2 (2068) TACAGGTGGTGTTGTTCAGTTGACT--TCGCAGTGGCTAACT-----AACATCTTTGGCACTGTTTATGAAAAACTCA--
Alternaria brassicicola endornavirus isolate 1 NC... (1700) ATAAAATCAGGCA----AGCACGCTTATTGGTGCCACTCGAAAGCGCAAAAT-TGTGGCATGGT-T---GACAAGTCGGG
                                                                      Section 28
                                              (2161) 2161     2170      2180      2190      2200      2210      2220      2230      2240
           SARS-CoV-2 Reference Genome NC_045512.2 (2139) AACCCGTCC--TTGATTGGCTTGAAGAGAAGTTTAAGGAAGGTGTAGAGTTTCTTAGAGACGGTTGGGAAATTGTTAAAT
Alternaria brassicicola endornavirus isolate 1 NC... (1771) AACCTCTTTTGGCGTGAGCATCGACACACTTTTTTATATGAAGCCTGAGGACATG-GTGGCCGTCATGAAGGCGCACAAC
```

Section 29

```
                                                          2241     2250      2260      2270      2280      2290      2300      2310     2320
                                                  (2241)
SARS-CoV-2 Reference Genome NC_045512.2           (2217)  TTATCTCAACCTGTGCTTGTGAAATTGTCGGTGGACAAATTGTCACCTGTGCAAAGGAAATTAAGGAGAGTGTTCAGACA
Alternaria brassicicola endornavirus isolate 1 NC...  (1850)  ATAATTTACGGCCTGCATGC--AATAATAGTTCCTCCGACATTCATGTATGAAACAAGTGGAAAGTTGGCTTTCAACGAA
```

Section 30

```
                                                          2321     2330      2340      2350      2360      2370      2380      2390     2400
                                                  (2321)
SARS-CoV-2 Reference Genome NC_045512.2           (2297)  TTCTTTAAGCTTGTAAATAAATTTTTGGCTTTGTGTGCTGACTCTATCATTATTGGTGGAGCTAAACTTAAAGCCTTGAA
Alternaria brassicicola endornavirus isolate 1 NC...  (1928)  GGCCAATGGCGGATAAGCAAAGGCACA--TTTGA-AATGGTCACCAATGGTTGCAGCGGCGCTTACCACAGCTCCGCGGC
```

Section 31

```
                                                          2401     2410      2420      2430      2440      2450      2460      2470     2480
                                                  (2401)
SARS-CoV-2 Reference Genome NC_045512.2           (2377)  TTTAGGTGAAACATTTGTCACGCACTCAAAGGGATTGTACAGAAAGTGTGTTAAATCCAGAGAAGAAACTGGCCTAC-TC
Alternaria brassicicola endornavirus isolate 1 NC...  (2005)  AGTCATGAAAACGTGGTTGACT-ACGCCCCTGTTTGTGGGCGAGGGTTTTGGCTTGTACGTGAGGAACGAAGGGTACATG
```

Section 32

```
                                                          2481     2490      2500      2510      2520      2530      2540      2550     2560
                                                  (2481)
SARS-CoV-2 Reference Genome NC_045512.2           (2456)  ATGCCTCTAAAAGCCCCAAAAGAAATTATCTTCTTAGAGGGAGAAACACTTCCCACAGAAGTGT--TAACAGAGGAAGTT
Alternaria brassicicola endornavirus isolate 1 NC...  (2084)  GGGTCTCACATGCTCCTCAAGATCATGAGGGTGCCATACGGAACTATCATGCAACACATGGTCAGGCACTGCCTTTGGTT
```

Section 33

```
                                                          2561     2570      2580      2590      2600      2610      2620      2630     2640
                                                  (2561)
SARS-CoV-2 Reference Genome NC_045512.2           (2534)  GTCTTGAAAACTGGTGATTTACAACCATTAGAACAACCTACTAGTGAAGCTGTTGAAGCTCCATTGGTTGGT---ACACC
Alternaria brassicicola endornavirus isolate 1 NC...  (2164)  GACGGGCAACCC--AGATGAGCAGATCTATAACATCCCAATTATAGATCCGGAAAAGCCGGTGACGTTGCTTTGGAAACA
```

Section 34

```
                                                          2641     2650      2660      2670      2680      2690      2700      2710     2720
                                                  (2641)
SARS-CoV-2 Reference Genome NC_045512.2           (2611)  AG--TTTGT-ATT-AACGGGCTTATGTTGCT----CGAAATCAAAGACACAGAAAAGTACTGTGCCCTTGCACCTAATAT
Alternaria brassicicola endornavirus isolate 1 NC...  (2242)  ACCGTTCACCATCGAACGGCTATGCGTCAACTCTACGATGCTCAGCATGCTAAAGGCAGACTGAGTATGGACGAAT-GT
```

Section 35

```
                                                          2721     2730      2740      2750      2760      2770      2780      2790     2800
                                                  (2721)
SARS-CoV-2 Reference Genome NC_045512.2           (2683)  GATGGTAACAAACAATACCTTCACACTCAAAGGCGGTGCACCAACAAAGGTTACTTTTGGTGATGACACTGTGATA-GAA
Alternaria brassicicola endornavirus isolate 1 NC...  (2321)  AATTGGACCAACTATCTATATACG---CACGTGGTTGCGCCATCAAGTTTACACCAGCCAAACAGGCCGGTCATGCA
```

Section 36

	(2801)	2801 2810 2820 2830 2840 2850 2860 2870 2880
SARS-CoV-2 Reference Genome NC_045512.2	(2762)	GTGCAAGGTTACAAGAGTGTGAATATCACTTTTGAACTT--GATGAAAGGATT--GATAAAGTACTTAATGAGAAGTGCT
Alternaria brassicicola endornavirus isolate 1 NC...	(2398)	ATTTAACATGAGCGAAGAG-GAGTGCCGCTTTCACATGATCTTAGCCATGATGGAGCACCACAAGCTCATGAGGCA-AAT

Section 37

	(2881)	2881 2890 2900 2910 2920 2930 2940 2950 2960
SARS-CoV-2 Reference Genome NC_045512.2	(2838)	CTGCCTATACAGTTGAACTCGGTACAGAAGTA------AATGAGTTCGCCTGTGTTGTGGCA-----GATGCTGTCATAA
Alternaria brassicicola endornavirus isolate 1 NC...	(2476)	CAGCCCATTG-ATTCCACACGTTGAGAACATCGGGAGCAAGGGCAATTGGTGGACACAGGCATTTAAGGTCATAAAGAAC

Section 38

	(2961)	2961 2970 2980 2990 3000 3010 3020 3030 3040
SARS-CoV-2 Reference Genome NC_045512.2	(2907)	AAACTTTGCAACCAGTATCTGAATTA---CTTACACCACTGGGCAT--TGATTTAGATGAGTGGAGTATGGCTA---CAT
Alternaria brassicicola endornavirus isolate 1 NC...	(2555)	ATGCTAATCAAAATGG-GCCGGACATTCGATCCAACTGATGATGAATACCTGTCAAAGGTCATGAAAGAGCATAACACTT

Section 39

	(3041)	3041 3050 3060 3070 3080 3090 3100 3110 3120
SARS-CoV-2 Reference Genome NC_045512.2	(2979)	ACTACTT--ATTTGATGAGTCTGGTGA--GTTTAAATTGGCTTCACA----TATGTATTGTTCTTTCTACCCTCCA---G
Alternaria brassicicola endornavirus isolate 1 NC...	(2634)	TAGACGAGTATAAGATGAGTCACGTGACTGTATCAGAAGTGTTGAAGGATGTAAGCATGTACACGTCGGGCATGCAGTCC

Section 40

	(3121)	3121 3130 3140 3150 3160 3170 3180 3190 3200
SARS-CoV-2 Reference Genome NC_045512.2	(3048)	ATGAGGATGAAGAAGAA-GGTG--AT-TGTGAAGAAGAAGAGTT-TGAGCCATCAACT-CAA-TATGAGTATGGTACTGA
Alternaria brassicicola endornavirus isolate 1 NC...	(2714)	ATGAGCAAGCAAAACAATGCGGCCATCAGTTACCACGGCCAAAATCGAATCAAC-GCGACGTGGGTGAGTAAGCTGTTGG

Section 41

	(3201)	3201 3210 3220 3230 3240 3250 3260 3270 3280
SARS-CoV-2 Reference Genome NC_045512.2	(3121)	AG-ATGA-TTACCAAGG-TAAACCTTTGGAATTTGGTGCCACTTCTGCTGCTCTTCAACCTGAAGAAGAGCAAGAAGAAG
Alternaria brassicicola endornavirus isolate 1 NC...	(2793)	ACTCACCAGAAACGAATTTCAACAATCCGAA--CGGCGCCA-AAGTGCACGCGCACTACCATAGTAAATTCAAAACCGAC

Section 42

	(3281)	3281 3290 3300 3310 3320 3330 3340 3350 3360
SARS-CoV-2 Reference Genome NC_045512.2	(3198)	ATTGGTTAGATGATGATAGTCAACAAACTGTTGGTCAACAAGACGG-CAGTGAGGACAATCAGACAACTAC-----TATT
Alternaria brassicicola endornavirus isolate 1 NC...	(2870)	CAAGCGACGCAGTGCAAA--CCGCAACTGGGCTGCC---CACACGACCACAGGGGAATGCACGAGCATGACATAGGGAGA

Section 43

(3361)		3361 3370 3380 3390 3400 3410 3420 3430 3440
SARS-CoV-2 Reference Genome NC_045512.2	(3272)	CAAACAATTGTTGAGGTTCAACCTCAATT-AGAGATGGAACTTACACCAGTTGTTCAG--ACTATTGAAGTGAATAGTTT
Alternaria brassicicola endornavirus isolate 1 NC...	(2945)	GAAGAAGCGAAGCGAGGCAATTGTTTATGTTGCGGCATCCTTAGTCCGCTTTTCGCCAACTTGTGCGAGGTGTGTCGTAC

Section 44

(3441)		3441 3450 3460 3470 3480 3490 3500 3510 3520
SARS-CoV-2 Reference Genome NC_045512.2	(3349)	TAGT-GGTTATTT-AAAACTTACT----GACAATGTATAC-ATTAAAAT------GCAGACATTGTG---GAAGAAGCT
Alternaria brassicicola endornavirus isolate 1 NC...	(3025)	GATAGAGAAATGTCATAACACAAGCAGCACGTGCAAACACCAGCATAACTTTGTCAACGAACATTGTTGCGGTGCTAGTG

Section 45

(3521)		3521 3530 3540 3550 3560 3570 3580 3590 3600
SARS-CoV-2 Reference Genome NC_045512.2	(3413)	AAA---AAGGTAAAACCAACAGTGGTTGTTAATGCAGCCAATGTTTACCTTAAACATGGAGGAGGTGT-TGCAGGAGCCT
Alternaria brassicicola endornavirus isolate 1 NC...	(3105)	ATTGCCAGTGCGTCAGAAACACGGTATGCATCTGTTGTGGGA---AACGGTCACTGTCCAGCCCGTGTTTGCTGTG--CG

Section 46

(3601)		3601 3610 3620 3630 3640 3650 3660 3670 3680
SARS-CoV-2 Reference Genome NC_045512.2	(3489)	TAAATAAGGCTACTAACAATGCCATGCAAGTTGAATCTGATGATTACATAGCTA-CTAATGGACCACTTAAAGTGGGTGG
Alternaria brassicicola endornavirus isolate 1 NC...	(3180)	ATGATTACCAACCACACGA------GGAAGCTGACTCTATAAACT--ATAGGGAACAAATCAAACAGGCAGGCGCCGAGA

Section 47

(3681)		3681 3690 3700 3710 3720 3730 3740 3750 3760
SARS-CoV-2 Reference Genome NC_045512.2	(3568)	TAGTTGTGTTTTAAGCGGACACAATCTTGCTAAACACTGTCTTCATGTTGTCGGCCCAAATGTTAACAAAGGTGAAGACA
Alternaria brassicicola endornavirus isolate 1 NC...	(3252)	TATTCCAAAACGCCAAAG-CAACGACTATCAAGACCATGTCG-CAACATAGCCGACC---CATTAGCGATTGGGCAGA--

Section 48

(3761)		3761 3770 3780 3790 3800 3810 3820 3830 3840
SARS-CoV-2 Reference Genome NC_045512.2	(3648)	TTCAACTTCTTAAGAGTGCTTATGAAAATTTTAATCAGCACGAAGTTCTACTTG-CACCATTATTATCAGCTGGTATTTT
Alternaria brassicicola endornavirus isolate 1 NC...	(3325)	--GCACGTCG-AAGAA--ATG--GAAGAGATGGAAC--------GAGA-ACCGGAAATCATGATCGAAACCACGGTTCAG

Section 49

(3841)		3841 3850 3860 3870 3880 3890 3900 3910 3920
SARS-CoV-2 Reference Genome NC_045512.2	(3727)	TGGTGCTGACCCTAT--ACAT-TCTTTAAGAGTTTGTGTAGAT-ACTGTTCGCACAAATGTCTACTTAGCTGTCTTTGAT
Alternaria brassicicola endornavirus isolate 1 NC...	(3389)	GGCGGTATAATGACGGAAGATGCCACGAAAACAGATAGGGTGAGGCTGTTGACATTGGCGGAGCCGCTTGAGGCCACAGT

Section 50

	(3921)	3921 3930 3940 3950 3960 3970 3980 3990 4000
SARS-CoV-2 Reference Genome NC_045512.2	(3803)	AAA-AATCTCTATGACAAACTTGTT-TCAAGCTTTTGGAAATGAAGAGTGAAAAG-CAAGTTGAACAA--AAGATCGCT
Alternaria brassicicola endornavirus isolate 1 NC...	(3469)	CACCAACATACACGGCGTTCACGTCGTGAAATATGT-GGACACGCCCGATGATGGCACTTGTGGTTTCACGGCGCTCGAG

Section 51

	(4001)	4001 4010 4020 4030 4040 4050 4060 4070 4080
SARS-CoV-2 Reference Genome NC_045512.2	(3878)	GAGATT-CCTAAA-GAGGAAGTTAAGCCATT-TATAACTGAAAGTAAACCTTCAGTTGAACAGAGAAAACAAGATGATAA
Alternaria brassicicola endornavirus isolate 1 NC...	(3548)	CAGCTGCCATACCCAAGCAAGCTAAACATGGCCGACCTTCAAAG--GA---TTAGC--CACAGAGACGATTGGTGGAGCG

Section 52

	(4081)	4081 4090 4100 4110 4120 4130 4140 4150 4160
SARS-CoV-2 Reference Genome NC_045512.2	(3955)	GAAAATCAAAGCTTGTGTTGAAGAAGTTACAACAACTCTGGAAGAAACTAAGTTCCTCACAGAAAACTTGTTACTTTATA
Alternaria brassicicola endornavirus isolate 1 NC...	(3621)	GCAACGACTTGGGTATGTTCTGCGTAAGCCAA--GGAGTGAACTTACTCGTGGTTGGAACAAGTACGTCCGCATATTATC

Section 53

	(4161)	4161 4170 4180 4190 4200 4210 4220 4230 4240
SARS-CoV-2 Reference Genome NC_045512.2	(4035)	T---TGACATTAATGGCAATCTTCATCCAGAT-TCTGCCACTCTTGTTAGTGA-CATTGACATCACTTTCTTAAAGAAAG
Alternaria brassicicola endornavirus isolate 1 NC...	(3699)	AAGGCGACACGAAGTCAAACCAATACGGCGCTATAGTTCACATGACCACTATATCCG-GCGAAGTGCAT-TGGAACGCAT

Section 54

	(4241)	4241 4250 4260 4270 4280 4290 4300 4310 4320
SARS-CoV-2 Reference Genome NC_045512.2	(4110)	ATGCTCCATATATAGTGGGTGATGTTGTTCAAGAGGGTGTTTTAACTGCTGTGGTTATACCTACTAAAAAGG-CTG-GTG
Alternaria brassicicola endornavirus isolate 1 NC...	(3777)	GTGAAGCGCGTATCATGAGTGTTAATGCAGACG-GCGACCGAGATTTAGATAGACGCAACATAATAGGAAGCACCGCCAA

Section 55

	(4321)	4321 4330 4340 4350 4360 4370 4380 4390 4400
SARS-CoV-2 Reference Genome NC_045512.2	(4188)	GCACTACTGAAATGCTAGCGAAAGC--TTTGAGA-AAAGTGCCAACAGACAATTATATAACCACTTACCCGGGTCAGGGT
Alternaria brassicicola endornavirus isolate 1 NC...	(3856)	AGACCGAACAGGCAA-AGAAATCGACGAAATGGACGTCGTGTCTAGAG--AAGAATTCGTGACCAATCTGTTGGCCGAAA

Section 56

	(4401)	4401 4410 4420 4430 4440 4450 4460 4470 4480
SARS-CoV-2 Reference Genome NC_045512.2	(4265)	TTAAATGGTTACACTGTAGAGGAGGCA-AAGACAGTGCTTAAA----AAGTGTAAAAGTGCCTTTTACATTCTACCATCT
Alternaria brassicicola endornavirus isolate 1 NC...	(3933)	AACAATGGCTATCAACGTTCCCAGCCTCAACAGAGCTAAACAGGCTGATGAGAACTAAATTAGTTAATGGGATGCTGGAA

```
                                                                     Section 57
                                       (4481) 4481      4490      4500      4510      4520      4530      4540      4550      4560
        SARS-CoV-2 Reference Genome NC_045512.2 (4340) ATTATCTCTAATGAGAA-GCAAGAAATTCTTGGAACTGTTTCT--TGGAATTTGCGAGAAATGCTTGCACATGCAGAAGA
Alternaria brassicicola endornavirus isolate 1 NC... (4013) GCGGAATCAGAGACGATCGGATTCCATTCAACGAATGGTATGAACTCGACCAAGCGGTTCACGGCTGATGCGGGAGTCGA
                                                                     Section 58
                                       (4561) 4561      4570      4580      4590      4600      4610      4620      4630      4640
        SARS-CoV-2 Reference Genome NC_045512.2 (4417) AACACGCAA----ATTAATG-CCTGTCTGTGTGGAAACTAAAGCCATAGTTTCAACTATACAGCGTAAATATAAGGTAT
Alternaria brassicicola endornavirus isolate 1 NC... (4093) GAAAGGCCTGCCCATTTACATAACGGACAACAGGATCCCGATGAATGGGTCTGATAAACCGACCTTCGTGAAATCAGTTA
                                                                     Section 59
                                       (4641) 4641      4650      4660      4670      4680      4690      4700      4710      4720
        SARS-CoV-2 Reference Genome NC_045512.2 (4492) TAAAATACAAGAGGGTGTGGTTGATTATGGTGCTAGATTTTACTTTTACACCAGTAAAACAACTGTAGCGTCACTTATCA
Alternaria brassicicola endornavirus isolate 1 NC... (4173) GCAGTTGTGACGACGTCATGTCATTTATGAGCCCCGGTGTTAAATTTTATTT--CTACGGA---TTTGAGATGCCGACTC
                                                                     Section 60
                                       (4721) 4721      4730      4740      4750      4760      4770      4780      4790      4800
        SARS-CoV-2 Reference Genome NC_045512.2 (4572) ACACACTTAACGATCTAAATGAAACTCTTGTTACAATGCCACTTGGCTATGTAACACATGGCTTAAATTTGGAAGAAGCT
Alternaria brassicicola endornavirus isolate 1 NC... (4248) CGACAAATGCCGCT-TACAT------CGTGCCA-GATGACGAAATCCAAGGCCTACAAGGGATAAACAAC--ACGGTAGT
                                                                     Section 61
                                       (4801) 4801      4810      4820      4830      4840      4850      4860      4870      4880
        SARS-CoV-2 Reference Genome NC_045512.2 (4652) GCTCGGTATATGAGATCTCTCAAAGTGCCAGCTACAGTTTCTGTTTCTTCACCTGATGCTGTTACAGCGTATAATGGTTA
Alternaria brassicicola endornavirus isolate 1 NC... (4318) GAACAACACAAAAAACGTCAGCGAGAACCA--AACGTGCACT----CTTGAGCTGTTCGAACTACAATCATGCCCCGCAA
                                                                     Section 62
                                       (4881) 4881      4890      4900      4910      4920      4930      4940      4950      4960
        SARS-CoV-2 Reference Genome NC_045512.2 (4732) TCTTACTTCTTCTTCTAAAACACCTGAAGAACATTTTATTGAAACCATCTCAC-TTGCTGGTTCCTATAAAGATTGGTCC
Alternaria brassicicola endornavirus isolate 1 NC... (4392) CA---GTCGTGCTGGGAATGGCCCGTGCCCATAG----GAGGATAGATTTCAACGAGCCAAACGCCATCGGCTTAGATAG
                                                                     Section 63
                                       (4961) 4961      4970      4980      4990      5000      5010      5020      5030      5040
        SARS-CoV-2 Reference Genome NC_045512.2 (4811) TATTCTGGACAATCTACACAACTAGGTATAGAATTTCTTAAGAGAGGTGATAAAAGTGTATATTACACTAGTAATCCTAC
Alternaria brassicicola endornavirus isolate 1 NC... (4465) GAAACGGCCAAATTGGAAAACATTTGTTGACATTGCCATAAG--TGCACGCAACCTTG-GCGTTAAAGAAGTTTACTTGC
```

Section 64

		5041 5050 5060 5070 5080 5090 5100 5110 5120
	(5041)	
SARS-CoV-2 Reference Genome NC_045512.2	(4891)	C-ACATTCCACCTAGATGGTGAAGTTATCACCTTTGACAATCTTAAGAC--ACTTCTTTCTTTGAGAGAAGTGAGGACTA
Alternaria brassicicola endornavirus isolate 1 NC...	(4542)	CTATGCTGGATGACGATTTCGACGTCGACGAATTTGACGCACTTGTGCTCAGCATGCTAAACGGTCGAAGTTTGCCACAT

Section 65

		5121 5130 5140 5150 5160 5170 5180 5190 5200
	(5121)	
SARS-CoV-2 Reference Genome NC_045512.2	(4968)	TTAAGGTGTTTACAACA---GTAGACAACATTAACCTCCACACGCAAGTTGTGGACATGTCAA------TGACATATGGA
Alternaria brassicicola endornavirus isolate 1 NC...	(4622)	CAAATCAAAGAAAAACGAGGCAAGGCGGTGCCAAAGATGGTTGTGCAACCATTGGCAAGGAAAGCCTTGAACCATTTGAT

Section 66

		5201 5210 5220 5230 5240 5250 5260 5270 5280
	(5201)	
SARS-CoV-2 Reference Genome NC_045512.2	(5039)	CAACAGTT-TGGTCCAACTTATTTGGATGGAGCTGATGTTACTAAAAT-AAAACCTCATAATTCACATGAAGGTAAAACA
Alternaria brassicicola endornavirus isolate 1 NC...	(4702)	CAATAACGCTGGGTTCGCTGACGTGGATATGGACATACTAAATTTCGTCAAGCGCGAGTCAGTAGGCATGAGCAAAGCTA

Section 67

		5281 5290 5300 5310 5320 5330 5340 5350 5360
	(5281)	
SARS-CoV-2 Reference Genome NC_045512.2	(5117)	TTTTATGTTTTACCTAATGATGACACTCTACGTGTTGAGGCTTTTGAGTACTACCACACAACTGATCCTAGTTTTCTGGG
Alternaria brassicicola endornavirus isolate 1 NC...	(4782)	GCGGCAAATTCATGAAGATAGTGAACTGCGGCAGCTGTGGGCTACGAATGCTGAAAATAACAGGAACAGAATGTAGCAGT

Section 68

		5361 5370 5380 5390 5400 5410 5420 5430 5440
	(5361)	
SARS-CoV-2 Reference Genome NC_045512.2	(5197)	TAGGTACATGTCAGCATTAAATCACACTAAAAAGTGGAAATACCCACAAGTTAATGGTTTAACT-TCTATTAAAT--GGG
Alternaria brassicicola endornavirus isolate 1 NC...	(4862)	T--GTGGGCCAACGTTTAATATGAAAGTAAGTGATGCGTTCAGCGAGGGTGAATTCATCATCATGCCCAGCAAAGTGGGG

Section 69

		5441 5450 5460 5470 5480 5490 5500 5510 5520
	(5441)	
SARS-CoV-2 Reference Genome NC_045512.2	(5274)	CAGATAACAACTGTTATCTTGCCACTGCATTGTTAACACTCCAACAAATAGAGTTGAAGTTT-AATCCACC---------
Alternaria brassicicola endornavirus isolate 1 NC...	(4940)	GTGGACAAAACCAGCAATTATTTGAATGGTAATTGGCCATGCACAGCAGACCCAGTTAGGCTGAATGGGGCATGCATGGA

Section 70

		5521 5530 5540 5550 5560 5570 5580 5590 5600
	(5521)	
SARS-CoV-2 Reference Genome NC_045512.2	(5344)	TGCTCTACAAGATGCTTATTA-CAGAGCAAGGGCTGG-TGAA-GCTGCTA--ACTTTTGTGCACTTATCTTAGCCTACTG
Alternaria brassicicola endornavirus isolate 1 NC...	(5020)	TTATTGGCGCGAACAATACCAAATGGGAACCAACAGCATGATTGAGGCCACCATGACGGTGCACGAA--AACGATTCGTA

Section 71

```
                                                    (5601) 5601     5610      5620      5630      5640      5650      5660      5670     5680
SARS-CoV-2 Reference Genome NC_045512.2             (5419) TAATAAGACAGTAGGTG-AGTTAGGTGATGT---TAGAGAAAC---------------------AATGAGTTACTTGTTT
Alternaria brassicicola endornavirus isolate 1 NC... (5098) CCACAAGAAGTCATGTGTAGTGGGATCATGTCACGTGAAAAACGGCCAACTAACCATACGCGCAAACGACATAAAAGAGT
```

Section 72

```
                                                    (5681) 5681     5690      5700      5710      5720      5730      5740      5750     5760
SARS-CoV-2 Reference Genome NC_045512.2             (5474) CA-------ACATGCCAATTTAGATTCTTGCAAAAGAGTCTTGAACGTGGTGTGTAAAACTTGTGGACAACAGCAGAC-A
Alternaria brassicicola endornavirus isolate 1 NC... (5178) GCAGCGCGTACGAGCCTTTTGTGGTGCAAACAGACAAGACTTGGTCGTGGGCATTCAGCATGCCCGACGGTACCATTGCA
```

Section 73

```
                                                    (5761) 5761     5770      5780      5790      5800      5810      5820      5830     5840
SARS-CoV-2 Reference Genome NC_045512.2             (5546) ACCCTTAAGGGTGTAGAAGCTGTTATGTACATGGGCACACTTTCTTATGAACAATTTAAGAAAGGTGTTCAGATACCTTG
Alternaria brassicicola endornavirus isolate 1 NC... (5258) ACAGCTGCTCGCCTCGACAATGGGCCAGTGCGGGTTATAGGGTC-TCAGGACAACATAGGGTAC-TGCTTGGTGATGGCC
```

Section 74

```
                                                    (5841) 5841     5850      5860      5870      5880      5890      5900      5910     5920
SARS-CoV-2 Reference Genome NC_045512.2             (5626) TACGTGTGGTAAACAAGCTACAAAATATCTAGTACAACAGGAGTCACCTTTTGTTATGATGTCAGCACCACCTGCTCAGT
Alternaria brassicicola endornavirus isolate 1 NC... (5336) TGCAAC--TTCGACTCCGGGAGGGGTTGCGTAACTGAGGAATTAAACCAGATGGTCAAGGGGGCAAAAGCGTTCGTCGGC
```

Section 75

```
                                                    (5921) 5921     5930      5940      5950      5960      5970      5980      5990     6000
SARS-CoV-2 Reference Genome NC_045512.2             (5706) ATGAACTTAAGCATGGTACATTTACTTGTGCTAGTGAGTACACTGGTAATTACCAGTGTGGTCACTATAAACATATAACT
Alternaria brassicicola endornavirus isolate 1 NC... (5414) GTGCCTGGAGGC--GGGAAAACAAGGGGTGCCAAAATCGCCTACCCCAAGGCC-ACGATAGTGACGAAACAAGCCGGCAT
```

Section 76

```
                                                    (6001) 6001     6010      6020      6030      6040      6050      6060      6070     6080
SARS-CoV-2 Reference Genome NC_045512.2             (5786) TCTAAAGAAACTTTGTATTGCATAGACGGTGCTTTACTTACAAAGTCCTCAGAATACAAAGGTCCTATTACGGATGTTTT
Alternaria brassicicola endornavirus isolate 1 NC... (5491) GTTAAAG--ACCTT---TCGAGCCGAGGGACGTGATGCTGTCACCCCAGCCCAATTGATAGCACGCAAAACACCCACCGA
```

Section 77

```
                                                    (6081) 6081     6090      6100      6110      6120      6130      6140      6150     6160
SARS-CoV-2 Reference Genome NC_045512.2             (5866) CTACAAAGAAAACAGTTACACAACAACCATAAAACCAGTTACTTATAAATTGGATGGTGTTGTTTGTACAGAAATTGACC
Alternaria brassicicola endornavirus isolate 1 NC... (5566) CTGCGTC-ATTCTAGACGAAGCGGGCCTGTGCAACTATACTGATCTTTGGCCAATGGTTGGGAAGGTCAACCAACTGATC
```

Section 78

	(6161)	6161 6170 6180 6190 6200 6210 6220 6230 6240
SARS-CoV-2 Reference Genome NC_045512.2	(5946)	CTAAGTTGGACAATTATTATAAGAAAGACAATTCTTATTTCACAGAGCAACCAATTGATCTTGTACCAAACCAACCATAT
Alternaria brassicicola endornavirus isolate 1 NC...	(5645)	ATAACAGCA--GATATAAACCAAAACAACACATCAT-TGATGGCCAGTAATGTATTGA--------CGGGT------GAT

Section 79

	(6241)	6241 6250 6260 6270 6280 6290 6300 6310 6320
SARS-CoV-2 Reference Genome NC_045512.2	(6026)	CCAAACGCAAGCTT-CGATAATTTTAAGTTTGTATGTGATAATATCAAATTTGCTGATGATTTAAACCAGTTAACTGGTT
Alternaria brassicicola endornavirus isolate 1 NC...	(5708)	CACACTTCGTGGTTAAGTGGAGTCCAAATTGAACAAGAATTTTTTAAGAGCT----ATCGATTTGGACCCAAAACTGCGC

Section 80

	(6321)	6321 6330 6340 6350 6360 6370 6380 6390 6400
SARS-CoV-2 Reference Genome NC_045512.2	(6105)	ATAAGAA-ACCTGCTTCAAGAGAGCTTAAAGTTACATTTTTCCCTGACTTAAATGGTGATGTGGTGGCTATTGA----TT
Alternaria brassicicola endornavirus isolate 1 NC...	(5784)	AATGGCTGAAACGATTCAACCCGAGG-ATAGAAGGCGTGAACTCAAACG-ATAAAGGAATAACATGGGTCCACACAACTG

Section 81

	(6401)	6401 6410 6420 6430 6440 6450 6460 6470 6480
SARS-CoV-2 Reference Genome NC_045512.2	(6180)	ATAAACAC--TACACACCCTCTTTTAAGAAAGGAGCTAAATTGTTACATAAA---CCTATTGTTTGGCATGTTAACAATG
Alternaria brassicicola endornavirus isolate 1 NC...	(5862)	CCAAACGAGTTGAAGACC---AGCTAAAAGAGCTGGTCGGCCGCAACGTGGATGTCGTCTTAGTGCCCACTAACGCGGAC

Section 82

	(6481)	6481 6490 6500 6510 6520 6530 6540 6550 6560
SARS-CoV-2 Reference Genome NC_045512.2	(6255)	CAACTAATAAAGCCACGTATAAACCAAATAC-C-TGGTGTATACGTTGTCTTTGGAGCACAA--AACCAGT--TGAAACA
Alternaria brassicicola endornavirus isolate 1 NC...	(5939)	AAGCTAAAACTGAGGAAGATAACCAACATCAAGATCGAGACCATGAAGAGGTTTCAAGGCCAGCAAGCAGACA-GAATTG

Section 83

	(6561)	6561 6570 6580 6590 6600 6610 6620 6630 6640
SARS-CoV-2 Reference Genome NC_045512.2	(6329)	TCAAATTCGTTTGATGTACTGAAGTCAGAGG--ACGCGCAGGGAATGGATAATCTT-GCCTGCGAAGATCTAAAACCAGT
Alternaria brassicicola endornavirus isolate 1 NC...	(6018)	GGGTGTTCGTG-ACAAAATTCAATCCAGCATTGACGAGCACGAAAGC---AATGTACACATGCCTA-AGCAGGCACAAGA

Section 84

	(6641)	6641 6650 6660 6670 6680 6690 6700 6710 6720
SARS-CoV-2 Reference Genome NC_045512.2	(6406)	CTCTGAAGAAGTAGTGGAAAATCCTAC-CATACAGAAAGACGTTCTTGAGTGTAATGTGA--AAACTACCGAAGTTGTAG
Alternaria brassicicola endornavirus isolate 1 NC...	(6093)	C--TGAATGCATAGTTGTGACCGGGCCACAAACTGCCAGCCTCAGCAGCTACATGTTCGGCCAAAATCTCGACCAGGTTG

Section 85

	(6721)	6721 6730 6740 6750 6760 6770 6780 6790 6800
SARS-CoV-2 Reference Genome NC_045512.2	(6483)	GAGACATTATACTTAAACCAGCAA-ATAATAGTTTAA-AAATTA-----CAGAAGAGGTTGGCCACACAGATCTAATGGC
Alternaria brassicicola endornavirus isolate 1 NC...	(6171)	CAGGGTCCGTCAGTAGACTGGCGTTTGAAAGATTTTTGAACTTACCCGGGCCAATAGTGTTCATAATCAAACTTGGAGCA

Section 86

	(6801)	6801 6810 6820 6830 6840 6850 6860 6870 6880
SARS-CoV-2 Reference Genome NC_045512.2	(6556)	TGCTTATGT-AGACAATTCTAGTCTTACTATTAAGAAACCTAATGAATTATCTAGAGT---ATTAGGTTTGAAAACCCTT
Alternaria brassicicola endornavirus isolate 1 NC...	(6251)	AAAATCACATGGGCCATACAAGGGCTGCTACGAGCATTGGCACGAAGTGCGCAACACAGCTACCAGGCCTTAAAGCGCAT

Section 87

	(6881)	6881 6890 6900 6910 6920 6930 6940 6950 6960
SARS-CoV-2 Reference Genome NC_045512.2	(6632)	GCTACTCATGGTTTAGCTGCTGTTAATAGTGTCCCTTGG-GATACTATAGCTAATTATGCTAAGCCTTTTCTTAAC--AA
Alternaria brassicicola endornavirus isolate 1 NC...	(6331)	---AATGAGCGAATTGTGGCCGCAAAGCGCTGACATCGATGACGATTTGCTATACGACTATGAGTTGGAGCGTGTTTTAA

Section 88

	(6961)	6961 6970 6980 6990 7000 7010 7020 7030 7040
SARS-CoV-2 Reference Genome NC_045512.2	(6709)	AGTTGTTAGTACAACTACTAACATAGTTACACGGTGTTTAAACCGTGTTTGTACTAATTATATGCCTTATTT--CTTTAC
Alternaria brassicicola endornavirus isolate 1 NC...	(6408)	GCCAGGAATCGAGGCTAATGAGCCAAGCAGACGACG--AAGAGCACGATGGATCCCAATTCGGGCCTGGGCCGACGATTC

Section 89

	(7041)	7041 7050 7060 7070 7080 7090 7100 7110 7120
SARS-CoV-2 Reference Genome NC_045512.2	(6787)	TTTATTGCTACAATTGTGTACTTTTACTAGAAGTACAAATTCTAGA--ATTAAAGCATCTATGCCGACTACTATAGCAAA
Alternaria brassicicola endornavirus isolate 1 NC...	(6486)	GAGAT-GACGCAACCAGCGTCGCGCCGAGTGACATTGAACTCAACGTGATTGATCCGACTC---------CTGAGTTGGA

Section 90

	(7121)	7121 7130 7140 7150 7160 7170 7180 7190 7200
SARS-CoV-2 Reference Genome NC_045512.2	(6865)	GAATACTGTTAAGAGTGTCGGTAAATTTTGTCTAGAGGCTTCATTTAATTATTTGAAGTCACCTAATTTTTTCTAAACTGA
Alternaria brassicicola endornavirus isolate 1 NC...	(6556)	GCAA-----GAAGAGTTCAAGGACTCGA-GGACAGAGGAATACGCACAAATGT--CAGCCAGAGGCTTGTTCTTATCACC

Section 91

	(7201)	7201 7210 7220 7230 7240 7250 7260 7270 7280
SARS-CoV-2 Reference Genome NC_045512.2	(6945)	TAAATATTATAATTTGGTTTTTACTATTAAGT-GTTTGCCTAGGTTCTTTAATCTACTCAACCGCTGCTTTAGGTGTTTT
Alternaria brassicicola endornavirus isolate 1 NC...	(6628)	AGATCAGCTGCCGCGACACACAACTCCCAAGGACTTGAGTGACGACGTGGGAGGTGCCAAAGTGGCGGA-ATGGTATACG

```
                                                          Section 92
                                  (7281) 7281     7290      7300      7310      7320      7330      7340      7350    7360
SARS-CoV-2 Reference Genome NC_045512.2 (7024) --AATGT-----CTAATTTAGGCATGCCTTCTTACTGTACTGGTTACAGAGA-AGGCTATTTGAACTCTACTAAT----G
Alternaria brassicicola endornavirus isolate 1 NC... (6707) AAAACGTATTTATGGTCAAAGGATCAAACCCGACAAGCAATTGGCAAGGAGTCAAGATTTGGAACATGGCGGATTCGTG
                                                          Section 93
                                  (7361) 7361     7370      7380      7390      7400      7410      7420      7430    7440
SARS-CoV-2 Reference Genome NC_045512.2 (7092) TCACTA--TTGCAA---CCTACTGTAC-TGGTTCTAT-ACCTTGTAGTGTTTGTCTTAGT--GGTTTAGATTCTTTAGAC
Alternaria brassicicola endornavirus isolate 1 NC... (6787) TGCGAACGGGGCAACGGAAGTCATGACGTGGATCGAAGACCTGGTAATGACGGTGTGGGACGGGTTGGTAAGATTTGTCG
                                                          Section 94
                                  (7441) 7441     7450      7460      7470      7480      7490      7500      7510    7520
SARS-CoV-2 Reference Genome NC_045512.2 (7163) ACCTATCCTTCTTTAGAA--ACTATACAAATTACCATTTCATCTTTTAAATG-GGATTTAACTGCTTTTGGCTTAGTTGC
Alternaria brassicicola endornavirus isolate 1 NC... (6867) GCATGATGTTGCAGCGAACTGGTCGAGCACTGAAAACAATCCGGCGGTGGTTCGCAAACGTGTTAAGCAGTGAAAGTCGA
                                                          Section 95
                                  (7521) 7521     7530      7540      7550      7560      7570      7580      7590    7600
SARS-CoV-2 Reference Genome NC_045512.2 (7240) AGAGTGGTTTTTGGCATATAT-TCTTTTC--ACTAGGTTTTTCTATGTACTTGGATTGGCTGC---AATCATGCAATTGT
Alternaria brassicicola endornavirus isolate 1 NC... (6947) AAGAGCGAAGAAGCAGCATGGGAGGCAGCGAAAAAGGAGGCTGAGCATAATAGGTTTGAGAGGGTGCCAGAAACCATTGC
                                                          Section 96
                                  (7601) 7601     7610      7620      7630      7640      7650      7660      7670    7680
SARS-CoV-2 Reference Genome NC_045512.2 (7314) TTTTCAGCTATTTTGCAGTACATTTT--ATTAGTAATTCTTGGCTTATGTGGTTAATAATTAATCTTGTACAAATGGCCC
Alternaria brassicicola endornavirus isolate 1 NC... (7027) TAT--AAGTACGGCGTCTGACATCGGCCAGATGCAACACGAGGC--AGGGTCATACAA--GAAGCGGCGGCCCATGCCAA
                                                          Section 97
                                  (7681) 7681     7690      7700      7710      7720      7730      7740      7750    7760
SARS-CoV-2 Reference Genome NC_045512.2 (7392) CGATTTCAGCTATGGTTAGAATGTACATC-----TTCTTTGCATCATTTTATTATGTATGGAAAGTTATGTG-CATGTT
Alternaria brassicicola endornavirus isolate 1 NC... (7101) CGAAAG-ACAAGTGGTT-GAAAGAGGAGGCCGGTTCAGTTGCCTCAACGAGAAATCCAAGCAGCAAAGGCCAGGCAAGCC
                                                          Section 98
                                  (7761) 7761     7770      7780      7790      7800      7810      7820      7830    7840
SARS-CoV-2 Reference Genome NC_045512.2 (7466) GTAGACGGTTGTAATTCATCAACTTGTATGATGTGTTACAAACGTAATAGAGCAACAAGAGTCGAATGTACAACTA-TTG
Alternaria brassicicola endornavirus isolate 1 NC... (7179) CGACAAGCGTGAAGAGCTTGGTTCAGA---ATATTGAGAAAGGGCGTGTCGGCCGCACCAG---AAAGTCGAGCAAAGTT
```

Section 99

(7841) 7841 7850 7860 7870 7880 7890 7900 7910 7920

SARS-CoV-2 Reference Genome NC_045512.2 (7545) TTAATGGTGTTAGAAGGTCCTTTTATGTCTATGCTAATGGAGGTAAAG--GCTTTTGCAAACTACACAATTGGAATTGTG

Alternaria brassicicola endornavirus isolate 1 NC... (7253) TTAAACGAAGCATTAGCCGCTTCCAGCGAAACACGCATGGAAGCAGAGAACCAGTTGTTTATTAAAATAGGCGAAA--AG

Section 100

(7921) 7921 7930 7940 7950 7960 7970 7980 7990 8000

SARS-CoV-2 Reference Genome NC_045512.2 (7623) TTAATTGTGATACATTCTGTGCTGGTAGTACATTTATTAGTGA---TGAAGTTGCGAGAGACTTGTCACTACAGTTTAAA

Alternaria brassicicola endornavirus isolate 1 NC... (7331) AAAGCTGGCCGCATGATCGAGATGAACTGGGACTTACTCCAAAACTTCGACTTTGAAGTCAGCTGGGAAGCATTTTGCAA

Section 101

(8001) 8001 8010 8020 8030 8040 8050 8060 8070 8080

SARS-CoV-2 Reference Genome NC_045512.2 (7700) AGACCAATAAATCCTACTGACCAGTCTTCTTACATCGTTGATAGTGTTACAGTGAAGAATGGTTCCATCCATCTTTACTT

Alternaria brassicicola endornavirus isolate 1 NC... (7411) GGAATGGGA-----TGCCGATCCATC-----A--TGCACGACTTTCTCAAAGC--------ACTCAAGCATGTTTAAAGG

Section 102

(8081) 8081 8090 8100 8110 8120 8130 8140 8150 8160

SARS-CoV-2 Reference Genome NC_045512.2 (7780) TGATAAAGCTGGTCAAAAGACTTATGAAAGACATTCT--CTCTCTCATTTTGTTAAC-----TTAGACAAC-CTGAGAGC

Alternaria brassicicola endornavirus isolate 1 NC... (7471) CAAGATAAACGCCGCCAAGCA---TGAAGACCACGATTACGAAAGCATTATAATAATGACATTTAGCAAACACATTGTCG

Section 103

(8161) 8161 8170 8180 8190 8200 8210 8220 8230 8240

SARS-CoV-2 Reference Genome NC_045512.2 (7852) TAATAACACTAAAGGTTCATTGCCTATTAATGTTATAGTTTTTGATGGTAAATCAAAATGTGAAGAATCATCTGCAAAAT

Alternaria brassicicola endornavirus isolate 1 NC... (7548) CCCTAATCGTGAAGCTGGACCAGACGTTTGTGTACTG-TCACGACGTACAAACAGCCCAGAATAGTGATAGCGTCGACGA

Section 104

(8241) 8241 8250 8260 8270 8280 8290 8300 8310 8320

SARS-CoV-2 Reference Genome NC_045512.2 (7932) CAGCGTCTGTTTACTACAGTCAGCTTATGTGTCAACCTATACTGTTA-CTAGATCAGGCATTAGTGTCTGATGTTGGTGA

Alternaria brassicicola endornavirus isolate 1 NC... (7627) AGTGCTCACATCATTAATGGAAGACCGAGCCGGGGCCAAGTCTGTCATGTCTATGATTTATGCTGCCCTGTGG--GGCCG

Section 105

(8321) 8321 8330 8340 8350 8360 8370 8380 8390 8400

SARS-CoV-2 Reference Genome NC_045512.2 (8011) TAGTGCGGAAGTTGCAGTTAAAATGTTTGATGCTTACGTTAATACGTTTTCATCAACTTTTAA-CGTAC-CAATGGAAAA

Alternaria brassicicola endornavirus isolate 1 NC... (7705) TATGAAGATAAGAATAATT----TATTGGCAATGGTATTTGCGCACATGGTT-TGATTTTTCATACAAAGCCATG-ATAA

```
                                                                   Section 106
                                              (8401) 8401     8410      8420      8430      8440      8450      8460      8470     8480
            SARS-CoV-2 Reference Genome NC_045512.2 (8089) ACTCAAAACACTAGTTGCAACTGCAGAAGCTGAACTTGCAAAGAATGTGTCCTTAGACA-ATGTCTTATCTACTTTTATT
Alternaria brassicicola endornavirus isolate 1 NC... (7779) GCGAAAAAATCGAGCTTGACGGG---GCTTTGAACTTGCAC-GAATGGGCTGCTAAAAACAGCTATTTGACAATATTGAA
                                                                   Section 107
                                              (8481) 8481     8490      8500      8510      8520      8530      8540      8550     8560
            SARS-CoV-2 Reference Genome NC_045512.2 (8168) TCAGCAGCTCGGC--AAGGGTTTGTTGATTCAGATGTAGAAACTAAAGATGTTGTTGAATGTCTTAA--ATTGTCACATC
Alternaria brassicicola endornavirus isolate 1 NC... (7855) GGCTAAGAAAAGGGTAACGGTTACCATAAGTGGAACCGACGCCCTGATA--TTGTTGAACGGTCAAGGGCTTAGCGGAAC
                                                                   Section 108
                                              (8561) 8561     8570      8580      8590      8600      8610      8620      8630     8640
            SARS-CoV-2 Reference Genome NC_045512.2 (8244) AATCTGACATAGAAGT-TACTGGCGATAGTTGTAATAACTATATGCTCACCTATAACAAAGTTGAAAACATG-ACACCCC
Alternaria brassicicola endornavirus isolate 1 NC... (7933) AAAGGGAGCGCGACCAATACACCTGATACGTCTGCGAAGGGG--GTCAACGGTGAACGAAGTCCTTGTCGAGGAAATAG-
                                                                   Section 109
                                              (8641) 8641     8650      8660      8670      8680      8690      8700      8710     8720
            SARS-CoV-2 Reference Genome NC_045512.2 (8322) GTGACCTTGGTGCTTGTATTGACTGTAGTGCGC--GTCATATTAATGCGCAGGTAGCAAAAAGTCACAACATTGCTTTGA
Alternaria brassicicola endornavirus isolate 1 NC... (8010) GTGACGTTAGAACTTCTGATTATGTTAGAGATACAGTCAAAAAATTGCTTTACCCAAGTTACTTCAGAAGACATGCCCGA
                                                                   Section 110
                                              (8721) 8721     8730      8740      8750      8760      8770      8780      8790     8800
            SARS-CoV-2 Reference Genome NC_045512.2 (8400) TATGGAACGTTAAAGATTTCATGTCATTGTCTGAACAACTAC-GAAAACAAATACGTAGTGCTGCTAAAAAGAATAACTT
Alternaria brassicicola endornavirus isolate 1 NC... (8090) ----GCACGGTCCAA--CGC--GTCGTTCGGAGGAGCAGTCGATATACTGGGAAAATACTGGTC---AAGGCTATTAGGA
                                                                   Section 111
                                              (8801) 8801     8810      8820      8830      8840      8850      8860      8870     8880
            SARS-CoV-2 Reference Genome NC_045512.2 (8479) ACCTTTTAAGTTGACATGTGCAACTACTAGACAAGTTGTTAATGTTGTAACAACAAAGATAGCACTTAAGGGTGGTAAAA
Alternaria brassicicola endornavirus isolate 1 NC... (8159) GC--CAGGAGTGGCAATCCCGTGCAGTTATCTGAAACATGGAACGAAATAAAACACAACCTGCCGCTCATGGTAGGTCAA
                                                                   Section 112
                                              (8881) 8881     8890      8900      8910      8920      8930      8940      8950     8960
            SARS-CoV-2 Reference Genome NC_045512.2 (8559) TTGTTAATAATTGGT-TGAAGCAGTTAATTAAAGTTACACTTGTGTTCCTTTTTGTTGCTGCTATTTTCTATTTAATAAC
Alternaria brassicicola endornavirus isolate 1 NC... (8237) GAGCATCAAAATAGTGTAAAACAAGGGAGAAAA--TACAAGGCATACCACACCATACGCGACACTAATAACCTGA--TAG
```

Section 113

	(8961)	8961 … 9040
SARS-CoV-2 Reference Genome NC_045512.2	(8638)	ACCTGTTCATGTCATGTCTAAACATACTGACTTTTCAAGTGAAATCATAGGATACAAGGCTATTGATGGTGGTGTCACTC
Alternaria brassicicola endornavirus isolate 1 NC...	(8313)	ACATCGACCCG------GTGACAGCAAGGGGCCT------GGTAGCAT--GCTGCATTCCAAGTGGAGCTG--GCAAAAC

Section 114

	(9041)	9041 … 9120
SARS-CoV-2 Reference Genome NC_045512.2	(8718)	GTGACATAGCATCTACAGATACTTGTTTTGCTAACAAACATGCTGATTTTGACACATGGTTTAGCCAGCGTGGTGGTAGT
Alternaria brassicicola endornavirus isolate 1 NC...	(8377)	CACTTTATGCACTAA-GGAGCCTGATAAATACCAAGATATTGATGACCTCATGACCACAGAAGACTTTGCTGACATAAAT

Section 115

	(9121)	9121 … 9200
SARS-CoV-2 Reference Genome NC_045512.2	(8798)	TATACTAATGACAA---AGCTTGCCCATTGATTGCTGCAGT---CATAACAAGAGAAGTGGGTTTTGTCGTGCCTGGTTT
Alternaria brassicicola endornavirus isolate 1 NC...	(8456)	GACATCAATTTGAATGAAGGAATGTTTTTGGTCACAAAAAGGTACAGATCAAAAGTTCAAGAGTGGATCATCAATAGGCC

Section 116

	(9201)	9201 … 9280
SARS-CoV-2 Reference Genome NC_045512.2	(8872)	GC-CTGGCACGATATTACGCACAACTAATGGTGACTTTTTGCATTTCTTACCTAGAGTTTTTAGTGCAGTTGGTAACATC
Alternaria brassicicola endornavirus isolate 1 NC...	(8536)	-CACGAAGAGGATCTT--GCTATGCCACCACCCAGAGCAGG--TTGAAGGCTTGATTAACAACGTTCTCGTAGTAATACC

Section 117

	(9281)	9281 … 9360
SARS-CoV-2 Reference Genome NC_045512.2	(8951)	TGTTACACACCATCAAAACTTATAGAGTACACTGACTTTGCAACATCAGCTTGTGTTT---TGGCTGCTGAATGT-ACAA
Alternaria brassicicola endornavirus isolate 1 NC...	(8611)	AAAGTGGGATCTCGGGAAAGCATGGAGCGACGTGAACAACCAAAGGCTGATCAAGTGCACAGGAGAGAAAATCGGCCCGT

Section 118

	(9361)	9361 … 9440
SARS-CoV-2 Reference Genome NC_045512.2	(9027)	TTTTTAAAGATGCTTCTGGTAAGCCAGTACCATATTGTTATGATACCAATG-TACTAGAAGGTTCTGTTGCTTATGAA-A
Alternaria brassicicola endornavirus isolate 1 NC...	(8691)	CAAACCATGATGAAATGGAGACGATGATAGATTTGTGGCGGAAAC-ACGGCTAC--GCCTGTCATTGAGAATGGGAACG

Section 119

	(9441)	9441 … 9520
SARS-CoV-2 Reference Genome NC_045512.2	(9105)	GTTTAC-GCCCTGACACACGTTATGTGCTCATGGATGGCTCTATTATTCAATTTCCTAACACCTACCTTGAAGGTTCTGT
Alternaria brassicicola endornavirus isolate 1 NC...	(8768)	TACGACAGCAACGAACCAAACGCCACGGCGATCGATTGGTTTCTGAA-CACCGACCAGACCGCGTACAACGAGATTAAGC

```
                                                                                    Section 120
                                                   (9521)  9521     9530      9540      9550      9560      9570      9580      9590     9600
SARS-CoV-2 Reference Genome NC_045512.2            (9184)  TAGAGTGGTAACAACTTTTGATTCTGAGTACTGTA-GGCACGGCACTTGTGAAA-GATCAGAAGCTGGTGTTTGTGTATC
Alternaria brassicicola endornavirus isolate 1 NC...  (8847)  TACCCAC-AAACGGTTCGACGAAATCACGCCTGCCTGTGTACGACAACGTGAAGCAGCATGTTACGGTGATTGGCCACGC
                                                                                    Section 121
                                                   (9601)  9601     9610      9620      9630      9640      9650      9660      9670     9680
SARS-CoV-2 Reference Genome NC_045512.2            (9262)  TACTAGTGGTAGATGG---GTACTTAACAATGATTATTACAGATCTTTACCAGGAGTTTTCTGTGGTGTAGATGCTGTAA
Alternaria brassicicola endornavirus isolate 1 NC...  (8926)  ACCACCTGTTACCAGACCGGTCATAGTGAAGTAC-ATCAATCAACTGTACAACTCGATCAC-GACGCGCATCCACGGAGA
                                                                                    Section 122
                                                   (9681)  9681     9690      9700      9710      9720      9730      9740      9750     9760
SARS-CoV-2 Reference Genome NC_045512.2            (9339)  ATT-TACTTACTAATATGTTTACACCACT-AATTCAACCTATTGGTGCTTTGGACATATCAGCATCTATAGTAGCTGGTG
Alternaria brassicicola endornavirus isolate 1 NC...  (9004)  AGTATCATACAGAAAACAAGTGGTTGATCCAGTGGAGAATTTTGAACGAGTCGCCAAAACATTCTTTAAGCCAG--GGTG
                                                                                    Section 123
                                                   (9761)  9761     9770      9780      9790      9800      9810      9820      9830     9840
SARS-CoV-2 Reference Genome NC_045512.2            (9417)  GTATTGTAGCTATCGTAGTAACATGCCTTGCCTACTATTTTATG--AGGTTTAGAAGAGCTTTTGGTGAATACAGTCATG
Alternaria brassicicola endornavirus isolate 1 NC...  (9082)  G----GAAGCCATGGTCGAGGAATTTGTTGAAAACCCAGTGCTGCCAGGATACAAATTGACCGAAGAATGGCTGAGCATG
                                                                                    Section 124
                                                   (9841)  9841     9850      9860      9870      9880      9890      9900      9910     9920
SARS-CoV-2 Reference Genome NC_045512.2            (9495)  TAGTTGCCTTTAATACTTTACTATTCCTTATGTCATTCACTGTACTCTGTTTAACACCAGTTTACT---CATTCTTACCT
Alternaria brassicicola endornavirus isolate 1 NC...  (9158)  AAGG-GCGATCCAGAACCGACAATGAAAGAAAT-----AAAGTTCAACATCTTGGACGACTTTAATCGCATGCCGTACAA
                                                                                    Section 125
                                                   (9921)  9921     9930      9940      9950      9960      9970      9980      9990    10000
SARS-CoV-2 Reference Genome NC_045512.2            (9572)  GGTGTTTATTCTGTTATTTACTTGTACTTGACATTT-TATCTTACTAATGATGTTTCTTTTTTAGCACATATTCAGTGGA
Alternaria brassicicola endornavirus isolate 1 NC...  (9232)  CAAATGCAAACTGCATTTAAAGTCGGAAACGCTTTTAAAAGAGCAGGTTGACAGGTTTGAAG-ACCAAATCGGTAGAGTG
                                                                                    Section 126
                                                  (10001)  10001    10010     10020     10030     10040     10050     10060     10070   10080
SARS-CoV-2 Reference Genome NC_045512.2            (9651)  TGGTTATGTTCACACCTTTAGTACCTTTCTGGATAACAATTGCTTATATCATTTGT---ATTTCCACAAAGCATTTCTAT
Alternaria brassicicola endornavirus isolate 1 NC...  (9311)  ATCGTGTGGCACCCCAAACCGTTCTGTGCAGTGTTATGTCCGGTGATCAACTTGATGAAAGAGCGATTTAAGTTGCTTCT
```

Section 127

		10081 … 10160
	(10081)	
SARS-CoV-2 Reference Genome NC_045512.2	(9728)	TGGTTCTTTAGTAATTACCTAAAGAGACGTGTAGTCTTTAATGGTGTTTCCTTTAGTACTTTTGAAGAAGCTGCGCTGTG
Alternaria brassicicola endornavirus isolate 1 NC...	(9391)	TGACAAAGAAAAAATAGTGTACACTGACGGCTGCGACATGAACGAGATTCAGTTCCACGTTCAGGACCTGCATCCAGA--

Section 128

		10161 … 10240
	(10161)	
SARS-CoV-2 Reference Genome NC_045512.2	(9808)	CACCTTTTGTTAAATAAAGAAATGTATCTAAAGTTGCGTAGTGATGTGCTATTACCTCTTACGCAATATAATAGATACT
Alternaria brassicicola endornavirus isolate 1 NC...	(9469)	CAAAGTCATCGAGATGGATTTGAAAAAACAAGACAGGCAAACTGACATGCACGAGCTTGAGAATGAATTCGTTTTAATGA

Section 129

		10241 … 10320
	(10241)	
SARS-CoV-2 Reference Genome NC_045512.2	(9888)	TAGCTCTTTATAATAAGTACAAG-TATTTTAGTGGAGCAATGGATACAACTAGCTACAGAGAAGCTGCTTGTTGTCATCT
Alternaria brassicicola endornavirus isolate 1 NC...	(9549)	CAGCCCTGGGTTTTCCTTCTTATCTAATGCCGCTGTGGCGCGCTTACAACGAAAATTGGCGTTACAAAGCCTCGGATATG

Section 130

		10321 … 10400
	(10321)	
SARS-CoV-2 Reference Genome NC_045512.2	(9967)	CGCAAAGGCTCTCAA--TGACTTCAGTAACTCAGGTTCTGATGTTCTTTACCAACCACCACAAACCTCTATCACCTCAGC
Alternaria brassicicola endornavirus isolate 1 NC...	(9629)	ACGGAGAGCTGGCAGGTTGGGAAAAGGAAAACCGGGGACGAAATGACCTCACTAGG--CAACACGTTCAAAAACATGAGC

Section 131

		10401 … 10480
	(10401)	
SARS-CoV-2 Reference Genome NC_045512.2	(10045)	TGTTTTGCAGAGTGGTTTTAGAAAAATGGCATTCCCATC-TGGTAAAGTTGAGGGTTGTATGGTACAAGTAACTTGTGGT
Alternaria brassicicola endornavirus isolate 1 NC...	(9707)	GGAATCGCCGAGCCGTTTGTGAGGTATAACACAGAAAGGGTTCTTATTTTGAGCGATGACATGA-----TAGCGTTTGGG

Section 132

		10481 … 10560
	(10481)	
SARS-CoV-2 Reference Genome NC_045512.2	(10124)	ACAACTACACTTAACGGTCTTTGGCTTGATGACGTAGTTTACTGTCCAAGACATGTGATCTGCACCTCTGAAGACATGCT
Alternaria brassicicola endornavirus isolate 1 NC...	(9782)	AGAACCGATTGGGACCAAAGGTGGGTTGAGGATCACTTAAGAGACAAATTCAATGTGCAATGTGAATGGGCTGGCGGGAA

Section 133

		10561 … 10640
	(10561)	
SARS-CoV-2 Reference Genome NC_045512.2	(10204)	TAACCCTAATTATGAAGATTTACTCATTCGTAAGTCTAATCATAATTTCTTGGTACAGGCTGGTAATGTTCAACTCAGGG
Alternaria brassicicola endornavirus isolate 1 NC...	(9862)	AAGCGGAAAATTTTGTCAGCTAGTC----GTCAGCCCTGTTCGAGGTTTTGGATTCATCGTGGCTGCAGATATAAAACGA

Section 134

```
                                                        (10641) 10641    10650     10660     10670     10680     10690     10700     10710    10720
SARS-CoV-2 Reference Genome NC_045512.2                 (10284) TTATTGGACATTCTATGCAAAATTGTGTACTTAAGCTTAAGGTT-GATACAGCCAATCCTAAGACACCTAAGTATAAGTT
Alternaria brassicicola endornavirus isolate 1 NC...    (9938)  TTAGCCGACAAGTTCAGAGTTGTTAAGAGCTCATTAACCAGGGAAGATGAAGACTGGGACGCTAGGTGCTACAGTTACTT
```

Section 135

```
                                                        (10721) 10721    10730     10740     10750     10760     10770     10780     10790    10800
SARS-CoV-2 Reference Genome NC_045512.2                 (10363) TGTTCGCATTCAACCAGGACAGACTTTTCAGTGTTAGCTTGTTACAATGGTTCACCATCTGGTGTTTACCAATGTGCTA
Alternaria brassicicola endornavirus isolate 1 NC...    (10018) AAGCCTGATAGGGCCTTGTGATGCTACCAACATCACAGCCAGCAAAATGAACTTGCCGAGACCGGTT---CAATGGGCAA
```

Section 136

```
                                                        (10801) 10801    10810     10820     10830     10840     10850     10860     10870    10880
SARS-CoV-2 Reference Genome NC_045512.2                 (10443) TGAGGCCCAATTTCACTATTAAGGGTTCATTCCTTAATGGTTCATGTGGTAGTGTTGGTTTTAACATAGATTATGACTGT
Alternaria brassicicola endornavirus isolate 1 NC...    (10095) GGTCGTGGGGCCTGAGGTTGCAAGCGAACGCCGAATACCATAAAGTCACTGGTGAGGAGTTGGACAGAGACGTAAAC---
```

Section 137

```
                                                        (10881) 10881    10890     10900     10910     10920     10930     10940     10950    10960
SARS-CoV-2 Reference Genome NC_045512.2                 (10523) GTCTCTTTTTGTTACATGCACCATATGGAATT--ACCAACTGGAGTTCATGCTGGCACAGACTTAGAAGGTAACTTTTAT
Alternaria brassicicola endornavirus isolate 1 NC...    (10172) GCCTTGTGCCACAACATGATGTATAGCGGTTTGGAAAAACGGACCTTCAACGTGTTGGGCACTCATAAGTTCAAAACCAA
```

Section 138

```
                                                        (10961) 10961    10970     10980     10990     11000     11010     11020     11030    11040
SARS-CoV-2 Reference Genome NC_045512.2                 (10601) GGACCTTTTGTTGACAGGCAAACAGCACAAGCAGCTGGTACGGACACAACTATTACAGTTAATGTTTTAGCTTGGTTGTA
Alternaria brassicicola endornavirus isolate 1 NC...    (10252) GTGAATGTTGTTGATGTTGGAGGAGGGGCCCCCCCCCC----------------------------------------
```

Section 139

```
                                                        (11041) 11041    11050     11060     11070     11080     11090     11100     11110    11120
SARS-CoV-2 Reference Genome NC_045512.2                 (10681) CGCTGCTGTTATAAATGGAGACAGGTGGTTTCTCAATCGATTTACCACAACTCTTAATGACTTTAACCTTGTGGCTATGA
Alternaria brassicicola endornavirus isolate 1 NC...    (10291) --------------------------------------------------------------------------------
```

Section 140

```
                                                        (11121) 11121    11130     11140     11150     11160     11170     11180     11190    11200
SARS-CoV-2 Reference Genome NC_045512.2                 (10761) AGTACAATTATGAACCTCTAACACAAGACCATGTTGACATACTAGGACCTCTTTCTGCTCAAACTGGAATTGCCGTTTTA
Alternaria brassicicola endornavirus isolate 1 NC...    (10291) --------------------------------------------------------------------------------
```

Section 141

(11201)		11201 11210 11220 11230 11240 11250 11260 11270 11280
SARS-CoV-2 Reference Genome NC_045512.2	(10841)	GATATGTGTGCTTCATTAAAAGAATTACTGCAAAATGGTATGAATGGACGTACCATATTGGGTAGTGCTTTATTAGAAGA
Alternaria brassicicola endornavirus isolate 1 NC...	(10291)	--

Section 142

(11281)		11281 11290 11300 11310 11320 11330 11340 11350 11360
SARS-CoV-2 Reference Genome NC_045512.2	(10921)	TGAATTTACACCTTTTGATGTTGTTAGACAATGCTCAGGTGTTACTTTCCAAAGTGCAGTGAAAAGAACAATCAAGGGTA
Alternaria brassicicola endornavirus isolate 1 NC...	(10291)	--

Section 143

(11361)		11361 11370 11380 11390 11400 11410 11420 11430 11440
SARS-CoV-2 Reference Genome NC_045512.2	(11001)	CACACCACTGGTTGTTACTCACAATTTTGACTTCACTTTTAGTTTTAGTCCAGAGTACTCAATGGTCTTTGTTCTTTTTT
Alternaria brassicicola endornavirus isolate 1 NC...	(10291)	--

Section 144

(11441)		11441 11450 11460 11470 11480 11490 11500 11510 11520
SARS-CoV-2 Reference Genome NC_045512.2	(11081)	TTGTATGAAAATGCCTTTTTACCTTTTGCTATGGGTATTATTGCTATGTCTGCTTTTGCAATGATGTTTGTCAAACATAA
Alternaria brassicicola endornavirus isolate 1 NC...	(10291)	--

Section 145

(11521)		11521 11530 11540 11550 11560 11570 11580 11590 11600
SARS-CoV-2 Reference Genome NC_045512.2	(11161)	GCATGCATTTCTCTGTTTGTTTTTGTTACCTTCTCTTGCCACTGTAGCTTATTTTAATATGGTCTATATGCCTGCTAGTT
Alternaria brassicicola endornavirus isolate 1 NC...	(10291)	--

Section 146

(11601)		11601 11610 11620 11630 11640 11650 11660 11670 11680
SARS-CoV-2 Reference Genome NC_045512.2	(11241)	GGGTGATGCGTATTATGACATGGTTGGATATGGTTGATACTAGTTTGTCTGGTTTTAAGCTAAAAGACTGTGTTATGTAT
Alternaria brassicicola endornavirus isolate 1 NC...	(10291)	--

Section 147

(11681)		11681 11690 11700 11710 11720 11730 11740 11750 11760
SARS-CoV-2 Reference Genome NC_045512.2	(11321)	GCATCAGCTGTAGTGTTACTAATCCTTATGACAGCAAGAACTGTGTATGATGATGGTGCTAGGAGAGTGTGGACACTTAT
Alternaria brassicicola endornavirus isolate 1 NC...	(10291)	--

```
                                                                                                                    Section 148
                                                  (11761) 11761     11770     11780     11790     11800     11810     11820     11830   11840
              SARS-CoV-2 Reference Genome NC_045512.2 (11401) GAATGTCTTGACACTCGTTTATAAAGTTTATTATGGTAATGCTTTAGATCAAGCCATTTCCATGTGGGCTCTTATAATCT
Alternaria brassicicola endornavirus isolate 1 NC... (10291) --------------------------------------------------------------------------------
                                                                                                                    Section 149
                                                  (11841) 11841     11850     11860     11870     11880     11890     11900     11910   11920
              SARS-CoV-2 Reference Genome NC_045512.2 (11481) CTGTTACTTCTAACTACTCAGGTGTAGTTACAACTGTCATGTTTTTGGCCAGAGGTATTGTTTTTATGTGTGTTGAGTAT
Alternaria brassicicola endornavirus isolate 1 NC... (10291) --------------------------------------------------------------------------------
                                                                                                                    Section 150
                                                  (11921) 11921     11930     11940     11950     11960     11970     11980     11990   12000
              SARS-CoV-2 Reference Genome NC_045512.2 (11561) TGCCCTATTTTCTTCATAACTGGTAATACACTTCAGTGTATAATGCTAGTTTATTGTTTCTTAGGCTATTTTTGTACTTG
Alternaria brassicicola endornavirus isolate 1 NC... (10291) --------------------------------------------------------------------------------
                                                                                                                    Section 151
                                                  (12001) 12001     12010     12020     12030     12040     12050     12060     12070   12080
              SARS-CoV-2 Reference Genome NC_045512.2 (11641) TTACTTTGGCCTCTTTTGTTTACTCAACCGCTACTTTAGACTGACTCTTGGTGTTTATGATTACTTAGTTTCTACACAGG
Alternaria brassicicola endornavirus isolate 1 NC... (10291) --------------------------------------------------------------------------------
                                                                                                                    Section 152
                                                  (12081) 12081     12090     12100     12110     12120     12130     12140     12150   12160
              SARS-CoV-2 Reference Genome NC_045512.2 (11721) AGTTTAGATATATGAATTCACAGGGACTACTCCCACCCAAGAATAGCATAGATGCCTTCAAACTCAACATTAAATTGTTG
Alternaria brassicicola endornavirus isolate 1 NC... (10291) --------------------------------------------------------------------------------
                                                                                                                    Section 153
                                                  (12161) 12161     12170     12180     12190     12200     12210     12220     12230   12240
              SARS-CoV-2 Reference Genome NC_045512.2 (11801) GGTGTTGGTGGCAAACCTTGTATCAAAGTAGCCACTGTACAGTCTAAAATGTCAGATGTAAAGTGCACATCAGTAGTCTT
Alternaria brassicicola endornavirus isolate 1 NC... (10291) --------------------------------------------------------------------------------
                                                                                                                    Section 154
                                                  (12241) 12241     12250     12260     12270     12280     12290     12300     12310   12320
              SARS-CoV-2 Reference Genome NC_045512.2 (11881) ACTCTCAGTTTTGCAACAACTCAGAGTAGAATCATCATCTAAATTGTGGGCTCAATGTGTCCAGTTACACAATGACATTC
Alternaria brassicicola endornavirus isolate 1 NC... (10291) --------------------------------------------------------------------------------
```

```
                                                                                          Section 155
                                                    (12321) 12321    12330     12340     12350     12360     12370     12380     12390   12400
           SARS-CoV-2 Reference Genome NC_045512.2 (11961) TCTTAGCTAAAGATACTACTGAAGCCTTTGAAAAAATGGTTTCACTACTTTCTGTTTTGCTTTCCATGCAGGGTGCTGTA
Alternaria brassicicola endornavirus isolate 1 NC... (10291) --------------------------------------------------------------------------------
                                                                                          Section 156
                                                    (12401) 12401    12410     12420     12430     12440     12450     12460     12470   12480
           SARS-CoV-2 Reference Genome NC_045512.2 (12041) GACATAAACAAGCTTTGTGAAGAAATGCTGGACAACAGGGCAACCTTACAAGCTATAGCCTCAGAGTTTAGTTCCCTTCC
Alternaria brassicicola endornavirus isolate 1 NC... (10291) --------------------------------------------------------------------------------
                                                                                          Section 157
                                                    (12481) 12481    12490     12500     12510     12520     12530     12540     12550   12560
           SARS-CoV-2 Reference Genome NC_045512.2 (12121) ATCATATGCAGCTTTTGCTACTGCTCAAGAAGCTTATGAGCAGGCTGTTGCTAATGGTGATTCTGAAGTTGTTCTTAAAA
Alternaria brassicicola endornavirus isolate 1 NC... (10291) --------------------------------------------------------------------------------
                                                                                          Section 158
                                                    (12561) 12561    12570     12580     12590     12600     12610     12620     12630   12640
           SARS-CoV-2 Reference Genome NC_045512.2 (12201) AGTTGAAGAAGTCTTTGAATGTGGCTAAATCTGAATTTGACCGTGATGCAGCCATGCAACGTAAGTTGGAAAAGATGGCT
Alternaria brassicicola endornavirus isolate 1 NC... (10291) --------------------------------------------------------------------------------
                                                                                          Section 159
                                                    (12641) 12641    12650     12660     12670     12680     12690     12700     12710   12720
           SARS-CoV-2 Reference Genome NC_045512.2 (12281) GATCAAGCTATGACCCAAATGTATAAACAGGCTAGATCTGAGGACAAGAGGGCAAAAGTTACTAGTGCTATGCAGACAAT
Alternaria brassicicola endornavirus isolate 1 NC... (10291) --------------------------------------------------------------------------------
                                                                                          Section 160
                                                    (12721) 12721    12730     12740     12750     12760     12770     12780     12790   12800
           SARS-CoV-2 Reference Genome NC_045512.2 (12361) GCTTTTCACTATGCTTAGAAAGTTGGATAATGATGCACTCAACAACATTATCAACAATGCAAGAGATGGTTGTGTTCCCT
Alternaria brassicicola endornavirus isolate 1 NC... (10291) --------------------------------------------------------------------------------
                                                                                          Section 161
                                                    (12801) 12801    12810     12820     12830     12840     12850     12860     12870   12880
           SARS-CoV-2 Reference Genome NC_045512.2 (12441) TGAACATAATACCTCTTACAACAGCAGCCAAACTAATGGTTGTCATACCAGACTATAACACATATAAAAATACGTGTGAT
Alternaria brassicicola endornavirus isolate 1 NC... (10291) --------------------------------------------------------------------------------
```

```
Section 162
                                               (12881) 12881    12890     12900     12910     12920     12930     12940     12950   12960
SARS-CoV-2 Reference Genome NC_045512.2        (12521) GGTACAACATTTACTTATGCATCAGCATTGTGGGAAATCCAACAGGTTGTAGATGCAGATAGTAAAATTGTTCAACTTAG
Alternaria brassicicola endornavirus isolate 1 NC... (10291) --------------------------------------------------------------------------------

Section 163
                                               (12961) 12961    12970     12980     12990     13000     13010     13020     13030   13040
SARS-CoV-2 Reference Genome NC_045512.2        (12601) TGAAATTAGTATGGACAATTCACCTAATTTAGCATGGCCTCTTATTGTAACAGCTTTAAGGGCCAATTCTGCTGTCAAAT
Alternaria brassicicola endornavirus isolate 1 NC... (10291) --------------------------------------------------------------------------------

Section 164
                                               (13041) 13041    13050     13060     13070     13080     13090     13100     13110   13120
SARS-CoV-2 Reference Genome NC_045512.2        (12681) TACAGAATAATGAGCTTAGTCCTGTTGCACTACGACAGATGTCTTGTGCTGCCGGTACTACACAAACTGCTTGCACTGAT
Alternaria brassicicola endornavirus isolate 1 NC... (10291) --------------------------------------------------------------------------------

Section 165
                                               (13121) 13121    13130     13140     13150     13160     13170     13180     13190   13200
SARS-CoV-2 Reference Genome NC_045512.2        (12761) GACAATGCGTTAGCTTACTACAACACAACAAAGGGAGGTAGGTTTGTACTTGCACTGTTATCCGATTTACAGGATTTGAA
Alternaria brassicicola endornavirus isolate 1 NC... (10291) --------------------------------------------------------------------------------

Section 166
                                               (13201) 13201    13210     13220     13230     13240     13250     13260     13270   13280
SARS-CoV-2 Reference Genome NC_045512.2        (12841) ATGGGCTAGATTCCCTAAGAGTGATGGAACTGGTACTATCTATACAGAACTGGAACCACCTTGTAGGTTTGTTACAGACA
Alternaria brassicicola endornavirus isolate 1 NC... (10291) --------------------------------------------------------------------------------

Section 167
                                               (13281) 13281    13290     13300     13310     13320     13330     13340     13350   13360
SARS-CoV-2 Reference Genome NC_045512.2        (12921) CACCTAAAGGTCCTAAAGTGAAGTATTTATACTTTATTAAAGGATTAAACAACCTAAATAGAGGTATGGTACTTGGTAGT
Alternaria brassicicola endornavirus isolate 1 NC... (10291) --------------------------------------------------------------------------------

Section 168
                                               (13361) 13361    13370     13380     13390     13400     13410     13420     13430   13440
SARS-CoV-2 Reference Genome NC_045512.2        (13001) TTAGCTGCCACAGTACGTCTACAAGCTGGTAATGCAACAGAAGTGCCTGCCAATTCAACTGTATTATCTTTCTGTGCTTT
Alternaria brassicicola endornavirus isolate 1 NC... (10291) --------------------------------------------------------------------------------
```

```
                                                                                                                    Section 169
                                                  (13441) 13441    13450     13460     13470     13480     13490     13500     13510   13520
            SARS-CoV-2 Reference Genome NC_045512.2 (13081) TGCTGTAGATGCTGCTAAAGCTTACAAAGATTATCTAGCTAGTGGGGGACAACCAATCACTAATTGTGTTAAGATGTTGT
Alternaria brassicicola endornavirus isolate 1 NC... (10291) --------------------------------------------------------------------------------
                                                                                                                    Section 170
                                                  (13521) 13521    13530     13540     13550     13560     13570     13580     13590   13600
            SARS-CoV-2 Reference Genome NC_045512.2 (13161) GTACACACACTGGTACTGGTCAGGCAATAACAGTTACACCGGAAGCCAATATGGATCAAGAATCCTTTGGTGGTGCATCG
Alternaria brassicicola endornavirus isolate 1 NC... (10291) --------------------------------------------------------------------------------
                                                                                                                    Section 171
                                                  (13601) 13601    13610     13620     13630     13640     13650     13660     13670   13680
            SARS-CoV-2 Reference Genome NC_045512.2 (13241) TGTTGTCTGTACTGCCGTTGCCACATAGATCATCCAAATCCTAAAGGATTTTGTGACTTAAAAGGTAAGTATGTACAAAT
Alternaria brassicicola endornavirus isolate 1 NC... (10291) --------------------------------------------------------------------------------
                                                                                                                    Section 172
                                                  (13681) 13681    13690     13700     13710     13720     13730     13740     13750   13760
            SARS-CoV-2 Reference Genome NC_045512.2 (13321) ACCTACAACTTGTGCTAATGACCCTGTGGGTTTTACACTTAAAAACACAGTCTGTACCGTCTGCGGTATGTGGAAAGGTT
Alternaria brassicicola endornavirus isolate 1 NC... (10291) --------------------------------------------------------------------------------
                                                                                                                    Section 173
                                                  (13761) 13761    13770     13780     13790     13800     13810     13820     13830   13840
            SARS-CoV-2 Reference Genome NC_045512.2 (13401) ATGGCTGTAGTTGTGATCAACTCCGCGAACCCATGCTTCAGTCAGCTGATGCACAATCGTTTTTAAACGGGTTTGCGGTG
Alternaria brassicicola endornavirus isolate 1 NC... (10291) --------------------------------------------------------------------------------
                                                                                                                    Section 174
                                                  (13841) 13841    13850     13860     13870     13880     13890     13900     13910   13920
            SARS-CoV-2 Reference Genome NC_045512.2 (13481) TAAGTGCAGCCCGTCTTACACCGTGCGGCACAGGCACTAGTACTGATGTCGTATACAGGGCTTTTGACATCTACAATGAT
Alternaria brassicicola endornavirus isolate 1 NC... (10291) --------------------------------------------------------------------------------
                                                                                                                    Section 175
                                                  (13921) 13921    13930     13940     13950     13960     13970     13980     13990   14000
            SARS-CoV-2 Reference Genome NC_045512.2 (13561) AAAGTAGCTGGTTTTGCTAAATTCCTAAAAACTAATTGTTGTCGCTTCCAAGAAAAGGACGAAGATGACAATTTAATTGA
Alternaria brassicicola endornavirus isolate 1 NC... (10291) --------------------------------------------------------------------------------
```

```
                                                                                Section 176
                                                        (14001) 14001    14010     14020     14030     14040     14050     14060     14070   14080
                    SARS-CoV-2 Reference Genome NC_045512.2 (13641) TTCTTACTTTGTAGTTAAGAGACACACTTTCTCTAACTACCAACATGAAGAAACAATTTATAATTTACTTAAGGATTGTC
Alternaria brassicicola endornavirus isolate 1 NC... (10291) --------------------------------------------------------------------------------
                                                                                Section 177
                                                        (14081) 14081    14090     14100     14110     14120     14130     14140     14150   14160
                    SARS-CoV-2 Reference Genome NC_045512.2 (13721) CAGCTGTTGCTAAACATGACTTCTTTAAGTTTAGAATAGACGGTGACATGGTACCACATATATCACGTCAACGTCTTACT
Alternaria brassicicola endornavirus isolate 1 NC... (10291) --------------------------------------------------------------------------------
                                                                                Section 178
                                                        (14161) 14161    14170     14180     14190     14200     14210     14220     14230   14240
                    SARS-CoV-2 Reference Genome NC_045512.2 (13801) AAATACACAATGGCAGACCTCGTCTATGCTTTAAGGCATTTTGATGAAGGTAATTGTGACACATTAAAAGAAATACTTGT
Alternaria brassicicola endornavirus isolate 1 NC... (10291) --------------------------------------------------------------------------------
                                                                                Section 179
                                                        (14241) 14241    14250     14260     14270     14280     14290     14300     14310   14320
                    SARS-CoV-2 Reference Genome NC_045512.2 (13881) CACATACAATTGTTGTGATGATGATTATTTCAATAAAAAGGACTGGTATGATTTTGTAGAAAACCCAGATATATTACGCG
Alternaria brassicicola endornavirus isolate 1 NC... (10291) --------------------------------------------------------------------------------
                                                                                Section 180
                                                        (14321) 14321    14330     14340     14350     14360     14370     14380     14390   14400
                    SARS-CoV-2 Reference Genome NC_045512.2 (13961) TATACGCCAACTTAGGTGAACGTGTACGCCAAGCTTTGTTAAAAACAGTACAATTCTGTGATGCCATGCGAAATGCTGGT
Alternaria brassicicola endornavirus isolate 1 NC... (10291) --------------------------------------------------------------------------------
                                                                                Section 181
                                                        (14401) 14401    14410     14420     14430     14440     14450     14460     14470   14480
                    SARS-CoV-2 Reference Genome NC_045512.2 (14041) ATTGTTGGTGTACTGACATTAGATAATCAAGATCTCAATGGTAACTGGTATGATTTCGGTGATTTCATACAAACCACGCC
Alternaria brassicicola endornavirus isolate 1 NC... (10291) --------------------------------------------------------------------------------
                                                                                Section 182
                                                        (14481) 14481    14490     14500     14510     14520     14530     14540     14550   14560
                    SARS-CoV-2 Reference Genome NC_045512.2 (14121) AGGTAGTGGAGTTCCTGTTGTAGATTCTTATTATTCATTGTTAATGCCTATATTAACCTTGACCAGGGCTTTAACTGCAG
Alternaria brassicicola endornavirus isolate 1 NC... (10291) --------------------------------------------------------------------------------
```

```
                                                                                Section 183
                                                  (14561) 14561    14570     14580     14590     14600     14610     14620     14630   14640
                 SARS-CoV-2 Reference Genome NC_045512.2 (14201) AGTCACATGTTGACACTGACTTAACAAAGCCTTACATTAAGTGGGATTTGTTAAAATATGACTTCACGGAAGAGAGGTTA
Alternaria brassicicola endornavirus isolate 1 NC... (10291) --------------------------------------------------------------------------------
                                                                                Section 184
                                                  (14641) 14641    14650     14660     14670     14680     14690     14700     14710   14720
                 SARS-CoV-2 Reference Genome NC_045512.2 (14281) AAACTCTTTGACCGTTATTTTAAATATTGGGATCAGACATACCACCCAAATTGTGTTAACTGTTTGGATGACAGATGCAT
Alternaria brassicicola endornavirus isolate 1 NC... (10291) --------------------------------------------------------------------------------
                                                                                Section 185
                                                  (14721) 14721    14730     14740     14750     14760     14770     14780     14790   14800
                 SARS-CoV-2 Reference Genome NC_045512.2 (14361) TCTGCATTGTGCAAACTTTAATGTTTTATTCTCTACAGTGTTCCCACCTACAAGTTTTGGACCACTAGTGAGAAAAATAT
Alternaria brassicicola endornavirus isolate 1 NC... (10291) --------------------------------------------------------------------------------
                                                                                Section 186
                                                  (14801) 14801    14810     14820     14830     14840     14850     14860     14870   14880
                 SARS-CoV-2 Reference Genome NC_045512.2 (14441) TTGTTGATGGTGTTCCATTTGTAGTTTCAACTGGATACCACTTCAGAGAGCTAGGTGTTGTACATAATCAGGATGTAAAC
Alternaria brassicicola endornavirus isolate 1 NC... (10291) --------------------------------------------------------------------------------
                                                                                Section 187
                                                  (14881) 14881    14890     14900     14910     14920     14930     14940     14950   14960
                 SARS-CoV-2 Reference Genome NC_045512.2 (14521) TTACATAGCTCTAGACTTAGTTTTAAGGAATTACTTGTGTATGCTGCTGACCCTGCTATGCACGCTGCTTCTGGTAATCT
Alternaria brassicicola endornavirus isolate 1 NC... (10291) --------------------------------------------------------------------------------
                                                                                Section 188
                                                  (14961) 14961    14970     14980     14990     15000     15010     15020     15030   15040
                 SARS-CoV-2 Reference Genome NC_045512.2 (14601) ATTACTAGATAAACGCACTACGTGCTTTTCAGTAGCTGCACTTACTAACAATGTTGCTTTTCAAACTGTCAAACCCGGTA
Alternaria brassicicola endornavirus isolate 1 NC... (10291) --------------------------------------------------------------------------------
                                                                                Section 189
                                                  (15041) 15041    15050     15060     15070     15080     15090     15100     15110   15120
                 SARS-CoV-2 Reference Genome NC_045512.2 (14681) ATTTTAACAAAGACTTCTATGACTTTGCTGTGTCTAAGGGTTTCTTTAAGGAAGGAAGTTCTGTTGAATTAAAACACTTC
Alternaria brassicicola endornavirus isolate 1 NC... (10291) --------------------------------------------------------------------------------
```

```
                                                                                                   Section 190
                                                      (15121) 15121    15130     15140     15150     15160     15170     15180     15190   15200
               SARS-CoV-2 Reference Genome NC_045512.2 (14761) TTCTTTGCTCAGGATGGTAATGCTGCTATCAGCGATTATGACTACTATCGTTATAATCTACCAACAATGTGTGATATCAG
Alternaria brassicicola endornavirus isolate 1 NC... (10291) --------------------------------------------------------------------------------
                                                                                                   Section 191
                                                      (15201) 15201    15210     15220     15230     15240     15250     15260     15270   15280
               SARS-CoV-2 Reference Genome NC_045512.2 (14841) ACAACTACTATTTGTAGTTGAAGTTGTTGATAAGTACTTTGATTGTTACGATGGTGGCTGTATTAATGCTAACCAAGTCA
Alternaria brassicicola endornavirus isolate 1 NC... (10291) --------------------------------------------------------------------------------
                                                                                                   Section 192
                                                      (15281) 15281    15290     15300     15310     15320     15330     15340     15350   15360
               SARS-CoV-2 Reference Genome NC_045512.2 (14921) TCGTCAACAACCTAGACAAATCAGCTGGTTTTCCATTTAATAAATGGGGTAAGGCTAGACTTTATTATGATTCAATGAGT
Alternaria brassicicola endornavirus isolate 1 NC... (10291) --------------------------------------------------------------------------------
                                                                                                   Section 193
                                                      (15361) 15361    15370     15380     15390     15400     15410     15420     15430   15440
               SARS-CoV-2 Reference Genome NC_045512.2 (15001) TATGAGGATCAAGATGCACTTTTCGCATATACAAAACGTAATGTCATCCCTACTATAACTCAAATGAATCTTAAGTATGC
Alternaria brassicicola endornavirus isolate 1 NC... (10291) --------------------------------------------------------------------------------
                                                                                                   Section 194
                                                      (15441) 15441    15450     15460     15470     15480     15490     15500     15510   15520
               SARS-CoV-2 Reference Genome NC_045512.2 (15081) CATTAGTGCAAAGAATAGAGCTCGCACCGTAGCTGGTGTCTCTATCTGTAGTACTATGACCAATAGACAGTTTCATCAAA
Alternaria brassicicola endornavirus isolate 1 NC... (10291) --------------------------------------------------------------------------------
                                                                                                   Section 195
                                                      (15521) 15521    15530     15540     15550     15560     15570     15580     15590   15600
               SARS-CoV-2 Reference Genome NC_045512.2 (15161) AATTATTGAAATCAATAGCCGCCACTAGAGGAGCTACTGTAGTAATTGGAACAAGCAAATTCTATGGTGGTTGGCACAAC
Alternaria brassicicola endornavirus isolate 1 NC... (10291) --------------------------------------------------------------------------------
                                                                                                   Section 196
                                                      (15601) 15601    15610     15620     15630     15640     15650     15660     15670   15680
               SARS-CoV-2 Reference Genome NC_045512.2 (15241) ATGTTAAAAACTGTTTATAGTGATGTAGAAAACCCTCACCTTATGGGTTGGGATTATCCTAAATGTGATAGAGCCATGCC
Alternaria brassicicola endornavirus isolate 1 NC... (10291) --------------------------------------------------------------------------------
```

```
                                                                                                                                             Section 197
                                                  (15681) 15681    15690     15700     15710     15720     15730     15740     15750    15760
                 SARS-CoV-2 Reference Genome NC_045512.2 (15321) TAACATGCTTAGAATTATGGCCTCACTTGTTCTTGCTCGCAAACATACAACGTGTTGTAGCTTGTCACACCGTTTCTATA
Alternaria brassicicola endornavirus isolate 1 NC... (10291) --------------------------------------------------------------------------------
                                                                                                                                             Section 198
                                                  (15761) 15761    15770     15780     15790     15800     15810     15820     15830    15840
                 SARS-CoV-2 Reference Genome NC_045512.2 (15401) GATTAGCTAATGAGTGTGCTCAAGTATTGAGTGAAATGGTCATGTGTGGCGGTTCACTATATGTTAAACCAGGTGGAACC
Alternaria brassicicola endornavirus isolate 1 NC... (10291) --------------------------------------------------------------------------------
                                                                                                                                             Section 199
                                                  (15841) 15841    15850     15860     15870     15880     15890     15900     15910    15920
                 SARS-CoV-2 Reference Genome NC_045512.2 (15481) TCATCAGGAGATGCCACAACTGCTTATGCTAATAGTGTTTTTAACATTTGTCAAGCTGTCACGGCCAATGTTAATGCACT
Alternaria brassicicola endornavirus isolate 1 NC... (10291) --------------------------------------------------------------------------------
                                                                                                                                             Section 200
                                                  (15921) 15921    15930     15940     15950     15960     15970     15980     15990    16000
                 SARS-CoV-2 Reference Genome NC_045512.2 (15561) TTTATCTACTGATGGTAACAAAATTGCCGATAAGTATGTCCGCAATTTACAACACAGACTTTATGAGTGTCTCTATAGAA
Alternaria brassicicola endornavirus isolate 1 NC... (10291) --------------------------------------------------------------------------------
                                                                                                                                             Section 201
                                                  (16001) 16001    16010     16020     16030     16040     16050     16060     16070    16080
                 SARS-CoV-2 Reference Genome NC_045512.2 (15641) ATAGAGATGTTGACACAGACTTTGTGAATGAGTTTTACGCATATTTGCGTAAACATTTCTCAATGATGATACTCTCTGAC
Alternaria brassicicola endornavirus isolate 1 NC... (10291) --------------------------------------------------------------------------------
                                                                                                                                             Section 202
                                                  (16081) 16081    16090     16100     16110     16120     16130     16140     16150    16160
                 SARS-CoV-2 Reference Genome NC_045512.2 (15721) GATGCTGTTGTGTGTTTCAATAGCACTTATGCATCTCAAGGTCTAGTGGCTAGCATAAAGAACTTTAAGTCAGTTCTTTA
Alternaria brassicicola endornavirus isolate 1 NC... (10291) --------------------------------------------------------------------------------
                                                                                                                                             Section 203
                                                  (16161) 16161    16170     16180     16190     16200     16210     16220     16230    16240
                 SARS-CoV-2 Reference Genome NC_045512.2 (15801) TTATCAAAACAATGTTTTTATGTCTGAAGCAAAATGTTGGACTGAGACTGACCTTACTAAAGGACCTCATGAATTTTGCT
Alternaria brassicicola endornavirus isolate 1 NC... (10291) --------------------------------------------------------------------------------
```

Section 204

(16241)	16241 16250 16260 16270 16280 16290 16300 16310 16320
SARS-CoV-2 Reference Genome NC_045512.2 (15881)	CTCAACATACAATGCTAGTTAAACAGGGTGATGATTATGTGTACCTTCCTTACCCAGATCCATCAAGAATCCTAGGGGCC
Alternaria brassicicola endornavirus isolate 1 NC... (10291)	--

Section 205

(16321)	16321 16330 16340 16350 16360 16370 16380 16390 16400
SARS-CoV-2 Reference Genome NC_045512.2 (15961)	GGCTGTTTTGTAGATGATATCGTAAAAACAGATGGTACACTTATGATTGAACGGTTCGTGTCTTTAGCTATAGATGCTTA
Alternaria brassicicola endornavirus isolate 1 NC... (10291)	--

Section 206

(16401)	16401 16410 16420 16430 16440 16450 16460 16470 16480
SARS-CoV-2 Reference Genome NC_045512.2 (16041)	CCCACTTACTAAACATCCTAATCAGGAGTATGCTGATGTCTTTCATTTGTACTTACAATACATAAGAAAGCTACATGATG
Alternaria brassicicola endornavirus isolate 1 NC... (10291)	--

Section 207

(16481)	16481 16490 16500 16510 16520 16530 16540 16550 16560
SARS-CoV-2 Reference Genome NC_045512.2 (16121)	AGTTAACAGGACACATGTTAGACATGTATTCTGTTATGCTTACTAATGATAACACTTCAAGGTATTGGGAACCTGAGTTT
Alternaria brassicicola endornavirus isolate 1 NC... (10291)	--

Section 208

(16561)	16561 16570 16580 16590 16600 16610 16620 16630 16640
SARS-CoV-2 Reference Genome NC_045512.2 (16201)	TATGAGGCTATGTACACACCGCATACAGTCTTACAGGCTGTTGGGGCTTGTGTTCTTTGCAATTCACAGACTTCATTAAG
Alternaria brassicicola endornavirus isolate 1 NC... (10291)	--

Section 209

(16641)	16641 16650 16660 16670 16680 16690 16700 16710 16720
SARS-CoV-2 Reference Genome NC_045512.2 (16281)	ATGTGGTGCTTGCATACGTAGACCATTCTTATGTTGTAAATGCTGTTACGACCATGTCATATCAACATCACATAAATTAG
Alternaria brassicicola endornavirus isolate 1 NC... (10291)	--

Section 210

(16721)	16721 16730 16740 16750 16760 16770 16780 16790 16800
SARS-CoV-2 Reference Genome NC_045512.2 (16361)	TCTTGTCTGTTAATCCGTATGTTTGCAATGCTCCAGGTTGTGATGTCACAGATGTGACTCAACTTTACTTAGGAGGTATG
Alternaria brassicicola endornavirus isolate 1 NC... (10291)	--

```
                                                                   Section 211
                                           (16801) 16801    16810     16820     16830     16840     16850     16860     16870    16880
SARS-CoV-2 Reference Genome NC_045512.2    (16441) AGCTATTATTGTAAATCACATAAACCACCCATTAGTTTTCCATTGTGTGCTAATGGACAAGTTTTTGGTTTATATAAAAA
Alternaria brassicicola endornavirus isolate 1 NC... (10291) --------------------------------------------------------------------------------
                                                                   Section 212
                                           (16881) 16881    16890     16900     16910     16920     16930     16940     16950    16960
SARS-CoV-2 Reference Genome NC_045512.2    (16521) TACATGTGTTGGTAGCGATAATGTTACTGACTTTAATGCAATTGCAACATGTGACTGGACAAATGCTGGTGATTACATTT
Alternaria brassicicola endornavirus isolate 1 NC... (10291) --------------------------------------------------------------------------------
                                                                   Section 213
                                           (16961) 16961    16970     16980     16990     17000     17010     17020     17030    17040
SARS-CoV-2 Reference Genome NC_045512.2    (16601) TAGCTAACACCTGTACTGAAAGACTCAAGCTTTTTGCAGCAGAAACGCTCAAAGCTACTGAGGAGACATTTAAACTGTCT
Alternaria brassicicola endornavirus isolate 1 NC... (10291) --------------------------------------------------------------------------------
                                                                   Section 214
                                           (17041) 17041    17050     17060     17070     17080     17090     17100     17110    17120
SARS-CoV-2 Reference Genome NC_045512.2    (16681) TATGGTATTGCTACTGTACGTGAAGTGCTGTCTGACAGAGAATTACATCTTTCATGGGAAGTTGGTAAACCTAGACCACC
Alternaria brassicicola endornavirus isolate 1 NC... (10291) --------------------------------------------------------------------------------
                                                                   Section 215
                                           (17121) 17121    17130     17140     17150     17160     17170     17180     17190    17200
SARS-CoV-2 Reference Genome NC_045512.2    (16761) ACTTAACCGAAATTATGTCTTTACTGGTTATCGTGTAACTAAAAACAGTAAAGTACAAATAGGAGAGTACACCTTTGAAA
Alternaria brassicicola endornavirus isolate 1 NC... (10291) --------------------------------------------------------------------------------
                                                                   Section 216
                                           (17201) 17201    17210     17220     17230     17240     17250     17260     17270    17280
SARS-CoV-2 Reference Genome NC_045512.2    (16841) AAGGTGACTATGGTGATGCTGTTGTTTACCGAGGTACAACAACTTACAAATTAAATGTTGGTGATTATTTTGTGCTGACA
Alternaria brassicicola endornavirus isolate 1 NC... (10291) --------------------------------------------------------------------------------
                                                                   Section 217
                                           (17281) 17281    17290     17300     17310     17320     17330     17340     17350    17360
SARS-CoV-2 Reference Genome NC_045512.2    (16921) TCACATACAGTAATGCCATTAAGTGCACCTACACTAGTGCCACAAGAGCACTATGTTAGAATTACTGGCTTATACCCAAC
Alternaria brassicicola endornavirus isolate 1 NC... (10291) --------------------------------------------------------------------------------
```

Section 218

```
(17361) 17361 17370 17380 17390 17400 17410 17420 17430 17440
SARS-CoV-2 Reference Genome NC_045512.2 (17001) ACTCAATATCTCAGATGAGTTTTCTAGCAATGTTGCAAATTATCAAAAGGTTGGTATGCAAAAGTATTCTACACTCCAGG
Alternaria brassicicola endornavirus isolate 1 NC... (10291) --------------------------------------------------------------------------------
```

Section 219

```
(17441) 17441 17450 17460 17470 17480 17490 17500 17510 17520
SARS-CoV-2 Reference Genome NC_045512.2 (17081) GACCACCTGGTACTGGTAAGAGTCATTTTGCTATTGGCCTAGCTCTCTACTACCCTTCTGCTCGCATAGTGTATACAGCT
Alternaria brassicicola endornavirus isolate 1 NC... (10291) --------------------------------------------------------------------------------
```

Section 220

```
(17521) 17521 17530 17540 17550 17560 17570 17580 17590 17600
SARS-CoV-2 Reference Genome NC_045512.2 (17161) TGCTCTCATGCCGCTGTTGATGCACTATGTGAGAAGGCATTAAAATATTTGCCTATAGATAAATGTAGTAGAATTATACC
Alternaria brassicicola endornavirus isolate 1 NC... (10291) --------------------------------------------------------------------------------
```

Section 221

```
(17601) 17601 17610 17620 17630 17640 17650 17660 17670 17680
SARS-CoV-2 Reference Genome NC_045512.2 (17241) TGCACGTGCTCGTGTAGAGTGTTTTGATAAATTCAAAGTGAATTCAACATTAGAACAGTATGTCTTTTGTACTGTAAATG
Alternaria brassicicola endornavirus isolate 1 NC... (10291) --------------------------------------------------------------------------------
```

Section 222

```
(17681) 17681 17690 17700 17710 17720 17730 17740 17750 17760
SARS-CoV-2 Reference Genome NC_045512.2 (17321) CATTGCCTGAGACGACAGCAGATATAGTTGTCTTTGATGAAATTTCAATGGCCACAAATTATGATTTGAGTGTTGTCAAT
Alternaria brassicicola endornavirus isolate 1 NC... (10291) --------------------------------------------------------------------------------
```

Section 223

```
(17761) 17761 17770 17780 17790 17800 17810 17820 17830 17840
SARS-CoV-2 Reference Genome NC_045512.2 (17401) GCCAGATTACGTGCTAAGCACTATGTGTACATTGGCGACCCTGCTCAATTACCTGCACCACGCACATTGCTAACTAAGGG
Alternaria brassicicola endornavirus isolate 1 NC... (10291) --------------------------------------------------------------------------------
```

Section 224

```
(17841) 17841 17850 17860 17870 17880 17890 17900 17910 17920
SARS-CoV-2 Reference Genome NC_045512.2 (17481) CACACTAGAACCAGAATATTTCAATTCAGTGTGTAGACTTATGAAAACTATAGGTCCAGACATGTTCCTCGGAACTTGTC
Alternaria brassicicola endornavirus isolate 1 NC... (10291) --------------------------------------------------------------------------------
```

```
                                                                                                                       Section 225
                                                     (17921) 17921    17930     17940     17950     17960     17970     17980     17990   18000
SARS-CoV-2 Reference Genome NC_045512.2             (17561) GGCGTTGTCCTGCTGAAATTGTTGACACTGTGAGTGCTTTGGTTTATGATAATAAGCTTAAAGCACATAAAGACAAATCA
Alternaria brassicicola endornavirus isolate 1 NC... (10291) --------------------------------------------------------------------------------
                                                                                                                       Section 226
                                                     (18001) 18001    18010     18020     18030     18040     18050     18060     18070   18080
SARS-CoV-2 Reference Genome NC_045512.2             (17641) GCTCAATGCTTTAAAATGTTTTATAAGGGTGTTATCACGCATGATGTTTCATCTGCAATTAACAGGCCACAAATAGGCGT
Alternaria brassicicola endornavirus isolate 1 NC... (10291) --------------------------------------------------------------------------------
                                                                                                                       Section 227
                                                     (18081) 18081    18090     18100     18110     18120     18130     18140     18150   18160
SARS-CoV-2 Reference Genome NC_045512.2             (17721) GGTAAGAGAATTCCTTACACGTAACCCTGCTTGGAGAAAAGCTGTCTTTATTTCACCTTATAATTCACAGAATGCTGTAG
Alternaria brassicicola endornavirus isolate 1 NC... (10291) --------------------------------------------------------------------------------
                                                                                                                       Section 228
                                                     (18161) 18161    18170     18180     18190     18200     18210     18220     18230   18240
SARS-CoV-2 Reference Genome NC_045512.2             (17801) CCTCAAAGATTTTGGGACTACCAACTCAAACTGTTGATTCATCACAGGGCTCAGAATATGACTATGTCATATTCACTCAA
Alternaria brassicicola endornavirus isolate 1 NC... (10291) --------------------------------------------------------------------------------
                                                                                                                       Section 229
                                                     (18241) 18241    18250     18260     18270     18280     18290     18300     18310   18320
SARS-CoV-2 Reference Genome NC_045512.2             (17881) ACCACTGAAACAGCTCACTCTTGTAATGTAAACAGATTTAATGTTGCTATTACCAGAGCAAAAGTAGGCATACTTTGCAT
Alternaria brassicicola endornavirus isolate 1 NC... (10291) --------------------------------------------------------------------------------
                                                                                                                       Section 230
                                                     (18321) 18321    18330     18340     18350     18360     18370     18380     18390   18400
SARS-CoV-2 Reference Genome NC_045512.2             (17961) AATGTCTGATAGAGACCTTTATGACAAGTTGCAATTTACAAGTCTTGAAATTCCACGTAGGAATGTGGCAACTTTACAAG
Alternaria brassicicola endornavirus isolate 1 NC... (10291) --------------------------------------------------------------------------------
                                                                                                                       Section 231
                                                     (18401) 18401    18410     18420     18430     18440     18450     18460     18470   18480
SARS-CoV-2 Reference Genome NC_045512.2             (18041) CTGAAAATGTAACAGGACTCTTTAAAGATTGTAGTAAGGTAATCACTGGGTTACATCCTACACAGGCACCTACACACCTC
Alternaria brassicicola endornavirus isolate 1 NC... (10291) --------------------------------------------------------------------------------
```

Section 232

```
(18481) 18481    18490     18500     18510     18520     18530     18540     18550   18560
SARS-CoV-2 Reference Genome NC_045512.2 (18121) AGTGTTGACACTAAATTCAAAACTGAAGGTTTATGTGTTGACATACCTGGCATACCTAAGGACATGACCTATAGAAGACT
Alternaria brassicicola endornavirus isolate 1 NC... (10291) --------------------------------------------------------------------------------
```

Section 233

```
(18561) 18561    18570     18580     18590     18600     18610     18620     18630   18640
SARS-CoV-2 Reference Genome NC_045512.2 (18201) CATCTCTATGATGGGTTTTAAAATGAATTATCAAGTTAATGGTTACCCTAACATGTTTATCACCCGCGAAGAAGCTATAA
Alternaria brassicicola endornavirus isolate 1 NC... (10291) --------------------------------------------------------------------------------
```

Section 234

```
(18641) 18641    18650     18660     18670     18680     18690     18700     18710   18720
SARS-CoV-2 Reference Genome NC_045512.2 (18281) GACATGTACGTGCATGGATTGGCTTCGATGTCGAGGGGTGTCATGCTACTAGAGAAGCTGTTGGTACCAATTTACCTTTA
Alternaria brassicicola endornavirus isolate 1 NC... (10291) --------------------------------------------------------------------------------
```

Section 235

```
(18721) 18721    18730     18740     18750     18760     18770     18780     18790   18800
SARS-CoV-2 Reference Genome NC_045512.2 (18361) CAGCTAGGTTTTTCTACAGGTGTTAACCTAGTTGCTGTACCTACAGGTTATGTTGATACACCTAATAATACAGATTTTTC
Alternaria brassicicola endornavirus isolate 1 NC... (10291) --------------------------------------------------------------------------------
```

Section 236

```
(18801) 18801    18810     18820     18830     18840     18850     18860     18870   18880
SARS-CoV-2 Reference Genome NC_045512.2 (18441) CAGAGTTAGTGCTAAACCACCGCCTGGAGATCAATTTAAACACCTCATACCACTTATGTACAAAGGACTTCCTTGGAATG
Alternaria brassicicola endornavirus isolate 1 NC... (10291) --------------------------------------------------------------------------------
```

Section 237

```
(18881) 18881    18890     18900     18910     18920     18930     18940     18950   18960
SARS-CoV-2 Reference Genome NC_045512.2 (18521) TAGTGCGTATAAAGATTGTACAAATGTTAAGTGACACACTTAAAAATCTCTCTGACAGAGTCGTATTTGTCTTATGGGCA
Alternaria brassicicola endornavirus isolate 1 NC... (10291) --------------------------------------------------------------------------------
```

Section 238

```
(18961) 18961    18970     18980     18990     19000     19010     19020     19030   19040
SARS-CoV-2 Reference Genome NC_045512.2 (18601) CATGGCTTTGAGTTGACATCTATGAAGTATTTTGTGAAAATAGGACCTGAGCGCACCTGTTGTCTATGTGATAGACGTGC
Alternaria brassicicola endornavirus isolate 1 NC... (10291) --------------------------------------------------------------------------------
```

```
                                                                                                       Section 239
                                               (19041) 19041    19050      19060      19070      19080      19090      19100      19110   19120
        SARS-CoV-2 Reference Genome NC_045512.2 (18681) CACATGCTTTTCCACTGCTTCAGACACTTATGCCTGTTGGCATCATTCTATTGGATTTGATTACGTCTATAATCCGTTTA
Alternaria brassicicola endornavirus isolate 1 NC... (10291) --------------------------------------------------------------------------------
                                                                                                       Section 240
                                               (19121) 19121    19130      19140      19150      19160      19170      19180      19190   19200
        SARS-CoV-2 Reference Genome NC_045512.2 (18761) TGATTGATGTTCAACAATGGGGTTTTACAGGTAACCTACAAAGCAACCATGATCTGTATTGTCAAGTCCATGGTAATGCA
Alternaria brassicicola endornavirus isolate 1 NC... (10291) --------------------------------------------------------------------------------
                                                                                                       Section 241
                                               (19201) 19201    19210      19220      19230      19240      19250      19260      19270   19280
        SARS-CoV-2 Reference Genome NC_045512.2 (18841) CATGTAGCTAGTTGTGATGCAATCATGACTAGGTGTCTAGCTGTCCACGAGTGCTTTGTTAAGCGTGTTGACTGGACTAT
Alternaria brassicicola endornavirus isolate 1 NC... (10291) --------------------------------------------------------------------------------
                                                                                                       Section 242
                                               (19281) 19281    19290      19300      19310      19320      19330      19340      19350   19360
        SARS-CoV-2 Reference Genome NC_045512.2 (18921) TGAATATCCTATAATTGGTGATGAACTGAAGATTAATGCGGCTTGTAGAAAGGTTCAACACATGGTTGTTAAAGCTGCAT
Alternaria brassicicola endornavirus isolate 1 NC... (10291) --------------------------------------------------------------------------------
                                                                                                       Section 243
                                               (19361) 19361    19370      19380      19390      19400      19410      19420      19430   19440
        SARS-CoV-2 Reference Genome NC_045512.2 (19001) TATTAGCAGACAAATTCCCAGTTCTTCACGACATTGGTAACCCTAAAGCTATTAAGTGTGTACCTCAAGCTGATGTAGAA
Alternaria brassicicola endornavirus isolate 1 NC... (10291) --------------------------------------------------------------------------------
                                                                                                       Section 244
                                               (19441) 19441    19450      19460      19470      19480      19490      19500      19510   19520
        SARS-CoV-2 Reference Genome NC_045512.2 (19081) TGGAAGTTCTATGATGCACAGCCTTGTAGTGACAAAGCTTATAAAATAGAAGAATTATTCTATTCTTATGCCACACATTC
Alternaria brassicicola endornavirus isolate 1 NC... (10291) --------------------------------------------------------------------------------
                                                                                                       Section 245
                                               (19521) 19521    19530      19540      19550      19560      19570      19580      19590   19600
        SARS-CoV-2 Reference Genome NC_045512.2 (19161) TGACAAATTCACAGATGGTGTATGCCTATTTTGGAATTGCAATGTCGATAGATATCCTGCTAATTCCATTGTTTGTAGAT
Alternaria brassicicola endornavirus isolate 1 NC... (10291) --------------------------------------------------------------------------------
```

```
                                                                                                                     Section 246
                                                      (19601) 19601     19610      19620      19630      19640      19650      19660      19670    19680
         SARS-CoV-2 Reference Genome NC_045512.2 (19241) TTGACACTAGAGTGCTATCTAACCTTAACTTGCCTGGTTGTGATGGTGGCAGTTTGTATGTAAATAAACATGCATTCCAC
Alternaria brassicicola endornavirus isolate 1 NC... (10291) --------------------------------------------------------------------------------
                                                                                                                     Section 247
                                                      (19681) 19681     19690      19700      19710      19720      19730      19740      19750    19760
         SARS-CoV-2 Reference Genome NC_045512.2 (19321) ACACCAGCTTTTGATAAAAGTGCTTTTGTTAATTTAAAACAATTACCATTTTTCTATTACTCTGACAGTCCATGTGAGTC
Alternaria brassicicola endornavirus isolate 1 NC... (10291) --------------------------------------------------------------------------------
                                                                                                                     Section 248
                                                      (19761) 19761     19770      19780      19790      19800      19810      19820      19830    19840
         SARS-CoV-2 Reference Genome NC_045512.2 (19401) TCATGGAAAACAAGTAGTGTCAGATATAGATTATGTACCACTAAAGTCTGCTACGTGTATAACACGTTGCAATTTAGGTG
Alternaria brassicicola endornavirus isolate 1 NC... (10291) --------------------------------------------------------------------------------
                                                                                                                     Section 249
                                                      (19841) 19841     19850      19860      19870      19880      19890      19900      19910    19920
         SARS-CoV-2 Reference Genome NC_045512.2 (19481) GTGCTGTCTGTAGACATCATGCTAATGAGTACAGATTGTATCTCGATGCTTATAACATGATGATCTCAGCTGGCTTTAGC
Alternaria brassicicola endornavirus isolate 1 NC... (10291) --------------------------------------------------------------------------------
                                                                                                                     Section 250
                                                      (19921) 19921     19930      19940      19950      19960      19970      19980      19990    20000
         SARS-CoV-2 Reference Genome NC_045512.2 (19561) TTGTGGGTTTACAAACAATTTGATACTTATAACCTCTGGAACACTTTTACAAGACTTCAGAGTTTAGAAAATGTGGCTTT
Alternaria brassicicola endornavirus isolate 1 NC... (10291) --------------------------------------------------------------------------------
                                                                                                                     Section 251
                                                      (20001) 20001     20010      20020      20030      20040      20050      20060      20070    20080
         SARS-CoV-2 Reference Genome NC_045512.2 (19641) TAATGTTGTAAATAAGGGACACTTTGATGGACAACAGGGTGAAGTACCAGTTTCTATCATTAATAACACTGTTTACACAA
Alternaria brassicicola endornavirus isolate 1 NC... (10291) --------------------------------------------------------------------------------
                                                                                                                     Section 252
                                                      (20081) 20081     20090      20100      20110      20120      20130      20140      20150    20160
         SARS-CoV-2 Reference Genome NC_045512.2 (19721) AAGTTGATGGTGTTGATGTAGAATTGTTTGAAAATAAAACAACATTACCTGTTAATGTAGCATTTGAGCTTTGGGCTAAG
Alternaria brassicicola endornavirus isolate 1 NC... (10291) --------------------------------------------------------------------------------
```

```
                                                                                                                      Section 253
                                                       (20161) 20161    20170      20180      20190      20200      20210      20220      20230 20240
           SARS-CoV-2 Reference Genome NC_045512.2 (19801) CGCAACATTAAACCAGTACCAGAGGTGAAAATACTCAATAATTTGGGTGTGGACATTGCTGCTAATACTGTGATCTGGGA
Alternaria brassicicola endornavirus isolate 1 NC... (10291) --------------------------------------------------------------------------------
                                                                                                                      Section 254
                                                       (20241) 20241    20250      20260      20270      20280      20290      20300      20310 20320
           SARS-CoV-2 Reference Genome NC_045512.2 (19881) CTACAAAAGAGATGCTCCAGCACATATATCTACTATTGGTGTTTGTTCTATGACTGACATAGCCAAGAAACCAACTGAAA
Alternaria brassicicola endornavirus isolate 1 NC... (10291) --------------------------------------------------------------------------------
                                                                                                                      Section 255
                                                       (20321) 20321    20330      20340      20350      20360      20370      20380      20390 20400
           SARS-CoV-2 Reference Genome NC_045512.2 (19961) CGATTTGTGCACCACTCACTGTCTTTTTTGATGGTAGAGTTGATGGTCAAGTAGACTTATTTAGAAATGCCCGTAATGGT
Alternaria brassicicola endornavirus isolate 1 NC... (10291) --------------------------------------------------------------------------------
                                                                                                                      Section 256
                                                       (20401) 20401    20410      20420      20430      20440      20450      20460      20470 20480
           SARS-CoV-2 Reference Genome NC_045512.2 (20041) GTTCTTATTACAGAAGGTAGTGTTAAAGGTTTACAACCATCTGTAGGTCCCAAACAAGCTAGTCTTAATGGAGTCACATT
Alternaria brassicicola endornavirus isolate 1 NC... (10291) --------------------------------------------------------------------------------
                                                                                                                      Section 257
                                                       (20481) 20481    20490      20500      20510      20520      20530      20540      20550 20560
           SARS-CoV-2 Reference Genome NC_045512.2 (20121) AATTGGAGAAGCCGTAAAAACACAGTTCAATTATTATAAGAAAGTTGATGGTGTTGTCCAACAATTACCTGAAACTTACT
Alternaria brassicicola endornavirus isolate 1 NC... (10291) --------------------------------------------------------------------------------
                                                                                                                      Section 258
                                                       (20561) 20561    20570      20580      20590      20600      20610      20620      20630 20640
           SARS-CoV-2 Reference Genome NC_045512.2 (20201) TTACTCAGAGTAGAAATTTACAAGAATTTAAACCCAGGAGTCAAATGGAAATTGATTTCTTAGAATTAGCTATGGATGAA
Alternaria brassicicola endornavirus isolate 1 NC... (10291) --------------------------------------------------------------------------------
                                                                                                                      Section 259
                                                       (20641) 20641    20650      20660      20670      20680      20690      20700      20710 20720
           SARS-CoV-2 Reference Genome NC_045512.2 (20281) TTCATTGAACGGTATAAATTAGAAGGCTATGCCTTCGAACATATCGTTTATGGAGATTTTAGTCATAGTCAGTTAGGTGG
Alternaria brassicicola endornavirus isolate 1 NC... (10291) --------------------------------------------------------------------------------
```

```
                                                              Section 260
                                            (20721) 20721    20730     20740     20750     20760     20770     20780     20790   20800
SARS-CoV-2 Reference Genome NC_045512.2     (20361) TTTACATCTACTGATTGGACTAGCTAAACGTTTTAAGGAATCACCTTTTGAATTAGAAGATTTTATTCCTATGGACAGTA
Alternaria brassicicola endornavirus isolate 1 NC... (10291) --------------------------------------------------------------------------------
                                                              Section 261
                                            (20801) 20801    20810     20820     20830     20840     20850     20860     20870   20880
SARS-CoV-2 Reference Genome NC_045512.2     (20441) CAGTTAAAAACTATTTCATAACAGATGCGCAAACAGGTTCATCTAAGTGTGTGTGTTCTGTTATTGATTTATTACTTGAT
Alternaria brassicicola endornavirus isolate 1 NC... (10291) --------------------------------------------------------------------------------
                                                              Section 262
                                            (20881) 20881    20890     20900     20910     20920     20930     20940     20950   20960
SARS-CoV-2 Reference Genome NC_045512.2     (20521) GATTTTGTTGAAATAATAAAATCCCAAGATTTATCTGTAGTTTCTAAGGTTGTCAAAGTGACTATTGACTATACAGAAAT
Alternaria brassicicola endornavirus isolate 1 NC... (10291) --------------------------------------------------------------------------------
                                                              Section 263
                                            (20961) 20961    20970     20980     20990     21000     21010     21020     21030   21040
SARS-CoV-2 Reference Genome NC_045512.2     (20601) TTCATTTATGCTTTGGTGTAAAGATGGCCATGTAGAAACATTTTACCCAAAATTACAATCTAGTCAAGCGTGGCAACCGG
Alternaria brassicicola endornavirus isolate 1 NC... (10291) --------------------------------------------------------------------------------
                                                              Section 264
                                            (21041) 21041    21050     21060     21070     21080     21090     21100     21110   21120
SARS-CoV-2 Reference Genome NC_045512.2     (20681) GTGTTGCTATGCCTAATCTTTACAAAATGCAAAGAATGCTATTAGAAAAGTGTGACCTTCAAAATTATGGTGATAGTGCA
Alternaria brassicicola endornavirus isolate 1 NC... (10291) --------------------------------------------------------------------------------
                                                              Section 265
                                            (21121) 21121    21130     21140     21150     21160     21170     21180     21190   21200
SARS-CoV-2 Reference Genome NC_045512.2     (20761) ACATTACCTAAAGGCATAATGATGAATGTCGCAAAATATACTCAACTGTGTCAATATTTAAACACATTAACATTAGCTGT
Alternaria brassicicola endornavirus isolate 1 NC... (10291) --------------------------------------------------------------------------------
                                                              Section 266
                                            (21201) 21201    21210     21220     21230     21240     21250     21260     21270   21280
SARS-CoV-2 Reference Genome NC_045512.2     (20841) ACCCTATAATATGAGAGTTATACATTTTGGTGCTGGTTCTGATAAAGGAGTTGCACCAGGTACAGCTGTTTTAAGACAGT
Alternaria brassicicola endornavirus isolate 1 NC... (10291) --------------------------------------------------------------------------------
```

Section 267

```
                                                     (21281) 21281    21290     21300     21310     21320     21330     21340     21350  21360
             SARS-CoV-2 Reference Genome NC_045512.2 (20921) GGTTGCCTACGGGTACGCTGCTTGTCGATTCAGATCTTAATGACTTTGTCTCTGATGCAGATTCAACTTTGATTGGTGAT
Alternaria brassicicola endornavirus isolate 1 NC... (10291) --------------------------------------------------------------------------------
```

Section 268

```
                                                     (21361) 21361    21370     21380     21390     21400     21410     21420     21430  21440
             SARS-CoV-2 Reference Genome NC_045512.2 (21001) TGTGCAACTGTACATACAGCTAATAAATGGGATCTCATTATTAGTGATATGTACGACCCTAAGACTAAAAATGTTACAAA
Alternaria brassicicola endornavirus isolate 1 NC... (10291) --------------------------------------------------------------------------------
```

Section 269

```
                                                     (21441) 21441    21450     21460     21470     21480     21490     21500     21510  21520
             SARS-CoV-2 Reference Genome NC_045512.2 (21081) AGAAAATGACTCTAAAGAGGGTTTTTCACTTACATTTGTGGGTTTATACAACAAAAGCTAGCTCTTGGAGGTTCCGTGG
Alternaria brassicicola endornavirus isolate 1 NC... (10291) --------------------------------------------------------------------------------
```

Section 270

```
                                                     (21521) 21521    21530     21540     21550     21560     21570     21580     21590  21600
             SARS-CoV-2 Reference Genome NC_045512.2 (21161) CTATAAAGATAACAGAACATTCTTGGAATGCTGATCTTTATAAGCTCATGGGACACTTCGCATGGTGGACAGCCTTTGTT
Alternaria brassicicola endornavirus isolate 1 NC... (10291) --------------------------------------------------------------------------------
```

Section 271

```
                                                     (21601) 21601    21610     21620     21630     21640     21650     21660     21670  21680
             SARS-CoV-2 Reference Genome NC_045512.2 (21241) ACTAATGTGAATGCGTCATCATCTGAAGCATTTTTAATTGGATGTAATTATCTTGGCAAACCACGCGAACAAATAGATGG
Alternaria brassicicola endornavirus isolate 1 NC... (10291) --------------------------------------------------------------------------------
```

Section 272

```
                                                     (21681) 21681    21690     21700     21710     21720     21730     21740     21750  21760
             SARS-CoV-2 Reference Genome NC_045512.2 (21321) TTATGTCATGCATGCAAATTACATATTTTGGAGGAATACAAATCCAATTCAGTTGTCTTCCTATTCTTTATTTGACATGA
Alternaria brassicicola endornavirus isolate 1 NC... (10291) --------------------------------------------------------------------------------
```

Section 273

```
                                                     (21761) 21761    21770     21780     21790     21800     21810     21820     21830  21840
             SARS-CoV-2 Reference Genome NC_045512.2 (21401) GTAAATTTCCCCTTAAATTAAGGGGTACTGCTGTTATGTCTTTAAAAGAAGGTCAAATCAATGATATGATTTTATCTCTT
Alternaria brassicicola endornavirus isolate 1 NC... (10291) --------------------------------------------------------------------------------
```

Section 274

```
                                                     (21841) 21841    21850     21860     21870     21880     21890     21900     21910   21920
SARS-CoV-2 Reference Genome NC_045512.2              (21481) CTTAGTAAAGGTAGACTTATAATTAGAGAAAACAACAGAGTTGTTATTTCTAGTGATGTTCTTGTTAACAACTAAACGAA
Alternaria brassicicola endornavirus isolate 1 NC... (10291) --------------------------------------------------------------------------------
```

Section 275

```
                                                     (21921) 21921    21930     21940     21950     21960     21970     21980     21990   22000
SARS-CoV-2 Reference Genome NC_045512.2              (21561) CAATGTTTGTTTTTCTTGTTTTATTGCCACTAGTCTCTAGTCAGTGTGTTAATCTTACAACCAGAACTCAATTACCCCCT
Alternaria brassicicola endornavirus isolate 1 NC... (10291) --------------------------------------------------------------------------------
```

Section 276

```
                                                     (22001) 22001    22010     22020     22030     22040     22050     22060     22070   22080
SARS-CoV-2 Reference Genome NC_045512.2              (21641) GCATACACTAATTCTTTCACACGTGGTGTTTATTACCCTGACAAAGTTTTCAGATCCTCAGTTTTACATTCAACTCAGGA
Alternaria brassicicola endornavirus isolate 1 NC... (10291) --------------------------------------------------------------------------------
```

Section 277

```
                                                     (22081) 22081    22090     22100     22110     22120     22130     22140     22150   22160
SARS-CoV-2 Reference Genome NC_045512.2              (21721) CTTGTTCTTACCTTTCTTTTCCAATGTTACTTGGTTCCATGCTATACATGTCTCTGGGACCAATGGTACTAAGAGGTTTG
Alternaria brassicicola endornavirus isolate 1 NC... (10291) --------------------------------------------------------------------------------
```

Section 278

```
                                                     (22161) 22161    22170     22180     22190     22200     22210     22220     22230   22240
SARS-CoV-2 Reference Genome NC_045512.2              (21801) ATAACCCTGTCCTACCATTTAATGATGGTGTTTATTTTGCTTCCACTGAGAAGTCTAACATAATAAGAGGCTGGATTTTT
Alternaria brassicicola endornavirus isolate 1 NC... (10291) --------------------------------------------------------------------------------
```

Section 279

```
                                                     (22241) 22241    22250     22260     22270     22280     22290     22300     22310   22320
SARS-CoV-2 Reference Genome NC_045512.2              (21881) GGTACTACTTTAGATTCGAAGACCCAGTCCCTACTTATTGTTAATAACGCTACTAATGTTGTTATTAAAGTCTGTGAATT
Alternaria brassicicola endornavirus isolate 1 NC... (10291) --------------------------------------------------------------------------------
```

Section 280

```
                                                     (22321) 22321    22330     22340     22350     22360     22370     22380     22390   22400
SARS-CoV-2 Reference Genome NC_045512.2              (21961) TCAATTTTGTAATGATCCATTTTTGGGTGTTTATTACCACAAAAACAACAAAAGTTGGATGGAAAGTGAGTTCAGAGTTT
Alternaria brassicicola endornavirus isolate 1 NC... (10291) --------------------------------------------------------------------------------
```

```
                                                                                                                                          Section 281
                                                           (22401) 22401    22410    22420    22430    22440    22450    22460    22470   22480
                 SARS-CoV-2 Reference Genome NC_045512.2 (22041) ATTCTAGTGCGAATAATTGCACTTTTGAATATGTCTCTCAGCCTTTTCTTATGGACCTTGAAGGAAAACAGGGTAATTTC
Alternaria brassicicola endornavirus isolate 1 NC... (10291) --------------------------------------------------------------------------------
                                                                                                                                          Section 282
                                                           (22481) 22481    22490    22500    22510    22520    22530    22540    22550   22560
                 SARS-CoV-2 Reference Genome NC_045512.2 (22121) AAAAATCTTAGGGAATTTGTGTTTAAGAATATTGATGGTTATTTTAAAATATATTCTAAGCACACGCCTATTAATTTAGT
Alternaria brassicicola endornavirus isolate 1 NC... (10291) --------------------------------------------------------------------------------
                                                                                                                                          Section 283
                                                           (22561) 22561    22570    22580    22590    22600    22610    22620    22630   22640
                 SARS-CoV-2 Reference Genome NC_045512.2 (22201) GCGTGATCTCCCTCAGGGTTTTTCGGCTTTAGAACCATTGGTAGATTTGCCAATAGGTATTAACATCACTAGGTTTCAAA
Alternaria brassicicola endornavirus isolate 1 NC... (10291) --------------------------------------------------------------------------------
                                                                                                                                          Section 284
                                                           (22641) 22641    22650    22660    22670    22680    22690    22700    22710   22720
                 SARS-CoV-2 Reference Genome NC_045512.2 (22281) CTTTACTTGCTTTACATAGAAGTTATTTGACTCCTGGTGATTCTTCTTCAGGTTGGACAGCTGGTGCTGCAGCTTATTAT
Alternaria brassicicola endornavirus isolate 1 NC... (10291) --------------------------------------------------------------------------------
                                                                                                                                          Section 285
                                                           (22721) 22721    22730    22740    22750    22760    22770    22780    22790   22800
                 SARS-CoV-2 Reference Genome NC_045512.2 (22361) GTGGGTTATCTTCAACCTAGGACTTTTCTATTAAAATATAATGAAAATGGAACCATTACAGATGCTGTAGACTGTGCACT
Alternaria brassicicola endornavirus isolate 1 NC... (10291) --------------------------------------------------------------------------------
                                                                                                                                          Section 286
                                                           (22801) 22801    22810    22820    22830    22840    22850    22860    22870   22880
                 SARS-CoV-2 Reference Genome NC_045512.2 (22441) TGACCCTCTCTCAGAAACAAAGTGTACGTTGAAATCCTTCACTGTAGAAAAAGGAATCTATCAAACTTCTAACTTTAGAG
Alternaria brassicicola endornavirus isolate 1 NC... (10291) --------------------------------------------------------------------------------
                                                                                                                                          Section 287
                                                           (22881) 22881    22890    22900    22910    22920    22930    22940    22950   22960
                 SARS-CoV-2 Reference Genome NC_045512.2 (22521) TCCAACCAACAGAATCTATTGTTAGATTTCCTAATATTACAAACTTGTGCCCTTTTGGTGAAGTTTTTAACGCCACCAGA
Alternaria brassicicola endornavirus isolate 1 NC... (10291) --------------------------------------------------------------------------------
```

```
                                                                                                   Section 288
                                               (22961) 22961    22970     22980     22990     23000     23010     23020     23030 23040
SARS-CoV-2 Reference Genome NC_045512.2        (22601) TTTGCATCTGTTTATGCTTGGAACAGGAAGAGAATCAGCAACTGTGTTGCTGATTATTCTGTCCTATATAATTCCGCATC
Alternaria brassicicola endornavirus isolate 1 NC... (10291) --------------------------------------------------------------------------------
                                                                                                   Section 289
                                               (23041) 23041    23050     23060     23070     23080     23090     23100     23110 23120
SARS-CoV-2 Reference Genome NC_045512.2        (22681) ATTTTCCACTTTTAAGTGTTATGGAGTGTCTCCTACTAAATTAAATGATCTCTGCTTTACTAATGTCTATGCAGATTCAT
Alternaria brassicicola endornavirus isolate 1 NC... (10291) --------------------------------------------------------------------------------
                                                                                                   Section 290
                                               (23121) 23121    23130     23140     23150     23160     23170     23180     23190 23200
SARS-CoV-2 Reference Genome NC_045512.2        (22761) TTGTAATTAGAGGTGATGAAGTCAGACAAATCGCTCCAGGGCAAACTGGAAAGATTGCTGATTATAATTATAAATTACCA
Alternaria brassicicola endornavirus isolate 1 NC... (10291) --------------------------------------------------------------------------------
                                                                                                   Section 291
                                               (23201) 23201    23210     23220     23230     23240     23250     23260     23270 23280
SARS-CoV-2 Reference Genome NC_045512.2        (22841) GATGATTTTACAGGCTGCGTTATAGCTTGGAATTCTAACAATCTTGATTCTAAGGTTGGTGGTAATTATAATTACCTGTA
Alternaria brassicicola endornavirus isolate 1 NC... (10291) --------------------------------------------------------------------------------
                                                                                                   Section 292
                                               (23281) 23281    23290     23300     23310     23320     23330     23340     23350 23360
SARS-CoV-2 Reference Genome NC_045512.2        (22921) TAGATTGTTTAGGAAGTCTAATCTCAAACCTTTTGAGAGAGATATTTCAACTGAAATCTATCAGGCCGGTAGCACACCTT
Alternaria brassicicola endornavirus isolate 1 NC... (10291) --------------------------------------------------------------------------------
                                                                                                   Section 293
                                               (23361) 23361    23370     23380     23390     23400     23410     23420     23430 23440
SARS-CoV-2 Reference Genome NC_045512.2        (23001) GTAATGGTGTTGAAGGTTTTAATTGTTACTTTCCTTTACAATCATATGGTTTCCAACCCACTAATGGTGTTGGTTACCAA
Alternaria brassicicola endornavirus isolate 1 NC... (10291) --------------------------------------------------------------------------------
                                                                                                   Section 294
                                               (23441) 23441    23450     23460     23470     23480     23490     23500     23510 23520
SARS-CoV-2 Reference Genome NC_045512.2        (23081) CCATACAGAGTAGTAGTACTTTCTTTTGAACTTCTACATGCACCAGCAACTGTTTGTGGACCTAAAAAGTCTACTAATTT
Alternaria brassicicola endornavirus isolate 1 NC... (10291) --------------------------------------------------------------------------------
```

```
                                                                                                                 Section 295
                                                     (23521) 23521    23530     23540     23550     23560     23570     23580     23590   23600
             SARS-CoV-2 Reference Genome NC_045512.2 (23161) GGTTAAAAACAAATGTGTCAATTTCAACTTCAATGGTTTAACAGGCACAGGTGTTCTTACTGAGTCTAACAAAAAGTTTC
Alternaria brassicicola endornavirus isolate 1 NC... (10291) --------------------------------------------------------------------------------
                                                                                                                 Section 296
                                                     (23601) 23601    23610     23620     23630     23640     23650     23660     23670   23680
             SARS-CoV-2 Reference Genome NC_045512.2 (23241) TGCCTTTCCAACAATTTGGCAGAGACATTGCTGACACTACTGATGCTGTCCGTGATCCACAGACACTTGAGATTCTTGAC
Alternaria brassicicola endornavirus isolate 1 NC... (10291) --------------------------------------------------------------------------------
                                                                                                                 Section 297
                                                     (23681) 23681    23690     23700     23710     23720     23730     23740     23750   23760
             SARS-CoV-2 Reference Genome NC_045512.2 (23321) ATTACACCATGTTCTTTTGGTGGTGTCAGTGTTATAACACCAGGAACAAATACTTCTAACCAGGTTGCTGTTCTTTATCA
Alternaria brassicicola endornavirus isolate 1 NC... (10291) --------------------------------------------------------------------------------
                                                                                                                 Section 298
                                                     (23761) 23761    23770     23780     23790     23800     23810     23820     23830   23840
             SARS-CoV-2 Reference Genome NC_045512.2 (23401) GGATGTTAACTGCACAGAAGTCCCTGTTGCTATTCATGCAGATCAACTTACTCCTACTTGGCGTGTTTATTCTACAGGTT
Alternaria brassicicola endornavirus isolate 1 NC... (10291) --------------------------------------------------------------------------------
                                                                                                                 Section 299
                                                     (23841) 23841    23850     23860     23870     23880     23890     23900     23910   23920
             SARS-CoV-2 Reference Genome NC_045512.2 (23481) CTAATGTTTTTCAAACACGTGCAGGCTGTTTAATAGGGGCTGAACATGTCAACAACTCATATGAGTGTGACATACCCATT
Alternaria brassicicola endornavirus isolate 1 NC... (10291) --------------------------------------------------------------------------------
                                                                                                                 Section 300
                                                     (23921) 23921    23930     23940     23950     23960     23970     23980     23990   24000
             SARS-CoV-2 Reference Genome NC_045512.2 (23561) GGTGCAGGTATATGCGCTAGTTATCAGACTCAGACTAATTCTCCTCGGCGGGCACGTAGTGTAGCTAGTCAATCCATCAT
Alternaria brassicicola endornavirus isolate 1 NC... (10291) --------------------------------------------------------------------------------
                                                                                                                 Section 301
                                                     (24001) 24001    24010     24020     24030     24040     24050     24060     24070   24080
             SARS-CoV-2 Reference Genome NC_045512.2 (23641) TGCCTACACTATGTCACTTGGTGCAGAAAATTCAGTTGCTTACTCTAATAACTCTATTGCCATACCCACAAATTTTACTA
Alternaria brassicicola endornavirus isolate 1 NC... (10291) --------------------------------------------------------------------------------
```

```
Section 302
                                                   (24081) 24081    24090     24100     24110     24120     24130     24140     24150   24160
SARS-CoV-2 Reference Genome NC_045512.2            (23721) TTAGTGTTACCACAGAAATTCTACCAGTGTCTATGACCAAGACATCAGTAGATTGTACAATGTACATTTGTGGTGATTCA
Alternaria brassicicola endornavirus isolate 1 NC... (10291) --------------------------------------------------------------------------------

Section 303
                                                   (24161) 24161    24170     24180     24190     24200     24210     24220     24230   24240
SARS-CoV-2 Reference Genome NC_045512.2            (23801) ACTGAATGCAGCAATCTTTTGTTGCAATATGGCAGTTTTTGTACACAATTAAACCGTGCTTTAACTGGAATAGCTGTTGA
Alternaria brassicicola endornavirus isolate 1 NC... (10291) --------------------------------------------------------------------------------

Section 304
                                                   (24241) 24241    24250     24260     24270     24280     24290     24300     24310   24320
SARS-CoV-2 Reference Genome NC_045512.2            (23881) ACAAGACAAAAACACCCAAGAAGTTTTTGCACAAGTCAAACAAATTTACAAAACACCACCAATTAAAGATTTTGGTGGTT
Alternaria brassicicola endornavirus isolate 1 NC... (10291) --------------------------------------------------------------------------------

Section 305
                                                   (24321) 24321    24330     24340     24350     24360     24370     24380     24390   24400
SARS-CoV-2 Reference Genome NC_045512.2            (23961) TTAATTTTTCACAAATATTACCAGATCCATCAAAACCAAGCAAGAGGTCATTTATTGAAGATCTACTTTTCAACAAAGTG
Alternaria brassicicola endornavirus isolate 1 NC... (10291) --------------------------------------------------------------------------------

Section 306
                                                   (24401) 24401    24410     24420     24430     24440     24450     24460     24470   24480
SARS-CoV-2 Reference Genome NC_045512.2            (24041) ACACTTGCAGATGCTGGCTTCATCAAACAATATGGTGATTGCCTTGGTGATATTGCTGCTAGAGACCTCATTTGTGCACA
Alternaria brassicicola endornavirus isolate 1 NC... (10291) --------------------------------------------------------------------------------

Section 307
                                                   (24481) 24481    24490     24500     24510     24520     24530     24540     24550   24560
SARS-CoV-2 Reference Genome NC_045512.2            (24121) AAAGTTTAACGGCCTTACTGTTTTGCCACCTTTGCTCACAGATGAAATGATTGCTCAATACACTTCTGCACTGTTAGCGG
Alternaria brassicicola endornavirus isolate 1 NC... (10291) --------------------------------------------------------------------------------

Section 308
                                                   (24561) 24561    24570     24580     24590     24600     24610     24620     24630   24640
SARS-CoV-2 Reference Genome NC_045512.2            (24201) GTACAATCACTTCTGGTTGGACCTTTGGTGCAGGTGCTGCATTACAAATACCATTTGCTATGCAAATGGCTTATAGGTTT
Alternaria brassicicola endornavirus isolate 1 NC... (10291) --------------------------------------------------------------------------------
```

Section 309

```
(24641) 24641    24650    24660    24670    24680    24690    24700    24710  24720
SARS-CoV-2 Reference Genome NC_045512.2 (24281) AATGGTATTGGAGTTACACAGAATGTTCTCTATGAGAACCAAAAATTGATTGCCAACCAATTTAATAGTGCTATTGGCAA
Alternaria brassicicola endornavirus isolate 1 NC... (10291) --------------------------------------------------------------------------------
```

Section 310

```
(24721) 24721    24730    24740    24750    24760    24770    24780    24790  24800
SARS-CoV-2 Reference Genome NC_045512.2 (24361) AATTCAAGACTCACTTTCTTCCACAGCAAGTGCACTTGGAAAACTTCAAGATGTGGTCAACCAAAATGCACAAGCTTTAA
Alternaria brassicicola endornavirus isolate 1 NC... (10291) --------------------------------------------------------------------------------
```

Section 311

```
(24801) 24801    24810    24820    24830    24840    24850    24860    24870  24880
SARS-CoV-2 Reference Genome NC_045512.2 (24441) ACACGCTTGTTAAACAACTTAGCTCCAATTTTGGTGCAATTTCAAGTGTTTTAAATGATATCCTTTCACGTCTTGACAAA
Alternaria brassicicola endornavirus isolate 1 NC... (10291) --------------------------------------------------------------------------------
```

Section 312

```
(24881) 24881    24890    24900    24910    24920    24930    24940    24950  24960
SARS-CoV-2 Reference Genome NC_045512.2 (24521) GTTGAGGCTGAAGTGCAAATTGATAGGTTGATCACAGGCAGACTTCAAAGTTTGCAGACATATGTGACTCAACAATTAAT
Alternaria brassicicola endornavirus isolate 1 NC... (10291) --------------------------------------------------------------------------------
```

Section 313

```
(24961) 24961    24970    24980    24990    25000    25010    25020    25030  25040
SARS-CoV-2 Reference Genome NC_045512.2 (24601) TAGAGCTGCAGAAATCAGAGCTTCTGCTAATCTTGCTGCTACTAAAATGTCAGAGTGTGTACTTGGACAATCAAAAAGAG
Alternaria brassicicola endornavirus isolate 1 NC... (10291) --------------------------------------------------------------------------------
```

Section 314

```
(25041) 25041    25050    25060    25070    25080    25090    25100    25110  25120
SARS-CoV-2 Reference Genome NC_045512.2 (24681) TTGATTTTTGTGGAAAGGGCTATCATCTTATGTCCTTCCCTCAGTCAGCACCTCATGGTGTAGTCTTCTTGCATGTGACT
Alternaria brassicicola endornavirus isolate 1 NC... (10291) --------------------------------------------------------------------------------
```

Section 315

```
(25121) 25121    25130    25140    25150    25160    25170    25180    25190  25200
SARS-CoV-2 Reference Genome NC_045512.2 (24761) TATGTCCCTGCACAAGAAAAGAACTTCACAACTGCTCCTGCCATTTGTCATGATGGAAAAGCACACTTTCCTCGTGAAGG
Alternaria brassicicola endornavirus isolate 1 NC... (10291) --------------------------------------------------------------------------------
```

```
                                                                                                                  Section 316
                                                     (25201) 25201    25210     25220     25230     25240     25250     25260     25270   25280
                 SARS-CoV-2 Reference Genome NC_045512.2 (24841) TGTCTTTGTTTCAAATGGCACACACTGGTTTGTAACACAAAGGAATTTTTATGAACCACAAATCATTACTACAGACAACA
Alternaria brassicicola endornavirus isolate 1 NC... (10291) --------------------------------------------------------------------------------
                                                                                                                  Section 317
                                                     (25281) 25281    25290     25300     25310     25320     25330     25340     25350   25360
                 SARS-CoV-2 Reference Genome NC_045512.2 (24921) CATTTGTGTCTGGTAACTGTGATGTTGTAATAGGAATTGTCAACAACACAGTTTATGATCCTTTGCAACCTGAATTAGAC
Alternaria brassicicola endornavirus isolate 1 NC... (10291) --------------------------------------------------------------------------------
                                                                                                                  Section 318
                                                     (25361) 25361    25370     25380     25390     25400     25410     25420     25430   25440
                 SARS-CoV-2 Reference Genome NC_045512.2 (25001) TCATTCAAGGAGGAGTTAGATAAATATTTTAAGAATCATACATCACCAGATGTTGATTTAGGTGACATCTCTGGCATTAA
Alternaria brassicicola endornavirus isolate 1 NC... (10291) --------------------------------------------------------------------------------
                                                                                                                  Section 319
                                                     (25441) 25441    25450     25460     25470     25480     25490     25500     25510   25520
                 SARS-CoV-2 Reference Genome NC_045512.2 (25081) TGCTTCAGTTGTAAACATTCAAAAAGAAATTGACCGCCTCAATGAGGTTGCCAAGAATTTAAATGAATCTCTCATCGATC
Alternaria brassicicola endornavirus isolate 1 NC... (10291) --------------------------------------------------------------------------------
                                                                                                                  Section 320
                                                     (25521) 25521    25530     25540     25550     25560     25570     25580     25590   25600
                 SARS-CoV-2 Reference Genome NC_045512.2 (25161) TCCAAGAACTTGGAAAGTATGAGCAGTATATAAAATGGCCATGGTACATTTGGCTAGGTTTTATAGCTGGCTTGATTGCC
Alternaria brassicicola endornavirus isolate 1 NC... (10291) --------------------------------------------------------------------------------
                                                                                                                  Section 321
                                                     (25601) 25601    25610     25620     25630     25640     25650     25660     25670   25680
                 SARS-CoV-2 Reference Genome NC_045512.2 (25241) ATAGTAATGGTGACAATTATGCTTTGCTGTATGACCAGTTGCTGTAGTTGTCTCAAGGGCTGTTGTTCTTGTGGATCCTG
Alternaria brassicicola endornavirus isolate 1 NC... (10291) --------------------------------------------------------------------------------
                                                                                                                  Section 322
                                                     (25681) 25681    25690     25700     25710     25720     25730     25740     25750   25760
                 SARS-CoV-2 Reference Genome NC_045512.2 (25321) CTGCAAATTTGATGAAGACGACTCTGAGCCAGTGCTCAAAGGAGTCAAATTACATTACACATAAACGAACTTATGGATTT
Alternaria brassicicola endornavirus isolate 1 NC... (10291) --------------------------------------------------------------------------------
```

```
                                                                                                Section 323
                                                 (25761) 25761    25770     25780     25790     25800     25810     25820     25830     25840
             SARS-CoV-2 Reference Genome NC_045512.2 (25401) GTTTATGAGAATCTTCACAATTGGAACTGTAACTTTGAAGCAAGGTGAAATCAAGGATGCTACTCCTTCAGATTTTGTTC
Alternaria brassicicola endornavirus isolate 1 NC... (10291) --------------------------------------------------------------------------------
                                                                                                Section 324
                                                 (25841) 25841    25850     25860     25870     25880     25890     25900     25910     25920
             SARS-CoV-2 Reference Genome NC_045512.2 (25481) GCGCTACTGCAACGATACCGATACAAGCCTCACTCCCTTTCGGATGGCTTATTGTTGGCGTTGCACTTCTTGCTGTTTTT
Alternaria brassicicola endornavirus isolate 1 NC... (10291) --------------------------------------------------------------------------------
                                                                                                Section 325
                                                 (25921) 25921    25930     25940     25950     25960     25970     25980     25990     26000
             SARS-CoV-2 Reference Genome NC_045512.2 (25561) CAGAGCGCTTCCAAAATCATAACCCTCAAAAAGAGATGGCAACTAGCACTCTCCAAGGGTGTTCACTTTGTTTGCAACTT
Alternaria brassicicola endornavirus isolate 1 NC... (10291) --------------------------------------------------------------------------------
                                                                                                Section 326
                                                 (26001) 26001    26010     26020     26030     26040     26050     26060     26070     26080
             SARS-CoV-2 Reference Genome NC_045512.2 (25641) GCTGTTGTTGTTTGTAACAGTTTACTCACACCTTTTGCTCGTTGCTGCTGGCCTTGAAGCCCCTTTTCTCTATCTTTATG
Alternaria brassicicola endornavirus isolate 1 NC... (10291) --------------------------------------------------------------------------------
                                                                                                Section 327
                                                 (26081) 26081    26090     26100     26110     26120     26130     26140     26150     26160
             SARS-CoV-2 Reference Genome NC_045512.2 (25721) CTTTAGTCTACTTCTTGCAGAGTATAAACTTTGTAAGAATAATAATGAGGCTTTGGCTTTGCTGGAAATGCCGTTCCAAA
Alternaria brassicicola endornavirus isolate 1 NC... (10291) --------------------------------------------------------------------------------
                                                                                                Section 328
                                                 (26161) 26161    26170     26180     26190     26200     26210     26220     26230     26240
             SARS-CoV-2 Reference Genome NC_045512.2 (25801) AACCCATTACTTTATGATGCCAACTATTTTCTTTGCTGGCATACTAATTGTTACGACTATTGTATACCTTACAATAGTGT
Alternaria brassicicola endornavirus isolate 1 NC... (10291) --------------------------------------------------------------------------------
                                                                                                Section 329
                                                 (26241) 26241    26250     26260     26270     26280     26290     26300     26310     26320
             SARS-CoV-2 Reference Genome NC_045512.2 (25881) AACTTCTTCAATTGTCATTACTTCAGGTGATGGCACAACAAGTCCTATTTCTGAACATGACTACCAGATTGGTGGTTATA
Alternaria brassicicola endornavirus isolate 1 NC... (10291) --------------------------------------------------------------------------------
```

Section 330

(26321) 26321 26330 26340 26350 26360 26370 26380 26390 26400
SARS-CoV-2 Reference Genome NC_045512.2 (25961) CTGAAAAATGGGAATCTGGAGTAAAAGACTGTGTTGTATTACACAGTTACTTCACTTCAGACTATTACCAGCTGTACTCA
Alternaria brassicicola endornavirus isolate 1 NC... (10291) --

Section 331

(26401) 26401 26410 26420 26430 26440 26450 26460 26470 26480
SARS-CoV-2 Reference Genome NC_045512.2 (26041) ACTCAATTGAGTACAGACACTGGTGTTGAACATGTTACCTTCTTCATCTACAATAAAATTGTTGATGAGCCTGAAGAACA
Alternaria brassicicola endornavirus isolate 1 NC... (10291) --

Section 332

(26481) 26481 26490 26500 26510 26520 26530 26540 26550 26560
SARS-CoV-2 Reference Genome NC_045512.2 (26121) TGTCCAAATTCACACAATCGACGGTTCATCCGGAGTTGTTAATCCAGTAATGGAACCAATTTATGATGAACCGACGACGA
Alternaria brassicicola endornavirus isolate 1 NC... (10291) --

Section 333

(26561) 26561 26570 26580 26590 26600 26610 26620 26630 26640
SARS-CoV-2 Reference Genome NC_045512.2 (26201) CTACTAGCGTGCCTTTGTAAGCACAAGCTGATGAGTACGAACTTATGTACTCATTCGTTTCGGAAGAGACAGGTACGTTA
Alternaria brassicicola endornavirus isolate 1 NC... (10291) --

Section 334

(26641) 26641 26650 26660 26670 26680 26690 26700 26710 26720
SARS-CoV-2 Reference Genome NC_045512.2 (26281) ATAGTTAATAGCGTACTTCTTTTTCTTGCTTTCGTGGTATTCTTGCTAGTTACACTAGCCATCCTTACTGCGCTTCGATT
Alternaria brassicicola endornavirus isolate 1 NC... (10291) --

Section 335

(26721) 26721 26730 26740 26750 26760 26770 26780 26790 26800
SARS-CoV-2 Reference Genome NC_045512.2 (26361) GTGTGCGTACTGCTGCAATATTGTTAACGTGAGTCTTGTAAAACCTTCTTTTTACGTTTACTCTCGTGTTAAAAATCTGA
Alternaria brassicicola endornavirus isolate 1 NC... (10291) --

Section 336

(26801) 26801 26810 26820 26830 26840 26850 26860 26870 26880
SARS-CoV-2 Reference Genome NC_045512.2 (26441) ATTCTTCTAGAGTTCCTGATCTTCTGGTCTAAACGAACTAAATATTATATTAGTTTTTCTGTTTGGAACTTTAATTTTAG
Alternaria brassicicola endornavirus isolate 1 NC... (10291) --

Section 337

```
                                                             (26881) 26881    26890     26900     26910     26920     26930     26940     26950   26960
           SARS-CoV-2 Reference Genome NC_045512.2 (26521) CCATGGCAGATTCCAACGGTACTATTACCGTTGAAGAGCTTAAAAAGCTCCTTGAACAATGGAACCTAGTAATAGGTTTC
Alternaria brassicicola endornavirus isolate 1 NC... (10291) --------------------------------------------------------------------------------
```

Section 338

```
                                                             (26961) 26961    26970     26980     26990     27000     27010     27020     27030   27040
           SARS-CoV-2 Reference Genome NC_045512.2 (26601) CTATTCCTTACATGGATTTGTCTTCTACAATTTGCCTATGCCAACAGGAATAGGTTTTTGTATATAATTAAGTTAATTTT
Alternaria brassicicola endornavirus isolate 1 NC... (10291) --------------------------------------------------------------------------------
```

Section 339

```
                                                             (27041) 27041    27050     27060     27070     27080     27090     27100     27110   27120
           SARS-CoV-2 Reference Genome NC_045512.2 (26681) CCTCTGGCTGTTATGGCCAGTAACTTTAGCTTGTTTTGTGCTTGCTGCTGTTTACAGAATAAATTGGATCACCGGTGGAA
Alternaria brassicicola endornavirus isolate 1 NC... (10291) --------------------------------------------------------------------------------
```

Section 340

```
                                                             (27121) 27121    27130     27140     27150     27160     27170     27180     27190   27200
           SARS-CoV-2 Reference Genome NC_045512.2 (26761) TTGCTATCGCAATGGCTTGTCTTGTAGGCTTGATGTGGCTCAGCTACTTCATTGCTTCTTTCAGACTGTTTGCGCGTACG
Alternaria brassicicola endornavirus isolate 1 NC... (10291) --------------------------------------------------------------------------------
```

Section 341

```
                                                             (27201) 27201    27210     27220     27230     27240     27250     27260     27270   27280
           SARS-CoV-2 Reference Genome NC_045512.2 (26841) CGTTCCATGTGGTCATTCAATCCAGAAACTAACATTCTTCTCAACGTGCCACTCCATGGCACTATTCTGACCAGACCGCT
Alternaria brassicicola endornavirus isolate 1 NC... (10291) --------------------------------------------------------------------------------
```

Section 342

```
                                                             (27281) 27281    27290     27300     27310     27320     27330     27340     27350   27360
           SARS-CoV-2 Reference Genome NC_045512.2 (26921) TCTAGAAAGTGAACTCGTAATCGGAGCTGTGATCCTTCGTGGACATCTTCGTATTGCTGGACACCATCTAGGACGCTGTG
Alternaria brassicicola endornavirus isolate 1 NC... (10291) --------------------------------------------------------------------------------
```

Section 343

```
                                                             (27361) 27361    27370     27380     27390     27400     27410     27420     27430   27440
           SARS-CoV-2 Reference Genome NC_045512.2 (27001) ACATCAAGGACCTGCCTAAAGAAATCACTGTTGCTACATCACGAACGCTTTCTTATTACAAATTGGGAGCTTCGCAGCGT
Alternaria brassicicola endornavirus isolate 1 NC... (10291) --------------------------------------------------------------------------------
```

```
                                                                                                  Section 344
                                                      (27441) 27441    27450     27460     27470     27480     27490     27500     27510   27520
           SARS-CoV-2 Reference Genome NC 045512.2 (27081) GTAGCAGGTGACTCAGGTTTTGCTGCATACAGTCGCTACAGGATTGGCAACTATAAATTAAACACAGACCATTCCAGTAG
Alternaria brassicicola endornavirus isolate 1 NC... (10291) --------------------------------------------------------------------------------
                                                                                                  Section 345
                                                      (27521) 27521    27530     27540     27550     27560     27570     27580     27590   27600
           SARS-CoV-2 Reference Genome NC 045512.2 (27161) CAGTGACAATATTGCTTTGCTTGTACAGTAAGTGACAACAGATGTTTCATCTCGTTGACTTTCAGGTTACTATAGCAGAG
Alternaria brassicicola endornavirus isolate 1 NC... (10291) --------------------------------------------------------------------------------
                                                                                                  Section 346
                                                      (27601) 27601    27610     27620     27630     27640     27650     27660     27670   27680
           SARS-CoV-2 Reference Genome NC 045512.2 (27241) ATATTACTAATTATTATGAGGACTTTTAAAGTTTCCATTTGGAATCTTGATTACATCATAAACCTCATAATTAAAAATTT
Alternaria brassicicola endornavirus isolate 1 NC... (10291) --------------------------------------------------------------------------------
                                                                                                  Section 347
                                                      (27681) 27681    27690     27700     27710     27720     27730     27740     27750   27760
           SARS-CoV-2 Reference Genome NC 045512.2 (27321) ATCTAAGTCACTAACTGAGAATAAATATTCTCAATTAGATGAAGAGCAACCAATGGAGATTGATTAAACGAACATGAAAA
Alternaria brassicicola endornavirus isolate 1 NC... (10291) --------------------------------------------------------------------------------
                                                                                                  Section 348
                                                      (27761) 27761    27770     27780     27790     27800     27810     27820     27830   27840
           SARS-CoV-2 Reference Genome NC 045512.2 (27401) TTATTCTTTTCTTGGCACTGATAACACTCGCTACTTGTGAGCTTTATCACTACCAAGAGTGTGTTAGAGGTACAACAGTA
Alternaria brassicicola endornavirus isolate 1 NC... (10291) --------------------------------------------------------------------------------
                                                                                                  Section 349
                                                      (27841) 27841    27850     27860     27870     27880     27890     27900     27910   27920
           SARS-CoV-2 Reference Genome NC 045512.2 (27481) CTTTTAAAAGAACCTTGCTCTTCTGGAACATACGAGGGCAATTCACCATTTCATCCTCTAGCTGATAACAAATTTGCACT
Alternaria brassicicola endornavirus isolate 1 NC... (10291) --------------------------------------------------------------------------------
                                                                                                  Section 350
                                                      (27921) 27921    27930     27940     27950     27960     27970     27980     27990   28000
           SARS-CoV-2 Reference Genome NC 045512.2 (27561) GACTTGCTTTAGCACTCAATTTGCTTTTGCTTGTCCTGACGGCGTAAAACACGTCTATCAGTTACGTGCCAGATCAGTTT
Alternaria brassicicola endornavirus isolate 1 NC... (10291) --------------------------------------------------------------------------------
```

```
                                                              Section 351
                                             (28001) 28001    28010     28020     28030     28040     28050     28060     28070   28080
          SARS-CoV-2 Reference Genome NC 045512.2 (27641) CACCTAAACTGTTCATCAGACAAGAGGAAGTTCAAGAACTTTACTCTCCAATTTTTCTTATTGTTGCGGCAATAGTGTTT
Alternaria brassicicola endornavirus isolate 1 NC... (10291) --------------------------------------------------------------------------------
                                                              Section 352
                                             (28081) 28081    28090     28100     28110     28120     28130     28140     28150   28160
          SARS-CoV-2 Reference Genome NC 045512.2 (27721) ATAACACTTTGCTTCACACTCAAAAGAAAGACAGAATGATTGAACTTTCATTAATTGACTTCTATTTGTGCTTTTTAGCC
Alternaria brassicicola endornavirus isolate 1 NC... (10291) --------------------------------------------------------------------------------
                                                              Section 353
                                             (28161) 28161    28170     28180     28190     28200     28210     28220     28230   28240
          SARS-CoV-2 Reference Genome NC 045512.2 (27801) TTTCTGCTATTCCTTGTTTTAATTATGCTTATTATCTTTTGGTTCTCACTTGAACTGCAAGATCATAATGAAACTTGTCA
Alternaria brassicicola endornavirus isolate 1 NC... (10291) --------------------------------------------------------------------------------
                                                              Section 354
                                             (28241) 28241    28250     28260     28270     28280     28290     28300     28310   28320
          SARS-CoV-2 Reference Genome NC 045512.2 (27881) CGCCTAAACGAACATGAAATTTCTTGTTTTCTTAGGAATCATCACAACTGTAGCTGCATTTCACCAAGAATGTAGTTTAC
Alternaria brassicicola endornavirus isolate 1 NC... (10291) --------------------------------------------------------------------------------
                                                              Section 355
                                             (28321) 28321    28330     28340     28350     28360     28370     28380     28390   28400
          SARS-CoV-2 Reference Genome NC 045512.2 (27961) AGTCATGTACTCAACATCAACCATATGTAGTTGATGACCCGTGTCCTATTCACTTCTATTCTAAATGGTATATTAGAGTA
Alternaria brassicicola endornavirus isolate 1 NC... (10291) --------------------------------------------------------------------------------
                                                              Section 356
                                             (28401) 28401    28410     28420     28430     28440     28450     28460     28470   28480
          SARS-CoV-2 Reference Genome NC 045512.2 (28041) GGAGCTAGAAAATCAGCACCTTTAATTGAATTGTGCGTGGATGAGGCTGGTTCTAAATCACCCATTCAGTACATCGATAT
Alternaria brassicicola endornavirus isolate 1 NC... (10291) --------------------------------------------------------------------------------
                                                              Section 357
                                             (28481) 28481    28490     28500     28510     28520     28530     28540     28550   28560
          SARS-CoV-2 Reference Genome NC 045512.2 (28121) CGGTAATTATACAGTTTCCTGTTTACCTTTTACAATTAATTGCCAGGAACCTAAATTGGGTAGTCTTGTAGTGCGTTGTT
Alternaria brassicicola endornavirus isolate 1 NC... (10291) --------------------------------------------------------------------------------
```

```
                                                                                  Section 358
                                                    (28561) 28561    28570    28580    28590    28600    28610    28620    28630   28640
          SARS-CoV-2 Reference Genome NC_045512.2 (28201) CGTTCTATGAAGACTTTTTAGAGTATCATGACGTTCGTGTTGTTTTAGATTTCATCTAAACGAACAAACTAAAATGTCTG
Alternaria brassicicola endornavirus isolate 1 NC... (10291) --------------------------------------------------------------------------------
                                                                                  Section 359
                                                    (28641) 28641    28650    28660    28670    28680    28690    28700    28710   28720
          SARS-CoV-2 Reference Genome NC_045512.2 (28281) ATAATGGACCCCAAAATCAGCGAAATGCACCCCGCATTACGTTTGGTGGACCCTCAGATTCAACTGGCAGTAACCAGAAT
Alternaria brassicicola endornavirus isolate 1 NC... (10291) --------------------------------------------------------------------------------
                                                                                  Section 360
                                                    (28721) 28721    28730    28740    28750    28760    28770    28780    28790   28800
          SARS-CoV-2 Reference Genome NC_045512.2 (28361) GGAGAACGCAGTGGGGCGCGATCAAAACAACGTCGGCCCCAAGGTTTACCCAATAATACTGCGTCTTGGTTCACCGCTCT
Alternaria brassicicola endornavirus isolate 1 NC... (10291) --------------------------------------------------------------------------------
                                                                                  Section 361
                                                    (28801) 28801    28810    28820    28830    28840    28850    28860    28870   28880
          SARS-CoV-2 Reference Genome NC_045512.2 (28441) CACTCAACATGGCAAGGAAGACCTTAAATTCCCTCGAGGACAAGGCGTTCCAATTAACACCAATAGCAGTCCAGATGACC
Alternaria brassicicola endornavirus isolate 1 NC... (10291) --------------------------------------------------------------------------------
                                                                                  Section 362
                                                    (28881) 28881    28890    28900    28910    28920    28930    28940    28950   28960
          SARS-CoV-2 Reference Genome NC_045512.2 (28521) AAATTGGCTACTACCGAAGAGCTACCAGACGAATTCGTGGTGGTGACGGTAAAATGAAAGATCTCAGTCCAAGATGGTAT
Alternaria brassicicola endornavirus isolate 1 NC... (10291) --------------------------------------------------------------------------------
                                                                                  Section 363
                                                    (28961) 28961    28970    28980    28990    29000    29010    29020    29030   29040
          SARS-CoV-2 Reference Genome NC_045512.2 (28601) TTCTACTACCTAGGAACTGGGCCAGAAGCTGGACTTCCCTATGGTGCTAACAAAGACGGCATCATATGGGTTGCAACTGA
Alternaria brassicicola endornavirus isolate 1 NC... (10291) --------------------------------------------------------------------------------
                                                                                  Section 364
                                                    (29041) 29041    29050    29060    29070    29080    29090    29100    29110   29120
          SARS-CoV-2 Reference Genome NC_045512.2 (28681) GGGAGCCTTGAATACACCAAAAGATCACATTGGCACCCGCAATCCTGCTAACAATGCTGCAATCGTGCTACAACTTCCTC
Alternaria brassicicola endornavirus isolate 1 NC... (10291) --------------------------------------------------------------------------------
```

Section 365

```
                                              (29121) 29121    29130     29140     29150     29160     29170     29180     29190  29200
           SARS-CoV-2 Reference Genome NC_045512.2 (28761) AAGGAACAACATTGCCAAAAGGCTTCTACGCAGAAGGGAGCAGAGGCGGCAGTCAAGCCTCTTCTCGTTCCTCATCACGT
Alternaria brassicicola endornavirus isolate 1 NC... (10291) --------------------------------------------------------------------------------
```

Section 366

```
                                              (29201) 29201    29210     29220     29230     29240     29250     29260     29270  29280
           SARS-CoV-2 Reference Genome NC_045512.2 (28841) AGTCGCAACAGTTCAAGAAATTCAACTCCAGGCAGCAGTAGGGGAACTTCTCCTGCTAGAATGGCTGGCAATGGCGGTGA
Alternaria brassicicola endornavirus isolate 1 NC... (10291) --------------------------------------------------------------------------------
```

Section 367

```
                                              (29281) 29281    29290     29300     29310     29320     29330     29340     29350  29360
           SARS-CoV-2 Reference Genome NC_045512.2 (28921) TGCTGCTCTTGCTTTGCTGCTGCTTGACAGATTGAACCAGCTTGAGAGCAAAATGTCTGGTAAAGGCCAACAACAACAAG
Alternaria brassicicola endornavirus isolate 1 NC... (10291) --------------------------------------------------------------------------------
```

Section 368

```
                                              (29361) 29361    29370     29380     29390     29400     29410     29420     29430  29440
           SARS-CoV-2 Reference Genome NC_045512.2 (29001) GCCAAACTGTCACTAAGAAATCTGCTGCTGAGGCTTCTAAGAAGCCTCGGCAAAAACGTACTGCCACTAAAGCATACAAT
Alternaria brassicicola endornavirus isolate 1 NC... (10291) --------------------------------------------------------------------------------
```

Section 369

```
                                              (29441) 29441    29450     29460     29470     29480     29490     29500     29510  29520
           SARS-CoV-2 Reference Genome NC_045512.2 (29081) GTAACACAAGCTTTCGGCAGACGTGGTCCAGAACAAACCCAAGGAAATTTTGGGGACCAGGAACTAATCAGACAAGGAAC
Alternaria brassicicola endornavirus isolate 1 NC... (10291) --------------------------------------------------------------------------------
```

Section 370

```
                                              (29521) 29521    29530     29540     29550     29560     29570     29580     29590  29600
           SARS-CoV-2 Reference Genome NC_045512.2 (29161) TGATTACAAACATTGGCCGCAAATTGCACAATTTGCCCCCAGCGCTTCAGCGTTCTTCGGAATGTCGCGCATTGGCATGG
Alternaria brassicicola endornavirus isolate 1 NC... (10291) --------------------------------------------------------------------------------
```

Section 371

```
                                              (29601) 29601    29610     29620     29630     29640     29650     29660     29670  29680
           SARS-CoV-2 Reference Genome NC_045512.2 (29241) AAGTCACACCTTCGGGAACGTGGTTGACCTACACAGGTGCCATCAAATTGGATGACAAAGATCCAAATTTCAAAGATCAA
Alternaria brassicicola endornavirus isolate 1 NC... (10291) --------------------------------------------------------------------------------
```

```
                                                                                                         Section 372
                                                        (29681) 29681    29690     29700     29710     29720     29730     29740     29750   29760
            SARS-CoV-2 Reference Genome NC_045512.2 (29321) GTCATTTTGCTGAATAAGCATATTGACGCATACAAAACATTCCCACCAACAGAGCCTAAAAAGGACAAAAAGAAGAAGGC
Alternaria brassicicola endornavirus isolate 1 NC... (10291) --------------------------------------------------------------------------------
                                                                                                         Section 373
                                                        (29761) 29761    29770     29780     29790     29800     29810     29820     29830   29840
            SARS-CoV-2 Reference Genome NC_045512.2 (29401) TGATGAAACTCAAGCCTTACCGCAGAGACAGAAGAAACAGCAAACTGTGACTCTTCTTCCTGCTGCAGATTTGGATGATT
Alternaria brassicicola endornavirus isolate 1 NC... (10291) --------------------------------------------------------------------------------
                                                                                                         Section 374
                                                        (29841) 29841    29850     29860     29870     29880     29890     29900     29910   29920
            SARS-CoV-2 Reference Genome NC_045512.2 (29481) TCTCCAAACAATTGCAACAATCCATGAGCAGTGCTGACTCAACTCAGGCCTAAACTCATGCAGACCACACAAGGCAGATG
Alternaria brassicicola endornavirus isolate 1 NC... (10291) --------------------------------------------------------------------------------
                                                                                                         Section 375
                                                        (29921) 29921    29930     29940     29950     29960     29970     29980     29990   30000
            SARS-CoV-2 Reference Genome NC_045512.2 (29561) GGCTATATAAACGTTTTCGCTTTTCCGTTTACGATATATAGTCTACTCTTGTGCAGAATGAATTCTCGTAACTACATAGC
Alternaria brassicicola endornavirus isolate 1 NC... (10291) --------------------------------------------------------------------------------
                                                                                                         Section 376
                                                        (30001) 30001    30010     30020     30030     30040     30050     30060     30070   30080
            SARS-CoV-2 Reference Genome NC_045512.2 (29641) ACAAGTAGATGTAGTTAACTTTAATCTCACATAGCAATCTTTAATCAGTGTGTAACATTAGGGAGGACTTGAAAGAGCCA
Alternaria brassicicola endornavirus isolate 1 NC... (10291) --------------------------------------------------------------------------------
                                                                                                         Section 377
                                                        (30081) 30081    30090     30100     30110     30120     30130     30140     30150   30160
            SARS-CoV-2 Reference Genome NC_045512.2 (29721) CCACATTTTCACCGAGGCCACGCGGAGTACGATCGAGTGTACAGTGAACAATGCTAGGGAGAGCTGCCTATATGGAAGAG
Alternaria brassicicola endornavirus isolate 1 NC... (10291) --------------------------------------------------------------------------------
                                                                                                         Section 378
                                                        (30161) 30161    30170     30180     30190     30200     30210     30220     30230   30240
            SARS-CoV-2 Reference Genome NC_045512.2 (29801) CCCTAATGTGTAAAATTAATTTTAGTAGTGCTATCCCCATGTGATTTTAATAGCTTCTTAGGAGAATGACAAAAAAAAAA
Alternaria brassicicola endornavirus isolate 1 NC... (10291) --------------------------------------------------------------------------------
```

	(30241)	30241 30250 30263
SARS-CoV-2 Reference Genome NC_045512.2	(29881)	AAAAAAAAAAAAAAAAAAAAAAA
Alternaria brassicicola endornavirus isolate 1 NC...	(10291)	-----------------------
