# Supplementary material for: Ubiquitous Micro-Modular Homologies among Genomes from Viruses to Bacteria to Human Mitochondrial DNA: Platforms for Recombination during Evolution?
Source: Viruses. 2022 Apr 24;14(5):885. doi: 10.3390/v14050885 (PMC9147251; doi:10.3390/v14050885)
Supplement: Supplementary file 1 [file viruses-14-00885-s001.zip › Fig. S3B, SARS-CoV-2 & Autographa californica nucleopolyhedrovirus DNA Alignment.pdf]

```
                                                              Section 1
                                           (1) 1         11         22         33         44         55         66         77
Autographa californica nucleopolyhedrovirus NC_0... (1) GAATTCTACCCGTAAAGCGAGTTTAGTTTTGAAAAACAAATGACATCATTTGTATAATGACATCATCCCCTGATTGT
                         SARS-CoV-2 NC_045512.2 (1) -----------------------------------------------------------------------------
                                                              Section 2
                                          (78) 78        88         99         110        121        132        143        154
Autographa californica nucleopolyhedrovirus NC_0... (78) GTTTTACAAGTAGAATTCTATCCGTAAAGCGAGTTCAGTTTTGAAAACAAATGAGTCATACCTAAACACGTTAATAA
                         SARS-CoV-2 NC_045512.2 (1) -----------------------------------------------------------------------------
                                                              Section 3
                                         (155) 155       165        176        187        198        209        220        231
Autographa californica nucleopolyhedrovirus NC_0... (155) TCTTCTGATATCAGCTTATGACTCAAGTTATGAGCCGTGTGCAAAACATGAGATAAGTTTATGACATCATCCACTGA
                         SARS-CoV-2 NC_045512.2 (1) -----------------------------------------------------------------------------
                                                              Section 4
                                         (232) 232       242        253        264        275        286        297        308
Autographa californica nucleopolyhedrovirus NC_0... (232) TCGTGCGTTACAAGTAGAATTCTACTCGTAAAGCCAGTTCGGTTATGAGCCGTGTGCAAAACATGACATCAGCTTAT
                         SARS-CoV-2 NC_045512.2 (1) -----------------------------------------------------------------------------
                                                              Section 5
                                         (309) 309       319        330        341        352        363        374        385
Autographa californica nucleopolyhedrovirus NC_0... (309) GACTCATACTTGATTGTGTTTTACGCGTAGAATTCTACTCGTAAAGCGAGTTCGGTTATGAGCCGTGTGCAAAACAT
                         SARS-CoV-2 NC_045512.2 (1) -----------------------------------------------------------------------------
                                                              Section 6
                                         (386) 386       396        407        418        429        440        451        462
Autographa californica nucleopolyhedrovirus NC_0... (386) GACATCAGCTTATGAGTCATAATTAATCGTGCGTTACAAGTAGAATTCTACTCGTAAAGCGAGTTGAAGGATCATAT
                         SARS-CoV-2 NC_045512.2 (1) -----------------------------------------------------------------------------
                                                              Section 7
                                         (463) 463       473        484        495        506        517        528        539
Autographa californica nucleopolyhedrovirus NC_0... (463) TTAGTTGCGTTTATGAGATAAGATTGAAAGCACGTGTAAAATGTTTCCCGCGCGTTGGCACAACTATTTACAATGCG
                         SARS-CoV-2 NC_045512.2 (1) -----------------------------------------------------------------------------
```

```
                                                                                     Section 8
                                               (540) 540      550        561        572        583        594        605        616
Autographa californica nucleopolyhedrovirus NC_0... (540) GCCAAGTTATAAAAGATTCTAATCTGATATGTTTTAAAACACCTTTGCGGCCCGAGTTGTTTGCGTACGTGACTAGC
                          SARS-CoV-2 NC_045512.2 (1) -----------------------------------------------------------------------------
                                                                                     Section 9
                                               (617) 617      627        638        649        660        671        682        693
Autographa californica nucleopolyhedrovirus NC_0... (617) GAAGAAGATGTGTGGACCGCAGAACAGATAGTAAAACAAAACCCTAGTATTGGAGCAATAATCGATTTAACCAACAC
                          SARS-CoV-2 NC_045512.2 (1) -----------------------------------------------------------------------------
                                                                                     Section 10
                                               (694) 694      704        715        726        737        748        759        770
Autographa californica nucleopolyhedrovirus NC_0... (694) GTCTAAATATTATGATGGTGTGCATTTTTTGCGGGCGGGCCTGTTATACAAAAAAATTCAAGTACCTGGCCAGACTT
                          SARS-CoV-2 NC_045512.2 (1) -----------------------------------------------------------------------------
                                                                                     Section 11
                                               (771) 771      781        792        803        814        825        836        847
Autographa californica nucleopolyhedrovirus NC_0... (771) TGCCGCCTGAAAGCATAGTTCAAGAATTTATTGACACGGTAAAAGAATTTACAGAAAAGTGTCCCGGCATGTTGGTG
                          SARS-CoV-2 NC_045512.2 (1) -----------------------------------------------------------------------------
                                                                                     Section 12
                                               (848) 848      858        869        880        891        902        913        924
Autographa californica nucleopolyhedrovirus NC_0... (848) GGCGTGCACTGCACACACGGTATTAATCGCACCGGTTACATGGTGTGCAGATATTTAATGCACACCCTGGGTATTGC
                          SARS-CoV-2 NC_045512.2 (1) -----------------------------------------------------------------------------
                                                                                     Section 13
                                               (925) 925      935        946        957        968        979        990        1001
Autographa californica nucleopolyhedrovirus NC_0... (925) GCCGCAGGAAGCCATAGATAGATTCGAAAAAGCCAGAGGTCACAAAATTGAAAGACAAAATTACGTTCAAGATTTAT
                          SARS-CoV-2 NC_045512.2 (1) -----------------------------------------------------------------------------
                                                                                     Section 14
                                              (1002) 1002     1012       1023       1034       1045       1056       1067       1078
Autographa californica nucleopolyhedrovirus NC_0... (1002) TAATTTAATTAATATTATTTGCATTCTTTAACAAATACTTTATCCTATTTTCAAATTGTTGCGCTTCTTCCAGCGAA
                          SARS-CoV-2 NC_045512.2 (1) -----------------------------------------------------------------------------
```

```
                                                                                                       Section 15
                                                     (1079) 1079      1089       1100       1111       1122       1133       1144      1155
Autographa californica nucleopolyhedrovirus NC_0...  (1079) CCAAAACTATGCTTCGCTTGCTCCGTTTAGCTTGTAGCCGATCAGTGGCGTTGTTCCAATCGACGGTAGGATTAGGC
                           SARS-CoV-2 NC_045512.2       (1) -----------------------------------------------------------------------------
                                                                                                       Section 16
                                                     (1156) 1156      1166       1177       1188       1199       1210       1221      1232
Autographa californica nucleopolyhedrovirus NC_0...  (1156) CGGATATTCTCCACCACAATGTTGGCAACGTTGATGTTACGTTTATGCTTTTGGTTTTCCACGTACGTCTTTTGGCC
                           SARS-CoV-2 NC_045512.2       (1) -----------------------------------------------------------------------------
                                                                                                       Section 17
                                                     (1233) 1233      1243       1254       1265       1276       1287       1298      1309
Autographa californica nucleopolyhedrovirus NC_0...  (1233) GGTAATAGCCGTAAACGTAGTGCCGTCGCGCGTCACGCACAACACCGGATGTTTGCGCTTGTCCGCGGGGTATTGAA
                           SARS-CoV-2 NC_045512.2       (1) -----------------------------------------------------------------------------
                                                                                                       Section 18
                                                     (1310) 1310      1320       1331       1342       1353       1364       1375      1386
Autographa californica nucleopolyhedrovirus NC_0...  (1310) CCGCGCGATCCGACAAATCCACCACTTTGGCAACTAAATCGGTGACCTGCGCGTCTTTTTTCTGCATTATTTCGTCT
                           SARS-CoV-2 NC_045512.2       (1) -----------------------------------------------------------------------------
                                                                                                       Section 19
                                                     (1387) 1387      1397       1408       1419       1430       1441       1452      1463
Autographa californica nucleopolyhedrovirus NC_0...  (1387) TTCTTTTGCATGGTTTCCTGGAAGCCGGTGTACATGCGGTTTAGATCAGTCATGACGCGCGTGACCTGCAAATCTTT
                           SARS-CoV-2 NC_045512.2       (1) -----------------------------------------------------------------------------
                                                                                                       Section 20
                                                     (1464) 1464      1474       1485       1496       1507       1518       1529      1540
Autographa californica nucleopolyhedrovirus NC_0...  (1464) GGCCTCGATCTGCTTGTCCTTGATGGCAACGATGCGTTCAATAAACTCTTGTTTTTAACAAGTTCCTCGGTTTTTT
                           SARS-CoV-2 NC_045512.2       (1) -----------------------------------------------------------------------------
                                                                                                       Section 21
                                                     (1541) 1541      1551       1562       1573       1584       1595       1606      1617
Autographa californica nucleopolyhedrovirus NC_0...  (1541) GCGCCACCACCGCTTGCAGCGCGTTTGTGTGCTCGGTGAATGTCGCAATCAGCTTAGTCACCAACTGTTTGCTCTCC
                           SARS-CoV-2 NC_045512.2       (1) -----------------------------------------------------------------------------
```

```
                                                                                                    Section 22
                                                  (1618) 1618      1628      1639      1650      1661      1672      1683      1694
Autographa californica nucleopolyhedrovirus NC 0... (1618) TCCTCCCGTTGTTTGATCGCGGGATCGTACTTGCCGGTGCAGAGCACTTGAGGAATTACTTCTTCTAAAAGCCATTC
                          SARS-CoV-2 NC_045512.2      (1) -----------------------------------------------------------------------------
                                                                                                    Section 23
                                                  (1695) 1695      1705      1716      1727      1738      1749      1760      1771
Autographa californica nucleopolyhedrovirus NC 0... (1695) TTGTAATTCTATGGCGTAAGGCAATTTGGACTTCATAATCAGCTGAATCACGCCGGATTTAGTAATGAGCACTGTAT
                          SARS-CoV-2 NC_045512.2      (1) -----------------------------------------------------------------------------
                                                                                                    Section 24
                                                  (1772) 1772      1782      1793      1804      1815      1826      1837      1848
Autographa californica nucleopolyhedrovirus NC 0... (1772) GCGGCTGCAAATACAGCGGGTCGCCCCTTTTCACGACGCTGTTAGAGGTAGGGCCCCCATTTTGGATGGTCTGCTCA
                          SARS-CoV-2 NC_045512.2      (1) -----------------------------------------------------------------------------
                                                                                                    Section 25
                                                  (1849) 1849      1859      1870      1881      1892      1903      1914      1925
Autographa californica nucleopolyhedrovirus NC 0... (1849) AATAACGATTTGTATTTATTGTCTACATGAACACGTATAGCTTTATCACAAACTGTATATTTTAAACTGTTAGCGAC
                          SARS-CoV-2 NC_045512.2      (1) -----------------------------------------------------------------------------
                                                                                                    Section 26
                                                  (1926) 1926      1936      1947      1958      1969      1980      1991      2002
Autographa californica nucleopolyhedrovirus NC 0... (1926) GTCCTTGGCCACGAACCGGACCTGTTGGTCGCGCTCTAGCACGTACCGCAGGTTGAACGTATCTTCTCCAAATTTAA
                          SARS-CoV-2 NC_045512.2      (1) -----------------------------------------------------------------------------
                                                                                                    Section 27
                                                  (2003) 2003      2013      2024      2035      2046      2057      2068      2079
Autographa californica nucleopolyhedrovirus NC 0... (2003) ATTCTCCAATTTTAACGCGAGCCATTTTGATACACGTGTGTCGATTTTGCAACAACTATTGTTTTTTAACGCAAACT
                          SARS-CoV-2 NC_045512.2      (1) -----------------------------------------------------------------------------
                                                                                                    Section 28
                                                  (2080) 2080      2090      2101      2112      2123      2134      2145      2156
Autographa californica nucleopolyhedrovirus NC 0... (2080) AAACTTATTGTGGTAAGCAATAATTAAATATGGGGAACATGCGCCGCTACAACACTCGTCGTTATGAACGCAGACG
                          SARS-CoV-2 NC_045512.2      (1) -----------------------------------------------------------------------------
```

```
                                                                                                                      Section 29
                                             (2157) 2157      2167       2178       2189       2200       2211       2222       2233
Autographa californica nucleopolyhedrovirus NC_0... (2157) GCGCCGGTCTCGGCGCAAGCGGCTAAAACGTGTTGCGCGTTCAACGCGGCAAACATCGCAAAAGCCAATAGTACAGT
                          SARS-CoV-2 NC_045512.2    (1) -----------------------------------------------------------------------------
                                                                                                                      Section 30
                                             (2234) 2234      2244       2255       2266       2277       2288       2299       2310
Autographa californica nucleopolyhedrovirus NC_0... (2234) TTTGATTTGCATATTAACGGCGATTTTTTAAATTATCTTATTTAATAAATAGTTATGACGCCTACAACTCCCCGCCC
                          SARS-CoV-2 NC_045512.2    (1) -----------------------------------------------------------------------------
                                                                                                                      Section 31
                                             (2311) 2311      2321       2332       2343       2354       2365       2376       2387
Autographa californica nucleopolyhedrovirus NC_0... (2311) GCGTTGACTCGCTGCACCTCGAGCAGTTCGTTGACGCCTTCCTCCGTGTGGCCGAACACGTCGAGCGGGTGGTCGAT
                          SARS-CoV-2 NC_045512.2    (1) -----------------------------------------------------------------------------
                                                                                                                      Section 32
                                             (2388) 2388      2398       2409       2420       2431       2442       2453       2464
Autographa californica nucleopolyhedrovirus NC_0... (2388) GACCAGCGGCGTGCCGCACGCGACGCACAAGTATCTGTACACCGAATGATCGTCGGGCGAAGGCACGTCGGCCTCCA
                          SARS-CoV-2 NC_045512.2    (1) -----------------------------------------------------------------------------
                                                                                                                      Section 33
                                             (2465) 2465      2475       2486       2497       2508       2519       2530       2541
Autographa californica nucleopolyhedrovirus NC_0... (2465) AGTGGCAATATTGGCAAATTCGAAAATATATACAGTTGGGTTGTTTGCGCATATCTATCGTGGCGTTGGGCATGTAC
                          SARS-CoV-2 NC_045512.2    (1) -----------------------------------------------------------------------------
                                                                                                                      Section 34
                                             (2542) 2542      2552       2563       2574       2585       2596       2607       2618
Autographa californica nucleopolyhedrovirus NC_0... (2542) GTCCGAACGTTGATTTGCATGCAAGCCGAAATTAAATCATTGCGATTAGTGCGATTAAAACGTTGTACATCCTCGCT
                          SARS-CoV-2 NC_045512.2    (1) -----------------------------------------------------------------------------
                                                                                                                      Section 35
                                             (2619) 2619      2629       2640       2651       2662       2673       2684       2695
Autographa californica nucleopolyhedrovirus NC_0... (2619) TTTAATCATGCCGTCGATTAAATCGCGCAATCGAGTCAAGTGATCAAAGTGTGGAATAATGTTTTCTTTGTATTCCC
                          SARS-CoV-2 NC_045512.2    (1) -----------------------------------------------------------------------------
```

Section 36

```
                                                      (2696) 2696      2706       2717       2728       2739       2750       2761       2772
Autographa californica nucleopolyhedrovirus  NC_0...  (2696) GAGTCAAGCGCAGCGCGTATTTTAACAAACTAGCCATCTTGTAAGTTAGTTTCATTTAATGCAACTTTATCCAATAA
                          SARS-CoV-2 NC_045512.2      (1)    -----------------------------------------------------------------------------
```

Section 37

```
                                                      (2773) 2773      2783       2794       2805       2816       2827       2838       2849
Autographa californica nucleopolyhedrovirus  NC_0...  (2773) TATATTATGTATCGCACGTCAAGAATTAACAATGCGCCCGTTGTCGCATCTCAACACGACTATGATAGAGATCAAAT
                          SARS-CoV-2 NC_045512.2      (1)    -----------------------------------------------------------------------------
```

Section 38

```
                                                      (2850) 2850      2860       2871       2882       2893       2904       2915       2926
Autographa californica nucleopolyhedrovirus  NC_0...  (2850) AAAGCGCGAATTAAATAGCTTGCGACGCAACGTGCACGATCTGTGCACGCGTTCCGGCACGAGCTTTGATTGTAATA
                          SARS-CoV-2 NC_045512.2      (1)    -----------------------------------------------------------------------------
```

Section 39

```
                                                      (2927) 2927      2937       2948       2959       2970       2981       2992       3003
Autographa californica nucleopolyhedrovirus  NC_0...  (2927) AGTTTTTACGAAGCGATGACATGACCCCCGTAGTGACAACGATCACGCCCAAAAGAACTGCCGACTACAAAATTACC
                          SARS-CoV-2 NC_045512.2      (1)    -----------------------------------------------------------------------------
```

Section 40

```
                                                      (3004) 3004      3014       3025       3036       3047       3058       3069       3080
Autographa californica nucleopolyhedrovirus  NC_0...  (3004) GAGTATGTCGGTGACGTTAAAACTATTAAGCCATCCAATCGACCGTTAGTCGAATCAGGACCGCTGGTGCGAGAAGC
                          SARS-CoV-2 NC_045512.2      (1)    -----------------------------------------------------------------------------
```

Section 41

```
                                                      (3081) 3081      3091       3102       3113       3124       3135       3146       3157
Autographa californica nucleopolyhedrovirus  NC_0...  (3081) CGCGAAGTATGGCGAATGCATCGTATAACGTGTGGAGTCCGCTCATTAGAGCGTCATGTTTAGACAAGAAAGCTACA
                          SARS-CoV-2 NC_045512.2      (1)    -----------------------------------------------------------------------------
```

Section 42

```
                                                      (3158) 3158      3168       3179       3190       3201       3212       3223       3234
Autographa californica nucleopolyhedrovirus  NC_0...  (3158) TATTTAATTGATCCCGATGATTTTATTGATAAATTGACCCTAACTCCATACACGGTATTCTACAATGGCGGGGTTTT
                          SARS-CoV-2 NC_045512.2      (1)    -----------------------------------------------------------------------------
```

```
                                                                                   Section 43
                                                   (3235) 3235      3245       3256       3267       3278       3289       3300       3311
Autographa californica nucleopolyhedrovirus  NC_0... (3235) GGTCAAAATTTCCGGACTGCGATTGTACATGCTGTTAACGGCTCCGCCCACTATTAATGAAATTAAAAATTCCAATT
                          SARS-CoV-2 NC_045512.2      (1) -----------------------------------------------------------------------------
                                                                                   Section 44
                                                   (3312) 3312      3322       3333       3344       3355       3366       3377       3388
Autographa californica nucleopolyhedrovirus  NC_0... (3312) TTAAAAAACGCAGCAAGAGAAACATTTGTATGAAAGAATGCGTAGAAGGAAAGAAAAATGTCGTCGACATGCTGAAC
                          SARS-CoV-2 NC_045512.2      (1) -----------------------------------------------------------------------------
                                                                                   Section 45
                                                   (3389) 3389      3399       3410       3421       3432       3443       3454       3465
Autographa californica nucleopolyhedrovirus  NC_0... (3389) AACAAGATTAATATGCCTCCGTGTATAAAAAAAATATTGAACGATTTGAAAGAAAACAATGTACCGCGCGGCGGTAT
                          SARS-CoV-2 NC_045512.2      (1) -----------------------------------------------------------------------------
                                                                                   Section 46
                                                   (3466) 3466      3476       3487       3498       3509       3520       3531       3542
Autographa californica nucleopolyhedrovirus  NC_0... (3466) GTACAGGAAGAGGTTTATACTAAACTGTTACATTGCAAACGTGGTTTCGTGTGCCAAGTGTGAAAACCGATGTTTAA
                          SARS-CoV-2 NC_045512.2      (1) -----------------------------------------------------------------------------
                                                                                   Section 47
                                                   (3543) 3543      3553       3564       3575       3586       3597       3608       3619
Autographa californica nucleopolyhedrovirus  NC_0... (3543) TCAAGGCTCTGACGCATTTCTACAACCACGACTCCAAGTGTGTGGGTGAAGTCATGCATCTTTTAATCAAATCCCAA
                          SARS-CoV-2 NC_045512.2      (1) -----------------------------------------------------------------------------
                                                                                   Section 48
                                                   (3620) 3620      3630       3641       3652       3663       3674       3685       3696
Autographa californica nucleopolyhedrovirus  NC_0... (3620) GATGTGTATAAACCACCAAACTGCCAAAAAAATGAAAACTGTCGACAAGCTCTGTCCGTTTGCTGGCAACTGCAAGGG
                          SARS-CoV-2 NC_045512.2      (1) -----------------------------------------------------------------------------
                                                                                   Section 49
                                                   (3697) 3697      3707       3718       3729       3740       3751       3762       3773
Autographa californica nucleopolyhedrovirus  NC_0... (3697) TCTCAATCCTATTTGTAATTATTGAATAATAAAACAATTATAAATGCTAAATTTGTTTTTTATTAACGATACAAACC
                          SARS-CoV-2 NC_045512.2      (1) -----------------------------------------------------------------------------
```

```
                                                                                          Section 50
                                               (3774) 3774      3784       3795       3806       3817       3828       3839       3850
Autographa californica nucleopolyhedrovirus NC_0... (3774) AAACGCAACAAGAACATTTGTAGTATTATCTATAATTGAAAACGCGTAGTTATAATCGCTGAGGTAATATTTAAAAT
                     SARS-CoV-2 NC_045512.2    (1) -----------------------------------------------------------------------------
                                                                                          Section 51
                                               (3851) 3851      3861       3872       3883       3894       3905       3916       3927
Autographa californica nucleopolyhedrovirus NC_0... (3851) CATTTTCAAATGATTCACAGTTAATTTGCGACAATATAATTTTATTTTCACATAAACTAGACGCCTTGTCGTCTTCT
                     SARS-CoV-2 NC_045512.2    (1) -----------------------------------------------------------------------------
                                                                                          Section 52
                                               (3928) 3928      3938       3949       3960       3971       3982       3993       4004
Autographa californica nucleopolyhedrovirus NC_0... (3928) TCTTCGTATTCCTTCTCTTTTTCATTTTTCTCCTCATAAAAATTAACATAGTTATTATCGTATCCATATATGTATCT
                     SARS-CoV-2 NC_045512.2    (1) -----------------------------------------------------------------------------
                                                                                          Section 53
                                               (4005) 4005      4015       4026       4037       4048       4059       4070       4081
Autographa californica nucleopolyhedrovirus NC_0... (4005) ATCGTATAGAGTAAATTTTTTGTTGTCATAAATATATATGTCTTTTTTAATGGGGTGTATAGTACCGCTGCGCATAG
                     SARS-CoV-2 NC_045512.2    (1) -----------------------------------------------------------------------------
                                                                                          Section 54
                                               (4082) 4082      4092       4103       4114       4125       4136       4147       4158
Autographa californica nucleopolyhedrovirus NC_0... (4082) TTTTTCTGTAATTTACAACAGTGCTATTTTCTGGTAGTTCTTCGGAGTGTGTTGCTTTAATTATTAAATTTATATAA
                     SARS-CoV-2 NC_045512.2    (1) -----------------------------------------------------------------------------
                                                                                          Section 55
                                               (4159) 4159      4169       4180       4191       4202       4213       4224       4235
Autographa californica nucleopolyhedrovirus NC_0... (4159) TCAATGAATTTGGGATCGTCGGTTTTGTACAATATGTTGCCGGCATAGTACGCAGCTTCTTCTAGTTCAATTACACC
                     SARS-CoV-2 NC_045512.2    (1) -----------------------------------------------------------------------------
                                                                                          Section 56
                                               (4236) 4236      4246       4257       4268       4279       4290       4301       4312
Autographa californica nucleopolyhedrovirus NC_0... (4236) ATTTTTTAGCAGCACCGGATTAACATAACTTTCCAAAATGTTGTACGAACCGTTAAACAAAAACAGTTCACCTCCCT
                     SARS-CoV-2 NC_045512.2    (1) -----------------------------------------------------------------------------
```

```
                                                                                                                    Section 57
                                        (4313) 4313      4323       4334       4345       4356       4367       4378      4389
Autographa californica nucleopolyhedrovirus NC_0... (4313) TTTCTATACTATTGTCTGCGAGCAGTTGTTTGTTGTTAAAAATAACAGCCATTGTAATGAGACGCACAAACTAATAT
                         SARS-CoV-2 NC_045512.2 (1) -----------------------------------------------------------------------------
                                                                                                                    Section 58
                                        (4390) 4390      4400       4411       4422       4433       4444       4455      4466
Autographa californica nucleopolyhedrovirus NC_0... (4390) CACAAACTGGAAATGTCTATCAATATATAGTTGCTGATATCATGGAGATAATTAAAATGATAACCATCTCGCAAATA
                         SARS-CoV-2 NC_045512.2 (1) -----------------------------------------------------------------------------
                                                                                                                    Section 59
                                        (4467) 4467      4477       4488       4499       4510       4521       4532      4543
Autographa californica nucleopolyhedrovirus NC_0... (4467) AATAAGTATTTTACTGTTTTCGTAACAGTTTTGTAATAAAAAAACCTATAAATATGCCGGATTATTCATACCGTCCC
                         SARS-CoV-2 NC_045512.2 (1) -----------------------------------------------------------------------------
                                                                                                                    Section 60
                                        (4544) 4544      4554       4565       4576       4587       4598       4609      4620
Autographa californica nucleopolyhedrovirus NC_0... (4544) ACCATCGGGCGTACCTACGTGTACGACAACAAGTACTACAAAAATTTAGGTGCCGTTATCAAGAACGCTAAGCGCAA
                         SARS-CoV-2 NC_045512.2 (1) -----------------------------------------------------------------------------
                                                                                                                    Section 61
                                        (4621) 4621      4631       4642       4653       4664       4675       4686      4697
Autographa californica nucleopolyhedrovirus NC_0... (4621) GAAGCACTTCGCCGAACATGAGATCGAAGAGGCTACCCTCGACCCCCTAGACAACTACCTAGTGGCTGAGGATCCTT
                         SARS-CoV-2 NC_045512.2 (1) -----------------------------------------------------------------------------
                                                                                                                    Section 62
                                        (4698) 4698      4708       4719       4730       4741       4752       4763      4774
Autographa californica nucleopolyhedrovirus NC_0... (4698) TCCTGGGACCCGGCAAGAACCAAAAACTCACTCTCTTCAAGGAAATCCGTAATGTTAAACCCGACACGATGAAGCTT
                         SARS-CoV-2 NC_045512.2 (1) -----------------------------------------------------------------------------
                                                                                                                    Section 63
                                        (4775) 4775      4785       4796       4807       4818       4829       4840      4851
Autographa californica nucleopolyhedrovirus NC_0... (4775) GTCGTTGGATGGAAAGGAAAAGAGTTCTACAGGGAAACTTGGACCCGCTTCATGGAAGACAGCTTCCCCATTGTTAA
                         SARS-CoV-2 NC_045512.2 (1) -----------------------------------------------------------------------------
```

Section 64

```
                                                       (4852) 4852      4862       4873       4884       4895       4906       4917       4928
Autographa californica nucleopolyhedrovirus NC_0...  (4852) CGACCAAGAAGTGATGGATGTTTTCCTTGTTGTCAACATGCGTCCCACTAGACCCAACCGTTGTTACAAATTCCTGG
                               SARS-CoV-2 NC_045512.2    (1) -----------------------------------------------------------------------------
```

Section 65

```
                                                       (4929) 4929      4939       4950       4961       4972       4983       4994       5005
Autographa californica nucleopolyhedrovirus NC_0...  (4929) CCCAACACGCTCTGCGTTGCGACCCCGACTATGTACCTCATGACGTGATTAGGATCGTCGAGCCTTCATGGGTGGGC
                               SARS-CoV-2 NC_045512.2    (1) -----------------------------------------------------------------------------
```

Section 66

```
                                                       (5006) 5006      5016       5027       5038       5049       5060       5071       5082
Autographa californica nucleopolyhedrovirus NC_0...  (5006) AGCAACAACGAGTACCGCATCAGCCTGGCTAAGAAGGGCGGCGGCTGCCCAATAATGAACCTTCACTCTGAGTACAC
                               SARS-CoV-2 NC_045512.2    (1) -----------------------------------------------------------------------------
```

Section 67

```
                                                       (5083) 5083      5093       5104       5115       5126       5137       5148       5159
Autographa californica nucleopolyhedrovirus NC_0...  (5083) CAACTCGTTCGAACAGTTCATCGATCGTGTCATCTGGGAGAACTTCTACAAGCCCATCGTTTACATCGGTACCGACT
                               SARS-CoV-2 NC_045512.2    (1) -----------------------------------------------------------------------------
```

Section 68

```
                                                       (5160) 5160      5170       5181       5192       5203       5214       5225       5236
Autographa californica nucleopolyhedrovirus NC_0...  (5160) CTGCTGAAGAGGAGGAAATTCTCCTTGAAGTTTCCCTGGTGTTCAAAGTAAAGGAGTTTGCACCAGACGCACCTCTG
                               SARS-CoV-2 NC_045512.2    (1) -----------------------------------------------------------------------------
```

Section 69

```
                                                       (5237) 5237      5247       5258       5269       5280       5291       5302       5313
Autographa californica nucleopolyhedrovirus NC_0...  (5237) TTCACTGGTCCGGCGTATTAAAACACGATACATTGTTATTAGTACATTTATTAAGCGCTAGATTCTGTGCGTTGTTG
                               SARS-CoV-2 NC_045512.2    (1) -----------------------------------------------------------------------------
```

Section 70

```
                                                       (5314) 5314      5324       5335       5346       5357       5368       5379       5390
Autographa californica nucleopolyhedrovirus NC_0...  (5314) ATTTACAGACAATTGTTGTACGTATTTTAATAATTCATTAAATTTATAATCTTTAGGGTGGTATGTTAGAGCGAAAA
                               SARS-CoV-2 NC_045512.2    (1) -----------------------------------------------------------------------------
```

```
                                                                                    Section 71
                                             (5391) 5391      5401       5412       5423       5434       5445       5456       5467
Autographa californica nucleopolyhedrovirus NC 0... (5391) TCAAATGATTTTCAGCGTCTTTATATCTGAATTTAAATATTAAATCCTCAATAGATTTGTAAAATAGGTTTCGATTA
                          SARS-CoV-2 NC_045512.2    (1) -----------------------------------------------------------------------------
                                                                                    Section 72
                                             (5468) 5468      5478       5489       5500       5511       5522       5533       5544
Autographa californica nucleopolyhedrovirus NC 0... (5468) GTTTCAAACAAGGGTTGTTTTTCCGAACCGATGGCTGGACTATCTAATGGATTTTCGCTCAACGCCACAAAACTTGC
                          SARS-CoV-2 NC_045512.2    (1) -----------------------------------------------------------------------------
                                                                                    Section 73
                                             (5545) 5545      5555       5566       5577       5588       5599       5610       5621
Autographa californica nucleopolyhedrovirus NC 0... (5545) CAAATCTTGTAGCAGCAATCTAGCTTTGTCGATATTCGTTTGTGTTTTGTTTTGTAATAAAGGTTCGACGTCGTTCA
                          SARS-CoV-2 NC_045512.2    (1) -----------------------------------------------------------------------------
                                                                                    Section 74
                                             (5622) 5622      5632       5643       5654       5665       5676       5687       5698
Autographa californica nucleopolyhedrovirus NC 0... (5622) AAATATTATGCGCTTTTGTATTTCTTTCATCACTGTCGTTAGTGTACAATTGACTCGACGTAAACACGTTAAATAAA
                          SARS-CoV-2 NC_045512.2    (1) -----------------------------------------------------------------------------
                                                                                    Section 75
                                             (5699) 5699      5709       5720       5731       5742       5753       5764       5775
Autographa californica nucleopolyhedrovirus NC 0... (5699) GCTTGGACATATTTAACATCGGGCGTGTTAGCTTTATTAGGCCGATTATCGTCGTCGTCCCAACCCTCGTCGTTAGA
                          SARS-CoV-2 NC_045512.2    (1) -----------------------------------------------------------------------------
                                                                                    Section 76
                                             (5776) 5776      5786       5797       5808       5819       5830       5841       5852
Autographa californica nucleopolyhedrovirus NC 0... (5776) AGTTGCTTCCGAAGACGATTTTGCCATAGCCACACGACGCCTATTAATTGTGTCGGCTAACACGTCCGCGATCAAAT
                          SARS-CoV-2 NC_045512.2    (1) -----------------------------------------------------------------------------
                                                                                    Section 77
                                             (5853) 5853      5863       5874       5885       5896       5907       5918       5929
Autographa californica nucleopolyhedrovirus NC 0... (5853) TTGTAGTTGAGCTTTTTGGAATTATTTCTGATTGCGGGCGTTTTTGGGCGGGTTTCAATCTAACTGTGCCCGATTTT
                          SARS-CoV-2 NC_045512.2    (1) -----------------------------------------------------------------------------
```

Section 78

	(5930)	5930 5940 5951 5962 5973 5984 5995 6006
Autographa californica nucleopolyhedrovirus NC_0...	(5930)	AATTCAGACAACACGTTAGAAAGCGATGGTGCAGGCGGTGGTAACATTTCAGACGGCAAATCTACTAATGGCGGCGG
SARS-CoV-2 NC_045512.2	(1)	---

Section 79

	(6007)	6007 6017 6028 6039 6050 6061 6072 6083
Autographa californica nucleopolyhedrovirus NC_0...	(6007)	TGGTGGAGCTGATGATAAATCTACCATCGGTGGAGGCGCAGGCGGGGCTGGCGGCGGAGGCGGAGGCGGAGGTGGTG
SARS-CoV-2 NC_045512.2	(1)	---

Section 80

	(6084)	6084 6094 6105 6116 6127 6138 6149 6160
Autographa californica nucleopolyhedrovirus NC_0...	(6084)	GCGGTGATGCAGACGGCGGTTTAGGCTCAAATGTCTCTTTAGGCAACACAGTCGGCACCTCAACTATTGTACTGGTT
SARS-CoV-2 NC_045512.2	(1)	---

Section 81

	(6161)	6161 6171 6182 6193 6204 6215 6226 6237
Autographa californica nucleopolyhedrovirus NC_0...	(6161)	TCGGGCGCCGTTTTTGGTTTGACCGGTCTGAGACGAGTGCGATTTTTTTCGTTTCTAATAGCTTCCAACAATTGTTG
SARS-CoV-2 NC_045512.2	(1)	---

Section 82

	(6238)	6238 6248 6259 6270 6281 6292 6303 6314
Autographa californica nucleopolyhedrovirus NC_0...	(6238)	TCTGTCGTCTAAAGGTGCAGCGGGTTGAGGTTCCGTCGGCATTGGTGGAGCGGGCGGCAATTCAGACATCGATGGTG
SARS-CoV-2 NC_045512.2	(1)	---

Section 83

	(6315)	6315 6325 6336 6347 6358 6369 6380 6391
Autographa californica nucleopolyhedrovirus NC_0...	(6315)	GTGGTGGTGGTGGAGGCGCTGGAATGTTAGGCACGGGAGAAGGTGGTGGCGGCGGTGCCGCCGGTATAATTTGTTCT
SARS-CoV-2 NC_045512.2	(1)	---

Section 84

	(6392)	6392 6402 6413 6424 6435 6446 6457 6468
Autographa californica nucleopolyhedrovirus NC_0...	(6392)	GGTTTAGTTTGTTCGCGCACGATTGTGGGCACCGGCGCAGGCGCCGCTGGCTGCACAACGGAAGGTCGTCTGCTTCG
SARS-CoV-2 NC_045512.2	(1)	---

```
                                                                                          Section 85
                                              (6469) 6469      6479       6490       6501       6512       6523       6534      6545
Autographa californica nucleopolyhedrovirus  NC_0... (6469) AGGCAGCGCTTGGGGTGGTGGCAATTCAATATTATAATTGGAATACAAATCGTAAAAATCTGCTATAAGCATTGTAA
                            SARS-CoV-2 NC_045512.2    (1) -----------------------------------------------------------------------------
                                                                                          Section 86
                                              (6546) 6546      6556       6567       6578       6589       6600       6611      6622
Autographa californica nucleopolyhedrovirus  NC_0... (6546) TTTCGCTATCGTTTACCGTGCCGATATTTAACAACCGCTCAATGTAAGCAATTGTATTGTAAAGAGATTGTCTCAAG
                            SARS-CoV-2 NC_045512.2    (1) -----------------------------------------------------------------------------
                                                                                          Section 87
                                              (6623) 6623      6633       6644       6655       6666       6677       6688      6699
Autographa californica nucleopolyhedrovirus  NC_0... (6623) CTCGGATCCCGCACGCCGATAACAAGCCTTTTCATTTTTACTACAGCATTGTAGTGGCGAGACACTTCGCTGTCGTC
                            SARS-CoV-2 NC_045512.2    (1) -----------------------------------------------------------------------------
                                                                                          Section 88
                                              (6700) 6700      6710       6721       6732       6743       6754       6765      6776
Autographa californica nucleopolyhedrovirus  NC_0... (6700) GACGTACATGTATGCTTTGTTGTCAAAAACGTCGTTGGCAAGCTTTAAAATATTTAAAAGAACATCTCTGTTCAGCA
                            SARS-CoV-2 NC_045512.2    (1) -----------------------------------------------------------------------------
                                                                                          Section 89
                                              (6777) 6777      6787       6798       6809       6820       6831       6842      6853
Autographa californica nucleopolyhedrovirus  NC_0... (6777) CCACTGTGTTGTCGTAAATGTTGTTTTTGATAATTTGCGCTTCCGCAGTATCGACACGTTCAAAAAATTGATGCGCA
                            SARS-CoV-2 NC_045512.2    (1) -----------------------------------------------------------------------------
                                                                                          Section 90
                                              (6854) 6854      6864       6875       6886       6897       6908       6919      6930
Autographa californica nucleopolyhedrovirus  NC_0... (6854) TCAATTTTGTTGTTCCTATTATTGAATAAATAAGATTGTACAGATTCATATCTACGATTCGTCATGGCCACCACAAA
                            SARS-CoV-2 NC_045512.2    (1) -----------------------------------------------------------------------------
                                                                                          Section 91
                                              (6931) 6931      6941       6952       6963       6974       6985       6996      7007
Autographa californica nucleopolyhedrovirus  NC_0... (6931) TGCTACGCTGCAAACGCTGGTACAATTTTACGAAAACTGCAAAAACGTCAAAACTCGGTATAAAATAATCAACGGGC
                            SARS-CoV-2 NC_045512.2    (1) -----------------------------------------------------------------------------
```

```
                                                                                                    Section 92
                                                         (7008) 7008      7018       7029       7040       7051       7062       7073       7084
Autographa californica nucleopolyhedrovirus  NC_0...     (7008) GCTTTGGCAAAATATCTATTTTATCGCACAAGCCCACTAGCAAATTGTATTTGCAGAAAACAATTTCGGCGCACAAT
                          SARS-CoV-2 NC_045512.2            (1) -----------------------------------------------------------------------------
                                                                                                    Section 93
                                                         (7085) 7085      7095       7106       7117       7128       7139       7150       7161
Autographa californica nucleopolyhedrovirus  NC_0...     (7085) TTTAACGCTGACGAAATAAAAGTTCACCAGTTAATGAGCGACCACCCAAATTTTATAAAAATCTATTTTAATCACGG
                          SARS-CoV-2 NC_045512.2            (1) -----------------------------------------------------------------------------
                                                                                                    Section 94
                                                         (7162) 7162      7172       7183       7194       7205       7216       7227       7238
Autographa californica nucleopolyhedrovirus  NC_0...     (7162) TTCCATCAACAACCAAGTGATCGTGATGGACTACATTGACTGTCCCGATTTATTTGAAACACTACAAATTAAAGGCG
                          SARS-CoV-2 NC_045512.2            (1) -----------------------------------------------------------------------------
                                                                                                    Section 95
                                                         (7239) 7239      7249       7260       7271       7282       7293       7304       7315
Autographa californica nucleopolyhedrovirus  NC_0...     (7239) AGCTTTCGTACCAACTTGTTAGCAATATTATTAGACAGCTGTGTGAAGCGCTCAACGATTTGCACAAGCACAATTTC
                          SARS-CoV-2 NC_045512.2            (1) -----------------------------------------------------------------------------
                                                                                                    Section 96
                                                         (7316) 7316      7326       7337       7348       7359       7370       7381       7392
Autographa californica nucleopolyhedrovirus  NC_0...     (7316) ATACACAACGACATAAAACTCGAAAATGTCTTATATTTCGAAGCACTTGATCGCGTGTATGTTTGCGATTACGGATT
                          SARS-CoV-2 NC_045512.2            (1) -----------------------------------------------------------------------------
                                                                                                    Section 97
                                                         (7393) 7393      7403       7414       7425       7436       7447       7458       7469
Autographa californica nucleopolyhedrovirus  NC_0...     (7393) GTGCAAACACGAAAACTCACTTAGCGTGCACGACGGCACGTTGGAGTATTTTAGTCCGGAAAAAATTCGACACACAA
                          SARS-CoV-2 NC_045512.2            (1) -----------------------------------------------------------------------------
                                                                                                    Section 98
                                                         (7470) 7470      7480       7491       7502       7513       7524       7535       7546
Autographa californica nucleopolyhedrovirus  NC_0...     (7470) CTATGCACGTTTCGTTTGACTGGTACGCCGTCGGCGTGTTAACATACAAGTTGCTAACCGGCGGCCGACACCCATTT
                          SARS-CoV-2 NC_045512.2            (1) -----------------------------------------------------------------------------
```

```
                                                                                                           Section 99
                                                      (7547) 7547      7557       7568       7579       7590       7601       7612      7623
Autographa californica nucleopolyhedrovirus NC 0...  (7547) GAAAAAAGCGAAGACGAAATGTTGGACTTGAATAGCATGAAGCGTCGTCAGCAATACAATGACATTGGCGTTTTAAA
                           SARS-CoV-2 NC_045512.2       (1) -----------------------------------------------------------------------------
                                                                                                          Section 100
                                                      (7624) 7624      7634       7645       7656       7667       7678       7689      7700
Autographa californica nucleopolyhedrovirus NC 0...  (7624) ACACGTTCGTAACGTTAACGCTCGTGACTTTGTGTACTGCCTAACAAGATACAACATAGATTGTAGACTCACAAATT
                           SARS-CoV-2 NC_045512.2       (1) -----------------------------------------------------------------------------
                                                                                                          Section 101
                                                      (7701) 7701      7711       7722       7733       7744       7755       7766      7777
Autographa californica nucleopolyhedrovirus NC 0...  (7701) ACAAACAAATTATAAAACATGAGTTTTTGTCGTAAAAATGCCACTTGTTTTACGAGTAGAATTCTACGTGTAACACA
                           SARS-CoV-2 NC_045512.2       (1) -----------------------------------------------------------------------------
                                                                                                          Section 102
                                                      (7778) 7778      7788       7799       7810       7821       7832       7843      7854
Autographa californica nucleopolyhedrovirus NC 0...  (7778) CGATCTAAAAGATGATGTCATTTTTTATCAATGACTCATTTGTTTTAAAACAGACTTGTTTTACGAGTAGAATTCTA
                           SARS-CoV-2 NC_045512.2       (1) -----------------------------------------------------------------------------
                                                                                                          Section 103
                                                      (7855) 7855      7865       7876       7887       7898       7909       7920      7931
Autographa californica nucleopolyhedrovirus NC 0...  (7855) CGTGTAAAGCATGATCGTGAGTGGTGTTAATAAAATCATAAAAATTATTGTAAATGTTTATTATTTAAAAACGATTC
                           SARS-CoV-2 NC_045512.2       (1) -----------------------------------------------------------------------------
                                                                                                          Section 104
                                                      (7932) 7932      7942       7953       7964       7975       7986       7997      8008
Autographa californica nucleopolyhedrovirus NC 0...  (7932) AAATATATAATAAAAACAATCTACATCTATTTCTTCACAATCCATAACACACAACAGGTCCATCAATGAGTTTTTGT
                           SARS-CoV-2 NC_045512.2       (1) -----------------------------------------------------------------------------
                                                                                                          Section 105
                                                      (8009) 8009      8019       8030       8041       8052       8063       8074      8085
Autographa californica nucleopolyhedrovirus NC 0...  (8009) CTTTATCCGACATACTATGTGCATGTAACAAATCAAATACATCTTTTAAATTTTTATACACATCTTTACATTGTCTA
                           SARS-CoV-2 NC_045512.2       (1) -----------------------------------------------------------------------------
```

Section 106

```
                                                  (8086) 8086      8096       8107       8118       8129       8140       8151       8162
Autographa californica nucleopolyhedrovirus NC 0... (8086) CCAAAATCTTTAATAACCCTATAACAAGGAAAAGACTTTTCTTCTTGCGTGGTTTTGCCGCGCAGATATTGAAATAA
                         SARS-CoV-2 NC_045512.2    (1) -----------------------------------------------------------------------------
```

Section 107

```
                                                  (8163) 8163      8173       8184       8195       8206       8217       8228       8239
Autographa californica nucleopolyhedrovirus NC 0... (8163) AATGTGCATGCACGACAACTTGTGTTTACTAAAATGCTCCTTGCCTATACCGCAAAACCGGCCATACATTTCGGCGA
                         SARS-CoV-2 NC_045512.2    (1) -----------------------------------------------------------------------------
```

Section 108

```
                                                  (8240) 8240      8250       8261       8272       8283       8294       8305       8316
Autographa californica nucleopolyhedrovirus NC 0... (8240) TTACACGCGGACAATTGTACGATTCGTCTACGTGTAAACGATCATCATAATCACTCTTGCGCAAACGAATAAATTTT
                         SARS-CoV-2 NC_045512.2    (1) -----------------------------------------------------------------------------
```

Section 109

```
                                                  (8317) 8317      8327       8338       8349       8360       8371       8382       8393
Autographa californica nucleopolyhedrovirus NC 0... (8317) TTCACCGCTTCCGACAAACGAGGCACCAATTCGGCGGGCACGCTTCGATACATTATTCTGTGCACATAAGTTACCAC
                         SARS-CoV-2 NC_045512.2    (1) -----------------------------------------------------------------------------
```

Section 110

```
                                                  (8394) 8394      8404       8415       8426       8437       8448       8459       8470
Autographa californica nucleopolyhedrovirus NC 0... (8394) ACAAAATTTATTGTACCACCATCCGACAACGTCGTTATTAGGGTTGAACACGTTGGCGATGCGCAGCAGTTTCCCGT
                         SARS-CoV-2 NC_045512.2    (1) -----------------------------------------------------------------------------
```

Section 111

```
                                                  (8471) 8471      8481       8492       8503       8514       8525       8536       8547
Autographa californica nucleopolyhedrovirus NC 0... (8471) TTCTCATGAAATATTCAAAGCGGCCCAAAATAATTTGCAAGCAATCCAACATGTCTTGAGAAATTTCTCGTTCAAAA
                         SARS-CoV-2 NC_045512.2    (1) -----------------------------------------------------------------------------
```

Section 112

```
                                                  (8548) 8548      8558       8569       8580       8591       8602       8613       8624
Autographa californica nucleopolyhedrovirus NC 0... (8548) TTGTTCAAAGAGAATATCTGCCATCCGTTTTGAACGCGCACGCTGACGGGAACCACCGCATCGATTTGCTCCAACAC
                         SARS-CoV-2 NC_045512.2    (1) -----------------------------------------------------------------------------
```

Section 113

```
                                                  8625       8635       8646       8657       8668       8679       8690       8701
                                          (8625)
Autographa californica nucleopolyhedrovirus NC 0... (8625) TTCACGGACGTTATCGTCGATGCCCATCGTTTCGCTGGTGCTGAACCAATGGGAAAGGCTCTTGATGGAATCGCCCG
                         SARS-CoV-2 NC_045512.2 (1) -----------------------------------------------------------------------------
```

Section 114

```
                                                  8702       8712       8723       8734       8745       8756       8767       8778
                                          (8702)
Autographa californica nucleopolyhedrovirus NC 0... (8702) CGTCTATCATCTTGACCGCTTCGTCAAAGGTGCAACTGCCGCTCTTCAAACGCCGCATAGCGGTCACGTCCCGCTCT
                         SARS-CoV-2 NC_045512.2 (1) -----------------------------------------------------------------------------
```

Section 115

```
                                                  8779       8789       8800       8811       8822       8833       8844       8855
                                          (8779)
Autographa californica nucleopolyhedrovirus NC 0... (8779) ATGCACGACATACCGTTTACGTACGATTCTGATAGGTATTCCTGAACTATACGGTAATGGTGATACGACTCGCCATA
                         SARS-CoV-2 NC_045512.2 (1) -----------------------------------------------------------------------------
```

Section 116

```
                                                  8856       8866       8877       8888       8899       8910       8921       8932
                                          (8856)
Autographa californica nucleopolyhedrovirus NC 0... (8856) CACGTCGTGCACCTCATTGTATTTAGCATAATAATTGTAAATTATTAACTTTGCAGCGAGAGACATGTTGTCAGTAA
                         SARS-CoV-2 NC_045512.2 (1) -----------------------------------------------------------------------------
```

Section 117

```
                                                  8933       8943       8954       8965       8976       8987       8998       9009
                                          (8933)
Autographa californica nucleopolyhedrovirus NC 0... (8933) AGCGGTGCTAGGCTCAATAATACTGATGTACAGGCACGCGTGCTATTTATATATAATTTCGCAAGGAGGGGAGCTGT
                         SARS-CoV-2 NC_045512.2 (1) -----------------------------------------------------------------------------
```

Section 118

```
                                                  9010       9020       9031       9042       9053       9064       9075       9086
                                          (9010)
Autographa californica nucleopolyhedrovirus NC 0... (9010) TATCGGTTGCTATTATTAAAGAATGGCCGTCTGTTTTTATCACAAGCTTGGCAGCCTCAACCATGAAGCGTCGTCAT
                         SARS-CoV-2 NC_045512.2 (1) -----------------------------------------------------------------------------
```

Section 119

```
                                                  9087       9097       9108       9119       9130       9141       9152       9163
                                          (9087)
Autographa californica nucleopolyhedrovirus NC 0... (9087) TGTAAATTAAATTCTCTGCCTCAAGAATTATTTGACAAGATTGTCGAGTATTTATCTTTATCTGATTACTGCAATTT
                         SARS-CoV-2 NC_045512.2 (1) -----------------------------------------------------------------------------
```

```
                                                       Section 120
                                              (9164) 9164      9174       9185       9196       9207       9218       9229      9240
Autographa californica nucleopolyhedrovirus NC 0... (9164) GGTGCTTGTCTGTAAAAGACCTTCTAGTAAATATAACGTGATATTTGATAGTACTAATCACCAACATTTGAAAGGCG
                      SARS-CoV-2 NC_045512.2    (1) -----------------------------------------------------------------------------
                                                       Section 121
                                              (9241) 9241      9251       9262       9273       9284       9295       9306      9317
Autographa californica nucleopolyhedrovirus NC 0... (9241) TGTACAAAAAGACAGACGTGCAAATAACAAGCTACAACGAATACATCAACTGTATTTGCAACGAACTGAGACAAGAC
                      SARS-CoV-2 NC_045512.2    (1) -----------------------------------------------------------------------------
                                                       Section 122
                                              (9318) 9318      9328       9339       9350       9361       9372       9383      9394
Autographa californica nucleopolyhedrovirus NC 0... (9318) GAATTCTATGCCAAATCATCATGGATTGCGAGTATTTGCGGTCACCAGAGAGCGACAATTTTTAGTGTAACAAATAA
                      SARS-CoV-2 NC_045512.2    (1) -----------------------------------------------------------------------------
                                                       Section 123
                                              (9395) 9395      9405       9416       9427       9438       9449       9460      9471
Autographa californica nucleopolyhedrovirus NC 0... (9395) ACAAGTAGAAATGAAATATCATTTGTATAATATAGCAATTGTGGAAAGTGAAGATTGCAACGGATTTTACCCATTTG
                      SARS-CoV-2 NC_045512.2    (1) -----------------------------------------------------------------------------
                                                       Section 124
                                              (9472) 9472      9482       9493       9504       9515       9526       9537      9548
Autographa californica nucleopolyhedrovirus NC 0... (9472) AGCCAACGCGCGATTGTTTAATATGCAAACAAAAAAACCAATGTCCTCGTAATTCATTTATTGTTTCGTTGTGTAAA
                      SARS-CoV-2 NC_045512.2    (1) -----------------------------------------------------------------------------
                                                       Section 125
                                              (9549) 9549      9559       9570       9581       9592       9603       9614      9625
Autographa californica nucleopolyhedrovirus NC 0... (9549) TATTTAGAAAAACAAAATGTACAATCAAACTTTATATATTATTTATACGAAATAAATACATAATAATAACTATTATA
                      SARS-CoV-2 NC_045512.2    (1) -----------------------------------------------------------------------------
                                                       Section 126
                                              (9626) 9626      9636       9647       9658       9669       9680       9691      9702
Autographa californica nucleopolyhedrovirus NC 0... (9626) CATGTTTTTATTTTACAATACTTCCTGTATAACCTCTCTAACTACATTAGGAGTACAATCCACGTCAATTACACGTT
                      SARS-CoV-2 NC_045512.2    (1) -----------------------------------------------------------------------------
```

```
                                                                                      Section 127
                                                      (9703) 9703      9713       9724       9735       9746       9757       9768       9779
Autographa californica nucleopolyhedrovirus NC 0...  (9703) TAGCTATTTTTCTAATTTTGTAATGTTTATCGTAGAGTTTTTCGTTAATACATTGAATAGCCAACAAGGGATTTGGG
                            SARS-CoV-2 NC_045512.2      (1) -----------------------------------------------------------------------------
                                                                                      Section 128
                                                      (9780) 9780      9790       9801       9812       9823       9834       9845       9856
Autographa californica nucleopolyhedrovirus NC 0...  (9780) TGCACACCGTCATAGAGTACTTCCATGTCGTCTTCAAAGCGCATTTTTCGCTTGCGAAAATGCCGCTCTTGGCCCAA
                            SARS-CoV-2 NC_045512.2      (1) -----------------------------------------------------------------------------
                                                                                      Section 129
                                                      (9857) 9857      9867       9878       9889       9900       9911       9922       9933
Autographa californica nucleopolyhedrovirus NC 0...  (9857) AACAAAAGCGAGTTTGATGCGGTCGTCGATGCGTTCCGAAAATACGGCCAAATGCTGGTGTTTGGTGATGTCGCGCG
                            SARS-CoV-2 NC_045512.2      (1) -----------------------------------------------------------------------------
                                                                                      Section 130
                                                      (9934) 9934      9944       9955       9966       9977       9988       9999       10010
Autographa californica nucleopolyhedrovirus NC 0...  (9934) GAAACGTCACCGTGCCATTTTTGCTTTCCGCCACGACGGCGGTTTTCAATTTTTCGGCCGACTGCAGCATGTTAAGT
                            SARS-CoV-2 NC_045512.2      (1) -----------------------------------------------------------------------------
                                                                                      Section 131
                                                     (10011) 10011     10021      10032      10043      10054      10065      10076      10087
Autographa californica nucleopolyhedrovirus NC 0... (10011) TTGGCGTCGAGTTCGTGCAAACGCAATTCAAACTGCTCAAACCTGTTGCCCACCTCGTTCTTGAACGTCTCGTGGGT
                            SARS-CoV-2 NC_045512.2      (1) -----------------------------------------------------------------------------
                                                                                      Section 132
                                                     (10088) 10088     10098      10109      10120      10131      10142      10153      10164
Autographa californica nucleopolyhedrovirus NC 0... (10088) GACCATAAATTTTTCGCTGTTTGCATTCAGTTTCTTTACATGTTTTAAAACAGATTCAATCTTGTCGCGCAAATCAT
                            SARS-CoV-2 NC_045512.2      (1) -----------------------------------------------------------------------------
                                                                                      Section 133
                                                     (10165) 10165     10175      10186      10197      10208      10219      10230      10241
Autographa californica nucleopolyhedrovirus NC 0... (10165) CACGCTCGCCTTCAGTTTGAATGTGCAGCAACGCGTTGCTTTTGTTGGCAAAATTTAACCGCATCAAAATTTCCAAC
                            SARS-CoV-2 NC_045512.2      (1) -----------------------------------------------------------------------------
```

```
                                                                                                   Section 134
                                                (10242) 10242     10252      10263      10274      10285      10296      10307     10318
Autographa californica nucleopolyhedrovirus NC 0... (10242) AACCCGTGCTTGGTCGCGAACAATGCGCCCAACGAGTTGAGATCGCGTTTGGATCTCTGTTTGTGAAAAACAATTTC
                          SARS-CoV-2 NC_045512.2 (1) -----------------------------------------------------------------------------
                                                                                                   Section 135
                                                (10319) 10319     10329      10340      10351      10362      10373      10384     10395
Autographa californica nucleopolyhedrovirus NC 0... (10319) GTTTAAATGGTAAACTTGATCGCCGTCCCAATTGCAATCAAGTATGTCGTCGTGCGCAATTTCAAGACCTTTGCAAA
                          SARS-CoV-2 NC_045512.2 (1) -----------------------------------------------------------------------------
                                                                                                   Section 136
                                                (10396) 10396     10406      10417      10428      10439      10450      10461     10472
Autographa californica nucleopolyhedrovirus NC 0... (10396) AATCTATCACATTGTAGCATTTTGCGTTCGTGTCGCTGTGCACGTATCTGTACTTGAAACTGTGCGTGTTGCATTTG
                          SARS-CoV-2 NC_045512.2 (1) -----------------------------------------------------------------------------
                                                                                                   Section 137
                                                (10473) 10473     10483      10494      10505      10516      10527      10538     10549
Autographa californica nucleopolyhedrovirus NC 0... (10473) AATGAGTCCCATTTAACGATGTGCGACCATTGTTGGGCGTTTATGTGGTACTTTTTGTAGTCGTCTGCATTGAACCG
                          SARS-CoV-2 NC_045512.2 (1) -----------------------------------------------------------------------------
                                                                                                   Section 138
                                                (10550) 10550     10560      10571      10582      10593      10604      10615     10626
Autographa californica nucleopolyhedrovirus NC 0... (10550) ATCTTCGGCGGCGATGGCGTCGTTGTCGTTGTCACCGGACCACATCCACCAGTTCCATAACCAGGATAGCATTGCTT
                          SARS-CoV-2 NC_045512.2 (1) -----------------------------------------------------------------------------
                                                                                                   Section 139
                                                (10627) 10627     10637      10648      10659      10670      10681      10692     10703
Autographa californica nucleopolyhedrovirus NC 0... (10627) TAGCTTGTCTAGCAATTCCTTTGTTATACAACGAGAAAATTTCGTTCCCTTATAATTATAGCTGTACGGTGCGCGTA
                          SARS-CoV-2 NC_045512.2 (1) -----------------------------------------------------------------------------
                                                                                                   Section 140
                                                (10704) 10704     10714      10725      10736      10747      10758      10769     10780
Autographa californica nucleopolyhedrovirus NC 0... (10704) TTTGTTTGTTAACGTTACAAAAAATATCCCTGTCCACGTCCGGCCAATACTGCAACGTGAGCGCGTCCAAGTTTGAA
                          SARS-CoV-2 NC_045512.2 (1) -----------------------------------------------------------------------------
```

```
                                                                                                              Section 141
                                                     (10781) 10781     10791      10802      10813      10824      10835      10846      10857
Autographa californica nucleopolyhedrovirus NC 0...  (10781) TCTTGCATATGCGGAACGTACAAACGTACGGCCTCTCTCACACAATGCGCAAAACTGCCCGGCTGAATGTAATCACT
                          SARS-CoV-2 NC_045512.2         (1) -----------------------------------------------------------------------------
                                                                                                              Section 142
                                                     (10858) 10858     10868      10879      10890      10901      10912      10923      10934
Autographa californica nucleopolyhedrovirus NC 0...  (10858) GTCCAACTTTGCAGGTTTCTCGAAAGCCTTGTACCGATGCACGCGAACATTTTGAGCGGACGTGATTTTAAACTTGT
                          SARS-CoV-2 NC_045512.2         (1) -----------------------------------------------------------------------------
                                                                                                              Section 143
                                                     (10935) 10935     10945      10956      10967      10978      10989      11000      11011
Autographa californica nucleopolyhedrovirus NC 0...  (10935) CGGTGAATTTTAACCACAAATGAAATCCACGGTTGCCGGTATACATGACTCTTGACACGTTCTCTTCCGTGTAAAAC
                          SARS-CoV-2 NC_045512.2         (1) -----------------------------------------------------------------------------
                                                                                                              Section 144
                                                     (11012) 11012     11022      11033      11044      11055      11066      11077      11088
Autographa californica nucleopolyhedrovirus NC 0...  (11012) AACAGAAACGCCGTGGCGCCAATGTAAATTTTCAGCATTAAATCGTGTTCGTCAACATAATTTTTGTAATCGGCGTC
                          SARS-CoV-2 NC_045512.2         (1) -----------------------------------------------------------------------------
                                                                                                              Section 145
                                                     (11089) 11089     11099      11110      11121      11132      11143      11154      11165
Autographa californica nucleopolyhedrovirus NC 0...  (11089) TACGACCCATTCCCTGCCGCCGCCGTCGTCCAACGGTTTGACGTGCACGTCGGACACTTTGTTTTGCACAATATAAC
                          SARS-CoV-2 NC_045512.2         (1) -----------------------------------------------------------------------------
                                                                                                              Section 146
                                                     (11166) 11166     11176      11187      11198      11209      11220      11231      11242
Autographa californica nucleopolyhedrovirus NC 0...  (11166) TATACAATTGTGCGGAGGTATCAAAATATCTGTCGGCGTGAATCCAGCGCGCGTTGACCGTCATGAACGCGTACTTG
                          SARS-CoV-2 NC_045512.2         (1) -----------------------------------------------------------------------------
                                                                                                              Section 147
                                                     (11243) 11243     11253      11264      11275      11286      11297      11308      11319
Autographa californica nucleopolyhedrovirus NC 0...  (11243) CGGCTGTCGTTGTACGCAATGGCGTCCCACATCATGTCGACGCGCTTCTGCGTATAATTGCACACTAACATGTTGCC
                          SARS-CoV-2 NC_045512.2         (1) -----------------------------------------------------------------------------
```

Section 148

	(11320)	11320 11330 11341 11352 11363 11374 11385 11396
Autographa californica nucleopolyhedrovirus NC 0...	(11320)	CTTTGAACTTGACCTCGATTGTGTTAATTTTTGGCTATAAAAAGGTCACCCTTTAAAATTTGTTACATAATCAAATT
SARS-CoV-2 NC_045512.2	(1)	---

Section 149

	(11397)	11397 11407 11418 11429 11440 11451 11462 11473
Autographa californica nucleopolyhedrovirus NC 0...	(11397)	ACCAGTACAGTTATTCGGTTTGAAGCAAAATGACTATTCTCTGCTGGCTTGCACTGCTGTCTACGCTTACTGCTGTA
SARS-CoV-2 NC_045512.2	(1)	---

Section 150

	(11474)	11474 11484 11495 11506 11517 11528 11539 11550
Autographa californica nucleopolyhedrovirus NC 0...	(11474)	AATGCGGCCAATATATTGGCCGTGTTTCCTACGCCAGCTTACAGCCACCATATAGTGTACAAAGTGTATATTGAAGC
SARS-CoV-2 NC_045512.2	(1)	---

Section 151

	(11551)	11551 11561 11572 11583 11594 11605 11616 11627
Autographa californica nucleopolyhedrovirus NC 0...	(11551)	CCTTGCCGAAAAATGTCACAACGTTACGGTCGTCAAGCCCAAACTGTTTGCGTATTCAACTAAAACTTATTGCGGTA
SARS-CoV-2 NC_045512.2	(1)	---

Section 152

	(11628)	11628 11638 11649 11660 11671 11682 11693 11704
Autographa californica nucleopolyhedrovirus NC 0...	(11628)	ATATCACGGAAATTAATGCCGACATGTCTGTTGAGCAATACAAAAAACTAGTGGCGAATTCGGCAATGTTTAGAAAG
SARS-CoV-2 NC_045512.2	(1)	---

Section 153

	(11705)	11705 11715 11726 11737 11748 11759 11770 11781
Autographa californica nucleopolyhedrovirus NC 0...	(11705)	CGCGGAGTGGTGTCCGATACAGACACGGTAACCGCCGCTAACTACCTAGGCTTGATTGAAATGTTCAAAGACCAGTT
SARS-CoV-2 NC_045512.2	(1)	---

Section 154

	(11782)	11782 11792 11803 11814 11825 11836 11847 11858
Autographa californica nucleopolyhedrovirus NC 0...	(11782)	TGACAATATCAACGTGCGCAATCTCATTGCCAACAACCAGACGTTTGATTTAGTCGTCGTGGAAGCGTTTGCCGATT
SARS-CoV-2 NC_045512.2	(1)	---

```
                                                                                                          Section 155
                                                  (11859) 11859     11869      11880      11891      11902      11913      11924      11935
Autographa californica nucleopolyhedrovirus NC 0... (11859) ATGCGTTGGTGTTTGGTCACTTGTACGATCCGGCGCCCGTAATTCAAATCGCGCCTGGCTACGGTTTGGCGGAAAAC
                             SARS-CoV-2 NC_045512.2     (1) -----------------------------------------------------------------------------
                                                                                                          Section 156
                                                  (11936) 11936     11946      11957      11968      11979      11990      12001      12012
Autographa californica nucleopolyhedrovirus NC 0... (11936) TTTGACACGGTCGGCGCCGTGGCGCGGCACCCCGTCCACCATCCTAACATTTGGCGCAGCAATTTCGACGACACGGA
                             SARS-CoV-2 NC_045512.2     (1) -----------------------------------------------------------------------------
                                                                                                          Section 157
                                                  (12013) 12013     12023      12034      12045      12056      12067      12078      12089
Autographa californica nucleopolyhedrovirus NC 0... (12013) GGCAAACGTGATGACGGAAATGCGTTTGTATAAAGAATTTAAAATTTTGGCCAACATGTCCAACGCGTTGCTCAAAC
                             SARS-CoV-2 NC_045512.2     (1) -----------------------------------------------------------------------------
                                                                                                          Section 158
                                                  (12090) 12090     12100      12111      12122      12133      12144      12155      12166
Autographa californica nucleopolyhedrovirus NC 0... (12090) AACAGTTTGGACCCAACACACCGACAATTGAAAAACTACGCAACAAGGTGCAATTGCTTTTGCTAAACCTGCATCCC
                             SARS-CoV-2 NC_045512.2     (1) -----------------------------------------------------------------------------
                                                                                                          Section 159
                                                  (12167) 12167     12177      12188      12199      12210      12221      12232      12243
Autographa californica nucleopolyhedrovirus NC 0... (12167) ATATTTGACAACAACCGACCCGTGCCGCCCAGCGTGCAGTATCTTGGCGGAGGAATCCATCTTGTAAAGAGCGCGCC
                             SARS-CoV-2 NC_045512.2     (1) -----------------------------------------------------------------------------
                                                                                                          Section 160
                                                  (12244) 12244     12254      12265      12276      12287      12298      12309      12320
Autographa californica nucleopolyhedrovirus NC 0... (12244) GTTGACCAAATTAAGTCCGGTCATCAACGCGCAAATGAACAAGTCAAAAAGCGGAACGATTTACGTAAGTTTTGGGT
                             SARS-CoV-2 NC_045512.2     (1) -----------------------------------------------------------------------------
                                                                                                          Section 161
                                                  (12321) 12321     12331      12342      12353      12364      12375      12386      12397
Autographa californica nucleopolyhedrovirus NC 0... (12321) CGAGCATTGACACCAAATCGTTTGCAAACGAGTTTCTTTACATGTTAATCAATACGTTCAAAACGTTGGATAATTAC
                             SARS-CoV-2 NC_045512.2     (1) -----------------------------------------------------------------------------
```

```
                                                                                                    Section 162
                                          (12398) 12398     12408      12419      12430      12441      12452      12463      12474
Autographa californica nucleopolyhedrovirus  NC_0... (12398) ACCATATTATGGAAAATTGACGACGAAGTAGTAAAAAACATAACGTTGCCCGCCAACGTAATCACGCAAAATTGGTT
                            SARS-CoV-2 NC_045512.2     (1) -----------------------------------------------------------------------------
                                                                                                    Section 163
                                          (12475) 12475     12485      12496      12507      12518      12529      12540      12551
Autographa californica nucleopolyhedrovirus  NC_0... (12475) TAATCAACGCGCCGTGCTGCGTCATAAAAAAATGGCGGCGTTTATTACGCAAGGCGGACTACAATCGAGCGACGAGG
                            SARS-CoV-2 NC_045512.2     (1) -----------------------------------------------------------------------------
                                                                                                    Section 164
                                          (12552) 12552     12562      12573      12584      12595      12606      12617      12628
Autographa californica nucleopolyhedrovirus  NC_0... (12552) CCTTGGAAGCCGGGATACCCATGGTGTGTCTGCCCATGATGGGCGACCAGTTTTACCATGCGCACAAATTACAGCAA
                            SARS-CoV-2 NC_045512.2     (1) -----------------------------------------------------------------------------
                                                                                                    Section 165
                                          (12629) 12629     12639      12650      12661      12672      12683      12694      12705
Autographa californica nucleopolyhedrovirus  NC_0... (12629) CTCGGCGTAGCCCGCGCCTTGGACACTGTTACCGTTTCCAGCGATCAACTACTAGTGGCGATAAACGACGTGTTGTT
                            SARS-CoV-2 NC_045512.2     (1) -----------------------------------------------------------------------------
                                                                                                    Section 166
                                          (12706) 12706     12716      12727      12738      12749      12760      12771      12782
Autographa californica nucleopolyhedrovirus  NC_0... (12706) TAACGCGCCTACCTACAAAAAACACATGGCCGAGTTATATGCGCTCATCAATCATGATAAAGCAACGTTTCCGCCTC
                            SARS-CoV-2 NC_045512.2     (1) -----------------------------------------------------------------------------
                                                                                                    Section 167
                                          (12783) 12783     12793      12804      12815      12826      12837      12848      12859
Autographa californica nucleopolyhedrovirus  NC_0... (12783) TAGATAAAGCCATCAAATTCACAGAACGCGTAATTCGATATAGACATGACATCAGTCGTCAATTGTATTCATTAAAA
                            SARS-CoV-2 NC_045512.2     (1) -----------------------------------------------------------------------------
                                                                                                    Section 168
                                          (12860) 12860     12870      12881      12892      12903      12914      12925      12936
Autographa californica nucleopolyhedrovirus  NC_0... (12860) ACAACAGCTGCCAATGTACCGTATTCAAATTACTACATGTATAAATCTGTGTTTTCTATTGTAATGAATCACTTAAC
                            SARS-CoV-2 NC_045512.2     (1) -----------------------------------------------------------------------------
```

```
                                                                                                                 Section 169
                                                  (12937) 12937     12947      12958      12969      12980      12991      13002      13013
Autographa californica nucleopolyhedrovirus NC 0... (12937) ACACTTTTAATTACGTCAATAAATGTTATTCACCATTATTTACCTGGTTTTTTTGAGAGGGGCTTTGTGCGACTGCG
                              SARS-CoV-2 NC_045512.2 (1) -----------------------------------------------------------------------------
                                                                                                                 Section 170
                                                  (13014) 13014     13024      13035      13046      13057      13068      13079      13090
Autographa californica nucleopolyhedrovirus NC 0... (13014) CACTTCCAGCCTTTATAAACGCTCACCAACCAAAGCAGGTCATTATTGTGCCAGGACGTTCAAAGGCGAAACATCGA
                              SARS-CoV-2 NC_045512.2 (1) -----------------------------------------------------------------------------
                                                                                                                 Section 171
                                                  (13091) 13091     13101      13112      13123      13134      13145      13156      13167
Autographa californica nucleopolyhedrovirus NC 0... (13091) AATGGAGTCTGTTCAAACGCGCTTATGTGCCAGTAGCAATCAATTTGCTCCGTTCAAAAAGCGCCAGCTTGCCGTGC
                              SARS-CoV-2 NC_045512.2 (1) -----------------------------------------------------------------------------
                                                                                                                 Section 172
                                                  (13168) 13168     13178      13189      13200      13211      13222      13233      13244
Autographa californica nucleopolyhedrovirus NC 0... (13168) CGGTCGGTTCTGTGAACAGTTTGACACACACCATCACCTCCACCACCGTCACCAGCGTGATTCCAAAAAATTATCAA
                              SARS-CoV-2 NC_045512.2 (1) -----------------------------------------------------------------------------
                                                                                                                 Section 173
                                                  (13245) 13245     13255      13266      13277      13288      13299      13310      13321
Autographa californica nucleopolyhedrovirus NC 0... (13245) GAAAAACGTCAGAAAATATGCCACATAATATCTTCGTTGCGTAACACGCACTTGAATTTCAATAAGATACAGTCTGT
                              SARS-CoV-2 NC_045512.2 (1) -----------------------------------------------------------------------------
                                                                                                                 Section 174
                                                  (13322) 13322     13332      13343      13354      13365      13376      13387      13398
Autographa californica nucleopolyhedrovirus NC 0... (13322) ACATAAAAAGAAACTGCGGCATTTGCAAAATTTGCTAAGAAAAAAGAACGAAATTATTGCCGAGTTGGTTAGAAAAC
                              SARS-CoV-2 NC_045512.2 (1) -----------------------------------------------------------------------------
                                                                                                                 Section 175
                                                  (13399) 13399     13409      13420      13431      13442      13453      13464      13475
Autographa californica nucleopolyhedrovirus NC 0... (13399) TTGAAAGTGCACAGAAGAAGACAACGCACAGAAATATTAGTAAACCAGCTCATTGGAAATACTTTGGAGTAGTCAGA
                              SARS-CoV-2 NC_045512.2 (1) -----------------------------------------------------------------------------
```

```
                                                                                                                    Section 176
                                                  (13476) 13476     13486      13497      13508      13519      13530      13541  13552
Autographa californica nucleopolyhedrovirus NC 0... (13476) TGTGACAACACAATTCGCACAATTATTGGCAACGAAAAGTTTGTAAGGAGACGTTTGGCCGAGCTGTGCACATTGTA
                              SARS-CoV-2 NC_045512.2     (1) -----------------------------------------------------------------------------
                                                                                                                    Section 177
                                                  (13553) 13553     13563      13574      13585      13596      13607      13618  13629
Autographa californica nucleopolyhedrovirus NC 0... (13553) CAACGCCGAGTACGTGTTTTGCCAAGCACGCGCCGATGGAGACAAAGATCGACAGGCACTAGCGAGTCTGCTGACGG
                              SARS-CoV-2 NC_045512.2     (1) -----------------------------------------------------------------------------
                                                                                                                    Section 178
                                                  (13630) 13630     13640      13651      13662      13673      13684      13695  13706
Autographa californica nucleopolyhedrovirus NC 0... (13630) CGGCGTTTGGTTCGCGAGTCATAGTTTATGAAAATAGTCGCCGGTTCGAGTTTATAAATCCGGACGAGATTGCTAGT
                              SARS-CoV-2 NC_045512.2     (1) -----------------------------------------------------------------------------
                                                                                                                    Section 179
                                                  (13707) 13707     13717      13728      13739      13750      13761      13772  13783
Autographa californica nucleopolyhedrovirus NC 0... (13707) GGTAAACGTTTAATAATTAAACATTTGCAAGATGAATCTCAAAGTGATATTAACGCCTATTAATTTGAAAGGTGAGG
                              SARS-CoV-2 NC_045512.2     (1) -----------------------------------------------------------------------------
                                                                                                                    Section 180
                                                  (13784) 13784     13794      13805      13816      13827      13838      13849  13860
Autographa californica nucleopolyhedrovirus NC 0... (13784) AAGAGCCCAATTGCGTTGAGCGCATTACCATAATGCCATGTATTTTAATAGATACTGAGATCTGTTTAAATGTCAGA
                              SARS-CoV-2 NC_045512.2     (1) -----------------------------------------------------------------------------
                                                                                                                    Section 181
                                                  (13861) 13861     13871      13882      13893      13904      13915      13926  13937
Autographa californica nucleopolyhedrovirus NC 0... (13861) TGCCGTTCTCCTTTTGCCAAATTCAAAGTATTGATTATTGTAGATGGCTTTGATAGCGCTTATATTCAGGCTACCTT
                              SARS-CoV-2 NC_045512.2     (1) -----------------------------------------------------------------------------
                                                                                                                    Section 182
                                                  (13938) 13938     13948      13959      13970      13981      13992      14003  14014
Autographa californica nucleopolyhedrovirus NC 0... (13938) TTGTAGCATTAGCGATAGTGTAACAATTGTTAACAAATCTAACGAAAAGCATGTAACGTTTGACGGGTTTGTAAGGC
                              SARS-CoV-2 NC_045512.2     (1) -----------------------------------------------------------------------------
```

```
                                                                                    Section 183
                                                (14015) 14015     14025      14036      14047      14058      14069      14080   14091
Autographa californica nucleopolyhedrovirus NC 0... (14015) CGGACGATGAAGGTACAACAATGCCTTATGTCATTGGACCATTATATTCTGTCGACGCTGCTGTCGCCGACCGTAAA
                              SARS-CoV-2 NC_045512.2 (1) -----------------------------------------------------------------------------
                                                                                    Section 184
                                                (14092) 14092     14102      14113      14124      14135      14146      14157   14168
Autographa californica nucleopolyhedrovirus NC 0... (14092) GTGAAGGACGTGGTGGATTCAATTCAAAACCAACAGACAATGTTAAAAGTATTTATTAACGAGGCTAATGTGTATAA
                              SARS-CoV-2 NC_045512.2 (1) -----------------------------------------------------------------------------
                                                                                    Section 185
                                                (14169) 14169     14179      14190      14201      14212      14223      14234   14245
Autographa californica nucleopolyhedrovirus NC 0... (14169) CAAATGGAATATGCTTAAAGGTTTAATTTATAATAATAACAATGAATCTGTTTTAGTAAAATAATGTAGTAAAATTT
                              SARS-CoV-2 NC_045512.2 (1) -----------------------------------------------------------------------------
                                                                                    Section 186
                                                (14246) 14246     14256      14267      14278      14289      14300      14311   14322
Autographa californica nucleopolyhedrovirus NC 0... (14246) ATAAAGGTAGATAAAAATTATAATATTAATAAAAAAAATAATGTTACTAAATGGGTTCCTGCGTTAAATTATTTTAC
                              SARS-CoV-2 NC_045512.2 (1) -----------------------------------------------------------------------------
                                                                                    Section 187
                                                (14323) 14323     14333      14344      14355      14366      14377      14388   14399
Autographa californica nucleopolyhedrovirus NC 0... (14323) GGGTAGACAGCTATTAACTATTTTATTTATTTTTAAATTTAAATAAATGTATTGTTAGAAAATTGTGTTGTTTTATT
                              SARS-CoV-2 NC_045512.2 (1) -----------------------------------------------------------------------------
                                                                                    Section 188
                                                (14400) 14400     14410      14421      14432      14443      14454      14465   14476
Autographa californica nucleopolyhedrovirus NC 0... (14400) AGTATAACGAAAAAATACATGACATAAACCGCTTCCAATTTTGGTCACACAAACTCTTGTGTGGATAGTTTACGTAA
                              SARS-CoV-2 NC_045512.2 (1) -----------------------------------------------------------------------------
                                                                                    Section 189
                                                (14477) 14477     14487      14498      14509      14520      14531      14542   14553
Autographa californica nucleopolyhedrovirus NC 0... (14477) TGAGTTAAATAGGCGGGCAGTTGTCCGCTAAACGTGTCGGTGGTCAAGTAGATGTGCATTAATTTACGACAACCCAA
                              SARS-CoV-2 NC_045512.2 (1) -----------------------------------------------------------------------------
```

```
                                                                                                    Section 190
                                            (14554) 14554     14564      14575      14586      14597      14608      14619    14630
Autographa californica nucleopolyhedrovirus NC 0... (14554) AGCGGGGCCGCTTATGTCAAGTATTTTTTTCACAAAATTGGTAATGGTTTCGTTTTGTTCCTTGTACAAACACATGT
                             SARS-CoV-2 NC_045512.2     (1) -----------------------------------------------------------------------------
                                                                                                    Section 191
                                            (14631) 14631     14641      14652      14663      14674      14685      14696    14707
Autographa californica nucleopolyhedrovirus NC 0... (14631) CGGTGTGATCGTTGACGCACGAGTTGTACGATTCCGCCGGCAGGTTGGCAAACAAGCGCTTGAGATGCTTGAGTCTG
                             SARS-CoV-2 NC_045512.2     (1) -----------------------------------------------------------------------------
                                                                                                    Section 192
                                            (14708) 14708     14718      14729      14740      14751      14762      14773    14784
Autographa californica nucleopolyhedrovirus NC 0... (14708) CGTTCAATTTTATAATCAAACTTGTTGGTGAAAATGTCTTTCAGCAAGCACATTAACTGGTCGTTCAAAACGCGCTG
                             SARS-CoV-2 NC_045512.2     (1) -----------------------------------------------------------------------------
                                                                                                    Section 193
                                            (14785) 14785     14795      14806      14817      14828      14839      14850    14861
Autographa californica nucleopolyhedrovirus NC 0... (14785) CAACGACGACACCAACACATGATATTCGTTTCCAAAAAGCGAAAAATTTTTGATGCAGCGGTCCGCGTTGAAGGGTC
                             SARS-CoV-2 NC_045512.2     (1) -----------------------------------------------------------------------------
                                                                                                    Section 194
                                            (14862) 14862     14872      14883      14894      14905      14916      14927    14938
Autographa californica nucleopolyhedrovirus NC 0... (14862) GTTTCATAATGCGCACGTTGACAAAAAACACGTTGAAAGACAGCGGGGCTGTGGTTATTTTAACGCCGTTGTCGGTA
                             SARS-CoV-2 NC_045512.2     (1) -----------------------------------------------------------------------------
                                                                                                    Section 195
                                            (14939) 14939     14949      14960      14971      14982      14993      15004    15015
Autographa californica nucleopolyhedrovirus NC 0... (14939) TACTCGTCGACGCCGTCTGCGCTTGTTATGTCAATTTGTAGCGCAAATCTAACCAAATCAAACTCATCGTTGTACTG
                             SARS-CoV-2 NC_045512.2     (1) -----------------------------------------------------------------------------
                                                                                                    Section 196
                                            (15016) 15016     15026      15037      15048      15059      15070      15081    15092
Autographa californica nucleopolyhedrovirus NC 0... (15016) TGTCTTTATGCATTTTATATGGCGGTTTAAGTGCAAGTTGATTTGGCCGTTTAATCTATAGGCTCCGTTTTGATAAC
                             SARS-CoV-2 NC_045512.2     (1) -----------------------------------------------------------------------------
```

```
                                                                                                    Section 197
                                                 (15093) 15093     15103      15114      15125      15136      15147      15158      15169
Autographa californica nucleopolyhedrovirus NC 0... (15093) ATTTCAGCACTACCAACGGATCCGACATGTAAACTTGACGCGTTAGCACGTCCAATTCAGCGTAATGTTGGTCGACG
                          SARS-CoV-2 NC_045512.2     (1) -----------------------------------------------------------------------------
                                                                                                    Section 198
                                                 (15170) 15170     15180      15191      15202      15213      15224      15235      15246
Autographa californica nucleopolyhedrovirus NC 0... (15170) CATTTTTGTAAATTAGTTTGCAGGTTGCAAAACATTTTTGCGCAAAAGCCGTAATAGTCAAAATCTATGCATTTTAA
                          SARS-CoV-2 NC_045512.2     (1) -----------------------------------------------------------------------------
                                                                                                    Section 199
                                                 (15247) 15247     15257      15268      15279      15290      15301      15312      15323
Autographa californica nucleopolyhedrovirus NC 0... (15247) TGCGCTTCTGTCGTCGTCAATATGGCATGTCACGGCTGCGCCTCCAGTTAACACGAATAAACCGCCGTTTTCGCAAA
                          SARS-CoV-2 NC_045512.2     (1) -----------------------------------------------------------------------------
                                                                                                    Section 200
                                                 (15324) 15324     15334      15345      15356      15367      15378      15389      15400
Autographa californica nucleopolyhedrovirus NC 0... (15324) CTACGGCTTCGAAACAATCTTTGATAAATGCCAACTTTGCTTTAGCCACAATTTTATCGCGCAGGCGATCTTCAATA
                          SARS-CoV-2 NC_045512.2     (1) -----------------------------------------------------------------------------
                                                                                                    Section 201
                                                 (15401) 15401     15411      15422      15433      15444      15455      15466      15477
Autographa californica nucleopolyhedrovirus NC 0... (15401) TCCTTTGTCGTAATATAAGGTAGGACGCCAAGATTTAGTTGATTCAACAAACGTTCCATAATGAATAGCGGCGACGC
                          SARS-CoV-2 NC_045512.2     (1) -----------------------------------------------------------------------------
                                                                                                    Section 202
                                                 (15478) 15478     15488      15499      15510      15521      15532      15543      15554
Autographa californica nucleopolyhedrovirus NC 0... (15478) AACACGACTACACTGTTCAAATGCGCACGCAAAACAAACCCTTGCAACTTTATTTGCCAATCGTAATCACAGTAGTT
                          SARS-CoV-2 NC_045512.2     (1) -----------------------------------------------------------------------------
                                                                                                    Section 203
                                                 (15555) 15555     15565      15576      15587      15598      15609      15620      15631
Autographa californica nucleopolyhedrovirus NC 0... (15555) TTTACGAGTACGCCATCGCGTTTGTAAGCACATTGCTTTTTAAAAATAATTTAAATTTAATGACCGCGTGCAATTTG
                          SARS-CoV-2 NC_045512.2     (1) -----------------------------------------------------------------------------
```

```
                                                          Section 204
                                                (15632) 15632     15642      15653      15664      15675      15686      15697      15708
Autographa californica nucleopolyhedrovirus NC 0... (15632) ATCAACTCGTTGATCAACTTTGAACTCAACATGTTTGGTAAAAGTTTATTGCTAAATGGATTTGTTAATTTCTGCAT
                         SARS-CoV-2 NC_045512.2     (1) -----------------------------------------------------------------------------
                                                          Section 205
                                                (15709) 15709     15719      15730      15741      15752      15763      15774      15785
Autographa californica nucleopolyhedrovirus NC 0... (15709) TGCTAACAGCGACGGGGTTACGATTCAACATAAAATGTTAACCAACGTGTTAAGTTTTTTGTTGGAAAAATATTATT
                         SARS-CoV-2 NC_045512.2     (1) -----------------------------------------------------------------------------
                                                          Section 206
                                                (15786) 15786     15796      15807      15818      15829      15840      15851      15862
Autographa californica nucleopolyhedrovirus NC 0... (15786) AAAAATAAATAAATAAACTTGTTCAGTTCTAATTATTGTTTTATTTTTTATAAAATAATACAATTTTATTTATACAT
                         SARS-CoV-2 NC_045512.2     (1) -----------------------------------------------------------------------------
                                                          Section 207
                                                (15863) 15863     15873      15884      15895      15906      15917      15928      15939
Autographa californica nucleopolyhedrovirus NC 0... (15863) TAATACTTTGGTATTTATTAATACAATTATTTACAATACTTTATTTACACTATAATACTTTATTTACATTAGTACTA
                         SARS-CoV-2 NC_045512.2     (1) -----------------------------------------------------------------------------
                                                          Section 208
                                                (15940) 15940     15950      15961      15972      15983      15994      16005      16016
Autographa californica nucleopolyhedrovirus NC 0... (15940) AATTAATACTAAATTACGCTAATACTAAATTAATACTTTATATAATCAAAAATAATACTTTATATAATACTTTCTAA
                         SARS-CoV-2 NC_045512.2     (1) -----------------------------------------------------------------------------
                                                          Section 209
                                                (16017) 16017     16027      16038      16049      16060      16071      16082      16093
Autographa californica nucleopolyhedrovirus NC 0... (16017) TCATCATAAACGGGTAATAGTTTTTTCTCTTGAAATTTACGCTGCAACTCTTCGCTAAAACACATGGGCGGTGGAGT
                         SARS-CoV-2 NC_045512.2     (1) -----------------------------------------------------------------------------
                                                          Section 210
                                                (16094) 16094     16104      16115      16126      16137      16148      16159      16170
Autographa californica nucleopolyhedrovirus NC 0... (16094) GGGAGCGGGTGGAGTAGGAGTCCTTACGGGTTTGATGGGCGACAGTTCTCTGGACTTGCGGAACAGCTTGGGCGAAA
                         SARS-CoV-2 NC_045512.2     (1) -----------------------------------------------------------------------------
```

```
                                                                                                  Section 211
                                                   (16171) 16171     16181      16192      16203      16214      16225      16236      16247
Autographa californica nucleopolyhedrovirus NC_0... (16171) ACGTCGGCGTGCGCCGACTAATGATTTCTTCATCGCACGAGGCGTCGCACATTGTGCACGCGTCCGGTGAGGTACAC
                           SARS-CoV-2 NC_045512.2     (1) -----------------------------------------------------------------------------
                                                                                                  Section 212
                                                   (16248) 16248     16258      16269      16280      16291      16302      16313      16324
Autographa californica nucleopolyhedrovirus NC_0... (16248) AAAACTTTCTTGGGCACGCTGTACACCGGCTTGGGCACGCTATATGTGTTGCCAAAACTAGAACTCGTTGTGGTTGC
                           SARS-CoV-2 NC_045512.2     (1) -----------------------------------------------------------------------------
                                                                                                  Section 213
                                                   (16325) 16325     16335      16346      16357      16368      16379      16390      16401
Autographa californica nucleopolyhedrovirus NC_0... (16325) CGAACGGAGACGATGGGTGTGAAGACGGCGATGGCTGTGAAGACAAGTCCGAAGGCGCGATAAAAGATGAAAGTGTT
                           SARS-CoV-2 NC_045512.2     (1) -----------------------------------------------------------------------------
                                                                                                  Section 214
                                                   (16402) 16402     16412      16423      16434      16445      16456      16467      16478
Autographa californica nucleopolyhedrovirus NC_0... (16402) TCTGAAACCGAAGTGGTGGTAGAAGTGGTAGAAGGCGGGTGCGTTACGGCAACCACGCTGCTGCTATTTCTGCCTTC
                           SARS-CoV-2 NC_045512.2     (1) -----------------------------------------------------------------------------
                                                                                                  Section 215
                                                   (16479) 16479     16489      16500      16511      16522      16533      16544      16555
Autographa californica nucleopolyhedrovirus NC_0... (16479) GGAGACCACTTCCAGCAATCTAGAGTTACTCTCTCGTTCTTCGCGGCGATAGTCAATGTCGCAATAATGTTCATAAG
                           SARS-CoV-2 NC_045512.2     (1) -----------------------------------------------------------------------------
                                                                                                  Section 216
                                                   (16556) 16556     16566      16577      16588      16599      16610      16621      16632
Autographa californica nucleopolyhedrovirus NC_0... (16556) ATGCCTTTTCGGCTTCGGCGCGCCTTTTCATGTATATGTTGTGACGCATCTCCTTTAACTGCACGTACAAATTCCAG
                           SARS-CoV-2 NC_045512.2     (1) -----------------------------------------------------------------------------
                                                                                                  Section 217
                                                   (16633) 16633     16643      16654      16665      16676      16687      16698      16709
Autographa californica nucleopolyhedrovirus NC_0... (16633) CATTGCACAGCCAGTATCGTAAGCACGCCCATTATGATTACGGGATAATTTTGATTAAACACGGTCGGCTCGTGATC
                           SARS-CoV-2 NC_045512.2     (1) -----------------------------------------------------------------------------
```

```
                                                                                                              Section 218
                                               (16710) 16710     16720      16731      16742      16753      16764      16775  16786
Autographa californica nucleopolyhedrovirus NC 0... (16710) GCTTACAATCGCTCGGCACATGATGCATTTTTTGTAAATGTTCACATACACACAGTTTTGGCTCAAGGTTTCGGTAT
                        SARS-CoV-2 NC_045512.2     (1) -----------------------------------------------------------------------------
                                                                                                              Section 219
                                               (16787) 16787     16797      16808      16819      16830      16841      16852  16863
Autographa californica nucleopolyhedrovirus NC 0... (16787) TTGCGTAGTCAATTTCCAGATACACGATAGAGTTCCAGCACATTGATTCCAAATCGTAGTGACGATATAAAACATCT
                        SARS-CoV-2 NC_045512.2     (1) -----------------------------------------------------------------------------
                                                                                                              Section 220
                                               (16864) 16864     16874      16885      16896      16907      16918      16929  16940
Autographa californica nucleopolyhedrovirus NC 0... (16864) AGCGCCGGTAGATGACCATTTTTGAACACGTAGATTTGAAACGCGGCAAACAGCATCCAACACAGCCCAGTGATCAC
                        SARS-CoV-2 NC_045512.2     (1) -----------------------------------------------------------------------------
                                                                                                              Section 221
                                               (16941) 16941     16951      16962      16973      16984      16995      17006  17017
Autographa californica nucleopolyhedrovirus NC 0... (16941) GTTTACCATAATACACGTGATAGCGACGTAAAAGTTTTCTTTCGCATTGAAATTTACATTTGTGTTTGAAGAGCTGC
                        SARS-CoV-2 NC_045512.2     (1) -----------------------------------------------------------------------------
                                                                                                              Section 222
                                               (17018) 17018     17028      17039      17050      17061      17072      17083  17094
Autographa californica nucleopolyhedrovirus NC 0... (17018) TGCGATTTTTCGTCCACACGATAATCTTCCATATAAAATAAAACATGTAAAATAATATCCACATGCCGAACGCCAGC
                        SARS-CoV-2 NC_045512.2     (1) -----------------------------------------------------------------------------
                                                                                                              Section 223
                                               (17095) 17095     17105      17116      17127      17138      17149      17160  17171
Autographa californica nucleopolyhedrovirus NC 0... (17095) ATTATCGGTATAGATAGATTGATAACCGATTGCTTTCCTTCAATTTCCAGCAAAAACGCGTATCTGCTGTCTATCAC
                        SARS-CoV-2 NC_045512.2     (1) -----------------------------------------------------------------------------
                                                                                                              Section 224
                                               (17172) 17172     17182      17193      17204      17215      17226      17237  17248
Autographa californica nucleopolyhedrovirus NC 0... (17172) TCCCATTATAGATAACACAAACACTATCAGATATGCTAATAATAATGAGGCATTAAGCCCGAATTGTAAAACTGCAG
                        SARS-CoV-2 NC_045512.2     (1) -----------------------------------------------------------------------------
```

```
                                                                                Section 225
                                              (17249) 17249     17259      17270      17281      17292      17303      17314     17325
Autographa californica nucleopolyhedrovirus NC 0... (17249) TGATTTTATTTAACATTTTGAATATTTAATTCAACAACTAAGTAATGGCAATATGTATCGAGTACTGATCGTGTTTT
                              SARS-CoV-2 NC_045512.2 (1) -----------------------------------------------------------------------------
                                                                                Section 226
                                              (17326) 17326     17336      17347      17358      17369      17380      17391     17402
Autographa californica nucleopolyhedrovirus NC 0... (17326) TCCTGTTCGTGTTTCTTTATATAGTGTACCAGCCCTTTTATCAGGCATACTTGCATATCGGACATGCCCAACAAGAT
                              SARS-CoV-2 NC_045512.2 (1) -----------------------------------------------------------------------------
                                                                                Section 227
                                              (17403) 17403     17413      17424      17435      17446      17457      17468     17479
Autographa californica nucleopolyhedrovirus NC 0... (17403) TACAATGACACGTTGGACGATAGGATGGATTACATTGAATCCGTAATGCGTAGAAGGCACTACGTGCCGATTGAAGC
                              SARS-CoV-2 NC_045512.2 (1) -----------------------------------------------------------------------------
                                                                                Section 228
                                              (17480) 17480     17490      17501      17512      17523      17534      17545     17556
Autographa californica nucleopolyhedrovirus NC 0... (17480) GTTGCCCGCAATCAGGTTTGATACTAATCTCGGCACGTTGGCCGGTGACACGATTAAATGCATGTCGGTGCCTTTGT
                              SARS-CoV-2 NC_045512.2 (1) -----------------------------------------------------------------------------
                                                                                Section 229
                                              (17557) 17557     17567      17578      17589      17600      17611      17622     17633
Autographa californica nucleopolyhedrovirus NC 0... (17557) TTGTTAGTGACATTGACCTGCCGATGTTTGATTGTAGTCAGATATGCGATAACCCGTCTGCGGCGTATTTCTTTGTC
                              SARS-CoV-2 NC_045512.2 (1) -----------------------------------------------------------------------------
                                                                                Section 230
                                              (17634) 17634     17644      17655      17666      17677      17688      17699     17710
Autographa californica nucleopolyhedrovirus NC 0... (17634) AACGAAACGGATGTGTTTGTGGTCAACGGCCACAGACTGACGGTGGGCGGATACTGCTCCACTAATAGTTTGCCCCG
                              SARS-CoV-2 NC_045512.2 (1) -----------------------------------------------------------------------------
                                                                                Section 231
                                              (17711) 17711     17721      17732      17743      17754      17765      17776     17787
Autographa californica nucleopolyhedrovirus NC 0... (17711) CAACTGTAATCGCGAGACGAGCGTCATTTTAATGAGTCTCAATCAGTGGACGTGCATAGCCGAGGACCCGCGTTACT
                              SARS-CoV-2 NC_045512.2 (1) -----------------------------------------------------------------------------
```

Section 232

```
                                                  (17788) 17788     17798      17809      17820      17831      17842      17853      17864
Autographa californica nucleopolyhedrovirus  NC_0... (17788) ATGCGGGCACAGATAACATGACGCAACTCGCAGGCAGACAACACTTTGACCGCATTATGCCCGGACAGAGTGATAGG
                          SARS-CoV-2 NC_045512.2     (1) -----------------------------------------------------------------------------
```

Section 233

```
                                                  (17865) 17865     17875      17886      17897      17908      17919      17930      17941
Autographa californica nucleopolyhedrovirus  NC_0... (17865) AACGTCCTGTTTGACCGATTACTAGGCCGAGAGGTGAACGTGACCACTAACACGTTTCGCCGCAGCTGGGACGAGTT
                          SARS-CoV-2 NC_045512.2     (1) -----------------------------------------------------------------------------
```

Section 234

```
                                                  (17942) 17942     17952      17963      17974      17985      17996      18007      18018
Autographa californica nucleopolyhedrovirus  NC_0... (17942) GCTGGAGGACGGCACTAGGCGGTTCGAAATGCGCTGCAACGCCCGAGATAACAACAATAATCTCATGTTTGTTAATC
                          SARS-CoV-2 NC_045512.2     (1) -----------------------------------------------------------------------------
```

Section 235

```
                                                  (18019) 18019     18029      18040      18051      18062      18073      18084      18095
Autographa californica nucleopolyhedrovirus  NC_0... (18019) CGCTTAATCCCCTCGAGTGTCTCCCGAACGTGTGCACTAACGTTAGCAACGTGCACACCAGTGTTAGACCCGTATTT
                          SARS-CoV-2 NC_045512.2     (1) -----------------------------------------------------------------------------
```

Section 236

```
                                                  (18096) 18096     18106      18117      18128      18139      18150      18161      18172
Autographa californica nucleopolyhedrovirus  NC_0... (18096) GAAACGGGAGAGTGTGACTGCGGCGACGAAGCGGTCACGCGTGTTACGCACATTGTGCCGGGGGACAGGACCTCTAT
                          SARS-CoV-2 NC_045512.2     (1) -----------------------------------------------------------------------------
```

Section 237

```
                                                  (18173) 18173     18183      18194      18205      18216      18227      18238      18249
Autographa californica nucleopolyhedrovirus  NC_0... (18173) GTGTGCCAGCATTATAGATGGCCTGGATAAAAGTACGGCATCATATAGATATCGCGTAGAGTGCGTTAATCTGTACA
                          SARS-CoV-2 NC_045512.2     (1) -----------------------------------------------------------------------------
```

Section 238

```
                                                  (18250) 18250     18260      18271      18282      18293      18304      18315      18326
Autographa californica nucleopolyhedrovirus  NC_0... (18250) CCTCTATTCTAAATTATTCTAATAACAAATTGTTATGTCCCAGTGACACTTTTGATAGTAACACGGACGCAGCTTTT
                          SARS-CoV-2 NC_045512.2     (1) -----------------------------------------------------------------------------
```

```
                                                                                                              Section 239
                                                   (18327) 18327     18337      18348      18359      18370      18381      18392     18403
Autographa californica nucleopolyhedrovirus NC 0... (18327) GCCTTTGAAGTGCCCGGCTCCTACCCTTTATCGCGCAACGGCATCAACGAGCCAACTTATCGCTTTTATCTTGATAC
                               SARS-CoV-2 NC_045512.2 (1) -----------------------------------------------------------------------------
                                                                                                              Section 240
                                                   (18404) 18404     18414      18425      18436      18447      18458      18469     18480
Autographa californica nucleopolyhedrovirus NC 0... (18404) CAGATCTCGAGTTAATTACAATGACGTCAGAGGGCAGTTATCTTAATTGTGATAACACAAACAATAAGTCATTTAAA
                               SARS-CoV-2 NC_045512.2 (1) -----------------------------------------------------------------------------
                                                                                                              Section 241
                                                   (18481) 18481     18491      18502      18513      18524      18535      18546     18557
Autographa californica nucleopolyhedrovirus NC 0... (18481) TGTTACGTCAGTAGTTAGTATATAAGCCGTACATGTTGGCTTGCAAATTCAGTCAATATCAGGCTTTTATCATGGAC
                               SARS-CoV-2 NC_045512.2 (1) -----------------------------------------------------------------------------
                                                                                                              Section 242
                                                   (18558) 18558     18568      18579      18590      18601      18612      18623     18634
Autographa californica nucleopolyhedrovirus NC 0... (18558) GGTGTAAAGCTGCTAGGGACGTGCGCGCTAATAATTTTGTTATCGACGACGAGTACAGTTGTCGGGCGTGACCGTAT
                               SARS-CoV-2 NC_045512.2 (1) -----------------------------------------------------------------------------
                                                                                                              Section 243
                                                   (18635) 18635     18645      18656      18667      18678      18689      18700     18711
Autographa californica nucleopolyhedrovirus NC 0... (18635) CACGTTTACGCCGATAGAAGATAGCGCAGGCCTCATGTTTGAACGCATGTACGGCTTGCGACATCATACAGACGACA
                               SARS-CoV-2 NC_045512.2 (1) -----------------------------------------------------------------------------
                                                                                                              Section 244
                                                   (18712) 18712     18722      18733      18744      18755      18766      18777     18788
Autographa californica nucleopolyhedrovirus NC 0... (18712) GATTTGTGTTTGTGAAAAAATTCAATTTTGTTTCGGTGCTGCAAGAGCTCAATAATATCAAATCTAAAATTGAATTA
                               SARS-CoV-2 NC_045512.2 (1) -----------------------------------------------------------------------------
                                                                                                              Section 245
                                                   (18789) 18789     18799      18810      18821      18832      18843      18854     18865
Autographa californica nucleopolyhedrovirus NC 0... (18789) TATGAAGCGCAAGTTTCAACTTGCACAAACGTCAGACAAATAAAACAGAACAGATCGAGTATCATCAAAGCTCGCAT
                               SARS-CoV-2 NC_045512.2 (1) -----------------------------------------------------------------------------
```

```
                                                                                              Section 246
                                                    (18866) 18866     18876      18887      18898      18909      18920      18931      18942
Autographa californica nucleopolyhedrovirus NC 0... (18866) TGAAAATCAGCTGCAGTTTTTGACGCAACTAAACAAAAATCTCATCACATACTCTGTGGAAAGCAGCATTTTAAGCA
                             SARS-CoV-2 NC_045512.2     (1) -----------------------------------------------------------------------------
                                                                                              Section 247
                                                    (18943) 18943     18953      18964      18975      18986      18997      19008      19019
Autographa californica nucleopolyhedrovirus NC 0... (18943) ACGACGTGCTGGACAACATCGATCTGGAATATGACGACAGCGGTGAGTTTGACGTTTACGACGAATACGAACAGCCT
                             SARS-CoV-2 NC_045512.2     (1) -----------------------------------------------------------------------------
                                                                                              Section 248
                                                    (19020) 19020     19030      19041      19052      19063      19074      19085      19096
Autographa californica nucleopolyhedrovirus NC 0... (19020) TCGCATTGGAGCAACATGACTGTATCCGACGCGCAAGCTTTGCTCCGAAACCCGCCCAAAGACAGAGTAATGTTTTT
                             SARS-CoV-2 NC_045512.2     (1) -----------------------------------------------------------------------------
                                                                                              Section 249
                                                    (19097) 19097     19107      19118      19129      19140      19151      19162      19173
Autographa californica nucleopolyhedrovirus NC 0... (19097) GGACACGGTTACCACCAGCGACGTGAGCAGCAAATACGAAGAATACATAAACTGCATTGTGAGCAACCGTACCGTTG
                             SARS-CoV-2 NC_045512.2     (1) -----------------------------------------------------------------------------
                                                                                              Section 250
                                                    (19174) 19174     19184      19195      19206      19217      19228      19239      19250
Autographa californica nucleopolyhedrovirus NC 0... (19174) AAAACGAGTGCATGTTTTTAGCCAACATGATGAACGTGCTCAACGACAAATTGGACGACGCAGCAGCTTTGGCCAAG
                             SARS-CoV-2 NC_045512.2     (1) -----------------------------------------------------------------------------
                                                                                              Section 251
                                                    (19251) 19251     19261      19272      19283      19294      19305      19316      19327
Autographa californica nucleopolyhedrovirus NC 0... (19251) ATGCTGGAGCGAATAGTAAAACAAACGCGAAAGAACAAACTCAACATCTCCAACACGGTTATAGACGACGACACGCT
                             SARS-CoV-2 NC_045512.2     (1) -----------------------------------------------------------------------------
                                                                                              Section 252
                                                    (19328) 19328     19338      19349      19360      19371      19382      19393      19404
Autographa californica nucleopolyhedrovirus NC 0... (19328) GCTAACGGAAATGAAAAAATTAACACAAACTTTATACAACCAAAACCGCGTGTGGGTAGTGGATTTTAACAAGGACA
                             SARS-CoV-2 NC_045512.2     (1) -----------------------------------------------------------------------------
```

```
                                                                                                   Section 253
                                            (19405) 19405     19415      19426      19437      19448      19459      19470      19481
Autographa californica nucleopolyhedrovirus NC_0... (19405) TGAATAGTTATTTCGATTTGTCGCAAGCGTATAAATTGCATTTATATGTTGATTTAAACACGGTCATTATGTTTATT
                              SARS-CoV-2 NC_045512.2    (1) -----------------------------------------------------------------------------
                                                                                                   Section 254
                                            (19482) 19482     19492      19503      19514      19525      19536      19547      19558
Autographa californica nucleopolyhedrovirus NC_0... (19482) ACCATGCCATTGTTAAAATCCACCGCCGTTTCGTTTAATTTGTATCGCGTCATGACGGTGCCTTTTTGCAGGGGCAA
                              SARS-CoV-2 NC_045512.2    (1) -----------------------------------------------------------------------------
                                                                                                   Section 255
                                            (19559) 19559     19569      19580      19591      19602      19613      19624      19635
Autographa californica nucleopolyhedrovirus NC_0... (19559) AATGTGTCTGCTTATCATTTCGGGCAATGAATACTTTGGGATTACAGACAGCAAAAACTATTATGTGCCCGTATCTG
                              SARS-CoV-2 NC_045512.2    (1) -----------------------------------------------------------------------------
                                                                                                   Section 256
                                            (19636) 19636     19646      19657      19668      19679      19690      19701      19712
Autographa californica nucleopolyhedrovirus NC_0... (19636) ATAACTTTAGACAAGATTGCCAAGAGTTTACGGGCTACAATGAGTTTTTGTGTCCCGAAACTGAGCCGATTGCCACT
                              SARS-CoV-2 NC_045512.2    (1) -----------------------------------------------------------------------------
                                                                                                   Section 257
                                            (19713) 19713     19723      19734      19745      19756      19767      19778      19789
Autographa californica nucleopolyhedrovirus NC_0... (19713) ATGAACTCGAAAGTGTGCGAGATTGAAATGTTTATGGGTCGATATAGCGACGACGTGGACAACATGTGCGACATTAG
                              SARS-CoV-2 NC_045512.2    (1) -----------------------------------------------------------------------------
                                                                                                   Section 258
                                            (19790) 19790     19800      19811      19822      19833      19844      19855      19866
Autographa californica nucleopolyhedrovirus NC_0... (19790) GGTGGCCAATTATAATCCCAAAAAAGCTTACGTGAACACTTTAATAGACTACCGAAAATGGTTGTACATTTTTCCAA
                              SARS-CoV-2 NC_045512.2    (1) -----------------------------------------------------------------------------
                                                                                                   Section 259
                                            (19867) 19867     19877      19888      19899      19910      19921      19932      19943
Autographa californica nucleopolyhedrovirus NC_0... (19867) ACACGACCGTGTCCGTCCACTATTATTGTCACGACGCGCTTGTAGAAGTTGATACAAAAGTTTCGCCCGGCGTTGGT
                              SARS-CoV-2 NC_045512.2    (1) -----------------------------------------------------------------------------
```

```
                                                                                                    Section 260
                                              (19944) 19944     19954      19965      19976      19987      19998      20009  20020
Autographa californica nucleopolyhedrovirus NC 0... (19944) GTTATGTTTTCGACTATGGCGCAAACGTGTTCGATTAGAATAACGTATGATGTGACCATAACTGTAGATTCGCGATT
                              SARS-CoV-2 NC_045512.2 (1) -----------------------------------------------------------------------------
                                                                                                    Section 261
                                              (20021) 20021     20031      20042      20053      20064      20075      20086  20097
Autographa californica nucleopolyhedrovirus NC 0... (20021) TTATGTCAGCCATTCAACTACATACTGGCCTAAAAAGAAATTTAATTTTAACAACTACATCGACCAAATGTTGCTTG
                              SARS-CoV-2 NC_045512.2 (1) -----------------------------------------------------------------------------
                                                                                                    Section 262
                                              (20098) 20098     20108      20119      20130      20141      20152      20163  20174
Autographa californica nucleopolyhedrovirus NC 0... (20098) AAAAAGCGACCACCAGTTTTATACCGACTGTTGACAATTTTACCCGGCCCGTTTTATTGCAACTTCCTCATAAATTT
                              SARS-CoV-2 NC_045512.2 (1) -----------------------------------------------------------------------------
                                                                                                    Section 263
                                              (20175) 20175     20185      20196      20207      20218      20229      20240  20251
Autographa californica nucleopolyhedrovirus NC 0... (20175) CACATTAAAGATTACACATCGACGCCCCATCATTTTTTCCATCAGTCTAAAATTTACACCAACAGCGCGGCGCCCGA
                              SARS-CoV-2 NC_045512.2 (1) -----------------------------------------------------------------------------
                                                                                                    Section 264
                                              (20252) 20252     20262      20273      20284      20295      20306      20317  20328
Autographa californica nucleopolyhedrovirus NC 0... (20252) CGAAGACTCGCAAGACGACAGTAATACCACCGTGGTTATTATCGCTATTGTCGCTGCAATGATCCTATTCTGTGGAT
                              SARS-CoV-2 NC_045512.2 (1) -----------------------------------------------------------------------------
                                                                                                    Section 265
                                              (20329) 20329     20339      20350      20361      20372      20383      20394  20405
Autographa californica nucleopolyhedrovirus NC 0... (20329) TATTGTTATTTTTGTTTTGCTGTATAAAAAAACGGTGTCATCAATCAAATAACGTGGTTGTGCAATACAAAAATAAC
                              SARS-CoV-2 NC_045512.2 (1) -----------------------------------------------------------------------------
                                                                                                    Section 266
                                              (20406) 20406     20416      20427      20438      20449      20460      20471  20482
Autographa californica nucleopolyhedrovirus NC 0... (20406) AATGAATTTGTCACAATTTGCAATAATTTAGAAGACAATCGAGCATACATTAATTTACCTAATGAATACGATAGCGA
                              SARS-CoV-2 NC_045512.2 (1) -----------------------------------------------------------------------------
```

```
                                                                                                    Section 267
                                                 (20483) 20483     20493      20504      20515      20526      20537      20548      20559
Autographa californica nucleopolyhedrovirus NC 0... (20483) TGATATGCCAAAACCATTGTACCCTTTACTTGGCTTTAATGATGATTTGTTAAAAGATGATAAACCTGTGTTGTACC
                              SARS-CoV-2 NC_045512.2     (1) -----------------------------------------------------------------------------
                                                                                                    Section 268
                                                 (20560) 20560     20570      20581      20592      20603      20614      20625      20636
Autographa californica nucleopolyhedrovirus NC 0... (20560) CTATGATTATAGAAAGAATAAAATAAAACATGTATAATTGAAATAAATATATTATTTAATAAAATGTTTTTTATTTA
                              SARS-CoV-2 NC_045512.2     (1) -----------------------------------------------------------------------------
                                                                                                    Section 269
                                                 (20637) 20637     20647      20658      20669      20680      20691      20702      20713
Autographa californica nucleopolyhedrovirus NC 0... (20637) TATACTATTTTCTATTACATATTCCAATGCACACAAATGTTTAATGGCTATCAGTTTTAATTTTACTAATTCGTCTA
                              SARS-CoV-2 NC_045512.2     (1) -----------------------------------------------------------------------------
                                                                                                    Section 270
                                                 (20714) 20714     20724      20735      20746      20757      20768      20779      20790
Autographa californica nucleopolyhedrovirus NC 0... (20714) AACAAAAATTATTCACTTGCTGTTTTTCATCCATTTGACATATGGCGTTTATAAATAATTCGCTGTGTTTTATGAAC
                              SARS-CoV-2 NC_045512.2     (1) -----------------------------------------------------------------------------
                                                                                                    Section 271
                                                 (20791) 20791     20801      20812      20823      20834      20845      20856      20867
Autographa californica nucleopolyhedrovirus NC 0... (20791) GAATCGTAAACCGCTGCCTGGGCCTTCAGCACGGTCGGCGCATTGTATTTTTGGGTAAAGTACGCAATATTTTTAGT
                              SARS-CoV-2 NC_045512.2     (1) -----------------------------------------------------------------------------
                                                                                                    Section 272
                                                 (20868) 20868     20878      20889      20900      20911      20922      20933      20944
Autographa californica nucleopolyhedrovirus NC 0... (20868) CAAACACAGAGATTTTAAATCTTTTTCATTTATATCCAAGTCGGAACAATCGTATACAAAATCTAGCTTTTCACTTT
                              SARS-CoV-2 NC_045512.2     (1) -----------------------------------------------------------------------------
                                                                                                    Section 273
                                                 (20945) 20945     20955      20966      20977      20988      20999      21010      21021
Autographa californica nucleopolyhedrovirus NC 0... (20945) CGGGCGCGCCCAGATACTGGTTTACGAGTTCGAGCTGCTCCACTTGGCCTTTGATATCGGCCGCTATGCACAACATT
                              SARS-CoV-2 NC_045512.2     (1) -----------------------------------------------------------------------------
```

```
                                                                                                          Section 274
                                               (21022) 21022     21032      21043      21054      21065      21076      21087  21098
Autographa californica nucleopolyhedrovirus NC 0... (21022) TTGTCGATTGCAGTTTCATTGTTTTTAACATAATAATTTTTAACTTTTTTATTTTGCAATTTAATCAAACTATTTAA
                          SARS-CoV-2 NC_045512.2 (1) -----------------------------------------------------------------------------
                                                                                                          Section 275
                                               (21099) 21099     21109      21120      21131      21142      21153      21164  21175
Autographa californica nucleopolyhedrovirus NC 0... (21099) ATTCGCTTGACCTTTCTTACAAAGCGCAGTTAATATGCAAGACATTTTGACTTATAATAAAAAACAAAACTTTTATA
                          SARS-CoV-2 NC_045512.2 (1) -----------------------------------------------------------------------------
                                                                                                          Section 276
                                               (21176) 21176     21186      21197      21208      21219      21230      21241  21252
Autographa californica nucleopolyhedrovirus NC 0... (21176) TATTCATTTATTGTTCAATAATAACAAATATTCCAGGCTTAAAAGCTAACGAATAGGGCTTTTCGGTAATTTTCTTA
                          SARS-CoV-2 NC_045512.2 (1) -----------------------------------------------------------------------------
                                                                                                          Section 277
                                               (21253) 21253     21263      21274      21285      21296      21307      21318  21329
Autographa californica nucleopolyhedrovirus NC 0... (21253) TTATTCATGTCCGTCATCTGCATCTCTTTGCCGTACTTGACGCCGTCAATGGTGCCCATCATGTACATTTTAATCTC
                          SARS-CoV-2 NC_045512.2 (1) -----------------------------------------------------------------------------
                                                                                                          Section 278
                                               (21330) 21330     21340      21351      21362      21373      21384      21395  21406
Autographa californica nucleopolyhedrovirus NC 0... (21330) CTCCGAAGGTCCGTCTATTTTGTCCATTTCGAACAATCTATCAAAATCTTCAACGCTCATTCTCTGCATATCAAGAG
                          SARS-CoV-2 NC_045512.2 (1) -----------------------------------------------------------------------------
                                                                                                          Section 279
                                               (21407) 21407     21417      21428      21439      21450      21461      21472  21483
Autographa californica nucleopolyhedrovirus NC 0... (21407) GAACGTTTCTGATCTTTCCGGTGGCGTAAATTGATCCGTTGTTGTCACGGTTGATTATGTAAAACCGACGAATCAAC
                          SARS-CoV-2 NC_045512.2 (1) -----------------------------------------------------------------------------
                                                                                                          Section 280
                                               (21484) 21484     21494      21505      21516      21527      21538      21549  21560
Autographa californica nucleopolyhedrovirus NC 0... (21484) ATGTCGCGCTCGCTAGTTTTGTTCTTATCCGGCAAATGAATGCACACGTTTGGTTCCATCTTCAAAGGAAAATCGCT
                          SARS-CoV-2 NC_045512.2 (1) -----------------------------------------------------------------------------
```

Section 281

	(21561)	21561 21571 21582 21593 21604 21615 21626 21637
Autographa californica nucleopolyhedrovirus NC 0...	(21561)	TTGCAAGTGTTTTTGCAAAATGTTGCCAAATATATTGTTGTGTTTGTGAATGTCTCCGTATTGAATGCTAAAAAACT
SARS-CoV-2 NC_045512.2	(1)	---

Section 282

	(21638)	21638 21648 21659 21670 21681 21692 21703 21714
Autographa californica nucleopolyhedrovirus NC 0...	(21638)	GGCCAAAGTTGCTTTTGGCACGTTTTATGGTTCCAAAGTCGGAAAACCAAAATCCGCAGGGCTTGCCCTGCACTCTT
SARS-CoV-2 NC_045512.2	(1)	---

Section 283

	(21715)	21715 21725 21736 21747 21758 21769 21780 21791
Autographa californica nucleopolyhedrovirus NC 0...	(21715)	GGACCGATGGTGTACGTAGTCTTGCCGTTGGCCGGCTCCAACACCACGATATTTTTATCGGGCTCGGGATACAACTT
SARS-CoV-2 NC_045512.2	(1)	---

Section 284

	(21792)	21792 21802 21813 21824 21835 21846 21857 21868
Autographa californica nucleopolyhedrovirus NC 0...	(21792)	GTCTTCCCATTCGTGCAAACTGTTCAAATTAGACAGTCGACAAAATTCGTTTTTCAAAAATCTGCCTTCGAAACAAC
SARS-CoV-2 NC_045512.2	(1)	---

Section 285

	(21869)	21869 21879 21890 21901 21912 21923 21934 21945
Autographa californica nucleopolyhedrovirus NC 0...	(21869)	TACAATTCAGTATTGAAAAGTTGCCTCGTTTCACATTAATCGCCATCTGCTCCTGCCACAACATCTTCGTCAACTCG
SARS-CoV-2 NC_045512.2	(1)	---

Section 286

	(21946)	21946 21956 21967 21978 21989 22000 22011 22022
Autographa californica nucleopolyhedrovirus NC 0...	(21946)	TGTGGCTCCAATTGAATGGACGACGGCGTAAAATAGCACATTACGCCCGTTTCGTCGTGTTTCACGTTAAAAGCGCC
SARS-CoV-2 NC_045512.2	(1)	---

Section 287

	(22023)	22023 22033 22044 22055 22066 22077 22088 22099
Autographa californica nucleopolyhedrovirus NC 0...	(22023)	GCTGTTGTACGGCACCAGCTGCTGGTCCTCACCACCTTCCGATCTTTCCCGCTTCGGCTGGTTGTCGTCGCTGCTCG
SARS-CoV-2 NC_045512.2	(1)	---

```
                                                                                                                    Section 288
                                               (22100) 22100     22110      22121      22132      22143      22154      22165  22176
Autographa californica nucleopolyhedrovirus NC 0... (22100) AATATCCATCGCCAATCTTGCGTTTAGTTGCCATGCTACCGACGTGCGCTGTCTGCTGTGGTTCAAGTCTAATTGAA
                             SARS-CoV-2 NC_045512.2 (1) -----------------------------------------------------------------------------
                                                                                                                    Section 289
                                               (22177) 22177     22187      22198      22209      22220      22231      22242  22253
Autographa californica nucleopolyhedrovirus NC 0... (22177) GTGTTTCACAGAATATAAGATATATAATAAATATGGACGACTCTGTTGCCAGCATGTGCGTAGACAACGCGTTTGCG
                             SARS-CoV-2 NC_045512.2 (1) -----------------------------------------------------------------------------
                                                                                                                    Section 290
                                               (22254) 22254     22264      22275      22286      22297      22308      22319  22330
Autographa californica nucleopolyhedrovirus NC 0... (22254) TACACTACTGACGATTTATTGAAAAATATTCCTTTTAGTCATTCCAAATGCGCCCCTTTCAAGCTACAAAATTACAC
                             SARS-CoV-2 NC_045512.2 (1) -----------------------------------------------------------------------------
                                                                                                                    Section 291
                                               (22331) 22331     22341      22352      22363      22374      22385      22396  22407
Autographa californica nucleopolyhedrovirus NC 0... (22331) CGTTTTGAAGCGGTTGAGCAACGGGTTTATCGACAAGTATGTGGACGTGTGCTCTATCAGCGAGTTGCAAAAGTTTA
                             SARS-CoV-2 NC_045512.2 (1) -----------------------------------------------------------------------------
                                                                                                                    Section 292
                                               (22408) 22408     22418      22429      22440      22451      22462      22473  22484
Autographa californica nucleopolyhedrovirus NC 0... (22408) ATTTTAAGATAGATCGGCTAACCAACTACATATCAAACATTTTCGAGTACGAGTTTGTAGTTTTAGAACACGATTTG
                             SARS-CoV-2 NC_045512.2 (1) -----------------------------------------------------------------------------
                                                                                                                    Section 293
                                               (22485) 22485     22495      22506      22517      22528      22539      22550  22561
Autographa californica nucleopolyhedrovirus NC 0... (22485) TCCACAGTGCACGTCATTAACGCCGAAACAAAAACCAAACTGGGCCATATAAACGTGTCGCTAAACCAAAACGACGC
                             SARS-CoV-2 NC_045512.2 (1) -----------------------------------------------------------------------------
                                                                                                                    Section 294
                                               (22562) 22562     22572      22583      22594      22605      22616      22627  22638
Autographa californica nucleopolyhedrovirus NC 0... (22562) AAACGTGCTCATTTTGACCGTAACTTTAACGAGCTAAAATGAACGAGGACACGCCCCCGTTTTATTTTATCAGCGTG
                             SARS-CoV-2 NC_045512.2 (1) -----------------------------------------------------------------------------
```

```
                                                                                                    Section 295
                                              (22639) 22639     22649      22660      22671      22682      22693      22704  22715
Autographa californica nucleopolyhedrovirus NC 0... (22639) TGTGACAACTTTCGCGACAACACCGCCGAACACGTATTCGACATGTTAATAGAAAGACATAGTTCGTTTGAAAATTA
                              SARS-CoV-2 NC_045512.2 (1) -----------------------------------------------------------------------------
                                                                                                    Section 296
                                              (22716) 22716     22726      22737      22748      22759      22770      22781  22792
Autographa californica nucleopolyhedrovirus NC 0... (22716) TCCCATTGAAAACACGGCGTTTATTAACAGCTTGATCGTTAACGGGTTTAAATACAATCAAGTTGACGATCACGTTG
                              SARS-CoV-2 NC_045512.2 (1) -----------------------------------------------------------------------------
                                                                                                    Section 297
                                              (22793) 22793     22803      22814      22825      22836      22847      22858  22869
Autographa californica nucleopolyhedrovirus NC 0... (22793) TGTGCGAGTATTGCGAAGCAGAAATAAAAAATTGGTCCGAAGACGAGTGTATTGAATATGCACACGTAACCTTGTCG
                              SARS-CoV-2 NC_045512.2 (1) -----------------------------------------------------------------------------
                                                                                                    Section 298
                                              (22870) 22870     22880      22891      22902      22913      22924      22935  22946
Autographa californica nucleopolyhedrovirus NC 0... (22870) CCGTATTGCGCGTATGCTAACAAGATCGCCGAGCGTGAATCGTTTGGCGACAACATTACCATCAACGCTGTACTAGT
                              SARS-CoV-2 NC_045512.2 (1) -----------------------------------------------------------------------------
                                                                                                    Section 299
                                              (22947) 22947     22957      22968      22979      22990      23001      23012  23023
Autographa californica nucleopolyhedrovirus NC 0... (22947) GAAAGAAGGCAAACCCAAGTGTGTGTACAGATGCATGTCCAATTTACAGTCGCGTATGGATACGTTTGTTAACTTTT
                              SARS-CoV-2 NC_045512.2 (1) -----------------------------------------------------------------------------
                                                                                                    Section 300
                                              (23024) 23024     23034      23045      23056      23067      23078      23089  23100
Autographa californica nucleopolyhedrovirus NC 0... (23024) GGCCTGCCGCATTGCGTGACATGATTACAAACATTGCGGAAGCGGGACTTTTTTACACGGGTCGCGGAGACGAAACT
                              SARS-CoV-2 NC_045512.2 (1) -----------------------------------------------------------------------------
                                                                                                    Section 301
                                              (23101) 23101     23111      23122      23133      23144      23155      23166  23177
Autographa californica nucleopolyhedrovirus NC 0... (23101) GTGTGTTTCTTTTGCGACTGTTGCGTACGTGATTGGCATACTAATGAAGACACCTGGCAGCGACACGCCGCCGAAAA
                              SARS-CoV-2 NC_045512.2 (1) -----------------------------------------------------------------------------
```

```
                                                                                                                            Section 302
                                                (23178) 23178     23188      23199      23210      23221      23232      23243      23254
Autographa californica nucleopolyhedrovirus NC 0... (23178) CCCGCAATGTTATTTTGTATTGTCGGTGAAAGGTAAAGAATTTTGTCAAAACTCAATTACTGTCACTCACGTTGATA
                            SARS-CoV-2 NC_045512.2      (1) -----------------------------------------------------------------------------
                                                                                                                            Section 303
                                                (23255) 23255     23265      23276      23287      23298      23309      23320      23331
Autographa californica nucleopolyhedrovirus NC 0... (23255) AACGTGACGACGACAATTTAAACGAAAACGCCGACGACATTGAGGAAAAATATGAATGCAAAGTCTGTCTCGAACGC
                            SARS-CoV-2 NC_045512.2      (1) -----------------------------------------------------------------------------
                                                                                                                            Section 304
                                                (23332) 23332     23342      23353      23364      23375      23386      23397      23408
Autographa californica nucleopolyhedrovirus NC 0... (23332) CAACGCGACGCCGTGCTTATGCCGTGTCGGCATTTTTGCGTTTGCGTTCAGTGTTATTTTGGATTAGATCAAAAGTG
                            SARS-CoV-2 NC_045512.2      (1) -----------------------------------------------------------------------------
                                                                                                                            Section 305
                                                (23409) 23409     23419      23430      23441      23452      23463      23474      23485
Autographa californica nucleopolyhedrovirus NC 0... (23409) TCCGACGTGTCGTCAGGACGTCACCGATTTTATAAAAATATTTGTGGTGTAATAAAATGGTGTTCAACGTGTACTAC
                            SARS-CoV-2 NC_045512.2      (1) -----------------------------------------------------------------------------
                                                                                                                            Section 306
                                                (23486) 23486     23496      23507      23518      23529      23540      23551      23562
Autographa californica nucleopolyhedrovirus NC 0... (23486) AACGGCTATTATGTGGAAAAAAAATTCTCCAAGGAGTTTTTAATTCATATTGCGCCTGATTTGAAAAACAGCGTCGA
                            SARS-CoV-2 NC_045512.2      (1) -----------------------------------------------------------------------------
                                                                                                                            Section 307
                                                (23563) 23563     23573      23584      23595      23606      23617      23628      23639
Autographa californica nucleopolyhedrovirus NC 0... (23563) CTGGAACGGCAGCACGCGCAAACAGCTGCGCGTTCTAGACAAGCGCGCCTACAGGCAGGTGTTGCACTGCAACGGCA
                            SARS-CoV-2 NC_045512.2      (1) -----------------------------------------------------------------------------
                                                                                                                            Section 308
                                                (23640) 23640     23650      23661      23672      23683      23694      23705      23716
Autographa californica nucleopolyhedrovirus NC 0... (23640) GATACTACTGGCCCGATGGCACAAAGTTTGTCTCTCATCCGTACAACAAATCTATTCGCACGCACAGCGCAACAGTC
                            SARS-CoV-2 NC_045512.2      (1) -----------------------------------------------------------------------------
```

```
                                                                                                                Section 309
                                                    (23717) 23717      23727      23738      23749      23760      23771      23782     23793
Autographa californica nucleopolyhedrovirus NC_0... (23717) AAACGGACCGACAGCTCGCATCGATTAAAAAGCCACGTGGTCGACAAACGACCGCGCCGCTCTTTAGATTCTCCTCG
                             SARS-CoV-2 NC_045512.2     (1) -----------------------------------------------------------------------------
                                                                                                                Section 310
                                                    (23794) 23794      23804      23815      23826      23837      23848      23859     23870
Autographa californica nucleopolyhedrovirus NC_0... (23794) CTTGGACGGATATGTTTTGGCATCGTCGCCCATACCACACAGCGACTGGAATGAAGAACTAAAGCTGTACGCCCAGA
                             SARS-CoV-2 NC_045512.2     (1) -----------------------------------------------------------------------------
                                                                                                                Section 311
                                                    (23871) 23871      23881      23892      23903      23914      23925      23936     23947
Autographa californica nucleopolyhedrovirus NC_0... (23871) GCCACGGCTACGACGACTACGACGACAATTTAGAAGATGGCGAAATCGACGAACGTGACTCTTTAAAAAGTTTAAAT
                             SARS-CoV-2 NC_045512.2     (1) -----------------------------------------------------------------------------
                                                                                                                Section 312
                                                    (23948) 23948      23958      23969      23980      23991      24002      24013     24024
Autographa californica nucleopolyhedrovirus NC_0... (23948) AATCATCTAGACGACTTGAATGTATTAGAAAAACAATAAAACATGTATTAAAAATAATAATAATAAAACTATATTTT
                             SARS-CoV-2 NC_045512.2     (1) -----------------------------------------------------------------------------
                                                                                                                Section 313
                                                    (24025) 24025      24035      24046      24057      24068      24079      24090     24101
Autographa californica nucleopolyhedrovirus NC_0... (24025) GTAATATATAATGTATTTTATTTAAAAATTGTCTATTCCGTAGTTGAGAAAGTTTTGTCTTGACTTCATAACTCTCT
                             SARS-CoV-2 NC_045512.2     (1) -----------------------------------------------------------------------------
                                                                                                                Section 314
                                                    (24102) 24102      24112      24123      24134      24145      24156      24167     24178
Autographa californica nucleopolyhedrovirus NC_0... (24102) TCTCCATATTCTGCAGCTCGTTTACGTTTTTTGTGACGCTTTTAATTTTCTCAAAATGCTGGCTGTCAATAGTTATT
                             SARS-CoV-2 NC_045512.2     (1) -----------------------------------------------------------------------------
                                                                                                                Section 315
                                                    (24179) 24179      24189      24200      24211      24222      24233      24244     24255
Autographa californica nucleopolyhedrovirus NC_0... (24179) TTTTGCTTTTGTCTATTAATTTCTTCCAATTGAGATTTTAAATCTCGCTGAGATTGAGATGCGTTGTAATTCCTTGA
                             SARS-CoV-2 NC_045512.2     (1) -----------------------------------------------------------------------------
```

Section 316
```
                                                (24256) 24256     24266      24277      24288      24299      24310      24321      24332
Autographa californica nucleopolyhedrovirus NC 0... (24256) GAACATCTTGAGAAAACATACAGATGAGGTAAAACAGCATCTTTTATCCAAATTAGGAGTTAATTATTATTCATTTG
SARS-CoV-2 NC_045512.2 (1) -----------------------------------------------------------------------------
```

Section 317
```
                                                (24333) 24333     24343      24354      24365      24376      24387      24398      24409
Autographa californica nucleopolyhedrovirus NC 0... (24333) TATCGCGACCATTTGCTCGTACACATCTTCCATAAAATGGTTATTTTTATTGCGATAAGTGTTGGCATTGACATTTT
SARS-CoV-2 NC_045512.2 (1) -----------------------------------------------------------------------------
```

Section 318
```
                                                (24410) 24410     24420      24431      24442      24453      24464      24475      24486
Autographa californica nucleopolyhedrovirus NC 0... (24410) GCAAATGTCGTAGGTTAAAGGGGCAAATGGGCTGCGTGGCCGATAAAAGATTCCAGTTCAACAATCCCTCTTCGCCC
SARS-CoV-2 NC_045512.2 (1) -----------------------------------------------------------------------------
```

Section 319
```
                                                (24487) 24487     24497      24508      24519      24530      24541      24552      24563
Autographa californica nucleopolyhedrovirus NC 0... (24487) CCGTTTAACTTGAAAATGGCGCTACACGTTTCTACGCTATCGTGTTCCTGTTGAGTGGCGCACGGTTCGACCAGTAT
SARS-CoV-2 NC_045512.2 (1) -----------------------------------------------------------------------------
```

Section 320
```
                                                (24564) 24564     24574      24585      24596      24607      24618      24629      24640
Autographa californica nucleopolyhedrovirus NC 0... (24564) CATCTTGTGATATGCGGTTTTGACATTCATGTGCAACGGAATAACTTGCGGGTCATCGCATTCGTCGGAATTAAGCT
SARS-CoV-2 NC_045512.2 (1) -----------------------------------------------------------------------------
```

Section 321
```
                                                (24641) 24641     24651      24662      24673      24684      24695      24706      24717
Autographa californica nucleopolyhedrovirus NC 0... (24641) TTAAATGGCGTCCGTATGCTTTCCAAAGTTTTTCGTCGTCGAACCGCGGCACTGCTTGCAAGTCGACGCGGGGAAAC
SARS-CoV-2 NC_045512.2 (1) -----------------------------------------------------------------------------
```

Section 322
```
                                                (24718) 24718     24728      24739      24750      24761      24772      24783      24794
Autographa californica nucleopolyhedrovirus NC 0... (24718) GGCGCTCTGTACAAAACGCCTAAATTCAAAAACTGATTGCATTGTTGCAGCTCTGTCCAATCGACGCGATTTTTGTA
SARS-CoV-2 NC_045512.2 (1) -----------------------------------------------------------------------------
```

```
                                                                                                       Section 323
                                                 (24795) 24795     24805      24816      24827      24838      24849      24860     24871
Autographa californica nucleopolyhedrovirus NC 0... (24795) ATTTTGAAACAGCATCAGGTTGAACGCCGCGCTGGCGCGCACGTTTGTAATCACTGTGTAATTGATCAGCTTGTGCC
                          SARS-CoV-2 NC_045512.2 (1) -----------------------------------------------------------------------------
                                                                                                       Section 324
                                                 (24872) 24872     24882      24893      24904      24915      24926      24937     24948
Autographa californica nucleopolyhedrovirus NC 0... (24872) AATACTGGGCATTGAAATTTTCTTCAAACTCATTTCTAAACTCTGGATGCGCAAACATGTGTCTAATGTAGTACGCG
                          SARS-CoV-2 NC_045512.2 (1) -----------------------------------------------------------------------------
                                                                                                       Section 325
                                                 (24949) 24949     24959      24970      24981      24992      25003      25014     25025
Autographa californica nucleopolyhedrovirus NC 0... (24949) GGCGGGGCGTTGAACGCAGTCCATTTGTCAATACACTTCCAGTCTGAATGTAACGTGTTCACCAAACCGGGATATTC
                          SARS-CoV-2 NC_045512.2 (1) -----------------------------------------------------------------------------
                                                                                                       Section 326
                                                 (25026) 25026     25036      25047      25058      25069      25080      25091     25102
Autographa californica nucleopolyhedrovirus NC 0... (25026) GTCAAACACGAGCATGTGATCCGACCACGGTATGCTGTGGGCGATCAATTTTAGTTCTTGCACGCGGCCTTCGCGTA
                          SARS-CoV-2 NC_045512.2 (1) -----------------------------------------------------------------------------
                                                                                                       Section 327
                                                 (25103) 25103     25113      25124      25135      25146      25157      25168     25179
Autographa californica nucleopolyhedrovirus NC 0... (25103) AGCAATACAAAATGAGCGCGTCGCTGATCTTGACACAGTCTTGCATGTACGCGGACAAATTAACGTTTTCCATACAG
                          SARS-CoV-2 NC_045512.2 (1) -----------------------------------------------------------------------------
                                                                                                       Section 328
                                                 (25180) 25180     25190      25201      25212      25223      25234      25245     25256
Autographa californica nucleopolyhedrovirus NC 0... (25180) CTCACATTGTTTATTAGCGCCGTGTTCAAGTGTTTGTATTTGGACACATAATCGTAGTTGATGTACTGTTTAATGGG
                          SARS-CoV-2 NC_045512.2 (1) -----------------------------------------------------------------------------
                                                                                                       Section 329
                                                 (25257) 25257     25267      25278      25289      25300      25311      25322     25333
Autographa californica nucleopolyhedrovirus NC 0... (25257) TTCTTGAAACCATTCTTTTAGTAGTATGTGACTGGCCACTATGCGTTTCCAATTTAATTTGTGTGCGTATTTTTGCT
                          SARS-CoV-2 NC_045512.2 (1) -----------------------------------------------------------------------------
```

```
                                                                                                       Section 330
                                          (25334) 25334     25344      25355      25366      25377      25388      25399   25410
Autographa californica nucleopolyhedrovirus NC 0... (25334) GCACCGACAACGAGAGGTTATTGTAATTTTTGGATATTTCTTCCATGTCCAACAAGTCCCCAAACGCGAGTATAAAA
                          SARS-CoV-2 NC_045512.2      (1) -----------------------------------------------------------------------------
                                                                                                       Section 331
                                          (25411) 25411     25421      25432      25443      25454      25465      25476   25487
Autographa californica nucleopolyhedrovirus NC 0... (25411) TCTTGCGTCAAAAATTTTTGCTCAGACACCAACGACCAGATCAAATGTGATTTAAACCTGTTGGCGATTGTTATCGA
                          SARS-CoV-2 NC_045512.2      (1) -----------------------------------------------------------------------------
                                                                                                       Section 332
                                          (25488) 25488     25498      25509      25520      25531      25542      25553   25564
Autographa californica nucleopolyhedrovirus NC 0... (25488) CAACGGCGAAATTGAAATAATTTTCCAATCCAACTTGTTGCGAAACACGTGAATAAAATCGACGCGTCCGTAACATT
                          SARS-CoV-2 NC_045512.2      (1) -----------------------------------------------------------------------------
                                                                                                       Section 333
                                          (25565) 25565     25575      25586      25597      25608      25619      25630   25641
Autographa californica nucleopolyhedrovirus NC 0... (25565) CGCGCGATATGCGCTTCCAAAACGTGTCATCTTGCAAATTAAGCAAATAGACACGATTGTTGGGAGATTTGACGGCC
                          SARS-CoV-2 NC_045512.2      (1) -----------------------------------------------------------------------------
                                                                                                       Section 334
                                          (25642) 25642     25652      25663      25674      25685      25696      25707   25718
Autographa californica nucleopolyhedrovirus NC 0... (25642) AATTCAATTATTTTTATATATTCTTTTTGCTTTAAAGCGCGTTGTAGCACTTGGGTTGGAGCCATGTCGACTGAAGC
                          SARS-CoV-2 NC_045512.2      (1) -----------------------------------------------------------------------------
                                                                                                       Section 335
                                          (25719) 25719     25729      25740      25751      25762      25773      25784   25795
Autographa californica nucleopolyhedrovirus NC 0... (25719) TCCACGCTGTTTGAAGCAAGGTGACCGTTTTGGTCGGCATGTTCAAACGTCGATTACATGTTTGCTTTGCATCAAAA
                          SARS-CoV-2 NC_045512.2      (1) -----------------------------------------------------------------------------
                                                                                                       Section 336
                                          (25796) 25796     25806      25817      25828      25839      25850      25861   25872
Autographa californica nucleopolyhedrovirus NC 0... (25796) TGGCGTAATTAATTAAGAAACAACATGAAAGCCATCTGCATCATTAGCGGCGATGTTCATGGAAAAATTTATTTTCA
                          SARS-CoV-2 NC_045512.2      (1) -----------------------------------------------------------------------------
```

```
                                                                                     Section 337
                                             (25873) 25873     25883      25894      25905      25916      25927      25938      25949
Autographa californica nucleopolyhedrovirus NC 0... (25873) ACAAGAATCAGCGAATCAACCGCTTAAAATTAGCGGCTATTTGTTAAATTTGCCTCGAGGTTTGCACGGCTTTCACG
                         SARS-CoV-2 NC_045512.2 (1) -----------------------------------------------------------------------------
                                                                                     Section 338
                                             (25950) 25950     25960      25971      25982      25993      26004      26015      26026
Autographa californica nucleopolyhedrovirus NC 0... (25950) TGCACGAATATGGCGACACGAGCAACGGTTGCACGTCGGCCGGTGAGCACTTTAATCCCACCAATGAGGACCACGGC
                         SARS-CoV-2 NC_045512.2 (1) -----------------------------------------------------------------------------
                                                                                     Section 339
                                             (26027) 26027     26037      26048      26059      26070      26081      26092      26103
Autographa californica nucleopolyhedrovirus NC 0... (26027) GCTCCCGATGCTGAAATTAGGCATGTTGGCGACTTGGGCAACATAAAATCGGCTGGCTACAATTCACTGACCGAAGT
                         SARS-CoV-2 NC_045512.2 (1) -----------------------------------------------------------------------------
                                                                                     Section 340
                                             (26104) 26104     26114      26125      26136      26147      26158      26169      26180
Autographa californica nucleopolyhedrovirus NC 0... (26104) AAACATGATGGACAACGTTATGTCTCTATATGGCCCGCATAATATTATCGGAAGAAGTTTGGTCGTGCACACGGACA
                         SARS-CoV-2 NC_045512.2 (1) -----------------------------------------------------------------------------
                                                                                     Section 341
                                             (26181) 26181     26191      26202      26213      26224      26235      26246      26257
Autographa californica nucleopolyhedrovirus NC 0... (26181) AAGACGATTTGGGCCTTACCGATCATCCGTTGAGCAAAACAACCGGCAATTCTGGCGGCCGTTTGGGATGCGGAATA
                         SARS-CoV-2 NC_045512.2 (1) -----------------------------------------------------------------------------
                                                                                     Section 342
                                             (26258) 26258     26268      26279      26290      26301      26312      26323      26334
Autographa californica nucleopolyhedrovirus NC 0... (26258) ATTGCCATATGTAAATGATGTCATCGTTCTAACTCGCTTTACGAGTAGAATTCTACGTGTAAAACATAATCAAGAGA
                         SARS-CoV-2 NC_045512.2 (1) -----------------------------------------------------------------------------
                                                                                     Section 343
                                             (26335) 26335     26345      26356      26367      26378      26389      26400      26411
Autographa californica nucleopolyhedrovirus NC 0... (26335) TGATGTCATTTGTTTTTCAAAACTGAACTCAAGAAATGATGTCATTTGTTTTTCAAAACTGAACTGGCTTTACGAGT
                         SARS-CoV-2 NC_045512.2 (1) -----------------------------------------------------------------------------
```

Section 344

```
                                                  26412      26422      26433      26444      26455      26466      26477      26488
Autographa californica nucleopolyhedrovirus NC 0... (26412) AGAATTCTACTTGTAACGCATGATCAAGGGATGATGTCATTTGTTTTTCAAAACCGAACTCGCTTTACGAGTAGAAT
                            SARS-CoV-2 NC_045512.2      (1) -----------------------------------------------------------------------------
```

Section 345

```
                                                  26489      26499      26510      26521      26532      26543      26554      26565
Autographa californica nucleopolyhedrovirus NC 0... (26489) TCTACTTGTAAAACATAATCGAAAGATGATGTCATTTGTTTTTTAAAATTGAACTGGCTTTACGAGTAGAATTCTAC
                            SARS-CoV-2 NC_045512.2      (1) -----------------------------------------------------------------------------
```

Section 346

```
                                                  26566      26576      26587      26598      26609      26620      26631      26642
Autographa californica nucleopolyhedrovirus NC 0... (26566) TTGTAAAACACAATCGAGAGATGATGTCATATTTTGCACACGGCTCTAATTAAACTCGCTTTACGAGTAAAATTCTA
                            SARS-CoV-2 NC_045512.2      (1) -----------------------------------------------------------------------------
```

Section 347

```
                                                  26643      26653      26664      26675      26686      26697      26708      26719
Autographa californica nucleopolyhedrovirus NC 0... (26643) CTTGTAACGCATGATCAAGGGATGATGTATTGGATGAGTCATTTGTTTTTCAAAACTAAACTCGCTTTACGAGTAGA
                            SARS-CoV-2 NC_045512.2      (1) -----------------------------------------------------------------------------
```

Section 348

```
                                                  26720      26730      26741      26752      26763      26774      26785      26796
Autographa californica nucleopolyhedrovirus NC 0... (26720) ATTCTACTTGTAACGCACGCCCAAGGGATGATGTCATTTATTTGTGCAAAGCTGATGTCATCTTTTGCACACGATTA
                            SARS-CoV-2 NC_045512.2      (1) -----------------------------------------------------------------------------
```

Section 349

```
                                                  26797      26807      26818      26829      26840      26851      26862      26873
Autographa californica nucleopolyhedrovirus NC 0... (26797) TAAACACAATCAAATAATGACTCATTTGTTTTTCAAAACTGAACTCGCTTTACGAGTAGAATTCTACTTGTAAAACA
                            SARS-CoV-2 NC_045512.2      (1) -----------------------------------------------------------------------------
```

Section 350

```
                                                  26874      26884      26895      26906      26917      26928      26939      26950
Autographa californica nucleopolyhedrovirus NC 0... (26874) CAATCAAGCGATGATGTCATTTTAAAAATGATGTCATTTGTTTTTCAAAACTAAACTCGCTTTACGAGTAGAATTCT
                            SARS-CoV-2 NC_045512.2      (1) -----------------------------------------------------------------------------
```

Section 351

(26951) 26951 26961 26972 26983 26994 27005 27016 27027

Autographa californica nucleopolyhedrovirus NC_0... (26951) ACGTGTAAAACACAATCAAGGGATGATGTCATTTACTAAAATAAAATAATTATTTAAATAAAAATGTTTTTATTGTA

SARS-CoV-2 NC_045512.2 (1) ---

Section 352

(27028) 27028 27038 27049 27060 27071 27082 27093 27104

Autographa californica nucleopolyhedrovirus NC_0... (27028) AAATACACATTGATTACACGTGACATTTACGATGGCGAACAATAATTTCACTTTTTATATTAGGACACGACGTGTAT

SARS-CoV-2 NC_045512.2 (1) ---

Section 353

(27105) 27105 27115 27126 27137 27148 27159 27170 27181

Autographa californica nucleopolyhedrovirus NC_0... (27105) ATAGGAAAGCTTAAGCGTTTCAATAAAGCCATGGCGTACACGCTAAGCTTGCCCAGCTTGCGGCTCTTTGAAATCTG

SARS-CoV-2 NC_045512.2 (1) ---

Section 354

(27182) 27182 27192 27203 27214 27225 27236 27247 27258

Autographa californica nucleopolyhedrovirus NC_0... (27182) TAGTTTTCGGGGAGTACCGTCGTTCTTCAGTGCCACATACGTCAACTTGCGATCGTACACTTTATAATACGTGTTGT

SARS-CoV-2 NC_045512.2 (1) ---

Section 355

(27259) 27259 27269 27280 27291 27302 27313 27324 27335

Autographa californica nucleopolyhedrovirus NC_0... (27259) AGTTATTTTTTTCCAGAAATTCCCTCATAAAGCAATCCTTGGATAAAGTTTTTGATCCGTACAGTTGGCCACACCGG

SARS-CoV-2 NC_045512.2 (1) ---

Section 356

(27336) 27336 27346 27357 27368 27379 27390 27401 27412

Autographa californica nucleopolyhedrovirus NC_0... (27336) TCCATGCACAGGTACACACACGTGATGGCGTTTTGAATGACGATGCGATTTCTGTCAACGGCAACGCGCTTGAATAT

SARS-CoV-2 NC_045512.2 (1) ---

Section 357

(27413) 27413 27423 27434 27445 27456 27467 27478 27489

Autographa californica nucleopolyhedrovirus NC_0... (27413) GGTGTCGACGTTGTCCGATTCAATGGTTCCGTAAACAGCTCCGTCTGGATTTACTGCCAAAAACTGCCGGTTAATAA

SARS-CoV-2 NC_045512.2 (1) ---

```
                                                                                                                  Section 358
                                                   (27490) 27490     27500      27511      27522      27533      27544      27555      27566
Autographa californica nucleopolyhedrovirus NC_0... (27490) ACAGCTGGCCGGGAATAGACGTGCCCGTGATGTGTGTCAGCAGAGCTGAGCAGTCAGCCATAGAGGCTAGAGCTACA
                            SARS-CoV-2 NC_045512.2     (1) -----------------------------------------------------------------------------
                                                                                                                  Section 359
                                                   (27567) 27567     27577      27588      27599      27610      27621      27632      27643
Autographa californica nucleopolyhedrovirus NC_0... (27567) AGTGCCAGCAAGCGATACATGATGAACTTTAAGTCCCCACAGCAAACTGGCGCTTTTATATAAAAATTTGGGCCATT
                            SARS-CoV-2 NC_045512.2     (1) -----------------------------------------------------------------------------
                                                                                                                  Section 360
                                                   (27644) 27644     27654      27665      27676      27687      27698      27709      27720
Autographa californica nucleopolyhedrovirus NC_0... (27644) TTTGGCGATTAGATAATTTTTGAAGATTAGATAATATTGAGATTAGTTAATAATTTGTGTGATTAGATAACTTTTTA
                            SARS-CoV-2 NC_045512.2     (1) -----------------------------------------------------------------------------
                                                                                                                  Section 361
                                                   (27721) 27721     27731      27742      27753      27764      27775      27786      27797
Autographa californica nucleopolyhedrovirus NC_0... (27721) GGGTATTGCGCATTATAAATCAAGGTCGAGTTGTATAAACTGCTCTGGCGTGTAAAACTGCAGACTTAAGTTTTTTG
                            SARS-CoV-2 NC_045512.2     (1) -----------------------------------------------------------------------------
                                                                                                                  Section 362
                                                   (27798) 27798     27808      27819      27830      27841      27852      27863      27874
Autographa californica nucleopolyhedrovirus NC_0... (27798) CAAACACTCGGTCTGAATCGCTAAAATCTTTCTGACCGGTGGTTAGATTAATTCGGCCAGCCGCGTCGCCCACATAA
                            SARS-CoV-2 NC_045512.2     (1) -----------------------------------------------------------------------------
                                                                                                                  Section 363
                                                   (27875) 27875     27885      27896      27907      27918      27929      27940      27951
Autographa californica nucleopolyhedrovirus NC_0... (27875) AAAGATTGTTCCTTGTCAATATGCGTAAACTGTTTGGCCATCTCGCGCCACATTCCCGTGTCGGGCTTTCGATGCTC
                            SARS-CoV-2 NC_045512.2     (1) -----------------------------------------------------------------------------
                                                                                                                  Section 364
                                                   (27952) 27952     27962      27973      27984      27995      28006      28017      28028
Autographa californica nucleopolyhedrovirus NC_0... (27952) ATCCTTGTTGGGCGACACATAAAACGATATGGGCACGCCAGTAGCTTTTTTAATATTCTCTAATTTATATAATAAAT
                            SARS-CoV-2 NC_045512.2     (1) -----------------------------------------------------------------------------
```

```
                                                                                                  Section 365
                                               (28029) 28029     28039      28050      28061      28072      28083      28094  28105
Autographa californica nucleopolyhedrovirus NC 0... (28029) CGCTCGCTTTGATTTTGCCGGAACCTAAATGGGCTTGGTTCGTAAAAACAACTAAATCGTAGCCTAATTCGTACAAA
                             SARS-CoV-2 NC_045512.2     (1) -----------------------------------------------------------------------------
                                                                                                  Section 366
                                               (28106) 28106     28116      28127      28138      28149      28160      28171  28182
Autographa californica nucleopolyhedrovirus NC 0... (28106) CGCTTTAGCTTGTGTGCGCACGGAAGGAGCTGCCAGTCGTCTGGGTTTTTTGGAAATTTGGACCGTGTCTTTGAGCT
                             SARS-CoV-2 NC_045512.2     (1) -----------------------------------------------------------------------------
                                                                                                  Section 367
                                               (28183) 28183     28193      28204      28215      28226      28237      28248  28259
Autographa californica nucleopolyhedrovirus NC 0... (28183) AATTAGCGTGCCGTCCAAATCAAAAGCCGCAATTTTGGTTCTTTTAGCGCCGTCATGAACCGCGTACGCATACAAAT
                             SARS-CoV-2 NC_045512.2     (1) -----------------------------------------------------------------------------
                                                                                                  Section 368
                                               (28260) 28260     28270      28281      28292      28303      28314      28325  28336
Autographa californica nucleopolyhedrovirus NC 0... (28260) CGGGCTGCTGTAACGTCCACATGGTGAATGCATCTTACTCAAAGTCCATCAATTCGTACGCGTTTGTGTCCAGGTCG
                             SARS-CoV-2 NC_045512.2     (1) -----------------------------------------------------------------------------
                                                                                                  Section 369
                                               (28337) 28337     28347      28358      28369      28380      28391      28402  28413
Autographa californica nucleopolyhedrovirus NC 0... (28337) GGCGTTGAAAAATTGTAGCTTGCCATTAGATCGGATAGCGATTCAAATTTTGTAAGCGTTTGTAGCGCACGTTTGGC
                             SARS-CoV-2 NC_045512.2     (1) -----------------------------------------------------------------------------
                                                                                                  Section 370
                                               (28414) 28414     28424      28435      28446      28457      28468      28479  28490
Autographa californica nucleopolyhedrovirus NC 0... (28414) ATCTTGTTTAAAATTACACGACGACAGACAGTAAAAATATTCCTCGATAAGCATGACTACACCCATATCACTGTTTA
                             SARS-CoV-2 NC_045512.2     (1) -----------------------------------------------------------------------------
                                                                                                  Section 371
                                               (28491) 28491     28501      28512      28523      28534      28545      28556  28567
Autographa californica nucleopolyhedrovirus NC 0... (28491) AGTGCTCGACGTAGTTGTTGCATGTTATGTCGCGTGTGCCGCGATACGCGTGATTTCGGTGAAAATCACACCACAAC
                             SARS-CoV-2 NC_045512.2     (1) -----------------------------------------------------------------------------
```

Section 372

(28568) 28568 28578 28589 28600 28611 28622 28633 28644
Autographa californica nucleopolyhedrovirus NC_0... (28568) CAGTCGGCGTGCGTGTAACAAAGTCGACAGCGAAACAATTTATCGTTTTCCAAAAAATTTAAATACTCGACAGTTTT
SARS-CoV-2 NC_045512.2 (1) ---

Section 373

(28645) 28645 28655 28666 28677 28688 28699 28710 28721
Autographa californica nucleopolyhedrovirus NC_0... (28645) GCAGCTTAGATTCCGCGTTTGATTCACCTTAAAATCGTCGTCAGCCTCTATAATCTCGGGCAACAGCTTGCCTTGTT
SARS-CoV-2 NC_045512.2 (1) ---

Section 374

(28722) 28722 28732 28743 28754 28765 28776 28787 28798
Autographa californica nucleopolyhedrovirus NC_0... (28722) GCCCCATCGTATCGATCACCTCCCCCAAGTGGCCCGGTGTTATATTAAGTCGTTTAAAATCATTTATTGCTTCCTGC
SARS-CoV-2 NC_045512.2 (1) ---

Section 375

(28799) 28799 28809 28820 28831 28842 28853 28864 28875
Autographa californica nucleopolyhedrovirus NC_0... (28799) ACGTCGGCCTGGTAATTTTTGACCACGGGCGTGGAAATCAATTGCCGTTGAAGGGAAATAATTCGTGGTGTGGGTAT
SARS-CoV-2 NC_045512.2 (1) ---

Section 376

(28876) 28876 28886 28897 28908 28919 28930 28941 28952
Autographa californica nucleopolyhedrovirus NC_0... (28876) CGGCCGCCTGTTGCACAATTCCACCAGCGGTGGAGGCAAGGGCGCATTCACAGCAACCGTTGTCATTTATAAGTAAT
SARS-CoV-2 NC_045512.2 (1) ---

Section 377

(28953) 28953 28963 28974 28985 28996 29007 29018 29029
Autographa californica nucleopolyhedrovirus NC_0... (28953) AGTGTAAAAATGCAAATATTCATCAAAACATTGACGGGCAAAACCATTACCGCCGAAACGGAACCCGCAGAGACGGT
SARS-CoV-2 NC_045512.2 (1) ---

Section 378

(29030) 29030 29040 29051 29062 29073 29084 29095 29106
Autographa californica nucleopolyhedrovirus NC_0... (29030) GGCCGATCTTAAGCAAAAAATTGCCGATAAAGAAGGTGTGCCCGTAGATCAACAAAGACTTATCTTTGCGGGCAAAC
SARS-CoV-2 NC_045512.2 (1) ---

```
Section 379
                                                   (29107) 29107     29117     29128     29139     29150     29161     29172     29183
Autographa californica nucleopolyhedrovirus  NC_0... (29107) AACTGGAAGATTCCAAAACTATGGCCGATTACAATATTCAGAAGGAATCTACTCTTCACATGGTGTTACGATTACGA
                              SARS-CoV-2 NC_045512.2     (1) -----------------------------------------------------------------------------

Section 380
                                                   (29184) 29184     29194     29205     29216     29227     29238     29249     29260
Autographa californica nucleopolyhedrovirus  NC_0... (29184) GGAGGGTATTAATAATAACAATAATAAAAACCATTAAATATACATAAAAGTTTTTTATTTAATCTGACATATTTGTA
                              SARS-CoV-2 NC_045512.2     (1) -----------------------------------------------------------------------------

Section 381
                                                   (29261) 29261     29271     29282     29293     29304     29315     29326     29337
Autographa californica nucleopolyhedrovirus  NC_0... (29261) TCTTGTGTATTATCGCTAACCATTAAAAGTGCTGGAGCCACAGTGTTGCGGCGAGTCTTTATAGAAGATCGTTGTTT
                              SARS-CoV-2 NC_045512.2     (1) -----------------------------------------------------------------------------

Section 382
                                                   (29338) 29338     29348     29359     29370     29381     29392     29403     29414
Autographa californica nucleopolyhedrovirus  NC_0... (29338) GGCTGGAACTGAGCTTTTCCTTTTCCTGCTGCCGCTAATGGGAGTGGGCACGTACTCTGTAGTAGACGGTGCAACGG
                              SARS-CoV-2 NC_045512.2     (1) -----------------------------------------------------------------------------

Section 383
                                                   (29415) 29415     29425     29436     29447     29458     29469     29480     29491
Autographa californica nucleopolyhedrovirus  NC_0... (29415) GCAACTTGAGCGCTACCGTCTTAAATTTGGCCATACTTTTAGTGATGAAATCGCGCGTTAACACTTCGTCGTAAATG
                              SARS-CoV-2 NC_045512.2     (1) -----------------------------------------------------------------------------

Section 384
                                                   (29492) 29492     29502     29513     29524     29535     29546     29557     29568
Autographa californica nucleopolyhedrovirus  NC_0... (29492) TTACTTAGCAGAGGCGCAACATTGTGATTAAATGTCTCGTTTAACAAGCTGTAAAACTCCGAATAAAGCTTATCGCG
                              SARS-CoV-2 NC_045512.2     (1) -----------------------------------------------------------------------------

Section 385
                                                   (29569) 29569     29579     29590     29601     29612     29623     29634     29645
Autographa californica nucleopolyhedrovirus  NC_0... (29569) CATTTCGCAGCTCTCCTTCAATTCTGCCAAATTTGCGTTGGTAAGCACCACAGTCTGTCTTTTTTTGCTCGCTGGAA
                              SARS-CoV-2 NC_045512.2     (1) -----------------------------------------------------------------------------
```

```
                                                                                                                       Section 386
                                                  (29646) 29646      29656      29667      29678      29689      29700      29711      29722
Autographa californica nucleopolyhedrovirus NC 0... (29646) TTGCTGCGTTCTCGCTTGAAGACGACGATGTCGATCGGTCGGCCATTTTTTTGCCCAGCTTTTCAGTGTGATCAAAA
                            SARS-CoV-2 NC_045512.2      (1) -----------------------------------------------------------------------------
                                                                                                                       Section 387
                                                  (29723) 29723      29733      29744      29755      29766      29777      29788      29799
Autographa californica nucleopolyhedrovirus NC 0... (29723) ATGAACACAAAATCTGCCAATTCGGGCTTGTTTTTCACCAAATCCCACATGGCCGGGCTACTAGGCCACTCGGGCTG
                            SARS-CoV-2 NC_045512.2      (1) -----------------------------------------------------------------------------
                                                                                                                       Section 388
                                                  (29800) 29800      29810      29821      29832      29843      29854      29865      29876
Autographa californica nucleopolyhedrovirus NC 0... (29800) CTTGATCTTAGTGTACCAACTGTTAAACAAAATGTATTTATTGTTGTTAATCACTTTCTTCTTGCGTTTGGACATTT
                            SARS-CoV-2 NC_045512.2      (1) -----------------------------------------------------------------------------
                                                                                                                       Section 389
                                                  (29877) 29877      29887      29898      29909      29920      29931      29942      29953
Autographa californica nucleopolyhedrovirus NC 0... (29877) TGCGTTCGTCTTGCATGACAGGCACCACGTTAAGGATATAGTTAATGTTCTTTCTTTCCAAGAAATTTACAATAACG
                            SARS-CoV-2 NC_045512.2      (1) -----------------------------------------------------------------------------
                                                                                                                       Section 390
                                                  (29954) 29954      29964      29975      29986      29997      30008      30019      30030
Autographa californica nucleopolyhedrovirus NC 0... (29954) GCCAGCTGGTCCATGTTGGATTTGTTGTAAGAGCTCGATTCCAGTTTATTCAACAGCTTTTCATTTTTGCACACGGC
                            SARS-CoV-2 NC_045512.2      (1) -----------------------------------------------------------------------------
                                                                                                                       Section 391
                                                  (30031) 30031      30041      30052      30063      30074      30085      30096      30107
Autographa californica nucleopolyhedrovirus NC 0... (30031) CGCAGTCTCCGGAGATTGTTGCTCCGGCACGTTTACCATGTTTGCTTCTTGTAAACCTTTGAAACAACCCGTTTGTA
                            SARS-CoV-2 NC_045512.2      (1) -----------------------------------------------------------------------------
                                                                                                                       Section 392
                                                  (30108) 30108      30118      30129      30140      30151      30162      30173      30184
Autographa californica nucleopolyhedrovirus NC 0... (30108) TTCTTGATGATATATTTTTTTAATGCCCAACAACCTGGCAATTCGTTTGTGATGAAGACACACCTTACGCTTCGAAC
                            SARS-CoV-2 NC_045512.2      (1) -----------------------------------------------------------------------------
```

Section 393

(30185) 30185 30195 30206 30217 30228 30239 30250 30261

Autographa californica nucleopolyhedrovirus NC 0... (30185) ATTTGTCGGTGATTACTGTGAAATGGCCTAAATTAGCTCTTATATATTCTTTTATACGCTCAAACGACACGATGTCC

SARS-CoV-2 NC_045512.2 (1) ---

Section 394

(30262) 30262 30272 30283 30294 30305 30316 30327 30338

Autographa californica nucleopolyhedrovirus NC 0... (30262) AACATGTGCGCGCAGACGTTTTCTGTGTTCATCGTGTGCTTGAGCGTGTTGATGGCTTCCCTGAACAGCGCTTGTAT

SARS-CoV-2 NC_045512.2 (1) ---

Section 395

(30339) 30339 30349 30360 30371 30382 30393 30404 30415

Autographa californica nucleopolyhedrovirus NC 0... (30339) TTCGCTGCGAGTCAAGCAGTCCGAATCACACCCGCCTAAGTGCGTGCAATTTTTGGGGGGCATCGTTGTCTATCTTT

SARS-CoV-2 NC_045512.2 (1) ---

Section 396

(30416) 30416 30426 30437 30448 30459 30470 30481 30492

Autographa californica nucleopolyhedrovirus NC 0... (30416) TTCAGAGTGGCGTAGAAAAAGTCCTGCAATTGCCTATTATCAAAACGCGCCTTGACGCTGCGCACAAAATCAAAAAA

SARS-CoV-2 NC_045512.2 (1) ---

Section 397

(30493) 30493 30503 30514 30525 30536 30547 30558 30569

Autographa californica nucleopolyhedrovirus NC 0... (30493) TTCAATGTAATTGCTGTAATCGTACGTGATCAGTTGTTTGTCGTTCATATAATTAAAGTATTTGTTGAGCGGCACGA

SARS-CoV-2 NC_045512.2 (1) ---

Section 398

(30570) 30570 30580 30591 30602 30613 30624 30635 30646

Autographa californica nucleopolyhedrovirus NC 0... (30570) TGGCCAGGCTGCGCGCTATTTCGCAATTGAAGCGTCGCGGTTTTAACATTATACGGTAGTCATTGCCAAACGTGCCC

SARS-CoV-2 NC_045512.2 (1) ---

Section 399

(30647) 30647 30657 30668 30679 30690 30701 30712 30723

Autographa californica nucleopolyhedrovirus NC 0... (30647) GGCAACAACTTCACGGTGTACGTGTTGGGTTTGGCGTTCACGTTAATCAAGTTGCCGCGCACGACGCCTACGTATAT

SARS-CoV-2 NC_045512.2 (1) ---

```
                                                                                                                Section 400
                                              (30724) 30724     30734      30745      30756      30767      30778      30789   30800
Autographa californica nucleopolyhedrovirus NC 0... (30724) CAAATACTTGTAGGTGACGCCGTCATCTTTCCATTGTAACGTAAATGGCAACTTGTAGATGAACGCGCTGTCAAAAA
                          SARS-CoV-2 NC_045512.2     (1) -----------------------------------------------------------------------------
                                                                                                                Section 401
                                              (30801) 30801     30811      30822      30833      30844      30855      30866   30877
Autographa californica nucleopolyhedrovirus NC 0... (30801) ACCGGCCAGTTTCTTCCACAAACTCGCGCACGGCTGTCTCGTAAACTTTTGCGTCGCAACAATCGCGATGACCTCGT
                          SARS-CoV-2 NC_045512.2     (1) -----------------------------------------------------------------------------
                                                                                                                Section 402
                                              (30878) 30878     30888      30899      30910      30921      30932      30943   30954
Autographa californica nucleopolyhedrovirus NC 0... (30878) GGTATGGAAATTTTTTCTAAAAAAGTGTCGTTCATGTCGGCGGCGGGCGCGTTCGCGCTCCGGTACGCGCGACGGGC
                          SARS-CoV-2 NC_045512.2     (1) -----------------------------------------------------------------------------
                                                                                                                Section 403
                                              (30955) 30955     30965      30976      30987      30998      31009      31020   31031
Autographa californica nucleopolyhedrovirus NC 0... (30955) ACACAGCAGGACAGCCTTGTCCGGCTCGATTATCATAAACAATCCTGCAGCGTTTCGCATTTTACATATTTGACACT
                          SARS-CoV-2 NC_045512.2     (1) -----------------------------------------------------------------------------
                                                                                                                Section 404
                                              (31032) 31032     31042      31053      31064      31075      31086      31097   31108
Autographa californica nucleopolyhedrovirus NC 0... (31032) TAAAAAATTGCGCACACGAGCACCATCGTTTGATACCTAATTGCAACTATTTACAATTTATCAGTTTACGTTGAACC
                          SARS-CoV-2 NC_045512.2     (1) -----------------------------------------------------------------------------
                                                                                                                Section 405
                                              (31109) 31109     31119      31130      31141      31152      31163      31174   31185
Autographa californica nucleopolyhedrovirus NC 0... (31109) CGTTTTAATTTTTTAGATCCGTCCTTGTTCAGTTGCAAGTTGACTAAATGACAAAATTTTTCGGTTCTGCAAAACCG
                          SARS-CoV-2 NC_045512.2     (1) -----------------------------------------------------------------------------
                                                                                                                Section 406
                                              (31186) 31186     31196      31207      31218      31229      31240      31251   31262
Autographa californica nucleopolyhedrovirus NC 0... (31186) CCCTTGTCTGTTCCACCCGTTGTATTTGAAAAAACTTTTTTTCACGCGGCGACAACTGCTTGTATAATATTGCCCAA
                          SARS-CoV-2 NC_045512.2     (1) -----------------------------------------------------------------------------
```

Section 407

```
                                                    31263     31273      31284      31295      31306      31317      31328   31339
                                          (31263)
Autographa californica nucleopolyhedrovirus NC 0... (31263) TGTAAACATGCAAAATTTTGTTACTCTCGTCAAAACAGCGGTTGGCGTTCCATTCCATAATTTTTTTATTATTTATC
                               SARS-CoV-2 NC_045512.2 (1) -----------------------------------------------------------------------------
```

Section 408

```
                                                    31340     31350      31361      31372      31383      31394      31405   31416
                                          (31340)
Autographa californica nucleopolyhedrovirus NC 0... (31340) AACGATGGCCATTGTAAATTGTCGTCATTTATACGCATCATATGATTTAACAAAAGCTTTTCGTATAGCGGAACTTC
                               SARS-CoV-2 NC_045512.2 (1) -----------------------------------------------------------------------------
```

Section 409

```
                                                    31417     31427      31438      31449      31460      31471      31482   31493
                                          (31417)
Autographa californica nucleopolyhedrovirus NC 0... (31417) AATTCCCTTGGAACATTTTTCAAACGATAATTTAATTTGTTTCTCGGTTGGCAGCATTTCATGCTTGATTAACAATC
                               SARS-CoV-2 NC_045512.2 (1) -----------------------------------------------------------------------------
```

Section 410

```
                                                    31494     31504      31515      31526      31537      31548      31559   31570
                                          (31494)
Autographa californica nucleopolyhedrovirus NC 0... (31494) GCCTGACTTTTATAGCCACGTTTATGTCTTTGCACAGCAAATGTGGGTTGTCGACAATGTAATAGTGCAAAGCATTT
                               SARS-CoV-2 NC_045512.2 (1) -----------------------------------------------------------------------------
```

Section 411

```
                                                    31571     31581      31592      31603      31614      31625      31636   31647
                                          (31571)
Autographa californica nucleopolyhedrovirus NC 0... (31571) GTTACGGCAAATGCGTAGTTTGATTTGACGACGCCCTTTTTCTTGACGGGCATTGCGGCTTTTAAAATTACTTGCAA
                               SARS-CoV-2 NC_045512.2 (1) -----------------------------------------------------------------------------
```

Section 412

```
                                                    31648     31658      31669      31680      31691      31702      31713   31724
                                          (31648)
Autographa californica nucleopolyhedrovirus NC 0... (31648) GCATTGTACGAATACCTCTTTGTGTTTAAACAATAATATGGACAAACATCGGCGAAACAATTTGTAATAATTATGAA
                               SARS-CoV-2 NC_045512.2 (1) -----------------------------------------------------------------------------
```

Section 413

```
                                                    31725     31735      31746      31757      31768      31779      31790   31801
                                          (31725)
Autographa californica nucleopolyhedrovirus NC 0... (31725) ATCCCAAATTGCAGGTTTTAAACTTCTTTGTTACTTGTTTTATAATAAATAAAATTTGCTGACCCATGTCTGCGCCC
                               SARS-CoV-2 NC_045512.2 (1) -----------------------------------------------------------------------------
```

```
                                                                                                       Section 414
                                                 (31802) 31802     31812      31823      31834      31845      31856      31867     31878
Autographa californica nucleopolyhedrovirus NC 0... (31802) ACAACTTTAATTAACCATTTGTGCGCATATTGATTGTCTCGTTGTTCCCAACCGGAAAATTGATTGATCTCGAGCCA
                          SARS-CoV-2 NC_045512.2        (1) -----------------------------------------------------------------------------
                                                                                                       Section 415
                                                 (31879) 31879     31889      31900      31911      31922      31933      31944     31955
Autographa californica nucleopolyhedrovirus NC 0... (31879) CCGGCATTGGTCGTTTGATACCGTCGTTAACGCCGACGCTCCTGCCTGTTTGATTACGGGTTCTAAAAGACGAAACA
                          SARS-CoV-2 NC_045512.2        (1) -----------------------------------------------------------------------------
                                                                                                       Section 416
                                                 (31956) 31956     31966      31977      31988      31999      32010      32021     32032
Autographa californica nucleopolyhedrovirus NC 0... (31956) GCAGCGTAAATTTGTTTTTGCGTCGGTAGTATTTTGGCAGGCAATAATCAAAAAAATCCGTAAGCAATTCTCTGCAT
                          SARS-CoV-2 NC_045512.2        (1) -----------------------------------------------------------------------------
                                                                                                       Section 417
                                                 (32033) 32033     32043      32054      32065      32076      32087      32098     32109
Autographa californica nucleopolyhedrovirus NC 0... (32033) CTATTAATATTCGTTGCGTACGAATCGAGTTTTTCAAAAATTACTTTGTTTGTATGAAAATAACGTTTGGGCTTCTC
                          SARS-CoV-2 NC_045512.2        (1) -----------------------------------------------------------------------------
                                                                                                       Section 418
                                                 (32110) 32110     32120      32131      32142      32153      32164      32175     32186
Autographa californica nucleopolyhedrovirus NC 0... (32110) ACAATAATAATCTTCGTTGTAGAACAGAAACGGTTTGCGAGAATTGGCACGTTTGTCCATGATTGGCTCAGTGTAAC
                          SARS-CoV-2 NC_045512.2        (1) -----------------------------------------------------------------------------
                                                                                                       Section 419
                                                 (32187) 32187     32197      32208      32219      32230      32241      32252     32263
Autographa californica nucleopolyhedrovirus NC 0... (32187) GATTGATTCAAATCAAAATTGACAACACGTTTGCCGTAATGTGCACCGGTTCGCACACGTTTGCCGCGTATGTAATC
                          SARS-CoV-2 NC_045512.2        (1) -----------------------------------------------------------------------------
                                                                                                       Section 420
                                                 (32264) 32264     32274      32285      32296      32307      32318      32329     32340
Autographa californica nucleopolyhedrovirus NC 0... (32264) CATGTTTATTTCGCTGTCGCAATTGATTACACGATTGTGTTGGGCGGCGCGTTTTATTGAATTTAGGCGACGCGTCG
                          SARS-CoV-2 NC_045512.2        (1) -----------------------------------------------------------------------------
```

Section 421

```
                                                   (32341) 32341     32351      32362      32373      32384      32395      32406   32417
Autographa californica nucleopolyhedrovirus NC 0... (32341) ACAACTCCAAAGGATTGTAAAGCGCAGATTTTTCCAGAGTAAACGAGTTTAAGTGGCCACCGTTGAACCATTCCAGA
                              SARS-CoV-2 NC_045512.2 (1) -----------------------------------------------------------------------------
```

Section 422

```
                                                   (32418) 32418     32428      32439      32450      32461      32472      32483   32494
Autographa californica nucleopolyhedrovirus NC 0... (32418) GCCACGATTGTGTACAGCAAAAAGAATATTTCTTTGTCGACGTTTTCAAACGCAAACTTGTTTTTTAGGCAATAGTA
                              SARS-CoV-2 NC_045512.2 (1) -----------------------------------------------------------------------------
```

Section 423

```
                                                   (32495) 32495     32505      32516      32527      32538      32549      32560   32571
Autographa californica nucleopolyhedrovirus NC 0... (32495) GTAAAATTTTAACGAATTGTATAAATAAAACATAAAATTGCCATTTTTAAAGTAAAATTCTACATCCGTGACGAACA
                              SARS-CoV-2 NC_045512.2 (1) -----------------------------------------------------------------------------
```

Section 424

```
                                                   (32572) 32572     32582      32593      32604      32615      32626      32637   32648
Autographa californica nucleopolyhedrovirus NC 0... (32572) AAAGGTTTACTATTTTGTTCTCCAACAAGTGTGCCAATTTTCTTAAGTACACCATTGAATTTTTGTCGTCGTCCATC
                              SARS-CoV-2 NC_045512.2 (1) -----------------------------------------------------------------------------
```

Section 425

```
                                                   (32649) 32649     32659      32670      32681      32692      32703      32714   32725
Autographa californica nucleopolyhedrovirus NC 0... (32649) TCGATCAACAACACGTACGGCGTTTTGGAATTTAAAATTATTCTAAAATTTTCCTGTTGCAACGATTCCACAGCGTC
                              SARS-CoV-2 NC_045512.2 (1) -----------------------------------------------------------------------------
```

Section 426

```
                                                   (32726) 32726     32736      32747      32758      32769      32780      32791   32802
Autographa californica nucleopolyhedrovirus NC 0... (32726) CGACCAATATGACGCTGCCACCTCTAGACAGATGTATTTCTTGGAAAACACGTGTCGTTTGATAACCTCGCTGATGG
                              SARS-CoV-2 NC_045512.2 (1) -----------------------------------------------------------------------------
```

Section 427

```
                                                   (32803) 32803     32813      32824      32835      32846      32857      32868   32879
Autographa californica nucleopolyhedrovirus NC 0... (32803) ACGTGATCGATTGTAAATACTTTTCAAACGTCGCGTCTTCCCAACCACGCACCGAAACGGGCGCTGTCGTGTCGGGC
                              SARS-CoV-2 NC_045512.2 (1) -----------------------------------------------------------------------------
```

```
                                                                                                                      Section 428
                                              (32880) 32880     32890      32901      32912      32923      32934      32945  32956
Autographa californica nucleopolyhedrovirus NC 0... (32880) TGATGTTTGAAATCCAAACCACTCTGAATTAACTTGGTTGTGATTCGTATGCTCAACTGTTGACCCAACGTGTAGTG
                            SARS-CoV-2 NC_045512.2     (1) -----------------------------------------------------------------------------
                                                                                                                      Section 429
                                              (32957) 32957     32967      32978      32989      33000      33011      33022  33033
Autographa californica nucleopolyhedrovirus NC 0... (32957) ATCTTCGTAGGCGCGCTCCCACATCACGTTACACACAAATTTGACGAGATCATCAACGTCTTTCTGTTGCAAAATTC
                            SARS-CoV-2 NC_045512.2     (1) -----------------------------------------------------------------------------
                                                                                                                      Section 430
                                              (33034) 33034     33044      33055      33066      33077      33088      33099  33110
Autographa californica nucleopolyhedrovirus NC 0... (33034) GCCGCAAACGCGCCACATCGCCCTTGTACCACCGATCTCGGCACACAAGCTGTAGCATTTTTAAATCGTGATCGCTC
                            SARS-CoV-2 NC_045512.2     (1) -----------------------------------------------------------------------------
                                                                                                                      Section 431
                                              (33111) 33111     33121      33132      33143      33154      33165      33176  33187
Autographa californica nucleopolyhedrovirus NC 0... (33111) AAGCTATTAATTCTGGTTAGATTTATATAGTCGTCAATATCCTCGGGCGTGGTTTGCGTCATGTCTGTAAAACGTGC
                            SARS-CoV-2 NC_045512.2     (1) -----------------------------------------------------------------------------
                                                                                                                      Section 432
                                              (33188) 33188     33198      33209      33220      33231      33242      33253  33264
Autographa californica nucleopolyhedrovirus NC 0... (33188) AAAATCAAACATTTTTATGTTGTAGTCGAATCTAACAAATCCATCGGCGTTCACTTGCACTTCGCGCTTTACAAAAC
                            SARS-CoV-2 NC_045512.2     (1) -----------------------------------------------------------------------------
                                                                                                                      Section 433
                                              (33265) 33265     33275      33286      33297      33308      33319      33330  33341
Autographa californica nucleopolyhedrovirus NC 0... (33265) GAGGTAGCGTGTAATCGAACCCGTTTAAATAGATTGCGTACAAAACCAGCACTTCATCTTCCAGTTTGCACGCTTGC
                            SARS-CoV-2 NC_045512.2     (1) -----------------------------------------------------------------------------
                                                                                                                      Section 434
                                              (33342) 33342     33352      33363      33374      33385      33396      33407  33418
Autographa californica nucleopolyhedrovirus NC 0... (33342) GGCAAAAATTGTGTGGTGTGCTCCAACCGGGTGACAAACATGACTATGGAAAATAACGCGGAATTCAACAGACGACT
                            SARS-CoV-2 NC_045512.2     (1) -----------------------------------------------------------------------------
```

Section 435

	(33419)	33419 33429 33440 33451 33462 33473 33484 33495
Autographa californica nucleopolyhedrovirus NC_0...	(33419)	AGAGTACGTGGGCACGATCGCCACAATGATGAAACGAACATTGAACGTTTTACGACAGCAGGGCTATTGCACGCAAC
SARS-CoV-2 NC_045512.2	(1)	---

Section 436

	(33496)	33496 33506 33517 33528 33539 33550 33561 33572
Autographa californica nucleopolyhedrovirus NC_0...	(33496)	AGGATGCGGATTCTTTGTGCGTGTCAGACGACACGGCGGCCTGGTTATGCGGCCGTTTGCCGACCTGCAATTTTGTA
SARS-CoV-2 NC_045512.2	(1)	---

Section 437

	(33573)	33573 33583 33594 33605 33616 33627 33638 33649
Autographa californica nucleopolyhedrovirus NC_0...	(33573)	TCGTTCCGCGTGCACATCGACCAGTTTGAGCATCCAAATCCGGCGTTGGAATATTTTAAATTTGAAGAAAGTCTGGC
SARS-CoV-2 NC_045512.2	(1)	---

Section 438

	(33650)	33650 33660 33671 33682 33693 33704 33715 33726
Autographa californica nucleopolyhedrovirus NC_0...	(33650)	GCAACGCCAACACGTGGGCCCGCGTTACACGTACATGAATTACACGCTTTTTAAAAACGTCGTGGCCCTCAAATTGG
SARS-CoV-2 NC_045512.2	(1)	---

Section 439

	(33727)	33727 33737 33748 33759 33770 33781 33792 33803
Autographa californica nucleopolyhedrovirus NC_0...	(33727)	TCGTGTACACGCGCACGCTACAAGCTAACATGTACGCGGACGGGTTGCCGTATTTTGTGCAAAATTTTTCAGAAACA
SARS-CoV-2 NC_045512.2	(1)	---

Section 440

	(33804)	33804 33814 33825 33836 33847 33858 33869 33880
Autographa californica nucleopolyhedrovirus NC_0...	(33804)	AGCTACAAACATGTTCGTGTGTATGTTAGAAAACTTGGTGCGATACAAGTAGCGACATTATCAGTTTACGAACAAAT
SARS-CoV-2 NC_045512.2	(1)	---

Section 441

	(33881)	33881 33891 33902 33913 33924 33935 33946 33957
Autographa californica nucleopolyhedrovirus NC_0...	(33881)	TATTGAAGATACAATAAATGAACTCGTCGTCAATCACGTTGATTAGATAATGTCCGTGTTAAATGTGATATCTTAGA
SARS-CoV-2 NC_045512.2	(1)	---

Section 442

	(33958)	33958 33968 33979 33990 34001 34012 34023 34034
Autographa californica nucleopolyhedrovirus NC 0...	(33958)	TTACGAGCGCGCAATAACCATAGTTTAATCGAAGAGAATAGCCGTCGCCACAATGGATAATTACAAATTGCAATTGC
SARS-CoV-2 NC_045512.2	(1)	---

Section 443

	(34035)	34035 34045 34056 34067 34078 34089 34100 34111
Autographa californica nucleopolyhedrovirus NC 0...	(34035)	AAGAATTTTTTGACCAAGCGCCCGACAACGACGATCCCAACTTTGAACATCAAACGCCCAATCTATTGGCGCATCAG
SARS-CoV-2 NC_045512.2	(1)	---

Section 444

	(34112)	34112 34122 34133 34144 34155 34166 34177 34188
Autographa californica nucleopolyhedrovirus NC 0...	(34112)	AAAAAAGGCATACAGTGGATGATTAACAGAGAAAAAAACGGCCGGCCCAACGGCGGCGTGCTTGCCGACGACATGGG
SARS-CoV-2 NC_045512.2	(1)	---

Section 445

	(34189)	34189 34199 34210 34221 34232 34243 34254 34265
Autographa californica nucleopolyhedrovirus NC 0...	(34189)	ACTCGGCAAAACGCTCTCTGTGCTAATGTTAATCGCAAAAAACAACTCTCTACAATTGAAAACTCTAATAGTGTGTC
SARS-CoV-2 NC_045512.2	(1)	---

Section 446

	(34266)	34266 34276 34287 34298 34309 34320 34331 34342
Autographa californica nucleopolyhedrovirus NC 0...	(34266)	CTTTGTCTTTAATCAATCATTGGGTAACCGAAAACAAGAAGCATGATTTAAATTTTAACATTTTAAAGTATTACAAA
SARS-CoV-2 NC_045512.2	(1)	---

Section 447

	(34343)	34343 34353 34364 34375 34386 34397 34408 34419
Autographa californica nucleopolyhedrovirus NC 0...	(34343)	TCTTTGGATGCCGACACGGTTGAGCATTACCACATTGTGGTGACCACGTACGACGTTTTATTGGCACATTTCAAATT
SARS-CoV-2 NC_045512.2	(1)	---

Section 448

	(34420)	34420 34430 34441 34452 34463 34474 34485 34496
Autographa californica nucleopolyhedrovirus NC 0...	(34420)	GATCAAACAAAATAAACAGTCAAGTCTGTTTTCAACCCGCTGGCATCGAGTTGTTCTAGATGAAGCGCATATTATCA
SARS-CoV-2 NC_045512.2	(1)	---

```
                                                                                                   Section 449
                                              (34497) 34497     34507      34518      34529      34540      34551      34562  34573
Autographa californica nucleopolyhedrovirus NC 0... (34497) AAAACTGCAAGACGGGCGTGCACAACGCCGCGTGCGCTTTGACCGCAACAAACCGATGGTGCATTACCGGCACACCG
                            SARS-CoV-2 NC_045512.2      (1) -----------------------------------------------------------------------------
                                                                                                   Section 450
                                              (34574) 34574     34584      34595      34606      34617      34628      34639  34650
Autographa californica nucleopolyhedrovirus NC 0... (34574) ATCCACAACAAGCATTGGGACATGTACTCGATGATTAATTTTTTGCAATGTCGTCCTTTTAACAATCCAAGAGTGTG
                            SARS-CoV-2 NC_045512.2      (1) -----------------------------------------------------------------------------
                                                                                                   Section 451
                                              (34651) 34651     34661      34672      34683      34694      34705      34716  34727
Autographa californica nucleopolyhedrovirus NC 0... (34651) GAAAATGTTAAATAAAAACAACGACTCTACAAATCGCATAAAAAGTATTATTAAAAAAATTGTTTTAAAACGCGACA
                            SARS-CoV-2 NC_045512.2      (1) -----------------------------------------------------------------------------
                                                                                                   Section 452
                                              (34728) 34728     34738      34749      34760      34771      34782      34793  34804
Autographa californica nucleopolyhedrovirus NC 0... (34728) AATCTGAAATTTCTTCTAACATTCCTAAACACACGGTTGAGTATGTACATGTTAATTTTAATGAAGAAGAAAAAACG
                            SARS-CoV-2 NC_045512.2      (1) -----------------------------------------------------------------------------
                                                                                                   Section 453
                                              (34805) 34805     34815      34826      34837      34848      34859      34870  34881
Autographa californica nucleopolyhedrovirus NC 0... (34805) TTGTACGATAAATTAAAGTGTGAATCGGAAGAGGCGTATGTGAAGGCTGTGGCAGCGCGTGAAAACGAAAACGCACT
                            SARS-CoV-2 NC_045512.2      (1) -----------------------------------------------------------------------------
                                                                                                   Section 454
                                              (34882) 34882     34892      34903      34914      34925      34936      34947  34958
Autographa californica nucleopolyhedrovirus NC 0... (34882) AAGCCGATTGCAGCAAATGCAGCACGTGTTATGGCTAATACTGAAATTGAGGCAAATCTGCTGCCACCCGTATTTGG
                            SARS-CoV-2 NC_045512.2      (1) -----------------------------------------------------------------------------
                                                                                                   Section 455
                                              (34959) 34959     34969      34980      34991      35002      35013      35024  35035
Autographa californica nucleopolyhedrovirus NC 0... (34959) CCATGCACGGTAAAAATATTTTGGAAACAAACGACTGTTTTAAAATGGATTATATGAGCAGCAAGTGCAAACGAGTG
                            SARS-CoV-2 NC_045512.2      (1) -----------------------------------------------------------------------------
```

Section 456

	(35036)	35036 35046 35057 35068 35079 35090 35101 35112
Autographa californica nucleopolyhedrovirus NC 0...	(35036)	CTCGACTTGGTAGACGACATTTTGAACACAAGCAACGACAAGATAATATTGGTTTCGCAATGGGTGGAATATTTAAA
SARS-CoV-2 NC_045512.2	(1)	---

Section 457

	(35113)	35113 35123 35134 35145 35156 35167 35178 35189
Autographa californica nucleopolyhedrovirus NC 0...	(35113)	AATATTTGAAAACTTTTTTAAACAAAAAAACATTGCTACGTTAATGTACACGGGCCAATTAAAAGTGGAAGACAGGA
SARS-CoV-2 NC_045512.2	(1)	---

Section 458

	(35190)	35190 35200 35211 35222 35233 35244 35255 35266
Autographa californica nucleopolyhedrovirus NC 0...	(35190)	TTTTGGCCGAGACGACATTCAATGATGCTGCCAATACTCAACATCGAATTTTGCTGCTTTCCATTAAGTGCGGCGGC
SARS-CoV-2 NC_045512.2	(1)	---

Section 459

	(35267)	35267 35277 35288 35299 35310 35321 35332 35343
Autographa californica nucleopolyhedrovirus NC 0...	(35267)	GTCGGGTTAAACTTAATAGGCGGAAACCACATTGTAATGTTGGAGCCTCATTGGAACCCGCAAATTGAATTGCAGGC
SARS-CoV-2 NC_045512.2	(1)	---

Section 460

	(35344)	35344 35354 35365 35376 35387 35398 35409 35420
Autographa californica nucleopolyhedrovirus NC 0...	(35344)	GCAAGACCGAATCAGTCGTATGGGACAAACAAAAAACACGTACGTGTACAAGATGCTAAATGTGGAAGACAACAGCA
SARS-CoV-2 NC_045512.2	(1)	---

Section 461

	(35421)	35421 35431 35442 35453 35464 35475 35486 35497
Autographa californica nucleopolyhedrovirus NC 0...	(35421)	TCGAAAAATACATTAAACAACGCCAAGACAAAAAGATTGCGTTTGTCAACACGGTCTTTGAAGAGACTCTGCTCAAT
SARS-CoV-2 NC_045512.2	(1)	---

Section 462

	(35498)	35498 35508 35519 35530 35541 35552 35563 35574
Autographa californica nucleopolyhedrovirus NC 0...	(35498)	TACGAAGACATTAAAAAATTTTTCAACTTGTAGCTGGTAAGTCGTCATGAACACCCGATATGCTACTTGCTATGTTT
SARS-CoV-2 NC_045512.2	(1)	---

```
                                                                                                Section 463
                                              (35575) 35575     35585      35596      35607      35618      35629      35640      35651
Autographa californica nucleopolyhedrovirus NC_0... (35575) GCGACGAGTTGGTGTACTTGTTTAAGAAAACGTTTAGTAACATGTCCCCTTCGGCCGCTGCGTTTTACCAACGGCGC
                              SARS-CoV-2 NC_045512.2 (1) -----------------------------------------------------------------------------
                                                                                                Section 464
                                              (35652) 35652     35662      35673      35684      35695      35706      35717      35728
Autographa californica nucleopolyhedrovirus NC_0... (35652) ATGGCCATTGTTAAAAACGGTATCGTGCTGTGCCCACGTTGTTCGTCGGAACTAAAAATTGGCAACGGCGTTTCGAT
                              SARS-CoV-2 NC_045512.2 (1) -----------------------------------------------------------------------------
                                                                                                Section 465
                                              (35729) 35729     35739      35750      35761      35772      35783      35794      35805
Autographa californica nucleopolyhedrovirus NC_0... (35729) TCCAATTTACCCCCACCGCGCTCAACAACATGCACGACGGTCGCGTTAAGACGCAAGCGCTTCGAGTTTTGGCCCGC
                              SARS-CoV-2 NC_045512.2 (1) -----------------------------------------------------------------------------
                                                                                                Section 466
                                              (35806) 35806     35816      35827      35838      35849      35860      35871      35882
Autographa californica nucleopolyhedrovirus NC_0... (35806) TCGCTACCTCCGCTGTACGACTCGACCGTCGATCGACACGGCTGCAAGGTGTTCACGGTGCGGCGCTACAACAGACG
                              SARS-CoV-2 NC_045512.2 (1) -----------------------------------------------------------------------------
                                                                                                Section 467
                                              (35883) 35883     35893      35904      35915      35926      35937      35948      35959
Autographa californica nucleopolyhedrovirus NC_0... (35883) CGTAATCGACTTTGCGGGCATTCGCAACAAAACGCTGGAAATCATTAAAACGGATAGAAACTTGCCGCTCAACACAG
                              SARS-CoV-2 NC_045512.2 (1) -----------------------------------------------------------------------------
                                                                                                Section 468
                                              (35960) 35960     35970      35981      35992      36003      36014      36025      36036
Autographa californica nucleopolyhedrovirus NC_0... (35960) AATGCAATGTGAAAGTTGTCGACAGTGCATGCATGCGTTGCAGAAAAAGTTTCGCAGTTTACCCCGCCGTTACCTAT
                              SARS-CoV-2 NC_045512.2 (1) -----------------------------------------------------------------------------
                                                                                                Section 469
                                              (36037) 36037     36047      36058      36069      36080      36091      36102      36113
Autographa californica nucleopolyhedrovirus NC_0... (36037) CTGCATTGCGGACATTCGTGTCTGTGCACCGACTGCGACGAAACGGTAAACGTGGACAACACGTGTCCTAAATGTAA
                              SARS-CoV-2 NC_045512.2 (1) -----------------------------------------------------------------------------
```

Section 470

```
                                               (36114) 36114     36124      36135      36146      36157      36168      36179      36190
Autographa californica nucleopolyhedrovirus NC 0... (36114) AAGCGGCATTAGATATAAATTAAAATACAAAACTTTGTAACATGTTGCCCTACGAAATGGTGATTGCCGTGTTGGTT
                            SARS-CoV-2 NC_045512.2 (1) -----------------------------------------------------------------------------
```

Section 471

```
                                               (36191) 36191     36201      36212      36223      36234      36245      36256      36267
Autographa californica nucleopolyhedrovirus NC 0... (36191) TACTTGTCGCCGGCGCAGATTCTAAATTTAAACCTTCCTTTTGCATACCAAAAAAGTGTGCTGTTTGCCAGCAACTC
                            SARS-CoV-2 NC_045512.2 (1) -----------------------------------------------------------------------------
```

Section 472

```
                                               (36268) 36268     36278      36289      36300      36311      36322      36333      36344
Autographa californica nucleopolyhedrovirus NC 0... (36268) TGCAAAAGTTAACGAACGCATCAGGCGGCGAGCGCGTGACGACAACGACGACGACCCCTATTTTTACTACAAACAGT
                            SARS-CoV-2 NC_045512.2 (1) -----------------------------------------------------------------------------
```

Section 473

```
                                               (36345) 36345     36355      36366      36377      36388      36399      36410      36421
Autographa californica nucleopolyhedrovirus NC 0... (36345) TCATAAAGATTAATTTTTTAACTAAAAAAATAATAAATGTTTATAATAAAACTGAAAAGTGTATTAGAGCGACGTTT
                            SARS-CoV-2 NC_045512.2 (1) -----------------------------------------------------------------------------
```

Section 474

```
                                               (36422) 36422     36432      36443      36454      36465      36476      36487      36498
Autographa californica nucleopolyhedrovirus NC 0... (36422) GATGGTCGGTATGTGGTTACACGCGACGTTTTAATGTGCTTTGTAAACAAGAGTTATATGAAGCAATTGCTGCGCGA
                            SARS-CoV-2 NC_045512.2 (1) -----------------------------------------------------------------------------
```

Section 475

```
                                               (36499) 36499     36509      36520      36531      36542      36553      36564      36575
Autographa californica nucleopolyhedrovirus NC 0... (36499) GGTTGACACTCGCATTACACTACAGCAACTTGTTAAAATGTATAGTCCAGAATTTGGTTTTTATGTAAATAGCAAAA
                            SARS-CoV-2 NC_045512.2 (1) -----------------------------------------------------------------------------
```

Section 476

```
                                               (36576) 36576     36586      36597      36608      36619      36630      36641      36652
Autographa californica nucleopolyhedrovirus NC 0... (36576) TTATGTTTGTGTTAACTGAATCGGTGTTGGCGTCTATTTGTTTAAAACACTCGTTCGGCAAATGCGAGTGGTTGGAC
                            SARS-CoV-2 NC_045512.2 (1) -----------------------------------------------------------------------------
```

```
                                                                                                                    Section 477
                                                  (36653) 36653     36663      36674      36685      36696      36707      36718      36729
Autographa californica nucleopolyhedrovirus NC_0... (36653) AAAAATATAAAAACTGTGTGTTTACAATTAAGAAAAATTTGTATTAATAATAAGCAACATTCGACATGTCTATCGTA
                             SARS-CoV-2 NC_045512.2 (1)     -----------------------------------------------------------------------------
                                                                                                                    Section 478
                                                  (36730) 36730     36740      36751      36762      36773      36784      36795      36806
Autographa californica nucleopolyhedrovirus NC_0... (36730) TTGATTATTGTCATAGTTGTAATATTTTTAATATGTTTTTTGTACCTATCAAATAGCAATAATAAAAATGATGCCAA
                             SARS-CoV-2 NC_045512.2 (1)     -----------------------------------------------------------------------------
                                                                                                                    Section 479
                                                  (36807) 36807     36817      36828      36839      36850      36861      36872      36883
Autographa californica nucleopolyhedrovirus NC_0... (36807) TAAAAACAATGCTTTTATTGATCTCAATCCCTTGCCGCTCAATGCTACAACCGCTACTACTACCACTGCCGTTGCTA
                             SARS-CoV-2 NC_045512.2 (1)     -----------------------------------------------------------------------------
                                                                                                                    Section 480
                                                  (36884) 36884     36894      36905      36916      36927      36938      36949      36960
Autographa californica nucleopolyhedrovirus NC_0... (36884) CCACCACTACCAACAACAACAACAGCATAGTGGCCTTTCGGCAAAACAACATTCAAGAACTACAAAACTTTGAACGA
                             SARS-CoV-2 NC_045512.2 (1)     -----------------------------------------------------------------------------
                                                                                                                    Section 481
                                                  (36961) 36961     36971      36982      36993      37004      37015      37026      37037
Autographa californica nucleopolyhedrovirus NC_0... (36961) TGGTTCAAAAATAATCTCTCATATTCGTTTAGCCAAAAAGCTGAAAAGGTGGTAAATCCCAATAGAAATTGGAACGA
                             SARS-CoV-2 NC_045512.2 (1)     -----------------------------------------------------------------------------
                                                                                                                    Section 482
                                                  (37038) 37038     37048      37059      37070      37081      37092      37103      37114
Autographa californica nucleopolyhedrovirus NC_0... (37038) CAACACGGTATTTGACAATTTGAGTCCGTGGACAAGCGTTCCGGACTTTGGTACCGTGTGCCACACGCTCATAGGGT
                             SARS-CoV-2 NC_045512.2 (1)     -----------------------------------------------------------------------------
                                                                                                                    Section 483
                                                  (37115) 37115     37125      37136      37147      37158      37169      37180      37191
Autographa californica nucleopolyhedrovirus NC_0... (37115) ATTGCGTACGCTACAACAACACCAGCGACACGTTATACCAGAACCCTGAATTGGCTTACAATCTCATTAACGGGCTG
                             SARS-CoV-2 NC_045512.2 (1)     -----------------------------------------------------------------------------
```

Section 484

```
                                                 (37192) 37192     37202      37213      37224      37235      37246      37257   37268
Autographa californica nucleopolyhedrovirus NC 0... (37192) CGCATCATTTGCAGCAAACTGCCCGATCCGCCGCCGCACCAACAAGCGCCCTGGGGCCCGGTCGCCGATTGGTACCA
                            SARS-CoV-2 NC_045512.2     (1) -----------------------------------------------------------------------------
```

Section 485

```
                                                 (37269) 37269     37279      37290      37301      37312      37323      37334   37345
Autographa californica nucleopolyhedrovirus NC 0... (37269) TTTCACAATCACAATGCCCGAGGTGTTTATGAACATTACCATTGTGCTAAACGAAACGCAGCATTACGACGAAGCTG
                            SARS-CoV-2 NC_045512.2     (1) -----------------------------------------------------------------------------
```

Section 486

```
                                                 (37346) 37346     37356      37367      37378      37389      37400      37411   37422
Autographa californica nucleopolyhedrovirus NC 0... (37346) CGTCCCTCACGCGTTACTGGCTCGGCTTGTATCTGCCCACGGCCGTCAACTCGATGGGCTGGCACCGGACGGCAGGC
                            SARS-CoV-2 NC_045512.2     (1) -----------------------------------------------------------------------------
```

Section 487

```
                                                 (37423) 37423     37433      37444      37455      37466      37477      37488   37499
Autographa californica nucleopolyhedrovirus NC 0... (37423) AACTCAATGCGCATGGGTGTGCCCTACACGTACAGTCAAATCTTGCGCGGATATTCATTGGCGCAAATTAGGCAAGA
                            SARS-CoV-2 NC_045512.2     (1) -----------------------------------------------------------------------------
```

Section 488

```
                                                 (37500) 37500     37510      37521      37532      37543      37554      37565   37576
Autographa californica nucleopolyhedrovirus NC 0... (37500) GCAGGGAATACAAGAAATCCTAAACACGATCGCGTTTCCGTACGTGACTCAAGGCAACGGCTTGCACGTCGATTCGA
                            SARS-CoV-2 NC_045512.2     (1) -----------------------------------------------------------------------------
```

Section 489

```
                                                 (37577) 37577     37587      37598      37609      37620      37631      37642   37653
Autographa californica nucleopolyhedrovirus NC 0... (37577) TATACATCGATCACATTGACGTGCGCGCTTACGGCTATTTGATAAATTCATACTTTACGTTTGCCTATTACACGTAC
                            SARS-CoV-2 NC_045512.2     (1) -----------------------------------------------------------------------------
```

Section 490

```
                                                 (37654) 37654     37664      37675      37686      37697      37708      37719   37730
Autographa californica nucleopolyhedrovirus NC 0... (37654) TATTTTGGAGACGAGGTAATCAACACGGTGGGTTTGACGAGAGCCATCGAAAACGTGGGCAGTCCCGAGGGAGTTGT
                            SARS-CoV-2 NC_045512.2     (1) -----------------------------------------------------------------------------
```

Section 491

(37731) 37731 37741 37752 37763 37774 37785 37796 37807
Autographa californica nucleopolyhedrovirus NC 0... (37731) GGTGCCAGGCGTCATGTCTCGAAACGGCACGTTGTACTCTAACGTGATAGGCAACTTTATTACGTATCCGTTGGCCG
SARS-CoV-2 NC_045512.2 (1) ---

Section 492

(37808) 37808 37818 37829 37840 37851 37862 37873 37884
Autographa californica nucleopolyhedrovirus NC 0... (37808) TCCATTCGGCCGATTACTCCAAAGTGTTGACCAAACTTTCAAAAACATATTACGGTTCGGTTGTGGGCGTAACGAAT
SARS-CoV-2 NC_045512.2 (1) ---

Section 493

(37885) 37885 37895 37906 37917 37928 37939 37950 37961
Autographa californica nucleopolyhedrovirus NC 0... (37885) AGGTTGGCTTACTACGAATCCGATCCCACAAACAACATTCAAGCGCCCCTGTGGACCATGGCGCGGCGCATTTGGAA
SARS-CoV-2 NC_045512.2 (1) ---

Section 494

(37962) 37962 37972 37983 37994 38005 38016 38027 38038
Autographa californica nucleopolyhedrovirus NC 0... (37962) TCGGCGCGGCAGAATTATCAACTATAATGCCAACACGGTGTCGTTTGAGTCGGGTATTATTTTGCAAAGTTTGAACG
SARS-CoV-2 NC_045512.2 (1) ---

Section 495

(38039) 38039 38049 38060 38071 38082 38093 38104 38115
Autographa californica nucleopolyhedrovirus NC 0... (38039) GAATCATGCGCATCCCGTCGGGCACCACGTCCACGCAGTCGTTCAGACCGACCATTGGCCAAACGGCTATAGCCAAA
SARS-CoV-2 NC_045512.2 (1) ---

Section 496

(38116) 38116 38126 38137 38148 38159 38170 38181 38192
Autographa californica nucleopolyhedrovirus NC 0... (38116) ACCGACACGGCCGGCGCCATTTTGGTGTACGCCAAGTTTGCGGAAATGAACAATTTGCAATTTAAATCGTGCACGTT
SARS-CoV-2 NC_045512.2 (1) ---

Section 497

(38193) 38193 38203 38214 38225 38236 38247 38258 38269
Autographa californica nucleopolyhedrovirus NC 0... (38193) GTTCTACGATCACGGCATGTTCCAGCTATATTACAACATTGGCGTGGAACCAAACTCGCTCAACAACACAAACGGGC
SARS-CoV-2 NC_045512.2 (1) ---

```
                                                                                                                Section 498
                                            (38270) 38270     38280      38291      38302      38313      38324      38335      38346
Autographa californica nucleopolyhedrovirus NC 0... (38270) GGGTGATTGTGCTAAGCAGAGACACGTCGGTCAACACCAACGATTTGTCATTTGAAGCGCAAAGAATTAACAACAAC
                             SARS-CoV-2 NC_045512.2 (1) -----------------------------------------------------------------------------
                                                                                                                Section 499
                                            (38347) 38347     38357      38368      38379      38390      38401      38412      38423
Autographa californica nucleopolyhedrovirus NC 0... (38347) AACTCGTCGGAAGGCACCACGTTCAACGGTGTGGTCTGTCATCGCGTTCCTATCACAAACATCAACGTGCCTTCTCT
                             SARS-CoV-2 NC_045512.2 (1) -----------------------------------------------------------------------------
                                                                                                                Section 500
                                            (38424) 38424     38434      38445      38456      38467      38478      38489      38500
Autographa californica nucleopolyhedrovirus NC 0... (38424) GACCGTTCGAAGTCCCAATTCTAGCGTCGAACTAGTCGAGCAGATAATTAGTTTTCAAACAATGTACACGGCCACGG
                             SARS-CoV-2 NC_045512.2 (1) -----------------------------------------------------------------------------
                                                                                                                Section 501
                                            (38501) 38501     38511      38522      38533      38544      38555      38566      38577
Autographa californica nucleopolyhedrovirus NC 0... (38501) CTTCGGCCTGTTACAAATTAAACGTCGAAGGTCATTCGGATTCCCTGAGAGCTTTTAGAGTTAATTCCGACGAAAAC
                             SARS-CoV-2 NC_045512.2 (1) -----------------------------------------------------------------------------
                                                                                                                Section 502
                                            (38578) 38578     38588      38599      38610      38621      38632      38643      38654
Autographa californica nucleopolyhedrovirus NC 0... (38578) ATTTATGTAAACGTGGGCAACGGCGTTAAAGCCCTGTTTAATTATCCCTGGGTAATGGTCAAAGAAAATAACAAAGT
                             SARS-CoV-2 NC_045512.2 (1) -----------------------------------------------------------------------------
                                                                                                                Section 503
                                            (38655) 38655     38665      38676      38687      38698      38709      38720      38731
Autographa californica nucleopolyhedrovirus NC 0... (38655) GTCTTTCATGTCGGCTAACGAAGACACTACTATACCATTTAGCGTTATAATGAATTCCTTCACCTCTATCGGCGAAC
                             SARS-CoV-2 NC_045512.2 (1) -----------------------------------------------------------------------------
                                                                                                                Section 504
                                            (38732) 38732     38742      38753      38764      38775      38786      38797      38808
Autographa californica nucleopolyhedrovirus NC 0... (38732) CAGCTTTGCAATACTCTCCATCAAATTGCTTTGTGTATGGAAACGGTTTCAAATTGAACAACAGCACGTTTGATTTA
                             SARS-CoV-2 NC_045512.2 (1) -----------------------------------------------------------------------------
```

```
                                                                                         Section 505
                                                     (38809) 38809     38819      38830      38841      38852      38863      38874      38885
Autographa californica nucleopolyhedrovirus  NC_0... (38809) CAATTTATTTTTGAAATTGTGTAATTATATTTAGGGAGAATGTGATATTCAAAAGACTGACTGTTAACACAAAAGAC
                              SARS-CoV-2 NC_045512.2     (1) -----------------------------------------------------------------------------
                                                                                         Section 506
                                                     (38886) 38886     38896      38907      38918      38929      38940      38951      38962
Autographa californica nucleopolyhedrovirus  NC_0... (38886) TGATATTGTTGTTGTTACAAAATAGATAATAAAACAAAAAATAAATTAAATATTATTTATTTATTAAACTGTTTAAT
                              SARS-CoV-2 NC_045512.2     (1) -----------------------------------------------------------------------------
                                                                                         Section 507
                                                     (38963) 38963     38973      38984      38995      39006      39017      39028      39039
Autographa californica nucleopolyhedrovirus  NC_0... (38963) TTTAATGCTAACGCGTACAAATCACGCTGTTCCGACGTGGACATGGAATTGCGCAGAAAAGTCTTGATAGTGTCGAT
                              SARS-CoV-2 NC_045512.2     (1) -----------------------------------------------------------------------------
                                                                                         Section 508
                                                     (39040) 39040     39050      39061      39072      39083      39094      39105      39116
Autographa californica nucleopolyhedrovirus  NC_0... (39040) TTCTTCGCCGTCATCCACTTCCATATATTTGATTTCTTCCTCGATTTGCATTTCCAAGTTTGCGTATTCTTGCAAAT
                              SARS-CoV-2 NC_045512.2     (1) -----------------------------------------------------------------------------
                                                                                         Section 509
                                                     (39117) 39117     39127      39138      39149      39160      39171      39182      39193
Autographa californica nucleopolyhedrovirus  NC_0... (39117) AATAATCTAGTCGTTGGGCGACCTCGCCAATTTTAAATAATACATTATCCGACACCAAATGCCAGCGAGTGACTGTG
                              SARS-CoV-2 NC_045512.2     (1) -----------------------------------------------------------------------------
                                                                                         Section 510
                                                     (39194) 39194     39204      39215      39226      39237      39248      39259      39270
Autographa californica nucleopolyhedrovirus  NC_0... (39194) CGCTCCATCATCCTGGCACTTTTTAATGTGAATATTAAAAGGTTGTTGCATATATATCGTTAAACGTTTATGTTTAC
                              SARS-CoV-2 NC_045512.2     (1) -----------------------------------------------------------------------------
                                                                                         Section 511
                                                     (39271) 39271     39281      39292      39303      39314      39325      39336      39347
Autographa californica nucleopolyhedrovirus  NC_0... (39271) TTTCACGTTAGCTCGTTTCATTGATGTAAACATTTAGTTTTATAACAGCGTCGGTAATTTTATTTTTTAAAGTAAAC
                              SARS-CoV-2 NC_045512.2     (1) -----------------------------------------------------------------------------
```

```
                                                                                                   Section 512
                                                    (39348) 39348     39358      39369      39380      39391      39402      39413      39424
Autographa californica nucleopolyhedrovirus NC 0... (39348) AGACCAAAATCAAAGGTGTCTTCGACAGGTACGATTATTTTCCCATTGACACTGTTTTCGTGCACAGATATAATTTT
                            SARS-CoV-2 NC_045512.2      (1) -----------------------------------------------------------------------------
                                                                                                   Section 513
                                                    (39425) 39425     39435      39446      39457      39468      39479      39490      39501
Autographa californica nucleopolyhedrovirus NC 0... (39425) ATCACCGTTTATTATTTTGCCCAAACACACGTACTCGTTTCTTCTCAAGCCAACTATTTCTAAACAATTCACTTTTC
                            SARS-CoV-2 NC_045512.2      (1) -----------------------------------------------------------------------------
                                                                                                   Section 514
                                                    (39502) 39502     39512      39523      39534      39545      39556      39567      39578
Autographa californica nucleopolyhedrovirus NC 0... (39502) TATTATCGTGTACGCAATTAAAAGTAAACGAAGCGCTACAATTGTCGTATTCTATTACAATTCTGCGGCATTTATAA
                            SARS-CoV-2 NC_045512.2      (1) -----------------------------------------------------------------------------
                                                                                                   Section 515
                                                    (39579) 39579     39589      39600      39611      39622      39633      39644      39655
Autographa californica nucleopolyhedrovirus NC 0... (39579) AATTTATTAATGTTGACGCAAATTCCATGCAGCGCATCCATTTCGTACTGCAAATGCGGCGCAATTAAAAAATTTCC
                            SARS-CoV-2 NC_045512.2      (1) -----------------------------------------------------------------------------
                                                                                                   Section 516
                                                    (39656) 39656     39666      39677      39688      39699      39710      39721      39732
Autographa californica nucleopolyhedrovirus NC 0... (39656) TCGTCGTTGTTAACAATCTTGGGCGCTAAAAAGCACGCCAACACGCCCACGTCTTTAATGCAATATTCCAATTTGAA
                            SARS-CoV-2 NC_045512.2      (1) -----------------------------------------------------------------------------
                                                                                                   Section 517
                                                    (39733) 39733     39743      39754      39765      39776      39787      39798      39809
Autographa californica nucleopolyhedrovirus NC 0... (39733) CGGCAGTTCCTCGGACATGTATATTGTCACGGTGGGCGCCAAAGGAGCGGCTTTAGCAAAATGACACAAGTAATCGC
                            SARS-CoV-2 NC_045512.2      (1) -----------------------------------------------------------------------------
                                                                                                   Section 518
                                                    (39810) 39810     39820      39831      39842      39853      39864      39875      39886
Autographa californica nucleopolyhedrovirus NC 0... (39810) CCGCAAAAGTGTGCGTTACGGTTTGCTTTGCTTTGAGAACGGAAAAGTTTTCGTTGTCCGCGCTCATCTGCACGTCC
                            SARS-CoV-2 NC_045512.2      (1) -----------------------------------------------------------------------------
```

```
                                                                                                      Section 519
                                                    (39887) 39887     39897      39908      39919      39930      39941      39952    39963
Autographa californica nucleopolyhedrovirus NC 0... (39887) GCCGAGCCAATGTCGCCATTTGCTCTAAACTGCAGACCCTTCTTGGAACACGACACAATAATATCGTGGTCGAATTG
                             SARS-CoV-2 NC_045512.2     (1) -----------------------------------------------------------------------------
```

```
                                                                                                      Section 520
                                                    (39964) 39964     39974      39985      39996      40007      40018      40029    40040
Autographa californica nucleopolyhedrovirus NC 0... (39964) CGTCATGTCTTTGCACACCTGCGCAAACTCGACGCTCGACATGTGGACGACGCAATCGTAATCGCTATCCGGAATTC
                             SARS-CoV-2 NC_045512.2     (1) -----------------------------------------------------------------------------
```

```
                                                                                                      Section 521
                                                    (40041) 40041     40051      40062      40073      40084      40095      40106    40117
Autographa californica nucleopolyhedrovirus NC 0... (40041) CCAAATGTTCCACGTCGATGCACATCAACTTGAGCGTGTACGTGCAGATTCTATTGTCGTTGTTGAACACGAACGCC
                             SARS-CoV-2 NC_045512.2     (1) -----------------------------------------------------------------------------
```

```
                                                                                                      Section 522
                                                    (40118) 40118     40128      40139      40150      40161      40172      40183    40194
Autographa californica nucleopolyhedrovirus NC 0... (40118) ATCACATCGCCCTGATCTTCCGCTTTCATCAGTACAGAGCTGCGCTCGTTAACGCATTTGACAATTTTACTTAAACT
                             SARS-CoV-2 NC_045512.2     (1) -----------------------------------------------------------------------------
```

```
                                                                                                      Section 523
                                                    (40195) 40195     40205      40216      40227      40238      40249      40260    40271
Autographa californica nucleopolyhedrovirus NC 0... (40195) GTTTATGGACACGTTGAGCGGCACGTTGCGGTCACATCTATATTTTTTGAAACCCTCGGCGTGTAGTTGCAACGACA
                             SARS-CoV-2 NC_045512.2     (1) -----------------------------------------------------------------------------
```

```
                                                                                                      Section 524
                                                    (40272) 40272     40282      40293      40304      40315      40326      40337    40348
Autographa californica nucleopolyhedrovirus NC 0... (40272) CGAGCGCGACATGCGAGGTGTCCATAACCTGCATGCTTACGCCTCGATTATCACAATCAAAAGTAGCGTGCGGCAGC
                             SARS-CoV-2 NC_045512.2     (1) -----------------------------------------------------------------------------
```

```
                                                                                                      Section 525
                                                    (40349) 40349     40359      40370      40381      40392      40403      40414    40425
Autographa californica nucleopolyhedrovirus NC 0... (40349) AGATCCTTAAAAGTTTCCACCAGCCTCTTCAAAACTGCGCCGGTTTTAAATTCCGCTTCGAACATTTTTAGCAGTGA
                             SARS-CoV-2 NC_045512.2     (1) -----------------------------------------------------------------------------
```

Section 526

```
                                               (40426) 40426     40436      40447      40458      40469      40480      40491  40502
Autographa californica nucleopolyhedrovirus NC 0... (40426) TTCTAATTGCAGCTGCTCTTTGATACAACTAATTTTACGACGACGATGCGAGCTTTTATTCAACCGAGCGTGCATGT
                             SARS-CoV-2 NC_045512.2     (1) -----------------------------------------------------------------------------
```

Section 527

```
                                               (40503) 40503     40513      40524      40535      40546      40557      40568  40579
Autographa californica nucleopolyhedrovirus NC 0... (40503) TTGCAATCGTGCAAGCGTTATCAATTTTTCATTATCGTATTGTTGCACATCAACAGGCTGGACACCACGTTGAACTC
                             SARS-CoV-2 NC_045512.2     (1) -----------------------------------------------------------------------------
```

Section 528

```
                                               (40580) 40580     40590      40601      40612      40623      40634      40645  40656
Autographa californica nucleopolyhedrovirus NC 0... (40580) GCCGCAGTTTTGCGGCAAGTTGGACCCGCCGCGCATCCAATGCAAACTTTCCGACATTCTGTTGCCTACGAACGATT
                             SARS-CoV-2 NC_045512.2     (1) -----------------------------------------------------------------------------
```

Section 529

```
                                               (40657) 40657     40667      40678      40689      40700      40711      40722  40733
Autographa californica nucleopolyhedrovirus NC 0... (40657) GATTCTTTGTCCATTGATCGAAGCGAGTGCCTTCGACTTTTTCGTGTCCAGTGTGGCTTGTTTTAATAAATTCTTTG
                             SARS-CoV-2 NC_045512.2     (1) -----------------------------------------------------------------------------
```

Section 530

```
                                               (40734) 40734     40744      40755      40766      40777      40788      40799  40810
Autographa californica nucleopolyhedrovirus NC 0... (40734) AAAATATTGTCGGGTGTATTATTAAATAGCATGTATGGTATGTTGAAGATGGGATAACGCTTGGCGTGCGGGTCGTC
                             SARS-CoV-2 NC_045512.2     (1) -----------------------------------------------------------------------------
```

Section 531

```
                                               (40811) 40811     40821      40832      40843      40854      40865      40876  40887
Autographa californica nucleopolyhedrovirus NC 0... (40811) ATGATTTCCACCGCGCACCACATATTTGCGCTCAATTTTATCAAAATTGGACTGGCGAGACAAAAACGAGACGGGCG
                             SARS-CoV-2 NC_045512.2     (1) -----------------------------------------------------------------------------
```

Section 532

```
                                               (40888) 40888     40898      40909      40920      40931      40942      40953  40964
Autographa californica nucleopolyhedrovirus NC 0... (40888) ACAGGCATATTTGGGCGTGCGTACCATCTTCGGCCATCCACTCGGTCAGGTCTTCGCTGCGGTTAAACACACCTTTC
                             SARS-CoV-2 NC_045512.2     (1) -----------------------------------------------------------------------------
```

Section 533

```
                                                   (40965) 40965     40975      40986      40997      41008      41019      41030  41041
Autographa californica nucleopolyhedrovirus NC 0... (40965) TGACCGTGAATGCCACATATTTTTATTCCTTCCAAATCGTTGGTGGACGTGACTATGACTATTTTAAGCATAACGTT
                           SARS-CoV-2 NC_045512.2     (1) -----------------------------------------------------------------------------
```

Section 534

```
                                                   (41042) 41042     41052      41063      41074      41085      41096      41107  41118
Autographa californica nucleopolyhedrovirus NC 0... (41042) GTCGCCGTTAACCACCATGCTGGCGTCGAGTTTTTCAATTTTTTGATTTTTAATTTGTCTAAAGTAAACGTACACTT
                           SARS-CoV-2 NC_045512.2     (1) -----------------------------------------------------------------------------
```

Section 535

```
                                                   (41119) 41119     41129      41140      41151      41162      41173      41184  41195
Autographa californica nucleopolyhedrovirus NC 0... (41119) TGTAAACGTTAAAATTGCCGTTGGTGCACGTTTCAATTTTGTACCGTCGGCCGTCGTACACCCAATTAATCTTTGCG
                           SARS-CoV-2 NC_045512.2     (1) -----------------------------------------------------------------------------
```

Section 536

```
                                                   (41196) 41196     41206      41217      41228      41239      41250      41261  41272
Autographa californica nucleopolyhedrovirus NC 0... (41196) TTGCTCACCAACACACCGGCCATGTACAGCACAAGTCCGTCGTCTAGCGCAACGTAATTTTTGTCGCTACTATTCGT
                           SARS-CoV-2 NC_045512.2     (1) -----------------------------------------------------------------------------
```

Section 537

```
                                                   (41273) 41273     41283      41294      41305      41316      41327      41338  41349
Autographa californica nucleopolyhedrovirus NC 0... (41273) AAACTTTACTAAACACGACTGCTTGGGGCCGACCACAAGCTTGCCCTTCAATTTGTTCACTTTGTTGTTGTATAAAC
                           SARS-CoV-2 NC_045512.2     (1) -----------------------------------------------------------------------------
```

Section 538

```
                                                   (41350) 41350     41360      41371      41382      41393      41404      41415  41426
Autographa californica nucleopolyhedrovirus NC 0... (41350) AAATGGGCAGCGCAATGTGCGGAATGTACGGATCTTCGGCGGTCATGAGTTTATTGTCTCGCACCAACGTCCACAAT
                           SARS-CoV-2 NC_045512.2     (1) -----------------------------------------------------------------------------
```

Section 539

```
                                                   (41427) 41427     41437      41448      41459      41470      41481      41492  41503
Autographa californica nucleopolyhedrovirus NC 0... (41427) TTAAACATTTTATTGTTGAGCAAAATGGACTTGTTTACCGCCACAGAGTAGCCATTTGGTAAACCCGATACGCAATT
                           SARS-CoV-2 NC_045512.2     (1) -----------------------------------------------------------------------------
```

```
                                                                                                   Section 540
                                                   (41504) 41504     41514      41525      41536      41547      41558      41569      41580
Autographa californica nucleopolyhedrovirus NC 0... (41504) TTCCTCTTTGTACTCAAACACGGGCATGGCATTCTTTAGATTGGTTAGGGACACAATCAATTTGGGTACGGGCGTGG
                           SARS-CoV-2 NC_045512.2      (1) -----------------------------------------------------------------------------
                                                                                                   Section 541
                                                   (41581) 41581     41591      41602      41613      41624      41635      41646      41657
Autographa californica nucleopolyhedrovirus NC 0... (41581) TATGAAATAAATGTATAAAATTACGATAATAATACTGCTCCAACTTGGACATGAGCGATTTGACGTCATCGTTTTCT
                           SARS-CoV-2 NC_045512.2      (1) -----------------------------------------------------------------------------
                                                                                                   Section 542
                                                   (41658) 41658     41668      41679      41690      41701      41712      41723      41734
Autographa californica nucleopolyhedrovirus NC 0... (41658) ACGATCGTACACTGAATAATGGGATTATAGTATATAGAATGTTTATAGTGGTATTCGTAGGGTGTCAACAATACGTT
                           SARS-CoV-2 NC_045512.2      (1) -----------------------------------------------------------------------------
                                                                                                   Section 543
                                                   (41735) 41735     41745      41756      41767      41778      41789      41800      41811
Autographa californica nucleopolyhedrovirus NC 0... (41735) AATGTCGGCTTCGTTGTTCACCCGCAACTTTTTTTTGATGCATATCATTCCTTCGTGATGATTAACGTAAAGTATTC
                           SARS-CoV-2 NC_045512.2      (1) -----------------------------------------------------------------------------
                                                                                                   Section 544
                                                   (41812) 41812     41822      41833      41844      41855      41866      41877      41888
Autographa californica nucleopolyhedrovirus NC 0... (41812) TGTCTGTAATCTTCAATTCGATGGGCGCCATGTTTCTTTTCATAGTGTACACGATAAACGACGTGTTTGATTTTAAA
                           SARS-CoV-2 NC_045512.2      (1) -----------------------------------------------------------------------------
                                                                                                   Section 545
                                                   (41889) 41889     41899      41910      41921      41932      41943      41954      41965
Autographa californica nucleopolyhedrovirus NC 0... (41889) CATTTTAAATTTGTGGGTCTATCATTAAACGCGATCAGCAACGAGTCGTCTTGAACGTCGTTGAGGTCGTCCACGAA
                           SARS-CoV-2 NC_045512.2      (1) -----------------------------------------------------------------------------
                                                                                                   Section 546
                                                   (41966) 41966     41976      41987      41998      42009      42020      42031      42042
Autographa californica nucleopolyhedrovirus NC 0... (41966) CGCGACCAGATTGTGTTTTAGCAAATATTGAAATTTTTGCGCAACCATTTCGTAGTCCACGTTGGGCAAACATGCGT
                           SARS-CoV-2 NC_045512.2      (1) -----------------------------------------------------------------------------
```

Section 547

(42043) 42043 42053 42064 42075 42086 42097 42108 42119

Autographa californica nucleopolyhedrovirus NC_0... (42043) TGCGGCAAAGGAAAAACTTTTTGCCCGCCACGGTCATTTCGCCGTGAAAAAAACTGCCAATAAATTTCACAAAATCC

SARS-CoV-2 NC_045512.2 (1) ---

Section 548

(42120) 42120 42130 42141 42152 42163 42174 42185 42196

Autographa californica nucleopolyhedrovirus NC_0... (42120) TTTTTTTGCTTCAACATTTTCTGGCGCATGCTGTCGTTGGTGATTCGCGCCACCTCGTTGCCGACGCGATATTTTAA

SARS-CoV-2 NC_045512.2 (1) ---

Section 549

(42197) 42197 42207 42218 42229 42240 42251 42262 42273

Autographa californica nucleopolyhedrovirus NC_0... (42197) CACGGGCAACGAAATTTCAATATTGTTATTGCTGCTGTTGTCCTGTTGATTGGGAAAGACTTTGCGTTGCTTGCTAA

SARS-CoV-2 NC_045512.2 (1) ---

Section 550

(42274) 42274 42284 42295 42306 42317 42328 42339 42350

Autographa californica nucleopolyhedrovirus NC_0... (42274) AAGTTTTCGATACGCAATATATGAGACGCCCGTTGACTATACAATCGACAATCTTTTTCGACTCTTTGTTGTACAAG

SARS-CoV-2 NC_045512.2 (1) ---

Section 551

(42351) 42351 42361 42372 42383 42394 42405 42416 42427

Autographa californica nucleopolyhedrovirus NC_0... (42351) ACGCTTTGAATTTTACGACGCTTGTTCGCCACCGTGTACGCGTCGTCGTCGGCCGTCTTGTCGAGAACTCGTTGATA

SARS-CoV-2 NC_045512.2 (1) ---

Section 552

(42428) 42428 42438 42449 42460 42471 42482 42493 42504

Autographa californica nucleopolyhedrovirus NC_0... (42428) GTTTTGCAAAATTGTCGAAGTTAATAACAGTTCTATCAAATAGGCGTGCTTGTATACAATTTTGTTGGCCAAACTGT

SARS-CoV-2 NC_045512.2 (1) ---

Section 553

(42505) 42505 42515 42526 42537 42548 42559 42570 42581

Autographa californica nucleopolyhedrovirus NC_0... (42505) CTATAGAATAGTTTATGTCGTGATTCATAATAATTTTTATGTGTTCCACGAGTTGTTGCTTGTGAAGCGTGTTGTAT

SARS-CoV-2 NC_045512.2 (1) ---

Section 554

(42582) 42582 42592 42603 42614 42625 42636 42647 42658
Autographa californica nucleopolyhedrovirus NC_0... (42582) TCGAAGAGAAAATCGAGCGGTTTCCATTTGCCGCTGTTGGCCAGATATGTTTCCAGCACAGAATTTAAATCTTCCGT
SARS-CoV-2 NC_045512.2 (1) ---

Section 555

(42659) 42659 42669 42680 42691 42702 42713 42724 42735
Autographa californica nucleopolyhedrovirus NC_0... (42659) CACTACGTAATCGCTAGCGTACACGTCTCGAGCAAACAGGACGTCGTCTTGTTTGTCGTAAACTAGTTGGATTGCGC
SARS-CoV-2 NC_045512.2 (1) ---

Section 556

(42736) 42736 42746 42757 42768 42779 42790 42801 42812
Autographa californica nucleopolyhedrovirus NC_0... (42736) GATTGATGTGCTTCTCTTGATCCACGTTGCCGTACAAAAACATGCGTTTGCAATGTTTGGCGTATAGCTTGTCGTAG
SARS-CoV-2 NC_045512.2 (1) ---

Section 557

(42813) 42813 42823 42834 42845 42856 42867 42878 42889
Autographa californica nucleopolyhedrovirus NC_0... (42813) AAATTGTGCACCAAAACGTTGTTGTTCATCATTATGTTGGGAAAACTCAAAAATCTGCCGTCCAGCATAAAAGTTCC
SARS-CoV-2 NC_045512.2 (1) ---

Section 558

(42890) 42890 42900 42911 42922 42933 42944 42955 42966
Autographa californica nucleopolyhedrovirus NC_0... (42890) GTTAATATTGTTGTTTGCGTCGACATCGTCCGTTTCTCTAAATTGCTTGTCTAAGCGCGTGCCGAATATAACGGGCA
SARS-CoV-2 NC_045512.2 (1) ---

Section 559

(42967) 42967 42977 42988 42999 43010 43021 43032 43043
Autographa californica nucleopolyhedrovirus NC_0... (42967) CACATTTATGCATTACGCAACTGAGCTGTTCATTAAGAGCGCAACACAAATAAGACTTGCGTTCTTGAATAGCGCAA
SARS-CoV-2 NC_045512.2 (1) ---

Section 560

(43044) 43044 43054 43065 43076 43087 43098 43109 43120
Autographa californica nucleopolyhedrovirus NC_0... (43044) AAAAGCATACGTTCATTGCTGTTTGTAGCGCAATCAAAAGTATATTTTAATTTGTATTTATTTTCAATTCTATCGTA
SARS-CoV-2 NC_045512.2 (1) ---

Section 561

		43121 43131 43142 43153 43164 43175 43186 43197
Autographa californica nucleopolyhedrovirus NC_0...	(43121)	CAACTCGTTGAAATCTTGAACCACGTCCGTCATCGTGAAGCGATTACTGCGCACTAATTATGTCTAAACGTGTTCGT
SARS-CoV-2 NC_045512.2	(1)	---

Section 562

		43198 43208 43219 43230 43241 43252 43263 43274
Autographa californica nucleopolyhedrovirus NC_0...	(43198)	GAACGGTCGGTTGTTTCGGATGAAACGGCCAAACGCATTCGACAAAACGAACACTGTCATGCCAAAAATGAATCTTT
SARS-CoV-2 NC_045512.2	(1)	---

Section 563

		43275 43285 43296 43307 43318 43329 43340 43351
Autographa californica nucleopolyhedrovirus NC_0...	(43275)	TTTGGGGTTTTGCAACTTGGAAGAAATTGATTATTATCAATGTTTAAAAATGCAATACGTTCCGGACCAAAAGTTTG
SARS-CoV-2 NC_045512.2	(1)	---

Section 564

		43352 43362 43373 43384 43395 43406 43417 43428
Autographa californica nucleopolyhedrovirus NC_0...	(43352)	ACAACGATTTTATTTTAACAGTGTACAGAATGGCCAACGTGGTGACGAAACAAGTTAGACCGTATAACAGTATCGAC
SARS-CoV-2 NC_045512.2	(1)	---

Section 565

		43429 43439 43450 43461 43472 43483 43494 43505
Autographa californica nucleopolyhedrovirus NC_0...	(43429)	GAAAAGCACCATTACAACACGGTGCGTAACGTGTTGATTTTAATAAAAAATGCGCGTTTAGTGCTTAGTAATAGTGT
SARS-CoV-2 NC_045512.2	(1)	---

Section 566

		43506 43516 43527 43538 43549 43560 43571 43582
Autographa californica nucleopolyhedrovirus NC_0...	(43506)	CAAAAAGCAATACTATGACGATGTGTTAAAATTGAAAAAAAATACAGACTTGGAATCGTACGATCCATTGATTACGG
SARS-CoV-2 NC_045512.2	(1)	---

Section 567

		43583 43593 43604 43615 43626 43637 43648 43659
Autographa californica nucleopolyhedrovirus NC_0...	(43583)	TCTTTTTACAAATTGGCGAATCTGTAAATGAAGAAATACAAAAACTCAGAAAAGCTTTGGTCAATATTTTTACTAAT
SARS-CoV-2 NC_045512.2	(1)	---

Section 568

	(43660)	43660 43670 43681 43692 43703 43714 43725 43736
Autographa californica nucleopolyhedrovirus NC_0...	(43660)	AAACCCGACAAGTCGGATATAAACAACCCAGATGTAGTTTCGTATCAATTTATTTTTGGCAGAGTACAAAAATTGTA
SARS-CoV-2 NC_045512.2	(1)	---

Section 569

	(43737)	43737 43747 43758 43769 43780 43791 43802 43813
Autographa californica nucleopolyhedrovirus NC_0...	(43737)	TAACAGGGCAATTAAACAAAAAACTAAAACTATAATTGTAAAACGTCCTACAACTATGAACAGAATTCAAATAGATT
SARS-CoV-2 NC_045512.2	(1)	---

Section 570

	(43814)	43814 43824 43835 43846 43857 43868 43879 43890
Autographa californica nucleopolyhedrovirus NC_0...	(43814)	GGAAAACTCTTTCCGAAGACGAACAAAAAATGACTAGACAAGAAATTGCCGAAAAAATTGTAAAGCCTTGTTTTGAG
SARS-CoV-2 NC_045512.2	(1)	---

Section 571

	(43891)	43891 43901 43912 43923 43934 43945 43956 43967
Autographa californica nucleopolyhedrovirus NC_0...	(43891)	CAATTTGGCACTATATTACACATATACGTATGTCCTTTAAAACACAACCGAATTATTGTCGAGTATGCAAACTCAGA
SARS-CoV-2 NC_045512.2	(1)	---

Section 572

	(43968)	43968 43978 43989 44000 44011 44022 44033 44044
Autographa californica nucleopolyhedrovirus NC_0...	(43968)	GTCGGTACAAAAAGCCATGACTGTAAATGACGACACTCGATTTACAGTTACAGAGTTTTCCGTGGTTCAGTACTACA
SARS-CoV-2 NC_045512.2	(1)	---

Section 573

	(44045)	44045 44055 44066 44077 44088 44099 44110 44121
Autographa californica nucleopolyhedrovirus NC_0...	(44045)	ACGTGGCCAAAACAGAAATGGTGAACCAGCGAATTGACATAATAAGCAAGGACATTGAGGATTTAAGAAACGCTTTA
SARS-CoV-2 NC_045512.2	(1)	---

Section 574

	(44122)	44122 44132 44143 44154 44165 44176 44187 44198
Autographa californica nucleopolyhedrovirus NC_0...	(44122)	AAATCTTACACATAAATTAAAATATCGAACAAAGGAAAAAAACAATTGTAACAAAAATAATTTACATTAAAATTTAC
SARS-CoV-2 NC_045512.2	(1)	---

```
                                                                                                    Section 575
                                                  (44199) 44199     44209      44220      44231      44242      44253      44264      44275
Autographa californica nucleopolyhedrovirus NC 0... (44199) AAGTTTTTTTCTAGTGTCGTACTTTTTTACAATGCGTCTGTTGTCCGTCGAGCATTGCAAACATATTGTGGACGGCG
                           SARS-CoV-2 NC_045512.2     (1) -----------------------------------------------------------------------------
                                                                                                    Section 576
                                                  (44276) 44276     44286      44297      44308      44319      44330      44341      44352
Autographa californica nucleopolyhedrovirus NC 0... (44276) CAAAATAGCAAACAAAAGGCACGTCCGCGCTCTCCCACGCTATTCTAAAACGATGAATCCATATTAATTTTTCATTG
                           SARS-CoV-2 NC_045512.2     (1) -----------------------------------------------------------------------------
                                                                                                    Section 577
                                                  (44353) 44353     44363      44374      44385      44396      44407      44418      44429
Autographa californica nucleopolyhedrovirus NC 0... (44353) TCGCCAAACGTCGCTCCGCTGCCTCCTTCCAATAACAAATACTCAGAAACACAAACATGTACAATTGCTGTCGCGGC
                           SARS-CoV-2 NC_045512.2     (1) -----------------------------------------------------------------------------
                                                                                                    Section 578
                                                  (44430) 44430     44440      44451      44462      44473      44484      44495      44506
Autographa californica nucleopolyhedrovirus NC 0... (44430) GTTAATTGTCGCTGTTTTTCCAAATAGTCTATTATGGGAAACAAACACTTGTCACAACACAAATACTCGTTAATTGT
                           SARS-CoV-2 NC_045512.2     (1) -----------------------------------------------------------------------------
                                                                                                    Section 579
                                                  (44507) 44507     44517      44528      44539      44550      44561      44572      44583
Autographa californica nucleopolyhedrovirus NC 0... (44507) CACAACCGACAAGCACATTTGGCAAAATGCGTCGCAATTTTTGTACGGACGAGATTCTATGCGAAGTTCGTTGTCCA
                           SARS-CoV-2 NC_045512.2     (1) -----------------------------------------------------------------------------
                                                                                                    Section 580
                                                  (44584) 44584     44594      44605      44616      44627      44638      44649      44660
Autographa californica nucleopolyhedrovirus NC 0... (44584) TGACGTCTTGGGTCCACTTTTTCAACAAGACACTTTTATATTTGTGATTTGTACAACTTTGGTACGTGTTAGAGTGT
                           SARS-CoV-2 NC_045512.2     (1) -----------------------------------------------------------------------------
                                                                                                    Section 581
                                                  (44661) 44661     44671      44682      44693      44704      44715      44726      44737
Autographa californica nucleopolyhedrovirus NC 0... (44661) TTTTGATAAGCTTTGATAAGTTTAAAACTGTTGGAGTAAGGCCACGTCATTATGTTCTGCACCTTTTGTTTAAAAGA
                           SARS-CoV-2 NC_045512.2     (1) -----------------------------------------------------------------------------
```

```
                                                                 Section 582
                                          (44738) 44738     44748      44759      44770      44781      44792      44803      44814
Autographa californica nucleopolyhedrovirus  NC_0... (44738) CAGAAATTACTATATGTTCAAACTATTTAAAGATTATTGGCCAACGTGCACGACAGAATGCCAGATATGTCTTGAGA
                              SARS-CoV-2 NC_045512.2     (1) -----------------------------------------------------------------------------
```

```
                                                                 Section 583
                                          (44815) 44815     44825      44836      44847      44858      44869      44880      44891
Autographa californica nucleopolyhedrovirus  NC_0... (44815) AAATTGACGATAACGGGGGCATAGTGGCAATGCCCGACACTGGCATGTTAAACTTGGAAAAGATGTTTCACGAACAA
                              SARS-CoV-2 NC_045512.2     (1) -----------------------------------------------------------------------------
```

```
                                                                 Section 584
                                          (44892) 44892     44902      44913      44924      44935      44946      44957      44968
Autographa californica nucleopolyhedrovirus  NC_0... (44892) TGTATTCAGCGTTGGCGTCGCGAACATACTCGAGATCCCTTTAATCGTGTTATAAAATATTATTTTAACTTTCCCCC
                              SARS-CoV-2 NC_045512.2     (1) -----------------------------------------------------------------------------
```

```
                                                                 Section 585
                                          (44969) 44969     44979      44990      45001      45012      45023      45034      45045
Autographa californica nucleopolyhedrovirus  NC_0... (44969) AAAAACACTAGAGGAGTGCAACGTGATGCTTCGAGAAACTAAAGGGTCTATAGGCGATCACGAAATTGATCGCGTTT
                              SARS-CoV-2 NC_045512.2     (1) -----------------------------------------------------------------------------
```

```
                                                                 Section 586
                                          (45046) 45046     45056      45067      45078      45089      45100      45111      45122
Autographa californica nucleopolyhedrovirus  NC_0... (45046) ACAAACGCGTTTATCAACGCGTTACACAGGAAGACGCCCTGGACATTGAACTCGATTTTAGGCATTTTTTTAAAATG
                              SARS-CoV-2 NC_045512.2     (1) -----------------------------------------------------------------------------
```

```
                                                                 Section 587
                                          (45123) 45123     45133      45144      45155      45166      45177      45188      45199
Autographa californica nucleopolyhedrovirus  NC_0... (45123) CAATCATGACGAACGTATGGTTCGCGACGGACGTCAACCTGATCAATTGTGTACTGAAAGATAATTTATTTTTGATA
                              SARS-CoV-2 NC_045512.2     (1) -----------------------------------------------------------------------------
```

```
                                                                 Section 588
                                          (45200) 45200     45210      45221      45232      45243      45254      45265      45276
Autographa californica nucleopolyhedrovirus  NC_0... (45200) GATAATAATTACATTATTTTAAATGTGTTCGACCAAGAAACCGATCAAGTTAGACCTCTGTGCCTCGGTGAAATTAA
                              SARS-CoV-2 NC_045512.2     (1) -----------------------------------------------------------------------------
```

Section 589

	(45277)	45277 45287 45298 45309 45320 45331 45342 45353
Autographa californica nucleopolyhedrovirus NC 0...	(45277)	CGCCCTTCAAACCGATGCGGCCGCCCAAGCCGATGCAATGCTGGATACATCCTCGACGAGCGAATTGCAAAGTAACG
SARS-CoV-2 NC_045512.2	(1)	--

Section 590

	(45354)	45354 45364 45375 45386 45397 45408 45419 45430
Autographa californica nucleopolyhedrovirus NC 0...	(45354)	CGTCCACGTAACAATTATTCAGATCCCGATAACGAAAACGACATGTTGCACATGACCGTGTTAAACAGCGTGTTTTT
SARS-CoV-2 NC_045512.2	(1)	--

Section 591

	(45431)	45431 45441 45452 45463 45474 45485 45496 45507
Autographa californica nucleopolyhedrovirus NC 0...	(45431)	GAACGAGCACGCGAAATTGTATTATCGGCACTTGTTGCGCAACGATCAAGCCGAGGCGAGAAAAACAATTCTCAACG
SARS-CoV-2 NC_045512.2	(1)	--

Section 592

	(45508)	45508 45518 45529 45540 45551 45562 45573 45584
Autographa californica nucleopolyhedrovirus NC 0...	(45508)	CCGACAGCGTGTACGAGTGCATGTTAATTAGACCAATTCGTACGGAACATTTTAGAAGCGTCGACGAGGCTGGCGAA
SARS-CoV-2 NC_045512.2	(1)	--

Section 593

	(45585)	45585 45595 45606 45617 45628 45639 45650 45661
Autographa californica nucleopolyhedrovirus NC 0...	(45585)	CACAACATGAGCGTTTTAAAGATCATCATCGATGCGGTCATCAAGTACATTGGCAAACTGGCCGACGACGAGTACAT
SARS-CoV-2 NC_045512.2	(1)	--

Section 594

	(45662)	45662 45672 45683 45694 45705 45716 45727 45738
Autographa californica nucleopolyhedrovirus NC 0...	(45662)	TTTGATAGCGGACCGCATGTATGTCGATTTAATCTATTCCGAATTTAGGGCCATTATTTTGCCTCAAAGCGCGTACA
SARS-CoV-2 NC_045512.2	(1)	--

Section 595

	(45739)	45739 45749 45760 45771 45782 45793 45804 45815
Autographa californica nucleopolyhedrovirus NC 0...	(45739)	TTATCAAAGGAGATTACGCAGAAAGCGATAGTGAAAGCGGGCAAAGTGTCGACGTTTGTAATGAACTCGAATATCCT
SARS-CoV-2 NC_045512.2	(1)	--

```
Section 596
                                                (45816) 45816     45826      45837      45848      45859      45870      45881      45892
Autographa californica nucleopolyhedrovirus  NC_0... (45816) TGGAAATTAATTACGGCGAACAATTGTATTGTTTCTACGGACGAGTCACGTCAGTCGCAATACATTTATCGCACTTT
                       SARS-CoV-2 NC_045512.2     (1) -----------------------------------------------------------------------------
Section 597
                                                (45893) 45893     45903      45914      45925      45936      45947      45958      45969
Autographa californica nucleopolyhedrovirus  NC_0... (45893) TCTTTTGTACAATACAGTCTTGACCGCAATTCTTAAACAAAACAATCCATTCGACGTAATTGCCGAAAATACTTCTA
                       SARS-CoV-2 NC_045512.2     (1) -----------------------------------------------------------------------------
Section 598
                                                (45970) 45970     45980      45991      46002      46013      46024      46035      46046
Autographa californica nucleopolyhedrovirus  NC_0... (45970) TTTCAATTATAGTCAGGAATTTGGGCAGCTGTCCAAACAATAAAGATCGGGTAAAGTGCTGCGATCTTAATTACGGC
                       SARS-CoV-2 NC_045512.2     (1) -----------------------------------------------------------------------------
Section 599
                                                (46047) 46047     46057      46068      46079      46090      46101      46112      46123
Autographa californica nucleopolyhedrovirus  NC_0... (46047) GGCGTCCCGCCGGGACATGTCATGTGCCCGCCGCGTGAGATCACCAAAAAATTTTTTCATTACGCAAAGTGGGTTCG
                       SARS-CoV-2 NC_045512.2     (1) -----------------------------------------------------------------------------
Section 600
                                                (46124) 46124     46134      46145      46156      46167      46178      46189      46200
Autographa californica nucleopolyhedrovirus  NC_0... (46124) AAATCCCAACAAGTACAAACGATACAGCGAGTTAATCGCGCGCCAATCAGAAACCGGCGGCGGATCTGCGAGTTTAC
                       SARS-CoV-2 NC_045512.2     (1) -----------------------------------------------------------------------------
Section 601
                                                (46201) 46201     46211      46222      46233      46244      46255      46266      46277
Autographa californica nucleopolyhedrovirus  NC_0... (46201) GCGAAAACGTAAACAACCAGCTACACGCTCGAGATGTGTCTCAATTACATTTATTGGATTGGGAAAACTTTATGGGT
                       SARS-CoV-2 NC_045512.2     (1) -----------------------------------------------------------------------------
Section 602
                                                (46278) 46278     46288      46299      46310      46321      46332      46343      46354
Autographa californica nucleopolyhedrovirus  NC_0... (46278) GAATTCAGCAGTTATTTTGGTCTGCACGCACACAACGTGTAGCATCGCCAGTATTTAACAGCTGACCTATTTGTTAA
                       SARS-CoV-2 NC_045512.2     (1) -----------------------------------------------------------------------------
```

```
                                                                                                                        Section 603
                                              (46355) 46355     46365      46376      46387      46398      46409      46420      46431
Autographa californica nucleopolyhedrovirus NC_0... (46355) ACAAGCATTCTTATCTCAATAATTGGTCCGACGTGGTGACAATTGTATCCACAATCATGAAAAAAGTAGCGCTTGGA
                              SARS-CoV-2 NC_045512.2    (1) -----------------------------------------------------------------------------
                                                                                                                        Section 604
                                              (46432) 46432     46442      46453      46464      46475      46486      46497      46508
Autographa californica nucleopolyhedrovirus NC_0... (46432) AAAATTATCGAAAACACAGTAGAAAGCAAATATAAAAGCAACAGTGTGTCGTCGTCATTGTCAACGGGCGCCAGTGC
                              SARS-CoV-2 NC_045512.2    (1) -----------------------------------------------------------------------------
                                                                                                                        Section 605
                                              (46509) 46509     46519      46530      46541      46552      46563      46574      46585
Autographa californica nucleopolyhedrovirus NC_0... (46509) AAAATTGAGTTTAAGCGAATATTACAAAACTTTTGAAGCAAATAAAGTGGGCCAGCACACTACGTACGACGTGGTCG
                              SARS-CoV-2 NC_045512.2    (1) -----------------------------------------------------------------------------
                                                                                                                        Section 606
                                              (46586) 46586     46596      46607      46618      46629      46640      46651      46662
Autographa californica nucleopolyhedrovirus NC_0... (46586) GCAAGCGAGATTACACGAAATTTGACAAATTGGTGAAAAAATATTGACATGCTGCGATCAATCATGCGACGTTTCAA
                              SARS-CoV-2 NC_045512.2    (1) -----------------------------------------------------------------------------
                                                                                                                        Section 607
                                              (46663) 46663     46673      46684      46695      46706      46717      46728      46739
Autographa californica nucleopolyhedrovirus NC_0... (46663) GAGTACAAACAATCTCAGCAAAAAACCCTCCGATTATTATGTAGTGTTATGTCCAAAGTGTTATTTTGTGACGTCGG
                              SARS-CoV-2 NC_045512.2    (1) -----------------------------------------------------------------------------
                                                                                                                        Section 608
                                              (46740) 46740     46750      46761      46772      46783      46794      46805      46816
Autographa californica nucleopolyhedrovirus NC_0... (46740) CCGAAGTGAGCGTGGCTGAATACATAGAAATGCATAAAAATTTTAACACGAAATTCGCCGATCGGTGCCCTAACGAT
                              SARS-CoV-2 NC_045512.2    (1) -----------------------------------------------------------------------------
                                                                                                                        Section 609
                                              (46817) 46817     46827      46838      46849      46860      46871      46882      46893
Autographa californica nucleopolyhedrovirus NC_0... (46817) TTTATTGTGACCAACTCTAAAAGTTGGAATAATCATGAAAATTGTTCTGCCCTATTTTACCCTCTGTGTTAATAAAG
                              SARS-CoV-2 NC_045512.2    (1) -----------------------------------------------------------------------------
```

```
                                                                                                           Section 610
                                                      (46894) 46894     46904      46915      46926      46937      46948      46959  46970
Autographa californica nucleopolyhedrovirus  NC_0...  (46894) TTTGTTGTTTGTATTTTGTGGTTTTATTTATTTACGCTAGATATTGGGTTTAAGGTTCTTAGAAATAGAGTTGTATT
                              SARS-CoV-2 NC_045512.2      (1) -----------------------------------------------------------------------------
                                                                                                           Section 611
                                                      (46971) 46971     46981      46992      47003      47014      47025      47036  47047
Autographa californica nucleopolyhedrovirus  NC_0...  (46971) TTCCCTACCAAAAGGGATTTGAGCTTCATATAAATACAATATTCGCTCGACAAGCGGTTTATTTCACTCGGAGGTAT
                              SARS-CoV-2 NC_045512.2      (1) -----------------------------------------------------------------------------
                                                                                                           Section 612
                                                      (47048) 47048     47058      47069      47080      47091      47102      47113  47124
Autographa californica nucleopolyhedrovirus  NC_0...  (47048) TATATCAGGCAGTCGAACGTGCGCGATGAAACATCCCGTTTACGCTAGATATTTGGAGTTTGATGATGTAGTGTTAG
                              SARS-CoV-2 NC_045512.2      (1) -----------------------------------------------------------------------------
                                                                                                           Section 613
                                                      (47125) 47125     47135      47146      47157      47168      47179      47190  47201
Autographa californica nucleopolyhedrovirus  NC_0...  (47125) ATTTGACTAGTTTAATATTTTTAGAGTTTGATAACGCTCAAAATGAAGAGTACATTATTTTTATGAATGTAAAAAAG
                              SARS-CoV-2 NC_045512.2      (1) -----------------------------------------------------------------------------
                                                                                                           Section 614
                                                      (47202) 47202     47212      47223      47234      47245      47256      47267  47278
Autographa californica nucleopolyhedrovirus  NC_0...  (47202) GCGTTTTACAAAAACTTTCACATTACTTGTGATCTGTCGCTTGAAACGCTGACCGTGTTGGTGTACGAAAAAGCTCG
                              SARS-CoV-2 NC_045512.2      (1) -----------------------------------------------------------------------------
                                                                                                           Section 615
                                                      (47279) 47279     47289      47300      47311      47322      47333      47344  47355
Autographa californica nucleopolyhedrovirus  NC_0...  (47279) CCTAATTGTGAAACAAATGGAGTTTGAGCAGCCGCCAAACTTTGTTAATTTTATCAGTTTCAACGCGACCGACAACG
                              SARS-CoV-2 NC_045512.2      (1) -----------------------------------------------------------------------------
                                                                                                           Section 616
                                                      (47356) 47356     47366      47377      47388      47399      47410      47421  47432
Autographa californica nucleopolyhedrovirus  NC_0...  (47356) ACAACTCCATGATAATAGACTTGTGTTCCGACGCGCGCATAATCGTGGCCAAGAAGCTGACGCCCGACGAAACGTAT
                              SARS-CoV-2 NC_045512.2      (1) -----------------------------------------------------------------------------
```

Section 617

```
                                              (47433) 47433     47443      47454      47465      47476      47487      47498     47509
Autographa californica nucleopolyhedrovirus NC_0... (47433) CATCAGCGCGTGTCCGGATTTTTGGATTTTCAAAAACGTAACTGCATACCTCGGCCCCCAATCGAGTCGGACCCAAA
                             SARS-CoV-2 NC_045512.2 (1) -----------------------------------------------------------------------------
```

Section 618

```
                                              (47510) 47510     47520      47531      47542      47553      47564      47575     47586
Autographa californica nucleopolyhedrovirus NC_0... (47510) AGTGCGAGACGCCTTGGATCGTGAACTAGAAATAAAACTATACAAGTAGAAAAAAATTAATTTATTAATAGTTGTAA
                             SARS-CoV-2 NC_045512.2 (1) -----------------------------------------------------------------------------
```

Section 619

```
                                              (47587) 47587     47597      47608      47619      47630      47641      47652     47663
Autographa californica nucleopolyhedrovirus NC_0... (47587) TAATTATCTTCGTCCTCATCTTCGCTGGTGTCATAATGCGGTGGTGTGTTTGTGTTTTGTTTTAATCGTTTGCGCGT
                             SARS-CoV-2 NC_045512.2 (1) -----------------------------------------------------------------------------
```

Section 620

```
                                              (47664) 47664     47674      47685      47696      47707      47718      47729     47740
Autographa californica nucleopolyhedrovirus NC_0... (47664) CGACACCACTTCGCCGATAGGAAATTTTTTGGATTTCGCATTAAATGCCCTCTTAGCGACGCGCCGTTTACGACTAC
                             SARS-CoV-2 NC_045512.2 (1) -----------------------------------------------------------------------------
```

Section 621

```
                                              (47741) 47741     47751      47762      47773      47784      47795      47806     47817
Autographa californica nucleopolyhedrovirus NC_0... (47741) TAAACATGTTGACGCGCTCGTCGTCTTCAGTGTCATAATCCGTGCTAGTGTTTTCGTTGTTATTTTCTATGAGACGA
                             SARS-CoV-2 NC_045512.2 (1) -----------------------------------------------------------------------------
```

Section 622

```
                                              (47818) 47818     47828      47839      47850      47861      47872      47883     47894
Autographa californica nucleopolyhedrovirus NC_0... (47818) TCGTTTGATTTAGTTTTCGTAGAATTGTCCGCGTTATCGTCGCTTTCGTCGATGTCGTCCCTAACTATCTCGTAGGC
                             SARS-CoV-2 NC_045512.2 (1) -----------------------------------------------------------------------------
```

Section 623

```
                                              (47895) 47895     47905      47916      47927      47938      47949      47960     47971
Autographa californica nucleopolyhedrovirus NC_0... (47895) GGCTTTGCGCGGAATCCAAGATTTTGCAATGTATCTATTTTAACGTACTTTTCTTCGAGCGCTTTTCTAGCTTTATG
                             SARS-CoV-2 NC_045512.2 (1) -----------------------------------------------------------------------------
```

Section 624

	(47972)	47972 47982 47993 48004 48015 48026 48037 48048
Autographa californica nucleopolyhedrovirus NC 0...	(47972)	CATAGCAATGTCTTCGTCGCCGCCGTTCATTTTATGATACTTTGTAAACGTCTCGACGAATAACTTTTTGGCGCGAG
SARS-CoV-2 NC_045512.2	(1)	---

Section 625

	(48049)	48049 48059 48070 48081 48092 48103 48114 48125
Autographa californica nucleopolyhedrovirus NC 0...	(48049)	GAGGCATTTTTTCATTGTATAACATATCGGGAATTTGATACATTGTAATTAGAATTAAGCAAGTTCGTCTTCGGTTG
SARS-CoV-2 NC_045512.2	(1)	---

Section 626

	(48126)	48126 48136 48147 48158 48169 48180 48191 48202
Autographa californica nucleopolyhedrovirus NC 0...	(48126)	TACTGTATTCGGTTTCTGTATCTGTAGTGGAATCCTCTGTACTAGTAGTAGTGTCGCTATTGTTGGCGTCAGGCCTT
SARS-CoV-2 NC_045512.2	(1)	---

Section 627

	(48203)	48203 48213 48224 48235 48246 48257 48268 48279
Autographa californica nucleopolyhedrovirus NC 0...	(48203)	GGCTGCCATTTACCGTCTATCAACATGTATTTTTTCCTAACAGCACAACATGCTAGCTTGGTAGCTATCTGTGTCGA
SARS-CoV-2 NC_045512.2	(1)	---

Section 628

	(48280)	48280 48290 48301 48312 48323 48334 48345 48356
Autographa californica nucleopolyhedrovirus NC 0...	(48280)	CTTATATTTTTGTAAACTACGATCGTAGAATTTTTCAAATATCCTCTTACCGTTATAGGGAAGGTTTTGATAATATT
SARS-CoV-2 NC_045512.2	(1)	---

Section 629

	(48357)	48357 48367 48378 48389 48400 48411 48422 48433
Autographa californica nucleopolyhedrovirus NC 0...	(48357)	TAGGCAACATATCAATAAAAGACAATATAAAAACTTTGTGTTTGTGTTTTATTTATCACATAAAATGGACGTCTGGC
SARS-CoV-2 NC_045512.2	(1)	---

Section 630

	(48434)	48434 48444 48455 48466 48477 48488 48499 48510
Autographa californica nucleopolyhedrovirus NC 0...	(48434)	AAGAATCACAACCAATATTAGTGTTTTTTTTCTTACATTACGAGATTCAACTTGATACTAAAATTAATTATTAATTA
SARS-CoV-2 NC_045512.2	(1)	---

```
                                                                                                   Section 631
                                                (48511) 48511     48521      48532      48543      48554      48565      48576   48587
Autographa californica nucleopolyhedrovirus NC 0... (48511) AATTAAATTAAATTTTGAAGCATTTTTTCGCTATCGTTTTCAGACTCAAAATTATCGACGCTATCGCTATGAAAAGC
                          SARS-CoV-2 NC_045512.2     (1) -----------------------------------------------------------------------------
                                                                                                   Section 632
                                                (48588) 48588     48598      48609      48620      48631      48642      48653   48664
Autographa californica nucleopolyhedrovirus NC 0... (48588) GTAATATTTGTTGGCTTTGAGATATTCTATATTTTGCTCATTTTTAACAATAAACACGCGACTCTTTTCGTCGCGTC
                          SARS-CoV-2 NC_045512.2     (1) -----------------------------------------------------------------------------
                                                                                                   Section 633
                                                (48665) 48665     48675      48686      48697      48708      48719      48730   48741
Autographa californica nucleopolyhedrovirus NC 0... (48665) TCACCATAACACCGTTTTTACAAATGGAAATGTATTTGTAAAACGGCAACAGAGCGTCGCGAGTTTTTTTAAGTAAC
                          SARS-CoV-2 NC_045512.2     (1) -----------------------------------------------------------------------------
                                                                                                   Section 634
                                                (48742) 48742     48752      48763      48774      48785      48796      48807   48818
Autographa californica nucleopolyhedrovirus NC 0... (48742) AGCTTTTGCTCCGCTGTGGCGGCCACAAATATTTTTACGGGCCCGTCGTAATTAATGTTTAAATTAAAATTTTTAAG
                          SARS-CoV-2 NC_045512.2     (1) -----------------------------------------------------------------------------
                                                                                                   Section 635
                                                (48819) 48819     48829      48840      48851      48862      48873      48884   48895
Autographa californica nucleopolyhedrovirus NC 0... (48819) TCGACGCTCGCGCGACTTGGTTTGCCATTCTTTAGCGCGCGTCGCGTCACACAGCTTGGCCACAATGTGGTTTTTGT
                          SARS-CoV-2 NC_045512.2     (1) -----------------------------------------------------------------------------
                                                                                                   Section 636
                                                (48896) 48896     48906      48917      48928      48939      48950      48961   48972
Autographa californica nucleopolyhedrovirus NC 0... (48896) CAAACGAAGATTCTATGACGTGTTTAAAGTTTAGGTCGAGTAAAGCGCAAATCTTTTTTAAATAATAGTTTCTAATT
                          SARS-CoV-2 NC_045512.2     (1) -----------------------------------------------------------------------------
                                                                                                   Section 637
                                                (48973) 48973     48983      48994      49005      49016      49027      49038   49049
Autographa californica nucleopolyhedrovirus NC 0... (48973) TTTTTATTATTCAGCCTGCTGTCGTGAATACCGTATATCTCAACGCTGTCTGTGAGATTGTCGTATTCTAGCCTTTT
                          SARS-CoV-2 NC_045512.2     (1) -----------------------------------------------------------------------------
```

```
                                                                                                   Section 638
                                                  (49050) 49050     49060      49071      49082      49093      49104      49115     49126
Autographa californica nucleopolyhedrovirus  NC_0... (49050) TAGTTTTTCGCTCATCGACTTGATATTGTCCGACACATTTTCGTCGATTTGCGTTTTGATCAACGACTTGAGCAGAG
                             SARS-CoV-2 NC_045512.2      (1) -----------------------------------------------------------------------------
                                                                                                   Section 639
                                                  (49127) 49127     49137      49148      49159      49170      49181      49192     49203
Autographa californica nucleopolyhedrovirus  NC_0... (49127) ACACGTTAATCAACTGTTCAAATTGATCCATATTAACTATATCAACCCGATGCGTATATGGTGCGTAAAATATATTT
                             SARS-CoV-2 NC_045512.2      (1) -----------------------------------------------------------------------------
                                                                                                   Section 640
                                                  (49204) 49204     49214      49225      49236      49247      49258      49269     49280
Autographa californica nucleopolyhedrovirus  NC_0... (49204) TTTAACCCTCTTATACTTTGCACTCTGCGTTAATACGCGTTCGTGTACAGACGTAATCATGTTTTCTTTTTTGGATA
                             SARS-CoV-2 NC_045512.2      (1) -----------------------------------------------------------------------------
                                                                                                   Section 641
                                                  (49281) 49281     49291      49302      49313      49324      49335      49346     49357
Autographa californica nucleopolyhedrovirus  NC_0... (49281) AAACTCCTACTGAGTTTGACCTCATATTAGACCCTCACAAGTTGCAAAACGTGGCATTTTTTACCAATGAAGAATTT
                             SARS-CoV-2 NC_045512.2      (1) -----------------------------------------------------------------------------
                                                                                                   Section 642
                                                  (49358) 49358     49368      49379      49390      49401      49412      49423     49434
Autographa californica nucleopolyhedrovirus  NC_0... (49358) AAAGTTATTTTAAAAAATTTCATCACAGATTTAAAGAAGAACCAAAAATTAAATTATTTCAACAGTTTAATCGACCA
                             SARS-CoV-2 NC_045512.2      (1) -----------------------------------------------------------------------------
                                                                                                   Section 643
                                                  (49435) 49435     49445      49456      49467      49478      49489      49500     49511
Autographa californica nucleopolyhedrovirus  NC_0... (49435) ATTAATCAACGTGTACACAGACGCGTCGGTGAAAAACACGCAGCCCGACGTGTTGGCTAAAATTATCAAATCAACTT
                             SARS-CoV-2 NC_045512.2      (1) -----------------------------------------------------------------------------
                                                                                                   Section 644
                                                  (49512) 49512     49522      49533      49544      49555      49566      49577     49588
Autographa californica nucleopolyhedrovirus  NC_0... (49512) GTGTTATAGTCACAGATTTGCCGTCCAACGTGTTTCTCAAAAAGTTGAAGACCAACAAGTTTACAGACACTATTAAT
                             SARS-CoV-2 NC_045512.2      (1) -----------------------------------------------------------------------------
```

```
                                                                                                       Section 645
                                               (49589) 49589     49599      49610      49621      49632      49643      49654   49665
Autographa californica nucleopolyhedrovirus NC 0... (49589) TATTTAATTTTGCCCCACTTTATTTTGTGGGATCACAATTTTGTTATATTTTTAAACAAAGCTTTCAATTCTAAACA
                             SARS-CoV-2 NC_045512.2     (1) -----------------------------------------------------------------------------
                                                                                                       Section 646
                                               (49666) 49666     49676      49687      49698      49709      49720      49731   49742
Autographa californica nucleopolyhedrovirus NC 0... (49666) TGAAAACGATCTGGTTGACATTTCGGGCGCTCTGCAGAAAATCAAACTTACACACGGTGTCATCAAAGATCAGTTGC
                             SARS-CoV-2 NC_045512.2     (1) -----------------------------------------------------------------------------
                                                                                                       Section 647
                                               (49743) 49743     49753      49764      49775      49786      49797      49808   49819
Autographa californica nucleopolyhedrovirus NC 0... (49743) AGAGCAAAAACGGGTACGCGGTCCAATACTTGTACGCGACGTTTCTCAACACGGCCTCGTTCTACGCCAACGTGCAA
                             SARS-CoV-2 NC_045512.2     (1) -----------------------------------------------------------------------------
                                                                                                       Section 648
                                               (49820) 49820     49830      49841      49852      49863      49874      49885   49896
Autographa californica nucleopolyhedrovirus NC 0... (49820) TGTTTAAATGGTGTCAACGAAATTATGCCGCCGCGGAGCAGCGTAAAGCGCTATTATGGACGTGATGTGGACAACGT
                             SARS-CoV-2 NC_045512.2     (1) -----------------------------------------------------------------------------
                                                                                                       Section 649
                                               (49897) 49897     49907      49918      49929      49940      49951      49962   49973
Autographa californica nucleopolyhedrovirus NC 0... (49897) GCGTGCATGGACCACGCGTCATCCCAACATTAGCCAGCTGAGTACGCAAGTCTCGGACGTCCACATTAACGAGTCAT
                             SARS-CoV-2 NC_045512.2     (1) -----------------------------------------------------------------------------
                                                                                                       Section 650
                                               (49974) 49974     49984      49995      50006      50017      50028      50039   50050
Autographa californica nucleopolyhedrovirus NC 0... (49974) CTACCGACTGGAATGTAAAAGTGGGTCTGGGAATATTTCCCGGCGCTAACACAGACTGCGACGGTGACAAAAAAATT
                             SARS-CoV-2 NC_045512.2     (1) -----------------------------------------------------------------------------
                                                                                                       Section 651
                                               (50051) 50051     50061      50072      50083      50094      50105      50116   50127
Autographa californica nucleopolyhedrovirus NC 0... (50051) ATTACATTTTTACCCAAACCTAATTCCCTAATCGACTCGGAATGCCTTTTGTACGGCGACCCTCGGTTTAATTTCAT
                             SARS-CoV-2 NC_045512.2     (1) -----------------------------------------------------------------------------
```

Section 652

(50128) 50128 50138 50149 50160 50171 50182 50193 50204
Autographa californica nucleopolyhedrovirus NC_0... (50128) TTGCTTTGACAAAAACCGTTTGTCGTTTGTGTCACAACAAATTTATTATTTGTACAAAAATATTGACGCAATGGAGG
SARS-CoV-2 NC_045512.2 (1) --

Section 653

(50205) 50205 50215 50226 50237 50248 50259 50270 50281
Autographa californica nucleopolyhedrovirus NC_0... (50205) CGTTGTTTAAATCTACACCATTGGTTTACGCGCTGTGGCAAAAACATAAACATGAGCAGTTTGCACAGAGGCTAGAG
SARS-CoV-2 NC_045512.2 (1) --

Section 654

(50282) 50282 50292 50303 50314 50325 50336 50347 50358
Autographa californica nucleopolyhedrovirus NC_0... (50282) ATGTTGTTGCGTGATTTTTGCTTAATTGCCAGTTCAAACGCTAGTTATTTACTTTTTAAACAGCTTACACAGCTCAT
SARS-CoV-2 NC_045512.2 (1) --

Section 655

(50359) 50359 50369 50380 50391 50402 50413 50424 50435
Autographa californica nucleopolyhedrovirus NC_0... (50359) AGCTAACGAAGAAATGGTGTGCGGAGATGAAGAAATATTCAATTTAGGCGGCCAATTTGTAGACATGATTAAAAGCG
SARS-CoV-2 NC_045512.2 (1) --

Section 656

(50436) 50436 50446 50457 50468 50479 50490 50501 50512
Autographa californica nucleopolyhedrovirus NC_0... (50436) GTGCTAAAGGCAGTCAAAATCTGATTAAAAGCACGCAACAATACCGACAGACTTTAAATACAGATATTGAAACTGTG
SARS-CoV-2 NC_045512.2 (1) --

Section 657

(50513) 50513 50523 50534 50545 50556 50567 50578 50589
Autographa californica nucleopolyhedrovirus NC_0... (50513) TCTTCACGAGCCACCACCAGTTTAAATAGTTACATATCTTCTCACAATAAGGTAAAAGTGTGTGGCGCCGACATATA
SARS-CoV-2 NC_045512.2 (1) --

Section 658

(50590) 50590 50600 50611 50622 50633 50644 50655 50666
Autographa californica nucleopolyhedrovirus NC_0... (50590) TCATAACACGGTTGTGTTACAGAGCGTGTTTATTAAAAATAACTATGTTTGTTACAAAAACGACGAACGTACAATCA
SARS-CoV-2 NC_045512.2 (1) --

```
                                                                                Section 659
                                          (50667) 50667     50677      50688      50699      50710      50721      50732      50743
Autographa californica nucleopolyhedrovirus NC 0... (50667) TGAATATTTGCGCTTTGCCCTCTGAGTTTCTGTTTCCAGAACATTTGCTCGACATGTTCATTGAATGATAATATAAA
                           SARS-CoV-2 NC_045512.2      (1) -----------------------------------------------------------------------------
                                                                                Section 660
                                          (50744) 50744     50754      50765      50776      50787      50798      50809      50820
Autographa californica nucleopolyhedrovirus NC 0... (50744) TAGAGCGCATTTGATTGCATGCAATCAGTGTTTTATTAATTTTAGAGCAACATGTACGATAAATTTATGATCTATCT
                           SARS-CoV-2 NC_045512.2      (1) -----------------------------------------------------------------------------
                                                                                Section 661
                                          (50821) 50821     50831      50842      50853      50864      50875      50886      50897
Autographa californica nucleopolyhedrovirus NC 0... (50821) TCACTTGAATGGGCTGCACGGAGAAGCAAAATACTACAAATATTTAATGTCTCAAATGGATTTTGAAAATCAAGTAG
                           SARS-CoV-2 NC_045512.2      (1) -----------------------------------------------------------------------------
                                                                                Section 662
                                          (50898) 50898     50908      50919      50930      50941      50952      50963      50974
Autographa californica nucleopolyhedrovirus NC 0... (50898) CCGATGAAATCAAGCGGTTTTGTGAAACTCGTCTGAAACCGGCAATCAGTTGCAACACTTTAACTGCGGAAAGTCTC
                           SARS-CoV-2 NC_045512.2      (1) -----------------------------------------------------------------------------
                                                                                Section 663
                                          (50975) 50975     50985      50996      51007      51018      51029      51040      51051
Autographa californica nucleopolyhedrovirus NC 0... (50975) AATACGCTCGTAGACAGCGTAGTCTGCAAAAATGGACTGTTAAATCCTTACGCCAAAGAAGTACAGTTTGCTTTGCA
                           SARS-CoV-2 NC_045512.2      (1) -----------------------------------------------------------------------------
                                                                                Section 664
                                          (51052) 51052     51062      51073      51084      51095      51106      51117      51128
Autographa californica nucleopolyhedrovirus NC 0... (51052) ATATCTTTTTGACGATGACGAAATATCCAAACGAGATCAAGATGGCTTTAAACTATTTTTATTACATAATTATGACA
                           SARS-CoV-2 NC_045512.2      (1) -----------------------------------------------------------------------------
                                                                                Section 665
                                          (51129) 51129     51139      51150      51161      51172      51183      51194      51205
Autographa californica nucleopolyhedrovirus NC 0... (51129) GGTGTGAAAATATGGAAGAATATTTTTTAATTAACAATTTTAGCATAGCAGACTACGAATTTGAAGACATGTTTGAA
                           SARS-CoV-2 NC_045512.2      (1) -----------------------------------------------------------------------------
```

```
                                                                                                  Section 666
                                          (51206) 51206     51216      51227      51238      51249      51260      51271    51282
Autographa californica nucleopolyhedrovirus NC 0... (51206) ATTGTTCGTATTGATTGTAGAGATCTGTTATTACTTCTTGCTAAATATAATATGTAATTAAAATTTTGTTTGTTTTA
                              SARS-CoV-2 NC_045512.2 (1) -----------------------------------------------------------------------------
                                                                                                  Section 667
                                          (51283) 51283     51293      51304      51315      51326      51337      51348    51359
Autographa californica nucleopolyhedrovirus NC 0... (51283) TTAAAATCCTGGATTAAAAAATGACGAATAATTTGATTTGCGTGCACGCCAACAAGATTCTTCGTCATTATGATCAA
                              SARS-CoV-2 NC_045512.2 (1) -----------------------------------------------------------------------------
                                                                                                  Section 668
                                          (51360) 51360     51370      51381      51392      51403      51414      51425    51436
Autographa californica nucleopolyhedrovirus NC 0... (51360) TGCGTGCATCAAGTTTATGCTTTTGTAATTGGCTTCTGACCACTTTAGCCATTTGAGCGTATCTGCATTCGTCGTCT
                              SARS-CoV-2 NC_045512.2 (1) -----------------------------------------------------------------------------
                                                                                                  Section 669
                                          (51437) 51437     51447      51458      51469      51480      51491      51502    51513
Autographa californica nucleopolyhedrovirus NC 0... (51437) AGAGTTTCAAACACCAGATCGGCGCAATTATAAAATCCTTCACCCACGGGATCTATGCGCTGCCAACGCACATACAT
                              SARS-CoV-2 NC_045512.2 (1) -----------------------------------------------------------------------------
                                                                                                  Section 670
                                          (51514) 51514     51524      51535      51546      51557      51568      51579    51590
Autographa californica nucleopolyhedrovirus NC 0... (51514) TACAAATTGATTTGACCTGTACGGTATTACTACGGGTATAGAATAGACTAGACTGTTGTCACATAATGAATCGCCCG
                              SARS-CoV-2 NC_045512.2 (1) -----------------------------------------------------------------------------
                                                                                                  Section 671
                                          (51591) 51591     51601      51612      51623      51634      51645      51656    51667
Autographa californica nucleopolyhedrovirus NC 0... (51591) GATTTGGAATTAAATTTGAATCGTTACCACCTATGTATTCTAATTCGTTCCAAGTTATTGGATTGCGACGATCCCAG
                              SARS-CoV-2 NC_045512.2 (1) -----------------------------------------------------------------------------
                                                                                                  Section 672
                                          (51668) 51668     51678      51689      51700      51711      51722      51733    51744
Autographa californica nucleopolyhedrovirus NC 0... (51668) TTTGATTTAGTAATAAACACTTCAAAATAACTGGGCTCGTGTATGGCTGTTGGACAAAAATGAACATTCATCTGATA
                              SARS-CoV-2 NC_045512.2 (1) -----------------------------------------------------------------------------
```

Section 673

```
                                                  (51745) 51745     51755      51766      51777      51788      51799      51810   51821
Autographa californica nucleopolyhedrovirus NC_0... (51745) AACCGGTTGATAGCGATTTAAATATAGCGTATTTGGCCTCCAGTTGTTAAAAGGTTCGTCCATTCCGCTTTTATCAC
                              SARS-CoV-2 NC_045512.2 (1) -----------------------------------------------------------------------------
```

Section 674

```
                                                  (51822) 51822     51832      51843      51854      51865      51876      51887   51898
Autographa californica nucleopolyhedrovirus NC_0... (51822) CAAACACAGAATTGCGATCGTTTGAACCGGCACCGCAAAGTGTGTGCGGCACAACCCTTTGTTTGATTAGGTCAAAA
                              SARS-CoV-2 NC_045512.2 (1) -----------------------------------------------------------------------------
```

Section 675

```
                                                  (51899) 51899     51909      51920      51931      51942      51953      51964   51975
Autographa californica nucleopolyhedrovirus NC_0... (51899) TCGTCATAATTAGGACCGGCCACAGCCGCGTATTCCATATACTGTTGAAACATGTATTGCGCTGTGGAAGCGGCCGC
                              SARS-CoV-2 NC_045512.2 (1) -----------------------------------------------------------------------------
```

Section 676

```
                                                  (51976) 51976     51986      51997      52008      52019      52030      52041   52052
Autographa californica nucleopolyhedrovirus NC_0... (51976) CCCGGATTCTAAATCGAGAGCTCGATATTTATAATAGACTGATTTGTAAGCATTGCGGCACGCGGCGTCGGGAATGT
                              SARS-CoV-2 NC_045512.2 (1) -----------------------------------------------------------------------------
```

Section 677

```
                                                  (52053) 52053     52063      52074      52085      52096      52107      52118   52129
Autographa californica nucleopolyhedrovirus NC_0... (52053) TATCGCCATTGTCGGGCCAATAAAAGTTTCCATCTTTAAAACATTTATATTGACGGGCCGTCGGCACGGACAAATAG
                              SARS-CoV-2 NC_045512.2 (1) -----------------------------------------------------------------------------
```

Section 678

```
                                                  (52130) 52130     52140      52151      52162      52173      52184      52195   52206
Autographa californica nucleopolyhedrovirus NC_0... (52130) CCGTGAGAGCGCACTGCCGGCGCGTGAATCGCAGCAAACAATGCAATTAATAATGCAATCATTATGATTATACTTAT
                              SARS-CoV-2 NC_045512.2 (1) -----------------------------------------------------------------------------
```

Section 679

```
                                                  (52207) 52207     52217      52228      52239      52250      52261      52272   52283
Autographa californica nucleopolyhedrovirus NC_0... (52207) AGAACACTAATCGGAATAATAACCGCTGTCGTAATCTTGGTCAAAAACGTTATGTTGAAACATAATAACACCTTACA
                              SARS-CoV-2 NC_045512.2 (1) -----------------------------------------------------------------------------
```

Section 680

	(52284)	52284 52294 52305 52316 52327 52338 52349 52360
Autographa californica nucleopolyhedrovirus NC 0...	(52284)	GTAACATACAATAAAACAACATAGTATCGTATATAATTATAAACTTTATTTTTTCATTTTATACAAACAAAATTTAT
SARS-CoV-2 NC_045512.2	(1)	---

Section 681

	(52361)	52361 52371 52382 52393 52404 52415 52426 52437
Autographa californica nucleopolyhedrovirus NC 0...	(52361)	ACGTATTGTTAGCACATTGAGTGTCATTTTCGCTGTCTGAACTATCACAATCATCGTCATCATCATCATCATTGTCA
SARS-CoV-2 NC_045512.2	(1)	---

Section 682

	(52438)	52438 52448 52459 52470 52481 52492 52503 52514
Autographa californica nucleopolyhedrovirus NC 0...	(52438)	TCGTCGTCGTCACGTTTGCGTTTGACACTGCATTTTTTTTGGTTAATTTTCACTAACACTGGTTCTTTTCGATCGTA
SARS-CoV-2 NC_045512.2	(1)	---

Section 683

	(52515)	52515 52525 52536 52547 52558 52569 52580 52591
Autographa californica nucleopolyhedrovirus NC 0...	(52515)	CAATTGATTCTGCATGTACTTTTGCATGATCGCGGTAAAACACTTTGCAATTTTATCCTTTTGTTCGTCGCCAAATA
SARS-CoV-2 NC_045512.2	(1)	---

Section 684

	(52592)	52592 52602 52613 52624 52635 52646 52657 52668
Autographa californica nucleopolyhedrovirus NC 0...	(52592)	TTTCCAGCAACTCGTTCATAAATGTGCACAAAATGCCCATGTGTTTTATCCAGCTGATTCGCATTTTCACTGGATCG
SARS-CoV-2 NC_045512.2	(1)	---

Section 685

	(52669)	52669 52679 52690 52701 52712 52723 52734 52745
Autographa californica nucleopolyhedrovirus NC 0...	(52669)	AACAAACGCAAGGGGTACGCTTTTTCTGTTACCTTGCCTTCGATGTCTATCAAAAGGTACGGGATACGATCTCCGTT
SARS-CoV-2 NC_045512.2	(1)	---

Section 686

	(52746)	52746 52756 52767 52778 52789 52800 52811 52822
Autographa californica nucleopolyhedrovirus NC 0...	(52746)	GCCGGGCACAAAATCCGTGCCTTTGTTAACCAAAATTTCTCTACAATGCCTAGCCACCGTAATCACGCGTCTTTTGG
SARS-CoV-2 NC_045512.2	(1)	---

Section 687

	(52823)	52823 52833 52844 52855 52866 52877 52888 52899
Autographa californica nucleopolyhedrovirus NC_0...	(52823)	GTGACGGACCCTCATTATCGTCAGTTGATTTGCGTTTTTTGCCCGGGTTATCGTTATAGGTCATACTAAAGCTGTAG
SARS-CoV-2 NC_045512.2	(1)	---

Section 688

	(52900)	52900 52910 52921 52932 52943 52954 52965 52976
Autographa californica nucleopolyhedrovirus NC_0...	(52900)	TCGGTCAACGATTTTGATTTGGCAAACTCATCATAGTATTCATAAAAACTAGTCTGTAAACTTTGCAAACATTTGTC
SARS-CoV-2 NC_045512.2	(1)	---

Section 689

	(52977)	52977 52987 52998 53009 53020 53031 53042 53053
Autographa californica nucleopolyhedrovirus NC_0...	(52977)	CATGTCCAAATGACGCAATATTTGTTCCACTGCCGTCCTAAACGCGATTCTCATAAAAACGGGCATATCCTTTTTAA
SARS-CoV-2 NC_045512.2	(1)	---

Section 690

	(53054)	53054 53064 53075 53086 53097 53108 53119 53130
Autographa californica nucleopolyhedrovirus NC_0...	(53054)	CTAACCAACCCTTGTATACGATTTTATTCTCACTGTTGAGATAGCAATATTTTTTCTTTTTTAATAGTATTAAAACT
SARS-CoV-2 NC_045512.2	(1)	---

Section 691

	(53131)	53131 53141 53152 53163 53174 53185 53196 53207
Autographa californica nucleopolyhedrovirus NC_0...	(53131)	TTCATTAAATTTTCAAATGCCATTTTGTAACCGTCCGTGAATGAGTTATTAACGCGTGTCTCAACATGTGTGCATAT
SARS-CoV-2 NC_045512.2	(1)	---

Section 692

	(53208)	53208 53218 53229 53240 53251 53262 53273 53284
Autographa californica nucleopolyhedrovirus NC_0...	(53208)	TTGTTTTAATGTGTCGGTTTCGTTGGATATTTCGTTATAGTTAAATGTGGGCAAAACAAATGTAGAATCTGTGTCGC
SARS-CoV-2 NC_045512.2	(1)	---

Section 693

	(53285)	53285 53295 53306 53317 53328 53339 53350 53361
Autographa californica nucleopolyhedrovirus NC_0...	(53285)	CGTACACAACTTTAAAAGTGATGCTGCCCAGATTGAATTTTTCTAAAATCTCAGGGTCGTTGCTCAAACCTTCAATC
SARS-CoV-2 NC_045512.2	(1)	---

```
                                                                                                    Section 694
                                                  (53362) 53362     53372      53383      53394      53405      53416      53427      53438
Autographa californica nucleopolyhedrovirus NC 0... (53362) AGAGAAATGGCCAGCCGCAACTGATTGCGACCAACTCTAGTGATGTAGTTTGCAAGCACTTTGTAAAAAATGCCATA
                             SARS-CoV-2 NC_045512.2     (1) -----------------------------------------------------------------------------
                                                                                                    Section 695
                                                  (53439) 53439     53449      53460      53471      53482      53493      53504      53515
Autographa californica nucleopolyhedrovirus NC 0... (53439) ATAACCGTATATGCTATTGGCGGTGCGCTTCACGGAATTTTGTTTTTGATCGTACAGATCGTACAAGAATGCCGATT
                             SARS-CoV-2 NC_045512.2     (1) -----------------------------------------------------------------------------
                                                                                                    Section 696
                                                  (53516) 53516     53526      53537      53548      53559      53570      53581      53592
Autographa californica nucleopolyhedrovirus NC 0... (53516) CGCTTTGATTGTCGCGATTCTTTTTAAATTTGCACCTTTCGCTTAACAATTTTAATAGCAATTTAACAACTATTGCA
                             SARS-CoV-2 NC_045512.2     (1) -----------------------------------------------------------------------------
                                                                                                    Section 697
                                                  (53593) 53593     53603      53614      53625      53636      53647      53658      53669
Autographa californica nucleopolyhedrovirus NC 0... (53593) CGCGAATTGTGGTTCAAATACACGTTGCCGTCTTCGCATAAAATTAAATTGGACAAACAAGCACAAATGGCTATCAT
                             SARS-CoV-2 NC_045512.2     (1) -----------------------------------------------------------------------------
                                                                                                    Section 698
                                                  (53670) 53670     53680      53691      53702      53713      53724      53735      53746
Autographa californica nucleopolyhedrovirus NC 0... (53670) TATAGTCAAGTACAAAGAATTAAAATCGAGAGAAAACGCGTTCTTGTAAATGCCTGCACGAGGTTTTAACACTTTGC
                             SARS-CoV-2 NC_045512.2     (1) -----------------------------------------------------------------------------
                                                                                                    Section 699
                                                  (53747) 53747     53757      53768      53779      53790      53801      53812      53823
Autographa californica nucleopolyhedrovirus NC 0... (53747) CGCCTTTGTACTTGACCGTTTGATTGGCGGGTCCCAAATTGATGGCATCTTTAGGTATGTTTTTTAGAGGTATCAAT
                             SARS-CoV-2 NC_045512.2     (1) -----------------------------------------------------------------------------
                                                                                                    Section 700
                                                  (53824) 53824     53834      53845      53856      53867      53878      53889      53900
Autographa californica nucleopolyhedrovirus NC 0... (53824) TTTCTTTTGAGATTAGAAATACCCGCTGCGGCTTTGTCGGCTTTGAATTGGCCCGATATTATTGACAGATCGTTTTT
                             SARS-CoV-2 NC_045512.2     (1) -----------------------------------------------------------------------------
```

```
                                                                Section 701
                                          (53901) 53901     53911      53922      53933      53944      53955      53966      53977
Autographa californica nucleopolyhedrovirus  NC_0... (53901) GTTAAAAAAATACGGGTCAGGCTCCTCTTTGCCGGTGCTCTCGTTAATGCGCGTGTTTGTGATGGCTGCGTAAAAGC
                          SARS-CoV-2 NC_045512.2 (1) -----------------------------------------------------------------------------
                                                                Section 702
                                          (53978) 53978     53988      53999      54010      54021      54032      54043      54054
Autographa californica nucleopolyhedrovirus  NC_0... (53978) ACGCCACGCTAATCAAATGCGAAATATTACATATCACGTCGTCTGTACACAAACGATGCAATATACATTGCGAATAT
                          SARS-CoV-2 NC_045512.2 (1) -----------------------------------------------------------------------------
                                                                Section 703
                                          (54055) 54055     54065      54076      54087      54098      54109      54120      54131
Autographa californica nucleopolyhedrovirus  NC_0... (54055) ACAGAATCGGCCATTTTCAATTTGACAAACAATTTTATCGGCAACATGCAATCCTGCACGTTGTACTTGGCAATCAC
                          SARS-CoV-2 NC_045512.2 (1) -----------------------------------------------------------------------------
                                                                Section 704
                                          (54132) 54132     54142      54153      54164      54175      54186      54197      54208
Autographa californica nucleopolyhedrovirus  NC_0... (54132) GTCCAGCCGTCGAGTGTTGTACATCTTGACCATTTCGGTCCAAGGCAAATCGATTTTGTTTTCACCCAAATAGTAAC
                          SARS-CoV-2 NC_045512.2 (1) -----------------------------------------------------------------------------
                                                                Section 705
                                          (54209) 54209     54219      54230      54241      54252      54263      54274      54285
Autographa californica nucleopolyhedrovirus  NC_0... (54209) TACTGATTGTGTTCAATTGAAAGTTTTCAACTTTATGCTGATTAGAATCGCTGCTGAAAAATTTATACAAATCAATG
                          SARS-CoV-2 NC_045512.2 (1) -----------------------------------------------------------------------------
                                                                Section 706
                                          (54286) 54286     54296      54307      54318      54329      54340      54351      54362
Autographa californica nucleopolyhedrovirus  NC_0... (54286) TGAATGTAATAGTTAAAATAATACGTGTCCACTTTGTTGCCCAACTTGTTTATAAACAGCTTTGTCGTCGGCGCCGC
                          SARS-CoV-2 NC_045512.2 (1) -----------------------------------------------------------------------------
                                                                Section 707
                                          (54363) 54363     54373      54384      54395      54406      54417      54428      54439
Autographa californica nucleopolyhedrovirus  NC_0... (54363) AGCCGGCAAATCGTAACGCTTTAATAGCATTTTGGTTTTATTCAATCGTCCAAGTATATAGGGCAGATCAAATACGT
                          SARS-CoV-2 NC_045512.2 (1) -----------------------------------------------------------------------------
```

```
                                                                                                                   Section 708
                                                    (54440) 54440     54450      54461      54472      54483      54494      54505 54516
Autographa californica nucleopolyhedrovirus NC_0... (54440) CTCCGTTAAAATCCAAAATCACATCGGGATTTGTAATTTTTATCATGTCAAAAAACGCTGTAATCATGTCGATTTCA
                            SARS-CoV-2 NC_045512.2      (1) -----------------------------------------------------------------------------
                                                                                                                   Section 709
                                                    (54517) 54517     54527      54538      54549      54560      54571      54582 54593
Autographa californica nucleopolyhedrovirus NC_0... (54517) TTTTGAAACATGACCACATACGTGTCATCGTCATAGGTCTCTGGAATCTGGGTCGGCAGCTTGTGATACATAAAACA
                            SARS-CoV-2 NC_045512.2      (1) -----------------------------------------------------------------------------
                                                                                                                   Section 710
                                                    (54594) 54594     54604      54615      54626      54637      54648      54659 54670
Autographa californica nucleopolyhedrovirus NC_0... (54594) AAATTTTGCATACTCGTCGTTTTTGTACACCACAAATCCTATAGACATTATGCAATCAACCGATGCTTTCGACATGT
                            SARS-CoV-2 NC_045512.2      (1) -----------------------------------------------------------------------------
                                                                                                                   Section 711
                                                    (54671) 54671     54681      54692      54703      54714      54725      54736 54747
Autographa californica nucleopolyhedrovirus NC_0... (54671) TGTGGCCGTCCGAATGAGTCTCAATGTCATAGCACGACAAAACGGGCATGATGCCGCTGGTTAAAGTCATTTCATCG
                            SARS-CoV-2 NC_045512.2      (1) -----------------------------------------------------------------------------
                                                                                                                   Section 712
                                                    (54748) 54748     54758      54769      54780      54791      54802      54813 54824
Autographa californica nucleopolyhedrovirus NC_0... (54748) ACCAACTCAAAGTCTTCATTAAAATGTTGCAAATTAAACATGCGCGTCGTCGATCCACCGACATAGTTATTTTGGCA
                            SARS-CoV-2 NC_045512.2      (1) -----------------------------------------------------------------------------
                                                                                                                   Section 713
                                                    (54825) 54825     54835      54846      54857      54868      54879      54890 54901
Autographa californica nucleopolyhedrovirus NC_0... (54825) GCGTTGTGTTTTCTTGAATCGCATATAGGCGCCTTCCACAAACGGCGTTTGCATGTGTACGCGATTAACGTTGTGAA
                            SARS-CoV-2 NC_045512.2      (1) -----------------------------------------------------------------------------
                                                                                                                   Section 714
                                                    (54902) 54902     54912      54923      54934      54945      54956      54967 54978
Autographa californica nucleopolyhedrovirus NC_0... (54902) GAAACTTGTCCAAACACGCCGCGTTGTCCGATGGCGCTGCTTTGTTTCTTTCGTATTTAATCACGTTTATCTTGTTC
                            SARS-CoV-2 NC_045512.2      (1) -----------------------------------------------------------------------------
```

```
                                                                                                        Section 715
                                              (54979) 54979     54989      55000      55011      55022      55033      55044   55055
Autographa californica nucleopolyhedrovirus NC 0... (54979) AAATAATTTCCTTCCACGCCCGGCGCCACAAACGTGGTGTAGCTGATGCACTTGTTGCGGCAAGACGGAAATATGTG
                             SARS-CoV-2 NC_045512.2     (1) -----------------------------------------------------------------------------
                                                                                                        Section 716
                                              (55056) 55056     55066      55077      55088      55099      55110      55121   55132
Autographa californica nucleopolyhedrovirus NC 0... (55056) CTTGTCGTAGCATTGTTTGTAAGAATACAAATTTAGTTTTACTTTAAAGTAAAACTGCAGCACTCGTTCTTTGATAT
                             SARS-CoV-2 NC_045512.2     (1) -----------------------------------------------------------------------------
                                                                                                        Section 717
                                              (55133) 55133     55143      55154      55165      55176      55187      55198   55209
Autographa californica nucleopolyhedrovirus NC 0... (55133) TTGTATTACAAAATGCAAACAAGCAACCTTGTTTTTCATCGTAATGCAAACGAATGATACGAAACGTATCGGCTGAA
                             SARS-CoV-2 NC_045512.2     (1) -----------------------------------------------------------------------------
                                                                                                        Section 718
                                              (55210) 55210     55220      55231      55242      55253      55264      55275   55286
Autographa californica nucleopolyhedrovirus NC 0... (55210) GTAATATTGAATTCTCCTGGTTTTGCATATTCTGCAAAGCGCGTTTTGAGTTCATTGTAAGGATATATTTTCATTTT
                             SARS-CoV-2 NC_045512.2     (1) -----------------------------------------------------------------------------
                                                                                                        Section 719
                                              (55287) 55287     55297      55308      55319      55330      55341      55352   55363
Autographa californica nucleopolyhedrovirus NC 0... (55287) TAAATATGCAGCGATGGCCCAAATATGGAGGCACAGACGTCAACACGCGCACTGTACACGATTTGTTAAACACCATA
                             SARS-CoV-2 NC_045512.2     (1) -----------------------------------------------------------------------------
                                                                                                        Section 720
                                              (55364) 55364     55374      55385      55396      55407      55418      55429   55440
Autographa californica nucleopolyhedrovirus NC 0... (55364) AACACCATGAGTGCTCGAATCAAAACTCTGGAGCGGTATGAGCACGCTTTGCGAGAGATTCACAAAGTCGTTGTAAT
                             SARS-CoV-2 NC_045512.2     (1) -----------------------------------------------------------------------------
                                                                                                        Section 721
                                              (55441) 55441     55451      55462      55473      55484      55495      55506   55517
Autographa californica nucleopolyhedrovirus NC 0... (55441) TTTGAAACCGTCCGCGAACACACATAGCTTTGAACCCGACGCTCTGCCGGCGTTGATTATGCAATTTTTATCGGATT
                             SARS-CoV-2 NC_045512.2     (1) -----------------------------------------------------------------------------
```

```
                                                                                                      Section 722
                                                 (55518) 55518     55528      55539      55550      55561      55572      55583      55594
Autographa californica nucleopolyhedrovirus NC 0... (55518) TCGCCGGCCGAGATATCAACACGTTGACGCACAACATCAACTACAAGTACGATTACAATTATCCGCCGGCGCCCGTG
                            SARS-CoV-2 NC_045512.2      (1) -----------------------------------------------------------------------------
                                                                                                      Section 723
                                                 (55595) 55595     55605      55616      55627      55638      55649      55660      55671
Autographa californica nucleopolyhedrovirus NC 0... (55595) CCCGCGATGCAACCACCGCCACCGCCTCCTCAACCCCCCGCGCCACCTCAACCACCGTATTACAACAATTATCCGTA
                            SARS-CoV-2 NC_045512.2      (1) -----------------------------------------------------------------------------
                                                                                                      Section 724
                                                 (55672) 55672     55682      55693      55704      55715      55726      55737      55748
Autographa californica nucleopolyhedrovirus NC 0... (55672) TTATCCGCCGTATCCGTTTTCGACACCGCCGCCAACACAGCCGCCAGAATCGAACGTCGCGGGCGTCGGCGGCTCGC
                            SARS-CoV-2 NC_045512.2      (1) -----------------------------------------------------------------------------
                                                                                                      Section 725
                                                 (55749) 55749     55759      55770      55781      55792      55803      55814      55825
Autographa californica nucleopolyhedrovirus NC 0... (55749) AAAGTTTGAATCAAATCACGTTGACTAACGAGGAGGAGTCTGAACTGGCGGCTTTATTTAAAAACATGCAAACGAAC
                            SARS-CoV-2 NC_045512.2      (1) -----------------------------------------------------------------------------
                                                                                                      Section 726
                                                 (55826) 55826     55836      55847      55858      55869      55880      55891      55902
Autographa californica nucleopolyhedrovirus NC 0... (55826) ATGACTTGGGAACTTGTTCAAAATTTCGTTGAAGTGTTAATCAGGATCGTACGCGTGCACGTAGTAAACAACGTGAC
                            SARS-CoV-2 NC_045512.2      (1) -----------------------------------------------------------------------------
                                                                                                      Section 727
                                                 (55903) 55903     55913      55924      55935      55946      55957      55968      55979
Autographa californica nucleopolyhedrovirus NC 0... (55903) CATGATTAACGTTATATCGTCTATAACTTCCGTTCGAACATTAATTGATTACAATTTTACAGAATTTATTAGATGCG
                            SARS-CoV-2 NC_045512.2      (1) -----------------------------------------------------------------------------
                                                                                                      Section 728
                                                 (55980) 55980     55990      56001      56012      56023      56034      56045      56056
Autographa californica nucleopolyhedrovirus NC 0... (55980) TATACCAAAAAACAAACATACGTTTTGCAATAGATCAGTATCTGTGCACTAACATAGTTACGTTTATAGATTTTTTT
                            SARS-CoV-2 NC_045512.2      (1) -----------------------------------------------------------------------------
```

Section 729

```
                                              (56057) 56057     56067      56078      56089      56100      56111      56122   56133
Autographa californica nucleopolyhedrovirus NC 0... (56057) ACTAGAGTCTTTTATTTGGTGATGCGAACAAATTTTCAGTTCACCACTTTTGACCAATTGACCCAATACTCTAACGA
                             SARS-CoV-2 NC_045512.2 (1) -----------------------------------------------------------------------------
```

Section 730

```
                                              (56134) 56134     56144      56155      56166      56177      56188      56199   56210
Autographa californica nucleopolyhedrovirus NC 0... (56134) ACTTTACACAAGAATTCAAACGAGCATACTTCAAAGCGCGGCTCCTCTTTCTCCTCCGACCGTGGAAACGGTCAACA
                             SARS-CoV-2 NC_045512.2 (1) -----------------------------------------------------------------------------
```

Section 731

```
                                              (56211) 56211     56221      56232      56243      56254      56265      56276   56287
Autographa californica nucleopolyhedrovirus NC 0... (56211) GCGATATCGTCATTTCAAATTTGCAAGAACAATTAAAAAGAGAACGCGCTTTGATGCAACAAATCAGCGAGCAACAT
                             SARS-CoV-2 NC_045512.2 (1) -----------------------------------------------------------------------------
```

Section 732

```
                                              (56288) 56288     56298      56309      56320      56331      56342      56353   56364
Autographa californica nucleopolyhedrovirus NC 0... (56288) AGAATTGCAAACGAAAGAGTGGAAACTCTGCAATCGCAATACGACGAGTTGGATTTAAAGTATAAAGAGATATTTGA
                             SARS-CoV-2 NC_045512.2 (1) -----------------------------------------------------------------------------
```

Section 733

```
                                              (56365) 56365     56375      56386      56397      56408      56419      56430   56441
Autographa californica nucleopolyhedrovirus NC 0... (56365) AGACAAAAGTGAATTCGCACAACAAAAAAGTGAAAACGTGCGAAAAATTAAACAATTAGAGAGATCCAACAAAGAAC
                             SARS-CoV-2 NC_045512.2 (1) -----------------------------------------------------------------------------
```

Section 734

```
                                              (56442) 56442     56452      56463      56474      56485      56496      56507   56518
Autographa californica nucleopolyhedrovirus NC 0... (56442) TCAACGACACCGTACAGAAATTGAGAGATGAAAATGCCGAAAGATTGTCTGAAATACAATTGCAAAAAGGCGATTTG
                             SARS-CoV-2 NC_045512.2 (1) -----------------------------------------------------------------------------
```

Section 735

```
                                              (56519) 56519     56529      56540      56551      56562      56573      56584   56595
Autographa californica nucleopolyhedrovirus NC 0... (56519) GACGAATATAAAAACATGAATCGCCAGTTGAACGAGGACATTTATAAACTCAAAAGAAGAATAGAATCGACATTTGA
                             SARS-CoV-2 NC_045512.2 (1) -----------------------------------------------------------------------------
```

```
                                                               Section 736
                                           (56596) 56596     56606      56617      56628      56639      56650      56661      56672
Autographa californica nucleopolyhedrovirus NC 0... (56596) TAAAGATTACGTCGAAACCTTGAACGATAAAATTGAATCGTTGGAAAAGCAATTGGATGATAAACAAAATTTAAACC
                          SARS-CoV-2 NC_045512.2 (1) -----------------------------------------------------------------------------
                                                               Section 737
                                           (56673) 56673     56683      56694      56705      56716      56727      56738      56749
Autographa californica nucleopolyhedrovirus NC 0... (56673) GGGAACTAAGAAGCAGCATTTCAAAAATAGACGAAACTACACAGAGGTACAAACTTGACGCCAAAGATATTATGGAA
                          SARS-CoV-2 NC_045512.2 (1) -----------------------------------------------------------------------------
                                                               Section 738
                                           (56750) 56750     56760      56771      56782      56793      56804      56815      56826
Autographa californica nucleopolyhedrovirus NC 0... (56750) CTCAAACAGTCGGTATCGATTAAAGATCAAGAAATTGCCATGAAAAACGCTCAATATTTAGAATTGAGTGCTATATA
                          SARS-CoV-2 NC_045512.2 (1) -----------------------------------------------------------------------------
                                                               Section 739
                                           (56827) 56827     56837      56848      56859      56870      56881      56892      56903
Autographa californica nucleopolyhedrovirus NC 0... (56827) TCAACAAACTGTAAATGAATTAACTGCAACTAAAAATGAATTGTCTCAAGTCGCGACAACCAATCAAAGTTTATTTG
                          SARS-CoV-2 NC_045512.2 (1) -----------------------------------------------------------------------------
                                                               Section 740
                                           (56904) 56904     56914      56925      56936      56947      56958      56969      56980
Autographa californica nucleopolyhedrovirus NC 0... (56904) CAGAAAATGAAGAATCTAAAGTGCTTTTAGAAGGCACGTTGGCGTTTATAGATAGCTTTTATCAAATAATTATGCAG
                          SARS-CoV-2 NC_045512.2 (1) -----------------------------------------------------------------------------
                                                               Section 741
                                           (56981) 56981     56991      57002      57013      57024      57035      57046      57057
Autographa californica nucleopolyhedrovirus NC 0... (56981) ATTGAAAAACCTGATTACGTGCCGATTTCTAAACCACAGCTTACAGCACAAGAAAGTATATATCAAACGGATTATAT
                          SARS-CoV-2 NC_045512.2 (1) -----------------------------------------------------------------------------
                                                               Section 742
                                           (57058) 57058     57068      57079      57090      57101      57112      57123      57134
Autographa californica nucleopolyhedrovirus NC 0... (57058) CAAAGATTGGTTGCAAAAATTGAGGTCTAAACTGTCAAACGCCGACGTTGCCAATTTGCAATCAGTTTCCGAATTGA
                          SARS-CoV-2 NC_045512.2 (1) -----------------------------------------------------------------------------
```

Section 743

	(57135)	57135 57145 57156 57167 57178 57189 57200 57211
Autographa californica nucleopolyhedrovirus NC 0...	(57135)	GTGATTTAAAAAGTCAAATAATTTCTATTGTACCACGAAATATTGTAAATCGAATTTTAAAAGAAAATTATAAAGTA
SARS-CoV-2 NC_045512.2	(1)	---

Section 744

	(57212)	57212 57222 57233 57244 57255 57266 57277 57288
Autographa californica nucleopolyhedrovirus NC 0...	(57212)	AAAGTAGAAAATGTCAATGCAGAATTACTGGAAAGTGTTGCTGTCACAAGTGCTGTAAGCGCTTTAGTACAGCAATA
SARS-CoV-2 NC_045512.2	(1)	---

Section 745

	(57289)	57289 57299 57310 57321 57332 57343 57354 57365
Autographa californica nucleopolyhedrovirus NC 0...	(57289)	TGAACGATCAGAAAAGCAAAACGTTAAACTTAGACAAGAATTCGAAATAAAATTAAACGATTTACAAAGATTATTGG
SARS-CoV-2 NC_045512.2	(1)	---

Section 746

	(57366)	57366 57376 57387 57398 57409 57420 57431 57442
Autographa californica nucleopolyhedrovirus NC 0...	(57366)	AGCAAAATCAGACTGATTTTGAGTCAATATCAGAGTTTATCTCACGAGATCCGGCTTTCAACAGAAATTTAAATGAC
SARS-CoV-2 NC_045512.2	(1)	---

Section 747

	(57443)	57443 57453 57464 57475 57486 57497 57508 57519
Autographa californica nucleopolyhedrovirus NC 0...	(57443)	GAGCGATTCCAAAACTTGAGGCAACAATACGACGAAATGTCTAGTAAATATTCAGCCTTGGAAACGACTAAAATTAA
SARS-CoV-2 NC_045512.2	(1)	---

Section 748

	(57520)	57520 57530 57541 57552 57563 57574 57585 57596
Autographa californica nucleopolyhedrovirus NC 0...	(57520)	AGAGATGGAGTCTATTGCAGATCAGGCTGTCAAATCTGAAATGAGTAAATTAAACACACAACTAGATGAATTAAACT
SARS-CoV-2 NC_045512.2	(1)	---

Section 749

	(57597)	57597 57607 57618 57629 57640 57651 57662 57673
Autographa californica nucleopolyhedrovirus NC 0...	(57597)	CTTTATTTGTTAAATATAATCGTAAAGCTCAAGACATATTTGAGTGGAAAACTAGCATGCTTAAAAGGTACGAAACG
SARS-CoV-2 NC_045512.2	(1)	---

Section 750

	(57674)	57674 57684 57695 57706 57717 57728 57739 57750
Autographa californica nucleopolyhedrovirus NC 0...	(57674)	TTGGCGCGAACAACAGCGGCCAGCGTTCAACCAAACGTCGAATAGAATTACAAAAATTTATATTCATTTTCATCTTC
SARS-CoV-2 NC_045512.2	(1)	---

Section 751

	(57751)	57751 57761 57772 57783 57794 57805 57816 57827
Autographa californica nucleopolyhedrovirus NC 0...	(57751)	GTCATACTTCAACAGTCCCAACACGTTCATGTTGTGATTCTCGCCGTTCTCGACAGTTACGTAAATAGTTACTTTGA
SARS-CoV-2 NC_045512.2	(1)	---

Section 752

	(57828)	57828 57838 57849 57860 57871 57882 57893 57904
Autographa californica nucleopolyhedrovirus NC 0...	(57828)	TTAAATTATCTTCCAGCAGCATTGAGATTTGATTGAAATCCGCACATAGCTTTTGTAGCGAATCCGCTTCGGTTTTT
SARS-CoV-2 NC_045512.2	(1)	---

Section 753

	(57905)	57905 57915 57926 57937 57948 57959 57970 57981
Autographa californica nucleopolyhedrovirus NC 0...	(57905)	TTATTTGTGTTGACGTAGAAAACAGATTTGTTCCATTTGCCCAAGTCGGAAGAGGTAGAACAGTCATCCGAATCGGC
SARS-CoV-2 NC_045512.2	(1)	---

Section 754

	(57982)	57982 57992 58003 58014 58025 58036 58047 58058
Autographa californica nucleopolyhedrovirus NC 0...	(57982)	AATGTTCAACTCGTCGCTTTTAAACTGCACAATAAACTTGTTATCGCCCATGTCATTTTCTTCCAATTCGCTTTTTA
SARS-CoV-2 NC_045512.2	(1)	---

Section 755

	(58059)	58059 58069 58080 58091 58102 58113 58124 58135
Autographa californica nucleopolyhedrovirus NC 0...	(58059)	ACACATTTACATTGTACGAAGCAACGTGTTTGTTCGATCGACTAATGTTGATCTTTGCGTTTGTGCAATTTTGCAAA
SARS-CoV-2 NC_045512.2	(1)	---

Section 756

	(58136)	58136 58146 58157 58168 58179 58190 58201 58212
Autographa californica nucleopolyhedrovirus NC 0...	(58136)	TTTGAATATGCTTCGCTTTCTTTAGCCTCGCACAATTCGATGCGCGTAGAGTTGACCACGTTCCAATTCATGTACAC
SARS-CoV-2 NC_045512.2	(1)	---

Section 757

	(58213)	58213 58223 58234 58245 58256 58267 58278 58289
Autographa californica nucleopolyhedrovirus NC_0...	(58213)	GTTTGATCCATTAAAAATTTGTTGACACTTTATACTGTAAATGGTAAAGATTTGGTTTTCATTGTCTTTTAAATATT
SARS-CoV-2 NC_045512.2	(1)	---

Section 758

	(58290)	58290 58300 58311 58322 58333 58344 58355 58366
Autographa californica nucleopolyhedrovirus NC_0...	(58290)	TAAACACCTCATTGATGTCGTCAGACCCCTTTATATTGTTCTTGAATAGATTTATTAGTGTTTTCGCATTGACAGAA
SARS-CoV-2 NC_045512.2	(1)	---

Section 759

	(58367)	58367 58377 58388 58399 58410 58421 58432 58443
Autographa californica nucleopolyhedrovirus NC_0...	(58367)	CATTCCACTTGAACCACGTCGGGATCGTCGTTGAGATTTTTGTACACAACCTCAAAAACAACTTTGTACAAACCGCT
SARS-CoV-2 NC_045512.2	(1)	---

Section 760

	(58444)	58444 58454 58465 58476 58487 58498 58509 58520
Autographa californica nucleopolyhedrovirus NC_0...	(58444)	GTTGATTTTCTTGTAGATAAATTTGTACTTTACAATAATATTGACGCCATCTTCATTTTCAAAATGTTTGTTAGTCA
SARS-CoV-2 NC_045512.2	(1)	---

Section 761

	(58521)	58521 58531 58542 58553 58564 58575 58586 58597
Autographa californica nucleopolyhedrovirus NC_0...	(58521)	AATAGTCGCTCATGGGGGTTGCAGTTTCAATTTCCATTTCACATTCTTTGTATTCGTTGATCTGAATCATTTGACTA
SARS-CoV-2 NC_045512.2	(1)	---

Section 762

	(58598)	58598 58608 58619 58630 58641 58652 58663 58674
Autographa californica nucleopolyhedrovirus NC_0...	(58598)	AACTTTGTTTTCACATAATTTAAACTAATGTCATAGCACTTGCCTTCTTCCATGTCTTTGAAAGATTGCGAATCGCC
SARS-CoV-2 NC_045512.2	(1)	---

Section 763

	(58675)	58675 58685 58696 58707 58718 58729 58740 58751
Autographa californica nucleopolyhedrovirus NC_0...	(58675)	GTAGTATTCTTGAATTTTGTTGTCGGACATTATTCGAAAAGTGTAATGGTATTCATTATCGATACTCAACGTCATTT
SARS-CoV-2 NC_045512.2	(1)	---

Section 764

	(58752)	58752 58762 58773 58784 58795 58806 58817 58828
Autographa californica nucleopolyhedrovirus NC 0...	(58752)	TGCTCATCAATTTACCACTAATCCTTTTGTAATTTTCTCTAATCTTCTTGGGGCTACTGGCCATAGCCATGCGTTTT
SARS-CoV-2 NC_045512.2	(1)	---

Section 765

	(58829)	58829 58839 58850 58861 58872 58883 58894 58905
Autographa californica nucleopolyhedrovirus NC 0...	(58829)	ATAAGCGGCTCACCGCTACTTTCTCCAGACAAAGATCTTTTGGTCGCCATATTGCTGTTGTCGATATGTGGGAATCT
SARS-CoV-2 NC_045512.2	(1)	---

Section 766

	(58906)	58906 58916 58927 58938 58949 58960 58971 58982
Autographa californica nucleopolyhedrovirus NC 0...	(58906)	ATCCGATGGCAAATACTGAATGGCGACGAAATCGAAGTGTCGCCAGAGCACCGTTCGTTAGCGTGGAGGGAGTTGAT
SARS-CoV-2 NC_045512.2	(1)	---

Section 767

	(58983)	58983 58993 59004 59015 59026 59037 59048 59059
Autographa californica nucleopolyhedrovirus NC 0...	(58983)	TATAAACGTGGCCAGCAACACGCCGCTCGACAACACGTTCAGAACAATGTTTCAAAAAGCCGATTTTGAAAATTTCG
SARS-CoV-2 NC_045512.2	(1)	---

Section 768

	(59060)	59060 59070 59081 59092 59103 59114 59125 59136
Autographa californica nucleopolyhedrovirus NC 0...	(59060)	ACTACAACACGCCGATTGTGTACAATTTAAAAACAAAAACTTTAACAATGTACAACGAGAGAATAAGAGCGGCTCTG
SARS-CoV-2 NC_045512.2	(1)	---

Section 769

	(59137)	59137 59147 59158 59169 59180 59191 59202 59213
Autographa californica nucleopolyhedrovirus NC 0...	(59137)	AACAGACCCGTCCGATTTAACGATCAAACGGTCAATGTTAATATTGCGTACGTATTTTTGTTCTTTATTTGTATAGT
SARS-CoV-2 NC_045512.2	(1)	---

Section 770

	(59214)	59214 59224 59235 59246 59257 59268 59279 59290
Autographa californica nucleopolyhedrovirus NC 0...	(59214)	TTTGCTGAGCGTGTTGGCCGTCTTTTTCGACACAAACATTGCGACCGACACGAAGAGTAAAAATGTTGCAGCAAAAA
SARS-CoV-2 NC_045512.2	(1)	---

Section 771

(59291) 59291 59301 59312 59323 59334 59345 59356 59367
Autographa californica nucleopolyhedrovirus NC_0... (59291) TTAAATAAACTCAAAGATGGTTTGAACACGTTCAGCAGCAAGTCGGTGGTTTGCGCTCGCTCAAAATTATTTGACAA
SARS-CoV-2 NC_045512.2 (1) ---

Section 772

(59368) 59368 59378 59389 59400 59411 59422 59433 59444
Autographa californica nucleopolyhedrovirus NC_0... (59368) ACGCCCAACGCGCAGACCTAGATGTTGGCGAAAACTATCAGAGATCGACAAAAAGTTTCACGTTTGCCGACACGTTG
SARS-CoV-2 NC_045512.2 (1) ---

Section 773

(59445) 59445 59455 59466 59477 59488 59499 59510 59521
Autographa californica nucleopolyhedrovirus NC_0... (59445) ACACGTTTTTGGATTTGTGCGGCGGACCGGGCGAGTTTGCCAACTATACCATGTCGTTGAACCCGCTTTGCAAAGCG
SARS-CoV-2 NC_045512.2 (1) ---

Section 774

(59522) 59522 59532 59543 59554 59565 59576 59587 59598
Autographa californica nucleopolyhedrovirus NC_0... (59522) TATGGCGTCACGTTGACAAACAACTCGGTGTGCGTGTACAAACCGACAGTGCGCAAACGCAAAAATTTCACAACCAT
SARS-CoV-2 NC_045512.2 (1) ---

Section 775

(59599) 59599 59609 59620 59631 59642 59653 59664 59675
Autographa californica nucleopolyhedrovirus NC_0... (59599) TACGGGGCCCGACAAGTCAGGCGACGTGTTTGATAAAAATGTTGTATTTGAGATTAGCATCAAGTGTGGCAACGCGT
SARS-CoV-2 NC_045512.2 (1) ---

Section 776

(59676) 59676 59686 59697 59708 59719 59730 59741 59752
Autographa californica nucleopolyhedrovirus NC_0... (59676) GCGATCTGGTGTTGGCAGATGGCTCGGTTGACGTTAATGGACGCGAAAACGAACAAGAACGTCTCAACTTTGATTTG
SARS-CoV-2 NC_045512.2 (1) ---

Section 777

(59753) 59753 59763 59774 59785 59796 59807 59818 59829
Autographa californica nucleopolyhedrovirus NC_0... (59753) ATCATGTGCGAGACGCAGCTAATTTTAATTTGCCTGCGTCCCGGCGGCAATTGCGTTTTAAAAGTTTTCGACGCGTT
SARS-CoV-2 NC_045512.2 (1) ---

Section 778

	(59830)	59830 59840 59851 59862 59873 59884 59895 59906
Autographa californica nucleopolyhedrovirus NC_0...	(59830)	TGAACACGAAACGATCCAAATGCTAAACAAGTTTGTTAACCATTTCGAAAAATGGGTTTTATACAAACCGCCTTCTT
SARS-CoV-2 NC_045512.2	(1)	---

Section 779

	(59907)	59907 59917 59928 59939 59950 59961 59972 59983
Autographa californica nucleopolyhedrovirus NC_0...	(59907)	CTCGGCCTGCCAATTCCGAACGCTATTTAATTTGTTTCAATAAATTAGTTAGACCGTATTGTAACAATTATGTCAAC
SARS-CoV-2 NC_045512.2	(1)	---

Section 780

	(59984)	59984 59994 60005 60016 60027 60038 60049 60060
Autographa californica nucleopolyhedrovirus NC_0...	(59984)	GAGTTGGAAAAACAGTTTGAAAAATATTATCGCATACAATTAAAAAACTTAAACAAGTTGATAAACTTGTTGAAAAT
SARS-CoV-2 NC_045512.2	(1)	---

Section 781

	(60061)	60061 60071 60082 60093 60104 60115 60126 60137
Autographa californica nucleopolyhedrovirus NC_0...	(60061)	ATAACGTGTGTATAAAAAGCCAGCGGCTTCAAATCAGGCATCATTCAACATGGATTCGCTAGCCAATTTGTGCTTGA
SARS-CoV-2 NC_045512.2	(1)	---

Section 782

	(60138)	60138 60148 60159 60170 60181 60192 60203 60214
Autographa californica nucleopolyhedrovirus NC_0...	(60138)	AAACCCTGCCTTACAAGTTTGAGCCGCCTAAGTTTTTACGAACAAAATATTGCGACGCATGTCGCTACAGATTTTTA
SARS-CoV-2 NC_045512.2	(1)	---

Section 783

	(60215)	60215 60225 60236 60247 60258 60269 60280 60291
Autographa californica nucleopolyhedrovirus NC_0...	(60215)	CCAAAATTTTCTGATGAAAAATTTTGTGGACAATGCATATGCAACATATGCAACAATCCAAAAAATATAGATTGTCC
SARS-CoV-2 NC_045512.2	(1)	---

Section 784

	(60292)	60292 60302 60313 60324 60335 60346 60357 60368
Autographa californica nucleopolyhedrovirus NC_0...	(60292)	ATCATCATATATATCGAAAATTAAACCGAAGAAAGAAAACAAAGAAATATATATTACCAGCAACAAGTTTAATAAAA
SARS-CoV-2 NC_045512.2	(1)	---

```
                                                                                                  Section 785
                                              (60369) 60369     60379      60390      60401      60412      60423      60434  60445
Autographa californica nucleopolyhedrovirus NC_0... (60369) CGTGCAAAAACGAATGTAATCAACAATCAAACCGGAGATGTTTAATTTCCTATTTTACAAATGAAAGTTGTAAAGAG
                           SARS-CoV-2 NC_045512.2 (1) -----------------------------------------------------------------------------
                                                                                                  Section 786
                                              (60446) 60446     60456      60467      60478      60489      60500      60511  60522
Autographa californica nucleopolyhedrovirus NC_0... (60446) CTCAATTGTTGTTGGTTTAATAAAAACTGTTACATGTGTTTGGAATATAAAAAGAATTTATACAATGTAAATTTGTA
                           SARS-CoV-2 NC_045512.2 (1) -----------------------------------------------------------------------------
                                                                                                  Section 787
                                              (60523) 60523     60533      60544      60555      60566      60577      60588  60599
Autographa californica nucleopolyhedrovirus NC_0... (60523) TACGATTGATGGTCATTGTCCTTCGTTTAAAGCCGTTTGTTTTTCATGTATAAAAAGAATCAAAACGTGCCAAGTTT
                           SARS-CoV-2 NC_045512.2 (1) -----------------------------------------------------------------------------
                                                                                                  Section 788
                                              (60600) 60600     60610      60621      60632      60643      60654      60665  60676
Autographa californica nucleopolyhedrovirus NC_0... (60600) GCAATCAACCTTTATTGAAAATGTACAAAGAGAAGCAAGAAGAGCGTTTGAAGATGCAGTCGCTGTACGCAACGTTG
                           SARS-CoV-2 NC_045512.2 (1) -----------------------------------------------------------------------------
                                                                                                  Section 789
                                              (60677) 60677     60687      60698      60709      60720      60731      60742  60753
Autographa californica nucleopolyhedrovirus NC_0... (60677) GCCGATGTAGATTTAAAAATATTAGACATTTACGATGTCGACAATTATTCTAGAAAAATGATATTGTGTGCTCAATG
                           SARS-CoV-2 NC_045512.2 (1) -----------------------------------------------------------------------------
                                                                                                  Section 790
                                              (60754) 60754     60764      60775      60786      60797      60808      60819  60830
Autographa californica nucleopolyhedrovirus NC_0... (60754) TCATATATTTGCACGCTGTTTTTGTACCAATACCATGCAATGTTTTTGTCCTCGACAGGGTTATAAGTGTGAATGTA
                           SARS-CoV-2 NC_045512.2 (1) -----------------------------------------------------------------------------
                                                                                                  Section 791
                                              (60831) 60831     60841      60852      60863      60874      60885      60896  60907
Autographa californica nucleopolyhedrovirus NC_0... (60831) TATGCCGACGATCTAAATATTTTAAAAATAATGTATTGTGTGTTAAAAGTAAAGCGGCTTGTTTTAATAAAATGAAA
                           SARS-CoV-2 NC_045512.2 (1) -----------------------------------------------------------------------------
```

Section 792

(60908) 60908 60918 60929 60940 60951 60962 60973 60984
Autographa californica nucleopolyhedrovirus NC_0... (60908) ATAAAACGTGTTCCAAAATGGAAGCATAGTGTAGATTATACTTTCAAAAGTATATACAAGTTAATAAATGTTTAATT
SARS-CoV-2 NC_045512.2 (1) ---

Section 793

(60985) 60985 60995 61006 61017 61028 61039 61050 61061
Autographa californica nucleopolyhedrovirus NC_0... (60985) TTAAGGATATTGTTATGGAATAAACTATAAAATGAATTTGATGCAATTTAATTTTTTGATACTTTCCACAGACGGTA
SARS-CoV-2 NC_045512.2 (1) ---

Section 794

(61062) 61062 61072 61083 61094 61105 61116 61127 61138
Autographa californica nucleopolyhedrovirus NC_0... (61062) GATTCAGAACGATGGCAAACATGTCGCTAGACAATGAGTACAAACTTGAATTGGCCAAAACGGGGCTGTTTTCTCAC
SARS-CoV-2 NC_045512.2 (1) ---

Section 795

(61139) 61139 61149 61160 61171 61182 61193 61204 61215
Autographa californica nucleopolyhedrovirus NC_0... (61139) AATAACCTGATTAAATGTATAGGCTGTCGCACGATTTTGGACAAGATTAACGCCAAGCAAATTAAACGACACACGTA
SARS-CoV-2 NC_045512.2 (1) ---

Section 796

(61216) 61216 61226 61237 61248 61259 61270 61281 61292
Autographa californica nucleopolyhedrovirus NC_0... (61216) TTCGAATTATTGCATATCGTCAACCAACGCGTTGATGTTCAATGAATCGATGAGAAAAAAATCATTTACGAGTTTTA
SARS-CoV-2 NC_045512.2 (1) ---

Section 797

(61293) 61293 61303 61314 61325 61336 61347 61358 61369
Autographa californica nucleopolyhedrovirus NC_0... (61293) AAAGCTCTCGGCGTCAGTTTGCATCACAATCCGTGGTCGTTGACATGTTGGCTCGTCGCGGCTTCTATTATTTTGGC
SARS-CoV-2 NC_045512.2 (1) ---

Section 798

(61370) 61370 61380 61391 61402 61413 61424 61435 61446
Autographa californica nucleopolyhedrovirus NC_0... (61370) AAAGCCGGCCATTTGCGTTGTTCCGGATGCCATATAGTTTTTAAATATAAAAGCGTAGACGACGCCCAACGCCGGCA
SARS-CoV-2 NC_045512.2 (1) ---

```
                                                                                                              Section 799
                                                 (61447) 61447     61457      61468      61479      61490      61501      61512    61523
Autographa californica nucleopolyhedrovirus NC_0... (61447) CAAACAAAATTGCAAGTTTCTCAACGCAATAGAAGACTATTCCGTCAATGAACAATTTGGCAAACTCGATGTTGCGG
                             SARS-CoV-2 NC_045512.2     (1) -----------------------------------------------------------------------------
                                                                                                              Section 800
                                                 (61524) 61524     61534      61545      61556      61567      61578      61589    61600
Autographa californica nucleopolyhedrovirus NC_0... (61524) AAAAAGAAATACTGGCTGCCGATTTGATTCCTCCGCGGCTAAGCGTTAAACCTTCGGCGCCGCCCGCCGAACCGCTA
                             SARS-CoV-2 NC_045512.2     (1) -----------------------------------------------------------------------------
                                                                                                              Section 801
                                                 (61601) 61601     61611      61622      61633      61644      61655      61666    61677
Autographa californica nucleopolyhedrovirus NC_0... (61601) ACTCAACAGGTCTCCGAATGCAAAGTTTGTTTTGATAGAGAAAAATCGGTGTGTTTCATGCCGTGCCGTCACCTGGC
                             SARS-CoV-2 NC_045512.2     (1) -----------------------------------------------------------------------------
                                                                                                              Section 802
                                                 (61678) 61678     61688      61699      61710      61721      61732      61743    61754
Autographa californica nucleopolyhedrovirus NC_0... (61678) TGTGTGCACGGAATGTTCGCGTCGGTGCAAGCGTTGTTGTGTGTGCAACGCAAAAATTATGCAGCGCATCGAAACAT
                             SARS-CoV-2 NC_045512.2     (1) -----------------------------------------------------------------------------
                                                                                                              Section 803
                                                 (61755) 61755     61765      61776      61787      61798      61809      61820    61831
Autographa californica nucleopolyhedrovirus NC_0... (61755) TACCTCAGTAAACATTGCAAACGACTACGACATTCTTTAAAAATAAGCTATATATAAATATTGCATTGTATGACAAA
                             SARS-CoV-2 NC_045512.2     (1) -----------------------------------------------------------------------------
                                                                                                              Section 804
                                                 (61832) 61832     61842      61853      61864      61875      61886      61897    61908
Autographa californica nucleopolyhedrovirus NC_0... (61832) AAAATTATTAACCTACTGCAAAGTAAAACTTGTAAAAGGCTTTTCAAAAAAATTTGCGAGTTTATTTTGTCGCTGCG
                             SARS-CoV-2 NC_045512.2     (1) -----------------------------------------------------------------------------
                                                                                                              Section 805
                                                 (61909) 61909     61919      61930      61941      61952      61963      61974    61985
Autographa californica nucleopolyhedrovirus NC_0... (61909) TCGTGTCGCATCTAAGCGACGAAGACGACAGCGACGGTGATCGCTATTATCAGTATAATAACAATTGTAATTTCATA
                             SARS-CoV-2 NC_045512.2     (1) -----------------------------------------------------------------------------
```

```
                                                                                                   Section 806
                                              (61986) 61986     61996      62007      62018      62029      62040      62051   62062
Autographa californica nucleopolyhedrovirus NC 0... (61986) TACATAAATATTGTAAAATAAAAGACATATTATTGTACATAATGTTTTATTGTAATTAAATTAATACACCAATTTAA
                              SARS-CoV-2 NC_045512.2 (1) -----------------------------------------------------------------------------
                                                                                                   Section 807
                                              (62063) 62063     62073      62084      62095      62106      62117      62128   62139
Autographa californica nucleopolyhedrovirus NC 0... (62063) ACACATGTTGATGTTGTTGTGAATAATTTTTAAATTTTTACTTTTTTCGTCAAACACTATGGCGTTGCTTTCGATTA
                              SARS-CoV-2 NC_045512.2 (1) -----------------------------------------------------------------------------
                                                                                                   Section 808
                                              (62140) 62140     62150      62161      62172      62183      62194      62205   62216
Autographa californica nucleopolyhedrovirus NC 0... (62140) GTTTTTTCGTTAGCATTTCATCTAAAAAATCAAACTGTTTGCCCGGCGCGTTTAGGGATTCTATGGTGTAGTCGGGC
                              SARS-CoV-2 NC_045512.2 (1) -----------------------------------------------------------------------------
                                                                                                   Section 809
                                              (62217) 62217     62227      62238      62249      62260      62271      62282   62293
Autographa californica nucleopolyhedrovirus NC 0... (62217) GTGTCGCTGTTTAGATATTGGTCCACTTCGCGCATTATGTCCAAGACGTTGTTCTGCAAATGAATGAGCTTTGTCAC
                              SARS-CoV-2 NC_045512.2 (1) -----------------------------------------------------------------------------
                                                                                                   Section 810
                                              (62294) 62294     62304      62315      62326      62337      62348      62359   62370
Autographa californica nucleopolyhedrovirus NC 0... (62294) CACGTCCACGGACGTGTTCATGTTTCTTTTTTGAAAACTAAATTGCAACAATTGTACGTGTCCACTATACAATTCGG
                              SARS-CoV-2 NC_045512.2 (1) -----------------------------------------------------------------------------
                                                                                                   Section 811
                                              (62371) 62371     62381      62392      62403      62414      62425      62436   62447
Autographa californica nucleopolyhedrovirus NC 0... (62371) CTTAATATACTCGTCGGCGCAATCGTATTTGCAATCCAATTTCGTGTTCAACAAATTGGTGATGATATCTTTGAACG
                              SARS-CoV-2 NC_045512.2 (1) -----------------------------------------------------------------------------
                                                                                                   Section 812
                                              (62448) 62448     62458      62469      62480      62491      62502      62513   62524
Autographa californica nucleopolyhedrovirus NC 0... (62448) TGCACGTTTTCAATTTGTCCTTATCGGCCAACGCAAGTTTCAATTCGCTCTGTAAAGTTTCTAAAATTTTGTCTTTA
                              SARS-CoV-2 NC_045512.2 (1) -----------------------------------------------------------------------------
```

Section 813

```
                                                   (62525) 62525     62535      62546      62557      62568      62579      62590   62601
Autographa californica nucleopolyhedrovirus NC_0... (62525) TTGTTGTCAAATTCGTGCGTGTTGCGTTCCAACCACAATTTGAACGGCTCGTCGACAAAAATGCTGCGCAACACCTC
                            SARS-CoV-2 NC_045512.2     (1) -----------------------------------------------------------------------------
```

Section 814

```
                                                   (62602) 62602     62612      62623      62634      62645      62656      62667   62678
Autographa californica nucleopolyhedrovirus NC_0... (62602) GTACAACTGTCTGCCTAACGTGTACACTTGCTCGTATTCTTTCATGCTGACCTCTTTGCTAACGTACATTACTAAAA
                            SARS-CoV-2 NC_045512.2     (1) -----------------------------------------------------------------------------
```

Section 815

```
                                                   (62679) 62679     62689      62700      62711      62722      62733      62744   62755
Autographa californica nucleopolyhedrovirus NC_0... (62679) AATCTACAAGTATTTTCAAACATTTGTAATAGGCGACGTATTTTGATTTAAGTTTTAAACCGTCCACCGTGTATTCG
                            SARS-CoV-2 NC_045512.2     (1) -----------------------------------------------------------------------------
```

Section 816

```
                                                   (62756) 62756     62766      62777      62788      62799      62810      62821   62832
Autographa californica nucleopolyhedrovirus NC_0... (62756) TCCACGTTCGCATCGACCACTTTTCGATTATTATCGCCGCTTGTTGCCGGCGCGTCGGCCTGTTCGGTTTTAACTAT
                            SARS-CoV-2 NC_045512.2     (1) -----------------------------------------------------------------------------
```

Section 817

```
                                                   (62833) 62833     62843      62854      62865      62876      62887      62898   62909
Autographa californica nucleopolyhedrovirus NC_0... (62833) ATCCGGTTCAATATTTAAAGTTTCAAAAGATTTAATGGCATTCATAAAATCATCTTTTTGCTTTGGCGTGGTCAATG
                            SARS-CoV-2 NC_045512.2     (1) -----------------------------------------------------------------------------
```

Section 818

```
                                                   (62910) 62910     62920      62931      62942      62953      62964      62975   62986
Autographa californica nucleopolyhedrovirus NC_0... (62910) GTAAATCTATCGAGGAGTTGTCGTCCGTGTGCTCTTCGGGCACGCTGTTCAGACGTAACGTAATCTTTTTGGGATCG
                            SARS-CoV-2 NC_045512.2     (1) -----------------------------------------------------------------------------
```

Section 819

```
                                                   (62987) 62987     62997      63008      63019      63030      63041      63052   63063
Autographa californica nucleopolyhedrovirus NC_0... (62987) TCTTCATCGGGTATCAAATCGGCTTTAATTTTATTAGAATTGAGCAACGACATGGTGGTCGCTTGTAAATTTAATAA
                            SARS-CoV-2 NC_045512.2     (1) -----------------------------------------------------------------------------
```

```
                                                                                                                    Section 820
                                                    (63064) 63064     63074      63085      63096      63107      63118      63129  63140
Autographa californica nucleopolyhedrovirus NC 0... (63064) ATTAATTAAAGACTGAAATTGTATATTGCACAAATTTATTTTCATTTTTATTGATCTTACTATTAATACGCTGGCAG
                               SARS-CoV-2 NC_045512.2   (1) -----------------------------------------------------------------------------
                                                                                                                    Section 821
                                                    (63141) 63141     63151      63162      63173      63184      63195      63206  63217
Autographa californica nucleopolyhedrovirus NC 0... (63141) TTGGTATGCTTCATCCATTTTTGTGACTAGAAAATTTGCTAAAAAACTGAGCTCGTCCTGTGTTAAAACGTTGTCGT
                               SARS-CoV-2 NC_045512.2   (1) -----------------------------------------------------------------------------
                                                                                                                    Section 822
                                                    (63218) 63218     63228      63239      63250      63261      63272      63283  63294
Autographa californica nucleopolyhedrovirus NC 0... (63218) CCACGAATCTATGCAATGTAAATGTTACACTGACATTGTTTAACAATGCATGTATTAAAAAATCAACCTGTCGCCTA
                               SARS-CoV-2 NC_045512.2   (1) -----------------------------------------------------------------------------
                                                                                                                    Section 823
                                                    (63295) 63295     63305      63316      63327      63338      63349      63360  63371
Autographa californica nucleopolyhedrovirus NC 0... (63295) CTGAGTTTATTAGAAGAGTCGACCGTTTCTACTAGTTTGTAGATTTTGTTATTTTCAATTTCATTGTTTAAAAACAT
                               SARS-CoV-2 NC_045512.2   (1) -----------------------------------------------------------------------------
                                                                                                                    Section 824
                                                    (63372) 63372     63382      63393      63404      63415      63426      63437  63448
Autographa californica nucleopolyhedrovirus NC 0... (63372) GTTAACTACTCGTTTGAGTTTAAGCGAAAAATCCTTGTCCGGATAGACTTGTTCGCACAGCCAATTGCTAAGAGTGG
                               SARS-CoV-2 NC_045512.2   (1) -----------------------------------------------------------------------------
                                                                                                                    Section 825
                                                    (63449) 63449     63459      63470      63481      63492      63503      63514  63525
Autographa californica nucleopolyhedrovirus NC 0... (63449) TTTTGACCACGGACACCTTGGTGGTGAACGTCGTCGATTTGACCAGTTCGGTGAAAAAGTTTTTCATTAAATTGGAC
                               SARS-CoV-2 NC_045512.2   (1) -----------------------------------------------------------------------------
                                                                                                                    Section 826
                                                    (63526) 63526     63536      63547      63558      63569      63580      63591  63602
Autographa californica nucleopolyhedrovirus NC 0... (63526) ATTTTAACAAACACTTATCAATCTATTGAGCTGGTATTTTTGTTTAGAATCGCATCAAGCGCTTGCTCGATCTCCAA
                               SARS-CoV-2 NC_045512.2   (1) -----------------------------------------------------------------------------
```

Section 827

(63603) 63603 63613 63624 63635 63646 63657 63668 63679

Autographa californica nucleopolyhedrovirus NC 0... (63603) TTTTTTTCGGACGCTCTTAGCTTTATGACTCGGTATGTCTTCTACGGTAGACTCGGTGTTCTTACTTATAATGGCCG

SARS-CoV-2 NC_045512.2 (1) ---

Section 828

(63680) 63680 63690 63701 63712 63723 63734 63745 63756

Autographa californica nucleopolyhedrovirus NC 0... (63680) GGCTGACGATAATAAACACGAGAAACAATATGAGCAGATACAAAAAGATGCTGTTTTCCTTTTTGTCATACACTAGG

SARS-CoV-2 NC_045512.2 (1) ---

Section 829

(63757) 63757 63767 63778 63789 63800 63811 63822 63833

Autographa californica nucleopolyhedrovirus NC 0... (63757) CTAAATATGGCCAGTGCGCCCAACAACAAATATAAATTCATTTTTATTCCCTTACTCTATTCGTTGCGATAGTACAA

SARS-CoV-2 NC_045512.2 (1) ---

Section 830

(63834) 63834 63844 63855 63866 63877 63888 63899 63910

Autographa californica nucleopolyhedrovirus NC 0... (63834) CAACGATTCTCCCGACGAACCGGACGAATTGCGATTATGCTGCGCGTCGTCGTCGTCGTTGTTGTTCTCCTCTTCGC

SARS-CoV-2 NC_045512.2 (1) ---

Section 831

(63911) 63911 63921 63932 63943 63954 63965 63976 63987

Autographa californica nucleopolyhedrovirus NC 0... (63911) TGCTCGTTTCGTCTAAACCTATATTGTATTTGTTCAAGTAATGTTTGGTGCTTGCGGAGGATTCGTGGTTCATTAAT

SARS-CoV-2 NC_045512.2 (1) ---

Section 832

(63988) 63988 63998 64009 64020 64031 64042 64053 64064

Autographa californica nucleopolyhedrovirus NC 0... (63988) TTGGCCACTTTTTGTAAAGGCACGCCGCTATTGTATAGGTTACTGCTCAAATAATGTCTTATCATGTTGCTGCGCGG

SARS-CoV-2 NC_045512.2 (1) ---

Section 833

(64065) 64065 64075 64086 64097 64108 64119 64130 64141

Autographa californica nucleopolyhedrovirus NC 0... (64065) CCGTTCCATCTCGACGCCCGACTCTTCAAGGAGTCGCCTGAAATCTTTGAAGGGCGTCGAGGTGTTTTTAGATATTT

SARS-CoV-2 NC_045512.2 (1) ---

```
                                                                                                  Section 834
                                            (64142) 64142     64152      64163      64174      64185      64196      64207  64218
Autographa californica nucleopolyhedrovirus NC 0... (64142) GCAAAATGGTCGGGTTTCGTGAATAAATCTCGCGTGCCAATTCCAACGGTTTCATTTTGATGTTGTTGAGTGTGTTA
                             SARS-CoV-2 NC_045512.2     (1) -----------------------------------------------------------------------------
                                                                                                  Section 835
                                            (64219) 64219     64229      64240      64251      64262      64273      64284  64295
Autographa californica nucleopolyhedrovirus NC 0... (64219) TTACGACTGCGTTTTCGCTTTAAATTAATCGTGTCGCTGTGCAGTTTTCCTCTTTTAATTAGCACGTTGAGATCGTC
                             SARS-CoV-2 NC_045512.2     (1) -----------------------------------------------------------------------------
                                                                                                  Section 836
                                            (64296) 64296     64306      64317      64328      64339      64350      64361  64372
Autographa californica nucleopolyhedrovirus NC 0... (64296) CACGCTGAGTTGGCGCGCTTCGTTGATTCGCATACCCGTCCCTAACATGATGCAAAACACTATCGCGCCCCTAATTA
                             SARS-CoV-2 NC_045512.2     (1) -----------------------------------------------------------------------------
                                                                                                  Section 837
                                            (64373) 64373     64383      64394      64405      64416      64427      64438  64449
Autographa californica nucleopolyhedrovirus NC 0... (64373) GACCGCGGTCGTGAACATAATCGCTGTTGAGCATTTTAATTTTATCATTAATAAAATTTAATATGGTATCTATTACG
                             SARS-CoV-2 NC_045512.2     (1) -----------------------------------------------------------------------------
                                                                                                  Section 838
                                            (64450) 64450     64460      64471      64482      64493      64504      64515  64526
Autographa californica nucleopolyhedrovirus NC 0... (64450) TTTTTAAGCATTAAATTCTTTTCCTTTTCCCTGATATTTTTGAGCTCCTTGTCGCGCGGCAGCATAACCATGCGGGG
                             SARS-CoV-2 NC_045512.2     (1) -----------------------------------------------------------------------------
                                                                                                  Section 839
                                            (64527) 64527     64537      64548      64559      64570      64581      64592  64603
Autographa californica nucleopolyhedrovirus NC 0... (64527) AATTTTGTATTCGGGCAAGTTCATCATGTTGGTGTAAAAGTTTATAGTCAACTGTAGTGTTTCTTTGGTGACCGAGC
                             SARS-CoV-2 NC_045512.2     (1) -----------------------------------------------------------------------------
                                                                                                  Section 840
                                            (64604) 64604     64614      64625      64636      64647      64658      64669  64680
Autographa californica nucleopolyhedrovirus NC 0... (64604) GAAGTTCGAGCATGCGCCTGCACAGTTCTTGGGGATCAATGAGAAGTGTTTGGTTTTCTATCGAGTCAAACTCCTTG
                             SARS-CoV-2 NC_045512.2     (1) -----------------------------------------------------------------------------
```

```
                                                                                                              Section 841
                                               (64681) 64681     64691      64702      64713      64724      64735      64746   64757
Autographa californica nucleopolyhedrovirus NC 0... (64681) TCCAACGAGTACGACATGTCTTCCAGGTGAACATCGTCTACCGAGCAGTACACAATTTTAATGAATCGAGACTTGTA
                           SARS-CoV-2 NC_045512.2     (1) -----------------------------------------------------------------------------
                                                                                                              Section 842
                                               (64758) 64758     64768      64779      64790      64801      64812      64823   64834
Autographa californica nucleopolyhedrovirus NC 0... (64758) ACTTTTTAAAGTGGTGGGCGCAAACGGTTTGGGGAACATGTACTTGCTCCACAGACTGTTGTTTTTCACCTCGTCGG
                           SARS-CoV-2 NC_045512.2     (1) -----------------------------------------------------------------------------
                                                                                                              Section 843
                                               (64835) 64835     64845      64856      64867      64878      64889      64900   64911
Autographa californica nucleopolyhedrovirus NC 0... (64835) GCGTGCATCGTTGCCGATCGGTGGCCAAATCGAACACGGACTCGAACCGGGGAGCGGATTGAATTTTTATTTTCCAA
                           SARS-CoV-2 NC_045512.2     (1) -----------------------------------------------------------------------------
                                                                                                              Section 844
                                               (64912) 64912     64922      64933      64944      64955      64966      64977   64988
Autographa californica nucleopolyhedrovirus NC 0... (64912) GAATTAAAATTGTTTTCGTTGCGAACATTAAAACCGTTCATTGTGGTTAATCAAATTTATTAAAAACAAAAGGAGAA
                           SARS-CoV-2 NC_045512.2     (1) -----------------------------------------------------------------------------
                                                                                                              Section 845
                                               (64989) 64989     64999      65010      65021      65032      65043      65054   65065
Autographa californica nucleopolyhedrovirus NC 0... (64989) TCGGTGTCAATACTATCCGAATATTGTTGTTGTTCTCTTAATATTACGAAATAATATATTACATACAGCAGTAAGAA
                           SARS-CoV-2 NC_045512.2     (1) -----------------------------------------------------------------------------
                                                                                                              Section 846
                                               (65066) 65066     65076      65087      65098      65109      65120      65131   65142
Autographa californica nucleopolyhedrovirus NC 0... (65066) TAAAGCTATAAAAGCGACTACACTAATTAAAATTATAATTCCCGCCGACACGTTGCTCGTCGTGTTGTCATAGCCCA
                           SARS-CoV-2 NC_045512.2     (1) -----------------------------------------------------------------------------
                                                                                                              Section 847
                                               (65143) 65143     65153      65164      65175      65186      65197      65208   65219
Autographa californica nucleopolyhedrovirus NC 0... (65143) CCATGTCGTTTATTGGCATTTTGTGAACGGGCTCGCTAAATTGTTGCGGTTCGCTGGCAGTATCGTCGTTGAGCGCC
                           SARS-CoV-2 NC_045512.2     (1) -----------------------------------------------------------------------------
```

```
                                                                                                                    Section 848
                                                      (65220) 65220     65230      65241      65252      65263      65274      65285   65296
Autographa californica nucleopolyhedrovirus  NC_0...  (65220) AATTTCAACGGGATGTATTCCACCTTTTCGTGGTTGCCCAACCGATAGTAGGGCACGTCCAAATTCATGTTTACAAC
                         SARS-CoV-2 NC_045512.2          (1) -----------------------------------------------------------------------------
                                                                                                                    Section 849
                                                      (65297) 65297     65307      65318      65329      65340      65351      65362   65373
Autographa californica nucleopolyhedrovirus  NC_0...  (65297) TTATTTGCTAACAGGAATTTATGCAACAAAAGTGGTTTGGCTTTGATGAGACGCAATTTGAAATACTTGCTGCATTT
                         SARS-CoV-2 NC_045512.2          (1) -----------------------------------------------------------------------------
                                                                                                                    Section 850
                                                      (65374) 65374     65384      65395      65406      65417      65428      65439   65450
Autographa californica nucleopolyhedrovirus  NC_0...  (65374) ACGCTTAAGATTGTATTCCATGCGGGCGGCGGTGTTGTAGTCGTACGCGCTCGCGCTGTGATACACGAGCCGTAAAT
                         SARS-CoV-2 NC_045512.2          (1) -----------------------------------------------------------------------------
                                                                                                                    Section 851
                                                      (65451) 65451     65461      65472      65483      65494      65505      65516   65527
Autographa californica nucleopolyhedrovirus  NC_0...  (65451) TGGTTGCGTTGCGCAAACACTTGGCGCCTTGTTTGTTCGAATGCTGTTTTATGCGTCTGTTAAGATTGCTCGTGATG
                         SARS-CoV-2 NC_045512.2          (1) -----------------------------------------------------------------------------
                                                                                                                    Section 852
                                                      (65528) 65528     65538      65549      65560      65571      65582      65593   65604
Autographa californica nucleopolyhedrovirus  NC_0...  (65528) CCCGTGTACAATTTTCCATTGTCTTGCCGCAGAATGTACACGCACCACACCTTGTTGGTGTACAGAGTCGTCGCCAT
                         SARS-CoV-2 NC_045512.2          (1) -----------------------------------------------------------------------------
                                                                                                                    Section 853
                                                      (65605) 65605     65615      65626      65637      65648      65659      65670   65681
Autographa californica nucleopolyhedrovirus  NC_0...  (65605) GATTATGCAGTGCGCCCTTTCGTGTTCGGCCGAGTGGCGTTAGGCGCAGCCGCGGCAATAATCGCGTTGGCGTCCTT
                         SARS-CoV-2 NC_045512.2          (1) -----------------------------------------------------------------------------
                                                                                                                    Section 854
                                                      (65682) 65682     65692      65703      65714      65725      65736      65747   65758
Autographa californica nucleopolyhedrovirus  NC_0...  (65682) GTTGTAATTTATTTGTTGAAAAATAAAACGTCTTAGAGTTTCGTTTTGGAACGCCAATTCGGTCAAGCTCTCCTGGC
                         SARS-CoV-2 NC_045512.2          (1) -----------------------------------------------------------------------------
```

Section 855

	(65759)	65759 65769 65780 65791 65802 65813 65824 65835
Autographa californica nucleopolyhedrovirus NC_0...	(65759)	AAGCGCTTTTGGTCAAATGAGCGGCCGGCGAATTGACCGCGTTGGCGGCCGACGTTAAGAAGGTGGCGTTCTGGAAC
SARS-CoV-2 NC_045512.2	(1)	---

Section 856

	(65836)	65836 65846 65857 65868 65879 65890 65901 65912
Autographa californica nucleopolyhedrovirus NC_0...	(65836)	ATGCTGGGCTGCTTGCCGGCTCGCGTCGCCAGCTCGGCCATGTAATTGAATATGTTGGCAGACGCAGATAGCGGCGC
SARS-CoV-2 NC_045512.2	(1)	---

Section 857

	(65913)	65913 65923 65934 65945 65956 65967 65978 65989
Autographa californica nucleopolyhedrovirus NC_0...	(65913)	CAAAAACGCAACGTTCTCTTTTAAACTCATGACTCGCGCCCTGTTTTTTTCGTTCAGCACGTAGTGGTAGTAATCGC
SARS-CoV-2 NC_045512.2	(1)	---

Section 858

	(65990)	65990 66000 66011 66022 66033 66044 66055 66066
Autographa californica nucleopolyhedrovirus NC_0...	(65990)	CGCCGCCGGCAAACAGATCGTCAATCACGGCGTTGATCAGATCGTTGATCATGTTGATGTGCGGAAAGCGACGCGAC
SARS-CoV-2 NC_045512.2	(1)	---

Section 859

	(66067)	66067 66077 66088 66099 66110 66121 66132 66143
Autographa californica nucleopolyhedrovirus NC_0...	(66067)	TCGACTGCGCTCTGTATGTTTGGCGGCAGAGTGGCGTGCTTGAGCAACAGAGTCATGTAATTGTTGGCCAGCTGCTG
SARS-CoV-2 NC_045512.2	(1)	---

Section 860

	(66144)	66144 66154 66165 66176 66187 66198 66209 66220
Autographa californica nucleopolyhedrovirus NC_0...	(66144)	ATTGAAAGGTAACGGAATGGGAATGTTGCACGTCACCGCTTCCGCCACCATGTACTGGACGGCCAGACTGAGTTGTT
SARS-CoV-2 NC_045512.2	(1)	---

Section 861

	(66221)	66221 66231 66242 66253 66264 66275 66286 66297
Autographa californica nucleopolyhedrovirus NC_0...	(66221)	TGGCGGCCTCGGCCAAAGCGTCTTTGCCCAACATATCAGCGCCACCGTTGTAAAACTTTTGCGCGTACGCCGGCAGC
SARS-CoV-2 NC_045512.2	(1)	---

Section 862

```
                                                      (66298) 66298     66308      66319      66330      66341      66352      66363      66374
Autographa californica nucleopolyhedrovirus  NC_0...  (66298) GAATTTAGCACAAACGATGGCTGAAATATATTTGAATCGCTCGACAGGGACTCGGCCGCGTTGCTCTGTCCCAACTC
                              SARS-CoV-2 NC_045512.2      (1) -----------------------------------------------------------------------------
```

Section 863

```
                                                      (66375) 66375     66385      66396      66407      66418      66429      66440      66451
Autographa californica nucleopolyhedrovirus  NC_0...  (66375) TTTTTGCAACCGAATCAGGTGGCGTATCATGGTTTCCTCCGATTCAAACCGCTTTACCACGTTTACGCTGATTGGGT
                              SARS-CoV-2 NC_045512.2      (1) -----------------------------------------------------------------------------
```

Section 864

```
                                                      (66452) 66452     66462      66473      66484      66495      66506      66517      66528
Autographa californica nucleopolyhedrovirus  NC_0...  (66452) TCGTGTCGATGCACATGTCACGAATAGTGTTTATAAAAAGAATCATGAGAGGACTAAGTTCTGACATGTCATTGCAC
                              SARS-CoV-2 NC_045512.2      (1) -----------------------------------------------------------------------------
```

Section 865

```
                                                      (66529) 66529     66539      66550      66561      66572      66583      66594      66605
Autographa californica nucleopolyhedrovirus  NC_0...  (66529) CTGTAATATCTAATAATCTTTTGAACAAAATCCACACATTTGTTGTACCAAATAGATTCACCGGCGTCGAGCGTCGG
                              SARS-CoV-2 NC_045512.2      (1) -----------------------------------------------------------------------------
```

Section 866

```
                                                      (66606) 66606     66616      66627      66638      66649      66660      66671      66682
Autographa californica nucleopolyhedrovirus  NC_0...  (66606) TTCTTTGCTCTTGTTGTACGGTGCAATCGCTACCGAGTTTGTGCTGTTGCTGCGGCTCGTGTAATCCATCCTGTTGT
                              SARS-CoV-2 NC_045512.2      (1) -----------------------------------------------------------------------------
```

Section 867

```
                                                      (66683) 66683     66693      66704      66715      66726      66737      66748      66759
Autographa californica nucleopolyhedrovirus  NC_0...  (66683) CGCGCGTGGCGACGGTCGTAGGCACCGTCGCCGGCGGCACGTACCCGGGCGCGTTGTAAGTTTGCGCGCTGGTGAAT
                              SARS-CoV-2 NC_045512.2      (1) -----------------------------------------------------------------------------
```

Section 868

```
                                                      (66760) 66760     66770      66781      66792      66803      66814      66825      66836
Autographa californica nucleopolyhedrovirus  NC_0...  (66760) ATGGCCGTTGCCGGATTAGAGGGATACCTCAGCGGCGGAGGGGTGTTGTAATAAAAATTGCCACGTTCATCTGTCAT
                              SARS-CoV-2 NC_045512.2      (1) -----------------------------------------------------------------------------
```

Section 869

(66837) 66837 66847 66858 66869 66880 66891 66902 66913
Autographa californica nucleopolyhedrovirus NC_0... (66837) ACTTTTTATTTGTACTCTTATGATTACAAAACTCAATATACGGATTACTTATAATATAGTTGTTGTGACAAAAAAGC
SARS-CoV-2 NC_045512.2 (1) ---

Section 870

(66914) 66914 66924 66935 66946 66957 66968 66979 66990
Autographa californica nucleopolyhedrovirus NC_0... (66914) GATAATAAAATTAACAAAATTATCAACAAGTTAATCATGGAAAATTTTTCAACGTTGAATAACAACAACAAAATGGC
SARS-CoV-2 NC_045512.2 (1) ---

Section 871

(66991) 66991 67001 67012 67023 67034 67045 67056 67067
Autographa californica nucleopolyhedrovirus NC_0... (66991) GCAGGTCAACAGCACCGTTTGAAAACTGACGCGCCGACACAAAATGCTTTCGCAATTTCTAAAAGCCACATTAAACG
SARS-CoV-2 NC_045512.2 (1) ---

Section 872

(67068) 67068 67078 67089 67100 67111 67122 67133 67144
Autographa californica nucleopolyhedrovirus NC_0... (67068) AATTTTCACCTTTGATATAATCACGCAGTTCTTTTTTACAACATTCGTCGCACAAAATTAACACCTTTATAATGAGG
SARS-CoV-2 NC_045512.2 (1) ---

Section 873

(67145) 67145 67155 67166 67177 67188 67199 67210 67221
Autographa californica nucleopolyhedrovirus NC_0... (67145) CCGTCGGTGTGTATCGTTTGAAATGTCCGCGGTTGACTGCCTGGATGAAATTCAAACGAGTACCCAGTGGACACGTG
SARS-CoV-2 NC_045512.2 (1) ---

Section 874

(67222) 67222 67232 67243 67254 67265 67276 67287 67298
Autographa californica nucleopolyhedrovirus NC_0... (67222) TATCTGTGCAAAATAATGGGCTAATATCGAGGCGCCCGTTTTTTTAACCTTTACTTTTGATATTTTAATAACATTAA
SARS-CoV-2 NC_045512.2 (1) ---

Section 875

(67299) 67299 67309 67320 67331 67342 67353 67364 67375
Autographa californica nucleopolyhedrovirus NC_0... (67299) TGTTGTTATTTGCGTAATCAGAGTTTTTATTGTGGTGATCATCGTACAAATAATGAAGCAACAGTTCACTATCGTAT
SARS-CoV-2 NC_045512.2 (1) ---

Section 876

	(67376)	67376 67386 67397 67408 67419 67430 67441 67452
Autographa californica nucleopolyhedrovirus NC 0...	(67376)	TTAATCTTGTTTAGCGTTGTCAAGTTTTTGTTTCTTAGGCGTTGGAGCGTCTCCGTCGTCGATATTTTCTTCGAAAT
SARS-CoV-2 NC_045512.2	(1)	--

Section 877

	(67453)	67453 67463 67474 67485 67496 67507 67518 67529
Autographa californica nucleopolyhedrovirus NC 0...	(67453)	CGAGTCCAACAACGTCGGCGTTTCCTTCTTGCTCATCGATAGCGGCGGCGGAGGCGGCCTCTCCGTCGTCGTCATTC
SARS-CoV-2 NC_045512.2	(1)	--

Section 878

	(67530)	67530 67540 67551 67562 67573 67584 67595 67606
Autographa californica nucleopolyhedrovirus NC 0...	(67530)	GCGGTTTCTACAGTGCGTTTGGGCGACGACGTGTGTACAGCAGCGTCCGTCTTACTATTATCGGACCGCCAAATTTT
SARS-CoV-2 NC_045512.2	(1)	--

Section 879

	(67607)	67607 67617 67628 67639 67650 67661 67672 67683
Autographa californica nucleopolyhedrovirus NC 0...	(67607)	TGTTTGAAATAACATTTGGCCCTTGTTCAACTTTATTTCGGCGCAGTTAAACATTATTGCATTAAGATCATATTCGC
SARS-CoV-2 NC_045512.2	(1)	--

Section 880

	(67684)	67684 67694 67705 67716 67727 67738 67749 67760
Autographa californica nucleopolyhedrovirus NC 0...	(67684)	CGTTTTGCACCAAATTGCACAAAACACCATAGTTGCCGCACGACACTGTAGAATAGGCGTTTTTGTACAACAATCTG
SARS-CoV-2 NC_045512.2	(1)	--

Section 881

	(67761)	67761 67771 67782 67793 67804 67815 67826 67837
Autographa californica nucleopolyhedrovirus NC 0...	(67761)	AGTTGCGGCGAGCTAGCCACCTTGATAATATGGGCGCCAACGCCCCGTTTTTTTAAGTAATATTCGTCTTCAATTAT
SARS-CoV-2 NC_045512.2	(1)	--

Section 882

	(67838)	67838 67848 67859 67870 67881 67892 67903 67914
Autographa californica nucleopolyhedrovirus NC 0...	(67838)	AAAATCTAGTACGTTTTCATCTTCACTGTTGATTTGGGCGTTCACGATGATGTCTGGCGTAATGTTGCTCATGCTTG
SARS-CoV-2 NC_045512.2	(1)	--

```
                                                                                                   Section 883
                                            (67915) 67915     67925      67936      67947      67958      67969      67980    67991
Autographa californica nucleopolyhedrovirus NC 0... (67915) CCATTTTTCTTATAATAGCGTTTACTTTAATGTATTTGGCAATTTATTTTGAATTTGACGAAACGACTTTCACCAAG
                             SARS-CoV-2 NC_045512.2     (1) -----------------------------------------------------------------------------
                                                                                                   Section 884
                                            (67992) 67992     68002      68013      68024      68035      68046      68057    68068
Autographa californica nucleopolyhedrovirus NC 0... (67992) CGGCTCCAAGTGATGACTGAATATGTGAAGCGCACCAACGCAGACGAACCCACACCCGACGTAATAGGCTACGTGTC
                             SARS-CoV-2 NC_045512.2     (1) -----------------------------------------------------------------------------
                                                                                                   Section 885
                                            (68069) 68069     68079      68090      68101      68112      68123      68134    68145
Autographa californica nucleopolyhedrovirus NC 0... (68069) GGATATTATGCAAAACACTTATATTGTAACGTGGTTCAACACCGTCGACCTTTCCACCTATCACGAAAGCGTGCATG
                             SARS-CoV-2 NC_045512.2     (1) -----------------------------------------------------------------------------
                                                                                                   Section 886
                                            (68146) 68146     68156      68167      68178      68189      68200      68211    68222
Autographa californica nucleopolyhedrovirus NC 0... (68146) ATGACCGGATTGAAATTTTTGATTTCTTAAATCAAAAATTTCAACCTGTTGATCGAATCGTACACGATCGCGTTAGA
                             SARS-CoV-2 NC_045512.2     (1) -----------------------------------------------------------------------------
                                                                                                   Section 887
                                            (68223) 68223     68233      68244      68255      68266      68277      68288    68299
Autographa californica nucleopolyhedrovirus NC 0... (68223) GCAAATGATGAAAATCCCAACGAGTTTATTTTGAGCGGCGACAAGGCCGACGTGACCATGAAATGCCCCGCATATTT
                             SARS-CoV-2 NC_045512.2     (1) -----------------------------------------------------------------------------
                                                                                                   Section 888
                                            (68300) 68300     68310      68321      68332      68343      68354      68365    68376
Autographa californica nucleopolyhedrovirus NC 0... (68300) TAACTTTGATTACGCACAACTAAAATGTGTTCCCGTGCCGCCGTGCGACAACAAGTCTGCCGGTCTTTATCCCATGG
                             SARS-CoV-2 NC_045512.2     (1) -----------------------------------------------------------------------------
                                                                                                   Section 889
                                            (68377) 68377     68387      68398      68409      68420      68431      68442    68453
Autographa californica nucleopolyhedrovirus NC 0... (68377) ACGAGCGTTTGCTGGACACGTTGGTGTTGAACCAACACTTGGACAAAGATTATTCTACCAACGCGCACTTGTATCAT
                             SARS-CoV-2 NC_045512.2     (1) -----------------------------------------------------------------------------
```

Section 890

	(68454)	68454 68464 68475 68486 68497 68508 68519 68530
Autographa californica nucleopolyhedrovirus NC 0...	(68454)	CCCACGTTCTATCTTAGGTGTTTTGCAAACGGAGCGCACGCAGTCGAAGAATGTCCAGATAATTACACGTTTGACGC
SARS-CoV-2 NC_045512.2	(1)	---

Section 891

	(68531)	68531 68541 68552 68563 68574 68585 68596 68607
Autographa californica nucleopolyhedrovirus NC 0...	(68531)	GGAAACCGGCCAGTGTAAAGTTAACGAATTGTGTGAAAACAGGCCAGACGGCTATATACTATCATACTTTCCCTCCA
SARS-CoV-2 NC_045512.2	(1)	---

Section 892

	(68608)	68608 68618 68629 68640 68651 68662 68673 68684
Autographa californica nucleopolyhedrovirus NC 0...	(68608)	ATTTGCTCGTCAACCAGTTTATGCAGTGCGTAAATGGGCGCCACGTGGTGGGCGAATGCCCCGCGAATAAAATATTT
SARS-CoV-2 NC_045512.2	(1)	---

Section 893

	(68685)	68685 68695 68706 68717 68728 68739 68750 68761
Autographa californica nucleopolyhedrovirus NC 0...	(68685)	GATCGCAACTTAATGTCGTGCGTGGAAGCGCATCCGTGCGCGTTTAACGGCGCCGGACACACGTACATAACGGCCGA
SARS-CoV-2 NC_045512.2	(1)	---

Section 894

	(68762)	68762 68772 68783 68794 68805 68816 68827 68838
Autographa californica nucleopolyhedrovirus NC 0...	(68762)	TATCGGCGACACGCAATATTTCAAATGTTTGAATAATAACGAGTCACAACTGATAACGTGCATCAACCGGATCAGAA
SARS-CoV-2 NC_045512.2	(1)	---

Section 895

	(68839)	68839 68849 68860 68871 68882 68893 68904 68915
Autographa californica nucleopolyhedrovirus NC 0...	(68839)	ACTCTGACAACCAGTACGAGTGTTCCGGCGACTCCAGATGCATAGATTTACCCAACGGTACGGGCCAACATGTATTC
SARS-CoV-2 NC_045512.2	(1)	---

Section 896

	(68916)	68916 68926 68937 68948 68959 68970 68981 68992
Autographa californica nucleopolyhedrovirus NC 0...	(68916)	AAACACGTTGACGACGATATTTCGTACAACAGTGGCCAATTGGTGTGCGATAATTTTGAAGTTATTTCCGACATCGA
SARS-CoV-2 NC_045512.2	(1)	---

Section 897

	(68993)	68993 69003 69014 69025 69036 69047 69058 69069
Autographa californica nucleopolyhedrovirus NC 0...	(68993)	ATGTGATCAATCAAACGTGTTTGAAAACGCGTTGTTTATGGACAAATTTAGATTAAACATGCAATTCCCAACTGAGG
SARS-CoV-2 NC_045512.2	(1)	---

Section 898

	(69070)	69070 69080 69091 69102 69113 69124 69135 69146
Autographa californica nucleopolyhedrovirus NC 0...	(69070)	TGTTTGACGGCACCGCGTGCGTGCCAGCCACCGCGGACAATGTCAACTTTTTACGTTCCACGTTTGCCATTGAAAAT
SARS-CoV-2 NC_045512.2	(1)	---

Section 899

	(69147)	69147 69157 69168 69179 69190 69201 69212 69223
Autographa californica nucleopolyhedrovirus NC 0...	(69147)	ATTCCAAACCATTATGGCATCGACATGCAAACCTCCATGTTGGGCACGACCGAAATGGTTAAACAGTTGGTTTCCAA
SARS-CoV-2 NC_045512.2	(1)	---

Section 900

	(69224)	69224 69234 69245 69256 69267 69278 69289 69300
Autographa californica nucleopolyhedrovirus NC 0...	(69224)	AGATTTGTCGTTAAACAACGACGCCATCTTTGCTCAATGGCTTTTGTATGCGAGAGACAAAGACGCCATCGGGCTTA
SARS-CoV-2 NC_045512.2	(1)	---

Section 901

	(69301)	69301 69311 69322 69333 69344 69355 69366 69377
Autographa californica nucleopolyhedrovirus NC 0...	(69301)	ACCCGTTCACCGGCGAGCCTATCGACTGTTTTGGAGACAACTTGTACGATGTGTTTGACGCTAGACGCGCAAACATT
SARS-CoV-2 NC_045512.2	(1)	---

Section 902

	(69378)	69378 69388 69399 69410 69421 69432 69443 69454
Autographa californica nucleopolyhedrovirus NC 0...	(69378)	TGTAACGATTCGGGAACGAGCGTTTTAAAAACGCTCAATTTTGGCGATGGCGAGTTTTTAAACGTATTGAGCAGCAC
SARS-CoV-2 NC_045512.2	(1)	---

Section 903

	(69455)	69455 69465 69476 69487 69498 69509 69520 69531
Autographa californica nucleopolyhedrovirus NC 0...	(69455)	GCTGACCGGAAAAGATGAGGATTATCGCCAATTTTGTGCTATATCCTACGAAAACGGCCAAAAAATCGTAGAAAACG
SARS-CoV-2 NC_045512.2	(1)	---

Section 904

```
                                                        (69532) 69532     69542      69553      69564      69575      69586      69597   69608
Autographa californica nucleopolyhedrovirus NC_0...    (69532) AACATTTTCAGCGACGTATATTGACAAATATACTACAGTCGGACGTTTGTGCCGACCTATATACTACACTTTACCAA
                              SARS-CoV-2 NC_045512.2    (1) -----------------------------------------------------------------------------
```

Section 905

```
                                                        (69609) 69609     69619      69630      69641      69652      69663      69674   69685
Autographa californica nucleopolyhedrovirus NC_0...    (69609) AAATATACTACACTAAACTCTAAATATACTACAACTCCACTTCAATATAACCACACTCTCGTAAAACGGCCCAAAAA
                              SARS-CoV-2 NC_045512.2    (1) -----------------------------------------------------------------------------
```

Section 906

```
                                                        (69686) 69686     69696      69707      69718      69729      69740      69751   69762
Autographa californica nucleopolyhedrovirus NC_0...    (69686) TATCGAAATATATGGGGCAAATACACGTTTAAAAAACGCTACGATTCCAAAAAACGCTGCAACTATTCCGCCCGTGT
                              SARS-CoV-2 NC_045512.2    (1) -----------------------------------------------------------------------------
```

Section 907

```
                                                        (69763) 69763     69773      69784      69795      69806      69817      69828   69839
Autographa californica nucleopolyhedrovirus NC_0...    (69763) TTAATCCCTTTGAAAACCAGCCAAATAACAGGCAAAACGATTCTATTCTACCCCTGTTTAACCCTTTTCAAACGACC
                              SARS-CoV-2 NC_045512.2    (1) -----------------------------------------------------------------------------
```

Section 908

```
                                                        (69840) 69840     69850      69861      69872      69883      69894      69905   69916
Autographa californica nucleopolyhedrovirus NC_0...    (69840) GACGCCGTATGGTACAGCGAACCAGGTGGCGACGACGACCATTGGGTAGTGGCGCCGCCAACCGCACCACCTCCACC
                              SARS-CoV-2 NC_045512.2    (1) -----------------------------------------------------------------------------
```

Section 909

```
                                                        (69917) 69917     69927      69938      69949      69960      69971      69982   69993
Autographa californica nucleopolyhedrovirus NC_0...    (69917) GCCCGAGCCAGAACCAGAGCCAGAACCCGAGCCAGAACCCGAGCCAGAGTTACCGTCACCGCTAATATTAGACAACA
                              SARS-CoV-2 NC_045512.2    (1) -----------------------------------------------------------------------------
```

Section 910

```
                                                        (69994) 69994     70004      70015      70026      70037      70048      70059   70070
Autographa californica nucleopolyhedrovirus NC_0...    (69994) AAGATTTATTTTATTCATGCCACTACTCGGTTCCGTTTTTCAAGCTAACCAGTTGTCATGCGGAAAATGACGTCATT
                              SARS-CoV-2 NC_045512.2    (1) -----------------------------------------------------------------------------
```

```
                                                                        Section 911
                                                   (70071) 70071     70081      70092      70103      70114      70125      70136   70147
Autographa californica nucleopolyhedrovirus NC_0... (70071) ATTGATGCTTTAAACGAGTTACGCAACAACGTTAAAGTGGACGCTGATTGCGAATTGGCCAAAGACCTATCGCACGT
                           SARS-CoV-2 NC_045512.2     (1) -----------------------------------------------------------------------------
                                                                        Section 912
                                                   (70148) 70148     70158      70169      70180      70191      70202      70213   70224
Autographa californica nucleopolyhedrovirus NC_0... (70148) TTTGAACGCGTACGCTTATGTGGGCAATGGGATTGGTTGTAGATCCGCGTACGACGGAGATGCGATAGTGGTAAAAA
                           SARS-CoV-2 NC_045512.2     (1) -----------------------------------------------------------------------------
                                                                        Section 913
                                                   (70225) 70225     70235      70246      70257      70268      70279      70290   70301
Autographa californica nucleopolyhedrovirus NC_0... (70225) AAGAAGCCGTGCCTAGTCACGTGTACGCCAACCTGAACACGCAATCCAACGACGGCGTCAAATACAACCGTTGGTTG
                           SARS-CoV-2 NC_045512.2     (1) -----------------------------------------------------------------------------
                                                                        Section 914
                                                   (70302) 70302     70312      70323      70334      70345      70356      70367   70378
Autographa californica nucleopolyhedrovirus NC_0... (70302) CACGTCAAAAACGGCCAATACATGGCGTGTCCCGAAGAATTGTACGATAACAACGAATTTAAATGTAACATAGAATC
                           SARS-CoV-2 NC_045512.2     (1) -----------------------------------------------------------------------------
                                                                        Section 915
                                                   (70379) 70379     70389      70400      70411      70422      70433      70444   70455
Autographa californica nucleopolyhedrovirus NC_0... (70379) GGATAAATTATACTATTTGGATAATTTACAAGAAGATTCCATTGTATAAACATTTTATGTCGAAAACAAATGACATC
                           SARS-CoV-2 NC_045512.2     (1) -----------------------------------------------------------------------------
                                                                        Section 916
                                                   (70456) 70456     70466      70477      70488      70499      70510      70521   70532
Autographa californica nucleopolyhedrovirus NC_0... (70456) ATTCCGGATCATGATTTACGCGTAGAATTCTACTTGTAAAGCAAGTTAAAATAAGCCGTGTGCAAAAATGACATCAG
                           SARS-CoV-2 NC_045512.2     (1) -----------------------------------------------------------------------------
                                                                        Section 917
                                                   (70533) 70533     70543      70554      70565      70576      70587      70598   70609
Autographa californica nucleopolyhedrovirus NC_0... (70533) ACAAATGACATCATCTACCTATCATGATCATGTTAATAATCATGTTTTAAAATGACATCAGCTTATGACTAATAATT
                           SARS-CoV-2 NC_045512.2     (1) -----------------------------------------------------------------------------
```

Section 918

(70610) 70610 70620 70631 70642 70653 70664 70675 70686

Autographa californica nucleopolyhedrovirus NC_0... (70610) GATCGTGCGTTACAAGTAGAATTCTACTCGTAAAGCGAGTTTAGTTTTGAAAAACAAATGAGTCATCATTAAACATG

SARS-CoV-2 NC_045512.2 (1) ---

Section 919

(70687) 70687 70697 70708 70719 70730 70741 70752 70763

Autographa californica nucleopolyhedrovirus NC_0... (70687) TTAATAATCGTGTATAAAGGATGACATCATCCACTAATCGTGCGTTACAAGTAGAATTCTACTCGTAAAGCGAGTTC

SARS-CoV-2 NC_045512.2 (1) ---

Section 920

(70764) 70764 70774 70785 70796 70807 70818 70829 70840

Autographa californica nucleopolyhedrovirus NC_0... (70764) GGTTTTGAAAAACAAATGACATCATTTCTTGATTGTGTTTTACACGTAGAATTCTACTCGTAAAGTATGTTCAGTTT

SARS-CoV-2 NC_045512.2 (1) ---

Section 921

(70841) 70841 70851 70862 70873 70884 70895 70906 70917

Autographa californica nucleopolyhedrovirus NC_0... (70841) AAAAAACAAATGACATCATTTTACAGATGACATCATTTCTTGATTATGTTTTACAAGTAGAATTCTACTCGTAAAGC

SARS-CoV-2 NC_045512.2 (1) ---

Section 922

(70918) 70918 70928 70939 70950 70961 70972 70983 70994

Autographa californica nucleopolyhedrovirus NC_0... (70918) AAGTTTAGTTTTAAAAAACAAATGACATCATCTCTTGATTATGTTTTACAAGTAGAATTCTACTCGTAAAGCGAGTT

SARS-CoV-2 NC_045512.2 (1) ---

Section 923

(70995) 70995 71005 71016 71027 71038 71049 71060 71071

Autographa californica nucleopolyhedrovirus NC_0... (70995) TAGTTTTGAAAAACAAATGACATCATCTCTTGATTATGTTTTACAAGTAGAATTCTACTCGTAAAGCGAGTTTAGTT

SARS-CoV-2 NC_045512.2 (1) ---

Section 924

(71072) 71072 71082 71093 71104 71115 71126 71137 71148

Autographa californica nucleopolyhedrovirus NC_0... (71072) TTCAAAAACAAATGACATCATCCCTTGATCATGCGTTACAAGTAGAATTCTACTCGTAAAGCGAGTTGAATTTTGAT

SARS-CoV-2 NC_045512.2 (1) ---

Section 925

	(71149)	71149 71159 71170 71181 71192 71203 71214 71225
Autographa californica nucleopolyhedrovirus NC 0...	(71149)	TACAAATATTTTGTTTATGATAGCAAGTATAAATAACCGCACAAAGTTAAATTTTTTTCATTTACTTGTCACCATGT
SARS-CoV-2 NC_045512.2	(1)	---

Section 926

	(71226)	71226 71236 71247 71258 71269 71280 71291 71302
Autographa californica nucleopolyhedrovirus NC 0...	(71226)	TTCGAATATACCCTAATAACACAACTGTGCCCGGTTGTTTAGTGGGTGACATTATTCAAGTTCGTTATAAAGATGTA
SARS-CoV-2 NC_045512.2	(1)	---

Section 927

	(71303)	71303 71313 71324 71335 71346 71357 71368 71379
Autographa californica nucleopolyhedrovirus NC 0...	(71303)	TCACATATTCGCTTTTTGTCAGATTATTTATCTTTGATGCCTAACGTTGCGATTGTAAACGAATATGGACCTAACAA
SARS-CoV-2 NC_045512.2	(1)	---

Section 928

	(71380)	71380 71390 71401 71412 71423 71434 71445 71456
Autographa californica nucleopolyhedrovirus NC 0...	(71380)	CCAGTTAGTAATAAAACGCAAAAACAAATCGCTGAAAAGCTTGCAAGATTTGTGTCTGGACAAAATAGCCGTTTCGC
SARS-CoV-2 NC_045512.2	(1)	---

Section 929

	(71457)	71457 71467 71478 71489 71500 71511 71522 71533
Autographa californica nucleopolyhedrovirus NC 0...	(71457)	TCAAGAAACCTTTTCGTCAGTTAAAATCGTTAAATGCTGTTTGTTTGATGCGAGACATTATATTTTCGCTGGGTTTA
SARS-CoV-2 NC_045512.2	(1)	---

Section 930

	(71534)	71534 71544 71555 71566 71577 71588 71599 71610
Autographa californica nucleopolyhedrovirus NC 0...	(71534)	CCAATTATTTTTAATCCGGCTTTGCTACAAAGAAAAGTGCCGCAGCGCAGCGTGGGATATTTCATGAATTCAAAATT
SARS-CoV-2 NC_045512.2	(1)	---

Section 931

	(71611)	71611 71621 71632 71643 71654 71665 71676 71687
Autographa californica nucleopolyhedrovirus NC 0...	(71611)	GGAAAGGTTTGCCAATTGTGATCGGGGTCATGTCGTTGAAGAGAAACAATTGCAGAGTAATTTGTATATAGATTATT
SARS-CoV-2 NC_045512.2	(1)	---

```
                                                                                                    Section 932
                                                  (71688) 71688     71698      71709      71720      71731      71742      71753   71764
Autographa californica nucleopolyhedrovirus NC 0... (71688) TTTGTATGATTTGTGGTTTAAATGTTTTTAAAATAAAAGAATAACAATTTACACATTGTTTTATTACATGGATAATG
                             SARS-CoV-2 NC_045512.2     (1) -----------------------------------------------------------------------------
                                                                                                    Section 933
                                                  (71765) 71765     71775      71786      71797      71808      71819      71830   71841
Autographa californica nucleopolyhedrovirus NC 0... (71765) TTGTTTGTTTGACATTAAAGGTTATCATGGTGCAATGATTAATAATAAAACAATATTATGACATTATTTTCCTGTTA
                             SARS-CoV-2 NC_045512.2     (1) -----------------------------------------------------------------------------
                                                                                                    Section 934
                                                  (71842) 71842     71852      71863      71874      71885      71896      71907   71918
Autographa californica nucleopolyhedrovirus NC 0... (71842) TTTTACAATATAAAATCACACCAATTGTGCAAAGTTTTATTATTTGTTTGTCGACGGTCGAGGGGTCAGCGGCGTGT
                             SARS-CoV-2 NC_045512.2     (1) -----------------------------------------------------------------------------
                                                                                                    Section 935
                                                  (71919) 71919     71929      71940      71951      71962      71973      71984   71995
Autographa californica nucleopolyhedrovirus NC 0... (71919) GCAACAATAAAAAACATGAAGCTGTTAACAATTTTGATTTTATTTTATTCATTTTTTATGAATTTGCAAGCGCTACC
                             SARS-CoV-2 NC_045512.2     (1) -----------------------------------------------------------------------------
                                                                                                    Section 936
                                                  (71996) 71996     72006      72017      72028      72039      72050      72061   72072
Autographa californica nucleopolyhedrovirus NC 0... (71996) AGATTACCATCAAGCAAATAGGTGTGTGTTGCTGGGAACTCGCATTGGATGGAACGATGACAATAGCCAAGATCCCA
                             SARS-CoV-2 NC_045512.2     (1) -----------------------------------------------------------------------------
                                                                                                    Section 937
                                                  (72073) 72073     72083      72094      72105      72116      72127      72138   72149
Autographa californica nucleopolyhedrovirus NC 0... (72073) ACGTATATTGGAAATGGTGTTAAATAAAAGTGAATATATTTTTTATAAAATTTTTTATTTAAAATTCCAAGTAATCC
                             SARS-CoV-2 NC_045512.2     (1) -----------------------------------------------------------------------------
                                                                                                    Section 938
                                                  (72150) 72150     72160      72171      72182      72193      72204      72215   72226
Autographa californica nucleopolyhedrovirus NC 0... (72150) CTGCAAACATTAAACACTGTAGGTATTTTTAAATCTTGCCACATGCGAACAACGCACGGCCTGTCGTCGAACACCGC
                             SARS-CoV-2 NC_045512.2     (1) -----------------------------------------------------------------------------
```

```
                                                                                                        Section 939
                                                   (72227) 72227     72237      72248      72259      72270      72281      72292    72303
Autographa californica nucleopolyhedrovirus NC 0... (72227) TATTACATTATATTTTCCTCTGATATAGTTGTTAAACAATTTTAATTTTAATAAATAATCTTTACAAGTATCGTCTG
                              SARS-CoV-2 NC_045512.2    (1) -----------------------------------------------------------------------------
                                                                                                        Section 940
                                                   (72304) 72304     72314      72325      72336      72347      72358      72369    72380
Autographa californica nucleopolyhedrovirus NC 0... (72304) AAGGCCTCATAAACAATTTATATGATTTAATATCAAAATACTTTTCAATCCAGTTTCGAGTGGGCTGTTCACAAATT
                              SARS-CoV-2 NC_045512.2    (1) -----------------------------------------------------------------------------
                                                                                                        Section 941
                                                   (72381) 72381     72391      72402      72413      72424      72435      72446    72457
Autographa californica nucleopolyhedrovirus NC 0... (72381) ACGCTTCTCCCGCTCATAAACACGATAATTGCGTCGTGGCAATTTGCCAAATACTTAACGCAAGTAATAACGTCTAA
                              SARS-CoV-2 NC_045512.2    (1) -----------------------------------------------------------------------------
                                                                                                        Section 942
                                                   (72458) 72458     72468      72479      72490      72501      72512      72523    72534
Autographa californica nucleopolyhedrovirus NC 0... (72458) GCGGGCTTCATCTTGAGCAACTCTATTATCAAAATCATAAAACGATCTATTTGTGGGCAAAGCTACTGTACCGTCTA
                              SARS-CoV-2 NC_045512.2    (1) -----------------------------------------------------------------------------
                                                                                                        Section 943
                                                   (72535) 72535     72545      72556      72567      72578      72589      72600    72611
Autographa californica nucleopolyhedrovirus NC 0... (72535) AATCACATAATACAGCGCGGGGAAATTTGTCGCCGACAGGAACGTAATATTCGAAATTATTTACCTTTAGAAACTTT
                              SARS-CoV-2 NC_045512.2    (1) -----------------------------------------------------------------------------
                                                                                                        Section 944
                                                   (72612) 72612     72622      72633      72644      72655      72666      72677    72688
Autographa californica nucleopolyhedrovirus NC 0... (72612) TTATATTGCTTTTTAATAGTTTCTGGATTTAATGGAAATTTATCAGAGCGTTTATAATTGCGTTCAAGAGCCGTTTC
                              SARS-CoV-2 NC_045512.2    (1) -----------------------------------------------------------------------------
                                                                                                        Section 945
                                                   (72689) 72689     72699      72710      72721      72732      72743      72754    72765
Autographa californica nucleopolyhedrovirus NC 0... (72689) CAAAGAAACGTCCATCAAACGCGTTAAAAAATGGTAATTATGCGTTGCGGCCATTTTTTGCCACATGTCCACCGATT
                              SARS-CoV-2 NC_045512.2    (1) -----------------------------------------------------------------------------
```

Section 946

(72766) 72766 72776 72787 72798 72809 72820 72831 72842
Autographa californica nucleopolyhedrovirus NC_0... (72766) GAGTGTTCAAATTAGTGTCGCTGACAACCACGTTGGCACCACATTTTGCGGCTTTTAAAAACTGTTCAATGCACATT
SARS-CoV-2 NC_045512.2 (1) ---

Section 947

(72843) 72843 72853 72864 72875 72886 72897 72908 72919
Autographa californica nucleopolyhedrovirus NC_0... (72843) TTGGTAATTTGTTCTTCTTTAGTTTGTCTACATTTCCGCGATTGGTTATAGAAAGCGTTCAGTTTTGTATAATCGCC
SARS-CoV-2 NC_045512.2 (1) ---

Section 948

(72920) 72920 72930 72941 72952 72963 72974 72985 72996
Autographa californica nucleopolyhedrovirus NC_0... (72920) GTTTAAAAACAACTTAACGCGCACGTCGTCTCTGTTGATTTCTGTATAGCCTTTTAAACTTTTGGCATACGTGCTTT
SARS-CoV-2 NC_045512.2 (1) ---

Section 949

(72997) 72997 73007 73018 73029 73040 73051 73062 73073
Autographa californica nucleopolyhedrovirus NC_0... (72997) TGCCCGAACCCGAAATGCCTATCAACACCAACAATTGTTTTGAAGAAGGCAATTTAATTGTTGGAGCAAGTTTATTA
SARS-CoV-2 NC_045512.2 (1) ---

Section 950

(73074) 73074 73084 73095 73106 73117 73128 73139 73150
Autographa californica nucleopolyhedrovirus NC_0... (73074) TTTAATGCCTGCTTAGTCGATACAAATTTTATAATATTTTTGATCATTTTAATTTTTTCAGGCTCGGTTAATTTTAA
SARS-CoV-2 NC_045512.2 (1) ---

Section 951

(73151) 73151 73161 73172 73183 73194 73205 73216 73227
Autographa californica nucleopolyhedrovirus NC_0... (73151) AAATTCGCTCTCCACATCGATCGTTTGTGCTTTACGACATCTGTACGCTAAACATTTCCACGGCAAAGTTTGCACCA
SARS-CoV-2 NC_045512.2 (1) ---

Section 952

(73228) 73228 73238 73249 73260 73271 73282 73293 73304
Autographa californica nucleopolyhedrovirus NC_0... (73228) GTTCGTTGAAACGCTGTTGATTCAAAGTCAAACCCGACACCATAATATTTATTGTAGACTCGTTGGTGAACGTGTTT
SARS-CoV-2 NC_045512.2 (1) ---

Section 953

```
                                              (73305) 73305     73315      73326      73337      73348      73359      73370   73381
Autographa californica nucleopolyhedrovirus NC_0... (73305) CTAGCATCAACGTACGGTTTAATGACACTTTTTAAATGCGGGAAAAGAGCTAGAAAGTCATCGTGTTCGCCATTTAT
                        SARS-CoV-2 NC_045512.2 (1) -----------------------------------------------------------------------------
```

Section 954

```
                                              (73382) 73382     73392      73403      73414      73425      73436      73447   73458
Autographa californica nucleopolyhedrovirus NC_0... (73382) AACAAGCTGCGCCAATTTAGTAGGATTTTCAGCACGGCTCTGATTTTTGTGCATGTTCAAATACACGTCGCTTTTAA
                        SARS-CoV-2 NC_045512.2 (1) -----------------------------------------------------------------------------
```

Section 955

```
                                              (73459) 73459     73469      73480      73491      73502      73513      73524   73535
Autographa californica nucleopolyhedrovirus NC_0... (73459) TCTTGCATAGTGGCGCGTTGTTTTTATCGTAAACTACAAATCCTTCTTCCAAATTTTTCAACTGGGC-CGCGTGTTC
                        SARS-CoV-2 NC_045512.2 (1) ------ATTAAAGGTTTATACCTTCCCAGGTAACAAACCAACCAAC-----TTTCGATCTCTTGTAGATCTGTTCTC
```

Section 956

```
                                              (73536) 73536     73546      73557      73568      73579      73590      73601   73612
Autographa californica nucleopolyhedrovirus NC_0... (73535) GACACATTCTTGCACAGACGTAAACTCGTAACATTTGGGTATTTGCAAAACGGCAAATTGGAACAGTAAAAATAAT
                        SARS-CoV-2 NC_045512.2 (67) TAAACGAACTTT---AAAATCTGTGTGGCTGTCACTCGGCTGCATGCTTAGTGCACT---CACGCAGTATAATTAAT
```

Section 957

```
                                              (73613) 73613     73623      73634      73645      73656      73667      73678   73689
Autographa californica nucleopolyhedrovirus NC_0... (73612) CGCCCGTTTCGTTGTTTCTGCTTGCCAAATACCACAACGTTGGCTGTTCATCGTAAACGGTTACAATTCTGTTGTGT
                        SARS-CoV-2 NC_045512.2 (138) AACTAATTACTGTCGTTGACAGGACACG--AGTAACTCGTCTAT----CTTCTGCAGGCTGC-TTACGGTTTCGTCC
```

Section 958

```
                                              (73690) 73690     73700      73711      73722      73733      73744      73755   73766
Autographa californica nucleopolyhedrovirus NC_0... (73689) TTGCTTGTTAACTCAAACATGTGAGTCGACGCGCAGTCTAAATATTCGTTACACAACGCTTGAAATTGATTGTGGGC
                        SARS-CoV-2 NC_045512.2 (208) GTG-T-TGCAGC---CGATCATC-AGCACATCTAGG----TTTC-GTCCGGGT---GTGACCGAAAGGTAAGA-TGG
```

Section 959

```
                                              (73767) 73767     73777      73788      73799      73810      73821      73832   73843
Autographa californica nucleopolyhedrovirus NC_0... (73766) CTCGTCAAGTTGAAGAGCTTGCAAAACTAAACGTTTAAACGTCACGTCTGACACGCAAAGGTTTTCTGCAAAAGCAC
                        SARS-CoV-2 NC_045512.2 (270) AG-AGCCT--TGTCCC-TGGTTTCAACG-AG-AAAACACACGTCCAACTCAGTTTGCCTGTTTACAGGTTCGCGAC
```

Section 960

		73844 73854 73865 73876 73887 73898 73909 73920
	(73844)	
Autographa californica nucleopolyhedrovirus NC_0...	(73843)	TTCCTCGGGTGCTGGCATGCCATTCGCCGTTGTACTTGTAGATTTAATTAAACTTCCGTCGATTTTTTCGTAAAAC
SARS-CoV-2 NC_045512.2	(341)	GTGCTCGT-ACGTGGCTTTGGAGACTCCGT-GGAGGAGGTCTTATCAGAGGCACGT-CAACAT-CTTAAAGATGGCA

Section 961

		73921 73931 73942 73953 73964 73975 73986 73997
	(73921)	
Autographa californica nucleopolyhedrovirus NC_0...	(73920)	TTAAAATTCTCCTTCGATTGGAACAGTTTGTGATGAGCATCTTCGCCGCCGATATTTGTAGCAATTCTTGAAAATT
SARS-CoV-2 NC_045512.2	(414)	CTTGTGGCTTAGTAGAAGTTGAAAAAGGCGTTTTGCCTCAACT-TGAACAGCCCTATGTGTTCATC-AAACGTT---

Section 962

		73998 74008 74019 74030 74041 74052 74063 74074
	(73998)	
Autographa californica nucleopolyhedrovirus NC_0...	(73997)	AAAGAAACGATCGAAAGAACGCGACACAACGGCGTACGTGCGGCTGTTAAGAATTAAACCGCGACATTCCACGACCA
SARS-CoV-2 NC_045512.2	(486)	---CGGATGCTCGAACTGCACCTCATGGTCATGTTATGGTTGAGCTGGTAG--CAGAACT-CGAAGGCATTCAGTAC

Section 963

		74075 74085 74096 74107 74118 74129 74140 74151
	(74075)	
Autographa californica nucleopolyhedrovirus NC_0...	(74074)	CAGGATGATCTCGATCGCGTTCAAACGATTCGTAATTAAGAACCATCAAATCGTGTTCGGTATAATTTTTAATTTTG
SARS-CoV-2 NC_045512.2	(557)	GGTCGTAGTGGTGAGACACTTGGTGTCCTTGTCCCTCATGT-------------GGGCGAA--ATACCAGTG----G

Section 964

		74152 74162 74173 74184 74195 74206 74217 74228
	(74152)	
Autographa californica nucleopolyhedrovirus NC_0...	(74151)	ACTTTAAACTTGTCACAAAGATTTTTCACTCCGCCGTTTGCAAGTAGACGCGAAACGTGCAACATGATTGCTGTTTA
SARS-CoV-2 NC_045512.2	(615)	C--TTACCGCA-AGGTTCTTC--TTCGTAAGAACGGTAATAAA-----GGAGCTGGTGGCCATAGTTACGGCGCCGA

Section 965

		74229 74239 74250 74261 74272 74283 74294 74305
	(74229)	
Autographa californica nucleopolyhedrovirus NC_0...	(74228)	ATAATGCATACCAATGCTAAACTGTCTATTATATAAAGTGCAGTGATAACTTTGTTATCAACGCGTTCGATGCCGAC
SARS-CoV-2 NC_045512.2	(682)	TCTAA-A--GTCATTTGACTTAGGCG-ACGAGCTTGGCACTGATCCTTATGAAGATTTTCAAGAAAACTGGAACACT

Section 966

		74306 74316 74327 74338 74349 74360 74371 74382
	(74306)	
Autographa californica nucleopolyhedrovirus NC_0...	(74305)	ATATATAAACGCAATGTAACAGTTTTTGCTAGTACCATCGCATACAACATTATGAATACAAGGGGTTGTG----TTA
SARS-CoV-2 NC_045512.2	(755)	AAACATAGCAGTGGTGTTAC---CCGTGAACTCATGCGTGAGCTTAACGGAGGGGCATACACTCGCTATGTCGATAA

Section 967

(74383) 74383 74393 74404 74415 74426 74437 74448 74459
Autographa californica nucleopolyhedrovirus NC 0... (74378) ATAATAATAAAATGATATTTATGAATGCTTTGGGCTTGCAACCTCAAAGTAAATTGAAAATTATTGCACATAAAATA
SARS-CoV-2 NC_045512.2 (829) CAACTT--CTGTGG-CCCTG---ATGGCTACCCT---CTTGAGTGCATTAAAGACCTTCTAGCACGTGCTGGTAAAG

Section 968

(74460) 74460 74470 74481 74492 74503 74514 74525 74536
Autographa californica nucleopolyhedrovirus NC 0... (74455) CTAGAAAAATGTAAACGTGACGCGTACACGCGTTTCAAGGGCGTAAAGGCGATCAAGAATGAACTAAAAACATACAA
SARS-CoV-2 NC_045512.2 (897) CTTCATGCACTTTGTCCGAACAACTGGACTTTATTGACACTA--AGAGGGGTGTATACTGCTGCCGTGAACATGAGC

Section 969

(74537) 74537 74547 74558 74569 74580 74591 74602 74613
Autographa californica nucleopolyhedrovirus NC 0... (74532) TCTTACGTTGCAACAATACAACGAGGCGCTCAATCAGTGCGCTTTAAACGATAGCCGATGGCGCGACACAAATAATT
SARS-CoV-2 NC_045512.2 (972) ATGAAATTGCTTGGTACACGGAACGTTCTGAAAAGAG--CTATGAATTGCAGACACCTTT----TGAAATTAAATTG

Section 970

(74614) 74614 74624 74635 74646 74657 74668 74679 74690
Autographa californica nucleopolyhedrovirus NC 0... (74609) GGCATCACGATATTGAAGAAGGTGTGAAAATAAACAAGAGACATATATATAGAGTTAATTTTAATTCTAAAACCCAA
SARS-CoV-2 NC_045512.2 (1043) GCAAAGAAATTTGACACCTTC-AATGGG--GAATGTCCAAATTTTGTAT------TTCCCTTAAATTCCATAATCAA

Section 971

(74691) 74691 74701 74712 74723 74734 74745 74756 74767
Autographa californica nucleopolyhedrovirus NC 0... (74686) GAAATTGAAGAATATTATTACATTAAAGTAGAATGTTATGTAAACAGTTAATTAATCTACATTTATTGTAACATTTG
SARS-CoV-2 NC_045512.2 (1111) GACTATTCAACCAAGGGTTGAAAAG----AAAAAGCTTGATGGCTTTATGGGTAGAATTC-GAT---CTGTCTATCC

Section 972

(74768) 74768 74778 74789 74800 74811 74822 74833 74844
Autographa californica nucleopolyhedrovirus NC 0... (74763) TGGTAATAGTGGCGTTGGTTATACATTTATATGATTGTAATGTTGTGTACTCGTTTTGTAATAAATTTTTGTGTTTA
SARS-CoV-2 NC_045512.2 (1180) AGTTGCGT-CACCAAATGAATGCAACCAAATGTGCCTTTCAACT---------CTCATGAAGTGTGATCAT-TGTGGT

Section 973

(74845) 74845 74855 74866 74877 74888 74899 74910 74921
Autographa californica nucleopolyhedrovirus NC 0... (74840) ATCAATTCAATATTTTTATTTGATAAAACCTTATTTTCGCTACTCAATTTGGCGTTTTTAGACGCAAGTTTTGCGTA
SARS-CoV-2 NC_045512.2 (1247) G--AAACTTCATGGCAGACGGGC---------GATTTTGTTAAAGCCACTT---------GCGAATTTTGTGGCACTGA

```
Section 974
                                                   (74922) 74922     74932      74943      74954      74965      74976      74987     74998
Autographa californica nucleopolyhedrovirus NC 0... (74917) ATCGTCATTGAGCGATTTTAGCGCCTTTTCAGTTGTAATTCGTTTCAGTTGCAATTCTTTAAAAGATTTATGCATGT
                         SARS-CoV-2 NC_045512.2 (1306) GAATTTGACTAAAGAAGGTGCCACTACTTGTGGTTACTTA----CCCCAAAATGCTGTTGTTAAAATTTA------T

Section 975
                                                   (74999) 74999     75009      75020      75031      75042      75053      75064     75075
Autographa californica nucleopolyhedrovirus NC 0... (74994) TGTTGTAGTCGCTTTTAATTTTGTCTAACTTTTCTTGCATAGAAACGCTTGTTTGTTGTAATTTGTCTAAATCTAAT
                         SARS-CoV-2 NC_045512.2 (1373) --T-GTCCAGCATG------TCACAATTCAGAAGTAGGACCTGAGCATAGTCTTGCCGAATACCAT-------AATG

Section 976
                                                   (75076) 75076     75086      75097      75108      75119      75130      75141     75152
Autographa californica nucleopolyhedrovirus NC 0... (75071) TGTTGTTTAATGTTGAGCTGCGTTTGTTCGGCAATGTCTACCTGTAGTTTTTTTAGTATCGCTTGTGCTTCAGACAG
                         SARS-CoV-2 NC_045512.2 (1434) AATCTGGCTTGAAAACCATTCTTCGTAAG-GGTGGTCGCACTATT----GCCTTTGGAGGCTGTGTGTTCTCTTATG

Section 977
                                                   (75153) 75153     75163      75174      75185      75196      75207      75218     75229
Autographa californica nucleopolyhedrovirus NC 0... (75148) CATAGTGTCGTCGGCATTTGCGTTGTTGTCTTCTGCGT---C--GTCCAACAGACTTTTTTCAAACAACACACTGGC
                         SARS-CoV-2 NC_045512.2 (1506) TTGGTTGCCATAACAAGTGTGCCTATTGGGTTCCACGTGCTAGCGCTAACATAGGTTGTAACCATACAGGTGTTGTT

Section 978
                                                   (75230) 75230     75240      75251      75262      75273      75284      75295     75306
Autographa californica nucleopolyhedrovirus NC 0... (75220) CAAAGAGGCCGCATCAAAATTAGCGTTTATTTTATTCCATTGTGCGACACTCGACGCGCTGCATTTAATCACATCCA
                         SARS-CoV-2 NC_045512.2 (1583) GGAGAAGGTTCCGAAGGTCTTAATGACAACCTTCTTGAAATACTCCAAAAAG--AGAAAGTCAAC--ATCAATATTG

Section 979
                                                   (75307) 75307     75317      75328      75339      75350      75361      75372     75383
Autographa californica nucleopolyhedrovirus NC 0... (75297) CAAC-GTTTCGGTTTACGCTGTAAACGTTGAAATGCAAACTTTCAACCCTACACAAGGGACATGGTACTTTTTTTCG
                         SARS-CoV-2 NC_045512.2 (1656) TTGGTGACTTTAAACTTAATGAAGA-GAT---CGCCATTATTTTGGCATCTTTT-TCTGCTTCC--ACAAGTGCTTT

Section 980
                                                   (75384) 75384     75394      75405      75416      75427      75438      75449     75460
Autographa californica nucleopolyhedrovirus NC 0... (75373) TTTTCTAATCTTGCGTATACACATTGAGCATAATTGATGTTTGCACGTGTCTAGTTCTAATACGGGTATTATAGTCA
                         SARS-CoV-2 NC_045512.2 (1726) TGTGGAAACTGTGAAAGGT---TTGGATTATAAAG-CATTCAA--A---CAAATTGTTGAATCCTGTGGTAATTTTA
```

Section 981

(75461) 75461 75471 75482 75493 75504 75515 75526 75537

Autographa californica nucleopolyhedrovirus NC 0... (75450) ATCTGTCTATTGGTT----GCAGAAAA--TAATTTTTAATTTCTGCAACCGAAAAACAAATGTTGCATTGCAATTTA

SARS-CoV-2 NC_045512.2 (1794) AAGTTACAAAAGGAAAAGCTAAAAAAGGTGCCTGGAATATTGGTGAACA-GAAATCAATACTGAGTCCTCTTTATGC

Section 982

(75538) 75538 75548 75559 75570 75581 75592 75603 75614

Autographa californica nucleopolyhedrovirus NC 0... (75521) ACAAACTCCATTTTTAGACGGCTATTCCTCCACCTGCTTCGCCTGCAACACCAGGCGCAGGACCTGCCACTGCGCCG

SARS-CoV-2 NC_045512.2 (1870) ATTTGCATCAGAGGCTGCTCGTGTTGTACGATCAATTTTCTCCCGCACT-CTTGA--AACTGCTCAA-AATTCTGTG

Section 983

(75615) 75615 75625 75636 75647 75658 75669 75680 75691

Autographa californica nucleopolyhedrovirus NC 0... (75598) CCGCCCAGAGTAGCGTTAGGATTTGCTCTTGGTATAAAGTCGTTGCGCAAAAAGTTGTTTTCTGAATTGATTATTTG

SARS-CoV-2 NC_045512.2 (1943) CGTGTTTTA--C-AGAAGGCCGCTATAACAATACTAGATGGAATTTCACAGT----ATTCA---CTGAGACTCATTG

Section 984

(75692) 75692 75702 75713 75724 75735 75746 75757 75768

Autographa californica nucleopolyhedrovirus NC 0... (75675) GTATCCCAAAAACAGCGGAACGTACGTCGGGTATTCTTCGTATCCGCTAAGCGTTCTGTCCAGCTCACGTGTGTCGC

SARS-CoV-2 NC_045512.2 (2010) A--TGCTATGATGTTCAC-ATCTGATTTGGCTACTAACAATCTAGTTGTAATGGCCTACATTACAGGTGGTGTTGTT

Section 985

(75769) 75769 75779 75790 75801 75812 75823 75834 75845

Autographa californica nucleopolyhedrovirus NC 0... (75752) CTTCAAATTTCAAAACGTTTCTAATTTGCAAACGATTGGGTTGACTTCTCATAATGTCACTGCTTCTTATCGGGTTG

SARS-CoV-2 NC_045512.2 (2084) CAGTTGACTTCG-CA---GTGGCTAACTAACAT-CTTTGG-CA-----CTGTTTATGAAAAC-----TCAAACCCG

Section 986

(75846) 75846 75856 75867 75878 75889 75900 75911 75922

Autographa californica nucleopolyhedrovirus NC 0... (75829) TACAACTCGGGGCCGTCGGGCACAGACGCGACCAGACCCGTTTCGTCAATTATACACGTGGCGCAATTTCTAAACCT

SARS-CoV-2 NC_045512.2 (2145) TCCTTGATTGGCTTGAAG---AGAAGTTT-AAGGAAGGTGTAGAGTTTCTTAGAGACGGTTGGGAAATTGTTAAATT

Section 987

(75923) 75923 75933 75944 75955 75966 75977 75988 75999

Autographa californica nucleopolyhedrovirus NC 0... (75906) CAATTCCTCCGTGTCGATTTGCAAGTACTCGGGCGCTACTGCGCGTCGAATCAAATTTTGCAAAAATC----CACTG

SARS-CoV-2 NC_045512.2 (2218) TATCTCAACCTGTGCTTGTGAAATTGTCGGTGGACAAATT-GTCACCTGTGCAAAGGAAATTAAGGAGAGTGTTCAG

```
                                                                                                   Section 988
                                        (76000) 76000     76010      76021      76032      76043      76054      76065   76076
Autographa californica nucleopolyhedrovirus  NC_0... (75979) TAATTGTTAAATAATTGATCGCCAGCACCGCCTCGAAGCGCTCGGGCGTTGGTCACGTCAAAGAAACGCAATTCGTC
                          SARS-CoV-2 NC_045512.2  (2294) ACATTC--TTTAAGCTTGTAAATAAATTTTTGGCTTTG-TGTG--CTGACTCTATCATTATTGGTGGAGCTAAACTT
                                                                                                   Section 989
                                        (76077) 76077     76087      76098      76109      76120      76131      76142   76153
Autographa californica nucleopolyhedrovirus  NC_0... (76056) TCGCGACACCCGCGAACAAAACGTGTTCGGGTTTGTGGTGTCCAGAATGCTTTTTGTAGTTGCGTAAACGCTGTGTA
                          SARS-CoV-2 NC_045512.2  (2366) AAAGCCT----TGAATTTAGGTGAAACATTTGTCACGCACTCAAGGGATTGTACAGAAAGTGT-GTTAAATC----
                                                                                                   Section 990
                                        (76154) 76154     76164      76175      76186      76197      76208      76219   76230
Autographa californica nucleopolyhedrovirus  NC_0... (76133) TAACGCGTTGCGTGTTGCTTGTGAAACCTTCGGTATATTTTAGATTGTCGCATATAGTGTTAACTGCGTTTTCGTTG
                          SARS-CoV-2 NC_045512.2  (2434) ---CAGAGAAGAAA-CT-GGCCTACTCATGCCTCTAAAAGCCCCAAA---AGAAATTATCTTCTTAGA---GGGAGA
                                                                                                   Section 991
                                        (76231) 76231     76241      76252      76263      76274      76285      76296   76307
Autographa californica nucleopolyhedrovirus  NC_0... (76210) TTATATATCAAATGAAAGATTAGCTGTTCGGCTTGCATCATACTGTTTAGATTAAACACGTCTT-----GGTAATTG
                          SARS-CoV-2 NC_045512.2  (2500) AACACTTCCCACAGAAGTGTTAACAGAGGAAGTTGTCTTGAAAACTGGTGATTTACAACCATTAGAACAACCTACTA
                                                                                                   Section 992
                                        (76308) 76308     76318      76329      76340      76351      76362      76373   76384
Autographa californica nucleopolyhedrovirus  NC_0... (76282) GTTGCGCTTGGAATTAAAATTCGCTTGATACCTCTTTCTT-TATTTCCAACTAAATGCCTAGCGATCGTCATTTTGA
                          SARS-CoV-2 NC_045512.2  (2577) GTGAAGCTGTTGAAGCTCCATTGGTTGGTA-CACCAGTTTGTATTAACGGGCTTA----TGTTGCTCGAAATCAAAG
                                                                                                   Section 993
                                        (76385) 76385     76395      76406      76417      76428      76439      76450   76461
Autographa californica nucleopolyhedrovirus  NC_0... (76358) ATTGATTGTCGTCTTCGTCGAAAATGGGCAAAACCATTTTTGA-CATTTTAAAACGTTTTATGAGGTGGTTGTTGCA
                          SARS-CoV-2 NC_045512.2  (2649) ACACAGAAAAGTACTGTGCCCT--TGCACCTAATATGATGGTAACAAACAATACCTTCACA-CTCAAAGGCGGTGC-
                                                                                                   Section 994
                                        (76462) 76462     76472      76483      76494      76505      76516      76527   76538
Autographa californica nucleopolyhedrovirus  NC_0... (76434) AATAAACCATCCATCGTCATGATACGCGTCGGGCGAACACGGCGATTTGTATGTTATGCACGCGTCGAACGACACGA
                          SARS-CoV-2 NC_045512.2  (2722) ACCAACAAAGGTTACTTTTGGTGATGACAC-TGTGATAGAAG-TGCAAGGTTACAAGAGTGTGAATATCACTT---T
```

Section 995

(76539) 76539 76549 76560 76571 76582 76593 76604 76615

Autographa californica nucleopolyhedrovirus NC_0... (76511) TGGACGCGAAAATGCAGCGATTAACTCTCATTTGTCGCGGCGCCATACCCACGGGCACTAGCGCCATATTGTTGCCG

SARS-CoV-2 NC_045512.2 (2794) TGAACTTGATGAAAGGATTGATAAAGTACTTAATGAGAAG--TGCTCTGCCTATACAGTTGAACTCGGTACAGAAGT

Section 996

(76616) 76616 76626 76637 76648 76659 76670 76681 76692

Autographa californica nucleopolyhedrovirus NC_0... (76588) TTATAAATATGGACTACGGCGATTTTGTGATTGAGAAAGAAATCTCTTATTCAATAAATTTTAGCCAAGATTTGTTG

SARS-CoV-2 NC_045512.2 (2869) AAATGAGTTCGC-CTG---TGTTGTGGCAGATGCTGTCATAAAAACTTTGCAACCAGTATCTGA-ATTACT----TA

Section 997

(76693) 76693 76703 76714 76725 76736 76747 76758 76769

Autographa californica nucleopolyhedrovirus NC_0... (76665) TATAAAATTTTAAATTCTTATATTGTTCCTAATTATTCGCTGGCACAACAATATTTCGATTTGTACGACGAAAACGG

SARS-CoV-2 NC_045512.2 (2937) CACCA-CTGGGCATTGAT-TTAGATGAGTGG-AGTATGGCTACATACTACTTATT--TGATGAG------TCTGGTG

Section 998

(76770) 76770 76780 76791 76802 76813 76824 76835 76846

Autographa californica nucleopolyhedrovirus NC_0... (76742) CTTTCGCACTCGTATACCTATTCAGAGCGCTTGCAATAACATAATATCAAGCGTGAAAAAGACTAATTCCAAACACA

SARS-CoV-2 NC_045512.2 (3003) AGTTTAAATTGGCTTCA--CATATGTATTGTTCTTTCTACCC-TCCAGATGAGGATGAAGAAGAAGGTGATTGTGAA

Section 999

(76847) 76847 76857 76868 76879 76890 76901 76912 76923

Autographa californica nucleopolyhedrovirus NC_0... (76819) AAAAATTTGTTTATTGGCCTAAAGATACCAACGCGTTGGTGCCGTTGGTGTGGAGAGAAAGCAAAGAAATCAAACTG

SARS-CoV-2 NC_045512.2 (3077) GAAGAA---GAGTT-TGAGCCATCAACTCAATATGAGTATGGTACTGAAGATGATTACCAAGGTAA--ACC--TTTG

Section 1000

(76924) 76924 76934 76945 76956 76967 76978 76989 77000

Autographa californica nucleopolyhedrovirus NC_0... (76896) CCTTACAAGACTCTTTCGCACAACTTGAGTAAAATAATTAAAGTGTACGTTTACCAACACGATAAAATTGAAATCAA

SARS-CoV-2 NC_045512.2 (3146) GAATTTGGTGCCA-----CTTCTGCTG------CTCTTCAACCTG-AA---GAAGAGCAAGAAGAAGATTGGTTAGA

Section 1001

(77001) 77001 77011 77022 77033 77044 77055 77066 77077

Autographa californica nucleopolyhedrovirus NC_0... (76973) ATTTGAACATGTATATTTTTCGAAAAGTGACATTGATCTATTTGATTCCACGATGGCGAACAAGATATCCAAACTGC

SARS-CoV-2 NC_045512.2 (3208) TGATGATAGTCAA-CAAACTGTTGGTCAACAA--GACGGCAG---TG--AGGACAATCAGACAA--CTACTATTCAA

Section 1002

```
                                               (77078) 77078     77088      77099      77110      77121      77132      77143      77154
Autographa californica nucleopolyhedrovirus NC_0... (77050) TGACTTTGTTGGAAAATGGGGACGCTTCAGAGACGCTGCAAAACTCGCAAGTGGGCAGCGATGAAATTTTGGCCCGC
                          SARS-CoV-2 NC_045512.2 (3275) ACAATTGTT--GAGGTTCAACCTCAATTAGA-GATGGAACTTACACCAGTTGTTCAGACTATTGAAGTGAATAG---
```

Section 1003

```
                                               (77155) 77155     77165      77176      77187      77198      77209      77220      77231
Autographa californica nucleopolyhedrovirus NC_0... (77127) ATACGTCTCGAATATGAATTTGACGACGACGCGCCCGACGACGCGCAGCTAAACGTGATGTGCAACATAATTGCGGA
                          SARS-CoV-2 NC_045512.2 (3346) TTTTAGTGGTTATTTAAAACTTACTGA------CAATGTATACATTAAAAATGCAGACATTGT-GGAAGAAGCTAAA
```

Section 1004

```
                                               (77232) 77232     77242      77253      77264      77275      77286      77297      77308
Autographa californica nucleopolyhedrovirus NC_0... (77204) CATGGAAGCGTTAACCGACGCGCAAAACATATCACCGTTCGTGCCGTTGACCACGTTGATTGACAAGATGGCCCCTC
                          SARS-CoV-2 NC_045512.2 (3416) AAGGTAAA---ACCAACAG-TGGTTGTTAATGC--AGCCAATGTTTACCTTAAACATGGAGGAGGTGTTGCAGGAGC
```

Section 1005

```
                                               (77309) 77309     77319      77330      77341      77352      77363      77374      77385
Autographa californica nucleopolyhedrovirus NC_0... (77281) GAAAATTTGAACGGGAACAAAAAATAGTGTACGGCGACGACGCGTTCGACAACGCGTCCGTAAAAAAATGGGCGCTC
                          SARS-CoV-2 NC_045512.2 (3487) CTTAAA---TAAGGCTACTAACAATG---C-CATGCAA---GTTGAATCTG-ATGATTACATAGCTACTAATGGACC
```

Section 1006

```
                                               (77386) 77386     77396      77407      77418      77429      77440      77451      77462
Autographa californica nucleopolyhedrovirus NC_0... (77358) AAATTGGACGGTATGCGGGGCAGAGGTCTGTTTATGCGCAATTTTTGCATTATTCAAACCGACGATATGCAATTCTA
                          SARS-CoV-2 NC_045512.2 (3553) ACTTAAAGTGGGTGGTAGT--TGTGTTTTAAGCGGACACAATCTTGCTAAACACTGTCTTCA-TGTTGTCGGC-CCA
```

Section 1007

```
                                               (77463) 77463     77473      77484      77495      77506      77517      77528      77539
Autographa californica nucleopolyhedrovirus NC_0... (77435) CAAAACCAAAATGGCCAATCTGTTTGCGCTAAACAACATTGTGGCCTTTCAATGCGAGGTTATGGACAAACAAAAGA
                          SARS-CoV-2 NC_045512.2 (3626) AATGTTAACAAAGGTGAAGACATTCAACTTCTT--AAGAGTGCTTATG----AAAATTTTAATCAGCACGAAGTTCT
```

Section 1008

```
                                               (77540) 77540     77550      77561      77572      77583      77594      77605      77616
Autographa californica nucleopolyhedrovirus NC_0... (77512) TTTACATTACAGATTTGCTGCAAGTGTTTAAATACAAATACAACAATCGAACACAGTACGAATGCGGCGTGAACGCG
                          SARS-CoV-2 NC_045512.2 (3697) ACTTGCACCATTATTATCAGCTGGTATTTTTG--GTGCTGACCCTATACAT-TCTTT-AAGAGTTTG-TGTAGATAC
```

Section 1009

	(77617)	77617 77627 77638 77649 77660 77671 77682 77693
Autographa californica nucleopolyhedrovirus NC 0...	(77589)	TCATACGCTATAGATCCGGTGACGGCCATCGAATGTATAAACTACATGAACAACAACGTGCAAAGCGTCACGTTGAC
SARS-CoV-2 NC_045512.2	(3769)	TGTTC-GCACAAATGTCTACTTAGCTGTCTTT-GATAAAAATCTCTATGACAAACTTGTTTCAAGCTTTTT--GGA-

Section 1010

	(77694)	77694 77704 77715 77726 77737 77748 77759 77770
Autographa californica nucleopolyhedrovirus NC 0...	(77666)	CGACACTTGCCCCGCAA----TTGAATTGCGGTTTCAGCAATTTTTTGAT--CCACCGCTACAGCAGAGCAATTACA
SARS-CoV-2 NC_045512.2	(3841)	-AATGAAGAGTGAAAAGCAAGTTGAACA--AAAGATCGCTGAGATTCCTAAAGAGGAAGTTAAGC--CATTTATAAC

Section 1011

	(77771)	77771 77781 77792 77803 77814 77825 77836 77847
Autographa californica nucleopolyhedrovirus NC 0...	(77737)	TGACCGTGTCCGTGGACGGGTATGTCGTGCTCGACACCGAGTTGAGATACGTCAAATATAAATGGATGCCAACAACC
SARS-CoV-2 NC_045512.2	(3913)	TGAAAGTAAAC-CTTCAGTTGAACAGAGAAAACAAGATG-ATAAGAAAAT-CAAAGCTTGTGTTGAAGA-------A

Section 1012

	(77848)	77848 77858 77869 77880 77891 77902 77913 77924
Autographa californica nucleopolyhedrovirus NC 0...	(77814)	GAGTTAGAGTATGACGCCGTGAATAAGTCGTTTAACA-CACTCAATGGGCCATTGAACGGTCTCATGATTTTAACCG
SARS-CoV-2 NC_045512.2	(3980)	GTT--ACA--AC--AACTCTGGAAGAAACTAAGTTCCTCACAGAAAACTTGTTACTTT-ATATTGACATTAATGGCA

Section 1013

	(77925)	77925 77935 77946 77957 77968 77979 77990 78001
Autographa californica nucleopolyhedrovirus NC 0...	(77890)	ACTTGCCGGAGTTACTGCACGAAAACATTTACGAATGTGTAATCACGGACACGACAATAAACGTGTTGAAACATCGT
SARS-CoV-2 NC_045512.2	(4050)	A-T------CTTCATCCAGATTCTGCCACTCTTGTTAGTGACATTGA----CAT-CACTTTCTTAAA------GAAA

Section 1014

	(78002)	78002 78012 78023 78034 78045 78056 78067 78078
Autographa californica nucleopolyhedrovirus NC 0...	(77967)	CGCGACCGAATCGTGCCAAATTAAAGCACGTTAAGCGGATACAACGGGCAGTCCGAGCTGTTAAAGTCAATACAACC
SARS-CoV-2 NC_045512.2	(4109)	GATGCTCCAT-ATATAGTGGGTGATGTTGTTCAAGAGGGTGTTT-----------TAACT-GCTGTGGTTATACCTA

Section 1015

	(78079)	78079 78089 78100 78111 78122 78133 78144 78155
Autographa californica nucleopolyhedrovirus NC 0...	(78044)	ATCGTTAACAAACGAATACGCATTGTTGTGACAGCTGAGGATATAAAAAGGAATAGAGAAGTAATTGCAATGAAATA
SARS-CoV-2 NC_045512.2	(4173)	C-----TAAAAAGGCTGGTGGCACTACTGAAATGCTAGCGAAAGCTTTGAGAAAAGTG---CCAACAGACAATTATA

```
                                                                  Section 1016
                                       (78156) 78156     78166      78177      78188      78199      78210      78221   78232
Autographa californica nucleopolyhedrovirus_NC_0... (78121) TCCCGTTACAATTCCACGGCACAGCGTATGTTGCTCGAGTTCTATCAGTTGCACACAACGGCCTAAGAAAATTTATT
                      SARS-CoV-2 NC_045512.2 (4242) TAACCACTTACCCGGGTCAGGGTTTAAATGGTTACACTGTAGAGGAGG-CAAAGACAGTGCTTAAAA-AGTGTAAAA

                                                                  Section 1017
                                       (78233) 78233     78243      78254      78265      78276      78287      78298   78309
Autographa californica nucleopolyhedrovirus_NC_0... (78198) AATGCTTCATTTGTATCTATATTAGAAGGATAATACATAGGTTCGCCCAAAGGACTGGGAGAAGGCGGCGGCGAAGG
                      SARS-CoV-2 NC_045512.2 (4317) GTGCCT-----TTTACATTCTACCA-TCTATTATCTCT-AATGAGAAGCAAGAAATTCTTGGAACT-----------

                                                                  Section 1018
                                       (78310) 78310     78320      78331      78342      78353      78364      78375   78386
Autographa californica nucleopolyhedrovirus_NC_0... (78275) TGTAGGTGTAGGAGGAATAGGAGAAGGCGGCGGCGAAGGTGTAGGTGTTGGAGGAATAGGAGAAGGCGGCGGCGAAG
                      SARS-CoV-2 NC_045512.2 (4376) -GTTTCTTGGAATTTGCGAGAAATGCTTGCACATGCAGAAGAAACACGCAAATTAATGCCTGTCTGTGTGGAA---A

                                                                  Section 1019
                                       (78387) 78387     78397      78408      78419      78430      78441      78452   78463
Autographa californica nucleopolyhedrovirus_NC_0... (78352) GTGTAGGTGTAGGAGGAATAGGAGAAGGTGGAGGTGTAGGTGTAGGTGTTGGAGGTATAGGTGTTGGAGGAGGTGTA
                      SARS-CoV-2 NC_045512.2 (4449) CTAAAGCCATAGTT-TCAACTATAC-AGCGTAAATATAAGGGTATTAAAATACAAGAGGGTGTGG---------TTG

                                                                  Section 1020
                                       (78464) 78464     78474      78485      78496      78507      78518      78529   78540
Autographa californica nucleopolyhedrovirus_NC_0... (78429) GGTGTAGGTGTTGGAGGTATAGGTGTTGGAGGAGGTGTAGGCGAAGGTGGAGAAGGTGTAGGAGTAGGTGGAGGTGT
                      SARS-CoV-2 NC_045512.2 (4515) ATT----ATGGTGCT------AGATTTTACTTTTACACCAG--TAAAACAACTGTAGCGTCACTTATCAACACACTT

                                                                  Section 1021
                                       (78541) 78541     78551      78562      78573      78584      78595      78606   78617
Autographa californica nucleopolyhedrovirus_NC_0... (78506) AGGTAACGGTACAATTGGTGGAGATGTAGGTGGTGGTACAATTGGTGGATTTGGATACAATTCCTGAATGTCGTCTA
                      SARS-CoV-2 NC_045512.2 (4580) AACGATCTAAATGAAACT-----------C----TTGTTACAATGCCACTT-GGCTATGTA---ACACATGGCTTAA

                                                                  Section 1022
                                       (78618) 78618     78628      78639      78650      78661      78672      78683   78694
Autographa californica nucleopolyhedrovirus_NC_0... (78583) ATATTTTTAAAGTTAATAAAATTATTATAAATAAATTTAATATTATTATTATTATTATTATCACAATAATGTACCAC
                      SARS-CoV-2 NC_045512.2 (4638) ATTTGGAAGAAGCTGCTC--------GGTATATGAGATCTCTCAAAGTGCCAGCTACAGTTTCTGTTTCTTCACCTGA
```

Section 1023

	(78695)	78695 78705 78716 78727 78738 78749 78760 78771
Autographa californica nucleopolyhedrovirus NC_0...	(78660)	ATGTTGCTTAAATATAAAAATTAAACAAAGAATGTTGTATTATTGCAAATTTAACAATTTTTTGTATTCTCCCCATG
SARS-CoV-2 NC_045512.2	(4708)	TGCTGTTAC----AGC--GTATA-ATGGTTATCTTACTTCT-TCTTC---TAAAA---CACCTGAAGAACATTTTAT

Section 1024

	(78772)	78772 78782 78793 78804 78815 78826 78837 78848
Autographa californica nucleopolyhedrovirus NC_0...	(78737)	TCATGCGTTCGTAATGAGCGGGCGGTTTTTATTTCTTTGTATCCACTTGTAATCGTTAATGTGGTTGTGAAAAGTC
SARS-CoV-2 NC_045512.2	(4771)	TGAAACCATCTCAC-------TTGCTGGTTCCTATAAAGATTGGTCCT-ATTCTGGACAATCTACA----CAACTA-

Section 1025

	(78849)	78849 78859 78870 78881 78892 78903 78914 78925
Autographa californica nucleopolyhedrovirus NC_0...	(78814)	-ATACTGACGTAGGCCATTAAATTTTTCATGAGCATATTATTTGACACAACTGCAACATCTGCGCCTGCCGTTTCTT
SARS-CoV-2 NC_045512.2	(4835)	GGTATAGA---ATTTCTTAAGAGAGGTGATAAAAGTGTATATTACACTAG----TAATCCTACCACATTCCACCTAG

Section 1026

	(78926)	78926 78936 78947 78958 78969 78980 78991 79002
Autographa californica nucleopolyhedrovirus NC_0...	(78890)	GCTGGTACGAATCGACAAACGTAATGTCTGTGCCGTATTTTTCTTTGTCAAGTGCAATTTCTATAAGCTCAATGTGG
SARS-CoV-2 NC_045512.2	(4905)	ATGGTGAAGTTATCACCTTTGACAATCTTAAGACACTTCTTTCTTTGAGAGAAGTGAGGACTATTA-AGGTGTTTA-

Section 1027

	(79003)	79003 79013 79024 79035 79046 79057 79068 79079
Autographa californica nucleopolyhedrovirus NC_0...	(78967)	TAAATGATGAAACCTTTGACGTTCATATAATGATCGCGGCACATGGCGCACTGTAGTATGAAAAATACGTTGTAAAA
SARS-CoV-2 NC_045512.2	(4980)	CAACAGTAGACA-----ACATTAACCTCCAC--ACGCAAGTTGTGGACATGTCAATG---ACATAT---GGACAA-C

Section 1028

	(79080)	79080 79090 79101 79112 79123 79134 79145 79156
Autographa californica nucleopolyhedrovirus NC_0...	(79044)	TAGCACCTTCATTGTTTTCAACTGCTGCATGACAAAATCTAACTGCTTTTGTCTCGCGTATACACCATATCGTCGA
SARS-CoV-2 NC_045512.2	(5043)	AGTTTGGTCCAACTTATTTGGA---TGGAGCTGATGTTACTAAAATAAAACCTCA---------T------AATTCA

Section 1029

	(79157)	79157 79167 79178 79189 79200 79211 79222 79233
Autographa californica nucleopolyhedrovirus NC_0...	(79121)	TGATGAGACTGAGAAAGTGCATGGTGTCCCATATGGTAGTAAACGTGTAAGTAAAACTCTTGGGCTGGCACGAACGC
SARS-CoV-2 NC_045512.2	(5102)	CA-TGAAG-GTAAAACATTTTATGTTTTACCTAAT---GATGACACTCTACGT-----GTTGAGGCTTTT----GAG

Section 1030

		79234 79244 79255 79266 79277 79288 79299 79310
	(79234)	
Autographa californica nucleopolyhedrovirus NC 0...	(79198)	AAATTGAGTTCTGTGGTTTTGTCCATAAATTCTATGCGAAACTGTTGCAAGTCCATGTCGGGGGATGCGTTAATGGC
SARS-CoV-2 NC_045512.2	(5165)	TACTACCACACAACTGATCCTAGTTTTC--TGG-------GTAGGTACATGTCAGCATTAAATCACACTAAAAAGTG

Section 1031

		79311 79321 79332 79343 79354 79365 79376 79387
	(79311)	
Autographa californica nucleopolyhedrovirus NC 0...	(79275)	CCATTCGATCAACTGCTGCACCTCGTACTTTTGAATGTCTTTGTATTTCATCAAACACGCAAAATGGTATAAGTAAG
SARS-CoV-2 NC_045512.2	(5233)	GAAATAC-CCACAAGTTAATGGTTTAACTT-----CTATTAAATGGGCAGA----TAACAACTGTTATCTTGCCACT

Section 1032

		79388 79398 79409 79420 79431 79442 79453 79464
	(79388)	
Autographa californica nucleopolyhedrovirus NC 0...	(79352)	TTGCTTGCGAAGACAACAGTTTGGTGAGGTGCGTCGATTTAGAGGCTCGCAAAAGGTCTATGAGACGAAACGAATAC
SARS-CoV-2 NC_045512.2	(5300)	GCATTGTT------AACACTCCAACAAATAGAGTTGAAGT--------------TTAATCCACCTGC-----TCTAC

Section 1033

		79465 79475 79486 79497 79508 79519 79530 79541
	(79465)	
Autographa californica nucleopolyhedrovirus NC 0...	(79429)	AACAGATAGCTGTCTTTGTAACGAGAAAAAAGCGGCGTCAGCGGTATCATGGCGACTAGCAAAACGATCGTGCTGTA
SARS-CoV-2 NC_045512.2	(5352)	AAGA-TG------CTTATTACAGAGCAAGGGCTGGTGAAGCTGC--TAACTTTTGTGCACTTAT-CTTAGCCTA-CT

Section 1034

		79542 79552 79563 79574 79585 79596 79607 79618
	(79542)	
Autographa californica nucleopolyhedrovirus NC 0...	(79506)	CTTGTGTCAGGCGCCGGCCACAGCGTCGTTGTACGTTAGCGCAGACACGGACGCCGACGAGCCTATTATTTATTTCG
SARS-CoV-2 NC_045512.2	(5418)	GTAATAAGACAGTAGGTGA-GTTAGGTGAT-GTTAGAGAAACAATGAGTTACTTGT---TTCAACATGCCAATTTAG

Section 1035

		79619 79629 79640 79651 79662 79673 79684 79695
	(79619)	
Autographa californica nucleopolyhedrovirus NC 0...	(79583)	AAAATATTACAGAATGTCTTACGGACGACCAATGCGACAAGTTTACTTATTTTGCTGAACTCAAACAGGAGCAAGCC
SARS-CoV-2 NC_045512.2	(5490)	ATTCTTGCAA--------AAGAGTC---TTGAACGTGGTGTGTAAAACTTGTGGACAACAGCAGA------CAACCCT

Section 1036

		79696 79706 79717 79728 79739 79750 79761 79772
	(79696)	
Autographa californica nucleopolyhedrovirus NC 0...	(79660)	TTATTTATGAAAAAAGTATACAAACACTTGGTGCTTAAAAACGAGGGTGCTTTTAACAAACACCACGTATTGTTCGA
SARS-CoV-2 NC_045512.2	(5551)	TAAGGGTGTAGAAGCTGTT------A-TGTACATGGGCACACTTTCTTATGAACAA---TTTAAGAAA-GGTGTTCA

Section 1037

		79773 79783 79794 79805 79816 79827 79838 79849
	(79773)	
Autographa californica nucleopolyhedrovirus NC_0...	(79737)	TGCAATGATTATGTATAAGACATATGTGCATTTGGTCGACGAGTCTGCGTTCGGAAGCAACGTTATCAACTATTGCG
SARS-CoV-2 NC_045512.2	(5617)	GATACC-TTGTACGTG--TGGTAAACAAGCTACAAAATATCTAG-TACAACAGGAGTCACCTTTTGTTATGATG-TC

Section 1038

		79850 79860 79871 79882 79893 79904 79915 79926
	(79850)	
Autographa californica nucleopolyhedrovirus NC_0...	(79814)	AACAGTTTATCACGGCCATTTTTGAAATTTTTACGCTCAGCAGTAAAATCGTCGTGGCCGTGCCCGTCAATTGGGAA
SARS-CoV-2 NC_045512.2	(5689)	AGCACCACCTGC---TCAGTATGAACTTAAG------CATGGTACATTTAC------------TTGTGCTAG-TGAG

Section 1039

		79927 79937 79948 79959 79970 79981 79992 80003
	(79927)	
Autographa californica nucleopolyhedrovirus NC_0...	(79891)	AACGATAATTTAAGTGTACTTTTGAAACATTTGCACAACCTAAATCTCATTGGAATTGAAATTGTAAATTAAAACAA
SARS-CoV-2 NC_045512.2	(5744)	TACACTGGT--AATTACCAG----TGTGGTCACT-ATAAACATATAACTTCTAAGAAACTTTGTATTGCATAGACG

Section 1040

		80004 80014 80025 80036 80047 80058 80069 80080
	(80004)	
Autographa californica nucleopolyhedrovirus NC_0...	(79968)	ATCATGTGGGGAATCGTGTTACTTATCGTTTTGCTCATACTGTTTTATCTTTATTGGACGAATGCATTAAATTTCAA
SARS-CoV-2 NC_045512.2	(5814)	GTGCTTTACTT--ACAAAGTCCTCAGAATACAAAGGTCCTATT--ACGGATGTTTTCTACAAAGAAAACAGTTACAC

Section 1041

		80081 80091 80102 80113 80124 80135 80146 80157
	(80081)	
Autographa californica nucleopolyhedrovirus NC_0...	(80045)	TTCCTTAACCGAGTCGTCGCCCAGTTTAGGGCAGAGCAGCGACTCGGTGGAATTAGACGAGAACAAACAATTAAACG
SARS-CoV-2 NC_045512.2	(5887)	AACAACCATAAAACCAGT-TACT------TATAA-ATTGG----------------------------------ATG

Section 1042

		80158 80168 80179 80190 80201 80212 80223 80234
	(80158)	
Autographa californica nucleopolyhedrovirus NC_0...	(80122)	TAAAGCTGAATAACGGCCGGGTGGCCAACTTGCGCATCGCACACGGCGATAATAAATTGAGCCAAGTGTATATTGCC
SARS-CoV-2 NC_045512.2	(5922)	GTGTTG-------TTTGT---A---------CAGAAATTGACCCTA----AGTTGGA---C-AATTATTATAAGAAA

Section 1043

		80235 80245 80256 80267 80278 80289 80300 80311
	(80235)	
Autographa californica nucleopolyhedrovirus NC_0...	(80199)	GAAAAACCGCTATCTATAGACGACATAGTCAAAGAGGGCTCCAACAAGGTGGGCACTAACAGCGTTTTTCTGGGCAC
SARS-CoV-2 NC_045512.2	(5972)	GACAATTCTTATTTCACAGAGCAACCAATTGATCTTGTACCAAACCAACCATATCC--AAACGCAAGCTTCGA--TA

```
                                                                                              Section 1044
                                              (80312) 80312     80322      80333      80344      80355      80366      80377  80388
Autographa californica nucleopolyhedrovirus_NC_0... (80276) CGTATACGACTATGGAATCAAATCACCAAACGCGGCCAGCACATCTAGTAATGTAACCATGACGCGCGGCGCCGCAA
                     SARS-CoV-2 NC_045512.2 (6045) ATTTTAAGTTTGT--ATGTGATAATATCAAATTTGCTGATGATTTAAACCAGTTAAC--TGGTTATAAGA-----AA
                                                                                              Section 1045
                                              (80389) 80389     80399      80410      80421      80432      80443      80454  80465
Autographa californica nucleopolyhedrovirus_NC_0... (80353) ACTTTGATATCAAGGAATTCAAGTCCATGTTTATCGTATTCAAGGGTGTGACGCCCACTAAAACTGTAGAGGACAAT
                     SARS-CoV-2 NC_045512.2 (6113) CCTGCTTCAAGAGAGC-TTAAAGTTACATTTTTCCCTGACTTAAATGGTGATGTGG-TGGCTATTGATTATAAACAC
                                                                                              Section 1046
                                              (80466) 80466     80476      80487      80498      80509      80520      80531  80542
Autographa californica nucleopolyhedrovirus_NC_0... (80430) GGCATGTTGCGATTCGAAGTCGACAACATGATTGTGTGTTTGATCGACCCCAACACGGCGCCGCTGTCCGAACGAGA
                     SARS-CoV-2 NC_045512.2 (6188) T-AC-------ACACCCT--CTTTTAAGAAAGGAGCTAAATT--GTTACATAAACC---TATTGTTTGGCATGTTAA
                                                                                              Section 1047
                                              (80543) 80543     80553      80564      80575      80586      80597      80608  80619
Autographa californica nucleopolyhedrovirus_NC_0... (80507) GGTGCGCGAATTGCGCAAATCTAATTGCACTTTGGTGTACACAAGAAACGCGGCAGCTCAGCAAGTTTTATTGGAAA
                     SARS-CoV-2 NC_045512.2 (6250) CAAT--GC--AACTAA--T---AAAGCCACGT-----ATAAACCAAAT--AC-CTGGTG-----------TATAC-G
                                                                                              Section 1048
                                              (80620) 80620     80630      80641      80652      80663      80674      80685  80696
Autographa californica nucleopolyhedrovirus_NC_0... (80584) ATAACTTTACCGTCATTAATGCTGAACAAACCGCCTATCTCAAAAACTATAAATCATACAGAGAAATGAATTAATAA
                     SARS-CoV-2 NC_045512.2 (6298) TTGTCTTTGGAGCA-CAAAACCAGT-TGAA------ACATCAAATTCGTTTGA---TGTACTGAA------GTC---
                                                                                              Section 1049
                                              (80697) 80697     80707      80718      80729      80740      80751      80762  80773
Autographa californica nucleopolyhedrovirus_NC_0... (80661) AACAAAAAGTCTATTTATATAATATATTATTTATTAACATACAAAATTTGGTACACTAGTGTTCAAATCGTTTCTGT
                     SARS-CoV-2 NC_045512.2 (6355) AGAGGACGCGCAGGGAATGGATAATCTTGCCTGCGAAG--ATCTAAAACCAGTCTCTGAAGAAGTAG-TGGAAA--A
                                                                                              Section 1050
                                              (80774) 80774     80784      80795      80806      80817      80828      80839  80850
Autographa californica nucleopolyhedrovirus_NC_0... (80738) TCAACGCCATTGTCATGTTATAAAACACATTTGTAGTTTTATTGTAATTATTTTTAAATTTATTTTTAA-TTTGCTG
                     SARS-CoV-2 NC_045512.2 (6427) -TCCTACCA---TACAGAAAGACGTTCTT---GAGTGTAATGTGAAAACTACCGAAGTTGTAGGAGACATTATACTT
```

Section 1051

	(80851)	80851 80861 80872 80883 80894 80905 80916 80927
Autographa californica nucleopolyhedrovirus NC_0...	(80814)	TAATAAAACTTGTTCATTAAATACAAAAGACTTTGAACTACTTGCGTTTATATTCTTTTTATAATTGTACTGAACAA
SARS-CoV-2 NC_045512.2	(6497)	AAAC-CAGCAAATAA----TAGTTTAAAAATTACAGAAGAGGTTGG------CCACACAGATCTAATGGCTGCTTAT

Section 1052

	(80928)	80928 80938 80949 80960 80971 80982 80993 81004
Autographa californica nucleopolyhedrovirus NC_0...	(80891)	ACGAGGGGTGCAAAAAGTTTTGAAATGCTGCACGGCAATACCTATCATCTCCTCCATTTTGTCCTCTCCTATTGTA
SARS-CoV-2 NC_045512.2	(6563)	GTAGACAATTCTAGTCTTACTA-----------------TT-------------------------------AAGAA

Section 1053

	(81005)	81005 81015 81026 81037 81048 81059 81070 81081
Autographa californica nucleopolyhedrovirus NC_0...	(80968)	ATAGTGGCACTGCGCACCGTTTTAATGTTTAGAATGTAAATGAGCGCATACAGCGGACTATTGTTGGTGCTCAAGCA
SARS-CoV-2 NC_045512.2	(6592)	ACCTAAT--------------GAATTATCTAG---------------A-------------------------GTAT

Section 1054

	(81082)	81082 81092 81103 81114 81125 81136 81147 81158
Autographa californica nucleopolyhedrovirus NC_0...	(81045)	CATTAGGTTGTGCTTATGCATAGGGTCGTTGCTCAGCAGCGTTTTGTATACTACAAAGCCCGTTTTGGGGTCGCGTC
SARS-CoV-2 NC_045512.2	(6615)	TAGGTT-----------TGAAAC-------CCTTGCTACTCATGGTTTAGCTGCTGTTAATAGTGTCC-CTTG----

Section 1055

	(81159)	81159 81169 81180 81191 81202 81213 81224 81235
Autographa californica nucleopolyhedrovirus NC_0...	(81122)	TGTACATTAGTACGTGCGACAAAAA-CAAACGCACCGGCGTCACAAGCGACTCGTAATACATGCTTTCTATCGGAAA
SARS-CoV-2 NC_045512.2	(6670)	--GGATAC-------------TATAGC--TAATTATGCTAAGCCT-----------------------TTTCTTAAC

Section 1056

	(81236)	81236 81246 81257 81268 81279 81290 81301 81312
Autographa californica nucleopolyhedrovirus NC_0...	(81198)	CTGTTTGGACTTGATGTGTTCGTACACGGAGCCGGCAAACTTGACGCTGTCTACAAACTTATGGTTCGTGTAAACAA
SARS-CoV-2 NC_045512.2	(6707)	AAAGTTGTTA-------GTACAA--CTACTAA---CATAG-----TTACACGGT-----------GTTTAAACCGTG

Section 1057

	(81313)	81313 81323 81334 81345 81356 81367 81378 81389
Autographa californica nucleopolyhedrovirus NC_0...	(81275)	TCAAAAATCTGTCTTGTACACCGTCGTCATAATCGTCCACGTACAGCGGCTTGTTGTTAACAATTAACATTTTGTAG
SARS-CoV-2 NC_045512.2	(6756)	TTT-GTACTAATTATATGC-----CTTATTTCTTTACTTTA---TTGCTACAATTGT-GTACTTT------TACTAG

```
                                                                    Section 1058
                                                   (81390) 81390     81400      81411      81422      81433      81444      81455      81466
Autographa californica nucleopolyhedrovirus_NC_0... (81352) TTGGCTTCATACTTTAGCAGCCCTTGGTATTTTCTGCTCTTGGAATCGCTCTTGCTCGAATCGGCATGCTTCTTAAA
                             SARS-CoV-2 NC_045512.2 (6817)  AAGTACA-------AATTCTAGAA--TTAAAGCATCTATGCCGACTACTATAGCAAAGAATACTGTTAAG-AGTGTC
                                                                    Section 1059
                                                   (81467) 81467     81477      81488      81499      81510      81521      81532      81543
Autographa californica nucleopolyhedrovirus_NC_0... (81429) GTACGACTCGCTGCATTGTTTCAACTCGTTGATAGTGTACAACTGCGAGTTGAGTTTGCTCACTTCCTTGTCGCTCG
                             SARS-CoV-2 NC_045512.2 (6884)  GGTA--AATTTTGTCTAG----AGGCTTCATTT-----AATTATTTGAAGTC-ACCTAATTTTTCTAAACTG-ATAA
                                                                    Section 1060
                                                   (81544) 81544     81554      81565      81576      81587      81598      81609      81620
Autographa californica nucleopolyhedrovirus_NC_0... (81506) TTTCCTTGTTGGACTCTCCGCTGTGGTTGTCATCGTCAAACTTGTGCATCAACACCAAATAGTCCAACAGCTCAAAA
                             SARS-CoV-2 NC_045512.2 (6948)  ATATTATAATTTGGTTTTTACTATTAAGTGTTTGCCT-AGGTTCTTTA-ATCTACTCAACCGCTGCTTTAGGT--GT
                                                                    Section 1061
                                                   (81621) 81621     81631      81642      81653      81664      81675      81686      81697
Autographa californica nucleopolyhedrovirus_NC_0... (81583) AACGACGACTTGCCCGAACCCGGTTCGCCGGGCATGTAAATAGCCTTCTTTCCGTAATCTACGGGAATGGCCAAACT
                             SARS-CoV-2 NC_045512.2 (7021)  TTTAATGTCTAATTTAGG--CATGC-----CTTCTTACTGT--ACTGGTTACAGAGAAGGCTATTTGAACTCTACTA
                                                                    Section 1062
                                                   (81698) 81698     81708      81719      81730      81741      81752      81763      81774
Autographa californica nucleopolyhedrovirus_NC_0... (81660) AGCGGCGAAATGCATCAACATAATCGCGTTCGCGTGATTAAAATTGGTGAAGCGTTTAAAGTACAAATAGCCTTCGA
                             SARS-CoV-2 NC_045512.2 (7089)  ATGTCACTATTGCAACCTACTGTACTGGTTCTATACCTTGTAGTGTTTGTCTTAGTGGTTTAGATTCTTTAGACACC
                                                                    Section 1063
                                                   (81775) 81775     81785      81796      81807      81818      81829      81840      81851
Autographa californica nucleopolyhedrovirus_NC_0... (81737) CAATCTTTTTCAAATAATTGTACGAGTACTCCTTCAAGTCCACTTTGGACATGATGATGCGCATGTAGAATCGAGTC
                             SARS-CoV-2 NC_045512.2 (7166)  TATCCTTCTTTAGAAACTA-TACAAATTA-----CCATTT----CATC-TTTT-AAATGGGATTTAACTG--CTTTT
                                                                    Section 1064
                                                   (81852) 81852     81862      81873      81884      81895      81906      81917      81928
Autographa californica nucleopolyhedrovirus_NC_0... (81814) AGCCAAGTGGGCAAATCGTCCGTGCTGCGCGCCAATATGATTTTGTCCCACCACACA-TTGTACTTCTTCAAGATCA
                             SARS-CoV-2 NC_045512.2 (7229)  GGCTTAGTTGCAGAGTGGTTTTGGCATATATTCTTTTCACTAGGTTTTTCTATGTACTTGGATTGGCTGCA----A
```

Section 1065

	(81929)	81929 81939 81950 81961 81972 81983 81994 82005
Autographa californica nucleopolyhedrovirus NC_0...	(81890)	TTAACGCGTCGGCGTGGTGCGTGTAAAATTTGGAAATGTTATCCGATTCTTCAAACTGAACATCGGGTTCACGTGCA
SARS-CoV-2 NC_045512.2	(7302)	TCATGCAATTGTTTTTCAGCTATTTTGC--AGTACATTTTATTAGT---AATTCTTGGCTTATGTGGTT--AATAA-

Section 1066

	(82006)	82006 82016 82027 82038 82049 82060 82071 82082
Autographa californica nucleopolyhedrovirus NC_0...	(81967)	ACATCATCGCGCAATTCGGTTAAAAACAAACGTTTATCATTAAACTTGTCCATCAACATGTCGACATATTCGATTTT
SARS-CoV-2 NC_045512.2	(7371)	-------------TTAATCTTGTAC-AAATGGCCCCGATTTCAGCT-ATGGTTAGAATGTACAT----C--------

Section 1067

	(82083)	82083 82093 82104 82115 82126 82137 82148 82159
Autographa californica nucleopolyhedrovirus NC_0...	(82044)	GTGAATTGTTCGATACAAGTACTGAATAATTTTGTTGTGTTCTTTGGAAAAAAACTCTCCGTGTTGGTTAACAAATT
SARS-CoV-2 NC_045512.2	(7421)	-------------TTCT-TTGCAT---------C--ATTTTATTATGTATGGAA-AAGTTATGTGCATGTTGTAGAC

Section 1068

	(82160)	82160 82170 82181 82192 82203 82214 82225 82236
Autographa californica nucleopolyhedrovirus NC_0...	(82121)	CGCTGTTCGTGCGAATCAACGTGGTCGACACGTACGTTTTGTTAGTAAAAATTAGCATCCAAATCAATTCGCTCAAT
SARS-CoV-2 NC_045512.2	(7472)	GGTTGTAATTCATCAA-CTTGTATGATGTGT-TACAAACGTAATAGAGCAACAAGAGTCGAATGTACAACTATTGTT

Section 1069

	(82237)	82237 82247 82258 82269 82280 82291 82302 82313
Autographa californica nucleopolyhedrovirus NC_0...	(82198)	TCTGCATCGTTACCGAACATGTC-CGCCATCAAGCAGACTTTTAGCGCTTTTCTATTGATCTTTATTTTCTTGTAGC
SARS-CoV-2 NC_045512.2	(7547)	AATGGTGTTAGAAGGTCCTTTTATGTCTATG---CTAATGGAGGTAAAGGCTTTTGCAAAC----TACACAATTGGA

Section 1070

	(82314)	82314 82324 82335 82346 82357 82368 82379 82390
Autographa californica nucleopolyhedrovirus NC_0...	(82274)	ATTTGCATTTTGGTCGAGATCCCGATACCGTTGACCGACACGGTTTGCATTTTAGGTTGTGCAACATGTCGGAAACC
SARS-CoV-2 NC_045512.2	(7617)	ATTGTGTTAATTGTGATACATTCTGTGCTGGTAGTACATTTATTAGTGATGAAGTTGCGAGAGACTTGTCACTA--C

Section 1071

	(82391)	82391 82401 82412 82423 82434 82445 82456 82467
Autographa californica nucleopolyhedrovirus NC_0...	(82351)	CTGTTCTTGTTTACGTACAGAGCGAGCGTAATCAGATTTTCATCGTCCAAATTCCACAAATCGCGAAACAGGTTGTT
SARS-CoV-2 NC_045512.2	(7692)	AGTT------TAAAAGACCAATAAATCCT------ACTGA--CCAGTCTTCT------TACATCGTTGAT-AGT-GT

Section 1072

(82468) 82468 82478 82489 82500 82511 82522 82533 82544

Autographa californica nucleopolyhedrovirus NC_0... (82428) TAACGCGACTCGCATATCGGCTTGGCATGTGTTGCAATTGCCCATGTAGTTAACTATGGCCGTGTTAGTTTTTAGCA

SARS-CoV-2 NC_045512.2 (7747) TACAGTGAAGAATGGT------TCCATCC-ATCTTTACTTTG-ATAAAGCTGGTCAAAAGACTT--A---TGAAAGA

Section 1073

(82545) 82545 82555 82566 82577 82588 82599 82610 82621

Autographa californica nucleopolyhedrovirus NC_0... (82505) TTTTTACATCTCGGCACATTTTGGCGATGTGATAAGTTCTATAAATGCTGAGCTCGTCGGCGCTAGTAGATAGCATG

SARS-CoV-2 NC_045512.2 (7811) CATTCTCTCTCTCATTTTGTTA-AC--TTAGACAA-CCTGAGAGCTAATAACACTAAAGGTTCAT--TGCCTATTAA

Section 1074

(82622) 82622 82632 82643 82654 82665 82676 82687 82698

Autographa californica nucleopolyhedrovirus NC_0... (82582) TAATTAAACGCGTCCTCGGGCAAATACTTTTCGTCGGTGGGCTTCTTGAATGTCTGCGGCAACGTGGTGCCCAACAA

SARS-CoV-2 NC_045512.2 (7882) TGTTATAGTTTTTGATGGTA----AATCAAAATGTGAAGAATCATCTGCAAAATCAGCGTCTGTTTAC--TACAGT-

Section 1075

(82699) 82699 82709 82720 82731 82742 82753 82764 82775

Autographa californica nucleopolyhedrovirus NC_0... (82659) AAATGGACAGCTCGAATGAAAGCTGTTGGTGAACACGTTGTACACACCGTGCGTTGTCAAGTACAAGTATTTCCAAT

SARS-CoV-2 NC_045512.2 (7952) ---CAGC-TTATGTGTCAACCTATACTG------TTACTAGATCA---GGCATTAGT--GTCTGATGTTGGTGATAG

Section 1076

(82776) 82776 82786 82797 82808 82819 82830 82841 82852

Autographa californica nucleopolyhedrovirus NC_0... (82736) TGTTAAATTTTATGTTGCTCAACTTGTAACAATTGCTTTTGGTCAATTTGAATAGGTCATCCTCTTTCTTTACAATT

SARS-CoV-2 NC_045512.2 (8014) TGCGGAAGTT---GCAGTTAAAATGTTTGATGCTTACGTTAATACGTTT-T----CATCAA-------CTTTTA---

Section 1077

(82853) 82853 82863 82874 82885 82896 82907 82918 82929

Autographa californica nucleopolyhedrovirus NC_0... (82813) TGATAATGTTTGCCGTTGAAAACCAAATTGACTCCGGTCACTACGTTTTCCAATTTTCTAAAGAATCCTTTACACAC

SARS-CoV-2 NC_045512.2 (8073) ACGTACCAAT---GGAAAAACTCAAAACACTAGTTGCA--ACTGCAGAAGCTGAACTTGCAAAGAATGTGTCCTTAG

Section 1078

(82930) 82930 82940 82951 82962 82973 82984 82995 83006

Autographa californica nucleopolyhedrovirus NC_0... (82890) AATGTCAGGCGGCAAGTTTAGCGCCATCACATTCTCGTACGTGTACGCCCACAATTCATCGTGATCCAAAATTTCGT

SARS-CoV-2 NC_045512.2 (8145) ACAATGTCTTATCT-ACTTTTATTTCAGCAGCTCGGCAAGGGTTTGTTGATTCAGATGTA--GAAACTAAAGATGTT

Section 1079

```
                                          (83007) 83007     83017      83028      83039      83050      83061      83072      83083
Autographa californica nucleopolyhedrovirus NC_0... (82967) TTTTAGCCGACTGAGTCAAATATATCATGTAGTGTATGCCAAAATAATAGCCCAACGATACGCACAATTTGGTATCG
                            SARS-CoV-2 NC_045512.2 (8219) GTTGAATGTCTTAAAT-T-GTCACATCAATCTGACATAGAAG--TTACTGGCGATAGTTGTAATAACTATATGCTCA
```

Section 1080

```
                                          (83084) 83084     83094      83105      83116      83127      83138      83149      83160
Autographa californica nucleopolyhedrovirus NC_0... (83044) TCAAAGTCAAACCAATGATTGCAGGCCCTATTAAACACTATTTTCTCTTGTTTTTTGTAAGGCTCACATCGCTTCAA
                            SARS-CoV-2 NC_045512.2 (8292) CCTATAACAAAGTTGAAAACATGACAC-CCCGTGAC---CTTGGTGCTTGTATTGACTGTAGTGCGCGTCATATTAA
```

Section 1081

```
                                          (83161) 83161     83171      83182      83193      83204      83215      83226      83237
Autographa californica nucleopolyhedrovirus NC_0... (83121) AGCTTCATTCAAAGCTTCTTTGTCGCAGGCAAATAATGATTCACACAAAAGTTCCAAAAACAGTTTGATGTCGGTTT
                            SARS-CoV-2 NC_045512.2 (8365) TGC-GCAGGTAGCAAAAAGTCACAACATTGCTTTGATATGGAACGTTAAAGATTTCATGTCATTGTCTGAACAA---
```

Section 1082

```
                                          (83238) 83238     83248      83259      83270      83281      83292      83303      83314
Autographa californica nucleopolyhedrovirus NC_0... (83198) CTCTGTACGAGAAATTT-TCGTTCTTGGTCAATATCTTCCACAGTACATAGATTAAAAAATCAAAATTTTTAAATTT
                            SARS-CoV-2 NC_045512.2 (8438) -CTACGAAAACAAATACGTAGTGCTGCTAAAAAGAATAACTTACCTTTT-----A---AGTTGAC-ATGTGCAAC--
```

Section 1083

```
                                          (83315) 83315     83325      83336      83347      83358      83369      83380      83391
Autographa californica nucleopolyhedrovirus NC_0... (83274) GCTTTTTTCAAAGTATTGTTGTAGAAGGTTTGGATCGTTGGCTCGTTCGTGGGTCGCCAAAACTTTAACCATGTTCT
                            SARS-CoV-2 NC_045512.2 (8503) ---------TA--------CTAGACAAGTTGTTAATGTT-----GTAACAACAAAGATAGCACTTAAGGGTGGT---
```

Section 1084

```
                                          (83392) 83392     83402      83413      83424      83435      83446      83457      83468
Autographa californica nucleopolyhedrovirus NC_0... (83351) CGTGAATTGCTATAAGCCCCAAATTGATTTGCGTTTGAATGTAGTCTGCATTTTCGCTGCTCGCCGATATAATGGGT
                            SARS-CoV-2 NC_045512.2 (8555) ----A---AAATTGTTAATAA-TTGG---TTGA--AGCAGTT-AATTAAAGTTACACTTGTGTTCCTTTTTGTTGCT
```

Section 1085

```
                                          (83469) 83469     83479      83490      83501      83512      83523      83534      83545
Autographa californica nucleopolyhedrovirus NC_0... (83428) ACGATGCGCGGTTTTCTGGAACGCGTGTCGCTCAAGTCCACGTCGTTTTTGTCAAAATTGTTGTTCTCGAACACTCT
                            SARS-CoV-2 NC_045512.2 (8618) GCTATTTTCTATTTAATAACACCTGTTCATGTCATGTCTAAACATACTGACTTTTC---AAGTGAAATCA--TAGGA
```

```
                                                                   Section 1086
                                         (83546) 83546     83556      83567      83578      83589      83600      83611      83622
Autographa californica nucleopolyhedrovirus  NC_0... (83505) GAGGCTTTTGAGGTTGACGTTGACGATATGCTTGTACTTGGGCACCGTAATGCATTCCTCCAAATTAATGTCGTCCC
                      SARS-CoV-2 NC_045512.2 (8690)  TACAAGGCTATTGATGGTGGTGTCACTCGTGACATAGCATCTAC-AGATACTTGTTTTGCTAACA----AACATGCT

                                                                   Section 1087
                                         (83623) 83623     83633      83644      83655      83666      83677      83688      83699
Autographa californica nucleopolyhedrovirus  NC_0... (83582) TAATGTAATTGAAAAAATTTTTATCCGAATTGACCAGCTCGCCATTAACTTTGCACGTGGCCACAGTGCCGTCGGCC
                      SARS-CoV-2 NC_045512.2 (8762)  GATTTTGACAC---ATGGTTTAGCCAG------CGTGGTGGTAGTTA--TACTAA------TGACAAAGCTTGCCCA

                                                                   Section 1088
                                         (83700) 83700     83710      83721      83732      83743      83754      83765      83776
Autographa californica nucleopolyhedrovirus  NC_0... (83659) ATTTTGAGTATAAACAAGTCTTCGTGAGAATCGTCAAACTTGGTTTTTCCATTTACAAACAGCGTTTGCGGCGGATC
                      SARS-CoV-2 NC_045512.2 (8822)  TT---------GA--TTGCT--GCA----GTCATAACAAG---A-GAAGT-GGGTTTTGTCGTGCCTGGTTTGCCTG

                                                                   Section 1089
                                         (83777) 83777     83787      83798      83809      83820      83831      83842      83853
Autographa californica nucleopolyhedrovirus  NC_0... (83736) GTGATTCGTGCGCAGGCTGAGCTCGACGTTGAGAAAACATTTAGGGTCAAACACAAACAAATCCACAGGGCCTAGTT
                      SARS-CoV-2 NC_045512.2 (8877)  GCACGATATTACGCACA-ACTAATG--GTGACTTTTTGCATTTCTTACCTAGAGTTTTTAG--TGCAGTTGGTAACA

                                                                   Section 1090
                                         (83854) 83854     83864      83875      83886      83897      83908      83919      83930
Autographa californica nucleopolyhedrovirus  NC_0... (83813) TTTTGTTGTGTATGATTGGTATCGTGGGTTCGATGACAATTCCAAATTTTATATTTAAAAACAGCTGCCATCCGTTA
                      SARS-CoV-2 NC_045512.2 (8949)  ---TCTGTTACACACCATCAAAACTTATAG-A-GTACAC--TGACT---TTGCAACATCAGCTT----GTGTTTTGG

                                                                   Section 1091
                                         (83931) 83931     83941      83952      83963      83974      83985      83996      84007
Autographa californica nucleopolyhedrovirus  NC_0... (83890) AAAGAGAAAGCTTGCTTTTTGGGCCAGTTGGGCCAATAATAGTAATCGCCCGCTTGCACGCATTTGTTAATGTATCC
                      SARS-CoV-2 NC_045512.2 (9012)  C--TGCTGAATGTACAATTTTTAAAGATGCTTCTGGTAAGC--C--AG-----TACCATATTGT-TATG--ATAC--

                                                                   Section 1092
                                         (84008) 84008     84018      84029      84040      84051      84062      84073      84084
Autographa californica nucleopolyhedrovirus  NC_0... (83967) AGGGTCGGTGCTCTTGAAAAAATCTTCAAAATTAATATACTTTTGTATGATGTCATAGTGCTTCTTCAAAATGAAAG
                      SARS-CoV-2 NC_045512.2 (9073)  -CAATGTACTA----GAAGGTTCTGTTGCTTATGAAAG-----TTTACGCCCTGACACACGTTATGTGCTCA----T
```

Section 1093

(84085) 84085 84095 84106 84117 84128 84139 84150 84161
Autographa californica nucleopolyhedrovirus NC 0... (84044) GTTTTACAAAAATGCAAAAATCGTTACTTTCCAACACCCAGTCGTGGCCGTCTAATGTTTGAGCTGCGTGTTTCTCT
SARS-CoV-2 NC_045512.2 (9136) GGATGGCT------CTATTATT---------CAATTTCCTAAC---------------------ACCTACC----TT

Section 1094

(84162) 84162 84172 84183 84194 84205 84216 84227 84238
Autographa californica nucleopolyhedrovirus NC 0... (84121) GCAGGTT--CTTCGGTGTCTTCGCAAGATGCGCCCATGTCGTGTTTCGCGCACGGACCGTTAAAGTTGTTTCTAATT
SARS-CoV-2 NC_045512.2 (9173) GAAGGTTCTGTTAGAGTGGTAACAACTTTTGATTCT------GAGTACTGTAGGCACGGCACTTG-TGAAAGATCAG

Section 1095

(84239) 84239 84249 84260 84271 84282 84293 84304 84315
Autographa californica nucleopolyhedrovirus NC 0... (84196) GTGTTTAAGAACTGTTGAAAGTTGTTGACGTACTCAAACAATCTACGTGTTCCTGTTCGCGTGTTTCTAATGATTAA
SARS-CoV-2 NC_045512.2 (9243) AAGCTGGTGTTTGTGTATCTACTA--GTGGTAGATGGGTACTTAACA----ATGATTATTACAGATCTTTACCAGGA

Section 1096

(84316) 84316 84326 84337 84348 84359 84370 84381 84392
Autographa californica nucleopolyhedrovirus NC 0... (84273) ATGATTTGCATCTTGCAAGTTGTTAATCTCGTACGTTTTGTCTTGAGGCACGTTTTTCAAAAAAATTGTAAAATGT
SARS-CoV-2 NC_045512.2 (9314) ---GTTT----TCTG--TGGTGTAGAT-----------------GCTGTAAATTTACTTACTAATATGTTTA-----

Section 1097

(84393) 84393 84403 84414 84425 84436 84447 84458 84469
Autographa californica nucleopolyhedrovirus NC 0... (84350) TGTCAATCATGTTGGCTATCGTGTTTGTACTTTTCGTGTTAATTTATTTAATAATTTCGATCAAAAATCACCATCCA
SARS-CoV-2 NC_045512.2 (9360) --CACCACTAAT---TCAACCTA--TT--GGTGC----------TTTGGACA-TATCAG--CA----TC-TATAGTA

Section 1098

(84470) 84470 84480 84491 84502 84513 84524 84535 84546
Autographa californica nucleopolyhedrovirus NC 0... (84427) TTCTTACATAGAATAGAAACGCTAATACAAGATTTCAACAACACATTGTTGTTTGGCGCGTATGTACAGATTTACGA
SARS-CoV-2 NC_045512.2 (9410) GCTGGTGGT---ATTGTAGCTATCGTAGTA-ACATGCCTTGCCTACT-----------ATTTTATGAGGTT--TAGA

Section 1099

(84547) 84547 84557 84568 84579 84590 84601 84612 84623
Autographa californica nucleopolyhedrovirus NC 0... (84504) TTTAAGCACGCCCGCCCGCACCGAACGATTGTTATTATTGCGCCCGAAAATGTGGTGTTGTATAATTTTAACAAAA
SARS-CoV-2 NC_045512.2 (9470) AGAGCTTTTGGTGAATACAGTCATGTAGTTGCCTTTAATA-CTTT----ACTATTC-CTTATGTCATTCACTGTACT

Section 1100

		84624 84634 84645 84656 84667 84678 84689 84700
	(84624)	
Autographa californica nucleopolyhedrovirus NC_0...	(84581)	CGCTCTATTATTACTTGGACTCGGCGAACGTGTTTTGTCCCAACGAGTTTAGCGTGACCACGTTCACGCAATCCACT
SARS-CoV-2 NC_045512.2	(9541)	----CTGTTTAACACC--AGTT---TACTCATTCTTACCTGGTGTTTATTCTGTTAT------------------TT

Section 1101

		84701 84711 84722 84733 84744 84755 84766 84777
	(84701)	
Autographa californica nucleopolyhedrovirus NC_0...	(84658)	ATTAAAACGATCAACGAGACGGGAATATATGCCACCGCATGCACGCCGGTCAGCAGCTTGACGCTAATTGAACATTT
SARS-CoV-2 NC_045512.2	(9591)	ACTTGTACT-----TG--ACATT--TT-ATCTTAC------TAATGATGTTTCTTTTTTAGCACATATTCAGTGGAT

Section 1102

		84778 84788 84799 84810 84821 84832 84843 84854
	(84778)	
Autographa californica nucleopolyhedrovirus NC_0...	(84735)	TGCAACATTAAAAATAACGTGC----CCGATCACACGCTCGTTCTCGATGTGGTCGACCAACAGATTCAGTTTTCA
SARS-CoV-2 NC_045512.2	(9652)	GGTTATGTTCACACCTTTAGTACCTTTCTGGATAACAATTGCT--TATATCATTTGTATTT-----CCA----CAAA

Section 1103

		84855 84865 84876 84887 84898 84909 84920 84931
	(84855)	
Autographa californica nucleopolyhedrovirus NC_0...	(84808)	ATACTCGACATTATCAATTATTTGATTTACAATGGCTACGTGGATTTGTTGGCCGAATAACGCGTATATAGACGCTT
SARS-CoV-2 NC_045512.2	(9718)	GC-ATTTCTATTGGTT--C-TTTAGTAATTACCTAAAG---AGACGTGTAGTCTTTAATGGTGTTTCCTTTAGTACT

Section 1104

		84932 84942 84953 84964 84975 84986 84997 85008
	(84932)	
Autographa californica nucleopolyhedrovirus NC_0...	(84885)	GTACGTTCATCGTAGTAATCATTTTAATACATTTGATTGAACTAAACATACATCTGCAATGGGTGAAAGAGTCACTA
SARS-CoV-2 NC_045512.2	(9788)	TTTGAAGAAGCTGCGCTGTGCACCTTTTTGT-TAAATAAAGAAATGTATCTAAAGT-TGCGTAGTGATGTGCTATTA

Section 1105

		85009 85019 85030 85041 85052 85063 85074 85085
	(85009)	
Autographa californica nucleopolyhedrovirus NC_0...	(84962)	AATTTTGCAATGGAAAACGGCGATAAAGAAGACAGCGACAATGAATAGAGTTTATATTTTTATTTAATAAAATATTG
SARS-CoV-2 NC_045512.2	(9863)	CCTCTTACGCA--ATATAATAGATACTTAGCTCTTTATAATAAGTACAAGTATTTTAGTGG-AGCAATGGATACAAC

Section 1106

		85086 85096 85107 85118 85129 85140 85151 85162
	(85086)	
Autographa californica nucleopolyhedrovirus NC_0...	(85039)	TTCGTAATCCATAATGTTTTGTATTATTTCATTGTGATAATGTTCCCAATCTTGCACGGGGGTGGGGCATCGTTTGA
SARS-CoV-2 NC_045512.2	(9937)	TAGCTACAGAGA---AGCTGCTTGTT-GTCATCTCGCAAGGCTCTCAATGACTTCAGTAACTCAGGTTCTGAT-GT

Section 1107

```
                                                    (85163) 85163     85173      85184      85195      85206      85217      85228     85239
Autographa californica nucleopolyhedrovirus NC_0... (85116) CTTTGACGTAGAAATCGTACGCGTAGTTATTAGTTGGCAGATCGTCGACAAGTGTGAT-CGACTTGAAAAAGTTTAC
                             SARS-CoV-2 NC_045512.2 (10009) TCTTTAC----CAACCACC-ACAAACCTCTATCACCTCAGCTGT-TTTGCAGAGTGGTTTTAGA-AAAATGGCATT-
```

Section 1108

```
                                                    (85240) 85240     85250      85261      85272      85283      85294      85305     85316
Autographa californica nucleopolyhedrovirus NC_0... (85192) ATTTTTATCGCTCAAATATTTAATTACAATTTTTGGCGATTTGGGTATATTGTTGTCGGATCGATGATTGTGAATGT
                             SARS-CoV-2 NC_045512.2 (10078) --CCCATCTGGTAAAGTTGAGGGTTGTATGG-TACAAGTAACTTGT--GG----TACAACTACAC----TTAACGGT
```

Section 1109

```
                                                    (85317) 85317     85327      85338      85349      85360      85371      85382     85393
Autographa californica nucleopolyhedrovirus NC_0... (85269) CAAAAACAAATTTATTTTCAATGAAACGCTTTTTTAAATTGTAATCTACAATAGCGTTGTGTGAATTTTGAACTAAA
                             SARS-CoV-2 NC_045512.2 (10142) CTTTGGCTTGATGACGTAGTTTACTGTCCAAGA--CATGTGATCTGCACCTCT--GAAGACATGC-TTAACCCTAAT
```

Section 1110

```
                                                    (85394) 85394     85404      85415      85426      85437      85448      85459     85470
Autographa californica nucleopolyhedrovirus NC_0... (85346) TCAGAGCGTTCTTCTTGAACGGTGGAACCTTCGCTGATAATGATATCAAAATAGCCTTCCAAATCGACGTCTCGCAT
                             SARS-CoV-2 NC_045512.2 (10214) TATGAAG-----ATTTACTCATTCGTAAGTCTAATCATAATTTCTTGGTACAGGCTGGTAATGTTCAACTCAGGGTT
```

Section 1111

```
                                                    (85471) 85471     85481      85492      85503      85514      85525      85536     85547
Autographa californica nucleopolyhedrovirus NC_0... (85423) CGAGTGTGCTACATGATCTCTACTGCCATACGACCACAAGACTAAAACGCAACCCATCTCGTGCAACTCCTGCAAGC
                             SARS-CoV-2 NC_045512.2 (10286) -------ATTGGACATTCTATGCAAAATTGTGTACTTAAGCTTAAGGTTGATACAGCCAATCCTAAGACA-CC--TA
```

Section 1112

```
                                                    (85548) 85548     85558      85569      85580      85591      85602      85613     85624
Autographa californica nucleopolyhedrovirus NC_0... (85500) TGTCATACACAAACGGATCTCGAATCTCAACTTGCTCCTCTTCGGTTATGAGAGTGCTGTCCAAATCAAACACGACC
                             SARS-CoV-2 NC_045512.2 (10353) AGTATAAGTTTGTTCGCATTCAACCAGGACAGACTTTTTCAGTG-----TTAGCTTGTTAC-AATGGTTCAC-CATC
```

Section 1113

```
                                                    (85625) 85625     85635      85646      85657      85668      85679      85690     85701
Autographa californica nucleopolyhedrovirus NC_0... (85577) ACGTGCGGAAATCCCCACGTCAAAGATTCGCTTTTGAGAGAGACCACTTTGTAGTGTGGCAATAGAAACCATTCTTT
                             SARS-CoV-2 NC_045512.2 (10423) TGGTGTTTAC---CAATGTG----CTATGAG----GCCCAATTTCACTATTAAGGGTTCATTC-----------C-
```

Section 1114

```
                                                  (85702) 85702     85712      85723      85734      85745      85756      85767     85778
Autographa californica nucleopolyhedrovirus NC 0... (85654) AAGAAACGAATACATTGGCGGTTTGTTGCTAAGCACGCACATGTGGCCCAACACTGGCGTTTTGAATGCGCGTTTAA
                              SARS-CoV-2 NC_045512.2 (10476) ----TTAATGGTTCATGTGGTAGTGTTGGTTTTA--ACAT--AGATTATGACTGTGTC--TCTTTTTGTTACATGCA
```

Section 1115

```
                                                  (85779) 85779     85789      85800      85811      85822      85833      85844     85855
Autographa californica nucleopolyhedrovirus NC 0... (85731) TATTGTGCCTGATGTCGCGCATGTCGTCGGCGGGCGCTTTGAATATTTGCATACAGTAATTGTAATTGTTTTCTATG
                              SARS-CoV-2 NC_045512.2 (10543) CCATA---TGGAATTACCAACTGGAGTTCATGCTG-GCA--------CAGACTTAGAAG-GTAACTTTTATGG----
```

Section 1116

```
                                                  (85856) 85856     85866      85877      85888      85899      85910      85921     85932
Autographa californica nucleopolyhedrovirus NC 0... (85808) ATCTTGCACAGCTGCGGGTCGTTGCAAAATTGAAATATTACATATTCAAAAAATTTATACTTTTCAAAGCCAAGGTA
                              SARS-CoV-2 NC_045512.2 (10603) ----ACCTTTTG---TTGACAGGCAAACAGC--ACAAGCAGCTGGTACGGACACAACTATTACAGTTAATGTTTTAG
```

Section 1117

```
                                                  (85933) 85933     85943      85954      85965      85976      85987      85998     86009
Autographa californica nucleopolyhedrovirus NC 0... (85885) TTTGAGGTCGGCGTACTCGCTTAAAACGAGAACATGTCGTTTGATGATGGCGTCGTTAAGGCGCAAACAGATCCATT
                              SARS-CoV-2 NC_045512.2 (10671) CTTGGTTGTACGCTGCTGTTATAAATGGAGACAGGTGGTTTCTCAATCGATT------TACCACAACTCTTAATGAC
```

Section 1118

```
                                                  (86010) 86010     86020      86031      86042      86053      86064      86075     86086
Autographa californica nucleopolyhedrovirus NC 0... (85962) TGCTTTGAAGCGAGGAGGCCATAATGTACAAAAATGGACCAGTTACGCCTTATTTAAACTGTTTAAAGAGTTTCGTA
                              SARS-CoV-2 NC_045512.2 (10742) TTT--A--ACCTTGTGGCTATGAA-GTACAATTATGAACCTCTAACACAAGA------CCATGTTGACATACTAGGA
```

Section 1119

```
                                                  (86087) 86087     86097      86108      86119      86130      86141      86152     86163
Autographa californica nucleopolyhedrovirus NC 0... (86039) TAAACAAAAACTACTCTAAACTAATAGATTTCTTAACAGAAAATTTTCCCAACAACGTCAAAAAACAAAACGTTCAAC
                              SARS-CoV-2 NC_045512.2 (10808) CCTCTTTCTGC-TCAA-----ACTGGAATTGCCGTTTTAGATATGTGTGCTTCA-TTAAAAGAATTA-------CTG
```

Section 1120

```
                                                  (86164) 86164     86174      86185      86196      86207      86218      86229     86240
Autographa californica nucleopolyhedrovirus NC 0... (86116) TTTTCGTCTACCGGCCATCTGTTTCACTCGTTGCACG-CGTACGTGCCCAGCGTCAGTGATTTGGTGAAAGAGCGCA
                              SARS-CoV-2 NC_045512.2 (10871) CAAAATGGTATGAATGGACGT-ACCA--TATTGGGTAGTGCT-TTATTAGAAGATGAATTTACACCTTTTGATGTT-
```

Section 1121

	(86241)	86241 86251 86262 86273 86284 86295 86306 86317
Autographa californica nucleopolyhedrovirus NC_0...	(86192)	AACAAATTCGATTGCAGACAGAATATTTGGCAAAGCTGTTCAACAACACAATAAACGATTTCAAACTGTACACTGAG
SARS-CoV-2 NC_045512.2	(10943)	--GTTAGACAATGCTCAGGTGTTACTTT-----CCAAAGTGCAGTGAAAA-GAACA----ATCAAGGGTACACACCA

Section 1122

	(86318)	86318 86328 86339 86350 86361 86372 86383 86394
Autographa californica nucleopolyhedrovirus NC_0...	(86269)	CTGTACGAGTTTATCGAACGGACCGAAGGCGTCGATTGCTGTTGTCCGTGCCAGCTATTGCACAAGAGTCTACTCAA
SARS-CoV-2 NC_045512.2	(11008)	CTGGTTGTTACTCACAATTTTGACTTCACTTTTAGT-TTTAGTCCAGA-GTACTCAAT-GGTCTTTGTTCTTTTTTT

Section 1123

	(86395)	86395 86405 86416 86427 86438 86449 86460 86471
Autographa californica nucleopolyhedrovirus NC_0...	(86346)	CACCAAAAATTACGTGGAAAACTTAAATTGCAAACTGTTTGACATAAAGCCGCCCAAATTTAAAAAGGAACCTTTTG
SARS-CoV-2 NC_045512.2	(11082)	TGTATGAAAATGCCTTTTTACCTTTTGCTATGG-GTATTATTGCTATGTCTGCTTTTGCAA------TGATGTTTGT

Section 1124

	(86472)	86472 86482 86493 86504 86515 86526 86537 86548
Autographa californica nucleopolyhedrovirus NC_0...	(86423)	ACAACATTCTTTACAAGTATTCCCTAAATTACAAAAGTTTGTTGTTGAAAAAAAAGGAAAAACATACCAGCACTGGG
SARS-CoV-2 NC_045512.2	(11152)	CA----AACATAAGCATGCATTTCTCTGTTTGTTTTTGTTACCTTCTCTTGCCACTGTAGCTTATTTTAATATGGTC

Section 1125

	(86549)	86549 86559 86570 86581 86592 86603 86614 86625
Autographa californica nucleopolyhedrovirus NC_0...	(86500)	TGTACACGCAAAAAGAAAATCAAACACAGGCAAATATTGAATGATAAAGTTATTTATTTACAAAACAGTAATAAAAA
SARS-CoV-2 NC_045512.2	(11225)	TA--TATGCCTGCTAGTTGGGTGATGCGTATTATGACATGGTTGGATATG---GTTGATAC----T----AGTTTGT

Section 1126

	(86626)	86626 86636 86647 86658 86669 86680 86691 86702
Autographa californica nucleopolyhedrovirus NC_0...	(86577)	TAAACTATTTGAGCTTAGCGGGCTTAGTTTAAAATCTTGCAGACATGATTTTGTAACAGTCGAAAGCCAAACGAGGG
SARS-CoV-2 NC_045512.2	(11289)	CTGGTTTTAAGCTAAAAGACTGTGT----------TATGTATGCATCAGCTGTA-----GTG--TTACTAATCCTTA

Section 1127

	(86703)	86703 86713 86724 86735 86746 86757 86768 86779
Autographa californica nucleopolyhedrovirus NC_0...	(86654)	CAGGCGACGAAATCGCTTCGTTCATTCGCTACTGTCGGCTGTGTGGAATGTCTGGTTGTTAATAGTAGCGTGTTCTG
SARS-CoV-2 NC_045512.2	(11349)	TGACAGCAAGAACTGTGTATGATGATGG-TGCTAG--GAGAGT-GTGGACACTTAT-------G---AATGTCT-TG

Section 1128

	(86780)	86780 86790 86801 86812 86823 86834 86845 86856
Autographa californica nucleopolyhedrovirus NC_0...	(86731)	TAACTTCGGCGACCTGTCGATGAACGGCTCCTGGATCTTCTGTATGTGCGGGGTCTACCCGGGCGGCGTCTGTAACC
SARS-CoV-2 NC_045512.2	(11411)	ACACTCGTTTATAA-AGTT-TATTATGGT----AATGCTTTAG--------ATCAAGCCATTTCCATGTGGGCTCTT

Section 1129

	(86857)	86857 86867 86878 86889 86900 86911 86922 86933
Autographa californica nucleopolyhedrovirus NC_0...	(86808)	CGAGCTTCTGCGCCTGCGTGTCGAACCATATGTGGTACCGGTTGAAGAACGGCGACGGCGACGATAAACCATGTTTA
SARS-CoV-2 NC_045512.2	(11474)	ATAA--TCTCTGTT-ACTTCTAACTACTCAGGTGTAG-----TTAC-AACTGTCATGTTTTTGGCCAGAGGTATTGT

Section 1130

	(86934)	86934 86944 86955 86966 86977 86988 86999 87010
Autographa californica nucleopolyhedrovirus NC_0...	(86885)	AATTGTGTAATTTATGTAGCTGTAATTTTTACCTTATTAATATTTTTTACGCTTTGCATTCGACGACTGAACTCCCA
SARS-CoV-2 NC_045512.2	(11542)	TTTTAT-G----TGTGTTGAGTATTGCCCTATTTTTCTTCATA-ACTGGTAATACACTTCAG-TGTATAATGCTAGTT

Section 1131

	(87011)	87011 87021 87032 87043 87054 87065 87076 87087
Autographa californica nucleopolyhedrovirus NC_0...	(86962)	AATATATGTTTAACTCGTCTTGGTCGTTTGAATTTTTGTTGCTGTGTTTCCTAATATTTTCCATCACCTTAAATATG
SARS-CoV-2 NC_045512.2	(11612)	TATTGTTTCTTAGGCTATTTTTGTACTTGTTA-CTTTGGCCTCTTTTGTTTACTCAACCG--CTACTTTAGACTGAC

Section 1132

	(87088)	87088 87098 87109 87120 87131 87142 87153 87164
Autographa californica nucleopolyhedrovirus NC_0...	(87039)	TTATTGTAATCCTCAATGTTGAACTTGCAATTGGACACGGCATAGTTTTCCATAGTCGTGTAAAACATGGTATTGGC
SARS-CoV-2 NC_045512.2	(11686)	T---CTT-GGTGT--TTATGATTACTTAGTTTCTACACAGGAGTTTAGATATATGAATTCACA-GGGACTACTCCCA

Section 1133

	(87165)	87165 87175 87186 87197 87208 87219 87230 87241
Autographa californica nucleopolyhedrovirus NC_0...	(87116)	TGCATTGTAATACATCCGACTGAGCGGGTACGGATCTATGTGTTTGAGCAGCCTGTTCAAAAACTCTGCATCGTCGC
SARS-CoV-2 NC_045512.2	(11756)	--CCCAAGAATAGCATAG---ATG--CCTTCAAAC-TCAACATTAAATTGTTGGGTGTTGGTGGCAAACCTTGTATC

Section 1134

	(87242)	87242 87252 87263 87274 87285 87296 87307 87318
Autographa californica nucleopolyhedrovirus NC_0...	(87193)	AAAACGGAATTTCGGTACCGCTGTTGATGTATTGTTGCGGCTGCAACATTTGTATCTTTTCGCCGCGCTCGATCAAC
SARS-CoV-2 NC_045512.2	(11825)	AAAGTAGCCACT-GTACAGTCTAAAA-----TGTCAG-ATGTAAAGTGCACATCAGTAGTCTTACTCTCAGTTTTGC

Section 1135

	(87319)	87319 87329 87340 87351 87362 87373 87384 87395
Autographa californica nucleopolyhedrovirus NC_0...	(87270)	AATTCTTCAAGAGTGGTGCGTTTGTCGCGCTGTAAAGCCACGTTTTGTAACAGCACTATTTTCGCATATCTCATAAT
SARS-CoV-2 NC_045512.2	(11895)	AACA-ACTCAGAGTAGAATCATCATCTAAATTGTGGGCTCA---ATGTGTCCAGTTACACAATGACATTCTCTTAGC

Section 1136

	(87396)	87396 87406 87417 87428 87439 87450 87461 87472
Autographa californica nucleopolyhedrovirus NC_0...	(87347)	CGGACTGTTGAAACAGCGTGCAAACGACGACCGCATAATATCGACGGTCGTCAAGTCGATTGTGGTCGAAGGCATCT
SARS-CoV-2 NC_045512.2	(11968)	T------AAAGATACTACTGAAG-CCTT-TGAAAAAATGGTTTCACTACTTT-CTGTTTTGCTTTCC-ATGCAGGGT

Section 1137

	(87473)	87473 87483 87494 87505 87516 87527 87538 87549
Autographa californica nucleopolyhedrovirus NC_0...	(87424)	CCAACAGAGATCGCACGGCGTCCAACAGCGTGTCCGTTTGAACCTGCGTCATTTGCGGTCTGCACGTGTAGTCGTCA
SARS-CoV-2 NC_045512.2	(12035)	GCTGTAGACATAAA---CAAGCTTTGTGAAGAA----ATGCTGGA------CAACAGGGCAACCTTACAAGCTAT--

Section 1138

	(87550)	87550 87560 87571 87582 87593 87604 87615 87626
Autographa californica nucleopolyhedrovirus NC_0...	(87501)	AACGTGGTTTCGAGCAGTTTGAACAACGAATGATACTTTTCCGATCGCAGCAAAAATATCATGGTCATGACCACGTC
SARS-CoV-2 NC_045512.2	(12097)	AGCCTCA-GAGTTTAGTTCCCTT--CCATCATATGCAGCTTT----TGCTACTGCTCAAGAAGCTTATGAGCAGGCT

Section 1139

	(87627)	87627 87637 87648 87659 87670 87681 87692 87703
Autographa californica nucleopolyhedrovirus NC_0...	(87578)	GCTGATTTTGTATTCTGTAGAACTGGTGCTGTTCAACGAATAGTGATGGATTAGTTTGCGAGCAGCATTTCTGTATC
SARS-CoV-2 NC_045512.2	(12167)	GTTGCTAAT--GGTG-ATTCTGAAGTTGTTCTTAAAAAGTTGAAGAAG-----TCTT---TG---AATGTGGCTA--

Section 1140

	(87704)	87704 87714 87725 87736 87747 87758 87769 87780
Autographa californica nucleopolyhedrovirus NC_0...	(87655)	GGCGCATGTTGATCAACTCTTCGGAAGGCTGCGCGGGCGCGGCGGCGTTGGCTCGCGCAAACAAATTTATTACGGGA
SARS-CoV-2 NC_045512.2	(12228)	---------AATCTGAATTTGACCGT---GATGCAGCCATGCAACGTAAGTTGGAA--AAGATGGCT-GATCAAGCT

Section 1141

	(87781)	87781 87791 87802 87813 87824 87835 87846 87857
Autographa californica nucleopolyhedrovirus NC_0...	(87732)	CGCGGCGTAGGCTGCGCGGACGCTGGCGCGGCGACGACGTCCGCGTTTCCCGCCGCGTACTGAGACGCTATGGCAGC
SARS-CoV-2 NC_045512.2	(12290)	ATGACCCAAATGTATAAACAGGCTAGATCTGAGGACAAGAGG----GCAAAAGTTACTAGTGCTATGCAGACAATGC

```
Section 1142
                                                   (87858) 87858     87868      87879      87890      87901      87912      87923 87934
Autographa californica nucleopolyhedrovirus NC 0... (87809) GTTGTTATTTAAAATTGTGTTTTGCGATTTGCGAGCCACGTGCATCATAAAATTTATCAACACGTCGGTGTTCAACT
                            SARS-CoV-2 NC_045512.2 (12363) TTTTCA-CTATGCTTAG---AAAGTTGGATAATGATGCACTCAACAA---CATTATCAACAATGCAAGAGATGGTTG

Section 1143
                                                   (87935) 87935     87945      87956      87967      87978      87989      88000 88011
Autographa californica nucleopolyhedrovirus NC 0... (87886) GCACGCTTTGATGTTCGTCGCAGAGCAAAGGAAATAGCTGGGGCCATATCGCCAATTGCATAGGCTCGTCTATTTTT
                            SARS-CoV-2 NC_045512.2 (12433) TGTTCCC-TTGAACATAATACCTCTTA-CAACAGCAGCCAAACTAATGGTT-----GTCATACCAGACTATA-ACAC

Section 1144
                                                   (88012) 88012     88022      88033      88044      88055      88066      88077 88088
Autographa californica nucleopolyhedrovirus NC 0... (87963) AACCGCAATTTGTTTATTTCCAAATACAACGCGATAGCGCTCATCGTGACCGACGACGCACACTTACTCTGTAACTA
                            SARS-CoV-2 NC_045512.2 (12502) ATATAAAAATACGTGTGATGGTACAACATTTACTTATGCATCAGCAT--TGTGGGAAATCCAACAGGTTGTAGATGC

Section 1145
                                                   (88089) 88089     88099      88110      88121      88132      88143      88154 88165
Autographa californica nucleopolyhedrovirus NC 0... (88040) TCACTTGGATCGTGTTGTCGTAAACGCTTCCCAAAAAGTCTAACACGTTGACCGTTTCGATTCTATTCAACTTAATT
                            SARS-CoV-2 NC_045512.2 (12577) AGATAGTAAAATTGTTCAACTTAGTGAAATT---AGTATGGAC-AATTCACCTAATTTAGC--ATGGCCTCTTATTG

Section 1146
                                                   (88166) 88166     88176      88187      88198      88209      88220      88231 88242
Autographa californica nucleopolyhedrovirus NC 0... (88117) GTGGACGCGTTGGCTTGCATCGGTTCCAACAGACTGCGCGCTCCGACAGATTGAGTAGACAAAATTTTTAAACTTTC
                            SARS-CoV-2 NC_045512.2 (12648) -TAACAGCTTTAAGGGCCAATTCTGCTGTCAAATTACAGAATAATGAGCTTAGT-CCTGTTGCACTACGACAGATGT

Section 1147
                                                   (88243) 88243     88253      88264      88275      88286      88297      88308 88319
Autographa californica nucleopolyhedrovirus NC 0... (88194) CGTCTTATTGGGCGTAATGTCGTTGATTAACAACGACGCAGCCGTTTGAGAGGCCGCAGTGTTGATGGTTTGCAACA
                            SARS-CoV-2 NC_045512.2 (12723) CTTGTGCTGCCGGTACTACACAAACTGCTTGCACTGATGACA-----ATGCGTTAGCTTACTACA-----ACACAAC

Section 1148
                                                   (88320) 88320     88330      88341      88352      88363      88374      88385 88396
Autographa californica nucleopolyhedrovirus NC 0... (88271) TGTCGACGGCCGCCATTTGCGT-TTGCGCCGAAGGTCTTGCTGGCGGCCTGTTGCGGCGGTTTCTTCGTGCTTGCGA
                            SARS-CoV-2 NC_045512.2 (12790) AAAGGGAGGTAGGTTTGTACTTGCACTGTTATCCGATTTACAGGA---------TTTGAAATGGGCTA-GATTCCCT
```

Section 1149

(88397) 88397 88407 88418 88429 88440 88451 88462 88473
Autographa californica nucleopolyhedrovirus NC_0... (88347) CATGTTGTCGTCAGTGTCCATATCGGTATCATTTATTGAAGCAATCATGGTTGAGTTCGATAAGCAGAGATATTTCG
SARS-CoV-2 NC_045512.2 (12857) AAGAGTGATGGAACTGGT--ACTATCTATACAGAA---CTGGAACCACCTTGTAGGTTTGTTACAGACACACCTAAA

Section 1150

(88474) 88474 88484 88495 88506 88517 88528 88539 88550
Autographa californica nucleopolyhedrovirus NC_0... (88424) TTGTCCAATTGGTACTTGGTAATGATGTGCCTTATAAATGTTTCGGGCACAATCATTTCTGTCATTAGCACGTTACA
SARS-CoV-2 NC_045512.2 (12929) GG-TCCTA-AAGTGAAGTATTTATACTTTATTA------AAGGA----TTAAACAACC--------TAAATAGAGGT

Section 1151

(88551) 88551 88561 88572 88583 88594 88605 88616 88627
Autographa californica nucleopolyhedrovirus NC_0... (88501) AATATCTATTTTGATCAATTTCAATTTATGAATTAACAGATTAATGTTTTCGTCCGAGTACTTGCTCATGATGAAAC
SARS-CoV-2 NC_045512.2 (12986) ATGGTACTTGGTAGTTTAGCTGCCACAGTACGTCTACAAGCTGG--TAATGCAACAGAAGTGCCTGCCAATTCAA--

Section 1152

(88628) 88628 88638 88649 88660 88671 88682 88693 88704
Autographa californica nucleopolyhedrovirus NC_0... (88578) GACAAACGTTGCGGAGTTCCAACTCCGCTACCGGATACGCTTTGTTGGGCAAACTCTCTAAATAGTGTCTCAAATAA
SARS-CoV-2 NC_045512.2 (13059) -----CTGTATTATCTTTCTGTGCTTTTGCTGTAGATGCTGCTAAAGCTTACAAAGATTATCTAGCTAGTGGGGGAC

Section 1153

(88705) 88705 88715 88726 88737 88748 88759 88770 88781
Autographa californica nucleopolyhedrovirus NC_0... (88655) AAGCCGATCAATACGGTGG-ACGCTATTTTGTTAACCTTTTTCATTTTAGTATTGCGGCCCATTTCTATCATGAAGT
SARS-CoV-2 NC_045512.2 (13131) AACCAATCACTAATTGTGTTAAGATGTTGTGTACACACACTGGTA-CTGGTCAGGCAAT-AACAGTTACACCGGAAG

Section 1154

(88782) 88782 88792 88803 88814 88825 88836 88847 88858
Autographa californica nucleopolyhedrovirus NC_0... (88731) TTTTAAACGGTAGCAACAGCCTGTCTCCGTTAGCAACAGTGGAGCAGCCGTTGCATTGCGCGCTCAAAATACTCAAC
SARS-CoV-2 NC_045512.2 (13206) CCAATATGG--ATC-AAGAATCC-TTTGGTGGTGCATCGTGTTGTCTGTACTGCCGTTGCCAC-ATAGATCATCCAA

Section 1155

(88859) 88859 88869 88880 88891 88902 88913 88924 88935
Autographa californica nucleopolyhedrovirus NC_0... (88808) ACGCGCTCGTGATCTTCTTGGCGCAATCCGACGGTTGCTTTTTTGCATTCTTTGACAAATGGCACGCACATGTCGCG
SARS-CoV-2 NC_045512.2 (13278) ATCCTAAA---GGATTTTGTGACTTAAAAGG---TAAGTATGTACAAATACCTA-----------CAACTTGTGCTA

```
Section 1156
                                                    (88936) 88936     88946      88957      88968      88979      88990      89001      89012
Autographa californica nucleopolyhedrovirus NC 0... (88885) TTTCGTGTACAAAGAATACGCTTTGTCGCAA-ATCAAGTTATAGAAAAATTGCACAAATAT----CTGCGTAATCAA
                         SARS-CoV-2 NC_045512.2 (13338) ATGACCCTGTGGGTTTTAC----ACTTAAAAACA-CAGTCTGTACCGTCTGCGGTATGTGGAAAGGTTATGGCTGTA

Section 1157
                                                    (89013) 89013     89023      89034      89045      89056      89067      89078      89089
Autographa californica nucleopolyhedrovirus NC 0... (88957) GTTGTTTTCGTTAATAATGTCACTTTCGTTTTTGTAATCGGTTCGAAGCAACACGTACAACATCAGAGGCATGCCGA
                         SARS-CoV-2 NC_045512.2 (13410) GTTGTG-ATCAACTCCGCGAACCCATGCTTCAGTCAGCTG--ATGCACAATCGTTTTTAAACGGGTTTGCGGTGTAA

Section 1158
                                                    (89090) 89090     89100      89111      89122      89133      89144      89155      89166
Autographa californica nucleopolyhedrovirus NC 0... (89034) ACATGGGTCTTAAAAAAATGTCCCAACCATTTTGCAAGCCCGCGTCGAGGGTGCTCAGCGAGGACGCCAAGTATTTG
                         SARS-CoV-2 NC_045512.2 (13484) GTGCAGCCCGTCTTACACCGTGCGGCACAGGCACT-AGT---ACTGATGTCG----------TATACAGGGCTTTTG

Section 1159
                                                    (89167) 89167     89177      89188      89199      89210      89221      89232      89243
Autographa californica nucleopolyhedrovirus NC 0... (89111) CATTTGCACTCAAAACATTGAATTTTGTTTGCGGGCTTGCACGACTGACACATGATCGCATCCACGTCGGGTGCCGG
                         SARS-CoV-2 NC_045512.2 (13547) ACATCTACAATGATAAA-GTAGCT---------GGTTTTGCTAAATTCCTAAAAAC-T----A-ATTGTTGTCGCTTC

Section 1160
                                                    (89244) 89244     89254      89265      89276      89287      89298      89309      89320
Autographa californica nucleopolyhedrovirus NC 0... (89188) CGTCGGATTGTAATATTTTTGCAAGTATTGCATAATGGTCCTAAAATGGGGTACCTGTTTGATAAACTCGTCGCGCA
                         SARS-CoV-2 NC_045512.2 (13609) CAAGAAAAGGACGAAG-----ATGACAATTTAATTGATTCTTACTTTGTAGTT-AAGAGACACACTT-TCTCTAACT

Section 1161
                                                    (89321) 89321     89331      89342      89353      89364      89375      89386      89397
Autographa californica nucleopolyhedrovirus NC 0... (89265) AAAATATCGAAAAAATGTTTTTTACATTGTGTATGTTGTCTGTGTTG-TTGGCTTGATTCTCAAAACTACTCTTTAT
                         SARS-CoV-2 NC_045512.2 (13679) ACCAACATGAAGAAACAATTTATAATTTACTTAAG-GATTGTCCAG-CTGTTGCTAAACATGACTTCTTTAAGTTTA

Section 1162
                                                    (89398) 89398     89408      89419      89430      89441      89452      89463      89474
Autographa californica nucleopolyhedrovirus NC 0... (89341) GGAAACAATACATTTGTTAAATTCTGTGAAA-AAAGTAAGACCTTTACTGTCCACGATCAAGCTTTGGTTGAAATAT
                         SARS-CoV-2 NC_045512.2 (13754) GAATAGAC-----GGTGACATG-GTACCACATATATCACGT-CAACGTCTTACTAAAT-ACACAATGGCAGACCTCG
```

Section 1163

(89475) 89475 89485 89496 89507 89518 89529 89540 89551
Autographa californica nucleopolyhedrovirus NC 0... (89417) TTTGAAAATAAAAAACACAACGAATCGATTTCATCTTGTAACAATTGCGCTTCAAAACACA---CGTTTTCAA-AGC
SARS-CoV-2 NC_045512.2 (13823) -TCTATGCTTTAAGGCATTTTGATGAAGGTAATTGTGACACATTAAAAGAAATACTTGTCACATACAATTGTTGTGA

Section 1164

(89552) 89552 89562 89573 89584 89595 89606 89617 89628
Autographa californica nucleopolyhedrovirus NC 0... (89490) GGTCGTAAATGTTAAACCTTAAACTGTATTGTAATCTGTAAGCGCACATGGTGCATTCGATATAACCTTATAATATG
SARS-CoV-2 NC_045512.2 (13899) TGATGATTATT-TCAATAAAAAGGACTGG--------TATGATTTTGTAGAAAA--CCCAGATATATT-A-------

Section 1165

(89629) 89629 89639 89650 89661 89672 89683 89694 89705
Autographa californica nucleopolyhedrovirus NC 0... (89567) AACGATTCCAATTCTCTGTTGATTACGCGTTTGGCAGCGCAAATACTGTCCAGAAACATGCAAACGGTGGATGTGAT
SARS-CoV-2 NC_045512.2 (13957) -CGCGTATACGCCAACTTAGGTGAACGTGTACGCCAAGCTTT-------GTTAAAAACAGTACAATT---CTGTGAT

Section 1166

(89706) 89706 89716 89727 89738 89749 89760 89771 89782
Autographa californica nucleopolyhedrovirus NC 0... (89644) TGTTGACGACAAAACGCTCAGTTTGGAAGAAAA--AATAGACACGTTGACCAGCATGGTGTTGGCTGTAAATAGCC-
SARS-CoV-2 NC_045512.2 (14023) GCCATGCGAAATGCTGGTATTGTTGGTGTACTGACATTAGATAA--TCAAGATCTCAATGGTAACTGGTATGAT--T

Section 1167

(89783) 89783 89793 89804 89815 89826 89837 89848 89859
Autographa californica nucleopolyhedrovirus NC 0... (89718) -CGCCGCAATCGCCGCCGCGGGTAACATCCAGCGACCTGGCCGCATCGATCATTAAAAATAACAGCAAAATGGTGGG
SARS-CoV-2 NC_045512.2 (14096) TCGGTGATTTCATACAAACCACGCCAGGTAGTGGAGTTCCTGTTGTAGATTCTTATTATTCATTGTTAATGCCTA--

Section 1168

(89860) 89860 89870 89881 89892 89903 89914 89925 89936
Autographa californica nucleopolyhedrovirus NC 0... (89794) CAACGATTTTGAAATGCGATACAACGTGTTGCGTATGGCCGTCGTTTTTGTTAAGCATTATCCCAAGTATTACAACG
SARS-CoV-2 NC_045512.2 (14171) TATTAACCT------TGACCAGGGCTTTAACTGCA-GAGTCACATGTTGACACTGACTT-AACAAAGCCTTACATTA

Section 1169

(89937) 89937 89947 89958 89969 89980 89991 90002 90013
Autographa californica nucleopolyhedrovirus NC 0... (89871) AGACGACCGCCGGTTTAGTTGCCGAAATAGAAAGTAATCTGTTGCAATATCAAAATTATGTAAACCAAGGCAATTAT
SARS-CoV-2 NC_045512.2 (14240) AGTGGGATTTGTTAAAATATGACTTCACGGAAGAGAGGTTAAAACTCTTTGACCGTTATTTT---AAATATTGGGAT

Section 1170

(90014) 90014 90024 90035 90046 90057 90068 90079 90090
Autographa californica nucleopolyhedrovirus NC 0... (89948) CAGAACATTGAGGGTTACGATAGTTTATTAAATAAGGCGGAAGAGTGTTATGTTAAAATTGATAGACTATTTAAAGA
SARS-CoV-2 NC_045512.2 (14314) CAGACATACCACCC-AAATTGTGTTAACTGTTTGGATGACAGATGCATTCT--GCATTGTGCAAACTTTAATGTTTT

Section 1171

(90091) 90091 90101 90112 90123 90134 90145 90156 90167
Autographa californica nucleopolyhedrovirus NC 0... (90025) GAGCATTAAAAAAATCATGGACGACA--CGGAAGCGTTCGAAAGAGAACAGGAAGCGGAGAGATTGAGGGCCGAACA
SARS-CoV-2 NC_045512.2 (14388) ATTCTCTACA-GTGTTCCCACCTACAAGTTTTGGACCACTAGTGAGAAAAATAT-----TTGTTGATGGTGTTCCA-

Section 1172

(90168) 90168 90178 90189 90200 90211 90222 90233 90244
Autographa californica nucleopolyhedrovirus NC 0... (90100) AACTGCC--GCAAACGCTCTTCTGGAGAGGCGAGCGCAGACGTCCGCAGACGATGTCGTTAATCGTGCCGACGCCAA
SARS-CoV-2 NC_045512.2 (14458) --TTTGTAGTTTCAACTGGATACCACTTCA-GAGAGCTAGGTGTTGTACATAATCAGGATGTAAACTTAC---ATAG

Section 1173

(90245) 90245 90255 90266 90277 90288 90299 90310 90321
Autographa californica nucleopolyhedrovirus NC 0... (90175) TATTCCCACGGCATTTAGCGATCCGCTTCCAGGCCCCAGCGCGCCGCGGTACATGTACGAAAGTTCAGAGTCGGACA
SARS-CoV-2 NC_045512.2 (14529) CTCTAGACT--TAGT-TTTAAGGAATTA--CTTGTGTAT-GCTGCT--GACCCT-GCTATGCACGCTGCTTCTG---

Section 1174

(90322) 90322 90332 90343 90354 90365 90376 90387 90398
Autographa californica nucleopolyhedrovirus NC 0... (90252) CGTACATGGAAACCGCCCGA--CGTACCGCCGAACATTACACCGATCAGGACAAAGACTACAACGCGGCGTACACTG
SARS-CoV-2 NC_045512.2 (14594) GTAATCTATT--ACTAGATAAACGCACTACGTGCTTTTCAGTAGCTGCACTTACTAACAATGTTGCTTTTCAAACTG

Section 1175

(90399) 90399 90409 90420 90431 90442 90453 90464 90475
Autographa californica nucleopolyhedrovirus NC 0... (90327) CCGACGAGTACAATTCCCTGGTCAAGACGGTTCTTTTGCGTTTAATCGAAAAGGCGCTGGCCACTCTAAAAAATCGG
SARS-CoV-2 NC_045512.2 (14669) TCAAACCCGGTAA---------------TTTTAACAAAGACTTCTATGACT--TTGCTGTGTCT----AAGGGTTTC

Section 1176

(90476) 90476 90486 90497 90508 90519 90530 90541 90552
Autographa californica nucleopolyhedrovirus NC 0... (90404) TTGCACATAACAACTATTGATCAATTGAAAAAGTTTAGAGATTATCTGAATAGCGATGCTGATGCTGGAGAATTTCA
SARS-CoV-2 NC_045512.2 (14725) TTTAA-----------GGAAGGAAGTTCTGTTGAAT--------TAAAACACTTCTTC--TTTGCTCAG--GATGGT

```
                                                                   Section 1177
                                                   (90553) 90553     90563      90574      90585      90596      90607      90618    90629
Autographa californica nucleopolyhedrovirus NC 0... (90481) AATATTTTTAAACCAGGAAGATTGTGTGATACTGAAAAATTTGTCAAATTTAGCGTCAAAGTTTTTCAACGTTCGTT
                            SARS-CoV-2 NC_045512.2 (14779) AATGCTGCTATCAGCGATTA---TGACTACTATCGTTATAATCTACCAACAA-------TGTGTGATATCAGACAAC
```

```
                                                                   Section 1178
                                                   (90630) 90630     90640      90651      90662      90673      90684      90695    90706
Autographa californica nucleopolyhedrovirus NC 0... (90558) GCGTGGCCGACACGTTAGAGGTAATGTTGGAAG--CGCTTCGCAATAAT-ATTGAGTTGGTGCAGCCTGAAAGCGAT
                            SARS-CoV-2 NC_045512.2 (14846) TACTATTTGTAGTT--GAAGTTGTTGATAAGTACTTTGATTGTTACGATG-GTGGCTGTATTAATGCTAA----CCA
```

```
                                                                   Section 1179
                                                   (90707) 90707     90717      90728      90739      90750      90761      90772    90783
Autographa californica nucleopolyhedrovirus NC 0... (90632) GCCGTACGGCGAATAGTCATAAAAATGACGCAAGAAATTAAAGATTCGAGCACGCCGCTGTACAACATTGCCATGTA
                            SARS-CoV-2 NC_045512.2 (14916) AGTCA-T--CGTCAACAACCT------AGACAAATCAG------CTGGT-TTTCCATTTAATAAATGGGGTAAG--G
```

```
                                                                   Section 1180
                                                   (90784) 90784     90794      90805      90816      90827      90838      90849    90860
Autographa californica nucleopolyhedrovirus NC 0... (90709) CAAAGCGATTATGACGCCATAAAAA-CAAAAACATTAAAACCTTGTTCGACTTGTACAACGACAGGCTGCCAATC
                            SARS-CoV-2 NC_045512.2 (14975) CTA--GACTTTATTATGATTCAATGAGTTATGAGG------ATCAAGATGCA------CTTTTCGCATATACAAAAC
```

```
                                                                   Section 1181
                                                   (90861) 90861     90871      90882      90893      90904      90915      90926    90937
Autographa californica nucleopolyhedrovirus NC 0... (90785) AATTTCTTGGACACGTCCGCAACCAGTCCAGTTCGCAAAACTTCCGGCAAGAGATCTGCGGAAGACG-ACTTGTTGC
                            SARS-CoV-2 NC_045512.2 (15038) GTAATGT-------CATCCCTACTATA------ACTCAAATGAATC---TTAAGTATGCCATTAGTGCAAAGAATAG
```

```
                                                                   Section 1182
                                                   (90938) 90938     90948      90959      90970      90981      90992      91003    91014
Autographa californica nucleopolyhedrovirus NC 0... (90861) CGACTCGCAGCAGCAAACGTGCCAATAGACCCGAAATTAATGTAATATCGTCAGAAGACGAGCAGGAAGATGATGAC
                            SARS-CoV-2 NC_045512.2 (15099) AGCTCGCACCGTAGCT-GGTGTCTC-TATCTGTAGTACTATGACCAATAGACAGTTTCAT--------CAAAAA-TT
```

```
                                                                   Section 1183
                                                   (91015) 91015     91025      91036      91047      91058      91069      91080    91091
Autographa californica nucleopolyhedrovirus NC 0... (90938) GTTGAAGATGTCGACTACGAAAAAGAAAGTAAACGCAGAAAATTAGAAGACGAAGATTTTCTCAAATTAAAAGCATT
                            SARS-CoV-2 NC_045512.2 (15165) ATT-----GAAATCAATAGCCGCC-ACT--AGAG-GAGCTAC--TGTA-----GTAATTGGAACAAGCAAATTCTAT
```

Section 1184

```
                                          (91092) 91092     91102      91113      91124      91135      91146      91157      91168
Autographa californica nucleopolyhedrovirus NC_0... (91015) AGAATTTAGC-AAGGACATTGTCAACGAAAAGCTTCAAAAAATTATTGTGGTCACCGACGGTATGAAACGGCTGTAC
                         SARS-CoV-2 NC_045512.2 (15226) GGTGGTTGGCACAACATGTTAAAAACTGTT--------TATAGTGATGTAGAAAACCCTC-ACCTTATGGGT-----
```

Section 1185

```
                                          (91169) 91169     91179      91190      91201      91212      91223      91234      91245
Autographa californica nucleopolyhedrovirus NC_0... (91091) GAATACTGCAACTGCAAAAATTCTTTAGAGACTTTACCGAGCGCCGCTAACTATGGCAGCTTGCTCAAAAGGCTAAA
                         SARS-CoV-2 NC_045512.2 (15289) -----TGGGATTATCCTAAATGTGATAGAGCCATGCCTAACAT--GCTTAGAATTATGGCCT-CACTTGTTCTTGCT
```

Section 1186

```
                                          (91246) 91246     91256      91267      91278      91289      91300      91311      91322
Autographa californica nucleopolyhedrovirus NC_0... (91168) CCTGTACAATCTCGATCATATCGAAATGAATGTAAATTTTTACGAGTTGCT--GTTTCCATTGACACTGTACAATGA
                         SARS-CoV-2 NC_045512.2 (15358) CGCAAACATACAACGTGTTGT----A--GCTTGTC---ACACCGTTTCTATAGATTAGCTAATGAGTGTGCTCAAGT
```

Section 1187

```
                                          (91323) 91323     91333      91344      91355      91366      91377      91388      91399
Autographa californica nucleopolyhedrovirus NC_0... (91243) CAATGATAACAGTGACAAAACGCTTTCTCATCAATTGGTAAATTACATATTTTTGGCCAGTAACTATTTTCAAAACT
                         SARS-CoV-2 NC_045512.2 (15426) ATTGAGTGAAATGGTCATGTGTGGCG---GTTCACTATATGTTAAACCAGGTGGAACCTCATCAGGAGATGCCACAA
```

Section 1188

```
                                          (91400) 91400     91410      91421      91432      91443      91454      91465      91476
Autographa californica nucleopolyhedrovirus NC_0... (91320) GCGCTAAAAACTTCAACTATATGCGCGAAACTTTTAACGTGTTTGGCCCGTTTAAACAAATCGACTTTATGGTCATG
                         SARS-CoV-2 NC_045512.2 (15500) CTGCTTAT-GCTAATAGTGTTTT-TAACATTTGTCAAGCTG--TCACG--GCCAATGTT-AATGCACTTTTATCTAC
```

Section 1189

```
                                          (91477) 91477     91487      91498      91509      91520      91531      91542      91553
Autographa californica nucleopolyhedrovirus NC_0... (91397) -TTTGTTATAAAATTTAACTTTTTATGCGACATGCGTAATTTTGCCAAATTAATCGACGAGCTGGTGCCCAACAAAC
                         SARS-CoV-2 NC_045512.2 (15570) TGATGGTAACAAAATTGCCGATAAGTATGTCCGCAATTTACAACACAGACTTTATGAGTGTCTCTATAGAAATAGAG
```

Section 1190

```
                                          (91554) 91554     91564      91575      91586      91597      91608      91619      91630
Autographa californica nucleopolyhedrovirus NC_0... (91473) AGCCCAACATGAGAATTCACAGCGTGTTGGT----CATGCGGGATAAAATTGTTAAACTAGCTTTTAGTAATTTACA
                         SARS-CoV-2 NC_045512.2 (15647) ATGTTGACACAGACTTTGTGAATGAGTTTTACGCATATTTGCGTAAACATTTCTCAATGATGATACTCTCTGACGAT
```

Section 1191

	(91631)	91631 91641 91652 91663 91674 91685 91696 91707
Autographa californica nucleopolyhedrovirus_NC_0...	(91546)	ATTTCAAACCTTTTCAAAGAAAGACAAGTCGCGCAACACAAAACATTTGCAAAGACTAATAATGTTGATGAA--C--
SARS-CoV-2 NC_045512.2	(15724)	GCTGTTGTGTGTTTCAATAGCACTTATGCATCTCAAGGTCTAGTGGCTAGCATAAAGAA--CTTTAAGTCAGTTCTT

Section 1192

	(91708)	91708 91718 91729 91740 91751 91762 91773 91784
Autographa californica nucleopolyhedrovirus_NC_0...	(91619)	GCAAACTACAATGTTATATAATAAAAAATTATAAAATATTTTAATTTTTATTTATATTCAGTACATTTACACATAT
SARS-CoV-2 NC_045512.2	(15799)	TATTA-TCAAAACAATGTTTTTATGTCTGAAGCA-AA--ATGTTGGACTGAGACTGACCT--TACTAAAGGACCTCA

Section 1193

	(91785)	91785 91795 91806 91817 91828 91839 91850 91861
Autographa californica nucleopolyhedrovirus_NC_0...	(91696)	TAACATATTGTTTATACAAATTCTTATA-ATCATTATGATTTAAATTGAATTGTTGTCTAAACAAATTAAACACTTT
SARS-CoV-2 NC_045512.2	(15870)	TGAATTTTGCTCTCAACATACAATGCTAGTTAAACAGGGTGATGATT-ATGTGTACCTT--CCTTACCCAGATCCAT

Section 1194

	(91862)	91862 91872 91883 91894 91905 91916 91927 91938
Autographa californica nucleopolyhedrovirus_NC_0...	(91772)	ATTAAACAATAACTTTTCGTTG------TAATTTTTTAC-TTTGCACATGTTATAACAAAAAATTAAAATTTTCATC
SARS-CoV-2 NC_045512.2	(15944)	CAAGAATCCTAGGGGCCGGCTGTTTTGTAGAT-GATATCGTAAAAACAGATGGTACACTTATGATTGAACG-GTTCG

Section 1195

	(91939)	91939 91949 91960 91971 91982 91993 92004 92015
Autographa californica nucleopolyhedrovirus_NC_0...	(91842)	ATGTCTGATTTGTCTATGGCGTCACAGTTGCTTTTAATGTAATCGCAAGTTAACCACTCAAAAGGACCCTTTTCTAT
SARS-CoV-2 NC_045512.2	(16019)	TGTCTTTAGCTATAGATGCTTACCCACTTA------CTAAAC--------A-TCCTAATCAGGAGTATGCTGATGTCT

Section 1196

	(92016)	92016 92026 92037 92048 92059 92070 92081 92092
Autographa californica nucleopolyhedrovirus_NC_0...	(91919)	TT-TTAATTTGTTT--AAATCTTTATAATCAGACTTCAGTTTGTAAATTAGATTTCCACATCGA--ATAATAAATCC
SARS-CoV-2 NC_045512.2	(16082)	TTCATTTGTACTTACAATACATAAGAAAGCTACATGATGAGTTAACAGGACACATGTTAGAC--ATGTATTCTGTTA

Section 1197

	(92093)	92093 92103 92114 92125 92136 92147 92158 92169
Autographa californica nucleopolyhedrovirus_NC_0...	(91991)	TTCCAGCGGGCTTTGGGGAA--ACATTAAAGACTTGAAATTTAACCTTTCTACAAAATCGTTGTACAAATA--TTTG
SARS-CoV-2 NC_045512.2	(16157)	TGCTTACTAAT-----GATAACACTTCAAGG--------------------------TATTGGGAACCTGAGTTTTA

```
Section 1198
                                               (92170) 92170     92180      92191      92202      92213      92224      92235   92246
Autographa californica nucleopolyhedrovirus NC 0... (92064) TGACACGGAATAGTATTAAACCCCACGTTAGTCAACAACTCTTGCGCCTCCACAAAGGGCACAAACTCCCCGCCGTA
                      SARS-CoV-2 NC_045512.2 (16203) TGAGGCTATGTACACACCGCATACAGTCTTACAGGCTGTTGGGGCTTG--TGTTCTTTGCAATTCACAGACTTCATT

Section 1199
                                               (92247) 92247     92257      92268      92279      92290      92301      92312   92323
Autographa californica nucleopolyhedrovirus NC 0... (92141) TAATTGAATTTCGTAAGCGTAGTATTTCAAACTCTCTTTCTGGTCCACGTAGTTAATTACGTTAATGGGTGTCGTTT
                      SARS-CoV-2 NC_045512.2 (16278) AAGAT----GTGGTGCTTGCATACGTAG---ACCATTCTTATGTTGTAAATGCTGTTACGACCATG---------TC

Section 1200
                                               (92324) 92324     92334      92345      92356      92367      92378      92389   92400
Autographa californica nucleopolyhedrovirus NC 0... (92218) TTGCGTCGTCTTTCCAACCCATTAATTCGCCGTAGACAATAAAACCGTCATTGAACCGCGCCTGAAGCGATCGCATG
                      SARS-CoV-2 NC_045512.2 (16339) ----ATATCAACATCAC---ATAAATTAGTCTTGTCTGTTAATCCGTATGTTT-----------GCAA---------

Section 1201
                                               (92401) 92401     92411      92422      92433      92444      92455      92466   92477
Autographa californica nucleopolyhedrovirus NC 0... (92295) CACGTTTCTAAATCTTTTCGAATGCGG--TAATAATTCATAAAATTGCCGTCCGGTCTGTAAGTGTTTCTTGACCCG
                      SARS-CoV-2 NC_045512.2 (16389) TGCTCCAGGTTGTGATGTCACAGATGTGACTCAACTTTACTTAG--G-AGGTATGAGCTATTATTGTAA--ATCACA

Section 1202
                                               (92478) 92478     92488      92499      92510      92521      92532      92543   92554
Autographa californica nucleopolyhedrovirus NC 0... (92370) TACGTAATTTTATTTTGGTTGCAAATGA---TTCTGAAATTACAACCGTCCAACTTTTCTTGAACAATAATTTCTTT
                      SARS-CoV-2 NC_045512.2 (16461) TA--AACCACCCATTAGTTTTCCATT--GTGTGCTAATGGACAAGTTTTTGGT--TTATATAAAAATACATGTGTTG

Section 1203
                                               (92555) 92555     92565      92576      92587      92598      92609      92620   92631
Autographa californica nucleopolyhedrovirus NC 0... (92444) GTCGGCCAACGTACCTTTTTTACCTTGATCTAGATGCGACACAGATGGATAAATTTGATACACAATTTTATTCTCAT
                      SARS-CoV-2 NC_045512.2 (16532) GT--AGCGATAA-TGTTACTGAC-TTTAA----TGCAATTG--CAACATGTGACTGGACAAAT-GCTGGTGATTACA

Section 1204
                                               (92632) 92632     92642      92653      92664      92675      92686      92697   92708
Autographa californica nucleopolyhedrovirus NC 0... (92521) CTTCGGGCATTACGGGTCCGCGTTCATTTAACGCGTACATGACAATGTTGTGGCGAATGTCGGTGCGCTCCGGCGGT
                      SARS-CoV-2 NC_045512.2 (16598) TTTTAGCTAACACCTG-TACTGAAAGACTCAAGCTTTTTGCAGCAGAAACGCTCAAAGCTACTGA-------GGAGA
```

Section 1205

```
                                                   (92709) 92709     92719       92730       92741       92752       92763       92774   92785
Autographa californica nucleopolyhedrovirus NC_0... (92598) TCTGGCACGTGGTGCAGTCTGTCCTGCAATTGTTGCTTCCATTGTTGAAAATATTCGGTCCATTCTTGTTGATACTC
                             SARS-CoV-2 NC_045512.2 (16667) CATTTAAACTGTCTTATGGTATT-------------GCTACTGTACG--TGAAGTGCTGTCTGACAGAGAATTACATC
```

Section 1206

```
                                                   (92786) 92786     92796       92807       92818       92829       92840       92851   92862
Autographa californica nucleopolyhedrovirus NC_0... (92675) GCCGCGTTGCATGAGTTTTACGTACAGTTTTAAAAGTTTGACATTCTTTACAAATAACGTTAGAGTTTCGTCGATTT
                             SARS-CoV-2 NC_045512.2 (16730) TTTCATGGGAAGTTGGTAAACCTAGAC-----CACCACTTAACCGA-----AATTATGTCTTTACTGGTTATCGTGT
```

Section 1207

```
                                                   (92863) 92863     92873       92884       92895       92906       92917       92928   92939
Autographa californica nucleopolyhedrovirus NC_0... (92752) TGTATCCTCCATTATTTTTGTTTAAATCCAATACATTTAAATCGTTCACTACCAGTTGATTGTTTTTATCCATCGTA
                             SARS-CoV-2 NC_045512.2 (16797) AACTAAAAACAGTAAAGTACAAATAGGA--GA--G--TACA---CCT---------TTGAAAAAGGTGAC-TATGGTG
```

Section 1208

```
                                                   (92940) 92940     92950       92961       92972       92983       92994       93005   93016
Autographa californica nucleopolyhedrovirus NC_0... (92829) ATTTTTATCTCATCGCCCACGTTGAACAACATGTTTAAAATTTTGGTGGATTTCGGCGCACGTTTATAATCTAAATA
                             SARS-CoV-2 NC_045512.2 (16856) ATGCTGTTGTTTACCGAGGT---ACA-ACAACTTACAAATTAAATGTTGGT-GATTATTTTGTGCTG-ACATCACAT
```

Section 1209

```
                                                   (93017) 93017     93027       93038       93049       93060       93071       93082   93093
Autographa californica nucleopolyhedrovirus NC_0... (92906) ATATTCAACGTACACGTAATTGAACATGAGCTGCAACAATCCTTTGGCATTGTTCAAAATTTTGTATCTCATCAAAG
                             SARS-CoV-2 NC_045512.2 (16927) ACAGTAATGC-------------CATTA--AGTGCAC-----CTACACTA-GTGCCACAAGAGCAC-TATGTTAGAAT
```

Section 1210

```
                                                   (93094) 93094     93104       93115       93126       93137       93148       93159   93170
Autographa californica nucleopolyhedrovirus NC_0... (92983) TATAAATAATTTTCACCATCGACACCGTCATCAACTTGGTTACAAACTCGTACAATTGCAAGTTTTCAATACCGTAT
                             SARS-CoV-2 NC_045512.2 (16983) T---------ACTGGC-TTATACCCAACACTCAATATCTCA--G--ATG--AGTTTTCTAGCAATGT---TGCAAAT
```

Section 1211

```
                                                   (93171) 93171     93181       93192       93203       93214       93225       93236   93247
Autographa californica nucleopolyhedrovirus NC_0... (93060) TTGTCTTTAAAATCTTCACGTTTACTGAACATGCTTAATTCGGGAGATTTTCCAGTCAAAATGCCAATTAATCCCGT
                             SARS-CoV-2 NC_045512.2 (17041) TATCAA-------AAGGTTGGTATGCAA--AAGTATTCTACACTCC--AGGGACCACCTGGTACTGGTAAGAGTCAT
```

```
                                                                                                           Section 1212
                                                  (93248) 93248     93258      93269      93280      93291      93302      93313      93324
Autographa californica nucleopolyhedrovirus  NC_0... (93137) GTACAAGTCAACGTATTTGACATCG----TTGCCCGATTCATCTTTTGCAT---GTCGATTTTTCAAAA---GCTCT
                          SARS-CoV-2 NC_045512.2 (17107) TTTGCTATTGGCCTAGCTCTCTACTACCCTTCTGCTCG-CATAGTGTATACAGCTTGCTCTCATGCCGCTGTTGATG
                                                                                                           Section 1213
                                                  (93325) 93325     93335      93346      93357      93368      93379      93390      93401
Autographa californica nucleopolyhedrovirus  NC_0... (93204) TTATTGTCGATAAATTTTTCAAAGGTCTCTCGATCACATTTAGTGTAAATATGGTAGTCAGTGTCGCTGCTTTCGAC
                          SARS-CoV-2 NC_045512.2 (17183) CACTATGTGAGAAGGCATT-AAAATAT--TTGCCTATAGATAAATGTAGTAGAATTATACCTGCACGTGCTCGTGTA
                                                                                                           Section 1214
                                                  (93402) 93402     93412      93423      93434      93445      93456      93467      93478
Autographa californica nucleopolyhedrovirus  NC_0... (93281) CGCGTATCCCTTGGCATGGCTGCCCGTATCAATGCAAATGTACACCATGTTAGAATGTGCTGCTTACTGTGCCTGTA
                          SARS-CoV-2 NC_045512.2 (17257) GAGTGTTTTGATAAATTCAAAGTGAATTCAACATTAG--A-ACAGTAT---GTCTTTTGTACTGTAA-ATGCATTGC
                                                                                                           Section 1215
                                                  (93479) 93479     93489      93500      93511      93522      93533      93544      93555
Autographa californica nucleopolyhedrovirus  NC_0... (93358) TCAAGCCTTATATACCTCAAAATATTTCACATTTTTGCATCATCGTAAAATATACATGCATATAATTGTGTACAAAA
                          SARS-CoV-2 NC_045512.2 (17327) CTGAGAC---GACAGCA---GATATAGTTGTCT-TTGATGAA------ATTTCAATGGCCACAAATTATGAT--TTG
                                                                                                           Section 1216
                                                  (93556) 93556     93566      93577      93588      93599      93610      93621      93632
Autographa californica nucleopolyhedrovirus  NC_0... (93435) TATGACTCATTAATCGATCGTGCGTTACAAGTAGAATTCTACTGGTAAAGCAAGTTCGGTTGTGAGCCGTGTGCAAA
                          SARS-CoV-2 NC_045512.2 (17389) AGTGTTGTCAATGCCAGATTACGTGCTAAGCACTATGTGTACA-TTGGCGAC--CCTGCTCAATTACCTGCACCACG
                                                                                                           Section 1217
                                                  (93633) 93633     93643      93654      93665      93676      93687      93698      93709
Autographa californica nucleopolyhedrovirus  NC_0... (93512) ACATGACATCATAACTAATCATGTTTATAATCATGTGCAAAATATGACATCATCCGACGATTGTGTTTTACAAGTAG
                          SARS-CoV-2 NC_045512.2 (17463) C----ACATTGC--TAACTAAGGGCACACTAGAACCAGAATATTTCA-ATTC-AGTGTGTAG--ACTTA------TG
                                                                                                           Section 1218
                                                  (93710) 93710     93720      93731      93742      93753      93764      93775      93786
Autographa californica nucleopolyhedrovirus  NC_0... (93589) AATTCTACTCGTAAAGCGAGTTTAAAAATTTTGTGACGTCAATGAAACAACGTGTAATATTTTTTACAATATTTAAG
                          SARS-CoV-2 NC_045512.2 (17524) ---AAAACTATAGGTCCAGACATGTTCCTCGGAACTTGTCGGCGTTGT--CCTGCTGAAATTGTTGACACTGTGA--
```

Section 1219

	(93787)	93787 93797 93808 93819 93830 93841 93852 93863
Autographa californica nucleopolyhedrovirus NC_0...	(93666)	TGAAACATTATGACTTCCAATAAT--TTTGTGGATGTGGATACGTTTGCAAGACAATTGATTACAGATAAATGTAGT
SARS-CoV-2 NC_045512.2	(17594)	-GTGC--T----TTGGTTTATGATAATAAGCTTAAAGCACATAAAG-ACAAATCAGCTCAATGCTTTAAAATGTTTT

Section 1220

	(93864)	93864 93874 93885 93896 93907 93918 93929 93940
Autographa californica nucleopolyhedrovirus NC_0...	(93741)	GCTCTA-ATCAAAGTGCGGATCTGTTGCCGGCAAACATTTTAGAGATTGTAGAGA--------AGGCCAGAGACAAG
SARS-CoV-2 NC_045512.2	(17663)	ATAAGGGTGT-TATCACGCATGATGTTTCATCTGCAATTAACAGGCCA---CAAATAGGCGTGGTAAGAGAATTCCT

Section 1221

	(93941)	93941 93951 93962 93973 93984 93995 94006 94017
Autographa californica nucleopolyhedrovirus NC_0...	(93809)	TATTTTGAGGGCCAACTCAAAAAAACTATGAATACATTAAAAAATTATTTTTACGAAAAAATATATGGACGATTCGA
SARS-CoV-2 NC_045512.2	(17736)	TA------CACGT---------AACCC-TGCTTGGAGAAAAGCTGTCTTTATTTCACCTTATAATTCACAGAATGCT

Section 1222

	(94018)	94018 94028 94039 94050 94061 94072 94083 94094
Autographa californica nucleopolyhedrovirus NC_0...	(93886)	TAGATTATAAAGATTTTAACAGACGCATCCTATTGATAGTTTTAAATTCGCTTTAAACAAGAGCACAATACTTTCC
SARS-CoV-2 NC_045512.2	(17797)	GTAGCCTCAAAGATTTT----GGGACTACCAACTCAAACTGTTGATTCATCACAGGGCTCAGAATATGACTAT-GTC

Section 1223

	(94095)	94095 94105 94116 94127 94138 94149 94160 94171
Autographa californica nucleopolyhedrovirus NC_0...	(93963)	ATCGTACAAAGAGATCATCGAGTGGCCATTAAACGTTTAAACAAAATTAACCCCGATTTAAAGAGTTCTCCGCGCAA
SARS-CoV-2 NC_045512.2	(17869)	ATATTCACTCAAACCACTGAAACAGCTCACTCTTGTAATGTAAACA-----GATTTAATGTT-----GCTATTACCA

Section 1224

	(94172)	94172 94182 94193 94204 94215 94226 94237 94248
Autographa californica nucleopolyhedrovirus NC_0...	(94040)	TGCTTCAGCATTACAATGAATGTTGGAAAATCTAGACAATCCAGTCACGGACGAACATCATTTGTTGACAAAAGAG
SARS-CoV-2 NC_045512.2	(17936)	-----GAGCAAAAGTAGGCATACTTTGCAT-----AATGTCTGATAGAGACCTTTATGACAAG--TTGCAATTTACA

Section 1225

	(94249)	94249 94259 94270 94281 94292 94303 94314 94325
Autographa californica nucleopolyhedrovirus NC_0...	(94117)	TTGCTACAAAAATATTTATCGAAGCGTTTGAATACAGTTACACCAACACTAATGCCA-TCAGCATGGACAAAACAGA
SARS-CoV-2 NC_045512.2	(18001)	AGTCTTGA-AATTCCACGT--AGG---AATGTGGCAACTTTACAAGCTGAAATGTAACAGGAC---TCTTTAAAGA

Section 1226

(94326) 94326 94336 94347 94358 94369 94380 94391 94402
Autographa californica nucleopolyhedrovirus NC 0... (94193) -TGAATTTGATTTTATTAAACCGGCATTGAAACCTTTGCCAGATGCAAGACCGCCATCGCTTTTGGCCAACGTGATG
SARS-CoV-2 NC_045512.2 (18069) TTGTAGT--AAGGTAATCACTGGGTT------ACATCCTACACAGGC------AC------CTACACACCTC---AG

Section 1227

(94403) 94403 94413 94424 94435 94446 94457 94468 94479
Autographa californica nucleopolyhedrovirus NC 0... (94269) AACGAACGTAAAAGAAAATTACAAAACACCAACTCAACGGCAAAATGTTTGCTACCAGCACCACCGCCAC-AATTGC
SARS-CoV-2 NC_045512.2 (18123) TGTTGACACTAAATTCAAAACT------GAAGGTTTATGT-----------GTTGACAT--ACCTGGCATACCTAAG

Section 1228

(94480) 94480 94490 94501 94512 94523 94534 94545 94556
Autographa californica nucleopolyhedrovirus NC 0... (94345) GTAAACTTGAAAAAAAGAATCATTTATTGCCTTTGTTTTCTTTGTAATTATATTGTTGCATTTCTATTTCTAATATC
SARS-CoV-2 NC_045512.2 (18181) GACATGACCTATAGAAGACT-CATCTCTATGATGGGTTTTAAAATGAATTA--TCAAGTTAATGGTTACCCTA----

Section 1229

(94557) 94557 94567 94578 94589 94600 94611 94622 94633
Autographa californica nucleopolyhedrovirus NC 0... (94422) ATAGTTTTCTAATAAAGTAGTTTCATATTTT----TGTTTTTGTACAGTAATTGTTTCTTGGTTTAACAAGATCACA
SARS-CoV-2 NC_045512.2 (18251) A---CAT---GTT-TATCACCCGCGAAGAAGCTATAAGACATGTACGTGCATGGATTGGCTTCGATGTCGAG--GGG

Section 1230

(94634) 94634 94644 94655 94666 94677 94688 94699 94710
Autographa californica nucleopolyhedrovirus NC 0... (94495) ACCAATAACATAAAGAATAACACAATCATAACAAAAATTAAAAAGCCGCATACTACTAGAACAAATTCTTTAATTAG
SARS-CoV-2 NC_045512.2 (18319) TGTCA-TGCTACTAGAGAAGCTGTTGGTACCAATTT-----------ACCTTTACAGCTAGGTTTTTCT--ACAGG-

Section 1231

(94711) 94711 94721 94732 94743 94754 94765 94776 94787
Autographa californica nucleopolyhedrovirus NC 0... (94572) CGATCGGTTTCTATTTACAAATTGGCCGAGCTGATCGCCTTCAGTCGGCGAGTTGTGGGCTTGGATGATGTCGACGA
SARS-CoV-2 NC_045512.2 (18381) TGTTAACC----TAGTTGCTGT--ACCTACAGGTTATGTTGAT-ACACCTAATAATA----CAGATTTTTCCAGAGT

Section 1232

(94788) 94788 94798 94809 94820 94831 94842 94853 94864
Autographa californica nucleopolyhedrovirus NC 0... (94649) TATTGTTGCCGGCGCGACCGCCTGTCGCTCTCGATATAATGTCGGCCGCCGTCGGTTTCATGATGTGCTTAACTACA
SARS-CoV-2 NC_045512.2 (18447) TAG---TGCTAAACC-ACCGCCTGGAGATCA-------ATTTAAACACCTCA----TACCACTTAT-----GTACAA

Section 1233

	(94865)	94865 94875 94886 94897 94908 94919 94930 94941
Autographa californica nucleopolyhedrovirus NC_0...	(94726)	AATAATAGTTGTACTTGACGGGCGTCACCGTGATGCCGCTGCTAAAACCTCCGTCCGTTAAGACGCGTTGCGTTACA
SARS-CoV-2 NC_045512.2	(18504)	AGGACTTCCTTGGAATGTAGTGCGTATAA---AGATTGTAC----AAATGTTAAGTGACACAC------TTAA----

Section 1234

	(94942)	94942 94952 94963 94974 94985 94996 95007 95018
Autographa californica nucleopolyhedrovirus NC_0...	(94803)	AAATTAATGTTTGTCCGATTAGCGTAGTCGGAATAATCAAACGTGTTGGGCGGACTAAAATCGGGCATG-TTGATGG
SARS-CoV-2 NC_045512.2	(18564)	----AAATCTCTCTGACAGAGTCGTATTTGT-----CTTATGG-----G-CACATGGCTTTGA-GTTGACATC-TAT

Section 1235

	(95019)	95019 95029 95040 95051 95062 95073 95084 95095
Autographa californica nucleopolyhedrovirus NC_0...	(94879)	GCACAATGCCGCTGGAGCTGATAGCAATGCTG--TCGTTCTTGCAAAACAGCCGAATTTTTTTGTAGGGCTCTGCTT
SARS-CoV-2 NC_045512.2	(18624)	GAAGTATTTTGTGAAAA----TAGGAC--CTGAGCGCACCTGTTGTCTATGTGATAGACGTGCCACATGCTTT-TCC

Section 1236

	(95096)	95096 95106 95117 95128 95139 95150 95161 95172
Autographa californica nucleopolyhedrovirus NC_0...	(94954)	TATTCGGCGCAGACGACACCATCTGGTCAAAGTTGTTCAATTTTATGATTACGTTGGGTACCAATTGATAGGGGAAA
SARS-CoV-2 NC_045512.2	(18694)	ACTGCTTCAGACACTTATGCC-----TGTTGGC-AT-CATTCTATTGGATTTGATTACGTCTATAATCCGTTTATGA

Section 1237

	(95173)	95173 95183 95194 95205 95216 95227 95238 95249
Autographa californica nucleopolyhedrovirus NC_0...	(95031)	ATTATTTTCTGGAACATTTTGACAAAGTCCACAACCGTTTGGCTATAGTCGGGAATGCCGAGCAAAGACTGCGCCTG
SARS-CoV-2 NC_045512.2	(18764)	TTGATGTTCAACAATGGGGTTTTACAGG--TAACCTA----CAAAGCAACCATGATCTGTATTGTCAAGTCCATGGT

Section 1238

	(95250)	95250 95260 95271 95282 95293 95304 95315 95326
Autographa californica nucleopolyhedrovirus NC_0...	(95108)	TTTAATGTATTTGAGACTGGAGCGGTTTACTGTAGCGCAATTGGATGGCACGTCGCCCTTCATAAGCCGGCGCGTTC
SARS-CoV-2 NC_045512.2	(18835)	AATG---CACATGTAGCTAGTTGTGATGCAATCATGACTAGG--TGTCTA-GCTGTCCACGAGTGCTTTGTTAAGCG

Section 1239

	(95327)	95327 95337 95348 95359 95370 95381 95392 95403
Autographa californica nucleopolyhedrovirus NC_0...	(95185)	TCTCCCAATTCAATTTGTTGTACAAATTATCAATCTCCTCGTGCGGCAGATTGATTACATAGCGCGCGGGCTGTTTG
SARS-CoV-2 NC_045512.2	(18906)	TGTTGACTGGACTATTGAATATCCTATAATTGGTGATGAACTGAAGATTAATGCGGCT-TGTAGAAAGG-TTCAACA

Section 1240

```
                                                 (95404) 95404     95414      95425      95436      95447      95458      95469      95480
Autographa californica nucleopolyhedrovirus NC_0... (95262) CGATATTGAAAGATGCAAAAAATGCGTTTCAACGACAATATCTTCA-----CCATGGTGGACGT-TTCCAGATTGAA
                     SARS-CoV-2 NC_045512.2 (18981) CATGGTTGTTAAAGCTGCATTATTAGCAGACAAATTCCCAGTTCTTCACGACATTGGTAACCCTAAAGCTATTAAGT
```

Section 1241

```
                                                 (95481) 95481     95491      95502      95513      95524      95535      95546      95557
Autographa californica nucleopolyhedrovirus NC_0... (95333) ACATAACAAAAAGTCATTGCTTTCCACCAATTCTTTAAAATGAGACAGCGGAATTTCACAAGCGATCGGTCGCAAAT
                     SARS-CoV-2 NC_045512.2 (19058) GTGTA-CCTCAAGCTGATGTAGAATGGAAGTTCTATGATGCACAGCCTTG--T-------AGTGACAAAGC------
```

Section 1242

```
                                                 (95558) 95558     95568      95579      95590      95601      95612      95623      95634
Autographa californica nucleopolyhedrovirus NC_0... (95410) TGCTTTTTATTGGAGGCGGAACGCTTTGACCGTTGCGGTTTTTTA-GTAACGCGCTGCACGCA-GATTGCATGTCCG
                     SARS-CoV-2 NC_045512.2 (19119) ---TTATAAAATAGAAGAATTATTCTA--TTCTTATGCCACACA-TTCTGACAAATTCACAGATG-GTGTATGCCTA
```

Section 1243

```
                                                 (95635) 95635     95645      95656      95667      95678      95689      95700      95711
Autographa californica nucleopolyhedrovirus NC_0... (95485) TTTCGGGATACGTAAACTCGATGGGACATTTGGGGTTTTCATGGTGAACGATCA-TAGTGT-----TGCAATAAAAC
                     SARS-CoV-2 NC_045512.2 (19189) TTTTG------------GAATTGCAATGTCGATAGATATCCTGCTAA---TTCCATTGTTTGTAGATT---TGACAC
```

Section 1244

```
                                                 (95712) 95712     95722      95733      95744      95755      95766      95777      95788
Autographa californica nucleopolyhedrovirus NC_0... (95556) AAGTTGTTGGTCAGGAGCA----CGCTAAAAACACGCGTTTCGCCCGCACCGATTTCGGTGATGGGTACCAACGGGT
                     SARS-CoV-2 NC_045512.2 (19248) TA-------GAGTGCTATCTAACCTTAACTTGCCTGGTTGTGAT-GGTGGCAGTTTGTATGTAA--ATAAACATGCA
```

Section 1245

```
                                                 (95789) 95789     95799      95810      95821      95832      95843      95854      95865
Autographa californica nucleopolyhedrovirus NC_0... (95629) TCCAGTAGACTATGGTGGCGGACGCTGTTTTTTTTGGCGATCGACTGTCTATGTTAACATCATGCTCGTGCCTGTAC
                     SARS-CoV-2 NC_045512.2 (19315) TTCC--ACACACCAGCTTTTGATAAAAGTGCTTTTGTTAATTTAAAACAATTACCATT---TTTCTATTACTCTGAC
```

Section 1246

```
                                                 (95866) 95866     95876      95887      95898      95909      95920      95931      95942
Autographa californica nucleopolyhedrovirus NC_0... (95706) ACTAGCACAGAATTGAATTTTGGAAATTGTTTTTTGTCAATGTACAACCGGTCGTCGTCTGTGGGCACGTAC----A
                     SARS-CoV-2 NC_045512.2 (19387) AGT--CCATGTGA-GTCTCATGGAAAACAAGTAGTGTCAGATA--TAGATT-----ATGTACCACTAAAGTCTGCTA
```

```
                                                                                        Section 1247
                                                (95943) 95943     95953      95964      95975      95986      95997      96008   96019
Autographa californica nucleopolyhedrovirus NC_0... (95779) CGATCAAGTT----TTCGATT----AATTTGTTGCCTACGTCGCTTTGCGGTTCCACCAAATTGTGAGGGAACGC-A
                        SARS-CoV-2 NC_045512.2 (19454) CGTGTATAACACGTTGCAATTTAGGTGGTGCTGTCTGTAGACATCATGCTAATGAGTACAGATTGTATCTCGATGCT
                                                                                        Section 1248
                                                (96020) 96020     96030      96041      96052      96063      96074      96085   96096
Autographa californica nucleopolyhedrovirus NC_0... (95847) AAAAAGCGATCGCTAATACAAACTTGAATCTGAAACGGGCACTCCAT-CGTGATG-TATATGTCTTACTTCATTAGA
                        SARS-CoV-2 NC_045512.2 (19531) TATAACATGATGATCTCAGCTGGCTTTAGCTTGTGGGTTTACAAACAATTTGAT-ACTTATAACCT-CTGGA-ACAC
                                                                                        Section 1249
                                                (96097) 96097     96107      96118      96129      96140      96151      96162   96173
Autographa californica nucleopolyhedrovirus NC_0... (95922) C-TTTAGATTATTTTAATTTGTGAACTCGTACCGTATTCAATAGGGTGTCGGGCACGTAATTGTAATGGTAAAACAG
                        SARS-CoV-2 NC_045512.2 (19605) TTTTACAAGACTTCAGAGTTTAGAA--AATGTGGCTTTTAATGTTGT-AAATAAGGGACACTTTGATGGACAACAGG
                                                                                        Section 1250
                                                (96174) 96174     96184      96195      96206      96217      96228      96239   96250
Autographa californica nucleopolyhedrovirus NC_0... (95998) ATCCTGTTGAACAC--G-TGCGTTGTTCACTACGATTGAAATGCAAAAATACATCAAGTACATAAACA-CTATGATT
                        SARS-CoV-2 NC_045512.2 (19679) GTGAAGTACCAGTTTCTATCATTA--ATAACACTGTTTACACAAAAGTTGATGGTGTTGATGTAGAATTGTTTGAA-
                                                                                        Section 1251
                                                (96251) 96251     96261      96272      96283      96294      96305      96316   96327
Autographa californica nucleopolyhedrovirus NC_0... (96071) AGAAAGG--TAGCAGACAGAAAATATTTCATCTTTAAATCTTATGCTAGTTGAATAAAATA----CATAGTACTTTT
                        SARS-CoV-2 NC_045512.2 (19753) AATAAAACAACATTACCTGTTAATGTAG--CATTTGAGCTTTGGGCTAAGCG-CAACATTAAACCAGTACCAGAGG-
                                                                                        Section 1252
                                                (96328) 96328     96338      96349      96360      96371      96382      96393   96404
Autographa californica nucleopolyhedrovirus NC_0... (96142) -ATACGTTTATTTATATTTGTTTTCTTTGTTATAACCGTAATTGTAAAACTTGTGATCGTGCTCGCCAGGCATAATT
                        SARS-CoV-2 NC_045512.2 (19826) TGAAAATACTCAATAA-TT-TGGGTGTGGACATTGCTGCTAATACTGTGATCTGGGACTACAAAAGAGATGCTCCAG
                                                                                        Section 1253
                                                (96405) 96405     96415      96426      96437      96448      96459      96470   96481
Autographa californica nucleopolyhedrovirus NC_0... (96218) TCTTTGCACATCAGCTTGCGAATATATGTGACATCTTCGTACACCGATTTCTTGATGTTACCATCGTGAAGCGTTGT
                        SARS-CoV-2 NC_045512.2 (19901) CACATATATCTA---CTATTGGTGTT-TGTTCTATGACTGACAT---AGCCAAGAAACCAACTGAAACGATTTGTGC
```

Section 1254

	(96482)	96482 96492 96503 96514 96525 96536 96547 96558
Autographa californica nucleopolyhedrovirus NC_0...	(96295)	CGGCTTGAGAGGTTTGCGGTCGTTGTTGTAAAAATTTTGCACCGAATAATTATCCATAGTGCAGCACAGGCAATGTC
SARS-CoV-2 NC_045512.2	(19971)	ACCACTCACTGTCTTTT-TTGATGGTAGAGTTGATGGTCAA-GTAGACTTATTTAGAAATGCCCGTAATGGTGTTCT

Section 1255

	(96559)	96559 96569 96580 96591 96602 96613 96624 96635
Autographa californica nucleopolyhedrovirus NC_0...	(96372)	ACTGATGCATATGCTTTAATTTTTTATTGCATTCAGTTATTATATGATTTAATAAACGTACACAATAGCACGTTTAT
SARS-CoV-2 NC_045512.2	(20046)	TATTACAGA---AGGTAGTGTTA--AAGGTTTACAA----CCATC-TGTAGGTCCCAAA---CAAGCTAGTCTT---

Section 1256

	(96636)	96636 96646 96657 96668 96679 96690 96701 96712
Autographa californica nucleopolyhedrovirus NC_0...	(96449)	CGGTTAAAGATAACTTTCAATATATAAA-AGTGTTTGAATTGCGAGACCGTCAACATAACGTTTATCAACGCGATGA
SARS-CoV-2 NC_045512.2	(20107)	---AATGGAGTCACATTAATTGGAGAAGCCGTAAAAACACAGTTCAAT-TATTATAAGAAAGT--TGATGGTGTT-G

Section 1257

	(96713)	96713 96723 96734 96745 96756 96767 96778 96789
Autographa californica nucleopolyhedrovirus NC_0...	(96525)	CTAAACGACAATTTGCTTTGCT-GTTTGTGTGGCACCACGACAACCAATTTGTTTGCAACACGGACGAATACCCGTT
SARS-CoV-2 NC_045512.2	(20177)	TCCAACAATTACCTGAAACTTACTTTACTCAGAGTAGAAATTTA-CAAGAATTT-A-------AACCCAGGAGTCAA

Section 1258

	(96790)	96790 96800 96811 96822 96833 96844 96855 96866
Autographa californica nucleopolyhedrovirus NC_0...	(96601)	TTGGCACAACATT--GAATACCATGCACGGCGCTATAAATGCATCGTTTTGTACTGTGTGGAAAACGACGGATCGCT
SARS-CoV-2 NC_045512.2	(20245)	ATGGAAATTG---ATTTCTTAGAATTAGCTATG-GATGA--------------ATTCATTGAA-CGG---TATAAAT

Section 1259

	(96867)	96867 96877 96888 96899 96910 96921 96932 96943
Autographa californica nucleopolyhedrovirus NC_0...	(96676)	ACAACTGCCCGTTTGCAAAAACATAAATCTCA---TAAATTATAAAAAGCGTATCCTCATTATTATGGAAACTGTG
SARS-CoV-2 NC_045512.2	(20300)	TAGAAGGCTATGCCTTCGAACATATCGTTTA-TGGAGATTTTAGTCATAGTCAGT-TAGGTG-----GTTTACATCT

Section 1260

	(96944)	96944 96954 96965 96976 96987 96998 97009 97020
Autographa californica nucleopolyhedrovirus NC_0...	(96750)	T---TGACAGTATAGTGAAACGTGCTGGCAAAAATTGATTATATGAAAGTAACTGCAATGTTAAACCCCCACCTGTT
SARS-CoV-2 NC_045512.2	(20370)	ACTGATTGGACTAGCTAAACGTTTTAAGGAATCACCTTTTGAAT-TAGAAGATTTTATTCCT--ATGGACAGTACAG

Section 1261

(97021) 97021 97031 97042 97053 97064 97075 97086 97097
Autographa californica nucleopolyhedrovirus NC 0... (96824) GGACGTCGCGTACAATTATTTGCTGTTGATGGACATGGATTGTGTGGTGCAAAGCGTGCAATGGAAACAATTGTCAA
SARS-CoV-2 NC_045512.2 (20444) T---TAAAAACTATTTCATA-ACAGATGCGCAAACAGGTTCATCTAAGTGTGTGTGTTCTGTTATTG--ATTTATTA

Section 1262

(97098) 97098 97108 97119 97130 97141 97152 97163 97174
Autographa californica nucleopolyhedrovirus NC 0... (96901) CCGACACGTATTGTTTTGAGCCGTTTTACGACTCTCAAATTAAATGGTTGTACGCGCCCAAAAGCGGACAAAGTTTT
SARS-CoV-2 NC_045512.2 (20515) CTTGATGATTTTGTTGAAATA-ATAAAATCCC---AAGATTTATCTG-----TAGTTTCTAAGGTTG---------T

Section 1263

(97175) 97175 97185 97196 97207 97218 97229 97240 97251
Autographa californica nucleopolyhedrovirus NC 0... (96978) GATAGTTATCTTGAAAACTATGCAACTCTAATTCGAGTCAAACAAGTGCAGCAACATCGAAAAGAATTAATACTGCA
SARS-CoV-2 NC_045512.2 (20574) CAAAGTGACTATTGACTATACAGAAATTTCATT---------TATGCTTTGGTGTA--AAGATGGCCATGT-----A

Section 1264

(97252) 97252 97262 97273 97284 97295 97306 97317 97328
Autographa californica nucleopolyhedrovirus NC 0... (97055) TTGTGTGGATTTTCTTACAATGAAAGCAAATGACAATTTTATGGTGTTCAAAAATTATATTAACATGATTATAAAAG
SARS-CoV-2 NC_045512.2 (20635) ----GAAACAT--TTTACCCAAAATTACAAT--CTAGTCA----AGCGTGGCAACCGGGT----GTTGCT----ATG

Section 1265

(97329) 97329 97339 97350 97361 97372 97383 97394 97405
Autographa californica nucleopolyhedrovirus NC 0... (97132) TGTATTTGCAATTTTACAATTACAGATTTCCCATCAATTTTGAGGACAACACGATGAAACCTTGTGTAAATTTAACT
SARS-CoV-2 NC_045512.2 (20692) CCTA--AT---CTTTACAAAATGC-------AAAGAATGCTATTAGAAAAGTGTGACCTTC-----------AAAAT

Section 1266

(97406) 97406 97416 97427 97438 97449 97460 97471 97482
Autographa californica nucleopolyhedrovirus NC 0... (97209) TTTAGA-CGTGGCGGCAGTTGGAAAACTCAACTGCAACCCGTATGCAATTATGTTTACAAAAGTAAAAATATGCCAA
SARS-CoV-2 NC_045512.2 (20746) TATGGTGATAGTGCAACA-----TTACCTAAAGGC---ATAATGATGAAT----GTCGC--AA-AATATACTCAACT

Section 1267

(97483) 97483 97493 97504 97515 97526 97537 97548 97559
Autographa californica nucleopolyhedrovirus NC 0... (97285) AATTTATTAAATAAAACAAATTAATTTAAACAAGCGTTTTTATTGACAATACTCACATTTGATATTATTTATAATCA
SARS-CoV-2 NC_045512.2 (20808) GTGTCAATATTT---AAACACATTAACATTAGCTGTACCCTATAATATGAGAGTTATACATTTTGGTGCTG-GTTCT

```
                                                                                                              Section 1268
                                                 (97560) 97560     97570      97581      97592      97603      97614      97625  97636
Autographa californica nucleopolyhedrovirus NC 0... (97362) AGAAATGATGTCATTTGTTTTCAAAATTGAACTGGCTTTACGAGTAGAATTTTACTTGTAAAACACAATCAAGAAAT
                            SARS-CoV-2 NC_045512.2 (20881) GATAAAGGAGTTGCACCAGGT-ACAGCTG---T-TTTAAGACAGTGGTTGCCTACG--GGTACG--CTGCTTGTCGA
                                                                                                              Section 1269
                                                 (97637) 97637     97647      97658      97669      97680      97691      97702  97713
Autographa californica nucleopolyhedrovirus NC 0... (97439) GATGTCATTTTTGTACGTGATTATAAACATGTTTAAACATGGTACATTGAACTTAATTTTTGCAAGTTGATAAACA-
                            SARS-CoV-2 NC_045512.2 (20949) TTCAGATCTTAATGACTT------TGTCTCTGATGCAGATTCAACTTTGATTGGTGATTGTGCAACTGTACATACAG
                                                                                                              Section 1270
                                                 (97714) 97714     97724      97735      97746      97757      97768      97779  97790
Autographa californica nucleopolyhedrovirus NC 0... (97515) TGATTAATGTACGACTCATTTGTTTGTGCAAGTTGATAAACGTGATTAATATATGACTCATATGTTTGTGCAAAAAT
                            SARS-CoV-2 NC_045512.2 (21020) CTAATAAATGGGATCTCATTATTAGTGATATGTACGACCCTAAGACTAAAAATG-TTACAAAAGAAAATGACTCTA-
                                                                                                              Section 1271
                                                 (97791) 97791     97801      97812      97823      97834      97845      97856  97867
Autographa californica nucleopolyhedrovirus NC 0... (97592) GATGTCATCGTACAAACTCGCTTTACGAGTAGAATTCTACTTGTAACGCATGATCAAGGGATGATGTCATTTGTTTT
                            SARS-CoV-2 NC_045512.2 (21095) -AAGAGGGTTTTTTCACTTACATTTGTGGGTTTATACAACAAAAGCTAGCTCTTGGAGGT-TCCGTGGCTATAAAGA
                                                                                                              Section 1272
                                                 (97868) 97868     97878      97889      97900      97911      97922      97933  97944
Autographa californica nucleopolyhedrovirus NC 0... (97669) TTTAAAATTCAACTCGCTTTACGAGTAGAATTCTACTTGTAAAACACAATCGAGGGATGATGTCATTTGTAGAATGA
                            SARS-CoV-2 NC_045512.2 (21170) TAACAGAACATTCTTG-GAATGCTGAT-CTTTATAAGCTCATGGGACACTTCGCAT-----GGTGGACAGCCTTTGT
                                                                                                              Section 1273
                                                 (97945) 97945     97955      97966      97977      97988      97999      98010  98021
Autographa californica nucleopolyhedrovirus NC 0... (97746) TGTCATTTGTTTTTCAAAACCGAACTCGCTTTACGAGTAGAATTCTACTTGTAACGCAAGATCGGTGGATGATGTCA
                            SARS-CoV-2 NC_045512.2 (21240) TACTAATGTGAA---------TGCGTCATCATCTGAAGCATTTTTA-ATTGG----------ATGTAATTATCTTGG
                                                                                                              Section 1274
                                                 (98022) 98022     98032      98043      98054      98065      98076      98087  98098
Autographa californica nucleopolyhedrovirus NC 0... (97823) TTTTAAAAATGATGTCATCGTACAAACTCGCTTTACGAGTAGAATTCTACGTGTAAAACACGATTACAGCACTTCGT
                            SARS-CoV-2 NC_045512.2 (21297) CAAACCACGCGAACAAATAGATGGTT---ATGT----CATGCATGC----------AAATTACATAT-----TTTGG
```

Section 1275

(98099) 98099 98109 98120 98131 98142 98153 98164 98175
Autographa californica nucleopolyhedrovirus NC_0... (97900) AGTTGTATCGAAAATTGTTCAATGGCTCTTTGTTAATGTCGTAATTGATTAATATGTCGTACAATTTGGCGGCGTTG
SARS-CoV-2 NC_045512.2 (21352) AGGAA------TACAAATCCAATTCAGTTG-TCTTCCT---ATTCT-TTATTTGACATGAGTAAATTTCCCCTTAAA

Section 1276

(98176) 98176 98186 98197 98208 98219 98230 98241 98252
Autographa californica nucleopolyhedrovirus NC_0... (97977) TGTTTGCACACGACCGTTTTTAGTTCTTGAAACATTTTTTCGTGTATGTTTAGCATGTTGTATTTCAGAGTGCGATG
SARS-CoV-2 NC_045512.2 (21418) TTAAGGG---GTACTGCTGTTATGTCTTTAAA--AGAAGGTCAAATCAATGATATG--------------ATTTTAT

Section 1277

(98253) 98253 98263 98274 98285 98296 98307 98318 98329
Autographa californica nucleopolyhedrovirus NC_0... (98054) TGTAATGCTGGTGACGAGCATCAAAATGATAAAATCTAAAGCGGCTAATTTGTAATCCCGTTCATACGCTCTGTAAT
SARS-CoV-2 NC_045512.2 (21476) CTCTTCTTAGTAAAGGTAGA-----CTTATAATTAGAGA-----AAACAACAGAGTTGTTATTTCTAGTGATGTTCT

Section 1278

(98330) 98330 98340 98351 98362 98373 98384 98395 98406
Autographa californica nucleopolyhedrovirus NC_0... (98131) CGCCAACAACTCTGTGGCCAGATCTTTTTAGATTTTGACAGGCGTTATGGTACGAATTGATAATATTTACTATAGTT
SARS-CoV-2 NC_045512.2 (21543) TGTTAACAACT---AAACGAACAATGT------------T---TGTTTTTCTT-------------GTTTTATTGC-

Section 1279

(98407) 98407 98417 98428 98439 98450 98461 98472 98483
Autographa californica nucleopolyhedrovirus NC_0... (98208) TCTCTTGTTATCGGTTTGTCGATTAAACTGTTAACAAACATCACGTTGCCCAAGCGCGACGGTTTAGACACCGACTT
SARS-CoV-2 NC_045512.2 (21588) -CACTAGTCTCTAGTCAGTGT---GTTAATCTTACA-----ACCAGAACTCAATTACCCC-----CTGCATACAC--

Section 1280

(98484) 98484 98494 98505 98516 98527 98538 98549 98560
Autographa californica nucleopolyhedrovirus NC_0... (98285) GTTTTTTGTCTGTTCAAATTTGTACAAATTAAAAACGCTCATAGACTGGTCGTCAGGCAGTGTGTCGTTATACAAAC
SARS-CoV-2 NC_045512.2 (21649) ------TAATTCTTT-----CACACGT------------GGTGTTTATTACCCTGACAAAGTTTTCAG-ATCCT---

Section 1281

(98561) 98561 98571 98582 98593 98604 98615 98626 98637
Autographa californica nucleopolyhedrovirus NC_0... (98362) AAAATGGTAAAACGTTTAATTCGACAAACGACGAGCACATTAAAGTTTGTTGGCTGTTAACGTCCTGGGGATGTAAA
SARS-CoV-2 NC_045512.2 (21699) CAGTTTTACATTC-------------AACTCA----GGACTTGTTCT------TA----------CCTTTCTTTTCCAA

Section 1282

		98638 98648 98659 98670 98681 98692 98703 98714
	(98638)	
Autographa californica nucleopolyhedrovirus_NC_0...	(98439)	CTGTTATTCATAACGTAACACACTTCAATGTCGGAATGCTTGTTTTCAAATTTGTCCTTGTCTACAGTTTCAATGGT
SARS-CoV-2 NC_045512.2	(21745)	TGTTACTTGGTTCCATGCTATACATG-TC-TCTGGGACCAATGGTACTAAGAG------GTTT--------GATAAC

Section 1283

		98715 98725 98736 98747 98758 98769 98780 98791
	(98715)	
Autographa californica nucleopolyhedrovirus_NC_0...	(98516)	GATTGAGCGAGGTTTGAGTTTATTTTCTAAATTCATTTGGATATTTTCAATATGGTATACCACCGACACGTTGTGAG
SARS-CoV-2 NC_045512.2	(21806)	CC-TGTCCTACCATTTAATGAT------------------GGTGTTTATTTTGC-TTCCACTGAG-----AAGTCTA

Section 1284

		98792 98802 98813 98824 98835 98846 98857 98868
	(98792)	
Autographa californica nucleopolyhedrovirus_NC_0...	(98593)	CCAGCGATCCTTGATTGGTTTTAATCATATTCAAAATATTCATGATATGGTTGAAAAAAGAGTCTGTCAAAACGTTT
SARS-CoV-2 NC_045512.2	(21858)	ACATAAT-AAGAGGCTGGATTTTTGGTACTACTT-------------------------TAGATTCGAAGACC-CAG

Section 1285

		98869 98879 98890 98901 98912 98923 98934 98945
	(98869)	
Autographa californica nucleopolyhedrovirus_NC_0...	(98670)	GTGTCGTTGTTAAATATCGCTTTCCAGGGTTTACTGTTGCGTGACTCAACGACGGCCGTGTAACATAACAAGCGCGC
SARS-CoV-2 NC_045512.2	(21908)	T---CCCTACTTATTGTTAATAA----CGCT-ACTAATGTTGTTATTAAAGTCTGTGAATTTCAATTTT-GTAATGA

Section 1286

		98946 98956 98967 98978 98989 99000 99011 99022
	(98946)	
Autographa californica nucleopolyhedrovirus_NC_0...	(98747)	CAGTTGCATGTGCGACAACTTAATGTTATCAATGTCGGTGATGTTTGGCACCAGATTTTCATTGCCGTCTTCCAGTA
SARS-CoV-2 NC_045512.2	(21976)	TCCAT------------TTTTG--G--------GTGTTTATTACCACAAAAACAACAA---AAGTTGGA----TGGA

Section 1287

		99023 99033 99044 99055 99066 99077 99088 99099
	(99023)	
Autographa californica nucleopolyhedrovirus_NC_0...	(98824)	GCGTGCTCAGTTCGGTCGAGTAGTTATTCAACGATCGATTGTGCGATTCAAACAAGTTTACTATCGCAGGTTGTACA
SARS-CoV-2 NC_045512.2	(22024)	AAGTGAGTTCAGAGTTTAT-TCT-AGTGCG--AATAATT---GCACTTT--TGAATATGTCTCTCA--GCCTTTTC-

Section 1288

		99100 99110 99121 99132 99143 99154 99165 99176
	(99100)	
Autographa californica nucleopolyhedrovirus_NC_0...	(98901)	TAGTTTTTTATGTCGTCAAATTGAATTATATCGATCTTGTCCTTGTTCTCCAGCATAAACGACAAATTTTTTAGGTC
SARS-CoV-2 NC_045512.2	(22089)	TTATGGACCTTGAAGGAAAACAGGGTAATTT--C---AAAAATCTTAGGGAATTT--GTGTT-TAAGAATATTGATG

Section 1289

```
                                              99177     99187      99198      99209      99220      99231      99242     99253
                                     (99177)
Autographa californica nucleopolyhedrovirus NC 0... (98978) GAAT-TTAATATTTGGCGCGTTTTCGTTGGACTTTTTGTAATTTAACAACATCGCCAACAGTTTGTGTAACTCGCCG
                     SARS-CoV-2 NC_045512.2 (22158) GTTATTTTAAAATATATTCTAAGCACACGCC---TATTAA--TT-TAGTGCGTGATC---TCCCTCAGGGTTTTTCG
```

Section 1290

```
                                              99254     99264      99275      99286      99297      99308      99319     99330
                                     (99254)
Autographa californica nucleopolyhedrovirus NC 0... (99054) TTAGCTTGATCTTTGCTAAACAGTTTATTGGTAGCGTAATTCACGTTGTCGTTCAAAAACAGCAACTCGTTGATGAT
                     SARS-CoV-2 NC_045512.2 (22226) GCTTTAGAACCATTGGTAGATTT-GCC---AATAGGTATTAACATCACTAGGTTTCAAACTTTACTTG-CTTTACAT
```

Section 1291

```
                                              99331     99341      99352      99363      99374      99385      99396     99407
                                     (99331)
Autographa californica nucleopolyhedrovirus NC 0... (99131) CATTTTTTGTAAAAGCGCGTACTTGCTCATGTTGACAGAATCTCTTACATTTCAGTTGTAAACGCGTCTGTACAAAT
                     SARS-CoV-2 NC_045512.2 (22298) --AGAAGTT--ATTTGACTCCTGGTGATTCTTCTTCAGGTTGGACAGC-TGG-TGCTGCA--GCTTATTATGT-GGG
```

Section 1292

```
                                              99408     99418      99429      99440      99451      99462      99473     99484
                                     (99408)
Autographa californica nucleopolyhedrovirus NC 0... (99208) TGGCCATGCGATTCGGAATGCACACGGGGATCGTGCGAGCCAGTGCCGTTTGGCGAAATAGCATTTTTTCATAGCCG
                     SARS-CoV-2 NC_045512.2 (22366) TTATCTTCAACCTAGGACTTTTCTATTAAAATAT---A------------ATGAAAATGGAACCATTACAGATGCT-G
```

Section 1293

```
                                              99485     99495      99506      99517      99528      99539      99550     99561
                                     (99485)
Autographa californica nucleopolyhedrovirus NC 0... (99285) CTCGAACAATCGCACGCGTCCGGCGAAAATTGCACCGTGTTCAAATTCATATTCAACCGGCCGTCGTTGCATAGATA
                     SARS-CoV-2 NC_045512.2 (22428) T-AGACTGTGC--------------ACTTGACCCTCTCT--CAGAAACAAAGTGTACGTTGAAATCCTTCACTGTAG
```

Section 1294

```
                                              99562     99572      99583      99594      99605      99616      99627     99638
                                     (99562)
Autographa californica nucleopolyhedrovirus NC 0... (99362) AGGCCTCGGTGTTCCCGTATCGTCCACCAAGTCTCTGTACGTGCTCACGCATGTTTGAGACACGACAAAATCTCCGC
                     SARS-CoV-2 NC_045512.2 (22488) AAA---AAGGA---ATCTATCAAACT-------T-----CTAACTTTAGAG---TCCAACCAA--CAGAATCTATTG
```

Section 1295

```
                                              99639     99649      99660      99671      99682      99693      99704     99715
                                     (99639)
Autographa californica nucleopolyhedrovirus NC 0... (99439) CGGCGGAGAAAACGTGAACCAAGCCCAGTGCGGGATCGCATTCTATCAAGTCCGGAGCCTGCGCGTTTACCAAAGCG
                     SARS-CoV-2 NC_045512.2 (22542) TTA----------GATTTCCTAAT--ATTACAAACT--TGTGCCTTTTGG-----------TGAAGTTTTTAA--CG
```

Section 1296

(99716) 99716 99726 99737 99748 99759 99770 99781 99792
Autographa californica nucleopolyhedrovirus NC_0... (99516) TCGGAGGCGTTGCAAAAGCCATCCTGGCAGGTCAACTCGTTTGCAGCGCTGGAGATCACGCAGTTGTCTCTACACTG
SARS-CoV-2 NC_045512.2 (22593) CCACCAGATTTGCAT-CTGTT--TATGCTTGGAACAGGAAGAGAATCAGCAACTGTGTTGCTGAT-TATTCTGTCCT

Section 1297

(99793) 99793 99803 99814 99825 99836 99847 99858 99869
Autographa californica nucleopolyhedrovirus NC_0... (99593) CTGATCCGTCACGCACGGTAACCGGTTCAATGAACAATCTACGCCTCGATTGCGCTGAAACGTAAAATTTAACGGCG
SARS-CoV-2 NC_045512.2 (22666) ATATAATTC--CGCATCATTTTCCACTTTTA--AGT-GTTATGGA--GTGTCT-CCTACTAAATTAAAT-------G

Section 1298

(99870) 99870 99880 99891 99902 99913 99924 99935 99946
Autographa californica nucleopolyhedrovirus NC_0... (99670) GCGCTTCCAACTCGTTAATGTGCATGTATGCATCTTGCAAAATAAATTTTTGAACAAATTTAAACGTGTACATGTAC
SARS-CoV-2 NC_045512.2 (22728) AT------CTCTGCTTTACTAATGTC-TATGCAGATTCATTTGTAATTAGAGGTGAT--GAAGTCAGACAAATCGCT

Section 1299

(99947) 99947 99957 99968 99979 99990 100001 100012 100023
Autographa californica nucleopolyhedrovirus NC_0... (99747) ACGATTAGTATAATTACCAGTAGAATAAGTATTTGCCAAAAGTTCAACATGATCGTCTTAACTGAGTGTGAAAAGCG
SARS-CoV-2 NC_045512.2 (22796) CCAGGGCAAACTGG-AAAGATTGCTGATTATAATTATAAA---TTACCAGATGATTTTA---CAGGCTGCG------

Section 1300

(100024) 100024 100034 100045 100056 100067 100078 100089 100100
Autographa californica nucleopolyhedrovirus NC_0... (99824) TGGTGTGACGCACGAAATGACTGGTTGCGCAAAAAATAAACCGGGGTCTATATAACTCGGCGTCGACCGCGTTCATT
SARS-CoV-2 NC_045512.2 (22860) --TTATA--GCTTGGAATTCTAACAATCTTG---------------ATTCTAAGGTTGGTGGTAATTATA------A

Section 1301

(100101) 100101 100111 100122 100133 100144 100155 100166 100177
Autographa californica nucleopolyhedrovirus NC_0... (99901) TTTACCGTCATGCATCTGACGGCTAATGTATTGCTCGTTCCTAACGCGCTCAAAAAGCGGGACGTGAAATACATTT-
SARS-CoV-2 NC_045512.2 (22912) TTACCTGTATA--GATTGT-----TT-AGGAAGTCTAATCTCAAACCTTTTGAGAGAGAT--------ATTTCAACT

Section 1302

(100178) 100178 100188 100199 100210 100221 100232 100243 100254
Autographa californica nucleopolyhedrovirus NC_0... (99977) -ATAATACCTATTTGAAAAATTACAGTGTAATTGAAGGTGTGATGTGTTGCAATGGCGATTGTTTGGCCGTGGTGGT
SARS-CoV-2 NC_045512.2 (22973) GAAATCTATCAGGCCGGTAGCACACCTTGTA--AT-G--GTGTTGAAGGTTTAATTGTTACTTTCCTTTACAATCA

Section 1303

```
                                                    (100255) 100255    100265     100276     100287     100298     100309     100320  100331
Autographa californica nucleopolyhedrovirus  NC_0... (100053) GTTGGACCGAAATCAGCTGCAAAACACGGACATGG--AAGTGTTGGAGAGTTTAGAATACACTAGTGACAACATTGA
                              SARS-CoV-2 NC_045512.2  (23045) TATGGTTT---CCAACCCACTAATGGTGTTGGTTACCAACCATACAGAGTAGTAGTACTT-TCTTT-TGAACTTCTA
```

Section 1304

```
                                                    (100332) 100332    100342     100353     100364     100375     100386     100397  100408
Autographa californica nucleopolyhedrovirus  NC_0... (100128) ACTGTTATGCGAAAAAATATGTGTGATAGTTGATAATTACGACAAGTATTACCAAAAAAATTGTGTATAAATAAAAT
                              SARS-CoV-2 NC_045512.2  (23117) CATGCACCA--GCAACTGTTTGTGGACCTAA-AAAGTC--TACTAATTTGG------TTAAAAACA------AATGT
```

Section 1305

```
                                                    (100409) 100409    100419     100430     100441     100452     100463     100474  100485
Autographa californica nucleopolyhedrovirus  NC_0... (100205) ACCAAATTTTATTATATCATTTTGTTTTATTTAATAATTAAAGAATACAACGCCACATCTATTCCTAGTACAACAAA
                              SARS-CoV-2 NC_045512.2  (23177) G-TCAATTTCAACTTCAATGG--TTTAACAGGCACAGGT-GTTCTTACTGAG-TCTAACAAAAGTT--TCTGC---
```

Section 1306

```
                                                    (100486) 100486    100496     100507     100518     100529     100540     100551  100562
Autographa californica nucleopolyhedrovirus  NC_0... (100282) TAATTTGATTATTATTTTTGAGTGCACATTAAAAAATAACAAACAGTGTAAAAATACTACAGAATAATACAATACAT
                              SARS-CoV-2 NC_045512.2  (23244) CTTTC--CAACAATTTG-GCAGAGA-CATTGCTGACACTACTGATGCTGTCCGTGA--TCCACAGACACTTGAGATT
```

Section 1307

```
                                                    (100563) 100563    100573     100584     100595     100606     100617     100628  100639
Autographa californica nucleopolyhedrovirus  NC_0... (100359) AAATATTATAGTAAATAGCTGCAATTTTGATAGCGTAATTTATACTTTGATATTTTTCAACGTACAACGTTAAATGT
                              SARS-CoV-2 NC_045512.2  (23315) CTTGACATTAC-----ACCATGTTCTTTTGGTGGTGTCAGTGTTATAACACCAGGAAC---AAATACTTCTA--AC-
```

Section 1308

```
                                                    (100640) 100640    100650     100661     100672     100683     100694     100705  100716
Autographa californica nucleopolyhedrovirus  NC_0... (100436) TGATACGCATTATTCACAAATAACAAAATTTTTCTAATATGCCATTTGTCCGCAATTGTTTTTGCGATATCAAAGCC
                              SARS-CoV-2 NC_045512.2  (23381) -CAG--GTTGC-TGTTC--TTT-----AT----C-AGGATGTTAACTGCAC------------AGAAGTCCCTGTT-
```

Section 1309

```
                                                    (100717) 100717    100727     100738     100749     100760     100771     100782  100793
Autographa californica nucleopolyhedrovirus  NC_0... (100513) TTTTTCAAACAATTGAAAAATTGCAAACAAAACC--ACGTACATGACGTTATACATAGTGTTAAAGTTTTTACATAA
                              SARS-CoV-2 NC_045512.2  (23429) ------------GCTATTCATGCAGATCAACTTACTCCTACTTGG--CGTGTTTAT---TCTACAGGTTCTAATGTT
```

Section 1310

		100794 100804 100815 100826 100837 100848 100859 100870
	(100794)	
Autographa californica nucleopolyhedrovirus NC 0...	(100588)	CAATTCTATAATGAAGAAAATTGCTAAACACGGCATGAGCGCGCACATAATCGCGTTGGCCGCAAATATCTCGTACG
SARS-CoV-2 NC_045512.2	(23489)	TTTCAA------ACACGTGCAGGCTGTT--TAA------TAGGGGCTGAACA-TGTCAAC--------AACTCATATG

Section 1311

		100871 100881 100892 100903 100914 100925 100936 100947
	(100871)	
Autographa californica nucleopolyhedrovirus NC 0...	(100665)	TACAAAAATACTCGGACATTCTCCAATAAGTAAAATGCATTTTGCTATTATACTGTTGTTTCTTCTAGTGATTATTG
SARS-CoV-2 NC_045512.2	(23544)	-A-GTGTGACATACCCATTGGTG---CAGGTATATGCGCT-------A---GTTATCAGACTCAGACTAATTCTCCTC

Section 1312

		100948 100958 100969 100980 100991 101002 101013 101024
	(100948)	
Autographa californica nucleopolyhedrovirus NC 0...	(100742)	CAATAGTGTACACGTATGTAGACTTGATAGATGTGCACCATGAAGAGGTGCGTTATCCTATTACGGTTTTTGACAAC
SARS-CoV-2 NC_045512.2	(23607)	GGCGGGCACGTAG-----TGTAGCTAGTCAATCCATC-----ATTGCCTACACTATGTCACT--TGGTGCAG-----

Section 1313

		101025 101035 101046 101057 101068 101079 101090 101101
	(101025)	
Autographa californica nucleopolyhedrovirus NC 0...	(100819)	ACACGCGCGCCGCTTATTGAACCGCCGTCCGAAATAGTAATCGAAGGCAATGCACACGAATGTCACAAAACTTTGAC
SARS-CoV-2 NC_045512.2	(23667)	AAAATTCAGTTGCTTACTCTAATAACTCTATTGCCATACCC-----ACAAATTT-TACTATTAG----TGTTACCAC

Section 1314

		101102 101112 101123 101134 101145 101156 101167 101178
	(101102)	
Autographa californica nucleopolyhedrovirus NC 0...	(100896)	GCCGTGCTTCACACACGGCGATTGCGATCTGTGCCGCGAAGGATTAGCCAACTGCCAGTTGTTTGACGAAGATACAA
SARS-CoV-2 NC_045512.2	(23734)	AGAAATTCT-ACCAGTGTCTATGACC-AAGACATCAGTAGA-TTGTACAATGTACATTTGTGGTGATTCAACT---G

Section 1315

		101179 101189 101200 101211 101222 101233 101244 101255
	(101179)	
Autographa californica nucleopolyhedrovirus NC 0...	(100973)	TAGTCAAGATGCGTGGAGATGACGGCCAAGAACACGAGACGCTTATT---CGAGCGGGAGAAGCGTACTGCTTGGCT
SARS-CoV-2 NC_045512.2	(23805)	AATGCAGCAATCTTT-TG-TTGC---AAT------ATGGCAGTTTTTGTAC--ACAATTAAACCGTGCTTTA---AC

Section 1316

		101256 101266 101277 101288 101299 101310 101321 101332
	(101256)	
Autographa californica nucleopolyhedrovirus NC 0...	(101047)	TTGGATCGAGAACGCGCCCGATCGTGTAACCCCAACACGGGTGTGTGGTTGTTGGCCGAAACTGAAACTGGTTTCGC
SARS-CoV-2 NC_045512.2	(23866)	TGGAATAG-CTGTTGAACAAG--ACAAAAACACCCAAGAAGTTT--TTGCACAAGTCAAA---CAAATTTACAAAAC

Section 1317

```
                                             (101333) 101333    101343     101354     101365     101376     101387     101398 101409
Autographa californica nucleopolyhedrovirus NC_0... (101124) TC--TTTTGTGCAACTGCTTACGGCCCGGACTTGTTACGCAGCTCAACATGTACGAAGACTGCAACGTGCCCGTGGG
                          SARS-CoV-2 NC_045512.2 (23935) ACCACCAATTAAAGATTTTGGTGGTTTTAATTTTT----CACAAATATTACC----AGATCCATCA--AAACCAAGC
```

Section 1318

```
                                             (101410) 101410    101420     101431     101442     101453     101464     101475 101486
Autographa californica nucleopolyhedrovirus NC_0... (101199) CTGCGCGCCTCACGGCCGTATCGACAATATCAACAGCGCTTCGATCCGGTGCGTGTGCGACGACGGGTACGTGAGCG
                          SARS-CoV-2 NC_045512.2 (24002) AAGAGGTCAT-------TTATTGAAGATCTACTTTTCAACAAAGTGACACTTGCA---------G--ATGCTGGCTT
```

Section 1319

```
                                             (101487) 101487    101497     101508     101519     101530     101541     101552 101563
Autographa californica nucleopolyhedrovirus NC_0... (101276) ACTATAACGCCGACACCGAAACTCCGTATTGCCGTCCGCGCACCGTGCGCGACGTAATGTACGACGAGAGTTTTTTT
                          SARS-CoV-2 NC_045512.2 (24061) CAT--CAAACAATATGGTG--------ATTGCCTTGGTGATATTGCTGCTAGAGACCTCAT--TTGTGCACAAAAGT
```

Section 1320

```
                                             (101564) 101564    101574     101585     101596     101607     101618     101629 101640
Autographa californica nucleopolyhedrovirus NC_0... (101353) CCGCGGGCGCCATGCGCAGACGGCCAAGTTCGTCTGGATCATCCGGCGCTCAATGATTTTTACCGCAGACACTTTAG
                          SARS-CoV-2 NC_045512.2 (24126) TTAACGGCCTTA----CTGTTTTGCCACCTTTGCTCACAGA-TGAAATGATT--G-------CTCAATACACT----
```

Section 1321

```
                                             (101641) 101641    101651     101662     101673     101684     101695     101706 101717
Autographa californica nucleopolyhedrovirus NC_0... (101430) ACTCGAAGACATTTGCGTGATCGACCCTTGCTCGGTGGACCCGATTAGCGGGCAACGCACATCGGGACGCTTATTTC
                          SARS-CoV-2 NC_045512.2 (24185) ---T-CTGCACTGTT---AGC-GGGTACAATCACTTCTGGTTGGACCTTTG--GTGCAG----GTGCTGCATTA--C
```

Section 1322

```
                                             (101718) 101718    101728     101739     101750     101761     101772     101783 101794
Autographa californica nucleopolyhedrovirus NC_0... (101507) ACCAACCAACCGTAAATGGTGTGGGAATCAACGGATGCAATTGTCCGGCCGATGACGGGTTACTGCCCGTGTTTAAT
                          SARS-CoV-2 NC_045512.2 (24246) AAATACCATTTGCTATGCAAATGGCTTATAGGTTTAATGGTATTGGAGTTACACAGAATGTTCTCTATGAGAACCAA
```

Section 1323

```
                                             (101795) 101795    101805     101816     101827     101838     101849     101860 101871
Autographa californica nucleopolyhedrovirus NC_0... (101584) CGACACACCGCCGACACGGGCATGGTTAGACAAAGCGACCGCACCGTCGCGAACGCTTGCTTGCAGCCGTTTAACGT
                          SARS-CoV-2 NC_045512.2 (24323) AAATTGATTGCCAACCAATTTAATAGTGCTATTGGCAAAATTCAAGACTCACTTTC--TTCCACAGCAAGTGCACTT
```

```
                                                                                                              Section 1324
                                                    (101872) 101872    101882     101893     101904     101915     101926     101937  101948
Autographa californica nucleopolyhedrovirus_NC_0... (101661) GCACATGTTATCGTTGCGTCATGTGGATTACAAATTTTTCTGGGGCCGCAGCGACCACACCGAGTTTGCCGACGCGG
                             SARS-CoV-2 NC_045512.2  (24398) GGAAAACTTCAAGATGT--GGTCAACCAAAATGCACAAGCTTTAAACACGCTTGTTAAACAACTTAG---CTCCAAT
                                                                                                              Section 1325
                                                    (101949) 101949    101959     101970     101981     101992     102003     102014  102025
Autographa californica nucleopolyhedrovirus_NC_0... (101738) ACATGGTGTTTCAAGCGAATGTCAACCAACTCAGTCACGAACGGTATCGAGCGATTTTGTACTCGTTGCTCGAGTCG
                             SARS-CoV-2 NC_045512.2  (24470) TTTGGTGCAATTTCA---AGTGTTTTAAATGATATCCTTTCACGTCTTGACAAAGTTGAGGCTGAAGTGCAAATTGA
                                                                                                              Section 1326
                                                    (102026) 102026    102036     102047     102058     102069     102080     102091  102102
Autographa californica nucleopolyhedrovirus_NC_0... (101815) CACCCGGACGTAACAGAAATCGTAACAGTCAACATGGGTGTCATGAAAATTTCCGTGTCATACGATACCACATTGAA
                             SARS-CoV-2 NC_045512.2  (24544) TAGGTTG-ATCACAGGCAGACTTCAAAGTTTGCAGACATATGTGACTCAACAA-----TTAATTAGAGCTGCAGAAA
                                                                                                              Section 1327
                                                    (102103) 102103    102113     102124     102135     102146     102157     102168  102179
Autographa californica nucleopolyhedrovirus_NC_0... (101892) AAATATACTATTACCATCTTCTGTTTTTAGGCTATTTAGATTTAAAGAAAGTGGCACTGCTCAGCCGGTATGCTTCT
                             SARS-CoV-2 NC_045512.2  (24615) TCA------GAGCTTCT-GCTAATCTTGCTGCTACTAAAATGTCAGAGTGTGTACTTGGACAATCAAAAAGAG----
                                                                                                              Section 1328
                                                    (102180) 102180    102190     102201     102212     102223     102234     102245  102256
Autographa californica nucleopolyhedrovirus_NC_0... (101969) TTCCAGGCGTAGGACGGTGCATAACCGTCAATTCCGATTCGTGCATCAGGCGACACGCTGGTGGTCAAGTGTGGACC
                             SARS-CoV-2 NC_045512.2  (24681) -----TTGATTTTTGTGGAAAGG-GCTATCATCTTATGTCCT------TCCCTCAGTCAGCACCTCA--TGGTGTAG
                                                                                                              Section 1329
                                                    (102257) 102257    102267     102278     102289     102300     102311     102322  102333
Autographa californica nucleopolyhedrovirus_NC_0... (102046) GCAGAAACGTTCACCAACTCGTGGTGTGTACTGAGTCGTGAAGGTACGCATATAAAAGTTTGGAGTCGCGCGTCACG
                             SARS-CoV-2 NC_045512.2  (24744) TCTTCTTGCATGT-GACTTATGTCCCTGCACAAGAA--AAGAACTTCACAACT----GCTCCTGCCATT-TGTCATG
                                                                                                              Section 1330
                                                    (102334) 102334    102344     102355     102366     102377     102388     102399  102410
Autographa californica nucleopolyhedrovirus_NC_0... (102123) ATATCCACGCGGAGACGCGCCTGCAGCGTTAAGATTGCGCGGCTTCTTTCTGAACAACGATCGCGAACGAAACACAA
                             SARS-CoV-2 NC_045512.2  (24813) ATGGAAAAGCACACTTTCCTCGTGAAGGTGTCTTTGTTTCAAATGGCACA------C-ACTG--GTTTGTAACACAA
```

Section 1331

	(102411)	102411 102421 102432 102443 102454 102465 102476 102487
Autographa californica nucleopolyhedrovirus_NC_0...	(102200)	TAAGAGCGGTCACTACAGGCGACATGACCCAAGGGCAACAAATAGACGCATTAACCCAAATACTTGAAACTTACCCC
SARS-CoV-2 NC_045512.2	(24881)	-AGGAATTTTTATGAACCACAAATCATTACTACAGACAACACATTTGTGTCTGGTA-----ACTGTGATGTTGTAA-

Section 1332

	(102488)	102488 102498 102509 102520 102531 102542 102553 102564
Autographa californica nucleopolyhedrovirus_NC_0...	(102277)	AACTACTCTGTATAACAACATGAGCATTTTAAAAGTTGTAGAAGCGTGCAATTTGGCACACACTTTTTTAAAATTGG
SARS-CoV-2 NC_045512.2	(24951)	-----TAGGAATTGTCAACAACAC--AGTTTATGATCC---TTTGCAACCTGAATTAGAC---------TCATTCAA

Section 1333

	(102565)	102565 102575 102586 102597 102608 102619 102630 102641
Autographa californica nucleopolyhedrovirus_NC_0...	(102354)	GTTATTTATTTAGGGCCAAGACTTGTTTGGATATCGCTTTAGATAATTTGGAACTATTGCGTCGAAAGACTAACATA
SARS-CoV-2 NC_045512.2	(25009)	GGAGG---AGTTAGATAAATA-----TTTTAAGAATCAT-ACATCACCAGA-TGTTGATTTAGGTGACATCTCTGGC

Section 1334

	(102642)	102642 102652 102663 102674 102685 102696 102707 102718
Autographa californica nucleopolyhedrovirus_NC_0...	(102431)	AAAGAAGTGGCAGTCATGTTAAACAAGAAAACTACAGAGTGTTTGCAATTGAAACGAAAAATAGATAAAAAAATTGC
SARS-CoV-2 NC_045512.2	(25076)	ATTAATGCTTCAGTTGTAAACATTCAAAAAGAAATTG-ACCGCCTCAATGAGGTTGCCAA---GAAT--TTAAATGA

Section 1335

	(102719)	102719 102729 102740 102751 102762 102773 102784 102795
Autographa californica nucleopolyhedrovirus_NC_0...	(102508)	ACAACGTGTTTTAATAAAAATTTACACTATCAAATGATGACATCATAACGGGTTCAATATTCTGTGTGCAAAAATAA
SARS-CoV-2 NC_045512.2	(25147)	ATCTCTCATC-GATCTCCAAGAACTTGGAAAGTATGAGCAGTATATAAAA-TGG--CCATGGTACATTTGGCTAGGT

Section 1336

	(102796)	102796 102806 102817 102828 102839 102850 102861 102872
Autographa californica nucleopolyhedrovirus_NC_0...	(102585)	ATGACATCATATTTCAAACTTGTTTTACGCGTAAAATTCTACTGGTAAAACAAGTTTGAGATATGATGTCATCATCA
SARS-CoV-2 NC_045512.2	(25220)	TTTATAGCTGGCTT--------GATTGCCATAGTAAT-GGTGACAATTATGCTTT-------GCTGTATGACCAGTT

Section 1337

	(102873)	102873 102883 102894 102905 102916 102927 102938 102949
Autographa californica nucleopolyhedrovirus_NC_0...	(102662)	CAAATAATAGTATGTAATAAAATAAACATATTTGTGTGTAAATATAATTTATTACAAATAAATTTTACATTGAATCA
SARS-CoV-2 NC_045512.2	(25281)	GCTGTAG------TTGTCTCAAGGGCTGTTGTTCT-TGTGGAT-CCT---GCTGCAAATTTGATGAAGA------CG

```
                                                        Section 1338
                                            (102950) 102950    102960     102971     102982     102993     103004     103015 103026
Autographa californica nucleopolyhedrovirus NC_0... (102739) ATCTGTCTTCGTGTTTGTTGTAAGGTCTTCGAATCTTGTGTTTCAGCCCCTCGGGATGGTCAAAATGCGCCGTAGTA
                             SARS-CoV-2 NC_045512.2  (25341) ACT-----CTGAG---CCAGT-----GCTCAAAGGAGTCAAAT--TACATTACACATAAACGAACTTATGGATTTGT
                                                        Section 1339
                                            (103027) 103027    103037     103048     103059     103070     103081     103092 103103
Autographa californica nucleopolyhedrovirus NC_0... (102816) ATTGTTAATGGATCTTTCAACGATTTTTTGCCCATGGCGAGTGTGACAAACGCGGCCACGACAAACAGCAGGATAAT
                             SARS-CoV-2 NC_045512.2  (25403) TTATGAGAATCTTCAC---------AATTGGAACTGTAA--CTTTGAAGCAAG--GTGAAATCAAGGATGCTACTCCT
                                                        Section 1340
                                            (103104) 103104    103114     103125     103136     103147     103158     103169 103180
Autographa californica nucleopolyhedrovirus NC_0... (102893) CAGTTTCATGGTGTTCTATATTCGACAATATATGGGTCGCTTCTAAATCACCTTGTCCCCAAAAGCCTCTTTTATAG
                             SARS-CoV-2 NC_045512.2  (25468) TCAGAT-TTTGTTCGCGC--TACTGCAACGATACCGATACAAGCCTCAC--TCCCTTTCGGATGGC-TTATTGTTGG
                                                        Section 1341
                                            (103181) 103181    103191     103202     103213     103224     103235     103246 103257
Autographa californica nucleopolyhedrovirus NC_0... (102970) TTTTTTAGAACACGTTGTGTATTCCAACAGTAATTGTTCCATCTCTTTCAACAGCCATTCAGCATCCGGTCGTTGAC
                             SARS-CoV-2 NC_045512.2  (25539) CGTTGCACTTC--TTGCTGTTTTTCAGAGCGCTTCCAAAATCATAACCCTCAAAAAGAGATGGCAA--CTAGCACTC
                                                        Section 1342
                                            (103258) 103258    103268     103279     103290     103301     103312     103323 103334
Autographa californica nucleopolyhedrovirus NC_0... (103047) TGTAATCATGCTGAATTAATTTACAAACAATTTCGGTCAATTTAGGATGGCCTTGGGATAAACTTGCCGGCATTTGC
                             SARS-CoV-2 NC_045512.2  (25612) TCCAAGGGTGTTCACTTTGTTTGCAACTTGCTGTTGTTGTTTGTAACAGTT-TACTCA------CACCTTTTGCTCG
                                                        Section 1343
                                            (103335) 103335    103345     103356     103367     103378     103389     103400 103411
Autographa californica nucleopolyhedrovirus NC_0... (103124) TG------TACATTGTTTCTAAAGTTAGTTAGCGTAGTTTCGCGTTCCAAAGCAGTCTTGAAGGGCATTATCAATTC
                             SARS-CoV-2 NC_045512.2  (25682) TTGCTGCTGGCCTTGAAGCCCCTTTTCTCTATCTTTATGCTTTAGTCTACTTCTTGCAGAGTATAAACTTTGTAAGA
                                                        Section 1344
                                            (103412) 103412    103422     103433     103444     103455     103466     103477 103488
Autographa californica nucleopolyhedrovirus NC_0... (103195) GAATAAAACAATGCCCAAACTATACATGTCATTTTTGGGGGTGTACACTTTTTTTGATTTGTTCTGGTGCAGCGTACA
                             SARS-CoV-2 NC_045512.2  (25759) ATAATAATGAG-GCTTTGGCT-TTGCTGGAAATGCCGTTCCAAAAACCCAT----TACTT----TATGATGCCAACT
```

Section 1345

	(103489)	103489 103499 103510 103521 103532 103543 103554 103565
Autographa californica nucleopolyhedrovirus NC_0...	(103272)	AAGTTATATTTTGAGGGTTGTTTTTGATAAACGTTTTGTATAGACTGCCAAACATGCCGCCCACATACAAATCAAAG
SARS-CoV-2 NC_045512.2	(25826)	ATTTTCTTTGCTGGCATACTAATTGTTACG-ACTATTGTATACCTTACAATAG-TGTAACTTCTTCAATTGTC-ATT

Section 1346

	(103566)	103566 103576 103587 103598 103609 103620 103631 103642
Autographa californica nucleopolyhedrovirus NC_0...	(103349)	TCGGGCCCAGTCATGAAAATATCTTCGGGATTAATATTGTGGTGCACGATATTTACGGAATGAATCGCTTTCACGGC
SARS-CoV-2 NC_045512.2	(25900)	ACTTCAGGTGATGGCACAACAAGTCCTATTTTCTGAACATGACTACCAGATTGGTGGTTATACTGAAAAATGGGAATC

Section 1347

	(103643)	103643 103653 103664 103675 103686 103697 103708 103719
Autographa californica nucleopolyhedrovirus NC_0...	(103426)	GCTCACCAAATCAACAAACTTGCTAATATAAAAGCCAAAATCCGCCGGAACTTTAATGTTGGTCTTTGCAAAAGTTT
SARS-CoV-2 NC_045512.2	(25977)	----TGGAGTAAAAGACTGTGTTGTATTACACAGT--TACTTCACTTCAGACTATT-ACCAGCTGTACTCAACTCA-

Section 1348

	(103720)	103720 103730 103741 103752 103763 103774 103785 103796
Autographa californica nucleopolyhedrovirus NC_0...	(103503)	GCAAATTGCGTTGTTTCAAATAGTCGCTCAACATGTACTCGTTTAGAGGCGACGCAATATATATGCGGTGCTGCCGC
SARS-CoV-2 NC_045512.2	(26046)	----ATTGAGTACAGACACTGGTG-TTGAACATGTTACCTTCTTCATCTACAATAAAATTGTTGATGAGCCTGAAGA

Section 1349

	(103797)	103797 103807 103818 103829 103840 103851 103862 103873
Autographa californica nucleopolyhedrovirus NC_0...	(103580)	GGATTCAAATAAACCAATTGTTCGGGTTTCATG-GTATACAGTTAAGTGTTAACGCGTCACTAAATTCAGACACGAG
SARS-CoV-2 NC_045512.2	(26118)	ACATGTCCAAATTCACACAATCGACGGTTCATCCGGAGTTGTTAATCCAGTAATGGAACCAATTTATGATGAACCGA

Section 1350

	(103874)	103874 103884 103895 103906 103917 103928 103939 103950
Autographa californica nucleopolyhedrovirus NC_0...	(103656)	CGCACGCCCTATATACATACAATTTATCGCACAAGATGCTTAACGCGATCTGTTTATAAACTAAAACGCACTGCAAT
SARS-CoV-2 NC_045512.2	(26195)	CGACGACTACTAGCGTGC-----CTTTGTAAGCACAAGCTGATG-------AGTACGAACTTATGTACTCATTCGTT

Section 1351

	(103951)	103951 103961 103972 103983 103994 104005 104016 104027
Autographa californica nucleopolyhedrovirus NC_0...	(103733)	AAATTTTAGCAAGCATTTGTATTTAATCAATCGAACCGTGCACTGATATAAGAATTAAAAATGGGTTTGTTTGCGTG
SARS-CoV-2 NC_045512.2	(26260)	TCGGAAGAGACAGGTACGTTAATAGTTAATAGCGTACTTCTTTTTCTTGCTTTCGTGGTATTCTTGCTAGTTACACT

Section 1352

(104028) 104028 104038 104049 104060 104071 104082 104093 104104
Autographa californica nucleopolyhedrovirus NC_0... (103810) TTGCACAAAATACACAAGGCTGTCGACCGACACAAAAATGAAGTTTCCCTATGTTGCGTTGTCGTACATCAACGTGA
SARS-CoV-2 NC_045512.2 (26337) AGCCATCCTTACTGCGCTTCGATT-GTGTGCGTACTGCTGCAATATTGTTAACGTGAGTCTTGTAAAACCTTCTTTT

Section 1353

(104105) 104105 104115 104126 104137 104148 104159 104170 104181
Autographa californica nucleopolyhedrovirus NC_0... (103887) CGCTGTGCACCTACACCGCCATGTTGG-TGGGATACATGGTAACATTCAATGACTCCAGCGAATTGAAATATTTACA
SARS-CoV-2 NC_045512.2 (26413) TACGTTTACTCTCGTGTTAAAAATCTGAATTCTTCTAGAGTTCCTGATCTTCTGGTCTAAAC---GAACTAAATATT

Section 1354

(104182) 104182 104192 104203 104214 104225 104236 104247 104258
Autographa californica nucleopolyhedrovirus NC_0... (103963) ATACTGGTTGCTGTTGTCGTTTTTGATGTCCGTGGTGCTAAACGCTCCGACTCTGTGGACGATGCTCAAAACCACAG
SARS-CoV-2 NC_045512.2 (26487) ATATT------------AGTTTTTCTGTTTGGAAC-TTTAATTTTAG------CCATGGCAGATTCCAACGGTACTA

Section 1355

(104259) 104259 104269 104280 104291 104302 104313 104324 104335
Autographa californica nucleopolyhedrovirus NC_0... (104040) AAGCCCATGAAGTAATTTACGAAATGAAGCTGTTCCACGCCATGTACTTTAGTAACGTGCTGTTGAATTATGTGGTG
SARS-CoV-2 NC_045512.2 (26545) TTACCGTTGAAGAG--CTTAAAAA-GCTCCTT-----GAACAATGG--AACCTAGT--AATAGGTTTCCTATTC---

Section 1356

(104336) 104336 104346 104357 104368 104379 104390 104401 104412
Autographa californica nucleopolyhedrovirus NC_0... (104117) TTTTTGGACAATCAAATGGGTACAAATTTTGTTTTTGTTAACAATTTAATTCACTGTTGTGTACTTTTTATGATATT
SARS-CoV-2 NC_045512.2 (26607) -CTTACATGGAT--TTGTCTTCTACAATTTGCCTATGCCAACAG--GAATAGGTTTTGTATATAATTAAGT---TA

Section 1357

(104413) 104413 104423 104434 104445 104456 104467 104478 104489
Autographa californica nucleopolyhedrovirus NC_0... (104194) TGTTGAATTGCTTATCCTGTTGGGCCACACAATGGGCACGTACACGGATTATCAATATGTCAAATCGTGTTATATGG
SARS-CoV-2 NC_045512.2 (26676) ATTTTCCTCTGGCTGTTATGGCCAGTAACTTTAGCTTGTTTTGTGCTTGCTGCT--GTTTACAGAATAAATTGGAT-

Section 1358

(104490) 104490 104500 104511 104522 104533 104544 104555 104566
Autographa californica nucleopolyhedrovirus NC_0... (104271) TTATATTGTTTGTTTCAGTTATGAGTGTTACTATTGTTATGG-GTTTAGAGTGTTTGAAAACGAAACTAATTGATAA
SARS-CoV-2 NC_045512.2 (26750) -----CAC-CGGTGGAATTGCTATCGCAATGGCTTGTCTTGTAGGCT------TGATGTGGCTCAGCTACTTCA---

```
                                                              Section 1359
                                    (104567) 104567    104577    104588    104599    104610    104621    104632    104643
Autographa californica nucleopolyhedrovirus_NC_0... (104347) CAGTTTGATGTTTAACGCGTTTGTGTGCGCTTTGTACATTGTGATTGCAATAATGTGGTCTTTAAAAAATAATTTGA
                         SARS-CoV-2 NC_045512.2 (26812) -------TTGCT-----TCTTTCAGACTG-----TTTGCGCG-TACGCG--TTCCATGTGGTCAT----TCAATCCA

                                                              Section 1360
                                    (104644) 104644    104654    104665    104676    104687    104698    104709    104720
Autographa californica nucleopolyhedrovirus_NC_0... (104424) CTAGTTATTACGTTTCAAATTTACAAAGTATTCAAGTTGTTCCGTTTTCATACAACGATCCGCCGCCACCGTTCTCT
                         SARS-CoV-2 NC_045512.2 (26865) ---GAAA--CTAACATTCTTCTC----AACGTGCCACTCCATGGCACTATTCTGACCA-GACCGCTTCTAGAAAGTG

                                                              Section 1361
                                    (104721) 104721    104731    104742    104753    104764    104775    104786    104797
Autographa californica nucleopolyhedrovirus_NC_0... (104501) AACATTGTAATGGATGACATAAAAAATAAAAAATAATTTATAAAAATGTTTTTTATTCTTTCACAATTCTGTAAATT
                         SARS-CoV-2 NC_045512.2 (26932) AACTCGTAATCGGAGCTGTGATCCTTCGTGGACATCTTCGTATTGCTGGACACCA-TCTAGGACGCTGT--GACATC

                                                              Section 1362
                                    (104798) 104798    104808    104819    104830    104841    104852    104863    104874
Autographa californica nucleopolyhedrovirus_NC_0... (104578) CTAAACA--AAAAATATAAATACAAACTTATTATGTTGTCGTCTAAATAAACATCAATTTGTAAATCTGGACACCTA
                         SARS-CoV-2 NC_045512.2 (27006) AAGGACCTGCCTAA-AGAAATCACTGTTGCTACATCACGAACGCTTTCTTATTACAAATTGGGAG-CTTCGCAGCGT

                                                              Section 1363
                                    (104875) 104875    104885    104896    104907    104918    104929    104940    104951
Autographa californica nucleopolyhedrovirus_NC_0... (104653) TTCATA----TCATTGATATTACAGTCTACTATACAACAATTAAAACTAACCAAATTATCTTTACAACAATTAAAGC
                         SARS-CoV-2 NC_045512.2 (27081) GTAGCAGGTGACTCAGGTTTTGCTGC--ATACAGTCGCTACAGGATTGGCAACT---ATAAATTAAAC--ACAGACC

                                                              Section 1364
                                    (104952) 104952    104962    104973    104984    104995    105006    105017    105028
Autographa californica nucleopolyhedrovirus_NC_0... (104726) AATTAAAACAATTTAAATAATCTTCATTGTCGTCGTATAAGTTTATTTGCACTGTAGACGGTGTTACACAGCGATCC
                         SARS-CoV-2 NC_045512.2 (27151) ATTCC--AG---TAGC---AGTGACAAT---ATTGCTTTGCTTGTACAGTAAGTGACAA---------CAGATGTTTC

                                                              Section 1365
                                    (105029) 105029    105039    105050    105061    105072    105083    105094    105105
Autographa californica nucleopolyhedrovirus_NC_0... (104803) ATTCGACGTTCGTGTTCGATCAACTTTCTCGCCAACTTGTACCATAAAAATTGTTTGGACAAAAGTTTTCCAACAA
                         SARS-CoV-2 NC_045512.2 (27209) ATCTC-----GTTGA-CTTTCA-GGTTACTAT----AGCAGAGATATTACTAATTATTATGAG-----GACTTTTAA
```

Section 1366

```
                                          (105106) 105106    105116     105127     105138     105149     105160     105171  105182
Autographa californica nucleopolyhedrovirus NC 0... (104880) TGGTAACGGCCAATTCAACGTGACGATGCGCACGTCCTCGGGTATGCATTTGTTAAAAAACACA-CAGCTCGCTTTA
                    SARS-CoV-2 NC_045512.2 (27270) AGTTTCCAT--TTGGAATCTTGATT-ACATCATAAACCTCATAATTAAAAATTTATCTAAGTCACTAACTGAGAA--
```

Section 1367

```
                                          (105183) 105183    105193     105204     105215     105226     105237     105248  105259
Autographa californica nucleopolyhedrovirus NC 0... (104956) CCAAACGAAAGCAAGGTACTAAATATGGCGCCATTGGCTGATTTGTTATTCCAAGATAATTACAAATAAACTGATC
                    SARS-CoV-2 NC_045512.2 (27342) -TAAA-TATTCTCAATTAGATGA--AGAGCAACCAATGGAGATT----GATTAAACGAACATGAAAAT-TATTCTTT
```

Section 1368

```
                                          (105260) 105260    105270     105281     105292     105303     105314     105325  105336
Autographa californica nucleopolyhedrovirus NC 0... (105033) CGTCGTGGGGTGATAACTGGCAGGTGTCAGCTTTAAATAATCTTCAACGTTGTTGTCGCGCAAAAGTCTGCATTTTA
                    SARS-CoV-2 NC_045512.2 (27410) TCTTGGCACTGATAACACTCGCTACTTGTGAGCTTTATCACTACCAAGAGTGTGTTAGAGGTACAA-CAGTACTTTT
```

Section 1369

```
                                          (105337) 105337    105347     105358     105369     105380     105391     105402  105413
Autographa californica nucleopolyhedrovirus NC 0... (105110) CACGCGTTGTTAATCCCACGACTTTTGCATGTAAAATCGGATCCAAATACTGCAGAATCG-TGTCTATAATTTCTAA
                    SARS-CoV-2 NC_045512.2 (27486) AAAAGAACCTTGCTC----TTCTGGAACATACGAGGGCAATTCACCATTTCATCCTCTAGCTGATAACAAATTTGCA
```

Section 1370

```
                                          (105414) 105414    105424     105435     105446     105457     105468     105479  105490
Autographa californica nucleopolyhedrovirus NC 0... (105186) TGGTAAACGTATGCGTTTTGCTCGTGGGCGCTTTGTAACGCTCGACATCCTAATAACAACTAACACAAAACTAAAAT
                    SARS-CoV-2 NC_045512.2 (27559) CTGACTTGCTTTAGCACTCAATTTGCTTTTGCTTGTCCTGACGGCGTAAAACACGTCTATCAGTTACGTGCCAGATC
```

Section 1371

```
                                          (105491) 105491    105501     105512     105523     105534     105545     105556  105567
Autographa californica nucleopolyhedrovirus NC 0... (105263) GATACTCAATATATTGCTTTTACAGTTCATCTTTAGGTTTAAACTGTGCGTTTATCGCGTTGAGCAAGTCGCCGT-T
                    SARS-CoV-2 NC_045512.2 (27636) AGTTTCACCTAAACTGTTCATCAGACA--AGAGGA---AGTTCAAGAACTTTACTCTCCAAT---TTTTCTTATTGT
```

Section 1372

```
                                          (105568) 105568    105578     105589     105600     105611     105622     105633  105644
Autographa californica nucleopolyhedrovirus NC 0... (105339) ATCGGCATCAATCTCCCAAGCAAACAGGCCGCCCAATTTATTTCGGTCGACATATTTAACTTTTCCTAACACAGAGT
                    SARS-CoV-2 NC_045512.2 (27705) TGCGGCAATAGTGTTATAACACTTTGCTTCACACTCAAAAGAAAGACAGAATGAT-TGAACTTTCAT-TAATTGAC
```

```
                                                                                                                Section 1373
                                                  (105645) 105645    105655     105666     105677     105688     105699     105710 105721
Autographa californica nucleopolyhedrovirus_NC_0... (105416) CGACGCTGTCAAACGAAATCAAATCACCTTTACTTTTATCGAAAACGTACGACGCTTGAGCGGCGCTGTCAAACGTG
                              SARS-CoV-2 NC_045512.2 (27780) TTCTATTTGTGCTTTT-----T----AGCCTT----TCTGCTA------------------------TTCC------
                                                                                                                Section 1374
                                                  (105722) 105722    105732     105743     105754     105765     105776     105787 105798
Autographa californica nucleopolyhedrovirus_NC_0... (105493) TACACATAATTGTTGAGATCTTTTTGAATTTGACGATAATCTACAACACCGTCCTCCCACGTGCCCGACCCCGGCCC
                              SARS-CoV-2 NC_045512.2 (27814) ------TTGTTTTAATTA--TGCT-------TATTATCTTTTGGTTCTCACTTGAACTGCAAGATCATAATGA-AAC
                                                                                                                Section 1375
                                                  (105799) 105799    105809     105820     105831     105842     105853     105864 105875
Autographa californica nucleopolyhedrovirus_NC_0... (105570) GTTGCCAGTGCCGGAAAAATAGTTGTCATTCGTATAATTTGTTACGCCGGTCCAGCCGCGGCCGTACATGGCGACGC
                              SARS-CoV-2 NC_045512.2 (27875) TTGTCACGCCTAAACGAA--CATGAAATTTCTTGTTT---TCTTAG--GAATCATCACAACTGTAGCTGCATTTCAC
                                                                                                                Section 1376
                                                  (105876) 105876    105886     105897     105908     105919     105930     105941 105952
Autographa californica nucleopolyhedrovirus_NC_0... (105647) CCACAATTATTTTGTTGGGATCGACGCCTTGTTTCAGTAACGCATCGACAGCGTAGTGTGTAGTGTATAGCTCTTCC
                              SARS-CoV-2 NC_045512.2 (27945) CAAGAATG-------TAGTTTACAGTCATGTACTCAACATCA--ACCATATGTAGTTGATGACCCGTGTCCTATTCA
                                                                                                                Section 1377
                                                  (105953) 105953    105963     105974     105985     105996     106007     106018 106029
Autographa californica nucleopolyhedrovirus_NC_0... (105724) GAGTTCCAACTTGGCGCGTAGACTGTTGTTTGGTAGCCCAAATCCGTGTTTGACCAAGCCCCTTTAAAATCGTAACT
                              SARS-CoV-2 NC_045512.2 (28013) CTTCTATTCTAAATGGTATATTAGAGTAGGAGCTAG---AAAATCAGCACCTTTAATTGAATTGTGCGTGGATGAG-
                                                                                                                Section 1378
                                                  (106030) 106030    106040     106051     106062     106073     106084     106095 106106
Autographa californica nucleopolyhedrovirus_NC_0... (105801) CATGAGAAATATTTTGCCTAATGACTTTTG--CGCTTCGGCGTAGTTTACCACGGCAATCTTGTCGTAACCCGCGCT
                              SARS-CoV-2 NC_045512.2 (28086) --GCTGGTTCTAAATCACC-CATTCAGTACATCGATATCGGT--AATTATAC----------AGTTT------C-CT
                                                                                                                Section 1379
                                                  (106107) 106107    106117     106128     106139     106150     106161     106172 106183
Autographa californica nucleopolyhedrovirus_NC_0... (105876) TATAGCGCTTGTTAATTCGTAAACCCTGCCGGTTTGCGCTTCGAGGTCGTCTAGCATTGCGCGCAGCTCCTCCAACA
                              SARS-CoV-2 NC_045512.2 (28141) GTTTACCTTT-----TACAA----TTAATTGCCAGGAAC-----CTAAATTGGGTAGT----CTTGTAGTGCGTTGT
```

Section 1380

(106184) 106184 106194 106205 106216 106227 106238 106249 106260
Autographa californica nucleopolyhedrovirus NC 0... (105953) ACAAAATGTATGTTTTGGCGTCACCGTCCGCATCGCCCAACGACGGGTTAGCCCCTTTGCCGCCCGGAAACTCCCAA
SARS-CoV-2 NC_045512.2 (28200) TC----G-----TTCTATGAAG-ACTTTTTAGAGTATCATGACGTT-CGTGTTGTTTTAGATTTCATCTAAACGAAC

Section 1381

(106261) 106261 106271 106282 106293 106304 106315 106326 106337
Autographa californica nucleopolyhedrovirus NC 0... (106030) TCGATGTCTACACCGTCAAAGAATTTCCACACTTGCAGAAATTCCTTAACCGAATCTACAAAAACGTTTCTTTTTTC
SARS-CoV-2 NC_045512.2 (28266) AAACTAAAATGTCTG----ATAATGGACCCCAAAATCAGCGAAATGCACCCCGCATTACGTTTGGTGGACCCTCAGA

Section 1382

(106338) 106338 106348 106359 106370 106381 106392 106403 106414
Autographa californica nucleopolyhedrovirus NC 0... (106107) AACATCGTGCATAAAATAAAATGGGTCTGATAGAGTCCAGCCTCCTATTGAAGGAAGAATTTTTA-AATGGGGGTTT
SARS-CoV-2 NC_045512.2 (28339) TTCAAC-TG--GCA----GTAACCAGA-----ATGGAGAACGCAGTGGGGCG-------CGATCAAAACAACGTCGG

Section 1383

(106415) 106415 106425 106436 106447 106458 106469 106480 106491
Autographa californica nucleopolyhedrovirus NC 0... (106183) GCTAATTTTGCCGCCATCAACTGTCCAAAATTGCCTTTATACGGCTCGTTCCAAGCGGACACACCTTTTTGGGGTTT
SARS-CoV-2 NC_045512.2 (28397) CCCCAAGGTTTACCCAATAATACTGCGTCTTGGTTCACCGCTCTCACTCAACATGGCAAGGAAGACCTTAAATTCCC

Section 1384

(106492) 106492 106502 106513 106524 106535 106546 106557 106568
Autographa californica nucleopolyhedrovirus NC 0... (106260) TTGTACGGCGGCCCACGGATCGTGAATGGCAACTTTGAAATCTTCGCGTCCCTTGCACGATCTTTGCAAAGATTCAA
SARS-CoV-2 NC_045512.2 (28474) TCGAGGA--CAAGGCGTTCCAATTAACACCAATAGCAGTCCAGATGACCAAATTGGCTACTAC-CGA-AG---AGCT

Section 1385

(106569) 106569 106579 106590 106601 106612 106623 106634 106645
Autographa californica nucleopolyhedrovirus NC 0... (106337) AGCTTCCGGGTATCGTTTTGAGGGCGTCGTTTATTCCATCGCCGCCGCAGATGGGTATGAAACCATACAACAAGTGT
SARS-CoV-2 NC_045512.2 (28544) ACCAGACGAATTCG-TGGTGGTGA--CGGTAAAATGAAAGATCTCAGTCCAAGATGGTATTTCTACTACCTAGGAAC

Section 1386

(106646) 106646 106656 106667 106678 106689 106700 106711 106722
Autographa californica nucleopolyhedrovirus NC 0... (106414) GATAAATTTGGCAAGGGAACTTTGTCTACGGGAAAGTTGCGCCCGTAC-ACACCCCACTCAACAAAGTACGCAGCGA
SARS-CoV-2 NC_045512.2 (28618) -TGGGCCAGAAGCTG--GACTTCCC--TATGGTGCTAACAAAGACGGCATCATATGGGTTGC-AACTGAGGGAGCCT

Section 1387

```
                                                     (106723) 106723    106733     106744     106755     106766     106777     106788    106799
Autographa californica nucleopolyhedrovirus NC_0... (106490) CAATTTTATCCTCTCTCCTGCCAGGTTTGTTGTTTTCCAGCCATGTGTATTCGAGCGGTGCCAGATGGCCGCCGTCG
                          SARS-CoV-2 NC_045512.2  (28689) TGA-ATACACCAAAAGATCACATTGGCACCCG-----CAATCCTGCTAAC----AATGCT---GCAATCGTGCTACA
```

Section 1388

```
                                                     (106800) 106800    106810     106821     106832     106843     106854     106865    106876
Autographa californica nucleopolyhedrovirus NC_0... (106567) GTGTCTGCGACTTTGACCAACACGGGATCGCTCACGGAACAGCCGTCCTCATTGCAAAGTTTGACACGCATGTTAAA
                          SARS-CoV-2 NC_045512.2  (28753) ACTTCCTCAAGGAACAACATTGCCA-----AAAGGCTTCTACGCAG---AAGG--------GAGCAGAGGCGGC---
```

Section 1389

```
                                                     (106877) 106877    106887     106898     106909     106920     106931     106942    106953
Autographa californica nucleopolyhedrovirus NC_0... (106644) TTGCCCGCTCACAAGAACTTTAATGGTAGCCCTTTTACTTTCGGCGTCGCCTTTCCATACCTGCTGCTCGTCAAACA
                          SARS-CoV-2 NC_045512.2  (28811) ------AGTCAA---GCCTCTTCTCGTTCC-----------TCATCACGTAG-TCGC--AACAGTTCAAGAA-----
```

Section 1390

```
                                                     (106954) 106954    106964     106975     106986     106997     107008     107019    107030
Autographa californica nucleopolyhedrovirus NC_0... (106721) ACACGTACGCTATGTCGCCAATGTCGCCGTTCCAGACGTTCCAACTGACTTGAACGTCGACTTGTTCTTTAGGCTTT
                          SARS-CoV-2 NC_045512.2  (28860) ----ATTCAACT---C---CAGGCAG-----CAGTAGGGGAACTTCTCCTG---------CTAGAATGGCTGGCAAT
```

Section 1391

```
                                                     (107031) 107031    107041     107052     107063     107074     107085     107096    107107
Autographa californica nucleopolyhedrovirus NC_0... (106798) ATTAAATTTTCGTAAGCGGTGGCCTCGTAATTTATTTCTACGAGCGCATAATTGCGATCGGCCCAATCGATCACCGG
                          SARS-CoV-2 NC_045512.2  (28913) GGCGGTGATGCTGCT---CTTGCTTTGCTGCTGCTT------GACAGATTGAACCAGCTTGAGAGCAAAATGTCTGG
```

Section 1392

```
                                                     (107108) 107108    107118     107129     107140     107151     107162     107173    107184
Autographa californica nucleopolyhedrovirus NC_0... (106875) CGTGCCGGGAATCGCGTTAGAAACGGCGACCAACCACAAAACGTTTAACAATTTGTACAACATTTTAATTTATCTTA
                          SARS-CoV-2 NC_045512.2  (28981) TAAAGGCC-------AACAACAACAAGG-------CCAAACTGTCA--CTAAGAAATCTGCTGCTGAGGCTTCTAAG
```

Section 1393

```
                                                     (107185) 107185    107195     107206     107217     107228     107239     107250    107261
Autographa californica nucleopolyhedrovirus NC_0... (106952) ATTTTAAGTTGTAATTATTTTATGTAAAAAAATGAACAAATTTTGTTTTATTTGTTTGTGTACGGCGTTGTAAACA
                          SARS-CoV-2 NC_045512.2  (29042) AAGCCTCGGCAAAAACGTACTGC-CA--CTAAAGCATACAATGTAACACAAGCTTTCGGCAGACGTGGTCCAGAACA
```

Section 1394

```
                                                   (107262) 107262    107272    107283     107294     107305     107316     107327 107338
Autographa californica nucleopolyhedrovirus NC 0... (107029) GCGCGGCGTACGACCTTTTGAAAGCGCCTAATTATTTTGAAGAATTTGTTCATCGATTCAACAAAGATTATGGTAGC
                          SARS-CoV-2 NC_045512.2   (29116) AACCCAAGGAAATTTTGGGGACCAGGAACTAATCAGACAAGGAA-CTGATTACAAACATTGGCCGCAAATTGCACAA
```

Section 1395

```
                                                   (107339) 107339    107349    107360     107371     107382     107393     107404 107415
Autographa californica nucleopolyhedrovirus NC 0... (107106) GAAGTTGAAAAATTGCGAAGATTCAAAATTTTCCAACACAATTTAAATGAAATTATTAATAAAAACCAAAACGATTC
                          SARS-CoV-2 NC_045512.2   (29192) TTTGCCCCCAGCGC-TTCA-GCGTTCTTCGGAATGTCGCGCATTGGCATG--GAAGTCACACCTTCGG--GAACGTG
```

Section 1396

```
                                                   (107416) 107416    107426    107437     107448     107459     107470     107481 107492
Autographa californica nucleopolyhedrovirus NC 0... (107183) GGCCAAATATGAAATAAACAAATTCTCGGATTTGTCCAAAGACGAAACTATCGCAAAATACACAGGTTTGTCTTTGC
                          SARS-CoV-2 NC_045512.2   (29263) GTTGACCTACACA---G---GTGCCATCAAATTGGATGACAAAGATCCAAATTTCAAAGATCAAGTCATTTTGCTGA
```

Section 1397

```
                                                   (107493) 107493    107503    107514     107525     107536     107547     107558 107569
Autographa californica nucleopolyhedrovirus NC 0... (107260) CTATTCAGACTCAAATTTTTGCAAAGTAATAGTCCTAGACCAGCCACCGGGCAAAGGGCCCCTTGAATTCGACTGG
                          SARS-CoV-2 NC_045512.2   (29334) ATAAGCATATTGACGCA-TACAAAA--CATTCCCACCAACAGAGCCT----AAAAAGGACAAAAAGAAGAAGG--CT
```

Section 1398

```
                                                   (107570) 107570    107580    107591     107602     107613     107624     107635 107646
Autographa californica nucleopolyhedrovirus NC 0... (107337) CGTCGTCTCAACAAAGTCACTAGCGTAAAAAATCAGGGCATGTGTGGCGCCTGCTGGGCGTTTGCCACTCTGGCTAG
                          SARS-CoV-2 NC_045512.2   (29402) GATGAAACTCAAGCCTT-ACCGCAGAGACAGAAGAAACAGCAAACTGTGA--CTCTTCTTCCTGCTGCA-GATTTGG
```

Section 1399

```
                                                   (107647) 107647    107657    107668     107679     107690     107701     107712 107723
Autographa californica nucleopolyhedrovirus NC 0... (107414) TTTGGAAAGTCAATTTGCAATCAAACATAACCAGTTGATTAATCTGTCGGAGCAGCAAATGATCGATTGTGATTTTG
                          SARS-CoV-2 NC_045512.2   (29475) ATG--ATTTCTCCAAACAATTG---CAACAATC---CATGA-GCAGTGCTGACTCAAC----TCAGGCCTAAACTCA
```

Section 1400

```
                                                   (107724) 107724    107734    107745     107756     107767     107778     107789 107800
Autographa californica nucleopolyhedrovirus NC 0... (107491) TCGACGCTGGCTGTAACGGCGGCTTGTTGCACACAGCGTTCGAAGCCATCATTAAAATGGGCGGCGTACAGCTGGAA
                          SARS-CoV-2 NC_045512.2   (29539) T-GCAGACCACACAAG-GCAGATGGGCTATATAAACGT--TT----------TCGCTTTTCCGTTTACGAT--ATAT
```

```
                                                                                                       Section 1401
                                                    (107801) 107801    107811     107822     107833     107844     107855     107866   107877
Autographa californica nucleopolyhedrovirus  NC_0... (107568) AGCGACTATCCATACGAAGCAGACAATAACAATTGCCGTATGAACTCCAATAAGTTTCTAGTTCAAGTAAAAG--AT
                             SARS-CoV-2 NC_045512.2  (29600) AGTCTACTCTTGTGCAGAATGAATTCTCGTAACTACATAGCACAAGTAGATGTAG-----TTAACTTTAATCTCACA
                                                                                                       Section 1402
                                                    (107878) 107878    107888     107899     107910     107921     107932     107943   107954
Autographa californica nucleopolyhedrovirus  NC_0... (107643) TGTTATAGATACATTACCGTGTACGAGGAAAAACTTAAAGATTTGTTACGCCTTGTCGGCCCTATTCCTATGGCCAT
                             SARS-CoV-2 NC_045512.2  (29672) TAGCAATCTT--TAATCAGTGTGTAACATT---AGGGAGGACTTG-AAAGAGCCACCA---CATTTT--CACCGAGG
                                                                                                       Section 1403
                                                    (107955) 107955    107965     107976     107987     107998     108009     108020   108031
Autographa californica nucleopolyhedrovirus  NC_0... (107720) AGACGCTGCCGACATTGTTAACTATAAACAGGGTATTATAAAATATTGTTTCAACAGCGGTCTAAACCATGCGGTTC
                             SARS-CoV-2 NC_045512.2  (29738) CCAC---GCGGAGTACGATCG-----AGTG----TACAGTGAACAATGCTAGGGAGAGCTGCCTATATGGAAGAGCC
                                                                                                       Section 1404
                                                    (108032) 108032    108042     108053     108064     108075     108086     108097   108108
Autographa californica nucleopolyhedrovirus  NC_0... (107797) TTTTAGTGGGTTATGGTGTTGAAAACAACATTCCATATTGGACCTTTAAAAACACTTGGGGCACGGATTGGGGAGAG
                             SARS-CoV-2 NC_045512.2  (29803) CTAATGTGTAAAATTAATTTT--AG--TAGTGCTATCCCCA--TGTGATTTTAATAGCTTCTTAGGAGAATGACAAA
                                                                                                       Section 1405
                                                    (108109) 108109    108119     108130     108141     108152     108163     108174   108185
Autographa californica nucleopolyhedrovirus  NC_0... (107874) GACGGATTTTTCAGGGTACAACAAAACATAAACGCCTGTGGTATGAGAAACGAACTTGCGTCTACTGCAGTCATTTA
                             SARS-CoV-2 NC_045512.2  (29874) AAAAAAAAAAAAAAAAAAAAAAAAAAAAAA-----------------------------------------------
                                                                                                       Section 1406
                                                    (108186) 108186    108196     108207     108218     108229     108240     108251   108262
Autographa californica nucleopolyhedrovirus  NC_0... (107951) TTAATCTCAACACACTCGCTATTTGGAACATAATCATATCGTCTCAGTAGCTCAAGGTAGAGCGTAGCGCTCTGGAT
                             SARS-CoV-2 NC_045512.2  (29904) -----------------------------------------------------------------------------
                                                                                                       Section 1407
                                                    (108263) 108263    108273     108284     108295     108306     108317     108328   108339
Autographa californica nucleopolyhedrovirus  NC_0... (108028) CGTATAGATCTTGCTAAGGTTGTGAGTTCAAGTCTCGCCTGAGATATTAAAAAACTTTGTAATTTTAAAAATTTTAT
                             SARS-CoV-2 NC_045512.2  (29904) -----------------------------------------------------------------------------
```

Section 1408

(108340) 108340 108350 108361 108372 108383 108394 108405 108416
Autographa californica nucleopolyhedrovirus NC 0... (108105) TTTATAATATACAATTAAAAACTATACAATTTTTTATTATTACATTAATAATGATACAATTTTTATTATTACATTTA
SARS-CoV-2 NC_045512.2 (29904) ---

Section 1409

(108417) 108417 108427 108438 108449 108460 108471 108482 108493
Autographa californica nucleopolyhedrovirus NC 0... (108182) ATATTGTCTATTACGGTTTCTAATCATACAGTACAAAAATAAAATCACAATTAATATAATTACAAAGTTAACTACAT
SARS-CoV-2 NC_045512.2 (29904) ---

Section 1410

(108494) 108494 108504 108515 108526 108537 108548 108559 108570
Autographa californica nucleopolyhedrovirus NC 0... (108259) GACCAAACATGAACGAAGTCAATTTAGCGGCCAATTCGCCTTCAGCCATGGAAGTGATGTCGCTCAGACTGGTGCCG
SARS-CoV-2 NC_045512.2 (29904) ---

Section 1411

(108571) 108571 108581 108592 108603 108614 108625 108636 108647
Autographa californica nucleopolyhedrovirus NC 0... (108336) ACGCCGCCAAACTTGGTGTTCTCCATGGTGGTTATGAGGTTGCTTTTTTGTTGGGCAATAAACGACCAGCCGCTGGC
SARS-CoV-2 NC_045512.2 (29904) ---

Section 1412

(108648) 108648 108658 108669 108680 108691 108702 108713 108724
Autographa californica nucleopolyhedrovirus NC 0... (108413) ATCTTTCCAACTGTCGTGATAGGTCGTGTTGCCGATGGTCGGGATCCAAAACTCGACGTCGTCGTCAATTGCTAGTT
SARS-CoV-2 NC_045512.2 (29904) ---

Section 1413

(108725) 108725 108735 108746 108757 108768 108779 108790 108801
Autographa californica nucleopolyhedrovirus NC 0... (108490) CCTTGTAGTTGCTAAAATCTATGCATTGCGACGAGTCCGTGTTGGCCACCCAACGCCCTTCTTTGTAGATGCTGTTG
SARS-CoV-2 NC_045512.2 (29904) ---

Section 1414

(108802) 108802 108812 108823 108834 108845 108856 108867 108878
Autographa californica nucleopolyhedrovirus NC 0... (108567) TTGTAGCAATTACTGGTGTGTGCCGGCGGATTGGTGCACGGCATCAGCAAAAACGTGTCGTCCGACAAAAATGTTGA
SARS-CoV-2 NC_045512.2 (29904) ---

```
                                                         Section 1415
                                        (108879) 108879    108889     108900     108911     108922     108933     108944    108955
Autographa californica nucleopolyhedrovirus NC 0... (108644) AGAAACAGAGTTGTTCATGAGATTGCCAATCAAACGCTCGTCCACCTTGGCCACGGAGACTATCAGGTCGTGCAGCA
                           SARS-CoV-2 NC_045512.2  (29904) -----------------------------------------------------------------------------
                                                         Section 1416
                                        (108956) 108956    108966     108977     108988     108999     109010     109021    109032
Autographa californica nucleopolyhedrovirus NC 0... (108721) TATTGTTTAGCTTGTTGATGTGCGCATGCATCAGCTCAATGTTCATTTTCAGCAAATCGTTTTCGTACATCAGCTCC
                           SARS-CoV-2 NC_045512.2  (29904) -----------------------------------------------------------------------------
                                                         Section 1417
                                        (109033) 109033    109043     109054     109065     109076     109087     109098    109109
Autographa californica nucleopolyhedrovirus NC 0... (108798) TCTTGAATATGCATCAGGTCGCCTTTGGTGGCAGTGTCTCCCTCTGTGTACTTGGCTCTAACGTTGTGGCGCCAAGT
                           SARS-CoV-2 NC_045512.2  (29904) -----------------------------------------------------------------------------
                                                         Section 1418
                                        (109110) 109110    109120     109131     109142     109153     109164     109175    109186
Autographa californica nucleopolyhedrovirus NC 0... (108875) GGGCGGCCGCTTCTTGACTCGGTGCTCGACTTTGCGTTTAATGCATCTGTTAAACTTGCAGTTCCACGTGTTTTTAG
                           SARS-CoV-2 NC_045512.2  (29904) -----------------------------------------------------------------------------
                                                         Section 1419
                                        (109187) 109187    109197     109208     109219     109230     109241     109252    109263
Autographa californica nucleopolyhedrovirus NC 0... (108952) AAAGATCATATATATCATTGTCAATCAAACAGTGTTCGCGTGTCACCGACTCGGGGTTATTTTTGTCATCTTTAATG
                           SARS-CoV-2 NC_045512.2  (29904) -----------------------------------------------------------------------------
                                                         Section 1420
                                        (109264) 109264    109274     109285     109296     109307     109318     109329    109340
Autographa californica nucleopolyhedrovirus NC 0... (109029) AGCAGACACGCAGCTTTTATTTGGCGCGTGGTGAACGTAGACTTTTGTTTGAGAATCATACTCACGCCGTCTCGATG
                           SARS-CoV-2 NC_045512.2  (29904) -----------------------------------------------------------------------------
                                                         Section 1421
                                        (109341) 109341    109351     109362     109373     109384     109395     109406    109417
Autographa californica nucleopolyhedrovirus NC 0... (109106) AAGCACAGTGTCCACGGTCACGTTGATGGGGTTGCCCTCAGCGTCCAAAATGTATACCTGGCACTCGTCCGTGTCGT
                           SARS-CoV-2 NC_045512.2  (29904) -----------------------------------------------------------------------------
```

Section 1422

	(109418)	109418 109428 109439 109450 109461 109472 109483 109494
Autographa californica nucleopolyhedrovirus NC 0...	(109183)	CCTGGCACTCGAGCCTGCTGTACATTTTCGAAGTGGAAATGCCGCATCGCCACGATTTGTTGCACGTGTGGTGCGCA
SARS-CoV-2 NC_045512.2	(29904)	--

Section 1423

	(109495)	109495 109505 109516 109527 109538 109549 109560 109571
Autographa californica nucleopolyhedrovirus NC 0...	(109260)	AAGTGATTGTTATTCTGCCGCTTCACCAACTCTTTGCCTTTGACCCACTGGCCGCGGCCCTCGTTGTCGCGAAAACA
SARS-CoV-2 NC_045512.2	(29904)	--

Section 1424

	(109572)	109572 109582 109593 109604 109615 109626 109637 109648
Autographa californica nucleopolyhedrovirus NC 0...	(109337)	GTCGTCGCTGTCACTGCCCCAACGGTCGATCAGCTCTTCGCCCACCTCGCACTGCTGCCTGATGCTCCACATAAGCA
SARS-CoV-2 NC_045512.2	(29904)	--

Section 1425

	(109649)	109649 109659 109670 109681 109692 109703 109714 109725
Autographa californica nucleopolyhedrovirus NC 0...	(109414)	AATCCTCTTTGCCCACATTCAGCGTTTTCATGGTTTCTTCGACGCGTGTGTTGGGATCCAGCGAGCCGCCGTTGTAC
SARS-CoV-2 NC_045512.2	(29904)	--

Section 1426

	(109726)	109726 109736 109747 109758 109769 109780 109791 109802
Autographa californica nucleopolyhedrovirus NC 0...	(109491)	GCATACGCCTGGTAGTACCCCTTGTAGCCGATAATCACGTTTTCGTTGTAGTCCGTCTCCACGATGGTGATTTCCAC
SARS-CoV-2 NC_045512.2	(29904)	--

Section 1427

	(109803)	109803 109813 109824 109835 109846 109857 109868 109879
Autographa californica nucleopolyhedrovirus NC 0...	(109568)	GTCCTTTTGCAGCGTTTCCTTGGGCGGGGTAATGTCCAAGTTTTTAATCTTGTACGGACCCGTCTTCATTTGCGCGT
SARS-CoV-2 NC_045512.2	(29904)	--

Section 1428

	(109880)	109880 109890 109901 109912 109923 109934 109945 109956
Autographa californica nucleopolyhedrovirus NC 0...	(109645)	TGCAGTGCTCCGCCGCAAAGGCAGAATGCGCCGCCGCCGCCAAAAGCACATATAAAACAATAGCGCTTACCATCTTG
SARS-CoV-2 NC_045512.2	(29904)	--

```
                                                          Section 1429
                                               (109957) 109957    109967     109978     109989     110000     110011     110022  110033
Autographa californica nucleopolyhedrovirus_NC_0... (109722) CTTGTGTGTTCCTTATTGAAGCCTTGGTGTGACTGATTTACTAGTAGCATTGAGGCATCTTATATACCCGACCGTTA
                          SARS-CoV-2 NC_045512.2  (29904) -----------------------------------------------------------------------------
                                                          Section 1430
                                               (110034) 110034    110044     110055     110066     110077     110088     110099  110110
Autographa californica nucleopolyhedrovirus_NC_0... (109799) TCTGGCCTACGTGACACAAGGCACGTTGTTAGATTAATAATCTTATCTTTTTATCTTAATTGATAAGATTATTTTTA
                          SARS-CoV-2 NC_045512.2  (29904) -----------------------------------------------------------------------------
                                                          Section 1431
                                               (110111) 110111    110121     110132     110143     110154     110165     110176  110187
Autographa californica nucleopolyhedrovirus_NC_0... (109876) TCTGGCTGTTATAAAAACGGGATCATGAACACGGACGCTCAGTCGACATCGAACACGCGCAACTTCATGTACTCTCC
                          SARS-CoV-2 NC_045512.2  (29904) -----------------------------------------------------------------------------
                                                          Section 1432
                                               (110188) 110188    110198     110209     110220     110231     110242     110253  110264
Autographa californica nucleopolyhedrovirus_NC_0... (109953) CGACAGCAGTCTGGAGGTGGTCATCATTACCAATTCGGACGGCGATCACGATGGCTATCTGGAACTAACCGCCGCCG
                          SARS-CoV-2 NC_045512.2  (29904) -----------------------------------------------------------------------------
                                                          Section 1433
                                               (110265) 110265    110275     110286     110297     110308     110319     110330  110341
Autographa californica nucleopolyhedrovirus_NC_0... (110030) CCAAAGTCATGTCACCTTTTCTTAGCAACGGCAGTTCGGCCGTGTGGACCAACGCGGCGCCCTCGCACAAATTGATT
                          SARS-CoV-2 NC_045512.2  (29904) -----------------------------------------------------------------------------
                                                          Section 1434
                                               (110342) 110342    110352     110363     110374     110385     110396     110407  110418
Autographa californica nucleopolyhedrovirus_NC_0... (110107) AAAAACAATAAAAATTATATTCATGTGTTTGGTTTATTTAAATATCTGTCAAATTACAATTTAAATAATAAAAAGCG
                          SARS-CoV-2 NC_045512.2  (29904) -----------------------------------------------------------------------------
                                                          Section 1435
                                               (110419) 110419    110429     110440     110451     110462     110473     110484  110495
Autographa californica nucleopolyhedrovirus_NC_0... (110184) TCCTAAAGAGTATTACACCCTTAAATCGATTATTAGCGACTTGCTTATGGGCGCTCAAGGCAAAGTATTTGATCCGC
                          SARS-CoV-2 NC_045512.2  (29904) -----------------------------------------------------------------------------
```

```
                                                                                    Section 1436
                                             (110496) 110496    110506          110517          110528          110539          110550          110561   110572
Autographa californica nucleopolyhedrovirus NC 0... (110261) TTTGCGAAGTAAAAACGCAACTGTGTGCGATTCAGGAGAGTCTCAACGAGGCTATTTCGATTTTGAACGTTCATAGC
                         SARS-CoV-2 NC_045512.2 (29904) -----------------------------------------------------------------------------
                                                                                    Section 1437
                                             (110573) 110573    110583          110594          110605          110616          110627          110638   110649
Autographa californica nucleopolyhedrovirus NC 0... (110338) AACGATGCGGCCGCCAACCCGCCTGCGCCAGACATTAACAAGTTGCAAGAACTGATACAAGATTTGCAGTCTGAATA
                         SARS-CoV-2 NC_045512.2 (29904) -----------------------------------------------------------------------------
                                                                                    Section 1438
                                             (110650) 110650    110660          110671          110682          110693          110704          110715   110726
Autographa californica nucleopolyhedrovirus NC 0... (110415) CAATAAAAAAATTACCTTTACCACTGATACAATTTTGGAGAATTTAAAAAATATAAAGGATTTAATGTGCCTGAATA
                         SARS-CoV-2 NC_045512.2 (29904) -----------------------------------------------------------------------------
                                                                                    Section 1439
                                             (110727) 110727    110737          110748          110759          110770          110781          110792   110803
Autographa californica nucleopolyhedrovirus NC 0... (110492) AATAATAATAAGGGTTTTGTACGATTTCAACAATGAACTTTTGGGCCACGTTTAGCATTTGTCTGGTGGGTTATTTG
                         SARS-CoV-2 NC_045512.2 (29904) -----------------------------------------------------------------------------
                                                                                    Section 1440
                                             (110804) 110804    110814          110825          110836          110847          110858          110869   110880
Autographa californica nucleopolyhedrovirus NC 0... (110569) GTGTACGCGGGACACTTGAATAACGAGCTACAAGAAATAAAATCAATATTAGTGGTCATGTACGAATCTATGGAAAA
                         SARS-CoV-2 NC_045512.2 (29904) -----------------------------------------------------------------------------
                                                                                    Section 1441
                                             (110881) 110881    110891          110902          110913          110924          110935          110946   110957
Autographa californica nucleopolyhedrovirus NC 0... (110646) GCATTTTTCCAATGTGGTAGACGAAATTGATTCTCTTAAAACGGACACGTTTATGATGTTGAGCAACTTGCAAAATA
                         SARS-CoV-2 NC_045512.2 (29904) -----------------------------------------------------------------------------
                                                                                    Section 1442
                                             (110958) 110958    110968          110979          110990          111001          111012          111023   111034
Autographa californica nucleopolyhedrovirus NC 0... (110723) ACACGATTCGAACGTGGGACGCAGTTGTAAAAAATGGCAAAAAAATATCCAATCTCGACGAAAAAATTAACGTGTTA
                         SARS-CoV-2 NC_045512.2 (29904) -----------------------------------------------------------------------------
```

```
Section 1443
                                                 (111035) 111035    111045     111056     111067     111078     111089     111100  111111
Autographa californica nucleopolyhedrovirus NC 0... (110800) TTAACAAAAAACGGGGTAGTTAACAACGTGCTAAACGTTCAATAAACGCTTATCACTAAGTTAATATACTAAAAATC
                          SARS-CoV-2 NC_045512.2  (29904) -----------------------------------------------------------------------------
```

```
Section 1444
                                                 (111112) 111112    111122     111133     111144     111155     111166     111177  111188
Autographa californica nucleopolyhedrovirus NC 0... (110877) ACATAGTCACTACAATATTTCAAAATATGAAGCCGACGAATAACGTTATGTTCGACGACGCGTCGGTCCTTTGGATC
                          SARS-CoV-2 NC_045512.2  (29904) -----------------------------------------------------------------------------
```

```
Section 1445
                                                 (111189) 111189    111199     111210     111221     111232     111243     111254  111265
Autographa californica nucleopolyhedrovirus NC 0... (110954) GACACGGACTACATTTATCAAAATTTAAAAATGCCTTTGCAGGCGTTTCAACAACTTTTGTTCACCATTCCATCTAA
                          SARS-CoV-2 NC_045512.2  (29904) -----------------------------------------------------------------------------
```

```
Section 1446
                                                 (111266) 111266    111276     111287     111298     111309     111320     111331  111342
Autographa californica nucleopolyhedrovirus NC 0... (111031) ACATAGAAAAATGATCAACGATGCGGGCGGATCGTGTCATAACACGGTCAAATACATGGTGGACATTTACGGAGCGG
                          SARS-CoV-2 NC_045512.2  (29904) -----------------------------------------------------------------------------
```

```
Section 1447
                                                 (111343) 111343    111353     111364     111375     111386     111397     111408  111419
Autographa californica nucleopolyhedrovirus NC 0... (111108) CCGTTCTGGTTTTGCGAACGCCTTGCTCGTTCGCCGACCAGTTGTTGAGCACATTTATTGCAAACAATTATTTGTGC
                          SARS-CoV-2 NC_045512.2  (29904) -----------------------------------------------------------------------------
```

```
Section 1448
                                                 (111420) 111420    111430     111441     111452     111463     111474     111485  111496
Autographa californica nucleopolyhedrovirus NC 0... (111185) TACTTTTACCGTCGTCGCCGATCACGATCACGCTCACGATCACGCTCGCGATCACGTTCTCCTCATTGCAGACCTCG
                          SARS-CoV-2 NC_045512.2  (29904) -----------------------------------------------------------------------------
```

```
Section 1449
                                                 (111497) 111497    111507     111518     111529     111540     111551     111562  111573
Autographa californica nucleopolyhedrovirus NC 0... (111262) TTCGCGCTCTCCTCATTGCAGACCTCGTTCGCGATCTCGGTCCCGGTCTAGATCGCGGTCACGTTCATCGTCTCCCA
                          SARS-CoV-2 NC_045512.2  (29904) -----------------------------------------------------------------------------
```

Section 1450

(111574) 111574 111584 111595 111606 111617 111628 111639 111650
Autographa californica nucleopolyhedrovirus NC_0... (111339) GGCGAGGGCGTCGACAAATATTCGACGCGCTGGAAAAGATTCGTCATCAAAACGACATGTTGATGAGCAACGTCAAC
SARS-CoV-2 NC_045512.2 (29904) --

Section 1451

(111651) 111651 111661 111672 111683 111694 111705 111716 111727
Autographa californica nucleopolyhedrovirus NC_0... (111416) CAAATAAATCTCAACCAAACTAATCAATTTTTAGAATTGTCCAACATGATGACGGGCGTGCGCAATCAAAACGTGCA
SARS-CoV-2 NC_045512.2 (29904) --

Section 1452

(111728) 111728 111738 111749 111760 111771 111782 111793 111804
Autographa californica nucleopolyhedrovirus NC_0... (111493) GCTCCTCGCGGCGTTGGAAACCGCTAAAGATGTTATTTTGACCAGATTAAACACATTGCTTGCCGAGATTACAGACT
SARS-CoV-2 NC_045512.2 (29904) --

Section 1453

(111805) 111805 111815 111826 111837 111848 111859 111870 111881
Autographa californica nucleopolyhedrovirus NC_0... (111570) CGTTACCCGACTTGACGTCCATGTTAGATAAATTAGCTGAACAATTGTTGGACGCCATCAACACGGTGCAGCAAACC
SARS-CoV-2 NC_045512.2 (29904) --

Section 1454

(111882) 111882 111892 111903 111914 111925 111936 111947 111958
Autographa californica nucleopolyhedrovirus NC_0... (111647) TGCGCAACGAGTTGAACAACACCAACTCTATTTTGACCAATTTAGCGTCAAGCGTCACAAACATCAACGGTACGCTC
SARS-CoV-2 NC_045512.2 (29904) --

Section 1455

(111959) 111959 111969 111980 111991 112002 112013 112024 112035
Autographa californica nucleopolyhedrovirus NC_0... (111724) AACAATTTGCTAGCCGCTATCGAAAACTTAGTAGGCGGCGGCGGCGGTGGCAATTTTAACGAAGCCGACAGACAAAA
SARS-CoV-2 NC_045512.2 (29904) --

Section 1456

(112036) 112036 112046 112057 112068 112079 112090 112101 112112
Autographa californica nucleopolyhedrovirus NC_0... (111801) ACTGGACCTCGTGTACACTTTGGTTAACGAAATCAAAAATATACTCACGGGAACGCTGACAAAAAAATAAGCATGTC
SARS-CoV-2 NC_045512.2 (29904) --

```
Section 1457
(112113) 112113    112123    112134    112145    112156    112167    112178    112189
Autographa californica nucleopolyhedrovirus NC 0... (111878) CGACAAAACACCAACAAAAAAGGGTGGCAGCCATGCCATGACGTTGCGAGAGCGCGGCGTAACAAAACCCCCAAAAA
SARS-CoV-2 NC_045512.2 (29904) -----------------------------------------------------------------------------

Section 1458
(112190) 112190    112200    112211    112222    112233    112244    112255    112266
Autographa californica nucleopolyhedrovirus NC 0... (111955) AGTCTGAAAAGTTGCAGCAATACAAGAAAGCCATCGCTGCCGAGCAAACGCTGCGCACCACAGCAGATGTTTCTTCT
SARS-CoV-2 NC_045512.2 (29904) -----------------------------------------------------------------------------

Section 1459
(112267) 112267    112277    112288    112299    112310    112321    112332    112343
Autographa californica nucleopolyhedrovirus NC 0... (112032) TTGCAGAACCCCGGGGAGAGTGCCGTTTTTCAAGAGTTGGAAAGATTAGAGAATGCAGTTGTAGTATTAGAAAATGA
SARS-CoV-2 NC_045512.2 (29904) -----------------------------------------------------------------------------

Section 1460
(112344) 112344    112354    112365    112376    112387    112398    112409    112420
Autographa californica nucleopolyhedrovirus NC 0... (112109) ACAAAAACGATTGTATCCCATATTAGATACGCCTCTTGATAATTTTATTGTCGCATTCGTGAATCCGACGTATCCCA
SARS-CoV-2 NC_045512.2 (29904) -----------------------------------------------------------------------------

Section 1461
(112421) 112421    112431    112442    112453    112464    112475    112486    112497
Autographa californica nucleopolyhedrovirus NC 0... (112186) TGGCCTATTTTGTCAATACCGATTACAAATTAAAACTAGAATGTGCCAGAATCAGAAGCGATTTACTTTACAAAAAC
SARS-CoV-2 NC_045512.2 (29904) -----------------------------------------------------------------------------

Section 1462
(112498) 112498    112508    112519    112530    112541    112552    112563    112574
Autographa californica nucleopolyhedrovirus NC 0... (112263) AAAAACGAAGTCGCTATCAACAGGCCTAAGATATCGTCTTTTAAATTGCAATTGAACAACGTAATTTTAGACACTAT
SARS-CoV-2 NC_045512.2 (29904) -----------------------------------------------------------------------------

Section 1463
(112575) 112575    112585    112596    112607    112618    112629    112640    112651
Autographa californica nucleopolyhedrovirus NC 0... (112340) AGAAACTATTGAATACGATTTACAAAATAAAGTTCTCACAATTACTGCACCTGTTCAAGATCAAGAACTAAGAAAAT
SARS-CoV-2 NC_045512.2 (29904) -----------------------------------------------------------------------------
```

```
                                                                                                          Section 1464
                                                  (112652) 112652    112662          112673          112684          112695          112706          112717   112728
Autographa californica nucleopolyhedrovirus  NC_0... (112417) CCATTATTTATTTTAATATTTTAAATAGTGACAGTTGGGAAGTACCAAAGTATATGAAAAAATTGTTTGATGAAATG
                               SARS-CoV-2 NC_045512.2  (29904) -----------------------------------------------------------------------------
                                                                                                          Section 1465
                                                  (112729) 112729    112739          112750          112761          112772          112783          112794   112805
Autographa californica nucleopolyhedrovirus  NC_0... (112494) CAATTGGAACCTCCCGTCATTTTACCATTAGGTCTTTAGATTTGGTAAGGCTAGCACGTCGACATCATGTTTGCGTC
                               SARS-CoV-2 NC_045512.2  (29904) -----------------------------------------------------------------------------
                                                                                                          Section 1466
                                                  (112806) 112806    112816          112827          112838          112849          112860          112871   112882
Autographa californica nucleopolyhedrovirus  NC_0... (112571) GTTGACCTCAGAGCAAAAGCTGTTATTAAAAAAATATAAATTTAACAATTATGTGAAAACGATCGAGTTGAGTCAAG
                               SARS-CoV-2 NC_045512.2  (29904) -----------------------------------------------------------------------------
                                                                                                          Section 1467
                                                  (112883) 112883    112893          112904          112915          112926          112937          112948   112959
Autographa californica nucleopolyhedrovirus  NC_0... (112648) CGCAGTTGGCTCATTGGCGTTCAAACAAAGATATTCAGCCAAAACCTTTGGATCGTGCAGAAATTTTACGTGTCGAA
                               SARS-CoV-2 NC_045512.2  (29904) -----------------------------------------------------------------------------
                                                                                                          Section 1468
                                                  (112960) 112960    112970          112981          112992          113003          113014          113025   113036
Autographa californica nucleopolyhedrovirus  NC_0... (112725) AAGGCCACCAGGGGACAAAGCAAAAATGAGCTGTGGACGCTATTGCGTTTGGATCGCAACACAGCGTCTGCATCGTC
                               SARS-CoV-2 NC_045512.2  (29904) -----------------------------------------------------------------------------
                                                                                                          Section 1469
                                                  (113037) 113037    113047          113058          113069          113080          113091          113102   113113
Autographa californica nucleopolyhedrovirus  NC_0... (112802) CAACTCGTCCGGCAACATGTTACAACGACCAGCGCTTTTGTTTGGAAACGCGCAAGAAAGTCACGTCAAAGAAACCA
                               SARS-CoV-2 NC_045512.2  (29904) -----------------------------------------------------------------------------
                                                                                                          Section 1470
                                                  (113114) 113114    113124          113135          113146          113157          113168          113179   113190
Autographa californica nucleopolyhedrovirus  NC_0... (112879) ACGGCATCATGTTAGACCACATGCGCGAAATCATAGAAAGTAAAATTATGAGCGCGGTCGTTGAAACGGTTTTGGAT
                               SARS-CoV-2 NC_045512.2  (29904) -----------------------------------------------------------------------------
```

```
                                                                                                                            Section 1471
                                                  (113191) 113191     113201     113212     113223     113234     113245     113256  113267
Autographa californica nucleopolyhedrovirus NC 0... (112956) TGCGGCATGTTCTTTAGCCCCTTGGGTTTGCACGCCGCTTCGCCCGATGCGTATTTTTCTCTCGCCGACGGAACGTG
                            SARS-CoV-2 NC_045512.2  (29904) -----------------------------------------------------------------------------
                                                                                                                            Section 1472
                                                  (113268) 113268     113278     113289     113300     113311     113322     113333  113344
Autographa californica nucleopolyhedrovirus NC 0... (113033) GATCCCAGTGGAAATAAAATGTCCGTACAATTACCGAGACACGACCGTGGAGCAGATGCGTGTCGAGTTGGGGAACG
                            SARS-CoV-2 NC_045512.2  (29904) -----------------------------------------------------------------------------
                                                                                                                            Section 1473
                                                  (113345) 113345     113355     113366     113377     113388     113399     113410  113421
Autographa californica nucleopolyhedrovirus NC 0... (113110) GCAATCGCAAGTATCGCGTGAAACACACCGCGCTGTTGGTTAACAAGAAAGGCACGCCCCAGTTCGAAATGGTCAAA
                            SARS-CoV-2 NC_045512.2  (29904) -----------------------------------------------------------------------------
                                                                                                                            Section 1474
                                                  (113422) 113422     113432     113443     113454     113465     113476     113487  113498
Autographa californica nucleopolyhedrovirus NC 0... (113187) ACGGATGCGCATTACAAGCAAATGCAACGGCAGATGTATGTGATGAACGCGCCTATGGGCTTTTACGTGGTCAAATT
                            SARS-CoV-2 NC_045512.2  (29904) -----------------------------------------------------------------------------
                                                                                                                            Section 1475
                                                  (113499) 113499     113509     113520     113531     113542     113553     113564  113575
Autographa californica nucleopolyhedrovirus NC 0... (113264) CAAACAAAATTTGGTGGTGGTTTCTGTGCCGCGCGACGAAACGTTCTGCAACAAAGAACTGTCTACGGAAAACAACG
                            SARS-CoV-2 NC_045512.2  (29904) -----------------------------------------------------------------------------
                                                                                                                            Section 1476
                                                  (113576) 113576     113586     113597     113608     113619     113630     113641  113652
Autographa californica nucleopolyhedrovirus NC 0... (113341) CGTACGTGGCGTTTGCCGTGGAAAACTCCAACTGCGCGCGCTACCAATGCGCCGACAAGCGACGGCTTTCATTCAAA
                            SARS-CoV-2 NC_045512.2  (29904) -----------------------------------------------------------------------------
                                                                                                                            Section 1477
                                                  (113653) 113653     113663     113674     113685     113696     113707     113718  113729
Autographa californica nucleopolyhedrovirus NC 0... (113418) ACGCACAGCTGCAATCACAACTATAGTGGTCAAGAAATCGATGCTATGGTCGATCGCGGAATATATTTAGATTATGG
                            SARS-CoV-2 NC_045512.2  (29904) -----------------------------------------------------------------------------
```

```
                                                                                                                   Section 1478
                                                  (113730) 113730    113740     113751     113762     113773     113784     113795 113806
Autographa californica nucleopolyhedrovirus NC 0... (113495) ACATTTAAAATGTGCGTACTGTGATTTTAGCTCAGACAGTCGGGAAACGTGCGATTCTGTTTTAAAACGCGAGCACA
                           SARS-CoV-2 NC_045512.2 (29904) -----------------------------------------------------------------------------
                                                                                                                   Section 1479
                                                  (113807) 113807    113817     113828     113839     113850     113861     113872 113883
Autographa californica nucleopolyhedrovirus NC 0... (113572) CCAACTGCAAAAGTTTTAACTTGAAACATAAAAACTTTGACAATCCTACATACTTTGATTATGTTAAAAGATTGCAA
                           SARS-CoV-2 NC_045512.2 (29904) -----------------------------------------------------------------------------
                                                                                                                   Section 1480
                                                  (113884) 113884    113894     113905     113916     113927     113938     113949 113960
Autographa californica nucleopolyhedrovirus NC 0... (113649) AGTTTGCTAAAGAGTCACCACTTTAGAAACGACGCTAAAACACTTGCCTATTTTGGTTACTATTTAACTCATACAGG
                           SARS-CoV-2 NC_045512.2 (29904) -----------------------------------------------------------------------------
                                                                                                                   Section 1481
                                                  (113961) 113961    113971     113982     113993     114004     114015     114026 114037
Autographa californica nucleopolyhedrovirus NC 0... (113726) AACCCTGAAGACCTTTTGCTGCGGATCGCAAAACTCGTCGCCCACCAAACACGATCATTTAAACGACTGTGTATATT
                           SARS-CoV-2 NC_045512.2 (29904) -----------------------------------------------------------------------------
                                                                                                                   Section 1482
                                                  (114038) 114038    114048     114059     114070     114081     114092     114103 114114
Autographa californica nucleopolyhedrovirus NC 0... (113803) ATTTGGAAATAAAATAAACCTTTATATTATATATAATTCTTTTATTTATACATTTGTTTATACAATTTTATTTACGA
                           SARS-CoV-2 NC_045512.2 (29904) -----------------------------------------------------------------------------
                                                                                                                   Section 1483
                                                  (114115) 114115    114125     114136     114147     114158     114169     114180 114191
Autographa californica nucleopolyhedrovirus NC 0... (113880) CAAATATTGACTCGTTGTTCAGAAAGTTTAATAAGCTTGTCAATTTCTTCGGCTTGCAAAGGGCTGCCAACGCGTTC
                           SARS-CoV-2 NC_045512.2 (29904) -----------------------------------------------------------------------------
                                                                                                                   Section 1484
                                                  (114192) 114192    114202     114213     114224     114235     114246     114257 114268
Autographa californica nucleopolyhedrovirus NC 0... (113957) GTTTTGAATGCGCGTAATCCGGTTTACGGTATTGTTGGCGCGAACAATAAACTCCTCAACTGGCAAATTAACAATTT
                           SARS-CoV-2 NC_045512.2 (29904) -----------------------------------------------------------------------------
```

Section 1485

```
                                                     (114269) 114269    114279     114290     114301     114312     114323     114334    114345
Autographa californica nucleopolyhedrovirus NC 0... (114034) TGTTTGCGTACTCATTGTGCACTGCGGCCAGGTTTTGTAGAATGTTTTCGGGAAAAATGGCAATTCTATTAAATTTG
                             SARS-CoV-2 NC_045512.2  (29904) -----------------------------------------------------------------------------
```

Section 1486

```
                                                     (114346) 114346    114356     114367     114378     114389     114400     114411    114422
Autographa californica nucleopolyhedrovirus NC 0... (114111) ACATGTTTTTGATTGTATACATAGTTTTGATATTCTTCCAGCGTAGGATATTTGTTTAAACTCTTGACGCATTCAAT
                             SARS-CoV-2 NC_045512.2  (29904) -----------------------------------------------------------------------------
```

Section 1487

```
                                                     (114423) 114423    114433     114444     114455     114466     114477     114488    114499
Autographa californica nucleopolyhedrovirus NC 0... (114188) GTACAATTTGTGCAGTGACAAAATTCTGTTAAAATCCAAACGAGAACATTTCTCAAAAGTTATTTCTTGACCGTTGA
                             SARS-CoV-2 NC_045512.2  (29904) -----------------------------------------------------------------------------
```

Section 1488

```
                                                     (114500) 114500    114510     114521     114532     114543     114554     114565    114576
Autographa californica nucleopolyhedrovirus NC 0... (114265) AATGTACACTTTGCAATTGTTTCAATAAACTGTCGTAAAAAGTTTTTCCTTCTTCAAGCACAAACGCGGGGCGCATC
                             SARS-CoV-2 NC_045512.2  (29904) -----------------------------------------------------------------------------
```

Section 1489

```
                                                     (114577) 114577    114587     114598     114609     114620     114631     114642    114653
Autographa californica nucleopolyhedrovirus NC 0... (114342) GTGTTATCTACAACGCTTATGTACTTGTCAAAATCTTCAATTATATGATAGAAATACAAATATCTCTCCGCGTTTAT
                             SARS-CoV-2 NC_045512.2  (29904) -----------------------------------------------------------------------------
```

Section 1490

```
                                                     (114654) 114654    114664     114675     114686     114697     114708     114719    114730
Autographa californica nucleopolyhedrovirus NC 0... (114419) GGACGTGTCGTTTAAAACATGTTCGTCAACAACTCCGTTATGATTTACTTTCAAAAATTTCAAATCTTGCAAAGCGT
                             SARS-CoV-2 NC_045512.2  (29904) -----------------------------------------------------------------------------
```

Section 1491

```
                                                     (114731) 114731    114741     114752     114763     114774     114785     114796    114807
Autographa californica nucleopolyhedrovirus NC 0... (114496) CCGCGTTGGTCAACTTGTTGATAATAAATTTGTCTTTGCATTCAAACGCTCTGTTTGCAATCCACTCCACAGCGTCC
                             SARS-CoV-2 NC_045512.2  (29904) -----------------------------------------------------------------------------
```

```
                                                                                                    Section 1492
                                          (114808) 114808    114818     114829     114840     114851     114862     114873    114884
Autographa californica nucleopolyhedrovirus NC 0... (114573) AAAACGGACATGCGTTTAAACATGTTGATACGTTTTAGACAATACGCTCGTTTTTTTACCGCCTCAACGTTCACGTC
                            SARS-CoV-2 NC_045512.2  (29904) -----------------------------------------------------------------------------
                                                                                                    Section 1493
                                          (114885) 114885    114895     114906     114917     114928     114939     114950    114961
Autographa californica nucleopolyhedrovirus NC 0... (114650) CGTGTAGTCGCACCATTGCAGGATTTGCAACATGTCCTCGGCAAAATGCGCGAACTGCCGCAGCTTTTCCTTTCCAA
                            SARS-CoV-2 NC_045512.2  (29904) -----------------------------------------------------------------------------
                                                                                                    Section 1494
                                          (114962) 114962    114972     114983     114994     115005     115016     115027    115038
Autographa californica nucleopolyhedrovirus NC 0... (114727) AATGTTGATTGTCGTGTTTAAAAAGCAACGTTGAAATTTCCGAGACATACCACAAAGCCGTGGGCAATTTTACTTTG
                            SARS-CoV-2 NC_045512.2  (29904) -----------------------------------------------------------------------------
                                                                                                    Section 1495
                                          (115039) 115039    115049     115060     115071     115082     115093     115104    115115
Autographa californica nucleopolyhedrovirus NC 0... (114804) ATCAGCGGCTCCATAGCCAGGTTGCTGAACCCGATCATGCATTCCGTGTTGTTAATGCGGTAAATGACATAGCGTTT
                            SARS-CoV-2 NC_045512.2  (29904) -----------------------------------------------------------------------------
                                                                                                    Section 1496
                                          (115116) 115116    115126     115137     115148     115159     115170     115181    115192
Autographa californica nucleopolyhedrovirus NC 0... (114881) AAAGTAGTCCTTTACATTATCGTCAATGTATTCTGCGTCGTTTATGTGCTTGTACAGCAAATAGTACATAAGGCCCG
                            SARS-CoV-2 NC_045512.2  (29904) -----------------------------------------------------------------------------
                                                                                                    Section 1497
                                          (115193) 115193    115203     115214     115225     115236     115247     115258    115269
Autographa californica nucleopolyhedrovirus NC 0... (114958) CGTTAAACGCGACCTTTTTAGCGTCAAAATACGTGCACGCCAACACGTAATCGTTGTATTCGTCGAATTGCTCGTTG
                            SARS-CoV-2 NC_045512.2  (29904) -----------------------------------------------------------------------------
                                                                                                    Section 1498
                                          (115270) 115270    115280     115291     115302     115313     115324     115335    115346
Autographa californica nucleopolyhedrovirus NC 0... (115035) GGCACTATGGCGCCCGTAAAAGGGCGTCTGCTGCGCGGTGACAAACGCGTTCCATGCTGAATCAACTGCTTCAAACT
                            SARS-CoV-2 NC_045512.2  (29904) -----------------------------------------------------------------------------
```

Section 1499

```
                                                  (115347) 115347    115357     115368     115379     115390     115401     115412     115423
Autographa californica nucleopolyhedrovirus NC_0... (115112) TTCCAAATTATAACAATATTCAATTGAATTTTTAATCTCTTTATTTTGGCTCCATAAAAGAGGAAACTCGAGTCGGC
                          SARS-CoV-2 NC_045512.2  (29904) -----------------------------------------------------------------------------
```

Section 1500

```
                                                  (115424) 115424    115434     115445     115456     115467     115478     115489     115500
Autographa californica nucleopolyhedrovirus NC_0... (115189) TTTTAAACTTGGTCAAACTGCCCTGAATTGTTTCAAACAAGTTGTAATGTGTTAACAATATGGCCGGCACACCGCTA
                          SARS-CoV-2 NC_045512.2  (29904) -----------------------------------------------------------------------------
```

Section 1501

```
                                                  (115501) 115501    115511     115522     115533     115544     115555     115566     115577
Autographa californica nucleopolyhedrovirus NC_0... (115266) TCGTTGGCTAAAATACAATCGGGGAATCGAATATTTTCTACGTTGCTGTAATCGTACGCTTCGTCGTCGTCGTTGGC
                          SARS-CoV-2 NC_045512.2  (29904) -----------------------------------------------------------------------------
```

Section 1502

```
                                                  (115578) 115578    115588     115599     115610     115621     115632     115643     115654
Autographa californica nucleopolyhedrovirus NC_0... (115343) AACAACATCGTCGGTTTCGGCGTTAACGCTCGCTAACTTGTTCTGATAGTGTAAATTTTTCATTACATCAAAAGCGT
                          SARS-CoV-2 NC_045512.2  (29904) -----------------------------------------------------------------------------
```

Section 1503

```
                                                  (115655) 115655    115665     115676     115687     115698     115709     115720     115731
Autographa californica nucleopolyhedrovirus NC_0... (115420) ATGACTTGTTGCGATTGTGCAAATAATTTATGGCCGTGCTAATGGTGCTGTCGATAATTTTATCAAAATTGAGAACA
                          SARS-CoV-2 NC_045512.2  (29904) -----------------------------------------------------------------------------
```

Section 1504

```
                                                  (115732) 115732    115742     115753     115764     115775     115786     115797     115808
Autographa californica nucleopolyhedrovirus NC_0... (115497) TCGGCGTTATACAACGTTTTATAAAATTCTGTTGACTTGAACGTGTTTACAAACTCATTTTTATTTTTAATCTGGTC
                          SARS-CoV-2 NC_045512.2  (29904) -----------------------------------------------------------------------------
```

Section 1505

```
                                                  (115809) 115809    115819     115830     115841     115852     115863     115874     115885
Autographa californica nucleopolyhedrovirus NC_0... (115574) AAAATTCATACTAGAATTGTTAGTTTGTTTGATTTCGCTGAATAGCCGCTGGCGGAGACGCTTCAGCTTGTCCACCT
                          SARS-CoV-2 NC_045512.2  (29904) -----------------------------------------------------------------------------
```

```
                                                                                                    Section 1506
                                              (115886) 115886    115896      115907      115918      115929      115940      115951  115962
Autographa californica nucleopolyhedrovirus_NC_0... (115651) CGTTTAACACGTTGGCGTCCGTCGGCATGGAATTGATAAATTTGAACCGAACAAAAGACAGCAGTTCATCTTTTTTC
                        SARS-CoV-2 NC_045512.2 (29904) -----------------------------------------------------------------------------
                                                                                                    Section 1507
                                              (115963) 115963    115973      115984      115995      116006      116017      116028  116039
Autographa californica nucleopolyhedrovirus_NC_0... (115728) GATATAAAATTTTCGGTTGTAATGATATCGTAGTTAAATTCTTTGGTTAAATTGACCCATTCGACCATTTCATCGTT
                        SARS-CoV-2 NC_045512.2 (29904) -----------------------------------------------------------------------------
                                                                                                    Section 1508
                                              (116040) 116040    116050      116061      116072      116083      116094      116105  116116
Autographa californica nucleopolyhedrovirus_NC_0... (115805) GCGATAAATCTTGCAGTCCGAGTTGTTGACAAACGCCGAGGCAACGGACAAATCAATCTGTTCCGTGTTATTATTGA
                        SARS-CoV-2 NC_045512.2 (29904) -----------------------------------------------------------------------------
                                                                                                    Section 1509
                                              (116117) 116117    116127      116138      116149      116160      116171      116182  116193
Autographa californica nucleopolyhedrovirus_NC_0... (115882) TGGCATAAAACACAATGCGTTCGAAACTAAACGGTTTTTCGTTTAGCAAATTTTTGCAAACGTTTGCCTCATTTTTG
                        SARS-CoV-2 NC_045512.2 (29904) -----------------------------------------------------------------------------
                                                                                                    Section 1510
                                              (116194) 116194    116204      116215      116226      116237      116248      116259  116270
Autographa californica nucleopolyhedrovirus_NC_0... (115959) GAAATTTGGCCGTCGGTCACCATGTACAAAAGTTTCAACTTGCCGTCGAGCAAGTTTATATTCTTGTGAATCCACTT
                        SARS-CoV-2 NC_045512.2 (29904) -----------------------------------------------------------------------------
                                                                                                    Section 1511
                                              (116271) 116271    116281      116292      116303      116314      116325      116336  116347
Autographa californica nucleopolyhedrovirus_NC_0... (116036) TATGAATTCGCTGGGCCTGGTGTCAGTACCCTCGCCATTGCGGCGCAAATAACGACTCTTGACGTCTCCGATTTCTT
                        SARS-CoV-2 NC_045512.2 (29904) -----------------------------------------------------------------------------
                                                                                                    Section 1512
                                              (116348) 116348    116358      116369      116380      116391      116402      116413  116424
Autographa californica nucleopolyhedrovirus_NC_0... (116113) TTTGGCGGCAATAAGCACTCCAATGCAAATACAAAACTTTGTCGCAACTACTGATGTTTTCGATTTCATTCTGAAAT
                        SARS-CoV-2 NC_045512.2 (29904) -----------------------------------------------------------------------------
```

Section 1513

(116425) 116425 116435 116446 116457 116468 116479 116490 116501
Autographa californica nucleopolyhedrovirus NC_0... (116190) TGTTCTAAAGTTTGTAACGCGTTCTTGTTAAAGTAATAGTCCGAGTTTGTCGACAAGGAATCGTCGGTGGCGTACAC
SARS-CoV-2 NC_045512.2 (29904) --

Section 1514

(116502) 116502 116512 116523 116534 116545 116556 116567 116578
Autographa californica nucleopolyhedrovirus NC_0... (116267) GTAGTAGTTAATCATCTTGTTGATTGATATTTAATTTTGGCGACGGATTTTTATATACACGAGCGGAGCGGTCACGT
SARS-CoV-2 NC_045512.2 (29904) --

Section 1515

(116579) 116579 116589 116600 116611 116622 116633 116644 116655
Autographa californica nucleopolyhedrovirus NC_0... (116344) TCTGTAACATGAGTGATCGTGTGTGTGTTATCTCTGGCAGCGCGATAGTGGTCGCGAAAATTACACGCGCGTCGTAA
SARS-CoV-2 NC_045512.2 (29904) --

Section 1516

(116656) 116656 116666 116677 116688 116699 116710 116721 116732
Autographa californica nucleopolyhedrovirus NC_0... (116421) CGTGAACGTTTATATTATAAATATTCAACGTTGCTTGTATTAAGTGAGCATTTGAGCTTTACCATTGCAAAATGTGT
SARS-CoV-2 NC_045512.2 (29904) --

Section 1517

(116733) 116733 116743 116754 116765 116776 116787 116798 116809
Autographa californica nucleopolyhedrovirus NC_0... (116498) GTAATTTTTCCGGTAGAAATCGACGTGTCCCAGACGATTATTCGAGATTGTCAGGTGGACAAACAAACCAGAGAGTT
SARS-CoV-2 NC_045512.2 (29904) --

Section 1518

(116810) 116810 116820 116831 116842 116853 116864 116875 116886
Autographa californica nucleopolyhedrovirus NC_0... (116575) GGTGTACATTAACAAGATTATGAACACGCAATTGACAAAACCCGTTCTCATGATGTTTAACATTTCGGGTCCTATAC
SARS-CoV-2 NC_045512.2 (29904) --

Section 1519

(116887) 116887 116897 116908 116919 116930 116941 116952 116963
Autographa californica nucleopolyhedrovirus NC_0... (116652) GAAGCGTTACGCGCAAGAACAACAATTTGCGCGACAGAATAAAATCAAAAGTCGATGAACAATTTGATCAACTAGAA
SARS-CoV-2 NC_045512.2 (29904) --

Section 1520

	(116964)	116964 116974 116985 116996 117007 117018 117029 117040
Autographa californica nucleopolyhedrovirus NC 0...	(116729)	CGCGATTACAGCGATCAAATGGATGGATTCCACGATAGCATCAAGTATTTTAAAGATGAACACTATTCGGTAAGTTG
SARS-CoV-2 NC_045512.2	(29904)	---

Section 1521

	(117041)	117041 117051 117062 117073 117084 117095 117106 117117
Autographa californica nucleopolyhedrovirus NC 0...	(116806)	CCAAAATGGCAGCGTGTTGAAAAGCAAGTTTGCTAAAATTTTAAAGAGTCATGATTATACCGATAAAAAGTCTATTG
SARS-CoV-2 NC_045512.2	(29904)	---

Section 1522

	(117118)	117118 117128 117139 117150 117161 117172 117183 117194
Autographa californica nucleopolyhedrovirus NC 0...	(116883)	AAGCTTACGAGAAATACTGTTTGCCCAAATTGGTCGACGAACGCAACGACTACTACGTGGCGGTATGCGTGTTGAAG
SARS-CoV-2 NC_045512.2	(29904)	---

Section 1523

	(117195)	117195 117205 117216 117227 117238 117249 117260 117271
Autographa californica nucleopolyhedrovirus NC 0...	(116960)	CCGGGATTTGAGAACGGCAGCAACCAAGTGCTATCTTTCGAGTACAACCCGATTGGTAACAAAGTTATTGTGCCGTT
SARS-CoV-2 NC_045512.2	(29904)	---

Section 1524

	(117272)	117272 117282 117293 117304 117315 117326 117337 117348
Autographa californica nucleopolyhedrovirus NC 0...	(117037)	TGCTCACGAAATTAACGACACGGGACTTTACGAGTACGACGTCGTAGCTTACGTGGACAGTGTGCAGTTTGATGGCG
SARS-CoV-2 NC_045512.2	(29904)	---

Section 1525

	(117349)	117349 117359 117370 117381 117392 117403 117414 117425
Autographa californica nucleopolyhedrovirus NC 0...	(117114)	AACAATTTGAAGAGTTTGTGCAGAGTTTAATATTGCCGTCGTCGTTCAAAAATTCGGAAAAGGTTTTATATTACAAC
SARS-CoV-2 NC_045512.2	(29904)	---

Section 1526

	(117426)	117426 117436 117447 117458 117469 117480 117491 117502
Autographa californica nucleopolyhedrovirus NC 0...	(117191)	GAAGCGTCGAAAAACAAAAGCATGATCTACAAGGCTTTAGAGTTTACTACAGAATCGAGCTGGGGCAAATCCGAAAA
SARS-CoV-2 NC_045512.2	(29904)	---

```
                                                                                         Section 1527
                                             (117503) 117503    117513     117524     117535     117546     117557     117568   117579
Autographa californica nucleopolyhedrovirus  NC_0... (117268) GTATAATTGGAAAATTTTTTGTAACGGTTTTATTTATGATAAAAAATCAAAAGTGTTGTATGTTAAATTGCACAATG
                          SARS-CoV-2 NC_045512.2  (29904) -----------------------------------------------------------------------------
                                                                                         Section 1528
                                             (117580) 117580    117590     117601     117612     117623     117634     117645   117656
Autographa californica nucleopolyhedrovirus  NC_0... (117345) TAACTAGTGCACTCAACAAAAATGTAATATTAAACACAATTAAATAAATGTTAAAATTTATTGCCTAATATTATTTT
                          SARS-CoV-2 NC_045512.2  (29904) -----------------------------------------------------------------------------
                                                                                         Section 1529
                                             (117657) 117657    117667     117678     117689     117700     117711     117722   117733
Autographa californica nucleopolyhedrovirus  NC_0... (117422) GTCATTGCTTGTCATTTATTAATTTGGATGATGTCATTTGTTTTTAAAATTGAACTGGCTTTACGAGTAGAATTCTA
                          SARS-CoV-2 NC_045512.2  (29904) -----------------------------------------------------------------------------
                                                                                         Section 1530
                                             (117734) 117734    117744     117755     117766     117777     117788     117799   117810
Autographa californica nucleopolyhedrovirus  NC_0... (117499) CGCGTAAAACACAATCAAGTATGAGTCATAATCTGATGTCATGTTTTGTACACGGCTCATAACCGAACTGGCTTTAC
                          SARS-CoV-2 NC_045512.2  (29904) -----------------------------------------------------------------------------
                                                                                         Section 1531
                                             (117811) 117811    117821     117832     117843     117854     117865     117876   117887
Autographa californica nucleopolyhedrovirus  NC_0... (117576) GAGTAGAATTCTACTTGTAATGCACGATCAGTGGATGATGTCATTTGTTTTTCAAATCGAGATGATGTCATGTTTTG
                          SARS-CoV-2 NC_045512.2  (29904) -----------------------------------------------------------------------------
                                                                                         Section 1532
                                             (117888) 117888    117898     117909     117920     117931     117942     117953   117964
Autographa californica nucleopolyhedrovirus  NC_0... (117653) CACACGGCTCATAAACTCGCTTTACGAGTAGAATTCTACGTGTAACGCACGATCGATTGATGAGTCATTTGTTTTGC
                          SARS-CoV-2 NC_045512.2  (29904) -----------------------------------------------------------------------------
                                                                                         Section 1533
                                             (117965) 117965    117975     117986     117997     118008     118019     118030   118041
Autographa californica nucleopolyhedrovirus  NC_0... (117730) AATATGATATCATACAATATGACTCATTTGTTTTCAAAACCGAACTTGATTTACGGGTAGAATTCTACTTGTAAAG
                          SARS-CoV-2 NC_045512.2  (29904) -----------------------------------------------------------------------------
```

Section 1534

(118042) 118042 118052 118063 118074 118085 118096 118107 118118
Autographa californica nucleopolyhedrovirus NC_0... (117807) CACAATCAAAAAGATGATGTCATTTGTTTTTCAAAACTGAACTCGCTTTACGAGTAGAATTCTACGTGTAAAACACA
SARS-CoV-2 NC_045512.2 (29904) ---

Section 1535

(118119) 118119 118129 118140 118151 118162 118173 118184 118195
Autographa californica nucleopolyhedrovirus NC_0... (117884) ATCAAGAAATGATGTCATTTGTTATAAAAATAAAAGCTGATGTCATGTTTTGCACATGGCTCATAACTAAACTCGCT
SARS-CoV-2 NC_045512.2 (29904) ---

Section 1536

(118196) 118196 118206 118217 118228 118239 118250 118261 118272
Autographa californica nucleopolyhedrovirus NC_0... (117961) TTACGGGTAGAATTCTACGCGTAAAACATGATTGATAATTAAATAATTCATTTGCAAGCTATACGTTAAATCAAACG
SARS-CoV-2 NC_045512.2 (29904) ---

Section 1537

(118273) 118273 118283 118294 118305 118316 118327 118338 118349
Autographa californica nucleopolyhedrovirus NC_0... (118038) GACGTTATGGAATTGTATAATATTAAATATGCAATTGATCCAACAAATAAAATTGTAATAGAGCAAGTCGACAATGT
SARS-CoV-2 NC_045512.2 (29904) ---

Section 1538

(118350) 118350 118360 118371 118382 118393 118404 118415 118426
Autographa californica nucleopolyhedrovirus NC_0... (118115) GGACGCGTTTGTGCATATTTTAGAACCGGGTCAAGAAGTGTTCGACGAAACGCTAAGCCAGTACCACCAATTTCCTG
SARS-CoV-2 NC_045512.2 (29904) ---

Section 1539

(118427) 118427 118437 118448 118459 118470 118481 118492 118503
Autographa californica nucleopolyhedrovirus NC_0... (118192) GCGTCGTTAGTTCGATTATTTTCCCGCAACTCGTGTTAAACACAATAATTAGCGTTTTGAGCGAAGACGGCAGTTTG
SARS-CoV-2 NC_045512.2 (29904) ---

Section 1540

(118504) 118504 118514 118525 118536 118547 118558 118569 118580
Autographa californica nucleopolyhedrovirus NC_0... (118269) CTCACGTTGAAACTCGAAAACACTTGTTTTAATTTTCACGTGTGCAATAAACGCTTTGTGTTTGGCAATTTGCCAGC
SARS-CoV-2 NC_045512.2 (29904) ---

```
Section 1541
                                                   (118581) 118581     118591     118602     118613     118624     118635     118646  118657
Autographa californica nucleopolyhedrovirus NC_0... (118346) GGCGGTCGTGAATAATGAAACGAAGCAAAAACTGCGCATTGGAGCTCCAATTTTTGCCGGCAAAAAGCTGGTTTCGG
                             SARS-CoV-2 NC_045512.2 (29904) -----------------------------------------------------------------------------

Section 1542
                                                   (118658) 118658     118668     118679     118690     118701     118712     118723  118734
Autographa californica nucleopolyhedrovirus NC_0... (118423) TCGTGACGGCGTTTCATCGTGTTGGCGAAAACGAATGGCTGTTACCGGTGACGGGAATTCGAGAGGCGTCCCAGCTG
                             SARS-CoV-2 NC_045512.2 (29904) -----------------------------------------------------------------------------

Section 1543
                                                   (118735) 118735     118745     118756     118767     118778     118789     118800  118811
Autographa californica nucleopolyhedrovirus NC_0... (118500) TCGGGACATATGAAGGTGCTGAACGGCGTCCGTGTTGAAAAATGGCGACCCAACATGTCCGTCTACGGGACTGTGCA
                             SARS-CoV-2 NC_045512.2 (29904) -----------------------------------------------------------------------------

Section 1544
                                                   (118812) 118812     118822     118833     118844     118855     118866     118877  118888
Autographa californica nucleopolyhedrovirus NC_0... (118577) ATTGCCGTACGATAAAATTAAACAGCATGCGCTCGAGCAAGAAAATAAAACGCCAAACGCGTTGGAGTCTTGTGTGC
                             SARS-CoV-2 NC_045512.2 (29904) -----------------------------------------------------------------------------

Section 1545
                                                   (118889) 118889     118899     118910     118921     118932     118943     118954  118965
Autographa californica nucleopolyhedrovirus NC_0... (118654) TATTTTACAAAGATTCAGAAATACGCATCACTTACAACAAGGGGGACTATGAAATTATGCATTTGAGGATGCCGGGA
                             SARS-CoV-2 NC_045512.2 (29904) -----------------------------------------------------------------------------

Section 1546
                                                   (118966) 118966     118976     118987     118998     119009     119020     119031  119042
Autographa californica nucleopolyhedrovirus NC_0... (118731) CCTTTAATTCAACCCAACACAATATATTATAGTTAAATAAGAATTATTATCAAATCATTTGTATATTAATTAAAATA
                             SARS-CoV-2 NC_045512.2 (29904) -----------------------------------------------------------------------------

Section 1547
                                                   (119043) 119043     119053     119064     119075     119086     119097     119108  119119
Autographa californica nucleopolyhedrovirus NC_0... (118808) CTATACTGTAAATTACATTTTATTTACAATCATGTCAAAGCCTAACGTTTTGACGCAAATTTTAGACGCCGTTACGG
                             SARS-CoV-2 NC_045512.2 (29904) -----------------------------------------------------------------------------
```

Section 1548

(119120) 119120 119130 119141 119152 119163 119174 119185 119196
Autographa californica nucleopolyhedrovirus NC_0... (118885) AAACTAACACAAAGGTTGACAGTGTTCAAACTCAGTTAAACGGGCTGGAAGAATCATTCCAGCTTTTGGACGGTTTG
SARS-CoV-2 NC_045512.2 (29904) ---

Section 1549

(119197) 119197 119207 119218 119229 119240 119251 119262 119273
Autographa californica nucleopolyhedrovirus NC_0... (118962) CCCGCTCAATTGACCGATCTTAACACTAAGATCTCAGAAATTCAATCCATATTGACCGGCGACATTGTTCCGGATCT
SARS-CoV-2 NC_045512.2 (29904) ---

Section 1550

(119274) 119274 119284 119295 119306 119317 119328 119339 119350
Autographa californica nucleopolyhedrovirus NC_0... (119039) TCCAGACTCACTAAAGCCTAAGCTGAAAACCCAAGCTTTTGAACTCGATTCAGACGCTCGTCGTGGTAAACGCAGTT
SARS-CoV-2 NC_045512.2 (29904) ---

Section 1551

(119351) 119351 119361 119372 119383 119394 119405 119416 119427
Autographa californica nucleopolyhedrovirus NC_0... (119116) CCAAGTAAATGAATCGTTTTTAAAATAACAAATCAATTGTTTTATAATATTCGTACGATTCTTTGATTATGTAATAA
SARS-CoV-2 NC_045512.2 (29904) ---

Section 1552

(119428) 119428 119438 119449 119460 119471 119482 119493 119504
Autographa californica nucleopolyhedrovirus NC_0... (119193) AATGTGATCATTAGGAAGATTACGAAAAATATAAAAAATATGAGTTCTGTGTGTATAACAAATGCTGTAAACGCCAC
SARS-CoV-2 NC_045512.2 (29904) ---

Section 1553

(119505) 119505 119515 119526 119537 119548 119559 119570 119581
Autographa californica nucleopolyhedrovirus NC_0... (119270) AATTGTGTTTGTTGCAAATAAACCCAGTATTATTTGATTAAAATTGTTGTTTTCTTTGTTCATAGACAATAGTGTGT
SARS-CoV-2 NC_045512.2 (29904) ---

Section 1554

(119582) 119582 119592 119603 119614 119625 119636 119647 119658
Autographa californica nucleopolyhedrovirus NC_0... (119347) TTTGCCTAAACGTGTACTGCATAAACTCCATGCGAGTGTATAGCGAGCTAGTGGCTAACGCTTGCCCCACCAAAGTA
SARS-CoV-2 NC_045512.2 (29904) ---

```
                                                                                                                        Section 1555
                                              (119659) 119659    119669     119680     119691     119702     119713     119724  119735
Autographa californica nucleopolyhedrovirus NC 0... (119424) GATTCGTCAAAATCCTCAATTTCATCACCCTCCTCCAAGTTTAACATTTGGCCGTCGGAATTAACTTCTAAAGATGC
                              SARS-CoV-2 NC_045512.2 (29904) -----------------------------------------------------------------------------
                                                                                                                        Section 1556
                                              (119736) 119736    119746     119757     119768     119779     119790     119801  119812
Autographa californica nucleopolyhedrovirus NC 0... (119501) CACATAATCTAATAAATGAAATAGAGATTCAAACGTGGCGTCATCGTCCGTTTCGACCATTTCCGAAAAGAACTCGG
                              SARS-CoV-2 NC_045512.2 (29904) -----------------------------------------------------------------------------
                                                                                                                        Section 1557
                                              (119813) 119813    119823     119834     119845     119856     119867     119878  119889
Autographa californica nucleopolyhedrovirus NC 0... (119578) GCATAAACTCTATGATTTCTCTGGACGTGGTGTTGTCGAAACTCTCAAAGTACGCAGTCAGGAACGTGCGCGACATG
                              SARS-CoV-2 NC_045512.2 (29904) -----------------------------------------------------------------------------
                                                                                                                        Section 1558
                                              (119890) 119890    119900     119911     119922     119933     119944     119955  119966
Autographa californica nucleopolyhedrovirus NC 0... (119655) TCGTCGGGAAACTCGCGCGGAAACATGTTGTTGTAACCGAACGGGTCCCATAGCGCCAAAACCAAATCTGCCAGCGT
                              SARS-CoV-2 NC_045512.2 (29904) -----------------------------------------------------------------------------
                                                                                                                        Section 1559
                                              (119967) 119967    119977     119988     119999     120010     120021     120032  120043
Autographa californica nucleopolyhedrovirus NC 0... (119732) CAATAGAATGAGCACGATGCCGACAATGGAGCTGGCTTGGATAGCGATTCGAGTTAACGCTTTGGCAGTCACGGTCA
                              SARS-CoV-2 NC_045512.2 (29904) -----------------------------------------------------------------------------
                                                                                                                        Section 1560
                                              (120044) 120044    120054     120065     120076     120087     120098     120109  120120
Autographa californica nucleopolyhedrovirus NC 0... (119809) GCGTTTTGATGGCGATCACGTTGAGCGAGTGCACTAACGCGGCTTTGTAAGTCTCTCCCAACATGCGCACGGTCACG
                              SARS-CoV-2 NC_045512.2 (29904) -----------------------------------------------------------------------------
                                                                                                                        Section 1561
                                              (120121) 120121    120131     120142     120153     120164     120175     120186  120197
Autographa californica nucleopolyhedrovirus NC 0... (119886) CGCCGAGTCGTGCTAAGCAACATGTGTTTCATGGCCGGAATGAGAGAAGTGTTAATTTTTTTCAACATGCTTTTAAA
                              SARS-CoV-2 NC_045512.2 (29904) -----------------------------------------------------------------------------
```

```
                                                                                          Section 1562
                                        (120198) 120198    120208    120219    120230    120241    120252    120263 120274
Autographa californica nucleopolyhedrovirus NC 0... (119963) CCCGGACATTAGCATATCAAAGCCAATGTCCGTAGCAATACCGAAAACGAGCGCGTAATCTTCCAAAAACGATGTTA
                       SARS-CoV-2 NC_045512.2 (29904) -----------------------------------------------------------------------------
                                                                                          Section 1563
                                        (120275) 120275    120285    120296    120307    120318    120329    120340 120351
Autographa californica nucleopolyhedrovirus NC 0... (120040) TAATTGACTCCAAGTCTTGGTCGCTGATTGAACGGTCGAGCGCCTCGAAATGTTCGACACGTGCACGTTCGTTACCG
                       SARS-CoV-2 NC_045512.2 (29904) -----------------------------------------------------------------------------
                                                                                          Section 1564
                                        (120352) 120352    120362    120373    120384    120395    120406    120417 120428
Autographa californica nucleopolyhedrovirus NC 0... (120117) CGGTAATTGTATGCGATCGGAGTTTTAGTAAAGCCGGTTTCGGCCGTGTACGTGATCTGGACGGGCGACCCGTTGAC
                       SARS-CoV-2 NC_045512.2 (29904) -----------------------------------------------------------------------------
                                                                                          Section 1565
                                        (120429) 120429    120439    120450    120461    120472    120483    120494 120505
Autographa californica nucleopolyhedrovirus NC 0... (120194) GATCATGCCCAAATCGTTTAGTGTTGGATTTTTGTTAAAAAGTTTTTCAAATTCCAAGTCTGTGGCGTTATCGCGCA
                       SARS-CoV-2 NC_045512.2 (29904) -----------------------------------------------------------------------------
                                                                                          Section 1566
                                        (120506) 120506    120516    120527    120538    120549    120560    120571 120582
Autographa californica nucleopolyhedrovirus NC 0... (120271) CGCTGCGCCATTGCGCTAGTATTGCGTTGGAGTCCACGTTGGGTCGTGGCGGTAGTATGCTGGAAGGCGCTTTGTAA
                       SARS-CoV-2 NC_045512.2 (29904) -----------------------------------------------------------------------------
                                                                                          Section 1567
                                        (120583) 120583    120593    120604    120615    120626    120637    120648 120659
Autographa californica nucleopolyhedrovirus NC 0... (120348) TCAAAATCGCGCAGTTCGCTAAAAATGTTGTTGGCCAGCATTTTGAAAGTGACAAAGATCGTGTCGCCCAGCACGAA
                       SARS-CoV-2 NC_045512.2 (29904) -----------------------------------------------------------------------------
                                                                                          Section 1568
                                        (120660) 120660    120670    120681    120692    120703    120714    120725 120736
Autographa californica nucleopolyhedrovirus NC 0... (120425) TCCGATGAGCGATTCCCACCATCTAAACGAACAACCGCCGTTGAATAGCTCTCTGCCGAAACGTCGACAGTAGGCTT
                       SARS-CoV-2 NC_045512.2 (29904) -----------------------------------------------------------------------------
```

```
                                                                                                              Section 1569
                                                    (120737) 120737    120747     120758     120769     120780     120791     120802  120813
Autographa californica nucleopolyhedrovirus NC 0... (120502) CGTTGAATTCGCCTTTAAAGCGTTCGGGAAACAAGGGGTCGGGATCGGGCCGAACGTTAAAAGCCGGCACATCGTCC
                         SARS-CoV-2 NC_045512.2     (29904)  -----------------------------------------------------------------------------
                                                                                                              Section 1570
                                                    (120814) 120814    120824     120835     120846     120857     120868     120879  120890
Autographa californica nucleopolyhedrovirus NC 0... (120579) ACGCCCATGATCGTGTGTTCTTCGGTGCGCAAGTATGGGCTGTTAAAGTACATTTTGGACAGCGAGTCCACTAAGAT
                         SARS-CoV-2 NC_045512.2     (29904)  -----------------------------------------------------------------------------
                                                                                                              Section 1571
                                                    (120891) 120891    120901     120912     120923     120934     120945     120956  120967
Autographa californica nucleopolyhedrovirus NC 0... (120656) GCATTTGTTGTCGAGCGTGTATCTAAACTCGGCAGACTGAACTTGGGTTTCGGCGCCTTCACGCATGGCCGCCGCCC
                         SARS-CoV-2 NC_045512.2     (29904)  -----------------------------------------------------------------------------
                                                                                                              Section 1572
                                                    (120968) 120968    120978     120989     121000     121011     121022     121033  121044
Autographa californica nucleopolyhedrovirus NC 0... (120733) TGTCCAGGTGGTAGCACGCGGGCTGCGCGTAACCCACGCTAGTCTCGGAGGTCTGCATGTACATGAACGGCGTCGTG
                         SARS-CoV-2 NC_045512.2     (29904)  -----------------------------------------------------------------------------
                                                                                                              Section 1573
                                                    (121045) 121045    121055     121066     121077     121088     121099     121110  121121
Autographa californica nucleopolyhedrovirus NC 0... (120810) TTGGACACGACGCCGGTTTCGTGAAACGGATAGCAGCTCATGCTTACACACCCGCGCTTGCTGAAAGCCAGTTTGAC
                         SARS-CoV-2 NC_045512.2     (29904)  -----------------------------------------------------------------------------
                                                                                                              Section 1574
                                                    (121122) 121122    121132     121143     121154     121165     121176     121187  121198
Autographa californica nucleopolyhedrovirus NC 0... (120887) GGCCAGCGCTTTGTCGGCCAATTTCGGCGGCACATAATAATCGTCGTCACTTGACGCGGGACGCAGCGTGTAGTCGA
                         SARS-CoV-2 NC_045512.2     (29904)  -----------------------------------------------------------------------------
                                                                                                              Section 1575
                                                    (121199) 121199    121209     121220     121231     121242     121253     121264  121275
Autographa californica nucleopolyhedrovirus NC 0... (120964) TTAGTATATGCGGAAACCTGGTGCGCCATCTCGAAATAAACTCGAGACGATGCATATGTATGGCATACCTACTGGCA
                         SARS-CoV-2 NC_045512.2     (29904)  -----------------------------------------------------------------------------
```

```
                                                                                                              Section 1576
                                        (121276) 121276    121286     121297     121308     121319     121330     121341  121352
Autographa californica nucleopolyhedrovirus  NC_0... (121041) TTAGTTAAATCGACGGCTGTTAAAACCGCCATGTTATATAGGACTTAAAATAAACAACAATATATAATGAAATATTT
                             SARS-CoV-2 NC_045512.2  (29904) -----------------------------------------------------------------------------
                                                                                                              Section 1577
                                        (121353) 121353    121363     121374     121385     121396     121407     121418  121429
Autographa californica nucleopolyhedrovirus  NC_0... (121118) ATTAGATTATATTATAGCAATACATTTACATTTATTATAACAATACTTTTTATTTAATCTGATTATATTATAACGAT
                             SARS-CoV-2 NC_045512.2  (29904) -----------------------------------------------------------------------------
                                                                                                              Section 1578
                                        (121430) 121430    121440     121451     121462     121473     121484     121495  121506
Autographa californica nucleopolyhedrovirus  NC_0... (121195) ACATTTTTATTTAGACATTGTTATTTACAATATTAATTAACTTTTTATACATTTTTAAATCATAATATATAATCATT
                             SARS-CoV-2 NC_045512.2  (29904) -----------------------------------------------------------------------------
                                                                                                              Section 1579
                                        (121507) 121507    121517     121528     121539     121550     121561     121572  121583
Autographa californica nucleopolyhedrovirus  NC_0... (121272) TCGTTGTGCATTTCAAAGCTTTTGATAGCTTCAAAGTAATACATGAATTTAGAGTATTCAGGAAAATGATAAACGTT
                             SARS-CoV-2 NC_045512.2  (29904) -----------------------------------------------------------------------------
                                                                                                              Section 1580
                                        (121584) 121584    121594     121605     121616     121627     121638     121649  121660
Autographa californica nucleopolyhedrovirus  NC_0... (121349) GGTAAACCCGCATTTGGTACAATATAACACGGGATTTTTATAATACAGTTTAGTTTTTTTACACAATTTGCAATAGT
                             SARS-CoV-2 NC_045512.2  (29904) -----------------------------------------------------------------------------
                                                                                                              Section 1581
                                        (121661) 121661    121671     121682     121693     121704     121715     121726  121737
Autographa californica nucleopolyhedrovirus  NC_0... (121426) TGTTAGTTGTAGGTTTCAAAGGAAACGTGATTGCGCCGTCCAATACCTGGGTAAACTTTTTGACTTTAACAGTGGCA
                             SARS-CoV-2 NC_045512.2  (29904) -----------------------------------------------------------------------------
                                                                                                              Section 1582
                                        (121738) 121738    121748     121759     121770     121781     121792     121803  121814
Autographa californica nucleopolyhedrovirus  NC_0... (121503) AACACGGTTCCTTTGATACCCGAAAATCGGTTGTCTTGCAGAGCGGCCATCATTTCGCTTGGCTCTTGAAGTATAAA
                             SARS-CoV-2 NC_045512.2  (29904) -----------------------------------------------------------------------------
```

```
                                                                                                                Section 1583
                                                  (121815) 121815    121825          121836          121847          121858          121869          121880    121891
Autographa californica nucleopolyhedrovirus  NC_0... (121580) ACAGTTGACGTCATCCACCACGTCGGGTCTGGTGCACATGCTTCGGTAGCGCTGCAACACTATATTGGTGTATGTTT
                            SARS-CoV-2 NC_045512.2  (29904) -----------------------------------------------------------------------------
                                                                                                                Section 1584
                                                  (121892) 121892    121902          121913          121924          121935          121946          121957    121968
Autographa californica nucleopolyhedrovirus  NC_0... (121657) CCCTGAGAACGAGACCGCCGGTGGTGCTAAGATCGATTGTTTGAATGCGCTCGTTGGGCTCTTTGTGATTTCGAATT
                            SARS-CoV-2 NC_045512.2  (29904) -----------------------------------------------------------------------------
                                                                                                                Section 1585
                                                  (121969) 121969    121979          121990          122001          122012          122023          122034    122045
Autographa californica nucleopolyhedrovirus  NC_0... (121734) ATGCGCCGAATTATTTCAAACACTTTGCAGTTGTGATCGTCAATTCTCAATTCTTTAACTTCCGTCGTGTGCTCTAA
                            SARS-CoV-2 NC_045512.2  (29904) -----------------------------------------------------------------------------
                                                                                                                Section 1586
                                                  (122046) 122046    122056          122067          122078          122089          122100          122111    122122
Autographa californica nucleopolyhedrovirus  NC_0... (121811) ACTTACAGGGAAAATGTATTGGTAAAAAAACCTCTCTCTGGCTAAATAGCTGAGGTCGACCAAATTGATAGAAGGAT
                            SARS-CoV-2 NC_045512.2  (29904) -----------------------------------------------------------------------------
                                                                                                                Section 1587
                                                  (122123) 122123    122133          122144          122155          122166          122177          122188    122199
Autographa californica nucleopolyhedrovirus  NC_0... (121888) ATATTTCGTACGAGGTTTTTGGAACGTTGTGATATAGATAGCATTTTTGACAGCAGATGTCTATGCGGTCAGGATCG
                            SARS-CoV-2 NC_045512.2  (29904) -----------------------------------------------------------------------------
                                                                                                                Section 1588
                                                  (122200) 122200    122210          122221          122232          122243          122254          122265    122276
Autographa californica nucleopolyhedrovirus  NC_0... (121965) TCCAACGGCTTTTCGATGTGAACCACAACATACAAAAACCATTCGCGCGTGTTGTCTTTGAATCTATAATTGCAAGT
                            SARS-CoV-2 NC_045512.2  (29904) -----------------------------------------------------------------------------
                                                                                                                Section 1589
                                                  (122277) 122277    122287          122298          122309          122320          122331          122342    122353
Autographa californica nucleopolyhedrovirus  NC_0... (122042) GGTGCATCGCGAATCGCTCATGTGCTCCATAGTCTTCTTGTATTTCACAGGCCTGCTTGCAAATTTGCCCGTCATGC
                            SARS-CoV-2 NC_045512.2  (29904) -----------------------------------------------------------------------------
```

```
                                                                                                      Section 1590
                                             (122354) 122354    122364     122375     122386     122397     122408     122419  122430
Autographa californica nucleopolyhedrovirus NC_0... (122119) GCATATCTTTGCTGTTTATGTAGCCCATAATGTAATTGGTGGAAAATTTTAGCGTGGCTTTCATGATGTCGCGTTCT
                            SARS-CoV-2 NC_045512.2  (29904) -----------------------------------------------------------------------------
                                                                                                      Section 1591
                                             (122431) 122431    122441     122452     122463     122474     122485     122496  122507
Autographa californica nucleopolyhedrovirus NC_0... (122196) AAATCGCTCATGAAATGCATACGTAGATCGCGCTCTTGTTTGAAATCCAGTTTGTCGCTGTACGCGGGCAAACCTTC
                            SARS-CoV-2 NC_045512.2  (29904) -----------------------------------------------------------------------------
                                                                                                      Section 1592
                                             (122508) 122508    122518     122529     122540     122551     122562     122573  122584
Autographa californica nucleopolyhedrovirus NC_0... (122273) AAACTTGTTCCCAAACTCGGGCGGCACAAAATATCCATCTTTTCTGTTGACGACTGGTTTTTTACTTACAATGCTGC
                            SARS-CoV-2 NC_045512.2  (29904) -----------------------------------------------------------------------------
                                                                                                      Section 1593
                                             (122585) 122585    122595     122606     122617     122628     122639     122650  122661
Autographa californica nucleopolyhedrovirus NC_0... (122350) TGTGCTCCAACGGCTTGGCCGGAGAGGTGCACATAGGCTGTTTAGGCGGAGAGATGCGCGTAGGTGGTTTGATGTTA
                            SARS-CoV-2 NC_045512.2  (29904) -----------------------------------------------------------------------------
                                                                                                      Section 1594
                                             (122662) 122662    122672     122683     122694     122705     122716     122727  122738
Autographa californica nucleopolyhedrovirus NC_0... (122427) GATTTTGGCGGCGGACGAACAGGCGACGGCGGCGAGTTGGCGGCAGGCGCTGGCAAAGATTTGGCACGACCCTTGCC
                            SARS-CoV-2 NC_045512.2  (29904) -----------------------------------------------------------------------------
                                                                                                      Section 1595
                                             (122739) 122739    122749     122760     122771     122782     122793     122804  122815
Autographa californica nucleopolyhedrovirus NC_0... (122504) CCCGGTCCTTGGCGCGTCAAAAATGTTATTCTCTCGAAAAAAACGGTTCATTGTAACTGTTAGTTAGCACTCAGAAA
                            SARS-CoV-2 NC_045512.2  (29904) -----------------------------------------------------------------------------
                                                                                                      Section 1596
                                             (122816) 122816    122826     122837     122848     122859     122870     122881  122892
Autographa californica nucleopolyhedrovirus NC_0... (122581) TCAACACGATACTGTGCACGTTCAGCCATCGAGAGGCTTTATATATGGAAACCTTATCTATAGAGATAAGATTGTAT
                            SARS-CoV-2 NC_045512.2  (29904) -----------------------------------------------------------------------------
```

Section 1597

(122893) 122893 122903 122914 122925 122936 122947 122958 122969
Autographa californica nucleopolyhedrovirus NC 0... (122658) ATGCGTAGGAGAGCCTGGTCACGTAGGCACTTTGCGCACGGCACTAGGGCTGTGGAGGGGACAGGCTATATAAAGCC
SARS-CoV-2 NC_045512.2 (29904) ---

Section 1598

(122970) 122970 122980 122991 123002 123013 123024 123035 123046
Autographa californica nucleopolyhedrovirus NC 0... (122735) CGTTTGCCCAACTCGTAAATCAGTATCAATTGTGCTCCGGCGCACACGCTCGCTTGCGCGCCGGATAGTATAAGTAA
SARS-CoV-2 NC_045512.2 (29904) ---

Section 1599

(123047) 123047 123057 123068 123079 123090 123101 123112 123123
Autographa californica nucleopolyhedrovirus NC 0... (122812) TTGATAACGGGCAACGCAACATGATAAGAACCAGCAGTCACGTGCTGAACGTCCAGGAAAATATAATGACGTCAAAC
SARS-CoV-2 NC_045512.2 (29904) ---

Section 1600

(123124) 123124 123134 123145 123156 123167 123178 123189 123200
Autographa californica nucleopolyhedrovirus NC 0... (122889) TGTGCGTCATCGCCATATTCGTGCGAGGCAACGTCCGCTTGCGCAGAAGCTCAGCAGGTAATGATCGATAACTTTGT
SARS-CoV-2 NC_045512.2 (29904) ---

Section 1601

(123201) 123201 123211 123222 123233 123244 123255 123266 123277
Autographa californica nucleopolyhedrovirus NC 0... (122966) TTTCTTTCACATGTACAACGCCGACATACAAATTGACGCAAAGCTGCAATGCGGCGTGCGCTCGGCCGCGTTTGCAA
SARS-CoV-2 NC_045512.2 (29904) ---

Section 1602

(123278) 123278 123288 123299 123310 123321 123332 123343 123354
Autographa californica nucleopolyhedrovirus NC 0... (123043) TGATCGACGATAAACATTTGGAAATGTACAAGCATAGAATAGAGAATAAATTTTTTTATTACTATGATCAATGTGCC
SARS-CoV-2 NC_045512.2 (29904) ---

Section 1603

(123355) 123355 123365 123376 123387 123398 123409 123420 123431
Autographa californica nucleopolyhedrovirus NC 0... (123120) GACATTGCCAAACCCGACCGTCTGCCCGATGACGACGGCGCGTGCTGTCACCATTTTATTTTTGATGCCCAACGTAT
SARS-CoV-2 NC_045512.2 (29904) ---

```
                                                       Section 1604
                                          (123432) 123432    123442     123453     123464     123475     123486     123497  123508
Autographa californica nucleopolyhedrovirus NC 0... (123197) TATTCAATGTATTAAAGAGATTGAAAGCGCGTACGGCGTGCGTGATCGCGGCAATGTAATAGTGTTTTATCCGTACT
                          SARS-CoV-2 NC_045512.2 (29904) -----------------------------------------------------------------------------
                                                       Section 1605
                                          (123509) 123509    123519     123530     123541     123552     123563     123574  123585
Autographa californica nucleopolyhedrovirus NC 0... (123274) TGAAACAGTTGCGAGACGCGTTGAAGCTAATTAAAAACTCTTTTGCGTGTTGTTTTAAAATTATAAATTCTATGCAA
                          SARS-CoV-2 NC_045512.2 (29904) -----------------------------------------------------------------------------
                                                       Section 1606
                                          (123586) 123586    123596     123607     123618     123629     123640     123651  123662
Autographa californica nucleopolyhedrovirus NC 0... (123351) ATGTACGTGAACGAGTTAATATCAAATTGCCTGTTGTTTATTGAAAAGCTGGAAACTATTAATAAAACTGTTAAAGT
                          SARS-CoV-2 NC_045512.2 (29904) -----------------------------------------------------------------------------
                                                       Section 1607
                                          (123663) 123663    123673     123684     123695     123706     123717     123728  123739
Autographa californica nucleopolyhedrovirus NC 0... (123428) TATGAATTTGTTTGTAGACAATTTGGTTTTGTACGAATGCAATGTTTGTAAAGAAATATCTACGGATGAAAGATTTT
                          SARS-CoV-2 NC_045512.2 (29904) -----------------------------------------------------------------------------
                                                       Section 1608
                                          (123740) 123740    123750     123761     123772     123783     123794     123805  123816
Autographa californica nucleopolyhedrovirus NC 0... (123505) TAAAGCCAAAAGAATGTTGCGAATACGCTATATGCAACGCGTGCTGCGTTAACATGTGGAAGACGGCCACCACGCAC
                          SARS-CoV-2 NC_045512.2 (29904) -----------------------------------------------------------------------------
                                                       Section 1609
                                          (123817) 123817    123827     123838     123849     123860     123871     123882  123893
Autographa californica nucleopolyhedrovirus NC 0... (123582) GCAAAATGTCCAGCGTGCAGGACATCGTATAAATAAGCACGCAACGCAAAATGAGTGGTGGCGGCAACTTGTTGACT
                          SARS-CoV-2 NC_045512.2 (29904) -----------------------------------------------------------------------------
                                                       Section 1610
                                          (123894) 123894    123904     123915     123926     123937     123948     123959  123970
Autographa californica nucleopolyhedrovirus NC 0... (123659) CTGGAAAGAGATCATTTTAAATATTTATTTTTGACCAGCTATTTTGATTTAAAAGATAATGAACATGTTCCTTCAGA
                          SARS-CoV-2 NC_045512.2 (29904) -----------------------------------------------------------------------------
```

```
                                                               Section 1611
                                           (123971) 123971    123981     123992     124003     124014     124025     124036  124047
Autographa californica nucleopolyhedrovirus  NC_0... (123736) GCCTATGGCATTTATTCGCAATTACTTGAATTGCACGTTTGATTTGCTAGACGATGCCGTGCTCATGAACTATTTCA
                          SARS-CoV-2 NC_045512.2  (29904) -----------------------------------------------------------------------------
                                                               Section 1612
                                           (124048) 124048    124058     124069     124080     124091     124102     124113  124124
Autographa californica nucleopolyhedrovirus  NC_0... (123813) ATTACTTGCAAAGCATGCAATTGAAACATTTGGTGGGCAGCACGTCGACAAACATTTTCAAGTTTGTAAAGCCACAA
                          SARS-CoV-2 NC_045512.2  (29904) -----------------------------------------------------------------------------
                                                               Section 1613
                                           (124125) 124125    124135     124146     124157     124168     124179     124190  124201
Autographa californica nucleopolyhedrovirus  NC_0... (123890) TTTAGATTTGTGTGCGATCGCACAACTGTGGACATTTTAGAATTTGACACGCGCATGTACATAAAACCCGGCACGCC
                          SARS-CoV-2 NC_045512.2  (29904) -----------------------------------------------------------------------------
                                                               Section 1614
                                           (124202) 124202    124212     124223     124234     124245     124256     124267  124278
Autographa californica nucleopolyhedrovirus  NC_0... (123967) CGTGTACGCCACGAACCTGTTCACGTCCAATCCCCGCAAGATGATGGCTTTCCTGTACGCTGAATTTGGCAAGGTGT
                          SARS-CoV-2 NC_045512.2  (29904) -----------------------------------------------------------------------------
                                                               Section 1615
                                           (124279) 124279    124289     124300     124311     124322     124333     124344  124355
Autographa californica nucleopolyhedrovirus  NC_0... (124044) TTAAAAATAAAATATTCGTAAACATCAACAACTACGGCTGCGTGTTGGCGGGCAGTGCCGGTTTCTTGTTCGACGAT
                          SARS-CoV-2 NC_045512.2  (29904) -----------------------------------------------------------------------------
                                                               Section 1616
                                           (124356) 124356    124366     124377     124388     124399     124410     124421  124432
Autographa californica nucleopolyhedrovirus  NC_0... (124121) GCGTACGTGGATTGGAATGGTGTGCGAATGTGTGCGGCGCCGCGATTAGATAACAACATGCATCCGTTCCGACTGTA
                          SARS-CoV-2 NC_045512.2  (29904) -----------------------------------------------------------------------------
                                                               Section 1617
                                           (124433) 124433    124443     124454     124465     124476     124487     124498  124509
Autographa californica nucleopolyhedrovirus  NC_0... (124198) TCTACTGGGCGAGGACATGGCTAAGCACTTTGTCGATAATAATATACTACCGCCGCACCCTTCTAACGCAAAGACTC
                          SARS-CoV-2 NC_045512.2  (29904) -----------------------------------------------------------------------------
```

Section 1618

(124510) 124510 124520 124531 124542 124553 124564 124575 124586
Autographa californica nucleopolyhedrovirus NC_0... (124275) GCAAAATCAACAATTCAATGTTTATGCTGAAAAACTTTTACAAAGGTCTGCCGCTGTTCAAATCAAAGTACACGGTG
SARS-CoV-2 NC_045512.2 (29904) ---

Section 1619

(124587) 124587 124597 124608 124619 124630 124641 124652 124663
Autographa californica nucleopolyhedrovirus NC_0... (124352) GTGAACAGCACTAAAATCGTGACCCGAAAACCCAACGATATATTTAATGAGATAGATAAAGAATTAAATGGCAACTG
SARS-CoV-2 NC_045512.2 (29904) ---

Section 1620

(124664) 124664 124674 124685 124696 124707 124718 124729 124740
Autographa californica nucleopolyhedrovirus NC_0... (124429) TCCGTTTATCAAGTTTATTCAGCGCGACTACATATTCGACGCCCAGTTTCCGCCAGATTTGCTTGATTTGCTAAACG
SARS-CoV-2 NC_045512.2 (29904) ---

Section 1621

(124741) 124741 124751 124762 124773 124784 124795 124806 124817
Autographa californica nucleopolyhedrovirus NC_0... (124506) AATACATGACCAAAAGCTCGATCATGAAAATAATTACCAAGTTTGTGATTGAAGAAAACCCCGCTATGAGCGGTGAA
SARS-CoV-2 NC_045512.2 (29904) ---

Section 1622

(124818) 124818 124828 124839 124850 124861 124872 124883 124894
Autographa californica nucleopolyhedrovirus NC_0... (124583) ATGTCTCGCGAGATTATTCTTGATCGCTACTCAGTAGACAATTATCGCAAGCTGTACATAAAAATGGAAATAACCAA
SARS-CoV-2 NC_045512.2 (29904) ---

Section 1623

(124895) 124895 124905 124916 124927 124938 124949 124960 124971
Autographa californica nucleopolyhedrovirus NC_0... (124660) CCAGTTTCCTGTCATGTACGATCATGAATCGTCGTACATTTTTGTGAGCAAAGACTTTTTGCAATTGAAAGGCACTA
SARS-CoV-2 NC_045512.2 (29904) ---

Section 1624

(124972) 124972 124982 124993 125004 125015 125026 125037 125048
Autographa californica nucleopolyhedrovirus NC_0... (124737) TGAACGCGTTCTACGCGCCCAAGCAGCGTATATTAAGTATTTTGGCGGTGAATCGTTTGTTTGGCGCCACGGAAACG
SARS-CoV-2 NC_045512.2 (29904) ---

```
                                                          Section 1625
                                            (125049) 125049    125059     125070     125081     125092     125103     125114 125125
Autographa californica nucleopolyhedrovirus NC 0... (124814) ATCGACTTTCATCCCAACCTGCTCGTGTACCGGCAGAGTTCGCCGCCGGTCCGTTTGACGGGCGACGTGTATGTTGT
                             SARS-CoV-2 NC_045512.2 (29904) -----------------------------------------------------------------------------
```

```
                                                          Section 1626
                                            (125126) 125126    125136     125147     125158     125169     125180     125191 125202
Autographa californica nucleopolyhedrovirus NC 0... (124891) TGATAAGAACGAAAAAGTTTTTTTGGTCAAACACGTGTTCTCAAACACGGTGCCTGCATATCTTTTAATAAGAGGTG
                             SARS-CoV-2 NC_045512.2 (29904) -----------------------------------------------------------------------------
```

```
                                                          Section 1627
                                            (125203) 125203    125213     125224     125235     125246     125257     125268 125279
Autographa californica nucleopolyhedrovirus NC 0... (124968) ATTACGAAAGTTCGTCTGACTTGAAATCCCTTCGCGATTTGAATCCGTGGGTTCAGAACACGCTTCTCAAATTATTA
                             SARS-CoV-2 NC_045512.2 (29904) -----------------------------------------------------------------------------
```

```
                                                          Section 1628
                                            (125280) 125280    125290     125301     125312     125323     125334     125345 125356
Autographa californica nucleopolyhedrovirus NC 0... (125045) ATCCCCGACTCGGTACAATAATATGATTTACACTGATCCCACTACTGGCGCTACGACTAGCACAGACGTCGTCCGTC
                             SARS-CoV-2 NC_045512.2 (29904) -----------------------------------------------------------------------------
```

```
                                                          Section 1629
                                            (125357) 125357    125367     125378     125389     125400     125411     125422 125433
Autographa californica nucleopolyhedrovirus NC 0... (125122) CACAAACTATTTAAACAGGCTAACTCCAAACATGTTCTTGACCATCTTGGCTGTAGTAGTAATTATTGCTTTAATAA
                             SARS-CoV-2 NC_045512.2 (29904) -----------------------------------------------------------------------------
```

```
                                                          Section 1630
                                            (125434) 125434    125444     125455     125466     125477     125488     125499 125510
Autographa californica nucleopolyhedrovirus NC 0... (125199) TTATATTTGTTCAATCTAGCAGTAATGGAAACAGCTCGGGGGGTAATGTACCTCCAAACGCCCTGGGGGGTTTTGTA
                             SARS-CoV-2 NC_045512.2 (29904) -----------------------------------------------------------------------------
```

```
                                                          Section 1631
                                            (125511) 125511    125521     125532     125543     125554     125565     125576 125587
Autographa californica nucleopolyhedrovirus NC 0... (125276) AATCCTTTAAACGCTACCATGCGAGCTAATCCCTTTATGAACACGCCTCAAAGGCAAATGTTGTAGATAAGTGTATA
                             SARS-CoV-2 NC_045512.2 (29904) -----------------------------------------------------------------------------
```

Section 1632

(125588) 125588 125598 125609 125620 125631 125642 125653 125664
Autographa californica nucleopolyhedrovirus NC_0... (125353) AAAAATGAAACGTATCAAATGCAACAAAGTTCGAACGGTCACCGAGATTGTAAACAGCGATGAAAAAATCCAAAAGA
SARS-CoV-2 NC_045512.2 (29904) ---

Section 1633

(125665) 125665 125675 125686 125697 125708 125719 125730 125741
Autographa californica nucleopolyhedrovirus NC_0... (125430) CCTACGAATTGGCTGAATTTGATTTAAAAAATCTAAGCAGTTTAGAAAGCTATGAAACTCTAAAAATTAAATTGGCG
SARS-CoV-2 NC_045512.2 (29904) ---

Section 1634

(125742) 125742 125752 125763 125774 125785 125796 125807 125818
Autographa californica nucleopolyhedrovirus NC_0... (125507) CTCAGCAAATACATGGCTATGCTCAGCACCCTGGAAATGACTCAACCGCTGTTGGAAATATTTAGAAACAAAGCAGA
SARS-CoV-2 NC_045512.2 (29904) ---

Section 1635

(125819) 125819 125829 125840 125851 125862 125873 125884 125895
Autographa californica nucleopolyhedrovirus NC_0... (125584) CACTCGGCAGATTGCCGCCGTGGTGTTTAGCACATTAGCTTTTATACACAATAGATTCCATCCCCTTGTTACTAATT
SARS-CoV-2 NC_045512.2 (29904) ---

Section 1636

(125896) 125896 125906 125917 125928 125939 125950 125961 125972
Autographa californica nucleopolyhedrovirus NC_0... (125661) TTACTAACAAAATGGAGTTTGTGGTCACTGAAACCAACGACACAAGCATTCCCGGAGAACCCATTTTGTTTACGGAA
SARS-CoV-2 NC_045512.2 (29904) ---

Section 1637

(125973) 125973 125983 125994 126005 126016 126027 126038 126049
Autographa californica nucleopolyhedrovirus NC_0... (125738) AACGAAGGTGTGCTGCTGTGTTCCGTGGACAGACCGTCTATCGTTAAAATGCTAAGCCGCGAGTTTGACACCGAGGC
SARS-CoV-2 NC_045512.2 (29904) ---

Section 1638

(126050) 126050 126060 126071 126082 126093 126104 126115 126126
Autographa californica nucleopolyhedrovirus NC_0... (125815) TTTAGTAAACTTTGAAAACGACAACTGCAACGTGCGGATAGCCAAGACGTTTGGCGCCTCTAAGCGCAAAAACACGA
SARS-CoV-2 NC_045512.2 (29904) ---

```
                                                                                          Section 1639
                                                       (126127) 126127    126137     126148     126159     126170     126181     126192 126203
Autographa californica nucleopolyhedrovirus NC_0... (125892) CGCGCAGCGATGATTACGAGTCAAATAAACAACCCAATTACGATATGGATTTGAGCGATTTTAGCATAACTGAGGTT
                              SARS-CoV-2 NC_045512.2 (29904) -----------------------------------------------------------------------------
                                                                                          Section 1640
                                                       (126204) 126204    126214     126225     126236     126247     126258     126269 126280
Autographa californica nucleopolyhedrovirus NC_0... (125969) GAAGCCACTCAATATTTAACTCTGTTGCTGACCGTCGAACATGCCTATTTACATTATTATATTTTTAAAAATTACGG
                              SARS-CoV-2 NC_045512.2 (29904) -----------------------------------------------------------------------------
                                                                                          Section 1641
                                                       (126281) 126281    126291     126302     126313     126324     126335     126346 126357
Autographa californica nucleopolyhedrovirus NC_0... (126046) GGTGTTTGAATATTGCAAATCGCTAACGGACCATTCGCTTTTTACCAACAAATTGCGATCGACAATGAGCACAAAAA
                              SARS-CoV-2 NC_045512.2 (29904) -----------------------------------------------------------------------------
                                                                                          Section 1642
                                                       (126358) 126358    126368     126379     126390     126401     126412     126423 126434
Autographa californica nucleopolyhedrovirus NC_0... (126123) CGTCTAATTTACTGTTAAGCAAATTCAAATTTACCATTGAAGATTTTGACAAAATAAACTCAAATTCTGTAACATCA
                              SARS-CoV-2 NC_045512.2 (29904) -----------------------------------------------------------------------------
                                                                                          Section 1643
                                                       (126435) 126435    126445     126456     126467     126478     126489     126500 126511
Autographa californica nucleopolyhedrovirus NC_0... (126200) GGGTTTAATATATATAATTTTAATAAATAATTAAATAATATACAATGTTTTTATTAATTATATTTTTAATATTAATT
                              SARS-CoV-2 NC_045512.2 (29904) -----------------------------------------------------------------------------
                                                                                          Section 1644
                                                       (126512) 126512    126522     126533     126544     126555     126566     126577 126588
Autographa californica nucleopolyhedrovirus NC_0... (126277) AAAAGTATTAATATTTAAAAAAATGAATCAAATTCATCTAAAGTGTCACAGCGATAAAATTTGTCCTAAAGGGTATT
                              SARS-CoV-2 NC_045512.2 (29904) -----------------------------------------------------------------------------
                                                                                          Section 1645
                                                       (126589) 126589    126599     126610     126621     126632     126643     126654 126665
Autographa californica nucleopolyhedrovirus NC_0... (126354) TTGGCCTCAACGCCGATCCCTATGATTGCACGGCGTATTATCTGTGTCCGCATAAAGTGCAAATGTTTTGCGAATTA
                              SARS-CoV-2 NC_045512.2 (29904) -----------------------------------------------------------------------------
```

```
                                                             Section 1646
                                              (126666) 126666    126676     126687     126698     126709     126720     126731 126742
Autographa californica nucleopolyhedrovirus NC 0... (126431) AATCACGAATTTGACTTGGACTCCGCCAGCTGCAAGCCTATCGTGTACGATCACACGGGCAGCGGGTGTACGGCTCG
                              SARS-CoV-2 NC_045512.2 (29904) -----------------------------------------------------------------------------
                                                             Section 1647
                                              (126743) 126743    126753     126764     126775     126786     126797     126808 126819
Autographa californica nucleopolyhedrovirus NC 0... (126508) CATGTATAGAAACTTGTTACTATGAAGAGCGGGTTTCCAGTTGCACAACACTATTATCGATTTGCAGTTCGGGACAT
                              SARS-CoV-2 NC_045512.2 (29904) -----------------------------------------------------------------------------
                                                             Section 1648
                                              (126820) 126820    126830     126841     126852     126863     126874     126885 126896
Autographa californica nucleopolyhedrovirus NC 0... (126585) AAATGTTTAAATATATCGATGTCTTTGTGATGCGCGCGACATTTTTGTAGGTTATTGATAAAATGAACGGATACGTT
                              SARS-CoV-2 NC_045512.2 (29904) -----------------------------------------------------------------------------
                                                             Section 1649
                                              (126897) 126897    126907     126918     126929     126940     126951     126962 126973
Autographa californica nucleopolyhedrovirus NC 0... (126662) GCCCGACATTATCATTAAATCCTTGGCGTAGAATTTGTCGGGTCCATTGTCCGTGTGCGCTAGCATGCCCGTAACGG
                              SARS-CoV-2 NC_045512.2 (29904) -----------------------------------------------------------------------------
                                                             Section 1650
                                              (126974) 126974    126984     126995     127006     127017     127028     127039 127050
Autographa californica nucleopolyhedrovirus NC 0... (126739) ACCTCGTACTTTTGGCTTCAAAGGTTTTGCGCACAGACAAAATGTGCCACACTTGCAGCTCTGCATGTGTGCGCGTT
                              SARS-CoV-2 NC_045512.2 (29904) -----------------------------------------------------------------------------
                                                             Section 1651
                                              (127051) 127051    127061     127072     127083     127094     127105     127116 127127
Autographa californica nucleopolyhedrovirus NC 0... (126816) ACCACAAATCCCAACGGCGCAGTGTACTTGTTGTATGCAAATAAATCTCGATAAAGGCGCGGCGCGCGAATGCAGCT
                              SARS-CoV-2 NC_045512.2 (29904) -----------------------------------------------------------------------------
                                                             Section 1652
                                              (127128) 127128    127138     127149     127160     127171     127182     127193 127204
Autographa californica nucleopolyhedrovirus NC 0... (126893) GATCACGTACGCTCCTCGTGTTCCGTTCAAGGACGGTGTTATCGACCTCAGATTAATGTTTATCGGCCGACTGTTTT
                              SARS-CoV-2 NC_045512.2 (29904) -----------------------------------------------------------------------------
```

```
                                                                                    Section 1653
                                        (127205) 127205    127215    127226    127237    127248    127259    127270    127281
Autographa californica nucleopolyhedrovirus NC 0... (126970) CGTATCCGCTCACCAAACGCGTTTTTGCATTAACATTGTATGTCGGCGGATGTTCTATATCTAATTTGAATAAATAA
                    SARS-CoV-2 NC_045512.2 (29904) -----------------------------------------------------------------------------
                                                                                    Section 1654
                                        (127282) 127282    127292    127303    127314    127325    127336    127347    127358
Autographa californica nucleopolyhedrovirus NC 0... (127047) ACGATAACCGCGTTGGTTTTAGAGGGCATAATAAAAGAAATATTGTTATCGTGTTCGCCATTAGGGCAGTATAAATT
                    SARS-CoV-2 NC_045512.2 (29904) -----------------------------------------------------------------------------
                                                                                    Section 1655
                                        (127359) 127359    127369    127380    127391    127402    127413    127424    127435
Autographa californica nucleopolyhedrovirus NC 0... (127124) GACGTTCATGTTGGATATTGTTTCAGTTGCAAGTTGACACTGGCGGCGACAAGATCGTGAACAACCAAGTGACTATG
                    SARS-CoV-2 NC_045512.2 (29904) -----------------------------------------------------------------------------
                                                                                    Section 1656
                                        (127436) 127436    127446    127457    127468    127479    127490    127501    127512
Autographa californica nucleopolyhedrovirus NC 0... (127201) ACGCAAATTAATTTTAACGCGTCGTACACCAGCGCTTCGACGCCGTCCCGAGCGTCGTTCGACAACAGCTATTCAGA
                    SARS-CoV-2 NC_045512.2 (29904) -----------------------------------------------------------------------------
                                                                                    Section 1657
                                        (127513) 127513    127523    127534    127545    127556    127567    127578    127589
Autographa californica nucleopolyhedrovirus NC 0... (127278) GTTTTGTGATAAACAACCCAACGACTATTTAAGTTATTATAACCATCCCACCCCGGATGGAGCCGACACGGTGATAT
                    SARS-CoV-2 NC_045512.2 (29904) -----------------------------------------------------------------------------
                                                                                    Section 1658
                                        (127590) 127590    127600    127611    127622    127633    127644    127655    127666
Autographa californica nucleopolyhedrovirus NC 0... (127355) CTGACAGCGAGACTGCGGCAGCTTCAAACTTTTTGGCAAGCGTCAACTCGTTAACTGATAATGATTTAGTGGAATGT
                    SARS-CoV-2 NC_045512.2 (29904) -----------------------------------------------------------------------------
                                                                                    Section 1659
                                        (127667) 127667    127677    127688    127699    127710    127721    127732    127743
Autographa californica nucleopolyhedrovirus NC 0... (127432) TTGCTCAAGACCACTGATAATCTCGAAGAAGCAGTTAGTTCTGCTTATTATTCGGAATCCCTTGAGCAGCCTGTTGT
                    SARS-CoV-2 NC_045512.2 (29904) -----------------------------------------------------------------------------
```

```
                                                                                              Section 1660
                                        (127744) 127744    127754      127765      127776      127787      127798      127809  127820
Autographa californica nucleopolyhedrovirus NC 0... (127509) GGAGCAACCATCGCCCAGTTCTGCTTATCATGCGGAATCTTTTGAGCATTCTGCTGGTGTGAACCAACCATCGGCAA
                     SARS-CoV-2 NC_045512.2 (29904) -----------------------------------------------------------------------------
                                                                                              Section 1661
                                        (127821) 127821    127831      127842      127853      127864      127875      127886  127897
Autographa californica nucleopolyhedrovirus NC 0... (127586) CTGGAACTAAACGGAAGCTGGACGAATACTTGGACAATTCACAAGGTGTGGTGGGCCAGTTTAACAAAATTAAATTG
                     SARS-CoV-2 NC_045512.2 (29904) -----------------------------------------------------------------------------
                                                                                              Section 1662
                                        (127898) 127898    127908      127919      127930      127941      127952      127963  127974
Autographa californica nucleopolyhedrovirus NC 0... (127663) AGGCCTAAATACAAGAAAAGCACAATTCAAAGCTGTGCAACCCTTGAACAGACAATTAATCACAACACGAACATTTG
                     SARS-CoV-2 NC_045512.2 (29904) -----------------------------------------------------------------------------
                                                                                              Section 1663
                                        (127975) 127975    127985      127996      128007      128018      128029      128040  128051
Autographa californica nucleopolyhedrovirus NC 0... (127740) CACGGTCGCTTCAACTCAAGAAATTACGCATTATTTTACTAATGATTTTGCGCCGTATTTAATGCGTTTCGACGACA
                     SARS-CoV-2 NC_045512.2 (29904) -----------------------------------------------------------------------------
                                                                                              Section 1664
                                        (128052) 128052    128062      128073      128084      128095      128106      128117  128128
Autographa californica nucleopolyhedrovirus NC 0... (127817) ACGACTACAATTCCAACAGGTTCTCCGACCATATGTCCGAAACTGGTTATTACATGTTTGTGGTTAAAAAAAGTGAA
                     SARS-CoV-2 NC_045512.2 (29904) -----------------------------------------------------------------------------
                                                                                              Section 1665
                                        (128129) 128129    128139      128150      128161      128172      128183      128194  128205
Autographa californica nucleopolyhedrovirus NC 0... (127894) GTGAAGCCGTTTGAAATTATATTTGCCAAGTACGTGAGCAATGTGGTTTACGAATATACAAACAATTATTACATGGT
                     SARS-CoV-2 NC_045512.2 (29904) -----------------------------------------------------------------------------
                                                                                              Section 1666
                                        (128206) 128206    128216      128227      128238      128249      128260      128271  128282
Autographa californica nucleopolyhedrovirus NC 0... (127971) AGATAATCGCGTGTTTGTGGTAACTTTTGATAAAATTAGGTTTATGATTTCGTACAATTTGGTTAAAGAAACCGGCA
                     SARS-CoV-2 NC_045512.2 (29904) -----------------------------------------------------------------------------
```

Section 1667

	(128283)	128283 128293 128304 128315 128326 128337 128348 128359
Autographa californica nucleopolyhedrovirus NC 0...	(128048)	TAGAAATTCCTCATTCTCAAGATGTGTGCAACGACGAGACGGCTGCACAAAATTGTAAAAAATGCCATTTCGTCGAT
SARS-CoV-2 NC_045512.2	(29904)	---

Section 1668

	(128360)	128360 128370 128381 128392 128403 128414 128425 128436
Autographa californica nucleopolyhedrovirus NC 0...	(128125)	GTGCACCACACGTTTAAAGCTGCTCTGACTTCATATTTTAATTTAGATATGTATTACGCGCAAACCACATTTGTGAC
SARS-CoV-2 NC_045512.2	(29904)	---

Section 1669

	(128437)	128437 128447 128458 128469 128480 128491 128502 128513
Autographa californica nucleopolyhedrovirus NC 0...	(128202)	TTTGTTACAATCGTTGGGCGAAAGAAAATGTGGGTTTCTTTTGAGCAAGTTGTACGAAATGTATCAAGATAAAAATT
SARS-CoV-2 NC_045512.2	(29904)	---

Section 1670

	(128514)	128514 128524 128535 128546 128557 128568 128579 128590
Autographa californica nucleopolyhedrovirus NC 0...	(128279)	TATTTACTTTGCCTATTATGCTTAGTCGTAAAGAGAGTAATGAAATTGAGACTGCATCTAATAATTTCTTTGTATCG
SARS-CoV-2 NC_045512.2	(29904)	---

Section 1671

	(128591)	128591 128601 128612 128623 128634 128645 128656 128667
Autographa californica nucleopolyhedrovirus NC 0...	(128356)	CCGTATGTGAGTCAAATATTAAAGTATTCGGAAAGTGTGCAGTTTCCCGACAATCCCCCAAACAAATATGTGGTGGA
SARS-CoV-2 NC_045512.2	(29904)	---

Section 1672

	(128668)	128668 128678 128689 128700 128711 128722 128733 128744
Autographa californica nucleopolyhedrovirus NC 0...	(128433)	CAATTTAAATTTAATTGTTAACAAAAAAAGTACGCTCACGTACAAATACAGCAGCGTCGCTAATCTTTTGTTTAATA
SARS-CoV-2 NC_045512.2	(29904)	---

Section 1673

	(128745)	128745 128755 128766 128777 128788 128799 128810 128821
Autographa californica nucleopolyhedrovirus NC 0...	(128510)	ATTATAAATATCATGACAATATTGCGAGTAATAATAACGCAGAAAATTTAAAAAAGGTTAAGAAGGAGGACGGCAGC
SARS-CoV-2 NC_045512.2	(29904)	---

Section 1674

(128822) 128822 128832 128843 128854 128865 128876 128887 128898
Autographa californica nucleopolyhedrovirus NC 0... (128587) ATGCACATTGTCGAACAGTATTTGACTCAGAATGTAGATAATGTAAAGGGTCACAATTTTATAGTATTGTCTTTCAA
SARS-CoV-2 NC_045512.2 (29904) ---

Section 1675

(128899) 128899 128909 128920 128931 128942 128953 128964 128975
Autographa californica nucleopolyhedrovirus NC 0... (128664) AAACGAGGAGCGATTGACTATAGCTAAGAAAAACAAAGAGTTTTATTGGATTTCTGGCGAAATTAAAGATGTAGACG
SARS-CoV-2 NC_045512.2 (29904) ---

Section 1676

(128976) 128976 128986 128997 129008 129019 129030 129041 129052
Autographa californica nucleopolyhedrovirus NC 0... (128741) TTAGTCAAGTAATTCAAAAATATAATAGATTTAAGCATCACATGTTTGTAATCGGTAAAGTGAACCGAAGAGAGAGC
SARS-CoV-2 NC_045512.2 (29904) ---

Section 1677

(129053) 129053 129063 129074 129085 129096 129107 129118 129129
Autographa californica nucleopolyhedrovirus NC 0... (128818) ACTACATTGCACAATAATTTGTTAAAATTGTTAGCTTTAATATTACAGGGTCTGGTTCCGTTGTCCGACGCTATAAC
SARS-CoV-2 NC_045512.2 (29904) ---

Section 1678

(129130) 129130 129140 129151 129162 129173 129184 129195 129206
Autographa californica nucleopolyhedrovirus NC 0... (128895) GTTTGCGGAACAAAAACTAAATTGTAAATATAAAAAATTCGAATTTAATTAATTATACATATATTTTGAATTTAATT
SARS-CoV-2 NC_045512.2 (29904) ---

Section 1679

(129207) 129207 129217 129228 129239 129250 129261 129272 129283
Autographa californica nucleopolyhedrovirus NC 0... (128972) AATTATACATATATTTTATATTATTTTTGTCTTTTATTATCGAGGGGCCGTTGTTGGTGTGGGGTTTTGCATAGAAA
SARS-CoV-2 NC_045512.2 (29904) ---

Section 1680

(129284) 129284 129294 129305 129316 129327 129338 129349 129360
Autographa californica nucleopolyhedrovirus NC 0... (129049) TAACAATGGGAGTTGGCGACGTTGCTGCGCCAACACCACCTCCTCCTCCTCCTTTCATCATGTATCTGTAGATAAAA
SARS-CoV-2 NC_045512.2 (29904) ---

Section 1681

```
                                                   (129361) 129361    129371     129382     129393     129404     129415     129426 129437
Autographa californica nucleopolyhedrovirus NC 0... (129126) TAAAATATTAAACCTAAAAACAAGACCGCGCCTATCAACAAAATGATAGGCATTAACTTGCCGCTGACGCTGTCACT
                         SARS-CoV-2 NC_045512.2   (29904) -----------------------------------------------------------------------------
```

Section 1682

```
                                                   (129438) 129438    129448     129459     129470     129481     129492     129503 129514
Autographa californica nucleopolyhedrovirus NC 0... (129203) AACGTTGGACGATTTGCCGACTAAACCTTCATCGCCCAGTAACCAATCTAGACCCAAGTCGCCAACTAAATCACCAA
                         SARS-CoV-2 NC_045512.2   (29904) -----------------------------------------------------------------------------
```

Section 1683

```
                                                   (129515) 129515    129525     129536     129547     129558     129569     129580 129591
Autographa californica nucleopolyhedrovirus NC 0... (129280) ACGAGTAAGGTTCGATGCACATGAGTGTTTGGCCCGCAGGAAGATCGCTAATATCTACGTATTGAGGCGAATCTGGG
                         SARS-CoV-2 NC_045512.2   (29904) -----------------------------------------------------------------------------
```

Section 1684

```
                                                   (129592) 129592    129602     129613     129624     129635     129646     129657 129668
Autographa californica nucleopolyhedrovirus NC 0... (129357) TCGGCGGACGGATCGCTGCCGCGACAAACTGTTTTTTCTACTTCATAGTTGAATCCTTGGCACATGTTGGTTAGTTC
                         SARS-CoV-2 NC_045512.2   (29904) -----------------------------------------------------------------------------
```

Section 1685

```
                                                   (129669) 129669    129679     129690     129701     129712     129723     129734 129745
Autographa californica nucleopolyhedrovirus NC 0... (129434) GGGCGGATTGTTAGGCAACAAGGGGTCGAATGGGCAAATGGTAACATCCGACTGATTTAGATTGGGGTCTTGACGAC
                         SARS-CoV-2 NC_045512.2   (29904) -----------------------------------------------------------------------------
```

Section 1686

```
                                                   (129746) 129746    129756     129767     129778     129789     129800     129811 129822
Autographa californica nucleopolyhedrovirus NC 0... (129511) AAGTGCGCTGCAATAACAAGCAGGCCTCGGCGATTTCTCCGGCGTCTTTACCTTGCACATAATAACTTCCGCCGGTG
                         SARS-CoV-2 NC_045512.2   (29904) -----------------------------------------------------------------------------
```

Section 1687

```
                                                   (129823) 129823    129833     129844     129855     129866     129877     129888 129899
Autographa californica nucleopolyhedrovirus NC 0... (129588) TTATTGATGGCGTTGATTATATCTTGTACTAGTGTGGCGGCGCTAAACAAGAAATAGCCGCCGGTGGCCAAGAGTAT
                         SARS-CoV-2 NC_045512.2   (29904) -----------------------------------------------------------------------------
```

```
                                                                                        Section 1688
                                       (129900) 129900    129910     129921     129932     129943     129954     129965  129976
Autographa californica nucleopolyhedrovirus NC 0... (129665) GCCCGTTCCTCCTACTTTTAAGCTTTGCATGTAACTATGTAGACGGGGGTTTTGCTGCAGTGCGTTTTGAACACCTT
                          SARS-CoV-2 NC_045512.2 (29904) -----------------------------------------------------------------------------
                                                                                        Section 1689
                                       (129977) 129977    129987     129998     130009     130020     130031     130042  130053
Autographa californica nucleopolyhedrovirus NC 0... (129742) CGGGCGTGCGCACGTTGGTTTCCGGGAAGTTTTGTTTGACTGCATTGGATCGCGTCTGCTTGGTGTGGTAATTAAAG
                          SARS-CoV-2 NC_045512.2 (29904) -----------------------------------------------------------------------------
                                                                                        Section 1690
                                       (130054) 130054    130064     130075     130086     130097     130108     130119  130130
Autographa californica nucleopolyhedrovirus NC 0... (129819) TCTGGCACGTTGTCCACGCGCCGCAATTGGCTCAATGAGTTTATTTGAGGGTCTGAAATGCCCTGAAATACTCCGCG
                          SARS-CoV-2 NC_045512.2 (29904) -----------------------------------------------------------------------------
                                                                                        Section 1691
                                       (130131) 130131    130141     130152     130163     130174     130185     130196  130207
Autographa californica nucleopolyhedrovirus NC 0... (129896) TATGTTGGGGACATCATTGTTACGAGTAATTCTGTTTATGTCTGAAGTGCTCACAAACTGGTTGTTAGATAGTTGAT
                          SARS-CoV-2 NC_045512.2 (29904) -----------------------------------------------------------------------------
                                                                                        Section 1692
                                       (130208) 130208    130218     130229     130240     130251     130262     130273  130284
Autographa californica nucleopolyhedrovirus NC 0... (129973) AGCCCGGCTGAAATCTGTTGTTTCCAATGTTGCGTACACTGGGCGCGTTGAGCACATTTGTGAAACCGGCGGGAGTG
                          SARS-CoV-2 NC_045512.2 (29904) -----------------------------------------------------------------------------
                                                                                        Section 1693
                                       (130285) 130285    130295     130306     130317     130328     130339     130350  130361
Autographa californica nucleopolyhedrovirus NC 0... (130050) CTTGTTAAAAGACGCGTATTATCAGTAATAAAACTGGCCTGATTAGGATACAATTTATTGACTGCGCGAAGATTTGA
                          SARS-CoV-2 NC_045512.2 (29904) -----------------------------------------------------------------------------
                                                                                        Section 1694
                                       (130362) 130362    130372     130383     130394     130405     130416     130427  130438
Autographa californica nucleopolyhedrovirus NC 0... (130127) AAAAAAACTCATTTTAAAGCAAACTTATTTAATAAATATATCACAGTAAAGGTTTTGCAAAACTGCCGTCGTCAATA
                          SARS-CoV-2 NC_045512.2 (29904) -----------------------------------------------------------------------------
```

Section 1695

	(130439)	130439 130449 130460 130471 130482 130493 130504 130515
Autographa californica nucleopolyhedrovirus NC_0...	(130204)	CAACACGGCAGCGGCGTCATGTTGGTAAAATCTAATCTTCTCCTTGCTTTAGATTCTGGGCGAGAAGGCGCATTTGT
SARS-CoV-2 NC_045512.2	(29904)	---

Section 1696

	(130516)	130516 130526 130537 130548 130559 130570 130581 130592
Autographa californica nucleopolyhedrovirus NC_0...	(130281)	TGTGTAAGTTATTTCGACGTCTGCATTATTTGTTGTGTAAGGTATCTCGACGTATGAAGCAACTTTAACATTGTTAT
SARS-CoV-2 NC_045512.2	(29904)	---

Section 1697

	(130593)	130593 130603 130614 130625 130636 130647 130658 130669
Autographa californica nucleopolyhedrovirus NC_0...	(130358)	AATTTTTTTTAAATATTGATGCGCTCCACGGCGCGCGTTGATACGGATGATATCTCTCCATTGTATGATCGCTAAAT
SARS-CoV-2 NC_045512.2	(29904)	---

Section 1698

	(130670)	130670 130680 130691 130702 130713 130724 130735 130746
Autographa californica nucleopolyhedrovirus NC_0...	(130435)	TTATATACCGTTTCAATAAATATGTTAAAACCCAACATGTTAATTATAATATTCATAATAGTTTGTTTGTTTTCAAT
SARS-CoV-2 NC_045512.2	(29904)	---

Section 1699

	(130747)	130747 130757 130768 130779 130790 130801 130812 130823
Autographa californica nucleopolyhedrovirus NC_0...	(130512)	AATTATTTTTACTGTTTTGAAATCTAAAAGAGGTGACGATGACGAATCAGACGACGGGTTCAGTTGCTATAACAAAC
SARS-CoV-2 NC_045512.2	(29904)	---

Section 1700

	(130824)	130824 130834 130845 130856 130867 130878 130889 130900
Autographa californica nucleopolyhedrovirus NC_0...	(130589)	CAATTGGAGTAAATTTTCCGCATCCTACTAGATGTGACGCTTTCTACATGTGTGTCGGTTTAAATCAAAAATTAGAG
SARS-CoV-2 NC_045512.2	(29904)	---

Section 1701

	(130901)	130901 130911 130922 130933 130944 130955 130966 130977
Autographa californica nucleopolyhedrovirus NC_0...	(130666)	TTAATCTGCCCTGAAGGATTTGAATTTGATCCAGATGTTAAAAATTGTGTTCCTATATCAGATTATGGATGTACCGC
SARS-CoV-2 NC_045512.2	(29904)	---

Section 1702

(130978) 130978 130988 130999 131010 131021 131032 131043 131054

Autographa californica nucleopolyhedrovirus NC_0... (130743) TAACCAAAACTAAAAATAAAATAAAATTTATATAGATTAATGAAATAAAATTTATATAGATTAATAAAATAAAATTT

SARS-CoV-2 NC_045512.2 (29904) ---

Section 1703

(131055) 131055 131065 131076 131087 131098 131109 131120 131131

Autographa californica nucleopolyhedrovirus NC_0... (130820) ATTTAATATATTATACTATTTATATTATTTACAACACTTAACGTCTAGACATAACAGTTTGTAACTTAGAAACTAAA

SARS-CoV-2 NC_045512.2 (29904) ---

Section 1704

(131132) 131132 131142 131153 131164 131175 131186 131197 131208

Autographa californica nucleopolyhedrovirus NC_0... (130897) TCAGAGTTACTGCGCTCAAACTCTGAAAATTTGGCTTGAGACTCGGCCACCTGCTTACGCAATTGTTCTTGCAGATT

SARS-CoV-2 NC_045512.2 (29904) ---

Section 1705

(131209) 131209 131219 131230 131241 131252 131263 131274 131285

Autographa californica nucleopolyhedrovirus NC_0... (130974) ATTCACAGTCGATTGCAACTCTTCTGATTTCTTGGTAGATTCTTGCAAGTCATAGTTTGCCTTTTGTAAATCTAATT

SARS-CoV-2 NC_045512.2 (29904) ---

Section 1706

(131286) 131286 131296 131307 131318 131329 131340 131351 131362

Autographa californica nucleopolyhedrovirus NC_0... (131051) CGGCGACAGCATGCTTGTGTTTAAGCATAATGTAGTCGCTGTTTAACATGGTCATTTTATGTTCAACTTGGCTGGTC

SARS-CoV-2 NC_045512.2 (29904) ---

Section 1707

(131363) 131363 131373 131384 131395 131406 131417 131428 131439

Autographa californica nucleopolyhedrovirus NC_0... (131128) TTGGCTCGCAGCTCGGACAGTTCTTTTTGCAATTGCTCCACATAGTTCAAGTCCGTGGTGTGATTGTTGACCGTGTT

SARS-CoV-2 NC_045512.2 (29904) ---

Section 1708

(131440) 131440 131450 131461 131472 131483 131494 131505 131516

Autographa californica nucleopolyhedrovirus NC_0... (131205) ATTTTCTAAAAGCTCGCGCCAATGCTGTTTGATGGAATCCTGGTTACGAGTGACGTTAATGGGCATAAATTCTACAT

SARS-CoV-2 NC_045512.2 (29904) ---

```
                                                               Section 1709
                                        (131517) 131517    131527     131538     131549     131560     131571     131582 131593
Autographa californica nucleopolyhedrovirus NC_0... (131282) ACCCGTGCTTATTGTACACGCGACAATCTGATGAAGTAGCGCTGCAAAAACATTTGTACACAGAATTGTCCATAATT
                  SARS-CoV-2 NC_045512.2 (29904) -----------------------------------------------------------------------------
                                                               Section 1710
                                        (131594) 131594    131604     131615     131626     131637     131648     131659 131670
Autographa californica nucleopolyhedrovirus NC_0... (131359) ATCTTGACATAACACTTGAAACACACAGCATGGTTACAATGAATCGAAGTCACAAACGAGGAATTTACGTTTTTAGT
                  SARS-CoV-2 NC_045512.2 (29904) -----------------------------------------------------------------------------
                                                               Section 1711
                                        (131671) 131671    131681     131692     131703     131714     131725     131736 131747
Autographa californica nucleopolyhedrovirus NC_0... (131436) GTCTTTAAAAGTAGTAAAACAAATATTACACGAAACCTCTACTTCTTCTTCGGGTTCTGATTGCTGCTGCTGCTGCT
                  SARS-CoV-2 NC_045512.2 (29904) -----------------------------------------------------------------------------
                                                               Section 1712
                                        (131748) 131748    131758     131769     131780     131791     131802     131813 131824
Autographa californica nucleopolyhedrovirus NC_0... (131513) GCTGCGGCTGCGGAGACTGCGGCGAGGCAAACAAATCTGGCGACTGTGGTATTACGTAATTCGGCGAATAAGATGGA
                  SARS-CoV-2 NC_045512.2 (29904) -----------------------------------------------------------------------------
                                                               Section 1713
                                        (131825) 131825    131835     131846     131857     131868     131879     131890 131901
Autographa californica nucleopolyhedrovirus NC_0... (131590) CTATAAGTGGGAGACCTTGGGGCAATCTCATTCATCAGCTGAGCCTCAAGATCTAAACCTCGTTGCAGAGCCCTCTG
                  SARS-CoV-2 NC_045512.2 (29904) -----------------------------------------------------------------------------
                                                               Section 1714
                                        (131902) 131902    131912     131923     131934     131945     131956     131967 131978
Autographa californica nucleopolyhedrovirus NC_0... (131667) CGCAGCTGTCTCCGACGCAATGTTATCCTGGTACTGCTGGGCAGTGATGTCGGGAAACCGTTCACGATCCACATTTT
                  SARS-CoV-2 NC_045512.2 (29904) -----------------------------------------------------------------------------
                                                               Section 1715
                                        (131979) 131979    131989     132000     132011     132022     132033     132044 132055
Autographa californica nucleopolyhedrovirus NC_0... (131744) CACTATTAATTAGTATGACGTCATCCTCTTGACTTAATAGCGGATCGTCATTGCTAATGTTAACCTGACCGTGCACG
                  SARS-CoV-2 NC_045512.2 (29904) -----------------------------------------------------------------------------
```

```
                                                              Section 1716
                                  (132056) 132056    132066     132077     132088     132099     132110     132121   132132
Autographa californica nucleopolyhedrovirus  NC_0... (131821) TAATACGTGACACCCTGACGATGGTAGGTGCGCGTCAACGGCTCGTTGACGTTCCCGATAATCTGCACGTTTTCTTC
                    SARS-CoV-2 NC_045512.2  (29904) -----------------------------------------------------------------------------
                                                              Section 1717
                                  (132133) 132133    132143     132154     132165     132176     132187     132198   132209
Autographa californica nucleopolyhedrovirus  NC_0... (131898) GCTGACACGCTGCTCCTGACGCCGCTCCTGACGGCGATGGCTGCGACTGCTTGAAGACGGCTGGCTGCGACTGCTTG
                    SARS-CoV-2 NC_045512.2  (29904) -----------------------------------------------------------------------------
                                                              Section 1718
                                  (132210) 132210    132220     132231     132242     132253     132264     132275   132286
Autographa californica nucleopolyhedrovirus  NC_0... (131975) AAGACGGCTGGGCTTCGGGAGATGTTGTAAAGTTGATGCGGCGACGGCTGAGAGACAGCCTGTGGCGGCGGCTGCTG
                    SARS-CoV-2 NC_045512.2  (29904) -----------------------------------------------------------------------------
                                                              Section 1719
                                  (132287) 132287    132297     132308     132319     132330     132341     132352   132363
Autographa californica nucleopolyhedrovirus  NC_0... (132052) CTGGGAGTGGCGGCGTTGATTTGGCGACTCATGGCTGGGCTGGTAGGATACTGTTCACTAGGCTGTGAGGCTTGAAC
                    SARS-CoV-2 NC_045512.2  (29904) -----------------------------------------------------------------------------
                                                              Section 1720
                                  (132364) 132364    132374     132385     132396     132407     132418     132429   132440
Autographa californica nucleopolyhedrovirus  NC_0... (132129) TGTGCTTACGAGTAGAACGGCAGCTGTATTTATACTGTTTATCAGTACTGCACGACTGATAAGACAATAGTGGTGGG
                    SARS-CoV-2 NC_045512.2  (29904) -----------------------------------------------------------------------------
                                                              Section 1721
                                  (132441) 132441    132451     132462     132473     132484     132495     132506   132517
Autographa californica nucleopolyhedrovirus  NC_0... (132206) GGAACTTGCCAGGCAAAAATGAACTTTTTTGTAATGCAAAAAAGTTGATAGTGTAGTAGTATATTGGGAGCGTATCG
                    SARS-CoV-2 NC_045512.2  (29904) -----------------------------------------------------------------------------
                                                              Section 1722
                                  (132518) 132518    132528     132539     132550     132561     132572     132583   132594
Autographa californica nucleopolyhedrovirus  NC_0... (132283) TACAGTGTAGACTATTCTAATAAAATAGTCTACGATTTGTAGAGATTGTACTGTATATGGAGTGTCAGGCAAAAGTG
                    SARS-CoV-2 NC_045512.2  (29904) -----------------------------------------------------------------------------
```

Section 1723

	(132595)	132595 132605 132616 132627 132638 132649 132660 132671
Autographa californica nucleopolyhedrovirus NC 0...	(132360)	AACTTTTTTGCATTGCAAAAAAATTCATTTTAAATTTATCATATCACAGGCTGCAGTTTCTGTTATCTGTCCCCCAC
SARS-CoV-2 NC_045512.2	(29904)	---

Section 1724

	(132672)	132672 132682 132693 132704 132715 132726 132737 132748
Autographa californica nucleopolyhedrovirus NC 0...	(132437)	TCAGGCGTGCAGCTATAAAAGCAGGCACTCACCAACTCGTAAGCACAGTTCGTTGTGAAGTGAACACGGAGAGCCTG
SARS-CoV-2 NC_045512.2	(29904)	---

Section 1725

	(132749)	132749 132759 132770 132781 132792 132803 132814 132825
Autographa californica nucleopolyhedrovirus NC 0...	(132514)	CCAATAAGCAAAATGCCAAGGGACACCAACAATCGCCACCGGTCTACGCCATATGAACGTCCTACGCTTGAAGATCT
SARS-CoV-2 NC_045512.2	(29904)	---

Section 1726

	(132826)	132826 132836 132847 132858 132869 132880 132891 132902
Autographa californica nucleopolyhedrovirus NC 0...	(132591)	CCGCAGACAGTTGCAAGACAATTTGGACAGCATAAACCGCCGAGACAGAATGCAAGAAGAACAAGAAGAAAACCTGC
SARS-CoV-2 NC_045512.2	(29904)	---

Section 1727

	(132903)	132903 132913 132924 132935 132946 132957 132968 132979
Autographa californica nucleopolyhedrovirus NC 0...	(132668)	GCTATCAAGTGCGTAGAAGGCAGCGTCAAAACCAGCTCCGCTCCATACAAATGGAACAGCAGCGAATGATGGCGGAA
SARS-CoV-2 NC_045512.2	(29904)	---

Section 1728

	(132980)	132980 132990 133001 133012 133023 133034 133045 133056
Autographa californica nucleopolyhedrovirus NC 0...	(132745)	TTAAACAACGAGCCGGTGATTAATTTTAAATTTGAGTGTAGTGTGTGTTTAGAAACATATTCCCAACAATCTAACGA
SARS-CoV-2 NC_045512.2	(29904)	---

Section 1729

	(133057)	133057 133067 133078 133089 133100 133111 133122 133133
Autographa californica nucleopolyhedrovirus NC 0...	(132822)	TACTTGTCCTTTTTTGATTCCGACTACGTGCGACCACGGTTTTTGTTTCAAATGCGTCATCAATCTGCAAAGCAACG
SARS-CoV-2 NC_045512.2	(29904)	---

```
                                                                                Section 1730
                                           (133134) 133134    133144     133155     133166     133177     133188     133199  133210
Autographa californica nucleopolyhedrovirus NC 0... (132899) CGATGAATATTCCGCATTCCACTGTGTGCTGTCCATTGTGCAATACCCAGGTAAAAATGTGGCGTTCCTTAAAGCCT
                             SARS-CoV-2 NC_045512.2 (29904) -----------------------------------------------------------------------------
                                                                                Section 1731
                                           (133211) 133211    133221     133232     133243     133254     133265     133276  133287
Autographa californica nucleopolyhedrovirus NC 0... (132976) AACGCTGTTGTGACGTGTAAGTTTTACAAGAAAACTCAAGAAAGAGTTCCGCCCGTGCAGCAGTATAAAAACATTAT
                             SARS-CoV-2 NC_045512.2 (29904) -----------------------------------------------------------------------------
                                                                                Section 1732
                                           (133288) 133288    133298     133309     133320     133331     133342     133353  133364
Autographa californica nucleopolyhedrovirus NC 0... (133053) TAAAGTGCTACAAGAACGGAGCGTGATTAGTGTCGAAGACAACGACAATAATTGTGACATAAATATGGAGAATCAGG
                             SARS-CoV-2 NC_045512.2 (29904) -----------------------------------------------------------------------------
                                                                                Section 1733
                                           (133365) 133365    133375     133386     133397     133408     133419     133430  133441
Autographa californica nucleopolyhedrovirus NC 0... (133130) CAAAGATAGCTGCTTTGGAAGCTGAATTGGAAGAAGAAAAAAATCACAGTGATCAAGTAGCTTCTGAAAACCGACAG
                             SARS-CoV-2 NC_045512.2 (29904) -----------------------------------------------------------------------------
                                                                                Section 1734
                                           (133442) 133442    133452     133463     133474     133485     133496     133507  133518
Autographa californica nucleopolyhedrovirus NC 0... (133207) CTGATAGAAGAAAATACTCGTCTCAATGAACAGATTCAAGAGTTGCAGCATCAGGTGAGGACATTGGTGCCGCAACG
                             SARS-CoV-2 NC_045512.2 (29904) -----------------------------------------------------------------------------
                                                                                Section 1735
                                           (133519) 133519    133529     133540     133551     133562     133573     133584  133595
Autographa californica nucleopolyhedrovirus NC 0... (133284) TGGCATTACGGTTAATCAGCAAATTGGCCGTGACGACAGTGCGCCAGCCGAGCTGAACGAGCGTTTTCGCTCACTTG
                             SARS-CoV-2 NC_045512.2 (29904) -----------------------------------------------------------------------------
                                                                                Section 1736
                                           (133596) 133596    133606     133617     133628     133639     133650     133661  133672
Autographa californica nucleopolyhedrovirus NC 0... (133361) TCTATTCGACTATTTCAGAGCTGTTTATTGAAAATGGCGTTCATAGTATTCAAAATTATGTTTATGCCGGAACTTCT
                             SARS-CoV-2 NC_045512.2 (29904) -----------------------------------------------------------------------------
```

```
                                                                                        Section 1737
                                              (133673) 133673    133683     133694     133705     133716     133727     133738  133749
Autographa californica nucleopolyhedrovirus  NC_0... (133438) GCTGCTAGTTCATGTGATGTAAATGTTACTGTTAATTTTGGGTTTGAAAATTAATGTGATATGAAATGTATATATAA
                             SARS-CoV-2 NC_045512.2   (29904) -----------------------------------------------------------------------------
                                                                                        Section 1738
                                              (133750) 133750    133760     133771     133782     133793     133804     133815  133826
Autographa californica nucleopolyhedrovirus  NC_0... (133515) AAATGATGGAATAAATAATAAACATTTTTATACTTTTTATGTTTTTTTTATTTCATGTGATTAAGAAACTTTTAAGA
                             SARS-CoV-2 NC_045512.2   (29904) -----------------------------------------------------------------------------
                                                                                        Section 1739
                                              (133827) 133827    133837     133848     133859     133870     133881     133892  133903
Autographa californica nucleopolyhedrovirus  NC_0... (133592) TGGATAGTAGTAATTGTATTAAAATAGATGTAAAATACGATATGCCGTTACATTATCAATGTGACAATAACGCAGAT
                             SARS-CoV-2 NC_045512.2   (29904) -----------------------------------------------------------------------------
                                                                                        Section 1740
                                              (133904) 133904    133914     133925     133936     133947     133958     133969  133980
Autographa californica nucleopolyhedrovirus  NC_0... (133669) AAAGACGTTGTAAATGCGTATGACACTATCGATGTTGACCCCAACAAAAGATTTATAATTAATCATAATCACGAACA
                             SARS-CoV-2 NC_045512.2   (29904) -----------------------------------------------------------------------------
                                                                                        Section 1741
                                              (133981) 133981    133991     134002     134013     134024     134035     134046  134057
Autographa californica nucleopolyhedrovirus  NC_0... (133746) ACAACAAGTCAATGAAACAAATAAACAAGTTGTCGATAAAACATTCATAAATGACACAGCAACATACAATTCTTGCA
                             SARS-CoV-2 NC_045512.2   (29904) -----------------------------------------------------------------------------
                                                                                        Section 1742
                                              (134058) 134058    134068     134079     134090     134101     134112       134129
Autographa californica nucleopolyhedrovirus  NC_0... (133823) TAATAAAAATTTAAATGACATCATATTTGAGAATAACAAATGACATTATCCCTCGATTGTGTTTTACAAGTA
                             SARS-CoV-2 NC_045512.2   (29904) ------------------------------------------------------------------------
```
