# Supplementary material for: Ubiquitous Micro-Modular Homologies among Genomes from Viruses to Bacteria to Human Mitochondrial DNA: Platforms for Recombination during Evolution?
Source: Viruses. 2022 Apr 24;14(5):885. doi: 10.3390/v14050885 (PMC9147251; doi:10.3390/v14050885)
Supplement: Supplementary file 1 [file viruses-14-00885-s001.zip › Fig. S4A, SARS-CoV-2 & Bakterien, Carsonella ruddii.pdf]

```
                                                                                                     Section 1
                                                          (1) 1         11         22         33         44         55         66         77
Candidatus Carsonella ruddii strain BT chromosome...      (1) AAATAATTAATATTAAAGAAATAATTAATATTAAAAAAATACATAATGTAATTTCTTATATTAATAAAATTTTAATT
         Candidatus Carsonella 135557 - 167442nt          (1) -----------------------------------------------------------------------------
    SARS-CoV-2 Reference Genome NC_045512.2               (1) -----------------------------------------------------------------------------
                                                                                                     Section 2
                                                         (78) 78        88         99         110        121        132        143        154
Candidatus Carsonella ruddii strain BT chromosome...     (78) TATAATAATTCAATAAAACAAATAGAAAATATTATTTTTTGGTTGTTTCCAATTATACCAAATATTTCTAAAATTTT
         Candidatus Carsonella 135557 - 167442nt          (1) -----------------------------------------------------------------------------
    SARS-CoV-2 Reference Genome NC_045512.2               (1) -----------------------------------------------------------------------------
                                                                                                     Section 3
                                                        (155) 155       165        176        187        198        209        220        231
Candidatus Carsonella ruddii strain BT chromosome...    (155) TTGGTTTAAAATTGGAAATTTATTTCCAATTGAAAAATTTAAAAATCAAAATTTAATATTAAAAAAATTTAAAATTT
         Candidatus Carsonella 135557 - 167442nt          (1) -----------------------------------------------------------------------------
    SARS-CoV-2 Reference Genome NC_045512.2               (1) -----------------------------------------------------------------------------
                                                                                                     Section 4
                                                        (232) 232       242        253        264        275        286        297        308
Candidatus Carsonella ruddii strain BT chromosome...    (232) ATTATAAACAAAAATTTATTAAGTCTATTAATAATATAGAAATAAAAATAATTTCTAATAAAAATATTTATATTCAA
         Candidatus Carsonella 135557 - 167442nt          (1) -----------------------------------------------------------------------------
    SARS-CoV-2 Reference Genome NC_045512.2               (1) -----------------------------------------------------------------------------
                                                                                                     Section 5
                                                        (309) 309       319        330        341        352        363        374        385
Candidatus Carsonella ruddii strain BT chromosome...    (309) AAAATTATATTTTCAATGGATAAAATTTCTATTTTAATAGAATAATATCTTCTAAATTATTAAAAATTAAAAATTTT
         Candidatus Carsonella 135557 - 167442nt          (1) -----------------------------------------------------------------------------
    SARS-CoV-2 Reference Genome NC_045512.2               (1) -----------------------------------------------------------------------------
                                                                                                     Section 6
                                                        (386) 386       396        407        418        429        440        451        462
Candidatus Carsonella ruddii strain BT chromosome...    (386) TTTTTTATTTTTTTTAATTTTTTTGTAATATATCATTATTTTTATTATTTATAGCTCCAGCATGCCCCATAATTTTTTC
         Candidatus Carsonella 135557 - 167442nt          (1) -----------------------------------------------------------------------------
    SARS-CoV-2 Reference Genome NC_045512.2               (1) -----------------------------------------------------------------------------
```

Section 7

	(463)	463 473 484 495 506 517 528 539
Candidatus Carsonella ruddii strain BT chromosome...	(463)	TAATGGAGAAAAAACACCAGAAATAAAAAAAAACATTGGTTTTTTTTTAAATTTTGAAAATAAAATTTCTATATTTC
Candidatus Carsonella 135557 - 167442nt	(1)	---
SARS-CoV-2 Reference Genome NC_045512.2	(1)	---

Section 8

	(540)	540 550 561 572 583 594 605 616
Candidatus Carsonella ruddii strain BT chromosome...	(540)	CTCCAATTTCTCCTATTATTAAAATTTTTTTTGTAAAATTTAAACTAAATAATGGTAAAAATTTTTTAAAATTAAAA
Candidatus Carsonella 135557 - 167442nt	(1)	---
SARS-CoV-2 Reference Genome NC_045512.2	(1)	---

Section 9

	(617)	617 627 638 649 660 671 682 693
Candidatus Carsonella ruddii strain BT chromosome...	(617)	TTTATGACTACATCACCTCCTATTCCTAAACATAAAGATTGACCTATATTTAATTTATTAGATATTTTTATAGCTTC
Candidatus Carsonella 135557 - 167442nt	(1)	---
SARS-CoV-2 Reference Genome NC_045512.2	(1)	---

Section 10

	(694)	694 704 715 726 737 748 759 770
Candidatus Carsonella ruddii strain BT chromosome...	(694)	ATATGTTAAAGTTCCAGATCTTGAAATAATTGCTAAATATCCTTTTTTTATATATTTTAAAGGAAAAATACCAAGTC
Candidatus Carsonella 135557 - 167442nt	(1)	---
SARS-CoV-2 Reference Genome NC_045512.2	(1)	---

Section 11

	(771)	771 781 792 803 814 825 836 847
Candidatus Carsonella ruddii strain BT chromosome...	(771)	TAATATTAAAATATGGTAAAATAATTCCTGGAGAATTAGGACCTATAAATATTATGTTATATTTATTACAATAATAT
Candidatus Carsonella 135557 - 167442nt	(1)	---
SARS-CoV-2 Reference Genome NC_045512.2	(1)	---

Section 12

	(848)	848 858 869 880 891 902 913 924
Candidatus Carsonella ruddii strain BT chromosome...	(848)	TTTATTTTTAAAATATCAAAAATTGATATATTTTCAGTTATGCATATTATAATTTTTATACCTGAAAATATATTTTC
Candidatus Carsonella 135557 - 167442nt	(1)	---
SARS-CoV-2 Reference Genome NC_045512.2	(1)	---

Section 13

		925 935 946 957 968 979 990 1001
	(925)	
Candidatus Carsonella ruddii strain BT chromosome...	(925)	TAATATAATGTTTTTACAAAATAAAAACGGAACAAATATAATTGAAATTTTACATTTAGTTATTTTTAAAGCTTTTA
Candidatus Carsonella 135557 - 167442nt	(1)	---
SARS-CoV-2 Reference Genome NC_045512.2	(1)	---

Section 14

		1002 1012 1023 1034 1045 1056 1067 1078
	(1002)	
Candidatus Carsonella ruddii strain BT chromosome...	(1002)	AACTAGAAGAAAAAATTGGTAAATTTAAAATTAAATAACCCTTTTTTTTATAATTTAAACCACAAGAAAAAAATGTT
Candidatus Carsonella 135557 - 167442nt	(1)	---
SARS-CoV-2 Reference Genome NC_045512.2	(1)	---

Section 15

		1079 1089 1100 1111 1122 1133 1144 1155
	(1079)	
Candidatus Carsonella ruddii strain BT chromosome...	(1079)	CCATAATTAATAGAAATTTTAGTATGAAAAAAACCAAATTTACCAGTTAAACCACAAGATAAAATTTTTTTATTTAA
Candidatus Carsonella 135557 - 167442nt	(1)	---
SARS-CoV-2 Reference Genome NC_045512.2	(1)	---

Section 16

		1156 1166 1177 1188 1199 1210 1221 1232
	(1156)	
Candidatus Carsonella ruddii strain BT chromosome...	(1156)	CATTTGATAAAATTATACATTTTTTAATACAATTATTTAAATTTTCGATAATTATTAAATTTTTAAATTTTATTAAT
Candidatus Carsonella 135557 - 167442nt	(1)	---
SARS-CoV-2 Reference Genome NC_045512.2	(1)	---

Section 17

		1233 1243 1254 1265 1276 1287 1298 1309
	(1233)	
Candidatus Carsonella ruddii strain BT chromosome...	(1233)	TTTTTTTTAGAATAATTAGAAAAAGCTCCATCTAATCTAATTATTATTTTATGATTAAAATTCAAATACATATAATT
Candidatus Carsonella 135557 - 167442nt	(1)	---
SARS-CoV-2 Reference Genome NC_045512.2	(1)	---

Section 18

		1310 1320 1331 1342 1353 1364 1375 1386
	(1310)	
Candidatus Carsonella ruddii strain BT chromosome...	(1310)	TAATAAAGAGTTTAATATTTTTTTACAAGAAACTATTCCTCCAAATAAATTTATAAACAAAATTTTAATATTTTTAG
Candidatus Carsonella 135557 - 167442nt	(1)	---
SARS-CoV-2 Reference Genome NC_045512.2	(1)	---

Section 19

		1387 1397 1408 1419 1430 1441 1452 1463
	(1387)	
Candidatus Carsonella ruddii strain BT chromosome...	(1387)	TTAAAACAAAATTAAATAAATTATTTATTTGCAAATCTTTAATTTGACCGCTTAAATCTATAAAATTAAAACATTTA
Candidatus Carsonella 135557 - 167442nt	(1)	---
SARS-CoV-2 Reference Genome NC_045512.2	(1)	---

Section 20

		1464 1474 1485 1496 1507 1518 1529 1540
	(1464)	
Candidatus Carsonella ruddii strain BT chromosome...	(1464)	GAATTATTATAACAAAAAAAATCTAAAGTTTTTAAAGCTAAACCTGCTCCATTTACTATACAACAAATTTTACCTTT
Candidatus Carsonella 135557 - 167442nt	(1)	---
SARS-CoV-2 Reference Genome NC_045512.2	(1)	---

Section 21

		1541 1551 1562 1573 1584 1595 1606 1617
	(1541)	
Candidatus Carsonella ruddii strain BT chromosome...	(1541)	TAATTGAATATAATTTATTTTTAATAAATTTGAAATTTTTTCAGAAAAATTTAAAATACTTTTTTTTTCAATAACAA
Candidatus Carsonella 135557 - 167442nt	(1)	---
SARS-CoV-2 Reference Genome NC_045512.2	(1)	---

Section 22

		1618 1628 1639 1650 1661 1672 1683 1694
	(1618)	
Candidatus Carsonella ruddii strain BT chromosome...	(1618)	TTTTACAATCTAATATATAAATATTGTTATTATTTATTATTAAAGGATTAATTTCTATTAATAAAATATTTTTTATA
Candidatus Carsonella 135557 - 167442nt	(1)	---
SARS-CoV-2 Reference Genome NC_045512.2	(1)	---

Section 23

		1695 1705 1716 1727 1738 1749 1760 1771
	(1695)	
Candidatus Carsonella ruddii strain BT chromosome...	(1695)	AATAAAATTTTGTATATATTATAAATTATATTAATAATTTTTTTTATAGAATTATTATTAAAATAACAATTTGATAA
Candidatus Carsonella 135557 - 167442nt	(1)	---
SARS-CoV-2 Reference Genome NC_045512.2	(1)	---

Section 24

		1772 1782 1793 1804 1815 1826 1837 1848
	(1772)	
Candidatus Carsonella ruddii strain BT chromosome...	(1772)	ATAATCAAAAATAGAAAAATTAATAATTAATAAATTTATTTTTAATTTTAAAAAATTAATTTTTTTTTGTAGTTCTA
Candidatus Carsonella 135557 - 167442nt	(1)	---
SARS-CoV-2 Reference Genome NC_045512.2	(1)	---

Section 25

	(1849)	1849 1859 1870 1881 1892 1903 1914 1925
Candidatus Carsonella ruddii strain BT chromosome...	(1849)	TATTTATACCACCTTTTTCTGATAAAATAAAAATTAAAAAATTATTATTAAAAAAAAACGAAATATATAATTCATTT
Candidatus Carsonella 135557 - 167442nt	(1)	---
SARS-CoV-2 Reference Genome NC_045512.2	(1)	---

Section 26

	(1926)	1926 1936 1947 1958 1969 1980 1991 2002
Candidatus Carsonella ruddii strain BT chromosome...	(1926)	TCTATTAAAATATAATTTTCAATTAAAAAATTTTAATTTTTTTTTTAAATAAAAAAGAATTTTTCCATTTTTTTAAA
Candidatus Carsonella 135557 - 167442nt	(1)	---
SARS-CoV-2 Reference Genome NC_045512.2	(1)	---

Section 27

	(2003)	2003 2013 2024 2035 2046 2057 2068 2079
Candidatus Carsonella ruddii strain BT chromosome...	(2003)	AAACGATAAACAATATTTAAAAGTGTTTATTAAAATTACACCATTATTTTTTTTTCTATATCCGCTATGAACTTGTA
Candidatus Carsonella 135557 - 167442nt	(1)	---
SARS-CoV-2 Reference Genome NC_045512.2	(1)	---

Section 28

	(2080)	2080 2090 2101 2112 2123 2134 2145 2156
Candidatus Carsonella ruddii strain BT chromosome...	(2080)	TTTTATATATTTTGTTTTTTAAAAAAAAAAAATTATTAACTAAATAAGAATTTAAAATTAAAAAATTAAATTTTCTT
Candidatus Carsonella 135557 - 167442nt	(1)	---
SARS-CoV-2 Reference Genome NC_045512.2	(1)	---

Section 29

	(2157)	2157 2167 2178 2189 2200 2211 2222 2233
Candidatus Carsonella ruddii strain BT chromosome...	(2157)	AAAATTTTTTTAGCTTCGTATTCATATAAATTCATATTTTTTTATTTTTAAAATTAAAATTTTCATTTTTTGAAAAA
Candidatus Carsonella 135557 - 167442nt	(1)	---
SARS-CoV-2 Reference Genome NC_045512.2	(1)	---

Section 30

	(2234)	2234 2244 2255 2266 2277 2288 2299 2310
Candidatus Carsonella ruddii strain BT chromosome...	(2234)	AAAAAATATTTTTAATATTTTTTAATATTTTTTGAAAAAAATTTAAATTTGAAAAATCAATATTTAATTGTCTAAAA
Candidatus Carsonella 135557 - 167442nt	(1)	---
SARS-CoV-2 Reference Genome NC_045512.2	(1)	---

Section 31

	(2311)	2311 2321 2332 2343 2354 2365 2376 2387
Candidatus Carsonella ruddii strain BT chromosome...	(2311)	TGTTTTAAAAAATTTATTTTTAATGAAATTAAATTATAAATTTCTTTAAAATTAGTATTTTTAATTAAATAAATTAA
Candidatus Carsonella 135557 - 167442nt	(1)	---
SARS-CoV-2 Reference Genome NC_045512.2	(1)	---

Section 32

	(2388)	2388 2398 2409 2420 2431 2442 2453 2464
Candidatus Carsonella ruddii strain BT chromosome...	(2388)	ATTTATCATAATATAGTTATTTATTTTTTTAATATATTTCTTTTTTTTTTCTATTATTAAATACTTTATATCTTTTA
Candidatus Carsonella 135557 - 167442nt	(1)	---
SARS-CoV-2 Reference Genome NC_045512.2	(1)	---

Section 33

	(2465)	2465 2475 2486 2497 2508 2519 2530 2541
Candidatus Carsonella ruddii strain BT chromosome...	(2465)	TACTATTTAAAATTTTTTTTATTTTTTTAAAAAAAAAAAAAATATTAATATTATTTATTAAATAAAAATTTCTTTTT
Candidatus Carsonella 135557 - 167442nt	(1)	---
SARS-CoV-2 Reference Genome NC_045512.2	(1)	---

Section 34

	(2542)	2542 2552 2563 2574 2585 2596 2607 2618
Candidatus Carsonella ruddii strain BT chromosome...	(2542)	GATAATTTTTTACCATTTAAATAATTAATGTTAGGTAAATGATTAAAATTGAAAAAAAAATTATATTTTTTACATAA
Candidatus Carsonella 135557 - 167442nt	(1)	---
SARS-CoV-2 Reference Genome NC_045512.2	(1)	---

Section 35

	(2619)	2619 2629 2640 2651 2662 2673 2684 2695
Candidatus Carsonella ruddii strain BT chromosome...	(2619)	AAATAATTGAAAAGGAATTTGATTTAACCATTCTTTACCCCTTATAATAATAAGAGTATTATTATAATAATCATTAA
Candidatus Carsonella 135557 - 167442nt	(1)	---
SARS-CoV-2 Reference Genome NC_045512.2	(1)	---

Section 36

	(2696)	2696 2706 2717 2728 2739 2750 2761 2772
Candidatus Carsonella ruddii strain BT chromosome...	(2696)	ATAATGTAGAAAAATTATATGTAGGTATACCTATTTTTTTAATTAAAATTGTTTTTTCTAATTGTATTTTATAATTT
Candidatus Carsonella 135557 - 167442nt	(1)	---
SARS-CoV-2 Reference Genome NC_045512.2	(1)	---

Section 37

```
                                                        2773       2783       2794       2805       2816       2827       2838       2849
Candidatus Carsonella ruddii strain BT chromosome... (2773) AATAAACCATAATTATTATCAAAATAATTAATTTTACAATTTTTTAAAATTATAAAAAAAGAATAATTAATGTTTTT
      Candidatus Carsonella 135557 - 167442nt           (1) -----------------------------------------------------------------------------
  SARS-CoV-2 Reference Genome NC_045512.2               (1) -----------------------------------------------------------------------------
```

Section 38

```
                                                        2850       2860       2871       2882       2893       2904       2915       2926
Candidatus Carsonella ruddii strain BT chromosome... (2850) ATTTTTTATTTTATTGTTAAAAAATATTTTTTTAAAACTATTTTTTTTAAAATTAATAATATTTTTTATATATAAAC
      Candidatus Carsonella 135557 - 167442nt           (1) -----------------------------------------------------------------------------
  SARS-CoV-2 Reference Genome NC_045512.2               (1) -----------------------------------------------------------------------------
```

Section 39

```
                                                        2927       2937       2948       2959       2970       2981       2992       3003
Candidatus Carsonella ruddii strain BT chromosome... (2927) TATTAAAATCTGTTTGCTTTTTTATTTTTTTAATAAAAATACCATTATTTTTTAATTTTTTTAAAATATAAAATTTA
      Candidatus Carsonella 135557 - 167442nt           (1) -----------------------------------------------------------------------------
  SARS-CoV-2 Reference Genome NC_045512.2               (1) -----------------------------------------------------------------------------
```

Section 40

```
                                                        3004       3014       3025       3036       3047       3058       3069       3080
Candidatus Carsonella ruddii strain BT chromosome... (3004) TTTATTAATTTATTTTTTTTTTCATTTGTGTTATCAAATCTTAAAGTTATATTACCGATAATTTTATTTTTTAAAAT
      Candidatus Carsonella 135557 - 167442nt           (1) -----------------------------------------------------------------------------
  SARS-CoV-2 Reference Genome NC_045512.2               (1) -----------------------------------------------------------------------------
```

Section 41

```
                                                        3081       3091       3102       3113       3124       3135       3146       3157
Candidatus Carsonella ruddii strain BT chromosome... (3081) ATAATTTGTTAATAACACATATATATTACCTATATGTGGTACTCCACTTGGAGTTATAGCAATTCTAAAATTATACA
      Candidatus Carsonella 135557 - 167442nt           (1) -----------------------------------------------------------------------------
  SARS-CoV-2 Reference Genome NC_045512.2               (1) -----------------------------------------------------------------------------
```

Section 42

```
                                                        3158       3168       3179       3190       3201       3212       3223       3234
Candidatus Carsonella ruddii strain BT chromosome... (3158) TTTTTTTTTTTTAAAAAATGGGTTTAGTAGGACTTGAACCTACAACTAGAGGATTATGAGTCCTATACTCTAACCAA
      Candidatus Carsonella 135557 - 167442nt           (1) -----------------------------------------------------------------------------
  SARS-CoV-2 Reference Genome NC_045512.2               (1) -----------------------------------------------------------------------------
```

Section 43

		3235 3245 3256 3267 3278 3289 3300 3311
	(3235)	
Candidatus Carsonella ruddii strain BT chromosome...	(3235)	TTGAGTTATAAACCCTTTATAAATTTAATTTTTTAATAAATAATATTATTAACAAAAAAAATTAAATCATTGTTATC
Candidatus Carsonella 135557 - 167442nt	(1)	---
SARS-CoV-2 Reference Genome NC_045512.2	(1)	---

Section 44

		3312 3322 3333 3344 3355 3366 3377 3388
	(3312)	
Candidatus Carsonella ruddii strain BT chromosome...	(3312)	ATAAATAATATAATATATATGAAAAAAATTTTAATAACAACAGCTTTACCATATATTAATGGAGTTATACATATTGG
Candidatus Carsonella 135557 - 167442nt	(1)	---
SARS-CoV-2 Reference Genome NC_045512.2	(1)	---

Section 45

		3389 3399 3410 3421 3432 3443 3454 3465
	(3389)	
Candidatus Carsonella ruddii strain BT chromosome...	(3389)	GCATATTTATGAAATGTATATATCTAATATGTATTATTTTATTATAAAAAAATATTATAATTGTAAAAATTTTTCTG
Candidatus Carsonella 135557 - 167442nt	(1)	---
SARS-CoV-2 Reference Genome NC_045512.2	(1)	---

Section 46

		3466 3476 3487 3498 3509 3520 3531 3542
	(3466)	
Candidatus Carsonella ruddii strain BT chromosome...	(3466)	GTTTAGATTGTCATGGTTTAATTAAAAATAATAATTTATTAAAAATTGAATTTTTTAATAAAAAAAAAATTTTTTTT
Candidatus Carsonella 135557 - 167442nt	(1)	---
SARS-CoV-2 Reference Genome NC_045512.2	(1)	---

Section 47

		3543 3553 3564 3575 3586 3597 3608 3619
	(3543)	
Candidatus Carsonella ruddii strain BT chromosome...	(3543)	TATAATTTAAAATTAAATCAAAAAAAAACTATTTCTTTTATTAATAAAAGATTTTGTAATTGGGTTTTTATATTATT
Candidatus Carsonella 135557 - 167442nt	(1)	---
SARS-CoV-2 Reference Genome NC_045512.2	(1)	---

Section 48

		3620 3630 3641 3652 3663 3674 3685 3696
	(3620)	
Candidatus Carsonella ruddii strain BT chromosome...	(3620)	ATCAGATAAAAATAAAATATTTAGTAAAATTACTTATAAATTTTTTAATAAAAAAAAAAAATTTTTTTATACCTGATA
Candidatus Carsonella 135557 - 167442nt	(1)	---
SARS-CoV-2 Reference Genome NC_045512.2	(1)	---

Section 49

	(3697)	3697 3707 3718 3729 3740 3751 3762 3773
Candidatus Carsonella ruddii strain BT chromosome...	(3697)	AATATATAAAAATATTATGTAAAAAATGTAATTTTGAAATGGAAAATTTTTATTGTACAAATTGTTTATATTATAAA
Candidatus Carsonella 135557 - 167442nt	(1)	---
SARS-CoV-2 Reference Genome NC_045512.2	(1)	---

Section 50

	(3774)	3774 3784 3795 3806 3817 3828 3839 3850
Candidatus Carsonella ruddii strain BT chromosome...	(3774)	AAATTTTATATTGTTTCAAAATTTAAAAAAAAAAATATAAAATTAAAAAAAATAAATAGCATTTTTTTAAAAAATTA
Candidatus Carsonella 135557 - 167442nt	(1)	---
SARS-CoV-2 Reference Genome NC_045512.2	(1)	---

Section 51

	(3851)	3851 3861 3872 3883 3894 3905 3916 3927
Candidatus Carsonella ruddii strain BT chromosome...	(3851)	TATTTTTAAAGATTGGGATATTTCAAGATATAAAAAATATAATGGTTTTAAAATTATATCAAAAAAAAAAATTTTTT
Candidatus Carsonella 135557 - 167442nt	(1)	---
SARS-CoV-2 Reference Genome NC_045512.2	(1)	---

Section 52

	(3928)	3928 3938 3949 3960 3971 3982 3993 4004
Candidatus Carsonella ruddii strain BT chromosome...	(3928)	TTTATGTTTGGTATGATGCTATTATAAGTTATATTTCTAATAATTTAAATTTTATAAAAAATAATTTTTATAATTTT
Candidatus Carsonella 135557 - 167442nt	(1)	---
SARS-CoV-2 Reference Genome NC_045512.2	(1)	---

Section 53

	(4005)	4005 4015 4026 4037 4048 4059 4070 4081
Candidatus Carsonella ruddii strain BT chromosome...	(4005)	GTTTTTTTCCAAATTATTGGTAAAGATATTATTTATTTTCATAAATTATTTTATATAATTTTAAAATTTTTAAATTT
Candidatus Carsonella 135557 - 167442nt	(1)	---
SARS-CoV-2 Reference Genome NC_045512.2	(1)	---

Section 54

	(4082)	4082 4092 4103 4114 4125 4136 4147 4158
Candidatus Carsonella ruddii strain BT chromosome...	(4082)	TAAAAAATATAATTTTTTAATTCATGGTTTTATTTTTTATAATAACAAAATTTCTAAATCTAAAATAATTAAAATTA
Candidatus Carsonella 135557 - 167442nt	(1)	---
SARS-CoV-2 Reference Genome NC_045512.2	(1)	---

Section 55

		4159 4169 4180 4191 4202 4213 4224 4235
	(4159)	
Candidatus Carsonella ruddii strain BT chromosome...	(4159)	AGAAAATAAAAAAAAATAAATTGTTATTTTTTTTTTAATTAATAATAAAAAATTATAAAGAAGATATACATTATGAT
Candidatus Carsonella 135557 - 167442nt	(1)	---
SARS-CoV-2 Reference Genome NC_045512.2	(1)	---

Section 56

		4236 4246 4257 4268 4279 4290 4301 4312
	(4236)	
Candidatus Carsonella ruddii strain BT chromosome...	(4236)	TATCTATTAATTAATAATTATTATAAAAAAATATTTTTTAATAAAATTTAAATTTATTTTTTTAGAATAAGAACAAT
Candidatus Carsonella 135557 - 167442nt	(1)	---
SARS-CoV-2 Reference Genome NC_045512.2	(1)	---

Section 57

		4313 4323 4334 4345 4356 4367 4378 4389
	(4313)	
Candidatus Carsonella ruddii strain BT chromosome...	(4313)	TTTAAAAAAATATGATAATAAAATTATTGATTTTTTTAAATTTAAAAAAAATCATTTTTATGAATTTAATTTTTTAA
Candidatus Carsonella 135557 - 167442nt	(1)	---
SARS-CoV-2 Reference Genome NC_045512.2	(1)	---

Section 58

		4390 4400 4411 4422 4433 4444 4455 4466
	(4390)	
Candidatus Carsonella ruddii strain BT chromosome...	(4390)	AAAAAAAAAAATTAAACAATTTTTATTATAAAAAAATAAAAAATATATTAAATATAAACAAAAATATAAATAAAAAT
Candidatus Carsonella 135557 - 167442nt	(1)	---
SARS-CoV-2 Reference Genome NC_045512.2	(1)	---

Section 59

		4467 4477 4488 4499 4510 4521 4532 4543
	(4467)	
Candidatus Carsonella ruddii strain BT chromosome...	(4467)	AAATTTTGGAAAAAAAAAAATAATTTTTTAATTCATTTTTATTATTCAAAAATTATTTTTTTTTATATAAAAAATTT
Candidatus Carsonella 135557 - 167442nt	(1)	---
SARS-CoV-2 Reference Genome NC_045512.2	(1)	---

Section 60

		4544 4554 4565 4576 4587 4598 4609 4620
	(4544)	
Candidatus Carsonella ruddii strain BT chromosome...	(4544)	AAATATTTTAATTGAAATAATTAACAATAAAATTATTAAAAACAATTTATATAAAAATTGTAAAATATTTAATTTAA
Candidatus Carsonella 135557 - 167442nt	(1)	---
SARS-CoV-2 Reference Genome NC_045512.2	(1)	---

Section 61

	(4621)	4621 4631 4642 4653 4664 4675 4686 4697
Candidatus Carsonella ruddii strain BT chromosome...	(4621)	AAAAAATTAATGAAATTTAAAAATTTTATTTTATATAATATTAAAAAAAATATTAAAAACTGTATAAATTGTAATTT
Candidatus Carsonella 135557 - 167442nt	(1)	---
SARS-CoV-2 Reference Genome NC_045512.2	(1)	---

Section 62

	(4698)	4698 4708 4719 4730 4741 4752 4763 4774
Candidatus Carsonella ruddii strain BT chromosome...	(4698)	TTGTATATTTTTTTGTCCAAAATTTAAAATTAACAAAAAAAAAAATAAAATAAAAATTAAAAATTGTAATTTTTGTA
Candidatus Carsonella 135557 - 167442nt	(1)	---
SARS-CoV-2 Reference Genome NC_045512.2	(1)	---

Section 63

	(4775)	4775 4785 4796 4807 4818 4829 4840 4851
Candidatus Carsonella ruddii strain BT chromosome...	(4775)	ATTATTGTAATATTTTTTGTAAAATTAATTGTATTAAATAATTAATCTTAAACTATTAATATTTATAAAACCTTGAG
Candidatus Carsonella 135557 - 167442nt	(1)	---
SARS-CoV-2 Reference Genome NC_045512.2	(1)	---

Section 64

	(4852)	4852 4862 4873 4884 4895 4906 4917 4928
Candidatus Carsonella ruddii strain BT chromosome...	(4852)	AATCAATTTGTTCAAATTTTTTGCTAATTTCATCAAAAGAAGAATTTTTTTTATCATATAAAGAATTAAAAGAATAT
Candidatus Carsonella 135557 - 167442nt	(1)	---
SARS-CoV-2 Reference Genome NC_045512.2	(1)	---

Section 65

	(4929)	4929 4939 4950 4961 4972 4983 4994 5005
Candidatus Carsonella ruddii strain BT chromosome...	(4929)	CTATTTACAATAGTAATACATCCTTTATAAATTTTTATTTTAATAATACCATTTAAATATTTTTGTATATTATTTAT
Candidatus Carsonella 135557 - 167442nt	(1)	---
SARS-CoV-2 Reference Genome NC_045512.2	(1)	---

Section 66

	(5006)	5006 5016 5027 5038 5049 5060 5071 5082
Candidatus Carsonella ruddii strain BT chromosome...	(5006)	AATATTTTGAATTAAAATTCTTTCAGGCGACCACCAATAACCATTGTAAACTAAATTTGTATATTTTATTGATAAAG
Candidatus Carsonella 135557 - 167442nt	(1)	---
SARS-CoV-2 Reference Genome NC_045512.2	(1)	---

```
                                                                                                                                          Section 67
                                                          (5083) 5083      5093       5104       5115       5126       5137       5148       5159
Candidatus Carsonella ruddii strain BT chromosome...      (5083) ATTCTTTAAAAAAAAATATTTCTTTATCTAATACAAAACTTTCTAAATGTTTTCTAGAAAACATTATTATTGTTGCA
         Candidatus Carsonella 135557 - 167442nt             (1) -----------------------------------------------------------------------------
    SARS-CoV-2 Reference Genome NC_045512.2                  (1) -----------------------------------------------------------------------------
                                                                                                                                          Section 68
                                                          (5160) 5160      5170       5181       5192       5203       5214       5225       5236
Candidatus Carsonella ruddii strain BT chromosome...      (5160) CCTGGTGATTCATAACATCCTCTAGATTTTATTCCTATATATCTATTTTCTATTATATCTATTCTACCAATACCACA
         Candidatus Carsonella 135557 - 167442nt             (1) -----------------------------------------------------------------------------
    SARS-CoV-2 Reference Genome NC_045512.2                  (1) -----------------------------------------------------------------------------
                                                                                                                                          Section 69
                                                          (5237) 5237      5247       5258       5269       5280       5291       5302       5313
Candidatus Carsonella ruddii strain BT chromosome...      (5237) AATTGATCCAATTTTATTTAATTTTAAAAATAAATTTTCATTATTATAAAAATTATTATTTATTTTAATTGGATCAC
         Candidatus Carsonella 135557 - 167442nt             (1) -----------------------------------------------------------------------------
    SARS-CoV-2 Reference Genome NC_045512.2                  (1) -----------------------------------------------------------------------------
                                                                                                                                          Section 70
                                                          (5314) 5314      5324       5335       5346       5357       5368       5379       5390
Candidatus Carsonella ruddii strain BT chromosome...      (5314) CATTTTTAAAAGTTATAGATAAATAAATTGGATAATCAATACTAATTTCATTACTTAAAGTATTTTCCCACATATTT
         Candidatus Carsonella 135557 - 167442nt             (1) -----------------------------------------------------------------------------
    SARS-CoV-2 Reference Genome NC_045512.2                  (1) -----------------------------------------------------------------------------
                                                                                                                                          Section 71
                                                          (5391) 5391      5401       5412       5423       5434       5445       5456       5467
Candidatus Carsonella ruddii strain BT chromosome...      (5391) TCTTCAGCTTCAAATAATAAATTATCCAATTCACCTCCTTCATAAGAATTATGAAATAAATTTTTATCTATAGAATA
         Candidatus Carsonella 135557 - 167442nt             (1) -----------------------------------------------------------------------------
    SARS-CoV-2 Reference Genome NC_045512.2                  (1) -----------------------------------------------------------------------------
                                                                                                                                          Section 72
                                                          (5468) 5468      5478       5489       5500       5511       5522       5533       5544
Candidatus Carsonella ruddii strain BT chromosome...      (5468) TTTTTTTGTTTTTTTATTAATTTTAATATTATTTTTAATACAAAAATCTAATAATTTATTTCTTGAATTTAAATCCC
         Candidatus Carsonella 135557 - 167442nt             (1) -----------------------------------------------------------------------------
    SARS-CoV-2 Reference Genome NC_045512.2                  (1) -----------------------------------------------------------------------------
```

```
                                                                                         Section 73
                                                     (5545) 5545      5555        5566        5577        5588        5599        5610     5621
Candidatus Carsonella ruddii strain BT chromosome... (5545) AAATTCTCCATGGTGCTATAACTTTAATATTTGAATTAAAATATTTAAAACCTAATTCAAAACGAATTTGGTCATTT
          Candidatus Carsonella 135557 - 167442nt       (1) -----------------------------------------------------------------------------
     SARS-CoV-2 Reference Genome NC_045512.2            (1) -----------------------------------------------------------------------------
                                                                                         Section 74
                                                     (5622) 5622      5632        5643        5654        5665        5676        5687     5698
Candidatus Carsonella ruddii strain BT chromosome... (5622) CCCTTTCCTGTTGCTCCATGTGCAACGTAATTAGAATTAGTATAATAAGCAATTTTCATAATTTCTTTAGAAATTAA
          Candidatus Carsonella 135557 - 167442nt       (1) -----------------------------------------------------------------------------
     SARS-CoV-2 Reference Genome NC_045512.2            (1) -----------------------------------------------------------------------------
                                                                                         Section 75
                                                     (5699) 5699      5709        5720        5731        5742        5753        5764     5775
Candidatus Carsonella ruddii strain BT chromosome... (5699) AGGTCTAGCAATTGCTGTACCTAATAAATAAAAATTTTCATATAAAAAACTAGATCTTAAATATGGAAAAACGTAAT
          Candidatus Carsonella 135557 - 167442nt       (1) -----------------------------------------------------------------------------
     SARS-CoV-2 Reference Genome NC_045512.2            (1) -----------------------------------------------------------------------------
                                                                                         Section 76
                                                     (5776) 5776      5786        5797        5808        5819        5830        5841     5852
Candidatus Carsonella ruddii strain BT chromosome... (5776) TTTTAATAAATTCATTTTTTAAATTTTTAATAAAAATATTTTTAACATTTAATTTTTTTGCTCTTTCTTTAGCAATT
          Candidatus Carsonella 135557 - 167442nt       (1) -----------------------------------------------------------------------------
     SARS-CoV-2 Reference Genome NC_045512.2            (1) -----------------------------------------------------------------------------
                                                                                         Section 77
                                                     (5853) 5853      5863        5874        5885        5896        5907        5918     5929
Candidatus Carsonella ruddii strain BT chromosome... (5853) TTAATTTCGTCTCCTTGACCTATATCAGCAGTAAATGTTATAACTTCAAAATTTTGTTCTTGTAACCATTTAATTAT
          Candidatus Carsonella 135557 - 167442nt       (1) -----------------------------------------------------------------------------
     SARS-CoV-2 Reference Genome NC_045512.2            (1) -----------------------------------------------------------------------------
                                                                                         Section 78
                                                     (5930) 5930      5940        5951        5962        5973        5984        5995     6006
Candidatus Carsonella ruddii strain BT chromosome... (5930) AACTGAAGTATCTAATCCTCCAGAATAAGCTAATACAATTTTTTTTTTTATCATATTGACATTTATATATAATAATA
          Candidatus Carsonella 135557 - 167442nt       (1) -----------------------------------------------------------------------------
     SARS-CoV-2 Reference Genome NC_045512.2            (1) -----------------------------------------------------------------------------
```

Section 79

```
                                                    (6007) 6007      6017       6028       6039       6050       6061       6072      6083
Candidatus Carsonella ruddii strain BT chromosome... (6007) TAAAATAATTAGGCTAAATAACATATTGGATATGTAATGGGTTGCAAACCCGTATAAACTGGTTCGATTCCAGTTTT
             Candidatus Carsonella 135557 - 167442nt   (1) -----------------------------------------------------------------------------
        SARS-CoV-2 Reference Genome NC_045512.2        (1) -----------------------------------------------------------------------------
```

Section 80

```
                                                    (6084) 6084      6094       6105       6116       6127       6138       6149      6160
Candidatus Carsonella ruddii strain BT chromosome... (6084) AGCTTAATAAAAATGCCCATATGGCGGAATGGTAGACGCAAGGGACTTAAAATCCCTAGTTGAAAAACGTGTCAGTT
             Candidatus Carsonella 135557 - 167442nt   (1) -----------------------------------------------------------------------------
        SARS-CoV-2 Reference Genome NC_045512.2        (1) -----------------------------------------------------------------------------
```

Section 81

```
                                                    (6161) 6161      6171       6182       6193       6204       6215       6226      6237
Candidatus Carsonella ruddii strain BT chromosome... (6161) CAAGTCTGACTTTGGGTAAAGTAAAATTTTTCTACACCAAATAATAATGAATTAAAAATTCCTTTTATTAAAAAAAC
             Candidatus Carsonella 135557 - 167442nt   (1) -----------------------------------------------------------------------------
        SARS-CoV-2 Reference Genome NC_045512.2        (1) -----------------------------------------------------------------------------
```

Section 82

```
                                                    (6238) 6238      6248       6259       6270       6281       6292       6303      6314
Candidatus Carsonella ruddii strain BT chromosome... (6238) TTTGTATAAAAAAAATTTTATCAAAATAGGATTAATATGAAAATTTAATTTATTAATTAAAAAATTTAATTTTTTGT
             Candidatus Carsonella 135557 - 167442nt   (1) -----------------------------------------------------------------------------
        SARS-CoV-2 Reference Genome NC_045512.2        (1) -----------------------------------------------------------------------------
```

Section 83

```
                                                    (6315) 6315      6325       6336       6347       6358       6369       6380      6391
Candidatus Carsonella ruddii strain BT chromosome... (6315) TTATAAAAAAAAAAATTTCTAGGTATAATATTGTTAAAAACAGATATTGTACCATAATAATTATTTTTTAAACAAAAT
             Candidatus Carsonella 135557 - 167442nt   (1) -----------------------------------------------------------------------------
        SARS-CoV-2 Reference Genome NC_045512.2        (1) -----------------------------------------------------------------------------
```

Section 84

```
                                                    (6392) 6392      6402       6413       6424       6435       6446       6457      6468
Candidatus Carsonella ruddii strain BT chromosome... (6392) AATAAATTTTTATCTTCTCCAGAAAATAAAATTATATTTTTATTTTTATATATCAAAGTGTTTTTAATATTATTTAT
             Candidatus Carsonella 135557 - 167442nt   (1) -----------------------------------------------------------------------------
        SARS-CoV-2 Reference Genome NC_045512.2        (1) -----------------------------------------------------------------------------
```

Section 85

		6469 6479 6490 6501 6512 6523 6534 6545
	(6469)	
Candidatus Carsonella ruddii strain BT chromosome...	(6469)	AGAATTTTTCATATAAATTAAAAAATTTTTTTTTAATAATTTATTAATAATATTATAATTTAAAATAATATTATTTC
Candidatus Carsonella 135557 - 167442nt	(1)	---
SARS-CoV-2 Reference Genome NC_045512.2	(1)	---

Section 86

		6546 6556 6567 6578 6589 6600 6611 6622
	(6546)	
Candidatus Carsonella ruddii strain BT chromosome...	(6546)	TTTTTGGTATATTATATAATATATTTTTTAAACCAAATTTATTAATTATTTTAAAATATTTAAATAAATTTAAATTA
Candidatus Carsonella 135557 - 167442nt	(1)	---
SARS-CoV-2 Reference Genome NC_045512.2	(1)	---

Section 87

		6623 6633 6644 6655 6666 6677 6688 6699
	(6623)	
Candidatus Carsonella ruddii strain BT chromosome...	(6623)	TTAATTAAAATATAATTTGGTATATTAATTAATACTTTAATATTATTATTTATTTTTAATAAAAAAAATAAATAAAA
Candidatus Carsonella 135557 - 167442nt	(1)	---
SARS-CoV-2 Reference Genome NC_045512.2	(1)	---

Section 88

		6700 6710 6721 6732 6743 6754 6765 6776
	(6700)	
Candidatus Carsonella ruddii strain BT chromosome...	(6700)	AATTTTTTTAATGTTAATTTTATTTATATTAAAAAAAAATTTAATATTATATTTTTTAATATATAATAAGATATTTA
Candidatus Carsonella 135557 - 167442nt	(1)	---
SARS-CoV-2 Reference Genome NC_045512.2	(1)	---

Section 89

		6777 6787 6798 6809 6820 6831 6842 6853
	(6777)	
Candidatus Carsonella ruddii strain BT chromosome...	(6777)	TATAATCTTTTGTTTTAAAATTATGAGATTCACCTGTTGTACCTAAAAATAAAAAATTTTTAATTTTGTTAAAAATA
Candidatus Carsonella 135557 - 167442nt	(1)	---
SARS-CoV-2 Reference Genome NC_045512.2	(1)	---

Section 90

		6854 6864 6875 6886 6897 6908 6919 6930
	(6854)	
Candidatus Carsonella ruddii strain BT chromosome...	(6854)	TAAAAATTTATTAAAAAATTTAAATTTAACCAATTTATTTTTCCGTTTTTTTTAAAAGGAGTAACAATTGCAATTGT
Candidatus Carsonella 135557 - 167442nt	(1)	---
SARS-CoV-2 Reference Genome NC_045512.2	(1)	---

Section 91

	(6931)	6931 6941 6952 6963 6974 6985 6996 7007
Candidatus Carsonella ruddii strain BT chromosome...	(6931)	ATTGTACATTTTAAATATATAAAATATTGAGTTGTGTAGGATTTGAACCTACGACCAATTGGTTAAAAGCCAATTGC
Candidatus Carsonella 135557 - 167442nt	(1)	---
SARS-CoV-2 Reference Genome NC_045512.2	(1)	---

Section 92

	(7008)	7008 7018 7029 7040 7051 7062 7073 7084
Candidatus Carsonella ruddii strain BT chromosome...	(7008)	TCTACCTGTCTGAGCTAACAACCCATTTTTATTGTTTAACAATAATATTAATTCTATTTTTAAAAAAAATCAATTTT
Candidatus Carsonella 135557 - 167442nt	(1)	---
SARS-CoV-2 Reference Genome NC_045512.2	(1)	---

Section 93

	(7085)	7085 7095 7106 7117 7128 7139 7150 7161
Candidatus Carsonella ruddii strain BT chromosome...	(7085)	TTTATTTTAAAAAAATGTTTAACATTTCTATAATTGGTGCTGGTGTTATTAGTGCTATAACAAGTTTAATGATAAAT
Candidatus Carsonella 135557 - 167442nt	(1)	---
SARS-CoV-2 Reference Genome NC_045512.2	(1)	---

Section 94

	(7162)	7162 7172 7183 7194 7205 7216 7227 7238
Candidatus Carsonella ruddii strain BT chromosome...	(7162)	TTAATTAATCAAAAATTAAAAATATTTATTTTTGAAATTAACAATAAAGTTGCAATAGAAAATTCTAAAGCAACAAA
Candidatus Carsonella 135557 - 167442nt	(1)	---
SARS-CoV-2 Reference Genome NC_045512.2	(1)	---

Section 95

	(7239)	7239 7249 7260 7271 7282 7293 7304 7315
Candidatus Carsonella ruddii strain BT chromosome...	(7239)	CAATGCTGGTACAGGTCATGAAGGAATGTGTGAAAATAATTATGTTAAACTATTAAATAAAAATTATTATATTAAAA
Candidatus Carsonella 135557 - 167442nt	(1)	---
SARS-CoV-2 Reference Genome NC_045512.2	(1)	---

Section 96

	(7316)	7316 7326 7337 7348 7359 7370 7381 7392
Candidatus Carsonella ruddii strain BT chromosome...	(7316)	AAAATATAAATATTTATTTTAAATTTAATTTAACAAAAAATTTTTTTTCATGGATTAAATTTTTAAATATATTTAAT
Candidatus Carsonella 135557 - 167442nt	(1)	---
SARS-CoV-2 Reference Genome NC_045512.2	(1)	---

Section 97

```
                                                      (7393) 7393      7403       7414       7425       7436       7447       7458      7469
Candidatus Carsonella ruddii strain BT chromosome...  (7393) TTTAAAAAAGTTATTTCTAAAACTCCACATGTTTCTTTTTTTTTTTTTAAAAATAATATAAAGTTAAAAAAAATTTT
          Candidatus Carsonella 135557 - 167442nt     (1)    -----------------------------------------------------------------------------
       SARS-CoV-2 Reference Genome NC_045512.2        (1)    -----------------------------------------------------------------------------
```

Section 98

```
                                                      (7470) 7470      7480       7491       7502       7513       7524       7535      7546
Candidatus Carsonella ruddii strain BT chromosome...  (7470) TAATAAAATTAGAATTTTTAATAAATCTATAAAATTTACTAATAATAATTTTTTTTTAAATAAATTTTCATTGATAA
          Candidatus Carsonella 135557 - 167442nt     (1)    -----------------------------------------------------------------------------
       SARS-CoV-2 Reference Genome NC_045512.2        (1)    -----------------------------------------------------------------------------
```

Section 99

```
                                                      (7547) 7547      7557       7568       7579       7590       7601       7612      7623
Candidatus Carsonella ruddii strain BT chromosome...  (7547) TTAAAAAAAAAAAAATAAAAAAAAAATTTAGTATAACATATTACAAATATGGTTTTGATATAAATTACCAATATTTA
          Candidatus Carsonella 135557 - 167442nt     (1)    -----------------------------------------------------------------------------
       SARS-CoV-2 Reference Genome NC_045512.2        (1)    -----------------------------------------------------------------------------
```

Section 100

```
                                                      (7624) 7624      7634       7645       7656       7667       7678       7689      7700
Candidatus Carsonella ruddii strain BT chromosome...  (7624) TCTTTGAAAATTTTTCATTATTTAACAAAAAAAAATAAAATAAATTTATTTTTATCTTGTAAAATTATTAAAATTAA
          Candidatus Carsonella 135557 - 167442nt     (1)    -----------------------------------------------------------------------------
       SARS-CoV-2 Reference Genome NC_045512.2        (1)    -----------------------------------------------------------------------------
```

Section 101

```
                                                      (7701) 7701      7711       7722       7733       7744       7755       7766      7777
Candidatus Carsonella ruddii strain BT chromosome...  (7701) AAATTACAAAAAACAATTTATTTTAACATTATTTAAAAAAAAATTTTTTAGAATATTAACTGATTATGTTTTTGTTT
          Candidatus Carsonella 135557 - 167442nt     (1)    -----------------------------------------------------------------------------
       SARS-CoV-2 Reference Genome NC_045512.2        (1)    -----------------------------------------------------------------------------
```

Section 102

```
                                                      (7778) 7778      7788       7799       7810       7821       7832       7843      7854
Candidatus Carsonella ruddii strain BT chromosome...  (7778) GTGCAGGTGGTAGTAGTTTAAGTATATTAAAAAAATTTAATAAAAAAATTTCAAAAAATTATTTTGAATTACCAATA
          Candidatus Carsonella 135557 - 167442nt     (1)    -----------------------------------------------------------------------------
       SARS-CoV-2 Reference Genome NC_045512.2        (1)    -----------------------------------------------------------------------------
```

Section 103

	(7855)	7855 7865 7876 7887 7898 7909 7920 7931
Candidatus Carsonella ruddii strain BT chromosome...	(7855)	AATGGAACATGGTTAATTTCTGAAGAAAAAAATATTATTAAAAAACATAATTTAAAAATTTATAGTGAAACGATAAA
Candidatus Carsonella 135557 - 167442nt	(1)	---
SARS-CoV-2 Reference Genome NC_045512.2	(1)	---

Section 104

	(7932)	7932 7942 7953 7964 7975 7986 7997 8008
Candidatus Carsonella ruddii strain BT chromosome...	(7932)	AGGTAACCCACCAATGAGTATTCCTCATTTAGATATGAGAACAATTTTTAATGAGAAAAAAATATTTTTTGGTCCTT
Candidatus Carsonella 135557 - 167442nt	(1)	---
SARS-CoV-2 Reference Genome NC_045512.2	(1)	---

Section 105

	(8009)	8009 8019 8030 8041 8052 8063 8074 8085
Candidatus Carsonella ruddii strain BT chromosome...	(8009)	TTGCTGGATTAACTTTTAAAATTTTAATAAACACTAAAAAAAATATAATAGAAAGTATTAATTTAAAACATTTTATT
Candidatus Carsonella 135557 - 167442nt	(1)	---
SARS-CoV-2 Reference Genome NC_045512.2	(1)	---

Section 106

	(8086)	8086 8096 8107 8118 8129 8140 8151 8162
Candidatus Carsonella ruddii strain BT chromosome...	(8086)	AATTTAATTATTTTTTCATATGAAAACAGGTATATTATTAATTATTTAATAAATGAAAATTTAAAAACTAAAAAAAA
Candidatus Carsonella 135557 - 167442nt	(1)	---
SARS-CoV-2 Reference Genome NC_045512.2	(1)	---

Section 107

	(8163)	8163 8173 8184 8195 8206 8217 8228 8239
Candidatus Carsonella ruddii strain BT chromosome...	(8163)	AAAAATATTTGATATGTTTCCTTTTTGTAAAGTTATAAAAAATACTTATTGTAAAATAGCAGGAAAAAGATTGCAAA
Candidatus Carsonella 135557 - 167442nt	(1)	---
SARS-CoV-2 Reference Genome NC_045512.2	(1)	---

Section 108

	(8240)	8240 8250 8261 8272 8283 8294 8305 8316
Candidatus Carsonella ruddii strain BT chromosome...	(8240)	TTTTAAAAAAAAAAAACAAAAAATTAAAATTAAATTTTGGTACAAAATTAATTTTTAATAAAAATAAAAATTTTGCA
Candidatus Carsonella 135557 - 167442nt	(1)	---
SARS-CoV-2 Reference Genome NC_045512.2	(1)	---

Section 109

	(8317)	8317 8327 8338 8349 8360 8371 8382 8393
Candidatus Carsonella ruddii strain BT chromosome...	(8317)	ACATTATTAGGAGCATCGCCTGGAGCTTCTATTTCTGTAAATATTGCTAAAAATTTAATTAAAAAGTGGTTTTTTTT
Candidatus Carsonella 135557 - 167442nt	(1)	---
SARS-CoV-2 Reference Genome NC_045512.2	(1)	---

Section 110

	(8394)	8394 8404 8415 8426 8437 8448 8459 8470
Candidatus Carsonella ruddii strain BT chromosome...	(8394)	TCCTAAAAAATTTTTATTTAATTATAAAAAAAATAATAAAAAAATCTAAAATATTTAATTTTTTATTGTATTTGAAAG
Candidatus Carsonella 135557 - 167442nt	(1)	---
SARS-CoV-2 Reference Genome NC_045512.2	(1)	---

Section 111

	(8471)	8471 8481 8492 8503 8514 8525 8536 8547
Candidatus Carsonella ruddii strain BT chromosome...	(8471)	GAATTGAACCTTTTACTGTTTGATTCGTAATCAAAAACTCTATCCGATGAGTTACAAATACTTAAAAATAGAGAAAG
Candidatus Carsonella 135557 - 167442nt	(1)	---
SARS-CoV-2 Reference Genome NC_045512.2	(1)	---

Section 112

	(8548)	8548 8558 8569 8580 8591 8602 8613 8624
Candidatus Carsonella ruddii strain BT chromosome...	(8548)	TAGGATTCGAACCTACGATAATAAATATACTCCTTTAGCAGAGGAGTGTCTTAAGCCACTCGACCATTTCTCCTAAA
Candidatus Carsonella 135557 - 167442nt	(1)	---
SARS-CoV-2 Reference Genome NC_045512.2	(1)	---

Section 113

	(8625)	8625 8635 8646 8657 8668 8679 8690 8701
Candidatus Carsonella ruddii strain BT chromosome...	(8625)	CATAAAATTTTTATTAAACATTAAATATAATTCTTTAATAGTTTTTATGGTATCTTTATCTTTAATAACTACAGATA
Candidatus Carsonella 135557 - 167442nt	(1)	---
SARS-CoV-2 Reference Genome NC_045512.2	(1)	---

Section 114

	(8702)	8702 8712 8723 8734 8745 8756 8767 8778
Candidatus Carsonella ruddii strain BT chromosome...	(8702)	TTTTTGTTTCTGAAGTAGAAATTAAAATAATATTTATTTTTAATTTAGACATGCAATATAATATTTTGTCTATAATA
Candidatus Carsonella 135557 - 167442nt	(1)	---
SARS-CoV-2 Reference Genome NC_045512.2	(1)	---

```
                                                                                 Section 115
                                                    (8779) 8779      8789       8800       8811       8822       8833       8844      8855
Candidatus Carsonella ruddii strain BT chromosome... (8779) TTATTATGAGTTCTTAATCCTATACCAACAACAGAAATTTTTGTTATATTTAATTCATATTCTACTATTCCTCCTAA
         Candidatus Carsonella 135557 - 167442nt    (1)    -----------------------------------------------------------------------------
     SARS-CoV-2 Reference Genome NC_045512.2        (1)    -----------------------------------------------------------------------------
                                                                                 Section 116
                                                    (8856) 8856      8866       8877       8888       8899       8910       8921      8932
Candidatus Carsonella ruddii strain BT chromosome... (8856) TTTAGGAATAATATATTTATTTATTAAATATTTAGTTTTTTTACAAAATTTATTTTCAACTAAAAAAGTAAAATTTG
         Candidatus Carsonella 135557 - 167442nt    (1)    -----------------------------------------------------------------------------
     SARS-CoV-2 Reference Genome NC_045512.2        (1)    -----------------------------------------------------------------------------
                                                                                 Section 117
                                                    (8933) 8933      8943       8954       8965       8976       8987       8998      9009
Candidatus Carsonella ruddii strain BT chromosome... (8933) TATTTTTTGCATTAATAATAGAATTTTGAATAACCATATCTACACATATATTATTACATATAATAGGTCCTAAAATT
         Candidatus Carsonella 135557 - 167442nt    (1)    -----------------------------------------------------------------------------
     SARS-CoV-2 Reference Genome NC_045512.2        (1)    -----------------------------------------------------------------------------
                                                                                 Section 118
                                                    (9010) 9010      9020       9031       9042       9053       9064       9075      9086
Candidatus Carsonella ruddii strain BT chromosome... (9010) TTCGATGCTACTCCTGCTGTATTTGGCAAATTTTTTACAGTAATTTTTGCTTCATTATTAGAATGAGTTATACCAGA
         Candidatus Carsonella 135557 - 167442nt    (1)    -----------------------------------------------------------------------------
     SARS-CoV-2 Reference Genome NC_045512.2        (1)    -----------------------------------------------------------------------------
                                                                                 Section 119
                                                    (9087) 9087      9097       9108       9119       9130       9141       9152      9163
Candidatus Carsonella ruddii strain BT chromosome... (9087) AATCATTATACGTTCCATAGAATTAGAAAATTTTTTTTTATTTGAAATTAATGTTCCTTTATTTTTAAGAAAAGAAG
         Candidatus Carsonella 135557 - 167442nt    (1)    -----------------------------------------------------------------------------
     SARS-CoV-2 Reference Genome NC_045512.2        (1)    -----------------------------------------------------------------------------
                                                                                 Section 120
                                                    (9164) 9164      9174       9185       9196       9207       9218       9229      9240
Candidatus Carsonella ruddii strain BT chromosome... (9164) ATAAAATTCTAATGTTAACATTATATTTTATTGCTATTTCAATTGATCTAACATATAAAACTTTTGAACCAGAACTA
         Candidatus Carsonella 135557 - 167442nt    (1)    -----------------------------------------------------------------------------
     SARS-CoV-2 Reference Genome NC_045512.2        (1)    -----------------------------------------------------------------------------
```

Section 121

	(9241)	9241 9251 9262 9273 9284 9295 9306 9317
Candidatus Carsonella ruddii strain BT chromosome...	(9241)	GATAATTCTAAAATATTTTCAAAAGGAACAAATTCAATTTTAAAATTTTTACAAATTCTTGGATCAGAAAAAAAAAT
Candidatus Carsonella 135557 - 167442nt	(1)	---
SARS-CoV-2 Reference Genome NC_045512.2	(1)	---

Section 122

	(9318)	9318 9328 9339 9350 9361 9372 9383 9394
Candidatus Carsonella ruddii strain BT chromosome...	(9318)	TGAATCAACATCAGTATAAATTTGACATTCTTTTGCTTTTAAACATACTGCAATTGAAATAGCAGTTGTATCTGAAC
Candidatus Carsonella 135557 - 167442nt	(1)	---
SARS-CoV-2 Reference Genome NC_045512.2	(1)	---

Section 123

	(9395)	9395 9405 9416 9427 9438 9449 9460 9471
Candidatus Carsonella ruddii strain BT chromosome...	(9395)	CACCTCTTCCTAAAGTAGTAAAATTTCCAGATTTTGTAATTCCTTGAAAACCTGCTAAAATTGGAATTTTACCTTTT
Candidatus Carsonella 135557 - 167442nt	(1)	---
SARS-CoV-2 Reference Genome NC_045512.2	(1)	---

Section 124

	(9472)	9472 9482 9493 9504 9515 9526 9537 9548
Candidatus Carsonella ruddii strain BT chromosome...	(9472)	AATAAATTTTTTTTTATAATAAAATAATTTTTAATATTTATTATTCTAGCATTATTATATTCTTTATTTGTTATTAA
Candidatus Carsonella 135557 - 167442nt	(1)	---
SARS-CoV-2 Reference Genome NC_045512.2	(1)	---

Section 125

	(9549)	9549 9559 9570 9581 9592 9603 9614 9625
Candidatus Carsonella ruddii strain BT chromosome...	(9549)	ACCTATTTGTATACTAGTTAAAGATTTTGATTTTATTTTTATACTATTTAACAAAATACTAAATAAAGAAACACTCA
Candidatus Carsonella 135557 - 167442nt	(1)	---
SARS-CoV-2 Reference Genome NC_045512.2	(1)	---

Section 126

	(9626)	9626 9636 9647 9658 9669 9680 9691 9702
Candidatus Carsonella ruddii strain BT chromosome...	(9626)	TTTGCTCTCCTATACATAATAAACAATCTAATTCTTTTAAATTTTTAAAAGAAAAATATTTGGCTAATCTTATCATT
Candidatus Carsonella 135557 - 167442nt	(1)	---
SARS-CoV-2 Reference Genome NC_045512.2	(1)	---

```
                                                                                                   Section 127
                                                (9703) 9703      9713       9724       9735       9746       9757       9768       9779
Candidatus Carsonella ruddii strain BT chromosome... (9703) TTAGTTGTTTCTCCACTCATTGCAGATAATATCATTACTGTATAAATTTTTAATTTTTTATATTTTTTTTATTATATT
      Candidatus Carsonella 135557 - 167442nt (1) -----------------------------------------------------------------------------
   SARS-CoV-2 Reference Genome NC_045512.2 (1) -----------------------------------------------------------------------------
                                                                                                   Section 128
                                                (9780) 9780      9790       9801       9812       9823       9834       9845       9856
Candidatus Carsonella ruddii strain BT chromosome... (9780) TTTAAGAAATTTAATTCTTTTTTTTAGAACCAATTGAAGTACCTCCAAATTTTTGAATTATTATCATTTATAAATATT
      Candidatus Carsonella 135557 - 167442nt (1) -----------------------------------------------------------------------------
   SARS-CoV-2 Reference Genome NC_045512.2 (1) -----------------------------------------------------------------------------
                                                                                                   Section 129
                                                (9857) 9857      9867       9878       9889       9900       9911       9922       9933
Candidatus Carsonella ruddii strain BT chromosome... (9857) TTTAATTATTAAATATATTATTTTATATGATATTCCAAATGTTTCAAAAAAAAAATTTCATTTGTATTAAATTTATTT
      Candidatus Carsonella 135557 - 167442nt (1) -----------------------------------------------------------------------------
   SARS-CoV-2 Reference Genome NC_045512.2 (1) -----------------------------------------------------------------------------
                                                                                                   Section 130
                                                (9934) 9934      9944       9955       9966       9977       9988       9999       10010
Candidatus Carsonella ruddii strain BT chromosome... (9934) TTTTTAATTTTATTTTTTTAAAATCAAAAAAAAAAACTATATTCTTTTTTTATTAAAAAAAAATAAAAAAATTTTTAACA
      Candidatus Carsonella 135557 - 167442nt (1) -----------------------------------------------------------------------------
   SARS-CoV-2 Reference Genome NC_045512.2 (1) -----------------------------------------------------------------------------
                                                                                                   Section 131
                                                (10011) 10011     10021      10032      10043      10054      10065      10076      10087
Candidatus Carsonella ruddii strain BT chromosome... (10011) TTATTTTTAAAATAAAATTTTTTATTTTTTTTAAAAAAAAAAAATAAATATATTAAATATATTTATTTTTTTTTTTAAA
      Candidatus Carsonella 135557 - 167442nt (1) -----------------------------------------------------------------------------
   SARS-CoV-2 Reference Genome NC_045512.2 (1) -----------------------------------------------------------------------------
                                                                                                   Section 132
                                                (10088) 10088     10098      10109      10120      10131      10142      10153      10164
Candidatus Carsonella ruddii strain BT chromosome... (10088) AATTAAATTTAAAATTATTTTAAATAATTTATTTAATATAAAATTATGATATTTTGTAAAATTTAAATTAACGTTAT
      Candidatus Carsonella 135557 - 167442nt (1) -----------------------------------------------------------------------------
   SARS-CoV-2 Reference Genome NC_045512.2 (1) -----------------------------------------------------------------------------
```

Section 133

	(10165)	10165 10175 10186 10197 10208 10219 10230 10241
Candidatus Carsonella ruddii strain BT chromosome...	(10165)	TTTTTATTAAAAATATTGAATTGCACAAATCATTTAATTTTAAATTTTTTTTAAAAAATAAAAAATTTAACCTATCA
Candidatus Carsonella 135557 - 167442nt	(1)	---
SARS-CoV-2 Reference Genome NC_045512.2	(1)	---

Section 134

	(10242)	10242 10252 10263 10274 10285 10296 10307 10318
Candidatus Carsonella ruddii strain BT chromosome...	(10242)	TATCCTAAACCTGAATCTATTATATTATTTTTTAATTTAATAACTTTTTTATTATTTTTATAATATTTAATATTAAC
Candidatus Carsonella 135557 - 167442nt	(1)	---
SARS-CoV-2 Reference Genome NC_045512.2	(1)	---

Section 135

	(10319)	10319 10329 10340 10351 10362 10373 10384 10395
Candidatus Carsonella ruddii strain BT chromosome...	(10319)	AATATTCCATATTTCTAACATAATATTTTTAAAAAAATAATATATTTCTAAACAAAAACCTAAAAATCCATTTTTAT
Candidatus Carsonella 135557 - 167442nt	(1)	---
SARS-CoV-2 Reference Genome NC_045512.2	(1)	---

Section 136

	(10396)	10396 10406 10417 10428 10439 10450 10461 10472
Candidatus Carsonella ruddii strain BT chromosome...	(10396)	TAATTTTCCAAATATTTTTTTTTGTAAAAATTATATTTTTAATATTTATTTTTAATATTAAAACTATTAATATAGTT
Candidatus Carsonella 135557 - 167442nt	(1)	---
SARS-CoV-2 Reference Genome NC_045512.2	(1)	---

Section 137

	(10473)	10473 10483 10494 10505 10516 10527 10538 10549
Candidatus Carsonella ruddii strain BT chromosome...	(10473)	AAAAAATCAACAATATTTAAAGAAAAAAAAATTTTTTTTTTATTTAATTTCTTAAAATAAAATAAATATTGTATAGA
Candidatus Carsonella 135557 - 167442nt	(1)	---
SARS-CoV-2 Reference Genome NC_045512.2	(1)	---

Section 138

	(10550)	10550 10560 10571 10582 10593 10604 10615 10626
Candidatus Carsonella ruddii strain BT chromosome...	(10550)	TATATTTAATAAAAAAAAAATGTTTGTTTTACTTAAAAAATTACCTAACATTATAAATGAAGTTTTATGTATACCAT
Candidatus Carsonella 135557 - 167442nt	(1)	---
SARS-CoV-2 Reference Genome NC_045512.2	(1)	---

```
                                                                                                                              Section 139
                                                   (10627) 10627     10637      10648      10659      10670      10681      10692       10703
Candidatus Carsonella ruddii strain BT chromosome... (10627) CATTATTTAAATTTAAATCATTAAAAATACCTTTTAATCTTACACAATATTGAAAAGAGCAAAAATTATTTTTAAAT
           Candidatus Carsonella 135557 - 167442nt (1) -----------------------------------------------------------------------------
          SARS-CoV-2 Reference Genome NC_045512.2 (1) -----------------------------------------------------------------------------
                                                                                                                              Section 140
                                                   (10704) 10704     10714      10725      10736      10747      10758      10769       10780
Candidatus Carsonella ruddii strain BT chromosome... (10704) GAATTTAGAATTATTTTTTTAATTGATGAAAAACCAGAATTGTTAAACAATAAAGTTTTACAATTTGAAATAATATT
           Candidatus Carsonella 135557 - 167442nt (1) -----------------------------------------------------------------------------
          SARS-CoV-2 Reference Genome NC_045512.2 (1) -----------------------------------------------------------------------------
                                                                                                                              Section 141
                                                   (10781) 10781     10791      10802      10813      10824      10835      10846       10857
Candidatus Carsonella ruddii strain BT chromosome... (10781) TTGTGTTCCTAATATTTTAAAATTATAAATATTATAATATTTTAAATAATTAATCATATTTTTTAATTAAAATTTCT
           Candidatus Carsonella 135557 - 167442nt (1) -----------------------------------------------------------------------------
          SARS-CoV-2 Reference Genome NC_045512.2 (1) -----------------------------------------------------------------------------
                                                                                                                              Section 142
                                                   (10858) 10858     10868      10879      10890      10901      10912      10923       10934
Candidatus Carsonella ruddii strain BT chromosome... (10858) ATATTTTTTAAAAATTTTTTTTCAAAAGACATTTTTTTAATTAATTCATTTTTCTCATATAATTTTTGATTAAATAA
           Candidatus Carsonella 135557 - 167442nt (1) -----------------------------------------------------------------------------
          SARS-CoV-2 Reference Genome NC_045512.2 (1) -----------------------------------------------------------------------------
                                                                                                                              Section 143
                                                   (10935) 10935     10945      10956      10967      10978      10989      11000       11011
Candidatus Carsonella ruddii strain BT chromosome... (10935) AAATTTATTTTTTTTTTTTTCAAATATTTTTAATTTAATTGAAAAATTAAAAATTTCATTAATTTTATAAATTCCAT
           Candidatus Carsonella 135557 - 167442nt (1) -----------------------------------------------------------------------------
          SARS-CoV-2 Reference Genome NC_045512.2 (1) -----------------------------------------------------------------------------
                                                                                                                              Section 144
                                                   (11012) 11012     11022      11033      11044      11055      11066      11077       11088
Candidatus Carsonella ruddii strain BT chromosome... (11012) ATCCAAAAATTAATTGTATTTTAGTTTCTCTGTAAGGTTTTGATAATTTATTTTTTAAAATTAAAACTTTAATTTCT
           Candidatus Carsonella 135557 - 167442nt (1) -----------------------------------------------------------------------------
          SARS-CoV-2 Reference Genome NC_045512.2 (1) -----------------------------------------------------------------------------
```

Section 145

	(11089)	11089 11099 11110 11121 11132 11143 11154 11165
Candidatus Carsonella ruddii strain BT chromosome...	(11089)	TGACCAATAATATTATTTTTACTTTTTAAAAAACCAATTCTTTTTAATTCTATTCTTATAGAAGAATAAAATTTAAC
Candidatus Carsonella 135557 - 167442nt	(1)	---
SARS-CoV-2 Reference Genome NC_045512.2	(1)	---

Section 146

	(11166)	11166 11176 11187 11198 11209 11220 11231 11242
Candidatus Carsonella ruddii strain BT chromosome...	(11166)	AGAATTTCCACCTGTAGAAATTTCTTTTGAAAAAAAATTAGAAATTTTAACTCTTACTTGATTAATTAATATAAATA
Candidatus Carsonella 135557 - 167442nt	(1)	---
SARS-CoV-2 Reference Genome NC_045512.2	(1)	---

Section 147

	(11243)	11243 11253 11264 11275 11286 11297 11308 11319
Candidatus Carsonella ruddii strain BT chromosome...	(11243)	AAATATTATTTTTTCTTATATTAGGTATAATTTTTTTTAAACTTTTTGAAATTAATCTAGCATGACAACCTATTAAA
Candidatus Carsonella 135557 - 167442nt	(1)	---
SARS-CoV-2 Reference Genome NC_045512.2	(1)	---

Section 148

	(11320)	11320 11330 11341 11352 11363 11374 11385 11396
Candidatus Carsonella ruddii strain BT chromosome...	(11320)	TTATCTTGAGATATTATTTCATTTTGAGATACAATTGCTGCTATTGAATCAATTATAATAACATTTACTAATTTTGT
Candidatus Carsonella 135557 - 167442nt	(1)	---
SARS-CoV-2 Reference Genome NC_045512.2	(1)	---

Section 149

	(11397)	11397 11407 11418 11429 11440 11451 11462 11473
Candidatus Carsonella ruddii strain BT chromosome...	(11397)	ATTTATTAATTTAAAACATATTTCAAATACATTTTCACCATTATTTGGTTGAAATATTAATAATTTAGAAATGTTTA
Candidatus Carsonella 135557 - 167442nt	(1)	---
SARS-CoV-2 Reference Genome NC_045512.2	(1)	---

Section 150

	(11474)	11474 11484 11495 11506 11517 11528 11539 11550
Candidatus Carsonella ruddii strain BT chromosome...	(11474)	TCCCCAAATTTTCTAAGTAATTTACATCTATACAATGTTCAACATCTATATACGCACAAATATCTCCAACTTTTTGT
Candidatus Carsonella 135557 - 167442nt	(1)	---
SARS-CoV-2 Reference Genome NC_045512.2	(1)	---

Section 151

```
                                                     (11551) 11551     11561      11572      11583      11594      11605      11616      11627
Candidatus Carsonella ruddii strain BT chromosome... (11551) GCTTCTTTAATTATCGATAAAGATAAAGTTGTTTTTCCTGATGATTCTTGACCAAATATTTCAATAATTCTACCATA
         Candidatus Carsonella 135557 - 167442nt         (1) -----------------------------------------------------------------------------
     SARS-CoV-2 Reference Genome NC_045512.2             (1) -----------------------------------------------------------------------------
```

Section 152

```
                                                     (11628) 11628     11638      11649      11660      11671      11682      11693      11704
Candidatus Carsonella ruddii strain BT chromosome... (11628) AGGTAATCCACCGATACCCAAAATATTATCTATGTTTAAAGAACCAGTTGATAAACATTCAATTTTTCTAAGATGAT
         Candidatus Carsonella 135557 - 167442nt         (1) -----------------------------------------------------------------------------
     SARS-CoV-2 Reference Genome NC_045512.2             (1) -----------------------------------------------------------------------------
```

Section 153

```
                                                     (11705) 11705     11715      11726      11737      11748      11759      11770      11781
Candidatus Carsonella ruddii strain BT chromosome... (11705) CAAAATTCATATTTTTTTAAAAAAAAAATTATTTTTTATAATTTTACCACCTCCAAATATTACAGGAGAAATGCATA
         Candidatus Carsonella 135557 - 167442nt         (1) -----------------------------------------------------------------------------
     SARS-CoV-2 Reference Genome NC_045512.2             (1) -----------------------------------------------------------------------------
```

Section 154

```
                                                     (11782) 11782     11792      11803      11814      11825      11836      11847      11858
Candidatus Carsonella ruddii strain BT chromosome... (11782) TAGGTCATTTTTTTCAATATTTTATTATAGATATAATAATAAAATGGAAAATTTTACAAGAATATAAAATTAATTAT
         Candidatus Carsonella 135557 - 167442nt         (1) -----------------------------------------------------------------------------
     SARS-CoV-2 Reference Genome NC_045512.2             (1) -----------------------------------------------------------------------------
```

Section 155

```
                                                     (11859) 11859     11869      11880      11891      11902      11913      11924      11935
Candidatus Carsonella ruddii strain BT chromosome... (11859) ATTTTTGGTTTAGATCATGCTGGAATTTCAGCAATTATAAAATTTAAAAATATTAAAAACATAATTAAAAAAAAAAA
         Candidatus Carsonella 135557 - 167442nt         (1) -----------------------------------------------------------------------------
     SARS-CoV-2 Reference Genome NC_045512.2             (1) -----------------------------------------------------------------------------
```

Section 156

```
                                                     (11936) 11936     11946      11957      11968      11979      11990      12001      12012
Candidatus Carsonella ruddii strain BT chromosome... (11936) ACAAATAATTATTAATTTTTTTAATCAATTAAAATATATAAACTTTTTTATAATAAATAAAAAAATATTATTTACAT
         Candidatus Carsonella 135557 - 167442nt         (1) -----------------------------------------------------------------------------
     SARS-CoV-2 Reference Genome NC_045512.2             (1) -----------------------------------------------------------------------------
```

Section 157

	(12013)	12013 12023 12034 12045 12056 12067 12078 12089
Candidatus Carsonella ruddii strain BT chromosome...	(12013)	TAAATAATAAATATTCTAATTTTATAAAAAAAAAATTTATATTTTTTTATAAAAAAAAATTTATATATATAGATAAA
Candidatus Carsonella 135557 - 167442nt	(1)	---
SARS-CoV-2 Reference Genome NC_045512.2	(1)	---

Section 158

	(12090)	12090 12100 12111 12122 12133 12144 12155 12166
Candidatus Carsonella ruddii strain BT chromosome...	(12090)	AAAATTTTAAATTTTAATAAAAATAATATAATTTCAGATTTTGAAATTTTTAAAAAAATATATAATAAATATTTTTA
Candidatus Carsonella 135557 - 167442nt	(1)	---
SARS-CoV-2 Reference Genome NC_045512.2	(1)	---

Section 159

	(12167)	12167 12177 12188 12199 12210 12221 12232 12243
Candidatus Carsonella ruddii strain BT chromosome...	(12167)	TTATATAAAATATAAAAATAAAAAAAATTTTATAATTTCAACTTCAAATTTAAAATCTTTATTATATAATACTTTTT
Candidatus Carsonella 135557 - 167442nt	(1)	---
SARS-CoV-2 Reference Genome NC_045512.2	(1)	---

Section 160

	(12244)	12244 12254 12265 12276 12287 12298 12309 12320
Candidatus Carsonella ruddii strain BT chromosome...	(12244)	GTTGTTTAAATTTTAAATTTTATAATAAAAAAATAAAATCTCCTTTTAAAATTAAAACTTATATTTATTATTTAAAA
Candidatus Carsonella 135557 - 167442nt	(1)	---
SARS-CoV-2 Reference Genome NC_045512.2	(1)	---

Section 161

	(12321)	12321 12331 12342 12353 12364 12375 12386 12397
Candidatus Carsonella ruddii strain BT chromosome...	(12321)	AAAAAAATTAATAATTTTTCAAAAATAATAAAATTATCACCAAATATAAATAATTATGATTATTTAATTTGTGATAA
Candidatus Carsonella 135557 - 167442nt	(1)	---
SARS-CoV-2 Reference Genome NC_045512.2	(1)	---

Section 162

	(12398)	12398 12408 12419 12430 12441 12452 12463 12474
Candidatus Carsonella ruddii strain BT chromosome...	(12398)	AATAAAAGAAAATATATTTTTAAAAAATAAAAAAAATATTTTTTTGTATAAAATTAATAATTTTCATAAAAAAATTA
Candidatus Carsonella 135557 - 167442nt	(1)	---
SARS-CoV-2 Reference Genome NC_045512.2	(1)	---

Section 163

	(12475)	12475 12485 12496 12507 12518 12529 12540 12551
Candidatus Carsonella ruddii strain BT chromosome...	(12475)	AAAAAGTATTTTTTTTATAATAAAATTAATATTAATAATATTTTTTTTTTTTAAAAAAAAACAATTATATTATTTGT
Candidatus Carsonella 135557 - 167442nt	(1)	---
SARS-CoV-2 Reference Genome NC_045512.2	(1)	---

Section 164

	(12552)	12552 12562 12573 12584 12595 12606 12617 12628
Candidatus Carsonella ruddii strain BT chromosome...	(12552)	ATTAAAAAATATAAATCTTTTAAAAATTTACATAAATTTAAAAATTTAGAAATTAAAAATTCTATATTTGATCAATG
Candidatus Carsonella 135557 - 167442nt	(1)	---
SARS-CoV-2 Reference Genome NC_045512.2	(1)	---

Section 165

	(12629)	12629 12639 12650 12661 12672 12683 12694 12705
Candidatus Carsonella ruddii strain BT chromosome...	(12629)	GTTTTTAAAAATTAAAAAAATAATATATTTTAAAAAAATAATTAATTCAATTAATATTAAACCAGATAAATATAAAA
Candidatus Carsonella 135557 - 167442nt	(1)	---
SARS-CoV-2 Reference Genome NC_045512.2	(1)	---

Section 166

	(12706)	12706 12716 12727 12738 12749 12760 12771 12782
Candidatus Carsonella ruddii strain BT chromosome...	(12706)	AAATATTTTTAATTTGGATAAAAAATATGTCAAATTGGTGTATTTCAAGAAAAATTAAATGGGGAACAAAAATACCA
Candidatus Carsonella 135557 - 167442nt	(1)	---
SARS-CoV-2 Reference Genome NC_045512.2	(1)	---

Section 167

	(12783)	12783 12793 12804 12815 12826 12837 12848 12859
Candidatus Carsonella ruddii strain BT chromosome...	(12783)	ATTTTTTATGATAAAGAAAAAAATTTATTTTTAAAAAAAAAATTTAGATTTTTTAAATATTACAAATTATTTGAAGT
Candidatus Carsonella 135557 - 167442nt	(1)	---
SARS-CoV-2 Reference Genome NC_045512.2	(1)	---

Section 168

	(12860)	12860 12870 12881 12892 12903 12914 12925 12936
Candidatus Carsonella ruddii strain BT chromosome...	(12860)	TTTTGATACTTGGTTTAATTCATCTTTTTGGATATTATTTTTATATTATAAATATAAAAAATTCCAAGATATTATTT
Candidatus Carsonella 135557 - 167442nt	(1)	---
SARS-CoV-2 Reference Genome NC_045512.2	(1)	---

Section 169

	(12937)	12937 12947 12958 12969 12980 12991 13002 13013
Candidatus Carsonella ruddii strain BT chromosome...	(12937)	TATCTGGTTTTGATATTATTTTTTATTGGATAATAAAAATGATTATTAATAATATTTTTTTTAAAAAAAAAATATTA
Candidatus Carsonella 135557 - 167442nt	(1)	---
SARS-CoV-2 Reference Genome NC_045512.2	(1)	---

Section 170

	(13014)	13014 13024 13035 13046 13057 13068 13079 13090
Candidatus Carsonella ruddii strain BT chromosome...	(13014)	ATTAAAAAAATAATAATTCATGGAATATTAAGAGATAATAATAAAAAAAAAATATCAAAAACAACTAATAATGTAAT
Candidatus Carsonella 135557 - 167442nt	(1)	---
SARS-CoV-2 Reference Genome NC_045512.2	(1)	---

Section 171

	(13091)	13091 13101 13112 13123 13134 13145 13156 13167
Candidatus Carsonella ruddii strain BT chromosome...	(13091)	AAATTTTATACATATTAAAAAAAACATAAATAAAATAAAAATTATGTTATTAAATAATATTTTTGATGATAATGATT
Candidatus Carsonella 135557 - 167442nt	(1)	---
SARS-CoV-2 Reference Genome NC_045512.2	(1)	---

Section 172

	(13168)	13168 13178 13189 13200 13211 13222 13233 13244
Candidatus Carsonella ruddii strain BT chromosome...	(13168)	TAAAAAATATTAATTTTTTTTTAAAAAAAAAATATTTATTAAAAAATACAAATAAAATTAATATAATTTTTCAAATT
Candidatus Carsonella 135557 - 167442nt	(1)	---
SARS-CoV-2 Reference Genome NC_045512.2	(1)	---

Section 173

	(13245)	13245 13255 13266 13277 13288 13299 13310 13321
Candidatus Carsonella ruddii strain BT chromosome...	(13245)	TTTTTATTTTATAAAATTAAAAAACAATATATATTGAAATTTAATAATAATTATATAAATAATGTAAATTTTTT
Candidatus Carsonella 135557 - 167442nt	(1)	---
SARS-CoV-2 Reference Genome NC_045512.2	(1)	---

Section 174

	(13322)	13322 13332 13343 13354 13365 13376 13387 13398
Candidatus Carsonella ruddii strain BT chromosome...	(13322)	TTTAAAAAAAAATAACCTATCAAAAAAAATTTATATATTATTAATTAAATTTTTTTTATCCTAAAAAAATGTTTTAA
Candidatus Carsonella 135557 - 167442nt	(1)	---
SARS-CoV-2 Reference Genome NC_045512.2	(1)	---

Section 175

```
                                                    (13399) 13399     13409      13420      13431      13442      13453      13464      13475
Candidatus Carsonella ruddii strain BT chromosome... (13399) ATGTTAAAAATTGGAATTTTTCAATTAATAATTATGTTTATAAAATTAAATTTAATTATTTTTTATTAATAAAAATT
         Candidatus Carsonella 135557 - 167442nt     (1) -----------------------------------------------------------------------------
        SARS-CoV-2 Reference Genome NC_045512.2     (1) -----------------------------------------------------------------------------
```

Section 176

```
                                                    (13476) 13476     13486      13497      13508      13519      13530      13541      13552
Candidatus Carsonella ruddii strain BT chromosome... (13476) AAAAAAAAACTTTTCTTAATTAAAAAAATTTTTTTTTTAAATTATTTTGAAATATTATATAATATAAAAATAATATT
         Candidatus Carsonella 135557 - 167442nt     (1) -----------------------------------------------------------------------------
        SARS-CoV-2 Reference Genome NC_045512.2     (1) -----------------------------------------------------------------------------
```

Section 177

```
                                                    (13553) 13553     13563      13574      13585      13596      13607      13618      13629
Candidatus Carsonella ruddii strain BT chromosome... (13553) TTATGTTAAATACAAGAATAAAACCTTTTAAATCAATAGCTTATTTTGAAAAAAAATTTTTTGAAATAAATGAAAAA
         Candidatus Carsonella 135557 - 167442nt     (1) -----------------------------------------------------------------------------
        SARS-CoV-2 Reference Genome NC_045512.2     (1) -----------------------------------------------------------------------------
```

Section 178

```
                                                    (13630) 13630     13640      13651      13662      13673      13684      13695      13706
Candidatus Carsonella ruddii strain BT chromosome... (13630) TATTTAAAAAATAAATGGAATGTTTTGTTTTTTTTTCCTTATTCATACTCGTTTATTTGTCCTACAGAATTAAAAGA
         Candidatus Carsonella 135557 - 167442nt     (1) -----------------------------------------------------------------------------
        SARS-CoV-2 Reference Genome NC_045512.2     (1) -----------------------------------------------------------------------------
```

Section 179

```
                                                    (13707) 13707     13717      13728      13739      13750      13761      13772      13783
Candidatus Carsonella ruddii strain BT chromosome... (13707) TATTTCAAATAATATTTATAATTTTAATAAAAATAATATAAATATTTATGCAATATCAACTGATAGTCATTTTTCAC
         Candidatus Carsonella 135557 - 167442nt     (1) -----------------------------------------------------------------------------
        SARS-CoV-2 Reference Genome NC_045512.2     (1) -----------------------------------------------------------------------------
```

Section 180

```
                                                    (13784) 13784     13794      13805      13816      13827      13838      13849      13860
Candidatus Carsonella ruddii strain BT chromosome... (13784) AAAAAAATTGGATTGAACAAAAATTAAACTTCATAAATTTTCCATTTATATCAGATTTTAATCATCAAATTTCTAAA
         Candidatus Carsonella 135557 - 167442nt     (1) -----------------------------------------------------------------------------
        SARS-CoV-2 Reference Genome NC_045512.2     (1) -----------------------------------------------------------------------------
```

Section 181

(13861) 13861 13871 13882 13893 13904 13915 13926 13937
Candidatus Carsonella ruddii strain BT chromosome... (13861) AATTTAAATATTTTAAATAAAAAAGATGGAAACTGTTTTAGATCAACAATAATTTTAGATCCAAATTTAATTATAAA
Candidatus Carsonella 135557 - 167442nt (1) ---
SARS-CoV-2 Reference Genome NC_045512.2 (1) ---

Section 182

(13938) 13938 13948 13959 13970 13981 13992 14003 14014
Candidatus Carsonella ruddii strain BT chromosome... (13938) ATCAATAGATATTATTGATTCAAATATTTCTAGATGTATAAAAAATATATTGGAAAGATTAAATATGTTAATTTATA
Candidatus Carsonella 135557 - 167442nt (1) ---
SARS-CoV-2 Reference Genome NC_045512.2 (1) ---

Section 183

(14015) 14015 14025 14036 14047 14058 14069 14080 14091
Candidatus Carsonella ruddii strain BT chromosome... (14015) CTAATTTAAAAAACAATGAATTATGTCCTTATAGTTGGAATAACGACAAAATTAGTATTAAAATTGATATTTAAAAA
Candidatus Carsonella 135557 - 167442nt (1) ---
SARS-CoV-2 Reference Genome NC_045512.2 (1) ---

Section 184

(14092) 14092 14102 14113 14124 14135 14146 14157 14168
Candidatus Carsonella ruddii strain BT chromosome... (14092) TTATTAATTATTAAAGATAATAAATTTATTAAATTATTATGATCTTTTTCAAATTGTTTAATTCCATTAAATAATAA
Candidatus Carsonella 135557 - 167442nt (1) ---
SARS-CoV-2 Reference Genome NC_045512.2 (1) ---

Section 185

(14169) 14169 14179 14190 14201 14212 14223 14234 14245
Candidatus Carsonella ruddii strain BT chromosome... (14169) TATATGAGAAATATTGTTTAATATACTAAAATTAAAATTTTTAAAAAATATTTTTTTTTTATAAATATTATAATTAA
Candidatus Carsonella 135557 - 167442nt (1) ---
SARS-CoV-2 Reference Genome NC_045512.2 (1) ---

Section 186

(14246) 14246 14256 14267 14278 14289 14300 14311 14322
Candidatus Carsonella ruddii strain BT chromosome... (14246) TATTTTTTTTTAAAAGTTCATCAAAAAAAGATGGAGAAATTGTTAAATAATCACAAAAAAATAAATTTAAAATTTGA
Candidatus Carsonella 135557 - 167442nt (1) ---
SARS-CoV-2 Reference Genome NC_045512.2 (1) ---

Section 187

		14323 14333 14344 14355 14366 14377 14388 14399
	(14323)	
Candidatus Carsonella ruddii strain BT chromosome...	(14323)	TTTATATTTCTAAAACTTGCTGCCATTATTTTTGTTTTAAAATGAAAAGATTGTTTATAATCATAAATATTTTTAAC
Candidatus Carsonella 135557 - 167442nt	(1)	---
SARS-CoV-2 Reference Genome NC_045512.2	(1)	---

Section 188

		14400 14410 14421 14432 14443 14454 14465 14476
	(14400)	
Candidatus Carsonella ruddii strain BT chromosome...	(14400)	AAAATTAACTCCAGAATCTAATTTACAAAAAATTTTATCACTTATTCTTCCTACAAATGGAGATATTAAATATATGC
Candidatus Carsonella 135557 - 167442nt	(1)	---
SARS-CoV-2 Reference Genome NC_045512.2	(1)	---

Section 189

		14477 14487 14498 14509 14520 14531 14542 14553
	(14477)	
Candidatus Carsonella ruddii strain BT chromosome...	(14477)	CATTATCGAAACATTTTTTAACTTGATTCATATCAAAAATTAATGTTAAATTACATTCTATACCAAAATTTTTTAAT
Candidatus Carsonella 135557 - 167442nt	(1)	---
SARS-CoV-2 Reference Genome NC_045512.2	(1)	---

Section 190

		14554 14564 14575 14586 14597 14608 14619 14630
	(14554)	
Candidatus Carsonella ruddii strain BT chromosome...	(14554)	TTACTAGCAGCAATAATACCTGAATCTGTTGCAGGAATTTTAATTAATATTTTTTTATTATTTAAACCAAATAAATT
Candidatus Carsonella 135557 - 167442nt	(1)	---
SARS-CoV-2 Reference Genome NC_045512.2	(1)	---

Section 191

		14631 14641 14652 14663 14674 14685 14696 14707
	(14631)	
Candidatus Carsonella ruddii strain BT chromosome...	(14631)	ACATAAATAAATCAATTTTTTAGAATAAATTATAATTTTTTTATAATTATAAGATATTCTTGCTGGAATTTCTAAAG
Candidatus Carsonella 135557 - 167442nt	(1)	---
SARS-CoV-2 Reference Genome NC_045512.2	(1)	---

Section 192

		14708 14718 14729 14740 14751 14762 14773 14784
	(14708)	
Candidatus Carsonella ruddii strain BT chromosome...	(14708)	AAATTTTATCTTTAATAAAAGGAATTAAATTCCCTGCAATAAAAATTAATAAATTATCATAAAAACTAATTTCTTCA
Candidatus Carsonella 135557 - 167442nt	(1)	---
SARS-CoV-2 Reference Genome NC_045512.2	(1)	---

Section 193

	(14785)	14785 14795 14806 14817 14828 14839 14850 14861
Candidatus Carsonella ruddii strain BT chromosome...	(14785)	TCTGAATCTAATTTTATATTTTTATTAAATTTTTTAAAAAAATTTATTAAAATTTTATCATAAGATTTATTTATAAT
Candidatus Carsonella 135557 - 167442nt	(1)	---
SARS-CoV-2 Reference Genome NC_045512.2	(1)	---

Section 194

	(14862)	14862 14872 14883 14894 14905 14916 14927 14938
Candidatus Carsonella ruddii strain BT chromosome...	(14862)	TGAAGATAAAATTAAACTTGGATTACTTGTTAAAGAATCAAAATTATACATTTTAACAAAATTAATGTTTGCGCTAT
Candidatus Carsonella 135557 - 167442nt	(1)	---
SARS-CoV-2 Reference Genome NC_045512.2	(1)	---

Section 195

	(14939)	14939 14949 14960 14971 14982 14993 15004 15015
Candidatus Carsonella ruddii strain BT chromosome...	(14939)	CTATTGATATTTTTGTATTTTTTTTTAAAAAATTAAATAAATTCATTTATATTTTAATAATAATTGATATTAATTTG
Candidatus Carsonella 135557 - 167442nt	(1)	---
SARS-CoV-2 Reference Genome NC_045512.2	(1)	---

Section 196

	(15016)	15016 15026 15037 15048 15059 15070 15081 15092
Candidatus Carsonella ruddii strain BT chromosome...	(15016)	ATAATTTTAAAAATTTTTTTATAATTATAATAATATTTATTTATTAAATAAATAATAAAACTTCCGATAACAAAATT
Candidatus Carsonella 135557 - 167442nt	(1)	---
SARS-CoV-2 Reference Genome NC_045512.2	(1)	---

Section 197

	(15093)	15093 15103 15114 15125 15136 15147 15158 15169
Candidatus Carsonella ruddii strain BT chromosome...	(15093)	TTTTACTTTTTTAACATATATTAATAATTTAATATTAGACAAACCAAATCCAAATATAAAATTATTTTTAATTTTAT
Candidatus Carsonella 135557 - 167442nt	(1)	---
SARS-CoV-2 Reference Genome NC_045512.2	(1)	---

Section 198

	(15170)	15170 15180 15191 15202 15213 15224 15235 15246
Candidatus Carsonella ruddii strain BT chromosome...	(15170)	TTAAATATTTATTTAAATATTTAATATTAATTTTTTTATCTCCAGTAATTCCTTTTATATATGAAAAATAATTGTAT
Candidatus Carsonella 135557 - 167442nt	(1)	---
SARS-CoV-2 Reference Genome NC_045512.2	(1)	---

Section 199

		15247 15257 15268 15279 15290 15301 15312 15323
	(15247)	
Candidatus Carsonella ruddii strain BT chromosome...	(15247)	TTTATAAAAAAATTATTTAAAAAAATATTTTTATTATTTGAAATATTGCTAGTTATAAAATTAATAAAATTTATATT
Candidatus Carsonella 135557 - 167442nt	(1)	---
SARS-CoV-2 Reference Genome NC_045512.2	(1)	---

Section 200

		15324 15334 15345 15356 15367 15378 15389 15400
	(15324)	
Candidatus Carsonella ruddii strain BT chromosome...	(15324)	TTTATTTTTAATTAAAAAAAATAAATTATAATTACATTCTAAATTATAATCTATTAATAAAATTGAATCAATTTTAA
Candidatus Carsonella 135557 - 167442nt	(1)	---
SARS-CoV-2 Reference Genome NC_045512.2	(1)	---

Section 201

		15401 15411 15422 15433 15444 15455 15466 15477
	(15401)	
Candidatus Carsonella ruddii strain BT chromosome...	(15401)	CTATATTTAATAAATAAGAAAAATTGAATTCTCCATATTTTAAAATAGAATTATAATAACTCATTAATATTAATAAA
Candidatus Carsonella 135557 - 167442nt	(1)	---
SARS-CoV-2 Reference Genome NC_045512.2	(1)	---

Section 202

		15478 15488 15499 15510 15521 15532 15543 15554
	(15478)	
Candidatus Carsonella ruddii strain BT chromosome...	(15478)	CTTGCATTATTTAATAATTTAAAATTAAAAATATTATTTAAATATTTTTCAATATTATAATTTTTTATTATATATTT
Candidatus Carsonella 135557 - 167442nt	(1)	---
SARS-CoV-2 Reference Genome NC_045512.2	(1)	---

Section 203

		15555 15565 15576 15587 15598 15609 15620 15631
	(15555)	
Candidatus Carsonella ruddii strain BT chromosome...	(15555)	ATTACTATCATAAATAATATTTCCATCTGCAATTGAATCAACATTGGGTAAACCTACTTCAATAGAATCTATTCCAT
Candidatus Carsonella 135557 - 167442nt	(1)	---
SARS-CoV-2 Reference Genome NC_045512.2	(1)	---

Section 204

		15632 15642 15653 15664 15675 15686 15697 15708
	(15632)	
Candidatus Carsonella ruddii strain BT chromosome...	(15632)	ATTTAAAAATTTTTTTAATAAAATTAAAAAAAAATAATTTATTAGGATAATTTAAAGGTAAAAATAAAATTATTTTT
Candidatus Carsonella 135557 - 167442nt	(1)	---
SARS-CoV-2 Reference Genome NC_045512.2	(1)	---

```
                                                                                                    Section 205
                                                       (15709) 15709     15719      15730      15741      15752      15763      15774    15785
Candidatus Carsonella ruddii strain BT chromosome...   (15709) TTCATAAATCTTTATCTCCTCTTCCACTCATATTAATTAAAATTAAATCTTTATAATTTAAAAATTTAGAAAGTTTA
            Candidatus Carsonella 135557 - 167442nt        (1) -----------------------------------------------------------------------------
         SARS-CoV-2 Reference Genome NC_045512.2           (1) -----------------------------------------------------------------------------
                                                                                                    Section 206
                                                       (15786) 15786     15796      15807      15818      15829      15840      15851    15862
Candidatus Carsonella ruddii strain BT chromosome...   (15786) AAAGATAAAAATAATGAATGAGAAGTTTCTAAAGCAGGAATTATTCCTTCTATTTTTGAAAAAAATTTAAATATATA
            Candidatus Carsonella 135557 - 167442nt        (1) -----------------------------------------------------------------------------
         SARS-CoV-2 Reference Genome NC_045512.2           (1) -----------------------------------------------------------------------------
                                                                                                    Section 207
                                                       (15863) 15863     15873      15884      15895      15906      15917      15928    15939
Candidatus Carsonella ruddii strain BT chromosome...   (15863) TAAAACTTCATTATTAAAAATAGGAATAAAAAATATTTTATTTTTAGAAAATAAAAAAGAAATTTCTGGACCAATTC
            Candidatus Carsonella 135557 - 167442nt        (1) -----------------------------------------------------------------------------
         SARS-CoV-2 Reference Genome NC_045512.2           (1) -----------------------------------------------------------------------------
                                                                                                    Section 208
                                                       (15940) 15940     15950      15961      15972      15983      15994      16005    16016
Candidatus Carsonella ruddii strain BT chromosome...   (15940) CTGGATAATCTAAACCAGCAGAAATAGAATTGGTTTTTTTTATATTTCCATATTTATCTTGTAAAATATAACTATAA
            Candidatus Carsonella 135557 - 167442nt        (1) -----------------------------------------------------------------------------
         SARS-CoV-2 Reference Genome NC_045512.2           (1) -----------------------------------------------------------------------------
                                                                                                    Section 209
                                                       (16017) 16017     16027      16038      16049      16060      16071      16082    16093
Candidatus Carsonella ruddii strain BT chromosome...   (16017) TTACCATGTAATATACCAATGTTTCCAAAAAAAAATACTAGAAGCTGTTTTTTTTTGTGAAATTCCTCCAGATTCTAC
            Candidatus Carsonella 135557 - 167442nt        (1) -----------------------------------------------------------------------------
         SARS-CoV-2 Reference Genome NC_045512.2           (1) -----------------------------------------------------------------------------
                                                                                                    Section 210
                                                       (16094) 16094     16104      16115      16126      16137      16148      16159    16170
Candidatus Carsonella ruddii strain BT chromosome...   (16094) AGCTAATAATTTAAATTTTGTATTTATAAAAGGAAAAAAAAATCCTAAACAATTAGAACCACCACCAACACATGCAA
            Candidatus Carsonella 135557 - 167442nt        (1) -----------------------------------------------------------------------------
         SARS-CoV-2 Reference Genome NC_045512.2           (1) -----------------------------------------------------------------------------
```

Section 211

	(16171)	16171 16181 16192 16203 16214 16225 16236 16247
Candidatus Carsonella ruddii strain BT chromosome...	(16171)	TTAAAAATTTTTTTTTATAAAATTTAAAATTAATTTGTTGAAAAATTTCATATCCTATAATAGATTGAAAATCTCTT
Candidatus Carsonella 135557 - 167442nt	(1)	---
SARS-CoV-2 Reference Genome NC_045512.2	(1)	---

Section 212

	(16248)	16248 16258 16269 16280 16291 16302 16313 16324
Candidatus Carsonella ruddii strain BT chromosome...	(16248)	ACAATTAAAGGATATGGGTGTGGACCCGAAGCTGTTCCAATTAAATAATATGAACTATTTATATTGTTGCACCAATC
Candidatus Carsonella 135557 - 167442nt	(1)	---
SARS-CoV-2 Reference Genome NC_045512.2	(1)	---

Section 213

	(16325)	16325 16335 16346 16357 16368 16379 16390 16401
Candidatus Carsonella ruddii strain BT chromosome...	(16325)	TTTTATTGCTTCATTCATTGCTTCTTTTAAATTTCCATTTGAAATAAGAAAAATTTTACCTCCTAAAAGTTTAATTT
Candidatus Carsonella 135557 - 167442nt	(1)	---
SARS-CoV-2 Reference Genome NC_045512.2	(1)	---

Section 214

	(16402)	16402 16412 16423 16434 16445 16456 16467 16478
Candidatus Carsonella ruddii strain BT chromosome...	(16402)	TTTTAACATTTATATTTTGTCTTTTATAATCATTTTCTCCCATATATATTATTGATTCTAAATTAAACAAAGAACAT
Candidatus Carsonella 135557 - 167442nt	(1)	---
SARS-CoV-2 Reference Genome NC_045512.2	(1)	---

Section 215

	(16479)	16479 16489 16500 16511 16522 16533 16544 16555
Candidatus Carsonella ruddii strain BT chromosome...	(16479)	GAAGTAGAAGTTGCAACACCGTGCATTCCAGCACCAGTTTCACATATAATTCTTTTTTTTTTAAGAAATTTAGCTAA
Candidatus Carsonella 135557 - 167442nt	(1)	---
SARS-CoV-2 Reference Genome NC_045512.2	(1)	---

Section 216

	(16556)	16556 16566 16577 16588 16599 16610 16621 16632
Candidatus Carsonella ruddii strain BT chromosome...	(16556)	TAAAATTTGTCCTATTGAATTATTTATTTTATGAGCTCCAGTAAAATTTAAATCTTCTCTTTTTAATAAAATTTTTG
Candidatus Carsonella 135557 - 167442nt	(1)	---
SARS-CoV-2 Reference Genome NC_045512.2	(1)	---

Section 217

	(16633)	16633 16643 16654 16665 16676 16687 16698 16709
Candidatus Carsonella ruddii strain BT chromosome...	(16633)	ATCCTTTTAATATATTAGAAATATTTTTACAATAATAAATAGGTGTTGGTCTTCCAATAAATAAATTAAAAAATTTT
Candidatus Carsonella 135557 - 167442nt	(1)	---
SARS-CoV-2 Reference Genome NC_045512.2	(1)	---

Section 218

	(16710)	16710 16720 16731 16742 16753 16764 16775 16786
Candidatus Carsonella ruddii strain BT chromosome...	(16710)	AAAATTTCTATTTTAAATTTTTTTTTTTTTATTATTTTTAAATAAATTTTAAATAAAGAATTTAATAATGGTTGTAA
Candidatus Carsonella 135557 - 167442nt	(1)	---
SARS-CoV-2 Reference Genome NC_045512.2	(1)	---

Section 219

	(16787)	16787 16797 16808 16819 16830 16841 16852 16863
Candidatus Carsonella ruddii strain BT chromosome...	(16787)	CAATTCTGGTAAATATTTACCTCCAAAAATATTAAACATTCCTAAATTATTTGGATAAATCATAATAAATTATCTAA
Candidatus Carsonella 135557 - 167442nt	(1)	---
SARS-CoV-2 Reference Genome NC_045512.2	(1)	---

Section 220

	(16864)	16864 16874 16885 16896 16907 16918 16929 16940
Candidatus Carsonella ruddii strain BT chromosome...	(16864)	TTACAACTTATTCTCACATAAAATGCTACCATAAGTATTAAATAGTTTCACTTTTGAGTTCGGAAAGAGATCATGTG
Candidatus Carsonella 135557 - 167442nt	(1)	---
SARS-CoV-2 Reference Genome NC_045512.2	(1)	---

Section 221

	(16941)	16941 16951 16962 16973 16984 16995 17006 17017
Candidatus Carsonella ruddii strain BT chromosome...	(16941)	TTTCCTATTTATTAAATAATTAGATTATAATTATTAAAATTTATAGATTAAAAACTCACGATAAATTAGTATACATT
Candidatus Carsonella 135557 - 167442nt	(1)	---
SARS-CoV-2 Reference Genome NC_045512.2	(1)	---

Section 222

	(17018)	17018 17028 17039 17050 17061 17072 17083 17094
Candidatus Carsonella ruddii strain BT chromosome...	(17018)	AGCTAAAATATTACTATTTTTACACATTGTATCTATTAAACTTATTATATTTAAGTTATCTTAAGAAGAGCAACAAA
Candidatus Carsonella 135557 - 167442nt	(1)	---
SARS-CoV-2 Reference Genome NC_045512.2	(1)	---

Section 223

```
                                                                   17095      17105      17116      17127      17138      17149      17160      17171
                                                          (17095)
Candidatus Carsonella ruddii strain BT chromosome...      (17095) CTCTTTGGAGATTTTTTTTAAGATATGTTTCCTACTTATATGCTTTCAGTAGTTATCATTTAAAACTTAGCTAACCG
         Candidatus Carsonella 135557 - 167442nt              (1) -----------------------------------------------------------------------------
      SARS-CoV-2 Reference Genome NC_045512.2                 (1) -----------------------------------------------------------------------------
```

Section 224

```
                                                                   17172      17182      17193      17204      17215      17226      17237      17248
                                                          (17172)
Candidatus Carsonella ruddii strain BT chromosome...      (17172) ACTATGCTATTGGTATAACAATCGGAACACCATTGGTTTTACCATCTTGGTCCTCTCGTACTAAAGATAGATTCTTT
         Candidatus Carsonella 135557 - 167442nt              (1) -----------------------------------------------------------------------------
      SARS-CoV-2 Reference Genome NC_045512.2                 (1) -----------------------------------------------------------------------------
```

Section 225

```
                                                                   17249      17259      17270      17281      17292      17303      17314      17325
                                                          (17249)
Candidatus Carsonella ruddii strain BT chromosome...      (17249) CAAAATCTCTACATTTACGGTAGATAGGGACCGAACTGTCTCACGACGTTCTAAACCCAGCTCGCGTACCACTTTAA
         Candidatus Carsonella 135557 - 167442nt              (1) -----------------------------------------------------------------------------
      SARS-CoV-2 Reference Genome NC_045512.2                 (1) -----------------------------------------------------------------------------
```

Section 226

```
                                                                   17326      17336      17347      17358      17369      17380      17391      17402
                                                          (17326)
Candidatus Carsonella ruddii strain BT chromosome...      (17326) ATGGCGAACAGCCATACCCTTGGGACCAACTCCAGCCCCAGGATGTGATGAGCCGACATCGAGGTGCCAAACACTGC
         Candidatus Carsonella 135557 - 167442nt              (1) -----------------------------------------------------------------------------
      SARS-CoV-2 Reference Genome NC_045512.2                 (1) -----------------------------------------------------------------------------
```

Section 227

```
                                                                   17403      17413      17424      17435      17446      17457      17468      17479
                                                          (17403)
Candidatus Carsonella ruddii strain BT chromosome...      (17403) CGTCGATATGGACTCTTGGGCAGTATTAGCCTGTTATCCCCGGAGTACCTTTTATTCGTTGAGCGATAACCTTTCCA
         Candidatus Carsonella 135557 - 167442nt              (1) -----------------------------------------------------------------------------
      SARS-CoV-2 Reference Genome NC_045512.2                 (1) -----------------------------------------------------------------------------
```

Section 228

```
                                                                   17480      17490      17501      17512      17523      17534      17545      17556
                                                          (17480)
Candidatus Carsonella ruddii strain BT chromosome...      (17480) TACAGAATTATCGGATCACTAGAACCTACTTTCGTATCTGTTTGATATGTCTATCTTACAGTTAAGCACTCATATGC
         Candidatus Carsonella 135557 - 167442nt              (1) -----------------------------------------------------------------------------
      SARS-CoV-2 Reference Genome NC_045512.2                 (1) -----------------------------------------------------------------------------
```

```
                                                                                                                          Section 229
                                                         (17557) 17557     17567      17578      17589      17600      17611      17622      17633
Candidatus Carsonella ruddii strain BT chromosome...     (17557) TCTTATGCTCAACATACGTTTTCCAAACGTATTGAGAGTACCTTTGTACTCCTCCGTTACTTTTTAGGAGGAGACCG
                 Candidatus Carsonella 135557 - 167442nt     (1) ----------------------------------------------------------------------------------------------------------------------------------------------------------
       SARS-CoV-2 Reference Genome NC_045512.2               (1) -----------------------------------------------------------------------------
                                                                                                                          Section 230
                                                         (17634) 17634     17644      17655      17666      17677      17688      17699      17710
Candidatus Carsonella ruddii strain BT chromosome...     (17634) CCCCAGTCAAACTACCCACCACACATTTTTTATATAATGGATTACAAAAAATATATATTAGAATATCAAAAATTTTA
                 Candidatus Carsonella 135557 - 167442nt     (1) -----------------------------------------------------------------------------
       SARS-CoV-2 Reference Genome NC_045512.2               (1) -----------------------------------------------------------------------------
                                                                                                                          Section 231
                                                         (17711) 17711     17721      17732      17743      17754      17765      17776      17787
Candidatus Carsonella ruddii strain BT chromosome...     (17711) GACTGGTATTTCAAGGTCGACTACTTATAAACTTGGATTTATAGATTAACATCTCCCAGTTATCCTACACAAAAATT
                 Candidatus Carsonella 135557 - 167442nt     (1) -----------------------------------------------------------------------------
       SARS-CoV-2 Reference Genome NC_045512.2               (1) -----------------------------------------------------------------------------
                                                                                                                          Section 232
                                                         (17788) 17788     17798      17809      17820      17831      17842      17853      17864
Candidatus Carsonella ruddii strain BT chromosome...     (17788) TCCAATATTCAATGTGAAGTTATAGTAAAGGTTCACGGGGTCTTTCCGTCTAGCCGTAAATACACTGCATCTTCACA
                 Candidatus Carsonella 135557 - 167442nt     (1) -----------------------------------------------------------------------------
       SARS-CoV-2 Reference Genome NC_045512.2               (1) -----------------------------------------------------------------------------
                                                                                                                          Section 233
                                                         (17865) 17865     17875      17886      17897      17908      17919      17930      17941
Candidatus Carsonella ruddii strain BT chromosome...     (17865) GCAAATTCAATTTCACTGAGTTCTAAATGGAGACAGCGTGACCATCATTACGCCATTCGTGCAGGTCGGAACTTACC
                 Candidatus Carsonella 135557 - 167442nt     (1) -----------------------------------------------------------------------------
       SARS-CoV-2 Reference Genome NC_045512.2               (1) -----------------------------------------------------------------------------
                                                                                                                          Section 234
                                                         (17942) 17942     17952      17963      17974      17985      17996      18007      18018
Candidatus Carsonella ruddii strain BT chromosome...     (17942) CGACAAGGAATTTCGCTACCTTTGGACCGTTATAGTTACGGCCGCCGTTTACTGGGGCTTTTATTAAATGCTTCATT
                 Candidatus Carsonella 135557 - 167442nt     (1) -----------------------------------------------------------------------------
       SARS-CoV-2 Reference Genome NC_045512.2               (1) -----------------------------------------------------------------------------
```

```
                                                             Section 235
                                         (18019) 18019     18029      18040      18051      18062      18073      18084   18095
Candidatus Carsonella ruddii strain BT chromosome... (18019) TTTATATTAACATTCTCAATTAACCTACCAGCACCGGGCAGGCGTCACACCCTATACATCCGTTTGCACGTTAGCAG
           Candidatus Carsonella 135557 - 167442nt (1) -----------------------------------------------------------------------------
           SARS-CoV-2 Reference Genome NC_045512.2 (1) -----------------------------------------------------------------------------
                                                             Section 236
                                         (18096) 18096     18106      18117      18128      18139      18150      18161   18172
Candidatus Carsonella ruddii strain BT chromosome... (18096) AGTGCTGTGTTTTTAATAAACAGTTGCAGTCACCTTACCTCTTAGACTAGTTCTTTATAAAAAAATTTACAAATATT
           Candidatus Carsonella 135557 - 167442nt (1) -----------------------------------------------------------------------------
           SARS-CoV-2 Reference Genome NC_045512.2 (1) -----------------------------------------------------------------------------
                                                             Section 237
                                         (18173) 18173     18183      18194      18205      18216      18227      18238   18249
Candidatus Carsonella ruddii strain BT chromosome... (18173) CTAGTGTATCTTCTCCAAAAGTTACGATACTATTTTGCCTAGTTCCTTCATTTAGATTATCTCATACGCCTTAGTAT
           Candidatus Carsonella 135557 - 167442nt (1) -----------------------------------------------------------------------------
           SARS-CoV-2 Reference Genome NC_045512.2 (1) -----------------------------------------------------------------------------
                                                             Section 238
                                         (18250) 18250     18260      18271      18282      18293      18304      18315   18326
Candidatus Carsonella ruddii strain BT chromosome... (18250) ACTCTACTTAATTACCTGTGTCGGTTTACGGTACGTACATTAATATGTATTATATTAGAAATTTTTCTTGTTAATTT
           Candidatus Carsonella 135557 - 167442nt (1) -----------------------------------------------------------------------------
           SARS-CoV-2 Reference Genome NC_045512.2 (1) -----------------------------------------------------------------------------
                                                             Section 239
                                         (18327) 18327     18337      18348      18359      18370      18381      18392   18403
Candidatus Carsonella ruddii strain BT chromosome... (18327) AATTTTATTGATTTTTTTTTATTAATTAAATAAAATTAACTAGATTTACTTGGTTAATTATTCTTTTAATTTTAAAC
           Candidatus Carsonella 135557 - 167442nt (1) -----------------------------------------------------------------------------
           SARS-CoV-2 Reference Genome NC_045512.2 (1) -----------------------------------------------------------------------------
                                                             Section 240
                                         (18404) 18404     18414      18425      18436      18447      18458      18469   18480
Candidatus Carsonella ruddii strain BT chromosome... (18404) CTAATATTCCTTTTTTTAGGATCAAAAAACAAATTAAGACATTCCATAATTAATATTAATGGTAAAGAAATATTAATC
           Candidatus Carsonella 135557 - 167442nt (1) -----------------------------------------------------------------------------
           SARS-CoV-2 Reference Genome NC_045512.2 (1) -----------------------------------------------------------------------------
```

Section 241

		18481 18491 18502 18513 18524 18535 18546 18557
	(18481)	
Candidatus Carsonella ruddii strain BT chromosome...	(18481)	TTTTTTCCATCGACTACGTTTTTCAACCTCATCTTAGGTTACGACTAACTCTATTTCGATCATCGTTGAATAGAAAA
Candidatus Carsonella 135557 - 167442nt	(1)	---
SARS-CoV-2 Reference Genome NC_045512.2	(1)	---

Section 242

		18558 18568 18579 18590 18601 18612 18623 18634
	(18558)	
Candidatus Carsonella ruddii strain BT chromosome...	(18558)	CCTTAATTTTTCGGCGGAAATGATTTTCACATTTCATTTTGTTACTTATGTCAGCATTCGCACTTGTGATGTTTCCA
Candidatus Carsonella 135557 - 167442nt	(1)	---
SARS-CoV-2 Reference Genome NC_045512.2	(1)	---

Section 243

		18635 18645 18656 18667 18678 18689 18700 18711
	(18635)	
Candidatus Carsonella ruddii strain BT chromosome...	(18635)	ATTTATTTTAAAATAAATCTTCTTTAACTTACACAACGCTCCTCTACCACAATAATAATGTCCATAATTTCGGTACT
Candidatus Carsonella 135557 - 167442nt	(1)	---
SARS-CoV-2 Reference Genome NC_045512.2	(1)	---

Section 244

		18712 18722 18733 18744 18755 18766 18777 18788
	(18712)	
Candidatus Carsonella ruddii strain BT chromosome...	(18712)	TATTTTAGCCCCGTTAAATTTTACGAATAAATCATCTATATCAATGAGCTATTACGCTTTCTTTAAAGGATGGCTGC
Candidatus Carsonella 135557 - 167442nt	(1)	---
SARS-CoV-2 Reference Genome NC_045512.2	(1)	---

Section 245

		18789 18799 18810 18821 18832 18843 18854 18865
	(18789)	
Candidatus Carsonella ruddii strain BT chromosome...	(18789)	TTCTAAGCCCACCTTTTGATTGTCAAAGATTATTAAATTCATTTTCCACTTAAATAAGATTTAGGGACCTTAATTTA
Candidatus Carsonella 135557 - 167442nt	(1)	---
SARS-CoV-2 Reference Genome NC_045512.2	(1)	---

Section 246

		18866 18876 18887 18898 18909 18920 18931 18942
	(18866)	
Candidatus Carsonella ruddii strain BT chromosome...	(18866)	TGGTCTGGGTTGTTTCCCTTTTCACTATGGATGTTATCACCCATAGTGTGTCTCCTATAATAAAAAAATTAATATTC
Candidatus Carsonella 135557 - 167442nt	(1)	---
SARS-CoV-2 Reference Genome NC_045512.2	(1)	---

Section 247

```
                                                      (18943) 18943     18953      18964      18975      18986      18997      19008      19019
Candidatus Carsonella ruddii strain BT chromosome...  (18943) ATAGTTTGTTATGATTCAGAATAATCTTATTCAAAACAGTGCTTTACCATTAATTTTTACTTATAAGGCGCTACCTA
          Candidatus Carsonella 135557 - 167442nt         (1) -----------------------------------------------------------------------------
        SARS-CoV-2 Reference Genome NC_045512.2           (1) -----------------------------------------------------------------------------
```

Section 248

```
                                                      (19020) 19020     19030      19041      19052      19063      19074      19085      19096
Candidatus Carsonella ruddii strain BT chromosome...  (19020) AATAGCTTTCGAGGAGAACCAGCTATCTCCGAGCTTGATTAGCCTTTCACCCCTATCCACAAATCATCTGAATCTTT
          Candidatus Carsonella 135557 - 167442nt         (1) -----------------------------------------------------------------------------
        SARS-CoV-2 Reference Genome NC_045512.2           (1) -----------------------------------------------------------------------------
```

Section 249

```
                                                      (19097) 19097     19107      19118      19129      19140      19151      19162      19173
Candidatus Carsonella ruddii strain BT chromosome...  (19097) TGCAACAGATACCAGTTCGGTCCTTCAGTAATTTTTACTTTACCTTCAACCTGTTTATAGATAGATCGCTCGGTTTC
          Candidatus Carsonella 135557 - 167442nt         (1) -----------------------------------------------------------------------------
        SARS-CoV-2 Reference Genome NC_045512.2           (1) -----------------------------------------------------------------------------
```

Section 250

```
                                                      (19174) 19174     19184      19195      19206      19217      19228      19239      19250
Candidatus Carsonella ruddii strain BT chromosome...  (19174) GGGTCTATTTTTTATAACTTTCGCCCTTTCAGACTAGATTTCTCTGCGCCTACTTTAAATTAAGCTTGCTAAAAAAA
          Candidatus Carsonella 135557 - 167442nt         (1) -----------------------------------------------------------------------------
        SARS-CoV-2 Reference Genome NC_045512.2           (1) -----------------------------------------------------------------------------
```

Section 251

```
                                                      (19251) 19251     19261      19272      19283      19294      19305      19316      19327
Candidatus Carsonella ruddii strain BT chromosome...  (19251) ATAAGTCGCTGACCCATTATACAAAAGGTATATAGTTACTTTTCAGTTTCTATTGCTTTTACGTATATAATTTCAGG
          Candidatus Carsonella 135557 - 167442nt         (1) -----------------------------------------------------------------------------
        SARS-CoV-2 Reference Genome NC_045512.2           (1) -----------------------------------------------------------------------------
```

Section 252

```
                                                      (19328) 19328     19338      19349      19360      19371      19382      19393      19404
Candidatus Carsonella ruddii strain BT chromosome...  (19328) TTCTATTTCACTCCTATTTAAAGGTTCTATTTCATCTTTCCCTCACGGTACTAGTTCACTATCGGTTAATTATTAGT
          Candidatus Carsonella 135557 - 167442nt         (1) -----------------------------------------------------------------------------
        SARS-CoV-2 Reference Genome NC_045512.2           (1) -----------------------------------------------------------------------------
```

Section 253

	(19405)	19405 19415 19426 19437 19448 19459 19470 19481
Candidatus Carsonella ruddii strain BT chromosome...	(19405)	ATTTAGCCTTAGAGGATGGTCCCCCTTTATTCTGTTAAGATTTCACGTGTCTAAACATACTCATAATTAAAAAAATA
Candidatus Carsonella 135557 - 167442nt	(1)	---
SARS-CoV-2 Reference Genome NC_045512.2	(1)	---

Section 254

	(19482)	19482 19492 19503 19514 19525 19536 19547 19558
Candidatus Carsonella ruddii strain BT chromosome...	(19482)	AAAAATTATTTAAATAAAAGACTATTACTTTTTATGTTAATTATTCAAAATTATTTTAAAGTTTTATTTTTTTTTTT
Candidatus Carsonella 135557 - 167442nt	(1)	---
SARS-CoV-2 Reference Genome NC_045512.2	(1)	---

Section 255

	(19559)	19559 19569 19580 19591 19602 19613 19624 19635
Candidatus Carsonella ruddii strain BT chromosome...	(19559)	GGGCTTTTCTCATTTCGCTCGCCACTACTATGAGAATCTCATTTGATTTCTTTTCCTTGGATTACTTAGATGTTTCA
Candidatus Carsonella 135557 - 167442nt	(1)	---
SARS-CoV-2 Reference Genome NC_045512.2	(1)	---

Section 256

	(19636)	19636 19646 19657 19668 19679 19690 19701 19712
Candidatus Carsonella ruddii strain BT chromosome...	(19636)	GTTCATCCAGTTTGCGTATTATATACACTAATTTTAATTAGTAGGTTTCCCTATTAAGATACCTTAAAAGATTATCG
Candidatus Carsonella 135557 - 167442nt	(1)	---
SARS-CoV-2 Reference Genome NC_045512.2	(1)	---

Section 257

	(19713)	19713 19723 19734 19745 19756 19767 19778 19789
Candidatus Carsonella ruddii strain BT chromosome...	(19713)	CATTTTAGCGTCTGTCATCGCCTATAATTACCTAGACATCCTTTATATACGTTTATTTATTTTAATCTATAAATTAA
Candidatus Carsonella 135557 - 167442nt	(1)	---
SARS-CoV-2 Reference Genome NC_045512.2	(1)	---

Section 258

	(19790)	19790 19800 19811 19822 19833 19844 19855 19866
Candidatus Carsonella ruddii strain BT chromosome...	(19790)	TTTTATCATTAAAAAAATTTAAAAATATATTTATATATCCAGCCACAGGTTCCCCTACAGCTACCTTGTTACGACTT
Candidatus Carsonella 135557 - 167442nt	(1)	---
SARS-CoV-2 Reference Genome NC_045512.2	(1)	---

```
                                                                                   Section 259
                                              (19867) 19867     19877      19888      19899      19910      19921      19932      19943
Candidatus Carsonella ruddii strain BT chromosome... (19867) CACCCCAGTTACAAATCATACCGTTATAATAATGAAATTATTTATGATACAATTTACTTCCATGGTGTGACGGGCGG
Candidatus Carsonella 135557 - 167442nt                  (1) -----------------------------------------------------------------------------
SARS-CoV-2 Reference Genome NC_045512.2                  (1) -----------------------------------------------------------------------------
                                                                                   Section 260
                                              (19944) 19944     19954      19965      19976      19987      19998      20009      20020
Candidatus Carsonella ruddii strain BT chromosome... (19944) TGTGTACAAGGCTCGAGAACGTATTCACCGTAACATTCTGATTTACGATTACTAGCGATTCCAACTTCATAAAATCG
Candidatus Carsonella 135557 - 167442nt                  (1) -----------------------------------------------------------------------------
SARS-CoV-2 Reference Genome NC_045512.2                  (1) -----------------------------------------------------------------------------
                                                                                   Section 261
                                              (20021) 20021     20031      20042      20053      20064      20075      20086      20097
Candidatus Carsonella ruddii strain BT chromosome... (20021) AGTTACAGATTTTAATCCGAACTAAGAGTATGTTTTAAGATTAGCATTATGTTACCATATAGCACAACTTTTTGTTT
Candidatus Carsonella 135557 - 167442nt                  (1) -----------------------------------------------------------------------------
SARS-CoV-2 Reference Genome NC_045512.2                  (1) -----------------------------------------------------------------------------
                                                                                   Section 262
                                              (20098) 20098     20108      20119      20130      20141      20152      20163      20174
Candidatus Carsonella ruddii strain BT chromosome... (20098) TACTCATTGTAGCACGTGTGTAGCCCTACTCATAAGGGCCATGATGACTTGACGTCGTCCTCACCTTCCTCCAATTT
Candidatus Carsonella 135557 - 167442nt                  (1) -----------------------------------------------------------------------------
SARS-CoV-2 Reference Genome NC_045512.2                  (1) -----------------------------------------------------------------------------
                                                                                   Section 263
                                              (20175) 20175     20185      20196      20207      20218      20229      20240      20251
Candidatus Carsonella ruddii strain BT chromosome... (20175) ATCATAGGCAGTCTCTTATTAGTTCTAAGATTATTTTAGTAATATAAGATAAGGGTTGCGCTCGTTATAGGACTTAA
Candidatus Carsonella 135557 - 167442nt                  (1) -----------------------------------------------------------------------------
SARS-CoV-2 Reference Genome NC_045512.2                  (1) -----------------------------------------------------------------------------
                                                                                   Section 264
                                              (20252) 20252     20262      20273      20284      20295      20306      20317      20328
Candidatus Carsonella ruddii strain BT chromosome... (20252) CCCAACATTTCACAACACGAGCTGACGACAGCCATGCAGCACCTGTCTCAAAGCTTAAAAGCTTTACTATTTCTAGT
Candidatus Carsonella 135557 - 167442nt                  (1) -----------------------------------------------------------------------------
SARS-CoV-2 Reference Genome NC_045512.2                  (1) -----------------------------------------------------------------------------
```

```
                                                                  Section 265
                                           (20329) 20329     20339      20350      20361      20372      20383      20394      20405
Candidatus Carsonella ruddii strain BT chromosome... (20329) AAATTCTTTGGATGTCAAGAGTAGGTAAGATTTTTCGTGTTGTATCGAATTAAACCACATGCTCCACCGCTTGTGCG
        Candidatus Carsonella 135557 - 167442nt     (1) -----------------------------------------------------------------------------
      SARS-CoV-2 Reference Genome NC_045512.2       (1) -----------------------------------------------------------------------------
                                                                  Section 266
                                           (20406) 20406     20416      20427      20438      20449      20460      20471      20482
Candidatus Carsonella ruddii strain BT chromosome... (20406) AGCCCCCGTCAATTCATTTGAGTTTTAACCTTGCGGTCGTAATCCCCAGGCGGTCAACTTAACGCGTTAGCTTTTTC
        Candidatus Carsonella 135557 - 167442nt     (1) -----------------------------------------------------------------------------
      SARS-CoV-2 Reference Genome NC_045512.2       (1) -----------------------------------------------------------------------------
                                                                  Section 267
                                           (20483) 20483     20493      20504      20515      20526      20537      20548      20559
Candidatus Carsonella ruddii strain BT chromosome... (20483) ACTAAAAATACATTTTTGAATTAAAAATATTTTTCAAAATATGTTATTTAATAAATAGTTGACATCGTTTACTGCGT
        Candidatus Carsonella 135557 - 167442nt     (1) -----------------------------------------------------------------------------
      SARS-CoV-2 Reference Genome NC_045512.2       (1) -----------------------------------------------------------------------------
                                                                  Section 268
                                           (20560) 20560     20570      20581      20592      20603      20614      20625      20636
Candidatus Carsonella ruddii strain BT chromosome... (20560) GGACTACCAGGGTATCTAATCCTGTTTGCTCCCCACGCTTTCGTGTATCAGTGTCAGTATTAAAATAGAAAAACGCC
        Candidatus Carsonella 135557 - 167442nt     (1) -----------------------------------------------------------------------------
      SARS-CoV-2 Reference Genome NC_045512.2       (1) -----------------------------------------------------------------------------
                                                                  Section 269
                                           (20637) 20637     20647      20658      20669      20680      20691      20702      20713
Candidatus Carsonella ruddii strain BT chromosome... (20637) TTCGCCACTAGTATTCTTTCAGATATCTGAGCATTTCACTGCTACTCCCGAAATTCTAATTTCTTCTTTTATACTCA
        Candidatus Carsonella 135557 - 167442nt     (1) -----------------------------------------------------------------------------
      SARS-CoV-2 Reference Genome NC_045512.2       (1) -----------------------------------------------------------------------------
                                                                  Section 270
                                           (20714) 20714     20724      20735      20746      20757      20768      20779      20790
Candidatus Carsonella ruddii strain BT chromosome... (20714) AGTTTATTAGTATTAATTGCTTTATTAAATTACTTTAATAATTTTAACAAAATTAACTTTTATAAACAACCTACACA
        Candidatus Carsonella 135557 - 167442nt     (1) -----------------------------------------------------------------------------
      SARS-CoV-2 Reference Genome NC_045512.2       (1) -----------------------------------------------------------------------------
```

Section 271

		(20791) 20791 20801 20812 20823 20834 20845 20856 20867
Candidatus Carsonella ruddii strain BT chromosome...	(20791)	CCCTTTACGCCCAATAATTTCGATTAACGCTTGCACCCCTCGTATTACCGCGGCTGCTGGCACGAAGTTAGCCGGTG
Candidatus Carsonella 135557 - 167442nt	(1)	---
SARS-CoV-2 Reference Genome NC_045512.2	(1)	---

Section 272

		(20868) 20868 20878 20889 20900 20911 20922 20933 20944
Candidatus Carsonella ruddii strain BT chromosome...	(20868)	CTTCTTTTACAAATATCGTCAAAAAAAAAAATTTTTTAAATTTTTTATTCTTCTTTCATTGTTGAAAGTGTTTTACA
Candidatus Carsonella 135557 - 167442nt	(1)	---
SARS-CoV-2 Reference Genome NC_045512.2	(1)	---

Section 273

		(20945) 20945 20955 20966 20977 20988 20999 21010 21021
Candidatus Carsonella ruddii strain BT chromosome...	(20945)	ACCCTAAGGCCTTCTTCACACACGCGACATAGCTGGATCAAGCTTTCGCTCATTGTCCAATATTCCCCACTGCTGCC
Candidatus Carsonella 135557 - 167442nt	(1)	---
SARS-CoV-2 Reference Genome NC_045512.2	(1)	---

Section 274

		(21022) 21022 21032 21043 21054 21065 21076 21087 21098
Candidatus Carsonella ruddii strain BT chromosome...	(21022)	TTCCTTAGAAGTTTGGGCCGTGTCTCAGTCCCAATGTGGTTGTTCATCCTCTAAGATCAACTACGGATCATAGTCTT
Candidatus Carsonella 135557 - 167442nt	(1)	---
SARS-CoV-2 Reference Genome NC_045512.2	(1)	---

Section 275

		(21099) 21099 21109 21120 21131 21142 21153 21164 21175
Candidatus Carsonella ruddii strain BT chromosome...	(21099)	GTTAAACCTTTACTTTAACAACTAACTAATCCGATACAAGCTCTTCTTTTAGCATATGACTATCTCATACTTTATCC
Candidatus Carsonella 135557 - 167442nt	(1)	---
SARS-CoV-2 Reference Genome NC_045512.2	(1)	---

Section 276

		(21176) 21176 21186 21197 21208 21219 21230 21241 21252
Candidatus Carsonella ruddii strain BT chromosome...	(21176)	ATTAGGATTTATATCGAATTACTATACATTTCTATATACTTTTCGTTACTAAAAGGCAGATTCTTGCATATTACTCA
Candidatus Carsonella 135557 - 167442nt	(1)	---
SARS-CoV-2 Reference Genome NC_045512.2	(1)	---

Section 277

```
                                                        (21253) 21253     21263      21274      21285      21296      21307      21318   21329
Candidatus Carsonella ruddii strain BT chromosome...    (21253) CCCGTTCGCTGCTAATATTTAAATTCGCACAACTTGCATGTGTTAAGCTTGTCGCCAGCGTTTAATCTGAGCTATGA
          Candidatus Carsonella 135557 - 167442nt           (1) -----------------------------------------------------------------------------
       SARS-CoV-2 Reference Genome NC_045512.2              (1) -----------------------------------------------------------------------------
```

Section 278

```
                                                        (21330) 21330     21340      21351      21362      21373      21384      21395   21406
Candidatus Carsonella ruddii strain BT chromosome...    (21330) TCAAACTCGTAAAAACTTTTTTATTATTTAAAAAAATTATTTTAATAAAAATTTATTTATTAAAAAATTCTTATAAA
          Candidatus Carsonella 135557 - 167442nt           (1) -----------------------------------------------------------------------------
       SARS-CoV-2 Reference Genome NC_045512.2              (1) -----------------------------------------------------------------------------
```

Section 279

```
                                                        (21407) 21407     21417      21428      21439      21450      21461      21472   21483
Candidatus Carsonella ruddii strain BT chromosome...    (21407) GTTAACATTATAAGTTAAAATTTTTAAATAAATTAAAAATATTATTGTTAATAATATTTTTATAATAATTATTGTTT
          Candidatus Carsonella 135557 - 167442nt           (1) -----------------------------------------------------------------------------
       SARS-CoV-2 Reference Genome NC_045512.2              (1) -----------------------------------------------------------------------------
```

Section 280

```
                                                        (21484) 21484     21494      21505      21516      21527      21538      21549   21560
Candidatus Carsonella ruddii strain BT chromosome...    (21484) AAATTTAATTTTAATATTAAACTATCAATATTACTTAAATTAAAAATAAATTTTTTTTTGAAAAATAAAAATTTAGT
          Candidatus Carsonella 135557 - 167442nt           (1) -----------------------------------------------------------------------------
       SARS-CoV-2 Reference Genome NC_045512.2              (1) -----------------------------------------------------------------------------
```

Section 281

```
                                                        (21561) 21561     21571      21582      21593      21604      21615      21626   21637
Candidatus Carsonella ruddii strain BT chromosome...    (21561) ATATATTAATTCAGAAATATTAAAAATAAAATTATTTTGTTTTAAATTAAAAAACATTTTTTTTATTATAATATTAT
          Candidatus Carsonella 135557 - 167442nt           (1) -----------------------------------------------------------------------------
       SARS-CoV-2 Reference Genome NC_045512.2              (1) -----------------------------------------------------------------------------
```

Section 282

```
                                                        (21638) 21638     21648      21659      21670      21681      21692      21703   21714
Candidatus Carsonella ruddii strain BT chromosome...    (21638) TTAAAATATTAATTGAAAAATTTAATAAAGATTTCTTTTTTTTTTTTGTAAATTTAAATTTTTTAAAAAAAAAAAAA
          Candidatus Carsonella 135557 - 167442nt           (1) -----------------------------------------------------------------------------
       SARS-CoV-2 Reference Genome NC_045512.2              (1) -----------------------------------------------------------------------------
```

Section 283

	(21715)	21715 21725 21736 21747 21758 21769 21780 21791
Candidatus Carsonella ruddii strain BT chromosome...	(21715)	ATTTTTTTAAAATTAGAAAAAATAAATAAATCATTTTTATTTGTCTTAGACATTTTTTTTTTATTAAAACTATATAA
Candidatus Carsonella 135557 - 167442nt	(1)	---
SARS-CoV-2 Reference Genome NC_045512.2	(1)	---

Section 284

	(21792)	21792 21802 21813 21824 21835 21846 21857 21868
Candidatus Carsonella ruddii strain BT chromosome...	(21792)	AATATTGTTATTTATAATAAAATTAGAATTTTTAATTATTTTAAAATTTAATAAATAATTAATTTTGTTTTTAATTT
Candidatus Carsonella 135557 - 167442nt	(1)	---
SARS-CoV-2 Reference Genome NC_045512.2	(1)	---

Section 285

	(21869)	21869 21879 21890 21901 21912 21923 21934 21945
Candidatus Carsonella ruddii strain BT chromosome...	(21869)	TTTTATATAATTCAATATGTTGCAATTGATCTATACCTACAAAAATATATTTGTTGTTATTACAAATAATATCTGAA
Candidatus Carsonella 135557 - 167442nt	(1)	---
SARS-CoV-2 Reference Genome NC_045512.2	(1)	---

Section 286

	(21946)	21946 21956 21967 21978 21989 22000 22011 22022
Candidatus Carsonella ruddii strain BT chromosome...	(21946)	CACATTAAAATAGGATAACAAATTTTACCAAATGAAATTATTTTTTTTTTATATAATGAATGAAAAATTTTCATTTT
Candidatus Carsonella 135557 - 167442nt	(1)	---
SARS-CoV-2 Reference Genome NC_045512.2	(1)	---

Section 287

	(22023)	22023 22033 22044 22055 22066 22077 22088 22099
Candidatus Carsonella ruddii strain BT chromosome...	(22023)	ATTTTTATTATAAATACATAATAATAACCAAAAAAAATTTAAAAATTTTTCATTAAAAGATTGATAAAAATATAAAT
Candidatus Carsonella 135557 - 167442nt	(1)	---
SARS-CoV-2 Reference Genome NC_045512.2	(1)	---

Section 288

	(22100)	22100 22110 22121 22132 22143 22154 22165 22176
Candidatus Carsonella ruddii strain BT chromosome...	(22100)	TTTTAAAAAAAGATAAAAAAATAGAAATTAATATTATTTTATTTTTTAATATATTTAAAATAGATATGTTTTTTGAT
Candidatus Carsonella 135557 - 167442nt	(1)	---
SARS-CoV-2 Reference Genome NC_045512.2	(1)	---

Section 289

```
                                                          22177      22187       22198       22209       22220       22231       22242      22253
                                             (22177)
Candidatus Carsonella ruddii strain BT chromosome... (22177) AAACTATGTAAATCTGCTATAAAAATATTTCTATTTTTAAAAAAAAAAATAGGTTTAATAATACTTAAATAATTTCC
         Candidatus Carsonella 135557 - 167442nt     (1) -----------------------------------------------------------------------------
     SARS-CoV-2 Reference Genome NC_045512.2         (1) -----------------------------------------------------------------------------
```

Section 290

```
                                                          22254      22264       22275       22286       22297       22308       22319      22330
                                             (22254)
Candidatus Carsonella ruddii strain BT chromosome... (22254) AAAGTGTAATAAACCACTTGAATTTATTCCTACAAAAATCATAATTTTTCTTCTATAAATAAAACTTTTTTTTTTAT
         Candidatus Carsonella 135557 - 167442nt     (1) -----------------------------------------------------------------------------
     SARS-CoV-2 Reference Genome NC_045512.2         (1) -----------------------------------------------------------------------------
```

Section 291

```
                                                          22331      22341       22352       22363       22374       22385       22396      22407
                                             (22331)
Candidatus Carsonella ruddii strain BT chromosome... (22331) AATTGGATCAAATTTAATAATTTTAATTTTTTTATTATTTTTTTATTTTTTTTTTTTAATATAAAAATAACAACTTA
         Candidatus Carsonella 135557 - 167442nt     (1) -----------------------------------------------------------------------------
     SARS-CoV-2 Reference Genome NC_045512.2         (1) -----------------------------------------------------------------------------
```

Section 292

```
                                                          22408      22418       22429       22440       22451       22462       22473      22484
                                             (22408)
Candidatus Carsonella ruddii strain BT chromosome... (22408) AACTACTTTTCAATTTTATTAAAATATACATTTTTTAATTATTTTTATTGATTTAGAAGAAATTTTAATTTTGTTAA
         Candidatus Carsonella 135557 - 167442nt     (1) -----------------------------------------------------------------------------
     SARS-CoV-2 Reference Genome NC_045512.2         (1) -----------------------------------------------------------------------------
```

Section 293

```
                                                          22485      22495       22506       22517       22528       22539       22550      22561
                                             (22485)
Candidatus Carsonella ruddii strain BT chromosome... (22485) ATTTATTTTTTACCCAAATAATTTGTTTGTTAAAATTAATTTTAGAAAAACATTTATTTTTAATATTAGAATGAGAA
         Candidatus Carsonella 135557 - 167442nt     (1) -----------------------------------------------------------------------------
     SARS-CoV-2 Reference Genome NC_045512.2         (1) -----------------------------------------------------------------------------
```

Section 294

```
                                                          22562      22572       22583       22594       22605       22616       22627      22638
                                             (22562)
Candidatus Carsonella ruddii strain BT chromosome... (22562) ACTTTACATTTTGTAATTTTTTTTTTTTTTGTTAAAATACAAATATTAGACATTTTTTTTTTTTTTAATTCTTATTT
         Candidatus Carsonella 135557 - 167442nt     (1) -----------------------------------------------------------------------------
     SARS-CoV-2 Reference Genome NC_045512.2         (1) -----------------------------------------------------------------------------
```

Section 295

	(22639)	22639 22649 22660 22671 22682 22693 22704 22715
Candidatus Carsonella ruddii strain BT chromosome...	(22639)	TTTCTATAGACATATTAAAAATATAACTTAATTCTTTATATGGAATTTTATATTCTTTAAAATTTCTGTATAAAAAA
Candidatus Carsonella 135557 - 167442nt	(1)	---
SARS-CoV-2 Reference Genome NC_045512.2	(1)	---

Section 296

	(22716)	22716 22726 22737 22748 22759 22770 22781 22792
Candidatus Carsonella ruddii strain BT chromosome...	(22716)	ACTTCTTTTTCAAATTTAAATACTTTTTTTTTATTAAAATAATTTTTTGTTTTATTAAAATAATTTTTTATTTTATC
Candidatus Carsonella 135557 - 167442nt	(1)	---
SARS-CoV-2 Reference Genome NC_045512.2	(1)	---

Section 297

	(22793)	22793 22803 22814 22825 22836 22847 22858 22869
Candidatus Carsonella ruddii strain BT chromosome...	(22793)	AAAAAAAGTATTTATTTTTATTCCATAAAATGGATTAAAATAATTTAACGATTTTTTAAAATTGTTATAATAATTTA
Candidatus Carsonella 135557 - 167442nt	(1)	---
SARS-CoV-2 Reference Genome NC_045512.2	(1)	---

Section 298

	(22870)	22870 22880 22891 22902 22913 22924 22935 22946
Candidatus Carsonella ruddii strain BT chromosome...	(22870)	AAATATCTATTTTTTTCATTTTTAATATTTTTAAATTTTTCGTATATAATTTTGTTAAAAAAAATTTTATTTTTAAAT
Candidatus Carsonella 135557 - 167442nt	(1)	---
SARS-CoV-2 Reference Genome NC_045512.2	(1)	---

Section 299

	(22947)	22947 22957 22968 22979 22990 23001 23012 23023
Candidatus Carsonella ruddii strain BT chromosome...	(22947)	AAATAAAAATTTAAATTTAATAAATATTCATTATATTTCATAAAATTTTTTTAATATGTTTTATTTTAAATAAAATC
Candidatus Carsonella 135557 - 167442nt	(1)	---
SARS-CoV-2 Reference Genome NC_045512.2	(1)	---

Section 300

	(23024)	23024 23034 23045 23056 23067 23078 23089 23100
Candidatus Carsonella ruddii strain BT chromosome...	(23024)	ATAATAATTCTATCGATTCCCACACCAACTCCATTAGTATTATATAAACAATTTTTTAATTTAATTAAAAAATCTTT
Candidatus Carsonella 135557 - 167442nt	(1)	---
SARS-CoV-2 Reference Genome NC_045512.2	(1)	---

Section 301

```
                                                       (23101) 23101     23111      23122      23133      23144      23155      23166   23177
Candidatus Carsonella ruddii strain BT chromosome...   (23101) ATTTTTTTTAAATAAATTTTTTTTTTGTATATAATAATCATTTAATTCATCAAATCCATTAGAAATTTCTATTCCTG
          Candidatus Carsonella 135557 - 167442nt          (1) -----------------------------------------------------------------------------
     SARS-CoV-2 Reference Genome NC_045512.2               (1) -----------------------------------------------------------------------------
```

Section 302

```
                                                       (23178) 23178     23188      23199      23210      23221      23232      23243   23254
Candidatus Carsonella ruddii strain BT chromosome...   (23178) AAATAAATAATTCAAATCTTTTTGAATAATTTAAATTTGTTATAATATTTTTTGTTAAAGGACTACTTTTTGTAGGA
          Candidatus Carsonella 135557 - 167442nt          (1) -----------------------------------------------------------------------------
     SARS-CoV-2 Reference Genome NC_045512.2               (1) -----------------------------------------------------------------------------
```

Section 303

```
                                                       (23255) 23255     23265      23276      23287      23298      23309      23320   23331
Candidatus Carsonella ruddii strain BT chromosome...   (23255) AAAAATTTAATAAAAATAGGAAAAATTAATTTTTTTTTTATTTTATCATCAAAAATTTTAAATAAAATATCACTTAA
          Candidatus Carsonella 135557 - 167442nt          (1) -----------------------------------------------------------------------------
     SARS-CoV-2 Reference Genome NC_045512.2               (1) -----------------------------------------------------------------------------
```

Section 304

```
                                                       (23332) 23332     23342      23353      23364      23375      23386      23397   23408
Candidatus Carsonella ruddii strain BT chromosome...   (23332) AAAATAATTTTTTATATTGTTTATTCCTTTATAATAACAAAATAAAAAAAATTTTTTTTTATTTATTAAAAAAAATT
          Candidatus Carsonella 135557 - 167442nt          (1) -----------------------------------------------------------------------------
     SARS-CoV-2 Reference Genome NC_045512.2               (1) -----------------------------------------------------------------------------
```

Section 305

```
                                                       (23409) 23409     23419      23430      23441      23452      23463      23474   23485
Candidatus Carsonella ruddii strain BT chromosome...   (23409) TTTTTTTTTTAAAATAAATAAAAATAATTTCTAATAAACTTATTTTTTTAAAAAATAAATTATAATTAAAAATTAAA
          Candidatus Carsonella 135557 - 167442nt          (1) -----------------------------------------------------------------------------
     SARS-CoV-2 Reference Genome NC_045512.2               (1) -----------------------------------------------------------------------------
```

Section 306

```
                                                       (23486) 23486     23496      23507      23518      23529      23540      23551   23562
Candidatus Carsonella ruddii strain BT chromosome...   (23486) TTAAAAATTTCTATAAAATAATAATTTGTTATTAAAAATAAATTTTTTAAAATTTTTTCAACAAAAAACAAAGAATC
          Candidatus Carsonella 135557 - 167442nt          (1) -----------------------------------------------------------------------------
     SARS-CoV-2 Reference Genome NC_045512.2               (1) -----------------------------------------------------------------------------
```

Section 307

		23563 23573 23584 23595 23606 23617 23628 23639
	(23563)	
Candidatus Carsonella ruddii strain BT chromosome...	(23563)	TTTAAAATTAAAATTAGATGAATAATATTCTAACATATTAAATTCAAAATAATGAATATTAGAACATCCTTCATTTC
Candidatus Carsonella 135557 - 167442nt	(1)	---
SARS-CoV-2 Reference Genome NC_045512.2	(1)	---

Section 308

		23640 23650 23661 23672 23683 23694 23705 23716
	(23640)	
Candidatus Carsonella ruddii strain BT chromosome...	(23640)	TATAACATTTTGTTATATCAAAAAGTCTATTATAATTATTTGATAAAATTTTTTTTATATAAAATTCTGGAGAAATT
Candidatus Carsonella 135557 - 167442nt	(1)	---
SARS-CoV-2 Reference Genome NC_045512.2	(1)	---

Section 309

		23717 23727 23738 23749 23760 23771 23782 23793
	(23717)	
Candidatus Carsonella ruddii strain BT chromosome...	(23717)	TTTAAATAAAAAAATTTTTTTTTACAAAAATTATAAGTTTTAAATATATTAGAATATGAATTTGATTTATTTTTACA
Candidatus Carsonella 135557 - 167442nt	(1)	---
SARS-CoV-2 Reference Genome NC_045512.2	(1)	---

Section 310

		23794 23804 23815 23826 23837 23848 23859 23870
	(23794)	
Candidatus Carsonella ruddii strain BT chromosome...	(23794)	AAAATTACTAGAATTAATATTTATAAACATAAATTTTTTAAAATAATTGTTTAAAAAATTAATAACACAAAATTTTT
Candidatus Carsonella 135557 - 167442nt	(1)	---
SARS-CoV-2 Reference Genome NC_045512.2	(1)	---

Section 311

		23871 23881 23892 23903 23914 23925 23936 23947
	(23871)	
Candidatus Carsonella ruddii strain BT chromosome...	(23871)	TTTTTATTAAAAAATTATTACTTTTATGAAAAGATAAAGTTTTTACGACTAACTTAATAAAAAACGGTATTATAAAA
Candidatus Carsonella 135557 - 167442nt	(1)	---
SARS-CoV-2 Reference Genome NC_045512.2	(1)	---

Section 312

		23948 23958 23969 23980 23991 24002 24013 24024
	(23948)	
Candidatus Carsonella ruddii strain BT chromosome...	(23948)	ATTATTTTTTTTTTTGTTTTTTTAATTTTACCATAAACAATTAATATATCACCAATTGAAAATTTTTCATTAGAATA
Candidatus Carsonella 135557 - 167442nt	(1)	---
SARS-CoV-2 Reference Genome NC_045512.2	(1)	---

```
                                                                                                              Section 313
                                                       (24025) 24025     24035      24046      24057      24068      24079      24090  24101
Candidatus Carsonella ruddii strain BT chromosome...   (24025) AATTTGTATTATTCCTGAATAATCTTTTAATTCTATAAAAGGTTTTTTGATTCTATTTACTTTTCCGTAAATAATTT
             Candidatus Carsonella 135557 - 167442nt       (1) -----------------------------------------------------------------------------
        SARS-CoV-2 Reference Genome NC_045512.2            (1) -----------------------------------------------------------------------------
                                                                                                              Section 314
                                                       (24102) 24102     24112      24123      24134      24145      24156      24167  24178
Candidatus Carsonella ruddii strain BT chromosome...   (24102) TAAATTTTTTAAAATTTATAATCATATTATTATAAAATTTATTTCATATTTAAAAAATTTATTTAAATTATATTTTT
             Candidatus Carsonella 135557 - 167442nt       (1) -----------------------------------------------------------------------------
        SARS-CoV-2 Reference Genome NC_045512.2            (1) -----------------------------------------------------------------------------
                                                                                                              Section 315
                                                       (24179) 24179     24189      24200      24211      24222      24233      24244  24255
Candidatus Carsonella ruddii strain BT chromosome...   (24179) GATTATTAATATGATTTATAATAAAATTATTTTCAAAATGATAAGTTTTTATATATTTTTTTTCTAAATTTTTAAAA
             Candidatus Carsonella 135557 - 167442nt       (1) -----------------------------------------------------------------------------
        SARS-CoV-2 Reference Genome NC_045512.2            (1) -----------------------------------------------------------------------------
                                                                                                              Section 316
                                                       (24256) 24256     24266      24277      24288      24299      24310      24321  24332
Candidatus Carsonella ruddii strain BT chromosome...   (24256) TAATTATTAAAATTTAAATTTTTTTTCAAAAAAAATTTAAATTCTAAAATTTTTAAAGCAAAATTTTTATTTTTTAA
             Candidatus Carsonella 135557 - 167442nt       (1) -----------------------------------------------------------------------------
        SARS-CoV-2 Reference Genome NC_045512.2            (1) -----------------------------------------------------------------------------
                                                                                                              Section 317
                                                       (24333) 24333     24343      24354      24365      24376      24387      24398  24409
Candidatus Carsonella ruddii strain BT chromosome...   (24333) TTGAGATCTTTCAGATTGACAACTAACAACAATATTTGTAGGAATATGTTTTATTCTTATTGCAGAATTTGTATTAT
             Candidatus Carsonella 135557 - 167442nt       (1) -----------------------------------------------------------------------------
        SARS-CoV-2 Reference Genome NC_045512.2            (1) -----------------------------------------------------------------------------
                                                                                                              Section 318
                                                       (24410) 24410     24420      24431      24442      24453      24464      24475  24486
Candidatus Carsonella ruddii strain BT chromosome...   (24410) TTACATGTTGTCCTCCTGGACCTTTAGATTTAAAACTTTCAATAATAATATCTTCTTTATTAAAAAAATTTTTTTCT
             Candidatus Carsonella 135557 - 167442nt       (1) -----------------------------------------------------------------------------
        SARS-CoV-2 Reference Genome NC_045512.2            (1) -----------------------------------------------------------------------------
```

Section 319

	(24487)	24487 24497 24508 24519 24530 24541 24552 24563
Candidatus Carsonella ruddii strain BT chromosome...	(24487)	TTATTAATTTTAGGAATAACAATTATATTTATATATGAAGTTTGTATTTTATTATTTGTTGTTATAGGATTTTTTCT
Candidatus Carsonella 135557 - 167442nt	(1)	---
SARS-CoV-2 Reference Genome NC_045512.2	(1)	---

Section 320

	(24564)	24564 24574 24585 24596 24607 24618 24629 24640
Candidatus Carsonella ruddii strain BT chromosome...	(24564)	CATTATTCTATGTATACCAGATTCATTTTTTAATAATAAAAAGGAATAAATATCATCTATAAAAATTAAAGATTTTT
Candidatus Carsonella 135557 - 167442nt	(1)	---
SARS-CoV-2 Reference Genome NC_045512.2	(1)	---

Section 321

	(24641)	24641 24651 24662 24673 24684 24695 24706 24717
Candidatus Carsonella ruddii strain BT chromosome...	(24641)	TATATCCATAATCATTATTTTCAAAATAAATAATTTCCACATTAAAATTATTTTTTTTTAACCATTTATAATAAAAA
Candidatus Carsonella 135557 - 167442nt	(1)	---
SARS-CoV-2 Reference Genome NC_045512.2	(1)	---

Section 322

	(24718)	24718 24728 24739 24750 24761 24772 24783 24794
Candidatus Carsonella ruddii strain BT chromosome...	(24718)	TCTGTAAAAAATTTAACTAATTGGTATGTTTCTATTCCTCCAGCATTAGGATAAATTTCTATATAACATTTACAAAT
Candidatus Carsonella 135557 - 167442nt	(1)	---
SARS-CoV-2 Reference Genome NC_045512.2	(1)	---

Section 323

	(24795)	24795 24805 24816 24827 24838 24849 24860 24871
Candidatus Carsonella ruddii strain BT chromosome...	(24795)	TGAATAATTTTCAATTTTTTTTTCAATATAAGATTTAATGTTATTATAATCATTAATATTATATACAGAAAAATTAT
Candidatus Carsonella 135557 - 167442nt	(1)	---
SARS-CoV-2 Reference Genome NC_045512.2	(1)	---

Section 324

	(24872)	24872 24882 24893 24904 24915 24926 24937 24948
Candidatus Carsonella ruddii strain BT chromosome...	(24872)	TTTTAAAAATAAATTCAATATATTTATTTTTAATTAAATTATAATACATTACAATTGTTTTTTTATAAAATTTATTA
Candidatus Carsonella 135557 - 167442nt	(1)	---
SARS-CoV-2 Reference Genome NC_045512.2	(1)	---

```
                                                                                                                      Section 325
                                                         (24949) 24949     24959      24970      24981      24992      25003      25014      25025
Candidatus Carsonella ruddii strain BT chromosome...     (24949) AAATATTAAAATTTTGAGAAAAAATTTTATATTTTTCATTTAACAAAAATAATTTTTTAATATTTAATTTAATTTTT
Candidatus Carsonella 135557 - 167442nt                  (1)     -----------------------------------------------------------------------------
SARS-CoV-2 Reference Genome NC_045512.2                  (1)     -----------------------------------------------------------------------------
                                                                                                                      Section 326
                                                         (25026) 25026     25036      25047      25058      25069      25080      25091      25102
Candidatus Carsonella ruddii strain BT chromosome...     (25026) AAATTAGGTATTATTTTTTTAATCGCAAAAATAAATTTAATAGGATAAGAGGTAGAAACAACAATATTATTTTTTTT
Candidatus Carsonella 135557 - 167442nt                  (1)     -----------------------------------------------------------------------------
SARS-CoV-2 Reference Genome NC_045512.2                  (1)     -----------------------------------------------------------------------------
                                                                                                                      Section 327
                                                         (25103) 25103     25113      25124      25135      25146      25157      25168      25179
Candidatus Carsonella ruddii strain BT chromosome...     (25103) TTCATTTTTAATTAATGATGTTAATGATGTAATGGTATGAGGATCGTGAATTTTTTTATATTTATTATAAAAATATT
Candidatus Carsonella 135557 - 167442nt                  (1)     -----------------------------------------------------------------------------
SARS-CoV-2 Reference Genome NC_045512.2                  (1)     -----------------------------------------------------------------------------
                                                                                                                      Section 328
                                                         (25180) 25180     25190      25201      25212      25223      25234      25245      25256
Candidatus Carsonella ruddii strain BT chromosome...     (25180) CAATAGAATTAATAATAATTTTATTATTAATTTTATCAGAAAAAAAACTTTTATTAATATTATTCATTTGATTATTT
Candidatus Carsonella 135557 - 167442nt                  (1)     -----------------------------------------------------------------------------
SARS-CoV-2 Reference Genome NC_045512.2                  (1)     -----------------------------------------------------------------------------
                                                                                                                      Section 329
                                                         (25257) 25257     25267      25278      25289      25300      25311      25322      25333
Candidatus Carsonella ruddii strain BT chromosome...     (25257) TTTAAATTATTATTATTAACTAATCTTAAAAAATTAGATGGAATAGAAATATCTATTGATGGTGAAATTGTTTTTTT
Candidatus Carsonella 135557 - 167442nt                  (1)     -----------------------------------------------------------------------------
SARS-CoV-2 Reference Genome NC_045512.2                  (1)     -----------------------------------------------------------------------------
                                                                                                                      Section 330
                                                         (25334) 25334     25344      25355      25366      25377      25388      25399      25410
Candidatus Carsonella ruddii strain BT chromosome...     (25334) TAAAGAATAATTTTTAATTATATTATTTTTAATAAAATTATCTAAAAAATTATTATCATTTGTACAAGTTATAATTT
Candidatus Carsonella 135557 - 167442nt                  (1)     -----------------------------------------------------------------------------
SARS-CoV-2 Reference Genome NC_045512.2                  (1)     -----------------------------------------------------------------------------
```

Section 331

	(25411)	25411 25421 25432 25443 25454 25465 25476 25487
Candidatus Carsonella ruddii strain BT chromosome...	(25411)	TTTCAATTGGAAAACCTAATTTTTTAGCTATAAAAGCAGATAGAGAATTACCAAAATTTCCAGATGGAATTATAAAA
Candidatus Carsonella 135557 - 167442nt	(1)	---
SARS-CoV-2 Reference Genome NC_045512.2	(1)	---

Section 332

	(25488)	25488 25498 25509 25520 25531 25542 25553 25564
Candidatus Carsonella ruddii strain BT chromosome...	(25488)	TTTACATAATTATTTTTATAAACATTTAATGAACAATAACAATAATAAATTGTTTGAAAAATTATTCTAAACCAATT
Candidatus Carsonella 135557 - 167442nt	(1)	---
SARS-CoV-2 Reference Genome NC_045512.2	(1)	---

Section 333

	(25565)	25565 25575 25586 25597 25608 25619 25630 25641
Candidatus Carsonella ruddii strain BT chromosome...	(25565)	TATAGAATTAACAGATATTAAAAAAATTTTTTTATTATTTTTAATATTTTCAAATATTTTTTTAATTAAAAATTGAG
Candidatus Carsonella 135557 - 167442nt	(1)	---
SARS-CoV-2 Reference Genome NC_045512.2	(1)	---

Section 334

	(25642)	25642 25652 25663 25674 25685 25696 25707 25718
Candidatus Carsonella ruddii strain BT chromosome...	(25642)	AAGTATCAAAATTTCCTAAAATTGAAATATTTTTAATATTATTTTTAATTAAAGTAGTAATTTGTTTTCTTTGAATA
Candidatus Carsonella 135557 - 167442nt	(1)	---
SARS-CoV-2 Reference Genome NC_045512.2	(1)	---

Section 335

	(25719)	25719 25729 25740 25751 25762 25773 25784 25795
Candidatus Carsonella ruddii strain BT chromosome...	(25719)	TTTGAAATCATATTAAATGGATAAAAAGTAAATAATTTAATTTTTTCAAAATTTTTAAAAGAATTATTAGCTGAGGA
Candidatus Carsonella 135557 - 167442nt	(1)	---
SARS-CoV-2 Reference Genome NC_045512.2	(1)	---

Section 336

	(25796)	25796 25806 25817 25828 25839 25850 25861 25872
Candidatus Carsonella ruddii strain BT chromosome...	(25796)	ACCAGTATCTCCTGAAGTAGCACAAAAAATTAAAAATTTTTTATTAATTTTTTTTGAAAAAAAATTTAATATATTTC
Candidatus Carsonella 135557 - 167442nt	(1)	---
SARS-CoV-2 Reference Genome NC_045512.2	(1)	---

Section 337

```
                                                (25873) 25873     25883      25894      25905      25916      25927      25938      25949
Candidatus Carsonella ruddii strain BT chromosome... (25873) CTAAAGGAATCATAGCTATATCTTTAAAAGTTAAAGTAGGGCCATTATTTAAATTTAATAAAAAATTATTATTAAAT
          Candidatus Carsonella 135557 - 167442nt (1) -----------------------------------------------------------------------------
       SARS-CoV-2 Reference Genome NC_045512.2 (1) -----------------------------------------------------------------------------
```

Section 338

```
                                                (25950) 25950     25960      25971      25982      25993      26004      26015      26026
Candidatus Carsonella ruddii strain BT chromosome... (25950) ATTTTTTCAATATTTAAAACTAAATTAAAATTAAAATTAAAATAACTTTTATTAATTATATTATATAAATTTTCTTC
          Candidatus Carsonella 135557 - 167442nt (1) -----------------------------------------------------------------------------
       SARS-CoV-2 Reference Genome NC_045512.2 (1) -----------------------------------------------------------------------------
```

Section 339

```
                                                (26027) 26027     26037      26048      26059      26070      26081      26092      26103
Candidatus Carsonella ruddii strain BT chromosome... (26027) TGAAATATTATTTATAAATAATCTTGATATTTTAAAAGCAAAATTAACATAATTTAAATTTTTTAAACTAAAAATTT
          Candidatus Carsonella 135557 - 167442nt (1) -----------------------------------------------------------------------------
       SARS-CoV-2 Reference Genome NC_045512.2 (1) -----------------------------------------------------------------------------
```

Section 340

```
                                                (26104) 26104     26114      26125      26136      26147      26158      26169      26180
Candidatus Carsonella ruddii strain BT chromosome... (26104) GATTTTTTTTTAATTTTAATATTTTAAAAGGAAAAAATAAAGTTTTGTTTTTAGGAATATTTGAAAAAAAAATATCT
          Candidatus Carsonella 135557 - 167442nt (1) -----------------------------------------------------------------------------
       SARS-CoV-2 Reference Genome NC_045512.2 (1) -----------------------------------------------------------------------------
```

Section 341

```
                                                (26181) 26181     26191      26202      26213      26224      26235      26246      26257
Candidatus Carsonella ruddii strain BT chromosome... (26181) GAAAATTTTTTCACATCTAATTTATTGTTAATACTGTTATAAAACATTATAATAATTTATAAATAAAAAATTTTTTT
          Candidatus Carsonella 135557 - 167442nt (1) -----------------------------------------------------------------------------
       SARS-CoV-2 Reference Genome NC_045512.2 (1) -----------------------------------------------------------------------------
```

Section 342

```
                                                (26258) 26258     26268      26279      26290      26301      26312      26323      26334
Candidatus Carsonella ruddii strain BT chromosome... (26258) TTTTTAAAAAAATATAAAAAAAACAATATTTTCTTATAATAAATTTTTTTAGTTTTTATTAAAATATATTTATTAAA
          Candidatus Carsonella 135557 - 167442nt (1) -----------------------------------------------------------------------------
       SARS-CoV-2 Reference Genome NC_045512.2 (1) -----------------------------------------------------------------------------
```

Section 343

```
                                                       (26335) 26335     26345      26356      26367      26378      26389      26400   26411
Candidatus Carsonella ruddii strain BT chromosome...  (26335) GTAAAATTTTAAAAAATTTATTTTTAAAATATTTAAAAAATAAAAAATATTGTAATCAAATTTAAATTTAATTAAAT
          Candidatus Carsonella 135557 - 167442nt      (1) -----------------------------------------------------------------------------
      SARS-CoV-2 Reference Genome NC_045512.2          (1) -----------------------------------------------------------------------------
```

Section 344

```
                                                       (26412) 26412     26422      26433      26444      26455      26466      26477   26488
Candidatus Carsonella ruddii strain BT chromosome...  (26412) AATTTAAACATAAAAAATTTTTATTGATTATTTTTACTTTTTTAAAACAATTATATTTAAAAAAAATTTTTTTTTCA
          Candidatus Carsonella 135557 - 167442nt      (1) -----------------------------------------------------------------------------
      SARS-CoV-2 Reference Genome NC_045512.2          (1) -----------------------------------------------------------------------------
```

Section 345

```
                                                       (26489) 26489     26499      26510      26521      26532      26543      26554   26565
Candidatus Carsonella ruddii strain BT chromosome...  (26489) AAAAATTGATAAAAATTACTAACTACAGAAGCAGCAGTGGATAAACCGCCAGCTCCTAAAGATGTTAAAACTGTTTT
          Candidatus Carsonella 135557 - 167442nt      (1) -----------------------------------------------------------------------------
      SARS-CoV-2 Reference Genome NC_045512.2          (1) -----------------------------------------------------------------------------
```

Section 346

```
                                                       (26566) 26566     26576      26587      26598      26609      26620      26631   26642
Candidatus Carsonella ruddii strain BT chromosome...  (26566) TTTAGAATTTTTACAATTTAAAATAATAGTATTTAAAGAATTTTTGCTTTTTATAAAAAATATATTTTTTGTTAAAA
          Candidatus Carsonella 135557 - 167442nt      (1) -----------------------------------------------------------------------------
      SARS-CoV-2 Reference Genome NC_045512.2          (1) -----------------------------------------------------------------------------
```

Section 347

```
                                                       (26643) 26643     26653      26664      26675      26686      26697      26708   26719
Candidatus Carsonella ruddii strain BT chromosome...  (26643) ATAAAGATACATTTATAAATAAATAATTGTTTATATTTAAATAAAAAGATAAAAATTTTTTTTTAAAAAATATTTTT
          Candidatus Carsonella 135557 - 167442nt      (1) -----------------------------------------------------------------------------
      SARS-CoV-2 Reference Genome NC_045512.2          (1) -----------------------------------------------------------------------------
```

Section 348

```
                                                       (26720) 26720     26730      26741      26752      26763      26774      26785   26796
Candidatus Carsonella ruddii strain BT chromosome...  (26720) CTTATATAATTAGATAAATTAAAAATACTTTCTAAATAAAAAATATTACAACAAATGTAAAAATTTTTTATTTTAGA
          Candidatus Carsonella 135557 - 167442nt      (1) -----------------------------------------------------------------------------
      SARS-CoV-2 Reference Genome NC_045512.2          (1) -----------------------------------------------------------------------------
```

Section 349

```
                                                          (26797) 26797     26807      26818      26829      26840      26851      26862      26873
Candidatus Carsonella ruddii strain BT chromosome...      (26797) AAGTAAAATTGAAAATTTAAATAAAGTATCAAACCCAAAAATATCTTTTTCATAGTTTTTTTCTGATATACCTTTTT
Candidatus Carsonella 135557 - 167442nt                   (1)     -----------------------------------------------------------------------------
SARS-CoV-2 Reference Genome NC_045512.2                   (1)     -----------------------------------------------------------------------------
```

Section 350

```
                                                          (26874) 26874     26884      26895      26906      26917      26928      26939      26950
Candidatus Carsonella ruddii strain BT chromosome...      (26874) TTATTGATATTTTTATTAATTTTAAAAAAGACATTTTTTCAATATTTGTTAAAATATAATTACAAGTACCATTTAAA
Candidatus Carsonella 135557 - 167442nt                   (1)     -----------------------------------------------------------------------------
SARS-CoV-2 Reference Genome NC_045512.2                   (1)     -----------------------------------------------------------------------------
```

Section 351

```
                                                          (26951) 26951     26961      26972      26983      26994      27005      27016      27027
Candidatus Carsonella ruddii strain BT chromosome...      (26951) ATACCAAAAAAATAATTAATATTTTCTTTTGAATAAAATTTATCTAATAAATTTATTATTGGTAAAGAACCACAAAC
Candidatus Carsonella 135557 - 167442nt                   (1)     -----------------------------------------------------------------------------
SARS-CoV-2 Reference Genome NC_045512.2                   (1)     -----------------------------------------------------------------------------
```

Section 352

```
                                                          (27028) 27028     27038      27049      27060      27071      27082      27093      27104
Candidatus Carsonella ruddii strain BT chromosome...      (27028) AGATGCTTCATAATAAATTTTTTTATTGTTTATTTTAAACAAATAATTTATAAAAAATAAATATTTAGAAATTAAAT
Candidatus Carsonella 135557 - 167442nt                   (1)     -----------------------------------------------------------------------------
SARS-CoV-2 Reference Genome NC_045512.2                   (1)     -----------------------------------------------------------------------------
```

Section 353

```
                                                          (27105) 27105     27115      27126      27137      27148      27159      27170      27181
Candidatus Carsonella ruddii strain BT chromosome...      (27105) CTTTATTTGCAGAAATATAATTATTTTTTTTTTTTAAACAATTTAATATTATTTCTATAACTACATGTATATTACCT
Candidatus Carsonella 135557 - 167442nt                   (1)     -----------------------------------------------------------------------------
SARS-CoV-2 Reference Genome NC_045512.2                   (1)     -----------------------------------------------------------------------------
```

Section 354

```
                                                          (27182) 27182     27192      27203      27214      27225      27236      27247      27258
Candidatus Carsonella ruddii strain BT chromosome...      (27182) ATTAATTCTATAAATAAATTTTTATTTTTAAATAATTTTTCATATTTATTTTTATTAAATTTATTTAAGCAATTAAA
Candidatus Carsonella 135557 - 167442nt                   (1)     -----------------------------------------------------------------------------
SARS-CoV-2 Reference Genome NC_045512.2                   (1)     -----------------------------------------------------------------------------
```

Section 355

	(27259)	27259 27269 27280 27291 27302 27313 27324 27335
Candidatus Carsonella ruddii strain BT chromosome...	(27259)	TCTATTATTTTTTGAAAAAGTTAAAATTTTATTTTTTTTATTTAATATATTAAAAACATTATTTCCTACATTTCCTA
Candidatus Carsonella 135557 - 167442nt	(1)	---
SARS-CoV-2 Reference Genome NC_045512.2	(1)	---

Section 356

	(27336)	27336 27346 27357 27368 27379 27390 27401 27412
Candidatus Carsonella ruddii strain BT chromosome...	(27336)	AACCAAAAATTGAAAAATCCATAATTAAATATTTTTTAATGAAATTAAATTTGGATTTTTAATAAAAAAAATTTTAT
Candidatus Carsonella 135557 - 167442nt	(1)	---
SARS-CoV-2 Reference Genome NC_045512.2	(1)	---

Section 357

	(27413)	27413 27423 27434 27445 27456 27467 27478 27489
Candidatus Carsonella ruddii strain BT chromosome...	(27413)	TTAATTTTATTCCTTGTATTTTTTTTTCTACATTAAAAGTAAATTTTTTAATTTTAATAATTTTACCAAAAAAAATA
Candidatus Carsonella 135557 - 167442nt	(1)	---
SARS-CoV-2 Reference Genome NC_045512.2	(1)	---

Section 358

	(27490)	27490 27500 27511 27522 27533 27544 27555 27566
Candidatus Carsonella ruddii strain BT chromosome...	(27490)	GAAATTTTATTATTAACTAAATAAGAAACTGTAATATTATTATTTTTTTTAAAAAATTTCATATATTGAAAAATTTT
Candidatus Carsonella 135557 - 167442nt	(1)	---
SARS-CoV-2 Reference Genome NC_045512.2	(1)	---

Section 359

	(27567)	27567 27577 27588 27599 27610 27621 27632 27643
Candidatus Carsonella ruddii strain BT chromosome...	(27567)	TTTTTAAAAGTGGAAATCTAATTATTATTTTTTTTTTAAAAACAATATAATTATTAGGAATAAAATATTTAAAATTG
Candidatus Carsonella 135557 - 167442nt	(1)	---
SARS-CoV-2 Reference Genome NC_045512.2	(1)	---

Section 360

	(27644)	27644 27654 27665 27676 27687 27698 27709 27720
Candidatus Carsonella ruddii strain BT chromosome...	(27644)	AAATTAATTATTAAAAAACATTTCTTTGTGTTAAAAATTTTAAATATAAAATATTTTTTATTAAAATAACAATTTGT
Candidatus Carsonella 135557 - 167442nt	(1)	---
SARS-CoV-2 Reference Genome NC_045512.2	(1)	---

Section 361

	(27721)	27721 27731 27742 27753 27764 27775 27786 27797
Candidatus Carsonella ruddii strain BT chromosome...	(27721)	TATTAGTTTCATATTTTTAAAATTTCAAATAATCTTTTACTTAATTTAGCTCCTTTTTTAATATAAAAAAATAACAA
Candidatus Carsonella 135557 - 167442nt	(1)	---
SARS-CoV-2 Reference Genome NC_045512.2	(1)	---

Section 362

	(27798)	27798 27808 27819 27830 27841 27852 27863 27874
Candidatus Carsonella ruddii strain BT chromosome...	(27798)	ATTATAATTAACAAAAAAACTTTTTCCATTATTTATATTAGAATTAAAAAAACCAATTCTTTTAATAATTTTTCCAA
Candidatus Carsonella 135557 - 167442nt	(1)	---
SARS-CoV-2 Reference Genome NC_045512.2	(1)	---

Section 363

	(27875)	27875 27885 27896 27907 27918 27929 27940 27951
Candidatus Carsonella ruddii strain BT chromosome...	(27875)	AAACAGAATTTTTTCTATAAATAACATTAATATAAAACAAATTTTCTAAGTTATTTTTTTTTTGCAATCTGACAACA
Candidatus Carsonella 135557 - 167442nt	(1)	---
SARS-CoV-2 Reference Genome NC_045512.2	(1)	---

Section 364

	(27952)	27952 27962 27973 27984 27995 28006 28017 28028
Candidatus Carsonella ruddii strain BT chromosome...	(27952)	ATCATTTTAAAATACATTTAAAAAAAAAAAATATTTCAACATAATCTTAATGTTTGCGGGGTAGAGCAGTTGGTAGCT
Candidatus Carsonella 135557 - 167442nt	(1)	---
SARS-CoV-2 Reference Genome NC_045512.2	(1)	---

Section 365

	(28029)	28029 28039 28050 28061 28072 28083 28094 28105
Candidatus Carsonella ruddii strain BT chromosome...	(28029)	CGTCGGGCTCATAATCCGAAGGACGTTGGTTCAAATCCAATCCCCGCAATTTTTAATTAATTAATTATTTTTTTATG
Candidatus Carsonella 135557 - 167442nt	(1)	---
SARS-CoV-2 Reference Genome NC_045512.2	(1)	---

Section 366

	(28106)	28106 28116 28127 28138 28149 28160 28171 28182
Candidatus Carsonella ruddii strain BT chromosome...	(28106)	TTTCCAATTAAACATTTTTCTTATATTATCTCCAACATTTTCTATTAAATGATTTTTATAATAATCTCTAAATAAAT
Candidatus Carsonella 135557 - 167442nt	(1)	---
SARS-CoV-2 Reference Genome NC_045512.2	(1)	---

Section 367

		28183 28193 28204 28215 28226 28237 28248 28259
	(28183)	
Candidatus Carsonella ruddii strain BT chromosome...	(28183)	TTAATTTAATAAAATTTGATTTTTTATCAAGAATAAAATTTTTAGAAAAATTACCATTTTGAATATTTTTTAATATT
Candidatus Carsonella 135557 - 167442nt	(1)	---
SARS-CoV-2 Reference Genome NC_045512.2	(1)	---

Section 368

		28260 28270 28281 28292 28303 28314 28325 28336
	(28260)	
Candidatus Carsonella ruddii strain BT chromosome...	(28260)	TTTTTCATTTTTTTTTTTAAAATATTATAATAATATTCGTTCATAACATAATAATCACCATATTCTGCAGTATTAGA
Candidatus Carsonella 135557 - 167442nt	(1)	---
SARS-CoV-2 Reference Genome NC_045512.2	(1)	---

Section 369

		28337 28347 28358 28369 28380 28391 28402 28413
	(28337)	
Candidatus Carsonella ruddii strain BT chromosome...	(28337)	TATTGAATATCTCATATTTTTAATACCTCCTTCATATATTAAATCAACAATTAATTTCATTTCATGTAAACATTCAA
Candidatus Carsonella 135557 - 167442nt	(1)	---
SARS-CoV-2 Reference Genome NC_045512.2	(1)	---

Section 370

		28414 28424 28435 28446 28457 28468 28479 28490
	(28414)	
Candidatus Carsonella ruddii strain BT chromosome...	(28414)	AATATGAAAGATCTGGTGAATATCCAGCTTCTACTAAAGTTTCAAATCCACAAATTATAAGAGAACTTACACCTCCA
Candidatus Carsonella 135557 - 167442nt	(1)	---
SARS-CoV-2 Reference Genome NC_045512.2	(1)	---

Section 371

		28491 28501 28512 28523 28534 28545 28556 28567
	(28491)	
Candidatus Carsonella ruddii strain BT chromosome...	(28491)	CATAAAACTGATTGTTCTCCAAATAAATCAGTTTCAGTTTCTTCTTTAAAACTTGTAGAAATTATTCCAACCCTACC
Candidatus Carsonella 135557 - 167442nt	(1)	---
SARS-CoV-2 Reference Genome NC_045512.2	(1)	---

Section 372

		28568 28578 28589 28600 28611 28622 28633 28644
	(28568)	
Candidatus Carsonella ruddii strain BT chromosome...	(28568)	ACTACCTATACACATTGAATAAGATAATGCAATATTTAATGATTCTTTATTATAATCTTGATAAATAGCTATTAAAG
Candidatus Carsonella 135557 - 167442nt	(1)	---
SARS-CoV-2 Reference Genome NC_045512.2	(1)	---

```
                                                                                         Section 373
                                                          (28645) 28645     28655      28666      28677      28688      28699      28710      28721
Candidatus Carsonella ruddii strain BT chromosome...      (28645) AAGGAACTCCACTTCCTTCACAAAATGTTGATCTTACTGTATGACCAGGAGCTTTAGGAGCAACTAAAATAATATTT
        Candidatus Carsonella 135557 - 167442nt               (1) -----------------------------------------------------------------------------
     SARS-CoV-2 Reference Genome NC_045512.2                  (1) -----------------------------------------------------------------------------
                                                                                         Section 374
                                                          (28722) 28722     28732      28743      28754      28765      28776      28787      28798
Candidatus Carsonella ruddii strain BT chromosome...      (28722) AAATTATTTTTTGGAATTATTTGTTTATAATGTATATTAAAACCATGGGAAAAAATTAACGTTGTTCCCTCTTTAAT
        Candidatus Carsonella 135557 - 167442nt               (1) -----------------------------------------------------------------------------
     SARS-CoV-2 Reference Genome NC_045512.2                  (1) -----------------------------------------------------------------------------
                                                                                         Section 375
                                                          (28799) 28799     28809      28820      28831      28842      28853      28864      28875
Candidatus Carsonella ruddii strain BT chromosome...      (28799) ATTTTTTTCTATATAATCATAATATAAATCTCTTTGATCTTCATCAGGAATTAAAATAACTAATATATCAGATTTTA
        Candidatus Carsonella 135557 - 167442nt               (1) -----------------------------------------------------------------------------
     SARS-CoV-2 Reference Genome NC_045512.2                  (1) -----------------------------------------------------------------------------
                                                                                         Section 376
                                                          (28876) 28876     28886      28897      28908      28919      28930      28941      28952
Candidatus Carsonella ruddii strain BT chromosome...      (28876) ATAAAGCTTCCTTAATATCAATTACTTTAAAACCGTCTTTTTTTGCTTTTTCTATTTTTTTAGAATTATTTTTTAAA
        Candidatus Carsonella 135557 - 167442nt               (1) -----------------------------------------------------------------------------
     SARS-CoV-2 Reference Genome NC_045512.2                  (1) -----------------------------------------------------------------------------
                                                                                         Section 377
                                                          (28953) 28953     28963      28974      28985      28996      29007      29018      29029
Candidatus Carsonella ruddii strain BT chromosome...      (28953) CAAACATATGTTTCAATACCTGAATCTTTTAAATTTTGAGCTTGTGCATGACCTTGAGAACCATATCCTATTACTGT
        Candidatus Carsonella 135557 - 167442nt               (1) -----------------------------------------------------------------------------
     SARS-CoV-2 Reference Genome NC_045512.2                  (1) -----------------------------------------------------------------------------
                                                                                         Section 378
                                                          (29030) 29030     29040      29051      29062      29073      29084      29095      29106
Candidatus Carsonella ruddii strain BT chromosome...      (29030) TATAATTTTATTTTTTAATAATAATTTATTACAATCTTTTTCGTAAAACATAATTTTATAATATATTTTTTATAATA
        Candidatus Carsonella 135557 - 167442nt               (1) -----------------------------------------------------------------------------
     SARS-CoV-2 Reference Genome NC_045512.2                  (1) -----------------------------------------------------------------------------
```

Section	Sequence	Start	Alignment
Section 379		(29107)	29107 29117 29128 29139 29150 29161 29172 29183
	Candidatus Carsonella ruddii strain BT chromosome...	(29107)	GAGAAAATTATTCAATATACTAAAAACATAATTTATTTTTTTTGAATTAAAAAAAAAATAAATAATTTTTAAAAAAT
	Candidatus Carsonella 135557 - 167442nt	(1)	---
	SARS-CoV-2 Reference Genome NC_045512.2	(1)	---
Section 380		(29184)	29184 29194 29205 29216 29227 29238 29249 29260
	Candidatus Carsonella ruddii strain BT chromosome...	(29184)	TTACAAACATAAAATTTTTATAAAATTTAATTTTAATTTTATTAAAAATATTATTATAAATAATAATTTTAATATTT
	Candidatus Carsonella 135557 - 167442nt	(1)	---
	SARS-CoV-2 Reference Genome NC_045512.2	(1)	---
Section 381		(29261)	29261 29271 29282 29293 29304 29315 29326 29337
	Candidatus Carsonella ruddii strain BT chromosome...	(29261)	TTAACAAAATAAATTTTATTTATATTTAAACATTTTGATAAAGATTTTAAAAATTGATTAATTTTAAAATATGAATC
	Candidatus Carsonella 135557 - 167442nt	(1)	---
	SARS-CoV-2 Reference Genome NC_045512.2	(1)	---
Section 382		(29338)	29338 29348 29359 29370 29381 29392 29403 29414
	Candidatus Carsonella ruddii strain BT chromosome...	(29338)	ATTAATAAAAATAATTTTTAAATGATTTTTAATATTGCTTTTAATTTTAAATAATTCTATTTTTAAATTAATTTGAG
	Candidatus Carsonella 135557 - 167442nt	(1)	---
	SARS-CoV-2 Reference Genome NC_045512.2	(1)	---
Section 383		(29415)	29415 29425 29436 29447 29458 29469 29480 29491
	Candidatus Carsonella ruddii strain BT chromosome...	(29415)	TTAAAAAATTATAAAAATTACTAACACAATTAATTTTATTATGAATTAAAAAAATAAATTTTTTTTTCATATTTTTT
	Candidatus Carsonella 135557 - 167442nt	(1)	---
	SARS-CoV-2 Reference Genome NC_045512.2	(1)	---
Section 384		(29492)	29492 29502 29513 29524 29535 29546 29557 29568
	Candidatus Carsonella ruddii strain BT chromosome...	(29492)	TATTGAAAAAAAAGTAATTAATTCACACATAGATTGATTATAAATTTGTATAGGAAATACATTTTCTTTATGATTAA
	Candidatus Carsonella 135557 - 167442nt	(1)	---
	SARS-CoV-2 Reference Genome NC_045512.2	(1)	---

Section 385

```
                                                       (29569) 29569     29579      29590      29601      29612      29623      29634  29645
Candidatus Carsonella ruddii strain BT chromosome...   (29569) TATAAATATTTATAAAACAAAATATATCTACAAATATTAAATTTATAAAAAAAAAATAAAATTTTTTAATATTATAA
            Candidatus Carsonella 135557 - 167442nt        (1) -----------------------------------------------------------------------------
         SARS-CoV-2 Reference Genome NC_045512.2           (1) -----------------------------------------------------------------------------
```

Section 386

```
                                                       (29646) 29646     29656      29667      29678      29689      29700      29711  29722
Candidatus Carsonella ruddii strain BT chromosome...   (29646) AAATTAAAAGAATATAAATTATAAGAATTAATTAATTTTTTAAAATTTGGAAGAGAATAAATATATGAATGTGAAAA
            Candidatus Carsonella 135557 - 167442nt        (1) -----------------------------------------------------------------------------
         SARS-CoV-2 Reference Genome NC_045512.2           (1) -----------------------------------------------------------------------------
```

Section 387

```
                                                       (29723) 29723     29733      29744      29755      29766      29777      29788  29799
Candidatus Carsonella ruddii strain BT chromosome...   (29723) TCTATTATTATAGTTTAATTCTTGCCATTGTTTAACCATACCTAAACTTTGATTATTTAAATTAACGATAATAATGT
            Candidatus Carsonella 135557 - 167442nt        (1) -----------------------------------------------------------------------------
         SARS-CoV-2 Reference Genome NC_045512.2           (1) -----------------------------------------------------------------------------
```

Section 388

```
                                                       (29800) 29800     29810      29821      29832      29843      29854      29865  29876
Candidatus Carsonella ruddii strain BT chromosome...   (29800) TTATTTTATATTGTTTACAAGTTGATAATTCTTGCATCATCATTTGAAAACTACCTTCTCCAGTTATTAATAATACT
            Candidatus Carsonella 135557 - 167442nt        (1) -----------------------------------------------------------------------------
         SARS-CoV-2 Reference Genome NC_045512.2           (1) -----------------------------------------------------------------------------
```

Section 389

```
                                                       (29877) 29877     29887      29898      29909      29920      29931      29942  29953
Candidatus Carsonella ruddii strain BT chromosome...   (29877) TTTTTATTTTTATTTGCAAATTTTATTCCTATTGCAGAAGGAAAACCAAACCCCATTGTTCCTAATCCTCCTGATGA
            Candidatus Carsonella 135557 - 167442nt        (1) -----------------------------------------------------------------------------
         SARS-CoV-2 Reference Genome NC_045512.2           (1) -----------------------------------------------------------------------------
```

Section 390

```
                                                       (29954) 29954     29964      29975      29986      29997      30008      30019  30030
Candidatus Carsonella ruddii strain BT chromosome...   (29954) AATAAATTTTTTGTAATTAAAATAATAATATTTTGCTGCAAACATTTGATGTTGTCCTACATCAGTAGTTAAAAAAA
            Candidatus Carsonella 135557 - 167442nt        (1) -----------------------------------------------------------------------------
         SARS-CoV-2 Reference Genome NC_045512.2           (1) -----------------------------------------------------------------------------
```

```
                                                                                                                Section 391
                                                        (30031) 30031     30041      30052      30063      30074      30085      30096   30107
Candidatus Carsonella ruddii strain BT chromosome...    (30031) ACTTTCCTTTACTAATAATAAAAATTAATTCTATTATTTGTTGAGGCTTACAAAAAAAAAAATCATTTTCATATTTT
            Candidatus Carsonella 135557 - 167442nt         (1) -----------------------------------------------------------------------------
         SARS-CoV-2 Reference Genome NC_045512.2            (1) -----------------------------------------------------------------------------
                                                                                                                Section 392
                                                        (30108) 30108     30118      30129      30140      30151      30162      30173   30184
Candidatus Carsonella ruddii strain BT chromosome...    (30108) TTATTAAAAAATTTTTTAAAAAAAATTATTATTTTCCACCAAAATTTTATATTTTTGTTAAAAAAATTATTTTTTAA
            Candidatus Carsonella 135557 - 167442nt         (1) -----------------------------------------------------------------------------
         SARS-CoV-2 Reference Genome NC_045512.2            (1) -----------------------------------------------------------------------------
                                                                                                                Section 393
                                                        (30185) 30185     30195      30206      30217      30228      30239      30250   30261
Candidatus Carsonella ruddii strain BT chromosome...    (30185) AAAAAATTCAATATCACATAAAAAAAATTTTTCACAATAAATATTTTTAGAAATTGAATTTAAATCAATATCAATAT
            Candidatus Carsonella 135557 - 167442nt         (1) -----------------------------------------------------------------------------
         SARS-CoV-2 Reference Genome NC_045512.2            (1) -----------------------------------------------------------------------------
                                                                                                                Section 394
                                                        (30262) 30262     30272      30283      30294      30305      30316      30327   30338
Candidatus Carsonella ruddii strain BT chromosome...    (30262) GAATAATACTTGAATATGGAGAAAATTTTTCATTATTATTTATTATTCTATCATCTAATCTAGTTCCTAAACAAATA
            Candidatus Carsonella 135557 - 167442nt         (1) -----------------------------------------------------------------------------
         SARS-CoV-2 Reference Genome NC_045512.2            (1) -----------------------------------------------------------------------------
                                                                                                                Section 395
                                                        (30339) 30339     30349      30360      30371      30382      30393      30404   30415
Candidatus Carsonella ruddii strain BT chromosome...    (30339) ATAATATCAGAATAATGAATTATATTATTAACACATGCATCGCCATGCATACCTAACCAACCTAAATAATACGGATT
            Candidatus Carsonella 135557 - 167442nt         (1) -----------------------------------------------------------------------------
         SARS-CoV-2 Reference Genome NC_045512.2            (1) -----------------------------------------------------------------------------
                                                                                                                Section 396
                                                        (30416) 30416     30426      30437      30448      30459      30470      30481   30492
Candidatus Carsonella ruddii strain BT chromosome...    (30416) TCTGTAATTAAAACCTCCTAAACCCATAAAAGTTGATATAAAAGGTATTTTATTTTTTTTAATAAAAACATCTAATA
            Candidatus Carsonella 135557 - 167442nt         (1) -----------------------------------------------------------------------------
         SARS-CoV-2 Reference Genome NC_045512.2            (1) -----------------------------------------------------------------------------
```

```
                                                                                  Section 397
                                                  (30493) 30493     30503      30514      30525      30536      30547      30558     30569
Candidatus Carsonella ruddii strain BT chromosome... (30493) ATTTTATATAATTTTTTTTTAACAAACCTCCTCCAATTAAAATAATAGGTCTTTTATATTTTAAATTAATAAAAAAA
           Candidatus Carsonella 135557 - 167442nt       (1) -----------------------------------------------------------------------------
       SARS-CoV-2 Reference Genome NC_045512.2           (1) -----------------------------------------------------------------------------
```

```
                                                                                  Section 398
                                                  (30570) 30570     30580      30591      30602      30613      30624      30635     30646
Candidatus Carsonella ruddii strain BT chromosome... (30570) ATATTTTTATTTTTATTTTTTTTTATTTTTATAAATGGATATTTATTTCTTAATATATTTAAAGAAGAAGTTAGATC
           Candidatus Carsonella 135557 - 167442nt       (1) -----------------------------------------------------------------------------
       SARS-CoV-2 Reference Genome NC_045512.2           (1) -----------------------------------------------------------------------------
```

```
                                                                                  Section 399
                                                  (30647) 30647     30657      30668      30679      30690      30701      30712     30723
Candidatus Carsonella ruddii strain BT chromosome... (30647) TTTTGGAAAATCTATTACTACTGGTCCTTTTTTAAAACAATCATTTTTATAAAATGATTTTGAAATAATATATTGAA
           Candidatus Carsonella 135557 - 167442nt       (1) -----------------------------------------------------------------------------
       SARS-CoV-2 Reference Genome NC_045512.2           (1) -----------------------------------------------------------------------------
```

```
                                                                                  Section 400
                                                  (30724) 30724     30734      30745      30756      30767      30778      30789     30800
Candidatus Carsonella ruddii strain BT chromosome... (30724) TATTATAATAACAATTTAAAGAATAAAATTTTTTTATTACAGAATTAGATAAATTTAAGTTATCTAATTCTTGAAAA
           Candidatus Carsonella 135557 - 167442nt       (1) -----------------------------------------------------------------------------
       SARS-CoV-2 Reference Genome NC_045512.2           (1) -----------------------------------------------------------------------------
```

```
                                                                                  Section 401
                                                  (30801) 30801     30811      30822      30833      30844      30855      30866     30877
Candidatus Carsonella ruddii strain BT chromosome... (30801) GAATTTTGAGCTATCATTTTTTTTAATTACTTGCCCACATAAAATAATAATTGAAGAATTATCAAAATTAGCTGTTGC
           Candidatus Carsonella 135557 - 167442nt       (1) -----------------------------------------------------------------------------
       SARS-CoV-2 Reference Genome NC_045512.2           (1) -----------------------------------------------------------------------------
```

```
                                                                                  Section 402
                                                  (30878) 30878     30888      30899      30910      30921      30932      30943     30954
Candidatus Carsonella ruddii strain BT chromosome... (30878) TAATCCAGTAATACAATTTGTAAATCCAGGTCCAGAAGTTACTAAAACTACACCTGTTAAATTTGATGATCTTGAAT
           Candidatus Carsonella 135557 - 167442nt       (1) -----------------------------------------------------------------------------
       SARS-CoV-2 Reference Genome NC_045512.2           (1) -----------------------------------------------------------------------------
```

Section 403

```
                                                   (30955) 30955     30965      30976      30987      30998      31009      31020      31031
Candidatus Carsonella ruddii strain BT chromosome... (30955) ATCCATCAGCCATGTGTACTGCAGCTTGTTCGTGTCTTACTAATATATGTTTTATTTTTGATGTAAATATTGCATCA
            Candidatus Carsonella 135557 - 167442nt (1) -----------------------------------------------------------------------------
         SARS-CoV-2 Reference Genome NC_045512.2 (1) -----------------------------------------------------------------------------
```

Section 404

```
                                                   (31032) 31032     31042      31053      31064      31075      31086      31097      31108
Candidatus Carsonella ruddii strain BT chromosome... (31032) TAAATATGTAAAACTGCACCTCCAGGATATCCGAAAACAAATTCAATATTTTCTAACAATAATGATTCTATTATTAT
            Candidatus Carsonella 135557 - 167442nt (1) -----------------------------------------------------------------------------
         SARS-CoV-2 Reference Genome NC_045512.2 (1) -----------------------------------------------------------------------------
```

Section 405

```
                                                   (31109) 31109     31119      31130      31141      31152      31163      31174      31185
Candidatus Carsonella ruddii strain BT chromosome... (31109) TTTTGAAGATGAAATCTTCATTTTAAAATATTTATTAAAAAGATTTTTTTCATTTCTGTTGGTATATTACAATATAA
            Candidatus Carsonella 135557 - 167442nt (1) -----------------------------------------------------------------------------
         SARS-CoV-2 Reference Genome NC_045512.2 (1) -----------------------------------------------------------------------------
```

Section 406

```
                                                   (31186) 31186     31196      31207      31218      31229      31240      31251      31262
Candidatus Carsonella ruddii strain BT chromosome... (31186) AAAAAAATTTTTCATAATTAAAGGATAAAAAAAAGAAATTTTTTTATGATGCAATGTATTTATTAATTTTAAGTTTT
            Candidatus Carsonella 135557 - 167442nt (1) -----------------------------------------------------------------------------
         SARS-CoV-2 Reference Genome NC_045512.2 (1) -----------------------------------------------------------------------------
```

Section 407

```
                                                   (31263) 31263     31273      31284      31295      31306      31317      31328      31339
Candidatus Carsonella ruddii strain BT chromosome... (31263) TGTTATAATAAAATTCATTTTTTATTATTATATTAGAAAAATTTAAATGTTTTCTAATTTGATGAGTTCTTCCAGTT
            Candidatus Carsonella 135557 - 167442nt (1) -----------------------------------------------------------------------------
         SARS-CoV-2 Reference Genome NC_045512.2 (1) -----------------------------------------------------------------------------
```

Section 408

```
                                                   (31340) 31340     31350      31361      31372      31383      31394      31405      31416
Candidatus Carsonella ruddii strain BT chromosome... (31340) TTTATATAAATATTATAAATATTGTTTTTATATGAATTTTTTATTTTTTTATAAAAAGTAATACTTTTTTTAAAAAA
            Candidatus Carsonella 135557 - 167442nt (1) -----------------------------------------------------------------------------
         SARS-CoV-2 Reference Genome NC_045512.2 (1) -----------------------------------------------------------------------------
```

Section 409

```
                                              (31417) 31417     31427      31438      31449      31460      31471      31482      31493
Candidatus Carsonella ruddii strain BT chromosome... (31417) TTTTTTTATTAAAATTTTACGTTTTTTATAAATTGATAAATTAATAAAACCACTATTATTATTTTTTTCTACTATTG
          Candidatus Carsonella 135557 - 167442nt     (1) -----------------------------------------------------------------------------
       SARS-CoV-2 Reference Genome NC_045512.2     (1) -----------------------------------------------------------------------------
```

Section 410

```
                                              (31494) 31494     31504      31515      31526      31537      31548      31559      31570
Candidatus Carsonella ruddii strain BT chromosome... (31494) TTATATAATTTTTTTTAATAATTTTTTTTAAAATAATTTTTTTATAAAAAAAATAAAACATTAAATTTCTTGCTATT
          Candidatus Carsonella 135557 - 167442nt     (1) -----------------------------------------------------------------------------
       SARS-CoV-2 Reference Genome NC_045512.2     (1) -----------------------------------------------------------------------------
```

Section 411

```
                                              (31571) 31571     31581      31592      31603      31614      31625      31636      31647
Candidatus Carsonella ruddii strain BT chromosome... (31571) AAAATTATACCAGATGTATATTTATCTAATCTATTTAAAATTCCTTTATTTGGTAAATTATTTTTTATTTTATTTTT
          Candidatus Carsonella 135557 - 167442nt     (1) -----------------------------------------------------------------------------
       SARS-CoV-2 Reference Genome NC_045512.2     (1) -----------------------------------------------------------------------------
```

Section 412

```
                                              (31648) 31648     31658      31669      31680      31691      31702      31713      31724
Candidatus Carsonella ruddii strain BT chromosome... (31648) TAACAAACTAAATATTTCTACACCTACTGGTTTATTTATAATAATTATATTCTTGTCATAATATTCAATTTTTATTT
          Candidatus Carsonella 135557 - 167442nt     (1) -----------------------------------------------------------------------------
       SARS-CoV-2 Reference Genome NC_045512.2     (1) -----------------------------------------------------------------------------
```

Section 413

```
                                              (31725) 31725     31735      31746      31757      31768      31779      31790      31801
Candidatus Carsonella ruddii strain BT chromosome... (31725) TATGCATAAAATCTAATTTCTTCTAAATTATGAAATTTATTTATATAACAAATATTACAAATATTTTTATTATATAT
          Candidatus Carsonella 135557 - 167442nt     (1) -----------------------------------------------------------------------------
       SARS-CoV-2 Reference Genome NC_045512.2     (1) -----------------------------------------------------------------------------
```

Section 414

```
                                              (31802) 31802     31812      31823      31834      31845      31856      31867      31878
Candidatus Carsonella ruddii strain BT chromosome... (31802) ATGTTTTTTAATATTTTTATAATCTTTTGAAATTAAACAAAATAATTTTTTTTTTATTTTGTAAAAATAATATTTAT
          Candidatus Carsonella 135557 - 167442nt     (1) -----------------------------------------------------------------------------
       SARS-CoV-2 Reference Genome NC_045512.2     (1) -----------------------------------------------------------------------------
```

Section 415

	(31879)	31879 31889 31900 31911 31922 31933 31944 31955
Candidatus Carsonella ruddii strain BT chromosome...	(31879)	AATTATACCAATTCCAAAAAAAATTATTAAAAAAAAATTTCTTTTAAAAAAAATATTAAAAAAATTCATTTTTAATTTTA
Candidatus Carsonella 135557 - 167442nt	(1)	---
SARS-CoV-2 Reference Genome NC_045512.2	(1)	---

Section 416

	(31956)	31956 31966 31977 31988 31999 32010 32021 32032
Candidatus Carsonella ruddii strain BT chromosome...	(31956)	TTTAATAAATAAAAATTTTTAAAAAAAAATAATATCTTTTTTTAATAATATTTTAATTTTTAAAAAAATAGAAAAAAA
Candidatus Carsonella 135557 - 167442nt	(1)	---
SARS-CoV-2 Reference Genome NC_045512.2	(1)	---

Section 417

	(32033)	32033 32043 32054 32065 32076 32087 32098 32109
Candidatus Carsonella ruddii strain BT chromosome...	(32033)	AACATTATTGTTTGCTATTGTTTTGTAACTTTTTTTAAAAAAAAAATTTAATTCTTTTGAAGAATAATATAAAATAG
Candidatus Carsonella 135557 - 167442nt	(1)	---
SARS-CoV-2 Reference Genome NC_045512.2	(1)	---

Section 418

	(32110)	32110 32120 32131 32142 32153 32164 32175 32186
Candidatus Carsonella ruddii strain BT chromosome...	(32110)	GAAATAAATTTGCTTTTAAAAAGTTTAAAATAAAATATAATGAAAATAAACAACTATTTCTTATTAAAGAATTAATT
Candidatus Carsonella 135557 - 167442nt	(1)	---
SARS-CoV-2 Reference Genome NC_045512.2	(1)	---

Section 419

	(32187)	32187 32197 32208 32219 32230 32241 32252 32263
Candidatus Carsonella ruddii strain BT chromosome...	(32187)	TTGCATATATATAAATTATATTTAATATAATTAATATATAAATCATTTATAAAAGTAATATATAATTTTATTATTTT
Candidatus Carsonella 135557 - 167442nt	(1)	---
SARS-CoV-2 Reference Genome NC_045512.2	(1)	---

Section 420

	(32264)	32264 32274 32285 32296 32307 32318 32329 32340
Candidatus Carsonella ruddii strain BT chromosome...	(32264)	AATAACATTGAAAAAATTAATATTTAAAAATAATTTTTCAATTATTTTGTTTAAATATTTTGTTTTTTTAATAATCC
Candidatus Carsonella 135557 - 167442nt	(1)	---
SARS-CoV-2 Reference Genome NC_045512.2	(1)	---

Section 421

```
                                                          (32341) 32341     32351      32362      32373      32384      32395      32406      32417
Candidatus Carsonella ruddii strain BT chromosome...     (32341) AATAATCAAAAAATATTTTTTTAGTATTATAAAAATTAAATTTGTAAAAATTATTTAAAATAAATTTAAAAAAATTT
           Candidatus Carsonella 135557 - 167442nt           (1) -----------------------------------------------------------------------------
        SARS-CoV-2 Reference Genome NC_045512.2              (1) -----------------------------------------------------------------------------
```

Section 422

```
                                                          (32418) 32418     32428      32439      32450      32461      32472      32483      32494
Candidatus Carsonella ruddii strain BT chromosome...     (32418) CTTATTAATTTATAAGTAAAATTTGTATTTTCAAAATTAACAATATTAAAAATTATATTTTTAAAATAATCATGTTC
           Candidatus Carsonella 135557 - 167442nt           (1) -----------------------------------------------------------------------------
        SARS-CoV-2 Reference Genome NC_045512.2              (1) -----------------------------------------------------------------------------
```

Section 423

```
                                                          (32495) 32495     32505      32516      32527      32538      32549      32560      32571
Candidatus Carsonella ruddii strain BT chromosome...     (32495) TAATAAATACAATTTAATAATTTCATTACTATTTATTTTTAATATATCATTTATTTTTTTAAAATTTTTAGAAGATT
           Candidatus Carsonella 135557 - 167442nt           (1) -----------------------------------------------------------------------------
        SARS-CoV-2 Reference Genome NC_045512.2              (1) -----------------------------------------------------------------------------
```

Section 424

```
                                                          (32572) 32572     32582      32593      32604      32615      32626      32637      32648
Candidatus Carsonella ruddii strain BT chromosome...     (32572) TTGATAATTTATTTCCTTTTATATCTATACAAAACCCATGAGAAATGATAATATTTAAATTTAAATTAAAATAAATT
           Candidatus Carsonella 135557 - 167442nt           (1) -----------------------------------------------------------------------------
        SARS-CoV-2 Reference Genome NC_045512.2              (1) -----------------------------------------------------------------------------
```

Section 425

```
                                                          (32649) 32649     32659      32670      32681      32692      32703      32714      32725
Candidatus Carsonella ruddii strain BT chromosome...     (32649) AAAAAATTTATAATTATACAAGATTGATACCAACCTCTAATTTGATCTTTACCTTCTACAATAATTTTATTATTTTT
           Candidatus Carsonella 135557 - 167442nt           (1) -----------------------------------------------------------------------------
        SARS-CoV-2 Reference Genome NC_045512.2              (1) -----------------------------------------------------------------------------
```

Section 426

```
                                                          (32726) 32726     32736      32747      32758      32769      32780      32791      32802
Candidatus Carsonella ruddii strain BT chromosome...     (32726) AAAAAATAAAAATAAAATAGATGAATCAAACCAAACGTCAAAAATATCTTTTTTAATTTTTTCTATAAAAAAAATAA
           Candidatus Carsonella 135557 - 167442nt           (1) -----------------------------------------------------------------------------
        SARS-CoV-2 Reference Genome NC_045512.2              (1) -----------------------------------------------------------------------------
```

Section 427

	(32803)	32803 32813 32824 32835 32846 32857 32868 32879
Candidatus Carsonella ruddii strain BT chromosome...	(32803)	TATGAGAACTAAATGTTTTGATTAAATTTTTATAAAAAGAAATTTTTTTATTTATTAATATAATAGGAACACCCCAA
Candidatus Carsonella 135557 - 167442nt	(1)	---
SARS-CoV-2 Reference Genome NC_045512.2	(1)	---

Section 428

	(32880)	32880 32890 32901 32912 32923 32934 32945 32956
Candidatus Carsonella ruddii strain BT chromosome...	(32880)	TATCTTTGTCTAGAAATACACCAATTATTTCTAAAATTAATCATATTAAATAAAGTTTTTTTAATAAAAAAAGGAAA
Candidatus Carsonella 135557 - 167442nt	(1)	---
SARS-CoV-2 Reference Genome NC_045512.2	(1)	---

Section 429

	(32957)	32957 32967 32978 32989 33000 33011 33022 33033
Candidatus Carsonella ruddii strain BT chromosome...	(32957)	AAAAGAAATTTTCTTCATTTTTTTAAGTAAAATTTTTTTTATATTAATGTTTTTAAATTTAAAATTTAAATCTATAA
Candidatus Carsonella 135557 - 167442nt	(1)	---
SARS-CoV-2 Reference Genome NC_045512.2	(1)	---

Section 430

	(33034)	33034 33044 33055 33066 33077 33088 33099 33110
Candidatus Carsonella ruddii strain BT chromosome...	(33034)	AAATTTGATTACTTAAATAATAAAAAATTGGTTTTTATGTCTCCAACAAAACATATAATTATGATTTATTTTTTTCA
Candidatus Carsonella 135557 - 167442nt	(1)	---
SARS-CoV-2 Reference Genome NC_045512.2	(1)	---

Section 431

	(33111)	33111 33121 33132 33143 33154 33165 33176 33187
Candidatus Carsonella ruddii strain BT chromosome...	(33111)	ATTTTTAAAATTATTTTTCTTTTTTTTAAAATTTAAAAATAATTTTATTAAAATAAAAAAATATTAATATTATTTAA
Candidatus Carsonella 135557 - 167442nt	(1)	---
SARS-CoV-2 Reference Genome NC_045512.2	(1)	---

Section 432

	(33188)	33188 33198 33209 33220 33231 33242 33253 33264
Candidatus Carsonella ruddii strain BT chromosome...	(33188)	AATTTTAATTAATTTAATAAAAAACTTTTTATTAAAAAAATTTATATTGTTTTTTTTATTATAAATTTCATAATCTT
Candidatus Carsonella 135557 - 167442nt	(1)	---
SARS-CoV-2 Reference Genome NC_045512.2	(1)	---

Section 433

	(33265)	33265 33275 33286 33297 33308 33319 33330 33341
Candidatus Carsonella ruddii strain BT chromosome...	(33265)	CTAATCCATTAGAAGGAGCACAATGAATAAAACCGCTTCCTATATTTATATCTATAAAATTATTATTAATAAAAAGA
Candidatus Carsonella 135557 - 167442nt	(1)	---
SARS-CoV-2 Reference Genome NC_045512.2	(1)	---

Section 434

	(33342)	33342 33352 33363 33374 33385 33396 33407 33418
Candidatus Carsonella ruddii strain BT chromosome...	(33342)	TTTTTTTTATTATAATATAAAAAAATATTTTTTAAAATAAAAAAATATTTTTTATTAAAAAAAAAATTTCCATTAAT
Candidatus Carsonella 135557 - 167442nt	(1)	---
SARS-CoV-2 Reference Genome NC_045512.2	(1)	---

Section 435

	(33419)	33419 33429 33440 33451 33462 33473 33484 33495
Candidatus Carsonella ruddii strain BT chromosome...	(33419)	AAAAGAAAAAATTTTTCCTTTTATTTTTAAAATTTTTATTATTTTATATATTAAAAAATTAGAAAAAATTAATTTGC
Candidatus Carsonella 135557 - 167442nt	(1)	---
SARS-CoV-2 Reference Genome NC_045512.2	(1)	---

Section 436

	(33496)	33496 33506 33517 33528 33539 33550 33561 33572
Candidatus Carsonella ruddii strain BT chromosome...	(33496)	TATTTTTAGTTTTAAAAATAACATAAATTTGATTTTTTAAAAAAAAAATAGCTTGATTATTAATAATAGACCATAAA
Candidatus Carsonella 135557 - 167442nt	(1)	---
SARS-CoV-2 Reference Genome NC_045512.2	(1)	---

Section 437

	(33573)	33573 33583 33594 33605 33616 33627 33638 33649
Candidatus Carsonella ruddii strain BT chromosome...	(33573)	GTTGTTGTCCATATAATTATTAAAAATTTTAATAATTTAATTTTTATATAAAAAGAATAAGAATTTATTTTTTTATA
Candidatus Carsonella 135557 - 167442nt	(1)	---
SARS-CoV-2 Reference Genome NC_045512.2	(1)	---

Section 438

	(33650)	33650 33660 33671 33682 33693 33704 33715 33726
Candidatus Carsonella ruddii strain BT chromosome...	(33650)	TAAAATTTCAGAATATGATAAAGAAGAACAACATTTAAAACACCATAAAATTGGTATTTTTTCTTTTTTTATTATTT
Candidatus Carsonella 135557 - 167442nt	(1)	---
SARS-CoV-2 Reference Genome NC_045512.2	(1)	---

Section 439

	(33727)	33727 33737 33748 33759 33770 33781 33792 33803
Candidatus Carsonella ruddii strain BT chromosome...	(33727)	TATTTAAATATAAATAATTAAATATTTTATATTGAAAAGATTCATATCTTGGTTGCATTGTATTGTATGAATAATAA
Candidatus Carsonella 135557 - 167442nt	(1)	---
SARS-CoV-2 Reference Genome NC_045512.2	(1)	---

Section 440

	(33804)	33804 33814 33825 33836 33847 33858 33869 33880
Candidatus Carsonella ruddii strain BT chromosome...	(33804)	TTATTAAAATAATTAAAATTAAAAAAATCTTTTTTTTGTTTATTAATTATATTACAAACAAATTTTCTACATTTAAT
Candidatus Carsonella 135557 - 167442nt	(1)	---
SARS-CoV-2 Reference Genome NC_045512.2	(1)	---

Section 441

	(33881)	33881 33891 33902 33913 33924 33935 33946 33957
Candidatus Carsonella ruddii strain BT chromosome...	(33881)	AAAAAAAAAATTATTTATATAATTTAATTGTTCAATAGGTAAACCATGACAATCCCATCCTAAATTTGATAACAATA
Candidatus Carsonella 135557 - 167442nt	(1)	---
SARS-CoV-2 Reference Genome NC_045512.2	(1)	---

Section 442

	(33958)	33958 33968 33979 33990 34001 34012 34023 34034
Candidatus Carsonella ruddii strain BT chromosome...	(33958)	AAAAATTTTTATTATAAAAAAATTTAATTAAAGAATCTTTTATAGTTTTGTTTAATATATGACCAATATGAATTTCG
Candidatus Carsonella 135557 - 167442nt	(1)	---
SARS-CoV-2 Reference Genome NC_045512.2	(1)	---

Section 443

	(34035)	34035 34045 34056 34067 34078 34089 34100 34111
Candidatus Carsonella ruddii strain BT chromosome...	(34035)	CCATTAGCAAAAGGTGGACCATCCAATAAAAAAACATTTATTACTTTATTATTTAAAATATAATGAATATTATTTTT
Candidatus Carsonella 135557 - 167442nt	(1)	---
SARS-CoV-2 Reference Genome NC_045512.2	(1)	---

Section 444

	(34112)	34112 34122 34133 34144 34155 34166 34177 34188
Candidatus Carsonella ruddii strain BT chromosome...	(34112)	AATATTTATTGTTTTTTTTAAATTTGCTTTCATTAAATATTTTTTTCTAGGCAAATTTAACATATTTAAAATATTTT
Candidatus Carsonella 135557 - 167442nt	(1)	---
SARS-CoV-2 Reference Genome NC_045512.2	(1)	---

Section 445

	(34189)	34189 34199 34210 34221 34232 34243 34254 34265
Candidatus Carsonella ruddii strain BT chromosome...	(34189)	GATTTGTATTTAGAAGATAATTTTTTATTATTTATTTTTTTATCGAAAAAAGAATAAATTTCATTTAAATTTTCAAT
Candidatus Carsonella 135557 - 167442nt	(1)	---
SARS-CoV-2 Reference Genome NC_045512.2	(1)	---

Section 446

	(34266)	34266 34276 34287 34298 34309 34320 34331 34342
Candidatus Carsonella ruddii strain BT chromosome...	(34266)	TTTTTTATTTTTTATTTTTCTTATAAATTTTTTTATTAACAATTTTTCCATAGTTTTAAAAAATGAAAGAATTTTTT
Candidatus Carsonella 135557 - 167442nt	(1)	---
SARS-CoV-2 Reference Genome NC_045512.2	(1)	---

Section 447

	(34343)	34343 34353 34364 34375 34386 34397 34408 34419
Candidatus Carsonella ruddii strain BT chromosome...	(34343)	ATAACCTAAATTAAAAACACTAGATACCATAAAATAAGGTGTTATTATTAAATTTATTTTTTTTATTTTACTAAAAA
Candidatus Carsonella 135557 - 167442nt	(1)	---
SARS-CoV-2 Reference Genome NC_045512.2	(1)	---

Section 448

	(34420)	34420 34430 34441 34452 34463 34474 34485 34496
Candidatus Carsonella ruddii strain BT chromosome...	(34420)	AAAAAACATTTTCAATTCCATCTATTATAATCCAATAATTTATATTATTTAAATTTTTAATATATAAAAAATATAAA
Candidatus Carsonella 135557 - 167442nt	(1)	---
SARS-CoV-2 Reference Genome NC_045512.2	(1)	---

Section 449

	(34497)	34497 34507 34518 34529 34540 34551 34562 34573
Candidatus Carsonella ruddii strain BT chromosome...	(34497)	AAAAATTTTATTAATTTAAAAAATATTTTAAAAAATAATAAATTTATATAAAAAATATAAATTTTAGAAATAAAATT
Candidatus Carsonella 135557 - 167442nt	(1)	---
SARS-CoV-2 Reference Genome NC_045512.2	(1)	---

Section 450

	(34574)	34574 34584 34595 34606 34617 34628 34639 34650
Candidatus Carsonella ruddii strain BT chromosome...	(34574)	ATTTTTATAATTAAAATTGTTTAATAAAAAATTTTCAGAAATATTAATATAACATTTATAATAAAAATATTTGAAAT
Candidatus Carsonella 135557 - 167442nt	(1)	---
SARS-CoV-2 Reference Genome NC_045512.2	(1)	---

Section 451

	(34651)	34651 34661 34672 34683 34694 34705 34716 34727
Candidatus Carsonella ruddii strain BT chromosome...	(34651)	TAATTTTTATAAAAATAATTTTACCTTGTTTTCCAAAATTAGCAATTTTTGAATTATAAAAATTTTTATAACAAAAA
Candidatus Carsonella 135557 - 167442nt	(1)	---
SARS-CoV-2 Reference Genome NC_045512.2	(1)	---

Section 452

	(34728)	34728 34738 34749 34760 34771 34782 34793 34804
Candidatus Carsonella ruddii strain BT chromosome...	(34728)	TTTCCATTACCTCCTTTTCCTCCTTTTAATATTTTAATAAAATAATTGTTTTTAACAATATAAATTTTTTTTTATT
Candidatus Carsonella 135557 - 167442nt	(1)	---
SARS-CoV-2 Reference Genome NC_045512.2	(1)	---

Section 453

	(34805)	34805 34815 34826 34837 34848 34859 34870 34881
Candidatus Carsonella ruddii strain BT chromosome...	(34805)	TAATGTTATTATTACACCTAAAGATATTTTTATTATTAAATCTTTACCTTTTTTTCCTTTTTTGTTATTTTTTTCAC
Candidatus Carsonella 135557 - 167442nt	(1)	---
SARS-CoV-2 Reference Genome NC_045512.2	(1)	---

Section 454

	(34882)	34882 34892 34903 34914 34925 34936 34947 34958
Candidatus Carsonella ruddii strain BT chromosome...	(34882)	CATTTTTACCATTTTTTGAAATAAATAAATTATTATTTGGTAATTTAAAATTATTATTAGATAATAAATATAAATCA
Candidatus Carsonella 135557 - 167442nt	(1)	---
SARS-CoV-2 Reference Genome NC_045512.2	(1)	---

Section 455

	(34959)	34959 34969 34980 34991 35002 35013 35024 35035
Candidatus Carsonella ruddii strain BT chromosome...	(34959)	CCACCATCACCACCATCACCTCCATTAGCATAAAATTTTCCATTTAAAAATTTATAACTAATACAACCATTTCCTCC
Candidatus Carsonella 135557 - 167442nt	(1)	---
SARS-CoV-2 Reference Genome NC_045512.2	(1)	---

Section 456

	(35036)	35036 35046 35057 35068 35079 35090 35101 35112
Candidatus Carsonella ruddii strain BT chromosome...	(35036)	TTTACCACTTTTTAAAAGTAAATATTTTTCAATAAACATTATTAACTTCTATAAAAGTTTATTTTTTTTTTTTTTTA
Candidatus Carsonella 135557 - 167442nt	(1)	---
SARS-CoV-2 Reference Genome NC_045512.2	(1)	---

Section 457

	(35113)	35113 35123 35134 35145 35156 35167 35178 35189
Candidatus Carsonella ruddii strain BT chromosome...	(35113)	ATTAATACTATTCCATTTATTAATGACTGTATATAATAATTTTTTGAATAAAAAGTATTTTTTCCTGAAATTATTTT
Candidatus Carsonella 135557 - 167442nt	(1)	---
SARS-CoV-2 Reference Genome NC_045512.2	(1)	---

Section 458

	(35190)	35190 35200 35211 35222 35233 35244 35255 35266
Candidatus Carsonella ruddii strain BT chromosome...	(35190)	AGAACCATTTTGTTTAATTATTATAGATCCTTTATTAACATAATTATTATTATAAATTTTTAATCCTAATCTTTTAG
Candidatus Carsonella 135557 - 167442nt	(1)	---
SARS-CoV-2 Reference Genome NC_045512.2	(1)	---

Section 459

	(35267)	35267 35277 35288 35299 35310 35321 35332 35343
Candidatus Carsonella ruddii strain BT chromosome...	(35267)	AAATAGAATCTCTTCCATTTCTCGTACTACCTCCAGCTTTTTTTTGTGCCATATTAAATTATATTTTGTAAAAATAA
Candidatus Carsonella 135557 - 167442nt	(1)	---
SARS-CoV-2 Reference Genome NC_045512.2	(1)	---

Section 460

	(35344)	35344 35354 35365 35376 35387 35398 35409 35420
Candidatus Carsonella ruddii strain BT chromosome...	(35344)	TAAACTTTTTTTTTTATAATTTATATTAGTTTTTAAAAAATTTTTTCTTCTTTTTTTTTTTATAGATATAGATTTTA
Candidatus Carsonella 135557 - 167442nt	(1)	---
SARS-CoV-2 Reference Genome NC_045512.2	(1)	---

Section 461

	(35421)	35421 35431 35442 35453 35464 35475 35486 35497
Candidatus Carsonella ruddii strain BT chromosome...	(35421)	TATAAAAATGTTTTAAAATTATAAAACATGCTTTTAAATTTAAACCTTTTTTATCTAAAATTATTTTTTTATTAAAA
Candidatus Carsonella 135557 - 167442nt	(1)	---
SARS-CoV-2 Reference Genome NC_045512.2	(1)	---

Section 462

	(35498)	35498 35508 35519 35530 35541 35552 35563 35574
Candidatus Carsonella ruddii strain BT chromosome...	(35498)	AAAAAAAATATTTTTTTAAAAAAAATTTTTTTACCAATATTTTTATTTATATAATCAACAATTAAAAAATTATTTAA
Candidatus Carsonella 135557 - 167442nt	(1)	---
SARS-CoV-2 Reference Genome NC_045512.2	(1)	---

Section 463		
	(35575)	35575 35585 35596 35607 35618 35629 35640 35651
Candidatus Carsonella ruddii strain BT chromosome...	(35575)	TTTTGAAAAATAAATTTTTTTACCTATACAAAAAGATAACAAATTATTTAATTTTATCATTTTTTAAAATATTATTA
Candidatus Carsonella 135557 - 167442nt	(1)	---
SARS-CoV-2 Reference Genome NC_045512.2	(1)	---

Section 464		
	(35652)	35652 35662 35673 35684 35695 35706 35717 35728
Candidatus Carsonella ruddii strain BT chromosome...	(35652)	TTATAATAATTAAAAACACCAAGTTCACCAATTAAAGAACAATCACCTTTTCTATATCCTATTTTTATTAAATAAAA
Candidatus Carsonella 135557 - 167442nt	(1)	---
SARS-CoV-2 Reference Genome NC_045512.2	(1)	---

Section 465		
	(35729)	35729 35739 35750 35761 35772 35783 35794 35805
Candidatus Carsonella ruddii strain BT chromosome...	(35729)	AAAAATTTTTTTTTTAAAATAATTATTTAAAAGAATAAAAATTTTAATTTTATTTTTTTTTAATTTAAAATAATTAC
Candidatus Carsonella 135557 - 167442nt	(1)	---
SARS-CoV-2 Reference Genome NC_045512.2	(1)	---

Section 466		
	(35806)	35806 35816 35827 35838 35849 35860 35871 35882
Candidatus Carsonella ruddii strain BT chromosome...	(35806)	TTTTAATTTTACCAAATTTTATTAAATTAAAAAAACAATTATTTATAAATTTTTTTTTAAAATTGTATTTCATATTC
Candidatus Carsonella 135557 - 167442nt	(1)	---
SARS-CoV-2 Reference Genome NC_045512.2	(1)	---

Section 467		
	(35883)	35883 35893 35904 35915 35926 35937 35948 35959
Candidatus Carsonella ruddii strain BT chromosome...	(35883)	AATTTTTACATTTAATTTTAATTTTTTGTTTTTTAAAGCATTTGATATTTCTAAAAATACAAATTTATTCATATTTT
Candidatus Carsonella 135557 - 167442nt	(1)	---
SARS-CoV-2 Reference Genome NC_045512.2	(1)	---

Section 468		
	(35960)	35960 35970 35981 35992 36003 36014 36025 36036
Candidatus Carsonella ruddii strain BT chromosome...	(35960)	TTAATTTAAATAAATCAAATTCTGATAATTTAATCAAGTCTCCTATTAAAAAAATGTTGTTTCTTTTTAATATATTT
Candidatus Carsonella 135557 - 167442nt	(1)	---
SARS-CoV-2 Reference Genome NC_045512.2	(1)	---

```
                                                                                                                    Section 469
                                                  (36037) 36037     36047      36058      36069      36080      36091      36102     36113
Candidatus Carsonella ruddii strain BT chromosome... (36037) GAAGTTTTTATACTTAATTCTAAATTATCTACTGATCTAATAAATATAGGATTAATACTTAAAAAATTTTTTTTTT
        Candidatus Carsonella 135557 - 167442nt (1) -----------------------------------------------------------------------------
     SARS-CoV-2 Reference Genome NC_045512.2 (1) -----------------------------------------------------------------------------
                                                                                                                    Section 470
                                                  (36114) 36114     36124      36135      36146      36157      36168      36179     36190
Candidatus Carsonella ruddii strain BT chromosome... (36114) AATTTTTTTTGTTTTTTTTTTTCCTAAAACTGAAAAAAAAACATCAAAATATTTTTTTATATAAAATATACAATTAT
        Candidatus Carsonella 135557 - 167442nt (1) -----------------------------------------------------------------------------
     SARS-CoV-2 Reference Genome NC_045512.2 (1) -----------------------------------------------------------------------------
                                                                                                                    Section 471
                                                  (36191) 36191     36201      36212      36223      36234      36245      36256     36267
Candidatus Carsonella ruddii strain BT chromosome... (36191) TAAAACATTCTTCAGGTTTAATAATTCCGTCAGTTTCAATATCAAAAAATAAATTTTTAATTTTTTTATTAAAAAAT
        Candidatus Carsonella 135557 - 167442nt (1) -----------------------------------------------------------------------------
     SARS-CoV-2 Reference Genome NC_045512.2 (1) -----------------------------------------------------------------------------
                                                                                                                    Section 472
                                                  (36268) 36268     36278      36289      36300      36311      36322      36333     36344
Candidatus Carsonella ruddii strain BT chromosome... (36268) TTTTTATGTATAAAATAATTTATGTTTTTTAAAGAAGACTTTAAAAAATTAATTTTAATAACTTTTGAATTAAAAAT
        Candidatus Carsonella 135557 - 167442nt (1) -----------------------------------------------------------------------------
     SARS-CoV-2 Reference Genome NC_045512.2 (1) -----------------------------------------------------------------------------
                                                                                                                    Section 473
                                                  (36345) 36345     36355      36366      36377      36388      36399      36410     36421
Candidatus Carsonella ruddii strain BT chromosome... (36345) TTTATTAATAATATTATTATTAGTAAAATTATAACTAGTATTAATACATTTCATTAAAACAAAAAAAATTATATTAT
        Candidatus Carsonella 135557 - 167442nt (1) -----------------------------------------------------------------------------
     SARS-CoV-2 Reference Genome NC_045512.2 (1) -----------------------------------------------------------------------------
                                                                                                                    Section 474
                                                  (36422) 36422     36432      36443      36454      36465      36476      36487     36498
Candidatus Carsonella ruddii strain BT chromosome... (36422) CTGTTATATTTGCTATTATTATATCTGGATTATAAATAATAATATGTTTATCAGAAAAAATATCTTTTGCTTTAACA
        Candidatus Carsonella 135557 - 167442nt (1) -----------------------------------------------------------------------------
     SARS-CoV-2 Reference Genome NC_045512.2 (1) -----------------------------------------------------------------------------
```

Section 475

		36499 36509 36520 36531 36542 36553 36564 36575
	(36499)	
Candidatus Carsonella ruddii strain BT chromosome...	(36499)	ATACAAGGTCCTTTTTTTTTTATAATTAAAAAAGCAGTTATTGAATTTTTAATTTTTATTAAAATATTATTTATATT
Candidatus Carsonella 135557 - 167442nt	(1)	---
SARS-CoV-2 Reference Genome NC_045512.2	(1)	---

Section 476

		36576 36586 36597 36608 36619 36630 36641 36652
	(36576)	
Candidatus Carsonella ruddii strain BT chromosome...	(36576)	ATTAATAATTTTTAAAGTATTTTCTTTTATACCTTTTATATTAGAAAATTCAGAATTAATATTAAAAAATTTAACAT
Candidatus Carsonella 135557 - 167442nt	(1)	---
SARS-CoV-2 Reference Genome NC_045512.2	(1)	---

Section 477

		36653 36663 36674 36685 36696 36707 36718 36729
	(36653)	
Candidatus Carsonella ruddii strain BT chromosome...	(36653)	ATATTATTTTAAAACTAAAAGTTGTTAAAAAAATAGTTCTTCTTAAAAAATTTCCTAAAGAATCACAATATGAATTA
Candidatus Carsonella 135557 - 167442nt	(1)	---
SARS-CoV-2 Reference Genome NC_045512.2	(1)	---

Section 478

		36730 36740 36751 36762 36773 36784 36795 36806
	(36730)	
Candidatus Carsonella ruddii strain BT chromosome...	(36730)	TTAAAAGATTCAATTTTTATTAAACTTCTAAAAAAAGAAATTTTTTTAAAACTAATTTTATTTAAAAAAAAAATTTT
Candidatus Carsonella 135557 - 167442nt	(1)	---
SARS-CoV-2 Reference Genome NC_045512.2	(1)	---

Section 479

		36807 36817 36828 36839 36850 36861 36872 36883
	(36807)	
Candidatus Carsonella ruddii strain BT chromosome...	(36807)	TTTCTGAAAATTTATCATTTATAAATATCTAAAATTAAATTTTTATTAATATTAAATTTAAAAATATTAGGAAGAAA
Candidatus Carsonella 135557 - 167442nt	(1)	---
SARS-CoV-2 Reference Genome NC_045512.2	(1)	---

Section 480

		36884 36894 36905 36916 36927 36938 36949 36960
	(36884)	
Candidatus Carsonella ruddii strain BT chromosome...	(36884)	TAAAAAAATTCCAAATCCATAAAAATAATTTGTAAATAACCAATTTAAATTGAAAAAATTTTTACAATTATAATTAA
Candidatus Carsonella 135557 - 167442nt	(1)	---
SARS-CoV-2 Reference Genome NC_045512.2	(1)	---

Section 481

	(36961)	36961 36971 36982 36993 37004 37015 37026 37037
Candidatus Carsonella ruddii strain BT chromosome...	(36961)	AATTTTCAAATAAAAAATTTTCAACATAAATTGAATCTCCAGGAGATAAAATAAAAGATGGAAATTTTATTTTTTTA
Candidatus Carsonella 135557 - 167442nt	(1)	---
SARS-CoV-2 Reference Genome NC_045512.2	(1)	---

Section 482

	(37038)	37038 37048 37059 37070 37081 37092 37103 37114
Candidatus Carsonella ruddii strain BT chromosome...	(37038)	AAATTAACATATATAAAACCATGTAAAATTTTTTGTCTAGAATTTTTTCTAGAAATTGAAAAATTCATTCTATAAAC
Candidatus Carsonella 135557 - 167442nt	(1)	---
SARS-CoV-2 Reference Genome NC_045512.2	(1)	---

Section 483

	(37115)	37115 37125 37136 37147 37158 37169 37180 37191
Candidatus Carsonella ruddii strain BT chromosome...	(37115)	AAGATTATCTAATCTTCTTTCTAAAAAATTTATTAAATCTTCACATAAAAAAAATTTTTTTGATATTTTTTTTATATA
Candidatus Carsonella 135557 - 167442nt	(1)	---
SARS-CoV-2 Reference Genome NC_045512.2	(1)	---

Section 484

	(37192)	37192 37202 37213 37224 37235 37246 37257 37268
Candidatus Carsonella ruddii strain BT chromosome...	(37192)	TTAATTTAAATTTTTTTTCAGAAATAATATAATATCTTTTAATTTTTTGTTTAATTCTTAAAATTATACCATAATAA
Candidatus Carsonella 135557 - 167442nt	(1)	---
SARS-CoV-2 Reference Genome NC_045512.2	(1)	---

Section 485

	(37269)	37269 37279 37290 37301 37312 37323 37334 37345
Candidatus Carsonella ruddii strain BT chromosome...	(37269)	GATAATTTATTTTGATCAACTCCATGTTCACCAGGAGTTAATAATGATCTAGATTTAGAAAAAAAATTTTTTTCTGA
Candidatus Carsonella 135557 - 167442nt	(1)	---
SARS-CoV-2 Reference Genome NC_045512.2	(1)	---

Section 486

	(37346)	37346 37356 37367 37378 37389 37400 37411 37422
Candidatus Carsonella ruddii strain BT chromosome...	(37346)	AAAAAACTCTAAATTTTCTCCTTCTCTTCTACATATTTTATTTCTTTTAATTTTCATTAAACTCTTCTTTTTTTTTT
Candidatus Carsonella 135557 - 167442nt	(1)	---
SARS-CoV-2 Reference Genome NC_045512.2	(1)	---

Section 487

```
                                                  (37423) 37423     37433     37444      37455      37466      37477      37488     37499
Candidatus Carsonella ruddii strain BT chromosome... (37423) TTTTCTACAACCATTATAAGGTAATGGAGTAATATCTGTTATACTCCTTATATAAATTCCACAATTATTTATTATTC
            Candidatus Carsonella 135557 - 167442nt     (1) -----------------------------------------------------------------------------
          SARS-CoV-2 Reference Genome NC_045512.2       (1) -----------------------------------------------------------------------------
```

Section 488

```
                                                  (37500) 37500     37510     37521      37532      37543      37554      37565     37576
Candidatus Carsonella ruddii strain BT chromosome... (37500) TTAATATTGTTTCTTTTCCAATGCTTATTCCTTTTATATATATATCAACAATTTTTATATTTTTTTTATAAGCAAGT
            Candidatus Carsonella 135557 - 167442nt     (1) -----------------------------------------------------------------------------
          SARS-CoV-2 Reference Genome NC_045512.2       (1) -----------------------------------------------------------------------------
```

Section 489

```
                                                  (37577) 37577     37587     37598      37609      37620      37631      37642     37653
Candidatus Carsonella ruddii strain BT chromosome... (37577) ATAGATATTTTTTCACTTATTGATTGAGATGCTAAAGATGTACTTTTTTTAGGACCTTTAAAACCTAAAACACCTGA
            Candidatus Carsonella 135557 - 167442nt     (1) -----------------------------------------------------------------------------
          SARS-CoV-2 Reference Genome NC_045512.2       (1) -----------------------------------------------------------------------------
```

Section 490

```
                                                  (37654) 37654     37664     37675      37686      37697      37708      37719     37730
Candidatus Carsonella ruddii strain BT chromosome... (37654) TGAAAACCAACCTATTGTATTTCCTTTTAAATCAGTTAAAGTAGAAACAGTATTATTAAAAGTTGAATTAATATATA
            Candidatus Carsonella 135557 - 167442nt     (1) -----------------------------------------------------------------------------
          SARS-CoV-2 Reference Genome NC_045512.2       (1) -----------------------------------------------------------------------------
```

Section 491

```
                                                  (37731) 37731     37741     37752      37763      37774      37785      37796     37807
Candidatus Carsonella ruddii strain BT chromosome... (37731) TTATTCCATAATTATAATCATATATTTTCATGATTCATTTTTTTTCTAGTTTTTGCATTTGTTTTTGTTCTTTGTCC
            Candidatus Carsonella 135557 - 167442nt     (1) -----------------------------------------------------------------------------
          SARS-CoV-2 Reference Genome NC_045512.2       (1) -----------------------------------------------------------------------------
```

Section 492

```
                                                  (37808) 37808     37818     37829      37840      37851      37862      37873     37884
Candidatus Carsonella ruddii strain BT chromosome... (37808) TCTGCAAGGTAATTTGTTTTTATGTCTTAAACTTTTAAAACTATTTAAGTTTAATATTCTTTTAAGATTTTCTTTTA
            Candidatus Carsonella 135557 - 167442nt     (1) -----------------------------------------------------------------------------
          SARS-CoV-2 Reference Genome NC_045512.2       (1) -----------------------------------------------------------------------------
```

Section 493

	(37885)	37885 37895 37906 37917 37928 37939 37950 37961
Candidatus Carsonella ruddii strain BT chromosome...	(37885)	TTAACATTTTTAAATCATTTTCTATTGTTAATGTTCTTATAGTATTTATCAATTTTTGTTTTTCATATTCATTTAAT
Candidatus Carsonella 135557 - 167442nt	(1)	---
SARS-CoV-2 Reference Genome NC_045512.2	(1)	---

Section 494

	(37962)	37962 37972 37983 37994 38005 38016 38027 38038
Candidatus Carsonella ruddii strain BT chromosome...	(37962)	TTTATTAATTTTTTGTTTTGAATATTTAATTTTTTACAAATTTTTATAGAAGTTTTTATACCTATTCCAAAAATTTT
Candidatus Carsonella 135557 - 167442nt	(1)	---
SARS-CoV-2 Reference Genome NC_045512.2	(1)	---

Section 495

	(38039)	38039 38049 38060 38071 38082 38093 38104 38115
Candidatus Carsonella ruddii strain BT chromosome...	(38039)	AGTTATACTAAAAGTAACGTTTTTGTAAGCTTGAATATTTACACCACAAATATTAATTTTCATAATTGTCTTTGTTT
Candidatus Carsonella 135557 - 167442nt	(1)	---
SARS-CoV-2 Reference Genome NC_045512.2	(1)	---

Section 496

	(38116)	38116 38126 38137 38148 38159 38170 38181 38192
Candidatus Carsonella ruddii strain BT chromosome...	(38116)	ATGTTTTTTAAAATAACATATAACGAAAAAAATTTTTTTTCTTTTTATTATTATACATTTACTACATATTTTTTTAA
Candidatus Carsonella 135557 - 167442nt	(1)	---
SARS-CoV-2 Reference Genome NC_045512.2	(1)	---

Section 497

	(38193)	38193 38203 38214 38225 38236 38247 38258 38269
Candidatus Carsonella ruddii strain BT chromosome...	(38193)	TTGAATTTTTAACTTTCATATAAAAAAATAATATTTTTTTTATTTTTTTTATTTCTTATTTTTTGTTTAATTTTTGG
Candidatus Carsonella 135557 - 167442nt	(1)	---
SARS-CoV-2 Reference Genome NC_045512.2	(1)	---

Section 498

	(38270)	38270 38280 38291 38302 38313 38324 38335 38346
Candidatus Carsonella ruddii strain BT chromosome...	(38270)	TTTTCTTTTAAATATATTTGTTTGACCTCCTTCAAATAATTTAGGAATATTGTATCCTGATCTTGATTTTTGTCCTT
Candidatus Carsonella 135557 - 167442nt	(1)	---
SARS-CoV-2 Reference Genome NC_045512.2	(1)	---

Section 499

		38347 38357 38368 38379 38390 38401 38412 38423
	(38347)	
Candidatus Carsonella ruddii strain BT chromosome...	(38347)	TATGTCCTCTGCCACATGTTTTACCTTTTCCAGAAGAAAAACCTCTTCCAATTCTTTTTTTTTTTGTATTAAAAATA
Candidatus Carsonella 135557 - 167442nt	(1)	---
SARS-CoV-2 Reference Genome NC_045512.2	(1)	---

Section 500

		38424 38434 38445 38456 38467 38478 38489 38500
	(38424)	
Candidatus Carsonella ruddii strain BT chromosome...	(38424)	TTAAAAATTTCGATAATCATTATTTTAAAAATGTTAATGCTTTAATTGTACAAATTGTTACATTAATTGTATTTGAA
Candidatus Carsonella 135557 - 167442nt	(1)	---
SARS-CoV-2 Reference Genome NC_045512.2	(1)	---

Section 501

		38501 38511 38522 38533 38544 38555 38566 38577
	(38501)	
Candidatus Carsonella ruddii strain BT chromosome...	(38501)	GAACCATGAATTTTTGAAAAAACACTTTTAATACCAGCAGAAATTAAAACTTGTTTTACAACATTACTACAAATTAA
Candidatus Carsonella 135557 - 167442nt	(1)	---
SARS-CoV-2 Reference Genome NC_045512.2	(1)	---

Section 502

		38578 38588 38599 38610 38621 38632 38643 38654
	(38578)	
Candidatus Carsonella ruddii strain BT chromosome...	(38578)	ACCAATTATTTTTGATGCAGGAAACAAATATATTTTTGTTGCAGAATGTTTAGCATATAAAAAATGAGGAATTGAAT
Candidatus Carsonella 135557 - 167442nt	(1)	---
SARS-CoV-2 Reference Genome NC_045512.2	(1)	---

Section 503

		38655 38665 38676 38687 38698 38709 38720 38731
	(38655)	
Candidatus Carsonella ruddii strain BT chromosome...	(38655)	TATTTAATATATTTAATTTTATAATATTATTTCTAGCTTTTAAATATGATTTTTTTATAGATTCTAAAATTTCTCTT
Candidatus Carsonella 135557 - 167442nt	(1)	---
SARS-CoV-2 Reference Genome NC_045512.2	(1)	---

Section 504

		38732 38742 38753 38764 38775 38786 38797 38808
	(38732)	
Candidatus Carsonella ruddii strain BT chromosome...	(38732)	GATTTACCTCTTCCAAATCCAATTAAATAATTTTTATTTCCACAAATAGATGTAGACGTATAAGAAAAAATTCTTCC
Candidatus Carsonella 135557 - 167442nt	(1)	---
SARS-CoV-2 Reference Genome NC_045512.2	(1)	---

Section 505

```
                                                    (38809) 38809     38819      38830      38841      38852      38863      38874  38885
Candidatus Carsonella ruddii strain BT chromosome... (38809) TCCTTTAACTACTTTTGTAACTCTATGAATTTTAACCAATCTGTCTTCCATTTTTATTATTAATAAAATTTATTATT
             Candidatus Carsonella 135557 - 167442nt (1) -----------------------------------------------------------------------------
           SARS-CoV-2 Reference Genome NC_045512.2 (1) -----------------------------------------------------------------------------
```

Section 506

```
                                                    (38886) 38886     38896      38907      38918      38929      38940      38951  38962
Candidatus Carsonella ruddii strain BT chromosome... (38886) TTTTTATAATATCCATTATAATTTTTTTTATTATAAAAAATTTTCTTATTAAAAAATAATATTATTTTTTAATTTT
             Candidatus Carsonella 135557 - 167442nt (1) -----------------------------------------------------------------------------
           SARS-CoV-2 Reference Genome NC_045512.2 (1) -----------------------------------------------------------------------------
```

Section 507

```
                                                    (38963) 38963     38973      38984      38995      39006      39017      39028  39039
Candidatus Carsonella ruddii strain BT chromosome... (38963) TTCAAATAAAAATAAAAAATTTTTTTTTTTATTTTTTATAAAATAAATATTTTTATTATTATAAAATAAATAAATTA
             Candidatus Carsonella 135557 - 167442nt (1) -----------------------------------------------------------------------------
           SARS-CoV-2 Reference Genome NC_045512.2 (1) -----------------------------------------------------------------------------
```

Section 508

```
                                                    (39040) 39040     39050      39061      39072      39083      39094      39105  39116
Candidatus Carsonella ruddii strain BT chromosome... (39040) AAAAATTTTATTTGTTTTTTTTTAAAATTATTTTCATTGTTTTTTTTTTGAAGATTTTTTTAAAATTGGATTTTCTA
             Candidatus Carsonella 135557 - 167442nt (1) -----------------------------------------------------------------------------
           SARS-CoV-2 Reference Genome NC_045512.2 (1) -----------------------------------------------------------------------------
```

Section 509

```
                                                    (39117) 39117     39127      39138      39149      39160      39171      39182  39193
Candidatus Carsonella ruddii strain BT chromosome... (39117) AAAACATTCCTTTTTTTTTATATGGATTAAATTTTTTTATTTTTAATAATTTATTTACAAAAATACCAATTTTATCT
             Candidatus Carsonella 135557 - 167442nt (1) -----------------------------------------------------------------------------
           SARS-CoV-2 Reference Genome NC_045512.2 (1) -----------------------------------------------------------------------------
```

Section 510

```
                                                    (39194) 39194     39204      39215      39226      39237      39248      39259  39270
Candidatus Carsonella ruddii strain BT chromosome... (39194) TTAAAAACAGATTTTAAAACAATTTTTTCTTTAAAAATTTCAATAGTAATATAATTTGGAATTACTAAAATTTTTGA
             Candidatus Carsonella 135557 - 167442nt (1) -----------------------------------------------------------------------------
           SARS-CoV-2 Reference Genome NC_045512.2 (1) -----------------------------------------------------------------------------
```

```
                                                                                                Section 511
                                                         (39271) 39271     39281      39292      39303      39314      39325      39336   39347
Candidatus Carsonella ruddii strain BT chromosome...     (39271) ATCAGAAAAACCTAAATTTAAATAAATTTCATTTTTTTTAATAAAAATTTTATATCCAATTCCTGAAAAAAAAATTG
              Candidatus Carsonella 135557 - 167442nt        (1) -----------------------------------------------------------------------------
          SARS-CoV-2 Reference Genome NC_045512.2            (1) -----------------------------------------------------------------------------
                                                                                                Section 512
                                                         (39348) 39348     39358      39369      39380      39391      39402      39413   39424
Candidatus Carsonella ruddii strain BT chromosome...     (39348) TTTTTTGCCATAAATTATTTATACCAATTAATAAATTTTTTATTGTTTTTAAAAAAGAAATTTCATTTTTTTTTTTA
              Candidatus Carsonella 135557 - 167442nt        (1) -----------------------------------------------------------------------------
          SARS-CoV-2 Reference Genome NC_045512.2            (1) -----------------------------------------------------------------------------
                                                                                                Section 513
                                                         (39425) 39425     39435      39446      39457      39468      39479      39490   39501
Candidatus Carsonella ruddii strain BT chromosome...     (39425) AAACTTATTGATGATAAAAAAATATTATTTTCTTTTAAATTTAAAATTATATCATTTGGTATTTTTATTTTTCCAAA
              Candidatus Carsonella 135557 - 167442nt        (1) -----------------------------------------------------------------------------
          SARS-CoV-2 Reference Genome NC_045512.2            (1) -----------------------------------------------------------------------------
                                                                                                Section 514
                                                         (39502) 39502     39512      39523      39534      39545      39556      39567   39578
Candidatus Carsonella ruddii strain BT chromosome...     (39502) ATATTTATTTTTAACAAATAAAAAACCATTTTTAATTAAAACTTTTTCTTTTAAAATTATATTTTTCATAAAATTAA
              Candidatus Carsonella 135557 - 167442nt        (1) -----------------------------------------------------------------------------
          SARS-CoV-2 Reference Genome NC_045512.2            (1) -----------------------------------------------------------------------------
                                                                                                Section 515
                                                         (39579) 39579     39589      39600      39611      39622      39633      39644   39655
Candidatus Carsonella ruddii strain BT chromosome...     (39579) AACAATTAAAAAACCACTTATTCCAATTTGTTTACATTCTTTTAAAGTTAATAAACCTAAATTTGTATTAATTATAC
              Candidatus Carsonella 135557 - 167442nt        (1) -----------------------------------------------------------------------------
          SARS-CoV-2 Reference Genome NC_045512.2            (1) -----------------------------------------------------------------------------
                                                                                                Section 516
                                                         (39656) 39656     39666      39677      39688      39699      39710      39721   39732
Candidatus Carsonella ruddii strain BT chromosome...     (39656) ATAAACTTGTATTTAATAAAAAATTATTTATTTTTTTATTTTTAATTGAAATTTTTCTTGAAGGTTTAGAAAAAAAT
              Candidatus Carsonella 135557 - 167442nt        (1) -----------------------------------------------------------------------------
          SARS-CoV-2 Reference Genome NC_045512.2            (1) -----------------------------------------------------------------------------
```

Section 517

	(39733)	39733 39743 39754 39765 39776 39787 39798 39809
Candidatus Carsonella ruddii strain BT chromosome...	(39733)	TTAATATAATAAATGTTTTTCACAAAAACAACTATATATTTTTTTTTTTTTAATTATTAAAAAAAAATAATTTTCAAT
Candidatus Carsonella 135557 - 167442nt	(1)	---
SARS-CoV-2 Reference Genome NC_045512.2	(1)	---

Section 518

	(39810)	39810 39820 39831 39842 39853 39864 39875 39886
Candidatus Carsonella ruddii strain BT chromosome...	(39810)	AAAATTATATTCTAAAAATATTTCAAATATTAACATAGTAAAATTAGAATAATTACAAAAACTTATTTTTTTACCAG
Candidatus Carsonella 135557 - 167442nt	(1)	---
SARS-CoV-2 Reference Genome NC_045512.2	(1)	---

Section 519

	(39887)	39887 39897 39908 39919 39930 39941 39952 39963
Candidatus Carsonella ruddii strain BT chromosome...	(39887)	AATTATAAGAATTAAAAATTCTATTTATACTATCAAAATAATTTTTCATTACCAACTAGATTTTTCTAAACCTACAA
Candidatus Carsonella 135557 - 167442nt	(1)	---
SARS-CoV-2 Reference Genome NC_045512.2	(1)	---

Section 520

	(39964)	39964 39974 39985 39996 40007 40018 40029 40040
Candidatus Carsonella ruddii strain BT chromosome...	(39964)	TATTTCCATATCCGCCTATTTTTCTTATTAAATTTCTATTTAAATAAAATTTACTATAAACACTTCTGTTTCTACCA
Candidatus Carsonella 135557 - 167442nt	(1)	---
SARS-CoV-2 Reference Genome NC_045512.2	(1)	---

Section 521

	(40041)	40041 40051 40062 40073 40084 40095 40106 40117
Candidatus Carsonella ruddii strain BT chromosome...	(40041)	CTTATGTAACATCTTTTTTTAAATTTAACAAATAATTTTTTTTTTTTTAAATTAAATAATTTAAAATAATTTTCTAA
Candidatus Carsonella 135557 - 167442nt	(1)	---
SARS-CoV-2 Reference Genome NC_045512.2	(1)	---

Section 522

	(40118)	40118 40128 40139 40150 40161 40172 40183 40194
Candidatus Carsonella ruddii strain BT chromosome...	(40118)	AGAAGAATTTAAAAATAAATTTTGTTTTATTTTTATAATATTTTTAATATTAAATAAACTTTTCTTATAAATTATAA
Candidatus Carsonella 135557 - 167442nt	(1)	---
SARS-CoV-2 Reference Genome NC_045512.2	(1)	---

```
                                                                                                     Section 523
                                                    (40195) 40195     40205      40216      40227      40238      40249      40260    40271
Candidatus Carsonella ruddii strain BT chromosome... (40195) TATTTTTAAAAATCAAACTTTTTTTTGCCATTTTATTTTATAGGAAAATTAATTATTTTATAAAAATTTTTAAAATT
           Candidatus Carsonella 135557 - 167442nt       (1) -----------------------------------------------------------------------------
        SARS-CoV-2 Reference Genome NC_045512.2          (1) -----------------------------------------------------------------------------
                                                                                                     Section 524
                                                    (40272) 40272     40282      40293      40304      40315      40326      40337    40348
Candidatus Carsonella ruddii strain BT chromosome... (40272) GTTTTTTAAATTTTTAACAATAATACTAATATTAAAACCAATATTAAATTCTTTATAAAAATCAGGAAAAACTGATA
           Candidatus Carsonella 135557 - 167442nt       (1) -----------------------------------------------------------------------------
        SARS-CoV-2 Reference Genome NC_045512.2          (1) -----------------------------------------------------------------------------
                                                                                                     Section 525
                                                    (40349) 40349     40359      40370      40381      40392      40403      40414    40425
Candidatus Carsonella ruddii strain BT chromosome... (40349) TATCAGTTATACCAAAATGAATATTACCAAATCTATCAATTGATGTTTCATTTATTCCTTTAAATTCTTTTATTCTT
           Candidatus Carsonella 135557 - 167442nt       (1) -----------------------------------------------------------------------------
        SARS-CoV-2 Reference Genome NC_045512.2          (1) -----------------------------------------------------------------------------
                                                                                                     Section 526
                                                    (40426) 40426     40436      40447      40458      40469      40480      40491    40502
Candidatus Carsonella ruddii strain BT chromosome... (40426) GGTATAACAACACAAATTAATTTATGATAAAATTCCCACATTTTTATTTTTCTTAATGTTGTATAAACTCCAGCAAT
           Candidatus Carsonella 135557 - 167442nt       (1) -----------------------------------------------------------------------------
        SARS-CoV-2 Reference Genome NC_045512.2          (1) -----------------------------------------------------------------------------
                                                                                                     Section 527
                                                    (40503) 40503     40513      40524      40535      40546      40557      40568    40579
Candidatus Carsonella ruddii strain BT chromosome... (40503) TTCACCAATCTTAGATTTAAAATTAGAAATAGATTTTTTAATTTTAGAAAAAACTGGTTTTTGTCCAGATATATATT
           Candidatus Carsonella 135557 - 167442nt       (1) -----------------------------------------------------------------------------
        SARS-CoV-2 Reference Genome NC_045512.2          (1) -----------------------------------------------------------------------------
                                                                                                     Section 528
                                                    (40580) 40580     40590      40601      40612      40623      40634      40645    40656
Candidatus Carsonella ruddii strain BT chromosome... (40580) CTATAGAATCAAAAACATTTTTAATAAATATTTTATCTTTATTGTTTTTTGCTACACCAGAATGTATTATTATTTTT
           Candidatus Carsonella 135557 - 167442nt       (1) -----------------------------------------------------------------------------
        SARS-CoV-2 Reference Genome NC_045512.2          (1) -----------------------------------------------------------------------------
```

```
                                                                                                        Section 529
                                                    (40657) 40657     40667      40678      40689      40700      40711      40722      40733
Candidatus Carsonella ruddii strain BT chromosome... (40657) TCTAATTTTATATTATAAATTTTGTTTTTTAAAGAAAATTCTTCTATTTTTAATTTATATAAATTCATACTAATTCA
         Candidatus Carsonella 135557 - 167442nt     (1)     -----------------------------------------------------------------------------
     SARS-CoV-2 Reference Genome NC_045512.2         (1)     -----------------------------------------------------------------------------
                                                                                                        Section 530
                                                    (40734) 40734     40744      40755      40766      40777      40788      40799      40810
Candidatus Carsonella ruddii strain BT chromosome... (40734) TTTGATAATGATAATAATTTTTTAAAATGAAAAAATTTAATTTCTCTAGCTAAACAACCGAAAACACGCGTTCCAAT
         Candidatus Carsonella 135557 - 167442nt     (1)     -----------------------------------------------------------------------------
     SARS-CoV-2 Reference Genome NC_045512.2         (1)     -----------------------------------------------------------------------------
                                                                                                        Section 531
                                                    (40811) 40811     40821      40832      40843      40854      40865      40876      40887
Candidatus Carsonella ruddii strain BT chromosome... (40811) CATTTGTTTATTATTATTTAATAATAGAACCGCATTATCACTAAAACGTAAATAAGTACCATCTTGTCTTCTATAAG
         Candidatus Carsonella 135557 - 167442nt     (1)     -----------------------------------------------------------------------------
     SARS-CoV-2 Reference Genome NC_045512.2         (1)     -----------------------------------------------------------------------------
                                                                                                        Section 532
                                                    (40888) 40888     40898      40909      40920      40931      40942      40953      40964
Candidatus Carsonella ruddii strain BT chromosome... (40888) GAAATTTAGTTCTAACTATAATAGCATTTAATACTTGACCTTTTTTAACTTTAGAATTAAAATTTGAACTTTTAACA
         Candidatus Carsonella 135557 - 167442nt     (1)     -----------------------------------------------------------------------------
     SARS-CoV-2 Reference Genome NC_045512.2         (1)     -----------------------------------------------------------------------------
                                                                                                        Section 533
                                                    (40965) 40965     40975      40986      40997      41008      41019      41030      41041
Candidatus Carsonella ruddii strain BT chromosome... (40965) ACTACTTTAATTATATCTCCAATTTTAGCAAAAATTTTTTTTGAACCATTTAAAACTTTTATACATTTAACAATTTT
         Candidatus Carsonella 135557 - 167442nt     (1)     -----------------------------------------------------------------------------
     SARS-CoV-2 Reference Genome NC_045512.2         (1)     -----------------------------------------------------------------------------
                                                                                                        Section 534
                                                    (41042) 41042     41052      41063      41074      41085      41096      41107      41118
Candidatus Carsonella ruddii strain BT chromosome... (41042) TCCACCACTATTATCAATAACTTTTAATTTAGTTTGTTCTTGTATCATATTAATTTTACTTTTTCAATAATTTTTGA
         Candidatus Carsonella 135557 - 167442nt     (1)     -----------------------------------------------------------------------------
     SARS-CoV-2 Reference Genome NC_045512.2         (1)     -----------------------------------------------------------------------------
```

```
                                                                                                   Section 535
                                                   (41119) 41119     41129      41140      41151      41162      41173      41184      41195
Candidatus Carsonella ruddii strain BT chromosome... (41119) TACTATCCAAAAAATTGTTTTAGTTAAAGGTCTAGATTTTGTTACAATAATATAATCTCCAATATTTGTTTCATTAA
            Candidatus Carsonella 135557 - 167442nt (1) -----------------------------------------------------------------------------
         SARS-CoV-2 Reference Genome NC_045512.2 (1) -----------------------------------------------------------------------------
                                                                                                   Section 536
                                                   (41196) 41196     41206      41217      41228      41239      41250      41261      41272
Candidatus Carsonella ruddii strain BT chromosome... (41196) AAATGTCATATGATATTAATTTTTTATAATAAAAAATTCTTTTATTTAAAATAGGTAAAATATAACTTTTTTTAATT
            Candidatus Carsonella 135557 - 167442nt (1) -----------------------------------------------------------------------------
         SARS-CoV-2 Reference Genome NC_045512.2 (1) -----------------------------------------------------------------------------
                                                                                                   Section 537
                                                   (41273) 41273     41283      41294      41305      41316      41327      41338      41349
Candidatus Carsonella ruddii strain BT chromosome... (41273) ATAATAATAATTTTTTTAAATTTTTTTTTAATAACTCTTCCAAATGTTTTATTCATAACAAATTTCAGTTTTAATAG
            Candidatus Carsonella 135557 - 167442nt (1) -----------------------------------------------------------------------------
         SARS-CoV-2 Reference Genome NC_045512.2 (1) -----------------------------------------------------------------------------
                                                                                                   Section 538
                                                   (41350) 41350     41360      41371      41382      41393      41404      41415      41426
Candidatus Carsonella ruddii strain BT chromosome... (41350) ATAATTTTTGAGCGGCACAAAAAAACGATTTTTTTACAATATCAAAATTTTCATATTTAACTTCAAAAATAATTATT
            Candidatus Carsonella 135557 - 167442nt (1) -----------------------------------------------------------------------------
         SARS-CoV-2 Reference Genome NC_045512.2 (1) -----------------------------------------------------------------------------
                                                                                                   Section 539
                                                   (41427) 41427     41437      41448      41459      41470      41481      41492      41503
Candidatus Carsonella ruddii strain BT chromosome... (41427) CCAGGTTTAATAACAGATACCCAATCATGTATATTTCCCTTACCTTTACCCATTCTAACTTCTAAAGGTTTTTTAGT
            Candidatus Carsonella 135557 - 167442nt (1) -----------------------------------------------------------------------------
         SARS-CoV-2 Reference Genome NC_045512.2 (1) -----------------------------------------------------------------------------
                                                                                                   Section 540
                                                   (41504) 41504     41514      41525      41536      41547      41558      41569      41580
Candidatus Carsonella ruddii strain BT chromosome... (41504) AATTACTTTATCTGGAAAAATTCTAATAAGAATTTTATTATTTTTCCCTAAATTATGAACTAAAGATTTTCTAGCAG
            Candidatus Carsonella 135557 - 167442nt (1) -----------------------------------------------------------------------------
         SARS-CoV-2 Reference Genome NC_045512.2 (1) -----------------------------------------------------------------------------
```

```
                                                                                    Section 541
                                                     (41581) 41581     41591      41602      41613      41624      41635      41646      41657
Candidatus Carsonella ruddii strain BT chromosome... (41581) ATTCAATCTGCTTAAAATTTAAAAATCCGTTATCTAATGATTTTAATCCATAATTACCAAAAATTAAAATATTTCTT
          Candidatus Carsonella 135557 - 167442nt        (1) -----------------------------------------------------------------------------
      SARS-CoV-2 Reference Genome NC_045512.2            (1) -----------------------------------------------------------------------------
                                                                                    Section 542
                                                     (41658) 41658     41668      41679      41690      41701      41712      41723      41734
Candidatus Carsonella ruddii strain BT chromosome... (41658) TTACAAGAAAAACCTTTATTTCTATTTTTTTGATATTTTAAAAATTTTAATTGATTAGGTTTTTGTTTCATATTTTT
          Candidatus Carsonella 135557 - 167442nt        (1) -----------------------------------------------------------------------------
      SARS-CoV-2 Reference Genome NC_045512.2            (1) -----------------------------------------------------------------------------
                                                                                    Section 543
                                                     (41735) 41735     41745      41756      41767      41778      41789      41800      41811
Candidatus Carsonella ruddii strain BT chromosome... (41735) TATTTTTTTATTTATTAAAAATCCAAGTTTTAATACCTAAAATACCATATTGTGATATAAATTCACAAAAATAATAA
          Candidatus Carsonella 135557 - 167442nt        (1) -----------------------------------------------------------------------------
      SARS-CoV-2 Reference Genome NC_045512.2            (1) -----------------------------------------------------------------------------
                                                                                    Section 544
                                                     (41812) 41812     41822      41833      41844      41855      41866      41877      41888
Candidatus Carsonella ruddii strain BT chromosome... (41812) TCTAATTTACATCTAATTGTATGTAATGGTATACTACCAAATAAATACCATTCTTTTCTAGCTATATCTACTCCTTC
          Candidatus Carsonella 135557 - 167442nt        (1) -----------------------------------------------------------------------------
      SARS-CoV-2 Reference Genome NC_045512.2            (1) -----------------------------------------------------------------------------
                                                                                    Section 545
                                                     (41889) 41889     41899      41910      41921      41932      41943      41954      41965
Candidatus Carsonella ruddii strain BT chromosome... (41889) TAATCTTCCACTTATTTGAACTTTACATCCATTAAATTGTTTATTAAATTTTAATATTTCTTTTTTTATAATTCTTT
          Candidatus Carsonella 135557 - 167442nt        (1) -----------------------------------------------------------------------------
      SARS-CoV-2 Reference Genome NC_045512.2            (1) -----------------------------------------------------------------------------
                                                                                    Section 546
                                                     (41966) 41966     41976      41987      41998      42009      42020      42031      42042
Candidatus Carsonella ruddii strain BT chromosome... (41966) TTGTAGATGATTTATTATCTAATTGATTTAATATATTTAAAGATAATAATTTAGCATTTATAAATTCTTCTAAACAA
          Candidatus Carsonella 135557 - 167442nt        (1) -----------------------------------------------------------------------------
      SARS-CoV-2 Reference Genome NC_045512.2            (1) -----------------------------------------------------------------------------
```

Section 547

	(42043)	42043 42053 42064 42075 42086 42097 42108 42119
Candidatus Carsonella ruddii strain BT chromosome...	(42043)	AAATTAATAATAATATTTATTTTTAAAATTTTAGAAAATTCTAAAATAAAAAAATCAATATAAAAATAAAAATCTTC
Candidatus Carsonella 135557 - 167442nt	(1)	---
SARS-CoV-2 Reference Genome NC_045512.2	(1)	---

Section 548

	(42120)	42120 42130 42141 42152 42163 42174 42185 42196
Candidatus Carsonella ruddii strain BT chromosome...	(42120)	AATTTTTTCTAAATCATTTAAATATATATTTAATTTTAAAAAATTTGAAATAATTATATCTATACAACTTATATTAA
Candidatus Carsonella 135557 - 167442nt	(1)	---
SARS-CoV-2 Reference Genome NC_045512.2	(1)	---

Section 549

	(42197)	42197 42207 42218 42229 42240 42251 42262 42273
Candidatus Carsonella ruddii strain BT chromosome...	(42197)	TAAAAAAAAAATTTTTTTTTAAAATTTCTTTTATTAAAATATTAATTCTTAATTTTTTTGAATATTTTTTTTTGTTT
Candidatus Carsonella 135557 - 167442nt	(1)	---
SARS-CoV-2 Reference Genome NC_045512.2	(1)	---

Section 550

	(42274)	42274 42284 42295 42306 42317 42328 42339 42350
Candidatus Carsonella ruddii strain BT chromosome...	(42274)	TCATACCATAAACTGTTATAACAAACAGATTTTCCTAAACGAAAAAAAATAGGATTAATTTTTTTACCCATAAATTA
Candidatus Carsonella 135557 - 167442nt	(1)	---
SARS-CoV-2 Reference Genome NC_045512.2	(1)	---

Section 551

	(42351)	42351 42361 42372 42383 42394 42405 42416 42427
Candidatus Carsonella ruddii strain BT chromosome...	(42351)	CATTTATTATAATATTTGAATATCTTTTAATAATAAAATTTATTCTTCCTTTAGCTCTAAAATTTATTTTTTTTATA
Candidatus Carsonella 135557 - 167442nt	(1)	---
SARS-CoV-2 Reference Genome NC_045512.2	(1)	---

Section 552

	(42428)	42428 42438 42449 42460 42471 42482 42493 42504
Candidatus Carsonella ruddii strain BT chromosome...	(42428)	ATTAAACTTTTTCCTACTAAAAATTGTTTTATAAAAACATTAGTAGTTTTACAATTATTAATAATTAAATTAACAAT
Candidatus Carsonella 135557 - 167442nt	(1)	---
SARS-CoV-2 Reference Genome NC_045512.2	(1)	---

Section 553

		42505 42515 42526 42537 42548 42559 42570 42581
	(42505)	
Candidatus Carsonella ruddii strain BT chromosome...	(42505)	TTGTTTTAAATAAAAATCTATTTTTTTAAAAAAAATATTATTTATATAAAATAATATAGGTTTGTTAGATAAAAATT
Candidatus Carsonella 135557 - 167442nt	(1)	---
SARS-CoV-2 Reference Genome NC_045512.2	(1)	---

Section 554

		42582 42592 42603 42614 42625 42636 42647 42658
	(42582)	
Candidatus Carsonella ruddii strain BT chromosome...	(42582)	TAGAATATAAACTTAATTTTTTGTAAGAAATAGGAATAAAATTCCATTTAAATTTAAAATTATTTAACATTTTTATT
Candidatus Carsonella 135557 - 167442nt	(1)	---
SARS-CoV-2 Reference Genome NC_045512.2	(1)	---

Section 555

		42659 42669 42680 42691 42702 42713 42724 42735
	(42659)	
Candidatus Carsonella ruddii strain BT chromosome...	(42659)	TTTAATTTTTTTTGTATGTGATTTAAAATTTCTTGTAAAAGAAAATTCTCCTAATTTATGTCCAATCATGTTTTCGT
Candidatus Carsonella 135557 - 167442nt	(1)	---
SARS-CoV-2 Reference Genome NC_045512.2	(1)	---

Section 556

		42736 42746 42757 42768 42779 42790 42801 42812
	(42736)	
Candidatus Carsonella ruddii strain BT chromosome...	(42736)	TTATATAAACTTTAATAAATCTTTTTCCATTATGAACATATATTGTAAAATCAACCATATCAGGTATGATTGTAGAA
Candidatus Carsonella 135557 - 167442nt	(1)	---
SARS-CoV-2 Reference Genome NC_045512.2	(1)	---

Section 557

		42813 42823 42834 42845 42856 42867 42878 42889
	(42813)	
Candidatus Carsonella ruddii strain BT chromosome...	(42813)	TTTCTAGACCAAGTTTTTATATTATTTTTAGAAAATTTTAAAAATAATGATTTATTTATAAAAATTCCTTTTTTTAA
Candidatus Carsonella 135557 - 167442nt	(1)	---
SARS-CoV-2 Reference Genome NC_045512.2	(1)	---

Section 558

		42890 42900 42911 42922 42933 42944 42955 42966
	(42890)	
Candidatus Carsonella ruddii strain BT chromosome...	(42890)	TGATCTAATCATTTTTTAGTTTTATATCCTTTTGATTTTTTTCCTTTAGGAGAACAAGGATGTCTACCACCACTAGT
Candidatus Carsonella 135557 - 167442nt	(1)	---
SARS-CoV-2 Reference Genome NC_045512.2	(1)	---

		Section 559
	(42967)	42967 42977 42988 42999 43010 43021 43032 43043
Candidatus Carsonella ruddii strain BT chromosome...	(42967)	TTTACCTTCTCCACCACCTAATGGATGATCGACTGGATTCATCGCAACACCTCTTACTGTAGGTCTAATTCCTTTCC
Candidatus Carsonella 135557 - 167442nt	(1)	---
SARS-CoV-2 Reference Genome NC_045512.2	(1)	---
		Section 560
	(43044)	43044 43054 43065 43076 43087 43098 43109 43120
Candidatus Carsonella ruddii strain BT chromosome...	(43044)	ATCTATTTTGACCAGCTTTATATAATTTTTTTTTATGTTTATTAAAAATTTTTCCTAATGTAGCCATACAATTTAAA
Candidatus Carsonella 135557 - 167442nt	(1)	---
SARS-CoV-2 Reference Genome NC_045512.2	(1)	---
		Section 561
	(43121)	43121 43131 43142 43153 43164 43175 43186 43197
Candidatus Carsonella ruddii strain BT chromosome...	(43121)	GATATTTTTATTTTTTTGAAAGATGAAAGTTTTAAAATACAATATTTACTATCTTTAAAAATTAATTCAGAGTTTGT
Candidatus Carsonella 135557 - 167442nt	(1)	---
SARS-CoV-2 Reference Genome NC_045512.2	(1)	---
		Section 562
	(43198)	43198 43208 43219 43230 43241 43252 43263 43274
Candidatus Carsonella ruddii strain BT chromosome...	(43198)	TCCGGAACTTCTAGAAAAAATTGCTCCAGATTGAGGCTTTTTTTCTATACAATTTAAAAAAGAACCTATTGGAAAAT
Candidatus Carsonella 135557 - 167442nt	(1)	---
SARS-CoV-2 Reference Genome NC_045512.2	(1)	---
		Section 563
	(43275)	43275 43285 43296 43307 43318 43329 43340 43351
Candidatus Carsonella ruddii strain BT chromosome...	(43275)	TTTTTATTCTGGTAGAATTACCAGAATTGAAAACAGGATTATTTATACTTGAAATTATTTGATCATTTATAAACATT
Candidatus Carsonella 135557 - 167442nt	(1)	---
SARS-CoV-2 Reference Genome NC_045512.2	(1)	---
		Section 564
	(43352)	43352 43362 43373 43384 43395 43406 43417 43428
Candidatus Carsonella ruddii strain BT chromosome...	(43352)	TCATTAGTTCTTAATATATATCTATATTCTCCATCTAAATATTTTAATAAAGCTAAATCAACATTTCTATTTGGGTC
Candidatus Carsonella 135557 - 167442nt	(1)	---
SARS-CoV-2 Reference Genome NC_045512.2	(1)	---

Section 565

		43429 43439 43450 43461 43472 43483 43494 43505
	(43429)	
Candidatus Carsonella ruddii strain BT chromosome...	(43429)	ATAATCAAATCTTATTATTTTTGAAGGTATATCAAATTTATTTCTTAAAAAATCTATTTTTCTATACAATTTTTTAT
Candidatus Carsonella 135557 - 167442nt	(1)	---
SARS-CoV-2 Reference Genome NC_045512.2	(1)	---

Section 566

		43506 43516 43527 43538 43549 43560 43571 43582
	(43506)	
Candidatus Carsonella ruddii strain BT chromosome...	(43506)	GTTTATTTCCAATATGTCTTACTGAAATTTTACCATGACTTCTTCCAGAATTTTGTTTTTTAAAAAAACACATTTTT
Candidatus Carsonella 135557 - 167442nt	(1)	---
SARS-CoV-2 Reference Genome NC_045512.2	(1)	---

Section 567

		43583 43593 43604 43615 43626 43637 43648 43659
	(43583)	
Candidatus Carsonella ruddii strain BT chromosome...	(43583)	TTAAAAATATTCATTTAAAAATATTATTTATTAAATAAAAAAAAGATTTTTTTTCAATAAAGATTATTTCAAATTTT
Candidatus Carsonella 135557 - 167442nt	(1)	---
SARS-CoV-2 Reference Genome NC_045512.2	(1)	---

Section 568

		43660 43670 43681 43692 43703 43714 43725 43736
	(43660)	
Candidatus Carsonella ruddii strain BT chromosome...	(43660)	AAAAAATCATAAACTGATATTTTTTTAATATCTTTAAAAAAAAACATTATTTAAATTATTAAAAATTAAATTTTTTTT
Candidatus Carsonella 135557 - 167442nt	(1)	---
SARS-CoV-2 Reference Genome NC_045512.2	(1)	---

Section 569

		43737 43747 43758 43769 43780 43791 43802 43813
	(43737)	
Candidatus Carsonella ruddii strain BT chromosome...	(43737)	ATTTCTAAAAATAAAAATAAAAAACAACAAATTTTTTTCATAAATATAAATTTTATTATTTATTAAAAATAAAAAAA
Candidatus Carsonella 135557 - 167442nt	(1)	---
SARS-CoV-2 Reference Genome NC_045512.2	(1)	---

Section 570

		43814 43824 43835 43846 43857 43868 43879 43890
	(43814)	
Candidatus Carsonella ruddii strain BT chromosome...	(43814)	ATAAATTTTCAAATATTTTTTTAATATTTTTTTTTTTTTTAGAAAATAAATAATTTGAAAAAGTTACTCCACCACCT
Candidatus Carsonella 135557 - 167442nt	(1)	---
SARS-CoV-2 Reference Genome NC_045512.2	(1)	---

Section 571

(43891) 43891 43901 43912 43923 43934 43945 43956 43967
Candidatus Carsonella ruddii strain BT chromosome... (43891) CTCCAAATTGGAGATTTTTTATCTCCAGCTCTTGCTCTTCCAGTACCTTTTTGAGGCCAAGGTTTTTTTCCACTTCC
Candidatus Carsonella 135557 - 167442nt (1) ---
SARS-CoV-2 Reference Genome NC_045512.2 (1) ---

Section 572

(43968) 43968 43978 43989 44000 44011 44022 44033 44044
Candidatus Carsonella ruddii strain BT chromosome... (43968) TTTTACCAATCTTTTATTTTTTTTAAATTTTTTTTGTTTAATTTTATTTTTTAAAAAAATATAAATTAAATTTATAT
Candidatus Carsonella 135557 - 167442nt (1) ---
SARS-CoV-2 Reference Genome NC_045512.2 (1) ---

Section 573

(44045) 44045 44055 44066 44077 44088 44099 44110 44121
Candidatus Carsonella ruddii strain BT chromosome... (44045) TTAATTCTTTTATATAAATATTTAAATATTTAGGTAAAAAACTAAAAATGGGTAGTTTCATAATATTTTTCCATATA
Candidatus Carsonella 135557 - 167442nt (1) ---
SARS-CoV-2 Reference Genome NC_045512.2 (1) ---

Section 574

(44122) 44122 44132 44143 44154 44165 44176 44187 44198
Candidatus Carsonella ruddii strain BT chromosome... (44122) TAAAATCATTTTTTTTACCAGGTAATATTTTTGAAAAATAAAAATAATTTTTATTTCTTAAAAAAATTTTTAAATAT
Candidatus Carsonella 135557 - 167442nt (1) ---
SARS-CoV-2 Reference Genome NC_045512.2 (1) ---

Section 575

(44199) 44199 44209 44220 44231 44242 44253 44264 44275
Candidatus Carsonella ruddii strain BT chromosome... (44199) GTAGAAAAAATAATATCACCCATTTTTCCAGGCATTTTTTTCCCTTTAAAAACTTTGCCAGGATCTTGACATTGTCC
Candidatus Carsonella 135557 - 167442nt (1) ---
SARS-CoV-2 Reference Genome NC_045512.2 (1) ---

Section 576

(44276) 44276 44286 44297 44308 44319 44330 44341 44352
Candidatus Carsonella ruddii strain BT chromosome... (44276) AGTAGATCCCATAGTTCTATATGATAAAGAACAGCCATGACTTTTATCTTTTGTTTTAAAACCCCATTTTTTTATAA
Candidatus Carsonella 135557 - 167442nt (1) ---
SARS-CoV-2 Reference Genome NC_045512.2 (1) ---

```
                                                                                                                     Section 577
                                                       (44353) 44353     44363      44374      44385      44396      44407      44418   44429
Candidatus Carsonella ruddii strain BT chromosome...   (44353) CTCCTTGAAAACCTTTACCATTACTTATAGATTTTAATAAAAGATAATTAAAAGAAAAATTTAATAAAATTTTGTTT
           Candidatus Carsonella 135557 - 167442nt         (1) -----------------------------------------------------------------------------
        SARS-CoV-2 Reference Genome NC_045512.2            (1) -----------------------------------------------------------------------------
                                                                                                                     Section 578
                                                       (44430) 44430     44440      44451      44462      44473      44484      44495   44506
Candidatus Carsonella ruddii strain BT chromosome...   (44430) AAATTAATATATTTTATTATATTTAATAATATTATTTTTTTATTAATAAAAAATTGTTTAATTCCTTTATTAATGAA
           Candidatus Carsonella 135557 - 167442nt         (1) -----------------------------------------------------------------------------
        SARS-CoV-2 Reference Genome NC_045512.2            (1) -----------------------------------------------------------------------------
                                                                                                                     Section 579
                                                       (44507) 44507     44517      44528      44539      44550      44561      44572   44583
Candidatus Carsonella ruddii strain BT chromosome...   (44507) AATCATAAAAACAAATAATTTAATAATAAACCATCTATATTTCTATTTTTAAAAAGAAAATTTATTAAGTTTAAATC
           Candidatus Carsonella 135557 - 167442nt         (1) -----------------------------------------------------------------------------
        SARS-CoV-2 Reference Genome NC_045512.2            (1) -----------------------------------------------------------------------------
                                                                                                                     Section 580
                                                       (44584) 44584     44594      44605      44616      44627      44638      44649   44660
Candidatus Carsonella ruddii strain BT chromosome...   (44584) AAAATTTTTAATAAAAAAAAAAACTTTAAAATATTTAATTTGTATTTGATCTCTTGCATTTTTATCAACGTGTGGTG
           Candidatus Carsonella 135557 - 167442nt         (1) -----------------------------------------------------------------------------
        SARS-CoV-2 Reference Genome NC_045512.2            (1) -----------------------------------------------------------------------------
                                                                                                                     Section 581
                                                       (44661) 44661     44671      44682      44693      44704      44715      44726   44737
Candidatus Carsonella ruddii strain BT chromosome...   (44661) AAACTAATAAAGTAAATTTTTCAATTTTTTTAGGCATATAAAATGGACCAATTATCTTATATTTTTTTTTTATATTT
           Candidatus Carsonella 135557 - 167442nt         (1) -----------------------------------------------------------------------------
        SARS-CoV-2 Reference Genome NC_045512.2            (1) -----------------------------------------------------------------------------
                                                                                                                     Section 582
                                                       (44738) 44738     44748      44759      44770      44781      44792      44803   44814
Candidatus Carsonella ruddii strain BT chromosome...   (44738) TTTAATAAAAAAAATAAAAAAGATTTTATCTTAAAAAAAAAAAATGATTTAATAATAATTTTAATCATTTAATATTT
           Candidatus Carsonella 135557 - 167442nt         (1) -----------------------------------------------------------------------------
        SARS-CoV-2 Reference Genome NC_045512.2            (1) -----------------------------------------------------------------------------
```

```
                                                                                                  Section 583
                                                  (44815) 44815     44825      44836      44847      44858      44869      44880      44891
Candidatus Carsonella ruddii strain BT chromosome... (44815) TAGTAACTATTCCGGCACCAACAGTTTTTCCACCTTCTCTAATAGCAAATCTTAAACCAGCTTCTATCGCTATAGAA
         Candidatus Carsonella 135557 - 167442nt     (1) -----------------------------------------------------------------------------
        SARS-CoV-2 Reference Genome NC_045512.2      (1) -----------------------------------------------------------------------------
```

```
                                                                                                  Section 584
                                                  (44892) 44892     44902      44913      44924      44935      44946      44957      44968
Candidatus Carsonella ruddii strain BT chromosome... (44892) GAAAGCAACGAAACATTTAATTTAATATTATCTCCAGGTAATACCATTTCAACAGAAGAAGGTAGTTTACAAATACC
         Candidatus Carsonella 135557 - 167442nt     (1) -----------------------------------------------------------------------------
        SARS-CoV-2 Reference Genome NC_045512.2      (1) -----------------------------------------------------------------------------
```

```
                                                                                                  Section 585
                                                  (44969) 44969     44979      44990      45001      45012      45023      45034      45045
Candidatus Carsonella ruddii strain BT chromosome... (44969) AGTAACATCTGTTGTTCTAAAATAAAATTGAGGTTTATATCCATTAAAAAATGGTGTGTGTCTTCCACCTTCTTCTT
         Candidatus Carsonella 135557 - 167442nt     (1) -----------------------------------------------------------------------------
        SARS-CoV-2 Reference Genome NC_045512.2      (1) -----------------------------------------------------------------------------
```

```
                                                                                                  Section 586
                                                  (45046) 45046     45056      45067      45078      45089      45100      45111      45122
Candidatus Carsonella ruddii strain BT chromosome... (45046) TAGATAATATATATACTTCACATTCAAAATTAGTATATGATTTTATACTTCCAAGTTTAGCAACAACTTGACCTCTT
         Candidatus Carsonella 135557 - 167442nt     (1) -----------------------------------------------------------------------------
        SARS-CoV-2 Reference Genome NC_045512.2      (1) -----------------------------------------------------------------------------
```

```
                                                                                                  Section 587
                                                  (45123) 45123     45133      45144      45155      45166      45177      45188      45199
Candidatus Carsonella ruddii strain BT chromosome... (45123) TCTACTTCTTCTCTTTTTACACTTCTTAATAAAATTCCAACATTTTCTCCAGCTATTCCTTCTTCTAAAGTTTTTTT
         Candidatus Carsonella 135557 - 167442nt     (1) -----------------------------------------------------------------------------
        SARS-CoV-2 Reference Genome NC_045512.2      (1) -----------------------------------------------------------------------------
```

```
                                                                                                  Section 588
                                                  (45200) 45200     45210      45221      45232      45243      45254      45265      45276
Candidatus Carsonella ruddii strain BT chromosome... (45200) AAACATTTCTATACCAGTAATTGTTGTTTTTAAAGTTGGTTTTAATCCAACAATTTCTATTTCTTCACCATTTTTAA
         Candidatus Carsonella 135557 - 167442nt     (1) -----------------------------------------------------------------------------
        SARS-CoV-2 Reference Genome NC_045512.2      (1) -----------------------------------------------------------------------------
```

```
                                                                    Section 589
                                                   (45277) 45277     45287      45298      45309      45320      45331      45342      45353
Candidatus Carsonella ruddii strain BT chromosome... (45277) CAATACCTCTTTCAATTTTACCAGTTACAACAGTTCCTCTACCTGATATAGAAAATACATCTTCAATTGGCATTAAA
         Candidatus Carsonella 135557 - 167442nt (1) -----------------------------------------------------------------------------
       SARS-CoV-2 Reference Genome NC_045512.2 (1) -----------------------------------------------------------------------------
                                                                    Section 590
                                                   (45354) 45354     45364      45375      45386      45397      45408      45419      45430
Candidatus Carsonella ruddii strain BT chromosome... (45354) AAAGGTTGATCTGTAATTCTATTTGGAACAGGAATATTTTTATCTAATACATTTAACAAATTTTTTATTGATGTAGT
         Candidatus Carsonella 135557 - 167442nt (1) -----------------------------------------------------------------------------
       SARS-CoV-2 Reference Genome NC_045512.2 (1) -----------------------------------------------------------------------------
                                                                    Section 591
                                                   (45431) 45431     45441      45452      45463      45474      45485      45496      45507
Candidatus Carsonella ruddii strain BT chromosome... (45431) TCCTAATTGATTATCATCTTTTCCTTCTAATGCTAATAATGCAGATCCTATTATAATTTTAACATTATTACCATCAT
         Candidatus Carsonella 135557 - 167442nt (1) -----------------------------------------------------------------------------
       SARS-CoV-2 Reference Genome NC_045512.2 (1) -----------------------------------------------------------------------------
                                                                    Section 592
                                                   (45508) 45508     45518      45529      45540      45551      45562      45573      45584
Candidatus Carsonella ruddii strain BT chromosome... (45508) AATCATATTCTGATAACAATTCTCTTATCTCCATTTCAACTAAATCTAAAAGTTCTTTATCTTGAACACAATCAGCT
         Candidatus Carsonella 135557 - 167442nt (1) -----------------------------------------------------------------------------
       SARS-CoV-2 Reference Genome NC_045512.2 (1) -----------------------------------------------------------------------------
                                                                    Section 593
                                                   (45585) 45585     45595      45606      45617      45628      45639      45650      45661
Candidatus Carsonella ruddii strain BT chromosome... (45585) TTATTTAAAAAAACAATTATATTAGGAACACCTACTTGTCTTGCTAAAAGAATATGCTCTCTTGTTTGAGGCATAGG
         Candidatus Carsonella 135557 - 167442nt (1) -----------------------------------------------------------------------------
       SARS-CoV-2 Reference Genome NC_045512.2 (1) -----------------------------------------------------------------------------
                                                                    Section 594
                                                   (45662) 45662     45672      45683      45694      45705      45716      45727      45738
Candidatus Carsonella ruddii strain BT chromosome... (45662) ACCATCGATAGCTGAACACACTAAAATTGCTCCATCCATTTGTGCTGCTCCTGTTATCATATTTTTAATATAATCAG
         Candidatus Carsonella 135557 - 167442nt (1) -----------------------------------------------------------------------------
       SARS-CoV-2 Reference Genome NC_045512.2 (1) -----------------------------------------------------------------------------
```

```
                                                                                                              Section 595
                                                   (45739) 45739     45749      45760      45771      45782      45793      45804     45815
Candidatus Carsonella ruddii strain BT chromosome... (45739) CATGACCAGGACAATCTACATGAGCATAATGTTTAGTATCAGATTCATATTCTACATGAGAAGTAGAAATAGTTATA
          Candidatus Carsonella 135557 - 167442nt      (1) -----------------------------------------------------------------------------
       SARS-CoV-2 Reference Genome NC_045512.2         (1) -----------------------------------------------------------------------------
                                                                                                              Section 596
                                                   (45816) 45816     45826      45837      45848      45859      45870      45881     45892
Candidatus Carsonella ruddii strain BT chromosome... (45816) CCTCTTTCTTTTTCTTCTGGAGCATTATCAATGCTATCAAAAGCTCTAATTTCTCCTCCATAAATTTCTGAAGATAC
          Candidatus Carsonella 135557 - 167442nt      (1) -----------------------------------------------------------------------------
       SARS-CoV-2 Reference Genome NC_045512.2         (1) -----------------------------------------------------------------------------
                                                                                                              Section 597
                                                   (45893) 45893     45903      45914      45925      45936      45947      45958     45969
Candidatus Carsonella ruddii strain BT chromosome... (45893) TTTAGTTAAAGCAGCAGTTAAAGTTGTTTTACCATGATCAACATGTCCTATTGTTCCAACATTAATATGTATTTTAT
          Candidatus Carsonella 135557 - 167442nt      (1) -----------------------------------------------------------------------------
       SARS-CoV-2 Reference Genome NC_045512.2         (1) -----------------------------------------------------------------------------
                                                                                                              Section 598
                                                   (45970) 45970     45980      45991      46002      46013      46024      46035     46046
Candidatus Carsonella ruddii strain BT chromosome... (45970) TTCTATTAAATTTTATTTTAGCCATATTTTTAATTATTTTTAATTATTTTTTCAACTATATAATTTGGAAGTTCATT
          Candidatus Carsonella 135557 - 167442nt      (1) -----------------------------------------------------------------------------
       SARS-CoV-2 Reference Genome NC_045512.2         (1) -----------------------------------------------------------------------------
                                                                                                              Section 599
                                                   (46047) 46047     46057      46068      46079      46090      46101      46112     46123
Candidatus Carsonella ruddii strain BT chromosome... (46047) ATAATTATGAAATTCCATATTATAACTTGCTCTACCTTTTGTATTAGATCTTAATTCAGTAGAATAACCAAATAATT
          Candidatus Carsonella 135557 - 167442nt      (1) -----------------------------------------------------------------------------
       SARS-CoV-2 Reference Genome NC_045512.2         (1) -----------------------------------------------------------------------------
                                                                                                              Section 600
                                                   (46124) 46124     46134      46145      46156      46167      46178      46189     46200
Candidatus Carsonella ruddii strain BT chromosome... (46124) CTCTTAAAGGTAAAAACGAATTGATAACTTTAAAATTATTATAATCTATAACATTATTTATAATACCTCTTTTTTTA
          Candidatus Carsonella 135557 - 167442nt      (1) -----------------------------------------------------------------------------
       SARS-CoV-2 Reference Genome NC_045512.2         (1) -----------------------------------------------------------------------------
```

```
                                                                                                                                      Section 601
                                                           (46201) 46201     46211      46222      46233      46244      46255      46266      46277
Candidatus Carsonella ruddii strain BT chromosome... (46201) TTAATATCTGCAACAACAATTCCTAAATATTCACTAGGTAAATTAACTTCTACTTTCATAATAGGTTCAAGAACATA
            Candidatus Carsonella 135557 - 167442nt      (1) -----------------------------------------------------------------------------
        SARS-CoV-2 Reference Genome NC_045512.2      (1) -----------------------------------------------------------------------------
                                                                                                                                      Section 602
                                                           (46278) 46278     46288      46299      46310      46321      46332      46343      46354
Candidatus Carsonella ruddii strain BT chromosome... (46278) AGAATTTGCTTTAATTAAACCTTCTTTTATTGCTATAGAAGAAGCTGCTTTAAAAGCATATTCACTTGAATCAACAG
            Candidatus Carsonella 135557 - 167442nt      (1) -----------------------------------------------------------------------------
        SARS-CoV-2 Reference Genome NC_045512.2      (1) -----------------------------------------------------------------------------
                                                                                                                                      Section 603
                                                           (46355) 46355     46365      46376      46387      46398      46409      46420      46431
Candidatus Carsonella ruddii strain BT chromosome... (46355) AATGATAAGAACCATCTAAAACACTTATTTTAATTTTTATTACTGGATAACCGAGTATTACTCCTTGATTTAATTGT
            Candidatus Carsonella 135557 - 167442nt      (1) -----------------------------------------------------------------------------
        SARS-CoV-2 Reference Genome NC_045512.2      (1) -----------------------------------------------------------------------------
                                                                                                                                      Section 604
                                                           (46432) 46432     46442      46453      46464      46475      46486      46497      46508
Candidatus Carsonella ruddii strain BT chromosome... (46432) TGAATTACTCCTTTTTCAATAGAAGGAATATATTCTTTTGGTATAGATCCACCAATTATTTCAGATTTAAAAATAAA
            Candidatus Carsonella 135557 - 167442nt      (1) -----------------------------------------------------------------------------
        SARS-CoV-2 Reference Genome NC_045512.2      (1) -----------------------------------------------------------------------------
                                                                                                                                      Section 605
                                                           (46509) 46509     46519      46530      46541      46552      46563      46574      46585
Candidatus Carsonella ruddii strain BT chromosome... (46509) ATTTTCTTCTTTATCAAAAGATATTGGTTCTAATTTTAAAAAAACATGACCGTATTGTCCTTTTCCTCCAGATTGTT
            Candidatus Carsonella 135557 - 167442nt      (1) -----------------------------------------------------------------------------
        SARS-CoV-2 Reference Genome NC_045512.2      (1) -----------------------------------------------------------------------------
                                                                                                                                      Section 606
                                                           (46586) 46586     46596      46607      46618      46629      46640      46651      46662
Candidatus Carsonella ruddii strain BT chromosome... (46586) TTATATATTTTCCTTCTTGAATTATTGTTTTTTTTATACTTTCTTTATAAGAAACTTGAGGTTTGCTAATTTTAGTT
            Candidatus Carsonella 135557 - 167442nt      (1) -----------------------------------------------------------------------------
        SARS-CoV-2 Reference Genome NC_045512.2      (1) -----------------------------------------------------------------------------
```

```
                                                                    Section 607
                                                     (46663) 46663     46673      46684      46695      46706      46717      46728     46739
Candidatus Carsonella ruddii strain BT chromosome... (46663) TTTATATTATAATCTCTATTAATTCTATCTACAATAATTTCTAAATGTAATTCTCCCATTCCTGAAATAAGAGTTTC
          Candidatus Carsonella 135557 - 167442nt        (1) -----------------------------------------------------------------------------
         SARS-CoV-2 Reference Genome NC_045512.2         (1) -----------------------------------------------------------------------------
```

```
                                                                    Section 608
                                                     (46740) 46740     46750      46761      46772      46783      46794      46805     46816
Candidatus Carsonella ruddii strain BT chromosome... (46740) TCCTGTTAAATTATTATTATTTAAAATTAAAGATGGATCTTCTTTACAAATTTTTTGTATTATATTTAATAATTTTT
          Candidatus Carsonella 135557 - 167442nt        (1) -----------------------------------------------------------------------------
         SARS-CoV-2 Reference Genome NC_045512.2         (1) -----------------------------------------------------------------------------
```

```
                                                                    Section 609
                                                     (46817) 46817     46827      46838      46849      46860      46871      46882     46893
Candidatus Carsonella ruddii strain BT chromosome... (46817) CTTGATCATTTTTAAATATTGGTTCTATTGAAATTGAAATTACAGGTATTGGTATATTAATTTTTTCTAATTGAAAA
          Candidatus Carsonella 135557 - 167442nt        (1) -----------------------------------------------------------------------------
         SARS-CoV-2 Reference Genome NC_045512.2         (1) -----------------------------------------------------------------------------
```

```
                                                                    Section 610
                                                     (46894) 46894     46904      46915      46926      46937      46948      46959     46970
Candidatus Carsonella ruddii strain BT chromosome... (46894) TTTTCATTTAAAGAACAAAGTGTATCACCAGTTGTAGATTCTTTTAATCCTATTAAAACAACAATATCACCACAATT
          Candidatus Carsonella 135557 - 167442nt        (1) -----------------------------------------------------------------------------
         SARS-CoV-2 Reference Genome NC_045512.2         (1) -----------------------------------------------------------------------------
```

```
                                                                    Section 611
                                                     (46971) 46971     46981      46992      47003      47014      47025      47036     47047
Candidatus Carsonella ruddii strain BT chromosome... (46971) TGCTTTATTAATATCTTTTTTAGAATTTGCAAACATTCTTATAATTCTAAAAATTTTTTCTTTAATTTTTTTTGTAG
          Candidatus Carsonella 135557 - 167442nt        (1) -----------------------------------------------------------------------------
         SARS-CoV-2 Reference Genome NC_045512.2         (1) -----------------------------------------------------------------------------
```

```
                                                                    Section 612
                                                     (47048) 47048     47058      47069      47080      47091      47102      47113     47124
Candidatus Carsonella ruddii strain BT chromosome... (47048) AATTATAAATAATGTCACCACTTTTAATTGAACCAGAATAAATTCTTATAAAAGATAATGAACCTAAAAATGGATCA
          Candidatus Carsonella 135557 - 167442nt        (1) -----------------------------------------------------------------------------
         SARS-CoV-2 Reference Genome NC_045512.2         (1) -----------------------------------------------------------------------------
```

Section 613

	(47125)	47125 47135 47146 47157 47168 47179 47190 47201
Candidatus Carsonella ruddii strain BT chromosome...	(47125)	TTATGTATTTTAAACAATAAAGATAAAAATTTTTTATTAATAGATACATTAAAATTTAAATTATTACATCCTTTAAT
Candidatus Carsonella 135557 - 167442nt	(1)	---
SARS-CoV-2 Reference Genome NC_045512.2	(1)	---

Section 614

	(47202)	47202 47212 47223 47234 47245 47256 47267 47278
Candidatus Carsonella ruddii strain BT chromosome...	(47202)	TCCTATATCAATCGGAGAAGGTAAAAAATTACAAATACTATTTAATAAATATTCTATACCTTTATTTTTTAAAGAAG
Candidatus Carsonella 135557 - 167442nt	(1)	---
SARS-CoV-2 Reference Genome NC_045512.2	(1)	---

Section 615

	(47279)	47279 47289 47300 47311 47322 47333 47344 47355
Candidatus Carsonella ruddii strain BT chromosome...	(47279)	AACCACACATAATTGGTATAATTTTATTATTTATAACTAAATTTCTGATAGATTTTTTAATATCTAATAAATTAAAT
Candidatus Carsonella 135557 - 167442nt	(1)	---
SARS-CoV-2 Reference Genome NC_045512.2	(1)	---

Section 616

	(47356)	47356 47366 47377 47388 47399 47410 47421 47432
Candidatus Carsonella ruddii strain BT chromosome...	(47356)	TCATTATTTATATATTTAATTAAAAATTCTTCATCATTATTAGATAACAATTCTAACAAATAATTTCTATAAAAATT
Candidatus Carsonella 135557 - 167442nt	(1)	---
SARS-CoV-2 Reference Genome NC_045512.2	(1)	---

Section 617

	(47433)	47433 47443 47454 47465 47476 47487 47498 47509
Candidatus Carsonella ruddii strain BT chromosome...	(47433)	TGCAATTTTTAAATATTCATTATTTATATTTTCTATATATAAAATATTATTTTTCCAAATGTTAATTTTCATTTCTA
Candidatus Carsonella 135557 - 167442nt	(1)	---
SARS-CoV-2 Reference Genome NC_045512.2	(1)	---

Section 618

	(47510)	47510 47520 47531 47542 47553 47564 47575 47586
Candidatus Carsonella ruddii strain BT chromosome...	(47510)	TTATATCAATAAATCCTAAAAAATTATTTTCTGAACCTATATTAAAATTTAAAGGTGCTAAAACACAATTAAATTTT
Candidatus Carsonella 135557 - 167442nt	(1)	---
SARS-CoV-2 Reference Genome NC_045512.2	(1)	---

Section 619

```
                                            (47587) 47587     47597      47608      47619      47630      47641      47652      47663
Candidatus Carsonella ruddii strain BT chromosome... (47587) TTTTTAATATTATCTATACAATTTTTATAATTTGCTCCTATTCTATCCATTTTATTAACAAATAAAATTTTTGGTAT
         Candidatus Carsonella 135557 - 167442nt     (1) -----------------------------------------------------------------------------
      SARS-CoV-2 Reference Genome NC_045512.2     (1) -----------------------------------------------------------------------------
```

Section 620

```
                                            (47664) 47664     47674      47685      47696      47707      47718      47729      47740
Candidatus Carsonella ruddii strain BT chromosome... (47664) TTTATATTTTTGAGATTGTAACCAAACGGTTTCAGTTTGTGGTTGAATTCCAGAAGATGCACATACAATTATTACAG
         Candidatus Carsonella 135557 - 167442nt     (1) -----------------------------------------------------------------------------
      SARS-CoV-2 Reference Genome NC_045512.2     (1) -----------------------------------------------------------------------------
```

Section 621

```
                                            (47741) 47741     47751      47762      47773      47784      47795      47806      47817
Candidatus Carsonella ruddii strain BT chromosome... (47741) CCCCATCTAATACTCTTAATGATCTTTCAACTTCAATAGTAAAATCAACATGTCCTGGAGTATCTATTATATTTATA
         Candidatus Carsonella 135557 - 167442nt     (1) -----------------------------------------------------------------------------
      SARS-CoV-2 Reference Genome NC_045512.2     (1) -----------------------------------------------------------------------------
```

Section 622

```
                                            (47818) 47818     47828      47839      47850      47861      47872      47883      47894
Candidatus Carsonella ruddii strain BT chromosome... (47818) ATATATTGTATATCATTTATTTTCCAATATATTGTTACTGATGCTGATGTTATTGTTATTCCTCTTTCTTGTTCTTG
         Candidatus Carsonella 135557 - 167442nt     (1) -----------------------------------------------------------------------------
      SARS-CoV-2 Reference Genome NC_045512.2     (1) -----------------------------------------------------------------------------
```

Section 623

```
                                            (47895) 47895     47905      47916      47927      47938      47949      47960      47971
Candidatus Carsonella ruddii strain BT chromosome... (47895) TTTCATCCAATCAGTAACAGTATTTCCATGATGTACTTCTCCTATTTTATGTGAAACTCCTGAGTAAAACAATATTC
         Candidatus Carsonella 135557 - 167442nt     (1) -----------------------------------------------------------------------------
      SARS-CoV-2 Reference Genome NC_045512.2     (1) -----------------------------------------------------------------------------
```

Section 624

```
                                            (47972) 47972     47982      47993      48004      48015      48026      48037      48048
Candidatus Carsonella ruddii strain BT chromosome... (47972) TTTCTGTAGTAGTAGTTTTTCCAGCATCTACATGTGCTATTATACCTATATTTCTTAAATATAAAATATTATTCATA
         Candidatus Carsonella 135557 - 167442nt     (1) -----------------------------------------------------------------------------
      SARS-CoV-2 Reference Genome NC_045512.2     (1) -----------------------------------------------------------------------------
```

Section 625

		(48049) 48049 48059 48070 48081 48092 48103 48114 48125
Candidatus Carsonella ruddii strain BT chromosome...	(48049)	TTTTAAAATTAGAATAAGCTTTATTTTGTTCTGAAATTTTTAAAATATTTTCTTTTTGTTTTGTTGATAAAGAATTA
Candidatus Carsonella 135557 - 167442nt	(1)	---
SARS-CoV-2 Reference Genome NC_045512.2	(1)	---

Section 626

		(48126) 48126 48136 48147 48158 48169 48180 48191 48202
Candidatus Carsonella ruddii strain BT chromosome...	(48126)	TTATAATAAGTATCTATCATTTCACCTACTAATTTATTTTTAAAACCATTTTCATTTCTTTTTCTTGAATTAAACAC
Candidatus Carsonella 135557 - 167442nt	(1)	---
SARS-CoV-2 Reference Genome NC_045512.2	(1)	---

Section 627

		(48203) 48203 48213 48224 48235 48246 48257 48268 48279
Candidatus Carsonella ruddii strain BT chromosome...	(48203)	AATCCATTTCATTGCAAACATTAAACTTCTTTTTAAATTTATTTTAAAAGGGATATTATAATTCATTCCTCCTATTT
Candidatus Carsonella 135557 - 167442nt	(1)	---
SARS-CoV-2 Reference Genome NC_045512.2	(1)	---

Section 628

		(48280) 48280 48290 48301 48312 48323 48334 48345 48356
Candidatus Carsonella ruddii strain BT chromosome...	(48280)	TTCTTTTTTTAATTTCAAATATTGGTTGAACATTTAATAATATTTTTTAATTAAAAAAAATGGATTTTTTTTTAAT
Candidatus Carsonella 135557 - 167442nt	(1)	---
SARS-CoV-2 Reference Genome NC_045512.2	(1)	---

Section 629

		(48357) 48357 48367 48378 48389 48400 48411 48422 48433
Candidatus Carsonella ruddii strain BT chromosome...	(48357)	TTAATTGTAATTAAATTTATACTATAATAAAAAATTTTTTGAGCTATTTGTTTTTTTCCATTTTTCATAATATAATT
Candidatus Carsonella 135557 - 167442nt	(1)	---
SARS-CoV-2 Reference Genome NC_045512.2	(1)	---

Section 630

		(48434) 48434 48444 48455 48466 48477 48488 48499 48510
Candidatus Carsonella ruddii strain BT chromosome...	(48434)	TATAAATTTAGAAAATAAATAACTTCCATATTTAATATCATTAAATATTATTATTTTTAAATATTTTTTTTTCCTAG
Candidatus Carsonella 135557 - 167442nt	(1)	---
SARS-CoV-2 Reference Genome NC_045512.2	(1)	---

Section 631

	(48511)	48511 48521 48532 48543 48554 48565 48576 48587
Candidatus Carsonella ruddii strain BT chromosome...	(48511)	ACATTTTTTGTTCCATATTTTGATCTAGAATTTTTTCTATTAGAAACACCTGATAAATCGTAAACATTTCTTATAGC
Candidatus Carsonella 135557 - 167442nt	(1)	---
SARS-CoV-2 Reference Genome NC_045512.2	(1)	---

Section 632

	(48588)	48588 48598 48609 48620 48631 48642 48653 48664
Candidatus Carsonella ruddii strain BT chromosome...	(48588)	ATGATATTTTACTCCTGGTAAATCTTTAACTCTACCTCCTCTTACTAAAATATTTGAATGTTCTTGTAAATTATGTC
Candidatus Carsonella 135557 - 167442nt	(1)	---
SARS-CoV-2 Reference Genome NC_045512.2	(1)	---

Section 633

	(48665)	48665 48675 48686 48697 48708 48719 48730 48741
Candidatus Carsonella ruddii strain BT chromosome...	(48665)	CTTCACCAGGTATGTAAACAGTTATTTCATTTTTATTTGATAATTTTACTCTACAAACTTTTCTTAAGGCAGAATTT
Candidatus Carsonella 135557 - 167442nt	(1)	---
SARS-CoV-2 Reference Genome NC_045512.2	(1)	---

Section 634

	(48742)	48742 48752 48763 48774 48785 48796 48807 48818
Candidatus Carsonella ruddii strain BT chromosome...	(48742)	GGTTTTTTTGGTGTAGTTGTATATACTTTAATGCATATTCCTTTTTTTTGTGGAGAGCTTAATAAAGCTGGTGTTTT
Candidatus Carsonella 135557 - 167442nt	(1)	---
SARS-CoV-2 Reference Genome NC_045512.2	(1)	---

Section 635

	(48819)	48819 48829 48840 48851 48862 48873 48884 48895
Candidatus Carsonella ruddii strain BT chromosome...	(48819)	TATTTTTTTTTTATTTTTTTTTCTTCCTAATCTAATAATTTGATTTATAGTCATTTAATTATATAAATAATATTTAA
Candidatus Carsonella 135557 - 167442nt	(1)	---
SARS-CoV-2 Reference Genome NC_045512.2	(1)	---

Section 636

	(48896)	48896 48906 48917 48928 48939 48950 48961 48972
Candidatus Carsonella ruddii strain BT chromosome...	(48896)	AATTTTTTCTTTTTACAACATCATTTTTTTTTGTTTTTTTTTCTAAAAAAAAAATATGATTTATAAGTCCTGTACCA
Candidatus Carsonella 135557 - 167442nt	(1)	---
SARS-CoV-2 Reference Genome NC_045512.2	(1)	---

```
                                                                                              Section 637
                                               (48973) 48973     48983      48994      49005      49016      49027      49038   49049
Candidatus Carsonella ruddii strain BT chromosome... (48973) GCTGGTATTAATTTACCTATTACTACATTTTCTTTTAATCCTAATAAATAATCTATTCTATTTCTAATAGCAGAATC
           Candidatus Carsonella 135557 - 167442nt (1) -----------------------------------------------------------------------------
           SARS-CoV-2 Reference Genome NC_045512.2 (1) -----------------------------------------------------------------------------
                                                                                              Section 638
                                               (49050) 49050     49060      49071      49082      49093      49104      49115   49126
Candidatus Carsonella ruddii strain BT chromosome... (49050) AACTAAAATTCTTGTTGTTTCTTGAAATGAAGCTGCAGAAAAAAAAGATGTTGATTCTAATGAAACTTTTGTAATAC
           Candidatus Carsonella 135557 - 167442nt (1) -----------------------------------------------------------------------------
           SARS-CoV-2 Reference Genome NC_045512.2 (1) -----------------------------------------------------------------------------
                                                                                              Section 639
                                               (49127) 49127     49137      49148      49159      49170      49181      49192   49203
Candidatus Carsonella ruddii strain BT chromosome... (49127) CAAATATTATTCTTTCATATAAAGAAATTTTTTTAGAATATTTTAATGTATTAATATTTTCAACTAATACATCTTCT
           Candidatus Carsonella 135557 - 167442nt (1) -----------------------------------------------------------------------------
           SARS-CoV-2 Reference Genome NC_045512.2 (1) -----------------------------------------------------------------------------
                                                                                              Section 640
                                               (49204) 49204     49214      49225      49236      49247      49258      49269   49280
Candidatus Carsonella ruddii strain BT chromosome... (49204) AAATATAAAATATCGTTTTGCATAAAAAAACAATCACCTGAATAAAGGATTTTTACTTTTTTTGTCATTTGTTTTAA
           Candidatus Carsonella 135557 - 167442nt (1) -----------------------------------------------------------------------------
           SARS-CoV-2 Reference Genome NC_045512.2 (1) -----------------------------------------------------------------------------
                                                                                              Section 641
                                               (49281) 49281     49291      49302      49313      49324      49335      49346   49357
Candidatus Carsonella ruddii strain BT chromosome... (49281) AACTAATTCAATATGTTTATCATTAATAAAAATTCCTTGTGGTTCATAAATAATATTTATTTCTTCTACAAAAAAGT
           Candidatus Carsonella 135557 - 167442nt (1) -----------------------------------------------------------------------------
           SARS-CoV-2 Reference Genome NC_045512.2 (1) -----------------------------------------------------------------------------
                                                                                              Section 642
                                               (49358) 49358     49368      49379      49390      49401      49412      49423   49434
Candidatus Carsonella ruddii strain BT chromosome... (49358) CTATTAAATAATCTATACCTATTATTTTAATTATATCATTTAAATCAATTTTTCCTTCTGAAATAATATCACCTATT
           Candidatus Carsonella 135557 - 167442nt (1) -----------------------------------------------------------------------------
           SARS-CoV-2 Reference Genome NC_045512.2 (1) -----------------------------------------------------------------------------
```

Section 643

		49435 49445 49456 49467 49478 49489 49500 49511
	(49435)	
Candidatus Carsonella ruddii strain BT chromosome...	(49435)	TTTACATAATCTCCATTACTAATATATATTTTTGTTAAACAATCTAAAATATATTGTTTATAAAATCCATATTTTGA
Candidatus Carsonella 135557 - 167442nt	(1)	---
SARS-CoV-2 Reference Genome NC_045512.2	(1)	---

Section 644

		49512 49522 49533 49544 49555 49566 49577 49588
	(49512)	
Candidatus Carsonella ruddii strain BT chromosome...	(49512)	AGTAATAATAATATTAATTTTACAATTTTTATCAAAAAAAATTTGTACTATTCCATCTATTTCAGATAATAAAGATT
Candidatus Carsonella 135557 - 167442nt	(1)	---
SARS-CoV-2 Reference Genome NC_045512.2	(1)	---

Section 645

		49589 49599 49610 49621 49632 49643 49654 49665
	(49589)	
Candidatus Carsonella ruddii strain BT chromosome...	(49589)	TTTTTTTTGGAACTCTTGCTTCAAAAAGTTCACTTAATCTAGGTAAACCACCTATTATACTTGATTTATAAATATTT
Candidatus Carsonella 135557 - 167442nt	(1)	---
SARS-CoV-2 Reference Genome NC_045512.2	(1)	---

Section 646

		49666 49676 49687 49698 49709 49720 49731 49742
	(49666)	
Candidatus Carsonella ruddii strain BT chromosome...	(49666)	TGTTTTACAATTTTTGCTATTATTGAACCTGGTAATAAAAAATCATAATTTTCCACTATAATAAAAAAATTTTTAGG
Candidatus Carsonella 135557 - 167442nt	(1)	---
SARS-CoV-2 Reference Genome NC_045512.2	(1)	---

Section 647

		49743 49753 49764 49775 49786 49797 49808 49819
	(49743)	
Candidatus Carsonella ruddii strain BT chromosome...	(49743)	TATACAATATTTTTTAATTTTATTTTTATTTTTTATAAAAATAATACAATTTTCTAAAGATTTATAATAAAATTTAT
Candidatus Carsonella 135557 - 167442nt	(1)	---
SARS-CoV-2 Reference Genome NC_045512.2	(1)	---

Section 648

		49820 49830 49841 49852 49863 49874 49885 49896
	(49820)	
Candidatus Carsonella ruddii strain BT chromosome...	(49820)	TATCAGAAATACATAAAACTTTGTTTAAAATACCTTTAATTAAAACATAACCTATATTTTCTGTATATATATTAAAA
Candidatus Carsonella 135557 - 167442nt	(1)	---
SARS-CoV-2 Reference Genome NC_045512.2	(1)	---

Section 649

		49897 49907 49918 49929 49940 49951 49962 49973
	(49897)	
Candidatus Carsonella ruddii strain BT chromosome...	(49897)	TCATTTTCAAAATTTTTAATTTTAGTATTTTTTTTTATAAAAATACCATTTCTAATAGGAATTGTATCACCATAATA
Candidatus Carsonella 135557 - 167442nt	(1)	---
SARS-CoV-2 Reference Genome NC_045512.2	(1)	---

Section 650

		49974 49984 49995 50006 50017 50028 50039 50050
	(49974)	
Candidatus Carsonella ruddii strain BT chromosome...	(49974)	TAATTTATAAATTAAATTTTCTTTATTAAATTTTTCAATAATTAAAATACTATAATTATTTAATATAATAAATTCTC
Candidatus Carsonella 135557 - 167442nt	(1)	---
SARS-CoV-2 Reference Genome NC_045512.2	(1)	---

Section 651

		50051 50061 50072 50083 50094 50105 50116 50127
	(50051)	
Candidatus Carsonella ruddii strain BT chromosome...	(50051)	CATTACTATTTAACAAACATTTACTATTTTTAAAGTATACAAAACCTGATATATTTGATTTTAAATATTCAAAACTA
Candidatus Carsonella 135557 - 167442nt	(1)	---
SARS-CoV-2 Reference Genome NC_045512.2	(1)	---

Section 652

		50128 50138 50149 50160 50171 50182 50193 50204
	(50128)	
Candidatus Carsonella ruddii strain BT chromosome...	(50128)	GAAAAATAAGTAGCAACTCCACCAGTATGAAAAGTTCTCATTGTTAATTGTGTTCCTGGTTCTCCAATTGATTGAGC
Candidatus Carsonella 135557 - 167442nt	(1)	---
SARS-CoV-2 Reference Genome NC_045512.2	(1)	---

Section 653

		50205 50215 50226 50237 50248 50259 50270 50281
	(50205)	
Candidatus Carsonella ruddii strain BT chromosome...	(50205)	AGATAAAACACCAATTGGAACACCAATTAAAACTAATTTATTAGTACTAAAATCAATTCCATAACAAAAACTACATA
Candidatus Carsonella 135557 - 167442nt	(1)	---
SARS-CoV-2 Reference Genome NC_045512.2	(1)	---

Section 654

		50282 50292 50303 50314 50325 50336 50347 50358
	(50282)	
Candidatus Carsonella ruddii strain BT chromosome...	(50282)	CACCTCTAGATGATATACAATGTATAACACTTCTTATAATTAAATAATCAATTTTTTTTTAATAATTAAAAAAATT
Candidatus Carsonella 135557 - 167442nt	(1)	---
SARS-CoV-2 Reference Genome NC_045512.2	(1)	---

Section 655

	(50359)	50359 50369 50380 50391 50402 50413 50424 50435
Candidatus Carsonella ruddii strain BT chromosome...	(50359)	ATTTTATTATCTATAAAAGTATTTTCTTTTATTAATAATTTATTTTTATTGTAAATATTAAATAATAATACTCTACC
Candidatus Carsonella 135557 - 167442nt	(1)	---
SARS-CoV-2 Reference Genome NC_045512.2	(1)	---

Section 656

	(50436)	50436 50446 50457 50468 50479 50490 50501 50512
Candidatus Carsonella ruddii strain BT chromosome...	(50436)	AAATATTTTTTTATATAATAAATAAATATTTTTATAAAAATAAATAAAAATTTCTATACCAATTTTTGTAAAACAAT
Candidatus Carsonella 135557 - 167442nt	(1)	---
SARS-CoV-2 Reference Genome NC_045512.2	(1)	---

Section 657

	(50513)	50513 50523 50534 50545 50556 50567 50578 50589
Candidatus Carsonella ruddii strain BT chromosome...	(50513)	CAATTTTATAAATAACAATATCTTGCATAACATCTACTAATTTTCTGGTCAAATATCCTGAATTTGCTGTTTTTAAA
Candidatus Carsonella 135557 - 167442nt	(1)	---
SARS-CoV-2 Reference Genome NC_045512.2	(1)	---

Section 658

	(50590)	50590 50600 50611 50622 50633 50644 50655 50666
Candidatus Carsonella ruddii strain BT chromosome...	(50590)	GAAGTATCTGTTAAACCTTTTCTAGCACCAAAAGAAGATATAAAAAAATTTTTCATACTTAAACCATTTTTTAAATT
Candidatus Carsonella 135557 - 167442nt	(1)	---
SARS-CoV-2 Reference Genome NC_045512.2	(1)	---

Section 659

	(50667)	50667 50677 50688 50699 50710 50721 50732 50743
Candidatus Carsonella ruddii strain BT chromosome...	(50667)	ATCTAATATAGGATCAATAATAATATCTCCATTAGATTTTGAAAAAAATCCTCTAAAAGTTATTAATTGTTTAATTT
Candidatus Carsonella 135557 - 167442nt	(1)	---
SARS-CoV-2 Reference Genome NC_045512.2	(1)	---

Section 660

	(50744)	50744 50754 50765 50776 50787 50798 50809 50820
Candidatus Carsonella ruddii strain BT chromosome...	(50744)	GTAACATACTTCCTCTTGAACCAGAATCTAACATAATAAATAAATTATTTAAATAAAAATTATTTTTTTTTAAATTT
Candidatus Carsonella 135557 - 167442nt	(1)	---
SARS-CoV-2 Reference Genome NC_045512.2	(1)	---

Section 661

		50821 50831 50842 50853 50864 50875 50886 50897
	(50821)	
Candidatus Carsonella ruddii strain BT chromosome...	(50821)	AATTTTTTAATTATTAAATTTAAAAAAATTTTTTCTAAAACATTAATAACATCAAATATGTTATTTTTATATTTAAT
Candidatus Carsonella 135557 - 167442nt	(1)	---
SARS-CoV-2 Reference Genome NC_045512.2	(1)	---

Section 662

		50898 50908 50919 50930 50941 50952 50963 50974
	(50898)	
Candidatus Carsonella ruddii strain BT chromosome...	(50898)	TTTTTTTATTATTTTAAAAATACAATTTCTATTGTTTAATAAAATTAAATCATTATAACTTATAGAAATACCAAAAA
Candidatus Carsonella 135557 - 167442nt	(1)	---
SARS-CoV-2 Reference Genome NC_045512.2	(1)	---

Section 663

		50975 50985 50996 51007 51018 51029 51040 51051
	(50975)	
Candidatus Carsonella ruddii strain BT chromosome...	(50975)	AAGTAGAAAATAAAAAACCTATTTTTTTAATTTTATCTAATATTTTTATAGTTTGTTCTAAACCAAAAAAATCAAAA
Candidatus Carsonella 135557 - 167442nt	(1)	---
SARS-CoV-2 Reference Genome NC_045512.2	(1)	---

Section 664

		51052 51062 51073 51084 51095 51106 51117 51128
	(51052)	
Candidatus Carsonella ruddii strain BT chromosome...	(51052)	ATATATTTTATTATATAAGAAAGTTCTTTTTTTTTTAAAATTTTATTTAAAAATTTTATTTTAATAATTTTTTTTAA
Candidatus Carsonella 135557 - 167442nt	(1)	---
SARS-CoV-2 Reference Genome NC_045512.2	(1)	---

Section 665

		51129 51139 51150 51161 51172 51183 51194 51205
	(51129)	
Candidatus Carsonella ruddii strain BT chromosome...	(51129)	AAAAGAATAAAAAATTATTCTACCCATAGTAGAAAAAATTATTTTTTTTTTTTTAATTTTTATATTTTTTAAAATAT
Candidatus Carsonella 135557 - 167442nt	(1)	---
SARS-CoV-2 Reference Genome NC_045512.2	(1)	---

Section 666

		51206 51216 51227 51238 51249 51260 51271 51282
	(51206)	
Candidatus Carsonella ruddii strain BT chromosome...	(51206)	TTTTATTATTTTTATAATAATATAAAATTGCTTCTTTAAAAGAATTAAAAGAACAACAATTATAATTATATTTATAC
Candidatus Carsonella 135557 - 167442nt	(1)	---
SARS-CoV-2 Reference Genome NC_045512.2	(1)	---

Section 667

	(51283)	51283 51293 51304 51315 51326 51337 51348 51359
Candidatus Carsonella ruddii strain BT chromosome...	(51283)	GTTAAACAATAAATACCCATTACTATATCTTGAGTTGGAATAATAATAGGATCTCCATTAGAAGGAGAAATAATATT
Candidatus Carsonella 135557 - 167442nt	(1)	---
SARS-CoV-2 Reference Genome NC_045512.2	(1)	---

Section 668

	(51360)	51360 51370 51381 51392 51403 51414 51425 51436
Candidatus Carsonella ruddii strain BT chromosome...	(51360)	ATTTATTGATAATAGTAAATAATTTGACTCCATTTGAGAATTTATAGTTAAAGGTAAATGAATAGCCATTTGATCAC
Candidatus Carsonella 135557 - 167442nt	(1)	---
SARS-CoV-2 Reference Genome NC_045512.2	(1)	---

Section 669

	(51437)	51437 51447 51458 51469 51480 51491 51502 51513
Candidatus Carsonella ruddii strain BT chromosome...	(51437)	CATCAAAATCTGCATTATAACTTAAACATACTAAAGGATGTAATTTTATTGTTTTATCTTGAGTTAATAAGATTTTA
Candidatus Carsonella 135557 - 167442nt	(1)	---
SARS-CoV-2 Reference Genome NC_045512.2	(1)	---

Section 670

	(51514)	51514 51524 51535 51546 51557 51568 51579 51590
Candidatus Carsonella ruddii strain BT chromosome...	(51514)	AAAGATTGAATACCCATTCTATGTAATGTTGGAGCACGATTTAAAATTATTGTATTTTTTTTACATATATAATTTAA
Candidatus Carsonella 135557 - 167442nt	(1)	---
SARS-CoV-2 Reference Genome NC_045512.2	(1)	---

Section 671

	(51591)	51591 51601 51612 51623 51634 51645 51656 51667
Candidatus Carsonella ruddii strain BT chromosome...	(51591)	ATATTCTATTGATTTTTTTTTATTTTGTTTATAATAATCATCTATAAAACCAATATTTGTAATTTGTTTATTATTTT
Candidatus Carsonella 135557 - 167442nt	(1)	---
SARS-CoV-2 Reference Genome NC_045512.2	(1)	---

Section 672

	(51668)	51668 51678 51689 51700 51711 51722 51733 51744
Candidatus Carsonella ruddii strain BT chromosome...	(51668)	TTAATATATTATAAAGAAAAGGTTTAAATAATTCTAATAATATATAAATAGGTAATTCACACTGATAAAGTTTTAAT
Candidatus Carsonella 135557 - 167442nt	(1)	---
SARS-CoV-2 Reference Genome NC_045512.2	(1)	---

```
                                                                                                                Section 673
                                                    (51745) 51745     51755      51766      51777      51788      51799      51810    51821
Candidatus Carsonella ruddii strain BT chromosome... (51745) TCTGGCCCTACTGATATTACTGTTCTTCCTGAAAAATCAACTCTTTTTCCTAATAAATTTTGTCTAAATCTACCATA
           Candidatus Carsonella 135557 - 167442nt     (1) -----------------------------------------------------------------------------
         SARS-CoV-2 Reference Genome NC_045512.2      (1) -----------------------------------------------------------------------------
                                                                                                                Section 674
                                                    (51822) 51822     51832      51843      51854      51865      51876      51887    51898
Candidatus Carsonella ruddii strain BT chromosome... (51822) TTTTCCTCTAATTGAATTAGAAAAAGATTTTAAAATTCTTTTTGAAGTTGTTAAAATAGGGTTTTCAATTTTTTCAT
           Candidatus Carsonella 135557 - 167442nt     (1) -----------------------------------------------------------------------------
         SARS-CoV-2 Reference Genome NC_045512.2      (1) -----------------------------------------------------------------------------
                                                                                                                Section 675
                                                    (51899) 51899     51909      51920      51931      51942      51953      51964    51975
Candidatus Carsonella ruddii strain BT chromosome... (51899) TATCAAATAAAGCATTAACTGATTCTTGAAGAAGAATTCTTTCATTTATAATAATTTGTTTAGGTATTCTTAATATT
           Candidatus Carsonella 135557 - 167442nt     (1) -----------------------------------------------------------------------------
         SARS-CoV-2 Reference Genome NC_045512.2      (1) -----------------------------------------------------------------------------
                                                                                                                Section 676
                                                    (51976) 51976     51986      51997      52008      52019      52030      52041    52052
Candidatus Carsonella ruddii strain BT chromosome... (51976) TTTAAATTTTTTAATCTTAAATTTCTATCTATTATTTTTTTATATAATTCGTTTAAATCTGAAGTTGCAAATTTTCC
           Candidatus Carsonella 135557 - 167442nt     (1) -----------------------------------------------------------------------------
         SARS-CoV-2 Reference Genome NC_045512.2      (1) -----------------------------------------------------------------------------
                                                                                                                Section 677
                                                    (52053) 52053     52063      52074      52085      52096      52107      52118    52129
Candidatus Carsonella ruddii strain BT chromosome... (52053) TACACTTAATGGTATTAAAGGTCTCATTTTTGGAGGAATAACAGGTAAAATTTTTAAACAAAACCAACTAGGTTTAT
           Candidatus Carsonella 135557 - 167442nt     (1) -----------------------------------------------------------------------------
         SARS-CoV-2 Reference Genome NC_045512.2      (1) -----------------------------------------------------------------------------
                                                                                                                Section 678
                                                    (52130) 52130     52140      52151      52162      52173      52184      52195    52206
Candidatus Carsonella ruddii strain BT chromosome... (52130) TTCCTGATAAATAAAATATATATATTTTATTTATTTTATCTAAAAAAGAAAATAATGAATTAAAAGATTTACAATTT
           Candidatus Carsonella 135557 - 167442nt     (1) -----------------------------------------------------------------------------
         SARS-CoV-2 Reference Genome NC_045512.2      (1) -----------------------------------------------------------------------------
```

```
                                                                                                                    Section 679
                                                 (52207) 52207     52217      52228      52239      52250      52261      52272      52283
Candidatus Carsonella ruddii strain BT chromosome... (52207) AAAATTTTTTGTTTTACAATTTTACAATCTAATAAAATTTCATTATCTAATAATAATTTTAGAATTGCTTTTGTTCC
        Candidatus Carsonella 135557 - 167442nt     (1) -----------------------------------------------------------------------------
     SARS-CoV-2 Reference Genome NC_045512.2     (1) -----------------------------------------------------------------------------
                                                                                                                    Section 680
                                                 (52284) 52284     52294      52305      52316      52327      52338      52349      52360
Candidatus Carsonella ruddii strain BT chromosome... (52284) TGATAATATATAAAAATCATTTTGTTGTTTTTTATTAAATACAACAGAATATTTTTTAAATTTTTTGTTAAAAGATT
        Candidatus Carsonella 135557 - 167442nt     (1) -----------------------------------------------------------------------------
     SARS-CoV-2 Reference Genome NC_045512.2     (1) -----------------------------------------------------------------------------
                                                                                                                    Section 681
                                                 (52361) 52361     52371      52382      52393      52404      52415      52426      52437
Candidatus Carsonella ruddii strain BT chromosome... (52361) TTATAACAATATTAATTTTAAAATTTAATATTTTATCTAATAATTTATAGGTGATGTTTATTATATTAGAAACATTA
        Candidatus Carsonella 135557 - 167442nt     (1) -----------------------------------------------------------------------------
     SARS-CoV-2 Reference Genome NC_045512.2     (1) -----------------------------------------------------------------------------
                                                                                                                    Section 682
                                                 (52438) 52438     52448      52459      52470      52481      52492      52503      52514
Candidatus Carsonella ruddii strain BT chromosome... (52438) TTAGGTAATGTTTTTATAAACCAAATATGAAGTACAGGATAATTTAAAATTATATGCCCAAATCTATATGAAGAATG
        Candidatus Carsonella 135557 - 167442nt     (1) -----------------------------------------------------------------------------
     SARS-CoV-2 Reference Genome NC_045512.2     (1) -----------------------------------------------------------------------------
                                                                                                                    Section 683
                                                 (52515) 52515     52525      52536      52547      52558      52569      52580      52591
Candidatus Carsonella ruddii strain BT chromosome... (52515) AGATTTTTTTAAATTTAAATATTTATTTGATTTATTACAATCACATTTATATATATTACAACATATATTATAAGAAC
        Candidatus Carsonella 135557 - 167442nt     (1) -----------------------------------------------------------------------------
     SARS-CoV-2 Reference Genome NC_045512.2     (1) -----------------------------------------------------------------------------
                                                                                                                    Section 684
                                                 (52592) 52592     52602      52613      52624      52635      52646      52657      52668
Candidatus Carsonella ruddii strain BT chromosome... (52592) TAAAAATTTTTAAACAAAAAAGACCACTTAATTCAGGTTTAAAATTTTTAAAATTTATATAAATAGATTTTTTAACT
        Candidatus Carsonella 135557 - 167442nt     (1) -----------------------------------------------------------------------------
     SARS-CoV-2 Reference Genome NC_045512.2     (1) -----------------------------------------------------------------------------
```

```
                                                                                                                                 Section 685
                                                  (52669) 52669     52679      52690      52701      52712      52723      52734      52745
Candidatus Carsonella ruddii strain BT chromosome... (52669) TCTCCATAAGACCATTCTAAAATTTTTTCAGAAGAAGCAATTTTAATTGATATTTTTTTAAAAAATTTTTTTTTCAT
            Candidatus Carsonella 135557 - 167442nt     (1) -----------------------------------------------------------------------------
         SARS-CoV-2 Reference Genome NC_045512.2     (1) -----------------------------------------------------------------------------
                                                                                                                                 Section 686
                                                  (52746) 52746     52756      52767      52778      52789      52800      52811      52822
Candidatus Carsonella ruddii strain BT chromosome... (52746) ATTGTTTTTATATCAAAACATAAAGCTTTTATCTCATTTAATAAAACTTGAAATGATTCTGGTATTCCAGAATCAAC
            Candidatus Carsonella 135557 - 167442nt     (1) -----------------------------------------------------------------------------
         SARS-CoV-2 Reference Genome NC_045512.2     (1) -----------------------------------------------------------------------------
                                                                                                                                 Section 687
                                                  (52823) 52823     52833      52844      52855      52866      52877      52888      52899
Candidatus Carsonella ruddii strain BT chromosome... (52823) GTTATGTATACCTTTCATAATATTTTTAAATAATAAAGTTCTTCCTTCTATATCATCAGATTTTACTGTTAACATTT
            Candidatus Carsonella 135557 - 167442nt     (1) -----------------------------------------------------------------------------
         SARS-CoV-2 Reference Genome NC_045512.2     (1) -----------------------------------------------------------------------------
                                                                                                                                 Section 688
                                                  (52900) 52900     52910      52921      52932      52943      52954      52965      52976
Candidatus Carsonella ruddii strain BT chromosome... (52900) CTTTTAAAATATGAGAAGCACCATAAGCTTCTATAGCCCAAACTTCCATTTCACCTAATCTTTGTCCTCCAAAATTT
            Candidatus Carsonella 135557 - 167442nt     (1) -----------------------------------------------------------------------------
         SARS-CoV-2 Reference Genome NC_045512.2     (1) -----------------------------------------------------------------------------
                                                                                                                                 Section 689
                                                  (52977) 52977     52987      52998      53009      53020      53031      53042      53053
Candidatus Carsonella ruddii strain BT chromosome... (52977) GATTTTCCTCCTAATGGTTGTTGTGTTATCATTGAATAAGGACCTATTGATCTAGCATAAATTTTATCTGAAACTAA
            Candidatus Carsonella 135557 - 167442nt     (1) -----------------------------------------------------------------------------
         SARS-CoV-2 Reference Genome NC_045512.2     (1) -----------------------------------------------------------------------------
                                                                                                                                 Section 690
                                                  (53054) 53054     53064      53075      53086      53097      53108      53119      53130
Candidatus Carsonella ruddii strain BT chromosome... (53054) ATGATTTAATTTCATAAAATATATATATCCAACATTAATAGGTTTTATATATTTAATACCAGTAATTCCATCAAATA
            Candidatus Carsonella 135557 - 167442nt     (1) -----------------------------------------------------------------------------
         SARS-CoV-2 Reference Genome NC_045512.2     (1) -----------------------------------------------------------------------------
```

```
                                                                                                                    Section 691
                                                   (53131) 53131     53141      53152      53163      53174      53185      53196   53207
Candidatus Carsonella ruddii strain BT chromosome... (53131) AAAAAATTTTTCCGTTTTGAGAAAATCCTAATTTGTTTATTATATTTTTAATAATTAAATCGTTTATTTTATAAAAA
          Candidatus Carsonella 135557 - 167442nt       (1) -----------------------------------------------------------------------------
         SARS-CoV-2 Reference Genome NC_045512.2        (1) -----------------------------------------------------------------------------
                                                                                                                    Section 692
                                                   (53208) 53208     53218      53229      53240      53251      53262      53273   53284
Candidatus Carsonella ruddii strain BT chromosome... (53208) TTATGTACGCAAATTTTTAATTTTAATTTAAAATTTAAAAAAATTTTATAACAATCAAAATTATTTAAATTTTCAGT
          Candidatus Carsonella 135557 - 167442nt       (1) -----------------------------------------------------------------------------
         SARS-CoV-2 Reference Genome NC_045512.2        (1) -----------------------------------------------------------------------------
                                                                                                                    Section 693
                                                   (53285) 53285     53295      53306      53317      53328      53339      53350   53361
Candidatus Carsonella ruddii strain BT chromosome... (53285) TTTTATATTTTTATTAAAAATTATTTTTATAATTATTTTTATAATCATTTTTAATTTGTAAAAAGAAAAATTATAAA
          Candidatus Carsonella 135557 - 167442nt       (1) -----------------------------------------------------------------------------
         SARS-CoV-2 Reference Genome NC_045512.2        (1) -----------------------------------------------------------------------------
                                                                                                                    Section 694
                                                   (53362) 53362     53372      53383      53394      53405      53416      53427   53438
Candidatus Carsonella ruddii strain BT chromosome... (53362) TATTAATTTTGTTAAATATATTTTTTATTTCTTTTATAGAAGAACTTAAATAAACTTCTAATAATTGACCAACATTC
          Candidatus Carsonella 135557 - 167442nt       (1) -----------------------------------------------------------------------------
         SARS-CoV-2 Reference Genome NC_045512.2        (1) -----------------------------------------------------------------------------
                                                                                                                    Section 695
                                                   (53439) 53439     53449      53460      53471      53482      53493      53504   53515
Candidatus Carsonella ruddii strain BT chromosome... (53439) ATTCTAGAAGGAACACCTAATGGATTTAGGATTAACTCAATATGTTTTCCATATTTATCATAAGGCATATTAATTTC
          Candidatus Carsonella 135557 - 167442nt       (1) -----------------------------------------------------------------------------
         SARS-CoV-2 Reference Genome NC_045512.2        (1) -----------------------------------------------------------------------------
                                                                                                                    Section 696
                                                   (53516) 53516     53526      53537      53548      53559      53570      53581   53592
Candidatus Carsonella ruddii strain BT chromosome... (53516) ATCAACAATATTTGAAATTACTCCTTTATTACCATGTCTTCCAGACATTTTATCTCCAATTTTTAATTTTTTTTTAA
          Candidatus Carsonella 135557 - 167442nt       (1) -----------------------------------------------------------------------------
         SARS-CoV-2 Reference Genome NC_045512.2        (1) -----------------------------------------------------------------------------
```

Section 697

	(53593)	53593 53603 53614 53625 53636 53647 53658 53669
Candidatus Carsonella ruddii strain BT chromosome...	(53593)	CAATAATTTTAATTTTAATAATTCTTATTATATTATTATCAAAATCATTATAATTTAAAAAATTTTTTTTTTTAATT
Candidatus Carsonella 135557 - 167442nt	(1)	---
SARS-CoV-2 Reference Genome NC_045512.2	(1)	---

Section 698

	(53670)	53670 53680 53691 53702 53713 53724 53735 53746
Candidatus Carsonella ruddii strain BT chromosome...	(53670)	ATAAATATTTTTTTTTTTTTTAATATTATTTTATTAAAATTTTTTTAAATATTTTTATTTTATAATTATTTTTTTTT
Candidatus Carsonella 135557 - 167442nt	(1)	---
SARS-CoV-2 Reference Genome NC_045512.2	(1)	---

Section 699

	(53747)	53747 53757 53768 53779 53790 53801 53812 53823
Candidatus Carsonella ruddii strain BT chromosome...	(53747)	ATTTGTATATTTTAAATTTAGTAAAATATTAATATTGTTATTAAAAATAAATATTTTATTATTTATAAAAACTTTTT
Candidatus Carsonella 135557 - 167442nt	(1)	---
SARS-CoV-2 Reference Genome NC_045512.2	(1)	---

Section 700

	(53824)	53824 53834 53845 53856 53867 53878 53889 53900
Candidatus Carsonella ruddii strain BT chromosome...	(53824)	TATTAAAAGAATTTTTTTAATTATTAAAAAAATAAAATAAAATAAATTCATATAAAAAATTATTCATATTTTTTGTT
Candidatus Carsonella 135557 - 167442nt	(1)	---
SARS-CoV-2 Reference Genome NC_045512.2	(1)	---

Section 701

	(53901)	53901 53911 53922 53933 53944 53955 53966 53977
Candidatus Carsonella ruddii strain BT chromosome...	(53901)	ACATATTTTAATTGTTCTAAATTTAAAATTTTAATAATATTTTTTTTAAAAGAATAAAGTTTAAAATCATCAATATT
Candidatus Carsonella 135557 - 167442nt	(1)	---
SARS-CoV-2 Reference Genome NC_045512.2	(1)	---

Section 702

	(53978)	53978 53988 53999 54010 54021 54032 54043 54054
Candidatus Carsonella ruddii strain BT chromosome...	(53978)	TAATACAGTACCATTAATATTTTTAGGTAAACATAAAGGTTGTTCAAAATAATTAAAATTATTATCTGAAAAAACAA
Candidatus Carsonella 135557 - 167442nt	(1)	---
SARS-CoV-2 Reference Genome NC_045512.2	(1)	---

Section 703

```
                                                        (54055) 54055     54065      54076      54087      54098      54109      54120      54131
Candidatus Carsonella ruddii strain BT chromosome...    (54055) TTTTAAATAATTTTTCTTCTGGTGAAAAATTACTTTTATTTTTAGGACACATTTTTCCTATTAAAACTTCTTTTGAA
Candidatus Carsonella 135557 - 167442nt                 (1)     -----------------------------------------------------------------------------
SARS-CoV-2 Reference Genome NC_045512.2                 (1)     -----------------------------------------------------------------------------
```

Section 704

```
                                                        (54132) 54132     54142      54153      54164      54175      54186      54197      54208
Candidatus Carsonella ruddii strain BT chromosome...    (54132) TTAACAAATTCGCCAATTTTAATTATACCAGATTTAATTTTTTTATTTAAAATTTGATCTTTATTAAATACATCATT
Candidatus Carsonella 135557 - 167442nt                 (1)     -----------------------------------------------------------------------------
SARS-CoV-2 Reference Genome NC_045512.2                 (1)     -----------------------------------------------------------------------------
```

Section 705

```
                                                        (54209) 54209     54219      54230      54241      54252      54263      54274      54285
Candidatus Carsonella ruddii strain BT chromosome...    (54209) TGATAAAATATCAAATCCATTTTCATTATATTTTAAAACACATACATATTCATAAATATGAAAAGAAGAAAACGAAT
Candidatus Carsonella 135557 - 167442nt                 (1)     -----------------------------------------------------------------------------
SARS-CoV-2 Reference Genome NC_045512.2                 (1)     -----------------------------------------------------------------------------
```

Section 706

```
                                                        (54286) 54286     54296      54307      54318      54329      54340      54351      54362
Candidatus Carsonella ruddii strain BT chromosome...    (54286) GTTTTTTTAATATTTTATCAGAAATTAATATTGAATCTTCAAAATTATAACCATACCAACTCATAAAAGCTACACGA
Candidatus Carsonella 135557 - 167442nt                 (1)     -----------------------------------------------------------------------------
SARS-CoV-2 Reference Genome NC_045512.2                 (1)     -----------------------------------------------------------------------------
```

Section 707

```
                                                        (54363) 54363     54373      54384      54395      54406      54417      54428      54439
Candidatus Carsonella ruddii strain BT chromosome...    (54363) ATATTTTTACCTAAACTAATTTCCCCATTTTTTGTAACATTACTATCAGCTAAAATTGATCCTTTATTTATAATATT
Candidatus Carsonella 135557 - 167442nt                 (1)     -----------------------------------------------------------------------------
SARS-CoV-2 Reference Genome NC_045512.2                 (1)     -----------------------------------------------------------------------------
```

Section 708

```
                                                        (54440) 54440     54450      54461      54472      54483      54494      54505      54516
Candidatus Carsonella ruddii strain BT chromosome...    (54440) TCCTATATTAATTTTTATTTGTTGATTTATAACAGTATTTTGATTTGTTCTTAAAAAATTTATAATTTTATATTTTT
Candidatus Carsonella 135557 - 167442nt                 (1)     -----------------------------------------------------------------------------
SARS-CoV-2 Reference Genome NC_045512.2                 (1)     -----------------------------------------------------------------------------
```

Section 709

		54517 54527 54538 54549 54560 54571 54582 54593
	(54517)	
Candidatus Carsonella ruddii strain BT chromosome...	(54517)	TAATTTTAAAATTATTTTTAACTATAATAGAATAATTATCTAAATATAATATATATCCTTTTAAATCTGATAAAATA
Candidatus Carsonella 135557 - 167442nt	(1)	---
SARS-CoV-2 Reference Genome NC_045512.2	(1)	---

Section 710

		54594 54604 54615 54626 54637 54648 54659 54670
	(54594)	
Candidatus Carsonella ruddii strain BT chromosome...	(54594)	TTGTACCTTGTGTTTAACCCAATAATTAATTCGTTTCCTGTTCCTACTATTGAATTATCTGAATTAATTAATGGAAC
Candidatus Carsonella 135557 - 167442nt	(1)	---
SARS-CoV-2 Reference Genome NC_045512.2	(1)	---

Section 711

		54671 54681 54692 54703 54714 54725 54736 54747
	(54671)	
Candidatus Carsonella ruddii strain BT chromosome...	(54671)	AGCTTGTCTTTGCATATTACTTCCCATTAAACATCTATTTGCGTCATTATTTGACAAAAATGGTATTAAAGATGCAC
Candidatus Carsonella 135557 - 167442nt	(1)	---
SARS-CoV-2 Reference Genome NC_045512.2	(1)	---

Section 712

		54748 54758 54769 54780 54791 54802 54813 54824
	(54748)	
Candidatus Carsonella ruddii strain BT chromosome...	(54748)	CCAAAGAAACAATTTGATCTCCTGAAATTTCAATTAAATCAATAAAAAAAAATTTTTTTTTATTATAATTATTTTTT
Candidatus Carsonella 135557 - 167442nt	(1)	---
SARS-CoV-2 Reference Genome NC_045512.2	(1)	---

Section 713

		54825 54835 54846 54857 54868 54879 54890 54901
	(54825)	
Candidatus Carsonella ruddii strain BT chromosome...	(54825)	TTTCTAGTTTCTAAAAAAGGAAATTTAAAAATTTCTCCATATATTGTTTTAACAGTTGATAAATAATTTGTTATTAT
Candidatus Carsonella 135557 - 167442nt	(1)	---
SARS-CoV-2 Reference Genome NC_045512.2	(1)	---

Section 714

		54902 54912 54923 54934 54945 54956 54967 54978
	(54902)	
Candidatus Carsonella ruddii strain BT chromosome...	(54902)	TTTTAATTTTTCTTTTTTATAATCTAAAAAAATAACACCAATAATATTTCCTATATATGATATTTTATATATTGTTG
Candidatus Carsonella 135557 - 167442nt	(1)	---
SARS-CoV-2 Reference Genome NC_045512.2	(1)	---

```
                                                                                        Section 715
                                          (54979) 54979     54989      55000      55011      55022      55033      55044  55055
Candidatus Carsonella ruddii strain BT chromosome... (54979) AAATATAATTATATTTATTAATTTTTGAATAAAATGAAAAAGAATTAATTAATCCAATATTATGTCCTTCTGGTGTA
          Candidatus Carsonella 135557 - 167442nt (1) -----------------------------------------------------------------------------
       SARS-CoV-2 Reference Genome NC_045512.2 (1) -----------------------------------------------------------------------------

                                                                                        Section 716
                                          (55056) 55056     55066      55077      55088      55099      55110      55121  55132
Candidatus Carsonella ruddii strain BT chromosome... (55056) TCTATTGGACAAATTTTGCAATAATGAGAAATATGTAAATCTCTAATATCAAAACCACAATTTTCTTTTTTTATATT
          Candidatus Carsonella 135557 - 167442nt (1) -----------------------------------------------------------------------------
       SARS-CoV-2 Reference Genome NC_045512.2 (1) -----------------------------------------------------------------------------

                                                                                        Section 717
                                          (55133) 55133     55143      55154      55165      55176      55187      55198  55209
Candidatus Carsonella ruddii strain BT chromosome... (55133) ATTACCTATAAGAGAAATTTTACGTAAATGTGAAATTTCTGATAAAGGATTATTTTGATCTAAAAATTGAGATAATT
          Candidatus Carsonella 135557 - 167442nt (1) -----------------------------------------------------------------------------
       SARS-CoV-2 Reference Genome NC_045512.2 (1) -----------------------------------------------------------------------------

                                                                                        Section 718
                                          (55210) 55210     55220      55231      55242      55253      55264      55275  55286
Candidatus Carsonella ruddii strain BT chromosome... (55210) CATTATTACAAAAATAATCCTTTAATTCTATTGTAATAATATTTTTTTCTATAATATATTCTAAATCATTAATATTG
          Candidatus Carsonella 135557 - 167442nt (1) -----------------------------------------------------------------------------
       SARS-CoV-2 Reference Genome NC_045512.2 (1) -----------------------------------------------------------------------------

                                                                                        Section 719
                                          (55287) 55287     55297      55308      55319      55330      55341      55352  55363
Candidatus Carsonella ruddii strain BT chromosome... (55287) TTAAAATTTTTAACTTTAAATTTAATATTTTTAATAATTTTTATTAAACTTTTTTCAAATTTTAAAGATAATAATTT
          Candidatus Carsonella 135557 - 167442nt (1) -----------------------------------------------------------------------------
       SARS-CoV-2 Reference Genome NC_045512.2 (1) -----------------------------------------------------------------------------

                                                                                        Section 720
                                          (55364) 55364     55374      55385      55396      55407      55418      55429  55440
Candidatus Carsonella ruddii strain BT chromosome... (55364) ACCAGAACTATAAATATATTTATTTTCTAAACTTTCAAAAGAATCATTTATAATATCAAATTTTAAAAATTTTAAAA
          Candidatus Carsonella 135557 - 167442nt (1) -----------------------------------------------------------------------------
       SARS-CoV-2 Reference Genome NC_045512.2 (1) -----------------------------------------------------------------------------
```

Section 721

	(55441)	55441 55451 55462 55473 55484 55495 55506 55517
Candidatus Carsonella ruddii strain BT chromosome...	(55441)	ATTTTTTTATAATTTCAAATATAGTTAAAATATTAATATTATTGTTTTTTTCATATAAAATTTTTTTTACTCTTTTA
Candidatus Carsonella 135557 - 167442nt	(1)	---
SARS-CoV-2 Reference Genome NC_045512.2	(1)	---

Section 722

	(55518)	55518 55528 55539 55550 55561 55572 55583 55594
Candidatus Carsonella ruddii strain BT chromosome...	(55518)	AAACCTATTTCTAAAAAATTATAATTTTTTTTTTTAAATATTTTTTTTAAAAAATTAAAAATTTTCCTTTTTTTATT
Candidatus Carsonella 135557 - 167442nt	(1)	---
SARS-CoV-2 Reference Genome NC_045512.2	(1)	---

Section 723

	(55595)	55595 55605 55616 55627 55638 55649 55660 55671
Candidatus Carsonella ruddii strain BT chromosome...	(55595)	TAAAAAATTAGTTTCTTCAAAATTAAATTTATAATTTTTGTATATTTTTATTATACTAATATTTTCTATTTCTTTTT
Candidatus Carsonella 135557 - 167442nt	(1)	---
SARS-CoV-2 Reference Genome NC_045512.2	(1)	---

Section 724

	(55672)	55672 55682 55693 55704 55715 55726 55737 55748
Candidatus Carsonella ruddii strain BT chromosome...	(55672)	TAAAAATTATTATTATTAAAAATAAAATTTTTTTAAATAAAATAAAATCAATTATTTTATTATTTAATAATTTAAAT
Candidatus Carsonella 135557 - 167442nt	(1)	---
SARS-CoV-2 Reference Genome NC_045512.2	(1)	---

Section 725

	(55749)	55749 55759 55770 55781 55792 55803 55814 55825
Candidatus Carsonella ruddii strain BT chromosome...	(55749)	TTTTTAAAAAAATTATTTTTTAGAAAAATATATTCTCCTAAATAAAAATTTTTAATATTTTTAAATGAAAAACAAAA
Candidatus Carsonella 135557 - 167442nt	(1)	---
SARS-CoV-2 Reference Genome NC_045512.2	(1)	---

Section 726

	(55826)	55826 55836 55847 55858 55869 55880 55891 55902
Candidatus Carsonella ruddii strain BT chromosome...	(55826)	ATATTTTTTATATTTTTTTTTAAAAAAAATTTTTTTATTTATTAAAAAAAATTTTTTTTTTTTGCCTTTTATTATTT
Candidatus Carsonella 135557 - 167442nt	(1)	---
SARS-CoV-2 Reference Genome NC_045512.2	(1)	---

Section 727

	(55903)	55903 55913 55924 55935 55946 55957 55968 55979
Candidatus Carsonella ruddii strain BT chromosome...	(55903)	TTAATTTTATTTTAAAAAAAAAATATTTAAAAAAAAAATTTTTGTTTAAACCTAAACTAATTAATAAATTTTTAACA
Candidatus Carsonella 135557 - 167442nt	(1)	---
SARS-CoV-2 Reference Genome NC_045512.2	(1)	---

Section 728

	(55980)	55980 55990 56001 56012 56023 56034 56045 56056
Candidatus Carsonella ruddii strain BT chromosome...	(55980)	ATAAATGATATTTTTTTATCAAAAAAAAAATAAATAATATTATTATTATTAATAATTTCTATCCAATTTCCATAATA
Candidatus Carsonella 135557 - 167442nt	(1)	---
SARS-CoV-2 Reference Genome NC_045512.2	(1)	---

Section 729

	(56057)	56057 56067 56078 56089 56100 56111 56122 56133
Candidatus Carsonella ruddii strain BT chromosome...	(56057)	AGGAATAATTTTGCAATATTTATATTTTTCTATAAAAAAAAATATACCATGTGCTTTATTAAATTGAGAAATTAAAA
Candidatus Carsonella 135557 - 167442nt	(1)	---
SARS-CoV-2 Reference Genome NC_045512.2	(1)	---

Section 730

	(56134)	56134 56144 56155 56166 56177 56188 56199 56210
Candidatus Carsonella ruddii strain BT chromosome...	(56134)	CTCTGTTTGTACCATTAATAATAAAATTACCTTTATTAGTTATATACGGAATATAACCTAATTCAAATTTTTTAATA
Candidatus Carsonella 135557 - 167442nt	(1)	---
SARS-CoV-2 Reference Genome NC_045512.2	(1)	---

Section 731

	(56211)	56211 56221 56232 56243 56254 56265 56276 56287
Candidatus Carsonella ruddii strain BT chromosome...	(56211)	AAAATTAAATTTTTATTAATTATATATATTTTAAAATAAACATAAATAATAGTATAAAAGTGAATATTTTTTTTTTT
Candidatus Carsonella 135557 - 167442nt	(1)	---
SARS-CoV-2 Reference Genome NC_045512.2	(1)	---

Section 732

	(56288)	56288 56298 56309 56320 56331 56342 56353 56364
Candidatus Carsonella ruddii strain BT chromosome...	(56288)	ACAATATTCTTCATTAAAAAATGGTTTTTTAATAATTAATTTAAAAAATTTTAAAAAAACGTTTTTTTTTATATTAA
Candidatus Carsonella 135557 - 167442nt	(1)	---
SARS-CoV-2 Reference Genome NC_045512.2	(1)	---

Section 733

	(56365)	56365 56375 56386 56397 56408 56419 56430 56441
Candidatus Carsonella ruddii strain BT chromosome...	(56365)	ATAAAGGAAAGTGTTTTTTAAATATATTTTTAATACTAAAATTTTTGTATTTTGATTTTAATAAATTATTATAAGAA
Candidatus Carsonella 135557 - 167442nt	(1)	---
SARS-CoV-2 Reference Genome NC_045512.2	(1)	---

Section 734

	(56442)	56442 56452 56463 56474 56485 56496 56507 56518
Candidatus Carsonella ruddii strain BT chromosome...	(56442)	TTTATTTGTATATGATTAAAATATGGTTTATAACATTTATGAATAAATTTTTTTTTAGATAAACAATATCTATTAAA
Candidatus Carsonella 135557 - 167442nt	(1)	---
SARS-CoV-2 Reference Genome NC_045512.2	(1)	---

Section 735

	(56519)	56519 56529 56540 56551 56562 56573 56584 56595
Candidatus Carsonella ruddii strain BT chromosome...	(56519)	ATTAATAAACGAAATCATTTAATTTCAACTTTTCCTCCTACACTATTAATTTTTTTAACAATTTCATCACATTGTTC
Candidatus Carsonella 135557 - 167442nt	(1)	---
SARS-CoV-2 Reference Genome NC_045512.2	(1)	---

Section 736

	(56596)	56596 56606 56617 56628 56639 56650 56661 56672
Candidatus Carsonella ruddii strain BT chromosome...	(56596)	TTTATCTAAACCACTTTTTATTAAAGAATTTTTTTTTTCTACTAAATCTTTTGATTCTTTTAAACTTAAATTAGTAA
Candidatus Carsonella 135557 - 167442nt	(1)	---
SARS-CoV-2 Reference Genome NC_045512.2	(1)	---

Section 737

	(56673)	56673 56683 56694 56705 56716 56727 56738 56749
Candidatus Carsonella ruddii strain BT chromosome...	(56673)	TTTCTTTAATAACTTTTATTAAATTAATTTTTTTTATCTCCTATTTCTTTTAAAAAAATATCAAATTTATTTTTTTT
Candidatus Carsonella 135557 - 167442nt	(1)	---
SARS-CoV-2 Reference Genome NC_045512.2	(1)	---

Section 738

	(56750)	56750 56760 56771 56782 56793 56804 56815 56826
Candidatus Carsonella ruddii strain BT chromosome...	(56750)	ATATTTTCAACAATATTTTCATTTTTTAAATTAAATTTTTGTTCAATTTTAATTATAAGATCACTTAATTCTAACAA
Candidatus Carsonella 135557 - 167442nt	(1)	---
SARS-CoV-2 Reference Genome NC_045512.2	(1)	---

```
                                                                                 Section 739
                                              (56827) 56827     56837      56848      56859      56870      56881      56892  56903
Candidatus Carsonella ruddii strain BT chromosome... (56827) ATTTAATTTTGAAATTAAATCTAAAATATTTTCAATTATTATATTTTTCATATTTGTTTTAATAATTTTATTAATCT
       Candidatus Carsonella 135557 - 167442nt (1) -----------------------------------------------------------------------------
  SARS-CoV-2 Reference Genome NC_045512.2 (1) -----------------------------------------------------------------------------
                                                                                 Section 740
                                              (56904) 56904     56914      56925      56936      56947      56958      56969  56980
Candidatus Carsonella ruddii strain BT chromosome... (56904) AAAAAATTTTAATTTTAAAAAATTAATAAATTCTGAAATTAAATTTTCTTTTGATAAATTTGAGAATTCTTTAATAG
       Candidatus Carsonella 135557 - 167442nt (1) -----------------------------------------------------------------------------
  SARS-CoV-2 Reference Genome NC_045512.2 (1) -----------------------------------------------------------------------------
                                                                                 Section 741
                                              (56981) 56981     56991      57002      57013      57024      57035      57046  57057
Candidatus Carsonella ruddii strain BT chromosome... (56981) GTATTTTTTTAATAAAACAATTATTAGAAAATAAAAAAATTGGTTTTTTTTCTAAAAAATTTAAAATATTAATTAAT
       Candidatus Carsonella 135557 - 167442nt (1) -----------------------------------------------------------------------------
  SARS-CoV-2 Reference Genome NC_045512.2 (1) -----------------------------------------------------------------------------
                                                                                 Section 742
                                              (57058) 57058     57068      57079      57090      57101      57112      57123  57134
Candidatus Carsonella ruddii strain BT chromosome... (57058) ATATTTTTTATTAACAATGTATCATTTTTTTATAAATAAAAAATATAAATTATTATATTCAGACAAATTAAAAAAATA
       Candidatus Carsonella 135557 - 167442nt (1) -----------------------------------------------------------------------------
  SARS-CoV-2 Reference Genome NC_045512.2 (1) -----------------------------------------------------------------------------
                                                                                 Section 743
                                              (57135) 57135     57145      57156      57167      57178      57189      57200  57211
Candidatus Carsonella ruddii strain BT chromosome... (57135) TAATTCTTTACTATTTAAATAAGTAATTCTAAAATGTGTTTTTTTTTAAAATTAAATTTAATAAAGTTTTTTTAACAT
       Candidatus Carsonella 135557 - 167442nt (1) -----------------------------------------------------------------------------
  SARS-CoV-2 Reference Genome NC_045512.2 (1) -----------------------------------------------------------------------------
                                                                                 Section 744
                                              (57212) 57212     57222      57233      57244      57255      57266      57277  57288
Candidatus Carsonella ruddii strain BT chromosome... (57212) TATTTAAATTATAAGATATAATTATATAATTATTATTTATTATTTTTTTAATATTTTTAAATTTATTATAAAATTTA
       Candidatus Carsonella 135557 - 167442nt (1) -----------------------------------------------------------------------------
  SARS-CoV-2 Reference Genome NC_045512.2 (1) -----------------------------------------------------------------------------
```

```
                                                                                Section 745
                                                (57289) 57289     57299      57310      57321      57332      57343      57354   57365
Candidatus Carsonella ruddii strain BT chromosome... (57289) TTCATTTTATTATATAACTTTTACTCATTGTTGTATTTATATATATATTTTCTATAATTATATTATTTTTAAAAAAA
            Candidatus Carsonella 135557 - 167442nt     (1) -----------------------------------------------------------------------------
        SARS-CoV-2 Reference Genome NC_045512.2         (1) -----------------------------------------------------------------------------
                                                                                Section 746
                                                (57366) 57366     57376      57387      57398      57409      57420      57431   57442
Candidatus Carsonella ruddii strain BT chromosome... (57366) ATTTTTTTAATATAATTTATAATGTAAAAATAATTTTTTTCAATCATATTGCTATTAAAATTTATATTTCCAATTTT
            Candidatus Carsonella 135557 - 167442nt     (1) -----------------------------------------------------------------------------
        SARS-CoV-2 Reference Genome NC_045512.2         (1) -----------------------------------------------------------------------------
                                                                                Section 747
                                                (57443) 57443     57453      57464      57475      57486      57497      57508   57519
Candidatus Carsonella ruddii strain BT chromosome... (57443) AAAATTTAAAAAATTATTTTTATTAATTATAATTTTAATAATTTTATTTTTTAATTCATTTATTTTTAATTCATAAT
            Candidatus Carsonella 135557 - 167442nt     (1) -----------------------------------------------------------------------------
        SARS-CoV-2 Reference Genome NC_045512.2         (1) -----------------------------------------------------------------------------
                                                                                Section 748
                                                (57520) 57520     57530      57541      57552      57563      57574      57585   57596
Candidatus Carsonella ruddii strain BT chromosome... (57520) TATTTATTAAATTTTTTTTTTTAGAAAACTTTTTAACTAAATTATGTTTTTTTATTAAGAAAAAATTATCTTTATTA
            Candidatus Carsonella 135557 - 167442nt     (1) -----------------------------------------------------------------------------
        SARS-CoV-2 Reference Genome NC_045512.2         (1) -----------------------------------------------------------------------------
                                                                                Section 749
                                                (57597) 57597     57607      57618      57629      57640      57651      57662   57673
Candidatus Carsonella ruddii strain BT chromosome... (57597) TAAAAAATTTTATCAAAAAAAATTTTTTTTTCCAAAAAATTATTAAAATAATTATTACCTATATAGATATTATCTTT
            Candidatus Carsonella 135557 - 167442nt     (1) -----------------------------------------------------------------------------
        SARS-CoV-2 Reference Genome NC_045512.2         (1) -----------------------------------------------------------------------------
                                                                                Section 750
                                                (57674) 57674     57684      57695      57706      57717      57728      57739   57750
Candidatus Carsonella ruddii strain BT chromosome... (57674) TACTGAATTTTTATCTGATAAAAATAAAATTTTTTTATTATCATTAACAGAATAAAATAAATTTATAAAAAAATTAA
            Candidatus Carsonella 135557 - 167442nt     (1) -----------------------------------------------------------------------------
        SARS-CoV-2 Reference Genome NC_045512.2         (1) -----------------------------------------------------------------------------
```

```
                                                                    Section 751
                                                (57751) 57751     57761      57772      57783      57794      57805      57816   57827
Candidatus Carsonella ruddii strain BT chromosome... (57751) AATAATTTTTTTTTTTTTATTATTAAATATAATATTTAGATCAATAGATTCAGTAAAGTTAAATTTTTTATTTAAAAAA
          Candidatus Carsonella 135557 - 167442nt (1) -----------------------------------------------------------------------------
         SARS-CoV-2 Reference Genome NC_045512.2 (1) -----------------------------------------------------------------------------

                                                                    Section 752
                                                (57828) 57828     57838      57849      57860      57871      57882      57893   57904
Candidatus Carsonella ruddii strain BT chromosome... (57828) AAATTACAAAAATTAATCAAAGAATTATTCATATTTAATACCTAAACTATAAAAAGTACCTAAAATCATTTTTCTAA
          Candidatus Carsonella 135557 - 167442nt (1) -----------------------------------------------------------------------------
         SARS-CoV-2 Reference Genome NC_045512.2 (1) -----------------------------------------------------------------------------

                                                                    Section 753
                                                (57905) 57905     57915      57926      57937      57948      57959      57970   57981
Candidatus Carsonella ruddii strain BT chromosome... (57905) TAGAAGAAATTTTTTTTTTTTCATAAAAAATTCTATTTTTAGAAATAATATTAATAATATCAAAATTATTATAAACA
          Candidatus Carsonella 135557 - 167442nt (1) -----------------------------------------------------------------------------
         SARS-CoV-2 Reference Genome NC_045512.2 (1) -----------------------------------------------------------------------------

                                                                    Section 754
                                                (57982) 57982     57992      58003      58014      58025      58036      58047   58058
Candidatus Carsonella ruddii strain BT chromosome... (57982) ATAGTTTCTATTCCTGGTTTTTTAGAAAATTTTTCTATATTTGTAATTTTTTTTATTACATTAGTTAAAGGTTCTAA
          Candidatus Carsonella 135557 - 167442nt (1) -----------------------------------------------------------------------------
         SARS-CoV-2 Reference Genome NC_045512.2 (1) -----------------------------------------------------------------------------

                                                                    Section 755
                                                (58059) 58059     58069      58080      58091      58102      58113      58124   58135
Candidatus Carsonella ruddii strain BT chromosome... (58059) ACCTAATATTATTTCATAATTTAAATTTTGAAAAATTATTATTTTAACATGAATAAATTCATTAATATTATTTTTAG
          Candidatus Carsonella 135557 - 167442nt (1) -----------------------------------------------------------------------------
         SARS-CoV-2 Reference Genome NC_045512.2 (1) -----------------------------------------------------------------------------

                                                                    Section 756
                                                (58136) 58136     58146      58157      58168      58179      58190      58201   58212
Candidatus Carsonella ruddii strain BT chromosome... (58136) TTAATTCATTAAATTTATTACAAAAAGTCATTAAATTTAAACCATATTGACCTAATATTGGACCAATAGGTGGTGTA
          Candidatus Carsonella 135557 - 167442nt (1) -----------------------------------------------------------------------------
         SARS-CoV-2 Reference Genome NC_045512.2 (1) -----------------------------------------------------------------------------
```

Section 757

		58213 58223 58234 58245 58256 58267 58278 58289
	(58213)	
Candidatus Carsonella ruddii strain BT chromosome...	(58213)	GGTGTTGCTTTTCCTGGTTTTAAAATTAATTTTAATTTATATTTAACAATCATTATTTAATACAAAAAATAAATTTC
Candidatus Carsonella 135557 - 167442nt	(1)	---
SARS-CoV-2 Reference Genome NC_045512.2	(1)	---

Section 758

		58290 58300 58311 58322 58333 58344 58355 58366
	(58290)	
Candidatus Carsonella ruddii strain BT chromosome...	(58290)	AAGTCAAAGAGGAATTGAACCTCTATTAACAGTTTTGGAAACTATTACTTTACCTTTAAGTTATTGACTTATTAATA
Candidatus Carsonella 135557 - 167442nt	(1)	---
SARS-CoV-2 Reference Genome NC_045512.2	(1)	---

Section 759

		58367 58377 58388 58399 58410 58421 58432 58443
	(58367)	
Candidatus Carsonella ruddii strain BT chromosome...	(58367)	ATAAAGCGGAAAAAGGGAATTGAACCCTTTACATTAACTTGGAAAGATAATATTTTACCAATAAACTATTCCCGCAC
Candidatus Carsonella 135557 - 167442nt	(1)	---
SARS-CoV-2 Reference Genome NC_045512.2	(1)	---

Section 760

		58444 58454 58465 58476 58487 58498 58509 58520
	(58444)	
Candidatus Carsonella ruddii strain BT chromosome...	(58444)	AAAAAATGAAGGAAAGAGGATTCGAACCTCTGAAGCTTTCGCAGCAGATTTACAGTCTGTCCCTTTTGACCGCTCAG
Candidatus Carsonella 135557 - 167442nt	(1)	---
SARS-CoV-2 Reference Genome NC_045512.2	(1)	---

Section 761

		58521 58531 58542 58553 58564 58575 58586 58597
	(58521)	
Candidatus Carsonella ruddii strain BT chromosome...	(58521)	GAATTCCTTCTTAAGAATTTTATTTTTTTTAACAAATAAAAATTCAAATTCAAATTTTTTTTATTAAATAATAACAT
Candidatus Carsonella 135557 - 167442nt	(1)	---
SARS-CoV-2 Reference Genome NC_045512.2	(1)	---

Section 762

		58598 58608 58619 58630 58641 58652 58663 58674
	(58598)	
Candidatus Carsonella ruddii strain BT chromosome...	(58598)	TATTTTTTAAAATTTATCATAATCCAGTTAATACCTAAAAATAAAATTGATATAGTAAAAATCAAAAAAATAATTAA
Candidatus Carsonella 135557 - 167442nt	(1)	---
SARS-CoV-2 Reference Genome NC_045512.2	(1)	---

Section 763

```
                                                            (58675) 58675     58685      58696      58707      58718      58729      58740      58751
Candidatus Carsonella ruddii strain BT chromosome...        (58675) AATTAAGTAAATTTTATTTGTAAAATTTTTTATTTTTAAAAAAAATAATATTTGTAATAATATTTGAAAAACAAAAT
Candidatus Carsonella 135557 - 167442nt                     (1)     -----------------------------------------------------------------------------
SARS-CoV-2 Reference Genome NC_045512.2                     (1)     -----------------------------------------------------------------------------
```

Section 764

```
                                                            (58752) 58752     58762      58773      58784      58795      58806      58817      58828
Candidatus Carsonella ruddii strain BT chromosome...        (58752) TATAAAATATTATTTTTATATTTTTAATAATAAAAAAATGTATAGTTAAACAAAATAATAAAAAAAAAATTTTTTTT
Candidatus Carsonella 135557 - 167442nt                     (1)     -----------------------------------------------------------------------------
SARS-CoV-2 Reference Genome NC_045512.2                     (1)     -----------------------------------------------------------------------------
```

Section 765

```
                                                            (58829) 58829     58839      58850      58861      58872      58883      58894      58905
Candidatus Carsonella ruddii strain BT chromosome...        (58829) TCTTTTAGTAAAATTATTTTTTTATATAAATTATTAATATAAGACATAACCATACAATGTGAATAAAATGCCAAAAT
Candidatus Carsonella 135557 - 167442nt                     (1)     -----------------------------------------------------------------------------
SARS-CoV-2 Reference Genome NC_045512.2                     (1)     -----------------------------------------------------------------------------
```

Section 766

```
                                                            (58906) 58906     58916      58927      58938      58949      58960      58971      58982
Candidatus Carsonella ruddii strain BT chromosome...        (58906) AAAGAAAATATTATATTGTTAATTTTAAATTTTTTATTTATAAAGTTTAATAATAATAAATTTAAAGAAATAAAAAT
Candidatus Carsonella 135557 - 167442nt                     (1)     -----------------------------------------------------------------------------
SARS-CoV-2 Reference Genome NC_045512.2                     (1)     -----------------------------------------------------------------------------
```

Section 767

```
                                                            (58983) 58983     58993      59004      59015      59026      59037      59048      59059
Candidatus Carsonella ruddii strain BT chromosome...        (58983) AGATATTAAAACATGAAATGCGTGAAACATAATAATAAAATAATAATTAGATAAATAATTATTTGTGTTAAAATTTA
Candidatus Carsonella 135557 - 167442nt                     (1)     -----------------------------------------------------------------------------
SARS-CoV-2 Reference Genome NC_045512.2                     (1)     -----------------------------------------------------------------------------
```

Section 768

```
                                                            (59060) 59060     59070      59081      59092      59103      59114      59125      59136
Candidatus Carsonella ruddii strain BT chromosome...        (59060) AATTAATTTTATTTAAATGATAAAAATCTTTAAATTCTATAATTAAAAATAATATAGAAAAAATTAAATTTAAATAA
Candidatus Carsonella 135557 - 167442nt                     (1)     -----------------------------------------------------------------------------
SARS-CoV-2 Reference Genome NC_045512.2                     (1)     -----------------------------------------------------------------------------
```

```
                                                                                                                   Section 769
                                                   (59137) 59137     59147      59158      59169      59180      59191      59202  59213
Candidatus Carsonella ruddii strain BT chromosome... (59137) CAATATTTATTTTTAAAATTATTAATATTTTTTAAAGATAAAAAACTACAAAAAAGAAGTAAAAGCGTTTCTATTAA
          Candidatus Carsonella 135557 - 167442nt   (1) -----------------------------------------------------------------------------
       SARS-CoV-2 Reference Genome NC_045512.2      (1) -----------------------------------------------------------------------------
                                                                                                                   Section 770
                                                   (59214) 59214     59224      59235      59246      59257      59268      59279  59290
Candidatus Carsonella ruddii strain BT chromosome... (59214) TAAAATTCTATAATTAAAAATAATTTTTTTTAAAAAAAAATTATTAGATGATAAAAATGAAATAAATAAAACTGCAA
          Candidatus Carsonella 135557 - 167442nt   (1) -----------------------------------------------------------------------------
       SARS-CoV-2 Reference Genome NC_045512.2      (1) -----------------------------------------------------------------------------
                                                                                                                   Section 771
                                                   (59291) 59291     59301      59312      59323      59334      59345      59356  59367
Candidatus Carsonella ruddii strain BT chromosome... (59291) ACATTATACAATCACTTATCATATATAACCAAAAACCTAACAATCTTTTATTTATTTTATTCATATAAAAATAAAAT
          Candidatus Carsonella 135557 - 167442nt   (1) -----------------------------------------------------------------------------
       SARS-CoV-2 Reference Genome NC_045512.2      (1) -----------------------------------------------------------------------------
                                                                                                                   Section 772
                                                   (59368) 59368     59378      59389      59400      59411      59422      59433  59444
Candidatus Carsonella ruddii strain BT chromosome... (59368) TATATTAATTATAATAAAAATAAATGATAAAAAAGTAATCTTCCACATCCACCAAATAATTGAAAATGAAAAAATAA
          Candidatus Carsonella 135557 - 167442nt   (1) -----------------------------------------------------------------------------
       SARS-CoV-2 Reference Genome NC_045512.2      (1) -----------------------------------------------------------------------------
                                                                                                                   Section 773
                                                   (59445) 59445     59455      59466      59477      59488      59499      59510  59521
Candidatus Carsonella ruddii strain BT chromosome... (59445) ATAAAGATAAAGAAATTAAAAATGGAAAAATATTTTTTTTATGATTTATTTTAATAAAATTATTTTTTAAAAAAAAT
          Candidatus Carsonella 135557 - 167442nt   (1) -----------------------------------------------------------------------------
       SARS-CoV-2 Reference Genome NC_045512.2      (1) -----------------------------------------------------------------------------
                                                                                                                   Section 774
                                                   (59522) 59522     59532      59543      59554      59565      59576      59587  59598
Candidatus Carsonella ruddii strain BT chromosome... (59522) TTTTTATATAAAAAATCATTATTTTGATTTATAATATTATTATTAACAAAAAATTGTTCATAATTATTTATAAAATT
          Candidatus Carsonella 135557 - 167442nt   (1) -----------------------------------------------------------------------------
       SARS-CoV-2 Reference Genome NC_045512.2      (1) -----------------------------------------------------------------------------
```

```
                                                                                  Section 775
                                            (59599) 59599     59609      59620      59631      59642      59653      59664   59675
Candidatus Carsonella ruddii strain BT chromosome... (59599) ATAATTTTTTTTAGCAAATATTAAATTTATAATTTGAATTAAAATAGATAAAAAAATTATCAAACTACCTATAAATG
         Candidatus Carsonella 135557 - 167442nt     (1) -----------------------------------------------------------------------------
        SARS-CoV-2 Reference Genome NC_045512.2      (1) -----------------------------------------------------------------------------
                                                                                  Section 776
                                            (59676) 59676     59686      59697      59708      59719      59730      59741   59752
Candidatus Carsonella ruddii strain BT chromosome... (59676) AAATTAATAATAATTTTGACCATAAAAAATTATAATAAATTATAGTTCTTCTATTCATTCCATAATAACCTAATAAA
         Candidatus Carsonella 135557 - 167442nt     (1) -----------------------------------------------------------------------------
        SARS-CoV-2 Reference Genome NC_045512.2      (1) -----------------------------------------------------------------------------
                                                                                  Section 777
                                            (59753) 59753     59763      59774      59785      59796      59807      59818   59829
Candidatus Carsonella ruddii strain BT chromosome... (59753) TAAAAAGGAAAAAAAGTTATTGTAACTCCTAAAAACCATAATAAAATATTTATATAAGAAAAAAATTCTGATAATTT
         Candidatus Carsonella 135557 - 167442nt     (1) -----------------------------------------------------------------------------
        SARS-CoV-2 Reference Genome NC_045512.2      (1) -----------------------------------------------------------------------------
                                                                                  Section 778
                                            (59830) 59830     59840      59851      59862      59873      59884      59895   59906
Candidatus Carsonella ruddii strain BT chromosome... (59830) TTTATTAAAAATAATAGAAAACCAATATTGAAATCCAGCAAAATATCCAAATAATACTCCTCCTATAATAACTGAAT
         Candidatus Carsonella 135557 - 167442nt     (1) -----------------------------------------------------------------------------
        SARS-CoV-2 Reference Genome NC_045512.2      (1) -----------------------------------------------------------------------------
                                                                                  Section 779
                                            (59907) 59907     59917      59928      59939      59950      59961      59972   59983
Candidatus Carsonella ruddii strain BT chromosome... (59907) GAAAATGAGCAATTAAAAACATTGAATTATGAAAAATATAATCTAAATTTGGAATAGATAAAATAATACCTGAAAAA
         Candidatus Carsonella 135557 - 167442nt     (1) -----------------------------------------------------------------------------
        SARS-CoV-2 Reference Genome NC_045512.2      (1) -----------------------------------------------------------------------------
                                                                                  Section 780
                                            (59984) 59984     59994      60005      60016      60027      60038      60049   60060
Candidatus Carsonella ruddii strain BT chromosome... (59984) CCTCCAATAGAAAATATTATTATAAATGATATAAACCAAAATAATAAAGATGTTTTTTTTTTTTTACTAAATAAAAT
         Candidatus Carsonella 135557 - 167442nt     (1) -----------------------------------------------------------------------------
        SARS-CoV-2 Reference Genome NC_045512.2      (1) -----------------------------------------------------------------------------
```

```
                                                                                       Section 781
                                                    (60061) 60061     60071      60082      60093      60104      60115      60126      60137
Candidatus Carsonella ruddii strain BT chromosome... (60061) TGTAAATAACCAATTATAAATTTTAACTCCGGTAGGTATTGCTATTACCATAGTTGATATACTAAAAAATATATTAG
          Candidatus Carsonella 135557 - 167442nt      (1) -----------------------------------------------------------------------------
       SARS-CoV-2 Reference Genome NC_045512.2         (1) -----------------------------------------------------------------------------
                                                                                       Section 782
                                                    (60138) 60138     60148      60159      60170      60181      60192      60203      60214
Candidatus Carsonella ruddii strain BT chromosome... (60138) TAGTATAACCAGAACCCATTGTAAAAAAATGATGTAACCAAACTAAAAAAGATAAAATAGTTATTGATAATGTAGCA
          Candidatus Carsonella 135557 - 167442nt      (1) -----------------------------------------------------------------------------
       SARS-CoV-2 Reference Genome NC_045512.2         (1) -----------------------------------------------------------------------------
                                                                                       Section 783
                                                    (60215) 60215     60225      60236      60247      60258      60269      60280      60291
Candidatus Carsonella ruddii strain BT chromosome... (60215) TAAAGTAATGAATTATAGGAAAATAATTGTTTTTCACATATATTTGTTAATATTTCAGAAAATATTCCAAAAGAAGG
          Candidatus Carsonella 135557 - 167442nt      (1) -----------------------------------------------------------------------------
       SARS-CoV-2 Reference Genome NC_045512.2         (1) -----------------------------------------------------------------------------
                                                                                       Section 784
                                                    (60292) 60292     60302      60313      60324      60335      60346      60357      60368
Candidatus Carsonella ruddii strain BT chromosome... (60292) TAAAATTAAAATATAAACTTCTGGGTGTCCCCATGCCCATATTAAATTAATATATAACATTTGTTGTCCACCATAAA
          Candidatus Carsonella 135557 - 167442nt      (1) -----------------------------------------------------------------------------
       SARS-CoV-2 Reference Genome NC_045512.2         (1) -----------------------------------------------------------------------------
                                                                                       Section 785
                                                    (60369) 60369     60379      60390      60401      60412      60423      60434      60445
Candidatus Carsonella ruddii strain BT chromosome... (60369) ATGATGTAAAAAAATGAAAATTAAAATTTCTATCTAAAAATAATAAAAATATTATTATATTTAAGATAGGAAAAGAA
          Candidatus Carsonella 135557 - 167442nt      (1) -----------------------------------------------------------------------------
       SARS-CoV-2 Reference Genome NC_045512.2         (1) -----------------------------------------------------------------------------
                                                                                       Section 786
                                                    (60446) 60446     60456      60467      60478      60489      60500      60511      60522
Candidatus Carsonella ruddii strain BT chromosome... (60446) ATTAAAATTAATATATTTGAACATAAACATGTCCAAATAAAAATTGGTAATTTTAAAAAAAATAAATTTTTTTTTCT
          Candidatus Carsonella 135557 - 167442nt      (1) -----------------------------------------------------------------------------
       SARS-CoV-2 Reference Genome NC_045512.2         (1) -----------------------------------------------------------------------------
```

```
                                                                                                Section 787
                                               (60523) 60523     60533      60544      60555      60566      60577      60588      60599
Candidatus Carsonella ruddii strain BT chromosome... (60523) ACATTTTAAAATTGTTGTTATAAAATTTATAGATGTTATTAAAGTTGAAATACCAGAAATCTGTAAACTCCAAATCC
            Candidatus Carsonella 135557 - 167442nt (1) -----------------------------------------------------------------------------
         SARS-CoV-2 Reference Genome NC_045512.2 (1) -----------------------------------------------------------------------------
                                                                                                Section 788
                                               (60600) 60600     60610      60621      60632      60643      60654      60665      60676
Candidatus Carsonella ruddii strain BT chromosome... (60600) AATAATCAACTCCTACCCAAGGACTAAATGTTTTTTCTGAAAGAGGAGGATATGCTAACCATCCTATTTTAGAAAAC
            Candidatus Carsonella 135557 - 167442nt (1) -----------------------------------------------------------------------------
         SARS-CoV-2 Reference Genome NC_045512.2 (1) -----------------------------------------------------------------------------
                                                                                                Section 789
                                               (60677) 60677     60687      60698      60709      60720      60731      60742      60753
Candidatus Carsonella ruddii strain BT chromosome... (60677) TCACCAAATAATAAAGATATGTTAATTAATAATACTGAAAAAAAATTTAGCCAAAAACTTAGTAAATTCAAAGAAGG
            Candidatus Carsonella 135557 - 167442nt (1) -----------------------------------------------------------------------------
         SARS-CoV-2 Reference Genome NC_045512.2 (1) -----------------------------------------------------------------------------
                                                                                                Section 790
                                               (60754) 60754     60764      60775      60786      60797      60808      60819      60830
Candidatus Carsonella ruddii strain BT chromosome... (60754) AAAAGCAACATCTATAGATCCTATTTGTAAAGGAATAATTATATTCATTAAACCAATCATAAACGGCATTGCAACAA
            Candidatus Carsonella 135557 - 167442nt (1) -----------------------------------------------------------------------------
         SARS-CoV-2 Reference Genome NC_045512.2 (1) -----------------------------------------------------------------------------
                                                                                                Section 791
                                               (60831) 60831     60841      60852      60863      60874      60885      60896      60907
Candidatus Carsonella ruddii strain BT chromosome... (60831) AAAAAATCATGATTACTCCATGAGCTGTAAAAATTTGACTATAATGGTTTGAAGATAAATATCCTTCATGATTGTAA
            Candidatus Carsonella 135557 - 167442nt (1) -----------------------------------------------------------------------------
         SARS-CoV-2 Reference Genome NC_045512.2 (1) -----------------------------------------------------------------------------
                                                                                                Section 792
                                               (60908) 60908     60918      60929      60940      60951      60962      60973      60984
Candidatus Carsonella ruddii strain BT chromosome... (60908) CTTAATAATTGTTGTAATCTCATCATTAAAGCATCAACAAAACCTCTAATTAACATAATAAATGATATTAATAAATA
            Candidatus Carsonella 135557 - 167442nt (1) -----------------------------------------------------------------------------
         SARS-CoV-2 Reference Genome NC_045512.2 (1) -----------------------------------------------------------------------------
```

```
                                                                                                              Section 793
                                                            (60985) 60985     60995      61006      61017      61028      61039      61050   61061
Candidatus Carsonella ruddii strain BT chromosome...       (60985) TAATTTTCCTATTTTTTTTATGATCAAAAGAAACAGAATAATTTTTTATTTTTTTTATAAAAAGGTTTTAAAAATGGAA
              Candidatus Carsonella 135557 - 167442nt          (1) -----------------------------------------------------------------------------
          SARS-CoV-2 Reference Genome NC_045512.2              (1) -----------------------------------------------------------------------------
                                                                                                              Section 794
                                                            (61062) 61062     61072      61083      61094      61105      61116      61127   61138
Candidatus Carsonella ruddii strain BT chromosome...       (61062) TTGAAATAAATAAAAATATTATTATTGTTATAAATAAAAATATTATTATTGGTTCTGTTATAGGAACAGAATTAAAA
              Candidatus Carsonella 135557 - 167442nt          (1) -----------------------------------------------------------------------------
          SARS-CoV-2 Reference Genome NC_045512.2              (1) -----------------------------------------------------------------------------
                                                                                                              Section 795
                                                            (61139) 61139     61149      61160      61171      61182      61193      61204   61215
Candidatus Carsonella ruddii strain BT chromosome...       (61139) TTTAATTTTCCTAAAATCATTTTTTTTTAATTATTAAATAAAATATTTTATTATTTCTTATTTTAAAAAATTTATTT
              Candidatus Carsonella 135557 - 167442nt          (1) -----------------------------------------------------------------------------
          SARS-CoV-2 Reference Genome NC_045512.2              (1) -----------------------------------------------------------------------------
                                                                                                              Section 796
                                                            (61216) 61216     61226      61237      61248      61259      61270      61281   61292
Candidatus Carsonella ruddii strain BT chromosome...       (61216) ACAAAACTTCCTTCTTTTATTAATAAATTATATTTTTTAATATTAAAAAAAATTGATTTTTTAATATTGTTAACCCA
              Candidatus Carsonella 135557 - 167442nt          (1) -----------------------------------------------------------------------------
          SARS-CoV-2 Reference Genome NC_045512.2              (1) -----------------------------------------------------------------------------
                                                                                                              Section 797
                                                            (61293) 61293     61303      61314      61325      61336      61347      61358   61369
Candidatus Carsonella ruddii strain BT chromosome...       (61293) ATAAAAAAAATTTTTTTTAATTAATAAAAAAAATATTTATTCTTAATTTATTAAATCCTATTCCACTATAATTTGAAG
              Candidatus Carsonella 135557 - 167442nt          (1) -----------------------------------------------------------------------------
          SARS-CoV-2 Reference Genome NC_045512.2              (1) -----------------------------------------------------------------------------
                                                                                                              Section 798
                                                            (61370) 61370     61380      61391      61402      61413      61424      61435   61446
Candidatus Carsonella ruddii strain BT chromosome...       (61370) AAAAACCATGACAAAAACCATGTTTTAAAATTAAAAAATTTATATATTTATGAGAATTACTCATACAATAAAATTGA
              Candidatus Carsonella 135557 - 167442nt          (1) -----------------------------------------------------------------------------
          SARS-CoV-2 Reference Genome NC_045512.2              (1) -----------------------------------------------------------------------------
```

```
                                                                               Section 799
                                                   (61447) 61447     61457      61468      61479      61490      61501      61512     61523
Candidatus Carsonella ruddii strain BT chromosome... (61447) AATCCAAATTTTGGAATACATAAAGTATTTAAAACATTGTTAGAAGATAAAAAAATTTTTATAGGTATTAATATAGG
               Candidatus Carsonella 135557 - 167442nt (1) -----------------------------------------------------------------------------
           SARS-CoV-2 Reference Genome NC_045512.2 (1) -----------------------------------------------------------------------------
                                                                               Section 800
                                                   (61524) 61524     61534      61545      61556      61567      61578      61589     61600
Candidatus Carsonella ruddii strain BT chromosome... (61524) TAAGCAAAATTCGTTAAAAGTTAAAATTTTTTGAATAGGAAAAATAATCATCCATTTCCAATTCATTGATATAATTT
               Candidatus Carsonella 135557 - 167442nt (1) -----------------------------------------------------------------------------
           SARS-CoV-2 Reference Genome NC_045512.2 (1) -----------------------------------------------------------------------------
                                                                               Section 801
                                                   (61601) 61601     61611      61622      61633      61644      61655      61666     61677
Candidatus Carsonella ruddii strain BT chromosome... (61601) CTAATATTAATGGTTTTAAATTTGAATATAAAATTTTTATAGGATTAATGTAAAAATTACTTTTTATTGTGTAAATA
               Candidatus Carsonella 135557 - 167442nt (1) -----------------------------------------------------------------------------
           SARS-CoV-2 Reference Genome NC_045512.2 (1) -----------------------------------------------------------------------------
                                                                               Section 802
                                                   (61678) 61678     61688      61699      61710      61721      61732      61743     61754
Candidatus Carsonella ruddii strain BT chromosome... (61678) CTTAATATTAAAATTATTAAACTAGGAAAAATAAAAGCGATAAATTCTATTAAATTTGAATCAATTAAAAAAGGAAT
               Candidatus Carsonella 135557 - 167442nt (1) -----------------------------------------------------------------------------
           SARS-CoV-2 Reference Genome NC_045512.2 (1) -----------------------------------------------------------------------------
                                                                               Section 803
                                                   (61755) 61755     61765      61776      61787      61798      61809      61820     61831
Candidatus Carsonella ruddii strain BT chromosome... (61755) TTTTTTCATATTTTTAATTATAAATAAACTTACTAATAGAGTAAATATTGATATTAATAATATTAAATAAATTGTAT
               Candidatus Carsonella 135557 - 167442nt (1) -----------------------------------------------------------------------------
           SARS-CoV-2 Reference Genome NC_045512.2 (1) -----------------------------------------------------------------------------
                                                                               Section 804
                                                   (61832) 61832     61842      61853      61864      61875      61886      61897     61908
Candidatus Carsonella ruddii strain BT chromosome... (61832) TTAATAACAATTGATTCTCAATTAATCTATTCAAACTTAAAAAATGGTTTTTAATAATATTTATCATATTTTAAACA
               Candidatus Carsonella 135557 - 167442nt (1) -----------------------------------------------------------------------------
           SARS-CoV-2 Reference Genome NC_045512.2 (1) -----------------------------------------------------------------------------
```

Section 805

	(61909)	61909 61919 61930 61941 61952 61963 61974 61985
Candidatus Carsonella ruddii strain BT chromosome...	(61909)	TTTTTTATAAATTTTCAAAAAAAAATTATATCAAACTTATTAAATTTGGAACAAATAGTAAAAATTGTTTTATTAAA
Candidatus Carsonella 135557 - 167442nt	(1)	---
SARS-CoV-2 Reference Genome NC_045512.2	(1)	---

Section 806

	(61986)	61986 61996 62007 62018 62029 62040 62051 62062
Candidatus Carsonella ruddii strain BT chromosome...	(61986)	AAAAATAATGTTTTTTTTTTTTATTCCATTATAAAAAAAAATTTTATAAAAATAAACAATATTAAAATATTTCAAAA
Candidatus Carsonella 135557 - 167442nt	(1)	---
SARS-CoV-2 Reference Genome NC_045512.2	(1)	---

Section 807

	(62063)	62063 62073 62084 62095 62106 62117 62128 62139
Candidatus Carsonella ruddii strain BT chromosome...	(62063)	TAATTTTAATTTATTAACTAAAATATTATTTTTTTGTAAAAATATTTTATTTTATAAAAAAATTTATGGAAAAATAA
Candidatus Carsonella 135557 - 167442nt	(1)	---
SARS-CoV-2 Reference Genome NC_045512.2	(1)	---

Section 808

	(62140)	62140 62150 62161 62172 62183 62194 62205 62216
Candidatus Carsonella ruddii strain BT chromosome...	(62140)	TATTTAATTTTTTTTTAAATATAATTTCTAAAATTTCAAATTTTATATCTATTATTAATAAAAAAAAATTTTTTTTA
Candidatus Carsonella 135557 - 167442nt	(1)	---
SARS-CoV-2 Reference Genome NC_045512.2	(1)	---

Section 809

	(62217)	62217 62227 62238 62249 62260 62271 62282 62293
Candidatus Carsonella ruddii strain BT chromosome...	(62217)	AAATTTTATAATATTAATATATATTTTATAAAAAAAAAAAATTTCTTGTTTAAAAAAATTTTTTTTTTAAAAAATAT
Candidatus Carsonella 135557 - 167442nt	(1)	---
SARS-CoV-2 Reference Genome NC_045512.2	(1)	---

Section 810

	(62294)	62294 62304 62315 62326 62337 62348 62359 62370
Candidatus Carsonella ruddii strain BT chromosome...	(62294)	TTTTATTTTTGAATCATTAATTTTAAACCATAATGATTTATTTAAAGATAATATATTATTTTTTGGAAATAAGATAT
Candidatus Carsonella 135557 - 167442nt	(1)	---
SARS-CoV-2 Reference Genome NC_045512.2	(1)	---

Section 811

```
                                                         62371     62381      62392      62403      62414      62425      62436    62447
Candidatus Carsonella ruddii strain BT chromosome... (62371) CATCTATTATTGATTTTAATAATTATTGTTTTTTATCAATAAATAATGATTTAATTAATTTAATTTTAGAATTTACA
         Candidatus Carsonella 135557 - 167442nt     (1) -----------------------------------------------------------------------------
      SARS-CoV-2 Reference Genome NC_045512.2        (1) -----------------------------------------------------------------------------
```

Section 812

```
                                                         62448     62458      62469      62480      62491      62502      62513    62524
Candidatus Carsonella ruddii strain BT chromosome... (62448) GAAAATTATTTAATTAAAAAAAACATAATATTAGAAAATTATTACAAAAAAAAACAAAATATTAAAATAAAAGATTT
         Candidatus Carsonella 135557 - 167442nt     (1) -----------------------------------------------------------------------------
      SARS-CoV-2 Reference Genome NC_045512.2        (1) -----------------------------------------------------------------------------
```

Section 813

```
                                                         62525     62535      62546      62557      62568      62579      62590    62601
Candidatus Carsonella ruddii strain BT chromosome... (62525) TTTTATATTAAAAATTTTTATATTAAGAAAAAAAAAAAATGATATTATTTAAAAAAACAAAAAATCCAAATAATTATT
         Candidatus Carsonella 135557 - 167442nt     (1) -----------------------------------------------------------------------------
      SARS-CoV-2 Reference Genome NC_045512.2        (1) -----------------------------------------------------------------------------
```

Section 814

```
                                                         62602     62612      62623      62634      62645      62656      62667    62678
Candidatus Carsonella ruddii strain BT chromosome... (62602) TAAAAAAATATTTTTATGAAAAAAATTTTTGAATATAAAAAATTAAAATATAGATATATTAAAAAATGGAATTTAAT
         Candidatus Carsonella 135557 - 167442nt     (1) -----------------------------------------------------------------------------
      SARS-CoV-2 Reference Genome NC_045512.2        (1) -----------------------------------------------------------------------------
```

Section 815

```
                                                         62679     62689      62700      62711      62722      62733      62744    62755
Candidatus Carsonella ruddii strain BT chromosome... (62679) TTGGAATTGCGGTTTTTATATTAAAAAAAGTTTATTATTAATTAATGAAGGATCTTCATCAATAAATTATTCACAAC
         Candidatus Carsonella 135557 - 167442nt     (1) -----------------------------------------------------------------------------
      SARS-CoV-2 Reference Genome NC_045512.2        (1) -----------------------------------------------------------------------------
```

Section 816

```
                                                         62756     62766      62777      62788      62799      62810      62821    62832
Candidatus Carsonella ruddii strain BT chromosome... (62756) AATGTTTTGAAGGTGTTAAATGTTTTTTAAAAAAAAAAAATTTTTTTCATTTTAGAATTAATTTAAATTCTTATAGA
         Candidatus Carsonella 135557 - 167442nt     (1) -----------------------------------------------------------------------------
      SARS-CoV-2 Reference Genome NC_045512.2        (1) -----------------------------------------------------------------------------
```

```
                                                                   Section 817
                                                  (62833) 62833     62843      62854      62865      62876      62887      62898    62909
Candidatus Carsonella ruddii strain BT chromosome... (62833) TTTCAAAAAAGTTGTAAATATTTATTTTTACCAATAATAAATAATTTTATTTTTTTAAAATCTATAAAAATAACATC
        Candidatus Carsonella 135557 - 167442nt     (1) -----------------------------------------------------------------------------
    SARS-CoV-2 Reference Genome NC_045512.2         (1) -----------------------------------------------------------------------------
                                                                   Section 818
                                                  (62910) 62910     62920      62931      62942      62953      62964      62975    62986
Candidatus Carsonella ruddii strain BT chromosome... (62910) ATATTTAAATTTCAAATATATACCAAATTTAAAAATTGGTTTTTTATATATTAGACCTTTATTAATTGGTGTTGGAA
        Candidatus Carsonella 135557 - 167442nt     (1) -----------------------------------------------------------------------------
    SARS-CoV-2 Reference Genome NC_045512.2         (1) -----------------------------------------------------------------------------
                                                                   Section 819
                                                  (62987) 62987     62997      63008      63019      63030      63041      63052    63063
Candidatus Carsonella ruddii strain BT chromosome... (62987) AAAATATAGGAGTAAAAACTTCAAAAAAATTTATTTTTACAATTTTTTGTACACCTTCTTTTTTAACATTTAAAAAA
        Candidatus Carsonella 135557 - 167442nt     (1) -----------------------------------------------------------------------------
    SARS-CoV-2 Reference Genome NC_045512.2         (1) -----------------------------------------------------------------------------
                                                                   Section 820
                                                  (63064) 63064     63074      63085      63096      63107      63118      63129    63140
Candidatus Carsonella ruddii strain BT chromosome... (63064) CAATTTATAAAATCAATATTTTTAAAAAGAATTATAGAAAATAATGGACATTATAAAATAGGTTGTAATTATATAAC
        Candidatus Carsonella 135557 - 167442nt     (1) -----------------------------------------------------------------------------
    SARS-CoV-2 Reference Genome NC_045512.2         (1) -----------------------------------------------------------------------------
                                                                   Section 821
                                                  (63141) 63141     63151      63162      63173      63184      63195      63206    63217
Candidatus Carsonella ruddii strain BT chromosome... (63141) AAATATATATAATAATTTTTTTTTAAAAAATATAATTTTGATGATTATATTCATTTAAATAATAATTATTTTGAAG
        Candidatus Carsonella 135557 - 167442nt     (1) -----------------------------------------------------------------------------
    SARS-CoV-2 Reference Genome NC_045512.2         (1) -----------------------------------------------------------------------------
                                                                   Section 822
                                                  (63218) 63218     63228      63239      63250      63261      63272      63283    63294
Candidatus Carsonella ruddii strain BT chromosome... (63218) AAATAGGAACTTCAAATATAATTTTATATTTAAAAAAATTTTTTTTATCTCCTTCTAATAAAAATATACTTCCTGGA
        Candidatus Carsonella 135557 - 167442nt     (1) -----------------------------------------------------------------------------
    SARS-CoV-2 Reference Genome NC_045512.2         (1) -----------------------------------------------------------------------------
```

Section 823

(63295) 63295 63305 63316 63327 63338 63349 63360 63371
Candidatus Carsonella ruddii strain BT chromosome... (63295) ATAAACAAATTTTCTTTTTTAACTTTTTTTAATAAAAAAAAATTTTTTTTAAATATAAATATATCATTTAATATAAT
Candidatus Carsonella 135557 - 167442nt (1) ---
SARS-CoV-2 Reference Genome NC_045512.2 (1) ---

Section 824

(63372) 63372 63382 63393 63404 63415 63426 63437 63448
Candidatus Carsonella ruddii strain BT chromosome... (63372) AAATAATAGCGTTAATATTATTTCTTGTGGAACAGCAATTTTTTATAAAATAATAAATTCAATTTTATTTAAAAATA
Candidatus Carsonella 135557 - 167442nt (1) ---
SARS-CoV-2 Reference Genome NC_045512.2 (1) ---

Section 825

(63449) 63449 63459 63470 63481 63492 63503 63514 63525
Candidatus Carsonella ruddii strain BT chromosome... (63449) AAATTATAAAATATAAAAAAAGTTTTTTGTTTTTTTTTATAAATTTTTTTTTTAAAAAAATATATAATTGTTTATAT
Candidatus Carsonella 135557 - 167442nt (1) ---
SARS-CoV-2 Reference Genome NC_045512.2 (1) ---

Section 826

(63526) 63526 63536 63547 63558 63569 63580 63591 63602
Candidatus Carsonella ruddii strain BT chromosome... (63526) AAAATTAATAAATGGATTTTTTAAAATTAATTAAAAATAATTTTAAAAATTTTAAAAAAAATATTTTTTTTTTAAAA
Candidatus Carsonella 135557 - 167442nt (1) ---
SARS-CoV-2 Reference Genome NC_045512.2 (1) ---

Section 827

(63603) 63603 63613 63624 63635 63646 63657 63668 63679
Candidatus Carsonella ruddii strain BT chromosome... (63603) AAAATAAAAAAAAAAAAGTTTTTTTTTTATAAATTTTAATAATTAAAAAAATATTATTTATTTTATTACAAATATAA
Candidatus Carsonella 135557 - 167442nt (1) ---
SARS-CoV-2 Reference Genome NC_045512.2 (1) ---

Section 828

(63680) 63680 63690 63701 63712 63723 63734 63745 63756
Candidatus Carsonella ruddii strain BT chromosome... (63680) AATTAAACATTATAAAAATATAAAAATTTTTTTTATAAAAAAAAGAAAATTAACAACAATTAAAAAAATATTAAAAA
Candidatus Carsonella 135557 - 167442nt (1) ---
SARS-CoV-2 Reference Genome NC_045512.2 (1) ---

		Section 829
	(63757)	63757 63767 63778 63789 63800 63811 63822 63833
Candidatus Carsonella ruddii strain BT chromosome...	(63757)	TATTATGCTTTTAAAAATATTAAGTATAAAAGATAAAAGATTAAGAAAAAAATCAAAAAAAATGTTTTTTTATAATA
Candidatus Carsonella 135557 - 167442nt	(1)	---
SARS-CoV-2 Reference Genome NC_045512.2	(1)	---

		Section 830
	(63834)	63834 63844 63855 63866 63877 63888 63899 63910
Candidatus Carsonella ruddii strain BT chromosome...	(63834)	AAATTAAAAAAATATTTTTAATTAAAAGAATGATTTTTACAATGTATGAAAAAAAGGGAATTGGAATTTCTTCTACT
Candidatus Carsonella 135557 - 167442nt	(1)	---
SARS-CoV-2 Reference Genome NC_045512.2	(1)	---

		Section 831
	(63911)	63911 63921 63932 63943 63954 63965 63976 63987
Candidatus Carsonella ruddii strain BT chromosome...	(63911)	CAAATTAATTTTAAAAATAAAATAATTATAGTTGATTTAAATAAAAAAAATAAACCAAGTATAATTATAAATCCTTC
Candidatus Carsonella 135557 - 167442nt	(1)	---
SARS-CoV-2 Reference Genome NC_045512.2	(1)	---

		Section 832
	(63988)	63988 63998 64009 64020 64031 64042 64053 64064
Candidatus Carsonella ruddii strain BT chromosome...	(63988)	AGTAATAAAAAATAGTAGTTTAATTACTTTAAGTACTGAAGGTTGTTTATCTATTAAAAATTTTTTTATAAATATTC
Candidatus Carsonella 135557 - 167442nt	(1)	---
SARS-CoV-2 Reference Genome NC_045512.2	(1)	---

		Section 833
	(64065)	64065 64075 64086 64097 64108 64119 64130 64141
Candidatus Carsonella ruddii strain BT chromosome...	(64065)	CTAGACCTGAAAAAATATTTTTAAATTTTGTAAATTTATATAATAAAATTAAAAAAAAAATTTTTAATAATATAAAT
Candidatus Carsonella 135557 - 167442nt	(1)	---
SARS-CoV-2 Reference Genome NC_045512.2	(1)	---

		Section 834
	(64142)	64142 64152 64163 64174 64185 64196 64207 64218
Candidatus Carsonella ruddii strain BT chromosome...	(64142)	TCAAGATGTTTACAACATGAGATAGATCATTTAAATGGTATATTAATAATAGATTATAATAAAATAATAAAAAC
Candidatus Carsonella 135557 - 167442nt	(1)	---
SARS-CoV-2 Reference Genome NC_045512.2	(1)	---

```
                                                                                                    Section 835
                                               (64219) 64219     64229      64240      64251      64262      64273      64284     64295
Candidatus Carsonella ruddii strain BT chromosome... (64219) ATGAATAAATTATGGGGAGGAAGATTTGAAAATCAATTAGATTATGATATTGAGATTTATTTAAATTCATTTTATAT
           Candidatus Carsonella 135557 - 167442nt     (1) -----------------------------------------------------------------------------
          SARS-CoV-2 Reference Genome NC_045512.2     (1) -----------------------------------------------------------------------------
                                                                                                    Section 836
                                               (64296) 64296     64306      64317      64328      64339      64350      64361     64372
Candidatus Carsonella ruddii strain BT chromosome... (64296) AGATAAATATTTATTTAATTATGAAATAAAAGTAATTAGTGCACATGTAAAAAGTTTATATTTGTGTAATTATATAA
           Candidatus Carsonella 135557 - 167442nt     (1) -----------------------------------------------------------------------------
          SARS-CoV-2 Reference Genome NC_045512.2     (1) -----------------------------------------------------------------------------
                                                                                                    Section 837
                                               (64373) 64373     64383      64394      64405      64416      64427      64438     64449
Candidatus Carsonella ruddii strain BT chromosome... (64373) AATTAAATGAAAAAAAACAAATTTTAAATTCTTTGTTTTTAATTAAAAATAATTTTAGAAAATTAAATGTAGAAGAT
           Candidatus Carsonella 135557 - 167442nt     (1) -----------------------------------------------------------------------------
          SARS-CoV-2 Reference Genome NC_045512.2     (1) -----------------------------------------------------------------------------
                                                                                                    Section 838
                                               (64450) 64450     64460      64471      64482      64493      64504      64515     64526
Candidatus Carsonella ruddii strain BT chromosome... (64450) ATACATTCTTATTTAGAATTAGAATTAATAAAGTATAATGGTGATTTAGGAAAAAAAATATATATTGGAAAATCAAG
           Candidatus Carsonella 135557 - 167442nt     (1) -----------------------------------------------------------------------------
          SARS-CoV-2 Reference Genome NC_045512.2     (1) -----------------------------------------------------------------------------
                                                                                                    Section 839
                                               (64527) 64527     64537      64548      64559      64570      64581      64592     64603
Candidatus Carsonella ruddii strain BT chromosome... (64527) AAATGATCAAATCACTACTTTAAATAAATTAATGTTATTGGATGAAATTGAAAAAATTAATATTTTATTAATATTCA
           Candidatus Carsonella 135557 - 167442nt     (1) -----------------------------------------------------------------------------
          SARS-CoV-2 Reference Genome NC_045512.2     (1) -----------------------------------------------------------------------------
                                                                                                    Section 840
                                               (64604) 64604     64614      64625      64636      64647      64658      64669     64680
Candidatus Carsonella ruddii strain BT chromosome... (64604) TAAGAAAAACAATTATTAATTTAGCAGAAATTGAATTTAATACACCTGTACCTGGTTTTACTCATTTACAAATAGCT
           Candidatus Carsonella 135557 - 167442nt     (1) -----------------------------------------------------------------------------
          SARS-CoV-2 Reference Genome NC_045512.2     (1) -----------------------------------------------------------------------------
```

```
                                                                                                    Section 841
                                                      (64681) 64681     64691      64702      64713      64724      64735      64746     64757
Candidatus Carsonella ruddii strain BT chromosome...  (64681) CAACCTATAACATTAGGACATTATTTATTATCCTGGAATGAAATGTTAAAAAGAGATCATATAAATCTTTTAAATTG
                Candidatus Carsonella 135557 - 167442nt   (1) -----------------------------------------------------------------------------
        SARS-CoV-2 Reference Genome NC_045512.2           (1) -----------------------------------------------------------------------------

                                                                                                    Section 842
                                                      (64758) 64758     64768      64779      64790      64801      64812      64823     64834
Candidatus Carsonella ruddii strain BT chromosome...  (64758) TAAAAAAAACACAAATTTTTGTCCTTTAGGATCAGCAGCATTAGCTGGGCATAATTATAAAATTAATAGAAATATTG
                Candidatus Carsonella 135557 - 167442nt   (1) -----------------------------------------------------------------------------
        SARS-CoV-2 Reference Genome NC_045512.2           (1) -----------------------------------------------------------------------------

                                                                                                    Section 843
                                                      (64835) 64835     64845      64856      64867      64878      64889      64900     64911
Candidatus Carsonella ruddii strain BT chromosome...  (64835) TTAAAAAAATATTAAATTTTAAATATTTAACAGAAAATTCAATAGATGCAGTTAGTGATAGAGATTATGTTATATTA
                Candidatus Carsonella 135557 - 167442nt   (1) -----------------------------------------------------------------------------
        SARS-CoV-2 Reference Genome NC_045512.2           (1) -----------------------------------------------------------------------------

                                                                                                    Section 844
                                                      (64912) 64912     64922      64933      64944      64955      64966      64977     64988
Candidatus Carsonella ruddii strain BT chromosome...  (64912) TTTTCTCAATTTTGTAATATTCTAATTATTCATTTATCTAGAATTTGTGAAGATATGATTATTTGGAGTAGTAATAA
                Candidatus Carsonella 135557 - 167442nt   (1) -----------------------------------------------------------------------------
        SARS-CoV-2 Reference Genome NC_045512.2           (1) -----------------------------------------------------------------------------

                                                                                                    Section 845
                                                      (64989) 64989     64999      65010      65021      65032      65043      65054     65065
Candidatus Carsonella ruddii strain BT chromosome...  (64989) ATTTAATTTTTTAAAATTATCAGACAAGATATCTTCTGGATCTTCTATTATGCCTCAAAAAAAAAATCCAGATTTAT
                Candidatus Carsonella 135557 - 167442nt   (1) -----------------------------------------------------------------------------
        SARS-CoV-2 Reference Genome NC_045512.2           (1) -----------------------------------------------------------------------------

                                                                                                    Section 846
                                                      (65066) 65066     65076      65087      65098      65109      65120      65131     65142
Candidatus Carsonella ruddii strain BT chromosome...  (65066) TTGAATTAATTAGATCAAAATCTGGTAGAATTATGGGAAATTCAGTTAATATTTTAACTATTTTAAAATCACAAGCA
                Candidatus Carsonella 135557 - 167442nt   (1) -----------------------------------------------------------------------------
        SARS-CoV-2 Reference Genome NC_045512.2           (1) -----------------------------------------------------------------------------
```

```
                                                                                    Section 847
                                                   (65143) 65143     65153      65164      65175      65186      65197      65208      65219
Candidatus Carsonella ruddii strain BT chromosome... (65143) TTATCTTATAATAAAGATAATCAAGAAGATAAAGAAAGTTTATTTGATAATATTTATACAATAAAAAATACTTTAAA
          Candidatus Carsonella 135557 - 167442nt      (1) -----------------------------------------------------------------------------
       SARS-CoV-2 Reference Genome NC_045512.2         (1) -----------------------------------------------------------------------------
                                                                                    Section 848
                                                   (65220) 65220     65230      65241      65252      65263      65274      65285      65296
Candidatus Carsonella ruddii strain BT chromosome... (65220) TTGTTTTAGAAAATGCATTTTAGTATTAAAATTTAATAAAAAAAATATGTATTTATCTACTTTAAAAAATTATTCTA
          Candidatus Carsonella 135557 - 167442nt      (1) -----------------------------------------------------------------------------
       SARS-CoV-2 Reference Genome NC_045512.2         (1) -----------------------------------------------------------------------------
                                                                                    Section 849
                                                   (65297) 65297     65307      65318      65329      65340      65351      65362      65373
Candidatus Carsonella ruddii strain BT chromosome... (65297) CCGCAACTGATATGGCTGATTATTTAGTTAAAAAAGGTCTAACTTTTAGAGATTCTCATAAAATTGTAGGTAATTGT
          Candidatus Carsonella 135557 - 167442nt      (1) -----------------------------------------------------------------------------
       SARS-CoV-2 Reference Genome NC_045512.2         (1) -----------------------------------------------------------------------------
                                                                                    Section 850
                                                   (65374) 65374     65384      65395      65406      65417      65428      65439      65450
Candidatus Carsonella ruddii strain BT chromosome... (65374) ATTAAATATTGTGAAAATAATGATATTAATTTTTTTAATATTTCATTAAAAGAATTAAAAATTATTTGTAATTTATT
          Candidatus Carsonella 135557 - 167442nt      (1) -----------------------------------------------------------------------------
       SARS-CoV-2 Reference Genome NC_045512.2         (1) -----------------------------------------------------------------------------
                                                                                    Section 851
                                                   (65451) 65451     65461      65472      65483      65494      65505      65516      65527
Candidatus Carsonella ruddii strain BT chromosome... (65451) TGAAAATGATATTTTTTATTTTTTATCTATTGAAGGATCAATTAAAAATAAAAATACTTATGGGAGTACTTCTCCTA
          Candidatus Carsonella 135557 - 167442nt      (1) -----------------------------------------------------------------------------
       SARS-CoV-2 Reference Genome NC_045512.2         (1) -----------------------------------------------------------------------------
                                                                                    Section 852
                                                   (65528) 65528     65538      65549      65560      65571      65582      65593      65604
Candidatus Carsonella ruddii strain BT chromosome... (65528) ATCAAGTTTTAAATTCTATTCAAAGAGCAAAATTTTTTTTAAATAATATTATTTTAAAATTTAAATGATAGAAATTA
          Candidatus Carsonella 135557 - 167442nt      (1) -----------------------------------------------------------------------------
       SARS-CoV-2 Reference Genome NC_045512.2         (1) -----------------------------------------------------------------------------
```

```
                                                                                           Section 853
                                              (65605) 65605     65615      65626      65637      65648      65659      65670  65681
Candidatus Carsonella ruddii strain BT chromosome... (65605) AAAATATTGAAAATAAAATATTAATTAATAAAATTTTAATAAATGTATTTTTTCAAAAATTTGAATTACCTATTTAT
        Candidatus Carsonella 135557 - 167442nt      (1) -----------------------------------------------------------------------------
    SARS-CoV-2 Reference Genome NC_045512.2          (1) -----------------------------------------------------------------------------
                                                                                           Section 854
                                              (65682) 65682     65692      65703      65714      65725      65736      65747  65758
Candidatus Carsonella ruddii strain BT chromosome... (65682) GTTTATGATTATAAAAAAATAATTTTTAATTGTTTTTTTATAAAAAAATTAATTTTTTTTGTTTTTTATTCAATAAA
        Candidatus Carsonella 135557 - 167442nt      (1) -----------------------------------------------------------------------------
    SARS-CoV-2 Reference Genome NC_045512.2          (1) -----------------------------------------------------------------------------
                                                                                           Section 855
                                              (65759) 65759     65769      65780      65791      65802      65813      65824  65835
Candidatus Carsonella ruddii strain BT chromosome... (65759) ATCTAATGATAATTTATTTTTATTAAAAATAATAAACAATATAATTAATAAATTTGATATTGTTTCAATTGGAGAAC
        Candidatus Carsonella 135557 - 167442nt      (1) -----------------------------------------------------------------------------
    SARS-CoV-2 Reference Genome NC_045512.2          (1) -----------------------------------------------------------------------------
                                                                                           Section 856
                                              (65836) 65836     65846      65857      65868      65879      65890      65901  65912
Candidatus Carsonella ruddii strain BT chromosome... (65836) TTAAAAAAATTTTATTAATTTCTATTAAAAAACCTTATATTATTTTTTCTGGAAGTGGTAAAAATATATCAGAATTA
        Candidatus Carsonella 135557 - 167442nt      (1) -----------------------------------------------------------------------------
    SARS-CoV-2 Reference Genome NC_045512.2          (1) -----------------------------------------------------------------------------
                                                                                           Section 857
                                              (65913) 65913     65923      65934      65945      65956      65967      65978  65989
Candidatus Carsonella ruddii strain BT chromosome... (65913) TTAATTTCATTAAATTTAAACATATTTTGTTTAAATATTGAGTCAATACAAGAATTTATAAAAATTTTTTTTTTAAT
        Candidatus Carsonella 135557 - 167442nt      (1) -----------------------------------------------------------------------------
    SARS-CoV-2 Reference Genome NC_045512.2          (1) -----------------------------------------------------------------------------
                                                                                           Section 858
                                              (65990) 65990     66000      66011      66022      66033      66044      66055  66066
Candidatus Carsonella ruddii strain BT chromosome... (65990) AAAAAAATATAAAAAAAAAATTAATTTAATGATTAGAATTAATCCTAATATTGATGCTAAAACTCATGAAAAAATTA
        Candidatus Carsonella 135557 - 167442nt      (1) -----------------------------------------------------------------------------
    SARS-CoV-2 Reference Genome NC_045512.2          (1) -----------------------------------------------------------------------------
```

```
                                                                                                    Section 859
                                                        (66067) 66067     66077      66088      66099      66110      66121      66132    66143
Candidatus Carsonella ruddii strain BT chromosome...   (66067) GTACTGGTATTAAAGTTAATAAATTTGGAATTTCAGTAATAAATTTAAAAAATTTATTTATTTTAAATAAAATTACA
         Candidatus Carsonella 135557 - 167442nt           (1) -----------------------------------------------------------------------------
       SARS-CoV-2 Reference Genome NC_045512.2             (1) -----------------------------------------------------------------------------
                                                                                                    Section 860
                                                        (66144) 66144     66154      66165      66176      66187      66198      66209    66220
Candidatus Carsonella ruddii strain BT chromosome...   (66144) AAATTAAATATTATTGGTTATGATTTTCATATAGGTTCTCAAATCGAAAAAATATCTCCTATTAAAAAATTATTAAA
         Candidatus Carsonella 135557 - 167442nt           (1) -----------------------------------------------------------------------------
       SARS-CoV-2 Reference Genome NC_045512.2             (1) -----------------------------------------------------------------------------
                                                                                                    Section 861
                                                        (66221) 66221     66231      66242      66253      66264      66275      66286    66297
Candidatus Carsonella ruddii strain BT chromosome...   (66221) TTTAATTATTAATTTATATAAAATTAAAAAATTTGATTATTTAGATATAGGTGGAGGAATAGGTGTTAATTATTATA
         Candidatus Carsonella 135557 - 167442nt           (1) -----------------------------------------------------------------------------
       SARS-CoV-2 Reference Genome NC_045512.2             (1) -----------------------------------------------------------------------------
                                                                                                    Section 862
                                                        (66298) 66298     66308      66319      66330      66341      66352      66363    66374
Candidatus Carsonella ruddii strain BT chromosome...   (66298) TTAATAAAAATATTATTAAATTTTCTAATTATTATAATATAATAATTAAAATATTAAAAAAAAAAAAAATTAATTTA
         Candidatus Carsonella 135557 - 167442nt           (1) -----------------------------------------------------------------------------
       SARS-CoV-2 Reference Genome NC_045512.2             (1) -----------------------------------------------------------------------------
                                                                                                    Section 863
                                                        (66375) 66375     66385      66396      66407      66418      66429      66440    66451
Candidatus Carsonella ruddii strain BT chromosome...   (66375) AAAATTTTTTTAGAAATTGGAAGATATTTTTTAACAAATTCATGTATATTATTTTCAAAAATTAATTACATAAAATT
         Candidatus Carsonella 135557 - 167442nt           (1) -----------------------------------------------------------------------------
       SARS-CoV-2 Reference Genome NC_045512.2             (1) -----------------------------------------------------------------------------
                                                                                                    Section 864
                                                        (66452) 66452     66462      66473      66484      66495      66506      66517    66528
Candidatus Carsonella ruddii strain BT chromosome...   (66452) TAATAAATCTAATAATTTAACTATTTTAAATTTAGGAATGAATGATATAATCAGACCATCTTTGTATGATTCTTATC
         Candidatus Carsonella 135557 - 167442nt           (1) -----------------------------------------------------------------------------
       SARS-CoV-2 Reference Genome NC_045512.2             (1) -----------------------------------------------------------------------------
```

Section 865

	(66529)	66529 66539 66550 66561 66572 66583 66594 66605
Candidatus Carsonella ruddii strain BT chromosome...	(66529)	ATAAAATTGAAAATAATAATATAGGTAATAAAATAAAAAATTTTTTTGGTCCTATATGTGAATCAAGTGATAAATTT
Candidatus Carsonella 135557 - 167442nt	(1)	---
SARS-CoV-2 Reference Genome NC_045512.2	(1)	---

Section 866

	(66606)	66606 66616 66627 66638 66649 66660 66671 66682
Candidatus Carsonella ruddii strain BT chromosome...	(66606)	ATAAATAATAAATTTTTAAATGTTAAAAATAATTCAATTATTTTTTTTTATTCTAGTGGAAGTTATTGTAAAGTTAT
Candidatus Carsonella 135557 - 167442nt	(1)	---
SARS-CoV-2 Reference Genome NC_045512.2	(1)	---

Section 867

	(66683)	66683 66693 66704 66715 66726 66737 66748 66759
Candidatus Carsonella ruddii strain BT chromosome...	(66683)	GAGTAATAATTATAATTCTAAATTTAAAATTTATGAAATTATTGTTTATAAAAATAAAATTAAAATAATATTTAAAA
Candidatus Carsonella 135557 - 167442nt	(1)	---
SARS-CoV-2 Reference Genome NC_045512.2	(1)	---

Section 868

	(66760)	66760 66770 66781 66792 66803 66814 66825 66836
Candidatus Carsonella ruddii strain BT chromosome...	(66760)	TTGAAAAATTTAAAAATATAATAAAAAATTATGCTTAAATTTTTAAAATTACATGCTAATGGTAATGATTTTTTAAT
Candidatus Carsonella 135557 - 167442nt	(1)	---
SARS-CoV-2 Reference Genome NC_045512.2	(1)	---

Section 869

	(66837)	66837 66847 66858 66869 66880 66891 66902 66913
Candidatus Carsonella ruddii strain BT chromosome...	(66837)	CATTTTTAGTTTAAAAAAAAATAATTTTTTAAAAAAATATTTAAATAAAAAAACTGGAATAAGTTGTGATCAATTAT
Candidatus Carsonella 135557 - 167442nt	(1)	---
SARS-CoV-2 Reference Genome NC_045512.2	(1)	---

Section 870

	(66914)	66914 66924 66935 66946 66957 66968 66979 66990
Candidatus Carsonella ruddii strain BT chromosome...	(66914)	TTATTATAAAAAGTATAAATTATAAAAATAAATTTATAAATATAAAAATTTTTAATAATAATTTAATTGAAGCTAAA
Candidatus Carsonella 135557 - 167442nt	(1)	---
SARS-CoV-2 Reference Genome NC_045512.2	(1)	---

```
                                                                                                                           Section 871
                                                       (66991) 66991     67001      67012      67023      67034      67045      67056      67067
Candidatus Carsonella ruddii strain BT chromosome...   (66991) AATTGTGGAAATGGTATAAGATGTTTATCTTGGTTTTTTTTATTAAAATTAAAAAAATTTATTTTTATTAATTTTAT
          Candidatus Carsonella 135557 - 167442nt      (1)     -----------------------------------------------------------------------------
      SARS-CoV-2 Reference Genome NC_045512.2          (1)     -----------------------------------------------------------------------------
                                                                                                                           Section 872
                                                       (67068) 67068     67078      67089      67100      67111      67122      67133      67144
Candidatus Carsonella ruddii strain BT chromosome...   (67068) ATTAAATAAAAATTTTATTTATTCTTGGAAAACTAAAAAAAACTTTTTTACTTCTTATAAAATTCCAAAAATAAATA
          Candidatus Carsonella 135557 - 167442nt      (1)     -----------------------------------------------------------------------------
      SARS-CoV-2 Reference Genome NC_045512.2          (1)     -----------------------------------------------------------------------------
                                                                                                                           Section 873
                                                       (67145) 67145     67155      67166      67177      67188      67199      67210      67221
Candidatus Carsonella ruddii strain BT chromosome...   (67145) TAAAAATAATTTTTAGATTTGAAAATATTTTTTTAAAATCTTGTTTTGTAAATTTATTGAATTTACACATTATTTTT
          Candidatus Carsonella 135557 - 167442nt      (1)     -----------------------------------------------------------------------------
      SARS-CoV-2 Reference Genome NC_045512.2          (1)     -----------------------------------------------------------------------------
                                                                                                                           Section 874
                                                       (67222) 67222     67232      67243      67254      67265      67276      67287      67298
Candidatus Carsonella ruddii strain BT chromosome...   (67222) ATTGTTAAAAATATTAATTCTAATAATTTAATTTTTTTAAAAAAAAAAAATAATTTTTCATTTTAAAGATAATTATAA
          Candidatus Carsonella 135557 - 167442nt      (1)     -----------------------------------------------------------------------------
      SARS-CoV-2 Reference Genome NC_045512.2          (1)     -----------------------------------------------------------------------------
                                                                                                                           Section 875
                                                       (67299) 67299     67309      67320      67331      67342      67353      67364      67375
Candidatus Carsonella ruddii strain BT chromosome...   (67299) TATTGAATTTATTCAAATAATAAAAAAATAATAATTTTTATATCAGAATTTTTGAAAAAAATGTAGGAGAAACATATT
          Candidatus Carsonella 135557 - 167442nt      (1)     -----------------------------------------------------------------------------
      SARS-CoV-2 Reference Genome NC_045512.2          (1)     -----------------------------------------------------------------------------
                                                                                                                           Section 876
                                                       (67376) 67376     67386      67397      67408      67419      67430      67441      67452
Candidatus Carsonella ruddii strain BT chromosome...   (67376) CTTGTGGATCAGGAATTATAAGTTCATGTTGTTATATTAAAAAAATTTTTTTTTAAATTCAAATATTTTTATAAAT
          Candidatus Carsonella 135557 - 167442nt      (1)     -----------------------------------------------------------------------------
      SARS-CoV-2 Reference Genome NC_045512.2          (1)     -----------------------------------------------------------------------------
```

Section 877

	(67453)	67453 67463 67474 67485 67496 67507 67518 67529
Candidatus Carsonella ruddii strain BT chromosome...	(67453)	TCTTTAGGAGGAAATTGTATTGTTAGTTTTAGTAAAAAATTTGTTATTATTAATGGTAAAGTTAATTTTTGTTTTTA
Candidatus Carsonella 135557 - 167442nt	(1)	---
SARS-CoV-2 Reference Genome NC_045512.2	(1)	---

Section 878

	(67530)	67530 67540 67551 67562 67573 67584 67595 67606
Candidatus Carsonella ruddii strain BT chromosome...	(67530)	CGGTTATATATAATGTTTAAAAAAATTTTATATTATTGGAAAAATAAGTATTTTTTTTTAATAAAAAAAAAAGAAAA
Candidatus Carsonella 135557 - 167442nt	(1)	---
SARS-CoV-2 Reference Genome NC_045512.2	(1)	---

Section 879

	(67607)	67607 67617 67628 67639 67650 67661 67672 67683
Candidatus Carsonella ruddii strain BT chromosome...	(67607)	TAAATCTGGTGCAGCAACTTATAATTATATAAATGTTAATTTTTTAATTAAAAAAAAAATCATTCAAAATTTATTTTA
Candidatus Carsonella 135557 - 167442nt	(1)	---
SARS-CoV-2 Reference Genome NC_045512.2	(1)	---

Section 880

	(67684)	67684 67694 67705 67716 67727 67738 67749 67760
Candidatus Carsonella ruddii strain BT chromosome...	(67684)	AACAAAATTGTTATCGTCAATTTGATTCTTACTTGTTTAAAAAAAATAAATTATCAATTCATAATCAATTACAAATA
Candidatus Carsonella 135557 - 167442nt	(1)	---
SARS-CoV-2 Reference Genome NC_045512.2	(1)	---

Section 881

	(67761)	67761 67771 67782 67793 67804 67815 67826 67837
Candidatus Carsonella ruddii strain BT chromosome...	(67761)	ATTTGTAAACCTATTCCAATTAATTTTTTAACAATTTATAAAAATTCAATATTTTTTTTAAAAAATATTTTTTTTAA
Candidatus Carsonella 135557 - 167442nt	(1)	---
SARS-CoV-2 Reference Genome NC_045512.2	(1)	---

Section 882

	(67838)	67838 67848 67859 67870 67881 67892 67903 67914
Candidatus Carsonella ruddii strain BT chromosome...	(67838)	AAAAGACAATTGGAAATCTCCTTTATTAGGAGCAAAAGGAATAGGTTATGAAGTTATTAGTAATAATATAGAAATTT
Candidatus Carsonella 135557 - 167442nt	(1)	---
SARS-CoV-2 Reference Genome NC_045512.2	(1)	---

Section 883

	(67915)	67915 67925 67936 67947 67958 67969 67980 67991
Candidatus Carsonella ruddii strain BT chromosome...	(67915)	CACAAATAACAATTTTTATTTTTTTTGGTAATAAAAAATTAAACATTCCTATTTTAGAAATTACTTATGGTATTGAA
Candidatus Carsonella 135557 - 167442nt	(1)	---
SARS-CoV-2 Reference Genome NC_045512.2	(1)	---

Section 884

	(67992)	67992 68002 68013 68024 68035 68046 68057 68068
Candidatus Carsonella ruddii strain BT chromosome...	(67992)	AGAATTAATAGTTTAAAAAATGTAAATTTTATAAACGAAAAATTTATATCTATAAATAATATTTTTAATAAATTTTC
Candidatus Carsonella 135557 - 167442nt	(1)	---
SARS-CoV-2 Reference Genome NC_045512.2	(1)	---

Section 885

	(68069)	68069 68079 68090 68101 68112 68123 68134 68145
Candidatus Carsonella ruddii strain BT chromosome...	(68069)	TATTAAAAAAATTATATTATTATATAATCAATGTTTATTAAATTTAAAAAAAAAAAATACATTTTTATCATATTTTT
Candidatus Carsonella 135557 - 167442nt	(1)	---
SARS-CoV-2 Reference Genome NC_045512.2	(1)	---

Section 886

	(68146)	68146 68156 68167 68178 68189 68200 68211 68222
Candidatus Carsonella ruddii strain BT chromosome...	(68146)	TTTTTATAAAAATAACAAATTATTTTAATTTAATTGATGAATTTTATTTAATTAATAATTATAATAGAATTAAAATA
Candidatus Carsonella 135557 - 167442nt	(1)	---
SARS-CoV-2 Reference Genome NC_045512.2	(1)	---

Section 887

	(68223)	68223 68233 68244 68255 68266 68277 68288 68299
Candidatus Carsonella ruddii strain BT chromosome...	(68223)	ATTAATTTGATTAAAATTATTTCTAATAATATAATAAAAAAAATTAATGTTTGTTAAAAATATTATAAAAAAAATAT
Candidatus Carsonella 135557 - 167442nt	(1)	---
SARS-CoV-2 Reference Genome NC_045512.2	(1)	---

Section 888

	(68300)	68300 68310 68321 68332 68343 68354 68365 68376
Candidatus Carsonella ruddii strain BT chromosome...	(68300)	TTAAAATAATTTTTAAAAAAATTTTTTTAATATATTTTTTTTATTTTAAAAAATATATAATTAAAAAAATTAAAAAA
Candidatus Carsonella 135557 - 167442nt	(1)	---
SARS-CoV-2 Reference Genome NC_045512.2	(1)	---

Section 889

		68377 68387 68398 68409 68420 68431 68442 68453
	(68377)	
Candidatus Carsonella ruddii strain BT chromosome...	(68377)	AAAATCAATATAAAATTAAAAAATATTTTTTATAAGTATAAAAATTTAATTAATAATAAAAATATTTTTTTTTTATC
Candidatus Carsonella 135557 - 167442nt	(1)	---
SARS-CoV-2 Reference Genome NC_045512.2	(1)	---

Section 890

		68454 68464 68475 68486 68497 68508 68519 68530
	(68454)	
Candidatus Carsonella ruddii strain BT chromosome...	(68454)	ACTTTTTTTTAAAAAAATAAAAAATATTTTTTTAATAAAAAATATTTTAAATTTAAAAAAAAAATTTTTAATATTAA
Candidatus Carsonella 135557 - 167442nt	(1)	---
SARS-CoV-2 Reference Genome NC_045512.2	(1)	---

Section 891

		68531 68541 68552 68563 68574 68585 68596 68607
	(68531)	
Candidatus Carsonella ruddii strain BT chromosome...	(68531)	AAGAAAATTTTAAATTTTTAAGAAAAATAAAAAATTTTTTATTTTTTAAAAAAAAAATTTTTTTTATAAATTTTAAA
Candidatus Carsonella 135557 - 167442nt	(1)	---
SARS-CoV-2 Reference Genome NC_045512.2	(1)	---

Section 892

		68608 68618 68629 68640 68651 68662 68673 68684
	(68608)	
Candidatus Carsonella ruddii strain BT chromosome...	(68608)	ATTTATAAAAAGAATAAAAAAAGATTTTTTAATTTTAATAATAATAATAAAGAAACACCCAATTTAATTTTTTTTAA
Candidatus Carsonella 135557 - 167442nt	(1)	---
SARS-CoV-2 Reference Genome NC_045512.2	(1)	---

Section 893

		68685 68695 68706 68717 68728 68739 68750 68761
	(68685)	
Candidatus Carsonella ruddii strain BT chromosome...	(68685)	ATATTATTGTTATTTAAATTATTTTTTAATAAAAAAAAAATATAAAAAAATTATAAATATTAGATTATCATCTTTTA
Candidatus Carsonella 135557 - 167442nt	(1)	---
SARS-CoV-2 Reference Genome NC_045512.2	(1)	---

Section 894

		68762 68772 68783 68794 68805 68816 68827 68838
	(68762)	
Candidatus Carsonella ruddii strain BT chromosome...	(68762)	ATAAAATTATATTTTATAACAATTTTTTAATATTAAAAATTATTTAATACCTATAGAACACTATAGGAAATTAATTA
Candidatus Carsonella 135557 - 167442nt	(1)	---
SARS-CoV-2 Reference Genome NC_045512.2	(1)	---

Section 895

```
                                                     (68839) 68839     68849      68860      68871      68882      68893      68904   68915
Candidatus Carsonella ruddii strain BT chromosome... (68839) AATAATTTTAATATTCGATGCTTGTAATCCTTTTTTACCTTGAGTAACATCAAAACTCACTCTTTGTCCATCTTGTA
               Candidatus Carsonella 135557 - 167442nt   (1) -----------------------------------------------------------------------------
            SARS-CoV-2 Reference Genome NC_045512.2   (1) -----------------------------------------------------------------------------
```

Section 896

```
                                                     (68916) 68916     68926      68937      68948      68959      68970      68981   68992
Candidatus Carsonella ruddii strain BT chromosome... (68916) ATGATTTAAATCCATCAAGTTTTATTTCTGAAAAATGAACAAATAAATCATCACCTCCATCATCAGGAGATATAAAA
               Candidatus Carsonella 135557 - 167442nt   (1) -----------------------------------------------------------------------------
            SARS-CoV-2 Reference Genome NC_045512.2   (1) -----------------------------------------------------------------------------
```

Section 897

```
                                                     (68993) 68993     69003      69014      69025      69036      69047      69058   69069
Candidatus Carsonella ruddii strain BT chromosome... (68993) CCGAATCCTTTTGTATCATTAAACCATTTTACTGTTCCAGTTGCCATATTTTATAGAAAAACGAAAAAAATTTTTTT
               Candidatus Carsonella 135557 - 167442nt   (1) -----------------------------------------------------------------------------
            SARS-CoV-2 Reference Genome NC_045512.2   (1) -----------------------------------------------------------------------------
```

Section 898

```
                                                     (69070) 69070     69080      69091      69102      69113      69124      69135   69146
Candidatus Carsonella ruddii strain BT chromosome... (69070) TAATTTTTTGTTAAAAATACAGAAAAACAATGTTATATATATCCTATAGTTTTAAAACAATATTCTATATTTTTAAA
               Candidatus Carsonella 135557 - 167442nt   (1) -----------------------------------------------------------------------------
            SARS-CoV-2 Reference Genome NC_045512.2   (1) -----------------------------------------------------------------------------
```

Section 899

```
                                                     (69147) 69147     69157      69168      69179      69190      69201      69212   69223
Candidatus Carsonella ruddii strain BT chromosome... (69147) TTTATAAGAATGTTTTTTAAAAACAACTAATTTTTTATTTATAAAATAATTTAAAAATATATATTTATTTTTTTTGT
               Candidatus Carsonella 135557 - 167442nt   (1) -----------------------------------------------------------------------------
            SARS-CoV-2 Reference Genome NC_045512.2   (1) -----------------------------------------------------------------------------
```

Section 900

```
                                                     (69224) 69224     69234      69245      69256      69267      69278      69289   69300
Candidatus Carsonella ruddii strain BT chromosome... (69224) TTTTCAATTTAAAAATATATTTTGAATTAAAACAAAAAAAAAAAATTACCTTTTTTAAAATAAATTTTAATTTTTAAA
               Candidatus Carsonella 135557 - 167442nt   (1) -----------------------------------------------------------------------------
            SARS-CoV-2 Reference Genome NC_045512.2   (1) -----------------------------------------------------------------------------
```

Section 901

		69301 69311 69322 69333 69344 69355 69366 69377
	(69301)	
Candidatus Carsonella ruddii strain BT chromosome...	(69301)	AATAAATTATTTTTTTCCATTGTTTTAAAAAATAAATAATTTCTGTTTAAAGAAATTATTATTTTTTTTTCTTTTTT
Candidatus Carsonella 135557 - 167442nt	(1)	---
SARS-CoV-2 Reference Genome NC_045512.2	(1)	---

Section 902

		69378 69388 69399 69410 69421 69432 69443 69454
	(69378)	
Candidatus Carsonella ruddii strain BT chromosome...	(69378)	TAAAAAAGAAAAAAAAATATTTTTTTTTTTTTTTATATTTATTAATATAATATTTTTTTTTTTTAAAAATAAAAAAA
Candidatus Carsonella 135557 - 167442nt	(1)	---
SARS-CoV-2 Reference Genome NC_045512.2	(1)	---

Section 903

		69455 69465 69476 69487 69498 69509 69520 69531
	(69455)	
Candidatus Carsonella ruddii strain BT chromosome...	(69455)	AATTCAATTCTTCTTGAACATAAAATTGATTTTCTTTTTTTGATAAAATAAAAATATTATTATTTATAAAAGAAATA
Candidatus Carsonella 135557 - 167442nt	(1)	---
SARS-CoV-2 Reference Genome NC_045512.2	(1)	---

Section 904

		69532 69542 69553 69564 69575 69586 69597 69608
	(69532)	
Candidatus Carsonella ruddii strain BT chromosome...	(69532)	TTTTTTTTTTTTAAAAAAAAATCATTTAAAAAATTTATTTTTAATAAAAAATTAACGTAAATTAATTTAAAAAAAAA
Candidatus Carsonella 135557 - 167442nt	(1)	---
SARS-CoV-2 Reference Genome NC_045512.2	(1)	---

Section 905

		69609 69619 69630 69641 69652 69663 69674 69685
	(69609)	
Candidatus Carsonella ruddii strain BT chromosome...	(69609)	TAATTTTAAAATATTTTTTTCCAAACTTAAAATAAAAAATTTATTTTTTAAAAATATAATTATATTGTTTTTTTTTA
Candidatus Carsonella 135557 - 167442nt	(1)	---
SARS-CoV-2 Reference Genome NC_045512.2	(1)	---

Section 906

		69686 69696 69707 69718 69729 69740 69751 69762
	(69686)	
Candidatus Carsonella ruddii strain BT chromosome...	(69686)	ATTTAATATTATTTTTATTTTTAAAATTGTTTTTACAATAAAAATTATAATATTCATTAAATATTAAATGTTTTTCG
Candidatus Carsonella 135557 - 167442nt	(1)	---
SARS-CoV-2 Reference Genome NC_045512.2	(1)	---

```
                                                                                    Section 907
                                                  (69763) 69763     69773      69784      69795      69806      69817      69828      69839
Candidatus Carsonella ruddii strain BT chromosome... (69763) TATTTAATATTTAAAATTTTTTCTATAAAATATTCAACATTTGTAAAAAAAATATTTTTTTTAAAAACAACATTTTT
             Candidatus Carsonella 135557 - 167442nt     (1) -----------------------------------------------------------------------------
         SARS-CoV-2 Reference Genome NC_045512.2     (1) -----------------------------------------------------------------------------
                                                                                    Section 908
                                                  (69840) 69840     69850      69861      69872      69883      69894      69905      69916
Candidatus Carsonella ruddii strain BT chromosome... (69840) AATTTTTAAAATATTTTTATTTTTATTTTTTATATTTATTTTTAAAATATTAAAATAAAAATTAATTTTTTTTAATTA
             Candidatus Carsonella 135557 - 167442nt     (1) -----------------------------------------------------------------------------
         SARS-CoV-2 Reference Genome NC_045512.2     (1) -----------------------------------------------------------------------------
                                                                                    Section 909
                                                  (69917) 69917     69927      69938      69949      69960      69971      69982      69993
Candidatus Carsonella ruddii strain BT chromosome... (69917) AAATTTGAAAATTAAAATATTTTTCTAAAAAACAATTTTCATTTAACATAAAAAAATATGAAAAAAATAATTGTTGC
             Candidatus Carsonella 135557 - 167442nt     (1) -----------------------------------------------------------------------------
         SARS-CoV-2 Reference Genome NC_045512.2     (1) -----------------------------------------------------------------------------
                                                                                    Section 910
                                                  (69994) 69994     70004      70015      70026      70037      70048      70059      70070
Candidatus Carsonella ruddii strain BT chromosome... (69994) TCAAGCAACTTCAGAAGGTTATGCATCAGTTTGCATAGTAAGAATTTCAGGTGAACATGTAAAACAATTTATTGAAC
             Candidatus Carsonella 135557 - 167442nt     (1) -----------------------------------------------------------------------------
         SARS-CoV-2 Reference Genome NC_045512.2     (1) -----------------------------------------------------------------------------
                                                                                    Section 911
                                                  (70071) 70071     70081      70092      70103      70114      70125      70136      70147
Candidatus Carsonella ruddii strain BT chromosome... (70071) CTTTAATAAAAGTAAAATTAAAAACTCAAAAATTAACATATACAAGATTATATGATTTAAATAATAATTTTATAGAT
             Candidatus Carsonella 135557 - 167442nt     (1) -----------------------------------------------------------------------------
         SARS-CoV-2 Reference Genome NC_045512.2     (1) -----------------------------------------------------------------------------
                                                                                    Section 912
                                                  (70148) 70148     70158      70169      70180      70191      70202      70213      70224
Candidatus Carsonella ruddii strain BT chromosome... (70148) TTTATATTAATAGTTTATTTTAAAGGACCTAAATCTTTAACAGGAGATGATATTATAGAATTTCATTTACATGGAAG
             Candidatus Carsonella 135557 - 167442nt     (1) -----------------------------------------------------------------------------
         SARS-CoV-2 Reference Genome NC_045512.2     (1) -----------------------------------------------------------------------------
```

```
                                                                                      Section 913
                                                  (70225) 70225     70235      70246      70257      70268      70279      70290      70301
Candidatus Carsonella ruddii strain BT chromosome... (70225) TATTTTATTATCTAAAAAATTAATAAAAATATTATTGTTAATGGGTGCTAAAGTAGCAAAACCAGGAGAATTTTTAG
           Candidatus Carsonella 135557 - 167442nt      (1) -----------------------------------------------------------------------------
           SARS-CoV-2 Reference Genome NC_045512.2      (1) -----------------------------------------------------------------------------
                                                                                      Section 914
                                                  (70302) 70302     70312      70323      70334      70345      70356      70367      70378
Candidatus Carsonella ruddii strain BT chromosome... (70302) AAAAAAGATATTTAAATGGTAAAATTTCATTAATAGAATGTGAAATTATTAACAACAAAATTTCTTTTTATAATGAA
           Candidatus Carsonella 135557 - 167442nt      (1) -----------------------------------------------------------------------------
           SARS-CoV-2 Reference Genome NC_045512.2      (1) -----------------------------------------------------------------------------
                                                                                      Section 915
                                                  (70379) 70379     70389      70400      70411      70422      70433      70444      70455
Candidatus Carsonella ruddii strain BT chromosome... (70379) AATATGTTTAATTTAAATTTAAATTCACAAAAAAATATTTTTTTATCTATAATTAAAAATTCAAAATTTAGATTAAA
           Candidatus Carsonella 135557 - 167442nt      (1) -----------------------------------------------------------------------------
           SARS-CoV-2 Reference Genome NC_045512.2      (1) -----------------------------------------------------------------------------
                                                                                      Section 916
                                                  (70456) 70456     70466      70477      70488      70499      70510      70521      70532
Candidatus Carsonella ruddii strain BT chromosome... (70456) TATGTTAATAATTTGTTTAGAAAAATCTTTTATAAATGAAAAAGATACATTTTTAAATGATTTTATTTTTTTAAAAA
           Candidatus Carsonella 135557 - 167442nt      (1) -----------------------------------------------------------------------------
           SARS-CoV-2 Reference Genome NC_045512.2      (1) -----------------------------------------------------------------------------
                                                                                      Section 917
                                                  (70533) 70533     70543      70554      70565      70576      70587      70598      70609
Candidatus Carsonella ruddii strain BT chromosome... (70533) AATTTTTAAAAAAATTTAAAAAATTAATAAATATATTATTAAATAAAATAAATAAAATAAAATATTTAAAAACAAAT
           Candidatus Carsonella 135557 - 167442nt      (1) -----------------------------------------------------------------------------
           SARS-CoV-2 Reference Genome NC_045512.2      (1) -----------------------------------------------------------------------------
                                                                                      Section 918
                                                  (70610) 70610     70620      70631      70642      70653      70664      70675      70686
Candidatus Carsonella ruddii strain BT chromosome... (70610) TTTGAAATATTAATAATGGGAAAGAGAAATGTTGGAAAATCAACATTATTTAATAAAATGTGTTTAAATTATTATTC
           Candidatus Carsonella 135557 - 167442nt      (1) -----------------------------------------------------------------------------
           SARS-CoV-2 Reference Genome NC_045512.2      (1) -----------------------------------------------------------------------------
```

```
                                                            Section 919
                                               (70687) 70687     70697      70708      70719      70730      70741      70752  70763
Candidatus Carsonella ruddii strain BT chromosome... (70687) TATTGTTACAAATATACCAGGAACTACAAAAAACATTATTTCAAAAGAAATTTTTTTTTCTTCTAAAAATATTAATT
         Candidatus Carsonella 135557 - 167442nt (1) -----------------------------------------------------------------------------
      SARS-CoV-2 Reference Genome NC_045512.2 (1) -----------------------------------------------------------------------------
                                                            Section 920
                                               (70764) 70764     70774      70785      70796      70807      70818      70829  70840
Candidatus Carsonella ruddii strain BT chromosome... (70764) TAAATGATACAGCAGGATTAAAATTAAAAACTAAAAATTTTATTGAAAAAATAGGAATTTTAAGAAATATTAATAAA
         Candidatus Carsonella 135557 - 167442nt (1) -----------------------------------------------------------------------------
      SARS-CoV-2 Reference Genome NC_045512.2 (1) -----------------------------------------------------------------------------
                                                            Section 921
                                               (70841) 70841     70851      70862      70873      70884      70895      70906  70917
Candidatus Carsonella ruddii strain BT chromosome... (70841) ATATTAAAAAGCGATTTAATTATTTATATAGTAGATAAATTTAATATTAAAAATATTTTTTACAATATGCCTTTAAA
         Candidatus Carsonella 135557 - 167442nt (1) -----------------------------------------------------------------------------
      SARS-CoV-2 Reference Genome NC_045512.2 (1) -----------------------------------------------------------------------------
                                                            Section 922
                                               (70918) 70918     70928      70939      70950      70961      70972      70983  70994
Candidatus Carsonella ruddii strain BT chromosome... (70918) TTTTTTAAAAAAAATAAAAAATAATCTTTTAATTATTGTTATTAATAAATGTGATTTATTTGGAATACCAGAAGGTT
         Candidatus Carsonella 135557 - 167442nt (1) -----------------------------------------------------------------------------
      SARS-CoV-2 Reference Genome NC_045512.2 (1) -----------------------------------------------------------------------------
                                                            Section 923
                                               (70995) 70995     71005      71016      71027      71038      71049      71060  71071
Candidatus Carsonella ruddii strain BT chromosome... (70995) TTTATAAAATGAAATCTATTATTGTTATTTTTTTAAATGCTAAAAATAGTGTTATTATTAATAATTTAAAATGTTTT
         Candidatus Carsonella 135557 - 167442nt (1) -----------------------------------------------------------------------------
      SARS-CoV-2 Reference Genome NC_045512.2 (1) -----------------------------------------------------------------------------
                                                            Section 924
                                               (71072) 71072     71082      71093      71104      71115      71126      71137  71148
Candidatus Carsonella ruddii strain BT chromosome... (71072) ATAAGTAAAATAATAACAAATAAAAAAATTTTAGATAATAATTTACATTATACTAATATATATACATTATATAATAA
         Candidatus Carsonella 135557 - 167442nt (1) -----------------------------------------------------------------------------
      SARS-CoV-2 Reference Genome NC_045512.2 (1) -----------------------------------------------------------------------------
```

```
                                                                   Section 925
                                          (71149) 71149     71159      71170      71181      71192      71203      71214      71225
Candidatus Carsonella ruddii strain BT chromosome... (71149) ATGTAGTAAATTTTATGAAAATTTTTTTTGTAGTTATGATTTAATATTAGAAAACATAATTAATTTTCAAAAAAAAA
         Candidatus Carsonella 135557 - 167442nt (1) -----------------------------------------------------------------------------
      SARS-CoV-2 Reference Genome NC_045512.2 (1) -----------------------------------------------------------------------------
                                                                   Section 926
                                          (71226) 71226     71236      71247      71258      71269      71280      71291      71302
Candidatus Carsonella ruddii strain BT chromosome... (71226) TTTTAAAAATATCAGGAGAATACACAAATAACGTAATTTTAAATTCTATTTTTAGAAATTTTTGTATGGGTAAATAA
         Candidatus Carsonella 135557 - 167442nt (1) -----------------------------------------------------------------------------
      SARS-CoV-2 Reference Genome NC_045512.2 (1) -----------------------------------------------------------------------------
                                                                   Section 927
                                          (71303) 71303     71313      71324      71335      71346      71357      71368      71379
Candidatus Carsonella ruddii strain BT chromosome... (71303) TGATATTTAATATAATTATTATAGGTGGAGGACATTCAGGAATAGAAGCATGTTTGTCTTCTTCAAAAATTTGTAAT
         Candidatus Carsonella 135557 - 167442nt (1) -----------------------------------------------------------------------------
      SARS-CoV-2 Reference Genome NC_045512.2 (1) -----------------------------------------------------------------------------
                                                                   Section 928
                                          (71380) 71380     71390      71401      71412      71423      71434      71445      71456
Candidatus Carsonella ruddii strain BT chromosome... (71380) AAAACAAAATTAATTTCATCAAATTTAGAAAATTTAGGAATGTTATCATGTAATCCTTCAATAGGTGGTGTTGGAAA
         Candidatus Carsonella 135557 - 167442nt (1) -----------------------------------------------------------------------------
      SARS-CoV-2 Reference Genome NC_045512.2 (1) -----------------------------------------------------------------------------
                                                                   Section 929
                                          (71457) 71457     71467      71478      71489      71500      71511      71522      71533
Candidatus Carsonella ruddii strain BT chromosome... (71457) AAGTCATTTAATAAAAGAATTAGAAATTTTAGGTGGAATAATATCTGAAGCTTCTGATTATTCTAGAATTCATTCTA
         Candidatus Carsonella 135557 - 167442nt (1) -----------------------------------------------------------------------------
      SARS-CoV-2 Reference Genome NC_045512.2 (1) -----------------------------------------------------------------------------
                                                                   Section 930
                                          (71534) 71534     71544      71555      71566      71577      71588      71599      71610
Candidatus Carsonella ruddii strain BT chromosome... (71534) AAATTTTAAATTTTAAAAAAGGGGAATCTATTCATTCTTTAAGGTATCAAATAGATAGAATTTTATATAAAAATTAT
         Candidatus Carsonella 135557 - 167442nt (1) -----------------------------------------------------------------------------
      SARS-CoV-2 Reference Genome NC_045512.2 (1) -----------------------------------------------------------------------------
```

Section 931

```
                                                        71611     71621      71632      71643      71654      71665      71676    71687
                                              (71611)
Candidatus Carsonella ruddii strain BT chromosome... (71611) ATTTTAAAAATTTTATTTATTAAAAAAAAATATTTTAATTGAACAAAATGATATTAAAAATATTATAAGATTTAAAAA
        Candidatus Carsonella 135557 - 167442nt      (1) -----------------------------------------------------------------------------
     SARS-CoV-2 Reference Genome NC_045512.2         (1) -----------------------------------------------------------------------------
```

Section 932

```
                                                        71688     71698      71709      71720      71731      71742      71753    71764
                                              (71688)
Candidatus Carsonella ruddii strain BT chromosome... (71688) AAAAATATTATTAATAGATAAATTTAATTGTATACATATTACAAAAAATATAATACTATGTGCTGGAACTTTTATTA
        Candidatus Carsonella 135557 - 167442nt      (1) -----------------------------------------------------------------------------
     SARS-CoV-2 Reference Genome NC_045512.2         (1) -----------------------------------------------------------------------------
```

Section 933

```
                                                        71765     71775      71786      71797      71808      71819      71830    71841
                                              (71765)
Candidatus Carsonella ruddii strain BT chromosome... (71765) ATTCAAAAATTTGTATAGGTAAAAATATTTTTAAATATGGAAGATATAATGAAAAGACATTTTATTTATCTAAATCA
        Candidatus Carsonella 135557 - 167442nt      (1) -----------------------------------------------------------------------------
     SARS-CoV-2 Reference Genome NC_045512.2         (1) -----------------------------------------------------------------------------
```

Section 934

```
                                                        71842     71852      71863      71874      71885      71896      71907    71918
                                              (71842)
Candidatus Carsonella ruddii strain BT chromosome... (71842) TTAAAAAAAATTAATTTATTTATAAATAAATTAAAAACAGGAACACCACCTAGAATAGATATAAATCATATTAATTA
        Candidatus Carsonella 135557 - 167442nt      (1) -----------------------------------------------------------------------------
     SARS-CoV-2 Reference Genome NC_045512.2         (1) -----------------------------------------------------------------------------
```

Section 935

```
                                                        71919     71929      71940      71951      71962      71973      71984    71995
                                              (71919)
Candidatus Carsonella ruddii strain BT chromosome... (71919) TAAAAAATTAATTATACAATATAGTGATTATTCTTATTATTTTGGTAAAAATTTTAATTTTAATAATAATATTAAAT
        Candidatus Carsonella 135557 - 167442nt      (1) -----------------------------------------------------------------------------
     SARS-CoV-2 Reference Genome NC_045512.2         (1) -----------------------------------------------------------------------------
```

Section 936

```
                                                        71996     72006      72017      72028      72039      72050      72061    72072
                                              (71996)
Candidatus Carsonella ruddii strain BT chromosome... (71996) GTTTTGTTACTAATACAAATTTAAAATTACATAATTTTATTAAAAAAAAAATTAAATCAACATCATTATATATAGGA
        Candidatus Carsonella 135557 - 167442nt      (1) -----------------------------------------------------------------------------
     SARS-CoV-2 Reference Genome NC_045512.2         (1) -----------------------------------------------------------------------------
```

Section 937

	(72073)	72073 72083 72094 72105 72116 72127 72138 72149
Candidatus Carsonella ruddii strain BT chromosome...	(72073)	AATATATCTAGTTTAGGACCAAGATATTGTCCTTCTATAGAAGATAAAATATATAAATTTTTATCAAAAAATAATCA
Candidatus Carsonella 135557 - 167442nt	(1)	---
SARS-CoV-2 Reference Genome NC_045512.2	(1)	---

Section 938

	(72150)	72150 72160 72171 72182 72193 72204 72215 72226
Candidatus Carsonella ruddii strain BT chromosome...	(72150)	TCAAATTTTTTTAGAACCTGAAGGATTTTATAGTAAAGAAATATATTTAAATGGTTTATCTACATCTTTATCTTTGA
Candidatus Carsonella 135557 - 167442nt	(1)	---
SARS-CoV-2 Reference Genome NC_045512.2	(1)	---

Section 939

	(72227)	72227 72237 72248 72259 72270 72281 72292 72303
Candidatus Carsonella ruddii strain BT chromosome...	(72227)	ATAATCAAAAAAAAATAATAAAAAAAATAAAAGGATTAGAAAAATCTTTAATTATTAGATTTGCATATAATATTGAA
Candidatus Carsonella 135557 - 167442nt	(1)	---
SARS-CoV-2 Reference Genome NC_045512.2	(1)	---

Section 940

	(72304)	72304 72314 72325 72336 72347 72358 72369 72380
Candidatus Carsonella ruddii strain BT chromosome...	(72304)	TATGATTATTTTGATCCAAGATGTTTAAATATTACTTTAAATATTAAATTTGCAAATAATATTTTTTTATGTGGACA
Candidatus Carsonella 135557 - 167442nt	(1)	---
SARS-CoV-2 Reference Genome NC_045512.2	(1)	---

Section 941

	(72381)	72381 72391 72402 72413 72424 72435 72446 72457
Candidatus Carsonella ruddii strain BT chromosome...	(72381)	AATAAATGGATCAACAGGTTATGAAGAAGCAGCAGTACAAGGTTTTGTAGCAGGTGTAAATGCTGCTAGAAAAAGTA
Candidatus Carsonella 135557 - 167442nt	(1)	---
SARS-CoV-2 Reference Genome NC_045512.2	(1)	---

Section 942

	(72458)	72458 72468 72479 72490 72501 72512 72523 72534
Candidatus Carsonella ruddii strain BT chromosome...	(72458)	TTAATTTATTATTATGGAAACCAAAAAAATGGAATTCATATATTGGAGTTTTAATAAATGATCTTATTAATATTGGT
Candidatus Carsonella 135557 - 167442nt	(1)	---
SARS-CoV-2 Reference Genome NC_045512.2	(1)	---

```
                                                                                                                    Section 943
                                                          (72535) 72535     72545      72556      72567      72578      72589      72600      72611
Candidatus Carsonella ruddii strain BT chromosome...      (72535) ATTAGTGAACCATATAGAATTTTTACTTCTAAATCAAAATTTAGATTATTTTTAAGAATGGATAATTCTTTATATAG
         Candidatus Carsonella 135557 - 167442nt              (1) -----------------------------------------------------------------------------
      SARS-CoV-2 Reference Genome NC_045512.2                 (1) -----------------------------------------------------------------------------
                                                                                                                    Section 944
                                                          (72612) 72612     72622      72633      72644      72655      72666      72677      72688
Candidatus Carsonella ruddii strain BT chromosome...      (72612) ATTAACAAATTTAGGTTTTTTAATTGGAACTATTACTAAATTTAAATTTAAATTTTATAATAATTATATATATAATA
         Candidatus Carsonella 135557 - 167442nt              (1) -----------------------------------------------------------------------------
      SARS-CoV-2 Reference Genome NC_045512.2                 (1) -----------------------------------------------------------------------------
                                                                                                                    Section 945
                                                          (72689) 72689     72699      72710      72721      72732      72743      72754      72765
Candidatus Carsonella ruddii strain BT chromosome...      (72689) CTTACAATAATTTATTAGATTTTTTAAAATATAATTTAAAAAAAAATTTTAATATTTATAAAAATATTATAATAATG
         Candidatus Carsonella 135557 - 167442nt              (1) -----------------------------------------------------------------------------
      SARS-CoV-2 Reference Genome NC_045512.2                 (1) -----------------------------------------------------------------------------
                                                                                                                    Section 946
                                                          (72766) 72766     72776      72787      72798      72809      72820      72831      72842
Candidatus Carsonella ruddii strain BT chromosome...      (72766) TCTAAATATTATGGATATATTAAAAAATATTTATTTAAGTTTTAATTTTATTAAAAATAATTCATTATTTGATAATG
         Candidatus Carsonella 135557 - 167442nt              (1) -----------------------------------------------------------------------------
      SARS-CoV-2 Reference Genome NC_045512.2                 (1) -----------------------------------------------------------------------------
                                                                                                                    Section 947
                                                          (72843) 72843     72853      72864      72875      72886      72897      72908      72919
Candidatus Carsonella ruddii strain BT chromosome...      (72843) ATATTAATTTAATTTCTTTAGTATTTAAAATATTTATATTTTATTTATTTATTAAATTAAACATATCTAAAGTATTT
         Candidatus Carsonella 135557 - 167442nt              (1) -----------------------------------------------------------------------------
      SARS-CoV-2 Reference Genome NC_045512.2                 (1) -----------------------------------------------------------------------------
                                                                                                                    Section 948
                                                          (72920) 72920     72930      72941      72952      72963      72974      72985      72996
Candidatus Carsonella ruddii strain BT chromosome...      (72920) ATTTATTTTAAAAATTTTATAAATAAATCAATTAATAAAGAAAATAGAAAAATTTTACTTTTTTTTATAAGTTTAAT
         Candidatus Carsonella 135557 - 167442nt              (1) -----------------------------------------------------------------------------
      SARS-CoV-2 Reference Genome NC_045512.2                 (1) -----------------------------------------------------------------------------
```

Section 949

		72997 73007 73018 73029 73040 73051 73062 73073
	(72997)	
Candidatus Carsonella ruddii strain BT chromosome...	(72997)	TTTTTTTAATATATTAATTTATAATTTATCAGATATTTTACCTAATGATTTATTAAAAAATATTTTTAAAAAAGAAT
Candidatus Carsonella 135557 - 167442nt	(1)	--
SARS-CoV-2 Reference Genome NC_045512.2	(1)	--

Section 950

		73074 73084 73095 73106 73117 73128 73139 73150
	(73074)	
Candidatus Carsonella ruddii strain BT chromosome...	(73074)	TTAATATTGTTGCTACTTCTAATATAAATATTACTTTTAGTTTTTCTATAATTTCTTTTTTAATAATAAATTATTTA
Candidatus Carsonella 135557 - 167442nt	(1)	--
SARS-CoV-2 Reference Genome NC_045512.2	(1)	--

Section 951

		73151 73161 73172 73183 73194 73205 73216 73227
	(73151)	
Candidatus Carsonella ruddii strain BT chromosome...	(73151)	TCAATTAAAAAAAATGGTATCTCAAATTTTATTTTGTACTTTTTAAAAAATCCAATAAAAAAAAAATATATGTTTTT
Candidatus Carsonella 135557 - 167442nt	(1)	--
SARS-CoV-2 Reference Genome NC_045512.2	(1)	--

Section 952

		73228 73238 73249 73260 73271 73282 73293 73304
	(73228)	
Candidatus Carsonella ruddii strain BT chromosome...	(73228)	TTTTAATTTTATAATTGAATTTATTTCATTTGTAATGAAACCTATATCATTATCTTTAAGATTATTTGGTAATATTT
Candidatus Carsonella 135557 - 167442nt	(1)	--
SARS-CoV-2 Reference Genome NC_045512.2	(1)	--

Section 953

		73305 73315 73326 73337 73348 73359 73370 73381
	(73305)	
Candidatus Carsonella ruddii strain BT chromosome...	(73305)	TTTCATCAGAAATAATATTTAATTTAATAAATAATATGAATATTTTTTTCAACATATTATTAAATTTATTATGGGGA
Candidatus Carsonella 135557 - 167442nt	(1)	--
SARS-CoV-2 Reference Genome NC_045512.2	(1)	--

Section 954

		73382 73392 73403 73414 73425 73436 73447 73458
	(73382)	
Candidatus Carsonella ruddii strain BT chromosome...	(73382)	ATATTTCATTATTTAGTTTTACCTTTACAAGCTTATATTTTTACTACATTGATTACTATATATGTTTCACAATCATT
Candidatus Carsonella 135557 - 167442nt	(1)	--
SARS-CoV-2 Reference Genome NC_045512.2	(1)	--

Section 955

		73459 73469 73480 73491 73502 73513 73524 73535
Candidatus Carsonella ruddii strain BT chromosome...	(73459)	AAAACATTAAATATATGGAAAAAATTTTAATATTATCATCTGCAATAATGATGGGATTAGCATCAGTAGGTACTGGA
Candidatus Carsonella 135557 - 167442nt	(1)	---
SARS-CoV-2 Reference Genome NC_045512.2	(1)	---

Section 956

		73536 73546 73557 73568 73579 73590 73601 73612
Candidatus Carsonella ruddii strain BT chromosome...	(73536)	ATTGGATTTGGTTTTTTAGGAGGTAAATTGTTAGAATCTATTTCAAGACAACCTGAATTAGATAATTTATTGTTAAC
Candidatus Carsonella 135557 - 167442nt	(1)	---
SARS-CoV-2 Reference Genome NC_045512.2	(1)	---

Section 957

		73613 73623 73634 73645 73656 73667 73678 73689
Candidatus Carsonella ruddii strain BT chromosome...	(73613)	TAGAACATTTTTAATAACTGGTTTGCTAGATGCAGTACCAATGATAAGTGTAGGTATAGGATTGTATATTATTTTTA
Candidatus Carsonella 135557 - 167442nt	(1)	---
SARS-CoV-2 Reference Genome NC_045512.2	(1)	---

Section 958

		73690 73700 73711 73722 73733 73744 73755 73766
Candidatus Carsonella ruddii strain BT chromosome...	(73690)	TATTATCAAATAAATAATATGAATATAAATTTTACATTAATTAATGAATTTTTATCTTTTTTAATTTTTTTTGTTTT
Candidatus Carsonella 135557 - 167442nt	(1)	---
SARS-CoV-2 Reference Genome NC_045512.2	(1)	---

Section 959

		73767 73777 73788 73799 73810 73821 73832 73843
Candidatus Carsonella ruddii strain BT chromosome...	(73767)	TTCATTTATTTTTATTTTTCCTAAAATTATAAATATTATAAATTATAATGATTTAAATGATTTTAAAAACAAAATTT
Candidatus Carsonella 135557 - 167442nt	(1)	---
SARS-CoV-2 Reference Genome NC_045512.2	(1)	---

Section 960

		73844 73854 73865 73876 73887 73898 73909 73920
Candidatus Carsonella ruddii strain BT chromosome...	(73844)	TTTTAAATTATAACAAAACATTAGAAAATAATTTAATTAAAAAAATATTTATAATAGAATTTGAAATTCAAAAACAA
Candidatus Carsonella 135557 - 167442nt	(1)	---
SARS-CoV-2 Reference Genome NC_045512.2	(1)	---

Section 961

	(73921)	73921 73931 73942 73953 73964 73975 73986 73997
Candidatus Carsonella ruddii strain BT chromosome...	(73921)	TTTGACAAAAATATTAAAAATATAAATAATAATATATTAAAAAAAAAAAATTTTTTACTTGATATTATAATATTAGA
Candidatus Carsonella 135557 - 167442nt	(1)	---
SARS-CoV-2 Reference Genome NC_045512.2	(1)	---

Section 962

	(73998)	73998 74008 74019 74030 74041 74052 74063 74074
Candidatus Carsonella ruddii strain BT chromosome...	(73998)	AAAAAATTTTTTTATTAAAAAAATTAAAAAAAATTTTAATTTAATAAATTATAATTATATTAAAAATTTTTATAATG
Candidatus Carsonella 135557 - 167442nt	(1)	---
SARS-CoV-2 Reference Genome NC_045512.2	(1)	---

Section 963

	(74075)	74075 74085 74096 74107 74118 74129 74140 74151
Candidatus Carsonella ruddii strain BT chromosome...	(74075)	AATTAAAAATAAATTTTGTACAAATTTTTAAAAAAATTTTTAATAATATTATAAATTATAACAAAGAATTTATTATA
Candidatus Carsonella 135557 - 167442nt	(1)	---
SARS-CoV-2 Reference Genome NC_045512.2	(1)	---

Section 964

	(74152)	74152 74162 74173 74184 74195 74206 74217 74228
Candidatus Carsonella ruddii strain BT chromosome...	(74152)	ATATGAATATTTTTTTTTTTAAAAATATAAATAAAAAATTATCAAAAAGAATTTTTTTTTTGTTTTTTATAAGTTTA
Candidatus Carsonella 135557 - 167442nt	(1)	---
SARS-CoV-2 Reference Genome NC_045512.2	(1)	---

Section 965

	(74229)	74229 74239 74250 74261 74272 74283 74294 74305
Candidatus Carsonella ruddii strain BT chromosome...	(74229)	ATATTATTTAAAAAAAATATAAAAAAGAATATTAAAAAAAAAAATAAAAAAATTATATTTAATAATAAAATTGAAAA
Candidatus Carsonella 135557 - 167442nt	(1)	---
SARS-CoV-2 Reference Genome NC_045512.2	(1)	---

Section 966

	(74306)	74306 74316 74327 74338 74349 74360 74371 74382
Candidatus Carsonella ruddii strain BT chromosome...	(74306)	ATATTTAATAAAAATTGTAGTTAAAAAATATGAATAATTATGGTATTATTAATAAAATATATGATGGAATAGTAGAA
Candidatus Carsonella 135557 - 167442nt	(1)	---
SARS-CoV-2 Reference Genome NC_045512.2	(1)	---

```
                                                                                         Section 967
                                                  (74383) 74383     74393      74404      74415      74426      74437      74448  74459
Candidatus Carsonella ruddii strain BT chromosome... (74383) GTAAATGGATTAAATTCCGCAAAATATGGTGAAATGATTCTTTTTGAAAACGATCAAAAAGGAATAGTTTTTAGTTT
            Candidatus Carsonella 135557 - 167442nt (1) -----------------------------------------------------------------------------
            SARS-CoV-2 Reference Genome NC_045512.2 (1) -----------------------------------------------------------------------------
                                                                                         Section 968
                                                  (74460) 74460     74470      74481      74492      74503      74514      74525  74536
Candidatus Carsonella ruddii strain BT chromosome... (74460) AAACAAAAATAATGTTAATATTGTATTATTAAATAATTTTGAAAATTTAAAACAAGGACAAAAATGTTTTTGTACTA
            Candidatus Carsonella 135557 - 167442nt (1) -----------------------------------------------------------------------------
            SARS-CoV-2 Reference Genome NC_045512.2 (1) -----------------------------------------------------------------------------
                                                                                         Section 969
                                                  (74537) 74537     74547      74558      74569      74580      74591      74602  74613
Candidatus Carsonella ruddii strain BT chromosome... (74537) ATAAAATATTTGAAGTACCTGTTGGTTATGAATTAATTGGAAGAATTATAAATTCAAAAGGAGAGTTTATAGATAAT
            Candidatus Carsonella 135557 - 167442nt (1) -----------------------------------------------------------------------------
            SARS-CoV-2 Reference Genome NC_045512.2 (1) -----------------------------------------------------------------------------
                                                                                         Section 970
                                                  (74614) 74614     74624      74635      74646      74657      74668      74679  74690
Candidatus Carsonella ruddii strain BT chromosome... (74614) AAAAAAAATATTAATATTTTTAAAAAAGACATAATTGAAAAAATAGCTCCAGGAGTAATGGAAAGAGAATCTGTGAA
            Candidatus Carsonella 135557 - 167442nt (1) -----------------------------------------------------------------------------
            SARS-CoV-2 Reference Genome NC_045512.2 (1) -----------------------------------------------------------------------------
                                                                                         Section 971
                                                  (74691) 74691     74701      74712      74723      74734      74745      74756  74767
Candidatus Carsonella ruddii strain BT chromosome... (74691) TGAACCATTATTAACAGGAATAAAATCTATTGATTGTATGATACCAATAGGTAAAGGACAAAGAGAATTAATTATAG
            Candidatus Carsonella 135557 - 167442nt (1) -----------------------------------------------------------------------------
            SARS-CoV-2 Reference Genome NC_045512.2 (1) -----------------------------------------------------------------------------
                                                                                         Section 972
                                                  (74768) 74768     74778      74789      74800      74811      74822      74833  74844
Candidatus Carsonella ruddii strain BT chromosome... (74768) GAGATAGACAAACCGGGAAAACAACAATAGCTATTGATTCTATTATTAATCAAAAAAATAAAAATGTAATTTGTATA
            Candidatus Carsonella 135557 - 167442nt (1) -----------------------------------------------------------------------------
            SARS-CoV-2 Reference Genome NC_045512.2 (1) -----------------------------------------------------------------------------
```

```
                                                                                    Section 973
                                                   (74845) 74845     74855      74866      74877      74888      74899      74910    74921
Candidatus Carsonella ruddii strain BT chromosome... (74845) TATGTATGTATAGGTCAAAAAATTTCTTCATTAATAAATGTAATTAATAAACTTAAAAAATTTAATTGTTTAGAATA
           Candidatus Carsonella 135557 - 167442nt      (1) -----------------------------------------------------------------------------
          SARS-CoV-2 Reference Genome NC_045512.2       (1) -----------------------------------------------------------------------------
                                                                                    Section 974
                                                   (74922) 74922     74932      74943      74954      74965      74976      74987    74998
Candidatus Carsonella ruddii strain BT chromosome... (74922) TACAATTATTATAGCAGCTACTGCTGCTGATTGCGCAGCGGAACAATATATAGCTCCATATACAGGTTGTACAATAG
           Candidatus Carsonella 135557 - 167442nt      (1) -----------------------------------------------------------------------------
          SARS-CoV-2 Reference Genome NC_045512.2       (1) -----------------------------------------------------------------------------
                                                                                    Section 975
                                                   (74999) 74999     75009      75020      75031      75042      75053      75064    75075
Candidatus Carsonella ruddii strain BT chromosome... (74999) GAGAATATTTCAGAGATTTAGGAAAAGATTGTTTAATTATTTATGATGATTTAACTAAACATGCTTGGTCTTATAGA
           Candidatus Carsonella 135557 - 167442nt      (1) -----------------------------------------------------------------------------
          SARS-CoV-2 Reference Genome NC_045512.2       (1) -----------------------------------------------------------------------------
                                                                                    Section 976
                                                   (75076) 75076     75086      75097      75108      75119      75130      75141    75152
Candidatus Carsonella ruddii strain BT chromosome... (75076) CAAATATCTCTTTTATTAAAAAGACCTCCTGGAAGGGAAGCATATCCAGGTGATGTTTTTTATTTACATTCTAGATT
           Candidatus Carsonella 135557 - 167442nt      (1) -----------------------------------------------------------------------------
          SARS-CoV-2 Reference Genome NC_045512.2       (1) -----------------------------------------------------------------------------
                                                                                    Section 977
                                                   (75153) 75153     75163      75174      75185      75196      75207      75218    75229
Candidatus Carsonella ruddii strain BT chromosome... (75153) ATTAGAAAGATCTTCTAAAGTTAATAAAATATTTTTAGAAAAAAAAAATATTTTTAACAAAAATAATGGTACTCTTA
           Candidatus Carsonella 135557 - 167442nt      (1) -----------------------------------------------------------------------------
          SARS-CoV-2 Reference Genome NC_045512.2       (1) -----------------------------------------------------------------------------
                                                                                    Section 978
                                                   (75230) 75230     75240      75251      75262      75273      75284      75295    75306
Candidatus Carsonella ruddii strain BT chromosome... (75230) CTGCTTTTCCAATTATAGAAACTTTAGAAGGTGATGTAACTTCATTTATACCTACTAATGTTATTTCTATTACTGAT
           Candidatus Carsonella 135557 - 167442nt      (1) -----------------------------------------------------------------------------
          SARS-CoV-2 Reference Genome NC_045512.2       (1) -----------------------------------------------------------------------------
```

```
                                                                  Section 979
                                           (75307) 75307     75317      75328      75339      75350      75361      75372     75383
Candidatus Carsonella ruddii strain BT chromosome... (75307) GGACAAATTTTTTTAGATACTAATTTATTTAATTCAGGAATAAGACCTGCTATTAATGTTGGTTTATCAGTTTCAAG
             Candidatus Carsonella 135557 - 167442nt (1) -----------------------------------------------------------------------------
          SARS-CoV-2 Reference Genome NC_045512.2 (1) -----------------------------------------------------------------------------
                                                                  Section 980
                                           (75384) 75384     75394      75405      75416      75427      75438      75449     75460
Candidatus Carsonella ruddii strain BT chromosome... (75384) AGTAGGAGGCTCTGCTCAATTTAAAATTATAAAAAAATTAAGTGGAGATATTAGAATAATGTTAGCTCAATATCGAG
             Candidatus Carsonella 135557 - 167442nt (1) -----------------------------------------------------------------------------
          SARS-CoV-2 Reference Genome NC_045512.2 (1) -----------------------------------------------------------------------------
                                                                  Section 981
                                           (75461) 75461     75471      75482      75493      75504      75515      75526     75537
Candidatus Carsonella ruddii strain BT chromosome... (75461) AATTAGAATCTTTTTCTAAATTTTCTTCAGATTTAGATATAGAAACTAAAAATCAATTGTTTAATGGGGAAAAAATA
             Candidatus Carsonella 135557 - 167442nt (1) -----------------------------------------------------------------------------
          SARS-CoV-2 Reference Genome NC_045512.2 (1) -----------------------------------------------------------------------------
                                                                  Section 982
                                           (75538) 75538     75548      75559      75570      75581      75592      75603     75614
Candidatus Carsonella ruddii strain BT chromosome... (75538) ACTTTATTAATGAAACAAAAACCTAACTTTAATTATTCTATTATTGAATTAACATTAGTTTTATTAATTATTAAAAA
             Candidatus Carsonella 135557 - 167442nt (1) -----------------------------------------------------------------------------
          SARS-CoV-2 Reference Genome NC_045512.2 (1) -----------------------------------------------------------------------------
                                                                  Section 983
                                           (75615) 75615     75625      75636      75647      75658      75669      75680     75691
Candidatus Carsonella ruddii strain BT chromosome... (75615) TAAATATTTTGTTAAAATACCAGTTAATCAAATAGAATATTTTGAATATAATATTATAAAATATTTAAGAAATTTAA
             Candidatus Carsonella 135557 - 167442nt (1) -----------------------------------------------------------------------------
          SARS-CoV-2 Reference Genome NC_045512.2 (1) -----------------------------------------------------------------------------
                                                                  Section 984
                                           (75692) 75692     75702      75713      75724      75735      75746      75757     75768
Candidatus Carsonella ruddii strain BT chromosome... (75692) ATTTTGAAAAAATTATAAATATTAATAATAAAAATTTAGAAAATTATTTATTAGAAATAATAAATTATTTCATATTT
             Candidatus Carsonella 135557 - 167442nt (1) -----------------------------------------------------------------------------
          SARS-CoV-2 Reference Genome NC_045512.2 (1) -----------------------------------------------------------------------------
```

Section 985

		75769 75779 75790 75801 75812 75823 75834 75845
Candidatus Carsonella ruddii strain BT chromosome...	(75769)	GGAAGTTTATTATGAATATAAGAGATATTAAAAATAAAATTAATATTTTATTTAATATAAATAAATTAACAAATACA
Candidatus Carsonella 135557 - 167442nt	(1)	---
SARS-CoV-2 Reference Genome NC_045512.2	(1)	---

Section 986

		75846 75856 75867 75878 75889 75900 75911 75922
Candidatus Carsonella ruddii strain BT chromosome...	(75846)	ATGAGTATGATTTCATTTTCAAAAATGAAAAAAATTTTTAAAAAATGTTTAATACTTAATAAATTATATTTAGAAAC
Candidatus Carsonella 135557 - 167442nt	(1)	---
SARS-CoV-2 Reference Genome NC_045512.2	(1)	---

Section 987

		75923 75933 75944 75955 75966 75977 75988 75999
Candidatus Carsonella ruddii strain BT chromosome...	(75923)	AAAAAAAATAATTTTTGAAATTTATAATTTTAAAAACAATAATTTTTTTTGTTGTGTATTAATTACAACAAATAAAG
Candidatus Carsonella 135557 - 167442nt	(1)	---
SARS-CoV-2 Reference Genome NC_045512.2	(1)	---

Section 988

		76000 76010 76021 76032 76043 76054 76065 76076
Candidatus Carsonella ruddii strain BT chromosome...	(76000)	GTTTTTGTGGAAATATTAATAGTGAAATTATTAAATATTGTTTAAAATTTTTAAAAAATAATATAAATTTAGATTTA
Candidatus Carsonella 135557 - 167442nt	(1)	---
SARS-CoV-2 Reference Genome NC_045512.2	(1)	---

Section 989

		76077 76087 76098 76109 76120 76131 76142 76153
Candidatus Carsonella ruddii strain BT chromosome...	(76077)	ATAATAATTGGTAAAAAAGCTATTGATTTTTTTAATAAAAAAAATATATATATTAAAAAAAAAATTGTTTTTAATGA
Candidatus Carsonella 135557 - 167442nt	(1)	---
SARS-CoV-2 Reference Genome NC_045512.2	(1)	---

Section 990

		76154 76164 76175 76186 76197 76208 76219 76230
Candidatus Carsonella ruddii strain BT chromosome...	(76154)	AAAAAATGAAATTTTTTTTTCAAATGAAATTTTAAACATTTTAAAATATTATGAAAATGTTTTTTTTTTAAGTTCAA
Candidatus Carsonella 135557 - 167442nt	(1)	---
SARS-CoV-2 Reference Genome NC_045512.2	(1)	---

Section 991

```
                                                          (76231) 76231     76241      76252      76263      76274      76285      76296      76307
Candidatus Carsonella ruddii strain BT chromosome...      (76231) AAATTATTAATAATAGTATTAAAATAATAAAGACTAATTTATATGAAAAAATTAAAAAAAATTTTTATGAAATTAAT
Candidatus Carsonella 135557 - 167442nt                   (1)     -----------------------------------------------------------------------------
SARS-CoV-2 Reference Genome NC_045512.2                   (1)     -----------------------------------------------------------------------------
```

Section 992

```
                                                          (76308) 76308     76318      76329      76340      76351      76362      76373      76384
Candidatus Carsonella ruddii strain BT chromosome...      (76308) ATAAATTATAATGATATAATTAATAATTATTTAAATTTTGTTTTAAATTATTTATATTCAGAAAATTATTTTTCTGA
Candidatus Carsonella 135557 - 167442nt                   (1)     -----------------------------------------------------------------------------
SARS-CoV-2 Reference Genome NC_045512.2                   (1)     -----------------------------------------------------------------------------
```

Section 993

```
                                                          (76385) 76385     76395      76406      76417      76428      76439      76450      76461
Candidatus Carsonella ruddii strain BT chromosome...      (76385) ATTAAAAATTAGAATGACTACTATGAGATCAGCTGCTGATAGTTCAAAAAAAATTATAAAAAACATGAATTTAATTA
Candidatus Carsonella 135557 - 167442nt                   (1)     -----------------------------------------------------------------------------
SARS-CoV-2 Reference Genome NC_045512.2                   (1)     -----------------------------------------------------------------------------
```

Section 994

```
                                                          (76462) 76462     76472      76483      76494      76505      76516      76527      76538
Candidatus Carsonella ruddii strain BT chromosome...      (76462) AAAATAAAATTAGACAATTCAAAGTTACTCAAGAAATGTTAGAAATAATAAATAGTATTAATTTATGATAGGAAGAA
Candidatus Carsonella 135557 - 167442nt                   (1)     -----------------------------------------------------------------------------
SARS-CoV-2 Reference Genome NC_045512.2                   (1)     -----------------------------------------------------------------------------
```

Section 995

```
                                                          (76539) 76539     76549      76560      76571      76582      76593      76604      76615
Candidatus Carsonella ruddii strain BT chromosome...      (76539) TAATACAAGTATTAGGTTCAATTTTAGATGTTGAATTTAAAAAAAAAAATATACCAAAAATTTATAATGCTTTATTT
Candidatus Carsonella 135557 - 167442nt                   (1)     -----------------------------------------------------------------------------
SARS-CoV-2 Reference Genome NC_045512.2                   (1)     -----------------------------------------------------------------------------
```

Section 996

```
                                                          (76616) 76616     76626      76637      76648      76659      76670      76681      76692
Candidatus Carsonella ruddii strain BT chromosome...      (76616) ATACCTGAAGTAAATATTTTTTTAGAAGTTCAACAACAAATTGGACAGAATATTGTCAGAACTATTGCTTTTGGTAA
Candidatus Carsonella 135557 - 167442nt                   (1)     -----------------------------------------------------------------------------
SARS-CoV-2 Reference Genome NC_045512.2                   (1)     -----------------------------------------------------------------------------
```

```
                                                                                    Section 997
                                        (76693) 76693     76703      76714      76725      76736      76747      76758    76769
Candidatus Carsonella ruddii strain BT chromosome... (76693) TACTAATGGAATTAAAAGAAATTCTATTGTTTTAGATACAAACAAACCAATAATGACACCTGTAGGTGATTGTACTC
        Candidatus Carsonella 135557 - 167442nt (1) -----------------------------------------------------------------------------
     SARS-CoV-2 Reference Genome NC_045512.2 (1) -----------------------------------------------------------------------------
                                                                                    Section 998
                                        (76770) 76770     76780      76791      76802      76813      76824      76835    76846
Candidatus Carsonella ruddii strain BT chromosome... (76770) TCGGAAGAATAATTAATGTATTAGGAGAACCAATAGATAATAAAGGAAATTTAAAATTTTTAAAAAAAAAACCAATT
        Candidatus Carsonella 135557 - 167442nt (1) -----------------------------------------------------------------------------
     SARS-CoV-2 Reference Genome NC_045512.2 (1) -----------------------------------------------------------------------------
                                                                                    Section 999
                                        (76847) 76847     76857      76868      76879      76890      76901      76912    76923
Candidatus Carsonella ruddii strain BT chromosome... (76847) CATTTAAATCCTCCTAAATTTTCAGATCAGATTTTTACAAATAATATATTAGAAACTGGAATTAAAATAATAGATTT
        Candidatus Carsonella 135557 - 167442nt (1) -----------------------------------------------------------------------------
     SARS-CoV-2 Reference Genome NC_045512.2 (1) -----------------------------------------------------------------------------
                                                                                    Section 1000
                                        (76924) 76924     76934      76945      76956      76967      76978      76989    77000
Candidatus Carsonella ruddii strain BT chromosome... (76924) ATTATGTCCTTTTTTAAAAGGAGGTAAAATTGGTTTATTTGGTGGTGCTGGAGTTGGAAAAACTGTTAATATGATGG
        Candidatus Carsonella 135557 - 167442nt (1) -----------------------------------------------------------------------------
     SARS-CoV-2 Reference Genome NC_045512.2 (1) -----------------------------------------------------------------------------
                                                                                    Section 1001
                                        (77001) 77001     77011      77022      77033      77044      77055      77066    77077
Candidatus Carsonella ruddii strain BT chromosome... (77001) AATTAATAAGAAATATAGCAATAGAACATAGTGGATGCTCTGTTTTTATTGGAGTAGGAGAAAGATCTCGGGAAGGA
        Candidatus Carsonella 135557 - 167442nt (1) -----------------------------------------------------------------------------
     SARS-CoV-2 Reference Genome NC_045512.2 (1) -----------------------------------------------------------------------------
                                                                                    Section 1002
                                        (77078) 77078     77088      77099      77110      77121      77132      77143    77154
Candidatus Carsonella ruddii strain BT chromosome... (77078) AATGATTTTTATCATGAAATGAAAGATTCAAATGTATTAGATAAAGTTTCATTAATATATGGACAAATGAACGAACC
        Candidatus Carsonella 135557 - 167442nt (1) -----------------------------------------------------------------------------
     SARS-CoV-2 Reference Genome NC_045512.2 (1) -----------------------------------------------------------------------------
```

```
                                                                 Section 1003
                                                          (77155) 77155     77165      77176      77187      77198      77209      77220      77231
Candidatus Carsonella ruddii strain BT chromosome...      (77155) ATCAGGAAATAGATTAAGAGTTGCTTTAACAGGATTAAGTATAGCTGAAGAATTTAGAAATTCTGGAAAAGATGTTT
Candidatus Carsonella 135557 - 167442nt                   (1)     -----------------------------------------------------------------------------
SARS-CoV-2 Reference Genome NC_045512.2                   (1)     -----------------------------------------------------------------------------

                                                                 Section 1004
                                                          (77232) 77232     77242      77253      77264      77275      77286      77297      77308
Candidatus Carsonella ruddii strain BT chromosome...      (77232) TATTATTTATAGATAATATTTATAGATTTACTTTAGCAGGTACAGAAGTTTCAGCAATGTTAGGAAGAACTCCATCT
Candidatus Carsonella 135557 - 167442nt                   (1)     -----------------------------------------------------------------------------
SARS-CoV-2 Reference Genome NC_045512.2                   (1)     -----------------------------------------------------------------------------

                                                                 Section 1005
                                                          (77309) 77309     77319      77330      77341      77352      77363      77374      77385
Candidatus Carsonella ruddii strain BT chromosome...      (77309) GCTGTAGGTTATCAACCTACTCTTGCTGAAGAAATGGAAAATTACAAGAAAGAATATCTTCAACAAAAAATGGAAG
Candidatus Carsonella 135557 - 167442nt                   (1)     -----------------------------------------------------------------------------
SARS-CoV-2 Reference Genome NC_045512.2                   (1)     -----------------------------------------------------------------------------

                                                                 Section 1006
                                                          (77386) 77386     77396      77407      77418      77429      77440      77451      77462
Candidatus Carsonella ruddii strain BT chromosome...      (77386) TATAACTTCTGTTCAAGCTATATATGTTCCAGCAGATGATTTAACAGATCCATCTCCAAGTACTACTTTTTCTCATT
Candidatus Carsonella 135557 - 167442nt                   (1)     -----------------------------------------------------------------------------
SARS-CoV-2 Reference Genome NC_045512.2                   (1)     -----------------------------------------------------------------------------

                                                                 Section 1007
                                                          (77463) 77463     77473      77484      77495      77506      77517      77528      77539
Candidatus Carsonella ruddii strain BT chromosome...      (77463) TAGATTCAACAATAGTTTTATCAAGACAAATAGCTGAATTAGGAATTTATCCTTCTATAGATCCTTTAGAATCTTAT
Candidatus Carsonella 135557 - 167442nt                   (1)     -----------------------------------------------------------------------------
SARS-CoV-2 Reference Genome NC_045512.2                   (1)     -----------------------------------------------------------------------------

                                                                 Section 1008
                                                          (77540) 77540     77550      77561      77572      77583      77594      77605      77616
Candidatus Carsonella ruddii strain BT chromosome...      (77540) TCAAAGCAATTAGATCCTTATATAGTTGGAGAAGAACATTACGATATCGCAAACAAAGTAAAATATTATTTGCAAAA
Candidatus Carsonella 135557 - 167442nt                   (1)     -----------------------------------------------------------------------------
SARS-CoV-2 Reference Genome NC_045512.2                   (1)     -----------------------------------------------------------------------------
```

```
                                                                                                                                 Section 1009
                                          (77617) 77617     77627      77638      77649      77660      77671      77682       77693
Candidatus Carsonella ruddii strain BT chromosome... (77617) ATATAAAGAATTAAAAGATACAATTGCTATTTTAGGAATAGATGAATTATCTGAATTCGATAAAACAATTGTATCAA
        Candidatus Carsonella 135557 - 167442nt (1) -----------------------------------------------------------------------------
      SARS-CoV-2 Reference Genome NC_045512.2 (1) -----------------------------------------------------------------------------
                                                                                                                                 Section 1010
                                          (77694) 77694     77704      77715      77726      77737      77748      77759       77770
Candidatus Carsonella ruddii strain BT chromosome... (77694) GAGCAAGAAAATTACAAAAATTTTTTCACAACCTTTTTTTGTTGGAGAAATTTTTACTAATATTAAAGGTAAATAT
        Candidatus Carsonella 135557 - 167442nt (1) -----------------------------------------------------------------------------
      SARS-CoV-2 Reference Genome NC_045512.2 (1) -----------------------------------------------------------------------------
                                                                                                                                 Section 1011
                                          (77771) 77771     77781      77792      77803      77814      77825      77836       77847
Candidatus Carsonella ruddii strain BT chromosome... (77771) GTTAAAATAAAAGATTCTTTAAAATGTTTTAAAAATATAATAAACGGTGTTTATGATAATTTAAACGAAAAAAATTT
        Candidatus Carsonella 135557 - 167442nt (1) -----------------------------------------------------------------------------
      SARS-CoV-2 Reference Genome NC_045512.2 (1) -----------------------------------------------------------------------------
                                                                                                                                 Section 1012
                                          (77848) 77848     77858      77869      77880      77891      77902      77913       77924
Candidatus Carsonella ruddii strain BT chromosome... (77848) TTATATGATTGGAAAAATATGAATGTTTTTATTTATAGTTTTGTATCAAATATAAATTATAATAATGTTAAAATTAT
        Candidatus Carsonella 135557 - 167442nt (1) -----------------------------------------------------------------------------
      SARS-CoV-2 Reference Genome NC_045512.2 (1) -----------------------------------------------------------------------------
                                                                                                                                 Section 1013
                                          (77925) 77925     77935      77946      77957      77968      77979      77990       78001
Candidatus Carsonella ruddii strain BT chromosome... (77925) AAAAATATTTGAAAATAATAATAATTCATATTCTATAATGAATAATCATATTTCATCAATAAAAAATTTAAATGTAA
        Candidatus Carsonella 135557 - 167442nt (1) -----------------------------------------------------------------------------
      SARS-CoV-2 Reference Genome NC_045512.2 (1) -----------------------------------------------------------------------------
                                                                                                                                 Section 1014
                                          (78002) 78002     78012      78023      78034      78045      78056      78067       78078
Candidatus Carsonella ruddii strain BT chromosome... (78002) TTTTATTTTATTATTTTAAAAAAGTTATAAAAATTAATTTTTTTTAATGGTTTTTATATTCAAAAAAAAAAAAATATT
        Candidatus Carsonella 135557 - 167442nt (1) -----------------------------------------------------------------------------
      SARS-CoV-2 Reference Genome NC_045512.2 (1) -----------------------------------------------------------------------------
```

```
                                                                                      Section 1015
                                                 (78079) 78079     78089      78100      78111      78122      78133      78144      78155
Candidatus Carsonella ruddii strain BT chromosome... (78079) AATATAATATGTAAAAAATATGAATTTTTATAACAAACATTTATTAAATGATTTCAATTTAAAAAAATACGAAATTT
        Candidatus Carsonella 135557 - 167442nt (1) -----------------------------------------------------------------------------
    SARS-CoV-2 Reference Genome NC_045512.2 (1) -----------------------------------------------------------------------------
                                                                                      Section 1016
                                                 (78156) 78156     78166      78177      78188      78199      78210      78221      78232
Candidatus Carsonella ruddii strain BT chromosome... (78156) TATCTATATTAGAAACTATAAAAAAATCTCATTGTTTATCATTTTTTAATAAAAAAATATGTATTATAAATGAAAAA
        Candidatus Carsonella 135557 - 167442nt (1) -----------------------------------------------------------------------------
    SARS-CoV-2 Reference Genome NC_045512.2 (1) -----------------------------------------------------------------------------
                                                                                      Section 1017
                                                 (78233) 78233     78243      78254      78265      78276      78287      78298      78309
Candidatus Carsonella ruddii strain BT chromosome... (78233) CAATCATTAAGAACTATTAATTCACTAATAAATTCTTTAAATTTTTTAAATTTAAAATTTACTATAATTAATAACAA
        Candidatus Carsonella 135557 - 167442nt (1) -----------------------------------------------------------------------------
    SARS-CoV-2 Reference Genome NC_045512.2 (1) -----------------------------------------------------------------------------
                                                                                      Section 1018
                                                 (78310) 78310     78320      78331      78342      78353      78364      78375      78386
Candidatus Carsonella ruddii strain BT chromosome... (78310) ACATAATATTAAAAAAGAAAATATAAAAGATTTTTCAAAAACATTAGGATTATTATATGATTATATTTTTATAAGAA
        Candidatus Carsonella 135557 - 167442nt (1) -----------------------------------------------------------------------------
    SARS-CoV-2 Reference Genome NC_045512.2 (1) -----------------------------------------------------------------------------
                                                                                      Section 1019
                                                 (78387) 78387     78397      78408      78419      78430      78441      78452      78463
Candidatus Carsonella ruddii strain BT chromosome... (78387) GTAATAATGATAATATTGTTAAAATTATTTCTAAAAACTCAGGTTCAATAATAATAAATATTTTAAGTAATGGTTTT
        Candidatus Carsonella 135557 - 167442nt (1) -----------------------------------------------------------------------------
    SARS-CoV-2 Reference Genome NC_045512.2 (1) -----------------------------------------------------------------------------
                                                                                      Section 1020
                                                 (78464) 78464     78474      78485      78496      78507      78518      78529      78540
Candidatus Carsonella ruddii strain BT chromosome... (78464) CACCCAATACAAGCAATTGGTGATTTATATTTTTTAAAAAAAAAAATAATTTATTTATTTGGTGATTTTAATTCTAA
        Candidatus Carsonella 135557 - 167442nt (1) -----------------------------------------------------------------------------
    SARS-CoV-2 Reference Genome NC_045512.2 (1) -----------------------------------------------------------------------------
```

		Section 1021
	(78541)	78541 78551 78562 78573 78584 78595 78606 78617
Candidatus Carsonella ruddii strain BT chromosome...	(78541)	TGTTTTTAGATCAATTATAATTAATTTATCTAAATTAAATAAAAATATAATCATATTTTCTCCAAAAATATATTGGA
Candidatus Carsonella 135557 - 167442nt	(1)	---
SARS-CoV-2 Reference Genome NC_045512.2	(1)	---
		Section 1022
	(78618)	78618 78628 78639 78650 78661 78672 78683 78694
Candidatus Carsonella ruddii strain BT chromosome...	(78618)	ATTTTATTTTTTTAAAAAAAATATTTAATAAAAAAAAAATTATAATAACCGAAAAATTAATAATTTCTAAAAAAAAA
Candidatus Carsonella 135557 - 167442nt	(1)	---
SARS-CoV-2 Reference Genome NC_045512.2	(1)	---
		Section 1023
	(78695)	78695 78705 78716 78727 78738 78749 78760 78771
Candidatus Carsonella ruddii strain BT chromosome...	(78695)	TGTATTATTTATAATGATGTTTGGTTCTCAATGGGTGAAAGTGATAATTTTATTAATAAAATTAAAAAATTAATTAA
Candidatus Carsonella 135557 - 167442nt	(1)	---
SARS-CoV-2 Reference Genome NC_045512.2	(1)	---
		Section 1024
	(78772)	78772 78782 78793 78804 78815 78826 78837 78848
Candidatus Carsonella ruddii strain BT chromosome...	(78772)	TTTTCAAATTAATAAAAAAATTTTAAAAATTTTAAAAATTGAAAAAATTTTTCACTGTCTTCCATGTTTTAAAAATA
Candidatus Carsonella 135557 - 167442nt	(1)	---
SARS-CoV-2 Reference Genome NC_045512.2	(1)	---
		Section 1025
	(78849)	78849 78859 78870 78881 78892 78903 78914 78925
Candidatus Carsonella ruddii strain BT chromosome...	(78849)	TTAAAATAAAAAAAACAAAATTTGAATCAGAATTAACAAAAAATGTTTTTGAATCAAATTTTTCTTTTTTTGAAAAA
Candidatus Carsonella 135557 - 167442nt	(1)	---
SARS-CoV-2 Reference Genome NC_045512.2	(1)	---
		Section 1026
	(78926)	78926 78936 78947 78958 78969 78980 78991 79002
Candidatus Carsonella ruddii strain BT chromosome...	(78926)	CAAATAATTAAAAAAATATCGATTATAAAATCATATATTATATTAGGAGAAAAAACTTTTTTTAAAGTTTTAAATTA
Candidatus Carsonella 135557 - 167442nt	(1)	---
SARS-CoV-2 Reference Genome NC_045512.2	(1)	---

Section 1027

	(79003)	79003 79013 79024 79035 79046 79057 79068 79079
Candidatus Carsonella ruddii strain BT chromosome...	(79003)	AAAATTATTTAATATTTTTACAAATTTTATTTTTTGAAATTTGATTTATAAAATCAAATATATTTTTTTTTTTAATA
Candidatus Carsonella 135557 - 167442nt	(1)	---
SARS-CoV-2 Reference Genome NC_045512.2	(1)	---

Section 1028

	(79080)	79080 79090 79101 79112 79123 79134 79145 79156
Candidatus Carsonella ruddii strain BT chromosome...	(79080)	AATAAATATTTTAATAACATAAATCATAAATATAATTAATAGCATTATAATAACCATTATATTTCATACCTGAAATT
Candidatus Carsonella 135557 - 167442nt	(1)	---
SARS-CoV-2 Reference Genome NC_045512.2	(1)	---

Section 1029

	(79157)	79157 79167 79178 79189 79200 79211 79222 79233
Candidatus Carsonella ruddii strain BT chromosome...	(79157)	ATAATATTTGAATTAAGAGATATTAAAGATTTATTTCTAAAATATTCTCTTTTATATAAATTTGTAATATGAACTTC
Candidatus Carsonella 135557 - 167442nt	(1)	---
SARS-CoV-2 Reference Genome NC_045512.2	(1)	---

Section 1030

	(79234)	79234 79244 79255 79266 79277 79288 79299 79310
Candidatus Carsonella ruddii strain BT chromosome...	(79234)	TATAATTGTTCCTTTAAATATTTTTAAACAATCTAATATTGATAGAGAATAATGACTAAACGATGCAGGATTAATTA
Candidatus Carsonella 135557 - 167442nt	(1)	---
SARS-CoV-2 Reference Genome NC_045512.2	(1)	---

Section 1031

	(79311)	79311 79321 79332 79343 79354 79365 79376 79387
Candidatus Carsonella ruddii strain BT chromosome...	(79311)	TCAACCAATCTATTTTGTAATTTTTTTGTAAAAAATTTACAATTTTTCCTTCACAATTTGAATTAAAAAATATCAAT
Candidatus Carsonella 135557 - 167442nt	(1)	---
SARS-CoV-2 Reference Genome NC_045512.2	(1)	---

Section 1032

	(79388)	79388 79398 79409 79420 79431 79442 79453 79464
Candidatus Carsonella ruddii strain BT chromosome...	(79388)	TTAAAATATTTTTTAGAATAATTTAATAATTCTTTTTTTAATTTTTTAAAAGAAATATTAGAATAAATTTTCTCTCT
Candidatus Carsonella 135557 - 167442nt	(1)	---
SARS-CoV-2 Reference Genome NC_045512.2	(1)	---

```
                                                                                                                     Section 1033
                                                      (79465) 79465     79475      79486      79497      79508      79519      79530   79541
Candidatus Carsonella ruddii strain BT chromosome...  (79465) TTTTTGTAAGAAATTTAAATTTGGTCCATTAACAATTAAAATTTTTACAATTTTATTATATATAAACATAATTTTAT
            Candidatus Carsonella 135557 - 167442nt       (1) -----------------------------------------------------------------------------
         SARS-CoV-2 Reference Genome NC_045512.2          (1) -----------------------------------------------------------------------------
                                                                                                                     Section 1034
                                                      (79542) 79542     79552      79563      79574      79585      79596      79607   79618
Candidatus Carsonella ruddii strain BT chromosome...  (79542) TAAATTTTTTTTTGTAAAATTAAAATATTTTTTTAAATCTTTTTCTGATGATGATTTACCATATTTATTAATATTAA
            Candidatus Carsonella 135557 - 167442nt       (1) -----------------------------------------------------------------------------
         SARS-CoV-2 Reference Genome NC_045512.2          (1) -----------------------------------------------------------------------------
                                                                                                                     Section 1035
                                                      (79619) 79619     79629      79640      79651      79662      79673      79684   79695
Candidatus Carsonella ruddii strain BT chromosome...  (79619) AAATAAATAAATAATTAATATTATATTTATTTAATAAAATATCATTAGAAGATTCAAAAAATAATGTTTTTTTATTA
            Candidatus Carsonella 135557 - 167442nt       (1) -----------------------------------------------------------------------------
         SARS-CoV-2 Reference Genome NC_045512.2          (1) -----------------------------------------------------------------------------
                                                                                                                     Section 1036
                                                      (79696) 79696     79706      79717      79728      79739      79750      79761   79772
Candidatus Carsonella ruddii strain BT chromosome...  (79696) AATATATTGTTTTTATAATTTTTATTTTGTTTATTAAATAAAAATATACAATATAAAGAAATTATTTTTATATTAAA
            Candidatus Carsonella 135557 - 167442nt       (1) -----------------------------------------------------------------------------
         SARS-CoV-2 Reference Genome NC_045512.2          (1) -----------------------------------------------------------------------------
                                                                                                                     Section 1037
                                                      (79773) 79773     79783      79794      79805      79816      79827      79838   79849
Candidatus Carsonella ruddii strain BT chromosome...  (79773) ATATTTTTTTAAAAAAAAATAACATTCAAATATACATTCTAAATCAGAACCTGAAGATACTATTGTTAAATCACATT
            Candidatus Carsonella 135557 - 167442nt       (1) -----------------------------------------------------------------------------
         SARS-CoV-2 Reference Genome NC_045512.2          (1) -----------------------------------------------------------------------------
                                                                                                                     Section 1038
                                                      (79850) 79850     79860      79871      79882      79893      79904      79915   79926
Candidatus Carsonella ruddii strain BT chromosome...  (79850) TTTTTTTAATTGAATTTATCAAATATCCTCCTTTAATAATTTTATTTATATTTATAAATTTTAAATTATTATTCTTA
            Candidatus Carsonella 135557 - 167442nt       (1) -----------------------------------------------------------------------------
         SARS-CoV-2 Reference Genome NC_045512.2          (1) -----------------------------------------------------------------------------
```

Section 1039

```
                                                          79927     79937      79948      79959      79970      79981      79992      80003
Candidatus Carsonella ruddii strain BT chromosome... (79927) AAATTTTGTCTTGATAATATTAATGAAGAAGAATTTTTTTCTAATTTTATAATTATATACCAAGAAACAATTAATTC
          Candidatus Carsonella 135557 - 167442nt    (1)     -----------------------------------------------------------------------------
       SARS-CoV-2 Reference Genome NC_045512.2       (1)     -----------------------------------------------------------------------------
```

Section 1040

```
                                                          80004     80014      80025      80036      80047      80058      80069      80080
Candidatus Carsonella ruddii strain BT chromosome... (80004) AATTAAATTATAAGGTCTAAAAACAAAAAATCTAGGAAAAGACCTTAAATTTTTTAATTGTTCAATAGGTTGATGTG
          Candidatus Carsonella 135557 - 167442nt    (1)     -----------------------------------------------------------------------------
       SARS-CoV-2 Reference Genome NC_045512.2       (1)     -----------------------------------------------------------------------------
```

Section 1041

```
                                                          80081     80091      80102      80113      80124      80135      80146      80157
Candidatus Carsonella ruddii strain BT chromosome... (80081) TTGGACCATCTTCACCAACATAAAATGAATCATGTGTAAAAATAAAAATATTTTTTAATTTAGAAATATTAAAATTT
          Candidatus Carsonella 135557 - 167442nt    (1)     -----------------------------------------------------------------------------
       SARS-CoV-2 Reference Genome NC_045512.2       (1)     -----------------------------------------------------------------------------
```

Section 1042

```
                                                          80158     80168      80179      80190      80201      80212      80223      80234
Candidatus Carsonella ruddii strain BT chromosome... (80158) CTTATAGCGCTATACATATAATTAGAAAAAACTAAAAAAGTAGAACAAAATGTGATCATATTTTTTTCGGAACAAAT
          Candidatus Carsonella 135557 - 167442nt    (1)     -----------------------------------------------------------------------------
       SARS-CoV-2 Reference Genome NC_045512.2       (1)     -----------------------------------------------------------------------------
```

Section 1043

```
                                                          80235     80245      80256      80267      80278      80289      80300      80311
Candidatus Carsonella ruddii strain BT chromosome... (80235) TCCATAATTAATAAGAGACATACAAAATTCTCTTACTCCATAATTTAAAAATCTATTATTAAATTTTTTTTTTGAAA
          Candidatus Carsonella 135557 - 167442nt    (1)     -----------------------------------------------------------------------------
       SARS-CoV-2 Reference Genome NC_045512.2       (1)     -----------------------------------------------------------------------------
```

Section 1044

```
                                                          80312     80322      80333      80344      80355      80366      80377      80388
Candidatus Carsonella ruddii strain BT chromosome... (80312) TTGAATTAATTTTTTTTCCTTTTGTTAAATTTGATGAAGTTAAATCAGCACTTCCACCTAAAAATTCATTTAATTGA
          Candidatus Carsonella 135557 - 167442nt    (1)     -----------------------------------------------------------------------------
       SARS-CoV-2 Reference Genome NC_045512.2       (1)     -----------------------------------------------------------------------------
```

```
                                                                                          Section 1045
                                                      (80389) 80389     80399      80410      80421      80432      80443      80454  80465
Candidatus Carsonella ruddii strain BT chromosome...  (80389) GATAAATCGTTAATTATAAGATTTGAAATAAATCTTGTAGATTTATTTAAAAATTTTTTTTCATTTTTTAAATAAAG
          Candidatus Carsonella 135557 - 167442nt         (1) -----------------------------------------------------------------------------
       SARS-CoV-2 Reference Genome NC_045512.2            (1) -----------------------------------------------------------------------------
                                                                                          Section 1046
                                                      (80466) 80466     80476      80487      80498      80509      80520      80531  80542
Candidatus Carsonella ruddii strain BT chromosome...  (80466) CTTAATTAAATTTATTTTAATTATTATATTATTTAAAATTCTTATTAATTCTAAAAATGAAAAATAATAATTTTTAA
          Candidatus Carsonella 135557 - 167442nt         (1) -----------------------------------------------------------------------------
       SARS-CoV-2 Reference Genome NC_045512.2            (1) -----------------------------------------------------------------------------
                                                                                          Section 1047
                                                      (80543) 80543     80553      80564      80575      80586      80597      80608  80619
Candidatus Carsonella ruddii strain BT chromosome...  (80543) TATAATATAATATATATTTTTTTTTAATATCAAATAATTTTTTTTTAATATATAAATATTTAATAGTATAATTTTTA
          Candidatus Carsonella 135557 - 167442nt         (1) -----------------------------------------------------------------------------
       SARS-CoV-2 Reference Genome NC_045512.2            (1) -----------------------------------------------------------------------------
                                                                                          Section 1048
                                                      (80620) 80620     80630      80641      80652      80663      80674      80685  80696
Candidatus Carsonella ruddii strain BT chromosome...  (80620) TAAATATTAATTATTTGTTTTTTATTAAATATTGTTCCATGAACAGATTCAGAATATGATTTATCAGATGACAAAAA
          Candidatus Carsonella 135557 - 167442nt         (1) -----------------------------------------------------------------------------
       SARS-CoV-2 Reference Genome NC_045512.2            (1) -----------------------------------------------------------------------------
                                                                                          Section 1049
                                                      (80697) 80697     80707      80718      80729      80740      80751      80762  80773
Candidatus Carsonella ruddii strain BT chromosome...  (80697) ACCAATTATTGTTTTAAAATAAATTAAAATAGGAAAATAACTTAAAATAGATTTTTTAATAGATTTTTTAATTAATT
          Candidatus Carsonella 135557 - 167442nt         (1) -----------------------------------------------------------------------------
       SARS-CoV-2 Reference Genome NC_045512.2            (1) -----------------------------------------------------------------------------
                                                                                          Section 1050
                                                      (80774) 80774     80784      80795      80806      80817      80828      80839  80850
Candidatus Carsonella ruddii strain BT chromosome...  (80774) TATAATCATGTCCGTTTATTGGACCTATTACATCCCAATTCATAGAATTAAATTTTTGTTTAATATTTTCAAAAAAA
          Candidatus Carsonella 135557 - 167442nt         (1) -----------------------------------------------------------------------------
       SARS-CoV-2 Reference Genome NC_045512.2            (1) -----------------------------------------------------------------------------
```

Section 1051

		80851 80861 80872 80883 80894 80905 80916 80927
	(80851)	
Candidatus Carsonella ruddii strain BT chromosome...	(80851)	TAATTTTTTACTATTCCATCAATTGATATGTTATTACTATCATAAATTAATATAATATTGTTAACATTTAAAGATCC
Candidatus Carsonella 135557 - 167442nt	(1)	---
SARS-CoV-2 Reference Genome NC_045512.2	(1)	---

Section 1052

		80928 80938 80949 80960 80971 80982 80993 81004
	(80928)	
Candidatus Carsonella ruddii strain BT chromosome...	(80928)	ACAAAATGAAATTGATTCGTTTCCAACTCCTTCCATTAAACATCCATCTCCACAAAATATCCAAATTTTATTATTAA
Candidatus Carsonella 135557 - 167442nt	(1)	---
SARS-CoV-2 Reference Genome NC_045512.2	(1)	---

Section 1053

		81005 81015 81026 81037 81048 81059 81070 81081
	(81005)	
Candidatus Carsonella ruddii strain BT chromosome...	(81005)	AAATATTAATTTTTTTATTAAATTTATTTTTAAAAATTTTATTTTTAATACCATTTCCTATTCCTATTCCTATTCCT
Candidatus Carsonella 135557 - 167442nt	(1)	---
SARS-CoV-2 Reference Genome NC_045512.2	(1)	---

Section 1054

		81082 81092 81103 81114 81125 81136 81147 81158
	(81082)	
Candidatus Carsonella ruddii strain BT chromosome...	(81082)	TGACCTAATGGTCCAGTAGATGCATCTATAAAATTTGATAATTCTGGATGAGCTGGTGTTAAAGAATTAAATTTTCT
Candidatus Carsonella 135557 - 167442nt	(1)	---
SARS-CoV-2 Reference Genome NC_045512.2	(1)	---

Section 1055

		81159 81169 81180 81191 81202 81213 81224 81235
	(81159)	
Candidatus Carsonella ruddii strain BT chromosome...	(81159)	TAATTTTTTTAAATCTATAATTTTAAATATTTTTGATAAATATAACATAACGTAATTTATAATTACTCCATGACCAT
Candidatus Carsonella 135557 - 167442nt	(1)	---
SARS-CoV-2 Reference Genome NC_045512.2	(1)	---

Section 1056

		81236 81246 81257 81268 81279 81290 81301 81312
	(81236)	
Candidatus Carsonella ruddii strain BT chromosome...	(81236)	TAGATATTATTATTTTATCTTTATTTATATTTTTAATATTTTTAAAATTATTTTTATAAAAAAAATACCATAAAAAA
Candidatus Carsonella 135557 - 167442nt	(1)	---
SARS-CoV-2 Reference Genome NC_045512.2	(1)	---

```
                                                                                                                  Section 1057
                                                         (81313) 81313     81323      81334      81345      81356      81367      81378   81389
Candidatus Carsonella ruddii strain BT chromosome...    (81313) GTAAATATATCTGTTATACCTAAAGGCATACCAGGATGACCTGATTTTGATTTTTGTATTGATTTAATACATATAAG
             Candidatus Carsonella 135557 - 167442nt        (1) -----------------------------------------------------------------------------
         SARS-CoV-2 Reference Genome NC_045512.2            (1) -----------------------------------------------------------------------------
                                                                                                                  Section 1058
                                                         (81390) 81390     81400      81411      81422      81433      81444      81455   81466
Candidatus Carsonella ruddii strain BT chromosome...    (81390) TCTAATATTATTTATTATATTGTATAACATGTTAAAGATAAAAATATTATTGTTAAAAATTTATTCTTTTAGTAATT
             Candidatus Carsonella 135557 - 167442nt        (1) -----------------------------------------------------------------------------
         SARS-CoV-2 Reference Genome NC_045512.2            (1) -----------------------------------------------------------------------------
                                                                                                                  Section 1059
                                                         (81467) 81467     81477      81488      81499      81510      81521      81532   81543
Candidatus Carsonella ruddii strain BT chromosome...    (81467) ATAAAACAAATATTTTTTTTTTTTTAAAAAAAATAAAAAAAAAATTTTTTATAAAAATAAATTATATAAATTTTATT
             Candidatus Carsonella 135557 - 167442nt        (1) -----------------------------------------------------------------------------
         SARS-CoV-2 Reference Genome NC_045512.2            (1) -----------------------------------------------------------------------------
                                                                                                                  Section 1060
                                                         (81544) 81544     81554      81565      81576      81587      81598      81609   81620
Candidatus Carsonella ruddii strain BT chromosome...    (81544) ATTAATAATTTAAATTTTTATAATAAGAAGAAAATGAGTTTTTTATTATATTTATTTATTTGTGAAATAATTTCTAA
             Candidatus Carsonella 135557 - 167442nt        (1) -----------------------------------------------------------------------------
         SARS-CoV-2 Reference Genome NC_045512.2            (1) -----------------------------------------------------------------------------
                                                                                                                  Section 1061
                                                         (81621) 81621     81631      81642      81653      81664      81675      81686   81697
Candidatus Carsonella ruddii strain BT chromosome...    (81621) TTTAAATATTAATAAAAATAATTATTTTTTATTTAATAAAAAAAATATTTTTTTAAAAGAAATAAAAAATAATTACT
             Candidatus Carsonella 135557 - 167442nt        (1) -----------------------------------------------------------------------------
         SARS-CoV-2 Reference Genome NC_045512.2            (1) -----------------------------------------------------------------------------
                                                                                                                  Section 1062
                                                         (81698) 81698     81708      81719      81730      81741      81752      81763   81774
Candidatus Carsonella ruddii strain BT chromosome...    (81698) CATTATTTGAAATTTTGTTTTTTTTATTTTTAACTAAAATTAAAAAAATATTTAATTTAAAAAAAATAAAAAAAATT
             Candidatus Carsonella 135557 - 167442nt        (1) -----------------------------------------------------------------------------
         SARS-CoV-2 Reference Genome NC_045512.2            (1) -----------------------------------------------------------------------------
```

```
                                                                                              Section 1063
                                              (81775) 81775     81785      81796      81807      81818      81829      81840  81851
Candidatus Carsonella ruddii strain BT chromosome... (81775) TTTACAAATGTATTAGTAAAAATAAAAAATGAAGTTTATGAAAGTTTAAAAGAAAGATATATTTCTACTTTTTTATA
        Candidatus Carsonella 135557 - 167442nt (1) -----------------------------------------------------------------------------
      SARS-CoV-2 Reference Genome NC_045512.2 (1) -----------------------------------------------------------------------------
                                                                                              Section 1064
                                              (81852) 81852     81862      81873      81884      81895      81906      81917  81928
Candidatus Carsonella ruddii strain BT chromosome... (81852) TATATTTATAAATGAACAAATTAAAATAAAAAATATTTTAAAATTGTATTTAAATTATATAGATTACAACTTTTTTT
        Candidatus Carsonella 135557 - 167442nt (1) -----------------------------------------------------------------------------
      SARS-CoV-2 Reference Genome NC_045512.2 (1) -----------------------------------------------------------------------------
                                                                                              Section 1065
                                              (81929) 81929     81939      81950      81961      81972      81983      81994  82005
Candidatus Carsonella ruddii strain BT chromosome... (81929) ATTTTTTAAAAAATATTAAAAAAGTTTGTTTATTAATTTTTTCTAATAAAGAAGAATTTAGTAAAAAATTATATATT
        Candidatus Carsonella 135557 - 167442nt (1) -----------------------------------------------------------------------------
      SARS-CoV-2 Reference Genome NC_045512.2 (1) -----------------------------------------------------------------------------
                                                                                              Section 1066
                                              (82006) 82006     82016      82027      82038      82049      82060      82071  82082
Candidatus Carsonella ruddii strain BT chromosome... (82006) GGTATGTTAAAAAATAATAATAATAATTTAAATTATTATTTAATTTTTTTAATTAATAATTTAATAAAAAAAAATAT
        Candidatus Carsonella 135557 - 167442nt (1) -----------------------------------------------------------------------------
      SARS-CoV-2 Reference Genome NC_045512.2 (1) -----------------------------------------------------------------------------
                                                                                              Section 1067
                                              (82083) 82083     82093      82104      82115      82126      82137      82148  82159
Candidatus Carsonella ruddii strain BT chromosome... (82083) ATATAAAATTAGTATTTCATTTGAAATTTATAATTTTGATATATTAAAAATAATTAAAAATATAAAAATATTTAAAA
        Candidatus Carsonella 135557 - 167442nt (1) -----------------------------------------------------------------------------
      SARS-CoV-2 Reference Genome NC_045512.2 (1) -----------------------------------------------------------------------------
                                                                                              Section 1068
                                              (82160) 82160     82170      82181      82192      82203      82214      82225  82236
Candidatus Carsonella ruddii strain BT chromosome... (82160) AATTAAAAATTAATATAATAATAGGAATACCAGATTTATTTATTGAATATATTATCAAAAAAAAAAATTGGTATTTA
        Candidatus Carsonella 135557 - 167442nt (1) -----------------------------------------------------------------------------
      SARS-CoV-2 Reference Genome NC_045512.2 (1) -----------------------------------------------------------------------------
```

Section 1069

	(82237)	82237 82247 82258 82269 82280 82291 82302 82313
Candidatus Carsonella ruddii strain BT chromosome...	(82237)	ATAGATTCTAATTGTATTAAAAAAAATTATGAATATTATTTACAAAGTTATTATGATGAATATATAGGATATGGAAC
Candidatus Carsonella 135557 - 167442nt	(1)	---
SARS-CoV-2 Reference Genome NC_045512.2	(1)	---

Section 1070

	(82314)	82314 82324 82335 82346 82357 82368 82379 82390
Candidatus Carsonella ruddii strain BT chromosome...	(82314)	ATTAAGAGAAAAATATAAAAATATTTTAAAAATAAAAAATATTTTAAAAATATCTATAAATTCAAAAAAATTATTTT
Candidatus Carsonella 135557 - 167442nt	(1)	---
SARS-CoV-2 Reference Genome NC_045512.2	(1)	---

Section 1071

	(82391)	82391 82401 82412 82423 82434 82445 82456 82467
Candidatus Carsonella ruddii strain BT chromosome...	(82391)	TAAATATTTTAAAAAATAAAATAAAAATTATTTATTCAGATAAAAAAAATAGAAATAATATTAATAAACATATAGGT
Candidatus Carsonella 135557 - 167442nt	(1)	---
SARS-CoV-2 Reference Genome NC_045512.2	(1)	---

Section 1072

	(82468)	82468 82478 82489 82500 82511 82522 82533 82544
Candidatus Carsonella ruddii strain BT chromosome...	(82468)	TGTTTAATTTCAAATTTTTATCAAAATAAAAATTTTGATAGAAATAATTATTTTAATAATAAAATTTATTATATTAA
Candidatus Carsonella 135557 - 167442nt	(1)	---
SARS-CoV-2 Reference Genome NC_045512.2	(1)	---

Section 1073

	(82545)	82545 82555 82566 82577 82588 82599 82610 82621
Candidatus Carsonella ruddii strain BT chromosome...	(82545)	AAATACAAATGTTATTATTAAAAGAAATAATAGTTTTTATGTAGAAGTAAAATTAGAACAAAATTTTTTTGTTTGTA
Candidatus Carsonella 135557 - 167442nt	(1)	---
SARS-CoV-2 Reference Genome NC_045512.2	(1)	---

Section 1074

	(82622)	82622 82632 82643 82654 82665 82676 82687 82698
Candidatus Carsonella ruddii strain BT chromosome...	(82622)	AAAATAATATAAAAAAATTTTTTTTAAAATTTAAAATTTTAAATTCTAAAAATAATTTTTTATTTGTTCAAAGAGAA
Candidatus Carsonella 135557 - 167442nt	(1)	---
SARS-CoV-2 Reference Genome NC_045512.2	(1)	---

```
                                                                                                                                        Section 1075
                                                      (82699) 82699     82709      82720      82731      82742      82753      82764      82775
Candidatus Carsonella ruddii strain BT chromosome...  (82699) ATGAATTTTCATTTAAATTTTAGAATAAAACCTATAATATTTAATATAAATTATTTAAATAAAAATTTTTATAATTA
             Candidatus Carsonella 135557 - 167442nt      (1) -----------------------------------------------------------------------------
           SARS-CoV-2 Reference Genome NC_045512.2        (1) -----------------------------------------------------------------------------
                                                                                                                                        Section 1076
                                                      (82776) 82776     82786      82797      82808      82819      82830      82841      82852
Candidatus Carsonella ruddii strain BT chromosome...  (82776) TTTAATGTTTAATTTAATAAAAATAAATTCATTTTACTCTAAATTATATATGCCTTATATATGGTATCCTGGAAGTG
             Candidatus Carsonella 135557 - 167442nt      (1) -----------------------------------------------------------------------------
           SARS-CoV-2 Reference Genome NC_045512.2        (1) -----------------------------------------------------------------------------
                                                                                                                                        Section 1077
                                                      (82853) 82853     82863      82874      82885      82896      82907      82918      82929
Candidatus Carsonella ruddii strain BT chromosome...  (82853) ATATATATTATAAAGATTATTATTATAAAAATCATTTTTTTTCTAAACATTGGAAATTATTATCCAATTATACAGAT
             Candidatus Carsonella 135557 - 167442nt      (1) -----------------------------------------------------------------------------
           SARS-CoV-2 Reference Genome NC_045512.2        (1) -----------------------------------------------------------------------------
                                                                                                                                        Section 1078
                                                      (82930) 82930     82940      82951      82962      82973      82984      82995      83006
Candidatus Carsonella ruddii strain BT chromosome...  (82930) AATAATAAGTTATTATGTTCTATAAATTTAATTATAAAAATAAAAAAAAAATTTATTACAACATTTGATAATTATTT
             Candidatus Carsonella 135557 - 167442nt      (1) -----------------------------------------------------------------------------
           SARS-CoV-2 Reference Genome NC_045512.2        (1) -----------------------------------------------------------------------------
                                                                                                                                        Section 1079
                                                      (83007) 83007     83017      83028      83039      83050      83061      83072      83083
Candidatus Carsonella ruddii strain BT chromosome...  (83007) TTTAAAATTATTAGTAATATTATGGCAAATACAAGATGAAAAATATTAGTTTTGAAATTTTTCCAAACAAAAATATA
             Candidatus Carsonella 135557 - 167442nt      (1) -----------------------------------------------------------------------------
           SARS-CoV-2 Reference Genome NC_045512.2        (1) -----------------------------------------------------------------------------
                                                                                                                                        Section 1080
                                                      (83084) 83084     83094      83105      83116      83127      83138      83149      83160
Candidatus Carsonella ruddii strain BT chromosome...  (83084) AAAGAAAGTATTAAATTTGTAAAAATTATAGAAAAAAAAAAAACCTGTTTTTATTTCTATAACATGTAGTAAAATTAA
             Candidatus Carsonella 135557 - 167442nt      (1) -----------------------------------------------------------------------------
           SARS-CoV-2 Reference Genome NC_045512.2        (1) -----------------------------------------------------------------------------
```

Section 1081

```
                                                        (83161) 83161     83171      83182      83193      83204      83215      83226      83237
Candidatus Carsonella ruddii strain BT chromosome...    (83161) TAATTTTGATTTTGTTAAAACTATTAAGAATAATACTAATATTAAAATTATACCACATATAATTTGTGACAATTTAT
Candidatus Carsonella 135557 - 167442nt                 (1)     -----------------------------------------------------------------------------
SARS-CoV-2 Reference Genome NC_045512.2                 (1)     -----------------------------------------------------------------------------
```

Section 1082

```
                                                        (83238) 83238     83248      83259      83270      83281      83292      83303      83314
Candidatus Carsonella ruddii strain BT chromosome...    (83238) ATAATATTGTTTTAAAAATAATTAATTTTATTAAAATTAAAATTTTTAATTTTATAATAATTACTGGAGATAATATT
Candidatus Carsonella 135557 - 167442nt                 (1)     -----------------------------------------------------------------------------
SARS-CoV-2 Reference Genome NC_045512.2                 (1)     -----------------------------------------------------------------------------
```

Section 1083

```
                                                        (83315) 83315     83325      83336      83347      83358      83369      83380      83391
Candidatus Carsonella ruddii strain BT chromosome...    (83315) TCTAATAATTCTTTTTTAACAATTAAAAAAATTAGATTTTTATTTGGACATATAATTAAAATTTTTTCTGGATCTTA
Candidatus Carsonella 135557 - 167442nt                 (1)     -----------------------------------------------------------------------------
SARS-CoV-2 Reference Genome NC_045512.2                 (1)     -----------------------------------------------------------------------------
```

Section 1084

```
                                                        (83392) 83392     83402      83413      83424      83435      83446      83457      83468
Candidatus Carsonella ruddii strain BT chromosome...    (83392) TTTAGAAGAACATAAATTATCATCTAATATAAATAAAGAAATTCTTTTTATTTATAAAAAAAAAAAAATAGGTATTA
Candidatus Carsonella 135557 - 167442nt                 (1)     -----------------------------------------------------------------------------
SARS-CoV-2 Reference Genome NC_045512.2                 (1)     -----------------------------------------------------------------------------
```

Section 1085

```
                                                        (83469) 83469     83479      83490      83501      83512      83523      83534      83545
Candidatus Carsonella ruddii strain BT chromosome...    (83469) ATTTATTTTTAACACAATTTTTTTATAATTTTAATATAATAAATTATTATTTAAATAAAATCAAAAAAACATATATA
Candidatus Carsonella 135557 - 167442nt                 (1)     -----------------------------------------------------------------------------
SARS-CoV-2 Reference Genome NC_045512.2                 (1)     -----------------------------------------------------------------------------
```

Section 1086

```
                                                        (83546) 83546     83556      83567      83578      83589      83600      83611      83622
Candidatus Carsonella ruddii strain BT chromosome...    (83546) AATAAAAATTTTATTCCTGGTATTTTTCCTAAAAAAAATATAAAAGAAATTTTATTATTTATAAATAAATGTAAAAT
Candidatus Carsonella 135557 - 167442nt                 (1)     -----------------------------------------------------------------------------
SARS-CoV-2 Reference Genome NC_045512.2                 (1)     -----------------------------------------------------------------------------
```

Section 1087

(83623) 83623 83633 83644 83655 83666 83677 83688 83699

Candidatus Carsonella ruddii strain BT chromosome... (83623) TGAAATTCCAATTTGGATAATAAAAAATTATGATTTTATTAATATAAAAAATTATTTATTAAAAAATTTAAATATTT

Candidatus Carsonella 135557 - 167442nt (1) ---

SARS-CoV-2 Reference Genome NC_045512.2 (1) ---

Section 1088

(83700) 83700 83710 83721 83732 83743 83754 83765 83776

Candidatus Carsonella ruddii strain BT chromosome... (83700) TTAAACATTTTCATTTTTATACTTTTAATAAAATTAATTTTGTAGATTATTATTTAAAGTAAATTTTTTTGTTATAA

Candidatus Carsonella 135557 - 167442nt (1) ---

SARS-CoV-2 Reference Genome NC_045512.2 (1) ---

Section 1089

(83777) 83777 83787 83798 83809 83820 83831 83842 83853

Candidatus Carsonella ruddii strain BT chromosome... (83777) TTTATAAAAAAAAATTTATTAAAAATTATTTTAAATTTATTTTTTTTTGTATTGATATTTAAAAAAAATATTTTAAT

Candidatus Carsonella 135557 - 167442nt (1) ---

SARS-CoV-2 Reference Genome NC_045512.2 (1) ---

Section 1090

(83854) 83854 83864 83875 83886 83897 83908 83919 83930

Candidatus Carsonella ruddii strain BT chromosome... (83854) TTTTAAAAAAAAATTTTTTATTATTAATAAATAGTTTTTTTTATTCATAATTTTTTATTATTAATTGTTTTTTCTAA

Candidatus Carsonella 135557 - 167442nt (1) ---

SARS-CoV-2 Reference Genome NC_045512.2 (1) ---

Section 1091

(83931) 83931 83941 83952 83963 83974 83985 83996 84007

Candidatus Carsonella ruddii strain BT chromosome... (83931) AAAAAAATTAAAATTTTGATAAAAAAAGTTAAAATAAATTTATTTTAAAAAAAAAAATTTGATTTTTTTTACAATTA

Candidatus Carsonella 135557 - 167442nt (1) ---

SARS-CoV-2 Reference Genome NC_045512.2 (1) ---

Section 1092

(84008) 84008 84018 84029 84040 84051 84062 84073 84084

Candidatus Carsonella ruddii strain BT chromosome... (84008) TATGTTTTTTTACAATTGGGATTATAGCTCAGTTGGTTAGAGCATGCCTTTGATAAGGGCAGGGTCAATAGTTCAAA

Candidatus Carsonella 135557 - 167442nt (1) ---

SARS-CoV-2 Reference Genome NC_045512.2 (1) ---

Section 1093

		84085 84095 84106 84117 84128 84139 84150 84161
	(84085)	
Candidatus Carsonella ruddii strain BT chromosome...	(84085)	TCTATTTAATCCCATTTGTAAAAATATTATAAATAATACTTAATATATATAAATCATCAAAAAAATTAAATTCATTA
Candidatus Carsonella 135557 - 167442nt	(1)	---
SARS-CoV-2 Reference Genome NC_045512.2	(1)	---

Section 1094

		84162 84172 84183 84194 84205 84216 84227 84238
	(84162)	
Candidatus Carsonella ruddii strain BT chromosome...	(84162)	ATTTTATGAATTGTTTTATTTAATAATCCTAATTCAAAATAATTTGATTTATTATAAAAATAATAACAATATCTTAA
Candidatus Carsonella 135557 - 167442nt	(1)	---
SARS-CoV-2 Reference Genome NC_045512.2	(1)	---

Section 1095

		84239 84249 84260 84271 84282 84293 84304 84315
	(84239)	
Candidatus Carsonella ruddii strain BT chromosome...	(84239)	ATCAGAAGTACCACCAAAAAAATTTATTTTTGAAAATATATTTTGAATATAATAAATTGAATTAAAAATTTTTTTTA
Candidatus Carsonella 135557 - 167442nt	(1)	---
SARS-CoV-2 Reference Genome NC_045512.2	(1)	---

Section 1096

		84316 84326 84337 84348 84359 84370 84381 84392
	(84316)	
Candidatus Carsonella ruddii strain BT chromosome...	(84316)	AATAAAAATTTTTATAACAAAAAAAGGGAATACCACTAAAAATCCAATTAAAATAAGAGTTTTTAAATTTTAAAAAA
Candidatus Carsonella 135557 - 167442nt	(1)	---
SARS-CoV-2 Reference Genome NC_045512.2	(1)	---

Section 1097

		84393 84403 84414 84425 84436 84447 84458 84469
	(84393)	
Candidatus Carsonella ruddii strain BT chromosome...	(84393)	TTAAATAAATTTTTTTTAAAAAAAATTATATTTTTATAAAAAAAAAAATCTATAATTAATTTTAACAAAAAAAATAAT
Candidatus Carsonella 135557 - 167442nt	(1)	---
SARS-CoV-2 Reference Genome NC_045512.2	(1)	---

Section 1098

		84470 84480 84491 84502 84513 84524 84535 84546
	(84470)	
Candidatus Carsonella ruddii strain BT chromosome...	(84470)	TGGTGTTAAATTATCTAATTTTGATATACTAAAAACATTATTTAATTCAAAAATTTCTTTTTTATCAAAAACATAAT
Candidatus Carsonella 135557 - 167442nt	(1)	---
SARS-CoV-2 Reference Genome NC_045512.2	(1)	---

Section 1099

```
                                                    84547     84557      84568      84579      84590      84601      84612      84623
Candidatus Carsonella ruddii strain BT chromosome... (84547) TTTTAAAATTATGTATTTTTTGTAAAGAAAAATTTATTATGTTTTTACCAATATAAGCGCAATGTTTTTGTTTACCG
        Candidatus Carsonella 135557 - 167442nt     (1)     -----------------------------------------------------------------------------
    SARS-CoV-2 Reference Genome NC_045512.2         (1)     -----------------------------------------------------------------------------
```

Section 1100

```
                                                    84624     84634      84645      84656      84667      84678      84689      84700
Candidatus Carsonella ruddii strain BT chromosome... (84624) ATAAAAATTAATTTAAAATTATAAGATCCTCTTCTTGATATTTTAATATAATCTCCTATTATTTCTTTTGAAGTAGG
        Candidatus Carsonella 135557 - 167442nt     (1)     -----------------------------------------------------------------------------
    SARS-CoV-2 Reference Genome NC_045512.2         (1)     -----------------------------------------------------------------------------
```

Section 1101

```
                                                    84701     84711      84722      84733      84744      84755      84766      84777
Candidatus Carsonella ruddii strain BT chromosome... (84701) TTCAGCGCCAAAAAACAAATTAAAAACATAATTTCTTTTTTTTAAAATATTTATTGAATATTGAATTCCATAAATAG
        Candidatus Carsonella 135557 - 167442nt     (1)     -----------------------------------------------------------------------------
    SARS-CoV-2 Reference Genome NC_045512.2         (1)     -----------------------------------------------------------------------------
```

Section 1102

```
                                                    84778     84788      84799      84810      84821      84832      84843      84854
Candidatus Carsonella ruddii strain BT chromosome... (84778) ATAAATTTTCTTCATCATTTGTTAAAATAAAATTATTATTTTTAGATAAAAAAAAAAATGAACAAAAACCACCTTTC
        Candidatus Carsonella 135557 - 167442nt     (1)     -----------------------------------------------------------------------------
    SARS-CoV-2 Reference Genome NC_045512.2         (1)     -----------------------------------------------------------------------------
```

Section 1103

```
                                                    84855     84865      84876      84887      84898      84909      84920      84931
Candidatus Carsonella ruddii strain BT chromosome... (84855) ATATCTGTAATTCCTCTACAAAAAATTTTTTTTTTAAAACAAATACAACTAAAAGGTGGTTTAATCCATTTAATATA
        Candidatus Carsonella 135557 - 167442nt     (1)     -----------------------------------------------------------------------------
    SARS-CoV-2 Reference Genome NC_045512.2         (1)     -----------------------------------------------------------------------------
```

Section 1104

```
                                                    84932     84942      84953      84964      84975      84986      84997      85008
Candidatus Carsonella ruddii strain BT chromosome... (84932) TCCTTCATTAACTACATCTAAATGAGAAATAAAAATTTCTTTAGATTTTGTAAAAAATAACAAATTAATTACATTTC
        Candidatus Carsonella 135557 - 167442nt     (1)     -----------------------------------------------------------------------------
    SARS-CoV-2 Reference Genome NC_045512.2         (1)     -----------------------------------------------------------------------------
```

```
                                                                                                                  Section 1105
                                                        (85009) 85009     85019      85030      85041      85052      85063      85074 85085
Candidatus Carsonella ruddii strain BT chromosome...    (85009) TAATTTTAATAATTTCAAAACGATTTAATTTCAAATATTTTAATATTATAAAAAATATATTTTCATAACAATAAGAA
           Candidatus Carsonella 135557 - 167442nt          (1) -----------------------------------------------------------------------------
         SARS-CoV-2 Reference Genome NC_045512.2            (1) -----------------------------------------------------------------------------
                                                                                                                  Section 1106
                                                        (85086) 85086     85096      85107      85118      85129      85140      85151 85162
Candidatus Carsonella ruddii strain BT chromosome...    (85086) AAAGTTTTTTTTATCAAAAAAAATTTTAAAAAAAAAAAAAAATTAATTAGCATTAATTATTGTGTAAAATTTTATTT
           Candidatus Carsonella 135557 - 167442nt          (1) -----------------------------------------------------------------------------
         SARS-CoV-2 Reference Genome NC_045512.2            (1) -----------------------------------------------------------------------------
                                                                                                                  Section 1107
                                                        (85163) 85163     85173      85184      85195      85206      85217      85228 85239
Candidatus Carsonella ruddii strain BT chromosome...    (85163) ATATTAATTGTTTTTGTAAAATTTTTACAACATTCTATCTTTCCATTTAAAGAATTTCTATAAAATATTAAATTATT
           Candidatus Carsonella 135557 - 167442nt          (1) -----------------------------------------------------------------------------
         SARS-CoV-2 Reference Genome NC_045512.2            (1) -----------------------------------------------------------------------------
                                                                                                                  Section 1108
                                                        (85240) 85240     85250      85261      85272      85283      85294      85305 85316
Candidatus Carsonella ruddii strain BT chromosome...    (85240) AATAAAAGAAAGTTTTTTAGCTTTTATAATAATAAAATTATTATTTAAAAATAAAAAAATTTTTGTACCAGCAGTTA
           Candidatus Carsonella 135557 - 167442nt          (1) -----------------------------------------------------------------------------
         SARS-CoV-2 Reference Genome NC_045512.2            (1) -----------------------------------------------------------------------------
                                                                                                                  Section 1109
                                                        (85317) 85317     85327      85338      85349      85360      85371      85382 85393
Candidatus Carsonella ruddii strain BT chromosome...    (85317) TATATAATCCTGCCTCAATAATACAATTATTTCCTAAACTAAATCCAATTCCTGAATTAGCACCTAATAAACAATTA
           Candidatus Carsonella 135557 - 167442nt          (1) -----------------------------------------------------------------------------
         SARS-CoV-2 Reference Genome NC_045512.2            (1) -----------------------------------------------------------------------------
                                                                                                                  Section 1110
                                                        (85394) 85394     85404      85415      85426      85437      85448      85459 85470
Candidatus Carsonella ruddii strain BT chromosome...    (85394) TTTCCTAAACTAATAATATTTTTTCCTCCACCAGATAATGTTCCCATTATTGAAGCAGATCCACCTATATCTGTATT
           Candidatus Carsonella 135557 - 167442nt          (1) -----------------------------------------------------------------------------
         SARS-CoV-2 Reference Genome NC_045512.2            (1) -----------------------------------------------------------------------------
```

```
                                                                                                    Section 1111
                                                      (85471) 85471     85481      85492      85503      85514      85525      85536    85547
Candidatus Carsonella ruddii strain BT chromosome...  (85471) ATTTGAGATATAAACTCCAGAAGATATTCTTCCTTCAATCATACAATTATTACCTATAAATGTATTAAAATTTACAT
            Candidatus Carsonella 135557 - 167442nt       (1) -----------------------------------------------------------------------------
           SARS-CoV-2 Reference Genome NC_045512.2        (1) -----------------------------------------------------------------------------
                                                                                                    Section 1112
                                                      (85548) 85548     85558      85569      85580      85591      85602      85613    85624
Candidatus Carsonella ruddii strain BT chromosome...  (85548) AACCTTCAGACATAATAGTTGTTCCTTTTGATAAAAAAGCTCCTAATCTTACTCTATTTGTGTTAGATAATCTAATA
            Candidatus Carsonella 135557 - 167442nt       (1) -----------------------------------------------------------------------------
           SARS-CoV-2 Reference Genome NC_045512.2        (1) -----------------------------------------------------------------------------
                                                                                                    Section 1113
                                                      (85625) 85625     85635      85646      85657      85668      85679      85690    85701
Candidatus Carsonella ruddii strain BT chromosome...  (85625) TTACTTGGTAAATAAAATTTTGTAAGATAAGGTATTTTATCAATACTTTCAATTATTAAATTAATTTTGTTTATATT
            Candidatus Carsonella 135557 - 167442nt       (1) -----------------------------------------------------------------------------
           SARS-CoV-2 Reference Genome NC_045512.2        (1) -----------------------------------------------------------------------------
                                                                                                    Section 1114
                                                      (85702) 85702     85712      85723      85734      85745      85756      85767    85778
Candidatus Carsonella ruddii strain BT chromosome...  (85702) TATTTTTGTAAAATCTTCAATATAATAACAACCTATATTTGTCCAAATTAAATTTATTAATTTTTCAAATATATTGT
            Candidatus Carsonella 135557 - 167442nt       (1) -----------------------------------------------------------------------------
           SARS-CoV-2 Reference Genome NC_045512.2        (1) -----------------------------------------------------------------------------
                                                                                                    Section 1115
                                                      (85779) 85779     85789      85800      85811      85822      85833      85844    85855
Candidatus Carsonella ruddii strain BT chromosome...  (85779) TTAAATTAATAAAATTACAAAGAAGTATTCCAATACTAATTAAATATAGTTTAAATAAACCATCTATATTTGATATT
            Candidatus Carsonella 135557 - 167442nt       (1) -----------------------------------------------------------------------------
           SARS-CoV-2 Reference Genome NC_045512.2        (1) -----------------------------------------------------------------------------
                                                                                                    Section 1116
                                                      (85856) 85856     85866      85877      85888      85899      85910      85921    85932
Candidatus Carsonella ruddii strain BT chromosome...  (85856) ATATTATTAATAAATATTGTTGTTATTAAAATAACATTTTTAAAATTTATTTTTTTCCAAAAATTAAATATAAACAT
            Candidatus Carsonella 135557 - 167442nt       (1) -----------------------------------------------------------------------------
           SARS-CoV-2 Reference Genome NC_045512.2        (1) -----------------------------------------------------------------------------
```

**Section 1117**	(85933)	85933 85943 85954 85965 85976 85987 85998 86009
Candidatus Carsonella ruddii strain BT chromosome...	(85933)	TAAATAAATAATTTGTTTTATATTTAACATTTTTTTAAATAATTTTTTTTTTATTTTTAAAAAAAAAAATTTTTTTT
Candidatus Carsonella 135557 - 167442nt	(1)	---
SARS-CoV-2 Reference Genome NC_045512.2	(1)	---
**Section 1118**	(86010)	86010 86020 86031 86042 86053 86064 86075 86086
Candidatus Carsonella ruddii strain BT chromosome...	(86010)	TAATAGCTATAAAATTACAAATTATTATTTTTTTTTTTTTTATATTTGTTTTATAAATTATTAAAGAATTATATAAC
Candidatus Carsonella 135557 - 167442nt	(1)	---
SARS-CoV-2 Reference Genome NC_045512.2	(1)	---
**Section 1119**	(86087)	86087 86097 86108 86119 86130 86141 86152 86163
Candidatus Carsonella ruddii strain BT chromosome...	(86087)	ATATTTCATTTTTTCTTAATGTTGTTAAAATTATTTTTTTATTATAAATATACAATGTATGTTCCCATTGAAAACTT
Candidatus Carsonella 135557 - 167442nt	(1)	---
SARS-CoV-2 Reference Genome NC_045512.2	(1)	---
**Section 1120**	(86164)	86164 86174 86185 86196 86207 86218 86229 86240
Candidatus Carsonella ruddii strain BT chromosome...	(86164)	AAATTATTTTTTTTTGTAAAAAAAATATTTAAAAAATCAATTCCTTTTTTGTTTTTAAAAATAAACATAGGTTCTAT
Candidatus Carsonella 135557 - 167442nt	(1)	---
SARS-CoV-2 Reference Genome NC_045512.2	(1)	---
**Section 1121**	(86241)	86241 86251 86262 86273 86284 86295 86306 86317
Candidatus Carsonella ruddii strain BT chromosome...	(86241)	AGTAAAACTATCAAAATTTTTTATTTTTTTACTATTTTTATTAAAATTATGTTTTATAATTAATTTATTATGTAATT
Candidatus Carsonella 135557 - 167442nt	(1)	---
SARS-CoV-2 Reference Genome NC_045512.2	(1)	---
**Section 1122**	(86318)	86318 86328 86339 86350 86361 86372 86383 86394
Candidatus Carsonella ruddii strain BT chromosome...	(86318)	TTTTAAAAATTCCATGACTACAATATTCTTCATTAATAAAAAAATTTTTTTTTAAAAATAATATTATTTTATTAAAA
Candidatus Carsonella 135557 - 167442nt	(1)	---
SARS-CoV-2 Reference Genome NC_045512.2	(1)	---

Section 1123

	(86395)	86395 86405 86416 86427 86438 86449 86460 86471
Candidatus Carsonella ruddii strain BT chromosome...	(86395)	AAAGAAAAAGATACATTTATTTTAATATAATTTAATATATTATAAAAAAAATTTTTAAATAAAAAATTTTTTTTAAC
Candidatus Carsonella 135557 - 167442nt	(1)	---
SARS-CoV-2 Reference Genome NC_045512.2	(1)	---

Section 1124

	(86472)	86472 86482 86493 86504 86515 86526 86537 86548
Candidatus Carsonella ruddii strain BT chromosome...	(86472)	ATAATTAAAATTATTTACATAACAACTATCACTGTGTAAATTATTAAATTTTAAAGAAATATCTAATTTAAAATAAT
Candidatus Carsonella 135557 - 167442nt	(1)	---
SARS-CoV-2 Reference Genome NC_045512.2	(1)	---

Section 1125

	(86549)	86549 86559 86570 86581 86592 86603 86614 86625
Candidatus Carsonella ruddii strain BT chromosome...	(86549)	TATATTTATAAAATTCAACGATAGGTATTCCATGACAAATTATATTTTCAATAGAAATACATGATGAAAATTTATAA
Candidatus Carsonella 135557 - 167442nt	(1)	---
SARS-CoV-2 Reference Genome NC_045512.2	(1)	---

Section 1126

	(86626)	86626 86636 86647 86658 86669 86680 86691 86702
Candidatus Carsonella ruddii strain BT chromosome...	(86626)	TTATAAAAATTTATAGTTGAAGATTTACATTTTATTTTTATAATAAAATATAAACTAATAAAATCTAATTCATATAA
Candidatus Carsonella 135557 - 167442nt	(1)	---
SARS-CoV-2 Reference Genome NC_045512.2	(1)	---

Section 1127

	(86703)	86703 86713 86724 86735 86746 86757 86768 86779
Candidatus Carsonella ruddii strain BT chromosome...	(86703)	ATTTATTTTTTTTGAATAATTACTTAACAAATTTATAATAAAATTATTATAAAAACCACATTTTAAAAATGATTTTA
Candidatus Carsonella 135557 - 167442nt	(1)	---
SARS-CoV-2 Reference Genome NC_045512.2	(1)	---

Section 1128

	(86780)	86780 86790 86801 86812 86823 86834 86845 86856
Candidatus Carsonella ruddii strain BT chromosome...	(86780)	TATGAAAATTTTTCATGTTTTAAAAAATAAAATTTATATAGGTAATAAAATTGAAAAAAGAAATAATTTATTAAAAA
Candidatus Carsonella 135557 - 167442nt	(1)	---
SARS-CoV-2 Reference Genome NC_045512.2	(1)	---

Section 1129

```
                                                     (86857) 86857     86867      86878      86889      86900      86911      86922      86933
Candidatus Carsonella ruddii strain BT chromosome... (86857) ATTTTTTAGTAGCAAACATTAATAAATTTAATATTATAAATGTTAAAAAAATTTCAATAAAGTTATTTTTATATAAA
            Candidatus Carsonella 135557 - 167442nt      (1) -----------------------------------------------------------------------------
         SARS-CoV-2 Reference Genome NC_045512.2         (1) -----------------------------------------------------------------------------
```

Section 1130

```
                                                     (86934) 86934     86944      86955      86966      86977      86988      86999      87010
Candidatus Carsonella ruddii strain BT chromosome... (86934) AAATATTTACAAAAAATTTTTTTTGAAAAAAAAAAAATTTTATTTATAAGTACAAAAAATTTTTTAAGAGAAATTAT
            Candidatus Carsonella 135557 - 167442nt      (1) -----------------------------------------------------------------------------
         SARS-CoV-2 Reference Genome NC_045512.2         (1) -----------------------------------------------------------------------------
```

Section 1131

```
                                                     (87011) 87011     87021      87032      87043      87054      87065      87076      87087
Candidatus Carsonella ruddii strain BT chromosome... (87011) TTATAAATATGCAAAAAGTTTACAACAATATTTTATTTGCACAAAATGGATAGCTGGAACTTTAACAAATTCTATAA
            Candidatus Carsonella 135557 - 167442nt      (1) -----------------------------------------------------------------------------
         SARS-CoV-2 Reference Genome NC_045512.2         (1) -----------------------------------------------------------------------------
```

Section 1132

```
                                                     (87088) 87088     87098      87109      87120      87131      87142      87153      87164
Candidatus Carsonella ruddii strain BT chromosome... (87088) ATTATAAAAAAATGTTAAATAAATCTATTTATTTAAAAAAAAAACAAAAAAATGAATTTTACAAAAAAGAAAAAAAT
            Candidatus Carsonella 135557 - 167442nt      (1) -----------------------------------------------------------------------------
         SARS-CoV-2 Reference Genome NC_045512.2         (1) -----------------------------------------------------------------------------
```

Section 1133

```
                                                     (87165) 87165     87175      87186      87197      87208      87219      87230      87241
Candidatus Carsonella ruddii strain BT chromosome... (87165) ATGTTTTTAAAAGAAGAATTAAAAATAGAAACAATTTATGGAGGATTAAGAGGAATGGTTGAAAATCCAGATTTAAT
            Candidatus Carsonella 135557 - 167442nt      (1) -----------------------------------------------------------------------------
         SARS-CoV-2 Reference Genome NC_045512.2         (1) -----------------------------------------------------------------------------
```

Section 1134

```
                                                     (87242) 87242     87252      87263      87274      87285      87296      87307      87318
Candidatus Carsonella ruddii strain BT chromosome... (87242) AATAATTACTGATATTAAAAAAGATAGAGTTGCAATTAATGAAGCTAAAAAATTAAAAATTAAAATTTTAGCATTGT
            Candidatus Carsonella 135557 - 167442nt      (1) -----------------------------------------------------------------------------
         SARS-CoV-2 Reference Genome NC_045512.2         (1) -----------------------------------------------------------------------------
```

```
                                                                      Section 1135
                                                 (87319) 87319     87329      87340      87351      87362      87373      87384      87395
Candidatus Carsonella ruddii strain BT chromosome... (87319) TAGATACAGATGATTCTCCTAAAGGAATTGATTATTTTATTCTTTGTAACAATAATTCAATTATTTGTGTTAAAAAT
             Candidatus Carsonella 135557 - 167442nt (1) -----------------------------------------------------------------------------
         SARS-CoV-2 Reference Genome NC_045512.2 (1) -----------------------------------------------------------------------------
                                                                      Section 1136
                                                 (87396) 87396     87406      87417      87428      87439      87450      87461      87472
Candidatus Carsonella ruddii strain BT chromosome... (87396) ATATTAAAAATATTATTTAGTGAAATATGTTAAATATAAATTTAATTTTAAAAATTAAAAAAGAATATAATTTCAAT
             Candidatus Carsonella 135557 - 167442nt (1) -----------------------------------------------------------------------------
         SARS-CoV-2 Reference Genome NC_045512.2 (1) -----------------------------------------------------------------------------
                                                                      Section 1137
                                                 (87473) 87473     87483      87494      87505      87516      87527      87538      87549
Candidatus Carsonella ruddii strain BT chromosome... (87473) ATTGGAAGTTGTAAAAAATTATTGGAAAATAATAATTGGGATTATAAAAAAACTATTTTGTATATAAAAAATAGTTT
             Candidatus Carsonella 135557 - 167442nt (1) -----------------------------------------------------------------------------
         SARS-CoV-2 Reference Genome NC_045512.2 (1) -----------------------------------------------------------------------------
                                                                      Section 1138
                                                 (87550) 87550     87560      87571      87582      87593      87604      87615      87626
Candidatus Carsonella ruddii strain BT chromosome... (87550) TAAAAAAATTAATTTTACAAATTTTAAATATTATTCTATAATAAATATAGAAAAAAAAAATAAAATTTTTATAATTA
             Candidatus Carsonella 135557 - 167442nt (1) -----------------------------------------------------------------------------
         SARS-CoV-2 Reference Genome NC_045512.2 (1) -----------------------------------------------------------------------------
                                                                      Section 1139
                                                 (87627) 87627     87637      87648      87659      87670      87681      87692      87703
Candidatus Carsonella ruddii strain BT chromosome... (87627) AAATATGTTATAATTCAACTATTATTGATAATTCAACTATTATTGAAAATTTTAAAAAAAAATTAATTTTAATAAAA
             Candidatus Carsonella 135557 - 167442nt (1) -----------------------------------------------------------------------------
         SARS-CoV-2 Reference Genome NC_045512.2 (1) -----------------------------------------------------------------------------
                                                                      Section 1140
                                                 (87704) 87704     87714      87725      87736      87747      87758      87769      87780
Candidatus Carsonella ruddii strain BT chromosome... (87704) ATTGATATTAAAATTTTAAAAAATGAAATAAATTTATTATCTTTTATATTAAAAGATAAAATTTTTTTAAATAAATT
             Candidatus Carsonella 135557 - 167442nt (1) -----------------------------------------------------------------------------
         SARS-CoV-2 Reference Genome NC_045512.2 (1) -----------------------------------------------------------------------------
```

```
                                                                                         Section 1141
                                                  (87781) 87781     87791      87802      87813      87824      87835      87846      87857
Candidatus Carsonella ruddii strain BT chromosome... (87781) TTTTATTTTTATAAATAAAAATATTTTTTTTTATAATCATAAAAATATTTTTTTTTGTTTAATAAATTATAAAAAAT
         Candidatus Carsonella 135557 - 167442nt      (1) -----------------------------------------------------------------------------
       SARS-CoV-2 Reference Genome NC_045512.2        (1) -----------------------------------------------------------------------------
                                                                                         Section 1142
                                                  (87858) 87858     87868      87879      87890      87901      87912      87923      87934
Candidatus Carsonella ruddii strain BT chromosome... (87858) TAATTTTAAATATTTGTTGTCATATAATATTCAAAAAGATTAATTTTTTTAATTTAAATATTTGTAAAAATAATTTG
         Candidatus Carsonella 135557 - 167442nt      (1) -----------------------------------------------------------------------------
       SARS-CoV-2 Reference Genome NC_045512.2        (1) -----------------------------------------------------------------------------
                                                                                         Section 1143
                                                  (87935) 87935     87945      87956      87967      87978      87989      88000      88011
Candidatus Carsonella ruddii strain BT chromosome... (87935) ATGAAACAAAAATATATAAAAAACGAAACATTATTTATTAATGATATAATCAAAAATAATTTTATAAATTATATTTT
         Candidatus Carsonella 135557 - 167442nt      (1) -----------------------------------------------------------------------------
       SARS-CoV-2 Reference Genome NC_045512.2        (1) -----------------------------------------------------------------------------
                                                                                         Section 1144
                                                  (88012) 88012     88022      88033      88044      88055      88066      88077      88088
Candidatus Carsonella ruddii strain BT chromosome... (88012) TTTAATAAATAAAAATAATATATATTTTTATTATGAATAATTTTAATATTTTTCAAGAAATATTAAATAATTATAAT
         Candidatus Carsonella 135557 - 167442nt      (1) -----------------------------------------------------------------------------
       SARS-CoV-2 Reference Genome NC_045512.2        (1) -----------------------------------------------------------------------------
                                                                                         Section 1145
                                                  (88089) 88089     88099      88110      88121      88132      88143      88154      88165
Candidatus Carsonella ruddii strain BT chromosome... (88089) TTTTTATTTAAAAATTTTAACATTAAAAATTTAGGAATTAAAACATTAAGTAATTTAAAAATAAATATTAATAAAAC
         Candidatus Carsonella 135557 - 167442nt      (1) -----------------------------------------------------------------------------
       SARS-CoV-2 Reference Genome NC_045512.2        (1) -----------------------------------------------------------------------------
                                                                                         Section 1146
                                                  (88166) 88166     88176      88187      88198      88209      88220      88231      88242
Candidatus Carsonella ruddii strain BT chromosome... (88166) ATATTTTAATTTAGAAAATATTTGTATTATTAAAAATATTAATGATAATAATTATTTATTACAATTTAAAGATACAA
         Candidatus Carsonella 135557 - 167442nt      (1) -----------------------------------------------------------------------------
       SARS-CoV-2 Reference Genome NC_045512.2        (1) -----------------------------------------------------------------------------
```

Section 1147

```
                                                  (88243) 88243     88253      88264      88275      88286      88297      88308      88319
Candidatus Carsonella ruddii strain BT chromosome... (88243) ACTTACTAAAACAAATAATTAAAAAAAATTTTTTTGAAAATTATGGTTTTCAAATAAAACAATCAAAAAATAATTTA
           Candidatus Carsonella 135557 - 167442nt (1) -----------------------------------------------------------------------------
        SARS-CoV-2 Reference Genome NC_045512.2 (1) -----------------------------------------------------------------------------
```

Section 1148

```
                                                  (88320) 88320     88330      88341      88352      88363      88374      88385      88396
Candidatus Carsonella ruddii strain BT chromosome... (88320) GAATTAATAATTCCAAAATTAAGTGTAGAATTTAGAAATAATGTATTAAAAATTTTAAAAAATGAATATTTCATTCA
           Candidatus Carsonella 135557 - 167442nt (1) -----------------------------------------------------------------------------
        SARS-CoV-2 Reference Genome NC_045512.2 (1) -----------------------------------------------------------------------------
```

Section 1149

```
                                                  (88397) 88397     88407      88418      88429      88440      88451      88462      88473
Candidatus Carsonella ruddii strain BT chromosome... (88397) CAAAGATAATATTGAAAATTATAGAAAAAAATTTTTATTAAATATAAAAAGAAATTTTAAGAGTATAGATGAAATAA
           Candidatus Carsonella 135557 - 167442nt (1) -----------------------------------------------------------------------------
        SARS-CoV-2 Reference Genome NC_045512.2 (1) -----------------------------------------------------------------------------
```

Section 1150

```
                                                  (88474) 88474     88484      88495      88506      88517      88528      88539      88550
Candidatus Carsonella ruddii strain BT chromosome... (88474) ATAATTTAGAAAAAAAATTAAATAAAGAAATTTTGTTTTGTAAAAATAAATTAAAAAATTTATTTGAAAATTTTTCT
           Candidatus Carsonella 135557 - 167442nt (1) -----------------------------------------------------------------------------
        SARS-CoV-2 Reference Genome NC_045512.2 (1) -----------------------------------------------------------------------------
```

Section 1151

```
                                                  (88551) 88551     88561      88572      88583      88594      88605      88616      88627
Candidatus Carsonella ruddii strain BT chromosome... (88551) AATAAAATATTAAATGAATAATTTTACTCATTTAAATTTACATACTGAATATTCATTTATAGATAGTATAATTAAAG
           Candidatus Carsonella 135557 - 167442nt (1) -----------------------------------------------------------------------------
        SARS-CoV-2 Reference Genome NC_045512.2 (1) -----------------------------------------------------------------------------
```

Section 1152

```
                                                  (88628) 88628     88638      88649      88660      88671      88682      88693      88704
Candidatus Carsonella ruddii strain BT chromosome... (88628) TTGGACAAATTATAGCGTTATATAAAGAGTATAAAATTAATACGATTTGTGTTACTGATTTATTATCAATGTCTTCA
           Candidatus Carsonella 135557 - 167442nt (1) -----------------------------------------------------------------------------
        SARS-CoV-2 Reference Genome NC_045512.2 (1) -----------------------------------------------------------------------------
```

```
                                                                    Section 1153
                                                   (88705) 88705     88715      88726      88737      88748      88759      88770      88781
Candidatus Carsonella ruddii strain BT chromosome... (88705) TTTTATGAATATTATTTTTTTTGTTATGATAATAATGTAAAACCTTTAATAGGTACAGAATGTTTTATTTTACATAA
          Candidatus Carsonella 135557 - 167442nt      (1) -----------------------------------------------------------------------------
        SARS-CoV-2 Reference Genome NC_045512.2        (1) -----------------------------------------------------------------------------
                                                                    Section 1154
                                                   (88782) 88782     88792      88803      88814      88825      88836      88847      88858
Candidatus Carsonella ruddii strain BT chromosome... (88782) TGGTAATATTTTAGGTTGTATTTTATTAGCAAAAAATTTTGAAGGATATAAAGAAATTATGCATATTTTATCAAATG
          Candidatus Carsonella 135557 - 167442nt      (1) -----------------------------------------------------------------------------
        SARS-CoV-2 Reference Genome NC_045512.2        (1) -----------------------------------------------------------------------------
                                                                    Section 1155
                                                   (88859) 88859     88869      88880      88891      88902      88913      88924      88935
Candidatus Carsonella ruddii strain BT chromosome... (88859) CTTGGAGATATGGTAATATAAATAATGGTGTTTTTATTAAAATACAATGGATATTAAATTTTAATAAAAATTTAATA
          Candidatus Carsonella 135557 - 167442nt      (1) -----------------------------------------------------------------------------
        SARS-CoV-2 Reference Genome NC_045512.2        (1) -----------------------------------------------------------------------------
                                                                    Section 1156
                                                   (88936) 88936     88946      88957      88968      88979      88990      89001      89012
Candidatus Carsonella ruddii strain BT chromosome... (88936) ATTATTTTAAATATTAGATATTATTTTTTATCAGAATTTAGTTTTATTAAACAAGAATTTATTTTTTTAATAAAATA
          Candidatus Carsonella 135557 - 167442nt      (1) -----------------------------------------------------------------------------
        SARS-CoV-2 Reference Genome NC_045512.2        (1) -----------------------------------------------------------------------------
                                                                    Section 1157
                                                   (89013) 89013     89023      89034      89045      89056      89067      89078      89089
Candidatus Carsonella ruddii strain BT chromosome... (89013) TATTTTATTTTTATTTGAAAATGATTGTTATTTTGAAATTAATAAAGTAAATTTACCTTTTGAAGATTATATTAATA
          Candidatus Carsonella 135557 - 167442nt      (1) -----------------------------------------------------------------------------
        SARS-CoV-2 Reference Genome NC_045512.2        (1) -----------------------------------------------------------------------------
                                                                    Section 1158
                                                   (89090) 89090     89100      89111      89122      89133      89144      89155      89166
Candidatus Carsonella ruddii strain BT chromosome... (89090) ATAAAATTTTTTTTTATTCAAAAAAATTTAAAATTAAATTAATAGCTACTAATAGTGTTAAGTTTTTATTCAAAAAT
          Candidatus Carsonella 135557 - 167442nt      (1) -----------------------------------------------------------------------------
        SARS-CoV-2 Reference Genome NC_045512.2        (1) -----------------------------------------------------------------------------
```

Section 1159

	(89167)	89167 89177 89188 89199 89210 89221 89232 89243
Candidatus Carsonella ruddii strain BT chromosome...	(89167)	GATTATGCTTCTAGTATTGGAAAAATTTTATTAAATCAAGAAGATTTAGATGATAATGAATTATATTTTGAATATAC
Candidatus Carsonella 135557 - 167442nt	(1)	---
SARS-CoV-2 Reference Genome NC_045512.2	(1)	---

Section 1160

	(89244)	89244 89254 89265 89276 89287 89298 89309 89320
Candidatus Carsonella ruddii strain BT chromosome...	(89244)	TGATAAACAATATTTAAAAAATTATTTTGAAATGAAAAAAATTTTTCAAAATAATTTAGAATGTTTAAATAACATTC
Candidatus Carsonella 135557 - 167442nt	(1)	---
SARS-CoV-2 Reference Genome NC_045512.2	(1)	---

Section 1161

	(89321)	89321 89331 89342 89353 89364 89375 89386 89397
Candidatus Carsonella ruddii strain BT chromosome...	(89321)	AAATTATTATTAATGATTGTAATATAAATTTTAAATGTTATGGTTTTAATTTGCCTAAAATTAAAATTGAAAAATTT
Candidatus Carsonella 135557 - 167442nt	(1)	---
SARS-CoV-2 Reference Genome NC_045512.2	(1)	---

Section 1162

	(89398)	89398 89408 89419 89430 89441 89452 89463 89474
Candidatus Carsonella ruddii strain BT chromosome...	(89398)	TCAATAAGAAAAAAAATTTTTAACAATTTATTAAAAAAAGGTATTAAAAACAGAATTATTAAAAAAAAAAGAAAAAA
Candidatus Carsonella 135557 - 167442nt	(1)	---
SARS-CoV-2 Reference Genome NC_045512.2	(1)	---

Section 1163

	(89475)	89475 89485 89496 89507 89518 89529 89540 89551
Candidatus Carsonella ruddii strain BT chromosome...	(89475)	ATTTTTACCATATATAAATAGAATAAATAAAGAATCTATTTTAATTAAAAAAATGGGTTTGATAGATTATTTTTTAA
Candidatus Carsonella 135557 - 167442nt	(1)	---
SARS-CoV-2 Reference Genome NC_045512.2	(1)	---

Section 1164

	(89552)	89552 89562 89573 89584 89595 89606 89617 89628
Candidatus Carsonella ruddii strain BT chromosome...	(89552)	TAATTTATGAATTTATTTTTTGGTCAAAAAAAAAAAATATTCCTTCTGGACCAGGAAGAGGTTCTGGAGCTTCATCT
Candidatus Carsonella 135557 - 167442nt	(1)	---
SARS-CoV-2 Reference Genome NC_045512.2	(1)	---

Section 1165

	(89629)	89629 89639 89650 89661 89672 89683 89694 89705
Candidatus Carsonella ruddii strain BT chromosome...	(89629)	TTAGTTTGTTATTCTTTGTATATAACAGATATAGATCCAGTAAATGAAAATTTATTATTTGAAAGATTTTTAACATC
Candidatus Carsonella 135557 - 167442nt	(1)	---
SARS-CoV-2 Reference Genome NC_045512.2	(1)	---

Section 1166

	(89706)	89706 89716 89727 89738 89749 89760 89771 89782
Candidatus Carsonella ruddii strain BT chromosome...	(89706)	TGAAAGATTTAGTATGCCTGATTTAGATTTAGATTTTTGTGTTTTAGAAAGAGACGCATTAATTGATCATATGTTTG
Candidatus Carsonella 135557 - 167442nt	(1)	---
SARS-CoV-2 Reference Genome NC_045512.2	(1)	---

Section 1167

	(89783)	89783 89793 89804 89815 89826 89837 89848 89859
Candidatus Carsonella ruddii strain BT chromosome...	(89783)	AGTATTATAATTATAATAATACATCACAAATAATAACATTTCATAATTTATCTACAAAATCTTTAATAAGAGATTTA
Candidatus Carsonella 135557 - 167442nt	(1)	---
SARS-CoV-2 Reference Genome NC_045512.2	(1)	---

Section 1168

	(89860)	89860 89870 89881 89892 89903 89914 89925 89936
Candidatus Carsonella ruddii strain BT chromosome...	(89860)	TCTAGAGCAATAGGTTTAGACTATTTATCTGGTGATAGATTTTCTAAATCAATTCCCTTTTCTTTAGAATTATCGAT
Candidatus Carsonella 135557 - 167442nt	(1)	---
SARS-CoV-2 Reference Genome NC_045512.2	(1)	---

Section 1169

	(89937)	89937 89947 89958 89969 89980 89991 90002 90013
Candidatus Carsonella ruddii strain BT chromosome...	(89937)	GGAAGATATCTTTAGAAAAAGTTTATCAGTTAGAAGTTATGTTTCAAGTAACGAAAAAAGTTTAGATATATGGAAAA
Candidatus Carsonella 135557 - 167442nt	(1)	---
SARS-CoV-2 Reference Genome NC_045512.2	(1)	---

Section 1170

	(90014)	90014 90024 90035 90046 90057 90068 90079 90090
Candidatus Carsonella ruddii strain BT chromosome...	(90014)	TATCTACTAAAATTGAAGGTGTATTTAGTAAAATTGGTAAACATGCTGGAGGAGTTGTTATTGCTAAAAATGGTATT
Candidatus Carsonella 135557 - 167442nt	(1)	---
SARS-CoV-2 Reference Genome NC_045512.2	(1)	---

Section 1171

	(90091)	90091 90101 90112 90123 90134 90145 90156 90167
Candidatus Carsonella ruddii strain BT chromosome...	(90091)	TATAATATTACTCCAATTATGTTTGATAATGATGACTGTATTACTCAATATGAAAAAAATATTTTACAAGAAATTGG
Candidatus Carsonella 135557 - 167442nt	(1)	---
SARS-CoV-2 Reference Genome NC_045512.2	(1)	---

Section 1172

	(90168)	90168 90178 90189 90200 90211 90222 90233 90244
Candidatus Carsonella ruddii strain BT chromosome...	(90168)	TTTATTAAAATTTGATTTTTTAGGATTAAAAACTATATCAATTATACATATTTCTTTAAATATGATACCTGAAAAAA
Candidatus Carsonella 135557 - 167442nt	(1)	---
SARS-CoV-2 Reference Genome NC_045512.2	(1)	---

Section 1173

	(90245)	90245 90255 90266 90277 90288 90299 90310 90321
Candidatus Carsonella ruddii strain BT chromosome...	(90245)	CAACAGGTGAATTTAATATAGATGATTATCATACATATCAAATGTTGAATAATTTAGAAACTGAATTAGTTTTTCAA
Candidatus Carsonella 135557 - 167442nt	(1)	---
SARS-CoV-2 Reference Genome NC_045512.2	(1)	---

Section 1174

	(90322)	90322 90332 90343 90354 90365 90376 90387 90398
Candidatus Carsonella ruddii strain BT chromosome...	(90322)	TTAGAATCATATGGAATGAAAAAAATGTTAAGAAGATCACCAATTAATAATATTTTTGATATTATTTTATATTTATC
Candidatus Carsonella 135557 - 167442nt	(1)	---
SARS-CoV-2 Reference Genome NC_045512.2	(1)	---

Section 1175

	(90399)	90399 90409 90420 90431 90442 90453 90464 90475
Candidatus Carsonella ruddii strain BT chromosome...	(90399)	TTTATTTAGACCAGGACCTATTCAATCAGGATGTGTTAATATTTTTTTAAATAGAAAAAATAATTTATTAAAAACTT
Candidatus Carsonella 135557 - 167442nt	(1)	---
SARS-CoV-2 Reference Genome NC_045512.2	(1)	---

Section 1176

	(90476)	90476 90486 90497 90508 90519 90530 90541 90552
Candidatus Carsonella ruddii strain BT chromosome...	(90476)	TTTTTCCATATGAAAATAACAATTATTATTTTTTAAAAATTTTTTTATCATATACACATGGTATAATGATATTTCAA
Candidatus Carsonella 135557 - 167442nt	(1)	---
SARS-CoV-2 Reference Genome NC_045512.2	(1)	---

```
Section 1177
                                                   (90553) 90553     90563      90574      90585      90596      90607      90618      90629
Candidatus Carsonella ruddii strain BT chromosome... (90553) GAACAAATAATATTGATTATACATACTTTTTTTGAATGTACAATATATGATTCTGAAAAAATTTTTAATTCAATGAT
        Candidatus Carsonella 135557 - 167442nt        (1) -----------------------------------------------------------------------------
   SARS-CoV-2 Reference Genome NC_045512.2              (1) -----------------------------------------------------------------------------
Section 1178
                                                   (90630) 90630     90640      90651      90662      90673      90684      90695      90706
Candidatus Carsonella ruddii strain BT chromosome... (90630) TTCTAATTCTATTAAAGAATTAAATATTCTCAATAAAATTTTTAAAAAAAAATGTTTAAAATTTAATTTATCTGATT
        Candidatus Carsonella 135557 - 167442nt        (1) -----------------------------------------------------------------------------
   SARS-CoV-2 Reference Genome NC_045512.2              (1) -----------------------------------------------------------------------------
Section 1179
                                                   (90707) 90707     90717      90728      90739      90750      90761      90772      90783
Candidatus Carsonella ruddii strain BT chromosome... (90707) ATGAATCTAATGAATTATTTTATTTAATTGTAACTTTTGCATGTTATAGTTTTAATAGAACTCATGCACATTCATAT
        Candidatus Carsonella 135557 - 167442nt        (1) -----------------------------------------------------------------------------
   SARS-CoV-2 Reference Genome NC_045512.2              (1) -----------------------------------------------------------------------------
Section 1180
                                                   (90784) 90784     90794      90805      90816      90827      90838      90849      90860
Candidatus Carsonella ruddii strain BT chromosome... (90784) GCTAAAATTGTTTATCAAACAGCTTTTTTAAAAGCAAATTATTTATTAGAATTTTGTTTAGCAAATATTTATGTTGA
        Candidatus Carsonella 135557 - 167442nt        (1) -----------------------------------------------------------------------------
   SARS-CoV-2 Reference Genome NC_045512.2              (1) -----------------------------------------------------------------------------
Section 1181
                                                   (90861) 90861     90871      90882      90893      90904      90915      90926      90937
Candidatus Carsonella ruddii strain BT chromosome... (90861) TCAATTATTAGGATTTGATTTAGATAGAATTATTTTTAATTTAAAAATTATAGGTGTTTATTTTTACAAACCAGACA
        Candidatus Carsonella 135557 - 167442nt        (1) -----------------------------------------------------------------------------
   SARS-CoV-2 Reference Genome NC_045512.2              (1) -----------------------------------------------------------------------------
Section 1182
                                                   (90938) 90938     90948      90959      90970      90981      90992      91003      91014
Candidatus Carsonella ruddii strain BT chromosome... (90938) TTAATATTTCTGATGAAAATTTTAAAATTTATAAAAAAGGTATTTTATTTGGTTTTTCTGTAGTTAAATTTATAGAT
        Candidatus Carsonella 135557 - 167442nt        (1) -----------------------------------------------------------------------------
   SARS-CoV-2 Reference Genome NC_045512.2              (1) -----------------------------------------------------------------------------
```

Section 1183

	(91015)	91015 91025 91036 91047 91058 91069 91080 91091
Candidatus Carsonella ruddii strain BT chromosome...	(91015)	GATTATTTTATAGATAGAATCTTATATTATAGAAATAAAATATTTTTTTATAATAGTTTTGAAATATTTTGTAAAAT
Candidatus Carsonella 135557 - 167442nt	(1)	---
SARS-CoV-2 Reference Genome NC_045512.2	(1)	---

Section 1184

	(91092)	91092 91102 91113 91124 91135 91146 91157 91168
Candidatus Carsonella ruddii strain BT chromosome...	(91092)	ATTTAGTATATTTAAAATTAAAAATAAAAAAGTAATAGAAAATTTAATTTTTTGTGGTTTTTTTGATTGTTATAGAA
Candidatus Carsonella 135557 - 167442nt	(1)	---
SARS-CoV-2 Reference Genome NC_045512.2	(1)	---

Section 1185

	(91169)	91169 91179 91190 91201 91212 91223 91234 91245
Candidatus Carsonella ruddii strain BT chromosome...	(91169)	TTAATAGAATATATTTATTTATTAATTTTCAATTTTTATATGAAAAAATCATTTTATTAAAAAAAGAATATAATAGA
Candidatus Carsonella 135557 - 167442nt	(1)	---
SARS-CoV-2 Reference Genome NC_045512.2	(1)	---

Section 1186

	(91246)	91246 91256 91267 91278 91289 91300 91311 91322
Candidatus Carsonella ruddii strain BT chromosome...	(91246)	TCGTTAATTTATAAATTAATAGAATATATTGATTATTCTAAATTATCTATTATAAAATTTAAAATTTTAAATGTATA
Candidatus Carsonella 135557 - 167442nt	(1)	---
SARS-CoV-2 Reference Genome NC_045512.2	(1)	---

Section 1187

	(91323)	91323 91333 91344 91355 91366 91377 91388 91399
Candidatus Carsonella ruddii strain BT chromosome...	(91323)	TTATTTAGATTTATTAAATATAGAAAAAAAATTATTAAAATTTTTCGTTTCTAATCAACCATCTGATTTTTTTATTT
Candidatus Carsonella 135557 - 167442nt	(1)	---
SARS-CoV-2 Reference Genome NC_045512.2	(1)	---

Section 1188

	(91400)	91400 91410 91421 91432 91443 91454 91465 91476
Candidatus Carsonella ruddii strain BT chromosome...	(91400)	CAAAATTTATAGGTCATAATTTATATAATTCAATAAATAGAGTTGAAAAAAAAAAAATTAATATTTTAATTTCTACA
Candidatus Carsonella 135557 - 167442nt	(1)	---
SARS-CoV-2 Reference Genome NC_045512.2	(1)	---

Section 1189

```
                                                   (91477) 91477     91487      91498      91509      91520      91531      91542   91553
Candidatus Carsonella ruddii strain BT chromosome... (91477) CCTAAAAAAAAACTTTTTTTTGAAAAACATGTAAATATTTTTAGAATAATTAATAATAATAAAATTAAAGTTTTAGA
            Candidatus Carsonella 135557 - 167442nt (1) -----------------------------------------------------------------------------
          SARS-CoV-2 Reference Genome NC_045512.2 (1) -----------------------------------------------------------------------------
```

Section 1190

```
                                                   (91554) 91554     91564      91575      91586      91597      91608      91619   91630
Candidatus Carsonella ruddii strain BT chromosome... (91554) TGTTTTTTCTAGATTTATACCTTTACCTGATATTGAAATACCTTTTAGTTTTTTATTATTATATAATAATGAAACAA
            Candidatus Carsonella 135557 - 167442nt (1) -----------------------------------------------------------------------------
          SARS-CoV-2 Reference Genome NC_045512.2 (1) -----------------------------------------------------------------------------
```

Section 1191

```
                                                   (91631) 91631     91641      91652      91663      91674      91685      91696   91707
Candidatus Carsonella ruddii strain BT chromosome... (91631) AAACTAATCAAATTATTAAATGTTATAATATAAATCATTTCTGTTATTCCATAGGATGTGTTTATTATATATATATA
            Candidatus Carsonella 135557 - 167442nt (1) -----------------------------------------------------------------------------
          SARS-CoV-2 Reference Genome NC_045512.2 (1) -----------------------------------------------------------------------------
```

Section 1192

```
                                                   (91708) 91708     91718      91729      91740      91751      91762      91773   91784
Candidatus Carsonella ruddii strain BT chromosome... (91708) AAAAATACTTTAAATTTAAAAAATATTATTTATTTGTTATTTAATAATTTTAATTTTATCGGTAGCAAAGTTTATAT
            Candidatus Carsonella 135557 - 167442nt (1) -----------------------------------------------------------------------------
          SARS-CoV-2 Reference Genome NC_045512.2 (1) -----------------------------------------------------------------------------
```

Section 1193

```
                                                   (91785) 91785     91795      91806      91817      91828      91839      91850   91861
Candidatus Carsonella ruddii strain BT chromosome... (91785) TAAATATAAAAATAAAATTTTTTCATCGAAAATTTGTGTTTTAATGGATGAAAAAATAATGTTACATTTAAAATTAT
            Candidatus Carsonella 135557 - 167442nt (1) -----------------------------------------------------------------------------
          SARS-CoV-2 Reference Genome NC_045512.2 (1) -----------------------------------------------------------------------------
```

Section 1194

```
                                                   (91862) 91862     91872      91883      91894      91905      91916      91927   91938
Candidatus Carsonella ruddii strain BT chromosome... (91862) TTGATATTACTAAAATAGTTTGTTTTACATGTATTAAATGAAAAATTTATTTTTAATAAAAAATATTTATTTATATT
            Candidatus Carsonella 135557 - 167442nt (1) -----------------------------------------------------------------------------
          SARS-CoV-2 Reference Genome NC_045512.2 (1) -----------------------------------------------------------------------------
```

		Section 1195
	(91939)	91939 91949 91960 91971 91982 91993 92004 92015
Candidatus Carsonella ruddii strain BT chromosome...	(91939)	TTTCATTTGGTAAAGATAGTTATTATTCATTATTTAATTTTTTTTTATAAAAAAAAAAAAAATTATTTTTTACAATTAT
Candidatus Carsonella 135557 - 167442nt	(1)	---
SARS-CoV-2 Reference Genome NC_045512.2	(1)	---
		Section 1196
	(92016)	92016 92026 92037 92048 92059 92070 92081 92092
Candidatus Carsonella ruddii strain BT chromosome...	(92016)	AATAAAAAAAATATTTTTAAAAAAATAAATACAAATTCTTTTATTTTTTTATAAAAAATTTTCTAAAAATGAATT
Candidatus Carsonella 135557 - 167442nt	(1)	---
SARS-CoV-2 Reference Genome NC_045512.2	(1)	---
		Section 1197
	(92093)	92093 92103 92114 92125 92136 92147 92158 92169
Candidatus Carsonella ruddii strain BT chromosome...	(92093)	ATTATTAAGAAGAACTAGATTTTTAATCAAAAAAAAAAAATATTTTTTTTATAAAAAATCATCATTTAATAGATAAAA
Candidatus Carsonella 135557 - 167442nt	(1)	---
SARS-CoV-2 Reference Genome NC_045512.2	(1)	---
		Section 1198
	(92170)	92170 92180 92191 92202 92213 92224 92235 92246
Candidatus Carsonella ruddii strain BT chromosome...	(92170)	TTGAATTTTTAATTTTTAAATTTTTAAAAAAAAATTTTTTTTTAAATTATAATAATATATGGATTAATAAAAAAAAT
Candidatus Carsonella 135557 - 167442nt	(1)	---
SARS-CoV-2 Reference Genome NC_045512.2	(1)	---
		Section 1199
	(92247)	92247 92257 92268 92279 92290 92301 92312 92323
Candidatus Carsonella ruddii strain BT chromosome...	(92247)	TTTTTAATTAAACCTTTTATAAATAAATTTATAAAAAAATATATTAATATTAATGATAAAACTAATAAGTTTATAAA
Candidatus Carsonella 135557 - 167442nt	(1)	---
SARS-CoV-2 Reference Genome NC_045512.2	(1)	---
		Section 1200
	(92324)	92324 92334 92345 92356 92367 92378 92389 92400
Candidatus Carsonella ruddii strain BT chromosome...	(92324)	AATAAGTAGAAATTTTATAAGATGTTTGATAAGTAAATTTTTATAAAAGAAATATTTTTTTAAATAATAACAATAATA
Candidatus Carsonella 135557 - 167442nt	(1)	---
SARS-CoV-2 Reference Genome NC_045512.2	(1)	---

```
                                                                 Section 1201
                                              (92401) 92401     92411      92422      92433      92444      92455      92466    92477
Candidatus Carsonella ruddii strain BT chromosome... (92401) ATTATGATAAACTCTTATTGTATAAATTCACCAAATATTGGAAAATATAGAGAATTAAAAAAATATACTGAAAAATA
         Candidatus Carsonella 135557 - 167442nt (1) -----------------------------------------------------------------------------
      SARS-CoV-2 Reference Genome NC_045512.2 (1) -----------------------------------------------------------------------------
                                                                 Section 1202
                                              (92478) 92478     92488      92499      92510      92521      92532      92543    92554
Candidatus Carsonella ruddii strain BT chromosome... (92478) TTGGAAAAATCAAAACGTAGAAAATTTAAAAAAATTAATAATGATATCTAAAAAAATTAAATTTGAAAAAATTTTAA
         Candidatus Carsonella 135557 - 167442nt (1) -----------------------------------------------------------------------------
      SARS-CoV-2 Reference Genome NC_045512.2 (1) -----------------------------------------------------------------------------
                                                                 Section 1203
                                              (92555) 92555     92565      92576      92587      92598      92609      92620    92631
Candidatus Carsonella ruddii strain BT chromosome... (92555) ATCAAACAAATAATAATTTTTTTATATATTAATTTTGGAGATTTTAGTTTTTATGATTATGTTTTTGACATAAGTTGT
         Candidatus Carsonella 135557 - 167442nt (1) -----------------------------------------------------------------------------
      SARS-CoV-2 Reference Genome NC_045512.2 (1) -----------------------------------------------------------------------------
                                                                 Section 1204
                                              (92632) 92632     92642      92653      92664      92675      92686      92697    92708
Candidatus Carsonella ruddii strain BT chromosome... (92632) TTAATAAATTCTTTTAATAATAATTCTATTATAAATATTAAAAAATATTTTTCATTTATTAGAGGTACAAATTATAC
         Candidatus Carsonella 135557 - 167442nt (1) -----------------------------------------------------------------------------
      SARS-CoV-2 Reference Genome NC_045512.2 (1) -----------------------------------------------------------------------------
                                                                 Section 1205
                                              (92709) 92709     92719      92730      92741      92752      92763      92774    92785
Candidatus Carsonella ruddii strain BT chromosome... (92709) TTTACCTTGTAAAATGACAAAATTTTTTGATACCAATTATCATTACATTATTCCTTATGATTTAAATAATTTAAAAT
         Candidatus Carsonella 135557 - 167442nt (1) -----------------------------------------------------------------------------
      SARS-CoV-2 Reference Genome NC_045512.2 (1) -----------------------------------------------------------------------------
                                                                 Section 1206
                                              (92786) 92786     92796      92807      92818      92829      92840      92851    92862
Candidatus Carsonella ruddii strain BT chromosome... (92786) TATTAAATAATTTTTTTTTAAATGATTTAAAAAAAATAATTAAAATAGGTATTTTACCTACACCTATAATATTAGGT
         Candidatus Carsonella 135557 - 167442nt (1) -----------------------------------------------------------------------------
      SARS-CoV-2 Reference Genome NC_045512.2 (1) -----------------------------------------------------------------------------
```

```
                                                                                   Section 1207
                                                     (92863) 92863     92873      92884      92895      92906      92917      92928      92939
Candidatus Carsonella ruddii strain BT chromosome... (92863) CCTTTAAGTTATTTGTTTTTATGTATAAAACAACAAAAATACTTTAAAAAATTATTTGAAATTTATATATTAATTAT
         Candidatus Carsonella 135557 - 167442nt         (1) -----------------------------------------------------------------------------
      SARS-CoV-2 Reference Genome NC_045512.2            (1) -----------------------------------------------------------------------------
                                                                                   Section 1208
                                                     (92940) 92940     92950      92961      92972      92983      92994      93005      93016
Candidatus Carsonella ruddii strain BT chromosome... (92940) TAAAAAAATAAAAAAAAATAATATACAAGAAATTTTTTTTGATGAACCAATATTATGTTGTAAAATAAATTCAAATT
         Candidatus Carsonella 135557 - 167442nt         (1) -----------------------------------------------------------------------------
      SARS-CoV-2 Reference Genome NC_045512.2            (1) -----------------------------------------------------------------------------
                                                                                   Section 1209
                                                     (93017) 93017     93027      93038      93049      93060      93071      93082      93093
Candidatus Carsonella ruddii strain BT chromosome... (93017) GGAAAAAATTATTCAATTTATTTTATAATAAAACTAAAAAAATAATTAAAATAATTATAATTTCTTCATTTAATTTT
         Candidatus Carsonella 135557 - 167442nt         (1) -----------------------------------------------------------------------------
      SARS-CoV-2 Reference Genome NC_045512.2            (1) -----------------------------------------------------------------------------
                                                                                   Section 1210
                                                     (93094) 93094     93104      93115      93126      93137      93148      93159      93170
Candidatus Carsonella ruddii strain BT chromosome... (93094) AATAAAAATAATTTTGATATTTTAAATAATTTTGAATATAATTTAAATTTAGAATTTAAAAAAAATAATTTATTAGA
         Candidatus Carsonella 135557 - 167442nt         (1) -----------------------------------------------------------------------------
      SARS-CoV-2 Reference Genome NC_045512.2            (1) -----------------------------------------------------------------------------
                                                                                   Section 1211
                                                     (93171) 93171     93181      93192      93203      93214      93225      93236      93247
Candidatus Carsonella ruddii strain BT chromosome... (93171) ATTTATAAAAAAAAATAATTTTAATATTATACAAAATAATTTAAATAAAACTAATATTTTATTTTTATTTAATTTTT
         Candidatus Carsonella 135557 - 167442nt         (1) -----------------------------------------------------------------------------
      SARS-CoV-2 Reference Genome NC_045512.2            (1) -----------------------------------------------------------------------------
                                                                                   Section 1212
                                                     (93248) 93248     93258      93269      93280      93291      93302      93313      93324
Candidatus Carsonella ruddii strain BT chromosome... (93248) TTATAAATAAAAATATTGTTTCAATCACAAGTAATTTAAAATACATACCTTATAATTTATATATAGAAAATTTTGAA
         Candidatus Carsonella 135557 - 167442nt         (1) -----------------------------------------------------------------------------
      SARS-CoV-2 Reference Genome NC_045512.2            (1) -----------------------------------------------------------------------------
```

Section 1213

	(93325)	93325 93335 93346 93357 93368 93379 93390 93401
Candidatus Carsonella ruddii strain BT chromosome...	(93325)	AACAAAAAATATTTATCTTTTTTTTTTCAAAAAATAAATGAATTAAAATTAATAAAAGAAATTTTAAATAAAAATTT
Candidatus Carsonella 135557 - 167442nt	(1)	---
SARS-CoV-2 Reference Genome NC_045512.2	(1)	---

Section 1214

	(93402)	93402 93412 93423 93434 93445 93456 93467 93478
Candidatus Carsonella ruddii strain BT chromosome...	(93402)	AAATAGTTATAACTTATTATATTTAATTAATTTTAAAATTTATAATAAAAAACTTATTATAAATAACAAATTTAATA
Candidatus Carsonella 135557 - 167442nt	(1)	---
SARS-CoV-2 Reference Genome NC_045512.2	(1)	---

Section 1215

	(93479)	93479 93489 93500 93511 93522 93533 93544 93555
Candidatus Carsonella ruddii strain BT chromosome...	(93479)	AAATTTTAAAAAAAAGCAATAAATTAAATAAAAAATTATTAATAAATAATACTATAGGATCTTTTCCACAAAATAAA
Candidatus Carsonella 135557 - 167442nt	(1)	---
SARS-CoV-2 Reference Genome NC_045512.2	(1)	---

Section 1216

	(93556)	93556 93566 93577 93588 93599 93610 93621 93632
Candidatus Carsonella ruddii strain BT chromosome...	(93556)	GAAATTAAAATTTCTAGAAAACAAAAAAATTTTTTTTTATTAAATGAAAAAGAATATAATAATATTTTAAAAGAAAA
Candidatus Carsonella 135557 - 167442nt	(1)	---
SARS-CoV-2 Reference Genome NC_045512.2	(1)	---

Section 1217

	(93633)	93633 93643 93654 93665 93676 93687 93698 93709
Candidatus Carsonella ruddii strain BT chromosome...	(93633)	TATTTATTTATGTTTTATTAAACAAATAAAATTAAAACATCAAATTTTAGTTAACGGTGAATTTGAAAGAACAGATA
Candidatus Carsonella 135557 - 167442nt	(1)	---
SARS-CoV-2 Reference Genome NC_045512.2	(1)	---

Section 1218

	(93710)	93710 93720 93731 93742 93753 93764 93775 93786
Candidatus Carsonella ruddii strain BT chromosome...	(93710)	TGGTAGAATTTTTTGCAAAAAATATAAAAGGAATTAATGTTACTAAAAATGGATGGGTACAAAGTTATGGAACTAGA
Candidatus Carsonella 135557 - 167442nt	(1)	---
SARS-CoV-2 Reference Genome NC_045512.2	(1)	---

Section 1219

		93787 93797 93808 93819 93830 93841 93852 93863
Candidatus Carsonella ruddii strain BT chromosome...	(93787)	TATGTTAAACCTCCTATAATATTAAATATCGAAAATAGTTTAAATATAACTAATAATTGGTATATTTTTACTAAAAA
Candidatus Carsonella 135557 - 167442nt	(1)	---
SARS-CoV-2 Reference Genome NC_045512.2	(1)	---

Section 1220

		93864 93874 93885 93896 93907 93918 93929 93940
Candidatus Carsonella ruddii strain BT chromosome...	(93864)	AATAACAAAAAAAAAAATTAAAATTATTGTTTCAGGACCTATAACAATGTTAAAATGGTCTTTTTTTGATAATAATA
Candidatus Carsonella 135557 - 167442nt	(1)	---
SARS-CoV-2 Reference Genome NC_045512.2	(1)	---

Section 1221

		93941 93951 93962 93973 93984 93995 94006 94017
Candidatus Carsonella ruddii strain BT chromosome...	(93941)	AAATTAAATTTTTATTTTGTTTTAAATTATCAGAAATAATTAGTTTAGAATTATTAAAATTACAAAATAATGGTTTT
Candidatus Carsonella 135557 - 167442nt	(1)	---
SARS-CoV-2 Reference Genome NC_045512.2	(1)	---

Section 1222

		94018 94028 94039 94050 94061 94072 94083 94094
Candidatus Carsonella ruddii strain BT chromosome...	(94018)	AAAATATTTCAAATAGATGAACCTACTATAAAAGAATGTTTAAATATAAACAAAAAGTATTGGAAATATGAAATAAA
Candidatus Carsonella 135557 - 167442nt	(1)	---
SARS-CoV-2 Reference Genome NC_045512.2	(1)	---

Section 1223

		94095 94105 94116 94127 94138 94149 94160 94171
Candidatus Carsonella ruddii strain BT chromosome...	(94095)	TAATTTTTTGTTCTGTTTTAATAATTGTACTAAATTTATTAAAAAAAATAATGAAATACATTCACATGTTTGTTATT
Candidatus Carsonella 135557 - 167442nt	(1)	---
SARS-CoV-2 Reference Genome NC_045512.2	(1)	---

Section 1224

		94172 94182 94193 94204 94215 94226 94237 94248
Candidatus Carsonella ruddii strain BT chromosome...	(94172)	CTATTTTTAACGATATAATAAAATTTATTGAAAAAATAAATATTGATATATTATCTATAGAATCTACTAGAGAAAAA
Candidatus Carsonella 135557 - 167442nt	(1)	---
SARS-CoV-2 Reference Genome NC_045512.2	(1)	---

Section 1225

(94249) 94249 94259 94270 94281 94292 94303 94314 94325
Candidatus Carsonella ruddii strain BT chromosome... (94249) ATGGAAAATTTAAATATATTTAAAAAAACTAAATTAAATTTAGGTGCTGGAATTTATGATGTTCATTCTCCAATTAT
Candidatus Carsonella 135557 - 167442nt (1) ---
SARS-CoV-2 Reference Genome NC_045512.2 (1) ---

Section 1226

(94326) 94326 94336 94347 94358 94369 94380 94391 94402
Candidatus Carsonella ruddii strain BT chromosome... (94326) ACCATATAAAAAAAAAATAAAAAAAAATATTTTTTTTTTTGTTAATAAAATTAATTTTTTAAAAATTTTAATTAATC
Candidatus Carsonella 135557 - 167442nt (1) ---
SARS-CoV-2 Reference Genome NC_045512.2 (1) ---

Section 1227

(94403) 94403 94413 94424 94435 94446 94457 94468 94479
Candidatus Carsonella ruddii strain BT chromosome... (94403) CTGATTGTGGATTAAAAACTAGAAATTGGTACGAATTAAATTTTACTTTTTTTAATATTAATAATATATTAAATTAT
Candidatus Carsonella 135557 - 167442nt (1) ---
SARS-CoV-2 Reference Genome NC_045512.2 (1) ---

Section 1228

(94480) 94480 94490 94501 94512 94523 94534 94545 94556
Candidatus Carsonella ruddii strain BT chromosome... (94480) ATAAAAAATTTTTATTATTAACGAAATTGTTTTTCTTTAAATCCAGAAATTTTTTCTCTTCTTATAAATTCTTTTTC
Candidatus Carsonella 135557 - 167442nt (1) ---
SARS-CoV-2 Reference Genome NC_045512.2 (1) ---

Section 1229

(94557) 94557 94567 94578 94589 94600 94611 94622 94633
Candidatus Carsonella ruddii strain BT chromosome... (94557) TATTTCAAAATAAGAAATATTATTAAATATAATAAATATTATTATATGATATAATAGATCACAAATTTCTTTTATTA
Candidatus Carsonella 135557 - 167442nt (1) ---
SARS-CoV-2 Reference Genome NC_045512.2 (1) ---

Section 1230

(94634) 94634 94644 94655 94666 94677 94688 94699 94710
Candidatus Carsonella ruddii strain BT chromosome... (94634) AAAAATATCTATTATAACAATTTTTTAATAAATAATATTTATTAAAAGAAATATAAATTTCAAATATTTCTTCAAAA
Candidatus Carsonella 135557 - 167442nt (1) ---
SARS-CoV-2 Reference Genome NC_045512.2 (1) ---

```
                                                          Section 1231
                                               (94711) 94711     94721      94732      94743      94754      94765      94776      94787
Candidatus Carsonella ruddii strain BT chromosome... (94711) ATTTTTTTAAATAAAAATTCAAAATTATTTAATAATATATATTTTGAGTAAGATTTACTTACTATGAATTTATTTTT
       Candidatus Carsonella 135557 - 167442nt       (1) -----------------------------------------------------------------------------
    SARS-CoV-2 Reference Genome NC_045512.2          (1) -----------------------------------------------------------------------------
                                                          Section 1232
                                               (94788) 94788     94798      94809      94820      94831      94842      94853      94864
Candidatus Carsonella ruddii strain BT chromosome... (94788) TATAATTTTTATTATTTTTTTAATCATGAATAAAATATATTTTAATTGTATTTGTAATGAAAAATATTATTTGTTTT
       Candidatus Carsonella 135557 - 167442nt       (1) -----------------------------------------------------------------------------
    SARS-CoV-2 Reference Genome NC_045512.2          (1) -----------------------------------------------------------------------------
                                                          Section 1233
                                               (94865) 94865     94875      94886      94897      94908      94919      94930      94941
Candidatus Carsonella ruddii strain BT chromosome... (94865) CTTTTTTTAAAAAAAATATAAATATAAATATTTGTGTTAATTGTCATCCTTTTTATACAAAAAAAAAAAATTTATTT
       Candidatus Carsonella 135557 - 167442nt       (1) -----------------------------------------------------------------------------
    SARS-CoV-2 Reference Genome NC_045512.2          (1) -----------------------------------------------------------------------------
                                                          Section 1234
                                               (94942) 94942     94952      94963      94974      94985      94996      95007      95018
Candidatus Carsonella ruddii strain BT chromosome... (94942) GATAATTCTAAAAGAGCTAAAAATTTTAATAATAAATATGCAATTTTTTTTAAAAAATAAATTTATATATGGAGATT
       Candidatus Carsonella 135557 - 167442nt       (1) -----------------------------------------------------------------------------
    SARS-CoV-2 Reference Genome NC_045512.2          (1) -----------------------------------------------------------------------------
                                                          Section 1235
                                               (95019) 95019     95029      95040      95051      95062      95073      95084      95095
Candidatus Carsonella ruddii strain BT chromosome... (95019) CATGTTCAGGTAAAACTTATATATATAAAAAAATAAAACTTAATAAATTTGATACTGATTATTATTTAAAATATAAA
       Candidatus Carsonella 135557 - 167442nt       (1) -----------------------------------------------------------------------------
    SARS-CoV-2 Reference Genome NC_045512.2          (1) -----------------------------------------------------------------------------
                                                          Section 1236
                                               (95096) 95096     95106      95117      95128      95139      95150      95161      95172
Candidatus Carsonella ruddii strain BT chromosome... (95096) AAAATTATTTTAAAATATGAATTTTTTTTTAGAATTATTGAAATAAAAATTTTATTATTAATTTTTAAATTAAAAAA
       Candidatus Carsonella 135557 - 167442nt       (1) -----------------------------------------------------------------------------
    SARS-CoV-2 Reference Genome NC_045512.2          (1) -----------------------------------------------------------------------------
```

Section 1237

	(95173)	95173 95183 95194 95205 95216 95227 95238 95249
Candidatus Carsonella ruddii strain BT chromosome...	(95173)	ATATTTTTTTTTAGGAGGTGGTTTAATTGTTAAAAATTGTTTTTTAATATATAAAAACAATTCTATAATTGATCAAA
Candidatus Carsonella 135557 - 167442nt	(1)	---
SARS-CoV-2 Reference Genome NC_045512.2	(1)	---

Section 1238

	(95250)	95250 95260 95271 95282 95293 95304 95315 95326
Candidatus Carsonella ruddii strain BT chromosome...	(95250)	AAAAAAGATTAATATTAAATAATTTTAATCGTCCAACATTAAAAAATAATATTTTTCTAAAAATAAGATTTTGTATA
Candidatus Carsonella 135557 - 167442nt	(1)	---
SARS-CoV-2 Reference Genome NC_045512.2	(1)	---

Section 1239

	(95327)	95327 95337 95348 95359 95370 95381 95392 95403
Candidatus Carsonella ruddii strain BT chromosome...	(95327)	AGAAAAAAATTTTATATAAAAATTTATAACATTTGTATTACAAAATGTTTAAAATGTAATATTTTATTATTATGATA
Candidatus Carsonella 135557 - 167442nt	(1)	---
SARS-CoV-2 Reference Genome NC_045512.2	(1)	---

Section 1240

	(95404)	95404 95414 95425 95436 95447 95458 95469 95480
Candidatus Carsonella ruddii strain BT chromosome...	(95404)	TTAATAAAAATTAAAAAAAAATATATAACAAATATATATATTAGTAATTCTTATAAAAAAATTTATAATTTTTTAAA
Candidatus Carsonella 135557 - 167442nt	(1)	---
SARS-CoV-2 Reference Genome NC_045512.2	(1)	---

Section 1241

	(95481)	95481 95491 95502 95513 95524 95535 95546 95557
Candidatus Carsonella ruddii strain BT chromosome...	(95481)	CAAAAAAAAATATTTTTTAATTTTTGATTATAATATATATTTTTTATTTTTTAAATTTATTAAATTTAATTTTAAAA
Candidatus Carsonella 135557 - 167442nt	(1)	---
SARS-CoV-2 Reference Genome NC_045512.2	(1)	---

Section 1242

	(95558)	95558 95568 95579 95590 95601 95612 95623 95634
Candidatus Carsonella ruddii strain BT chromosome...	(95558)	ATTATAAATTTATTATTCTTCCTTATGGAGAAAATGTTAAAAATATTAATTCTTTAAAAATAATTTGGAAATATATG
Candidatus Carsonella 135557 - 167442nt	(1)	---
SARS-CoV-2 Reference Genome NC_045512.2	(1)	---

```
                                                                 Section 1243
                                            (95635) 95635     95645      95656      95667      95678      95689      95700   95711
Candidatus Carsonella ruddii strain BT chromosome... (95635) ATTAAAAATCAAATAAATAAAAATTATAATACTTTTATAATAGGAGGTGGAGTAATAGGAGATTTAGTTAGTTATGT
        Candidatus Carsonella 135557 - 167442nt     (1) -----------------------------------------------------------------------------
     SARS-CoV-2 Reference Genome NC_045512.2       (1) -----------------------------------------------------------------------------
                                                                 Section 1244
                                            (95712) 95712     95722      95733      95744      95755      95766      95777   95788
Candidatus Carsonella ruddii strain BT chromosome... (95712) TTGTTCTTCATATTTTAGAGGAATAAATTATTGTTTGTTACCTACATCTTTATTATCTCAAATAGATTCTTCTATAG
        Candidatus Carsonella 135557 - 167442nt     (1) -----------------------------------------------------------------------------
     SARS-CoV-2 Reference Genome NC_045512.2       (1) -----------------------------------------------------------------------------
                                                                 Section 1245
                                            (95789) 95789     95799      95810      95821      95832      95843      95854   95865
Candidatus Carsonella ruddii strain BT chromosome... (95789) GTGGAAAAAATGCAATAAATTTTTTTTCAAAAAATACTTTAGGAATTATTAATTGTCCTATTTTTATATTTATTAAT
        Candidatus Carsonella 135557 - 167442nt     (1) -----------------------------------------------------------------------------
     SARS-CoV-2 Reference Genome NC_045512.2       (1) -----------------------------------------------------------------------------
                                                                 Section 1246
                                            (95866) 95866     95876      95887      95898      95909      95920      95931   95942
Candidatus Carsonella ruddii strain BT chromosome... (95866) TATTTAATTATTTTTTACATGAAAAAAAAAGAATTTAAAGATGGATATGCAGAAATAATTAAATATTCTTTATTAAA
        Candidatus Carsonella 135557 - 167442nt     (1) -----------------------------------------------------------------------------
     SARS-CoV-2 Reference Genome NC_045512.2       (1) -----------------------------------------------------------------------------
                                                                 Section 1247
                                            (95943) 95943     95953      95964      95975      95986      95997      96008   96019
Candidatus Carsonella ruddii strain BT chromosome... (95943) TAATAAAAAATTTTTTTTCTATTTGTTTAAAAAAAAAAAAATAAAAAAAATAATAATAAGATCATGTTATATTAAAA
        Candidatus Carsonella 135557 - 167442nt     (1) -----------------------------------------------------------------------------
     SARS-CoV-2 Reference Genome NC_045512.2       (1) -----------------------------------------------------------------------------
                                                                 Section 1248
                                            (96020) 96020     96030      96041      96052      96063      96074      96085   96096
Candidatus Carsonella ruddii strain BT chromosome... (96020) GTAAAATAATTTCTCAAGATTATAAAGAAATAAATTTTAGATCTGTTTTAAATTTAGGACATACTTATGCTCATTGT
        Candidatus Carsonella 135557 - 167442nt     (1) -----------------------------------------------------------------------------
     SARS-CoV-2 Reference Genome NC_045512.2       (1) -----------------------------------------------------------------------------
```

Section 1249

```
                                                        (96097) 96097     96107      96118      96129      96140      96151      96162      96173
Candidatus Carsonella ruddii strain BT chromosome...    (96097) ATAGAAAATAATAAAATAAAAAAATTTTCACATGGTGAATCTGTTACTATCGGTACTATTTTTGTTGTATTTTTATC
Candidatus Carsonella 135557 - 167442nt                 (1)     -----------------------------------------------------------------------------
SARS-CoV-2 Reference Genome NC_045512.2                 (1)     -----------------------------------------------------------------------------
```

Section 1250

```
                                                        (96174) 96174     96184      96195      96206      96217      96228      96239      96250
Candidatus Carsonella ruddii strain BT chromosome...    (96174) AAGTTTTTTTTATTTTTTTTGTAATAAAATTATTAAAAAAATTTTGAATTTATTTTTAAAATTTAATTTTTTTATTA
Candidatus Carsonella 135557 - 167442nt                 (1)     -----------------------------------------------------------------------------
SARS-CoV-2 Reference Genome NC_045512.2                 (1)     -----------------------------------------------------------------------------
```

Section 1251

```
                                                        (96251) 96251     96261      96272      96283      96294      96305      96316      96327
Candidatus Carsonella ruddii strain BT chromosome...    (96251) AAAATAAGATAAAATTTTCTAAAAAAATGATTAATAAAATTATTTTAGATAAAAAATTTGATAATAAAATAAATTAT
Candidatus Carsonella 135557 - 167442nt                 (1)     -----------------------------------------------------------------------------
SARS-CoV-2 Reference Genome NC_045512.2                 (1)     -----------------------------------------------------------------------------
```

Section 1252

```
                                                        (96328) 96328     96338      96349      96360      96371      96382      96393      96404
Candidatus Carsonella ruddii strain BT chromosome...    (96328) ATTATAATGAGAAAAATTGGTTTTTGTATAAAAAAAAAATTAAAAAAAAATTTTTTAATTATATTTTTAAAAAAATT
Candidatus Carsonella 135557 - 167442nt                 (1)     -----------------------------------------------------------------------------
SARS-CoV-2 Reference Genome NC_045512.2                 (1)     -----------------------------------------------------------------------------
```

Section 1253

```
                                                        (96405) 96405     96415      96426      96437      96448      96459      96470      96481
Candidatus Carsonella ruddii strain BT chromosome...    (96405) TTATGAAATCTAAATTAATAACTAAATGGCCAGAAAAAGCTTCAAATAGATCAATGTTAAGAGCTGTAGGCTACACT
Candidatus Carsonella 135557 - 167442nt                 (1)     -----------------------------------------------------------------------------
SARS-CoV-2 Reference Genome NC_045512.2                 (1)     -----------------------------------------------------------------------------
```

Section 1254

```
                                                        (96482) 96482     96492      96503      96514      96525      96536      96547      96558
Candidatus Carsonella ruddii strain BT chromosome...    (96482) GATTATGATTTTAATAAATTTCAAGTAGGAATAGCATCAACATGGAGTAATATAACTCCATGTAATAATCATATAAA
Candidatus Carsonella 135557 - 167442nt                 (1)     -----------------------------------------------------------------------------
SARS-CoV-2 Reference Genome NC_045512.2                 (1)     -----------------------------------------------------------------------------
```

```
                                                                                                              Section 1255
                                                 (96559) 96559     96569      96580      96591      96602      96613      96624  96635
Candidatus Carsonella ruddii strain BT chromosome... (96559) TGTATTATCTAAAGCAGTAGAATTTGGAGTTAATAAATTTTGTAAATCTACAATATTTAATACAATTACTGTATCTG
     Candidatus Carsonella 135557 - 167442nt    (1) -----------------------------------------------------------------------------
 SARS-CoV-2 Reference Genome NC_045512.2        (1) -----------------------------------------------------------------------------
                                                                                                              Section 1256
                                                 (96636) 96636     96646      96657      96668      96679      96690      96701  96712
Candidatus Carsonella ruddii strain BT chromosome... (96636) ATGGTATTTCAAATGGAAATTTAGGTATGAAATTTTCTTTATTATCAAGAGAAATAATTTCTTCTTCTATTGAAACA
     Candidatus Carsonella 135557 - 167442nt    (1) -----------------------------------------------------------------------------
 SARS-CoV-2 Reference Genome NC_045512.2        (1) -----------------------------------------------------------------------------
                                                                                                              Section 1257
                                                 (96713) 96713     96723      96734      96745      96756      96767      96778  96789
Candidatus Carsonella ruddii strain BT chromosome... (96713) GTTTGTAATGCTCAAAGTTTTGATGGTTTAGTTAGTATCGGAGGATGTGATAAAAATATACCAGGTTGTATATTAGG
     Candidatus Carsonella 135557 - 167442nt    (1) -----------------------------------------------------------------------------
 SARS-CoV-2 Reference Genome NC_045512.2        (1) -----------------------------------------------------------------------------
                                                                                                              Section 1258
                                                 (96790) 96790     96800      96811      96822      96833      96844      96855  96866
Candidatus Carsonella ruddii strain BT chromosome... (96790) TATGTGTAGATTAAATATACCATCTATATTTATTTATGGAGGTACTATTTTGCCAGGAAAAAATAGAACAGATATTA
     Candidatus Carsonella 135557 - 167442nt    (1) -----------------------------------------------------------------------------
 SARS-CoV-2 Reference Genome NC_045512.2        (1) -----------------------------------------------------------------------------
                                                                                                              Section 1259
                                                 (96867) 96867     96877      96888      96899      96910      96921      96932  96943
Candidatus Carsonella ruddii strain BT chromosome... (96867) TATCTGTTTTTGAATCATTAGGTAAATTTTTTTCTAAAAAAATAAATGAAAAAGAATTATTAGAAATAGAAAAAAAT
     Candidatus Carsonella 135557 - 167442nt    (1) -----------------------------------------------------------------------------
 SARS-CoV-2 Reference Genome NC_045512.2        (1) -----------------------------------------------------------------------------
                                                                                                              Section 1260
                                                 (96944) 96944     96954      96965      96976      96987      96998      97009  97020
Candidatus Carsonella ruddii strain BT chromosome... (96944) TCTATCATTGGATCTGGATCATGTAGTGGTATGTATACAGCAAATTCAATGGCAATTGTTGCTGAAGTTCTTGGAAT
     Candidatus Carsonella 135557 - 167442nt    (1) -----------------------------------------------------------------------------
 SARS-CoV-2 Reference Genome NC_045512.2        (1) -----------------------------------------------------------------------------
```

Section 1261

```
                                                     (97021) 97021     97031      97042      97053      97064      97075      97086      97097
Candidatus Carsonella ruddii strain BT chromosome... (97021) TAGTTTACCAAATTCTTCTATTCAAAATGCACAATCAATTAATAAAATAATAAATTGTTTAAATTCTGGTAAATTAA
           Candidatus Carsonella 135557 - 167442nt       (1) -----------------------------------------------------------------------------
      SARS-CoV-2 Reference Genome NC_045512.2            (1) -----------------------------------------------------------------------------
```

Section 1262

```
                                                     (97098) 97098     97108      97119      97130      97141      97152      97163      97174
Candidatus Carsonella ruddii strain BT chromosome... (97098) TTTTTAATTTATTAAAAAATAATATTAAACCTAAAAATATAATAAATATAAATTCTATAAAAAATGCAATTAAAGTA
           Candidatus Carsonella 135557 - 167442nt       (1) -----------------------------------------------------------------------------
      SARS-CoV-2 Reference Genome NC_045512.2            (1) -----------------------------------------------------------------------------
```

Section 1263

```
                                                     (97175) 97175     97185      97196      97207      97218      97229      97240      97251
Candidatus Carsonella ruddii strain BT chromosome... (97175) ATATCTTTTTTAGGTGGTTCTACCAATTGTATTCTTCATATATTAAGTTTTATAGATTGTTTAAAAATAAATTTTTC
           Candidatus Carsonella 135557 - 167442nt       (1) -----------------------------------------------------------------------------
      SARS-CoV-2 Reference Genome NC_045512.2            (1) -----------------------------------------------------------------------------
```

Section 1264

```
                                                     (97252) 97252     97262      97273      97284      97295      97306      97317      97328
Candidatus Carsonella ruddii strain BT chromosome... (97252) TTTACAACAAATAAAAGAATTTTCAAATAATTTACCAAATATTTCTGATTTAAAACCAAGTGGAAAATTTTTTATTT
           Candidatus Carsonella 135557 - 167442nt       (1) -----------------------------------------------------------------------------
      SARS-CoV-2 Reference Genome NC_045512.2            (1) -----------------------------------------------------------------------------
```

Section 1265

```
                                                     (97329) 97329     97339      97350      97361      97372      97383      97394      97405
Candidatus Carsonella ruddii strain BT chromosome... (97329) CTGATTTAATAAATTCTGGAGGAATACAAAAATTTTTAAAATTTTTAATAGAAGAAAATATTATTGATGGTAATACT
           Candidatus Carsonella 135557 - 167442nt       (1) -----------------------------------------------------------------------------
      SARS-CoV-2 Reference Genome NC_045512.2            (1) -----------------------------------------------------------------------------
```

Section 1266

```
                                                     (97406) 97406     97416      97427      97438      97449      97460      97471      97482
Candidatus Carsonella ruddii strain BT chromosome... (97406) TTAACCATTACTGGTTATTCATTAAAAGAAAATTTAAAATTTATAAAAACAAATTATATTAATAAATTATTAAAATG
           Candidatus Carsonella 135557 - 167442nt       (1) -----------------------------------------------------------------------------
      SARS-CoV-2 Reference Genome NC_045512.2            (1) -----------------------------------------------------------------------------
```

Section 1267

```
                                                      (97483) 97483     97493      97504      97515      97526      97537      97548      97559
Candidatus Carsonella ruddii strain BT chromosome...  (97483) TTTAAATTTTCCTATAAAAAAAACAAATCAAATAAAAATTTTATTTGGAAATTTAGCACAAAAGGGTTGTGTTGCAA
         Candidatus Carsonella 135557 - 167442nt          (1) -----------------------------------------------------------------------------
        SARS-CoV-2 Reference Genome NC_045512.2           (1) -----------------------------------------------------------------------------
```

Section 1268

```
                                                      (97560) 97560     97570      97581      97592      97603      97614      97625      97636
Candidatus Carsonella ruddii strain BT chromosome...  (97560) AAATTTCAGGTAAAGAAGGTGAAACTTTTTTAGGAAAAACAAAAGTTTTTAATTCAGAAGAAGAATCAGTAGAATTT
         Candidatus Carsonella 135557 - 167442nt          (1) -----------------------------------------------------------------------------
        SARS-CoV-2 Reference Genome NC_045512.2           (1) -----------------------------------------------------------------------------
```

Section 1269

```
                                                      (97637) 97637     97647      97658      97669      97680      97691      97702      97713
Candidatus Carsonella ruddii strain BT chromosome...  (97637) ATTTACAAAAACAAAATTTATTCAAAAACAATAATAATTATAAGATTTGAAGGAGTTAAAGGTGGTCCTGGAATGAG
         Candidatus Carsonella 135557 - 167442nt          (1) -----------------------------------------------------------------------------
        SARS-CoV-2 Reference Genome NC_045512.2           (1) -----------------------------------------------------------------------------
```

Section 1270

```
                                                      (97714) 97714     97724      97735      97746      97757      97768      97779      97790
Candidatus Carsonella ruddii strain BT chromosome...  (97714) AGAAATGCTTACACCTACTTCTGCTTTAATAGGTGTTGGAGTAAAAAAAAATATTGCTTTAATTACAGATGGGAGAT
         Candidatus Carsonella 135557 - 167442nt          (1) -----------------------------------------------------------------------------
        SARS-CoV-2 Reference Genome NC_045512.2           (1) -----------------------------------------------------------------------------
```

Section 1271

```
                                                      (97791) 97791     97801      97812      97823      97834      97845      97856      97867
Candidatus Carsonella ruddii strain BT chromosome...  (97791) TTTCTGGTGGAAGTCATGGATTTGTTGTTGGACACGTATCTCCTGAAGCGTATGAAAAAGGAGAAATATCACTTGTT
         Candidatus Carsonella 135557 - 167442nt          (1) -----------------------------------------------------------------------------
        SARS-CoV-2 Reference Genome NC_045512.2           (1) -----------------------------------------------------------------------------
```

Section 1272

```
                                                      (97868) 97868     97878      97889      97900      97911      97922      97933      97944
Candidatus Carsonella ruddii strain BT chromosome...  (97868) AAAGATAATGAAATTATTATTATTGATTCAATTAATAATAATTTAATATTTTTTATAAATAAAAAAAAATTGTTAAA
         Candidatus Carsonella 135557 - 167442nt          (1) -----------------------------------------------------------------------------
        SARS-CoV-2 Reference Genome NC_045512.2           (1) -----------------------------------------------------------------------------
```

Section 1273

	(97945)	97945 97955 97966 97977 97988 97999 98010 98021
Candidatus Carsonella ruddii strain BT chromosome...	(97945)	AAGAAAACAAAATTTTATAAATTATAGTAAAATATCTTTTGGTATATTAAATTTATATAGAAAAATGTCTTTAGATT
Candidatus Carsonella 135557 - 167442nt	(1)	---
SARS-CoV-2 Reference Genome NC_045512.2	(1)	---

Section 1274

	(98022)	98022 98032 98043 98054 98065 98076 98087 98098
Candidatus Carsonella ruddii strain BT chromosome...	(98022)	CTTCTAAAGGTTCAGGTTTTAATTATTAAAAAAACTAATAAAATTAAATTTAGTTATATTTTTTTTTTTGAAAATGT
Candidatus Carsonella 135557 - 167442nt	(1)	---
SARS-CoV-2 Reference Genome NC_045512.2	(1)	---

Section 1275

	(98099)	98099 98109 98120 98131 98142 98153 98164 98175
Candidatus Carsonella ruddii strain BT chromosome...	(98099)	TAAAATTTTTAAGATTAAAAATTAATTTAAAAAAAAATAAAGAAGTTTAATATAGAAAAATTTTGATATAAAAATATT
Candidatus Carsonella 135557 - 167442nt	(1)	---
SARS-CoV-2 Reference Genome NC_045512.2	(1)	---

Section 1276

	(98176)	98176 98186 98197 98208 98219 98230 98241 98252
Candidatus Carsonella ruddii strain BT chromosome...	(98176)	ATTAAAAAATTAATTAAAAATTAATTTATAACAAAAAAAAATAAATAAAAATGAATAAAAAAATGAATAAAAAAATTT
Candidatus Carsonella 135557 - 167442nt	(1)	---
SARS-CoV-2 Reference Genome NC_045512.2	(1)	---

Section 1277

	(98253)	98253 98263 98274 98285 98296 98307 98318 98329
Candidatus Carsonella ruddii strain BT chromosome...	(98253)	TAATGTTAAAAAAAGAAATTACAATTTCAATATTATTTTTTAAAAATTTAATTTTAGAAATAAATAATAATGTTGAT
Candidatus Carsonella 135557 - 167442nt	(1)	---
SARS-CoV-2 Reference Genome NC_045512.2	(1)	---

Section 1278

	(98330)	98330 98340 98351 98362 98373 98384 98395 98406
Candidatus Carsonella ruddii strain BT chromosome...	(98330)	TTATTAATAAATAATATCATAGATAATTTAAAAATATTTTTTTCATTGTCTTTTTTTAAAAATTTATTTTTAATTAT
Candidatus Carsonella 135557 - 167442nt	(1)	---
SARS-CoV-2 Reference Genome NC_045512.2	(1)	---

```
                                                                                   Section 1279
                                                 (98407) 98407     98417      98428      98439      98450      98461      98472   98483
Candidatus Carsonella ruddii strain BT chromosome... (98407) AAATGAAAATAAAAATGAAATTAAAGAATATGTATTTTTTTCTATAATAATGTTATTTATGATTATAATAAAATCAC
            Candidatus Carsonella 135557 - 167442nt (1) -----------------------------------------------------------------------------
         SARS-CoV-2 Reference Genome NC_045512.2 (1) -----------------------------------------------------------------------------
                                                                                   Section 1280
                                                 (98484) 98484     98494      98505      98516      98527      98538      98549   98560
Candidatus Carsonella ruddii strain BT chromosome... (98484) TTTTATTTGTTAAAAATTATTATAAATTATTTAAAAATAAAAAAAAAAACATAATTAAAAATATTAAAATAACATAT
            Candidatus Carsonella 135557 - 167442nt (1) -----------------------------------------------------------------------------
         SARS-CoV-2 Reference Genome NC_045512.2 (1) -----------------------------------------------------------------------------
                                                                                   Section 1281
                                                 (98561) 98561     98571      98582      98593      98604      98615      98626   98637
Candidatus Carsonella ruddii strain BT chromosome... (98561) TTATTTATAAAAAATTTTACTTTTATTTTGATAGAAAATTTATATGTTTTATTTTCTTTTTTTATAAATTATTTTTT
            Candidatus Carsonella 135557 - 167442nt (1) -----------------------------------------------------------------------------
         SARS-CoV-2 Reference Genome NC_045512.2 (1) -----------------------------------------------------------------------------
                                                                                   Section 1282
                                                 (98638) 98638     98648      98659      98670      98681      98692      98703   98714
Candidatus Carsonella ruddii strain BT chromosome... (98638) ATTTTTTTTAAATAAATTTATTATTTTTATTTATTTTTTATTTATAAAAACATTTTTTTGTATAAAATTAATAAAAA
            Candidatus Carsonella 135557 - 167442nt (1) -----------------------------------------------------------------------------
         SARS-CoV-2 Reference Genome NC_045512.2 (1) -----------------------------------------------------------------------------
                                                                                   Section 1283
                                                 (98715) 98715     98725      98736      98747      98758      98769      98780   98791
Candidatus Carsonella ruddii strain BT chromosome... (98715) TTTCAATTTTAAATATAATTGAAAATTTTTTTATTGTTTTATTAGAAATTTTTAATAAAAATTTAATTAATTTTAAA
            Candidatus Carsonella 135557 - 167442nt (1) -----------------------------------------------------------------------------
         SARS-CoV-2 Reference Genome NC_045512.2 (1) -----------------------------------------------------------------------------
                                                                                   Section 1284
                                                 (98792) 98792     98802      98813      98824      98835      98846      98857   98868
Candidatus Carsonella ruddii strain BT chromosome... (98792) AAAAAATTATTGTATTTTGAAATAAAATTTTGTTGTTTTTTATCAAATTTAATTTAAAAATGAATTTTTATAATTTT
            Candidatus Carsonella 135557 - 167442nt (1) -----------------------------------------------------------------------------
         SARS-CoV-2 Reference Genome NC_045512.2 (1) -----------------------------------------------------------------------------
```

Section 1285

	(98869)	98869 98879 98890 98901 98912 98923 98934 98945
Candidatus Carsonella ruddii strain BT chromosome...	(98869)	ATAAATTTAATTAAGTTAGAATCTATAAAACAAGAAAAAACATTAAATTTAATTGCTTCTGAAAATTACTCAAGTTT
Candidatus Carsonella 135557 - 167442nt	(1)	---
SARS-CoV-2 Reference Genome NC_045512.2	(1)	---

Section 1286

	(98946)	98946 98956 98967 98978 98989 99000 99011 99022
Candidatus Carsonella ruddii strain BT chromosome...	(98946)	ATCAACAATAATTTATTCATCTTCTTGTTATATAAATAAATATACAGAAGGTTATCCCAAAAATAGATATTATTCTG
Candidatus Carsonella 135557 - 167442nt	(1)	---
SARS-CoV-2 Reference Genome NC_045512.2	(1)	---

Section 1287

	(99023)	99023 99033 99044 99055 99066 99077 99088 99099
Candidatus Carsonella ruddii strain BT chromosome...	(99023)	GTTGTAAAATATTTGATATTTTTGAAAACAAAAGTATAATTGAATCTCAATTATTATTTTCTTCATTATTTTCAAAT
Candidatus Carsonella 135557 - 167442nt	(1)	---
SARS-CoV-2 Reference Genome NC_045512.2	(1)	---

Section 1288

	(99100)	99100 99110 99121 99132 99143 99154 99165 99176
Candidatus Carsonella ruddii strain BT chromosome...	(99100)	GTACAACCACATTCTGGAGCACAAGCAAATTTTGCTGTTTTCCAAACTTTATTAAACAAAAATGATAAAATTCTTTC
Candidatus Carsonella 135557 - 167442nt	(1)	---
SARS-CoV-2 Reference Genome NC_045512.2	(1)	---

Section 1289

	(99177)	99177 99187 99198 99209 99220 99231 99242 99253
Candidatus Carsonella ruddii strain BT chromosome...	(99177)	TTTAGATTTAAAATCAGGAGGACATTTAACACATGGTTTTATTAAGAATTTTTCTGCTAAAATCTTTAATATTATAA
Candidatus Carsonella 135557 - 167442nt	(1)	---
SARS-CoV-2 Reference Genome NC_045512.2	(1)	---

Section 1290

	(99254)	99254 99264 99275 99286 99297 99308 99319 99330
Candidatus Carsonella ruddii strain BT chromosome...	(99254)	ATTATTGTTTAGATAAAAATAATTTAATTGATAATAACTATATTAATTTAATAATTAAAAAAGAAAAACCAAAATTA
Candidatus Carsonella 135557 - 167442nt	(1)	---
SARS-CoV-2 Reference Genome NC_045512.2	(1)	---

Section 1291

```
                                                    (99331) 99331     99341      99352      99363      99374      99385      99396     99407
Candidatus Carsonella ruddii strain BT chromosome... (99331) TTAATTTTAGGATATTCTTCTTATCAAGAGTTAATAGATTGGAAAAAATTAAATATTATTTCTAAAAAAAAATAATTG
          Candidatus Carsonella 135557 - 167442nt   (1) -----------------------------------------------------------------------------
      SARS-CoV-2 Reference Genome NC_045512.2       (1) -----------------------------------------------------------------------------
```

Section 1292

```
                                                    (99408) 99408     99418      99429      99440      99451      99462      99473     99484
Candidatus Carsonella ruddii strain BT chromosome... (99408) TTATATATTATCTGATATTAGTCATATTTCTGGTTTTATATGTTCTGGTTTAATAAAAAATCCTATTAATTATTCAG
          Candidatus Carsonella 135557 - 167442nt   (1) -----------------------------------------------------------------------------
      SARS-CoV-2 Reference Genome NC_045512.2       (1) -----------------------------------------------------------------------------
```

Section 1293

```
                                                    (99485) 99485     99495      99506      99517      99528      99539      99550     99561
Candidatus Carsonella ruddii strain BT chromosome... (99485) ATGTTGTTTCTTCAACAACTCATAAAACTTTAAGAGGTATTAAAGGAGGAATACTTTTAAGTAACAATAAAAATATT
          Candidatus Carsonella 135557 - 167442nt   (1) -----------------------------------------------------------------------------
      SARS-CoV-2 Reference Genome NC_045512.2       (1) -----------------------------------------------------------------------------
```

Section 1294

```
                                                    (99562) 99562     99572      99583      99594      99605      99616      99627     99638
Candidatus Carsonella ruddii strain BT chromosome... (99562) TTTAATAAAATTAATTCATCTGTTTTTCCAGGTCAACAAGGTGGTTCTATAAGTAATAATATTATTAGTAAATATGT
          Candidatus Carsonella 135557 - 167442nt   (1) -----------------------------------------------------------------------------
      SARS-CoV-2 Reference Genome NC_045512.2       (1) -----------------------------------------------------------------------------
```

Section 1295

```
                                                    (99639) 99639     99649      99660      99671      99682      99693      99704     99715
Candidatus Carsonella ruddii strain BT chromosome... (99639) TTCTTTTAAAGAAGCAAATAATTTTAATTTTATAAAATATACAAAACAAATTTTAATAAATTCTAAAATAATGTTAA
          Candidatus Carsonella 135557 - 167442nt   (1) -----------------------------------------------------------------------------
      SARS-CoV-2 Reference Genome NC_045512.2       (1) -----------------------------------------------------------------------------
```

Section 1296

```
                                                    (99716) 99716     99726      99737      99748      99759      99770      99781     99792
Candidatus Carsonella ruddii strain BT chromosome... (99716) AAACTTTTTTTTTATAGAGGTTATAAATCTTCTACAAAAGAAACAATAAATCATATGTTTTTAATAAATTTATTAAAT
          Candidatus Carsonella 135557 - 167442nt   (1) -----------------------------------------------------------------------------
      SARS-CoV-2 Reference Genome NC_045512.2       (1) -----------------------------------------------------------------------------
```

Section 1297

		99793 99803 99814 99825 99836 99847 99858 99869
	(99793)	
Candidatus Carsonella ruddii strain BT chromosome...	(99793)	AAATGTACTTTATTAATTGAAAAAATATTAGAAAAAAATGGAATCCTTTTAAACAGGAATTTTTTTCCTAATGATAT
Candidatus Carsonella 135557 - 167442nt	(1)	---
SARS-CoV-2 Reference Genome NC_045512.2	(1)	---

Section 1298

		99870 99880 99891 99902 99913 99924 99935 99946
	(99870)	
Candidatus Carsonella ruddii strain BT chromosome...	(99870)	TAAATTTTCTAAAAATCCAAGTGCGATTAGAATCGGATTATCGTCTGTTACAACAAGAAAATTAAAAAATTTAGAAG
Candidatus Carsonella 135557 - 167442nt	(1)	---
SARS-CoV-2 Reference Genome NC_045512.2	(1)	---

Section 1299

		99947 99957 99968 99979 99990 100001 100012 100023
	(99947)	
Candidatus Carsonella ruddii strain BT chromosome...	(99947)	TTGAATTAATTTCTAACTATATTTGTGATATAATTGAAAATTATAAAAAAAATTATAAAAATCAAATAAAAATATTA
Candidatus Carsonella 135557 - 167442nt	(1)	---
SARS-CoV-2 Reference Genome NC_045512.2	(1)	---

Section 1300

		100024 100034 100045 100056 100067 100078 100089 100100
	(100024)	
Candidatus Carsonella ruddii strain BT chromosome...	(100024)	TGTTTAAAATTTCCAATTTATAAATAAATTTTTTATTAAAAAAAATAATAAAATTTATTTATAAATATTATATTTTT
Candidatus Carsonella 135557 - 167442nt	(1)	---
SARS-CoV-2 Reference Genome NC_045512.2	(1)	---

Section 1301

		100101 100111 100122 100133 100144 100155 100166 100177
	(100101)	
Candidatus Carsonella ruddii strain BT chromosome...	(100101)	TATTATAAGTATATTAATATTTTTTTATTAAAAAAATAATAAAATTTATTTATAAATATTATATTTTTTATTATAAG
Candidatus Carsonella 135557 - 167442nt	(1)	---
SARS-CoV-2 Reference Genome NC_045512.2	(1)	---

Section 1302

		100178 100188 100199 100210 100221 100232 100243 100254
	(100178)	
Candidatus Carsonella ruddii strain BT chromosome...	(100178)	TATATTAATATTTTTTTATTAAAAAAATAATAAAATTTATTTATAAATATTATTTCTATTAATAAATTTTGTATTTT
Candidatus Carsonella 135557 - 167442nt	(1)	---
SARS-CoV-2 Reference Genome NC_045512.2	(1)	---

Section 1303

(100255) 100255 100265 100276 100287 100298 100309 100320 100331

Candidatus Carsonella ruddii strain BT chromosome... (100255) TAAACAAATATAATTTTAAATCTGAATTATTAATGATGTTATTTTTATTATAAAAAAAAAAATAAATATCATTTATA

Candidatus Carsonella 135557 - 167442nt (1) ---

SARS-CoV-2 Reference Genome NC_045512.2 (1) ---

Section 1304

(100332) 100332 100342 100353 100364 100375 100386 100397 100408

Candidatus Carsonella ruddii strain BT chromosome... (100332) GAATCTGATATAAAAATTTTTTTTTCATTTTTAATTTTTAATGAGTTTAAAATTTTATTATTTTTAATATTTATTTT

Candidatus Carsonella 135557 - 167442nt (1) ---

SARS-CoV-2 Reference Genome NC_045512.2 (1) ---

Section 1305

(100409) 100409 100419 100430 100441 100452 100463 100474 100485

Candidatus Carsonella ruddii strain BT chromosome... (100409) TTTTATATTTGAACAAAATAAATAATTTTTATATATTTTAAATTTATTATATAATATATAATAATTAGTTGAAGTTG

Candidatus Carsonella 135557 - 167442nt (1) ---

SARS-CoV-2 Reference Genome NC_045512.2 (1) ---

Section 1306

(100486) 100486 100496 100507 100518 100529 100540 100551 100562

Candidatus Carsonella ruddii strain BT chromosome... (100486) AAACTATATTTTTTTTATTATTTCATAAAAATTATAATTGTAAAAAATTTTTTTTAAATAATTAAAAATTATTTTT

Candidatus Carsonella 135557 - 167442nt (1) ---

SARS-CoV-2 Reference Genome NC_045512.2 (1) ---

Section 1307

(100563) 100563 100573 100584 100595 100606 100617 100628 100639

Candidatus Carsonella ruddii strain BT chromosome... (100563) ATTTTTTTAATTTTTAAAAAAAAAAAATTAAATAATTTTAAATAACAAAAATTATTTATTATAATTTTTTCATATAA

Candidatus Carsonella 135557 - 167442nt (1) ---

SARS-CoV-2 Reference Genome NC_045512.2 (1) ---

Section 1308

(100640) 100640 100650 100661 100672 100683 100694 100705 100716

Candidatus Carsonella ruddii strain BT chromosome... (100640) ATAATAAAAAAAATTATTTATTTTTTTTTTTTTAAAATTAAAAATATTTAATTTATATAATAAATAACCCCATAAAT

Candidatus Carsonella 135557 - 167442nt (1) ---

SARS-CoV-2 Reference Genome NC_045512.2 (1) ---

```
                                                                                            Section 1309
                                              (100717) 100717    100727    100738     100749     100760     100771     100782  100793
Candidatus Carsonella ruddii strain BT chromosome... (100717) AATCACTATCACAATTTACGATTGTATTATCATAATCAAAAAATAATTTTATCATGTTTTTAAAATTTATTAATAAT
         Candidatus Carsonella 135557 - 167442nt (1) -----------------------------------------------------------------------------
        SARS-CoV-2 Reference Genome NC_045512.2 (1) -----------------------------------------------------------------------------
                                                                                            Section 1310
                                              (100794) 100794    100804    100815     100826     100837     100848     100859  100870
Candidatus Carsonella ruddii strain BT chromosome... (100794) ATTAATTATTTTTTTTTAAATATTTATAAAAAAAAAATAAAAAAAAAAATTTTAAATAAATTTATTTTTACAAAAAT
         Candidatus Carsonella 135557 - 167442nt (1) -----------------------------------------------------------------------------
        SARS-CoV-2 Reference Genome NC_045512.2 (1) -----------------------------------------------------------------------------
                                                                                            Section 1311
                                              (100871) 100871    100881    100892     100903     100914     100925     100936  100947
Candidatus Carsonella ruddii strain BT chromosome... (100871) TAAAAATTTATACAAATTAATAAACAAATTATGAATTTATCTATAATTGGATTACAATGGGGTGATGAAGGAAAGGG
         Candidatus Carsonella 135557 - 167442nt (1) -----------------------------------------------------------------------------
        SARS-CoV-2 Reference Genome NC_045512.2 (1) -----------------------------------------------------------------------------
                                                                                            Section 1312
                                              (100948) 100948    100958    100969     100980     100991     101002     101013  101024
Candidatus Carsonella ruddii strain BT chromosome... (100948) TAAAATTATAGATTTATTGTCTAATTATTCAGATTTAAATATTAGATATAATGGAGGACACAATGCTGGACATATTA
         Candidatus Carsonella 135557 - 167442nt (1) -----------------------------------------------------------------------------
        SARS-CoV-2 Reference Genome NC_045512.2 (1) -----------------------------------------------------------------------------
                                                                                            Section 1313
                                              (101025) 101025    101035    101046     101057     101068     101079     101090  101101
Candidatus Carsonella ruddii strain BT chromosome... (101025) TATATGTTAAAAACAAAAAAAAAAGTATTAGAATATTGCCATCTGGCATATTTAAAAAAAATTGTTTATGTTTATTA
         Candidatus Carsonella 135557 - 167442nt (1) -----------------------------------------------------------------------------
        SARS-CoV-2 Reference Genome NC_045512.2 (1) -----------------------------------------------------------------------------
                                                                                            Section 1314
                                              (101102) 101102    101112    101123     101134     101145     101156     101167  101178
Candidatus Carsonella ruddii strain BT chromosome... (101102) TCTAATAATGCTTTGTTATCATTATTTAATTTATTAAAAGAATTAAAAAATATAAAAAATAATTTAATAATTTCAGA
         Candidatus Carsonella 135557 - 167442nt (1) -----------------------------------------------------------------------------
        SARS-CoV-2 Reference Genome NC_045512.2 (1) -----------------------------------------------------------------------------
```

```
                                                                                  Section 1315
                                                  (101179) 101179    101189     101200     101211     101222     101233     101244  101255
Candidatus Carsonella ruddii strain BT chromosome... (101179) AAATGTATATATTATTAATAAACTTGATGAATTTATTGATTATTTAATTGATAAAATTATTAATATAGGAACAACTT
            Candidatus Carsonella 135557 - 167442nt (1) -----------------------------------------------------------------------------
         SARS-CoV-2 Reference Genome NC_045512.2 (1) -----------------------------------------------------------------------------
```

```
                                                                                  Section 1316
                                                  (101256) 101256    101266     101277     101288     101299     101310     101321  101332
Candidatus Carsonella ruddii strain BT chromosome... (101256) ATAGTGGTATAAGTATTTGTTATTTAAAAAAATATATAAAATGTTGTTTAAATATAAGAAATGTTTATAAAATAAAT
            Candidatus Carsonella 135557 - 167442nt (1) -----------------------------------------------------------------------------
         SARS-CoV-2 Reference Genome NC_045512.2 (1) -----------------------------------------------------------------------------
```

```
                                                                                  Section 1317
                                                  (101333) 101333    101343     101354     101365     101376     101387     101398  101409
Candidatus Carsonella ruddii strain BT chromosome... (101333) TATTTTAATTTAATAAAAAAAAATATTTTTTTTTATAATTTATTTTTTATTTTTTTTAAAATAAAATTAAAAATAAA
            Candidatus Carsonella 135557 - 167442nt (1) -----------------------------------------------------------------------------
         SARS-CoV-2 Reference Genome NC_045512.2 (1) -----------------------------------------------------------------------------
```

```
                                                                                  Section 1318
                                                  (101410) 101410    101420     101431     101442     101453     101464     101475  101486
Candidatus Carsonella ruddii strain BT chromosome... (101410) TTTTTATTTTTTTTCAAAAAATATTAAAAAAAATGATTTTTTTTTTTAAAGAAAAAATAATTAAAAATGATATATTTA
            Candidatus Carsonella 135557 - 167442nt (1) -----------------------------------------------------------------------------
         SARS-CoV-2 Reference Genome NC_045512.2 (1) -----------------------------------------------------------------------------
```

```
                                                                                  Section 1319
                                                  (101487) 101487    101497     101508     101519     101530     101541     101552  101563
Candidatus Carsonella ruddii strain BT chromosome... (101487) TAAATAATAAAAATAATAAAATTTTTGAAAGTTCACAAGGAACTTTATTAGATTTTGATTTAGGTACTTATCCTTTT
            Candidatus Carsonella 135557 - 167442nt (1) -----------------------------------------------------------------------------
         SARS-CoV-2 Reference Genome NC_045512.2 (1) -----------------------------------------------------------------------------
```

```
                                                                                  Section 1320
                                                  (101564) 101564    101574     101585     101596     101607     101618     101629  101640
Candidatus Carsonella ruddii strain BT chromosome... (101564) GTTACTTGTTCAAATACAGTTAATAAATCAATTTTTATAAATTCAAATTTTTTTTTAAAAAAAATAAAATTTATTGG
            Candidatus Carsonella 135557 - 167442nt (1) -----------------------------------------------------------------------------
         SARS-CoV-2 Reference Genome NC_045512.2 (1) -----------------------------------------------------------------------------
```

Section 1321

	(101641)	101641 101651 101662 101673 101684 101695 101706 101717
Candidatus Carsonella ruddii strain BT chromosome...	(101641)	TATAATAAAATCATATATTACTAGAGTTGGAAACGGTTATTTAATATCTAAAATTAAAAAATTCAAAAATAATTTAT
Candidatus Carsonella 135557 - 167442nt	(1)	---
SARS-CoV-2 Reference Genome NC_045512.2	(1)	---

Section 1322

	(101718)	101718 101728 101739 101750 101761 101772 101783 101794
Candidatus Carsonella ruddii strain BT chromosome...	(101718)	TTTCTATATTAACTAAAGAATTTGGAACAAATTCATTGAGATTAAGAAAAACAGGTTGGTTGGATTTATTTTCTTTA
Candidatus Carsonella 135557 - 167442nt	(1)	---
SARS-CoV-2 Reference Genome NC_045512.2	(1)	---

Section 1323

	(101795)	101795 101805 101816 101827 101838 101849 101860 101871
Candidatus Carsonella ruddii strain BT chromosome...	(101795)	ATTAATATGATAAAAATAAATAATATTTCAAATATTATTTTAACAAAAATGGATTCATTTATGTTTATTAAATATAT
Candidatus Carsonella 135557 - 167442nt	(1)	---
SARS-CoV-2 Reference Genome NC_045512.2	(1)	---

Section 1324

	(101872)	101872 101882 101893 101904 101915 101926 101937 101948
Candidatus Carsonella ruddii strain BT chromosome...	(101872)	AAAAATAGTAATAAATTATAAATCAAAAAATATTTTTAATAATTATAAAAATAATTTATTTTGTAAAGAAAAATATT
Candidatus Carsonella 135557 - 167442nt	(1)	---
SARS-CoV-2 Reference Genome NC_045512.2	(1)	---

Section 1325

	(101949)	101949 101959 101970 101981 101992 102003 102014 102025
Candidatus Carsonella ruddii strain BT chromosome...	(101949)	TATTTTTTTTTTCTTGGAAAAAAGTACATTTAAAATATTTTTTAAATGAAAAAAATTTTCATTTTTATATTAGATTT
Candidatus Carsonella 135557 - 167442nt	(1)	---
SARS-CoV-2 Reference Genome NC_045512.2	(1)	---

Section 1326

	(102026)	102026 102036 102047 102058 102069 102080 102091 102102
Candidatus Carsonella ruddii strain BT chromosome...	(102026)	ATTGAAAAAATTATAAATACTCCTATTTCAATTATTTCTATAGGTAAAAATAATAAAGATATTATTATTTTATGAAT
Candidatus Carsonella 135557 - 167442nt	(1)	---
SARS-CoV-2 Reference Genome NC_045512.2	(1)	---

Section 1327

(102103) 102103 102113 102124 102135 102146 102157 102168 102179
Candidatus Carsonella ruddii strain BT chromosome... (102103) AAATTTAATTATTATAGTATAATAAATTCATATCAAATAGAAGAAAAATTTTGTGAAAATTTAATAAAAGAAATTTC
Candidatus Carsonella 135557 - 167442nt (1) ---
SARS-CoV-2 Reference Genome NC_045512.2 (1) ---

Section 1328

(102180) 102180 102190 102201 102212 102223 102234 102245 102256
Candidatus Carsonella ruddii strain BT chromosome... (102180) ACGTGTAAATAATGAACCAATTTGGATGCTTTCATTTAGATTAAATTCTTTTAATTTTTTGAAAAAAATTAAAATTC
Candidatus Carsonella 135557 - 167442nt (1) ---
SARS-CoV-2 Reference Genome NC_045512.2 (1) ---

Section 1329

(102257) 102257 102267 102278 102289 102300 102311 102322 102333
Candidatus Carsonella ruddii strain BT chromosome... (102257) CAAATTGGGGAAATTTTTTTTTAAAAAAAATTTTACTTTATAATTCTTGTTATTATAGTTTTCATGATAATAAAAAT
Candidatus Carsonella 135557 - 167442nt (1) ---
SARS-CoV-2 Reference Genome NC_045512.2 (1) ---

Section 1330

(102334) 102334 102344 102355 102366 102377 102388 102399 102410
Candidatus Carsonella ruddii strain BT chromosome... (102334) AATTATTCTATTAAAAAAACTTTTCAAAAAATTATTAAAAATAAAAAACCAATAGATTTTATTTATAATTCTATATC
Candidatus Carsonella 135557 - 167442nt (1) ---
SARS-CoV-2 Reference Genome NC_045512.2 (1) ---

Section 1331

(102411) 102411 102421 102432 102443 102454 102465 102476 102487
Candidatus Carsonella ruddii strain BT chromosome... (102411) AATTAAAACTACTATGAAAAAAAAATTATTTAATAAAGGAATTATTTTTTGTTCATTTAATGAAGCAATAAAAAATT
Candidatus Carsonella 135557 - 167442nt (1) ---
SARS-CoV-2 Reference Genome NC_045512.2 (1) ---

Section 1332

(102488) 102488 102498 102509 102520 102531 102542 102553 102564
Candidatus Carsonella ruddii strain BT chromosome... (102488) TTAAACATATTATTTTAAAATATTTAGGAAGTGTTGTAAAACCATATGATAATTTTTTTTCTTGTTTAAATTCTTCA
Candidatus Carsonella 135557 - 167442nt (1) ---
SARS-CoV-2 Reference Genome NC_045512.2 (1) ---

Section 1333

	(102565)	102565 102575 102586 102597 102608 102619 102630 102641
Candidatus Carsonella ruddii strain BT chromosome...	(102565)	ATTTTTAGTGATGGAACTTTTGTTTATATTCCTAAAAATACTATATGTCCTATAGAATTATCATCTTATTTTAGAAT
Candidatus Carsonella 135557 - 167442nt	(1)	---
SARS-CoV-2 Reference Genome NC_045512.2	(1)	---

Section 1334

	(102642)	102642 102652 102663 102674 102685 102696 102707 102718
Candidatus Carsonella ruddii strain BT chromosome...	(102642)	AAATGATGAGGTTGGGCAATTTGAAAGAACATTAATAATATGTGAAGAGAATTCATCAGTTTCTTATTTAGAAGGTT
Candidatus Carsonella 135557 - 167442nt	(1)	---
SARS-CoV-2 Reference Genome NC_045512.2	(1)	---

Section 1335

	(102719)	102719 102729 102740 102751 102762 102773 102784 102795
Candidatus Carsonella ruddii strain BT chromosome...	(102719)	GTACAGCTTCTATTAAAAAAAAAAAATCAATTACATACAGCTATTGTTGAAATTATTATTAAAAAAAACTCAACTGTT
Candidatus Carsonella 135557 - 167442nt	(1)	---
SARS-CoV-2 Reference Genome NC_045512.2	(1)	---

Section 1336

	(102796)	102796 102806 102817 102828 102839 102850 102861 102872
Candidatus Carsonella ruddii strain BT chromosome...	(102796)	AAATATTCAACTGTTCAAAATTGGTATTCAGGAAATAAATTTAATTTAAATGGAATTTATAATTTTGTAACAAAAAG
Candidatus Carsonella 135557 - 167442nt	(1)	---
SARS-CoV-2 Reference Genome NC_045512.2	(1)	---

Section 1337

	(102873)	102873 102883 102894 102905 102916 102927 102938 102949
Candidatus Carsonella ruddii strain BT chromosome...	(102873)	AGGAATTTGCTATGGAAATAATTCAAATTTAATTTGGATTCAAATAGAAACTGGATCTTCTATTACTTGGAAATATC
Candidatus Carsonella 135557 - 167442nt	(1)	---
SARS-CoV-2 Reference Genome NC_045512.2	(1)	---

Section 1338

	(102950)	102950 102960 102971 102982 102993 103004 103015 103026
Candidatus Carsonella ruddii strain BT chromosome...	(102950)	CTTCTTCTATTTTAAAAGGTAATTTTACAAATAGTGAATTTTATTCTATAAGTATAAGCAATAATTATCAACAAATA
Candidatus Carsonella 135557 - 167442nt	(1)	---
SARS-CoV-2 Reference Genome NC_045512.2	(1)	---

Section 1339

```
                                                  (103027) 103027    103037     103048     103059     103070     103081     103092     103103
Candidatus Carsonella ruddii strain BT chromosome... (103027) GATACTGGAACAAAAATGTATTATTTAGGAAATAACGCTAATTCTTATATTAATTCTAAAAGTATATCATTAAATAA
           Candidatus Carsonella 135557 - 167442nt      (1) -----------------------------------------------------------------------------
         SARS-CoV-2 Reference Genome NC_045512.2      (1) -----------------------------------------------------------------------------
```

Section 1340

```
                                                  (103104) 103104    103114     103125     103136     103147     103158     103169     103180
Candidatus Carsonella ruddii strain BT chromosome... (103104) TTCTATACAAACTTATAGAGGATATATAAAAGTTTTGGAAAATTCTAAAAATTCTAAAAATTATACTTCTTGTGATT
           Candidatus Carsonella 135557 - 167442nt      (1) -----------------------------------------------------------------------------
         SARS-CoV-2 Reference Genome NC_045512.2      (1) -----------------------------------------------------------------------------
```

Section 1341

```
                                                  (103181) 103181    103191     103202     103213     103224     103235     103246     103257
Candidatus Carsonella ruddii strain BT chromosome... (103181) CGATAATTATGGATAATGGTAAAATTTTTACGTTTCCAATAAATTCTATTTTAAATTATAATTCAATTATAGAACAT
           Candidatus Carsonella 135557 - 167442nt      (1) -----------------------------------------------------------------------------
         SARS-CoV-2 Reference Genome NC_045512.2      (1) -----------------------------------------------------------------------------
```

Section 1342

```
                                                  (103258) 103258    103268     103279     103290     103301     103312     103323     103334
Candidatus Carsonella ruddii strain BT chromosome... (103258) GAAGCTAGTGTTACTAAAATTTCATTAAATGAAATTATTTTATTAAAAAGTAAAGGATTATGGAATCATCAATGTTA
           Candidatus Carsonella 135557 - 167442nt      (1) -----------------------------------------------------------------------------
         SARS-CoV-2 Reference Genome NC_045512.2      (1) -----------------------------------------------------------------------------
```

Section 1343

```
                                                  (103335) 103335    103345     103356     103367     103378     103389     103400     103411
Candidatus Carsonella ruddii strain BT chromosome... (103335) TAATATGTTAATTAATAATTTTTGTTACAGTATTATTAAAAAACTTCCTTTAGAATTTTTTTCTGAAATTAATGATT
           Candidatus Carsonella 135557 - 167442nt      (1) -----------------------------------------------------------------------------
         SARS-CoV-2 Reference Genome NC_045512.2      (1) -----------------------------------------------------------------------------
```

Section 1344

```
                                                  (103412) 103412    103422     103433     103444     103455     103466     103477     103488
Candidatus Carsonella ruddii strain BT chromosome... (103412) TAATAAGTTCTATAATAAATTATTCTATAATATGATTAAAATTATTAATTTATCAATAAAATGTGAAAATTTTTTCA
           Candidatus Carsonella 135557 - 167442nt      (1) -----------------------------------------------------------------------------
         SARS-CoV-2 Reference Genome NC_045512.2      (1) -----------------------------------------------------------------------------
```

Section 1345

	(103489)	103489 103499 103510 103521 103532 103543 103554 103565
Candidatus Carsonella ruddii strain BT chromosome...	(103489)	TTTTTAAAAATATTAATTTTTTAATAAATAAAAATAAAATTTATGTTATTATTGGAAATAATGGAATAGGAAAATCT
Candidatus Carsonella 135557 - 167442nt	(1)	---
SARS-CoV-2 Reference Genome NC_045512.2	(1)	---

Section 1346

	(103566)	103566 103576 103587 103598 103609 103620 103631 103642
Candidatus Carsonella ruddii strain BT chromosome...	(103566)	ACTTTGTTAAAATCTTTTATAAAAGATGAAAATTATTTTTTTAGTGGAAAAATTATTTTTAATAAAATTAATATTTT
Candidatus Carsonella 135557 - 167442nt	(1)	---
SARS-CoV-2 Reference Genome NC_045512.2	(1)	---

Section 1347

	(103643)	103643 103653 103664 103675 103686 103697 103708 103719
Candidatus Carsonella ruddii strain BT chromosome...	(103643)	AAATTATAAAACAGATTATATTTCTAGATTTGGTATTTTTTATTCTTTTCAAAATTCTATAGAAATTTATAATATTA
Candidatus Carsonella 135557 - 167442nt	(1)	---
SARS-CoV-2 Reference Genome NC_045512.2	(1)	---

Section 1348

	(103720)	103720 103730 103741 103752 103763 103774 103785 103796
Candidatus Carsonella ruddii strain BT chromosome...	(103720)	AAAATATTTTTTTTTTAAAAACTTGTTATAATATATTTAATTTAAATAATATTTTTTTTTTTAAATTATTAAAATTT
Candidatus Carsonella 135557 - 167442nt	(1)	---
SARS-CoV-2 Reference Genome NC_045512.2	(1)	---

Section 1349

	(103797)	103797 103807 103818 103829 103840 103851 103862 103873
Candidatus Carsonella ruddii strain BT chromosome...	(103797)	TATCAAGAAAAAATAAATTTTAAAAAAAAACTTTTTTTTAGAAGTTATAATTATGGTTTTTCTGGTGGTGAAAAAAA
Candidatus Carsonella 135557 - 167442nt	(1)	---
SARS-CoV-2 Reference Genome NC_045512.2	(1)	---

Section 1350

	(103874)	103874 103884 103895 103906 103917 103928 103939 103950
Candidatus Carsonella ruddii strain BT chromosome...	(103874)	AAAAAATGATTTTTTGTTTTTAATAATAATTAATCCATGTTTTATTTTATTAGATGAAATAGATTCAGGATTAGATT
Candidatus Carsonella 135557 - 167442nt	(1)	---
SARS-CoV-2 Reference Genome NC_045512.2	(1)	---

```
                                                                    Section 1351
                                            (103951) 103951    103961     103972     103983     103994     104005     104016     104027
Candidatus Carsonella ruddii strain BT chromosome... (103951) ATCAATCTTTTTTATTTATTGTTAATTACTTAAATTTAATAAAAAAAAATAAATTTATAATTTTAATTACTCATAAT
                 Candidatus Carsonella 135557 - 167442nt (1) -----------------------------------------------------------------------------
             SARS-CoV-2 Reference Genome NC_045512.2 (1) -----------------------------------------------------------------------------
                                                                    Section 1352
                                            (104028) 104028    104038     104049     104060     104071     104082     104093     104104
Candidatus Carsonella ruddii strain BT chromosome... (104028) AAAAATATTGGACAATTTTTAATTGTTGATTTTTTTATATTAATTAAAAATAATAATATTAATATATTATCATGTAT
                 Candidatus Carsonella 135557 - 167442nt (1) -----------------------------------------------------------------------------
             SARS-CoV-2 Reference Genome NC_045512.2 (1) -----------------------------------------------------------------------------
                                                                    Section 1353
                                            (104105) 104105    104115     104126     104137     104148     104159     104170     104181
Candidatus Carsonella ruddii strain BT chromosome... (104105) AAAAAATATTTATTAAAAAAAAAAACTTTTTTTTATATTTTAAAAAAATTTTATCATAAAAATAAGTACTTAAATTT
                 Candidatus Carsonella 135557 - 167442nt (1) -----------------------------------------------------------------------------
             SARS-CoV-2 Reference Genome NC_045512.2 (1) -----------------------------------------------------------------------------
                                                                    Section 1354
                                            (104182) 104182    104192     104203     104214     104225     104236     104247     104258
Candidatus Carsonella ruddii strain BT chromosome... (104182) TTTTTTTTATAAAATTTTAAAAAGAATTTTTTTTAAAAAAATTAAAATATTTAATTTAAATTGTATAATTATTAAAA
                 Candidatus Carsonella 135557 - 167442nt (1) -----------------------------------------------------------------------------
             SARS-CoV-2 Reference Genome NC_045512.2 (1) -----------------------------------------------------------------------------
                                                                    Section 1355
                                            (104259) 104259    104269     104280     104291     104302     104313     104324     104335
Candidatus Carsonella ruddii strain BT chromosome... (104259) ATTTTACAAATAAAATTTTTAATTATTTTTTTTTAAAAGAAATAAAAATAATTTTAAAAAATAATATTTTTAATATT
                 Candidatus Carsonella 135557 - 167442nt (1) -----------------------------------------------------------------------------
             SARS-CoV-2 Reference Genome NC_045512.2 (1) -----------------------------------------------------------------------------
                                                                    Section 1356
                                            (104336) 104336    104346     104357     104368     104379     104390     104401     104412
Candidatus Carsonella ruddii strain BT chromosome... (104336) TTATTTTTAATATTTAATATTAATTATTTATTACTGTTAGTTAAAAAATCTTTTTTATATATAAAAATTTTAAACAA
                 Candidatus Carsonella 135557 - 167442nt (1) -----------------------------------------------------------------------------
             SARS-CoV-2 Reference Genome NC_045512.2 (1) -----------------------------------------------------------------------------
```

Section 1357

	(104413)	104413 104423 104434 104445 104456 104467 104478 104489
Candidatus Carsonella ruddii strain BT chromosome...	(104413)	TATTAAAATTGAATTTTTTTTTATTTTTTCAAAAATTTTTATTTTTATTAACAATAATAAAAATATTTTTTTTTTAA
Candidatus Carsonella 135557 - 167442nt	(1)	---
SARS-CoV-2 Reference Genome NC_045512.2	(1)	---

Section 1358

	(104490)	104490 104500 104511 104522 104533 104544 104555 104566
Candidatus Carsonella ruddii strain BT chromosome...	(104490)	TAAAATATAATCATGTATTTAAAAAATTTTTTTTAAAAAAAAAAAATAATAAATTTTTAAACAATATTTTTTTTATA
Candidatus Carsonella 135557 - 167442nt	(1)	---
SARS-CoV-2 Reference Genome NC_045512.2	(1)	---

Section 1359

	(104567)	104567 104577 104588 104599 104610 104621 104632 104643
Candidatus Carsonella ruddii strain BT chromosome...	(104567)	CTAGAAAATAATTTTTTATATATATATATTAAAATTAAAAATTTTAAACATTTATTTATTTTTTTAAAATAAAAATTCA
Candidatus Carsonella 135557 - 167442nt	(1)	---
SARS-CoV-2 Reference Genome NC_045512.2	(1)	---

Section 1360

	(104644)	104644 104654 104665 104676 104687 104698 104709 104720
Candidatus Carsonella ruddii strain BT chromosome...	(104644)	AATTTATGAAAAAAAAAATTTTTATATTGAATTTTTTTTAATAAAAGGATATTTTTATTTTTCTTTAATATATAATA
Candidatus Carsonella 135557 - 167442nt	(1)	---
SARS-CoV-2 Reference Genome NC_045512.2	(1)	---

Section 1361

	(104721)	104721 104731 104742 104753 104764 104775 104786 104797
Candidatus Carsonella ruddii strain BT chromosome...	(104721)	AAATATATATTAAAAAAAAAATTAAAAATATATTTATTTATCAAAATGCAGAATTTATATTTAACAAATATAGTTTT
Candidatus Carsonella 135557 - 167442nt	(1)	---
SARS-CoV-2 Reference Genome NC_045512.2	(1)	---

Section 1362

	(104798)	104798 104808 104819 104830 104841 104852 104863 104874
Candidatus Carsonella ruddii strain BT chromosome...	(104798)	TCTATAATTAACTTAAATATTAATTTATATACTAAAAAAATTTTTTGCAAACATAAAATTTCTTTTTTATTGTTTAA
Candidatus Carsonella 135557 - 167442nt	(1)	---
SARS-CoV-2 Reference Genome NC_045512.2	(1)	---

Section 1363

```
                                                          (104875) 104875    104885     104896     104907     104918     104929     104940     104951
Candidatus Carsonella ruddii strain BT chromosome...      (104875) TAAAAAATTTTTTTTTATTAAAAATAAATTAATAAATAAAATATAATGTTTTTTTTTTTAATAAAATATAAAAAAAT
Candidatus Carsonella 135557 - 167442nt                   (1)      -----------------------------------------------------------------------------
SARS-CoV-2 Reference Genome NC_045512.2                   (1)      -----------------------------------------------------------------------------
```

Section 1364

```
                                                          (104952) 104952    104962     104973     104984     104995     105006     105017     105028
Candidatus Carsonella ruddii strain BT chromosome...      (104952) TATTTATTTTGATAATTCTTCTACAAATCAAAAACCTAATATTTTATTTAAATCTACTATTAATATTTTACAAAAAA
Candidatus Carsonella 135557 - 167442nt                   (1)      -----------------------------------------------------------------------------
SARS-CoV-2 Reference Genome NC_045512.2                   (1)      -----------------------------------------------------------------------------
```

Section 1365

```
                                                          (105029) 105029    105039     105050     105061     105072     105083     105094     105105
Candidatus Carsonella ruddii strain BT chromosome...      (105029) AAAATTTTAATATAAATAAAGGAAATTATAATTTATTAGATAAAAATTTAATTTTATTCAAAAAATTAAAATTATTA
Candidatus Carsonella 135557 - 167442nt                   (1)      -----------------------------------------------------------------------------
SARS-CoV-2 Reference Genome NC_045512.2                   (1)      -----------------------------------------------------------------------------
```

Section 1366

```
                                                          (105106) 105106    105116     105127     105138     105149     105160     105171     105182
Candidatus Carsonella ruddii strain BT chromosome...      (105106) ATAAAAAAAATATTACAATGTAATTTTATAGAAGAAATTATTTTTTTTTTTAATTCTACTTTTTCTATTAATTTTAT
Candidatus Carsonella 135557 - 167442nt                   (1)      -----------------------------------------------------------------------------
SARS-CoV-2 Reference Genome NC_045512.2                   (1)      -----------------------------------------------------------------------------
```

Section 1367

```
                                                          (105183) 105183    105193     105204     105215     105226     105237     105248     105259
Candidatus Carsonella ruddii strain BT chromosome...      (105183) TTTTTTAAATTTAATAAATTTTATTAAATTAACAAATGAAATTTTAATTTCAAATAAAGAACATAATTCAATATTTA
Candidatus Carsonella 135557 - 167442nt                   (1)      -----------------------------------------------------------------------------
SARS-CoV-2 Reference Genome NC_045512.2                   (1)      -----------------------------------------------------------------------------
```

Section 1368

```
                                                          (105260) 105260    105270     105281     105292     105303     105314     105325     105336
Candidatus Carsonella ruddii strain BT chromosome...      (105260) TTTCTTTATTACAATTATTTAAATTTAAAAAATTAAAAACAATAATTTTTCCAACTATTAAACAAAAAATTTTTAAA
Candidatus Carsonella 135557 - 167442nt                   (1)      -----------------------------------------------------------------------------
SARS-CoV-2 Reference Genome NC_045512.2                   (1)      -----------------------------------------------------------------------------
```

Section 1369

	(105337)	105337 105347 105358 105369 105380 105391 105402 105413
Candidatus Carsonella ruddii strain BT chromosome...	(105337)	AATATTTTTTGTAATTATATTAATAATACTTTTTGTTGTATAATAAATCAAATTTCAAATAATTATGGAATTTTAAA
Candidatus Carsonella 135557 - 167442nt	(1)	---
SARS-CoV-2 Reference Genome NC_045512.2	(1)	---

Section 1370

	(105414)	105414 105424 105435 105446 105457 105468 105479 105490
Candidatus Carsonella ruddii strain BT chromosome...	(105414)	TCATATTAAACATTTAATAAAAATTTGTAATTATAACAATATAATTACAATATTAGATTGTACACAATCTATTTCTT
Candidatus Carsonella 135557 - 167442nt	(1)	---
SARS-CoV-2 Reference Genome NC_045512.2	(1)	---

Section 1371

	(105491)	105491 105501 105512 105523 105534 105545 105556 105567
Candidatus Carsonella ruddii strain BT chromosome...	(105491)	TTAATAATATAAATATAAAAAAAATAAAACCAACTTTTTTATTTTTTTCTTTACACAAATTATATTCTTTAACAGGT
Candidatus Carsonella 135557 - 167442nt	(1)	---
SARS-CoV-2 Reference Genome NC_045512.2	(1)	---

Section 1372

	(105568)	105568 105578 105589 105600 105611 105622 105633 105644
Candidatus Carsonella ruddii strain BT chromosome...	(105568)	ATTTCAATTTTATACTATAATATTAATTTTTTATCAAAAATTAAACCGATTTTTGTAGGTGGTGGTTCATCTTATAT
Candidatus Carsonella 135557 - 167442nt	(1)	---
SARS-CoV-2 Reference Genome NC_045512.2	(1)	---

Section 1373

	(105645)	105645 105655 105666 105677 105688 105699 105710 105721
Candidatus Carsonella ruddii strain BT chromosome...	(105645)	TAATAATTATTTAATCAAATATAAAAAATTTAATGAAAAATTTTATTTTGGAACACAAAATTTTTTTTCTTTATTAT
Candidatus Carsonella 135557 - 167442nt	(1)	---
SARS-CoV-2 Reference Genome NC_045512.2	(1)	---

Section 1374

	(105722)	105722 105732 105743 105754 105765 105776 105787 105798
Candidatus Carsonella ruddii strain BT chromosome...	(105722)	CTAGTTATTATTCATTAAAATGGTTTTTAAAAAATAAAAAATTTATATTTTATATTAATTATTATTTAAAAAAAAAT
Candidatus Carsonella 135557 - 167442nt	(1)	---
SARS-CoV-2 Reference Genome NC_045512.2	(1)	---

```
                                                                    Section 1375
                                          (105799) 105799    105809     105820     105831     105842     105853     105864 105875
Candidatus Carsonella ruddii strain BT chromosome... (105799) ATTTTTTTTTATTTTTTAAAAAAAAAAAATTATTTTTCAAATACTTTATTATTAAAAAATAAAAATATAGAAAATTT
          Candidatus Carsonella 135557 - 167442nt (1) -----------------------------------------------------------------------------
          SARS-CoV-2 Reference Genome NC_045512.2 (1) -----------------------------------------------------------------------------
                                                                    Section 1376
                                          (105876) 105876    105886     105897     105908     105919     105930     105941 105952
Candidatus Carsonella ruddii strain BT chromosome... (105876) TAATAATTATTTAGATATTAATAAAATAATTTTAAGATGTGATAATTTATGTAATTTTATGAAAAATATGTTTATTA
          Candidatus Carsonella 135557 - 167442nt (1) -----------------------------------------------------------------------------
          SARS-CoV-2 Reference Genome NC_045512.2 (1) -----------------------------------------------------------------------------
                                                                    Section 1377
                                          (105953) 105953    105963     105974     105985     105996     106007     106018 106029
Candidatus Carsonella ruddii strain BT chromosome... (105953) ACAAAAAAAAAAATTGTAGAATTTCTTTTAATTTTTTAATAAATTAAAAGAAATTTTAAAAATAAAATTTTTAATT
          Candidatus Carsonella 135557 - 167442nt (1) -----------------------------------------------------------------------------
          SARS-CoV-2 Reference Genome NC_045512.2 (1) -----------------------------------------------------------------------------
                                                                    Section 1378
                                          (106030) 106030    106040     106051     106062     106073     106084     106095 106106
Candidatus Carsonella ruddii strain BT chromosome... (106030) TTTTTTTATAATTTTAGAATTAAAAATTGCTAAATCATTATAAAATAATAATATATTTTTTTCTAAATATTTTTTTT
          Candidatus Carsonella 135557 - 167442nt (1) -----------------------------------------------------------------------------
          SARS-CoV-2 Reference Genome NC_045512.2 (1) -----------------------------------------------------------------------------
                                                                    Section 1379
                                          (106107) 106107    106117     106128     106139     106150     106161     106172 106183
Candidatus Carsonella ruddii strain BT chromosome... (106107) TTTTATAAAAAACAACAAATTTTTTATTTTTAAATTTTATTAATTTTCCTATTATTTTAATACTTTGAGATTTTGTT
          Candidatus Carsonella 135557 - 167442nt (1) -----------------------------------------------------------------------------
          SARS-CoV-2 Reference Genome NC_045512.2 (1) -----------------------------------------------------------------------------
                                                                    Section 1380
                                          (106184) 106184    106194     106205     106216     106227     106238     106249 106260
Candidatus Carsonella ruddii strain BT chromosome... (106184) TTTAAAAAAATATTTATATTATTACATAAAAAATTAATTTTAATTATTTTAAAAAATAAATTTTTATTTTTTATAAT
          Candidatus Carsonella 135557 - 167442nt (1) -----------------------------------------------------------------------------
          SARS-CoV-2 Reference Genome NC_045512.2 (1) -----------------------------------------------------------------------------
```

Section 1381

```
                                                    (106261) 106261    106271     106282     106293     106304     106315     106326     106337
Candidatus Carsonella ruddii strain BT chromosome... (106261) ATAAATATTATTTTTTTTTATTTTAATAATTTTAAAAAAAATAATTTTTTTTCCTAAATTTAAATAATTTTTAGTTT
              Candidatus Carsonella 135557 - 167442nt (1) -----------------------------------------------------------------------------
           SARS-CoV-2 Reference Genome NC_045512.2 (1) -----------------------------------------------------------------------------
```

Section 1382

```
                                                    (106338) 106338    106348     106359     106370     106381     106392     106403     106414
Candidatus Carsonella ruddii strain BT chromosome... (106338) TTAAAATATTAAAAATCATTTTATTTTCTAATATAAAAATAAAAAGTTTTTTTTTATTTTTAAATTTAAAACAAATA
              Candidatus Carsonella 135557 - 167442nt (1) -----------------------------------------------------------------------------
           SARS-CoV-2 Reference Genome NC_045512.2 (1) -----------------------------------------------------------------------------
```

Section 1383

```
                                                    (106415) 106415    106425     106436     106447     106458     106469     106480     106491
Candidatus Carsonella ruddii strain BT chromosome... (106415) CCTGTAGTACTTTTTTTTTATTTTGCATAAAAAATTATTTTTAAAATTTAAAAAATTAATATAAAATTTATTATAATA
              Candidatus Carsonella 135557 - 167442nt (1) -----------------------------------------------------------------------------
           SARS-CoV-2 Reference Genome NC_045512.2 (1) -----------------------------------------------------------------------------
```

Section 1384

```
                                                    (106492) 106492    106502     106513     106524     106535     106546     106557     106568
Candidatus Carsonella ruddii strain BT chromosome... (106492) ACCTATAAAAGAATAAATATTATTTTTAAAATTTAAAAAAAAAAATTTGATCTTTTTTTTGATCATTTGAAGAAATAA
              Candidatus Carsonella 135557 - 167442nt (1) -----------------------------------------------------------------------------
           SARS-CoV-2 Reference Genome NC_045512.2 (1) -----------------------------------------------------------------------------
```

Section 1385

```
                                                    (106569) 106569    106579     106590     106601     106612     106623     106634     106645
Candidatus Carsonella ruddii strain BT chromosome... (106569) AAAATTTTTTTATTTTTTTAAAATAATGACCAGTAAATATAATTTTTTTTTTATAAATTTTTTTAATTAAATAAATT
              Candidatus Carsonella 135557 - 167442nt (1) -----------------------------------------------------------------------------
           SARS-CoV-2 Reference Genome NC_045512.2 (1) -----------------------------------------------------------------------------
```

Section 1386

```
                                                    (106646) 106646    106656     106667     106678     106689     106700     106711     106722
Candidatus Carsonella ruddii strain BT chromosome... (106646) TTAATTAATTTATTACAATAAAAATCTATATTTATATTTTTAAAAATAAAATAATTTAAAATTTTTTTATATTCTAA
              Candidatus Carsonella 135557 - 167442nt (1) -----------------------------------------------------------------------------
           SARS-CoV-2 Reference Genome NC_045512.2 (1) -----------------------------------------------------------------------------
```

```
                                                                                                              Section 1387
                                                   (106723) 106723    106733     106744     106755     106766     106777     106788  106799
Candidatus Carsonella ruddii strain BT chromosome... (106723) TTTTAAATTAATTGAAAATAATTTTTTATTTAAATTTTTAATATTTAAAATTGAATATTTTTTATCAATATAATTAT
             Candidatus Carsonella 135557 - 167442nt      (1) -----------------------------------------------------------------------------
          SARS-CoV-2 Reference Genome NC_045512.2      (1) -----------------------------------------------------------------------------
```

```
                                                                                                              Section 1388
                                                   (106800) 106800    106810     106821     106832     106843     106854     106865  106876
Candidatus Carsonella ruddii strain BT chromosome... (106800) TACAATTGTTAATTTTTATAAATAAATTATAATGATTAAAATTATAACAACAAAAATTTGAATCTTTTCCTCCAGAA
             Candidatus Carsonella 135557 - 167442nt      (1) -----------------------------------------------------------------------------
          SARS-CoV-2 Reference Genome NC_045512.2      (1) -----------------------------------------------------------------------------
```

```
                                                                                                              Section 1389
                                                   (106877) 106877    106887     106898     106909     106920     106931     106942  106953
Candidatus Carsonella ruddii strain BT chromosome... (106877) ATTAACAATATATTATACATTATAATCTATGTATAATTCTTCCTTTAGTTAAATCATAATAAGAAATTAAAATTTTA
             Candidatus Carsonella 135557 - 167442nt      (1) -----------------------------------------------------------------------------
          SARS-CoV-2 Reference Genome NC_045512.2      (1) -----------------------------------------------------------------------------
```

```
                                                                                                              Section 1390
                                                   (106954) 106954    106964     106975     106986     106997     107008     107019  107030
Candidatus Carsonella ruddii strain BT chromosome... (106954) ACTCTATCTCCTATCATTATTTTTATAAAATTTTTTCTAATTTTTCCTGAAATATAAGAATTTATTAAAAAACCATT
             Candidatus Carsonella 135557 - 167442nt      (1) -----------------------------------------------------------------------------
          SARS-CoV-2 Reference Genome NC_045512.2      (1) -----------------------------------------------------------------------------
```

```
                                                                                                              Section 1391
                                                   (107031) 107031    107041     107052     107063     107074     107085     107096  107107
Candidatus Carsonella ruddii strain BT chromosome... (107031) TTCTAATTTTACTTTAAAAGTAGCATTAGGCATTGTATTGATAACAACACCTTCCATTTCAAATACATTATTATTCA
             Candidatus Carsonella 135557 - 167442nt      (1) -----------------------------------------------------------------------------
          SARS-CoV-2 Reference Genome NC_045512.2      (1) -----------------------------------------------------------------------------
```

```
                                                                                                              Section 1392
                                                   (107108) 107108    107118     107129     107140     107151     107162     107173  107184
Candidatus Carsonella ruddii strain BT chromosome... (107108) TTTTAAAAATAAATTATATATTAAATATGTCTCGTTCGTCTATAGGTAGGACCTTGCTCTTTCACGGCGAAAAAAAG
             Candidatus Carsonella 135557 - 167442nt      (1) -----------------------------------------------------------------------------
          SARS-CoV-2 Reference Genome NC_045512.2      (1) -----------------------------------------------------------------------------
```

Section 1393

		107185 107195 107206 107217 107228 107239 107250 107261
Candidatus Carsonella ruddii strain BT chromosome...	(107185)	GGTTCGAATCCCTTACGAGATAGCGAAAGTATCTTAATGGTAAAGTATCACCTTGCCATGGTGAAAGTTGCGAGTTC
Candidatus Carsonella 135557 - 167442nt	(1)	---
SARS-CoV-2 Reference Genome NC_045512.2	(1)	---

Section 1394

		107262 107272 107283 107294 107305 107316 107327 107338
Candidatus Carsonella ruddii strain BT chromosome...	(107262)	AATTCTCGTCTTTCGCTAGAGATATGTCTGAGCGGTTTAAAGAATATGCTTGGAAAGCATAGAAAGCTTTTGCTTTC
Candidatus Carsonella 135557 - 167442nt	(1)	---
SARS-CoV-2 Reference Genome NC_045512.2	(1)	---

Section 1395

		107339 107349 107360 107371 107382 107393 107404 107415
Candidatus Carsonella ruddii strain BT chromosome...	(107339)	ATGGGTTCGAATCCCATTATCTCTTAAATAAAAAATATTTCTTTTAAAATATCTTTAATTGAAACCAAACCATTTAA
Candidatus Carsonella 135557 - 167442nt	(1)	---
SARS-CoV-2 Reference Genome NC_045512.2	(1)	---

Section 1396

		107416 107426 107437 107448 107459 107470 107481 107492
Candidatus Carsonella ruddii strain BT chromosome...	(107416)	TATTATTTTATTTTTAAAATCAAAAATAATAGTAGGAATTACTCTAATTCCTAATTCAATAGAATATTTTTGATGAT
Candidatus Carsonella 135557 - 167442nt	(1)	---
SARS-CoV-2 Reference Genome NC_045512.2	(1)	---

Section 1397

		107493 107503 107514 107525 107536 107547 107558 107569
Candidatus Carsonella ruddii strain BT chromosome...	(107493)	AATCAATATTTATTAAAAAAAATAATATATTTATTTTTTTTTCAATTTTTTTTAATTTTTTTTTTAATATATTACAA
Candidatus Carsonella 135557 - 167442nt	(1)	---
SARS-CoV-2 Reference Genome NC_045512.2	(1)	---

Section 1398

		107570 107580 107591 107602 107613 107624 107635 107646
Candidatus Carsonella ruddii strain BT chromosome...	(107570)	GGACTACACCAATTTGCTGTAAAAACTAAAATTTTTTTTTTATTGTTTATTTTTTTTAATATATTATTTTTAACAAT
Candidatus Carsonella 135557 - 167442nt	(1)	---
SARS-CoV-2 Reference Genome NC_045512.2	(1)	---

Section 1399

```
                                                     (107647) 107647    107657     107668     107679     107690     107701     107712  107723
Candidatus Carsonella ruddii strain BT chromosome... (107647) TATCATATAAATTTTATAAAATGATTACCAAAATAATTTCTTTCAAATTGAATTAATTTAAAATTGTAATTTCTATA
         Candidatus Carsonella 135557 - 167442nt          (1) -----------------------------------------------------------------------------
      SARS-CoV-2 Reference Genome NC_045512.2            (1) -----------------------------------------------------------------------------
```

Section 1400

```
                                                     (107724) 107724    107734     107745     107756     107767     107778     107789  107800
Candidatus Carsonella ruddii strain BT chromosome... (107724) ATAAATAATTAATAAAAAATTGTAACAAGATAAAATATTAAAAATTACAAAATTTAATTTTAATAAAATAGTAATAA
         Candidatus Carsonella 135557 - 167442nt          (1) -----------------------------------------------------------------------------
      SARS-CoV-2 Reference Genome NC_045512.2            (1) -----------------------------------------------------------------------------
```

Section 1401

```
                                                     (107801) 107801    107811     107822     107833     107844     107855     107866  107877
Candidatus Carsonella ruddii strain BT chromosome... (107801) TTTTTTTAATTAAATGAATTTTTTTTAAATAAATAAAAAATATTTTTATTAAAAAAAAGTTTTTTTTAAAATTTAAC
         Candidatus Carsonella 135557 - 167442nt          (1) -----------------------------------------------------------------------------
      SARS-CoV-2 Reference Genome NC_045512.2            (1) -----------------------------------------------------------------------------
```

Section 1402

```
                                                     (107878) 107878    107888     107899     107910     107921     107932     107943  107954
Candidatus Carsonella ruddii strain BT chromosome... (107878) AAATAAAATAATATATTAGAATCAATTATACAACTGTTTAAATAAGTTTTTATTAAATTAGAAAAATTAATTTTCCA
         Candidatus Carsonella 135557 - 167442nt          (1) -----------------------------------------------------------------------------
      SARS-CoV-2 Reference Genome NC_045512.2            (1) -----------------------------------------------------------------------------
```

Section 1403

```
                                                     (107955) 107955    107965     107976     107987     107998     108009     108020  108031
Candidatus Carsonella ruddii strain BT chromosome... (107955) ATTATATTTTTTTGAAATTTTTATTAATTGATTGAAACCTTGAATATAACATAATAATTTAGAATAAAAAAAAAACAT
         Candidatus Carsonella 135557 - 167442nt          (1) -----------------------------------------------------------------------------
      SARS-CoV-2 Reference Genome NC_045512.2            (1) -----------------------------------------------------------------------------
```

Section 1404

```
                                                     (108032) 108032    108042     108053     108064     108075     108086     108097  108108
Candidatus Carsonella ruddii strain BT chromosome... (108032) TTTTTATCATTTCTAAAAATATTGATATTTTAATTTTAATTTTTATATTATTATTATATTTTAATATATTTCTTAAA
         Candidatus Carsonella 135557 - 167442nt          (1) -----------------------------------------------------------------------------
      SARS-CoV-2 Reference Genome NC_045512.2            (1) -----------------------------------------------------------------------------
```

Section 1405

(108109) 108109 108119 108130 108141 108152 108163 108174 108185

Candidatus Carsonella ruddii strain BT chromosome... (108109) AAAATATTTTTAGATATAATTCTTATTATTAATGCTTCAAAAATTGAATTAATATTAATATTATTTTTAATTGAATT

Candidatus Carsonella 135557 - 167442nt (1) ---

SARS-CoV-2 Reference Genome NC_045512.2 (1) ---

Section 1406

(108186) 108186 108196 108207 108218 108229 108240 108251 108262

Candidatus Carsonella ruddii strain BT chromosome... (108186) TAATACTGTATTACTTCCTGTACCTTTATGATCAATAATATCATTAATATATAATTTTTTTTTTATTAAAATATTAA

Candidatus Carsonella 135557 - 167442nt (1) ---

SARS-CoV-2 Reference Genome NC_045512.2 (1) ---

Section 1407

(108263) 108263 108273 108284 108295 108306 108317 108328 108339

Candidatus Carsonella ruddii strain BT chromosome... (108263) ATAATATTTTTAATAAATAACAAGAAAAATCAGTTTTATTCCAATTTATTAATATATTAATTATTTTTTTTTTTTTT

Candidatus Carsonella 135557 - 167442nt (1) ---

SARS-CoV-2 Reference Genome NC_045512.2 (1) ---

Section 1408

(108340) 108340 108350 108361 108372 108383 108394 108405 108416

Candidatus Carsonella ruddii strain BT chromosome... (108340) ATTATAAAAACTTTTAATATATAATATATTTCTGATATTAATTGTAATATTCCATATTCAATTCCATTATGAATCAT

Candidatus Carsonella 135557 - 167442nt (1) ---

SARS-CoV-2 Reference Genome NC_045512.2 (1) ---

Section 1409

(108417) 108417 108427 108438 108449 108460 108471 108482 108493

Candidatus Carsonella ruddii strain BT chromosome... (108417) TTTAATAAAATGAGAACTAGCAAAACCTAAGGTTATACAACAACTAAATCTTTTTTTTTTTAAAAAAAAAATGTTTT

Candidatus Carsonella 135557 - 167442nt (1) ---

SARS-CoV-2 Reference Genome NC_045512.2 (1) ---

Section 1410

(108494) 108494 108504 108515 108526 108537 108548 108559 108570

Candidatus Carsonella ruddii strain BT chromosome... (108494) TTAATAAAAAAAATAATTTTTTATAATCTTTTATTAAACAATCTATCATACAACACAAACCATTTAAAGCTCCTAAA

Candidatus Carsonella 135557 - 167442nt (1) ---

SARS-CoV-2 Reference Genome NC_045512.2 (1) ---

```
                                                                                     Section 1411
                                           (108571) 108571    108581     108592     108603     108614     108625     108636  108647
Candidatus Carsonella ruddii strain BT chromosome... (108571) CATCCACCTGATATTCCAGAAGAAATAAAAATAAATTTTTTGTTAGTAATTTTATAATTTAAAAATGTTTCTTTAAA
        Candidatus Carsonella 135557 - 167442nt      (1) -----------------------------------------------------------------------------
     SARS-CoV-2 Reference Genome NC_045512.2         (1) -----------------------------------------------------------------------------

                                                                                     Section 1412
                                           (108648) 108648    108658     108669     108680     108691     108702     108713  108724
Candidatus Carsonella ruddii strain BT chromosome... (108648) AAAAGAATTTCCAAAATCAATAACAATATCATTTTTAGATAATTTATATTTTAATCTTAAAATTATTTTTTTAATAG
        Candidatus Carsonella 135557 - 167442nt      (1) -----------------------------------------------------------------------------
     SARS-CoV-2 Reference Genome NC_045512.2         (1) -----------------------------------------------------------------------------

                                                                                     Section 1413
                                           (108725) 108725    108735     108746     108757     108768     108779     108790  108801
Candidatus Carsonella ruddii strain BT chromosome... (108725) GTTTTCCTGTTTTAATTAATAAAAAAATAATTTTTTTTAATGGTAAAGAAAAAAGAAATTTTTTTAAACAATTAGTA
        Candidatus Carsonella 135557 - 167442nt      (1) -----------------------------------------------------------------------------
     SARS-CoV-2 Reference Genome NC_045512.2         (1) -----------------------------------------------------------------------------

                                                                                     Section 1414
                                           (108802) 108802    108812     108823     108834     108845     108856     108867  108878
Candidatus Carsonella ruddii strain BT chromosome... (108802) ATTATTTGTATATTATATATTTTTAAATTTAAAATAATTTTTTCTTTATTAAAAATTGAAATAATATTATCTTTTTT
        Candidatus Carsonella 135557 - 167442nt      (1) -----------------------------------------------------------------------------
     SARS-CoV-2 Reference Genome NC_045512.2         (1) -----------------------------------------------------------------------------

                                                                                     Section 1415
                                           (108879) 108879    108889     108900     108911     108922     108933     108944  108955
Candidatus Carsonella ruddii strain BT chromosome... (108879) AATAAGATTAATAGTAATATTTTTGGACATTGATCCATAACCTATTATTCCAATATGATTTTTCATAAATTTAAAAA
        Candidatus Carsonella 135557 - 167442nt      (1) -----------------------------------------------------------------------------
     SARS-CoV-2 Reference Genome NC_045512.2         (1) -----------------------------------------------------------------------------

                                                                                     Section 1416
                                           (108956) 108956    108966     108977     108988     108999     109010     109021  109032
Candidatus Carsonella ruddii strain BT chromosome... (108956) AATTTTAATATTTAAAAAATTTAAAATAAATTCATTCGTAAAACAAATTTTGTTAAAAAATTTTTTTTGTTTAAAAA
        Candidatus Carsonella 135557 - 167442nt      (1) -----------------------------------------------------------------------------
     SARS-CoV-2 Reference Genome NC_045512.2         (1) -----------------------------------------------------------------------------
```

Section 1417

(109033) 109033 109043 109054 109065 109076 109087 109098 109109
Candidatus Carsonella ruddii strain BT chromosome... (109033) AACAAAATATTATTTTTGTATCAAATTATTTTAATATAAATATATTTTTTAATAATATATATATATATAATAATAAA
Candidatus Carsonella 135557 - 167442nt (1) ---
SARS-CoV-2 Reference Genome NC_045512.2 (1) ---

Section 1418

(109110) 109110 109120 109131 109142 109153 109164 109175 109186
Candidatus Carsonella ruddii strain BT chromosome... (109110) AAAACTATAATATCTAATAATATTAATCATTATATTAAAAAAAAAAATTACAACAATAAAAAAAAATTTAGATTTTT
Candidatus Carsonella 135557 - 167442nt (1) ---
SARS-CoV-2 Reference Genome NC_045512.2 (1) ---

Section 1419

(109187) 109187 109197 109208 109219 109230 109241 109252 109263
Candidatus Carsonella ruddii strain BT chromosome... (109187) ATTTTCTTTTTTTAATTTTATTAATTTTAATTTTTTTAAAAAAAAATATTAAAAAGTATAAAAATATAAAAAATTTGT
Candidatus Carsonella 135557 - 167442nt (1) ---
SARS-CoV-2 Reference Genome NC_045512.2 (1) ---

Section 1420

(109264) 109264 109274 109285 109296 109307 109318 109329 109340
Candidatus Carsonella ruddii strain BT chromosome... (109264) TTTTTTTTCCAAAAAATGTTTTTTTTTTAAAAAAAAAAAAATAAAATTATTTATTTATTTAGTATTATTAAAAATAAT
Candidatus Carsonella 135557 - 167442nt (1) ---
SARS-CoV-2 Reference Genome NC_045512.2 (1) ---

Section 1421

(109341) 109341 109351 109362 109373 109384 109395 109406 109417
Candidatus Carsonella ruddii strain BT chromosome... (109341) AACAATTTTAATATTTTTTCAAAATTTTTAAATTTTTTTTTATTGAAAATAAAAAATTCTTTTTTAAATAAAAAATGT
Candidatus Carsonella 135557 - 167442nt (1) ---
SARS-CoV-2 Reference Genome NC_045512.2 (1) ---

Section 1422

(109418) 109418 109428 109439 109450 109461 109472 109483 109494
Candidatus Carsonella ruddii strain BT chromosome... (109418) TATTAAAAAAAAATATTAATATTAATTTTTTTATTAAAAAAAAAATATTTTTTTAAAAATATTTGTAAAATAAAAAAAA
Candidatus Carsonella 135557 - 167442nt (1) ---
SARS-CoV-2 Reference Genome NC_045512.2 (1) ---

```
                                                                                                   Section 1423
                                                   (109495) 109495    109505     109516     109527     109538     109549     109560  109571
Candidatus Carsonella ruddii strain BT chromosome... (109495) ATATTAATATAGGAAATTTAATTCAATGTGTAATTTCTAACATTTTAATAATAAAGAATTTAAAAAATAAAAAAATA
                Candidatus Carsonella 135557 - 167442nt      (1) -----------------------------------------------------------------------------
              SARS-CoV-2 Reference Genome NC_045512.2        (1) -----------------------------------------------------------------------------
                                                                                                   Section 1424
                                                   (109572) 109572    109582     109593     109604     109615     109626     109637  109648
Candidatus Carsonella ruddii strain BT chromosome... (109572) ATTAATAAAACATATTATAATTTTTATTTAAATTATAAAAATTTAATAATATTATTAAATTCTCCTGAATTTTTAAT
                Candidatus Carsonella 135557 - 167442nt      (1) -----------------------------------------------------------------------------
              SARS-CoV-2 Reference Genome NC_045512.2        (1) -----------------------------------------------------------------------------
                                                                                                   Section 1425
                                                   (109649) 109649    109659     109670     109681     109692     109703     109714  109725
Candidatus Carsonella ruddii strain BT chromosome... (109649) AAGAAAAGAAAACAATAAATATTATACAAAACCAATAGCAGGAACAACAGATACTAATTTTAATTTTTTAAAAATTT
                Candidatus Carsonella 135557 - 167442nt      (1) -----------------------------------------------------------------------------
              SARS-CoV-2 Reference Genome NC_045512.2        (1) -----------------------------------------------------------------------------
                                                                                                   Section 1426
                                                   (109726) 109726    109736     109747     109758     109769     109780     109791  109802
Candidatus Carsonella ruddii strain BT chromosome... (109726) TAAAAAATAAAAAAGAATTATCAGAACATTTAATGTTATTAGATTTAAGTTTAAATGATGTTTTTAAAAATAATATT
                Candidatus Carsonella 135557 - 167442nt      (1) -----------------------------------------------------------------------------
              SARS-CoV-2 Reference Genome NC_045512.2        (1) -----------------------------------------------------------------------------
                                                                                                   Section 1427
                                                   (109803) 109803    109813     109824     109835     109846     109857     109868  109879
Candidatus Carsonella ruddii strain BT chromosome... (109803) AAATTATTTTTTTTAAAAAAAATGTTTTATTGTGAAAAAAATAAAAGTTTAACTCATATTATATCTGAAATATTTTT
                Candidatus Carsonella 135557 - 167442nt      (1) -----------------------------------------------------------------------------
              SARS-CoV-2 Reference Genome NC_045512.2        (1) -----------------------------------------------------------------------------
                                                                                                   Section 1428
                                                   (109880) 109880    109890     109901     109912     109923     109934     109945  109956
Candidatus Carsonella ruddii strain BT chromosome... (109880) TAAAAATTATAAAAAAAATAATTTATTTTTATTAGAAAATTTTTTCCCAGCTGGTACTTTATCTGGTACTCCTAAAA
                Candidatus Carsonella 135557 - 167442nt      (1) -----------------------------------------------------------------------------
              SARS-CoV-2 Reference Genome NC_045512.2        (1) -----------------------------------------------------------------------------
```

Section 1429

	(109957)	109957 109967 109978 109989 110000 110011 110022 110033
Candidatus Carsonella ruddii strain BT chromosome...	(109957)	ATGATTGTATAAAAATAATCAATATAATAGAAAAACAAAATAGATTTTTATATGGTGGTGTTTTTAATTTTTTTAAT
Candidatus Carsonella 135557 - 167442nt	(1)	---
SARS-CoV-2 Reference Genome NC_045512.2	(1)	---

Section 1430

	(110034)	110034 110044 110055 110066 110077 110088 110099 110110
Candidatus Carsonella ruddii strain BT chromosome...	(110034)	AAAAATCATATAGAATCTTGTATTTTAATAAGAATAAAAATTTTTTTTTTAAACAATATTTATTTAGAATCAGGTGC
Candidatus Carsonella 135557 - 167442nt	(1)	---
SARS-CoV-2 Reference Genome NC_045512.2	(1)	---

Section 1431

	(110111)	110111 110121 110132 110143 110154 110165 110176 110187
Candidatus Carsonella ruddii strain BT chromosome...	(110111)	AGGATTAATTAATAAATCAATAAATTTTTTAGAATGGTTAGAAACTATAATTAAAAAACAATTTTTTTTAAAATGAT
Candidatus Carsonella 135557 - 167442nt	(1)	---
SARS-CoV-2 Reference Genome NC_045512.2	(1)	---

Section 1432

	(110188)	110188 110198 110209 110220 110231 110242 110253 110264
Candidatus Carsonella ruddii strain BT chromosome...	(110188)	TTTATTTTTAGATAATTTTGATAGTTTTTCAAATAATATAATAAAAAAAATAATTTTAAAAAAAATAAAAATTAATT
Candidatus Carsonella 135557 - 167442nt	(1)	---
SARS-CoV-2 Reference Genome NC_045512.2	(1)	---

Section 1433

	(110265)	110265 110275 110286 110297 110308 110319 110330 110341
Candidatus Carsonella ruddii strain BT chromosome...	(110265)	GTTTTACTTTAAATATAAAAAAAAATTATATAAATTTAATAATTGAAAAAATAATAATTTTAGGCCCAGGTCCAAAC
Candidatus Carsonella 135557 - 167442nt	(1)	---
SARS-CoV-2 Reference Genome NC_045512.2	(1)	---

Section 1434

	(110342)	110342 110352 110363 110374 110385 110396 110407 110418
Candidatus Carsonella ruddii strain BT chromosome...	(110342)	AATATAATTAATAGTAATTTAAATACTATTGTTTTAGATAAATTTATAGGTAAAAAAAAATTAATAGGAATATGTTT
Candidatus Carsonella 135557 - 167442nt	(1)	---
SARS-CoV-2 Reference Genome NC_045512.2	(1)	---

Section 1435

	(110419)	110419 110429 110440 110451 110462 110473 110484 110495
Candidatus Carsonella ruddii strain BT chromosome...	(110419)	GGGCCATCAAATAATATCTTGTTATTTAAATTATAAAATTTTTAAATTAAATAATGTTTTTCACGGTCAACTTAAAA
Candidatus Carsonella 135557 - 167442nt	(1)	---
SARS-CoV-2 Reference Genome NC_045512.2	(1)	---

Section 1436

	(110496)	110496 110506 110517 110528 110539 110550 110561 110572
Candidatus Carsonella ruddii strain BT chromosome...	(110496)	AAATTATTATTTTTTTAAACAAATATAAAATTTTTATAATTAAAAAAATAATTTTTTATAATTCTTTATCATGTATA
Candidatus Carsonella 135557 - 167442nt	(1)	---
SARS-CoV-2 Reference Genome NC_045512.2	(1)	---

Section 1437

	(110573)	110573 110583 110594 110605 110616 110627 110638 110649
Candidatus Carsonella ruddii strain BT chromosome...	(110573)	GGTTATTATAGAACAAATATTATTAGTAAAAATAGATTAGAATTATATATATTAAACAATAATAATTTAAATATGAA
Candidatus Carsonella 135557 - 167442nt	(1)	---
SARS-CoV-2 Reference Genome NC_045512.2	(1)	---

Section 1438

	(110650)	110650 110660 110671 110682 110693 110704 110715 110726
Candidatus Carsonella ruddii strain BT chromosome...	(110650)	AAGTTTTCAATTTCATTCAGAATCTATTTTATCAAATAAAAATTTTTTTATATGATTAAAAATAAATTAAAAAAAAT
Candidatus Carsonella 135557 - 167442nt	(1)	---
SARS-CoV-2 Reference Genome NC_045512.2	(1)	---

Section 1439

	(110727)	110727 110737 110748 110759 110770 110781 110792 110803
Candidatus Carsonella ruddii strain BT chromosome...	(110727)	TATATCTAATAATATTTTTATTAATAATAAATATTTATTATTTAAATTATTTTTTGTAAAAAAACATTTTTCTAATC
Candidatus Carsonella 135557 - 167442nt	(1)	---
SARS-CoV-2 Reference Genome NC_045512.2	(1)	---

Section 1440

	(110804)	110804 110814 110825 110836 110847 110858 110869 110880
Candidatus Carsonella ruddii strain BT chromosome...	(110804)	AAAAAAATTTTTTTTATAAAATTTTAATAAATTTAGAAATTATTAAAAAAAAATTTATTAATTAAAAAAAAATATTT
Candidatus Carsonella 135557 - 167442nt	(1)	---
SARS-CoV-2 Reference Genome NC_045512.2	(1)	---

Section 1441

(110881) 110881 110891 110902 110913 110924 110935 110946 110957

Candidatus Carsonella ruddii strain BT chromosome... (110881) AATAATTTAAACATTTGTGGAAGTGGTGGTGATAATATAATTTTACCAAATATATCAACTTCAGATTGTTTAATTTT
Candidatus Carsonella 135557 - 167442nt (1) ---
SARS-CoV-2 Reference Genome NC_045512.2 (1) ---

Section 1442

(110958) 110958 110968 110979 110990 111001 111012 111023 111034

Candidatus Carsonella ruddii strain BT chromosome... (110958) TTATTTTTTAAAAAAAAATTTAATTAAAAATTCTGGAAATTCATTTTTATTAAAAAATGGAAGTATAGATTACTTAA
Candidatus Carsonella 135557 - 167442nt (1) ---
SARS-CoV-2 Reference Genome NC_045512.2 (1) ---

Section 1443

(111035) 111035 111045 111056 111067 111078 111089 111100 111111

Candidatus Carsonella ruddii strain BT chromosome... (111035) ATAAATTTAAATTTTTAAATAATTATATTTTAAAAAAAAAAACAAATATTTTTTTATATATTAAAAATTTTTACAAT
Candidatus Carsonella 135557 - 167442nt (1) ---
SARS-CoV-2 Reference Genome NC_045512.2 (1) ---

Section 1444

(111112) 111112 111122 111133 111144 111155 111166 111177 111188

Candidatus Carsonella ruddii strain BT chromosome... (111112) TATAATTTTAATTTTAGAAAAAAAATTAAATTAAATAATATTTTTAATATACTTAATTCTATATTAAATATTTTATT
Candidatus Carsonella 135557 - 167442nt (1) ---
SARS-CoV-2 Reference Genome NC_045512.2 (1) ---

Section 1445

(111189) 111189 111199 111210 111221 111232 111243 111254 111265

Candidatus Carsonella ruddii strain BT chromosome... (111189) AAATATTAAAAATATAATAGGAATTTATTCAATAAAAATAATTTTTTTATATAAATTTTTAAATATTAATAATTTAA
Candidatus Carsonella 135557 - 167442nt (1) ---
SARS-CoV-2 Reference Genome NC_045512.2 (1) ---

Section 1446

(111266) 111266 111276 111287 111298 111309 111320 111331 111342

Candidatus Carsonella ruddii strain BT chromosome... (111266) TAATTTTTAATAATAAAAGTGATGATATTATTCAAAATAAAGAAATTTTTTTTTTTGAAAAAAAAATATTTTTTTTA
Candidatus Carsonella 135557 - 167442nt (1) ---
SARS-CoV-2 Reference Genome NC_045512.2 (1) ---

```
                                                                                Section 1447
                                               (111343) 111343    111353     111364     111375     111386     111397     111408     111419
Candidatus Carsonella ruddii strain BT chromosome... (111343) AATAATAATCAATATAATTTACATAATTTTTTTTTTAATAAAAATATTTTTTTTGAAAATTTAAAATTTCAATTATT
            Candidatus Carsonella 135557 - 167442nt      (1) -----------------------------------------------------------------------------
          SARS-CoV-2 Reference Genome NC_045512.2       (1) -----------------------------------------------------------------------------
                                                                                Section 1448
                                               (111420) 111420    111430     111441     111452     111463     111474     111485     111496
Candidatus Carsonella ruddii strain BT chromosome... (111420) TAATATAAAATTAAAAAATAATATTTTTATTTATAGTAAAATAATATTATTAAATAATTTTTTATTTATTAACAAAA
            Candidatus Carsonella 135557 - 167442nt      (1) -----------------------------------------------------------------------------
          SARS-CoV-2 Reference Genome NC_045512.2       (1) -----------------------------------------------------------------------------
                                                                                Section 1449
                                               (111497) 111497    111507     111518     111529     111540     111551     111562     111573
Candidatus Carsonella ruddii strain BT chromosome... (111497) AAAAACCATTTTTTTATTTTTATTTATTAATAAATAATTACTTTTACATTTTTTTAAAAAAAATAATTAAAAAAAAT
            Candidatus Carsonella 135557 - 167442nt      (1) -----------------------------------------------------------------------------
          SARS-CoV-2 Reference Genome NC_045512.2       (1) -----------------------------------------------------------------------------
                                                                                Section 1450
                                               (111574) 111574    111584     111595     111606     111617     111628     111639     111650
Candidatus Carsonella ruddii strain BT chromosome... (111574) AATGAATAAAATTTTATTTAACAAAAAAAAATATAAATTTTTTTATAATTTTATTATAAATAATTTTAATATTAAAA
            Candidatus Carsonella 135557 - 167442nt      (1) -----------------------------------------------------------------------------
          SARS-CoV-2 Reference Genome NC_045512.2       (1) -----------------------------------------------------------------------------
                                                                                Section 1451
                                               (111651) 111651    111661     111672     111683     111694     111705     111716     111727
Candidatus Carsonella ruddii strain BT chromosome... (111651) AAAATATTTTTAAAAAAGAAATTTTAAAAAAATTTAAAAAAAACAACATTTCTTTTATTTTAGAATATAAAAAACAA
            Candidatus Carsonella 135557 - 167442nt      (1) -----------------------------------------------------------------------------
          SARS-CoV-2 Reference Genome NC_045512.2       (1) -----------------------------------------------------------------------------
                                                                                Section 1452
                                               (111728) 111728    111738     111749     111760     111771     111782     111793     111804
Candidatus Carsonella ruddii strain BT chromosome... (111728) TCTCCATATAAAGGTTTATATAAAAAAAATATTTTAAAACAAATTAAAAAATATGAAATTTTAGGTTGTATATGTTT
            Candidatus Carsonella 135557 - 167442nt      (1) -----------------------------------------------------------------------------
          SARS-CoV-2 Reference Genome NC_045512.2       (1) -----------------------------------------------------------------------------
```

Section 1453

```
                                                        (111805) 111805    111815     111826     111837     111848     111859     111870    111881
Candidatus Carsonella ruddii strain BT chromosome...    (111805) ATCAATATTAAACGAATGTGTTCATTTTAAATGTAATTATATTGATTTTTTATTTATAAAAAAAAATACCAATCTTT
                Candidatus Carsonella 135557 - 167442nt (1)      -----------------------------------------------------------------------------
             SARS-CoV-2 Reference Genome NC_045512.2    (1)      -----------------------------------------------------------------------------
```

Section 1454

```
                                                        (111882) 111882    111892     111903     111914     111925     111936     111947    111958
Candidatus Carsonella ruddii strain BT chromosome...    (111882) GTATTCTTAAAAAAGATTTTATTTTTAATGAATTTCAAGTAATTCATTCTTATATACTAGGTTATAATATAATTTTG
                Candidatus Carsonella 135557 - 167442nt (1)      -----------------------------------------------------------------------------
             SARS-CoV-2 Reference Genome NC_045512.2    (1)      -----------------------------------------------------------------------------
```

Section 1455

```
                                                        (111959) 111959    111969     111980     111991     112002     112013     112024    112035
Candidatus Carsonella ruddii strain BT chromosome...    (111959) TTATTATCTAGTATTAAATTACACATATTACAAAAAATATATTTTATTTGTAAAAAATTAAAAATTTTTATTATTTT
                Candidatus Carsonella 135557 - 167442nt (1)      -----------------------------------------------------------------------------
             SARS-CoV-2 Reference Genome NC_045512.2    (1)      -----------------------------------------------------------------------------
```

Section 1456

```
                                                        (112036) 112036    112046     112057     112068     112079     112090     112101    112112
Candidatus Carsonella ruddii strain BT chromosome...    (112036) AGAAATTAATAATTTAAAAGAAATAAAAAAAATAAAAAATATAAAACCATTATTTTTAGGAATAAATTGTAGAAATT
                Candidatus Carsonella 135557 - 167442nt (1)      -----------------------------------------------------------------------------
             SARS-CoV-2 Reference Genome NC_045512.2    (1)      -----------------------------------------------------------------------------
```

Section 1457

```
                                                        (112113) 112113    112123     112134     112145     112156     112167     112178    112189
Candidatus Carsonella ruddii strain BT chromosome...    (112113) TAAAAAATTTAATAATAAACAAAAAAAAAATAAAATTATTAATTAAAAAAATACCAAAAAATTGTTTTATAATTGCA
                Candidatus Carsonella 135557 - 167442nt (1)      -----------------------------------------------------------------------------
             SARS-CoV-2 Reference Genome NC_045512.2    (1)      -----------------------------------------------------------------------------
```

Section 1458

```
                                                        (112190) 112190    112200     112211     112222     112233     112244     112255    112266
Candidatus Carsonella ruddii strain BT chromosome...    (112190) GAATCAGGTATTGTAGATTATAATGAATTATTTTTTTTTATTGATAACAATATTAGAATATTTTTAATAGGTGATTA
                Candidatus Carsonella 135557 - 167442nt (1)      -----------------------------------------------------------------------------
             SARS-CoV-2 Reference Genome NC_045512.2    (1)      -----------------------------------------------------------------------------
```

Section 1459

	(112267)	112267 112277 112288 112299 112310 112321 112332 112343
Candidatus Carsonella ruddii strain BT chromosome...	(112267)	TTTTTTAAATAAAATAAATTGTTTTAAATATTATTTTAAATTATTAAATTAATTTCCTTGGTAGCTCAGTTGGTAGA
Candidatus Carsonella 135557 - 167442nt	(1)	---
SARS-CoV-2 Reference Genome NC_045512.2	(1)	---

Section 1460

	(112344)	112344 112354 112365 112376 112387 112398 112409 112420
Candidatus Carsonella ruddii strain BT chromosome...	(112344)	GCAAGTGACTGTTAATCATTTGGTCACAGGTTCAAGTCCTGTCTAAGGAGTAGTTATATCCTGATTCGAACAGGAGA
Candidatus Carsonella 135557 - 167442nt	(1)	---
SARS-CoV-2 Reference Genome NC_045512.2	(1)	---

Section 1461

	(112421)	112421 112431 112442 112453 112464 112475 112486 112497
Candidatus Carsonella ruddii strain BT chromosome...	(112421)	CCTAATCATCATGAATGATTTGCTCTACCAACTAAGCTATATAACCAACAAAAATATACAAATTTTTATAAAAAATG
Candidatus Carsonella 135557 - 167442nt	(1)	---
SARS-CoV-2 Reference Genome NC_045512.2	(1)	---

Section 1462

	(112498)	112498 112508 112519 112530 112541 112552 112563 112574
Candidatus Carsonella ruddii strain BT chromosome...	(112498)	TCAAATTTGTTAAAAAATAATTTATTTTTTTTTTTTTAAAAAAGAAAAAAAAGAAAAATTTTTTTCAGAATAAAATT
Candidatus Carsonella 135557 - 167442nt	(1)	---
SARS-CoV-2 Reference Genome NC_045512.2	(1)	---

Section 1463

	(112575)	112575 112585 112596 112607 112618 112629 112640 112651
Candidatus Carsonella ruddii strain BT chromosome...	(112575)	TTTTTTTAAAAATAATATTTAAATTTATTGATCTAGGATAAAAATTGTTATTAATAAAATTTATTGTTTTTATATTC
Candidatus Carsonella 135557 - 167442nt	(1)	---
SARS-CoV-2 Reference Genome NC_045512.2	(1)	---

Section 1464

	(112652)	112652 112662 112673 112684 112695 112706 112717 112728
Candidatus Carsonella ruddii strain BT chromosome...	(112652)	TTTATATTATTGTTTTTATAATAAATTTTAAATTTTTTTTTAAACGTTTCTAATATAAAATATTTAATTTTATTATT
Candidatus Carsonella 135557 - 167442nt	(1)	---
SARS-CoV-2 Reference Genome NC_045512.2	(1)	---

Section 1465

		112729 112739 112750 112761 112772 112783 112794 112805
Candidatus Carsonella ruddii strain BT chromosome...	(112729)	TATAGTAGAATATACTACTTTTTTAATAAAAGTAAAATAAATTTTATTTAACAAAACAACAATTAAAAAATTATTTT
Candidatus Carsonella 135557 - 167442nt	(1)	---
SARS-CoV-2 Reference Genome NC_045512.2	(1)	---

Section 1466

		112806 112816 112827 112838 112849 112860 112871 112882
Candidatus Carsonella ruddii strain BT chromosome...	(112806)	TTAATATTTTTTTTAAATAGTTATTATTAAATGTATAAATATTAATAATTTTATTATTATTTATAAAATCTCCAAGA
Candidatus Carsonella 135557 - 167442nt	(1)	---
SARS-CoV-2 Reference Genome NC_045512.2	(1)	---

Section 1467

		112883 112893 112904 112915 112926 112937 112948 112959
Candidatus Carsonella ruddii strain BT chromosome...	(112883)	ATAATTTTATTATTATTTTTTATTTTATTAAAAATTTTAATTTTATATTTAAATTTCATATTTTTCTAATATAATTT
Candidatus Carsonella 135557 - 167442nt	(1)	---
SARS-CoV-2 Reference Genome NC_045512.2	(1)	---

Section 1468

		112960 112970 112981 112992 113003 113014 113025 113036
Candidatus Carsonella ruddii strain BT chromosome...	(112960)	TTTTAATTTCTTCTATAAATAAATATTTAGTAGTTATAATTTTAATTTTATCATTAAAATAATTAAAATTAATAGTG
Candidatus Carsonella 135557 - 167442nt	(1)	---
SARS-CoV-2 Reference Genome NC_045512.2	(1)	---

Section 1469

		113037 113047 113058 113069 113080 113091 113102 113113
Candidatus Carsonella ruddii strain BT chromosome...	(113037)	TCTGTTACTATTAATTTATTTATTTTAGAATTATAAATTTTTATAAAAGATTTTTCAGAAAAAATAGAATGAGTAAT
Candidatus Carsonella 135557 - 167442nt	(1)	---
SARS-CoV-2 Reference Genome NC_045512.2	(1)	---

Section 1470

		113114 113124 113135 113146 113157 113168 113179 113190
Candidatus Carsonella ruddii strain BT chromosome...	(113114)	ATAAACAATTACAGTATTTATTTTATTAAAAAATAAAAAATTAGTAACTTCTATTATAGAATTTCCAGTATCAATAA
Candidatus Carsonella 135557 - 167442nt	(1)	---
SARS-CoV-2 Reference Genome NC_045512.2	(1)	---

Section 1471

	(113191)	113191 113201 113212 113223 113234 113245 113256 113267
Candidatus Carsonella ruddii strain BT chromosome...	(113191)	TATCATCAACAACAATACATTTTTTAAATTCTACATTTCCAATAATACAAAAAATTTTTATATAATTTGAAATTGGT
Candidatus Carsonella 135557 - 167442nt	(1)	---
SARS-CoV-2 Reference Genome NC_045512.2	(1)	---

Section 1472

	(113268)	113268 113278 113289 113300 113311 113322 113333 113344
Candidatus Carsonella ruddii strain BT chromosome...	(113268)	CTTCTTTTATCAATTATCACTAATTCTAAATTTAAATAATTTGCTAAATTTTTCGCTCTTAAAACTCCACCTATATC
Candidatus Carsonella 135557 - 167442nt	(1)	---
SARS-CoV-2 Reference Genome NC_045512.2	(1)	---

Section 1473

	(113345)	113345 113355 113366 113377 113388 113399 113410 113421
Candidatus Carsonella ruddii strain BT chromosome...	(113345)	AGGTGAAACAATAATATCAAAACTAATATTATTTTTAACAATATCATTAACAAATAAAGATGTAGATTTTAATATTT
Candidatus Carsonella 135557 - 167442nt	(1)	---
SARS-CoV-2 Reference Genome NC_045512.2	(1)	---

Section 1474

	(113422)	113422 113432 113443 113454 113465 113476 113487 113498
Candidatus Carsonella ruddii strain BT chromosome...	(113422)	TTAAAGGTGTATTAAAACAACATTCAAAATTACTAGAATGTGGATCTAATGTAATAATTTTGTTTACATTAAAAAAA
Candidatus Carsonella 135557 - 167442nt	(1)	---
SARS-CoV-2 Reference Genome NC_045512.2	(1)	---

Section 1475

	(113499)	113499 113509 113520 113531 113542 113553 113564 113575
Candidatus Carsonella ruddii strain BT chromosome...	(113499)	TCAAATAAATTAGAAATTAATTTAATAGAAATAGAAGAATTTTCGTTAAATATTTTGTCTTGCCTAGAATAACAAAA
Candidatus Carsonella 135557 - 167442nt	(1)	---
SARS-CoV-2 Reference Genome NC_045512.2	(1)	---

Section 1476

	(113576)	113576 113586 113597 113608 113619 113630 113641 113652
Candidatus Carsonella ruddii strain BT chromosome...	(113576)	ATAAGGTATTATTAAATATATTTTTTTAATACATATTTTTTTTAAAGAATCAATTATTAAAAATAAATTTATTAAAT
Candidatus Carsonella 135557 - 167442nt	(1)	---
SARS-CoV-2 Reference Genome NC_045512.2	(1)	---

Section 1477

```
                                                         (113653) 113653    113663     113674     113685     113696     113707     113718     113729
Candidatus Carsonella ruddii strain BT chromosome... (113653) TATCATGAATAGGATTAGATAAACTTTGTAAAATAAAAATAATTTTTTCATTAAATTTTTTTAAAAATTGAATTTTA
          Candidatus Carsonella 135557 - 167442nt (1) -----------------------------------------------------------------------------
          SARS-CoV-2 Reference Genome NC_045512.2 (1) -----------------------------------------------------------------------------
```

Section 1478

```
                                                         (113730) 113730    113740     113751     113762     113773     113784     113795     113806
Candidatus Carsonella ruddii strain BT chromosome... (113730) TATTCATTATCTTTAAATTTTTCAATTTTAATATAATTTATTTTTATATTAAAAATTTTTAGCAAACTTTTAAAAAA
          Candidatus Carsonella 135557 - 167442nt (1) -----------------------------------------------------------------------------
          SARS-CoV-2 Reference Genome NC_045512.2 (1) -----------------------------------------------------------------------------
```

Section 1479

```
                                                         (113807) 113807    113817     113828     113839     113850     113861     113872     113883
Candidatus Carsonella ruddii strain BT chromosome... (113807) ATTTAAGTTTGAATCTCCAAATATAATATTTGGTTTTGAAATTTTCATATGAAAAATAATTTAATTTTAGAAATTAG
          Candidatus Carsonella 135557 - 167442nt (1) -----------------------------------------------------------------------------
          SARS-CoV-2 Reference Genome NC_045512.2 (1) -----------------------------------------------------------------------------
```

Section 1480

```
                                                         (113884) 113884    113894     113905     113916     113927     113938     113949     113960
Candidatus Carsonella ruddii strain BT chromosome... (113884) ACAAGGAATAGGAGGAGATGAATCCATCCTTTTTTTAAAAGATTTAAATAAAATGTATATTAAATTTTTTCAAAAAA
          Candidatus Carsonella 135557 - 167442nt (1) -----------------------------------------------------------------------------
          SARS-CoV-2 Reference Genome NC_045512.2 (1) -----------------------------------------------------------------------------
```

Section 1481

```
                                                         (113961) 113961    113971     113982     113993     114004     114015     114026     114037
Candidatus Carsonella ruddii strain BT chromosome... (113961) AAAAAATTAAATTTAATATATTTTTAAAAAAATAATAAAGAAATTATTATTAAAATAGAAAATAATAATTATTCTAAT
          Candidatus Carsonella 135557 - 167442nt (1) -----------------------------------------------------------------------------
          SARS-CoV-2 Reference Genome NC_045512.2 (1) -----------------------------------------------------------------------------
```

Section 1482

```
                                                         (114038) 114038    114048     114059     114070     114081     114092     114103     114114
Candidatus Carsonella ruddii strain BT chromosome... (114038) TTTTTTTTAAATGAATCTGGAATTCATAGAGTTCAAAGAATACCAAAAAAATGAAACACAAGGTAAAATACATACATC
          Candidatus Carsonella 135557 - 167442nt (1) -----------------------------------------------------------------------------
          SARS-CoV-2 Reference Genome NC_045512.2 (1) -----------------------------------------------------------------------------
```

```
                                                                                              Section 1483
                                             (114115) 114115    114125     114136     114147     114158     114169     114180 114191
Candidatus Carsonella ruddii strain BT chromosome... (114115) CACTTGTAGTGTTTTTGTTTCTAATGAAACAGATATAAAACATATTATTTTAGAAAATCAAGATTTAAAAATAGAAA
            Candidatus Carsonella 135557 - 167442nt       (1) -----------------------------------------------------------------------------
        SARS-CoV-2 Reference Genome NC_045512.2           (1) -----------------------------------------------------------------------------
                                                                                              Section 1484
                                             (114192) 114192    114202     114213     114224     114235     114246     114257 114268
Candidatus Carsonella ruddii strain BT chromosome... (114192) CTTGTAAATCAAGTGGATCAGGTGGACAACATGTAAATACAACAAATTCTTCTATTAAAATTACACATTTACCTACA
            Candidatus Carsonella 135557 - 167442nt       (1) -----------------------------------------------------------------------------
        SARS-CoV-2 Reference Genome NC_045512.2           (1) -----------------------------------------------------------------------------
                                                                                              Section 1485
                                             (114269) 114269    114279     114290     114301     114312     114323     114334 114345
Candidatus Carsonella ruddii strain BT chromosome... (114269) AATATTTATGTTGAATGTAGTGATGAACGTTCACAATTTTTAAATAAAAATAAAGCTTTAGAAATTTTAAATATAAA
            Candidatus Carsonella 135557 - 167442nt       (1) -----------------------------------------------------------------------------
        SARS-CoV-2 Reference Genome NC_045512.2           (1) -----------------------------------------------------------------------------
                                                                                              Section 1486
                                             (114346) 114346    114356     114367     114378     114389     114400     114411 114422
Candidatus Carsonella ruddii strain BT chromosome... (114346) ATTAAATAAATTAAAAAATGAAAAATTCAATAATGAATTAAATTTTATTAGAAAAAATATTATTTCTAAATCAGAAA
            Candidatus Carsonella 135557 - 167442nt       (1) -----------------------------------------------------------------------------
        SARS-CoV-2 Reference Genome NC_045512.2           (1) -----------------------------------------------------------------------------
                                                                                              Section 1487
                                             (114423) 114423    114433     114444     114455     114466     114477     114488 114499
Candidatus Carsonella ruddii strain BT chromosome... (114423) GATCAAAAAAAATAAGAACATATAATTTTCCAAATAATAAAATAATAGATCATGTTAAAAATAATTGTTATTTTCAA
            Candidatus Carsonella 135557 - 167442nt       (1) -----------------------------------------------------------------------------
        SARS-CoV-2 Reference Genome NC_045512.2           (1) -----------------------------------------------------------------------------
                                                                                              Section 1488
                                             (114500) 114500    114510     114521     114532     114543     114554     114565 114576
Candidatus Carsonella ruddii strain BT chromosome... (114500) TTAAATAAAATTTTAAATGGTGATTTAGATATTATGTTTAAAAATTAAAAATAAACAAATTTTTTATTTTAAAAAAA
            Candidatus Carsonella 135557 - 167442nt       (1) -----------------------------------------------------------------------------
        SARS-CoV-2 Reference Genome NC_045512.2           (1) -----------------------------------------------------------------------------
```

```
                                                                                      Section 1489
                                                  (114577) 114577    114587     114598     114609     114620     114631     114642     114653
Candidatus Carsonella ruddii strain BT chromosome... (114577) ATTTTTTTAAATAAATTTAATTTTAAAAAGAAAAAAAATTTTTTTTTATTTTTATATATAAAAATAAATATATTATA
                  Candidatus Carsonella 135557 - 167442nt (1) -----------------------------------------------------------------------------
               SARS-CoV-2 Reference Genome NC_045512.2 (1) -----------------------------------------------------------------------------
                                                                                      Section 1490
                                                  (114654) 114654    114664     114675     114686     114697     114708     114719     114730
Candidatus Carsonella ruddii strain BT chromosome... (114654) ATAAAAAAAAAATTTATTTAAAAAATATTTATTTTTATTAAAAAAAAATATGTTTATTATTAATTTTATATTTTTTTT
                  Candidatus Carsonella 135557 - 167442nt (1) -----------------------------------------------------------------------------
               SARS-CoV-2 Reference Genome NC_045512.2 (1) -----------------------------------------------------------------------------
                                                                                      Section 1491
                                                  (114731) 114731    114741     114752     114763     114774     114785     114796     114807
Candidatus Carsonella ruddii strain BT chromosome... (114731) TAGGAAATTTTTTAAAAAAATAACCAATTTTTTTTTTTTTAAAAATTATTTCAAAATCAAAATTTATAATATAACCA
                  Candidatus Carsonella 135557 - 167442nt (1) -----------------------------------------------------------------------------
               SARS-CoV-2 Reference Genome NC_045512.2 (1) -----------------------------------------------------------------------------
                                                                                      Section 1492
                                                  (114808) 114808    114818     114829     114840     114851     114862     114873     114884
Candidatus Carsonella ruddii strain BT chromosome... (114808) ATATCATAATCAATAAAATAATTATAATTTTTTGAAAAACATTTTTTTCTTAATTTAGATGTATAATTAATATTAAA
                  Candidatus Carsonella 135557 - 167442nt (1) -----------------------------------------------------------------------------
               SARS-CoV-2 Reference Genome NC_045512.2 (1) -----------------------------------------------------------------------------
                                                                                      Section 1493
                                                  (114885) 114885    114895     114906     114917     114928     114939     114950     114961
Candidatus Carsonella ruddii strain BT chromosome... (114885) ATTTCTAGTTTGTGATAATAAATTATTTTTTTTTTCTAAATTTATTATTTTTCTAAAAATTTCATATTTTATAGATT
                  Candidatus Carsonella 135557 - 167442nt (1) -----------------------------------------------------------------------------
               SARS-CoV-2 Reference Genome NC_045512.2 (1) -----------------------------------------------------------------------------
                                                                                      Section 1494
                                                  (114962) 114962    114972     114983     114994     115005     115016     115027     115038
Candidatus Carsonella ruddii strain BT chromosome... (114962) TCTTAATATTTTCATATGAATTTAAATTTTTAATTTCTGTTTTTTTACTTTTTTTAAAATTAAAATTATTTAAAACA
                  Candidatus Carsonella 135557 - 167442nt (1) -----------------------------------------------------------------------------
               SARS-CoV-2 Reference Genome NC_045512.2 (1) -----------------------------------------------------------------------------
```

Section 1495

	(115039)	115039 115049 115060 115071 115082 115093 115104 115115
Candidatus Carsonella ruddii strain BT chromosome...	(115039)	GAAATATTTAAATCAATACGCATTTCTCCTAAATGCATTTTTAAATTAGAAATTTTATGTTTAATAAATTTTTTTTT
Candidatus Carsonella 135557 - 167442nt	(1)	---
SARS-CoV-2 Reference Genome NC_045512.2	(1)	---

Section 1496

	(115116)	115116 115126 115137 115148 115159 115170 115181 115192
Candidatus Carsonella ruddii strain BT chromosome...	(115116)	TATTTTTTTTAAGAAAAAATATAAACAATTAAAATTTGAAAAATTTGGTTCAGTAACTAATTCAAATAAACCATTTC
Candidatus Carsonella 135557 - 167442nt	(1)	---
SARS-CoV-2 Reference Genome NC_045512.2	(1)	---

Section 1497

	(115193)	115193 115203 115214 115225 115236 115247 115258 115269
Candidatus Carsonella ruddii strain BT chromosome...	(115193)	CACTTCTTTTATAATTTATTTTTAAAAAATTACTTTCTTTTTTAGAAGAAGCAGCATCTTCTTCTAAATGAATTTTT
Candidatus Carsonella 135557 - 167442nt	(1)	---
SARS-CoV-2 Reference Genome NC_045512.2	(1)	---

Section 1498

	(115270)	115270 115280 115291 115302 115313 115324 115335 115346
Candidatus Carsonella ruddii strain BT chromosome...	(115270)	TTAATAACAATTTTTTTTTTACATTTATTATTAATTACTAAATTAAATAAATGTTGAAAATTATTTTGAGTTAATTG
Candidatus Carsonella 135557 - 167442nt	(1)	---
SARS-CoV-2 Reference Genome NC_045512.2	(1)	---

Section 1499

	(115347)	115347 115357 115368 115379 115390 115401 115412 115423
Candidatus Carsonella ruddii strain BT chromosome...	(115347)	ATAATTTTTTGATAAATCATAATAAAAATAATTTTTTCTTTCAATAATAGATAGTAAAAAACAACGACTTATTAATA
Candidatus Carsonella 135557 - 167442nt	(1)	---
SARS-CoV-2 Reference Genome NC_045512.2	(1)	---

Section 1500

	(115424)	115424 115434 115445 115456 115467 115478 115489 115500
Candidatus Carsonella ruddii strain BT chromosome...	(115424)	TTTTATTTATTAAAAATATTAAATTAATAATTTTAATGTTAAATATTGGTAATTTTCCTGGATATGCAATATCATAT
Candidatus Carsonella 135557 - 167442nt	(1)	---
SARS-CoV-2 Reference Genome NC_045512.2	(1)	---

```
                                                                                                         Section 1501
                                                   (115501) 115501    115511      115522      115533      115544      115555      115566  115577
Candidatus Carsonella ruddii strain BT chromosome... (115501) AAATTAAAATTATTATTAGAAAAAATTTTATTATTAATTAAACTTATATGTATTTCACAACCTATAAATATTTTATA
                Candidatus Carsonella 135557 - 167442nt (1) -----------------------------------------------------------------------------
            SARS-CoV-2 Reference Genome NC_045512.2 (1) -----------------------------------------------------------------------------
                                                                                                         Section 1502
                                                   (115578) 115578    115588      115599      115610      115621      115632      115643  115654
Candidatus Carsonella ruddii strain BT chromosome... (115578) ATATTTTTTAATTAAAAACATAATTTTTAAAATTAAAAGATTCGTATAAAATTGATAATTCTAATAAATCTAAATCA
                Candidatus Carsonella 135557 - 167442nt (1) -----------------------------------------------------------------------------
            SARS-CoV-2 Reference Genome NC_045512.2 (1) -----------------------------------------------------------------------------
                                                                                                         Section 1503
                                                   (115655) 115655    115665      115676      115687      115698      115709      115720  115731
Candidatus Carsonella ruddii strain BT chromosome... (115655) TTATTTTTATTACAAATTATTTGAAATCCTATAGGTGAATGATTAATAAATCCTATTCTTAAATTAATAGTAGGAAA
                Candidatus Carsonella 135557 - 167442nt (1) -----------------------------------------------------------------------------
            SARS-CoV-2 Reference Genome NC_045512.2 (1) -----------------------------------------------------------------------------
                                                                                                         Section 1504
                                                   (115732) 115732    115742      115753      115764      115775      115786      115797  115808
Candidatus Carsonella ruddii strain BT chromosome... (115732) ACCTAAAATATTAGATATCATTGTAAATAAATCAACATATTCTGAATAAATATTATTTTTTTTTTCAAAAATATCTA
                Candidatus Carsonella 135557 - 167442nt (1) -----------------------------------------------------------------------------
            SARS-CoV-2 Reference Genome NC_045512.2 (1) -----------------------------------------------------------------------------
                                                                                                         Section 1505
                                                   (115809) 115809    115819      115830      115841      115852      115863      115874  115885
Candidatus Carsonella ruddii strain BT chromosome... (115809) AATTCGTTGGTAATAATATAAAATTTGATTTTTCAAAACAATTTTGAAAAATTTTTTCAATTTTTTTAATTTCTAAA
                Candidatus Carsonella 135557 - 167442nt (1) -----------------------------------------------------------------------------
            SARS-CoV-2 Reference Genome NC_045512.2 (1) -----------------------------------------------------------------------------
                                                                                                         Section 1506
                                                   (115886) 115886    115896      115907      115918      115929      115940      115951  115962
Candidatus Carsonella ruddii strain BT chromosome... (115886) ATATTAAAATTTTTTTTAAATATAAAAAAATTATTACCTTTAATAATTTTATTTTTACAATCTTTAGAAAATATTCT
                Candidatus Carsonella 135557 - 167442nt (1) -----------------------------------------------------------------------------
            SARS-CoV-2 Reference Genome NC_045512.2 (1) -----------------------------------------------------------------------------
```

Section 1507

	(115963)	115963 115973 115984 115995 116006 116017 116028 116039
Candidatus Carsonella ruddii strain BT chromosome...	(115963)	ATTTAATTTTATAAAATCATTTATATTTTTATAAAAAAAATTATTAAATCCGTACTTTATACCATCAAATTTACATG
Candidatus Carsonella 135557 - 167442nt	(1)	---
SARS-CoV-2 Reference Genome NC_045512.2	(1)	---

Section 1508

	(116040)	116040 116050 116061 116072 116083 116094 116105 116116
Candidatus Carsonella ruddii strain BT chromosome...	(116040)	TATTTGAAAAAAAATCTTTAGATGAAATTGAATTATATAAATTTAAAAAAGAACAAAAATTTATATTTTCATTAATT
Candidatus Carsonella 135557 - 167442nt	(1)	---
SARS-CoV-2 Reference Genome NC_045512.2	(1)	---

Section 1509

	(116117)	116117 116127 116138 116149 116160 116171 116182 116193
Candidatus Carsonella ruddii strain BT chromosome...	(116117)	ATATTAAAATTTAATTCTTCAAAATTTTTAATAATTTTTTCAAATATTATTTTTAATTCATTTGAAGAAAAAAATTC
Candidatus Carsonella 135557 - 167442nt	(1)	---
SARS-CoV-2 Reference Genome NC_045512.2	(1)	---

Section 1510

	(116194)	116194 116204 116215 116226 116237 116248 116259 116270
Candidatus Carsonella ruddii strain BT chromosome...	(116194)	TTTGTAATTTAAAATTAAAATTTTTTTTTTTTTTTTAAAATTAATAAATGTTTTTTTAATATTAGAAAATAAATCTA
Candidatus Carsonella 135557 - 167442nt	(1)	---
SARS-CoV-2 Reference Genome NC_045512.2	(1)	---

Section 1511

	(116271)	116271 116281 116292 116303 116314 116325 116336 116347
Candidatus Carsonella ruddii strain BT chromosome...	(116271)	AATTTTTATATCTTAAAATATTAAATAAAAATTTACAATCTTCTGCATAATTTGTTATAATAGAACAATTATCTAAA
Candidatus Carsonella 135557 - 167442nt	(1)	---
SARS-CoV-2 Reference Genome NC_045512.2	(1)	---

Section 1512

	(116348)	116348 116358 116369 116380 116391 116402 116413 116424
Candidatus Carsonella ruddii strain BT chromosome...	(116348)	AATGATGAATAAGGAATCATACCGTTTCTTGTAAAAATACCGTTTGTAGGTTTAAATCCTATTATATTAGAAAATGA
Candidatus Carsonella 135557 - 167442nt	(1)	---
SARS-CoV-2 Reference Genome NC_045512.2	(1)	---

Section 1513

		116425 116435 116446 116457 116468 116479 116490 116501
	(116425)	
Candidatus Carsonella ruddii strain BT chromosome...	(116425)	TGAAGGAGTTCTACTCGAACCACCTGTATCACTTCCTAATGATAATAAATTACAACCATAAGAAATATTTAAACAAG
Candidatus Carsonella 135557 - 167442nt	(1)	---
SARS-CoV-2 Reference Genome NC_045512.2	(1)	---

Section 1514

		116502 116512 116523 116534 116545 116556 116567 116578
	(116502)	
Candidatus Carsonella ruddii strain BT chromosome...	(116502)	AACCAGTTGATGATCCACCAATTATATTTTTTTTATTAAATAAATTTTTTAAATTTAAGTTATTTATTCCATCACTA
Candidatus Carsonella 135557 - 167442nt	(1)	---
SARS-CoV-2 Reference Genome NC_045512.2	(1)	---

Section 1515

		116579 116589 116600 116611 116622 116633 116644 116655
	(116579)	
Candidatus Carsonella ruddii strain BT chromosome...	(116579)	CCTAAACAAAATTCATCTAATAAATCTATTGATAAAATATTAAAATTATTTATTTTTAAAATTTTTACAATATCTGA
Candidatus Carsonella 135557 - 167442nt	(1)	---
SARS-CoV-2 Reference Genome NC_045512.2	(1)	---

Section 1516

		116656 116666 116677 116688 116699 116710 116721 116732
	(116656)	
Candidatus Carsonella ruddii strain BT chromosome...	(116656)	ATTATATTTTGAATTATGTTTTTTTAAAATATTTGAATTTGATGTTAATTTTTTGTTTTTAATTGAAAAAATATTTT
Candidatus Carsonella 135557 - 167442nt	(1)	---
SARS-CoV-2 Reference Genome NC_045512.2	(1)	---

Section 1517

		116733 116743 116754 116765 116776 116787 116798 116809
	(116733)	
Candidatus Carsonella ruddii strain BT chromosome...	(116733)	TTAATGATATAGGTATAGCAAATGTTTTTTTATTATCTAATTGTTTAGCAAAATTTAATGATTCTTTATAAAAAAAA
Candidatus Carsonella 135557 - 167442nt	(1)	---
SARS-CoV-2 Reference Genome NC_045512.2	(1)	---

Section 1518

		116810 116820 116831 116842 116853 116864 116875 116886
	(116810)	
Candidatus Carsonella ruddii strain BT chromosome...	(116810)	TCAATAATAAAATTATTTTTTTTATTAATTTTTATTAAATTATTTAAACAACATTTAGTTAATTCATAATTAGATAT
Candidatus Carsonella 135557 - 167442nt	(1)	---
SARS-CoV-2 Reference Genome NC_045512.2	(1)	---

Section 1519

	(116887)	116887 116897 116908 116919 116930 116941 116952 116963
Candidatus Carsonella ruddii strain BT chromosome...	(116887)	TATTTTATTTTTTAACAGTAAAAATATTTTTTTAATACCAATTTTACACAAAAAATTCATTTTTTTTATATTTTTTA
Candidatus Carsonella 135557 - 167442nt	(1)	---
SARS-CoV-2 Reference Genome NC_045512.2	(1)	---

Section 1520

	(116964)	116964 116974 116985 116996 117007 117018 117029 117040
Candidatus Carsonella ruddii strain BT chromosome...	(116964)	TTATATTTTTTATATATTTTTATTTTATAAAAATTTTTTAATAAATTTTTTTTATTATAATTATTTATAAAAATTTT
Candidatus Carsonella 135557 - 167442nt	(1)	---
SARS-CoV-2 Reference Genome NC_045512.2	(1)	---

Section 1521

	(117041)	117041 117051 117062 117073 117084 117095 117106 117117
Candidatus Carsonella ruddii strain BT chromosome...	(117041)	CTTTAAAGATTTTATGTTAAAATTATTAAAATTTATGATTTTAGAACTATTGTTTAAATTTTTGAAATATAAAAAAT
Candidatus Carsonella 135557 - 167442nt	(1)	---
SARS-CoV-2 Reference Genome NC_045512.2	(1)	---

Section 1522

	(117118)	117118 117128 117139 117150 117161 117172 117183 117194
Candidatus Carsonella ruddii strain BT chromosome...	(117118)	ATTTAATGATATTCATATCTATTCCAAAAGGTAGAAATTTTTTGTTTTCATTATTAATATTAAAAAAAATAAAAATT
Candidatus Carsonella 135557 - 167442nt	(1)	---
SARS-CoV-2 Reference Genome NC_045512.2	(1)	---

Section 1523

	(117195)	117195 117205 117216 117227 117238 117249 117260 117271
Candidatus Carsonella ruddii strain BT chromosome...	(117195)	ATTTTATTAGAAATTATATATAGAAAAATAATATTAAAAACAAATAGAAAAAATATTTTTATAATTATTTTAAAAAA
Candidatus Carsonella 135557 - 167442nt	(1)	---
SARS-CoV-2 Reference Genome NC_045512.2	(1)	---

Section 1524

	(117272)	117272 117282 117293 117304 117315 117326 117337 117348
Candidatus Carsonella ruddii strain BT chromosome...	(117272)	TAAAGATTTACAAAAAATATTAAATTATGGTTTAACAAATTTAAGTATTATTGGTTCAGATTTATTTAATAAATAATT
Candidatus Carsonella 135557 - 167442nt	(1)	---
SARS-CoV-2 Reference Genome NC_045512.2	(1)	---

Section 1525

		117349 117359 117370 117381 117392 117403 117414 117425
	(117349)	
Candidatus Carsonella ruddii strain BT chromosome...	(117349)	ATAAAAAAATAATAAAATTAAAAAAAATAAATAGTTATTTGTTTTTAAATAATAATAATATTATTGTGACTAAATTT
Candidatus Carsonella 135557 - 167442nt	(1)	---
SARS-CoV-2 Reference Genome NC_045512.2	(1)	---

Section 1526

		117426 117436 117447 117458 117469 117480 117491 117502
	(117426)	
Candidatus Carsonella ruddii strain BT chromosome...	(117426)	TTTAATTTGTGTAATTATTATAATATAAAAAAATTTAATTTAAAATTAAATGGAGTTTTAGAATTTTTTTCTAATTT
Candidatus Carsonella 135557 - 167442nt	(1)	---
SARS-CoV-2 Reference Genome NC_045512.2	(1)	---

Section 1527

		117503 117513 117524 117535 117546 117557 117568 117579
	(117503)	
Candidatus Carsonella ruddii strain BT chromosome...	(117503)	TAAAAATTATGGATTATTGGATATAACAGAAACTAATAAAACTCTTTATGAAAATAAATTATTTCCTAAAAAATTTT
Candidatus Carsonella 135557 - 167442nt	(1)	---
SARS-CoV-2 Reference Genome NC_045512.2	(1)	---

Section 1528

		117580 117590 117601 117612 117623 117634 117645 117656
	(117580)	
Candidatus Carsonella ruddii strain BT chromosome...	(117580)	TACAAAAAATAAATTTATTTATAATTTTAAAAAATTTAAAAAAAAAAATAATAAAAATTATTAATAAAAATGTTAAA
Candidatus Carsonella 135557 - 167442nt	(1)	---
SARS-CoV-2 Reference Genome NC_045512.2	(1)	---

Section 1529

		117657 117667 117678 117689 117700 117711 117722 117733
	(117657)	
Candidatus Carsonella ruddii strain BT chromosome...	(117657)	AATTTTAGATAATATTATTTTTATAAAAAAAAATATTTTTAAAATTAAAGATAAAGTAAAAATAATTATTAAAGACA
Candidatus Carsonella 135557 - 167442nt	(1)	---
SARS-CoV-2 Reference Genome NC_045512.2	(1)	---

Section 1530

		117734 117744 117755 117766 117777 117788 117799 117810
	(117734)	
Candidatus Carsonella ruddii strain BT chromosome...	(117734)	TTAAAAAATATACAGAAAATGCATTAATTTATTATATTAAAAAAATAGATGGTATTTGTTTAAATAATATTAAAAAA
Candidatus Carsonella 135557 - 167442nt	(1)	---
SARS-CoV-2 Reference Genome NC_045512.2	(1)	---

Section 1531

	(117811)	117811 117821 117832 117843 117854 117865 117876 117887
Candidatus Carsonella ruddii strain BT chromosome...	(117811)	ATAATATTAAATAATAATGTATTAAAAAAAACATATAAAAAAATTAATATTTATAAAAAAAAAAATATTAAAAGAAAT
Candidatus Carsonella 135557 - 167442nt	(1)	---
SARS-CoV-2 Reference Genome NC_045512.2	(1)	---

Section 1532

	(117888)	117888 117898 117909 117920 117931 117942 117953 117964
Candidatus Carsonella ruddii strain BT chromosome...	(117888)	ATATATAAGGCTTAAAAGGTATCATTTTCATAATTTTATTAATTCATGGCAAATAAATAACAAAAAAGATATTATAT
Candidatus Carsonella 135557 - 167442nt	(1)	---
SARS-CoV-2 Reference Genome NC_045512.2	(1)	---

Section 1533

	(117965)	117965 117975 117986 117997 118008 118019 118030 118041
Candidatus Carsonella ruddii strain BT chromosome...	(117965)	ATGGTCAAAAAATAAATTGTATTGAAAAAATTTGTATTTATGTTCCTGGTGGAAATACAATTTATCCATCTTCAATT
Candidatus Carsonella 135557 - 167442nt	(1)	---
SARS-CoV-2 Reference Genome NC_045512.2	(1)	---

Section 1534

	(118042)	118042 118052 118063 118074 118085 118096 118107 118118
Candidatus Carsonella ruddii strain BT chromosome...	(118042)	TTTATGAATTGTTCTCCATCTTTAATATATAAACCAAAAATAATTTTTATGATCTCTCCAATAAAAAATGGTTTAAT
Candidatus Carsonella 135557 - 167442nt	(1)	---
SARS-CoV-2 Reference Genome NC_045512.2	(1)	---

Section 1535

	(118119)	118119 118129 118140 118151 118162 118173 118184 118195
Candidatus Carsonella ruddii strain BT chromosome...	(118119)	TTCAAATTTAATTTTATCTATTTCATATTTAAATAATATAAAAAAAATAATTTTTTCAGGTGGTGTTCATACTTTAT
Candidatus Carsonella 135557 - 167442nt	(1)	---
SARS-CoV-2 Reference Genome NC_045512.2	(1)	---

Section 1536

	(118196)	118196 118206 118217 118228 118239 118250 118261 118272
Candidatus Carsonella ruddii strain BT chromosome...	(118196)	CTTGTGTTGTATTCGGAACAAAAAATATAATAAAAGTTGATAAAATAACTGGTCCTGGAAATTATTTTGTTACAAAA
Candidatus Carsonella 135557 - 167442nt	(1)	---
SARS-CoV-2 Reference Genome NC_045512.2	(1)	---

```
                                                                                                                   Section 1537
                                                         (118273) 118273    118283     118294     118305     118316     118327     118338  118349
Candidatus Carsonella ruddii strain BT chromosome... (118273) GCTAAAAAAATATTTTTTGGTAATATTGGTATAGATATGCTTGCTGGACCATCAGAATTAATAATAGTTTGTGACTC
                 Candidatus Carsonella 135557 - 167442nt      (1) -----------------------------------------------------------------------------
              SARS-CoV-2 Reference Genome NC_045512.2      (1) -----------------------------------------------------------------------------
                                                                                                                   Section 1538
                                                         (118350) 118350    118360     118371     118382     118393     118404     118415  118426
Candidatus Carsonella ruddii strain BT chromosome... (118350) TAATATTCATCCATATATAATATCTTTAGATTTTTTTTCTCAATTAGAACATGATATTTATGCATCTTGTATTGTTT
                 Candidatus Carsonella 135557 - 167442nt      (1) -----------------------------------------------------------------------------
              SARS-CoV-2 Reference Genome NC_045512.2      (1) -----------------------------------------------------------------------------
                                                                                                                   Section 1539
                                                         (118427) 118427    118437     118448     118459     118470     118481     118492  118503
Candidatus Carsonella ruddii strain BT chromosome... (118427) TATCTAATAATAAAAAATATTTAAATTTATTATATAAAACTATATATAAATTATTAAAAATTCAAAAAAATAAAAAA
                 Candidatus Carsonella 135557 - 167442nt      (1) -----------------------------------------------------------------------------
              SARS-CoV-2 Reference Genome NC_045512.2      (1) -----------------------------------------------------------------------------
                                                                                                                   Section 1540
                                                         (118504) 118504    118514     118525     118536     118547     118558     118569  118580
Candidatus Carsonella ruddii strain BT chromosome... (118504) ATAATTTTTAAATCTTTAAAAAATAGTTTTTTATTATTTAAAAAAATAATAAATATAATTGAAATAATAAATTTTTT
                 Candidatus Carsonella 135557 - 167442nt      (1) -----------------------------------------------------------------------------
              SARS-CoV-2 Reference Genome NC_045512.2      (1) -----------------------------------------------------------------------------
                                                                                                                   Section 1541
                                                         (118581) 118581    118591     118602     118613     118624     118635     118646  118657
Candidatus Carsonella ruddii strain BT chromosome... (118581) TTCTCCTGAACATTTAATGTTATGTATTAAAAATTCAAATAAAATATTAAAATATATTAAAAATTGTGGAGCAATTT
                 Candidatus Carsonella 135557 - 167442nt      (1) -----------------------------------------------------------------------------
              SARS-CoV-2 Reference Genome NC_045512.2      (1) -----------------------------------------------------------------------------
                                                                                                                   Section 1542
                                                         (118658) 118658    118668     118679     118690     118701     118712     118723  118734
Candidatus Carsonella ruddii strain BT chromosome... (118658) TTTTAAACTATAATACAAGCGAAACTTATGGTGATTATATGATAGGACCAAGTCACGTTATTCCAACTAATAAAACA
                 Candidatus Carsonella 135557 - 167442nt      (1) -----------------------------------------------------------------------------
              SARS-CoV-2 Reference Genome NC_045512.2      (1) -----------------------------------------------------------------------------
```

Section 1543

(118735) 118735 118745 118756 118767 118778 118789 118800 118811
Candidatus Carsonella ruddii strain BT chromosome... (118735) TCTAAATTTTTTTCTTCATTAAATATTAATGATTTTTGTAAAAAAATAGGAATTATTAAAATTATTAATAACAATTA
Candidatus Carsonella 135557 - 167442nt (1) ---
SARS-CoV-2 Reference Genome NC_045512.2 (1) ---

Section 1544

(118812) 118812 118822 118833 118844 118855 118866 118877 118888
Candidatus Carsonella ruddii strain BT chromosome... (118812) TTCAAAAAAAATATCTTCATTTATTTCAAAAATAGAACATTTCTTTTCTCATTCATATTCTTCTATAATTAGAAAAT
Candidatus Carsonella 135557 - 167442nt (1) ---
SARS-CoV-2 Reference Genome NC_045512.2 (1) ---

Section 1545

(118889) 118889 118899 118910 118921 118932 118943 118954 118965
Candidatus Carsonella ruddii strain BT chromosome... (118889) GATTTTAATAAATTGTAAAAATAAAATTTTAGGAAGGATAAGTTCAATAATTAGTAAACTTATAATTAATTTTAATT
Candidatus Carsonella 135557 - 167442nt (1) ---
SARS-CoV-2 Reference Genome NC_045512.2 (1) ---

Section 1546

(118966) 118966 118976 118987 118998 119009 119020 119031 119042
Candidatus Carsonella ruddii strain BT chromosome... (118966) TTTTTAAAAAAAATTTTTTTTTTTTTTTATTTAATGTTAATTATATTTTAATTAAAAAAAATCATTTTTTTTCACAT
Candidatus Carsonella 135557 - 167442nt (1) ---
SARS-CoV-2 Reference Genome NC_045512.2 (1) ---

Section 1547

(119043) 119043 119053 119064 119075 119086 119097 119108 119119
Candidatus Carsonella ruddii strain BT chromosome... (119043) TCTGGTTATATAGGTAATGAAAAAATTAAAAAAAAAAATAATTTACAAATTTTAAAAAAATGTATTTTTAGAATGTT
Candidatus Carsonella 135557 - 167442nt (1) ---
SARS-CoV-2 Reference Genome NC_045512.2 (1) ---

Section 1548

(119120) 119120 119130 119141 119152 119163 119174 119185 119196
Candidatus Carsonella ruddii strain BT chromosome... (119120) ACCTAAAAACAAAAAAAGAATATTTCTTATGAAAAAAATATTTTATTTTGAAAACAAAATTATATGAAATATATATC
Candidatus Carsonella 135557 - 167442nt (1) ---
SARS-CoV-2 Reference Genome NC_045512.2 (1) ---

Section 1549

	(119197)	119197 119207 119218 119229 119240 119251 119262 119273
Candidatus Carsonella ruddii strain BT chromosome...	(119197)	ATTTTCTAAAAAAAAAACTTCTAGAAGTAAAGTAATTATGAAATTTGGAATTGGAATTATTTTAATAAATAATAAAA
Candidatus Carsonella 135557 - 167442nt	(1)	---
SARS-CoV-2 Reference Genome NC_045512.2	(1)	---

Section 1550

	(119274)	119274 119284 119295 119306 119317 119328 119339 119350
Candidatus Carsonella ruddii strain BT chromosome...	(119274)	CAATTTTAGATTATTTTGGAAAAAATTTTAATAATAATATAATTATATTTCCTTTATTAATATCAAATTTAAAAAAT
Candidatus Carsonella 135557 - 167442nt	(1)	---
SARS-CoV-2 Reference Genome NC_045512.2	(1)	---

Section 1551

	(119351)	119351 119361 119372 119383 119394 119405 119416 119427
Candidatus Carsonella ruddii strain BT chromosome...	(119351)	TTTAATTTTTTAATAAAAGTAAAAGGAGGTGGAAAAAATTCACAAATATTTTGTATTAAAATTTCTATTTGTAAATG
Candidatus Carsonella 135557 - 167442nt	(1)	---
SARS-CoV-2 Reference Genome NC_045512.2	(1)	---

Section 1552

	(119428)	119428 119438 119449 119460 119471 119482 119493 119504
Candidatus Carsonella ruddii strain BT chromosome...	(119428)	TATATTATTATTTAATTATAAACTAAAAAAAAAATTTAGAAAATTAAATTTTTTTTCTGTAGATGATAGAATTGTGG
Candidatus Carsonella 135557 - 167442nt	(1)	---
SARS-CoV-2 Reference Genome NC_045512.2	(1)	---

Section 1553

	(119505)	119505 119515 119526 119537 119548 119559 119570 119581
Candidatus Carsonella ruddii strain BT chromosome...	(119505)	AAAGAAAAAAATATGGTAAAAAAAAATCAAGAAAAACTGAACAATATTCAAAAAGATAATTTTTTTTTATTAATATA
Candidatus Carsonella 135557 - 167442nt	(1)	---
SARS-CoV-2 Reference Genome NC_045512.2	(1)	---

Section 1554

	(119582)	119582 119592 119603 119614 119625 119636 119647 119658
Candidatus Carsonella ruddii strain BT chromosome...	(119582)	TAATATATGTATTACATAATATAGGAAAGTTAAAAAAAATTATTTTTAAACAGAAAAAAAATTTTTTTTAATAATTT
Candidatus Carsonella 135557 - 167442nt	(1)	---
SARS-CoV-2 Reference Genome NC_045512.2	(1)	---

Section 1555

	(119659)	119659 119669 119680 119691 119702 119713 119724 119735
Candidatus Carsonella ruddii strain BT chromosome...	(119659)	GAAAAAAAAAAAAAATACTTAAAATTGGTTAGTTTAAAAAGATAAATTTTAACACTTAATTTAACTTATGAATATGA
Candidatus Carsonella 135557 - 167442nt	(1)	---
SARS-CoV-2 Reference Genome NC_045512.2	(1)	---

Section 1556

	(119736)	119736 119746 119757 119768 119779 119790 119801 119812
Candidatus Carsonella ruddii strain BT chromosome...	(119736)	TAAATAAACATATAACTTTTTTTGCTAATAAATTTAATAAAAATATAAAATGTATTGATTTGACGTTTGGTTTTGGT
Candidatus Carsonella 135557 - 167442nt	(1)	---
SARS-CoV-2 Reference Genome NC_045512.2	(1)	---

Section 1557

	(119813)	119813 119823 119834 119845 119856 119867 119878 119889
Candidatus Carsonella ruddii strain BT chromosome...	(119813)	GGGTATAATTTTATTTATAATATTTACAATTTAATTTCAATAGATAAAAATTTATCTTCTATATTAATTTCTAGAAA
Candidatus Carsonella 135557 - 167442nt	(1)	---
SARS-CoV-2 Reference Genome NC_045512.2	(1)	---

Section 1558

	(119890)	119890 119900 119911 119922 119933 119944 119955 119966
Candidatus Carsonella ruddii strain BT chromosome...	(119890)	AATATTTAAGAAAAATTTTTTTATTTTTAATATAAAAATAAAAAATTTTTTTTTTATATTAAAAAAATTTAATTTCA
Candidatus Carsonella 135557 - 167442nt	(1)	---
SARS-CoV-2 Reference Genome NC_045512.2	(1)	---

Section 1559

	(119967)	119967 119977 119988 119999 120010 120021 120032 120043
Candidatus Carsonella ruddii strain BT chromosome...	(119967)	AAAAAAGCAACTTATTAATTTATGATCAAGGTTTTAATAGTTGTGAAATAAAAAATTTTTTTTATTATTTTAAAAAA
Candidatus Carsonella 135557 - 167442nt	(1)	---
SARS-CoV-2 Reference Genome NC_045512.2	(1)	---

Section 1560

	(120044)	120044 120054 120065 120076 120087 120098 120109 120120
Candidatus Carsonella ruddii strain BT chromosome...	(120044)	AAAAAATATAATTTTGAAAAAAATAAAATAAATAATAATTTATATTTTATATTTTATAAAATTATTTTATATTTAGA
Candidatus Carsonella 135557 - 167442nt	(1)	---
SARS-CoV-2 Reference Genome NC_045512.2	(1)	---

Section 1561

```
                                                 (120121) 120121    120131     120142     120153     120164     120175     120186     120197
Candidatus Carsonella ruddii strain BT chromosome... (120121) AAAAAAATTTTTTATTGCATTATCAGTTTTTAATTTTTACGATTATTATAAAACAATATTATTTATAAAAAAAATTA
               Candidatus Carsonella 135557 - 167442nt (1) -----------------------------------------------------------------------------
          SARS-CoV-2 Reference Genome NC_045512.2 (1) -----------------------------------------------------------------------------
```

Section 1562

```
                                                 (120198) 120198    120208     120219     120230     120241     120252     120263     120274
Candidatus Carsonella ruddii strain BT chromosome... (120198) AAATTTTTAAATTTAAAATTTTTAAATTAAATTTGTTTGAAAAAAGTTTAAATAACGGAATTAAAAATTCAATTTTT
               Candidatus Carsonella 135557 - 167442nt (1) -----------------------------------------------------------------------------
          SARS-CoV-2 Reference Genome NC_045512.2 (1) -----------------------------------------------------------------------------
```

Section 1563

```
                                                 (120275) 120275    120285     120296     120307     120318     120329     120340     120351
Candidatus Carsonella ruddii strain BT chromosome... (120275) ATTATTTTATATGTTTAAAAAAAAAATTAAAGTTAAAATTATTTTTTATTTAATAAAATCATTAAATAATATTAAAA
               Candidatus Carsonella 135557 - 167442nt (1) -----------------------------------------------------------------------------
          SARS-CoV-2 Reference Genome NC_045512.2 (1) -----------------------------------------------------------------------------
```

Section 1564

```
                                                 (120352) 120352    120362     120373     120384     120395     120406     120417     120428
Candidatus Carsonella ruddii strain BT chromosome... (120352) AAAAAAAAATTTCAATTTTAGGTCCAATTGGAAATTACAGTTATAATATTTTATGTAAAAAAATAAGTAAAAAATTT
               Candidatus Carsonella 135557 - 167442nt (1) -----------------------------------------------------------------------------
          SARS-CoV-2 Reference Genome NC_045512.2 (1) -----------------------------------------------------------------------------
```

Section 1565

```
                                                 (120429) 120429    120439     120450     120461     120472     120483     120494     120505
Candidatus Carsonella ruddii strain BT chromosome... (120429) AATCTATTTCCTTTAATAAATATTAAATCTTTGAAAAATAAAAAAAATATAATAATACCATATGAAAATAACAACGG
               Candidatus Carsonella 135557 - 167442nt (1) -----------------------------------------------------------------------------
          SARS-CoV-2 Reference Genome NC_045512.2 (1) -----------------------------------------------------------------------------
```

Section 1566

```
                                                 (120506) 120506    120516     120527     120538     120549     120560     120571     120582
Candidatus Carsonella ruddii strain BT chromosome... (120506) TGGTATTGTAAGAGATTGTTTGGAATTATTATTTAAAAAAAAGATATTTATAAATAAAATAGTTATAATAAATATAA
               Candidatus Carsonella 135557 - 167442nt (1) -----------------------------------------------------------------------------
          SARS-CoV-2 Reference Genome NC_045512.2 (1) -----------------------------------------------------------------------------
```

Section 1567

	(120583)	120583 120593 120604 120615 120626 120637 120648 120659
Candidatus Carsonella ruddii strain BT chromosome...	(120583)	AACATAATTTTTTTTTTATATAAAAAAAAAAAAGTTTTTTTTTTACATAAACAGTCATATAAACAAATAAAAAAAAAT
Candidatus Carsonella 135557 - 167442nt	(1)	---
SARS-CoV-2 Reference Genome NC_045512.2	(1)	---

Section 1568

	(120660)	120660 120670 120681 120692 120703 120714 120725 120736
Candidatus Carsonella ruddii strain BT chromosome...	(120660)	TTAATTTTATTTTTTAAAAAATTAAAAATCATATTTAATAATTCTAATTCAAATATTAGTAATGGTATAAATATATG
Candidatus Carsonella 135557 - 167442nt	(1)	---
SARS-CoV-2 Reference Genome NC_045512.2	(1)	---

Section 1569

	(120737)	120737 120747 120758 120769 120780 120791 120802 120813
Candidatus Carsonella ruddii strain BT chromosome...	(120737)	TAATTATAACACAAAAAAAATATTTCCTATAAATATAAAAAATATAAATTTAAAAGATAATTTTATAAATAAAACAA
Candidatus Carsonella 135557 - 167442nt	(1)	---
SARS-CoV-2 Reference Genome NC_045512.2	(1)	---

Section 1570

	(120814)	120814 120824 120835 120846 120857 120868 120879 120890
Candidatus Carsonella ruddii strain BT chromosome...	(120814)	AATTTATTATAAATAAAAATTCTTTTTGTAAAAAAAAAATATTAAGTTTTTTTGTTAATAATTATTTTTTTTTTGAA
Candidatus Carsonella 135557 - 167442nt	(1)	---
SARS-CoV-2 Reference Genome NC_045512.2	(1)	---

Section 1571

	(120891)	120891 120901 120912 120923 120934 120945 120956 120967
Candidatus Carsonella ruddii strain BT chromosome...	(120891)	AAAAAAATAACAATATATAAAAAAAAAAAATTATTTTATTTTGAAATATTTATTAATAGTTATAGAAAACTTTTATT
Candidatus Carsonella 135557 - 167442nt	(1)	---
SARS-CoV-2 Reference Genome NC_045512.2	(1)	---

Section 1572

	(120968)	120968 120978 120989 121000 121011 121022 121033 121044
Candidatus Carsonella ruddii strain BT chromosome...	(120968)	TTCAATGAAAAATATTAAAAATAAAATTAAAATTTTGTTTACAGGTTTTTATAATATATTATGAAATCATATTTTAA
Candidatus Carsonella 135557 - 167442nt	(1)	---
SARS-CoV-2 Reference Genome NC_045512.2	(1)	---

Section 1573

	(121045)	121045 121055 121066 121077 121088 121099 121110 121121
Candidatus Carsonella ruddii strain BT chromosome...	(121045)	AATAAAAAATATAAATATAAAAAAAAATTTTTTAAATTCTGGAGATAAATCAATTTCTCATAGATCCATTATTTATT
Candidatus Carsonella 135557 - 167442nt	(1)	---
SARS-CoV-2 Reference Genome NC_045512.2	(1)	---

Section 1574

	(121122)	121122 121132 121143 121154 121165 121176 121187 121198
Candidatus Carsonella ruddii strain BT chromosome...	(121122)	CATTAATTAAAAATTGTATAATAGAAATTAAAAATTTATTAGAATCTTTTGATATAATATCAACAATAAATTTATGT
Candidatus Carsonella 135557 - 167442nt	(1)	---
SARS-CoV-2 Reference Genome NC_045512.2	(1)	---

Section 1575

	(121199)	121199 121209 121220 121231 121242 121253 121264 121275
Candidatus Carsonella ruddii strain BT chromosome...	(121199)	AAAAATTTAGGAATTTATATTTATGGACCAATAAATTCTTACGTTTTAATTTCTTCTTTAAAAAAAAAAAAAAAAAA
Candidatus Carsonella 135557 - 167442nt	(1)	---
SARS-CoV-2 Reference Genome NC_045512.2	(1)	---

Section 1576

	(121276)	121276 121286 121297 121308 121319 121330 121341 121352
Candidatus Carsonella ruddii strain BT chromosome...	(121276)	AAATAAAATTAATTTTATAGGTAATTCAGGAACAACAATAAGAATAAGTTTATCTTTGTTAATAACTAATAATATCA
Candidatus Carsonella 135557 - 167442nt	(1)	---
SARS-CoV-2 Reference Genome NC_045512.2	(1)	---

Section 1577

	(121353)	121353 121363 121374 121385 121396 121407 121418 121429
Candidatus Carsonella ruddii strain BT chromosome...	(121353)	TTTTAGGTGATAAATCTTTAAATAAGAGAACAATGTATAGAATAATTAAACCATTAACTTTATTAGGTTTTATTATA
Candidatus Carsonella 135557 - 167442nt	(1)	---
SARS-CoV-2 Reference Genome NC_045512.2	(1)	---

Section 1578

	(121430)	121430 121440 121451 121462 121473 121484 121495 121506
Candidatus Carsonella ruddii strain BT chromosome...	(121430)	CAATGTAAAAAAAATTTCTTTTCTCCTATAATAATTTTTAAAAAAAATAATTTAGGATTAAAATATAATCTTGTAAA
Candidatus Carsonella 135557 - 167442nt	(1)	---
SARS-CoV-2 Reference Genome NC_045512.2	(1)	---

```
                                                                         Section 1579
                                              (121507) 121507    121517     121528     121539     121550     121561     121572   121583
Candidatus Carsonella ruddii strain BT chromosome... (121507) CGCAAGTTCTCAAATAAAATCTTGTTTAATTTTATCATCAATTAATTCTTTTATAAATATATATATAAAAGAAAATA
          Candidatus Carsonella 135557 - 167442nt        (1) -----------------------------------------------------------------------------
      SARS-CoV-2 Reference Genome NC_045512.2            (1) -----------------------------------------------------------------------------
                                                                         Section 1580
                                              (121584) 121584    121594     121605     121616     121627     121638     121649   121660
Candidatus Carsonella ruddii strain BT chromosome... (121584) AAAAAACAAGAGATCATAGTGAAAGATTTTTAAATATTTTAATTAAAAAAAAAAATGATTTTTTAATAAAAATATTA
          Candidatus Carsonella 135557 - 167442nt        (1) -----------------------------------------------------------------------------
      SARS-CoV-2 Reference Genome NC_045512.2            (1) -----------------------------------------------------------------------------
                                                                         Section 1581
                                              (121661) 121661    121671     121682     121693     121704     121715     121726   121737
Candidatus Carsonella ruddii strain BT chromosome... (121661) AATGATTTTTCATCATTTTCATTTATAATTAGTTATTATTTAATTAAAAAAAAAAATTTTATTTTAAATTTTTAATTT
          Candidatus Carsonella 135557 - 167442nt        (1) -----------------------------------------------------------------------------
      SARS-CoV-2 Reference Genome NC_045512.2            (1) -----------------------------------------------------------------------------
                                                                         Section 1582
                                              (121738) 121738    121748     121759     121770     121781     121792     121803   121814
Candidatus Carsonella ruddii strain BT chromosome... (121738) TAATAAATTAAGAATAGGTTTTTTTGAATATTTAATAAAAATAGGATTTTTTTTTTTATATTATTAAAAAAAAAATTA
          Candidatus Carsonella 135557 - 167442nt        (1) -----------------------------------------------------------------------------
      SARS-CoV-2 Reference Genome NC_045512.2            (1) -----------------------------------------------------------------------------
                                                                         Section 1583
                                              (121815) 121815    121825     121836     121847     121858     121869     121880   121891
Candidatus Carsonella ruddii strain BT chromosome... (121815) TTAATAATGAACATTATGTAAAAATTTTAATTTTTAATAAAATAATAAAAAATAGTATAATTTATTCAAAAACAATA
          Candidatus Carsonella 135557 - 167442nt        (1) -----------------------------------------------------------------------------
      SARS-CoV-2 Reference Genome NC_045512.2            (1) -----------------------------------------------------------------------------
                                                                         Section 1584
                                              (121892) 121892    121902     121913     121924     121935     121946     121957   121968
Candidatus Carsonella ruddii strain BT chromosome... (121892) TCTAAATTAATCGATGAAATTCCAGTTTTAATTTCTTTAATTATAAATTATAAAAATAAAACAAAAATTTATGGTTT
          Candidatus Carsonella 135557 - 167442nt        (1) -----------------------------------------------------------------------------
      SARS-CoV-2 Reference Genome NC_045512.2            (1) -----------------------------------------------------------------------------
```

Section 1585

	(121969)	121969 121979 121990 122001 122012 122023 122034 122045
Candidatus Carsonella ruddii strain BT chromosome...	(121969)	AGAAGAATTAAAATTTAAAGAATCTAATAGATATAATAGTATGTATTATAATTTATTATTATTAGGTATAAAAATAA
Candidatus Carsonella 135557 - 167442nt	(1)	---
SARS-CoV-2 Reference Genome NC_045512.2	(1)	---

Section 1586

	(122046)	122046 122056 122067 122078 122089 122100 122111 122122
Candidatus Carsonella ruddii strain BT chromosome...	(122046)	TTAAAAAAAAAAATTATTTAATTTTTAAAAATAATAAAATTCATCCAAATTTAATTTTTTCTCAATCAGATCACAGA
Candidatus Carsonella 135557 - 167442nt	(1)	---
SARS-CoV-2 Reference Genome NC_045512.2	(1)	---

Section 1587

	(122123)	122123 122133 122144 122155 122166 122177 122188 122199
Candidatus Carsonella ruddii strain BT chromosome...	(122123)	TTATTTATGTCATTTTATATAATTAATTTAAAAAATTTAAAAATTTCAAATCCTGAAAACACATTATCTTCTTTTCC
Candidatus Carsonella 135557 - 167442nt	(1)	---
SARS-CoV-2 Reference Genome NC_045512.2	(1)	---

Section 1588

	(122200)	122200 122210 122221 122232 122243 122254 122265 122276
Candidatus Carsonella ruddii strain BT chromosome...	(122200)	TAATTTTTTAAAATATTTTAATAATAAAAACAATAAATTATATGTATTTAATTAATAATTTTATTAAAAGTAATATA
Candidatus Carsonella 135557 - 167442nt	(1)	---
SARS-CoV-2 Reference Genome NC_045512.2	(1)	---

Section 1589

	(122277)	122277 122287 122298 122309 122320 122331 122342 122353
Candidatus Carsonella ruddii strain BT chromosome...	(122277)	ATAATTTTTTTTGAAAATTTTTTAATAACAAATTTTTTAAAAACAATATTGATAGAAAAAATTTCTAAAAATTTTAT
Candidatus Carsonella 135557 - 167442nt	(1)	---
SARS-CoV-2 Reference Genome NC_045512.2	(1)	---

Section 1590

	(122354)	122354 122364 122375 122386 122397 122408 122419 122430
Candidatus Carsonella ruddii strain BT chromosome...	(122354)	AAAAAAAAATGTAATTTTTTTCAAGAAAAAAGAAATTATTATAAATAATTATTTTTTTAGTTTTTTAACAAAAAAAA
Candidatus Carsonella 135557 - 167442nt	(1)	---
SARS-CoV-2 Reference Genome NC_045512.2	(1)	---

Section 1591

		122431 122441 122452 122463 122474 122485 122496 122507
Candidatus Carsonella ruddii strain BT chromosome...	(122431)	TAAAATTTGGTTATTTTATAAATAATAACTTTAATATATTTTTACCTAATCATTTATTAAATACTAAAAAAATAATA
Candidatus Carsonella 135557 - 167442nt	(1)	---
SARS-CoV-2 Reference Genome NC_045512.2	(1)	---

Section 1592

		122508 122518 122529 122540 122551 122562 122573 122584
Candidatus Carsonella ruddii strain BT chromosome...	(122508)	TTTCCTAAAAAAATTTTTTATTTTGAAATTTTATTTAAAAAATTTAATATAGAAAAAAAAATTTTTGTTAGTAGAAA
Candidatus Carsonella 135557 - 167442nt	(1)	---
SARS-CoV-2 Reference Genome NC_045512.2	(1)	---

Section 1593

		122585 122595 122606 122617 122628 122639 122650 122661
Candidatus Carsonella ruddii strain BT chromosome...	(122585)	ATTTAATTTTAAAAAAAAAAAAAAAAATTATATTAAAATAAATAAAAATTATTTAGGAATTATTAAAAATGTAATAA
Candidatus Carsonella 135557 - 167442nt	(1)	---
SARS-CoV-2 Reference Genome NC_045512.2	(1)	---

Section 1594

		122662 122672 122683 122694 122705 122716 122727 122738
Candidatus Carsonella ruddii strain BT chromosome...	(122662)	ATTACGGAATTTTTATTGATATTGGACAAGCAGATGGATTACTTCATGTTTCAGATATTCCATTTTTTAAGAAAATA
Candidatus Carsonella 135557 - 167442nt	(1)	---
SARS-CoV-2 Reference Genome NC_045512.2	(1)	---

Section 1595

		122739 122749 122760 122771 122782 122793 122804 122815
Candidatus Carsonella ruddii strain BT chromosome...	(122739)	TATAATAAATTGATAACAAAAAATATTATTTCTATTAAAGTAATAAAATTTGATAAAAAAATTAAAAAAGTATCTTT
Candidatus Carsonella 135557 - 167442nt	(1)	---
SARS-CoV-2 Reference Genome NC_045512.2	(1)	---

Section 1596

		122816 122826 122837 122848 122859 122870 122881 122892
Candidatus Carsonella ruddii strain BT chromosome...	(122816)	AAGTTTAAAAAAAAATTATAAAAAAAATTATGAAAAATTTTTCAATTTAAATATAATAAAATGTTATGCTAAACAAT
Candidatus Carsonella 135557 - 167442nt	(1)	---
SARS-CoV-2 Reference Genome NC_045512.2	(1)	---

Section 1597

	(122893)	122893 122903 122914 122925 122936 122947 122958 122969
Candidatus Carsonella ruddii strain BT chromosome...	(122893)	TTATTAATAATGGAGTAATTTGTTACAACAATTTAAGTAAAAAAATTATTTTATTTAATAAATTTATAAAATTTAAA
Candidatus Carsonella 135557 - 167442nt	(1)	---
SARS-CoV-2 Reference Genome NC_045512.2	(1)	---

Section 1598

	(122970)	122970 122980 122991 123002 123013 123024 123035 123046
Candidatus Carsonella ruddii strain BT chromosome...	(122970)	AAAAAAGATACTTTAATTGCTTATTTTTTAAAAAAAACAGAAAAATTTATATATTTAAATTTTAATTATAAATTAAT
Candidatus Carsonella 135557 - 167442nt	(1)	---
SARS-CoV-2 Reference Genome NC_045512.2	(1)	---

Section 1599

	(123047)	123047 123057 123068 123079 123090 123101 123112 123123
Candidatus Carsonella ruddii strain BT chromosome...	(123047)	AAAAAACAATTTAAAATATCAAAAATTAATAATAAAATATAAATTTAATAATTTTTTTTTATTATCTTGTAAAAAGT
Candidatus Carsonella 135557 - 167442nt	(1)	---
SARS-CoV-2 Reference Genome NC_045512.2	(1)	---

Section 1600

	(123124)	123124 123134 123145 123156 123167 123178 123189 123200
Candidatus Carsonella ruddii strain BT chromosome...	(123124)	TAAAAATTTTATATAAAAAAATAATTTTTAATATTAAAATTAAAAATATTAATTTTTATAAATTTTTTATATTTAAT
Candidatus Carsonella 135557 - 167442nt	(1)	---
SARS-CoV-2 Reference Genome NC_045512.2	(1)	---

Section 1601

	(123201)	123201 123211 123222 123233 123244 123255 123266 123277
Candidatus Carsonella ruddii strain BT chromosome...	(123201)	TTAATTAATAATAATTTTAATAAAATTGTTTTTATAAATAATAACTATTATTTAAAAAACAACAATTATTATTTAAA
Candidatus Carsonella 135557 - 167442nt	(1)	---
SARS-CoV-2 Reference Genome NC_045512.2	(1)	---

Section 1602

	(123278)	123278 123288 123299 123310 123321 123332 123343 123354
Candidatus Carsonella ruddii strain BT chromosome...	(123278)	ATTAAAAAATAAAAAAAATTTTTTTTTAAACATGTCAAAATTTTTAATATTTAAAAAAAAAATTTTTTGTTTTATAA
Candidatus Carsonella 135557 - 167442nt	(1)	---
SARS-CoV-2 Reference Genome NC_045512.2	(1)	---

```
                                                                                                         Section 1603
                                                  (123355) 123355    123365     123376     123387     123398     123409     123420  123431
Candidatus Carsonella ruddii strain BT chromosome... (123355) GAAAATATTAATTTTTTGTTTCATCAAAATCTGCAATCATAGCTTCTGTTGTGATTAATAATCCTCCTATTGATCCT
            Candidatus Carsonella 135557 - 167442nt      (1) -----------------------------------------------------------------------------
         SARS-CoV-2 Reference Genome NC_045512.2      (1) -----------------------------------------------------------------------------
                                                                                                         Section 1604
                                                  (123432) 123432    123442     123453     123464     123475     123486     123497  123508
Candidatus Carsonella ruddii strain BT chromosome... (123432) GCTGATTGTAAAGCACTTCTTGTAACTTTAACAGGATCTATTATTCCCATTTTAAACATATCACCATAAAATCCTGT
            Candidatus Carsonella 135557 - 167442nt      (1) -----------------------------------------------------------------------------
         SARS-CoV-2 Reference Genome NC_045512.2      (1) -----------------------------------------------------------------------------
                                                                                                         Section 1605
                                                  (123509) 123509    123519     123530     123541     123552     123563     123574  123585
Candidatus Carsonella ruddii strain BT chromosome... (123509) TGCAGCGTTATAACCAAAATTTTTTGAAGAAGATTTAATATTATTTAAAACTATTGATGGTTCTTCACCAGAATTTT
            Candidatus Carsonella 135557 - 167442nt      (1) -----------------------------------------------------------------------------
         SARS-CoV-2 Reference Genome NC_045512.2      (1) -----------------------------------------------------------------------------
                                                                                                         Section 1606
                                                  (123586) 123586    123596     123607     123618     123629     123640     123651  123662
Candidatus Carsonella ruddii strain BT chromosome... (123586) TTACTATTTGTTTTAATGGAGTTTCCAAAGATTTAAGAGCGATATAAATACCATAATTTTGATCGTTGTTTTCTCCT
            Candidatus Carsonella 135557 - 167442nt      (1) -----------------------------------------------------------------------------
         SARS-CoV-2 Reference Genome NC_045512.2      (1) -----------------------------------------------------------------------------
                                                                                                         Section 1607
                                                  (123663) 123663    123673     123684     123695     123706     123717     123728  123739
Candidatus Carsonella ruddii strain BT chromosome... (123663) TTTAAATTTTTTAATTTTTCTAAAATTCTAACTAAAGCAACTCCTCCACCAATTACTACTCCTTCTTCAACTGCTGC
            Candidatus Carsonella 135557 - 167442nt      (1) -----------------------------------------------------------------------------
         SARS-CoV-2 Reference Genome NC_045512.2      (1) -----------------------------------------------------------------------------
                                                                                                         Section 1608
                                                  (123740) 123740    123750     123761     123772     123783     123794     123805  123816
Candidatus Carsonella ruddii strain BT chromosome... (123740) TCTAGTTGAATGTAAAGCATCTTCAATTCTTGCTTTTTTTTCTTTCATTTCAATTTCAGTAGCAGCACCTACTCTTA
            Candidatus Carsonella 135557 - 167442nt      (1) -----------------------------------------------------------------------------
         SARS-CoV-2 Reference Genome NC_045512.2      (1) -----------------------------------------------------------------------------
```

```
                                                                                  Section 1609
                                              (123817) 123817    123827     123838     123849     123860     123871     123882     123893
Candidatus Carsonella ruddii strain BT chromosome... (123817) TTACAGCAACTCCACCAGATAATTTAGCCATTCTTTCTTGTAATTTTTCTTTATCATAATCAGAAGTTGATTCTGAA
             Candidatus Carsonella 135557 - 167442nt      (1) -----------------------------------------------------------------------------
           SARS-CoV-2 Reference Genome NC_045512.2      (1) -----------------------------------------------------------------------------
                                                                                  Section 1610
                                              (123894) 123894    123904     123915     123926     123937     123948     123959     123970
Candidatus Carsonella ruddii strain BT chromosome... (123894) ATTTGTTTTTTAATATTTTTAATTCTTTCTTTAATTTTTAATTCATTACCCGATCCTCCAACTATAATAGTGTTTTC
             Candidatus Carsonella 135557 - 167442nt      (1) -----------------------------------------------------------------------------
           SARS-CoV-2 Reference Genome NC_045512.2      (1) -----------------------------------------------------------------------------
                                                                                  Section 1611
                                              (123971) 123971    123981     123992     124003     124014     124025     124036     124047
Candidatus Carsonella ruddii strain BT chromosome... (123971) TTTATTTGATGTAATTTTTTTAGCAAATCCTAAATCTTCAATTTTAATTTTATCTAATTGTATTCCAATTTCATCTG
             Candidatus Carsonella 135557 - 167442nt      (1) -----------------------------------------------------------------------------
           SARS-CoV-2 Reference Genome NC_045512.2      (1) -----------------------------------------------------------------------------
                                                                                  Section 1612
                                              (124048) 124048    124058     124069     124080     124091     124102     124113     124124
Candidatus Carsonella ruddii strain BT chromosome... (124048) TAATAAAATTAGCACCAGTTAAAATTGAAATATCTTTTAAAATATCTTTTCTTCTATCACCAAAACCAGGTGCTTTA
             Candidatus Carsonella 135557 - 167442nt      (1) -----------------------------------------------------------------------------
           SARS-CoV-2 Reference Genome NC_045512.2      (1) -----------------------------------------------------------------------------
                                                                                  Section 1613
                                              (124125) 124125    124135     124146     124157     124168     124179     124190     124201
Candidatus Carsonella ruddii strain BT chromosome... (124125) ACAGCACCTAATTTTATTATTCCTCTAATATTATTTATAACTAATGTAGCTAAAGCTTCACCTTCTATATCTTCAGC
             Candidatus Carsonella 135557 - 167442nt      (1) -----------------------------------------------------------------------------
           SARS-CoV-2 Reference Genome NC_045512.2      (1) -----------------------------------------------------------------------------
                                                                                  Section 1614
                                              (124202) 124202    124212     124223     124234     124245     124256     124267     124278
Candidatus Carsonella ruddii strain BT chromosome... (124202) AATAATAAAAAGTGATTTATTTTTTTTTGAAATTAATTCTAAAATATTAACTATATCTCTAATATTATTTATTTTTT
             Candidatus Carsonella 135557 - 167442nt      (1) -----------------------------------------------------------------------------
           SARS-CoV-2 Reference Genome NC_045512.2      (1) -----------------------------------------------------------------------------
```

Section 1615

	(124279)	124279 124289 124300 124311 124322 124333 124344 124355
Candidatus Carsonella ruddii strain BT chromosome...	(124279)	TATCTGTTATTAAAACAAAACAATTTTCTAAGATTAAAGACATATTTTCTTGATTATTAATAAAATAAGGAGAAATA
Candidatus Carsonella 135557 - 167442nt	(1)	---
SARS-CoV-2 Reference Genome NC_045512.2	(1)	---

Section 1616

	(124356)	124356 124366 124377 124388 124399 124410 124421 124432
Candidatus Carsonella ruddii strain BT chromosome...	(124356)	TAACCTCTATCAAATTGCATACCTTCTACTACTTCTAATTCATCTTCAATTCCTTTTCCTTCATCAACTGTAATTAC
Candidatus Carsonella 135557 - 167442nt	(1)	---
SARS-CoV-2 Reference Genome NC_045512.2	(1)	---

Section 1617

	(124433)	124433 124443 124454 124465 124476 124487 124498 124509
Candidatus Carsonella ruddii strain BT chromosome...	(124433)	TCCATTTTTTCCAACTCTATTCATAGCATCAGAAATAATTTTTCCAATTATTTTTTCTCCATTAGCAGAAATTGTTC
Candidatus Carsonella 135557 - 167442nt	(1)	---
SARS-CoV-2 Reference Genome NC_045512.2	(1)	---

Section 1618

	(124510)	124510 124520 124531 124542 124553 124564 124575 124586
Candidatus Carsonella ruddii strain BT chromosome...	(124510)	CTACTTGTGAAATTGATAAAGTATCTAAACATGGAATAGAAATTTTTTTTAATTCTAAAACAGCATGATAAATTGCT
Candidatus Carsonella 135557 - 167442nt	(1)	---
SARS-CoV-2 Reference Genome NC_045512.2	(1)	---

Section 1619

	(124587)	124587 124597 124608 124619 124630 124641 124652 124663
Candidatus Carsonella ruddii strain BT chromosome...	(124587)	TTATCAATGCCTCTTTTTAAATCCATCGGATTCATTCCGGATATAACAGCTTTTATACCTTCATTTACTATTGATTG
Candidatus Carsonella 135557 - 167442nt	(1)	---
SARS-CoV-2 Reference Genome NC_045512.2	(1)	---

Section 1620

	(124664)	124664 124674 124685 124696 124707 124718 124729 124740
Candidatus Carsonella ruddii strain BT chromosome...	(124664)	TGCTAAAACTGTAGCAGTTGTTGTTCCATCTCCTGCAACATCAGAAGTTTTAGATGCAACTTCTTTAACCATTTGAG
Candidatus Carsonella 135557 - 167442nt	(1)	---
SARS-CoV-2 Reference Genome NC_045512.2	(1)	---

Section 1621

(124741) 124741 124751 124762 124773 124784 124795 124806 124817
Candidatus Carsonella ruddii strain BT chromosome... (124741) CTCCCATATTTTCAAATTTATCTTTTAATTCTATTTCTTTTGCAACAGAAACTCCATCTTTAGTTACTAATGGAGAA
Candidatus Carsonella 135557 - 167442nt (1) ---
SARS-CoV-2 Reference Genome NC_045512.2 (1) ---

Section 1622

(124818) 124818 124828 124839 124850 124861 124872 124883 124894
Candidatus Carsonella ruddii strain BT chromosome... (124818) CTAAAATTTTTATCTAAAATAACATTTCTTCCTTTTGGACCAAGAGTAGTTTTAACCGCATTTGCTAATAAATTTAC
Candidatus Carsonella 135557 - 167442nt (1) ---
SARS-CoV-2 Reference Genome NC_045512.2 (1) ---

Section 1623

(124895) 124895 124905 124916 124927 124938 124949 124960 124971
Candidatus Carsonella ruddii strain BT chromosome... (124895) ACCATTTGCTAAATTTTTTCTAGCTTCATCACCAAATTTAATTTTTTTAAAACCCATTTTATTTGATAATAGAAATT
Candidatus Carsonella 135557 - 167442nt (1) ---
SARS-CoV-2 Reference Genome NC_045512.2 (1) ---

Section 1624

(124972) 124972 124982 124993 125004 125015 125026 125037 125048
Candidatus Carsonella ruddii strain BT chromosome... (124972) ATATCATTTTCTTTAAGAAAAAAATACTCTTTTTCATTTACTTTATATTTTTCTATATTATAACTATCTTTAAAAAT
Candidatus Carsonella 135557 - 167442nt (1) ---
SARS-CoV-2 Reference Genome NC_045512.2 (1) ---

Section 1625

(125049) 125049 125059 125070 125081 125092 125103 125114 125125
Candidatus Carsonella ruddii strain BT chromosome... (125049) TATTATATCTCCAATATTAACAATTAATGGTTTTAAAACTCCATTTTCTAATAGTTTTCCACATCCAATTTTTATTA
Candidatus Carsonella 135557 - 167442nt (1) ---
SARS-CoV-2 Reference Genome NC_045512.2 (1) ---

Section 1626

(125126) 125126 125136 125147 125158 125169 125180 125191 125202
Candidatus Carsonella ruddii strain BT chromosome... (125126) TTTTTCCTTTTAAAATATTATTTTCATTTTTAGGTATATAAATACTTCCTATTTTATTTTCTTCATCTAATTTTATT
Candidatus Carsonella 135557 - 167442nt (1) ---
SARS-CoV-2 Reference Genome NC_045512.2 (1) ---

Section 1627

	(125203)	125203 125213 125224 125235 125246 125257 125268 125279
Candidatus Carsonella ruddii strain BT chromosome...	(125203)	ACAACAATTTTATCATATAAAGGTATAAAATCCATTTTTTTTAAAAAAAAATATTTTTTAATAATTAAAAATTAAT
Candidatus Carsonella 135557 - 167442nt	(1)	---
SARS-CoV-2 Reference Genome NC_045512.2	(1)	---

Section 1628

	(125280)	125280 125290 125301 125312 125323 125334 125345 125356
Candidatus Carsonella ruddii strain BT chromosome...	(125280)	AAAAAAATAACAGATATTTTTCAATAATAACGTTATTTTTTAATTTTTATTATTTTGACATATTATGAAAAATTAAT
Candidatus Carsonella 135557 - 167442nt	(1)	---
SARS-CoV-2 Reference Genome NC_045512.2	(1)	---

Section 1629

	(125357)	125357 125367 125378 125389 125400 125411 125422 125433
Candidatus Carsonella ruddii strain BT chromosome...	(125357)	AAGAAATTAAAATAGGTAATTAACTCAATAGGTAGAGTATCAGTTTTACATACTGAAAGTTACAAGTTCAAATCTTG
Candidatus Carsonella 135557 - 167442nt	(1)	---
SARS-CoV-2 Reference Genome NC_045512.2	(1)	---

Section 1630

	(125434)	125434 125444 125455 125466 125477 125488 125499 125510
Candidatus Carsonella ruddii strain BT chromosome...	(125434)	TATTACCTATTTTTGGATTGATAGTTTAGTTGGTTAAAATGTTGGCCTGTCACGCTGAATATCACGGGTTCAAATCC
Candidatus Carsonella 135557 - 167442nt	(1)	---
SARS-CoV-2 Reference Genome NC_045512.2	(1)	---

Section 1631

	(125511)	125511 125521 125532 125543 125554 125565 125576 125587
Candidatus Carsonella ruddii strain BT chromosome...	(125511)	CGTTCAATCCGTAATTTATTTAAAAAATAATATAATAATTATAAATTTTAGAAAAATTTTAAAAAAAATAAAAATTT
Candidatus Carsonella 135557 - 167442nt	(1)	---
SARS-CoV-2 Reference Genome NC_045512.2	(1)	---

Section 1632

	(125588)	125588 125598 125609 125620 125631 125642 125653 125664
Candidatus Carsonella ruddii strain BT chromosome...	(125588)	TAATTAAATTAAAAATTAAATTTTTTTTTTTAAATTTATTTTTAAAAAAAAAATACTTTTTAAAAAATAATTTTTT
Candidatus Carsonella 135557 - 167442nt	(1)	---
SARS-CoV-2 Reference Genome NC_045512.2	(1)	---

Section 1633

```
                                                    (125665) 125665    125675     125686     125697     125708     125719     125730     125741
Candidatus Carsonella ruddii strain BT chromosome... (125665) TGTATTTTTTTTAAAAATAAAATAAGAAAAAAATTTTTTAATAAAAATTATGTAGTTTTATTTTTATTAAAAAATAA
              Candidatus Carsonella 135557 - 167442nt (1)      -----------------------------------------------------------------------------
           SARS-CoV-2 Reference Genome NC_045512.2 (1)      -----------------------------------------------------------------------------
```

Section 1634

```
                                                    (125742) 125742    125752     125763     125774     125785     125796     125807     125818
Candidatus Carsonella ruddii strain BT chromosome... (125742) CGGAAATTTATATTCATGTATAAGAACTTGTTATATATATAATATAATACCTGTTTTAAATTATAAAAAAAATAAAA
              Candidatus Carsonella 135557 - 167442nt (1)      -----------------------------------------------------------------------------
           SARS-CoV-2 Reference Genome NC_045512.2 (1)      -----------------------------------------------------------------------------
```

Section 1635

```
                                                    (125819) 125819    125829     125840     125851     125862     125873     125884     125895
Candidatus Carsonella ruddii strain BT chromosome... (125819) TAATAAATTCTTATTTTAATAATATTTTATTTATAAAAAATATTTATTTTTTTACAAAATATATAAAAAATAAAATT
              Candidatus Carsonella 135557 - 167442nt (1)      -----------------------------------------------------------------------------
           SARS-CoV-2 Reference Genome NC_045512.2 (1)      -----------------------------------------------------------------------------
```

Section 1636

```
                                                    (125896) 125896    125906     125917     125928     125939     125950     125961     125972
Candidatus Carsonella ruddii strain BT chromosome... (125896) ATTATAAGTTTAACAATTAATTCAAAAACTTTATTAAAAAAAATAGTTTTAAAAATTAAATTTATTATTTTTATAGG
              Candidatus Carsonella 135557 - 167442nt (1)      -----------------------------------------------------------------------------
           SARS-CoV-2 Reference Genome NC_045512.2 (1)      -----------------------------------------------------------------------------
```

Section 1637

```
                                                    (125973) 125973    125983     125994     126005     126016     126027     126038     126049
Candidatus Carsonella ruddii strain BT chromosome... (125973) AAATGAAAAAAAATCATTGTCTAAAAAAATACTTAAAAAAAGTGATATTTTACTTAAAATTAATACTTATAGAAAAA
              Candidatus Carsonella 135557 - 167442nt (1)      -----------------------------------------------------------------------------
           SARS-CoV-2 Reference Genome NC_045512.2 (1)      -----------------------------------------------------------------------------
```

Section 1638

```
                                                    (126050) 126050    126060     126071     126082     126093     126104     126115     126126
Candidatus Carsonella ruddii strain BT chromosome... (126050) AAAGTTTAAATGTTAATGTTGTTAATGGAATTATATTAAATTTTTTAAAATGAATAAATATAATATATATATAATTT
              Candidatus Carsonella 135557 - 167442nt (1)      -----------------------------------------------------------------------------
           SARS-CoV-2 Reference Genome NC_045512.2 (1)      -----------------------------------------------------------------------------
```

Section 1639

	(126127)	126127 126137 126148 126159 126170 126181 126192 126203
Candidatus Carsonella ruddii strain BT chromosome...	(126127)	TTGAAAAAAAAAAAATAGATTCATATTCAAATTTCTTAAAAAGTTTTTCTTCTTTTTTTTTAAGATCAAAAAGTAAT
Candidatus Carsonella 135557 - 167442nt	(1)	---
SARS-CoV-2 Reference Genome NC_045512.2	(1)	---

Section 1640

	(126204)	126204 126214 126225 126236 126247 126258 126269 126280
Candidatus Carsonella ruddii strain BT chromosome...	(126204)	ATTTTAAAAATTTTAGATTTTGGTAATTATTTTTGTAATAAAAAAATAAAAAGAATTTTTTTTTTAGAAATAAATTG
Candidatus Carsonella 135557 - 167442nt	(1)	---
SARS-CoV-2 Reference Genome NC_045512.2	(1)	---

Section 1641

	(126281)	126281 126291 126302 126313 126324 126335 126346 126357
Candidatus Carsonella ruddii strain BT chromosome...	(126281)	TAAAAAAAAAGTATTAAACAAAATACCTAAACTTTTAAAAACAAAAAAAGAATTAATAATTTTTTTTTTAATTTTAA
Candidatus Carsonella 135557 - 167442nt	(1)	---
SARS-CoV-2 Reference Genome NC_045512.2	(1)	---

Section 1642

	(126358)	126358 126368 126379 126390 126401 126412 126423 126434
Candidatus Carsonella ruddii strain BT chromosome...	(126358)	AAAATAATATAAACAAATATTTATTAAATATATTTTTTTTAAAAAAATATATTTCAAAAAATTTTAGATTAAAATCT
Candidatus Carsonella 135557 - 167442nt	(1)	---
SARS-CoV-2 Reference Genome NC_045512.2	(1)	---

Section 1643

	(126435)	126435 126445 126456 126467 126478 126489 126500 126511
Candidatus Carsonella ruddii strain BT chromosome...	(126435)	TCTATTTTAACTAAAATAAAATATAGTAAACATAATTATTATTCAAAAATAATAAAAAATTTAAAAATAATAAATAT
Candidatus Carsonella 135557 - 167442nt	(1)	---
SARS-CoV-2 Reference Genome NC_045512.2	(1)	---

Section 1644

	(126512)	126512 126522 126533 126544 126555 126566 126577 126588
Candidatus Carsonella ruddii strain BT chromosome...	(126512)	TATACCAATTAATTATATTTTTTACATAAAAAAAATAAATTTTTATTATGATAAATAATATTTCTACATTAAGTTCA
Candidatus Carsonella 135557 - 167442nt	(1)	---
SARS-CoV-2 Reference Genome NC_045512.2	(1)	---

```
                                                                                       Section 1645
                                             (126589) 126589    126599     126610     126621     126632     126643     126654    126665
Candidatus Carsonella ruddii strain BT chromosome... (126589) GAATTTATTATAATAAACTTTATATTTAGTAATTTAAAATTATCAAAAAATGTATTTGATATTTTAAAAGAAAATGA
               Candidatus Carsonella 135557 - 167442nt (1) -----------------------------------------------------------------------------
           SARS-CoV-2 Reference Genome NC_045512.2 (1) -----------------------------------------------------------------------------
                                                                                       Section 1646
                                             (126666) 126666    126676     126687     126698     126709     126720     126731    126742
Candidatus Carsonella ruddii strain BT chromosome... (126666) TTTTTTTTATGAAACAACTAAAGAATATTTTTTAAAAAAAAAATTATTTTATAAAATAAAATTTATTGATGAAAAAA
               Candidatus Carsonella 135557 - 167442nt (1) -----------------------------------------------------------------------------
           SARS-CoV-2 Reference Genome NC_045512.2 (1) -----------------------------------------------------------------------------
                                                                                       Section 1647
                                             (126743) 126743    126753     126764     126775     126786     126797     126808    126819
Candidatus Carsonella ruddii strain BT chromosome... (126743) TTATTTTTTCTAATTTAAAAAATTTATTAGAAAAATCTAAAAAAAGAAAAATGTTAAAAATTTTATTTAATATTTCT
               Candidatus Carsonella 135557 - 167442nt (1) -----------------------------------------------------------------------------
           SARS-CoV-2 Reference Genome NC_045512.2 (1) -----------------------------------------------------------------------------
                                                                                       Section 1648
                                             (126820) 126820    126830     126841     126852     126863     126874     126885    126896
Candidatus Carsonella ruddii strain BT chromosome... (126820) TTTGAATTGATTTTTAATGAACATATAGATGAAAAAATATTTGAAAAAATTAATTTTTTACAAAATTTTAAAAAAAA
               Candidatus Carsonella 135557 - 167442nt (1) -----------------------------------------------------------------------------
           SARS-CoV-2 Reference Genome NC_045512.2 (1) -----------------------------------------------------------------------------
                                                                                       Section 1649
                                             (126897) 126897    126907     126918     126929     126940     126951     126962    126973
Candidatus Carsonella ruddii strain BT chromosome... (126897) TAATCTTTTTGTTAAAAAAATAAATTATGTAAAATTATTAAAAGAATATTTTTCAAAAAAAGAATTAATTTTATTTA
               Candidatus Carsonella 135557 - 167442nt (1) -----------------------------------------------------------------------------
           SARS-CoV-2 Reference Genome NC_045512.2 (1) -----------------------------------------------------------------------------
                                                                                       Section 1650
                                             (126974) 126974    126984     126995     127006     127017     127028     127039    127050
Candidatus Carsonella ruddii strain BT chromosome... (126974) CTGGTTATAAAAATTTTGATATTTTATTAAATGGATTACAAAAAGGAAATTTAATTGTATTGGCAGGAAGACCTTCT
               Candidatus Carsonella 135557 - 167442nt (1) -----------------------------------------------------------------------------
           SARS-CoV-2 Reference Genome NC_045512.2 (1) -----------------------------------------------------------------------------
```

```
Section 1651
(127051) 127051 127061 127072 127083 127094 127105 127116 127127
Candidatus Carsonella ruddii strain BT chromosome... (127051) ATAGGTAAAACCTCATTTTCTTTAAATATAATAAAAAATTTAATTTTATTAAATAAAAAAATAATAATTTTTTCATT
Candidatus Carsonella 135557 - 167442nt (1) -----------------------------------------------------------------------------
SARS-CoV-2 Reference Genome NC_045512.2 (1) -----------------------------------------------------------------------------

Section 1652
(127128) 127128 127138 127149 127160 127171 127182 127193 127204
Candidatus Carsonella ruddii strain BT chromosome... (127128) AGAAATGACGACTATACAAATTTATATTAGATTAATGTCTATTATATTAGAAATTAATCAAAATAACTTTATTAAAA
Candidatus Carsonella 135557 - 167442nt (1) -----------------------------------------------------------------------------
SARS-CoV-2 Reference Genome NC_045512.2 (1) -----------------------------------------------------------------------------

Section 1653
(127205) 127205 127215 127226 127237 127248 127259 127270 127281
Candidatus Carsonella ruddii strain BT chromosome... (127205) ATAATTTTAATAAAAATGATATGAAAAAAATTTCATTTTTTATAAAAAATATAAATTTTGATTCTTTAATAATAAAA
Candidatus Carsonella 135557 - 167442nt (1) -----------------------------------------------------------------------------
SARS-CoV-2 Reference Genome NC_045512.2 (1) -----------------------------------------------------------------------------

Section 1654
(127282) 127282 127292 127303 127314 127325 127336 127347 127358
Candidatus Carsonella ruddii strain BT chromosome... (127282) GATTGTTCTAATTTATCTTCAAATGATATAGAATCTCAATTAGAGTATTATAAAAAAAATAATATAAATATAGAAAT
Candidatus Carsonella 135557 - 167442nt (1) -----------------------------------------------------------------------------
SARS-CoV-2 Reference Genome NC_045512.2 (1) -----------------------------------------------------------------------------

Section 1655
(127359) 127359 127369 127380 127391 127402 127413 127424 127435
Candidatus Carsonella ruddii strain BT chromosome... (127359) AATAGTTATAGATTATATTCAATTAATGAAATCTGAATTTTATTCAAATAATAGAGTATTGGAAATTTCTGATATAT
Candidatus Carsonella 135557 - 167442nt (1) -----------------------------------------------------------------------------
SARS-CoV-2 Reference Genome NC_045512.2 (1) -----------------------------------------------------------------------------

Section 1656
(127436) 127436 127446 127457 127468 127479 127490 127501 127512
Candidatus Carsonella ruddii strain BT chromosome... (127436) CTAGATCATTAAAATTAATAGCTAAACATTTTAATTGTGTAGTAATTGCTTTATCTCAATTAAATAGATTAATAGAA
Candidatus Carsonella 135557 - 167442nt (1) -----------------------------------------------------------------------------
SARS-CoV-2 Reference Genome NC_045512.2 (1) -----------------------------------------------------------------------------
```

Section 1657

(127513) 127513 127523 127534 127545 127556 127567 127578 127589
Candidatus Carsonella ruddii strain BT chromosome... (127513) TATAGAGTTGAAAAAATTCCTATGCTTTCAGATTTAAGAGATTCAGGATCAATAGAGCAAGATGCTGATATAGTTAT
Candidatus Carsonella 135557 - 167442nt (1) ---
SARS-CoV-2 Reference Genome NC_045512.2 (1) ---

Section 1658

(127590) 127590 127600 127611 127622 127633 127644 127655 127666
Candidatus Carsonella ruddii strain BT chromosome... (127590) AATTTTACATAAAAAAAATTTTAATTTTATAGATGTTTTAATTCTTAAAAATAGAAATGGTCCTTTAGGTGTTATTA
Candidatus Carsonella 135557 - 167442nt (1) ---
SARS-CoV-2 Reference Genome NC_045512.2 (1) ---

Section 1659

(127667) 127667 127677 127688 127699 127710 127721 127732 127743
Candidatus Carsonella ruddii strain BT chromosome... (127667) ATTTTATTTTTAAAAATGAATATACAAAATTTATACAAATTTAAAAAAAAAATTGGACAAAATTATTTAATTTTTAA
Candidatus Carsonella 135557 - 167442nt (1) ---
SARS-CoV-2 Reference Genome NC_045512.2 (1) ---

Section 1660

(127744) 127744 127754 127765 127776 127787 127798 127809 127820
Candidatus Carsonella ruddii strain BT chromosome... (127744) TTTAAAATTTAAATTTGATTTATGTTCAGGTTATAATTATTTTAATATTTTTAATAATGAATTAGATTTTTTAAAAA
Candidatus Carsonella 135557 - 167442nt (1) ---
SARS-CoV-2 Reference Genome NC_045512.2 (1) ---

Section 1661

(127821) 127821 127831 127842 127853 127864 127875 127886 127897
Candidatus Carsonella ruddii strain BT chromosome... (127821) GCAAGTTTTTAAATAATAAAATTTATTTATTAAAAAATAAAAATTTATATTTAAATTTTTTATATTTTAATTTTAAT
Candidatus Carsonella 135557 - 167442nt (1) ---
SARS-CoV-2 Reference Genome NC_045512.2 (1) ---

Section 1662

(127898) 127898 127908 127919 127930 127941 127952 127963 127974
Candidatus Carsonella ruddii strain BT chromosome... (127898) ATTTTAAAATTAATAAATATAAATTTACCTTTTAATATTATAAAAATTTTTTTTAAAAAAATAAAAAAAATAAAAAG
Candidatus Carsonella 135557 - 167442nt (1) ---
SARS-CoV-2 Reference Genome NC_045512.2 (1) ---

Section 1663

(127975) 127975 127985 127996 128007 128018 128029 128040 128051

Candidatus Carsonella ruddii strain BT chromosome... (127975) ATTTTTTTTTAAAATAATTGTTAATTTTATAAATTTTTTAATTTTTAAAAAAAAAATATTAATTTTTAAAAAAAAAT
Candidatus Carsonella 135557 - 167442nt (1) ---
SARS-CoV-2 Reference Genome NC_045512.2 (1) ---

Section 1664

(128052) 128052 128062 128073 128084 128095 128106 128117 128128

Candidatus Carsonella ruddii strain BT chromosome... (128052) TTTTTTTAAAAAAAATTTTTTTCCCTTATCCTAAAATTAATATAATAAAAATTTATTTTAAAAAAAAAAAAAATAAA
Candidatus Carsonella 135557 - 167442nt (1) ---
SARS-CoV-2 Reference Genome NC_045512.2 (1) ---

Section 1665

(128129) 128129 128139 128150 128161 128172 128183 128194 128205

Candidatus Carsonella ruddii strain BT chromosome... (128129) AGTATTAATTTTTTTTTAAATATTATTAAATTAAAACAAAATAAATCAGAAATAACAAAGTTTTTTGTAAAAAAAAT
Candidatus Carsonella 135557 - 167442nt (1) ---
SARS-CoV-2 Reference Genome NC_045512.2 (1) ---

Section 1666

(128206) 128206 128216 128227 128238 128249 128260 128271 128282

Candidatus Carsonella ruddii strain BT chromosome... (128206) AATTTTAATAATTAAAAAAAATGTTTTTTTCTATTAGATTTAAAAAAGAAAATTTTGATTTTTTTTTAAAAAGATTT
Candidatus Carsonella 135557 - 167442nt (1) ---
SARS-CoV-2 Reference Genome NC_045512.2 (1) ---

Section 1667

(128283) 128283 128293 128304 128315 128326 128337 128348 128359

Candidatus Carsonella ruddii strain BT chromosome... (128283) AAAAAAAATATTGAAAAACAAAATAAAATTTGTAAAAAAGAAAAAAAAAAAATTTTAAATAAAATAAAAAAATGAAT
Candidatus Carsonella 135557 - 167442nt (1) ---
SARS-CoV-2 Reference Genome NC_045512.2 (1) ---

Section 1668

(128360) 128360 128370 128381 128392 128403 128414 128425 128436

Candidatus Carsonella ruddii strain BT chromosome... (128360) TTAAAATGTCCATTTCACAATGATAATAATGCATCTTTTTCTATAAGAAAAAATAAATTTATTTGTTATGGATGTAA
Candidatus Carsonella 135557 - 167442nt (1) ---
SARS-CoV-2 Reference Genome NC_045512.2 (1) ---

Section 1669

	(128437)	128437 128447 128458 128469 128480 128491 128502 128513
Candidatus Carsonella ruddii strain BT chromosome...	(128437)	TTTATATGGTTTTGTTAAAAATTCATTTAAAATTAAAAAATTTGATTGTTTTTTTAATAAAAAAATTATATTATATT
Candidatus Carsonella 135557 - 167442nt	(1)	---
SARS-CoV-2 Reference Genome NC_045512.2	(1)	---

Section 1670

	(128514)	128514 128524 128535 128546 128557 128568 128579 128590
Candidatus Carsonella ruddii strain BT chromosome...	(128514)	CAAAAAGAAATTTATATATTAAAAAAAATTTTTGGTTAAAATATTTATTATTAAGAAATATAAATATGAATACATCA
Candidatus Carsonella 135557 - 167442nt	(1)	---
SARS-CoV-2 Reference Genome NC_045512.2	(1)	---

Section 1671

	(128591)	128591 128601 128612 128623 128634 128645 128656 128667
Candidatus Carsonella ruddii strain BT chromosome...	(128591)	TTAAAATTTAATTTAGGTGTATTAAATTTTACTCAAAAAAAAGTAAATAACATTATTTTAAATAGATTAATTTTTCC
Candidatus Carsonella 135557 - 167442nt	(1)	---
SARS-CoV-2 Reference Genome NC_045512.2	(1)	---

Section 1672

	(128668)	128668 128678 128689 128700 128711 128722 128733 128744
Candidatus Carsonella ruddii strain BT chromosome...	(128668)	AATACTAAATGATAAAAATATATTGTTAGGTATAGGTTTAAAAACAAACAATAAAAAAAATAAATATATAAATTTAT
Candidatus Carsonella 135557 - 167442nt	(1)	---
SARS-CoV-2 Reference Genome NC_045512.2	(1)	---

Section 1673

	(128745)	128745 128755 128766 128777 128788 128799 128810 128821
Candidatus Carsonella ruddii strain BT chromosome...	(128745)	TAAAAAACAACATTAATGAAAATGTTTTTTTTGGTATTAATAATATAAAAAATTTTAATTTTATAATTGTAGTAGAA
Candidatus Carsonella 135557 - 167442nt	(1)	---
SARS-CoV-2 Reference Genome NC_045512.2	(1)	---

Section 1674

	(128822)	128822 128832 128843 128854 128865 128876 128887 128898
Candidatus Carsonella ruddii strain BT chromosome...	(128822)	GGTTACTTTGATTTATTAAGTATTTATAAAAATAATTTTTTAAATGTAATTTCTATATTAGGTTGTAATATTAATTA
Candidatus Carsonella 135557 - 167442nt	(1)	---
SARS-CoV-2 Reference Genome NC_045512.2	(1)	---

Section 1675

(128899) 128899 128909 128920 128931 128942 128953 128964 128975
Candidatus Carsonella ruddii strain BT chromosome... (128899) TGAAAAAATAAATTTTTTATTGTTTAAATTTAAAAAAATTTATTTTTGTTTAGATGGAGATTATTCTGGTTATAATT
Candidatus Carsonella 135557 - 167442nt (1) ---
SARS-CoV-2 Reference Genome NC_045512.2 (1) ---

Section 1676

(128976) 128976 128986 128997 129008 129019 129030 129041 129052
Candidatus Carsonella ruddii strain BT chromosome... (128976) CTTCTGAAAAAATTAAAAAAATATTTAAAAATTATTATAAAAAAAGAATTTTTATTAAAAATATGCCATTTAATTTT
Candidatus Carsonella 135557 - 167442nt (1) ---
SARS-CoV-2 Reference Genome NC_045512.2 (1) ---

Section 1677

(129053) 129053 129063 129074 129085 129096 129107 129118 129129
Candidatus Carsonella ruddii strain BT chromosome... (129053) GATCCTGATAGTTTTATAAATTTTTATGGAATTAAATCTTTTTTAAAATTTTTAATAAAATGAATATATTTTTTTTA
Candidatus Carsonella 135557 - 167442nt (1) ---
SARS-CoV-2 Reference Genome NC_045512.2 (1) ---

Section 1678

(129130) 129130 129140 129151 129162 129173 129184 129195 129206
Candidatus Carsonella ruddii strain BT chromosome... (129130) TTTTTAAACAAAAATTTGTTTCCTTTTTTAAAAATTATAAAAAAAAAATTTTTTTATATTTTTAAAAACAAAATAAA
Candidatus Carsonella 135557 - 167442nt (1) ---
SARS-CoV-2 Reference Genome NC_045512.2 (1) ---

Section 1679

(129207) 129207 129217 129228 129239 129250 129261 129272 129283
Candidatus Carsonella ruddii strain BT chromosome... (129207) AAAAAAAATTAATATTAAAAATAATTTTTTTTTTTTTTTTAAAAAAATAAATAAAAAAATTTATTTAAAAAAAAAAA
Candidatus Carsonella 135557 - 167442nt (1) ---
SARS-CoV-2 Reference Genome NC_045512.2 (1) ---

Section 1680

(129284) 129284 129294 129305 129316 129327 129338 129349 129360
Candidatus Carsonella ruddii strain BT chromosome... (129284) TAATTATTATTAATTTAATAAATTCTTTTTTTTTTAATATTTATTTAATCAAAAATTTTATTTTGAAAATTTTTTTT
Candidatus Carsonella 135557 - 167442nt (1) ---
SARS-CoV-2 Reference Genome NC_045512.2 (1) ---

Section 1681

	(129361)	129361 129371 129382 129393 129404 129415 129426 129437
Candidatus Carsonella ruddii strain BT chromosome...	(129361)	TATTTCGATTTTTTTTTTACAAAAAAAAAATTTTATAAAAAAAATTTTAATTTTTATAAAAAAAAAATTAAAATAAA
Candidatus Carsonella 135557 - 167442nt	(1)	---
SARS-CoV-2 Reference Genome NC_045512.2	(1)	---

Section 1682

	(129438)	129438 129448 129459 129470 129481 129492 129503 129514
Candidatus Carsonella ruddii strain BT chromosome...	(129438)	AAATTTTTTAAATCATATTGAAAATAAAATTTTCTATATAAATAAAAATTTAATATTAATTAAAAATAAAAAAAATA
Candidatus Carsonella 135557 - 167442nt	(1)	---
SARS-CoV-2 Reference Genome NC_045512.2	(1)	---

Section 1683

	(129515)	129515 129525 129536 129547 129558 129569 129580 129591
Candidatus Carsonella ruddii strain BT chromosome...	(129515)	ATATAGAACAATTAATAAACATAAATTTAATTAAAACAATTATTTATCTTTCATTTATAAAAAAAAAAATAAATGAA
Candidatus Carsonella 135557 - 167442nt	(1)	---
SARS-CoV-2 Reference Genome NC_045512.2	(1)	---

Section 1684

	(129592)	129592 129602 129613 129624 129635 129646 129657 129668
Candidatus Carsonella ruddii strain BT chromosome...	(129592)	AATATTTTAAAAAATAATATAAAAATTTTAAAAAAAATTTTAAAAAAATATAAAAATAAACAAATTTACAATGATTT
Candidatus Carsonella 135557 - 167442nt	(1)	---
SARS-CoV-2 Reference Genome NC_045512.2	(1)	---

Section 1685

	(129669)	129669 129679 129690 129701 129712 129723 129734 129745
Candidatus Carsonella ruddii strain BT chromosome...	(129669)	ATATCAAGAATGTTATTATGAATTTAAAAATGTAATTTTTGATAATAATTATTGTAATAATAATTTTTATAAATTTT
Candidatus Carsonella 135557 - 167442nt	(1)	---
SARS-CoV-2 Reference Genome NC_045512.2	(1)	---

Section 1686

	(129746)	129746 129756 129767 129778 129789 129800 129811 129822
Candidatus Carsonella ruddii strain BT chromosome...	(129746)	GTTATTGGAATATTAAAAAAAAAATGATAAGCATAATAAATTTTAAAAAAATAATTTTTAAAAATCAAATTTTATCA
Candidatus Carsonella 135557 - 167442nt	(1)	---
SARS-CoV-2 Reference Genome NC_045512.2	(1)	---

```
                                                                                  Section 1687
                                                (129823) 129823    129833     129844     129855     129866     129877     129888  129899
Candidatus Carsonella ruddii strain BT chromosome... (129823) ATTGATAAACCTTTATTTTATCATTCTTTAAAATATTTTATATCAGAAAATGATAGATCGTTTCTTTTAGAATATAC
            Candidatus Carsonella 135557 - 167442nt (1) -----------------------------------------------------------------------------
        SARS-CoV-2 Reference Genome NC_045512.2 (1) -----------------------------------------------------------------------------
```

```
                                                                                  Section 1688
                                                (129900) 129900    129910     129921     129932     129943     129954     129965  129976
Candidatus Carsonella ruddii strain BT chromosome... (129900) AAGAAATTCTTTAAAAAATTTAATAAGAGAATTAATTTCTAGTTTACCTCAAAGAGAACAAAAAATAATAAGATTAA
            Candidatus Carsonella 135557 - 167442nt (1) -----------------------------------------------------------------------------
        SARS-CoV-2 Reference Genome NC_045512.2 (1) -----------------------------------------------------------------------------
```

```
                                                                                  Section 1689
                                                (129977) 129977    129987     129998     130009     130020     130031     130042  130053
Candidatus Carsonella ruddii strain BT chromosome... (129977) GATTTGGAATAGGTCACCAAAAATATTATACTCTTGAAGAAATAGGAATTTTACATTATTTAACAAAAGAAAGAATA
            Candidatus Carsonella 135557 - 167442nt (1) -----------------------------------------------------------------------------
        SARS-CoV-2 Reference Genome NC_045512.2 (1) -----------------------------------------------------------------------------
```

```
                                                                                  Section 1690
                                                (130054) 130054    130064     130075     130086     130097     130108     130119  130130
Candidatus Carsonella ruddii strain BT chromosome... (130054) AGACAAATTGAGTTAGCTATCATATTAAAAATAAGAAAACCTTTAATAATTGAATTATTAAAACCTTATGTAAAATT
            Candidatus Carsonella 135557 - 167442nt (1) -----------------------------------------------------------------------------
        SARS-CoV-2 Reference Genome NC_045512.2 (1) -----------------------------------------------------------------------------
```

```
                                                                                  Section 1691
                                                (130131) 130131    130141     130152     130163     130174     130185     130196  130207
Candidatus Carsonella ruddii strain BT chromosome... (130131) ATTAAAAGCTTTAGAATAATGGGGTTATAATTTAACTGGTAAAATATTTGCTTTGCAAGCAAAAATAAAGGGTTCAA
            Candidatus Carsonella 135557 - 167442nt (1) -----------------------------------------------------------------------------
        SARS-CoV-2 Reference Genome NC_045512.2 (1) -----------------------------------------------------------------------------
```

```
                                                                                  Section 1692
                                                (130208) 130208    130218     130229     130240     130251     130262     130273  130284
Candidatus Carsonella ruddii strain BT chromosome... (130208) TTCCCTTTAACTCCATTTTTTTAAATTATTTAAAATTATTTTAAATATTTTATATTTTGAAACTGTTATTCTTAAAA
            Candidatus Carsonella 135557 - 167442nt (1) -----------------------------------------------------------------------------
        SARS-CoV-2 Reference Genome NC_045512.2 (1) -----------------------------------------------------------------------------
```

Section 1693

(130285) 130285 130295 130306 130317 130328 130339 130350 130361
Candidatus Carsonella ruddii strain BT chromosome... (130285) TGTTATTTTTAAAATATCTAAAAAAAAAATTTTTTTTTTTAAAAAAAATAAAAAAATCTATTTTTTTTTTTAAACAA
Candidatus Carsonella 135557 - 167442nt (1) ---
SARS-CoV-2 Reference Genome NC_045512.2 (1) ---

Section 1694

(130362) 130362 130372 130383 130394 130405 130416 130427 130438
Candidatus Carsonella ruddii strain BT chromosome... (130362) ATAAAATTTCCTTTTTGTTTTTTTTTAAAAATAAAATTAAATAATAATTTTAAATAATTGATTTTTTGTATATTTAA
Candidatus Carsonella 135557 - 167442nt (1) ---
SARS-CoV-2 Reference Genome NC_045512.2 (1) ---

Section 1695

(130439) 130439 130449 130460 130471 130482 130493 130504 130515
Candidatus Carsonella ruddii strain BT chromosome... (130439) AAATAAAAAATGTTTATCTTTTAAACATTCAATAGATATAGAATTTGATACAATATCTATTGTATAAGAATTAAAAC
Candidatus Carsonella 135557 - 167442nt (1) ---
SARS-CoV-2 Reference Genome NC_045512.2 (1) ---

Section 1696

(130516) 130516 130526 130537 130548 130559 130570 130581 130592
Candidatus Carsonella ruddii strain BT chromosome... (130516) ATTGTTTTAAATATTTAATTTTATTAATTATTTTTTTATTTGAAAATATTAATCCTATTCTTATTCCAGCAATACTA
Candidatus Carsonella 135557 - 167442nt (1) ---
SARS-CoV-2 Reference Genome NC_045512.2 (1) ---

Section 1697

(130593) 130593 130603 130614 130625 130636 130647 130658 130669
Candidatus Carsonella ruddii strain BT chromosome... (130593) AAAGCTTTAGATAATGTATTTATAATAATTAAATTATTTTTAATTTTAATTTTTTTTTTTAAACCTAAATTATAAAA
Candidatus Carsonella 135557 - 167442nt (1) ---
SARS-CoV-2 Reference Genome NC_045512.2 (1) ---

Section 1698

(130670) 130670 130680 130691 130702 130713 130724 130735 130746
Candidatus Carsonella ruddii strain BT chromosome... (130670) TTCTGAATAAGCTTCATCTATAATTAAAAAATTTTTTTTTTTTATCTTTTTTATTAAAAAAAAATTTTTAAAAAATT
Candidatus Carsonella 135557 - 167442nt (1) ---
SARS-CoV-2 Reference Genome NC_045512.2 (1) ---

Section 1699

	(130747)	130747 130757 130768 130779 130790 130801 130812 130823
Candidatus Carsonella ruddii strain BT chromosome...	(130747)	CACCAGAAGGTGAATTTGGGTTTGGAAAAATTATATTTTTATATTTTAAAATATTTTTATATTTAACTTCTTTTGTG
Candidatus Carsonella 135557 - 167442nt	(1)	---
SARS-CoV-2 Reference Genome NC_045512.2	(1)	---

Section 1700

	(130824)	130824 130834 130845 130856 130867 130878 130889 130900
Candidatus Carsonella ruddii strain BT chromosome...	(130824)	TATATTTTAAAAAAATTAGAATATATTTTATAAAAAGGATAACTTTGTTTAGGAATTTTTATTTTAAAATTTTTAAA
Candidatus Carsonella 135557 - 167442nt	(1)	---
SARS-CoV-2 Reference Genome NC_045512.2	(1)	---

Section 1701

	(130901)	130901 130911 130922 130933 130944 130955 130966 130977
Candidatus Carsonella ruddii strain BT chromosome...	(130901)	CAAAAAAAATAAAAAAAATAACCCTTCATCTGAACCATTAGATATAAATATATTATTTTTATTTAAATTATATATTT
Candidatus Carsonella 135557 - 167442nt	(1)	---
SARS-CoV-2 Reference Genome NC_045512.2	(1)	---

Section 1702

	(130978)	130978 130988 130999 131010 131021 131032 131043 131054
Candidatus Carsonella ruddii strain BT chromosome...	(130978)	TACTAATAATTTTTGTTAAATGTAAATTTTCAGAATTTGGGTAAAATTTAAATTTATTAAAATTTTTAGAAATAATT
Candidatus Carsonella 135557 - 167442nt	(1)	---
SARS-CoV-2 Reference Genome NC_045512.2	(1)	---

Section 1703

	(131055)	131055 131065 131076 131087 131098 131109 131120 131131
Candidatus Carsonella ruddii strain BT chromosome...	(131055)	TTTAATAAAAAAAAACAGTTTTTACTTGAAGAATAAATATTTTCATTAGTATTTAATTTATAGCCTTTATAACACCT
Candidatus Carsonella 135557 - 167442nt	(1)	---
SARS-CoV-2 Reference Genome NC_045512.2	(1)	---

Section 1704

	(131132)	131132 131142 131153 131164 131175 131186 131197 131208
Candidatus Carsonella ruddii strain BT chromosome...	(131132)	TTCACCAGGTATATATTTTTTCATATTAATTTTATATATTTTTTAAAATAATTAGGTACTAAATAATTAAAATGAAA
Candidatus Carsonella 135557 - 167442nt	(1)	---
SARS-CoV-2 Reference Genome NC_045512.2	(1)	---

Section 1705

```
                                                    (131209) 131209    131219     131230     131241     131252     131263     131274     131285
Candidatus Carsonella ruddii strain BT chromosome... (131209) AACACTAGCAATTAAAAAACTATTAATATTACAATATTTATAAATATAATAAATATCATTTATACTTCCTAAACCAC
             Candidatus Carsonella 135557 - 167442nt (1) -----------------------------------------------------------------------------
          SARS-CoV-2 Reference Genome NC_045512.2 (1) -----------------------------------------------------------------------------
```

Section 1706

```
                                                    (131286) 131286    131296     131307     131318     131329     131340     131351     131362
Candidatus Carsonella ruddii strain BT chromosome... (131286) CAGATATAATTATAGAAATATCTATATTTTTTTAACATAAATAATAGATTTAATATCATATCCTTTTTTTAAACCA
             Candidatus Carsonella 135557 - 167442nt (1) -----------------------------------------------------------------------------
          SARS-CoV-2 Reference Genome NC_045512.2 (1) -----------------------------------------------------------------------------
```

Section 1707

```
                                                    (131363) 131363    131373     131384     131395     131406     131417     131428     131439
Candidatus Carsonella ruddii strain BT chromosome... (131363) TCATTTTCAATAGATGTAATTAATATTTCTCCACAACCTATTTTTTTAACTATTATAACCCAATCTAGTAATTTAAA
             Candidatus Carsonella 135557 - 167442nt (1) -----------------------------------------------------------------------------
          SARS-CoV-2 Reference Genome NC_045512.2 (1) -----------------------------------------------------------------------------
```

Section 1708

```
                                                    (131440) 131440    131450     131461     131472     131483     131494     131505     131516
Candidatus Carsonella ruddii strain BT chromosome... (131440) ATTAGTTTTAATAAAACCACTGTTTTTATAAGTAAAAAAAAAATTGTTTTTAAAAAATATATCAATAGAAACAATTA
             Candidatus Carsonella 135557 - 167442nt (1) -----------------------------------------------------------------------------
          SARS-CoV-2 Reference Genome NC_045512.2 (1) -----------------------------------------------------------------------------
```

Section 1709

```
                                                    (131517) 131517    131527     131538     131549     131560     131571     131582     131593
Candidatus Carsonella ruddii strain BT chromosome... (131517) ATGCTTGAGATCCATATAAATTTGAAATCTTTTTTAAAAATACTATGTTATTATAACAAGAAGAATTTAAAACAATT
             Candidatus Carsonella 135557 - 167442nt (1) -----------------------------------------------------------------------------
          SARS-CoV-2 Reference Genome NC_045512.2 (1) -----------------------------------------------------------------------------
```

Section 1710

```
                                                    (131594) 131594    131604     131615     131626     131637     131648     131659     131670
Candidatus Carsonella ruddii strain BT chromosome... (131594) CTGTCAGCTCCTAATTTAAATAATTTAGAAATTTTATTTAAATTTTTTATACCTCCTCCAATAGAAATAGGAATATT
             Candidatus Carsonella 135557 - 167442nt (1) -----------------------------------------------------------------------------
          SARS-CoV-2 Reference Genome NC_045512.2 (1) -----------------------------------------------------------------------------
```

```
                                                                  Section 1711
                                             (131671) 131671    131681     131692     131703     131714     131725     131736     131747
Candidatus Carsonella ruddii strain BT chromosome... (131671) AATATTTTTAGAAATTAATTTTATATTTTTATAAGATAGACTTTTTTTAGTATAATTAGAAGTTATATCTAAAATTA
             Candidatus Carsonella 135557 - 167442nt      (1) -----------------------------------------------------------------------------
          SARS-CoV-2 Reference Genome NC_045512.2         (1) -----------------------------------------------------------------------------
                                                                  Section 1712
                                             (131748) 131748    131758     131769     131780     131791     131802     131813     131824
Candidatus Carsonella ruddii strain BT chromosome... (131748) TAATTTCATCAGTTTTATAAAATTCATATTTTTTACATAATAAATAAGGATAATTAATAATTTTTAATTTTTTAAAA
             Candidatus Carsonella 135557 - 167442nt      (1) -----------------------------------------------------------------------------
          SARS-CoV-2 Reference Genome NC_045512.2         (1) -----------------------------------------------------------------------------
                                                                  Section 1713
                                             (131825) 131825    131835     131846     131857     131868     131879     131890     131901
Candidatus Carsonella ruddii strain BT chromosome... (131825) CTTATACCTTTTACAACTAAATTATTTTTAATATCTAAACAACTTATAATTCTAATCATAAATTTTTTATTTTAATA
             Candidatus Carsonella 135557 - 167442nt      (1) -----------------------------------------------------------------------------
          SARS-CoV-2 Reference Genome NC_045512.2         (1) -----------------------------------------------------------------------------
                                                                  Section 1714
                                             (131902) 131902    131912     131923     131934     131945     131956     131967     131978
Candidatus Carsonella ruddii strain BT chromosome... (131902) CTAAATTTATATAAAGACAATCCTATTATAAATTTAAAATTGCATTTTTTATTAATTTTTTTAATATCATCTATATT
             Candidatus Carsonella 135557 - 167442nt      (1) -----------------------------------------------------------------------------
          SARS-CoV-2 Reference Genome NC_045512.2         (1) -----------------------------------------------------------------------------
                                                                  Section 1715
                                             (131979) 131979    131989     132000     132011     132022     132033     132044     132055
Candidatus Carsonella ruddii strain BT chromosome... (131979) TGATATACCTCCTGAGATAATAAAATTACATAAATTTTTTTTATTTAATATAAAACATAATTCTTCAATTTTTATTC
             Candidatus Carsonella 135557 - 167442nt      (1) -----------------------------------------------------------------------------
          SARS-CoV-2 Reference Genome NC_045512.2         (1) -----------------------------------------------------------------------------
                                                                  Section 1716
                                             (132056) 132056    132066     132077     132088     132099     132110     132121     132132
Candidatus Carsonella ruddii strain BT chromosome... (132056) CAGACAAAGTACCATCTTTAATTATATTAGTGTATATTATTTTTTTAATATTTATATCAAATAAATATATAAAAATT
             Candidatus Carsonella 135557 - 167442nt      (1) -----------------------------------------------------------------------------
          SARS-CoV-2 Reference Genome NC_045512.2         (1) -----------------------------------------------------------------------------
```

Section 1717

	(132133)	132133 132143 132154 132165 132176 132187 132198 132209
Candidatus Carsonella ruddii strain BT chromosome...	(132133)	TTTTCAAAAGAAATTTTTGTTTGAATAATCCAACCATTAATTGTGATAAAATTATTTAAACAATCTAAACTTATTAA
Candidatus Carsonella 135557 - 167442nt	(1)	---
SARS-CoV-2 Reference Genome NC_045512.2	(1)	---

Section 1718

	(132210)	132210 132220 132231 132242 132253 132264 132275 132286
Candidatus Carsonella ruddii strain BT chromosome...	(132210)	TATTTTTTTTTTATAAAAAGATAAAATTTTTTTAAATTCTTTTTTATTTGTAAACAAAATACTTCCTAAAACAAATT
Candidatus Carsonella 135557 - 167442nt	(1)	---
SARS-CoV-2 Reference Genome NC_045512.2	(1)	---

Section 1719

	(132287)	132287 132297 132308 132319 132330 132341 132352 132363
Candidatus Carsonella ruddii strain BT chromosome...	(132287)	TTACAAAATTTTGTTTAAAATAAAAATCTATTATTTTACATGAATAAATTCCTCCTCCAATTTGAGCATTTATATTT
Candidatus Carsonella 135557 - 167442nt	(1)	---
SARS-CoV-2 Reference Genome NC_045512.2	(1)	---

Section 1720

	(132364)	132364 132374 132385 132396 132407 132418 132429 132440
Candidatus Carsonella ruddii strain BT chromosome...	(132364)	TTATTAATTTTAATTTTTTTTTGTTTTTTTAAAAATATATTATCTAATGAAACAATATGTATATTTTTAATATTTAT
Candidatus Carsonella 135557 - 167442nt	(1)	---
SARS-CoV-2 Reference Genome NC_045512.2	(1)	---

Section 1721

	(132441)	132441 132451 132462 132473 132484 132495 132506 132517
Candidatus Carsonella ruddii strain BT chromosome...	(132441)	AAAATAATAAAATTCTATATATTCAAGATAATATCCATAAAAAAAACATTTATTAATATCTCCTTTATATAATCTAA
Candidatus Carsonella 135557 - 167442nt	(1)	---
SARS-CoV-2 Reference Genome NC_045512.2	(1)	---

Section 1722

	(132518)	132518 132528 132539 132550 132561 132572 132583 132594
Candidatus Carsonella ruddii strain BT chromosome...	(132518)	CAACTTTATTTTTATAAATATCTATTGATGATATTATCATAAATTTAAAAAATTATTTAATAATAATAATCCAATTT
Candidatus Carsonella 135557 - 167442nt	(1)	---
SARS-CoV-2 Reference Genome NC_045512.2	(1)	---

```
                                                                                          Section 1723
                                                   (132595) 132595    132605     132616     132627     132638     132649     132660  132671
Candidatus Carsonella ruddii strain BT chromosome... (132595) TACTACTTTTTTCTGGATGAAATTGAAATAAAAATTTATTTTCTTTTATAATTATAGAATTAAAAATTATATTGTTA
                 Candidatus Carsonella 135557 - 167442nt (1) -----------------------------------------------------------------------------
                 SARS-CoV-2 Reference Genome NC_045512.2 (1) -----------------------------------------------------------------------------
                                                                                          Section 1724
                                                   (132672) 132672    132682     132693     132704     132715     132726     132737  132748
Candidatus Carsonella ruddii strain BT chromosome... (132672) AAAATTGAAGTTGCATAAGAAAATTTTTGATTTTTAAAAATAGAAGAATAACTATGCATAAAATATTGTTTAAAATT
                 Candidatus Carsonella 135557 - 167442nt (1) -----------------------------------------------------------------------------
                 SARS-CoV-2 Reference Genome NC_045512.2 (1) -----------------------------------------------------------------------------
                                                                                          Section 1725
                                                   (132749) 132749    132759     132770     132781     132792     132803     132814  132825
Candidatus Carsonella ruddii strain BT chromosome... (132749) TAATGGTAATTTTTTAAAAATTTTATGATTTTTAATAATATTAACATTATTCCAATTAATATTAGGATTTGAAGTAT
                 Candidatus Carsonella 135557 - 167442nt (1) -----------------------------------------------------------------------------
                 SARS-CoV-2 Reference Genome NC_045512.2 (1) -----------------------------------------------------------------------------
                                                                                          Section 1726
                                                   (132826) 132826    132836     132847     132858     132869     132880     132891  132902
Candidatus Carsonella ruddii strain BT chromosome... (132826) TGTAACAGCAATTTTTTGCTATTAAATCAATATTTTCATTAATAATATTTAAACAATTAATTTTTTCATTTTCTATA
                 Candidatus Carsonella 135557 - 167442nt (1) -----------------------------------------------------------------------------
                 SARS-CoV-2 Reference Genome NC_045512.2 (1) -----------------------------------------------------------------------------
                                                                                          Section 1727
                                                   (132903) 132903    132913     132924     132935     132946     132957     132968  132979
Candidatus Carsonella ruddii strain BT chromosome... (132903) CTTTTTTTAAAAAAAATGTGGTATCCTATACATATTCCTAAAATATAACTATTTGATAAATAATTTTTTAAATAATA
                 Candidatus Carsonella 135557 - 167442nt (1) -----------------------------------------------------------------------------
                 SARS-CoV-2 Reference Genome NC_045512.2 (1) -----------------------------------------------------------------------------
                                                                                          Section 1728
                                                   (132980) 132980    132990     133001     133012     133023     133034     133045  133056
Candidatus Carsonella ruddii strain BT chromosome... (132980) AATTTTTGATAAAAATTTTATACAATTAGTTATGTGTCCTTGACCTGGAAAAATAATTTTATCGTAATGTTTTTTTT
                 Candidatus Carsonella 135557 - 167442nt (1) -----------------------------------------------------------------------------
                 SARS-CoV-2 Reference Genome NC_045512.2 (1) -----------------------------------------------------------------------------
```

```
                                                                                                    Section 1729
                                                   (133057) 133057    133067     133078     133089     133100     133111     133122  133133
Candidatus Carsonella ruddii strain BT chromosome... (133057) CAAAATTATTTATAAAAACTTTTATTTTTTTTACTTTTTTTAAAGCATTATAAATTGAATTTATATTACTTGTTCCA
             Candidatus Carsonella 135557 - 167442nt      (1) -----------------------------------------------------------------------------
          SARS-CoV-2 Reference Genome NC_045512.2      (1) -----------------------------------------------------------------------------
                                                                                                    Section 1730
                                                   (133134) 133134    133144     133155     133166     133177     133188     133199  133210
Candidatus Carsonella ruddii strain BT chromosome... (133134) TAATTAATTAATAAAATATTCATAAAAATCCTTTTGTAGATTGTAAAATATTACAATTTTTAGATTTATTAAAGATA
             Candidatus Carsonella 135557 - 167442nt      (1) -----------------------------------------------------------------------------
          SARS-CoV-2 Reference Genome NC_045512.2      (1) -----------------------------------------------------------------------------
                                                                                                    Section 1731
                                                   (133211) 133211    133221     133232     133243     133254     133265     133276  133287
Candidatus Carsonella ruddii strain BT chromosome... (133211) ATACCAAATAATTTAAAAATTGATTCAATTAAATGATGTTTATTAAAATAATTAATATAATATAAATAAAAACAACA
             Candidatus Carsonella 135557 - 167442nt      (1) -----------------------------------------------------------------------------
          SARS-CoV-2 Reference Genome NC_045512.2      (1) -----------------------------------------------------------------------------
                                                                                                    Section 1732
                                                   (133288) 133288    133298     133309     133320     133331     133342     133353  133364
Candidatus Carsonella ruddii strain BT chromosome... (133288) TTTATAATTTTTTGAAAAAGAAGAAAAAAAATTCTCTTATAATTTGTAAATCTAAATCTAAATTTAAAAAATTATCGT
             Candidatus Carsonella 135557 - 167442nt      (1) -----------------------------------------------------------------------------
          SARS-CoV-2 Reference Genome NC_045512.2      (1) -----------------------------------------------------------------------------
                                                                                                    Section 1733
                                                   (133365) 133365    133375     133386     133397     133408     133419     133430  133441
Candidatus Carsonella ruddii strain BT chromosome... (133365) AATAAAATATTCTATTACATGCATCAATACTCATTAAAACAAAAGAATCATCCATACAAATATAATAACTATTATAT
             Candidatus Carsonella 135557 - 167442nt      (1) -----------------------------------------------------------------------------
          SARS-CoV-2 Reference Genome NC_045512.2      (1) -----------------------------------------------------------------------------
                                                                                                    Section 1734
                                                   (133442) 133442    133452     133463     133474     133485     133496     133507  133518
Candidatus Carsonella ruddii strain BT chromosome... (133442) CTATTTATTTTTTGTTTTTTTAATAATAAACTTAAACTTATTGAAATATCTTCTATACAATGATGATTATCAATTTT
             Candidatus Carsonella 135557 - 167442nt      (1) -----------------------------------------------------------------------------
          SARS-CoV-2 Reference Genome NC_045512.2      (1) -----------------------------------------------------------------------------
```

```
                                                                                                 Section 1735
                                            (133519) 133519    133529     133540     133551     133562     133573     133584   133595
Candidatus Carsonella ruddii strain BT chromosome... (133519) AAGGTCTGAATAACATCTTAAATAACAATCAAATAAACTATAATTTATAAATTGTGATATAATATGATCAAAATAAA
             Candidatus Carsonella 135557 - 167442nt (1) -----------------------------------------------------------------------------
         SARS-CoV-2 Reference Genome NC_045512.2 (1) -----------------------------------------------------------------------------
                                                                                                 Section 1736
                                            (133596) 133596    133606     133617     133628     133639     133650     133661   133672
Candidatus Carsonella ruddii strain BT chromosome... (133596) AAATTGAAGTTTTAATTTTAAAAATACCAATACCAAAAAAATTTAATTTAATTTCAATATCAGATTCTAATGATTTT
             Candidatus Carsonella 135557 - 167442nt (1) -----------------------------------------------------------------------------
         SARS-CoV-2 Reference Genome NC_045512.2 (1) -----------------------------------------------------------------------------
                                                                                                 Section 1737
                                            (133673) 133673    133683     133694     133705     133716     133727     133738   133749
Candidatus Carsonella ruddii strain BT chromosome... (133673) CTTCTTAATTGGTAAATTTTCATATTTTAAGGCTAAATAGCTCAGTAGGCAGAGCAAAGGACTGAAAATCCTTGTGT
             Candidatus Carsonella 135557 - 167442nt (1) -----------------------------------------------------------------------------
         SARS-CoV-2 Reference Genome NC_045512.2 (1) -----------------------------------------------------------------------------
                                                                                                 Section 1738
                                            (133750) 133750    133760     133771     133782     133793     133804     133815   133826
Candidatus Carsonella ruddii strain BT chromosome... (133750) CGGTGGTTCGATTCCACCTTTAGCTATGAGATATAGCCAAGTGGTAAGGCATTGGTCTTTGACTCCAAAATCCTAGG
             Candidatus Carsonella 135557 - 167442nt (1) -----------------------------------------------------------------------------
         SARS-CoV-2 Reference Genome NC_045512.2 (1) -----------------------------------------------------------------------------
                                                                                                 Section 1739
                                            (133827) 133827    133837     133848     133859     133870     133881     133892   133903
Candidatus Carsonella ruddii strain BT chromosome... (133827) TTCAATTCCTAGTATCTCAGAGCTTTTGTAGCTTAAAAGGTAAAGCTGTTGATTTGTAATCAGCTGTTCCGGGTTCA
             Candidatus Carsonella 135557 - 167442nt (1) -----------------------------------------------------------------------------
         SARS-CoV-2 Reference Genome NC_045512.2 (1) -----------------------------------------------------------------------------
                                                                                                 Section 1740
                                            (133904) 133904    133914     133925     133936     133947     133958     133969   133980
Candidatus Carsonella ruddii strain BT chromosome... (133904) ATTCCTGGCGAAAGTATTTTATTATGGAGATGATGGGAATTGAACCCATATCCGTTTTAAAAAATATAAAAATATAA
             Candidatus Carsonella 135557 - 167442nt (1) -----------------------------------------------------------------------------
         SARS-CoV-2 Reference Genome NC_045512.2 (1) -----------------------------------------------------------------------------
```

Section 1741

	(133981)	133981 133991 134002 134013 134024 134035 134046 134057
Candidatus Carsonella ruddii strain BT chromosome...	(133981)	TACATGATTTATTAGTTAGATATTTTTATTATTTAATATTATATATTTTTTTAGTATGATTTACAATTTTTTTTTAT
Candidatus Carsonella 135557 - 167442nt	(1)	---
SARS-CoV-2 Reference Genome NC_045512.2	(1)	---

Section 1742

	(134058)	134058 134068 134079 134090 134101 134112 134123 134134
Candidatus Carsonella ruddii strain BT chromosome...	(134058)	TCATGTCAAAAAATTCATTCTTCATAACGTCAACACCTTTCATCCCCTTGTATTTTTTTTTTACTAAAGAAATTTTT
Candidatus Carsonella 135557 - 167442nt	(1)	---
SARS-CoV-2 Reference Genome NC_045512.2	(1)	---

Section 1743

	(134135)	134135 134145 134156 134167 134178 134189 134200 134211
Candidatus Carsonella ruddii strain BT chromosome...	(134135)	ATTTTAAAAAAAGATTTTATCTTAAATAAAAAAATAGGAATACATTTATAATTTTTATTTTTTAAAACGTTTAAAAT
Candidatus Carsonella 135557 - 167442nt	(1)	---
SARS-CoV-2 Reference Genome NC_045512.2	(1)	---

Section 1744

	(134212)	134212 134222 134233 134244 134255 134266 134277 134288
Candidatus Carsonella ruddii strain BT chromosome...	(134212)	AAAAAAAATTTCTTTTTTATTTAATAAAAGAAATCTATTATTATTATTAAAACAAAAACAAACGTTATTGTTATCTA
Candidatus Carsonella 135557 - 167442nt	(1)	---
SARS-CoV-2 Reference Genome NC_045512.2	(1)	---

Section 1745

	(134289)	134289 134299 134310 134321 134332 134343 134354 134365
Candidatus Carsonella ruddii strain BT chromosome...	(134289)	TTTTAATAAAACTTGAATTAAAATTTAATTTATTAAATTTAATAATATTTATGTCTTTTTTATTTAATTTTATACCA
Candidatus Carsonella 135557 - 167442nt	(1)	---
SARS-CoV-2 Reference Genome NC_045512.2	(1)	---

Section 1746

	(134366)	134366 134376 134387 134398 134409 134420 134431 134442
Candidatus Carsonella ruddii strain BT chromosome...	(134366)	GAAATAAAATTTTTTACAAAATAATACTTATCTAAAAAAATTTTACTTATTTTATACATATGGAAAAAGAATTTTCG
Candidatus Carsonella 135557 - 167442nt	(1)	---
SARS-CoV-2 Reference Genome NC_045512.2	(1)	---

Section 1747

	(134443)	134443 134453 134464 134475 134486 134497 134508 134519
Candidatus Carsonella ruddii strain BT chromosome...	(134443)	TTGTTAAATTTTTTAGAAAGAGTAACAATATTAGAAGAAAAATTCTTCTTCTTTATAACAAATGCAAAAAAAATTTA
Candidatus Carsonella 135557 - 167442nt	(1)	---
SARS-CoV-2 Reference Genome NC_045512.2	(1)	---

Section 1748

	(134520)	134520 134530 134541 134552 134563 134574 134585 134596
Candidatus Carsonella ruddii strain BT chromosome...	(134520)	TAAACATAATTTAGAAGAATTCAATAATCTTAAAGAATCTTTAATAAATAATATATTATCAGAATTGATACCAATTA
Candidatus Carsonella 135557 - 167442nt	(1)	---
SARS-CoV-2 Reference Genome NC_045512.2	(1)	---

Section 1749

	(134597)	134597 134607 134618 134629 134640 134651 134662 134673
Candidatus Carsonella ruddii strain BT chromosome...	(134597)	CTGATACGTTAGAAATGTTTTCAAAAAATTTTACTTCAAATCAAAATAATGAAATTGAAATTTTAGTATTAATTTTT
Candidatus Carsonella 135557 - 167442nt	(1)	---
SARS-CoV-2 Reference Genome NC_045512.2	(1)	---

Section 1750

	(134674)	134674 134684 134695 134706 134717 134728 134739 134750
Candidatus Carsonella ruddii strain BT chromosome...	(134674)	AAATTAATAAAAAAATTTTTTTTAAAATTTAATATAAAACAAATTTCAAAAATTGGTATTTTATTTAATCCTGAAAT
Candidatus Carsonella 135557 - 167442nt	(1)	---
SARS-CoV-2 Reference Genome NC_045512.2	(1)	---

Section 1751

	(134751)	134751 134761 134772 134783 134794 134805 134816 134827
Candidatus Carsonella ruddii strain BT chromosome...	(134751)	ACATGAAGCAATAGGAATTTATCCTACAGAAAGTAACAAAAAAGGTATTATTAAAAGTATTTTACAAACAGGATATA
Candidatus Carsonella 135557 - 167442nt	(1)	---
SARS-CoV-2 Reference Genome NC_045512.2	(1)	---

Section 1752

	(134828)	134828 134838 134849 134860 134871 134882 134893 134904
Candidatus Carsonella ruddii strain BT chromosome...	(134828)	CAAAGAATGGTATTCTTATTAGACCTGCTTTAGTTGTTATTTACAATTAAACAATAAACAATTGAAAATAAGAAAAA
Candidatus Carsonella 135557 - 167442nt	(1)	---
SARS-CoV-2 Reference Genome NC_045512.2	(1)	---

```
                                                                                Section 1753
                                               (134905) 134905    134915     134926     134937     134948     134959     134970     134981
Candidatus Carsonella ruddii strain BT chromosome... (134905) AAAAAATATAATTATATTAAAAAATATGAGTAAAATAATTGGAATAGATTTAGGAACAACAAATTCTTGTGTAGCTG
            Candidatus Carsonella 135557 - 167442nt (1) -----------------------------------------------------------------------------
           SARS-CoV-2 Reference Genome NC_045512.2 (1) -----------------------------------------------------------------------------
```

```
                                                                                Section 1754
                                               (134982) 134982    134992     135003     135014     135025     135036     135047     135058
Candidatus Carsonella ruddii strain BT chromosome... (134982) TTTTAAACAATGGTAAACCACAAGTAATTGAAAATTCAGAAGGAGGACGTACAACACCGTCAGTAGTAGGATATACA
            Candidatus Carsonella 135557 - 167442nt (1) -----------------------------------------------------------------------------
           SARS-CoV-2 Reference Genome NC_045512.2 (1) -----------------------------------------------------------------------------
```

```
                                                                                Section 1755
                                               (135059) 135059    135069     135080     135091     135102     135113     135124     135135
Candidatus Carsonella ruddii strain BT chromosome... (135059) GATGATAATAGAATTATTGTTGGATTACCAGCAAAAAGACAAGCTATAACTAATCCTAAAAATACTTTATATGCTAT
            Candidatus Carsonella 135557 - 167442nt (1) -----------------------------------------------------------------------------
           SARS-CoV-2 Reference Genome NC_045512.2 (1) -----------------------------------------------------------------------------
```

```
                                                                                Section 1756
                                               (135136) 135136    135146     135157     135168     135179     135190     135201     135212
Candidatus Carsonella ruddii strain BT chromosome... (135136) TAAAAGATTAATAGGAAGAAAATATACAGATGATATAGTACAAAAAGATATTAAAATGGTACCATATAATATTATAT
            Candidatus Carsonella 135557 - 167442nt (1) -----------------------------------------------------------------------------
           SARS-CoV-2 Reference Genome NC_045512.2 (1) -----------------------------------------------------------------------------
```

```
                                                                                Section 1757
                                               (135213) 135213    135223     135234     135245     135256     135267     135278     135289
Candidatus Carsonella ruddii strain BT chromosome... (135213) CATCTGAAAACGGGGATGCATGGGTTGAAGTAAAAGATAAAAAATTAGCTCCACCACAAATAAGTGCAGAAATTTTA
            Candidatus Carsonella 135557 - 167442nt (1) -----------------------------------------------------------------------------
           SARS-CoV-2 Reference Genome NC_045512.2 (1) -----------------------------------------------------------------------------
```

```
                                                                                Section 1758
                                               (135290) 135290    135300     135311     135322     135333     135344     135355     135366
Candidatus Carsonella ruddii strain BT chromosome... (135290) AAAAAAATGAAATTAACAGCTGAATCATATTTAAATGAAAAAATTTATAAAGCAGTAATTACTGTACCAGCATATTT
            Candidatus Carsonella 135557 - 167442nt (1) -----------------------------------------------------------------------------
           SARS-CoV-2 Reference Genome NC_045512.2 (1) -----------------------------------------------------------------------------
```

```
                                                         Section 1759
                                                (135367) 135367    135377     135388     135399     135410     135421     135432  135443
Candidatus Carsonella ruddii strain BT chromosome... (135367) TAATGATTCGCAAAGACAAGCAACAAAAGATGCTGGAAAAATAGCAGGATTAGATGTACTAAGAATAATAAATGAAC
        Candidatus Carsonella 135557 - 167442nt      (1) -----------------------------------------------------------------------------
     SARS-CoV-2 Reference Genome NC_045512.2         (1) -----------------------------------------------------------------------------

                                                         Section 1760
                                                (135444) 135444    135454     135465     135476     135487     135498     135509  135520
Candidatus Carsonella ruddii strain BT chromosome... (135444) CAACTGCTGCAGCATTAGCATATGGTTTAGATAAAAAAAAAGAAGATAGAATTATAGTAGTATATGATTTAGGAGGT
        Candidatus Carsonella 135557 - 167442nt      (1) -----------------------------------------------------------------------------
     SARS-CoV-2 Reference Genome NC_045512.2         (1) -----------------------------------------------------------------------------

                                                         Section 1761
                                                (135521) 135521    135531     135542     135553     135564     135575     135586  135597
Candidatus Carsonella ruddii strain BT chromosome... (135521) GGAACTTTTGATATTTCTATTATTGAAATTGCTAATGTTGAAGGAGAAACTCAATTTGAAGTTTTATCCACAAATGG
        Candidatus Carsonella 135557 - 167442nt      (1) ------------------------------------GTTGAAGGAGAAACTCAATTTGAAGTTTTATCCACAAATGG
     SARS-CoV-2 Reference Genome NC_045512.2         (1) ------------------------------------ATTAAAGGTTTATACC--TTCCCAGGTA-----ACAAACCA

                                                         Section 1762
                                                (135598) 135598    135608     135619     135630     135641     135652     135663  135674
Candidatus Carsonella ruddii strain BT chromosome... (135598) AGATACTTTTTTAGGAGGTGAAGATTTTGATATGAGAATAATTAATCATTTAATTTATGAATTTAAAATTGAAAATG
        Candidatus Carsonella 135557 - 167442nt     (42) AGATACTTTTTTAGGAGGTGAAGATTTTGATATGAGAATAATTAATCATTTAATTTATGAATTTAAAATTGAAAATG
     SARS-CoV-2 Reference Genome NC_045512.2        (35) ACCAACTTTCG-ATCTCTTGTAGATCT-GTTCTC----TAAACGAAC-TTTAAAATCTGTGT----GGCTGTCACTC

                                                         Section 1763
                                                (135675) 135675    135685     135696     135707     135718     135729     135740  135751
Candidatus Carsonella ruddii strain BT chromosome... (135675) GAATTGATT-TAAGTGGAGATTCTCTAGCAATGCAAAGATTAAAAGAAT-CTGCTGAAAAAGCAAAAATAG-AATTA
        Candidatus Carsonella 135557 - 167442nt    (119) GAATTGATT-TAAGTGGAGATTCTCTAGCAATGCAAAGATTAAAAGAAT-CTGCTGAAAAAGCAAAAATAG-AATTA
     SARS-CoV-2 Reference Genome NC_045512.2       (101) GGCTGCATGCTTAGTGCA--CTCAC--GCAGTATAATTAATAACTAATTACTGTCGTTGACAGGACACGAGTAACTC

                                                         Section 1764
                                                (135752) 135752    135762     135773     135784     135795     135806     135817  135828
Candidatus Carsonella ruddii strain BT chromosome... (135749) TCAAATACAGAACAAACTGACATTAATTTACCATATATTACAGCTGATAAAAACGGTCCTAAACATTTAAATATTAA
        Candidatus Carsonella 135557 - 167442nt    (193) TCAAATACAGAACAAACTGACATTAATTTACCATATATTACAGCTGATAAAAACGGTCCTAAACATTTAAATATTAA
     SARS-CoV-2 Reference Genome NC_045512.2       (174) GTCTATCTTCTGCAGGCTGCTTACGGTTTCGTCCGTGTTGCAGCCGATCATCA-GCACATCTAGGTTTCGTCCGGGT
```

Section 1765

```
                                                         (135829) 135829    135839     135850     135861     135872     135883     135894    135905
Candidatus Carsonella ruddii strain BT chromosome...     (135826) AATAACTAGATCTAAATTAGAATCTTTAGTAGAAGATTTAATTATTAAATCAT---TAAAACCATGTGAAATTGCTT
Candidatus Carsonella 135557 - 167442nt                  (270)    AATAACTAGATCTAAATTAGAATCTTTAGTAGAAGATTTAATTATTAAATCAT---TAAAACCATGTGAAATTGCTT
SARS-CoV-2 Reference Genome NC_045512.2                  (250)    GTGACCGAAAGGTAAGATGGAGAGCCTTGTCCCTGGTTTCAACGAGAAAACACACGTCCAACTCAGTTTGCCTGTTT
```

Section 1766

```
                                                         (135906) 135906    135916     135927     135938     135949     135960     135971    135982
Candidatus Carsonella ruddii strain BT chromosome...     (135900) TAAATGATGCAAAAATAGACAAAACTAAAATTGATGAAATTATTTTAGTAGGAGGACAAACAAGAATGCCACTTGTG
Candidatus Carsonella 135557 - 167442nt                  (344)    TAAATGATGCAAAAATAGACAAAACTAAAATTGATGAAATTATTTTAGTAGGAGGACAAACAAGAATGCCACTTGTG
SARS-CoV-2 Reference Genome NC_045512.2                  (327)    TACAGGTTCGCGACGTGCTCGTACGTGGCTTTG--GAGACTCCGT--GGAGGAGGTCTTATCAGA--GGCACGTCAA
```

Section 1767

```
                                                         (135983) 135983    135993     136004     136015     136026     136037     136048    136059
Candidatus Carsonella ruddii strain BT chromosome...     (135977) CA----AAAAATGGTAAGTGATTTTTTTGAAAAAGAAGTTAAAAAAGATATTAATCCAGATGAAGCAGTAGCAGTAG
Candidatus Carsonella 135557 - 167442nt                  (421)    CA----AAAAATGGTAAGTGATTTTTTTGAAAAAGAAGTTAAAAAAGATATTAATCCAGATGAAGCAGTAGCAGTAG
SARS-CoV-2 Reference Genome NC_045512.2                  (398)    CATCTTAAAGATGGCACTTGTGGCTT----AGTAGAAGTTGAAAAAGGCGTTTTGCCTCAACTTG-AACAGCCCTAT
```

Section 1768

```
                                                         (136060) 136060    136070     136081     136092     136103     136114     136125    136136
Candidatus Carsonella ruddii strain BT chromosome...     (136050) GCG--CATCA---GTAC--AAGCTGGAGTTTTAAGT---GGTGTAGTTAAAGATGTTTTATTATTAGATGTT-ACTC
Candidatus Carsonella 135557 - 167442nt                  (494)    GCG--CATCA---GTAC--AAGCTGGAGTTTTAAGT---GGTGTAGTTAAAGATGTTTTATTATTAGATGTT-ACTC
SARS-CoV-2 Reference Genome NC_045512.2                  (470)    GTGTTCATCAAACGTTCGGATGCTCGAACTGCACCTCATGGTCATGTTATGGTTGAGCTGGTAGCAGAACTCGAAGG
```

Section 1769

```
                                                         (136137) 136137    136147     136158     136169     136180     136191     136202    136213
Candidatus Carsonella ruddii strain BT chromosome...     (136116) CATTAA---CGTTAG--GTATAGAAACAATGGGTGGAATAATGACACCTTTAATAGAAAAAAACACAACTATACCTA
Candidatus Carsonella 135557 - 167442nt                  (560)    CATTAA---CGTTAG--GTATAGAAACAATGGGTGGAATAATGACACCTTTAATAGAAAAAAACACAACTATACCTA
SARS-CoV-2 Reference Genome NC_045512.2                  (547)    CATTCAGTACGGTCGTAGTGGTGAGACACTTGGTGTCCTTGTCCCTCATGTGGGCGAA--A----------TACC-A
```

Section 1770

```
                                                         (136214) 136214    136224     136235     136246     136257     136268     136279    136290
Candidatus Carsonella ruddii strain BT chromosome...     (136188) CAAGAAAAACTCAAGTTTTTTCTACTGCAGAA-GATAATCAAACTGCAGTTACAATTCATACTCTTCAAGGAGAAAG
Candidatus Carsonella 135557 - 167442nt                  (632)    CAAGAAAAACTCAAGTTTTTTCTACTGCAGAA-GATAATCAAACTGCAGTTACAATTCATACTCTTCAAGGAGAAAG
SARS-CoV-2 Reference Genome NC_045512.2                  (611)    GTGGCTTACCGCAAGGTTCTTCTTCGTAAGAACGGTAATAAA---GGAGCTGGTGGCCATAGT-TACGGCGCCGATC
```

Section 1771

		136291 … 136367
	(136291)	
Candidatus Carsonella ruddii strain BT chromosome...	(136264)	AAAAAAAGCTTCACAAAATAAATCATTAGGAAAATTTGATTTAAATGATATTTCTCCAGCACCAAGAGGTGTTCCTC
Candidatus Carsonella 135557 - 167442nt	(708)	AAAAAAAGCTTCACAAAATAAATCATTAGGAAAATTTGATTTAAATGATATTTCTCCAGCACCAAGAGGTGTTCCTC
SARS-CoV-2 Reference Genome NC_045512.2	(684)	TAAAGTCATTTGACTTAGGCGACGAGCTTGGCACT--GATCCTTATGAAGATTTTCAAGAA--AACTGGAACACTAA

Section 1772

		136368 … 136444
	(136368)	
Candidatus Carsonella ruddii strain BT chromosome...	(136341)	AAATAGAAGTTTCTTTTGATTTAGACGCTAATGGTATTTTAAACGTTACAGCTAAAGATAAAAAAACAGGAAAAGAA
Candidatus Carsonella 135557 - 167442nt	(785)	AAATAGAAGTTTCTTTTGATTTAGACGCTAATGGTATTTTAAACGTTACAGCTAAAGATAAAAAAACAGGAAAAGAA
SARS-CoV-2 Reference Genome NC_045512.2	(757)	ACATAGCAGTG------GTGTTACCCGTGAA--C---T-CATGCGTG--AGCTTAACGGAG-------GGGCATACA

Section 1773

		136445 … 136521
	(136445)	
Candidatus Carsonella ruddii strain BT chromosome...	(136418)	CAATCTATAATTATAAAATCTTCTGGAGGATTATCTGAAATTGAAATTGAAAATATGATAAAAGATGCAGAAGCTAA
Candidatus Carsonella 135557 - 167442nt	(862)	CAATCTATAATTATAAAATCTTCTGGAGGATTATCTGAAATTGAAATTGAAAATATGATAAAAGATGCAGAAGCTAA
SARS-CoV-2 Reference Genome NC_045512.2	(813)	CTCGCTATGTCGATAACAACTTCTGTGGC----CCTGATGGCTACCCTCTTGAGTGCATTAAAGACCTTCTAGCACG

Section 1774

		136522 … 136598
	(136522)	
Candidatus Carsonella ruddii strain BT chromosome...	(136495)	TTCTGAATTGGATAAAA--ACTTTGAA-GAATTAGTAAAATGTAA--AAATGAAGCTGATAGTACAATTTCAATAGT
Candidatus Carsonella 135557 - 167442nt	(939)	TTCTGAATTGGATAAAA--ACTTTGAA-GAATTAGTAAAATGTAA--AAATGAAGCTGATAGTACAATTTCAATAGT
SARS-CoV-2 Reference Genome NC_045512.2	(886)	TGCTGGTAAAGCTTCATGCACTTTGTCCGAACAACTGGACTTTATTGACACTAAGAGGGGTGTATACTG-CTGCCGT

Section 1775

		136599 … 136675
	(136599)	
Candidatus Carsonella ruddii strain BT chromosome...	(136567)	AAGAAAAAATTTAAAAGATGAAAATACAAAAATAAGTGATGAAGAAAAAGAATCAATTGAAAAATCAATATCAAATT
Candidatus Carsonella 135557 - 167442nt	(1011)	AAGAAAAAATTTAAAAGATGAAAATACAAAAATAAGTGATGAAGAAAAAGAATCAATTGAAAAATCAATATCAAATT
SARS-CoV-2 Reference Genome NC_045512.2	(962)	GAACATGAGCATGAAATTGCTTGGTACACGGA-ACGTTCTGAA-AAGAGCTATGAATTGCAGACACCTTTTGAAATT

Section 1776

		136676 … 136752
	(136676)	
Candidatus Carsonella ruddii strain BT chromosome...	(136644)	TAGAATTATTAATAAAAGGTGATGATTTAACATCAATTAAAAAAG--CAAATGAAG-ATTTACTAAAAATAAGTGAA
Candidatus Carsonella 135557 - 167442nt	(1088)	TAGAATTATTAATAAAAGGTGATGATTTAACATCAATTAAAAAAG--CAAATGAAG-ATTTACTAAAAATAAGTGAA
SARS-CoV-2 Reference Genome NC_045512.2	(1037)	AA--ATTGGCAAAGAAATTTGAC-----ACCTTCAATGGGGAATGTCCAAATTTTGTATTTCCCTTAAAT---TCCA

Section 1777

		136753 136763 136774 136785 136796 136807 136818 136829
	(136753)	
Candidatus Carsonella ruddii strain BT chromosome...	(136718)	AATATAATTAAAAAACAATAAAAATAATAAAAGCGCTATCGTTAATGTTTTAACGATAGCGTAATAAATTAATGATT
Candidatus Carsonella 135557 - 167442nt	(1162)	AATATAATTAAAAAACAATAAAAATAATAAAAGCGCTATCGTTAATGTTTTAACGATAGCGTAATAAATTAATGATT
SARS-CoV-2 Reference Genome NC_045512.2	(1104)	TA-ATCAAGACTATTCAACCAAGGGTTGAAAAGAAAAAGCTTGATGGCTTTATGGGTAG-------AATT--CGATC

Section 1778

		136830 136840 136851 136862 136873 136884 136895 136906
	(136830)	
Candidatus Carsonella ruddii strain BT chromosome...	(136795)	AAAATATTTATTTTTGGAATAACAGGAAAAATGGGAAAATCAATTTTAAATTTTTTAAAATTAAATAAAATTTTTAA
Candidatus Carsonella 135557 - 167442nt	(1239)	AAAATATTTATTTTTGGAATAACAGGAAAAATGGGAAAATCAATTTTAAATTTTTTAAAATTAAATAAAATTTTTAA
SARS-CoV-2 Reference Genome NC_045512.2	(1171)	TGTCTATCCAGTTGCG---TCACCAAATGAATGC---AACCAAATGTGCCTTTC---AACTCTCATGAAGTGTGA--

Section 1779

		136907 136917 136928 136939 136950 136961 136972 136983
	(136907)	
Candidatus Carsonella ruddii strain BT chromosome...	(136872)	TTTATTAGGTGGAATAAATAAAAAAAATTCTTTAAATTTATTTAAAAATAAATATAATTTAATTTTTAAAAACATGA
Candidatus Carsonella 135557 - 167442nt	(1316)	TTTATTAGGTGGAATAAATAAAAAAAATTCTTTAAATTTATTTAAAAATAAATATAATTTAATTTTTAAAAACATGA
SARS-CoV-2 Reference Genome NC_045512.2	(1237)	-TCATT--GTGGTGAAACTTCATGGCAGACGGGCGATTTTGTTAAAGCCACTTGCGA----ATTTTGTGGCAC-TGA

Section 1780

		136984 136994 137005 137016 137027 137038 137049 137060
	(136984)	
Candidatus Carsonella ruddii strain BT chromosome...	(136949)	GTAAAAATAGTATATTAATAGATTTTTCAAATTTTAATTTATTAAAA-AAAATTCTTTTAATATCGTTTTTTTTTAA
Candidatus Carsonella 135557 - 167442nt	(1393)	GTAAAAATAGTATATTAATAGATTTTTCAAATTTTAATTTATTAAAA-AAAATTCTTTTAATATCGTTTTTTTTTAA
SARS-CoV-2 Reference Genome NC_045512.2	(1306)	G---AATTTGACTAA-AGAAGGTGCCACTACTTGTGGTTACTTACCCCAAAATGCTGTTGTTAAAATTTATTGTCCA

Section 1781

		137061 137071 137082 137093 137104 137115 137126 137137
	(137061)	
Candidatus Carsonella ruddii strain BT chromosome...	(137025)	AATTCCATTAATAATTGGAACTACAGGATTAAATATAGTTTTATTAAATTATATAAAATATGTATCTTTATATGTTT
Candidatus Carsonella 135557 - 167442nt	(1469)	AATTCCATTAATAATTGGAACTACAGGATTAAATATAGTTTTATTAAATTATATAAAATATGTATCTTTATATGTTT
SARS-CoV-2 Reference Genome NC_045512.2	(1379)	GCAT--GTCACAATTCAGAAGTAG-GACCTGAGCATAGTCTTGCCGAATACCATAA----TGAATCTG-----GCTT

Section 1782

		137138 137148 137159 137170 137181 137192 137203 137214
	(137138)	
Candidatus Carsonella ruddii strain BT chromosome...	(137102)	CAATATTATTATCTTATAATATGAGTATTGGTTTAAATTTAATTAATTATTTTTTATCAAATTTAAATATTTATTTA
Candidatus Carsonella 135557 - 167442nt	(1546)	CAATATTATTATCTTATAATATGAGTATTGGTTTAAATTTAATTAATTATTTTTTATCAAATTTAAATATTTATTTA
SARS-CoV-2 Reference Genome NC_045512.2	(1444)	GAAAACCATTCT----TCGTAAGGGTGGTCGCACTATTGCCTTTGGAGGCTGTGTGTTCTCTT---ATGTTGGTTGC

Section 1783

		137215 137225 137236 137247 137258 137269 137280 137291
	(137215)	
Candidatus Carsonella ruddii strain BT chromosome...	(137179)	AAAATTTATAATTTTAATTCTTTTATTTTAGACATTCATCATGATAAAAAAATAGATTCT--CCAT-CAGGTACATC
Candidatus Carsonella 135557 - 167442nt	(1623)	AAAATTTATAATTTTAATTCTTTTATTTTAGACATTCATCATGATAAAAAAATAGATTCT--CCAT-CAGGTACATC
SARS-CoV-2 Reference Genome NC_045512.2	(1514)	CATA---ACAAGTGTGC--CTATTGGGTTCCACGTGC-TAGCGCTAACA---TAGGTTGTAACCATACAGGTG--TT

Section 1784

		137292 137302 137313 137324 137335 137346 137357 137368
	(137292)	
Candidatus Carsonella ruddii strain BT chromosome...	(137253)	TTTAATGATTTTTTCTAAAATTAAAAATATTAATAAAAATATTTTTAGTATAAGAATTAAAAATATAATAGGAAACC
Candidatus Carsonella 135557 - 167442nt	(1697)	TTTAATGATTTTTTCTAAAATTAAAAATATTAATAAAAATATTTTTAGTATAAGAATTAAAAATATAATAGGAAACC
SARS-CoV-2 Reference Genome NC_045512.2	(1580)	GTTGGAGAAGGTTCCGAAGGTC-------TTAATGACAACCTTCTTGAAATACTC--CAAAAAGAGAA-AGTCAAC-

Section 1785

		137369 137379 137390 137401 137412 137423 137434 137445
	(137369)	
Candidatus Carsonella ruddii strain BT chromosome...	(137330)	ATGTTATTTATTTAATTTCTAATAATGAAATTATTAAAATAGAACATTTAATATTAAATAGAAATATTTTTATATTG
Candidatus Carsonella 135557 - 167442nt	(1774)	ATGTTATTTATTTAATTTCTAATAATGAAATTATTAAAATAGAACATTTAATATTAAATAGAAATATTTTTATATTG
SARS-CoV-2 Reference Genome NC_045512.2	(1646)	-----ATCAATATTGTTGGTGACTTTAAACTTAATGAAG-AGATC----GCCATTATTTTGGCATCTTTTTCTGCT-

Section 1786

		137446 137456 137467 137478 137489 137500 137511 137522
	(137446)	
Candidatus Carsonella ruddii strain BT chromosome...	(137407)	GGAATATTTTATTCAATATTATGGTTATTGAATAAAAAAAAAGGTTGTTTTTCAATGTATGATATTTATTTTTTATG
Candidatus Carsonella 135557 - 167442nt	(1851)	GGAATATTTTATTCAATATTATGGTTATTGAATAAAAAAAAAGGTTGTTTTTCAATGTATGATATTTATTTTTTATG
SARS-CoV-2 Reference Genome NC_045512.2	(1712)	-----------TCCACAAGTGCTTTTGTGGAAACTGTGAAAGGTTTGGATTATAAAGCATTCAAACAAATTGTT---

Section 1787

		137523 137533 137544 137555 137566 137577 137588 137599
	(137523)	
Candidatus Carsonella ruddii strain BT chromosome...	(137484)	TTAATAATAGAAAGTGGTTATTTTTTTAAATGCAAAATTGTTAATTATAAAAAAAGTTGTGGTGAATTAATTTTTAA
Candidatus Carsonella 135557 - 167442nt	(1928)	TTAATAATAGAAAGTGGTTATTTTTTTAAATGCAAAATTGTTAATTATAAAAAAAGTTGTGGTGAATTAATTTTTAA
SARS-CoV-2 Reference Genome NC_045512.2	(1775)	-GAATCCT-----GTGGTAATTTT---AAAGTTACAAAAGGAAAAGCTAAAAAAGGTGCC--TGGAATATTGGTGAA

Section 1788

		137600 137610 137621 137632 137643 137654 137665 137676
	(137600)	
Candidatus Carsonella ruddii strain BT chromosome...	(137561)	TTCTTCAAATTACGGATA-----TAATGAAATTATATCAGATCCATCATATAAATCACAATTTTTAATCTTCTCTAA
Candidatus Carsonella 135557 - 167442nt	(2005)	TTCTTCAAATTACGGATA-----TAATGAAATTATATCAGATCCATCATATAAATCACAATTTTTAATCTTCTCTAA
SARS-CoV-2 Reference Genome NC_045512.2	(1841)	CAGAAATCAATACTGAGTCCTCTTTATGCATTTGCATCAGAGGCTGCTCGTGTTGTACGAT---CAATTTTCTC---

```
                                                                                                            Section 1789
                                                 (137677) 137677    137687     137698     137709     137720     137731     137742 137753
Candidatus Carsonella ruddii strain BT chromosome... (137633) TCCACA-TATTGGAAATGTTGGAATAATAGAACAAGATTTGCAATTATTTAAAATAAAATTAAATGCAATTATAATT
              Candidatus Carsonella 135557 - 167442nt (2077) TCCACA-TATTGGAAATGTTGGAATAATAGAACAAGATTTGCAATTATTTAAAATAAAATTAAATGCAATTATAATT
         SARS-CoV-2 Reference Genome NC_045512.2 (1912) -CCGCACTCTTGAAACTGCTCAAATTCTGTGCGTGTTTACAGAAGGCCGCTATAACAATA---CTAGATGGAATT
                                                                                                            Section 1790
                                                 (137754) 137754    137764     137775     137786     137797     137808     137819 137830
Candidatus Carsonella ruddii strain BT chromosome... (137709) AAAAATTATTCAATATCTAGTAATTTTAGAAGTAATTTTAAAATAA-ATACTTTTTTAAAAATTCAAAAAAAAATAA
              Candidatus Carsonella 135557 - 167442nt (2153) AAAAATTATTCAATATCTAGTAATTTTAGAAGTAATTTTAAAATAA-ATACTTTTTTAAAAATTCAAAAAAAAATAA
         SARS-CoV-2 Reference Genome NC_045512.2 (1985) TCACAGTATTCACTGAG-ACTCATTGATGCTATGATGTTCACATCTGATTTGGCTACTAACAATCTAGTTGTAATGG
                                                                                                            Section 1791
                                                 (137831) 137831    137841     137852     137863     137874     137885     137896 137907
Candidatus Carsonella ruddii strain BT chromosome... (137785) TTTTAATTGGAATTGATACTAGATATTTAATTTCA-AGTG--TAAGAACATTGGTGTTCAATTCGCAATTATTATT
              Candidatus Carsonella 135557 - 167442nt (2229) TTTTAATTGGAATTGATACTAGATATTTAATTTCA-AGTG--TAAGAACATTGGTGTTCAATTCGCAATTATTATT
         SARS-CoV-2 Reference Genome NC_045512.2 (2061) CCTACATTACAGGTGGTGTTGTTCAGTTGACTTCGCAGTGGCTAACTAACATCT---------TTGGCACTGTTTATG
                                                                                                            Section 1792
                                                 (137908) 137908    137918     137929     137940     137951     137962     137973 137984
Candidatus Carsonella ruddii strain BT chromosome... (137859) AAAAATATTGATAATAAAAATATTATTATTAAATATTTAAGATTTTTAAACAATCAAAAAA-TGTATTAT-GATTAA
              Candidatus Carsonella 135557 - 167442nt (2303) AAAAATATTGATAATAAAAATATTATTATTAAATATTTAAGATTTTTAAACAATCAAAAAA-TGTATTAT-GATTAA
         SARS-CoV-2 Reference Genome NC_045512.2 (2130) AAAAAC-----TCAAACCCGTCCT-TGATTGGCTTGAAGAGAAGTTTAAGGAAGGTGTAGAGTTCTTAGAGACGGT
                                                                                                            Section 1793
                                                 (137985) 137985    137995     138006     138017     138028     138039     138050 138061
Candidatus Carsonella ruddii strain BT chromosome... (137934) TATTTTATTAATAAATTTAGGATGTAAACTTGGTATTTTTAAACTTTTAATTAAAAATAATTGTTTTATTTACGAAT
              Candidatus Carsonella 135557 - 167442nt (2378) TATTTTATTAATAAATTTAGGATGTAAACTTGGTATTTTTAAACTTTTAATTAAAAATAATTGTTTTATTTACGAAT
         SARS-CoV-2 Reference Genome NC_045512.2 (2201) TGGGAAATTGTTAAATTTA---TCTCAACCTGTGCTTGTGAAATTGTCGGTGGACA--AATTGTCACCTGTGCAAAG
                                                                                                            Section 1794
                                                 (138062) 138062    138072     138083     138094     138105     138116     138127 138138
Candidatus Carsonella ruddii strain BT chromosome... (138011) TAAAAAAACAAACAAATGTAAAAATT-TTAAAAAAAATAGATGGAGTATTTATTTCGAATGGGCCAGGTAATCCTTT
              Candidatus Carsonella 135557 - 167442nt (2455) TAAAAAAACAAACAAATGTAAAAATT-TTAAAAAAAATAGATGGAGTATTTATTTCGAATGGGCCAGGTAATCCTTT
         SARS-CoV-2 Reference Genome NC_045512.2 (2273) GAAATTAAGGAGAGTGTTCAGACATTCTTTAAGCTTGTAAATAAATTTTTGGCTTTGTGTGCTGACTCTATCATTAT
```

Section 1795

	(138139)	138139 138149 138160 138171 138182 138193 138204 138215
Candidatus Carsonella ruddii strain BT chromosome...	(138087)	TAAATATTCTAAATTTTATAATGTGATATTATTTTTAATATATATTAAAGTTCCAACTATTAGTATTTGTTTAGGTC
Candidatus Carsonella 135557 - 167442nt	(2531)	TAAATATTCTAAATTTTATAATGTGATATTATTTTTAATATATATTAAAGTTCCAACTATTAGTATTTGTTTAGGTC
SARS-CoV-2 Reference Genome NC_045512.2	(2350)	TGGTGGAGCTAAACTTAAAGCCTTGAATTTAGGTGAAACATTTGTC--A---CGCACTCAAAGGGATTGTACAG---

Section 1796

	(138216)	138216 138226 138237 138248 138259 138270 138281 138292
Candidatus Carsonella ruddii strain BT chromosome...	(138164)	ATCAAATATTAGGTATTTTAAATAATTTTCATTTTTTTAAACTTAAAAATGGACATCATGGTGTAAATCATCCTATT
Candidatus Carsonella 135557 - 167442nt	(2608)	ATCAAATATTAGGTATTTTAAATAATTTTCATTTTTTTAAACTTAAAAATGGACATCATGGTGTAAATCATCCTATT
SARS-CoV-2 Reference Genome NC_045512.2	(2419)	AAAGTGTGTTAAATCCAGAGAAGAAACTGGCCTACTCATGCCTCTAAAAGCCCCAAAAG-----AAATTATCTTCTT

Section 1797

	(138293)	138293 138303 138314 138325 138336 138347 138358 138369
Candidatus Carsonella ruddii strain BT chromosome...	(138241)	TATAATAAATATAATAAAAAAATTTTTATAAGTTCACAGAATCATAATTTTAATTTAAAAAATAATAAAAAAAAAAA
Candidatus Carsonella 135557 - 167442nt	(2685)	TATAATAAATATAATAAAAAAATTTTTATAAGTTCACAGAATCATAATTTTAATTTAAAAAATAATAAAAAAAAAAA
SARS-CoV-2 Reference Genome NC_045512.2	(2491)	AGAGGGAGAAACACTTCCCACAG----AAGTGTTAACAGAGG---AAGTTGTCTTGAAAACTGGTGATTTACAACCA

Section 1798

	(138370)	138370 138380 138391 138402 138413 138424 138435 138446
Candidatus Carsonella ruddii strain BT chromosome...	(138318)	TTTTAATAATTCTTATTATTCATTATTTGATAATACTATTCAAAGCATTAACTCGGTTTTTCAACCACTATTTAGTT
Candidatus Carsonella 135557 - 167442nt	(2762)	TTTTAATAATTCTTATTATTCATTATTTGATAATACTATTCAAAGCATTAACTCGGTTTTTCAACCACTATTTAGTT
SARS-CoV-2 Reference Genome NC_045512.2	(2561)	TTAGAACAA--CCTACTAGTGA--AGCTGTTGAAGCTC-------CATT-----GGTTGGTACACCAGTTTGTA-TT

Section 1799

	(138447)	138447 138457 138468 138479 138490 138501 138512 138523
Candidatus Carsonella ruddii strain BT chromosome...	(138395)	TTCAGGGTCATCCTGAAGGATGTGCGGGTCCAAATGATTTAATTTCATTTTTTAATTTTTATAATAAAATTTATGAA
Candidatus Carsonella 135557 - 167442nt	(2839)	TTCAGGGTCATCCTGAAGGATGTGCGGGTCCAAATGATTTAATTTCATTTTTTAATTTTTATAATAAAATTTATGAA
SARS-CoV-2 Reference Genome NC_045512.2	(2621)	AACGGGCTTATGTTGCTCGAAATCAAAGACACAGAAAAGTACTGTGCCCTTGCACCTAATATGATGGTAACAAACAA

Section 1800

	(138524)	138524 138534 138545 138556 138567 138578 138589 138600
Candidatus Carsonella ruddii strain BT chromosome...	(138472)	TAAAATTTTAGTAATAGGTGCAGGACCAATAATTGTTGGACAAGCT--TGCGAATTTGATTA---TTCAGGAACACA
Candidatus Carsonella 135557 - 167442nt	(2916)	TAAAATTTTAGTAATAGGTGCAGGACCAATAATTGTTGGACAAGCT--TGCGAATTTGATTA---TTCAGGAACACA
SARS-CoV-2 Reference Genome NC_045512.2	(2698)	TACCTTCACACTCAAAGGCGGTGCACCAACAAAGGTTACTTTTGGTGATGACACTGTGATAGAAGTGCAAGGTTACA

Section 1801

		138601 138611 138622 138633 138644 138655 138666 138677
	(138601)	
Candidatus Carsonella ruddii strain BT chromosome...	(138544)	AGCTTGTAAA----ACTTTAAAAGAAGAAAAATATCATGTTATTTTATTAAATTCTAATC---CTGCA---ACAATT
Candidatus Carsonella 135557 - 167442nt	(2988)	AGCTTGTAAA----ACTTTAAAAGAAGAAAAATATCATGTTATTTTATTAAATTCTAATC---CTGCA---ACAATT
SARS-CoV-2 Reference Genome NC_045512.2	(2775)	AGAGTGTGAATATCACTTTTGAACTTGATGAAAGGATTGATAAAGTACTTAATGAGAAGTGCTCTGCCTATACAGTT

Section 1802

		138678 138688 138699 138710 138721 138732 138743 138754
	(138678)	
Candidatus Carsonella ruddii strain BT chromosome...	(138611)	ATGACTGATTATGAAATAGCAGATTCAATCTATATTGAA-CAAATAAA-TAAAGATAATTTAATAAAAATAATTTTA
Candidatus Carsonella 135557 - 167442nt	(3055)	ATGACTGATTATGAAATAGCAGATTCAATCTATATTGAA-CAAATAAA-TAAAGATAATTTAATAAAAATAATTTTA
SARS-CoV-2 Reference Genome NC_045512.2	(2852)	GAACTCGGTACAGAAGTAAATGAGTTCGCCTGTGTTGTGGCAGATGCTGTCATAAAAACTTTGCAACCAGTATCTGA

Section 1803

		138755 138765 138776 138787 138798 138809 138820 138831
	(138755)	
Candidatus Carsonella ruddii strain BT chromosome...	(138686)	AT-AGAAAAACCAAATTTTATTTTACCAACTATGGGTGGTCAAACTGCTTTAAATTGTATTTATGATTTTA-TGAAT
Candidatus Carsonella 135557 - 167442nt	(3130)	AT-AGAAAAACCAAATTTTATTTTACCAACTATGGGTGGTCAAACTGCTTTAAATTGTATTTATGATTTTA-TGAAT
SARS-CoV-2 Reference Genome NC_045512.2	(2929)	ATTACTTACACCACTGGGCATTGATTTAGATGAGTGGAGTATGGCTACATACTACTTATTTGATGAGTCTGGTGAGT

Section 1804

		138832 138842 138853 138864 138875 138886 138897 138908
	(138832)	
Candidatus Carsonella ruddii strain BT chromosome...	(138761)	TCAAATTTTTATTTTCCAAAAGAAAATATTTTAGGAATAAATAAAAAAACATTAATTAATGCTGAAAGTAGAAATTC
Candidatus Carsonella 135557 - 167442nt	(3205)	TCAAATTTTTATTTTCCAAAAGAAAATATTTTAGGAATAAATAAAAAAACATTAATTAATGCTGAAAGTAGAAATTC
SARS-CoV-2 Reference Genome NC_045512.2	(3006)	TTAAATTGG--CTTCACATATG----TATTGTT-------CTTTCTACCCTCCAGATGAGGATGAA-GAAGAAGGTG

Section 1805

		138909 138919 138930 138941 138952 138963 138974 138985
	(138909)	
Candidatus Carsonella ruddii strain BT chromosome...	(138838)	TTTTTATAATTTAATAAAAAAAATTGGATTTAAATGTCCAGATTCAACTATTATAAATAATATTAATAATAGTAACT
Candidatus Carsonella 135557 - 167442nt	(3282)	TTTTTATAATTTAATAAAAAAAATTGGATTTAAATGTCCAGATTCAACTATTATAAATAATATTAATAATAGTAACT
SARS-CoV-2 Reference Genome NC_045512.2	(3069)	ATTGTG------AAGAAGAAGAGTTTGAGCCATCAACTCA-ATATGAGTATGGTACTGAAGATGATTACCA---AGG

Section 1806

		138986 138996 139007 139018 139029 139040 139051 139062
	(138986)	
Candidatus Carsonella ruddii strain BT chromosome...	(138915)	TTAATCATAAAATAGAATTTCCTTGTGTTATAAGACCATCTTTTACATTAGGTGGACTAGGAAGTGGTATTGCATAT
Candidatus Carsonella 135557 - 167442nt	(3359)	TTAATCATAAAATAGAATTTCCTTGTGTTATAAGACCATCTTTTACATTAGGTGGACTAGGAAGTGGTATTGCATAT
SARS-CoV-2 Reference Genome NC_045512.2	(3136)	TAAACCTT----TGGAATTTGGT-GCCACTTCTGCTGCTCTTCAACCTGAAGAAGAGCAAGAAGAAG-ATTGGTTAG

Section 1807

		139063 139073 139084 139095 139106 139117 139128 139139
	(139063)	
Candidatus Carsonella ruddii strain BT chromosome...	(138992)	AATAAAAAA---AATTTTTTTTTAATTATAAAAAATGCTTTTTCTTTT-TCAAACGAAATTACTATAGATAAATCTA
Candidatus Carsonella 135557 - 167442nt	(3436)	AATAAAAAA---AATTTTTTTTTAATTATAAAAAATGCTTTTTCTTTT-TCAAACGAAATTACTATAGATAAATCTA
SARS-CoV-2 Reference Genome NC_045512.2	(3207)	ATGATGATAGTCAACAAACTGTTGGTCAACAAGACGGCAGTGAGGACAATCAGAC--AACTACTATTCAAACAATTG

Section 1808

		139140 139150 139161 139172 139183 139194 139205 139216
	(139140)	
Candidatus Carsonella ruddii strain BT chromosome...	(139065)	TAATAGGATGGAAAG-AATACGAATTAGAATTATTAATAGATAATTATTATAATATTATTGTAATATGTTGTATTGA
Candidatus Carsonella 135557 - 167442nt	(3509)	TAATAGGATGGAAAG-AATACGAATTAGAATTATTAATAGATAATTATTATAATATTATTGTAATATGTTGTATTGA
SARS-CoV-2 Reference Genome NC_045512.2	(3282)	TTG-AGGTTCAACCTCAATTAGAGATGGAACTTACACCAGTTGTTCAG---ACTATTGAAGTGAATAGTTTTAGTGG

Section 1809

		139217 139227 139238 139249 139260 139271 139282 139293
	(139217)	
Candidatus Carsonella ruddii strain BT chromosome...	(139141)	AAATTTAGATCCTGTTGGAATTCATACAGGTGATTCTATTACATTAACACCTTCACAAACTTTATCTGA--CAAAGA
Candidatus Carsonella 135557 - 167442nt	(3585)	AAATTTAGATCCTGTTGGAATTCATACAGGTGATTCTATTACATTAACACCTTCACAAACTTTATCTGA--CAAAGA
SARS-CoV-2 Reference Genome NC_045512.2	(3355)	TTATTTAAAACTTACTG--------ACA-----ATGTAT-ACATTAA-AAATGCAGACATTGTGGAAGAAGCTAAAA

Section 1810

		139294 139304 139315 139326 139337 139348 139359 139370
	(139294)	
Candidatus Carsonella ruddii strain BT chromosome...	(139216)	ATATGAAAACATAAGAGATTGTTCTTTTATTATTTTAAAATCTGTAGGTTTAAAA-GGAGGAGGTGCAAATATACAA
Candidatus Carsonella 135557 - 167442nt	(3660)	ATATGAAAACATAAGAGATTGTTCTTTTATTATTTTAAAATCTGTAGGTTTAAAA-GGAGGAGGTGCAAATATACAA
SARS-CoV-2 Reference Genome NC_045512.2	(3417)	AGGTAAAACCAACAGTGGTTGTTAATGCAGCC------AATGTTTACCTTAAACATGGAGGAGGTGT---TGCAGGA

Section 1811

		139371 139381 139392 139403 139414 139425 139436 139447
	(139371)	
Candidatus Carsonella ruddii strain BT chromosome...	(139292)	TTTGCTATTAATCCTATTAATGGAGATATAATTATAATAGAAATGAATCCTAGAATTTCTAGATCTGCTGCTTTAGC
Candidatus Carsonella 135557 - 167442nt	(3736)	TTTGCTATTAATCCTATTAATGGAGATATAATTATAATAGAAATGAATCCTAGAATTTCTAGATCTGCTGCTTTAGC
SARS-CoV-2 Reference Genome NC_045512.2	(3485)	GCCTTAAATAAGGCTACTAAC--------AATGCCATGCAAGTTGAATCTGATGATTAC-ATAGCTACTAATGGACC

Section 1812

		139448 139458 139469 139480 139491 139502 139513 139524
	(139448)	
Candidatus Carsonella ruddii strain BT chromosome...	(139369)	TTCAAAAGCTACCGGATATCCTATAGCAAAAATATCTACAAAATTATCTATTGGTTATAATTTAATAAATTTACTTA
Candidatus Carsonella 135557 - 167442nt	(3813)	TTCAAAAGCTACCGGATATCCTATAGCAAAAATATCTACAAAATTATCTATTGGTTATAATTTAATAAATTTACTTA
SARS-CoV-2 Reference Genome NC_045512.2	(3553)	ACTTAAAGTGGGTGGTAGTTGTGTTTTAAGCGGAC--ACAATCTTG-CTAAACACTGTCTTCATGTTGTCGGCCCAA

```
                                                                                 Section 1813
                                          (139525) 139525    139535     139546     139557     139568     139579     139590  139601
Candidatus Carsonella ruddii strain BT chromosome... (139446) ATGATATTACATGTGGAAATTTACCAGCTTCATTTGAACCGTCAATTGATTATATTGTTTTGAAATTTCCTAAATTT
               Candidatus Carsonella 135557 - 167442nt (3890) ATGATATTACATGTGGAAATTTACCAGCTTCATTTGAACCGTCAATTGATTATATTGTTTTGAAATTTCCTAAATTT
         SARS-CoV-2 Reference Genome NC_045512.2 (3627) ATGTTAACAAGGTGAAGACATTC-AACTTCTTAAGAG---------TGCTTATGAAAATTTTAATCAGCACGAAGTT
                                                                                 Section 1814
                                          (139602) 139602    139612     139623     139634     139645     139656     139667  139678
Candidatus Carsonella ruddii strain BT chromosome... (139523) AATTTTGAAAAATTTAATAGTATAAATTATTTAAATACTGTAATGAAATCTATCGGAGAAATTATGAGTTTAGG---
               Candidatus Carsonella 135557 - 167442nt (3967) AATTTTGAAAAATTTAATAGTATAAATTATTTAAATACTGTAATGAAATCTATCGGAGAAATTATGAGTTTAGG---
         SARS-CoV-2 Reference Genome NC_045512.2 (3695) CTACTTGCACCATT-ATTATCAGCTGGTATTTTT----GGTGCTGACC-CTATAC-ATTCTTTAAGAGTTTGTGTAG
                                                                                 Section 1815
                                          (139679) 139679    139689     139700     139711     139722     139733     139744  139755
Candidatus Carsonella ruddii strain BT chromosome... (139597) ATCTTCTTTTCAAGAATCTTTTATAAAAGCAATTTATTCAATAACAGAAACAGAAACAA--TTCCTTCTTTTATAAA
               Candidatus Carsonella 135557 - 167442nt (4041) ATCTTCTTTTCAAGAATCTTTTATAAAAGCAATTTATTCAATAACAGAAACAGAAACAA--TTCCTTCTTTTATAAA
         SARS-CoV-2 Reference Genome NC_045512.2 (3765) ATACTGTTCGCACAAATGTCTACTTAGCTGTCTTTGATAAAAATCTCTATGACAAACTTGTTTCAAGCTTTTTGGAA
                                                                                 Section 1816
                                          (139756) 139756    139766     139777     139788     139799     139810     139821  139832
Candidatus Carsonella ruddii strain BT chromosome... (139672) GGGAAAATATTATAATAATTATAAATTTAAAATAATTAAAAAAATATTAAATCCTAATAGTAAAAGAATATTTTATA
               Candidatus Carsonella 135557 - 167442nt (4116) GGGAAAATATTATAATAATTATAAATTTAAAATAATTAAAAAAATATTAAATCCTAATAGTAAAAGAATATTTTATA
         SARS-CoV-2 Reference Genome NC_045512.2 (3842) ATGAAGAGTGAAAAGCAAGTTGAA---CAAAAGATCGCTGAGATTCCTAAAGAGGAA--GTTAAGCCATTTATAACT
                                                                                 Section 1817
                                          (139833) 139833    139843     139854     139865     139876     139887     139898  139909
Candidatus Carsonella ruddii strain BT chromosome... (139749) TAATAGATGCTTTTAGATTAAATTTTAACATAAAATTAATTTTTTGTTTATCAAAAATTGATCCTTGGTTTTTGTAT
               Candidatus Carsonella 135557 - 167442nt (4193) TAATAGATGCTTTTAGATTAAATTTTAACATAAAATTAATTTTTTGTTTATCAAAAATTGATCCTTGGTTTTTGTAT
         SARS-CoV-2 Reference Genome NC_045512.2 (3914) GAAAGTAAACCTTCAGTTGAACAGAGAAAACAAGATGA---TAA-GAAAATCAAAGCTTG---------TGTTGAAG
                                                                                 Section 1818
                                          (139910) 139910    139920     139931     139942     139953     139964     139975  139986
Candidatus Carsonella ruddii strain BT chromosome... (139826) TATATAAAAGAAATTATTGATTATGAAAAAAAATTTTATTATATTATAAATAAAAAAAAATTATATTAAAACAAAT
               Candidatus Carsonella 135557 - 167442nt (4270) TATATAAAAGAAATTATTGATTATGAAAAAAAATTTTATTATATTATAAATAAAAAAAAATTATATTAAAACAAAT
         SARS-CoV-2 Reference Genome NC_045512.2 (3978) AAGTTACAACAACTC--------TGGAAGAAACTAAGTTCCTCACAGAAA-ACTTGTTACTTTATATTGACATTAAT
```

Section 1819

		139987 … 140063
	(139987)	139987 139997 140008 140019 140030 140041 140052 140063
Candidatus Carsonella ruddii strain BT chromosome...	(139903)	AG-ATTCTTGTTCTAATGAATTTGTTAGTCCAACTTCTTATTTATATTCTTCTAATTATTTTTATAATGAAATTAGA
Candidatus Carsonella 135557 - 167442nt	(4347)	AG-ATTCTTGTTCTAATGAATTTGTTAGTCCAACTTCTTATTTATATTCTTCTAATTATTTTTATAATGAAATTAGA
SARS-CoV-2 Reference Genome NC_045512.2	(4046)	GGCAATCTTCATCCA--GATTCTGCCACTC----TTGTTAGTGACATTG---ACATCACTTTCTTAAAGAAA---GA

Section 1820

		140064 … 140140
	(140064)	140064 140074 140085 140096 140107 140118 140129 140140
Candidatus Carsonella ruddii strain BT chromosome...	(139979)	TATACTATTAATAAAAAAATATTAATTTTAGGAAGTGGTTTTAATAGAATAGG----ACAAAGTATAGAATTTGATT
Candidatus Carsonella 135557 - 167442nt	(4423)	TATACTATTAATAAAAAAATATTAATTTTAGGAAGTGGTTTTAATAGAATAGG----ACAAAGTATAGAATTTGATT
SARS-CoV-2 Reference Genome NC_045512.2	(4111)	TGCTCCATATATAGTGGGTGATGTTGTTCAAGAGGGTGTTTTAACTGCTGTGGTTATACCTACTAAAAAGGCTGGTG

Section 1821

		140141 … 140217
	(140141)	140141 140151 140162 140173 140184 140195 140206 140217
Candidatus Carsonella ruddii strain BT chromosome...	(140052)	ACTGTTGTACAAAAGCT-GCTAAATTTATTAAAAAAATAGGTTTTGATTCAATAATCATAAATTGTAATCCAGAAAC
Candidatus Carsonella 135557 - 167442nt	(4496)	ACTGTTGTACAAAAGCT-GCTAAATTTATTAAAAAAATAGGTTTTGATTCAATAATCATAAATTGTAATCCAGAAAC
SARS-CoV-2 Reference Genome NC_045512.2	(4188)	GCACTACTG-AAATGCTAGCGAAAGCTTTGAGAAAAGT--GCCAACAGACAATTAT-ATAACCACTTA-CCCGGGTC

Section 1822

		140218 … 140294
	(140218)	140218 140228 140239 140250 140261 140272 140283 140294
Candidatus Carsonella ruddii strain BT chromosome...	(140128)	AGTTTCTACAGATTATGATTCTAGTT----CACAATTATTTTTTGACCCTATTACTATTACTTATTTAAATAATTTA
Candidatus Carsonella 135557 - 167442nt	(4572)	AGTTTCTACAGATTATGATTCTAGTT----CACAATTATTTTTTGACCCTATTACTATTACTTATTTAAATAATTTA
SARS-CoV-2 Reference Genome NC_045512.2	(4260)	AGGGTTTAAATGGTTACACTGTAGAGGAGGCAAAGACAGTGCTTAAAAAGTGTAAAAGTGCCT-TTTACATTCTACC

Section 1823

		140295 … 140371
	(140295)	140295 140305 140316 140327 140338 140349 140360 140371
Candidatus Carsonella ruddii strain BT chromosome...	(140201)	ATTTATAATTTTAAACCAATTTTAATAATTTGTC--AATTAGGTGGTC-AATTACCTTTAAATTATTTAAGATTTAA
Candidatus Carsonella 135557 - 167442nt	(4645)	ATTTATAATTTTAAACCAATTTTAATAATTTGTC--AATTAGGTGGTC-AATTACCTTTAAATTATTTAAGATTTAA
SARS-CoV-2 Reference Genome NC_045512.2	(4336)	ATCTATTATCTCTAATGAGAAGCAAGAAATTCTTGGAACTGTTTCTTGGAATTTGCGAGAAATGCTTGCACATGCAG

Section 1824

		140372 … 140448
	(140372)	140372 140382 140393 140404 140415 140426 140437 140448
Candidatus Carsonella ruddii strain BT chromosome...	(140275)	TAATAATAAAAATAATAATAAAATTTTAGGAATAAATAAAATTAATAATAATATTTGTGGATC-TAAAAATAAATTT
Candidatus Carsonella 135557 - 167442nt	(4719)	TAATAATAAAAATAATAATAAAATTTTAGGAATAAATAAAATTAATAATAATATTTGTGGATC-TAAAAATAAATTT
SARS-CoV-2 Reference Genome NC_045512.2	(4413)	AAGAAACACGCAAATTAATGCCTGTCTGTGTGGAAACTAAAGCCATAGTTTCAACTATACAGCGTAAATATAAGGGT

Section 1825

	(140449)	140449 140459 140470 140481 140492 140503 140514 140525
Candidatus Carsonella ruddii strain BT chromosome...	(140351)	AATAAAATATTAAAAAATATAAATTTAAACATTTTTAAAAATTTTTTTTCTAAAAATATAAATGATTTAATATTTTA
Candidatus Carsonella 135557 - 167442nt	(4795)	AATAAAATATTAAAAAATATAAATTTAAACATTTTTAAAAATTTTTTTTCTAAAAATATAAATGATTTAATATTTTA
SARS-CoV-2 Reference Genome NC_045512.2	(4490)	ATTAAAATACAAGAGGGTGTGGTTGATTATGGTGCTAGATTTTACTTTTACACCAGTAAAACAACTGTAGCGTCACT

Section 1826

	(140526)	140526 140536 140547 140558 140569 140580 140591 140602
Candidatus Carsonella ruddii strain BT chromosome...	(140428)	TTCAAATATAATTAATAAACCTATAATATTAAGACCATCATATATTATTGGAGGAAGCAATATTAATATATTAATAA
Candidatus Carsonella 135557 - 167442nt	(4872)	TTCAAATATAATTAATAAACCTATAATATTAAGACCATCATATATTATTGGAGGAAGCAATATTAATATATTAATAA
SARS-CoV-2 Reference Genome NC_045512.2	(4567)	TATCAACACACTTAACGATC---TAA-ATGAAACTCTTGTTACA--ATGCCACTTGGCTATGT-AACACATGGCTTA

Section 1827

	(140603)	140603 140613 140624 140635 140646 140657 140668 140679
Candidatus Carsonella ruddii strain BT chromosome...	(140505)	ACAATA-ATGATTTAATAAAATATATAAATAATTTTAAAAAAAAAAATATT-TTTATATTTTTAGAAAAATTTTTAA
Candidatus Carsonella 135557 - 167442nt	(4949)	ACAATA-ATGATTTAATAAAATATATAAATAATTTTAAAAAAAAAAATATT-TTTATATTTTTAGAAAAATTTTTAA
SARS-CoV-2 Reference Genome NC_045512.2	(4637)	AATTTGGAAGAAGCTGCTCGGTATATGAGATCTCTCAAAGTGCCAGCTACAGTTTCTGTTTCTTCACCTGATGCTG-

Section 1828

	(140680)	140680 140690 140701 140712 140723 140734 140745 140756
Candidatus Carsonella ruddii strain BT chromosome...	(140580)	TTAATGCAAAAGAATTTGATTTAGATTTATTAGTAGAAAAAGGAAAAATAATAAAATTTTTTTATAATTGAACATATA
Candidatus Carsonella 135557 - 167442nt	(5024)	TTAATGCAAAAGAATTTGATTTAGATTTATTAGTAGAAAAAGGAAAAATAATAAAATTTTTTTATAATTGAACATATA
SARS-CoV-2 Reference Genome NC_045512.2	(4713)	TTACAGCGTATAATGGTTATCTT-ACTTCTTCTTCTAAAA---CACCTGAAGAACATTT--T---ATTGAAACCATC

Section 1829

	(140757)	140757 140767 140778 140789 140800 140811 140822 140833
Candidatus Carsonella ruddii strain BT chromosome...	(140657)	GAAAATGCTGGTATACATTCAGGA-GATTCTATGATGATTTATCCTACTTTTACTATTAATAAAAATATTAAAAAGA
Candidatus Carsonella 135557 - 167442nt	(5101)	GAAAATGCTGGTATACATTCAGGA-GATTCTATGATGATTTATCCTACTTTTACTATTAATAAAAATATTAAAAAGA
SARS-CoV-2 Reference Genome NC_045512.2	(4781)	TCACTTGCTGGTTCCTATAAAGATTGGTCCTATTCTGGACAATC-TACACA-ACTAGGTATAGAATTTCTTAAGAGA

Section 1830

	(140834)	140834 140844 140855 140866 140877 140888 140899 140910
Candidatus Carsonella ruddii strain BT chromosome...	(140733)	AA--ATTATATTTTATATATCTTAATA-TGTTATAA-ATTAAAAATTAATGG---AATTATAAATTTTCAAATTTG
Candidatus Carsonella 135557 - 167442nt	(5177)	AA--ATTATATTTTATATATCTTAATA-TGTTATAA-ATTAAAAATTAATGG---AATTATAAATTTTCAAATTTG
SARS-CoV-2 Reference Genome NC_045512.2	(4856)	GGTGATAAAGTGTATATTACACTAGTAATCCTACCACATTCCACCTAGATGGTGAAGTTATCACCTTTGACAA---

```
                                                                  Section 1831
                                                   (140911) 140911    140921     140932     140943     140954     140965     140976   140987
Candidatus Carsonella ruddii strain BT chromosome... (140803) TTTTAAAAAAAAAATTTATATAATAGAATGCAATCCTAGAAGTTCAAGAACAATACCATTTGTTTCTAAATCTTCTA
            Candidatus Carsonella 135557 - 167442nt  (5247) TTTTAAAAAAAAAATTTATATAATAGAATGCAATCCTAGAAGTTCAAGAACAATACCATTTGTTTCTAAATCTTCTA
        SARS-CoV-2 Reference Genome NC_045512.2      (4930) TCTTAAGACACTTCTTTCTTTGAGAGAAGTGAGGACT--A---TTAAGGTGTTTACAACAGTAGACAACAT----TA

                                                                  Section 1832
                                                   (140988) 140988    140998     141009     141020     141031     141042     141053   141064
Candidatus Carsonella ruddii strain BT chromosome... (140880) AATTTCCTATGATATATAATTATATATCAATTTTTTAGGATATAATATTTTTTATAAAGAAAAAATTAATAATTTT
            Candidatus Carsonella 135557 - 167442nt  (5324) AATTTCCTATGATATATAATTATATATCAATTTTTTAGGATATAATATTTTTTATAAAGAAAAAATTAATAATTTT
        SARS-CoV-2 Reference Genome NC_045512.2      (4998) ACCTCCACACGCAAGTTGTGGACATGTCAATGACATATGGACA--ACAGTTTGGTCCAACTTATTTGGATGGAGCTG

                                                                  Section 1833
                                                   (141065) 141065    141075     141086     141097     141108     141119     141130   141141
Candidatus Carsonella ruddii strain BT chromosome... (140957) TATTTTTTAAAAGAATCTATATTACCAACAAATAAATTTAAAATAGGTTTATTATCACCTGAA-ATGAAA-TCTAC-
            Candidatus Carsonella 135557 - 167442nt  (5401) TATTTTTTAAAAGAATCTATATTACCAACAAATAAATTTAAAATAGGTTTATTATCACCTGAA-ATGAAA-TCTAC-
        SARS-CoV-2 Reference Genome NC_045512.2      (5073) ATGTTACTAAAATAAAACCTCATAATT-CACATGAAGGTAAAACATTTTATGTTTTACCTAATGATGACACTCTACG

                                                                  Section 1834
                                                   (141142) 141142    141152     141163     141174     141185     141196     141207   141218
Candidatus Carsonella ruddii strain BT chromosome... (141031) TGGAGAAACTAT-GAGTATAGGGCTAACAATTCAAGAATGTTTTA-----AAAAATCTCAAAATATAAAATCAAAAA
            Candidatus Carsonella 135557 - 167442nt  (5475) TGGAGAAACTAT-GAGTATAGGGCTAACAATTCAAGAATGTTTTA-----AAAAATCTCAAAATATAAAATCAAAAA
        SARS-CoV-2 Reference Genome NC_045512.2      (5149) TGTTGAGGCTTTTGAGTACTAC-CACACAACTGATCCTAGTTTTCTGGGTAGGTACATGTCAGCATTAAATCACACT

                                                                  Section 1835
                                                   (141219) 141219    141229     141240     141251     141262     141273     141284   141295
Candidatus Carsonella ruddii strain BT chromosome... (141102) CAATATTTATTAATACTAAAAA---AAT--TTTTAGATATATTAAATATTTAAAAAATATAAAAAATATTAAAACTA
            Candidatus Carsonella 135557 - 167442nt  (5546) CAATATTTATTAATACTAAAAA---AAT--TTTTAGATATATTAAATATTTAAAAAATATAAAAAATATTAAAACTA
        SARS-CoV-2 Reference Genome NC_045512.2      (5225) -AAAAAGTGGAAATACCCACAAGTTAATGGTTTAACTTCTATTAAATGGGCAGATAACAACTGTTATCTTGCCACTG

                                                                  Section 1836
                                                   (141296) 141296    141306     141317     141328     141339     141350     141361   141372
Candidatus Carsonella ruddii strain BT chromosome... (141174) -ATAGTTTTTA-TAAAAAAATTTTTTTTGATCTTCATATTTGTTCATATGTTAGTAAAAAAAAAAAATATACATGTA
            Candidatus Carsonella 135557 - 167442nt  (5618) -ATAGTTTTTA-TAAAAAAATTTTTTTTGATCTTCATATTTGTTCATATGTTAGTAAAAAAAAAAAATATACATGTA
        SARS-CoV-2 Reference Genome NC_045512.2      (5301) CATTGTTAACACTCCAACAAATAGAGTTGA-----AGTTTAATCCACCTGCTCTACAAGATGCTTATTACAGAGCAA
```

Section 1837

		141373 141383 141394 141405 141416 141427 141438 141449
	(141373)	
Candidatus Carsonella ruddii strain BT chromosome...	(141249)	TTTTATCAAATAAAATAAATAATATTTGTAATAATTATACTTATTCAACTA-ATTCATCCATTAAATTATTTTTATC
Candidatus Carsonella 135557 - 167442nt	(5693)	TTTTATCAAATAAAATAAATAATATTTGTAATAATTATACTTATTCAACTA-ATTCATCCATTAAATTATTTTTATC
SARS-CoV-2 Reference Genome NC_045512.2	(5373)	GGG---CTGGTGAAGCTGCTAACTTTTGTGC-ACTTAT-CTTAGCCTACTGTAATAAGACAGTAGGTGAGTTAGGTG

Section 1838

		141450 141460 141471 141482 141493 141504 141515 141526
	(141450)	
Candidatus Carsonella ruddii strain BT chromosome...	(141325)	ATCTTTTAATAAAAATTA-----TTATATAATAAGAAAAATAAATAATATTTAAACCGAAATGGTGAAA-TGGTATA
Candidatus Carsonella 135557 - 167442nt	(5769)	ATCTTTTAATAAAAATTA-----TTATATAATAAGAAAAATAAATAATATTTAAACCGAAATGGTGAAA-TGGTATA
SARS-CoV-2 Reference Genome NC_045512.2	(5445)	ATGTTAGAGAAACAATGAGTTACTTGTTTCAACATGCCAATTTAGATTCTTGCAAAAGAG-TCTTGAACGTGGTGTG

Section 1839

		141527 141537 141548 141559 141570 141581 141592 141603
	(141527)	
Candidatus Carsonella ruddii strain BT chromosome...	(141396)	CACTTTACTTTGAGAGAGTAATGGATTTTTTATCCATACGGGTTCAATTCCCGTTTTCGGTAAAAAAAATATGAAAA
Candidatus Carsonella 135557 - 167442nt	(5840)	CACTTTACTTTGAGAGAGTAATGGATTTTTTATCCATACGGGTTCAATTCCCGTTTTCGGTAAAAAAAATATGAAAA
SARS-CoV-2 Reference Genome NC_045512.2	(5521)	TAAA--ACTTGTGGACAACAGCAGACA----ACCCTTAAGGGTGTAGAAGCTGTTAT--GTACA-----TGGGCACA

Section 1840

		141604 141614 141625 141636 141647 141658 141669 141680
	(141604)	
Candidatus Carsonella ruddii strain BT chromosome...	(141473)	ATTTTTATATCAATGAGATAATTTCTATTAAAGAATTATCTGAAATAATAGGAATAAGCAAATTAGATTTAATAAAA
Candidatus Carsonella 135557 - 167442nt	(5917)	ATTTTTATATCAATGAGATAATTTCTATTAAAGAATTATCTGAAATAATAGGAATAAGCAAATTAGATTTAATAAAA
SARS-CoV-2 Reference Genome NC_045512.2	(5585)	CTTTCT-T----ATGA-ACAATT--TAAGAAAGG-TGTTCAGATACCTTG----TACGTGTGGTAAACAAGCTACAA

Section 1841

		141681 141691 141702 141713 141724 141735 141746 141757
	(141681)	
Candidatus Carsonella ruddii strain BT chromosome...	(141550)	AATTTTTTTTTTAA-AGGTATAATAATTAAAAAAAATGAATTTTTAAAATTTTCAACAGTAAAAGATTTATG-TAAA
Candidatus Carsonella 135557 - 167442nt	(5994)	AATTTTTTTTTTAA-AGGTATAATAATTAAAAAAAATGAATTTTTAAAATTTTCAACAGTAAAAGATTTATG-TAAA
SARS-CoV-2 Reference Genome NC_045512.2	(5649)	AATATCTAGTACAACAGGAGTCACCTTTTGTTATGATGTCAGCACCACCTGCTCAGTATGAACTTAAGCATGGTACA

Section 1842

		141758 141768 141779 141790 141801 141812 141823 141834
	(141758)	
Candidatus Carsonella ruddii strain BT chromosome...	(141625)	AATATTTTTAATA-TAAATATTTTAAAAA--AAGAAAAAACAAATTTTAAAATAATACCATCTTTAAATAATTTTTT
Candidatus Carsonella 135557 - 167442nt	(6069)	AATATTTTTAATA-TAAATATTTTAAAAA--AAGAAAAAACAAATTTTAAAATAATACCATCTTTAAATAATTTTTT
SARS-CoV-2 Reference Genome NC_045512.2	(5726)	TTTACTTGTGCTAGTGAGTACACTGGTAATTACCAGTGTGGTCACTATAAACATATAACTTCTAAAGAAACTTTGTA

```
                                                                                      Section 1843
                                                   (141835) 141835    141845     141856     141867     141878     141889     141900 141911
Candidatus Carsonella ruddii strain BT chromosome... (141699) TTATATTTCTATAACTGGAAATGTTAATTC-TGGTAAAACAACTTTAATAGATTTTATATTTAAAAAAAATATTTCT
       Candidatus Carsonella 135557 - 167442nt       (6143) TTATATTTCTATAACTGGAAATGTTAATTC-TGGTAAAACAACTTTAATAGATTTTATATTTAAAAAAAATATTTCT
   SARS-CoV-2 Reference Genome NC_045512.2           (5803) TTGCATAGACGGTGCTTTACTTACAAGTCCTCAGAATACAAAGGTCCTATTACGGATGTTTTCTACAAAGAAAACA
```

```
                                                                                      Section 1844
                                                   (141912) 141912    141922     141933     141944     141955     141966     141977 141988
Candidatus Carsonella ruddii strain BT chromosome... (141775) TTTAATGAATTTGGTAAAATTACACAATTTGTTTCAATTTTTGAAACTTTTTTTTATAAAAAAAATATTTTTATTTT
       Candidatus Carsonella 135557 - 167442nt       (6219) TTTAATGAATTTGGTAAAATTACACAATTTGTTTCAATTTTTGAAACTTTTTTTTATAAAAAAAATATTTTTATTTT
   SARS-CoV-2 Reference Genome NC_045512.2           (5880) GTTACACAACA----ACCATAAAACCAGTTACTTATAAATTGGATGGTGTTGTTTGTACAGAAATTGACCCTAAGTT
```

```
                                                                                      Section 1845
                                                   (141989) 141989    141999     142010     142021     142032     142043     142054 142065
Candidatus Carsonella ruddii strain BT chromosome... (141852) CGATTTACCTGGTCACAAAATATTTAATAAAATAATAAAAACTTTTATTAAAATAAGTAAT-ATTTTATTTT-TAAT
       Candidatus Carsonella 135557 - 167442nt       (6296) CGATTTACCTGGTCACAAAATATTTAATAAAATAATAAAAACTTTTATTAAAATAAGTAAT-ATTTTATTTT-TAAT
   SARS-CoV-2 Reference Genome NC_045512.2           (5953) GGA------------CAA-----TTATTATAAGAAAGACAATTCTTATTTCACAGAGCAACCAATTGATCTTGTACC
```

```
                                                                                      Section 1846
                                                   (142066) 142066    142076     142087     142098     142109     142120     142131 142142
Candidatus Carsonella ruddii strain BT chromosome... (141927) AAT------ATCTTCAGAATCAGATAATGATAATGTATATGATAATATATTAGAA-ATAAAAAAACAAAATAATATT
       Candidatus Carsonella 135557 - 167442nt       (6371) AAT------ATCTTCAGAATCAGATAATGATAATGTATATGATAATATATTAGAA-ATAAAAAAACAAAATAATATT
   SARS-CoV-2 Reference Genome NC_045512.2           (6013) AAACCAACCATATCCAACGCAAGCTTCGATAATTT-TAAGTTTGTATGTGATAATATCAAATTTGCTGATGATTTA
```

```
                                                                                      Section 1847
                                                   (142143) 142143    142153     142164     142175     142186     142197     142208 142219
Candidatus Carsonella ruddii strain BT chromosome... (141997) AATATTATTATTTGTTTAAACAAAATAGATAAAAAAAATAAAAATTTTTATTTAAAAGAAAACAAAATTTTTAAAAT
       Candidatus Carsonella 135557 - 167442nt       (6441) AATATTATTATTTGTTTAAACAAAATAGATAAAAAAAATAAAAATTTTTATTTAAAAGAAAACAAAATTTTTAAAAT
   SARS-CoV-2 Reference Genome NC_045512.2           (6089) AACCAGTTAACTGGTTATAAGAAACCTGCTTCAAGAGAGCTTAAAGTTACATTTTTCCCTGACTTAAATGGTGATGT
```

```
                                                                                      Section 1848
                                                   (142220) 142220    142230     142241     142252     142263     142274     142285 142296
Candidatus Carsonella ruddii strain BT chromosome... (142074) TTCTGCTAAAAATGGATTTAATATTAAAAAAC---TTTTAATAGAATCTATTAATTCTTTTAATTTAATTAAAAATA
       Candidatus Carsonella 135557 - 167442nt       (6518) TTCTGCTAAAAATGGATTTAATATTAAAAAAC---TTTTAATAGAATCTATTAATTCTTTTAATTTAATTAAAAATA
   SARS-CoV-2 Reference Genome NC_045512.2           (6166) GGTGGCTATTGATTA--TAAACACTACACACCCTCTTTTAAGAAAGGAGCTAAATTG-------TTACATAAACCTA
```

Section 1849

	(142297)	142297 142307 142318 142329 142340 142351 142362 142373
Candidatus Carsonella ruddii strain BT chromosome...	(142148)	TTAATTTAATGGAAGAAGATTATAATGGAATTATTGTAAATAGTATTGTAAAAATAATTTTTTTATACAACTTTA
Candidatus Carsonella 135557 - 167442nt	(6592)	TTAATTTAATGGAAGAAGATTATAATGGAATTATTGTAAATAGTATTGTAAAAATAATTTTTTTATACAACTTTA
SARS-CoV-2 Reference Genome NC_045512.2	(6234)	TTG-TTT---GGCA--TGTTAACAATGCAACTAATAAAGCCACGTATAAACCAAATACCTGGTGT-ATACGT-TGTC

Section 1850

	(142374)	142374 142384 142395 142406 142417 142428 142439 142450
Candidatus Carsonella ruddii strain BT chromosome...	(142225)	TTTTTATTA-AAAGGTATTTTAAAAGTTAA--TAATTTTATTTATTTTAAAAATAAAAAAATATTAATTGATAATAT
Candidatus Carsonella 135557 - 167442nt	(6669)	TTTTTATTA-AAAGGTATTTTAAAAGTTAA--TAATTTTATTTATTTTAAAAATAAAAAAATATTAATTGATAATAT
SARS-CoV-2 Reference Genome NC_045512.2	(6303)	TTTGGAGCACAAAACCAGTTGAAACATCAAATTCGTTTGATGTACTG---AAGTCAGAGGACGCGCAGGGAATGGAT

Section 1851

	(142451)	142451 142461 142472 142483 142494 142505 142516 142527
Candidatus Carsonella ruddii strain BT chromosome...	(142299)	AATTGTTAACAATTTTAATGTAAAAGAATGTGTTTCTCCTTGTATATTTTTAGTTAAAAATATTAGTTTTCCAATTG
Candidatus Carsonella 135557 - 167442nt	(6743)	AATTGTTAACAATTTTAATGTAAAAGAATGTGTTTCTCCTTGTATATTTTTAGTTAAAAATATTAGTTTTCCAATTG
SARS-CoV-2 Reference Genome NC_045512.2	(6377)	AATC-TTGCCTGCGAAGATCTAAAACCAG----T-CTC--TGAAGAAG--TAGTGGAAAATCCTACCATACAGAAAG

Section 1852

	(142528)	142528 142538 142549 142560 142571 142582 142593 142604
Candidatus Carsonella ruddii strain BT chromosome...	(142376)	ATTTTTTTTTTAGTTTTAAAAAAAATAATGATATTTTTATATCTGAAGATATTTATAAAGAAAAATATCATATTTTT
Candidatus Carsonella 135557 - 167442nt	(6820)	ATTTTTTTTTTAGTTTTAAAAAAAATAATGATATTTTTATATCTGAAGATATTTATAAAGAAAAATATCATATTTTT
SARS-CoV-2 Reference Genome NC_045512.2	(6444)	ACGTTCTTGAGTGTAATGTGAAAACTACCGAAGTTGT---AGGAGACATTATACTTAAACCAGCAAATAATAGTTT-

Section 1853

	(142605)	142605 142615 142626 142637 142648 142659 142670 142681
Candidatus Carsonella ruddii strain BT chromosome...	(142453)	GATAACTTATATATAAAATCAGATAATCATACTATTTCTGTTGCTATAGAAGATTATTATAATAATTTAAAATTTAA
Candidatus Carsonella 135557 - 167442nt	(6897)	GATAACTTATATATAAAATCAGATAATCATACTATTTCTGTTGCTATAGAAGATTATTATAATAATTTAAAATTTAA
SARS-CoV-2 Reference Genome NC_045512.2	(6517)	-AAAAATTACAGAAGAGGTTGGCCACACAGA-TCTAATGGCTGCTTATGTAGACAATTCTAGTC--TTACTATTAAG

Section 1854

	(142682)	142682 142692 142703 142714 142725 142736 142747 142758
Candidatus Carsonella ruddii strain BT chromosome...	(142530)	TAATAAATTAAACTTATTAAAAATTTCTTTAGGTAATTTTACAATATCTGATTTTAAATATTGTTTAAATTTTAATT
Candidatus Carsonella 135557 - 167442nt	(6974)	TAATAAATTAAACTTATTAAAAATTTCTTTAGGTAATTTTACAATATCTGATTTTAAATATTGTTTAAATTTTAATT
SARS-CoV-2 Reference Genome NC_045512.2	(6590)	AAACCTAATGAA-TTATCTAGAGT---ATTAGGT----TTGAAAACCCT--TGCTACTCATGGTTTAGCTGCTGT-

Section 1855

Sequence	Position	142759 142769 142780 142791 142802 142813 142824 142835
	(142759)	
Candidatus Carsonella ruddii strain BT chromosome...	(142607)	GTAAAATATTATTAATTGGTGTTGAATTAGATTATATCATAAAAAAAGAAATTGTTAAAAAAAATATTTTTTATAAA
Candidatus Carsonella 135557 - 167442nt	(7051)	GTAAAATATTATTAATTGGTGTTGAATTAGATTATATCATAAAAAAAGAAATTGTTAAAAAAAATATTTTTTATAAA
SARS-CoV-2 Reference Genome NC_045512.2	(6656)	----AATAGTGTCCCTTGGATACTAT-AGCTAAT----TATGCTAAGCCTTTTCTTAACAAAGTTGTTAGTACAAC

Section 1856

Sequence	Position	142836 142846 142857 142868 142879 142890 142901 142912
	(142836)	
Candidatus Carsonella ruddii strain BT chromosome...	(142684)	GAATTTAAATTAATTA-ATGATGT--AATCGATTTTTTTAATAAAAATTATAATTGTAATAAATTAGAAATAATTAC
Candidatus Carsonella 135557 - 167442nt	(7128)	GAATTTAAATTAATTA-ATGATGT--AATCGATTTTTTTAATAAAAATTATAATTGTAATAAATTAGAAATAATTAC
SARS-CoV-2 Reference Genome NC_045512.2	(6724)	TAC--TAACATAGTTACACGGTGTTTAAACCGTGTTTGTACTAA---TTATATGCCTTATTTCTTT-ACTTTATTGC

Section 1857

Sequence	Position	142913 142923 142934 142945 142956 142967 142978 142989
	(142913)	
Candidatus Carsonella ruddii strain BT chromosome...	(142758)	TGGAGAATTAATTATAAAAGATATTTTTCCATGTGGTAAAAATAAAAAAATAGCAGGTTGTAAATTAATAAAAGGTT
Candidatus Carsonella 135557 - 167442nt	(7202)	TGGAGAATTAATTATAAAAGATATTTTTCCATGTGGTAAAAATAAAAAAATAGCAGGTTGTAAATTAATAAAAGGTT
SARS-CoV-2 Reference Genome NC_045512.2	(6795)	T--ACAATTGTGTACTTTTACTAGAAGTACAAATTCTAGAATTAAAGCATCTATGCCGACTACTATAGCAAAGAATA

Section 1858

Sequence	Position	142990 143000 143011 143022 143033 143044 143055 143066
	(142990)	
Candidatus Carsonella ruddii strain BT chromosome...	(142835)	ATTTAGAAATTGAAAATATAATAAAAATATATAAAGAATTAAAATTAATATTTCAAGGTTTTATAAATACATTAAAA
Candidatus Carsonella 135557 - 167442nt	(7279)	ATTTAGAAATTGAAAATATAATAAAAATATATAAAGAATTAAAATTAATATTTCAAGGTTTTATAAATACATTAAAA
SARS-CoV-2 Reference Genome NC_045512.2	(6870)	CTGTTAAGAGTGTCGGTAAATT----TTGTCTAGAGGCTTCATTTAATTATTTGAAG-TCACCTAATTTTTCTAAAC

Section 1859

Sequence	Position	143067 143077 143088 143099 143110 143121 143132 143143
	(143067)	
Candidatus Carsonella ruddii strain BT chromosome...	(142912)	ATA-AAAGATAAAAATGTAGTTAAAATTAATGAAAATGATGAATGTGGAATAAATTTTAAAAATTTTAACGATATCG
Candidatus Carsonella 135557 - 167442nt	(7356)	ATA-AAAGATAAAAATGTAGTTAAAATTAATGAAAATGATGAATGTGGAATAAATTTTAAAAATTTTAACGATATCG
SARS-CoV-2 Reference Genome NC_045512.2	(6942)	TGATAAATATTATAATTTGGTTTTTACTATT--AAGTGTT---TGCCTAGGTTCTTTAATCTACTCAACCGCT---G

Section 1860

Sequence	Position	143144 143154 143165 143176 143187 143198 143209 143220
	(143144)	
Candidatus Carsonella ruddii strain BT chromosome...	(142988)	AAATAGGATTAAAAATATTTTCAGTTAAATATGTCGATATTACATAATATTATAAAAAAAAAAGGATATGGTGATTT
Candidatus Carsonella 135557 - 167442nt	(7432)	AAATAGGATTAAAAATATTTTCAGTTAAATATGTCGATATTACATAATATTATAAAAAAAAAAGGATATGGTGATTT
SARS-CoV-2 Reference Genome NC_045512.2	(7011)	CTTTAGGTGTTTTAATGTCTA-ATTTAGGCATGCCT-TCTTACTGTACTGGTTACAGAGAAGGCTATTTGAACTCTA

Section 1861

	(143221)	143221 143231 143242 143253 143264 143275 143286 143297
Candidatus Carsonella ruddii strain BT chromosome...	(143065)	AAAAGTTCAAAATTATTTTTTAATAAAAAAATTAAAAAAAATAAAATTTCATTTTTTAAATAACAAAAAAGATTTAA
Candidatus Carsonella 135557 - 167442nt	(7509)	AAAAGTTCAAAATTATTTTTTAATAAAAAAATTAAAAAAAATAAAATTTCATTTTTTAAATAACAAAAAAGATTTAA
SARS-CoV-2 Reference Genome NC_045512.2	(7086)	CTAATGTCACTATTGCAACCTACTGTACTGGTTCTATACCTTGTAGTGT--TTGTCTTAGT---------GGTTTAG

Section 1862

	(143298)	143298 143308 143319 143330 143341 143352 143363 143374
Candidatus Carsonella ruddii strain BT chromosome...	(143142)	AATGTAAAATTAATATAAATAAAATAATTTTTAAAATTAAAAAAAATATTAATTT-TATGAAAAATTTATTATAATT
Candidatus Carsonella 135557 - 167442nt	(7586)	AATGTAAAATTAATATAAATAAAATAATTTTTAAAATTAAAAAAAATATTAATTT-TATGAAAAATTTATTATAATT
SARS-CoV-2 Reference Genome NC_045512.2	(7152)	ATTCT-----TTAGACACCTATCCTTCTTTAGAAACT-ATACAAATTACCATTTCATCTTTTAAATGGGATTTAACT

Section 1863

	(143375)	143375 143385 143396 143407 143418 143429 143440 143451
Candidatus Carsonella ruddii strain BT chromosome...	(143218)	TTTTTATGTTTTTTTTAAAAAAAATATTTAAAAAAATTATTGGATTTTTTAAATTTAAAAATAAAATAATTAAATTT
Candidatus Carsonella 135557 - 167442nt	(7662)	TTTTTATGTTTTTTTTAAAAAAAATATTTAAAAAAATTATTGGATTTTTTAAATTTAAAAATAAAATAATTAAATTT
SARS-CoV-2 Reference Genome NC_045512.2	(7223)	GCTTTTGGCTTAGTTGCAGAGTGGTTTTTGGCATA--TATTCTTTTCACTAGGTTTTTCTATG---TACTTGGATT-

Section 1864

	(143452)	143452 143462 143473 143484 143495 143506 143517 143528
Candidatus Carsonella ruddii strain BT chromosome...	(143295)	GGTATTGATCCTACATTT-TACTCAATTCATTTAGGTCATTTTTTTATTATAAATTTTTTTTTTTATTTGTTAAAAA
Candidatus Carsonella 135557 - 167442nt	(7739)	GGTATTGATCCTACATTT-TACTCAATTCATTTAGGTCATTTTTTTATTATAAATTTTTTTTTTTATTTGTTAAAAA
SARS-CoV-2 Reference Genome NC_045512.2	(7294)	GGCTGCAATCATGCAATTGTTTTTCAGCTATTTTGCAGTACATTTTATTAGTAATTCTTGGCTTATGTGGTTAATAA

Section 1865

	(143529)	143529 143539 143550 143561 143572 143583 143594 143605
Candidatus Carsonella ruddii strain BT chromosome...	(143371)	AAAATTTTTTATTTGTAATTATAATAGGTGATTTCACTACTATTCTTAAAAAAAAATAAAAAAATCAG-ATATTAT
Candidatus Carsonella 135557 - 167442nt	(7815)	AAAATTTTTTATTTGTAATTATAATAGGTGATTTCACTACTATTCTTAAAAAAAAATAAAAAAATCAG-ATATTAT
SARS-CoV-2 Reference Genome NC_045512.2	(7371)	TTAATCTTGTAC----AAATGGCCCCG---ATTTCA--GCTATGGTTAGAATGTACATCTTCTTTGCATCATTTTAT

Section 1866

	(143606)	143606 143616 143627 143638 143649 143660 143671 143682
Candidatus Carsonella ruddii strain BT chromosome...	(143447)	TTTAAATTCAATTTGTTTAAAATCTCAATTTTATAATATATTAGGAGATGTTTATATTTTTTTTAATTCAATATGGA
Candidatus Carsonella 135557 - 167442nt	(7891)	TTTAAATTCAATTTGTTTAAAATCTCAATTTTATAATATATTAGGAGATGTTTATATTTTTTTTAATTCAATATGGA
SARS-CoV-2 Reference Genome NC_045512.2	(7439)	TATGTATGGAAAA-GTT----ATGTGCATGTTGTAG-ACGGTTGTAATTCATCAACTTGTATGATGTGTTACAAACG

Section 1867

		143683 143693 143704 143715 143726 143737 143748 143759
	(143683)	
Candidatus Carsonella ruddii strain BT chromosome...	(143524)	TATATAAAAAAAAAATTTTTTTTTTTTTAAAAATATTTAATT-TATTAAATGTTAATAAATATATCAATAAAAATTT
Candidatus Carsonella 135557 - 167442nt	(7968)	TATATAAAAAAAAAATTTTTTTTTTTTTAAAAATATTTAATT-TATTAAATGTTAATAAATATATCAATAAAAATTT
SARS-CoV-2 Reference Genome NC_045512.2	(7510)	TA-ATAGAGCAACAAGAGTCGAATGTACAACTATTGTTAATGGTGTTAGAAGGTCCTTT-TATGTCTATGCTAATGG

Section 1868

		143760 143770 143781 143792 143803 143814 143825 143836
	(143760)	
Candidatus Carsonella ruddii strain BT chromosome...	(143600)	AAAAATAGATATTTTTTGCAAAAAAATTAACATAAATAATATTATTTATCCTTTATTTCAAGCATATGATTCATTAA
Candidatus Carsonella 135557 - 167442nt	(8044)	AAAAATAGATATTTTTTGCAAAAAAATTAACATAAATAATATTATTTATCCTTTATTTCAAGCATATGATTCATTAA
SARS-CoV-2 Reference Genome NC_045512.2	(7585)	AGGTAAAG---GCTTTTGCAAACTACACAATTGGAATTGTGTTAATTGTGATACATTCTGTGCTGGTAGTACATTTA

Section 1869

		143837 143847 143858 143869 143880 143891 143902 143913
	(143837)	
Candidatus Carsonella ruddii strain BT chromosome...	(143677)	TTATTAATT--GTTATATAGAAATAGGTGGTATAGATCAATTGTTGAATTG-TATTTGTGGTAGATTTATACAAAAA
Candidatus Carsonella 135557 - 167442nt	(8121)	TTATTAATT--GTTATATAGAAATAGGTGGTATAGATCAATTGTTGAATTG-TATTTGTGGTAGATTTATACAAAAA
SARS-CoV-2 Reference Genome NC_045512.2	(7659)	TTAGTGATGAAGTTGCGAGAGACTTGTCACTACAGTTTAAAAGACCAATAAATCCTACTGACCAGTCTTCTTACATC

Section 1870

		143914 143924 143935 143946 143957 143968 143979 143990
	(143914)	
Candidatus Carsonella ruddii strain BT chromosome...	(143751)	ATTTTTAAAAAAAAAAAACAAAATTTAATTTTGTTTTTTTTATTAGAAAAAAATAATGAAAAAATTTCAAAATCTAA
Candidatus Carsonella 135557 - 167442nt	(8195)	ATTTTTAAAAAAAAAAAACAAAATTTAATTTTGTTTTTTTTATTAGAAAAAAATAATGAAAAAATTTCAAAATCTAA
SARS-CoV-2 Reference Genome NC_045512.2	(7736)	GTTGATAGTGTTACAGTGAAGAATGGTTCCATCCATCTTTACTTTGATAAAGCTGGTCAAAAGACTTATGAAAGACA

Section 1871

		143991 144001 144012 144023 144034 144045 144056 144067
	(143991)	
Candidatus Carsonella ruddii strain BT chromosome...	(143828)	AAGTTTTT----TTTTTATAAATAAAAAAAATTTAGATTTAATTTTATTTATAAAAATTTTTTATAACATAAAAATA
Candidatus Carsonella 135557 - 167442nt	(8272)	AAGTTTTT----TTTTTATAAATAAAAAAAATTTAGATTTAATTTTATTTATAAAAATTTTTTATAACATAAAAATA
SARS-CoV-2 Reference Genome NC_045512.2	(7813)	TTCTCTCTCTCATTTTGTTAACTTAGACAACCTGAGAGCTAATAACACT---AAAGGTTCATTGCCTATTAATGTTA

Section 1872

		144068 144078 144089 144100 144111 144122 144133 144144
	(144068)	
Candidatus Carsonella ruddii strain BT chromosome...	(143901)	TT-TTTTAAAAGTTACAAAAAAA----AAAATTTATTTTTTAAAGGATATTATAATAA-TATTTTTTTTATAAATAA
Candidatus Carsonella 135557 - 167442nt	(8345)	TT-TTTTAAAAGTTACAAAAAAA----AAAATTTATTTTTTAAAGGATATTATAATAA-TATTTTTTTTATAAATAA
SARS-CoV-2 Reference Genome NC_045512.2	(7887)	TAGTTTTTGATGGTAAATCAAAATGTGAAGAATCATCTGCAAAATCAGCGTCTGTTTACTACAGTCAGCTTATGTGT

Section 1873

	(144145)	144145 144155 144166 144177 144188 144199 144210 144221
Candidatus Carsonella ruddii strain BT chromosome...	(143972)	TAAAAAATATTTTTTTTTTAAAAAAAAGAATATTTAAGTTTTATTTTAAAAAAATATTTTCAAATAATAAAAAATT
Candidatus Carsonella 135557 - 167442nt	(8416)	TAAAAAATATTTTTTTTTTAAAAAAAAGAATATTTAAGTTTTATTTTAAAAAAATATTTTCAAATAATAAAAAATT
SARS-CoV-2 Reference Genome NC_045512.2	(7964)	CAACCTATACTGTTACTAGATCAGGCATTAGTGTCTGA-TGTTGGTGATAGTGCGGAAGTTGCAGTTA--AAA----

Section 1874

	(144222)	144222 144232 144243 144254 144265 144276 144287 144298
Candidatus Carsonella ruddii strain BT chromosome...	(144049)	TGTTTAATAATAATATTTATAAAAAAAAATTATTTATTTT--TAAAAAATTAATAATAAAAAATTTTTTTATTAAAA
Candidatus Carsonella 135557 - 167442nt	(8493)	TGTTTAATAATAATATTTATAAAAAAAAATTATTTATTTT--TAAAAAATTAATAATAAAAAATTTTTTTATTAAAA
SARS-CoV-2 Reference Genome NC_045512.2	(8034)	TGTTTGATGCTTACGTTAATACGTTTTCATCAACTTTTAACGTACCAATGGAAAAACTCAAAACACTAGTTGCAACT

Section 1875

	(144299)	144299 144309 144320 144331 144342 144353 144364 144375
Candidatus Carsonella ruddii strain BT chromosome...	(144124)	AAAACAAATGTATAATTAAAAATTTTTTCAATATTATTATATATGATAAATAAAATTTTTACTTTTTCTAAAAAAAA
Candidatus Carsonella 135557 - 167442nt	(8568)	AAAACAAATGTATAATTAAAAATTTTTTCAATATTATTATATATGATAAATAAAATTTTTACTTTTTCTAAAAAAAA
SARS-CoV-2 Reference Genome NC_045512.2	(8111)	GCAGAAGCTGAACTTGCAAAGAATGTGTCCTTAG-ACAATGTCTTATCTACTTTTATTTCAGCAGCTCGGCAAGGGT

Section 1876

	(144376)	144376 144386 144397 144408 144419 144430 144441 144452
Candidatus Carsonella ruddii strain BT chromosome...	(144201)	ATCTTTTCTTAGGTTTC----CTCCAGATCCAAATGGATTTTTGCATATAGGACATGTTTTTAACATATTAATTAAT
Candidatus Carsonella 135557 - 167442nt	(8645)	ATCTTTTCTTAGGTTTC----CTCCAGATCCAAATGGATTTTTGCATATAGGACATGTTTTTAACATATTAATTAAT
SARS-CoV-2 Reference Genome NC_045512.2	(8187)	TTGTTGATTCAGATGTAGAAACTAAAGATGTTGTTGAATGTCTTAAATTGTCACATCAATCTGACATAGAAGTTACT

Section 1877

	(144453)	144453 144463 144474 144485 144496 144507 144518 144529
Candidatus Carsonella ruddii strain BT chromosome...	(144274)	TTTTATTTATCAATTAATAAAAAAGGAAAATTTATATTAAGATTTGATAACACAAATTTAAAAAATTATAATTTATT
Candidatus Carsonella 135557 - 167442nt	(8718)	TTTTATTTATCAATTAATAAAAAAGGAAAATTTATATTAAGATTTGATAACACAAATTTAAAAAATTATAATTTATT
SARS-CoV-2 Reference Genome NC_045512.2	(8264)	GGCGATAGTTGTAATAACTATAT--GCTCACCTATAACAA-AGTTGAAAACATGA---CACCCCGTGACCTTGGTGC

Section 1878

	(144530)	144530 144540 144551 144562 144573 144584 144595 144606
Candidatus Carsonella ruddii strain BT chromosome...	(144351)	TTTTTATAATTTTATACAATATGATTTATTATGGTTAGGTATAAAATGGAATAAAAATAATTATTTTTTAAATTATA
Candidatus Carsonella 135557 - 167442nt	(8795)	TTTTTATAATTTTATACAATATGATTTATTATGGTTAGGTATAAAATGGAATAAAAATAATTATTTTTTAAATTATA
SARS-CoV-2 Reference Genome NC_045512.2	(8335)	TTGTATTGACTGTAGTGCGCGTCATAT-TAATGCGCAGGTAGCAAAA--AGTCACAACATTGCTTTGATATGGAACG

```
                                                                                                                                              Section 1879
                                                  (144607) 144607    144617     144628     144639     144650     144661     144672 144683
Candidatus Carsonella ruddii strain BT chromosome... (144428) TAAATAAATTTTATTTCATTTTGTTTTTTTATTTTAAAAATAAAAAAATTTATTTTAAAAAAAAAATTAATTTAATT
                 Candidatus Carsonella 135557 - 167442nt (8872) TAAATAAATTTTATTTCATTTTGTTTTTTTATTTTAAAAATAAAAAAATTTATTTTAAAAAAAAAATTAATTTAATT
        SARS-CoV-2 Reference Genome NC_045512.2 (8409) TTAA-AGATTTCATGTCAT--TGTCTGAACAACTACGAAAACAAATACGTAGTGCTGCTAAAAAGAATAACTTACCT
                                                                                                                                              Section 1880
                                                  (144684) 144684    144694     144705     144716     144727     144738     144749 144760
Candidatus Carsonella ruddii strain BT chromosome... (144505) AGAATTAATTTTAATTATTTAAATTATAAAAAAATAATTAATTTTATTTTATTTAAAAATGATTTTTTTAAAAAAAA
                 Candidatus Carsonella 135557 - 167442nt (8949) AGAATTAATTTTAATTATTTAAATTATAAAAAAATAATTAATTTTATTTTATTTAAAAATGATTTTTTTAAAAAAAA
        SARS-CoV-2 Reference Genome NC_045512.2 (8483) T---TTAAGTTGACATGTGCAACTACTAGACAAGTTGTTAATGTTGTAACA----ACAAAGAT--------AGCACT
                                                                                                                                              Section 1881
                                                  (144761) 144761    144771     144782     144793     144804     144815     144826 144837
Candidatus Carsonella ruddii strain BT chromosome... (144582) TTATTTTGTTTTTTTTATTAAAAAAAAAATAATTTTTAGAATTAAAAAAAAAAAAAAATCATTGGAATATTTTTCCTA
                 Candidatus Carsonella 135557 - 167442nt (9026) TTATTTTGTTTTTTTTATTAAAAAAAAAATAATTTTTAGAATTAAAAAAAAAAAAAAATCATTGGAATATTTTTCCTA
        SARS-CoV-2 Reference Genome NC_045512.2 (8545) TAAGGGTGGTAAAATTGTTAAT-----AATTGGTTGAAGCAGTTAATTAAAGTTACA-CTTGTGTTCCTTTTTGTTG
                                                                                                                                              Section 1882
                                                  (144838) 144838    144848     144859     144870     144881     144892     144903 144914
Candidatus Carsonella ruddii strain BT chromosome... (144659) CTTATGATTTCACACAAGTATTAATGATTATTTTAATAATATAAATGTTTCTATTTGTACAAAAGAATTTTTAAAT
                 Candidatus Carsonella 135557 - 167442nt (9103) CTTATGATTTCACACAAGTATTAATGATTATTTTAATAATATAAATGTTTCTATTTGTACAAAAGAATTTTTAAAT
        SARS-CoV-2 Reference Genome NC_045512.2 (8616) CTGCTATTTTCTATTTAATAACACCTGTTCATGTCATGTCTAAACAT---ACTGACTTTTCAAGTGAAATC----AT
                                                                                                                                              Section 1883
                                                  (144915) 144915    144925     144936     144947     144958     144969     144980 144991
Candidatus Carsonella ruddii strain BT chromosome... (144736) AATGTTAAAATATATAACTATTTTTTAAAATATTTAAAAAAAAAATCTTATCAAATAGAATTTTCTAAAAAAAATTT
                 Candidatus Carsonella 135557 - 167442nt (9180) AATGTTAAAATATATAACTATTTTTTAAAATATTTAAAAAAAAAATCTTATCAAATAGAATTTTCTAAAAAAAATTT
        SARS-CoV-2 Reference Genome NC_045512.2 (8686) AGGATACAAGGCTATTGATGGTGGTGTCACTCGTGACATAGC--ATCTACAGATACTTGTTTTGCTAACAAACATGC
                                                                                                                                              Section 1884
                                                  (144992) 144992    145002     145013     145024     145035     145046     145057 145068
Candidatus Carsonella ruddii strain BT chromosome... (144813) TAAAAAAAATAAAATTTCTAAAGAAAATTAAAAAATATTTTTTTTTTAAATATATTTTATTTTAAAAAAATAGGTT
                 Candidatus Carsonella 135557 - 167442nt (9257) TAAAAAAAATAAAATTTCTAAAGAAAATTAAAAAATATTTTTTTTTTAAATATATTTTATTTTAAAAAAATAGGTT
        SARS-CoV-2 Reference Genome NC_045512.2 (8761) TGATTTTGACACATGGTTTAGCC-AGCGTGGTGGTAGTTATACTAATGACAAAGCTTGCCCATTGATTGCTGCAGTC
```

Section 1885

(145069) 145069 145079 145090 145101 145112 145123 145134 145145

Candidatus Carsonella ruddii strain BT chromosome... (144890) TTAATCCAAAAAATTTTTATTTTTTATGTAATATAATTGGTGTTTCAAAAAAAATATCTTTTATTGATTATAAAAAA
Candidatus Carsonella 135557 - 167442nt (9334) TTAATCCAAAAAATTTTTATTTTTTATGTAATATAATTGGTGTTTCAAAAAAAATATCTTTTATTGATTATAAAAAA
SARS-CoV-2 Reference Genome NC_045512.2 (8837) ATAA--CAAGAGAAGTGGGTTTTGTCG------TGCCTGGTTTGCCTGGCACGATAT----TACGCACAACTAATGG

Section 1886

(145146) 145146 145156 145167 145178 145189 145200 145211 145222

Candidatus Carsonella ruddii strain BT chromosome... (144967) ATTATTTTATCATTGAAAATTGAAAATAATTTTTTAGAAAAATGTTTTTTATTTGTTAAAAATTATATTAAAAATTT
Candidatus Carsonella 135557 - 167442nt (9411) ATTATTTTATCATTGAAAATTGAAAATAATTTTTTAGAAAAATGTTTTTTATTTGTTAAAAATTATATTAAAAATTT
SARS-CoV-2 Reference Genome NC_045512.2 (8902) TGACTTTTTGCATTTCTTACCTAGA----GTTTTTAGTGCAGTTGGTAACATCTGTTACACACCAT--CAAAACTTA

Section 1887

(145223) 145223 145233 145244 145255 145266 145277 145288 145299

Candidatus Carsonella ruddii strain BT chromosome... (145044) AAAAAAAAATTTAATAATTAGTATTTTGTTAAATGAAAAAAATATTAATTTAAAATTGAATAATTTTTTAATAAATA
Candidatus Carsonella 135557 - 167442nt (9488) AAAAAAAAATTTAATAATTAGTATTTTGTTAAATGAAAAAAATATTAATTTAAAATTGAATAATTTTTTAATAAATA
SARS-CoV-2 Reference Genome NC_045512.2 (8973) TAGAGTACACTGACTTTGCAACATCAGCTTG--TG-------TTTTGGCTGCTGAATGTACAATTTTT----AAAGA

Section 1888

(145300) 145300 145310 145321 145332 145343 145354 145365 145376

Candidatus Carsonella ruddii strain BT chromosome... (145121) AAATAATAAAAATAAGTTTAAATAAGTTTAATTATTCAATTTTAAAAAAAATTTTATTATTTAAAAAAAAAATATTT
Candidatus Carsonella 135557 - 167442nt (9565) AAATAATAAAAATAAGTTTAAATAAGTTTAATTATTCAATTTTAAAAAAAATTTTATTATTTAAAAAAAAAATATTT
SARS-CoV-2 Reference Genome NC_045512.2 (9037) TGCTTCTGGTAAGCCAGTACCATAT-TGTTATGATACCAATGTACTAGAAGGTTCTGTTGCTTATGAAAGTTTACGC

Section 1889

(145377) 145377 145387 145398 145409 145420 145431 145442 145453

Candidatus Carsonella ruddii strain BT chromosome... (145198) TTTAATAATTTTTTTTTTTTAAAACATAATTTTATTATATTAAAAAGAATATCAAAAAATAATAAAAAAAATTTTAA
Candidatus Carsonella 135557 - 167442nt (9642) TTTAATAATTTTTTTTTTTTAAAACATAATTTTATTATATTAAAAAGAATATCAAAAAATAATAAAAAAAATTTTAA
SARS-CoV-2 Reference Genome NC_045512.2 (9113) CCTGACACACGTTATGTGCTCATGGATGGCTCTATTATTC-------AATTTCCTAACACC-------TACCTTGAA

Section 1890

(145454) 145454 145464 145475 145486 145497 145508 145519 145530

Candidatus Carsonella ruddii strain BT chromosome... (145275) TATTAAAAAAAAACATTGTTTATATGAAAATATATTAAAATTTAAAAAAAATATTCTTAAAATAAAATTAAAATAAC
Candidatus Carsonella 135557 - 167442nt (9719) TATTAAAAAAAAACATTGTTTATATGAAAATATATTAAAATTTAAAAAAAATATTCTTAAAATAAAATTAAAATAAC
SARS-CoV-2 Reference Genome NC_045512.2 (9176) GGTTC-----------TGTTAGAGTGGTAACAACTTTTGATTCTGAGTAC-TGTAGGCACGGCACTTGTGAAAGATC

Section 1891

		145531 145541 145552 145563 145574 145585 145596 145607
	(145531)	
Candidatus Carsonella ruddii strain BT chromosome...	(145352)	GGAACATGGCGAAGTTTGGTATCGTATTTGCTTTGGGAGTAAGTGATCAAAGGTTCAAATCCTTTTGTTCCGAGCGT
Candidatus Carsonella 135557 - 167442nt	(9796)	GGAACATGGCGAAGTTTGGTATCGTATTTGCTTTGGGAGTAAGTGATCAAAGGTTCAAATCCTTTTGTTCCGAGCGT
SARS-CoV-2 Reference Genome NC_045512.2	(9241)	AGAAGCTGGTGT--TTGTGTATCTACTAGTGGTAGATGGGTACTTAACAATGATTATTACAGATCTTTACCAGGAGT

Section 1892

		145608 145618 145629 145640 145651 145662 145673 145684
	(145608)	
Candidatus Carsonella ruddii strain BT chromosome...	(145429)	TTATAGCTCAATGGATAGAGCAATGATCTTCTAAGTCAAAGGTTGTAAGTTC-AATTCTTACTAAACGTATGGTAAA
Candidatus Carsonella 135557 - 167442nt	(9873)	TTATAGCTCAATGGATAGAGCAATGATCTTCTAAGTCAAAGGTTGTAAGTTC-AATTCTTACTAAACGTATGGTAAA
SARS-CoV-2 Reference Genome NC_045512.2	(9316)	TTTCTGTGGTGTAGATGCTGTAA--ATTTACTTACTAATATGTTTACACCACTAATTCAACCTATTGGTGCTTTGGA

Section 1893

		145685 145695 145706 145717 145728 145739 145750 145761
	(145685)	
Candidatus Carsonella ruddii strain BT chromosome...	(145505)	TATAACTCAGTTGGTAGAGTAATGAATTGTGGATTCATATGTCAAGGGTTCAACCCCCTTTATTTACCCTAATTATA
Candidatus Carsonella 135557 - 167442nt	(9949)	TATAACTCAGTTGGTAGAGTAATGAATTGTGGATTCATATGTCAAGGGTTCAACCCCCTTTATTTACCCTAATTATA
SARS-CoV-2 Reference Genome NC_045512.2	(9391)	CATA--TCAGCATCTATAGTAGC---TGGTGGTATTGTAGCT-ATCGTAGTAACATGCCTTGCCTACT---ATTTTA

Section 1894

		145762 145772 145783 145794 145805 145816 145827 145838
	(145762)	
Candidatus Carsonella ruddii strain BT chromosome...	(145582)	TAAAAATAAAAAAATGATATTTATTCCTACTGTTATAGACAAAAATATAAAAGGAGATAGATTTTTAGATTTATTTT
Candidatus Carsonella 135557 - 167442nt	(10026)	TAAAAATAAAAAAATGATATTTATTCCTACTGTTATAGACAAAAATATAAAAGGAGATAGATTTTTAGATTTATTTT
SARS-CoV-2 Reference Genome NC_045512.2	(9459)	TGAGGTTTAGAAGA-GCTTTTGGTGAATACAGTCATGTAGTTGCCTTTAATACTTTACT-ATTCCTTATGTCATTCA

Section 1895

		145839 145849 145860 145871 145882 145893 145904 145915
	(145839)	
Candidatus Carsonella ruddii strain BT chromosome...	(145659)	CTAAAATGTTAAAAGAAAGAGTAATTTTTATTAATGGAGCTATAGATGATAATTTATCTGTTAT--AATAGTAGCTC
Candidatus Carsonella 135557 - 167442nt	(10103)	CTAAAATGTTAAAAGAAAGAGTAATTTTTATTAATGGAGCTATAGATGATAATTTATCTGTTAT--AATAGTAGCTC
SARS-CoV-2 Reference Genome NC_045512.2	(9534)	CTGTACTCTGTTTAACACCAGT----T-TACTCATT---CT-TACCTGGTGTTTATTCTGTTATTTACTTGTACTTG

Section 1896

		145916 145926 145937 145948 145959 145970 145981 145992
	(145916)	
Candidatus Carsonella ruddii strain BT chromosome...	(145734)	AATTATTATTTTTAGATTCAGATAATAATAAAGATATAATTTTATATATTAATTCACCTGGAGGTGTAGTTTCTTCA
Candidatus Carsonella 135557 - 167442nt	(10178)	AATTATTATTTTTAGATTCAGATAATAATAAAGATATAATTTTATATATTAATTCACCTGGAGGTGTAGTTTCTTCA
SARS-CoV-2 Reference Genome NC_045512.2	(9602)	ACATTTTATCTT---------AC--TAATGATG-TTTCTTTTTTAGCACATATTCAG-TGGATGGTTATGTTCA-CA

```
                                                                   Section 1897
                                              (145993) 145993    146003      146014      146025      146036      146047      146058   146069
Candidatus Carsonella ruddii strain BT chromosome... (145811) GGATTAAGTATATATGATACAATACAATTTATAAAATCAGATGTAGTTACTATTTGTATAGGTCAAGCAGCTAGTAT
       Candidatus Carsonella 135557 - 167442nt (10255) GGATTAAGTATATATGATACAATACAATTTATAAAATCAGATGTAGTTACTATTTGTATAGGTCAAGCAGCTAGTAT
    SARS-CoV-2 Reference Genome NC_045512.2 (9665) CC-TTTAGTACCT-TTCTGGATAACAATTGCTTATATC--A-----TTTGTATTTCCACA----AAGCAT-TTCTAT

                                                                   Section 1898
                                              (146070) 146070    146080      146091      146102      146113      146124      146135   146146
Candidatus Carsonella ruddii strain BT chromosome... (145888) GGGAGCTGTTTTATTATCATCTGGTAAAAAAGGGAAAAGATTTTCTTTACAAAATTCTAGAATAATGATTCATCAAC
       Candidatus Carsonella 135557 - 167442nt (10332) GGGAGCTGTTTTATTATCATCTGGTAAAAAAGGGAAAAGATTTTCTTTACAAAATTCTAGAATAATGATTCATCAAC
    SARS-CoV-2 Reference Genome NC_045512.2 (9728) TGGTTCT--TTAGTAATTACCT----AAA--GAGACGTG-TAGTCTTTA---------A---TGGTGTTTC------

                                                                   Section 1899
                                              (146147) 146147    146157      146168      146179      146190      146201      146212   146223
Candidatus Carsonella ruddii strain BT chromosome... (145965) CTTTAGGTTATGCACAAGGACAAGCTACAGATGTAGAAATACATGCAAGAGAAATGATTATTGTTAAAAATATATTA
       Candidatus Carsonella 135557 - 167442nt (10409) CTTTAGGTTATGCACAAGGACAAGCTACAGATGTAGAAATACATGCAAGAGAAATGATTATTGTTAAAAATATATTA
    SARS-CoV-2 Reference Genome NC_045512.2 (9778) CTTTAG----TACTTTTGAAGAAGCTGC-GCTGTGCACCTTTTTGTTAAATAAAGAAATGTATCTAAAGTTGCGTAG

                                                                   Section 1900
                                              (146224) 146224    146234      146245      146256      146267      146278      146289   146300
Candidatus Carsonella ruddii strain BT chromosome... (146042) TGTAGTATTTTATCTTCTCATACAA-ATAATAG-TGTTGAAAAAATTTTCAAAGATACCGATCGTGATAATTTTATG
       Candidatus Carsonella 135557 - 167442nt (10486) TGTAGTATTTTATCTTCTCATACAA-ATAATAG-TGTTGAAAAAATTTTCAAAGATACCGATCGTGATAATTTTATG
    SARS-CoV-2 Reference Genome NC_045512.2 (9850) TGATGTGCTATTACCTCTTACGCAATATAATAGATACTTAGCTCTTTATAATAAGTACAAGTATT--TTAGTGGA-G

                                                                   Section 1901
                                              (146301) 146301    146311      146322      146333      146344      146355      146366   146377
Candidatus Carsonella ruddii strain BT chromosome... (146117) GATTGTAAACAATCATTAAAATATGGAATAATAGATAATGTTTTGTATAAAAAAT-AATTATATTAATA--TTGATT
       Candidatus Carsonella 135557 - 167442nt (10561) GATTGTAAACAATCATTAAAATATGGAATAATAGATAATGTTTTGTATAAAAAAT-AATTATATTAATA--TTGATT
    SARS-CoV-2 Reference Genome NC_045512.2 (9924) CAATGGATACAACTAGCTACAGAGAAGCTGCTTGTTGTCATCTCGCAAAGGCTCTCAATGACTTCAGTAACTCAGGT

                                                                   Section 1902
                                              (146378) 146378    146388      146399      146410      146421      146432      146443   146454
Candidatus Carsonella ruddii strain BT chromosome... (146191) TTTTAAATTATTTAAACCCAATAAATATAAAAAATGAATTAGAT-AGATATATTATTG-GTCAAAATGAAACTAAAA
       Candidatus Carsonella 135557 - 167442nt (10635) TTTTAAATTATTTAAACCCAATAAATATAAAAAATGAATTAGAT-AGATATATTATTG-GTCAAAATGAAACTAAAA
    SARS-CoV-2 Reference Genome NC_045512.2 (10001) TCTGATGTTCTTTA---CCAACCACCACAAACC-TCTATCACCTCAGCTGTTTTGCAGAGTGGTTTTAGAAAAATGG
```

Section 1903

```
                                                    (146455) 146455    146465     146476     146487     146498     146509     146520 146531
Candidatus Carsonella ruddii strain BT chromosome... (146266) AAATAATATCTGTTGCTGTTTATAATC--ATTATAAAAGACTTTATCTTTTACAATCA-----AAAAATATTTATTT
       Candidatus Carsonella 135557 - 167442nt (10710) AAATAATATCTGTTGCTGTTTATAATC--ATTATAAAAGACTTTATCTTTTACAATCA-----AAAAATATTTATTT
   SARS-CoV-2 Reference Genome NC_045512.2 (10074) CATTCCCATCTGGTAAAGTTGAGGGTTGTATGGTACAAGTAACT-TGTGGTACAACTACACTTAACGGTCTTTGGCT
```

Section 1904

```
                                                    (146532) 146532    146542     146553     146564     146575     146586     146597 146608
Candidatus Carsonella ruddii strain BT chromosome... (146336) AGAAAAAAGTAATATAATTTT-AATAGGTCCTA-CAGGATGTGGTAAAACATTGATGGTTAAAACATTAGCTAAGAT
       Candidatus Carsonella 135557 - 167442nt (10780) AGAAAAAAGTAATATAATTTT-AATAGGTCCTA-CAGGATGTGGTAAAACATTGATGGTTAAAACATTAGCTAAGAT
   SARS-CoV-2 Reference Genome NC_045512.2 (10150) TGATGAC-GTAGTTTACTGTCCAAGACATGTGATCTGCACCTCTGAAGACAT----GCTTA--ACCCTAATTATGAA
```

Section 1905

```
                                                    (146609) 146609    146619     146630     146641     146652     146663     146674 146685
Candidatus Carsonella ruddii strain BT chromosome... (146411) AATTAATACTCCAATAATAGTTGTTGACGCTACTTCTTT-TACTGAAGCAGGTTATGTTGGAG--ATGATGTTGAAT
       Candidatus Carsonella 135557 - 167442nt (10855) AATTAATACTCCAATAATAGTTGTTGACGCTACTTCTTT-TACTGAAGCAGGTTATGTTGGAG--ATGATGTTGAAT
   SARS-CoV-2 Reference Genome NC_045512.2 (10220) GATT--TACTC-ATTCGTAAGTCTAATCATAATTTCTTGGTACAG--GCTGGTAATGTTCAACTCAGGGTTATTGGA
```

Section 1906

```
                                                    (146686) 146686    146696     146707     146718     146729     146740     146751 146762
Candidatus Carsonella ruddii strain BT chromosome... (146485) CAATTATTCAAAAATTGCTTCATGAATGTGAATATGATGTTGAATTAACTGAAAGAAGTATAATATATATTGATGAA
       Candidatus Carsonella 135557 - 167442nt (10929) CAATTATTCAAAAATTGCTTCATGAATGTGAATATGATGTTGAATTAACTGAAAGAAGTATAATATATATTGATGAA
   SARS-CoV-2 Reference Genome NC_045512.2 (10292) CATTCTATGCAAAATTG-----TGTACTTAAGCTTAAGGTTGAT---ACAGCCAATCCTAAGACACCTAAGTATAAG
```

Section 1907

```
                                                    (146763) 146763    146773     146784     146795     146806     146817     146828 146839
Candidatus Carsonella ruddii strain BT chromosome... (146562) ATTGATAAAATTTCTAAAAAAACAGATTTTAT-----TTCGG--GTAAAGATGTTTCAGGAGAAGGAGTTCAACAAT
       Candidatus Carsonella 135557 - 167442nt (11006) ATTGATAAAATTTCTAAAAAAACAGATTTTAT-----TTCGG--GTAAAGATGTTTCAGGAGAAGGAGTTCAACAAT
   SARS-CoV-2 Reference Genome NC_045512.2 (10361) TTTGTTCGCATT-CAACCAGGACAGACTTTTTCAGTGTTAGCTTGTTACAATGGTTCACCATCTGGTGTTTACCAAT
```

Section 1908

```
                                                    (146840) 146840    146850     146861     146872     146883     146894     146905 146916
Candidatus Carsonella ruddii strain BT chromosome... (146632) CTT-TATTAAAATTAATTGAAGGAATTAGCATTAATG--TTACTTCATTAATTGATAAAAAAAATCCTCAACAAAAT
       Candidatus Carsonella 135557 - 167442nt (11076) CTT-TATTAAAATTAATTGAAGGAATTAGCATTAATG--TTACTTCATTAATTGATAAAAAAAATCCTCAACAAAAT
   SARS-CoV-2 Reference Genome NC_045512.2 (10437) GTGCTATGAGGCCCAATTTCACTATTAAGGGTTCATTCCTTAATGGTTCATGTGGTAGTGTTGGTTTTAACATAGAT
```

Section 1909

	(146917)	146917 146927 146938 146949 146960 146971 146982 146993
Candidatus Carsonella ruddii strain BT chromosome...	(146706)	CCTCAA-GTTTTTAATGTAGATACAACAAATATATTATTTATAGCTGGTGGAGCTTTTTCAGGTATAGAAAATTTTA
Candidatus Carsonella 135557 - 167442nt	(11150)	CCTCAA-GTTTTTAATGTAGATACAACAAATATATTATTTATAGCTGGTGGAGCTTTTTCAGGTATAGAAAATTTTA
SARS-CoV-2 Reference Genome NC_045512.2	(10514)	TATGACTGTGTCTCTTTTTGTTACATGCACCATATGGAAT-TACCAACTGGAGTTCATGCTGGCACAGA--C-----

Section 1910

	(146994)	146994 147004 147015 147026 147037 147048 147059 147070
Candidatus Carsonella ruddii strain BT chromosome...	(146782)	TTTTAAATAGAATAGATAAAGAATATTCTTTTTTAGATAATAAAGATAATAAAAATATTATTGAAAAAACTGATACT
Candidatus Carsonella 135557 - 167442nt	(11226)	TTTTAAATAGAATAGATAAAGAATATTCTTTTTTAGATAATAAAGATAATAAAAATATTATTGAAAAAACTGATACT
SARS-CoV-2 Reference Genome NC_045512.2	(10583)	-TTAGAAGGTAACTTTTATGGACCTTTTGTTGACAGGCAA-ACAGCACAAGCAGCTGGTACGGACACAACTATTACA

Section 1911

	(147071)	147071 147081 147092 147103 147114 147125 147136 147147
Candidatus Carsonella ruddii strain BT chromosome...	(146859)	GAAGATTTAATAAATTTTGGTATTATTCCTGAATTTTTAGGTAGATTACCTATTTTAGCTAAATTTAGAGAATT-AA
Candidatus Carsonella 135557 - 167442nt	(11303)	GAAGATTTAATAAATTTTGGTATTATTCCTGAATTTTTAGGTAGATTACCTATTTTAGCTAAATTTAGAGAATT-AA
SARS-CoV-2 Reference Genome NC_045512.2	(10658)	GTTAATGTTTAGCT--TGGTTGTACGC-TGCTGTTATAAATGGAG-ACAGGTGGTTTCTCAATCGATTTACCACAA

Section 1912

	(147148)	147148 147158 147169 147180 147191 147202 147213 147224
Candidatus Carsonella ruddii strain BT chromosome...	(146935)	CTGAATTTGAATTTATTTATATATTAATAAAATCTAAAAATTCATTATTAAAACAATTTTGTTATTTATTTTTAATA
Candidatus Carsonella 135557 - 167442nt	(11379)	CTGAATTTGAATTTATTTATATATTAATAAAATCTAAAAATTCATTATTAAAACAATTTTGTTATTTATTTTTAATA
SARS-CoV-2 Reference Genome NC_045512.2	(10731)	CTCTTAATGACTTTAACCTTGTGGCTATGAAGTACAATTATGAACCTCTAACACAAGA---CCATGTTGACATACTA

Section 1913

	(147225)	147225 147235 147246 147257 147268 147279 147290 147301
Candidatus Carsonella ruddii strain BT chromosome...	(147012)	GAAGGAGTAAGTATTAAATTTACTTTTTCATCTATAAAAGAAATTGCTAAAATAGCTGT-TAATAAAAAAATTGGAG
Candidatus Carsonella 135557 - 167442nt	(11456)	GAAGGAGTAAGTATTAAATTTACTTTTTCATCTATAAAAGAAATTGCTAAAATAGCTGT-TAATAAAAAAATTGGAG
SARS-CoV-2 Reference Genome NC_045512.2	(10805)	GGACCTCTTTCTGCTCAAACTGGAATTGCCGTTTTAGATATGTGTGCTTCATTAAAAGAATTACTGCAAAATGGTAT

Section 1914

	(147302)	147302 147312 147323 147334 147345 147356 147367 147378
Candidatus Carsonella ruddii strain BT chromosome...	(147088)	CAAGAGGTTTAAAATCAATATTAGAAAATGTTCTTTTAAATACAATGTTTTTATTTCCTTCTAAAATTAATTTAAAA
Candidatus Carsonella 135557 - 167442nt	(11532)	CAAGAGGTTTAAAATCAATATTAGAAAATGTTCTTTTAAATACAATGTTTTTATTTCCTTCTAAAATTAATTTAAAA
SARS-CoV-2 Reference Genome NC_045512.2	(10882)	GAATGGACGTA----CCATATTGGGTAGTG--CTTTATTAGAAGATGAATTTACA-CCTTTTGATGTTGTTAGACAA

```
                                                                                                   Section 1915
                                                    (147379) 147379    147389     147400     147411     147422     147433     147444 147455
Candidatus Carsonella ruddii strain BT chromosome... (147165) TTAGTAGTAATTTATAAAGAAGTTATTACAAAAAATAAAAAACCAATATTTATTTATAATTAATGTTTTTTGTTTTG
       Candidatus Carsonella 135557 - 167442nt (11609) TTAGTAGTAATTTATAAAGAAGTTATTACAAAAAATAAAAAACCAATATTTATTTATAATTAATGTTTTTTGTTTTG
   SARS-CoV-2 Reference Genome NC_045512.2 (10952) TGCTCAGGTGTTACTTTCCAA---AGTGCAGTGA---AAAGAACAATCAAGGGTACACACCACTGGTTGTTACT---

                                                                                                   Section 1916
                                                    (147456) 147456    147466     147477     147488     147499     147510     147521 147532
Candidatus Carsonella ruddii strain BT chromosome... (147242) TTTCCACATTTTTTTTTATTTTTTTTTTTTTTTAAAAAAAAAAAAATAAATATTATTTTTTATAATAATAAAAAAAAA
       Candidatus Carsonella 135557 - 167442nt (11686) TTTCCACATTTTTTTTTATTTTTTTTTTTTTTTAAAAAAAAAAAAATAAATATTATTTTTTATAATAATAAAAAAAAA
   SARS-CoV-2 Reference Genome NC_045512.2 (11020) ----CACAATTTTGAC--TTCACTTTTAGTTTTAGTCCAGAGTACTCAATGGTCTTTGTTCTT----TT--------

                                                                                                   Section 1917
                                                    (147533) 147533    147543     147554     147565     147576     147587     147598 147609
Candidatus Carsonella ruddii strain BT chromosome... (147319) AATTTTTTTTTAAAATACAATTTT---TTTTTAAATGGTATTTTTTTTTTTAAATTTATTTAAATCTATTTTTTA
       Candidatus Carsonella 135557 - 167442nt (11763) AATTTTTTTTTAAAATACAATTTT---TTTTTAAATGGTATTTTTTTTTTTAAATTTATTTAAATCTATTTTTTA
   SARS-CoV-2 Reference Genome NC_045512.2 (11079) --TTTTGTATGAAAATGCCTTTTTACCTTTTGCTATGGGTATTATTGCTATGTCTGCTTTTGCAATGATGTTTGTCA

                                                                                                   Section 1918
                                                    (147610) 147610    147620     147631     147642     147653     147664     147675 147686
Candidatus Carsonella ruddii strain BT chromosome... (147393) TGCTAAAAAATATATTTTTTTTCCAATTTTATATAATTAAAAATGATTTTTTAGGAAAAAAATTTATAAATTTATTAA
       Candidatus Carsonella 135557 - 167442nt (11837) TGCTAAAAAATATATTTTTTTTCCAATTTTATATAATTAAAAATGATTTTTTAGGAAAAAAATTTATAAATTTATTAA
   SARS-CoV-2 Reference Genome NC_045512.2 (11154) AACATAAGCATGCATTTCT---CTGTTTGTTTTGTTACCTTCTCTTGCCACTGTAGCTTATTTTAATATGGTCT--

                                                                                                   Section 1919
                                                    (147687) 147687    147697     147708     147719     147730     147741     147752 147763
Candidatus Carsonella ruddii strain BT chromosome... (147470) AATATAAATCTTATTTATGTATTATTTATTTAATAATTGATAAAATTGGTTCTTTTAATTTTTTTTTATTTTTAAAA
       Candidatus Carsonella 135557 - 167442nt (11914) AATATAAATCTTATTTATGTATTATTTATTTAATAATTGATAAAATTGGTTCTTTTAATTTTTTTTTATTTTTAAAA
   SARS-CoV-2 Reference Genome NC_045512.2 (11226) -ATATGCCTGCTAGTTGGGTGATGCGTATTATGACATGGTTGGATATGGTTGATACTAGTTTGTCTGGTTTTAAGCT

                                                                                                   Section 1920
                                                    (147764) 147764    147774     147785     147796     147807     147818     147829 147840
Candidatus Carsonella ruddii strain BT chromosome... (147547) AATATTTTTTTTTATAATTTATAATAATAGTAAATTTTTGTTAAATATAAGAAATCATCAAAAAATTGTTTATATAGA
       Candidatus Carsonella 135557 - 167442nt (11991) AATATTTTTTTTTATAATTTATAATAATAGTAAATTTTTGTTAAATATAAGAAATCATCAAAAAATTGTTTATATAGA
   SARS-CoV-2 Reference Genome NC_045512.2 (11302) AAAAGACTGTGT-TATGTATGCATCAGCTGTAGTGTTACTAATCCTTATGACAGC----AAGAACTGTGTATGATGA
```

Section 1921

		147841 147851 147862 147873 147884 147895 147906 147917
	(147841)	
Candidatus Carsonella ruddii strain BT chromosome...	(147624)	TAATAAAATCTTGTGGATTGGAGGAAATAATATTGGTATAGAATATTTAGGAATAGATTCTAATTTTGGAATATGG-
Candidatus Carsonella 135557 - 167442nt	(12068)	TAATAAAATCTTGTGGATTGGAGGAAATAATATTGGTATAGAATATTTAGGAATAGATTCTAATTTTGGAATATGG-
SARS-CoV-2 Reference Genome NC_045512.2	(11374)	TGGT----GCTAGGAGAGTGTGGACACTTATGAATGTCTTGA--------CACTCGTTTATAAAGTTT-ATTATGGT

Section 1922

		147918 147928 147939 147950 147961 147972 147983 147994
	(147918)	
Candidatus Carsonella ruddii strain BT chromosome...	(147700)	AATGATATTCATTGTAAAATTAATAATGTTTATTTTAATTATTTTTTAAAAAAAATATTTACTTTTTAAAAATAATTT
Candidatus Carsonella 135557 - 167442nt	(12144)	AATGATATTCATTGTAAAATTAATAATGTTTATTTTAATTATTTTTTAAAAAAAATATTTACTTTTTAAAAATAATTT
SARS-CoV-2 Reference Genome NC_045512.2	(11438)	AATGCTTTAGATCAAGCCATTTCCA-TGTGGGCTCTTATAATCTCTG----------TTACTTCTAACTACTCAGGT

Section 1923

		147995 148005 148016 148027 148038 148049 148060 148071
	(147995)	
Candidatus Carsonella ruddii strain BT chromosome...	(147777)	TACAATTAAAAAAATGGAATTTTTAAAAAAAAATTTTTTTTTTGAAAATTATATAAATTTATTTAATGTTTTTAAAT
Candidatus Carsonella 135557 - 167442nt	(12221)	TACAATTAAAAAAATGGAATTTTTAAAAAAAAATTTTTTTTTTGAAAATTATATAAATTTATTTAATGTTTTTAAAT
SARS-CoV-2 Reference Genome NC_045512.2	(11504)	GT-AGTTACAACTGTCATGTTTTTGGCCAGAGGTATTGTTTTT-----------ATGTGTGTTGAGTATTGCCCTAT

Section 1924

		148072 148082 148093 148104 148115 148126 148137 148148
	(148072)	
Candidatus Carsonella ruddii strain BT chromosome...	(147854)	TAATTTTAAAAAAAAAAATAATAATAATTTCTCCTTATATAATTTTAGATTTAAAATTAATTTTTTTATTTAAAAAA
Candidatus Carsonella 135557 - 167442nt	(12298)	TAATTTTAAAAAAAAAAATAATAATAATTTCTCCTTATATAATTTTAGATTTAAAATTAATTTTTTTATTTAAAAAA
SARS-CoV-2 Reference Genome NC_045512.2	(11569)	TTTCTTCATAAC------TGGTAATACACT-TCAGTGTATAATGCTAG-TTTATTGTTTCTTAGGCTATTTTTGTAC

Section 1925

		148149 148159 148170 148181 148192 148203 148214 148225
	(148149)	
Candidatus Carsonella ruddii strain BT chromosome...	(147931)	TTAAAATACATACATATATATATATATGTAACATATAAATCAGATAATTTATATATACAAAATAGTTCGTTTATTTT
Candidatus Carsonella 135557 - 167442nt	(12375)	TTAAAATACATACATATATATATATATGTAACATATAAATCAGATAATTTATATATACAAAATAGTTCGTTTATTTT
SARS-CoV-2 Reference Genome NC_045512.2	(11638)	TTGTTACTTTGGCCTCT-TTTGTTTACTCAACCGCTACTTTAGACTGACTCTTGGTGTTTATGATTAC--TTAGTTT

Section 1926

		148226 148236 148247 148258 148269 148280 148291 148302
	(148226)	
Candidatus Carsonella ruddii strain BT chromosome...	(148008)	TTTAAAATTTTTGAAAAAATATAAAATAAAAATTTATTTTTTAAAAAAAGGTTTTTTCCATAGAAAAATTTATATTA
Candidatus Carsonella 135557 - 167442nt	(12452)	TTTAAAATTTTTGAAAAAATATAAAATAAAAATTTATTTTTTAAAAAAAGGTTTTTTCCATAGAAAAATTTATATTA
SARS-CoV-2 Reference Genome NC_045512.2	(11712)	CTACACAGGAGTTTAGATATATGAATTCACAGGGACTACTCCCACCCAAGAATA---GCATAGATGCCTTCAAACTC

Section 1927

		148303 148313 148324 148335 148346 148357 148368 148379
	(148303)	
Candidatus Carsonella ruddii strain BT chromosome...	(148085)	TAGAT-AATTTTTTTTTTTTAGGATCAAATAATTTTGACAATAGATCTATTTATATTAATAAAGAATGTTTGTTTTG
Candidatus Carsonella 135557 - 167442nt	(12529)	TAGAT-AATTTTTTTTTTTTAGGATCAAATAATTTTGACAATAGATCTATTTATATTAATAAAGAATGTTTGTTTTG
SARS-CoV-2 Reference Genome NC_045512.2	(11786)	AACATTAAATTGTTGGGTGTTGGTGGCAAACCTTGTATCAAAGTAGCCACTG-TAC-AGTCTAAAATGTCAGATGTA

Section 1928

		148380 148390 148401 148412 148423 148434 148445 148456
	(148380)	
Candidatus Carsonella ruddii strain BT chromosome...	(148161)	CTTTTTTGATAAAAA-TATTAAAAAAACTTTTTTTAAAAAAATTAAAAATTTTATATTTTACATTAATTACAAAAAA
Candidatus Carsonella 135557 - 167442nt	(12605)	CTTTTTTGATAAAAA-TATTAAAAAAACTTTTTTTAAAAAAATTAAAAATTTTATATTTTACATTAATTACAAAAAA
SARS-CoV-2 Reference Genome NC_045512.2	(11861)	AAGTGCACATCAGTAGTCTTACTCTCAGTTTTGCAACAACTCAGAGTAGAATCATCATCTAAATTGTGGGCTCAATG

Section 1929

		148457 148467 148478 148489 148500 148511 148522 148533
	(148457)	
Candidatus Carsonella ruddii strain BT chromosome...	(148237)	AAA--ATTTTTTTTATAAAATTTATTTATATAATTTCATTTTTAAATTATTTTTTTCAATGAAATTATTAACAAAAC
Candidatus Carsonella 135557 - 167442nt	(12681)	AAA--ATTTTTTTTATAAAATTTATTTATATAATTTCATTTTTAAATTATTTTTTTCAATGAAATTATTAACAAAAC
SARS-CoV-2 Reference Genome NC_045512.2	(11938)	TGTCCAGTTACACAATGACATTCTCTTAGCTAAAGATACTACTGAAGCCTTTGAAAAAATGGTTTCACTACTTTCTG

Section 1930

		148534 148544 148555 148566 148577 148588 148599 148610
	(148534)	
Candidatus Carsonella ruddii strain BT chromosome...	(148312)	ATTTTTTTTTATTTTTTAGTGTTATTAAAAAAATAAAAAAAATAGGAAAAATTATTTTTTTAAATTTGTATTATAAA
Candidatus Carsonella 135557 - 167442nt	(12756)	ATTTTTTTTTATTTTTTAGTGTTATTAAAAAAATAAAAAAAATAGGAAAAATTATTTTTTTAAATTTGTATTATAAA
SARS-CoV-2 Reference Genome NC_045512.2	(12015)	TTTTGCTTTCCATGCAGGGTGCTGT--A-GACATAAACAAG------------CTTTGTGAAGAAATGCTGGACAAC

Section 1931

		148611 148621 148632 148643 148654 148665 148676 148687
	(148611)	
Candidatus Carsonella ruddii strain BT chromosome...	(148389)	AAAAAAAAATTAAATTTTATAATAAAAAATTTTAATTTTAAAATAAAACCATCTTTAATAGGTATTTATTTTTTTAA
Candidatus Carsonella 135557 - 167442nt	(12833)	AAAAAAAAATTAAATTTTATAATAAAAAATTTTAATTTTAAAATAAAACCATCTTTAATAGGTATTTATTTTTTTAA
SARS-CoV-2 Reference Genome NC_045512.2	(12077)	AGGGCAACCTTACAAGCTATAGCCTCAGAGTTTAGTTCCCTT------CCATCATATGCAGCTTTTGCTACTGCTCA

Section 1932

		148688 148698 148709 148720 148731 148742 148753 148764
	(148688)	
Candidatus Carsonella ruddii strain BT chromosome...	(148466)	AAATGAAACAAATTTTATTATATTCGAAATTTGTTATTATAAAAATAAAATTAATAAGTTTAAAAATTATTTATATA
Candidatus Carsonella 135557 - 167442nt	(12910)	AAATGAAACAAATTTTATTATATTCGAAATTTGTTATTATAAAAATAAAATTAATAAGTTTAAAAATTATTTATATA
SARS-CoV-2 Reference Genome NC_045512.2	(12148)	A---GAAGCTTATGAGCAGGCTGTTGCTAATGGTGATTCTGAAGTTGTTCTTAAAAAGTTGAAGAAGTCTTTGAATG

Section 1933

	(148765)	148765 148775 148786 148797 148808 148819 148830 148841
Candidatus Carsonella ruddii strain BT chromosome...	(148543)	AAAATAAATCAAATATTTTATATTTTATAAAAAATTTTTTTTTTTCATTAAATTTTTTTGA--AATGGATGTTCCAA
Candidatus Carsonella 135557 - 167442nt	(12987)	AAAATAAATCAAATATTTTATATTTTATAAAAAATTTTTTTTTTTCATTAAATTTTTTTGA--AATGGATGTTCCAA
SARS-CoV-2 Reference Genome NC_045512.2	(12222)	TGGCTAAATCTGAATTTGACCGTGATGCAGCCATGCAACGTAAGTTGGAAAAGATGGCTGATCAAGCTATGACCCAA

Section 1934

	(148842)	148842 148852 148863 148874 148885 148896 148907 148918
Candidatus Carsonella ruddii strain BT chromosome...	(148618)	CTATAGTAAATTATTCTAAATCTGGTTCAAAACAATTTTTAATTATTAATAAAAAAAAAAAAAATGAATTTTTTTCT
Candidatus Carsonella 135557 - 167442nt	(13062)	CTATAGTAAATTATTCTAAATCTGGTTCAAAACAATTTTTAATTATTAATAAAAAAAAAAAAAATGAATTTTTTTCT
SARS-CoV-2 Reference Genome NC_045512.2	(12299)	ATGTA-TAAAC-AGGCTAGATCTGAGGACAAGAGGGCAAAAGTTACTAGTGCTATGCAGACAA-TGCTTTTCACTAT

Section 1935

	(148919)	148919 148929 148940 148951 148962 148973 148984 148995
Candidatus Carsonella ruddii strain BT chromosome...	(148695)	CTTTCACAATCTCCACAATGTATTAAACAATATTATATGTTTAATAACGTTAAAAAATATTTTAAAATAATTAATTG
Candidatus Carsonella 135557 - 167442nt	(13139)	CTTTCACAATCTCCACAATGTATTAAACAATATTATATGTTTAATAACGTTAAAAAATATTTTAAAATAATTAATTG
SARS-CoV-2 Reference Genome NC_045512.2	(12373)	GCTTAGAAAGTTGGATAATGATGCACTCAACA--ACATTATCAACAATGC-AAGAGATGGTTGTGTTCCCTTGAACA

Section 1936

	(148996)	148996 149006 149017 149028 149039 149050 149061 149072
Candidatus Carsonella ruddii strain BT chromosome...	(148772)	TTTTAGAGATGAAGATGAAAG---ATCTAACAGATTAAAAGAATTTACTCAATTAGATATAGAAATGTGTTTTGTTG
Candidatus Carsonella 135557 - 167442nt	(13216)	TTTTAGAGATGAAGATGAAAG---ATCTAACAGATTAAAAGAATTTACTCAATTAGATATAGAAATGTGTTTTGTTG
SARS-CoV-2 Reference Genome NC_045512.2	(12447)	TAATACCTCTTACAACAGCAGCCAAACTAATGG-TTGTCATACCAGACTATAACACATATAAAAATACGTGTGATGG

Section 1937

	(149073)	149073 149083 149094 149105 149116 149127 149138 149149
Candidatus Carsonella ruddii strain BT chromosome...	(148846)	AATTTTTAAAATTAAAAAAATTAATTAACTATTTATTAAAATCTATATTTTATTACTTTTTTAATAAAAAAATTTTT
Candidatus Carsonella 135557 - 167442nt	(13290)	AATTTTTAAAATTAAAAAAATTAATTAACTATTTATTAAAATCTATATTTTATTACTTTTTTAATAAAAAAATTTTT
SARS-CoV-2 Reference Genome NC_045512.2	(12523)	TAC----AACATTTACTTA-TGCATCAGCATTGTGGGAAATCCAACAGGTTGTAGATGCAG--ATAGTAAAATTGTT

Section 1938

	(149150)	149150 149160 149171 149182 149193 149204 149215 149226
Candidatus Carsonella ruddii strain BT chromosome...	(148923)	TTTTTAACTTTAAAATATAAATATATAAAAAATTTTTTTTTTGAAAAAAAAAAATTTTAAAAAACCATATATTTATAA
Candidatus Carsonella 135557 - 167442nt	(13367)	TTTTTAACTTTAAAATATAAATATATAAAAAATTTTTTTTTTGAAAAAAAAAAATTTTAAAAAACCATATATTTATAA
SARS-CoV-2 Reference Genome NC_045512.2	(12593)	----CAACTTAGTGAAATTAGTATGGACAA-------TT---CACCTAA----T-TTAGCATGGCCTCTTATTGTAA

Section 1939

(149227) 149227 149237 149248 149259 149270 149281 149292 149303

Candidatus Carsonella ruddii strain BT chromosome... (149000) AAAAATTAAATATAAAAATTTTATAATTTATTTGTTTAAATATAATTATTATAATTTTTTAATAAGAAAAAAATTTT

Candidatus Carsonella 135557 - 167442nt (13444) AAAAATTAAATATAAAAATTTTATAATTTATTTGTTTAAATATAATTATTATAATTTTTTAATAAGAAAAAAATTTT

SARS-CoV-2 Reference Genome NC_045512.2 (12651) CAGCTTTAAGGGCCA--ATTCTGCTGTCAAATTACAGAATAATGAGCTTAGTCCTGTTGCACTACGACAGATGTCTT

Section 1940

(149304) 149304 149314 149325 149336 149347 149358 149369 149380

Candidatus Carsonella ruddii strain BT chromosome... (149077) TTTTTTATAAATTTAAAAAATATATTTTAA-TAATATCAAAAAAAAAAATTGATTTTAATTTATGTTATTATTTTGA

Candidatus Carsonella 135557 - 167442nt (13521) TTTTTTATAAATTTAAAAAATATATTTTAA-TAATATCAAAAAAAAAAATTGATTTTAATTTATGTTATTATTTTGA

SARS-CoV-2 Reference Genome NC_045512.2 (12726) GTGCTGCCGGTACTACACAAACTGCTTGCACTGATGACAATGCGTTAGCTTACTACAACACAACAAAGGGAGGTAGG

Section 1941

(149381) 149381 149391 149402 149413 149424 149435 149446 149457

Candidatus Carsonella ruddii strain BT chromosome... (149153) TTTTTGTTTAAAATATTTAAATATTATTAATAATA-AAATAATTTTTTTTTGGTACGT-TAATTTTTTTTATTTTAA

Candidatus Carsonella 135557 - 167442nt (13597) TTTTTGTTTAAAATATTTAAATATTATTAATAATA-AAATAATTTTTTTTTGGTACGT-TAATTTTTTTTATTTTAA

SARS-CoV-2 Reference Genome NC_045512.2 (12803) TTTGTACTTGCACTGTTATCCGATTTACAGGATTTGAAATGGGCTAGATTCCCTAAGAGTGATGGAACTGGTACTAT

Section 1942

(149458) 149458 149468 149479 149490 149501 149512 149523 149534

Candidatus Carsonella ruddii strain BT chromosome... (149228) AAAAAAAAAAATA-AATCATCATCAATTTTCATCATATAAAAATAATTTTACTTCAATTTATAATTCTAAATCTTTA

Candidatus Carsonella 135557 - 167442nt (13672) AAAAAAAAAAATA-AATCATCATCAATTTTCATCATATAAAAATAATTTTACTTCAATTTATAATTCTAAATCTTTA

SARS-CoV-2 Reference Genome NC_045512.2 (12880) CTATACAGAACTGGAACCACCTTGTAGGTTTGTTACAGACACAC-------CTAAAGGTCCTAAAGTGAAGTATTTA

Section 1943

(149535) 149535 149545 149556 149567 149578 149589 149600 149611

Candidatus Carsonella ruddii strain BT chromosome... (149304) TCATATGATATAATTATAAATGGAATTGAAATAGGAGGAGGATCTATTAGAAATTTAAATTTTAAATTACAAAAAAA

Candidatus Carsonella 135557 - 167442nt (13748) TCATATGATATAATTATAAATGGAATTGAAATAGGAGGAGGATCTATTAGAAATTTAAATTTTAAATTACAAAAAAA

SARS-CoV-2 Reference Genome NC_045512.2 (12950) TACTTTATTAAAGGATTAAAC--AACCTAAATAGAGGTATGGTAC-TTGGTAGTTTAGCTGCCACAGTACGTCTACA

Section 1944

(149612) 149612 149622 149633 149644 149655 149666 149677 149688

Candidatus Carsonella ruddii strain BT chromosome... (149381) AAATTTTTTTTTATCAAAAGAAAATAAATCTAATTTTTTAAATTTTTTTAAAAAAAGTATTCCACAACATTGTGGTA

Candidatus Carsonella 135557 - 167442nt (13825) AAATTTTTTTTTATCAAAAGAAAATAAATCTAATTTTTTAAATTTTTTTAAAAAAAGTATTCCACAACATTGTGGTA

SARS-CoV-2 Reference Genome NC_045512.2 (13024) AGCTGGTAAT---GCAACAGAAGTGCCTGCCAATT----CAACTGTATTA-------TCTTTC------T-GTGCTT

Section 1945

		149689 149699 149710 149721 149732 149743 149754 149765
	(149689)	
Candidatus Carsonella ruddii strain BT chromosome...	(149458)	TTGCAATAGGTTTAGATAGATTATTTTTTATAATATTTAAAACAAATATTAAAAATATAATTACATATCAAAAATAT
Candidatus Carsonella 135557 - 167442nt	(13902)	TTGCAATAGGTTTAGATAGATTATTTTTTATAATATTTAAAACAAATATTAAAAATATAATTACATATCAAAAATAT
SARS-CoV-2 Reference Genome NC_045512.2	(13080)	TTGCTGTAGATGCTGCTA-------------AAGCTT---A-CAAAGATTATCTAGCTAGTGGGGGACAACCAATCA

Section 1946

		149766 149776 149787 149798 149809 149820 149831 149842
	(149766)	
Candidatus Carsonella ruddii strain BT chromosome...	(149535)	AAAAAAATAATAAAATCAA---TAAACATTAATGATATTAATTGAAAAAAAATATCAACAAATAAATTTAATTTTAA
Candidatus Carsonella 135557 - 167442nt	(13979)	AAAAAAATAATAAAATCAA---TAAACATTAATGATATTAATTGAAAAAAAATATCAACAAATAAATTTAATTTTAA
SARS-CoV-2 Reference Genome NC_045512.2	(13140)	CTAATTGTGTTAAGATGTTGTGTACACAC-ACTGGTACTGGTCAGGCAATAACAGTTACACCGGAAGCCAATATGGA

Section 1947

		149843 149853 149864 149875 149886 149897 149908 149919
	(149843)	
Candidatus Carsonella ruddii strain BT chromosome...	(149609)	AAAAAAATAAAATTAATAGTAAAATTAAAAATAATTTTTATTCTTTTTTTTTTTTAAAATATTAATAAATAAAAAT
Candidatus Carsonella 135557 - 167442nt	(14053)	AAAAAAATAAAATTAATAGTAAAATTAAAAATAATTTTTATTCTTTTTTTTTTTTAAAATATTAATAAATAAAAAT
SARS-CoV-2 Reference Genome NC_045512.2	(13216)	TCAAGAATCCT-TTGGTGGT-----------GCATCGTGTTGTCTGTACTGCCGTTGCCACATAGATCATCCAAATC

Section 1948

		149920 149930 149941 149952 149963 149974 149985 149996
	(149920)	
Candidatus Carsonella ruddii strain BT chromosome...	(149686)	ATAAAAATCAAAAATTTTATACAAATAAATTT--TAAAAACAAAAAAAAAAAAATTTTTTTTTTTCCACTTTTTTTT
Candidatus Carsonella 135557 - 167442nt	(14130)	ATAAAAATCAAAAATTTTATACAAATAAATTT--TAAAAACAAAAAAAAAAAAATTTTTTTTTTTCCACTTTTTTTT
SARS-CoV-2 Reference Genome NC_045512.2	(13281)	CTAAAGG-----ATTTTGTGACTTAAAAGGTAAGTATGTACAAATACCTACAACTTGTGCTAATGACCCTGTGGGTT

Section 1949

		149997 150007 150018 150029 150040 150051 150062 150073
	(149997)	
Candidatus Carsonella ruddii strain BT chromosome...	(149761)	ATTTTAAATAACATAAAAATAAAAAAAAATTTTTTTATTTTTATTTCAAATATTTATATTTATTAGGAAAGATGGTA
Candidatus Carsonella 135557 - 167442nt	(14205)	ATTTTAAATAACATAAAAATAAAAAAAAATTTTTTTATTTTTATTTCAAATATTTATATTTATTAGGAAAGATGGTA
SARS-CoV-2 Reference Genome NC_045512.2	(13353)	---TT--ACA-------CTTAAAAACACAGTCTGT--------------ACCGTCTGCGGTATGTGGAAAGGTTATG

Section 1950

		150074 150084 150095 150106 150117 150128 150139 150150
	(150074)	
Candidatus Carsonella ruddii strain BT chromosome...	(149838)	GA-GTGGTTTAATACATCGGTCTTGAAAACCGAAAAAGTTAATTCTTTCCAGGGTTCGAATCCCTGTCTTTCCAAAA
Candidatus Carsonella 135557 - 167442nt	(14282)	GA-GTGGTTTAATACATCGGTCTTGAAAACCGAAAAAGTTAATTCTTTCCAGGGTTCGAATCCCTGTCTTTCCAAAA
SARS-CoV-2 Reference Genome NC_045512.2	(13404)	GCTGTAGTT----------GTGATCAACTCCGCGAAC-----------CCATGCTTCAGTCAGCTGA--TGC-ACAA

Section 1951

(150151)	150151 150161 150172 150183 150194 150205 150216 150227
Candidatus Carsonella ruddii strain BT chromosome... (149914)	AAATTTTAATTTCTATTTTATCTATTAAAAAAGAATTTTTAAAAAAAATACTAATTAAAATAATTATAAGTAATTTT
Candidatus Carsonella 135557 - 167442nt (14358)	AAATTTTAATTTCTATTTTATCTATTAAAAAAGAATTTTTAAAAAAAATACTAATTAAAATAATTATAAGTAATTTT
SARS-CoV-2 Reference Genome NC_045512.2 (13457)	TCGTTTTTAAACGGGTTTGCGGTGTAAGTGCAGCCCGTCTTACACCG-TGCGG--CACAGGCACTAGTACTGATGTC

Section 1952

(150228)	150228 150238 150249 150260 150271 150282 150293 150304
Candidatus Carsonella ruddii strain BT chromosome... (149991)	TTATATTATCATATAGATTTAATGAATTTCAATTATGTTAATAATGATTCGTTTTGT---ATTAATGAAATTTTATT
Candidatus Carsonella 135557 - 167442nt (14435)	TTATATTATCATATAGATTTAATGAATTTCAATTATGTTAATAATGATTCGTTTTGT---ATTAATGAAATTTTATT
SARS-CoV-2 Reference Genome NC_045512.2 (13531)	GTAT------ACAGGGCTTTTGACATCTACAATGAT----AAAGTAGCTGGTTTTGCTAAATTCCTAAAAACTAATT

Section 1953

(150305)	150305 150315 150326 150337 150348 150359 150370 150381
Candidatus Carsonella ruddii strain BT chromosome... (150065)	TTTAATAATTTATGTTAAAAAATTTTTTAATATAAAAATAGAATTACATTTAATGAATAAAATATTAAATATAAAAA
Candidatus Carsonella 135557 - 167442nt (14509)	TTTAATAATTTATGTTAAAAAATTTTTTAATATAAAAATAGAATTACATTTAATGAATAAAATATTAAATATAAAAA
SARS-CoV-2 Reference Genome NC_045512.2 (13598)	GTTGTCGCTTC----CAAGAAAAGGACGAAGATGACAATTTAAT---------TGATTCTTACTTTGTAGTTAAGAG

Section 1954

(150382)	150382 150392 150403 150414 150425 150436 150447 150458
Candidatus Carsonella ruddii strain BT chromosome... (150142)	ATAAAATAAAAAAAATTTTTCATTTTGAAAATAATTTTTTAGGAAAAAATTTAACAATTGGTTATAATTTTTGTTGG
Candidatus Carsonella 135557 - 167442nt (14586)	ATAAAATAAAAAAAATTTTTCATTTTGAAAATAATTTTTTAGGAAAAAATTTAACAATTGGTTATAATTTTTGTTGG
SARS-CoV-2 Reference Genome NC_045512.2 (13662)	ACACACTTTCTCTAACTACCAACATGAAGAAACAATTTATA---ATTTACTTAAGGATTGTCCAGC-----TGTTGC

Section 1955

(150459)	150459 150469 150480 150491 150502 150513 150524 150535
Candidatus Carsonella ruddii strain BT chromosome... (150219)	TATAATATATTTTTTTTAAAAAAAAAATTATTAATAATGTCTGTGTTACCTGGATTTGGTAATCAAAAATTTTTAAA
Candidatus Carsonella 135557 - 167442nt (14663)	TATAATATATTTTTTTTAAAAAAAAAATTATTAATAATGTCTGTGTTACCTGGATTTGGTAATCAAAAATTTTTAAA
SARS-CoV-2 Reference Genome NC_045512.2 (13731)	TA-AACATGACTTCTTTA-------AGTTTAGAATA--GACGGTG--ACATGG--TACCACATATATCACGTCAACG

Section 1956

(150536)	150536 150546 150557 150568 150579 150590 150601 150612
Candidatus Carsonella ruddii strain BT chromosome... (150296)	AAAAACTAAATACATATATATTAAAAATCAAAATATAGATGGTGGCATT---AATAAAATAATTTATAAATTTTTAA
Candidatus Carsonella 135557 - 167442nt (14740)	AAAAACTAAATACATATATATTAAAAATCAAAATATAGATGGTGGCATT---AATAAAATAATTTATAAATTTTTAA
SARS-CoV-2 Reference Genome NC_045512.2 (13794)	TCTTACTAAATACACA-ATGGCAGACCTCGTC-TATGCTTTAAGGCATTTTGATGAAGGTAATTGTGACACATTAAA

```
                                                             Section 1957
                                            (150613) 150613    150623      150634      150645      150656      150667      150678 150689
Candidatus Carsonella ruddii strain BT chromosome... (150370) AAAATTATTTTAATAAAATAATTATAGGGTCTAATATTATAAATTTTAAAAAAATAAATTCGTTTTTTTTTTATAAT
           Candidatus Carsonella 135557 - 167442nt   (14814) AAAATTATTTTAATAAAATAATTATAGGGTCTAATATTATAAATTTTAAAAAAATAAATTCGTTTTTTTTTTATAAT
        SARS-CoV-2 Reference Genome NC_045512.2      (13869) AGAAATACTTGTCACATACAATTGTTGTGATGATGATTAT----TTCAATAAAAAGGACTGGTATGATT-------T

                                                             Section 1958
                                            (150690) 150690    150700      150711      150722      150733      150744      150755 150766
Candidatus Carsonella ruddii strain BT chromosome... (150447) TTTATAAAAAATGAATATTTAAATTAAATAAAAAATTTTATTTTAA-GTATTAACTA---TTTAATAATTCAATATT
           Candidatus Carsonella 135557 - 167442nt   (14891) TTTATAAAAAATGAATATTTAAATTAAATAAAAAATTTTATTTTAA-GTATTAACTA---TTTAATAATTCAATATT
        SARS-CoV-2 Reference Genome NC_045512.2      (13935) TGTAGAAAACCCAGATATATTACGCGTATACGCCAACTTAGGTGAACGTGTACGCCAAGCTTTGTTAAA--AACAGT

                                                             Section 1959
                                            (150767) 150767    150777      150788      150799      150810      150821      150832 150843
Candidatus Carsonella ruddii strain BT chromosome... (150520) ACCTCCTTGAGCTGCAATATTTATTGTTACTGTTTTTTCATTTATTAATGAATTAATATAATTACCACCTCCTGCTT
           Candidatus Carsonella 135557 - 167442nt   (14964) ACCTCCTTGAGCTGCAATATTTATTGTTACTGTTTTTTCATTTATTAATGAATTAATATAATTACCACCTCCTGCTT
        SARS-CoV-2 Reference Genome NC_045512.2      (14010) ACAATTCTGTGATGCCATGCGAAATGCTGGTATTGTTGG----TGTACTGACATTAGATAATCAAGATCTCAA---T

                                                             Section 1960
                                            (150844) 150844    150854      150865      150876      150887      150898      150909 150920
Candidatus Carsonella ruddii strain BT chromosome... (150597) TTGGTCCAGTACCAGATAAATTACATCCACCAAAAGGTTGCATACCAACCATTGCTCCTATAGTATTTCTATTTATA
           Candidatus Carsonella 135557 - 167442nt   (15041) TTGGTCCAGTACCAGATAAATTACATCCACCAAAAGGTTGCATACCAACCATTGCTCCTATAGTATTTCTATTTATA
        SARS-CoV-2 Reference Genome NC_045512.2      (14080) G-------GTAACTGGTA--TGATTTCGGT-----GATTTCATACAAACCACGCCAGGTAGTGGAGTTCCTGTTGTA

                                                             Section 1961
                                            (150921) 150921    150931      150942      150953      150964      150975      150986 150997
Candidatus Carsonella ruddii strain BT chromosome... (150674) TAAATATTTCCTATATTTAATTTATTAGCTAAATATTTATAAAAAGATTCATTTCTACTATGAATTCCTAAAGTTA-
           Candidatus Carsonella 135557 - 167442nt   (15118) TAAATATTTCCTATATTTAATTTATTAGCTAAATATTTATAAAAAGATTCATTTCTACTATGAATTCCTAAAGTTA-
        SARS-CoV-2 Reference Genome NC_045512.2      (14143) GA-----TTCTTATTATTCATT------GTTAATGCCTATATTAACCTTGACCAGGGCTTTAACTGC--AGAGTCAC

                                                             Section 1962
                                            (150998) 150998    151008      151019      151030      151041      151052      151063 151074
Candidatus Carsonella ruddii strain BT chromosome... (150750) ATCCATATTCTACATTATTAATATCTTTTATTATATCATTTAATTGTTCGTTTTTATAAATTGCTACATGCAATATA
           Candidatus Carsonella 135557 - 167442nt   (15194) ATCCATATTCTACATTATTAATATCTTTTATTATATCATTTAATTGTTCGTTTTTATAAATTGCTACATGCAATATA
        SARS-CoV-2 Reference Genome NC_045512.2      (14207) ATGTTGACACTGACTTAACAAAGCCTTA--------CATTAAGTGGGATTTGTTAAAATATGACTTCACGGAAGAGA
```

```
                                                                                    Section 1963
                                                  (151075) 151075    151085     151096     151107     151118     151129     151140 151151
Candidatus Carsonella ruddii strain BT chromosome... (150827) GGACCAAACTGTTCAGATTTTAAATCATTTATTCCATTTACTCTAATAATTGTAGGTTTTATAAAATTTCCATTTAT
        Candidatus Carsonella 135557 - 167442nt (15271) GGACCAAACTGTTCAGATTTTAAATCATTTATTCCATTTACTCTAATAATTGTAGGTTTTATAAAATTTCCATTTAT
   SARS-CoV-2 Reference Genome NC_045512.2 (14276) GGTTAAAACTCTTTGACCGTTATTTTAAATATTGGGATCAGACATACCACCCAAATTGTGTTAACTGTTTGGATGAC
                                                                                    Section 1964
                                                  (151152) 151152    151162     151173     151184     151195     151206     151217 151228
Candidatus Carsonella ruddii strain BT chromosome... (150904) TGGTA-ATTCTGTATTATTAAATTTTTTATCATTTTTTAT----ACTTTT------TAAATATTTTTTTAAATTATT
        Candidatus Carsonella 135557 - 167442nt (15348) TGGTA-ATTCTGTATTATTAAATTTTTTATCATTTTTTAT----ACTTTT------TAAATATTTTTTTAAATTATT
   SARS-CoV-2 Reference Genome NC_045512.2 (14353) AGATGCATTCTGCATTGTGCAAACTTTAATGTTTTATTCTCTACAGTGTTCCCACCTACAAGTTTTGGACCACTAGT
                                                                                    Section 1965
                                                  (151229) 151229    151239     151250     151261     151272     151283     151294 151305
Candidatus Carsonella ruddii strain BT chromosome... (150970) TAAATTTCTATTTGTTATAATTGGACCAATATCAGTTTCAAGATCCAACGGATCTCCTATTTTTAAATTATTTAAAC
        Candidatus Carsonella 135557 - 167442nt (15414) TAAATTTCTATTTGTTATAATTGGACCAATATCAGTTTCAAGATCCAACGGATCTCCTATTTTTAAATTATTTAAAC
   SARS-CoV-2 Reference Genome NC_045512.2 (14430) GAGAAAAATATTTGTTGATGGTGTTCCATTTGTAGTTTCAACTGGATACC-ACTTCAGAGAGCTAGGTGTTGTACAT
                                                                                    Section 1966
                                                  (151306) 151306    151316     151327     151338     151349     151360     151371 151382
Candidatus Carsonella ruddii strain BT chromosome... (151047) ATTCA--AATAAAATTTTAATAGTTGTTAAATAAATTGATTCATTTAAATATAAAAGTCTTAAAGCTGAACAT-CTT
        Candidatus Carsonella 135557 - 167442nt (15491) ATTCA--AATAAAATTTTAATAGTTGTTAAATAAATTGATTCATTTAAATATAAAAGTCTTAAAGCTGAACAT-CTT
   SARS-CoV-2 Reference Genome NC_045512.2 (14506) AATCAGGATGTAAACTTACATAGCTCTAGACT---TAGTTTTAAGGAATTACTTGTGTATGCT-GCTGACCCTGCTA
                                                                                    Section 1967
                                                  (151383) 151383    151393     151404     151415     151426     151437     151448 151459
Candidatus Carsonella ruddii strain BT chromosome... (151121) TGACCACAACTTTTAAAAGAAGATT-CAAGAATATCTATTACTAATTGTTCTATTAATGTAGAAGAATCAGCTATCA
        Candidatus Carsonella 135557 - 167442nt (15565) TGACCACAACTTTTAAAAGAAGATT-CAAGAATATCTATTACTAATTGTTCTATTAATGTAGAAGAATCAGCTATCA
   SARS-CoV-2 Reference Genome NC_045512.2 (14579) TGCACGCTGCTTCTGGTAATCTATTACTAGATAAACG--CACTACGTG--CTTTTCA-GTAGCTGCACTTACTAACA
                                                                                    Section 1968
                                                  (151460) 151460    151470     151481     151492     151503     151514     151525 151536
Candidatus Carsonella ruddii strain BT chromosome... (151197) TTGTGTTTAATCCTCCAGTTTCTGCTATTAATTTATGTATAGG--TCTATTATCT--CTCATAATAAGATTTTTATT
        Candidatus Carsonella 135557 - 167442nt (15641) TTGTGTTTAATCCTCCAGTTTCTGCTATTAATTTATGTATAGG--TCTATTATCT--CTCATAATAAGATTTTTATT
   SARS-CoV-2 Reference Genome NC_045512.2 (14651) ATGTTGCTTTTCAAACTGTCAAACCCGGTAATTTTAACAAAGACTTCTATGACTTTGCTGTGTCTAAGGGTT---TC
```

Section 1969

		(151537) 151537 151547 151558 151569 151580 151591 151602 151613
Candidatus Carsonella ruddii strain BT chromosome...	(151270)	TATAAGATTAGCAATTTTATTAGAACCAGTAAATATAACACCATATATATCTTTATGAAATGATATTAAATTACCAA
Candidatus Carsonella 135557 - 167442nt	(15714)	TATAAGATTAGCAATTTTATTAGAACCAGTAAATATAACACCATATATATCTTTATGAAATGATATTAAATTACCAA
SARS-CoV-2 Reference Genome NC_045512.2	(14725)	TTTAAGGAAGGAAGTTCTGTTG--------AATTAAAACAC----TTCTTCTTTGCTCAG-GATGGTAATGCTGCTA

Section 1970

		(151614) 151614 151624 151635 151646 151657 151668 151679 151690
Candidatus Carsonella ruddii strain BT chromosome...	(151347)	TAAAAGATCCTTTTCCTAAAATTATTTGACAAACACTAACAGGAATACCAGATTT-ATATAATAATTGTATTATTTT
Candidatus Carsonella 135557 - 167442nt	(15791)	TAAAAGATCCTTTTCCTAAAATTATTTGACAAACACTAACAGGAATACCAGATTT-ATATAATAATTGTATTATTTT
SARS-CoV-2 Reference Genome NC_045512.2	(14789)	TCAGCGAT--TATGACTACTATCGTT----ATAATCTACCAACAATGTGTGATATCAGACAACTACTAT-TTGTAGT

Section 1971

		(151691) 151691 151701 151712 151723 151734 151745 151756 151767
Candidatus Carsonella ruddii strain BT chromosome...	(151423)	AAAAGCTATTAATGATGTACTTTCTGCAGGTTTAGCTAACACAACATTTCCTGTAATTAATGCAGATATAGT--TTG
Candidatus Carsonella 135557 - 167442nt	(15867)	AAAAGCTATTAATGATGTACTTTCTGCAGGTTTAGCTAACACAACATTTCCTGTAATTAATGCAGATATAGT--TTG
SARS-CoV-2 Reference Genome NC_045512.2	(14859)	TGAAGTTGTTGATAA-GTACTTT--GATTGTT--------ACGATGGTGGCTGTA-TTAATGCTAACCAAGTCATCG

Section 1972

		(151768) 151768 151778 151789 151800 151811 151822 151833 151844
Candidatus Carsonella ruddii strain BT chromosome...	(151498)	ACCACAAAAAATTGCAATTGGAAAATT--CCAAGGACTAATTGTAGAAATTATACCTTTTCCTTCTATAAAATAAAT
Candidatus Carsonella 135557 - 167442nt	(15942)	ACCACAAAAAATTGCAATTGGAAAATT--CCAAGGACTAATTGTAGAAATTATACCTTTTCCTTCTATAAAATAAAT
SARS-CoV-2 Reference Genome NC_045512.2	(14924)	TCAACAACCTAGACAAATCAGCTGGTTTTCCATTTAATAAATGGGTAAGGCTAGACTTTATTATGATTCAATGAGT

Section 1973

		(151845) 151845 151855 151866 151877 151888 151899 151910 151921
Candidatus Carsonella ruddii strain BT chromosome...	(151573)	ATTGTTTTCTCCTGTATAATTAGGTAAATAAGAAATTTTGCAAATATTATATGCTTGATTTGTATAATATTTGCAAA
Candidatus Carsonella 135557 - 167442nt	(16017)	ATTGTTTTCTCCTGTATAATTAGGTAAATAAGAAATTTTGCAAATATTATATGCTTGATTTGTATAATATTTGCAAA
SARS-CoV-2 Reference Genome NC_045512.2	(15001)	TATGA--------GGATCAAGATGCACTTTTCGCATATACAAAACGTAATGT-CATCCCTACTATAACTCAAATGAA

Section 1974

		(151922) 151922 151932 151943 151954 151965 151976 151987 151998
Candidatus Carsonella ruddii strain BT chromosome...	(151650)	AATCTATTGCTTCTTTAATATCAGAAACTGAATCTAAAATTGTTTTTCCTGCTTCTTTACAACATAAAATTATTAAT
Candidatus Carsonella 135557 - 167442nt	(16094)	AATCTATTGCTTCTTTAATATCAGAAACTGAATCTAAAATTGTTTTTCCTGCTTCTTTACAACATAAAATTATTAAT
SARS-CoV-2 Reference Genome NC_045512.2	(15069)	TCTTAAGTATGCCATTAGTG-CAAAGAATAGAGCTCGCACCGTAGCTGGTGTCTCTATCTG---TAGTACTATGAC-

Section 1975

	(151999)	151999 152009 152020 152031 152042 152053 152064 152075
Candidatus Carsonella ruddii strain BT chromosome...	(151727)	TCAATAAAATTTTTTTGTAATAATGAAATAAATTTTTTAATAAATATAACTTTTTTATCAATGCTAATTAATTTCCA
Candidatus Carsonella 135557 - 167442nt	(16171)	TCAATAAAATTTTTTTGTAATAATGAAATAAATTTTTTAATAAATATAACTTTTTTATCAATGCTAATTAATTTCCA
SARS-CoV-2 Reference Genome NC_045512.2	(15141)	-CAATAGACAGTTTCATCAAAAATTATTGAAA----TCAATAGCCGCCACTAGAGGAGCTACTGTAGTAA--TTGGA

Section 1976

	(152076)	152076 152086 152097 152108 152119 152130 152141 152152
Candidatus Carsonella ruddii strain BT chromosome...	(151804)	AAATTTAAAAGATTGTTTTAATATTTTTATTGAATTATTTATACA-TTTATCATGGTATACAATACCATTAATAAAA
Candidatus Carsonella 135557 - 167442nt	(16248)	AAATTTAAAAGATTGTTTTAATATTTTTATTGAATTATTTATACA-TTTATCATGGTATACAATACCATTAATAAAA
SARS-CoV-2 Reference Genome NC_045512.2	(15211)	ACA---AGCAAATTCTATGGTGGTTGGCACAACATGTTAAAAACTGTTTATAGTGATGTAGAAAACCCTCACC----

Section 1977

	(152153)	152153 152163 152174 152185 152196 152207 152218 152229
Candidatus Carsonella ruddii strain BT chromosome...	(151880)	AATTTATTAGAAGGTGATATTGAAATTATTTTTTTATTTTTATTATTAAAAGAATTAGCAATATAATTTTTTTTATC
Candidatus Carsonella 135557 - 167442nt	(16324)	AATTTATTAGAAGGTGATATTGAAATTATTTTTTTATTTTTATTATTAAAAGAATTAGCAATATAATTTTTTTTATC
SARS-CoV-2 Reference Genome NC_045512.2	(15281)	------TTATGGGTTGGGATTATCCTAAATGTGATAGAGCCATGCCTAACATGCTTAGAATTAT----------GGC

Section 1978

	(152230)	152230 152240 152251 152262 152273 152284 152295 152306
Candidatus Carsonella ruddii strain BT chromosome...	(151957)	AAAAATTATTATTTTTTTAAAAAATACATAAAAATGACAAGAAATGTTTAAATTATAAAAATTAAAAATATCTCTTT
Candidatus Carsonella 135557 - 167442nt	(16401)	AAAAATTATTATTTTTTTAAAAAATACATAAAAATGACAAGAAATGTTTAAATTATAAAAATTAAAAATATCTCTTT
SARS-CoV-2 Reference Genome NC_045512.2	(15342)	CTCACTTGTTCTTGCTCGCAAACATACAACGTGTTGTAGCTTGTCACACCGTTTCTATAGATTAGCTA-ATGAGTGT

Section 1979

	(152307)	152307 152317 152328 152339 152350 152361 152372 152383
Candidatus Carsonella ruddii strain BT chromosome...	(152034)	TACCTCCAAATAAATCTATAGAAAATG-GTAATTTTTTATTATATTT---ATCATTTTTAATAA-ATAATGAATTAA
Candidatus Carsonella 135557 - 167442nt	(16478)	TACCTCCAAATAAATCTATAGAAAATG-GTAATTTTTTATTATATTT---ATCATTTTTAATAA-ATAATGAATTAA
SARS-CoV-2 Reference Genome NC_045512.2	(15418)	GCTCAAGTATTGAGTGAAATGGTCATGTGTGGCGGTTCACTATATGTTAAACCAGGTGGAACCTCATCAGGAGATGC

Section 1980

	(152384)	152384 152394 152405 152416 152427 152438 152449 152460
Candidatus Carsonella ruddii strain BT chromosome...	(152106)	TTAATATTTTTTTCATATTAGTATTTTTATTTACCACTTTGTTTACAAAAGATGAATTTGCACCATTTTCTAATAAT
Candidatus Carsonella 135557 - 167442nt	(16550)	TTAATATTTTTTTCATATTAGTATTTTTATTTACCACTTTGTTTACAAAAGATGAATTTGCACCATTTTCTAATAAT
SARS-CoV-2 Reference Genome NC_045512.2	(15495)	CACAACTGCTTATGCTAATAGTGTTTTTA----ACA-TTTGTC-----AAGCTGTCACGGC--CAATGT-TAATGCA

Section 1981

		152461 152471 152482 152493 152504 152515 152526 152537
	(152461)	
Candidatus Carsonella ruddii strain BT chromosome...	(152183)	CTTCTTACTAAATAAGGTAATAATTCTTTATATTTTCCTATAGGAGCATATTCTCTACATACTATA---ACTTT-TA
Candidatus Carsonella 135557 - 167442nt	(16627)	CTTCTTACTAAATAAGGTAATAATTCTTTATATTTTCCTATAGGAGCATATTCTCTACATACTATA---ACTTT-TA
SARS-CoV-2 Reference Genome NC_045512.2	(15559)	CTTTTATCTACTGATGGTAACAA---------AATTGCCGATA--AGTATGTCCGCAATTTACAACACAGACTTTATG

Section 1982

		152538 152548 152559 152570 152581 152592 152603 152614
	(152538)	
Candidatus Carsonella ruddii strain BT chromosome...	(152256)	ATTTTTTTAATGAGTTATAAACTACATCACCCATACCATATAATTTTTGAAATTCTAATTTTCTATCTTTTCCTAAA
Candidatus Carsonella 135557 - 167442nt	(16700)	ATTTTTTTAATGAGTTATAAACTACATCACCCATACCATATAATTTTTGAAATTCTAATTTTCTATCTTTTCCTAAA
SARS-CoV-2 Reference Genome NC_045512.2	(15626)	AGTGTCTCTAT-AGAAATAGAGATGTTGACACAGAC-------TTTGTGAA-TGAGTTTTACGCATATTTGCGTAAA

Section 1983

		152615 152625 152636 152647 152658 152669 152680 152691
	(152615)	
Candidatus Carsonella ruddii strain BT chromosome...	(152333)	ATTGTAATAA--ATGAAATTGTTTGAATATTATGAGTTGCAAACTGAGGTAAAATATTGTTTTTACAACATTTTGAT
Candidatus Carsonella 135557 - 167442nt	(16777)	ATTGTAATAA--ATGAAATTGTTTGAATATTATGAGTTGCAAACTGAGGTAAAATATTGTTTTTACAACATTTTGAT
SARS-CoV-2 Reference Genome NC_045512.2	(15694)	CATTTCTCAATGATGATACTCTCTGACGATGCTG--TTG-----TGTGTTTCAATAGCACTTATG---CATCTCAAG

Section 1984

		152692 152702 152713 152724 152735 152746 152757 152768
	(152692)	
Candidatus Carsonella ruddii strain BT chromosome...	(152408)	AATAAATACATTGAACAAAATAAATATGATAAATCAGTGCAAAATTTTTTAATATAAACTGGATACATATTTAAATT
Candidatus Carsonella 135557 - 167442nt	(16852)	AATAAATACATTGAACAAAATAAATATGATAAATCAGTGCAAAATTTTTTAATATAAACTGGATACATATTTAAATT
SARS-CoV-2 Reference Genome NC_045512.2	(15761)	GTCTAGTGGCTAGCATAAAGAACTT----TAAGTCAGTTCTTTATTATCAAA---ACAATGTTTTTATGTCTGAAGC

Section 1985

		152769 152779 152790 152801 152812 152823 152834 152845
	(152769)	
Candidatus Carsonella ruddii strain BT chromosome...	(152485)	TAAAAATTGTGC--ATTTTTAACTTCATAATCCCAATAAGCACCTTTAA-CTAATCTTACA--GGTATTTTT---GT
Candidatus Carsonella 135557 - 167442nt	(16929)	TAAAAATTGTGC--ATTTTTAACTTCATAATCCCAATAAGCACCTTTAA-CTAATCTTACA--GGTATTTTT---GT
SARS-CoV-2 Reference Genome NC_045512.2	(15831)	AAAATGTTGGACTGAGACTGACCTTACTAAAGGACCTCATGAATTTTGCTCTCAACATACAATGCTAGTTAAACAGG

Section 1986

		152846 152856 152867 152878 152889 152900 152911 152922
	(152846)	
Candidatus Carsonella ruddii strain BT chromosome...	(152554)	TTGTTTATCATAAGCAATTTTATTAATCCAATATAAAGCAGGTAAAGCTCTTTTAGAATAAGCTTGAATTACCAACC
Candidatus Carsonella 135557 - 167442nt	(16998)	TTGTTTATCATAAGCAATTTTATTAATCCAATATAAAGCAGGTAAAGCTCTTTTAGAATAAGCTTGAATTACCAACC
SARS-CoV-2 Reference Genome NC_045512.2	(15908)	GTGATGATTATGTGTACCTTCCTTA--CCCAGATCCATCAAG--AA--TC--TAGGGGCCGGCTGTTTTGTAGATG

Section 1987

	(152923)	152923 152933 152944 152955 152966 152977 152988 152999
Candidatus Carsonella ruddii strain BT chromosome...	(152631)	CAAAACTATTCCAATATAAACAATATTTTGTATAAAATATTTCACTAAATAAATATAAATTTAATTCCATTCTATCA
Candidatus Carsonella 135557 - 167442nt	(17075)	CAAAACTATTCCAATATAAACAATATTTTGTATAAAATATTTCACTAAATAAATATAAATTTAATTCCATTCTATCA
SARS-CoV-2 Reference Genome NC_045512.2	(15977)	ATATCGTAAAAACAGATGGTACAC-TTATGATTGAACGGTTCGTGTCTTTAGCTATAGATGCTTACCCACTTACTAA

Section 1988

	(153000)	153000 153010 153021 153032 153043 153054 153065 153076
Candidatus Carsonella ruddii strain BT chromosome...	(152708)	CATTCTTCAGCATCTATTGTTAAAGATGAATTTGCTTTTTTAACACCTATTACTAAAATTTTTAAAATAGGAATTAA
Candidatus Carsonella 135557 - 167442nt	(17152)	CATTCTTCAGCATCTATTGTTAAAGATGAATTTGCTTTTTTAACACCTATTACTAAAATTTTTAAAATAGGAATTAA
SARS-CoV-2 Reference Genome NC_045512.2	(16053)	ACATCCTAATCAGGAGTA--TGCTGATGTCTTT-CATTTGTACTTACAATACATAAGAAAGCTACATGATGAGTTAA

Section 1989

	(153077)	153077 153087 153098 153109 153120 153131 153142 153153
Candidatus Carsonella ruddii strain BT chromosome...	(152785)	ATCTCTATATACTATTTGTCTATTAGAAAAAGAATATTTAGGATTTAAAGCTGATAATTTAATAGAAACAGTTGGAA
Candidatus Carsonella 135557 - 167442nt	(17229)	ATCTCTATATACTATTTGTCTATTAGAAAAAGAATATTTAGGATTTAAAGCTGATAATTTAATAGAAACAGTTGGAA
SARS-CoV-2 Reference Genome NC_045512.2	(16127)	CAGGACACATGTTAGACATGTATTCTGTTATGCTTACTAATGAT--AACACTTC-AAGGTATTGGGAACCTGAGTTT

Section 1990

	(153154)	153154 153164 153175 153186 153197 153208 153219 153230
Candidatus Carsonella ruddii strain BT chromosome...	(152862)	TACGAT-CTATATTTAAACCTAAATAACTATTACTAATATTATTAATTATTTTTAAATATTGATTAAAAAATTTTTT
Candidatus Carsonella 135557 - 167442nt	(17306)	TACGAT-CTATATTTAAACCTAAATAACTATTACTAATATTATTAATTATTTTTAAATATTGATTAAAAAATTTTTT
SARS-CoV-2 Reference Genome NC_045512.2	(16201)	TATGAGGCTATGTACACACCGCATACAGTCTTAC--AGGCTGTTGGGGCTTGTGTTCTTTGCAATTCACAGACTT--

Section 1991

	(153231)	153231 153241 153252 153263 153274 153285 153296 153307
Candidatus Carsonella ruddii strain BT chromosome...	(152938)	TGCATCATAATATGTTAATGCTGCTTCACCTAACATATCAAAAGAATAAATATTTTTATCTGATAAAGATTTTTTTA
Candidatus Carsonella 135557 - 167442nt	(17382)	TGCATCATAATATGTTAATGCTGCTTCACCTAACATATCAAAAGAATAAATATTTTTATCTGATAAAGATTTTTTTA
SARS-CoV-2 Reference Genome NC_045512.2	(16274)	--CAT--TAAGATG----TGGTGCTTG------CATACGTAGACCAT------TCTTATGTTGTAA--ATGCTGTTA

Section 1992

	(153308)	153308 153318 153329 153340 153351 153362 153373 153384
Candidatus Carsonella ruddii strain BT chromosome...	(153015)	TAGCTGAATTTTCATTTGAAGAATAAACAAATTTTTTTCCTATATTTTTCATAACATAATTTGAAAAAAAAACAATT
Candidatus Carsonella 135557 - 167442nt	(17459)	TAGCTGAATTTTCATTTGAAGAATAAACAAATTTTTTTCCTATATTTTTCATAACATAATTTGAAAAAAAAACAATT
SARS-CoV-2 Reference Genome NC_045512.2	(16329)	CGACCA---TGTCATATCAACATCACATAAATTAGTCTTGTCTGTTAATC----CGTATGTTTGCAATGCTCCAGGT

Section 1993

		153385 153395 153406 153417 153428 153439 153450 153461
	(153385)	
Candidatus Carsonella ruddii strain BT chromosome...	(153092)	ATTTTTCTAAAAACAAATATAATTAAATTATAATTA-----TTTACATTTTTTTTGTAAAAAAAATTTGAAACAAAA
Candidatus Carsonella 135557 - 167442nt	(17536)	ATTTTTCTAAAAACAAATATAATTAAATTATAATTA-----TTTACATTTTTTTTGTAAAAAAAATTTGAAACAAAA
SARS-CoV-2 Reference Genome NC_045512.2	(16399)	TGTGATGTCA---CAGATGTGACTCAACTTTACTTAGGAGGTATGAGCTATTATTGTAAATCACATAAACCACCCAT

Section 1994

		153462 153472 153483 153494 153505 153516 153527 153538
	(153462)	
Candidatus Carsonella ruddii strain BT chromosome...	(153164)	TCTATTATCATATTATAAAAAATAATTTTCCAATATTCTGAAGA-ATAATAATATTTCCATTCTTCAAAAGATAATT
Candidatus Carsonella 135557 - 167442nt	(17608)	TCTATTATCATATTATAAAAAATAATTTTCCAATATTCTGAAGA-ATAATAATATTTCCATTCTTCAAAAGATAATT
SARS-CoV-2 Reference Genome NC_045512.2	(16473)	TAGTTTTCCATTGTGTG----CTAATGGACAAGTTTTTGGTTTATATAAAAATACATGTGTTGGT--AGCGATAATG

Section 1995

		153539 153549 153560 153571 153582 153593 153604 153615
	(153539)	
Candidatus Carsonella ruddii strain BT chromosome...	(153240)	TATCTTTTATAAAAGAATTAGATGTAAAATAATCTGGAATTCTTAATAA--AGATTCTGCTAAACACATCATTTGTA
Candidatus Carsonella 135557 - 167442nt	(17684)	TATCTTTTATAAAAGAATTAGATGTAAAATAATCTGGAATTCTTAATAA--AGATTCTGCTAAACACATCATTTGTA
SARS-CoV-2 Reference Genome NC_045512.2	(16544)	TTACTGACTTTAATGCAATTGCAACATGTGACTGGACAAATGCTGGTGATTACATTTTAGCTAACACCTGTACTGAA

Section 1996

		153616 153626 153637 153648 153659 153670 153681 153692
	(153616)	
Candidatus Carsonella ruddii strain BT chromosome...	(153315)	TTCCTTCCTTAGTACTTAAATTATAAATATTTAATA-TATTATCTATATTATCTAAAT---TTGTGTTTTTTCTACT
Candidatus Carsonella 135557 - 167442nt	(17759)	TTCCTTCCTTAGTACTTAAATTATAAATATTTAATA-TATTATCTATATTATCTAAAT---TTGTGTTTTTTCTACT
SARS-CoV-2 Reference Genome NC_045512.2	(16621)	AGACT--CAAGCTTTTTGCAGCAGAAACGCTCAAAGCTACTGAGGAGACATTTAAACTGTCTTATGGTATTGCTACT

Section 1997

		153693 153703 153714 153725 153736 153747 153758 153769
	(153693)	
Candidatus Carsonella ruddii strain BT chromosome...	(153388)	TT-CATTAATTAAACTATAAGAAAATGTTTCTACATTATCGAAAAAATCATTTGAAAAAGAACAATTTTTTAAA-AT
Candidatus Carsonella 135557 - 167442nt	(17832)	TT-CATTAATTAAACTATAAGAAAATGTTTCTACATTATCGAAAAAATCATTTGAAAAAGAACAATTTTTTAAA-AT
SARS-CoV-2 Reference Genome NC_045512.2	(16696)	GTACGTGAAGTGCTGTCTGACAGAGAATTACATCTTTCATGGGAAGTTGGTAA-ACCTAGACCACCACTTAACCGAA

Section 1998

		153770 153780 153791 153802 153813 153824 153835 153846
	(153770)	
Candidatus Carsonella ruddii strain BT chromosome...	(153463)	ATTAAATAAATAGATATTTTCATCTATTAAGTAATATTTACTTACTATGTTTAATAAAAAATTATAAGTCATATTTT
Candidatus Carsonella 135557 - 167442nt	(17907)	ATTAAATAAATAGATATTTTCATCTATTAAGTAATATTTACTTACTATGTTTAATAAAAAATTATAAGTCATATTTT
SARS-CoV-2 Reference Genome NC_045512.2	(16772)	ATTATGTCTTTACTGGTTATCGTGTAACTAAAAACAGTAAAGTACAAATAGGAGAGTACACCTTTGAAAAAGGTGAC

Section 1999

		153847 153857 153868 153879 153890 153901 153912 153923
	(153847)	
Candidatus Carsonella ruddii strain BT chromosome...	(153540)	TTTAAATTATAATAATGTTTAATTGGGTT--AGTAATTATATATATAAAGCTCCTATTTTTGTAAGTGAAGATTTGA
Candidatus Carsonella 135557 - 167442nt	(17984)	TTTAAATTATAATAATGTTTAATTGGGTT--AGTAATTATATATATAAAGCTCCTATTTTTGTAAGTGAAGATTTGA
SARS-CoV-2 Reference Genome NC_045512.2	(16849)	TATGG-TGATGCTGTTGTTTACCGAGGTACAACAACTTACAAATTAAATGTTGGTGATTAT-TTTGTGCTGACATCA

Section 2000

		153924 153934 153945 153956 153967 153978 153989 154000
	(153924)	
Candidatus Carsonella ruddii strain BT chromosome...	(153615)	GAGATGGAAATCAATCTTTAATTAATGGATATGATATTAAAAAAAAAATAGAAATTTGGAATATTCTTTTAAATATA
Candidatus Carsonella 135557 - 167442nt	(18059)	GAGATGGAAATCAATCTTTAATTAATGGATATGATATTAAAAAAAAAATAGAAATTTGGAATATTCTTTTAAATATA
SARS-CoV-2 Reference Genome NC_045512.2	(16924)	CATACAGTAATGCC------ATTAAGTGCACCTACACTAGTGCCACAAGAGCACTATGTTAGAATTACTGGCTTATA

Section 2001

		154001 154011 154022 154033 154044 154055 154066 154077
	(154001)	
Candidatus Carsonella ruddii strain BT chromosome...	(153692)	GGAATAAAACAA-ATCGTTATTGGGTTTCCTTCATCAAATAAACATGATTACAATTTTATAAAGTATTTAATTAATA
Candidatus Carsonella 135557 - 167442nt	(18136)	GGAATAAAACAA-ATCGTTATTGGGTTTCCTTCATCAAATAAACATGATTACAATTTTATAAAGTATTTAATTAATA
SARS-CoV-2 Reference Genome NC_045512.2	(16995)	CCCAACACTCAATATCTCAGATGAGTTTTTCTAGCAATGTTGCAAATTATCAAAGGTTGGTATGCA--AAAGTATTC

Section 2002

		154078 154088 154099 154110 154121 154132 154143 154154
	(154078)	
Candidatus Carsonella ruddii strain BT chromosome...	(153768)	ATAAATTAATTAAAAAAAATATAACTGTATCTGTATTAGTTCCAT-GTAAAAAAAATCTTATAGATTTAAGTTTTAA
Candidatus Carsonella 135557 - 167442nt	(18212)	ATAAATTAATTAAAAAAAATATAACTGTATCTGTATTAGTTCCAT-GTAAAAAAAATCTTATAGATTTAAGTTTTAA
SARS-CoV-2 Reference Genome NC_045512.2	(17070)	TACACTCCAGGGACCACCTGGTACTGGTAAGAGTCATTTTGCTATTGGCCTAGCTCTCTACTACCCTTCTGCTCGCA

Section 2003

		154155 154165 154176 154187 154198 154209 154220 154231
	(154155)	
Candidatus Carsonella ruddii strain BT chromosome...	(153844)	TA-TGATTAAAAATTTAGATAATGCAGTTTTACATTTATATAATTCTGTATCTAAAAATCAAAGAAAAAAAGTATTT
Candidatus Carsonella 135557 - 167442nt	(18288)	TA-TGATTAAAAATTTAGATAATGCAGTTTTACATTTATATAATTCTGTATCTAAAAATCAAAGAAAAAAAGTATTT
SARS-CoV-2 Reference Genome NC_045512.2	(17147)	TAGTGTATACAGCTTGCTCTCATGCCGCTGT---------TGATGCACTATGTGAGAAG----GCATTAAAATATTT

Section 2004

		154232 154242 154253 154264 154275 154286 154297 154308
	(154232)	
Candidatus Carsonella ruddii strain BT chromosome...	(153920)	AAATTTAATAAAAAAAAAACTTTGAAATTTTCATTAGAAAGTA-TAAAATATACTATTTCAATTATAAAAAAAAAATA
Candidatus Carsonella 135557 - 167442nt	(18364)	AAATTTAATAAAAAAAAAACTTTGAAATTTTCATTAGAAAGTA-TAAAATATACTATTTCAATTATAAAAAAAAAATA
SARS-CoV-2 Reference Genome NC_045512.2	(17211)	GCCT---ATAGATAAATGTAGTAGAATTATACCT--GCACGTGCTCGTGTAGAGTGTTT---TGATAAATTCAAAGT

Section 2005

(154309) 154309 154319 154330 154341 154352 154363 154374 154385
Candidatus Carsonella ruddii strain BT chromosome... (153996) AAAACATTCATTTTCAATATTCTC-CTGAAAGTTTTCAGATAGTGAATTATTTTTTCAAAAAAATTTCTTTTA
Candidatus Carsonella 135557 - 167442nt (18440) AAAACATTCATTTTCAATATTCTC-CTGAAAGTTTTCAGATAGTGAATTATTTTTTCAAAAAAATTTCTTTTA
SARS-CoV-2 Reference Genome NC_045512.2 (17280) GAA---------TTCAACATTAGAACAGTATGTCTTTTGTACTGTAAATGCATTGCCTGAGACGACAGCAGATAT-A

Section 2006

(154386) 154386 154396 154407 154418 154429 154440 154451 154462
Candidatus Carsonella ruddii strain BT chromosome... (154072) TTTTCTTATTTATGTTATTTAAATAAA-ATAAAATCTATTTTAAAT-TTACCAATAACAGTAGATAATATATTACC
Candidatus Carsonella 135557 - 167442nt (18516) TTTTCTTATTTATGTTATTTAAATAAA-ATAAAATCTATTTTAAAT-TTACCAATAACAGTAGATAATATATTACC
SARS-CoV-2 Reference Genome NC_045512.2 (17347) GTTGTCT--TTGATGAAATTTCAATGGCCACAAATTATGATTTGAGTGTTGTCAATGCCAG----------ATTACG

Section 2007

(154463) 154463 154473 154484 154495 154506 154517 154528 154539
Candidatus Carsonella ruddii strain BT chromosome... (154147) TAATAAATATATTAATTCAATTAAATATATTACAAAACATAATTTTAATTCTGTTTTATCTG-CTCATAT-ACATAA
Candidatus Carsonella 135557 - 167442nt (18591) TAATAAATATATTAATTCAATTAAATATATTACAAAACATAATTTTAATTCTGTTTTATCTG-CTCATAT-ACATAA
SARS-CoV-2 Reference Genome NC_045512.2 (17412) TGCTAAGCACTATGTGTACATTGGCGACCCTGCTCAAT-TACCTGCACCACGCACATTGCTAACTAAGGGCACACTA

Section 2008

(154540) 154540 154550 154561 154572 154583 154594 154605 154616
Candidatus Carsonella ruddii strain BT chromosome... (154222) TGATATGGGTAATGCAATTTCTAATTCAATTTTATCATTATTATCTAGTATAAAAAGAATAGAAGGAACTATTTT--
Candidatus Carsonella 135557 - 167442nt (18666) TGATATGGGTAATGCAATTTCTAATTCAATTTTATCATTATTATCTAGTATAAAAAGAATAGAAGGAACTATTTT--
SARS-CoV-2 Reference Genome NC_045512.2 (17488) GAACCAGAATATTTCAATTCAGTGTGTAGACTTATGAAACTATAG-GTCCAGACATGTTCCTCGGAACTTGTCGGC

Section 2009

(154617) 154617 154627 154638 154649 154660 154671 154682 154693
Candidatus Carsonella ruddii strain BT chromosome... (154297) -TGGTAATGGAGAAAGAGCAGGTAATGC-ATCATTATTAATTATAGCTTTAAATTTATATAATTTAGGTATTAATCC
Candidatus Carsonella 135557 - 167442nt (18741) -TGGTAATGGAGAAAGAGCAGGTAATGC-ATCATTATTAATTATAGCTTTAAATTTATATAATTTAGGTATTAATCC
SARS-CoV-2 Reference Genome NC_045512.2 (17564) GTTGTCCTGCTGAAATTGTTGACACTGTGAGTGCTTTGGTTTATGATAATAAGCTTAAAGCACATAAAGACAAATC-

Section 2010

(154694) 154694 154704 154715 154726 154737 154748 154759 154770
Candidatus Carsonella ruddii strain BT chromosome... (154372) TAAAATTAATATTTTCAAACACAATTTATTAAATTATTTTT-CAAATGAAAAAATATCTTTATTAAATAGATTACC-
Candidatus Carsonella 135557 - 167442nt (18816) TAAAATTAATATTTTCAAACACAATTTATTAAATTATTTTT-CAAATGAAAAAATATCTTTATTAAATAGATTACC-
SARS-CoV-2 Reference Genome NC_045512.2 (17640) --AGCTCAATGCTTT--AAAATGTTTTATAAGGGTGTTATCACGCATGATGTTTCATCTGCAATTAACAGGCCACAA

Section 2011

	(154771)	154771 154781 154792 154803 154814 154825 154836 154847
Candidatus Carsonella ruddii strain BT chromosome...	(154447)	-TTGGTTTAGTAGATTGAATTATT--CTGCTTTTTCTGGTAGTCATCAAGATGCTATTTTTAAATCATATTTTAAAA
Candidatus Carsonella 135557 - 167442nt	(18891)	-TTGGTTTAGTAGATTGAATTATT--CTGCTTTTTCTGGTAGTCATCAAGATGCTATTTTTAAATCATATTTTAAAA
SARS-CoV-2 Reference Genome NC_045512.2	(17713)	ATAGGCGTGGTA-AGAGAATTCCTTACACGTAACCCTGCTTGGAGAAAAGCTGTCTTTATTTCACCTTATAATTCAC

Section 2012

	(154848)	154848 154858 154869 154880 154891 154902 154913 154924
Candidatus Carsonella ruddii strain BT chromosome...	(154521)	AAAAATTTTTTTTATGGAAAGTATTATATATACCAATAAATCCTAAAATTTTTCATTTTTCTCATAAAAATATAATA
Candidatus Carsonella 135557 - 167442nt	(18965)	AAAAATTTTTTTTATGGAAAGTATTATATATACCAATAAATCCTAAAATTTTTCATTTTTCTCATAAAAATATAATA
SARS-CoV-2 Reference Genome NC_045512.2	(17789)	AGAATGCTGTAGCCTCAAAGATTTTGGGACTACCAA------CTCAAACTGTTGATTCATCACAGGGCTC-AGAATA

Section 2013

	(154925)	154925 154935 154946 154957 154968 154979 154990 155001
Candidatus Carsonella ruddii strain BT chromosome...	(154598)	AAAATAAATTCTCAATCAGGAAAAGGAGGTTTAAAATTTATTTTTTGGTATAATTATAATTTATTGTTAAATAATTT
Candidatus Carsonella 135557 - 167442nt	(19042)	AAAATAAATTCTCAATCAGGAAAAGGAGGTTTAAAATTTATTTTTTGGTATAATTATAATTTATTGTTAAATAATTT
SARS-CoV-2 Reference Genome NC_045512.2	(17859)	TGACTATGT-CATATTCACTCAAACCAC-TGAAACAGCTCACTCTTG---TAATGTAAACAGATT------TAATGT

Section 2014

	(155002)	155002 155012 155023 155034 155045 155056 155067 155078
Candidatus Carsonella ruddii strain BT chromosome...	(154675)	TATTTTTATAAAAATATATAATATTATTCAAGATTTATCTGAAATTTTAATGTGTGAAATTTATAAAGAAATGATAT
Candidatus Carsonella 135557 - 167442nt	(19119)	TATTTTTATAAAAATATATAATATTATTCAAGATTTATCTGAAATTTTAATGTGTGAAATTTATAAAGAAATGATAT
SARS-CoV-2 Reference Genome NC_045512.2	(17925)	TGCTATTACCAGA----GCAAAAGTAGGCAT-ACTTTGCATAATGTCTGATA-GAGACCTTTATGACAAGTTGCAAT

Section 2015

	(155079)	155079 155089 155100 155111 155122 155133 155144 155155
Candidatus Carsonella ruddii strain BT chromosome...	(154752)	TTTCTATATTTTATTTAAGATCAAATAATAT---AATTTTAAAAAAAATTAAAATAATAAATTTTTATTATTCTTTT
Candidatus Carsonella 135557 - 167442nt	(19196)	TTTCTATATTTTATTTAAGATCAAATAATAT---AATTTTAAAAAAAATTAAAATAATAAATTTTTATTATTCTTTT
SARS-CoV-2 Reference Genome NC_045512.2	(17996)	TTACAAGTCTTGAAATTCCACGTAGGAATGTGGCAACTTTACAAGCTGAAAATGTAACAGGACTCTTTAAAGATTGT

Section 2016

	(155156)	155156 155166 155177 155188 155199 155210 155221 155232
Candidatus Carsonella ruddii strain BT chromosome...	(154826)	ATTTTCAATTTTAAAATTATCGTTTTAATAATAAAAAAAAACAAAAAAAAAATAATAAAAAATATTATAATTAAAAA
Candidatus Carsonella 135557 - 167442nt	(19270)	ATTTTCAATTTTAAAATTATCGTTTTAATAATAAAAAAAAACAAAAAAAAAATAATAAAAAATATTATAATTAAAAA
SARS-CoV-2 Reference Genome NC_045512.2	(18073)	AGT---AAGGT--AATCACTGGGTTACATCCTACACAGGCACCTACACACCTCAGTGTTGAC-ACTAAATTCAAAAC

Section 2017

		155233 155243 155254 155265 155276 155287 155298 155309
	(155233)	
Candidatus Carsonella ruddii strain BT chromosome...	(154903)	TGAATAATACTTATGGAGAAATAATTAAAATTTCTACTTTTG-GAGAAAGTCATGGATTATTAATAGGAGCATTAAT
Candidatus Carsonella 135557 - 167442nt	(19347)	TGAATAATACTTATGGAGAAATAATTAAAATTTCTACTTTTG-GAGAAAGTCATGGATTATTAATAGGAGCATTAAT
SARS-CoV-2 Reference Genome NC_045512.2	(18144)	TGAAGGTTTATG-TGTTGACATACCTGGCATACCTAAGGACATGACCTATAGAAGACTCATCTCTATGATGGGTTTT

Section 2018

		155310 155320 155331 155342 155353 155364 155375 155386
	(155310)	
Candidatus Carsonella ruddii strain BT chromosome...	(154979)	AGATGGTTATTTTCACAATAAATATATTTTTGAAAAAACAATTCAAGAAAAATTAAATTTAAGAAAACCTTTTACAT
Candidatus Carsonella 135557 - 167442nt	(19423)	AGATGGTTATTTTCACAATAAATATATTTTTGAAAAAACAATTCAAGAAAAATTAAATTTAAGAAAACCTTTTACAT
SARS-CoV-2 Reference Genome NC_045512.2	(18220)	AAAATGA-ATTATCAAGTTAATGGTTACCCTAACATGTTTATCACCCGCGAAGAAGCTATAAGACATGTACGTGCAT

Section 2019

		155387 155397 155408 155419 155430 155441 155452 155463
	(155387)	
Candidatus Carsonella ruddii strain BT chromosome...	(155056)	CT-TTATTTTCTACACAAAGAAAAGAATTAGATAAAGTAAAAATTTTTACTGGAGTATTTAATTCTAAAACTACAGG
Candidatus Carsonella 135557 - 167442nt	(19500)	CT-TTATTTTCTACACAAAGAAAAGAATTAGATAAAGTAAAAATTTTTACTGGAGTATTTAATTCTAAAACTACAGG
SARS-CoV-2 Reference Genome NC_045512.2	(18296)	GGATTGGCTTCGATGTCGAGGGGTGTCAT-GCTACTAGAGAAGCTGTTGGTACCA-ATTTACCTTTACAGCT--AGG

Section 2020

		155464 155474 155485 155496 155507 155518 155529 155540
	(155464)	
Candidatus Carsonella ruddii strain BT chromosome...	(155132)	ATGTCCGATATTAATGATAATAAAAAATCATGATAATAAAAGTTTAGATTATAATAATATATCTAAAAATTTTAGAC
Candidatus Carsonella 135557 - 167442nt	(19576)	ATGTCCGATATTAATGATAATAAAAAATCATGATAATAAAAGTTTAGATTATAATAATATATCTAAAAATTTTAGAC
SARS-CoV-2 Reference Genome NC_045512.2	(18369)	TTTTTCTACAG--GTGTTAACCTAGT-TGCTGTACCTACAGGTTATGTTGATACACCTAATAATACAGATTTTTCCA

Section 2021

		155541 155551 155562 155573 155584 155595 155606 155617
	(155541)	
Candidatus Carsonella ruddii strain BT chromosome...	(155209)	CAGGACATGCTGATTTAAC------ATATCAATTAAAATA--TAAATTTAGAGAT-TATAGAGGAGGTGGTAG--AT
Candidatus Carsonella 135557 - 167442nt	(19653)	CAGGACATGCTGATTTAAC------ATATCAATTAAAATA--TAAATTTAGAGAT-TATAGAGGAGGTGGTAG--AT
SARS-CoV-2 Reference Genome NC_045512.2	(18443)	GAGTTAGTGCTAAACCACCGCCTGGAGATCAATTTAAACACCTCATACCACTTATGTACAAAGGACTTCCTTGGAAT

Section 2022

		155618 155628 155639 155650 155661 155672 155683 155694
	(155618)	
Candidatus Carsonella ruddii strain BT chromosome...	(155275)	CTAGTGCTAGAGAAACCGCTTGTAGAGTTGCTTCAGGAGCAATATTTACTAATATTTTGTTTAATAAAGGTATTT-T
Candidatus Carsonella 135557 - 167442nt	(19719)	CTAGTGCTAGAGAAACCGCTTGTAGAGTTGCTTCAGGAGCAATATTTACTAATATTTTGTTTAATAAAGGTATTT-T
SARS-CoV-2 Reference Genome NC_045512.2	(18520)	GTAGTGCGT-ATAAA--GATTGTACAAATG--TTAAGTGACACACTTAAAAATCTCTC-TGACAGAGTCGTATTTGT

```
                                                                                     Section 2023
                                                       (155695) 155695    155705     155716     155727     155738     155749     155760 155771
Candidatus Carsonella ruddii strain BT chromosome... (155351) AATAAGATCATATATAAAAAAAATTGGATTTTTAAAAATTAATTTTTATTATTGGAAAAATATTATAAATAGATTTT
               Candidatus Carsonella 135557 - 167442nt  (19795) AATAAGATCATATATAAAAAAAATTGGATTTTTAAAAATTAATTTTTATTATTGGAAAAATATTATAAATAGATTTT
         SARS-CoV-2 Reference Genome NC_045512.2  (18591) CTTATGGGCACATGGCTTTGAG--TTGACATCTATGAAGTATTTTGTGAAAATAGGACCTGAGCGCACCTGTTGTCT
                                                                                     Section 2024
                                                       (155772) 155772    155782     155793     155804     155815     155826     155837 155848
Candidatus Carsonella ruddii strain BT chromosome... (155428) TTGTAA-ATATTT-----ATTATATTAAAGAATTAAAAGAATATATTAATAATTTCAAAAATTCAA-GTAACTCATC
               Candidatus Carsonella 135557 - 167442nt  (19872) TTGTAA-ATATTT-----ATTATATTAAAGAATTAAAAGAATATATTAATAATTTCAAAAATTCAA-GTAACTCATC
         SARS-CoV-2 Reference Genome NC_045512.2  (18666) ATGTGATAGACGTGCCACATGCTTTTCCACTGCTTCAGACACTTATGCCTGTTGGCATCATTCTATTGGATTTGATT
                                                                                     Section 2025
                                                       (155849) 155849    155859     155870     155881     155892     155903     155914 155925
Candidatus Carsonella ruddii strain BT chromosome... (155498) AAGTGCAGAAATAATAACAATAATTAATGGAATCAAACATGGTTTAGGTGATCCATTATATAAAAAAATTGATTCTA
               Candidatus Carsonella 135557 - 167442nt  (19942) AAGTGCAGAAATAATAACAATAATTAATGGAATCAAACATGGTTTAGGTGATCCATTATATAAAAAAATTGATTCTA
         SARS-CoV-2 Reference Genome NC_045512.2  (18743) ACGTCTATAATCCGTTT--ATGATTGATGTTCAACAATGGGGTTTTACAGGTAACCTACAAAGCAACCATGAT-CTG
                                                                                     Section 2026
                                                       (155926) 155926    155936     155947     155958     155969     155980     155991 156002
Candidatus Carsonella ruddii strain BT chromosome... (155575) TTATTTCTGATTATATTTTAAGTATTAATGCTGTAAAAAGTATTTTTTTGGAATTAATTTTAAAAATAAAAATTCT
               Candidatus Carsonella 135557 - 167442nt  (20019) TTATTTCTGATTATATTTTAAGTATTAATGCTGTAAAAAGTATTTTTTTGGAATTAATTTTAAAAATAAAAATTCT
         SARS-CoV-2 Reference Genome NC_045512.2  (18817) T-ATTGTCAAGTCCATGGTAA-TGCACATGTAGCTAGTTGTGATGCAATCATGACTAGGTGTCTAGCTGTCCACGAG
                                                                                     Section 2027
                                                       (156003) 156003    156013     156024     156035     156046     156057     156068 156079
Candidatus Carsonella ruddii strain BT chromosome... (155652) TACAATATAAAAGATGAA-ATTTCAAAATTTGGTTTTTTATCAAATAATAAT--AGTGGAATTTTAGGTGGTAT-TA
               Candidatus Carsonella 135557 - 167442nt  (20096) TACAATATAAAAGATGAA-ATTTCAAAATTTGGTTTTTTATCAAATAATAAT--AGTGGAATTTTAGGTGGTAT-TA
         SARS-CoV-2 Reference Genome NC_045512.2  (18892) TGCTTTGTTAAGCGTGTTGACTGGACTATTGAATATCCTAT-AATTGGTGATGAACTGAAGATTAATGCGGCTTGTA
                                                                                     Section 2028
                                                       (156080) 156080    156090     156101     156112     156123     156134     156145 156156
Candidatus Carsonella ruddii strain BT chromosome... (155725) CCAATGGTCAACCAATAATATTAAAA-----ATTGTTTTTAAACCTACTTCTAGCACTTCAAAAAAAATAAAAACAA
               Candidatus Carsonella 135557 - 167442nt  (20169) CCAATGGTCAACCAATAATATTAAAA-----ATTGTTTTTAAACCTACTTCTAGCACTTCAAAAAAAATAAAAACAA
         SARS-CoV-2 Reference Genome NC_045512.2  (18968) GAAAGGTTCAACACATGGTTGTTAAAGCTGCATTATTAGCAGACAAATTCCCAGTTCTTCACGACAT-TGGTAACCC
```

Section 2029

	(156157)	156157 156167 156178 156189 156200 156211 156222 156233
Candidatus Carsonella ruddii strain BT chromosome...	(155797)	TAAATCAAGATAATAAAGAAATAATTTCAAAAACTTATGGAAGACATGATCCATG-TGTTG-GTTTAAGAGCAATAC
Candidatus Carsonella 135557 - 167442nt	(20241)	TAAATCAAGATAATAAAGAAATAATTTCAAAAACTTATGGAAGACATGATCCATG-TGTTG-GTTTAAGAGCAATAC
SARS-CoV-2 Reference Genome NC_045512.2	(19044)	TAAAGCTA--TT---AAGTGTGTACCTCAAGCTGATGTAGAATGGAAGTTCTATGATGCACAGCCTTGTAGTGACAA

Section 2030

	(156234)	156234 156244 156255 156266 156277 156288 156299 156310
Candidatus Carsonella ruddii strain BT chromosome...	(155872)	CAATAATAGAATCAATGATTTCTACTATTTTAATAAATAAAATTTTAATAAATAAAATTTATGACTAATACTTTATA
Candidatus Carsonella 135557 - 167442nt	(20316)	CAATAATAGAATCAATGATTTCTACTATTTTAATAAATAAAATTTTAATAAATAAAATTTATGACTAATACTTTATA
SARS-CoV-2 Reference Genome NC_045512.2	(19116)	AGCTTATAAAATAGAAGAATTATTCTATTCTTATGCCACACATTCTGACAAAT--------TCACAGATGGTGTA--

Section 2031

	(156311)	156311 156321 156332 156343 156354 156365 156376 156387
Candidatus Carsonella ruddii strain BT chromosome...	(155949)	TGACAATATAATAAATAATCATATTGTTAATATTTATGATGATTTATATCTTTTATATATAGATAAAATTTTATTAC
Candidatus Carsonella 135557 - 167442nt	(20393)	TGACAATATAATAAATAATCATATTGTTAATATTTATGATGATTTATATCTTTTATATATAGATAAAATTTTATTAC
SARS-CoV-2 Reference Genome NC_045512.2	(19183)	TGCCTATTTTGGAATTG----CAATGTCGATAGATATCCTGCT--AATTCCATTGTTTGTAGAT-----TTGACACT

Section 2032

	(156388)	156388 156398 156409 156420 156431 156442 156453 156464
Candidatus Carsonella ruddii strain BT chromosome...	(156026)	ATGAAGTTACATCACCACAAGCTTTTAATTTATTAAAAAATAAAATTATTTGGAAAAATAAAAGTATTTTAT----C
Candidatus Carsonella 135557 - 167442nt	(20470)	ATGAAGTTACATCACCACAAGCTTTTAATTTATTAAAAAATAAAATTATTTGGAAAAATAAAAGTATTTTAT----C
SARS-CoV-2 Reference Genome NC_045512.2	(19249)	AGAGTGCTATCTAACCTTAACTTGCCTGGTTGTGATGGTGGCAGTTTGTATGTAAATAAACATGCATTCCACACACC

Section 2033

	(156465)	156465 156475 156486 156497 156508 156519 156530 156541
Candidatus Carsonella ruddii strain BT chromosome...	(156099)	AACATCTGATCATAATGTATCAACTGATAAAAAACAAAGATTTTTTTT-TAATAAAAAATAAAACAATTAAATTGT
Candidatus Carsonella 135557 - 167442nt	(20543)	AACATCTGATCATAATGTATCAACTGATAAAAAACAAAGATTTTTTTT-TAATAAAAAATAAAACAATTAAATTGT
SARS-CoV-2 Reference Genome NC_045512.2	(19326)	AGCTTTTGATAAAAGTGCTTTTGTTAATTTAAAACAATTACCATTTTTCTATTACTCTGACAGTCCATGTGAGTCTC

Section 2034

	(156542)	156542 156552 156563 156574 156585 156596 156607 156618
Candidatus Carsonella ruddii strain BT chromosome...	(156175)	ATAAAAAAAAA-TAAAGAAAAATATTTTTTTAAATATTATGATTTAAATAATCCTAAACAAGGTATAATACATATTA
Candidatus Carsonella 135557 - 167442nt	(20619)	ATAAAAAAAAA-TAAAGAAAAATATTTTTTTAAATATTATGATTTAAATAATCCTAAACAAGGTATAATACATATTA
SARS-CoV-2 Reference Genome NC_045512.2	(19403)	ATGGAAAACAAGTAGTGTCAGATAT--------AGATTATGTACCACTAAAGTCTGCTACGTGTATAACACGTTGCA

Section 2035

```
                                                        156619    156629     156640     156651     156662     156673     156684  156695
                                                       (156619)
Candidatus Carsonella ruddii strain BT chromosome... (156251) TAGCAACAGAATCTATGTTTTTATTTCCAGGTA-TGATTACAGTTTGTGGTGATTCTCATACAACAACAAATGGAAG
      Candidatus Carsonella 135557 - 167442nt (20695) TAGCAACAGAATCTATGTTTTTATTTCCAGGTA-TGATTACAGTTTGTGGTGATTCTCATACAACAACAAATGGAAG
  SARS-CoV-2 Reference Genome NC_045512.2 (19472) ATTTAGGTGGTGCTGTCTGTAGACATCATGCTAATGAGTACAGATTGTATCTCGATGCTTATAACAT---------G
```

Section 2036

```
                                                        156696    156706     156717     156728     156739     156750     156761  156772
                                                       (156696)
Candidatus Carsonella ruddii strain BT chromosome... (156327) TTTATCATTATTAGCTATTGGTATTGGTACTTCAGATTTAAACATAGTTATATCTACTCAA-----TGTATAATACA
      Candidatus Carsonella 135557 - 167442nt (20771) TTTATCATTATTAGCTATTGGTATTGGTACTTCAGATTTAAACATAGTTATATCTACTCAA-----TGTATAATACA
  SARS-CoV-2 Reference Genome NC_045512.2 (19540) ATGATCTCAGCTGGCTTTAGCTTGTGGGTTTACAACAATTTGATACTTATAACCTCTGGAACACTTTTACAAGACT
```

Section 2037

```
                                                        156773    156783     156794     156805     156816     156827     156838  156849
                                                       (156773)
Candidatus Carsonella ruddii strain BT chromosome... (156399) AAAAAAATTAAAAAATATGAATATTTTATTATATAATAAAATTAATAAAATATTTTCAAAAGATTTAA-TACTA---
      Candidatus Carsonella 135557 - 167442nt (20843) AAAAAAATTAAAAAATATGAATATTTTATTATATAATAAAATTAATAAAATATTTTCAAAAGATTTAA-TACTA---
  SARS-CoV-2 Reference Genome NC_045512.2 (19617) TCAGAGTTTAGAAAATGTGGCT-TTTAATGTTGTAAATAAGGGACACTTTGATGGACAACAGGGTGAAGTACCAGTT
```

Section 2038

```
                                                        156850    156860     156871     156882     156893     156904     156915  156926
                                                       (156850)
Candidatus Carsonella ruddii strain BT chromosome... (156472) TATATAATAAAAAAAATTTCTTCAAAAGGTGGTACAGGATATTGTATTGAATTTAGTGGAAATTGTATTGAAAACTT
      Candidatus Carsonella 135557 - 167442nt (20916) TATATAATAAAAAAAATTTCTTCAAAAGGTGGTACAGGATATTGTATTGAATTTAGTGGAAATTGTATTGAAAACTT
  SARS-CoV-2 Reference Genome NC_045512.2 (19693) TCTATCATTAATAACACTGTTTACACAAAAGTTGATGGTGTTGATGTAGAATTGTTTGAAAAT-------AAAAC--
```

Section 2039

```
                                                        156927    156937     156948     156959     156970     156981     156992  157003
                                                       (156927)
Candidatus Carsonella ruddii strain BT chromosome... (156549) ATCAATATCTGAAAAAATGACAATTTGTAATATGTCTATAGAAGCTGGTTCTAAATCTAGTTTAATTTCACCAGATA
      Candidatus Carsonella 135557 - 167442nt (20993) ATCAATATCTGAAAAAATGACAATTTGTAATATGTCTATAGAAGCTGGTTCTAAATCTAGTTTAATTTCACCAGATA
  SARS-CoV-2 Reference Genome NC_045512.2 (19761) AACATTACCTGTTAATGTAGCATTTGAGCTTTGGGCTA----AGCGCAACATTAAACCAGT---------ACCAGAGG
```

Section 2040

```
                                                        157004    157014     157025     157036     157047     157058     157069  157080
                                                       (157004)
Candidatus Carsonella ruddii strain BT chromosome... (156626) TAAAATTAATTAATTTTTATAAAAAAAAAATTATTGATATTAAAAAATTTTATTTTTTA-TAAATAAAATAAAATC
      Candidatus Carsonella 135557 - 167442nt (21070) TAAAATTAATTAATTTTTATAAAAAAAAAATTATTGATATTAAAAAATTTTATTTTTTA-TAAATAAAATAAAATC
  SARS-CoV-2 Reference Genome NC_045512.2 (19826) TGAAAATACTCAATAATTTGGGTGTGGACATTGCTGCTAATAC-----TGTGATCTGGGACTACAAAGAGATGCTC
```

Section 2041

	(157081)	157081 157091 157102 157113 157124 157135 157146 157157
Candidatus Carsonella ruddii strain BT chromosome...	(156702)	TAATAAAAAT-TCTTTTTATGAAAAACGTTATATTTTTGATGTATCTAAAATAAAACCACACTTAACATGGGGTACA
Candidatus Carsonella 135557 - 167442nt	(21146)	TAATAAAAAT-TCTTTTTATGAAAAACGTTATATTTTTGATGTATCTAAAATAAAACCACACTTAACATGGGGTACA
SARS-CoV-2 Reference Genome NC_045512.2	(19898)	CAGCACATATATCTACTATTGGTGTTTGTTCTATGACTGACATAGCCAAGA---AACCA-ACTGAA--------ACG

Section 2042

	(157158)	157158 157168 157179 157190 157201 157212 157223 157234
Candidatus Carsonella ruddii strain BT chromosome...	(156778)	AATTTAGATACAATAATTGAACTTGATGAAAATATTAAAACTAATGATATTAATATATTAAATTATATGAATTTAAA
Candidatus Carsonella 135557 - 167442nt	(21222)	AATTTAGATACAATAATTGAACTTGATGAAAATATTAAAACTAATGATATTAATATATTAAATTATATGAATTTAAA
SARS-CoV-2 Reference Genome NC_045512.2	(19963)	ATTTGTGCACCACTCACTGTCTTTTTTGA---------------TGGTA----------GAGTTG------------

Section 2043

	(157235)	157235 157245 157256 157267 157278 157289 157300 157311
Candidatus Carsonella ruddii strain BT chromosome...	(156855)	AAATAATCAAAAAATTACTAATATTAAAATTCAAAAAATGTTTTTAGGATCATGTACAAATTCAAGATTTGAAGATT
Candidatus Carsonella 135557 - 167442nt	(21299)	AAATAATCAAAAAATTACTAATATTAAAATTCAAAAAATGTTTTTAGGATCATGTACAAATTCAAGATTTGAAGATT
SARS-CoV-2 Reference Genome NC_045512.2	(20003)	--ATGGTCAAGTA--GACTTATTTAGAAATGCCCGTAATGGTGTTCTTAT----TACAGAAGGTAGTGTTAAAGGTT

Section 2044

	(157312)	157312 157322 157333 157344 157355 157366 157377 157388
Candidatus Carsonella ruddii strain BT chromosome...	(156932)	TATTAATTTGTTGTTTATTGTTATTAAAAATTAATAAAAAAATTAATAAAAATGTTATTGGATTAATTGTTCCTGGA
Candidatus Carsonella 135557 - 167442nt	(21376)	TATTAATTTGTTGTTTATTGTTATTAAAAATTAATAAAAAAATTAATAAAAATGTTATTGGATTAATTGTTCCTGGA
SARS-CoV-2 Reference Genome NC_045512.2	(20072)	TACAACCATCT--------GT-------AGGTCCCAAACAAGCTAGTCTTAATGGAGTCACATTAATTGGA---GAA

Section 2045

	(157389)	157389 157399 157410 157421 157432 157443 157454 157465
Candidatus Carsonella ruddii strain BT chromosome...	(157009)	ACTGAATATATAAGATTAAAATCAGAATTTTATGGTATAAATAAAATTTTTATTTTTTTGGTTATAATTGGAAAAA
Candidatus Carsonella 135557 - 167442nt	(21453)	ACTGAATATATAAGATTAAAATCAGAATTTTATGGTATAAATAAAATTTTTATTTTTTTGGTTATAATTGGAAAAA
SARS-CoV-2 Reference Genome NC_045512.2	(20131)	GCCG------------TAAAAACACAGTTCAAT--TATTATAAGAAAGTTGATGGTGTTGTCCAACAATTACCTGAA

Section 2046

	(157466)	157466 157476 157487 157498 157509 157520 157531 157542
Candidatus Carsonella ruddii strain BT chromosome...	(157086)	TTCTGGTTGTTCTATGTGTTTAGCAATGAATGAAGATAAACTTTTACCTGAAGAAAGATGTGTTTCTACATCTAATA
Candidatus Carsonella 135557 - 167442nt	(21530)	TTCTGGTTGTTCTATGTGTTTAGCAATGAATGAAGATAAACTTTTACCTGAAGAAAGATGTGTTTCTACATCTAATA
SARS-CoV-2 Reference Genome NC_045512.2	(20194)	A-CTTACTTTACTCAGAGTAGAAATTTACAAGAATTTAAAC-----CCAGGAGTCAAATGGAAATTGATTTCT--TA

		Section 2047
	(157543)	157543 157553 157564 157575 157586 157597 157608 157619
Candidatus Carsonella ruddii strain BT chromosome...	(157163)	GAAATTTTATAGGAAGACAAGGTTATAAAAGTAGAACACATTTAGTAAGTCCTGTTTTAGCAATAATTATTTCTATT
Candidatus Carsonella 135557 - 167442nt	(21607)	GAAATTTTATAGGAAGACAAGGTTATAAAAGTAGAACACATTTAGTAAGTCCTGTTTTAGCAATAATTATTTCTATT
SARS-CoV-2 Reference Genome NC_045512.2	(20263)	GAA-TTAGCTATGGATGAATTCATTGAACGGTATA----AATTAGAAGGCTATGCCTTCG-----AACATATCG-TT
		Section 2048
	(157620)	157620 157630 157641 157652 157663 157674 157685 157696
Candidatus Carsonella ruddii strain BT chromosome...	(157240)	TATGGCGAATTTGTTAATTATAATTTATATAATAAATTAATAAATGAAATTAATTTCTAATTATATTTATTTAAATA
Candidatus Carsonella 135557 - 167442nt	(21684)	TATGGCGAATTTGTTAATTATAATTTATATAATAAATTAATAAATGAAATTAATTTCTAATTATATTTATTTAAATA
SARS-CoV-2 Reference Genome NC_045512.2	(20329)	TATGGAGATTTTAGTCATAGTCAGTTAGGTGGTTTACATCTAC-TGATTGGACTAGCTAAACGTTTTAAGGAATCAC
		Section 2049
	(157697)	157697 157707 157718 157729 157740 157751 157762 157773
Candidatus Carsonella ruddii strain BT chromosome...	(157317)	TAGATAATATAGATACTGATATTATAATTCCTAAACAATTTTTAAATACTTTGAAAAAAAATGGTTTTGAAAAATGT
Candidatus Carsonella 135557 - 167442nt	(21761)	TAGATAATATAGATACTGATATTATAATTCCTAAACAATTTTTAAATACTTTGAAAAAAAATGGTTTTGAAAAATGT
SARS-CoV-2 Reference Genome NC_045512.2	(20405)	CTTTTGAATTAGAAGATTTTATTCCTATGGACAGTACAGTTAAAAACTATTTCATAACAGATGCG---CAAACAGGT
		Section 2050
	(157774)	157774 157784 157795 157806 157817 157828 157839 157850
Candidatus Carsonella ruddii strain BT chromosome...	(157394)	TTATTTTTTGATTTAAGATATTTTATAAATAACAATAATTTATTATTAAATAATGATTTTAATTTAAATAAAAAAAA
Candidatus Carsonella 135557 - 167442nt	(21838)	TTATTTTTTGATTTAAGATATTTTATAAATAACAATAATTTATTATTAAATAATGATTTTAATTTAAATAAAAAAAA
SARS-CoV-2 Reference Genome NC_045512.2	(20479)	TCATCTAAGTGTGTGTGTTCTGTTAT---------TGATTTATTACT---TGATGATTTTG-TTGAAATAATAAAAT
		Section 2051
	(157851)	157851 157861 157872 157883 157894 157905 157916 157927
Candidatus Carsonella ruddii strain BT chromosome...	(157471)	AACTAAAGTATTAATTTCAAAAAAAAATTTTGGATGTGGAAGTTCC-AGAGAACATGCAGTTTGGGCATTAAAAAAT
Candidatus Carsonella 135557 - 167442nt	(21915)	AACTAAAGTATTAATTTCAAAAAAAAATTTTGGATGTGGAAGTTCC-AGAGAACATGCAGTTTGGGCATTAAAAAAT
SARS-CoV-2 Reference Genome NC_045512.2	(20543)	--CCCAAGATTTATCTGTAGTTTCTAA----GGTTGTCAAAGTGACTATTGACTATACAGAAATTTCATTTATGC--
		Section 2052
	(157928)	157928 157938 157949 157960 157971 157982 157993 158004
Candidatus Carsonella ruddii strain BT chromosome...	(157547)	TTTGGTTTTAAAATAATAATATCAGAAAGTTTTAGTGATATATTTTATGATAATTCTATGAAAAATAATTTGTTTTT
Candidatus Carsonella 135557 - 167442nt	(21991)	TTTGGTTTTAAAATAATAATATCAGAAAGTTTTAGTGATATATTTTATGATAATTCTATGAAAAATAATTTGTTTTT
SARS-CoV-2 Reference Genome NC_045512.2	(20612)	TTTGGTGTAAAGATGGCCATGTAGAAACATTTTACCCAAA------ATTACAAT-CTAGTCAAG--C----GTGGCA

```
                                                                                                    Section 2053
                                                      (158005) 158005    158015     158026     158037     158048     158059     158070   158081
Candidatus Carsonella ruddii strain BT chromosome... (157624) AATAGAATTTACAAAAAAACAAATTGAAAAAATTTTAAATTATAATTATATATATTTTAATATAGAAAAACAATATA
          Candidatus Carsonella 135557 - 167442nt    (22068) AATAGAATTTACAAAAAAACAAATTGAAAAAATTTTAAATTATAATTATATATATTTTAATATAGAAAAACAATATA
    SARS-CoV-2 Reference Genome NC_045512.2          (20676) ACCGGGTGTTGCTATGCCTAATCTTTACAAAATGCAAAG----AATGCTAT-----------TAGAAAAGTGTGACC
                                                                                                    Section 2054
                                                      (158082) 158082    158092     158103     158114     158125     158136     158147   158158
Candidatus Carsonella ruddii strain BT chromosome... (157701) TTAAATTTGATAAATTATATTATTATTTTTTAATTAATAAATTATATAAAAATATTTTTATAAATAAATTTTTATTA
          Candidatus Carsonella 135557 - 167442nt    (22145) TTAAATTTGATAAATTATATTATTATTTTTTAATTAATAAATTATATAAAAATATTTTTATAAATAAATTTTTATTA
    SARS-CoV-2 Reference Genome NC_045512.2          (20738) TTCAA-------AATTATGGTGATAGTGCAACATTACCTAAAGGCATAATGATGAATGTCGCAAAATATACTCA---
                                                                                                    Section 2055
                                                      (158159) 158159    158169     158180     158191     158202     158213     158224   158235
Candidatus Carsonella ruddii strain BT chromosome... (157778) ACTGATTTTATATTAAGAAAAAAAAAAGAAATAATTTATTATTTAA-ATAAATGTTAAATATTTTGGTTTTACCTGG
          Candidatus Carsonella 135557 - 167442nt    (22222) ACTGATTTTATATTAAGAAAAAAAAAAGAAATAATTTATTATTTAA-ATAAATGTTAAATATTTTGGTTTTACCTGG
    SARS-CoV-2 Reference Genome NC_045512.2          (20805) ACTGTGTCAATATTTA-AACACATTAACATTAGCTGTACCCTATAATATGAGAGTTATACATTTTGGT----GCTGG
                                                                                                    Section 2056
                                                      (158236) 158236    158246     158257     158268     158279     158290     158301   158312
Candidatus Carsonella ruddii strain BT chromosome... (157854) TGATGGTATAGGTCCAGAAATAATGAAACAAGCTATTAAAATTATTAAAACATGTATTTATTTAGGATATAAAATTA
          Candidatus Carsonella 135557 - 167442nt    (22298) TGATGGTATAGGTCCAGAAATAATGAAACAAGCTATTAAAATTATTAAAACATGTATTTATTTAGGATATAAAATTA
    SARS-CoV-2 Reference Genome NC_045512.2          (20877) TTCTGATAAAGGAGTTGCACCA--GGTACA-GCTGTT-------TTAAGACA-GTGGTTGCCTACGGG-TACGCTGC
                                                                                                    Section 2057
                                                      (158313) 158313    158323     158334     158345     158356     158367     158378   158389
Candidatus Carsonella ruddii strain BT chromosome... (157931) ATATTTATTATAATTATATTGGTGGTATATCTATTGATAAAAATAAAAATCCTTTAACAGAAAATACAATTAAAATT
          Candidatus Carsonella 135557 - 167442nt    (22375) ATATTTATTATAATTATATTGGTGGTATATCTATTGATAAAAATAAAAATCCTTTAACAGAAAATACAATTAAAATT
    SARS-CoV-2 Reference Genome NC_045512.2          (20942) TTGTCGATTCAGATCTTAATGACTTTGTCTCT---GATGCAGATTCAACTTTGATTGGTGATTGTGCAACTGTACAT
                                                                                                    Section 2058
                                                      (158390) 158390    158400     158411     158422     158433     158444     158455   158466
Candidatus Carsonella ruddii strain BT chromosome... (158008) TGTTTAAATTCAGATGCTATCTTATTAGGT-TGTGTTGGAGGATATAAATGGAATAATTCTTTTTTAAAACCTGAAC
          Candidatus Carsonella 135557 - 167442nt    (22452) TGTTTAAATTCAGATGCTATCTTATTAGGT-TGTGTTGGAGGATATAAATGGAATAATTCTTTTTTAAAACCTGAAC
    SARS-CoV-2 Reference Genome NC_045512.2          (21016) ACAGCTAAT--AAATGGGATCTCATTATTAGTGATATGTACGACCCTAA--GACTAAAAATGTTACAAAA-------
```

Section 2059

		158467 158477 158488 158499 158510 158521 158532 158543
	(158467)	
Candidatus Carsonella ruddii strain BT chromosome...	(158084)	AAGGTTTGTTAAAATTAAGAAAAAAATTAAATTTATTTGCAAATATTAGACCTATTAAATGTCCTTTTAAAAATATA
Candidatus Carsonella 135557 - 167442nt	(22528)	AAGGTTTGTTAAAATTAAGAAAAAAATTAAATTTATTTGCAAATATTAGACCTATTAAATGTCCTTTTAAAAATATA
SARS-CoV-2 Reference Genome NC_045512.2	(21082)	--G-------AAAATGACTCTAAAGAGGG--TTTTTTCACTTACATTTGTGGGTTTA----TACAAC--AAAAGCTA

Section 2060

		158544 158554 158565 158576 158587 158598 158609 158620
	(158544)	
Candidatus Carsonella ruddii strain BT chromosome...	(158161)	GATTTTTTAATTTTAAGAGAATTAAATGGAGGAATTTATTATGGAAAACCAAAAGGTATTTCTAAACAAATAATTAA
Candidatus Carsonella 135557 - 167442nt	(22605)	GATTTTTTAATTTTAAGAGAATTAAATGGAGGAATTTATTATGGAAAACCAAAAGGTATTTCTAAACAAATAATTAA
SARS-CoV-2 Reference Genome NC_045512.2	(21142)	GCTCTTGGAGGTTCCGTGGCTATAAAGATAACAGAACATTCTTGGAATGCT---GATCTTTATAAGC---TCATGGG

Section 2061

		158621 158631 158642 158653 158664 158675 158686 158697
	(158621)	
Candidatus Carsonella ruddii strain BT chromosome...	(158238)	TAATATACCAACTTGGTTTTCTTATGATACAGAAATTTATAATGAACAAGAATTAATAAGA-GTAATTAAATTAGGT
Candidatus Carsonella 135557 - 167442nt	(22682)	TAATATACCAACTTGGTTTTCTTATGATACAGAAATTTATAATGAACAAGAATTAATAAGA-GTAATTAAATTAGGT
SARS-CoV-2 Reference Genome NC_045512.2	(21213)	ACACTTCGCATGGTGGACAGCCTTTGTTAC-------TAATGTGAATGCGTCATCATCTGAAGCATTTTTAATTGGA

Section 2062

		158698 158708 158719 158730 158741 158752 158763 158774
	(158698)	
Candidatus Carsonella ruddii strain BT chromosome...	(158314)	TTTAATTTATCTATGAATAGAAAAAAAAAAATTACTTCTATAGATAAATCTAATGTATTAATTTCTTCTAAATTATG
Candidatus Carsonella 135557 - 167442nt	(22758)	TTTAATTTATCTATGAATAGAAAAAAAAAAATTACTTCTATAGATAAATCTAATGTATTAATTTCTTCTAAATTATG
SARS-CoV-2 Reference Genome NC_045512.2	(21283)	TGTAATT-ATCTTGGCAAACCACGCGAACAAATAGATGGTTATGTCATGCATGCAAATTACATATTTTGGAGGAATA

Section 2063

		158775 158785 158796 158807 158818 158829 158840 158851
	(158775)	
Candidatus Carsonella ruddii strain BT chromosome...	(158391)	GAGAAAAATTGTAAATTATATTTCTAATTTTT--TTAGCAATATTAAAGTTAATCATTTATATATTGACTATGCTAC
Candidatus Carsonella 135557 - 167442nt	(22835)	GAGAAAAATTGTAAATTATATTTCTAATTTTT--TTAGCAATATTAAAGTTAATCATTTATATATTGACTATGCTAC
SARS-CoV-2 Reference Genome NC_045512.2	(21359)	CAAATCCAAT-TCAGTTGTCTTCCTATTCTTTATTTGACATGAGTAAATTTCCCCTTAAATTAAGGGGTACTGCTGT

Section 2064

		158852 158862 158873 158884 158895 158906 158917 158928
	(158852)	
Candidatus Carsonella ruddii strain BT chromosome...	(158466)	AATTGAATTAATAAAAAATTATAATAATTTTGATATAATTTTAACTTCAAATATATTTGGT-GATATATTATCTGAT
Candidatus Carsonella 135557 - 167442nt	(22910)	AATTGAATTAATAAAAAATTATAATAATTTTGATATAATTTTAACTTCAAATATATTTGGT-GATATATTATCTGAT
SARS-CoV-2 Reference Genome NC_045512.2	(21435)	TATGTCTTTAAAAGAAGGTCAAATCAA---TGATATGATTTTATCTCTTCTTAGTAAAGGTAGACTTATAATTAGAG

Section 2065

(158929) 158929 158939 158950 158961 158972 158983 158994 159005

Candidatus Carsonella ruddii strain BT chromosome... (158542) CAATGTTCTTTATTAACTGGTTCATTAGGAATGTTGCCTTCAATTTCTATAAATAATAAATCAATTTGTTTATTTGA
Candidatus Carsonella 135557 - 167442nt (22986) CAATGTTCTTTATTAACTGGTTCATTAGGAATGTTGCCTTCAATTTCTATAAATAATAAATCAATTTGTTTATTTGA
SARS-CoV-2 Reference Genome NC_045512.2 (21509) AAAACA-----ACAGAGTTGTT-ATTTCTAGTGATG--TTCT-TGTTAACAACTAAACGAACAATGT-TTGTTTTT-

Section 2066

(159006) 159006 159016 159027 159038 159049 159060 159071 159082

Candidatus Carsonella ruddii strain BT chromosome... (158619) ACCATGTCATGGAAGTGCGCCAGATATAGCTGGTAAAAACATAGCAAATCCAATAGGGATTTTATTATCTTTAGTTA
Candidatus Carsonella 135557 - 167442nt (23063) ACCATGTCATGGAAGTGCGCCAGATATAGCTGGTAAAAACATAGCAAATCCAATAGGGATTTTATTATCTTTAGTTA
SARS-CoV-2 Reference Genome NC_045512.2 (21575) -CT-TGTTTT--AT-TG--CCACTAGTCTCTAGTCAGTGTGTTAATCTTACAACCAGAACTCAATTACCCCCTGCA-

Section 2067

(159083) 159083 159093 159104 159115 159126 159137 159148 159159

Candidatus Carsonella ruddii strain BT chromosome... (158696) TGTTATTTGAATATTTATTAAATAATAAAAAATTATCTAATAAATTATTTT-ATTCT-ATTTATAAAGTTTTATCAT
Candidatus Carsonella 135557 - 167442nt (23140) TGTTATTTGAATATTTATTAAATAATAAAAAATTATCTAATAAATTATTTT-ATTCT-ATTTATAAAGTTTTATCAT
SARS-CoV-2 Reference Genome NC_045512.2 (21644) --TACACTAATTCTTTCACACGTGGTGTTTATTACCCTGACAAAGTTTTCAGATCCTCAGTTTTACATTCAACTCAG

Section 2068

(159160) 159160 159170 159181 159192 159203 159214 159225 159236

Candidatus Carsonella ruddii strain BT chromosome... (158771) TTGGTTTTGTACAATTGATATGAAAAATTTTTTAAAAAAAAAAAAAATAATTTCTACAGAAGAATTTGGAGATTTA
Candidatus Carsonella 135557 - 167442nt (23215) TTGGTTTTGTACAATTGATATGAAAAATTTTTTAAAAAAAAAAAAAATAATTTCTACAGAAGAATTTGGAGATTTA
SARS-CoV-2 Reference Genome NC_045512.2 (21719) GACTTGTTCTTACCTTTCTTTTCCAATGTTACTTGGTTC----CA-------TGCTATACATGTCTCTGG-GACCAA

Section 2069

(159237) 159237 159247 159258 159269 159280 159291 159302 159313

Candidatus Carsonella ruddii strain BT chromosome... (158848) GTAAATTATTATTTTTTATATGATTAATTTAGGAATAATTGGTTGGAGAGGTTTAGTTGGAAGTGTTTTAATAAATA
Candidatus Carsonella 135557 - 167442nt (23292) GTAAATTATTATTTTTTATATGATTAATTTAGGAATAATTGGTTGGAGAGGTTTAGTTGGAAGTGTTTTAATAAATA
SARS-CoV-2 Reference Genome NC_045512.2 (21784) TGGTACTAAGAGGTTTGATAACCCTGTCCTACCATTTAATGATGGTGTTTATTTTGCTTCCACTG---AGAAGTCTA

Section 2070

(159314) 159314 159324 159335 159346 159357 159368 159379 159390

Candidatus Carsonella ruddii strain BT chromosome... (158925) GAATAAAAAATAGCTTTATTTATAAATATTGTAATATATATTTATTTTCTACAAATAAAATAATAATAAAAAATGAT
Candidatus Carsonella 135557 - 167442nt (23369) GAATAAAAAATAGCTTTATTTATAAATATTGTAATATATATTTATTTTCTACAAATAAAATAATAATAAAAAATGAT
SARS-CoV-2 Reference Genome NC_045512.2 (21858) ACATAATAAGAGGCTGGATTTTT--G----GTACTA---CTTTAGATTCGAAGACCCAGTCCCTACTTAT---TGTT

Section 2071

	(159391)	159391 159401 159412 159423 159434 159445 159456 159467
Candidatus Carsonella ruddii strain BT chromosome...	(159002)	ATAAACGATGCATATAATATAAAATTATTAAAAAATATGAATATAATAATTTGTTGTCAAGGAAGTAATTTTACAAA
Candidatus Carsonella 135557 - 167442nt	(23446)	ATAAACGATGCATATAATATAAAATTATTAAAAAATATGAATATAATAATTTGTTGTCAAGGAAGTAATTTTACAAA
SARS-CoV-2 Reference Genome NC_045512.2	(21923)	AATAACGCTAC---TAATGTTG--TTATTAAAGTCTGTGAATTTCA-ATTTTGT----AATGATCCATTTTTGGGTG

Section 2072

	(159468)	159468 159478 159489 159500 159511 159522 159533 159544
Candidatus Carsonella ruddii strain BT chromosome...	(159079)	AAAAATTTTAAAAAAATAAAAAATATTTATTGGAAAGGTTATTGGATAGATGCTTCTAGTTTTTTAAGAATGAATA
Candidatus Carsonella 135557 - 167442nt	(23523)	AAAAATTTTAAAAAAATAAAAAATATTTATTGGAAAGGTTATTGGATAGATGCTTCTAGTTTTTTAAGAATGAATA
SARS-CoV-2 Reference Genome NC_045512.2	(21990)	TTTA-TTACCACAAAAACAACAAAAGTTGGATGGAAAG-TGAGTTCAGAGTTTATTCTAGTGC-----GAAT-AATT

Section 2073

	(159545)	159545 159555 159566 159577 159588 159599 159610 159621
Candidatus Carsonella ruddii strain BT chromosome...	(159156)	ACGAATGTTCTTTAATATTAGATCTTTTAAATAAAGATGATTTAATAAAAAAAATTAAATATATTAAATTTTTTTCT
Candidatus Carsonella 135557 - 167442nt	(23600)	ACGAATGTTCTTTAATATTAGATCTTTTAAATAAAGATGATTTAATAAAAAAAATTAAATATATTAAATTTTTTTCT
SARS-CoV-2 Reference Genome NC_045512.2	(22059)	GCACTTTTGAATATGTCTCTCAGCCTTTTCTTATGGACC-TTGAAGGAAAACAGGGTAATTTCAAAAATCTT-----

Section 2074

	(159622)	159622 159632 159643 159654 159665 159676 159687 159698
Candidatus Carsonella ruddii strain BT chromosome...	(159233)	GGAGCTAATTGTACTGTTAGTTTAGCTTTAATTTTATTAAAAAATTTATTAAAATTAAACTTAATAGAATGGATTTC
Candidatus Carsonella 135557 - 167442nt	(23677)	GGAGCTAATTGTACTGTTAGTTTAGCTTTAATTTTATTAAAAAATTTATTAAAATTAAACTTAATAGAATGGATTTC
SARS-CoV-2 Reference Genome NC_045512.2	(22130)	--AGGGAATTTGTGTTTAAGAATA--TTGATGGTTATTTTAAAATATATTCTAAGCACACGCCTATTAATTTAGTGC

Section 2075

	(159699)	159699 159709 159720 159731 159742 159753 159764 159775
Candidatus Carsonella ruddii strain BT chromosome...	(159310)	CACATCTAGTTATCAAGCTATATCTGGAGCAGGTTCAAAATTAGTAACAAAATTATTAAATAATATTAATTTATCAT
Candidatus Carsonella 135557 - 167442nt	(23754)	CACATCTAGTTATCAAGCTATATCTGGAGCAGGTTCAAAATTAGTAACAAAATTATTAAATAATATTAATTTATCAT
SARS-CoV-2 Reference Genome NC_045512.2	(22203)	GTGATCTCCCT--CAGGGTTTTTCGGCTTTAGAACCATTGGTAGATTTGCCAATAGGTATTAACATCACTAGGTTTC

Section 2076

	(159776)	159776 159786 159797 159808 159819 159830 159841 159852
Candidatus Carsonella ruddii strain BT chromosome...	(159387)	ATAATTTAGAAAATAATTTATTAAAATTGAAAAAAATATTAATTTTTTTTTTAAAAAAAATA-ATCCAATATTATA
Candidatus Carsonella 135557 - 167442nt	(23831)	ATAATTTAGAAAATAATTTATTAAAATTGAAAAAAATATTAATTTTTTTTTTAAAAAAAATA-ATCCAATATTATA
SARS-CoV-2 Reference Genome NC_045512.2	(22278)	AAACTTTA---CTTGCTTTACATAGAAGTTATTTGACTCCTGGTGATTCTTCTTCAGGTTGGACAGCTGGTGCTGCA

Section 2077

	(159853)	159853 159863 159874 159885 159896 159907 159918 159929
Candidatus Carsonella ruddii strain BT chromosome...	(159463)	TAATGCTATACCTTGGATAGATAAAAAAGTAAATTT-TAGTCAAACAAAAGAAGAATGGAAATCATCTTCTGAAGCT
Candidatus Carsonella 135557 - 167442nt	(23907)	TAATGCTATACCTTGGATAGATAAAAAAGTAAATTT-TAGTCAAACAAAAGAAGAATGGAAATCATCTTCTGAAGCT
SARS-CoV-2 Reference Genome NC_045512.2	(22352)	GCTTATTATGTGGGTTATCTTCAACCTAGGACTTTTCTATTAAAATATAATGAAAATGGAAC-CAT-TACAGATGCT

Section 2078

	(159930)	159930 159940 159951 159962 159973 159984 159995 160006
Candidatus Carsonella ruddii strain BT chromosome...	(159539)	TCTAAAATATTAAATAAAAAAATTATAATTGATTCAAATTGTGTAAGAATAGCATCTTTAAGATGTCATTCACAGTA
Candidatus Carsonella 135557 - 167442nt	(23983)	TCTAAAATATTAAATAAAAAAATTATAATTGATTCAAATTGTGTAAGAATAGCATCTTTAAGATGTCATTCACAGTA
SARS-CoV-2 Reference Genome NC_045512.2	(22427)	G-TAGACTGTGCACTTGACCCTCTCTCA--GAAACAAA--GTGTA----------CGTT--GAAATCCTTCACTGTA

Section 2079

	(160007)	160007 160017 160028 160039 160050 160061 160072 160083
Candidatus Carsonella ruddii strain BT chromosome...	(159616)	TTTTTTAATAAAATTTAATAAAAATATTAATATTAATAATATATATGAAATTATACATCATAAATTTGTTTATATAG
Candidatus Carsonella 135557 - 167442nt	(24060)	TTTTTTAATAAAATTTAATAAAAATATTAATATTAATAATATATATGAAATTATACATCATAAATTTGTTTATATAG
SARS-CoV-2 Reference Genome NC_045512.2	(22487)	G----AAAAAGGAATCTATCAAACTTCTAAC-TTTAGAGTCCAACCAACAGAATCTATTGTTAGATTTCCTA-ATAT

Section 2080

	(160084)	160084 160094 160105 160116 160127 160138 160149 160160
Candidatus Carsonella ruddii strain BT chromosome...	(159693)	TTAAAAATAATGAAAAAAATTCTAATTTAATTTTAACACCATATAATGTATCTGGAAAATTAAAAATTTTTGTAGGT
Candidatus Carsonella 135557 - 167442nt	(24137)	TTAAAAATAATGAAAAAAATTCTAATTTAATTTTAACACCATATAATGTATCTGGAAAATTAAAAATTTTTGTAGGT
SARS-CoV-2 Reference Genome NC_045512.2	(22558)	TACAAACTTGTGCCCTTTTGGTGAAGTTTTTAACGCCACCAGATT-TGCATCTG-----TTTATGCTTGGAACAGGA

Section 2081

	(160161)	160161 160171 160182 160193 160204 160215 160226 160237
Candidatus Carsonella ruddii strain BT chromosome...	(159770)	AGA--ATTAAAAAAAGTTTAATTAATAATAAAATTTTTACAATGTTTAGCATAGGAGATCAATTATT-GTGGGGTGC
Candidatus Carsonella 135557 - 167442nt	(24214)	AGA--ATTAAAAAAAGTTTAATTAATAATAAAATTTTTACAATGTTTAGCATAGGAGATCAATTATT-GTGGGGTGC
SARS-CoV-2 Reference Genome NC_045512.2	(22629)	AGAGAATCAGCAACTGTGTTGCTGATTATTCTGTCCT-ATATAATTCCGCATCATTTTCCACTTTTAAGTGTTATGG

Section 2082

	(160238)	160238 160248 160259 160270 160281 160292 160303 160314
Candidatus Carsonella ruddii strain BT chromosome...	(159844)	AGCTGAACCTATTAAAAGATTTTTAGAAATATTAATAAGTAATATATTATGAAAATTAAATTTTGTGGTGTTAAAAA
Candidatus Carsonella 135557 - 167442nt	(24288)	AGCTGAACCTATTAAAAGATTTTTAGAAATATTAATAAGTAATATATTATGAAAATTAAATTTTGTGGTGTTAAAAA
SARS-CoV-2 Reference Genome NC_045512.2	(22705)	AGTGTCTCCTACTAAA---TTAAATGATCTCTGCTTTACTAATGTCT-ATGCAGATTCA--TTTGTAA--TTAGAGG

Section 2083

(160315) 160315 160325 160336 160347 160358 160369 160380 160391
Candidatus Carsonella ruddii strain BT chromosome... (159921) TAAAAAGATATTAAAAAAGCTATTTTATGTAATGCTAATTTTATAGGTTTTAATTTTTATAAAAATAGTAAAAGAT
Candidatus Carsonella 135557 - 167442nt (24365) TAAAAAGATATTAAAAAAGCTATTTTATGTAATGCTAATTTTATAGGTTTTAATTTTTATAAAAATAGTAAAAGAT
SARS-CoV-2 Reference Genome NC_045512.2 (22774) TGATGAAGTCAGACAAATCGCTCCAGGGCAAACTGGAAAGATTGCTGATTATAATTAT----AAATTACCAGATGAT

Section 2084

(160392) 160392 160402 160413 160424 160435 160446 160457 160468
Candidatus Carsonella ruddii strain BT chromosome... (159998) TTTTACAAGATA-GTTTTA--TTCCATTAATTAAAAT---GATTCC-----TTCTTATATTTATAA--AGTTGCTTT
Candidatus Carsonella 135557 - 167442nt (24442) TTTTACAAGATA-GTTTTA--TTCCATTAATTAAAAT---GATTCC-----TTCTTATATTTATAA--AGTTGCTTT
SARS-CoV-2 Reference Genome NC_045512.2 (22847) TTT-ACAGGCTGCGTTATAGCTTGGAATTCTAACAATCTTGATTCTAAGGTTGGTGGTAATTATAATTACCTGTATA

Section 2085

(160469) 160469 160479 160490 160501 160512 160523 160534 160545
Candidatus Carsonella ruddii strain BT chromosome... (160062) ATTTGTTAATGAAGAATATAATATAATTAATAAAATTAAAAATTATTTTGATTTAATTCAATTTCATGGAAACGAAA
Candidatus Carsonella 135557 - 167442nt (24506) ATTTGTTAATGAAGAATATAATATAATTAATAAAATTAAAAATTATTTTGATTTAATTCAATTTCATGGAAACGAAA
SARS-CoV-2 Reference Genome NC_045512.2 (22923) GATTGTTTAGGAAG--TCTAATCTCAACCTTTTGAGAGAGAT-ATTTCAACTGAAATCTATCAGGCCGGTAGCACA

Section 2086

(160546) 160546 160556 160567 160578 160589 160600 160611 160622
Candidatus Carsonella ruddii strain BT chromosome... (160139) GTTTTTTTTTTTGT-GAATTATTTAATAAAAAATATA--TTAAAACTATAAGGTTAAATAAAAAAACAGATTTTAAT
Candidatus Carsonella 135557 - 167442nt (24583) GTTTTTTTTTTTGT-GAATTATTTAATAAAAAATATA--TTAAAACTATAAGGTTAAATAAAAAAACAGATTTTAAT
SARS-CoV-2 Reference Genome NC_045512.2 (22997) CCTTGTAATGGTGTTGAAGGTTTTAATTGTTACTTTCCTTTACAATCATATGGTTTC--CAACCCACTAATGGTGTT

Section 2087

(160623) 160623 160633 160644 160655 160666 160677 160688 160699
Candidatus Carsonella ruddii strain BT chromosome... (160213) ATTTTTATT-ATTCAAA-TAATTTTTTTCTTTTTAATTGATAAAAAAATATTAATTATGGTGGTTTTGGTGAA
Candidatus Carsonella 135557 - 167442nt (24657) ATTTTTATT-ATTCAAA-TAATTTTTTTCTTTTTAATTGATAAAAAAATATTAATTATGGTGGTTTTGGTGAA
SARS-CoV-2 Reference Genome NC_045512.2 (23072) GGTTACCAACCATACAGAGTAGTAGTACTTTCTTTTGAACTTCTACATGCACCAGCAAC--TGTTTGTGGACCTAAA

Section 2088

(160700) 160700 160710 160721 160732 160743 160754 160765 160776
Candidatus Carsonella ruddii strain BT chromosome... (160288) CAATATAAAAAATTTAAATTTAATAAAAAAAATTCATGTTTTATTTC--TGGT------GGAATTAATTTATTTAAT
Candidatus Carsonella 135557 - 167442nt (24732) CAATATAAAAAATTTAAATTTAATAAAAAAAATTCATGTTTTATTTC--TGGT------GGAATTAATTTATTTAAT
SARS-CoV-2 Reference Genome NC_045512.2 (23147) AAGTCTACTAA--TTTGGTTAAAAACAAATGTGTCAATTTCAACTTCAATGGTTTAACAGGCACAGGTGTTCTTACT

Section 2089

	(160777)	160777 160787 160798 160809 160820 160831 160842 160853
Candidatus Carsonella ruddii strain BT chromosome...	(160357)	TA---TAAAAAAAA--TTCTTATTTTTCAAATTGTTTTAATATAGATATTTGTAGTAGTTTAGAATATAAACAATTA
Candidatus Carsonella 135557 - 167442nt	(24801)	TA---TAAAAAAAA--TTCTTATTTTTCAAATTGTTTTAATATAGATATTTGTAGTAGTTTAGAATATAAACAATTA
SARS-CoV-2 Reference Genome NC_045512.2	(23222)	GAGTCTAACAAAAAGTTTCTGCCTTTCCAACA--ATTTGGCAGAGACATTGCTGACACTACTGATGCTGTCCGTGAT

Section 2090

	(160854)	160854 160864 160875 160886 160897 160908 160919 160930
Candidatus Carsonella ruddii strain BT chromosome...	(160429)	AAAAATATTAAAAAAATGATAAATTTTATATAATGTTTTTTTTATTAAATAAAATATTAAGAAAAATAAAAATAAAT
Candidatus Carsonella 135557 - 167442nt	(24873)	AAAAATATTAAAAAAATGATAAATTTTATATAATGTTTTTTTTATTAAATAAAATATTAAGAAAAATAAAAATAAAT
SARS-CoV-2 Reference Genome NC_045512.2	(23297)	CCACAGACACTTGAGATTCTTGACATTACACCATGTTCTTTTGGTGGTGTCAG-TGTTAT-AACACCAGGAACAAAT

Section 2091

	(160931)	160931 160941 160952 160963 160974 160985 160996 161007
Candidatus Carsonella ruddii strain BT chromosome...	(160506)	ATTGTTTTTTTAAACAAAATATTTTTATTGGAAAAAAAAATAAATAAAATAAAAAAAAAAAAAATAATTTTTTTTC
Candidatus Carsonella 135557 - 167442nt	(24950)	ATTGTTTTTTTAAACAAAATATTTTTATTGGAAAAAAAAATAAATAAAATAAAAAAAAAAAAAATAATTTTTTTTC
SARS-CoV-2 Reference Genome NC_045512.2	(23372)	AC-----TTCTAACCAGGTTGCTGTTCTTTATCAGGATGTTAACT----------GCACAGAAGTCCCTGTTGCTAT

Section 2092

	(161008)	161008 161018 161029 161040 161051 161062 161073 161084
Candidatus Carsonella ruddii strain BT chromosome...	(160583)	TAATTTAGTTTATTTTAATAATTATTACAATAATTATAATAGAATAATTTATTATAGT-AAGTATATTAAAAATTAT
Candidatus Carsonella 135557 - 167442nt	(25027)	TAATTTAGTTTATTTTAATAATTATTACAATAATTATAATAGAATAATTTATTATAGT-AAGTATATTAAAAATTAT
SARS-CoV-2 Reference Genome NC_045512.2	(23434)	TCATGCAGATCAACTTACTCCTACTTGGCGTGTTTATTCTACAGGTTCTAATGTTTTTCAAACACGTGCAGGCTGTT

Section 2093

	(161085)	161085 161095 161106 161117 161128 161139 161150 161161
Candidatus Carsonella ruddii strain BT chromosome...	(160659)	AAATTTATTCTTATACCTATTATTAATTTAAATTGTAAAATAGATAAAATAATAAGTTCTATTAAATCAACT--TTG
Candidatus Carsonella 135557 - 167442nt	(25103)	AAATTTATTCTTATACCTATTATTAATTTAAATTGTAAAATAGATAAAATAATAAGTTCTATTAAATCAACT--TTG
SARS-CoV-2 Reference Genome NC_045512.2	(23511)	TAATAGGGGCTGA-ACATGTCAACAACTCATATG---AGTGTGACATACCCATTGGTGCAGGTATATGCGCTAGTTA

Section 2094

	(161162)	161162 161172 161183 161194 161205 161216 161227 161238
Candidatus Carsonella ruddii strain BT chromosome...	(160734)	TTAGATAAAATTTATTGCTTATTAGA----AAAAAGTTTAGAAATATTTATTATAAAAATTCAACAATTAAATTTTT
Candidatus Carsonella 135557 - 167442nt	(25178)	TTAGATAAAATTTATTGCTTATTAGA----AAAAAGTTTAGAAATATTTATTATAAAAATTCAACAATTAAATTTTT
SARS-CoV-2 Reference Genome NC_045512.2	(23584)	TCAGACTCAGACTAATTCTCCTCGGCGGGCACGTAGTGTAG--CTAGTCAATCCATCATTGCCTACACTATGTCACT

Section 2095

		161239 161249 161260 161271 161282 161293 161304 161315
	(161239)	
Candidatus Carsonella ruddii strain BT chromosome...	(160807)	TTTACGAAGAAATAACTATACCTTTATTTATAAATTATTCAAATTTATTATATTCAGGACAAATACCTAAATTTTAT
Candidatus Carsonella 135557 - 167442nt	(25251)	TTTACGAAGAAATAACTATACCTTTATTTATAAATTATTCAAATTTATTATATTCAGGACAAATACCTAAATTTTAT
SARS-CoV-2 Reference Genome NC_045512.2	(23659)	TGGT-GCAGAAA-----ATTCAGTTGCTTA--C-----TCTAATA-ACTCTATT---GCCATACCCACAAATTTTAC

Section 2096

		161316 161326 161337 161348 161359 161370 161381 161392
	(161316)	
Candidatus Carsonella ruddii strain BT chromosome...	(160884)	AATAATTTATTTAAAATAGAAAAAATTAATTCTTTTTAATTCCAACTTCAGAAGTAATTTTAAATTGTTTATCTTT
Candidatus Carsonella 135557 - 167442nt	(25328)	AATAATTTATTTAAAATAGAAAAAATTAATTCTTTTTAATTCCAACTTCAGAAGTAATTTTAAATTGTTTATCTTT
SARS-CoV-2 Reference Genome NC_045512.2	(23719)	TATTAGTG--TTACCACAGAAATTCTACCAGTGTCTATGACCAAGACATCAGTAG--ATTGTACAATGTACATTTGT

Section 2097

		161393 161403 161414 161425 161436 161447 161458 161469
	(161393)	
Candidatus Carsonella ruddii strain BT chromosome...	(160961)	TTTTATAAAAAATAATTTTTTACCAATAAAATTAATATGTAATAGTTATTGTTTTAGAAAAGAAATAGGTAATCTTT
Candidatus Carsonella 135557 - 167442nt	(25405)	TTTTATAAAAAATAATTTTTTACCAATAAAATTAATATGTAATAGTTATTGTTTTAGAAAAGAAATAGGTAATCTTT
SARS-CoV-2 Reference Genome NC_045512.2	(23792)	GGTGATTCAACTGAATG------CAGCAATCTTTGTTGCAATA-TGGCAGTTTTTGTACACAATTAAACCGTGCTT

Section 2098

		161470 161480 161491 161502 161513 161524 161535 161546
	(161470)	
Candidatus Carsonella ruddii strain BT chromosome...	(161038)	GTAAAAATAATTTAGGTATAAAAAGACAACAACAATTTAAAAAAATTGAAATTTTTCAATTTTTTAAAAAAAATGAA
Candidatus Carsonella 135557 - 167442nt	(25482)	GTAAAAATAATTTAGGTATAAAAAGACAACAACAATTTAAAAAAATTGAAATTTTTCAATTTTTTAAAAAAAATGAA
SARS-CoV-2 Reference Genome NC_045512.2	(23862)	-TAACTGGAATAGCTGTTGAACAAGACAAAAACACCCAAGAAGTTTTTGCACAAGTCAAACAAATTTACAAAACACC

Section 2099

		161547 161557 161568 161579 161590 161601 161612 161623
	(161547)	
Candidatus Carsonella ruddii strain BT chromosome...	(161115)	TCTTTTGAAAGATTTTATTCTATATGTTCTAATATAATTTATATATTA--AAATTATTAGAAATAAAATTTAGAATA
Candidatus Carsonella 135557 - 167442nt	(25559)	TCTTTTGAAAGATTTTATTCTATATGTTCTAATATAATTTATATATTA--AAATTATTAGAAATAAAATTTAGAATA
SARS-CoV-2 Reference Genome NC_045512.2	(23938)	ACCAATTAAAGATTTTGGT-----GTTTTAATTTTTCACAAATATTACCAGATCCATCAAACCAAGCAAGAGGTC

Section 2100

		161624 161634 161645 161656 161667 161678 161689 161700
	(161624)	
Candidatus Carsonella ruddii strain BT chromosome...	(161190)	ATAAAATTAAATTCT---TATGAATTAAATTTAAATTCTTTTTTAACCTTTGATTTTGAAATTTGGAG-TTATATTG
Candidatus Carsonella 135557 - 167442nt	(25634)	ATAAAATTAAATTCT---TATGAATTAAATTTAAATTCTTTTTTAACCTTTGATTTTGAAATTTGGAG-TTATATTG
SARS-CoV-2 Reference Genome NC_045512.2	(24010)	ATTTATTGAAGATCTACTTTCAACAAAGTGACACTTGCAGATGCTGGCTTCATCAAACAATATGGTGATTGCCTTG

Section 2101

	(161701)	161701 161711 161722 161733 161744 161755 161766 161777
Candidatus Carsonella ruddii strain BT chromosome...	(161263)	ATAATTCTTG-GATAGAAATTTCTTCTTTGTCGTTATGTTTAAA----TAAATCTTTTAATATTTTTTTAAAAAAAA
Candidatus Carsonella 135557 - 167442nt	(25707)	ATAATTCTTG-GATAGAAATTTCTTCTTTGTCGTTATGTTTAAA----TAAATCTTTTAATATTTTTTTAAAAAAAA
SARS-CoV-2 Reference Genome NC_045512.2	(24087)	GTGATATTGCTGCTAGAGACCTCATTTGTGCACAAAAGTTTAACGGCCTTACTGTTTTGCCACCTTTGCTCACAGAT

Section 2102

	(161778)	161778 161788 161799 161810 161821 161832 161843 161854
Candidatus Carsonella ruddii strain BT chromosome...	(161335)	AAAATATTTATTTAATAAATGCTTCCTGCCTCCCTTTAGGAAGATTAATATATATTATTTTTTATACTTTTAGAATT
Candidatus Carsonella 135557 - 167442nt	(25779)	AAAATATTTATTTAATAAATGCTTCCTGCCTCCCTTTAGGAAGATTAATATATATTATTTTTTATACTTTTAGAATT
SARS-CoV-2 Reference Genome NC_045512.2	(24164)	GAAATGATTGCTCAATACA--CTTC-TGCA--CTGTTAGCGGGTACAATC---ACTTCTGGTTGGACCTTT-GGT--

Section 2103

	(161855)	161855 161865 161876 161887 161898 161909 161920 161931
Candidatus Carsonella ruddii strain BT chromosome...	(161412)	AGTAATTGTTTTATTAAAATTCCTAAAAAAATTAATAAAATTTTAACCGATCTGTTGAAATGGTAAACAAACAAGAA
Candidatus Carsonella 135557 - 167442nt	(25856)	AGTAATTGTTTTATTAAAATTCCTAAAAAAATTAATAAAATTTTAACCGATCTGTTGAAATGGTAAACAAACAAGAA
SARS-CoV-2 Reference Genome NC_045512.2	(24230)	-GCAGGTGCTGCATTACAA---------------ATACCATTTG-------CTATGCAAATGG----CTTATAGGTT

Section 2104

	(161932)	161932 161942 161953 161964 161975 161986 161997 162008
Candidatus Carsonella ruddii strain BT chromosome...	(161489)	TTAGAATCTTGTGCTTATGCATAGGGGTTCAAATCCCCTGTTCGGTAAAAAAAATATATGAAAAATTTTTTAATTAA
Candidatus Carsonella 135557 - 167442nt	(25933)	TTAGAATCTTGTGCTTATGCATAGGGGTTCAAATCCCCTGTTCGGTAAAAAAAATATATGAAAAATTTTTTAATTAA
SARS-CoV-2 Reference Genome NC_045512.2	(24280)	TAATGGTATTGGAGTTACACAGAATGTT------C-TCTATGAGAACCAAAAATTGATTGCCAACCAATTTAAT--A

Section 2105

	(162009)	162009 162019 162030 162041 162052 162063 162074 162085
Candidatus Carsonella ruddii strain BT chromosome...	(161566)	AATTAGATATGGTATTAATAATGAGAAAATTTTTTTATTAATTAAAAAATTACTAAAAAAAAGAAAAAATATTTTTT
Candidatus Carsonella 135557 - 167442nt	(26010)	AATTAGATATGGTATTAATAATGAGAAAATTTTTTTATTAATTAAAAAATTACTAAAAAAAAGAAAAAATATTTTTT
SARS-CoV-2 Reference Genome NC_045512.2	(24348)	-GTGCTAT-TGGCAAAATTCAAGACTCACTTTCTTCCACAG---CAAGTGCACTTGGAAAACTTCAAGATGTGGTC-

Section 2106

	(162086)	162086 162096 162107 162118 162129 162140 162151 162162
Candidatus Carsonella ruddii strain BT chromosome...	(161643)	TTATAATAATTATTATAAATAACAATTTTACATA--TAATATAAAATTAAAAAAAAATTTTTTTTTATGCAAAAATT
Candidatus Carsonella 135557 - 167442nt	(26087)	TTATAATAATTATTATAAATAACAATTTTACATA--TAATATAAAATTAAAAAAAAATTTTTTTTTATGCAAAAATT
SARS-CoV-2 Reference Genome NC_045512.2	(24419)	-------AACCAAAATGCACAAGCTTTAAACACGCTTGTTAAACAACTTAGCTCCAATTTTGG----TGCAA----T

Section 2107

	(162163)	162163 162173 162184 162195 162206 162217 162228 162239
Candidatus Carsonella ruddii strain BT chromosome...	(161718)	ATTTATTTTTTAAAATTATATTAATATGTGG-TGGAGATGGAAGTATTATAAATAGTTTTAAATATTTTTATAATAG
Candidatus Carsonella 135557 - 167442nt	(26162)	ATTTATTTTTTAAAATTATATTAATATGTGG-TGGAGATGGAAGTATTATAAATAGTTTTAAATATTTTTATAATAG
SARS-CoV-2 Reference Genome NC_045512.2	(24481)	TTCAAGTGTTTTAAATGATATCCTTTCACGTCTTGACAAAGTTGAGGCTGAAGTGCAAATTGATAGGTTGATCACAG

Section 2108

	(162240)	162240 162250 162261 162272 162283 162294 162305 162316
Candidatus Carsonella ruddii strain BT chromosome...	(161794)	AAAAATATTTATTAAAAATTTAAATTTTGGTAATTTAGGATTTTTAACAAATAATAATATTAAAAAAAATTT---T
Candidatus Carsonella 135557 - 167442nt	(26238)	AAAAATATTTATTAAAAATTTAAATTTTGGTAATTTAGGATTTTTAACAAATAATAATATTAAAAAAAATTT---T
SARS-CoV-2 Reference Genome NC_045512.2	(24558)	GCAGAC------------TTCAAAGTTTGCAGACATATGTGACTCAACAATTAATTAGAGCTGCAGAAATCAGAGCT

Section 2109

	(162317)	162317 162327 162338 162349 162360 162371 162382 162393
Candidatus Carsonella ruddii strain BT chromosome...	(161868)	TATTAAAATCTTG-TATTGGTAATATATTAA--TTTTTAGTAAAAATATTAATTTTTCTTTATTATTTTTGAATGAA
Candidatus Carsonella 135557 - 167442nt	(26312)	TATTAAAATCTTG-TATTGGTAATATATTAA--TTTTTAGTAAAAATATTAATTTTTCTTTATTATTTTTGAATGAA
SARS-CoV-2 Reference Genome NC_045512.2	(24623)	TCTGCTAATCTTGCTGCTACTAAAATGTCAGAGTGTGTACTTGGACAATCAAAAAGAGTTGATTTTTGTGGAAAGGG

Section 2110

	(162394)	162394 162404 162415 162426 162437 162448 162459 162470
Candidatus Carsonella ruddii strain BT chromosome...	(161942)	ATATTAATTAAAAATAAAAGTTTAAAAATAAAAATTAAAAACAATTTTTTTTATTCTGATTTAATTTTAATATCATT
Candidatus Carsonella 135557 - 167442nt	(26386)	ATATTAATTAAAAATAAAAGTTTAAAAATAAAAATTAAAAACAATTTTTTTTATTCTGATTTAATTTTAATATCATT
SARS-CoV-2 Reference Genome NC_045512.2	(24700)	CTATCATCTTATGTCCTTCCCTCAGTCAGCACCTCATGGTGTAGTCTTCTTGCATGTGACTTA--TGTCCCTGCACA

Section 2111

	(162471)	162471 162481 162492 162503 162514 162525 162536 162547
Candidatus Carsonella ruddii strain BT chromosome...	(162019)	AGATAATGGAT---CAACAGGATATATGTTTTCTAATAAT---AAAATACAATTTAAAGGTAATTATTTAACATTTT
Candidatus Carsonella 135557 - 167442nt	(26463)	AGATAATGGAT---CAACAGGATATATGTTTTCTAATAAT---AAAATACAATTTAAAGGTAATTATTTAACATTTT
SARS-CoV-2 Reference Genome NC_045512.2	(24775)	AGAAAAGAACTTCACAACTGCTCCTGCCATTTGTCATGATGGAAAAGCACACTTTCCTCGTGAAGGTGTC---TTTG

Section 2112

	(162548)	162548 162558 162569 162580 162591 162602 162613 162624
Candidatus Carsonella ruddii strain BT chromosome...	(162090)	TATTAATAAGATCTCATACAAATAAAAAATTTATTTTTTTTTTTAAAAAAATAAAAATTAAAATTAATTCTAAAATA
Candidatus Carsonella 135557 - 167442nt	(26534)	TATTAATAAGATCTCATACAAATAAAAAATTTATTTTTTTTTTTAAAAAAATAAAAATTAAAATTAATTCTAAAATA
SARS-CoV-2 Reference Genome NC_045512.2	(24849)	TTTCAAATGGCACACACTGGTTTGTAACACAAAGGAATTTTTATGAACCACAAATCATTACTACAGACAACACATTT

Section 2113

		162625 162635 162646 162657 162668 162679 162690 162701
	(162625)	
Candidatus Carsonella ruddii strain BT chromosome...	(162167)	AATTTAATTTTAGATTATGTTCAAAAATTTTTTTTAAAAAACAATATTTTAATAATTTTTATTAAAAAAAAAAATAT
Candidatus Carsonella 135557 - 167442nt	(26611)	AATTTAATTTTAGATTATGTTCAAAAATTTTTTTTAAAAAACAATATTTTAATAATTTTTATTAAAAAAAAAAATAT
SARS-CoV-2 Reference Genome NC_045512.2	(24926)	GTGTCTGGTAACTGTGATGTTGTAATAGGAATTGTCAACAACACAGTTT--ATGATCCTTTGCAACCTGAATTAGAC

Section 2114

		162702 162712 162723 162734 162745 162756 162767 162778
	(162702)	
Candidatus Carsonella ruddii strain BT chromosome...	(162244)	TTTTTTAA--ATGATTTAAAAATAAAATTATATGATAAATAAAATAAAAAAAGTTAAAAAAAAATTTTTTAAAAATA
Candidatus Carsonella 135557 - 167442nt	(26688)	TTTTTTAA--ATGATTTAAAAATAAAATTATATGATAAATAAAATAAAAAAAGTTAAAAAAAAATTTTTTAAAAATA
SARS-CoV-2 Reference Genome NC_045512.2	(25001)	TCATTCAAGGAGGAGTTAGATA-AATATTTTAAGAATCATACATCACCAGATGTTGATTTAGGTGACATCTCTGGCA

Section 2115

		162779 162789 162800 162811 162822 162833 162844 162855
	(162779)	
Candidatus Carsonella ruddii strain BT chromosome...	(162319)	AAAATATTTATAATTTTTTAATTAAAATAGGAATACAAAAAAAAAATTT--TTTAAAAGAAAATTTTTTAATAAATA
Candidatus Carsonella 135557 - 167442nt	(26763)	AAAATATTTATAATTTTTTAATTAAAATAGGAATACAAAAAAAAAATTT--TTTAAAAGAAAATTTTTTAATAAATA
SARS-CoV-2 Reference Genome NC_045512.2	(25077)	TTAATGCTTCAGTTGTAAACATTCAAAAAGAAATTGACCGCCTCAATGAGGTTGCCAAGAATTTAAATGAATCTCTC

Section 2116

		162856 162866 162877 162888 162899 162910 162921 162932
	(162856)	
Candidatus Carsonella ruddii strain BT chromosome...	(162394)	ATTGTTTAATAAATACTTGGATAAAA--ATTATTAAAAAAAAATTTTTTTTTTTTTTTGGTTATTCAAATTCTTTAT
Candidatus Carsonella 135557 - 167442nt	(26838)	ATTGTTTAATAAATACTTGGATAAAA--ATTATTAAAAAAAAATTTTTTTTTTTTTTTGGTTATTCAAATTCTTTAT
SARS-CoV-2 Reference Genome NC_045512.2	(25154)	ATCGATCTCCAAGAACTTGGAAAGTATGAGCAGTATATAAAATGGCCATGGTACATTTGGCTAGGT----------T

Section 2117

		162933 162943 162954 162965 162976 162987 162998 163009
	(162933)	
Candidatus Carsonella ruddii strain BT chromosome...	(162469)	TTTTATCTGGAATAATTAAAATATTTTTTTTAATATTAAATAAAAATTTATTAAATAATATTAAAATATTATTTAAA
Candidatus Carsonella 135557 - 167442nt	(26913)	TTTTATCTGGAATAATTAAAATATTTTTTTTAATATTAAATAAAAATTTATTAAATAATATTAAAATATTATTTAAA
SARS-CoV-2 Reference Genome NC_045512.2	(25221)	TTATAGCTGGCTTGATTGCCATAGTAATGGTGACA----ATTATGCTTTGCT-----GTATGACCAGTTGCTGTAGT

Section 2118

		163010 163020 163031 163042 163053 163064 163075 163086
	(163010)	
Candidatus Carsonella ruddii strain BT chromosome...	(162546)	TTTAATATTTTAAAAATTTTAAAAATTGAAAAAAAAATAACAAATTTTAGAAAAGAATCTTTTAATAATATTTTATA
Candidatus Carsonella 135557 - 167442nt	(26990)	TTTAATATTTTAAAAATTTTAAAAATTGAAAAAAAAATAACAAATTTTAGAAAAGAATCTTTTAATAATATTTTATA
SARS-CoV-2 Reference Genome NC_045512.2	(25289)	T-----GTCTCAAGGGCTGTTGTTCTTGTGGATCCTGCTGCAAATTTGATGAAGACGACTCTGAGCCAGTGCTCAAA

Section 2119

		163087 163097 163108 163119 163130 163141 163152 163163
	(163087)	
Candidatus Carsonella ruddii strain BT chromosome...	(162623)	TTATATATATAATTTTATAATAAATAATTAAAACAACTTATTAAATTATA-ATGACAA-CTAAATAATTT-AATAAA
Candidatus Carsonella 135557 - 167442nt	(27067)	TTATATATATAATTTTATAATAAATAATTAAAACAACTTATTAAATTATA-ATGACAA-CTAAATAATTT-AATAAA
SARS-CoV-2 Reference Genome NC_045512.2	(25361)	GGAGTCAAA-----TTACATTACACA--TAAACGAACTTATGGATTTGTTTATGAGAATCTTCACAATTGGAACTGT

Section 2120

		163164 163174 163185 163196 163207 163218 163229 163240
	(163164)	
Candidatus Carsonella ruddii strain BT chromosome...	(162697)	AATTATAAATATAAATGAA-TCTATATCACAATCAAAAAATATTTTTTTTATAATTTGAATATTATTAGAATAATAT
Candidatus Carsonella 135557 - 167442nt	(27141)	AATTATAAATATAAATGAA-TCTATATCACAATCAAAAAATATTTTTTTTATAATTTGAATATTATTAGAATAATAT
SARS-CoV-2 Reference Genome NC_045512.2	(25431)	AACTTGAAGCAAGGTGAAATCAAGGATGCTACTCCTTCAGATTTTGTTCGCGCTACTGCAACGATACCGATACAAG

Section 2121

		163241 163251 163262 163273 163284 163295 163306 163317
	(163241)	
Candidatus Carsonella ruddii strain BT chromosome...	(162773)	CCTTTTCTCCAAAAAATTAATTTACTTCTAGACCAATAACAAGGTTTTTTATAAAAAAAAGATTCAATAAAACAAAA
Candidatus Carsonella 135557 - 167442nt	(27217)	CCTTTTCTCCAAAAAATTAATTTACTTCTAGACCAATAACAAGGTTTTTTATAAAAAAAAGATTCAATAAAACAAAA
SARS-CoV-2 Reference Genome NC_045512.2	(25508)	CCTCACTCCTTTCGGATGGCTTA-TTGTTGGCGTTGCACTTCTTGCTGTTTTTCAGAGCGCTTC-------CAAAA

Section 2122

		163318 163328 163339 163350 163361 163372 163383 163394
	(163318)	
Candidatus Carsonella ruddii strain BT chromosome...	(162850)	TTTATTAATCCAAACTAAAGATAAAATTTTTTTATTTAAAAAATTTTGTAATATTATAGGAAATAATTTTTTTTTCC
Candidatus Carsonella 135557 - 167442nt	(27294)	TTTATTAATCCAAACTAAAGATAAAATTTTTTTATTTAAAAAATTTTGTAATATTATAGGAAATAATTTTTTTTTCC
SARS-CoV-2 Reference Genome NC_045512.2	(25577)	TC-ATAACCCTCAAAAAGAGATGGCAACTA-----------------GCACTCTCCAAGGTGTTCACTTTGTTTGC

Section 2123

		163395 163405 163416 163427 163438 163449 163460 163471
	(163395)	
Candidatus Carsonella ruddii strain BT chromosome...	(162927)	AAATAACTAAAAATAATATTTTATAAAAAAAATTATTCATTTAATTTTATAATTTTTATTAATTTTATTTTTTTAAA
Candidatus Carsonella 135557 - 167442nt	(27371)	AAATAACTAAAAATAATATTTTATAAAAAAAATTATTCATTTAATTTTATAATTTTTATTAATTTTATTTTTTTAAA
SARS-CoV-2 Reference Genome NC_045512.2	(25636)	AACTTGC------TGTTGTTGTTTGTAACAGTTTACTCACACCTTTTGCTCGTTGCTGCTGGCCTT--G-----AAG

Section 2124

		163472 163482 163493 163504 163515 163526 163537 163548
	(163472)	
Candidatus Carsonella ruddii strain BT chromosome...	(163004)	AATTTTTAAAACAAATTTATTAAAAGTTTTTAAAGATAAATTACAATTTACACAATCTCCATAAAAAATAAACATA
Candidatus Carsonella 135557 - 167442nt	(27448)	AATTTTTAAAACAAATTTATTAAAAGTTTTTAAAGATAAATTACAATTTACACAATCTCCATAAAAAATAAACATA
SARS-CoV-2 Reference Genome NC_045512.2	(25700)	CCCCTTTTCTCTATCTTTAT-----GCTTTAGTCTACTTCTTGCAGAGTATAAACTTTGTA-AGAATAATAATGAGG

```
                                                            Section 2125
                                                        (163549) 163549    163559     163570     163581     163592     163603     163614 163625
Candidatus Carsonella ruddii strain BT chromosome... (163081) ATGTTTTTTTATTAAAATTAAAATAATTTATTTTTAATAAACCATTGTGTAAAATTAATTTTTTATTTATTATATAA
            Candidatus Carsonella 135557 - 167442nt  (27525) ATGTTTTTTTATTAAAATTAAAATAATTTATTTTTAATAAACCATTGTGTAAAATTAATTTTTTATTTATTATATAA
       SARS-CoV-2 Reference Genome NC_045512.2  (25771) CTTTGGCTTTGCTGGAA----A----TGCCGTTCCAAAAACCCATT----ACT-TTATGATGCCAACTATTTTCTTT
                                                            Section 2126
                                                        (163626) 163626    163636     163647     163658     163669     163680     163691 163702
Candidatus Carsonella ruddii strain BT chromosome... (163158) TAAAGTAAAAAAATTATTTTACTTAGTATTTTATTTTCTAAAAATAAATTTGTAAAAGAATTTGGTGAATTAATTAT
            Candidatus Carsonella 135557 - 167442nt  (27602) TAAAGTAAAAAAATTATTTTACTTAGTATTTTATTTTCTAAAAATAAATTTGTAAAAGAATTTGGTGAATTAATTAT
       SARS-CoV-2 Reference Genome NC_045512.2  (25835) GCTGGCATACTAATTGT--TACG-ACTATTGTATAC-CTTACAATAG---TGTAAC-----TTCTTCAATTG-TCAT
                                                            Section 2127
                                                        (163703) 163703    163713     163724     163735     163746     163757     163768 163779
Candidatus Carsonella ruddii strain BT chromosome... (163235) TAATTTTTTTTTATAATGTATACAAGTATTATTTAAAAAACATAAACTTAAAATATCTATTAAAATGTTTATTTCTT
            Candidatus Carsonella 135557 - 167442nt  (27679) TAATTTTTTTTTATAATGTATACAAGTATTATTTAAAAAACATAAACTTAAAATATCTATTAAAATGTTTATTTCTT
       SARS-CoV-2 Reference Genome NC_045512.2  (25899) TACTTCAGGTG-ATGGCACA-ACAAGTCCTATTT------------CTGAACATGACTACCAGATTGGTGGTTATAC
                                                            Section 2128
                                                        (163780) 163780    163790     163801     163812     163823     163834     163845 163856
Candidatus Carsonella ruddii strain BT chromosome... (163312) TTATTTTTATAAATTT--ATTAAAAATATTTTTTTTATAAGTTGAATAATATATTTTTGTTTTTGAAAAAACATT-T
            Candidatus Carsonella 135557 - 167442nt  (27756) TTATTTTTATAAATTT--ATTAAAAATATTTTTTTTATAAGTTGAATAATATATTTTTGTTTTTGAAAAAACATT-T
       SARS-CoV-2 Reference Genome NC_045512.2  (25962) TGAAAAATGGGAATCTGGAGTAAAAGACTGTGTTGTATTACACAGTTACTTCACTTCAGACTATTACCAGCTGTACT
                                                            Section 2129
                                                        (163857) 163857    163867     163878     163889     163900     163911     163922 163933
Candidatus Carsonella ruddii strain BT chromosome... (163386) C--CTTTATTTTTTATAAAAATTT-TATAAATCATTTTATTTTTTTTGT-TATTATATAAATATTTATAACAAGAAT
            Candidatus Carsonella 135557 - 167442nt  (27830) C--CTTTATTTTTTATAAAAATTT-TATAAATCATTTTATTTTTTTTGT-TATTATATAAATATTTATAACAAGAAT
       SARS-CoV-2 Reference Genome NC_045512.2  (26039) CAACTCAATTGAGTACAGACACTGGTGTTGAACATGTTACCTTCTTCATCTACAATAAAATTGTTGAT-----GAGC
                                                            Section 2130
                                                        (163934) 163934    163944     163955     163966     163977     163988     163999 164010
Candidatus Carsonella ruddii strain BT chromosome... (163459) CAGTTAAATAAATATTATAATTATATATTTTAACAATATACA-TAAAAATTAAATCATTTTTTTATCAAAAAAACCC
            Candidatus Carsonella 135557 - 167442nt  (27903) CAGTTAAATAAATATTATAATTATATATTTTAACAATATACA-TAAAAATTAAATCATTTTTTTATCAAAAAAACCC
       SARS-CoV-2 Reference Genome NC_045512.2  (26111) CTG--AAGAACATGTCCAAATTCAC-------ACAATCGACGGTTCATCCGGAGTTGTTAATCCAGTAATGGAACCA
```

Section 2131

(164011)	164011 164021 164032 164043 164054 164065 164076 164087
Candidatus Carsonella ruddii strain BT chromosome... (163535)	ATTTTTATAATTAAAATAGAAAGCAACCAAGCATCTAATATTGCTGAATGGTTTTTTCTTATTTCTTCTATATATAT
Candidatus Carsonella 135557 - 167442nt (27979)	ATTTTTATAATTAAAATAGAAAGCAACCAAGCATCTAATATTGCTGAATGGTTTTTTCTTATTTCTTCTATATATAT
SARS-CoV-2 Reference Genome NC_045512.2 (26179)	ATTT--ATGATGAACCGACGACGACTACTAGCGTGCCTTTGTAAGCACAAGCTGATG---AGTACGAACTTATGTAC

Section 2132

(164088)	164088 164098 164109 164120 164131 164142 164153 164164
Candidatus Carsonella ruddii strain BT chromosome... (163612)	TTCATATCTTTTACATAATGAATCTAAATCATTCCTTTTTCTAGGATGTAATTTTCTAAATATTTCTAAAGAATCTA
Candidatus Carsonella 135557 - 167442nt (28056)	TTCATATCTTTTACATAATGAATCTAAATCATTCCTTTTTCTAGGATGTAATTTTCTAAATATTTCTAAAGAATCTA
SARS-CoV-2 Reference Genome NC_045512.2 (26251)	T-CATTCGTTTCGGAAGAGACAGGTACGTTAATAGTTAATAGCGTACTTCTTTTTCTTGC--TTTCGTGGTATTCTT

Section 2133

(164165)	164165 164175 164186 164197 164208 164219 164230 164241
Candidatus Carsonella ruddii strain BT chromosome... (163689)	AAAAATAACATATAGAATATAAACGACTCATACTTCCACCTGAATAATTTATTTCATAATGTAAAAAATTAATATCA
Candidatus Carsonella 135557 - 167442nt (28133)	AAAAATAACATATAGAATATAAACGACTCATACTTCCACCTGAATAATTTATTTCATAATGTAAAAAATTAATATCA
SARS-CoV-2 Reference Genome NC_045512.2 (26325)	GCTAGTTACA-------------CTAGCCATCCTTACTGCGCTTCGATTGTGTGCGTACTGCTGCAA--TATTGTTA

Section 2134

(164242)	164242 164252 164263 164274 164285 164296 164307 164318
Candidatus Carsonella ruddii strain BT chromosome... (163766)	AATTTAGCATTATGAGCTATAATAATAGAATTATTTACAAAACCTAAAAATTCATCTAATTTTTCACAAAAAAGAGG
Candidatus Carsonella 135557 - 167442nt (28210)	AATTTAGCATTATGAGCTATAATAATAGAATTATTTACAAAACCTAAAAATTCATCTAATTTTTCACAAAAAAGAGG
SARS-CoV-2 Reference Genome NC_045512.2 (26387)	ACGTGAGTCTTGT-AA-------AA---CCTTCTTT--------TTACGTTTACTCTCGTGTT----AAAAATCTGA

Section 2135

(164319)	164319 164329 164340 164351 164362 164373 164384 164395
Candidatus Carsonella ruddii strain BT chromosome... (163843)	TTTATTTTTAAGAAAATTATTATTTATACCATGTATATTAAAAGCTTCTTCACTAATTCTTGTTTTTGTA----TAA
Candidatus Carsonella 135557 - 167442nt (28287)	TTTATTTTTAAGAAAATTATTATTTATACCATGTATATTAAAAGCTTCTTCACTAATTCTTGTTTTTGTA----TAA
SARS-CoV-2 Reference Genome NC_045512.2 (26441)	ATTCTTCTAGAGTTCCTGATCTTCTGGTCTAAACGAACTAAA---TATTATATTAGTTTTTCTGTTTGGAACTTTAA

Section 2136

(164396)	164396 164406 164417 164428 164439 164450 164461 164472
Candidatus Carsonella ruddii strain BT chromosome... (163916)	AAATAAGAATGAAAAATTCTACCTGTAATAAAACCATTAATTACTTCAACACAACCAATTTCAATAATTCTATCTCC
Candidatus Carsonella 135557 - 167442nt (28360)	AAATAAGAATGAAAAATTCTACCTGTAATAAAACCATTAATTACTTCAACACAACCAATTTCAATAATTCTATCTCC
SARS-CoV-2 Reference Genome NC_045512.2 (26515)	TTTTAGCCATGGCAGATTCCAACGGTACTATTACCGTTGAAGAGCTTAAAA--AGCTCCTTGAACAATGGAACC---

Section 2137

(164473)		164473 164483 164494 164505 164516 164527 164538 164549
Candidatus Carsonella ruddii strain BT chromosome...	(163993)	TTTATGTGGAGATAATCCAGTTGTTTCAACATCTAAAAAAACATGTCTTTTCATATATTTATTTTTATATTAAATTT
Candidatus Carsonella 135557 - 167442nt	(28437)	TTTATGTGGAGATAATCCAGTTGTTTCAACATCTAAAAAAACATGTCTTTTCATATATTTATTTTTATATTAAATTT
SARS-CoV-2 Reference Genome NC_045512.2	(26587)	-T-------AG-TAAT--AG--GTTTCCTATTCCTTACATGGATTTGTCTTC-TACAATT-TGCCTATGCCAACAGG

Section 2138

(164550)		164550 164560 164571 164582 164593 164604 164615 164626
Candidatus Carsonella ruddii strain BT chromosome...	(164070)	AAAATATTTTTTTTTAAAGATAAACAAATTTATATTTTTTATTTTACACAGGAAATTAACATTTCCATTTATTTTTT
Candidatus Carsonella 135557 - 167442nt	(28514)	AAAATATTTTTTTTTAAAGATAAACAAATTTATATTTTTTATTTTACACAGGAAATTAACATTTCCATTTATTTTTT
SARS-CoV-2 Reference Genome NC_045512.2	(26649)	AATAGGTTTTTGTATATAATTAAGTTAATTT-TCCTCTGGCTGTTAT---GGCCAGTAACTTTAGC--TTGTTTTGT

Section 2139

(164627)		164627 164637 164648 164659 164670 164681 164692 164703
Candidatus Carsonella ruddii strain BT chromosome...	(164147)	TTTTATTAAAAAAAATTTTTTTTAAAAAAAAAATTTTATTATAATTTAAAATATAATTTAAATAATTATTAAAAATT
Candidatus Carsonella 135557 - 167442nt	(28591)	TTTTATTAAAAAAAATTTTTTTTAAAAAAAAAATTTTATTATAATTTAAAATATAATTTAAATAATTATTAAAAATT
SARS-CoV-2 Reference Genome NC_045512.2	(26720)	GCTT---------GCTGCTGTTTACAGAATAAATTGGATCACCGGTGGAATTGCTATCGCAATGGCTTGTCTTG-TA

Section 2140

(164704)		164704 164714 164725 164736 164747 164758 164769 164780
Candidatus Carsonella ruddii strain BT chromosome...	(164224)	ATTTTTATTTTTATC-GATATTTTTTTATTATAAAAAAAATTTTTTTTTATATTTTTTAAATAAAATTCAAATATTT
Candidatus Carsonella 135557 - 167442nt	(28668)	ATTTTTATTTTTATC-GATATTTTTTTATTATAAAAAAAATTTTTTTTTATATTTTTTAAATAAAATTCAAATATTT
SARS-CoV-2 Reference Genome NC_045512.2	(26787)	GGCTTGATGTGGCTCAGCTACTTCATTGCTTCTTTCAGACTGTTTGCGCGTACGCGTTCCATGTGGT-CA-----TT

Section 2141

(164781)		164781 164791 164802 164813 164824 164835 164846 164857
Candidatus Carsonella ruddii strain BT chromosome...	(164300)	TTTTATTTAAAAAATTTTTTTTATTAATATAAAAACCTAAATAATAAATTTTATTGTTTTTAAAAATGAAAAATATT
Candidatus Carsonella 135557 - 167442nt	(28744)	TTTTATTTAAAAAATTTTTTTTATTAATATAAAAACCTAAATAATAAATTTTATTGTTTTTAAAAATGAAAAATATT
SARS-CoV-2 Reference Genome NC_045512.2	(26858)	CAATCCAGAAACTAACATTCTTCTCAACGTGCCA--CTCCATGGCAC-TATTCTGACCAGACCGCTTCTAGAAAGTG

Section 2142

(164858)		164858 164868 164879 164890 164901 164912 164923 164934
Candidatus Carsonella ruddii strain BT chromosome...	(164377)	TTTTTTTTATAATAATTAAAATATTCAAAATATATATTTA-ATTCATAAAAAAAATTTAAAATTTCTTTTTTTACTT
Candidatus Carsonella 135557 - 167442nt	(28821)	TTTTTTTTATAATAATTAAAATATTCAAAATATATATTTA-ATTCATAAAAAAAATTTAAAATTTCTTTTTTTACTT
SARS-CoV-2 Reference Genome NC_045512.2	(26932)	AACTCGTAATCGGAGCTGTGATCCTTCGTGGACATCTTCGTATTGCTGGACACCATCTAGGACG----------CTG

Section 2143

(164935) 164935 164945 164956 164967 164978 164989 165000 165011
Candidatus Carsonella ruddii strain BT chromosome... (164453) TTAAATAATTTAATTTATTTAAAAAAACTATTTTTTTTTTATTTAAACAATTTTCAATTTCTAAAAAATTATAATTT
Candidatus Carsonella 135557 - 167442nt (28897) TTAAATAATTTAATTTATTTAAAAAAACTATTTTTTTTTTATTTAAACAATTTTCAATTTCTAAAAAATTATAATTT
SARS-CoV-2 Reference Genome NC_045512.2 (26999) TGACATCAAGGACCTGCCT-AAAGAAATCACTGTTGCTACATCACGAACGCTTTC--TTA-TTACAAATTGGGAGCT

Section 2144

(165012) 165012 165022 165033 165044 165055 165066 165077 165088
Candidatus Carsonella ruddii strain BT chromosome... (164530) TTAATTATATTATTTAAATTTTTAAATAATTTATCTTTAATAAAAATATTTTT--ATTAAAATTTATTATTTTATTT
Candidatus Carsonella 135557 - 167442nt (28974) TTAATTATATTATTTAAATTTTTAAATAATTTATCTTTAATAAAAATATTTTT--ATTAAAATTTATTATTTTATTT
SARS-CoV-2 Reference Genome NC_045512.2 (27072) T----CGCAGCGTGTAGCAGGTGACTCAGGTTTTGCTGCATACAGTCGCTACAGGATTGGCAACTATAAATTAAACA

Section 2145

(165089) 165089 165099 165110 165121 165132 165143 165154 165165
Candidatus Carsonella ruddii strain BT chromosome... (164605) TTTGTTTTTAAATTAGAAAATAAAAAATTA-TTTGTTTTTTTATAATTTTTAAAAAAAAAATTAATTT--TTAAATA
Candidatus Carsonella 135557 - 167442nt (29049) TTTGTTTTTAAATTAGAAAATAAAAAATTA-TTTGTTTTTTTATAATTTTTAAAAAAAAAATTAATTT--TTAAATA
SARS-CoV-2 Reference Genome NC_045512.2 (27145) CAGACCATTCCAGTAGCAGTGACAATATTGCTTTGCTTGTACAGTAAGTGACAACAGATGTTTCATCTCGTTGACTT

Section 2146

(165166) 165166 165176 165187 165198 165209 165220 165231 165242
Candidatus Carsonella ruddii strain BT chromosome... (164679) TCTTTTTAATAAAAAAAA-ATTTTTTTTTTTATAATAAAAATTTATAAATTTTTTTATTTTTTTAAT-ATTAAAACT
Candidatus Carsonella 135557 - 167442nt (29123) TCTTTTTAATAAAAAAAA-ATTTTTTTTTTTATAATAAAAATTTATAAATTTTTTTATTTTTTTAAT-ATTAAAACT
SARS-CoV-2 Reference Genome NC_045512.2 (27222) TCAGGTTACTATAGCAGAGATATTACTAATTATTATGAGGACTTTTAAAGTTTCCATTTGGAATCTTGATTACATCA

Section 2147

(165243) 165243 165253 165264 165275 165286 165297 165308 165319
Candidatus Carsonella ruddii strain BT chromosome... (164754) AAACCAATATGGAGTAAAATATGAAAAATATTTTTTTTTTTTAATAAATACAAAACCTATTAAATTTATTTTTATTT
Candidatus Carsonella 135557 - 167442nt (29198) AAACCAATATGGAGTAAAATATGAAAAATATTTTTTTTTTTTAATAAATACAAAACCTATTAAATTTATTTTTATTT
SARS-CoV-2 Reference Genome NC_045512.2 (27299) TAAACCTCATA-ATTAAAAATTTATCTAAGTCACTAACTGAGAATAAATATTC--TCAATTAGATGAAGAGCAACCA

Section 2148

(165320) 165320 165330 165341 165352 165363 165374 165385 165396
Candidatus Carsonella ruddii strain BT chromosome... (164831) TAGAATAAAAAACAAACTTTAATTTAAATAATGTTTTTTTTTA--TAAAAAATTTTTTTTTGTGTAATATTTATAA
Candidatus Carsonella 135557 - 167442nt (29275) TAGAATAAAAAACAAACTTTAATTTAAATAATGTTTTTTTTTA--TAAAAAATTTTTTTTTGTGTAATATTTATAA
SARS-CoV-2 Reference Genome NC_045512.2 (27373) ATGGAGATTGATTAAACGAACATGAAAATTATTCTTTTCTTGGCACTGATAACACTCGCTACTTGTGAGCTTTATCA

Section 2149

		165397 165407 165418 165429 165440 165451 165462 165473
	(165397)	
Candidatus Carsonella ruddii strain BT chromosome...	(164906)	AATTTTTAAATTTTATTTTTGAAAAAAAAATATTTTTATAATTTAAATTAAACAAATTATAATTTGATTTAAAAAAA
Candidatus Carsonella 135557 - 167442nt	(29350)	AATTTTTAAATTTTATTTTTGAAAAAAAAATATTTTTATAATTTAAATTAAACAAATTATAATTTGATTTAAAAAAA
SARS-CoV-2 Reference Genome NC_045512.2	(27450)	C-TACCAAGAGTGTGTTAGAGGTACAACAGTACTTTTA--A---------AAGAACCT-TGCTCT--TCTGGAACAT

Section 2150

		165474 165484 165495 165506 165517 165528 165539 165550
	(165474)	
Candidatus Carsonella ruddii strain BT chromosome...	(164983)	ATAATTTCTTTTTTTTTTAATTTAATATAATAGTTTAAAAAATATATATTTTTTTTTTTTTTTTTAAAAAATTTTTT
Candidatus Carsonella 135557 - 167442nt	(29427)	ATAATTTCTTTTTTTTTTAATTTAATATAATAGTTTAAAAAATATATATTTTTTTTTTTTTTTTTAAAAAATTTTTT
SARS-CoV-2 Reference Genome NC_045512.2	(27512)	ACGAGGGCAATTCACC---ATTTCATCCTCTAGCTGATAACAAATTTGCACTGACTTGCTTTAGCACTCAATTTGCT

Section 2151

		165551 165561 165572 165583 165594 165605 165616 165627
	(165551)	
Candidatus Carsonella ruddii strain BT chromosome...	(165060)	TAAAAAAAAAAATAAAAAAAAAAAAAATATCTTTTAAATAAAAAAAAATAAAATTTAAAAAAATAAATTTTTTTTA
Candidatus Carsonella 135557 - 167442nt	(29504)	TAAAAAAAAAAATAAAAAAAAAAAAAATATCTTTTAAATAAAAAAAAATAAAATTTAAAAAAATAAATTTTTTTTA
SARS-CoV-2 Reference Genome NC_045512.2	(27586)	TTTGCTTGTCC-TGACGGCGTAAAACACGTCTAT--CAGTTACGTGCCAGATCAGTTTCACCTA-AACTGTTCATCA

Section 2152

		165628 165638 165649 165660 165671 165682 165693 165704
	(165628)	
Candidatus Carsonella ruddii strain BT chromosome...	(165137)	AAAAATAATTATTAAAATATTTTTTTTTTGTAAAATTTTTGTTTGTTTTTTTAATAATAACAAAAATTTCAAAAAAA
Candidatus Carsonella 135557 - 167442nt	(29581)	AAAAATAATTATTAAAATATTTTTTTTTTGTAAAATTTTTGTTTGTTTTTTTAATAATAACAAAAATTTCAAAAAAA
SARS-CoV-2 Reference Genome NC_045512.2	(27659)	GACAAGAGGAAGTTCAAGAACTTTACTCTCCAATTTTTCTTATTGTTGCGGCAATAGTG-------TTT------A-

Section 2153

		165705 165715 165726 165737 165748 165759 165770 165781
	(165705)	
Candidatus Carsonella ruddii strain BT chromosome...	(165214)	AATAAATATTTTTTGAAAAATAAAAATAAAAA-ATTATTATTGTTTTTTT-TTAAAAAAGAAAAATTAATCATAAAT
Candidatus Carsonella 135557 - 167442nt	(29658)	AATAAATATTTTTTGAAAAATAAAAATAAAAA-ATTATTATTGTTTTTTT-TTAAAAAAGAAAAATTAATCATAAAT
SARS-CoV-2 Reference Genome NC_045512.2	(27722)	--TAACACTTTGCTTCACACTCAAAAGAAAGACAGAATGATTGAACTTTCATTAATTGACTTCTATTTGTGCTTTTT

Section 2154

		165782 165792 165803 165814 165825 165836 165847 165858
	(165782)	
Candidatus Carsonella ruddii strain BT chromosome...	(165289)	ATTTTTAATATGATTTTTTTTTTTAAAAA--CAAAAAATAATCTATCTAAACCTATACCTCCAGCAATCATTTTAAT
Candidatus Carsonella 135557 - 167442nt	(29733)	ATTTTTAATATGATTTTTTTTTTTAAAAA--CAAAAAATAATCTATCTAAACCTATACCTCCAGCAATCATTTTAAT
SARS-CoV-2 Reference Genome NC_045512.2	(27797)	AGCCTTTCTGCTATTCCTTGTTTTAATTATGCTTATTATCTTTTGGTTCTCACTTGAACTGCAAGA-TCATAATGAA

Section 2155

	(165859)	165859 165869 165880 165891 165902 165913 165924 165935
Candidatus Carsonella ruddii strain BT chromosome...	(165364)	ATTTATTTTA--TTATTTAATAAAATATTTTTATTTATCAAACCATACCCAGCTAATTCTATCCATTCTAATTTATT
Candidatus Carsonella 135557 - 167442nt	(29808)	ATTTATTTTA--TTATTTAATAAAATATTTTTATTTATCAAACCATACCCAGCTAATTCTATCCATTCTAATTTATT
SARS-CoV-2 Reference Genome NC_045512.2	(27873)	ACTTGTCACGCCTAAACGAACATGAAATTTCTTGTTTTCTTAGGA-ATCATCACAACTGTAGC--TGC--ATTTCAC

Section 2156

	(165936)	165936 165946 165957 165968 165979 165990 166001 166012
Candidatus Carsonella ruddii strain BT chromosome...	(165439)	TAATATATCTATTTCAAAAGAATTTATTGTAAA---AGGAAATTTAGTTTTTCTAATTTTAAAAAAAAACTTTTTTT
Candidatus Carsonella 135557 - 167442nt	(29883)	TAATATATCTATTTCAAAAGAATTTATTGTAAA---AGGAAATTTAGTTTTTCTAATTTTAAAAAAAAACTTTTTTT
SARS-CoV-2 Reference Genome NC_045512.2	(27945)	CAAGA-ATGTAGTTTACAGTCATGTACTCAACATCAACCATATGTAGTTGATGACCCGTGTCCTATTCACTTCTATT

Section 2157

	(166013)	166013 166023 166034 166045 166056 166067 166078 166089
Candidatus Carsonella ruddii strain BT chromosome...	(165513)	TTAAAGATTTAGAAAAAAAATTCAATAAAAATTCTAAAATTTTATTTTTTTTATTTATTATAAAATCTATTTGATAA
Candidatus Carsonella 135557 - 167442nt	(29957)	TTAAAGATTTAGAAAAAAAATTCAATAAAAATTCTAAAATTTTATTTTTTTTATTTATTATAAAATCTATTTGATAA
SARS-CoV-2 Reference Genome NC_045512.2	(28021)	CTAAATGGTATATTAGAGTAGGAGCTAGAAAATCAGCACCTTTAATTGAATTGTGCGTGGATGAGGCTGGTTCTAAA

Section 2158

	(166090)	166090 166100 166111 166122 166133 166144 166155 166166
Candidatus Carsonella ruddii strain BT chromosome...	(165590)	AAACAAGGTAAATGTGTTT-TATCG-TAATCATTTC-TATAAACTTTACCTATATTAAAATAATTATTAGTAAAATA
Candidatus Carsonella 135557 - 167442nt	(30034)	AAACAAGGTAAATGTGTTT-TATCG-TAATCATTTC-TATAAACTTTACCTATATTAAAATAATTATTAGTAAAATA
SARS-CoV-2 Reference Genome NC_045512.2	(28098)	TCACCCATTCAGTACATCGATATCGGTAATTATACAGTTTCCTGTTTACCTTT-TACAATTAATTGCCAGGAACCTA

Section 2159

	(166167)	166167 166177 166188 166199 166210 166221 166232 166243
Candidatus Carsonella ruddii strain BT chromosome...	(165664)	ATTTTTAAATAATCTATTTTGAAAACAAGAAGTATGAGTTCTTAAAATTTTATTTTTAAAAT-ATAAACTACCTTTT
Candidatus Carsonella 135557 - 167442nt	(30108)	ATTTTTAAATAATCTATTTTGAAAACAAGAAGTATGAGTTCTTAAAATTTTATTTTTAAAAT-ATAAACTACCTTTT
SARS-CoV-2 Reference Genome NC_045512.2	(28174)	AATTGGG--TAGTCT----TGTAGT---GCGTTGTTCGTTCTATGAAGACTTTTTAGAGTATCATGACGTTCGTGTT

Section 2160

	(166244)	166244 166254 166265 166276 166287 166298 166309 166320
Candidatus Carsonella ruddii strain BT chromosome...	(165740)	ATATCTTTTTTAAAAATATTTAATAATTTAAAAT-TATAATTATAATTTTCAAACTCAGTAGTATTTAATT-GTATA
Candidatus Carsonella 135557 - 167442nt	(30184)	ATATCTTTTTTAAAAATATTTAATAATTTAAAAT-TATAATTATAATTTTCAAACTCAGTAGTATTTAATT-GTATA
SARS-CoV-2 Reference Genome NC_045512.2	(28242)	GTTTTAGATTTCATCTAAACGAACAAACTAAAATGTCTGATAATGGACCCCAAAATCAGCGAAATGCACCCCGCATT

Section 2161

	(166321)	166321 166331 166342 166353 166364 166375 166386 166397
Candidatus Carsonella ruddii strain BT chromosome...	(165815)	AAATTATTTCTTAAAAAA-ATTTTTTTAGAATTTACTATAAAATTTGAATAAAAATTATTTT----TTAAAAAAAAA
Candidatus Carsonella 135557 - 167442nt	(30259)	AAATTATTTCTTAAAAAA-ATTTTTTTAGAATTTACTATAAAATTTGAATAAAAATTATTTT----TTAAAAAAAAA
SARS-CoV-2 Reference Genome NC_045512.2	(28319)	ACGTTTGGTGGACCCTCAGATTCAACTGGCAGTAACCAGAA---TGGAGAACGCAGTGGGGCGCGATCAAAACAACG

Section 2162

	(166398)	166398 166408 166419 166430 166441 166452 166463 166474
Candidatus Carsonella ruddii strain BT chromosome...	(165887)	TTTTTTTTTTTATTAAAAAAATAAAATTTTTTATTTTTTTTGTTTTCTATAAAGTTAATTTTATTAATAAAAAAATA
Candidatus Carsonella 135557 - 167442nt	(30331)	TTTTTTTTTTTATTAAAAAAATAAAATTTTTTATTTTTTTTGTTTTCTATAAAGTTAATTTTATTAATAAAAAAATA
SARS-CoV-2 Reference Genome NC_045512.2	(28393)	TCGGCCCCAAGGTTTACCCAATAATACTGCGTCTTGGTTCACCGCTCTC--A-CTCAAC-------ATGGCAAGGAA

Section 2163

	(166475)	166475 166485 166496 166507 166518 166529 166540 166551
Candidatus Carsonella ruddii strain BT chromosome...	(165964)	ATTTTTAATTTATTTATATTACAATAATTTTTTTTTAATAAAAATACTAATTTATTATAAGAAAAACCAAAATATA-
Candidatus Carsonella 135557 - 167442nt	(30408)	ATTTTTAATTTATTTATATTACAATAATTTTTTTTTAATAAAAATACTAATTTATTATAAGAAAAACCAAAATATA-
SARS-CoV-2 Reference Genome NC_045512.2	(28460)	GACCTTAAATTCCCTCGAGGACAAGGCGTTCCAATTAACACCAATAGCAGTCCAGATGACCAAATTGGCTACTACCG

Section 2164

	(166552)	166552 166562 166573 166584 166595 166606 166617 166628
Candidatus Carsonella ruddii strain BT chromosome...	(166040)	---ATCTAAAATAAAATTAATTATAATTATTTTTTTAATTGAAAAAAATCTTTTTTTAATTTTTTTC--CTATAT
Candidatus Carsonella 135557 - 167442nt	(30484)	---ATCTAAAATAAAATTAATTATAATTATTTTTTTAATTGAAAAAAATCTTTTTTTAATTTTTTTC--CTATAT
SARS-CoV-2 Reference Genome NC_045512.2	(28537)	AAGAGCTACCAGACGAATTCGTGGTGGTGACGGTA--AAATGAAAGATCTCAGTCCAAGATGGTATTTCTACTACCT

Section 2165

	(166629)	166629 166639 166650 166661 166672 166683 166694 166705
Candidatus Carsonella ruddii strain BT chromosome...	(166112)	A--ATTTTTACATAAAGATTTTATATAGTATTGTT-TAGATAATTTAAAACAATTTTTTTTTTTACCAAAAAATCCT
Candidatus Carsonella 135557 - 167442nt	(30556)	A--ATTTTTACATAAAGATTTTATATAGTATTGTT-TAGATAATTTAAAACAATTTTTTTTTTTACCAAAAAATCCT
SARS-CoV-2 Reference Genome NC_045512.2	(28612)	AGGAACTGGGCCAGAAGCTGGACTTCCCTATGGTGCTAACAAAGACGGCATCATATGGGTTGCAACTGAGGGAGCCT

Section 2166

	(166706)	166706 166716 166727 166738 166749 166760 166771 166782
Candidatus Carsonella ruddii strain BT chromosome...	(166186)	TTTGTATAATTTAAATATTTTTTTCTTTTTTTAATTTGTAATGATTTTACAGATCTTGTCATAATATTTTTTTAATT
Candidatus Carsonella 135557 - 167442nt	(30630)	TTTGTATAATTTAAATATTTTTTTCTTTTTTTAATTTGTAATGATTTTACAGATCTTGTCATAATATTTTTTTAATT
SARS-CoV-2 Reference Genome NC_045512.2	(28689)	T--GAATACACCAAAAG-----ATCACATTGGCACCCGCAATCCTGCTAACAATGCTGC---AATCGTGCTACAACT

```
                                                              Section 2167
                                          (166783) 166783    166793     166804     166815     166826     166837     166848 166859
Candidatus Carsonella ruddii strain BT chromosome... (166263) TTTTTAACAATTGAT-TTATTTAAATAATTTTTTG-ATAAATTAAAACTTTTTTTATTAGAAGATTTATTAATTAAT
           Candidatus Carsonella 135557 - 167442nt  (30707) TTTTTAACAATTGAT-TTATTTAAATAATTTTTTG-ATAAATTAAAACTTTTTTTATTAGAAGATTTATTAATTAAT
      SARS-CoV-2 Reference Genome NC_045512.2  (28756) TCCTCAAGGAACAACATTGCCAAAAGGCTTCTACGCAGAAGGGAGCAGAGGCGGCAGTCAAGCCTCTTCTCGTTCCT
```

```
                                                              Section 2168
                                          (166860) 166860    166870     166881     166892     166903     166914     166925 166936
Candidatus Carsonella ruddii strain BT chromosome... (166338) AA--ATGATGTTTATTTGCTTTTAATAATTTTATTTTTTTTTTAATTATTATT-CTTTTTTTTATAGATTTTATACT
           Candidatus Carsonella 135557 - 167442nt  (30782) AA--ATGATGTTTATTTGCTTTTAATAATTTTATTTTTTTTTTAATTATTATT-CTTTTTTTTATAGATTTTATACT
      SARS-CoV-2 Reference Genome NC_045512.2  (28833) CATCACGTAGTCGCAACAGTTCAAGAAATTCAACTCCAGGCAGCAGTAGGGGAACTTCTCCTGCTAGAATGGCTGGC
```

```
                                                              Section 2169
                                          (166937) 166937    166947     166958     166969     166980     166991     167002 167013
Candidatus Carsonella ruddii strain BT chromosome... (166412) TTTTTTTTTTATTTTCATTTTTAATTTTTGGTATAAAAATAGAAAATATATATTTTCCTTCAAATTCTATATCAGAA
           Candidatus Carsonella 135557 - 167442nt  (30856) TTTTTTTTTTATTTTCATTTTTAATTTTTGGTATAAAAATAGAAAATATATATTTTCCTTCAAATTCTATATCAGAA
      SARS-CoV-2 Reference Genome NC_045512.2  (28910) AATGGCGGTGATGCTGCTCTTGCTTTGCTGCTGCTTGAC-AGATTGAACCAGCTTGAGAGCAAAATGTCTGGTAAAG
```

```
                                                              Section 2170
                                          (167014) 167014    167024     167035     167046     167057     167068     167079 167090
Candidatus Carsonella ruddii strain BT chromosome... (166489) AATTTAAAATAAACTCCTTTAATATTATTTTGAAATTTTAAAATTAA-CTCAACACCTTTTTCTTTAAAAA--TAAT
           Candidatus Carsonella 135557 - 167442nt  (30933) AATTTAAAATAAACTCCTTTAATATTATTTTGAAATTTTAAAATTAA-CTCAACACCTTTTTCTTTAAAAA--TAAT
      SARS-CoV-2 Reference Genome NC_045512.2  (28986) GCCAACAACAACAAGGCCAAACTGTCACTAAGAAATCTGCTGCTGAGGCTTCTAAGAAGCCTCGGCAAAAACGTACT
```

```
                                                              Section 2171
                                          (167091) 167091    167101     167112     167123     167134     167145     167156 167167
Candidatus Carsonella ruddii strain BT chromosome... (166563) TTCTCTTC--CTTTAAAAATTACACAAACTTTTAAAGTATAACCTTCTAATAAAAAAAAATTAGCTTTTTTAATTTT
           Candidatus Carsonella 135557 - 167442nt  (31007) TTCTCTTC--CTTTAAAAATTACACAAACTTTTAAAGTATAACCTTCTAATAAAAAAAAATTAGCTTTTTTAATTTT
      SARS-CoV-2 Reference Genome NC_045512.2  (29063) GCCACTAAAGCATACAATGTAACACAAGCTTTCGGCAGACGTGGTCCAGAACAAACCCAAGGAAATTTTGGGGACC-
```

```
                                                              Section 2172
                                          (167168) 167168    167178     167189     167200     167211     167222     167233 167244
Candidatus Carsonella ruddii strain BT chromosome... (166638) AATATCATAATCTCTTTTATGAATATTTAAACCAATT-----CTAATTTCTTTTAATCTACCTACTTTGGATTTTTT
           Candidatus Carsonella 135557 - 167442nt  (31082) AATATCATAATCTCTTTTATGAATATTTAAACCAATT-----CTAATTTCTTTTAATCTACCTACTTTGGATTTTTT
      SARS-CoV-2 Reference Genome NC_045512.2  (29139) AGGAAC-TAATCAGACAAGGAACTGATTACAAACATTGGCCGCAAATTGCA--CAATTTGCCCCCAGCGCTTCAGCG
```

Section 2173

	(167245)	167245 167255 167266 167277 167288 167299 167310 167321
Candidatus Carsonella ruddii strain BT chromosome...	(166710)	TTTTACAATTTTTTTTTTCTATTATTTTTAAAAATTAAAAATAATTGATTTTTTTTTTTGTTAAAAAAAATAAAAG
Candidatus Carsonella 135557 - 167442nt	(31154)	TTTTACAATTTTTTTTTTCTATTATTTTTAAAAATTAAAAATAATTGATTTTTTTTTTTGTTAAAAAAAATAAAAG
SARS-CoV-2 Reference Genome NC_045512.2	(29213)	TTCTTCGGAATGTC----GCGCATTGGCATGGAAGTCACACCTTCGGGAACGTGGTTGACCTACACAGGTGCCATCA

Section 2174

	(167322)	167322 167332 167343 167354 167365 167376 167387 167398
Candidatus Carsonella ruddii strain BT chromosome...	(166787)	AATACTTTTTAGTTTTTTTAAAAATAAATAAAAAATTTATTTTTAATAAATTGTATAAAAAAGTTTTTATTAAAATT
Candidatus Carsonella 135557 - 167442nt	(31231)	AATACTTTTTAGTTTTTTTAAAAATAAATAAAAAATTTATTTTTAATAAATTGTATAAAAAAGTTTTTATTAAAATT
SARS-CoV-2 Reference Genome NC_045512.2	(29286)	AATTGGATGACAAAGATCCAAATTTCAAAGATCAAGTCATTTTGCTGAATAAGCATATTGACGCATACAAAACATTC

Section 2175

	(167399)	167399 167409 167420 167431 167442 167453 167464 167475
Candidatus Carsonella ruddii strain BT chromosome...	(166864)	ATATTAATTTTATTTAAAATTAA--AAAAGAAATTTTTTTATACATTTATTAATTATTTAAAATCAGATTTTGAAAT
Candidatus Carsonella 135557 - 167442nt	(31308)	ATATTAATTTTATTTAAAATTAA--AAAAGAAATTTTTTTATACATTTATTAATTATTTAAAATCAGATTTTGAAAT
SARS-CoV-2 Reference Genome NC_045512.2	(29363)	CCACCAACAGAGCCTAAAAAGGACAAAAAGAAGAAGGCTGATGAA---ACTCAAGCCTTACCG-CAGAGACAGAAGA

Section 2176

	(167476)	167476 167486 167497 167508 167519 167530 167541 167552
Candidatus Carsonella ruddii strain BT chromosome...	(166939)	CTGTTTTCAATTTCATACCAATTTATATTTTCTAAAAAATTGTATAAATATTGATTTTTATTGTTTAAATAATCTAA
Candidatus Carsonella 135557 - 167442nt	(31383)	CTGTTTTCAATTTCATACCAATTTATATTTTCTAAAAAATTGTATAAATATTGATTTTTATTGTTTAAATAATCTAA
SARS-CoV-2 Reference Genome NC_045512.2	(29436)	AACAGCAAACTGTGACTC----TTCTTCCTGCTGCAGATTTGGATG---ATTTCTCCAAACAATTGCAACAATCCA-

Section 2177

	(167553)	167553 167563 167574 167585 167596 167607 167618 167629
Candidatus Carsonella ruddii strain BT chromosome...	(167016)	ATAATAAGAATGTTCATGTAAATCAATACATATCAAAGGAACACTGTTAAAACCACCTAAGTCTATTGAGAATAATG
Candidatus Carsonella 135557 - 167442nt	(31460)	ATAATAAGAATGTTCATGTAAATCAATACATATCAAAGGAACACTGTTAAAACCACCTAAGTCTATTGAGAATAATG
SARS-CoV-2 Reference Genome NC_045512.2	(29505)	-TGAGCAG--TGCTGACTCAACTCAGGCCTAAACTCATGCAGACCACACAAGGCAGATG-GGCTAT-----ATAAAC

Section 2178

	(167630)	167630 167640 167651 167662 167673 167684 167695 167706
Candidatus Carsonella ruddii strain BT chromosome...	(167093)	GATTATTATTTTCATAAGTTTTTATCATACATAATCTATTATTGTTATACACTAACCATCCCCAACCATTAAAATTT
Candidatus Carsonella 135557 - 167442nt	(31537)	GATTATTATTTTCATAAGTTTTTATCATACATAATCTATTATTGTTATACACTAACCATCCCCAACCATTAAAATTT
SARS-CoV-2 Reference Genome NC_045512.2	(29573)	GTTTTCGCTTTTC-----CGTTTACGATATATAGTCTACTCTTGTG---CAGAATGAATTCTCGTAACTACATAGCA

Section 2179

(167707) 167707 167717 167728 167739 167750 167761 167772 167783

Candidatus Carsonella ruddii strain BT chromosome... (167170) TCAAAATTTTTAATAAAATTAAATTTAAAATTTTCATAATTATTATAAGTTTTTATAATTTTATTATAAATTTCACC
Candidatus Carsonella 135557 - 167442nt (31614) TCAAAATTTTTAATAAAATTAAATTTAAAATTTTCATAATTATTATAAGTTTTTATAATTTTATTATAAATTTCACC
SARS-CoV-2 Reference Genome NC_045512.2 (29642) CAAGTAGATGTAGTTAACTTTAATCTCACATAGCAATCTTTA--ATCAGTGTGTAACATTAGGGAGGACTTGAAAGA

Section 2180

(167784) 167784 167794 167805 167816 167827 167838 167849 167860

Candidatus Carsonella ruddii strain BT chromosome... (167247) AAAACAAAAATTTTTGTTAAAACA-----AATATTTTTAAAAAAATAATCATGATTTAAATAAATACCTAAAATATT
Candidatus Carsonella 135557 - 167442nt (31691) AAAACAAAAATTTTTGTTAAAACA-----AATATTTTTAAAAAAATAATCATGATTTAAATAAATACCTAAAATATT
SARS-CoV-2 Reference Genome NC_045512.2 (29717) GCCACCACATTTTCACCGAGGCCACGCGGAGTACGATCGAGTGTACAGTGAACAATGCTAGGGAGAGCTGCC-TATA

Section 2181

(167861) 167861 167871 167882 167893 167904 167915 167926 167937

Candidatus Carsonella ruddii strain BT chromosome... (167319) TGAAAAATAATTTTTATCAAATTCAGATAAATCATTTAATATATCTATTAATTCAAATTTATTTTTAAAAATAATAT
Candidatus Carsonella 135557 - 167442nt (31763) TGAAAAATAATTTTTATCAAATTCAGATAAATCATTTAATATATCTATTAATTCAAATTTATTTTTAAAAATAATAT
SARS-CoV-2 Reference Genome NC_045512.2 (29793) TGGAAGAGCCCTAATGTGTA----AAATTAAT--TTTAGTAGTGCTA----TCCCCATGTGATTTTAA---TAGCTT

Section 2182

(167938) 167938 167948 167959 167970 167981 167992 168003 168014

Candidatus Carsonella ruddii strain BT chromosome... (167396) TTTTTTTAAATAAAAAATTATTTAATTCAATTAAACTATTATGATAAATTTTTTTTTGCAATAAAAATTGTTTTTCA
Candidatus Carsonella 135557 - 167442nt (31840) TTTTTTTAAATAAAAAATTATTTAATTCAATTAAACTATTATGATAA------------------------------
SARS-CoV-2 Reference Genome NC_045512.2 (29857) CTTAGGAGAATGACAAAAAAAAAAAAAAAAAAAAAAAAAAAAAAAAA------------------------------

Section 2183

(168015) 168015 168025 168036 168047 168058 168069 168080 168091

Candidatus Carsonella ruddii strain BT chromosome... (167473) CTTAAAAAACTATTTTTTTTATAATTTATATTAAATATTTTAATCATATATTAAAAATATTAATTAACAAAATTAAA
Candidatus Carsonella 135557 - 167442nt (31887) ---
SARS-CoV-2 Reference Genome NC_045512.2 (29904) ---

Section 2184

(168092) 168092 168102 168113 168124 168135 168146 168157 168168

Candidatus Carsonella ruddii strain BT chromosome... (167550) AAACTATTGCATTAAAATAGTTTTTAATAAATTTTTATAAGAATTTTCTTCAGCTATAGTTCTATTTAAACCAATTC
Candidatus Carsonella 135557 - 167442nt (31887) ---
SARS-CoV-2 Reference Genome NC_045512.2 (29904) ---

Section 2185

	(168169)	168169 168179 168190 168201 168212 168223 168234 168245
Candidatus Carsonella ruddii strain BT chromosome...	(167627)	CATAATTTTTAACTATTTTTTTGTAAAATTTACAACATATAATAAAAGTATTATGATTTTTATTTCTTTTAATCTTT
Candidatus Carsonella 135557 - 167442nt	(31887)	---
SARS-CoV-2 Reference Genome NC_045512.2	(29904)	---

Section 2186

	(168246)	168246 168256 168267 168278 168289 168300 168311 168322
Candidatus Carsonella ruddii strain BT chromosome...	(167704)	GATAAAACAAAAGATATATTTTTAAAAGAAATTATTTTTAATATTTCTTTTTTTATATTTAATGTTATTATACTTTT
Candidatus Carsonella 135557 - 167442nt	(31887)	---
SARS-CoV-2 Reference Genome NC_045512.2	(29904)	---

Section 2187

	(168323)	168323 168333 168344 168355 168366 168377 168388 168399
Candidatus Carsonella ruddii strain BT chromosome...	(167781)	AATATAATTATTATAATTAAATAATATTAATAAAAAAGTTAATTTATTTCCAAATTTAATATACATTAAACATGTTG
Candidatus Carsonella 135557 - 167442nt	(31887)	---
SARS-CoV-2 Reference Genome NC_045512.2	(29904)	---

Section 2188

	(168400)	168400 168410 168421 168432 168443 168454 168465 168476
Candidatus Carsonella ruddii strain BT chromosome...	(167858)	AATAATAATAAAAAAAATTATTAAATTTAATTGTTTTTTTAAAAAAAAGAACTCTTTTTTTATAATAATTAAATTTA
Candidatus Carsonella 135557 - 167442nt	(31887)	---
SARS-CoV-2 Reference Genome NC_045512.2	(29904)	---

Section 2189

	(168477)	168477 168487 168498 168509 168520 168531 168542 168553
Candidatus Carsonella ruddii strain BT chromosome...	(167935)	TTTATTTTAAATTTTTTTTTTAAAATATGTAAAAAATTTATTAAATATAAAAATATTCCTAAATTATAAATTTTTTT
Candidatus Carsonella 135557 - 167442nt	(31887)	---
SARS-CoV-2 Reference Genome NC_045512.2	(29904)	---

Section 2190

	(168554)	168554 168564 168575 168586 168597 168608 168619 168630
Candidatus Carsonella ruddii strain BT chromosome...	(168012)	AAATGTTAGAAAATTCATTTTTAATTATATTTATTATTATTTTTTTACTTAAATAAAAATTTGATAATTTTTGCATA
Candidatus Carsonella 135557 - 167442nt	(31887)	---
SARS-CoV-2 Reference Genome NC_045512.2	(29904)	---

Section 2191

	(168631)	168631 168641 168652 168663 168674 168685 168696 168707
Candidatus Carsonella ruddii strain BT chromosome...	(168089)	ATTTGTTTACCATTATTTTGTTTATTTATTAATTTTATTTTTCTTGTTATATCACCACCATAACATTTATCTAAAAC
Candidatus Carsonella 135557 - 167442nt	(31887)	---
SARS-CoV-2 Reference Genome NC_045512.2	(29904)	---

Section 2192

	(168708)	168708 168718 168729 168740 168751 168762 168773 168784
Candidatus Carsonella ruddii strain BT chromosome...	(168166)	ATTTTTTTTATAAGAAAATATTTTTTTTGAAAATATTATTTTTTTTTTTATATAAAATTTTATATTTATATTAATTA
Candidatus Carsonella 135557 - 167442nt	(31887)	---
SARS-CoV-2 Reference Genome NC_045512.2	(29904)	---

Section 2193

	(168785)	168785 168795 168806 168817 168828 168839 168850 168861
Candidatus Carsonella ruddii strain BT chromosome...	(168243)	AATTATTTTTAATATTATTTTTTATTTTTTGAAAAAATAAATAACAATTATTTATTAAATCATTTTCTATAATAAAA
Candidatus Carsonella 135557 - 167442nt	(31887)	---
SARS-CoV-2 Reference Genome NC_045512.2	(29904)	---

Section 2194

	(168862)	168862 168872 168883 168894 168905 168916 168927 168938
Candidatus Carsonella ruddii strain BT chromosome...	(168320)	TTAAATTCTTCTATTATTTTATCATTTATAGAAACTTTAATAAAAAAAAGTTCACTTTTTAAAAAATGATGAAATTT
Candidatus Carsonella 135557 - 167442nt	(31887)	---
SARS-CoV-2 Reference Genome NC_045512.2	(29904)	---

Section 2195

	(168939)	168939 168949 168960 168971 168982 168993 169004 169015
Candidatus Carsonella ruddii strain BT chromosome...	(168397)	AAATTCATAAAATAAAAAACCATTTATTTTTTTATTTAAAATTAAAAAAAAATTATTAATAATTTTATATAAATTTA
Candidatus Carsonella 135557 - 167442nt	(31887)	---
SARS-CoV-2 Reference Genome NC_045512.2	(29904)	---

Section 2196

	(169016)	169016 169026 169037 169048 169059 169070 169081 169092
Candidatus Carsonella ruddii strain BT chromosome...	(168474)	TATAAAAAATAATAATTAATAAATTATCATATTTTTTTATTTTTATTTTATAATAATTAAAATTAAAAATATATTGT
Candidatus Carsonella 135557 - 167442nt	(31887)	---
SARS-CoV-2 Reference Genome NC_045512.2	(29904)	---

Section 2197

(169093)	169093 169103 169114 169125 169136 169147 169158 169169
Candidatus Carsonella ruddii strain BT chromosome... (168551)	ATTATTAAAAAAAAATTTTTTTTTAAAACATATAAATTAATAATTACAATTTGTTCATATTCAAAAAAATTTTTTTC
Candidatus Carsonella 135557 - 167442nt (31887)	---
SARS-CoV-2 Reference Genome NC_045512.2 (29904)	---

Section 2198

(169170)	169170 169180 169191 169202 169213 169224 169235 169246
Candidatus Carsonella ruddii strain BT chromosome... (168628)	ATTAAAAATATTTATTATCCAAATATTTTTTTTTATTAAATAAAAAAATATTATTGGATTTGTTATCCAAAATTTAG
Candidatus Carsonella 135557 - 167442nt (31887)	---
SARS-CoV-2 Reference Genome NC_045512.2 (29904)	---

Section 2199

(169247)	169247 169257 169268 169279 169290 169301 169312 169323
Candidatus Carsonella ruddii strain BT chromosome... (168705)	ATAAAATTTTCAATTTTTTAAAAAAAATTTCACTATGTAAATTTCCTATAAAACCTAAAAAAAAACAATTACCAAAC
Candidatus Carsonella 135557 - 167442nt (31887)	---
SARS-CoV-2 Reference Genome NC_045512.2 (29904)	---

Section 2200

(169324)	169324 169334 169345 169356 169367 169378 169389 169400
Candidatus Carsonella ruddii strain BT chromosome... (168782)	AAAATAGAATTTTTTCTCTCAAAAAAAATAGAGTAATCATTGTTTATTAATTTAAATAAAAAAAAATTATCTTTTAA
Candidatus Carsonella 135557 - 167442nt (31887)	---
SARS-CoV-2 Reference Genome NC_045512.2 (29904)	---

Section 2201

(169401)	169401 169411 169422 169433 169444 169455 169466 169477
Candidatus Carsonella ruddii strain BT chromosome... (168859)	ATAAATAGTAAAATATATATTAACATTAAATTTATAAAAAAAAAAAATATTTTTTTTATTATAAAAAAAAATATTTT
Candidatus Carsonella 135557 - 167442nt (31887)	---
SARS-CoV-2 Reference Genome NC_045512.2 (29904)	---

Section 2202

(169478)	169478 169488 169499 169510 169521 169532 169543 169554
Candidatus Carsonella ruddii strain BT chromosome... (168936)	TTTTATCATAATTATTATCAATAATAAAAAAATGAATACAATTAATAAAAGTTTTTTTAAGAAAAAAATTTTTTGGT
Candidatus Carsonella 135557 - 167442nt (31887)	---
SARS-CoV-2 Reference Genome NC_045512.2 (29904)	---

Section 2203

	(169555)	169555 169565 169576 169587 169598 169609 169620 169631
Candidatus Carsonella ruddii strain BT chromosome...	(169013)	AAAAAAATACCTATTTTAATAATTTTAATAAAATTATTATTTTTATTTTTTAAAATATCTTTAATATTAATTATACC
Candidatus Carsonella 135557 - 167442nt	(31887)	---
SARS-CoV-2 Reference Genome NC_045512.2	(29904)	---

Section 2204

	(169632)	169632 169642 169653 169664 169675 169686 169697 169708
Candidatus Carsonella ruddii strain BT chromosome...	(169090)	AGATAAAATTTTAACTATAATTATGTTTCCATATTTTAAATTATTCAAATATTCTAAAATAAAAATAACTTTTTCTT
Candidatus Carsonella 135557 - 167442nt	(31887)	---
SARS-CoV-2 Reference Genome NC_045512.2	(29904)	---

Section 2205

	(169709)	169709 169719 169730 169741 169752 169763 169774 169785
Candidatus Carsonella ruddii strain BT chromosome...	(169167)	TTTTAAAAATAATTTTTTTTTTTAAAAAAAATAAATTAATTAAACCAATACTATATTTAGAAGAAATAAAAAAAATT
Candidatus Carsonella 135557 - 167442nt	(31887)	---
SARS-CoV-2 Reference Genome NC_045512.2	(29904)	---

Section 2206

	(169786)	169786 169796 169807 169818 169829 169840 169851 169862
Candidatus Carsonella ruddii strain BT chromosome...	(169244)	TTTTGTTTTTTATTAAATAATGATAATTTATTTTTAATTTTTTTAAAATTAATATAAATATCTATTTTATTTAAAAA
Candidatus Carsonella 135557 - 167442nt	(31887)	---
SARS-CoV-2 Reference Genome NC_045512.2	(29904)	---

Section 2207

	(169863)	169863 169873 169884 169895 169906 169917 169928 169939
Candidatus Carsonella ruddii strain BT chromosome...	(169321)	AATAATAATAATTTTGTTAAATTTTTTAGACAAATTATAACAAAATAAAGTTTGTGGTTCTATACCTTTTAACACAT
Candidatus Carsonella 135557 - 167442nt	(31887)	---
SARS-CoV-2 Reference Genome NC_045512.2	(29904)	---

Section 2208

	(169940)	169940 169950 169961 169972 169983 169994 170005 170016
Candidatus Carsonella ruddii strain BT chromosome...	(169398)	CTATTAATATTAAACATATTTCACTACAAATTATAGATTTATATATTTCATTAATAAAATCAAAATGACCAGGACAA
Candidatus Carsonella 135557 - 167442nt	(31887)	---
SARS-CoV-2 Reference Genome NC_045512.2	(29904)	---

Section 2209

```
                                              (170017) 170017    170027     170038     170049     170060     170071     170082  170093
Candidatus Carsonella ruddii strain BT chromosome... (169475) TCAATAAAGTTTATTATTTTTTTATTTATTAAAACAGAATAAATATTTAATTTTATAGTATAAAAATAATTATTTAA
Candidatus Carsonella 135557 - 167442nt (31887) -----------------------------------------------------------------------------
SARS-CoV-2 Reference Genome NC_045512.2 (29904) -----------------------------------------------------------------------------
```

Section 2210

```
                                              (170094) 170094    170104     170115     170126     170137     170148     170159  170170
Candidatus Carsonella ruddii strain BT chromosome... (169552) AAAGTAAAATTTTTTTTTATAATTTAAAATTTTTTTACAAAAATTAATTAAAGAATTTAATAAAGTTGTTTTACCAT
Candidatus Carsonella 135557 - 167442nt (31887) -----------------------------------------------------------------------------
SARS-CoV-2 Reference Genome NC_045512.2 (29904) -----------------------------------------------------------------------------
```

Section 2211

```
                                              (170171) 170171    170181     170192     170203     170214     170225     170236  170247
Candidatus Carsonella ruddii strain BT chromosome... (169629) GATCAACATGAGCAATAATTGAAACATTATTTGCTAAATTCATTATTAATTTTATCAATTATTATTTTTAATATATT
Candidatus Carsonella 135557 - 167442nt (31887) -----------------------------------------------------------------------------
SARS-CoV-2 Reference Genome NC_045512.2 (29904) -----------------------------------------------------------------------------
```

Section 2212

```
                                              (170248) 170248    170258     170269     170280     170291     170302     170313  170324
Candidatus Carsonella ruddii strain BT chromosome... (169706) TACATTAATACAAGAATCTGTAATAGAAATATTTTTATAATAATTTTTAATTGATTGATTTCCATATTCTAAATAAG
Candidatus Carsonella 135557 - 167442nt (31887) -----------------------------------------------------------------------------
SARS-CoV-2 Reference Genome NC_045512.2 (29904) -----------------------------------------------------------------------------
```

Section 2213

```
                                              (170325) 170325    170335     170346     170357     170368     170379     170390  170401
Candidatus Carsonella ruddii strain BT chromosome... (169783) ATTCAAACATTAAACCATTTATATAAGATTTTAAATATTTAAATTGATTAAATATATTTTCAAAAATATAAATTTGA
Candidatus Carsonella 135557 - 167442nt (31887) -----------------------------------------------------------------------------
SARS-CoV-2 Reference Genome NC_045512.2 (29904) -----------------------------------------------------------------------------
```

Section 2214

```
                                              (170402) 170402    170412     170423     170434     170445     170456     170467  170478
Candidatus Carsonella ruddii strain BT chromosome... (169860) TTAATTGCATGTTTCATTGAATTAGAATGAGAACAATCAATTATTAATGAATTTTTAATATTTTTTACAGTATTATA
Candidatus Carsonella 135557 - 167442nt (31887) -----------------------------------------------------------------------------
SARS-CoV-2 Reference Genome NC_045512.2 (29904) -----------------------------------------------------------------------------
```

```
                                                                                                    Section 2215
                                                 (170479) 170479    170489     170500     170511     170522     170533     170544  170555
Candidatus Carsonella ruddii strain BT chromosome... (169937) ATTATAATTAGGAACAATTCCACCTCTTAAAACAAATTGACAATTTTTATTTCCTTTTGAAAATAAAATTTTTGTAT
                  Candidatus Carsonella 135557 - 167442nt (31887) -----------------------------------------------------------------------------
             SARS-CoV-2 Reference Genome NC_045512.2 (29904) -----------------------------------------------------------------------------
                                                                                                    Section 2216
                                                 (170556) 170556    170566     170577     170588     170599     170610     170621  170632
Candidatus Carsonella ruddii strain BT chromosome... (170014) TTTCAAAAAATGATAAAAAACAATGTTTATTTATAATAGATTTTAAAGAATCATTTAAAAAAATAATATTTCCAGAT
                  Candidatus Carsonella 135557 - 167442nt (31887) -----------------------------------------------------------------------------
             SARS-CoV-2 Reference Genome NC_045512.2 (29904) -----------------------------------------------------------------------------
                                                                                                    Section 2217
                                                 (170633) 170633    170643     170654     170665     170676     170687     170698  170709
Candidatus Carsonella ruddii strain BT chromosome... (170091) GAATCGTTTTTTATTCCAATTATATTATTTATATTAGAACAAAATTCTCTATGAATTTGAGAATTCATTGTTCTAGC
                  Candidatus Carsonella 135557 - 167442nt (31887) -----------------------------------------------------------------------------
             SARS-CoV-2 Reference Genome NC_045512.2 (29904) -----------------------------------------------------------------------------
                                                                                                    Section 2218
                                                 (170710) 170710    170720     170731     170742     170753     170764     170775  170786
Candidatus Carsonella ruddii strain BT chromosome... (170168) ACCTATACATACCCATATTATGCAATCTATAAAATAATTTAAAAAATAAAAATTTAAACATTCTGTAGCTATATTAA
                  Candidatus Carsonella 135557 - 167442nt (31887) -----------------------------------------------------------------------------
             SARS-CoV-2 Reference Genome NC_045512.2 (29904) -----------------------------------------------------------------------------
                                                                                                    Section 2219
                                                 (170787) 170787    170797     170808     170819     170830     170841     170852  170863
Candidatus Carsonella ruddii strain BT chromosome... (170245) TTTTTATATTAATTAAATCCAATAAAAATCTTCTTATAATATAAATTCCATCTAAAATACAGAAACTATTATTAATA
                  Candidatus Carsonella 135557 - 167442nt (31887) -----------------------------------------------------------------------------
             SARS-CoV-2 Reference Genome NC_045512.2 (29904) -----------------------------------------------------------------------------
                                                                                                    Section 2220
                                                 (170864) 170864    170874     170885     170896     170907     170918     170929  170940
Candidatus Carsonella ruddii strain BT chromosome... (170322) TAAGGATCATAAACATAACCTTTCCAACCTATATTTGTTCTTGGTTTTTCATAATAAACTCTAATTAAAAATTTCAA
                  Candidatus Carsonella 135557 - 167442nt (31887) -----------------------------------------------------------------------------
             SARS-CoV-2 Reference Genome NC_045512.2 (29904) -----------------------------------------------------------------------------
```

Section 2221

(170941) 170941 170951 170962 170973 170984 170995 171006 171017
Candidatus Carsonella ruddii strain BT chromosome... (170399) ATTATAAAAAAATTTTTTTATTTTATTTATATAAAAAAATAATTCTTTTATGTTACTAAGTGAACATGGACCTATTA
Candidatus Carsonella 135557 - 167442nt (31887) ---
SARS-CoV-2 Reference Genome NC_045512.2 (29904) ---

Section 2222

(171018) 171018 171028 171039 171050 171061 171072 171083 171094
Candidatus Carsonella ruddii strain BT chromosome... (170476) TTATTATTAATTTTTTTAAATTTTTTTTTATTATTTCTTTGTAAATTTTAATGTTTAAAACATTTTTATTAAACAAA
Candidatus Carsonella 135557 - 167442nt (31887) ---
SARS-CoV-2 Reference Genome NC_045512.2 (29904) ---

Section 2223

(171095) 171095 171105 171116 171127 171138 171149 171160 171171
Candidatus Carsonella ruddii strain BT chromosome... (170553) TCTTTTTTTAATCTTTTTAAATTTATTATTTTATACATATATTTTTTATATTTTTATTTAAAAAAAAATCATTTTTT
Candidatus Carsonella 135557 - 167442nt (31887) ---
SARS-CoV-2 Reference Genome NC_045512.2 (29904) ---

Section 2224

(171172) 171172 171182 171193 171204 171215 171226 171237 171248
Candidatus Carsonella ruddii strain BT chromosome... (170630) AAAAAAATTTTTTTTTTATTTATTTTTTTTACTCTTGATTTACTTTTAGATTTTTTAGTTTTTTGAACCGCCATGTT
Candidatus Carsonella 135557 - 167442nt (31887) ---
SARS-CoV-2 Reference Genome NC_045512.2 (29904) ---

Section 2225

(171249) 171249 171259 171270 171281 171292 171303 171314 171325
Candidatus Carsonella ruddii strain BT chromosome... (170707) TGATAAAATAAATATTTTTATAAAAACATTAGGATGCAACATAAATAATTTGGAATCTGAAAATTTTTTAAATTTTT
Candidatus Carsonella 135557 - 167442nt (31887) ---
SARS-CoV-2 Reference Genome NC_045512.2 (29904) ---

Section 2226

(171326) 171326 171336 171347 171358 171369 171380 171391 171402
Candidatus Carsonella ruddii strain BT chromosome... (170784) TAAAGTATTTTAAAATAAAATTAAAAAAAAAATTTTTTAATTCAAATATATTAATTATTAATAGTTGTATTATAAGA
Candidatus Carsonella 135557 - 167442nt (31887) ---
SARS-CoV-2 Reference Genome NC_045512.2 (29904) ---

Section 2227

```
                                                   (171403) 171403    171413     171424     171435     171446     171457     171468  171479
Candidatus Carsonella ruddii strain BT chromosome... (170861) AAAAATCCACAAATTAAAATTTTAAAAGAATTAAAAAAATGGACTTTTTTAAAAAAATATAAAAAAAGTATAATTTT
           Candidatus Carsonella 135557 - 167442nt  (31887) -----------------------------------------------------------------------------
        SARS-CoV-2 Reference Genome NC_045512.2    (29904) -----------------------------------------------------------------------------
```

Section 2228

```
                                                   (171480) 171480    171490     171501     171512     171523     171534     171545  171556
Candidatus Carsonella ruddii strain BT chromosome... (170938) TTTAACTGGTTGTTTATCTGAATTTGAAAATTTTAAAAAAATTAAATTAATAAAAATAGATATTATTATTAATTCTA
           Candidatus Carsonella 135557 - 167442nt  (31887) -----------------------------------------------------------------------------
        SARS-CoV-2 Reference Genome NC_045512.2    (29904) -----------------------------------------------------------------------------
```

Section 2229

```
                                                   (171557) 171557    171567     171578     171589     171600     171611     171622  171633
Candidatus Carsonella ruddii strain BT chromosome... (171015) ATTGTTACTTTTATTTAAAAAAAATAATATTATTTTATATTAAAAATAAAAAAAAAATTTATTTTATAAAAAAACAA
           Candidatus Carsonella 135557 - 167442nt  (31887) -----------------------------------------------------------------------------
        SARS-CoV-2 Reference Genome NC_045512.2    (29904) -----------------------------------------------------------------------------
```

Section 2230

```
                                                   (171634) 171634    171644     171655     171666     171677     171688     171699  171710
Candidatus Carsonella ruddii strain BT chromosome... (171092) AAAAAATTTTTTTTAAAAAATAAAATTAATAATTACTTTTTAATAATGAAAGGATGTAATCATAATTGCTCATATTG
           Candidatus Carsonella 135557 - 167442nt  (31887) -----------------------------------------------------------------------------
        SARS-CoV-2 Reference Genome NC_045512.2    (29904) -----------------------------------------------------------------------------
```

Section 2231

```
                                                   (171711) 171711    171721     171732     171743     171754     171765     171776  171787
Candidatus Carsonella ruddii strain BT chromosome... (171169) TGTAATACCTCAAATAAAAGGAAAAGAAGAATATTTTTCTTACAAAAAAATATTTTTTAATATTTTGAATAATATAA
           Candidatus Carsonella 135557 - 167442nt  (31887) -----------------------------------------------------------------------------
        SARS-CoV-2 Reference Genome NC_045512.2    (29904) -----------------------------------------------------------------------------
```

Section 2232

```
                                                   (171788) 171788    171798     171809     171820     171831     171842     171853  171864
Candidatus Carsonella ruddii strain BT chromosome... (171246) AAACATTTTTTTGTGAAATAACTTTATTAGGTCAAAATGTAAATTCTTATTATTATAAAAATATTGATTTTAATTCT
           Candidatus Carsonella 135557 - 167442nt  (31887) -----------------------------------------------------------------------------
        SARS-CoV-2 Reference Genome NC_045512.2    (29904) -----------------------------------------------------------------------------
```

```
                                                                                                   Section 2233
                                               (171865) 171865    171875     171886     171897     171908     171919     171930  171941
Candidatus Carsonella ruddii strain BT chromosome... (171323) TTAATATTTAATTTATCAAAAATTAAAAATATTAAAAGAATAAATTTTTTATCTTCAAATATAAAAGATTTTAATAA
              Candidatus Carsonella 135557 - 167442nt (31887) -----------------------------------------------------------------------------
           SARS-CoV-2 Reference Genome NC_045512.2 (29904) -----------------------------------------------------------------------------
                                                                                                   Section 2234
                                               (171942) 171942    171952     171963     171974     171985     171996     172007  172018
Candidatus Carsonella ruddii strain BT chromosome... (171400) AAATTTTTTTTTCTTATATAAAAATATTAATAAAATTTCATCACATATTCATTTACCAGTTCAATCTGGATCTAATA
              Candidatus Carsonella 135557 - 167442nt (31887) -----------------------------------------------------------------------------
           SARS-CoV-2 Reference Genome NC_045512.2 (29904) -----------------------------------------------------------------------------
                                                                                                   Section 2235
                                               (172019) 172019    172029     172040     172051     172062     172073     172084  172095
Candidatus Carsonella ruddii strain BT chromosome... (171477) AAATTTTAAAAAAAATGAATAGAAAATATACTATTAATGAGTATTTATATTTTATAAAAAAAATAAAAAAAATAAAA
              Candidatus Carsonella 135557 - 167442nt (31887) -----------------------------------------------------------------------------
           SARS-CoV-2 Reference Genome NC_045512.2 (29904) -----------------------------------------------------------------------------
                                                                                                   Section 2236
                                               (172096) 172096    172106     172117     172128     172139     172150     172161  172172
Candidatus Carsonella ruddii strain BT chromosome... (171554) AAAACATCTTTTTCAACTGATATTATTATATCTTTTCCTTATGAAAAATTAATAGATTTTGATTTATCAATTAAAAT
              Candidatus Carsonella 135557 - 167442nt (31887) -----------------------------------------------------------------------------
           SARS-CoV-2 Reference Genome NC_045512.2 (29904) -----------------------------------------------------------------------------
                                                                                                   Section 2237
                                               (172173) 172173    172183     172194     172205     172216     172227     172238  172249
Candidatus Carsonella ruddii strain BT chromosome... (171631) AATAAAAAAAATTAAATTTACAGATATTTTTTTTTTTTCTTATTCAAAAAGAATAAACACAATTTCTTTTAATTTTA
              Candidatus Carsonella 135557 - 167442nt (31887) -----------------------------------------------------------------------------
           SARS-CoV-2 Reference Genome NC_045512.2 (29904) -----------------------------------------------------------------------------
                                                                                                   Section 2238
                                               (172250) 172250    172260     172271     172282     172293     172304     172315  172326
Candidatus Carsonella ruddii strain BT chromosome... (171708) AAAAAAAAGATTTATTAAAAAAATTTAAATTGAAAATTTTTCAAAAAAATATTAAAAATAATTTTTTTTTTAATAAA
              Candidatus Carsonella 135557 - 167442nt (31887) -----------------------------------------------------------------------------
           SARS-CoV-2 Reference Genome NC_045512.2 (29904) -----------------------------------------------------------------------------
```

```
                                                                                                    Section 2239
                                            (172327) 172327    172337     172348     172359     172370     172381     172392  172403
Candidatus Carsonella ruddii strain BT chromosome... (171785) AACGAAAGAGTATTAGTTATTGGATATTTAAAAAAAAATATTTTTATAGGAAAAACTGATAGTTTAAAAATAGTTTT
         Candidatus Carsonella 135557 - 167442nt  (31887) -----------------------------------------------------------------------------
      SARS-CoV-2 Reference Genome NC_045512.2  (29904) -----------------------------------------------------------------------------
                                                                                                    Section 2240
                                            (172404) 172404    172414     172425     172436     172447     172458     172469  172480
Candidatus Carsonella ruddii strain BT chromosome... (171862) TTTTGAATATTTTAATTATAATATTATAGGAAAATTTTTATTAATAAAAATAATAAATATTAAAAAAAATTTTTTTT
         Candidatus Carsonella 135557 - 167442nt  (31887) -----------------------------------------------------------------------------
      SARS-CoV-2 Reference Genome NC_045512.2  (29904) -----------------------------------------------------------------------------
                                                                                                    Section 2241
                                            (172481) 172481    172491     172502     172513     172524     172535     172546  172557
Candidatus Carsonella ruddii strain BT chromosome... (171939) TAGGAATATATGAAAGTATATATTCAAATATATAGTTTTTACAAATTAAAATTTTTAATAAAATTTAAAACAAAAAA
         Candidatus Carsonella 135557 - 167442nt  (31887) -----------------------------------------------------------------------------
      SARS-CoV-2 Reference Genome NC_045512.2  (29904) -----------------------------------------------------------------------------
                                                                                                    Section 2242
                                            (172558) 172558    172568     172579     172590     172601     172612     172623  172634
Candidatus Carsonella ruddii strain BT chromosome... (172016) TATTTTAAAAATAATTATAATTATTAAAAAAAAAACCTTTAATTATTTTAATCATAAAAAAAATATTATTTTGTAATA
         Candidatus Carsonella 135557 - 167442nt  (31887) -----------------------------------------------------------------------------
      SARS-CoV-2 Reference Genome NC_045512.2  (29904) -----------------------------------------------------------------------------
                                                                                                    Section 2243
                                            (172635) 172635    172645     172656     172667     172678     172689     172700  172711
Candidatus Carsonella ruddii strain BT chromosome... (172093) ATTATTATAAAAAAAAAAAATTAATAATGATAATTCATTTTTGTAATTTTATTTTTTTTAATCACAGTATAAAACAT
         Candidatus Carsonella 135557 - 167442nt  (31887) -----------------------------------------------------------------------------
      SARS-CoV-2 Reference Genome NC_045512.2  (29904) -----------------------------------------------------------------------------
                                                                                                    Section 2244
                                            (172712) 172712    172722     172733     172744     172755     172766     172777  172788
Candidatus Carsonella ruddii strain BT chromosome... (172170) TTTTTAAAAATTAATCATAATTCAATTATAGAAAATAAAATTAATGATATATTTTAAAAATCCATTATTTATTGAAA
         Candidatus Carsonella 135557 - 167442nt  (31887) -----------------------------------------------------------------------------
      SARS-CoV-2 Reference Genome NC_045512.2  (29904) -----------------------------------------------------------------------------
```

Section 2245

	(172789)	172789 172799 172810 172821 172832 172843 172854 172865
Candidatus Carsonella ruddii strain BT chromosome...	(172247)	ATAAAATAAATAAAAATTTAAAAATTATAAAAAATTGTAAAAAATTTTTTTGTTTACCTATGTTTCCGTATCCTAGT
Candidatus Carsonella 135557 - 167442nt	(31887)	---
SARS-CoV-2 Reference Genome NC_045512.2	(29904)	---

Section 2246

	(172866)	172866 172876 172887 172898 172909 172920 172931 172942
Candidatus Carsonella ruddii strain BT chromosome...	(172324)	GGAAAATTACATTTAGGACACGTTAGAAATTATATTTTAACAGATATAATTTCTAAAATTAAAATTTTAGAAAAAAA
Candidatus Carsonella 135557 - 167442nt	(31887)	---
SARS-CoV-2 Reference Genome NC_045512.2	(29904)	---

Section 2247

	(172943)	172943 172953 172964 172975 172986 172997 173008 173019
Candidatus Carsonella ruddii strain BT chromosome...	(172401)	AACAGTAATACACCCTATGGGATGGGATTCATTTGGATTACCTGCAGAAAATGCTTCTAAAAAATTAAATTTAAATT
Candidatus Carsonella 135557 - 167442nt	(31887)	---
SARS-CoV-2 Reference Genome NC_045512.2	(29904)	---

Section 2248

	(173020)	173020 173030 173041 173052 173063 173074 173085 173096
Candidatus Carsonella ruddii strain BT chromosome...	(172478)	CTTTAAAATGGACATTATCAAATATAAAATTAATGAAAAACCAATTAAAAATTTTACAAATTAATTACTCTAAAAAA
Candidatus Carsonella 135557 - 167442nt	(31887)	---
SARS-CoV-2 Reference Genome NC_045512.2	(29904)	---

Section 2249

	(173097)	173097 173107 173118 173129 173140 173151 173162 173173
Candidatus Carsonella ruddii strain BT chromosome...	(172555)	TTAGAGTTTTTAACTTGTGATGTTAATTTTTATAAATGGGAATTTTGGATATTGTTATATTTTTATAAAAACAAATT
Candidatus Carsonella 135557 - 167442nt	(31887)	---
SARS-CoV-2 Reference Genome NC_045512.2	(29904)	---

Section 2250

	(173174)	173174 173184 173195 173206 173217 173228 173239 173250
Candidatus Carsonella ruddii strain BT chromosome...	(172632)	ATTATTAAAACAATATGATGAAATATATTGGGATAAAATTCAACAATGTATTTTATCTAATGAACAAATAAATGATA
Candidatus Carsonella 135557 - 167442nt	(31887)	---
SARS-CoV-2 Reference Genome NC_045512.2	(29904)	---

```
                                                                                 Section 2251
                                                  (173251) 173251    173261     173272     173283     173294     173305     173316  173327
Candidatus Carsonella ruddii strain BT chromosome... (172709) ATTTATGTTGGAGATCTAATTGTAAACCCATCAAAATTAAAAATTTTACTTGGTTTATAAATATTAAAAAATATACA
           Candidatus Carsonella 135557 - 167442nt (31887) -----------------------------------------------------------------------------
          SARS-CoV-2 Reference Genome NC_045512.2 (29904) -----------------------------------------------------------------------------
                                                                                 Section 2252
                                                  (173328) 173328    173338     173349     173360     173371     173382     173393  173404
Candidatus Carsonella ruddii strain BT chromosome... (172786) AAAAAATTAATTTTTAATTTAAAAAATATTAATTGGTCTAAAAAAATTAAAAATATACAAAAAAAATGGATAAATAT
           Candidatus Carsonella 135557 - 167442nt (31887) -----------------------------------------------------------------------------
          SARS-CoV-2 Reference Genome NC_045512.2 (29904) -----------------------------------------------------------------------------
                                                                                 Section 2253
                                                  (173405) 173405    173415     173426     173437     173448     173459     173470  173481
Candidatus Carsonella ruddii strain BT chromosome... (172863) AAAATTTTTATTAATTAGAAATATAAAATTTTATTCAATTTATAATTATTATTATATTAATATTAATAATATTAAAA
           Candidatus Carsonella 135557 - 167442nt (31887) -----------------------------------------------------------------------------
          SARS-CoV-2 Reference Genome NC_045512.2 (29904) -----------------------------------------------------------------------------
                                                                                 Section 2254
                                                  (173482) 173482    173492     173503     173514     173525     173536     173547  173558
Candidatus Carsonella ruddii strain BT chromosome... (172940) AAATAATAAATTTAAAAATATTATATTTATTAATTAAAAAAAAAAATAAATTTTTATTTAAAAAAAAAATATTTATA
           Candidatus Carsonella 135557 - 167442nt (31887) -----------------------------------------------------------------------------
          SARS-CoV-2 Reference Genome NC_045512.2 (29904) -----------------------------------------------------------------------------
                                                                                 Section 2255
                                                  (173559) 173559    173569     173580     173591     173602     173613     173624  173635
Candidatus Carsonella ruddii strain BT chromosome... (173017) AATGATAATTTATTAAAAATTAAAAAAAAAATAATTATTACTAATTTTAATTTAAATTTAATAAAAATTTTAAAAGA
           Candidatus Carsonella 135557 - 167442nt (31887) -----------------------------------------------------------------------------
          SARS-CoV-2 Reference Genome NC_045512.2 (29904) -----------------------------------------------------------------------------
                                                                                 Section 2256
                                                  (173636) 173636    173646     173657     173668     173679     173690     173701  173712
Candidatus Carsonella ruddii strain BT chromosome... (173094) AGAAATTAATTTTTTAATTTGTAAAAAAAAAATATTTTTTATAAAAAAAACAAATATTAATAATTGGGCTTTTATAA
           Candidatus Carsonella 135557 - 167442nt (31887) -----------------------------------------------------------------------------
          SARS-CoV-2 Reference Genome NC_045512.2 (29904) -----------------------------------------------------------------------------
```

Section 2257

	(173713)	173713 173723 173734 173745 173756 173767 173778 173789
Candidatus Carsonella ruddii strain BT chromosome...	(173171)	GACAACGTTTATGGGGATCTCCTTTTTTTTATAAAAAAATTAAAAACAAAAATTTTAAATCAAAATTGACAATTGAT
Candidatus Carsonella 135557 - 167442nt	(31887)	---
SARS-CoV-2 Reference Genome NC_045512.2	(29904)	---

Section 2258

	(173790)	173790 173800 173811 173822 173833 173844 173855 173866
Candidatus Carsonella ruddii strain BT chromosome...	(173248)	ACTTTTTTTCAATCTTCTTGGTATTATATTTATTATTTAAAATCATTAAATTTAACAAATAAATTTAAAAATGATTG
Candidatus Carsonella 135557 - 167442nt	(31887)	---
SARS-CoV-2 Reference Genome NC_045512.2	(29904)	---

Section 2259

	(173867)	173867 173877 173888 173899 173910 173921 173932 173943
Candidatus Carsonella ruddii strain BT chromosome...	(173325)	GTTTCCAATAAATATTTATGTTGGAGGAATAGAACATGCAAATTTACATTTACTTTATTTAAGAATAATAAATTTTA
Candidatus Carsonella 135557 - 167442nt	(31887)	---
SARS-CoV-2 Reference Genome NC_045512.2	(29904)	---

Section 2260

	(173944)	173944 173954 173965 173976 173987 173998 174009 174020
Candidatus Carsonella ruddii strain BT chromosome...	(173402)	TATTTTATGATTTAAATATTATTGATAAAAAAAATGTTATTTTAAATTTAATTAATCAAGGTTTTATAAATAATGAA
Candidatus Carsonella 135557 - 167442nt	(31887)	---
SARS-CoV-2 Reference Genome NC_045512.2	(29904)	---

Section 2261

	(174021)	174021 174031 174042 174053 174064 174075 174086 174097
Candidatus Carsonella ruddii strain BT chromosome...	(173479)	GTTTATTTTAAAATTAAAAATTTTAAAAAAATTTTTTGCAAAAAAAATAAAAAAGCATTTTTAGGTGGAATTGAAAA
Candidatus Carsonella 135557 - 167442nt	(31887)	---
SARS-CoV-2 Reference Genome NC_045512.2	(29904)	---

Section 2262

	(174098)	174098 174108 174119 174130 174141 174152 174163 174174
Candidatus Carsonella ruddii strain BT chromosome...	(173556)	AATGTCAAAATCAAAAAAAAATGGAATAAACCCAATATATTTAATAAATAAATTTGGATCAGATATATTAAAATTAT
Candidatus Carsonella 135557 - 167442nt	(31887)	---
SARS-CoV-2 Reference Genome NC_045512.2	(29904)	---

```
                                                                          Section 2263
                                              (174175) 174175    174185     174196     174207     174218     174229     174240 174251
Candidatus Carsonella ruddii strain BT chromosome... (173633) CTATTATTACAAATAAACCAATAGACAAAAATATAAATTGGGGAAATATAAATTTTTTTTCAATTAAAAAATTTATA
           Candidatus Carsonella 135557 - 167442nt  (31887) -----------------------------------------------------------------------------
          SARS-CoV-2 Reference Genome NC_045512.2  (29904) -----------------------------------------------------------------------------
                                                                          Section 2264
                                              (174252) 174252    174262     174273     174284     174295     174306     174317 174328
Candidatus Carsonella ruddii strain BT chromosome... (173710) TTAAAAATTAATAATTTAATAGAATTAAAAATAATTAATATTAAAGAAATAATTAATATTAAAGAAATAATTAATAT
           Candidatus Carsonella 135557 - 167442nt  (31887) -----------------------------------------------------------------------------
          SARS-CoV-2 Reference Genome NC_045512.2  (29904) -----------------------------------------------------------------------------
                                                                          Section 2265
                                              (174329) 174329    174339     174350     174361     174372     174383     174394 174405
Candidatus Carsonella ruddii strain BT chromosome... (173787) TAAAAAAATACATAATGTAATTTCTTATATTAATAAAATTTTAATTTATAATAATTCAATAAAACAAATAGAAAATA
           Candidatus Carsonella 135557 - 167442nt  (31887) -----------------------------------------------------------------------------
          SARS-CoV-2 Reference Genome NC_045512.2  (29904) -----------------------------------------------------------------------------
                                                                          Section 2266
                                              (174406) 174406    174416     174427              174446
Candidatus Carsonella ruddii strain BT chromosome... (173864) TTATTTTTTGGTTGTTTCCAATTATACCAAATATTTCTAAA
           Candidatus Carsonella 135557 - 167442nt  (31887) -----------------------------------------
          SARS-CoV-2 Reference Genome NC_045512.2  (29904) -----------------------------------------
```
