# Supplementary material for: Ubiquitous Micro-Modular Homologies among Genomes from Viruses to Bacteria to Human Mitochondrial DNA: Platforms for Recombination during Evolution?
Source: Viruses. 2022 Apr 24;14(5):885. doi: 10.3390/v14050885 (PMC9147251; doi:10.3390/v14050885)
Supplement: Supplementary file 1 [file viruses-14-00885-s001.zip › Fig. S4B, Sars-CoV-2 & E.coli K12 Zwischenstück 255.328 - 290.948.pdf]

Sars-CoV-2 & E.coli K12 Zwischenstückeapr.apr

Section 1

```
                                            (1)  1      10        20        30        40        50        60        70           83
         K12 Zwischenstück 255.328 - 290.948 (1)  TCGTCAGCCAGAACCGCCAGCGCAGAGGCCATACCAATGCCGTTATCCGCACCCAGCGTGGTGCCGCGCTTTAACCCATTC
SARS-CoV-2 Reference Genome NC_045512.2      (1)  -----------------------------------------------------------------------------------
                                   Consensus (1)
```

Section 2

```
                                            (84)  84    90        100       110       120       130       140       150         166
         K12 Zwischenstück 255.328 - 290.948 (84)  GCCATCAATATAAGGCTGGATAGGATCTTTCGTGAAGTCATGCACGGTGTCGTTATTTTTCTGCGGCACCATATCGAGGTGGG
SARS-CoV-2 Reference Genome NC_045512.2      (1)   -----------------------------------------------------------------------------------
                                   Consensus (84)
```

Section 3

```
                                            (167)  167         180       190       200       210       220       230               249
         K12 Zwischenstück 255.328 - 290.948 (167)  CCTGTAAGACGACCGGTTTACGATTTTCCATACCTGCGGTAGCAGGTTTACGAATCAGGATATTACCTACCTGATCGCGTTCG
SARS-CoV-2 Reference Genome NC_045512.2      (1)    -----------------------------------------------------------------------------------
                                   Consensus (167)
```

Section 4

```
                                            (250)  250       260       270       280       290       300       310       320         332
         K12 Zwischenstück 255.328 - 290.948 (250)  ACATGGAAACCTTTCTCTTTTGCCCAACCAACAATGTATTCAGCGAGTTGCTCTTCATGATAGGACGGGTGAGGAATAGAACA
SARS-CoV-2 Reference Genome NC_045512.2      (1)    -----------------------------------------------------------------------------------
                                   Consensus (250)
```

Section 5

```
                                            (333)  333    340       350       360       370       380       390       400           415
         K12 Zwischenstück 255.328 - 290.948 (333)  GATTTTGGCAAAAATATCCCACAGCGGCTGTGGAGATAATTGAGACAGTTCAGACACGTTAAGTCTCCTTGTCGATCACCCGC
SARS-CoV-2 Reference Genome NC_045512.2      (1)    -----------------------------------------------------------------------------------
                                   Consensus (333)
```

Section 6

```
                                            (416)  416           430       440       450       460       470       480               498
         K12 Zwischenstück 255.328 - 290.948 (416)  AAAACAGTATTGCAGGTCACAGGGTTAGCAGAAAATGTTGTCAACACAAGACAGGCTTGCGAGATATGTTTGAGAATACCACT
SARS-CoV-2 Reference Genome NC_045512.2      (1)    -----------------------------------------------------------------------------------
                                   Consensus (416)
```

Section 7

```
                                            (499)  499        510       520       530       540       550       560       570         581
         K12 Zwischenstück 255.328 - 290.948 (499)  TTATCCCGCGTCAGGGAGAGGCAGTGCGTAAAAAGACGCGGACTCATGTGAAATACTGGTTTTTAGTGCGCCAGATCTCTATA
SARS-CoV-2 Reference Genome NC_045512.2      (1)    -----------------------------------------------------------------------------------
                                   Consensus (499)
```

Section 8

```
                                            (582) 582     590            600            610            620            630            640            650            664
K12 Zwischenstück 255.328 - 290.948         (582) ATCTCGCGCAACCTATTTTCCCCTCGAACACTTTTTAAGCCGTAGATAAACAGGCTGGGACACTTCACATGAGCGAAAAATAC
SARS-CoV-2 Reference Genome NC_045512.2       (1) -----------------------------------------------------------------------------------
Consensus                                   (582)
```

Section 9

```
                                            (665) 665  670            680            690            700            710            720            730              747
K12 Zwischenstück 255.328 - 290.948         (665) ATCGTCACCTGGGACATGTTGCAGATCCATGCACGTAAACTCGCAAGCCGACTGATGCCTTCTGAACAATGGAAAGGCATTAT
SARS-CoV-2 Reference Genome NC_045512.2       (1) -----------------------------------------------------------------------------------
Consensus                                   (665)
```

Section 10

```
                                            (748) 748         760            770            780            790            800            810            820         830
K12 Zwischenstück 255.328 - 290.948         (748) TGCCGTAAGCCGTGGCGGTCTGGTACCGGGTGCGTTACTGGCGCGTGAACTGGGTATTCGTCATGTCGATACCGTTTGTATTT
SARS-CoV-2 Reference Genome NC_045512.2       (1) -----------------------------------------------------------------------------------
Consensus                                   (748)
```

Section 11

```
                                            (831) 831      840            850            860            870            880            890            900          913
K12 Zwischenstück 255.328 - 290.948         (831) CCAGCTACGATCACGACAACCAGCGCGAGCTTAAAGTGCTGAAACGCGCAGAAGGCGATGGCGAAGGCTTCATCGTTATTGAT
SARS-CoV-2 Reference Genome NC_045512.2       (1) -----------------------------------------------------------------------------------
Consensus                                   (831)
```

Section 12

```
                                            (914) 914   920            930            940            950            960            970            980              996
K12 Zwischenstück 255.328 - 290.948         (914) GACCTGGTGGATACCGGTGGTACTGCGGTTGCGATTCGTGAAATGTATCCAAAAGCGCACTTTGTCACCATCTTCGCAAAACC
SARS-CoV-2 Reference Genome NC_045512.2       (1) -----------------------------------------------------------------------------------
Consensus                                   (914)
```

Section 13

```
                                            (997) 997          1010           1020           1030           1040           1050           1060                1079
K12 Zwischenstück 255.328 - 290.948         (997) GGCTGGTCGTCCGCTGGTTGATGACTATGTTGTTGATATCCCGCAAGATACCTGGATTGAACAGCCGTGGGATATGGGCGTCG
SARS-CoV-2 Reference Genome NC_045512.2       (1) -----------------------------------------------------------------------------------
Consensus                                   (997)
```

Section 14

```
                                           (1080) 1080      1090           1100           1110           1120           1130           1140           1150        1162
K12 Zwischenstück 255.328 - 290.948        (1080) TATTCGTCCCGCCAATCTCCGGTCGCTAATCTTTTCAACGCCTGGCACTGCCGGGCGTTGTTCTTTTTAACTTCAGGCGGGTT
SARS-CoV-2 Reference Genome NC_045512.2       (1) -----------------------------------------------------------------------------------
Consensus                                  (1080)
```

Section 15

```
                                             (1163) 1163   1170      1180      1190      1200      1210      1220      1230          1245
        K12 Zwischenstück 255.328 - 290.948  (1163) ACAATAGTTTCCAGTAAGTATTCTGGAGGCTGCATCCATGACACAGGCAAACCTGAGCGAAACCCTGTTCAAACCCCGCTTTA
SARS-CoV-2 Reference Genome NC_045512.2         (1) -----------------------------------------------------------------------------------
                                   Consensus (1163)
```

Section 16

```
                                             (1246) 1246          1260      1270      1280      1290      1300      1310             1328
        K12 Zwischenstück 255.328 - 290.948  (1246) AACATCCTGAAACCTCGACGCTAGTCCGCCGCTTTAATCACGGCGCACAACCGCCTGTGCAGTCGGCCCTTGATGGTAAAACC
SARS-CoV-2 Reference Genome NC_045512.2         (1) -----------------------------------------------------------------------------------
                                   Consensus (1246)
```

Section 17

```
                                             (1329) 1329       1340      1350      1360      1370      1380      1390      1400     1411
        K12 Zwischenstück 255.328 - 290.948  (1329) ATCCCTCACTGGTATCGCATGATTAACCGTCTGATGTGGATCTGGCGCGGCATTGACCCACGCGAAATCCTCGACGTCCAGGC
SARS-CoV-2 Reference Genome NC_045512.2         (1) -----------------------------------------------------------------------------------
                                   Consensus (1329)
```

Section 18

```
                                             (1412) 1412    1420      1430      1440      1450      1460      1470      1480         1494
        K12 Zwischenstück 255.328 - 290.948  (1412) ACGTATTGTGATGAGCGATGCCGAACGTACCGACGATGATTTATACGATACGGTGATTGGCTACCGTGGCGGCAACTGGATTT
SARS-CoV-2 Reference Genome NC_045512.2         (1) -----------------------------------------------------------------------------------
                                   Consensus (1412)
```

Section 19

```
                                             (1495) 1495 1500      1510      1520      1530      1540      1550      1560             1577
        K12 Zwischenstück 255.328 - 290.948  (1495) ATGAGTGGGCCACCCAGGCGATGGTGTGGCAACAAAAAGCCTGTGCGGAAGACGATCCGCAACTCAGTGGTCGTCACTGGCTG
SARS-CoV-2 Reference Genome NC_045512.2         (1) -----------------------------------------------------------------------------------
                                   Consensus (1495)
```

Section 20

```
                                             (1578) 1578        1590      1600      1610      1620      1630      1640      1650      1660
        K12 Zwischenstück 255.328 - 290.948  (1578) CATGCGGCTACGTTGTACAACATTGCCGCCTATCCTCATCTGAAAGGAGATGACCTGGCCGAGCAAGCGCAGGCTTTGTCAAA
SARS-CoV-2 Reference Genome NC_045512.2         (1) -----------------------------------------------------------------ATTAAAGGTTTATACCTT
                                   Consensus (1578)                                                                  A    AGG TT   C
```

Section 21

```
                                             (1661) 1661     1670      1680      1690      1700      1710      1720      1730         1743
        K12 Zwischenstück 255.328 - 290.948  (1661) CCGCGCCTATGAAG-AGGCCGCTCAGCGTCTACCGG-GCACGATGCGGCAGATGGAGTTTACCGTACCCGGCGGTGCGCCCAT
SARS-CoV-2 Reference Genome NC_045512.2        (19) CCCAGGTAACAAACCAACCAACTTTCGATCTCTTGTAGATCTGTTCTCTAAACGAACTTTAAAAT------CTGTGTGGCTGT
                                   Consensus (1661) CC  G   A  AA  A  C  CT     TCT   G  G  C  T C   A A G A TTTA   T      C GTG G C  T
```

```
                                                       Section 22
                                         (1744) 1744 1750 1760 1770 1780 1790 1800 1810 1826
K12 Zwischenstück 255.328 - 290.948      (1742) CAC-CGGCTTTTTGCATATGCCGAAAGGCGATGGCCCGTTCCCGACGGTATTAATGTGTGGTGGTCTGGATGCGATGCAGACG
SARS-CoV-2 Reference Genome NC_045512.2  (96)   CACTCGGCTGCATGCTTAGTGC-ACTCACGCAGTATAATTAATAACTA-ATTACTGTC--GTTGACAGGACACGAGTAACTCG
Consensus                                (1744) CAC CGGCT   TGC TA   C A    CG  G     TT    AC   ATTA TGT   GT G C GGA  CGA   A  CG

                                                       Section 23
                                         (1827) 1827 1840 1850 1860 1870 1880 1890 1909
K12 Zwischenstück 255.328 - 290.948      (1824) ---GACTATTACAGCCTGTATGAACGTTATTTTGCGCCGCGCGGCATTGCGATGCTGACTATTGATATGCCGTCGGTGGGCTT
SARS-CoV-2 Reference Genome NC_045512.2  (175)  TCTATCTTCTGCAGGCTGCTT--ACGGTTTCGTCCGTGTTGCAGC----CGATCATCAGCACATCTAGGT--TTCGTCCGGGT
Consensus                                (1827)      CT  T CAG CTG  T  ACG T T  T CG    GC GC    CGAT  T A  A    TA G   T  GT  G  T

                                                       Section 24
                                         (1910) 1910 1920 1930 1940 1950 1960 1970 1980 1992
K12 Zwischenstück 255.328 - 290.948      (1904) TTCTTCAAAATGGAAGCTCACCCAGGACTCCAGCCTGTTGCATCAGCACGTCTTAAAGGCGCTGCCTAACGTACCGTGGGTGG
SARS-CoV-2 Reference Genome NC_045512.2  (250)  GTGACCGAAAGGTAAGATGGA----GAGCCTTGTCCCTGGTTTCAACGAGA---AAACACACGTCC-AACTCAGTTTGCCTGT
Consensus                                (1910)  T   C AAA G AAG T       GA  C  G C  T G  TCA C  G    AAA  C C  CC AAC  A   TG  TG

                                                       Section 25
                                         (1993) 1993 2000 2010 2020 2030 2040 2050 2060 2075
K12 Zwischenstück 255.328 - 290.948      (1987) ATCACAC--TCGCGTCGCGGCCTTTGGTTTCCGTTTCGGCGCTAACGTTGCCGTGCGTCTGGCATACCTTGAATCGCCGCGTC
SARS-CoV-2 Reference Genome NC_045512.2  (325)  TTTACAGGTTCGCGACGTGCTCGTACGTGGC---TTTGGAGACTCCGTGGAGGAG-GTCTT--ATCAGAGGCACGTCAACATC
Consensus                                (1993)  T ACA   TCGCG CG G  C T  GT  C   TT GG G    CGT G  G G GTCT   AT     G A   C  C TC

                                                       Section 26
                                         (2076) 2076 2090 2100 2110 2120 2130 2140 2158
K12 Zwischenstück 255.328 - 290.948      (2068) TGAAAGCGGTTGCCTGT--CTTGGTCCGGTAGTTCATA---CCCTGTTGAGTGATTTTAAGTGCCAGCAACAGGTGCCGGAAA
SARS-CoV-2 Reference Genome NC_045512.2  (402)  TTAAAGATGGCACTTGTGGCTTAGTA--GAAGTTGAAAAAGGCGTTTTGCCTCAACTTGAA---CAGCCCTATGTG-------
Consensus                                (2076) T AAAG  G   C TGT  CTT GT   G AGTT A A    C T TTG  T A  TT A    CAGC   A GTG

                                                       Section 27
                                         (2159) 2159 2170 2180 2190 2200 2210 2220 2230 2241
K12 Zwischenstück 255.328 - 290.948      (2146) TGTATCTTGACGTTCTGGCCAGTCGTTTGGGGATGCATGATGCTTCCGATGAAGCGTTGCGCGTGGAGCTGAATCGCTATTCA
SARS-CoV-2 Reference Genome NC_045512.2  (473)  TTCATCAA-ACGTTC-GGATGCTCGAACTGCACCTCATGGT-CATGTTATG----GTTGAGCTGGTAGCAGAACTCGAAGGCA
Consensus                                (2159) T  ATC   ACGTTC GG    TCG    G     CATG T C T   ATG    GTTG GC  G AGC GAA     A  CA

                                                       Section 28
                                         (2242) 2242 2250 2260 2270 2280 2290 2300 2310 2324
K12 Zwischenstück 255.328 - 290.948      (2229) TTAAAAGTGCAAGGATTGCTGGGACG-TCGCTGCCCAACGCCA--ATGTTATC---AGGCTACTGGAAGAACGATCCGTTCAG
SARS-CoV-2 Reference Genome NC_045512.2  (549)  TTCAGTACGGTCGTAGTGGTGAGACACTTGGTGTCCTTGTCCCTCATGTGGGCGAAATACCAGTGGCTTACCGCAAGGTTCTT
Consensus                                (2242) TT A    G   G A TG TG GAC  T G TG CC    CC   ATGT   C   A  C A TGG   A CG    GTTC
```

Section 29

```
                                              (2325) 2325 2330      2340      2350      2360      2370      2380      2390          2407
        K12 Zwischenstück 255.328 - 290.948 (2306) CC-CGGAAGAGGACTCACGCTTAATCACCTCATCATC-TGCTGACGGTA--AATTATTAGAGATCCCATTTAACCCG-GTGTA
SARS-CoV-2 Reference Genome NC_045512.2      (632) CTTCGTAAGAACGGTAA---TAAAGGAGCTGGTGGCCATAGTTACGGCGCCGATCTAAAGTCATTTGACTTAGGCGACGAGCT
                                  Consensus (2325) C  CG AAGA    T A   T AA  A CT  T   C T  T ACGG     AT    AG  AT   A TTA  C   G G
```

Section 30

```
                                              (2408) 2408        2420      2430      2440      2450      2460      2470      2480     2490
        K12 Zwischenstück 255.328 - 290.948 (2384) TCGGA---ATTTTGACAAAGGTCTTCAGGAAATCACCGACTGGATCGAAAAACGCTTGTGTTAAAAATTTGCTAAATTTTGCC
SARS-CoV-2 Reference Genome NC_045512.2      (712) TGGCACTGATCCTTATGAAGATTTTCAAGAAAACTGGAACACTAAACATAGCAG-TGGTGTTACCCGTGAACTCATGCGTGAG
                                  Consensus (2408) T G A   AT  T A  AAG T TTCA GAAA C    AC   A   A A   G T GTGTTA    T   CT A    TG
```

Section 31

```
                                              (2491) 2491     2500      2510      2520      2530      2540      2550      2560         2573
        K12 Zwischenstück 255.328 - 290.948 (2464) AATTTGGTAAAACAGTTGCATCACAACAGGAGATAGCAA-TGACGTTACCGAG-TGGACACCCGAA-GAGCAGATTGATCAAA
SARS-CoV-2 Reference Genome NC_045512.2      (794) CTTAACGGAGGGGCATACACTCGCTAT-GTCGATAACAACTTCTGTGGCCCTGATGGCTACCCTCTTGAGTGCATT----AAA
                                  Consensus (2491)   T   G A      T    TC C A  G  GATA CAA T   GT  CC  G TGG  ACCC    GAG   ATT    AAA
```

Section 32

```
                                              (2574) 2574  2580      2590      2600      2610      2620      2630      2640            2656
        K12 Zwischenstück 255.328 - 290.948 (2544) AAATTTACCGCACTAGGCCCGTATA--TTCGTGAAGGTAGG--TAATGACTCCAACTTATTGATAGTGTTTTATGTTCAGATA
SARS-CoV-2 Reference Genome NC_045512.2      (872) GACCTTCTAGCACGTG-CTGGTAAAGCTTCATGCACTTTGTCCGAACAACTGGACTTTATTGACACTAAGAGGGGTGTATACT
                                  Consensus (2574)  A  TT   GCAC  G C  GTA A  TTC TG A  T G    AA  ACT  A  TTATTGA A T       GT  A A
```

Section 33

```
                                              (2657) 2657         2670      2680      2690      2700      2710      2720            2739
        K12 Zwischenstück 255.328 - 290.948 (2623) ATGCCCGATGACTTTGTCATGCA-------GCTCCACCGA---TTTTGAGAACGACAGCGACTTCCGTCCCAGCCGTGCCAGG
SARS-CoV-2 Reference Genome NC_045512.2      (954) GCTGCCGTGAACATGAGCATGAAATTGCTTGGTACACGGAACGTTCTGAAAAGAGCTATGAATTGCAGAC-ACCTTTTGAAAT
                                  Consensus (2657)     CCG   AC T   CATG A       G T CAC GA   TT TGA AA   C   GA TT C   C A C  T   A
```

Section 34

```
                                              (2740) 2740      2750      2760      2770      2780      2790      2800      2810        2822
        K12 Zwischenstück 255.328 - 290.948 (2696) TGCTGCCTCAGATTCAGGTTATGCCGCTCAATTCGCTGCGTATATCGCTTGCTGATTACGTGCAGCTTTCCCTTCAGGCGGGA
SARS-CoV-2 Reference Genome NC_045512.2     (1036) TAAATTGGCAAAGAAATTTGACACCT-TCAATGGGGAATGTCCAAATTTTG-TATTTCCCTTAAATTCCATAATCAAGACT-A
                                  Consensus (2740) T       CA A   A  T A  CC  TCAAT  G    GT  A    TTG T  TT C T  A  T      TCA G    A
```

Section 35

```
                                              (2823) 2823   2830      2840      2850      2860      2870      2880      2890           2905
        K12 Zwischenstück 255.328 - 290.948 (2779) TTCATACAGCGGCCAGCCATCCGTCATCCATATCACCACGTCAAAGGGTGACAGCAGGCTCATAAGACGCCCCAGCGTCGCCA
SARS-CoV-2 Reference Genome NC_045512.2     (1116) TTCAACCAAGGGTT-GAAAAGAAAAAGCTTGATGGC----TTTATGGGT-AGAATTCGATCTGTCTATCCAGTTGCGTCACCA
                                  Consensus (2823) TTCA  CA  GG   G  A      A C   AT  C    T  A GGGT A A    G TC     A  C    GCGTC CCA
```

```
                                                                 Section 36
                                                (2906)  2906          2920      2930      2940      2950      2960      2970            2988
           K12 Zwischenstück 255.328 - 290.948  (2862)  TAGTGCGTTCACCGAATACGTGCGCAACAACCGTCTTCCGGAGACTG-TCATACGCGTAAAACAGCCAG-CGCTGGCGCGATT
SARS-CoV-2 Reference Genome NC_045512.2         (1193)  AA-TGAATGCAACCAA-ATGTGCCTTTCAACTCTCAT--GAAGTGTGATCATTGTGGTGAAACTTCATGGCAGACGGGCGATT
                                     Consensus  (2906)   A TG  T CA C AA A GTGC    CAAC  TC T  G AG  TG TCAT    GT AAAC  C  G C    G GCGATT
                                                                 Section 37
                                                (2989)  2989       3000      3010      3020      3030      3040      3050      3060      3071
           K12 Zwischenstück 255.328 - 290.948  (2943)  TAGCCCCGACATAGCCCCACTGTTCGTCCATTTCCGCGCAGACGATGACGTCACTGCCCGGCTGTATGCGCGAGGTTACCGAC
SARS-CoV-2 Reference Genome NC_045512.2         (1272)  TTGT-------TAAAGCCACT-TGCGAATTTTGTGGCACTGAGAATTTGACTAAAGAAGGTGCCACTACTTGTGGTTACTTAC
                                     Consensus  (2989)  T G        TA   CCACT T CG    TT   GC C GA  AT      A  G   G      T C  G GGTTAC  AC
                                                                 Section 38
                                                (3072)  3072    3080      3090      3100      3110      3120      3130      3140          3154
           K12 Zwischenstück 255.328 - 290.948  (3026)  TGCGGCCTGAGTTTTTTAAGTGACGTAAAATCGTGTTGAGGCCAACGCCCATAATGCGGGCTGTTGCCCGGCATCCAACGCCA
SARS-CoV-2 Reference Genome NC_045512.2         (1347)  CCCAAAATGCTGTTGTTAA-------AATTTATTGTCCAG--CATGTCACA--ATTCAGAAGTAGGACCTGAG--CATAGTCT
                                     Consensus  (3072)    C    TG   TT TTAA       AA  T  TGT  AG  CA   C CA  AT C G      G CC G    CA  G C
                                                                 Section 39
                                                (3155)  3155 3160      3170      3180      3190      3200      3210      3220          3237
           K12 Zwischenstück 255.328 - 290.948  (3109)  TTC--ATGGCCATATCAATGATTTTCTGGTGCGTACCGGGTTGAGAAGCGGTGTAAGTGAACTGCAGTTGCCATGTTTTACGG
SARS-CoV-2 Reference Genome NC_045512.2         (1417)  TGCCGAATACCATA---ATGAATCTGGCTTGAAAACCATTCTTCGTAAGGGTGGTCGC--ACTA---TTGCC---TTTGGAGG
                                     Consensus  (3155)  T C  A   CCATA   ATGA T T    TG   ACC    T  G A  GGTG   G   ACT    TTGCC   TTT   GG
                                                                 Section 40
                                                (3238)  3238         3250      3260      3270      3280      3290      3300      3310    3320
           K12 Zwischenstück 255.328 - 290.948  (3190)  CAGTGAGAGCAGAGATAGCGCT---GATGTCCGGCGGTGCTTTTGCCGTT-ACGCACCACCCCGTCAGTAGCTGAACAGGAGG
SARS-CoV-2 Reference Genome NC_045512.2         (1489)  CTGTGTGTTCTCTTATGTTGGTTGCCATAACAAGTGTGCCTATTGGGTTCCACGTGCTAGCGCTAACATAGGTTGTAACCAT-
                                     Consensus  (3238)  C GTG G  C    AT   G T    AT  C  G G   CT TTG   T  ACG  C A C C     TAG T    A  A
                                                                 Section 41
                                                (3321)  3321     3330      3340      3350      3360      3370      3380      3390        3403
           K12 Zwischenstück 255.328 - 290.948  (3269)  GACAGCTGATAGAAACAGAAGCCACTGGAGCACCTCAAAAACACCATCATACACTAAATCAGTAAG---TTGGCAGCATCACC
SARS-CoV-2 Reference Genome NC_045512.2         (1571)  -ACAGGTGTT-GTTGGAGAAGGTTCCGAAGGTCTTAATGACAACCTTCTTGAAATACTCCAAAAAGAGAAAGTCAACATCAAT
                                     Consensus  (3321)   ACAG TG T G    AGAAG   C G AG  C T A  A  ACC TC T  A TA   CA  AAG     G CA CATCA
                                                                 Section 42
                                                (3404)  3404  3410      3420      3430      3440      3450      3460      3470          3486
           K12 Zwischenstück 255.328 - 290.948  (3349)  --TGAAGGTAAGTGCAAAGATAATCGATTCTTTTTCGATTGTCTGGCTGTATGCGTCAACGTGAAACCGGCACCGGAAGTGCG
SARS-CoV-2 Reference Genome NC_045512.2         (1652)  ATTGTTGGTGACTTTAAACTTAAT-GAAGAGATCGCCATTATTTTGGCATCTTTTTCTGCTT----CCACAAGTGCTTTTGTG
                                     Consensus  (3404)    TG  GGT A T  AAA  TAAT GA     T  C ATT T T G   T T   TC  C T    CC   A  G    TG G
```

```
                                                                                                   Section 43
                                          (3487) 3487         3500      3510      3520      3530      3540      3550              3569
       K12 Zwischenstück 255.328 - 290.948 (3430) TGAATTCTG---GGGCTGGTGGATGGAGC-TGGAAGCGCAG----GAATCCCGTTTTACCT--ACAGTTACCAGTTTGGTCTG
SARS-CoV-2 Reference Genome NC_045512.2 (1730) GAAACTGTGAAAGGTTTGGATTATAAAGCATTCAAACAAATTGTTGAATCCTGTGGTAATTTTAAAGTTACAAAA--GGAAAA
                               Consensus (3487)   AA T TG   GG  TGG   AT  AGC T  AA C  A     GAATCC GT  TA  T  A AGTTAC A    GG
                                                                                                   Section 44
                                          (3570) 3570      3580      3590      3600      3610      3620      3630      3640         3652
       K12 Zwischenstück 255.328 - 290.948 (3503) TTCGATAAAGCAGGCGACTGGAAGAGTGTTC--CGGTAAAAGACACTGAAG--TGGTTGAACGACTGGAGCACACCCTGCGTG
SARS-CoV-2 Reference Genome NC_045512.2 (1811) ---GCTAAAAAAGGTGCCTGGAATATTGGTGAACAGAAATCAATACTGAGTCCTCTTTATGCATTTGCATCAGAGGCTGCTCG
                               Consensus (3570)    G TAAA  AGG G CTGGAA A TG T   C G AA   A ACTGA    T  TT   C   TG A CA A  CTGC  G
                                                                                                   Section 45
                                          (3653) 3653   3660      3670      3680      3690      3700      3710      3720           3735
       K12 Zwischenstück 255.328 - 290.948 (3582) AGTTTCACGAGAAGCTG---CGTGAACTGCTGACGACGCTGAA---TCTGAAGCTGG--AACCGGCGGATGATTTT-CGTGAC
SARS-CoV-2 Reference Genome NC_045512.2 (1891) TGTTGTACGATCAATTTTCTCCCGCACTCTTGAAACTGCTCAAAATTCTGTGCGTGTTTTACAGAAGGCCGCTATAACAATAC
                               Consensus (3653)  GTT  ACGA  A  T    C  G ACT  TGA    GCT AA   TCTG    TG    AC G  GG  G T T  C   AC
                                                                                                   Section 46
                                          (3736) 3736          3750      3760      3770      3780      3790      3800              3818
       K12 Zwischenstück 255.328 - 290.948 (3656) GAGCCGG----TGA-AGTTAACGGCGTGAGTGAAATGTGCCG-GATGCATCACATCCGGCAATATTCATTAAAACTGATACGT
SARS-CoV-2 Reference Genome NC_045512.2 (1974) TAGATGGAATTTCACAGTATTCACTGAGACTCATTGATGCTATGATGT-TCACATCTGATTTGGCTACTAACAATCTAGTTGT
                               Consensus (3736)  AG  GG    T A AGT   C   G GA T A    TGC   GATG  TCACATC G       T  T A AA   A   GT
                                                                                                   Section 47
                                          (3819) 3819       3830      3840      3850      3860      3870      3880      3890       3901
       K12 Zwischenstück 255.328 - 290.948 (3733) CATGCCAACCGCGACAATATCATCATTATTAATATTCAATTTGTTATCGCTATCCAG-----TTGGTTGATTTTATAATCAAC
SARS-CoV-2 Reference Genome NC_045512.2 (2056) AATGGCCTACATTACAGG-TGGTG-TTGTTCAG-TTGACTTCGCAGTGGCTAACTAACATCTTTGGCACTGTTTATGAAAAAC
                               Consensus (3819)  ATG C   C   ACA   T  T  TT TT A  TT A TT G   T GCTA C A      TTGG     TTTAT A  AAC
                                                                                                   Section 48
                                          (3902) 3902       3910      3920      3930      3940      3950      3960      3970       3984
       K12 Zwischenstück 255.328 - 290.948 (3811) --AAACGCTGACATATTTTTGTTGAAATAATACGTAGCACCGACGTCGATATAATTGACCAGATCTTCATCACCGATACCTTC
SARS-CoV-2 Reference Genome NC_045512.2 (2136) TCAAACCCGTCCTTGATTGGCTTGAAGAGAAGTTTAAGGAAGGTGTAGAGTTTCTTAGAGACGGTTGGGAAATTGTTAAATT-
                               Consensus (3902)   AAAC C   C T  TT   TTGAA   A    TA     G  GT GA   T TT    A    T     A  G TA  TT
                                                                                                   Section 49
                                          (3985) 3985 3990      4000      4010      4020      4030      4040      4050            4067
       K12 Zwischenstück 255.328 - 290.948 (3892) AATATCTTTCCCTTTCGATAAGACATAACCCAGCGATGGACGCA--GACCAAAGTCAAACTGGTATTGAGCGACCGCTTCAAA
SARS-CoV-2 Reference Genome NC_045512.2 (2218) --TATCTCAACCTGTGCTTGTGAAATTGTC----GGTGGACAAATTGTCACCTGTGCAAAGGAAATTAAGGAGAGTGTTCAGA
                               Consensus (3985)   TATCT   CCT T   T  GA AT   C    G TGGAC  A  G C    GT  AA  G  ATT AG       TTCA A
```

Section 50

```
                                        (4068) 4068        4080      4090      4100      4110      4120      4130      4140   4150
    K12 Zwischenstück 255.328 - 290.948 (3973) GTTCTGTGTCTTATTGGCAAAGCCGCCAGTTATTGGCGTCATTT--TGCGTGTTTCAGAATAGAAAGTTGCCAGATAAATATT
SARS-CoV-2 Reference Genome NC_045512.2 (2295) CAT-TCTTTAAGCTTGTAAATA-----AATTTTTGGCTTTGTGTGCTGACTCTATCATTATTG---GTGG--AGCTAAACTTA
                              Consensus (4068)   T T T T    TTG  AA       A TT TTGGC T  T T  TG  T T TCA  AT G   GT G  AG TAAA  T
```

Section 51

```
                                        (4151) 4151     4160      4170      4180      4190      4200      4210      4220      4233
    K12 Zwischenstück 255.328 - 290.948 (4054) ATTGGCATCGTATTTCAGACCTGTTGCCCATGCTTCTGCACGCTTGCCTGTGCCACGGCTTTGCAGGTTCTGCTCGTTGGTGC
SARS-CoV-2 Reference Genome NC_045512.2 (2367) AA--GCCTTGAATTT-AG----GTGAAACAT--TTGT-CACGCA---CT---CAAAGGGATTGTA----CAGAAAGTGTGTTA
                              Consensus (4151) A   GC T G ATTT AG    GT    CAT  TT T CACGC    CT   C A GG  TTG A    C G   GT  GT
```

Section 52

```
                                        (4234) 4234  4240      4250      4260      4270      4280      4290      4300         4316
    K12 Zwischenstück 255.328 - 290.948 (4137) GATCT-GAGTTGGTATAGGCCCCACTAATGGCGAAATCGCTGCCGCCAAAGTCATATGTCAATGACGTGCCGAAGCCATCGCC
SARS-CoV-2 Reference Genome NC_045512.2 (2430) AATCCAGAGAAGAAACTGGCCT-ACTCATGCCTCTAAAA--GCCCCAAAAGAAATTATCTTCTTAGAGGGAGAAACACTTCCC
                              Consensus (4234)  ATC  GAG  G  A  GGCC  ACT ATG C   A     GCC C AAAG  AT       T A   G  GAA C  T  CC
```

Section 53

```
                                        (4317) 4317         4330      4340      4350      4360      4370      4380            4399
    K12 Zwischenstück 255.328 - 290.948 (4219) GTTTTGCTTTTTAACGTCGCGGTTTTCGTTTTTCCCT-TGATATTGCAGGGTTAAGTTCAGGCCATCGATAACGCCGAAGAAG
SARS-CoV-2 Reference Genome NC_045512.2 (2510) ACAGAAGTGTTAACAGAGGAAGTTGTCTTGAAAACTGGTGAT-TTACAACCATTAGAACAACCTACTAGTGAAGCTGTTGAAG
                              Consensus (4317)        T TT A  G  G  GTT TC T     C   TGAT TT CA    T AG  CA  C A    T A GC G  GAAG
```

Section 54

```
                                        (4400) 4400      4410      4420      4430      4440      4450      4460      4470     4482
    K12 Zwischenstück 255.328 - 290.948 (4301) TCGGTGTTCCGATACGTCGCCAGACCGCTGGCGCGTTTGGTCATAAAGTTGTCGGTCTGCGCCGAGGAATCGCCACCAAATTC
SARS-CoV-2 Reference Genome NC_045512.2 (2592) C------TCC-ATTGGTTGGTACACCA-------GTTTG-TATTAACGGGCTTATGTTGC---------TCGAAATCAAAGAC
                              Consensus (4400)        TCC AT  GT G  A ACC        GTTTG T  TAA G   T     TGC         TCG  A CAAA  C
```

Section 55

```
                                        (4483) 4483   4490      4500      4510      4520      4530      4540      4550        4565
    K12 Zwischenstück 255.328 - 290.948 (4384) CGGGAACATATCGGTCCAGGCTTCCACGTCATACAACGCCCCCAGGTTACGACCATAATCGAAAGAACCCAAATCTTTATATT
SARS-CoV-2 Reference Genome NC_045512.2 (2651) ACAGAAAAGTACTGTGCC--CTTGCACCTAATATGATG-------GTAACAAACAATACCTTCACACTCAAAGGCGGTGCA--
                              Consensus (4483)    GAA A   C GT C   CTT CAC T ATA  A G       GT AC A CA  A C   A A  C AA  C  T  A
```

Section 56

```
                                        (4566) 4566          4580      4590      4600      4610      4620      4630           4648
    K12 Zwischenstück 255.328 - 290.948 (4467) TCAACCCGGCAAAAGCGAGACGCGTTTTTTGCTGTGCAGTATCACTCTCTGCTTT-ATTACCGGCAAACTCTGCTTCCCAACG
SARS-CoV-2 Reference Genome NC_045512.2 (2723) -----CCAACAAAGGTTACTTTTGGTGATGACACTGTGATAGAAGTGCAAGGTTACAAGAGTGTGAATATCACTTTTGAACTT
                              Consensus (4566)      CC  CAAA G  A     G T  T  C  TG   TA  A T    G TT  A  A  G  AA  TC   TT   A
```

Section 57

```
                                    (4649) 4649 4660 4670 4680 4690 4700 4710 4720 4731
K12 Zwischenstück 255.328 - 290.948 (4549) ACCATAACCAGTCAGTTGATCGTTAATT---TGTGTTTCGCCT------TTGAAACCAAAACGGATATAACTCTGGTCGCCAT
SARS-CoV-2 Reference Genome NC_045512.2 (2801) GATGAAAGGATTGATAAAGTACTTAATGAGAAGTGCTCTGCCTATACAGTTGAACTCGGTACAGAAGTAAATGAGTTCGCC-T
Consensus                           (4649)      AA  A T A     T  TTAAT     GTG T  GCCT      TTGAA  C   AC GA  TAA T  G TCGCC T
```

Section 58

```
                                    (4732) 4732 4740 4750 4760 4770 4780 4790 4800 4814
K12 Zwischenstück 255.328 - 290.948 (4623) CTTTACTGGCGTTATCACTCATATAATGCATGGCTTTAACTTTGCCATAGACATCCAGTTTATTACCGTCTTTATTATATATT
SARS-CoV-2 Reference Genome NC_045512.2 (2883) GTGTTGTGGCAGATGCTGTCATAAAA------------ACTTTGCAACCAGTATC---TGAATTACTTACACCACTGGGCATT
Consensus                           (4732)  T T  TGGC     C  TCATA AA            ACTTTGC A     ATC   T  ATTAC   C   A T    ATT
```

Section 59

```
                                    (4815) 4815 4820 4830 4840 4850 4860 4870 4880 4897
K12 Zwischenstück 255.328 - 290.948 (4706) TCTGCAGCCTGTACAGATGCAGATGCCACAATGCCCATCACCACTAATGCCAGAGTGCTCTTTTTCATTTTCATTCCTGATTT
SARS-CoV-2 Reference Genome NC_045512.2 (2951) GATTTAGA-TG---AGTGGAGTATGGCTACATACTACTTATT--TGATG--AGTCTGGTGAGTTTAAATTGGCTTCACATATG
Consensus                           (4815)   T  AG  TG   AG  G   ATG C   AT C   T A    T ATG  AG  TG T   TTT A TT   TTC     T
```

Section 60

```
                                    (4898) 4898 4910 4920 4930 4940 4950 4960 4970 4980
K12 Zwischenstück 255.328 - 290.948 (4789) TAATTAACGCGCGAATATTCAGCGGGAGAGTCCCGTTGAAAACAGGAAAGTTTTTAACCTGAGATTGTTAAAGATATATTACA
SARS-CoV-2 Reference Genome NC_045512.2 (3026) TATTGTTCTTTCTACCCTCCAGATGAGGAT----GAAGAAGA-AGGTGATTGTGAAGAAGAAGAGTTTGAGCCATCAACTCAA
Consensus                           (4898) TA T   C   C A   T CAG  G  GA     G  GAA A AGG  A T T  A     AGA T T A   AT  A T  A
```

Section 61

```
                                    (4981) 4981 4990 5000 5010 5020 5030 5040 5050 5063
K12 Zwischenstück 255.328 - 290.948 (4872) GATTAATAATATTCTTAAAATGTGGTAATTTATTAAATCTGTAATAAAAGCGTAAACAACTGCCGCTAGGCTTGCTGATCCCG
SARS-CoV-2 Reference Genome NC_045512.2 (3104) TATGAGTATGGTACTGAAGATGAT-TACCAAGGTAAACCTTTGGAATTTGGTGCCACTTCTGCTGCTCTTCAACCTGAAGAAG
Consensus                           (4981)  AT A TA   T CT AA ATG   TA      TAAA CT T   A   G     AC  CTGC GCT   C   CTGA    G
```

Section 62

```
                                    (5064) 5064 5070 5080 5090 5100 5110 5120 5130 5146
K12 Zwischenstück 255.328 - 290.948 (4955) CGCAACAAAACGCCATGCTTTGCTCGCAGATGGTTGGCAACCGACGACAGTCCTGCTAAAACGTTCGTTTGATATCATTTTTC
SARS-CoV-2 Reference Genome NC_045512.2 (3186) AGCAAGAAGAAGA-----TTGGTTAGATGATGATAGTCAACAAACTGTTGGTCAAC-AAGACGGCAGT--GAGGACAATCAGA
Consensus                           (5064)  GCAA AA A G      TT G T G  GATG T G CAAC  AC    G  C  C AA ACG   GT  GA   CA T
```

Section 63

```
                                    (5147) 5147 5160 5170 5180 5190 5200 5210 5229
K12 Zwischenstück 255.328 - 290.948 (5038) CTAAAATTGAATGGCAGAGAATCATGAGTGACAGCCAGACGCTGGTGGTAAAACTCGGCACCAGT-GTGCTAACAGGCGGATC
SARS-CoV-2 Reference Genome NC_045512.2 (3261) CAACTACT--ATTCAAACAATTGTTGAGGTTCAACCTCAAT-TAGAGATGGAACTTA-CACCAGTTGTTCAGACTATTGAAGT
Consensus                           (5147) C A  A T  AT   A   A T  TGAG   CA CC  A   T G G T  AACT   CACCAGT GT C  AC    G A
```

Section 64

```
                                      (5230) 5230      5240      5250      5260      5270      5280      5290      5300        5312
K12 Zwischenstück 255.328 - 290.948   (5120) GCGCCGTCTGAACCGTGCCCATATCGTTGAACTTGTTCGCCAGTGCGCGCAGTTACA-TGCCGCCGGGCATCGGATTGTTATT
SARS-CoV-2 Reference Genome NC 045512.2 (3340) GAATAGTTTTA---GTGGTTATTTAA---AACTTACT-GACAATGTATACATTAAAAATGCAGAC---------ATTGT----
Consensus                             (5230) G    GT T A   GTG   AT T     AACTT  T G CA TG    CA T A A TGC G C         ATTGT
```

Section 65

```
                                      (5313) 5313   5320      5330      5340      5350      5360      5370      5380         5395
K12 Zwischenstück 255.328 - 290.948   (5202) GTGACGTCGGGCGCGATCGCCGCCGGACGTGAGCACCTGGGTTACCCGGAACTGCCAGCGACCATCGCCTCGAAACAACTGCT
SARS-CoV-2 Reference Genome NC 045512.2 (3403) -----GGAAGAAGCTAAAAAGGTAAAACCA----ACAGTGGTT----GTTAATGCAGCCAATGTTTACCTT-AAACA--TGGA
Consensus                             (5313)      G   G  GC A     G    AC      AC   GGTT    G  A TGC   C A   T  CCT  AAACA  TG
```

Section 66

```
                                      (5396) 5396          5410      5420      5430      5440      5450      5460         5478
K12 Zwischenstück 255.328 - 290.948   (5285) GGCGGCGGTAGGGCAGAGTCGACTGATTCAACTGTGGGAACAGCTGTTTTCGATTTATGGCATTCACGTCGGGCAAATGCTGC
SARS-CoV-2 Reference Genome NC 045512.2 (3470) GGAGGTGTTGCAGGAGCCTTAAATAAGGCTACT-----AACA--------------ATGCCATGCAAGTTGA--ATCTGATGA
Consensus                             (5396) GG GG G T   G AG  T  A T A  C ACT     AACA              ATG CAT CA GT G   A  TG TG
```

Section 67

```
                                      (5479) 5479       5490      5500      5510      5520      5530      5540      5550      5561
K12 Zwischenstück 255.328 - 290.948   (5368) TGACCCGTGCTGATATGGAAGACCGTGAACGCTTCCTGAACGCCCGCGACACCCTGCGAGCGTTGCTCGATAACAATATCGTT
SARS-CoV-2 Reference Genome NC 045512.2 (3532) TTACATA-GCTACTAATG--GACCA----------CTTAAAGTGGGTGGTAGTT---GTGTTTTAAGCGGACACAATCTTGCT
Consensus                             (5479) T AC    GCT  TA  G  GACC           CT AA G   G G  A      G G  TT   CG   ACAAT T G T
```

Section 68

```
                                      (5562) 5562    5570      5580      5590      5600      5610      5620      5630         5644
K12 Zwischenstück 255.328 - 290.948   (5451) CCGGTAATCAATGAGAACGATGCTGTCGCTACGGCAGAGATTAAGGTCGGCGATAACGATAACCTTTCTGCGCTGGCGGCGAT
SARS-CoV-2 Reference Genome NC 045512.2 (3599) -----AAACACTGTCTTC-ATGTTGTCGGCCC---AAATGTTAACAAAGGTGAAGACATTCAACTT-----------------
Consensus                             (5562)      AA CA TG    C ATG TGTCG   C   A A  TTAA    GG GA  AC  T A CTT
```

Section 69

```
                                      (5645) 5645 5650      5660      5670      5680      5690      5700      5710         5727
K12 Zwischenstück 255.328 - 290.948   (5534) TCTTGCGGGTGCCGATAAACTGTTGCTGCTGACCGATCAAAAAGGTTTGTATACCGCTGACCCGCGCAGCAATCCGCAGGCAG
SARS-CoV-2 Reference Genome NC 045512.2 (3656) -CTTAAGAGTGCTTATGAAAATTTTAATCAGCACGAAGTTCTAC--TTGCACCATTATTATCAGCTG-GTATTTTTGGTGCTG
Consensus                             (5645)  CTT  G GTGC  AT AA   TT    C G  CGA      A   TTG A      T A C GC   G A T      GC G
```

Section 70

```
                                      (5728) 5728        5740      5750      5760      5770      5780      5790      5800  5810
K12 Zwischenstück 255.328 - 290.948   (5617) AACTGATTAAAGATGTTTACGGCATTGATGACGCACTGCGCGCGATTGCCGGTGACAGCGTTTCAGGCCTCGG-AACTGGCGG
SARS-CoV-2 Reference Genome NC 045512.2 (3735) ACCCTATACAT--TCTTTAAGAGTTTGTGTAGATACTGTTCGCA----CAAATGTCTACTTAGCTGTCTTTGATAAAAATCTC
Consensus                             (5728) A C  AT A    T TTTA G   TTG   A   ACTG  CGC     C   TG C  C T  C G C T G  AA    C
```

```
                                                                                                         Section 71
                                          (5811) 5811     5820      5830      5840      5850      5860      5870      5880     5893
         K12 Zwischenstück 255.328 - 290.948 (5699) CATGAGTACCAAATTGCAGGCCGCTGACGTGGCTTGCCGTGCGGGTATCGACACCATTATTGCCGCGGGCAGCAAGCCGGGCG
SARS-CoV-2 Reference Genome NC_045512.2 (3812) TATGACAAACTTGTTTCAAGCTTTTTG---GAAATGAAGAGTGAAAAGCAAGTTGAACAAAAGATCGCTGAG-ATTCC-----
                               Consensus (5811)  ATGA  A C   TT CA GC   T     G   TG  G G G   A C A    A  A      CG   AG A  CC

                                                                                                         Section 72
                                          (5894) 5894 5900      5910      5920      5930      5940      5950      5960      5976
         K12 Zwischenstück 255.328 - 290.948 (5782) TTATTGGTGATGTGATGGAAGGCATTTCCGTCGGTACGCTGTTCCATGCCCAGGCGACTCCGCTTGAAAACCGTAAACGCTGG
SARS-CoV-2 Reference Genome NC_045512.2 (3886) -TAAAGAGGAAGTTA----AGCCATTTATAACTGAAAG-TAAACCTT---CAG----------TTGAACAGAGAAAACAA--G
                               Consensus (5894)  TA  G  GA GT A    AG CATTT    C G A G T   CC T   CAG          TTGAA A  G AAAC    G

                                                                                                         Section 73
                                          (5977) 5977         5990      6000      6010      6020      6030      6040      6059
         K12 Zwischenstück 255.328 - 290.948 (5865) ATTTTCGGTGCGCCGCCGGCGGGTGAAATCACGGTAGATGAAGGGGCAACTGCCGCCATTCTGGAACGCGGCAGCTCCCTGTT
SARS-CoV-2 Reference Genome NC_045512.2 (3948) ATGATAAGA----------------AAATCAAGCTTGTGTTGAAGAAGTTACAACAACTCTGGAAGAAACTAAGTTCCTCA-
                               Consensus (5977) AT  T  G                 AAATCA  G    TG  G  G A  T C  C A TCTGGAA      A  T CCT

                                                                                                         Section 74
                                          (6060) 6060      6070      6080      6090      6100      6110      6120      6130     6142
         K12 Zwischenstück 255.328 - 290.948 (5948) GCCGAAAGGCATTAAAAGCGTGACTGGCAATTTCTCGCGTGGTGAAGTCATCCGCATT-TGCAACCTCGAAGGCCGCGATATC
SARS-CoV-2 Reference Genome NC_045512.2 (4014) -CAGAAAAC--TTGTTACTTTATATTGACATTAATGGCAATCT----TCATCCAGATTCTGCCACT--------CTTGTTAGT
                               Consensus (6060)  C GAAA    TT   A   T   T G  ATT  T GC    T    TCATCC  ATT TGC AC         C  G TA

                                                                                                         Section 75
                                          (6143) 6143   6150      6160      6170      6180      6190      6200      6210     6225
         K12 Zwischenstück 255.328 - 290.948 (6030) GCCCACGGCGTCAGTCGTTACAACAGCGATGCATTACGCCGTATTGCCGGACACCACTCGCAAGAAATTGATGCAA-TACTG-
SARS-CoV-2 Reference Genome NC_045512.2 (4082) GACATTGACATCACTTTCTTAAAGAAAGATGCTCCATA---TATAGTGGGTGATGTTGTTCAAGAGGGTGTTTTAACTGCTGT
                               Consensus (6143) G C   G C TCA T   T  AA A  GATGC   A     TAT G  GG  A       CAAGA   TG T  AA T CTG

                                                                                                         Section 76
                                          (6226) 6226             6240      6250      6260      6270      6280      6290     6308
         K12 Zwischenstück 255.328 - 290.948 (6111) GGATATGAATACGGCCCGGTTGCCGTTCACCGTGATGACATGATTACC----CGTTAAGGAGCAGGCTGATGCTGGAACAAAT
SARS-CoV-2 Reference Genome NC_045512.2 (4162) GGTTATACCTACTAAAAAG--GCTGGTGGCACTACTGAAATGCTAGCGAAAGCTTTGAGAAAAGTGCCAACAGACAATTATAT
                               Consensus (6226) GG TAT   TAC      G  GC G T  C  T  TGA ATG T  C     C TT AG A    GC  A      A  A AT

                                                                                                         Section 77
                                          (6309) 6309       6320      6330      6340      6350      6360      6370      6380     6391
         K12 Zwischenstück 255.328 - 290.948 (6190) GGGCATTGCCGCGAAGCAAGCCTCGTATAAATTAGCGCAACTCTCCAGCCGCGAAAAAAATCGCGTGCTGGAAAAAATCGCCG
SARS-CoV-2 Reference Genome NC_045512.2 (4243) AACCACTTACCCGGGTCAGG----GTTTAAATGGTTACA-CTGTAGAGGAGGCAAAGACA----GTGCTTAAAAAGTGTAAAA
                               Consensus (6309)    CA T  C CG   CA G    GT TAAAT     CA CT T  AG  G  AAA A A    GTGCT  AAAA
```

```
Section 78
                                        (6392) 6392    6400      6410      6420      6430      6440      6450      6460      6474
K12 Zwischenstück 255.328 - 290.948     (6273) ATGAACTGGAAGCACAAAGCGAAATCATCCTCAACGCTAACGCCCAGGATGTTGCTGACGCGCGAGCCAATGGCCTTAGCGAA
SARS-CoV-2 Reference Genome NC_045512.2 (4317) GTGCCTTTTACATTCTACCATCTATTATCTCTAATGAGAAG---CAAGAAATTCTTGGAACTGTTTCT--TGGAATTTGCGA-
Consensus                               (6392)  TG   T  A    C A       AT ATC   AA G  AA     CA GA  TT  TG   C     C   TGG  TT GCGA

Section 79
                                        (6475) 6475 6480      6490      6500      6510      6520      6530      6540            6557
K12 Zwischenstück 255.328 - 290.948     (6356) GCGATGCTTGACCGTCTGGCACTGACGCCCGCACGGCTGAAAGGCATTGCCGACGATGTACGTCAGGTGTGCAACCTCGCCGA
SARS-CoV-2 Reference Genome NC_045512.2 (4394) GAAATGCTTGCACATGCAGAAGAAACACGCAAATTAATGCCTGTCTGTGTGGAAACTAAA-GCCATAGTTTCAACT------A
Consensus                               (6475) G  ATGCTTG  C T   G A   AC C C  A     TG   G C  TG  GA   T  A G CA    T CAAC      A

Section 80
                                        (6558) 6558        6570      6580      6590      6600      6610      6620      6630      6640
K12 Zwischenstück 255.328 - 290.948     (6439) TCCGGTGGGGCAGGTAATCGATGGCGGCGTACTGGACAGCGGCCTGCGTCTTGAGCGTCGTCGCGTACCGCTGGGGGTTATTG
SARS-CoV-2 Reference Genome NC_045512.2 (4470) TACAGCGTA------AATATAAGG-GTATTAAAATACAAGAGGGTGTGG-TTGATTATGGT--------GCTAGATTTTACTT
Consensus                               (6558) T C G G      AAT  A GG G   TA    ACA   G  TG G TTGA   T GT        GCT G   TTA T

Section 81
                                        (6641) 6641     6650      6660      6670      6680      6690      6700      6710         6723
K12 Zwischenstück 255.328 - 290.948     (6522) GCGTGATTTATGAAGCGCGCCCGAACGTGACGGTTGATGTCGCTTCGCTGTGCCTGAAAACCGGTAATGCGGTGATCCTGCGC
SARS-CoV-2 Reference Genome NC_045512.2 (4537) T--TACACCAGTAAAACAACTGTAGCGTCACTTATCAACACACTTAACGAT--CTAAAT---GAAACTCTTGTTACAATGCCA
Consensus                               (6641)    T     A  AA      C   A CGT AC    T A    C CTT  C  T   CT AA     G  A T    GT A   TGC

Section 82
                                        (6724) 6724  6730      6740      6750      6760      6770      6780      6790            6806
K12 Zwischenstück 255.328 - 290.948     (6605) GGTGGCAAAGAAACGTGTCGCACTAACGCTGCAACGGTGGCGGTGATTCAGGACGCCCTGAAATCCTGCGGCTTACCGGCGGG
SARS-CoV-2 Reference Genome NC_045512.2 (4613) CTTGGCTATGTAACACATGGCTTAAATTTGGAAGAAGCTGCTCGGTATATGAGATCTCTCAAAG--TGCCAGCTACAGT----
Consensus                               (6724)   TGGC A G AAC    T GC    AA     G A    G  GC    G  T  G      C CT AAA    TGC     TAC G

Section 83
                                        (6807) 6807         6820      6830      6840      6850      6860      6870            6889
K12 Zwischenstück 255.328 - 290.948     (6688) TGCCGTGCAGGCGATTGATAATCCTG-ACCGTGCGCTGGTCAGTGAAATGCTGCGTATGGATAAATACATCGACATGCTGATC
SARS-CoV-2 Reference Genome NC_045512.2 (4690) TTCTGTTTCTTCACCTGATGCTGTTACAGCGTATAATGGTTATCTTACTTCTTCTTCTAAAACACCTGAAGAACATTTTATTG
Consensus                               (6807) T C GT      C   TGAT   T  T  A CGT    TGGT A     A T CT C T T   A  A    A   ACAT   T  T

Section 84
                                        (6890) 6890      6900      6910      6920      6930      6940      6950      6960         6972
K12 Zwischenstück 255.328 - 290.948     (6770) CCGCGTGGTGGC--GCTGGTTTGCATAAACTGTGCCGTGAACAGTCGACAATCCCGGTGATCACAGGT-GGTATAGGCGTATG
SARS-CoV-2 Reference Genome NC_045512.2 (4773) AAACCATCTCACTTGCTGGTTCCTATAAAGATTGGTCCTATTC-TGGACAATCTA------CACAACTAGGTATAGA---ATT
Consensus                               (6890)    C     T  C  GCTGGT   ATAAA    TG      A     T GACAATC        CACA  T GGTATAG     AT
```

```
                                                         Section 85
                                        (6973) 6973   6980      6990      7000      7010      7020      7030      7040     7055
    K12 Zwischenstück 255.328 - 290.948 (6850) CCATATTTACGTTGATGAAAGTGTAGAGATCGCTGAAGCATTAAAAGTGATCGTCAACGCGAAAACTCAGCGTCCGAGCACAT
SARS-CoV-2 Reference Genome NC_045512.2 (4846) TCTTAAGAGAGGTGATAAAAGTGTATATTACACT-----------AGTAATCCTA----CCACATTCCA----CCTAGATGGT
                              Consensus (6973)  C TA      G TGAT AAAGTGTA A    C CT           AGT ATC T     C A A   CA     CC AG     T
                                                         Section 86
                                        (7056) 7056          7070      7080      7090      7100      7110      7120     7138
    K12 Zwischenstück 255.328 - 290.948 (6933) GTAATACGGTTGAAACGTTGCTGGTGAATAAAAACATCGCCGATAGCTTCCTGCCCGCATTAAGCAAACAAATGGCGGAAAGC
SARS-CoV-2 Reference Genome NC_045512.2 (4910) GAA-----GTTATCACCTTT---GACAATCTTAAGA-CACTTCTTTCTT--TGAGAGAAGTGAGGACTATTAAGGTGTTTACA
                              Consensus (7056) G A      GTT    AC TT     G   AAT    AA A C C    T  CTT   TG    G A T AG A      A GG G    A
                                                         Section 87
                                        (7139) 7139       7150      7160      7170      7180      7190      7200      7210    7221
    K12 Zwischenstück 255.328 - 290.948 (7016) GGCGTGACATTACACGCAGATGCAGCTGCACTGGCGCAGTTGCAGGCAGGCCCTGCGAAGGTGGTTGCTGTTAAAGCCGAAGA
SARS-CoV-2 Reference Genome NC_045512.2 (4982) ------ACAGTAGACA-ACATTAACCTCCACACGCA-AGTTGTGGACATGTCAATGACATATGGACAACAGTTTGGTCCAACT
                              Consensus (7139)       ACA TA AC   A AT   A CT CAC  GC  AGTTG  G CA G C       A  TGG        T    G C AA
                                                         Section 88
                                        (7222) 7222     7230      7240      7250      7260      7270      7280      7290     7304
    K12 Zwischenstück 255.328 - 290.948 (7099) GTATGACGATG-AGTTTCTGTCATTAGATTTGAACGTCAAAATCGTCAGCGATCTTGACGATGCCATCGCCCATATTCGTGAA
SARS-CoV-2 Reference Genome NC_045512.2 (5057) -TATTTGGATGGAGCTGATGTTACTAAAATAAAACCTCATAATTCACATGAAGGTAAAACATTTTATGT----TTTACCTAAT
                              Consensus (7222)  TAT    GATG AG T   TGT A TA A T   AAC TCA AAT    CA    A  T  A   AT    AT         T T C T A
                                                         Section 89
                                        (7305) 7305 7310      7320      7330      7340      7350      7360      7370     7387
    K12 Zwischenstück 255.328 - 290.948 (7181) CACGGCACACAACACTCCGATGCGATCCTGACCCGCGATATGCGCAAC-GCCCAGCGTTTTGTTAACGAAGTGGATTCGTCCG
SARS-CoV-2 Reference Genome NC_045512.2 (5135) GATGACACTCTACGTGTTGAGGCTTTTGAGTACTACCACA----CAACTGATCCTAGTTTTCT---------GGGTAGGTACA
                              Consensus (7305)  A G CAC C AC      GA GC   T   G   C   C A A    CAAC G   C    GTTTT T          GG T   GT C
                                                         Section 90
                                        (7388) 7388        7400      7410      7420      7430      7440      7450      7460    7470
    K12 Zwischenstück 255.328 - 290.948 (7263) CTGTTTACGTTAACGC-CTCTACGCGTTTTACCGACGGCGGCCAGTT--TGGTCTGGGTGCG--GAAGTGGCGGTAAGCACAC
SARS-CoV-2 Reference Genome NC_045512.2 (5205) -TGTCAGCATTAAATCACACTAAAAAGTGGAAATAC--CCACAAGTTAATGGTTTAACTTCTATTAAATGG------GCAGAT
                              Consensus (7388)  TGT    C TTAA   C C CTA       T  A    AC   C   C AGTT   TGGT T    T C     AA TGG       GCA A
                                                         Section 91
                                        (7471) 7471     7480      7490      7500      7510      7520      7530      7540     7553
    K12 Zwischenstück 255.328 - 290.948 (7341) AAAAACTCCACGCGCGTGGCCCAATGGGGCTGGAAGCACTGACCACTTACAAGTGGATCGGCATTGGTGATTACACCATTCGT
SARS-CoV-2 Reference Genome NC_045512.2 (5279) AACAACTGTTATCTTG----CCACTGCAT-TGTTAACACT--CCA---ACAAATAGAG-----TTGAAGTTTAATCCA--CCT
                              Consensus (7471) AA AACT      C  G     CCA TG     TG   A CACT   CCA   ACAA T GA       TTG   G TTA   CCA   C T
```

```
                                                          Section 92
                                                 (7554) 7554  7560      7570      7580      7590      7600      7610      7620            7636
K12 Zwischenstück 255.328 - 290.948              (7424) GCGTAAATAAAACCGGGTGATGCAAAAGTAGCCATTTGATTCACAAGGCCATTGACGCATCGCCCGGTTAGTTTTAACCTTGT
SARS-CoV-2 Reference Genome NC_045512.2          (5345) GCTC---TACAAGATGCTTATTACAGAGCA--------------AGGGCTGGTGAAGCTGC------TAACTTTT------GT
Consensus                                        (7554) GC     TA AA   G T AT   A AG A              A GGC   TGA GC  C      T A TTTT      GT
                                                          Section 93
                                                 (7637) 7637         7650      7660      7670      7680      7690      7700                7719
K12 Zwischenstück 255.328 - 290.948              (7507) CCACCGTGATTCACGTTCGTGAACATGTCCTTTCAGGGCCGATATAGCTCAGTTGGTAGAGCAGCGCATTCGTAATGCGAAGG
SARS-CoV-2 Reference Genome NC_045512.2          (5399) GCACTTATCTTAGCCTACTGTAATAAGACAGT--AGGT--GAGTTAGGTGA--TGTTAGAGAAAC--------AATG---AGT
Consensus                                        (7637)  CAC     TT  C T C   AA A G C  T  AGG   GA  TAG T A  TG TAGAG A C        AATG   AG
                                                          Section 94
                                                 (7720) 7720      7730      7740      7750      7760      7770      7780      7790        7802
K12 Zwischenstück 255.328 - 290.948              (7590) TCGTAGGTTCGAC-TCCTATTATCGGCACCATTAAAATCAAATTGTTACGTAAGATCTTATCATTCTCC-CACCAAAAAATTA
SARS-CoV-2 Reference Genome NC_045512.2          (5465) TACTTGTTTCAACATGCCAATTTAGATTCTTGCAAAAGAGTCTTGA-ACGTGGTGTGTAAAACTTGTGGACAACAGCAGACAA
Consensus                                        (7720) T  T G TTC AC T C A T T G   C    AAAA     TTG  ACGT    T T A   TT T   CA CA  A A   A
                                                          Section 95
                                                 (7803) 7803   7810      7820      7830      7840      7850      7860      7870         7885
K12 Zwischenstück 255.328 - 290.948              (7671) TCTTAATGTAACAGCTGGTGTAAGTAAATTCTATCAACGAAGATCAATCTTATCTACTGACCAAAAAGGCCTGATAGGGCTTC
SARS-CoV-2 Reference Genome NC_045512.2          (5547) CCCTTAAG--------GGTGTA-GAAGCTGTTATGTAC-ATGGGCACACTT-TCTTATGAACAATTTAA---GAAAGGTGTTC
Consensus                                        (7803)  C T A G        GGTGTA G A  T  TAT  AC A G  CA  CTT TCT  TGA CAA        GA AGG  TTC
                                                          Section 96
                                                 (7886) 7886          7900      7910      7920      7930      7940      7950            7968
K12 Zwischenstück 255.328 - 290.948              (7754) GCTCACTATACATCCTTGGCTGCAGGTTTAGTTG-TACACCACTCCTAAATTTAATGTGTTGGCAATGTGTTCAATAAAGCTC
SARS-CoV-2 Reference Genome NC_045512.2          (5616) -------AGATACCTTGTACGTGTGGTAAACAAGCTACAAAATATCTAGTACAACAGGAGTCACCTTTTGTT--ATGATGTCA
Consensus                                        (7886)        A A A C T   C    GGT  A   G TACA  A   CTA     A  G   T  C  T TGTT  AT A G
                                                          Section 97
                                                 (7969) 7969       7980      7990      8000      8010      8020      8030      8040       8051
K12 Zwischenstück 255.328 - 290.948              (7836) GAACAAATTAGCTCATTATGATCGGTTAATACTTCAACTTCTGGTTGCATGATTGTTTGTCCGTAAAAAGATAACGCGCCTGC
SARS-CoV-2 Reference Genome NC_045512.2          (5690) GCACCACCT-GCTCAGTATGAAC--TTAAGCATGGTACATTTACTTG------TGCTAGTGAGTACACTGGTAAT--------
Consensus                                        (7969) G AC A  T GCTCA TATGA C  TTAA   T   AC T T  TTG      TG T GT  GTA A  G TAA
                                                          Section 98
                                                 (8052) 8052    8060      8070      8080      8090      8100      8110      8120          8134
K12 Zwischenstück 255.328 - 290.948              (7919) CGGGTAGTAGCAGGCGCATTACGCAATAGGTAAACAAGGGAGGAAGTTCAGAAATGTAAATCGGGAAGGTT--GTACGCAATG
SARS-CoV-2 Reference Genome NC_045512.2          (5756) ----TACCAGTGTGGTCACTATA-AACATATAACTTCTAAAGAAACTTT-GTATTGCATAGACGGTGCTTTACTTACAAAGTC
Consensus                                        (8052)     TA  AG   G  CA TA   AA A  TAA       AG AA TT  G A TG A A   GG    TT   TAC  A T
```

Section 99

```
                                          (8135) 8135 8140      8150      8160      8170      8180      8190      8200             8217
     K12 Zwischenstück 255.328 - 290.948  (8000) TTCATCGTACTACGTTG---TTACGGCTTTGCCGCAACAAGCCAGTTGCCTGCCGCGCTCGCAGAATGTCTGCAGCCCGGAGA
SARS-CoV-2 Reference Genome NC_045512.2  (5833) CTCAGAATACAAAGGTCCTATTACGGATGTTTTCTACAAAGAAAACAGT-TACACAACAACCATAAAACCAGTTACTTATAAA
                               Consensus  (8135)  TCA   TAC A G T    TTACGG T T     A  AAG  A   G  T C    C   CA AA   C G   C    A A
```

Section 100

```
                                          (8218) 8218        8230      8240      8250      8260      8270      8280      8290      8300
     K12 Zwischenstück 255.328 - 290.948  (8080) TAAGGAGATTGTTCCTGCCAGCTAAATCCCTTCCTGTCGATACGAACCAGCTCGTATTTTTCTACCAGAAAATTCACGGCATC
SARS-CoV-2 Reference Genome NC_045512.2  (5915) TT-GGATGGTGTTGTTTGTACAGAAAT-------TGACCCTA------AGTTGGACAATTATTATAAGAAAGA-CAATTCTTA
                               Consensus  (8218) T  GGA   TGTT  T   A   AAAT       TG C  TA      AG T G    TT  TA  AGAAA   CA   C T 
```

Section 101

```
                                          (8301) 8301     8310      8320      8330      8340      8350      8360      8370         8383
     K12 Zwischenstück 255.328 - 290.948  (8163) GGCTAGGGTGATACCGGCATCGATGTGTTCCTTAATCACAGCCTCATTGCAGAATGGCGTGTCGTTTATTGTCAGACCATAGT
SARS-CoV-2 Reference Genome NC_045512.2  (5983) TTTCACAGAGCAACCA--ATTGATCTTGTACCAAACCA-ACCATA--TCCAAACGCAAGCTTCGATAATTTTAAGTTTGTA-T
                               Consensus  (8301)     A  G G  ACC   AT GAT T  T C  AA CA A C T   T CA A     G  TCG T ATT T AG    TA T
```

Section 102

```
                                          (8384) 8384  8390      8400      8410      8420      8430      8440      8450            8466
     K12 Zwischenstück 255.328 - 290.948  (8246) GCTGTTCCAGCAGACGTGTCAGTAACATTTGCCAGACAGCCACGGGTGACAGGCAGGGCTTCACCGCCCGCTGAGTTATTGCA
SARS-CoV-2 Reference Genome NC_045512.2  (6060) GTGATAATATCAAATTTGCTGATGATTTAAACCAGTTA---ACTGGTTATAAGAAA----------CCTGCT---TCAAGAGA
                               Consensus  (8384) G   T   A CA A  TG    T A  T   CCAG  A   AC GGT A A G A          CC GCT   T A     A
```

Section 103

```
                                          (8467) 8467         8480      8490      8500      8510      8520      8530               8549
     K12 Zwischenstück 255.328 - 290.948  (8329) GGTAAAGTTTTCATGTTTGCTCTCGTGTAGGTAATTAACGCTGAGTGGGGTAA--ATGGCGATGTATACGTAGCCGCAACTGC
SARS-CoV-2 Reference Genome NC_045512.2  (6127) GCTTAAAGTTACATTTTTCC-CTG----ACTTAAATGGTGATGTGGTGGCTATTGATTATAAACACTACACACCCTCTTTTAA
                               Consensus  (8467) G T AA  TT CAT TTT C CT     A  TAA T   G TG G  GG TA   AT    A    TAC  A CC C    T  
```

Section 104

```
                                          (8550) 8550      8560      8570      8580      8590      8600      8610      8620        8632
     K12 Zwischenstück 255.328 - 290.948  (8410) CAAGGGTGTCGGCTTCGCAGGTTAAATCGTTGTGGTACAGGGTAACGCAGTGGGCATGGTGGGGGCT-GAGTTCACCGG----
SARS-CoV-2 Reference Genome NC_045512.2  (6205) GAAAGGAGCTAAATTGTTACATAAACCTATTGTTTGGCATGTTAAC--AATGCAACTAATAAAGCCACGTATAAACCAAATAC
                               Consensus  (8550)  AA GG G      TT   A  T AA    TTGT    CA G TAAC  A TG    T  T   G C  G  T  ACC      
```

Section 105

```
                                          (8633) 8633   8640      8650      8660      8670      8680      8690      8700           8715
     K12 Zwischenstück 255.328 - 290.948  (8488) -TGGTCAGCATCGATTCCATCTGGAGGATAAAGTGCGGGAATGTTTCATCCAGCTTCCGGCATTCGATGTCACTGAACTTGCC
SARS-CoV-2 Reference Genome NC_045512.2  (6286) CTGGT--GTATACGTTGTCTTTGGAGCACAAAACCAGT---TGAAACATCAAATT-----CGTTTGATGT-ACTGAAGT--CA
                               Consensus  (8633)  TGGT  G AT   TT   T TGGAG A AAA    G    TG   CATC A  T     C TT GATGT ACTGAA T  C 
```

Section 106

```
                                          (8716) 8716 8730 8740 8750 8760 8770 8780 8798
   K12 Zwischenstück 255.328 - 290.948    (8570) GGTGATGCTGGCCCGGTCAGCCAGATAATGCAGTCGGTTGCCTTCCTGCACCAGACGGGCTCCCAGGCGCAGTGTAATCTCCC
SARS-CoV-2 Reference Genome NC_045512.2   (6356) GAGGACGC--GCAGGGAATG---GATAATCTTGCCTGCGAAGATCTAAAACCAGTCT--CTGA-AGAAGTAGTGGAAAATCCT
                             Consensus    (8716) G  GA GC  GC  GG   G   GATAAT   G C G      TC    ACCAG C   CT   AG  G AGTG AA  TCC
```

Section 107

```
                                          (8799) 8799 8810 8820 8830 8840 8850 8860 8870 8881
   K12 Zwischenstück 255.328 - 290.948    (8653) GCTGCAGGCCCCTGGTAGG-GTTGCTCATTACAGT-TCTCCACTATTGTCAGTTCAGGGTGATGCTCATCAGGCAGGTATAGG
SARS-CoV-2 Reference Genome NC_045512.2   (6431) ACCATA-----CAGAAAGACGTTCTTGAGTGTAATGTGAAAACTACCGA-AGTTGTAGGAGA----CATTATACT--TAAACC
                             Consensus    (8799)  C   A     C G  AG  GTT  T A T  A T T    ACTA  G  AGTT   GG GA    CAT A  C   TA A
```

Section 108

```
                                          (8882) 8882 8890 8900 8910 8920 8930 8940 8950 8964
   K12 Zwischenstück 255.328 - 290.948    (8734) GCCCATTGCGGTCCTGGCGGCGTTCGGCGTATACCGCGAGGACTCCGGCGATATCCGGAACGTCCCTGCCGGTGTAATGACAG
SARS-CoV-2 Reference Genome NC_045512.2   (6502) AGCAAATAATA----------GTTTAAAAATTACAG--AAGAGGTTGGCCACACAGATCTAATGGCTGCTTATGTA--GACA-
                             Consensus    (8882)   C A T             GTT       TAC G  A GA    GGC A A         T  CTGC   TGTA  GACA
```

Section 109

```
                                          (8965) 8965 8970 8980 8990 9000 9010 9020 9030 9047
   K12 Zwischenstück 255.328 - 290.948    (8817) ACGCTACCGTGCCACTGGTATTTGCCGGTGCAGTAGCGAAAGATTCGGGACTCAGGATGCTGGCGGTATATCGTCATTGCCCT
SARS-CoV-2 Reference Genome NC_045512.2   (6570) ATTCTAGTCTTACTATTAAGAAACCTAATGAATTATCTAGAGTATTAGGTTTGAAAACCCTTGCTACTCATGGTTTAGCTGCT
                             Consensus    (8965) A  CTA   T  C  T        C   TG A TA C A AG  T  GG  T A  A  CT GC     AT GT       CT
```

Section 110

```
                                          (9048) 9048 9060 9070 9080 9090 9100 9110 9120 9130
   K12 Zwischenstück 255.328 - 290.948    (8900) GCGTTTACTGATAATTTTCATGTAATACCTCA--AAGCAGACCGTGTTCTGCGAACGAATAGATTTGCCTGCCACCGACAATC
SARS-CoV-2 Reference Genome NC_045512.2   (6653) GTTAATAGTGTCCCTTGGGATACTATAGCTAATTATGCTAAGCCTTTTCTT--AACAAAGTTGTTAGTA---CAACTACTAAC
                             Consensus    (9048) G    TA TG    TT   AT   ATA CT A  A GC  A C T TTCT   AAC AA    TT G     CA C AC A C
```

Section 111

```
                                          (9131) 9131 9140 9150 9160 9170 9180 9190 9200 9213
   K12 Zwischenstück 255.328 - 290.948    (8981) AGATGGTCAGGGACACGGATATCCACCAGCTGAAGCACCTGAACCAGTCGCTGCGTGAGGGTTTTGTCGGCCTGGCTAGGTGT
SARS-CoV-2 Reference Genome NC_045512.2   (6731) ATA------GTTACACGGTGTTTAAACCGTGTTTGTACTA-----ATTATATGCCTTA----TTTCTTTACTT----------
                             Consensus    (9131) A A      G  ACACGG   T  A C G     G AC       AT     TGC T A    TTT T   C T
```

Section 112

```
                                          (9214) 9214 9220 9230 9240 9250 9260 9270 9280 9296
   K12 Zwischenstück 255.328 - 290.948    (9064) CGTCTCGCCGGAAGGATGGTTATGCGCGAGTATCACCGCCGCCGCGTTGAAGTGCAGAGCACGTTTGACCACCTCCCGGGGAT
SARS-CoV-2 Reference Genome NC_045512.2   (6789) --TATTGCTACAATTGTGTACTTTTACTAGAAGTACAAATTC----TAGAATT--AAAGCATCTATGCCGACTACTATAGCAA
                             Consensus    (9214)   T T GC   AA   TG    T   C AG A  AC     C    T GAA T  A AGCA  T TG C AC  C    G A
```

Section 113

```
                                                (9297)  9297         9310      9320      9330      9340      9350      9360                 9379
K12 Zwischenstück 255.328 - 290.948             (9147)  GCACCTCGGTGCGGTTAATCGTGCCGGTGAAGAGCGTTTCATGGGCAATCAACTGATTCTGGTTGTCCAGATACAACACCCGG
SARS-CoV-2 Reference Genome NC 045512.2         (6864)  AGAA-----TACTGTTAAGAGTGTCGGTAAATTTTGTCT-AGAGGCT-TCATTTAATTAT--TTG---------AAGTCACCT
Consensus                                       (9297)    A      T C GTTAA  GTG CGGT AA    GT T A  GGC  TCA  T ATT T  TTG         AA  C C
```

Section 114

```
                                                (9380)  9380      9390      9400      9410      9420      9430      9440      9450        9462
K12 Zwischenstück 255.328 - 290.948             (9230)  AACTCTTCCCG-CTCAAGCGCGGCCATATGCAGTCGCAGCCATTCACGTACGGCGTGGGTAGAGGTGAAGGCTACGCCGGGCT
SARS-CoV-2 Reference Genome NC 045512.2         (6929)  AATTTTTCTAAACTGATAAATATTATAATTTGGTTTTTACTATTAAGTGTTTGCCTAGGTTC---TTTAATCTACTCAA--CC
Consensus                                       (9380)  AA T TTC    CT A           AT   GT     C ATT A      GC T GGT     T  A  CTAC C    C
```

Section 115

```
                                                (9463)  9463   9470      9480      9490      9500      9510      9520      9530           9545
K12 Zwischenstück 255.328 - 290.948             (9312)  CATGCAGGTGGCGGTCCAGAGCCCTGAGCGCCCGCTGAATGAGACGCCGGTCCTGTGGCGTCAT-CTCGCCGGGTAAAAAGGA
SARS-CoV-2 Reference Genome NC 045512.2         (7007)  GCTGCTTTAGGTGTTTTAATGTC------------TAATTTAGGCA---------TGCCTTCTTACTGTACTGGTTACAGAGA
Consensus                                       (9463)    TGC    GG G T  A  G C            T A T AG C         TG C TC T CT   C GGT A A  GA
```

Section 116

```
                                                (9546)  9546          9560      9570      9580      9590      9600      9610             9628
K12 Zwischenstück 255.328 - 290.948             (9394)  AAGCTGTTTCATCTGTTGCTCCTTCGGTCAGTCGATAATACGCAGAATGGCGTGAGCCTCTGGATGTTGCATGGCATACTCCC
SARS-CoV-2 Reference Genome NC 045512.2         (7069)  AGGCTATTTGAACT----CTACTAATGTCACT--ATTGCAACCT-ACTGTACTGGTTCTAT--ACCTTGTAGTGTTTGT---C
Consensus                                       (9546)  A GCT TTT A CT    CT CT   GTCA T  AT   A  C  A TG   TG   CT T  A  TTG A  G  T     C
```

Section 117

```
                                                (9629)  9629       9640      9650      9660      9670      9680      9690      9700      9711
K12 Zwischenstück 255.328 - 290.948             (9477)  GCAGGCGGTAATAGTGTGCGGTCATCGCGTCACATTCTGTACGGCAGGCATGGTGGCTATACGCAATCAGGCAGACAGCAATA
SARS-CoV-2 Reference Genome NC 045512.2         (7140)  TTAGTGGTTTAGATTCTTTAGACACC---TATCCTTCTTTA-----------GAAACTATACA-AATTACCATTTCATCTTTT
Consensus                                       (9629)    AG  G T A A T T   G CA C   T  C TTCT TA           G   CTATAC  AAT A      CA C  T
```

Section 118

```
                                                (9712)  9712    9720      9730      9740      9750      9760      9770      9780          9794
K12 Zwischenstück 255.328 - 290.948             (9560)  CCTGCTGCTTCTGCACTCATTTGGGCATCGTTACCGTTCAGGCAGTTAAACAGACGCCATGTCTCATCGTTGTCAGGCTCGGG
SARS-CoV-2 Reference Genome NC 045512.2         (7208)  AAATGGGATTTA--ACTGCTTTTGGCTTAGTTGCAGAGTGGTTTTTGGCATATATTCTTT-TCACTAGGTTTT-----TCTAT
Consensus                                       (9712)        G TT    ACT  TTT GGC T GTT C G    G    T   A A A  C  T TC C   GTT T     TC
```

Section 119

```
                                                (9795)  9795 9800      9810      9820      9830      9840      9850      9860            9877
K12 Zwischenstück 255.328 - 290.948             (9643)  GGACATAAATGCGCCGCCATTGCTGAGGGTGTAGAACGACCAGATACCACCGCTGTAGCCCTCACAGAAGCGGTCCATCCAGG
SARS-CoV-2 Reference Genome NC 045512.2         (7283)  GTACTTGGATTGGCTGCAATCA-TGCAATTGTTTTTCAGCTATTTTGCA-----GTA-----CATTTTATTAGTAATTCTTGG
Consensus                                       (9795)  G AC T  AT  GC GC AT   TG    TGT    C  C A  T  CA     GTA     CA    A   GT  TC   GG
```

```
                                                    Section 120
                                   (9878) 9878        9890      9900      9910      9920      9930      9940      9950    9960
K12 Zwischenstück 255.328 - 290.948 (9726) CGAAGATATGCGGCTCCAGGAGTAGCCACTGCGGGATAGCGCCAAAGT--ACTGTGGCCAGAAA-TCGATACGCTGTTCATCG
SARS-CoV-2 Reference Genome NC 045512.2 (7355) CT----TATGTGGTTA-ATAATTAATCTTGTACAAATGGCCCCGATTTCAGCTATGGTTAGAATGTACATCTTCTTGCATCA
                          Consensus (9878) C     TATG GG T  A  A TA  C         AT GC CC A  T   CT TGG  AGAA  T  AT   CT T CATC

                                                    Section 121
                                   (9961) 9961     9970      9980      9990      10000     10010     10020     10030    10043
K12 Zwischenstück 255.328 - 290.948 (9806) GGGACCGGCGTGACGGTCAGTTCAAATTCGGGTTGGTTAGCGGGTGCGAGGTCGTGCTGCGTCTGTGTTGTCATGGGTATGTC
SARS-CoV-2 Reference Genome NC 045512.2 (7433) TTTTATTATGTATGGAAAAGTT-ATGTGCATGTTG--TAGACGGTTGTAATTCAT-CAACTTGTATGATGTGTTACAAACGTA
                          Consensus (9961)          GT   G    AGTT A  T C  GTTG  TAG  GGT   A  TC T C  C T T TG TGT  T     A GT

                                                    Section 122
                                  (10044) 10044 10050     10060     10070     10080     10090     10100     10110    10126
K12 Zwischenstück 255.328 - 290.948 (9889) TCCGTCAATAAAAACGCCAGCGGCGATGGCTGGCGTATGGGGATATAAAGTG--TGTTCGGGGAGGTGAATGCGGGTAAATGC
SARS-CoV-2 Reference Genome NC 045512.2 (7512) ATAGAGCAACAAGAGTCGAATGTACAACTATTGTTAATGGTGTTAGAAGGTCCTTTTATGTCTATGCTAATGGAGGTAAAGGC
                         Consensus (10044)    G    A   AA A   C A   G    A     T G   ATGG G TA AA GT    T T   G    A G  AATG   GGTAAA GC

                                                    Section 123
                                  (10127) 10127        10140     10150     10160     10170     10180     10190        10209
K12 Zwischenstück 255.328 - 290.948 (9970) TTC-GCGATCAGCGGG--TGGC--CGTGTCTGTACGGATGCCTGAGGTGCGGATATAGCGGTTAAGACCTTCACCGGCATCCG
SARS-CoV-2 Reference Genome NC 045512.2 (7595) TTTTGCAAACTACACAATTGGAATTGTGTTAATTGTGATACATTCTGTGCTGGTAGTACATTTATTA---------GTGATG--
                         Consensus (10127) TT  GC A C  C       TGG     GTGT     T     GAT C T     GTGC G TA    C  TTA  A          G  AT

                                                    Section 124
                                  (10210) 10210     10220     10230     10240     10250     10260     10270     10280    10292
K12 Zwischenstück 255.328 - 290.948 (10048) GCTCAAAGTTCCATGCCCGCCAGACCATCCGGCCTTCAGTATCACGAACCACCAGACGGAAGTGACTGCCCTGGTCGTCTTCG
SARS-CoV-2 Reference Genome NC 045512.2 (7668) -----AAGTT---------GCGAGAGACTTGTCACTACAGTTTAAAAGACCAATAAATCCTACTGACCA-----GTCTTCTTAC
                         Consensus (10210)      AAGTT             GC AGA    T      CT CAGT T A    ACCA  A A     A TGAC         GTC TCTT

                                                    Section 125
                                  (10293) 10293 10300     10310     10320     10330     10340     10350     10360    10375
K12 Zwischenstück 255.328 - 290.948 (10131) AGTGT-GATATTGCTGTACGTGGTAGTGACCGCTTGCGCTTGTCTCCGGGTGAAAGGCCCCGGTGGCAGCAACACGGATTGG-
SARS-CoV-2 Reference Genome NC 045512.2 (7733) ATCGTTGATAGTGTTACAGTGAAGAATGGTTCCATCCATCTTTACTTTGATAAA-------GCTGGTCAAAAGACTTATGAAA
                         Consensus (10293) A  GT GATA TG T  A        A TG     C T C    T T      G T AA       G TGG     AA AC  AT

                                                    Section 126
                                  (10376) 10376         10390     10400     10410     10420     10430     10440        10458
K12 Zwischenstück 255.328 - 290.948 (10212) GTCATTTTCGGGCTCCTGATAAAAGAAAACCCCGGCAGCCTGCTAGCTG-TCGGGGTGAATTTGCTGGGGAAGATACTACTAT
SARS-CoV-2 Reference Genome NC 045512.2 (7809) GACATTCTCTCTCTCATTTT----GTTAACTTAGACAACCTGAGAGCTAATAACACTAAAGGTTCATTGCCTATTAATGTTAT
                         Consensus (10376) G CATT TC    CTC T  T     G  AAC    G CA CCTG  AGCT   T      T AA   T C    G       TA T  TAT
```

Section 127

```
(10459)                                          10459 10470 10480 10490 10500 10510 10520 10530 10541
K12 Zwischenstück 255.328 - 290.948 (10294)      CAGTCGTTGCTGCAGTCTCCGAGAGTGGACAGAACTTTCTCAGCGTTCTTCCGGTCCGCAGTGAAGGTCCCTGCTTTGTGGTC
SARS-CoV-2 Reference Genome NC_045512.2 (7888)   -AGTTTTTGATGGTAAATCAAAATGTGAA--GAATCATCTGCAAAATCAGCGTCTGTTTACTACAGTCAGCTTATGTGTCAAC
Consensus (10459)                                 AGT  TTG TG     TC  A  GTG A  GAA   TCT      TC  C   T    A T  AG    CT  T TGT   C
```

Section 128

```
(10542)                                          10542 10550 10560 10570 10580 10590 10600 10610 10624
K12 Zwischenstück 255.328 - 290.948 (10377)      ATTGACGTAGACGTCGAACTGCCCGGCCTCAGAGATATTGCTGATGAAGTCAAACCAGGCGTTATCGCCGTTACGCCAGCCCA
SARS-CoV-2 Reference Genome NC_045512.2 (7968)   CTATACTGTTAC-TAGATCAGGCATTAGTGTCTGATGTTGGTGAT--AGTG----CGGAAGTT---GCAGTTAAATGTTTGA
Consensus (10542)                                 T  AC    AC T GA C G C     T    GAT TTG TGAT  AGT     C G  GTT   GC GTTA         A
```

Section 129

```
(10625)                                          10625 10630 10640 10650 10660 10670 10680 10690 10707
K12 Zwischenstück 255.328 - 290.948 (10460)      GGCTGGACGGAATAATGTACTGCTGGTGATCCATCACTACGGTGATAGTAGTGTCGTCA-TCGTGCGAACTGACCATCTTGTC
SARS-CoV-2 Reference Genome NC_045512.2 (8041)   TGCTT-ACGTTAATACGTTTTCATCAACTTTTAACGTACCAATGGAAAAACTCAAAACACTAGTTGCAACTGCAGAAGCTGAA
Consensus (10625)                                 GCT  ACG  A  A GT  T  T     T  A C    C  TG  A  A T     CA T GT   AACTG   A   TG
```

Section 130

```
(10708)                                          10708 10720 10730 10740 10750 10760 10770 10780 10790
K12 Zwischenstück 255.328 - 290.948 (10542)      ATCGGCAAGGGTAAGAAAGACTGAATGCTGATAGAAACCATTCTGGTCCGGGTTCCCTGTGCAGTTGATGGTAAACGTCTTTC
SARS-CoV-2 Reference Genome NC_045512.2 (8123)   CTTGC----------AAAGAATGTGTCCTTAGACAATGTCTTATCTAC----TTTTATTT-CAGCAGCTCGGCAAGGGTTTGT
Consensus (10708)                                 T G           AAAGA TG  T CT A A AA    TT T   C    TT   T T CAG  G T G  AA G  TT
```

Section 131

```
(10791)                                          10791 10800 10810 10820 10830 10840 10850 10860 10873
K12 Zwischenstück 255.328 - 290.948 (10625)      CGCTGGCTTCGGTCACGCTGTATTCCGTATTGCCCTGACCGTAACCCTGCTGCCAGAACCCCGGGATAGCAGAGGCATTAAAG
SARS-CoV-2 Reference Genome NC_045512.2 (8191)   TGATTCAGATGTAGAAACTAAAGATGTTGTTGAA-TGTCTTAAA---TTGTCACATCAATCTGACATAGAAGTTACTGGCGAT
Consensus (10791)                                 G T      G   A  CT  A     T TTG   TG C   AA   T  T  CA  A  C G  ATAG AG   C     A
```

Section 132

```
(10874)                                          10874 10880 10890 10900 10910 10920 10930 10940 10956
K12 Zwischenstück 255.328 - 290.948 (10708)      C-TCGCGAGCAGTACACCCGCCAGCATAAACCGACTTAGTGAAAGTATTGTCATTCTTGTCTCCTTTGTCGTTGTTTTATTCC
SARS-CoV-2 Reference Genome NC_045512.2 (8270)   AGTTGTAATAACTATATGCTCACCTATAA-----CAAAGTTGAAAACATGACACCCC-GTGACCTTGGTGCTTGTATT-----
Consensus (10874)                                  T G  A  A TA A  C C    ATAA     C  AGT AA     TG CA  C  GT  CCTT GT  TTGT TT
```

Section 133

```
(10957)                                          10957 10970 10980 10990 11000 11010 11020 11039
K12 Zwischenstück 255.328 - 290.948 (10790)      TGATTGTCAGGGTTCGAGGGTATCAGTAGCTGCCCCATCAGTTTGCCGTCATGGGCGTACTCAAAGTATTTTTCTTTGGTATA
SARS-CoV-2 Reference Genome NC_045512.2 (8342)   -GACTGT-----------------AGTGCGCGTCATATTAATGCGCAGGTA-GCAAAAAGTCACAACATTG--CTTTGATAT-
Consensus (10957)                                 GA TGT                 AGT    G C  AT A T  GC G  A G     A TCA A  ATT   CTTTG TAT
```

```
                                                     Section 134
                                          (11040) 11040     11050     11060     11070     11080     11090     11100     11110    11122
K12 Zwischenstück 255.328 - 290.948     (10873) CGGGTCCGTCACCTCCTGGTATTCCAGTTTGATGTTATCGGCAATACACAGCGCATTCATCAGTGGCTGGACGGTTTTTTCCT
SARS-CoV-2 Reference Genome NC_045512.2  (8403) --GGAACGTTAAAG------ATTTCA---TGTCATTGTCTGAACAACTACGAAAACAAATACGTAGT-----GCTGCTAAAAA
Consensus                               (11040)   GG  CGT A         ATT CA   TG   TT TC G A  AC   G   A   AT  GT G      G T  T

                                                     Section 135
                                          (11123) 11123 11130     11140     11150     11160     11170     11180     11190    11205
K12 Zwischenstück 255.328 - 290.948     (10956) GCATATCCACGAGGTAGTAGTAACTGCCATCCTCGCAGCCATCGGGCGACTGGCGGGTACGTAAAACCTGCAAGGTGGGACCG
SARS-CoV-2 Reference Genome NC_045512.2  (8470) GAATAACTT--ACCTTTTA--AGTTGACATGT--GCAACTACTAGACAAGTTGTTAAT--GTTGTAACAACAAAGATAGCACT
Consensus                               (11123) G ATA C    A  T  TA  A  TG CAT    GCA C A   G C A T G    T  GT   A C  CAA G   G  C

                                                     Section 136
                                          (11206) 11206         11220     11230     11240     11250     11260     11270        11288
K12 Zwischenstück 255.328 - 290.948     (11039) GATAAGTAGTCAACCTGTGACCATTCTTCGCTGACATCATCCTGGTGGCTGACCACATCACTGAAACGCGGTGGGGTGAGGTC
SARS-CoV-2 Reference Genome NC_045512.2  (8545) TAAGGGTGGTAAAATTGTTA--ATAATTGGTTGA-AGCA-----GTTAATTAAAGTTACACT--------------TGTGTTC
Consensus                               (11206)  A   GT GT AA  TGT A  AT  TT G TGA A CA     GT   T A      CACT              TG G TC

                                                     Section 137
                                          (11289) 11289      11300     11310     11320     11330     11340     11350     11360   11371
K12 Zwischenstück 255.328 - 290.948     (11122) CTTAAATTTACTGATGGCCTTCAGGTCATCACTTCTGT-CATCGCATGCGATGAGAAACAGAGTGGTG----GCAACCAGCGC
SARS-CoV-2 Reference Genome NC_045512.2  (8606) CTTTTTGTTGCTGCTATTTTCTATTTAATAACACCTGTTCATGTCATGTCTAAACATACTGACTTTTCAAGTGAAATCATAGG
Consensus                               (11289) CTT    TT CTG T    T  A  T AT AC  CTGT CAT  CATG      A A AC GA T  T     G AA CA  G

                                                     Section 138
                                          (11372) 11372   11380     11390     11400     11410     11420     11430     11440    11454
K12 Zwischenstück 255.328 - 290.948     (11200) CAGCAGAGGCAGTGTTTTACGTTTCATTATTTTTTTCCTGAAATCAGACGAACCACTTTGGCAAAGACATAAATGCCCACGAA
SARS-CoV-2 Reference Genome NC_045512.2  (8689) ATACAAGGCTATTGATGGTGGTGTCACTCGTGACATAGCATCTACAGAT--ACTTGTTTTGCTAA--CAAACATGCTGATTTT
Consensus                               (11372)    CA  G  A TG T    GT TCA T  T    T        CAGA   AC   TTT GC AA  CA A ATGC  A

                                                     Section 139
                                          (11455) 11455 11460     11470     11480     11490     11500     11510     11520    11537
K12 Zwischenstück 255.328 - 290.948     (11283) AATACCCACCGGCACGCCGACGAACGGTGTCAGCGCGACACTGGCAGCACCGGCTGCGCCACCTCCCGTCAGCAGTGCGGCAA
SARS-CoV-2 Reference Genome NC_045512.2  (8768) GACAC--ATGGTTTAGCCAGCGT--GGTGGTAGTT--ATACTAATGACAAAGCTTGC-CCATTG---------ATTGCTGCAG
Consensus                               (11455)  A AC  A  G    GCC  CG   GGTG  AG    A ACT     CA  G  TGC CCA            A TGC GCA

                                                     Section 140
                                          (11538) 11538       11550     11560     11570     11580     11590     11600     11610 11620
K12 Zwischenstück 255.328 - 290.948     (11366) CAGTGGCAAGGGTCAGGGCCGCGAGACTGTCGGACACCCCGGTTTTGTTCAGAATGATGACGATGACAAC-AATGGCGATAAT
SARS-CoV-2 Reference Genome NC_045512.2  (8835) TCATAACAAGAGA-AGTG----GGTTTTGTCGTGC----CTGGTTTGCCTGGCACGATATTACGCACAACTAATGGTGACTTT
Consensus                               (11538)    T  CAAG G  AG G    G    TGTCG  C    C G TTTG    G A GAT       ACAAC AATGG GA   T
```

Section 141

```
                                        (11621) 11621 11630 11640 11650 11660 11670 11680 11690 11703
    K12 Zwischenstück 255.328 - 290.948 (11448) GGCAATATCCGGACTGATACTCCAGGTCCAGATAC-ATCAGCCGCTCACGAAAATCACGCCGCAGCGTGCGCACCGACACACC
 SARS-CoV-2 Reference Genome NC 045512.2 (8909) TTGCATTTCTTACCTAGAGTTTTTAGTGCAGTTGGTAACATCTGTTACACACCATCAAAACTTATAGAGTACACTGACTTTGC
                              Consensus (11621)     AT TC    CT     T    GT CAG T   A CA C G T    A  ATCA   C  A  G G  CAC GAC    C
```

Section 142

```
                                        (11704) 11704 11710 11720 11730 11740 11750 11760 11770 11786
    K12 Zwischenstück 255.328 - 290.948 (11530) AAACTCAGCGGCAAGCTTACGCACACTCAGCGTTTCCCCTGCCACCAGACGGCTGATTATCAGTGACAGCCTGACAGCCAGCC
 SARS-CoV-2 Reference Genome NC 045512.2 (8992) AACATCAGC-------TT-----------GTGTTTTGGCTGCTGAATGTACAATTTTTAAAGATG-CTTCTGGTAAGCCAGTA
                              Consensus (11704) AA  TCAGC       TT           G GTTT   CTGC     G     T  TTA    TG C  C  G  AGCCAG
```

Section 143

```
                                        (11787) 11787 11800 11810 11820 11830 11840 11850 11869
    K12 Zwischenstück 255.328 - 290.948 (11613) GGTCATGGCGGCGTTCTGCCTGTGTCATGAGACGTTCTCCG-----TGAAAGTTAACTGACTGAAAATGATGTGATTACTTTA
 SARS-CoV-2 Reference Genome NC 045512.2 (9056) C--CATATTGTTATGATACCAATGTACTAGAAGGTTCTGTTGCTTATGAAAGTTTACGCCCTGACAC--ACGTTATGTGCTCA
                              Consensus (11787)    CAT   G   T  T CC  TGT  T   A GTTCT        TGAAAGTT AC   CTGA A   A GT AT    T A
```

Section 144

```
                                        (11870) 11870 11880 11890 11900 11910 11920 11930 11940 11952
    K12 Zwischenstück 255.328 - 290.948 (11691) AAGAGGGGTCGGACAGGGTATGGACACCACAGAAACTATTTTTCAT-TTCTGCAAAGCCAGAGGCAGCGGGGGTTACAGGC
 SARS-CoV-2 Reference Genome NC 045512.2 (9135) TGGATGGC-TCTATTATTCAATTTCCT----AACACCTACCTTGAAGGTTCTGTTAGAGTGGTAACAACTTTTGATT------
                              Consensus (11870)   GA GG  TC    A    AT   C     A  A CTA  TT  A  TTCTG  A AG    A   A     G TT
```

Section 145

```
                                        (11953) 11953 11960 11970 11980 11990 12000 12010 12020 12035
    K12 Zwischenstück 255.328 - 290.948 (11773) CTTCCCTCGGTGAGTGAGGACCGTCAGCACGGTGCGATGCGGACAGGTG-GTGTCCGATATTAACAAAGCAGCAACATTAAAC
 SARS-CoV-2 Reference Genome NC 045512.2 (9207) CTGAGTACTGTAGGCACGGCACTTGTGAAAGATCAGAAGCTGGTGTTTGTGTATCTACTAGTGGTAGATGGGTACTTAACAAT
                              Consensus (11953) CT     C GT  G   GG  C T  G A G T  GA GC G     TG GT TC   TA T   A A   G A      AA
```

Section 146

```
                                        (12036) 12036 12050 12060 12070 12080 12090 12100 12118
    K12 Zwischenstück 255.328 - 290.948 (11855) GGTCACTGCAGTTGCGTGAGCAGGGCTTCCGCCATCACCCACAGTGCCCGGTTAAGTTTC--ACGTCCCCGTCGATACCGCGA
 SARS-CoV-2 Reference Genome NC 045512.2 (9290) GATTATTACAGAT-CTTTACCAGGAGTTTT-CTGTGGTGTAGA-TGCT--GTAAATTTACTTACTAATATGTTTACACCACTA
                              Consensus (12036) G T A T CAG T C T A CAGG  TT   C  T     A A TGC   GT AA TT C  AC      GT  A ACC C A
```

Section 147

```
                                        (12119) 12119 12130 12140 12150 12160 12170 12180 12190 12201
    K12 Zwischenstück 255.328 - 290.948 (11936) ACGGCACGGGTATGTGACCGTCCTCCTTTGGCATTACGGCCACTGAGCCCGCCCTTAATCAGGTTCTCCTGAATACGCTGGTA
 SARS-CoV-2 Reference Genome NC 045512.2 (9368) ATTCAACCTATTGGTG--------CTTTGGACATATCAGCATCTATAGTAGCT--------GGTGGTATTGTA---GCTAT--
                              Consensus (12119) A    AC   T  GTG        C TT G CAT  C GC  CT      GC         GGT  T  TG A   GCT
```

```
                                                                Section 148
                                              (12202) 12202   12210     12220     12230     12240     12250     12260     12270      12284
           K12 Zwischenstück 255.328 - 290.948 (12019) CGTGGTCCACAGGTCATTGCTCTCATCCTGCCAGCGGCGAGGGGAGAGGATCTGCGATTCAGTCACCGGCTGGTGGTCTTCAC
SARS-CoV-2 Reference Genome NC 045512.2  (9430) CGTAGTA-ACATGCCTTGCCTACTATTTTATGAG-GTTTAGAAGAGCTTTTGGTGAATACAGTCATG---TAGTTGCCTTTAA
                                    Consensus (12202) CGT GT  ACA G C T  CT   AT  T   AG G   AG  GAG    T     AT CAGTCA     T GT G CTT A
                                                                Section 149
                                              (12285) 12285 12290     12300     12310     12320     12330     12340     12350      12367
           K12 Zwischenstück 255.328 - 290.948 (12102) CAAAGCGGTATGTGAGGGCGGCTTTTGCCAGTGCCTGCTGTGCCGGGGGTGGCAACAGCAACGACTGCATGGCATCCCGTTTC
SARS-CoV-2 Reference Genome NC 045512.2  (9508) TACTTTACTATTC--------CTTATGTCATTCACTG---TACTCTGTTTAACACCAGTTT--ACT-CATTCTTACCTGGTGT
                                    Consensus (12285)  A      TAT          CTT TG CA T  CTG   T C   G  T  CA CAG     ACT CAT     CC G T
                                                                Section 150
                                              (12368) 12368       12380     12390     12400     12410     12420     12430     12440 12450
           K12 Zwischenstück 255.328 - 290.948 (12185) TCCTCCACCCGGTCAAAAATCCCCAGCACCTCATACGCGCCTTCAATCACCTGACTCACCACGTCCCCCTTGTGTGGCACCCG
SARS-CoV-2 Reference Genome NC 045512.2  (9577) TTATTCTGTTATTTACTTGTACTTGACATTTTATCTTACTAATGATGTTTCTTTTTTAGCACATATTCAGTGGATGGTTATGT
                                    Consensus (12368) T  T C      T A    T C    CA  T AT        T A     CT   T A CAC T   C  TG  TGG
                                                                Section 151
                                              (12451) 12451    12460     12470     12480     12490     12500     12510     12520     12533
           K12 Zwischenstück 255.328 - 290.948 (12268) CACCTCGCCAAACGACTCACCGCAGACGAGCCCGTTCTGACA-----AACCGCACGAAATAGTCCCGGCAACATCTGATACGA
SARS-CoV-2 Reference Genome NC 045512.2  (9660) TCACACCTTTAGTACCTTTCTGGATAACAATTGCTTATATCATTTGTATTTCCACAAAGCATTTCT------ATTGGTTCTTT
                                    Consensus (12451)    C C    A    CT  C G A A  A     TT T  CA     A    CAC AA  A T C       AT  G T
                                                                Section 152
                                              (12534) 12534 12540     12550     12560     12570     12580     12590     12600      12616
           K12 Zwischenstück 255.328 - 290.948 (12346) ACTGGTTCCATCGTGAGA---GTTGAGTAGAATAA--TTTCCGGCACCTGTTTACCGGTGATCTGCCCTTCCCGCCGCAGACG
SARS-CoV-2 Reference Genome NC 045512.2  (9737) AGTAATTACCTAAAGAGACGTGTAGTCTTTAATGGTGTTTCCTTTAGTACTTTTGAAGA-AGCTGCGCTGTGCACCTTTTTGT
                                    Consensus (12534) A T  TT C T   GAGA   GT G  T  AAT    TTTCC   A    TTT    G  A CTGC CT   C CC
                                                                Section 153
                                              (12617) 12617        12630     12640     12650     12660     12670     12680      12699
           K12 Zwischenstück 255.328 - 290.948 (12424) CAGCATATGCTTTGTATGTTCACGACGACCCGGGTCACGCACGCGGGTCTGACAGGCAAAGAATGGCTGGAAGCCTTCTCGCT
SARS-CoV-2 Reference Genome NC 045512.2  (9819) TAAATAAAGAAATGTATCTAAA-GTTGCGTAGTGATGTGCTATTACCTCTTAC-GCAATATAATAGATA-----CTTAGCTCT
                                    Consensus (12617)  A    A G   TGTAT T  A G  G    G G    GC       TCT AC G  A A AAT G T      CTT  C CT
                                                                Section 154
                                              (12700) 12700     12710     12720     12730     12740     12750     12760     12770      12782
           K12 Zwischenstück 255.328 - 290.948 (12507) GTAGGCTGTCGAGCAGGGAGATGGTGGGTATATAGGTATAACGCTCACTACGGGACTCGTGTTTGTCCTCACTGAATACACTG
SARS-CoV-2 Reference Genome NC 045512.2  (9895) TTATAATAAGTACAAGTATTTTAGTGGAGCAATGGATACAAC--TAGCTACAGAGAAGCTGCTTGTTGTCAT-----------
                                    Consensus (12700)  TA   T    A  AG     T GTGG    AT G TA AAC  T  CTAC G      TG TTGT  TCA
```

```
                                          Section 155
                                 (12783) 12783  12790     12800     12810     12820     12830     12840     12850     12865
K12 Zwischenstück 255.328 - 290.948 (12590) GGCACTACGCGAAACAGCTCTTCACGGGTTAACGGACGGTCGCGACGGATAAGGTTTGCTGCGCCAAAGCGCGAAGCCAGACG
SARS-CoV-2 Reference Genome NC_045512.2 (9965) -------CTCGCAAAGGCTCTCAATGACTTCA------GTAACTCAGGTTCTGATGTTCTTTACCAACCACCACAAACCTCTA
                         Consensus (12783)        C CG AA  GCTCT  A G  TT A      GT  C   GG T  G T T CT   CCAA    C  A  C

                                          Section 156
                                 (12866) 12866         12880     12890     12900     12910     12920     12930     12948
K12 Zwischenstück 255.328 - 290.948 (12673) GGTCATAAGCAGACTCCTCATAACGGGAAAACAAATAAAAGGGATCCCCGTC--GCATCGGCGACAGGGGCAGGG---AAGTA
SARS-CoV-2 Reference Genome NC_045512.2 (10035) TCACCTCAGCTGTTTTG-CAGAGTGGTTTTAGAAA--AATGGCATTCCCATCTGGTAAAGTTGAGGGTTGTATGGTACAAGTA
                         Consensus (12866)    C T AGC G  T   CA A  GG    A AAA  AA GG AT CCC TC  G A  G  GA  G  G A GG   AAGTA

                                          Section 157
                                 (12949) 12949      12960     12970     12980     12990     13000     13010     13020     13031
K12 Zwischenstück 255.328 - 290.948 (12751) ACAGGGATGGGTTAAATACTCAGAAGAAGAAATCCCA-GACGGCGCGGCCACTGAC-ACCACC-GTGGTGCGCACGGCCTGA
SARS-CoV-2 Reference Genome NC_045512.2 (10115) ACTTG--TGGTACAACTACACTTAACGGTCTTTGGCTTGATGACGTAGTTTACTGTCCAAGACATGTGATCTGCACCTC-TGA
                         Consensus (12949) AC  G  TGG   AA TAC C  AA        T  C   GA G CG  G    ACTG C A  AC  GTG T  GCAC  C TGA

                                          Section 158
                                 (13032) 13032   13040     13050     13060     13070     13080     13090     13100     13114
K12 Zwischenstück 255.328 - 290.948 (12831) ATGACGGCCCGCACC--------GGGGCGGGTATCAGGGGAAAGGC-ACTGATGCTATCGAG-TACGGCCCCGACGGTTTCAC
SARS-CoV-2 Reference Genome NC_045512.2 (10195) A-GACATGCTTAACCCTAATTATGAAGATTTACTCATTCGTAAGTCTAATCATAATTTCTTGGTACAGGCTGGTAATGTTCAA
                         Consensus (13032) A GAC   C   ACC        G  G       TCA   G AAG C A T AT   T TC  G TAC G C  G     TTCA

                                          Section 159
                                 (13115) 13115 13120     13130     13140     13150     13160     13170     13180     13197
K12 Zwischenstück 255.328 - 290.948 (12904) CAAAATCGCTACGAGCCTGTTCCCGGACTACCGTCGTGCGAAAGG--AAGGATGGAGTTGCGACACCACCGGGCTGGTGGCCT
SARS-CoV-2 Reference Genome NC_045512.2 (10277) CTCAGG-GTTATTGGACATTCTATGCAAAATTGT-GTACTTAAGCTTAAGGTTGATACAGCCAATCCTAAGACACCTAAGTAT
                         Consensus (13115) C  A    G TA   G C  T    G A  A  GT GT C  AAG   AAGG TG     GC A  CC   G         G T

                                          Section 160
                                 (13198) 13198       13210     13220     13230     13240     13250     13260     13270 13280
K12 Zwischenstück 255.328 - 290.948 (12985) CACGTGGCAGGCACTTAATCATCCGCTCGGCCATCACCTTAAGCCC----CCATTGTAGACGGACCGACACAGCACACACCGG
SARS-CoV-2 Reference Genome NC_045512.2 (10358) AAGTTGTTCGCATTCAACCAGGACAGACTTTTTCAGTGTTAGCTTGTTACAATGGTTCACCATCTGGTGTTTACCAATGTGC
                         Consensus (13198)  A  T G   GCA T AA CA              TCA   T AGC     C AT GT  AC   C G         CA    G

                                          Section 161
                                 (13281) 13281    13290     13300     13310     13320     13330     13340     13350     13363
K12 Zwischenstück 255.328 - 290.948 (13064) ATGGACGGGC--TGGAACAGCTCATGCAGCAGGCA--GATTTTGCGGCTGATATTCT--GCTTCTGCGCCGTGGACAACTGTC
SARS-CoV-2 Reference Genome NC_045512.2 (10441) TATGAGGCCCAATTTCACTATTAAGGGTTCATTCCTTAATGGTTCATGTGGTAGTGTTGGTTTAACATAGATTATGACTGTG
                         Consensus (13281)    GA G  C  T   AC   T A G  CA  C    AT  T C  TG TA T T  G TT   C   G   A  ACTGT
```

Section 162

```
                                        (13364) 13364 13370 13380 13390 13400 13410 13420 13430 13446
    K12 Zwischenstück 255.328 - 290.948 (13141) CCCCACCGCTGGTAGGTTCGGCCTTATCCGACTGGCTGATAACAAACAGCACCTTATGCCGGTATACCTCTCCAATCACCTGA
SARS-CoV-2 Reference Genome NC_045512.2 (10524) TCTCTTTT-TGTTACATGCA-CCATATG-GA-------ATTACCAACTGGAGTTCATGCTGGCACAG------ACTTA---GA
                              Consensus (13364)  C C     TG TA  T C  CC TAT  GA       AT AC AAC G A  T ATGC GG A A       A T A   GA
```

Section 163

```
                                        (13447) 13447 13460 13470 13480 13490 13500 13510 13529
    K12 Zwischenstück 255.328 - 290.948 (13224) TGATAAAAATGTTCATCCACCGTCAGCGCCCGGTCATCGGCCTTAATCAGCCACAGTAC---CAGGTCGAGGCGAGG-AAGCT
SARS-CoV-2 Reference Genome NC_045512.2 (10588) AGGTAACT---TTTATGGACCTTTTGTTGACAGGCAAACAGCACAAGCAGCTG--GTACGGACACAACTATTACAGTTAATGT
                              Consensus (13447)  G TAA     TT AT  ACC T  G    C G CA     C  AA CAGC    GTAC   CA   C A    AG  AA  T
```

Section 164

```
                                        (13530) 13530 13540 13550 13560 13570 13580 13590 13600 13612
    K12 Zwischenstück 255.328 - 290.948 (13303) GTTCGC--GGTACAGCGCAGCATACTCGGTATCGCGAGCGCCACTTTCGCCCACGCCGGGCAGATCCATCAGCGTTATATAGC
SARS-CoV-2 Reference Genome NC_045512.2 (10666) TTTAGCTTGGTTGTACGCTGC-TGTTATAAATGGAGACAGGTGGTTTCTCAATCG------ATTTACCACAACTCTTAATGAC
                              Consensus (13530)  TT GC  GGT    CGC GC T  T    AT G GA  G    TTTC C   CG      A  T C  CA C  T  AT  C
```

Section 165

```
                                        (13613) 13613 13620 13630 13640 13650 13660 13670 13680 13695
    K12 Zwischenstück 255.328 - 290.948 (13384) GGTCGCCGACTTGCAGGCGAAAGCGCAGTGGCTCACGTGTA-CAGGCCGCCACATCGCTGACCGGTGATATATCTCCGGCAAA
SARS-CoV-2 Reference Genome NC_045512.2 (10742) TTTAACCTTGTGGCTATG--AAGTACAATTATGAACCTCTAACACAAGACCATGTTGACATACTAGGACCTCTTTCTGCTCAA
                              Consensus (13613)   T  CC   T GC      AAG  CA T     AC T TA CA     CCA  T G     C   GA  T T TC G   AA
```

Section 166

```
                                        (13696) 13696 13710 13720 13730 13740 13750 13760 13778
    K12 Zwischenstück 255.328 - 290.948 (13466) CAGGGCATTGCAC----AGACTGCTCTTCC-CAACACCGGTTTTACC-CATAATGCCGATCACTGGCTCGTAGTTAGTTAACT
SARS-CoV-2 Reference Genome NC_045512.2 (10823) ACTGGAATTGCCGTTTTAGATATGTGTGCTTCATTAAAAGAATTACTGCAAAATGGT-ATGAATGGA-CGTACCATATTGGGT
                              Consensus (13696)    GG ATTGC      AGA    T T C  CA  A   G  TTAC  CA AATG   AT A TGG  CGTA     TT   T
```

Section 167

```
                                        (13779) 13779 13790 13800 13810 13820 13830 13840 13850 13861
    K12 Zwischenstück 255.328 - 290.948 (13543) GGTTAATTTGCTGCAGAAGCCGCTCTGATACCCATTGCGGCAGATCAGCAAGCGATTGCTGAAACGACTTCAAACCTTCAG--
SARS-CoV-2 Reference Genome NC_045512.2 (10904) AGTGC-TTTATT--AGAAGATGAATTTACACCTTTTGATGTTGTTAGACAATGCTCAGGTGTTACT--TTCCAAAGTGCAGTG
                              Consensus (13779)  GT   TTT  T  AGAAG  G   T A ACC  TTG  G  G T   CAA      G TG  AC   TTC AA  T CAG
```

Section 168

```
                                        (13862) 13862 13870 13880 13890 13900 13910 13920 13930 13944
    K12 Zwischenstück 255.328 - 290.948 (13624) -AATTATTCATCAC-----TACTCCTCTGAAAAATAATAAAAAA--CGGTAGAATCGTGAGATTCCGCCGTTAATTGCGTATG
SARS-CoV-2 Reference Genome NC_045512.2 (10982) AAAAGAACAATCAAGGGTACACACCACTGGTTGTTACTCACAATTTTGACTTCACTTTTAGTTTTAGTCCAGAGTACTCAATG
                              Consensus (13862)  AA  A   ATCA       AC CC CTG     TA T A AA    G     A   T AG TT  G C   A T     ATG
```

```
                                                Section 169
                                          (13945) 13945 13950 13960 13970 13980 13990 14000 14010 14027
         K12 Zwischenstück 255.328 - 290.948 (13699) TTCAGAGTGATGATATATATCTGAAGATTTTTTCAATCCTCTGCGCTTGAGGCAGCTGCGCGACTGCTGGCTCAGGCAATGAA
SARS-CoV-2 Reference Genome NC_045512.2 (11065) GTCTTTGTTCTTTTTTTTGTATGAAAATGCCTTTTTACCTTTT-GCTATGGGTATTATTGCTATGTCTG-CTTTTGCAATGAT
                                 Consensus (13945)  TC   GT  T  T T T T TGAA AT   TT    CCT T  GCT   GG A     GC A   CTG CT   GCAATGA 

                                                Section 170
                                          (14028) 14028 14040 14050 14060 14070 14080 14090 14100 14110
         K12 Zwischenstück 255.328 - 290.948 (13782) TGAGTTATAATAGCAGCAT---TTACTAACAGGGATTTATTGAGAGTATGA-GCCGCCGTGATACACCTTAAAAT---CTCAA
SARS-CoV-2 Reference Genome NC_045512.2 (11146) GTTTGTCAAACATAAGCATGCATTTCTCTGTTTGTTTTTGTTACCTTCTCTTGCCACTGTAGCTTATTTTAATATGGTCTATA
                                 Consensus (14028)      T  AA A  AGCAT   TT CT      G TTT  T A   T T   GCC C GT     A  TTAA AT   CT   A

                                                Section 171
                                          (14111) 14111 14120 14130 14140 14150 14160 14170 14180 14193
         K12 Zwischenstück 255.328 - 290.948 (13858) CCCAGCAAAGTTTCGGAGCCGCGAGCAAAAGTGAGAGCCTTCACAAAATAATGCTTAGTAATAAAGTTACTTTGAATTCAGAG
SARS-CoV-2 Reference Genome NC_045512.2 (11229) TGCCTGCTAGTTG-GGTGATGCGTATTA---TGACATGGTTGG----ATA-TGGTTGATACTA--GTTTGTCTGGTTTTA-AG
                                 Consensus (14111)   C     AGTT  GG G  GCG    A   TGA A   TT      ATA TG TT  TA TA  GTT  T TG  TT A AG

                                                Section 172
                                          (14194) 14194 14200 14210 14220 14230 14240 14250 14260 14276
         K12 Zwischenstück 255.328 - 290.948 (13941) CCCCGATTAATGTGAACATGATCAACGAATTTTACCCAGATTGAGTTCATTCAGTAGCCAGGTTTTGAACACATCAAGCGCAG
SARS-CoV-2 Reference Genome NC_045512.2 (11300) CTAAAAG-ACTGTG----TTATGTATGCAT------CAGCTGTAGTG--TTACTAATCC---TTATGA---CAGCAAGAACTG
                                 Consensus (14194) C    A  A TGTG    T AT  A G AT      CAG T  AGT   TT    A CC   TT TGA   CA CAAG  C G

                                                Section 173
                                          (14277) 14277 14290 14300 14310 14320 14330 14340 14359
         K12 Zwischenstück 255.328 - 290.948 (14024) AATGCATG-TGTTGGTGCTGGGGGAAAACCAAATAATGACCGATGTAATGTATTTCGCTGGTACTGTTTACAAGTGGGCATAC
SARS-CoV-2 Reference Genome NC_045512.2 (11364) --TGTATGATGATGGTGCTAGGAGAGTGTGGAC-ACTTATGAATGTCTTGACACTCGTTTATAAAGTTTATTA-TGGTAATGC
                                 Consensus (14277)   TG ATG TG TGGTGCT GG GA      A  A T A   ATGT  TG    TCG T  TA  GTTTA  A TGG  AT C

                                                Section 174
                                          (14360) 14360 14370 14380 14390 14400 14410 14420 14430 14442
         K12 Zwischenstück 255.328 - 290.948 (14106) CAGTTTACCGCTAGCGATTTCACGTTCAGCCAGTAGTTTTGATTCAAGCACAACTCCCAAGCCGTCAACTGCGGCAGCAATCG
SARS-CoV-2 Reference Genome NC_045512.2 (11443) ---TTTAGATCAAGCCATTTCCATGTGGGCTCTTA----TAATCTCTGTT--ACTTCTAACTACTCAGGTGTAGTTACAACTG
                                 Consensus (14360)    TTTA   C AGC ATTTC    T  GC   TA    T AT    G    ACT C AA    TCA  TG  G   CAA  G

                                                Section 175
                                          (14443) 14443 14450 14460 14470 14480 14490 14500 14510 14525
         K12 Zwischenstück 255.328 - 290.948 (14189) CCATAAAGCTACGGTCAAATCGAAGGCCATAATTATTGGGTGGCGTCATCTTATTCGCCTCAAACCATCCTTTCCACTG----
SARS-CoV-2 Reference Genome NC_045512.2 (11517) TCATGTTTTT--GGCCAGAGGTATTGTTTTTATGTGTGTTGAGTATTGCCCTATTTTCTTCATAACTGGTAATACACTTCAGT
                                 Consensus (14443)  CAT     T  GG CA A   A  G   T AT   TG    G  T   C TATT  C TCA A C      T CACT   
```

Section 176

```
                                          (14526) 14526         14540     14550     14560     14570     14580     14590             14608
       K12 Zwischenstück 255.328 - 290.948 (14268) GTACAACTGCACATCGCACTGAATCAATGTCAACGCATAGA---GATCCTCTGGTTTCTTTAGCCG-CTCAGCCAGTTGGGGA
SARS-CoV-2 Reference Genome NC_045512.2 (11598) GTATAA-TGCTAGTTT-ATTGTTTCTTAGGCTATTTTTGTACTTGTTACTTTGGCCTCTTTTGTTTACTCAACCGCTACTTTA
                                Consensus (14526) GTA AA TGC   T   A TG  TC   G C A    T  A   G T CT TGG  TCTTT G    CTCA CC  T     A
```

Section 177

```
                                          (14609) 14609      14620     14630     14640     14650     14660     14670     14680      14691
       K12 Zwischenstück 255.328 - 290.948 (14347) GA---ACACAGTGGCGTAAGTTCTTCAACAG---CAAGCGGGATCTTCTCATAGGGTGATGGGCGGGGTTCAC-CGTAGACTA
SARS-CoV-2 Reference Genome NC_045512.2 (11679) GACTGACTCT-TGGTGTTTATGATTACTTAGTTTCTACACAGGAGTTTAGATATATGAATTCACAGGGACTACTCCCACCCAA
                                Consensus (14609) GA   AC C  TGG GT   T  TT    AG   C A    G   TT   ATA     AT   C GGG   AC C  A  C A
```

Section 178

```
                                          (14692) 14692   14700     14710     14720     14730     14740     14750     14760         14774
       K12 Zwischenstück 255.328 - 290.948 (14423) TATCGAGATCAAAATCATCCTGTTCAAAACGTGCATATTCTGTGCTGGCTGAAAGTCGCAGATCGATGCTTGGGTTCTCACGT
SARS-CoV-2 Reference Genome NC_045512.2 (11761) GAATAGCATAGATGCCTTCAAACTCAACATTAAATTGTTGGGTGTTGGTGGCAAACCTTGTATCAAAGTAGCCACTGTACAGT
                                Consensus (14692)  A     AT  A   C TC    TCAA A      T TT  GTG TGG  G AA  C    ATC A G       T T   GT
```

Section 179

```
                                          (14775) 14775 14780     14790     14800     14810     14820     14830     14840            14857
       K12 Zwischenstück 255.328 - 290.948 (14506) ATGAACTTACCAAGACGTGGTAAAAGCCATTGATGGGCAAAACTCGGTGCAGTATGAAGGCGCAAAGGCCTGGATTCATCAGC
SARS-CoV-2 Reference Genome NC_045512.2 (11844) CTAAAATGTC--AGATGTA--AAGTGCACATCAGTAGTCTTACTCTCAGTTTTGCAACAACTCAGAG---TAGAATCATCATC
                                Consensus (14775)  T AA T  C  AGA GT   AA  GC   T A   G    ACTC   G   T   A   C CA AG   T GA TCATCA C
```

Section 180

```
                                          (14858) 14858       14870     14880     14890     14900     14910     14920     14930   14940
       K12 Zwischenstück 255.328 - 290.948 (14589) CGTCACTAATGC---CAACCCCTGCTGTAATTCG-TTAAATCCCC---GCTGTATGTGCTCAAGCAGTATTTCACCTTCTTTC
SARS-CoV-2 Reference Genome NC_045512.2 (11920) --TAAATTGTGGGCTCAATGTGTCCAGTTACACAATGACATTCTCTTAGCTAAAGATACTACTGAAGCCTTTGAAAAAATGGT
                                Consensus (14858)   T A T  TG    CAA    T C GT A  C  T A AT C C   GCT  A  T CT   G AG  TTT A     T   
```

Section 181

```
                                          (14941) 14941    14950     14960     14970     14980     14990     15000     15010        15023
       K12 Zwischenstück 255.328 - 290.948 (14665) GTCAGCGTAATTTCTCGCGTACTGCGCTGAAAAAGGCGTACATCAAGCAAGTTTTCCAGTTTGCGGATGGCATGGCTGATCGC
SARS-CoV-2 Reference Genome NC_045512.2 (12001) TTCAC--TACTTTCT---GTTTTGCTTTCCATGCAGGGTGCTGTAGACA--TAAACAAGCTTTGTGAAGAAATG-CTGGACA-
                                Consensus (14941)  TCA   TA TTTCT   GT  TGC  T  A    G GT C   A  CA  T   C AG TT   GA G  ATG CTG  C  
```

Section 182

```
                                          (15024) 15024 15030     15040     15050     15060     15070     15080     15090            15106
       K12 Zwischenstück 255.328 - 290.948 (14748) ACTGGGTGACAGTTCCAACTCAGAGGCGGCCAACGCAAAAGC--TCCGGTACGACCAGCCGCTTCAAAGGCGCGTAACAGATT
SARS-CoV-2 Reference Genome NC_045512.2 (12075) ACAGGGCAACCTTACAAGCT-ATAGCCTCAGAGTTTAGTTCCCTTCCATCATATGCAGCTTTTGCTACTGCTCAAGA-AGCTT
                                Consensus (15024) AC GGG  AC  T C A CT A AG C    A    A    C  TCC   A    CAGC   T C A  GC C   A AG TT
```

Section 183

```
                                            (15107) 15107 15120 15130 15140 15150 15160 15170 15189
        K12 Zwischenstück 255.328 - 290.948 (14829) TAGAGGTG-CTTTTGCTAAGAGCTTCATTCATGAATCCTTTTCACTAGGGATGAAGTGGTTTACTGAATTTGGCCACCTGAAC
SARS-CoV-2 Reference Genome NC_045512.2 (12156) ATGAGCAGGCTGTTGCTAATGG--TGATTCTGAAGTTGTTCTTAAAAAGT-TGAAGAAGTCTT-TGAATGTGGCTAAAT---C
                                  Consensus (15107)   GAG  G CT TTGCTAA  G  T ATTC   A T  TT T A  A G  TGAAG  GT T  TGAAT TGGC A  T   C
```

Section 184

```
                                            (15190) 15190 15200 15210 15220 15230 15240 15250 15260 15272
        K12 Zwischenstück 255.328 - 290.948 (14911) AGAGGTGATATGCTCACCTCAGAACAACACAGGTGTCATAATGAAAAAAAGAAATTTCAGCGCAGAGTTTAAACGCGAATCCG
SARS-CoV-2 Reference Genome NC_045512.2 (12232) TGA----ATTTG---ACCGTGATGCAGC-CATGCAACGTAAGTTGGAAAAGA--TGGCTGATCA-AGCTATGACCCAAATGTA
                                  Consensus (15190)  GA    AT TG   ACC      CA C CA G   C TAA     AAAAGA  T  C G  CA AG T   AC C AAT
```

Section 185

```
                                            (15273) 15273 15280 15290 15300 15310 15320 15330 15340 15355
        K12 Zwischenstück 255.328 - 290.948 (14994) CTCAACTGGTCGTTGACCAGAACTACACCGTGGCAGATGCAGCCAGCGCTATGGATGTCGGCCTTTCCACAATGACGCGATGG
SARS-CoV-2 Reference Genome NC_045512.2 (12304) -TAAACAGGC--TAGATCTGAGG-ACAAGAGGGCAAAAGTTACTAGTGCTATGCAGACAATGCTTTTCACTATGCTTAGAAAG
                                  Consensus (15273)  T AAC GG   T GA C GA   ACA    GGCA A G   C AG GCTATG A       CTTT CAC ATG    GA  G
```

Section 186

```
                                            (15356) 15356 15370 15380 15390 15400 15410 15420 15438
        K12 Zwischenstück 255.328 - 290.948 (15077) GTGAAACAATTACGTGATGAACGGCAGGG-CAAAACACCAAAAGCCTCCCCCATTACCCCGGAACAAATTGAAATCCGTGAGC
SARS-CoV-2 Reference Genome NC_045512.2 (12383) TTGGAT-AATGATGCACTCAACAACATTATCAACAATGCAAGAGATGGTTGTGTT-CCCTTGAACATAAT---ACCTCTTACA
                                  Consensus (15356)  TG A  AAT A G   T AAC  CA    CAA A   CAA AG         TT CCC  GAACA A T   A C  T A
```

Section 187

```
                                            (15439) 15439 15450 15460 15470 15480 15490 15500 15510 15521
        K12 Zwischenstück 255.328 - 290.948 (15159) TCAGGAAA-AAGCTACAACGTATTGAAATGGAAAATGAAATATTAAAAAAGGCTACTGTAGATTCAA--TTGGTCAACGCAAC
SARS-CoV-2 Reference Genome NC_045512.2 (12461) ACAGCAGCCAAACTAATGGTTGTCATACCAGACTATAACACATATAAAAATACGTGTGATGGTACAACATTTACTTATGCATC
                                  Consensus (15439)  CAG A   AA CTA     T T   A   GA  AT A A AT  AAAAA  C   TG  G T CAA  TT     A GCA C
```

Section 188

```
                                            (15522) 15522 15530 15540 15550 15560 15570 15580 15590 15604
        K12 Zwischenstück 255.328 - 290.948 (15239) AG--TTATGTGAAAAC-ATGGGGTTGCGGAGGTTTTTTG--AATGAGACGAAC--ATTTACAGCAGA--GGAAAAAGCCTCTG
SARS-CoV-2 Reference Genome NC_045512.2 (12544) AGCATTGTGGGAAATCCAACAGGTTGTAGATGCAGATAGTAAAATTGTTCAACTTAGTGAAATTAGTATGGACAATTCACCTA
                                  Consensus (15522) AG  TT TG GAAA C A   GGTTG  GA G     TG  AA   G   AAC  A T A A  AG   GGA AA  C  CT
```

Section 189

```
                                            (15605) 15605 15610 15620 15630 15640 15650 15660 15670 15687
        K12 Zwischenstück 255.328 - 290.948 (15313) TTTTTGAACTATGGAAGAACGGAACAGGCTTCAGTGAAATAGCGAATATCCTGGGTTCAAAACCCGGAACGATCTTCACTATG
SARS-CoV-2 Reference Genome NC_045512.2 (12627) ATTTAGCATGGCCTCTTATTGTAACAGCTTTAAGGGCCA--------ATTCTGCTGTCAAATTACAGAATAATGAGC------
                                  Consensus (15605)  TTT G A         A  G AACAG  TT AG G  A        AT CTG   TCAAA   C GAA  AT   C
```

Section 190

```
                                         (15688) 15688      15700       15710       15720       15730       15740       15750       15760   15770
       K12 Zwischenstück 255.328 - 290.948 (15396) TTAAGGGATACTGGCGGCATAAAACCCCATGAGCGTAAGCGGGCTGTAGCTCACCTGACACTGTCTGAGCGCGAGGAGATACG
SARS-CoV-2 Reference Genome NC_045512.2 (12696) TTAG----TCCTGTTGCACTACGACAG-ATGT-CTTGTGCTGCCGGTACTACAC---AAACTGCTTGCACT-GATGACA-ATG
                               Consensus (15688) TTA     T CTG  G   TA  AC   ATG  C T  GC G C GTA   CAC   A ACTG  TG  C  GA GA A A G
```

Section 191

```
                                         (15771) 15771    15780       15790       15800       15810       15820       15830       15840      15853
       K12 Zwischenstück 255.328 - 290.948 (15479) AGCTGGTTTGTCAGCCAAAATGAGCATTCGTGCGATAGCTACTGCGCTGAATCGCAGTCCTTCGACGATCTCACGTGAAGTTC
SARS-CoV-2 Reference Genome NC_045512.2 (12768) CGTTAGCTTACTA--CAACACAACAAA--GGGAGGTAGGTT-TGTACTT----GCACTG-TTATCCGATTT-ACAGGATTT--
                               Consensus (15771)  G T G TT   A  CAA A  A  A   G G G TAG T  TG  CT     GCA T  TT   CGAT T AC  GA  T
```

Section 192

```
                                         (15854) 15854 15860     15870       15880       15890       15900       15910       15920      15936
       K12 Zwischenstück 255.328 - 290.948 (15562) AGCGTAATCGGGGCAGACGCTATTACAAAGCTGTTGATGCTAATAACCGAGCCAACAGAATGGCGAAAAGGCCAAAACCGTGC
SARS-CoV-2 Reference Genome NC_045512.2 (12838) ---GAAAT-GGGCTAGATTCCCTAAGAGTGATGGAACTGGTACTATCTAT----ACAGAACTG-GAACCACCTTGTAGGTTTG
                               Consensus (15854)    G AAT GGG  AGA  C  T A A  G TG    TG TA TA C       ACAGAA  G GAA    C    A   T
```

Section 193

```
                                         (15937) 15937        15950       15960       15970       15980       15990       16000          16019
       K12 Zwischenstück 255.328 - 290.948 (15645) TTACTG---GATCAAAATTTACCATTGCGAAAGCTTGTTCTGGAAAAGCTGGAGATGAAATGGTCTCCAGAGCAAATAT-CAG
SARS-CoV-2 Reference Genome NC_045512.2 (12912) TTACAGACACACCTAAAGGTCCTAAAGTGAAGTATTTATACTTTATTAAAGGATTAAACAACCTAAATAGAGGTATGGTACTT
                               Consensus (15937) TTAC G    A C AAA  T C A  G GAA   TT  T     A     GGA    A A   T    AGAG  A   T C
```

Section 194

```
                                         (16020) 16020     16030       16040       16050       16060       16070       16080       16090    16102
       K12 Zwischenstück 255.328 - 290.948 (15724) GATGGTTAAGGCGAACAAAACCACGTCAAAAAACGCTGCGAATATCACCTGAGACAATTTATAAAACGCTGTACTTTCGTAGC
SARS-CoV-2 Reference Genome NC_045512.2 (12995) GGTAGTTTAGCTG--CCACAGTACGTCTACAA--GCTGGTAATGCAACAGAAGTGCCTGCCAATTCAACTGTATTATCTTT-C
                               Consensus (16020) G T GTT AG  G  C A A  ACGTC A AA  GCTG  AAT   AC   AG    T    A     CTGTA T TC T  C
```

Section 195

```
                                         (16103) 16103  16110       16120       16130       16140       16150       16160       16170      16185
       K12 Zwischenstück 255.328 - 290.948 (15807) CGTGAAGCGCTACACCACCTGAATATACAGCATCTGCGACGGTCGCATAGCCTTCGCCATGGCAGGCGTCATACCCGCAAAGG
SARS-CoV-2 Reference Genome NC_045512.2 (13073) TGTGCTTT--TGCTGTAGATGCTGCTAAAGCTTA--CAAAGATTATCTAGC--TAG---TGGGGGACAACCAATCACTAATTG
                               Consensus (16103)  GTG      T C   A  TG    TA AGC T   C A G T    TAGC  T G   TGG  G C  C  A C   AA  G
```

Section 196

```
                                         (16186) 16186            16200       16210       16220       16230       16240       16250        16268
       K12 Zwischenstück 255.328 - 290.948 (15890) CGA-AAGAGGTACGATTAACATAGTGA-ACGGAACACCAATTCACGAACGTTCCCGAA----ATATCGAT-AACAGACGCTCT
SARS-CoV-2 Reference Genome NC_045512.2 (13147) TGTTAAGATGTTGTGTACACACACTGGTACTGGTCAGGCAATAACAGTTACACCGGAAGCCAATATGGATCAAGAATCCTTTG
                               Consensus (16186)  G  AAGA GT    T  ACA A TG  AC G  CA   A T AC       CC GAA    ATAT GAT AA A  C  T
```

Section 197

```
                                                      (16269) 16269 16280 16290 16300 16310 16320 16330 16340 16351
            K12 Zwischenstück 255.328 - 290.948 (15966) CTGGGGCATTGGGAGGGCGATTTAGTCTCAGGTACAAAAAACTCTCATATAGCCAC--ACTTGTAGACCGAAAATCACGTTAT
SARS-CoV-2 Reference Genome NC_045512.2 (13230) GTGGTGCATCGTGTTGTCTGTACTGCCGTTGCCACATAGATCATCCAAATCCTAAAGGATTTTGTGACTTAAAAGGTAAGTAT
                                    Consensus (16269)  TGG GCAT G G  G C  T   G C   G  ACA A A C   CA AT    A   A TT   GAC  AAAA      TAT
```

Section 198

```
                                                      (16352) 16352 16360 16370 16380 16390 16400 16410 16420 16434
            K12 Zwischenstück 255.328 - 290.948 (16047) ACGATCATCGTTAGACTCAGGGGCAAAGATTCTGTCTCAGTAAATCAGGCTCTTACCGACAAATTCCTGAGTTTACCGTCAGA
SARS-CoV-2 Reference Genome NC_045512.2 (13313) GTACAAATACCTACAACTTGTGCTAATGACCCTGTGGGTTTTA------CACTTAAAAACACAGTC-----TGTACCGTCTGC
                                    Consensus (16352)       AT   TA A    G G  AA GA  CTGT     T A      C CTTA   ACA A TC     T TACCGTC G
```

Section 199

```
                                                      (16435) 16435 16440 16450 16460 16470 16480 16490 16500 16517
            K12 Zwischenstück 255.328 - 290.948 (16130) ACTCAGAAAATCACTGACATGGGACAGAGGAATGGAACTGGCCAGACATCTAGAATTTACTGTCAGCACCGGCGTTAAAGTTT
SARS-CoV-2 Reference Genome NC_045512.2 (13385) GGTATGTGGA--AAGGTTATGG--CTGTAGTTGTGATCAACTCCGCGAACCCATGCTT-CAGTCAGCTGATGCACAATCGTTT
                                    Consensus (16435)   T  G   A  A  G  ATGG  C G  G    GA C    C G  A C      TT C GTCAGC    GC   A  GTTT
```

Section 200

```
                                                      (16518) 16518 16530 16540 16550 16560 16570 16580 16590 16600
            K12 Zwischenstück 255.328 - 290.948 (16213) ACTTCTGCGATCCTCAGAGTCCTTGGCAGCGGGGAACAAATGAGAATACAAATGGGCTAATTCGGCAGTACTTTCCTAAAAAG
SARS-CoV-2 Reference Genome NC_045512.2 (13463) TTAAACGGGTTTG-CGGTGTAAGTG-CAGCCCGTCTTACACCGTGCGGCACAGGCACTAGTACTGATGTCGTAT---------
                                    Consensus (16518)       G G T   C G GT   TG CAGC  G    A A        CA A G  CTA T C G  GT  T T
```

Section 201

```
                                                      (16601) 16601 16610 16620 16630 16640 16650 16660 16670 16683
            K12 Zwischenstück 255.328 - 290.948 (16296) ACATGTCTTGCCCAATATACTCAACATGAACTAGATCTGGTTGCTGCTCAGCTAAACAACAGACCGAGAAAGACACTGAAGTT
SARS-CoV-2 Reference Genome NC_045512.2 (13535) ACAGGGCTTTTGACATCTACA-ATGATAAAGTAGCTGGTTTTGCTAAATTCCTAAA-AACTAATTGTTGTCGCTTCC-AAG--
                                    Consensus (16601) ACA G CTT     AT TAC  A  AT AA TAG T    TTGCT      CTAAA AAC  A  G     G   C  AAG
```

Section 202

```
                                                      (16684) 16684 16690 16700 16710 16720 16730 16740 16750 16766
            K12 Zwischenstück 255.328 - 290.948 (16379) CAAAACACCGAAAGAGATAATTGAAAGGGGTGTTGCATTGACAGATTGAATCTACAACCGCGCTCTTGATGTCAGACTCCCTG
SARS-CoV-2 Reference Genome NC_045512.2 (13613) -AAAAGGACGAAGATGACAATTTAATTGATTCTTACTTTGT---AGTTAAGAGACACACTTTCTCTAACTACCAACATGAAGA
                                    Consensus (16684)  AAAA   CGAA   GA AATT AA  G  T TT C TTG    A T AA   ACA  C   CTCT   T  CA   T
```

Section 203

```
                                                      (16767) 16767 16780 16790 16800 16810 16820 16830 16849
            K12 Zwischenstück 255.328 - 290.948 (16462) AACAGTTCTCGATAATCGGGAAACTCAGGGCGCGTTATCCTGTGGCCACTCTCTGCCATGTGTTCGGGGTTCATCGCAGCAGC
SARS-CoV-2 Reference Genome NC_045512.2 (13692) AACAATTT---ATAATTT----ACTTAAGGATTGTCCAGCTGTTGCTAAAC------ATGACTTCT---TTAAGTTTAGAATA
                                    Consensus (16767) AACA TT    ATAAT      ACT A GG   GT    CTGT GC A  C      ATG  TTC    TT A    AG A
```

Section 204

```
(16850)                                         16850 16860 16870 16880 16890 16900 16910 16920 16932
K12 Zwischenstück 255.328 - 290.948 (16545)     TACAAATACTGGAAAAACCGTCCTGAAAAGCCAGACGGCAGACGGGCTGTATTACGCAGTCAGGTACTTGAACTGCATGGCAT
SARS-CoV-2 Reference Genome NC 045512.2 (13759) GACGGTGACATGGTACCACATA-TATCACGTCA-ACGTCTTACTA-----AATACACAATGGCAGACCTCGTCT--ATGCTTT
Consensus (16850)                                AC    AC  G  A   C T  T   A G CA ACG C  AC       A TAC CA T     AC T   CT  ATG   T
```

Section 205

```
(16933)                                         16933 16940 16950 16960 16970 16980 16990 17000 17015
K12 Zwischenstück 255.328 - 290.948 (16628)     CAGCCACGGCTCTGCCGGAGCAAGAAGCATCGCCACAATGGCAACCCAGAGAGGATACCAGATGGGGCGCTGGCTTGCTGGCA
SARS-CoV-2 Reference Genome NC 045512.2 (13833) AAGGCAT---TTTGATGAAGGTAATTGTGACACATTAAAAGAAATACTTGTCACATACAATT------GTTGTGATGATGATT
Consensus (16933)                                AG CA    T TG  G AG  A   G   C C   AA  G AA  C       ATAC A        G TG   TG TG
```

Section 206

```
(17016)                                         17016 17030 17040 17050 17060 17070 17080 17098
K12 Zwischenstück 255.328 - 290.948 (16711)     GACTCATGAAAGAGCTGGGGCTGGTCAGTTGCCAGCAGCCGACTCACCGGTAT-AAGCGTGGCGGTCA--TGAGCACGTTGCT
SARS-CoV-2 Reference Genome NC 045512.2 (13907) ATTTCAATAAAAAG----GACTGGTATGATTTT-GTAGAAAACCCAGATATATTACGCGTATACGCCAACTTAGGTGAACGTG
Consensus (17016)                                  TCA  AAA AG    G CTGGT  G T    G AG   AC CA    TAT A GCGT    G CA  T AG      G
```

Section 207

```
(17099)                                         17099 17110 17120 17130 17140 17150 17160 17170 17181
K12 Zwischenstück 255.328 - 290.948 (16791)     ATCCCGAATCATCTTGAGCGACAGTTCG---CCGTAACGGAA--CCAAAT-CAGGTGTGGTGCGGTGATGTGACCTATAGTGT
SARS-CoV-2 Reference Genome NC 045512.2 (13985) TACGCCAAGCTTTGTTAAAAACAGTACAATTCTGTGATGCCATGCGAAATGCTGGTATTGTT-GGTGTACTGACAT-TAGATA
Consensus (17099)                                 C C AA C T  T A   ACAGT C    C GT A G  A  C AAAT C GGT T GT  GGTG   TGAC T TAG
```

Section 208

```
(17182)                                         17182 17190 17200 17210 17220 17230 17240 17250 17264
K12 Zwischenstück 255.328 - 290.948 (16868)     GCCCGGAGTTCAGGGCGGGCATGGATGCTTAAATGAACCGCGAGTCTGTCTGGAATATTGAACCGGTAAC-----TCACGATG
SARS-CoV-2 Reference Genome NC 045512.2 (14066) ATCAAGATCTCA--------ATGGTAACTGGTATGATTTCGGTGATTTCATACAAACCACGCCAGGTAGTGGAGTTCCTGTTG
Consensus (17182)                                 C  GA  TCA        ATGG   CT   ATGA     G G  T   T  AA       C GGTA       TC  G TG
```

Section 209

```
(17265)                                         17265 17270 17280 17290 17300 17310 17320 17330 17347
K12 Zwischenstück 255.328 - 290.948 (16946)     AGAAACCCAACAATCCCACCGGGTGTGACGGTGGAGAACCTGAGC-GGCAGTGACCTGCGGCATGCCCGCAGGGTGATGTAAC
SARS-CoV-2 Reference Genome NC 045512.2 (14141) TAGATTCTTATTATTC-ATTGTTAATGCCTATATTAACCTTGACCAGGGCTTTAACTGCAGAGT---CACATGTTGACACTGA
Consensus (17265)                                  A  C  A  AT C A  G    TG C  T    A C TGA C GG   T A CTGC G  T   C CA G TGA
```

Section 210

```
(17348)                                         17348 17360 17370 17380 17390 17400 17410 17420 17430
K12 Zwischenstück 255.328 - 290.948 (17028)     CCGCTGACAACGGGGATTGAGGCGAGATCACTAAGCCGAGATGATCCTCAAGGTTAAGTACTGAAAGGTTGAAGAACATGAAC
SARS-CoV-2 Reference Genome NC 045512.2 (14220) CTTAACAAAGCCTTACATTAAGTGGGATTTGTTAAA--ATATGA-CTTCACGG--AAG-----AGAGGTTAAAACTCTTTGAC
Consensus (17348)                               C     A A C      T A G G GAT   T A    A ATGA C TCA GG  AAG     A AGGTT AA   C T  AC
```

Section 211

```
                                          (17431) 17431    17440     17450     17460     17470     17480     17490     17500        17513
K12 Zwischenstück 255.328 - 290.948       (17111) CCGTTAATCCGCCTCTGTGGGTTGAAAACGTCACCACGGCCTACGTGATCTGACAGGCCGTGCAGGAGGAACTGGCAGTG---
SARS-CoV-2 Reference Genome NC_045512.2   (14293) C-GTTATTTTAAATATTGGGATCAGACATACCACC-CAAATTGTGTTAACTGTTTGGATGA-CAGATGCATTCTGCATTGTGC
Consensus                                 (17431) C GTTA T     T T  GG T   A A   CACC C    T  GT A CTG   GG  G  CAG  G A    GCA TG
```

Section 212

```
                                          (17514) 17514 17520     17530     17540     17550     17560     17570     17580           17596
K12 Zwischenstück 255.328 - 290.948       (17191) ATACGT-AAGCACTGCCGGTCGAAGGTGTTTTGACATGTA---TGCGAAACACCGGGGCAGCAGCGTCTATCACGCTTGCGTT
SARS-CoV-2 Reference Genome NC_045512.2   (14373) AAACTTTAATGTTTTATTCTCTACAGTGTTCCCACCTACAAGTTTTGGACCACTAGTG-AGAAAAATATTTGTTGATGGTGTT
Consensus                                 (17514) A AC T AA    T     TC A  GTGTT   AC T  A   T  G A CAC  G G AG A   T T T   G T G GTT
```

Section 213

```
                                          (17597) 17597        17610     17620     17630     17640     17650     17660             17679
K12 Zwischenstück 255.328 - 290.948       (17270) GC-TGACTTCTGCCAACTTGCGGCAAGCAAGGATAAAGAGTGCGACGGGCAG--CCTCCTCAGTATGCCTGAGTCCAGGCAGG
SARS-CoV-2 Reference Genome NC_045512.2   (14455) CCATTTGTAGTTTCAACT---GGATACCAC---TTCAGAGAGCTAGGTGTTGTACATAATCAGGATGTAAACTTACATAGCTC
Consensus                                 (17597)  C T   T  T  CAACT   GG  A CA    T  AGAG GC A G G  G  C T  TCAG ATG      T CA
```

Section 214

```
                                          (17680) 17680     17690     17700     17710     17720     17730     17740     17750       17762
K12 Zwischenstück 255.328 - 290.948       (17350) TAAACCGGGGAAGGTCAGCGACGGATGTTAAGGGGGCATGGCTCCGATGACGCGCTGGCTGGCGGAGCTTCCGTAGTAGTCCG
SARS-CoV-2 Reference Genome NC_045512.2   (14532) TAGACTT----AGTTTT---AAGGAA-TTACTTGTGTATGCTGCTGACCCTGCTATGCA---CGCTGCTTCTG--GTAATCTA
Consensus                                 (17680) TA AC      AG T     A GGA  TTA   G G ATG   C GA    GC  TG     CG  GCTTC G  GTA TC
```

Section 215

```
                                          (17763) 17763  17770     17780     17790     17800     17810     17820     17830          17845
K12 Zwischenstück 255.328 - 290.948       (17433) CGATGGGGAAAGCCCATTACATGGCGAAGGGAAGCAGTTTGAATGTGTTTGCGACGTGAATTAACTGACCTAACGAGGTGAAG
SARS-CoV-2 Reference Genome NC_045512.2   (14602) TTACTAGATAAACGCACTACGTGCTTTTCAGTAGC----TGCACTTACTAACAATGTTGCTTTTCAAACT-------GTCAAA
Consensus                                 (17763)   A   G  AA C CA TAC TG       G AGC    TG A  T  T  C A GT   TT  C  AC        GT AA
```

Section 216

```
                                          (17846) 17846          17860     17870     17880     17890     17900     17910           17928
K12 Zwischenstück 255.328 - 290.948       (17516) ACCTTTGATAATCAGCGAAATGCAACGCAAGCTTGCCACATGGGCAGCCACCGATCCGTCCCTACGGATTCAACGGCTGCTGC
SARS-CoV-2 Reference Genome NC_045512.2   (14674) CCCGGTAATTTTAAC--AAAGACTTCTATGACTTTGC---TGTGT--CTAAGGGTT--TCTTTAAGGAAGGAAGTTCTGTTGA
Consensus                                 (17846)  CC  T AT  T A   AAA  C  C     CTT  C   TG G   C A  G T   TC  TA GGA   AA   CTG TG
```

Section 217

```
                                          (17929) 17929      17940     17950     17960     17970     17980     17990     18000    18011
K12 Zwischenstück 255.328 - 290.948       (17599) GTCTGATAACACAACCAGAATGG-CTGGCTGAAGCGGCGCGGATCA-CGCTT-TCATCAAAGGGGGCCCATACCCCCGGCGTT
SARS-CoV-2 Reference Genome NC_045512.2   (14748) ATT--AAAACACTTCTTCTTTGCTCAGGATGGTAATGCTGCTATCAGCGATTATGACTACTATCGTTATAATCTACCAACAAT
Consensus                                 (17929)  T   A AACAC  C     TG  C GG TG     GC    ATCA CG TT T A  A     G    A  C  CC  C  T
```

```
                                                        Section 218
                                            (18012) 18012 18020 18030 18040 18050 18060 18070 18080 18094
        K12 Zwischenstück 255.328 - 290.948 (17679) GATGGCGTGAACAAAACAATGCTACAGGCCAGACTGGCTGTTGAGCTGCAAATCCTCAGGGATGAATTACTCTCAGGCCACTA
SARS-CoV-2 Reference Genome NC_045512.2 (14829) GT--GTGATATCAGACAACTACTATTTGTAGTTGAAGTTGTTGAT----AAGTACTTTGAT-TG--TTACGATGGTGGCTGTA
                                  Consensus (18012) G   G G  A CA A  A T CTA   G        G TGTTGA     AA T CT  G   TG  TTAC  T   G C  TA

                                                        Section 219
                                            (18095) 18095 18100 18110 18120 18130 18140 18150 18160 18177
        K12 Zwischenstück 255.328 - 290.948 (17762) CCAGCCCTTGCCCGCCAGACGGGTTTACATCCCTAAAAGCAACGGCAAACTGCGACCACTGGGTATCCCCGCGTTGCGGGATC
SARS-CoV-2 Reference Genome NC_045512.2 (14903) TTAA----TGCTAACCAA----GT---CATCGTCAACAACCTAGACAAAT-----CAGCTGGTTTTCCAT---TTAATAAATG
                                  Consensus (18095)   A     TGC   CCA     GT   CATC   AA A C   G CAAA      C  CTGG T TCC     TT     AT

                                                        Section 220
                                            (18178) 18178 18190 18200 18210 18220 18230 18240 18250 18260
        K12 Zwischenstück 255.328 - 290.948 (17845) GTATTGTTCAGCGGGCCATGCTGATGGCGATGGAGCCGATATG-GGAGAGTGATTTTCATACGCTCTCATATGGCTTCCGGCC
SARS-CoV-2 Reference Genome NC_045512.2 (14967) GG---GTAAGGCTAGACTTTATTATGAT--TCAATGAGTTATGAGGATCAAGATGCACTTT---TCGCATATA---------C
                                  Consensus (18178) G    GT   GC  G C T  T ATG    T  A   G TATG GGA    GAT   C T    TC CATAT          C

                                                        Section 221
                                            (18261) 18261 18270 18280 18290 18300 18310 18320 18330 18343
        K12 Zwischenstück 255.328 - 290.948 (17927) TGAGCGCAGTGTCCACCACGCGATCCGCACGGTGAAATTACAGCTCACAGACTGTGGTGAAACCCGGGGACGCTGGGTGATTG
SARS-CoV-2 Reference Genome NC_045512.2 (15033) AAAACGTAATGTCATCCCTACTATAACTCAAATGAATCTTAAGTATGCCATTAGTGCAAAGAATAGAGCTCGCACCGTAGCTG
                                  Consensus (18261)   A CG A TGTC  CC  C AT         TGAA  T  AG    C     GTG   A A   G G  CGC   GT   TG

                                                        Section 222
                                            (18344) 18344 18350 18360 18370 18380 18390 18400 18410 18426
        K12 Zwischenstück 255.328 - 290.948 (18010) AAGGCGACCTGTCCAGTTACTTCGAC-ACCGTACA---TCATCGACTGCTGATGAAGGCTGTA--CGCCGCAGGATCAGTGAC
SARS-CoV-2 Reference Genome NC_045512.2 (15116) GTGTCTCTATCTGTAGT-ACTATGACCAATAGACAGTTTCATCAAAAATTATTGAAATCAATAGCCGCCACTAGAGGAGCTAC
                                  Consensus (18344)   G C    T T  AGT ACT  GAC A    ACA   TCATC A    T  TGAA  C  TA  CGCC C  GA  AG  AC

                                                        Section 223
                                            (18427) 18427 18440 18450 18460 18470 18480 18490 18509
        K12 Zwischenstück 255.328 - 290.948 (18087) GCACGTTTCATGACTCTGCTGTGGAAAACCATCAAGGCGGGACATATCGATGTCGGTCTCTTTCGGGCGGCCAGTGAAGGTGT
SARS-CoV-2 Reference Genome NC_045512.2 (15198) TGTAGTAATTGGAACAAGCAAATTCTATGGTGGTTGGCACAACATGTTAAAAACTGTTTATA--GTGATGT-AGAAAACCCTC
                                  Consensus (18427)     GT     GA    GC       A        GGC   ACAT T  A   C GT T T   G G  G  AG  AA

                                                        Section 224
                                            (18510) 18510 18520 18530 18540 18550 18560 18570 18580 18592
        K12 Zwischenstück 255.328 - 290.948 (18170) GCCACAGGGCGGTGTTATATCGCCGCTATTATCGAACATCATGCTGAATGAGTTCGATCAATACCTGCATGAGCGCTACCTGA
SARS-CoV-2 Reference Genome NC_045512.2 (15278) ACCTTATGGGTTGGGATTATCCTAAATGTGATAGAGC--CATGCCTAACATGCTTAG--AATTATGGCCTCACTTGTTCTTGC
                                  Consensus (18510)  CC  A GG    G   TATC     T T AT GA C  CATGC  AA   G T     AAT    GC T A    T C TG
```

Section 225

```
                                        (18593) 18593 18600 18610 18620 18630 18640 18650 18660 18675
    K12 Zwischenstück 255.328 - 290.948 (18253) GCGGGAAAGCCAGAAAAGATCGGTGGTACTGGAATAACAGTATCCAACGGGGC-CGAAGTACGGCGGTCAGAGAAAACTGGCA
SARS-CoV-2 Reference Genome NC_045512.2 (15357) TCGCAAA---CATACAACGT-GTTGTAGCTTGTCACACCGTTTCTATAGATTAGCTAATGAGTGTGCTCAAGTATTGAGTGAA
                              Consensus (18593)  CG  AA   CA A AA  T G TG   CT G    AC GT TC A  G     C AA  A  G G TCA   A      G A
```

Section 226

```
                                        (18676) 18676 18690 18700 18710 18720 18730 18740 18758
    K12 Zwischenstück 255.328 - 290.948 (18335) GTGGAAACCCGCGGTGGCGTACTGCCGCTATGCCGATGATTTTGTCCTCATC-GTCAAAGGCACCAAAGCACAGGTGGAAGCC
SARS-CoV-2 Reference Genome NC_045512.2 (15436) ATGGTCATGTGTGGCGGTTCACTATATGTTAAACCAGG--TGGAACCTCATCAGGAGATGCCACAACTGCTTATGCTAATAGT
                              Consensus (18676)  TGG  A   G GG GG   ACT     T    C A G  T    CCTCATC G   A G CAC A  GC  A G   A
```

Section 227

```
                                        (18759) 18759 18770 18780 18790 18800 18810 18820 18830 18841
    K12 Zwischenstück 255.328 - 290.948 (18417) ATCAGGGAGGAGTGTCGGGGTGTGCTCGAAGGCAGTCTGAAACTCAGGCTGAAC-ATGGATAAGACTAAAATCCCCCATGTTA
SARS-CoV-2 Reference Genome NC_045512.2 (15517) GTTTTTAACATTTGTCAAGCTGT-CACG--GCCAATGTTAATG-CACTTTTATCTACTGATGGTAACAAAATTGCCGATAAGT
                              Consensus (18759)  T    A     TGTC  G TGT C CG  G CA T T AA   CA   T A C A  GAT   A  AAAAT  CC AT
```

Section 228

```
                                        (18842) 18842 18850 18860 18870 18880 18890 18900 18910 18924
    K12 Zwischenstück 255.328 - 290.948 (18499) ATGACGGCTTTATCTTTCTGGGGCACAGGCTCATTCGCAAACGCAGTCGTTATGGCGAGATGCGAGTGGTCTCAACGATCCCG
SARS-CoV-2 Reference Genome NC_045512.2 (15596) ATGTCCGCAAT---TTACAA---CACAGACTTTATGAGTGTCTCTATAGAAATAG--AGATG----TTGACACAGACTTTGTG
                              Consensus (18842) ATG C GC  T   TT C     CACAG CT   T      C C  T G  AT G  AGATG    T G C CA    T   G
```

Section 229

```
                                        (18925) 18925 18930 18940 18950 18960 18970 18980 18990 19007
    K12 Zwischenstück 255.328 - 290.948 (18582) CAGGAGAAAGCCAGAAACTTCGCCGCATCGCTGACAGCACTGTTATGGAAGGTGCGAATAAGCGGGGAAATTCTTCTCGGCTG
SARS-CoV-2 Reference Genome NC_045512.2 (15667) AATGAG------------TTTTACGCATATTTGCGTAAACATTTCTCAATGATG---ATACTCTCTGACGATGCTGTTGTGTG
                              Consensus (18925)  A GAG            TT   CGCAT   TG     AC  TT T  A G TG   ATA  C   GA   T  T T G  TG
```

Section 230

```
                                        (19008) 19008 19020 19030 19040 19050 19060 19070 19080 19090
    K12 Zwischenstück 255.328 - 290.948 (18665) ACTCAGTCATTTCATTTCTTCATGTTTGAGCCGATTTTTTCTCCCGTAAATGCCTTGAA-TCAGCCTATTTAGACCGTTTCTT
SARS-CoV-2 Reference Genome NC_045512.2 (15735) TTTCA---ATAGCACTTATGCATCTCAAGGTCTAGTGG--CTAGCATAAAGAACTTTAAGTCAGTTCTTTATTATCAAAACAA
                              Consensus (19008)   TCA   AT  CA TT T CAT T    G C A T    CT  C TAAA   CTT AA TCAG    TT   A C    C
```

Section 231

```
                                        (19091) 19091 19100 19110 19120 19130 19140 19150 19160 19173
    K12 Zwischenstück 255.328 - 290.948 (18747) CGCCATTTAAGGCGTTATCCCCAGTTTTTAGTGAGATCTCTCCCACTGACGTA--TCATTTGGTCCGCCCGAAACAGGTTGGC
SARS-CoV-2 Reference Genome NC_045512.2 (15813) TGTT-TTTATGTCTGAAGCAAAATGTTGGACTGAGACTGACCTTACTAAAGGACCTCATGAATTTTGCTCTCAACATA-----
                              Consensus (19091)  G   TTTA G C  A C    A  TT  A TGAGA     C  ACT A G A  TCAT    T  GC C  AACA
```

Section 232

```
                                              (19174) 19174 19180 19190 19200 19210 19220 19230 19240 19256
K12 Zwischenstück 255.328 - 290.948           (18828) CAGCGTGAATAACATCGCCAGTTGGTTATCGTT--TTTCAGCAACCCCTTGTATCTGGCTTTCACGAAGCCGAACTGTCGCTT
SARS-CoV-2 Reference Genome NC_045512.2       (15890) CAATGCTAGTTAAACAGGGTGATGATTATGTGTACCTTCCTTACCCAGATCCATCAAGAATCCTAGGGGCCGG-CTGTT--TT
Consensus                                     (19174) CA  G  A T A A  G   G TG TTAT   T   TTC   A CC   T  ATC  G  T C  G  GCCG  CTGT    TT
```

Section 233

```
                                              (19257) 19257 19270 19280 19290 19300 19310 19320 19339
K12 Zwischenstück 255.328 - 290.948           (18909) GATGATGCGAAATGGGTGCTCCACCCTGGCCCGGATGCTGGCTTTCATGTATTCGATGTTGATGGCCGTTTTGTTCT---TGC
SARS-CoV-2 Reference Genome NC_045512.2       (15970) GTAGATG--ATATCGTAAAAACA----------GATGGTACACTT-ATGATTGAACGGTTCGTGTC--TTTAGCTATAGATGC
Consensus                                     (19257) G  GATG  A AT G      CA          GATG T    TT ATG  T     GTT  TG C  TTT G T T   TGC
```

Section 234

```
                                              (19340) 19340 19350 19360 19370 19380 19390 19400 19410 19422
K12 Zwischenstück 255.328 - 290.948           (18989) GTGGATGCTGTTTCAAGGTTCTTACCTTGCCGGGGCGCTCGGCGATCAGCCAGTCCACATCCACCTCGGCCAGCTCCTCGCGC
SARS-CoV-2 Reference Genome NC_045512.2       (16038) TTACCCACTTACTAAACATCCTAATCAGGAGTATGCTGATGTCTTTCATTTGTACTTACAATACATAAGAAAGCTACATGATG
Consensus                                     (19340)  T     CT   T AA  T CT A C  G     GC    G C  TCA      C       AC T  G  AGCT C  G
```

Section 235

```
                                              (19423) 19423 19430 19440 19450 19460 19470 19480 19490 19505
K12 Zwischenstück 255.328 - 290.948           (19072) TGTGG---CGCCCCTTGGTAGCCG-GCATCGGCTGAGACAAATTGCTCCTCTCCATGCAGCAGATTACCCAGCTGATTGAGGT
SARS-CoV-2 Reference Genome NC_045512.2       (16121) AGTTAACAGGACACATGTTAGACATGTATTCTGTTATGCTTACTAATGATAACACTTCAAGGTATTGGGAACCTGAGTTTTAT
Consensus                                     (19423)  GT      G C C TG TAG C  G AT    T A  C  A T  T  T  C  T CA    ATT    A CTGA T    T
```

Section 236

```
                                              (19506) 19506 19520 19530 19540 19550 19560 19570 19588
K12 Zwischenstück 255.328 - 290.948           (19151) CATGCTCGTTGGCCGCGGTGGTGACCAGGCTGTGGGTCAGGCCACTCTTGGCATCGACACCAATGTGGGCCTTCATGCCAAAG
SARS-CoV-2 Reference Genome NC_045512.2       (16204) GAGGCTA--TGTACACACCGCATAC-AGTCTTACAGGCTGTTGGGGCTTGTGTTCTTTGCAATTCACAGACTTCATTAAGATG
Consensus                                     (19506)  A GCT   TG  C C   G   AC AG CT    G C G      CTTG   TC    C A T    G CTTCAT    A G
```

Section 237

```
                                              (19589) 19589 19600 19610 19620 19630 19640 19650 19660 19671
K12 Zwischenstück 255.328 - 290.948           (19234) TGCCACTGATTGCCTTTCTTGGTCTGATGCATCTCCGGATCGCGTTGCTGCTCTTTGTTCTTGGTCGAGCTGGGTGCCTCAAT
SARS-CoV-2 Reference Genome NC_045512.2       (16284) TGG---TGCTTGCATACGTAGACC--ATTCTT------ATGTTGTAAATGCTGTTA----------CGACCATGTCATATCAA-
Consensus                                     (19589) TG    TG TTGC T   T G  C  AT C T      AT   GT   TGCT TT          CGA C  G     TCAA
```

Section 238

```
                                              (19672) 19672 19680 19690 19700 19710 19720 19730 19740 19754
K12 Zwischenstück 255.328 - 290.948           (19317) GATGGTGGCATCG-ACCAAGGTGCCTTGAGTCATCATGACGCCTGCTTCGGCCAGCCAGCGATTGATGGTCTTGAACAATTGG
SARS-CoV-2 Reference Genome NC_045512.2       (16346) --------CATCACATAAATTAGTCTTGTCTGTTAATC-CGTATGTTT--GCAATGCTCCAGGTTGTGATGTCACAGATGTGA
Consensus                                     (19672)         CATC  A  AA   G CTTG  T  T AT  CG  TG TT  GC A  C  C   T  TG T T   A A  TG
```

```
                                                   Section 239
                                         (19755) 19755 19760 19770 19780 19790 19800 19810 19820 19837
   K12 Zwischenstück 255.328 - 290.948 (19399) CGGGCCAGTTGATGCTGCTCCAGCAGGTGGCGGAAATTCATGATGGTGGTGCGGTCCGGCAAGGCGCTATCCAGGGATAACCG
SARS-CoV-2 Reference Genome NC_045512.2 (16418) CT--CAACTTTA--CTT----AGGAGGTATGAGCTATTATTGTAAATCACATAAACCACCCATTAGTTTTCCATTGT-----G
                               Consensus (19755) C   C A TT A  CT     AG AGGT    G  ATT  TG    T        CC  C A   G T TCCA  G      G

                                                   Section 240
                                         (19838) 19838 19850 19860 19870 19880 19890 19900 19910 19920
   K12 Zwischenstück 255.328 - 290.948 (19482) GGCAACAGACGCATGGAGGCGATTTCGTACAGAGCATCTTCCATCGCGCCATCGCTCAGGTTGTACCAATGCTGCATGCAGT
SARS-CoV-2 Reference Genome NC_045512.2 (16488) TGCTAATGGACAAGTT-------TTTGGTTTATATAAAAATACAT---------GTGTTGGTAGCGATAATGTTAC-TGACTT
                               Consensus (19838)  GC AA  GAC   T        TTT GT  A A  A   T CAT         G    GGT G    AATG T C TG   T

                                                   Section 241
                                         (19921) 19921 19930 19940 19950 19960 19970 19980 19990 20003
   K12 Zwischenstück 255.328 - 290.948 (19565) GAATGCG-TAGCATGGTTTCCAGCGGATAAGGTCGCCGGCCATTACCAGCCTTGGGGTAAAACGGCTCGATGACTTCCACCAT
SARS-CoV-2 Reference Genome NC_045512.2 (16554) TAATGCAATTGCAACATGTG-ACTGGACAAAT--GCTGGTGATTACAT---TTTAGCTAACACCTGTAC-TGAAAGACTCAAG
                               Consensus (19921)  AATGC  T GCA   T T  A  GGA AA    GC GG  ATTAC     TT  G TAA AC   T   TGA    C C A

                                                   Section 242
                                         (20004) 20004 20010 20020 20030 20040 20050 20060 20070 20086
   K12 Zwischenstück 255.328 - 290.948 (19647) GTTTTGCCATGGCAGAATCTGCTCCATGC----GGG---ACAAGAAAATCTCTTTTCTGGTCTGACGGCGCTTACTGCTGAAT
SARS-CoV-2 Reference Genome NC_045512.2 (16630) CTTTTTGCA--GCAGAAAC-GCTCAAAGCTACTGAGGAGACATTTAAA-CTGTCTTATGGTATTGCTACTGT-ACG--TGAAG
                               Consensus (20004)  TTTT  CA  GCAGAA C GCTC A GC    G G   ACA   AAA CT T TT TGGT T  C  C  T AC   TGAA

                                                   Section 243
                                         (20087) 20087 20100 20110 20120 20130 20140 20150 20169
   K12 Zwischenstück 255.328 - 290.948 (19723) TCACTGTCGGCGAAGGTAAGTTGATGACTCATGATGAACCCTGT---TCTATGGCTCCAGATGACAAACATGATCTCATATCA
SARS-CoV-2 Reference Genome NC_045512.2 (16706) TG-CTGTCTGACAGAG-AATTACATCTTTCATGG-GAAGTTGGTAAACCTAGACCACCACTTAACCGAAATTATGTCTTTACT
                               Consensus (20087) T  CTGTC G  A  G AA T  AT   TCATG  GAA    GT    CTA   C CCA  T AC  A AT AT TC T  C

                                                   Section 244
                                         (20170) 20170 20180 20190 20200 20210 20220 20230 20240 20252
   K12 Zwischenstück 255.328 - 290.948 (19803) GGGACTTGT-TCGCACCTTCCGGAGGCGTTATGAGCTG-GCGGCCCTTTTTGTATCTGATTATTAATCCCCACCCGCTATTAA
SARS-CoV-2 Reference Genome NC_045512.2 (16786) GGTTATCGTGTAACTAAAAACAGTAAAGTACAAATAGGAGAGTACACCTTTGAAAAAGGTGACTATGGTGATGCTGTTGTTTA
                               Consensus (20170) GG   T GT T  C      C G    GT    A   G G G  C   TTTG A   G T A TA        C G T TT A

                                                   Section 245
                                         (20253) 20253 20260 20270 20280 20290 20300 20310 20320 20335
   K12 Zwischenstück 255.328 - 290.948 (19884) GCGCCCGGCGCGGGCATCTGCGTCTGGTGCAGG-GTTGACTTTGCATTCTGTTAACAAACGCGGTATAACAAACCTTCTTTGG
SARS-CoV-2 Reference Genome NC_045512.2 (16869) CCGAGGTACAACAACTTACAAATTAAATGTTGGTGATTATTTTG--TGCTGACATCACATACAGTAATGCCA---TTAAGTGC
                               Consensus (20253)  CG     C     C T     T    TG  GG G T A TTTG  T CTG  A CA A  C GTA   C A   TT   TG
```

Section 246

```
                                                 (20336) 20336         20350     20360     20370     20380     20390     20400           20418
               K12 Zwischenstück 255.328 - 290.948 (19966) ATGTTTAGATGT-CCATACGTTTAGAAGGTTATATGCAAACAACACAACAAAATGCGCCACTGAAGCGCACAATGAAAACGCG
SARS-CoV-2 Reference Genome NC_045512.2 (16947) ACCTACACTAGTGCCACAAGAGCACTATGTTAGA-----ATTACTGGCTTATACCCAACACTCAATATCTCA--GATGAGTTT
                                     Consensus (20336) A  T  A   GT CCA A G   A  A GTTA A     A  AC      A A  C  CACT AA   C CA  GA  A
```

Section 247

```
                                                 (20419) 20419      20430     20440     20450     20460     20470     20480     20490     20501
               K12 Zwischenstück 255.328 - 290.948 (20048) TCACCTGATTATGCTTTCCTTGGGCGGCGTGATTGGCACAGGATTATTCTTCAATACCGGGTACATCATTTCCACCACTGG-A
SARS-CoV-2 Reference Genome NC_045512.2 (17023) TCTAGCAATGTTGCAAATTATCAAAAGGTTGGTATGCAAAAG--TATTCTACACTCCAGGGACCACCTGGT-----ACTGGTA
                                     Consensus (20419) TC     AT  TGC      T     G  TG T  GCA A G  TATTCT CA T C GGG  CA C   T     ACTGG A
```

Section 248

```
                                                 (20502) 20502   20510     20520     20530     20540     20550     20560     20570        20584
               K12 Zwischenstück 255.328 - 290.948 (20130) GCGGCGGGAACGCTGCTGGCCTATCTGATTGGTGCGCTGGTGGTCTGGCTGGTTATGCAGTGTCTGGGCGAGCTGTCGGTCGC
SARS-CoV-2 Reference Genome NC_045512.2 (17099) AGAGTCATTTTGCTATTGGCCTAGCTCTCTACTACCCTTCTGCTCGCATAGTGTATACAG---CTTGCTCTCATGCCGCTGTT
                                     Consensus (20502)    G       GCT  TGGCCTA CT   T  T C CT  TG TC     G  TAT CAG   CT G      TG CG T
```

Section 249

```
                                                 (20585) 20585 20590     20600     20610     20620     20630     20640     20650         20667
               K12 Zwischenstück 255.328 - 290.948 (20213) GATGC-CGGAGACCGGAGCGTTTCACGTTTATGCCGCGCGCTATCT-TGGTCCGGCTAC-CGGGTATACCGTGGCCTGGCTTT
SARS-CoV-2 Reference Genome NC_045512.2 (17179) GATGCACTATGTGAGAAGGCATTAAAATATTTGCCTATAGATAAATGTAGTAGAATTATACCTGCACGTGCTCGTGTAGAGTG
                                     Consensus (20585) GATGC C   G   G AG   TT A  T T TGCC    G TA  T T GT     TA  C  G A     T G  T G  T
```

Section 250

```
                                                 (20668) 20668       20680     20690     20700     20710     20720     20730     20740 20750
               K12 Zwischenstück 255.328 - 290.948 (20293) ACTGGCTGACCTGGACCGTGGCGCTGGGTTCGAGCTTTACCGCCGCTGGATTCTGTATGCAGTACTGGTTTCCACAGGTGCCG
SARS-CoV-2 Reference Genome NC_045512.2 (17262) TTTTGATAAATTCAAAGTGAATTCAACATTAGAACAGTATGTCTTTTGTACTGTAAATGCATTGCCTGAGACGACAG---CAG
                                     Consensus (20668)   T G T A  T  A        C    TT GA C  TA   C   TG A T T  ATGCA T C  G   C ACAG   C G
```

Section 251

```
                                                 (20751) 20751    20760     20770     20780     20790     20800     20810     20820        20833
               K12 Zwischenstück 255.328 - 290.948 (20376) GTATGGGTCTGGTGCGTGGTGTTCTGC--GCGATTATTTTTGGTCTGAATGTTATCTCCACGCGCTTTTTTGCCGAAGGGGAG
SARS-CoV-2 Reference Genome NC_045512.2 (17342) ATATAGTTGTCTTTGATGAAATTTCAATGGCCACAAATTATGATTTGAGTGTTGTCAATGCCAGATTACGTGCTAA---GCAC
                                     Consensus (20751)  TAT G T T  T   TG   TT      GC A  A TT TG T TGA TGTT TC    C  G TT   TGC  A   G A
```

Section 252

```
                                                 (20834) 20834 20840     20850     20860     20870     20880     20890     20900          20916
               K12 Zwischenstück 255.328 - 290.948 (20457) TTCTGGTTCTCGCTGGTCAAAGTGGTCACTATCATCGCCTTTATCATCCTCGGTGGGGCGGCGATTTTCGGCTTTATTCCGAT
SARS-CoV-2 Reference Genome NC_045512.2 (17422) TATGTGTACAT--TGGCGACCCTGCTCA-----ATTACCTGCACCACGCACATTG------CTAACTAAGGGCACACTA-GAA
                                     Consensus (20834) T    GT C    TGG  A   TG TCA     AT  CCT  A CA  C C  TG      C A  T  GG    A T  GA
```

```
                                                                         Section 253
                                      (20917) 20917        20930     20940     20950     20960     20970     20980         20999
         K12 Zwischenstück 255.328 - 290.948 (20540) GCAGGATGGCTCG--CCCGCGCCGGGGCTGAGTAATATCACGGCAGAAGGCTGGTTCC-CGCACGGTGGCTTACCGATTTTGA
SARS-CoV-2 Reference Genome NC_045512.2 (17491) CCAGAATATTTCAATTCAGTGTGTAGACTTATGAAAACTATAGGTCCAGACATGTTCCTCGGAACTTGTCGGCGTTGTCCTGC
                                    Consensus (20917)  CAG AT   TC    C G G    G CT A  AA A  A  G    AG C  GTTCC CG A   TG C       T  TG
                                                                         Section 254
                                      (21000) 21000     21010     21020     21030     21040     21050     21060     21070    21082
         K12 Zwischenstück 255.328 - 290.948 (20620) TGACTATGGTGGCAGTGAACTTTGCTTTTTCGGGTACCGAGCTTATCGGCATTGCCGCCGGTGAAACGGAAAACCCGCGCAAA
SARS-CoV-2 Reference Genome NC_045512.2 (17574) TGAAATTGTTGACACTGTGAGT-GCTTT---GGTTTATGA--TAATAAGC-TTAAAGCACATAAA--GACAAATCAGCTCAAT
                                    Consensus (21000) TGA   TG TG CA TG    T GCTTT   GG T   GA  T AT  GC TT   GC   T AA  G  AAA C GC CAA
                                                                         Section 255
                                      (21083) 21083  21090     21100     21110     21120     21130     21140     21150        21165
         K12 Zwischenstück 255.328 - 290.948 (20703) G--TTATCCCGGTAGCGATTCGTACTACCATCGCGCGACTGATTATTTTC--TTTATCGGCACCGTGTTTGTGCTGGCAGCGC
SARS-CoV-2 Reference Genome NC_045512.2 (17648) GCTTTAAAATGTTTTATAAGGGTGTTATCA-CGCATGATGTTTCATCTGCAATTAACAGGCCACAAATAGGCGTGGTAAGAGA
                                    Consensus (21083) G  TTA    G T    A   GT  TA CA CGC  GA    T AT T C  TT A  GGC  C   T  G G  G  AG G
                                                                         Section 256
                                      (21166) 21166          21180     21190     21200     21210     21220     21230         21248
         K12 Zwischenstück 255.328 - 290.948 (20782) TGATCCCGATGCAGCAGGTGGGCGTGGAGAAAAGCCCGTTTGTGCTGGTATTTGAGAAAGTAGGGATCCCGTACGCCGCTGAT
SARS-CoV-2 Reference Genome NC_045512.2 (17730) --ATTCCTTACACGTAACCCTGCTTGGAGAAAAGCT-GTCTTTATTTCACCTTATAATTCACAGAATGCTGTA-GCCTCAAAG
                                    Consensus (21166)   AT CC      G A     GC TGGAGAAAAGC  GT T T  T     TT   A      G AT C GTA GCC C  A
                                                                         Section 257
                                      (21249) 21249       21260     21270     21280     21290     21300     21310     21320   21331
         K12 Zwischenstück 255.328 - 290.948 (20865) ATTTTTAACTTCGTGATCCTGACGGCTATTCTTTCTGCAGCGAACTCCGGGTTATATGCCTCCGGGCGCATGCTGTGGTCGTT
SARS-CoV-2 Reference Genome NC_045512.2 (17809) ATTTTGGGACTACCAACTCAAACTG---TTGATTCATCACAGGGCTCAGA---ATATGACT--------ATG------TCATA
                                    Consensus (21249) ATTTT     T    A  C  AC G   TT  TTC  CA  G  CTC G    ATATG CT        ATG      TC T
                                                                         Section 258
                                      (21332) 21332   21340     21350     21360     21370     21380     21390     21400        21414
         K12 Zwischenstück 255.328 - 290.948 (20948) GTCGAATGAACGTACGCTACCGGCCTGTTTTGCGCGAGTAACGAAAAACGGCGTGCCACTGACGGCGCTGTCGGTCAGTATGC
SARS-CoV-2 Reference Genome NC_045512.2 (17872) TTCACTCAAACCACTGAAACAGCTCACTCTTGTAATGTAAACAGATTTAATGTTGCTATTACCAGAGCAAAAG-TAGGCATAC
                                    Consensus (21332)  TC     AAC    G  AC G  C  T TTG       AAC  A        TGC A T  C G GC    G T  G AT C
                                                                         Section 259
                                      (21415) 21415 21420     21430     21440     21450     21460     21470     21480        21497
         K12 Zwischenstück 255.328 - 290.948 (21031) TCGGTGGTGTGCTGGCGCTGTTTTCCAGCGTGGTGGCCCCGGACACGGTATTTGTTGCGCTGTCGGCAATCTCCGGGTTTGCG
SARS-CoV-2 Reference Genome NC_045512.2 (17954) T---TTGCATAATGTC--TGATAGAGACCTTTATG-------ACAAGTTGCAATTTACAA-GTCTTGAAATTCCACGT-----
                                    Consensus (21415) T   T G  T  TG C  TG T    A C T  TG       ACA G T     TT C   GTC   AA  TCC  GT
```

Section 260

```
                                             (21498) 21498       21510     21520     21530     21540     21550     21560     21570   21580
        K12 Zwischenstück 255.328 - 290.948 (21114) GTGGTAGCGGTGTGGCTGAGTATCTGCGCCTCGCATTTTGTTTTTCGTCGCCGTCATCTGCAACAAGGTAAGGCATTGAGTGA
SARS-CoV-2 Reference Genome NC_045512.2 (18019) -----AGGAATGTGGCA-ACTTTACAAGCTGAAAAT---GTAACAGGACTCTTTAAAG---ATTGTAGTAAGGTAATCACTGG
                                   Consensus (21498)      AG   TGTGGC  A T T    GC     AT   GT     G C C  T A     A     GTAAGG A T A TG
```

Section 261

```
                                             (21581) 21581    21590     21600     21610     21620     21630     21640     21650    21663
        K12 Zwischenstück 255.328 - 290.948 (21197) ATTACATTATCGCGCGCCGTGGTATCCGCTGGTGCCAGTATTAGGTTTTGTGCTGTGCCTGGTGGCCTGTGTTGGGCTGGCAT
SARS-CoV-2 Reference Genome NC_045512.2 (18090) GTTACATCCTA-----CACAGGCACCTACACACCTCAGTGTTGACACTAAATTCAAAACTGAAGGTTTATGT---GTTGACAT
                                   Consensus (21581)  TTACAT  T      C   GG A C  C      CAGT TT     T          CTG  GG  T TGT   G TG CAT
```

Section 262

```
                                             (21664) 21664 21670     21680     21690     21700     21710     21720     21730      21746
        K12 Zwischenstück 255.328 - 290.948 (21280) TCGATCCAGCGCAGAGAAT-TGCGTTGTGGTGCGGGTTACCGTTTGTTGCGTTGTGCTATGGTGCTTATTTCCTTACTCA--A
SARS-CoV-2 Reference Genome NC_045512.2 (18165) ACCTGGCATACCTAAGGACATGACCTATAGAAGACTCATCTCTATGATGGGTTTTAAAATGAA--TTATCAAGTTAATGGTTA
                                   Consensus (21664)  C    CA   C  AG A  TG   T T G          C  T TG TG GTT T   ATG    TTAT    TTA T   A
```

Section 263

```
                                             (21747) 21747        21760     21770     21780     21790     21800     21810      21829
        K12 Zwischenstück 255.328 - 290.948 (21360) CCCCGAAACGCAAAACAGGAGCCAGAACATGTCGCAGAATAATCCGTTACGCGCTCTTCTT-GATAAACAGGA-TATCCTGCT
SARS-CoV-2 Reference Genome NC_045512.2 (18246) CCCTAACATGTTTATCACCCGCGAAGA-AGCTATAAGACATGTACGTGCATGGATTGGCTTCGATGTCGAGGGGTGTCATGCT
                                   Consensus (21747) CCC  A A G   A CA   GC A  A A  T   AGA    T CGT     G T   CTT GAT    AGG  T TC TGCT
```

Section 264

```
                                             (21830) 21830     21840     21850     21860     21870     21880     21890     21900   21912
        K12 Zwischenstück 255.328 - 290.948 (21441) GCTGGATGGCGCGATGGCGACGGAGCTGGAAGCGCGAGGGTGTAACTTAGCCGACAGCCTGTGGTCAGCCAAAGTGCTGGTAG
SARS-CoV-2 Reference Genome NC_045512.2 (18328) ACTAGA-GAAGCTGTTGGTACCAATTT---ACCTTTACAGC-TAGGTTTTTCTACAG---GTGTTAA-CCTAGTTGCTGTACC
                                   Consensus (21830)  CT GA G  GC  T G  AC  A  T   A C   A  G  TA  TT   C ACAG   GTG T A CC A  TGCTG
```

Section 265

```
                                             (21913) 21913  21920     21930     21940     21950     21960     21970     21980      21995
        K12 Zwischenstück 255.328 - 290.948 (21524) AAAACCCGGAGCTTATCCGCGAAGTGCATCTTGATTACTACCGGGCGGGGGCGCAATGCGCGATCACTGCCAGCT-ATCAGGC
SARS-CoV-2 Reference Genome NC_045512.2 (18402) TACAGGTTATGTTGATACACCTAATA-ATACAGATTTTTCCAGAGTTAGTGCTAAAC-------CACCGCCTGGAGATCAATT
                                   Consensus (21913)  A A      G T AT C C  A T  AT   GATT  T C G G   G GC  AA        CAC GCC G   ATCA
```

Section 266

```
                                             (21996) 21996          22010     22020     22030     22040     22050     22060      22078
        K12 Zwischenstück 255.328 - 290.948 (21606) GACGCCGGCGGGCTTCGCCGCGCGAGGTCTGGATGAAGCGCAGTCGAAAGCGCTGATTGGCAAAAGCGTGGAGCTGGCGCGTA
SARS-CoV-2 Reference Genome NC_045512.2 (18477) TAAACA------CCTCATACCACTTATGTACAAAGGACTTCCTTGGAATGTAGTGCGTATAAAGATTGTACAAATGTTAAGTG
                                   Consensus (21996)  A  C       C TC    C C         A G A   C  T GAA G   TG  T   AA A  GT  A  TG    GT
```

Section 267

```
                                         (22079) 22079      22090     22100     22110     22120     22130     22140     22150   22161
     K12 Zwischenstück 255.328 - 290.948 (21689) AAGCCCGTGAAGCG-TATCTGGCAGAGAACCCGCAGGCGGGAACGCTTCTGGTGGCG-GGATCCGTCGGGCCTTACGGCGCGT
SARS-CoV-2 Reference Genome NC_045512.2 (18554) ACACACTTAAAAATCTCTCTGACAGAGT---CGTATTTGT-----CTTATGGGCACATGGCTTTGAGTTGACATCTATGAAGT
                               Consensus (22079) A  C C T AA    T TCTG CAGAG    CG A   G      CTT TGG   C  GG T  G    G C T       GT
```

Section 268

```
                                         (22162) 22162   22170     22180     22190     22200     22210     22220     22230       22244
     K12 Zwischenstück 255.328 - 290.948 (21770) ATCTGGCGGATGGCTCTGAATACCGTGGCGATTATCATTGTAGCGTTGAGGCATTTCAGGCGTTTCATCGCCCGCGCGTGGAA
SARS-CoV-2 Reference Genome NC_045512.2 (18629) ATTTTGTGAAAATAGGACCTGAGCGCACCTGTTGTCTATGTGA--TAGACGTGCCACATGCTTTTCCACT-----GCTTCAGA
                               Consensus (22162) AT T G G A           A CG   C  TT TC  TGT    T GA G     CA GC TTTC  C      GC T   A
```

Section 269

```
                                         (22245) 22245 22250     22260     22270     22280     22290     22300     22310           22327
     K12 Zwischenstück 255.328 - 290.948 (21853) GCCTTGCTGGATGCCGGGGCCGATCTGCTGGCCTGCGAAACCCTGCCGAATTTTTCCGAGATTGAGGCGTTGGCCGAGCTGTT
SARS-CoV-2 Reference Genome NC_045512.2 (18705) CACTTA-TGCCTGTTGGCATCATTCTATTGGATTT-GATTACGTCTATAATCCGTTTATGATTGATGT-TCAACAATGGGGTT
                               Consensus (22245)   CTT  TG  TG  GG   C  TCT  TGG  T  GA   C T    AAT   T    GATTGA G  T   C   G  GTT
```

Section 270

```
                                         (22328) 22328       22340     22350     22360     22370     22380     22390     22400   22410
     K12 Zwischenstück 255.328 - 290.948 (21936) GACCGCATATCCGCGTGCGCGGGCGTGGT-TCTCATTTACCCTGCGCGACAGCGAACACCTGAGCGACGGTACGCCGCTGCGT
SARS-CoV-2 Reference Genome NC_045512.2 (18785) TTACAGGTAACCTACAAAGCAACCATGATCTGTATTGTCAAGTCCATGGTAATGCACATGT-AGCTA---------GTTGTGA
                               Consensus (22328)    C   TA CC      GC   C TG T T T  T T    T C  G  A  G ACA  T AGC A         G TG G
```

Section 271

```
                                         (22411) 22411    22420     22430     22440     22450     22460     22470     22480        22493
     K12 Zwischenstück 255.328 - 290.948 (22018) GACGTGGTTGCGTTGCTGGCGGGTTATCCGCAGGTGGTGGCGCTAG-GCATTAACTG---TATTGCGCTGGAAAACACCACCG
SARS-CoV-2 Reference Genome NC_045512.2 (18858) TGCAATCATGACTAGGTGTCTAGCTGTCCACGAGTGCTTTGTTAAGCGTGTTGACTGGACTATTGAATATCCTATAATTGGTG
                               Consensus (22411)   C     TG  T G TG C  G T TCC C  GTG T      AG G  TT ACTG   TATTG        A  A     G
```

Section 272

```
                                         (22494) 22494 22500     22510     22520     22530     22540     22550     22560           22576
     K12 Zwischenstück 255.328 - 290.948 (22097) CTGCGTTGCAGCATTTACACGGTTT--A--ACGGTGCTGCCGCTGGTGGTGTATCCGAACTCGGGCGAGCATTACGATGCCGT
SARS-CoV-2 Reference Genome NC_045512.2 (18941) ATGAACTGAAG-ATTAATGCGGCTTGTAGAAAGGTTCAACACATGGTTGTTAA----AGCT-------GCATTATTA-GCAGA
                               Consensus (22494)  TG   TG AG ATT A  CGG TT  A  A GGT C  C   TGGT GT  A    A CT       GCATTA  A GC G
```

Section 273

```
                                         (22577) 22577        22590     22600     22610     22620     22630     22640            22659
     K12 Zwischenstück 255.328 - 290.948 (22176) GAGCAAAACCTGGCATCATCACGGTGAACATTGCGCGCAGCTGGCGGATTATCTGCCGCAGTGGCAGGCCGCTGGCGCACGGT
SARS-CoV-2 Reference Genome NC_045512.2 (19011) ---CAAATTCCCAGTTCTTCACG----ACATTGGTAACCCTAAAGCTATTAAGTGTGTACCT--CAAGCTGATGTAGAATGGA
                               Consensus (22577)    CAAA  C     TC TCACG    ACATTG    C         ATTA  TG      T  CA GC G TG  G A GG
```

Section 274

```
                                            (22660) 22660     22670     22680     22690     22700     22710     22720     22730     22742
K12 Zwischenstück 255.328 - 290.948         (22259) TGATTGGCGGGTGCTGTCGCACCACGCCTGCGGATATCGCCGCGTTAAAAGCGCGAAGCTGAGGGTTTATCGGGTCTACATCG
SARS-CoV-2 Reference Genome NC_045512.2     (19085) AGTTCTA-------TGATGCACA--GCCTTGTAGTGACAAAGC-TTATAAAATAGAAGAA-----TTATTCTATTCTT-ATGC
Consensus                                   (22660)  G T          TG  GCAC   GCCT     T  C   GC TTA AA    GAAG       TT  TC   TCT  AT
```

Section 275

```
                                            (22743) 22743  22750     22760     22770     22780     22790     22800     22810     22825
K12 Zwischenstück 255.328 - 290.948         (22342) TTCATTGTAGGCCTGATAAGCGCAGCGCATCAGGC-ATTGCCGGATGGCGGCACAAGCGCCTTATCCGGCCTACAAAACCACA
SARS-CoV-2 Reference Genome NC_045512.2     (19152) CACACATTCTGACAAATTCACAGATGGTGTATGCCTATTTTGGAATTGC-----AATGTCGATAGATATCCTGCTAATTCC--
Consensus                                   (22743)   CA   T  G C  AT   C  A  G  T  G C ATT   G AT GC     AA   C  TA     CCT C AA  C
```

Section 276

```
                                            (22826) 22826         22840     22850     22860     22870     22880     22890     22908
K12 Zwischenstück 255.328 - 290.948         (22424) AAACGTTATGCCGCATCCGCCAGAACAAACATGCCGTACGGATGGATTTCAAGATAATACTGCTCGCCGACGTCCGGTTGCAG
SARS-CoV-2 Reference Genome NC_045512.2     (19228) -ATTGTT-TGTAG-ATTTGACACTAGAGTGCTATCTAACCT-TAACTTGCCTGGTTGTGATGGTGGCAG---TTTGTATGTAA
Consensus                                   (22826)  A  GTT TG  G AT  G CA  A A    T  C  AC   T   TT C  G T  T  TG T GC G   T  G  TG A
```

Section 277

```
                                            (22909) 22909      22920     22930     22940     22950     22960     22970     22980     22991
K12 Zwischenstück 255.328 - 290.948         (22507) ACGCGTAGCGTTGACCTGCAATAATATCTCCTGCCCGTGCCATTCCACCGTCACTTCATACTGCGGCCCCATATAGGCGACA-
SARS-CoV-2 Reference Genome NC_045512.2     (19304) ATAAACATGCATTCCACACACCAGCTTTTGATAAAAGTGCTTTTGT----TAATTTAAAACAATTACCATTTTTCTATTACTC
Consensus                                   (22909) A     A    T  C   CA  A   T T  T    GTGC  TT      T A TT A AC     CC   T T     AC
```

Section 278

```
                                            (22992) 22992   23000     23010     23020     23030     23040     23050     23060     23074
K12 Zwischenstück 255.328 - 290.948         (22589) TGGCGGATCACGCAGCGCTGGCTCTCTTCGCCG-CGATCGCTGAGCGTGATCGCTTCCGGGCGCACACCGACCATCCCTTCAC
SARS-CoV-2 Reference Genome NC_045512.2     (19383) TGACAGTCCATG------TGAGTCTCATGGAAAACAAGTAGTGTCAGATATAGATTATGT------ACCACTAAAGTCTGCTA
Consensus                                   (22992) TG C G  CA G      TG  TCTC T G    C A    TG   G  AT G TT  G       ACC    A   CT C
```

Section 279

```
                                            (23075) 23075 23080     23090     23100     23110     23120     23130     23140     23157
K12 Zwischenstück 255.328 - 290.948         (22671) CCTGTGTACCAAAGTGCAGCGGGCGCGGCAGATGATAGCCGTAGATATCAACGTATCCGTCGCTGAAGGTTGCCGGGAACAGG
SARS-CoV-2 Reference Genome NC_045512.2     (19454) CGTGTATAACACGTTGCAATTTAGGTGG----TGCTGTCTGTAGACATCATGCTAA---TGAGTACAGATTGT----ATCTCG
Consensus                                   (23075) C TGT TA CA   TGCA      G GG    TG T  C GTAGA ATCA   TA    T   T  AG TTG     A C  G
```

Section 280

```
                                            (23158) 23158       23170     23180     23190     23200     23210     23220     23230  23240
K12 Zwischenstück 255.328 - 290.948         (22754) TTGGCATCGCCCATAAAGCTCGCCATAAAGCGGGAGGCGGGCTGGCGGTAAAGATCCTGCGGTGAGCCGATCTGCATGATGTG
SARS-CoV-2 Reference Genome NC_045512.2     (19526) ATG-CTTATAACATGATGATCTC-----AGCTGGCTTTAGCTTGTGGGTTTACAAACAATT-TGATACTTATAACCTC-TGGA
Consensus                                   (23158)  TG C T    CAT A G TC C     AGC GG     G  TG  GGT  A A  C     TGA  C      C T  TG
```

```
                                                                Section 281
                                        (23241) 23241 23250 23260 23270 23280 23290 23300 23310 23323
          K12 Zwischenstück 255.328 - 290.948 (22837) TCCCTTGTTCATCACCAGCACAGTATCAGAAACCGCAAAGGCTTCGCTCTGATCGTGGGTGACGTACAGCGAGGTG-ATATCA
SARS-CoV-2 Reference Genome NC 045512.2 (19601) ACACTTTTACAAGACTT-CAGAGTTT-AGAAAATGT---GGCTTT--TAATGTTGTAAATAAGGGACACTTTGATGGACAACA
                                      Consensus (23241)  C CTT T CA  AC   CA AGT T AGAAA  G    GGCTT   T    T GT   T A G ACA    G TG A A CA

                                                                Section 282
                                        (23324) 23324 23330 23340 23350 23360 23370 23380 23390 23406
          K12 Zwischenstück 255.328 - 290.948 (22919) AACTGCTTTTGCAACTCGCGGATCTTGTCGCGCATGCTGCGACGCAGGT---TGGCGTCGAGGTT-ACTCAACGGCTCATCAA
SARS-CoV-2 Reference Genome NC 045512.2 (19677) GGGTGAAGTACCAGTTTCTATCATTAATAACAC-TGTTTACACAAAAGTTGATGGTGTTGATGTAGAATTGTTTGAAAATAAA
                                      Consensus (23324)    TG   T  CA  T        T  T  C C TG T   AC  A GT   TGG GT GA GT  A T     G   AT AA

                                                                Section 283
                                        (23407) 23407 23420 23430 23440 23450 23460 23470 23489
          K12 Zwischenstück 255.328 - 290.948 (22998) ACAGCAGCACTTTCGGCTTGAGGATCAGCGCGCGGGCC--AGCGCCACGCGCTGCTGCTGCCCGCCGGAGATCTGATCGACAA
SARS-CoV-2 Reference Genome NC 045512.2 (19759) ACAACATTACCTGTTAATGTAGCATTTGAGCTTTGGGCTAAGCGCAACATTAAACCAGTACCAGAGGTGAAAATACTCAATAA
                                      Consensus (23407) ACA CA  AC T     T  AG AT  G GC   GG C  AGCGC AC      C   T CC G  G   A  T  TC A AA

                                                                Section 284
                                        (23490) 23490 23500 23510 23520 23530 23540 23550 23560 23572
          K12 Zwischenstück 255.328 - 290.948 (23079) AGCGGTCTTCGAATCCTTCCAGATCCACCATCGCCAACGCCTCTTTGACGCGGGCTTTCAGCTCTGCGCGCGGTACGCCGAGC
SARS-CoV-2 Reference Genome NC 045512.2 (19842) TTTGGGTGTGGA----------------CATTGCTGCTAATACTGTGATCTGGGACTACAAAA------GAGATGCTCC-AGC
                                      Consensus (23490)    GG   T GA                CAT GC        CT TGA   GGG  T CA         G G T C CC AGC

                                                                Section 285
                                        (23573) 23573 23580 23590 23600 23610 23620 23630 23640 23655
          K12 Zwischenstück 255.328 - 290.948 (23162) ATTTTCAGGCCATAACCGACATTCT-CTCCCAGCGACATATGCGGGAA--CAGGGCATAGGACTGAAACACCATACAGATATC
SARS-CoV-2 Reference Genome NC 045512.2 (19902) ACATATATCTACTATTGGTGTTTGTTCTATGACTGACATAGCCAAGAAACCAACTGAAACGATTTGTGCACCACTCACTGTCT
                                      Consensus (23573) A  T  A     TA   G   TT T CT   A  GACATA  C  GAA  CA    A A GA T    CACCA  CA

                                                                Section 286
                                        (23656) 23656 23670 23680 23690 23700 23710 23720 23738
          K12 Zwischenstück 255.328 - 290.948 (23242) GCGCTGCTGAATAGAGCGATGGGTGACGTCTTCGCCATCAATGAAAATTTGCCCTTCGCTCGGTTTTTCCAGCCCGGCAACCA
SARS-CoV-2 Reference Genome NC 045512.2 (19985) TTTTTGATGG-TAGAGTTGATGGTCAAGTAGACTTATTTA--GAAA---TGCCCGTAATGGTGTTCTT--ATTACAGAAGGTA
                                      Consensus (23656)     TG TG  TAGAG     GGT A GT   C    T A  GAAA   TGCCC T      GTT TT  A   C G A   A

                                                                Section 287
                                        (23739) 23739 23750 23760 23770 23780 23790 23800 23810 23821
          K12 Zwischenstück 255.328 - 290.948 (23325) GGCGCAAAATAGTGGTTTTGCCGCAGCCGGACGGGCCGAGCA-GC--GTCACCATTTGCCCCTGCGGGATGGTGAGGTTG-AT
SARS-CoV-2 Reference Genome NC 045512.2 (20060) GTGTTAAA-----GGTTTACAACCATCTGTAGGTCCCAAACAAGCTAGTCTTAATGGAGTCACATTAATTGGAGAAGCCGTAA
                                      Consensus (23739) G    AAA     GGTTT     CA C G A G  CC A CA GC  GTC   AT     C        TGG GA G  G A
```

Section 288

```
                                     (23822) 23822   23830     23840     23850     23860     23870     23880     23890     23904
K12 Zwischenstück 255.328 - 290.948 (23404) ATTGTCGATTACCGTATTACTGCCAAATCGTTTAGTGACGTTGCGCAGTTCAACGAAATTTTTCTGAGTCATAGTGCGCTCCA
SARS-CoV-2 Reference Genome NC_045512.2 (20138) AAACACAGTTCAATTATTATAAGAAAGTTGATG-GTGTTGTCCAACAATTACCTGAAACTTACTTTACTCAGAGTAGA----A
                           Consensus (23822) A    C  TT    TATTA     AA T G T  GTG  GT    CA TT    GAAA TT   T A TCA AGT       A
```

Section 289

```
                                     (23905) 23905 23910     23920     23930     23940     23950     23960     23970     23987
K12 Zwischenstück 255.328 - 290.948 (23487) TTACGCCTGGTTTTTGGCTTTTGAA-CGGGAGGTACGTGATTCACCGATCAGCCAGTCAAAGATGAAAATAATCGCCAGCATC
SARS-CoV-2 Reference Genome NC_045512.2 (20216) ATTTACAAGAATTTAAACCCAGGAGTCAAATGGAAATTGATTT--CTTAGAATTAGCTATGGATGAATTCATTGAACGGTATA
                           Consensus (23905)  T   C  G  TTT   C    GA  C    GG A  TGATT   C    A   AG  A  GATGAA   A T   C G AT
```

Section 290

```
                                     (23988) 23988       24000     24010     24020     24030     24040     24050     24060  24070
K12 Zwischenstück 255.328 - 290.948 (23569) ACCACGATCAGAATGGAACCGTAGGCAATCGCTACACCG-TATTCGCCATCTTCCACGCGGTTGAGGATGTAAGCCGTCGCTA
SARS-CoV-2 Reference Genome NC_045512.2 (20297) AATTAGAAGGCTATGCCTTCGAACATATCGTTTATGGAGATTTTAGTCATAGTCAGTTAGGTGGTTTACATCTACTGATTGGA
                           Consensus (23988) A    GA     ATG    CG A   A     TA    G T TT G CAT  TC     GGT G   A  T   C G     A
```

Section 291

```
                                     (24071) 24071    24080     24090     24100     24110     24120     24130     24140     24153
K12 Zwischenstück 255.328 - 290.948 (23651) CGCGGGTATCCGGCGTGACGAGGAACACAATGGCGCTGACGGTGGTAATGGCGCGCACAAAGCTGTAAATCAGCGCCGAGAGG
SARS-CoV-2 Reference Genome NC_045512.2 (20380) CTAGC-TAAACGTTTTAAGGAATCACCTTTTGAATTAGAAGATTTTATTCCTATGGACAGTACAGTTAAAAA----CTATTTC
                           Consensus (24071) C  G  TA  CG   T A GA   AC    TG     GA G T  TA T     G ACA   C GT AA  A    C A
```

Section 292

```
                                     (24154) 24154 24160     24170     24180     24190     24200     24210     24220     24236
K12 Zwischenstück 255.328 - 290.948 (23734) ATCGCCGG-GCGCAG-CAGCGGCAGC--AGGATGTGCGTAATGGTACGCAGGGAACCGGCGCGCAGGCTGAGTGAGGCTTCA-
SARS-CoV-2 Reference Genome NC_045512.2 (20458) ATAACAGATGCGCAAACAGGTTCATCTAAGTGTGTGTTCTGTTATTGATTTATTACTTGATGATTTTGTTGAAATAATAAA
                           Consensus (24154) AT  C G  GCGCA  CAG   CA C  AG  TGTG GT  TG TA   A   A      G   A   TG    A    T A
```

Section 293

```
                                     (24237) 24237        24250     24260     24270     24280     24290     24300          24319
K12 Zwischenstück 255.328 - 290.948 (23812) -TCGAGCGATTTATCGATTTGGCCCGGTAATGACTCCAACTTATTGATAGTGTTTTATGTTCAGATAATGCCCGATGACTTTG
SARS-CoV-2 Reference Genome NC_045512.2 (20541) ATCCCAAGATTTATCTGTAGTTTC---TAAGGTTGTCAA---AGTGAC--TATTGACTATACAGAAATTTCATTTATGCTTTG
                           Consensus (24237)  TC    GATTTATC  T     C   TAA G    CAA   A TGA   T TT   T T CAGA A T C       CTTTG
```

Section 294

```
                                     (24320) 24320     24330     24340     24350     24360     24370     24380     24390     24402
K12 Zwischenstück 255.328 - 290.948 (23894) TCATGCTGCTCCACCGATTTTGAGAACGACAGCGACTTCCGTCCCAGCCGTGCCAGGTGCTGCCTCAGATTCAGGTTATGCCG
SARS-CoV-2 Reference Genome NC_045512.2 (20616) GTGTAAAGATGGCC--ATGT--AGAAACATTTTACCCAAAATTACAATCTAGTCAAGCGTGGCAACCGGGTGTTGCTATGCCT
                           Consensus (24320)    T   G T   C  AT T  AGAA  A      C     T  CA  C  G CA G G  GC  C G  T   G TATGCC
```

```
Section 295
                                        (24403) 24403 24410 24420 24430 24440 24450 24460 24470 24485
    K12 Zwischenstück 255.328 - 290.948 (23977) ---CTCAATTCGCTGCGTATATCGCTTGCTGATTACGTGCAGCTTTCCC--TTCAGGCGGGATTCATACAGCGGCCA--GCCA
SARS-CoV-2 Reference Genome NC 045512.2 (20695) AATCTTTACAAAATGCAAAGAATGCTATTAGAAAA-GTGTGACCTTCAAAATTATGGTGATAGTGCAACATTACCTAAAGGCA
                              Consensus (24403)    CT  A     TGC  A A  GCT    GA  A GTG   C TTC    TT  GG G  A T   ACA    C A  G CA

Section 296
                                        (24486) 24486 24500 24510 24520 24530 24540 24550 24568
    K12 Zwischenstück 255.328 - 290.948 (24053) TCCGTCATCCATATCACCACGTCAAAGGGTGACAGCAGGCTCATAAGACGCCCCAGCGTCGCCATAGTGCGTTCACCGAATAC
SARS-CoV-2 Reference Genome NC 045512.2 (20777) TAA-TGATGAATGTCGCAAAATATACT-----CAACTGTGTCAATATTTAAAC-ACATTAACATTAGCTGTACCCTATAATAT
                              Consensus (24486) T   T AT  AT TC C A  T  A       CA C G  TCA  A      C A   T  C  TAG      C    AATA

Section 297
                                        (24569) 24569 24580 24590 24600 24610 24620 24630 24640 24651
    K12 Zwischenstück 255.328 - 290.948 (24136) GTGCGCAACA-ACCGTCTTCCGGAGCCTGTCATACGCGTAAAAC-AGCCAGCGCTGGCGCGATTTAGCCCCGACGTATCCCCA
SARS-CoV-2 Reference Genome NC 045512.2 (20853) GAGAGTTATACATTTTGGTGCTGGTTCTGATAAAGGAGTTGCACCAGGTACAGCTGT-----TTTAAGACAGTGGTTGCCTAC
                              Consensus (24569) G G G  A A A   T  T C G   CTG  A A G GT   AC AG  A  GCTG      TTTA   C G  GT  CC

Section 298
                                        (24652) 24652 24660 24670 24680 24690 24700 24710 24720 24734
    K12 Zwischenstück 255.328 - 290.948 (24217) CTGTTCG-TCCATTTCCGCGCAGACGATGACGTCACTGCCCGGCTGTATGCGCGAGGTTACCGACTGCGGCCTGAGTTTTTA
SARS-CoV-2 Reference Genome NC 045512.2 (20931) GGGTACGCTGCTTGTCGATTCAGATCTTAATGACTTTGTCT--CTG-ATGC---AGATTCAACTTTGATTGGTGA-TTGTGCA
                              Consensus (24652)   GT CG T C T TC    CAGA   T A G C  TG C   CTG ATGC   AG TT      TG     TGA TT T  A

Section 299
                                        (24735) 24735 24740 24750 24760 24770 24780 24790 24800 24817
    K12 Zwischenstück 255.328 - 290.948 (24299) AATGGCGGAAAATCGTGTTGAGGCCAACGCCCATAATGCGGGCGGTTGCCCGGCATCCAACGCCATTCATGGCCATATCAA-T
SARS-CoV-2 Reference Genome NC 045512.2 (21007) ACTGTACATACAGCTAATAAATGGGATCTC--ATTATTAGTGATATGTACGACCCTAAGACTAAAAATGTTACAAAAGAAAAT
                              Consensus (24735) A TG     A A C   T  A G  A C C  AT AT  G G   T   C   C T   AC   A    T  C A A  AA T

Section 300
                                        (24818) 24818 24830 24840 24850 24860 24870 24880 24890 24900
    K12 Zwischenstück 255.328 - 290.948 (24381) GATTTTCTGGTGCGTAC---CGGGTTGAGAAGCGGTGTAAGTGAACTGCAGTTGCCATGTTTTACGG-CAGTGAGAGCAGAGA
SARS-CoV-2 Reference Genome NC 045512.2 (21088) GACTCTAAAGAGGGTTTTTTCACTTACATTTGTGGGTTTATACAACAAAAGCTAGC-TCTTGGAGGTTCCGTGGCTATAAAGA
                              Consensus (24818) GA T T   G G GT     C   T  A   G GG  T A   AAC   AG T  C T TT  A G  C GTG     A AGA

Section 301
                                        (24901) 24901 24910 24920 24930 24940 24950 24960 24970 24983
    K12 Zwischenstück 255.328 - 290.948 (24460) TAGCGCTGATGTCCGGCAGTGCTTTTGCCGTTACGCACCACCCCGTCAGTAGCTGAACAGGAGGGACAGCTGATAGAAACAGA
SARS-CoV-2 Reference Genome NC 045512.2 (21170) TAACAGAACATTCTTGGAATGCTGAT--CTTTATA----AGCTCATGGGACACTTCGCATGGTGGACAGCCTTT-GTTACTAA
                              Consensus (24901) TA C       TC  G A TGCT  T  C TTA      A C C T  G   CT   CA G  GGACAGC   T G  AC  A
```

Section 302

```
                                                 (24984) 24984 24990     25000     25010     25020     25030     25040     25050          25066
         K12 Zwischenstück 255.328 - 290.948 (24543) AGCCACTGGAGCA-CCTCAAAAACACCATCATACACTAAATCAGTAAGTTGGCAGCATCACCGAGAACACAAATGGGCTAATT
SARS-CoV-2 Reference Genome NC_045512.2 (21246) TGTGAATGCGTCATCATCTGAAGCATTTTTA-ATTGGATGTAATTATCTTGGCA--AACCACGCGAACAAATAGATGGTTAT-
                                       Consensus (24984)  G  A TG   CA C TC  AA CA   T A A    A  T A TA  TTGGCA  A C  CG GAACA A A   G T AT 
```

Section 303

```
                                                 (25067) 25067        25080     25090     25100     25110     25120     25130              25149
         K12 Zwischenstück 255.328 - 290.948 (24625) CGGCAGTACTTTCCTAAAAAGACATGTCTTGCCCAATATACTCAACATGAACTAGATCTGGTTGCTGCTCAGCTAAACAACAG
SARS-CoV-2 Reference Genome NC_045512.2 (21325) -GTCA-TGCATGC---AAATTACATATTTTGGAGGA-ATAC---AAATCCAATTCAGTTGTCTTCCTATTCTTTATTTGACAT
                                       Consensus (25067)  G CA T C T C   AAA  ACAT T TTG    A ATAC   A AT  A T  A  TG  T C   T    TA    ACA 
```

Section 304

```
                                                 (25150) 25150     25160     25170     25180     25190     25200     25210     25220       25232
         K12 Zwischenstück 255.328 - 290.948 (24708) ACCGAGAAAGACACTGAAGTTCAAAACACCGAAAGAGATAATTGAAAGGGGTGTTGCATTGACAGAT-TGAATCTACAGCAGC
SARS-CoV-2 Reference Genome NC_045512.2 (21399) GAGTAAATTTCCCCTTAAATTAAGGGGTACTGCTGTTATGTCTTTAAAAGAAGGTCAAATCAATGATATGATTTTATCTCTTC
                                       Consensus (25150)     A A    C CT AA TT A      C    G  AT   T  AA  G  G T  A T A  GAT TGA T TA   C  C
```

Section 305

```
                                                 (25233) 25233  25240     25250     25260     25270     25280     25290     25300          25315
         K12 Zwischenstück 255.328 - 290.948 (24790) TTTTTTTAATATGTCCCGTTCGTCGGTAACCCGTTTCAGCTCTTTCTGGAGACGGCGGATCTCGGCCTGAGCATCTGACTGTT
SARS-CoV-2 Reference Genome NC_045512.2 (21482) TTAGTAAAGGTAGACTTATAATTAGAGAAAACAACAGAGTTGTTATT------------TCTAG---TGATGTTCT---TGTT
                                       Consensus (25233) TT  T  A    G C   T   T G  AA  C     AG T TT  T            TCT G   TGA   TCT   TGTT
```

Section 306

```
                                                 (25316) 25316         25330     25340     25350     25360     25370     25380          25398
         K12 Zwischenstück 255.328 - 290.948 (24873) CTTTATTAGTGGAAGAATCCGGACCGTACTTCTT-TATCCAGGCATAAAGGCTGTGGGTGGTGATATCGAGACGTGTTGCAAC
SARS-CoV-2 Reference Genome NC_045512.2 (21547) AACAACTAAACGAACAAT---GTTTGTTTTTCTTGTTTTATTGCCACTAGTCTCTAGTCAGTG-TGTTAA----TCTTACAAC
                                       Consensus (25316)     A TA   GAA AAT   G   GT  TTCTT T T    GC    AG CT T G   GTG T T  A    T TT CAAC
```

Section 307

```
                                                 (25399) 25399      25410     25420     25430     25440     25450     25460     25470    25481
         K12 Zwischenstück 255.328 - 290.948 (24955) GCTGGCAACAGAATAACCGAGATCAACAACCTGTTTGACTGCTTCAGTTTTAAACTCTTCGGGATAACGCTTACCGCTCATGG
SARS-CoV-2 Reference Genome NC_045512.2 (21622) CAGAACTCAATTACCCCCTGCATACACTAATTCTTTCACACGTGGTGTTTATTACCCT----GACAAAGTTTCAGATC----
                                       Consensus (25399)      C   A  A   CC   AT  AC A  T TTT AC   T   GTTT   AC CT    GA AA G TT C G TC
```

Section 308

```
                                                 (25482) 25482   25490     25500     25510     25520     25530     25540     25550         25564
         K12 Zwischenstück 255.328 - 290.948 (25038) GCACCTCTCTTTAAGCCATTTTAAATGACTCGGAGGTGTCTGTTAAACCCGTGGCGATTCAAAATTGCGATATATACATACCC
SARS-CoV-2 Reference Genome NC_045512.2 (21697) ----CTCAGTTTTA--CATTC-AACTCA---GGACTTGTTCTTACCTTTCTTTTCCAATGTTACTTG-GTTCCATGC-TATAC
                                       Consensus (25482)     CTC  TTT A  CATT  AA T A   GGA  TGT   T      C T  C A T   A TTG G T  AT C TA  C
```

```
                                                             Section 309
                                          (25565) 25565 25570 25580 25590 25600 25610 25620 25630 25647
         K12 Zwischenstück 255.328 - 290.948 (25121) ATGACTGCCCAGCGTATCGGCTTCACCAGTCAGCCCGTTACGGTACAGTGTGACACAGTGCGCGTACCTGGGATTCAGCTCAC
SARS-CoV-2 Reference Genome NC_045512.2 (21768) ATGTCTCTGGGACCAATGGTACTAAGAGGTTTGA---TAACCCTGTCCTACCATTTAATGATGGTGTTT---ATTTTGCTTCC
                                Consensus (25565) ATG CT      C  AT G   T A   GT  G    T AC  T    T   A   A TG   GT   T   ATT  GCT   C
```

```
                                                             Section 310
                                          (25648) 25648 25660 25670 25680 25690 25700 25710 25720 25730
         K12 Zwischenstück 255.328 - 290.948 (25204) AGGAGAGCAGCATCAGCTCCAGATGTGCGACAAAATGCGGGAAGGCTTCATCCAGTTGTAGAGTCTGCACCTCACTGAATGAG
SARS-CoV-2 Reference Genome NC_045512.2 (21845) ACT-GAGAAGTCTAACATA-ATAAGAGGCTGGATTTTTGGTACTACTTTAG--ATTCGAAGACCCAGTCCCT-ACTTATTG--
                                Consensus (25648) A   GAG AG  T A  T  A A G G     A  T  GG A   CTT A   A T G AGA  C G  CCT ACT A TG
```

```
                                                             Section 311
                                          (25731) 25731 25740 25750 25760 25770 25780 25790 25800 25813
         K12 Zwischenstück 255.328 - 290.948 (25287) CCGGTGAATCCAGCCCGGTCAGCCAGAAAATGCAGACGATGACCTTCCTGCACCAGTCGTGCTCCGAAACACGGTGTGATATC
SARS-CoV-2 Reference Genome NC_045512.2 (21921) ----TTAATAACGCTAC-TAATGTTGTTATTAAAGTCTGTGAATTTCAATTTT--GTAATGATCCATTTTTGGGTGTTTATTA
                                Consensus (25731)     T AAT   GC    T A    G  A T  AG C  TGA  TTC        GT  TG TCC       GGTGT    T
```

```
                                                             Section 312
                                          (25814) 25814 25820 25830 25840 25850 25860 25870 25880 25896
         K12 Zwischenstück 255.328 - 290.948 (25370) TCGTTTAAGCCCCCAAGTTTGAGTAGAGTCTTTATTCATAATTTTATTCCTGTTA-AAGAAGACCATGTTCGGCGAATGAGAA
SARS-CoV-2 Reference Genome NC_045512.2 (21997) CCACAAAAACAACAAAAGTTGGATGGAAAGTGAGTTCAGAGTTT-ATTCTAGTGCGAATAATTGCACTTTTG---AATATGTC
                                Consensus (25814)  C    AA C  C AA  TTG  T GA   T   TTCA A TTT ATTC  GT   AA AA   CA  TT G   AAT  G
```

```
                                                             Section 313
                                          (25897) 25897 25910 25920 25930 25940 25950 25960 25979
         K12 Zwischenstück 255.328 - 290.948 (25452) TATTTGCCTGCCGCCGACAATCAGGTGGTCGGGGACATGGTGAACACTTTTTAACATCCTTTACCCATGGTGATCGACTTTTT
SARS-CoV-2 Reference Genome NC_045512.2 (22076) TCTCAGCCTTTTCTTATGGACCTTGAAG--GAAAACAGGGTAA----TTTCAAAAATCTTAGGGAATTTGTGTTTAAGAATAT
                                Consensus (25897) T T  GCCT          A C  G  G  G   ACA GGT A    TTT  AA ATC T       T GTG T  A   T T
```

```
                                                             Section 314
                                          (25980) 25980 25990 26000 26010 26020 26030 26040 26050 26062
         K12 Zwischenstück 255.328 - 290.948 (25535) CTTCAGGTCGATCACCCCCACTTTCGTGCTGTACCACCACACCTCGTAGCTGCCGTCTTCCTGCATCTCCTTCAGCCCGACCC
SARS-CoV-2 Reference Genome NC_045512.2 (22153) -TGATGGTTATTTTAAAATATATTC------TAAGCACACGCCTATTAATT----TAGTGCGTGATCTCCCTCAGGGTTT---
                                Consensus (25980)  T   GGT   T       A  TTC      TA    CAC CCT  TA  T    T  T C   ATCTCC TCAG
```

```
                                                             Section 315
                                          (26063) 26063 26070 26080 26090 26100 26110 26120 26130 26145
         K12 Zwischenstück 255.328 - 290.948 (25618) GTTCTCCCCTGAACGCCTTGCCTGCGCTCAGACTTACCCCTTTCACGCTCAGCTTTCCGCTGATATCCACTTTCCTGACCATC
SARS-CoV-2 Reference Genome NC_045512.2 (22222) -TTCGGCTTTAGAACCATTGGTAGATTTGCCAATAGGTATTAACATCACTAGGTTTCA----AACTTTACTTGCTTTACATAG
                                Consensus (26063)  TTC  C  T  A  C TTG   G   T   A T      T  CA     AG TTTC     A  T  ACTT C T AC
```

Section 316

```
                                                 (26146) 26146         26160     26170     26180     26190     26200     26210           26228
                K12 Zwischenstück 255.328 - 290.948 (25701) ACCCCTTCATCGTATTCCGGGGGCGTTGTGTTGCCGCTGTACTGCCGC-GCTGACGGCTGATACCGCGAGCCCGGTACCGCCA
SARS-CoV-2 Reference Genome NC_045512.2 (22300) AAG---TTATTTGACTCCTGGTGATTCTTCTTCAGGTTGGACAGCTGGTGCTG-CAGCTTATTATGTGGGTTATCTTCAACC-
                                  Consensus (26146) A     T AT   A TCC GG G  T  T TT   G TG AC GC G  GCTG C GCT AT   G G G     T C  CC
```

Section 317

```
                                                 (26229) 26229      26240     26250     26260     26270     26280     26290     26300   26311
                K12 Zwischenstück 255.328 - 290.948 (25783) TATCCAGCGCCTCGTGCGGGCGTTCAAGGTTATAGACCGTCCGCCAGTGGTCGAAGGCGCGCTGCAGTTCACCGCTGTCTGCG
SARS-CoV-2 Reference Genome NC_045512.2 (22378) --TAGGACTTTTCTATTAAAATATAATGAAAATGGAACCATTACAGATGCT-GTAGACTG--TGCACTTGACC-CTCTCTCAG
                                  Consensus (26229)   T    C   TC          T A G   AT GA C     C   TG T G AG C    TGCA TT ACC CT TCT  G
```

Section 318

```
                                                 (26312) 26312   26320     26330     26340     26350     26360     26370     26380     26394
                K12 Zwischenstück 255.328 - 290.948 (25866) AACCATTTTCCCTGCAGCACTTCCGCCTTCAGGCTGCGGTGAAAACGCTCCAGCTTCCCCTGCGTCTGCGGATGATAAGGCCG
SARS-CoV-2 Reference Genome NC_045512.2 (22455) AAACAAAGTGTACGTTGAAAT----CCTTCA--CTGTAGAAAAAGGAATCTATCAAACT-TCTAACTTTAGAGTCCAACCAAC
                                  Consensus (26312) AA CA   T    G  G A T    CCTTCA  CTG  G  AAA    TC A C   C  T    CT   GA    AA
```

Section 319

```
                                                 (26395) 26395 26400     26410     26420     26430     26440     26450     26460         26477
                K12 Zwischenstück 255.328 - 290.948 (25949) GGAGTGCCCCACCCGGATACCCAGGCGCATCAGCCACAGCTCCAGCGCCGTCCAGGTGCCGGTGGTGTCGCCCCACGGTGAGC
SARS-CoV-2 Reference Genome NC_045512.2 (22531) AGAATCTATTGTTAGATTTCCTA-----ATATTACAAACTT---GTGCCCTTTTGGTGAAGTTTTTAACGCCACCAGATTTGC
                                  Consensus (26395)  GA T         G  T CC A     AT    CA A  T   G GCC T   GGTG  G T  T  CGCC C  G T  GC
```

Section 320

```
                                                 (26478) 26478       26490     26500     26510     26520     26530     26540     26550 26560
                K12 Zwischenstück 255.328 - 290.948 (26032) CGTTATCCATGGTCATCCGGTCCGGCAGGCCGTAACGCTCAAACACGCTGACCAGCTGCTGCTGCACGGTCTCGCGCCGTTCA
SARS-CoV-2 Reference Genome NC_045512.2 (22606) ATCTGTTTATGCTTG---GAACAGGAAGAGAATCAG---CAACTGTGTTG--CTGATTATTCTGTCCTATATAATTCCG--CA
                                  Consensus (26478)    T T  ATG T     G  C GG AG    T A    CAA    G TG  C G T  T CTG  C  T T    CCG  CA
```

Section 321

```
                                                 (26561) 26561    26570     26580     26590     26600     26610     26620     26630     26643
                K12 Zwischenstück 255.328 - 290.948 (26115) TCGGTACAGTGCGCCAGGCACAGGGAAAAACGGGAGTGGTCGTCCAGCAGGGTGAGCGGATGGCAGCGTCCACCGCCAAAAGG
SARS-CoV-2 Reference Genome NC_045512.2 (22679) TCATT----TTCCACTTTTA-AGTGTTAT---GGAGTG-TC-TCCTACTAAATTAAATGATCTCTGCTTTA----CTAA----
                                  Consensus (26561) TC  T    T C  C    A AG G  A    GGAGTG TC TCC  C    T A   GAT  C GC T      C AA
```

Section 322

```
                                                 (26644) 26644 26650     26660     26670     26680     26690     26700     26710         26726
                K12 Zwischenstück 255.328 - 290.948 (26198) AAAGTGGCCCTTAAAATCCATCTGCCAGAGGCGGTTCGGCGCGTCGTGTTCGAACCGGCCCGTGGCGGGAATGCCCGGTGAAG
SARS-CoV-2 Reference Genome NC_045512.2 (22744) ----TGTCTATGCAGATTCATTTGTAATTAGAGGTGATGAA-GTCAGA--CAAATCGCTCCAGGGCA--AA----CTGGAAAG
                                  Consensus (26644)     TG C  T  A AT CAT TG  A   G GGT   G   GTC     C AA CG  CC  GGC   AA    C G  AAG
```

```
                                                           Section 323
                                           (26727) 26727        26740       26750       26760       26770       26780       26790         26809
          K12 Zwischenstück 255.328 - 290.948 (26281) CGCCCGGCAGCAGGCCATGGCGGGCCATCAGGTTATGGACGGTGCTGAAGGCGGGCATGGTGTGCCCCTGGTCCTCGAGCCAG
SARS-CoV-2 Reference Genome NC_045512.2 (22814) ATTGCTGATTATAATTATAAATTACCAGATGATTTTACAGGCTGC---------GTTATAG------CTTGGAATTCTAACAAT
                                 Consensus (26727)     C G          AT        CCA    G TT T   A G TGC         G  AT G      C TGG    TC A C A
                                                           Section 324
                                           (26810) 26810       26820       26830       26840       26850       26860       26870       26880      26892
          K12 Zwischenstück 255.328 - 290.948 (26364) CGCTTAATCTTGCGGGCTCCCCAGCGTTCATGACGGTCATGGGCCATACGCAGCAGGGCCGTGATGTCGTCAGATGAGCGGTT
SARS-CoV-2 Reference Genome NC_045512.2 (22883) C--TTGATTCTAAGGTTG-----GTGGTAATTA---TAATTACCTGTAT--AGATTGTTTAGGAAGTC-TAATCTCAAACCTT
                                 Consensus (26810) C  TT AT  T  GG        G G T AT A   T AT   C  TA   AG   G      GA GTC T A  T A     TT
                                                           Section 325
                                           (26893) 26893    26900       26910       26920       26930       26940       26950       26960      26975
          K12 Zwischenstück 255.328 - 290.948 (26447) CGGGGAATGGTGCGGAATGCGCGGGCGGTCCTGAAGACCGGCGGCACCTTCCTGAGCCCAGCGCTGGAGCCACTTGTAGCCGG
SARS-CoV-2 Reference Genome NC_045512.2 (22953) TTGAGAGAGAT----ATTTCAACTGAAATCTATCAGGCCGGTAGCACA--CCTTGTAATGGTGTTGAAGGTTTTAATTGTTAC
                                 Consensus (26893)   G GA  G T    A T C    G   TC    AG CCGG  GCAC   CCT         G G TG AG     T  T G
                                                           Section 326
                                           (26976) 26976          26990       27000       27010       27020       27030       27040         27058
          K12 Zwischenstück 255.328 - 290.948 (26530) TGGCAGGTGAAATGCCGAAGCGACGGCAGAGGGAACGGATGTTCGCCCCGTCCTGCGAGGCGAACAAAACAAACTCAGTACGT
SARS-CoV-2 Reference Genome NC_045512.2 (23030) TTTCCTTTACAAT--CATATGGTTTCCAACCC--ACTAATG--------GTGTTG-GTTACCAACCATACAGAGT-AGTA-GT
                                 Consensus (26976) T  C   T  AAT  C  A  G    CA      AC  ATG         GT  TG G   C AAC A ACA A T AGTA GT
                                                           Section 327
                                           (27059) 27059       27070       27080       27090       27100       27110       27120       27130      27141
          K12 Zwischenstück 255.328 - 290.948 (26613) AATGACATGGTATCTCTCGCATCCCAGGGCATAAGCGACTCCATAAACGGGTTCTTATGCCTTAGTTGTAAGTGT-CTACCAT
SARS-CoV-2 Reference Genome NC_045512.2 (23098) ACTTTCTTTGAACTTCTACAT------GCACCAGCAACT----------GTTTGTGGACCTAAA----AAGTCTACTAATTT
                                 Consensus (27059) A T  C T   A CT     CAT      GCA  AGC ACT          GTT  T    CCT A      AAGT T CTA    T
                                                           Section 328
                                           (27142) 27142    27150       27160       27170       27180       27190       27200       27210      27224
          K12 Zwischenstück 255.328 - 290.948 (26695) GTCCCCGAACAAGTGT-TCACTATGTCCCCGGACCGTACACCCCAAAGGGGAGAGGGGACTGCACCGAGCCATCT-TTTCCC
SARS-CoV-2 Reference Genome NC_045512.2 (23161) GGTTAAAAACAAATGTGTCAATTTCAACTTCAATGGTTTAACAGGCACAGGTGTTCTTACTGAGTCTAACAAAAGTTTCTGC
                                 Consensus (27142) G      AACAA TGT TCA T T    C     A  GT  A C     A  GG G      ACTG    C A C A      TTTC  C
                                                           Section 329
                                           (27225) 27225 27230       27240       27250       27260       27270       27280       27290      27307
          K12 Zwischenstück 255.328 - 290.948 (26776) CTCGCC--CCTTTGGGGAGAGGGCCGGGGTGAGGGGCAATATGTGATCCAGCTTAAATTTCCCGCACTCCCTCTTCCCTTCCG
SARS-CoV-2 Reference Genome NC_045512.2 (23244) CTTTCCAACAATTTGGCAGAGA-CATTGCTGA----CACTAC-TGATGCTGT---------CCGTGATCCACAGACACTTGAG
                                 Consensus (27225) CT  CC  C  TT GG AGAG  C   G TGA    CA TA  TGAT C G          CCG   TCC      C CTT  G
```

Section 330

```
                                        (27308) 27308       27320     27330     27340     27350     27360     27370     27380   27390
            K12 Zwischenstück 255.328 - 290.948 (26857) ATTTACCTCTCCTTGTTCTGCGTCATAGTATGATCGTTAAATAAACGAACGCTGTTCTATAATGTAGAACAAAATGATTCAGC
SARS-CoV-2 Reference Genome NC_045512.2 (23312) ATT--------CTTGA----CATTACACCATGTTCTTTTGGTGGTGTCAG--TGTTATAACACCAGGAACAAA-TACTTCT--
                                      Consensus (27308) ATT        CTTG     C T A A  ATG TC TT   T      A   TGTT TA A     GAACAAA T  TTC
```

Section 331

```
                                        (27391) 27391    27400     27410     27420     27430     27440     27450     27460       27473
            K12 Zwischenstück 255.328 - 290.948 (26940) AAGGAGATCTCATGCCGCAGTCCGCGTTGTTCACGGGA-ATCATTCCCCCTGTCTCCACCATTTTTACCGC-CGACGGCCAGC
SARS-CoV-2 Reference Genome NC_045512.2 (23378) AACCAGGTTGCTGTTCTTTATCAG-GATGTTAACTGCACAGAAGTCCCTGTTGCT-----ATTCATGCAGATCAACTTACTCC
                                      Consensus (27391) AA  AG T  C    C    TC G G TGTT AC G A A  A TCCC  T  CT     ATT  T C G  C AC   C  C
```

Section 332

```
                                        (27474) 27474 27480     27490     27500     27510     27520     27530     27540           27556
            K12 Zwischenstück 255.328 - 290.948 (27021) TCGATAAGCCGGGCACCGCCGCGCTGATCGACGATCTGATCAAAGCAGGCGTTGACGGCCTGTTCTTCCTGGGCAGCGGTGGC
SARS-CoV-2 Reference Genome NC_045512.2 (23455) TACTT--GGCGTGTTTATTCTACAGGTTCTA--ATGTTTTTCAAACACGTG----CAGGCTGTTTAATAGGGGC-----TGAA
                                      Consensus (27474) T   T  G CG G      C     G TC A  AT T  T  AA CA G G    C G CTGTT      GGGC     TG
```

Section 333

```
                                        (27557) 27557        27570     27580     27590     27600     27610     27620            27639
            K12 Zwischenstück 255.328 - 290.948 (27104) GAGTTCTCCCAGCTCGGCGCCGAAGAGCGTAAAGCCATTGCCCGCTTTGCTATCGATCATGTCGATCGTCGCGTGCCGGTG-C
SARS-CoV-2 Reference Genome NC_045512.2 (23525) CATGTCAACAA------CTCATATGAGTGTGA---CATACCCA---TTGGTGCAGGTATATGCGCTAGTTATCAGACTCAGAC
                                      Consensus (27557)  A  TC  C A      C C  A GAG GT A   CAT  CC    TTG T   G T     CG T GT     G C   G C
```

Section 334

```
                                        (27640) 27640     27650     27660     27670     27680     27690     27700     27710       27722
            K12 Zwischenstück 255.328 - 290.948 (27186) TGATCGGCACCGGCGG-CACCAACGCCCGGGAAACCATCGAACTCAGCCAGCACGCGCAGCAGGCGGGCGCGGACGGCATCGT
SARS-CoV-2 Reference Genome NC_045512.2 (23596) TAATTCTCCTCGGCGGGCACGTAGTGTAGCTAGTCAATCCATCATTGCCTACACTAT--GTCACTTGGTGCAGAAA--ATTCA
                                      Consensus (27640) T AT   C  CGGCGG CAC  A     G  A  C ATC A C   GCC  CAC     G      GG GC GA    AT
```

Section 335

```
                                        (27723) 27723 27730     27740     27750     27760     27770     27780     27790           27805
            K12 Zwischenstück 255.328 - 290.948 (27268) GGTGATCAACCCCTACTACTGGAAAGTGTCGGAAGCGAACCTGATCCGCTATTTCGAGCAGGTGGCCGACAGCGTCACGCTGC
SARS-CoV-2 Reference Genome NC_045512.2 (23675) GTTGCT----------TACTCTAATAACTCTATTGCCATACCCA-CAAATTTTACTATTAGTGTTACCACAGAA--ATTCTAC
                                      Consensus (27723) G TG T          TACT  AA    TC    GC A  C  A C   T TT C A  AG     C ACAG    A  CT C
```

Section 336

```
                                        (27806) 27806         27820     27830     27840     27850     27860     27870           27888
            K12 Zwischenstück 255.328 - 290.948 (27351) CGGTGATGCTCTATAACTT--CCCGGCGCTGACCGGGCAGGATCTGACTCCGGCGCTGGTGAAAACCCTCGCCGACTCGCGCA
SARS-CoV-2 Reference Genome NC_045512.2 (23745) CAGTG----TCTATGACCAAGACATCAGTAGATTGTACAATGTACATTTGTGGTGATTCAACTGAATGCAGCAATCTTTTGTT
                                      Consensus (27806) C GTG    TCTAT AC     C    G  GA  G  CA   T     T  GG G T       A     GC   CT   G
```

Section 337

```
                                        (27889) 27889      27900     27910     27920     27930     27940     27950     27960      27971
    K12 Zwischenstück 255.328 - 290.948 (27432) GCAATA------TTATCGGCATCAAAGACACCATCGACTCCGTCGCCCACCTGCGCAGCATGATCCATACCGTCAAAGGTGCC
SARS-CoV-2 Reference Genome NC_045512.2 (23824) GCAATATGGCAGTTTTTGTACACAATTAAACCGTGCTTTAACTGGAATAGCTGTTGAACAAGA-CAAAAACACCCAAGAAGTT
                              Consensus (27889) GCAATA      TT T G    CAA  A ACC T    T   T G   A CTG   A CA GA C A A C  C AAG  G
```

Section 338

```
                                        (27972) 27972   27980     27990     28000     28010     28020     28030     28040         28054
    K12 Zwischenstück 255.328 - 290.948 (27509) CATCCGCACTTCACCGTGCTCTGCGGCTACGACGATCATCTGTTCAATACCCTGCTGCTCGGCGGCGACGGGGCGATATCGGC
SARS-CoV-2 Reference Genome NC_045512.2 (23906) TTTGCACAAGTCAAACAAATTTAC----AAAAC-ACCACCAATTAAAGATTTTGGTGGTTTTAATTTTTCACAA-ATATTACC
                              Consensus (27972)   T C CA  TCA      T T C    A  AC A CA C  TT AA A   TG TG T               ATAT   C
```

Section 339

```
                                        (28055) 28055 28060     28070     28080     28090     28100     28110     28120          28137
    K12 Zwischenstück 255.328 - 290.948 (27592) GAGCGGCAACTTTGCCCCGCAGGTGTCGGTGAATCTTCTGAAAGCCTGGCGCGACGGGGACGTGGCGAAAGCGG--CCGGGTA
SARS-CoV-2 Reference Genome NC_045512.2 (23983) -AGATCCATCAAAACCAAGCAAGAG---GTCATTTAT-TGAAGATCTA-CTTTTCAACAAAGTGACACTTGCAGATGCTGGCT
                              Consensus (28055)  AG   CA C    CC  GCA G G   GT A T  T TGAA   CT  C    C    A GTG C    GC G   C GG
```

Section 340

```
                                        (28138) 28138       28150     28160     28170     28180     28190     28200     28210 28220
    K12 Zwischenstück 255.328 - 290.948 (27673) TCATCAGACCTTGCTGCAAATTCC--GCAGATGTATCAGCTGGATACGCCGTTTGTGAACGTGATTAAAGAGGCGATCGTGCT
SARS-CoV-2 Reference Genome NC_045512.2 (24060) TCATCAAACAATATGGTGATTGCCTTGGTGATATTGCTGCTAGAGACCTCATTTGTGCACAAAAGTTTAACGGCCTTACTGTT
                              Consensus (28138) TCATCA AC  T   G  A T CC  G  GAT T  C GCT GA AC  C TTTGTG AC   A T  A  GGC  T  TG T
```

Section 341

```
                                        (28221) 28221    28230     28240     28250     28260     28270     28280     28290      28303
    K12 Zwischenstück 255.328 - 290.948 (27754) CTGCGGTCGTCCTGTCTCCACGCACGTGCTGCC-GCCCGCCTCGCCGCTGGACGAGCCGCGCAAGGCGCAGCTGAAAACCCTG
SARS-CoV-2 Reference Genome NC_045512.2 (24143) TTGCCA----CCTTTGCTCACAGATGAAATGATTGCTCAATACACTTCTGCACTGTTAGCG---------GGTACAATCACTT
                              Consensus (28221)  TGC      CCT T   CAC  A G   TG   GC C    C C  CTG AC     GCG         G T  AA C CT
```

Section 342

```
                                        (28304) 28304 28310     28320     28330     28340     28350     28360     28370          28386
    K12 Zwischenstück 255.328 - 290.948 (27836) CTGCAACAGCTCAAGCTTTGCTG-AGCCGGACGATAACGATGACCATTGAGAAAATTTTCACCCCGCAGGACGACGCGTTTT-
SARS-CoV-2 Reference Genome NC_045512.2 (24213) CTG-----GTTGGACCTTTGGTGCAGGTGCTGCATTACAAATACCATTTGCTATGCAAATGGCTTATAGGTTTAATGGTATTG
                              Consensus (28304) CTG     G T  A CTTTG TG AG  G    AT AC A  ACCATT    A         C    AGG   A   GT TT
```

Section 343

```
                                        (28387) 28387        28400     28410     28420     28430     28440     28450          28469
    K12 Zwischenstück 255.328 - 290.948 (27917) -ATGCGGTGATCACCCACGCGGCGGGGCCGCAGGGCGCTCTGCCGCTGACCCCGCAGATGCTGATGG-AATCTCCCAGCGGCA
SARS-CoV-2 Reference Genome NC_045512.2 (24291) GAGTTACACAGAATGTTCTCTATGAGAACCAAAAATTGATTGCCAACCAATTTAATAGTGCTATTGGCAAAATTCAAGACTCA
                              Consensus (28387)  A       A  A    C C   G G  C  A        TGCC    A         TGCT  TGG AA  T C AG   CA
```

Section 344

```
(28470)                                         28470 28480 28490 28500 28510 28520 28530 28540 28552
K12 Zwischenstück 255.328 - 290.948 (27998)     ACCTGTTCGGCATGACG--CA---GAACGCCGGGATGGGCTGGGACGCCAACAAGCTCAC-CGGCAAAGAGG--TGCTGA---
SARS-CoV-2 Reference Genome NC_045512.2 (24374) CTTTCTTCCACAGCAAGTGCACTTGGAAAACTTCAAGATGTGGTCAACCAAAATGCACAAGCTTTAAACACGCTTGTTAAACA
Consensus (28470)                                  T TTC  CA  A G  CA   G A   C   A G   TGG    CCAA A GC CA  C   AAA A G  TG T A
```

Section 345

```
(28553)                                         28553 28560 28570 28580 28590 28600 28610 28620 28635
K12 Zwischenstück 255.328 - 290.948 (28070)     --TTATCGGCACTCAGGGCGGCAT--CCGCGCCGGAGACGGACGCCCAATCGCG-CTGGGCTACCACACCGGGCATTGGGAGA
SARS-CoV-2 Reference Genome NC_045512.2 (24457) ACTTAGCTCCAATTTTGGTGCAATTTCAAGTGTTTTAAATGATATCCTTTCACGTCTTGACAAAGTTGAGGCTGAAGTGCAAA
Consensus (28553)                                 TTA C  CA T   GG G  AT  C          A  GA   CC  TC CG CT G C A       G   A   G A A
```

Section 346

```
(28636)                                         28636 28650 28660 28670 28680 28690 28700 28718
K12 Zwischenstück 255.328 - 290.948 (28148)     TCGGCATGCAGATG-CAGGCGGCGGCGAAGGAGATCACCCGCAATGGCGGGATCCCGTTCGCGGCCTTCGTCAGCGATCCGTG
SARS-CoV-2 Reference Genome NC_045512.2 (24540) TTGATAGGTTGATCACAGGCAGACTTCAAAGTTTGCAGACATATGTGACTCAACAATTAATTAGAGCTG--CAGAAATCAGAG
Consensus (28636)                               T G  A G  GAT  CAGGC G     AA G    CA  C  A   G    A C   T     G   T   CAG  ATC G G
```

Section 347

```
(28719)                                         28719 28730 28740 28750 28760 28770 28780 28790 28801
K12 Zwischenstück 255.328 - 290.948 (28230)     CGACGGGCG--CTCGCAGGGCACGCACGGTATGTTCGATTCCCTGCCGTACCGCAACGACGCGGCGATCGTGTTTCGCCGCCT
SARS-CoV-2 Reference Genome NC_045512.2 (24621) CTTCTGCTAATCTTGCTGCTACTAAAATGTCAGAGTGTGTACTTGGACAATCAAAAAGAGTTGATTTTTGTGGAAAGGGCTAT
Consensus (28719)                               C  C G     CT GC G       A  GT  G   G  T C TG    A C  AA GA   G    T GTG    G     T
```

Section 348

```
(28802)                                         28802 28810 28820 28830 28840 28850 28860 28870 28884
K12 Zwischenstück 255.328 - 290.948 (28311)     GATCCGCTC-CCTGCCGACGCGGCGGGCGGTGATCGGCGTAGCGACCTGCGATAAAGGGCTGCCCGCCACCATGATTGCGCTG
SARS-CoV-2 Reference Genome NC_045512.2 (24704) CATCTTATGTCCTTCCCTCA-GTCAGCACCTCAT-GGTGTAGTCTTCT------------TGCATGTGAC--TTATGTCCCTG
Consensus (28802)                                ATC    T CCT CC  C  G C G    T AT GG GTAG    CT            TGC  G  AC  T AT  C CTG
```

Section 349

```
(28885)                                         28885 28890 28900 28910 28920 28930 28940 28950 28967
K12 Zwischenstück 255.328 - 290.948 (28393)     GCCGCGATGCACGACCTGCCGACTATTCTGGTGCCGGGCGGGGCGACGCTGCCGCCGACCGTCGGGGAAGACGCGGGCAAGGT
SARS-CoV-2 Reference Genome NC_045512.2 (24771) CACAAGAAA-AGAACTTCACAACTGCTCC--TGCCATTTGTCATGATGGAAAAGCACACTTTCCTCGTGAAGGTGTCTTTGTT
Consensus (28885)                                 C  GA   A  AC T  C ACT  TC   TGCC    G    GA G     GC  AC  TC   G   A G G     G T
```

Section 350

```
(28968)                                         28968 28980 28990 29000 29010 29020 29030 29040 29050
K12 Zwischenstück 255.328 - 290.948 (28476)     GCAGACCATCGGCGCGCGTTTCGCCAACCACGAACTCTCCCTGCAGGAGGCCGCCGAACTGGGCTGTCGCGCCTGCGCCTCGC
SARS-CoV-2 Reference Genome NC_045512.2 (24851) TCAAATGGCACACACTGGTTTGTAACACAAAGGAATTTTTATGAA-----CCACAAATCATTACTA-----------------
Consensus (28968)                                CA A       C C  GTTT     AC A G A T T   TG A     CC C  A C    CT
```

Section 351

```
                                        (29051) 29051 29060 29070 29080 29090 29100 29110 29120 29133
    K12 Zwischenstück 255.328 - 290.948 (28559) CGGGCGGCGGGTGTCAGTTCCTCGGCACGGCGGGCACCTCGCAGGTGGTCGCGGAGGCGCTGGGTCTGGCGCTGCCGCACTCC
SARS-CoV-2 Reference Genome NC_045512.2 (24912) CAGACAACACATTTGTGT--CTGGTAACTGTGATGTTGTAATAGGAATTGTCAACAACACAGTTTATGATCCTTT-GCAACCT
                              Consensus (29051) C G C  C   T T  GT  CT G  AC G G      T   AGG   T  C     C C G  T TG   CT   GCA  C
```

Section 352

```
                                        (29134) 29134 29140 29150 29160 29170 29180 29190 29200 29216
    K12 Zwischenstück 255.328 - 290.948 (28642) GCGCTGGCGCCGTCCGGGCAGGCGGTGTGGCTGGAGATCGCCCGCCAGTCGGCGCGCGCGGTCAGCGAGCTGGATAGCCGCGG
SARS-CoV-2 Reference Genome NC_045512.2 (24992) GAATTAGACTCATTCAAGGAGGAGTTA--GATAAATATTTTAAG--AATCATACATCAC---CAG--ATGTTGATTTAGGTGA
                              Consensus (29134) G   T G   C T C  G AGG G T   G T  A AT     G  A TC      C C   CAG  A  T GAT    G G
```

Section 353

```
                                        (29217) 29217 29230 29240 29250 29260 29270 29280 29299
    K12 Zwischenstück 255.328 - 290.948 (28725) CATCACCACGCGGGATATCCTCTCCGATAAAGCCATCGAAAACGCGATGGTGATCCACGCGGCGTTCGGCGGCTCCACCAATT
SARS-CoV-2 Reference Genome NC_045512.2 (25066) CATCTCTG-GCATTA-ATGCT-TCAGTTGTAAACATTCAAAAAGAAATTGACCGCCTCAATGAGGTTG------CCAAGAATT
                              Consensus (29217) CATC C   GC   A AT CT TC G T  A  CAT  AAAA G  AT G    CC C   G G T G      CCA  AATT
```

Section 354

```
                                        (29300) 29300 29310 29320 29330 29340 29350 29360 29370 29382
    K12 Zwischenstück 255.328 - 290.948 (28808) TACTGCTGCACATTCCGGCCATCGCCCACGCGGCGGGCTGCACGATCCCGGACGTTGAGCACTGGACGCGCATCAACCGTAAA
SARS-CoV-2 Reference Genome NC_045512.2 (25140) TAAA--TGAATCTCTCATCGATCTCCAAGAACTTGGAAAGTATGAGC----AGTATATAAAATGGCCATGGTACATTTGGCTA
                              Consensus (29300) TA    TG A  T  C  C ATC CC A      GG   G A GA C    A   T    A TGG C  G   CA   G   A
```

Section 355

```
                                        (29383) 29383 29390 29400 29410 29420 29430 29440 29450 29465
    K12 Zwischenstück 255.328 - 290.948 (28891) GTGCCGCGTCTGGTGAGCGTGCTGCCCAACGGCCCGGACTATCACCCGACCGTGCGCGCCTTCCTCGCGGGCGGCGTGCCGGA
SARS-CoV-2 Reference Genome NC_045512.2 (25217) GGTTT---TATAGCTGGCTTGATTGCCATAGTAATGGT---------GACAATTAT-GCTTTGCTGTATGACCAGTTGCTGTA
                              Consensus (29383) G       T T G   GC TG T  CCA  G    GG          GAC  T    GC TT CT    G C    TGC G A
```

Section 356

```
                                        (29466) 29466 29480 29490 29500 29510 29520 29530 29548
    K12 Zwischenstück 255.328 - 290.948 (28974) GGTGATGCTCCACCTGCGCGACCTCGGCCTGCTGCATCTGGACGCCATGACCGTGACCGGCCAGACGGTGGGCGAGAACCTTG
SARS-CoV-2 Reference Genome NC_045512.2 (25287) GTTGT--CTCAA-----GGGCTGTTGTTCTTGTGGATCCTGCTGCAAATTTGATGAA-GAC--GACTCTGAGCCAGTGC--TC
                              Consensus (29466) G TG   CTC A     G G   T G  CT  TG ATC  G  GC A      TGA  G C  GAC  TG GC AG  C  T
```

Section 357

```
                                        (29549) 29549 29560 29570 29580 29590 29600 29610 29620 29631
    K12 Zwischenstück 255.328 - 290.948 (29057) AATGGTGGCAGGCGTCCGAGCGCCGGG-CGCGCTTCCGCCAGTGCCTGCGCGAGCAGGACGGCGTAGAGCCGGATGACGTGAT
SARS-CoV-2 Reference Genome NC_045512.2 (25358) AAAGGAGTCAAATTACATTACACATAAACGAACTTATGGATTTGTTTATGAGAATCTTCACAATTGGAACTGTAACTTTGAAG
                              Consensus (29549) AA GG G CA     C    C C     CG  CTT  G    TG  T  G GA           T GA C G A       A
```

Section 358

```
                                                      (29632) 29632   29640     29650     29660     29670     29680     29690     29700        29714
          K12 Zwischenstück 255.328 - 290.948 (29139) CCTGCCGCCGG--AGAAGGCAAAAGCGAAAGGGCTGACCTCGACGGTCTGCTTCCCGACGGGCAACATCGCTCCGGAAGGTTC
SARS-CoV-2 Reference Genome NC_045512.2 (25441) CAAGGTGAAATCAAGGATGCTACTCCTTCAGATTTTGT-TCGCGCTACTGCAACGATACCGATACAAGC-CTCACTCCCTTTC
                                        Consensus (29632) C  G  G      AG A GC A   C   AG    T   TCG     CTGC  C   AC G  A  A C CTC       TTC
```

Section 359

```
                                                      (29715) 29715 29720     29730     29740     29750     29760     29770     29780        29797
          K12 Zwischenstück 255.328 - 290.948 (29220) GGTGATCAAGGCCACGGCGATCGACCCGTCGGTGGTGGGCGAAGATGGCGTATACCACCACACCGGCCGGGTGCGGGTGTTTG
SARS-CoV-2 Reference Genome NC_045512.2 (25522) GGATGGCTTATTGTTGGCGTTGCACTTCTTGCTGTTTTTC--AGAGCGCTTCCAAAATCATAACCCTCAAAAAGAGATGGCAA
                                        Consensus (29715) GG    C        GGCG T  AC   T G TG T   C  AGA  GC T  A  A CA A C   C       G TG    
```

Section 360

```
                                                      (29798) 29798       29810     29820     29830     29840     29850     29860     29870 29880
          K12 Zwischenstück 255.328 - 290.948 (29303) TCTCGGAAGCGC---AGGCGATCAAGGCGATCAAGCGGGAAGAGATTGTGCAGGGCGATATCATGGTGGTGATCGGCGGCGGG
SARS-CoV-2 Reference Genome NC_045512.2 (25603) -CTAGCACTCTCCAAGGGTGTTCACTTTGTTT-----GCAACTTGCTGTTGTTGTTTGTAACAGTTTACTCA-CACCTTTTGC
                                        Consensus (29798)  CT G A  C C    GG G TCA    G T      G AA     TGT    G    TA CA   T  T A C  C    G 
```

Section 361

```
                                                      (29881) 29881    29890     29900     29910     29920     29930     29940     29950        29963
          K12 Zwischenstück 255.328 - 290.948 (29383) CCGTCCGGCACCGGCATGGAAGAGACCTACCAGCTCACCTCCGCGCTAAAGCATATCTCGTGGGGCA-AGACGGTGTC-GCTC
SARS-CoV-2 Reference Genome NC_045512.2 (25679) TCGTT-GCTGCTGGCCTTGAAGCCCCTTTTCTCTATCTTTATGCTTTAGTCTACTTCTTGCAGAGTATAAACTTTGTAAGAAT
                                        Consensus (29881)  CGT  G   C GGC T GAAG   C T  C        T  GC  TA    A  TCT G  G G A A AC  TGT  G   
```

Section 362

```
                                                      (29964) 29964 29970     29980     29990     30000     30010     30020     30030        30046
          K12 Zwischenstück 255.328 - 290.948 (29464) ATCACCGATGCG--CGCTTCTC-GGGCGTGTCG--ACGGGCGCCTGCTTCGGCCACGTGTCGCCGGAGGCGCTGGCGGGCGGG
SARS-CoV-2 Reference Genome NC_045512.2 (25761) AATAATGAGGCTTTGGCTTTGCTGGAAATGCCGTTCCAAAAACCCATTACTTTATGATGCCAACTATTTTCTTTGCTGGCATA
                                        Consensus (29964) A  A  GA GC    GCTT  C GG   TG CG   C     CC   T C       TG C  C        T GC GGC   
```

Section 363

```
                                                      (30047) 30047       30060     30070     30080     30090     30100     30110        30129
          K12 Zwischenstück 255.328 - 290.948 (29542) CCGATTGGCAAG-CTGCGCGATAACG-ACATCATCGAGATTGCCGTGGATCGTC-TGACGTTAACTGG-----CAGCGTGAAC
SARS-CoV-2 Reference Genome NC_045512.2 (25844) CTAATTGTTACGACTATTGTATACCTTACAATAGTGTAACTTCT-TCAATTGTCATTACTTCAGGTGATGGCACAACAAGTCC
                                        Consensus (30047) C  ATTG  A G CT     ATA C  ACA  A  G  A T C  T  AT GTC T AC T A  TG      CA C  G  C
```

Section 364

```
                                                      (30130) 30130       30140     30150     30160     30170     30180     30190     30200    30212
          K12 Zwischenstück 255.328 - 290.948 (29617) TTCATCGGCACCGCGGACAACC-------CGCTGACGCCGGAAGAGGGCGCGCGCGAGCTGGCG-CGGCGGCAGACGCACCCG
SARS-CoV-2 Reference Genome NC_045512.2 (25926) TATTTCTGAACAT--GACTACCAGATTGGTGGTTATACTGAAAAATGGGAATCTGGAGTAAAAGACTGTGTTGTATTACACAG
                                        Consensus (30130) T   TC G AC    GAC ACC        G T A  C G AA A GG    C  GAG     G C G G    A     C G
```

```
                                                         Section 365
                                                   (30213) 30213  30220  30230  30240  30250  30260  30270  30280  30295
K12 Zwischenstück 255.328 - 290.948                (29692) GACCTGCACGCCC-ACGACTTTTTGCCGGACGACACCCGGCTGTGGGC-GGCACTGCAGTCG-----GTGAGCGGCGGCACCT
SARS-CoV-2 Reference Genome NC_045512.2            (26007) TTACTTCACTTCAGACTATTACCAGCTGTACTCAACTCAATTGAGTACAGACACTGGTGTTGAACATGTTACCTTCTTCATCT
Consensus                                          (30213)    CT CAC  C  AC A T    GC G AC   AC C   TG G  C G CACTG  GT G     GT A C  C  CA CT
```

```
                                                         Section 366
                                                   (30296) 30296  30310  30320  30330  30340  30350  30360  30378
K12 Zwischenstück 255.328 - 290.948                (29768) GGAAAGGCTGTATTTATGACACCGATAAAATTATCGAGGTAATTAACGCCGGTAAAAAAGCGCTCGGAATTTAATTATTTTAA
SARS-CoV-2 Reference Genome NC_045512.2            (26090) ACAATAAAATTGTTGATGAGCCTGAAGAACATGTCCA---AATTCACACAATCGACGGTTCATCCGGAGTTG--TTAATCCAG
Consensus                                          (30296)   AA      T TT ATGA  C GA  AA  T TC A   AATT AC C     A     C   CGGA TT   TTA T  A
```

```
                                                         Section 367
                                                   (30379) 30379  30390  30400  30410  30420  30430  30440  30450  30461
K12 Zwischenstück 255.328 - 290.948                (29851) GAGATAAAACCGTCTGCGGAATATTTCCCGCAGACGGCTTTGTTGTTTTTGAAATTTATTAATTTAAACAATTAGTTGAGATA
SARS-CoV-2 Reference Genome NC_045512.2            (26168) TA-ATGGAACCAATTTATGATGAACCGACGACGACTACTAGCGTGCCTTTGTAAGCAC--AAGCTGATGAGTACGAACTTATG
Consensus                                          (30379)  A AT  AACC   T   GA  A     CG  GAC  CT    TG  TTTG AA      AA  T A  A T  G     AT
```

```
                                                         Section 368
                                                   (30462) 30462  30470  30480  30490  30500  30510  30520  30530  30544
K12 Zwischenstück 255.328 - 290.948                (29934) TA-TCGTTGGCGTCACAAAAGCAAAATAACGTAATTCGGAAATAAGATATGACCATTGCTGG--TTAATTGAATA---GCTCA
SARS-CoV-2 Reference Genome NC_045512.2            (26248) TACTCATTCGTTTCGGAAGAGACAGGTA-CGTTAATAGTTAATAGCGTACTTCTTTTTCTTGCTTTCGTGGTATTCTTGCTAG
Consensus                                          (30462) TA TC TT G  TC  AA AG  A  TA CGT A T G  AATA   TA   C  TT CT G  TT  T G AT    GCT
```

```
                                                         Section 369
                                                   (30545) 30545  30550  30560  30570  30580  30590  30600  30610  30627
K12 Zwischenstück 255.328 - 290.948                (30011) TTACACT--CCATTAACACGATGTTGTAATTCGGCACACTACATAAGGGTGTAATTCTGATGACGCAATTAACCATGAAAGAC
SARS-CoV-2 Reference Genome NC_045512.2            (26330) TTACACTAGCCATCCTTACTGCGCTTCGATTGTG-----TGCGTACTGCTGCAATATTGTTAACGTGAGT---CTTGTAAAAC
Consensus                                          (30545) TTACACT  CCAT    AC   G T   ATT  G     T C TA  G TG AAT  TG T ACG  A T   C TG AA AC
```

```
                                                         Section 370
                                                   (30628) 30628  30640  30650  30660  30670  30680  30690  30700  30710
K12 Zwischenstück 255.328 - 290.948                (30092) AAAATTGGCTACGGG--C--TGGGAGACACCGCCTGCGGCTTCGTCTGGCAGGCCACGATGTTCCTGCTGGCCTATTTCTACA
SARS-CoV-2 Reference Genome NC_045512.2            (26405) CTTCTTTT-TACGTTTACTCTCGTGTTAAAAATCTGAA--TTCTTCTAG-AGTTCCTGATCTTC----TGGTCTA-------A
Consensus                                          (30628)     TT   TACG    C  T G     A    CTG    TTC TCT G AG  C  GAT TTC    TGG CTA       A
```

```
                                                         Section 371
                                                   (30711) 30711  30720  30730  30740  30750  30760  30770  30780  30793
K12 Zwischenstück 255.328 - 290.948                (30171) CCGACGTCTTCGGCCTGTCGGCGGGGATTATGGGCACGCTGTTTTTGGTCTCCCGCGTGCTCGACGCCGTCACCGACCCGCTG
SARS-CoV-2 Reference Genome NC_045512.2            (26473) ACGAACTAAATATTATATTAGTTTTTCTGTTTGGAACTTTAATTTTAGCCATGGCAGATTCCAACG--GTACTATTACCGTTG
Consensus                                          (30711)  CGA  T                    T  T GG AC  T  TTTT G C      G    C ACG  GT       CCG TG
```

Section 372

```
                                       (30794) 30794 30800     30810     30820     30830     30840     30850     30860     30876
K12 Zwischenstück 255.328 - 290.948    (30254) ATGGGGCTGCTGGTAGACCGCACCCGCACGCGGCACGGCCAGTTCCGCCCGTTCCTGCTGTGGGGGGCCATCCCGTTCGGCAT
SARS-CoV-2 Reference Genome NC_045512.2 (26554) AAGAG----CTTAAAAAGCTC-CTTGAACAATGGAA--CCTAGTAATAGGTTTCCT-------------ATTCCTTAC---AT
Consensus                              (30794) A G G    CT   A A C C C  G AC   G A   CC   T       TTCCT             AT CC T C   AT
```

Section 373

```
                                       (30877) 30877        30890     30900     30910     30920     30930     30940     30959
K12 Zwischenstück 255.328 - 290.948    (30337) CGTCTGCGTGCTGACCTTCTACACGCCGGACTTCTCCGCACAGGGCAAGATCATCTACGCCTGCGTGACCTACATTCTCCTGA
SARS-CoV-2 Reference Genome NC_045512.2 (26614) GGATTT-GT------CTTCTACAATTTG--CCTATGCCAACAGGAATAGGTTTT-TGTATATAATTAAGTTA-ATTTTCCTCT
Consensus                              (30877)  G  T  GT      CTTCTACA    G  C T T C  ACAGG   AG T  T T     T   T A  TA ATT TCCT
```

Section 374

```
                                       (30960) 30960     30970     30980     30990     31000     31010     31020     31030     31042
K12 Zwischenstück 255.328 - 290.948    (30420) CCCTGGTCTA--CACCTTCGTTAACGTGCCGTACTGCGCCATGCCGGGCGTCATCACCGCCGACCCGAAAGAGCGTCACGCCC
SARS-CoV-2 Reference Genome NC_045512.2 (26686) GGCTGTTATGGCCAGTAACTTTAGCTTG---TTTTGTGCT-TGCTGCTGTTTA------CAGAAT--AAATTGGATCACCGGT
Consensus                              (30960)   CTG T T   CA    C TTA C TG   T  TG GC  TGC G    T A      C GA    AAA  G  TCAC
```

Section 375

```
                                       (31043) 31043  31050     31060     31070     31080     31090     31100     31110     31125
K12 Zwischenstück 255.328 - 290.948    (30501) TGCAGTCCTGGCGCTTCTTCCTGGCGGCGGCGGGCTCGCTCGCTATCAGCGGCATCGCGCTGCCGCTGGT-GAGCATCATCGG
SARS-CoV-2 Reference Genome NC_045512.2 (26757) GGAATTGCTATCGCAATGGCTTGTCTT--GTAGGCTTGATGTGGCTCAGCTACTTCAT--TGCTTCTTTCAGACTGTTTGCGC
Consensus                              (31043)  G A T CT  CGC     C TG C    G  GGCT G T     TCAGC  C TC    TGC  CT    GA   T   CG
```

Section 376

```
                                       (31126) 31126         31140     31150     31160     31170     31180     31190     31208
K12 Zwischenstück 255.328 - 290.948    (30583) CAAGGGGGACGAGCAGGTGGGCTACTTCGGCGCC--ATGTGCGTGCTGGGGCTGAGCGGCGTGGTGCTGCTCTACGTCTGCTT
SARS-CoV-2 Reference Genome NC_045512.2 (26836) GTACGCGTTCCATGTGGTCATTCAATCCAGAAACTAACATTCTTCTCAACGTGCCACTCCATGGCACTATTCTGACCAGACCG
Consensus                              (31126)   A G G  C A   GGT     A T C G   C  A  T C T      G     C  C TGG  CT  TCT       C
```

Section 377

```
                                       (31209) 31209      31220     31230     31240     31250     31260     31270     31280  31291
K12 Zwischenstück 255.328 - 290.948    (30664) CTTCACGACCAAAGAGCGCTACACCTTTGAGGTGCAGCCGGGCTCGTCGGTGGCGAAAGACCTTAAGCTGCTGCTGGGCAACA
SARS-CoV-2 Reference Genome NC_045512.2 (26919) CTTCTAGA---AAGTG------AACTCGTAATCGGAGCTGTGATCCTTCGTGG----ACATCTTCGTAT--TGCTGGACACCA
Consensus                              (31209) CTTC  GA   AAG G      A CT   A   G AGC G G TC T  GTGG    A A CTT    T  TGCTGG CA CA
```

Section 378

```
                                       (31292) 31292    31300     31310     31320     31330     31340     31350     31360     31374
K12 Zwischenstück 255.328 - 290.948    (30747) GCCAGTGGCGCATCATGTGCGCGTTCAAGATGATGGCGACCTGCTCCAACGT-GGTGCGCGGCGGGGCGACGCTCTACT-TCG
SARS-CoV-2 Reference Genome NC_045512.2 (26987) TCTAG-GACGC------TGTGACATCAAGGACCTGCCTAAAGAAATCACTGTTGCTACATCACGA----ACGCTTTCTTATTA
Consensus                              (31292)  C AG G CGC      TG G   TCAAG    TG C A       CA  GT G T C    CG     ACGCT T  T T
```

```
                                                  Section 379
                                          (31375) 31375 31380 31390 31400 31410 31420 31430 31440 31457
       K12 Zwischenstück 255.328 - 290.948 (30828) TGAAATACGTGATGGATCACCCGGAGTTGGCGACCCAGTTTTTACTTTACGGCAGCCTCGCCACCATGTTCGGCTCGCTTTGC
SARS-CoV-2 Reference Genome NC_045512.2 (27059) CAAATTGGGAGCTTCGCAGCGTGTAGCAGGTGACTCAGGTTTTGCTGCATA-CAGTCGCTACAGGATTGGCAACTATAAATTA
                                Consensus (31375)   AA T  G G T      C  G AG  GG GAC CAG TTTT CT  A   CAG C C  CA  AT   C  CT     T

                                                  Section 380
                                          (31458) 31458 31470 31480 31490 31500 31510 31520 31530 31540
       K12 Zwischenstück 255.328 - 290.948 (30911) TCCTCACGCCTGCTGGGCCGCTTCGACCGCGTCACCGCCTTCAAGTGGATCATCGTCGCCTACTCGCTGATCAGCCTGCTGAT
SARS-CoV-2 Reference Genome NC_045512.2 (27141) AACACAGACCATTCCAGTAGCAGTGACAATATTGC----TTTGCTTGTACAGTAAGTGACAACAGATGTTTCATCTCGTTGAC
                                Consensus (31458)   C CA  CC      G  GC   GAC    T  C    TT    TG A   T    G C AC       TCA C  G TGA

                                                  Section 381
                                          (31541) 31541 31550 31560 31570 31580 31590 31600 31610 31623
       K12 Zwischenstück 255.328 - 290.948 (30994) TTTC--GTCACCCGGCGGAGCACATCGCGCTCATTTTTGCCCTCAACATCCTGTTCCTGTTCGTCT-TTAATACCACCACGC
SARS-CoV-2 Reference Genome NC_045512.2 (27220) TTTCAGGTTACTATAGCAGAG-ATATTACTAATTATTATGAGGACTTTTAAAGTTTCCATTTGGAATCTTGATTACATCATAA
                                Consensus (31541) TTTC  GT AC    GC GAG A AT  C      TT TG    C         TTCC  TT G  T TT AT  CA CA

                                                  Section 382
                                          (31624) 31624 31630 31640 31650 31660 31670 31680 31690 31706
       K12 Zwischenstück 255.328 - 290.948 (31074) CGCTGCAGTGGCTGATGGCTTCTG-ACGTGGTGGACTACGAGGAGAGCCGCAGCGGTCGCCGCCTCGACGGGCTGGTGTTCTC
SARS-CoV-2 Reference Genome NC_045512.2 (27302) ACCT-CA-TAATTAAAAATTTATCTAAGTCACTAACTGAGAATAAATATTCTCAATTAGATG-----AAGAGCAACCAATGGA
                                Consensus (31624)   CT CA T   T A    TT T  A GT     ACT  GA  A A    C     T G  G     A G GC      T

                                                  Section 383
                                          (31707) 31707 31720 31730 31740 31750 31760 31770 31789
       K12 Zwischenstück 255.328 - 290.948 (31156) CACCTACCTGTTCAGCCTGAAGATTGGCCTGGCGATTGGCGGGGCGGTGGTGGG-CTGGATCCTGGCGTACGTCAACTATTCC
SARS-CoV-2 Reference Genome NC_045512.2 (27378) GATTGATTAAACGAACATGAAAATTATTCT------TTTCTTGGCACTGATAACACTCGCTACTTGTG------AGCTTTATC
                                Consensus (31707)  A   A       A C TGAA ATT   CT      T  C  GGC  TG T    CT G T CT G G      A CT T  C

                                                  Section 384
                                          (31790) 31790 31800 31810 31820 31830 31840 31850 31860 31872
       K12 Zwischenstück 255.328 - 290.948 (31238) GCCAGCAGCAGCGTGCAGCCGGTTGAGGTGCT-CACCACCATCAAAATTCTGTTCTGCGTGGTGCCGGTGGTGCTCTACGCGG
SARS-CoV-2 Reference Genome NC_045512.2 (27449) ACTACCAAGAGTGTGT------TAGAGGTACAACAGTACTTTTAAAAGAACCTTGCTCTTC-------TGGAACA-TACGAGG
                                Consensus (31790)  C A CA  AG GTG       T GAGGT C  CA  AC  T AAAA     TT   C T        TGG  C  TACG GG

                                                  Section 385
                                          (31873) 31873 31880 31890 31900 31910 31920 31930 31940 31955
       K12 Zwischenstück 255.328 - 290.948 (31320) GCATGTTCATCATGCTGTCGCTCTACAAGCTCACCGATGCCCGCGTGGAGGCCATCAGCCGGCAGCT-GATTAAGCA---CCG
SARS-CoV-2 Reference Genome NC_045512.2 (27518) GCAA-TTCACCATTTCATC-CTCTAGCTGATAACAAATTTGCAC-TGACTTGCTTTAGCACTCAATTTGCTTTTGCTTGTCCT
                                Consensus (31873) GCA  TTCA CAT    TC CTCTA   G T AC  AT   C C TG     C T AGC   CA  T G TT  GC    CC
```

Section 386

```
                                                    31956                                                                                 32038
K12 Zwischenstück 255.328 - 290.948 (31399)         CGCGGCGCAGGGCGAGGCCGTTCCCGACGCCGCGACAGCCGCATCCATTAACCGG------AGGCAATATGGAAATCACTAAC
SARS-CoV-2 Reference Genome NC_045512.2 (27598)     GACGGCGTAAAACACGTCTATCAGTTACGT-GCCAGATCAGTTTCACCTAAACTGTTCATCAGACAAGAGGAAGTTCAAGAAC
Consensus (31956)                                     CGGCG A   C  G C  T     ACG  GC A A C G  TC C T AAC G      AG CAA A G A  TCA  AAC
```

Section 387

```
                                                    32039                                                                                 32121
K12 Zwischenstück 255.328 - 290.948 (31477)         CCGATACTCACCGGCTTCAACCCGGACCCGTCCCTGTGCCGCCAGGGCGAGGACTACTACATCGCCACCTCGACCTTCGAGTG
SARS-CoV-2 Reference Genome NC_045512.2 (27680)     TT--TACTCTCCAATTTTT---------CTTATTGTTGCGGCAATAGTGTTTATAAC-ACTTTGCTTC----ACACTCAAAAG
Consensus (32039)                                       TACTC CC   TT           C T     TGC GC A  G G   A  AC AC T GC  C    AC  TC A  G
```

Section 388

```
                                                    32122                                                                                 32204
K12 Zwischenstück 255.328 - 290.948 (31560)         GTTCCCGGGCGTGCGCATCTACCACTCCCGTGACCTGAAAAACTGGTCGCTGGTCAGCACC-CCGTTGGACCGCGTGTCGATG
SARS-CoV-2 Reference Genome NC_045512.2 (27747)     AAAGACAGAATGATTGAACTTTCATTAAT-TGACTTCTAT---TTGT-GCTTTTTAGCCTTTCTGCTATTCCTTGTTTTAATT
Consensus (32122)                                        C G        A CT  CA T    TGAC T  A    T GT GCT  T AGC    C G T   CC  GT T  AT
```

Section 389

```
                                                    32205                                                                                 32287
K12 Zwischenstück 255.328 - 290.948 (31642)         CTGGACATGAAGGGCAACCCGGACTCCGGCGGCATCTGGGCGCCGTGCCTGAGCTACGCCGACGGTAAATTCTGGCTGCTCTA
SARS-CoV-2 Reference Genome NC_045512.2 (27825)     ATGCTTATTAT---CTTTTGGTTCTC-------ACTTGAAC----TGCAAGATC-------ATAATGAAACTTGTCACGCCTA
Consensus (32205)                                    TG   AT A    C     G  CTC       A  TG  C    TGC  GA C       A   T AA   TG C    CTA
```

Section 390

```
                                                    32288                                                                                 32370
K12 Zwischenstück 255.328 - 290.948 (31725)         CACCGACGTGAAGATTGTCGACTCGCCGTGGAAAAACGGCCGCAACTTCCTCGTCACCGCGCCCTCCATCGAGGGGCCATGGA
SARS-CoV-2 Reference Genome NC_045512.2 (27887)     AACGAACATGAAATTTCTTGTTTT-CTTAGGAATCAT---CACAACTGTAGCTGCATTTCACCAAGAATGTAGTTTACAGTCA
Consensus (32288)                                    AC  AC TGAA  TT T G  T  C   GGAA  A    C CAACT    C  CA   C CC    AT  AG    CA   A
```

Section 391

```
                                                    32371                                                                                 32453
K12 Zwischenstück 255.328 - 290.948 (31808)         GCGAGCCAATCCCGATGGGCAACGGCGGGTTTGACCCGTCCCTGTTCCACGACGACGATGGCCGCAAATACTATATCTACCGC
SARS-CoV-2 Reference Genome NC_045512.2 (27966)     TGTACTCAACATCAACCAT--ATGTAGTTGATGACCCGTGTCCTATTCACTTCTATTCT------AAATGGTATAT-------
Consensus (32371)                                      A  CAA   C A      A G  G    TGACCCGT  C   T CAC  C A   T      AAAT  TATAT
```

Section 392

```
                                                    32454                                                                                 32536
K12 Zwischenstück 255.328 - 290.948 (31891)         CCGTGGGGGCCGCGCCACCACAGCAACCCGCACAACACCATCGTGT-TACAGGCGTTTGACCCGCAGACCGGCACGCTCTCGC
SARS-CoV-2 Reference Genome NC_045512.2 (28034)     ---TAGAGTAGGAGCTAGAAAATCAGCACCTTTAATTGAATTGTGCGTGGATGAGGCTGGTTCTAAATCACCCATTCAGTACA
Consensus (32454)                                      T G G   G GC A  A A CA C C    AA    AT GTG  T  A G G  TG   C  A  C   CA  C  T
```

Section 393

```
                                               (32537) 32537        32550     32560     32570     32580     32590     32600             32619
  K12 Zwischenstück 255.328 - 290.948 (31973) CCGAGCGCAAAACGCTGTTTACCGGCACGCCGCTCTGCTACACCGAA--GGCGCGCACCTGTATCGCCACGCGGGATGGTACT
SARS-CoV-2 Reference Genome NC_045512.2 (28114) TCGATATCGGTAAT-TATACAGTTTCCTGTTTACCTTTTACAATTAATTGCCAGGAACCTAAATTGG---GTAGTCTTGTAGT
                               Consensus (32537)  CGA   C   A   T T  A    C  G     CT  TACA   AA  G C  G ACCT  AT G    G  G  T GTA T
```

Section 394

```
                                               (32620) 32620    32630     32640     32650     32660     32670     32680     32690       32702
  K12 Zwischenstück 255.328 - 290.948 (32054) ACCTGATGGCCGCCGAGGGCGGCACCAGCTACGAGCACGCCGT-CGTGGTGCTGC--GTTCCAAAAATATCGACGGGCCGTAC
SARS-CoV-2 Reference Genome NC_045512.2 (28193) GCGTTGTT-CGTTCTATGAAGACTTTTTAGAGTATCATGACGTTCGTGTTGTTTTAGATTTCATCTAAACGAACAAACTAAAA
                               Consensus (32620)  C T  T  C   C A G  G C       A  A CA G CGT CGTG TG T     TT CA   A A   AC   C   A
```

Section 395

```
                                               (32703) 32703  32710     32720     32730     32740     32750     32760     32770          32785
  K12 Zwischenstück 255.328 - 290.948 (32134) GAGCTGCACCCGGACGTAACGATGATGACCAGCTGGCACCTGCCGGAGAACCCGCTGCAGAAGAGCGGCCACGGCTCGCTGCT
SARS-CoV-2 Reference Genome NC_045512.2 (28275) TGTCTGATAATGGACC---CCAAAATCAGCGAAATGCACC--CCGCATTACGTTTGGTGGACCCTCAGATTCAACTGGCAGTA
                               Consensus (32703)    CTG     GGAC    C A  AT A C     GCACC  CCG A  AC     G  GA    C G   C  CT GC G
```

Section 396

```
                                               (32786) 32786         32800     32810     32820     32830     32840     32850           32868
  K12 Zwischenstück 255.328 - 290.948 (32217) GCAGACGCATACGGGTGAATGGTACATGGC-CTACCTCACCAGC--CGCCCGCTGCGCCTGCCCGGCGTGCCGCTGCTGGCCT
SARS-CoV-2 Reference Genome NC_045512.2 (28353) AC-----CAGAATGGAGAACGCAGTGGGGCGCGATCAAAACAACGTCGGCCCCAAGGTTTACCCAA--TAATACTGC--GTCT
                               Consensus (32786)  C     CA A  GG GAA G      GGC C A C  A CA C  CG CC C   G  T CCC    T    CTGC  G CT
```

Section 397

```
                                               (32869) 32869      32880     32890     32900     32910     32920     32930     32940      32951
  K12 Zwischenstück 255.328 - 290.948 (32297) CCGGCGGACGCGGCTACTGCCCGCTGGGGCGCGAGACCGGCATCGCCCGCATTGA-ATGGCGCGACGGCTGGCCGTACGTGGA
SARS-CoV-2 Reference Genome NC_045512.2 (28427) TGGTTCACCGCTCTCACTCAACA-TGGCAAGGAAGACCTTAAATTCCCTCGAGGACAAGGCGTTCCAATTAACACCAATAGCA
                               Consensus (32869)   G     CGC    ACT   C  TGG   G  AGACC   A   CCC C   GA A GGCG   C   T  C   A   G A
```

Section 398

```
                                               (32952) 32952   32960     32970     32980     32990     33000     33010     33020         33034
  K12 Zwischenstück 255.328 - 290.948 (32379) AGGCGGCAAGCACGCGCAGCTGACCGTGAAAGGCCCGCAAGTAGCCGAGCAGCCTGCAGCCGTTCCGGGCAACTGGCGGGACG
SARS-CoV-2 Reference Genome NC_045512.2 (28509) ----GTCCAGATGACCAAATTGGCTACTACCGAAGAGCTACCAGACGAATT-CGTGGTG--GTGACGGTAAAATGA----AAG
                               Consensus (32952)     G C AG    C  A  TG C    A  G    GC A  AG CGA    C TG  G  GT  CGG  AA TG     A G
```

Section 399

```
                                               (33035) 33035 33040      33050     33060     33070     33080     33090     33100          33117
  K12 Zwischenstück 255.328 - 290.948 (32462) ATTTCGACGCCAGTTCGCTTGACCCGGAGCTGCAGACCCTGCGCATTCCGTTCGACGACACCCTCGGCTCGCTCACCGCGCGC
SARS-CoV-2 Reference Genome NC_045512.2 (28581) ATCTCAGTCCAAGATGGTATTTCTACTACCT--AGGAACTGGGCCAGAAGCTGGACTTC--CCTATGGT-GCTAACAAAGAC-
                               Consensus (33035) AT TC    C AG T G  T  C    A CT  AG   CTG GC     G T GAC  C  CCT  G T GCT AC   G
```

```
                                                                      Section 400
                                          (33118) 33118       33130     33140     33150     33160     33170     33180     33190     33200
   K12 Zwischenstück 255.328 - 290.948 (32545) CCGGGCTTCTTACGGCTCTATGGCAACGACTCGCTCAATTCGACCTTCACCCAATCGACCGTGGCGCGCCGCTGGCAGCACTT
SARS-CoV-2 Reference Genome NC_045512.2 (28658) ---GGCATCATATGGGTT----GCAACTGAGGGAGCCTT--GAA-TACACCAAAA-GATCA---------CATTGGCACC----
                                Consensus (33118)    GGC TC TA GG T    GCAAC     G  C  T  GA  T CACC AA  GA C         C  TGGCA C

                                                                      Section 401
                                          (33201) 33201     33210     33220     33230     33240     33250     33260     33270     33283
   K12 Zwischenstück 255.328 - 290.948 (32628) CGCCTTCCGGGCAGAAACGCGGATGGAGTTCTCGCCGGTGCACTTCCAGCAGAGCGCGGGGCTGACCTGCTACTACAACAGCA
SARS-CoV-2 Reference Genome NC_045512.2 (28718) CGCAATCCTGCTAACAATGCTGCAATCGTGCTACAACTTCC---TCAAGGA-ACAACATTGCCAAAAGGCTTCTAC----GCA
                                Consensus (33201) CGC  TCC G  A  AA GC G      GT CT       T C   TC AG A A   C   GC  A   GCT CTAC    GCA

                                                                      Section 402
                                          (33284) 33284 33290     33300     33310     33320     33330     33340     33350     33366
   K12 Zwischenstück 255.328 - 290.948 (32711) AAAACTGGAGCTACTGCTTTGTGGACTACGAGGAGGGACAGGGTAGAACCATCAAAGTTATC-CAGCTCGACCACAACGTGCC
SARS-CoV-2 Reference Genome NC_045512.2 (28793) GAAG--GGAGCAGAGGC-----GGCAGTCAAGCCTCTTCTCGTTCCT--CATCAC-GTAGTCGCAACAGTTCAAGAAATTCAA
                                Consensus (33284)  AA    GGAGC     GC     GG     C AG         C  G T      CATCA  GT   TC CA C     C A AA   T

                                                                      Section 403
                                          (33367) 33367       33380     33390     33400     33410     33420     33430     33449
   K12 Zwischenstück 255.328 - 290.948 (32793) GTCGTGGCCGCTGCACGAGCAGCCCATTCCGGTGCCGGAA---CATGCGGAGAGCGTCTGGCTGCGGGTGGACGTGGATACGC
SARS-CoV-2 Reference Genome NC_045512.2 (28866) CTCCAGGCAGCAGTAGGGGAAC----TTCTCCTGCTAGAATGGCTGGCAATGGCGGTGATGCTGCTCTTG--CTTTGCT--GC
                                Consensus (33367)  TC   GGC GC G A G G A      TTC   TGC   GAA    C  GC    G    GT    GCTGC    TG   C T G T   GC

                                                                      Section 404
                                          (33450) 33450     33460     33470     33480     33490     33500     33510     33520     33532
   K12 Zwischenstück 255.328 - 290.948 (32873) TGGTCTACCGCTACAGCTACTCGTTTGATGGCGAGACGTGGCACACCGTGCCGGTGACGTATGAGGCGTGGAAGCTGTCGGAC
SARS-CoV-2 Reference Genome NC_045512.2 (28941) TGCTTGACAGATTGAACCA---GCTTGAGAGCAAAATGTCTGGTAAAG-GCCAACAACA-ACAAGGCC---AAACTGTC--AC
                                Consensus (33450) TG T  AC G T  A C A   G TTGA  GC A A GT      A  G GCC    AC  A  AGGC    AA CTGTC  AC

                                                                      Section 405
                                          (33533) 33533 33540     33550     33560     33570     33580     33590     33600     33615
   K12 Zwischenstück 255.328 - 290.948 (32956) GACTACATCGGCGGCGCGGCTTCTTCACCGGCGCGTTTGTG--GGCCTGCACTGCGAGGACATCAGCGGCGACGGCTGCTAC
SARS-CoV-2 Reference Genome NC_045512.2 (29014) TAAGAAATCTGCTGCTGAGGCTTCTAAGAAGCCTCGGCAAAAACGTACTGCCACTAAAGCATACAATGTAACACAAGCTTTCG
                                Consensus (33533)  A  A ATC GC G  G GGCTTCT     G C CG         G  CTGC      AG A A  A      AC     T

                                                                      Section 406
                                          (33616) 33616         33630     33640     33650     33660     33670     33680     33698
   K12 Zwischenstück 255.328 - 290.948 (33037) GCGGACTTCGACTACTTCACCTACGAGCCGGTCTAACGGCTCAGGCCGGGTAGCCCAGCGCGCGCGAGAGCGCGAGCCCGGCC
SARS-CoV-2 Reference Genome NC_045512.2 (29097) GCAGACGTGGTCCAGAACAAACCCAAGGAAATTTTGGGGACCAGGAAC--TAATCAGACAAGGAACTGATTACAAACATTGGC
                                Consensus (33616) GC GAC T G C A    CA    C AG    T T   GG  CAGG      TA  C   C  G     GA   C A C    G C
```

Section 407

```
                                             (33699) 33699      33710      33720      33730      33740      33750      33760      33770     33781
        K12 Zwischenstück 255.328 - 290.948 (33120) TGCTGAAGCTGCTCGCGGAAATTAGCCAGCTCCGCGTCGTCCACG--CGGGAGGTCAGCGTCGACAGGCTCAGGGCGGCGATG
SARS-CoV-2 Reference Genome NC_045512.2 (29178) CGC--AAATTGCAC-----AATTTGCCCCCAGCGCTTCAGCGTTCTTCGGAATGTC-GCG----CATTGGCATGGAAGTCACA
                                   Consensus (33699)  GC  AA  TGC C     AATT GCC  C  CGC TC  C      CGG A GTC GCG    CA    CA GG  G  A
```

Section 408

```
                                             (33782) 33782   33790      33800      33810      33820      33830      33840      33850     33864
        K12 Zwischenstück 255.328 - 290.948 (33201) ACGCGGGACTCGTGGTT--CC-ACACCGGCACCGCCACGCAGCGCACGCCCTGCTCGTTCTCTTCGCTGTCCAGGGCGTAGCC
SARS-CoV-2 Reference Genome NC_045512.2 (29249) CCTTCGGGAACGTGGTTGACCTACACAGGTGCCATCAAATTG-GATGACAAAGATCCAAAT-TTCAAAGATCAAGTCATT---
                                   Consensus (33782)  C   GG   CGTGGTT  CC ACAC GG  CC  CA    G G    C   G TC    T TTC   G  CA G C T
```

Section 409

```
                                             (33865) 33865 33870     33880      33890      33900      33910      33920      33930     33947
        K12 Zwischenstück 255.328 - 290.948 (33281) TTGCTCGCGGGTCTGCGCCAGGGCGCTCATTAAGGCTTCGCGAGACGCGAGGGTGGCG-GGCGTAAAGGTAGTGTACTGATAG
SARS-CoV-2 Reference Genome NC_045512.2 (29327) TTGCT---GAATAAGCATATTGACGCATACAAAACATTCCCACCAACAGAGCCTAAAAAGGACAAAAAGAAGAAGGCTGATGA
                                   Consensus (33865) TTGCT   G  T  GC     G CGC  A  AA   TTC C   A   GAG  T     GG   AAA G AG    CTGAT
```

Section 410

```
                                             (33948) 33948       33960      33970      33980      33990      34000      34010      34020 34030
        K12 Zwischenstück 255.328 - 290.948 (33363) CCCTCCAGCAGGGCGTTCAGCTCGGCCTCGCCCAGCCAGGCAATCAACACCTTGCCGATGGCGGTGGCGTGCACCGGCAGGCG
SARS-CoV-2 Reference Genome NC_045512.2 (29407) AACTCAAGC----CTTACCGCAGAGACA-GAAGAAACAGCAAACTGTGACTCTTCTTCCTGCTGCAGATTT----GGATGATT
                                   Consensus (33948)   CTC AGC    C T C GC   G C  G   A  CAG  AA     AC  T C     GC G  G  T     GG  G
```

Section 411

```
                                             (34031) 34031    34040      34050      34060      34070      34080      34090      34100     34113
        K12 Zwischenstück 255.328 - 290.948 (33446) GCGGCCGATGCGTG-AATAGGCGATGGCGGCCAGCTTGCCTTCAATCTTCTC-GATATAGACCCCTTCACGCCCGTCCAGGAT
SARS-CoV-2 Reference Genome NC_045512.2 (29481) TCTCCAAACAATTGCAACAATCCATGAG---CAG--TGC--TGACTCAACTCAGGCCTAAACTCATGCAGACCACACAAGG--
                                   Consensus (34031)  C  C  A    TG AA A  C ATG     CAG  TGC  T A TC  CTC G   TA AC C T CA  CC   C AGG
```

Section 412

```
                                             (34114) 34114 34120      34130      34140      34150      34160      34170      34180     34196
        K12 Zwischenstück 255.328 - 290.948 (33527) CCCCAGATGGGTGGTCTGCCCGGTC-CGCCGGGACAGCTCCGTCAGCCAGCCTTTTGCCTTCTGCCGAATATCGATGGAGCCC
SARS-CoV-2 Reference Genome NC_045512.2 (29555) ---CAGATGGGCTATATAAACGTTTTCGCTTTTCCGTTTACGATATATAGTCTACT--CTTGTGCAGAATGA--ATT---CTC
                                   Consensus (34114)    CAGATGGG   T T   CG T  CGC     C   T CG  A   AG CT  T  CTT TGC GAAT    AT    C C
```

Section 413

```
                                             (34197) 34197        34210      34220      34230      34240      34250      34260     34279
        K12 Zwischenstück 255.328 - 290.948 (33609) ACGACAAAATGGCCGCGCTCGACCAGCTTCATGCCGAGGCGATACTTGCCGTTCTCCGGGTTCTGATCGATATAGCCGTGAAG
SARS-CoV-2 Reference Genome NC_045512.2 (29628) GTAACTACATAGC--------ACAAG-TAGATG---------TAGTTAACTTTAATC----TCACATAGCAAT---CTTTAAT
                                   Consensus (34197)    AC A AT GC        AC AG T  ATG         TA TT  C TT   C    TC  AT G  AT   C T AA
```

Section 414

```
                                         (34280) 34280     34290     34300     34310     34320     34330     34340     34350    34362
   K12 Zwischenstück 255.328 - 290.948 (33692) CTGCAGGGTTTTTAGCAGCGAGTGGAGGGTACTCTTGCTCAGCCCCATCAGTTTGCTGATGTCGGTGATCTTAAGCTCGGTGG
SARS-CoV-2 Reference Genome NC_045512.2 (29686) CAG-----TGTGTAACAT----TAGGGAGGACT-TGAAAGAGCCACCACATTTTC---ACCGAGGCCACGCGGAGTACGATCG
                              Consensus (34280) C G     T T TA CA     T G G G ACT T     AGCC C  CA TTT    A    GG  A     AG  CG T G
```

Section 415

```
                                         (34363) 34363  34370     34380     34390     34400     34410     34420     34430        34445
   K12 Zwischenstück 255.328 - 290.948 (33775) CCTGCTCGTTGAACAGGTCGAGGATCTGCAACGCACGTTCAACAGACTGAATAATCGGCAT-AATGCTGGCATGTCCACGCTG
SARS-CoV-2 Reference Genome NC_045512.2 (29756) AGTGTACAGTGAACAATGCTAGGGAGAGCTGCCTATATGGAAGAGCCCTAATGTGTAAAATTAATTTTAGTA-GTGCTATCCC
                              Consensus (34363)   TG  C  TGAACA   C AGG    GC  C  A  T  AA AG C  AAT       AT AAT  T G A GT C   C
```

Section 416

```
                                         (34446) 34446        34460     34470     34480     34490     34500     34510          34528
   K12 Zwischenstück 255.328 - 290.948 (33857) GAATTAAGGCGAAAACGTACCTTTTTCGGG--ATGAAAAATCAATGAAATGGAGCCGGTGTTCTCCCTCTCCCTGTGGGAGAG
SARS-CoV-2 Reference Genome NC_045512.2 (29838) CATGTGATTTTAA----TAGCTTCTTAGGAGAATGACAAAAAAAAAAAAAAAAAAAAAAAAAAAAAAAAA-------------
                              Consensus (34446)  A  T A    AA    TA CTT TT GG   ATGA AAA  AA  AAA   A
```

Section 417

```
                                         (34529) 34529      34540     34550     34560     34570     34580     34590     34600    34611
   K12 Zwischenstück 255.328 - 290.948 (33938) GGCCGGGGTGAGGGCACCAACGCGCAGCGGACCCAATCACTCCCCAAGCGTTGCCATCATCACCGCCTTAATCGTATGCATCC
SARS-CoV-2 Reference Genome NC_045512.2 (29904) -----------------------------------------------------------------------------------
                              Consensus (34529)
```

Section 418

```
                                         (34612) 34612   34620     34630     34640     34650     34660     34670     34680       34694
   K12 Zwischenstück 255.328 - 290.948 (34021) GGTTTTCCGCCTGGTCGAACACGATGCTCGCCGCCGACTCAAACACCTCGTCCGTCACCTCCATCCCGCCGTGCAGATCGAAC
SARS-CoV-2 Reference Genome NC_045512.2 (29904) -----------------------------------------------------------------------------------
                              Consensus (34612)
```

Section 419

```
                                         (34695) 34695 34700     34710     34720     34730     34740     34750     34760           34777
   K12 Zwischenstück 255.328 - 290.948 (34104) TCCTTCGCCATCTGCTTGCCGAGCGTAGTCTGGTCGTCATGGAACGCCGGCAGACAGTGCAGGAACTTCACGTTCGGGTTGTC
SARS-CoV-2 Reference Genome NC_045512.2 (29904) -----------------------------------------------------------------------------------
                              Consensus (34695)
```

Section 420

```
                                         (34778) 34778       34790     34800     34810     34820     34830     34840     34850  34860
   K12 Zwischenstück 255.328 - 290.948 (34187) GGTCAGCGCCATCATCTGCGCGTTCACCTGATACCCGCGCAGCAGCGCAATCCGCTCTGCCCACTTCTCTTTGGCCTCGCCCA
SARS-CoV-2 Reference Genome NC_045512.2 (29904) -----------------------------------------------------------------------------------
                              Consensus (34778)
```

Section 421

```
                                                     (34861) 34861    34870     34880     34890     34900     34910     34920     34930     34943
          K12 Zwischenstück 255.328 - 290.948 (34270) TCGACACCCACACGTCGGTATAGATAAAGTCCGCGCCCTTAACGCCTGCCGCCACGTCTTCCGTCAGAGTAATTTTCCCGCCG
SARS-CoV-2 Reference Genome NC_045512.2 (29904) -----------------------------------------------------------------------------------
                                           Consensus (34861)
```

Section 422

```
                                                     (34944) 34944 34950     34960     34970     34980     34990     35000     35010      35026
          K12 Zwischenstück 255.328 - 290.948 (34353) TGCTTCTCCGCCAGCGCGCTGCACTCCGCCACCAGGCTCTCTTCCGGCCAGCAGGCTTTCGGGGCCAACAGGCGCAGATCCAG
SARS-CoV-2 Reference Genome NC_045512.2 (29904) -----------------------------------------------------------------------------------
                                           Consensus (34944)
```

Section 423

```
                                                     (35027) 35027        35040     35050     35060     35070     35080     35090          35109
          K12 Zwischenstück 255.328 - 290.948 (34436) CCCGGTCAGCGCCGCCGCTTCCAGCATCGAGTTGCCCATGTTGTTGCGCGCATCGCCCGCGTAGACCAGCGTCATCTCGTTAA
SARS-CoV-2 Reference Genome NC_045512.2 (29904) -----------------------------------------------------------------------------------
                                           Consensus (35027)
```

Section 424

```
                                                     (35110) 35110     35120     35130     35140     35150     35160     35170     35180     35192
          K12 Zwischenstück 255.328 - 290.948 (34519) ACGCCTTGCCCGGCAGGTGCTCCTGCATGGTCATCAGGTCCGCCAGCAGCTGGGTCGGGTGGAACTCGTTGGTCAGCCCGTTC
SARS-CoV-2 Reference Genome NC_045512.2 (29904) -----------------------------------------------------------------------------------
                                           Consensus (35110)
```

Section 425

```
                                                     (35193) 35193  35200     35210     35220     35230     35240     35250     35260      35275
          K12 Zwischenstück 255.328 - 290.948 (34602) CACACCGGCACGCCCGCATACTGCGCCAGCGTTTCGACCACTTCCTGGCCGTGACCGCGATACTGAATGCCGTCATACATCCG
SARS-CoV-2 Reference Genome NC_045512.2 (29904) -----------------------------------------------------------------------------------
                                           Consensus (35193)
```

Section 426

```
                                                     (35276) 35276         35290     35300     35310     35320     35330     35340          35358
          K12 Zwischenstück 255.328 - 290.948 (34685) CCCGAGAACCCGCGCGGTGTCCTTAATTGACTCTTTATGCCCAATCTGGCTGCCGCTCGGCCCTAAATAGGTAACGCGCGCGC
SARS-CoV-2 Reference Genome NC_045512.2 (29904) -----------------------------------------------------------------------------------
                                           Consensus (35276)
```

Section 427

```
                                                     (35359) 35359      35370     35380     35390     35400     35410     35420     35430  35441
          K12 Zwischenstück 255.328 - 290.948 (34768) CCTGGTCAAATGCGGCAACTTCGAAAGAGCAACGGGTACGAGTCGAGTCTTTTTCGAAGATGAGCGCGATGTTTTTACCGGTA
SARS-CoV-2 Reference Genome NC_045512.2 (29904) -----------------------------------------------------------------------------------
                                           Consensus (35359)
```

Section 428

```
                                         (35442) 35442   35450     35460     35470     35480     35490     35500     35510     35524
          K12 Zwischenstück 255.328 - 290.948 (34851) AGCTTCTGTACTTCCTTGCCATTTTTTTTATCGGCTTTGAGCTGTGCGGCAAGGGTCAGCAGAGAAGTGAACTGTGCAGGGGT
SARS-CoV-2 Reference Genome NC_045512.2 (29904) -----------------------------------------------------------------------------------
                               Consensus (35442)
```

Section 429

```
                                         (35525) 35525 35530     35540     35550     35560     35570     35580     35590     35607
          K12 Zwischenstück 255.328 - 290.948 (34934) AAAGTCGAGCAGTTTCAGAAAGTGTTTTTTGTATAAATCGGACATTTTATCCTCGCATGGCGAACGCCACTTATTGAATTAAA
SARS-CoV-2 Reference Genome NC_045512.2 (29904) -----------------------------------------------------------------------------------
                               Consensus (35525)
```

Section 430

```
                                         (35608) 35608       35620     35630     35640     35650     35660     35670     35680 35690
          K12 Zwischenstück 255.328 - 290.948 (35017) ATTCACTTTATATGTGTAATTATTCATTTGCAACCCCATTTCACAATTCTTTCTTACAAAGGTGGAGGCAAACCCGTCCGTGT
SARS-CoV-2 Reference Genome NC_045512.2 (29904) -----------------------------------------------------------------------------------
                               Consensus (35608)
```

Section 431

```
                                         (35691) 35691    35700     35710     35720     35730     35740     35750     35760     35773
          K12 Zwischenstück 255.328 - 290.948 (35100) GTGAAAATAATCGTATCTGCCTCCGATTCTCTGCAGAAGCAGAAAGACATTGGATCGAATTCTACAACCAGGTCGAGTCAGAA
SARS-CoV-2 Reference Genome NC_045512.2 (29904) -----------------------------------------------------------------------------------
                               Consensus (35691)
```

Section 432

```
                                         (35774) 35774 35780     35790     35800     35810     35820     35830     35840     35856
          K12 Zwischenstück 255.328 - 290.948 (35183) ATGAGAATGATTGGCCTTCTTTATGATTTTAAGGATTATGCTTCTAAAATGGCGGAGAACATGGCGAGGCTTGCTGCCTTACT
SARS-CoV-2 Reference Genome NC_045512.2 (29904) -----------------------------------------------------------------------------------
                               Consensus (35774)
```

Section 433

```
                                         (35857) 35857        35870     35880     35890     35900     35910     35920       35939
          K12 Zwischenstück 255.328 - 290.948 (35266) TCATTACTTCAGCGGTGATGGAGGCGATATATCTGTTACCGGGTAATGACTCCAACTTATTGATAGTGTTTTATGTTCAGATA
SARS-CoV-2 Reference Genome NC_045512.2 (29904) -----------------------------------------------------------------------------------
                               Consensus (35857)
```

Section 434

```
                                         (35940) 35940     35950     35960     35970     35980     35990     36000     36010 36022
          K12 Zwischenstück 255.328 - 290.948 (35349) ATGCCCGATGACTTTGTCATGCTGCTCCACCGATTTTGAGAACGACAGCGACTTCCGTCCCAGCCGTGCCAGGTGCTGCCTCA
SARS-CoV-2 Reference Genome NC_045512.2 (29904) -----------------------------------------------------------------------------------
                               Consensus (35940)
```

Section 435

```
                                        (36023) 36023  36030     36040     36050     36060     36070     36080     36090          36105
         K12 Zwischenstück 255.328 - 290.948 (35432) GATTCAGGTTATGCCGCTCAATTCGCTGCGTATATCGCTTGCTGATTACGTGCAGCTTTCCCTTCAGGCGGGATTCATACAGC
SARS-CoV-2 Reference Genome NC_045512.2 (29904) -----------------------------------------------------------------------------------
                                     Consensus (36023)
```

Section 436

```
                                        (36106) 36106         36120     36130     36140     36150     36160     36170              36188
         K12 Zwischenstück 255.328 - 290.948 (35515) GGCCAGCCATCCGTCATCCATATCACCACGTCAAAGGGTGACAGCAGGCTCATAAGACGCCCCAGCGTCGCCATAGTGCGTTC
SARS-CoV-2 Reference Genome NC_045512.2 (29904) -----------------------------------------------------------------------------------
                                     Consensus (36106)
```

Section 437

```
                                        (36189) 36189      36200       36212
         K12 Zwischenstück 255.328 - 290.948 (35598) ACCGAATACGTGCGCAACAACCGT
SARS-CoV-2 Reference Genome NC_045512.2 (29904) ------------------------
                                     Consensus (36189)
```
