# Supplementary material for: Ubiquitous Micro-Modular Homologies among Genomes from Viruses to Bacteria to Human Mitochondrial DNA: Platforms for Recombination during Evolution?
Source: Viruses. 2022 Apr 24;14(5):885. doi: 10.3390/v14050885 (PMC9147251; doi:10.3390/v14050885)
Supplement: Supplementary file 1 [file viruses-14-00885-s001.zip › Fig. S4C, SARS-CoV-2 + human mtDNA Region 11.168-29.055nt.pdf]

Section 1

		(1) 1 … 85
SARS-CoV-2 Region 11168 - 29055 nt	(1)	TTTCTCTGTTTGTTTTTGTTACCTTCTCTTGCCACTGTAGCT-TATTTTAATATGGTCTATATGCCTGCTAGTTGGGTGATGCGT
Homo sapiens mitochondrion NC_012920.1	(1)	GATCACAGGTCTATCACCCTA--TTAACCACTCACGGGAGCTCTCCATGCATTTGGTATTTTCGTCTGG-----GGGGTATGCAC

Section 2

		(86) 86 … 170
SARS-CoV-2 Region 11168 - 29055 nt	(85)	ATTATGACATGGTTGGATATGGTTGATACTAGTTTGTCTGGTTTTAAGCTAAAAGACTGTGTTATGTATGCATCAGCTGTAGTGT
Homo sapiens mitochondrion NC_012920.1	(79)	GCGATAGCATTGC--GAGACGCTGGAGCCGGAGCACCCTATGTCGCAG-TATCTGTCTTTG--ATTCCTGCCTCATCC-TATTAT

Section 3

		(171) 171 … 255
SARS-CoV-2 Region 11168 - 29055 nt	(170)	TACTAATCCTTATGACAGCAAGAACTGTGTATGATGATGGTGCTAGGAGAGTGTGGACACTTATGAATGTCTTGACACTCGTTTA
Homo sapiens mitochondrion NC_012920.1	(158)	TTATCGCACCTACGTTCAATATTACAGGCGAACATACT-----TACTAAAGTGTGTTAATTAATTAATG-CTTG----TAGGACA

Section 4

		(256) 256 … 340
SARS-CoV-2 Region 11168 - 29055 nt	(255)	TAAAGTTTATTATGGTAATGCTTTAGATCAAGCCATTTCCATGTGGGC-TCTTATAATCTCTGTTACTTCTAACTACTCAGGTGT
Homo sapiens mitochondrion NC_012920.1	(233)	TAATAATAACAATTG-AATGTCT--GCACAGCCACTTTCCACACAGACATCATAACAAAAAATTTCCACCAAACCCCCC------

Section 5

		(341) 341 … 425
SARS-CoV-2 Region 11168 - 29055 nt	(339)	AGTTACAACTGTCATGTTTTTGGCCAGAGGTATTGTTTTTATGTGTGTTGAGTATTGCCCTATTTTCTTCATAACTGGTAATACA
Homo sapiens mitochondrion NC_012920.1	(309)	--------CTCCCCCGCTTCTGGCCACAGCACTTAAACACATCTCTGCCAAA-----CCCCAAAAACAA-AGAACCC-TAACACC

Section 6

		(426) 426 … 510
SARS-CoV-2 Region 11168 - 29055 nt	(424)	CTTCAGTGTATAATGCTAGTTTATTGTTTCTTAGGCTATTTTTGTACTTGTTACTTTGGCCTCTTTTGTTTACTCAACCGCTACT
Homo sapiens mitochondrion NC_012920.1	(379)	AGCCTAACCAGATTTCAAATTTTATCTTTTGGCGG-----TATGCACTTTTAACAGTCACCCCCCA-----ACTAACACATTATT

Section 7

		(511) 511 … 595
SARS-CoV-2 Region 11168 - 29055 nt	(509)	TTAGACTGACTCTTGG-TGTTTATGATTACTTAGTTTCTACACAGGAGTTTAGATATATGAATTCACAGGGACTACTCCCACCCA
Homo sapiens mitochondrion NC_012920.1	(454)	TTCCCCTCCCACTCCCATACTACTAATCTCATCAATACAACCCCCGCCCATCCTACCCAGCACACACAC--ACCGCTGCTAACCC

```
                                                                                                                      Section 8
                                               (596)  596           610       620       630       640       650       660       670      680
          SARS-CoV-2 Region 11168 - 29055 nt   (593)  AGAATAGCATAGATGCCTTCAAACTCAACATTAAATTGTTGG-GTGTTGGTGGCAAACCTTGTATCAAAGTAGC-CACTGTACA-
Homo sapiens mitochondrion NC_012920.1         (537)  C--ATACCCC-GAACCAACCAAACCCCAAAGACACCCCCCACAGTTTATGTAGCTTACCTCCT--CAAAGCAATACACTGAAAAT
                                                                                                                      Section 9
                                               (681)  681      690       700       710       720       730       740       750            765
          SARS-CoV-2 Region 11168 - 29055 nt   (675)  GTCTAAAATGTCAGATGTAAAGTGCACATCAG-TAGTCTTACTCTCAGTTTTGCAAC-AACTCAGAGTA-GAATCATCATCTAA-
Homo sapiens mitochondrion NC_012920.1         (617)  GTTTAGACGGGCTCACATCACCCCATAAACAAATAGGTTTGGTCCTAGCCTTTCTATTAGCTCTTAGTAAGATTACACATGCAAG
                                                                                                                      Section 10
                                               (766)  766           780       790       800       810       820       830       840      850
          SARS-CoV-2 Region 11168 - 29055 nt   (756)  -ATTGTGGGCTCAATGTGTCCAGTTACACAATGACATTCTCTTAGCTAAAGATACTAC--TGAAGCCTTTGAAAAAATGGTT-TC
Homo sapiens mitochondrion NC_012920.1         (702)  CATCCCCGTTCCAGTGAGTTCACCCTCTAAATCAC---CACG-ATCAAAAGGAACAAGCATCAAGCAC--GCAGCAATGCAGCTC
                                                                                                                      Section 11
                                               (851)  851      860       870       880       890       900       910       920            935
          SARS-CoV-2 Region 11168 - 29055 nt   (837)  ACTACTTTCTGTTTTGCTTTCCATGCAGGGTGCTGTAGACATAAACAAGCTTTGTGAAGAAATGCTGGAC-AACAGGGCAACCTT
Homo sapiens mitochondrion NC_012920.1         (781)  AAAACGCTTAGCCTAGCCACACCCCCACGG-GAAACAGCAGTGATTAACCTTTAGCAATAAACGAAAGTTTAACTAAGCTA---T
                                                                                                                      Section 12
                                               (936)  936           950       960       970       980       990       1000      1010    1020
          SARS-CoV-2 Region 11168 - 29055 nt   (921)  ACAAGCTATAGCCTCAGAGTTTAGTTCCCTTCCATCATATGCAGCTTTTGCTACTGCTCAAGAAGCTTATGAGCAGGCTGTTGCT
Homo sapiens mitochondrion NC_012920.1         (862)  ACTAACCCCAGGGTTGG---TCAATTTCGTGCCAGCCACCGCGG-TCACACGATTAACCCAAGTCAATAGAAGCCGGC-GTAAAG
                                                                                                                      Section 13
                                              (1021)  1021     1030      1040      1050      1060      1070      1080      1090          1105
          SARS-CoV-2 Region 11168 - 29055 nt  (1006)  AATGGTGATTCTGAAGTTGTTCTTAAAAAGTTGAAGAAGTCTTTGAATGTGGCTAAATCTGAATTTGACCGTGATGCAGCCATGC
Homo sapiens mitochondrion NC_012920.1         (942)  AGTGTTTTAGATCACCCCCTCCCCAATAAAGCTAAAACTCACCTGAGT-TGTAAAAAACTCCAGTTGACACAAAAT-----AGAC
                                                                                                                      Section 14
                                              (1106)  1106          1120      1130      1140      1150      1160      1170      1180    1190
          SARS-CoV-2 Region 11168 - 29055 nt  (1091)  AACGTAAGTTGGAA-AAGATGGCTGATCA------AGCTATGACCCAAAT---GTATAAACAGGCTAG-AT-CTGAGGACAAGAG
Homo sapiens mitochondrion NC_012920.1        (1021)  TACGAAAGTGGCTTTAACATATCTGAACACACAATAGCTAAGACCCAAACTGGGATTAGATACCCCACTATGCTTAGCCCTAAAC
```

Section 15

```
                                                 1191     1200      1210      1220      1230      1240      1250      1260          1275
                                         (1191)
SARS-CoV-2 Region 11168 - 29055 nt       (1164)  GGCAAAAGTTACTAGTGCTATGCAGACAATGCTTTTCACTATGCTTAGAAAGTTGGATAATGATGCACTCAACAACATTATCAAC
Homo sapiens mitochondrion NC_012920.1   (1106)  CTCAACAGTTAAATCAACAAAACTG-CTCGCCAGAACACTACGAGCCACAGCTTAAAACTCAAAGGACCTGGCGG--TGCTTCAT
```

Section 16

```
                                                 1276          1290      1300      1310      1320      1330      1340      1350    1360
                                         (1276)
SARS-CoV-2 Region 11168 - 29055 nt       (1249)  AATGCAAGAGATGGTTGTGTTCCTTGAACATAATACCTCTTACAACAGCAGCCAAACTAATGGTTGTCATACCAGACTATAACA
Homo sapiens mitochondrion NC_012920.1   (1188)  ATCCCTCTAGAGGAGCCTGTTCTGTAATCGATAA-ACCCCGATCAACCTCACC--ACCTCTTGCT---CAGCCTATATACCGCCA
```

Section 17

```
                                                 1361     1370      1380      1390      1400      1410      1420      1430          1445
                                         (1361)
SARS-CoV-2 Region 11168 - 29055 nt       (1334)  CATATAAAAATACGTG-TGATGGTACAACATTTACTTATGCATCAGCATTGTGGGAAATCCAACAGGTTGTAGATGCAGATAGTA
Homo sapiens mitochondrion NC_012920.1   (1267)  TCTTCAGCAAACCCTGATGAAGG--CTACA---AAGTAAGCGCAAGTACCCACGTAAAGACGTTAGGTCA-AGGTGTAGCCCATG
```

Section 18

```
                                                 1446          1460      1470      1480      1490      1500      1510      1520    1530
                                         (1446)
SARS-CoV-2 Region 11168 - 29055 nt       (1418)  AAATTGTTCAACTTAGTGAAATTAGTATGGACAATTCACCTAATTTAGCATGGCCTCTTATTGTAACAGCTTTAAGGCCAATTC
Homo sapiens mitochondrion NC_012920.1   (1346)  AGGTGGCAAGAAATGGGCTACATTTTCTACCCCAGAAAACTACGATAGCC---CTTATGAAACTTAAGGGTCGAAGGTGGATTTA
```

Section 19

```
                                                 1531     1540      1550      1560      1570      1580      1590      1600          1615
                                         (1531)
SARS-CoV-2 Region 11168 - 29055 nt       (1503)  TGCTGTCAAATTACAGAATAATGAGCTTAGT---------CC-TGTTGCACTACGACAGATGTCTTGTGCTGCCGGTACTACACA
Homo sapiens mitochondrion NC_012920.1   (1428)  -GCAGT-AAACTA-AGAGTAGAGTGCTTAGTTGAACAGGGCCCTGAAGCGCGTACACACCGCCCGTCACCCTCCTCAAGTATACT
```

Section 20

```
                                                 1616          1630      1640      1650      1660      1670      1680      1690    1700
                                         (1616)
SARS-CoV-2 Region 11168 - 29055 nt       (1578)  AACTGCTTGCACTGATGACAATGCGTTAGCTTACTACAACACAACAAAGGAGGTAGGTTTGTACTTGCACTGTTATCCG-ATTT
Homo sapiens mitochondrion NC_012920.1   (1510)  TCAAAGGACATTTAACTAAAACCCCTACGCATTTATATAGAGGAGACAAGTCG-TAACATGGTAAGTGTACTGGAAAGTGCACTT
```

Section 21

```
                                                 1701     1710      1720      1730      1740      1750      1760      1770          1785
                                         (1701)
SARS-CoV-2 Region 11168 - 29055 nt       (1662)  ACAGGATTTGAAATGGCTAGATTCCCTAAGAGTGATGGAACTGGTACTATCTATACAGAACTGGAACCACCTTGTAGGTT---T
Homo sapiens mitochondrion NC_012920.1   (1594)  GGACGAACCAGAGTG---TAGCTTAACACAAAGC-ACCCAACTTACACT---TAGG-AGATTTCAACTTAACTTGACCGCTCTGA
```

Section 22

```
                                               (1786) 1786          1800      1810      1820      1830      1840      1850      1860      1870
SARS-CoV-2 Region 11168 - 29055 nt             (1744) GTTACAGACACACCTAAAGGTCCTAAAGTGAAGTATTTATACTTTATTAAAGGATTAAACAACCTAAATAGAGGTATGGTACTTG
Homo sapiens mitochondrion NC_012920.1         (1671) GCTAAACCTAGCCCCAAACCCACTCCACCTTACTACC-AGACAACCTTAGCCAAACCATTTACCCAAATAAAG-TATAGGCGATA
```

Section 23

```
                                               (1871) 1871     1880      1890      1900      1910      1920      1930      1940          1955
SARS-CoV-2 Region 11168 - 29055 nt             (1829) GTAGTTTA--GCTGCCACAGTACGTCTACAAGCTGGTAATGCAACAGAAGTGCCTGCCAATTCAACTGTATTATCTTTCTGTGCT
Homo sapiens mitochondrion NC_012920.1         (1754) GAAATTGAAACCTGGCGCAATAGATATAGTACC--GCAAGGGAA-AGATGAAAAATTATAACCAAGCATAATATAGCAAGGACTA
```

Section 24

```
                                               (1956) 1956          1970      1980      1990      2000      2010      2020      2030      2040
SARS-CoV-2 Region 11168 - 29055 nt             (1912) TTTGCTGTAGATGCTGCTAAAGCTTACAAAGATTATCTAGCTAGTGGGGGACAACCAATCACTAATTGTGTTAAGATGTTGTGTA
Homo sapiens mitochondrion NC_012920.1         (1836) ACCCCTATACCTTCTGCATAA--TGA-----ATTAACTAGAAATAACTTTGCAAGGAGAGCCAAA----GCTAAGACCCCCGAAA
```

Section 25

```
                                               (2041) 2041     2050      2060      2070      2080      2090      2100      2110          2125
SARS-CoV-2 Region 11168 - 29055 nt             (1997) CACACACTGGTACTGGTCAGGCAATAACAGTTACACCGGAAGCCAATATGGATCAAGAATCCTTTGGTGGTGCATCGTGTTGTCT
Homo sapiens mitochondrion NC_012920.1         (1910) C-CAGACGAG--CTACCTAAG-AACAGCTAAAAGAGCACACCCGTCTATGTAGCAA-AATAGTGGGAAGATTTATAG-GTAGAGG
```

Section 26

```
                                               (2126) 2126          2140      2150      2160      2170      2180      2190      2200      2210
SARS-CoV-2 Region 11168 - 29055 nt             (2082) GTACTGCCGTTGCCACATAGATCATC-CAAATCCTAAAGGATTT--TGTGACTTAAAAGGTAAGTATGTACAAATACCTACAACT
Homo sapiens mitochondrion NC_012920.1         (1989) CGACAAACCTACCGAGCCTGGTGATAGCTGGTTGTCCAAGATAGAATCTTAGTTCAACTTTAAATTTGCCCACAGAAC--CCTCT
```

Section 27

```
                                               (2211) 2211     2220      2230      2240      2250      2260      2270      2280          2295
SARS-CoV-2 Region 11168 - 29055 nt             (2164) TGTGCTAATGACCCTGTGGGTTTTACACTTAA---AAACACAGTCTGTACCGTCTGCGGTATGTGGAAAGGTTATGGCTGTAGTT
Homo sapiens mitochondrion NC_012920.1         (2072) A-----AATCCCCTTGTAAATTTAACTGTTAGTCCAAAGAGGAACAGCTCTTTGGACACTAGGAAAAAACCTTGTAGAGAGAGTA
```

Section 28

```
                                               (2296) 2296          2310      2320      2330      2340      2350      2360      2370      2380
SARS-CoV-2 Region 11168 - 29055 nt             (2246) GTGATCAACTCCGCGAACCCATGCTTCAGTCAGCTGATGCACAATCGTTTTTAAACGGGTTTGCGGTGTAAGTGCAGCCCGTCTT
Homo sapiens mitochondrion NC_012920.1         (2152) ---AAAAATTTAAC--ACCCATAGT--AGGCCTAAAAGCAGCCACCAATTAAGAAAGCGTTCAAGCTCAACACCCA---CTACCT
```

Section 29

	(2381)	2381 2390 2400 2410 2420 2430 2440 2450 2465
SARS-CoV-2 Region 11168 - 29055 nt	(2331)	ACACCGTGCGGCACAGGCACTAGTACTGATGTCGTATACAGGGCTTTTGAC--ATCTACAATGATAAAGTAG--CTGGTTTTGCT
Homo sapiens mitochondrion NC_012920.1	(2227)	AAAAAATCCCAAACA-----TATAACTGAACTCCTC-ACACCCAATTGGACCAATCTATCACCCTATAGAAGAACTAATGTTAGT

Section 30

	(2466)	2466 2480 2490 2500 2510 2520 2530 2540 2550
SARS-CoV-2 Region 11168 - 29055 nt	(2412)	AAATTCCTAAAAACTAATTGTTGTCGCTTCCAAGAAAAGGACGA-AGATGACAATTTAATTGATTCTTACTTTGTAGTTAAGAGA
Homo sapiens mitochondrion NC_012920.1	(2306)	ATAAG--TAACATGAAAACATTCTC-CTCCGCATAAGCCTGCGTCAGATTAAAAC---ACTGAA-CTGAC-----AATTAACAGC

Section 31

	(2551)	2551 2560 2570 2580 2590 2600 2610 2620 2635
SARS-CoV-2 Region 11168 - 29055 nt	(2496)	CACACTTTCTCTAACTACCAACATGAAGAAACAATTTATAATTTACTTAAGGATTGTCCAGCTGTTGCTAAACATGACTTCTT-T
Homo sapiens mitochondrion NC_012920.1	(2379)	C-CAATATCT---ACAATCAACC------AACAAGTCATTATTACCCTCAC---TGTCAACC-----CAACACAGGCATGCTCAT

Section 32

	(2636)	2636 2650 2660 2670 2680 2690 2700 2710 2720
SARS-CoV-2 Region 11168 - 29055 nt	(2580)	AAGTTTAGAATAGACGGTG-ACATGGTACCA--CATATATCACGTCAACGTCTTACTAAATACACAATGGCAGACCTCGTCTATG
Homo sapiens mitochondrion NC_012920.1	(2446)	AAGGAAAGGTTAAAAAAAGTAAAAGGAACTCGGCAAATCTTACCCCGCCTGTTTACCAAAAACATCACCTCTAGCATCACCAGTA

Section 33

	(2721)	2721 2730 2740 2750 2760 2770 2780 2790 2805
SARS-CoV-2 Region 11168 - 29055 nt	(2662)	CTTTAAGGCATTTTGATGAAGGTAATTGTGACACATTAAAAG----AAATACTTGTCACATACAATTGTTGTGATGATGATTATT
Homo sapiens mitochondrion NC_012920.1	(2531)	-TTAGAGGCACCGCCT----GCCCAGTGACACATGTTTAACGGCCGCGGTACCCTAACCGTGCAAAGGTAGC-ATAATCACTTGT

Section 34

	(2806)	2806 2820 2830 2840 2850 2860 2870 2880 2890
SARS-CoV-2 Region 11168 - 29055 nt	(2743)	TCAATAAAAAGG-ACTGGTATGATTTTGTAGAAAAC---CCAGATATATTACGCGTATACGCCAACTTAGGTGAACGTGTACGCC
Homo sapiens mitochondrion NC_012920.1	(2610)	TCCTTAAATAGGGACCTGTATGAATGGCTCCACGAGGGTTCAGCTGTCTCTTACTTTTAACC--AGTGAAATTGACCTGCCCGTG

Section 35

	(2891)	2891 2900 2910 2920 2930 2940 2950 2960 2975
SARS-CoV-2 Region 11168 - 29055 nt	(2824)	AAGCTTTGTTAAAAACAGTACAATTCTGTGATGCCATGCGAAATGCTGGTATTGTTGGTGTACTGACATTAGATAATCAAGATCT
Homo sapiens mitochondrion NC_012920.1	(2693)	AAGAGGCGGGCATAACACAGCAAGACGAGAAGACCCTATGGAGCTTTAAT-TTATTAATGCAA--ACAGTACCTAACAAA---CC

Section 36

```
                                           (2976) 2976          2990      3000      3010      3020      3030      3040      3050    3060
SARS-CoV-2 Region 11168 - 29055 nt         (2909) CAATGGTAACTGGTATGATTTCGGTGATTTCATACAAACCACGCCAGGTAGTGGAGTTCCTGTTGTAGATTCTTATTATTCATTG
Homo sapiens mitochondrion NC_012920.1     (2772) CACAGGTCC----TAAACTACCAAACCTGCATTAAAAATTTCG----GTTGGGGCGACCTCGGAGCAGAACCCAACCTCCGAGCA
```

Section 37

```
                                           (3061) 3061     3070      3080      3090      3100      3110      3120      3130        3145
SARS-CoV-2 Region 11168 - 29055 nt         (2994) TTAATGCCTATATTAACCTTGACCAGGGCTTTAACTGCAGAGTCACATGTTGACACTGACTTAACAAAGCCTTACATTAAGTGGG
Homo sapiens mitochondrion NC_012920.1     (2849) GTACATGCTA----AGACTTCACCAG-----TCAAAGCGAACTACTAT------ACTCAATTGA--------TCCAATAACTTGA
```

Section 38

```
                                           (3146) 3146          3160      3170      3180      3190      3200      3210      3220    3230
SARS-CoV-2 Region 11168 - 29055 nt         (3079) ATTTGTTAAAATATGACTTCACGGAAGAGAGGTTAAAACTCTTTGACCGTTATTTTAAATATTGGGATCAGACATACCACCCAAA
Homo sapiens mitochondrion NC_012920.1     (2911) CCAACGGAACAAGTTACCCTAGGGATAACAGCGCAATCCTATTCTAGAGTCCATATCAACAATAGGGTT-----TACGACCTCGA
```

Section 39

```
                                           (3231) 3231     3240      3250      3260      3270      3280      3290      3300        3315
SARS-CoV-2 Region 11168 - 29055 nt         (3164) TTGTGTTAACTGTTTGGATGACAGATGCATTCTGCATTGTGCAAACTTTAATGTTTTATTCTCTACAGTGTTCCCACCTACAAGT
Homo sapiens mitochondrion NC_012920.1     (2991) TGTTGG-ATCAG-------GACATCCCGATGGTGCAGCC-GCTA--TTAAAGGTTCGTTTGTTCAACGATTAAAGTCCTACGTGA
```

Section 40

```
                                           (3316) 3316          3330      3340      3350      3360      3370      3380      3390    3400
SARS-CoV-2 Region 11168 - 29055 nt         (3249) TTTGGACCACTAGTGAGAAAAATATTTGTTGATGGTGTTCCATTTGTAGTTTCAACTGGATACCACTT---CAGAGAGCTA-GGT
Homo sapiens mitochondrion NC_012920.1     (3065) TCTGAGTTCAGACCG-GAGTAATCCAGGTCGGTTTCTATCTACNTTCAAATTCCTCCCTGTACGAAAGGACAAGAGAAATAAGGC
```

Section 41

```
                                           (3401) 3401     3410      3420      3430      3440      3450      3460      3470        3485
SARS-CoV-2 Region 11168 - 29055 nt         (3330) GTTGTACATAATCAGGATGTAAACTTACAT---AGC-TCTAGACTTAGTTTTAAGGAATTACTTGTGTATGCTGCTGACCCTGCT
Homo sapiens mitochondrion NC_012920.1     (3149) CTACTTCACAAAGCGCCTTCCCCCGTAAATGATATCATCTCAACTTAGTATTATACCCACACCCACCCAAGAA-CAGGGTTTGTT
```

Section 42

```
                                           (3486) 3486          3500      3510      3520      3530      3540      3550      3560    3570
SARS-CoV-2 Region 11168 - 29055 nt         (3411) ATGCACGCTGCTTCTGGTAATCTATTACTAGATAAACGCACTACGTGCTTTTCAGTAGCTGCACTTACTAACAATGTTGCTTTTC
Homo sapiens mitochondrion NC_012920.1     (3233) AAGATGGCAGAGCCCGGTAATCGCATAAAACTTAAAA-CTTTACAGTCAGAGGTTCAATTCCTCTTCTTAACAACATACCCATGG
```

Section 43

```
                                               (3571) 3571     3580      3590      3600      3610      3620      3630      3640           3655
              SARS-CoV-2 Region 11168 - 29055 nt (3496) AAACTGTCAAACCCGGTAATTTAACAAAGACTTCTA-TGACTTTGCTGTGTCTAAGGGTTTCTTTAAGGAAGGAAGTTCTGTTG
Homo sapiens mitochondrion NC_012920.1 (3317) CCAACCTCCTACTCC-TCATTGTACCCA----TTCTAATCGCAATGGCATTCCTAATGCTTACCG-AACGAA--AAATTCTAGGC
```

Section 44

```
                                               (3656) 3656          3670      3680      3690      3700      3710      3720      3730    3740
              SARS-CoV-2 Region 11168 - 29055 nt (3580) AATTAAACACTTCTTCTTTGCTC-AGGATGGTAATGCTGCTATCAGCGATTATGACT----ACTATCGTTATAATCTAC--CAA
Homo sapiens mitochondrion NC_012920.1 (3394) TATATACA-ACTACGCAAAGGCCCCAACGTTGTAG-GCCCCTACGGGCTACTACAACCCTTCGCTGACGCCATAAAACTCTTCAC
```

Section 45

```
                                               (3741) 3741     3750      3760      3770      3780      3790      3800      3810           3825
              SARS-CoV-2 Region 11168 - 29055 nt (3658) CAATGTG----TGATATCAG--ACAACTACTATTTGTAGTTGAAGTTGTTGATAAGTACTTTGATTGTTACGATGGTGGCTGTAT
Homo sapiens mitochondrion NC_012920.1 (3477) CAAAGAGCCCCTAAAACCCGCCACATCTACCATCACCCTCTACA-TCACCGCCCCGACCTTAGCTC-TCACCATCGCTCTTCTAC
```

Section 46

```
                                               (3826) 3826          3840      3850      3860      3870      3880      3890      3900    3910
              SARS-CoV-2 Region 11168 - 29055 nt (3737) TAATG-CTAACCAAGTCATCGTCAACAACCTAGACAAA-TCAGC--TGGTTTTCCATTTAATAAATGGGGT--AAGGCTAGAC-T
Homo sapiens mitochondrion NC_012920.1 (3560) TATGAACCCCCCTCCCCATACCCAACCCCCTGGTCAACCTCAACCTAGGCCTCCTATTTATTCTAGCCACCTCTAGCCTAGCCGT
```

Section 47

```
                                               (3911) 3911     3920      3930      3940      3950      3960      3970      3980           3995
              SARS-CoV-2 Region 11168 - 29055 nt (3815) TTATT--ATGATTCAATGAGTTATGAGGATCAAGATGCA-CTTTTCGCATATA----CAAAACGTAATGTCATCCCTACTATAAC
Homo sapiens mitochondrion NC_012920.1 (3645) TTACTCAATCCTCTGATCAGGG-TGAGCATCAAACTCAAACTACGCCCTGATCGGCGCACTGCGAGCAGTAGCCCAAACAAT--C
```

Section 48

```
                                               (3996) 3996          4010      4020      4030      4040      4050      4060      4070    4080
              SARS-CoV-2 Region 11168 - 29055 nt (3893) TCAAATGAATCTTAAGTATGCCATTAGTGCAAAGAATAGAGCTCGCACCGTAGCTGGTGTCTCTATCTGTAGTACTATGACCAAT
Homo sapiens mitochondrion NC_012920.1 (3727) TCATATGAAGTCACCCTA-GCCATCATTCTACTATCAACATTACTAA---TAAGTGGCTCCTTTAACCTCTCCACCCTTATCACA
```

Section 49

```
                                               (4081) 4081     4090      4100      4110      4120      4130      4140      4150           4165
              SARS-CoV-2 Region 11168 - 29055 nt (3978) AGACAGTTTCATCAAAAATTATT---GAAATCAATAGCC---GCCACTAGAGGAGCTA---CTGTAGTAATTG-GAACAAGCAAA
Homo sapiens mitochondrion NC_012920.1 (3808) ACACAAGAACACCTCTGATTACTCCTGCCATCATGACCCTTGGCCATAATATGATTTATCTCCACACTAGCAGAGACCAACCGAA
```

Section 50

```
                                          (4166) 4166          4180      4190      4200      4210      4220      4230      4240     4250
SARS-CoV-2 Region 11168 - 29055 nt        (4053) TTCTATG-GTGGTTGCACAACATGTTAAAAACTGTTTATAGTGATGTAGA--AAACCCTCACCTTATGGGTTGGATTATCCTA
Homo sapiens mitochondrion NC_012920.1    (3893) CCCCCTTCGACCTTGCCGAAGGGGAGTCCGAACTAGTCTCAGGCTTCAACATCGAATACGCCGCAGGCCCCTTCGCCCTATTCT-
```

Section 51

```
                                          (4251) 4251    4260      4270      4280      4290      4300      4310      4320           4335
SARS-CoV-2 Region 11168 - 29055 nt        (4135) AATGTGATAGAGCCATGC-CTAACATGCTTAGAATTATGGCC-TCACTTGTTCTTGCTCGCAAACATACAACGTGTTGTAGCTTG
Homo sapiens mitochondrion NC_012920.1    (3977) ----TCATAGCCGAATACACAAACATTATTATAATAAACACCCTCACCACTACAATCTTCCTAGGA-ACAACATAT-GACGCAC-
```

Section 52

```
                                          (4336) 4336          4350      4360      4370      4380      4390      4400      4410     4420
SARS-CoV-2 Region 11168 - 29055 nt        (4218) TCACACCGTTTCTATAGATTAGCTAATGAGTGTGCTCAAGTATTGAGTGAAATGGTCATGTGTGGCGGTTCACTATATGTTAAAC
Homo sapiens mitochondrion NC_012920.1    (4055) TCTCCCCTGAACTCTACACAACATATTT--TGTCACCAAGACCCTACTTCTAACCTCC-------CTGTTCT-TATGAATTCGAA
```

Section 53

```
                                          (4421) 4421    4430      4440      4450      4460      4470      4480      4490           4505
SARS-CoV-2 Region 11168 - 29055 nt        (4303) CAGGTGGAACCTCATCAGGAGATGCCACAACTGCTTATGCTAATAGTGTTTTTAACATTTGTCAAGCTGTCACGGCCAATGTTAA
Homo sapiens mitochondrion NC_012920.1    (4130) CAGCATACCCCCGATTCCGCTACGAC-CAACTCATACACCTCCTA-----TGAAAAACTTCCTACCACTCACC-CTAGCATTA-
```

Section 54

```
                                          (4506) 4506          4520      4530      4540      4550      4560      4570      4580     4590
SARS-CoV-2 Region 11168 - 29055 nt        (4388) TGCACTTTTATCTACTGATGGTAACAAAATTGCCGATAAGTATGTCCGCAATTTACAACACAGACTTTATGAGTGTCTCTATAGA
Homo sapiens mitochondrion NC_012920.1    (4207) ----CTTATATGAT---ATGTCTCCATA----CCCATTACAATCTCCAGCATTCCCC-CTCAAACCTAAGAAATATGTCTGATAA
```

Section 55

```
                                          (4591) 4591    4600      4610      4620      4630      4640      4650      4660           4675
SARS-CoV-2 Region 11168 - 29055 nt        (4473) AATAGAGATGTTGACACAGACTTTGTGAATGAGTTTTACGCATATTTGCGTAAACATTTCTCAATGATGATACTCTCTGACGATG
Homo sapiens mitochondrion NC_012920.1    (4280) AAGAGTTACTTTGATAGAGTAAATAATAGGAGCTTAAACCCCCTTATTTCTAGGACTATGAGAATCGAACCCATCCCTGAGAATC
```

Section 56

```
                                          (4676) 4676          4690      4700      4710      4720      4730      4740      4750     4760
SARS-CoV-2 Region 11168 - 29055 nt        (4558) CTGTTGTGTGTTTCAATAGCACTTATG-CATCTCAAGGTCTAGT--GGCTAGCATAAAGAACTTTAAGTCAGTTCTTTATTATCA
Homo sapiens mitochondrion NC_012920.1    (4365) CAAAATTCT----CCGTGCCACCTATCACACCCCATCCTAAAGTAAGGTCAGC-TAAATAAGCTA---TCGGGCCCATACCCCGA
```

```
                                                                                                                                Section 57
                                          (4761)  4761     4770      4780      4790      4800      4810      4820      4830          4845
SARS-CoV-2 Region 11168 - 29055 nt        (4640)  AAACAATGTTTTTATGTCTGAAGCAAAATGTTGGACTGAGACTGACCTTACTAAAGGACCTCATGAATTTGCTCTCAACATACA
Homo sapiens mitochondrion NC_012920.1    (4442)  AAATGTTGGTTATACCCTTCCCGTACTAATTAATCCC----CTGGCCCAACC------CGTCATCTACTCTAC---CATCTTTGC
                                                                                                                                Section 58
                                          (4846)  4846          4860      4870      4880      4890      4900      4910      4920     4930
SARS-CoV-2 Region 11168 - 29055 nt        (4725)  ATGCTAGTTAAACAGGGTGATGATTATGTGTACCTTCCTTACCCAGATCCATCAAGAATCCTAGGGGCCGGCTGTTTTGTAGATG
Homo sapiens mitochondrion NC_012920.1    (4514)  AGGCACACTCATCACAGCGCTAAGCTCGCACTGATTTTTTACCTGAGTAGGCCTAGAAATAAACATGCTAGCTTTTATTCCAGTT
                                                                                                                                Section 59
                                          (4931)  4931     4940      4950      4960      4970      4980      4990      5000          5015
SARS-CoV-2 Region 11168 - 29055 nt        (4810)  ATATCGTAAAAACAGATGGTACACTTATGATTGAACGGTTCGTGTCTTTAGCTATAGATGCTTACCCACTTACTAAACATCCTAA
Homo sapiens mitochondrion NC_012920.1    (4599)  CTAACCAAAAAAATAA----ACCCTCGT---TCCACAGAAGCTGCCATCAAGTAT------TTCCTCACGCAAGCAACCGCATC-
                                                                                                                                Section 60
                                          (5016)  5016          5030      5040      5050      5060      5070      5080      5090     5100
SARS-CoV-2 Region 11168 - 29055 nt        (4895)  TCAGGAGTATGCTGATGTCTTTCATTTGTACTTACAATACATAAGAAAGCTACATGATGAGTTAACAGGACACATGTTAGACATG
Homo sapiens mitochondrion NC_012920.1    (4670)  -CATAATCCTTCTAATAGCTATCCTCTTCA---ACAATATACTCTCCGGACA-ATGAACCATAACCAATACTACCAATCAATACT
                                                                                                                                Section 61
                                          (5101)  5101     5110      5120      5130      5140      5150      5160      5170          5185
SARS-CoV-2 Region 11168 - 29055 nt        (4980)  TATTCTGT-TATGCTTACTAATGATAACACTTCAAG--GTATTGGAACCTGAGTTTTATGAGGCTATGTACACACCGCATACAG
Homo sapiens mitochondrion NC_012920.1    (4750)  CATCATTAATAATCATAATAGCTATAGCAATAAAACTAGGAATAGCCCCCTTTCACTTCTGAGTCCCAGAGGTTACCCAAGGCAC
                                                                                                                                Section 62
                                          (5186)  5186          5200      5210      5220      5230      5240      5250      5260     5270
SARS-CoV-2 Region 11168 - 29055 nt        (5062)  TCTTACAGGCTGTTGGGCTTGTGTTCTTTGCAATTCACAGACTTCATTAAGATGTGGTGCTTGCATACGTAGACCAT-TCTTAT
Homo sapiens mitochondrion NC_012920.1    (4835)  CCCT-CTGACA--TCCGGCCTGC-TTCTTCTCACATGACAA-------AACTAGCCCCCATCTCAATCATATACCAAATCTCTC
                                                                                                                                Section 63
                                          (5271)  5271     5280      5290      5300      5310      5320      5330      5340          5355
SARS-CoV-2 Region 11168 - 29055 nt        (5146)  GTTGTAAATGCTGTTACGACCATGTCA-TATCAACATCACATAAATTAGTCTTGTCTGTTAATCCGTATGTTTGCAATGCTCCAG
Homo sapiens mitochondrion NC_012920.1    (4909)  CCTCACTAAACGTAAGCCTTCTCCTCACTCTCTCAATCTTATCCATCATAGCAGGCAGTTGAGGTGGATTAAACCAAACC--CAG
```

Section 64

		5356 5370 5380 5390 5400 5410 5420 5430 5440
	(5356)	
SARS-CoV-2 Region 11168 - 29055 nt	(5230)	GTT-GTGATGTCACAG-ATGTGACTCAACTTTACTTAGGAGGTATGAGCTATTATTGTAAATCACATAAACCACCCATTAGTTTT
Homo sapiens mitochondrion NC_012920.1	(4992)	CTACGCAAAATCTTAGCATACTCCTCAA--TTACCCACATAGGATGA---ATAATAGCAGTTCTACCGTACAACCC-TAACATAA

Section 65

		5441 5450 5460 5470 5480 5490 5500 5510 5525
	(5441)	
SARS-CoV-2 Region 11168 - 29055 nt	(5313)	CCATTGTGTGCTAATGGACAAGTTTTTGGTTTATATAAAAATACATGTGTTGGTAGCGATAATGTTACTGACTTTAATGCAATTG
Homo sapiens mitochondrion NC_012920.1	(5071)	CCATTCT----TAATTTA--ACTATTTATATTATCCTAAC-TAC---------TACCGC-ATTCCTACT-ACTCAACTTAAACTC

Section 66

		5526 5540 5550 5560 5570 5580 5590 5600 5610
	(5526)	
SARS-CoV-2 Region 11168 - 29055 nt	(5398)	CAACATGT-GACTGGACAAATGCTGGTGATTACATTTTAGCTAACACCTGTACTGAAAGACTCAAGCTTTTTGCAGCAGAAACGC
Homo sapiens mitochondrion NC_012920.1	(5138)	CAGCACCACGACCCTACTACTATCTCGCACCTGAAACAAGCTAACA--TG-ACTAACACCCTTAA-----TTCCATCC---ACCC

Section 67

		5611 5620 5630 5640 5650 5660 5670 5680 5695
	(5611)	
SARS-CoV-2 Region 11168 - 29055 nt	(5482)	TCAAAGCTACTGAGGAGACATTTAAACTGTCTTATGGTATTGCTACTGTACGTGAAGTGCTGTCTGACAGAGAATTACATCTTTC
Homo sapiens mitochondrion NC_012920.1	(5212)	TCCT--CTCCCTAGGAGGCCTGCCCCCG--CTAACCGGCTTTTTGCCCAA----ATGGGCCAT-TATCGAAGAATT-CACAAAAA

Section 68

		5696 5710 5720 5730 5740 5750 5760 5770 5780
	(5696)	
SARS-CoV-2 Region 11168 - 29055 nt	(5567)	ATGGGAAGTTGGTAAACCTAGACCACCA-CTTAACCGAAATTATG-TCTTTACTGGTTATCGTGTAACTAAAAACAGTAAAGTAC
Homo sapiens mitochondrion NC_012920.1	(5287)	ACAATAGCCTCATCATCC----CCACCATCATAGCCACCATCACCCTCCTTAACCTCTA-CTTCTACCTACGCCTAATCTACTCC

Section 69

		5781 5790 5800 5810 5820 5830 5840 5850 5865
	(5781)	
SARS-CoV-2 Region 11168 - 29055 nt	(5650)	AAAT--AGGAGAGTACACCT---TTG-AAAAAGGTGACTATGGTGATGCTGTTGTTTACCGAGGTACAACAACTTACAAATTAAA
Homo sapiens mitochondrion NC_012920.1	(5367)	ACCTCAATCACACTACTCCCCATATCTAACAACGTAAAAATAAAATGACAGTT-TGAACA----TACAAAACCCACCCCATTCC-

Section 70

		5866 5880 5890 5900 5910 5920 5930 5940 5950
	(5866)	
SARS-CoV-2 Region 11168 - 29055 nt	(5729)	TGTTGGTGATTATTTTGTGCTGACATCACATACAGTAATGCCATTAAGTGCACCTACACTAGTGCCACAAGAGCACTATGTTAGA
Homo sapiens mitochondrion NC_012920.1	(5446)	TCCCCACACTCATC--GCCCTTACCACGC-TAC--TCCTACCTATCTCCCCTTTTATACTAATAATCTTATAGAAATTTAGGTTA

Section 71

```
(5951) 5951     5960      5970      5980      5990      6000      6010      6020          6035
SARS-CoV-2 Region 11168 - 29055 nt          (5814) ATTACTGGCTTATACCAACACTCAATATCTCAGATGAGTTTTCTAGCAATGTTGCAAATTATCAAAAGGTT-GGTATGCAAAAG
Homo sapiens mitochondrion NC_012920.1      (5526) AATACAGACCAAGAGCCTTCA---AAGCCCTCAG-TAAGTT-----GCAATACTTAATTTCTGTAACAGCTAAGGACTGCAAAA-
```

Section 72

```
(6036) 6036          6050      6060      6070      6080      6090      6100      6110    6120
SARS-CoV-2 Region 11168 - 29055 nt          (5898) TATTCTACACTCCAGGGACCACCTGGTACTGGTAAGAGTCATTTTGCTATTGGCCTAGCTCTCTACTACCCTTCTGCTCGCATAG
Homo sapiens mitochondrion NC_012920.1      (5601) ----CCCCACTCT--GCATCAACTGA-ACGCAAATCAGCCACTTTAAT-TAAGCTAAGCCCT-TACTAGACCAATGGG-----AC
```

Section 73

```
(6121) 6121     6130      6140      6150      6160      6170      6180      6190          6205
SARS-CoV-2 Region 11168 - 29055 nt          (5983) TGTATACAGCTTGCTCTCATGCCGCTGTTGATGCACTATG-TGAGAAGGCATTAAAATATTT-----GCCTATAG--ATAAATGT
Homo sapiens mitochondrion NC_012920.1      (5672) TTAAACCCACAAACACTTAGTTAACAGCTAA-GCACCCTAATCAACTGGCTTCAATCTACTTCTCCCGCCGCCGGGAAAAAAGGC
```

Section 74

```
(6206) 6206          6220      6230      6240      6250      6260      6270      6280    6290
SARS-CoV-2 Region 11168 - 29055 nt          (6060) AGTAGAATTATACCTGCACGTGCTCGTGTAGAGTGTTTTGATAAATTCAAAGTGAATTCAACATTAGAACAGTATGTCTTTTGTA
Homo sapiens mitochondrion NC_012920.1      (5756) GGGAGAAGCC--CCGGCAGGTT----TGAAGCTGCTTCTTCGAATTTGCAA-----TTCAATATGAAAA-----TCACCTCGGAG
```

Section 75

```
(6291) 6291     6300      6310      6320      6330      6340      6350      6360          6375
SARS-CoV-2 Region 11168 - 29055 nt          (6145) CTGTAAATGCATTGCCTGAGACGACAGCAGATATAGTTGTCTTTGATGAAATTTCAATGGCCACAAATTATGATTTGAGTGTTGT
Homo sapiens mitochondrion NC_012920.1      (5825) CTGGTAAAAAGAGGCCTAACCCC--------------TGTCTTT----AGATTTACA-GTCCA------ATGCTTCAC------T
```

Section 76

```
(6376) 6376          6390      6400      6410      6420      6430      6440      6450    6460
SARS-CoV-2 Region 11168 - 29055 nt          (6230) CAATGCCAGATTACGTGCTAAGCACT-ATGTGTACATTGGCGACCCTGCTCAATTACCTGCACCACGCACATTGCTAACTAAGGG
Homo sapiens mitochondrion NC_012920.1      (5879) CA--GCCATTTTACCTCACCCCCACTGATGT-----TCGCCGACCGTTGACTATTCTCTACA--AACCACAAAG---AC-ATTGG
```

Section 77

```
(6461) 6461     6470      6480      6490      6500      6510      6520      6530          6545
SARS-CoV-2 Region 11168 - 29055 nt          (6314) CACACTAGAACCAGAATATTTCAATTCAGTGTGTAGACTTATGAAAACTATAGGTCCAGACATGTTCCTCGGAACTTGTCGGCGT
Homo sapiens mitochondrion NC_012920.1      (5951) AACACTATACC-----TATT---ATTCGGCGCATGAGCTG--GAGTCCTA--GGCACAGCTCTAAGCCTC----CTTAT------
```

Section 78

```
                                             (6546) 6546          6560      6570      6580      6590      6600      6610      6620     6630
SARS-CoV-2 Region 11168 - 29055 nt           (6399) TGTCCTGCTGAAATTGTTGACACTGTGAGTGCTTTGGTTTATGATAATAAGCTTAAAGCACATAAAGACAAATCAGCTCAATGCT
Homo sapiens mitochondrion NC_012920.1       (6014) --TCGAGCCGAGCTGGGCCAGCCAGGCAACCTTCTAGGTAACGACCACATCTACAACGT---TATCGTCACAGCCCATGCATTTG
```

Section 79

```
                                             (6631) 6631     6640      6650      6660      6670      6680      6690      6700          6715
SARS-CoV-2 Region 11168 - 29055 nt           (6484) TTAAAATGTTTTATAAGGGTGTTATCACGCATGAT--GTTTCATCTGCAATTAACAGG--CCACAAATAGGCGTGGTAAGAGAAT
Homo sapiens mitochondrion NC_012920.1       (6094) TAATAATCTTCT-TCATAGTAATACCCATCATAATCGGAGGCTTTGGCAACTGACTAGTTCCCCTAATAATCGGTGCCCCCGATA
```

Section 80

```
                                             (6716) 6716          6730      6740      6750      6760      6770      6780      6790     6800
SARS-CoV-2 Region 11168 - 29055 nt           (6565) TCCTTACACGTAACCCTGCTTGGAGAAAAGCTGTCTTTATTTCACCTTATAATTCACAGAATGCTGTAGCCTCAAAGATTTTGGG
Homo sapiens mitochondrion NC_012920.1       (6178) TGG-----CGTTTCCCCGCATAAACAACA------------TAAGCTTCTGACTCTTACCTCCCTCTCTCCTAC----TCCTG--
```

Section 81

```
                                             (6801) 6801     6810      6820      6830      6840      6850      6860      6870          6885
SARS-CoV-2 Region 11168 - 29055 nt           (6650) ACTACCAACTCAAACTGTTGATTCATCACAGGGCTCAGAATATGACTATGTCATATTCACTCAAACCACTGAAACAGCTCACTCT
Homo sapiens mitochondrion NC_012920.1       (6240) -CTCGCATCTGCTATAGTGGAGGCCGGAGCAGGAACAGGTT--GAACA-GTC-TACCCTCCCTTAGCAGGGAACTACTCCCACCC
```

Section 82

```
                                             (6886) 6886          6900      6910      6920      6930      6940      6950      6960     6970
SARS-CoV-2 Region 11168 - 29055 nt           (6735) TGTAATGTAAACAGATTTAATGTTGCTATTACCAGAGCAAAAGTAGGCATACTTTGCATAATGTCTGATAGAGACCTTTATGACA
Homo sapiens mitochondrion NC_012920.1       (6320) TGGAGCCTCCGTAGACCTAACCATCTTCTCCTTACACCTAGCAGGTGTCTCCTCTATCTTAGGGGCCATCAATTTCATCACAACA
```

Section 83

```
                                             (6971) 6971     6980      6990      7000      7010      7020      7030      7040          7055
SARS-CoV-2 Region 11168 - 29055 nt           (6820) AGTTGCAATTTACAAGTCT-TGAAATT-CCACGTAGGAATGTGGCAACTT--TACAAGCTGA---AAATGTAACAGGACTCTTTA
Homo sapiens mitochondrion NC_012920.1       (6405) ATTATCAATATAAAACCCCCTGCCATAACCCAATACCAAACGCCCCTCTTCGTCTGATCCGTCCTAATCACAGCAGTCCTACTTC
```

Section 84

```
                                             (7056) 7056          7070      7080      7090      7100      7110      7120      7130     7140
SARS-CoV-2 Region 11168 - 29055 nt           (6898) AAGATTGTAGTAAGGTAATCACTGGGTTACATC-CTACACAGGCACCTACACACCTCAGTGTTGACACTAAATTCAAAACTGAAG
Homo sapiens mitochondrion NC_012920.1       (6490) TCCTATCTCTCCCAGTCCTAGCTGC-TGGCATCACTATACT---ACTAACAGACCGCAACCTCAACACCACCTTCTTCGACCCCG
```

Section 85

```
                                              (7141) 7141     7150      7160      7170      7180      7190      7200      7210          7225
SARS-CoV-2 Region 11168 - 29055 nt            (6982) GTTTATGTGTTGACATACCTGGCATACCTAAGGACATGACCTATAGAAGACTCA-TCTCTATGATGGGTTTAAAATGAATTATC
Homo sapiens mitochondrion NC_012920.1        (6571) CCGGAGGAGGAGACCC-CATTCTATACCAAC--ACCTATTCT---GATTTTTCGGTCACCCTGAAGTTTATATTCTTATCCTACC
```

Section 86

```
                                              (7226) 7226          7240      7250      7260      7270      7280      7290      7300    7310
SARS-CoV-2 Region 11168 - 29055 nt            (7066) AAGTTAATGGTTACCCTAACATGTTTATCACCCGCGAAGAAGCTATAAGACATGTACGTGCATGGATTGGCTTCGATGTCGAGGG
Homo sapiens mitochondrion NC_012920.1        (6650) AGGCTTCGGAATAATCTCCCATATTG-TAACTTACTACTCCGGAAAAAAAGAACCATTTGGATACATAGGTAT-----------G
```

Section 87

```
                                              (7311) 7311     7320      7330      7340      7350      7360      7370      7380          7395
SARS-CoV-2 Region 11168 - 29055 nt            (7151) GTGTCATGCTACTAGAGAAGCTGTTGGTACCAATTTACCTTTACAGCTAGGTTTTTCTACAGGTGTTAACC---TAGTTGCTGTA
Homo sapiens mitochondrion NC_012920.1        (6723) GTCTGA-GCTA-------------TGATATCAATTGGCTTC-----CTAGGGTTTATCGTGTGAGCACACCATATATTTACAGTA
```

Section 88

```
                                              (7396) 7396          7410      7420      7430      7440      7450      7460      7470    7480
SARS-CoV-2 Region 11168 - 29055 nt            (7233) CCTACAGGTTATGTTGATACACCTA-ATAATACAGATTT--TTCCAGAGTTAGTGCTAACC-ACCGCCTGGAGATCAATTT---
Homo sapiens mitochondrion NC_012920.1        (6789) GGAATAG---ACGTAGACACACGAGCATATTTCACCTCCGCTACCATAATCATCGCTATCCCCACCGGCGTCAAAGTATTTAGCT
```

Section 89

```
                                              (7481) 7481     7490      7500      7510      7520      7530      7540      7550          7565
SARS-CoV-2 Region 11168 - 29055 nt            (7311) -AAACACCTCATACCACTTATGTACAAAGGACTTCCTTGGAATGTAGTGCGTATAAAGATTGTACAAATGTTAAGTGACACACTT
Homo sapiens mitochondrion NC_012920.1        (6871) GACTCGCCACACTCCACGGAAGCAATATGAAATGATCTGC--TGCAGTGCTCTGAGCCCTAGGATTCATCTTTCTTTTCACCGTA
```

Section 90

```
                                              (7566) 7566          7580      7590      7600      7610      7620      7630      7640    7650
SARS-CoV-2 Region 11168 - 29055 nt            (7395) AAAAATCTCTCTGACAGAGTCGTATTTGTCTTATGGGCACATGGCTTTG-AGTTGACATCTATGAAGTATTTTGTGAAAATAGGA
Homo sapiens mitochondrion NC_012920.1        (6954) GGTGGCCTGACTGGCATTGTATTAGCAAACTCAT---CACTAGACATCGTACTACACGAC-ACGTACTACGTTGT---------A
```

Section 91

```
                                              (7651) 7651     7660      7670      7680      7690      7700      7710      7720          7735
SARS-CoV-2 Region 11168 - 29055 nt            (7479) CCTGAGCGCACCTGTTGTCTATGTGATAGACGTGCCACATGCTTTTCCACT--GCTTCAGACACTTATGCCTGTTGGCATCATTC
Homo sapiens mitochondrion NC_012920.1        (7026) GCCCACTTCCACTATGTCCTATCA-ATAGGAGCTGTATTTGCCATCATAGGAGGCTTCATTCACTGATTTCCCCTATTCTCAGGC
```

```
                                                                                                    Section 92
                                             (7736) 7736          7750      7760      7770      7780      7790      7800      7810    7820
           SARS-CoV-2 Region 11168 - 29055 nt (7562) TAT----TGGATTTGAT-TACGTCTATAATCCGTTTATGATTGATGTTCAACAATGGGGTTTACAGGTAACCTACAAAGCAACC
Homo sapiens mitochondrion NC_012920.1 (7110) TACACCCTAGACCAAACCTACGCCAA-AATCCATTTCACTATCATATTCATC---GGCGT---AAATCTAACTTTCTTCCCA--C
                                                                                                    Section 93
                                             (7821) 7821     7830      7840      7850      7860      7870      7880      7890         7905
           SARS-CoV-2 Region 11168 - 29055 nt (7642) ATGATCTGTATTGTCAAGTCCATGGTAATGCACATGTAGCTAGTTGTGATGCAATCATGACTAGGTGTCTAGCTGTCCACGAGTG
Homo sapiens mitochondrion NC_012920.1 (7186) AACA-CTTTCTCGGCCTATCC---GGAATGCCC-GACGTTACTCGGACTACCCCGATGCATA---------CACCACATGAAAC
                                                                                                    Section 94
                                             (7906) 7906          7920      7930      7940      7950      7960      7970      7980    7990
           SARS-CoV-2 Region 11168 - 29055 nt (7727) CTTTGTTAAGCGTGTTGACTGGACTATTGAATATCCTATAATTGGTGATGAACTGAAGATTAATGCGGCTTGTAGAAAGGTTCAA
Homo sapiens mitochondrion NC_012920.1 (7257) ATCCTATCATC-TGTAGGCTC----ATTCATTTCTCTAACAGCAGTAATATTAATAATTTTCATGA---TTTGAGAAGCCTTCG-
                                                                                                    Section 95
                                             (7991) 7991     8000      8010      8020      8030      8040      8050      8060         8075
           SARS-CoV-2 Region 11168 - 29055 nt (7812) CACATGGTTGTTAAAGCTGCATTATTAGCAGACAAATTCCCAGTTCTTCACGACATTGGTAACCCTAAAGCTATTAAGTG--TGT
Homo sapiens mitochondrion NC_012920.1 (7333) --CTTCGAAGCGAAAAGTCC--TAATAGTAGAAGAA--CCC-----TCCA----------TAAACCTGGAGTGACTATATGGATGC
                                                                                                    Section 96
                                             (8076) 8076          8090      8100      8110      8120      8130      8140      8150    8160
           SARS-CoV-2 Region 11168 - 29055 nt (7895) ACCTCAAGCTGATGTAGAATGGAAGTTCTATGATGCACAGCCTTGTAGTGACAAAGCTTATAAAATAGAAGAATTATTCTATTCT
Homo sapiens mitochondrion NC_012920.1 (7398) CCCCCACCCTACCACACATTCGAAGAACCCGTATACATA-------------AAATCT-AGACAAAAAAGGAAGGAATCGAACCC
                                                                                                    Section 97
                                             (8161) 8161     8170      8180      8190      8200      8210      8220      8230         8245
           SARS-CoV-2 Region 11168 - 29055 nt (7980) TATGCCACACATTCTGACAAATTCACAG-ATGG--TGTATGCCTATTTTGGAATTGCAATGTCGATAGATATCCTGCTAATTCCA
Homo sapiens mitochondrion NC_012920.1 (7469) C---CCAAAGCTGGTTTCAAGCCAACCCCATGGCCTCCATGACT-TTTTCAAAAAGGTAT-TAGAAAAACCATTTCATAACTTTG
                                                                                                    Section 98
                                             (8246) 8246          8260      8270      8280      8290      8300      8310      8320    8330
           SARS-CoV-2 Region 11168 - 29055 nt (8062) TTGTTTGTAGATTTGACACTAGAGTGCTATCTAACCTTAACTTGCCTGGTTGTGATGGTGGCAGTTTGTATGTAAATAAACATGC
Homo sapiens mitochondrion NC_012920.1 (7549) TCAAAGTTAAATTATAGGCTAAA-TCCTATATATC-TTAATGGCACATGCAGCGCAAGTAG--GTCTACAAGACGCTACTTCCCC
```

```
                                                        Section 99
                                        (8331) 8331     8340      8350      8360      8370      8380      8390      8400          8415
              SARS-CoV-2 Region 11168 - 29055 nt (8147) ATTCCACACACCAGCTTTTGATAAAAGTGCTTTTGTTAATTTAAAACAATTACCATTTTTCTATTACTCTGAC-AGTCCATGTGA
Homo sapiens mitochondrion NC_012920.1 (7630) TATC-ATAGAAGAGCTTATCAC-------CTTTCATGATCACGCCCTCATAATCATTTTCCTTATCTGCTTCCTAGTCC-TGTAT
                                                        Section 100
                                        (8416) 8416          8430      8440      8450      8460      8470      8480      8490    8500
              SARS-CoV-2 Region 11168 - 29055 nt (8231) GTCTCATGGAAAACAAGTAGTGTCAGATATAGATTATGTACCACTAAAGTCTGCTACGTGTATAACAC-GTTGCAATTTAGGTGG
Homo sapiens mitochondrion NC_012920.1 (7706) GCCCTTTTCCTAACAC------TCACAACAAAACTAACTAATACTAACATCTCAGACGCTCAGGAAATAGAAACCGTCTGAACTA
                                                        Section 101
                                        (8501) 8501     8510      8520      8530      8540      8550      8560      8570          8585
              SARS-CoV-2 Region 11168 - 29055 nt (8315) TGCTGTCTGTAGACATCATGCTAATGAGTACAGATTGTATCTCGATGCTTA--TAACATGATGATCTCAGCTGGCTTTAGC--TT
Homo sapiens mitochondrion NC_012920.1 (7785) TCCTGCCCGC---CATCATCCTA----GTCCTCATCGCCCTCCCATCCCTACGCATCCTTTACATAACAGACGAGGTCAACGATC
                                                        Section 102
                                        (8586) 8586          8600      8610      8620      8630      8640      8650      8660    8670
              SARS-CoV-2 Region 11168 - 29055 nt (8396) GTGGGTTTACAAACAATTTGATACTTATAACCTCTGGAACACTTTTAC--AAGACTTCAGAGTTTAGAAAATGTGGCTTTTAATG
Homo sapiens mitochondrion NC_012920.1 (7863) CCTCCCTTACCATCAAATCAAT--TGGCCACCAATGGTAC---TGAACCTACGAGTACACCGACTACGGCGGACTAATCTTCAAC
                                                        Section 103
                                        (8671) 8671     8680      8690      8700      8710      8720      8730      8740          8755
              SARS-CoV-2 Region 11168 - 29055 nt (8479) TTGTAAATAAGGGACACTTTGATGG-ACAACAGGGTGAAGTACCAGTTTCTATCATTAATAACACTGTT-TACACAAAAGTTGAT
Homo sapiens mitochondrion NC_012920.1 (7943) TCCTACATACTTCCCCCATTATTCCTAGAACCAGGCGACCTGCGACTCCTTGACGTTGACAATCGAGTAGTACTCCCGA-TTGAA
                                                        Section 104
                                        (8756) 8756          8770      8780      8790      8800      8810      8820      8830    8840
              SARS-CoV-2 Region 11168 - 29055 nt (8562) GGTGTTGAT-GTAGAATTGTTTGAAATAAAACAACATTACCTGTTAATGTAGCATTTGAGCTTTGGGCTAAGCGCAACATTAAA
Homo sapiens mitochondrion NC_012920.1 (8027) GCCCCCATTCGTATAATAATTACATCACAAGACGTCTTGCACTCATGAGCTGTCCCCACA--TTAGGCTTAAA---AACAG-ATG
                                                        Section 105
                                        (8841) 8841     8850      8860      8870      8880      8890      8900      8910          8925
              SARS-CoV-2 Region 11168 - 29055 nt (8646) CCAGTACCAGAGGTGAAAATACTCAATAATTTGGGTG-TGGACATTGCTGCTAATACTGTGATCTGGGACTACAAAGAGATGCT
Homo sapiens mitochondrion NC_012920.1 (8106) CAATTCCCGGACGTCTAAAC-CAAACCACTTTCACCGCTACACGACCGGGGGTATACTACGGTCAATG-CTCTGAAATCTGTGGA
```

```
                                                                                                  Section 106
                                           (8926) 8926          8940      8950      8960      8970      8980      8990      9000     9010
          SARS-CoV-2 Region 11168 - 29055 nt (8730) CCAGCACATATATCTACTATTGGTGTTTGTTCTATGACTGACATAGCCAAGAAACC-----AACTGAAACGATTTGTGCACCACT
Homo sapiens mitochondrion NC_012920.1 (8189) GCAAACCACAGTTTCA----TGCCCATCGTCCTA-GAATTAATTCCCCTAAAAATCTTTGAAATAGGGCCCGTATTTACCCTATA
                                                                                                  Section 107
                                           (9011) 9011     9020      9030      9040      9050      9060      9070      9080          9095
          SARS-CoV-2 Region 11168 - 29055 nt (8810) -CACTGTCTTTTTTGATGGTAGAGTTGATGGTCAAGTAGACTTATTTAGAAATGCCCGTAATGGTGTTCTTATTACAGAAGGTAG
Homo sapiens mitochondrion NC_012920.1 (8269) GCACCCCCTCTACCCCCTCTAGAGCCCACTGTAAAG----CTAACTTAGCATTAACCTTTTAAGTTAAAGATTAAGAGAACCAAC
                                                                                                  Section 108
                                           (9096) 9096          9110      9120      9130      9140      9150      9160      9170     9180
          SARS-CoV-2 Region 11168 - 29055 nt (8894) TGTTAAAGGTTTACAACCATCTGTAGGTCCCAAACAAGCTAGT-CTTAATGGAGTCACATTAATTGGAGAAGCCGTAAAAACACA
Homo sapiens mitochondrion NC_012920.1 (8350) ACCTC----TTTACAGTGAAATG-----CCCCAACTAAATACTACCGTATGGCC-CACCATAATTACCCCCATACTCCTTACACT
                                                                                                  Section 109
                                           (9181) 9181     9190      9200      9210      9220      9230      9240      9250          9265
          SARS-CoV-2 Region 11168 - 29055 nt (8978) GTTCAATTATTATAAGAAAGTTGATGGTGTTGTCCAACAATTACCTGAAACTTACTTTACTCAGAGTAGAAATTTACAAGAATTT
Homo sapiens mitochondrion NC_012920.1 (8425) ATTCC----TCATCACCCAACTAAAAATATTAAACACAAACTACCACCTACCTCCCTCAC-CAAAGCCCATAAAAATAAAAAATT
                                                                                                  Section 110
                                           (9266) 9266          9280      9290      9300      9310      9320      9330      9340     9350
          SARS-CoV-2 Region 11168 - 29055 nt (9063) AAACCCAGGAGTCAAATGGAAATTGATTTCTTAGAATTAGCTATGGATGAATTCATTGAACGGTATAAATTAGAAGGCTATGCCT
Homo sapiens mitochondrion NC_012920.1 (8505) ATAACAAACCCTGAGAACCAAAATGAACG---AAAATCTGTTC--GCTTCATTCATTGCCCCCACAATCCTAGGCCTACCCGCCG
                                                                                                  Section 111
                                           (9351) 9351     9360      9370      9380      9390      9400      9410      9420          9435
          SARS-CoV-2 Region 11168 - 29055 nt (9148) TCGAACATATCGTT-TATGGAGATTT-TAGTCATAGTCAGTTAGGTGGTTTACATCTACTGATTGGACTAG-CTAAACGTTTTAA
Homo sapiens mitochondrion NC_012920.1 (8585) CAGTACTGATCATTCTATTTCCCCCTCTATTGATCCCCACCTCCAAATATCTCATCAAC--AACCGACTAATCACCACCCAACAA
                                                                                                  Section 112
                                           (9436) 9436          9450      9460      9470      9480      9490      9500      9510     9520
          SARS-CoV-2 Region 11168 - 29055 nt (9230) GGAATCACCTTTTGAA--TTAGAAGATTTTATTCCTATGGACAGTACAGTTAAAAACTATT-TCATAACAGATGC--GCAAACAG
Homo sapiens mitochondrion NC_012920.1 (8668) TGACTAATCAAACTAACCTCAAAACAAATGATAACCATACACA--ACACTAAAGGACGAACCTGATCTCTTATACTAGTATCCTT
```

```
                                                                                  Section 113
                                         (9521) 9521     9530      9540      9550      9560      9570      9580      9590       9605
          SARS-CoV-2 Region 11168 - 29055 nt (9310) GTTCATCTAAGTGTGTGTGTTCTGTTATTGATTTATTACTTGATGATTTTGTTGAAATAATAAAATCCCAAGATTTATCTGTAGT
 Homo sapiens mitochondrion NC_012920.1 (8751) AATCATTTTTAT-TGCCACAACTAACCTCCTCGGACTCCTGCCTCACTCATTTACACCAACCA---CCCAAC---TATCTATAAA
                                                                                  Section 114
                                         (9606) 9606          9620      9630      9640      9650      9660      9670      9680   9690
          SARS-CoV-2 Region 11168 - 29055 nt (9395) TTCTAAGGTTGTCAAAGTGACTATTGACTAT-ACAGAAATTTCATTTATGCTTTGGTGTAAAGATGGCCATGTAGAAACATTTTA
 Homo sapiens mitochondrion NC_012920.1 (8829) C-CTAGCCATGGCCATCCCCTTATGAGCGGGCACAGTGATTA----TAGGCTTTCGCTCTAAGAT-------TAAAAATGCCCTA
                                                                                  Section 115
                                         (9691) 9691     9700      9710      9720      9730      9740      9750      9760       9775
          SARS-CoV-2 Region 11168 - 29055 nt (9479) CCCAAAAT--TACAATCTAGTCAAGCGTGGCAACCGGGTGTTGCTATGCCTAATCTTTACAAAATGCAAAGAATGCTATTAGAAA
 Homo sapiens mitochondrion NC_012920.1 (8902) GCCCACTTCTTACCA-CAAGGCACACCTAC--ACCCCTTATCCCCATACTAGTTATTATCGAAACCATCAGCCTACTCATTCAAC
                                                                                  Section 116
                                         (9776) 9776          9790      9800      9810      9820      9830      9840      9850   9860
          SARS-CoV-2 Region 11168 - 29055 nt (9562) AGTGTGACCTTCAAAATTATGGTGATAGTGCAACATTACCTAAAGGCA--TAATGATGAATGTCGCAAAATATACTCAACTGTGT
 Homo sapiens mitochondrion NC_012920.1 (8984) CAATAGCCCTGGCCG--TACGCCTAACCGCTAACATTACTGCAGGCCACCTACTCATGCACCTA--ATTGGAAGCGCCACCCTAG
                                                                                  Section 117
                                         (9861) 9861     9870      9880      9890      9900      9910      9920      9930       9945
          SARS-CoV-2 Region 11168 - 29055 nt (9645) CAATATTTAAACACATTAACATTAGCTGTACCCT-ATAATATG-AGAGTTATACATTTTGGTGCTGGTTCT-GATAAAGGAGTTG
 Homo sapiens mitochondrion NC_012920.1 (9065) CAATATC--AAC-CATTAACCTTCCCTCTACACTTATCATCTTCACAATTCTA-ATTCTACTGACTATCCTAGAAATCGCTGTCG
                                                                                  Section 118
                                         (9946) 9946          9960      9970      9980      9990      10000     10010     10020 10030
          SARS-CoV-2 Region 11168 - 29055 nt (9727) CACCAGGTACAGC-TGTTTTAAGACAGTGGTTGCCTACGGGTACGCTGCTTGTCGATTCAGATCTTAATGACTTTGTCTCTGATG
 Homo sapiens mitochondrion NC_012920.1 (9146) CCTTAATCCAAGCCTACGTTTTCACACTTCTAGTAAGCCTCTAC-CTGCACGACAACACA-----TAATGACC----CACCAAT-
                                                                                  Section 119
                                        (10031) 10031    10040     10050     10060     10070     10080     10090     10100     10115
          SARS-CoV-2 Region 11168 - 29055 nt (9811) CAGATTCAACTTTGATTGGTGATTGTGCAACTGTACATACAGCTAATAAATGGGATCTCATTATTAGTGATATGTACGACCCTAA
 Homo sapiens mitochondrion NC_012920.1 (9220) CACATGC--CTATCAT-----ATAGTAAAACCCAGCCCATGACCCCTAACAGGGGCCCTCTCAGCCCTCCTA---ATGACCTCCG
```

Section 120

	(10116)	10116 10130 10140 10150 10160 10170 10180 10190 10200
SARS-CoV-2 Region 11168 - 29055 nt	(9896)	GACTAAAAATGTTACAAAAGAAAATGACTCTAAAGAGGGTTTTTTCACTTACATTTGTGGGTTTATACAACAAAAGCTAGCTCTT
Homo sapiens mitochondrion NC_012920.1	(9295)	GCCTAGCCATGTGATTTCAC----TTCCACTCCATAACGCTC-----CTCATACTAGGCCTACTAACCAACACA--CTAAC-CAT

Section 121

	(10201)	10201 10210 10220 10230 10240 10250 10260 10270 10285
SARS-CoV-2 Region 11168 - 29055 nt	(9981)	GGAGGTTCCGTGGCTATAAAGATAACAGAACATTCTTGGAATGCTGATCTTTATAAGCTCATGGGACACTTCGCATGGTGGACAG
Homo sapiens mitochondrion NC_012920.1	(9368)	ATACCAATGATGGCGCGATG--TAACACGA--------GAAAGCACATACC---AAGGCCACCACACACCACCTGTCCAAAAAGG

Section 122

	(10286)	10286 10300 10310 10320 10330 10340 10350 10360 10370
SARS-CoV-2 Region 11168 - 29055 nt	(10066)	CCTTTGTTAC----TAATGTGAATGCGTCATCATCTGAAGCATTTTTAATTGGATGTAATTATCTTGGCAAACCACGCGAACAAA
Homo sapiens mitochondrion NC_012920.1	(9440)	CCTTCGATACGGGATAATCCTATTTATTA--CCTCAGAAGTTTTTTTCTTCGCAGG-ATTTTTCTGAGC------CTTTTACCAC

Section 123

	(10371)	10371 10380 10390 10400 10410 10420 10430 10440 10455
SARS-CoV-2 Region 11168 - 29055 nt	(10147)	TAGATGGTTATGTCATGCATGCAAATTACATATTTTGGAGGAATACAAATCCAATTCAGTTGTCTTCCTATTCTTTATTTGACAT
Homo sapiens mitochondrion NC_012920.1	(9516)	TCCA-GCCTAGCCCCTACCCCCCAATTA--------GGAGGGC-ACTGGCCCCCAACAGGCATCACCCCG--CTAAATCCCCTAG

Section 124

	(10456)	10456 10470 10480 10490 10500 10510 10520 10530 10540
SARS-CoV-2 Region 11168 - 29055 nt	(10232)	GAGTAAATTTCCCCTTAAATTAAGGGGTACTGCTGTTATGTCTTTAAAAGAAGGTCAAATCAATGATATGATTTTATCTCTTCTT
Homo sapiens mitochondrion NC_012920.1	(9589)	AAGTCCCACTCC---TAAACACATCCGTATTACTCGCATC--------AGGAGT----ATCAATCACCTGAGCTCACCATAGTCT

Section 125

	(10541)	10541 10550 10560 10570 10580 10590 10600 10610 10625
SARS-CoV-2 Region 11168 - 29055 nt	(10317)	AGTAAAGGTAGACTTATAATTAGAGAAAACAACAGAGTTGTTATTTCTAGTGATGTTCTTGTTAACAACTAAACGAACAATGTTT
Homo sapiens mitochondrion NC_012920.1	(9659)	AATAGAAAACAACCGA-AACCAAATAATTCAAGC-ACTGCTTATTACAATTT---TACTGGGTCTCTATTTTACCCTCC------

Section 126

	(10626)	10626 10640 10650 10660 10670 10680 10690 10700 10710
SARS-CoV-2 Region 11168 - 29055 nt	(10402)	GTTTTTCTTGTTTTATTGCCACTAGTCTCTAGTCAGTGTGTTAATCTTACAACCAGAACTCAATTACCCCCTGCATACACTAATT
Homo sapiens mitochondrion NC_012920.1	(9733)	--TACAAGCCTCAGAGTACTTCGAGTCTCCCTTCACCATTTCCGACGG-CATCTACGGCTCAACATTTTTTGTAGCCAC--AGG

Section 127

		10711 … 10795
	(10711)	10711 10720 10730 10740 10750 10760 10770 10780 10795
SARS-CoV-2 Region 11168 - 29055 nt	(10487)	CTTTCAC--ACGTGGTGTTTATTACCCTGACAAAGTTTTCAGATCCTCAGTTTTACATTCAACTCAGGACTTGTTCTTACCTTTC
Homo sapiens mitochondrion NC_012920.1	(9812)	CTTCCACGGACTTCACGTC-ATTAT--TGGCTCAACTTTC-----CTCACTATCTGCTTCATCCGCCAACTAATATTTCACTTTA

Section 128

		10796 … 10880
	(10796)	10796 10810 10820 10830 10840 10850 10860 10870 10880
SARS-CoV-2 Region 11168 - 29055 nt	(10570)	TTTTCCAATGTTACTTGG--TTCCATGCTATA-CATGTCTCTGGGACCAATGGTACTAAGAGGTTTGA-TAACCCTGTCCTAC--
Homo sapiens mitochondrion NC_012920.1	(9889)	CATCCAAACATCACTTTGGCTTCGAAGCCGCCGCCTGATACTGGCATT--TTGTAG-ATGTGGTTTGACTATTTCTGTATGTCTC

Section 129

		10881 … 10965
	(10881)	10881 10890 10900 10910 10920 10930 10940 10950 10965
SARS-CoV-2 Region 11168 - 29055 nt	(10649)	CATTTAATGATGGTGTTTATTTTGCTTCCACTGAGAAGTCTAACATAATAAGAGGCTGGATTTTTGGTACTACTTTAGATTCGAA
Homo sapiens mitochondrion NC_012920.1	(9971)	CATCTATTGATGAGGGT-------CTTACTCT------TTTAGTATAAATAGTACCGTTAACTTCCAATTAACT--AGTTTTGAC

Section 130

		10966 … 11050
	(10966)	10966 10980 10990 11000 11010 11020 11030 11040 11050
SARS-CoV-2 Region 11168 - 29055 nt	(10734)	GACCCAGTCCCTACTTATTGTTAATAACGCTACTAATGTTGTTATTAAAGTCTGTGAATTTCAATTTTGTAATGATCCATTTTTTG
Homo sapiens mitochondrion NC_012920.1	(10041)	AAC--ATTCAAAAAAGAGTAATAAACTTCGCCTTAATTTTAATAATCAACACCCTCCTAGCC---TTACTACTAATA-ATTATTA

Section 131

		11051 … 11135
	(11051)	11051 11060 11070 11080 11090 11100 11110 11120 11135
SARS-CoV-2 Region 11168 - 29055 nt	(10819)	GGTGTTTATTACCACAAAAACAACAAAAGTTGGATGGAAAGTGAGTTCAGAGTTTATTCTAGTGCGAATAATTGCACTTTTGAAT
Homo sapiens mitochondrion NC_012920.1	(10120)	CATTTTGACTACCACAACT----CAACGGCTACATAGAAA---AATCCACCCCTTA--CGAGTGCGGCT--TCGACCCTATATCC

Section 132

		11136 … 11220
	(11136)	11136 11150 11160 11170 11180 11190 11200 11210 11220
SARS-CoV-2 Region 11168 - 29055 nt	(10904)	ATGTCTCTCAGCCTTTTCTTATGGACCTTGAAGGAAAACAGGGTAATTTCAAAAATCTTAGGGAATTTGTGTTTAAGAATATTGA
Homo sapiens mitochondrion NC_012920.1	(10194)	CCCGCCCGCGTCCCTTTCT------CCATAAAATTCTTCTTAGTAGCTATTACCTTCTTAT----TATTTGATCTAGAA-ATTGC

Section 133

		11221 … 11305
	(11221)	11221 11230 11240 11250 11260 11270 11280 11290 11305
SARS-CoV-2 Region 11168 - 29055 nt	(10989)	TGGTTATTTTAAAATATATTCTAAGCACACGCCTATTAATTTAGTGCGTGATCTCCCTCAGGGTTTTTCGGCTTTAGAACCATTG
Homo sapiens mitochondrion NC_012920.1	(10268)	CC--TCCTTTTACCCCTACCATGAGC-----CCTACAAACAACTAACCTGC---CACTAATAGTTATG-----TCATCCCTCTTA

```
                                                                                                   Section 134
                                            (11306) 11306         11320    11330    11340    11350    11360    11370    11380  11390
        SARS-CoV-2 Region 11168 - 29055 nt (11074) GTAGATTTGCCAATAGGTATTAACATCACTAGGTTTCAAACTTTACT-TGCTTTAC-ATAGAAGTTATTTGACTCCTGGTGATTC
Homo sapiens mitochondrion NC_012920.1 (10338) TTA---------------ATCATCATC-CTAGCCCTAAGTCTGGCCTATGAGTGACTACAAAAAGGATTAGACTGAACCGAATTG
                                                                                                   Section 135
                                            (11391) 11391    11400    11410    11420    11430    11440    11450    11460        11475
        SARS-CoV-2 Region 11168 - 29055 nt (11157) TTCTTCAGGTTGGACAGCTGGTGCTGCAGCTTATTATGTGGGTTATCTTCAACCTAGGACTTTTCTATTAAAATATAATGAAAAT
Homo sapiens mitochondrion NC_012920.1 (10407) GTATATAGTTTAAACAA----------AACGAATGATTTCGACT--CATTAAATTATGA------TAATCATATTTACCAAATGC
                                                                                                   Section 136
                                            (11476) 11476         11490    11500    11510    11520    11530    11540    11550  11560
        SARS-CoV-2 Region 11168 - 29055 nt (11242) GGAACCATTACAGATGCTGTAGACTGTGCACTTGACCCTCTCTCAGAAACAAAGTGTACGTTGAAATCCTTCACTGTAGAAAAAG
Homo sapiens mitochondrion NC_012920.1 (10474) CCCTCATTTACATAAATATTATACTA-GCATTT-ACCATCTCACTTCTAGGAA---TACTAGTATATCGCTCACACCTCATATCC
                                                                                                   Section 137
                                            (11561) 11561    11570    11580    11590    11600    11610    11620    11630        11645
        SARS-CoV-2 Region 11168 - 29055 nt (11327) GAATCTATCAAACTTCTAACTTTAGAGTCCAACCAACAGAATCTATTGTTAGATTTCCTAATATTACAAACTTGTGCCCTTTTGG
Homo sapiens mitochondrion NC_012920.1 (10554) TCC-CTACTATGCCTAGAAGGAATAATACTATCGCTGTTCAT-TATAGCTAC-TCTCATAACCCTCAACACCCACTCCCTCTTAG
                                                                                                   Section 138
                                            (11646) 11646         11660    11670    11680    11690    11700    11710    11720  11730
        SARS-CoV-2 Region 11168 - 29055 nt (11412) TGAAGTTTTTAACGCCACCAGATTTGCATCTGTTTATGCTTGGAACAGGAAGAGAATCAGCAACTGTGTTGCTGATTATTCTGTC
Homo sapiens mitochondrion NC_012920.1 (10636) CCAATATTGT---GCCT----ATTGCCATAC---TAGTCTTTG--CCGCCTGCGAAGCAGCG---GTG--------------GGC
                                                                                                   Section 139
                                            (11731) 11731    11740    11750    11760    11770    11780    11790    11800        11815
        SARS-CoV-2 Region 11168 - 29055 nt (11497) CTATATAATTCCGCATCATTTTCCACTTTTAAGTGTTATGGAGTGTCTCCTACTAAATTAAATGATCTCTGCTTTACTAATGTCT
Homo sapiens mitochondrion NC_012920.1 (10692) CTAG------CCCTACTAGTCTCAATCTCCAACACATATGGCCTAGA--CTACGTACATAACCTAAACCTACT---CCAATG-CT
                                                                                                   Section 140
                                            (11816) 11816         11830    11840    11850    11860    11870    11880    11890  11900
        SARS-CoV-2 Region 11168 - 29055 nt (11582) ATGCAGATTCATTTGTAATTAGAGGTGATGAAGTCAGACAAATCGCTCCAGGGCAAACTGGAAAGATTGCTGATTATAATTATAA
Homo sapiens mitochondrion NC_012920.1 (10765) AAAACTAATCGTCCCAACAATTATATTACTACCACTGACATGACTTTCCAAAAAACAC-----------------ATAATT-TGA
```

```
                                                    Section 141
                                         (11901) 11901    11910     11920     11930     11940     11950     11960     11970          11985
SARS-CoV-2 Region 11168 - 29055 nt       (11667) ATTACCAGATGATTTTACAGGCTGCGTTATAGCTTGGAATTCTAACAATCTTGATTCTAAGGTTGGTGGTAATTATAATTACCTG
Homo sapiens mitochondrion NC_012920.1   (10832) ATCAACACAACCACCCACAGCCT--------------AATTATTAGCATCATCCCTCTACTATTTTT--TAACCA-AATCAACA-
```

```
                                                    Section 142
                                         (11986) 11986            12000     12010     12020     12030     12040     12050     12060  12070
SARS-CoV-2 Region 11168 - 29055 nt       (11752) TATAGATTGTTTAGGAAGTCTAATCTCAAACCTTTTGAGAGAGATATTTCAACTGAAATCTATCAGGCCGGTAGCACACCTTGTA
Homo sapiens mitochondrion NC_012920.1   (10899) -ACAACCTATTTAG-----CTGTTCCCCAACCTTTTCC---------TCCGACCCC---CTAACAACCC-----CCCTCCTAATA
```

```
                                                    Section 143
                                         (12071) 12071    12080     12090     12100     12110     12120     12130     12140          12155
SARS-CoV-2 Region 11168 - 29055 nt       (11837) ATGGTGTTGAAGGTTTTAATTGTTACTTTCCTTTACAATCATATGGTTTCCAAC-CCACTAATGGTGTTGGTTACCAACCATACA
Homo sapiens mitochondrion NC_012920.1   (10961) CT-------AACTACCTGACTCCTAC---CCCTCACAATCATG-GCAAGCCAACGCCACTTATCCAGTG----AACCACTATCAC
```

```
                                                    Section 144
                                         (12156) 12156            12170     12180     12190     12200     12210     12220     12230  12240
SARS-CoV-2 Region 11168 - 29055 nt       (11921) GAGTAGTAGTACTTTCTTTTGAACTTCTACATGCACCAGCAACTGTTTGTGGACCTAAAAGTCTACTAATTTGGTTAAAAACAA
Homo sapiens mitochondrion NC_012920.1   (11031) GAAAAAAACT-CTACCTCTCTA--TACTA-ATCTCCCTACAAATCTCCTT--AATTATAACATTCACAGCCACAGAACTAATCAT
```

```
                                                    Section 145
                                         (12241) 12241    12250     12260     12270     12280     12290     12300     12310          12325
SARS-CoV-2 Region 11168 - 29055 nt       (12006) ATGTGTCAATTTCAACTTCAATGGTTTAACAGGCACAGGTGTTCTTACTGAGTCTAACAAAAAGTTTCTGCCTTTCCAACAATTT
Homo sapiens mitochondrion NC_012920.1   (11110) ATTTT---ATATCTTCTTCGA------AAC---CACACTTATCCCCACCTTGGCTATCA-------TCACCCGATGAGGCAACCA
```

```
                                                    Section 146
                                         (12326) 12326            12340     12350     12360     12370     12380     12390     12400  12410
SARS-CoV-2 Region 11168 - 29055 nt       (12091) GGCAGAGACATTGCTGACACTACTGATGCTGTCCGTGATCCACAGACACTTGAGATTCTTGACATTACACCATGTTCTTTTGGTG
Homo sapiens mitochondrion NC_012920.1   (11176) GCCAGA---ACGCCTGAACGCAGGCACATACTTCCTATTCTACACCCTAGTAGGCTCCCTTCCCCTACTCATCGCACTAAT----
```

```
                                                    Section 147
                                         (12411) 12411    12420     12430     12440     12450     12460     12470     12480          12495
SARS-CoV-2 Region 11168 - 29055 nt       (12176) GTGTCAGTGTTATAACACCAGGAACAAATACTTCTAACCAGGTTGCTGTTCTTTATCAGGATGTTAACTGCACAGAAGTCCCTGT
Homo sapiens mitochondrion NC_012920.1   (11254) -TTACACTCACAACACCCTAGGCTCA-------CTAAACATTCTACTACTCACTCTCA---------CTGCCCAAGAA-CTATCA
```

```
                                                                                                                Section 148
                                          (12496) 12496         12510     12520     12530     12540     12550     12560     12570   12580
          SARS-CoV-2 Region 11168 - 29055 nt (12261) TGCTATTCATGCAGATCAACTTACTCCTACTTGGCGTGTTTATTCTACAGGTTCTAATGTTTTCAAACACGTGCAGGCTGTTTA
Homo sapiens mitochondrion NC_012920.1 (11321) AACTCCTGAGCCAA--CAACTTAATATGACTAGC-----TTACACAATAGCTTTTATAGT-----AAAGA--TAC---CTCTTTA
                                                                                                                Section 149
                                          (12581) 12581    12590     12600     12610     12620     12630     12640     12650          12665
          SARS-CoV-2 Region 11168 - 29055 nt (12346) ATAGGGGCTGAACATGTCAACAACTCATATGAGTGT-GACATACCCATTGGTGCAGGTATATGCGCTAGTTATCAGACTCAGACT
Homo sapiens mitochondrion NC_012920.1 (11389) C---GGACTCCACTTATGACTCCCTAAAGCCCATGTCGAAGCCCCCATCGCTG--GGTCAATAG--TACTTGCCGCAGT---ACT
                                                                                                                Section 150
                                          (12666) 12666         12680     12690     12700     12710     12720     12730     12740   12750
          SARS-CoV-2 Region 11168 - 29055 nt (12430) AATTCTCCTCGGCGGGCACGTAGTGTAGCTAGTCAATCCATCATTGCCTACACTATGTCACTTGGTGCAGAAAATTCAGTTGCTT
Homo sapiens mitochondrion NC_012920.1 (11464) CTTAAAACTAGGCGGCTATG--GTATAATACGCCTCACACTCATTCTCAACCCCCTGACAAAACACATAGCCTACCCC-TTCCTT
                                                                                                                Section 151
                                          (12751) 12751    12760     12770     12780     12790     12800     12810     12820          12835
          SARS-CoV-2 Region 11168 - 29055 nt (12515) ACTCTAATAACTCTATT-GCCATACCCACAAATTTTACTATTAGTGTTACCACAGAAATTC-TACCAGTGTCTATGACCAAGACA
Homo sapiens mitochondrion NC_012920.1 (11546) G---TACTATCCCTATGAGGCATAATTATAACAAGCTCCATCTGCCT-ACGACAAACAGACCTAAAATCGCTCATTGCATACTCT
                                                                                                                Section 152
                                          (12836) 12836         12850     12860     12870     12880     12890     12900     12910   12920
          SARS-CoV-2 Region 11168 - 29055 nt (12598) TCAGTAGATTGTACAATGTACATTTGTGGTGAT--TCAA-CTGAATGCAGCAATCTTTTGTTGCAATATGGCAGTTTTTGTACAC
Homo sapiens mitochondrion NC_012920.1 (11627) TCA----ATCAGCCACATAGCCCTCGTAGTAACAGCCATTCTCATCCAAACCCCCTGAAGCTTCACCGGCGCAGTCATTCT-CAT
                                                                                                                Section 153
                                          (12921) 12921    12930     12940     12950     12960     12970     12980     12990          13005
          SARS-CoV-2 Region 11168 - 29055 nt (12680) AATTAAAC-CGTGCTTTAACTGGAATAGCTGTTGAACAAGACAAAAACACCCAAGAAGTTTTTGCACAAGTCAAACAAATTTACA
Homo sapiens mitochondrion NC_012920.1 (11707) AATCGCCCACGGGCTTACATCCTCATTACTATTCTGCCTAGCAAACTCAAACTACGAACGCACTCACAGTCGCATCATAAT--CC
                                                                                                                Section 154
                                          (13006) 13006         13020     13030     13040     13050     13060     13070     13080   13090
          SARS-CoV-2 Region 11168 - 29055 nt (12764) AAACACCACCAATTAAAGATTTTGGTGGTTTTAA----TTTTTCACAAATATTACCAGATCCATCAAAACCAAGCAAGAGGTCAT
Homo sapiens mitochondrion NC_012920.1 (11790) TCTCTCAAGGACTTCAA-ACTCTACTCCCACTAATAGCTTTTTGATGACTTCTAGCA-AGCCTCGCTAACCTCGCCTTACCCCCC
```

Section 155

```
                                        13091    13100           13110           13120           13130           13140           13150           13160          13175
                                (13091)
SARS-CoV-2 Region 11168 - 29055 nt (12845) T-TATTGAAGATCTACTTTTCAACAAAGTGACACTTGCAGATGCTGGCTTCATCAAA---CAATATGGTGATTGCCTTGGTGATA
Homo sapiens mitochondrion NC_012920.1 (11873) ACTATTAACCTACTGGGAGAACTCTCTGTGCTAGTAACC-ACGTTCTCCTGATCAAATATCACTCTCCTACTTACAGGACTCAAC
```

Section 156

```
                                        13176         13190           13200           13210           13220           13230           13240           13250   13260
                                (13176)
SARS-CoV-2 Region 11168 - 29055 nt (12926) TTGCTGCT-AGAGACCTCATTTGTGCACAAAAGTTTAACGGCCTTACTGTTTTGCCACCTTTGCTCACAGATGAAATGATTGCTC
Homo sapiens mitochondrion NC_012920.1 (11957) ATACTAGTCACAGCCCT-ATACTCCCTCTACATATTTACCACAACACAATGGGGCTCACTCACCCACCACATTAACAACAT----
```

Section 157

```
                                        13261    13270           13280           13290           13300           13310           13320           13330          13345
                                (13261)
SARS-CoV-2 Region 11168 - 29055 nt (13010) AATACACTTCTGCACTGTTAGCGGGTACAATCACTTCTGGTTGGACCTTTGGTGCAGG-TGCTGCATTACAAATACCATTTGCTA
Homo sapiens mitochondrion NC_012920.1 (12037) AAAACCCTCATTCACAC---GAGAAAACACCCTCATGTTCATACACCTATCCCCCATTCTCCTCCTATCCCTCAACCCCGACATC
```

Section 158

```
                                        13346         13360           13370           13380           13390           13400           13410           13420   13430
                                (13346)
SARS-CoV-2 Region 11168 - 29055 nt (13094) TGCAAATGGCTTATAGGTT-TAATGGTA-TTGGAGTTACACAGAATGTTCTCTATGAGAACCAAAAATTGATTGCCAACCAATTT
Homo sapiens mitochondrion NC_012920.1 (12119) ATTACCGGGTTTTCCTCTTGTAAATATAGTTTAACCAAAACATCAGATTGTGAATCTGA--CAACAGAGGCTTACGACCCC--TT
```

Section 159

```
                                        13431    13440           13450           13460           13470           13480           13490           13500          13515
                                (13431)
SARS-CoV-2 Region 11168 - 29055 nt (13177) AATAGTGCTATTGGCAAAATTCAAGACTCACTTTCTTCCACAGCAAGTGCACTTGGAAAACTTCAAGATGTGGTCAACCAA-AAT
Homo sapiens mitochondrion NC_012920.1 (12200) ATT-----TACCGAGAAAGCTCA-----CAAGAACTGCTAACTCATGCCCCCATGTCTAACAACATGGCTTTCTCAACTTTTAAA
```

Section 160

```
                                        13516         13530           13540           13550           13560           13570           13580           13590   13600
                                (13516)
SARS-CoV-2 Region 11168 - 29055 nt (13261) GCACAAGCTTTAAACACGCTTGTTAAACAACTTAGCTCCAATTTTGGTGCAATTTCAAGTGTTTTAAATGATATCCTTTCACGT-
Homo sapiens mitochondrion NC_012920.1 (12275) GGATAACAGCTATCCATTGGTCTTAGGCCCCAAA-----AATTTTGGTGCAACTCCAAATAA---AAGTAATAACCATGCACACT
```

Section 161

```
                                        13601    13610           13620           13630           13640           13650           13660           13670          13685
                                (13601)
SARS-CoV-2 Region 11168 - 29055 nt (13345) -CT-TGACAAAGTTGAGGCTGAAGTGCA-AATTGATAGGTTGATCACAGGCAGACTTCAAAGTTTGCAGACATATGTGACTCAAC
Homo sapiens mitochondrion NC_012920.1 (12352) ACTATAACCACCCTAACCCTGACTTCCCTAATTCCCCCCATCCTTACCACCCTCGTT-AACCCTAACAAAAAAAACTCATACCCC
```

```
                                                                                                     Section 162
                                           (13686) 13686         13700     13710     13720     13730     13740     13750     13760    13770
              SARS-CoV-2 Region 11168 - 29055 nt (13427) AATTAATTAGA----GCTGCAGAAATCAGAGCTTCTGCTAATCTTGCTGCTACTAAAATGTCAGAGTGTGTACTTGGACAATCAA
Homo sapiens mitochondrion NC_012920.1 (12436) CATTATGTAAAATCCATTGTCGCATCCACCTTTATTATCAGTCTCTTCCCCACAACAATATTCATGTGCCTA----GAC---CAA
                                                                                                     Section 163
                                           (13771) 13771    13780     13790     13800     13810     13820     13830     13840          13855
              SARS-CoV-2 Region 11168 - 29055 nt (13508) AAAGAGTTGATTTTTGTGGAAAGGCTATCATCTTATGTCCTTCCCTCAGTCAGCACCTCATGGTGTAGTCTTCTTGCATGTGAC
Homo sapiens mitochondrion NC_012920.1 (12514) GAAG---TTATTATC-TCGAACTGACACTGAGC-------CACAACCCAAACAACCCAGCTCTCCCTAAGCTTCAAACTAG--AC
                                                                                                     Section 164
                                           (13856) 13856         13870     13880     13890     13900     13910     13920     13930    13940
              SARS-CoV-2 Region 11168 - 29055 nt (13593) TTATGTCCCTGCACAAGAAA-AGAACTTCACAACTGCTCCTGCCATTTGTCATGATGGAAAAGCACACTTTCCTCGTGAAGGTGT
Homo sapiens mitochondrion NC_012920.1 (12586) TACTT---CTCCATAATATTCATCCCTGTAGCATTGTTCGTTACATGGTCCATCATAGAATT-CTCACTGTGATATATAAA----
                                                                                                     Section 165
                                           (13941) 13941    13950     13960     13970     13980     13990     14000     14010          14025
              SARS-CoV-2 Region 11168 - 29055 nt (13677) CTTTGTTTCAAATGGCACACACTGGTTTGTAACACAAAGGAATTT--TTATGAACC-ACAAATCATTACTACAGACAACA--CAT
Homo sapiens mitochondrion NC_012920.1 (12663) CTCAGACCCAAACATTAATCAGTTCTTCAAATATCTACTCATCTTCCTAATTACCATACTAATCTTAGTTACCGCTAACAACCTA
                                                                                                     Section 166
                                           (14026) 14026         14040     14050     14060     14070     14080     14090     14100    14110
              SARS-CoV-2 Region 11168 - 29055 nt (13757) TTGTGTCTGGTAA-CTGTGATGTTGTAATAGGAATTGTCAACAACACAGTTTATGATCCTTTGCAACCTGAATTAGACTCATTCA
Homo sapiens mitochondrion NC_012920.1 (12748) TTCCAACTGTTCATCGGCTGAGAGGGCGTAGGAATTATATCCTTC----TTGCTCATCAGTTGA----TGA-TACGCCCGAGCAG
                                                                                                     Section 167
                                           (14111) 14111    14120     14130     14140     14150     14160     14170     14180          14195
              SARS-CoV-2 Region 11168 - 29055 nt (13841) AGGAGGAGTTAGATAAATATTTTAAGAATCATACATCACCAGAT-GTTGATTTAGGTGACATCTCTGGCATTAATGCTTCAGTTG
Homo sapiens mitochondrion NC_012920.1 (12824) ATGCCAACACAGC-AGCCATTCAAGCAATCCTATACAACCGTATCGGCGATATCGGTTTCATC-CTCGCCTTA--GCATGATTTA
                                                                                                     Section 168
                                           (14196) 14196         14210     14220     14230     14240     14250     14260     14270    14280
              SARS-CoV-2 Region 11168 - 29055 nt (13925) TAA-ACATTCAAAAAGAAATTGACCGCCTCAATGAGGTTGCCAAGAATTTAAATGAATCTCTCATCGATCTCCAAGAACTTGGAA
Homo sapiens mitochondrion NC_012920.1 (12905) TCCTACACTCCAACTCATGAGACCCACAACAAATAGCCCTTCTAAACGCTAATCCAAGC-CTCACCCCACTACTAGGCCTCCTCC
```

Section 169

(14281) 14281 14290 14300 14310 14320 14330 14340 14350 14365
SARS-CoV-2 Region 11168 - 29055 nt (14009) AGTATGAGCAGTATATAAAATGGCCATGGTACATTTGGCTAGGTTTTATAGCTGGCTTGATTGCCATAGTAATGGTGACAATTAT
Homo sapiens mitochondrion NC_012920.1 (12989) --TAGCAGCAGCAGGCAAATCAGCCCAATTAGGTCT--CCACCCCTGA---CTCCCCTCA--GCCATAGAAGGCCCCACCCCAGT

Section 170

(14366) 14366 14380 14390 14400 14410 14420 14430 14440 14450
SARS-CoV-2 Region 11168 - 29055 nt (14094) GCTTTGCTGTATGAC---CAGTTGCTGTAGTTGTCTCAAGGGCTGTTGTTCTTGTGGATCCTGCTGCAAATTTGATGAAGACGAC
Homo sapiens mitochondrion NC_012920.1 (13065) -CTCAGCCCTACTCCACTCAAGCACTATAGTTGTAGCAGGAA----TCTTCTTACTCATCC-GCTTCCACCCCCTAGCAGAAAA-

Section 171

(14451) 14451 14460 14470 14480 14490 14500 14510 14520 14535
SARS-CoV-2 Region 11168 - 29055 nt (14176) TCTGAGCCAGTGCTCAAGGAGTCAAATTACA-TTACAC---ATAAACGAACT-TATGGATTTGTTTATGAGAATCTTCACAATT
Homo sapiens mitochondrion NC_012920.1 (13143) --TAGCCCACTAATCCAAACTCTAACACTATGCTTAGGCGCTATCACCACTCTGTTCGCAGCAGTCTGCGCCCTTACACAAAATG

Section 172

(14536) 14536 14550 14560 14570 14580 14590 14600 14610 14620
SARS-CoV-2 Region 11168 - 29055 nt (14256) GGAACTGTAACTTTGAAGCAAGGTGAAAT-CAAGGATGCTA---CTCCTTCAGATTTTGTTCGCGCTACTGCAACGATACCGATA
Homo sapiens mitochondrion NC_012920.1 (13226) ACATCAAAAAAATCGTAGCCTTCTCCACTTCAAGTCAACTAGGACTCATAATAGTTACAATCGGCATCAACCAACCACACCTAG-

Section 173

(14621) 14621 14630 14640 14650 14660 14670 14680 14690 14705
SARS-CoV-2 Region 11168 - 29055 nt (14337) CAAGCCT-CACTCCCTTTCGGATGGCTTATTGTTGGCGTTGCACTTCTTGCTGTTTTTCAGAGCGCT--TCCAAAATCATAACCC
Homo sapiens mitochondrion NC_012920.1 (13310) CATTCCTGCACATCTGTACCCACGCCTTCTTCAAAGCCATAC---TATTTATGTGCTCCGGGTCCATCATCCACAACCTTAACAA

Section 174

(14706) 14706 14720 14730 14740 14750 14760 14770 14780 14790
SARS-CoV-2 Region 11168 - 29055 nt (14419) TCAAAAAGAGAT--GGCAACTAGCA---CTCTCCAAGGGTGTTCACTTTGTTTGCAACTTGCTGTTG-TTGTTTG--TAACAGTT
Homo sapiens mitochondrion NC_012920.1 (13392) TGAACAAGATATTCGAAAAATAGGAGGACTACTCAAAACCATACCTCTCACTTCAACCTCCCTCACCATTGGCAGCCTAGCATTA

Section 175

(14791) 14791 14800 14810 14820 14830 14840 14850 14860 14875
SARS-CoV-2 Region 11168 - 29055 nt (14496) TACTCACACCTTTTGCTC----GTTGCTGCTGGCCTTGAAGCCCCTTTTCTCTATCTTTATGCTTTAGTCTACTTCTTGCAGAGT
Homo sapiens mitochondrion NC_012920.1 (13477) GCAGGAATACCTTTCCTCACAGGTTTCTACT--CCAAAGACCACATCATCGAAACCGCAAACATATCATACACAAACGCCTGAGC

```
                                                                                                              Section 176
                                              (14876) 14876         14890     14900     14910     14920     14930     14940     14950   14960
               SARS-CoV-2 Region 11168 - 29055 nt (14577) ATAAACTTGTAAGAATAATAATGAGGCTTGGCTTTGCTGGAAATGCCGTTCCAAAAACCCAT--TACTTTATGATGCCAACTA
Homo sapiens mitochondrion NC_012920.1 (13560) CCTATCTAT-TACTCTCATCGCTACCTCCCTGACAA-GC-GCCTATAGCACTCGAATAATTCTTCTCACCCTAACAGGTCAACCT
                                                                                                              Section 177
                                              (14961) 14961    14970     14980     14990     15000     15010     15020     15030          15045
               SARS-CoV-2 Region 11168 - 29055 nt (14660) TTTTCTTTGCTGGCATACTAATTGTTACGACTATTGTATACCTTACAATAGTGTAACTTCTTCAAT---TGTCATTACTTCAGGT
Homo sapiens mitochondrion NC_012920.1 (13642) CGCT-TCCCCACCCTTACTAACATTAACGAAAATA----ACCCCACCCTACTAAACCCCATTAAACGCCTGGCAGCCGGAAGCCT
                                                                                                              Section 178
                                              (15046) 15046         15060     15070     15080     15090     15100     15110     15120   15130
               SARS-CoV-2 Region 11168 - 29055 nt (14742) GATGGCACAACAAGTCCTATTTCTGAACATGACTACCAGATTGGTGGTTATACTGAAAAATGGGAATCTGGAGTAAAAGACTGTG
Homo sapiens mitochondrion NC_012920.1 (13722) ATTCGCAGGATTTCTCATTACTAACAACATTTCCCCCGCATCCCCC-TTCCAAACAACAATCCCCCTCTACC-TAAAACTCACAG
                                                                                                              Section 179
                                              (15131) 15131    15140     15150     15160     15170     15180     15190     15200          15215
               SARS-CoV-2 Region 11168 - 29055 nt (14827) TTGTATTACACAGTTACTTCACTTCAGACTATTACCAGCTGTACTCAACTCAATTGAGTACAGACACTGGTGTTGAACATGTTAC
Homo sapiens mitochondrion NC_012920.1 (13805) CCCT----CGCTGTCACTTTCCTAG-GACTTCTAACAGCCCTAGAC--CTCAACTACCTAACCA-ACAAACTTAAAATAAAATCC
                                                                                                              Section 180
                                              (15216) 15216         15230     15240     15250     15260     15270     15280     15290   15300
               SARS-CoV-2 Region 11168 - 29055 nt (14912) CTTCTTCATCTACAATAAAATTGTTGATGAGCCTGAAGAACATGTCCAAATTCACACAATCGACGGTTC---ATCC-GGAGTTGT
Homo sapiens mitochondrion NC_012920.1 (13882) CCACT--ATGCACATTTTATTTCTCCAACATACTCGGATTCTACCCTAGCATCACACACCGCACAATCCCCTATCTAGGCCTTCT
                                                                                                              Section 181
                                              (15301) 15301    15310     15320     15330     15340     15350     15360     15370          15385
               SARS-CoV-2 Region 11168 - 29055 nt (14993) TA--ATCCAGTAATGGAACCAATTTATGATGAACCGACGACGACTACTAGCGTGCCTTTGTAAGCACAAGCTGATGAGTACGAAC
Homo sapiens mitochondrion NC_012920.1 (13965) TACGAGCCAAAACCTGCCCCTACTCCTCCTAGACCTAACCTGACTAGAAAAGCTATTACCTAAA-ACAATTTCAC-AGCACCAAA
                                                                                                              Section 182
                                              (15386) 15386         15400     15410     15420     15430     15440     15450     15460   15470
               SARS-CoV-2 Region 11168 - 29055 nt (15076) TTATGTACTCATTCGTTTCGGAAGAGACAGGTACGTTAATAGTTAATAGCGTACTTCTTTTCTTGCTTTCGTGGTATTCTTGCT
Homo sapiens mitochondrion NC_012920.1 (14048) TCTCCACCTCCATCATCACCTCA-ACCCAAAAAGGC--ATAATTAA-ACTTTACTTCCTCT-CTTTCTT-CTTCCCACTCATCCT
```

```
                                                                                                   Section 183
                                              (15471) 15471    15480      15490      15500      15510      15520      15530      15540      15555
           SARS-CoV-2 Region 11168 - 29055 nt (15161) AGTTACACTAGCCATCCTTACTGCGCTTCGATTGTGTGCGTACTGCTGCAATATTGTTAACGTGAGTCT-TGTAAAACCTTCTTT
Homo sapiens mitochondrion NC_012920.1 (14127) AACCCTACTCCTAATCACATAACCTATTCCCCCGAGCAATCTCAATTACAATATATACACCAACAAACAATGTTCAACCAG-TAA
                                                                                                   Section 184
                                              (15556) 15556            15570      15580      15590      15600      15610      15620      15630  15640
           SARS-CoV-2 Region 11168 - 29055 nt (15245) TTACGTTTACTCT-CGTGT-TAAAAATCTGAATTCTTCTAGAGTTCCTGATCTTCTGGTCTAAAC---GAACTAAATATTATATT
Homo sapiens mitochondrion NC_012920.1 (14211) CTACTACTAATCAACGCCCATAATCATACAAAGCCCCCGCACCAATAGGATCCTCCCGAATCAACCCTGACCCCTCTCCTTCATA
                                                                                                   Section 185
                                              (15641) 15641    15650      15660      15670      15680      15690      15700      15710      15725
           SARS-CoV-2 Region 11168 - 29055 nt (15325) AGTTTTTCTGTTTGGAAC----TTTAATTTTAGCCATGGCAGATTCCAACGGTACTATTACCGTTGAAGAGCTTAAAAAGCTCCT
Homo sapiens mitochondrion NC_012920.1 (14296) AATTATTCAGCTTCCTACACTATTAAAGTTTACCACAACCACCACCCCATCATACTCTTTCACCC--ACAGCACCAATCCTACCT
                                                                                                   Section 186
                                              (15726) 15726            15740      15750      15760      15770      15780      15790      15800  15810
           SARS-CoV-2 Region 11168 - 29055 nt (15406) TGAACAATGGAACCTAGTAATAGGTTT-CCTATTCCTTA--CATGGATTTGTCTTCTACAATTTGCCTATGCCAACAGGAATAGG
Homo sapiens mitochondrion NC_012920.1 (14379) CCATCGCTA-ACCCCACTAAAACACTCACCAAGACCTCAACCCCTGACCCCCATGCCTCAGGATACTCCT--CAATAGCCATCGC
                                                                                                   Section 187
                                              (15811) 15811    15820      15830      15840      15850      15860      15870      15880      15895
           SARS-CoV-2 Region 11168 - 29055 nt (15488) TTTT-TGTATATAATTAAGTTAATTTTCCTCTGGCTGTTATGGCCAGTAACTTTAGCTTGTTTTGTGCTTGCTGCTGTTTACAGA
Homo sapiens mitochondrion NC_012920.1 (14461) TGTAGTATATCCAAAGACAACCATCATTCCCCCTAAATAAATTAAAAAAACTATTAAACCCATATAACCTCCCCCAAAATTCAGA
                                                                                                   Section 188
                                              (15896) 15896            15910      15920      15930      15940      15950      15960      15970  15980
           SARS-CoV-2 Region 11168 - 29055 nt (15572) ATAAATTGGATCACCGGTGGAATTGCTATCGCAATGGCTTGTCTTGTAGGCTTGATGTGGCTCAGCTACTTCATTGCTTCTTTCA
Homo sapiens mitochondrion NC_012920.1 (14546) ATAA-TAACACACCGACCACACCGCTAAC--AATCAATA---------------------CTAAACCCC--CATAAATAGG---A
                                                                                                   Section 189
                                              (15981) 15981    15990      16000      16010      16020      16030      16040      16050      16065
           SARS-CoV-2 Region 11168 - 29055 nt (15657) GACTGTTTGCGCGTACGCGTTCCATGTGGTCATTCAATCCAGAAACTAACATTCTTCTCAACGTGCCACTCCATGGCACTATTCT
Homo sapiens mitochondrion NC_012920.1 (14603) GAAGGCTTAGAAGAAAACCCCACAA------ACCCCATTACTAAACCCACACTCAACAGAAACAAAGCATACAT--CATTATTCT
```

Section 190

```
(16066)                                                   16066         16080     16090     16100     16110     16120     16130     16140     16150
SARS-CoV-2 Region 11168 - 29055 nt (15742)                GACCAGACCGCTTCTAGAAAGTGAACTCGTAATCGGAGCTGTGATCCTTCGTGGACATCTTCGTATTGCTGGACACCATCTAGGA
Homo sapiens mitochondrion NC_012920.1 (14680)            CGC---ACGGA--CTACAACCACGACCAATGATATGAA----AACCATCGTTGT-AT-TTCAACTACAAGAACACCAATGACCC
```

Section 191

```
(16151)                                                   16151    16160     16170     16180     16190     16200     16210     16220          16235
SARS-CoV-2 Region 11168 - 29055 nt (15827)                CGCTGTGACATCAAGGACCTGCCTAAAGAAATCACTGTTGCTACATCACGAACGCTTTCTTATTACAAATTGGGAGCTTCGCAGC
Homo sapiens mitochondrion NC_012920.1 (14754)            CAATACGCAA--AACTAACCCCCTAATAAAATTAAT-TAACCACTCATTCATCGACCTCCCCACCCCATCCAACATCTCCGCATG
```

Section 192

```
(16236)                                                   16236         16250     16260     16270     16280     16290     16300     16310     16320
SARS-CoV-2 Region 11168 - 29055 nt (15912)                GTGTAGCAGGTGACTCAGGTTTTGCTGCATACAGTCGCTACAGGATTGGCAACTATAAATTAAACACAG-ACCATTCCAGTAGCA
Homo sapiens mitochondrion NC_012920.1 (14836)            ATGAAACTTCGG-CTCACTCCTTGGCGC---------CTGCCTGATC-----CTCCAAATCAC-CACAGGACTATTCC--TAGCC
```

Section 193

```
(16321)                                                   16321    16330     16340     16350     16360     16370     16380     16390          16405
SARS-CoV-2 Region 11168 - 29055 nt (15996)                GTGACAATATTGCTTTGCTTGTACAGTAAGTGACAACAGATGTTTCATCTCGTTGACTTTCAGGTTACTATAGCAGAGA--TATT
Homo sapiens mitochondrion NC_012920.1 (14903)            ATG-CACTACTC----------ACCAGACGCCTCAACCGCCTTTCATCAA-TCGCC---CACATCACTCGAGACGTAAATTATG
```

Section 194

```
(16406)                                                   16406         16420     16430     16440     16450     16460     16470     16480     16490
SARS-CoV-2 Region 11168 - 29055 nt (16079)                ACTAATTATTATGAGGACTTTTAAAGTTTCCATTTGGAATCTTGATTACATCATAAACCTCATAATTAAAAATTTATCTAAGTCA
Homo sapiens mitochondrion NC_012920.1 (14973)            GCTGA--ATCATCCGCTACCTTCACGC--CAATGGCGCCTCAATATT-CTTTATCTGCCTCTTCCT-ACACATCGGGCGAGGCCT
```

Section 195

```
(16491)                                                   16491    16500     16510     16520     16530     16540     16550     16560          16575
SARS-CoV-2 Region 11168 - 29055 nt (16164)                CTAACTGAGAATAAATATTCTCAATTAGATGAAGAGCAACCAATGGAGATTGATTAAACGAACATGAAAATTATTCTTTTCTTGG
Homo sapiens mitochondrion NC_012920.1 (15052)            ATAT-TACGGATCATTTCTCT-ACTCAGA--------AACC---------TGA-------AACATCGGCATTATCCTCCTGCTTG
```

Section 196

```
(16576)                                                   16576         16590     16600     16610     16620     16630     16640     16650     16660
SARS-CoV-2 Region 11168 - 29055 nt (16249)                CACTGATAACACTCGCTACTTGTGAGCTTTATCACTACCAAGAGTGTGTTAGAGGTACAACAGTACTTTAAAAGAACCTTGCTC
Homo sapiens mitochondrion NC_012920.1 (15111)            CAACTATAGCAACAGCCT-TCATAGGCTATGTC-CTCCCGTGAGG------------CCAAATATCATTCTGAGGGCCACAGTA
```

Section 197

```
                                          16661    16670     16680     16690     16700     16710     16720     16730          16745
                                  (16661)
SARS-CoV-2 Region 11168 - 29055 nt (16334) TTCTGGAACATACGAGGGCAATTCACCATTTCATCCTCTAGCTGATAACAAATTTGCACTGACTTGCTTTAGCACTCAATTTGCT
Homo sapiens mitochondrion NC_012920.1 (15182) ATTACAAACTTACTA------TCCGCCATCCCATACATTGGGACAGACCTAGTT--CAATGAATC---TGAGGAGGCTACT--CA
```

Section 198

```
                                          16746         16760     16770     16780     16790     16800     16810     16820  16830
                                  (16746)
SARS-CoV-2 Region 11168 - 29055 nt (16419) TTTGCTTGTCCTGACGGCGTAAAACACGTCTATCAGTTACGTGCCAGATCAGTTTCACCTAAACTGTTCATCAGACAAGAGGAAG
Homo sapiens mitochondrion NC_012920.1 (15254) GTAGACAGTCC---CACCCTCACACGATTCT-----TTACCTTTCACTTCATCTTGCCCTTCATTATT-------------GCAG
```

Section 199

```
                                          16831    16840     16850     16860     16870     16880     16890     16900          16915
                                  (16831)
SARS-CoV-2 Region 11168 - 29055 nt (16504) TTCAAGAACTTTACTCTCCAATTTTTCTTATTGTTGCGGCAATAGTGTTTATAACACTTTGCTTCACACTCAAAAGAAAGACAGA
Homo sapiens mitochondrion NC_012920.1 (15318) CCCTAGCA----ACACTCCACCT---CCTATTCTTGCACGAAACGGGATCAAACAACCC--CCTAGGAATCACCTCCCATTCCGA
```

Section 200

```
                                          16916         16930     16940     16950     16960     16970     16980     16990  17000
                                  (16916)
SARS-CoV-2 Region 11168 - 29055 nt (16589) ATGATTGAACTTTCA---TTAATTGACTTCTATTTGTGCTTTTTAGCCTTTCTGCTATTCCTTGTTTTAATTATGCTTATTATCT
Homo sapiens mitochondrion NC_012920.1 (15394) TAAAATCACCTTCCACCCTTACTACACAATCAAAGACGCCCTC--GGCTTACTTCTCTTCCTTCTCTCCTTAATGAC-ATTAACA
```

Section 201

```
                                          17001    17010     17020     17030     17040     17050     17060     17070          17085
                                  (17001)
SARS-CoV-2 Region 11168 - 29055 nt (16671) TTTGGTTCTCACTTGAACTGCAAGATCATAATGAAACTTGTCACGCCTAAACGAACATGAAATTTCTTGTTTTCTTAGGAATCAT
Homo sapiens mitochondrion NC_012920.1 (15476) CTA--TTCTCACCAGACCTCCTAGGCGACCCAGACAATTAT-AC-CCTAGCCAACCCCTTAAACACCCCTCCCCACATCAAGC--
```

Section 202

```
                                          17086         17100     17110     17120     17130     17140     17150     17160  17170
                                  (17086)
SARS-CoV-2 Region 11168 - 29055 nt (16756) CACAACTGTAGCTGCATTTCACCAAGAATGTAGTTTACAGTCATGTACTCAACATCAACCATATGTAGTTGATGACCCGTGTCCT
Homo sapiens mitochondrion NC_012920.1 (15555) CCGAATGATATTTCCTATTCGCCTACAC---AATTCTCCGATCCGTCCCTAACA--AACTAGGAGGCGT-------CCTTGCCCT
```

Section 203

```
                                          17171    17180     17190     17200     17210     17220     17230     17240          17255
                                  (17171)
SARS-CoV-2 Region 11168 - 29055 nt (16841) ATTCACTTCTATTCTAAATGGTATATTAGAGTAGGAGCTAGAAAATCAGCACCTTTAATTGAATTGTGCGTGGATGAGGCTGGTT
Homo sapiens mitochondrion NC_012920.1 (15628) ATTACTATCCATCCTCATCC-----------TAGCA-----ATAATCCCCATCCTCCATATATCCAAACA---ACAAAGCA--TA
```

Section 204

(17256)	17256 17270 17280 17290 17300 17310 17320 17330 17340
SARS-CoV-2 Region 11168 - 29055 nt (16926)	CTAAATCACCCATTCAGTACATCGATATCGGTAATTATACAGTTTCCTGTTTACCTTTTACAATTAATTGCCAGGAACCTAAATT
Homo sapiens mitochondrion NC_012920.1 (15692)	ATATTTCGCCCACTAAGCCAATCACTTTATTGACTCCTAGCCGCAGACCTCCTCATTCTAACCTGAATCGG-AGGACAACCAGTA

Section 205

(17341)	17341 17350 17360 17370 17380 17390 17400 17410 17425
SARS-CoV-2 Region 11168 - 29055 nt (17011)	GGGTAGTCTTGTAGTGCGTTGTTCGTTCTATGAAGACTTTTTAGAGTATCATGACGTTCGTGTTGTTTAGATTTCATCTAAACG
Homo sapiens mitochondrion NC_012920.1 (15776)	AGCTACCCTTTTA----------CCATCATTGGACA---------AGTAGCATC-CGTACTA--TACTTCACAACAATCCTAATCC

Section 206

(17426)	17426 17440 17450 17460 17470 17480 17490 17500 17510
SARS-CoV-2 Region 11168 - 29055 nt (17096)	AACAAACTAAAATGTCTGATAATGGACCCCAAAATCAGCGAAATGCACCCCGCATTACGTTTGGTGGACCCTCAGATTCAACTGG
Homo sapiens mitochondrion NC_012920.1 (15840)	TAATACCAACTATCTCCC-TAATTGAAAACAAAATACTC-AAATGGGCCTGTCCTTG----TAGTATAAACTAATACACCAGTCT

Section 207

(17511)	17511 17520 17530 17540 17550 17560 17570 17580 17595
SARS-CoV-2 Region 11168 - 29055 nt (17181)	CAGTAACCAGAATGGAGAACGCAGTGG---GGCGCGATCA-AAACAACGTCG---GCCCCAAGGTT--TACCCAATAATACTGCG
Homo sapiens mitochondrion NC_012920.1 (15919)	TGTAAACCGGAGATGAAAACCTTTTTCCAAGGACAAATCAGAGAAAAAGTCTTTAACTCCACCATTAGCACCCAAAGCTAA-GAT

Section 208

(17596)	17596 17610 17620 17630 17640 17650 17660 17670 17680
SARS-CoV-2 Region 11168 - 29055 nt (17257)	TCTTGGTTCACCG---CTCTCACTCAACATGGCAAGGAAGACCTTAAATTCCCTCGAGGACAAGGCGTTCCAATTAACACCAATA
Homo sapiens mitochondrion NC_012920.1 (16003)	TCTAATTTAAACTATTCTCTGTTCTTTCATGGGGAAGCAGAT-TTGGGTACCACCCAAGTATTGACTCACCCATCAACAAC----

Section 209

(17681)	17681 17690 17700 17710 17720 17730 17740 17750 17765
SARS-CoV-2 Region 11168 - 29055 nt (17339)	GCAGTCCAGATGACCAAATTGGCTACTACCGAAGAGCTACCAGACGAATTCGTGGTGGTGACGGTAAAATGAAAGATCTCAGTCC
Homo sapiens mitochondrion NC_012920.1 (16083)	-CGCTATGTATTTCGTACAT---TACTGCC----AGCCACCAT--GAATA--TTGT----ACGGTACCAT-AAATACTTGACCAC

Section 210

(17766)	17766 17780 17790 17800 17810 17820 17830 17840 17850
SARS-CoV-2 Region 11168 - 29055 nt (17424)	AAGATGGTATTTCTACTACCTAGGAACTGGGCCAGAAGCTGGACTTCCCTATGGTG-CTAACAAAGACGGCATCATATGGGTTGC
Homo sapiens mitochondrion NC_012920.1 (16151)	CTG-TAGTACATAAAAACCCAATCCACAT---CAAAACCC---CCTCCCCATGCTTACAAGCAAGTACAGCA--ATCAACCCT-C

Section 211

```
                                              (17851) 17851    17860     17870     17880     17890     17900     17910     17920          17935
                SARS-CoV-2 Region 11168 - 29055 nt (17508) AACTGAGGGAGCCTTGAATACACCAAAAGATCACATTGGCACCCGCAATCCTGCTAACAATGCTGCAATCGTGCTACAACTTCCT
Homo sapiens mitochondrion NC_012920.1 (16226) AACTATCACA-CATCAACTGCAACTCCAAAGCCACCCCTCACCCACTAGGATACCAACAAACCTAC--------CCAC-----CCT
```

Section 212

```
                                              (17936) 17936         17950     17960     17970     17980     17990     18000     18010  18020
                SARS-CoV-2 Region 11168 - 29055 nt (17593) CAAGGAACAACATTGCCAAAAGCTTCTACGCAGAAGGGAGCAGAGGCGGCAGTCAAGCCTCTTCTCGTTCCTCATCACGTAGTC
Homo sapiens mitochondrion NC_012920.1 (16298) TAACAGTACATAGTACATAAAGCCATTTACC--GTACATAGCACATTA--CAGTCAAATCCCTTCTCGTCCC-CATGGATGACCC
```

Section 213

```
                                              (18021) 18021    18030     18040     18050     18060     18070     18080     18090          18105
                SARS-CoV-2 Region 11168 - 29055 nt (17678) GCAACAGTTCAAGAAATTCAACTCCAGGCAGCAGTAGGGGAACTTCTCCTGCTAGAATGGCTGGCAATGGCGGTGATGCTGCTCT
Homo sapiens mitochondrion NC_012920.1 (16378) CCCTCAGAT--AGGGGTCCCTTGACCACCATCCTCCGTGAAA--TC---------AATATCCCGCACAAGAG-TGCTACTCTCCT
```

Section 214

```
                                              (18106) 18106         18120     18130     18140     18150     18160     18170     18180  18190
                SARS-CoV-2 Region 11168 - 29055 nt (17763) TGCTTTGCTGCTGCTTGACAGATTGAACCAGCTTGAGAGCAAAATGTCTGGTAAAGGCCAACAACAACAAGGCCAAACTGTCACT
Homo sapiens mitochondrion NC_012920.1 (16449) CGCTCCGGGCC--CATAACACTTGGGGGTAGCTAAAGTGAACTGTATCCGACATCTGGTTCCTACTTCAGGGTCATAAAGCC--T
```

Section 215

```
                                              (18191) 18191    18200     18210     18220   18231
                SARS-CoV-2 Region 11168 - 29055 nt (17848) AAGAAATCTGCTGCTGAGGCTTCTAAGAAGCCTCGGCAAAA
Homo sapiens mitochondrion NC_012920.1 (16530) AAATAGCCCACACGTTCCCCTTA-AATAAGACATCACGATG
```
