# Supplementary material for: Ubiquitous Micro-Modular Homologies among Genomes from Viruses to Bacteria to Human Mitochondrial DNA: Platforms for Recombination during Evolution?
Source: Viruses. 2022 Apr 24;14(5):885. doi: 10.3390/v14050885 (PMC9147251; doi:10.3390/v14050885)
Supplement: Supplementary file 1 [file viruses-14-00885-s001.zip › Fig. S5C, SARS-CoV-2 & Fig badnavirus 1.pdf]

```
                                                     Section 1
                                          (1) 1     10        20        30        40        50        60        70          83
SARS-CoV-2 Reference Genome NC_045512.2   (1) ATTAAAGGTTTATACCTTCCCAGGTAACAAACCAACCAACTTTCGATCTCTTGTAGATCTGTTCTCTAAACGAACTTTAAAAT
        Fig badnavirus 1 NC_017830.1      (1) -----------------------------------------------------------------------------------
                                                     Section 2
                                         (84) 84    90        100       110       120       130       140       150        166
SARS-CoV-2 Reference Genome NC_045512.2  (84) CTGTGTGGCTGTCACTCGGCTGCATGCTTAGTGCACTCACGCAGTATAATTAATAACTAATTACTGTCGTTGACAGGACACGA
        Fig badnavirus 1 NC_017830.1      (1) -----------------------------------------------------------------------------------
                                                     Section 3
                                        (167) 167          180       190       200       210       220       230                249
SARS-CoV-2 Reference Genome NC_045512.2 (167) GTAACTCGTCTATCTTCTGCAGGCTGCTTACGGTTTCGTCCGTGTTGCAGCCGATCATCAGCACATCTAGGTTTCGTCCGGGT
        Fig badnavirus 1 NC_017830.1      (1) -----------------------------------------------------------------------------------
                                                     Section 4
                                        (250) 250       260       270       280       290       300       310       320        332
SARS-CoV-2 Reference Genome NC_045512.2 (250) GTGACCGAAAGGTAAGATGGAGAGCCTTGTCCCTGGTTTCAACGAGAAAACACACGTCCAACTCAGTTTGCCTGTTTTACAGG
        Fig badnavirus 1 NC_017830.1      (1) -----------------------------------------------------------------------------------
                                                     Section 5
                                        (333) 333    340       350       360       370       380       390       400         415
SARS-CoV-2 Reference Genome NC_045512.2 (333) TTCGCGACGTGCTCGTACGTGGCTTTGGAGACTCCGTGGAGGAGGTCTTATCAGAGGCACGTCAACATCTTAAAGATGGCACT
        Fig badnavirus 1 NC_017830.1      (1) -----------------------------------------------------------------------------------
                                                     Section 6
                                        (416) 416           430       440       450       460       470       480              498
SARS-CoV-2 Reference Genome NC_045512.2 (416) TGTGGCTTAGTAGAAGTTGAAAAAGGCGTTTTGCCTCAACTTGAACAGCCCTATGTGTTCATCAAACGTTCGGATGCTCGAAC
        Fig badnavirus 1 NC_017830.1      (1) -----------------------------------------------------------------------------------
                                                     Section 7
                                        (499) 499        510       520       530       540       550       560       570       581
SARS-CoV-2 Reference Genome NC_045512.2 (499) TGCACCTCATGGTCATGTTATGGTTGAGCTGGTAGCAGAACTCGAAGGCATTCAGTACGGTCGTAGTGGTGAGACACTTGGTG
        Fig badnavirus 1 NC_017830.1      (1) -----------------------------------------------------------------------------------
```

```
                                                                                                  Section 8
                                        (582) 582       590       600       610       620       630       640       650       664
SARS-CoV-2 Reference Genome NC 045512.2 (582) TCCTTGTCCCTCATGTGGGCGAAATACCAGTGGCTTACCGCAAGGTTCTTCTTCGTAAGAACGGTAATAAAGGAGCTGGTGGC
       Fig badnavirus 1 NC_017830.1       (1) -----------------------------------------------------------------------------------
                                                                                                  Section 9
                                        (665) 665  670       680       690       700       710       720       730       747
SARS-CoV-2 Reference Genome NC 045512.2 (665) CATAGTTACGGCGCCGATCTAAAGTCATTTGACTTAGGCGACGAGCTTGGCACTGATCCTTATGAAGATTTTCAAGAAAACTG
       Fig badnavirus 1 NC_017830.1       (1) -----------------------------------------------------------------------------------
                                                                                                  Section 10
                                        (748) 748         760       770       780       790       800       810       820       830
SARS-CoV-2 Reference Genome NC 045512.2 (748) GAACACTAAACATAGCAGTGGTGTTACCCGTGAACTCATGCGTGAGCTTAACGGAGGGGCATACACTCGCTATGTCGATAACA
       Fig badnavirus 1 NC_017830.1       (1) -----------------------------------------------------------------------------------
                                                                                                  Section 11
                                        (831) 831      840       850       860       870       880       890       900       913
SARS-CoV-2 Reference Genome NC 045512.2 (831) ACTTCTGTGGCCCTGATGGCTACCCTCTTGAGTGCATTAAAGACCTTCTAGCACGTGCTGGTAAAGCTTCATGCACTTTGTCC
       Fig badnavirus 1 NC_017830.1       (1) -----------------------------------------------------------------------------------
                                                                                                  Section 12
                                        (914) 914   920       930       940       950       960       970       980       996
SARS-CoV-2 Reference Genome NC 045512.2 (914) GAACAACTGGACTTTATTGACACTAAGAGGGGTGTATACTGCTGCCGTGAACATGAGCATGAAATTGCTTGGTACACGGAACG
       Fig badnavirus 1 NC_017830.1       (1) -----------------------------------------------------------------------------------
                                                                                                  Section 13
                                        (997) 997          1010      1020      1030      1040      1050      1060      1079
SARS-CoV-2 Reference Genome NC 045512.2 (997) TTCTGAAAAGAGCTATGAATTGCAGACACCTTTTGAAATTAAATTGGCAAAGAAATTTGACACCTTCAATGGGGAATGTCCAA
       Fig badnavirus 1 NC_017830.1       (1) -----------------------------------------------------------------------------------
                                                                                                  Section 14
                                       (1080) 1080      1090      1100      1110      1120      1130      1140      1150      1162
SARS-CoV-2 Reference Genome NC 045512.2 (1080) ATTTTGTATTTCCCTTAAATTCCATAATCAAGACTATTCAACCAAGGGTTGAAAAGAAAAAGCTTGATGGCTTTATGGGTAGA
       Fig badnavirus 1 NC_017830.1       (1) -----------------------------------------------------------------------------------
```

```
                                                                    Section 15
                                          (1163) 1163   1170      1180      1190      1200      1210      1220      1230        1245
SARS-CoV-2 Reference Genome NC_045512.2   (1163) ATTCGATCTGTCTATCCAGTTGCGTCACCAAATGAATGCAACCAAATGTGCCTTTCAACTCTCATGAAGTGTGATCATTGTGG
     Fig badnavirus 1 NC_017830.1            (1) -----------------------------------------------------------------------------------
                                                                    Section 16
                                          (1246) 1246          1260      1270      1280      1290      1300      1310           1328
SARS-CoV-2 Reference Genome NC_045512.2   (1246) TGAAACTTCATGGCAGACGGGCGATTTTGTTAAAGCCACTTGCGAATTTTGTGGCACTGAGAATTTGACTAAAGAAGGTGCCA
     Fig badnavirus 1 NC_017830.1            (1) -----------------------------------------------------------------------------------
                                                                    Section 17
                                          (1329) 1329       1340      1350      1360      1370      1380      1390      1400    1411
SARS-CoV-2 Reference Genome NC_045512.2   (1329) CTACTTGTGGTTACTTACCCCAAAATGCTGTTGTTAAAATTTATTGTCCAGCATGTCACAATTCAGAAGTAGGACCTGAGCAT
     Fig badnavirus 1 NC_017830.1            (1) -----------------------------------------------------------------------------------
                                                                    Section 18
                                          (1412) 1412    1420      1430      1440      1450      1460      1470      1480        1494
SARS-CoV-2 Reference Genome NC_045512.2   (1412) AGTCTTGCCGAATACCATAATGAATCTGGCTTGAAAACCATTCTTCGTAAGGGTGGTCGCACTATTGCCTTTGGAGGCTGTGT
     Fig badnavirus 1 NC_017830.1            (1) -----------------------------------------------------------------------------------
                                                                    Section 19
                                          (1495) 1495 1500      1510      1520      1530      1540      1550      1560          1577
SARS-CoV-2 Reference Genome NC_045512.2   (1495) GTTCTCTTATGTTGGTTGCCATAACAAGTGTGCCTATTGGGTTCCACGTGCTAGCGCTAACATAGGTTGTAACCATACAGGTG
     Fig badnavirus 1 NC_017830.1            (1) -----------------------------------------------------------------------------------
                                                                    Section 20
                                          (1578) 1578        1590      1600      1610      1620      1630      1640      1650    1660
SARS-CoV-2 Reference Genome NC_045512.2   (1578) TTGTTGGAGAAGGTTCCGAAGGTCTTAATGACAACCTTCTTGAAATACTCCAAAAAGAGAAAGTCAACATCAATATTGTTGGT
     Fig badnavirus 1 NC_017830.1            (1) -----------------------------------------------------------------------------------
                                                                    Section 21
                                          (1661) 1661     1670      1680      1690      1700      1710      1720      1730      1743
SARS-CoV-2 Reference Genome NC_045512.2   (1661) GACTTTAAACTTAATGAAGAGATCGCCATTATTTTGGCATCTTTTTCTGCTTCCACAAGTGCTTTTGTGGAAACTGTGAAAGG
     Fig badnavirus 1 NC_017830.1            (1) -----------------------------------------------------------------------------------
```

```
                                                  Section 22
                                     (1744) 1744 1750 1760 1770 1780 1790 1800 1810 1826
SARS-CoV-2 Reference Genome NC_045512.2 (1744) TTTGGATTATAAAGCATTCAAACAAATTGTTGAATCCTGTGGTAATTTTAAAGTTACAAAAGGAAAAGCTAAAAAAGGTGCCT
     Fig badnavirus 1 NC_017830.1    (1) -----------------------------------------------------------------------------------
                                                  Section 23
                                     (1827) 1827 1840 1850 1860 1870 1880 1890 1909
SARS-CoV-2 Reference Genome NC_045512.2 (1827) GGAATATTGGTGAACAGAAATCAATACTGAGTCCTCTTTATGCATTTGCATCAGAGGCTGCTCGTGTTGTACGATCAATTTTC
     Fig badnavirus 1 NC_017830.1    (1) -----------------------------------------------------------------------------------
                                                  Section 24
                                     (1910) 1910 1920 1930 1940 1950 1960 1970 1980 1992
SARS-CoV-2 Reference Genome NC_045512.2 (1910) TCCCGCACTCTTGAAACTGCTCAAAATTCTGTGCGTGTTTTACAGAAGGCCGCTATAACAATACTAGATGGAATTTCACAG-T
     Fig badnavirus 1 NC_017830.1    (1) --------------------------------------------------------------------TGGTA--TCAGAGCT
                                                  Section 25
                                     (1993) 1993 2000 2010 2020 2030 2040 2050 2060 2075
SARS-CoV-2 Reference Genome NC_045512.2 (1992) ATTCACTGAGACTCATTGATGCTATGATGTTCACATCTGATTTGGCTACTAACAATCTAGTTGTAATGGCCTACATTACAGGT
     Fig badnavirus 1 NC_017830.1   (14) AGTTA-TGAGTGAGTAGGTTCTTATAAGGT----ATTCGGGTAAACAGGGAAAGATTTATTATGCCTAGATTGATTTGATAAT
                                                  Section 26
                                     (2076) 2076 2090 2100 2110 2120 2130 2140 2158
SARS-CoV-2 Reference Genome NC_045512.2 (2075) GGTGTTGTTCAGTTGACT---TCGCAGTGGCTAACT-----AACATCTTTGGCACTGTTTATGAAAAACTCA--AACCCGTCC
     Fig badnavirus 1 NC_017830.1   (92) CTTGTTTCTCAGATCTCTGAGTTGTAGTATCTAAAAGGAAAAAGTCCTTGGGCACTATATCTGGTTAAGGCAGGAGGCCGT--
                                                  Section 27
                                     (2159) 2159 2170 2180 2190 2200 2210 2220 2230 2241
SARS-CoV-2 Reference Genome NC_045512.2 (2148) TTGATTGGCTTGAAGAGAAGTTTAAGGAAGGTGTAGAGTTTCTTAGAGACGGTTGGGAAATTGTTAAATTTATCTCAACCTGT
     Fig badnavirus 1 NC_017830.1  (173) GTGATACCAGTGAA----ACCCTGAGGTGATCCTGATGGATATGAGTCATGTCTGAGAAATGGGAAAGGTCAATTCAGGAT-T
                                                  Section 28
                                     (2242) 2242 2250 2260 2270 2280 2290 2300 2310 2324
SARS-CoV-2 Reference Genome NC_045512.2 (2231) GCTTGTGAAATTGTCGGTGGACAAATTGTCACCTGTGCAAAGGAAATTAAGGAGAGTGTTCAGACATTCTTTAAGCTTGTAAA
     Fig badnavirus 1 NC_017830.1  (251) GGTATAATAATTCTAG-------AACTGCGAACCTTG--------AATACCTTGATTTAGCAGAAAGGAAAAACC-----AA
```

```
                                                                                    Section 29
                                          (2325) 2325 2330      2340      2350      2360      2370      2380      2390            2407
SARS-CoV-2 Reference Genome NC_045512.2   (2314) TAAATTTTTGGCTTTGTGTGCTGACTCTATCATTATTGGTGGAGCTAAACTTAAAGCCTTGAATTTAGGTGAAACATTTGTCA
           Fig badnavirus 1 NC_017830.1    (314) CAAATTCTCACCTTT---ACCATAATCTAGCAGTAGTTTACGA--TAGACTAAA--CTTGCAATCTCGTGTTAACCTCAAAAA
                                                                                    Section 30
                                          (2408) 2408        2420      2430      2440      2450      2460      2470      2480     2490
SARS-CoV-2 Reference Genome NC_045512.2   (2397) CGCACTCAAAGGGATTGTACAGAAAGTGTGTTAAATCCAGAGAAGAAACTGGCCTACTCATGCCTCTAAAAGCCCCAAAAGAA
           Fig badnavirus 1 NC_017830.1    (390) C---CTTAAGGGTATTTT--AGAAAGAGTGGAAAAACAAGAGAGAAAACTTGAGAGTTTAGAACT-----AGCAGTAAGGAAC
                                                                                    Section 31
                                          (2491) 2491     2500      2510      2520      2530      2540      2550      2560         2573
SARS-CoV-2 Reference Genome NC_045512.2   (2480) ATTATCTTCTTAGAGGGAGAAACACTTCCCACAGAAGTGTTAACAGAGGAAGTTGTCTTGAAAACTGGTGATTTACAACCATT
           Fig badnavirus 1 NC_017830.1    (463) CTTACCCAAGTTTTTGTTGAAAATAAGCCTT------TAACAACAACTGAAGTTA----GAAGGCT--TGTTTACGAAATCTC
                                                                                    Section 32
                                          (2574) 2574  2580      2590      2600      2610      2620      2630      2640           2656
SARS-CoV-2 Reference Genome NC_045512.2   (2563) AGAACAACCTACTAGTGAAGCTGTTGAAGCTCCATTGGTTGGTACACCAGTTTGT-ATTAACGGGCTTATGTTGCT-CGAAAT
           Fig badnavirus 1 NC_017830.1    (534) TCAGCAACCAAAGCTTGTAGAGCAGGAGGCTCTAAGGTTAACTGAAGAACTTCGTCAAAAACTCGAAAGAGTTGAGGCGATAG
                                                                                    Section 33
                                          (2657) 2657         2670      2680      2690      2700      2710      2720            2739
SARS-CoV-2 Reference Genome NC_045512.2   (2644) CAAAGACACAGAAAAGTACTGTGCCCTTGCACCTAATATGATGGTAACAAACAATACCTTCACA-CTCAAGGCGGTGCACCA
           Fig badnavirus 1 NC_017830.1    (617) TAAAGAAAGTTGAAAGT--TGGACCTCTTCATGAGTAGTTATATTGTCTCCCAAGGGACAAATAGCTACAAGGAGG----CC-
                                                                                    Section 34
                                          (2740) 2740      2750      2760      2770      2780      2790      2800      2810        2822
SARS-CoV-2 Reference Genome NC_045512.2   (2726) ACAAAGGTTACTTTTGGTGATGACACTGTGATAGAAGTGCAAGGTTACAAGAGTGTGAATATCACTTTTGAACTT--GATGAA
           Fig badnavirus 1 NC_017830.1    (693) ATAAAAGCTACTGAAGGAATAGAATCCCCAGCAGCAGGTTCG-TGAGACCAGCC-GACTTCCAGGGAGGAACATCGGCAGCG
                                                                                    Section 35
                                          (2823) 2823   2830      2840      2850      2860      2870      2880      2890         2905
SARS-CoV-2 Reference Genome NC_045512.2   (2807) AGGATT--GATAAAGTACTTAATGAGAAGTGCTCTGCCTATACAGTTGAACTCGGTACAGAAGTAAATGAGTTCGCCTGTGTT
           Fig badnavirus 1 NC_017830.1    (774) AGGGTTCAGATTAAGCAGAATAATACCCTTATTCAGCTTCTA--ATCCAGATCGCTGAGTCCCTGAAGGACATC-CGAGAAGA
```

```
                                                         Section 36
                                         (2906) 2906          2920      2930      2940      2950      2960      2970           2988
SARS-CoV-2 Reference Genome NC_045512.2  (2888) GTGGCAGATGCTGTCATAAAAACTTTGCAACCAGTATCTGAATTA-----CTTACACCACTGGGCATTGATTTAGATGAGTGG
         Fig badnavirus 1 NC_017830.1    (854)  GCAGCGAGTCCTAAAGGAGGAGGTTAGGCAACTGCAGAAGGACAAAGCAGCTTCAATCACAGAAGAGCTTGTGGAAAGACTTC
                                                         Section 37
                                         (2989) 2989       3000      3010      3020      3030      3040      3050      3060     3071
SARS-CoV-2 Reference Genome NC_045512.2  (2966) AGTATGGCTACATACTACTT--ATTTGATGAGTCTGGTGAGTTTAAATTGGCTTCACA--TATGTATTGTTCTTTCTACCCTC
         Fig badnavirus 1 NC_017830.1    (937)  AAAGTCTCTCGTTAGGAGTCCCAGAAAAGAAGGTAGCTGAGAAGAAAAGAACTTTTAGGGTATTCAAAGACCCTTTAAGAATC
                                                         Section 38
                                         (3072) 3072    3080      3090      3100      3110      3120      3130      3140          3154
SARS-CoV-2 Reference Genome NC_045512.2  (3045) CA-GATGAGGATGAAGAAGAAGGTG--ATTGTGAAGAAGAAGAGTT---TGAGCCATCAACT-CAA--TATGAGTATGGTACT
         Fig badnavirus 1 NC_017830.1    (1020) CTTGAAGAGGAAAAAGAA-AAGCTGCCAAAATGAACAGGTCGAGAACGGTGACTCAGCAGCTGCCAGCTGTGAGTACCAGACG
                                                         Section 39
                                         (3155) 3155 3160      3170      3180      3190      3200      3210      3220             3237
SARS-CoV-2 Reference Genome NC_045512.2  (3119) GAAG-ATGA--TTACCAAGG-TAAACCTTTGGAATTTGGTGCCACTTCTGCTGCTCTTCAACCTGAAGAAGAGCAAGAAGAAG
         Fig badnavirus 1 NC_017830.1    (1102) GAGGGAAGAAATTAACAGGGATCAACCTCTG----TTCGAGG---ATCAGATCCGTGACTACCGGAGGAATCA-AAGGCGAAG
                                                         Section 40
                                         (3238) 3238        3250      3260      3270      3280      3290      3300      3310     3320
SARS-CoV-2 Reference Genome NC_045512.2  (3198) ATTGGTTAGATGATGATAGTCAACAAACTGTTGGTCAACAAGACGG-CAGTGAGGACAATCAGACAACTACTATTCAAACAAT
         Fig badnavirus 1 NC_017830.1    (1177) GTTTGT-AGCTGA--------A-CAAGCCGT--------AAGACGAACCATGGAAGAATGCGAGGACGACGATACAACCAAA
                                                         Section 41
                                         (3321) 3321     3330      3340      3350      3360      3370      3380      3390          3403
SARS-CoV-2 Reference Genome NC_045512.2  (3280) TGTTGAGGTTCAACCTCAATT-AGAGATGGAACTTACACCAGTTGTTCAG--ACTATTGAAGTGAATAGTTTAGTGGTTATT
         Fig badnavirus 1 NC_017830.1    (1242) CTCTGGAGCAGATCGTAGATCCAGAGGTAGAGCTGAACCAATCTATGCAGGAAAGAGCGAACCTAGTACCTGCAGAAGTTCTG
                                                         Section 42
                                         (3404) 3404  3410      3420      3430      3440      3450      3460      3470             3486
SARS-CoV-2 Reference Genome NC_045512.2  (3360) TAAAACTTACT----GACAATGTATAC-ATTAAAAATGCAGACATTGTG--GAAGAAGCTAAA--------AAGGTAAAACCA
         Fig badnavirus 1 NC_017830.1    (1325) TACCGCTCAAGAAGAGACAACATAAACCATCAGATCTATAATCATAGATCCGAGGAAGCGATGCTTTGTGTAGGAGAACAGCA
```

Section 43

	(3487)	3487 3500 3510 3520 3530 3540 3550 3569
SARS-CoV-2 Reference Genome NC_045512.2	(3428)	ACAGTGGTTGTTAATGCAGCCAAT--GTTTACCTTAAACATGGAGGAGGTGT-TGCAGGAGCCTTAAATAAGGCTACTAACAA
Fig badnavirus 1 NC_017830.1	(1408)	AGACAGGATGTTTATCCAACCTAACAGCTTCCAAGAACTACAGAGGAGTGGTATGCAGTT--CATACATCTGGGAGTTT----

Section 44

	(3570)	3570 3580 3590 3600 3610 3620 3630 3640 3652
SARS-CoV-2 Reference Genome NC_045512.2	(3508)	TGCCATGCAAGTT-GAATCTGATGATTACATAGCTACTAATGGACCACTTAAAGTGGGTGGTAGTTGTGTTTTAAGCGGACAC
Fig badnavirus 1 NC_017830.1	(1485)	-----TGCAAGTAAGACTCCAGATACTGCATAGGG-CAGATGGAGGAAC-AATGGCCTTGGTAGTCTT------------CAG

Section 45

	(3653)	3653 3660 3670 3680 3690 3700 3710 3720 3735
SARS-CoV-2 Reference Genome NC_045512.2	(3590)	AATCTTGCTAAACACTGTCTTCATGTTGTCGGCCCAAATGTTAACAAAGGTGAAGACATTCAACTTCTTAAGAGTGCTTATGA
Fig badnavirus 1 NC_017830.1	(1549)	A-----GATAACCGATGGC--AAGGAGATCAGTC--AATCTTCGCAATCT--CAGAAGTTGA-CTTGACTAGAG-GATC--GC

Section 46

	(3736)	3736 3750 3760 3770 3780 3790 3800 3818
SARS-CoV-2 Reference Genome NC_045512.2	(3673)	AAATTTTAATCAGCACGAAGTTCTACTTG-CACCATTATTATCAGCTGGTATTTTGGTGCTGACCCTATACAT-TCTTTAAG
Fig badnavirus 1 NC_017830.1	(1617)	AAATAGTATAC---------GTGATACCTGACACGATGATGATTATCGGTGACTTCTACAG--AAACGTTCAGATCTCTATCCA

Section 47

	(3819)	3819 3830 3840 3850 3860 3870 3880 3890 3901
SARS-CoV-2 Reference Genome NC_045512.2	(3754)	AGTTTGTGTAGAT-ACTGTTCGCACAAATGTCTACTTAGCTGTCTTTGATAAAAATCTCTATGACAAACTTGTT-TCAAGCTT
Fig badnavirus 1 NC_017830.1	(1690)	GACCAGTGGATATGAGAATTGGCAGAA-TGGAGAGGCAAATTTGCT-GATCACAAGAGGCATGACGGGAAGGTTATCCAACAC

Section 48

	(3902)	3902 3910 3920 3930 3940 3950 3960 3970 3984
SARS-CoV-2 Reference Genome NC_045512.2	(3835)	TTTGGAAATGAAGAGTGAAAAGCAAGTTGAACAAAAGATCGCTGAGATT-CCTAAAGAGGAAGTTAAGCCATT-TATAACTGA
Fig badnavirus 1 NC_017830.1	(1771)	TC--CAAATGTTG---GATTTGCTTACAGAATAAGCCATGTGACGGATTACCTAGCAAGCAGGGGAGTCCAAGCTTTGCCGGG

Section 49

	(3985)	3985 3990 4000 4010 4020 4030 4040 4050 4067
SARS-CoV-2 Reference Genome NC_045512.2	(3916)	AAGTAAACCTTCAGTTGAACAGAGAAAACAAGATGATAAGAAAATCAAAGCTTGTGTTGAAGAAGTTACAA-CAACTCTGGAA
Fig badnavirus 1 NC_017830.1	(1849)	AAG--AAGATACAG---ATCGGAGATGCTACGAGGA--AGAAACTGGATCATAAGGCAGCCACAGATCCAGGCGGCAATGATG

```
                                                                                  Section 50
                                      (4068) 4068        4080      4090      4100      4110      4120      4130      4140    4150
SARS-CoV-2 Reference Genome NC_045512.2 (3998) GAAACTAAGTTCCTCACAGAAAACTTGTTACTTTATATTGACATTAATGGCAATCTTCATCCAGATTCTGCCACTCTTGTTAG
       Fig badnavirus 1 NC_017830.1 (1925) CCAAGGAACGTGGAAACAAGGAACCTGATAGATG----GAACAATCTCTGCAAGAT--CTGCAGATTA---CACTCAGGCACC
```

```
                                                                                  Section 51
                                      (4151) 4151     4160      4170      4180      4190      4200      4210      4220         4233
SARS-CoV-2 Reference Genome NC_045512.2 (4081) TGA-CATTGACATCACTTTCTTAAAGAAAGATGCTCCATATATAGTGGGTGATGTTGTTCAAGAGGGTGTTTTAACTGCTGTG
       Fig badnavirus 1 NC_017830.1 (1999) AGAACCACGACAACCCCAT-TACAATGAGCAGGACGAAGAAGTGGCGAGTGATGAGG---AAGAGCTGGCAGAAACTCAACAG
```

```
                                                                                  Section 52
                                      (4234) 4234  4240      4250      4260      4270      4280      4290      4300          4316
SARS-CoV-2 Reference Genome NC_045512.2 (4163) GTTATACCTACTAAAAAGGCTG-GTGGCACTACTGAAATGCTAGCGAAAGC--TTTGAGA-AAAGTGCCAACAGACAATTATA
       Fig badnavirus 1 NC_017830.1 (2078) ----CACATAGTAGCAATGCTGCGATTTCCGGATTATGTGG-AGATACAGGAGTTTAAGCCAAAGTACAAAGAG-CAATACGA
```

```
                                                                                  Section 53
                                      (4317) 4317         4330      4340      4350      4360      4370      4380             4399
SARS-CoV-2 Reference Genome NC_045512.2 (4242) TAACCACTTACCCGGGTCAGGGTTTAAATGGTTACACTGTAGAGGAGGCAAAGACAGTGCTTAAAAAGTGTAAAAGTGCCTTT
       Fig badnavirus 1 NC_017830.1 (2155) CAAGTACA-AAAGGGAGCAGGGCT----GGGATACCCTG----GGAG---AACCAAGCGGAAAGTATGACTACTAC-GTCCGC
```

```
                                                                                  Section 54
                                      (4400) 4400      4410      4420      4430      4440      4450      4460      4470         4482
SARS-CoV-2 Reference Genome NC_045512.2 (4325) TACATTCTACCATCTATTATCTCTAATGAGAAGCAAGAAATTCTTGGAACTGTTTCTTGGAATTTGCGAGAAATGCTTGCACA
       Fig badnavirus 1 NC_017830.1 (2225) TACACTGCACCTCCAGCGA---CAACGCCGATAGAAGATATTCAGCCAACAGGCTGGGAGGAGGAGCCTAAATGGGATGATTC
```

```
                                                                                  Section 55
                                      (4483) 4483   4490      4500      4510      4520      4530      4540      4550           4565
SARS-CoV-2 Reference Genome NC_045512.2 (4408) TGCAGAAGAAACACGCAAATTAATGCCTGTCTGTGTGGAAACTAAAGCCATAGTTTCAACTATACAGCGTAAATATAAGGGTA
       Fig badnavirus 1 NC_017830.1 (2305) ACCCGACGAGAAATCATTAGCAACAATTCGATTCGAGGAATACAAGGAAGAAG-----AATCTCAATCGGATGAAGAACATTA
```

```
                                                                                  Section 56
                                      (4566) 4566          4580      4590      4600      4610      4620      4630             4648
SARS-CoV-2 Reference Genome NC_045512.2 (4491) TTAAAATACAAGAGGGTGTGGTTGATTATGG-TGCTAGATTTTACTTTTACACCAGTAAAACAACTGTAGCGTCACTTATCAA
       Fig badnavirus 1 NC_017830.1 (2383) TCAA----CCAGATGCTGAAGCAGAATTTGACTACCCGCTAAAAAGGATTCATTACCTAAATC-CTTTTTCGGAAGGTGGTGG
```

```
                                                Section 57
                                      (4649) 4649       4660      4670      4680      4690      4700      4710      4720     4731
SARS-CoV-2 Reference Genome NC_045512.2 (4573) CACACTTAACGATCTAAATGAAACTCTTGTTACAATGCCACTTGGCTATGTAACACATGGCTTAAATTTGGAAGAAGCTGCTC
     Fig badnavirus 1 NC_017830.1 (2461) GAAAGATGACCATTTAAAT-----TCTGAAGA-AATGG-ACTACCC---GCAAAGAATGAAGAAAATAGAAAAGTTACTA-TC
                                                Section 58
                                      (4732) 4732    4740      4750      4760      4770      4780      4790      4800     4814
SARS-CoV-2 Reference Genome NC_045512.2 (4656) GGTATATGAGATCTCTCAAAGTGCCAGCTACAGTTTCTGTTTCTTCACCTGATGCTGTTACAGCGTATAATGGTTATCTTACT
     Fig badnavirus 1 NC_017830.1 (2533) CACCAGTGAGGTAACT---AGT-CCA--TACAGAC-------CACCAGAAGATGCAGCCATGGGAAGACCAAGTTATC---CA
                                                Section 59
                                      (4815) 4815 4820      4830      4840      4850      4860      4870      4880     4897
SARS-CoV-2 Reference Genome NC_045512.2 (4739) TCTTCTTCTAAAACACCTGAAGAACATTTTATTGAAACCATCTCACTTGCTGGTTCCTATAAAGATTGGTCCTATTCTGGACA
     Fig badnavirus 1 NC_017830.1 (2600) CCAGCTAGGACAATACCAGGAGGA---------GGGGCAAGCTCAAGCGCTGAA-CCTCCAAGGTTTGATGC-AGCCAGAACA
                                                Section 60
                                      (4898) 4898        4910      4920      4930      4940      4950      4960      4970     4980
SARS-CoV-2 Reference Genome NC_045512.2 (4822) ATCTACACAACTAGGTATAGAATTTCTTAAGAGAGGTGATAAAAGTGTATATTACACTAGTAATCCTACCACATTCCACCTAG
     Fig badnavirus 1 NC_017830.1 (2672) AGATTTAAAG--GGGGATACAATGACGAGAT-GTGGACACTACCATCAGCCCAACAGAAG-AATGGAGCCATGTTCGTAATAC
                                                Section 61
                                      (4981) 4981     4990      5000      5010      5020      5030      5040      5050     5063
SARS-CoV-2 Reference Genome NC_045512.2 (4905) ATGGTGAAGTTATCACCTTTGACAATCTTAAGACACTTCTTTCTTTGAGAGAAGTGAGGACTATTAAGGTGTTTACAACAGTA
     Fig badnavirus 1 NC_017830.1 (2751) CAG--------AACAACTTGGGCTATTCAATGATGCTT-TCTCTAGATGGGAATCAGTCACTAAAAACCATGTGGCTACACAA
                                                Section 62
                                      (5064) 5064  5070      5080      5090      5100      5110      5120      5130     5146
SARS-CoV-2 Reference Genome NC_045512.2 (4988) GACAACATTAACCTCCACACGCAAGTTGTGGACATGTCAATGACATATGGACAACAGTTTGGTCCAACTTATTTGGATGGAGC
     Fig badnavirus 1 NC_017830.1 (2825) GGTTTTACTGATACCCGAGATAAAATAAAGTACATGGAAAATCTACTCGGAGAAA----TTGAGAAGCTGATTTGGATACAGT
                                                Section 63
                                      (5147) 5147          5160      5170      5180      5190      5200      5210     5229
SARS-CoV-2 Reference Genome NC_045512.2 (5071) TGATGTTACTAAAATAAAACCTCATAATTCACATGAAGGTAAAACATTTTATGTTTTACCTAATGATGACACTCTACGTGTTG
     Fig badnavirus 1 NC_017830.1 (2904) GGAG---AATGACGTATG-CCACGGAATAC----GAAGCCTTAGTAGCCATCGGAGAAGGCAGGGATGGTACTCA--------
```

```
                                                                                                    Section 64
                                         (5230) 5230                5240                5250                5260                5270                5280                5290                5300             5312
SARS-CoV-2 Reference Genome NC_045512.2  (5154) AGGCTTTTGAGTACTACCACACAACTGATCCTAGTTTTCTGGGTAGGTACATGTCAGCATTAAATCACACTAAAAAGTGGAAA
       Fig badnavirus 1 NC_017830.1      (2971) --------GAACATTCTCTCGCAAATGAGAAGAGTGTTTTCCTTAGAAGATCCGGCGCAGGGTTCGACAGTAATCCAGGATGA
                                                                                                    Section 65
                                         (5313) 5313        5320                5330                5340                5350                5360                5370                5380             5395
SARS-CoV-2 Reference Genome NC_045512.2  (5237) TACCCACAAGTTAATGGTTTAACT-TCTATTAAATGGGCAGATAACAACTGTTATCTTGCCACTGCATTGTTAACACTCCAAC
       Fig badnavirus 1 NC_017830.1      (3046) AGCCTACAGAGAGTTGGAAAGAATGTCTTGTACAGACGTTAAATACATCA-TTCCATTTCTAAATGAGTATATGCGTTTGGCA
                                                                                                    Section 66
                                         (5396) 5396                          5410                5420                5430                5440                5450                5460             5478
SARS-CoV-2 Reference Genome NC_045512.2  (5319) AAATAGAGTTGAAGTTTAATCCACCTGCTCTACAAGATGCTTATTACAGAGCAAGGCTGG-TGAAGCTGCTA----ACTTTT
       Fig badnavirus 1 NC_017830.1      (3128) GCAAAGACGGGAAGGCTATTC----TTAG---GAGGAGAACTAT--CTGAG-AAGATCTGGATGAAGATGCCCGGAGATTTAA
                                                                                                    Section 67
                                         (5479) 5479              5490                5500                5510                5520                5530                5540                5550             5561
SARS-CoV-2 Reference Genome NC_045512.2  (5397) GTGCACTTATCTTAGCCTACTGTAATAAGACAGTAGGTG-AGTTAGGTGATGTTAGAGAAACAATGAGTTACTTGTTTCAACA
       Fig badnavirus 1 NC_017830.1      (3201) GAAAAACAATAAAAGAAGACTTTGACAACAAATACCCAGGAGTCGTATCAGGGGTGGTACCCAGGGTATTATTTGCTT--ACA
                                                                                                    Section 68
                                         (5562) 5562        5570                5580                5590                5600                5610                5620                5630             5644
SARS-CoV-2 Reference Genome NC_045512.2  (5479) TGCCAATTTAGATTCTTGCAAAAGAGTCTTGAACGTGGTGTGTAAAACTTGTGGACAACAGCAGACAACCCTTAAGGGTGTAG
       Fig badnavirus 1 NC_017830.1      (3282) AG----------TTCTTGGAGAAG------GAATGCAGAGATGCAGCATTCAAGAGGTCCCTAAAGAACCTGTCATTTTGTAG
                                                                                                    Section 69
                                         (5645) 5645 5650                5660                5670                5680                5690                5700                5710             5727
SARS-CoV-2 Reference Genome NC_045512.2  (5562) AAGCTGTTATGTACATGGGCACACTTTCTTATGAACAATTTAAGAAAGGTGTTCAGATACCTTGTACGTGTGGTAAACAAGCT
       Fig badnavirus 1 NC_017830.1      (3349) TTCAATTCCTATACCAGGATAC-------TAT-AAAAATACAGGCAAGAAGTATGGA------GTGCGAA-GATCCAAGACGT
                                                                                                    Section 70
                                         (5728) 5728                5740                5750                5760                5770                5780                5790                5800             5810
SARS-CoV-2 Reference Genome NC_045512.2  (5645) ACAAAATATCTAGTACAACAGGAGTCACCTTTTGTTATGATGTCAGCACCACCTGCTCAGTATGAACTTAAGCATGGTACATT
       Fig badnavirus 1 NC_017830.1      (3417) ACAAAG--GCAAGCCTCACGAGAGCCATGCTAGGATTGAAAGCGAAAGCACCTGGTCCGCAACAAAGATGCAAGTGCTTCC
```

```
                                                                                          Section 71
                                      (5811) 5811     5820      5830      5840      5850      5860      5870      5880       5893
SARS-CoV-2 Reference Genome NC_045512.2 (5728) TACTTGTGCTAGTGAGTACACTGGTAATTACCAGTGTGGTCACTATAAACATATAACTTCTAAAGAAACTTTGTATTGCATAG
     Fig badnavirus 1 NC_017830.1 (3498) TATGTGGACAAGAAGGTCATTTTGCAAGAA--AGTGTCCTAATGATAAA---AGGAGCTCTAA----A---------------
                                                                                          Section 72
                                      (5894) 5894  5900      5910      5920      5930      5940      5950      5960       5976
SARS-CoV-2 Reference Genome NC_045512.2 (5811) ACGGTGCTTTACTTACAAAGTCCTCAGAATACAAAGGTCCTATTACGGATGTTTCTACAAAGAAAACAGTTACACAACAACC
     Fig badnavirus 1 NC_017830.1 (3557) AGGATGGCTATGTTTGAAAATCTGGAAATACCAGAAG----ATTGCG-ATATAGTCTCTGTAGAAGAAGG--AGAGGATATGA
                                                                                          Section 73
                                      (5977) 5977         5990      6000      6010      6020      6030      6040       6059
SARS-CoV-2 Reference Genome NC_045512.2 (5894) ATAAAACCAGTTACTTATAAATTGGATGGTGTTGTTTGTACAGAAATTGACCCTAAGTTGGACAATTATTATAAGAAAGACAA
     Fig badnavirus 1 NC_017830.1 (3633) GTGATGCCATATACTCA-ATATCCGAAGGAGAAGATGGAGCAGA---TGAGCTGCAGAAAT-CACTCCAAACATTGGAAATTG
                                                                                          Section 74
                                      (6060) 6060      6070      6080      6090      6100      6110      6120      6130       6142
SARS-CoV-2 Reference Genome NC_045512.2 (5977) TTCTTATTTCACAGAGCAACCAATTGATCTTGTACCAAACCAACCATATCCAAACGCAAGCTTCGATAATTTTAAGTTTGTAT
     Fig badnavirus 1 NC_017830.1 (3711) TTCTTATGTTAG-GAGAAATTGATGGAG--GGTACCGGCCG---CAGATCAAGCTTCCTGA--GGAGCAGATGAATTGTGTCC
                                                                                          Section 75
                                      (6143) 6143   6150      6160      6170      6180      6190      6200      6210       6225
SARS-CoV-2 Reference Genome NC_045512.2 (6060) GTGATAATATCAAATTTGCTGATGATTTAAACCAGTTAACTGGTTATAAGAAACCTGCTTCAAGAGAGCTTAAAGTTACATTT
     Fig badnavirus 1 NC_017830.1 (3786) ATAACTGGATACATAATGGGGATATCTTTGTACAAAGAGCTGTTCATTGCA-----GCTTCTGCAGAAGGGAAAGCATAAAAA
                                                                                          Section 76
                                      (6226) 6226           6240      6250      6260      6270      6280      6290       6308
SARS-CoV-2 Reference Genome NC_045512.2 (6143) TTCCCTGACTTAAATGGTGATGTGGTGGCTATTGA----TTATAAACACTACACACC---CTCTTTAAGAAAGGAGCTAAAT
     Fig badnavirus 1 NC_017830.1 (3864) GGGCAAGAAT-ACATTGTCCTTTG-TGTCTCCTAACAGCTTGCAATCTGTGCGGACCTTACTACTTGAAGATAGAAGTACCGG
                                                                                          Section 77
                                      (6309) 6309       6320      6330      6340      6350      6360      6370      6380       6391
SARS-CoV-2 Reference Genome NC_045512.2 (6219) TGTTACATAAACCTATTGTTTGGCATGTTAACAATGCAACTAATAAAGCCACGTATAAACCAAATACC-TGGTGTATACGTTG
     Fig badnavirus 1 NC_017830.1 (3945) TGGAACCAGCACC-ATCAACCCCGATAAATCCGAGAAGAATTATTCAGGAGCAGCACAACTACATCGGATGGTGTG-AAGTAG
```

```
                                                  Section 78
                                     (6392) 6392    6400      6410      6420      6430      6440      6450      6460      6474
SARS-CoV-2 Reference Genome NC_045512.2 (6301) TCTTTGGAGCACAAAACCAGT---TGAAACATCAAATTCGTTTGATGTACTGAAGTCAGAGGACGCGCAGGGAATGGATAATC
  Fig badnavirus 1 NC_017830.1 (4026) AAATAGAGAGACTAAACAAGGAAGTGAAGCACTACAAAGG---GCTATAC-GAAGCCTTACTAATGGA--GAAATCCTTAAGG

                                                  Section 79
                                     (6475) 6475 6480      6490      6500      6510      6520      6530      6540           6557
SARS-CoV-2 Reference Genome NC_045512.2 (6381) TT-GCCTGCGAAGATCTAAAACCAGTCTCTGAAGAAGTAGTGGAAAATCCTACCATACAGAAAGACGTTCTTGAGTGTAATGT
  Fig badnavirus 1 NC_017830.1 (4103) AAAGACTACGAGGAGCTGAAAGAAG----GAGATAAGGAGCGAAGAAAGGG--CATAATGATACAAGAACCAGA-----AGAA

                                                  Section 80
                                     (6558) 6558        6570      6580      6590      6600      6610      6620      6630  6640
SARS-CoV-2 Reference Genome NC_045512.2 (6463) GAAAACTACCGAAGTTGTAGGAGACATTATACTTAAACCAGCAA-ATAATAGTTTAA-AAATTACAGAAGAGGTTGGCCACAC
  Fig badnavirus 1 NC_017830.1 (4175) GTTAACTATCTGGGA-GAAGATGAGAAGATTCTTGCAGCAGGAACAGAGGAAAAGAAGAAATTCGTCAAGAATATG-CTTTAT

                                                  Section 81
                                     (6641) 6641     6650      6660      6670      6680      6690      6700      6710      6723
SARS-CoV-2 Reference Genome NC_045512.2 (6544) AGATCTAATGGCTGCTTATGTAGACAATTCTAGTCTTACTATTAAGAAACCTAATGAATTATCTAGAGTATTAGGTTTGAAAA
  Fig badnavirus 1 NC_017830.1 (4256) AACTTTGATAT-TGAATTTG-AGATTCCAGATGTCTCAAGATTCAGAGTAAGAGC-AATTCTTGATAC-AGGAGCTTCAACAT

                                                  Section 82
                                     (6724) 6724  6730      6740      6750      6760      6770      6780      6790           6806
SARS-CoV-2 Reference Genome NC_045512.2 (6627) CCCTTGCTACTCATGGTTTAGCTGCTGTTAATAGTGTCCCTTGGGATACTATAGCTAATTATGCTAAGCCTTTTCTTAACAAA
  Fig badnavirus 1 NC_017830.1 (4335) GCTGTATCAATGAAGGAGCAGTTCC----AAAAGAAGCCCTC--GAAGAAAGCCCTTATGAAGTTCAGCTGAATGGGGTCAAC

                                                  Section 83
                                     (6807) 6807         6820      6830      6840      6850      6860      6870           6889
SARS-CoV-2 Reference Genome NC_045512.2 (6710) GTTGTTAGTACAACTACTAACATAGTTACACGGTGTTTAACCGTGTTTGTACTAATTATATGCCTTATTTCTTTACTTTATT
  Fig badnavirus 1 NC_017830.1 (4412) TCTGTGCAGAAAACAAAGAAGA-AGCTCAAGTACGGGAGAATGATGATCGGGATGAATACGTTCAGAATCCCTTT----TAC-

                                                  Section 84
                                     (6890) 6890      6900      6910      6920      6930      6940      6950      6960      6972
SARS-CoV-2 Reference Genome NC_045512.2 (6793) GCTACAATTGTGTACTTTTACTAGAAGTACAAATTCTAGA--ATTAAAGCATCTATGCCGACTACTATAGCAAAGAATACTGT
  Fig badnavirus 1 NC_017830.1 (4489) -ATACAGTCTGCCACTGGTCATCGGAG-ATAACATCCAGATGATTGTAGGTTGTA--------ACTTCATCAGAGCAATGTAT
```

Section 85

```
                                         (6973) 6973                                                                             7055
SARS-CoV-2 Reference Genome NC_045512.2 (6874) TAAGAGTGTCGGTAAATTTTGTCTAGAGGCTTCATTTAATTATTTGAAGTCACCTAATTTTTCTAAACTGATAAATATTATAA
         Fig badnavirus 1 NC_017830.1 (4562) GGAG-GAGTGAGAATAGAAGGGAATGAGGTAACCTTTTACAAGA--ACCTCACGGATTAGCACATCCCCAGAAG--TCTCG
```

Section 86

```
                                         (7056) 7056                                                                             7138
SARS-CoV-2 Reference Genome NC_045512.2 (6957) TTTGGTTTTTACTATTAAGT-GTTTGCCTAGGTTCTTTAATCTACTCAACCGCTGCTTTAGGTGTTTT--AATGTCTAATTTA
         Fig badnavirus 1 NC_017830.1 (4640) GTGAATTCCCTGGATGAAGAAGTTTGCGAAGAA----GAATATTTGCAGGTGCAGGAGATGGTGATCTGCAACATTGGAGAAA
```

Section 87

```
                                         (7139) 7139                                                                             7221
SARS-CoV-2 Reference Genome NC_045512.2 (7037) GGCATGCCTTCTTACTGTACTGGTTACAGAGAAGGCTATTTGAACTCTACTAAT----GTCACTATTGCAACCTACTGTACTG
         Fig badnavirus 1 NC_017830.1 (4719) GCCGAAAATCATTTCTTAAGAAGTTCAGGCCTTTGCTGGAAGAACTTAAGGAAGCAGGGTTTATAGGAGAAAATCCATTACAG
```

Section 88

```
                                         (7222) 7222                                                                             7304
SARS-CoV-2 Reference Genome NC_045512.2 (7116) GTTCTATACCTTGTAGTGTTTGTCTTAGTGGTTTAGATTCTTTAGACACCTATCCTTCTTTAGAAACTATACAAATTACCATT
         Fig badnavirus 1 NC_017830.1 (4802) CATTGGAAGAAAAATGGGATTTTATGCCAACTTGATATCAAAAATCCTGATTTCATAATT--GAAGATAGGCCACTGAAGAAT
```

Section 89

```
                                         (7305) 7305                                                                             7387
SARS-CoV-2 Reference Genome NC_045512.2 (7199) TCATCTTTTAAATGGGATTTAACTGCTTTTGGCTTAGTTGCAGAGTGGTTTTTGGCATATATTCTTTTCACTAGGTTTTTCTA
         Fig badnavirus 1 NC_017830.1 (4883) TTAACGCCACAGATGAAG--GAGTCCTTCAAGAAGCATATCAAGGT----TTTGCTGGATTTAGGAGTCATCAGGGC-----A
```

Section 90

```
                                         (7388) 7388                                                                             7470
SARS-CoV-2 Reference Genome NC_045512.2 (7282) TGTACTTGGATTGGCTGCAATCATGCAATTGTTTTTCAGCTATTTTGCAGTACATTTTATTAGTAATTCTTGGCTTATGTGGT
         Fig badnavirus 1 NC_017830.1 (4955) AGTAAAAGCCGCCACAG-AACGACGGCAATGCTAGTCAACTCA--GGAACAACGGTGGATCCG-AAAACAGGG---AAGGAGA
```

Section 91

```
                                         (7471) 7471                                                                             7553
SARS-CoV-2 Reference Genome NC_045512.2 (7365) TAATAATTAATCTTGTACAAATGGCCCCGATTTCAGCTATGGTTAGAATGTACATC--TTCTTTGCATCATTTTATTATGTAT
         Fig badnavirus 1 NC_017830.1 (5031) TCAAAAGGAAG---GAAAGAATGGTCTTCAACT-ACAAAAGGTT-GAATG-ACATCACTCATAAGGATCAGTATAGTCTTCCA
```

```
                                                   Section 92
                                            (7554) 7554 7560 7570 7580 7590 7600 7610 7620 7636
SARS-CoV-2 Reference Genome NC_045512.2     (7446) GGAAAAGTTATGTGCATGTTGTAGACGGTTGTAATTCATCAACTTGTATGATGTGTTACAAACGTAATAGAGCAACAAGAGTC
Fig badnavirus 1 NC_017830.1                (5108) GGAATAAATACG---ATCCTCAAGAAGGTTGGAAAT-AGCAA---G----ATATTCAGCAAGTTTGATCTA---A--AGAG-C
                                                   Section 93
                                            (7637) 7637 7650 7660 7670 7680 7690 7700 7719
SARS-CoV-2 Reference Genome NC_045512.2     (7529) GAATGTACAACTATTGTTAATGGTGTTAGAAGGTCCTTTTATGTCTATGCTAATGGAGGTAAAGGCTTTTGCAAACTACACAA
Fig badnavirus 1 NC_017830.1                (5174) GGAT-TTCACCAAGTTGCTATG------------CATCCTGACTCAATAG-AATGGAC----AGCTTTTTGGGTCCCAGACGG
                                                   Section 94
                                            (7720) 7720 7730 7740 7750 7760 7770 7780 7790 7802
SARS-CoV-2 Reference Genome NC_045512.2     (7612) TTGGAATTGTGTTAATTGTGATACATTCTGTGCTGGTAGTACATTTATTAGTGATGAAGTTGCGAGAGACTTGTCACTACAGT
Fig badnavirus 1 NC_017830.1                (5239) ATTGTAT-GAGTGGCTTGTCATGCCATTCGGACTCAAAA-ACGCTCCTTC----TGTTTTTCAAAGAAAGATGGATGAATGCT
                                                   Section 95
                                            (7803) 7803 7810 7820 7830 7840 7850 7860 7870 7885
SARS-CoV-2 Reference Genome NC_045512.2     (7695) TTAAAAGACCAATAAATCCTACTGACCAGTCTTCTTACATCGTTGATAGTGTTACAGTGAAGAATGGTTCCATCCATCTTTAC
Fig badnavirus 1 NC_017830.1                (5316) TCAAAGGGACAGAAGATTTTATTG--CAGTC----TACATTGACGATATTCTT-------------GTTTTCTC-----TGAA
                                                   Section 96
                                            (7886) 7886 7900 7910 7920 7930 7940 7950 7968
SARS-CoV-2 Reference Genome NC_045512.2     (7778) TTTGATAAAGCTGGTCAAAAGACTTATGAAAGACATTCTCTCTCTCATTTTGTTAACTTAGACAAC-CTGAGAGCTAATAACA
Fig badnavirus 1 NC_017830.1                (5375) AACGAGAAAGATCATGCGAAGCACT-TGAAGGCCATGTTGGAGATCTGCAAAAGAAATGGGTTAGTTCTAAGCCCATCTAAAA
                                                   Section 97
                                            (7969) 7969 7980 7990 8000 8010 8020 8030 8040 8051
SARS-CoV-2 Reference Genome NC_045512.2     (7860) CTAAAGGTTCATTGCCTATTAATGTTATAGTTTTTGATGGTAAATCAAAATGTGAAGAATCATCTGCAAAATCAGCGTCTGTT
Fig badnavirus 1 NC_017830.1                (5457) -TGAAGATAGCAGTCCAAGAGGTGGAATT-TCTTGGAGCCCAAATCGGAAATCAAAGGATC-----CGACTTCAGCCACATGT
                                                   Section 98
                                            (8052) 8052 8060 8070 8080 8090 8100 8110 8120 8134
SARS-CoV-2 Reference Genome NC_045512.2     (7943) TACTACAGTCAGCTTATGTGTCAACCTATACTGTTACTAGATCAGGCATTAGTGTCTGATGTTGGTGATAGTGCGGAAGTTGC
Fig badnavirus 1 NC_017830.1                (5533) TA-TAAAGAAGATCGTTGAGTTCAATGAGGCTG--AGTTGAAGGAGAAAAAGGGCATGA----GATCATGGTTAGGAATTCTC
```

Section 99

```
                                          (8135) 8135 8140      8150      8160      8170      8180      8190      8200          8217
SARS-CoV-2 Reference Genome NC_045512.2   (8026) AGTTAAAATGTTTGATGCTTACGTTAATACGTTTTCATCAACTTTTAA-CGTAC-CAATGGAAAACTCAAAACACTAGTTGC
Fig badnavirus 1 NC_017830.1              (5609) AACT---ATGCTCGC-GCATACATTCCTAACTTGGGC-CGCCTTTTAAGCCCACTCTATGCCAAAAC----AAGTCCAACTGG
```

Section 100

```
                                          (8218) 8218        8230      8240      8250      8260      8270      8280      8290   8300
SARS-CoV-2 Reference Genome NC_045512.2   (8107) AACTGCAGAAGCTGAACTTGCAAAGAATGTGTCCTTAGACAATGTCTTATCTACTTTTATTTCAGCAGCTCGGCAAGGGTTTG
Fig badnavirus 1 NC_017830.1              (5683) AGATA-AGAGGATGAA--TGCCCAGGAT-------TGG--AAGCTCGTGGCCCAAATAAAAGAAGAAGTCCAGAA--GC-TGC
```

Section 101

```
                                          (8301) 8301     8310      8320      8330      8340      8350      8360      8370      8383
SARS-CoV-2 Reference Genome NC_045512.2   (8190) TTGATTCAGATGTAGAAACTAAAGATGTTGTTGAATGTCTTAAATTGTCACATCAATCTGACATAGAAGTTACTGGCGATAGT
Fig badnavirus 1 NC_017830.1              (5751) CAGATCTGGAGCTACCACCCGAGGA--TTGTTTCATC------ATTCTTGAAACAGACGGCTGTATGACTGGTTGGGGAGGAA
```

Section 102

```
                                          (8384) 8384  8390      8400      8410      8420      8430      8440      8450      8466
SARS-CoV-2 Reference Genome NC_045512.2   (8273) TGTAATAACTATATGCTCACCTATAACAAAGTTGAAAACATG-ACACCCCGTGACCTTGGTGCTTGTATTGACTGTAGTGCGC
Fig badnavirus 1 NC_017830.1              (5826) TTTG-TAAATGGAAGCCCAAAAGAAACGACCCAAGAAGGATGGAGAAAGTTTGCGCCTA-TGCTAGTGGGAAGTTTAGTCCAA
```

Section 103

```
                                          (8467) 8467         8480      8490      8500      8510      8520      8530          8549
SARS-CoV-2 Reference Genome NC_045512.2   (8355) ----GTCAT--ATTAATGCGCAGGTAGCAAAAGTCAC-AACATTGCTTTGATATGGAACGTTAAAGATTTCATGTCATTGTC
Fig badnavirus 1 NC_017830.1              (5907) TCAAGTCAACCATTGATGCCGAAATC-CATGCAGTCATGAACA---CTCTG----GAAAATTAAAGATTTATTATCTTGATA
```

Section 104

```
                                          (8550) 8550      8560      8570      8580      8590      8600      8610      8620        8632
SARS-CoV-2 Reference Genome NC_045512.2   (8431) TGAACAACTACGAAACAAATACGTAGTGCTGCTAAAAAGAATAACTTACCTTTTAAGTTGACATGTGCAACTACTAGACAAG
Fig badnavirus 1 NC_017830.1              (5982) AAAAGGAGTT-GATCATCAGAACTGACTGCC-----AAGCTATCATCAGCTTCTTCAAT--AAATCCGC----CCTAAATAAA
```

Section 105

```
                                          (8633) 8633   8640      8650      8660      8670      8680      8690      8700          8715
SARS-CoV-2 Reference Genome NC_045512.2   (8514) TTGTTAATGTTGTAACAACAAAGATAGCACTTAAGGGTGGTA--AAATTGTTAATAATTGGT-TGAAGCAGTTAATTAAAGTT
Fig badnavirus 1 NC_017830.1              (6053) CCCTCTAGAGTG-----AGATGGGTAGCCTTTACGGATTTTATCACAGGGATAGGAATTAATGTGGAGTTCGAGCATATAGAT
```

```
                                                                                                   Section 106
                                          (8716) 8716          8730      8740      8750      8760      8770      8780          8798
SARS-CoV-2 Reference Genome NC_045512.2  (8594) ACACTTGTGTTCCTTTTTGTTGCTGCTATTTTCTATTTAATAACACCTGTTCATGTCATGTCTAAACATACTGACTTTTCAAG
     Fig badnavirus 1 NC_017830.1  (6131) GGAAGGGATAATGTGCTTGCTGATAGCCTTTCAAGGTTAATTA-ACAGTTTC-TT-----TCTTACAGAATGGAGTCTGAAAA
                                                                                                   Section 107
                                          (8799) 8799       8810      8820      8830      8840      8850      8860      8870    8881
SARS-CoV-2 Reference Genome NC_045512.2  (8677) TGAAATCATAGGATACAAGGCTATTGATGGTGGTGTCACTCGTGACATAGCATCTA-CAGATACTTGTTTTGCTAACAAACAT
     Fig badnavirus 1 NC_017830.1  (6207) AGGAGGAAGCAGTAGCAGAGCTAGAGAAGCTTATGGAAGACAA-ACAATGCAGGAAGCAACTCCTACCATTAATCAACAAAAT
                                                                                                   Section 108
                                          (8882) 8882    8890      8900      8910      8920      8930      8940      8950      8964
SARS-CoV-2 Reference Genome NC_045512.2  (8759) GCTGATTTTGACACATGGTTTAGCCAGCGTGGTGGTAGTTATACTAATGACAAAGCTTGCCCATTGATTGCTGCAGTCATAAC
     Fig badnavirus 1 NC_017830.1  (6289) GACGAACTGTTTCAACAATACCAGAACCTTGCTG--------AGGAAAGAGAAAGGAT--CCTTCGAT--CAGGAGGAATATC
                                                                                                   Section 109
                                          (8965) 8965 8970      8980      8990      9000      9010      9020      9030          9047
SARS-CoV-2 Reference Genome NC_045512.2  (8842) AAGAGAAGTGGGTTTTGTCGTGCCTGGTTTGC-CTGGCACGATATTACGCACAACTAATGGTGACTTTTTGCATTTCTTACCT
     Fig badnavirus 1 NC_017830.1  (6360) TTTTGCCAGAGACAATGAATTCAGAAAACAACTCAGGGAGGTT-----GAAGAAGAATCTGCAAGAAGAGCCATTAAAGCCCT
                                                                                                   Section 110
                                          (9048) 9048        9060      9070      9080      9090      9100      9110      9120  9130
SARS-CoV-2 Reference Genome NC_045512.2  (8924) AGAGTTTTTAGTGCAGTTGGTAACATCTGTTACACACCATCAAAACTTATAGAGTACACTGACTTTGCAACATCAGCTTGTGT
     Fig badnavirus 1 NC_017830.1  (6438) GGAACAATACAGGCAGATTCACACACTTAAAACAGCCCAATGTGCATCATGGAGTTCACCCGGAAGGGATGGGAATTATTGGT
                                                                                                   Section 111
                                          (9131) 9131     9140      9150      9160      9170      9180      9190      9200     9213
SARS-CoV-2 Reference Genome NC_045512.2  (9007) TT---TGGCTGCTGAATGT-ACAATTTTTAAAGATGCTTCTGGTAAGCCAGTACCATATTGTTATGATACCAATG-TACTAGA
     Fig badnavirus 1 NC_017830.1  (6521) CTGATTGCTTGCCAAGCGTTAGGCGATATGACGCAAGATTAGAAAAGGTCCTACAAGACTTGAAGGACGCGTCAGCTGACAGC
                                                                                                   Section 112
                                          (9214) 9214  9220      9230      9240      9250      9260      9270      9280         9296
SARS-CoV-2 Reference Genome NC_045512.2  (9085) AGGTTCTGTTGCTTATGAAAGTTTACGCCCTGACACACGTTATGTGCTCATGGATGGCTCTATTATTCAATTTCCTAACACCT
     Fig badnavirus 1 NC_017830.1  (6604) CTAAAGTTCCGCATTTGAAGCATGATGGGCCCAGAAAAGTGGT----TGAGGCGTCTCACTATAAT-CGATGCCCTTATCTTT
```

```
                                                                                                   Section 113
                                       (9297) 9297         9310        9320        9330        9340        9350        9360           9379
SARS-CoV-2 Reference Genome NC_045512.2 (9168) ACCTTGAAGGTTCTGTTAGAGTGGTAACAACTTTTGATTCTGAGTACTGTAGGCACGGCACTTGTGAAA-GATCAGAAGCTGG
         Fig badnavirus 1 NC_017830.1 (6682) TCCTTTGTCATTTTGTAATAATGGAATCGTCTTTTTGACCCAAAGGCTGCCGTC------CATGTGCAGTGATTC----CTTT
                                                                                                   Section 114
                                       (9380) 9380      9390        9400        9410        9420        9430        9440        9450     9462
SARS-CoV-2 Reference Genome NC_045512.2 (9250) TGTTTGTGTATCTACTAGTGGTAGATGG---GTACTTAACAATGATTATTACAGATCTTTACCAGGAGTTTTCTGTGGTGTAG
         Fig badnavirus 1 NC_017830.1 (6755) AGTTTGCGTGTTTTATCCGCTTACACGGATTGTCCTTTGTAACCACTTTCACGTGGTTTTGC------TTTTCTTTAGTGCGC
                                                                                                   Section 115
                                       (9463) 9463    9470        9480        9490        9500        9510        9520        9530       9545
SARS-CoV-2 Reference Genome NC_045512.2 (9330) ATGCTGTAAATT-TACTTACTAATATGTTTACACCACTAATTCAACCTATTGGTGCTTTGGACATATCAGCATCTATAGTAGC
         Fig badnavirus 1 NC_017830.1 (6832) CTCTTTTGCGCCCTTGTTTTTAGCGTTAGTACGCTTTGAGTCAAGTTTCCTTATCATTTGGGAATCTTTTGAGCTGTCGATG-
                                                                                                   Section 116
                                       (9546) 9546            9560        9570        9580        9590        9600        9610         9628
SARS-CoV-2 Reference Genome NC_045512.2 (9412) TGGTGGTATTGTAGCTATCGTAGTAACATGCCTTGCCTACTATTTTATGAGGTTTAGAAGAGCTTTTGGTGAATACAGTCATG
         Fig badnavirus 1 NC_017830.1 (6914) -GGGCCCAATGAAGCACCCG-----AGCTCTTTTAATTCCTATATAAGGAGCTTTAGTTTCG-TTGCAAGGCACACAACAATC
                                                                                                   Section 117
                                       (9629) 9629        9640        9650        9660        9670        9680        9690        9700    9711
SARS-CoV-2 Reference Genome NC_045512.2 (9495) TAGTTGCCTTTAATACTTTACTATTCCTTATGTCATTCACTGTACTCTGTTTAACACCAGTTTACTCATTCTTACCTGGTGTT
         Fig badnavirus 1 NC_017830.1 (6990) AAGCAACCCTTGAGCCTT-ACTTCGAAAAGAGTCTGTAAAAGTTTAGAGTCTGTAATCAGTGTG--AAGTTTGAAGAAATAAA
                                                                                                   Section 118
                                       (9712) 9712     9720        9730        9740        9750        9760        9770        9780       9794
SARS-CoV-2 Reference Genome NC_045512.2 (9578) TATTCTGTTATTTACTTGTACTTGACATTT-TATCTTACTAATGATGTTTCTTTTTTAGCACATATTCAGTGGATGGTTATGT
         Fig badnavirus 1 NC_017830.1 (7070) TTAGAGGTAATTCAGTTTAAAATCTCTTTTATTTCTTGTTTTCGATCCATTTTATTTGGGGGACACACAAA------------
                                                                                                   Section 119
                                       (9795) 9795 9800        9810        9820        9830        9840        9850        9860           9877
SARS-CoV-2 Reference Genome NC_045512.2 (9660) TCACACCTTTAGTACCTTTCTGGATAACAATTGCTTATATCATTTGTATTTCCACAAAGCATTTCTATTGGTTCTTTAGTAAT
         Fig badnavirus 1 NC_017830.1 (7141) -----------------------------------------------------------------------------------
```

```
                                                              Section 120
                                          (9878)  9878       9890      9900      9910      9920      9930      9940      9950    9960
SARS-CoV-2 Reference Genome NC_045512.2  (9743)  TACCTAAAGAGACGTGTAGTCTTTAATGGTGTTTCCTTTAGTACTTTTGAAGAAGCTGCGCTGTGCACCTTTTTGTTAAATAA
       Fig badnavirus 1 NC_017830.1      (7141)  -----------------------------------------------------------------------------------
                                                              Section 121
                                          (9961)  9961     9970      9980      9990      10000     10010     10020     10030     10043
SARS-CoV-2 Reference Genome NC_045512.2  (9826)  AGAAATGTATCTAAAGTTGCGTAGTGATGTGCTATTACCTCTTACGCAATATAATAGATACTTAGCTCTTTATAATAAGTACA
       Fig badnavirus 1 NC_017830.1      (7141)  -----------------------------------------------------------------------------------
                                                              Section 122
                                         (10044)  10044 10050     10060     10070     10080     10090     10100     10110           10126
SARS-CoV-2 Reference Genome NC_045512.2  (9909)  AGTATTTTAGTGGAGCAATGGATACAACTAGCTACAGAGAAGCTGCTTGTTGTCATCTCGCAAAGGCTCTCAATGACTTCAGT
       Fig badnavirus 1 NC_017830.1      (7141)  -----------------------------------------------------------------------------------
                                                              Section 123
                                         (10127)  10127        10140     10150     10160     10170     10180     10190           10209
SARS-CoV-2 Reference Genome NC_045512.2  (9992)  AACTCAGGTTCTGATGTTCTTTACCAACCACCACAAACCTCTATCACCTCAGCTGTTTTGCAGAGTGGTTTTAGAAAAATGGC
       Fig badnavirus 1 NC_017830.1      (7141)  -----------------------------------------------------------------------------------
                                                              Section 124
                                         (10210)  10210     10220     10230     10240     10250     10260     10270     10280     10292
SARS-CoV-2 Reference Genome NC_045512.2 (10075)  ATTCCCATCTGGTAAAGTTGAGGGTTGTATGGTACAAGTAACTTGTGGTACAACTACACTTAACGGTCTTTGGCTTGATGACG
       Fig badnavirus 1 NC_017830.1      (7141)  -----------------------------------------------------------------------------------
                                                              Section 125
                                         (10293)  10293  10300     10310     10320     10330     10340     10350     10360           10375
SARS-CoV-2 Reference Genome NC_045512.2 (10158)  TAGTTTACTGTCCAAGACATGTGATCTGCACCTCTGAAGACATGCTTAACCCTAATTATGAAGATTTACTCATTCGTAAGTCT
       Fig badnavirus 1 NC_017830.1      (7141)  -----------------------------------------------------------------------------------
                                                              Section 126
                                         (10376)  10376         10390     10400     10410     10420     10430     10440           10458
SARS-CoV-2 Reference Genome NC_045512.2 (10241)  AATCATAATTTCTTGGTACAGGCTGGTAATGTTCAACTCAGGGTTATTGGACATTCTATGCAAAATTGTGTACTTAAGCTTAA
       Fig badnavirus 1 NC_017830.1      (7141)  -----------------------------------------------------------------------------------
```

```
                                                                                                  Section 127
                                         (10459) 10459     10470     10480     10490     10500     10510     10520     10530     10541
SARS-CoV-2 Reference Genome NC_045512.2 (10324) GGTTGATACAGCCAATCCTAAGACACCTAAGTATAAGTTTGTTCGCATTCAACCAGGACAGACTTTTTCAGTGTTAGCTTGTT
            Fig badnavirus 1 NC_017830.1  (7141) -----------------------------------------------------------------------------------
                                                                                                  Section 128
                                         (10542) 10542  10550     10560     10570     10580     10590     10600     10610          10624
SARS-CoV-2 Reference Genome NC_045512.2 (10407) ACAATGGTTCACCATCTGGTGTTTACCAATGTGCTATGAGGCCCAATTTCACTATTAAGGGTTCATTCCTTAATGGTTCATGT
            Fig badnavirus 1 NC_017830.1  (7141) -----------------------------------------------------------------------------------
                                                                                                  Section 129
                                         (10625) 10625 10630     10640     10650     10660     10670     10680     10690           10707
SARS-CoV-2 Reference Genome NC_045512.2 (10490) GGTAGTGTTGGTTTTAACATAGATTATGACTGTGTCTCTTTTTGTTACATGCACCATATGGAATTACCAACTGGAGTTCATGC
            Fig badnavirus 1 NC_017830.1  (7141) -----------------------------------------------------------------------------------
                                                                                                  Section 130
                                         (10708) 10708       10720     10730     10740     10750     10760     10770     10780   10790
SARS-CoV-2 Reference Genome NC_045512.2 (10573) TGGCACAGACTTAGAAGGTAACTTTTATGGACCTTTTGTTGACAGGCAAACAGCACAAGCAGCTGGTACGGACACAACTATTA
            Fig badnavirus 1 NC_017830.1  (7141) -----------------------------------------------------------------------------------
                                                                                                  Section 131
                                         (10791) 10791    10800     10810     10820     10830     10840     10850     10860        10873
SARS-CoV-2 Reference Genome NC_045512.2 (10656) CAGTTAATGTTTTAGCTTGGTTGTACGCTGCTGTTATAAATGGAGACAGGTGGTTTCTCAATCGATTTACCACAACTCTTAAT
            Fig badnavirus 1 NC_017830.1  (7141) -----------------------------------------------------------------------------------
                                                                                                  Section 132
                                         (10874) 10874 10880     10890     10900     10910     10920     10930     10940           10956
SARS-CoV-2 Reference Genome NC_045512.2 (10739) GACTTTAACCTTGTGGCTATGAAGTACAATTATGAACCTCTAACACAAGACCATGTTGACATACTAGGACCTCTTTCTGCTCA
            Fig badnavirus 1 NC_017830.1  (7141) -----------------------------------------------------------------------------------
                                                                                                  Section 133
                                         (10957) 10957        10970     10980     10990     11000     11010     11020              11039
SARS-CoV-2 Reference Genome NC_045512.2 (10822) AACTGGAATTGCCGTTTTAGATATGTGTGCTTCATTAAAAGAATTACTGCAAAATGGTATGAATGGACGTACCATATTGGGTA
            Fig badnavirus 1 NC_017830.1  (7141) -----------------------------------------------------------------------------------
```

```
                                                          Section 134
                                          (11040) 11040     11050     11060     11070     11080     11090     11100     11110      11122
SARS-CoV-2 Reference Genome NC_045512.2 (10905) GTGCTTTATTAGAAGATGAATTTACACCTTTTGATGTTGTTAGACAATGCTCAGGTGTTACTTTCCAAAGTGCAGTGAAAAGA
          Fig badnavirus 1 NC_017830.1   (7141) -----------------------------------------------------------------------------------

                                                          Section 135
                                          (11123) 11123  11130     11140     11150     11160     11170     11180     11190       11205
SARS-CoV-2 Reference Genome NC_045512.2 (10988) ACAATCAAGGGTACACACCACTGGTTGTTACTCACAATTTTGACTTCACTTTTAGTTTTAGTCCAGAGTACTCAATGGTCTTT
          Fig badnavirus 1 NC_017830.1   (7141) -----------------------------------------------------------------------------------

                                                          Section 136
                                          (11206) 11206         11220     11230     11240     11250     11260     11270           11288
SARS-CoV-2 Reference Genome NC_045512.2 (11071) GTTCTTTTTTTTGTATGAAAATGCCTTTTTACCTTTTGCTATGGGTATTATTGCTATGTCTGCTTTTGCAATGATGTTTGTCA
          Fig badnavirus 1 NC_017830.1   (7141) -----------------------------------------------------------------------------------

                                                          Section 137
                                          (11289) 11289      11300     11310     11320     11330     11340     11350     11360   11371
SARS-CoV-2 Reference Genome NC_045512.2 (11154) AACATAAGCATGCATTTCTCTGTTTGTTTTTGTTACCTTCTCTTGCCACTGTAGCTTATTTTAATATGGTCTATATGCCTGCT
          Fig badnavirus 1 NC_017830.1   (7141) -----------------------------------------------------------------------------------

                                                          Section 138
                                          (11372) 11372   11380     11390     11400     11410     11420     11430     11440       11454
SARS-CoV-2 Reference Genome NC_045512.2 (11237) AGTTGGGTGATGCGTATTATGACATGGTTGGATATGGTTGATACTAGTTTGTCTGGTTTTAAGCTAAAAGACTGTGTTATGTA
          Fig badnavirus 1 NC_017830.1   (7141) -----------------------------------------------------------------------------------

                                                          Section 139
                                          (11455) 11455 11460     11470     11480     11490     11500     11510     11520           11537
SARS-CoV-2 Reference Genome NC_045512.2 (11320) TGCATCAGCTGTAGTGTTACTAATCCTTATGACAGCAAGAACTGTGTATGATGATGGTGCTAGGAGAGTGTGGACACTTATGA
          Fig badnavirus 1 NC_017830.1   (7141) -----------------------------------------------------------------------------------

                                                          Section 140
                                          (11538) 11538        11550     11560     11570     11580     11590     11600     11610 11620
SARS-CoV-2 Reference Genome NC_045512.2 (11403) ATGTCTTGACACTCGTTTATAAAGTTTATTATGGTAATGCTTTAGATCAAGCCATTTCCATGTGGGCTCTTATAATCTCTGTT
          Fig badnavirus 1 NC_017830.1   (7141) -----------------------------------------------------------------------------------
```

```
                                                        Section 141
                                    (11621) 11621    11630     11640     11650     11660     11670     11680     11690       11703
SARS-CoV-2 Reference Genome NC_045512.2 (11486) ACTTCTAACTACTCAGGTGTAGTTACAACTGTCATGTTTTTGGCCAGAGGTATTGTTTTTATGTGTGTTGAGTATTGCCCTAT
             Fig badnavirus 1 NC_017830.1  (7141) -----------------------------------------------------------------------------------
                                                        Section 142
                                    (11704) 11704 11710     11720     11730     11740     11750     11760     11770          11786
SARS-CoV-2 Reference Genome NC_045512.2 (11569) TTTCTTCATAACTGGTAATACACTTCAGTGTATAATGCTAGTTTATTGTTTCTTAGGCTATTTTTGTACTTGTTACTTTGGCC
             Fig badnavirus 1 NC_017830.1  (7141) -----------------------------------------------------------------------------------
                                                        Section 143
                                    (11787) 11787        11800     11810     11820     11830     11840     11850           11869
SARS-CoV-2 Reference Genome NC_045512.2 (11652) TCTTTTGTTTACTCAACCGCTACTTTAGACTGACTCTTGGTGTTTATGATTACTTAGTTTCTACACAGGAGTTTAGATATATG
             Fig badnavirus 1 NC_017830.1  (7141) -----------------------------------------------------------------------------------
                                                        Section 144
                                    (11870) 11870     11880     11890     11900     11910     11920     11930     11940      11952
SARS-CoV-2 Reference Genome NC_045512.2 (11735) AATTCACAGGGACTACTCCCACCCAAGAATAGCATAGATGCCTTCAAACTCAACATTAAATTGTTGGGTGTTGGTGGCAAACC
             Fig badnavirus 1 NC_017830.1  (7141) -----------------------------------------------------------------------------------
                                                        Section 145
                                    (11953) 11953  11960     11970     11980     11990     12000     12010     12020         12035
SARS-CoV-2 Reference Genome NC_045512.2 (11818) TTGTATCAAAGTAGCCACTGTACAGTCTAAAATGTCAGATGTAAAGTGCACATCAGTAGTCTTACTCTCAGTTTTGCAACAAC
             Fig badnavirus 1 NC_017830.1  (7141) -----------------------------------------------------------------------------------
                                                        Section 146
                                    (12036) 12036         12050     12060     12070     12080     12090     12100          12118
SARS-CoV-2 Reference Genome NC_045512.2 (11901) TCAGAGTAGAATCATCATCTAAATTGTGGGCTCAATGTGTCCAGTTACACAATGACATTCTCTTAGCTAAAGATACTACTGAA
             Fig badnavirus 1 NC_017830.1  (7141) -----------------------------------------------------------------------------------
                                                        Section 147
                                    (12119) 12119     12130     12140     12150     12160     12170     12180     12190      12201
SARS-CoV-2 Reference Genome NC_045512.2 (11984) GCCTTTGAAAAAATGGTTTCACTACTTTCTGTTTTGCTTTCCATGCAGGGTGCTGTAGACATAAACAAGCTTTGTGAAGAAAT
             Fig badnavirus 1 NC_017830.1  (7141) -----------------------------------------------------------------------------------
```

```
                                                                                                                     Section 148
                                            (12202) 12202   12210     12220     12230     12240     12250     12260     12270      12284
SARS-CoV-2 Reference Genome NC_045512.2 (12067) GCTGGACAACAGGGCAACCTTACAAGCTATAGCCTCAGAGTTTAGTTCCCTTCCATCATATGCAGCTTTTGCTACTGCTCAAG
         Fig badnavirus 1 NC_017830.1   (7141) -----------------------------------------------------------------------------------
                                                                                                                     Section 149
                                            (12285) 12285 12290     12300     12310     12320     12330     12340     12350        12367
SARS-CoV-2 Reference Genome NC_045512.2 (12150) AAGCTTATGAGCAGGCTGTTGCTAATGGTGATTCTGAAGTTGTTCTTAAAAAGTTGAAGAAGTCTTTGAATGTGGCTAAATCT
         Fig badnavirus 1 NC_017830.1   (7141) -----------------------------------------------------------------------------------
                                                                                                                     Section 150
                                            (12368) 12368       12380     12390     12400     12410     12420     12430     12440 12450
SARS-CoV-2 Reference Genome NC_045512.2 (12233) GAATTTGACCGTGATGCAGCCATGCAACGTAAGTTGGAAAAGATGGCTGATCAAGCTATGACCCAAATGTATAAACAGGCTAG
         Fig badnavirus 1 NC_017830.1   (7141) -----------------------------------------------------------------------------------
                                                                                                                     Section 151
                                            (12451) 12451    12460     12470     12480     12490     12500     12510     12520      12533
SARS-CoV-2 Reference Genome NC_045512.2 (12316) ATCTGAGGACAAGAGGGCAAAAGTTACTAGTGCTATGCAGACAATGCTTTTCACTATGCTTAGAAAGTTGGATAATGATGCAC
         Fig badnavirus 1 NC_017830.1   (7141) -----------------------------------------------------------------------------------
                                                                                                                     Section 152
                                            (12534) 12534 12540     12550     12560     12570     12580     12590     12600          12616
SARS-CoV-2 Reference Genome NC_045512.2 (12399) TCAACAACATTATCAACAATGCAAGAGATGGTTGTGTTCCCTTGAACATAATACCTCTTACAACAGCAGCCAAACTAATGGTT
         Fig badnavirus 1 NC_017830.1   (7141) -----------------------------------------------------------------------------------
                                                                                                                     Section 153
                                            (12617) 12617        12630     12640     12650     12660     12670     12680             12699
SARS-CoV-2 Reference Genome NC_045512.2 (12482) GTCATACCAGACTATAACACATATAAAAATACGTGTGATGGTACAACATTTACTTATGCATCAGCATTGTGGGAAATCCAACA
         Fig badnavirus 1 NC_017830.1   (7141) -----------------------------------------------------------------------------------
                                                                                                                     Section 154
                                            (12700) 12700     12710     12720     12730     12740     12750     12760     12770      12782
SARS-CoV-2 Reference Genome NC_045512.2 (12565) GGTTGTAGATGCAGATAGTAAAATTGTTCAACTTAGTGAAATTAGTATGGACAATTCACCTAATTTAGCATGGCCTCTTATTG
         Fig badnavirus 1 NC_017830.1   (7141) -----------------------------------------------------------------------------------
```

```
                                                   Section 155
                                          (12783) 12783 12790     12800     12810     12820     12830     12840     12850          12865
SARS-CoV-2 Reference Genome NC_045512.2   (12648) TAACAGCTTTAAGGGCCAATTCTGCTGTCAAATTACAGAATAATGAGCTTAGTCCTGTTGCACTACGACAGATGTCTTGTGCT
Fig badnavirus 1 NC_017830.1               (7141) -----------------------------------------------------------------------------------
                                                   Section 156
                                          (12866) 12866         12880     12890     12900     12910     12920     12930              12948
SARS-CoV-2 Reference Genome NC_045512.2   (12731) GCCGGTACTACACAAACTGCTTGCACTGATGACAATGCGTTAGCTTACTACAACACAACAAAGGGAGGTAGGTTTGTACTTGC
Fig badnavirus 1 NC_017830.1               (7141) -----------------------------------------------------------------------------------
                                                   Section 157
                                          (12949) 12949      12960     12970     12980     12990     13000     13010     13020  13031
SARS-CoV-2 Reference Genome NC_045512.2   (12814) ACTGTTATCCGATTTACAGGATTTGAAATGGGCTAGATTCCCTAAGAGTGATGGAACTGGTACTATCTATACAGAACTGGAAC
Fig badnavirus 1 NC_017830.1               (7141) -----------------------------------------------------------------------------------
                                                   Section 158
                                          (13032) 13032   13040     13050     13060     13070     13080     13090     13100     13114
SARS-CoV-2 Reference Genome NC_045512.2   (12897) CACCTTGTAGGTTTGTTACAGACACACCTAAAGGTCCTAAAGTGAAGTATTTATACTTTATTAAAGGATTAAACAACCTAAAT
Fig badnavirus 1 NC_017830.1               (7141) -----------------------------------------------------------------------------------
                                                   Section 159
                                          (13115) 13115 13120     13130     13140     13150     13160     13170     13180          13197
SARS-CoV-2 Reference Genome NC_045512.2   (12980) AGAGGTATGGTACTTGGTAGTTTAGCTGCCACAGTACGTCTACAAGCTGGTAATGCAACAGAAGTGCCTGCCAATTCAACTGT
Fig badnavirus 1 NC_017830.1               (7141) -----------------------------------------------------------------------------------
                                                   Section 160
                                          (13198) 13198        13210     13220     13230     13240     13250     13260     13270  13280
SARS-CoV-2 Reference Genome NC_045512.2   (13063) ATTATCTTTCTGTGCTTTTGCTGTAGATGCTGCTAAAGCTTACAAAGATTATCTAGCTAGTGGGGGACAACCAATCACTAATT
Fig badnavirus 1 NC_017830.1               (7141) -----------------------------------------------------------------------------------
                                                   Section 161
                                          (13281) 13281    13290     13300     13310     13320     13330     13340     13350     13363
SARS-CoV-2 Reference Genome NC_045512.2   (13146) GTGTTAAGATGTTGTGTACACACACTGGTACTGGTCAGGCAATAACAGTTACACCGGAAGCCAATATGGATCAAGAATCCTTT
Fig badnavirus 1 NC_017830.1               (7141) -----------------------------------------------------------------------------------
```

```
                                                    Section 162
                                            (13364) 13364 13370     13380     13390     13400     13410     13420     13430           13446
SARS-CoV-2 Reference Genome NC_045512.2 (13229) GGTGGTGCATCGTGTTGTCTGTACTGCCGTTGCCACATAGATCATCCAAATCCTAAAGGATTTTGTGACTTAAAAGGTAAGTA
         Fig badnavirus 1 NC_017830.1    (7141) -----------------------------------------------------------------------------------
                                                    Section 163
                                            (13447) 13447        13460     13470     13480     13490     13500     13510              13529
SARS-CoV-2 Reference Genome NC_045512.2 (13312) TGTACAAATACCTACAACTTGTGCTAATGACCCTGTGGGTTTTACACTTAAAAACACAGTCTGTACCGTCTGCGGTATGTGGA
         Fig badnavirus 1 NC_017830.1    (7141) -----------------------------------------------------------------------------------
                                                    Section 164
                                            (13530) 13530     13540     13550     13560     13570     13580     13590     13600       13612
SARS-CoV-2 Reference Genome NC_045512.2 (13395) AAGGTTATGGCTGTAGTTGTGATCAACTCCGCGAACCCATGCTTCAGTCAGCTGATGCACAATCGTTTTTAAACGGGTTTGCG
         Fig badnavirus 1 NC_017830.1    (7141) -----------------------------------------------------------------------------------
                                                    Section 165
                                            (13613) 13613  13620     13630     13640     13650     13660     13670     13680          13695
SARS-CoV-2 Reference Genome NC_045512.2 (13478) GTGTAAGTGCAGCCCGTCTTACACCGTGCGGCACAGGCACTAGTACTGATGTCGTATACAGGGCTTTTGACATCTACAATGAT
         Fig badnavirus 1 NC_017830.1    (7141) -----------------------------------------------------------------------------------
                                                    Section 166
                                            (13696) 13696         13710     13720     13730     13740     13750     13760           13778
SARS-CoV-2 Reference Genome NC_045512.2 (13561) AAAGTAGCTGGTTTTGCTAAATTCCTAAAAACTAATTGTTGTCGCTTCCAAGAAAAGGACGAAGATGACAATTTAATTGATTC
         Fig badnavirus 1 NC_017830.1    (7141) -----------------------------------------------------------------------------------
                                                    Section 167
                                            (13779) 13779      13790     13800     13810     13820     13830     13840     13850   13861
SARS-CoV-2 Reference Genome NC_045512.2 (13644) TTACTTTGTAGTTAAGAGACACACTTTCTCTAACTACCAACATGAAGAAACAATTTATAATTTACTTAAGGATTGTCCAGCTG
         Fig badnavirus 1 NC_017830.1    (7141) -----------------------------------------------------------------------------------
                                                    Section 168
                                            (13862) 13862   13870     13880     13890     13900     13910     13920     13930         13944
SARS-CoV-2 Reference Genome NC_045512.2 (13727) TTGCTAAACATGACTTCTTTAAGTTTAGAATAGACGGTGACATGGTACCACATATATCACGTCAACGTCTTACTAAATACACA
         Fig badnavirus 1 NC_017830.1    (7141) -----------------------------------------------------------------------------------
```

```
                                                                                                                         Section 169
                                           (13945) 13945 13950     13960     13970     13980     13990     14000     14010            14027
SARS-CoV-2 Reference Genome NC_045512.2 (13810) ATGGCAGACCTCGTCTATGCTTTAAGGCATTTTGATGAAGGTAATTGTGACACATTAAAAGAAATACTTGTCACATACAATTG
        Fig badnavirus 1 NC_017830.1  (7141) -----------------------------------------------------------------------------------
                                                                                                                         Section 170
                                           (14028) 14028       14040     14050     14060     14070     14080     14090     14100  14110
SARS-CoV-2 Reference Genome NC_045512.2 (13893) TTGTGATGATGATTATTTCAATAAAAAGGACTGGTATGATTTTGTAGAAAACCCAGATATATTACGCGTATACGCCAACTTAG
        Fig badnavirus 1 NC_017830.1  (7141) -----------------------------------------------------------------------------------
                                                                                                                         Section 171
                                           (14111) 14111    14120     14130     14140     14150     14160     14170     14180        14193
SARS-CoV-2 Reference Genome NC_045512.2 (13976) GTGAACGTGTACGCCAAGCTTTGTTAAAAACAGTACAATTCTGTGATGCCATGCGAAATGCTGGTATTGTTGGTGTACTGACA
        Fig badnavirus 1 NC_017830.1  (7141) -----------------------------------------------------------------------------------
                                                                                                                         Section 172
                                           (14194) 14194 14200     14210     14220     14230     14240     14250     14260            14276
SARS-CoV-2 Reference Genome NC_045512.2 (14059) TTAGATAATCAAGATCTCAATGGTAACTGGTATGATTTCGGTGATTTCATACAAACCACGCCAGGTAGTGGAGTTCCTGTTGT
        Fig badnavirus 1 NC_017830.1  (7141) -----------------------------------------------------------------------------------
                                                                                                                         Section 173
                                           (14277) 14277        14290     14300     14310     14320     14330     14340            14359
SARS-CoV-2 Reference Genome NC_045512.2 (14142) AGATTCTTATTATTCATTGTTAATGCCTATATTAACCTTGACCAGGGCTTTAACTGCAGAGTCACATGTTGACACTGACTTAA
        Fig badnavirus 1 NC_017830.1  (7141) -----------------------------------------------------------------------------------
                                                                                                                         Section 174
                                           (14360) 14360     14370     14380     14390     14400     14410     14420     14430     14442
SARS-CoV-2 Reference Genome NC_045512.2 (14225) CAAAGCCTTACATTAAGTGGGATTTGTTAAAATATGACTTCACGGAAGAGAGGTTAAAACTCTTTGACCGTTATTTTAAATAT
        Fig badnavirus 1 NC_017830.1  (7141) -----------------------------------------------------------------------------------
                                                                                                                         Section 175
                                           (14443) 14443 14450     14460     14470     14480     14490     14500     14510            14525
SARS-CoV-2 Reference Genome NC_045512.2 (14308) TGGGATCAGACATACCACCCAAATTGTGTTAACTGTTTGGATGACAGATGCATTCTGCATTGTGCAAACTTTAATGTTTTATT
        Fig badnavirus 1 NC_017830.1  (7141) -----------------------------------------------------------------------------------
```

```
                                                                                                   Section 176
                                 (14526) 14526            14540     14550     14560     14570     14580     14590              14608
SARS-CoV-2 Reference Genome NC_045512.2 (14391) CTCTACAGTGTTCCCACCTACAAGTTTTGGACCACTAGTGAGAAAAATATTTGTTGATGGTGTTCCATTTGTAGTTTCAACTG
          Fig badnavirus 1 NC_017830.1   (7141) -----------------------------------------------------------------------------------
                                                                                                   Section 177
                                 (14609) 14609       14620     14630     14640     14650     14660     14670     14680   14691
SARS-CoV-2 Reference Genome NC_045512.2 (14474) GATACCACTTCAGAGAGCTAGGTGTTGTACATAATCAGGATGTAAACTTACATAGCTCTAGACTTAGTTTTAAGGAATTACTT
          Fig badnavirus 1 NC_017830.1   (7141) -----------------------------------------------------------------------------------
                                                                                                   Section 178
                                 (14692) 14692   14700     14710     14720     14730     14740     14750     14760         14774
SARS-CoV-2 Reference Genome NC_045512.2 (14557) GTGTATGCTGCTGACCCTGCTATGCACGCTGCTTCTGGTAATCTATTACTAGATAAACGCACTACGTGCTTTTCAGTAGCTGC
          Fig badnavirus 1 NC_017830.1   (7141) -----------------------------------------------------------------------------------
                                                                                                   Section 179
                                 (14775) 14775 14780     14790     14800     14810     14820     14830     14840             14857
SARS-CoV-2 Reference Genome NC_045512.2 (14640) ACTTACTAACAATGTTGCTTTTCAAACTGTCAAACCCGGTAATTTTAACAAAGACTTCTATGACTTTGCTGTGTCTAAGGGTT
          Fig badnavirus 1 NC_017830.1   (7141) -----------------------------------------------------------------------------------
                                                                                                   Section 180
                                 (14858) 14858        14870     14880     14890     14900     14910     14920     14930   14940
SARS-CoV-2 Reference Genome NC_045512.2 (14723) TCTTTAAGGAAGGAAGTTCTGTTGAATTAAAACACTTCTTCTTTGCTCAGGATGGTAATGCTGCTATCAGCGATTATGACTAC
          Fig badnavirus 1 NC_017830.1   (7141) -----------------------------------------------------------------------------------
                                                                                                   Section 181
                                 (14941) 14941    14950     14960     14970     14980     14990     15000     15010        15023
SARS-CoV-2 Reference Genome NC_045512.2 (14806) TATCGTTATAATCTACCAACAATGTGTGATATCAGACAACTACTATTTGTAGTTGAAGTTGTTGATAAGTACTTTGATTGTTA
          Fig badnavirus 1 NC_017830.1   (7141) -----------------------------------------------------------------------------------
                                                                                                   Section 182
                                 (15024) 15024 15030     15040     15050     15060     15070     15080     15090             15106
SARS-CoV-2 Reference Genome NC_045512.2 (14889) CGATGGTGGCTGTATTAATGCTAACCAAGTCATCGTCAACAACCTAGACAAATCAGCTGGTTTTCCATTTAATAAATGGGGTA
          Fig badnavirus 1 NC_017830.1   (7141) -----------------------------------------------------------------------------------
```

```
                                                         Section 183
                                          (15107) 15107        15120     15130     15140     15150     15160     15170              15189
SARS-CoV-2 Reference Genome NC_045512.2 (14972) AGGCTAGACTTTATTATGATTCAATGAGTTATGAGGATCAAGATGCACTTTTCGCATATACAAAACGTAATGTCATCCCTACT
           Fig badnavirus 1 NC_017830.1  (7141) -----------------------------------------------------------------------------------
                                                         Section 184
                                          (15190) 15190     15200     15210     15220     15230     15240     15250     15260     15272
SARS-CoV-2 Reference Genome NC_045512.2 (15055) ATAACTCAAATGAATCTTAAGTATGCCATTAGTGCAAAGAATAGAGCTCGCACCGTAGCTGGTGTCTCTATCTGTAGTACTAT
           Fig badnavirus 1 NC_017830.1  (7141) -----------------------------------------------------------------------------------
                                                         Section 185
                                          (15273) 15273  15280     15290     15300     15310     15320     15330     15340          15355
SARS-CoV-2 Reference Genome NC_045512.2 (15138) GACCAATAGACAGTTTCATCAAAAATTATTGAAATCAATAGCCGCCACTAGAGGAGCTACTGTAGTAATTGGAACAAGCAAAT
           Fig badnavirus 1 NC_017830.1  (7141) -----------------------------------------------------------------------------------
                                                         Section 186
                                          (15356) 15356         15370     15380     15390     15400     15410     15420          15438
SARS-CoV-2 Reference Genome NC_045512.2 (15221) TCTATGGTGGTTGGCACAACATGTTAAAAACTGTTTATAGTGATGTAGAAAACCCTCACCTTATGGGTTGGGATTATCCTAAA
           Fig badnavirus 1 NC_017830.1  (7141) -----------------------------------------------------------------------------------
                                                         Section 187
                                          (15439) 15439      15450     15460     15470     15480     15490     15500     15510  15521
SARS-CoV-2 Reference Genome NC_045512.2 (15304) TGTGATAGAGCCATGCCTAACATGCTTAGAATTATGGCCTCACTTGTTCTTGCTCGCAAACATACAACGTGTTGTAGCTTGTC
           Fig badnavirus 1 NC_017830.1  (7141) -----------------------------------------------------------------------------------
                                                         Section 188
                                          (15522) 15522   15530     15540     15550     15560     15570     15580     15590        15604
SARS-CoV-2 Reference Genome NC_045512.2 (15387) ACACCGTTTCTATAGATTAGCTAATGAGTGTGCTCAAGTATTGAGTGAAATGGTCATGTGTGGCGGTTCACTATATGTTAAAC
           Fig badnavirus 1 NC_017830.1  (7141) -----------------------------------------------------------------------------------
                                                         Section 189
                                          (15605) 15605 15610     15620     15630     15640     15650     15660     15670           15687
SARS-CoV-2 Reference Genome NC_045512.2 (15470) CAGGTGGAACCTCATCAGGAGATGCCACAACTGCTTATGCTAATAGTGTTTTTAACATTTGTCAAGCTGTCACGGCCAATGTT
           Fig badnavirus 1 NC_017830.1  (7141) -----------------------------------------------------------------------------------
```

Section 190

```
                                       (15688) 15688       15700     15710     15720     15730     15740     15750     15760   15770
SARS-CoV-2 Reference Genome NC_045512.2 (15553) AATGCACTTTTATCTACTGATGGTAACAAAATTGCCGATAAGTATGTCCGCAATTTACAACACAGACTTTATGAGTGTCTCTA
        Fig badnavirus 1 NC_017830.1  (7141) -----------------------------------------------------------------------------------
```

Section 191

```
                                       (15771) 15771    15780     15790     15800     15810     15820     15830     15840       15853
SARS-CoV-2 Reference Genome NC_045512.2 (15636) TAGAAATAGAGATGTTGACACAGACTTTGTGAATGAGTTTTACGCATATTTGCGTAAACATTTCTCAATGATGATACTCTCTG
        Fig badnavirus 1 NC_017830.1  (7141) -----------------------------------------------------------------------------------
```

Section 192

```
                                       (15854) 15854 15860     15870     15880     15890     15900     15910     15920           15936
SARS-CoV-2 Reference Genome NC_045512.2 (15719) ACGATGCTGTTGTGTGTTTCAATAGCACTTATGCATCTCAAGGTCTAGTGGCTAGCATAAAGAACTTTAAGTCAGTTCTTTAT
        Fig badnavirus 1 NC_017830.1  (7141) -----------------------------------------------------------------------------------
```

Section 193

```
                                       (15937) 15937        15950     15960     15970     15980     15990     16000           16019
SARS-CoV-2 Reference Genome NC_045512.2 (15802) TATCAAAACAATGTTTTTATGTCTGAAGCAAAATGTTGGACTGAGACTGACCTTACTAAAGGACCTCATGAATTTTGCTCTCA
        Fig badnavirus 1 NC_017830.1  (7141) -----------------------------------------------------------------------------------
```

Section 194

```
                                       (16020) 16020     16030     16040     16050     16060     16070     16080     16090     16102
SARS-CoV-2 Reference Genome NC_045512.2 (15885) ACATACAATGCTAGTTAAACAGGGTGATGATTATGTGTACCTTCCTTACCCAGATCCATCAAGAATCCTAGGGGCCGGCTGTT
        Fig badnavirus 1 NC_017830.1  (7141) -----------------------------------------------------------------------------------
```

Section 195

```
                                       (16103) 16103  16110     16120     16130     16140     16150     16160     16170           16185
SARS-CoV-2 Reference Genome NC_045512.2 (15968) TTGTAGATGATATCGTAAAAACAGATGGTACACTTATGATTGAACGGTTCGTGTCTTTAGCTATAGATGCTTACCCACTTACT
        Fig badnavirus 1 NC_017830.1  (7141) -----------------------------------------------------------------------------------
```

Section 196

```
                                       (16186) 16186         16200     16210     16220     16230     16240     16250             16268
SARS-CoV-2 Reference Genome NC_045512.2 (16051) AAACATCCTAATCAGGAGTATGCTGATGTCTTTCATTTGTACTTACAATACATAAGAAAGCTACATGATGAGTTAACAGGACA
        Fig badnavirus 1 NC_017830.1  (7141) -----------------------------------------------------------------------------------
```

Section 197

```
                                            (16269) 16269      16280     16290     16300     16310     16320     16330     16340     16351
SARS-CoV-2 Reference Genome NC_045512.2 (16134) CATGTTAGACATGTATTCTGTTATGCTTACTAATGATAACACTTCAAGGTATTGGGAACCTGAGTTTTATGAGGCTATGTACA
       Fig badnavirus 1 NC_017830.1  (7141) -----------------------------------------------------------------------------------
```

Section 198

```
                                            (16352) 16352    16360     16370     16380     16390     16400     16410     16420     16434
SARS-CoV-2 Reference Genome NC_045512.2 (16217) CACCGCATACAGTCTTACAGGCTGTTGGGGCTTGTGTTCTTTGCAATTCACAGACTTCATTAAGATGTGGTGCTTGCATACGT
       Fig badnavirus 1 NC_017830.1  (7141) -----------------------------------------------------------------------------------
```

Section 199

```
                                            (16435) 16435 16440     16450     16460     16470     16480     16490     16500          16517
SARS-CoV-2 Reference Genome NC_045512.2 (16300) AGACCATTCTTATGTTGTAAATGCTGTTACGACCATGTCATATCAACATCACATAAATTAGTCTTGTCTGTTAATCCGTATGT
       Fig badnavirus 1 NC_017830.1  (7141) -----------------------------------------------------------------------------------
```

Section 200

```
                                            (16518) 16518       16530     16540     16550     16560     16570     16580     16590   16600
SARS-CoV-2 Reference Genome NC_045512.2 (16383) TTGCAATGCTCCAGGTTGTGATGTCACAGATGTGACTCAACTTTACTTAGGAGGTATGAGCTATTATTGTAAATCACATAAAC
       Fig badnavirus 1 NC_017830.1  (7141) -----------------------------------------------------------------------------------
```

Section 201

```
                                            (16601) 16601    16610     16620     16630     16640     16650     16660     16670     16683
SARS-CoV-2 Reference Genome NC_045512.2 (16466) CACCCATTAGTTTTCCATTGTGTGCTAATGGACAAGTTTTTGGTTTATATAAAAATACATGTGTTGGTAGCGATAATGTTACT
       Fig badnavirus 1 NC_017830.1  (7141) -----------------------------------------------------------------------------------
```

Section 202

```
                                            (16684) 16684 16690     16700     16710     16720     16730     16740     16750          16766
SARS-CoV-2 Reference Genome NC_045512.2 (16549) GACTTTAATGCAATTGCAACATGTGACTGGACAAATGCTGGTGATTACATTTTAGCTAACACCTGTACTGAAAGACTCAAGCT
       Fig badnavirus 1 NC_017830.1  (7141) -----------------------------------------------------------------------------------
```

Section 203

```
                                            (16767) 16767        16780     16790     16800     16810     16820     16830          16849
SARS-CoV-2 Reference Genome NC_045512.2 (16632) TTTTGCAGCAGAAACGCTCAAAGCTACTGAGGAGACATTTAAACTGTCTTATGGTATTGCTACTGTACGTGAAGTGCTGTCTG
       Fig badnavirus 1 NC_017830.1  (7141) -----------------------------------------------------------------------------------
```

Section 204

```
                                           (16850) 16850     16860     16870     16880     16890     16900     16910     16920      16932
SARS-CoV-2 Reference Genome NC_045512.2 (16715) ACAGAGAATTACATCTTTCATGGGAAGTTGGTAAACCTAGACCACCACTTAACCGAAATTATGTCTTTACTGGTTATCGTGTA
        Fig badnavirus 1 NC_017830.1  (7141) -----------------------------------------------------------------------------------
```

Section 205

```
                                           (16933) 16933 16940     16950     16960     16970     16980     16990     17000          17015
SARS-CoV-2 Reference Genome NC_045512.2 (16798) ACTAAAAACAGTAAAGTACAAATAGGAGAGTACACCTTTGAAAAAGGTGACTATGGTGATGCTGTTGTTTACCGAGGTACAAC
        Fig badnavirus 1 NC_017830.1  (7141) -----------------------------------------------------------------------------------
```

Section 206

```
                                           (17016) 17016         17030     17040     17050     17060     17070     17080          17098
SARS-CoV-2 Reference Genome NC_045512.2 (16881) AACTTACAAATTAAATGTTGGTGATTATTTTGTGCTGACATCACATACAGTAATGCCATTAAGTGCACCTACACTAGTGCCAC
        Fig badnavirus 1 NC_017830.1  (7141) -----------------------------------------------------------------------------------
```

Section 207

```
                                           (17099) 17099      17110     17120     17130     17140     17150     17160     17170   17181
SARS-CoV-2 Reference Genome NC_045512.2 (16964) AAGAGCACTATGTTAGAATTACTGGCTTATACCCAACACTCAATATCTCAGATGAGTTTTCTAGCAATGTTGCAAATTATCAA
        Fig badnavirus 1 NC_017830.1  (7141) -----------------------------------------------------------------------------------
```

Section 208

```
                                           (17182) 17182   17190     17200     17210     17220     17230     17240     17250      17264
SARS-CoV-2 Reference Genome NC_045512.2 (17047) AAGGTTGGTATGCAAAAGTATTCTACACTCCAGGGACCACCTGGTACTGGTAAGAGTCATTTTGCTATTGGCCTAGCTCTCTA
        Fig badnavirus 1 NC_017830.1  (7141) -----------------------------------------------------------------------------------
```

Section 209

```
                                           (17265) 17265 17270     17280     17290     17300     17310     17320     17330          17347
SARS-CoV-2 Reference Genome NC_045512.2 (17130) CTACCCTTCTGCTCGCATAGTGTATACAGCTTGCTCTCATGCCGCTGTTGATGCACTATGTGAGAAGGCATTAAAATATTTGC
        Fig badnavirus 1 NC_017830.1  (7141) -----------------------------------------------------------------------------------
```

Section 210

```
                                           (17348) 17348       17360     17370     17380     17390     17400     17410     17420  17430
SARS-CoV-2 Reference Genome NC_045512.2 (17213) CTATAGATAAATGTAGTAGAATTATACCTGCACGTGCTCGTGTAGAGTGTTTTGATAAATTCAAAGTGAATTCAACATTAGAA
        Fig badnavirus 1 NC_017830.1  (7141) -----------------------------------------------------------------------------------
```

```
                                                                                                                   Section 211
                                        (17431) 17431    17440     17450     17460     17470     17480     17490     17500        17513
SARS-CoV-2 Reference Genome NC_045512.2 (17296) CAGTATGTCTTTTGTACTGTAAATGCATTGCCTGAGACGACAGCAGATATAGTTGTCTTTGATGAAATTTCAATGGCCACAAA
          Fig badnavirus 1 NC_017830.1   (7141) -----------------------------------------------------------------------------------
                                                                                                                   Section 212
                                        (17514) 17514 17520     17530     17540     17550     17560     17570     17580           17596
SARS-CoV-2 Reference Genome NC_045512.2 (17379) TTATGATTTGAGTGTTGTCAATGCCAGATTACGTGCTAAGCACTATGTGTACATTGGCGACCCTGCTCAATTACCTGCACCAC
          Fig badnavirus 1 NC_017830.1   (7141) -----------------------------------------------------------------------------------
                                                                                                                   Section 213
                                        (17597) 17597        17610     17620     17630     17640     17650     17660              17679
SARS-CoV-2 Reference Genome NC_045512.2 (17462) GCACATTGCTAACTAAGGGCACACTAGAACCAGAATATTTCAATTCAGTGTGTAGACTTATGAAAACTATAGGTCCAGACATG
          Fig badnavirus 1 NC_017830.1   (7141) -----------------------------------------------------------------------------------
                                                                                                                   Section 214
                                        (17680) 17680     17690     17700     17710     17720     17730     17740     17750     17762
SARS-CoV-2 Reference Genome NC_045512.2 (17545) TTCCTCGGAACTTGTCGGCGTTGTCCTGCTGAAATTGTTGACACTGTGAGTGCTTTGGTTTATGATAATAAGCTTAAAGCACA
          Fig badnavirus 1 NC_017830.1   (7141) -----------------------------------------------------------------------------------
                                                                                                                   Section 215
                                        (17763) 17763 17770     17780     17790     17800     17810     17820     17830           17845
SARS-CoV-2 Reference Genome NC_045512.2 (17628) TAAAGACAAATCAGCTCAATGCTTTAAAATGTTTTATAAGGGTGTTATCACGCATGATGTTTCATCTGCAATTAACAGGCCAC
          Fig badnavirus 1 NC_017830.1   (7141) -----------------------------------------------------------------------------------
                                                                                                                   Section 216
                                        (17846) 17846         17860     17870     17880     17890     17900     17910             17928
SARS-CoV-2 Reference Genome NC_045512.2 (17711) AAATAGGCGTGGTAAGAGAATTCCTTACACGTAACCCTGCTTGGAGAAAAGCTGTCTTTATTTCACCTTATAATTCACAGAAT
          Fig badnavirus 1 NC_017830.1   (7141) -----------------------------------------------------------------------------------
                                                                                                                   Section 217
                                        (17929) 17929      17940     17950     17960     17970     17980     17990     18000    18011
SARS-CoV-2 Reference Genome NC_045512.2 (17794) GCTGTAGCCTCAAAGATTTTGGACTACCAACTCAAACTGTTGATTCATCACAGGGCTCAGAATATGACTATGTCATATTCAC
          Fig badnavirus 1 NC_017830.1   (7141) -----------------------------------------------------------------------------------
```

```
                                                                                                    Section 218
                                               (18012) 18012 18020     18030     18040     18050     18060     18070     18080     18094
SARS-CoV-2 Reference Genome NC_045512.2 (17877) TCAAACCACTGAAACAGCTCACTCTTGTAATGTAAACAGATTTAATGTTGCTATTACCAGAGCAAAAGTAGGCATACTTTGCA
             Fig badnavirus 1 NC_017830.1  (7141) -----------------------------------------------------------------------------------
                                                                                                    Section 219
                                               (18095) 18095 18100     18110     18120     18130     18140     18150     18160     18177
SARS-CoV-2 Reference Genome NC_045512.2 (17960) TAATGTCTGATAGAGACCTTTATGACAAGTTGCAATTTACAAGTCTTGAAATTCCACGTAGGAATGTGGCAACTTTACAAGCT
             Fig badnavirus 1 NC_017830.1  (7141) -----------------------------------------------------------------------------------
                                                                                                    Section 220
                                               (18178) 18178 18190     18200     18210     18220     18230     18240     18250     18260
SARS-CoV-2 Reference Genome NC_045512.2 (18043) GAAAATGTAACAGGACTCTTTAAAGATTGTAGTAAGGTAATCACTGGGTTACATCCTACACAGGCACCTACACACCTCAGTGT
             Fig badnavirus 1 NC_017830.1  (7141) -----------------------------------------------------------------------------------
                                                                                                    Section 221
                                               (18261) 18261 18270     18280     18290     18300     18310     18320     18330     18343
SARS-CoV-2 Reference Genome NC_045512.2 (18126) TGACACTAAATTCAAAACTGAAGGTTTATGTGTTGACATACCTGGCATACCTAAGGACATGACCTATAGAAGACTCATCTCTA
             Fig badnavirus 1 NC_017830.1  (7141) -----------------------------------------------------------------------------------
                                                                                                    Section 222
                                               (18344) 18344 18350     18360     18370     18380     18390     18400     18410     18426
SARS-CoV-2 Reference Genome NC_045512.2 (18209) TGATGGGTTTTAAAATGAATTATCAAGTTAATGGTTACCCTAACATGTTTATCACCCGCGAAGAAGCTATAAGACATGTACGT
             Fig badnavirus 1 NC_017830.1  (7141) -----------------------------------------------------------------------------------
                                                                                                    Section 223
                                               (18427) 18427 18440     18450     18460     18470     18480     18490     18509
SARS-CoV-2 Reference Genome NC_045512.2 (18292) GCATGGATTGGCTTCGATGTCGAGGGGTGTCATGCTACTAGAGAAGCTGTTGGTACCAATTTACCTTTACAGCTAGGTTTTTC
             Fig badnavirus 1 NC_017830.1  (7141) -----------------------------------------------------------------------------------
                                                                                                    Section 224
                                               (18510) 18510 18520     18530     18540     18550     18560     18570     18580     18592
SARS-CoV-2 Reference Genome NC_045512.2 (18375) TACAGGTGTTAACCTAGTTGCTGTACCTACAGGTTATGTTGATACACCTAATAATACAGATTTTTCCAGAGTTAGTGCTAAAC
             Fig badnavirus 1 NC_017830.1  (7141) -----------------------------------------------------------------------------------
```

Section 225

```
                                       (18593) 18593 18600      18610      18620      18630      18640      18650      18660          18675
SARS-CoV-2 Reference Genome NC_045512.2 (18458) CACCGCCTGGAGATCAATTTAAACACCTCATACCACTTATGTACAAAGGACTTCCTTGGAATGTAGTGCGTATAAAGATTGTA
         Fig badnavirus 1 NC_017830.1  (7141) -----------------------------------------------------------------------------------
```

Section 226

```
                                       (18676) 18676          18690      18700      18710      18720      18730      18740          18758
SARS-CoV-2 Reference Genome NC_045512.2 (18541) CAAATGTTAAGTGACACACTTAAAAATCTCTCTGACAGAGTCGTATTTGTCTTATGGGCACATGGCTTTGAGTTGACATCTAT
         Fig badnavirus 1 NC_017830.1  (7141) -----------------------------------------------------------------------------------
```

Section 227

```
                                       (18759) 18759       18770      18780      18790      18800      18810      18820      18830  18841
SARS-CoV-2 Reference Genome NC_045512.2 (18624) GAAGTATTTTGTGAAAATAGGACCTGAGCGCACCTGTTGTCTATGTGATAGACGTGCCACATGCTTTTCCACTGCTTCAGACA
         Fig badnavirus 1 NC_017830.1  (7141) -----------------------------------------------------------------------------------
```

Section 228

```
                                       (18842) 18842   18850      18860      18870      18880      18890      18900      18910      18924
SARS-CoV-2 Reference Genome NC_045512.2 (18707) CTTATGCCTGTTGGCATCATTCTATTGGATTTGATTACGTCTATAATCCGTTTATGATTGATGTTCAACAATGGGGTTTTACA
         Fig badnavirus 1 NC_017830.1  (7141) -----------------------------------------------------------------------------------
```

Section 229

```
                                       (18925) 18925 18930      18940      18950      18960      18970      18980      18990          19007
SARS-CoV-2 Reference Genome NC_045512.2 (18790) GGTAACCTACAAAGCAACCATGATCTGTATTGTCAAGTCCATGGTAATGCACATGTAGCTAGTTGTGATGCAATCATGACTAG
         Fig badnavirus 1 NC_017830.1  (7141) -----------------------------------------------------------------------------------
```

Section 230

```
                                       (19008) 19008        19020      19030      19040      19050      19060      19070      19080 19090
SARS-CoV-2 Reference Genome NC_045512.2 (18873) GTGTCTAGCTGTCCACGAGTGCTTTGTTAAGCGTGTTGACTGGACTATTGAATATCCTATAATTGGTGATGAACTGAAGATTA
         Fig badnavirus 1 NC_017830.1  (7141) -----------------------------------------------------------------------------------
```

Section 231

```
                                       (19091) 19091    19100      19110      19120      19130      19140      19150      19160      19173
SARS-CoV-2 Reference Genome NC_045512.2 (18956) ATGCGGCTTGTAGAAAGGTTCAACACATGGTTGTTAAAGCTGCATTATTAGCAGACAAATTCCCAGTTCTTCACGACATTGGT
         Fig badnavirus 1 NC_017830.1  (7141) -----------------------------------------------------------------------------------
```

Section 232

(19174) 19174 19180 19190 19200 19210 19220 19230 19240 19256
SARS-CoV-2 Reference Genome NC_045512.2 (19039) AACCCTAAAGCTATTAAGTGTGTACCTCAAGCTGATGTAGAATGGAAGTTCTATGATGCACAGCCTTGTAGTGACAAAGCTTA
Fig badnavirus 1 NC_017830.1 (7141) ---

Section 233

(19257) 19257 19270 19280 19290 19300 19310 19320 19339
SARS-CoV-2 Reference Genome NC_045512.2 (19122) TAAAATAGAAGAATTATTCTATTCTTATGCCACACATTCTGACAAATTCACAGATGGTGTATGCCTATTTTGGAATTGCAATG
Fig badnavirus 1 NC_017830.1 (7141) ---

Section 234

(19340) 19340 19350 19360 19370 19380 19390 19400 19410 19422
SARS-CoV-2 Reference Genome NC_045512.2 (19205) TCGATAGATATCCTGCTAATTCCATTGTTTGTAGATTTGACACTAGAGTGCTATCTAACCTTAACTTGCCTGGTTGTGATGGT
Fig badnavirus 1 NC_017830.1 (7141) ---

Section 235

(19423) 19423 19430 19440 19450 19460 19470 19480 19490 19505
SARS-CoV-2 Reference Genome NC_045512.2 (19288) GGCAGTTTGTATGTAAATAAACATGCATTCCACACACCAGCTTTTGATAAAAGTGCTTTTGTTAATTTAAAACAATTACCATT
Fig badnavirus 1 NC_017830.1 (7141) ---

Section 236

(19506) 19506 19520 19530 19540 19550 19560 19570 19588
SARS-CoV-2 Reference Genome NC_045512.2 (19371) TTTCTATTACTCTGACAGTCCATGTGAGTCTCATGGAAAACAAGTAGTGTCAGATATAGATTATGTACCACTAAAGTCTGCTA
Fig badnavirus 1 NC_017830.1 (7141) ---

Section 237

(19589) 19589 19600 19610 19620 19630 19640 19650 19660 19671
SARS-CoV-2 Reference Genome NC_045512.2 (19454) CGTGTATAACACGTTGCAATTTAGGTGGTGCTGTCTGTAGACATCATGCTAATGAGTACAGATTGTATCTCGATGCTTATAAC
Fig badnavirus 1 NC_017830.1 (7141) ---

Section 238

(19672) 19672 19680 19690 19700 19710 19720 19730 19740 19754
SARS-CoV-2 Reference Genome NC_045512.2 (19537) ATGATGATCTCAGCTGGCTTTAGCTTGTGGGTTTACAAACAATTTGATACTTATAACCTCTGGAACACTTTTACAAGACTTCA
Fig badnavirus 1 NC_017830.1 (7141) ---

```
                                                Section 239
                                        (19755) 19755 19760 19770 19780 19790 19800 19810 19820 19837
SARS-CoV-2 Reference Genome NC_045512.2 (19620) GAGTTTAGAAAATGTGGCTTTTAATGTTGTAAATAAGGGACACTTTGATGGACAACAGGGTGAAGTACCAGTTTCTATCATTA
           Fig badnavirus 1 NC_017830.1  (7141) -----------------------------------------------------------------------------------

                                                Section 240
                                        (19838) 19838 19850 19860 19870 19880 19890 19900 19910 19920
SARS-CoV-2 Reference Genome NC_045512.2 (19703) ATAACACTGTTTACACAAAAGTTGATGGTGTTGATGTAGAATTGTTTGAAAATAAAACAACATTACCTGTTAATGTAGCATTT
           Fig badnavirus 1 NC_017830.1  (7141) -----------------------------------------------------------------------------------

                                                Section 241
                                        (19921) 19921 19930 19940 19950 19960 19970 19980 19990 20003
SARS-CoV-2 Reference Genome NC_045512.2 (19786) GAGCTTTGGGCTAAGCGCAACATTAAACCAGTACCAGAGGTGAAAATACTCAATAATTTGGGTGTGGACATTGCTGCTAATAC
           Fig badnavirus 1 NC_017830.1  (7141) -----------------------------------------------------------------------------------

                                                Section 242
                                        (20004) 20004 20010 20020 20030 20040 20050 20060 20070 20086
SARS-CoV-2 Reference Genome NC_045512.2 (19869) TGTGATCTGGGACTACAAAAGAGATGCTCCAGCACATATATCTACTATTGGTGTTTGTTCTATGACTGACATAGCCAAGAAAC
           Fig badnavirus 1 NC_017830.1  (7141) -----------------------------------------------------------------------------------

                                                Section 243
                                        (20087) 20087 20100 20110 20120 20130 20140 20150 20169
SARS-CoV-2 Reference Genome NC_045512.2 (19952) CAACTGAAACGATTTGTGCACCACTCACTGTCTTTTTTGATGGTAGAGTTGATGGTCAAGTAGACTTATTTAGAAATGCCCGT
           Fig badnavirus 1 NC_017830.1  (7141) -----------------------------------------------------------------------------------

                                                Section 244
                                        (20170) 20170 20180 20190 20200 20210 20220 20230 20240 20252
SARS-CoV-2 Reference Genome NC_045512.2 (20035) AATGGTGTTCTTATTACAGAAGGTAGTGTTAAAGGTTTACAACCATCTGTAGGTCCCAAACAAGCTAGTCTTAATGGAGTCAC
           Fig badnavirus 1 NC_017830.1  (7141) -----------------------------------------------------------------------------------

                                                Section 245
                                        (20253) 20253 20260 20270 20280 20290 20300 20310 20320 20335
SARS-CoV-2 Reference Genome NC_045512.2 (20118) ATTAATTGGAGAAGCCGTAAAAACACAGTTCAATTATTATAAGAAAGTTGATGGTGTTGTCCAACAATTACCTGAAACTTACT
           Fig badnavirus 1 NC_017830.1  (7141) -----------------------------------------------------------------------------------
```

```
                                                                                                                    Section 246
                                           (20336) 20336         20350     20360     20370     20380     20390     20400             20418
SARS-CoV-2 Reference Genome NC_045512.2 (20201) TTACTCAGAGTAGAAATTTACAAGAATTTAAACCCAGGAGTCAAATGGAAATTGATTTCTTAGAATTAGCTATGGATGAATTC
            Fig badnavirus 1 NC_017830.1  (7141) -----------------------------------------------------------------------------------
                                                                                                                    Section 247
                                           (20419) 20419      20430     20440     20450     20460     20470     20480     20490   20501
SARS-CoV-2 Reference Genome NC_045512.2 (20284) ATTGAACGGTATAAATTAGAAGGCTATGCCTTCGAACATATCGTTTATGGAGATTTTAGTCATAGTCAGTTAGGTGGTTTACA
            Fig badnavirus 1 NC_017830.1  (7141) -----------------------------------------------------------------------------------
                                                                                                                    Section 248
                                           (20502) 20502   20510     20520     20530     20540     20550     20560     20570         20584
SARS-CoV-2 Reference Genome NC_045512.2 (20367) TCTACTGATTGGACTAGCTAAACGTTTTAAGGAATCACCTTTTGAATTAGAAGATTTTATTCCTATGGACAGTACAGTTAAAA
            Fig badnavirus 1 NC_017830.1  (7141) -----------------------------------------------------------------------------------
                                                                                                                    Section 249
                                           (20585) 20585 20590     20600     20610     20620     20630     20640     20650             20667
SARS-CoV-2 Reference Genome NC_045512.2 (20450) ACTATTTCATAACAGATGCGCAAACAGGTTCATCTAAGTGTGTGTGTTCTGTTATTGATTTATTACTTGATGATTTTGTTGAA
            Fig badnavirus 1 NC_017830.1  (7141) -----------------------------------------------------------------------------------
                                                                                                                    Section 250
                                           (20668) 20668       20680     20690     20700     20710     20720     20730     20740   20750
SARS-CoV-2 Reference Genome NC_045512.2 (20533) ATAATAAAATCCCAAGATTTATCTGTAGTTTCTAAGGTTGTCAAAGTGACTATTGACTATACAGAAATTTCATTTATGCTTTG
            Fig badnavirus 1 NC_017830.1  (7141) -----------------------------------------------------------------------------------
                                                                                                                    Section 251
                                           (20751) 20751    20760     20770     20780     20790     20800     20810     20820       20833
SARS-CoV-2 Reference Genome NC_045512.2 (20616) GTGTAAAGATGGCCATGTAGAAACATTTTACCCAAAATTACAATCTAGTCAAGCGTGGCAACCGGGTGTTGCTATGCCTAATC
            Fig badnavirus 1 NC_017830.1  (7141) -----------------------------------------------------------------------------------
                                                                                                                    Section 252
                                           (20834) 20834 20840     20850     20860     20870     20880     20890     20900           20916
SARS-CoV-2 Reference Genome NC_045512.2 (20699) TTTACAAAATGCAAAGAATGCTATTAGAAAAGTGTGACCTTCAAAATTATGGTGATAGTGCAACATTACCTAAAGGCATAATG
            Fig badnavirus 1 NC_017830.1  (7141) -----------------------------------------------------------------------------------
```

```
                                                          Section 253
                                           (20917) 20917        20930     20940     20950     20960     20970     20980              20999
SARS-CoV-2 Reference Genome NC_045512.2 (20782) ATGAATGTCGCAAAATATACTCAACTGTGTCAATATTTAAACACATTAACATTAGCTGTACCCTATAATATGAGAGTTATACA
        Fig badnavirus 1 NC_017830.1  (7141) -----------------------------------------------------------------------------------
                                                          Section 254
                                           (21000) 21000     21010     21020     21030     21040     21050     21060     21070      21082
SARS-CoV-2 Reference Genome NC_045512.2 (20865) TTTTGGTGCTGGTTCTGATAAAGGAGTTGCACCAGGTACAGCTGTTTTAAGACAGTGGTTGCCTACGGGTACGCTGCTTGTCG
        Fig badnavirus 1 NC_017830.1  (7141) -----------------------------------------------------------------------------------
                                                          Section 255
                                           (21083) 21083  21090     21100     21110     21120     21130     21140     21150          21165
SARS-CoV-2 Reference Genome NC_045512.2 (20948) ATTCAGATCTTAATGACTTTGTCTCTGATGCAGATTCAACTTTGATTGGTGATTGTGCAACTGTACATACAGCTAATAAATGG
        Fig badnavirus 1 NC_017830.1  (7141) -----------------------------------------------------------------------------------
                                                          Section 256
                                           (21166) 21166         21180     21190     21200     21210     21220     21230              21248
SARS-CoV-2 Reference Genome NC_045512.2 (21031) GATCTCATTATTAGTGATATGTACGACCCTAAGACTAAAAATGTTACAAAAGAAAATGACTCTAAAGAGGGTTTTTTCACTTA
        Fig badnavirus 1 NC_017830.1  (7141) -----------------------------------------------------------------------------------
                                                          Section 257
                                           (21249) 21249      21260     21270     21280     21290     21300     21310     21320    21331
SARS-CoV-2 Reference Genome NC_045512.2 (21114) CATTTGTGGGTTTATACAACAAAAGCTAGCTCTTGGAGGTTCCGTGGCTATAAAGATAACAGAACATTCTTGGAATGCTGATC
        Fig badnavirus 1 NC_017830.1  (7141) -----------------------------------------------------------------------------------
                                                          Section 258
                                           (21332) 21332   21340     21350     21360     21370     21380     21390     21400        21414
SARS-CoV-2 Reference Genome NC_045512.2 (21197) TTTATAAGCTCATGGGACACTTCGCATGGTGGACAGCCTTTGTTACTAATGTGAATGCGTCATCATCTGAAGCATTTTTAATT
        Fig badnavirus 1 NC_017830.1  (7141) -----------------------------------------------------------------------------------
                                                          Section 259
                                           (21415) 21415 21420     21430     21440     21450     21460     21470     21480              21497
SARS-CoV-2 Reference Genome NC_045512.2 (21280) GGATGTAATTATCTTGGCAAACCACGCGAACAAATAGATGGTTATGTCATGCATGCAAATTACATATTTTGGAGGAATACAAA
        Fig badnavirus 1 NC_017830.1  (7141) -----------------------------------------------------------------------------------
```

```
                                                                                                    Section 260
                                         (21498) 21498       21510     21520     21530     21540     21550     21560     21570  21580
SARS-CoV-2 Reference Genome NC_045512.2 (21363) TCCAATTCAGTTGTCTTCCTATTCTTTATTTGACATGAGTAAATTTCCCCTTAAATTAAGGGGTACTGCTGTTATGTCTTTAA
            Fig badnavirus 1 NC_017830.1  (7141) -----------------------------------------------------------------------------------
                                                                                                    Section 261
                                         (21581) 21581    21590     21600     21610     21620     21630     21640     21650     21663
SARS-CoV-2 Reference Genome NC_045512.2 (21446) AAGAAGGTCAAATCAATGATATGATTTTATCTCTTCTTAGTAAAGGTAGACTTATAATTAGAGAAAACAACAGAGTTGTTATT
            Fig badnavirus 1 NC_017830.1  (7141) -----------------------------------------------------------------------------------
                                                                                                    Section 262
                                         (21664) 21664 21670     21680     21690     21700     21710     21720     21730           21746
SARS-CoV-2 Reference Genome NC_045512.2 (21529) TCTAGTGATGTTCTTGTTAACAACTAAACGAACAATGTTTGTTTTTCTTGTTTTATTGCCACTAGTCTCTAGTCAGTGTGTTA
            Fig badnavirus 1 NC_017830.1  (7141) -----------------------------------------------------------------------------------
                                                                                                    Section 263
                                         (21747) 21747        21760     21770     21780     21790     21800     21810           21829
SARS-CoV-2 Reference Genome NC_045512.2 (21612) ATCTTACAACCAGAACTCAATTACCCCCTGCATACACTAATTCTTTCACACGTGGTGTTTATTACCCTGACAAAGTTTTCAGA
            Fig badnavirus 1 NC_017830.1  (7141) -----------------------------------------------------------------------------------
                                                                                                    Section 264
                                         (21830) 21830     21840     21850     21860     21870     21880     21890     21900    21912
SARS-CoV-2 Reference Genome NC_045512.2 (21695) TCCTCAGTTTTACATTCAACTCAGGACTTGTTCTTACCTTTCTTTTCCAATGTTACTTGGTTCCATGCTATACATGTCTCTGG
            Fig badnavirus 1 NC_017830.1  (7141) -----------------------------------------------------------------------------------
                                                                                                    Section 265
                                         (21913) 21913  21920     21930     21940     21950     21960     21970     21980          21995
SARS-CoV-2 Reference Genome NC_045512.2 (21778) GACCAATGGTACTAAGAGGTTTGATAACCCTGTCCTACCATTTAATGATGGTGTTTATTTTGCTTCCACTGAGAAGTCTAACA
            Fig badnavirus 1 NC_017830.1  (7141) -----------------------------------------------------------------------------------
                                                                                                    Section 266
                                         (21996) 21996         22010     22020     22030     22040     22050     22060          22078
SARS-CoV-2 Reference Genome NC_045512.2 (21861) TAATAAGAGGCTGGATTTTTGGTACTACTTTAGATTCGAAGACCCAGTCCCTACTTATTGTTAATAACGCTACTAATGTTGTT
            Fig badnavirus 1 NC_017830.1  (7141) -----------------------------------------------------------------------------------
```

```
                                                                                    Section 267
                                        (22079) 22079      22090     22100     22110     22120     22130     22140     22150   22161
SARS-CoV-2 Reference Genome NC_045512.2 (21944) ATTAAAGTCTGTGAATTTCAATTTTGTAATGATCCATTTTTGGGTGTTTATTACCACAAAAACAACAAAAGTTGGATGGAAAG
           Fig badnavirus 1 NC_017830.1  (7141) -----------------------------------------------------------------------------------
                                                                                    Section 268
                                        (22162) 22162   22170     22180     22190     22200     22210     22220     22230        22244
SARS-CoV-2 Reference Genome NC_045512.2 (22027) TGAGTTCAGAGTTTATTCTAGTGCGAATAATTGCACTTTTGAATATGTCTCTCAGCCTTTTCTTATGGACCTTGAAGGAAAAC
           Fig badnavirus 1 NC_017830.1  (7141) -----------------------------------------------------------------------------------
                                                                                    Section 269
                                        (22245) 22245 22250     22260     22270     22280     22290     22300     22310          22327
SARS-CoV-2 Reference Genome NC_045512.2 (22110) AGGGTAATTTCAAAAATCTTAGGGAATTTGTGTTTAAGAATATTGATGGTTATTTTAAAATATATTCTAAGCACACGCCTATT
           Fig badnavirus 1 NC_017830.1  (7141) -----------------------------------------------------------------------------------
                                                                                    Section 270
                                        (22328) 22328       22340     22350     22360     22370     22380     22390     22400  22410
SARS-CoV-2 Reference Genome NC_045512.2 (22193) AATTTAGTGCGTGATCTCCCTCAGGGTTTTTCGGCTTTAGAACCATTGGTAGATTTGCCAATAGGTATTAACATCACTAGGTT
           Fig badnavirus 1 NC_017830.1  (7141) -----------------------------------------------------------------------------------
                                                                                    Section 271
                                        (22411) 22411    22420     22430     22440     22450     22460     22470     22480       22493
SARS-CoV-2 Reference Genome NC_045512.2 (22276) TCAAACTTTACTTGCTTTACATAGAAGTTATTTGACTCCTGGTGATTCTTCTTCAGGTTGGACAGCTGGTGCTGCAGCTTATT
           Fig badnavirus 1 NC_017830.1  (7141) -----------------------------------------------------------------------------------
                                                                                    Section 272
                                        (22494) 22494  22500     22510     22520     22530     22540     22550     22560         22576
SARS-CoV-2 Reference Genome NC_045512.2 (22359) ATGTGGGTTATCTTCAACCTAGGACTTTTCTATTAAAATATAATGAAAATGGAACCATTACAGATGCTGTAGACTGTGCACTT
           Fig badnavirus 1 NC_017830.1  (7141) -----------------------------------------------------------------------------------
                                                                                    Section 273
                                        (22577) 22577        22590     22600     22610     22620     22630     22640             22659
SARS-CoV-2 Reference Genome NC_045512.2 (22442) GACCCTCTCTCAGAAACAAAGTGTACGTTGAAATCCTTCACTGTAGAAAAAGGAATCTATCAAACTTCTAACTTTAGAGTCCA
           Fig badnavirus 1 NC_017830.1  (7141) -----------------------------------------------------------------------------------
```

```
                                                                                                              Section 274
                                          (22660) 22660     22670     22680     22690     22700     22710     22720     22730       22742
SARS-CoV-2 Reference Genome NC_045512.2 (22525) ACCAACAGAATCTATTGTTAGATTTCCTAATATTACAAACTTGTGCCCTTTTGGTGAAGTTTTTAACGCCACCAGATTTGCAT
         Fig badnavirus 1 NC_017830.1   (7141) -----------------------------------------------------------------------------------
                                                                                                              Section 275
                                          (22743) 22743  22750     22760     22770     22780     22790     22800     22810           22825
SARS-CoV-2 Reference Genome NC_045512.2 (22608) CTGTTTATGCTTGGAACAGGAAGAGAATCAGCAACTGTGTTGCTGATTATTCTGTCCTATATAATTCCGCATCATTTTCCACT
         Fig badnavirus 1 NC_017830.1   (7141) -----------------------------------------------------------------------------------
                                                                                                              Section 276
                                          (22826) 22826         22840     22850     22860     22870     22880     22890              22908
SARS-CoV-2 Reference Genome NC_045512.2 (22691) TTTAAGTGTTATGGAGTGTCTCCTACTAAATTAAATGATCTCTGCTTTACTAATGTCTATGCAGATTCATTTGTAATTAGAGG
         Fig badnavirus 1 NC_017830.1   (7141) -----------------------------------------------------------------------------------
                                                                                                              Section 277
                                          (22909) 22909      22920     22930     22940     22950     22960     22970     22980      22991
SARS-CoV-2 Reference Genome NC_045512.2 (22774) TGATGAAGTCAGACAAATCGCTCCAGGGCAAACTGGAAAGATTGCTGATTATAATTATAAATTACCAGATGATTTTACAGGCT
         Fig badnavirus 1 NC_017830.1   (7141) -----------------------------------------------------------------------------------
                                                                                                              Section 278
                                          (22992) 22992   23000     23010     23020     23030     23040     23050     23060         23074
SARS-CoV-2 Reference Genome NC_045512.2 (22857) GCGTTATAGCTTGGAATTCTAACAATCTTGATTCTAAGGTTGGTGGTAATTATAATTACCTGTATAGATTGTTTAGGAAGTCT
         Fig badnavirus 1 NC_017830.1   (7141) -----------------------------------------------------------------------------------
                                                                                                              Section 279
                                          (23075) 23075 23080     23090     23100     23110     23120     23130     23140            23157
SARS-CoV-2 Reference Genome NC_045512.2 (22940) AATCTCAAACCTTTTGAGAGAGATATTTCAACTGAAATCTATCAGGCCGGTAGCACACCTTGTAATGGTGTTGAAGGTTTTAA
         Fig badnavirus 1 NC_017830.1   (7141) -----------------------------------------------------------------------------------
                                                                                                              Section 280
                                          (23158) 23158       23170     23180     23190     23200     23210     23220     23230   23240
SARS-CoV-2 Reference Genome NC_045512.2 (23023) TTGTTACTTTCCTTTACAATCATATGGTTTCCAACCCACTAATGGTGTTGGTTACCAACCATACAGAGTAGTAGTACTTTCTT
         Fig badnavirus 1 NC_017830.1   (7141) -----------------------------------------------------------------------------------
```

```
                                                  Section 281
                                          (23241) 23241    23250     23260     23270     23280     23290     23300     23310     23323
SARS-CoV-2 Reference Genome NC_045512.2 (23106) TTGAACTTCTACATGCACCAGCAACTGTTTGTGGACCTAAAAAGTCTACTAATTTGGTTAAAAACAAATGTGTCAATTTCAAC
         Fig badnavirus 1 NC_017830.1  (7141) -----------------------------------------------------------------------------------
                                                  Section 282
                                          (23324) 23324 23330     23340     23350     23360     23370     23380     23390     23406
SARS-CoV-2 Reference Genome NC_045512.2 (23189) TTCAATGGTTTAACAGGCACAGGTGTTCTTACTGAGTCTAACAAAAAGTTTCTGCCTTTCCAACAATTTGGCAGAGACATTGC
         Fig badnavirus 1 NC_017830.1  (7141) -----------------------------------------------------------------------------------
                                                  Section 283
                                          (23407) 23407        23420     23430     23440     23450     23460     23470     23489
SARS-CoV-2 Reference Genome NC_045512.2 (23272) TGACACTACTGATGCTGTCCGTGATCCACAGACACTTGAGATTCTTGACATTACACCATGTTCTTTTGGTGGTGTCAGTGTTA
         Fig badnavirus 1 NC_017830.1  (7141) -----------------------------------------------------------------------------------
                                                  Section 284
                                          (23490) 23490     23500     23510     23520     23530     23540     23550     23560     23572
SARS-CoV-2 Reference Genome NC_045512.2 (23355) TAACACCAGGAACAAATACTTCTAACCAGGTTGCTGTTCTTTATCAGGATGTTAACTGCACAGAAGTCCCTGTTGCTATTCAT
         Fig badnavirus 1 NC_017830.1  (7141) -----------------------------------------------------------------------------------
                                                  Section 285
                                          (23573) 23573  23580     23590     23600     23610     23620     23630     23640     23655
SARS-CoV-2 Reference Genome NC_045512.2 (23438) GCAGATCAACTTACTCCTACTTGGCGTGTTTATTCTACAGGTTCTAATGTTTTTCAAACACGTGCAGGCTGTTTAATAGGGGC
         Fig badnavirus 1 NC_017830.1  (7141) -----------------------------------------------------------------------------------
                                                  Section 286
                                          (23656) 23656         23670     23680     23690     23700     23710     23720     23738
SARS-CoV-2 Reference Genome NC_045512.2 (23521) TGAACATGTCAACAACTCATATGAGTGTGACATACCCATTGGTGCAGGTATATGCGCTAGTTATCAGACTCAGACTAATTCTC
         Fig badnavirus 1 NC_017830.1  (7141) -----------------------------------------------------------------------------------
                                                  Section 287
                                          (23739) 23739      23750     23760     23770     23780     23790     23800     23810  23821
SARS-CoV-2 Reference Genome NC_045512.2 (23604) CTCGGCGGGCACGTAGTGTAGCTAGTCAATCCATCATTGCCTACACTATGTCACTTGGTGCAGAAAATTCAGTTGCTTACTCT
         Fig badnavirus 1 NC_017830.1  (7141) -----------------------------------------------------------------------------------
```

```
                                                          Section 288
                                          (23822) 23822   23830     23840     23850     23860     23870     23880     23890          23904
SARS-CoV-2 Reference Genome NC_045512.2   (23687) AATAACTCTATTGCCATACCCACAAATTTTACTATTAGTGTTACCACAGAAATTCTACCAGTGTCTATGACCAAGACATCAGT
Fig badnavirus 1 NC_017830.1              (7141)  -----------------------------------------------------------------------------------
                                                          Section 289
                                          (23905) 23905 23910     23920     23930     23940     23950     23960     23970            23987
SARS-CoV-2 Reference Genome NC_045512.2   (23770) AGATTGTACAATGTACATTTGTGGTGATTCAACTGAATGCAGCAATCTTTTGTTGCAATATGGCAGTTTTTGTACACAATTAA
Fig badnavirus 1 NC_017830.1              (7141)  -----------------------------------------------------------------------------------
                                                          Section 290
                                          (23988) 23988       24000     24010     24020     24030     24040     24050     24060   24070
SARS-CoV-2 Reference Genome NC_045512.2   (23853) ACCGTGCTTTAACTGGAATAGCTGTTGAACAAGACAAAAACACCCAAGAAGTTTTTGCACAAGTCAAACAAATTTACAAAACA
Fig badnavirus 1 NC_017830.1              (7141)  -----------------------------------------------------------------------------------
                                                          Section 291
                                          (24071) 24071    24080     24090     24100     24110     24120     24130     24140        24153
SARS-CoV-2 Reference Genome NC_045512.2   (23936) CCACCAATTAAAGATTTTGGTGGTTTTAATTTTTCACAAATATTACCAGATCCATCAAAACCAAGCAAGAGGTCATTTATTGA
Fig badnavirus 1 NC_017830.1              (7141)  -----------------------------------------------------------------------------------
                                                          Section 292
                                          (24154) 24154 24160     24170     24180     24190     24200     24210     24220          24236
SARS-CoV-2 Reference Genome NC_045512.2   (24019) AGATCTACTTTTCAACAAAGTGACACTTGCAGATGCTGGCTTCATCAAACAATATGGTGATTGCCTTGGTGATATTGCTGCTA
Fig badnavirus 1 NC_017830.1              (7141)  -----------------------------------------------------------------------------------
                                                          Section 293
                                          (24237) 24237        24250     24260     24270     24280     24290     24300           24319
SARS-CoV-2 Reference Genome NC_045512.2   (24102) GAGACCTCATTTGTGCACAAAAGTTTAACGGCCTTACTGTTTTGCCACCTTTGCTCACAGATGAAATGATTGCTCAATACACT
Fig badnavirus 1 NC_017830.1              (7141)  -----------------------------------------------------------------------------------
                                                          Section 294
                                          (24320) 24320     24330     24340     24350     24360     24370     24380     24390     24402
SARS-CoV-2 Reference Genome NC_045512.2   (24185) TCTGCACTGTTAGCGGGTACAATCACTTCTGGTTGGACCTTTGGTGCAGGTGCTGCATTACAAATACCATTTGCTATGCAAAT
Fig badnavirus 1 NC_017830.1              (7141)  -----------------------------------------------------------------------------------
```

Section 295

(24403) 24403 24410 24420 24430 24440 24450 24460 24470 24485
SARS-CoV-2 Reference Genome NC_045512.2 (24268) GGCTTATAGGTTTAATGGTATTGGAGTTACACAGAATGTTCTCTATGAGAACCAAAAATTGATTGCCAACCAATTTAATAGTG
Fig badnavirus 1 NC_017830.1 (7141) ---

Section 296

(24486) 24486 24500 24510 24520 24530 24540 24550 24568
SARS-CoV-2 Reference Genome NC_045512.2 (24351) CTATTGGCAAAATTCAAGACTCACTTTCTTCCACAGCAAGTGCACTTGGAAAACTTCAAGATGTGGTCAACCAAAATGCACAA
Fig badnavirus 1 NC_017830.1 (7141) ---

Section 297

(24569) 24569 24580 24590 24600 24610 24620 24630 24640 24651
SARS-CoV-2 Reference Genome NC_045512.2 (24434) GCTTTAAACACGCTTGTTAAACAACTTAGCTCCAATTTTGGTGCAATTTCAAGTGTTTTAAATGATATCCTTTCACGTCTTGA
Fig badnavirus 1 NC_017830.1 (7141) ---

Section 298

(24652) 24652 24660 24670 24680 24690 24700 24710 24720 24734
SARS-CoV-2 Reference Genome NC_045512.2 (24517) CAAAGTTGAGGCTGAAGTGCAAATTGATAGGTTGATCACAGGCAGACTTCAAAGTTTGCAGACATATGTGACTCAACAATTAA
Fig badnavirus 1 NC_017830.1 (7141) ---

Section 299

(24735) 24735 24740 24750 24760 24770 24780 24790 24800 24817
SARS-CoV-2 Reference Genome NC_045512.2 (24600) TTAGAGCTGCAGAAATCAGAGCTTCTGCTAATCTTGCTGCTACTAAAATGTCAGAGTGTGTACTTGGACAATCAAAAAGAGTT
Fig badnavirus 1 NC_017830.1 (7141) ---

Section 300

(24818) 24818 24830 24840 24850 24860 24870 24880 24890 24900
SARS-CoV-2 Reference Genome NC_045512.2 (24683) GATTTTTGTGGAAAGGGCTATCATCTTATGTCCTTCCCTCAGTCAGCACCTCATGGTGTAGTCTTCTTGCATGTGACTTATGT
Fig badnavirus 1 NC_017830.1 (7141) ---

Section 301

(24901) 24901 24910 24920 24930 24940 24950 24960 24970 24983
SARS-CoV-2 Reference Genome NC_045512.2 (24766) CCCTGCACAAGAAAAGAACTTCACAACTGCTCCTGCCATTTGTCATGATGGAAAAGCACACTTTCCTCGTGAAGGTGTCTTTG
Fig badnavirus 1 NC_017830.1 (7141) ---

```
                                                Section 302
                                   (24984) 24984 24990     25000     25010     25020     25030     25040     25050           25066
SARS-CoV-2 Reference Genome NC_045512.2 (24849) TTTCAAATGGCACACACTGGTTTGTAACACAAAGGAATTTTTATGAACCACAAATCATTACTACAGACAACACATTTGTGTCT
          Fig badnavirus 1 NC_017830.1   (7141) -----------------------------------------------------------------------------------
                                                Section 303
                                   (25067) 25067        25080     25090     25100     25110     25120     25130               25149
SARS-CoV-2 Reference Genome NC_045512.2 (24932) GGTAACTGTGATGTTGTAATAGGAATTGTCAACAACACAGTTTATGATCCTTTGCAACCTGAATTAGACTCATTCAAGGAGGA
          Fig badnavirus 1 NC_017830.1   (7141) -----------------------------------------------------------------------------------
                                                Section 304
                                   (25150) 25150     25160     25170     25180     25190     25200     25210     25220       25232
SARS-CoV-2 Reference Genome NC_045512.2 (25015) GTTAGATAAATATTTTAAGAATCATACATCACCAGATGTTGATTTAGGTGACATCTCTGGCATTAATGCTTCAGTTGTAAACA
          Fig badnavirus 1 NC_017830.1   (7141) -----------------------------------------------------------------------------------
                                                Section 305
                                   (25233) 25233  25240     25250     25260     25270     25280     25290     25300          25315
SARS-CoV-2 Reference Genome NC_045512.2 (25098) TTCAAAAAGAAATTGACCGCCTCAATGAGGTTGCCAAGAATTTAAATGAATCTCTCATCGATCTCCAAGAACTTGGAAAGTAT
          Fig badnavirus 1 NC_017830.1   (7141) -----------------------------------------------------------------------------------
                                                Section 306
                                   (25316) 25316         25330     25340     25350     25360     25370     25380              25398
SARS-CoV-2 Reference Genome NC_045512.2 (25181) GAGCAGTATATAAAATGGCCATGGTACATTTGGCTAGGTTTTATAGCTGGCTTGATTGCCATAGTAATGGTGACAATTATGCT
          Fig badnavirus 1 NC_017830.1   (7141) -----------------------------------------------------------------------------------
                                                Section 307
                                   (25399) 25399      25410     25420     25430     25440     25450     25460     25470       25481
SARS-CoV-2 Reference Genome NC_045512.2 (25264) TTGCTGTATGACCAGTTGCTGTAGTTGTCTCAAGGGCTGTTGTTCTTGTGGATCCTGCTGCAAATTTGATGAAGACGACTCTG
          Fig badnavirus 1 NC_017830.1   (7141) -----------------------------------------------------------------------------------
                                                Section 308
                                   (25482) 25482   25490     25500     25510     25520     25530     25540     25550         25564
SARS-CoV-2 Reference Genome NC_045512.2 (25347) AGCCAGTGCTCAAAGGAGTCAAATTACATTACACATAAACGAACTTATGGATTTGTTTATGAGAATCTTCACAATTGGAACTG
          Fig badnavirus 1 NC_017830.1   (7141) -----------------------------------------------------------------------------------
```

```
                                                   Section 309
                                         (25565) 25565 25570     25580     25590     25600     25610     25620     25630          25647
SARS-CoV-2 Reference Genome NC_045512.2 (25430) TAACTTTGAAGCAAGGTGAAATCAAGGATGCTACTCCTTCAGATTTTGTTCGCGCTACTGCAACGATACCGATACAAGCCTCA
          Fig badnavirus 1 NC_017830.1  (7141) -----------------------------------------------------------------------------------
                                                   Section 310
                                         (25648) 25648       25660     25670     25680     25690     25700     25710     25720  25730
SARS-CoV-2 Reference Genome NC_045512.2 (25513) CTCCCTTTCGGATGGCTTATTGTTGGCGTTGCACTTCTTGCTGTTTTTCAGAGCGCTTCCAAAATCATAACCCTCAAAAAGAG
          Fig badnavirus 1 NC_017830.1  (7141) -----------------------------------------------------------------------------------
                                                   Section 311
                                         (25731) 25731    25740     25750     25760     25770     25780     25790     25800        25813
SARS-CoV-2 Reference Genome NC_045512.2 (25596) ATGGCAACTAGCACTCTCCAAGGGTGTTCACTTTGTTTGCAACTTGCTGTTGTTGTTTGTAACAGTTTACTCACACCTTTTGC
          Fig badnavirus 1 NC_017830.1  (7141) -----------------------------------------------------------------------------------
                                                   Section 312
                                         (25814) 25814 25820     25830     25840     25850     25860     25870     25880          25896
SARS-CoV-2 Reference Genome NC_045512.2 (25679) TCGTTGCTGCTGGCCTTGAAGCCCCTTTTCTCTATCTTTATGCTTTAGTCTACTTCTTGCAGAGTATAAACTTTGTAAGAATA
          Fig badnavirus 1 NC_017830.1  (7141) -----------------------------------------------------------------------------------
                                                   Section 313
                                         (25897) 25897        25910     25920     25930     25940     25950     25960            25979
SARS-CoV-2 Reference Genome NC_045512.2 (25762) ATAATGAGGCTTTGGCTTTGCTGGAAATGCCGTTCCAAAAACCCATTACTTTATGATGCCAACTATTTTCTTTGCTGGCATAC
          Fig badnavirus 1 NC_017830.1  (7141) -----------------------------------------------------------------------------------
                                                   Section 314
                                         (25980) 25980     25990     26000     26010     26020     26030     26040     26050      26062
SARS-CoV-2 Reference Genome NC_045512.2 (25845) TAATTGTTACGACTATTGTATACCTTACAATAGTGTAACTTCTTCAATTGTCATTACTTCAGGTGATGGCACAACAAGTCCTA
          Fig badnavirus 1 NC_017830.1  (7141) -----------------------------------------------------------------------------------
                                                   Section 315
                                         (26063) 26063  26070     26080     26090     26100     26110     26120     26130          26145
SARS-CoV-2 Reference Genome NC_045512.2 (25928) TTTCTGAACATGACTACCAGATTGGTGGTTATACTGAAAAATGGGAATCTGGAGTAAAAGACTGTGTTGTATTACACAGTTAC
          Fig badnavirus 1 NC_017830.1  (7141) -----------------------------------------------------------------------------------
```

```
                                                    Section 316
                                        (26146) 26146         26160     26170     26180     26190     26200     26210             26228
SARS-CoV-2 Reference Genome NC_045512.2 (26011) TTCACTTCAGACTATTACCAGCTGTACTCAACTCAATTGAGTACAGACACTGGTGTTGAACATGTTACCTTCTTCATCTACAA
        Fig badnavirus 1 NC_017830.1    (7141) -----------------------------------------------------------------------------------
                                                    Section 317
                                        (26229) 26229      26240     26250     26260     26270     26280     26290     26300      26311
SARS-CoV-2 Reference Genome NC_045512.2 (26094) TAAAATTGTTGATGAGCCTGAAGAACATGTCCAAATTCACACAATCGACGGTTCATCCGGAGTTGTTAATCCAGTAATGGAAC
        Fig badnavirus 1 NC_017830.1    (7141) -----------------------------------------------------------------------------------
                                                    Section 318
                                        (26312) 26312   26320     26330     26340     26350     26360     26370     26380             26394
SARS-CoV-2 Reference Genome NC_045512.2 (26177) CAATTTATGATGAACCGACGACGACTACTAGCGTGCCTTTGTAAGCACAAGCTGATGAGTACGAACTTATGTACTCATTCGTT
        Fig badnavirus 1 NC_017830.1    (7141) -----------------------------------------------------------------------------------
                                                    Section 319
                                        (26395) 26395 26400     26410     26420     26430     26440     26450     26460             26477
SARS-CoV-2 Reference Genome NC_045512.2 (26260) TCGGAAGAGACAGGTACGTTAATAGTTAATAGCGTACTTCTTTTTCTTGCTTTCGTGGTATTCTTGCTAGTTACACTAGCCAT
        Fig badnavirus 1 NC_017830.1    (7141) -----------------------------------------------------------------------------------
                                                    Section 320
                                        (26478) 26478        26490     26500     26510     26520     26530     26540     26550      26560
SARS-CoV-2 Reference Genome NC_045512.2 (26343) CCTTACTGCGCTTCGATTGTGTGCGTACTGCTGCAATATTGTTAACGTGAGTCTTGTAAAACCTTCTTTTTACGTTTACTCTC
        Fig badnavirus 1 NC_017830.1    (7141) -----------------------------------------------------------------------------------
                                                    Section 321
                                        (26561) 26561    26570     26580     26590     26600     26610     26620     26630             26643
SARS-CoV-2 Reference Genome NC_045512.2 (26426) GTGTTAAAAATCTGAATTCTTCTAGAGTTCCTGATCTTCTGGTCTAAACGAACTAAATATTATATTAGTTTTTCTGTTTGGAA
        Fig badnavirus 1 NC_017830.1    (7141) -----------------------------------------------------------------------------------
                                                    Section 322
                                        (26644) 26644 26650     26660     26670     26680     26690     26700     26710             26726
SARS-CoV-2 Reference Genome NC_045512.2 (26509) CTTTAATTTTAGCCATGGCAGATTCCAACGGTACTATTACCGTTGAAGAGCTTAAAAAGCTCCTTGAACAATGGAACCTAGTA
        Fig badnavirus 1 NC_017830.1    (7141) -----------------------------------------------------------------------------------
```

```
                                                     Section 323
                                             (26727) 26727        26740      26750      26760      26770      26780      26790            26809
SARS-CoV-2 Reference Genome NC_045512.2 (26592) ATAGGTTTCCTATTCCTTACATGGATTTGTCTTCTACAATTTGCCTATGCCAACAGGAATAGGTTTTTGTATATAATTAAGTT
        Fig badnavirus 1 NC_017830.1    (7141) -----------------------------------------------------------------------------------
                                                     Section 324
                                             (26810) 26810      26820      26830      26840      26850      26860      26870      26880    26892
SARS-CoV-2 Reference Genome NC_045512.2 (26675) AATTTTCCTCTGGCTGTTATGGCCAGTAACTTTAGCTTGTTTTGTGCTTGCTGCTGTTTACAGAATAAATTGGATCACCGGTG
        Fig badnavirus 1 NC_017830.1    (7141) -----------------------------------------------------------------------------------
                                                     Section 325
                                             (26893) 26893   26900      26910      26920      26930      26940      26950      26960        26975
SARS-CoV-2 Reference Genome NC_045512.2 (26758) GAATTGCTATCGCAATGGCTTGTCTTGTAGGCTTGATGTGGCTCAGCTACTTCATTGCTTCTTTCAGACTGTTTGCGCGTACG
        Fig badnavirus 1 NC_017830.1    (7141) -----------------------------------------------------------------------------------
                                                     Section 326
                                             (26976) 26976          26990      27000      27010      27020      27030      27040            27058
SARS-CoV-2 Reference Genome NC_045512.2 (26841) CGTTCCATGTGGTCATTCAATCCAGAAACTAACATTCTTCTCAACGTGCCACTCCATGGCACTATTCTGACCAGACCGCTTCT
        Fig badnavirus 1 NC_017830.1    (7141) -----------------------------------------------------------------------------------
                                                     Section 327
                                             (27059) 27059       27070      27080      27090      27100      27110      27120      27130   27141
SARS-CoV-2 Reference Genome NC_045512.2 (26924) AGAAAGTGAACTCGTAATCGGAGCTGTGATCCTTCGTGGACATCTTCGTATTGCTGGACACCATCTAGGACGCTGTGACATCA
        Fig badnavirus 1 NC_017830.1    (7141) -----------------------------------------------------------------------------------
                                                     Section 328
                                             (27142) 27142    27150      27160      27170      27180      27190      27200      27210        27224
SARS-CoV-2 Reference Genome NC_045512.2 (27007) AGGACCTGCCTAAAGAAATCACTGTTGCTACATCACGAACGCTTTCTTATTACAAATTGGGAGCTTCGCAGCGTGTAGCAGGT
        Fig badnavirus 1 NC_017830.1    (7141) -----------------------------------------------------------------------------------
                                                     Section 329
                                             (27225) 27225 27230      27240      27250      27260      27270      27280      27290           27307
SARS-CoV-2 Reference Genome NC_045512.2 (27090) GACTCAGGTTTTGCTGCATACAGTCGCTACAGGATTGGCAACTATAAATTAAACACAGACCATTCCAGTAGCAGTGACAATAT
        Fig badnavirus 1 NC_017830.1    (7141) -----------------------------------------------------------------------------------
```

```
                                                                                  Section 330
                                        (27308) 27308      27320     27330     27340     27350     27360     27370     27380     27390
SARS-CoV-2 Reference Genome NC_045512.2 (27173) TGCTTTGCTTGTACAGTAAGTGACAACAGATGTTTCATCTCGTTGACTTTCAGGTTACTATAGCAGAGATATTACTAATTATT
          Fig badnavirus 1 NC_017830.1   (7141) -----------------------------------------------------------------------------------
                                                                                  Section 331
                                        (27391) 27391    27400     27410     27420     27430     27440     27450     27460        27473
SARS-CoV-2 Reference Genome NC_045512.2 (27256) ATGAGGACTTTTAAAGTTTCCATTTGGAATCTTGATTACATCATAAACCTCATAATTAAAAATTTATCTAAGTCACTAACTGA
          Fig badnavirus 1 NC_017830.1   (7141) -----------------------------------------------------------------------------------
                                                                                  Section 332
                                        (27474) 27474 27480     27490     27500     27510     27520     27530     27540           27556
SARS-CoV-2 Reference Genome NC_045512.2 (27339) GAATAAATATTCTCAATTAGATGAAGAGCAACCAATGGAGATTGATTAAACGAACATGAAAATTATTCTTTTCTTGGCACTGA
          Fig badnavirus 1 NC_017830.1   (7141) -----------------------------------------------------------------------------------
                                                                                  Section 333
                                        (27557) 27557        27570     27580     27590     27600     27610     27620              27639
SARS-CoV-2 Reference Genome NC_045512.2 (27422) TAACACTCGCTACTTGTGAGCTTTATCACTACCAAGAGTGTGTTAGAGGTACAACAGTACTTTTAAAAGAACCTTGCTCTTCT
          Fig badnavirus 1 NC_017830.1   (7141) -----------------------------------------------------------------------------------
                                                                                  Section 334
                                        (27640) 27640     27650     27660     27670     27680     27690     27700     27710        27722
SARS-CoV-2 Reference Genome NC_045512.2 (27505) GGAACATACGAGGGCAATTCACCATTTCATCCTCTAGCTGATAACAAATTTGCACTGACTTGCTTTAGCACTCAATTTGCTTT
          Fig badnavirus 1 NC_017830.1   (7141) -----------------------------------------------------------------------------------
                                                                                  Section 335
                                        (27723) 27723  27730     27740     27750     27760     27770     27780     27790           27805
SARS-CoV-2 Reference Genome NC_045512.2 (27588) TGCTTGTCCTGACGGCGTAAAACACGTCTATCAGTTACGTGCCAGATCAGTTTCACCTAAACTGTTCATCAGACAAGAGGAAG
          Fig badnavirus 1 NC_017830.1   (7141) -----------------------------------------------------------------------------------
                                                                                  Section 336
                                        (27806) 27806         27820     27830     27840     27850     27860     27870               27888
SARS-CoV-2 Reference Genome NC_045512.2 (27671) TTCAAGAACTTTACTCTCCAATTTTTCTTATTGTTGCGGCAATAGTGTTTATAACACTTTGCTTCACACTCAAAAGAAAGACA
          Fig badnavirus 1 NC_017830.1   (7141) -----------------------------------------------------------------------------------
```

```
                                                          Section 337
                                         (27889) 27889      27900     27910     27920     27930     27940     27950     27960     27971
SARS-CoV-2 Reference Genome NC_045512.2 (27754) GAATGATTGAACTTTCATTAATTGACTTCTATTTGTGCTTTTTAGCCTTTCTGCTATTCCTTGTTTTAATTATGCTTATTATC
          Fig badnavirus 1 NC_017830.1   (7141) -----------------------------------------------------------------------------------
                                                          Section 338
                                         (27972) 27972    27980     27990     28000     28010     28020     28030     28040     28054
SARS-CoV-2 Reference Genome NC_045512.2 (27837) TTTTGGTTCTCACTTGAACTGCAAGATCATAATGAAACTTGTCACGCCTAAACGAACATGAAATTTCTTGTTTTCTTAGGAAT
          Fig badnavirus 1 NC_017830.1   (7141) -----------------------------------------------------------------------------------
                                                          Section 339
                                         (28055) 28055 28060     28070     28080     28090     28100     28110     28120     28137
SARS-CoV-2 Reference Genome NC_045512.2 (27920) CATCACAACTGTAGCTGCATTTCACCAAGAATGTAGTTTACAGTCATGTACTCAACATCAACCATATGTAGTTGATGACCCGT
          Fig badnavirus 1 NC_017830.1   (7141) -----------------------------------------------------------------------------------
                                                          Section 340
                                         (28138) 28138       28150     28160     28170     28180     28190     28200     28210 28220
SARS-CoV-2 Reference Genome NC_045512.2 (28003) GTCCTATTCACTTCTATTCTAAATGGTATATTAGAGTAGGAGCTAGAAAATCAGCACCTTTAATTGAATTGTGCGTGGATGAG
          Fig badnavirus 1 NC_017830.1   (7141) -----------------------------------------------------------------------------------
                                                          Section 341
                                         (28221) 28221    28230     28240     28250     28260     28270     28280     28290     28303
SARS-CoV-2 Reference Genome NC_045512.2 (28086) GCTGGTTCTAAATCACCCATTCAGTACATCGATATCGGTAATTATACAGTTTCCTGTTTACCTTTTACAATTAATTGCCAGGA
          Fig badnavirus 1 NC_017830.1   (7141) -----------------------------------------------------------------------------------
                                                          Section 342
                                         (28304) 28304 28310     28320     28330     28340     28350     28360     28370     28386
SARS-CoV-2 Reference Genome NC_045512.2 (28169) ACCTAAATTGGGTAGTCTTGTAGTGCGTTGTTCGTTCTATGAAGACTTTTTAGAGTATCATGACGTTCGTGTTGTTTTAGATT
          Fig badnavirus 1 NC_017830.1   (7141) -----------------------------------------------------------------------------------
                                                          Section 343
                                         (28387) 28387        28400     28410     28420     28430     28440     28450     28469
SARS-CoV-2 Reference Genome NC_045512.2 (28252) TCATCTAAACGAACAAACTAAAATGTCTGATAATGGACCCCAAAATCAGCGAAATGCACCCCGCATTACGTTTGGTGGACCCT
          Fig badnavirus 1 NC_017830.1   (7141) -----------------------------------------------------------------------------------
```

```
                                                    Section 344
                                         (28470) 28470     28480     28490     28500     28510     28520     28530     28540     28552
SARS-CoV-2 Reference Genome NC_045512.2 (28335) CAGATTCAACTGGCAGTAACCAGAATGGAGAACGCAGTGGGGCGCGATCAAAACAACGTCGGCCCCAAGGTTTACCCAATAAT
          Fig badnavirus 1 NC_017830.1   (7141) -----------------------------------------------------------------------------------
                                                    Section 345
                                         (28553) 28553 28560     28570     28580     28590     28600     28610     28620          28635
SARS-CoV-2 Reference Genome NC_045512.2 (28418) ACTGCGTCTTGGTTCACCGCTCTCACTCAACATGGCAAGGAAGACCTTAAATTCCCTCGAGGACAAGGCGTTCCAATTAACAC
          Fig badnavirus 1 NC_017830.1   (7141) -----------------------------------------------------------------------------------
                                                    Section 346
                                         (28636) 28636         28650     28660     28670     28680     28690     28700          28718
SARS-CoV-2 Reference Genome NC_045512.2 (28501) CAATAGCAGTCCAGATGACCAAATTGGCTACTACCGAAGAGCTACCAGACGAATTCGTGGTGGTGACGGTAAAATGAAAGATC
          Fig badnavirus 1 NC_017830.1   (7141) -----------------------------------------------------------------------------------
                                                    Section 347
                                         (28719) 28719      28730     28740     28750     28760     28770     28780     28790   28801
SARS-CoV-2 Reference Genome NC_045512.2 (28584) TCAGTCCAAGATGGTATTTCTACTACCTAGGAACTGGGCCAGAAGCTGGACTTCCCTATGGTGCTAACAAAGACGGCATCATA
          Fig badnavirus 1 NC_017830.1   (7141) -----------------------------------------------------------------------------------
                                                    Section 348
                                         (28802) 28802   28810     28820     28830     28840     28850     28860     28870          28884
SARS-CoV-2 Reference Genome NC_045512.2 (28667) TGGGTTGCAACTGAGGGAGCCTTGAATACACCAAAAGATCACATTGGCACCCGCAATCCTGCTAACAATGCTGCAATCGTGCT
          Fig badnavirus 1 NC_017830.1   (7141) -----------------------------------------------------------------------------------
                                                    Section 349
                                         (28885) 28885 28890     28900     28910     28920     28930     28940     28950          28967
SARS-CoV-2 Reference Genome NC_045512.2 (28750) ACAACTTCCTCAAGGAACAACATTGCCAAAAGGCTTCTACGCAGAAGGGAGCAGAGGCGGCAGTCAAGCCTCTTCTCGTTCCT
          Fig badnavirus 1 NC_017830.1   (7141) -----------------------------------------------------------------------------------
                                                    Section 350
                                         (28968) 28968       28980     28990     29000     29010     29020     29030     29040   29050
SARS-CoV-2 Reference Genome NC_045512.2 (28833) CATCACGTAGTCGCAACAGTTCAAGAAATTCAACTCCAGGCAGCAGTAGGGGAACTTCTCCTGCTAGAATGGCTGGCAATGGC
          Fig badnavirus 1 NC_017830.1   (7141) -----------------------------------------------------------------------------------
```

```
Section 351
                                            (29051) 29051 29060 29070 29080 29090 29100 29110 29120 29133
SARS-CoV-2 Reference Genome NC_045512.2 (28916) GGTGATGCTGCTCTTGCTTTGCTGCTGCTTGACAGATTGAACCAGCTTGAGAGCAAAATGTCTGGTAAAGGCCAACAACAACA
          Fig badnavirus 1 NC_017830.1  (7141) -----------------------------------------------------------------------------------
Section 352
                                            (29134) 29134 29140 29150 29160 29170 29180 29190 29200 29216
SARS-CoV-2 Reference Genome NC_045512.2 (28999) AGGCCAAACTGTCACTAAGAAATCTGCTGCTGAGGCTTCTAAGAAGCCTCGGCAAAAACGTACTGCCACTAAAGCATACAATG
          Fig badnavirus 1 NC_017830.1  (7141) -----------------------------------------------------------------------------------
Section 353
                                            (29217) 29217 29230 29240 29250 29260 29270 29280 29299
SARS-CoV-2 Reference Genome NC_045512.2 (29082) TAACACAAGCTTTCGGCAGACGTGGTCCAGAACAAACCCAAGGAAATTTTGGGGACCAGGAACTAATCAGACAAGGAACTGAT
          Fig badnavirus 1 NC_017830.1  (7141) -----------------------------------------------------------------------------------
Section 354
                                            (29300) 29300 29310 29320 29330 29340 29350 29360 29370 29382
SARS-CoV-2 Reference Genome NC_045512.2 (29165) TACAAACATTGGCCGCAAATTGCACAATTTGCCCCCAGCGCTTCAGCGTTCTTCGGAATGTCGCGCATTGGCATGGAAGTCAC
          Fig badnavirus 1 NC_017830.1  (7141) -----------------------------------------------------------------------------------
Section 355
                                            (29383) 29383 29390 29400 29410 29420 29430 29440 29450 29465
SARS-CoV-2 Reference Genome NC_045512.2 (29248) ACCTTCGGGAACGTGGTTGACCTACACAGGTGCCATCAAATTGGATGACAAAGATCCAAATTTCAAAGATCAAGTCATTTTGC
          Fig badnavirus 1 NC_017830.1  (7141) -----------------------------------------------------------------------------------
Section 356
                                            (29466) 29466 29480 29490 29500 29510 29520 29530 29548
SARS-CoV-2 Reference Genome NC_045512.2 (29331) TGAATAAGCATATTGACGCATACAAAACATTCCCACCAACAGAGCCTAAAAAGGACAAAAAGAAGAAGGCTGATGAAACTCAA
          Fig badnavirus 1 NC_017830.1  (7141) -----------------------------------------------------------------------------------
Section 357
                                            (29549) 29549 29560 29570 29580 29590 29600 29610 29620 29631
SARS-CoV-2 Reference Genome NC_045512.2 (29414) GCCTTACCGCAGAGACAGAAGAAACAGCAAACTGTGACTCTTCTTCCTGCTGCAGATTTGGATGATTTCTCCAAACAATTGCA
          Fig badnavirus 1 NC_017830.1  (7141) -----------------------------------------------------------------------------------
```

```
                                                    Section 358
                                          (29632) 29632 29640 29650 29660 29670 29680 29690 29700 29714
SARS-CoV-2 Reference Genome NC_045512.2  (29497) ACAATCCATGAGCAGTGCTGACTCAACTCAGGCCTAAACTCATGCAGACCACACAAGGCAGATGGGCTATATAAACGTTTTCG
         Fig badnavirus 1 NC_017830.1     (7141) -----------------------------------------------------------------------------------
                                                    Section 359
                                          (29715) 29715 29720 29730 29740 29750 29760 29770 29780 29797
SARS-CoV-2 Reference Genome NC_045512.2  (29580) CTTTTCCGTTTACGATATATAGTCTACTCTTGTGCAGAATGAATTCTCGTAACTACATAGCACAAGTAGATGTAGTTAACTTT
         Fig badnavirus 1 NC_017830.1     (7141) -----------------------------------------------------------------------------------
                                                    Section 360
                                          (29798) 29798 29810 29820 29830 29840 29850 29860 29870 29880
SARS-CoV-2 Reference Genome NC_045512.2  (29663) AATCTCACATAGCAATCTTTAATCAGTGTGTAACATTAGGGAGGACTTGAAAGAGCCACCACATTTTCACCGAGGCCACGCGG
         Fig badnavirus 1 NC_017830.1     (7141) -----------------------------------------------------------------------------------
                                                    Section 361
                                          (29881) 29881 29890 29900 29910 29920 29930 29940 29950 29963
SARS-CoV-2 Reference Genome NC_045512.2  (29746) AGTACGATCGAGTGTACAGTGAACAATGCTAGGGAGAGCTGCCTATATGGAAGAGCCCTAATGTGTAAAATTAATTTTAGTAG
         Fig badnavirus 1 NC_017830.1     (7141) -----------------------------------------------------------------------------------
                                                    Section 362
                                          (29964) 29964 29970 29980 29990 30000 30010 30020 30038
SARS-CoV-2 Reference Genome NC_045512.2  (29829) TGCTATCCCCATGTGATTTTAATAGCTTCTTAGGAGAATGACAAAAAAAAAAAAAAAAAAAAAAAAAAAAAAAAA
         Fig badnavirus 1 NC_017830.1     (7141) ---------------------------------------------------------------------------
```
