# Supplementary material for: Ubiquitous Micro-Modular Homologies among Genomes from Viruses to Bacteria to Human Mitochondrial DNA: Platforms for Recombination during Evolution?
Source: Viruses. 2022 Apr 24;14(5):885. doi: 10.3390/v14050885 (PMC9147251; doi:10.3390/v14050885)
Supplement: Supplementary file 1 [file viruses-14-00885-s001.zip › Fig. S5E, SARS-CoV-2 & lambda DNA Alignment.pdf]

SARS-CoV-2 & lambda.apr

```
                                                                                          Section 1
                              (1) 1        10        20        30        40        50        60        70        80               97
lambda NC_001416.1            (1) GGGCGGCGACCTCGCGGGTTTTCGCTATTTATGAAAATTTTCCGGTTTAAGGCGTTTCCGTTCTTCTTCGTCATAACTTAATGTTTTTATTTAAAAT
SARS-CoV-2 NC_045512.2        (1) -------------------------------------------------------------------------------------------------
                                                                                          Section 2
                             (98) 98          110       120       130       140       150       160       170       180          194
lambda NC_001416.1           (98) ACCCTCTGAAAAGAAAGGAAACGACAGGTGCTGAAAGCGAGGCTTTTTGGCCTCTGTCGTTTCCTTTCTCTGTTTTTGTCCGTGGAATGAACAATGG
SARS-CoV-2 NC_045512.2        (1) -------------------------------------------------------------------------------------------------
                                                                                          Section 3
                            (195) 195  200       210       220       230       240       250       260       270       280       291
lambda NC_001416.1          (195) AAGTCAACAAAAAGCAGCTGGCTGACATTTTCGGTGCGAGTATCCGTACCATTCAGAACTGGCAGGAACAGGGAATGCCCGTTCTGCGAGGCGGTGG
SARS-CoV-2 NC_045512.2        (1) -------------------------------------------------------------------------------------------------
                                                                                          Section 4
                            (292) 292     300       310       320       330       340       350       360       370              388
lambda NC_001416.1          (292) CAAGGGTAATGAGGTGCTTTATGACTCTGCCGCCGTCATAAAATGGTATGCCGAAAGGGATGCTGAAATTGAGAACGAAAAGCTGCGCCGGGAGGTT
SARS-CoV-2 NC_045512.2        (1) -------------------------------------------------------------------------------------------------
                                                                                          Section 5
                            (389) 389        400       410       420       430       440       450       460       470           485
lambda NC_001416.1          (389) GAAGAACTGCGGCAGGCCAGCGAGGCAGATCTCCAGCCAGGAACTATTGAGTACGAACGCCATCGACTTACGCGTGCGCAGGCCGACGCACAGGAAC
SARS-CoV-2 NC_045512.2        (1) -------------------------------------------------------------------------------------------------
                                                                                          Section 6
                            (486) 486           500       510       520       530       540       550       560       570         582
lambda NC_001416.1          (486) TGAAGAATGCCAGAGACTCCGCTGAAGTGGTGGAAACCGCATTCTGTACTTTCGTGCTGTCGCGGATCGCAGGTGAAATTGCCAGTATTCTCGACGG
SARS-CoV-2 NC_045512.2        (1) -------------------------------------------------------------------------------------------------
                                                                                          Section 7
                            (583) 583    590       600       610       620       630       640       650       660                679
lambda NC_001416.1          (583) GCTCCCCCTGTCGGTGCAGCGGCGTTTTCCGGAACTGGAAAACCGACATGTTGATTTCCTGAAACGGGATATCATCAAAGCCATGAACAAAGCAGCC
SARS-CoV-2 NC_045512.2        (1) -------------------------------------------------------------------------------------------------
```

```
Section 8
                              (680)  680       690       700       710       720       730       740       750       760            776
lambda NC_001416.1            (680)  GCGCTGGATGAACTGATACCGGGGTTGCTGAGTGAATATATCGAACAGTCAGGTTAACAGGCTGCGGCATTTTGTCCGCGCCGGGCTTCGCTCACTG
SARS-CoV-2 NC_045512.2          (1)  -------------------------------------------------------------------------------------------------

Section 9
                              (777)  777          790       800       810       820       830       840       850       860         873
lambda NC_001416.1            (777)  TTCAGGCCGGAGCCACAGACCGCCGTTGAATGGGCGGATGCTAATTACTATCTCCCGAAAGAATCCGCATACCAGGAAGGGCGCTGGGAAACACTGC
SARS-CoV-2 NC_045512.2          (1)  -------------------------------------------------------------------------------------------------

Section 10
                              (874)  874   880       890       900       910       920       930       940       950       960       970
lambda NC_001416.1            (874)  CCTTTCAGCGGGCCATCATGAATGCGATGGGCAGCGACTACATCCGTGAGGTGAATGTGGTGAAGTCTGCCCGTGTCGGTTATTCCAAAATGCTGCT
SARS-CoV-2 NC_045512.2          (1)  -------------------------------------------------------------------------------------------------

Section 11
                              (971)  971      980       990       1000      1010      1020      1030      1040      1050          1067
lambda NC_001416.1            (971)  GGGTGTTTATGCCTACTTTATAGAGCATAAGCAGCGCAACACCCTTATCTGGTTGCCGACGGATGGTGATGCCGAGAACTTTATGAAAACCCACGTT
SARS-CoV-2 NC_045512.2          (1)  -------------------------------------------------------------------------------------------------

Section 12
                             (1068)  1068        1080      1090      1100      1110      1120      1130      1140      1150          1164
lambda NC_001416.1           (1068)  GAGCCGACTATTCGTGATATTCCGTCGCTGCTGGCGCTGGCCCCGTGGTATGGCAAAAAGCACCGGGATAACACGCTCACCATGAAGCGTTTCACTA
SARS-CoV-2 NC_045512.2          (1)  -------------------------------------------------------------------------------------------------

Section 13
                             (1165)  1165 1170      1180      1190      1200      1210      1220      1230      1240      1250      1261
lambda NC_001416.1           (1165)  ATGGGCGTGGCTTCTGGTGCCTGGGCGGTAAAGCGGCAAAAAACTACCGTGAAAAGTCGGTGGATGTGGCGGGTTATGATGAACTTGCTGCTTTTGA
SARS-CoV-2 NC_045512.2          (1)  -------------------------------------------------------------------------------------------------

Section 14
                             (1262)  1262   1270      1280      1290      1300      1310      1320      1330      1340              1358
lambda NC_001416.1           (1262)  TGATGATATTGAACAGGAAGGCTCTCCGACGTTCCTGGGTGACAAGCGTATTGAAGGCTCGGTCTGGCCAAAGTCCATCCGTGGCTCCACGCCAAAA
SARS-CoV-2 NC_045512.2          (1)  -------------------------------------------------------------------------------------------------
```

Section 15

```
                     (1359) 1359      1370      1380      1390      1400      1410      1420      1430      1440          1455
lambda NC_001416.1   (1359) GTGAGAGGCACCTGTCAGATTGAGCGTGCAGCCAGTGAATCCCCGCATTTTATGCGTTTTCATGTTGCCTGCCCGCATTGCGGGGAGGAGCAGTATC
SARS-CoV-2 NC_045512.2  (1) -------------------------------------------------------------------------------------------------
```

Section 16

```
                     (1456) 1456          1470      1480      1490      1500      1510      1520      1530      1540      1552
lambda NC_001416.1   (1456) TTAAATTTGGCGACAAAGAGACGCCGTTTGGCCTCAAATGGACGCCGGATGACCCCTCCAGCGTGTTTTATCTCTGCGAGCATAATGCCTGCGTCAT
SARS-CoV-2 NC_045512.2  (1) -------------------------------------------------------------------------------------------------
```

Section 17

```
                     (1553) 1553   1560      1570      1580      1590      1600      1610      1620      1630              1649
lambda NC_001416.1   (1553) CCGCCAGCAGGAGCTGGACTTTACTGATGCCCGTTATATCTGCGAAAAGACCGGGATCTGGACCCGTGATGGCATTCTCTGGTTTTCGTCATCCGGT
SARS-CoV-2 NC_045512.2  (1) -------------------------------------------------------------------------------------------------
```

Section 18

```
                     (1650) 1650      1660      1670      1680      1690      1700      1710      1720      1730          1746
lambda NC_001416.1   (1650) GAAGAGATTGAGCCACCTGACAGTGTGACCTTTCACATCTGGACAGCGTACAGCCCGTTCACCACCTGGGTGCAGATTGTCAAAGACTGGATGAAAA
SARS-CoV-2 NC_045512.2  (1) -------------------------------------------------------------------------------------------------
```

Section 19

```
                     (1747) 1747         1760      1770      1780      1790      1800      1810      1820      1830      1843
lambda NC_001416.1   (1747) CGAAAGGGGATACGGGAAAACGTAAAACCTTCGTAAACACCACGCTCGGTGAGACGTGGAGGCGAAAATTGGCGAACGTCCGGATGCTGAAGTGAT
SARS-CoV-2 NC_045512.2  (1) -------------------------------------------------------------------------------------------------
```

Section 20

```
                     (1844) 1844  1850      1860      1870      1880      1890      1900      1910      1920      1930    1940
lambda NC_001416.1   (1844) GGCAGAGCGGAAAGAGCATTATTCAGCGCCCGTTCCTGACCGTGTGGCTTACCTGACCGCCGGTATCGACTCCCAGCTGGACCGCTACGAAATGCGC
SARS-CoV-2 NC_045512.2  (1) -------------------------------------------------------------------------------------------------
```

Section 21

```
                     (1941) 1941     1950      1960      1970      1980      1990      2000      2010      2020           2037
lambda NC_001416.1   (1941) GTATGGGGATGGGGGCCGGGTGAGGAAAGCTGGCTGATTGACCGGCAGATTATTATGGGCCGCCACGACGATGAACAGACGCTGCTGCGTGTGGATG
SARS-CoV-2 NC_045512.2  (1) -------------------------------------------------------------------------------------------------
```

```
                                                                                                                      Section 22
                           (2038) 2038        2050      2060      2070      2080      2090      2100      2110      2120          2134
      lambda NC_001416.1   (2038) AGGCCATCAATAAAACCTATACCCGCCGGAATGGTGCAGAAATGTCGATATCCCGTATCTGCTGGGATACTGGCGGGATTGACCCGACCATTGTGTA
SARS-CoV-2 NC_045512.2        (1) -------------------------------------------------------------------------------------------------
                                                                                                                      Section 23
                           (2135) 2135 2140      2150      2160      2170      2180      2190      2200      2210      2220     2231
      lambda NC_001416.1   (2135) TGAACGCTCGAAAAAACATGGGCTGTTCCGGGTGATCCCCATTAAAGGGGCATCCGTCTACGGAAAGCCGGTGGCCAGCATGCCACGTAAGCGAAAC
SARS-CoV-2 NC_045512.2        (1) -------------------------------------------------------------------------------------------------
                                                                                                                      Section 24
                           (2232) 2232    2240      2250      2260      2270      2280      2290      2300      2310              2328
      lambda NC_001416.1   (2232) AAAAACGGGGTTTACCTTACCGAAATCGGTACGGATACCGCGAAAGAGCAGATTTATAACCGCTTCACACTGACGCCGGAAGGGGATGAACCGCTTC
SARS-CoV-2 NC_045512.2        (1) -------------------------------------------------------------------------------------------------
                                                                                                                      Section 25
                           (2329) 2329       2340      2350      2360      2370      2380      2390      2400      2410           2425
      lambda NC_001416.1   (2329) CCGGTGCCGTTCACTTCCCGAATAACCCGGATATTTTTGATCTGACCGAAGCGCAGCAGCTGACTGCTGAAGAGCAGGTCGAAAAATGGGTGGATGG
SARS-CoV-2 NC_045512.2        (1) -------------------------------------------------------------------------------------------------
                                                                                                                      Section 26
                           (2426) 2426          2440      2450      2460      2470      2480      2490      2500      2510      2522
      lambda NC_001416.1   (2426) CAGGAAAAAAATACTGTGGGACAGCAAAAAGCGACGCAATGAGGCACTCGACTGCTTCGTTTATGCGCTGGCGGCGCTGCGCATCAGTATTTCCCGC
SARS-CoV-2 NC_045512.2        (1) -------------------------------------------------------------------------------------------------
                                                                                                                      Section 27
                           (2523) 2523  2530      2540      2550      2560      2570      2580      2590      2600              2619
      lambda NC_001416.1   (2523) TGGCAGCTGGATCTCAGTGCGCTGCTGGCGAGCCTGCAGGAAGAGGATGGTGCAGCAACCAACAAGAAAACACTGGCAGATTACGCCCGTGCCTTAT
SARS-CoV-2 NC_045512.2        (1) -------------------------------------------------------------------------------------------------
                                                                                                                      Section 28
                           (2620) 2620      2630      2640      2650      2660      2670      2680      2690      2700            2716
      lambda NC_001416.1   (2620) CCGGAGAGGATGAATGACGCGACAGGAAGAACTTGCCGCTGCCCGTGCGGCACTGCATGACCTGATGACAGGTAAACGGGTGGCAACAGTACAGAAA
SARS-CoV-2 NC_045512.2        (1) -------------------------------------------------------------------------------------------------
```

```
                                                                                                  Section 29
                    (2717) 2717         2730      2740      2750      2760      2770      2780      2790      2800        2813
   lambda NC_001416.1 (2717) GACGGACGAAGGGTGGAGTTTACGGCCACTTCCGTGTCTGACCTGAAAAAATATATTGCAGAGCTGGAAGTGCAGACCGGCATGACACAGCGACGCA
SARS-CoV-2 NC_045512.2    (1) -------------------------------------------------------------------------------------------------
                                                                                                  Section 30
                    (2814) 2814  2820      2830      2840      2850      2860      2870      2880      2890      2900   2910
   lambda NC_001416.1 (2814) GGGGACCTGCAGGATTTTATGTATGAAAACGCCCACCATTCCCACCCTTCTGGGGCCGGACGGCATGACATCGCTGCGCGAATATGCCGGTTATCAC
SARS-CoV-2 NC_045512.2    (1) -------------------------------------------------------------------------------------------------
                                                                                                  Section 31
                    (2911) 2911     2920      2930      2940      2950      2960      2970      2980      2990          3007
   lambda NC_001416.1 (2911) GGCGGTGGCAGCGGATTTGGAGGGCAGTTGCGGTCGTGGAACCCACCGAGTGAAAGTGTGGATGCAGCCCTGTTGCCCAACTTTACCCGTGGCAATG
SARS-CoV-2 NC_045512.2    (1) -------------------------------------------------------------------------------------------------
                                                                                                  Section 32
                    (3008) 3008         3020      3030      3040      3050      3060      3070      3080      3090        3104
   lambda NC_001416.1 (3008) CCCGCGCAGACGATCTGGTACGCAATAACGGCTATGCCGCCAACGCCATCCAGCTGCATCAGGATCATATCGTCGGGTCTTTTTTCCGGCTCAGTCA
SARS-CoV-2 NC_045512.2    (1) -------------------------------------------------------------------------------------------------
                                                                                                  Section 33
                    (3105) 3105 3110      3120      3130      3140      3150      3160      3170      3180      3190      3201
   lambda NC_001416.1 (3105) TCGCCCAAGCTGGCGCTATCTGGGCATCGGGGAGGAAGAAGCCCGTGCCTTTTCCCGCGAGGTTGAAGCGGCATGGAAAGAGTTTGCCGAGGATGAC
SARS-CoV-2 NC_045512.2    (1) -------------------------------------------------------------------------------------------------
                                                                                                  Section 34
                    (3202) 3202    3210      3220      3230      3240      3250      3260      3270      3280           3298
   lambda NC_001416.1 (3202) TGCTGCTGCATTGACGTTGAGCGAAAACGCACGTTTACCATGATGATTCGGGAAGGTGTGGCCATGCACGCCTTTAACGGTGAACTGTTCGTTCAGG
SARS-CoV-2 NC_045512.2    (1) -------------------------------------------------------------------------------------------------
                                                                                                  Section 35
                    (3299) 3299       3310      3320      3330      3340      3350      3360      3370      3380        3395
   lambda NC_001416.1 (3299) CCACCTGGGATACCAGTTCGTCGCGGCTTTTCCGGACACAGTTCCGGATGGTCAGCCCGAAGCGCATCAGCAACCCGAACAATACCGGCGACAGCCG
SARS-CoV-2 NC_045512.2    (1) -------------------------------------------------------------------------------------------------
```

Section 36

```
                       (3396) 3396          3410      3420      3430      3440      3450      3460      3470      3480          3492
lambda NC_001416.1     (3396) GAACTGCCGTGCCGGTGTGCAGATTAATGACAGCGGTGCGGCGCTGGGATATTACGTCAGCGAGGACGGGTATCCTGGCTGGATGCCGCAGAAATGG
SARS-CoV-2 NC_045512.2    (1) -------------------------------------------------------------------------------------------------
```

Section 37

```
                       (3493) 3493   3500      3510      3520      3530      3540      3550      3560      3570               3589
lambda NC_001416.1     (3493) ACATGGATACCCCGTGAGTTACCCGGCGGGCGCGCCTCGTTCATTCACGTTTTTGAACCCGTGGAGGACGGGCAGACTCGCGGTGCAAATGTGTTTT
SARS-CoV-2 NC_045512.2    (1) -------------------------------------------------------------------------------------------------
```

Section 38

```
                       (3590) 3590      3600      3610      3620      3630      3640      3650      3660      3670              3686
lambda NC_001416.1     (3590) ACAGCGTGATGGAGCAGATGAAGATGCTCGACACGCTGCAGAACACGCAGCTGCAGAGCGCCATTGTGAAGGCGATGTATGCCGCCACCATTGAGAG
SARS-CoV-2 NC_045512.2    (1) -------------------------------------------------------------------------------------------------
```

Section 39

```
                       (3687) 3687         3700      3710      3720      3730      3740      3750      3760      3770         3783
lambda NC_001416.1     (3687) TGAGCTGGATACGCAGTCAGCGATGGATTTTATTCTGGGCGCGAACAGTCAGGAGCAGCGGGAAAGGCTGACCGGCTGGATTGGTGAAATTGCCGCG
SARS-CoV-2 NC_045512.2    (1) -------------------------------------------------------------------------------------------------
```

Section 40

```
                       (3784) 3784  3790      3800      3810      3820      3830      3840      3850      3860      3870      3880
lambda NC_001416.1     (3784) TATTACGCCGCAGCGCCGGTCCGGCTGGGAGGCGCAAAAGTACCGCACCTGATGCCGGGTGACTCACTGAACCTGCAGACGGCTCAGGATACGGATA
SARS-CoV-2 NC_045512.2    (1) -------------------------------------------------------------------------------------------------
```

Section 41

```
                       (3881) 3881     3890      3900      3910      3920      3930      3940      3950      3960             3977
lambda NC_001416.1     (3881) ACGGCTACTCCGTGTTTGAGCAGTCACTGCTGCGGTATATCGCTGCCGGGCTGGGTGTCTCGTATGAGCAGCTTTCCCGGAATTACGCCCAGATGAG
SARS-CoV-2 NC_045512.2    (1) -------------------------------------------------------------------------------------------------
```

Section 42

```
                       (3978) 3978        3990      4000      4010      4020      4030      4040      4050      4060          4074
lambda NC_001416.1     (3978) CTACTCCACGGCACGGGCCAGTGCGAACGAGTCGTGGGCGTACTTTATGGGGCGGCGAAAATTCGTCGCATCCCGTCAGGCGAGCCAGATGTTTCTG
SARS-CoV-2 NC_045512.2    (1) -------------------------------------------------------------------------------------------------
```

Section 43

	(4075)	4075 4080 4090 4100 4110 4120 4130 4140 4150 4160 4171
lambda NC_001416.1	(4075)	TGCTGGCTGGAAGAGGCCATCGTTCGCCGCGTGGTGACGTTACCTTCAAAAGCGCGCTTCAGTTTTCAGGAAGCCCGCAGTGCCTGGGGGAACTGCG
SARS-CoV-2 NC_045512.2	(1)	---

Section 44

	(4172)	4172 4180 4190 4200 4210 4220 4230 4240 4250 4268
lambda NC_001416.1	(4172)	ACTGGATAGGCTCCGGTCGTATGGCCATCGATGGTCTGAAAGAAGTTCAGGAAGCGGTGATGCTGATAGAAGCCGGACTGAGTACCTACGAGAAAGA
SARS-CoV-2 NC_045512.2	(1)	---

Section 45

	(4269)	4269 4280 4290 4300 4310 4320 4330 4340 4350 4365
lambda NC_001416.1	(4269)	GTGCGCAAAACGCGGTGACGACTATCAGGAAATTTTTGCCCAGCAGGTCCGTGAAACGATGGAGCGCCGTGCAGCCGGTCTTAAACCGCCCGCCTGG
SARS-CoV-2 NC_045512.2	(1)	---

Section 46

	(4366)	4366 4380 4390 4400 4410 4420 4430 4440 4450 4462
lambda NC_001416.1	(4366)	GCGGCTGCAGCATTTGAATCCGGGCTGCGACAATCAACAGAGGAGGAGAAGAGTGACAGCAGAGCTGCGTAATCTCCCGCATATTGCCAGCATGGCC
SARS-CoV-2 NC_045512.2	(1)	---

Section 47

	(4463)	4463 4470 4480 4490 4500 4510 4520 4530 4540 4559
lambda NC_001416.1	(4463)	TTTAATGAGCCGCTGATGCTTGAACCCGCCTATGCGCGGGTTTTCTTTTGTGCGCTTGCAGGCCAGCTTGGGATCAGCAGCCTGACGGATGCGGTGT
SARS-CoV-2 NC_045512.2	(1)	---

Section 48

	(4560)	4560 4570 4580 4590 4600 4610 4620 4630 4640 4656
lambda NC_001416.1	(4560)	CCGGCGACAGCCTGACTGCCCAGGAGGCACTCGCGACGCTGGCATTATCCGGTGATGATGACGGACCACGACAGGCCCGCAGTTATCAGGTCATGAA
SARS-CoV-2 NC_045512.2	(1)	---

Section 49

	(4657)	4657 4670 4680 4690 4700 4710 4720 4730 4740 4753
lambda NC_001416.1	(4657)	CGGCATCGCCGTGCTGCCGGTGTCCGGCA-----CGCTGGTCAGCCGGACGCGGG-C-GCTGCAGCCGTACTCGGGGATGACCGGTTACAACGGCAT
SARS-CoV-2 NC_045512.2	(1)	---------------ATTAAAGGTTTATACCTTCCCAGGTAACAAACCAACCAACTTTCGATCTCTTGTAGATCTGTTCTCTAAACGAACTTT---A

```
Section 50
                          (4754) 4754
lambda NC_001416.1        (4747) TATCGCCCGTCTGCAACAGGCTGCCAGCGATCCGATG----GTGGACGGCATTCTGCTCGATAT-------GGACACGCCCGGCGGGATGGTGGCGG
SARS-CoV-2 NC_045512.2    (80)   AAATCTGTGTGGCTGTCACTCGGCTGCATGCTTAGTGCACT-CACGCAGTATAATTAATAACTAATTACTGTCGTTGACAGGACACG--AGTAACTC

Section 51
                          (4851) 4851
lambda NC_001416.1        (4833) GGGCATTTGACTGCGCTGACAT--CATCGCCCGTGTGCGTGACATAAAACCGGTATGGGCGCTTGCCAACGACATGAACTGCAGTGCAGGTCAGTTG
SARS-CoV-2 NC_045512.2    (174)  GTCTAT----CTTCTGCAGGCTGCTTACGGTTTCGTCCGTG-T-TGCAGC---CGATCATC-AGCACATCTAGG----TTTC-GTCCGGGT---GTG

Section 52
                          (4948) 4948
lambda NC_001416.1        (4928) CTTGCCAGTGCCGCCTCCCGGCGTCTGGTCACGC-AGACCGCCCGGACAGGCTCCATCGGCGTCATGATGGC-----TCACAGTAATTACGGTGCTG
SARS-CoV-2 NC_045512.2    (253)  ACCGAAAGGTAAGA-TGGAG-AGCCT--TGTCCCTGGTTTCAACG-AG-AAAACACACGTCCAACTCAGTTTGCCTGTTTTACAGGTTCGCGACGTG

Section 53
                          (5045) 5045
lambda NC_001416.1        (5019) CGC---TGGAGAAACAGGGTGTGGAAATCACGCTGATTT-ACAGCGGCAGCCATAAGG-TGGATGGCAACCCCTACAGCCATCTTCCGGATG---AC
SARS-CoV-2 NC_045512.2    (344)  CTCGTACGTGGCTTTGGAGACTCCGT-GGAGGAGGTCTTATCAGAGGCACGT-CAACATCTTAAAGATGGCACTTGTGGCTTAGTAGAAGTTGAAAA

Section 54
                          (5142) 5142
lambda NC_001416.1        (5108) G-TCCGGGAGACACTGCAGTCCCGGATGGACGCA---ACCCGCCAGAT-GTTTGCGCAGAAGGTGTCGGCATATACCGGCCTGTCCGTGCAGGTTGT
SARS-CoV-2 NC_045512.2    (439)  AGGCGTTTTGCCTCAACTTGAACAGCCCTATGTGTTCATC-AAACGTTCGGATGCTCGAACTGCACCTCATGGTCATGTTATGGTTGAGCTGGTAG-

Section 55
                          (5239) 5239
lambda NC_001416.1        (5200) GCTGGATACCGAGGCTGCAGTGTACAGCGGTCAGGAGGCCATTGATGCCGGACTGGCTGATGAACTTGTTAACAGCACCGATGCGATCACCGTCATG
SARS-CoV-2 NC_045512.2    (534)  -CAGAACT-CGAAGGCATTCAGTACGGTCGTAGTGGTGAGACACTTGGTGTCCTTGTCCCTCATGT-------------GGGCGAA--AT--ACCAG

Section 56
                          (5336) 5336
lambda NC_001416.1        (5297) CGTGATGCACTGGATGCACGTAAATCCCGTCTCTCAGGAGGGC--G-AATGACCAAA----GAGACTCAATCAACAAC-TGTTTCAGCCAC----TG
SARS-CoV-2 NC_045512.2    (612)  TG----GC--TTACCGCAAGGTTCTTC--TTCGTAAGAACGGTAATAAA-----GGAGCTGGTGGCCATAGTTACGGCGCCGATCTAA-A-GTCATT
```

Section 57

```
                        (5433) 5433  5440      5450      5460      5470      5480      5490      5500      5510            5529
lambda NC_001416.1      (5382) CTTCGCAGGCTGACGTTACTGACGTGGTGCCAGCGACGGAGGGCGAGAACGCCAGCGCGGCGCAGCCGGACGTGAACGCGCAGATCACCGCAGCGGT
SARS-CoV-2 NC_045512.2  (694)  TGACTTAGGCG-ACGAGCTTGGCACTGATCCTTATGAAGATTTTCAAGAAAACTGGAACACTAAACATAGCAGTGGTGTTAC---CCGTGAACTCAT
```

Section 58

```
                        (5530) 5530      5540      5550      5560      5570      5580      5590      5600      5610            5626
lambda NC_001416.1      (5479) TGCGGCAGAAA----ACAGCCGCATTATGGGGATCCTCAACTGTGAGGAGGCTCACGGACGCGAAGAACAGGCACGCGTGCTGGCAGAAACCCCCGG
SARS-CoV-2 NC_045512.2  (787)  GCGTGAGCTTAACGGAGGGGCATACACTCGCTATG-TCGATAACAACTT--CTGTGG-CCCTG---ATGGCTACCCT--CTTGAGTGCATTAAAGAC
```

Section 59

```
                        (5627) 5627         5640      5650      5660      5670      5680      5690      5700      5710          5723
lambda NC_001416.1      (5572) TATGA---CCGTGAAAACGGCCCGCCGCATTCTGGC----CG-CAGCACCACAGAGTGCACAGG--CGCGCAGT--GACACTGCGCTGGATCGTCTG
SARS-CoV-2 NC_045512.2  (875)  CTTCTAGCACGTGCTGGTAAAGCTTCATGCACTTTGTCCGAACAACTGGACTTTATTGACACTAAGAGGGGTGTATACTGCTGCCGTGAACATGAGC
```

Section 60

```
                        (5724) 5724  5730      5740      5750      5760      5770      5780      5790      5800      5810      5820
lambda NC_001416.1      (5657) ATGCAGGGGGCACCGGCA-----CCGCTGGCTGCAGGTAACCCGGCATCTGATGCCGTTA--ACGATTTGCTGAACACACCAGTGTAAGGGATGTTT
SARS-CoV-2 NC_045512.2  (972)  ATGAAATTGCTTGGTACACGGAACGTTCTGAAAAGAG--CTATGAATTGCAGACACCTTTTGAAATTAAATTGGCAAAGAAATTTGACACCTTC---
```

Section 61

```
                        (5821) 5821     5830      5840      5850      5860      5870      5880      5890      5900            5917
lambda NC_001416.1      (5747) ATGACGAGCAAAGAAACCTTTACCCATTACCAGC-CGCAGGGCAACAG-TGACCCGGCTCATACCGCAACCGC-GCCCGGCGGATTGAGTGCGAAAG
SARS-CoV-2 NC_045512.2  (1064) AATGGG--GAATGTCCAAATTTTGTAT------TTCCCTTAAATTCCATAATCAAGACTATTCAACCAAGGGTTGAAAAG----AAAAAGCTTGATG
```

Section 62

```
                        (5918) 5918        5930      5940      5950      5960      5970      5980      5990      6000          6014
lambda NC_001416.1      (5841) CGCCTGCAATGACCCCGCTGATGCTGGACACCTCCAGCCGTAAGCTGGTTGCG-TGGGATGGCACCACCGACGG-TGCTGCCGTTGGCATTCTTGCG
SARS-CoV-2 NC_045512.2  (1149) GCTTTATGGGTAGAATTC-GAT---CTGTCTATCCAGTTGCGT-CACCAAATGAATGCAACCAAATGTGCCTTTCAACT--------CTCATGAAGT
```

Section 63

```
                        (6015) 6015 6020      6030      6040      6050      6060      6070      6080      6090      6100        6111
lambda NC_001416.1      (5936) GTTGCTGCTGACCAGACCAGCACCACGCTGACGTTCTACAAGTCCGGCACGTTC--CGTTATGAGGATGTGCTCTGGCCGGAGGCTGCCAGC--GAC
SARS-CoV-2 NC_045512.2  (1233) GTGATCATTGTGGTG-AAACTTCATGGCAGACGGGC-GATTTTGTTAAAGCCACTT--GCGAATTTTGTGGCACTGAGAATTTGACTAAAGAAGGTG
```

```
                                                                                                         Section 64
                     (6112) 6112    6120      6130      6140      6150      6160      6170      6180      6190      6208
       lambda NC_001416.1 (6029) GAGACGAAAAAACGGACCGCGTTTGCCGGAACGGCAATCAGCATCGTTTAACTTTACCCTTCATCACTAAAGGCCGCCTGTGCGGCTTTTTTTACGG
SARS-CoV-2 NC_045512.2 (1326) CCACTACTTGTGGTTACTTA----CCCCAAAATGCTGTTGTTAAAATTTA-T--T-GTCCAGCATG------TCACAATTCAGAAGTAGGACCTGAG
                                                                                                         Section 65
                     (6209) 6209       6220      6230      6240      6250      6260      6270      6280      6290      6305
       lambda NC_001416.1 (6126) GATTTTTTTA----TGTCGATGTACACAACCGCCCAA-CTGC----TGGCGGCAAATGAGCAGAAATTTAAGTTTGATCCGCTGTTTCT----GCGT
SARS-CoV-2 NC_045512.2 (1409) CATAGTCTTGCCGAATACCAT----AATGAATCTGGCTTGAAAACCATTCTTCGTAAG-GGTGGTCGCACTATT---GCCTTTGGAGGCTGTGTGTT
                                                                                                         Section 66
                     (6306) 6306          6320      6330      6340      6350      6360      6370      6380      6390      6402
       lambda NC_001416.1 (6210) CTCTTTT-------TCCGTGAGAGCTATCCCTTCACCACGGAGAA-------AGTCTATCTCT---CACAAATTCCGGGACTGGTAAACATGGCGCT
SARS-CoV-2 NC_045512.2 (1498) CTCTTATGTTGGTTGCCATAACAAGTGTGCCTATTGGGTTCCACGTGCTAGCGCTAACATAGGTTGTAACCATACAGGTGTTGTTGGAGAAGGTTCC
                                                                                                         Section 67
                     (6403) 6403   6410      6420      6430      6440      6450      6460      6470      6480      6499
       lambda NC_001416.1 (6290) GTACGTTTCGCCGATT---GTTTCCGGTGAGGTTATCCG------TTCCCGTGGCGGCTCCACCTCT-GAATTTACGCCGGGATATGTCAAG--CCG
SARS-CoV-2 NC_045512.2 (1595) GAAGGTCTTAATGACAACCTTCTTGAAATACTCCAAAAAGAGAAAGTCAAC--ATCAATATTGTTGGTGACTTTAAACTTAATGAAGA-GATCGCCA
                                                                                                         Section 68
                     (6500) 6500      6510      6520      6530      6540      6550      6560      6570      6580      6596
       lambda NC_001416.1 (6375) AAGCATGAAGTGAATCCGCAGATGACCCTGCGT-CGCCTGCCGGATGAAGATCCGCAGAATCTGGCGGACCCGGCTTACCGC-CGCCGTCGCATCAT
SARS-CoV-2 NC_045512.2 (1689) TTATTTTGGCATCTTTT-TCTGCTTCC--ACAAGTGCTTTTGTGGAAACTGTGAAAGGT-TTGGATTATAAAG-CATTCAA-A--CAAATTGTTGAA
                                                                                                         Section 69
                     (6597) 6597         6610      6620      6630      6640      6650      6660      6670      6680      6693
       lambda NC_001416.1 (6470) CATGCAGAACATGCGTGACGAAGAGCTGGCCA--TTGCTCAGGTCGAAGAGATGCAGGCAGTTTCTGCCGTGCTTAAGGGCAAATACACCATGACCG
SARS-CoV-2 NC_045512.2 (1778) TCCTGTGGTAATTTTAAAGTTACAAAAGGAAAAGCTAAAAAAGGTGCCTGGAATATTGGTGAACA-GAAATCAATACTGAGTCCTCTTTATGCATTT
                                                                                                         Section 70
                     (6694) 6694  6700      6710      6720      6730      6740      6750      6760      6770      6780     6790
       lambda NC_001416.1 (6565) GTGAAGCCTTCGATCCGGTTGAGGTGGATATGGGCCGCAGTGAGGAGAATAACATC-ACGCAGTCCGGCGGCACGGAGTGGAGCAAGCGTGACAAGT
SARS-CoV-2 NC_045512.2 (1874) GCATCAGAGGCTGCTCGTGTTGTACGATCAATTTTCTCCCGCACT-CTTGA--AACTGCTCAA-AATTCTGTGCGTGTTTA--C-AGAAGGCCGCT
```

```
                                                                                                        Section 71
                         (6791) 6791     6800      6810      6820      6830      6840      6850      6860      6870                    6887
lambda NC_001416.1       (6661) CCAC--GTATGACCCGACCGACGATATCGAAGCCTACGCGCTGAACGCCAGCGGTGTGGTGAA--TATCATCGTGTTCGATCCGAAAGGCTGGGCGC
SARS-CoV-2 NC_045512.2   (1964) ATAACAATACTAGATGGAATTTCACAGT----ATTCA---CTGAGACTCATTGATGCTATGATGTTCAC-ATCTGATTTGGCTACTAACAATCTAGT
                                                                                                        Section 72
                         (6888) 6888        6900      6910      6920      6930      6940      6950      6960      6970                 6984
lambda NC_001416.1       (6754) TGTTCCGTTCCTTCAAAGCCGTCAAGGAGAAGCTGGATACCCGTCGTGGCTCTAATTCCGAGCTGGAGACAGCGGTGAAAGACCTGGGCAAAGCGGT
SARS-CoV-2 NC_045512.2   (2053) TGTAATGGCCTACATTACAGGTGGTGTTGTTCAGTTGACTTCG-CA---GTGGCTAACTAACAT-CTTTGG-CA-----CTGTTTATGAAAAAC--T
                                                                                                        Section 73
                         (6985) 6985 6990      7000      7010      7020      7030      7040      7050      7060      7070                7081
lambda NC_001416.1       (6851) GTCCTATAAGGGGATGTATGGCGATGTGGCCATCGT---CGTGTATTCCGGA--CAGTACGTGGAAAACGGCGTCAAAAGAACTTCCT-GCC--GG
SARS-CoV-2 NC_045512.2   (2137) CAAACCCGTCCTTGATTGGCTTGAAG---AGAAGTTTAAGGAAGGTGTAGAGTTTCTTAGAGACGGTTGGAAATTGTTAAATTTATCTCAACCTGT
                                                                                                        Section 74
                         (7082) 7082     7090      7100      7110      7120      7130      7140      7150      7160                    7178
lambda NC_001416.1       (6940) ACAACACGATGGTGCTGGGGA----C---ACTCAGGC-ACGCGGTCTGCGCAC-CTATGGCTGCATTC----AGGATGCGGACGCACAGCGCGAAG
SARS-CoV-2 NC_045512.2   (2231) GCTTGTGAAATTGTCGGTGGACAAATTGTCACCTGTGCAAAGGAAATTAAGGAGAGTGTTCAGACATTCTTTAAGCTTGTAAATAAATTTTTGGCTT
                                                                                                        Section 75
                         (7179) 7179       7190      7200      7210      7220      7230      7240      7250      7260                 7275
lambda NC_001416.1       (7024) GCATTAACGCCTCTGCCCGTTACCCGAAAAACTGGGTGACCACCGGCGATCCGGCGCGTGAGTTCACCATGATTCAGTCAGCACCGCTGATGCTGCT
SARS-CoV-2 NC_045512.2   (2328) TG-TGTG--CTGACTCTATCATTATTGGTGGAGCTAAACTTAAAGCCT----TGAATTTAGGTGAAACATTTGTCACGCACTCAAAGGGATTGTACA
                                                                                                        Section 76
                         (7276) 7276           7290      7300      7310      7320      7330      7340      7350      7360               7372
lambda NC_001416.1       (7121) GGCTGACCCTGATGAGTTCGTGTCCGTACAACTGGCGTAATCATGGCCCTTCG----GGGCCATTGTTTCTCTGTGGAGGAGTCCATGACGAAAGAT
SARS-CoV-2 NC_045512.2   (2418) GAAAGTGT-GTTAAATC-------CAGAGAAGAAA-CT-GGCCTACTCATGCCTCTAAAAGCCCCAAA---AGAAATTATCTTCTTAGA---GGGAG
                                                                                                        Section 77
                         (7373) 7373   7380      7390      7400      7410      7420      7430      7440      7450                       7469
lambda NC_001416.1       (7214) GAACTGATTGCCCGTCTCCGCTC---GCTG--GGTGAACAACTGAACCGTGATGTCAGCCTGACGGGGACGAAAGAAGAACTGGCGCTCCGTGTGGC
SARS-CoV-2 NC_045512.2   (2499) AAACACTTCCCACAGAAGTGTTAACAGAGGAAGTTGTCTTGAAAACTGGTGATTTACAACCATTAG--AACAACCTACTAGTGAAGCTGTTGAAGCT
```

Section 78

```
                          (7470) 7470      7480      7490      7500      7510      7520      7530      7540      7550          7566
      lambda NC_001416.1  (7306) AGAGCTGAAAGAGGAGCTTGATG-ACACGGATGAAACTGCCGGTCAGGACACCCCTCTCAGCCGGGAAAATGTGCTGACCGGACATGAAAATGAGGT
SARS-CoV-2 NC_045512.2    (2594) CCATTGGTTGGTA-CACCAGTTTGTATTAACGGGCTTA----TGTTGCTCGAAATCAAAGACACAGAAAA-GTACTGTGCCCT--TGCACCTAATAT
```

Section 79

```
                          (7567) 7567        7580      7590      7600      7610      7620      7630      7640      7650      7663
      lambda NC_001416.1  (7402) GGGATCAGCGCAGCCGGATACCGTGAT-TCTGGATACGTCTGAACTGGTCACGGTCGTGGCACTGGTGAAGCTGCATACTGATGCACTTCAC--GCC
SARS-CoV-2 NC_045512.2    (2683) GATGGTA--ACAAACAATACCTTCACACTCAAAGGCGGTGC-ACCAACAAAGGTTACTTTTGGTGATGACAC-TGTGATAGAAG-TGCAAGGTTACA
```

Section 80

```
                          (7664) 7664  7670      7680      7690      7700      7710      7720      7730      7740      7750  7760
      lambda NC_001416.1  (7496) ACGCGGGATGAACCTGTGGCATTTGTGCTGCCG--GGAACGGCGTTTCGTGTCTCTG-CCGGTGTGGCAGCCGAAATGACA-GAGCGCGGCC-TGGC
SARS-CoV-2 NC_045512.2    (2775) AGAGTGTGAATATCACTT---TTGAACTTGATGAAAGGATTGATAAAGTACTTAATGAGAAG--TGCTCTGCCTATACAGTTGAACTCGGTACAGAA
```

Section 81

```
                          (7761) 7761     7770      7780      7790      7800      7810      7820      7830      7840          7857
      lambda NC_001416.1  (7588) CAGAATGCAATAACGGGAGGCGCTGTGGCTGATTTCGATAACCTGTTCGATGCTGCCATTGCCCGCGCCGATGAAACGATACGCGGTACATGGAA
SARS-CoV-2 NC_045512.2    (2867) GTAAATGAGTTCGC-CTG---TGTTGTGGCAGATGCTGTCATAAAAACTTTGCAACCAGTATCTGA-ATTACT--TACACCA-CTGGCATTGAT-T
```

Section 82

```
                          (7858) 7858        7870      7880      7890      7900      7910      7920      7930      7940      7954
      lambda NC_001416.1  (7685) CGTCAGCCACC--ATTACATCCGGTGAGCAGTCAGGTGCGGTGATACGTGGTGTTTTTGATGACCCTGAAAATATCAGCTATGCCGGACAGGGCGTG
SARS-CoV-2 NC_045512.2    (2955) TAGATGAGTGGAGTATGGCTACATACTACTTATT--TGATGAG---TCTGGTGAGTTTAAATTGGCTTCACATATGTATTGTTCTTTCTACCC-TCC
```

Section 83

```
                          (7955) 7955 7960      7970      7980      7990      8000      8010      8020      8030      8040     8051
      lambda NC_001416.1  (7780) CGCGTTGAAGGCTCCAGCCCGTCCCTGTTTGTCCGGACTGATGAGGTGCGGCAGCTGCGGCGTGGAGACACGCTGA-CCATCGGTGAGGAAAATTTC
SARS-CoV-2 NC_045512.2    (3046) AGATGAGGATGAAGAAGAAGGTGATTGTGAAGAAGAA---GAGTT-TGAGCCATCAACTCAATATGAGTATGGTACTGAAGATGATTACCAAGGTAA
```

Section 84

```
                          (8052) 8052    8060      8070      8080      8090      8100      8110      8120      8130          8148
      lambda NC_001416.1  (7876) TGGGTAGATCGGGTTTCGCCGGATGATGGCGGAAGTTGTCATCTCTGGCTTGGACGGGGCGTACCGCCTGCCGTTAACCGTCGCC-----GCTGAAA
SARS-CoV-2 NC_045512.2    (3139) --ACC--TTTGGAATTTGGTGCCA-----CTTCTGCTG---CTCTTCAACCTGAA---GAAGAGCAAGAAGAAGATTGGTTAGATGATGATAGTCAA
```

```
                                                                                                   Section 85
                              (8149) 8149      8160      8170      8180      8190      8200      8210      8220      8230          8245
          lambda NC_001416.1  (7968) --GGGGATGTATGGCCATAAAAGGTCTTGAGCAGGCCGTTGAAAACCTCAGCCGTATCAGCAAAACGGCGGTGCCTGGTGCCGCCGCAATGGCCAT
     SARS-CoV-2 NC_045512.2   (3221) CAAACTGTTGGTCAACAAGACGGCAG-TG--AGGACAATCAGACAACTACTATTCAACAATTGTT--GAGGTTCAACCTCAATTAGA-GATGGAAC
                                                                                                   Section 86
                              (8246) 8246          8260      8270      8280      8290      8300      8310      8320      8330        8342
          lambda NC_001416.1  (8063) TAACCGCGTTGCTTCATCCGCGATATCGCAGTCGGCGTCACAGGTTGCCCGTGAGACAAAGGTACGCCGGAAACTGGTAAAGGAAAGGGCCAGGCTG
     SARS-CoV-2 NC_045512.2   (3312) TTACACCAGTTGTTCAGACTATTGAAGTGAATAG---TTTTAGTGGTTATTTAAAACTTACTGA------CAATGTATACATTAAAAATGCAGACAT
                                                                                                   Section 87
                              (8343) 8343   8350      8360      8370      8380      8390      8400      8410      8420             8439
          lambda NC_001416.1  (8160) AAA--AGGGCCACGGTCAAAAATCCGCAGGCCAGAATCAAAGTTAACCGGGGGGATTTGCCCGTAATCAAGCTGGGTAATGCGCGGTTGTCCTTTC
     SARS-CoV-2 NC_045512.2   (3400) TGTGGAAGAAGCTAAAAAGGTAAA---ACCAACAG-TGGTTGTTAATGC--AGCCAATGTTTACCTTAAACATGGAGGAGGTGTTGCAGGAGCCTTA
                                                                                                   Section 88
                              (8440) 8440      8450      8460      8470      8480      8490      8500      8510      8520            8536
          lambda NC_001416.1  (8255) GCGCCGCAGGCGTCGTAAAAAGGGGCAGCGTTCATCCCTGAAAGGTGGCGGCAGCGTGCTTGTGGTGGGTAACCGTCGTATTCCCGGCGCGT-TTAT
     SARS-CoV-2 NC_045512.2   (3491) AA----TAAGGCTACTAACAATG---C-CATGCAA---GTTGAATCTG-ATGATTACATAGCTACTAATGGACCACTTAAAGTGGGTGGTAGTTGTG
                                                                                                   Section 89
                              (8537) 8537          8550      8560      8570      8580      8590      8600      8610      8620        8633
          lambda NC_001416.1  (8351) TCAGCAACTGAAAAATGGCCGGTGGCATGTCATGCAGCGTGTGGCTGGGAAAAACCGTTACCCCATTGATGTGGTGAAAATCCCGATGGCGGTGCCG
     SARS-CoV-2 NC_045512.2   (3576) TTTTAAGCGGACACAATCTTGCTAAACACTGTCTTCA-TGTTGTCGGC-CCAAATGTTAACAAAGGTGAAGACATTCAACTTCTT--AAGAGTGCTT
                                                                                                   Section 90
                              (8634) 8634  8640      8650      8660      8670      8680      8690      8700      8710      8720    8730
          lambda NC_001416.1  (8448) CTGACCACGGCGTTTAAACAAAATATTGAGCGGATACGGCGTGAACGTCTTCCGAAAGAGCTGG-GCTATGCGCTGCAGCATCAACTGAGGATGGTA
     SARS-CoV-2 NC_045512.2   (3669) ATG----AAAATTTTAATCAGCACGAAGTTCTACTTGCACCATTATTATCAGCTGGTATTTTTGGTGCTGACCCTATACAT-TCTTT-AAGAGTTTG
                                                                                                   Section 91
                              (8731) 8731     8740      8750      8760      8770      8780      8790      8800      8810               8827
          lambda NC_001416.1  (8544) ATAAAGCGATGAAACATACTGAACTCCGTGCAGCCGTACTGGATGCACTGGAGAAGCATGACACCGGGGCGACGTTTTTTGATGGTCGCCCCGCTGT
     SARS-CoV-2 NC_045512.2   (3760) -TGTAGATACTGTTC-GCACAAATGTCTACTTAGCTGTCTTT-GATAAAAATCTCTATGACAAACTTGTTTCAAGCTTTTT--GGA--AATGAAGAG
```

```
                                                                                                   Section 92
                        (8828) 8828        8840      8850      8860      8870      8880      8890      8900      8910         8924
        lambda NC_001416.1 (8641) TTTTGATGAGGCGGATTTTCCGGCAGTTGCC-GTTTATCTCACCGGCGCTGAATACACGGGCGAAGAGCTGGACAGCGATACCTGGCAGGCGGAGCT
 SARS-CoV-2 NC_045512.2 (3850) TGAAAAGCAAGTTGAACA--AAAGATCGCTGAGATTCCTAAA---GAGGAAGTTAAGC--CATTTATAACTGAAAGTAAAC-CTTCAGTTGAACAGA
                                                                                                   Section 93
                        (8925) 8925 8930      8940      8950      8960      8970      8980      8990      9000      9010      9021
        lambda NC_001416.1 (8737) GCATATCGAAGT-T---TTCCTGCCTGCTCAGGTGCCGGATTCAGAGCTGGATGCGTGGATGGAGTCCCGG--ATTTATCCGGTGATGAGCGATATC
 SARS-CoV-2 NC_045512.2 (3939) GAAAACAAGATGATAAGAAAAT-CAAAGCTTGTGTTGAAGA-------AGTT--ACA--AC--AACTCTGGAAGAAACTAAGTTCCTCACAGAAAAC
                                                                                                   Section 94
                        (9022) 9022    9030      9040      9050      9060      9070      9080      9090      9100          9118
        lambda NC_001416.1 (8828) CCGGCACTGTCAGATTT-GATCACCAGTATGGTGGCCAGCGGCTATGACTACCGGCGCGACGATGATGCGGGCTTGTGGAGTTCAGCCGATCTGACT
 SARS-CoV-2 NC_045512.2 (4022) TTGTTACTTT-ATATTGACATTAATGGCAA-T-----CTTCATCCAGATTCTGCCACTCTTGTTAGTGACATTGA----CAT-CACTTTCTTAAA--
                                                                                                   Section 95
                        (9119) 9119       9130      9140      9150      9160      9170      9180      9190      9200           9215
        lambda NC_001416.1 (8924) TATGTCATTACCTATGAAATGT-GAGGACGCTATGCCTGTACCAAATCCTACAATGCCGGTGAAAGGTGCCGGGACCACCCTGTGGGTTTATAAGGG
 SARS-CoV-2 NC_045512.2 (4105) -GAAAGATGCTCCAT-ATATAGTGGGTGATGTTGTTCAAGAGGGTGTTT-----------TAACT-GCTGTGGTTATACCTAC-----TAAAAAGGC
                                                                                                   Section 96
                        (9216) 9216          9230      9240      9250      9260      9270      9280      9290      9300       9312
        lambda NC_001416.1 (9020) GAGCGGTGACCCTTACGCGAATCCGC-TTTCAGACGTTGACTGGTCGCGTCTGGCAAAAGTTAAAGACCT--GACGCCCGGCGAACTGACCGCTGAG
 SARS-CoV-2 NC_045512.2 (4183) TGGTGGCACTACTGAAATGCTAGCGAAAGCTTTGAGAAAAGTG---CCAACAGACAATTATATAACCACTTACCCGGGTCAGGGTTTAAATGGTTAC
                                                                                                   Section 97
                        (9313) 9313  9320      9330      9340      9350      9360      9370      9380      9390             9409
        lambda NC_001416.1 (9114) TCCTATGACGACAGCTATCTCGATGATGAAGATGCAGACTGGACTGCGACCGGGCAGGGGCAGAAATCTGCCGGAGATACCAGCTTCACGCTGGCGT
 SARS-CoV-2 NC_045512.2 (4277) ACTGTAGAGGAGG-CAAAGACAGTGCTTAAAA-AGTGTAAAAGTGCCTTTTACATTCTACCA-TCTATTATCTCT-AATGAGAAGCAAGAAATTCTT
                                                                                                   Section 98
                        (9410) 9410      9420      9430      9440      9450      9460      9470      9480      9490          9506
        lambda NC_001416.1 (9211) GGATGCCCGGAGAGCAGGGGCAGCAGGCGCTGCTGGCGTGGTTTAATGAAGGCGATAC-CCGTGCCTAT--AAAATCCGCTTCCCGAACGGCACGGT
 SARS-CoV-2 NC_045512.2 (4370) GGAACT------------GTTTCTTGGAATTTGCGAGAAATGCTTGCACATGCAGAAGAAACACGCAAATTAATGCCTGTCTGTGTGGAA-ACTAAA
```

Section 99

```
                        (9507) 9507         9520      9530      9540      9550      9560      9570      9580      9590      9603
lambda NC_001416.1      (9305) CGATGTGTTCCGTGGC-TGGGTCAGCAGTATCGGTAAGGCGGTGACGGCGAAGGAAG--TGATCACCCGCACGGTGAAAGTCACCAATGTGGGACGT
SARS-CoV-2 NC_045512.2  (4454) GCCATAGTT-TCAACTATAC-AGCGTAAATATAAGGGTATTAAAATACAAGAGGGTGTGG---------TTGATT----ATGGTGCT------AGAT
```

Section 100

```
                        (9604) 9604  9610      9620      9630      9640      9650      9660      9670      9680      9690   9700
lambda NC_001416.1      (9399) CCGTCGATGGCAGAAGATCGCAGCACGGTAACAGCGGCAACCGGCATGACCGTGACGCCTGC-CAGCACCTCGGTGGTGAAAGGGCAGAGCACCACG
SARS-CoV-2 NC_045512.2  (4530) TTTACTTTTACACCAG--TAAAACAACTGTAGCGTCACTTATCAACACACTTAACGATCTAAATGAAACT-----------C----TTGTTACAATG
```

Section 101

```
                        (9701) 9701     9710      9720      9730      9740      9750      9760      9770      9780           9797
lambda NC_001416.1      (9495) CTGACCGTGGCCTTCCAGCCGGAGGGCGTAACCGACAAGAGCTTTCGTGCGGTGTCTGCGGATAAAACAAAAGCCACCGTGTCGGTCAGTGGTATGA
SARS-CoV-2 NC_045512.2  (4610) CCACTT-GGCTATGTAACACATGGCTTAAATTTGGAAGAAGCTGCTC---GGTATATGAGATCTCTCAAAGTGCCAGCTACAGTTTCTGTTTCTTCA
```

Section 102

```
                        (9798) 9798        9810      9820      9830      9840      9850      9860      9870      9880        9894
lambda NC_001416.1      (9592) CCATCACCGTGAACGGCGTTGCTGCAGGCAAGGTCAACATTCCGGTTGTATCCGGTAATGGTGAGTTTGCTGCGGTT-GCAGAAATTACCGTCACCG
SARS-CoV-2 NC_045512.2  (4703) CCTGATGCTGTTAC----AGC--GTATA-ATGGTTATCTTACTTCT-TCTTC---TAAAA-CACCTGAAGAACATTTTATTGAAACCATCTCAC---
```

Section 103

```
                        (9895) 9895 9900      9910      9920      9930      9940      9950      9960      9970      9980       9991
lambda NC_001416.1      (9688) CCAGTTAATCCGGAGAGTCAGCGATGTTCCTGAAAACCGAATCATTTG--AACAT---AACGGTGTGACCGTCACGCTTTCTGAACTGT-CAGCCCT
SARS-CoV-2 NC_045512.2  (4785) ----TTGCTGGTTCCTATAAAGATTGGTCCT-ATTCTGGACAATCTACACAACTAGGTATAGA---ATTTCTTAAGAGAGGTGATAAAAGTGTATAT
```

Section 104

```
                        (9992) 9992    10000     10010     10020     10030     10040     10050     10060     10070         10088
lambda NC_001416.1      (9779) GCAGCGCATTGAGCATCTCGCCCTGATGAAACGGCAGGCAGAACAGGCGGAGTCAGACAGCAACCGGAAGTTTACTGTGGAAGACGCCATCAGAACC
SARS-CoV-2 NC_045512.2  (4874) TACACTAG---TAATCCTACCACATTCCACCTAGATGGTGAAGTTATCACCTTTGACAATCTTAAGACACTTCTTTCTTTGAGAGAAGTGAGGACTA
```

Section 105

```
                       (10089) 10089     10100     10110     10120     10130     10140     10150     10160     10170        10185
lambda NC_001416.1      (9876) GGCGCGTTTCTGGTGGCGATGTCCCTGTGGCATAACCATCCGCAGAAGACGCAGATGCCGTCCATGAATGAAGCCGTTAAACAGATTGAGCAGGAAG
SARS-CoV-2 NC_045512.2  (4968) TTA-AGGTGTTTA-CAACAGTAGACA-----ACATTAACCTCCAC--ACGCAAGTTGTGGACATGTCAATG---ACATAT---GGACAA-CAGTTTG
```

```
                                                                                          Section 106
                         (10186) 10186         10200      10210      10220      10230      10240      10250      10260      10270    10282
      lambda NC_001416.1  (9973) TGCTTACCACCTGGCCCACGGAGGCAATTTCTCATGCTGAAAACGTGGTGTACCGGCTGTCTGGTATGTATGAGTTTGTGGTGAATAATGCCCCTGA
SARS-CoV-2 NC_045512.2  (5049) GTCCAACTTATTTGGA--TGGAGCTGATGTTACTAAAATAAAACCTCA---------T------AATTCACA-TGAAG-GTAAAACATTTTATGTTT
                                                                                          Section 107
                         (10283) 10283  10290      10300      10310      10320      10330      10340      10350      10360           10379
      lambda NC_001416.1 (10070) ACAGACAGAGGACGCCGGGCCCGCAGAGCCTGTTTCTGCGGGAAAGTGTTCGACGGTGAGCTGAGTTTTGCCCTGAAACTGGCGCGTGAGATGGGGC
SARS-CoV-2 NC_045512.2  (5127) TACCTAAT---GATGACACTCTACGT-----GTTGAGGCTTTT-GAGTACTACCACACAACTGATCCTAGTTTTC--TGG-------GTAGGTACAT
                                                                                          Section 108
                         (10380) 10380     10390      10400      10410      10420      10430      10440      10450      10460       10476
      lambda NC_001416.1 (10167) GACCCGACTGGCGTGCCATGCTTGCCGGGATGTCATCCACGGAGTATGCCGACTGGCACCGCTTTTACAGTACCCATTATTTTCATGATGT-TCTGC
SARS-CoV-2 NC_045512.2  (5206) GTCAGCATTAAATCACACTAAAAAGTGGAAATAC-CCACAAGTTAATGGTTTAACTT-----CTATTAAATGGGCAGA---TAACAACTGTTATCTT
                                                                                          Section 109
                         (10477) 10477        10490      10500      10510      10520      10530      10540      10550      10560     10573
      lambda NC_001416.1 (10263) TGGATATGCACTTTTCCGGGCTGACGT-ACACCGTGCTCAGCCTGTTTTTCAGCGATCCGGATATGCATCCGCTGGATTTCAGTCTGCTGAACCGGC
SARS-CoV-2 NC_045512.2  (5294) GCCACTGCATTGTT------AACACTCCAACAAATAGAGTTGAAGT--------------TTAATCCACCTGC-----TCTACAAGA-TG------C
                                                                                          Section 110
                         (10574) 10574 10580      10590      10600      10610      10620      10630      10640      10650      10660 10670
      lambda NC_001416.1 (10359) GCGAGGCTGACGAAGAGCCTGAAGATGATGTGCTGATGCAGAAAGCGGCAGGGCTTGCCGGAGGTGTCCGCTTTGGCCCGGACGGGAATGAAGTTAT
SARS-CoV-2 NC_045512.2  (5359) TTATTACAGAGCAAGGGCTGGTGAAGCTGC--TAACTTTTGTGCACTTAT-CTTAGCCTA-CTGTAATAAGACAGTAGGTGA-GTTAGGTGAT-GTT
                                                                                          Section 111
                         (10671) 10671    10680      10690      10700      10710      10720      10730      10740      10750          10767
      lambda NC_001416.1 (10456) CCCCGCTTCCCCGGATGTGGCGGACATGACGGAGGATGACGTAATGCTGATGACAGTATCAGAAGGGATCGCAGGAGGAGTCCGGTATGGCTGAACC
SARS-CoV-2 NC_045512.2  (5450) AGAGAAACAATGAGTTACTTGT---TTCAACATGCCAATTTAGATTCTTGCAA-------AAGAGTC---TTGAACGTGGTGTGTAAAACTTGTGGA
                                                                                          Section 112
                         (10768) 10768        10780      10790      10800      10810      10820      10830      10840      10850     10864
      lambda NC_001416.1 (10553) GGTAGGCGATCTGGTCGTTG-ATTTGAGTCTGGATGCGGCCAGATTTGACGAGC-AGATGGCCAGAGTCAGGCGTCATTTTTCTGGTACGGAAAGTG
SARS-CoV-2 NC_045512.2  (5534) CAACAGCAGA----CAACCCTTAAGGGTGTAGAAGCTGTT---A-TGTACATGGGCACACTTTCTTATGAACAATTTAAGAAA-GGTGTTCAGATAC
```

Section 113

```
                          (10865) 10865 10870     10880     10890     10900     10910     10920     10930     10940     10950     10961
lambda NC_001416.1        (10648) ATGCGAAAAAAACAGCGGCAGTCGTTGAACAGTCGCTGAGCCGACAGGCGCTGGCTG---CACAGAAAGCGGGGATTTCCGTCGGGCAGTATAAAGC
SARS-CoV-2 NC_045512.2    (5622)  C-TTGTACGTG--TGGTAAACAAGCTACAAAATATCTAGTACAACAGGAGTCACCTTTTGTTATGATGTCAGCACCACCTGC---TCAGTATGAACT
```

Section 114

```
                          (10962) 10962   10970     10980     10990     11000     11010     11020     11030     11040           11058
lambda NC_001416.1        (10742) CGCCATGCGTATGCTGCCTGCACAGTTCACCGACGTGGCCA-CGCAGCT-TGCAGGCGGGCAAAGTCCGTGGCTGATCCTGCTGCAACAGGGGGGC
SARS-CoV-2 NC_045512.2    (5713)  TAAG-----CATGGTACATTTAC----------TTGTGCTAGTGAGTACACTGGT--AATTACCAG--TGTGGTCACT-ATAAACATATAACTTCTA
```

Section 115

```
                          (11059) 11059      11070     11080     11090     11100     11110     11120     11130     11140          11155
lambda NC_001416.1        (10837) AGGTGAAGGACTCCTTCGGCGGGATGATCCCCATGTTCAGGGGGCTTGCCGGTGCGATCACCCTGCCGATGGTGGG---GGCCAC-----CTCGCTG
SARS-CoV-2 NC_045512.2    (5790)  AAGAAACTTTGTATTGCATAGACGGTGCTTTACTT--ACAAAGTCCTCAGAATACAAAGGTCCTATT--ACGGATGTTTTCTACAAAGAAAACAGTT
```

Section 116

```
                          (11156) 11156         11170     11180     11190     11200     11210     11220     11230     11240       11252
lambda NC_001416.1        (10926) GCGGTGGCGACCGGTGCGCTGGCGTATGCCTGGTATCAGGGCAACTCAACCCTGTCCGATTTCAACAAAACGCTGGTCCTTTCCGGCAATCAGGCGG
SARS-CoV-2 NC_045512.2    (5883)  ACACAACAACCATAAAACCAGT-TACT-TATAA-ATTGG------------------------ATGGTGTTG-------TTTGT---A---------
```

Section 117

```
                          (11253) 11253  11260     11270     11280     11290     11300     11310     11320     11330              11349
lambda NC_001416.1        (11023) GACTGACG-GCAGATCGTATGCTGGTCCTGTCCAGAGCCGGGCAGGCGGCAGGGCTGACGTTTA-ACCAGACCAGCGAGTCACTCAGCGCACTGGTT
SARS-CoV-2 NC_045512.2    (5934)  CAGAAATTGACCCTA----AGTTGGA---C-AATTATTATAAGAAAGACAATTCTTATTTCACAGAGCAACCAATTGATCTTGTACCAAACCAACCA
```

Section 118

```
                          (11350) 11350     11360     11370     11380     11390     11400     11410     11420     11430           11446
lambda NC_001416.1        (11118) AAGGCGGGGGTAAGCGGTGAGGCTCAGATT-GCGTCCATCAGCCAGAGTGTGGCGCGTTTCTCCTCTGCATCCGGCGTGGAGGTGGACAAGGTCGCT
SARS-CoV-2 NC_045512.2    (6023)  TATCC--AAACGCAAGCTTCGA--TAATTTTAAGTTTGT--ATGTGATAATATCAAATTTGCTGATGATTTAAACCAGTTAAC---TGGTTATAAGA
```

Section 119

```
                          (11447) 11447         11460     11470     11480     11490     11500     11510     11520     11530       11543
lambda NC_001416.1        (11214) GAAGCCTTCGGGAAGCTGACCACAGACCCGACGTCGGGGCTGACGGCGATGGCTCGCCAGTTCCATAACGTGTCGGCGGAGCAGATTGCGTATGTTG
SARS-CoV-2 NC_045512.2    (6111)  --AACCTGCTTCAAGAGAGC-TTAAAGTTACATTTTTCCCTGACTTAAATGGTGATGTGG-TGGCTATTGATTATAAACACTAC-------ACACCC
```

Section 120

```
                                  11544 11550     11560     11570     11580     11590     11600     11610     11620     11630  11640
                         (11544)
lambda NC_001416.1       (11311) CTCAGTTGCAGCGTTCCGGCGATGAAGCCGGGGCATTGCAGGCGGCGAACGAGGCCGCAACGAAAGGGTTTGATG-A-CCAGACCCGCCGCCTGAAA
SARS-CoV-2 NC_045512.2   (6197)  T--CTTTTAAGAAAGGAGCTAAATT--GTTACATAAACC---TATTGTTTGGCATGTTAACAAT--GC--AACTAA-T---AAAGCCACGT-----A
```

Section 121

```
                                  11641     11650     11660     11670     11680     11690     11700     11710     11720        11737
                         (11641)
lambda NC_001416.1       (11406) GAGAACATGGGCACGCTGGAG----------ACCTGGGCAGACAGG-ACTGCGCGGGCATTCAAATCCATGTGGGATGCG-GTGCTGGATATTGGTC
SARS-CoV-2 NC_045512.2   (6274)  TAAACCAAAT--AC-CTGGTGTATACGTTGTCTTTGGAGCA-CAAAACCAGT-TGAA------ACATCAAATTCGTTTGATGTACTGAA------GT
```

Section 122

```
                                  11738       11750     11760     11770     11780     11790     11800     11810     11820        11834
                         (11738)
lambda NC_001416.1       (11491) GTCCTGATACCGCGCAGG----AGATGCTGATTAAGGCAGAG-GCTGCGTATAAGAAAGCAGACGACATCTGGAATCTGCGCAAGGATGATTATTTT
SARS-CoV-2 NC_045512.2   (6354)  C---AGAGGACGCGCAGGGAATGGATAATCTTGCCTGCGAAGATCTAAAACCAGTCTCTGAAGAAGTAG-TGGAAA--A-TCCTACCA---TACAGA
```

Section 123

```
                                  11835 11840     11850     11860     11870     11880     11890     11900     11910     11920  11931
                         (11835)
lambda NC_001416.1       (11583) GTTAACGATGAAGCGCGGGCGCGTTACTGGGATGATCGTGAAAAGGCCCGTCTTGCGCTTGAAGCCGCCCGAAAGAAGGCTGAGCAGCAGACTCAAC
SARS-CoV-2 NC_045512.2   (6441)  AAGACGTTCTT---GAGTGTAATGTGAAAACTACCGAAGTTGTAGGAGACATTATACTTAAAC-CAGCAAATAA---TAGTTTAAAAATTACAGAAG
```

Section 124

```
                                  11932   11940     11950     11960     11970     11980     11990     12000     12010          12028
                         (11932)
lambda NC_001416.1       (11680) AGG---ACAAAAATGCGCAGCAGCAGAGCGATACCGAAGCGTCACGGCTGAAATATACCGAAGAGGCGCAGAAGGCTTACGAACGGCTGCAGACGCC
SARS-CoV-2 NC_045512.2   (6531)  AGGTTGG------CCACACAGATCT-AATGGCTGCTTATGTAGACAATTCTAGTCTTACTA---------------TT-------------------
```

Section 125

```
                                  12029      12040     12050     12060     12070     12080     12090     12100     12110        12125
                         (12029)
lambda NC_001416.1       (11774) GCTGGAGAAATATACCGCCCGTCAGGAAGAACTGAACAAGGCA-CTGAAA-GACGGGAAAATCCTGCAGGCGGATTACAACACGCTGATGGCGGCGG
SARS-CoV-2 NC_045512.2   (6587)  ------------AAGAAACCTAAT---------GAATTATCTAG------A-------------------------GTATTAGGTT-----------
```

Section 126

```
                                  12126         12140     12150     12160     12170     12180     12190     12200     12210  12222
                         (12126)
lambda NC_001416.1       (11869) CGAAAAAGGATTATGAAGCGACGC-TGAAAAAGCCGAAACAGTCCAGCGTGAAGGTGTCTGCGGGCGATCGTCAGGAAGACAG-TGCTCATGCTG--
SARS-CoV-2 NC_045512.2   (6621)  TGAAAAC----CCTTGCTACTCATGGTTTAGCTGCTGTTAATAGTGTCC-CTTG------GGATAC------------TATAGC---TAATTATGCT
```

Section 127

```
                       (12223) 12223 12230 12240 12250 12260 12270 12280 12290 12300 12319
lambda NC_001416.1     (11962) --CCCTGCTGACGCTTCAGGCAGAACTCCGGACGCTGGAGAAGCATGCCGGAG---CAAATGAGAAAATCAGCC---AGCAGCGCCGGGATTTGTGG
SARS-CoV-2 NC_045512.2 (6692)  AAGCCT----------------TTTCTTAACAAAGTTGTTA-----GTACAA-CTACTAA---CATAGTTACACGGT---GTTTAAACCGTGTTT-G
```

Section 128

```
                       (12320) 12320 12330 12340 12350 12360 12370 12380 12390 12400 12416
lambda NC_001416.1     (12051) AAGGCGGAG-----AGTCAGTTCGCGGTACTGGAG--GAGGCGGCGCAACGTCGCCAGCTGTCTGCACAGGAGAAATCCCTGCTGGCGCATAAAGAT
SARS-CoV-2 NC_045512.2 (6760)  TACTAATTATATGC-CTTATTTCTTTACTTTA---TTGCTACAATTGT-GTACTTT-----TACTAGAAGTACAAATTCTAGAA--TTAAAGCATCT
```

Section 129

```
                       (12417) 12417 12430 12440 12450 12460 12470 12480 12490 12500 12513
lambda NC_001416.1     (12141) --GAGACGCTGGAGT--ACAAACGCCAGCTGGCTGCACTTGGCGACAAGGTTACGTATCAGGAGCGCCTGAACGCGCTGGCGCAGCAGGCGGATAAA
SARS-CoV-2 NC_045512.2 (6845)  ATGCCGACTACTATAGCAAAGAATACTGTTAAG-AGTGTCGGTA-AATTTTGTCTAG-AGGCTTCATTT-AATTATTTGAAGTC-ACCTAATTTTTC
```

Section 130

```
                       (12514) 12514 12520 12530 12540 12550 12560 12570 12580 12590 12600 12610
lambda NC_001416.1     (12234) TTCGCACAGCAGCAACGGGCAAAACGGGCCGCCATTGATGC-GAAAAGCCGGGGGCTGACTGACCGGCAGGCAGAACGGGAA---GCCACGGAACAG
SARS-CoV-2 NC_045512.2 (6937)  TAAACTG-ATAAATATTATAATTTGGTTTTTACTATTAAGTGTTTGCCT-AGGTTCTTTA-ATCTACTCAACCGCTGCTTTAGGTGTTTTAATGTCT
```

Section 131

```
                       (12611) 12611 12620 12630 12640 12650 12660 12670 12680 12690 12707
lambda NC_001416.1     (12327) CGCCTGAA---GGAACAGTATGGCGATAATCCGCTGGCGCTGAATAACGTCATGTCAG-AGCAGAAAAAGACCTGGG--CGGCTGAAGACCA-----
SARS-CoV-2 NC_045512.2 (7031)  AATTTAGGCATGC---CTTCTTACTGT--ACTGGTTACAGAGAAGGCTATTTGAACTCTACTAATGTCACTATTGCAACCTACTGTACTGGTTCTAT
```

Section 132

```
                       (12708) 12708 12720 12730 12740 12750 12760 12770 12780 12790 12804
lambda NC_001416.1     (12413) ---GCTTCGC---GGGAAC--TGGATGGCAGGCCTGAAGTCCGGC----TGGAGTGAGTGGGAAGAGAGCGCCACG-GACAGTATGTCGCAGGTAAA
SARS-CoV-2 NC_045512.2 (7123)  ACCTTGTAGTGTTTGTCTTAGTGGTTTAGATTCTTTAGACACCTATCCTTCTTTAGAAACTA-TACAAATTA----CCATTT----CATC-TTTT-A
```

Section 133

```
                       (12805) 12805 12810 12820 12830 12840 12850 12860 12870 12880 12890 12901
lambda NC_001416.1     (12497) AAGTGCAGCCACGCAGACCTTTGATGGTATTGCACAGAATAT--GGCGGCGATGCTGACCGGCAGTGAGCAGAACTGG---CGCAGCTTCACCCGTT
SARS-CoV-2 NC_045512.2 (7209)  AATGGGATTTAACTG--CTTTTGGCTTAGTTGCAGAGTGGTTTTTGGCATATATTCTTTTCACTAGGTTTTTCTATGTACTTGGATTGGCTGCA---
```

```
                                                                                                   Section 134
                        (12902) 12902   12910            12920            12930            12940            12950            12960            12970            12980          12998
 lambda NC_001416.1     (12589) CCGTGCTGTCCATGATGACAGAAATTCTGC--------TTAAGCAGGCAATGGTGGGGATTGTCGGGAGTATCGGCAGCGCCATTGGCGGGGCTGTT
 SARS-CoV-2 NC_045512.2  (7301) -ATCATGCAATTGTTTTTCAGCTATTTTGCAGTACATTTTATTAGT---AATTCTTGGCTTATGTGGTT--AATAA-------TTAATCTTGTAC-A
                                                                                                   Section 135
                        (12999) 12999    13010            13020            13030            13040            13050            13060            13070            13080          13095
 lambda NC_001416.1     (12678) GGTGGCGGCGCATCCGCGTCAGGCGGTACAGCCATTCAGGCCGCTGCGGCGAAATTCCATTTTGCAACCGGAGGATTTACGGGAACCGGCGGCAAAT
 SARS-CoV-2 NC_045512.2  (7384) AATGGCCCCGATTTCAGCT---ATGGTTAGAATGTACAT----C----------TTCT-TTGCAT-----C--ATTTTATTATGTATGGAA-AAGTT
                                                                                                   Section 136
                        (13096) 13096             13110            13120            13130            13140            13150            13160            13170            13180     13192
 lambda NC_001416.1     (12775) ATGAGCCAGCGGGGATTGTTCACCGT-GGTGAGTTTGTCTTCACGAAGGAGGCAACCAGCCGGATTGGCGTGGGGAATCTTTACCGGCTGA---TGC
 SARS-CoV-2 NC_045512.2  (7455) ATGTGCATGTTGTAGACGGTTGTAATTCATCAA-CTTGTATGATGTGT-TACAAACGTAATAGAGCAACAAGAGTCGAATGTACAACTATTGTTAAT
                                                                                                   Section 137
                        (13193) 13193   13200            13210            13220            13230            13240            13250            13260            13270          13289
 lambda NC_001416.1     (12868) GCGGCTATG-CCACCGGCGGTTATGTCGGTACACCGGGCAGCATGG---CAGACAGCCGGTCGCAGGCGT---CCGGGACGTTTGAGCAGAATAACC
 SARS-CoV-2 NC_045512.2  (7550) GGTGTTAGAAGGTCCTTTTATGTCTATG---CTAATGGAGGTAAAGGCTTTTGCAAAC----TACACAATTGGAATTGTGTTAATTGTGATACATTC
                                                                                                   Section 138
                        (13290) 13290     13300            13310            13320            13330            13340            13350            13360            13370        13386
 lambda NC_001416.1     (12958) ATGTGGTGATT--AACAACGACGGCACGAACG-GGCAGATAGGTCCGGCTGCTCTGAAGGCGGTGTATGACATGGCCCGCAAGGGTGCCCGTGATGA
 SARS-CoV-2 NC_045512.2  (7640) TGTGCTGGTAGTACATTTATTAGTGATGAAGTTGCGAGAGACTTGTCACTA--CAGTT---TAAAAGACCAATAAATCCTACTGA--CCAGTCTTCT
                                                                                                   Section 139
                        (13387) 13387           13400            13410            13420            13430            13440            13450            13460            13470      13483
 lambda NC_001416.1     (13052) AATTCA---GACACAGATGCGTGATGGTGGCCTGTTCTCCGGAGGTGGACGATGAAGACCTTCCGCTGGA----AAGTGAAACCCGGTATGGATGTG
 SARS-CoV-2 NC_045512.2  (7730) ---TACATCGTTGAT-AGT-GTTACAGTGAAGAATGGT------TCCATCC-ATCTTTACTTTG-ATAAAGCTGGTCAAAAGACTT--ATGAAAGAC
                                                                                                   Section 140
                        (13484) 13484 13490            13500            13510            13520            13530            13540            13550            13560            13570 13580
 lambda NC_001416.1     (13142) GCTTCGGTCCCTTCTGTAAGAAAGGTGCGCTTTGGTGATGGCTATT--CTCAGCGAGCGCCTGCCG-GGCTGAATGCCAACCTGAAAACGTACAGCG
 SARS-CoV-2 NC_045512.2  (7812) ATTCTCTCTCTCATTTTGTTA-ACTTAGACAA-CCTGAGAGCTAATAACACTAAAGGTTCAT--TGCCTATTAATGTTATAGTTTTGATGGTA---
```

Section 141

```
                           13581    13590     13600     13610     13620     13630     13640     13650     13660           13677
                  (13581)  |        |         |         |         |         |         |         |         |               |
lambda NC_001416.1 (13236) TGACGCT--------------TTCTGTCCCCCGTGAGGAGGCCACGGTACTGGAGTCGTTTCTGGAAGAGCA---CGGG-GGCTGGAAAT--CCTTT
SARS-CoV-2 NC_045512.2 (7902) -AATCAAAATGTGAAGAATCATCTGCAAAATCAGCGTCTGTTTAC--TACAGT--CAGCTTATGTGTCAACCTATACTGTTACTAGATCAGGCATTA
```

Section 142

```
                           13678       13690     13700     13710     13720     13730     13740     13750     13760        13774
                  (13678)
lambda NC_001416.1 (13313) CTGTGGACGCCGCCTTATGAGTGGCGGCAGATAAAGGTGAC-CTGCGCAAAATGGTCGTCGCGGGTCAGTATGCTGCGTGTTGAGTTCAGCGCAGAG
SARS-CoV-2 NC_045512.2 (7994) GT--GTCTGATGTTGGTGATAGTGCGGAAGTT---GCAGTTAAAATGTTTGATGCTTACGTTAATACGTTT-T----CATCAA-----CTTTTA---
```

Section 143

```
                           13775 13780     13790     13800     13810     13820     13830     13840     13850     13860  13871
                  (13775)
lambda NC_001416.1 (13409) TTTGAACAGGTGGTGAACTGATGCAGG-ATATC------CGGCAGGAAACACTGAATGAATGCACCCGTGCGGAG-CAGTCGGCCAGC--GTGGTGC
SARS-CoV-2 NC_045512.2 (8073) -ACGTACCAAT--GGAAAAACTCAAAACACTAGTTGCAACTGCAGAAGCTGAACTTGCAAAGAATGTGTCCTTAGACAATGTCTTATCTACTTTTAT
```

Section 144

```
                           13872   13880     13890     13900     13910     13920     13930     13940     13950          13968
                  (13872)
lambda NC_001416.1 (13496) TCTGGGAAATCGACCT-GACAGAGGTCGGTGGAGAACGTTATTTTTTCTGTAATGAGCAGAACGAAAAAGGTGAGCCGGTCACCT--GG----CAG-
SARS-CoV-2 NC_045512.2 (8167) TTCAGCAGCTCGGCAAGGGTTTGTTGATTCAGATGTA--GAAACTAAAGATGTTGTTGAATGTCTTAAAT-T-GTCACATCAATCTGACATAGAAGT
```

Section 145

```
                           13969      13980     13990     14000     14010     14020     14030     14040     14050         14065
                  (13969)
lambda NC_001416.1 (13585) ----GGGCGACAGTATCAGC-CGTATC-CCATTCAGGGGAGCGGTTTTGAACTGAATGGCAAAGGCACCAGTACGCGCCCCACGCTGACGG----TT
SARS-CoV-2 NC_045512.2 (8260) TACTGGCGATAGTTGTAATAACTATATGCTCACCTATAACAAAGTTGAAAACATGACAC-CCCGTGAC---CTTGGTGCTTGTATTGACTGTAGTGC
```

Section 146

```
                           14066         14080     14090     14100     14110     14120     14130     14140     14150      14162
                  (14066)
lambda NC_001416.1 (13672) TCTAACCTGTACGGTATGGTCACCGGGATGGCGGAAGATATGCAGAGTCTGGT-CGGCGGAACGGTGGTCCGGCGTAAGGTTTACGCCCGTTTTCTG
SARS-CoV-2 NC_045512.2 (8353) GCGTCATATTAATGC-GCAGGTAGCAAA-AAGTCACAACATTGCTTTGATATGGAACGTTAAAGATTTCATGTCATTGTCTGAACAA----CTACGA
```

Section 147

```
                           14163  14170     14180     14190     14200     14210     14220     14230     14240             14259
                  (14163)
lambda NC_001416.1 (13768) GATGCGGTGAACTTCGTCAACGGAAACAGTTACGCCGATCCGGAGCAGGAGGTGATCAGCCGCTGGCGC-ATTGAGCAGTGCAGCGAACTGAGCGCG
SARS-CoV-2 NC_045512.2 (8444) AAACAAATACG---TAGTGCTGCTAAAAAGAATAACTTACCTTTT----A---AGTTGAC-ATGTGCAAC--------TA-----CTAGACAAGTTG
```

```
                                                                                              Section 148
                         (14260) 14260     14270     14280     14290     14300     14310     14320     14330     14340        14356
    lambda NC_001416.1   (13864) GTGAGTGCCTCCTTTGTACTGTCCACGCCGACGGAAACGGATGGCGCTGTTTTTCCGGGACGTATCATGCTGGCCAACACCTGCACCTGGACCTATC
 SARS-CoV-2 NC_045512.2   (8517) TTAATGTT----GTAACAACAAAGATAGCACTTAAGGGTGGTA---AAATTGTTAATAA-TTGG---TTGA--AGCAGTT-AATTAAAGTTACACTT
                                                                                              Section 149
                         (14357) 14357        14370     14380     14390     14400     14410     14420     14430     14440     14453
    lambda NC_001416.1   (13961) GCGGTGACGAGTGCGGTTATAGCGGTCCGGCTGTCGCGGATGAATATGA---CCAGCCAACGTCCGATATCACGAAGGATAAATGCAGCAAATGCCT
 SARS-CoV-2 NC_045512.2   (8600) GTGTTCCTTTTTGTTGCTGCTATT--TTCTATTTAATAACACCTGTTCATGTCATGTCTAAACATACTGACTTTTC---AAGTGAAATCA--TAGGA
                                                                                              Section 150
                         (14454) 14454 14460     14470     14480     14490     14500     14510     14520     14530     14540  14550
    lambda NC_001416.1   (14055) GA-GCGGTTGTAAGTTCCGCAATAACGTCGGCAACTTTGGCGGCTTCCTTTCCATTAACAAACTTTCGC-AGTAAATCCC-ATGACACAGACAGAAT
 SARS-CoV-2 NC_045512.2   (8690) TACAAGGCTATTGATGGTGGTGTCACTCGTGACATAGCATCTAC-AGATACTTGTTTTGCTAACA----AACATGCTGATTTTGACAC---ATGGTT
                                                                                              Section 151
                         (14551) 14551    14560     14570     14580     14590     14600     14610     14620     14630        14647
    lambda NC_001416.1   (14149) CAGCGATTCTGGCGCACGCCCGGCGATGTGCGCCAGCGGAGTCGTGCGGCTTCGTGGTAAGCACGCCGGAGGGGGAAAGATATTTCCCCTGCGT-GA
 SARS-CoV-2 NC_045512.2   (8779) TAGCCAG------CGTGGTGGTAGTTA--TACTAA------TGACAAAGCTTGCCCATT---------GA--TTGCT--GCAGTCATAACAAG-AGA
                                                                                              Section 152
                         (14648) 14648       14660     14670     14680     14690     14700     14710     14720     14730     14744
    lambda NC_001416.1   (14245) ATATCTCCGGTGAGCCGGAGGCTATTTCCGTATGTC--GCCGGAAGACTGGCTGCAGGCAGAAATGCAGGGTGAGATTGTGGCGCTGGTCCACAGCC
 SARS-CoV-2 NC_045512.2   (8848) AGT-GGGTTTTGTCGTGCCTGGTTTGCCTGGCACGATATTACGCACA-ACTAATG--GTGACTTTTTGCATTTCTTACCTAGAGTTTTTAG--TGCA
                                                                                              Section 153
                         (14745) 14745 14750     14760     14770     14780     14790     14800     14810     14820     14830  14841
    lambda NC_001416.1   (14340) ACCCCGGTGGTCTGCCCTGGCTGAGTGAGGCCGACCGGCGGCTGCAGGTGCAGAGTGATTTGCCGTGGTGGCTGGTCTGCCGGGGGACGATTCATAA
 SARS-CoV-2 NC_045512.2   (8939) GTTGGTAACA---TCTGTTACACACCATCAAAACTTATAG-A-GTACAC--TGACT---TTGCAACATCAGCTT----GTGTTTTGGC--TGCTGAA
                                                                                              Section 154
                         (14842) 14842   14850     14860     14870     14880     14890     14900     14910     14920        14938
    lambda NC_001416.1   (14437) GTTCCGCTGTGTGCCGCATCTCACCGGGCGGCGCTTTGAGCACGGTGTGACGGACTGTTACACACTGTTCCGGGATGCTTATCATCTGGCGGGGATT
 SARS-CoV-2 NC_045512.2   (9020) TGTACAATTTTTAAAGATGCTTCTGGTAAGC--C--AG-----TACCATATTGT-TATG--ATAC---CAATGTACTA----GAAGGTTCTGTTGCT
```

```
                                                                                         Section 155
                          14939     14950     14960     14970     14980     14990     15000     15010     15020          15035
                 (14939)
lambda NC_001416.1 (14534) GAGATGCCGGACTTTCATCGTGAGGATGACTGGTGGCGTAACGGCCAGAATCTCTATCTGGATAATCTGGAGGCGACGGGGCTGTATCAGGTGCCGT
SARS-CoV-2 NC_045512.2 (9098) TATGAAAG-----TTTACGCCCTGACACACGTTATGTGCTCA----TGGATGGCT---CTATTATT----------CAATTTCCTAAC---------
                                                                                         Section 156
                          15036         15050     15060     15070     15080     15090     15100     15110     15120     15132
                 (15036)
lambda NC_001416.1 (14631) TGTCAGCGGCACAGCCGGGCGATGTGCTGCTGTGCTGTTTTGG----TTCAT-CAGTGCCGAATCACGCCGCAATTTACTGCGGCGACGG-CGAGCT
SARS-CoV-2 NC_045512.2 (9164) ------------ACCTACC----TTGAAGGTTCTGTTAGAGTGGTAACAACTTTTGATTCT------GAGTACTGTAGGCACGGCACTTGTGAAAGA
                                                                                         Section 157
                          15133  15140     15150     15160     15170     15180     15190     15200     15210           15229
                 (15133)
lambda NC_001416.1 (14722) GCTGCACCATATTCCTGAACAACTGAGCAAACGAGAGAGGTAC--ACCGACAAATGGCAGCGACGCA-CACACTCCCTCTGGCGTCACCGGGCATGG
SARS-CoV-2 NC_045512.2 (9239) TCAGAAGCTGGTGTTTGTGTATCTACTAGTGGTAGATGGGTACTTAACA----ATGATTATTACAGATCTTTACCAGGA--GTTT---TCTG--TGG
                                                                                         Section 158
                          15230     15240     15250     15260     15270     15280     15290     15300     15310          15326
                 (15230)
lambda NC_001416.1 (14816) CGCGCATCTGCCTTTACGGGGATTTACAACGATTTGGTCGCCGCATCGACCT-TCGTGTGAAAACGGGGGCTGAAGCCATCCGGGCACTGGCCACAC
SARS-CoV-2 NC_045512.2 (9325) TGTAGAT-----GCTGTAAATTTACTTACTAATATGTTTA-------CACCACTAAT---TCAACCTA--TT--GGTGC----------TTTGGACA
                                                                                         Section 159
                          15327        15340     15350     15360     15370     15380     15390     15400     15410     15423
                 (15327)
lambda NC_001416.1 (14912) AGCTCCCGGCGTTTCGTCAGAAACTGAGCGACGGCTGGTATCAGGTACGGATTGCCGGGCGGGACGTCAGCACGT-CCGGGTTAACGGCGCAGTTAC
SARS-CoV-2 NC_045512.2 (9393) -----TATCAG--CA----TC-TATAGTAGCTGGTGGT---ATTGTAGCTATCGTAGTA-ACATGCCTTGCCTACT--------ATTTTATGAGGTT
                                                                                         Section 160
                          15424 15430     15440     15450     15460     15470     15480     15490     15500     15510 15520
                 (15424)
lambda NC_001416.1 (15008) ATGAG-ACTCTGCCTGATGGCG-CTGTAATTCATATTGTTCCCAGAGTCGCCGGGGCCAAGTCAGGTGGCGTATTCCAGATTGTCCTGGGGGCTGCC
SARS-CoV-2 NC_045512.2 (9466) --TAGAAGAGCTTTTGGTGAATACAGTCATGTAGTTGCCTTTAATA-CTTT---ACTATTC-CTTATGTCATTCACTGTACT---CTGTTTAACACC
                                                                                         Section 161
                          15521    15530     15540     15550     15560     15570     15580     15590     15600          15617
                 (15521)
lambda NC_001416.1 (15103) GCCATTGCCGGATCATTCTTTACCGCCGGAGCCACCCTTGCAGCATGGGGGCAGCCATTGGGGCCGGTGGTATGACCGGCATCCTGTT-TTCTCTC
SARS-CoV-2 NC_045512.2 (9553) --AGTT---TACTCATTCTTACCTGGTGTTTATTCTGTTAT--TTACTTGTACT-----TG--ACATT---TT-ATCTTAC-TAATGATGTTTCTTT
```

```
Section 162
                        (15618) 15618       15630     15640     15650     15660     15670     15680     15690     15700         15714
lambda NC_001416.1      (15199) GGTGCCAG-TATGGTGCTCGGTGGTGTGGCGCA--GATGCTGGC------ACCGAAAGCCAGAACTCCCCGTATACAGACAACGGATAACGGTAAGC
SARS-CoV-2 NC_045512.2   (9631) TTTAGCACATATTCAGTGGATGGTTATGTTCACACCTTTAGTACCTTTCTGGATAACAATTGCT--TATATCATTTGTATTT---CCA----CAAAG

Section 163
                        (15715) 15715 15720     15730     15740     15750     15760     15770     15780     15790     15800      15811
lambda NC_001416.1      (15287) AGAACACCTATTTCTCCTCACTGGATAACATGGTTGCCCAGGGCAATGTTC-TGCCTGTTCTGTACGGGGAAATGCGCGTGGGGTCACGCG-TGGTT
SARS-CoV-2 NC_045512.2   (9719) C-ATTTCTATTGGTT-C-TTTAGTAATTACCTAAAG--AGACGTGTAGTCTTTAATGGTGTTTCCTTTAGTACTTTTGAAGAAGCTGCGCTGTGCAC

Section 164
                        (15812) 15812   15820     15830     15840     15850     15860     15870     15880     15890             15908
lambda NC_001416.1      (15382) TCTCAGGAGATCAGCACGGCAGACGAAGGGGACGGTGGTCAGGTTGTGGTGATTGGTCGCTGATGCAAAATGTTTATGTGAAACCGC--CTGCGGG
SARS-CoV-2 NC_045512.2   (9811) CTTTTTGT-TAAATAAAGAAATGTATCTAAAGT-TGCGTAGTGATGTGCTATTACCTCTTACGCA--ATATAATAGATACTTAGCTCTTTATAATAA

Section 165
                        (15909) 15909      15920     15930     15940     15950     15960     15970     15980     15990          16005
lambda NC_001416.1      (15477) CGGTTTTG-TCATTTATGGAGCGTGAGGAATGGGTAAAGGAAGCAGTAAGGGGCATACCCCGCGCGAAGCGAAGGACAACCTGAAGTCCACGCAGTT
SARS-CoV-2 NC_045512.2   (9904) GTACAAGTATTTTAGTGG-AGCAATGGATACAACTAGCTACAGAGA-AGCTGCTTGTT-GTCATCTCGCAAAGGCTCTCAATGACTTCAGTAACTCA

Section 166
                        (16006) 16006         16020     16030     16040     16050     16060     16070     16080     16090      16102
lambda NC_001416.1      (15573) GCTGAGTGTGATCGATGCCATCAGCGAAGGGCCGATTGAAGGTCCGGTGGATGGCTTAAAAAGCGTGCTGCTGAACAGTACGCCGGTGCTGGACACT
SARS-CoV-2 NC_045512.2   (9998) GGTTCTGAT-GTTCTTTAC----CAACCACC-ACAAACCTCTATCACCTCAGCTGTTTTGCAGAGTGGT-TTTAGA-AAAATGGCATT---CCCATC

Section 167
                        (16103) 16103  16110     16120     16130     16140     16150     16160     16170     16180          16199
lambda NC_001416.1      (15670) GAGGGGAATACCAACATATCCGGTGTCACGGTGGTGTTCCGGGCTGGTGAGCAGGAGCAGACTCCGCCGGAGGGATTTGA-ATCCTCCGGCTCCGAG
SARS-CoV-2 NC_045512.2  (10084) TGGTAAAGTTGAGGGTTGTATGG-TACAAGTAACTTGT--GG----TACAACTACAC----TTAACGGTCTTTGGCTTGATGACGTAGTTTACTGTC

Section 168
                        (16200) 16200     16210     16220     16230     16240     16250     16260     16270     16280          16296
lambda NC_001416.1      (15766) ACGGTGCTGGGTACGGAAGTGAAATATGACACGCCGATCACCCGCACCATTACGTCTGCAAACATCGACCGTCTGCGCTTTACCTTCGGTGTACAGG
SARS-CoV-2 NC_045512.2  (10170) CAAGACATGTGATCTGCACCTCT--GAAGACATGC-TTAACCCTAATTATGAAG-----ATTTACTCATTCGTAAGTCTAATCATAATTTCTTGGTA
```

```
                                                                                              Section 169
                          (16297) 16297        16310       16320       16330       16340       16350       16360       16370       16380      16393
           lambda NC_001416.1 (15863) CACTGGTGGAAACCACCTCAAAGGGTGACAGGAATCCGTCGGAAGTCCGCCTGCTGGTTCAGA---TACAACGTAACGGTGGCTGGGTGACGGAAAA
   SARS-CoV-2 NC_045512.2 (10259) CAGGCTGGTAATGTTCAACTCAGGGTT-----ATTGGACATTCTATGCAAAATTGTGTACTTAAGCTTAAGGTTGATACAGCCAATCCTAAGACA-C
                                                                                              Section 170
                          (16394) 16394 16400       16410       16420       16430       16440       16450       16460       16470       16480  16490
           lambda NC_001416.1 (15957) A-GACATCACCATTAAGGGCAAAACCACCTC----GCAGTATCTGGCCTCGGTGGTGAT---GGGTAACCTGCCGCCGCGCCCGTTTAATATCCGGA
   SARS-CoV-2 NC_045512.2 (10350) CTAAGTATAAGTTTGTTCGCATTCAACCAGGACAGACTTTTTCAGTGTTAGCTTGTTACAATGGTTCACCATCTGGTGTTTAC---CAATGTG-CTA
                                                                                              Section 171
                          (16491) 16491     16500       16510       16520       16530       16540       16550       16560       16570         16587
           lambda NC_001416.1 (16046) TGCG---------CAGGATGACGCCGGACAGCACC---ACAGACC----A---GCTGCAGAACAAAACGCTCTGGTCGTCATACA-CTGAAATCATC
   SARS-CoV-2 NC_045512.2 (10443) TGAGGCCCAATTTCACTATTAAGGGTTCATTC---CTTAATGGTTCATGTGGTAGTGTTGGTTTTA--ACAT--AGATTATGACTGTGTC--TCTTT
                                                                                              Section 172
                          (16588) 16588        16600       16610       16620       16630       16640       16650       16660       16670      16684
           lambda NC_001416.1 (16123) ---GATGTGAAACAGTG-CTACCCGAACACGGCACTGGTCGGCGTGCAGGTGGACTCGGAGCAGTTCGGCAGCCAGCAGGTGAGCCGTAAT------
   SARS-CoV-2 NC_045512.2 (10531) TTGTTACATGCACCATATGGAATTACCAACTGGAGTTCATGCTG-GCA------CAGACTTAGAAG-GTAACTTTTATGG--------ACCTTTTGT
                                                                                              Section 173
                          (16685) 16685 16690       16700       16710       16720       16730       16740       16750       16760       16770  16781
           lambda NC_001416.1 (16210) TATCATCTGCGCGGCGTATTCTGCAGGTGCC--GTCGAACTATAACCCGCA-GACGCGGCAATACAGCGGTATCTGGGACGGAACGTTTAAACCGG
   SARS-CoV-2 NC_045512.2 (10612) TGACAGGCAAACAGC-ACAAGCAGCTGGTACGGACACAACTATTACAGTTAATGTTTTAGCTTGGTTGTACGCTGCTGTTATAAATGGAGACAGGTG
                                                                                              Section 174
                          (16782) 16782  16790       16800       16810       16820       16830       16840       16850       16860          16878
           lambda NC_001416.1 (16304) CATACAGCAACAACATGGCCTGGTGTCTGTGGGATATGCTGACCCATCCGCGCTACGGCATGGGGAAACGTCTTGGTGCGGCGGATGTGGATAAATG
   SARS-CoV-2 NC_045512.2 (10708) GTTTCTCAATCGATT------TACCACAACTCTTAATGACTTT--A--ACCTTGTGGCTATGAAGTACAATTATGAACCTCTAACACAAGACCATGT
                                                                                              Section 175
                          (16879) 16879      16890       16900       16910       16920       16930       16940       16950       16960         16975
           lambda NC_001416.1 (16401) GGCGCTGTATGTCATCGGCCAGTACTGCGACCAGTCAGTGCCGGACGGCTTTGGCGGCACGGAGCCGCGCATCACCTGTAATGCGTACCTGACCACA
   SARS-CoV-2 NC_045512.2 (10795) TGACATACTAGGACCTCTTTCTGC-TCAA-----ACTGGAATTGCCGTTTTAGATATGTGTGCTTCA-TTAAAAGAATTA-------CTGCAAAATG
```

Section 176

(16976) 16976 16990 17000 17010 17020 17030 17040 17050 17060 17072
lambda NC_001416.1 (16498) CAGCGTAAGGCGTG----GGATGTGCTCAGCGATTTCTGCTCGGCGATGCGCTGTATGCCGGTATGGAACGGGCAGACGCTGACGTTCGTGCAGGAC
SARS-CoV-2 NC_045512.2 (10878) GTATGAATGGACGTACCATATTGGGTAGTGCT-TTATTAGAAGATGAATTTACACCTTTTGATGTT---GTTAGACAATGCTCAGGTGTTACTTT--

Section 177

(17073) 17073 17080 17090 17100 17110 17120 17130 17140 17150 17169
lambda NC_001416.1 (16591) CGACCGTCGGATAAGACGTGGACCTATAACCGCAGTAATGTGGTGATGCCGGATGATGGCGCG--CCGTTCCGCTACAGCTTCAGCGCCC-----TG
SARS-CoV-2 NC_045512.2 (10969) ---CCAAAGTGCAGTGAAAAGAACA--ATCAAGGGTACACACCACTGGTTGTTACTCACAATTTTGACTTCACTTTTAGT-TTTAGTCCAGAGTACT

Section 178

(17170) 17170 17180 17190 17200 17210 17220 17230 17240 17250 17266
lambda NC_001416.1 (16681) AA--GGACCGCCATAATGCCGTTGAGGTGAACTGGATTGACCCGAACAACGGCTGGGAGACGGCGACAGAGCTTGTTGAAGATACGCAGGCCATTGC
SARS-CoV-2 NC_045512.2 (11060) CAAT-GGTCTTTGTTCTTTTTTTTGTATGAAAATGCCTTTTTACCTTTTGCTATGGGTATTATTGCTATGTCTGCTTTTGCAA------TGATGTTT

Section 179

(17267) 17267 17280 17290 17300 17310 17320 17330 17340 17350 17363
lambda NC_001416.1 (16776) CCGTTACGGTCGTAATGTTACGAAGATGGATGCC-TTTGGCTGTACCAGCCGGGGGCAGGCACACCGCGCCGGGCTGTGGCTGATTAAAACAGAACT
SARS-CoV-2 NC_045512.2 (11150) GTCA----AACATAAGCATGCATTTCTCTGTTTGTTTTTGTTACCTTCTCTTGCCACTGTAGCTTATTTTAATATGGTCTATATGCCTGCTAGTTGG

Section 180

(17364) 17364 17370 17380 17390 17400 17410 17420 17430 17440 17450 17460
lambda NC_001416.1 (16872) GCTGGAAACGCAGA-CCGTGGATTTCAGCGTCGGCGCAGAAGGGCTTCGCCATGTACCGGGCGATGTTATTGAAATCTGCG-ATGATGACTATGCCG
SARS-CoV-2 NC_045512.2 (11243) GTGATGCGTATTATGACATGGTTGGATATG---GTTGATAC----T----AGTTTGTCTGGTTTTAAGCTAAAAGACTGTGT--------TATGTAT

Section 181

(17461) 17461 17470 17480 17490 17500 17510 17520 17530 17540 17557
lambda NC_001416.1 (16967) GTATCAGCACCGGTGGTCGTGTGCTGG-CGGTGAACAGCCAGACCCGGACGCTGACGCTCGACCGTGAAATCACGCTGCCATCCTCCGGTACCGCGC
SARS-CoV-2 NC_045512.2 (11321) GCATCAGCTGTA---GTG--TTACTAATCCTTATGACAGCAAGAACTGTGTATGATGATGG-TGCTAG--GAGAGT-GTGGACACTTAT-------G

Section 182

(17558) 17558 17570 17580 17590 17600 17610 17620 17630 17640 17654
lambda NC_001416.1 (17063) TGATAAGCCTGGTTGACGGAAGTGGCAATCC-GGTCAGCGTGGAGGTTCAGTCCGTCACCGACGGCGTGAAGGTAAAAGTGAGC-CGTGTTCCTGAC
SARS-CoV-2 NC_045512.2 (11402) ---AATGTCT-TGACACTCGTTTATAAAGTTTATTATGGT----AATGCTTTAG--------ATCAAGCCATTTCCATGTGGGCTCTTATAATCTCT

Section 183

```
                        (17655) 17655 17660 17670 17680 17690 17700 17710 17720 17730 17740 17751
     lambda NC_001416.1 (17158) GGTGTTGCTGAATACAGCGTATGGGAGCTGAAGCTGCCGACGCTGCGCCAGCGACTGTTCCGCTGCGTGAGTATCCGTGAGAACGACGACGGCACGT
SARS-CoV-2 NC_045512.2 (11483) GTT-ACTTCTAACTACTCAGGTGTAG-----TTAC-AACTGTCATGTTTTTGGCCAGAGGTATTGTTTTTAT-G----TGTGTTGAGTATTGCCCTA
```

Section 184

```
                        (17752) 17752 17760 17770 17780 17790 17800 17810 17820 17830 17848
     lambda NC_001416.1 (17255) ATGCCATCACCGCCGTGCAGCATGTGCC-GGAAAAAGAGGCCATCGTGGATAACGGGGCGCACTTTGACGGCGAACAGAGTGGCACGGTGAATGGTG
SARS-CoV-2 NC_045512.2 (11568) TTTTCTTCATAACTGGTAATACACTTCAGTGTATAATGCTAGTTTATTGTTTCTTAGGCTATTTTTGTACTTGTTA-CTTTGGCCTCTTTTGTTTAC
```

Section 185

```
                        (17849) 17849 17860 17870 17880 17890 17900 17910 17920 17930 17945
     lambda NC_001416.1 (17351) TCACGCCGCCAGCGGTGCAGCACCTGACCGCAGAAGTCACTGCAGACAGCGG---GGAATATCAGGTGCTGGC-----GCGATGGGACACACCGAAG
SARS-CoV-2 NC_045512.2 (11664) TCAACCG--CTACTTTAGACTGACT---CTT-GGTGT--TTATGATTACTTAGTTTCTACACAGGAGTTTAGATATATGAATTCACA-GGGACTACT
```

Section 186

```
                        (17946) 17946 17960 17970 17980 17990 18000 18010 18020 18030 18042
     lambda NC_001416.1 (17440) GTGGTGAAGGGCGTGAGTTTC-CTGCTCCGTCTGACCGTAACAGCGGACGACGGCAGTGAGCGGCTGGTCAGCACGGCCCGGACGACGG-AAACCAC
SARS-CoV-2 NC_045512.2 (11752) CCCA--CCCAAGAATAGCATAGATG--CCTTCAAAC-TCAACATTAAATTGTTGGGTGTTGGTGGCAAACCTTGTATCAAAGTAGCCACTGTACAGT
```

Section 187

```
                        (18043) 18043 18050 18060 18070 18080 18090 18100 18110 18120 18139
     lambda NC_001416.1 (17535) ATA------CCGCTTCACGCAACTGGCGCT-----GGG-GAACTACAGGCTGACAGTCCGGGCGGTAAATGCGTGGGGGCAGCAGGGCGATCCGGCG
SARS-CoV-2 NC_045512.2 (11844) CTAAAATGTCAG-ATGTAAAGTGCACATCAGTAGTCTTACTCTCAGTTTTGCAACA-ACTCAGAGTAGAATCATCATCTAAATTGTGGGCTCA---A
```

Section 188

```
                        (18140) 18140 18150 18160 18170 18180 18190 18200 18210 18220 18236
     lambda NC_001416.1 (17620) TCGGTATCGTTCCGGATTGCCGCACCGGCAGCACCGTCGAGGATTGAGCTGACGCCGGGCTATTTTCAGATAACCGCCACGCCGCATCTTGCCGTTT
SARS-CoV-2 NC_045512.2 (11936) TGTGTCCAGTTACACAATGACATTCTCTTAGCT--AAAGATACTACTGAAG-CCTT-TGAAAAAATGGTTTCACTACTTT-CTGTTTTGCTTTCC-A
```

Section 189

```
                        (18237) 18237 18250 18260 18270 18280 18290 18300 18310 18320 18333
     lambda NC_001416.1 (17717) ATGACCCGACGGTACAGTTTGAGTTCTGGTTCTCGGAAAAGCAGATTGCGGATATCAGACAGGTTGAAACCAGCACGCGTTATCTTGGTACGGCGCT
SARS-CoV-2 NC_045512.2 (12027) TGCAGGGTGCTGTAGACATAAA---CAAGCTTTGTGAAGAA----ATGCTGGA------CAACAGGGCAACCTTACAAGCTAT--AGCCTCAGAGTT
```

```
                                                                                                        Section 190
                              (18334) 18334 18340     18350     18360     18370     18380     18390     18400     18410     18420     18430
          lambda NC_001416.1 (17814) GTACTGGATAGCCGCCAGTATCAATATCAAACCGGGCCATGATTATTACTTTTATAT--CCGCAGTGTGAACACCGTTGGCAAATCGGCATTCGTG-
     SARS-CoV-2 NC_045512.2 (12109) TAGTTCCCTT---CCATCATATGCAGCTTT----TGCTACTGCTCAAGAAGCTTATGAGCAGGCTGTTGCTAAT--GGTG-ATTCTGAAGTTGTTCT
                                                                                                        Section 191
                              (18431) 18431    18440     18450     18460     18470     18480     18490     18500     18510          18527
          lambda NC_001416.1 (17908) GAGGCCGTCGGTCGGGCGAGCGATGATGCGGAAGGTTACCTGGATTTTTTCAAAGGCA-AGATAACCGAATCCCATCTCGGC---AAGGAGCTGCTG
     SARS-CoV-2 NC_045512.2 (12196) TAAAAAGTTGAAGAAG---TCTT---TG---AATGTGGCTA-----------AATCTGAATTTGACCGT---GATGCAGCCATGCAACGTAAGTTGG
                                                                                                        Section 192
                              (18528) 18528       18540     18550     18560     18570     18580     18590     18600     18610     18624
          lambda NC_001416.1 (18001) GAAAAAGTCGAGCTGACGGAGGA-TAACGCCAGCAGACTGGAGGAGTTTTCGAAAGAGTGGAAGGATGCCAGTGA-TAAGTGGAATG--CCATGTGG
     SARS-CoV-2 NC_045512.2 (12270) AA--AAGATGGCT-GATCAAGCTATGACCCAAATGTATAAACAGGCTAGATCTGAGGACAAGAGG----GCAAAAGTTACTAGTGCTATGCAGACAA
                                                                                                        Section 193
                              (18625) 18625 18630     18640     18650     18660     18670     18680     18690     18700     18710     18721
          lambda NC_001416.1 (18094) GCTGTCAAAATTGAGCAGACCAAAGAC---GGCAAACATTATGTCGCGGGTATTGGCCTCA-GCATGGAGGACACGGAGGAAGGCAAACTGAGCCAG
     SARS-CoV-2 NC_045512.2 (12360) TGCTTTTCA-CTATGCTTAG---AAAGTTGGATAATGATGCACTCAACAA---CATTATCAACAATGCAAGAGATGGTTGTGTTCCC-TTGAACATA
                                                                                                        Section 194
                              (18722) 18722   18730     18740     18750     18760     18770     18780     18790     18800          18818
          lambda NC_001416.1 (18187) TTTCTGGTTGCCGCCAATCGTATCGCATTTATTGACCCGGCAAACGGGAATGAAACGCCGATGTTTGTGGCGCAGGGCAACCA-GATATTCATGAAC
     SARS-CoV-2 NC_045512.2 (12449) ATACCTCTTA-CAACAGCAGCCAAACTAATGGTT----GTCATACCAGACTATAACACATATAAAAATACGTGTGATGGTACAACATTTACTTATGC
                                                                                                        Section 195
                              (18819) 18819      18830     18840     18850     18860     18870     18880     18890     18900          18915
          lambda NC_001416.1 (18283) GACGTGTTCCTGAAGCGCCTGACGG----CCCCCACCATTACCAGCGGC--GGCAATCCTCCGGCCTTTTCCCTGACACCGGACGGAAAGCTGACCG
     SARS-CoV-2 NC_045512.2 (12541) ATCAGCAT--TGTGGGAAATCCAACAGGTTGTAGATGCAGATAGTAAAATTGTTCAACTTAGTGAAATT--AGTATGGAC-AATTCACCTAATTTAG
                                                                                                        Section 196
                              (18916) 18916         18930     18940     18950     18960     18970     18980     18990     19000     19012
          lambda NC_001416.1 (18374) CTAAAAATGCGGATATCAGTGGCAGTGTGAATGCGAACTCCGGGACGCTCAGTAATGTGACGATAGCTGAAAACTGTACGATAAACGGTACGCTGAG
     SARS-CoV-2 NC_045512.2 (12633) C--ATGGCCTCTTATTG-TAACAGCTTTAAGGGCCAATTCTGCTGTCAAATTACAGAATAATGAGCTTAGT-CCTGTTGCACTACGACAGATGTCTT
```

```
Section 197
(19013)                        19013  19020           19030           19040           19050           19060           19070           19080           19090                     19109
lambda NC_001416.1 (18471)     GGC------GGAAAAAATCGTCGGGGACAT----TGTAAAGGCGGCGAGCGCGGCTTTTCCGCGCCAGCGTGAAAGCAGTGTGGA-CTGGCCGTCAG
SARS-CoV-2 NC_045512.2 (12726) GTGCTGCCGGTACTACACAAACTGCTTGCACTGATGACA-----ATGCGTTAGCTTACTACA-ACACAACAAAGGGAGGTAGGTTTGTACTTGCACT

Section 198
(19110)                        19110 19120 19130 19140 19150 19160 19170 19180 19190 19206
lambda NC_001416.1 (18557)     GT-ACCCGTACTGTCACCGTGACCGATGACCATCCTTTTGATCGCCAGATAGTGGTGCTTCCGCTGACGTTTCGCGGAAGTAAGCGTACTGTCAGCG
SARS-CoV-2 NC_045512.2 (12817) GTTATCCGATTTACAGGA------TTTGAAATGGGCTA-GATTCCCTAAGAGTGATGGAACTGGT--ACTATCTATACAGAA---CTGGAACCACCT

Section 199
(19207)                        19207 19220 19230 19240 19250 19260 19270 19280 19290 19303
lambda NC_001416.1 (18653)     GCAGGACAACGTATTCGA--TGTGTTATCTGAAAGTACTGATGAACGGTGCGGTGATTTATGATGGCGCGGCGAACGAGGCGGTACAGGTGTTCTCC
SARS-CoV-2 NC_045512.2 (12902) TGTAGGTTTGTTACAGACACACCTAAAGGTCCTA-AAGTGAAGTATTTATACTTTATTA------AAGGA----TTAAACAACC-------TAAATA

Section 200
(19304)                        19304 19310 19320 19330 19340 19350 19360 19370 19380 19390 19400
lambda NC_001416.1 (18748)     CGTATTGTTGACATGCCAGCGGGTCGGGGAAA---CGTGATCCTGACGT--TCACGCTTACGTCCACACGGCATTCGGCAGATATTCCGCCGTATAC
SARS-CoV-2 NC_045512.2 (12981) GAGGTATGGTACTTGGTAGTTTAGCTGCCACAGTACGTCTACAAGCTGGTAATGCAACAGAAGTGCCTGCCAATTCAA------CTGTATTATCTTT

Section 201
(19401)                        19401 19410 19420 19430 19440 19450 19460 19470 19480 19497
lambda NC_001416.1 (18840)     GTTTGCCAGCGATGTGCAGGTTATGGTGATTAAGAAACAGGCGCTGGCATCAGCGTGGTCTGAGTGTGTTACAGAGGTTCGTCCGGGAACGGGCGT
SARS-CoV-2 NC_045512.2 (13072) CTGTGCTTTTGCTGTAGATGCTGCTAAAGCTTACAAAGATTATCTAGCTAGTGGGGACAACCAATCACTAATTGTGTTAAGATGTTGTGTACACAC

Section 202
(19498)                        19498 19510 19520 19530 19540 19550 19560 19570 19580 19594
lambda NC_001416.1 (18937)     TTT--A--TTATAAAACAGTGAGAGGTGAACG----AT-GCGTAATGTGTGTATTGCCGTTGCTGTCTTTGCCGCACTTGCGGTGACAGTCACTCCG
SARS-CoV-2 NC_045512.2 (13169) ACTGGTACTGGTCAGGCAAT-AACAGTTACACCGGAAGCCAATATGG-ATC-AAGAATCCTTTGGTGGTGCATCGTGTTGTCTGTACTGCCGTTGCC

Section 203
(19595)                        19595 19600 19610 19620 19630 19640 19650 19660 19670 19680 19691
lambda NC_001416.1 (19025)     GC-CCGTGCGGAAGG----TGGACA----TGGTACGTTTACGGTGGGC--TATTTTCAAGTGAAACCGGGTACATTGCCGTCGTTGTCGGGCGGGA
SARS-CoV-2 NC_045512.2 (13263) ACATAGATCATCCAAATCCTAAAGGATTTTGTGACTTAAAAGG---TAAGTATGTACAAATACCTA-------CAACTTGTGCTAATGACCCTGTGG
```

Section 204

```
                              (19692) 19692   19700     19710     19720     19730     19740     19750     19760     19770          19788
         lambda NC_001416.1 (19111) TAC----CGGTGTGAGTCATCTGAAAGG-GATTAACGTGAAGTACCGTTATGAGCTGACGGACAGTGTGGGGGTGATGGCTTCCCTGGGGTTCGCCG
SARS-CoV-2 NC_045512.2 (13350) GTTTTAC-ACTTAAAAACA-CAGTCTGTACCGTCTGCGGTATGTGGAAAGGTTATGGCTGTAGTTGTG-ATCAACTCCGCGAACCCATGCTTCAGTC
```

Section 205

```
                              (19789) 19789      19800     19810     19820     19830     19840     19850     19860     19870          19885
         lambda NC_001416.1 (19203) CGTCGAAAAAGAGCAGC--ACAGTGATGACCGGGGAGGATACGTTTCACTATG--AGAGCCTGCGTGGACGTTATGTGAGCG--TGATGGCCGGACC
SARS-CoV-2 NC_045512.2 (13444) AGCTG--ATGCACAATCGTTTTTAAACGGGTTTGCGGTGTAAGTGCAGCCCGTCTTACACCGTGCGGCACAGGCACT-AGT-ACTGATGTCG-----
```

Section 206

```
                              (19886) 19886             19900     19910     19920     19930     19940     19950     19960     19970     19982
         lambda NC_001416.1 (19294) GGTTTT--ACAAATCAGTAAGCAGGTCAGTGCGTAC-GCCATGGCCGGAGTGGCTCACAGTCGGTGGTCCGGCAGTACAATGGAT---TACCGTAAG
SARS-CoV-2 NC_045512.2 (13532) -----TATACAGGGCTTTTGACATCTACAATGATAAAGTAGCT---GGTTTTGCTAAATTCCTAAAAAC-T--A-ATTGTTGTCGCTTCCAAGAAAA
```

Section 207

```
                              (19983) 19983  19990     20000     20010     20020     20030     20040     20050     20060          20079
         lambda NC_001416.1 (19385) ACGGAAATCACTCCCG----GGTATATGAA-AGAGACGACCACTGCCAGGGACG-----------AAAGTGCAATGCGGCATACCTCAGT---GGCG
SARS-CoV-2 NC_045512.2 (13617) GGACGAAG--ATGACAATTTAATTGATTCTTACTTTGTAGTTAAGAGACACACTTTCTCTAACTACCAACATGAAGAAACAATTTATAATTTACTTA
```

Section 208

```
                              (20080) 20080     20090     20100     20110     20120     20130     20140     20150     20160          20176
         lambda NC_001416.1 (19463) TGGAGTGCAG-GTATACAGATTAATCCGGCAGCGTCCGTCGTTGTTGATATTGCTTATGAAGGCTCCGGCAGTGGCGACTGGCGTACTGACGGATTC
SARS-CoV-2 NC_045512.2 (13712) AGGATTGTCCAG----CTGTTGCTAAACATGACTTCTTTAAGTTTAGAATAGAC-----GGTGACATG-GTACCAC--ATATATCACGT-CAACGTC
```

Section 209

```
                              (20177) 20177        20190     20200     20210     20220     20230     20240     20250     20260          20273
         lambda NC_001416.1 (19559) ATCGTTGGGGTCGGTTAT------AAATTCTGATTAGCCAGGTAACACAG----TGTTATGACAGCCCGCCGGAAC---CGGTGGCT-----TTTT
SARS-CoV-2 NC_045512.2 (13796) TTACTAAATACACAATGGCAGACCTCG-TCTATGCTTTAAGGCATTTTGATGAAGGTAATTGTGACACATTAAAAGAAATACTTGTCACATACAATT
```

Section 210

```
                              (20274) 20274 20280     20290     20300     20310     20320     20330     20340     20350     20360 20370
         lambda NC_001416.1 (19638) TGTGGGGTGAATATGGCAGTAAA--GATTTCAGGAGTCCTGAAAGACGGCACAGGAAAACCGGTACAGAACTGCACCATTCAGCTGAAAGCCAGACG
SARS-CoV-2 NC_045512.2 (13892) GTTGTGATGATGATTATT-TCAATAAAAAGGACTGG--------TATGATTTTGTAGAAAA--CCCAGATATATT--A--------CGCGTATACGC
```

Section 211

(20371) 20371 20380 20390 20400 20410 20420 20430 20440 20450 20467
lambda NC_001416.1 (19733) TAACAGCACCACGGTGGTGGTGAACACGGTGGGCTCAGAGAATCCGGATGA-AGCCGGGCGTTACAGCA---------TGGATGTGGAGTA-CGGTC
SARS-CoV-2 NC_045512.2 (13968) CAACTTAGGTGAACGTGTACGCCAAGCTTT--GTTAAAAACAGTACAATT-CTGTGATGCCATGCGAAATGCTGGTATTGTTGGTGTACTGACATTA

Section 212

(20468) 20468 20480 20490 20500 20510 20520 20530 20540 20550 20564
lambda NC_001416.1 (19819) AGTACAGTGTCATCC-TGCAGGTTGACGGTTTTCCACCATCGCACGCCGGGACCAT----C---ACCGTGTATGAAGA-TTC---ACAA----CCGG
SARS-CoV-2 NC_045512.2 (14062) GATAA--TCAAGATCTCAATGGTAACTGGTATGAT------TTCGGTGATTTCATACAAACCACGCCAGGTAGTGGAGTTCCTGTTGTAGATTCTTA

Section 213

(20565) 20565 20570 20580 20590 20600 20610 20620 20630 20640 20650 20661
lambda NC_001416.1 (19900) GGACGCTGAATGATTTTCTCTGTGCCATGACGGAGGATGATGCCCGGCCGGAGGTGCTGCGTCG--TCTTGAACTGATGGT-GGAAGAGGTGGCGCG
SARS-CoV-2 NC_045512.2 (14151) TTATTCATTGTTAATGCCTA--TATTAACCT------TGACCAGGGCTTTAACTGCA-GAGTCACATGTTGACACTGACTTAACAAAGCCTTACATT

Section 214

(20662) 20662 20670 20680 20690 20700 20710 20720 20730 20740 20758
lambda NC_001416.1 (19994) TAACGCGTC-CGTGGTGGCACAGAGT-ACGGCAGACGCGAAGAAATCAGCCGGCGATGCCAGTGCATCAGCTGCTCAGGTCGCGGCCCTTGTGACTG
SARS-CoV-2 NC_045512.2 (14239) AAGTGGGATTTGTTAAAATATGACTTCACGGAAGAGAGGTTAAAACTCTTTGACCGTTATTTT--AAATATTGGGATCAGACATACCACCCAAATTG

Section 215

(20759) 20759 20770 20780 20790 20800 20810 20820 20830 20840 20855
lambda NC_001416.1 (20089) ATGCAACTGACTCAGCACGCGCCGCCAGCACGTCCGCCGGACAGGCTGCATCGTCAGCTCAGGAAGCGTCCTCCGGCG------CAGAAGCGGCATC
SARS-CoV-2 NC_045512.2 (14334) TGTTAACTGTTTGGATGACAGATGCATTCT--GCATTGTGCAAACTTTAATGTTTTATTCTCTACA-GTGTTCCCACCTACAAGTTTTGGACCACTA

Section 216

(20856) 20856 20870 20880 20890 20900 20910 20920 20930 20940 20952
lambda NC_001416.1 (20180) A-GCAAAGGCCACTGAAGCGGAAAAAAGTGCCGCAGCCGCAGAGTCCTCAAAAAACGCGGCGGCCACCAGTGCCGGTGCGGCGAAAACGT-CAGAAA
SARS-CoV-2 NC_045512.2 (14428) GTGAGAAAAATAT-----TTGTTGATGGTGTTCCA---TTTGTAG--TTTCAACTGGATACCACTTCAGAGAGCTAGGTGTTGTACATAATCAGGAT

Section 217

(20953) 20953 20960 20970 20980 20990 21000 21010 21020 21030 21049
lambda NC_001416.1 (20275) CGAATGCTGCAGCGTCACAACAATCAGCCGCCACGTCTGCCTCCACCGCGGCCACGAAAGCGTCAGAGGCCGCCACTTCAGCACGAGATGCGGTGGC
SARS-CoV-2 NC_045512.2 (14515) GTAAACTTAC---ATAGCTCTAGACT--TAGT-TTTAAGGAATTA--CTTGTGTAT-GCTGCT--GACCCT-GCTATGCACGCTGCTTCTG---GTA

```
                                                                                                    Section 218
                             (21050) 21050           21060           21070           21080           21090           21100           21110           21120           21130                21146
         lambda NC_001416.1 (20372) CTCAAAAGAGGCAGCAAA---ATCATCA---GAAACGAACGCATCATCAAGTGCCG---GTCGTGCAGCTTCCTCGGCAACGGC-GGCAGAAAATTC
SARS-CoV-2 NC_045512.2 (14597) ATCTATT--ACTAGATAAACGCACTACGTGCTTTTCAGTAGCTGCACTTACTAACAATGTTGCTTTTCAAACTGTCAAACCCGGTAA----------
                                                                                                    Section 219
                             (21147) 21147               21160           21170           21180           21190           21200           21210           21220           21230          21243
         lambda NC_001416.1 (20459) TGCCAGGGCGGCAAAAACGTCCGAGACGAATGCCAGGTCATCTGAAACAGCAGCGGAACGGAGCGCCTCTGCCGCGGCAGACGCAAAAACAGCGGCG
SARS-CoV-2 NC_045512.2 (14682) -----TTTTAACAAAGACTTCTATGACT--TTGCTGTGTCT---AAGGGTTTCTTTAA-----------GGAAGGAAGTTCTGTTGAAT--------
                                                                                                    Section 220
                             (21244) 21244    21250           21260           21270           21280           21290           21300           21310           21320           21330    21340
         lambda NC_001416.1 (20556) GCGGGGAGTGCGTCAACGGCATCCACGAAGGCGACAGAGGCTGCGGGAAGTGCGGTATCAGCATCGCAGAGCAAAAGTGCGGCAGAAGCGGCGGCAA
SARS-CoV-2 NC_045512.2 (14750) TAAAACACTTCTTC--TTTGCTCAG--GATGGTAATGCTGCTATCAGCGATTA---TGACTACTATCGTTATAATCTACCAACAA-------TGTGT
                                                                                                    Section 221
                             (21341) 21341         21350           21360           21370           21380           21390           21400           21410           21420                21437
         lambda NC_001416.1 (20653) TACGTGCAAAAAATTCGGCAAAACGTGCAGAAGATATAGCTTCAGCTGTCGCGCTTGAGGATGCGGACACAACGAGAAAGGGGATAGTGCAGCTCAG
SARS-CoV-2 NC_045512.2 (14833) GATATCAGACAACTACTATTTGTAGTT--GAAGTTGTTGATAAGTACTTTGATTGTTACGATG--GTGGCTGTATTAATGCTAA----CCAAGTCA-
                                                                                                    Section 222
                             (21438) 21438             21450           21460           21470           21480           21490           21500           21510           21520          21534
         lambda NC_001416.1 (20750) CAGTGCAACCAACAGCA--CGTCTGAAACGCTTGCTGCAACGCCAAAGGCGGTTAAGGTGGTAATGGATGAAACGAACAGAAAAG-CCCACTGGACA
SARS-CoV-2 NC_045512.2 (14921) T--CGTCAACAACCT--AGACAAATCAG------CTGGT-TTTCCATTTAATAAATGGGGTAAG--GCTA--GACTTTATTATGATTCAATGAG-TT
                                                                                                    Section 223
                             (21535) 21535 21540           21550           21560           21570           21580           21590           21600           21610           21620    21631
         lambda NC_001416.1 (20844) GTCCGGCACTGACCGGAACGCCAACAGCACCA-ACCGCGCTCAGGGGAACAAACAATACCCAGATTGCGAACACCGCTTTTGTACTGGCCGCGATTG
SARS-CoV-2 NC_045512.2 (15002) ATGAGG------ATCAAGATGCA------CTTTTCGCATATACAAAACGTAATGT-------CATCCCTACTATA------ACTCAAATGAATCTTA
                                                                                                    Section 224
                             (21632) 21632       21640           21650           21660           21670           21680           21690           21700           21710                21728
         lambda NC_001416.1 (20940) CAGATGTTATCGACGCGTCACCTGACGCACTGAATACGCTGAATGAACTGGCCGCAGCGCTCGGGAATGATCCAGATTTTGCTACCACCATGACTAA
SARS-CoV-2 NC_045512.2 (15074) AGTATGCCATTAGTGCAAAGAATAGAGCTCGCACCGTAGCT---GGTGTCTC-TATCTGTAGTACTATGACCAATAGACAGTTTCAT--------CA
```

```
                                                                                                Section 225
                         (21729) 21729      21740       21750       21760       21770       21780       21790       21800       21810        21825
      lambda NC_001416.1 (21037) CGCGCTTGCGGGTAAACAACCGAAGAATGCG---ACACTGACGGCGCTGGCAGGGCTTTCCACGGCGAAAAATAAATTACCGTATTTTGCGGAAAAT
SARS-CoV-2 NC_045512.2 (15159) AAAA-TTATT-----GAAATCAATAGCCGCCACTAGAG-GAGCTAC--TGTA-------GTAATTGGAACAAGCAAATTCTATGGTGGTTGGCACAA
                                                                                                Section 226
                         (21826) 21826             21840       21850       21860       21870       21880       21890       21900       21910      21922
      lambda NC_001416.1 (21131) GATGCCGCCAGCCTGACTGAACTGACTCAGGTTGGCAGGGATATTCTGGCAAAAAATTCCGTTGCAGATGT---TCTTGAATACCTTGGGGCCGGTG
SARS-CoV-2 NC_045512.2 (15240) CATGTTAAAAACTGTT-TATAGTGATGTAGAAAACCCTC-ACCTTATGGGT----------TGGGATTATCCTAAATGTGATAGAGCCATGCCTAAC
                                                                                                Section 227
                         (21923) 21923  21930       21940       21950       21960       21970       21980       21990       22000        22019
      lambda NC_001416.1 (21225) AGAATTCGGCCTTTCCGGCAGGTGCGCCGATCCCGTGGCCATCAGATATCGTTCCGTCT-GGCTACGTCCTGATGCAGGGGCAGGCGTTTGACAAAT
SARS-CoV-2 NC_045512.2 (15325) AT--GCTTAGAATTATGGCCT-CACTTGTTCTTGCTCGCAAACATACAACGTGTTGT--A--GCTTGTC---ACACCGTTTCTATAGATTAGCTAAT
                                                                                                Section 228
                         (22020) 22020      22030       22040       22050       22060       22070       22080       22090       22100        22116
      lambda NC_001416.1 (21321) CAGCCTA-----CCCAAAACTTGCTGTCGCGTATCCATCGGGTGTGCTTCCTGATATGCGAGGCT-GGACAATCAAGGGGAAACCCGCCAGCGGTCG
SARS-CoV-2 NC_045512.2 (15412) GAGTGTGCTCAAGTATTGAGTGAAATGGTCATGTGTGGCG---GTTCACTATATGTTAAACCAGGTGGAACCTCATCAGGAGATGCCACAACTGCTT
                                                                                                Section 229
                         (22117) 22117         22130       22140       22150       22160       22170       22180       22190       22200      22213
      lambda NC_001416.1 (21412) --TGC---TGTATTGTCTCAGGAACAGGATGGAATTAAGTCGCACACCCACAGTGCCAGTGCATCCGGTACGGATTTGGGGACGAAAACCACATCGT
SARS-CoV-2 NC_045512.2 (15506) ATGCTAATAGTGTTTTTAACATTTGTCAAGCTG--TCACG--GCCAATGTT-AATGCACTTTTATCTAC--TGATGGTAACAAAATTGCCGATAAGT
                                                                                                Section 230
                         (22214) 22214 22220       22230       22240       22250       22260       22270       22280       22290       22300 22310
      lambda NC_001416.1 (21504) CGTTTG--ATT----ACGGGACGAAAACAACAGGCAG--TTTCGATTACGGCACCAAATCGACGAATAACACGGGGGCTCATGCTCACAGTCTGAGC
SARS-CoV-2 NC_045512.2 (15596) ATGTCCGCAATTTACAACACAGACTTTATGAGTGTCTCTATAGAAATAGAGATGTTGACACAGACTTTGTGAATGAGTTTTACGCATATTTGCGTAA
                                                                                                Section 231
                         (22311) 22311    22320       22330       22340       22350       22360       22370       22380       22390        22407
      lambda NC_001416.1 (21593) --GGTTCAACAGGGGCCGCGGGTGCTCATGCCCACACAAGTGGTTTAAGG--ATGAACAGTTCTGGCTGGAG-TCAGTATGGAACAGCAACCATTAC
SARS-CoV-2 NC_045512.2 (15693) ACATTTCTCAATGATGATACTCTCTGACGATGCTGTTGTGTGTTTCAATAGCACTTATGCATCTCAAGGTCTAGTGGCTAGCATAAAGAA--CTTTA
```

Section 232

```
                       (22408) 22408       22420       22430       22440       22450       22460       22470       22480       22490       22504
lambda NC_001416.1     (21685) AGGAAGTTTATCCACAGTTAAAGGAACCAGC--ACACAGGTATTGCTTATTTATCGAAAACGGACAGTCAGGGCAGCCACAGTCACTCATTGTCCG
SARS-CoV-2 NC_045512.2 (15788) AGTCAGTTCTTTATTA-TCAAAACAATGTTTTTATGTCTGAAGCA-AA-ATGTTGGACTGAGACTGACCT--TACTAAAGGACCTCATGAATTTTGC
```

Section 233

```
                       (22505) 22505 22510       22520       22530       22540       22550       22560       22570       22580       22590 22601
lambda NC_001416.1     (21780) GTACAGCCGTGAGTGCCGGTGCA-CATGCGCAT-AC-AGTTGGTATTGGTGCGCACCAGCATCCGGTTGTTATCGGTGCTCATGCCCA--TTCTTTC
SARS-CoV-2 NC_045512.2 (15880) TCTCAACATACAATGCTAGTTAAACAGGGTGATGATTATGTGTACCTT--CCTTACCCAGATCCATCAAGAATCC----TAGGGGCCGGCTGTTTTG
```

Section 234

```
                       (22602) 22602   22610       22620       22630       22640       22650       22660       22670       22680       22698
lambda NC_001416.1     (21872) AGTATTGGTTCACACGGACACACCATCACCGTTAACGCTGCGGGTAAC--GCGGAAAACACCGTC-AAAAACATTGCATTTAACTATATTGTGAGGC
SARS-CoV-2 NC_045512.2 (15971) TAGAT-GATATC------GTAAAAACAGATGGTACACTTATGATTGAACGGTTCGTGTCTTTAGCTATAGATGCTTACCCACTTA-----CTAAAC-
```

Section 235

```
                       (22699) 22699     22710       22720       22730       22740       22750       22760       22770       22780       22795
lambda NC_001416.1     (21966) TTGCATAATGGCATTCAGAATGAGTGAACAACCACGGACCATAAAATTTATAAT-CTGCTGGCCGGAAC---TAATGAATTTATTGGTGAAGGTG-
SARS-CoV-2 NC_045512.2 (16055) ------A-TCCTAATCAGGAGTATGCTGATGTCTTTCATTTGTACTTACAATACATAAGAAAGCTACATGATGAGTTAACAGGACACATGTTAGACA
```

Section 236

```
                       (22796) 22796         22810       22820       22830       22840       22850       22860       22870       22880 22892
lambda NC_001416.1     (22058) -ACGCA--TATATTCCGCCTCATACCGGTCTGCCTGCAAACAGTACCGATATTGCACCGCCAG-----ATATTCCGGCTGGCTTTGTGGCTGTTTTC
SARS-CoV-2 NC_045512.2 (16145) TGTATTCTGTTATGCTTACTAAT---GATAACACTTCAAGG--------TATTGGAACCTGAGTTTTATGAGGCTATGTACACACCGCATACAGTC
```

Section 237

```
                       (22893) 22893  22900       22910       22920       22930       22940       22950       22960       22970       22989
lambda NC_001416.1     (22147) AACAGTGATGAGGCATC----GTGGCATCTCGTTGAAGACCATCGGGGTAAAACCGTCTATGACGTGGCTTCCGGCGACGCGTTATTTATTTCTGAA
SARS-CoV-2 NC_045512.2 (16231) TTACAGGCTGTTGGGGCTTGTGTTCTTTGCAATTCACAGACTTCATT--AAGAT----GTGGTGCTTGCATACGTAG---ACCATTCTTATGTTGTA
```

Section 238

```
                       (22990) 22990     23000       23010       23020       23030       23040       23050       23060       23070       23086
lambda NC_001416.1     (22240) CTCGGTCCGTTACCGGAAAATTTTA--CCTGGTTATCGCCGGGAGGGGAATATCAGAAGTGGAACGGCACAGCCTGGGTGAAGGATACGGAAGCAGA
SARS-CoV-2 NC_045512.2 (16319) AATGCTGTTACGACCATG-------TC---ATATCAACATCAC---ATAAATTAGTCTTGTCTGTTAATCCGTATGTTT-----GCAA--TGCTCCA
```

Section 239

```
                              23087        23100     23110     23120     23130     23140     23150     23160     23170     23183
                    (23087)
lambda NC_001416.1 (22335)    AAAACTGT-TCCGGATCCGGGAGGCGGAAGAAACAAAAAAAAGCCTGATGCAGGTAGCC--AGTGAGCATATTGCGCCGCTTCAGGATGCTGCAGAT
SARS-CoV-2 NC_045512.2 (16396) GGTTGTGATGTCACAGATGT-GACTCAACTTTACTTAG--G-AGGTATGAGCTATTATTGTAA--ATCACATA--AACCACCCATTAGTTTTCCATT
```

Section 240

```
                              23184 23190     23200     23210     23220     23230     23240     23250     23260     23270 23280
                    (23184)
lambda NC_001416.1 (22429)    --CTGGAAATT--GCAACGAAGGAAGAAACCTCGTTGCTGGAAGCCTGGAAGAAGTATCGGGTGTTGCTGAACCGTGTTGATACATC---AACTGCA
SARS-CoV-2 NC_045512.2 (16485) GTGTGCTAATGGACAAGTTTTTGGT--TTATATAAAAATACATGTGTTGGT--AGCGATAA-TGTTACTGAC-TTTAATGCAATTG-CAACATGTGA
```

Section 241

```
                              23281    23290     23300     23310     23320     23330     23340     23350     23360          23377
                    (23281)
lambda NC_001416.1 (22519)    CCTGATATTGAGTGGCCTGCTGTCCCTGT-TATGGAGTAATCGTTTTGTGAT-ATGCCGCAGAAACGTTGTATGAAATAACGTTCTGCGGTTAGTTA
SARS-CoV-2 NC_045512.2 (16575) CTGGACAAATGCTGGTGATTACATTTTAGCTAACACCTG-TACTGAAAGACTCAAGCTTTTTGCAGCAGAAACGCTCAAAGCTACTGAGGAGACATT
```

Section 242

```
                              23378       23390     23400     23410     23420     23430     23440     23450     23460     23474
                    (23378)
lambda NC_001416.1 (22614)    GTATATTGTAAAGCTGAGTATTGGTTTATTTGGCGATTATTATCTTCAGGAG-AATAATGGAAGTTCTATGACTCAATTGTTCATAGTGTTTACATC
SARS-CoV-2 NC_045512.2 (16671) TAAACTGTCTTATGGTATT----GCTACTGTACG--TGAAGTGCTGTCTGACAGAGAATTACATCTTTCATGGGAAGTTGGTAAACCTAGAC-----
```

Section 243

```
                              23475 23480     23490     23500     23510     23520     23530     23540     23550     23560 23571
                    (23475)
lambda NC_001416.1 (22710)    ACCGCCA--ATTGCTTTTAAGACTG---------AACGCATGAAA-TATGGTTTTTCGTCATGTTTTGAGTCTGCTGTTGATATTTCTAAAGTCGGT
SARS-CoV-2 NC_045512.2 (16757) CACCACTTAACCGA---AATTATGTCTTTACTGGTTATCGTGTAACTAAAAACAGTAAAGTACAAATAGGA--GA-G--TACA---CCT--------
```

Section 244

```
                              23572   23580     23590     23600     23610     23620     23630     23640     23650          23668
                    (23572)
lambda NC_001416.1 (22795)    TTTTTTTCTTCGTTTTCTCTAACTATTTTCCATGAAATACATTTTTGATTATTATTTGAATCAATT-CCAATTACCTGAAGTCTTTCATCTATAATT
SARS-CoV-2 NC_045512.2 (16835) TTGAAAAAGGTGAC-TATGGTGATGCTGTTGTTTACCGAGGT---ACAACAACTTACAAATTAAATGTTGGTGATTATTTTGTGCTG-ACATCACAT
```

Section 245

```
                              23669      23680     23690     23700     23710     23720     23730     23740     23750          23765
                    (23669)
lambda NC_001416.1 (22891)    GGCATTGTATGTATTGGTTTATTGGAGTAGATGCTTGCTTTTCTGAGCCATAGCTCTGATATCCAAATGAAGCCATAGGCATTTGTTATTTTGGCTC
SARS-CoV-2 NC_045512.2 (16927) ACAGTAATGC---------CATTA-AGTGCAC-----CTACACTA-GTGCCACAAGAGCAC---TATGTTAGAATT----ACTGGCTTATACCCAAC
```

```
                                                                                                  Section 246
                              23766         23780     23790     23800     23810     23820     23830     23840     23850     23862
                      (23766)
     lambda NC_001416.1 (22988) TGTCAGCTGCATAACG-CCAAAAAATATATTTATCTGCTTGATCTTCAAATGTTGTATTGATTAAATCAATTGGAT---GGAATTGTTTATCATAAA
SARS-CoV-2 NC_045512.2 (17001) ACTCAATATCTCA--GATG-AGTTTTCTAGCAATGTTGCAAATTATCAA-------AAGGTTGGTATGCAA--AAGTATTCTACACTCC--AGGGAC
                                                                                                  Section 247
                              23863   23870     23880     23890     23900     23910     23920     23930     23940         23959
                      (23863)
     lambda NC_001416.1 (23081) AAATTAATGTTTGAATGTGATAACCGTCCTTTAAAAAAGTCGTTTCTGCAAGCTTGGCTGTATAGTC-AACTAAC----TCTTCTGTCGAAGTGATA
SARS-CoV-2 NC_045512.2 (17084) CACCTGGTACTGGTAAGAGTCATTTTGCTATTGGCCTAGCTCTCTACTA-CCCTTCTGCTCG-CATAGTGTATACAGCTTGCTCTCATGCCGC-TGT
                                                                                                  Section 248
                              23960     23970     23980     23990     24000     24010     24020     24030     24040         24056
                      (23960)
     lambda NC_001416.1 (23173) TTTTTAGGCTTATCTACCAGTTTTAGACGCTCTTTAATATCTTCAG---GAATTATTTTATTT--GTCATATTGTATCATGCTAAATG---ACAATTT
SARS-CoV-2 NC_045512.2 (17178) TGATGCACTATGTGAGAAGGCATTAAAATAT-TTGCCTATAGATAAATGTAGTAGAATTATACCTGCACGTGCTCGTGTAGAGTGTTTTGATAAATT
                                                                                                  Section 249
                              24057         24070     24080     24090     24100     24110     24120     24130     24140     24153
                      (24057)
     lambda NC_001416.1 (23262) GCTTATGGAGTAATCTTTTA-ATTTTA-AATAAGTTATTCTCCTGGCTTCATCAAATAAAGAGTCGAATGATGTTGGC-GAAATCACATCGTCACCC
SARS-CoV-2 NC_045512.2 (17274) CAAAGTGAATTCAACATTAGAACAGTAT-GTCTTTTGTACTGTAA-ATGCATTGCCTGAGAC---GACAGCA---GATATAGTTGTCTTTGATGAA-
                                                                                                  Section 250
                              24154 24160     24170     24180     24190     24200     24210     24220     24230     24240  24250
                      (24154)
     lambda NC_001416.1 (23356) ATTGGATTGTTTATTTGTATGCCAAGAGAGTTACAGCAGTTA---TACATTCTGCCATAGATTATAGCTAAGGCATGTAATAATTCGTAATCTTTTA
SARS-CoV-2 NC_045512.2 (17362) ATTTCAATGGCCACAAATTATGAT--TTGAGTGTTGTCAATGCCAGATTACGTGCTAAGCACTATGTGTACA-TTGGCGAC--CCTGCTCAATTACC
                                                                                                  Section 251
                              24251     24260     24270     24280     24290     24300     24310     24320     24330         24347
                      (24251)
     lambda NC_001416.1 (23450) GCGTATTAGCGACCCATCGTCTTTCTGAT-TTAATAATAGATGATTCAGTTAAATATGAAGGTAATTTCTTTTGTGCAAGTCTGACTAACTTTTTTA
SARS-CoV-2 NC_045512.2 (17454) TGCACCACGC---ACATTGC-TAACTAAGGGCACACTAGAACCAGAATATTTCA-ATTC-AGTGTGTAG--ACTTA------TG---AAAACTATAG
                                                                                                  Section 252
                              24348       24360     24370     24380     24390     24400     24410     24420     24430       24444
                      (24348)
     lambda NC_001416.1 (23546) TACCAATGTTTAACATACTTTCATTTGTAATAAAC-TCAATGTCATTTTCTTCAATGTAA--------GATGAAATA----AGAGTAGCCTTTGCCT
SARS-CoV-2 NC_045512.2 (17534) GTCCAGACATGTTCCTCGGAACTTGTCGGCGTTGTCCTGCTGAAATTGTTGACACTGTGAGTGCTTTGGTTTATGATAATAAGCTTAAAGCACATAA
```

Section 253

```
                                (24445) 24445 24450 24460 24470 24480 24490 24500 24510 24520 24530 24541
     lambda NC_001416.1 (23630) CGCTATACATTTCTAAATCG----CCTTGTTTTTCTATCGTATTGCGAGAATTTTTAGCCCAAGCCATTAATGGATCATTTTTCCATT---TTTCAA
SARS-CoV-2 NC_045512.2 (17631) AG-ACAAATCAGCTCAATGCTTTAAAATGTTTTATAAGGGTGT-TATCACGCATGATGTTTCATCTGCAATTAACAGGCCA--CAAATAGGCGTGGT
```

Section 254

```
                                (24542) 24542 24550 24560 24570 24580 24590 24600 24610 24620 24638
     lambda NC_001416.1 (23720) TAACATTATTGTTATACCAAATGTCATATCCTATAATCTGGTTTTTGTTTTTTTGAATAATAAATGTTACTGTTCTTGCGGTTTGGAGGAATTGATT
SARS-CoV-2 NC_045512.2 (17724) AAGAGAATTCCTTA------CACGT---------AACCC-TGCTTGGA--------GAAAAGCTGTCTTTATTTCACCTTATAATTCACAGAATGCT
```

Section 255

```
                                (24639) 24639 24650 24660 24670 24680 24690 24700 24710 24720 24735
     lambda NC_001416.1 (23817) CAAATTCAAGCGAAATAATTCAGGGT----CAAAATATGTATCAATGCAGCATTTGAGCAAGTGCGATAAA--TCTTTAA---GTCTTCTTTCCC--
SARS-CoV-2 NC_045512.2 (17797) GTAGCCTCAAAGATTTT----GGGACTACCAACTCAAACTGTTGATTCATCACAGGGCTCAGAATATGACTATGTCATATTCACTCAAACCACTGAA
```

Section 256

```
                                (24736) 24736 24750 24760 24770 24780 24790 24800 24810 24820 24832
     lambda NC_001416.1 (23903) -ATGGTTTTTTA-GTCATAAAACTCTCCATTTTGATAGGTTGCATGCTA---GATGCTGATATATTTTAGAGGTGATAAAATTAACTGCTTAACTGT
SARS-CoV-2 NC_045512.2 (17890) ACAGCTCACTCTTGTAATGTAAACA-----GATTTAATGTT-GCTATTACCAGAGCAAAA---------GTAGGCATACTTTGCAT-----AATGTC
```

Section 257

```
                                (24833) 24833 24840 24850 24860 24870 24880 24890 24900 24910 24929
     lambda NC_001416.1 (23995) CAATGTAATACAAGTTGTTTGATCTTTGCAATGATTCTTATCAGAAACCATATAGTAAATTAGTTACACAGGAAATT--TTTAATATTATTATTATC
SARS-CoV-2 NC_045512.2 (17967) TGATAGAGACCTTTATGACAAG--TTGCAATTTACAAGTCTTGA---AATTCCACGT--AGG---AATGTGGCAACTTTACAAGCTGAAAATGTAAC
```

Section 258

```
                                (24930) 24930 24940 24950 24960 24970 24980 24990 25000 25010 25026
     lambda NC_001416.1 (24090) ATTCATTATGTATTAAAATTAGAGTTGTGGCTTGGCTCTGCTAACACGTTGCTCATAGGAGATATGGTAGAGCCGCAG-----ACACGTCGTATGCA
SARS-CoV-2 NC_045512.2 (18054) AGGAC--TCTTTAAAGATTGTAGT-AAGGTAATCACTGGGTT---ACATCCTACACAGGC-AC---CTACACACCTC-AGTGTTGACACTAAATTCA
```

Section 259

```
                                (25027) 25027 25040 25050 25060 25070 25080 25090 25100 25110 25123
     lambda NC_001416.1 (24182) GGAACGTGCTGCGGCTGGCTGGTGAACTTCCGATAGTGCGGGTGTTGA--ATGATT-TCCAGTTGCTACCGATTTTACATATTTTTTGCATGAGAGA
SARS-CoV-2 NC_045512.2 (18140) AAACT-----GAAGGTTTATGT---GTTGACAT-ACCTGGCATACCTAAGGACATGACCTATAGAAGACTCATCTCTATGA--TGGGTTTTAAAATG
```

```
                                                                                              Section 260
                            (25124) 25124 25130     25140     25150     25160     25170     25180     25190     25200     25210 25220
       lambda NC_001416.1 (24276) ATTTGTACCACCTCCCACCGACCATCTATGACTGTACGCCACTGTCCCTAGGACTGCTATG-TGCCGGAGCGGACATTACAAACGTCCTTCTCGGTG
SARS-CoV-2 NC_045512.2 (18226) AATTA-TCAAGTTAATGGTTACCCTA----A---CAT---GTT-TATCACCCGCGAAGAAGCTATAAGACATGTACGTGCATGGATTGGCTTCGATG
                                                                                              Section 261
                            (25221) 25221    25230     25240     25250     25260     25270     25280     25290     25300        25317
       lambda NC_001416.1 (24372) CATGCCACT-----GTTGCCAATGACCT----GCCTAGGAATTGGTTAGCAAGTTACTACCGGATTTTGTAAAACAGCCCTCCTCATATAAAAAGT
SARS-CoV-2 NC_045512.2 (18311) TCGAGGGGTGTCATGCTACTAGAGAAGCTGTTGGTACCAATTT------ACCTTTACAGCTAGGTTTTTCTACAGG-TGTTAACC----TAGTTGCT
                                                                                              Section 262
                            (25318) 25318       25330     25340     25350     25360     25370     25380     25390     25400     25414
       lambda NC_001416.1 (24460) ATTCGTTCACTTCCGATAAGCGTCGTAATTTTCTATCTTTCATCATATTCTAGATCCCT--CTGAAAAAATCTTCCGAGTTTGCTAGGCACTGATAC
SARS-CoV-2 NC_045512.2 (18397) GT--ACCTACAGGTTATGTTGATACACCTAATAATA--CAGATTTTTCCAGAGTTAG--TGCTAAACCACCGCCTGGAGATCA------ATTTAAAC
                                                                                              Section 263
                            (25415) 25415 25420     25430     25440     25450     25460     25470     25480     25490     25500  25511
       lambda NC_001416.1 (24555) ATAAC-TCTTTTCCAATAATTGGGGAAGTCATTCAAATCTATAATAGGTTTCAGATTTGCTTCAATAAATTCTGACTGTAGCTG-CTGAAACGTTGC
SARS-CoV-2 NC_045512.2 (18482) ACCTCATACCACTTATGTACAAAGGACTTCCTTGGAAT-GTAGTGCGTATAA--AGATTGTAC---AAATGTTAAGTGACACACTTAAAAATCTCTC
                                                                                              Section 264
                            (25512) 25512   25520     25530     25540     25550     25560     25570     25580     25590        25608
       lambda NC_001416.1 (24650) ---GGTTGAACTATATTTCCTTATAA-CTTTTACGAAAGAGTTTCTTTG-AGTAATCACTTCACTCAAG---TGCTTCC-----CTGCCTCCAAACG
SARS-CoV-2 NC_045512.2 (18573) TGACAGAGTCGTATTTGT-CTTATGGGCACATGGCTTTGAGTTGACATCTATGAAGTATTTTGTGAAAATAGGAC--CTGAGCGCACCTGTTGTCTA
                                                                                              Section 265
                            (25609) 25609      25620     25630     25640     25650     25660     25670     25680     25690        25705
       lambda NC_001416.1 (24734) ATACCTGTTAGCAATATTTAATAGCTTGAAATGATGAAGAGCTCTGTGTTTGTCTTCCTGCCTCCAGTTCGCCGGGCATTCAACATAAAAACTGATA
SARS-CoV-2 NC_045512.2 (18667) TGTGATAGACGTGCCACATGCTTTTCCACTGCTTCAGACACTTATGCC--TGTTGGCAT-CATTCTATTGGATTTGATTACGTCTATAATCCGTTTA
                                                                                              Section 266
                            (25706) 25706         25720     25730     25740     25750     25760     25770     25780     25790  25802
       lambda NC_001416.1 (24831) GCACCCGGAGTTCCGGAAACGAAATTT-GCATATACCCATTGCTCACGAAAAAAAATGTCCTTGTCGATATAGGGATGAATCGCTTGGTGTACCTCA
SARS-CoV-2 NC_045512.2 (18761) TGATT--GATGTTCAACAATGGGGTTTTACAGGTAACCTA-CAAAGCAACCATGATCTGTATTGTCAAGTCCATGGTAATG---CACATGTAGCTAG
```

```
                                                                                                      Section 267
                                   (25803) 25803  25810          25820          25830          25840          25850          25860          25870          25880               25899
lambda NC_001416.1 (24927)         -TCTACTGCGAAAACTTGACCTTTCTCTCCCATATTGCAGTCGCGGCACGATGGAACTAAATTAATAGGCATC------ACCGAAAATTCAGG-ATA
SARS-CoV-2 NC_045512.2 (18852)     TTGTGATGCAATCATGACTAGG----TGTCTA-GCTGTCCACGAGTGCTTTGTTAAGCGTGTTGACTGGACTATTGAATATCCTATAATTGGTGATG
                                                                                                      Section 268
                                   (25900) 25900          25910          25920          25930          25940          25950          25960          25970          25980          25996
lambda NC_001416.1 (25016)         ATGTGCAATAGGAAGAAAATGATCT--ATATTTTTTGTCTGTCCTATATCACCACAAAATGGACATTTTTCACCTGATGAAACAAGCATGTCATCGT
SARS-CoV-2 NC_045512.2 (18944)     AACTGAAGATTAATGCGGCTTGTAGAAAGG-TTCAACACATGGTTGTTAAAGCTGCATTATTAGCAGACAAATTCCCAGTTCTT-CACGACATTGGT
                                                                                                      Section 269
                                   (25997) 25997             26010          26020          26030          26040          26050          26060          26070          26080          26093
lambda NC_001416.1 (25111)         AATAT----GTTCTAGCGGGTTTGTTTTTATCTCGGAGATTATTTTCATAAAGCTTTTCTAATTTAACCTTTGTCAGGTTACCAACTAC--TAAGGT
SARS-CoV-2 NC_045512.2 (19039)     AACCCTAAAGCTATTAAGTGTGTACCTCAAGCTGATGTAGAATGGAAGTTCTATGATGCACAGCCTTG--T---------AGTGACAAAGC-----T
                                                                                                      Section 270
                                   (26094) 26094  26100          26110          26120          26130          26140          26150          26160          26170          26180   26190
lambda NC_001416.1 (25202)         TGTAGGCTCAAGAGGGTGTGTCCTGTCGTAGGTAAATAACTGACCTGTCGAGCTTAATATTCTATATTGTTGTTCTTTCTGCAAAAAAGTGGGGAAG
SARS-CoV-2 NC_045512.2 (19120)     TATAAAATAGAAGAATTATTCTA--TTCTTATGCCACACA--TTCTGACAAATTCACAGATGGTGTATGCCTATTTTG----------GAATTGCAA
                                                                                                      Section 271
                                   (26191) 26191     26200          26210          26220          26230          26240          26250          26260          26270               26287
lambda NC_001416.1 (25299)         TGAGTAATGAAA---TTATTTCTAACATTTATCTGCATCATACCTTCCGAGCATTTATTAAGCATTTCGCTATAAGTTCTCGCTGGAAGAGGTAGTT
SARS-CoV-2 NC_045512.2 (19203)     TGTCGATAGATATCCTGCTAA---TTCCATTGTTTGTAGATT---TGACACTA-----------GAGTGCTATC-----TAACCTTAACTTGCCTGG
                                                                                                      Section 272
                                   (26288) 26288          26300          26310          26320          26330          26340          26350          26360          26370          26384
lambda NC_001416.1 (25393)         TTTTCATTGTACTTTACCTTCATCTCTGTTCAT--TATCATCGC-TTTTAAAACGGT---TCGACCTTCTAATCCTATCTGACCATTATAATTTTTT
SARS-CoV-2 NC_045512.2 (19278)     TTGTGAT-GGTGGCAGTTTGTATGTAA--ATAAACATGCATTCCACACACCAGCTTTTGATAAAAGTGCTTTTGTTAATTTAAAACAATTACCATT-
                                                                                                      Section 273
                                   (26385) 26385 26390          26400          26410          26420          26430          26440          26450          26460          26470     26481
lambda NC_001416.1 (25484)         AGAATGGTT-TCATAAGAAAGCTCTGAATCAACGGACTGC-GAT-AATAAGTGGTGGTATCCAGAATTTGTCACTTCAAGTAAAAACACCTCACGAG
SARS-CoV-2 NC_045512.2 (19371)     TTTCTATTACTCTGACAGT--CCATGTGA-GTCTCATGGAAAACAAGTAGTGTCAGATATAGATT-----ATGTACCACTAAAGTCTGC--TACGTG
```

```
                                                                                                   Section 274
                        (26482) 26482   26490     26500     26510     26520     26530     26540     26550     26560        26578
     lambda NC_001416.1 (25578) TTAAAACAC-CTAAGTTCTCACCGAATGTCTCAATATCCGGACGGATAATATTTATTGCTTCTCTTGACCG--TAGGACTT---TCCACATGCAGGA
 SARS-CoV-2 NC_045512.2 (19458) TATAACACGTTGCAATTTAGGTGGTGCTGTCTGTAGACATCATGCTAATGAGTACAGATTGTATCTCGATGCTTATAACATGATGATCTCAGCTGGC
                                                                                                   Section 275
                        (26579) 26579      26590     26600     26610     26620     26630     26640     26650     26660        26675
     lambda NC_001416.1 (25669) TTTTGGAACCTCTTGCAGTA----C-TACTGGGGAATGAGTTGCA---ATTATTGCTACACCATTGCGTGCATCGAG--TAAGTCGCTTAATGTTCG
 SARS-CoV-2 NC_045512.2 (19555) TTTAGCTTGTGGGTTTACAAACAATTTGAT-ACTTATAACCT-CTGGAACACTTTTACAAGACTTCAGAGTTTAGAAAATGTGGCTTTTAATGTTGT
                                                                                                   Section 276
                        (26676) 26676        26690     26700     26710     26720     26730     26740     26750     26760    26772
     lambda NC_001416.1 (25756) TAAAAAAGCAGA-----GAGCAAAGGTGGATGCAGATGAACCTCTGGTTCATCGAATAAAACTAATGACT-TTTCGCCAACGACATCTACTAATCTT
 SARS-CoV-2 NC_045512.2 (19650) AAATAAGGGACACTTTGATGGACAACAGGGTGAAGTACCAGTT-TCTATCATTA-ATAACACTGTTTACACAAAAGTTGATGGTGTTGATGTAGAAT
                                                                                                   Section 277
                        (26773) 26773  26780     26790     26800     26810     26820     26830     26840     26850           26869
     lambda NC_001416.1 (25847) GTGATAGTAAATAAAACAATTGCATGTCCAGAGCTCATTCGAAGCAGATATTTCTGGATATTGTCATAAAACAATTTAGTGAATTTATCATCGTCCA
 SARS-CoV-2 NC_045512.2 (19745) TGTTTGAA-AATAAAAC-AACATTACCTGTTAATGTAG--CATTTGA----GCTTTGGGCTAAGCG--CAACATTAAA-CCAGTACCAGAGG-T-GA
                                                                                                   Section 278
                        (26870) 26870     26880     26890     26900     26910     26920     26930     26940     26950        26966
     lambda NC_001416.1 (25944) CTTGAATCTGTGGTTCATTACGTCTTAACTCTTCATATTTAGAAATGAGGCTGATGAGTTCCATATTTGAAAAGTTTTCATCACTA-CTTAGTTTTT
 SARS-CoV-2 NC_045512.2 (19829) AAATACTCAATAA-TT-TGGGTGTGGACATTGCTGCTAATACTGTGATCTGGGACTACAAAAGAGATGCTCCAGCACATATATCTACTATTGGTGTT
                                                                                                   Section 279
                        (26967) 26967        26980     26990     27000     27010     27020     27030     27040     27050    27063
     lambda NC_001416.1 (26040) TGATAGCTTCAAGCCAGAGTTGTCTTTTTCTATCTAC--TCTCATACAACCAATAAATGC------TGAAATGAATTCTAAGCGGAGATC--GCCTA
 SARS-CoV-2 NC_045512.2 (19924) TGTTCTATGACTGACAT---AGCCAAGAAACCAACTGAAACGATTTGTGCACCACTCACTGTCTTTTTTGAT--GGTAGAGTTGATGGTCAAGTAGA
                                                                                                   Section 280
                        (27064) 27064 27070     27080     27090     27100     27110     27120     27130     27140     27150 27160
     lambda NC_001416.1 (26127) GTGAT------TTTAAACTATTGCTGGCAGCATTCTTGAGTCCAATATAAAAGTATTGTGTACCTTTTGCTGGGTCAGGTTGTTCTTTAGGAGGAGT
 SARS-CoV-2 NC_045512.2 (20016) CTTATTTAGAAATGCCCGTAATGGTGTTCTTATTACAGA---AGGTAGTGTTA-AAGGTTTACAA---CCATC-TGTAGGTCCCAAA---CAAGCTA
```

Section 281

```
                            (27161) 27161    27170      27180      27190      27200      27210      27220      27230      27240        27257
      lambda NC_001416.1 (26218) AA---AAGG--ATCAAATGCACTAAACGAAACTGAAACAAGCGATCGAA----AATAT-CCCTTTGGGATT--CTTGACTCGATAAGTCTATTATTT
SARS-CoV-2 NC_045512.2 (20102) GTCTTAATGGAGTCACATTAATTGGAGAAGCCGTAAAAACACAGTTCAATTATTATAAGAAAGT--TGATGGTGTT-GTCCAACAATTACCTGAAAC
```

Section 282

```
                            (27258) 27258        27270      27280      27290      27300      27310      27320      27330      27340      27354
      lambda NC_001416.1 (26303) TCAGAGAAAA-AATATTCATTGTTTTCTGGGTTGGTGATTGC-ACCAATCATTCCATTCAAAATTG------TTGTTTTACCACACCCATTCCGCCC
SARS-CoV-2 NC_045512.2 (20196) TTACTTTACTCAGAGTAGAAATTTA-CAAGAATTT-A-----AACCCAGGAGTCAAATGGAAATTGATTTCTTAGAATTAGCTATG-GATGA-----
```

Section 283

```
                            (27355) 27355 27360      27370      27380      27390      27400      27410      27420      27430      27440  27451
      lambda NC_001416.1 (26392) GATAAAAGCATGAATGTTCGTGCTGGGCATAGAATTAACCGTCACCT--CAAAAGGTAT-----A-----GTTAAATCACTGAATCCGGGAGCACTT
SARS-CoV-2 NC_045512.2 (20280) -----ATTCATTGAA-CGG---TATAAATTAGAAGGCTATGCCTTCGAACATATCGTTTATGGAGATTTTAGTCATAGTCAGT-TAGGTG-----GT
```

Section 284

```
                            (27452) 27452   27460      27470      27480      27490      27500      27510      27520      27530        27548
      lambda NC_001416.1 (26477) TTTCTATTAAATGAAAAGTGGA-AATCTGACAATTCTGGCAAACC--ATTTAACACACGTGCGAACTGTCCATGAATTTCTGAAAGAGTTACCCCTC
SARS-CoV-2 NC_045512.2 (20362) TTACATCTA-------CTGATTGGACTAGCTAAACGTTTTAAGGAATCACCTTTTGAAT-TAGAAGATTTTATTCCTATGGACAGTACAGT---TAA
```

Section 285

```
                            (27549) 27549      27560      27570      27580      27590      27600      27610      27620      27630        27645
      lambda NC_001416.1 (26571) TAAGTA-ATGAGGTGTTAAGGA-CGCTTTCAT-TTT----------CAATG--TCGGCTAATCGATTTGGCCATACTACTAAATCCTGAATAGCTTT
SARS-CoV-2 NC_045512.2 (20448) AAACTATTTCATA-ACAGATGCGCAAACAGGTTCATCTAAGTGTGTGTGTTCTGTTATTGATTTATTACTTGATGATTTTGTTGAAATA-ATAAAAT
```

Section 286

```
                            (27646) 27646        27660      27670      27680      27690      27700      27710      27720      27730    27742
      lambda NC_001416.1 (26653) AAGAAGGTTAT----GTTTAAAACCATCGCTTAATTTGCTGAGATTAACATAGTAGTCAATGCTTTCACCTAAGGAAAAAAACATTTCAGGGAG-TT
SARS-CoV-2 NC_045512.2 (20543) CCCAAGATTTATCTG---TAGTTTCTAAGGTTG--TCAAAGTGAC---TATTGACTATACAGAAATTTCATT-------TATGCTTTGGTGTA-AAG
```

Section 287

```
                            (27743) 27743  27750      27760      27770      27780      27790      27800      27810      27820          27839
      lambda NC_001416.1 (26745) GACTGAATTTTTTATCTATTAATGAATAAGTGCTTACTTC-TTCTTTTTGAC-CTACAAAACCAATTTTAACATTTCCGATATCGCATTTTT--C-A
SARS-CoV-2 NC_045512.2 (20624) ATGGCCATGT-----AGAAACAT--TTTACCCAAAATTACAAT-CTAGTCA-AGCGTGGCAACCGGGT----GTTGCT----ATGCCTA-ATCTTTA
```

Section 288

```
                        (27840) 27840     27850     27860     27870     27880     27890     27900     27910     27920          27936
lambda NC_001416.1      (26837) CCATGCTCATCAAAGACAGTAAGATAAAACATTGTAACAAA-GGAATAGTCATTCCAACCATCTGCTCGTAGGAATGCCTTATTTTTTTCTACTGCA
SARS-CoV-2 NC_045512.2  (20703) CAAAATGC---AAAGAATGCTATTAGAAAAGTGTGACCTTCAAAATTATGGTGATAGTGCAACA--TTACCTAAAGGC--ATAATGATGAAT-GTCG
```

Section 289

```
                        (27937) 27937        27950     27960     27970     27980     27990     28000     28010     28020        28033
lambda NC_001416.1      (26933) G--GAATATACCCGCCTCTTTCAATAACACTAAACTCCAACATATAGTAACCCTTAATTTTATTAAAATAACCGCAATTTATTTGGCGGCAACACAG
SARS-CoV-2 NC_045512.2  (20792) CAA-AATATACTCAACTGTGTCAATATTTAAACACATTAACATTAGCTGTACCCTATAATATGAGAGTTATACATTTTGGTGCTGGTTCTGATAAAG
```

Section 290

```
                        (28034) 28034 28040     28050     28060     28070     28080     28090     28100     28110     28120 28130
lambda NC_001416.1      (27028) GATCT---------CTCTTTTAAGTTACTCTCTATTACATACGTTTTCCATCTAAA-AATTAGTAGTATT-GAACTTAACGGGGC-ATCGTATTGTA
SARS-CoV-2 NC_045512.2  (20888) GAGTTGCACCAGGTACAGCTG---T-TTTAAGACAGTGGTTGCCTACG--GGTACGCTGCTTGTCGATTCAGATCTTAATGACTTTGTCTCTGATGC
```

Section 291

```
                        (28131) 28131    28140     28150     28160     28170     28180     28190     28200     28210          28227
lambda NC_001416.1      (27113) GTTTTCCATATTTAGCTTTCTGCTTCCTTTTGGATAACCCACTGTTATTCATGTTGCATGGTGCA-----CTGTTTATACCAACGATATAGTCTATT
SARS-CoV-2 NC_045512.2  (20979) AGATTCAACTTTGATTGGTGATTGTGCAACTGTACATACAGCTAATAAATGGGATCTCATTATTAGTGATATGTACGACCCTAAGACTAAAAATG--
```

Section 292

```
                        (28228) 28228       28240     28250     28260     28270     28280     28290     28300     28310      28324
lambda NC_001416.1      (27205) AATGCATATATAGTATCGCCGAACGATTAGCTCTTCAG-GCTTCTGAAGAAGCGTTTCAAGTACTA----ATAAGCCGATAGATAGCCACGGACTTC
SARS-CoV-2 NC_045512.2  (21074) -TTACAAAAGAAA-ATGACTCTA--AAGAGGGTTTTTTCACTTACATTTGTGGGTTTATACAACAAAAGCTAGCTCTTGGAGGT-TCCGTGGCTATA
```

Section 293

```
                        (28325) 28325 28330     28340     28350     28360     28370     28380     28390     28400     28410  28421
lambda NC_001416.1      (27297) GTAGCCATTTTTCATAAGTGTTAACTTCCGC-TCCTCGCTCATAACAGAC-ATTCACTACAGTTATGGCGGAAAGGTATGCATGCTGGGTGTGGGGA
SARS-CoV-2 NC_045512.2  (21166) AAGATAACAGAACATTCTTG-GAATGCTGATCTTTATAAGCTCATGGGACACTTCGCAT--GGTGGACAGCCTTTGTTACTAATGTGAA--------
```

Section 294

```
                        (28422) 28422   28430     28440     28450     28460     28470     28480     28490     28500          28518
lambda NC_001416.1      (27392) AGTCGTGAAAG----AAAAGAAGTCAGCTGCGTCGTTTGACATCACTGCTATCTTCTTACTGGTTATGCAGGTCGTAGTGGGTGGCACACAAAGCTT
SARS-CoV-2 NC_045512.2  (21252) -TGCGTCATCATCTGAAGCATTTTTA-ATTGG----------ATGTAATTATCTTGGCAAACCACGCGAACAAATAGATGGTT---ATGT----CAT
```

```
                                                                                                                    Section 295
                           (28519) 28519      28530      28540      28550      28560      28570      28580      28590      28600      28615
       lambda NC_001416.1 (27485) TGCACTG-GATTGCGAGGCTTTGTGCTTCTCTGGAGTGCGACAGGTTTGATGACAAAAAATTAGCGCAAGAAGACAAAAATCACCTTGCGCTAATGC
SARS-CoV-2 NC_045512.2 (21330) GCATGC-AAATTACATATTTTGGAGGAA-----TACAAATCCAATTCAGTTG-TCTTCCT-ATTCT-TTATTTGACATGAGTAAATTTCCCCTTAAA
                                                                                                                    Section 296
                           (28616) 28616         28630      28640      28650      28660      28670      28680      28690      28700    28712
       lambda NC_001416.1 (27581) TCTGTTACAGGTCACTAATACCATCTAAGTAGTTGATTCATAGTGA-CTGCATATGTTGTGTTTTACAGTATTATGTAG-TCTGTTTTTTATGCAAA
SARS-CoV-2 NC_045512.2 (21418) TTAAGGG--GTACTGCTGTTATGTCTTTAAA--AGAAGGTCAAATCAATGATATG-------------ATTTTATCTCTTCTTAGTAAAGGTAGA-C
                                                                                                                    Section 297
                           (28713) 28713  28720      28730      28740      28750      28760      28770      28780      28790          28809
       lambda NC_001416.1 (27676) ATCTAATTTAATATATTGATATTTATATCATTTTACGTTTCTCGTTCAGCTTTTTTATACTAAGTTGGCATTATAAAAAGCATTGCTTATCAATTT
SARS-CoV-2 NC_045512.2 (21497) TTATAATTAGAGA-----AAACAACAGAGTTGTTATTTCTAGTGATGTTCTTGTTAACAACT--AAACGAACAATGT------------TTGTTTTT
                                                                                                                    Section 298
                           (28810) 28810      28820      28830      28840      28850      28860      28870      28880      28890      28906
       lambda NC_001416.1 (27773) GTTGCAACGAACAGGTCACTATCAGTCAAAATAAAATCATTATTTGATTTCAATTTTGTCCCACTCCCTGCC-TCTGTCATCACGATACTGTGATGC
SARS-CoV-2 NC_045512.2 (21575) CTT-------------GTTTTATTGC--CACTAGTCTCTAGTCAGTGT---GTTAATCTTACA-----ACCAGAACTCAATTACCCC-CTGCATACA
                                                                                                                    Section 299
                           (28907) 28907         28920      28930      28940      28950      28960      28970      28980      28990    29003
       lambda NC_001416.1 (27869) CATGGTGTCCGACTTATGCCCGAGAAGATGTTGAGCAA---ACTTATC--GCTTAT-CTGCTTCTCATAGAGTCTTGCAGACAAACTGCGCAACTCG
SARS-CoV-2 NC_045512.2 (21648) C------TAATTCTTT-----CACACGT----------GGTGTTTATTACCCTGACAAAGTTTTCAG-ATCCT---CAGTTTTACATTC-------A
                                                                                                                    Section 300
                           (29004) 29004 29010      29020      29030      29040      29050      29060      29070      29080      29090 29100
       lambda NC_001416.1 (27960) TGAAAGGTAGGCGGATCCCCTTCG-AAGGAAAGACCTGATG-CTTTTCG---------TGCGCGCATAAAATACCT-TGATACTGTGCCGGATGAAA
SARS-CoV-2 NC_045512.2 (21713) ACTCA----GGACTTGTTCT----TA-----------CCTTTCTTTTCCAATGTTACTTGGTTCCATGCTATACATGTC-TCTGGACCAATGGTAC
                                                                                                                    Section 301
                           (29101) 29101    29110      29120      29130      29140      29150      29160      29170      29180          29197
       lambda NC_001416.1 (28045) GCGGTTCGCGACGAGTAGATGCAATTATGGTTTCTCCGCCAAGAATCTCTTTGC---ATTTATCAAGTGTTTCCTTCATTGATATTCCGA-GAGCAT
SARS-CoV-2 NC_045512.2 (21790) TAAGAG------GTTT-------GATAACCC-TGTCCTACCATTTAATGAT---GGTGTTTATTTTGC-TTCCACTGAG----AAGTCTAACATAAT
```

Section 302

```
                        (29198) 29198       29210     29220     29230     29240     29250     29260     29270     29280      29294
      lambda NC_001416.1 (28138) CAATATGCAATGCTGTTGGGATGGCAATTTTTACGCCTGTTTTGCTTTGCTCGACATAAAGATATCCATCTACGATATC-AGAC-CACTTCATTTCG
SARS-CoV-2 NC_045512.2 (21865) -AAGAGGCTGGATTTTTGGTACTACTT-----------------------TAGATTCGAAGACC-CAGT---CCCTACTTATTGTTAATAA--CGCT
```

Section 303

```
                        (29295) 29295 29300     29310     29320     29330     29340     29350     29360     29370     29380      29391
      lambda NC_001416.1 (28233) CATAAATCACCAACTCGTTGCCCGGTAACAACAG--CCAGTTCCATTGCAAGTCTGAGCCAACATGGTGATGATTCTGCTGCTTGATAAATTTTCAG
SARS-CoV-2 NC_045512.2 (21932) -ACTAATGTTGTTATTAAAGTCTGTGAATTTCAATTTT-GTAATGATCCAT-----------TTTTG--G--------GTGTTTAT-TACCACAAAA
```

Section 304

```
                        (29392) 29392   29400     29410     29420     29430     29440     29450     29460     29470            29488
      lambda NC_001416.1 (28328) GTATTCGTCAGCCGTAAGTCTTG-ATCTCCTTACCTCTGATTTTGCTGCGCG-AGTGGC--AGCGACATGGTTTGTTGTTATATGGCCTTCAGCTAT
SARS-CoV-2 NC_045512.2 (22005) ACAACAA-AAGTTGGA----TGGAAAGTGAGTTCAGAGTTTAT-TCTAGTGCGAATAATTGCACTTT--TGAATATGTCTCTCA--GCCTTTCTTA
```

Section 305

```
                        (29489) 29489      29500     29510     29520     29530     29540     29550     29560     29570          29585
      lambda NC_001416.1 (28421) TGCCTCTCGGAATGCATCG---CTCAGT-GTTGATCTGATTAACTTGGC--TGACGCCGCCTTGCCCTCGTCTA-TGTATCCATTGAGCATT-GCCG
SARS-CoV-2 NC_045512.2 (22092) TGGACCTTGAAGGAAAACAGGGTAATTTC---AAAAATCTTAGGGAATTTGTGTT-TAAGAATATTGATGGTTATTTTAAAATATATTCTAAGCACA
```

Section 306

```
                        (29586) 29586         29600     29610     29620     29630     29640     29650     29660     29670      29682
      lambda NC_001416.1 (28510) CAATTTCTTTTGTGGTGATGTCTTCAAGTGGAGCATCAGGCAGACCCCTCCTTATTGCTTTAATTTTGCTCATGTAATTTATGAGTGTCTTCTGCTT
SARS-CoV-2 NC_045512.2 (22185) CGCC---TATTAA--TT-TAGTGCGTGATC---TCCCTCAGGGTTTTTCGGCTTTAGAACCATTGGTAGATTT-GCC-AATAGGTATTAACATCACT
```

Section 307

```
                        (29683) 29683  29690     29700     29710     29720     29730     29740     29750     29760            29779
      lambda NC_001416.1 (28607) GATTCCTCTGCTGGCCAGGATTTTTTCGTAGCGATCAAGCCATGAATGTAACGTAACGGAATTATCACTGTTGATTCTCGCTGTCAGAGGCTTGTGT
SARS-CoV-2 NC_045512.2 (22271) AGGTTTCAAACTTTACTTG-CTTTACAT--AGAAGTT--ATTTGACTCCTGGTGATTCTTCTTCAGGTTGGACAGC-TGG-TGCTGCA--GCTTATT
```

Section 308

```
                        (29780) 29780     29790     29800     29810     29820     29830     29840     29850     29860          29876
      lambda NC_001416.1 (28704) TTGTGTCCTGAAAATAACTCAATG-TTGG--CCTGTATAGCTTCAGTGATTGCGATTCGC-CTGTCTCTGCCTAATCCAAACTCTTTACCCGTCCTT
SARS-CoV-2 NC_045512.2 (22359) ATGT-GGGTTATCTTCAACCTAGGACTTTTCTATTAAAATAT---A---------ATGAAAATGGAACCATTACAGATGCT-GT-AGACTGTGC---
```

Section 309

```
(29877) 29877        29890     29900     29910     29920     29930     29940     29950     29960     29973
lambda NC_001416.1 (28797) GGGTCCCTGTAGCAGTAATATCCATT--GTTTCTTATATAAGGTTAGG----G-GGTAAATCCCGGC-GCTCATGACTTCGC-CTTCTTCCCATTT
SARS-CoV-2 NC_045512.2 (22438) -----------ACTTGACCCTCTCT-CAGAAACAAAGTGTACGTTGAAATCCTTCACTGTAGAAA---AAGGA---ATCTATCAAACT-------T-
```

Section 310

```
(29974) 29974 29980     29990     30000     30010     30020     30030     30040     30050     30060 30070
lambda NC_001416.1 (28885) CTGATCCTCTTCAAAGGCCACCTGTTACTGGTCGATTTAAGTCAACCTTTACCGCTGATTCGTGGAACAGATACTCTCTTCCA-TCCTTAACCGGA
SARS-CoV-2 NC_045512.2 (22509) ----CTAACTTTAGAGTCCAACCAA-CAGAATCTATTGTTA------GATTTCCTAAT--ATTACAAACT--TGTGCCCTTTTGG--------TGAA
```

Section 311

```
(30071) 30071    30080     30090     30100     30110     30120     30130     30140     30150          30167
lambda NC_001416.1 (28981) GGTGGGAATATCCTGCATTCCCGAACCCATCGACGAACT--GTTTCA-AGGCTTCTTGG--ACGTCGCTGGCGTGCGTTCCACTCCTGAAGTGTCAA
SARS-CoV-2 NC_045512.2 (22583) GTTTTTAACGCCACCAGATTTGCAT-CTGTT--TATGCTTGGAACAGGAAGAGAATCAGCAACTGTGTTGCTGAT-TATTCTGTCCTATATAATTC-
```

Section 312

```
(30168) 30168       30180     30190     30200     30210     30220     30230     30240     30250         30264
lambda NC_001416.1 (29073) GTACATCGCAAAGTCTCCGCAATTACACGCAAGAAAAAACCGCCATCAGGCGGCTTGGTGTTCTTTCAGTTCTTCAATTCGAATATTGGTTACGTCT
SARS-CoV-2 NC_045512.2 (22675) --CGCATCATTTTCCACTTTTA--AGT-GTTATGGA--GTGTCT-CCTACTAAATTAAAT-------GAT------CTCTGCTTTACTAATGTC-TA
```

Section 313

```
(30265) 30265 30270     30280     30290     30300     30310     30320     30330     30340     30350   30361
lambda NC_001416.1 (29170) GCATGTGCTATCTGCGCCCATATCATCCAGTGGTCGTAGCAGTCGTTGATGTTCTCCGCTTCGAT---AACTCTGTTGAATGGCTCTCCATTCCATT
SARS-CoV-2 NC_045512.2 (22750) TGCAGATTCATTTGTAATTAGAGGTGAT-GAAGTCAGACAAATCGCTCCAGGGCAAACTGG-AAAGATTGCTGATTATAATTATAAA---TTACCAG
```

Section 314

```
(30362) 30362    30370     30380     30390     30400     30410     30420     30430     30440              30458
lambda NC_001416.1 (29264) CTCCTGTGACTCGGAAGTGCATTTATCATCTCCATAAAACAAAACCCGCCGTAGCGAGTTCAGATAAAATAAATCCCCGCGAGTGCGAGGATTGTTA
SARS-CoV-2 NC_045512.2 (22842) ATGATTTTA---CAGGCTGCG--------TTATA--GCTTGGAATTCTAACAATCTTG---------------ATTCTAAGGTTGGTGGTAATTATA
```

Section 315

```
(30459) 30459       30470     30480     30490     30500     30510     30520     30530     30540         30555
lambda NC_001416.1 (29361) TGTAATATTGGGTTTAATCATCTATATGTTTTGTACAGAGAGGGCAAGTATCGTTTCCACCGTACTCGTGATAATAATTTTGCACGGTATCAGTCA-
SARS-CoV-2 NC_045512.2 (22911) ------ATTACCTGTATA----GATTGT-----TT-AGGAAGTCTAATCTCAAACCTTTTGAGAGAGAT-------ATTTCAACTGAAATCTATCAG
```

Section 316

```
                         (30556) 30556         30570     30580     30590     30600     30610     30620     30630     30640      30652
      lambda NC_001416.1 (29457) TTTCTCGCACATTGCAGAATGGGGATTTGTCTTCATTAGACTTATAAACCTTCATG---GAATATTTGTATGCCGACTCTATATCTATACCTTCATC
SARS-CoV-2 NC_045512.2 (22985) GCCGGTAGCACACCTTGTA--AT-G-GTGTTGAAGGTTTTAATTGTTACTTTCCTTTACAATCATATGGTTT---CCAACCCACTAATGGTGTTGGT
```

Section 317

```
                         (30653) 30653  30660     30670     30680     30690     30700     30710     30720     30730             30749
      lambda NC_001416.1 (29551) TACATAAACACCTTCGTGATGTCTGCATGGAGACAAGACACCGGATCTGCACAACATTGATAACGCCCAATCTTTTTGCTCAGACTCTAACTCATTG
SARS-CoV-2 NC_045512.2 (23075) TA---CCAACCATACAGAGTAGTAGTACTT-TCTTT-TGAACTTCTACATGCACCA--GCAACTGTTTGTGGACCTAA-AAAGTC--TACTAATTTG
```

Section 318

```
                         (30750) 30750     30760     30770     30780     30790     30800     30810     30820     30830           30846
      lambda NC_001416.1 (29648) ATACTCATTTATAAACTCCTTGCAATGTATGTCGTTTCAGCTAAACGGTATCAGCAATGTTTATGTAAAGAAACAGTAAGATAATACTCAACCCGAT
SARS-CoV-2 NC_045512.2 (23162) G------TTAAAAACA-----AATGTG---------TCAATTTCAACTTCAATGG--TTTAACAGGCACAGGT-GTTCTTACTGAG-TCTAACAAAA
```

Section 319

```
                         (30847) 30847        30860     30870     30880     30890     30900     30910     30920     30930        30943
      lambda NC_001416.1 (29745) GTTTGAGTACG--GTCATCATCTGACACTACAGACT--C-TGGCATCGCTGTGAAGACGACGCGAA---ATTCAGCATTT--TCA-CAAGCGTTATC
SARS-CoV-2 NC_045512.2 (23235) AGTT--TCTGCCTTTC--CAACAATTTG-GCAGAGACATTGCTGACACTACTGATGCTGTCCGTGATCCACAGACACTTGAGATTCTTGACATTAC-
```

Section 320

```
                         (30944) 30944 30950     30960     30970     30980     30990     31000     31010     31020     31030  31040
      lambda NC_001416.1 (29831) TTTTACAAAACCGATCTCACTCTCCTTTGATGCGAATGCCAGCGTC--AGACATCATATGCAGATACTCACCTGCATCCTGAACCCATTGACCTCCA
SARS-CoV-2 NC_045512.2 (23326) ----ACCATGTTCTTTTGGTGGTGTCAGTGTTATAACACCAGGAACAAATACTTCTA--ACCAG--GTTGC-TGTTC--TTT--AT-----C-AGGA
```

Section 321

```
                         (31041) 31041    31050     31060     31070     31080     31090     31100     31110     31120             31137
      lambda NC_001416.1 (29926) ACCCCGTAATAGC----G---ATG--CGTAATGATGTCGATAGTTACTAACGGGTCTTGTTCGATTAACTGCCGCAGAAACTCTTCCAGGTCACCAG
SARS-CoV-2 NC_045512.2 (23404) TGTTAACTGCAC-AGAAGTCCCTGTTGCTATTCATGCAGATCAACTTACTCCTACTTGG--CGTGTTTATTCTACAGGTTCTAATGTTTTTCAAACA
```

Section 322

```
                         (31138) 31138       31150     31160     31170     31180     31190     31200     31210     31220        31234
      lambda NC_001416.1 (30014) TGCAGTGCTTGATAACAGGAGTCTTCC--CAGGATGGCGAACAACAAGAAACTGGTTTCCGTCTTCACGGACTTCGTTGCTTT-CCAGTTTAGCAAT
SARS-CoV-2 NC_045512.2 (23498) CGTGCAGGCTGTT--TAA------TAGGGGCTGAACA-TGTCAAC----AACTCATATG-A-GTGTGACATACCCATTGGTG-CAGGTATATGCGCT
```

```
                                                                                                       Section 323
                              31235 31240     31250     31260     31270     31280     31290     31300     31310     31320   31331
                    (31235)
lambda NC_001416.1 (30108)    -ACGCTTACTCCCATCCGAGATAACACCTTCGTAATACTCACGCTGCTCGTTGAGTTTTGATTTTGCTGTTTCAAGCTCAACACGCAGTTTCCCTAC
SARS-CoV-2 NC_045512.2 (23579) A---GTTATCAGACTCAGACTAATTCTCCTCGGCGGGCACGTAG-----TGTAGCTAGTCAATCCATC-----ATTGCCTACACTATGTCACT--TG
                                                                                                       Section 324
                              31332   31340     31350     31360     31370     31380     31390     31400     31410           31428
                    (31332)
lambda NC_001416.1 (30204)    TGTTAGCGCAATATCCTCGTTCTCCTGGTCGCGGCGTTTGATGTATTGCTGGTTTCTTTCCCGTTCATCCAGCAGTTCCAGCACAATCGATGGTGTT
SARS-CoV-2 NC_045512.2 (23661) GTGCAG------AAAATTCAGTTGCT-TACTCTAATAACTCTATTGCCATACCC-----ACAAATTT--TACTATTAG----TGTTACCACAGAAAT
                                                                                                       Section 325
                              31429      31440     31450     31460     31470     31480     31490     31500     31510      31525
                    (31429)
lambda NC_001416.1 (30301)    ACCAATTCATGGAAAAGGTCTGCGTCAAATCCCCAGTCG----TCATGCATTGCCTGCTCTGCCGCTTCACGCAGTGCCTGAGAGTTAATTTCGCTC
SARS-CoV-2 NC_045512.2 (23740) TCT-ACCAGTGTCTATGACC-AAGACATCAGTAGA-TTGTACAATGTACATTTGTGGTGATTCAACT---GAATGCAGCAATCTTT-TG-TTGC---
                                                                                                       Section 326
                              31526         31540     31550     31560     31570     31580     31590     31600     31610    31622
                    (31526)
lambda NC_001416.1 (30394)    ACTTCGAACCTCTCTGTTTAC---TGATAAGTTCCAGATCCTCCTGGCAACTTGCACAAGTCCGACAACCCTGAACGACCAGGCGTCTTCGTTCATC
SARS-CoV-2 NC_045512.2 (23826) AAT------ATGGCAGTTTTTGTAC--ACAATTAAACCGTGCTTTA---ACTGGAATAG-CTGTTGAACAAG--ACAAAAACACCCAAGAAGTTT--
                                                                                                       Section 327
                              31623  31630     31640     31650     31660     31670     31680     31690     31700           31719
                    (31623)
lambda NC_001416.1 (30488)    TATCGGATCGCCACACTCACAACAATGAGTG----GCAGATATAGCC--TGGTGGTTCAGGCGGCGCATTTTTATTGCT-GTGTTGCGCTGTAATTC
SARS-CoV-2 NC_045512.2 (23907) TTGCACAAGTCAAA---CAAATTTACAAAACACCACCAATTAAAGATTTTGGTGGTTTTAATTTTTCACAAATATTACCAGATCCATCA--AAACCA
                                                                                                       Section 328
                              31720     31730     31740     31750     31760     31770     31780     31790     31800         31816
                    (31720)
lambda NC_001416.1 (30578)    TTCTATTTCTGATGCTGAATCAATGATGTCTGCCATCTTTCATTAATCCC---TGAACTGTTGGTTAATACGCTTGAGGGTGAATGCGAATAATAAA
SARS-CoV-2 NC_045512.2 (23999) AGCAAGAGGTCAT------TTATTGAAGATCTACTTTTCAACAAAGTGACACTTGCA---------G--ATGCTGGCTTCAT-CAAACAATATGGTG
                                                                                                       Section 329
                              31817        31830     31840     31850     31860     31870     31880     31890     31900     31913
                    (31817)
lambda NC_001416.1 (30672)    AAAGGAGCCTGTAGC-TCCCTGA-TGATTTTGCTTTTC-ATGTTCATCGT--------TCCTTAAAGACGCCGTTTAACATGCCGATTGCCAGGCTT
SARS-CoV-2 NC_045512.2 (24078) -----ATTGCCTTGGTGATATTGCTGCTAGAGACCTCATTTGTGCACAAAAGTTTAACGGCCTTA----CTGTTTGCCACCTTTGCTCACAGA-TG
```

```
                                                                                                     Section 330
                           (31914) 31914 31920          31930          31940          31950          31960          31970          31980          31990          32000    32010
        lambda NC_001416.1 (30758) AAATGAGTCGGTGTGAATCCCATCAGCGTTACCGTTTCGCGGTGCTTCTTCAGTACGCTACGGCAAATGTCATCGACGTTTTTATCCGGAAACTGCT
   SARS-CoV-2 NC_045512.2 (24165) AAATGATT--G--------CTCAATACACT--------T-CTGCACTGTT---AGC-GGGTACAATCACTTCTGGTTGGACCTTTG--GTGCAG----G
                                                                                                     Section 331
                           (32011) 32011        32020          32030          32040          32050          32060          32070          32080          32090           32107
        lambda NC_001416.1 (30855) GTCTGGCTTTTTTTGATTTCAGAATTAGCCTGACGGGCAATGCTGCGAAGGGCGTTTTCCTGCTGAGGTGTCATTGAACAAGTCCCATGTC-GGCAA
   SARS-CoV-2 NC_045512.2 (24235) TGCTGCATTA--CAAATACCATTTGCTATGCAAATGGCTTATAGGTTTAATGGTATTGGAGTTACACAGAATGTTCTCTATGAGAACCAAAAATTGA
                                                                                                     Section 332
                           (32108) 32108           32120          32130          32140          32150          32160          32170          32180          32190           32204
        lambda NC_001416.1 (30951) GCATAAGCACAC-AGAATATG-AAGCCCGCTGCCAGAAAAATGCATTCCGTGGTTGTCATACCTGGT-TTCTCTCATCTGCTTCTGCTTTCGCCACC
   SARS-CoV-2 NC_045512.2 (24330) TTGCCAACCAATTTAATAGTGCTATTGGCAAAATTCAAGACTCACTTTC--TTCCACAGCAAGTGCACTTGGAAAACTTCAAGATGT--GGTCAACC
                                                                                                     Section 333
                           (32205) 32205 32210          32220          32230          32240          32250          32260          32270          32280          32290    32301
        lambda NC_001416.1 (31045) ATCATTTCCAG-CTTTTGTGAAAGGGATGCGGCTAACGTA--TGAAA-TTCTTCGTCTGTTTCTACTGGTATTGGCACAAACCTGATTCCAATTTG-
   SARS-CoV-2 NC_045512.2 (24423) AAAATGCACAAGCTTTAAACACGCTTGTTAAACAACTTAGCTCCAATTTTGGTGCAATTTCA---AGTGTTTTAAATGATATCCTTTCACGTCTTGA
                                                                                                     Section 334
                           (32302) 32302     32310          32320          32330          32340          32350          32360          32370          32380                32398
        lambda NC_001416.1 (31137) -AGCAAGGCTATGT---GCCATCTCGAT--ACTCGTTCTTAA--CTCAACAGAAG--------ATGCTTTGTGCATACAG-------CCCCTCGTTT
   SARS-CoV-2 NC_045512.2 (24517) CAAAGTTGAGGCTGAAGTGCAAATTGATAGGTTG-ATCACAGGCAGACTTCAAAGTTTGCAGACATATGTGACTCAACAATTAATTAGAGCTGCAGA
                                                                                                     Section 335
                           (32399) 32399         32410          32420          32430          32440          32450          32460          32470          32480           32495
        lambda NC_001416.1 (31211) ATTATTTATCTCCTCAGCCAGCCGCTGTGCTTTCA-----GTGGATTTCGGATAACAGAAAGGCCGGGAAATACCCAGCCTCGCTTTGTAACGGAGT
   SARS-CoV-2 NC_045512.2 (24613) AATCA--GAGCTTCT-GCTAATCTTGCTGCTACTAAAATGTCAGAGTGTGTACTTGGACAATCAAAAAGAG---------TTGATTTTTGTGGAAAG
                                                                                                     Section 336
                           (32496) 32496             32510          32520          32530          32540          32550          32560          32570          32580    32592
        lambda NC_001416.1 (31303) AGACGAAAGTGATTGCGCCTACCCGGATATTATCGTG----AGGATGCGTCATCGCCATTGCTCCCCAAATACAAAACCAATTTCAGCCAGTGCCTC
   SARS-CoV-2 NC_045512.2 (24698) G-GC--TATCATCTTATGTCCT------TCCCTCAGTCAGCACCTCA--TGGTGTAGTCTTCTTGCATGT-GACTTATGTCCCTGCACAAGAA--AA
```

Section 337

```
                              32593  32600     32610     32620     32630     32640     32650     32660     32670             32689
(32593)
lambda NC_001416.1 (31396)    GTCCATTTTTTCGATGAACTCCGGCACGATCTCGTCAAAACT---CGCCATGTACTTTTCA--TCC-----CGCTCAATCACGACA-TAATGCAGGC
SARS-CoV-2 NC_045512.2 (24781) -GAACTTCACAACT--GCTCCTGCCATT-TGTCATGATGGAAAAGCACACTTTCCTCGTGAAGGTGTCTTTGTTTCAAATGGCACAC-ACTG--GTT
```

Section 338

```
                              32690     32700     32710     32720     32730     32740     32750     32760     32770          32786
(32690)
lambda NC_001416.1 (31482)    CTTCACGCTTCATACGCGGGTCATAGTTGGC--AAAGTACCAGGCATTTTTTCGCGTCACCCACATGCTGTACTGCACCTGGGCCATGTAAGCTGAC
SARS-CoV-2 NC_045512.2 (24871) TGTAACACAA-AGGAATTTTTATGAACCACAAATCATTACTACAGACAACACATTTGTGTCTGGTA-----ACTGTGATGTTGTAA-----TAGGAA
```

Section 339

```
                              32787        32800     32810     32820     32830     32840     32850     32860     32870        32883
(32787)
lambda NC_001416.1 (31577)    TTTATGGCCTCGAAACCACCGAGCCGGAACTTCATGAAATCCCGGGAGGTAAACGGGCATTTC--AGTTCAAGGCCGTTGCC---GTCACTGCATAA
SARS-CoV-2 NC_045512.2 (24957) TTGTCAACAACAC-AGTTTATGATCC---TTTGCAACCTGAATTAGAC-------TCATTCAAGGAGG---AGTTAGATAAATATTTTAAGAATCAT
```

Section 340

```
                              32884 32890     32900     32910     32920     32930     32940     32950     32960     32970 32980
(32884)
lambda NC_001416.1 (31669)    ACCATCGGGAGAGCAGGCGGTACGCATACT--TTCGTCGCGATAGATGATCGGGGATTCAGTAACAT-TCACGC-CGGAAGTGAATTCAAACAGGGT
SARS-CoV-2 NC_045512.2 (25040) -ACATCACCAGA-TGTTGATTTAGGTGACATCTCTGGCATTAATGCTTCAGTTGTAAACATTCAAAAAGAAATTGACCGCCTCAATGAGGTTGCCAA
```

Section 341

```
                              32981    32990     33000     33010     33020     33030     33040     33050     33060          33077
(32981)
lambda NC_001416.1 (31762)    TCTGG-CGTCGTTCTCGTACTGTTTTCCCCAGGCCAGTGCTTTAGCGTTAACTTCCG-GAGCCACACCGGTGCAAACCTCAGCA-AGCAGGGTGTGG
SARS-CoV-2 NC_045512.2 (25135) ---GAAT--TTAAATGAATCTCTCATC-GATCTCCAAGAACTTGGAAAGTATGAGCAGTATATAAAATGG--CCATGGTACATTTGGCTAGGTTTTA
```

Section 342

```
                              33078       33090     33100     33110     33120     33130     33140     33150     33160        33174
(33078)
lambda NC_001416.1 (31856)    AAGTAGGAC--ATTTTCATGT-CAGGCCACTTCTTTCCGGAGCGGGGTTTTGCTATCACGTTGTGAACTTCTGAAGCGGTGATGACGCCGAGCCGTA
SARS-CoV-2 NC_045512.2 (25224) TAGCTGGCTTGATTGCCATAGTAAT-GGTGACAATTATGCTTT--------GCTGTATGACCAGTTGCTGTAG------TTGTCTCAAGGGCTGTTG
```

Section 343

```
                              33175 33180     33190     33200     33210     33220     33230     33240     33250     33260    33271
(33175)
lambda NC_001416.1 (31950)    ATTTGTGCCAC-GCATCATCCCCCTGTTCGACAGCTCTCACATCGATCCCGGTACGCTGCAGGATAATGTCCGG-TGTCATG--CTGCCACCTTCTG
SARS-CoV-2 NC_045512.2 (25306) TTCT-TGTGGATCCT---GCTGCAAATTTGATGAAGACGACT-----CTGAG---CCAGT-----GCTCAAAGGAGTCAAATTACATTACACATAAA
```

```
                                                                                         Section 344
                      (33272) 33272   33280        33290        33300        33310        33320        33330        33340        33350           33368
      lambda NC_001416.1 (32043) CTCTGCGGCTTTCTGTTTCAGGA-ATCCAAGAGCTTTTACTGCTTCGGCCTGTGTCAGTTCTGACGATGCACGAATGTCGCGGCGAAATATCTGGGA
SARS-CoV-2 NC_045512.2 (25386) CGAACTTATGGATTTGTTTATGAGAATCTTCAC--------AATTGGAACTGTAA--CTTTGAAGCAAG--GTGAAATCAAGGATGCTACTCCTTCA
                                                                                         Section 345
                      (33369) 33369       33380        33390        33400        33410        33420        33430        33440        33450       33465
      lambda NC_001416.1 (32139) ACAGAGCGGCAATAAGTCGTCATCCCATGTTTTATCCAGGGCGATCAGCAGAGTGTTAATCTCCTGCATGGTTTCATCG--TTAACCGGAGTGATGT
SARS-CoV-2 NC_045512.2 (25471) GAT-TTTGTTCGCGC--TACTGCAACGATACCGATACAAGCCTCAC--TCCCTTTCGGATGGC-TTATTGTTGGCGTTGCACTTCTTGCTGTTTTTC
                                                                                         Section 346
                      (33466) 33466            33480        33490        33500        33510        33520        33530        33540        33550    33562
      lambda NC_001416.1 (32234) CGCGTTCCGGCTGACGTTCTGCAGTGTATGCAGTATTTTCG-----ACAATGCGCTCGGCTTCATCCTTGTCATAGATACCAGCAAATCCGAAGGCC
SARS-CoV-2 NC_045512.2 (25562) AGAGCGCTTCCAAAATCATAACCCTCAAAAAGAGATGGCAACTAGCACTCTCCAAGGGTGTTCACTTTGTTTGCAACTTGCTGTTGTTGTTTGTAAC
                                                                                         Section 347
                      (33563) 33563  33570        33580        33590        33600        33610        33620        33630        33640           33659
      lambda NC_001416.1 (32326) AGACGGGCACACTGAATCATGGCTTTATGACGTAACA----TCCGTTTGGGATGCGACTGCCACGGCCCCGTGATTTCTCTGCCTTCGCGAGTTT--
SARS-CoV-2 NC_045512.2 (25659) AGTT-TACTCA------CACCTTTTGCTCGTT-----GCTGCTGGCCTTGAAGCCCCTTTTCTCTATC----TTTATGCTTTAGTCTACTTCTTGCA
                                                                                         Section 348
                      (33660) 33660      33670        33680        33690        33700        33710        33720        33730        33740          33756
      lambda NC_001416.1 (32417) -TGAATGGTTCGCGGCGGCATTCATCCATC-CATTCGGTAACGCAGATCGGA--TGATTACGGTCCTTGCGGTAAATCCGGCATGTACAGGATTCAT
SARS-CoV-2 NC_045512.2 (25740) GAGTATAAACTTTGTAAGAATAATAATGAGGCTTTGGCT-TTGCTGGAAATGCCGTTCCAAAAACCCAT----TACTTTATGATGCCAACTATTTTC
                                                                                         Section 349
                      (33757) 33757            33770        33780        33790        33800        33810        33820        33830        33840    33853
      lambda NC_001416.1 (32510) TGTCCT---GCTCAAAGTCCATGCCATCAAACTGCTGGTTTTCATTGATGATGCGGGACCAGCCATCAACG--CCCACCACCGGAACGATGCCATTC
SARS-CoV-2 NC_045512.2 (25832) TTTGCTGGCATACTAATTGTTACG-ACTATTGTATACCTTACAATAG-TGTAACTTCTTCAATTGTC-ATTACTTCAGGTGATGGCACAACAAGTCC
                                                                                         Section 350
                      (33854) 33854 33860        33870        33880        33890        33900        33910        33920        33930        33940 33950
      lambda NC_001416.1 (32602) TGCTTATCAGGAAAGGCGTAAATTTCTTTCGTCCACGGATTAAGGCCGTACTGGTTGGCAACGATCAGTAATGCGATGAACTGCGC-ATCGCTGGCA
SARS-CoV-2 NC_045512.2 (25926) TATTTCTGAACATGACTACCAGATTGGTGGTTATACTGAAAAATGGGAATC----TGGAGTAAAAGACTGTGTTGTATTACACAGTTACTTCACTTC
```

```
                                                                                                  Section 351
                         (33951) 33951    33960      33970      33980      33990      34000      34010      34020      34030         34047
       lambda NC_001416.1 (32698) TCACCTTTAAATGCCGTCTGGCGAAGAG----TGGTGATCAGTTCCTGTGGGTCGACAGAATCCA---------TGCCGACACGTTCAG--CCAGCTT
SARS-CoV-2 NC_045512.2 (26019) AGACTATT-ACCAGCTGTACTCAACTCAATTGAGTACAGACACTGGTG-TTGAACATGTTACCTTCTTCATCTACAATAAAATTGTTGATGAGCCTG
                                                                                                  Section 352
                         (34048) 34048        34060      34070      34080      34090      34100      34110      34120      34130      34144
       lambda NC_001416.1 (32781) CCCAGCCAGCGTTGCGAGTGCAGTACTCATTCGTT-----TTATACCTCTGAATCAATATCAACCTGGTGGTGAGCAATGGTTTCAACCA-TGTACC
SARS-CoV-2 NC_045512.2 (26114) AAGAACATGTCCAAATTCACACAATCGACGGTTCATCCGGAGTTGTTAATCCAGTAATGGAACCAATTTATGATGAACCGACGACGACTACTAGCGT
                                                                                                  Section 353
                         (34145) 34145 34150     34160      34170      34180      34190      34200      34210      34220      34230  34241
       lambda NC_001416.1 (32872) GGATGTGTTCTGCCATGCGCTCCTGAAAC----TCAACATCGTCA---TCAAACGCACGGGTAATGGATTTTTTGCTGGCCCCGTGGCGTTG--CAA
SARS-CoV-2 NC_045512.2 (26211) GC-----CTTTGTAAGCACAAGCTGATG-AGTACGAACTTATGTACTCATTCGTTTCGGAAGAGACAGGTACGTTAATAGTTAATAGCGTACTTCTT
                                                                                                  Section 354
                         (34242) 34242   34250      34260      34270      34280      34290      34300      34310      34320         34338
       lambda NC_001416.1 (32960) ATGATCGATG-CATAGCGATTCAAACAGGTGCTGGGG------CAGGCCTTTTTCCATGT-----CGTCTGCC----AGTTCTGCCTCTTTCTCTTC
SARS-CoV-2 NC_045512.2 (26302) TTTCTTGCTTTCGTGGTATTCTTGCTAGTTACACTAGCCATCCTTACTGCGCTTCGATT-GTGTGCGTACTGCTGCAATATTGTTAACGTGAGTCTT
                                                                                                  Section 355
                         (34339) 34339       34350      34360      34370      34380      34390      34400      34410      34420      34435
       lambda NC_001416.1 (33041) ACGGGCGAGCTGCTGGTAGTGACGCGCCCAGCTCTGAGCCTCAAGACGATCCTGAATGTAATA------AGCGTTCATGGCTGAACTC-CTGAAATA
SARS-CoV-2 NC_045512.2 (26398) GTAAAACCTTCTTTTTACGTTTACTCTCGTGTTAAAAATCTG--AATTCTTCTAGAGTTCCTGATCTTCTGGTCTAAAC---GAACTAAATATTATA
                                                                                                  Section 356
                         (34436) 34436          34450      34460      34470      34480      34490      34500      34510      34520  34532
       lambda NC_001416.1 (33131) G-CTGTGAAAATATCGCCCGCGAAATGCCGGGCTGATTAGGAAAACAGGA-----AAGGGGGTTAGTGAATGCTTTTGCTTGATCTCAGTTTCAGTA
SARS-CoV-2 NC_045512.2 (26490) TT-AGTTTTTCTGTTTGGAAC-TTTAATTTTAG------CCATGGCAGATTCCAACGGTACTATTACCGTTGAAGAG--CTTAAAAA-GCTCCTT--
                                                                                                  Section 357
                         (34533) 34533  34540      34550      34560      34570      34580      34590      34600      34610         34629
       lambda NC_001416.1 (33222) TTAATATCCATTTTTTATAAGCGTCGACGGCTTCACGAAACATCTTTTCATCGCCAATAAAAGTGGCGATAGTGAATTTAGTCTGGATAGCCATAAG
SARS-CoV-2 NC_045512.2 (26574) ---GAACAATGG--AACCTAGT--AATAGGTTTCCTATTC----CTTACATGGATTTGTCTTCTACAATTTGCCTATGCCAACAG--GAATAGGTTT
```

```
                                                                                            Section 358
                                (34630) 34630     34640     34650     34660     34670     34680     34690     34700     34710        34726
          lambda NC_001416.1 (33319) TGTTTGATCCATTCTTTGGGACTCCTGGCTGATTA-AGTATGTCGATAAG-GCGTTTCCATCC------GTCACGTAATTTACGGGTG--ATTCGTT
 SARS-CoV-2 NC_045512.2 (26658) TTGTATATAATTAAGT---TAATTTTCCTCTGGCTGTTATGGCCAGTAACTTTAGCTTGTTTTGTGCTTGCTGCT--GTTTACAGAATAAATTGGAT
                                                                                            Section 359
                                (34727) 34727        34740     34750     34760     34770     34780     34790     34800     34810       34823
          lambda NC_001416.1 (33406) CAAGTAAAGATTCGGAAGGGCAGCCAGCAACAGGCCACCCTGCAATGGCATATTG--CATGGTGTGCTCCTTATTTATACATAACGAAAAACGCCTC
 SARS-CoV-2 NC_045512.2 (26750) ------CAC-CGGTGGAATTGCTATCGCAATGGCTTGTCTTGTAGGCT--TGATGTGGCTCAGCTACTTCA----------TTGCT-----TCTTTC
                                                                                            Section 360
                                (34824) 34824 34830     34840     34850     34860     34870     34880     34890     34900     34910 34920
          lambda NC_001416.1 (33501) GAGTGAAGCGTTATTGGTATGCGGTAAAACCGCACTCAGGCGGC-CTTGATAGTCATATCATCTGAATCAAATATTCCTGATGTATCGATATCGGTA
 SARS-CoV-2 NC_045512.2 (26823) AGACTG-----TTTGCGCG-TACGCG--TTCCATGTGGTCAT--TCAATCCA---GAAA--CTAACATTCTTCTC----AACGTGCCACTCCATGGC
                                                                                            Section 361
                                (34921) 34921    34930     34940     34950     34960     34970     34980     34990     35000        35017
          lambda NC_001416.1 (33597) ATTCTTATTCCTTCGCTACCATCCATTGGAGGCCATCCTTCCTGACCATT---TCCATC------ATTCCAGTCGAACTCACACACAACACCATATG
 SARS-CoV-2 NC_045512.2 (26901) ACTATTCTGACCA-GACCGCTTCTAGAAAGTGAACTCGTAATCGGAGCTGTGATCCTTCGTGGACATCTTCGTATTGCTGGACACCA-TCTAGGACG
                                                                                            Section 362
                                (35018) 35018       35030     35040     35050     35060     35070     35080     35090     35100       35114
          lambda NC_001416.1 (33685) CATTTAAGTCGCTTGAA---ATTGCTATAAGCAGAGCATGTTGCGCCAGCATGATTAATACAGCATTTAATACAGAGCCGTGTTTATTGAGTCGGT-
 SARS-CoV-2 NC_045512.2 (26996) CTGT-GACATCAAGGACCTGCCTAA-AGAAATCACTGTTGCTACATCACGAACGCTTTCTTATTACAAATTGGGAG-CTTCGCAGCGTGTAGCAGGT
                                                                                            Section 363
                                (35115) 35115 35120     35130     35140     35150     35160     35170     35180     35190     35200  35211
          lambda NC_001416.1 (33778) ATTCAGAGTCTGACCAGAAATTATTAATCTGGTGAAGTTTTTCCTCTGTCATTACGTCATG--GTCGATTTCAATTTCTATTGATGCTTTCCAGTCG
 SARS-CoV-2 NC_045512.2 (27090) GACTCAGGTTTTGCTGC--ATACAGTCGCTACAGGATTGGCAACT--ATAAATTAAAC--ACAGACCATTCC-AG---TAGC---AGTGACAAT---
                                                                                            Section 364
                                (35212) 35212   35220     35230     35240     35250     35260     35270     35280     35290          35308
          lambda NC_001416.1 (33873) TAATCAATGATGTATTTTTTGATGTTTGACATCTGTTCATATCCTCACAGATAAAAAATCGCC-----C--TCACACT--GGAGGGCAA--AGAAGA
 SARS-CoV-2 NC_045512.2 (27171) --ATTGCTTTGCTTGTACAGTAAGTGAC-AA-----CAGATGTTTCATCTC-----GTTGA-CTTTCA-GGTTACTATAGCAGAGATATTACTAATT
```

```
                                                                                               Section 365
                          (35309) 35309      35320      35330      35340      35350      35360      35370      35380      35390          35405
           lambda NC_001416.1 (33959) TT--TCCA--ATAATCAGAACAAGTCGGCTCCTGTTT--AGTTACGAGCGACATTGCTCCGTGTATTCA--CTCGTTGGAATGAATACACAGTGCAG
  SARS-CoV-2 NC_045512.2 (27253) ATTATGAGGACTTTTAAAGTTTCCAT--TTGGAATCTTGATT-ACATCATAAACCTCATAATTAAAAATTTATCTAAGTCACTAACTGAGAA--TAA
                                                                                               Section 366
                          (35406) 35406         35420      35430      35440      35450      35460      35470      35480      35490      35502
           lambda NC_001416.1 (34048) TGTTT----ATTCTGTT--ATTTATGCCAAAAATAA--AGGCCACTATCAGGCAGCTTTGT--TGTTCTGTTTACCAAGTTCTCTGGCAATCATTGC
  SARS-CoV-2 NC_045512.2 (27345) A-TATTCTCAATTAGATGAAGAGCAACCAATGGAGATT-GATTAAACGAACATGAAAAT-TATTCTTTTCTTGGCACTGATAACACTCGCTACTTGT
                                                                                               Section 367
                          (35503) 35503  35510      35520      35530      35540      35550      35560      35570      35580          35599
           lambda NC_001416.1 (34135) CGTC-GTTCGTATTGCCCATTTATCGACA--TATTTCCCATCTTCCATTACAGGAAACATTTCTTCAGGCTTAACCATGC-ATTCCGATTGCAGCTT
  SARS-CoV-2 NC_045512.2 (27439) GAGCTTTATCACTACCAAGAGTGTGTTAGAGGTACAA-CAGTACTTTTAAAAGAACCTTGCTC----TTCTGGAACATACGAGGGCAATTCACCATT
                                                                                               Section 368
                          (35600) 35600      35610      35620      35630      35640      35650      35660      35670      35680          35696
           lambda NC_001416.1 (34228) GCATCC---A---TTGCATCGCTT-GAATTGTCCACACCATTGATTTTTATCAATAGTCGTAGTCATACGGATAGTCCTGGTATTGTTCCATCACAT
  SARS-CoV-2 NC_045512.2 (27531) TCATCCTCTAGCTGATAACAAATTTGCACTGACTTGCTTTAGCACTCAATTTGCTTTTGCTTGTCCTGACGGCGTAAAACACGTCTATCAGTTACGT
                                                                                               Section 369
                          (35697) 35697        35710      35720      35730      35740      35750      35760      35770      35780      35793
           lambda NC_001416.1 (34318) CCTGAGGATGCTCTTCGAACTCTTCAAATTCTTCTTCCATATATCACCTTAAATAGTGGATTGCGGTAGTAAAGATTGTGCCTGTCTTT-TAACCAC
  SARS-CoV-2 NC_045512.2 (27628) GCCAGATCAGTTTCACCTAAACTGTTCATCAGACA--AGAGGA---AGTTCAAGAACTTTACTCTCCAAT---TTTTCTTATTGTTGCGGCAATAGT
                                                                                               Section 370
                          (35794) 35794 35800      35810      35820      35830      35840      35850      35860      35870      35880 35890
           lambda NC_001416.1 (34414) ATCAGGCTCGG--TGGTTCTCGTGTACCCCTACAGCGAGAAAT-CGGATAAACTATTACAACCCCTACAGTTTGATGAGTATAGAAATGGATCCACT
  SARS-CoV-2 NC_045512.2 (27717) GTTTATAACACTTTGCTTCACACTCAAAAGAAAGACAGAATGATTGAACTTTCAT-TAATTGACTTCTATTTGTGCTTTT-----T----AGCCTT-
                                                                                               Section 371
                          (35891) 35891    35900      35910      35920      35930      35940      35950      35960      35970          35987
           lambda NC_001416.1 (34508) CGTTATTCTCGGACGAGTGTTCAGTAATGAACCTCTGGAGAGAACCATGTATATGATCGTTATCTGGGTTGGACTTCTGCTTTTAAGCCCAGATAAC
  SARS-CoV-2 NC_045512.2 (27803) ---TCTGCTA-------------------TTCC------TTGTTTTAATTA--TGCT-------TATTATCTTTTGGTTCTCACTTGAACTGCAAGA
```

```
                                                                                                         Section 372
                          (35988) 35988      36000      36010      36020      36030      36040      36050      36060      36070       36084
        lambda NC_001416.1 (34605) TGGCCTGAATATGTTAATGAGAGAATCGGTA----TTCCTCATGTGTG-GCATGTTTTCGTCTTTGCTCTT---GCATTTTCGCTAGCAATTAATGT
SARS-CoV-2 NC_045512.2 (27863) TCATAATGA-AACTTGTCACGCCTAAACGAACATGAAATTTCTTGTTTTCTTAGGAATCATCACAACTGTAGCTGCATTTCACCAAGAATG------
                                                                                                         Section 373
                          (36085) 36085 36090      36100      36110      36120      36130      36140      36150      36160      36170  36181
        lambda NC_001416.1 (34694) GCATCGATTATCAGCTATTGCCAGCGCCAGATATAAGCGATTTAAGCTA-AGAAAACGCATTAAGATG----CAAAACGATAAAGTGCGATCAGTAA
SARS-CoV-2 NC_045512.2 (27953) -TAGTTTACAGTCATGTACTCAACATCA-ACCATATGTAGTTGATGACCCGTGTCCTATTCACTTCTATTCTAAATGGTATATTAGAGTAGGAGCTA
                                                                                                         Section 374
                          (36182) 36182    36190      36200      36210      36220      36230      36240      36250      36260           36278
        lambda NC_001416.1 (34786) TTCAAAACCTTACAG-AAGAGCAATCTATGGTTTTGTGCGC-AGCCCT--TAATGAAGGCAGGAAGTATGTGGTTACATCAAAACAATTCCCATACA
SARS-CoV-2 NC_045512.2 (28048) G---AAAATCAGCACCTTTAATTGAATTGTGCGTGGATGAGGCTGGTTCTAAATCACC-CATTCAGTAC-ATCGATATCGGT-AATTATAC-----A
                                                                                                         Section 375
                          (36279) 36279      36290      36300      36310      36320      36330      36340      36350      36360      36375
        lambda NC_001416.1 (34879) TTAGTGAGTTGATTGAGCTTGGTGTGTTGAACAAAACTTTTTCCCGATGGAATGGAAAGCATATATTATTCCCTATTGAGGATATTTACTGGACTGA
SARS-CoV-2 NC_045512.2 (28134) GTTT------C-CTGTTTACCTTT-----TACAA----TTAATTGCCAGGAAC---CTAAATTGGGTAGT---CTTGTAGTGCGTTGTTC----G--
                                                                                                         Section 376
                          (36376) 36376          36390      36400      36410      36420      36430      36440      36450      36460  36472
        lambda NC_001416.1 (34976) ATTAGTTGCCAGCTATGATCCATA-TAATATTGAG-------A-----TAAAGCCAAGGCCAATATCTAAGTAACTAGATAAGAGGAATCGATTTTC
SARS-CoV-2 NC_045512.2 (28203) ---TTCTATGAAG-ACTTTTTAGAGTATCATGACGTTCGTGTTGTTTTAGATTTCATCTAAACGAACAAACTAAAATGTCTG--ATAATGGACCCCA
                                                                                                         Section 377
                          (36473) 36473 36480      36490      36500      36510      36520      36530      36540      36550           36569
        lambda NC_001416.1 (35060) CCTTAATTTTCTGGCGTCCACTGCATGTTATGCCGCG----TTCGCCAGGCTTGCTGTACCATGTGCGCTGATTCTTGCGCTCAATACGTT---GCA
SARS-CoV-2 NC_045512.2 (28294) AAATCAGCGAAATGCACCCCGCATTACGTTTGGTGGACCCTCAGATTCAAC-TG--GCA----GTAACCAGA-----ATGGAGAACGCAGTGGGGCG
                                                                                                         Section 378
                          (36570) 36570      36580      36590      36600      36610      36620      36630      36640      36650       36666
        lambda NC_001416.1 (35150) GGTTGCTTTCAATCTGTTTGTGGTATTCAGCCAGCACTGTAAGG-TCTATC--GGATTTAGTGCGCTTTCTACTCGT---GATTTCGGTT---TGCG
SARS-CoV-2 NC_045512.2 (28379) ---CGATCAAAACAACGTCGGCCCCAAGGTTTACCCAATAATACTGCGTCTTGGTTCACCGCTCTCACTCAACATGGCAAGGAAGACCTTAAATTCC
```

```
                                              Section 379
                             (36667) 36667        36680      36690      36700      36710      36720      36730      36740      36750      36763
        lambda NC_001416.1 (35238) ATTCAG-CGAGAGAATAGGGCGGTTAACTGGTTTTGCGCTTACCCCAACCAACAGGG--GATTTGCTGCTTTCCATTGAGCCTGTTTCTCTGCGCGA
SARS-CoV-2 NC_045512.2 (28473) CTCGAGGA--CAAGGCGTTCCAATTAACACCAATAGCAGTCCAGATGACCAAATTGGCTACTAC-CGA-----AG-AGCTACCAGACGAATTCG-TG
                                              Section 380
                             (36764) 36764 36770      36780      36790      36800      36810      36820      36830      36840      36850 36860
        lambda NC_001416.1 (35332) CGTTCGCGGCGGCGTGTTTGTGCATCCATCTGGATTCTCCTGTCAGTTAGCTTTGGTGGTGTGTGGCAGTTGTAGTCCTGAACGAA-----AACCCC
SARS-CoV-2 NC_045512.2 (28560) GTGGTGA--CGGTAAAATGAAAGATCTCAGTCCAAGATGGTATTTCTACTACCTAGGAAC-TGGGCCAGAAGCTG--GACTTCCC-TATGGTGCTAA
                                              Section 381
                             (36861) 36861    36870      36880      36890      36900      36910      36920      36930      36940      36957
        lambda NC_001416.1 (35424) CCGCGAT--TGGCACATTGGCAGCTAATCCGGAATCGCACTTACGGCCAATGCTTCGTTTCGTATCACACACCCCAAAGCCTTCTGCTTTGAATGC-
SARS-CoV-2 NC_045512.2 (28651) CAAAGACGGCATCATATGGGTTGC-AACTGAGGGAGCCTTGA-ATACACCAAAAGATCACATTGGCACCCG----CAATCCTGCTAAC---AATGCT
                                              Section 382
                             (36958) 36958        36970      36980      36990      37000      37010      37020      37030      37040      37054
        lambda NC_001416.1 (35518) -TGCCCTTCTTC--AGGGCTTAA-TTTTTAAGAGCGTCACCTTCATGGTGGTCAGTGCGTCCTGCTGATGTGCTCAGTATCACCGCCAGTGGTATTT
SARS-CoV-2 NC_045512.2 (28739) GCAATCGTGCTACAACTTCCTCAAGGAACAACATTGCCA-----AAAGGCTTCTACGCAG---AAGG--------GAGCAGAGGCGGC---------
                                              Section 383
                             (37055) 37055 37060      37070      37080      37090      37100      37110      37120      37130      37140 37151
        lambda NC_001416.1 (35611) ATGTCAACACCGC--CAGAGATAATTTATCACCGCAGATGGTTATCTGTATGTTTTTTATATGAATTTATTTTTTGCAGGGGGGCATTGTTTGGTAG
SARS-CoV-2 NC_045512.2 (28811) AGTCAA---GCCTCTTCTCGTTCC--TCATCACGTAG-TCGC-AACAGTTCAAGAA---------ATTCAACT---C---CAGGCAG----CAGTAG
                                              Section 384
                             (37152) 37152   37160      37170      37180      37190      37200      37210      37220      37230      37248
        lambda NC_001416.1 (35706) GTGAGAGATCTGA-ATTGCTATGTTTAGTGAGTTGTATCTATTTATTTT---TCAATAAATACAAT---TGGTTATGTGTTTTGGGGGCGATCGTGA
SARS-CoV-2 NC_045512.2 (28882) GGGAACTTCTCCTGCTAGAATGGCTGGCAATGGCGGTGATGCTGCT-CTTGCTTTGCTGCTGCTT-GACAGATTGAACCAGCTTGAGAGCAAAATGT
                                              Section 385
                             (37249) 37249       37260      37270      37280      37290      37300      37310      37320      37330      37345
        lambda NC_001416.1 (35796) --GGCAAAGAAAACCCGGCGCTGAGGCCGGGTTATTCTTGTTCTCTGGTCAAATTATATAGTTG--GAAAACAAGGAT-GCATATATGAATGAACGA
SARS-CoV-2 NC_045512.2 (28977) CTGGTAAAGGCC-------AACAACAACAAGG---CCAAACTGTCA--CTAAGAAATCTGCTGCTGAGGCTTCTAAGAAGCCTCGGCAAAAACGTAC
```

```
Section 386
(37346) 37346         37360     37370     37380     37390     37400     37410     37420     37430    37442
lambda NC_001416.1 (35888) TGCAGAGGCAATGCCGATGGCGATAGTGGGTATCATGTAGCCGCTTATGCTGGAAAGAAGCAA-TAACCCGCAGAAAAACAAAGCTCCAAGCTCAAC
SARS-CoV-2 NC_045512.2 (29062) TGC-CA-CTAAAGCATACAATGTAACACAAGCTTTCGGCAGACGTGGTCCAGAACAAACCCAAGGAAATTTTGGGGACCAGGAACTAATCAGACAAG
```

```
Section 387
(37443) 37443  37450     37460     37470     37480     37490     37500     37510     37520           37539
lambda NC_001416.1 (35984) AAAACTAAGGGCATAGACAATAACTACCGATGTCA--TATACCCATACTCTCTAATCTTGGCCAGTCGGCGCGTTCTGCTTCCGAT--TAGAAACGT
SARS-CoV-2 NC_045512.2 (29157) GAA-CTGATTACAAACATTGGCCGCAAATTGCACAATTTGCCCCCAGCGC-TTCA-GCGTTCTTCGGAATGTCGCGCATTGGCATGGAAGTCACACC
```

```
Section 388
(37540) 37540     37550     37560     37570     37580     37590     37600     37610     37620           37636
lambda NC_001416.1 (36077) CAAGGCAGCAATCAG-GATTGCAATCATGGTTCCTGCATATGATGACAATGTCGCCCCAAGACCATCTCTATGAGCTGAAAAAGAAACACCAGGAAT
SARS-CoV-2 NC_045512.2 (29251) TTCGG--GAACGTGGTTGACCTACACA-G---GTGCCATCAAATTGGATGACAAAGATCCAAATTTCAAAGATCAAGTCATTTTGCTGAATAAGCAT
```

```
Section 389
(37637) 37637        37650     37660     37670     37680     37690     37700     37710     37720         37733
lambda NC_001416.1 (36173) GTAGTGG---CGGAAA--AGGAGATAGCAAATGCTTACGATAACG-TAAGGAATTATTACTATGTAAACACC--AGGC--ATGATTCTGTTCCGCAT
SARS-CoV-2 NC_045512.2 (29342) ATTGACGCATACAAAACATTCCCACCAACAGAGCCT---AAAAAGGACAAAAAGAAGAAGGCTGATGAAACTCAAGCCTTACCGCAGAGACAGAAGA
```

```
Section 390
(37734) 37734 37740     37750     37760     37770     37780     37790     37800     37810     37820 37830
lambda NC_001416.1 (36260) AATTACTCCTGATAATTAATCCTTAACTTTGCCCACCTGCCTTTTAAAACATTCCAGTATATCACTTTTCATTCTTGCGTAGCAATATGCCATCTCT
SARS-CoV-2 NC_045512.2 (29436) AACAGCAAACTGTGA-CTCTTCTTCCTGCTGCA-GATTTGGATG--ATTTCTCCAAACAATTG---CAACAATC---CATGA-GCAGTGCTGACTCA
```

```
Section 391
(37831) 37831    37840     37850     37860     37870     37880     37890     37900     37910           37927
lambda NC_001416.1 (36357) TCAGCTATCTCAG-CATTGGTGACCTTGTTCAGAGGCGCTGAGAGATGGCCTTTTTCTGATAGATAATGTTCTGTTAAAATATCTCCGGCCTCATCT
SARS-CoV-2 NC_045512.2 (29522) AC---TCAGGCCTAAACTCAT-GCAGACCACACAAG-GCAGATGGGCTATATAAACGT--TT----------TCGCTTTTCCGTTTACGAT--ATAT
```

```
Section 392
(37928) 37928       37940     37950     37960     37970     37980     37990     38000     38010           38024
lambda NC_001416.1 (36453) TTTGCCCGCAGGCTAATGTCTGAAAATTGAGGTGACGGGTTAAAAATAATATCCTTGGCAACCTTTTTTATAT--C---CCTTTTAA-ATTTTGGCT
SARS-CoV-2 NC_045512.2 (29600) AGTCTACTCTTGTGCAGAATGAATTCTCGTAACTACATAGCACAAGTAGATGTAG-----TTAACTTTAATCTCACATAGCAATCTTTAATCAGTGT
```

Section 393

```
                              38025 38030     38040     38050     38060     38070     38080     38090     38100     38110     38121
                     (38025)
lambda NC_001416.1   (36544)  TAATGACT-ATATCCAATGAGTCAAAAAGCTC-CCCTTCAATATCTGTTGCCCCTAAGACCTTTAATATATCGCCAAATACAGGTAGCTTGGCTTCT
SARS-CoV-2 NC_045512.2 (29692) GTAACATTAGGGAGGACTTG-AAAGAGCCACCA-CATTTT--CACCGAGGCCAC---GCGGAGTACGATCG-----AGTG----TACAGTGAACAAT
```

Section 394

```
                              38122   38130     38140     38150     38160     38170     38180     38190     38200           38218
                     (38122)
lambda NC_001416.1   (36639)  ACCTTCACCGTTGTTCGGCCGATGAAATG-CATATGCATAACATCGTCTTTGGTGGTTCCCCTCATCAGTGGCTCTATCTGAACGCGCTCTCCACTG
SARS-CoV-2 NC_045512.2 (29773) GCTAGGGAGAGCTGCCTATATGGAAGAGCCCTAATGTGTAAAATTAATTTT--AG--TAGTGCTATCCCCA--TGTGATTTTAATAGCTTCTTAGGA
```

Section 395

```
                              38219      38230     38240     38250     38260     38270     38280     38290     38300          38315
                     (38219)
lambda NC_001416.1   (36735)  CTT-AATGACATTCCT---TTCCCGATTAAAAAAT---C-TGTCAGATCGGATGTGGTCGGCCCGAAAACAGTTCTGGCAAAACCAATGGTGTCGCC
SARS-CoV-2 NC_045512.2 (29864) GAATGACAAAAAAAAAAAAAAAAAAAAAAAAAAAAAAAAA---------------------------------------------------------
```

Section 396

```
                              38316         38330     38340     38350     38360     38370     38380     38390     38400     38412
                     (38316)
lambda NC_001416.1   (36824)  TTCAACAAACAAAAAAGATGGGAATCCCAATGATTCGTCATCTGCGAGGCTGTTCTTAATATCTTCAACTGAAGCTTTAGAGCGATTTATCTTCTGA
SARS-CoV-2 NC_045512.2 (29904) -------------------------------------------------------------------------------------------------
```

Section 397

```
                              38413  38420     38430     38440     38450     38460     38470     38480     38490          38509
                     (38413)
lambda NC_001416.1   (36921)  ACCAGACTCTTGTCATTTGTTTTGGTAAAGAGAAAAGTTTTTCCATCGATTTTATGAATATACAAATAATTGGAGCCAACCTGCAGGTGATGATTAT
SARS-CoV-2 NC_045512.2 (29904) -------------------------------------------------------------------------------------------------
```

Section 398

```
                              38510     38520     38530     38540     38550     38560     38570     38580     38590          38606
                     (38510)
lambda NC_001416.1   (37018)  CAGCCAGCAGAGAATTAAGGAAAACAGACAGGTTTATTGAGCGCTTATCTTTCCCTTTATTTTTGCTGCGGTAAGTCGCATAAAAACCATTCTTCAT
SARS-CoV-2 NC_045512.2 (29904) -------------------------------------------------------------------------------------------------
```

Section 399

```
                              38607        38620     38630     38640     38650     38660     38670     38680     38690      38703
                     (38607)
lambda NC_001416.1   (37115)  AATTCAATCCATTTACTATGTTATGTTCTGAGGGGAGTGAAAATTCCCCTAATTCGATGAAGATTCTTGCTCAATTGTTATCAGCTATGCGCCGACC
SARS-CoV-2 NC_045512.2 (29904) -------------------------------------------------------------------------------------------------
```

```
                                                                                                                              Section 400
                          (38704) 38704 38710     38720     38730     38740     38750     38760     38770     38780     38790    38800
        lambda NC_001416.1 (37212) AGAACACCTTGCCGATCAGCCAAACGTCTCTTCAGGCCACTGACTAGCGATAACTTTCCCCACAACGGAACAACTCTCATTGCATGGGATCATTGGG
SARS-CoV-2 NC_045512.2 (29904) -------------------------------------------------------------------------------------------------
                                                                                                                              Section 401
                          (38801) 38801    38810     38820     38830     38840     38850     38860     38870     38880           38897
        lambda NC_001416.1 (37309) TACTGTGGGTTTAGTGGTTGTAAAAACACCTGACCGCTATCCCTGATCAGTTTCTTGAAGGTAAACTCATCACCCCCAAGTCTGGCTATGCAGAAAT
SARS-CoV-2 NC_045512.2 (29904) -------------------------------------------------------------------------------------------------
                                                                                                                              Section 402
                          (38898) 38898       38910     38920     38930     38940     38950     38960     38970     38980         38994
        lambda NC_001416.1 (37406) CACCTGGCTCAACAGCCTGCTCAGGGTCAACGAGAATTAACATTCCGTCAGGAAAGCTTGGCTTGGAGCCTGTTGGTGCGGTCATGGAATTACCTTC
SARS-CoV-2 NC_045512.2 (29904) -------------------------------------------------------------------------------------------------
                                                                                                                              Section 403
                          (38995) 38995 39000     39010     39020     39030     39040     39050     39060     39070     39080     39091
        lambda NC_001416.1 (37503) AACCTCAAGCCAGAATGCAGAATCACTGGCTTTTTTGGTTGTGCTTACCCATCTCTCCGCATCACCTTTGGTAAAGGTTCTAAGCTCAGGTGAGAAC
SARS-CoV-2 NC_045512.2 (29904) -------------------------------------------------------------------------------------------------
                                                                                                                              Section 404
                          (39092) 39092   39100     39110     39120     39130     39140     39150     39160     39170             39188
        lambda NC_001416.1 (37600) ATCCCTGCCTGAACATGAGAAAAAACAGGGTACTCATACTCACTTCTAAGTGACGGCTGCATACTAACCGCTTCATACATCTCGTAGATTTCTCTGG
SARS-CoV-2 NC_045512.2 (29904) -------------------------------------------------------------------------------------------------
                                                                                                                              Section 405
                          (39189) 39189      39200     39210     39220     39230     39240     39250     39260     39270          39285
        lambda NC_001416.1 (37697) CGATTGAAGGGCTAAATTCTTCAACGCTAACTTTGAGAATTTTTGCAAGCAATGCGGCGTTATAAGCATTTAATGCATTGATGCCATTAAATAAAGC
SARS-CoV-2 NC_045512.2 (29904) -------------------------------------------------------------------------------------------------
                                                                                                                              Section 406
                          (39286) 39286         39300     39310     39320     39330     39340     39350     39360     39370      39382
        lambda NC_001416.1 (37794) ACCAACGCCTGACTGCCCCATCCCCATCTTGTCTGCGACAGATTCCTGGGATAAGCCAAGTTCATTTTTCTTTTTTTCATAAATTGCTTTAAGGCGA
SARS-CoV-2 NC_045512.2 (29904) -------------------------------------------------------------------------------------------------
```

```
                                                                                                         Section 407
                           (39383) 39383  39390          39400          39410          39420          39430          39440          39450          39460               39479
          lambda NC_001416.1 (37891) CGTGCGTCCTCAAGCTGCTCTTGTGTTAATGGTTTCTTTTTTGTGCTCATACGTTAAATCTATCACCGCAAGGGATAAATATCTAACACCGTGCGTG
   SARS-CoV-2 NC_045512.2 (29904) -------------------------------------------------------------------------------------------------
                                                                                                         Section 408
                           (39480) 39480          39490          39500          39510          39520          39530          39540          39550          39560           39576
          lambda NC_001416.1 (37988) TTGACTATTTTACCTCTGGCGGTGATAATGGTTGCATGTACTAAGGAGGTTGTATGGAACAACGCATAACCCTGAAAGATTATGCAATGCGCTTTGG
   SARS-CoV-2 NC_045512.2 (29904) -------------------------------------------------------------------------------------------------
                                                                                                         Section 409
                           (39577) 39577             39590          39600          39610          39620          39630          39640          39650          39660       39673
          lambda NC_001416.1 (38085) GCAAACCAAGACAGCTAAAGATCTCGGCGTATATCAAAGCGCGATCAACAAGGCCATTCATGCAGGCCGAAAGATTTTTTTAACTATAAACGCTGAT
   SARS-CoV-2 NC_045512.2 (29904) -------------------------------------------------------------------------------------------------
                                                                                                         Section 410
                           (39674) 39674 39680          39690          39700          39710          39720          39730          39740          39750          39760 39770
          lambda NC_001416.1 (38182) GGAAGCGTTTATGCGGAAGAGGTAAAGCCCTTCCCGAGTAACAAAAAAACAACAGCATAAATAACCCCGCTCTTACACATTCCAGCCCTGAAAAAGG
   SARS-CoV-2 NC_045512.2 (29904) -------------------------------------------------------------------------------------------------
                                                                                                         Section 411
                           (39771) 39771       39780          39790          39800          39810          39820          39830          39840          39850             39867
          lambda NC_001416.1 (38279) GCATCAAATTAAACCACACCTATGGTGTATGCATTTATTTGCATACATTCAATCAATTGTTATCTAAGGAAATACTTACATATGGTTCGTGCAAACA
   SARS-CoV-2 NC_045512.2 (29904) -------------------------------------------------------------------------------------------------
                                                                                                         Section 412
                           (39868) 39868            39880          39890          39900          39910          39920          39930          39940          39950          39964
          lambda NC_001416.1 (38376) AACGCAACGAGGCTCTACGAATCGAGAGTGCGTTGCTTAACAAAATCGCAATGCTTGGAACTGAGAAGACAGCGGAAGCTGTGGGCGTTGATAAGTC
   SARS-CoV-2 NC_045512.2 (29904) -------------------------------------------------------------------------------------------------
                                                                                                         Section 413
                           (39965) 39965 39970          39980          39990          40000          40010          40020          40030          40040          40050 40061
          lambda NC_001416.1 (38473) GCAGATCAGCAGGTGGAAGAGGGACTGGATTCCAAAGTTCTCAATGCTGCTTGCTGTTCTTGAATGGGGGGTCGTTGACGACGACATGGCTCGATTG
   SARS-CoV-2 NC_045512.2 (29904) -------------------------------------------------------------------------------------------------
```

```
                                                                                               Section 414
                          (40062) 40062 40070     40080     40090     40100     40110     40120     40130     40140           40158
       lambda NC_001416.1 (38570) GCGCGACAAGTTGCTGCGATTCTCACCAATAAAAAACGCCCGGCGGCAACCGAGCGTTCTGAACAAATCCAGATGGAGTTCTGAGGTCATTACTGGA
SARS-CoV-2 NC_045512.2 (29904) -------------------------------------------------------------------------------------------------
                                                                                               Section 415
                          (40159) 40159      40170     40180     40190     40200     40210     40220     40230     40240      40255
       lambda NC_001416.1 (38667) TCTATCAACAGGAGTCATTATGACAAATACAGCAAAAATACTCAACTTCGGCAGAGGTAACTTTGCCGGACAGGAGCGTAATGTGGCAGATCTCGAT
SARS-CoV-2 NC_045512.2 (29904) -------------------------------------------------------------------------------------------------
                                                                                               Section 416
                          (40256) 40256         40270     40280     40290     40300     40310     40320     40330     40340   40352
       lambda NC_001416.1 (38764) GATGGTTACGCCAGACTATCAAATATGCTGCTTGAGGCTTATTCGGGCGCAGATCTGACCAAGCGACAGTTTAAAGTGCTGCTTGCCATTCTGCGTA
SARS-CoV-2 NC_045512.2 (29904) -------------------------------------------------------------------------------------------------
                                                                                               Section 417
                          (40353) 40353  40360     40370     40380     40390     40400     40410     40420     40430           40449
       lambda NC_001416.1 (38861) AAACCTATGGGTGGAATAAACCAATGGACAGAATCACCGATTCTCAACTTAGCGAGATTACAAAGTTACCTGTCAAACGGTGCAATGAAGCCAAGTT
SARS-CoV-2 NC_045512.2 (29904) -------------------------------------------------------------------------------------------------
                                                                                               Section 418
                          (40450) 40450     40460     40470     40480     40490     40500     40510     40520     40530       40546
       lambda NC_001416.1 (38958) AGAACTCGTCAGAATGAATATTATCAAGCAGCAAGGCGGCATGTTTGGACCAAATAAAAACATCTCAGAATGGTGCATCCCTCAAAACGAGGGAAAA
SARS-CoV-2 NC_045512.2 (29904) -------------------------------------------------------------------------------------------------
                                                                                               Section 419
                          (40547) 40547        40560     40570     40580     40590     40600     40610     40620     40630    40643
       lambda NC_001416.1 (39055) TCCCCTAAAACGAGGGATAAAACATCCCTCAAATTGGGGGATTGCTATCCCTCAAAACAGGGGGACACAAAAGACACTATTACAAAAGAAAAAAGAA
SARS-CoV-2 NC_045512.2 (29904) -------------------------------------------------------------------------------------------------
                                                                                               Section 420
                          (40644) 40644 40650     40660     40670     40680     40690     40700     40710     40720     40730 40740
       lambda NC_001416.1 (39152) AAGATTATTCGTCAGAGAATTCTGGCGAATCCTCTGACCAGCCAGAAAACGACCTTTCTGTGGTGAAACCGGATGCTGCAATTCAGAGCGGCAGCAA
SARS-CoV-2 NC_045512.2 (29904) -------------------------------------------------------------------------------------------------
```

```
                                                                                                   Section 421
                    (40741) 40741    40750     40760     40770     40780     40790     40800     40810     40820              40837
  lambda NC_001416.1 (39249) GTGGGGGACAGCAGAAGACCTGACCGCCGCAGAGTGGATGTTTGACATGGTGAAGACTATCGCACCATCAGCCAGAAAACCGAATTTTGCTGGGTGG
SARS-CoV-2 NC_045512.2 (29904) -------------------------------------------------------------------------------------------------
                                                                                                   Section 422
                    (40838) 40838       40850     40860     40870     40880     40890     40900     40910     40920         40934
  lambda NC_001416.1 (39346) GCTAACGATATCCGCCTGATGCGTGAACGTGACGGACGTAACCACCGCGACATGTGTGTGCTGTTCCGCTGGGCATGCCAGGACAACTTCTGGTCCG
SARS-CoV-2 NC_045512.2 (29904) -------------------------------------------------------------------------------------------------
                                                                                                   Section 423
                    (40935) 40935 40940     40950     40960     40970     40980     40990     41000     41010     41020     41031
  lambda NC_001416.1 (39443) GTAACGTGCTGAGCCCGGCCAAACTCCGCGATAAGTGGACCCAACTCGAAATCAACCGTAACAAGCAACAGGCAGGCGTGACAGCCAGCAAACCAAA
SARS-CoV-2 NC_045512.2 (29904) -------------------------------------------------------------------------------------------------
                                                                                                   Section 424
                    (41032) 41032   41040     41050     41060     41070     41080     41090     41100     41110               41128
  lambda NC_001416.1 (39540) ACTCGACCTGACAAACACAGACTGGATTTACGGGGTGGATCTATGAAAAACATCGCCGCACAGATGGTTAACTTTGACCGTGAGCAGATGCGTCGGA
SARS-CoV-2 NC_045512.2 (29904) -------------------------------------------------------------------------------------------------
                                                                                                   Section 425
                    (41129) 41129      41140     41150     41160     41170     41180     41190     41200     41210          41225
  lambda NC_001416.1 (39637) TCGCCAACAACATGCCGGAACAGTACGACGAAAAGCCGCAGGTACAGCAGGTAGCGCAGATCATCAACGGTGTGTTCAGCCAGTTACTGGCAACTTT
SARS-CoV-2 NC_045512.2 (29904) -------------------------------------------------------------------------------------------------
                                                                                                   Section 426
                    (41226) 41226         41240     41250     41260     41270     41280     41290     41300     41310       41322
  lambda NC_001416.1 (39734) CCCGGCGAGCCTGGCTAACCGTGACCAGAACGAAGTGAACGAAATCCGTCGCCAGTGGGTTCTGGCTTTTCGGGAAAACGGGATCACCACGATGGAA
SARS-CoV-2 NC_045512.2 (29904) -------------------------------------------------------------------------------------------------
                                                                                                   Section 427
                    (41323) 41323  41330     41340     41350     41360     41370     41380     41390     41400               41419
  lambda NC_001416.1 (39831) CAGGTTAACGCAGGAATGCGCGTAGCCCGTCGGCAGAATCGACCATTTCTGCCATCACCCGGGCAGTTTGTTGCATGGTGCCGGGAAGAAGCATCCG
SARS-CoV-2 NC_045512.2 (29904) -------------------------------------------------------------------------------------------------
```

```
                                                                                    Section 428
                            (41420) 41420     41430     41440     41450     41460     41470     41480     41490     41500     41516
         lambda NC_001416.1 (39928) TTACCGCCGGACTGCCAAACGTCAGCGAGCTGGTTGATATGGTTTACGAGTATTGCCGGAAGCGAGGCCTGTATCCGGATGCGGAGTCTTATCCGTG
SARS-CoV-2 NC_045512.2 (29904) -------------------------------------------------------------------------------------------------
                                                                                    Section 429
                            (41517) 41517        41530     41540     41550     41560     41570     41580     41590     41600     41613
         lambda NC_001416.1 (40025) GAAATCAAACGCGCACTACTGGCTGGTTACCAACCTGTATCAGAACATGCGGGCCAATGCGCTTACTGATGCGGAATTACGCCGTAAGGCCGCAGAT
SARS-CoV-2 NC_045512.2 (29904) -------------------------------------------------------------------------------------------------
                                                                                    Section 430
                            (41614) 41614 41620     41630     41640     41650     41660     41670     41680     41690     41700 41710
         lambda NC_001416.1 (40122) GAGCTTGTCCATATGACTGCGAGAATTAACCGTGGTGAGGCGATCCCTGAACCAGTAAAACAACTTCCTGTCATGGGCGGTAGACCTCTAAATCGTG
SARS-CoV-2 NC_045512.2 (29904) -------------------------------------------------------------------------------------------------
                                                                                    Section 431
                            (41711) 41711    41720     41730     41740     41750     41760     41770     41780     41790         41807
         lambda NC_001416.1 (40219) CACAGGCTCTGGCGAAGATCGCAGAAATCAAAGCTAAGTTCGGACTGAAAGGAGCAAGTGTATGACGGGCAAAGAGGCAATTATTCATTACCTGGGG
SARS-CoV-2 NC_045512.2 (29904) -------------------------------------------------------------------------------------------------
                                                                                    Section 432
                            (41808) 41808       41820     41830     41840     41850     41860     41870     41880     41890      41904
         lambda NC_001416.1 (40316) ACGCATAATAGCTTCTGTGCGCCGGACGTTGCCGCGCTAACAGGCGCAACAGTAACCAGCATAAATCAGGCCGCGGCTAAAATGGCACGGGCAGGTC
SARS-CoV-2 NC_045512.2 (29904) -------------------------------------------------------------------------------------------------
                                                                                    Section 433
                            (41905) 41905 41910     41920     41930     41940     41950     41960     41970     41980     41990 42001
         lambda NC_001416.1 (40413) TTCTGGTTATCGAAGGTAAGGTCTGGCGAACGGTGTATTACCGGTTTGCTACCAGGGAAGAACGGGAAGGAAAGATGAGCACGAACCTGGTTTTTAA
SARS-CoV-2 NC_045512.2 (29904) -------------------------------------------------------------------------------------------------
                                                                                    Section 434
                            (42002) 42002  42010     42020     42030     42040     42050     42060     42070     42080          42098
         lambda NC_001416.1 (40510) GGAGTGTCGCCAGAGTGCCGCGATGAAACGGGTATTGGCGGTATATGGAGTTAAAAGATGACCATCTACATTACTGAGCTAATAACAGGCCTGCTGG
SARS-CoV-2 NC_045512.2 (29904) -------------------------------------------------------------------------------------------------
```

Section 435

```
(42099) 42099      42110      42120      42130      42140      42150      42160      42170      42180      42195
lambda NC_001416.1 (40607) TAATCGCAGGCCTTTTTATTTGGGGGAGAGGGAAGTCATGAAAAAACTAACCTTTGAAATTCGATCTCCAGCACATCAGCAAAACGCTATTCACGCA
SARS-CoV-2 NC_045512.2 (29904) -------------------------------------------------------------------------------------------------
```

Section 436

```
(42196) 42196      42210      42220      42230      42240      42250      42260      42270      42280      42292
lambda NC_001416.1 (40704) GTACAGCAAATCCTTCCAGACCCAACCAAACCAATCGTAGTAACCATTCAGGAACGCAACCGCAGCTTAGACCAAAACAGGAAGCTATGGGCCTGCT
SARS-CoV-2 NC_045512.2 (29904) -------------------------------------------------------------------------------------------------
```

Section 437

```
(42293) 42293      42300      42310      42320      42330      42340      42350      42360      42370      42389
lambda NC_001416.1 (40801) TAGGTGACGTCTCTCGTCAGGTTGAATGGCATGGTCGCTGGCTGGATGCAGAAAGCTGGAAGTGTGTGTTTACCGCAGCATTAAAGCAGCAGGATGT
SARS-CoV-2 NC_045512.2 (29904) -------------------------------------------------------------------------------------------------
```

Section 438

```
(42390) 42390      42400      42410      42420      42430      42440      42450      42460      42470      42486
lambda NC_001416.1 (40898) TGTTCCTAACCTTGCCGGGAATGGCTTTGTGGTAATAGGCCAGTCAACCAGCAGGATGCGTGTAGGCGAATTTGCGGAGCTATTAGAGCTTATACAG
SARS-CoV-2 NC_045512.2 (29904) -------------------------------------------------------------------------------------------------
```

Section 439

```
(42487) 42487      42500      42510      42520      42530      42540      42550      42560      42570      42583
lambda NC_001416.1 (40995) GCATTCGGTACAGAGCGTGGCGTTAAGTGGTCAGACGAAGCGAGACTGGCTCTGGAGTGGAAAGCGAGATGGGGAGACAGGGCTGCATGATAAATGT
SARS-CoV-2 NC_045512.2 (29904) -------------------------------------------------------------------------------------------------
```

Section 440

```
(42584) 42584      42590      42600      42610      42620      42630      42640      42650      42660      42670      42680
lambda NC_001416.1 (41092) CGTTAGTTTCTCCGGTGGCAGGACGTCAGCATATTTGCTCTGGCTAATGGAGCAAAAGCGACGGGCAGGTAAAGACGTGCATTACGTTTTCATGGAT
SARS-CoV-2 NC_045512.2 (29904) -------------------------------------------------------------------------------------------------
```

Section 441

```
(42681) 42681      42690      42700      42710      42720      42730      42740      42750      42760      42777
lambda NC_001416.1 (41189) ACAGGTTGTGAACATCCAATGACATATCGGTTTGTCAGGGAAGTTGTGAAGTTCTGGGATATACCGCTCACCGTATTGCAGGTTGATATCAACCCGG
SARS-CoV-2 NC_045512.2 (29904) -------------------------------------------------------------------------------------------------
```

Section 442

```
(42778)                      42778       42790     42800     42810     42820     42830     42840     42850     42860          42874
lambda NC_001416.1 (41286)   AGCTTGGACAGCCAAATGGTTATACGGTATGGGAACCAAAGGATATTCAGACGCGAATGCCTGTTCTGAAGCCATTTATCGATATGGTAAAGAAATA
SARS-CoV-2 NC_045512.2 (29904) -------------------------------------------------------------------------------------------------
```

Section 443

```
(42875)                      42875 42880     42890     42900     42910     42920     42930     42940     42950     42960      42971
lambda NC_001416.1 (41383)   TGGCACTCCATACGTCGGCGGCGCGTTCTGCACTGACAGATTAAAACTCGTTCCCTTCACCAAATACTGTGATGACCATTTCGGGCGAGGGAATTAC
SARS-CoV-2 NC_045512.2 (29904) -------------------------------------------------------------------------------------------------
```

Section 444

```
(42972)                      42972   42980     42990     43000     43010     43020     43030     43040     43050              43068
lambda NC_001416.1 (41480)   ACCACGTGGATTGGCATCAGAGCTGATGAACCGAAGCGGCTAAAGCCAAAGCCTGGAATCAGATATCTTGCTGAACTGTCAGACTTTGAGAAGGAAG
SARS-CoV-2 NC_045512.2 (29904) -------------------------------------------------------------------------------------------------
```

Section 445

```
(43069)                      43069      43080     43090     43100     43110     43120     43130     43140     43150           43165
lambda NC_001416.1 (41577)   ATATCCTCGCATGGTGGAAGCAACAACCATTCGATTTGCAAATACCGGAACATCTCGGTAACTGCATATTCTGCATTAAAAAATCAACGCAAAAAAT
SARS-CoV-2 NC_045512.2 (29904) -------------------------------------------------------------------------------------------------
```

Section 446

```
(43166)                      43166         43180     43190     43200     43210     43220     43230     43240     43250      43262
lambda NC_001416.1 (41674)   CGGACTTGCCTGCAAAGATGAGGAGGGATTGCAGCGTGTTTTTAATGAGGTCATCACGGGATCCCATGTGCGTGACGGACATCGGGAAACGCCAAAG
SARS-CoV-2 NC_045512.2 (29904) -------------------------------------------------------------------------------------------------
```

Section 447

```
(43263)                      43263  43270     43280     43290     43300     43310     43320     43330     43340               43359
lambda NC_001416.1 (41771)   GAGATTATGTACCGAGGAAGAATGTCGCTGGACGGTATCGCGAAAATGTATTCAGAAAATGATTATCAAGCCCTGTATCAGGACATGGTACGAGCTA
SARS-CoV-2 NC_045512.2 (29904) -------------------------------------------------------------------------------------------------
```

Section 448

```
(43360)                      43360     43370     43380     43390     43400     43410     43420     43430     43440           43456
lambda NC_001416.1 (41868)   AAAGATTCGATACCGGCTCTTGTTCTGAGTCATGCGAAATATTTGGAGGGCAGCTTGATTTCGACTTCGGGAGGGAAGCTGCATGATGCGATGTTAT
SARS-CoV-2 NC_045512.2 (29904) -------------------------------------------------------------------------------------------------
```

```
                                                                                                   Section 449
                            (43457) 43457        43470     43480     43490     43500     43510     43520     43530     43540    43553
        lambda NC_001416.1 (41965) CGGTGCGGTGAATGCAAAGAAGATAACCGCTTCCGACCAAATCAACCTTACTGGAATCGATGGTGTCTCCGGTGTGAAAGAACACCAACAGGGGTGT
SARS-CoV-2 NC_045512.2 (29904) -------------------------------------------------------------------------------------------------
                                                                                                   Section 450
                            (43554) 43554 43560     43570     43580     43590     43600     43610     43620     43630     43640 43650
        lambda NC_001416.1 (42062) TACCACTACCGCAGGAAAAGGAGGACGTGTGGCGAGACAGCGACGAAGTATCACCGACATAATCTGCGAAAACTGCAAATACCTTCCAACGAAACGC
SARS-CoV-2 NC_045512.2 (29904) -------------------------------------------------------------------------------------------------
                                                                                                   Section 451
                            (43651) 43651    43660     43670     43680     43690     43700     43710     43720     43730          43747
        lambda NC_001416.1 (42159) ACCAGAAATAAACCCAAGCCAATCCCAAAAGAATCTGACGTAAAAACCTTCAACTACACGGCTCACCTGTGGGATATCCGGTGGCTAAGACGTCGTG
SARS-CoV-2 NC_045512.2 (29904) -------------------------------------------------------------------------------------------------
                                                                                                   Section 452
                            (43748) 43748       43760     43770     43780     43790     43800     43810     43820     43830        43844
        lambda NC_001416.1 (42256) CGAGGAAAACAAGGTGATTGACCAAAATCGAAGTTACGAACAAGAAAGCGTCGAGCGAGCTTTAACGTGCGCTAACTGCGGTCAGAAGCTGCATGTG
SARS-CoV-2 NC_045512.2 (29904) -------------------------------------------------------------------------------------------------
                                                                                                   Section 453
                            (43845) 43845 43850     43860     43870     43880     43890     43900     43910     43920     43930  43941
        lambda NC_001416.1 (42353) CTGGAAGTTCACGTGTGTGAGCACTGCTGCGCAGAACTGATGAGCGATCCGAATAGCTCGATGCACGAGGAAGAAGATGATGGCTAAACCAGCGCGA
SARS-CoV-2 NC_045512.2 (29904) -------------------------------------------------------------------------------------------------
                                                                                                   Section 454
                            (43942) 43942   43950     43960     43970     43980     43990     44000     44010     44020          44038
        lambda NC_001416.1 (42450) AGACGATGTAAAAACGATGAATGCCGGGAATGGTTTCACCCTGCATTCGCTAATCAGTGGTGGTGCTCTCCAGAGTGTGGAACCAAGATAGCACTCG
SARS-CoV-2 NC_045512.2 (29904) -------------------------------------------------------------------------------------------------
                                                                                                   Section 455
                            (44039) 44039      44050     44060     44070     44080     44090     44100     44110     44120          44135
        lambda NC_001416.1 (42547) AACGACGAAGTAAAGAACGCGAAAAAGCGGAAAAAGCAGCAGAGAAGAAACGACGACGAGAGGAGCAGAAACAGAAAGATAAACTTAAGATTCGAAA
SARS-CoV-2 NC_045512.2 (29904) -------------------------------------------------------------------------------------------------
```

```
                                                                                                   Section 456
                          (44136) 44136         44150     44160     44170     44180     44190     44200     44210     44220       44232
   lambda NC_001416.1 (42644) ACTCGCCTTAAAGCCCCGCAGTTACTGGATTAAACAAGCCCAACAAGCCGTAAACGCCTTCATCAGAGAAAGAGACCGCGACTTACCATGTATCTCG
SARS-CoV-2 NC_045512.2 (29904) -------------------------------------------------------------------------------------------------
                                                                                                   Section 457
                          (44233) 44233  44240     44250     44260     44270     44280     44290     44300     44310            44329
   lambda NC_001416.1 (42741) TGCGGAACGCTCACGTCTGCTCAGTGGGATGCCGGACATTACCGGACAACTGCTGCGGCACCTCAACTCCGATTTAATGAACGCAATATTCACAAGC
SARS-CoV-2 NC_045512.2 (29904) -------------------------------------------------------------------------------------------------
                                                                                                   Section 458
                          (44330) 44330     44340     44350     44360     44370     44380     44390     44400     44410           44426
   lambda NC_001416.1 (42838) AATGCGTGGTGTGCAACCAGCACAAAAGCGGAAATCTCGTTCCGTATCGCGTCGAACTGATTAGCCGCATCGGGCAGGAAGCAGTAGACGAAATCGA
SARS-CoV-2 NC_045512.2 (29904) -------------------------------------------------------------------------------------------------
                                                                                                   Section 459
                          (44427) 44427        44440     44450     44460     44470     44480     44490     44500     44510        44523
   lambda NC_001416.1 (42935) ATCAAACCATAACCGCCATCGCTGGACTATCGAAGAGTGCAAGGCGATCAAGGCAGAGTACCAACAGAAACTCAAAGACCTGCGAAATAGCAGAAGT
SARS-CoV-2 NC_045512.2 (29904) -------------------------------------------------------------------------------------------------
                                                                                                   Section 460
                          (44524) 44524 44530     44540     44550     44560     44570     44580     44590     44600     44610   44620
   lambda NC_001416.1 (43032) GAGGCCGCATGACGTTCTCAGTAAAAACCATTCCAGACATGCTCGTTGAAGCATACGGAAATCAGACAGAAGTAGCACGCAGACTGAAATGTAGTCG
SARS-CoV-2 NC_045512.2 (29904) -------------------------------------------------------------------------------------------------
                                                                                                   Section 461
                          (44621) 44621    44630     44640     44650     44660     44670     44680     44690     44700            44717
   lambda NC_001416.1 (43129) CGGTACGGTCAGAAAATACGTTGATGATAAAGACGGGAAAATGCACGCCATCGTCAACGACGTTCTCATGGTTCATCGCGGATGGAGTGAAAGAGAT
SARS-CoV-2 NC_045512.2 (29904) -------------------------------------------------------------------------------------------------
                                                                                                   Section 462
                          (44718) 44718       44730     44740     44750     44760     44770     44780     44790     44800         44814
   lambda NC_001416.1 (43226) GCGCTATTACGAAAAAATTGATGGCAGCAAATACCGAAATATTTGGGTAGTTGGCGATCTGCACGGATGCTACACGAACCTGATGAACAAACTGGAT
SARS-CoV-2 NC_045512.2 (29904) -------------------------------------------------------------------------------------------------
```

```
                                                                                                  Section 463
                             (44815) 44815 44820     44830     44840     44850     44860     44870     44880     44890     44900      44911
              lambda NC_001416.1 (43323) ACGATTGGATTCGACAACAAAAAAGACCTGCTTATCTCGGTGGGCGATTTGGTTGATCGTGGTGCAGAGAACGTTGAATGCCTGGAATTAATCACAT
        SARS-CoV-2 NC_045512.2 (29904) -------------------------------------------------------------------------------------------------
                                                                                                  Section 464
                             (44912) 44912   44920     44930     44940     44950     44960     44970     44980     44990            45008
              lambda NC_001416.1 (43420) TCCCCTGGTTCAGAGCTGTACGTGGAAACCATGAGCAAATGATGATTGATGGCTTATCAGAGCGTGGAAACGTTAATCACTGGCTGCTTAATGGCGG
        SARS-CoV-2 NC_045512.2 (29904) -------------------------------------------------------------------------------------------------
                                                                                                  Section 465
                             (45009) 45009      45020     45030     45040     45050     45060     45070     45080     45090         45105
              lambda NC_001416.1 (43517) TGGCTGGTTCTTTAATCTCGATTACGACAAAGAAATTCTGGCTAAAGCTCTTGCCCATAAAGCAGATGAACTTCCGTTAATCATCGAACTGGTGAGC
        SARS-CoV-2 NC_045512.2 (29904) -------------------------------------------------------------------------------------------------
                                                                                                  Section 466
                             (45106) 45106         45120     45130     45140     45150     45160     45170     45180     45190      45202
              lambda NC_001416.1 (43614) AAAGATAAAAAATATGTTATCTGCCACGCCGATTATCCCTTTGACGAATACGAGTTTGGAAAGCCAGTTGATCATCAGCAGGTAATCTGGAACCGCG
        SARS-CoV-2 NC_045512.2 (29904) -------------------------------------------------------------------------------------------------
                                                                                                  Section 467
                             (45203) 45203  45210     45220     45230     45240     45250     45260     45270     45280            45299
              lambda NC_001416.1 (43711) AACGAATCAGCAACTCACAAAACGGGATCGTGAAAGAAATCAAAGGCGCGGACACGTTCATCTTTGGTCATACGCCAGCAGTGAAACCACTCAAGTT
        SARS-CoV-2 NC_045512.2 (29904) -------------------------------------------------------------------------------------------------
                                                                                                  Section 468
                             (45300) 45300     45310     45320     45330     45340     45350     45360     45370     45380          45396
              lambda NC_001416.1 (43808) TGCCAACCAAATGTATATCGATACCGGCGCAGTGTTCTGCGGAAACCTAACATTGATTCAGGTACAGGGAGAAGGCGCATGAGACTCGAAAGCGTAG
        SARS-CoV-2 NC_045512.2 (29904) -------------------------------------------------------------------------------------------------
                                                                                                  Section 469
                             (45397) 45397        45410     45420     45430     45440     45450     45460     45470     45480       45493
              lambda NC_001416.1 (43905) CTAAATTTCATTCGCCAAAAAGCCCGATGATGAGCGACTCACCACGGGCCACGGCTTCTGACTCTCTTTCCGGTACTGATGTGATGGCTGCTATGGG
        SARS-CoV-2 NC_045512.2 (29904) -------------------------------------------------------------------------------------------------
```

Section 470

(45494) 45494 45500 45510 45520 45530 45540 45550 45560 45570 45580 45590
lambda NC_001416.1 (44002) GATGGCGCAATCACAAGCCGGATTCGGTATGGCTGCATTCTGCGGTAAGCACGAACTCAGCCAGAACGACAAACAAAAGGCTATCAACTATCTGATG
SARS-CoV-2 NC_045512.2 (29904) ---

Section 471

(45591) 45591 45600 45610 45620 45630 45640 45650 45660 45670 45687
lambda NC_001416.1 (44099) CAATTTGCACACAAGGTATCGGGGAAATACCGTGGTGTGGCAAAGCTTGAAGGAAATACTAAGGCAAAGGTACTGCAAGTGCTCGCAACATTCGCTT
SARS-CoV-2 NC_045512.2 (29904) ---

Section 472

(45688) 45688 45700 45710 45720 45730 45740 45750 45760 45770 45784
lambda NC_001416.1 (44196) ATGCGGATTATTGCCGTAGTGCCGCGACGCCGGGGGCAAGATGCAGAGATTGCCATGGTACAGGCCGTGCGGTTGATATTGCCAAAACAGAGCTGTG
SARS-CoV-2 NC_045512.2 (29904) ---

Section 473

(45785) 45785 45790 45800 45810 45820 45830 45840 45850 45860 45870 45881
lambda NC_001416.1 (44293) GGGGAGAGTTGTCGAGAAAGAGTGCGGAAGATGCAAAGGCGTCGGCTATTCAAGGATGCCAGCAAGCGCAGCATATCGCGCTGTGACGATGCTAATC
SARS-CoV-2 NC_045512.2 (29904) ---

Section 474

(45882) 45882 45890 45900 45910 45920 45930 45940 45950 45960 45978
lambda NC_001416.1 (44390) CCAAACCTTACCCAACCCACCTGGTCACGCACTGTTAAGCCGCTGTATGACGCTCTGGTGGTGCAATGCCACAAAGAAGAGTCAATCGCAGACAACA
SARS-CoV-2 NC_045512.2 (29904) ---

Section 475

(45979) 45979 45990 46000 46010 46020 46030 46040 46050 46060 46075
lambda NC_001416.1 (44487) TTTTGAATGCGGTCACACGTTAGCAGCATGATTGCCACGGATGGCAACATATTAACGGCATGATATTGACTTATTGAATAAAATTGGGTAAATTTGA
SARS-CoV-2 NC_045512.2 (29904) ---

Section 476

(46076) 46076 46090 46100 46110 46120 46130 46140 46150 46160 46172
lambda NC_001416.1 (44584) CTCAACGATGGGTTAATTCGCTCGTTGTGGTAGTGAGATGAAAAGAGGCGGCGCTTACTACCGATTCCGCCTAGTTGGTCACTTCGACGTATCGTCT
SARS-CoV-2 NC_045512.2 (29904) ---

```
Section 477
(46173) 46173 46180 46190 46200 46210 46220 46230 46240 46250 46269
lambda NC_001416.1 (44681) GGAACTCCAACCATCGCAGGCAGAGAGGTCTGCAAAATGCAATCCCGAAACAGTTCGCAGGTAATAGTTAGAGCCTGCATAACGGTTTCGGGATTTT
SARS-CoV-2 NC_045512.2 (29904) -------------------------------------------------------------------------------------------------

Section 478
(46270) 46270 46280 46290 46300 46310 46320 46330 46340 46350 46366
lambda NC_001416.1 (44778) TTATATCTGCACAACAGGTAAGAGCATTGAGTCGATAATCGTGAAGAGTCGGCGAGCCTGGTTAGCCAGTGCTCTTTCCGTTGTGCTGAATTAAGCG
SARS-CoV-2 NC_045512.2 (29904) -------------------------------------------------------------------------------------------------

Section 479
(46367) 46367 46380 46390 46400 46410 46420 46430 46440 46450 46463
lambda NC_001416.1 (44875) AATACCGGAAGCAGAACCGGATCACCAAATGCGTACAGGCGTCATCGCCGCCCAGCAACAGCACAACCCAAACTGAGCCGTAGCCACTGTCTGTCCT
SARS-CoV-2 NC_045512.2 (29904) -------------------------------------------------------------------------------------------------

Section 480
(46464) 46464 46470 46480 46490 46500 46510 46520 46530 46540 46550 46560
lambda NC_001416.1 (44972) GAATTCATTAGTAATAGTTACGCTGCGGCCTTTTACACATGACCTTCGTGAAAGCGGGTGGCAGGAGGTCGCGCTAACAACCTCCTGCCGTTTTGCC
SARS-CoV-2 NC_045512.2 (29904) -------------------------------------------------------------------------------------------------

Section 481
(46561) 46561 46570 46580 46590 46600 46610 46620 46630 46640 46657
lambda NC_001416.1 (45069) CGTGCATATCGGTCACGAACAAATCTGATTACTAAACACAGTAGCCTGGATTTGTTCTATCAGTAATCGACCTTATTCCTAATTAAATAGAGCAAAT
SARS-CoV-2 NC_045512.2 (29904) -------------------------------------------------------------------------------------------------

Section 482
(46658) 46658 46670 46680 46690 46700 46710 46720 46730 46740 46754
lambda NC_001416.1 (45166) CCCCTTATTGGGGGTAAGACATGAAGATGCCAGAAAAACATGACCTGTTGGCCGCCATTCTCGCGGCAAAGGAACAAGGCATCGGGGCAATCCTTGC
SARS-CoV-2 NC_045512.2 (29904) -------------------------------------------------------------------------------------------------

Section 483
(46755) 46755 46760 46770 46780 46790 46800 46810 46820 46830 46840 46851
lambda NC_001416.1 (45263) GTTTGCAATGGCGTACCTTCGCGGCAGATATAATGGCGGTGCGTTTACAAAAACAGTAATCGACGCAACGATGTGCGCCATTATCGCCTGGTTCATT
SARS-CoV-2 NC_045512.2 (29904) -------------------------------------------------------------------------------------------------
```

Section 484

(46852) 46852 46860 46870 46880 46890 46900 46910 46920 46930 46948
lambda NC_001416.1 (45360) CGTGACCTTCTCGACTTCGCCGGACTAAGTAGCAATCTCGCTTATATAACGAGCGTGTTTATCGGCTACATCGGTACTGACTCGATTGGTTCGCTTA
SARS-CoV-2 NC_045512.2 (29904) ---

Section 485

(46949) 46949 46960 46970 46980 46990 47000 47010 47020 47030 47045
lambda NC_001416.1 (45457) TCAAACGCTTCGCTGCTAAAAAAGCCGGAGTAGAAGATGGTAGAAATCAATAATCAACGTAAGGCGTTCCTCGATATGCTGGCGTGGTCGGAGGGAA
SARS-CoV-2 NC_045512.2 (29904) ---

Section 486

(47046) 47046 47060 47070 47080 47090 47100 47110 47120 47130 47142
lambda NC_001416.1 (45554) CTGATAACGGACGTCAGAAAACCAGAAATCATGGTTATGACGTCATTGTAGGCGGAGAGCTATTTACTGATTACTCCGATCACCCTCGCAAACTTGT
SARS-CoV-2 NC_045512.2 (29904) ---

Section 487

(47143) 47143 47150 47160 47170 47180 47190 47200 47210 47220 47239
lambda NC_001416.1 (45651) CACGCTAAACCCAAAACTCAAATCAACAGGCGCCGGACGCTACCAGCTTCTTTCCCGTTGGTGGGATGCCTACCGCAAGCAGCTTGGCCTGAAAGAC
SARS-CoV-2 NC_045512.2 (29904) ---

Section 488

(47240) 47240 47250 47260 47270 47280 47290 47300 47310 47320 47336
lambda NC_001416.1 (45748) TTCTCTCCGAAAAGTCAGGACGCTGTGGCATTGCAGCAGATTAAGGAGCGTGGCGCTTTACCTATGATTGATCGTGGTGATATCCGTCAGGCAATCG
SARS-CoV-2 NC_045512.2 (29904) ---

Section 489

(47337) 47337 47350 47360 47370 47380 47390 47400 47410 47420 47433
lambda NC_001416.1 (45845) ACCGTTGCAGCAATATCTGGGCTTCACTGCCGGGCGCTGGTTATGGTCAGTTCGAGCATAAGGCTGACAGCCTGATTGCAAAATTCAAAGAAGCGGG
SARS-CoV-2 NC_045512.2 (29904) ---

Section 490

(47434) 47434 47440 47450 47460 47470 47480 47490 47500 47510 47520 47530
lambda NC_001416.1 (45942) CGGAACGGTCAGAGAGATTGATGTATGAGCAGAGTCACCGCGATTATCTCCGCTCTGGTTATCTGCATCATCGTCTGCCTGTCATGGGCTGTTAATC
SARS-CoV-2 NC_045512.2 (29904) ---

```
                                                                                                    Section 491
                       (47531) 47531    47540     47550     47560     47570     47580     47590     47600     47610           47627
    lambda NC_001416.1 (46039) ATTACCGTGATAACGCCATTACCTACAAAGCCCAGCGCGACAAAAATGCCAGAGAACTGAAGCTGGCGAACGCGGCAATTACTGACATGCAGATGCG
SARS-CoV-2 NC_045512.2 (29904) -------------------------------------------------------------------------------------------------
                                                                                                    Section 492
                       (47628) 47628       47640     47650     47660     47670     47680     47690     47700     47710        47724
    lambda NC_001416.1 (46136) TCAGCGTGATGTTGCTGCGCTCGATGCAAAATACACGAAGGAGTTAGCTGATGCTAAAGCTGAAAATGATGCTCTGCGTGATGATGTTGCCGCTGGT
SARS-CoV-2 NC_045512.2 (29904) -------------------------------------------------------------------------------------------------
                                                                                                    Section 493
                       (47725) 47725 47730     47740     47750     47760     47770     47780     47790     47800     47810    47821
    lambda NC_001416.1 (46233) CGTCGTCGGTTGCACATCAAAGCAGTCTGTCAGTCAGTGCGTGAAGCCACCACCGCCTCCGGCGTGGATAATGCAGCCTCCCCCCGACTGGCAGACA
SARS-CoV-2 NC_045512.2 (29904) -------------------------------------------------------------------------------------------------
                                                                                                    Section 494
                       (47822) 47822   47830     47840     47850     47860     47870     47880     47890     47900            47918
    lambda NC_001416.1 (46330) CCGCTGAACGGGATTATTTCACCCTCAGAGAGAGGCTGATCACTATGCAAAAACAACTGGAAGGAACCCAGAAGTATATTAATGAGCAGTGCAGATA
SARS-CoV-2 NC_045512.2 (29904) -------------------------------------------------------------------------------------------------
                                                                                                    Section 495
                       (47919) 47919      47930     47940     47950     47960     47970     47980     47990     48000         48015
    lambda NC_001416.1 (46427) GAGTTGCCCATATCGATGGGCAACTCATGCAATTATTGTGAGCAATACACACGCGCTTCCAGCGGAGTATAAATGCCTAAAGTAATAAAACCGAGCA
SARS-CoV-2 NC_045512.2 (29904) -------------------------------------------------------------------------------------------------
                                                                                                    Section 496
                       (48016) 48016         48030     48040     48050     48060     48070     48080     48090     48100      48112
    lambda NC_001416.1 (46524) ATCCATTTACGAATGTTTGCTGGGTTTCTGTTTTAACAACATTTTCTGCGCCGCCACAAATTTTGGCTGCATCGACAGTTTTCTTCTGCCCAATTCC
SARS-CoV-2 NC_045512.2 (29904) -------------------------------------------------------------------------------------------------
                                                                                                    Section 497
                       (48113) 48113  48120     48130     48140     48150     48160     48170     48180     48190           48209
    lambda NC_001416.1 (46621) AGAAACGAAGAAATGATGGGTGATGGTTTCCTTTGGTGCTACTGCTGCCGGTTTGTTTTGAACAGTAAACGTCTGTTGAGCACATCCTGTAATAAGC
SARS-CoV-2 NC_045512.2 (29904) -------------------------------------------------------------------------------------------------
```

```
                                                                                        Section 498
                     (48210) 48210     48220     48230     48240     48250     48260     48270     48280     48290     48306
    lambda NC_001416.1 (46718) AGGGCCAGCGCAGTAGCGAGTAGCATTTTTTTCATGGTGTTATTCCCGATGCTTTTTGAAGTTCGCAGAATCGTATGTGTAGAAAATTAAACAAACC
SARS-CoV-2 NC_045512.2 (29904) -------------------------------------------------------------------------------------------------
                                                                                        Section 499
                     (48307) 48307        48320     48330     48340     48350     48360     48370     48380     48390     48403
    lambda NC_001416.1 (46815) CTAAACAATGAGTTGAAATTTCATATTGTTAATATTTATTAATGTATGTCAGGTGCGATGAATCGTCATTGTATTCCCGGATTAACTATGTCCACAG
SARS-CoV-2 NC_045512.2 (29904) -------------------------------------------------------------------------------------------------
                                                                                        Section 500
                     (48404) 48404 48410     48420     48430     48440     48450     48460     48470     48480     48490  48500
    lambda NC_001416.1 (46912) CCCTGACGGGGAACTTCTCTGCGGGAGTGTCCGGGAATAATTAAAACGATGCACACAGGGTTTAGCGCGTACACGTATTGCATTATGCCAACGCCCC
SARS-CoV-2 NC_045512.2 (29904) -------------------------------------------------------------------------------------------------
                                                                                        Section 501
                     (48501) 48501    48510     48520     48530     48540     48550     48560     48570     48580          48597
    lambda NC_001416.1 (47009) GGTGCTGACACGGAAGAAACCGGACGTTATGATTTAGCGTGGAAAGATTTGTGTAGTGTTCTGAATGCTCTCAGTAAATAGTAATGAATTATCAAAG
SARS-CoV-2 NC_045512.2 (29904) -------------------------------------------------------------------------------------------------
                                                                                        Section 502
                     (48598) 48598       48610     48620     48630     48640     48650     48660     48670     48680      48694
    lambda NC_001416.1 (47106) GTATAGTAATATCTTTTATGTTCATGGATATTTGTAACCCATCGGAAAACTCCTGCTTTAGCAAGATTTTCCCTGTATTGCTGAAATGTGATTTCTC
SARS-CoV-2 NC_045512.2 (29904) -------------------------------------------------------------------------------------------------
                                                                                        Section 503
                     (48695) 48695 48700     48710     48720     48730     48740     48750     48760     48770     48780  48791
    lambda NC_001416.1 (47203) TTGATTTCAACCTATCATAGGACGTTTCTATAAGATGCGTGTTTCTTGAGAATTTAACATTTACAACCTTTTTAAGTCCTTTTATTAACACGGTGTT
SARS-CoV-2 NC_045512.2 (29904) -------------------------------------------------------------------------------------------------
                                                                                        Section 504
                     (48792) 48792   48800     48810     48820     48830     48840     48850     48860     48870          48888
    lambda NC_001416.1 (47300) ATCGTTTTCTAACACGATGTGAATATTATCTGTGGCTAGATAGTAAATATAATGTGAGACGTTGTGACGTTTTAGTTCAGAATAAAACAATTCACAG
SARS-CoV-2 NC_045512.2 (29904) -------------------------------------------------------------------------------------------------
```

```
Section 505
(48889) 48889      48900      48910      48920      48930      48940      48950      48960      48970      48985
lambda NC_001416.1 (47397) TCTAAATCTTTTCGCACTTGATCGAATATTTCTTTAAAAATGGCAACCTGAGCCATTGGTAAAACCTTCCATGTGATACGAGGGCGCGTAGTTTGCA
SARS-CoV-2 NC_045512.2 (29904) -------------------------------------------------------------------------------------------------

Section 506
(48986) 48986      49000      49010      49020      49030      49040      49050      49060      49070      49082
lambda NC_001416.1 (47494) TTATCGTTTTTATCGTTTCAATCTGGTCTGACCTCCTTGTGTTTTGTTGATGATTTATGTCAAATATTAGGAATGTTTTCACTTAATAGTATTGGTT
SARS-CoV-2 NC_045512.2 (29904) -------------------------------------------------------------------------------------------------

Section 507
(49083) 49083 49090      49100      49110      49120      49130      49140      49150      49160      49179
lambda NC_001416.1 (47591) GCGTAACAAAGTGCGGTCCTGCTGGCATTCTGGAGGGAAATACAACCGACAGATGTATGTAAGGCCAACGTGCTCAAATCTTCATACAGAAAGATTT
SARS-CoV-2 NC_045512.2 (29904) -------------------------------------------------------------------------------------------------

Section 508
(49180) 49180      49190      49200      49210      49220      49230      49240      49250      49260      49276
lambda NC_001416.1 (47688) GAAGTAATATTTTAACCGCTAGATGAAGAGCAAGCGCATGGAGCGACAAAATGAATAAAGAACAATCTGCTGATGATCCCTCCGTGGATCTGATTCG
SARS-CoV-2 NC_045512.2 (29904) -------------------------------------------------------------------------------------------------

Section 509
(49277) 49277      49290      49300      49310      49320      49330      49340      49350      49360      49373
lambda NC_001416.1 (47785) TGTAAAAAATATGCTTAATAGCACCATTTCTATGAGTTACCCTGATGTTGTAATTGCATGTATAGAACATAAGGTGTCTCTGGAAGCATTCAGAGCA
SARS-CoV-2 NC_045512.2 (29904) -------------------------------------------------------------------------------------------------

Section 510
(49374) 49374 49380      49390      49400      49410      49420      49430      49440      49450      49460 49470
lambda NC_001416.1 (47882) ATTGAGGCAGCGTTGGTGAAGCACGATAATAATATGAAGGATTATTCCCTGGTGGTTGACTGATCACCATAACTGCTAATCATTCAAACTATTTAGT
SARS-CoV-2 NC_045512.2 (29904) -------------------------------------------------------------------------------------------------

Section 511
(49471) 49471      49480      49490      49500      49510      49520      49530      49540      49550      49567
lambda NC_001416.1 (47979) CTGTGACAGAGCCAACACGCAGTCTGTCACTGTCAGGAAAGTGGTAAAACTGCAACTCAATTACTGCAATGCCCTCGTAATTAAGTGAATTTACAAT
SARS-CoV-2 NC_045512.2 (29904) -------------------------------------------------------------------------------------------------
```

```
                                                                                                                   Section 512
                      (49568) 49568       49580     49590     49600     49610     49620     49630     49640     49650         49664
     lambda NC_001416.1 (48076) ATCGTCCTGTTCGGAGGGAAGAACGCGGGATGTTCATTCTTCATCACTTTTAATTGATGTATATGCTCTCTTTTCTGACGTTAGTCTCCGACGGCAG
SARS-CoV-2 NC_045512.2 (29904) -------------------------------------------------------------------------------------------------
                                                                                                                   Section 513
                      (49665) 49665 49670     49680     49690     49700     49710     49720     49730     49740     49750      49761
     lambda NC_001416.1 (48173) GCTTCAATGACCCAGGCTGAGAAATTCCCGGACCCTTTTTGCTCAAGAGCGATGTTAATTTGTTCAATCATTTGGTTAGGAAAGCGGATGTTGCGGG
SARS-CoV-2 NC_045512.2 (29904) -------------------------------------------------------------------------------------------------
                                                                                                                   Section 514
                      (49762) 49762   49770     49780     49790     49800     49810     49820     49830     49840            49858
     lambda NC_001416.1 (48270) TTGTTGTTCTGCGGGTTCTGTTCTTCGTTGACATGAGGTTGCCCCGTATTCAGTGTCGCTGATTTGTATTGTCTGAAGTTGTTTTTACGTTAAGTTG
SARS-CoV-2 NC_045512.2 (29904) -------------------------------------------------------------------------------------------------
                                                                                                                   Section 515
                      (49859) 49859      49870     49880     49890     49900     49910     49920     49930     49940          49955
     lambda NC_001416.1 (48367) ATGCAGATCAATTAATACGATACCTGCGTCATAATTGATTATTTGACGTGGTTTGATGGCCTCCACGCACGTTGTGATATGTAGATGATAATCATTA
SARS-CoV-2 NC_045512.2 (29904) -------------------------------------------------------------------------------------------------
                                                                                                                   Section 516
                      (49956) 49956         49970     49980      49994
     lambda NC_001416.1 (48464) TCACTTTACGGGTCCTTTCCGGTGATCCGACAGGTTACG
SARS-CoV-2 NC_045512.2 (29904) ---------------------------------------
```
