# Supplementary material for: Ubiquitous Micro-Modular Homologies among Genomes from Viruses to Bacteria to Human Mitochondrial DNA: Platforms for Recombination during Evolution?
Source: Viruses. 2022 Apr 24;14(5):885. doi: 10.3390/v14050885 (PMC9147251; doi:10.3390/v14050885)
Supplement: Supplementary file 1 [file viruses-14-00885-s001.zip › Fig. S5G, SARS-CoV-2 & Hepatitis B DNA Alignment.pdf]

```
                                                                                                                         Section 1
                             (1)  1        10        20        30        40        50        60        70        80           94
Hepatitis B virus NC  003977.2  (1)  ----------------------------------------------------------------------------------------------
   SARS-CoV-2 NC_045512.2   (1)  ATTAAAGGTTTATACCTTCCCAGGTAACAAACCAACCAACTTTCGATCTCTTGTAGATCTGTTCTCTAAACGAACTTTAAAATCTGTGTGGCTG
                                                                                                                         Section 2
                            (95)  95   100       110       120       130       140       150       160       170            188
Hepatitis B virus NC  003977.2  (1)  ----------------------------------------------------------------------------------------------
   SARS-CoV-2 NC_045512.2  (95)  TCACTCGGCTGCATGCTTAGTGCACTCACGCAGTATAATTAATAACTAATTACTGTCGTTGACAGGACACGAGTAACTCGTCTATCTTCTGCAG
                                                                                                                         Section 3
                           (189)  189        200       210       220       230       240       250       260       270      282
Hepatitis B virus NC  003977.2  (1)  ----------------------------------------------------------------------------------------------
   SARS-CoV-2 NC_045512.2 (189)  GCTGCTTACGGTTTCGTCCGTGTTGCAGCCGATCATCAGCACATCTAGGTTTCGTCCGGGTGTGACCGAAAGGTAAGATGGAGAGCCTTGTCCC
                                                                                                                         Section 4
                           (283)  283    290       300       310       320       330       340       350       360          376
Hepatitis B virus NC  003977.2  (1)  ----------------------------------------------------------------------------------------------
   SARS-CoV-2 NC_045512.2 (283)  TGGTTTCAACGAGAAAACACACGTCCAACTCAGTTTGCCTGTTTTACAGGTTCGCGACGTGCTCGTACGTGGCTTTGGAGACTCCGTGGAGGAG
                                                                                                                         Section 5
                           (377)  377          390       400       410       420       430       440       450       460      470
Hepatitis B virus NC  003977.2  (1)  ----------------------------------------------------------------------------------------------
   SARS-CoV-2 NC_045512.2 (377)  GTCTTATCAGAGGCACGTCAACATCTTAAAGATGGCACTTGTGGCTTAGTAGAAGTTGAAAAAGGCGTTTTGCCTCAACTTGAACAGCCCTATG
                                                                                                                         Section 6
                           (471)  471      480       490       500       510       520       530       540       550          564
Hepatitis B virus NC  003977.2  (1)  ----------------------------------------------------------------------------------------------
   SARS-CoV-2 NC_045512.2 (471)  TGTTCATCAAACGTTCGGATGCTCGAACTGCACCTCATGGTCATGTTATGGTTGAGCTGGTAGCAGAACTCGAAGGCATTCAGTACGGTCGTAG
                                                                                                                         Section 7
                           (565)  565  570       580       590       600       610       620       630       640               658
Hepatitis B virus NC  003977.2  (1)  ----------------------------------------------------------------------------------------------
   SARS-CoV-2 NC_045512.2 (565)  TGGTGAGACACTTGGTGTCCTTGTCCCTCATGTGGGCGAAATACCAGTGGCTTACCGCAAGGTTCTTCTTCGTAAGAACGGTAATAAAGGAGCT
```

Section 8

```
                                   (659) 659       670       680       690       700       710       720       730       740         752
Hepatitis B virus NC_003977.2        (1) ----------------------------------------------------------------------------------------------
       SARS-CoV-2 NC_045512.2      (659) GGTGGCCATAGTTACGGCGCCGATCTAAAGTCATTTGACTTAGGCGACGAGCTTGGCACTGATCCTTATGAAGATTTTCAAGAAAACTGGAACA
```

Section 9

```
                                   (753) 753    760       770       780       790       800       810       820       830             846
Hepatitis B virus NC_003977.2        (1) ----------------------------------------------------------------------------------------------
       SARS-CoV-2 NC_045512.2      (753) CTAAACATAGCAGTGGTGTTACCCGTGAACTCATGCGTGAGCTTAACGGAGGGGCATACACTCGCTATGTCGATAACAACTTCTGTGGCCCTGA
```

Section 10

```
                                   (847) 847          860       870       880       890       900       910       920       930       940
Hepatitis B virus NC_003977.2        (1) ----------------------------------------------------------------------------------------------
       SARS-CoV-2 NC_045512.2      (847) TGGCTACCCTCTTGAGTGCATTAAAGACCTTCTAGCACGTGCTGGTAAAGCTTCATGCACTTTGTCCGAACAACTGGACTTTATTGACACTAAG
```

Section 11

```
                                   (941) 941      950       960       970       980       990       1000      1010      1020         1034
Hepatitis B virus NC_003977.2        (1) ----------------------------------------------------------------------------------------------
       SARS-CoV-2 NC_045512.2      (941) AGGGGTGTATACTGCTGCCGTGAACATGAGCATGAAATTGCTTGGTACACGGAACGTTCTGAAAAGAGCTATGAATTGCAGACACCTTTTGAAA
```

Section 12

```
                                  (1035) 1035 1040      1050      1060      1070      1080      1090      1100      1110             1128
Hepatitis B virus NC_003977.2        (1) ----------------------------------------------------------------------------------------------
       SARS-CoV-2 NC_045512.2     (1035) TTAAATTGGCAAAGAAATTTGACACCTTCAATGGGGAATGTCCAAATTTTGTATTTCCCTTAAATTCCATAATCAAGACTATTCAACCAAGGGT
```

Section 13

```
                                  (1129) 1129       1140      1150      1160      1170      1180      1190      1200      1210       1222
Hepatitis B virus NC_003977.2        (1) ----------------------------------------------------------------------------------------------
       SARS-CoV-2 NC_045512.2     (1129) TGAAAAGAAAAAGCTTGATGGCTTTATGGGTAGAATTCGATCTGTCTATCCAGTTGCGTCACCAAATGAATGCAACCAAATGTGCCTTTCAACT
```

Section 14

```
                                  (1223) 1223   1230      1240      1250      1260      1270      1280      1290      1300             1316
Hepatitis B virus NC_003977.2        (1) ----------------------------------------------------------------------------------------------
       SARS-CoV-2 NC_045512.2     (1223) CTCATGAAGTGTGATCATTGTGGTGAAACTTCATGGCAGACGGGCGATTTTGTTAAAGCCACTTGCGAATTTTGTGGCACTGAGAATTTGACTA
```

```
                                                                                                    Section 15
                          (1317) 1317         1330      1340      1350      1360      1370      1380      1390      1400      1410
Hepatitis B virus NC_003977.2 (1) ----------------------------------------------------------------------------------------------
SARS-CoV-2 NC_045512.2   (1317) AAGAAGGTGCCACTACTTGTGGTTACTTACCCCAAAATGCTGTTGTTAAAATTTATTGTCCAGCATGTCACAATTCAGAAGTAGGACCTGAGCA
                                                                                                    Section 16
                          (1411) 1411     1420      1430      1440      1450      1460      1470      1480      1490          1504
Hepatitis B virus NC_003977.2 (1) ----------------------------------------------------------------------------------------------
SARS-CoV-2 NC_045512.2   (1411) TAGTCTTGCCGAATACCATAATGAATCTGGCTTGAAAACCATTCTTCGTAAGGGTGGTCGCACTATTGCCTTTGGAGGCTGTGTGTTCTCTTAT
                                                                                                    Section 17
                          (1505) 1505 1510      1520      1530      1540      1550      1560      1570      1580              1598
Hepatitis B virus NC_003977.2 (1) ----------------------------------------------------------------------------------------------
SARS-CoV-2 NC_045512.2   (1505) GTTGGTTGCCATAACAAGTGTGCCTATTGGGTTCCACGTGCTAGCGCTAACATAGGTTGTAACCATACAGGTGTTGTTGGAGAAGGTTCCGAAG
                                                                                                    Section 18
                          (1599) 1599       1610      1620      1630      1640      1650      1660      1670      1680        1692
Hepatitis B virus NC_003977.2 (1) ----------------------------------------------------------------------------------------------
SARS-CoV-2 NC_045512.2   (1599) GTCTTAATGACAACCTTCTTGAAATACTCCAAAAAGAGAAAGTCAACATCAATATTGTTGGTGACTTTAAACTTAATGAAGAGATCGCCATTAT
                                                                                                    Section 19
                          (1693) 1693  1700      1710      1720      1730      1740      1750      1760      1770             1786
Hepatitis B virus NC_003977.2 (1) ----------------------------------------------------------------------------------------------
SARS-CoV-2 NC_045512.2   (1693) TTTGGCATCTTTTTCTGCTTCCACAAGTGCTTTTGTGGAAACTGTGAAAGGTTTGGATTATAAAGCATTCAAACAAATTGTTGAATCCTGTGGT
                                                                                                    Section 20
                          (1787) 1787         1800      1810      1820      1830      1840      1850      1860      1870      1880
Hepatitis B virus NC_003977.2 (1) ----------------------------------------------------------------------------------------------
SARS-CoV-2 NC_045512.2   (1787) AATTTTAAAGTTACAAAAGGAAAAGCTAAAAAAGGTGCCTGGAATATTGGTGAACAGAAATCAATACTGAGTCCTCTTTATGCATTTGCATCAG
                                                                                                    Section 21
                          (1881) 1881     1890      1900      1910      1920      1930      1940      1950      1960          1974
Hepatitis B virus NC_003977.2 (1) ----------------------------------------------------------------------------------------------
SARS-CoV-2 NC_045512.2   (1881) AGGCTGCTCGTGTTGTACGATCAATTTTCTCCCGCACTCTTGAAACTGCTCAAAATTCTGTGCGTGTTTTACAGAAGGCCGCTATAACAATACT
```

```
                                                                                                        Section 22
                                (1975) 1975 1980      1990      2000      2010      2020      2030      2040      2050              2068
Hepatitis B virus NC_003977.2      (1) ----------------------------------------------------------------------------------------------
   SARS-CoV-2 NC_045512.2    (1975) AGATGGAATTTCACAGTATTCACTGAGACTCATTGATGCTATGATGTTCACATCTGATTTGGCTACTAACAATCTAGTTGTAATGGCCTACATT
                                                                                                        Section 23
                                (2069) 2069       2080      2090      2100      2110      2120      2130      2140      2150      2162
Hepatitis B virus NC_003977.2      (1) ----------------------------------------------------------------------------------------------
   SARS-CoV-2 NC_045512.2    (2069) ACAGGTGGTGTTGTTCAGTTGACTTCGCAGTGGCTAACTAACATCTTTGGCACTGTTTATGAAAAACTCAAACCCGTCCTTGATTGGCTTGAAG
                                                                                                        Section 24
                                (2163) 2163   2170      2180      2190      2200      2210      2220      2230      2240              2256
Hepatitis B virus NC_003977.2      (1) ----------------------------------------------------------------------------------------------
   SARS-CoV-2 NC_045512.2    (2163) AGAAGTTTAAGGAAGGTGTAGAGTTTCTTAGAGACGGTTGGGAAATTGTTAAATTTATCTCAACCTGTGCTTGTGAAATTGTCGGTGGACAAAT
                                                                                                        Section 25
                                (2257) 2257         2270      2280      2290      2300      2310      2320      2330      2340      2350
Hepatitis B virus NC_003977.2      (1) ----------------------------------------------------------------------------------------------
   SARS-CoV-2 NC_045512.2    (2257) TGTCACCTGTGCAAAGGAAATTAAGGAGAGTGTTCAGACATTCTTTAAGCTTGTAAATAAATTTTTGGCTTTGTGTGCTGACTCTATCATTATT
                                                                                                        Section 26
                                (2351) 2351     2360      2370      2380      2390      2400      2410      2420      2430          2444
Hepatitis B virus NC_003977.2      (1) ----------------------------------------------------------------------------------------------
   SARS-CoV-2 NC_045512.2    (2351) GGTGGAGCTAAACTTAAAGCCTTGAATTTAGGTGAAACATTTGTCACGCACTCAAAGGGATTGTACAGAAAGTGTGTTAAATCCAGAGAAGAAA
                                                                                                        Section 27
                                (2445) 2445 2450      2460      2470      2480      2490      2500      2510      2520              2538
Hepatitis B virus NC_003977.2      (1) ----------------------------------------------------------------------------------------------
   SARS-CoV-2 NC_045512.2    (2445) CTGGCCTACTCATGCCTCTAAAAGCCCCAAAAGAAATTATCTTCTTAGAGGGAGAAACACTTCCCACAGAAGTGTTAACAGAGGAAGTTGTCTT
                                                                                                        Section 28
                                (2539) 2539       2550      2560      2570      2580      2590      2600      2610      2620      2632
Hepatitis B virus NC_003977.2      (1) ----------------------------------------------------------------------------------------------
   SARS-CoV-2 NC_045512.2    (2539) GAAAACTGGTGATTTACAACCATTAGAACAACCTACTAGTGAAGCTGTTGAAGCTCCATTGGTTGGTACACCAGTTTGTATTAACGGGCTTATG
```

```
                                                                                                       Section 29
                               (2633) 2633  2640           2650           2660           2670           2680           2690           2700           2710           2726
Hepatitis B virus NC  003977.2    (1) ----------------------------------------------------------------------------------------------
     SARS-CoV-2 NC_045512.2  (2633) TTGCTCGAAATCAAAGACACAGAAAAGTACTGTGCCCTTGCACCTAATATGATGGTAACAAACAATACCTTCACACTCAAAGGCGGTGCACCAA
                                                                                                       Section 30
                               (2727) 2727          2740           2750           2760           2770           2780           2790           2800           2810   2820
Hepatitis B virus NC  003977.2    (1) ----------------------------------------------------------------------------------------------
     SARS-CoV-2 NC_045512.2  (2727) CAAAGGTTACTTTTGGTGATGACACTGTGATAGAAGTGCAAGGTTACAAGAGTGTGAATATCACTTTTGAACTTGATGAAAGGATTGATAAAGT
                                                                                                       Section 31
                               (2821) 2821     2830           2840           2850           2860           2870           2880           2890           2900           2914
Hepatitis B virus NC  003977.2    (1) ----------------------------------------------------------------------------------------------
     SARS-CoV-2 NC_045512.2  (2821) ACTTAATGAGAAGTGCTCTGCCTATACAGTTGAACTCGGTACAGAAGTAAATGAGTTCGCCTGTGTTGTGGCAGATGCTGTCATAAAAACTTTG
                                                                                                       Section 32
                               (2915) 2915 2920           2930           2940           2950           2960           2970           2980           2990           3008
Hepatitis B virus NC  003977.2    (1) ----------------------------------------------------------------------------------------------
     SARS-CoV-2 NC_045512.2  (2915) CAACCAGTATCTGAATTACTTACACCACTGGGCATTGATTTAGATGAGTGGAGTATGGCTACATACTACTTATTTGATGAGTCTGGTGAGTTTA
                                                                                                       Section 33
                               (3009) 3009        3020           3030           3040           3050           3060           3070           3080           3090      3102
Hepatitis B virus NC  003977.2    (1) ----------------------------------------------------------------------------------------------
     SARS-CoV-2 NC_045512.2  (3009) AATTGGCTTCACATATGTATTGTTCTTTCTACCCTCCAGATGAGGATGAAGAAGAAGGTGATTGTGAAGAAGAAGAGTTTGAGCCATCAACTCA
                                                                                                       Section 34
                               (3103) 3103  3110           3120           3130           3140           3150           3160           3170           3180           3196
Hepatitis B virus NC  003977.2    (1) ----------------------------------------------------------------------------------------------
     SARS-CoV-2 NC_045512.2  (3103) ATATGAGTATGGTACTGAAGATGATTACCAAGGTAAACCTTTGGAATTTGGTGCCACTTCTGCTGCTCTTCAACCTGAAGAAGAGCAAGAAGAA
                                                                                                       Section 35
                               (3197) 3197          3210           3220           3230           3240           3250           3260           3270           3280   3290
Hepatitis B virus NC  003977.2    (1) ----------------------------------------------------------------------------------------------
     SARS-CoV-2 NC_045512.2  (3197) GATTGGTTAGATGATGATAGTCAACAAACTGTTGGTCAACAAGACGGCAGTGAGGACAATCAGACAACTACTATTCAAACAATTGTTGAGGTTC
```

```
                                                                                                   Section 36
                              (3291) 3291     3300       3310       3320       3330       3340       3350       3360       3370         3384
Hepatitis B virus NC_003977.2    (1) ----------------------------------------------------------------------------------------------
     SARS-CoV-2 NC_045512.2 (3291) AACCTCAATTAGAGATGGAACTTACACCAGTTGTTCAGACTATTGAAGTGAATAGTTTTAGTGGTTATTTAAAACTTACTGACAATGTATACAT
                                                                                                   Section 37
                              (3385) 3385 3390       3400       3410       3420       3430       3440       3450       3460          3478
Hepatitis B virus NC_003977.2    (1) ----------------------------------------------------------------------------------------------
     SARS-CoV-2 NC_045512.2 (3385) TAAAAATGCAGACATTGTGGAAGAAGCTAAAAAGGTAAAACCAACAGTGGTTGTTAATGCAGCCAATGTTTACCTTAAACATGGAGGAGGTGTT
                                                                                                   Section 38
                              (3479) 3479        3490       3500       3510       3520       3530       3540       3550       3560     3572
Hepatitis B virus NC_003977.2    (1) ----------------------------------------------------------------------------------------------
     SARS-CoV-2 NC_045512.2 (3479) GCAGGAGCCTTAAATAAGGCTACTAACAATGCCATGCAAGTTGAATCTGATGATTACATAGCTACTAATGGACCACTTAAAGTGGGTGGTAGTT
                                                                                                   Section 39
                              (3573) 3573   3580       3590       3600       3610       3620       3630       3640       3650          3666
Hepatitis B virus NC_003977.2    (1) ----------------------------------------------------------------------------------------------
     SARS-CoV-2 NC_045512.2 (3573) GTGTTTTAAGCGGACACAATCTTGCTAAACACTGTCTTCATGTTGTCGGCCCAAATGTTAACAAAGGTGAAGACATTCAACTTCTTAAGAGTGC
                                                                                                   Section 40
                              (3667) 3667          3680       3690       3700       3710       3720       3730       3740       3750   3760
Hepatitis B virus NC_003977.2    (1) ----------------------------------------------------------------------------------------------
     SARS-CoV-2 NC_045512.2 (3667) TTATGAAAATTTTAATCAGCACGAAGTTCTACTTGCACCATTATTATCAGCTGGTATTTTTGGTGCTGACCCTATACATTCTTTAAGAGTTTGT
                                                                                                   Section 41
                              (3761) 3761     3770       3780       3790       3800       3810       3820       3830       3840         3854
Hepatitis B virus NC_003977.2    (1) ----------------------------------------------------------------------------------------------
     SARS-CoV-2 NC_045512.2 (3761) GTAGATACTGTTCGCACAAATGTCTACTTAGCTGTCTTTGATAAAAATCTCTATGACAAACTTGTTTCAAGCTTTTTGGAAATGAAGAGTGAAA
                                                                                                   Section 42
                              (3855) 3855 3860       3870       3880       3890       3900       3910       3920       3930          3948
Hepatitis B virus NC_003977.2    (1) ----------------------------------------------------------------------------------------------
     SARS-CoV-2 NC_045512.2 (3855) AGCAAGTTGAACAAAAGATCGCTGAGATTCCTAAAGAGGAAGTTAAGCCATTTATAACTGAAAGTAAACCTTCAGTTGAACAGAGAAAACAAGA
```

```
                                                                                                  Section 43
                             (3949) 3949       3960      3970      3980      3990      4000      4010      4020      4030     4042
Hepatitis B virus NC_003977.2   (1) ----------------------------------------------------------------------------------------------
    SARS-CoV-2 NC_045512.2   (3949) TGATAAGAAAATCAAAGCTTGTGTTGAAGAAGTTACAACAACTCTGGAAGAAACTAAGTTCCTCACAGAAAACTTGTTACTTTATATTGACATT
                                                                                                  Section 44
                             (4043) 4043   4050      4060      4070      4080      4090      4100      4110      4120          4136
Hepatitis B virus NC_003977.2   (1) ----------------------------------------------------------------------------------------------
    SARS-CoV-2 NC_045512.2   (4043) AATGGCAATCTTCATCCAGATTCTGCCACTCTTGTTAGTGACATTGACATCACTTTCTTAAAGAAAGATGCTCCATATATAGTGGGTGATGTTG
                                                                                                  Section 45
                             (4137) 4137         4150      4160      4170      4180      4190      4200      4210      4220     4230
Hepatitis B virus NC_003977.2   (1) ----------------------------------------------------------------------------------------------
    SARS-CoV-2 NC_045512.2   (4137) TTCAAGAGGGTGTTTTAACTGCTGTGGTTATACCTACTAAAAAGGCTGGTGGCACTACTGAAATGCTAGCGAAAGCTTTGAGAAAAGTGCCAAC
                                                                                                  Section 46
                             (4231) 4231     4240      4250      4260      4270      4280      4290      4300      4310          4324
Hepatitis B virus NC_003977.2   (1) ----------------------------------------------------------------------------------------------
    SARS-CoV-2 NC_045512.2   (4231) AGACAATTATATAACCACTTACCCGGGTCAGGGTTTAAATGGTTACACTGTAGAGGAGGCAAAGACAGTGCTTAAAAAGTGTAAAAGTGCCTTT
                                                                                                  Section 47
                             (4325) 4325 4330      4340      4350      4360      4370      4380      4390      4400              4418
Hepatitis B virus NC_003977.2   (1) ----------------------------------------------------------------------------------------------
    SARS-CoV-2 NC_045512.2   (4325) TACATTCTACCATCTATTATCTCTAATGAGAAGCAAGAAATTCTTGGAACTGTTTCTTGGAATTTGCGAGAAATGCTTGCACATGCAGAAGAAA
                                                                                                  Section 48
                             (4419) 4419       4430      4440      4450      4460      4470      4480      4490      4500     4512
Hepatitis B virus NC_003977.2   (1) ----------------------------------------------------------------------------------------------
    SARS-CoV-2 NC_045512.2   (4419) CACGCAAATTAATGCCTGTCTGTGTGGAAACTAAAGCCATAGTTTCAACTATACAGCGTAAATATAAGGGTATTAAAATACAAGAGGGTGTGGT
                                                                                                  Section 49
                             (4513) 4513   4520      4530      4540      4550      4560      4570      4580      4590          4606
Hepatitis B virus NC_003977.2   (1) ----------------------------------------------------------------------------------------------
    SARS-CoV-2 NC_045512.2   (4513) TGATTATGGTGCTAGATTTTACTTTTACACCAGTAAAACAACTGTAGCGTCACTTATCAACACACTTAACGATCTAAATGAAACTCTTGTTACA
```

```
                                                                                                  Section 50
                                 (4607) 4607        4620      4630      4640      4650      4660      4670      4680      4690      4700
Hepatitis B virus NC_003977.2       (1) ----------------------------------------------------------------------------------------------
      SARS-CoV-2 NC_045512.2     (4607) ATGCCACTTGGCTATGTAACACATGGCTTAAATTTGGAAGAAGCTGCTCGGTATATGAGATCTCTCAAAGTGCCAGCTACAGTTTCTGTTTCTT
                                                                                                  Section 51
                                 (4701) 4701     4710      4720      4730      4740      4750      4760      4770      4780          4794
Hepatitis B virus NC_003977.2       (1) ----------------------------------------------------------------------------------------------
      SARS-CoV-2 NC_045512.2     (4701) CACCTGATGCTGTTACAGCGTATAATGGTTATCTTACTTCTTCTTCTAAAACACCTGAAGAACATTTTATTGAAACCATCTCACTTGCTGGTTC
                                                                                                  Section 52
                                 (4795) 4795 4800      4810      4820      4830      4840      4850      4860      4870            4888
Hepatitis B virus NC_003977.2       (1) ----------------------------------------------------------------------------------------------
      SARS-CoV-2 NC_045512.2     (4795) CTATAAAGATTGGTCCTATTCTGGACAATCTACACAACTAGGTATAGAATTTCTTAAGAGAGGTGATAAAAGTGTATATTACACTAGTAATCCT
                                                                                                  Section 53
                                 (4889) 4889       4900      4910      4920      4930      4940      4950      4960      4970        4982
Hepatitis B virus NC_003977.2       (1) ----------------------------------------------------------------------------------------------
      SARS-CoV-2 NC_045512.2     (4889) ACCACATTCCACCTAGATGGTGAAGTTATCACCTTTGACAATCTTAAGACACTTCTTTCTTTGAGAGAAGTGAGGACTATTAAGGTGTTTACAA
                                                                                                  Section 54
                                 (4983) 4983   4990      5000      5010      5020      5030      5040      5050      5060            5076
Hepatitis B virus NC_003977.2       (1) ----------------------------------------------------------------------------------------------
      SARS-CoV-2 NC_045512.2     (4983) CAGTAGACAACATTAACCTCCACACGCAAGTTGTGGACATGTCAATGACATATGGACAACAGTTTGGTCCAACTTATTTGGATGGAGCTGATGT
                                                                                                  Section 55
                                 (5077) 5077        5090      5100      5110      5120      5130      5140      5150      5160      5170
Hepatitis B virus NC_003977.2       (1) ----------------------------------------------------------------------------------------------
      SARS-CoV-2 NC_045512.2     (5077) TACTAAAATAAAACCTCATAATTCACATGAAGGTAAAACATTTTATGTTTTACCTAATGATGACACTCTACGTGTTGAGGCTTTTGAGTACTAC
                                                                                                  Section 56
                                 (5171) 5171     5180      5190      5200      5210      5220      5230      5240      5250          5264
Hepatitis B virus NC_003977.2       (1) ----------------------------------------------------------------------------------------------
      SARS-CoV-2 NC_045512.2     (5171) CACACAACTGATCCTAGTTTTCTGGGTAGGTACATGTCAGCATTAAATCACACTAAAAAGTGGAAATACCCACAAGTTAATGGTTTAACTTCTA
```

```
                                                                                                                   Section 57
                            (5265) 5265 5270      5280      5290      5300      5310      5320      5330      5340              5358
Hepatitis B virus NC_003977.2  (1) ----------------------------------------------------------------------------------------------
   SARS-CoV-2 NC_045512.2 (5265) TTAAATGGGCAGATAACAACTGTTATCTTGCCACTGCATTGTTAACACTCCAACAAATAGAGTTGAAGTTTAATCCACCTGCTCTACAAGATGC
                                                                                                                   Section 58
                            (5359) 5359       5370      5380      5390      5400      5410      5420      5430      5440        5452
Hepatitis B virus NC_003977.2  (1) ----------------------------------------------------------------------------------------------
   SARS-CoV-2 NC_045512.2 (5359) TTATTACAGAGCAAGGGCTGGTGAAGCTGCTAACTTTTGTGCACTTATCTTAGCCTACTGTAATAAGACAGTAGGTGAGTTAGGTGATGTTAGA
                                                                                                                   Section 59
                            (5453) 5453   5460      5470      5480      5490      5500      5510      5520      5530            5546
Hepatitis B virus NC_003977.2  (1) ----------------------------------------------------------------------------------------------
   SARS-CoV-2 NC_045512.2 (5453) GAAACAATGAGTTACTTGTTTCAACATGCCAATTTAGATTCTTGCAAAAGAGTCTTGAACGTGGTGTGTAAAACTTGTGGACAACAGCAGACAA
                                                                                                                   Section 60
                            (5547) 5547         5560      5570      5580      5590      5600      5610      5620      5630      5640
Hepatitis B virus NC_003977.2  (1) ----------------------------------------------------------------------------------------------
   SARS-CoV-2 NC_045512.2 (5547) CCCTTAAGGGTGTAGAAGCTGTTATGTACATGGGCACACTTTCTTATGAACAATTTAAGAAAGGTGTTCAGATACCTTGTACGTGTGGTAAACA
                                                                                                                   Section 61
                            (5641) 5641     5650      5660      5670      5680      5690      5700      5710      5720          5734
Hepatitis B virus NC_003977.2  (1) ----------------------------------------------------------------------------------------------
   SARS-CoV-2 NC_045512.2 (5641) AGCTACAAAATATCTAGTACAACAGGAGTCACCTTTTGTTATGATGTCAGCACCACCTGCTCAGTATGAACTTAAGCATGGTACATTTACTTGT
                                                                                                                   Section 62
                            (5735) 5735 5740      5750      5760      5770      5780      5790      5800      5810              5828
Hepatitis B virus NC_003977.2  (1) ----------------------------------------------------------------------------------------------
   SARS-CoV-2 NC_045512.2 (5735) GCTAGTGAGTACACTGGTAATTACCAGTGTGGTCACTATAAACATATAACTTCTAAAGAAACTTTGTATTGCATAGACGGTGCTTTACTTACAA
                                                                                                                   Section 63
                            (5829) 5829       5840      5850      5860      5870      5880      5890      5900      5910        5922
Hepatitis B virus NC_003977.2  (1) ----------------------------------------------------------------------------------------------
   SARS-CoV-2 NC_045512.2 (5829) AGTCCTCAGAATACAAAGGTCCTATTACGGATGTTTTCTACAAAGAAAACAGTTACACAACAACCATAAAACCAGTTACTTATAAATTGGATGG
```

```
                                                                                                          Section 64
                             (5923) 5923  5930      5940      5950      5960      5970      5980      5990      6000         6016
 Hepatitis B virus NC  003977.2  (1) ----------------------------------------------------------------------------------------------
    SARS-CoV-2 NC_045512.2  (5923) TGTTGTTTGTACAGAAATTGACCCTAAGTTGGACAATTATTATAAGAAAGACAATTCTTATTTCACAGAGCAACCAATTGATCTTGTACCAAAC
                                                                                                          Section 65
                             (6017) 6017         6030      6040      6050      6060      6070      6080      6090      6100   6110
 Hepatitis B virus NC  003977.2  (1) ----------------------------------------------------------------------------------------------
    SARS-CoV-2 NC_045512.2  (6017) CAACCATATCCAAACGCAAGCTTCGATAATTTTAAGTTTGTATGTGATAATATCAAATTTGCTGATGATTTAAACCAGTTAACTGGTTATAAGA
                                                                                                          Section 66
                             (6111) 6111     6120      6130      6140      6150      6160      6170      6180      6190        6204
 Hepatitis B virus NC  003977.2  (1) ----------------------------------------------------------------------------------------------
    SARS-CoV-2 NC_045512.2  (6111) AACCTGCTTCAAGAGAGCTTAAAGTTACATTTTTCCCTGACTTAAATGGTGATGTGGTGGCTATTGATTATAAACACTACACACCCTCTTTTAA
                                                                                                          Section 67
                             (6205) 6205 6210      6220      6230      6240      6250      6260      6270      6280          6298
 Hepatitis B virus NC  003977.2  (1) ----------------------------------------------------------------------------------------------
    SARS-CoV-2 NC_045512.2  (6205) GAAAGGAGCTAAATTGTTACATAAACCTATTGTTTGGCATGTTAACAATGCAACTAATAAAGCCACGTATAAACCAAATACCTGGTGTATACGT
                                                                                                          Section 68
                             (6299) 6299        6310      6320      6330      6340      6350      6360      6370      6380     6392
 Hepatitis B virus NC  003977.2  (1) ----------------------------------------------------------------------------------------------
    SARS-CoV-2 NC_045512.2  (6299) TGTCTTTGGAGCACAAAACCAGTTGAAACATCAAATTCGTTTGATGTACTGAAGTCAGAGGACGCGCAGGGAATGGATAATCTTGCCTGCGAAG
                                                                                                          Section 69
                             (6393) 6393   6400      6410      6420      6430      6440      6450      6460      6470        6486
 Hepatitis B virus NC  003977.2  (1) ----------------------------------------------------------------------------------------------
    SARS-CoV-2 NC_045512.2  (6393) ATCTAAAACCAGTCTCTGAAGAAGTAGTGGAAAATCCTACCATACAGAAAGACGTTCTTGAGTGTAATGTGAAAACTACCGAAGTTGTAGGAGA
                                                                                                          Section 70
                             (6487) 6487         6500      6510      6520      6530      6540      6550      6560      6570   6580
 Hepatitis B virus NC  003977.2  (1) ----------------------------------------------------------------------------------------------
    SARS-CoV-2 NC_045512.2  (6487) CATTATACTTAAACCAGCAAATAATAGTTTAAAAATTACAGAAGAGGTTGGCCACACAGATCTAATGGCTGCTTATGTAGACAATTCTAGTCTT
```

```
                                                                                                          Section 71
                             (6581) 6581    6590      6600      6610      6620      6630      6640      6650      6660         6674
Hepatitis B virus NC_003977.2   (1) ----------------------------------------------------------------------------------------------
   SARS-CoV-2 NC_045512.2    (6581) ACTATTAAGAAACCTAATGAATTATCTAGAGTATTAGGTTTGAAAACCCTTGCTACTCATGGTTTAGCTGCTGTTAATAGTGTCCCTTGGGATA
                                                                                                          Section 72
                             (6675) 6675 6680      6690      6700      6710      6720      6730      6740      6750              6768
Hepatitis B virus NC_003977.2   (1) ----------------------------------------------------------------------------------------------
   SARS-CoV-2 NC_045512.2    (6675) CTATAGCTAATTATGCTAAGCCTTTTCTTAACAAAGTTGTTAGTACAACTACTAACATAGTTACACGGTGTTTAAACCGTGTTTGTACTAATTA
                                                                                                          Section 73
                             (6769) 6769       6780      6790      6800      6810      6820      6830      6840      6850      6862
Hepatitis B virus NC_003977.2   (1) ----------------------------------------------------------------------------------------------
   SARS-CoV-2 NC_045512.2    (6769) TATGCCTTATTTCTTTACTTTATTGCTACAATTGTGTACTTTTACTAGAAGTACAAATTCTAGAATTAAAGCATCTATGCCGACTACTATAGCA
                                                                                                          Section 74
                             (6863) 6863   6870      6880      6890      6900      6910      6920      6930      6940           6956
Hepatitis B virus NC_003977.2   (1) ----------------------------------------------------------------------------------------------
   SARS-CoV-2 NC_045512.2    (6863) AAGAATACTGTTAAGAGTGTCGGTAAATTTTGTCTAGAGGCTTCATTTAATTATTTGAAGTCACCTAATTTTTCTAAACTGATAAATATTATAA
                                                                                                          Section 75
                             (6957) 6957          6970      6980      6990      7000      7010      7020      7030      7040    7050
Hepatitis B virus NC_003977.2   (1) ----------------------------------------------------------------------------------------------
   SARS-CoV-2 NC_045512.2    (6957) TTTGGTTTTTACTATTAAGTGTTTGCCTAGGTTCTTTAATCTACTCAACCGCTGCTTTAGGTGTTTTAATGTCTAATTTAGGCATGCCTTCTTA
                                                                                                          Section 76
                             (7051) 7051    7060      7070      7080      7090      7100      7110      7120      7130         7144
Hepatitis B virus NC_003977.2   (1) ----------------------------------------------------------------------------------------------
   SARS-CoV-2 NC_045512.2    (7051) CTGTACTGGTTACAGAGAAGGCTATTTGAACTCTACTAATGTCACTATTGCAACCTACTGTACTGGTTCTATACCTTGTAGTGTTTGTCTTAGT
                                                                                                          Section 77
                             (7145) 7145 7150      7160      7170      7180      7190      7200      7210      7220              7238
Hepatitis B virus NC_003977.2   (1) ----------------------------------------------------------------------------------------------
   SARS-CoV-2 NC_045512.2    (7145) GGTTTAGATTCTTTAGACACCTATCCTTCTTTAGAAACTATACAAATTACCATTTCATCTTTTAAATGGGATTTAACTGCTTTTGGCTTAGTTG
```

```
                                                                                                                  Section 78
                             (7239) 7239       7250      7260      7270      7280      7290      7300      7310      7320        7332
Hepatitis B virus NC  003977.2  (1) ----------------------------------------------------------------------------------------------
   SARS-CoV-2 NC_045512.2    (7239) CAGAGTGGTTTTTGGCATATATTCTTTTCACTAGGTTTTTCTATGTACTTGGATTGGCTGCAATCATGCAATTGTTTTTCAGCTATTTTGCAGT
                                                                                                                  Section 79
                             (7333) 7333   7340      7350      7360      7370      7380      7390      7400      7410            7426
Hepatitis B virus NC  003977.2  (1) ----------------------------------------------------------------------------------------------
   SARS-CoV-2 NC_045512.2    (7333) ACATTTTATTAGTAATTCTTGGCTTATGTGGTTAATAATTAATCTTGTACAAATGGCCCCGATTTCAGCTATGGTTAGAATGTACATCTTCTTT
                                                                                                                  Section 80
                             (7427) 7427         7440      7450      7460      7470      7480      7490      7500      7510      7520
Hepatitis B virus NC  003977.2  (1) ----------------------------------------------------------------------------------------------
   SARS-CoV-2 NC_045512.2    (7427) GCATCATTTTATTATGTATGGAAAAGTTATGTGCATGTTGTAGACGGTTGTAATTCATCAACTTGTATGATGTGTTACAAACGTAATAGAGCAA
                                                                                                                  Section 81
                             (7521) 7521     7530      7540      7550      7560      7570      7580      7590      7600          7614
Hepatitis B virus NC  003977.2  (1) ----------------------------------------------------------------------------------------------
   SARS-CoV-2 NC_045512.2    (7521) CAAGAGTCGAATGTACAACTATTGTTAATGGTGTTAGAAGGTCCTTTTATGTCTATGCTAATGGAGGTAAAGGCTTTTGCAAACTACACAATTG
                                                                                                                  Section 82
                             (7615) 7615 7620      7630      7640      7650      7660      7670      7680      7690              7708
Hepatitis B virus NC  003977.2  (1) ----------------------------------------------------------------------------------------------
   SARS-CoV-2 NC_045512.2    (7615) GAATTGTGTTAATTGTGATACATTCTGTGCTGGTAGTACATTTATTAGTGATGAAGTTGCGAGAGACTTGTCACTACAGTTTAAAAGACCAATA
                                                                                                                  Section 83
                             (7709) 7709       7720      7730      7740      7750      7760      7770      7780      7790        7802
Hepatitis B virus NC  003977.2  (1) ----------------------------------------------------------------------------------------------
   SARS-CoV-2 NC_045512.2    (7709) AATCCTACTGACCAGTCTTCTTACATCGTTGATAGTGTTACAGTGAAGAATGGTTCCATCCATCTTTACTTTGATAAAGCTGGTCAAAAGACTT
                                                                                                                  Section 84
                             (7803) 7803   7810      7820      7830      7840      7850      7860      7870      7880            7896
Hepatitis B virus NC  003977.2  (1) ----------------------------------------------------------------------------------------------
   SARS-CoV-2 NC_045512.2    (7803) ATGAAAGACATTCTCTCTCATTTTGTTAACTTAGACAACCTGAGAGCTAATAACACTAAAGGTTCATTGCCTATTAATGTTATAGTTTTTGA
```

Section 85

	(7897)	7897 7910 7920 7930 7940 7950 7960 7970 7980 7990
Hepatitis B virus NC_003977.2	(1)	--
SARS-CoV-2 NC_045512.2	(7897)	TGGTAAATCAAAATGTGAAGAATCATCTGCAAAATCAGCGTCTGTTTACTACAGTCAGCTTATGTGTCAACCTATACTGTTACTAGATCAGGCA

Section 86

	(7991)	7991 8000 8010 8020 8030 8040 8050 8060 8070 8084
Hepatitis B virus NC_003977.2	(1)	--
SARS-CoV-2 NC_045512.2	(7991)	TTAGTGTCTGATGTTGGTGATAGTGCGGAAGTTGCAGTTAAAATGTTTGATGCTTACGTTAATACGTTTTCATCAACTTTTAACGTACCAATGG

Section 87

	(8085)	8085 8090 8100 8110 8120 8130 8140 8150 8160 8178
Hepatitis B virus NC_003977.2	(1)	--
SARS-CoV-2 NC_045512.2	(8085)	AAAAACTCAAAACACTAGTTGCAACTGCAGAAGCTGAACTTGCAAAGAATGTGTCCTTAGACAATGTCTTATCTACTTTTATTTCAGCAGCTCG

Section 88

	(8179)	8179 8190 8200 8210 8220 8230 8240 8250 8260 8272
Hepatitis B virus NC_003977.2	(1)	--
SARS-CoV-2 NC_045512.2	(8179)	GCAAGGGTTTGTTGATTCAGATGTAGAAACTAAAGATGTTGTTGAATGTCTTAAATTGTCACATCAATCTGACATAGAAGTTACTGGCGATAGT

Section 89

	(8273)	8273 8280 8290 8300 8310 8320 8330 8340 8350 8366
Hepatitis B virus NC_003977.2	(1)	--
SARS-CoV-2 NC_045512.2	(8273)	TGTAATAACTATATGCTCACCTATAACAAAGTTGAAAACATGACACCCCGTGACCTTGGTGCTTGTATTGACTGTAGTGCGCGTCATATTAATG

Section 90

	(8367)	8367 8380 8390 8400 8410 8420 8430 8440 8450 8460
Hepatitis B virus NC_003977.2	(1)	--
SARS-CoV-2 NC_045512.2	(8367)	CGCAGGTAGCAAAAAGTCACAACATTGCTTTGATATGGAACGTTAAAGATTTCATGTCATTGTCTGAACAACTACGAAAACAAATACGTAGTGC

Section 91

	(8461)	8461 8470 8480 8490 8500 8510 8520 8530 8540 8554
Hepatitis B virus NC_003977.2	(1)	--
SARS-CoV-2 NC_045512.2	(8461)	TGCTAAAAAGAATAACTTACCTTTTAAGTTGACATGTGCAACTACTAGACAAGTTGTTAATGTTGTAACAACAAAGATAGCACTTAAGGGTGGT

```
                                                                                                              Section 92
                           (8555) 8555 8560      8570      8580      8590      8600      8610      8620      8630              8648
Hepatitis B virus NC 003977.2 (1) ----------------------------------------------------------------------------------------------
   SARS-CoV-2 NC_045512.2 (8555) AAAATTGTTAATAATTGGTTGAAGCAGTTAATTAAAGTTACACTTGTGTTCCTTTTTGTTGCTGCTATTTTCTATTTAATAACACCTGTTCATG
                                                                                                              Section 93
                           (8649) 8649      8660      8670      8680      8690      8700      8710      8720      8730      8742
Hepatitis B virus NC 003977.2 (1) ----------------------------------------------------------------------------------------------
   SARS-CoV-2 NC_045512.2 (8649) TCATGTCTAAACATACTGACTTTTCAAGTGAAATCATAGGATACAAGGCTATTGATGGTGGTGTCACTCGTGACATAGCATCTACAGATACTTG
                                                                                                              Section 94
                           (8743) 8743   8750      8760      8770      8780      8790      8800      8810      8820           8836
Hepatitis B virus NC 003977.2 (1) ----------------------------------------------------------------------------------------------
   SARS-CoV-2 NC_045512.2 (8743) TTTTGCTAACAAACATGCTGATTTTGACACATGGTTTAGCCAGCGTGGTGGTAGTTATACTAATGACAAAGCTTGCCCATTGATTGCTGCAGTC
                                                                                                              Section 95
                           (8837) 8837         8850      8860      8870      8880      8890      8900      8910      8920    8930
Hepatitis B virus NC 003977.2 (1) ----------------------------------------------------------------------------------------------
   SARS-CoV-2 NC_045512.2 (8837) ATAACAAGAGAAGTGGGTTTTGTCGTGCCTGGTTTGCCTGGCACGATATTACGCACAACTAATGGTGACTTTTTGCATTTCTTACCTAGAGTTT
                                                                                                              Section 96
                           (8931) 8931     8940      8950      8960      8970      8980      8990      9000      9010          9024
Hepatitis B virus NC 003977.2 (1) ----------------------------------------------------------------------------------------------
   SARS-CoV-2 NC_045512.2 (8931) TTAGTGCAGTTGGTAACATCTGTTACACACCATCAAAACTTATAGAGTACACTGACTTTGCAACATCAGCTTGTGTTTTGGCTGCTGAATGTAC
                                                                                                              Section 97
                           (9025) 9025 9030      9040      9050      9060      9070      9080      9090      9100              9118
Hepatitis B virus NC 003977.2 (1) ----------------------------------------------------------------------------------------------
   SARS-CoV-2 NC_045512.2 (9025) AATTTTTAAAGATGCTTCTGGTAAGCCAGTACCATATTGTTATGATACCAATGTACTAGAAGGTTCTGTTGCTTATGAAAGTTTACGCCCTGAC
                                                                                                              Section 98
                           (9119) 9119      9130      9140      9150      9160      9170      9180      9190      9200      9212
Hepatitis B virus NC 003977.2 (1) ----------------------------------------------------------------------------------------------
   SARS-CoV-2 NC_045512.2 (9119) ACACGTTATGTGCTCATGGATGGCTCTATTATTCAATTTCCTAACACCTACCTTGAAGGTTCTGTTAGAGTGGTAACAACTTTTGATTCTGAGT
```

```
                                                                                                   Section 99
                            (9213) 9213  9220      9230      9240      9250      9260      9270      9280      9290         9306
Hepatitis B virus NC_003977.2  (1) ----------------------------------------------------------------------------------------------
  SARS-CoV-2 NC_045512.2    (9213) ACTGTAGGCACGGCACTTGTGAAAGATCAGAAGCTGGTGTTTGTGTATCTACTAGTGGTAGATGGGTACTTAACAATGATTATTACAGATCTTT
                                                                                                   Section 100
                            (9307) 9307         9320      9330      9340      9350      9360      9370      9380      9390    9400
Hepatitis B virus NC_003977.2  (1) ----------------------------------------------------------------------------------------------
  SARS-CoV-2 NC_045512.2    (9307) ACCAGGAGTTTTCTGTGGTGTAGATGCTGTAAATTTACTTACTAATATGTTTACACCACTAATTCAACCTATTGGTGCTTTGGACATATCAGCA
                                                                                                   Section 101
                            (9401) 9401     9410      9420      9430      9440      9450      9460      9470      9480          9494
Hepatitis B virus NC_003977.2  (1) ----------------------------------------------------------------------------------------------
  SARS-CoV-2 NC_045512.2    (9401) TCTATAGTAGCTGGTGGTATTGTAGCTATCGTAGTAACATGCCTTGCCTACTATTTTATGAGGTTTAGAAGAGCTTTTGGTGAATACAGTCATG
                                                                                                   Section 102
                            (9495) 9495 9500      9510      9520      9530      9540      9550      9560      9570              9588
Hepatitis B virus NC_003977.2  (1) ----------------------------------------------------------------------------------------------
  SARS-CoV-2 NC_045512.2    (9495) TAGTTGCCTTTAATACTTTACTATTCCTTATGTCATTCACTGTACTCTGTTTAACACCAGTTTACTCATTCTTACCTGGTGTTTATTCTGTTAT
                                                                                                   Section 103
                            (9589) 9589        9600      9610      9620      9630      9640      9650      9660      9670       9682
Hepatitis B virus NC_003977.2  (1) ----------------------------------------------------------------------------------------------
  SARS-CoV-2 NC_045512.2    (9589) TTACTTGTACTTGACATTTTATCTTACTAATGATGTTTCTTTTTTAGCACATATTCAGTGGATGGTTATGTTCACACCTTTAGTACCTTTCTGG
                                                                                                   Section 104
                            (9683) 9683  9690      9700      9710      9720      9730      9740      9750      9760         9776
Hepatitis B virus NC_003977.2  (1) ----------------------------------------------------------------------------------------------
  SARS-CoV-2 NC_045512.2    (9683) ATAACAATTGCTTATATCATTTGTATTTCCACAAAGCATTTCTATTGGTTCTTTAGTAATTACCTAAAGAGACGTGTAGTCTTTAATGGTGTTT
                                                                                                   Section 105
                            (9777) 9777         9790      9800      9810      9820      9830      9840      9850      9860    9870
Hepatitis B virus NC_003977.2  (1) ----------------------------------------------------------------------------------------------
  SARS-CoV-2 NC_045512.2    (9777) CCTTTAGTACTTTTGAAGAAGCTGCGCTGTGCACCTTTTTGTTAAATAAAGAAATGTATCTAAAGTTGCGTAGTGATGTGCTATTACCTCTTAC
```

```
                                                                                                  Section 106
                           (9871) 9871     9880      9890      9900      9910      9920      9930      9940      9950         9964
Hepatitis B virus NC_003977.2 (1) ----------------------------------------------------------------------------------------------
SARS-CoV-2 NC_045512.2     (9871) GCAATATAATAGATACTTAGCTCTTTATAATAAGTACAAGTATTTTAGTGGAGCAATGGATACAACTAGCTACAGAGAAGCTGCTTGTTGTCAT
                                                                                                  Section 107
                           (9965) 9965 9970      9980      9990      10000     10010     10020     10030     10040        10058
Hepatitis B virus NC_003977.2 (1) ----------------------------------------------------------------------------------------------
SARS-CoV-2 NC_045512.2     (9965) CTCGCAAAGGCTCTCAATGACTTCAGTAACTCAGGTTCTGATGTTCTTTACCAACCACCACAAACCTCTATCACCTCAGCTGTTTTGCAGAGTG
                                                                                                  Section 108
                          (10059) 10059      10070     10080     10090     10100     10110     10120     10130     10140   10152
Hepatitis B virus NC_003977.2 (1) ----------------------------------------------------------------------------------------------
SARS-CoV-2 NC_045512.2    (10059) GTTTTAGAAAAATGGCATTCCCATCTGGTAAAGTTGAGGGTTGTATGGTACAAGTAACTTGTGGTACAACTACACTTAACGGTCTTTGGCTTGA
                                                                                                  Section 109
                          (10153) 10153  10160     10170     10180     10190     10200     10210     10220     10230       10246
Hepatitis B virus NC_003977.2 (1) ----------------------------------------------------------------------------------------------
SARS-CoV-2 NC_045512.2    (10153) TGACGTAGTTTACTGTCCAAGACATGTGATCTGCACCTCTGAAGACATGCTTAACCCTAATTATGAAGATTTACTCATTCGTAAGTCTAATCAT
                                                                                                  Section 110
                          (10247) 10247        10260     10270     10280     10290     10300     10310     10320     10330 10340
Hepatitis B virus NC_003977.2 (1) ----------------------------------------------------------------------------------------------
SARS-CoV-2 NC_045512.2    (10247) AATTTCTTGGTACAGGCTGGTAATGTTCAACTCAGGGTTATTGGACATTCTATGCAAAATTGTGTACTTAAGCTTAAGGTTGATACAGCCAATC
                                                                                                  Section 111
                          (10341) 10341    10350     10360     10370     10380     10390     10400     10410     10420       10434
Hepatitis B virus NC_003977.2 (1) ----------------------------------------------------------------------------------------------
SARS-CoV-2 NC_045512.2    (10341) CTAAGACACCTAAGTATAAGTTTGTTCGCATTCAACCAGGACAGACTTTTTCAGTGTTAGCTTGTTACAATGGTTCACCATCTGGTGTTTACCA
                                                                                                  Section 112
                          (10435) 10435 10440     10450     10460     10470     10480     10490     10500     10510          10528
Hepatitis B virus NC_003977.2 (1) ----------------------------------------------------------------------------------------------
SARS-CoV-2 NC_045512.2    (10435) ATGTGCTATGAGGCCCAATTTCACTATTAAGGGTTCATTCCTTAATGGTTCATGTGGTAGTGTTGGTTTTAACATAGATTATGACTGTGTCTCT
```

```
                                                                                                                Section 113
                            (10529) 10529     10540     10550     10560     10570     10580     10590     10600     10610    10622
Hepatitis B virus NC_003977.2   (1) ----------------------------------------------------------------------------------------------
   SARS-CoV-2 NC_045512.2 (10529) TTTTGTTACATGCACCATATGGAATTACCAACTGGAGTTCATGCTGGCACAGACTTAGAAGGTAACTTTTATGGACCTTTTGTTGACAGGCAAA
                                                                                                                Section 114
                            (10623) 10623  10630     10640     10650     10660     10670     10680     10690     10700          10716
Hepatitis B virus NC_003977.2   (1) ----------------------------------------------------------------------------------------------
   SARS-CoV-2 NC_045512.2 (10623) CAGCACAAGCAGCTGGTACGGACACAACTATTACAGTTAATGTTTTAGCTTGGTTGTACGCTGCTGTTATAAATGGAGACAGGTGGTTTCTCAA
                                                                                                                Section 115
                            (10717) 10717        10730     10740     10750     10760     10770     10780     10790     10800   10810
Hepatitis B virus NC_003977.2   (1) ----------------------------------------------------------------------------------------------
   SARS-CoV-2 NC_045512.2 (10717) TCGATTTACCACAACTCTTAATGACTTTAACCTTGTGGCTATGAAGTACAATTATGAACCTCTAACACAAGACCATGTTGACATACTAGGACCT
                                                                                                                Section 116
                            (10811) 10811    10820     10830     10840     10850     10860     10870     10880     10890        10904
Hepatitis B virus NC_003977.2   (1) ----------------------------------------------------------------------------------------------
   SARS-CoV-2 NC_045512.2 (10811) CTTTCTGCTCAAACTGGAATTGCCGTTTTAGATATGTGTGCTTCATTAAAAGAATTACTGCAAAATGGTATGAATGGACGTACCATATTGGGTA
                                                                                                                Section 117
                            (10905) 10905 10910     10920     10930     10940     10950     10960     10970     10980           10998
Hepatitis B virus NC_003977.2   (1) ----------------------------------------------------------------------------------------------
   SARS-CoV-2 NC_045512.2 (10905) GTGCTTTATTAGAAGATGAATTTACACCTTTTGATGTTGTTAGACAATGCTCAGGTGTTACTTTCCAAAGTGCAGTGAAAAGAACAATCAAGGG
                                                                                                                Section 118
                            (10999) 10999     11010     11020     11030     11040     11050     11060     11070     11080    11092
Hepatitis B virus NC_003977.2   (1) ----------------------------------------------------------------------------------------------
   SARS-CoV-2 NC_045512.2 (10999) TACACACCACTGGTTGTTACTCACAATTTTGACTTCACTTTTAGTTTTAGTCCAGAGTACTCAATGGTCTTTGTTCTTTTTTTGTATGAAAAT
                                                                                                                Section 119
                            (11093) 11093  11100     11110     11120     11130     11140     11150     11160     11170          11186
Hepatitis B virus NC_003977.2   (1) ----------------------------------------------------------------------------------------------
   SARS-CoV-2 NC_045512.2 (11093) GCCTTTTTACCTTTTGCTATGGGTATTATTGCTATGTCTGCTTTTGCAATGATGTTTGTCAAACATAAGCATGCATTTCTCTGTTTGTTTTTGT
```

```
                                                                                                   Section 120
                             (11187) 11187        11200    11210    11220    11230    11240    11250    11260    11270  11280
Hepatitis B virus NC_003977.2    (1) ----------------------------------------------------------------------------------------------
SARS-CoV-2 NC_045512.2       (11187) TACCTTCTCTTGCCACTGTAGCTTATTTTAATATGGTCTATATGCCTGCTAGTTGGGTGATGCGTATTATGACATGGTTGGATATGGTTGATAC
                                                                                                   Section 121
                             (11281) 11281    11290    11300    11310    11320    11330    11340    11350    11360       11374
Hepatitis B virus NC_003977.2    (1) ----------------------------------------------------------------------------------------------
SARS-CoV-2 NC_045512.2       (11281) TAGTTTGTCTGGTTTTAAGCTAAAAGACTGTGTTATGTATGCATCAGCTGTAGTGTTACTAATCCTTATGACAGCAAGAACTGTGTATGATGAT
                                                                                                   Section 122
                             (11375) 11375 11380    11390    11400    11410    11420    11430    11440    11450          11468
Hepatitis B virus NC_003977.2    (1) ----------------------------------------------------------------------------------------------
SARS-CoV-2 NC_045512.2       (11375) GGTGCTAGGAGAGTGTGGACACTTATGAATGTCTTGACACTCGTTTATAAAGTTTATTATGGTAATGCTTTAGATCAAGCCATTTCCATGTGGG
                                                                                                   Section 123
                             (11469) 11469      11480    11490    11500    11510    11520    11530    11540    11550    11562
Hepatitis B virus NC_003977.2    (1) ----------------------------------------------------------------------------------------------
SARS-CoV-2 NC_045512.2       (11469) CTCTTATAATCTCTGTTACTTCTAACTACTCAGGTGTAGTTACAACTGTCATGTTTTTGGCCAGAGGTATTGTTTTTATGTGTGTTGAGTATTG
                                                                                                   Section 124
                             (11563) 11563 11570    11580    11590    11600    11610    11620    11630    11640          11656
Hepatitis B virus NC_003977.2    (1) ----------------------------------------------------------------------------------------------
SARS-CoV-2 NC_045512.2       (11563) CCCTATTTTCTTCATAACTGGTAATACACTTCAGTGTATAATGCTAGTTTATTGTTTCTTAGGCTATTTTTGTACTTGTTACTTTGGCCTCTTT
                                                                                                   Section 125
                             (11657) 11657        11670    11680    11690    11700    11710    11720    11730    11740  11750
Hepatitis B virus NC_003977.2    (1) ----------------------------------------------------------------------------------------------
SARS-CoV-2 NC_045512.2       (11657) TGTTTACTCAACCGCTACTTTAGACTGACTCTTGGTGTTTATGATTACTTAGTTTCTACACAGGAGTTTAGATATATGAATTCACAGGGACTAC
                                                                                                   Section 126
                             (11751) 11751    11760    11770    11780    11790    11800    11810    11820    11830       11844
Hepatitis B virus NC_003977.2    (1) ----------------------------------------------------------------------------------------------
SARS-CoV-2 NC_045512.2       (11751) TCCCACCCAAGAATAGCATAGATGCCTTCAAACTCAACATTAAATTGTTGGGTGTTGGTGGCAAACCTTGTATCAAAGTAGCCACTGTACAGTC
```

```
                                                                                                      Section 127
                            (11845) 11845 11850     11860     11870     11880     11890     11900     11910     11920            11938
Hepatitis B virus NC_003977.2   (1) ----------------------------------------------------------------------------------------------
SARS-CoV-2 NC_045512.2      (11845) TAAAATGTCAGATGTAAAGTGCACATCAGTAGTCTTACTCTCAGTTTTGCAACAACTCAGAGTAGAATCATCATCTAAATTGTGGGCTCAATGT
                                                                                                      Section 128
                            (11939) 11939      11950     11960     11970     11980     11990     12000     12010     12020       12032
Hepatitis B virus NC_003977.2   (1) ----------------------------------------------------------------------------------------------
SARS-CoV-2 NC_045512.2      (11939) GTCCAGTTACACAATGACATTCTCTTAGCTAAAGATACTACTGAAGCCTTTGAAAAAATGGTTTCACTACTTTCTGTTTTGCTTTCCATGCAGG
                                                                                                      Section 129
                            (12033) 12033  12040     12050     12060     12070     12080     12090     12100     12110           12126
Hepatitis B virus NC_003977.2   (1) ----------------------------------------------------------------------------------------------
SARS-CoV-2 NC_045512.2      (12033) GTGCTGTAGACATAAACAAGCTTTGTGAAGAAATGCTGGACAACAGGGCAACCTTACAAGCTATAGCCTCAGAGTTTAGTTCCCTTCCATCATA
                                                                                                      Section 130
                            (12127) 12127        12140     12150     12160     12170     12180     12190     12200     12210   12220
Hepatitis B virus NC_003977.2   (1) ----------------------------------------------------------------------------------------------
SARS-CoV-2 NC_045512.2      (12127) TGCAGCTTTTGCTACTGCTCAAGAAGCTTATGAGCAGGCTGTTGCTAATGGTGATTCTGAAGTTGTTCTTAAAAAGTTGAAGAAGTCTTTGAAT
                                                                                                      Section 131
                            (12221) 12221    12230     12240     12250     12260     12270     12280     12290     12300         12314
Hepatitis B virus NC_003977.2   (1) ----------------------------------------------------------------------------------------------
SARS-CoV-2 NC_045512.2      (12221) GTGGCTAAATCTGAATTTGACCGTGATGCAGCCATGCAACGTAAGTTGGAAAAGATGGCTGATCAAGCTATGACCCAAATGTATAAACAGGCTA
                                                                                                      Section 132
                            (12315) 12315 12320     12330     12340     12350     12360     12370     12380     12390            12408
Hepatitis B virus NC_003977.2   (1) ----------------------------------------------------------------------------------------------
SARS-CoV-2 NC_045512.2      (12315) GATCTGAGGACAAGAGGGCAAAAGTTACTAGTGCTATGCAGACAATGCTTTTCACTATGCTTAGAAAGTTGGATAATGATGCACTCAACAACAT
                                                                                                      Section 133
                            (12409) 12409      12420     12430     12440     12450     12460     12470     12480     12490       12502
Hepatitis B virus NC_003977.2   (1) ----------------------------------------------------------------------------------------------
SARS-CoV-2 NC_045512.2      (12409) TATCAACAATGCAAGAGATGGTTGTGTTCCCTTGAACATAATACCTCTTACAACAGCAGCCAAACTAATGGTTGTCATACCAGACTATAACACA
```

Section 134

```
                              (12503) 12503  12510     12520     12530     12540     12550     12560     12570     12580          12596
Hepatitis B virus NC_003977.2     (1) ----------------------------------------------------------------------------------------------
SARS-CoV-2 NC_045512.2        (12503) TATAAAAATACGTGTGATGGTACAACATTTACTTATGCATCAGCATTGTGGGAAATCCAACAGGTTGTAGATGCAGATAGTAAAATTGTTCAAC
```

Section 135

```
                              (12597) 12597        12610     12620     12630     12640     12650     12660     12670     12680     12690
Hepatitis B virus NC_003977.2     (1) ----------------------------------------------------------------------------------------------
SARS-CoV-2 NC_045512.2        (12597) TTAGTGAAATTAGTATGGACAATTCACCTAATTTAGCATGGCCTCTTATTGTAACAGCTTTAAGGGCCAATTCTGCTGTCAAATTACAGAATAA
```

Section 136

```
                              (12691) 12691    12700     12710     12720     12730     12740     12750     12760     12770         12784
Hepatitis B virus NC_003977.2     (1) ----------------------------------------------------------------------------------------------
SARS-CoV-2 NC_045512.2        (12691) TGAGCTTAGTCCTGTTGCACTACGACAGATGTCTTGTGCTGCCGGTACTACACAAACTGCTTGCACTGATGACAATGCGTTAGCTTACTACAAC
```

Section 137

```
                              (12785) 12785 12790     12800     12810     12820     12830     12840     12850     12860             12878
Hepatitis B virus NC_003977.2     (1) ----------------------------------------------------------------------------------------------
SARS-CoV-2 NC_045512.2        (12785) ACAACAAAGGGAGGTAGGTTTGTACTTGCACTGTTATCCGATTTACAGGATTTGAAATGGGCTAGATTCCCTAAGAGTGATGGAACTGGTACTA
```

Section 138

```
                              (12879) 12879      12890     12900     12910     12920     12930     12940     12950     12960       12972
Hepatitis B virus NC_003977.2     (1) ----------------------------------------------------------------------------------------------
SARS-CoV-2 NC_045512.2        (12879) TCTATACAGAACTGGAACCACCTTGTAGGTTTGTTACAGACACACCTAAAGGTCCTAAAGTGAAGTATTTATACTTTATTAAAGGATTAAACAA
```

Section 139

```
                              (12973) 12973  12980     12990     13000     13010     13020     13030     13040     13050          13066
Hepatitis B virus NC_003977.2     (1) ----------------------------------------------------------------------------------------------
SARS-CoV-2 NC_045512.2        (12973) CCTAAATAGAGGTATGGTACTTGGTAGTTTAGCTGCCACAGTACGTCTACAAGCTGGTAATGCAACAGAAGTGCCTGCCAATTCAACTGTATTA
```

Section 140

```
                              (13067) 13067        13080     13090     13100     13110     13120     13130     13140     13150     13160
Hepatitis B virus NC_003977.2     (1) ----------------------------------------------------------------------------------------------
SARS-CoV-2 NC_045512.2        (13067) TCTTTCTGTGCTTTTGCTGTAGATGCTGCTAAAGCTTACAAAGATTATCTAGCTAGTGGGGGACAACCAATCACTAATTGTGTTAAGATGTTGT
```

```
                                                                                                   Section 141
                            (13161) 13161    13170     13180     13190     13200     13210     13220     13230     13240        13254
  Hepatitis B virus NC_003977.2 (1) ----------------------------------------------------------------------------------------------
   SARS-CoV-2 NC_045512.2 (13161) GTACACACACTGGTACTGGTCAGGCAATAACAGTTACACCGGAAGCCAATATGGATCAAGAATCCTTTGGTGGTGCATCGTGTTGTCTGTACTG
                                                                                                   Section 142
                            (13255) 13255 13260     13270     13280     13290     13300     13310     13320     13330           13348
  Hepatitis B virus NC_003977.2 (1) ----------------------------------------------------------------------------------------------
   SARS-CoV-2 NC_045512.2 (13255) CCGTTGCCACATAGATCATCCAAATCCTAAAGGATTTTGTGACTTAAAAGGTAAGTATGTACAAATACCTACAACTTGTGCTAATGACCCTGTG
                                                                                                   Section 143
                            (13349) 13349      13360     13370     13380     13390     13400     13410     13420     13430     13442
  Hepatitis B virus NC_003977.2 (1) ----------------------------------------------------------------------------------------------
   SARS-CoV-2 NC_045512.2 (13349) GGTTTTACACTTAAAAACACAGTCTGTACCGTCTGCGGTATGTGGAAAGGTTATGGCTGTAGTTGTGATCAACTCCGCGAACCCATGCTTCAGT
                                                                                                   Section 144
                            (13443) 13443  13450     13460     13470     13480     13490     13500     13510     13520          13536
  Hepatitis B virus NC_003977.2 (1) ----------------------------------------------------------------------------------------------
   SARS-CoV-2 NC_045512.2 (13443) CAGCTGATGCACAATCGTTTTTAAACGGGTTTGCGGTGTAAGTGCAGCCCGTCTTACACCGTGCGGCACAGGCACTAGTACTGATGTCGTATAC
                                                                                                   Section 145
                            (13537) 13537        13550     13560     13570     13580     13590     13600     13610     13620   13630
  Hepatitis B virus NC_003977.2 (1) ----------------------------------------------------------------------------------------------
   SARS-CoV-2 NC_045512.2 (13537) AGGGCTTTTGACATCTACAATGATAAAGTAGCTGGTTTTGCTAAATTCCTAAAAACTAATTGTTGTCGCTTCCAAGAAAAGGACGAAGATGACA
                                                                                                   Section 146
                            (13631) 13631    13640     13650     13660     13670     13680     13690     13700     13710        13724
  Hepatitis B virus NC_003977.2 (1) ----------------------------------------------------------------------------------------------
   SARS-CoV-2 NC_045512.2 (13631) ATTTAATTGATTCTTACTTTGTAGTTAAGAGACACACTTTCTCTAACTACCAACATGAAGAAACAATTTATAATTTACTTAAGGATTGTCCAGC
                                                                                                   Section 147
                            (13725) 13725 13730     13740     13750     13760     13770     13780     13790     13800           13818
  Hepatitis B virus NC_003977.2 (1) ----------------------------------------------------------------------------------------------
   SARS-CoV-2 NC_045512.2 (13725) TGTTGCTAAACATGACTTCTTTAAGTTTAGAATAGACGGTGACATGGTACCACATATATCACGTCAACGTCTTACTAAATACACAATGGCAGAC
```

```
                                                                                                           Section 148
                          (13819) 13819      13830     13840     13850     13860     13870     13880     13890     13900      13912
Hepatitis B virus NC_003977.2 (1) ----------------------------------------------------------------------------------------------
SARS-CoV-2 NC_045512.2  (13819) CTCGTCTATGCTTTAAGGCATTTTGATGAAGGTAATTGTGACACATTAAAAGAAATACTTGTCACATACAATTGTTGTGATGATGATTATTTCA
                                                                                                           Section 149
                          (13913) 13913  13920     13930     13940     13950     13960     13970     13980     13990           14006
Hepatitis B virus NC_003977.2 (1) ----------------------------------------------------------------------------------------------
SARS-CoV-2 NC_045512.2  (13913) ATAAAAAGGACTGGTATGATTTTGTAGAAAACCCAGATATATTACGCGTATACGCCAACTTAGGTGAACGTGTACGCCAAGCTTTGTTAAAAAC
                                                                                                           Section 150
                          (14007) 14007        14020     14030     14040     14050     14060     14070     14080     14090    14100
Hepatitis B virus NC_003977.2 (1) ----------------------------------------------------------------------------------------------
SARS-CoV-2 NC_045512.2  (14007) AGTACAATTCTGTGATGCCATGCGAAATGCTGGTATTGTTGGTGTACTGACATTAGATAATCAAGATCTCAATGGTAACTGGTATGATTTCGGT
                                                                                                           Section 151
                          (14101) 14101   14110     14120     14130     14140     14150     14160     14170     14180          14194
Hepatitis B virus NC_003977.2 (1) ----------------------------------------------------------------------------------------------
SARS-CoV-2 NC_045512.2  (14101) GATTTCATACAAACCACGCCAGGTAGTGGAGTTCCTGTTGTAGATTCTTATTATTCATTGTTAATGCCTATATTAACCTTGACCAGGGCTTTAA
                                                                                                           Section 152
                          (14195) 14195 14200     14210     14220     14230     14240     14250     14260     14270            14288
Hepatitis B virus NC_003977.2 (1) ----------------------------------------------------------------------------------------------
SARS-CoV-2 NC_045512.2  (14195) CTGCAGAGTCACATGTTGACACTGACTTAACAAAGCCTTACATTAAGTGGGATTTGTTAAAATATGACTTCACGGAAGAGAGGTTAAAACTCTT
                                                                                                           Section 153
                          (14289) 14289      14300     14310     14320     14330     14340     14350     14360     14370      14382
Hepatitis B virus NC_003977.2 (1) ----------------------------------------------------------------------------------------------
SARS-CoV-2 NC_045512.2  (14289) TGACCGTTATTTTAAATATTGGGATCAGACATACCACCCAAATTGTGTTAACTGTTTGGATGACAGATGCATTCTGCATTGTGCAAACTTTAAT
                                                                                                           Section 154
                          (14383) 14383  14390     14400     14410     14420     14430     14440     14450     14460           14476
Hepatitis B virus NC_003977.2 (1) ----------------------------------------------------------------------------------------------
SARS-CoV-2 NC_045512.2  (14383) GTTTTATTCTCTACAGTGTTCCCACCTACAAGTTTTGGACCACTAGTGAGAAAAATATTTGTTGATGGTGTTCCATTTGTAGTTTCAACTGGAT
```

```
                                                                                                    Section 155
                           (14477) 14477       14490     14500     14510     14520     14530     14540     14550     14560   14570
Hepatitis B virus NC  003977.2 (1) ----------------------------------------------------------------------------------------------
 SARS-CoV-2 NC_045512.2 (14477) ACCACTTCAGAGAGCTAGGTGTTGTACATAATCAGGATGTAAACTTACATAGCTCTAGACTTAGTTTTAAGGAATTACTTGTGTATGCTGCTGA
                                                                                                    Section 156
                           (14571) 14571    14580     14590     14600     14610     14620     14630     14640     14650        14664
Hepatitis B virus NC  003977.2 (1) ----------------------------------------------------------------------------------------------
 SARS-CoV-2 NC_045512.2 (14571) CCCTGCTATGCACGCTGCTTCTGGTAATCTATTACTAGATAAACGCACTACGTGCTTTTCAGTAGCTGCACTTACTAACAATGTTGCTTTTCAA
                                                                                                    Section 157
                           (14665) 14665 14670     14680     14690     14700     14710     14720     14730     14740           14758
Hepatitis B virus NC  003977.2 (1) ----------------------------------------------------------------------------------------------
 SARS-CoV-2 NC_045512.2 (14665) ACTGTCAAACCCGGTAATTTTAACAAAGACTTCTATGACTTTGCTGTGTCTAAGGGTTTCTTTAAGGAAGGAAGTTCTGTTGAATTAAAACACT
                                                                                                    Section 158
                           (14759) 14759      14770     14780     14790     14800     14810     14820     14830     14840      14852
Hepatitis B virus NC  003977.2 (1) ----------------------------------------------------------------------------------------------
 SARS-CoV-2 NC_045512.2 (14759) TCTTCTTTGCTCAGGATGGTAATGCTGCTATCAGCGATTATGACTACTATCGTTATAATCTACCAACAATGTGTGATATCAGACAACTACTATT
                                                                                                    Section 159
                           (14853) 14853  14860     14870     14880     14890     14900     14910     14920     14930          14946
Hepatitis B virus NC  003977.2 (1) ----------------------------------------------------------------------------------------------
 SARS-CoV-2 NC_045512.2 (14853) TGTAGTTGAAGTTGTTGATAAGTACTTTGATTGTTACGATGGTGGCTGTATTAATGCTAACCAAGTCATCGTCAACAACCTAGACAAATCAGCT
                                                                                                    Section 160
                           (14947) 14947       14960     14970     14980     14990     15000     15010     15020     15030   15040
Hepatitis B virus NC  003977.2 (1) ----------------------------------------------------------------------------------------------
 SARS-CoV-2 NC_045512.2 (14947) GGTTTTCCATTTAATAAATGGGGTAAGGCTAGACTTTATTATGATTCAATGAGTTATGAGGATCAAGATGCACTTTTCGCATATACAAAACGTA
                                                                                                    Section 161
                           (15041) 15041    15050     15060     15070     15080     15090     15100     15110     15120        15134
Hepatitis B virus NC  003977.2 (1) ----------------------------------------------------------------------------------------------
 SARS-CoV-2 NC_045512.2 (15041) ATGTCATCCCTACTATAACTCAAATGAATCTTAAGTATGCCATTAGTGCAAAGAATAGAGCTCGCACCGTAGCTGGTGTCTCTATCTGTAGTAC
```

```
                                                                                                   Section 162
                       (15135) 15135 15140     15150     15160     15170     15180     15190     15200     15210          15228
Hepatitis B virus NC_003977.2     (1) ----------------------------------------------------------------------------------------------
SARS-CoV-2 NC_045512.2 (15135) TATGACCAATAGACAGTTTCATCAAAAATTATTGAAATCAATAGCCGCCACTAGAGGAGCTACTGTAGTAATTGGAACAAGCAAATTCTATGGT
                                                                                                   Section 163
                       (15229) 15229      15240     15250     15260     15270     15280     15290     15300     15310   15322
Hepatitis B virus NC_003977.2     (1) ----------------------------------------------------------------------------------------------
SARS-CoV-2 NC_045512.2 (15229) GGTTGGCACAACATGTTAAAAACTGTTTATAGTGATGTAGAAAACCCTCACCTTATGGGTTGGGATTATCCTAAATGTGATAGAGCCATGCCTA
                                                                                                   Section 164
                       (15323) 15323  15330     15340     15350     15360     15370     15380     15390     15400       15416
Hepatitis B virus NC_003977.2     (1) ----------------------------------------------------------------------------------------------
SARS-CoV-2 NC_045512.2 (15323) ACATGCTTAGAATTATGGCCTCACTTGTTCTTGCTCGCAAACATACAACGTGTTGTAGCTTGTCACACCGTTTCTATAGATTAGCTAATGAGTG
                                                                                                   Section 165
                       (15417) 15417        15430     15440     15450     15460     15470     15480     15490     15500 15510
Hepatitis B virus NC_003977.2     (1) ----------------------------------------------------------------------------------------------
SARS-CoV-2 NC_045512.2 (15417) TGCTCAAGTATTGAGTGAAATGGTCATGTGTGGCGGTTCACTATATGTTAAACCAGGTGGAACCTCATCAGGAGATGCCACAACTGCTTATGCT
                                                                                                   Section 166
                       (15511) 15511    15520     15530     15540     15550     15560     15570     15580     15590      15604
Hepatitis B virus NC_003977.2     (1) ----------------------------------------------------------------------------------------------
SARS-CoV-2 NC_045512.2 (15511) AATAGTGTTTTTAACATTTGTCAAGCTGTCACGGCCAATGTTAATGCACTTTTATCTACTGATGGTAACAAAATTGCCGATAAGTATGTCCGCA
                                                                                                   Section 167
                       (15605) 15605 15610     15620     15630     15640     15650     15660     15670     15680          15698
Hepatitis B virus NC_003977.2     (1) ----------------------------------------------------------------------------------------------
SARS-CoV-2 NC_045512.2 (15605) ATTTACAACACAGACTTTATGAGTGTCTCTATAGAAATAGAGATGTTGACACAGACTTTGTGAATGAGTTTTACGCATATTTGCGTAAACATTT
                                                                                                   Section 168
                       (15699) 15699      15710     15720     15730     15740     15750     15760     15770     15780   15792
Hepatitis B virus NC_003977.2     (1) ----------------------------------------------------------------------------------------------
SARS-CoV-2 NC_045512.2 (15699) CTCAATGATGATACTCTCTGACGATGCTGTTGTGTGTTTCAATAGCACTTATGCATCTCAAGGTCTAGTGGCTAGCATAAAGAACTTTAAGTCA
```

```
                                                                                                         Section 169
                           (15793) 15793 15800     15810     15820     15830     15840     15850     15860     15870         15886
Hepatitis B virus NC_003977.2  (1) ----------------------------------------------------------------------------------------------
  SARS-CoV-2 NC_045512.2 (15793) GTTCTTTATTATCAAAACAATGTTTTTATGTCTGAAGCAAAATGTTGGACTGAGACTGACCTTACTAAAGGACCTCATGAATTTTGCTCTCAAC
                                                                                                         Section 170
                           (15887) 15887        15900     15910     15920     15930     15940     15950     15960     15970 15980
Hepatitis B virus NC_003977.2  (1) ----------------------------------------------------------------------------------------------
  SARS-CoV-2 NC_045512.2 (15887) ATACAATGCTAGTTAAACAGGGTGATGATTATGTGTACCTTCCTTACCCAGATCCATCAAGAATCCTAGGGGCCGGCTGTTTTGTAGATGATAT
                                                                                                         Section 171
                           (15981) 15981   15990     16000     16010     16020     16030     16040     16050     16060         16074
Hepatitis B virus NC_003977.2  (1) ----------------------------------------------------------------------------------------------
  SARS-CoV-2 NC_045512.2 (15981) CGTAAAAACAGATGGTACACTTATGATTGAACGGTTCGTGTCTTTAGCTATAGATGCTTACCCACTTACTAAACATCCTAATCAGGAGTATGCT
                                                                                                         Section 172
                           (16075) 16075 16080     16090     16100     16110     16120     16130     16140     16150         16168
Hepatitis B virus NC_003977.2  (1) ----------------------------------------------------------------------------------------------
  SARS-CoV-2 NC_045512.2 (16075) GATGTCTTTCATTTGTACTTACAATACATAAGAAAGCTACATGATGAGTTAACAGGACACATGTTAGACATGTATTCTGTTATGCTTACTAATG
                                                                                                         Section 173
                           (16169) 16169      16180     16190     16200     16210     16220     16230     16240     16250     16262
Hepatitis B virus NC_003977.2  (1) ----------------------------------------------------------------------------------------------
  SARS-CoV-2 NC_045512.2 (16169) ATAACACTTCAAGGTATTGGGAACCTGAGTTTTATGAGGCTATGTACACACCGCATACAGTCTTACAGGCTGTTGGGGCTTGTGTTCTTTGCAA
                                                                                                         Section 174
                           (16263) 16263 16270     16280     16290     16300     16310     16320     16330     16340         16356
Hepatitis B virus NC_003977.2  (1) ----------------------------------------------------------------------------------------------
  SARS-CoV-2 NC_045512.2 (16263) TTCACAGACTTCATTAAGATGTGGTGCTTGCATACGTAGACCATTCTTATGTTGTAAATGCTGTTACGACCATGTCATATCAACATCACATAAA
                                                                                                         Section 175
                           (16357) 16357        16370     16380     16390     16400     16410     16420     16430     16440 16450
Hepatitis B virus NC_003977.2  (1) ----------------------------------------------------------------------------------------------
  SARS-CoV-2 NC_045512.2 (16357) TTAGTCTTGTCTGTTAATCCGTATGTTTGCAATGCTCCAGGTTGTGATGTCACAGATGTGACTCAACTTTACTTAGGAGGTATGAGCTATTATT
```

```
                                                                                                              Section 176
                           (16451) 16451    16460      16470      16480      16490      16500      16510      16520      16530      16544
Hepatitis B virus NC_003977.2  (1) ----------------------------------------------------------------------------------------------
   SARS-CoV-2 NC_045512.2 (16451) GTAAATCACATAAACCACCCATTAGTTTTCCATTGTGTGCTAATGGACAAGTTTTTGGTTTATATAAAAATACATGTGTTGGTAGCGATAATGT
                                                                                                              Section 177
                           (16545) 16545 16550      16560      16570      16580      16590      16600      16610      16620           16638
Hepatitis B virus NC_003977.2  (1) ----------------------------------------------------------------------------------------------
   SARS-CoV-2 NC_045512.2 (16545) TACTGACTTTAATGCAATTGCAACATGTGACTGGACAAATGCTGGTGATTACATTTTAGCTAACACCTGTACTGAAAGACTCAAGCTTTTTGCA
                                                                                                              Section 178
                           (16639) 16639      16650      16660      16670      16680      16690      16700      16710      16720      16732
Hepatitis B virus NC_003977.2  (1) ----------------------------------------------------------------------------------------------
   SARS-CoV-2 NC_045512.2 (16639) GCAGAAACGCTCAAAGCTACTGAGGAGACATTTAAACTGTCTTATGGTATTGCTACTGTACGTGAAGTGCTGTCTGACAGAGAATTACATCTTT
                                                                                                              Section 179
                           (16733) 16733 16740      16750      16760      16770      16780      16790      16800      16810           16826
Hepatitis B virus NC_003977.2  (1) ----------------------------------------------------------------------------------------------
   SARS-CoV-2 NC_045512.2 (16733) CATGGGAAGTTGGTAAACCTAGACCACCACTTAACCGAAATTATGTCTTTACTGGTTATCGTGTAACTAAAAACAGTAAAGTACAAATAGGAGA
                                                                                                              Section 180
                           (16827) 16827        16840      16850      16860      16870      16880      16890      16900      16910 16920
Hepatitis B virus NC_003977.2  (1) ----------------------------------------------------------------------------------------------
   SARS-CoV-2 NC_045512.2 (16827) GTACACCTTTGAAAAAGGTGACTATGGTGATGCTGTTGTTTACCGAGGTACAACAACTTACAAATTAAATGTTGGTGATTATTTTGTGCTGACA
                                                                                                              Section 181
                           (16921) 16921    16930      16940      16950      16960      16970      16980      16990      17000      17014
Hepatitis B virus NC_003977.2  (1) ----------------------------------------------------------------------------------------------
   SARS-CoV-2 NC_045512.2 (16921) TCACATACAGTAATGCCATTAAGTGCACCTACACTAGTGCCACAAGAGCACTATGTTAGAATTACTGGCTTATACCCAACACTCAATATCTCAG
                                                                                                              Section 182
                           (17015) 17015 17020      17030      17040      17050      17060      17070      17080      17090           17108
Hepatitis B virus NC_003977.2  (1) ----------------------------------------------------------------------------------------------
   SARS-CoV-2 NC_045512.2 (17015) ATGAGTTTTCTAGCAATGTTGCAAATTATCAAAAGGTTGGTATGCAAAAGTATTCTACACTCCAGGGACCACCTGGTACTGGTAAGAGTCATTT
```

Section 183

```
                            (17109) 17109      17120     17130     17140     17150     17160     17170     17180     17190    17202
Hepatitis B virus NC_003977.2   (1) ----------------------------------------------------------------------------------------------
SARS-CoV-2 NC_045512.2      (17109) TGCTATTGGCCTAGCTCTCTACTACCCTTCTGCTCGCATAGTGTATACAGCTTGCTCTCATGCCGCTGTTGATGCACTATGTGAGAAGGCATTA
```

Section 184

```
                            (17203) 17203  17210     17220     17230     17240     17250     17260     17270     17280         17296
Hepatitis B virus NC_003977.2   (1) ----------------------------------------------------------------------------------------------
SARS-CoV-2 NC_045512.2      (17203) AAATATTTGCCTATAGATAAATGTAGTAGAATTATACCTGCACGTGCTCGTGTAGAGTGTTTTGATAAATTCAAAGTGAATTCAACATTAGAAC
```

Section 185

```
                            (17297) 17297        17310     17320     17330     17340     17350     17360     17370     17380  17390
Hepatitis B virus NC_003977.2   (1) ----------------------------------------------------------------------------------------------
SARS-CoV-2 NC_045512.2      (17297) AGTATGTCTTTTGTACTGTAAATGCATTGCCTGAGACGACAGCAGATATAGTTGTCTTTGATGAAATTTCAATGGCCACAAATTATGATTTGAG
```

Section 186

```
                            (17391) 17391    17400     17410     17420     17430     17440     17450     17460     17470        17484
Hepatitis B virus NC_003977.2   (1) ----------------------------------------------------------------------------------------------
SARS-CoV-2 NC_045512.2      (17391) TGTTGTCAATGCCAGATTACGTGCTAAGCACTATGTGTACATTGGCGACCCTGCTCAATTACCTGCACCACGCACATTGCTAACTAAGGGCACA
```

Section 187

```
                            (17485) 17485 17490     17500     17510     17520     17530     17540     17550     17560           17578
Hepatitis B virus NC_003977.2   (1) ----------------------------------------------------------------------------------------------
SARS-CoV-2 NC_045512.2      (17485) CTAGAACCAGAATATTTCAATTCAGTGTGTAGACTTATGAAAACTATAGGTCCAGACATGTTCCTCGGAACTTGTCGGCGTTGTCCTGCTGAAA
```

Section 188

```
                            (17579) 17579      17590     17600     17610     17620     17630     17640     17650     17660    17672
Hepatitis B virus NC_003977.2   (1) ----------------------------------------------------------------------------------------------
SARS-CoV-2 NC_045512.2      (17579) TTGTTGACACTGTGAGTGCTTTGGTTTATGATAATAAGCTTAAAGCACATAAAGACAAATCAGCTCAATGCTTTAAAATGTTTTATAAGGGTGT
```

Section 189

```
                            (17673) 17673  17680     17690     17700     17710     17720     17730     17740     17750         17766
Hepatitis B virus NC_003977.2   (1) ----------------------------------------------------------------------------------------------
SARS-CoV-2 NC_045512.2      (17673) TATCACGCATGATGTTTCATCTGCAATTAACAGGCCACAAATAGGCGTGGTAAGAGAATTCCTTACACGTAACCCTGCTTGGAGAAAAGCTGTC
```

Section 190

```
                            (17767) 17767        17780     17790     17800     17810     17820     17830     17840     17850     17860
Hepatitis B virus NC_003977.2   (1) ----------------------------------------------------------------------------------------------
SARS-CoV-2 NC_045512.2      (17767) TTTATTTCACCTTATAATTCACAGAATGCTGTAGCCTCAAAGATTTTGGGACTACCAACTCAAACTGTTGATTCATCACAGGGCTCAGAATATG
```

Section 191

```
                            (17861) 17861    17870     17880     17890     17900     17910     17920     17930     17940         17954
Hepatitis B virus NC_003977.2   (1) ----------------------------------------------------------------------------------------------
SARS-CoV-2 NC_045512.2      (17861) ACTATGTCATATTCACTCAAACCACTGAAACAGCTCACTCTTGTAATGTAAACAGATTTAATGTTGCTATTACCAGAGCAAAAGTAGGCATACT
```

Section 192

```
                            (17955) 17955 17960     17970     17980     17990     18000     18010     18020     18030             18048
Hepatitis B virus NC_003977.2   (1) ---------------------------------------------------------------------------AATTCCACAACCTTCCACC
SARS-CoV-2 NC_045512.2      (17955) TTGCATAATGTCTGATAGAGACCTTTATGACAAGTTGCAATTTACAAGTCTTGAAATTCCACGTAGGAATGTGGCAACTTTACAAGCTGAAAAT
```

Section 193

```
                            (18049) 18049      18060     18070     18080     18090     18100     18110     18120     18130       18142
Hepatitis B virus NC_003977.2  (20) AAACTCTGCAAGATCCCAGAGTGAGAGGCCTGTATT-TCCCTGCTGG--TGGCTCCAGTTCAGGAACAGTAAACCCTGTT-CTGACTACTGCCT
SARS-CoV-2 NC_045512.2      (18049) GTAACAGGACTCTTTAAAGATTGTAGT-AAGGTAATCACTGGGTT--ACATCCTACACAGGC-AC---CTACACACCTC-AGTGTTGACACTAA
```

Section 194

```
                            (18143) 18143  18150     18160     18170     18180     18190     18200     18210     18220           18236
Hepatitis B virus NC_003977.2 (110) CTCCCTTATCGTCAATCTTCTCGAGGA-TTGGGGACCCTGCGCTGAACATGGAGAACATCACATCAGGATTCCTAGGACCCCTTCTCGTGTTAC
SARS-CoV-2 NC_045512.2      (18135) ATTCAAAACT-----GAAGGTTTATGTGTTGACATACCTGGCATACCTAAGGACATGACCTATAGAAGACT-CATCTCTATGA--TGGGTTTTA
```

Section 195

```
                            (18237) 18237        18250     18260     18270     18280     18290     18300     18310     18320   18330
Hepatitis B virus NC_003977.2 (203) AGGCGGGGTTTTTCTTGTTGACAAGAATCCTCA---CAATACCGCAGAGTCTAGACTCGTGGTGGACTT---CTCTCA-ATTTTCTAGGGGGAA
SARS-CoV-2 NC_045512.2      (18221) AAATGAATTA--TCAAGTTAATGGTTACCCTAACAT-GTT-TATCACCCGCGAAGAAGCTATAAGACATGTACGTGCATGGATTGGCTTCGATG
```

Section 196

```
                            (18331) 18331    18340     18350     18360     18370     18380     18390     18400     18410         18424
Hepatitis B virus NC_003977.2 (290) CTACCGTGTGTCTTGGCCAAAATTCGCAGTCCCCAACCTCCAATCACTCACCAACCTCTTGTCCTCCAACTTGTCCTGGTTATCGCTGGATGTG
SARS-CoV-2 NC_045512.2      (18311) TCGAG--GGGTGTCATGCTACTAGAGAAGCTGTTGGTACCAATTT----------ACCTTTACAGCTAGGTTTTTCTACAGG-TGTTAACC---
```

```
                                                                                              Section 197
                                   18425 18430      18440      18450      18460      18470      18480      18490      18500           18518
                          (18425)
Hepatitis B virus NC 003977.2 (384) TCTGCGGCGT---TTTATCATCTTCCTCTTCATCCTGCTGCTATGCCTCATCTTCTTGTTGGTTCTTCTGGACTATCAAGGTATGTTGCCCG-T
  SARS-CoV-2 NC_045512.2 (18389) TAGTTGCTGTACCTACAGGTTATGTTGATACACCTAATAATA----CAGATTTTTCCAGAGTTAG---TGCTAAACCACCGCCTGGAGATCAAT
                                                                                              Section 198
                                   18519      18530      18540      18550      18560      18570      18580      18590      18600      18612
                          (18519)
Hepatitis B virus NC 003977.2 (474) TTGTCCTCTAATTCCAGGATCCTCAACAACCAGCACGGGACCATGCCGGACCTGCATGACTACTGCTCAAGGAACCTCTATGTATCCCTCCTGT
  SARS-CoV-2 NC_045512.2 (18476) TTAAACACCTCATACCACTTATGTACAAAGGACTTCCTTGGAAT-GTAGTGCGTATAA-AGATTGTAC----AAATGTTAAGTGACACAC----
                                                                                              Section 199
                                   18613 18620      18630      18640      18650      18660      18670      18680      18690           18706
                          (18613)
Hepatitis B virus NC 003977.2 (568) TGCTGTACCAAACCTTCGGACGGAAATTGCACCTGTATTCCCATCCCATCATCCTGGGCTTTCGGAAAATTCCTATGGGAGTGGGCCTCAGCCC
  SARS-CoV-2 NC_045512.2 (18560) -TTAA----AAATCTCTCTGACAGAGTCGTATTTGT--------CTTATGG-----G-CACATGGCTTTGA-GTTGACATCTATGAAGTATTTT
                                                                                              Section 200
                                   18707           18720      18730      18740      18750      18760      18770      18780      18790 18800
                          (18707)
Hepatitis B virus NC 003977.2 (662) GTTTCTCCTGGCTCAGTTTACTAGTGCCATTTGTTCAGTGGTTCGTAGGGCTTTCCCCCACTGTTTGGCTTTCAGTTATATGGATGATGTGGTA
  SARS-CoV-2 NC_045512.2 (18634) GTGAAAA-TAGGAC--CTGAGCGCACCTGTTGTCTATGTGATAGACGTGCCACATGCTTTTCCACTGCTTCAGACACTTATGCC--------TG
                                                                                              Section 201
                                   18801      18810      18820      18830      18840      18850      18860      18870      18880      18894
                          (18801)
Hepatitis B virus NC 003977.2 (756) TTGGGGGCCAAGTCTGTACAGCATCTTGAGTCCCTTTTTACCGCTGTTACCAATTTTCTTTTGTCTTTGGGTATACATTTAAAC--CCTAACAA
  SARS-CoV-2 NC_045512.2 (18717) TTGGC-AT-CATTCTATTGGATTTGATTACGTCTATAATCCGTTTATGATTGATGTTCAACAATGGGGTTTTACAGG--TAACCTACAAAGCAA
                                                                                              Section 202
                                   18895 18900      18910      18920      18930      18940      18950      18960      18970           18988
                          (18895)
Hepatitis B virus NC 003977.2 (848) AACAAAGAGATGGGGTTACTCTCTAAATTTTATGGGTTATGTCATTGGATGTTATGGGTCCTTGCCACAAGAACACATCATACAAAAAATCAAA
  SARS-CoV-2 NC_045512.2 (18807) CCATGATCTGTATTGTCAAGTCCATGGTAATG---CACATGTAGCTAGTTGTGATGCAATCATGACTAGG-TGTCTA-GCTGTCCACGAGTGCT
                                                                                              Section 203
                                   18989      19000      19010      19020      19030      19040      19050      19060      19070      19082
                          (18989)
Hepatitis B virus NC 003977.2 (942) GAAT--GTTTTAGAAAACTTCCTATTAACAGGCCTATTGATTGG-AAAGTATGTCAACGAATTGTGGGTCTTTTGGGTTTTGCTGCCCCTTTTA
  SARS-CoV-2 NC_045512.2 (18896) TTGTTAAGCGTGTTGACTGGACTATTGAATATCCTATAATTGGTGATGAACTGAAGATTAATGCGGCTTGTAGAAAGG-TTCAACACATGGTTG
```

Section 204

```
                                 (19083) 19083  19090     19100     19110     19120     19130     19140     19150     19160     19176
Hepatitis B virus NC_003977.2     (1033) CACAATGTGGTTATCCTGCGTTGATGCCTTTGTATGCATGTATTCAATCTAAGCAGGCTTTCACTTTCTCGCCAACTTACAAGGCCTTTCTGTG
SARS-CoV-2 NC_045512.2           (18989) TTAAAGCTGCATTATTAGCAGACAAATTCCCAGTTCTT-CACGACATTGGTAACCCTAAAGCTATTAAGTGTGTA-CCTCAAGCTGATGTAGAA
```

Section 205

```
                                 (19177) 19177        19190     19200     19210     19220     19230     19240     19250     19260  19270
Hepatitis B virus NC_003977.2     (1127) TAAACAATACCTGAACCTTTACCCCGTTGCCCGGCAACGGCCAGGTCTGTGCCAAGTGTT-TGCTGACGCAACCCCCACTGGC--TGGGGCTTG
SARS-CoV-2 NC_045512.2           (19081) TGGAAGTTCTATGATGCACAGCCTTGT---------AGTGACAAAGC---------TTATAAAATAGAAGAATTATTCTA--TTCTTATGCCAC
```

Section 206

```
                                 (19271) 19271    19280     19290     19300     19310     19320     19330     19340     19350     19364
Hepatitis B virus NC_003977.2     (1218) GTCATGGGCCATCAGCGCATGCGTGGAACCTTTTCGGCTCCTCTGCCGATCCATACTGCGGAACTC--CTAG----CCGCTTGT-------TTT
SARS-CoV-2 NC_045512.2           (19155) ACA--TTCTGACAAATTCACAGATGGTGTATGCCTATTTTG----GAATTGCAATGTCGATAGATATCCTGCTAATTCCATTGTTTGTAGATT-
```

Section 207

```
                                 (19365) 19365 19370     19380     19390     19400     19410     19420     19430     19440     19458
Hepatitis B virus NC_003977.2     (1299) GCTCGCAGCAGGTCTGGAGCAAACATTATCGGGACTGATAACTCTGTTGTCCTATCCCGCAAATATACATCGTTTCCATGGCTGCT-AGGCTGT
SARS-CoV-2 NC_045512.2           (19242) --TGACACTA-GAGTGCTATC----TAACCTTAACTTGCCTGGTTGTGAT-GGTGGCAGTTTGTATGTAA--ATAAACATGCATTCCACACACC
```

Section 208

```
                                 (19459) 19459      19470     19480     19490     19500     19510     19520     19530     19540     19552
Hepatitis B virus NC_003977.2     (1392) GCTGCC-----AA-CTGGATCCTGCGCGGGACGTCCTTTGT--TTACGTCCCGTCGGCGCTGAATCCTGCGGACGACCCTTCTCGGGGTCGCTT
SARS-CoV-2 NC_045512.2           (19326) AGCTTTTGATAAAAGTGCTTTTGTTAATTTAAAACAATTACCATT-TTTCTATTACTCTGACAGT-CCATGTGA-GTCTCATGGAAAACAAGTA
```

Section 209

```
                                 (19553) 19553  19560     19570     19580     19590     19600     19610     19620     19630     19646
Hepatitis B virus NC_003977.2     (1478) GGGACTCTCTCGTCCCCTTCTCCGTCTGCCGTTCCGACCGACCACGGGGCGCAC----CTCTCTTTACGCGGACTCCCCGTCTGTGCCTTCTCA
SARS-CoV-2 NC_045512.2           (19417) GTGTCAGATA--TAGATT-----ATGTACCACTAAAGTCTGCTACGTGTATAACACGTTGCAATTTAGGTGGTGCTGTCTGTAGACATCATGCT
```

Section 210

```
                                 (19647) 19647        19660     19670     19680     19690     19700     19710     19720     19730  19740
Hepatitis B virus NC_003977.2     (1568) TCTGCCGGACCGTGTGCACTTCG----CTTCACCTCTGCACGTCGCATGGAGACCACCGTGAACGCCCACCA----AATATTGCCCAAGGTCTT
SARS-CoV-2 NC_045512.2           (19504) AATGAGTACAGATTGTATCTCGATGCTTATAACATGATGATCTCAGCTGGCTTTAGCTTG-TGGGTTTACAAACAATTTGAT-ACTTATAACCT
```

Section 211

```
                                   19741   19750     19760     19770     19780     19790     19800     19810     19820       19834
                          (19741)
Hepatitis B virus NC_003977.2 (1654) ACATAAGAGGACTCTTGGACTCTCAGCAATGTCAACGACCGACCTTGAGGCATACTTCAAAGACTGTT-----TGTTTAAAGACTGGGAGGAGT
SARS-CoV-2 NC_045512.2 (19596)      -CTGGA-ACACTTTTACAAGACTTCAGAGTTTAGAA--AATGTGGCTTTTAATGTTGTAAATAAGGGACACTTTGATGGACAACAGGGTGAAGT
```

Section 212

```
                                   19835 19840     19850     19860     19870     19880     19890     19900     19910         19928
                          (19835)
Hepatitis B virus NC_003977.2 (1743) TGGGGGA--GGAGATTAGGTTAAAGGTCTTTGTA----CTAGGAGGCTGTAGGCATAAATTGGTCTGCGCACCAGCACCATGCAACTTTTTCAC
SARS-CoV-2 NC_045512.2 (19686)      ACCAGTTTCTATCATTA--ATAACACTGTTTACACAAAAGTTGATGGTGTTGATGTAGAATTGTTTGAA-AATAAAAC-AACATTACCTGTTAA
```

Section 213

```
                                   19929      19940     19950     19960     19970     19980     19990     20000     20010     20022
                          (19929)
Hepatitis B virus NC_003977.2 (1831) CTCTGCCTAATCATCTCTTGTTCATGTCC-TACTGTT---CAAGCCTCCAAG---CTGTGCCTTGGGTGGCTTTGGGGCATGGACATCGACCCT
SARS-CoV-2 NC_045512.2 (19776)      TGTAG--CATTTGAGCTTTGGGCTAAGCGCAACATTAAACCAGTACCAGAGGTGAAAATACTCAATAA-TT-TGGGTGTGGACATTGCTGCTAA
```

Section 214

```
                                   20023  20030     20040     20050     20060     20070     20080     20090     20100         20116
                          (20023)
Hepatitis B virus NC_003977.2 (1918) TATAAAGAATTTGGAGCTACTGTGGAGTTACTCTCGTTTTTGCCTTCTGACTTCTTTCCTTCAGTACGAGATCTTCTAGATACCGCCTCAGCTC
SARS-CoV-2 NC_045512.2 (19866)      TACTGTGATCTGGGACTACAAAAGAGATGCTCCAGCACATATATCTA---CTATTGGTGTT-TGTTCTATGACTGACAT---AGCCAAGAAACC
```

Section 215

```
                                   20117        20130     20140     20150     20160     20170     20180     20190     20200  20210
                          (20117)
Hepatitis B virus NC_003977.2 (2012) TGTATCGGGAAGCCTTAGAGTCTCCTGAGCATTGTTCACCTCACCATACTGCACTCAGGCAAGCAATTCTTTGCTGGGGGGAACTAATGACTCT
SARS-CoV-2 NC_045512.2 (19953)      AACTGAAACGATTTGTGCACCACTCACTGTCTTTT-TTGAT--GGTAGAGTTGATGGTCAAGTAGACTTATTTAGAAATGCCCGTAATGGTGTT
```

Section 216

```
                                   20211   20220     20230     20240     20250     20260     20270     20280     20290       20304
                          (20211)
Hepatitis B virus NC_003977.2 (2106) AGCTACCTGGGTGGGTGTTAATTTGGAAGATCCAGCGTCTAGAGACCTAGTAGTCAGTTATGTCAACACTAAT--ATGGGCCTAAAGTTC---A
SARS-CoV-2 NC_045512.2 (20044)      CTTATTACAGA---AGGTAGTGTTA-AAGGTTTACAA----CCATC-TGTAGGTCCCAAA---CAAGCTAGTCTTAATGGAGTCACATTAATTG
```

Section 217

```
                                   20305 20310     20320     20330     20340     20350     20360     20370     20380         20398
                          (20305)
Hepatitis B virus NC_003977.2 (2195) GGCAACTCTT----GTGGTTTCACATTTCTTGTCTCACTTTTGGAAGAGAAACAGTTATAGAGTATTTGGTGTCTTTCGGAGTGTGGATTCGCA
SARS-CoV-2 NC_045512.2 (20126)      GAGAAGCCGTAAAAACACAGTTCAATTATTATAAGAAAGT--TGATGGTGTT-GTCCAACAATTACCTGAAACTTACTTTACTCAGAGTAGAAA
```

Section 218

```
                              20399      20410     20420     20430     20440     20450     20460     20470     20480        20492
                   (20399)
Hepatitis B virus NC 003977.2 (2285)  CTCCTCCAGCTTATAGACCACCAAATGCCCCTATCCTATCAAC---ACTTCCGGAGACTACTGTTGTTAGACGACGAGGCAGGTCCCCTAGAAG
SARS-CoV-2 NC_045512.2 (20217)        TTTA-CAAGAATTT-A---AACCCAGGAGTCAAATGGAAATTGATTTCTTAGAATTAGCTATG-GATGA--------------ATTCATTGAA-
```

Section 219

```
                              20493  20500     20510     20520     20530     20540     20550     20560     20570          20586
                   (20493)
Hepatitis B virus NC 003977.2 (2376)  AAGAACTCCCTCGCCTCGCAGACGAAGGTCTCAATCGCCGCGTCGC-AGAAGATCTCAATCTCGGGAATCTCAATGTTAGTATTCCTTGGA--C
SARS-CoV-2 NC_045512.2 (20290)        CGG---T--ATAAATTAGAAGGCTATGCCTTCGAACATATCGTTTATGGAGATTTTAGTCATAGTCAGT-TAGGTG---GTTTACATCTA-CTG
```

Section 220

```
                              20587        20600     20610     20620     20630     20640     20650     20660     20670   20680
                   (20587)
Hepatitis B virus NC 003977.2 (2467)  TCATAAGGTGGGGAACTTTACTGGGCTTTATTCTTCTACTGTACCTGTCTTTAATCCTCATTGGAAAACACCATCTTTTCCTAATA-TACATTT
SARS-CoV-2 NC_045512.2 (20374)        ATTGGACTAGCTAAACGTTTTAAGGAATCACCTTTTGAAT-TAGAAGATTTTATTCCT--ATGGACAGTACAGT---TAAAAACTATTTCATA-
```

Section 221

```
                              20681    20690     20700     20710     20720     20730     20740     20750     20760        20774
                   (20681)
Hepatitis B virus NC 003977.2 (2560)  ACACCAAGACATTATCAAAAAAT--GTGAACAGTTTGTAGGCCCACTCACAGTTAATGAGAAAAGAAGATTGCAATTGATTATGCCTGCCAGGT
SARS-CoV-2 NC_045512.2 (20461)        ACAGATGCGCAAACAGGTTCATCTAAGTGTGTGTGTTCTGTTATTG--ATTTATTACTTGATGATTTTGTTGAAATA-ATAAAATCCC-AAGAT
```

Section 222

```
                              20775 20780     20790     20800     20810     20820     20830     20840     20850           20868
                   (20775)
Hepatitis B virus NC 003977.2 (2652)  TTTATCCAAAGGTTA---CCAAATATTTACCATTGGATAAGGGTATTAAACCTTATTATCCAGAACATCTAGTTAATCATTACTTCCAAACTAG
SARS-CoV-2 NC_045512.2 (20551)        TTATCTG------TAGTTTCTAAGGTTG---------TCAAAGTGAC---TATTGACTATACAGAAATTTCATT-------TATGCTTTGGTGT
```

Section 223

```
                              20869      20880     20890     20900     20910     20920     20930     20940     20950        20962
                   (20869)
Hepatitis B virus NC 003977.2 (2743)  ACACTATTTACACACTCTATGGAAGGCGGGTATATTATATAAGAGAGAAACAACACATAGCGCCTCATTTTGTGGGTCACCATATTCTTGGGAA
SARS-CoV-2 NC_045512.2 (20620)        A-AAGATGGCCATGT-----AGAAACAT--TTTACCCAAAATTACAAT--CTAGTCA----AGCGTGGCAACCGGGT----GTTGCTATGCCTA
```

Section 224

```
                              20963  20970     20980     20990     21000     21010     21020     21030     21040          21056
                   (20963)
Hepatitis B virus NC 003977.2 (2837)  CAAGATCTACAGCATGGGGCAGAATCTTTTCCACCAGCAATCCTCTGGATTCTTTCCCGACC-ACCAGTTGGATCCAGCCTTCAGAGCAAACAC
SARS-CoV-2 NC_045512.2 (20696)        --AT--CTTTACAAAATGC---AAAGAATGCTATTAGAAAAGTGTGACCTTC----------AAAATTATGGTGATAGTGCAACA-----TTAC
```

```
                                                                                                  Section 225
                                   (21057) 21057        21070     21080     21090     21100     21110     21120     21130     21140  21150
Hepatitis B virus NC_003977.2       (2930) CGCAAATCCAGATTGGGACTTTCA--ATCCCAACAAGGACACCTGGCCAGACGCCAACAAGGTAGGAGCTGGAGCATTCGGGCTGGGTTTCACCC
SARS-CoV-2 NC_045512.2             (20768) CTAAAGGC-ATAATGATGAAT--GTCGC--AA-AATATACTCAACTGTGTCAATATTTAAACACATTAACATTAGCTGTACCCTATAATATGAG
                                                                                                  Section 226
                                   (21151) 21151    21160     21170     21180     21190     21200     21210     21220     21230      21244
Hepatitis B virus NC_003977.2       (3022) CACCGCACGGAGGCCTTTTGGGGTGGAGCCCTCAGGCT-CAGGGCATACTACAAACTTTGCCAGCAAATCCGCCTCCTGCCTCCACCAATCGCC
SARS-CoV-2 NC_045512.2             (20856) AGTTATACATTTTGGTGCTG-GTTCTGATAAAGGAGTTGCACCAGGTACAGCTG---T-TTTAAGACAGTGGTTGCCTACG--GGTACGCTGCT
                                                                                                  Section 227
                                   (21245) 21245 21250     21260     21270     21280     21290     21300     21310     21320         21338
Hepatitis B virus NC_003977.2       (3115) AGTCAGGAAGGCAGCCTACCCCGCTGTCTCCACCTTTGAGAAACACTCATCCTCAGGCCATGCAGTGG--------------------------
SARS-CoV-2 NC_045512.2             (20943) TGTCGATTCAGATCTTAATGACTT------TGTCTCTGATGCAGATTCAACTTTGATTGGTGATTGTGCAACTGTACATACAGCTAATAAATGG
                                                                                                  Section 228
                                   (21339) 21339      21350     21360     21370     21380     21390     21400     21410     21420    21432
Hepatitis B virus NC_003977.2       (3183) ----------------------------------------------------------------------------------------------
SARS-CoV-2 NC_045512.2             (21031) GATCTCATTATTAGTGATATGTACGACCCTAAGACTAAAAATGTTACAAAAGAAAATGACTCTAAAGAGGGTTTTTTCACTTACATTTGTGGGT
                                                                                                  Section 229
                                   (21433) 21433  21440     21450     21460     21470     21480     21490     21500     21510        21526
Hepatitis B virus NC_003977.2       (3183) ----------------------------------------------------------------------------------------------
SARS-CoV-2 NC_045512.2             (21125) TTATACAACAAAAGCTAGCTCTTGGAGGTTCCGTGGCTATAAAGATAACAGAACATTCTTGGAATGCTGATCTTTATAAGCTCATGGGACACTT
                                                                                                  Section 230
                                   (21527) 21527        21540     21550     21560     21570     21580     21590     21600     21610  21620
Hepatitis B virus NC_003977.2       (3183) ----------------------------------------------------------------------------------------------
SARS-CoV-2 NC_045512.2             (21219) CGCATGGTGGACAGCCTTTGTTACTAATGTGAATGCGTCATCATCTGAAGCATTTTTAATTGGATGTAATTATCTTGGCAAACCACGCGAACAA
                                                                                                  Section 231
                                   (21621) 21621    21630     21640     21650     21660     21670     21680     21690     21700      21714
Hepatitis B virus NC_003977.2       (3183) ----------------------------------------------------------------------------------------------
SARS-CoV-2 NC_045512.2             (21313) ATAGATGGTTATGTCATGCATGCAAATTACATATTTTGGAGGAATACAAATCCAATTCAGTTGTCTTCCTATTCTTTATTTGACATGAGTAAAT
```

```
                                                              Section 232
                               (21715) 21715 21720     21730     21740     21750     21760     21770     21780     21790        21808
Hepatitis B virus NC_003977.2   (3183) ----------------------------------------------------------------------------------------------
SARS-CoV-2 NC_045512.2         (21407) TTCCCCTTAAATTAAGGGGTACTGCTGTTATGTCTTTAAAAGAAGGTCAAATCAATGATATGATTTTATCTCTTCTTAGTAAAGGTAGACTTAT
                                                              Section 233
                               (21809) 21809      21820     21830     21840     21850     21860     21870     21880     21890    21902
Hepatitis B virus NC_003977.2   (3183) ----------------------------------------------------------------------------------------------
SARS-CoV-2 NC_045512.2         (21501) AATTAGAGAAAACAACAGAGTTGTTATTTCTAGTGATGTTCTTGTTAACAACTAAACGAACAATGTTTGTTTTTCTTGTTTTATTGCCACTAGT
                                                              Section 234
                               (21903) 21903  21910     21920     21930     21940     21950     21960     21970     21980        21996
Hepatitis B virus NC_003977.2   (3183) ----------------------------------------------------------------------------------------------
SARS-CoV-2 NC_045512.2         (21595) CTCTAGTCAGTGTGTTAATCTTACAACCAGAACTCAATTACCCCCTGCATACACTAATTCTTTCACACGTGGTGTTTATTACCCTGACAAAGTT
                                                              Section 235
                               (21997) 21997        22010     22020     22030     22040     22050     22060     22070     22080  22090
Hepatitis B virus NC_003977.2   (3183) ----------------------------------------------------------------------------------------------
SARS-CoV-2 NC_045512.2         (21689) TTCAGATCCTCAGTTTTACATTCAACTCAGGACTTGTTCTTACCTTTCTTTTCCAATGTTACTTGGTTCCATGCTATACATGTCTCTGGGACCA
                                                              Section 236
                               (22091) 22091    22100     22110     22120     22130     22140     22150     22160     22170        22184
Hepatitis B virus NC_003977.2   (3183) ----------------------------------------------------------------------------------------------
SARS-CoV-2 NC_045512.2         (21783) ATGGTACTAAGAGGTTTGATAACCCTGTCCTACCATTTAATGATGGTGTTTATTTTGCTTCCACTGAGAAGTCTAACATAATAAGAGGCTGGAT
                                                              Section 237
                               (22185) 22185 22190     22200     22210     22220     22230     22240     22250     22260        22278
Hepatitis B virus NC_003977.2   (3183) ----------------------------------------------------------------------------------------------
SARS-CoV-2 NC_045512.2         (21877) TTTTGGTACTACTTTAGATTCGAAGACCCAGTCCCTACTTATTGTTAATAACGCTACTAATGTTGTTATTAAAGTCTGTGAATTTCAATTTTGT
                                                              Section 238
                               (22279) 22279      22290     22300     22310     22320     22330     22340     22350     22360    22372
Hepatitis B virus NC_003977.2   (3183) ----------------------------------------------------------------------------------------------
SARS-CoV-2 NC_045512.2         (21971) AATGATCCATTTTTGGGTGTTTATTACCACAAAAACAACAAAAGTTGGATGGAAAGTGAGTTCAGAGTTTATTCTAGTGCGAATAATTGCACTT
```

```
                                                                                              Section 239
                            (22373) 22373  22380        22390        22400        22410        22420        22430        22440        22450          22466
 Hepatitis B virus NC_003977.2 (3183) ----------------------------------------------------------------------------------------------
    SARS-CoV-2 NC_045512.2 (22065) TTGAATATGTCTCTCAGCCTTTTCTTATGGACCTTGAAGGAAAACAGGGTAATTTCAAAAATCTTAGGGAATTTGTGTTTAAGAATATTGATGG
                                                                                              Section 240
                            (22467) 22467           22480        22490        22500        22510        22520        22530        22540        22550 22560
 Hepatitis B virus NC_003977.2 (3183) ----------------------------------------------------------------------------------------------
    SARS-CoV-2 NC_045512.2 (22159) TTATTTTAAAATATATTCTAAGCACACGCCTATTAATTTAGTGCGTGATCTCCCTCAGGGTTTTTCGGCTTTAGAACCATTGGTAGATTTGCCA
                                                                                              Section 241
                            (22561) 22561      22570        22580        22590        22600        22610        22620        22630        22640          22654
 Hepatitis B virus NC_003977.2 (3183) ----------------------------------------------------------------------------------------------
    SARS-CoV-2 NC_045512.2 (22253) ATAGGTATTAACATCACTAGGTTTCAAACTTTACTTGCTTTACATAGAAGTTATTTGACTCCTGGTGATTCTTCTTCAGGTTGGACAGCTGGTG
                                                                                              Section 242
                            (22655) 22655 22660        22670        22680        22690        22700        22710        22720        22730               22748
 Hepatitis B virus NC_003977.2 (3183) ----------------------------------------------------------------------------------------------
    SARS-CoV-2 NC_045512.2 (22347) CTGCAGCTTATTATGTGGGTTATCTTCAACCTAGGACTTTTCTATTAAAATATAATGAAAATGGAACCATTACAGATGCTGTAGACTGTGCACT
                                                                                              Section 243
                            (22749) 22749         22760        22770        22780        22790        22800        22810        22820        22830     22842
 Hepatitis B virus NC_003977.2 (3183) ----------------------------------------------------------------------------------------------
    SARS-CoV-2 NC_045512.2 (22441) TGACCCTCTCTCAGAAACAAAGTGTACGTTGAAATCCTTCACTGTAGAAAAAGGAATCTATCAAACTTCTAACTTTAGAGTCCAACCAACAGAA
                                                                                              Section 244
                            (22843) 22843  22850        22860        22870        22880        22890        22900        22910        22920          22936
 Hepatitis B virus NC_003977.2 (3183) ----------------------------------------------------------------------------------------------
    SARS-CoV-2 NC_045512.2 (22535) TCTATTGTTAGATTTCCTAATATTACAAACTTGTGCCCTTTTGGTGAAGTTTTTAACGCCACCAGATTTGCATCTGTTTATGCTTGGAACAGGA
                                                                                              Section 245
                            (22937) 22937           22950        22960        22970        22980        22990        23000        23010        23020 23030
 Hepatitis B virus NC_003977.2 (3183) ----------------------------------------------------------------------------------------------
    SARS-CoV-2 NC_045512.2 (22629) AGAGAATCAGCAACTGTGTTGCTGATTATTCTGTCCTATATAATTCCGCATCATTTTCCACTTTTAAGTGTTATGGAGTGTCTCCTACTAAATT
```

```
                                                                                                     Section 246
                              (23031) 23031    23040     23050     23060     23070     23080     23090     23100     23110     23124
Hepatitis B virus NC_003977.2  (3183) ----------------------------------------------------------------------------------------------
     SARS-CoV-2 NC_045512.2 (22723) AAATGATCTCTGCTTTACTAATGTCTATGCAGATTCATTTGTAATTAGAGGTGATGAAGTCAGACAAATCGCTCCAGGGCAAACTGGAAAGATT
                                                                                                     Section 247
                              (23125) 23125 23130     23140     23150     23160     23170     23180     23190     23200          23218
Hepatitis B virus NC_003977.2  (3183) ----------------------------------------------------------------------------------------------
     SARS-CoV-2 NC_045512.2 (22817) GCTGATTATAATTATAAATTACCAGATGATTTTACAGGCTGCGTTATAGCTTGGAATTCTAACAATCTTGATTCTAAGGTTGGTGGTAATTATA
                                                                                                     Section 248
                              (23219) 23219      23230     23240     23250     23260     23270     23280     23290     23300     23312
Hepatitis B virus NC_003977.2  (3183) ----------------------------------------------------------------------------------------------
     SARS-CoV-2 NC_045512.2 (22911) ATTACCTGTATAGATTGTTTAGGAAGTCTAATCTCAAACCTTTTGAGAGAGATATTTCAACTGAAATCTATCAGGCCGGTAGCACACCTTGTAA
                                                                                                     Section 249
                              (23313) 23313  23320     23330     23340     23350     23360     23370     23380     23390          23406
Hepatitis B virus NC_003977.2  (3183) ----------------------------------------------------------------------------------------------
     SARS-CoV-2 NC_045512.2 (23005) TGGTGTTGAAGGTTTTAATTGTTACTTTCCTTTACAATCATATGGTTTCCAACCCACTAATGGTGTTGGTTACCAACCATACAGAGTAGTAGTA
                                                                                                     Section 250
                              (23407) 23407        23420     23430     23440     23450     23460     23470     23480     23490 23500
Hepatitis B virus NC_003977.2  (3183) ----------------------------------------------------------------------------------------------
     SARS-CoV-2 NC_045512.2 (23099) CTTTCTTTTGAACTTCTACATGCACCAGCAACTGTTTGTGGACCTAAAAAGTCTACTAATTTGGTTAAAAACAAATGTGTCAATTTCAACTTCA
                                                                                                     Section 251
                              (23501) 23501    23510     23520     23530     23540     23550     23560     23570     23580     23594
Hepatitis B virus NC_003977.2  (3183) ----------------------------------------------------------------------------------------------
     SARS-CoV-2 NC_045512.2 (23193) ATGGTTTAACAGGCACAGGTGTTCTTACTGAGTCTAACAAAAAGTTTCTGCCTTTCCAACAATTTGGCAGAGACATTGCTGACACTACTGATGC
                                                                                                     Section 252
                              (23595) 23595 23600     23610     23620     23630     23640     23650     23660     23670          23688
Hepatitis B virus NC_003977.2  (3183) ----------------------------------------------------------------------------------------------
     SARS-CoV-2 NC_045512.2 (23287) TGTCCGTGATCCACAGACACTTGAGATTCTTGACATTACACCATGTTCTTTTGGTGGTGTCAGTGTTATAACACCAGGAACAAATACTTCTAAC
```

Section 253

```
                                 (23689) 23689      23700      23710      23720      23730      23740      23750      23760      23770      23782
Hepatitis B virus NC_003977.2    (3183)  ----------------------------------------------------------------------------------------------
SARS-CoV-2 NC_045512.2           (23381) CAGGTTGCTGTTCTTTATCAGGATGTTAACTGCACAGAAGTCCCTGTTGCTATTCATGCAGATCAACTTACTCCTACTTGGCGTGTTTATTCTA
```

Section 254

```
                                 (23783) 23783  23790      23800      23810      23820      23830      23840      23850      23860      23876
Hepatitis B virus NC_003977.2    (3183)  ----------------------------------------------------------------------------------------------
SARS-CoV-2 NC_045512.2           (23475) CAGGTTCTAATGTTTTTCAAACACGTGCAGGCTGTTTAATAGGGGCTGAACATGTCAACAACTCATATGAGTGTGACATACCCATTGGTGCAGG
```

Section 255

```
                                 (23877) 23877        23890      23900      23910      23920      23930      23940      23950      23960  23970
Hepatitis B virus NC_003977.2    (3183)  ----------------------------------------------------------------------------------------------
SARS-CoV-2 NC_045512.2           (23569) TATATGCGCTAGTTATCAGACTCAGACTAATTCTCCTCGGCGGGCACGTAGTGTAGCTAGTCAATCCATCATTGCCTACACTATGTCACTTGGT
```

Section 256

```
                                 (23971) 23971   23980      23990      24000      24010      24020      24030      24040      24050      24064
Hepatitis B virus NC_003977.2    (3183)  ----------------------------------------------------------------------------------------------
SARS-CoV-2 NC_045512.2           (23663) GCAGAAAATTCAGTTGCTTACTCTAATAACTCTATTGCCATACCCACAAATTTTACTATTAGTGTTACCACAGAAATTCTACCAGTGTCTATGA
```

Section 257

```
                                 (24065) 24065 24070      24080      24090      24100      24110      24120      24130      24140      24158
Hepatitis B virus NC_003977.2    (3183)  ----------------------------------------------------------------------------------------------
SARS-CoV-2 NC_045512.2           (23757) CCAAGACATCAGTAGATTGTACAATGTACATTTGTGGTGATTCAACTGAATGCAGCAATCTTTTGTTGCAATATGGCAGTTTTTGTACACAATT
```

Section 258

```
                                 (24159) 24159      24170      24180      24190      24200      24210      24220      24230      24240      24252
Hepatitis B virus NC_003977.2    (3183)  ----------------------------------------------------------------------------------------------
SARS-CoV-2 NC_045512.2           (23851) AAACCGTGCTTTAACTGGAATAGCTGTTGAACAAGACAAAAACACCCAAGAAGTTTTTGCACAAGTCAAACAAATTTACAAAACACCACCAATT
```

Section 259

```
                                 (24253) 24253  24260      24270      24280      24290      24300      24310      24320      24330      24346
Hepatitis B virus NC_003977.2    (3183)  ----------------------------------------------------------------------------------------------
SARS-CoV-2 NC_045512.2           (23945) AAAGATTTTGGTGGTTTTAATTTTTCACAAATATTACCAGATCCATCAAAACCAAGCAAGAGGTCATTTATTGAAGATCTACTTTTCAACAAAG
```

```
                                                                                          Section 260
                         (24347) 24347        24360     24370     24380     24390     24400     24410     24420     24430   24440
Hepatitis B virus NC_003977.2 (3183) ----------------------------------------------------------------------------------------------
SARS-CoV-2 NC_045512.2 (24039) TGACACTTGCAGATGCTGGCTTCATCAAACAATATGGTGATTGCCTTGGTGATATTGCTGCTAGAGACCTCATTTGTGCACAAAAGTTTAACGG
                                                                                          Section 261
                         (24441) 24441    24450     24460     24470     24480     24490     24500     24510     24520         24534
Hepatitis B virus NC_003977.2 (3183) ----------------------------------------------------------------------------------------------
SARS-CoV-2 NC_045512.2 (24133) CCTTACTGTTTTGCCACCTTTGCTCACAGATGAAATGATTGCTCAATACACTTCTGCACTGTTAGCGGGTACAATCACTTCTGGTTGGACCTTT
                                                                                          Section 262
                         (24535) 24535 24540     24550     24560     24570     24580     24590     24600     24610             24628
Hepatitis B virus NC_003977.2 (3183) ----------------------------------------------------------------------------------------------
SARS-CoV-2 NC_045512.2 (24227) GGTGCAGGTGCTGCATTACAAATACCATTTGCTATGCAAATGGCTTATAGGTTTAATGGTATTGGAGTTACACAGAATGTTCTCTATGAGAACC
                                                                                          Section 263
                         (24629) 24629      24640     24650     24660     24670     24680     24690     24700     24710       24722
Hepatitis B virus NC_003977.2 (3183) ----------------------------------------------------------------------------------------------
SARS-CoV-2 NC_045512.2 (24321) AAAAATTGATTGCCAACCAATTTAATAGTGCTATTGGCAAAATTCAAGACTCACTTTCTTCCACAGCAAGTGCACTTGGAAAACTTCAAGATGT
                                                                                          Section 264
                         (24723) 24723  24730     24740     24750     24760     24770     24780     24790     24800           24816
Hepatitis B virus NC_003977.2 (3183) ----------------------------------------------------------------------------------------------
SARS-CoV-2 NC_045512.2 (24415) GGTCAACCAAAATGCACAAGCTTTAAACACGCTTGTTAAACAACTTAGCTCCAATTTTGGTGCAATTTCAAGTGTTTTAAATGATATCCTTTCA
                                                                                          Section 265
                         (24817) 24817        24830     24840     24850     24860     24870     24880     24890     24900   24910
Hepatitis B virus NC_003977.2 (3183) ----------------------------------------------------------------------------------------------
SARS-CoV-2 NC_045512.2 (24509) CGTCTTGACAAAGTTGAGGCTGAAGTGCAAATTGATAGGTTGATCACAGGCAGACTTCAAAGTTTGCAGACATATGTGACTCAACAATTAATTA
                                                                                          Section 266
                         (24911) 24911    24920     24930     24940     24950     24960     24970     24980     24990         25004
Hepatitis B virus NC_003977.2 (3183) ----------------------------------------------------------------------------------------------
SARS-CoV-2 NC_045512.2 (24603) GAGCTGCAGAAATCAGAGCTTCTGCTAATCTTGCTGCTACTAAAATGTCAGAGTGTGTACTTGGACAATCAAAAAGAGTTGATTTTTGTGGAAA
```

Section 267

```
                          (25005) 25005 25010     25020     25030     25040     25050     25060     25070     25080             25098
Hepatitis B virus NC_003977.2 (3183) ----------------------------------------------------------------------------------------------
SARS-CoV-2 NC_045512.2 (24697) GGGCTATCATCTTATGTCCTTCCCTCAGTCAGCACCTCATGGTGTAGTCTTCTTGCATGTGACTTATGTCCCTGCACAAGAAAAGAACTTCACA
```

Section 268

```
                          (25099) 25099      25110     25120     25130     25140     25150     25160     25170     25180     25192
Hepatitis B virus NC_003977.2 (3183) ----------------------------------------------------------------------------------------------
SARS-CoV-2 NC_045512.2 (24791) ACTGCTCCTGCCATTTGTCATGATGGAAAAGCACACTTTCCTCGTGAAGGTGTCTTTGTTTCAAATGGCACACACTGGTTTGTAACACAAAGGA
```

Section 269

```
                          (25193) 25193  25200     25210     25220     25230     25240     25250     25260     25270           25286
Hepatitis B virus NC_003977.2 (3183) ----------------------------------------------------------------------------------------------
SARS-CoV-2 NC_045512.2 (24885) ATTTTTATGAACCACAAATCATTACTACAGACAACACATTTGTGTCTGGTAACTGTGATGTTGTAATAGGAATTGTCAACAACACAGTTTATGA
```

Section 270

```
                          (25287) 25287        25300     25310     25320     25330     25340     25350     25360     25370 25380
Hepatitis B virus NC_003977.2 (3183) ----------------------------------------------------------------------------------------------
SARS-CoV-2 NC_045512.2 (24979) TCCTTTGCAACCTGAATTAGACTCATTCAAGGAGGAGTTAGATAAATATTTTAAGAATCATACATCACCAGATGTTGATTTAGGTGACATCTCT
```

Section 271

```
                          (25381) 25381    25390     25400     25410     25420     25430     25440     25450     25460       25474
Hepatitis B virus NC_003977.2 (3183) ----------------------------------------------------------------------------------------------
SARS-CoV-2 NC_045512.2 (25073) GGCATTAATGCTTCAGTTGTAAACATTCAAAAAGAAATTGACCGCCTCAATGAGGTTGCCAAGAATTTAAATGAATCTCTCATCGATCTCCAAG
```

Section 272

```
                          (25475) 25475 25480     25490     25500     25510     25520     25530     25540     25550             25568
Hepatitis B virus NC_003977.2 (3183) ----------------------------------------------------------------------------------------------
SARS-CoV-2 NC_045512.2 (25167) AACTTGGAAAGTATGAGCAGTATATAAAATGGCCATGGTACATTTGGCTAGGTTTTATAGCTGGCTTGATTGCCATAGTAATGGTGACAATTAT
```

Section 273

```
                          (25569) 25569      25580     25590     25600     25610     25620     25630     25640     25650     25662
Hepatitis B virus NC_003977.2 (3183) ----------------------------------------------------------------------------------------------
SARS-CoV-2 NC_045512.2 (25261) GCTTTGCTGTATGACCAGTTGCTGTAGTTGTCTCAAGGGCTGTTGTTCTTGTGGATCCTGCTGCAAATTTGATGAAGACGACTCTGAGCCAGTG
```

```
                                                                                              Section 274
                                (25663) 25663 25670     25680     25690     25700     25710     25720     25730     25740          25756
Hepatitis B virus NC_003977.2    (3183) ----------------------------------------------------------------------------------------------
SARS-CoV-2 NC_045512.2          (25355) CTCAAAGGAGTCAAATTACATTACACATAAACGAACTTATGGATTTGTTTATGAGAATCTTCACAATTGGAACTGTAACTTTGAAGCAAGGTGA
                                                                                              Section 275
                                (25757) 25757        25770     25780     25790     25800     25810     25820     25830     25840  25850
Hepatitis B virus NC_003977.2    (3183) ----------------------------------------------------------------------------------------------
SARS-CoV-2 NC_045512.2          (25449) AATCAAGGATGCTACTCCTTCAGATTTTGTTCGCGCTACTGCAACGATACCGATACAAGCCTCACTCCCTTTCGGATGGCTTATTGTTGGCGTT
                                                                                              Section 276
                                (25851) 25851    25860     25870     25880     25890     25900     25910     25920     25930        25944
Hepatitis B virus NC_003977.2    (3183) ----------------------------------------------------------------------------------------------
SARS-CoV-2 NC_045512.2          (25543) GCACTTCTTGCTGTTTTTCAGAGCGCTTCCAAAATCATAACCCTCAAAAAGAGATGGCAACTAGCACTCTCCAAGGGTGTTCACTTTGTTTGCA
                                                                                              Section 277
                                (25945) 25945 25950     25960     25970     25980     25990     26000     26010     26020            26038
Hepatitis B virus NC_003977.2    (3183) ----------------------------------------------------------------------------------------------
SARS-CoV-2 NC_045512.2          (25637) ACTTGCTGTTGTTGTTTGTAACAGTTTACTCACACCTTTTGCTCGTTGCTGCTGGCCTTGAAGCCCCTTTTCTCTATCTTTATGCTTTAGTCTA
                                                                                              Section 278
                                (26039) 26039      26050     26060     26070     26080     26090     26100     26110     26120     26132
Hepatitis B virus NC_003977.2    (3183) ----------------------------------------------------------------------------------------------
SARS-CoV-2 NC_045512.2          (25731) CTTCTTGCAGAGTATAAACTTTGTAAGAATAATAATGAGGCTTTGGCTTTGCTGGAAATGCCGTTCCAAAAACCCATTACTTTATGATGCCAAC
                                                                                              Section 279
                                (26133) 26133 26140     26150     26160     26170     26180     26190     26200     26210          26226
Hepatitis B virus NC_003977.2    (3183) ----------------------------------------------------------------------------------------------
SARS-CoV-2 NC_045512.2          (25825) TATTTTCTTTGCTGGCATACTAATTGTTACGACTATTGTATACCTTACAATAGTGTAACTTCTTCAATTGTCATTACTTCAGGTGATGGCACAA
                                                                                              Section 280
                                (26227) 26227        26240     26250     26260     26270     26280     26290     26300     26310  26320
Hepatitis B virus NC_003977.2    (3183) ----------------------------------------------------------------------------------------------
SARS-CoV-2 NC_045512.2          (25919) CAAGTCCTATTTCTGAACATGACTACCAGATTGGTGGTTATACTGAAAAATGGGAATCTGGAGTAAAAGACTGTGTTGTATTACACAGTTACTT
```

```
                                                                                                    Section 281
                              (26321) 26321    26330     26340     26350     26360     26370     26380     26390     26400        26414
Hepatitis B virus NC_003977.2  (3183) ----------------------------------------------------------------------------------------------
   SARS-CoV-2 NC_045512.2    (26013) CACTTCAGACTATTACCAGCTGTACTCAACTCAATTGAGTACAGACACTGGTGTTGAACATGTTACCTTCTTCATCTACAATAAAATTGTTGAT
                                                                                                    Section 282
                              (26415) 26415 26420     26430     26440     26450     26460     26470     26480     26490             26508
Hepatitis B virus NC_003977.2  (3183) ----------------------------------------------------------------------------------------------
   SARS-CoV-2 NC_045512.2    (26107) GAGCCTGAAGAACATGTCCAAATTCACACAATCGACGGTTCATCCGGAGTTGTTAATCCAGTAATGGAACCAATTTATGATGAACCGACGACGA
                                                                                                    Section 283
                              (26509) 26509      26520     26530     26540     26550     26560     26570     26580     26590       26602
Hepatitis B virus NC_003977.2  (3183) ----------------------------------------------------------------------------------------------
   SARS-CoV-2 NC_045512.2    (26201) CTACTAGCGTGCCTTTGTAAGCACAAGCTGATGAGTACGAACTTATGTACTCATTCGTTTCGGAAGAGACAGGTACGTTAATAGTTAATAGCGT
                                                                                                    Section 284
                              (26603) 26603  26610     26620     26630     26640     26650     26660     26670     26680            26696
Hepatitis B virus NC_003977.2  (3183) ----------------------------------------------------------------------------------------------
   SARS-CoV-2 NC_045512.2    (26295) ACTTCTTTTTCTTGCTTTCGTGGTATTCTTGCTAGTTACACTAGCCATCCTTACTGCGCTTCGATTGTGTGCGTACTGCTGCAATATTGTTAAC
                                                                                                    Section 285
                              (26697) 26697        26710     26720     26730     26740     26750     26760     26770     26780 26790
Hepatitis B virus NC_003977.2  (3183) ----------------------------------------------------------------------------------------------
   SARS-CoV-2 NC_045512.2    (26389) GTGAGTCTTGTAAAACCTTCTTTTTACGTTTACTCTCGTGTTAAAAATCTGAATTCTTCTAGAGTTCCTGATCTTCTGGTCTAAACGAACTAAA
                                                                                                    Section 286
                              (26791) 26791    26800     26810     26820     26830     26840     26850     26860     26870        26884
Hepatitis B virus NC_003977.2  (3183) ----------------------------------------------------------------------------------------------
   SARS-CoV-2 NC_045512.2    (26483) TATTATATTAGTTTTTCTGTTTGGAACTTTAATTTTAGCCATGGCAGATTCCAACGGTACTATTACCGTTGAAGAGCTTAAAAAGCTCCTTGAA
                                                                                                    Section 287
                              (26885) 26885 26890     26900     26910     26920     26930     26940     26950     26960             26978
Hepatitis B virus NC_003977.2  (3183) ----------------------------------------------------------------------------------------------
   SARS-CoV-2 NC_045512.2    (26577) CAATGGAACCTAGTAATAGGTTTCCTATTCCTTACATGGATTTGTCTTCTACAATTTGCCTATGCCAACAGGAATAGGTTTTTGTATATAATTA
```

Section 288

(26979) 26979 26990 27000 27010 27020 27030 27040 27050 27060 27072
Hepatitis B virus NC_003977.2 (3183) --
SARS-CoV-2 NC_045512.2 (26671) AGTTAATTTTCCTCTGGCTGTTATGGCCAGTAACTTTAGCTTGTTTTGTGCTTGCTGCTGTTTACAGAATAAATTGGATCACCGGTGGAATTGC

Section 289

(27073) 27073 27080 27090 27100 27110 27120 27130 27140 27150 27166
Hepatitis B virus NC_003977.2 (3183) --
SARS-CoV-2 NC_045512.2 (26765) TATCGCAATGGCTTGTCTTGTAGGCTTGATGTGGCTCAGCTACTTCATTGCTTCTTTCAGACTGTTTGCGCGTACGCGTTCCATGTGGTCATTC

Section 290

(27167) 27167 27180 27190 27200 27210 27220 27230 27240 27250 27260
Hepatitis B virus NC_003977.2 (3183) --
SARS-CoV-2 NC_045512.2 (26859) AATCCAGAAACTAACATTCTTCTCAACGTGCCACTCCATGGCACTATTCTGACCAGACCGCTTCTAGAAAGTGAACTCGTAATCGGAGCTGTGA

Section 291

(27261) 27261 27270 27280 27290 27300 27310 27320 27330 27340 27354
Hepatitis B virus NC_003977.2 (3183) --
SARS-CoV-2 NC_045512.2 (26953) TCCTTCGTGGACATCTTCGTATTGCTGGACACCATCTAGGACGCTGTGACATCAAGGACCTGCCTAAAGAAATCACTGTTGCTACATCACGAAC

Section 292

(27355) 27355 27360 27370 27380 27390 27400 27410 27420 27430 27448
Hepatitis B virus NC_003977.2 (3183) --
SARS-CoV-2 NC_045512.2 (27047) GCTTTCTTATTACAAATTGGGAGCTTCGCAGCGTGTAGCAGGTGACTCAGGTTTTGCTGCATACAGTCGCTACAGGATTGGCAACTATAAATTA

Section 293

(27449) 27449 27460 27470 27480 27490 27500 27510 27520 27530 27542
Hepatitis B virus NC_003977.2 (3183) --
SARS-CoV-2 NC_045512.2 (27141) AACACAGACCATTCCAGTAGCAGTGACAATATTGCTTTGCTTGTACAGTAAGTGACAACAGATGTTTCATCTCGTTGACTTTCAGGTTACTATA

Section 294

(27543) 27543 27550 27560 27570 27580 27590 27600 27610 27620 27636
Hepatitis B virus NC_003977.2 (3183) --
SARS-CoV-2 NC_045512.2 (27235) GCAGAGATATTACTAATTATTATGAGGACTTTTAAAGTTTCCATTTGGAATCTTGATTACATCATAAACCTCATAATTAAAAATTTATCTAAGT

```
                                                                                                  Section 295
                        (27637) 27637       27650     27660     27670     27680     27690     27700     27710     27720  27730
Hepatitis B virus NC_003977.2 (3183) ----------------------------------------------------------------------------------------------
SARS-CoV-2 NC_045512.2 (27329) CACTAACTGAGAATAAATATTCTCAATTAGATGAAGAGCAACCAATGGAGATTGATTAAACGAACATGAAAATTATTCTTTTCTTGGCACTGAT
                                                                                                  Section 296
                        (27731) 27731    27740     27750     27760     27770     27780     27790     27800     27810       27824
Hepatitis B virus NC_003977.2 (3183) ----------------------------------------------------------------------------------------------
SARS-CoV-2 NC_045512.2 (27423) AACACTCGCTACTTGTGAGCTTTATCACTACCAAGAGTGTGTTAGAGGTACAACAGTACTTTTAAAAGAACCTTGCTCTTCTGGAACATACGAG
                                                                                                  Section 297
                        (27825) 27825 27830     27840     27850     27860     27870     27880     27890     27900          27918
Hepatitis B virus NC_003977.2 (3183) ----------------------------------------------------------------------------------------------
SARS-CoV-2 NC_045512.2 (27517) GGCAATTCACCATTTCATCCTCTAGCTGATAACAAATTTGCACTGACTTGCTTTAGCACTCAATTTGCTTTTGCTTGTCCTGACGGCGTAAAAC
                                                                                                  Section 298
                        (27919) 27919      27930     27940     27950     27960     27970     27980     27990     28000    28012
Hepatitis B virus NC_003977.2 (3183) ----------------------------------------------------------------------------------------------
SARS-CoV-2 NC_045512.2 (27611) ACGTCTATCAGTTACGTGCCAGATCAGTTTCACCTAAACTGTTCATCAGACAAGAGGAAGTTCAAGAACTTTACTCTCCAATTTTTCTTATTGT
                                                                                                  Section 299
                        (28013) 28013  28020     28030     28040     28050     28060     28070     28080     28090          28106
Hepatitis B virus NC_003977.2 (3183) ----------------------------------------------------------------------------------------------
SARS-CoV-2 NC_045512.2 (27705) TGCGGCAATAGTGTTTATAACACTTTGCTTCACACTCAAAAGAAAGACAGAATGATTGAACTTTCATTAATTGACTTCTATTTGTGCTTTTTAG
                                                                                                  Section 300
                        (28107) 28107       28120     28130     28140     28150     28160     28170     28180     28190  28200
Hepatitis B virus NC_003977.2 (3183) ----------------------------------------------------------------------------------------------
SARS-CoV-2 NC_045512.2 (27799) CCTTTCTGCTATTCCTTGTTTTAATTATGCTTATTATCTTTTGGTTCTCACTTGAACTGCAAGATCATAATGAAACTTGTCACGCCTAAACGAA
                                                                                                  Section 301
                        (28201) 28201    28210     28220     28230     28240     28250     28260     28270     28280       28294
Hepatitis B virus NC_003977.2 (3183) ----------------------------------------------------------------------------------------------
SARS-CoV-2 NC_045512.2 (27893) CATGAAATTTCTTGTTTTCTTAGGAATCATCACAACTGTAGCTGCATTTCACCAAGAATGTAGTTTACAGTCATGTACTCAACATCAACCATAT
```

```
                                                                                                              Section 302
                            (28295) 28295 28300     28310     28320     28330     28340     28350     28360     28370             28388
Hepatitis B virus NC_003977.2  (3183) ----------------------------------------------------------------------------------------------
SARS-CoV-2 NC_045512.2 (27987) GTAGTTGATGACCCGTGTCCTATTCACTTCTATTCTAAATGGTATATTAGAGTAGGAGCTAGAAAATCAGCACCTTTAATTGAATTGTGCGTGG
                                                                                                              Section 303
                            (28389) 28389      28400     28410     28420     28430     28440     28450     28460     28470    28482
Hepatitis B virus NC_003977.2  (3183) ----------------------------------------------------------------------------------------------
SARS-CoV-2 NC_045512.2 (28081) ATGAGGCTGGTTCTAAATCACCCATTCAGTACATCGATATCGGTAATTATACAGTTTCCTGTTTACCTTTTACAATTAATTGCCAGGAACCTAA
                                                                                                              Section 304
                            (28483) 28483  28490     28500     28510     28520     28530     28540     28550     28560          28576
Hepatitis B virus NC_003977.2  (3183) ----------------------------------------------------------------------------------------------
SARS-CoV-2 NC_045512.2 (28175) ATTGGGTAGTCTTGTAGTGCGTTGTTCGTTCTATGAAGACTTTTTAGAGTATCATGACGTTCGTGTTGTTTTAGATTTCATCTAAACGAACAAA
                                                                                                              Section 305
                            (28577) 28577        28590     28600     28610     28620     28630     28640     28650     28660 28670
Hepatitis B virus NC_003977.2  (3183) ----------------------------------------------------------------------------------------------
SARS-CoV-2 NC_045512.2 (28269) CTAAAATGTCTGATAATGGACCCCAAAATCAGCGAAATGCACCCCGCATTACGTTTGGTGGACCCTCAGATTCAACTGGCAGTAACCAGAATGG
                                                                                                              Section 306
                            (28671) 28671    28680     28690     28700     28710     28720     28730     28740     28750         28764
Hepatitis B virus NC_003977.2  (3183) ----------------------------------------------------------------------------------------------
SARS-CoV-2 NC_045512.2 (28363) AGAACGCAGTGGGGCGCGATCAAAACAACGTCGGCCCCAAGGTTTACCCAATAATACTGCGTCTTGGTTCACCGCTCTCACTCAACATGGCAAG
                                                                                                              Section 307
                            (28765) 28765 28770     28780     28790     28800     28810     28820     28830     28840             28858
Hepatitis B virus NC_003977.2  (3183) ----------------------------------------------------------------------------------------------
SARS-CoV-2 NC_045512.2 (28457) GAAGACCTTAAATTCCCTCGAGGACAAGGCGTTCCAATTAACACCAATAGCAGTCCAGATGACCAAATTGGCTACTACCGAAGAGCTACCAGAC
                                                                                                              Section 308
                            (28859) 28859      28870     28880     28890     28900     28910     28920     28930     28940    28952
Hepatitis B virus NC_003977.2  (3183) ----------------------------------------------------------------------------------------------
SARS-CoV-2 NC_045512.2 (28551) GAATTCGTGGTGGTGACGGTAAAATGAAAGATCTCAGTCCAAGATGGTATTTCTACTACCTAGGAACTGGGCCAGAAGCTGGACTTCCCTATGG
```

```
                                                                                                    Section 309
                          (28953) 28953 28960     28970     28980     28990     29000     29010     29020     29030          29046
Hepatitis B virus NC_003977.2 (3183) ----------------------------------------------------------------------------------------------
SARS-CoV-2 NC_045512.2 (28645) TGCTAACAAAGACGGCATCATATGGGTTGCAACTGAGGGAGCCTTGAATACACCAAAAGATCACATTGGCACCCGCAATCCTGCTAACAATGCT
                                                                                                    Section 310
                          (29047) 29047        29060     29070     29080     29090     29100     29110     29120     29130 29140
Hepatitis B virus NC_003977.2 (3183) ----------------------------------------------------------------------------------------------
SARS-CoV-2 NC_045512.2 (28739) GCAATCGTGCTACAACTTCCTCAAGGAACAACATTGCCAAAAGGCTTCTACGCAGAAGGGAGCAGAGGCGGCAGTCAAGCCTCTTCTCGTTCCT
                                                                                                    Section 311
                          (29141) 29141    29150     29160     29170     29180     29190     29200     29210     29220          29234
Hepatitis B virus NC_003977.2 (3183) ----------------------------------------------------------------------------------------------
SARS-CoV-2 NC_045512.2 (28833) CATCACGTAGTCGCAACAGTTCAAGAAATTCAACTCCAGGCAGCAGTAGGGGAACTTCTCCTGCTAGAATGGCTGGCAATGGCGGTGATGCTGC
                                                                                                    Section 312
                          (29235) 29235 29240     29250     29260     29270     29280     29290     29300     29310          29328
Hepatitis B virus NC_003977.2 (3183) ----------------------------------------------------------------------------------------------
SARS-CoV-2 NC_045512.2 (28927) TCTTGCTTTGCTGCTGCTTGACAGATTGAACCAGCTTGAGAGCAAAATGTCTGGTAAAGGCCAACAACAACAAGGCCAAACTGTCACTAAGAAA
                                                                                                    Section 313
                          (29329) 29329      29340     29350     29360     29370     29380     29390     29400     29410     29422
Hepatitis B virus NC_003977.2 (3183) ----------------------------------------------------------------------------------------------
SARS-CoV-2 NC_045512.2 (29021) TCTGCTGCTGAGGCTTCTAAGAAGCCTCGGCAAAAACGTACTGCCACTAAAGCATACAATGTAACACAAGCTTTCGGCAGACGTGGTCCAGAAC
                                                                                                    Section 314
                          (29423) 29423 29430     29440     29450     29460     29470     29480     29490     29500          29516
Hepatitis B virus NC_003977.2 (3183) ----------------------------------------------------------------------------------------------
SARS-CoV-2 NC_045512.2 (29115) AAACCCAAGGAAATTTTGGGGACCAGGAACTAATCAGACAAGGAACTGATTACAAACATTGGCCGCAAATTGCACAATTTGCCCCCAGCGCTTC
                                                                                                    Section 315
                          (29517) 29517        29530     29540     29550     29560     29570     29580     29590     29600 29610
Hepatitis B virus NC_003977.2 (3183) ----------------------------------------------------------------------------------------------
SARS-CoV-2 NC_045512.2 (29209) AGCGTTCTTCGGAATGTCGCGCATTGGCATGGAAGTCACACCTTCGGGAACGTGGTTGACCTACACAGGTGCCATCAAATTGGATGACAAAGAT
```

```
                                                                                                 Section 316
                              (29611) 29611    29620     29630     29640     29650     29660     29670     29680     29690         29704
Hepatitis B virus NC_003977.2  (3183) ----------------------------------------------------------------------------------------------
SARS-CoV-2 NC_045512.2        (29303) CCAAATTTCAAAGATCAAGTCATTTTGCTGAATAAGCATATTGACGCATACAAAACATTCCCACCAACAGAGCCTAAAAAGGACAAAAAGAAGA
                                                                                                 Section 317
                              (29705) 29705 29710     29720     29730     29740     29750     29760     29770     29780            29798
Hepatitis B virus NC_003977.2  (3183) ----------------------------------------------------------------------------------------------
SARS-CoV-2 NC_045512.2        (29397) AGGCTGATGAAACTCAAGCCTTACCGCAGAGACAGAAGAAACAGCAAACTGTGACTCTTCTTCCTGCTGCAGATTTGGATGATTTCTCCAAACA
                                                                                                 Section 318
                              (29799) 29799      29810     29820     29830     29840     29850     29860     29870     29880     29892
Hepatitis B virus NC_003977.2  (3183) ----------------------------------------------------------------------------------------------
SARS-CoV-2 NC_045512.2        (29491) ATTGCAACAATCCATGAGCAGTGCTGACTCAACTCAGGCCTAAACTCATGCAGACCACACAAGGCAGATGGGCTATATAAACGTTTTCGCTTTT
                                                                                                 Section 319
                              (29893) 29893  29900     29910     29920     29930     29940     29950     29960     29970          29986
Hepatitis B virus NC_003977.2  (3183) ----------------------------------------------------------------------------------------------
SARS-CoV-2 NC_045512.2        (29585) CCGTTTACGATATATAGTCTACTCTTGTGCAGAATGAATTCTCGTAACTACATAGCACAAGTAGATGTAGTTAACTTTAATCTCACATAGCAAT
                                                                                                 Section 320
                              (29987) 29987        30000     30010     30020     30030     30040     30050     30060     30070    30080
Hepatitis B virus NC_003977.2  (3183) ----------------------------------------------------------------------------------------------
SARS-CoV-2 NC_045512.2        (29679) CTTTAATCAGTGTGTAACATTAGGGAGGACTTGAAAGAGCCACCACATTTTCACCGAGGCCACGCGGAGTACGATCGAGTGTACAGTGAACAAT
                                                                                                 Section 321
                              (30081) 30081    30090     30100     30110     30120     30130     30140     30150     30160         30174
Hepatitis B virus NC_003977.2  (3183) ----------------------------------------------------------------------------------------------
SARS-CoV-2 NC_045512.2        (29773) GCTAGGGAGAGCTGCCTATATGGAAGAGCCCTAATGTGTAAAATTAATTTTAGTAGTGCTATCCCCATGTGATTTTAATAGCTTCTTAGGAGAA
                                                                                                 Section 322
                              (30175) 30175 30180     30190     30200     30211
Hepatitis B virus NC_003977.2  (3183) -------------------------------------
SARS-CoV-2 NC_045512.2        (29867) TGACAAAAAAAAAAAAAAAAAAAAAAAAAAAAAAAAA
```
