# Supplementary material for: Ubiquitous Micro-Modular Homologies among Genomes from Viruses to Bacteria to Human Mitochondrial DNA: Platforms for Recombination during Evolution?
Source: Viruses. 2022 Apr 24;14(5):885. doi: 10.3390/v14050885 (PMC9147251; doi:10.3390/v14050885)
Supplement: Supplementary file 1 [file viruses-14-00885-s001.zip › Fig. S7B, SARS-CoV-2 & T4 DNA Alignment.pdf]

SARS-CoV-2 & T4.apr

```
                                                                                                                    Section 1
                               (1)  1              11               22               33               44               55               66               77                      96
T4 NC 000866.4                 (1)  AATTTTCCTTATTAGGCCGCAAGGGCCTTCATAGTTTTAGCGATTTGGGAAACTTCATCATCACTTAAAGAGTTGCGATAACCGATGAAGTCGGAA
SARS-CoV-2 NC_045512.2         (1)  ------------------------------------------------------------------------------------------------
                                                                                                                    Section 2
                              (97)  97                  110              121              132              143              154              165              176                192
T4 NC 000866.4                (97)  ACAATACGGAATTTCTTGGTAAACTCAGCAACCATTTTATCACTGTTTTTTGAAGCATTATTTGATAATACATCAAAAAGATTAGTTACTGTCCAA
SARS-CoV-2 NC_045512.2         (1)  ------------------------------------------------------------------------------------------------
                                                                                                                    Section 3
                             (193)  193    198              209              220              231              242              253              264              275              288
T4 NC 000866.4               (193)  ATGTCATGACCGATGGTATCTTTTCCACCATTAAAATATACACCCTGTAATGAACTAACCATATTAGCGAGTCGTGTATATTCTTCAGAAACTTCA
SARS-CoV-2 NC_045512.2         (1)  ------------------------------------------------------------------------------------------------
                                                                                                                    Section 4
                             (289)  289        297              308              319              330              341              352              363                     384
T4 NC 000866.4               (289)  TCTATACTGAAGTACTTCATCATAAAATCTAACTCAGGATACTTGATAATTTTATCAATATATCGTTTAGCTGAACTTGAATAACCTACATACTTA
SARS-CoV-2 NC_045512.2         (1)  ------------------------------------------------------------------------------------------------
                                                                                                                    Section 5
                             (385)  385              396              407              418              429              440              451              462                480
T4 NC 000866.4               (385)  TCATAATCTACATCATCAAAAGCATCTACATATAAATCACGCAAAGCTTCAAAAATACATTGGCACTGACCGAGTTCTTTTACCTTTTTCTGTAAA
SARS-CoV-2 NC_045512.2         (1)  ------------------------------------------------------------------------------------------------
                                                                                                                    Section 6
                             (481)  481                 495              506              517              528              539              550              561                576
T4 NC 000866.4               (481)  AGCGGACGAATAACATAAAATTCATTAATGCCAATAAGATTAGCCATACGAATCAAAATATTCATAGATGGATGACAAAGAGATGTAGTACCATCC
SARS-CoV-2 NC_045512.2         (1)  ------------------------------------------------------------------------------------------------
                                                                                                                    Section 7
                             (577)  577     583              594              605              616              627              638              649              660              672
T4 NC 000866.4               (577)  ATAGAGAAAATATCAGAACGATGCATATACGCTACATAACCAGTAATTTCATCTGCTTCTGATGTGAGGCGTAAATAATTCCTCTTTTCCCAGCGC
SARS-CoV-2 NC_045512.2         (1)  ------------------------------------------------------------------------------------------------
```

```
                                                                                  Section 8
                          (673) 673      682        693        704        715        726        737        748             768
T4 NC_000866.4            (673) CCGTCTTTAATTTCAAACTTAAACGCTGTAGCAGCTTTAGGACGAGGAGCTTTACTTTTAACTACCTTTGGAATATAACTTTTTACTAAAGCTTCA
SARS-CoV-2 NC_045512.2      (1) ------------------------------------------------------------------------------------------------
                                                                                  Section 9
                          (769) 769         781        792        803        814        825        836        847              864
T4 NC_000866.4            (769) ATTTCTGACAAATAATGAATGTTAACTTCATCACTTTCAAACATCGCCATAATATCAGGAAGCAAATCAATCTGCGATTCTACTTCTGGATTAATA
SARS-CoV-2 NC_045512.2      (1) ------------------------------------------------------------------------------------------------
                                                                                  Section 10
                          (865) 865            880        891        902        913        924        935        946            960
T4 NC_000866.4            (865) AACAGAAGACGTTCGTTATGATGAATATTCAAAGTGTTATTAAATTCACTATCATCTAACGCACGTGCTAATCCACGGACAATATTAACACGATTT
SARS-CoV-2 NC_045512.2      (1) ------------------------------------------------------------------------------------------------
                                                                                  Section 11
                          (961) 961    968        979        990        1001       1012       1023       1034       1045      1056
T4 NC_000866.4            (961) TTAATATTATCAATAACGATATTAATTTTTGTTGTATTAATACCAAACAGACGATAACTTGATGCAACGGCTGAAGTTTCATGACTTTGCTTAATG
SARS-CoV-2 NC_045512.2      (1) ------------------------------------------------------------------------------------------------
                                                                                  Section 12
                         (1057) 1057      1067       1078       1089       1100       1111       1122       1133              1152
T4 NC_000866.4           (1057) CGCTTCAGTCGAGGGTCAAGATTTACTTCATACACAACTCCCGCGTTGCATAACTTACTGTCAGGTTCAAACATGCTCTGCATCTTTTTATATGAC
SARS-CoV-2 NC_045512.2      (1) ------------------------------------------------------------------------------------------------
                                                                                  Section 13
                         (1153) 1153         1166       1177       1188       1199       1210       1221       1232            1248
T4 NC_000866.4           (1153) AGATTTTTAGTCGTGAATTTGACTGAATTACTAATCATATAATCTCGAGCAGAATACCCCATCTTCATCAATTCACGATATGTGTGACGAGGAGAT
SARS-CoV-2 NC_045512.2      (1) ------------------------------------------------------------------------------------------------
                                                                                  Section 14
                         (1249) 1249 1254       1265       1276       1287       1298       1309       1320       1331      1344
T4 NC_000866.4           (1249) GTAGATTCTTTAAATCGTTTTACATCTTCATTAAATGCTTTCTCACTGAGTTCTTTAACTCGTTCAATAATATTTTTACGAGTGCGATCATCCAGT
SARS-CoV-2 NC_045512.2      (1) ------------------------------------------------------------------------------------------------
```

```
                                                                                                           Section 15
                              (1345) 1345    1353       1364       1375       1386       1397       1408       1419                1440
          T4 NC_000866.4      (1345) GAAAGAGCCTCGCGAGATGGAGCAATATCAAGTGAACCCATTGGAAACTTAATGTAATTCACTTCATTGCGAATGCTTAGCCAGTTACGGTCTCTA
  SARS-CoV-2 NC_045512.2         (1) ------------------------------------------------------------------------------------------------
                                                                                                           Section 16
                              (1441) 1441       1452       1463       1474       1485       1496       1507       1518               1536
          T4 NC_000866.4      (1441) ATAACACCATCGATAGGATAAACAATACCACCGTAGATAGCATATAATCCACCACGATCAGGCCAGTATCTTTCTGGATTTACACCGTAATAGTCA
  SARS-CoV-2 NC_045512.2         (1) ------------------------------------------------------------------------------------------------
                                                                                                           Section 17
                              (1537) 1537          1551       1562       1573       1584       1595       1606       1617            1632
          T4 NC_000866.4      (1537) TCAAAATCCGGAAAATAATCAATTTCGCGGTCAAGACCATTAATGATAGCCAAATCTTTGAACGGTCGCATGATATAAGAAACTTCATAAGCAAAG
  SARS-CoV-2 NC_045512.2         (1) ------------------------------------------------------------------------------------------------
                                                                                                           Section 18
                              (1633) 1633  1639       1650       1661       1672       1683       1694       1705       1716        1728
          T4 NC_000866.4      (1633) TTTCTAAAGTCTTTTTCTTCAACTGGAACTACGATTTCAATACCAGTTTTATCATCTGGACCCATTTCTTTTACGAATGTAGGTTTAATCTGTGGA
  SARS-CoV-2 NC_045512.2         (1) ------------------------------------------------------------------------------------------------
                                                                                                           Section 19
                              (1729) 1729     1738       1749       1760       1771       1782       1793       1804                1824
          T4 NC_000866.4      (1729) CCATCACCATCCATGTAAGCTACATAACCACGAATTTCACCTTTATGATACGAAGTAATACTAAACGTATCAGTATAACTAAACGGAGATTTAGAA
  SARS-CoV-2 NC_045512.2         (1) ------------------------------------------------------------------------------------------------
                                                                                                           Section 20
                              (1825) 1825        1837       1848       1859       1870       1881       1892       1903             1920
          T4 NC_000866.4      (1825) CCTAAACCAAATCCGCCAATAAAGTCATTAGATTCAGCTTTAGATGAACTGAAGTATGAATTATACAACCCAGGAGAATTATCATCACCTTGAATA
  SARS-CoV-2 NC_045512.2         (1) ------------------------------------------------------------------------------------------------
                                                                                                           Section 21
                              (1921) 1921           1936       1947       1958       1969       1980       1991       2002          2016
          T4 NC_000866.4      (1921) TCAAAATCACTCATACCCGGACCAAAATCTCGACAAACAAATCGTGGGTCTAAACGTCCAGGAACTTGTATGATAAATTTTTCAGGATTTCCATTA
  SARS-CoV-2 NC_045512.2         (1) ------------------------------------------------------------------------------------------------
```

```
                                                                                            Section 22
                         (2017) 2017  2024       2035       2046       2057       2068       2079       2090       2101     2112
T4 NC_000866.4           (2017) AGTGCATGAGCATCAATCATGTTAGTAATCAATTCACGGACTACTGCGCGAATCTTGTTTGTATACAAATCAGATGACAGAATTTTAAATACTTTA
SARS-CoV-2 NC_045512.2      (1) ------------------------------------------------------------------------------------------------
                                                                                            Section 23
                         (2113) 2113      2123       2134       2145       2156       2167       2178       2189              2208
T4 NC_000866.4           (2113) GGAGATGCTGTGATGCTAAATGCTTTTGATTTAGAACCATTACCAAGAATTGTTTCTTTTTCAGTGGTGATAATCATAATTTCCTCATTAATTCAT
SARS-CoV-2 NC_045512.2      (1) ------------------------------------------------------------------------------------------------
                                                                                            Section 24
                         (2209) 2209         2222       2233       2244       2255       2266       2277       2288            2304
T4 NC_000866.4           (2209) ATTACGCTTAATAACTTCAGCAACTTCTAGTAGTTCATCTTTAGTTGCGGTGTCGGATTGAATTTTATCTCTAATATCTTTAAAGCGGGTTTTAAA
SARS-CoV-2 NC_045512.2      (1) ------------------------------------------------------------------------------------------------
                                                                                            Section 25
                         (2305) 2305 2310       2321       2332       2343       2354       2365       2376       2387         2400
T4 NC_000866.4           (2305) TTCTTCGGCTTCTCCCATATCGAAAAAGCGTTGAATGATTCTATATTCTCGATGAACTGCTTTATCAAAAAGTTCTAAATTTACTTTATATGATTT
SARS-CoV-2 NC_045512.2      (1) ------------------------------------------------------------------------------------------------
                                                                                            Section 26
                         (2401) 2401    2409       2420       2431       2442       2453       2464       2475                2496
T4 NC_000866.4           (2401) CATTTCAATATCCTCATTTGCCCAATTAATTATACCACATCCTTGTGGTAAAGTAAACTACTGGCTCATCCATTCTTTACGAAGGTCAGCATTATC
SARS-CoV-2 NC_045512.2      (1) ------------------------------------------------------------------------------------------------
                                                                                            Section 27
                         (2497) 2497       2508       2519       2530       2541       2552       2563       2574             2592
T4 NC_000866.4           (2497) TCCCATGAGCATTTCAAAAAGCTCTTTCCAGTTCTCAGGAAGTTTAACAACATCATATACTGGGTTTTGAATCATCTCACGATATTCAGATTTTTC
SARS-CoV-2 NC_045512.2      (1) ------------------------------------------------------------------------------------------------
                                                                                            Section 28
                         (2593) 2593          2607       2618       2629       2640       2651       2662       2673            2688
T4 NC_000866.4           (2593) CAAAGAGCCAAGTCCCTTAATATAACGGATGCTATGTTTAGGTAGAGCATCTTTGGCACTCTCATATTCAGCGACTGTATAAAACCATTCTTGTTT
SARS-CoV-2 NC_045512.2      (1) ------------------------------------------------------------------------------------------------
```

```
                                                                                          Section 29
                          (2689) 2689  2695  2706  2717  2728  2739  2750  2761  2772  2784
T4 NC_000866.4            (2689) TTTACCGACCTGAGCGATGATTACAGGAGTTTTGACAAAGCGAATTCGTCCTTGCTCAAACAATTCTGGCCAATTACTAAAAAATCCGAGCAGAGA
SARS-CoV-2 NC_045512.2       (1) ------------------------------------------------------------------------------------------------
                                                                                          Section 30
                          (2785) 2785  2794  2805  2816  2827  2838  2849  2860  2880
T4 NC_000866.4            (2785) AGGATAAATAGAACCTAATCCAATAATCTCTTAATTATGAGGTATTTCTATAGATAGCCCGAAGGCTATCCATCGTGATCTGCGTCTGTCATAATA
SARS-CoV-2 NC_045512.2       (1) ------------------------------------------------------------------------------------------------
                                                                                          Section 31
                          (2881) 2881  2893  2904  2915  2926  2937  2948  2959  2976
T4 NC_000866.4            (2881) GCGACATTCGCATAGTTCATTGAAGAGGATTTAATAGAACGACGAACATTGTTCTGCAATTTATATTTTTTCATATCAACGCTAGAAGAATCAATT
SARS-CoV-2 NC_045512.2       (1) ------------------------------------------------------------------------------------------------
                                                                                          Section 32
                          (2977) 2977  2992  3003  3014  3025  3036  3047  3058  3072
T4 NC_000866.4            (2977) TTTACAAATTTCATTATACACCTCATAGAACTTTTCATCAGGAATCCAACCGCGTTTAAATTCATTAAATGCTCGGCCGAATAATTTTGAATTCAC
SARS-CoV-2 NC_045512.2       (1) ------------------------------------------------------------------------------------------------
                                                                                          Section 33
                          (3073) 3073  3080  3091  3102  3113  3124  3135  3146  3157  3168
T4 NC_000866.4            (3073) AGTTATATTATTAACTGATTTCCATTTAGCAACTCCCGTTCGTTTATAATGATCGGGGTCATATTTCGTGACGTACCATTCATATAAATTTGGTAT
SARS-CoV-2 NC_045512.2       (1) ------------------------------------------------------------------------------------------------
                                                                                          Section 34
                          (3169) 3169  3179  3190  3201  3212  3223  3234  3245  3264
T4 NC_000866.4            (3169) TAATTTTACAGCCTCTGGATTTGTCGCTGATTTATTATACCATGGTTTCGCTTTTTCAAGTTCAGCTGCTTTTGCAGTAGCAGCAACAGTATTTCC
SARS-CoV-2 NC_045512.2       (1) ------------------------------------------------------------------------------------------------
                                                                                          Section 35
                          (3265) 3265  3278  3289  3300  3311  3322  3333  3344  3360
T4 NC_000866.4            (3265) AACACCAACTAATTTTTCAGTTTTAATGCGTGGATCATTAACATGAAGACGAAAAACTTCGCCCTTTTCATTTTTAAAGCATGTCATGCCTTTAAT
SARS-CoV-2 NC_045512.2       (1) ------------------------------------------------------------------------------------------------
```

```
                                                                                                  Section 36
                          (3361) 3361 3366      3377      3388      3399      3410      3421      3432      3443      3456
        T4 NC_000866.4    (3361) TCCAGGAAGCTTAACACCTTTATTAACACCGCATCATTCCTTTGGGTTAAATGATCCTTTAATTAATAAGGCGCATTTACCCGATTTAACTACTTC
 SARS-CoV-2 NC_045512.2      (1) ------------------------------------------------------------------------------------------------
                                                                                                  Section 37
                          (3457) 3457    3465      3476      3487      3498      3509      3520      3531                3552
        T4 NC_000866.4    (3457) TCATTCAACAACTTTATCTTTCATAACGTTTTTTGACCATTCAGATACTGCTCTTTGATGGCTAAATTCTAGCAATTTCACTATAATTTGCACTAG
 SARS-CoV-2 NC_045512.2      (1) ------------------------------------------------------------------------------------------------
                                                                                                  Section 38
                          (3553) 3553       3564       3575       3586       3597       3608       3619       3630         3648
        T4 NC_000866.4    (3553) AACGTAAACTATTTTTCAGTGTTTCAACATTCTATTCATCGCATATGCCATTTCACGATTACGATGAATTTTATATAGTAGAAAATAGTGCTAAAA
 SARS-CoV-2 NC_045512.2      (1) ------------------------------------------------------------------------------------------------
                                                                                                  Section 39
                          (3649) 3649          3663       3674       3685       3696       3707       3718       3729         3744
        T4 NC_000866.4    (3649) AGTGTTCACGAAAAGTCATGTTTCACCAAATTTTCGTTATCATCAGAACCTCCCATTGATCTTGGAAGGATATGATGAATTTCACCCTTAAATTTA
 SARS-CoV-2 NC_045512.2      (1) ------------------------------------------------------------------------------------------------
                                                                                                  Section 40
                          (3745) 3745  3751       3762       3773       3784       3795       3806       3817       3828      3840
        T4 NC_000866.4    (3745) GAAACGCGGGTTTTACCCCGCACTATTAAGTCATTATAGATTTTTTCGTAATTCACCTACTGTTATCCATTTACCATTAATCTGTACTTCATCATT
 SARS-CoV-2 NC_045512.2      (1) ------------------------------------------------------------------------------------------------
                                                                                                  Section 41
                          (3841) 3841     3850       3861       3872       3883       3894       3905       3916             3936
        T4 NC_000866.4    (3841) TTCATTTACGATAATTGTATCGCCATTTAGCTCGAAAGTAAACCACTCGCCATCTTCTTTTTCTTCAAACGCTTTTTCACCGAGAACTAGACCAGT
 SARS-CoV-2 NC_045512.2      (1) ------------------------------------------------------------------------------------------------
                                                                                                  Section 42
                          (3937) 3937        3949       3960       3971       3982       3993       4004       4015             4032
        T4 NC_000866.4    (3937) GATTGCGCAAATATCAAATAGTTCTTTGTTTTTAAGCATATCTGCATAAGACATACCCCAACTGTTGAGAACTTTACCACGCAATGGATAACCACC
 SARS-CoV-2 NC_045512.2      (1) ------------------------------------------------------------------------------------------------
```

```
                                                                                             Section 43
                               (4033) 4033           4048       4059       4070       4081       4092       4103       4114           4128
T4 NC_000866.4                 (4033) GTGAAGTTCTTTATCACGAACATCAATAAGATATCCGATAGCCGAATCACCCTCAGTCAAGAAAAGAGTAGTATCAGCATCTTTACCGCAAAGATT
SARS-CoV-2 NC_045512.2            (1) ------------------------------------------------------------------------------------------------
                                                                                             Section 44
                               (4129) 4129   4136       4147       4158       4169       4180       4191       4202       4213       4224
T4 NC_000866.4                 (4129) CGCTTTGATATGTTTATGAACCTTAGCTTTAGAAGCCTTTTTAGCTGCTTTAGTTTCTGCTGCTTTTTCTGCCGCCAATTTACGAGCCAAAGCAGC
SARS-CoV-2 NC_045512.2            (1) ------------------------------------------------------------------------------------------------
                                                                                             Section 45
                               (4225) 4225      4235       4246       4257       4268       4279       4290       4301               4320
T4 NC_000866.4                 (4225) TTCAATAATCGGCATTAGAATTGCTTCATTATTTAGAATATCACGTGAAATCTTTTTAGCATCAAGTTGAATATGACTACGAATTTCGCCAAATGG
SARS-CoV-2 NC_045512.2            (1) ------------------------------------------------------------------------------------------------
                                                                                             Section 46
                               (4321) 4321         4334       4345       4356       4367       4378       4389       4400           4416
T4 NC_000866.4                 (4321) AGAAGTCAAACGCTCTTTAGTTTGACGAATCAATCGCATGTTTTTCATATCACGAACAAACATAACGATAGTCAAACATTCTTTGACACGTGCTTT
SARS-CoV-2 NC_045512.2            (1) ------------------------------------------------------------------------------------------------
                                                                                             Section 47
                               (4417) 4417 4422       4433       4444       4455       4466       4477       4488       4499         4512
T4 NC_000866.4                 (4417) AGTCACATCAATTTTGAACTTACGTTTGATTTGTGGAATAAGGTCTTCACAAATATCATCCATAGCGCAGTCAATGTGATGGCCACCATTCTTAGT
SARS-CoV-2 NC_045512.2            (1) ------------------------------------------------------------------------------------------------
                                                                                             Section 48
                               (4513) 4513    4521       4532       4543       4554       4565       4576       4587               4608
T4 NC_000866.4                 (4513) ATGAATGTTATTGACGTATGTTAATTGACGAAAACCATCCGGTGAACGACCAACCGCAATAGAACAATTTTCTTGCTCTTGAACAATAGCATGTTC
SARS-CoV-2 NC_045512.2            (1) ------------------------------------------------------------------------------------------------
                                                                                             Section 49
                               (4609) 4609       4620       4631       4642       4653       4664       4675       4686               4704
T4 NC_000866.4                 (4609) ATCATACTGCCGTGCATATTTCTTAAAATTGCCCTGAACCTTTTTACCATTAAAGGTAAATTGAATATCAGGATAAACTACAGCAAGTGTCTGGAG
SARS-CoV-2 NC_045512.2            (1) ------------------------------------------------------------------------------------------------
```

```
                                                                                                         Section 50
                         (4705) 4705          4719       4730       4741       4752       4763       4774       4785           4800
        T4 NC_000866.4   (4705) ACGATCCAGTGTAATGTCAAGATAAACTTGGGACAGCTCATTAGTTTCAAATGACATAAAATCAGGAATGAAAGTAACACGAGTTCCTTTCCATTT
SARS-CoV-2 NC_045512.2      (1) ------------------------------------------------------------------------------------------------
                                                                                                         Section 51
                         (4801) 4801  4807       4818       4829       4840       4851       4862       4873       4884        4896
        T4 NC_000866.4   (4801) TCCAGGAATATCTTCCCATGATTTATTTTCCATGCCATTTGAACAACGAACTACAATATTATTTTGACCGTCGCCAGTTTCACCGACAAACATCAC
SARS-CoV-2 NC_045512.2      (1) ------------------------------------------------------------------------------------------------
                                                                                                         Section 52
                         (4897) 4897     4906       4917       4928       4939       4950       4961       4972                4992
        T4 NC_000866.4   (4897) AGAAAAAATGTTTGTCAAACTAGAACCAACACCGTTCATACCGCCGGTGACGCGTTCTTTATCATCACCAAAGTTACCACCTGCTTTTGGAATAGT
SARS-CoV-2 NC_045512.2      (1) ------------------------------------------------------------------------------------------------
                                                                                                         Section 53
                         (4993) 4993        5005       5016       5027       5038       5049       5060       5071             5088
        T4 NC_000866.4   (4993) CCATGCGGCAACAGGACCAGGAATTTCTTCACCGGTAGGTGTTTTAACCATCGCTTGTGGAATACCGCGACCGTTATCTTCAACTGTTACTTGATT
SARS-CoV-2 NC_045512.2      (1) ------------------------------------------------------------------------------------------------
                                                                                                         Section 54
                         (5089) 5089           5104       5115       5126       5137       5148       5159       5170          5184
        T4 NC_000866.4   (5089) GTTTTTAATAGTAACATTAATTTTATTCGCGAATTTAAACTTAGTACGAATACCTTCATCTACTGAGTTATCGATAATTTCATCAATAAGCTTAAC
SARS-CoV-2 NC_045512.2      (1) ------------------------------------------------------------------------------------------------
                                                                                                         Section 55
                         (5185) 5185   5192       5203       5214       5225       5236       5247       5258       5269       5280
        T4 NC_000866.4   (5185) AAGACCAGGTACATACTGAACACTTTCCCATTTACCAAACATAAAGCGCTCATGCGTTTCATTAGCAGAAGAGCCAATGTACATGCCACTACGCTT
SARS-CoV-2 NC_045512.2      (1) ------------------------------------------------------------------------------------------------
                                                                                                         Section 56
                         (5281) 5281      5291       5302       5313       5324       5335       5346       5357               5376
        T4 NC_000866.4   (5281) TTTGATATGTTCAATATCGCTCAGAATTTTAATTTCATTCTTAATCATCACTTATCCTCGTTTGGTTTCGGGAATATTATACTCCGGTAATCATAA
SARS-CoV-2 NC_045512.2      (1) ------------------------------------------------------------------------------------------------
```

Section 57

```
                        (5377) 5377 5390 5401 5412 5423 5434 5445 5456 5472
T4 NC_000866.4          (5377) AGCTAAAGGCCCGAAGGCCTTTTATTTAAAACGAATAGTTGAATCCTTAAAGAACAGCCCAGAACATACTGTTCCTTCTACTTTCTGCCCGGTAGG
SARS-CoV-2 NC_045512.2     (1) ------------------------------------------------------------------------------------------------
```

Section 58

```
                        (5473) 5473 5478 5489 5500 5511 5522 5533 5544 5555 5568
T4 NC_000866.4          (5473) TCCAATAGCACGAAATCCAGTATGCTGGAAATCATTTTCAGAGCAACCGAACCAATTATATCCAGTGATTTCAATATTAGTAAAACCACTTGAAGA
SARS-CoV-2 NC_045512.2     (1) ------------------------------------------------------------------------------------------------
```

Section 59

```
                        (5569) 5569 5577 5588 5599 5610 5621 5632 5643 5664
T4 NC_000866.4          (5569) CAAAACTTTGGTTGCATTAATCAGCATCAGTACTATTAATTAAAGACACTGCTAATACTAATGCTGCAATTGAACGACTAATATATTTCATAACTA
SARS-CoV-2 NC_045512.2     (1) ------------------------------------------------------------------------------------------------
```

Section 60

```
                        (5665) 5665 5676 5687 5698 5709 5720 5731 5742 5760
T4 NC_000866.4          (5665) CCCTTTAAGCAAGTCGTAAAATCCATTATTCCCATGCTTAGGAAGCGGAAACTAACCGAACAGCCAGCCGATGACAATCAGGACATACACCAGTAT
SARS-CoV-2 NC_045512.2     (1) ------------------------------------------------------------------------------------------------
```

Section 61

```
                        (5761) 5761 5775 5786 5797 5808 5819 5830 5841 5856
T4 NC_000866.4          (5761) CTCTTCCAGAAATTTTCTTGATTTTTTCGTATTCTTTTGCACAGTCTTTGGATTGACATTTATAATCATAAAGCGGCATAATTATTCCTTAAAGTA
SARS-CoV-2 NC_045512.2     (1) ------------------------------------------------------------------------------------------------
```

Section 62

```
                        (5857) 5857 5863 5874 5885 5896 5907 5918 5929 5940 5952
T4 NC_000866.4          (5857) AGCTTTCAACATCTGATATAAAGACCACGCCTGATCATTATTTTCAATAGTAACTTTCATGACTGGGAATTCTGTGAAATCTTCTATTTGTTCTTG
SARS-CoV-2 NC_045512.2     (1) ------------------------------------------------------------------------------------------------
```

Section 63

```
                        (5953) 5953 5962 5973 5984 5995 6006 6017 6028 6048
T4 NC_000866.4          (5953) CTCTTTCTCTTCCTGCTCTTGCTCTTCAACCGCCTGATATGGATTTTCCACTTCATCAAAGAACCCAGCTTCGTTAGTAGAGAGCCAGATAAAGTT
SARS-CoV-2 NC_045512.2     (1) ------------------------------------------------------------------------------------------------
```

Section 64

```
                                 6049        6061       6072       6083       6094       6105       6116       6127             6144
                        (6049)
T4 NC_000866.4          (6049)   TTCGTCAAGGATATCACCGCCGGCACAACGTTTGAGTACACCTATGGATGTCATAATTTTAGTAGGACGCCCAAGATAATCAGCATCTAAAATTTT
SARS-CoV-2 NC_045512.2     (1)   ------------------------------------------------------------------------------------------------
```

Section 65

```
                                 6145           6160       6171       6182       6193       6204       6215       6226          6240
                        (6145)
T4 NC_000866.4          (6145)   AAAAGGCTCCATACCTAAACGTCGTGCATAGATTCCGTTATCAGTATGGTCTTTAATAAAATTTTCTTGAGCTTGTTTATTTTTAAATTGATACCA
SARS-CoV-2 NC_045512.2     (1)   ------------------------------------------------------------------------------------------------
```

Section 66

```
                                 6241   6248       6259       6270       6281       6292       6303       6314       6325       6336
                        (6241)
T4 NC_000866.4          (6241)   TTTATTAACTTCAAATTTAATAGCCATTAATAAATTTCCTTCCAGTAAGTTGTGCCGTCTTCAGTAATTTCACGAAATACACCATAAATTGGCTGT
SARS-CoV-2 NC_045512.2     (1)   ------------------------------------------------------------------------------------------------
```

Section 67

```
                                 6337      6347       6358       6369       6380       6391       6402       6413               6432
                        (6337)
T4 NC_000866.4          (6337)   TTATCACCGACTTTCTCATACACATAAACAGAAGTCAAGTGAGTAAACTTGCTAGTATGTTCCTTTTGAACTACTACCAAATTTGGATCAAATAAT
SARS-CoV-2 NC_045512.2     (1)   ------------------------------------------------------------------------------------------------
```

Section 68

```
                                 6433         6446       6457       6468       6479       6490       6501       6512            6528
                        (6433)
T4 NC_000866.4          (6433)   ACATCTTCAAATTCATCATTAGTGCAATTCTGAACAATTTTACGTTTCATTACAATTTCCTCATTAATTGAACAGTGGAGCGATACGTTTCAGAAG
SARS-CoV-2 NC_045512.2     (1)   ------------------------------------------------------------------------------------------------
```

Section 69

```
                                 6529 6534       6545       6556       6567       6578       6589       6600       6611         6624
                        (6529)
T4 NC_000866.4          (6529)   AGTATCAACACCTTTAGCGAATTTTCCATTTTATTCTCCAAGTTGTTTTCTGTATCAGTAGTTGATATTGATATAGTACCATAATCAACTACTGAT
SARS-CoV-2 NC_045512.2     (1)   ------------------------------------------------------------------------------------------------
```

Section 70

```
                                 6625    6633       6644       6655       6666       6677       6688       6699                 6720
                        (6625)
T4 NC_000866.4          (6625)   GTATATAGTTTTATGAAAAATTTAAACTTTATGCATAGAGAGCATTGCTATAGTGTTTAATCCAACTTTCAGGAATGACTTTGTATGTTCCTAAAA
SARS-CoV-2 NC_045512.2     (1)   ------------------------------------------------------------------------------------------------
```

```
                                                                                                             Section 71
                          (6721) 6721       6732       6743       6754       6765       6776       6787       6798              6816
T4 NC_000866.4            (6721) ATACCACGTTGTACAACTTAACACCATCTTCTACCCATTGATCGGTAATGTATCCACACATAGCGCGAGTATAAACAACCCTTCCATCATCTTTAA
SARS-CoV-2 NC_045512.2       (1) ------------------------------------------------------------------------------------------------
                                                                                                             Section 72
                          (6817) 6817          6831       6842       6853       6864       6875       6886       6897           6912
T4 NC_000866.4            (6817) TAAAGTTAAATTCACAAGGAGCAATGAACTTGATAGCCTGACCGAGTTTCCACTTAAAGTCTACACCTACATGCGAAGTATCAATCGTTTCAATTC
SARS-CoV-2 NC_045512.2       (1) ------------------------------------------------------------------------------------------------
                                                                                                             Section 73
                          (6913) 6913  6919       6930       6941       6952       6963       6974       6985       6996        7008
T4 NC_000866.4            (6913) CTTTAGCAGGAACAGCTTTTAAAAACGCAGACTCAAGAAATTTCGCACGAACATAGCCAAACTGGGGTTTAGACTTTCCATCTTTAGGAATGATAC
SARS-CoV-2 NC_045512.2       (1) ------------------------------------------------------------------------------------------------
                                                                                                             Section 74
                          (7009) 7009     7018       7029       7040       7051       7062       7073       7084                 7104
T4 NC_000866.4            (7009) GCACTTTTACTTCAGAATCTTCATCTTTAACACCATGCTTAAGCTGAATGCTTACAACTTCGACCAATTTTCCTGCTGCTTTAGAACGGGATTTAT
SARS-CoV-2 NC_045512.2       (1) ------------------------------------------------------------------------------------------------
                                                                                                             Section 75
                          (7105) 7105        7117       7128       7139       7150       7161       7172       7183             7200
T4 NC_000866.4            (7105) CAGATACACGAGCTAATTCACCAATATTAATAATCATAGTTATCTCTCACTTGTTAAAAAGATTTTATACTCCACGGGACCATTATACTCTGGTCC
SARS-CoV-2 NC_045512.2       (1) ------------------------------------------------------------------------------------------------
                                                                                                             Section 76
                          (7201) 7201           7216       7227       7238       7249       7260       7271       7282          7296
T4 NC_000866.4            (7201) CAAGAGTTTGTAAACTATTAATTCAAAATAGCTACCACTGCACTACGAGGAACTACGGAGTACTCTCCAGCATGAACTACGTTCAGAAGTTCAACG
SARS-CoV-2 NC_045512.2       (1) ------------------------------------------------------------------------------------------------
                                                                                                             Section 77
                          (7297) 7297   7304       7315       7326       7337       7348       7359       7370       7381       7392
T4 NC_000866.4            (7297) CCATCTTCCAATCCATTGGTCAGTTACCCAACCACCAATCGGATTCGCAAATGGACGACGAATGTAAACTGCCTTACACAACAAATCAGTCGGGTC
SARS-CoV-2 NC_045512.2       (1) ------------------------------------------------------------------------------------------------
```

```
                                                                                                    Section 78
                         (7393) 7393      7403       7414       7425       7436       7447       7458       7469            7488
T4 NC_000866.4           (7393) TTCAGGTTTTTCAATTTCTGTCAATAGATTAAAATCTTCTTCATAGATGATGAATGATGATACCCTTCCATAATAGTTTCTAATTTCCATGTACAC
SARS-CoV-2 NC_045512.2      (1) ------------------------------------------------------------------------------------------------
                                                                                                    Section 79
                         (7489) 7489         7502       7513       7524       7535       7546       7557       7568            7584
T4 NC_000866.4           (7489) TGTACCAATGAGAATTCCACTATTAACACTAATGACAGTAAAAGGATATCCGCCAGTTGTACCAAGAAGTTCCCAAAATTTGCTATCAGTCGTATT
SARS-CoV-2 NC_045512.2      (1) ------------------------------------------------------------------------------------------------
                                                                                                    Section 80
                         (7585) 7585 7590       7601       7612       7623       7634       7645       7656       7667         7680
T4 NC_000866.4           (7585) CGACATTGTCTGAAAATCAATTTCAGATGGTTTTTTCATTTGATACGCAGTATTAATTTTAATCATAATTTTCTCTTTAGTTTAAGGTAATAAAGC
SARS-CoV-2 NC_045512.2      (1) ------------------------------------------------------------------------------------------------
                                                                                                    Section 81
                         (7681) 7681    7689       7700       7711       7722       7733       7744       7755                 7776
T4 NC_000866.4           (7681) CTTTTAGTTCGGCATAGGATTTACGGAACATTACTTGATGCCCGCCAATGATAACTTGGTCATCTGGTACTTCGTATACAGCAAGATAAAATCCTT
SARS-CoV-2 NC_045512.2      (1) ------------------------------------------------------------------------------------------------
                                                                                                    Section 82
                         (7777) 7777       7788       7799       7810       7821       7832       7843       7854              7872
T4 NC_000866.4           (7777) TCGAAGCTAATTCTTCACGCTCTTCTCGTGTGAACCATTTCATCATATCATATTCGCTAGCAAAAGCAAAATGATAAAGAGCTACAAACCATCCGG
SARS-CoV-2 NC_045512.2      (1) ------------------------------------------------------------------------------------------------
                                                                                                    Section 83
                         (7873) 7873          7887       7898       7909       7920       7931       7942       7953            7968
T4 NC_000866.4           (7873) GAATATGATATTCTACTCCAACATAATCTTTCTTGAACTTAGTATTAATTACGATATTAGCATTTTTAACTAATAGTTTGTCTTCGTGCGGCACAG
SARS-CoV-2 NC_045512.2      (1) ------------------------------------------------------------------------------------------------
                                                                                                    Section 84
                         (7969) 7969  7975       7986       7997       8008       8019       8030       8041       8052        8064
T4 NC_000866.4           (7969) GAATTCTTTTATTATCATTACTATGATGCATAAAATTAGGTCTGTCATAACCTACATGTAATAACCACTCTTCACTCCATGAATCTATTATACTTC
SARS-CoV-2 NC_045512.2      (1) ------------------------------------------------------------------------------------------------
```

```
                                                                                                                  Section 85
                          (8065) 8065     8074       8085       8096       8107       8118       8129       8140            8160
        T4 NC_000866.4    (8065) TATACGGCGTTATTTGAACACAAAGATCTCGGCGTATTGTTATAGCGTCTTCATAATTAAGAATACTAAACGATGATTCAACACGATAAATTTTCA
  SARS-CoV-2 NC_045512.2     (1) ------------------------------------------------------------------------------------------------
                                                                                                                  Section 86
                          (8161) 8161        8173       8184       8195       8206       8217       8228       8239             8256
        T4 NC_000866.4    (8161) TTTTATTATCCTCAGTAGCTATGGTGTTATAGTACCACAACTAACCGAGGAAGTAAACAACTTTTTATCGTTTTGTTGGAAGAGATAGAGGATCGC
  SARS-CoV-2 NC_045512.2     (1) ------------------------------------------------------------------------------------------------
                                                                                                                  Section 87
                          (8257) 8257           8272       8283       8294       8305       8316       8327       8338          8352
        T4 NC_000866.4    (8257) ATTCTTCCTCTGATGGAGCATCTTCAAGACCCATAGCATATCGCAAAGCATACTTCATCATCAGGATGTCTTTCGCACAGTCATGAATAGAATCAT
  SARS-CoV-2 NC_045512.2     (1) ------------------------------------------------------------------------------------------------
                                                                                                                  Section 88
                          (8353) 8353   8360       8371       8382       8393       8404       8415       8426       8437       8448
        T4 NC_000866.4    (8353) GTGCAACGAATCCATCTAAAGTTCCCTTTGGAAGAGGACACGTTGTCATATCACGAACAAGCAGAAGTGCTTCAATTCTAGTACGAATATCACGCT
  SARS-CoV-2 NC_045512.2     (1) ------------------------------------------------------------------------------------------------
                                                                                                                  Section 89
                          (8449) 8449      8459       8470       8481       8492       8503       8514       8525              8544
        T4 NC_000866.4    (8449) GATTCCAAAATTTACAAGGTTCTAACTTAAATGTATCAAGCTCATTCTCGGAAACGCCGTTAAGACGTTGAATATCGCGAATAAGATCGACTAAAA
  SARS-CoV-2 NC_045512.2     (1) ------------------------------------------------------------------------------------------------
                                                                                                                  Section 90
                          (8545) 8545         8558       8569       8580       8591       8602       8613       8624            8640
        T4 NC_000866.4    (8545) TTGGAAAATCAAACGACATTCCACGGCACCAGCCTTGAGATTTCCAAGGATCGATATTATGTGCATTGATGTAATCATTAAATTTTGCAATACCGT
  SARS-CoV-2 NC_045512.2     (1) ------------------------------------------------------------------------------------------------
                                                                                                                  Section 91
                          (8641) 8641 8646       8657       8668       8679       8690       8701       8712       8723       8736
        T4 NC_000866.4    (8641) CGATAGTGCTTACATCTTCATCGGATGGTGCAATATTTTTTCGAGCTTCAGGAGATTGATTCTTCCACCATTCGATAGTACTTTTAGTAAAAAGAC
  SARS-CoV-2 NC_045512.2     (1) ------------------------------------------------------------------------------------------------
```

Section 92

```
                           (8737) 8737    8745        8756        8767        8778        8789        8800        8811                          8832
T4 NC_000866.4             (8737) GGTGTCCTTTTTGGCTTTTTAAATCAAATTTGATTTTAATGCCACGTGAAACTAATTCATCGAATGTTTCAACTACTTCTGGATTAGGGTCAAAAG
SARS-CoV-2 NC_045512.2     (1)    ------------------------------------------------------------------------------------------------
```

Section 93

```
                           (8833) 8833       8844        8855        8866        8877        8888        8899        8910                       8928
T4 NC_000866.4             (8833) CAATTACAGCCAAATCAATAACCGCTGCTTTTTCACCACTTCCCATTGTTTCAAAATCTATAATAAAATCAAACATTAAATTTTCCTCGCTAAATC
SARS-CoV-2 NC_045512.2     (1)    ------------------------------------------------------------------------------------------------
```

Section 94

```
                           (8929) 8929          8943        8954        8965        8976        8987        8998        9009                 9024
T4 NC_000866.4             (8929) ACGAATTTGACCTACAGTATAGTCTTGAATATAAACTTTATTAATAGGCTCATCAATAAATTTTGCCATAGATTCAATATCTTTTTGTATTTCTTC
SARS-CoV-2 NC_045512.2     (1)    ------------------------------------------------------------------------------------------------
```

Section 95

```
                           (9025) 9025  9031        9042        9053        9064        9075        9086        9097        9108        9120
T4 NC_000866.4             (9025) AAGACTGTATACTATCTTTGAAGCTTTTTCGCGAATAGTGATATTTTCAGGACCCGGATTTTCTTGAATGACAACTTTAACATTTGTCATAAGAGA
SARS-CoV-2 NC_045512.2     (1)    ------------------------------------------------------------------------------------------------
```

Section 96

```
                           (9121) 9121     9130        9141        9152        9163        9174        9185        9196                      9216
T4 NC_000866.4             (9121) TTTAAACTGGTACCAACTTAATTCAATCATTAATAATCGCCTCATAAAGATAGCTAATTTCGCCTAAAACATAATCATTGATTGTAACAGTTTTAA
SARS-CoV-2 NC_045512.2     (1)    ------------------------------------------------------------------------------------------------
```

Section 97

```
                           (9217) 9217        9229        9240        9251        9262        9273        9284        9295                 9312
T4 NC_000866.4             (9217) CTTCACCGCAAAAGAATTCTAACGCAATTAAATCTCGTTCAATTTCTTCTAATTGAAGCATCAACTTACTAGATTCAATTTTTACAGTTTCACGAT
SARS-CoV-2 NC_045512.2     (1)    ------------------------------------------------------------------------------------------------
```

Section 98

```
                           (9313) 9313           9328        9339        9350        9361        9372        9383        9394               9408
T4 NC_000866.4             (9313) TTTTGCTATAAGCTATTTCATAAATTTCGCTTACTTTATCTTGAAGAAGATAAAACTGATCTTTAGTTATTTCCACGAATAGCTTCCTCAAATTTA
SARS-CoV-2 NC_045512.2     (1)    ------------------------------------------------------------------------------------------------
```

```
                                                                                                   Section 99
                        (9409) 9409  9416       9427       9438       9449       9460       9471       9482       9493      9504
          T4 NC_000866.4 (9409) ATCATACATAAAACACATCATAACGACCACGGGTGACACCAACATAAAGAAGTTGTTGAGCTAATTCAACATCTGCATAATGAATACAAGGCGTAT
   SARS-CoV-2 NC_045512.2    (1) ------------------------------------------------------------------------------------------------
                                                                                                   Section 100
                        (9505) 9505      9515       9526       9537       9548       9559       9570       9581               9600
          T4 NC_000866.4 (9505) AAATGAAAGCACGGTCTACAGACATACCCTGCGCTTTATGGAATGTTGATGCAGGAAGTGCTTTCACTTTACTAAACTGTGATTTAGCATCCCAAA
   SARS-CoV-2 NC_045512.2    (1) ------------------------------------------------------------------------------------------------
                                                                                                   Section 101
                        (9601) 9601         9614       9625       9636       9647       9658       9669       9680            9696
          T4 NC_000866.4 (9601) AATCACTCCACGGAGCTTTTCCGCCTTTGTTCCAATTTTTATAAGTTTCTGCTGTTTTAGCTAAAAATAGGTTAAACTTATACAATTCTTCGTCAG
   SARS-CoV-2 NC_045512.2    (1) ------------------------------------------------------------------------------------------------
                                                                                                   Section 102
                        (9697) 9697 9702       9713       9724       9735       9746       9757       9768       9779         9792
          T4 NC_000866.4 (9697) ATGAAATTATTTTAATCTTTTCACGATAATATTCATCATCGCCATAAGTTTCTACTGTTAAATCCCAATGACGAATTAGATATTCTCCAGGAACAC
   SARS-CoV-2 NC_045512.2    (1) ------------------------------------------------------------------------------------------------
                                                                                                   Section 103
                        (9793) 9793    9801       9812       9823       9834       9845       9856       9867                 9888
          T4 NC_000866.4 (9793) CACGGGCTTTAACAAACGTTGATGTATACTCTGCTTCTATAATACGAACTAATTGTCCGTTATTAAAAATAATTTCTGACACAGGCTTTCCATCAA
   SARS-CoV-2 NC_045512.2    (1) ------------------------------------------------------------------------------------------------
                                                                                                   Section 104
                        (9889) 9889       9900       9911       9922       9933       9944       9955       9966               9984
          T4 NC_000866.4 (9889) TTTTATATGTTTTAAATAATGGTTCCTGCATTACAATAATTTCACCGACAATAAAATCTTTATCAGTTTCAAAAATCTTTTTACGAATAATGCTAT
   SARS-CoV-2 NC_045512.2    (1) ------------------------------------------------------------------------------------------------
                                                                                                   Section 105
                        (9985) 9985          9999       10010      10021      10032      10043      10054      10065         10080
          T4 NC_000866.4 (9985) TTAACTTGTCAACAGATTTATTCGTAAATGCCATTACGCGATTTTCAAACAAATCATCTAGTGATTTGACGATTGAAAAATAATTTACCATAAAAT
   SARS-CoV-2 NC_045512.2    (1) ------------------------------------------------------------------------------------------------
```

```
                                                                                                  Section 106
                          (10081) 10081 10087      10098      10109      10120      10131      10142      10153      10164      10176
          T4 NC_000866.4 (10081) CGCGTAAAGCGGTATCACCAGTAAATCCACGTACTCCATGCCCGTCAACAACTTTATCATAATTCCACTTACCGTTGCGAACGTCAGTAGCTACAT
    SARS-CoV-2 NC_045512.2     (1) ------------------------------------------------------------------------------------------------
                                                                                                  Section 107
                          (10177) 10177    10186      10197      10208      10219      10230      10241      10252                10272
          T4 NC_000866.4 (10177) CAATAATAGGAGCATTACTGCGTTTAACTTCAGTGAGTTCACACTGATAAAAATCTTTATGTGTAAAGAATGGACTGATATAAGCAGTATTTTCTC
    SARS-CoV-2 NC_045512.2     (1) ------------------------------------------------------------------------------------------------
                                                                                                  Section 108
                          (10273) 10273       10285      10296      10307      10318      10329      10340      10351            10368
          T4 NC_000866.4 (10273) CTGGTTCAACAGGTCTGATTTGCTTATTATCCCCTATTCCAATTATAGTACACCAAGGTGGAATAGTTGAAAGCAGAATTTTAAATAGCTTTCTAT
    SARS-CoV-2 NC_045512.2     (1) ------------------------------------------------------------------------------------------------
                                                                                                  Section 109
                          (10369) 10369          10384      10395      10406      10417      10428      10439      10450          10464
          T4 NC_000866.4 (10369) CATACATTGACACTTCGTCGCAGATTAATACTCTGCATTTGGCTAAATCAGGTACTTCTTTTTGTTCAAAAAGAACATTTTCTTCATATGTTACTG
    SARS-CoV-2 NC_045512.2     (1) ------------------------------------------------------------------------------------------------
                                                                                                  Section 110
                          (10465) 10465  10472      10483      10494      10505      10516      10527      10538      10549    10560
          T4 NC_000866.4 (10465) GGTTAATTTTAAGAATACTATGAATAGTACTCGCTTCTTTCCCTGATAGTTTTGAAAGAATCTTTTTAGCTGCATGTGTAGGAGCTGCTAAAATAA
    SARS-CoV-2 NC_045512.2     (1) ------------------------------------------------------------------------------------------------
                                                                                                  Section 111
                          (10561) 10561     10571      10582      10593      10604      10615      10626      10637                10656
          T4 NC_000866.4 (10561) TACCAGTTCCACCCGTAGATATTAAAGCTTCAATGATGAACTTAGTAAGAGTAGTCTTACCGGTACCAGCAGGTCCATTAATAGTTACATGATGTT
    SARS-CoV-2 NC_045512.2     (1) ------------------------------------------------------------------------------------------------
                                                                                                  Section 112
                          (10657) 10657        10670      10681      10692      10703      10714      10725      10736            10752
          T4 NC_000866.4 (10657) TCTTTTCTTTAATAGCCTTCATAACAATGTTAAAGGCATTTTTCTGGCCTTCGGTCAAATCATCAAATGTCATCGTAAATTCCCTGCAATTGGTAT
    SARS-CoV-2 NC_045512.2     (1) ------------------------------------------------------------------------------------------------
```

```
                                                                                                   Section 113
                       (10753) 10753 10758      10769      10780      10791      10802      10813      10824      10835        10848
T4 NC_000866.4         (10753) ACTAACAATACGCCCAGTATCTAAAATTCGCTGATATAATCTTTGCGTGTCTACGTCAGGCTTAACATGTTTAACTTCTATTTTATTAAACCAAAA
SARS-CoV-2 NC_045512.2     (1) ------------------------------------------------------------------------------------------------
                                                                                                   Section 114
                       (10849) 10849   10857      10868      10879      10890      10901      10912      10923                10944
T4 NC_000866.4         (10849) TTTACGTGGAGTCTCAACTAATCTTGGAATTCCCTTACCTAAAGCTAATCGATACTGCTCTTTAAGAGTGGTAAATACTTTATCAGCAATCTTCCA
SARS-CoV-2 NC_045512.2     (1) ------------------------------------------------------------------------------------------------
                                                                                                   Section 115
                       (10945) 10945      10956      10967      10978      10989      11000      11011      11022             11040
T4 NC_000866.4         (10945) TTCAAAAAATACAGCAGGACGATGTTCATCAAGCGGAACTGGCGCTGTAAATCCGTCTTTGTCTCGGTAAACTATCGCATATACATAAACCATATT
SARS-CoV-2 NC_045512.2     (1) ------------------------------------------------------------------------------------------------
                                                                                                   Section 116
                       (11041) 11041         11055      11066      11077      11088      11099      11110      11121          11136
T4 NC_000866.4         (11041) ATCCTCGGATAAGTTTAAAAATTGAACAATTTAGCGGATATCCTCTTTTCAGTTTAAGTTTATCAATAAAAGACAAATTTTGATACCGCTCTACAC
SARS-CoV-2 NC_045512.2     (1) ------------------------------------------------------------------------------------------------
                                                                                                   Section 117
                       (11137) 11137 11143      11154      11165      11176      11187      11198      11209      11220       11232
T4 NC_000866.4         (11137) CTTGAATAATTTTATCACACATATCATATTGCATTTCTGCTTCTGACAACTTTTTCACAATTTTCCAATCCGAGCCTTTAAGAAGAACGTTCAATT
SARS-CoV-2 NC_045512.2     (1) ------------------------------------------------------------------------------------------------
                                                                                                   Section 118
                       (11233) 11233    11242      11253      11264      11275      11286      11297      11308               11328
T4 NC_000866.4         (11233) TAACAACTTCAGCGCCTTCTGCTATGCGAGAACCATCAATACGTGCTTTAAGTGCTATAATTCTCAGCTTAATGTCAGAGGTCTGTTTTGATTTAG
SARS-CoV-2 NC_045512.2     (1) ------------------------------------------------------------------------------------------------
                                                                                                   Section 119
                       (11329) 11329       11341      11352      11363      11374      11385      11396      11407            11424
T4 NC_000866.4         (11329) AAAGCTGAGAAATGTGTTCAATTCGATTTTCACGTTTTTTCTGTATAGCTTTAATTTGATTATAAGTCTTTTTGATTTTAGCCCATTTCTTTTCAT
SARS-CoV-2 NC_045512.2     (1) ------------------------------------------------------------------------------------------------
```

```
Section 120
                          (11425) 11425          11440      11451      11462      11473      11484      11495      11506         11520
T4 NC_000866.4            (11425) CTAAATTTAGTTTATGAACTTTTTTCGCAGATGAACGACCAATTCGCAAAGCAAATAAATCACGCTTTTCAATCAACTCTTCTAAAGTATAATCAG
SARS-CoV-2 NC_045512.2        (1) ------------------------------------------------------------------------------------------------
Section 121
                          (11521) 11521  11528      11539      11550      11561      11572      11583      11594      11605   11616
T4 NC_000866.4            (11521) AACGAAATGTATTATACTTTTTCTTTACTGCAATAACATTCCCTTTAATGTATCCAACGTTATTATCAAAACGTTCTAATGATAATTTCTCTCCTT
SARS-CoV-2 NC_045512.2        (1) ------------------------------------------------------------------------------------------------
Section 122
                          (11617) 11617     11627      11638      11649      11660      11671      11682      11693              11712
T4 NC_000866.4            (11617) CAATACGATTATCAAAAGGTTCTCCCGAGTAAGCACAAACTTTTTGATCTAAAATGTTCTTAATGTAATTGAAGTCTAAGTTAAAATCTTTAGAAC
SARS-CoV-2 NC_045512.2        (1) ------------------------------------------------------------------------------------------------
Section 123
                          (11713) 11713        11726      11737      11748      11759      11770      11781      11792           11808
T4 NC_000866.4            (11713) GTCTTTTTGCAGATGCCTGAGTATGCTCTAAACGACGTTTAATTTTACGAATTTGGTTATTAGACAGCTTCATATTTTTCTCACATCTTACGGACG
SARS-CoV-2 NC_045512.2        (1) ------------------------------------------------------------------------------------------------
Section 124
                          (11809) 11809 11814      11825      11836      11847      11858      11869      11880      11891        11904
T4 NC_000866.4            (11809) GTTAACTACTTATACTATAACATTTTTACTTTAACTTGTAAACAACTTTATGAAAAATGCTTTAAAACTTTCATGGTATAATGAATCTAAGTCCTT
SARS-CoV-2 NC_045512.2        (1) ------------------------------------------------------------------------------------------------
Section 125
                          (11905) 11905   11913      11924      11935      11946      11957      11968      11979                12000
T4 NC_000866.4            (11905) CCATTATAGATTAAATCCTTCAAAATCAAGAGTATAGATAGTGTATGTTGAACACTTTTTATACTCATATCTATCTGCAATTCTAAATACACTTCC
SARS-CoV-2 NC_045512.2        (1) ------------------------------------------------------------------------------------------------
Section 126
                          (12001) 12001      12012      12023      12034      12045      12056      12067      12078              12096
T4 NC_000866.4            (12001) AGCTGGTATCATTACTTCTTGTTCATCTGAAACTAATTCCATATTACGATAACGATGACTATCCGGAAACTTAAAGTTTGGATTGTATTCTTTACA
SARS-CoV-2 NC_045512.2        (1) ------------------------------------------------------------------------------------------------
```

```
                                                                                                        Section 127
                         (12097) 12097         12111      12122      12133      12144      12155      12166      12177          12192
T4 NC_000866.4           (12097) GCGTAGAGCTTTTATAGCATACTCCTGGAAATTGAATACCATAGGAGCTTTGAATTCAAAAATAACTTGTGTGTTATACTCTAAACCAGAAGCAAA
SARS-CoV-2 NC_045512.2       (1) ------------------------------------------------------------------------------------------------
                                                                                                        Section 128
                         (12193) 12193 12199      12210      12221      12232      12243      12254      12265      12276       12288
T4 NC_000866.4           (12193) ATGTAGAGCTATATTTTTATCATATGAAGCTGATACGACTTTATCAAATGTAATAATATCAATTCCTTGATTTAATACTTGTTTAGTCTCAGCTGG
SARS-CoV-2 NC_045512.2       (1) ------------------------------------------------------------------------------------------------
                                                                                                        Section 129
                         (12289) 12289    12298      12309      12320      12331      12342      12353      12364               12384
T4 NC_000866.4           (12289) AACACCTCTCCAAAGAGGTTTATCGTTTGGAACCAAACGAGATTTGATTATTTCATTTAACCAAGAATGGTCATCTGGTTTATTAGTAATACAATG
SARS-CoV-2 NC_045512.2       (1) ------------------------------------------------------------------------------------------------
                                                                                                        Section 130
                         (12385) 12385       12397      12408      12419      12430      12441      12452      12463            12480
T4 NC_000866.4           (12385) AATTAAAAGTTCAATTTCAGATAAATTAAACCCTTCAGAAAGTAATTCTTCACGAATAGAAGCACGCACCGATGCATCCATTGATTTTATTTTAAA
SARS-CoV-2 NC_045512.2       (1) ------------------------------------------------------------------------------------------------
                                                                                                        Section 131
                         (12481) 12481          12496      12507      12518      12529      12540      12551      12562         12576
T4 NC_000866.4           (12481) ATCTTTTAGTTGCATTACTGAGTATTTCATTCAACTACCTCAATATCATAAACTTTAAATGTTCCAAATGAATCGTGTAATTTTTCTTTTGAAATA
SARS-CoV-2 NC_045512.2       (1) ------------------------------------------------------------------------------------------------
                                                                                                        Section 132
                         (12577) 12577  12584      12595      12606      12617      12628      12639      12650      12661      12672
T4 NC_000866.4           (12577) GAAGTTATTTTATACTTTCCAATTGGAATCATCCATTCTTGTTCACGCACAATCATCATTAAGTTATCAGTACGCTCTGAATCTAATCCATCAGTA
SARS-CoV-2 NC_045512.2       (1) ------------------------------------------------------------------------------------------------
                                                                                                        Section 133
                         (12673) 12673     12683      12694      12705      12716      12727      12738      12749              12768
T4 NC_000866.4           (12673) TCTTCATACGTGTACTTAAACTCAGTATTAGGAGAAGAAAGTATAATATCGCTGATATGGTCAGAATAATTAAAAGCTTTATCAGTTTTTAAACGA
SARS-CoV-2 NC_045512.2       (1) ------------------------------------------------------------------------------------------------
```

Section 134

```
                        (12769) 12769        12782      12793      12804      12815      12826      12837      12848           12864
T4 NC_000866.4          (12769) AGTATTGTTTCAGTGAAATATTCAGCATAAGAAAAAGAACACGCTGTATGCAAACTAGTAGTAAATGAATCTACCCTGTTCGTTGAAAACACTTCT
SARS-CoV-2 NC_045512.2      (1) ------------------------------------------------------------------------------------------------
```

Section 135

```
                        (12865) 12865 12870      12881      12892      12903      12914      12925      12936      12947      12960
T4 NC_000866.4          (12865) CCAACTTGTAAATCTTTAATGAGTTCTTTTGTCGATTTTGATATACCACGATATAATTGATAAGGCGATTTAGTTAAATGCTTTTTAATGATTTCA
SARS-CoV-2 NC_045512.2      (1) ------------------------------------------------------------------------------------------------
```

Section 136

```
                        (12961) 12961   12969      12980      12991      13002      13013      13024      13035                13056
T4 NC_000866.4          (12961) TTTAAATGCTTATGAAGAGCTTCATTCTTTTTGGCTTCCATACATTGCCAAAGAACAGACTGCTCAAAGTCAGTAAATTTTTCACAGACCTTTTTA
SARS-CoV-2 NC_045512.2      (1) ------------------------------------------------------------------------------------------------
```

Section 137

```
                        (13057) 13057      13068      13079      13090      13101      13112      13123      13134           13152
T4 NC_000866.4          (13057) TACATATCATATTGAAAATCAACGCTTTCAGCTTTTATAGATAACTGTTCAACATCTGCAAGATTAATAATCATGATAGCCTCCGTATACTTCAGA
SARS-CoV-2 NC_045512.2      (1) ------------------------------------------------------------------------------------------------
```

Section 138

```
                        (13153) 13153         13167      13178      13189      13200      13211      13222      13233          13248
T4 NC_000866.4          (13153) AGCTATCATATCATCGTTAGAAAGGAAAGTAAACAACTTTTTGAATTATTTTGCCCAGGGAGCCCAAGGCGGAGGGTCAAGATGGTATGAAGCTAG
SARS-CoV-2 NC_045512.2      (1) ------------------------------------------------------------------------------------------------
```

Section 139

```
                        (13249) 13249 13255      13266      13277      13288      13299      13310      13321      13332     13344
T4 NC_000866.4          (13249) TTCTTCTAGAAGAGCATCTGGGGCTTCAATTCCATAATTCTGTAATACTATACGGTACTCTTTCTTATAATCACTAGAATCATTCTGGTTATTCGT
SARS-CoV-2 NC_045512.2      (1) ------------------------------------------------------------------------------------------------
```

Section 140

```
                        (13345) 13345    13354      13365      13376      13387      13398      13409      13420               13440
T4 NC_000866.4          (13345) AGAATGATTATCTTCTAACATCTCAAATAAATCCATATTAATTCCTAGCGATAAAAACCAAATTTACGATTAGTTTCAATGATCTTTCTTTCTTCT
SARS-CoV-2 NC_045512.2      (1) ------------------------------------------------------------------------------------------------
```

```
                                                                                                                       Section 141
                           (13441) 13441       13453      13464      13475      13486      13497      13508      13519          13536
          T4 NC_000866.4   (13441) TCGGACATTCTCCAGCGTAGTCCAACATCAAAATGAGCCCAGACCATCCTAACAAATGCATTTATATCTTTAATATCTTCAATAATGAATGTTTTA
SARS-CoV-2 NC_045512.2         (1) ------------------------------------------------------------------------------------------------
                                                                                                                       Section 142
                           (13537) 13537          13552      13563      13574      13585      13596      13607      13618         13632
          T4 NC_000866.4   (13537) CTTTTAGAAGGTTGACTCGCTAATTTAACTTTATCGTCTTCAAACATGTCAAAAAGACCATATTCATGAGCTCTTTTATAGCCTTTAATAGTTAAA
SARS-CoV-2 NC_045512.2         (1) ------------------------------------------------------------------------------------------------
                                                                                                                       Section 143
                           (13633) 13633  13640      13651      13662      13673      13684      13695      13706      13717   13728
          T4 NC_000866.4   (13633) GCTTCAGAAGAAGAAAATGGTGAATCTGTATTCTGCAAAATATCTTCAGTATGCTTTATAATTAGAATAATATTTTCTGGATATTTTCTTTCTTTT
SARS-CoV-2 NC_045512.2         (1) ------------------------------------------------------------------------------------------------
                                                                                                                       Section 144
                           (13729) 13729     13739      13750      13761      13772      13783      13794      13805              13824
          T4 NC_000866.4   (13729) ATATCTTTAATTAAAAAATCCGGATTTTCTGCTAAAGGAATAATTAATGAGCAATTTTTATCAAGTGTATTTACTGATTTACCTTCTTTAGACATA
SARS-CoV-2 NC_045512.2         (1) ------------------------------------------------------------------------------------------------
                                                                                                                       Section 145
                           (13825) 13825        13838      13849      13860      13871      13882      13893      13904          13920
          T4 NC_000866.4   (13825) AATTCTATTGAATATAATTTTGCTACTTCAATCATGTGATTTCCTTTTGCCTACTAATGGACCGTCAGGAATTTTATTTTCCTGGATATATTTCTC
SARS-CoV-2 NC_045512.2         (1) ------------------------------------------------------------------------------------------------
                                                                                                                       Section 146
                           (13921) 13921 13926      13937      13948      13959      13970      13981      13992      14003      14016
          T4 NC_000866.4   (13921) ATTTTCTTCCATCATTTTACTGCCAATTTTAAGAAGCAAATCCATTGCTTCATTTGCTTTTGCTTTAGCTTCTTCTAACGTCATATCTTTGTTCAT
SARS-CoV-2 NC_045512.2         (1) ------------------------------------------------------------------------------------------------
                                                                                                                       Section 147
                           (14017) 14017   14025      14036      14047      14058      14069      14080      14091                14112
          T4 NC_000866.4   (14017) GATTTATCACCATAGATGTCTCTCATCAATTTAAGCGCTGAGCGTTCTAGTTTCTTTTCTTTCTCAGCACTAATCATTGATTTCATCCATTCTTCT
SARS-CoV-2 NC_045512.2         (1) ------------------------------------------------------------------------------------------------
```

```
                                                                                                                         Section 148
                             (14113) 14113      14124      14135      14146      14157      14168      14179      14190             14208
T4 NC_000866.4               (14113) GATTCATTCTGCATTTCTTTATTTGCTTGTTCAACCCAACCGTCATCAATATACATTGAGTTTGGTCTATTGAACCATTCAAGCATCTTCTTCAGA
SARS-CoV-2 NC_045512.2           (1) ------------------------------------------------------------------------------------------------
                                                                                                                         Section 149
                             (14209) 14209         14223      14234      14245      14256      14267      14278      14289          14304
T4 NC_000866.4               (14209) ACTTTCATTCGTTTTACCTAAAACAATAGTAGGAGCATCGTCAAATTTATGAATTTTTAGCAAATTTGGATTTAAATTATTCCATAAAGAGGTAAT
SARS-CoV-2 NC_045512.2           (1) ------------------------------------------------------------------------------------------------
                                                                                                                         Section 150
                             (14305) 14305 14311      14322      14333      14344      14355      14366      14377      14388      14400
T4 NC_000866.4               (14305) AAAATATGATAGCGCACTTTCGTCCGTAATTATAAATTTATTTCCTTTATCTATTTTCCAATCATATATTGAATCATATGAATAGAAGGATAACGG
SARS-CoV-2 NC_045512.2           (1) ------------------------------------------------------------------------------------------------
                                                                                                                         Section 151
                             (14401) 14401    14410      14421      14432      14443      14454      14465      14476               14496
T4 NC_000866.4               (14401) TTTATTATTATAATATGCTTCAGCAACATCAATATAGTTAGATTTAGTAAATGCTTGAATTGCCATAAATGGAGAATTTTGTACAGTTTCAATAAT
SARS-CoV-2 NC_045512.2           (1) ------------------------------------------------------------------------------------------------
                                                                                                                         Section 152
                             (14497) 14497       14509      14520      14531      14542      14553      14564      14575            14592
T4 NC_000866.4               (14497) TCCGATCTTTTTAAGTTTTATTTCAACATCTTCTGGAATTGGCATTGAAATAAAATCTTCTACTAGATACATATTATTGTCATATCTTTTATCAAT
SARS-CoV-2 NC_045512.2           (1) ------------------------------------------------------------------------------------------------
                                                                                                                         Section 153
                             (14593) 14593          14608      14619      14630      14641      14652      14663      14674         14688
T4 NC_000866.4               (14593) CATTACTGCTACAGTAATTGGAACATCCTTGACAAACGCCATACTAATACTATTGATAGACATTTCAAACAAAATTGCTTCCATAATTTTCCTCAA
SARS-CoV-2 NC_045512.2           (1) ------------------------------------------------------------------------------------------------
                                                                                                                         Section 154
                             (14689) 14689  14696      14707      14718      14729      14740      14751      14762      14773      14784
T4 NC_000866.4               (14689) TCACAAGATGTAGATGAACAACTAGAATCACAAGAACTTCCACATGAATCACCTGCCCATACATGAACAGGAACATTAGTATCATATGAATCAGAA
SARS-CoV-2 NC_045512.2           (1) ------------------------------------------------------------------------------------------------
```

```
                                                                                                                    Section 155
                           (14785) 14785     14795      14806      14817      14828      14839      14850      14861              14880
           T4 NC_000866.4  (14785) CTAGACTGTGTATTCTGTGTGTTAGATGATGTAGTAGGTGTTGACCAGCGCCAAGGATTTTTATAATATTCTTGGGCTTCTTCATAAGTCATAGTA
  SARS-CoV-2 NC_045512.2       (1) ------------------------------------------------------------------------------------------------
                                                                                                                    Section 156
                           (14881) 14881        14894      14905      14916      14927      14938      14949      14960           14976
           T4 NC_000866.4  (14881) ACTGCTTCTACTGTTCCATCTCCCATATAAACATATTCAACTACAGTTAAAGGAAGGTAGTCATTTGAAATAGGAACTACACCTTCCCCAGGAGTT
  SARS-CoV-2 NC_045512.2       (1) ------------------------------------------------------------------------------------------------
                                                                                                                    Section 157
                           (14977) 14977 14982     14993      15004      15015      15026      15037      15048      15059       15072
           T4 NC_000866.4  (14977) GTAGAGAAAAAATCCGTAAAGAAACTTTTAAACCAATTAAAGATAAACATTACAAAAAGCCTCTTTTGAATTCGACTTGCTTCTCACCATAATCAT
  SARS-CoV-2 NC_045512.2       (1) ------------------------------------------------------------------------------------------------
                                                                                                                    Section 158
                           (15073) 15073   15081      15092      15103      15114      15125      15136      15147                15168
           T4 NC_000866.4  (15073) ATCGAATCTCTACATTAAATTCGACAGAACCATCTGCGTACATCATAAATGAATGCACAACAACTTCTGTAGACCATGGTTGTAGTTCATATTTCT
  SARS-CoV-2 NC_045512.2       (1) ------------------------------------------------------------------------------------------------
                                                                                                                    Section 159
                           (15169) 15169      15180      15191      15202      15213      15224      15235      15246              15264
           T4 NC_000866.4  (15169) TCATTACATGTCGTGAAATGATAATATCTAAATCTTCATTTGGTTTAATCCAACGATTTAACATAGTACTCTCCTCTATAAGATAATTCTATTATA
  SARS-CoV-2 NC_045512.2       (1) ------------------------------------------------------------------------------------------------
                                                                                                                    Section 160
                           (15265) 15265         15279      15290      15301      15312      15323      15334      15345          15360
           T4 NC_000866.4  (15265) CCATACTCATTTTGGAAAGTAAACCATTTAAATGAAAAAAGGACTCCCGAAGGAGTCCTTGAGTTATTAACCAGTTACTTTCCACAAATCTTCATT
  SARS-CoV-2 NC_045512.2       (1) ------------------------------------------------------------------------------------------------
                                                                                                                    Section 161
                           (15361) 15361 15367      15378      15389      15400      15411      15422      15433      15444       15456
           T4 NC_000866.4  (15361) TGCAGCAATCCATTCAGTACGTTGATTTTCTTCATATACTGTAGAATATGCTGCTTTTTCTGAAGGGAATGTCTGGTAATGAGCGCCAGAAATTTT
  SARS-CoV-2 NC_045512.2       (1) ------------------------------------------------------------------------------------------------
```

```
                                                                                                                              Section 162
                           (15457) 15457    15466      15477      15488      15499      15510      15521      15532              15552
T4 NC_000866.4             (15457) GTGAACGTCAGAATAAAGAGGGAAAGCTACAGAAATTTCCTTTCCTTCAATTTTCTTATTTCCATCTAATTTCTGCTCAAATGTTTTGATATTAAC
SARS-CoV-2 NC_045512.2         (1) ------------------------------------------------------------------------------------------------
                                                                                                                              Section 163
                           (15553) 15553       15565      15576      15587      15598      15609      15620      15631            15648
T4 NC_000866.4             (15553) ATAACCGCGAGTACTAGCCATGTAATTCTCCTTTATTTAAATTACATGATTATTTATACATCTTCTTTTCTGAATAAGTAAATTAAATTCTTAAGA
SARS-CoV-2 NC_045512.2         (1) ------------------------------------------------------------------------------------------------
                                                                                                                              Section 164
                           (15649) 15649          15664      15675      15686      15697      15708      15719      15730         15744
T4 NC_000866.4             (15649) GCCGAACTTGTTACATCATATTTTCCTTTAAGCGCCTTTACAACCGGGCCTGTTGCTGGTTTACCTAAAGAAACCCATAACTCGTGTATTTCGCTT
SARS-CoV-2 NC_045512.2         (1) ------------------------------------------------------------------------------------------------
                                                                                                                              Section 165
                           (15745) 15745  15752      15763      15774      15785      15796      15807      15818      15829   15840
T4 NC_000866.4             (15745) TTAAGTGGTTCATGCCAATGCGGTGCTTTTCTTTGCGCCCTTGAAGTTCCTTCTGAAATCTTTTTATTTCCGCCATTCGAATAAAACTTTTTCATT
SARS-CoV-2 NC_045512.2         (1) ------------------------------------------------------------------------------------------------
                                                                                                                              Section 166
                           (15841) 15841     15851      15862      15873      15884      15895      15906      15917             15936
T4 NC_000866.4             (15841) ACTTCAGATTGCCGAGCTTTTCTTTCTGCCCTATTTTGGGCTATACGTTGTGAATTCTTCATCCGAGTTTTTGTTTCAGGATTGTTTAATCTAAGT
SARS-CoV-2 NC_045512.2         (1) ------------------------------------------------------------------------------------------------
                                                                                                                              Section 167
                           (15937) 15937        15950      15961      15972      15983      15994      16005      16016            16032
T4 NC_000866.4             (15937) TTATGTTCTAATCGTTGTTGCTCAGTCCATTTTCTTCCTTCTCCACCCTGACCACCAGGAGAAATATTAATGCAAGTATCTGGGTGTTTACGTTTT
SARS-CoV-2 NC_045512.2         (1) ------------------------------------------------------------------------------------------------
                                                                                                                              Section 168
                           (16033) 16033 16038      16049      16060      16071      16082      16093      16104      16115       16128
T4 NC_000866.4             (16033) AATGCAGATATTAGCTCACGTTCAACTTCATATGATTTTTCTCTAGAACCATGGCATTTTGACCATCGTATTTTATAATTAAATCCATACTTACGA
SARS-CoV-2 NC_045512.2         (1) ------------------------------------------------------------------------------------------------
```

Section 169

```
                        (16129) 16129 16137 16148 16159 16170 16181 16192 16203 16224
T4 NC_000866.4          (16129) TATATGTTCCATAGTATTTTACCTGAACCCGGATATTTATCATTATATGGATTTACAATAAATGATTCATGTTTCCCAGCGTACCAAAAGACGCCT
SARS-CoV-2 NC_045512.2      (1) ------------------------------------------------------------------------------------------------
```

Section 170

```
                        (16225) 16225 16236 16247 16258 16269 16280 16291 16302 16320
T4 NC_000866.4          (16225) TTCGGCGTCCTAACTTTTACTATATATGTTCTATAAAATTTTTGTTTAATATCCATTATCTTGACGTTCAAAATTATGTTTGTTCTTCAGATAATA
SARS-CoV-2 NC_045512.2      (1) ------------------------------------------------------------------------------------------------
```

Section 171

```
                        (16321) 16321 16335 16346 16357 16368 16379 16390 16401 16416
T4 NC_000866.4          (16321) AAGTTTAAAGATTTCTTCCGCATTCATTCCAAGGCCAACAAACATATTTAATACGAAATGAAATATATCCACAAGCTCAAATTTAATTTCGAGCTG
SARS-CoV-2 NC_045512.2      (1) ------------------------------------------------------------------------------------------------
```

Section 172

```
                        (16417) 16417 16423 16434 16445 16456 16467 16478 16489 16500 16512
T4 NC_000866.4          (16417) GTCTTCGGGGGACATTTCATCAATGCGTTTTTCTTGCGCTTCAATATAACGTGCTTTCCATTTTTTCCATACAGCAGAAGCTTCTTTTTCACCACG
SARS-CoV-2 NC_045512.2      (1) ------------------------------------------------------------------------------------------------
```

Section 173

```
                        (16513) 16513 16522 16533 16544 16555 16566 16577 16588 16608
T4 NC_000866.4          (16513) TGACATTTCACCAAGAGAAGTCAGAAGTTCGCGGAATTCATCATCAATACAGTCTTTTTGTTCACGCATCCAAGAAACAACATCACCGGCAGTTTC
SARS-CoV-2 NC_045512.2      (1) ------------------------------------------------------------------------------------------------
```

Section 174

```
                        (16609) 16609 16621 16632 16643 16654 16665 16676 16687 16704
T4 NC_000866.4          (16609) TAATTTATCTGGATGATAGCAGTATTCGCGGACATTAGCCAAACGAATCTGTAAAAACCGCTGCATATCAAGCATAACTTGCAGCGGATCTTTTTC
SARS-CoV-2 NC_045512.2      (1) ------------------------------------------------------------------------------------------------
```

Section 175

```
                        (16705) 16705 16720 16731 16742 16753 16764 16775 16786 16800
T4 NC_000866.4          (16705) ATCACCGAGAATATCCCAGTATTCATTTTGAGCTTTATCAACACCTTCGATCAAATGAGCACATTCATTAAAGTGAGCCATTAGTTTTCCTTTCAA
SARS-CoV-2 NC_045512.2      (1) ------------------------------------------------------------------------------------------------
```

```
                                                                                                   Section 176
                         (16801) 16801 16808      16819      16830      16841      16852      16863      16874      16885 16896
      T4 NC_000866.4     (16801) TTCATTAATAAGTTAAATAATTATATCATTTGAGTATGTAAGCAATTAATTAAAAATATATACTTCATCAGTTCCATTCTTTTCTTTGGAATGATA
SARS-CoV-2 NC_045512.2       (1) ------------------------------------------------------------------------------------------------
                                                                                                   Section 177
                         (16897) 16897     16907      16918      16929      16940      16951      16962      16973          16992
      T4 NC_000866.4     (16897) TATGTTAAAGACGTATTTTTTATTAAGATGCTTTACATTATATTTTTTAGACCATTCTTTAAGAAGAGTGTTTTCCTTTCCGTGGTGTTCTAAAAC
SARS-CoV-2 NC_045512.2       (1) ------------------------------------------------------------------------------------------------
                                                                                                   Section 178
                         (16993) 16993        17006      17017      17028      17039      17050      17061      17072          17088
      T4 NC_000866.4     (16993) ATTCGACTGCCCAAATTTTATTCCTCTATCATTTAAAGAATCTAAAAGATTTAAAAGGTCTTTTTCTTCATCTTCTGACCAAAATTTATTATAATC
SARS-CoV-2 NC_045512.2       (1) ------------------------------------------------------------------------------------------------
                                                                                                   Section 179
                         (17089) 17089 17094      17105      17116      17127      17138      17149      17160      17171     17184
      T4 NC_000866.4     (17089) AGCAACTGTTATGAGATACGGAGGATCTACATATACAAAATCGCCGTCTAAAATTTTAACATCTTTAAAATGCAATGAACTAAAGATTATTTTATC
SARS-CoV-2 NC_045512.2       (1) ------------------------------------------------------------------------------------------------
                                                                                                   Section 180
                         (17185) 17185   17193      17204      17215      17226      17237      17248      17259          17280
      T4 NC_000866.4     (17185) ACAATTTTGTTTAAAGTGATTATATTGTTTTTCACTATTTTTGTTTATAGTTCTTTTTCCAAACGGAGTAGTAAAATTTCCTTTATCGTTTATACG
SARS-CoV-2 NC_045512.2       (1) ------------------------------------------------------------------------------------------------
                                                                                                   Section 181
                         (17281) 17281      17292      17303      17314      17325      17336      17347      17358          17376
      T4 NC_000866.4     (17281) AATCATATTACTAAATCCGTGAAAATGAAGAACATAAAGTAAAAGAGGATCTCTAGTTTTATTATAATCTTCACGTAATTTCAAAAACTCTTCTTT
SARS-CoV-2 NC_045512.2       (1) ------------------------------------------------------------------------------------------------
                                                                                                   Section 182
                         (17377) 17377         17391      17402      17413      17424      17435      17446      17457     17472
      T4 NC_000866.4     (17377) TGATGTTTTTGATAGTTTGTATTGCTTTATTACTTTTAAAACGTCATCCCATGATACATTAATAAGACGCTTATACATTTCAATAATTGGTTCTTG
SARS-CoV-2 NC_045512.2       (1) ------------------------------------------------------------------------------------------------
```

Section 183

```
                        (17473) 17473 17479      17490      17501      17512      17523      17534      17545      17556      17568
         T4 NC_000866.4 (17473) AATATCATTGGCCAATACAGGGCCATTAACATTCAAAGACACTGATAAACCTCCACAAAATAAATCCACGAATCTGTTATATTTTGGAAAGTGAGA
SARS-CoV-2 NC_045512.2      (1) ------------------------------------------------------------------------------------------------
```

Section 184

```
                        (17569) 17569    17578      17589      17600      17611      17622      17633      17644                17664
         T4 NC_000866.4 (17569) TTTGAGTTCAGGTAATAATGATTGTTTATTACCTGTATACGCGATAGCTCCTAGCATTATATTCTCTCATTTATTGCAGCAAAAATGAATTATACA
SARS-CoV-2 NC_045512.2      (1) ------------------------------------------------------------------------------------------------
```

Section 185

```
                        (17665) 17665       17677      17688      17699      17710      17721      17732      17743            17760
         T4 NC_000866.4 (17665) ATTCTTCATCATATGTATTTGATAGTAATACTAACACGTTTTGCGATAAATATTAATTTAAAGGAGGATATATATGGTACAAAAATTAATGGCACT
SARS-CoV-2 NC_045512.2      (1) ------------------------------------------------------------------------------------------------
```

Section 186

```
                        (17761) 17761          17776      17787      17798      17809      17820      17831      17842         17856
         T4 NC_000866.4 (17761) TGTTAATGCCATAAAAGGTAATAAGAAACGTATAGCTTTTACTATTTCTACTATGGTAGGAATTTTACTCTGGAACTTTATTTTATCACCTGTTGC
SARS-CoV-2 NC_045512.2      (1) ------------------------------------------------------------------------------------------------
```

Section 187

```
                        (17857) 17857  17864      17875      17886      17897      17908      17919      17930      17941      17952
         T4 NC_000866.4 (17857) AATTGCACATGGTGTTAATATTCCAGTAGTTACTCTTGATACATTCGTAGATTTAGCATTTGCTTTAGTTGGGTTAATTTAAATCTTAGCATATTT
SARS-CoV-2 NC_045512.2      (1) ------------------------------------------------------------------------------------------------
```

Section 188

```
                        (17953) 17953     17963      17974      17985      17996      18007      18018      18029            18048
         T4 NC_000866.4 (17953) AGATAGCCGCATTTTAGCCATTAACCCCTGGGCAATATTATTTTTCATATATTCCATAATTTGTTCAGGGGTTGCACCTTCCTTTCTAATCATATC
SARS-CoV-2 NC_045512.2      (1) ------------------------------------------------------------------------------------------------
```

Section 189

```
                        (18049) 18049        18062      18073      18084      18095      18106      18117      18128         18144
         T4 NC_000866.4 (18049) ATTAACATCTTTTGATTTCCAGGGAGATTTATCCCAAAACATAACCCTTTCTCCTGCATCAACTAATTTAGTCATTCGTTTAATAGTGTCAGGGTG
SARS-CoV-2 NC_045512.2      (1) ------------------------------------------------------------------------------------------------
```

```
                                                                                                        Section 190
                       (18145) 18145 18150      18161      18172      18183      18194      18205      18216      18227      18240
        T4 NC_000866.4 (18145) ACGAGGTTCATTATCTAAGACCCACACACGTCTATCTTTAAATGGAACAACTTCTAGGTCTAATTGACCGCCCGTAATAGCTATACCATTTTCAAT
SARS-CoV-2 NC_045512.2     (1) ------------------------------------------------------------------------------------------------
                                                                                                        Section 191
                       (18241) 18241   18249      18260      18271      18282      18293      18304      18315                18336
        T4 NC_000866.4 (18241) AAAAAGTGAATCTATAGGTCCTTCTAGAACATATACATCACCATCTTTAACTCGTTCGACTCCATAGATTTTTGTTGCCTCAGGATAAGCTTCGAT
SARS-CoV-2 NC_045512.2     (1) ------------------------------------------------------------------------------------------------
                                                                                                        Section 192
                       (18337) 18337      18348      18359      18370      18381      18392      18403      18414                18432
        T4 NC_000866.4 (18337) GGTGATATATTTTTGAGGAGCATCTTTCTTTAATGCACGTCCTTGAAAAGACTCAGCTTTTCCATTAGCATTATAAATTGGAATAACAAGACGAGG
SARS-CoV-2 NC_045512.2     (1) ------------------------------------------------------------------------------------------------
                                                                                                        Section 193
                       (18433) 18433         18447      18458      18469      18480      18491      18502      18513           18528
        T4 NC_000866.4 (18433) CTCAGAAATTTCCTTTTTGTATGTTCCCGGTGCTATGCTATTAACTAATTTAGGCCATTCGGTTGTAAACCAAAGATATTTCCATTTATCCTTTGG
SARS-CoV-2 NC_045512.2     (1) ------------------------------------------------------------------------------------------------
                                                                                                        Section 194
                       (18529) 18529 18535      18546      18557      18568      18579      18590      18601      18612      18624
        T4 NC_000866.4 (18529) AATACAACGAGCTTTTACGTATTTTATAATTGGATGGTCTTCCGCCAGTTTATCTAATCTAACACATGACGGAAGAGATTTAATTATTTTCTTCTC
SARS-CoV-2 NC_045512.2     (1) ------------------------------------------------------------------------------------------------
                                                                                                        Section 195
                       (18625) 18625    18634      18645      18656      18667      18678      18689      18700                18720
        T4 NC_000866.4 (18625) GGGTTGTTTAGGAAGTTCTTTAGGTTTTTCTATTGGACGACTTTTACCTTTTTCTTTTCTTATTTCAAAGATATACTCACGATATAAATCGGGTTC
SARS-CoV-2 NC_045512.2     (1) ------------------------------------------------------------------------------------------------
                                                                                                        Section 196
                       (18721) 18721       18733      18744      18755      18766      18777      18788      18799                18816
        T4 NC_000866.4 (18721) AAACTCCTTTAAATATATTCCGATTGGTGCATGATAGTTACAGTTATAACAATGAATATTTCCTTCATTATTATCACCATAATACCATCCACGGGC
SARS-CoV-2 NC_045512.2     (1) ------------------------------------------------------------------------------------------------
```

```
                                                                                                                        Section 197
                       (18817) 18817          18832      18843      18854      18865      18876      18887      18898          18912
       T4 NC_000866.4  (18817) TTTATTCTGGTCGGTTTTTGAATCTCCACAAACAGGGCATCTAAACCGTAATTTAAAAGTTGAACTATTATTTACTTGTGTGAATTTAGGTAAATG
SARS-CoV-2 NC_045512.2     (1) ------------------------------------------------------------------------------------------------
                                                                                                                        Section 198
                       (18913) 18913  18920      18931      18942      18953      18964      18975      18986      18997      19008
       T4 NC_000866.4  (18913) AGCTAATGCACGGTATGCAAACTCATTATCAATCCAAGGTATTGATGACATTTTTACTCTTCTTTTTCTTTAGATTCCTCTTTTTTCTTTTTAGGA
SARS-CoV-2 NC_045512.2     (1) ------------------------------------------------------------------------------------------------
                                                                                                                        Section 199
                       (19009) 19009     19019      19030      19041      19052      19063      19074      19085          19104
       T4 NC_000866.4  (19009) ATCTGTTCAGGACCTTTATTTACTACAGCGCCTGATGTTGTTCCAATAGAGATATTTTCAGGATTACCACCTGAATCTCCAGCGACCATATCTTCT
SARS-CoV-2 NC_045512.2     (1) ------------------------------------------------------------------------------------------------
                                                                                                                        Section 200
                       (19105) 19105        19118      19129      19140      19151      19162      19173      19184          19200
       T4 NC_000866.4  (19105) TTGATAAATTCTTTAAATGTTTTCATATTAACCTCTATTCATAAAAGCATTAAAAATTTGGTCATCAATAGAAACATTTACTTTAGGCTGTTTTTC
SARS-CoV-2 NC_045512.2     (1) ------------------------------------------------------------------------------------------------
                                                                                                                        Section 201
                       (19201) 19201 19206      19217      19228      19239      19250      19261      19272      19283      19296
       T4 NC_000866.4  (19201) AGATGGCAATTCATATCCACATATTACAATTTTATGATCAATATCAAAATACACAGAAGCAATATGATTAATGATATTTTCAGTAAAGTCTAAATC
SARS-CoV-2 NC_045512.2     (1) ------------------------------------------------------------------------------------------------
                                                                                                                        Section 202
                       (19297) 19297   19305      19316      19327      19338      19349      19360      19371                19392
       T4 NC_000866.4  (19297) AACATCAATATCTTTTTGACCAAAGCCCAAAGGATAAATAATGCGAGTAATTCGATTATCTTTAACAAAGATTCCACCGACATACTCTGTGCTGCG
SARS-CoV-2 NC_045512.2     (1) ------------------------------------------------------------------------------------------------
                                                                                                                        Section 203
                       (19393) 19393      19404      19415      19426      19437      19448      19459      19470            19488
       T4 NC_000866.4  (19393) TTTAAAGTCTACACGCCGACGAAATGAAAAATATTCAGGCTCTTTATGAGCTCGGCTCATAGGACACAACGAATAACTAGAATAAGAGATGTCAAA
SARS-CoV-2 NC_045512.2     (1) ------------------------------------------------------------------------------------------------
```

```
                                                                                              Section 204
                        (19489) 19489         19503       19514       19525       19536       19547       19558       19569          19584
          T4 NC_000866.4 (19489) TCCTACGCCTTCAATATGAACTAAATTGTCATGATTAAACCAATTATAATCATATGCCAAGTCCATTAGATTGTCATATGTGAAAAGCACCGGATT
  SARS-CoV-2 NC_045512.2     (1) ------------------------------------------------------------------------------------------------
                                                                                              Section 205
                        (19585) 19585 19591       19602       19613       19624       19635       19646       19657       19668      19680
          T4 NC_000866.4 (19585) AACATCATTGGTCACAAGCATATAATTAGCAACTGCTATAATTTCATTATTTTTAACGAATACAAACCGTGATTGATGCGAATGGCCTGGACCTGC
  SARS-CoV-2 NC_045512.2     (1) ------------------------------------------------------------------------------------------------
                                                                                              Section 206
                        (19681) 19681    19690       19701       19712       19723       19734       19745       19756               19776
          T4 NC_000866.4 (19681) GTTTTCACGATTAATGATATAACACTGGGCACCTTTATATTTGTACGTGTCTTTACACTTGTGCATTTGATAAAGCATTATTCACCTACCACTTCA
  SARS-CoV-2 NC_045512.2     (1) ------------------------------------------------------------------------------------------------
                                                                                              Section 207
                        (19777) 19777       19789       19800       19811       19822       19833       19844       19855            19872
          T4 NC_000866.4 (19777) GCGATGATATTTTTGTTATTAAAGTTTTTATCGCAATACAGAACATAATTATACTGCATTACACCACCAGACTTAAGCTGTTTTTGCACTTCAGCT
  SARS-CoV-2 NC_045512.2     (1) ------------------------------------------------------------------------------------------------
                                                                                              Section 208
                        (19873) 19873          19888       19899       19910       19921       19932       19943       19954         19968
          T4 NC_000866.4 (19873) TTCATTTCAGGACGATCACGCTTAACGATATTCATAATATCTGCTTCAATTTGAGTTTCAACCTCAGTCTGATCCGCAGTCATAGACCATTCGCAC
  SARS-CoV-2 NC_045512.2     (1) ------------------------------------------------------------------------------------------------
                                                                                              Section 209
                        (19969) 19969  19976       19987       19998       20009       20020       20031       20042       20053    20064
          T4 NC_000866.4 (19969) AAATCTTTATCATAACCTGCCATAGCAGGCTGAGCAGCACAAGAAGCTAAAGCAAAAATTGTAGCAAAGATGAATTTTTTCATGATAATCTCCTCA
  SARS-CoV-2 NC_045512.2     (1) ------------------------------------------------------------------------------------------------
                                                                                              Section 210
                        (20065) 20065     20075       20086       20097       20108       20119       20130       20141              20160
          T4 NC_000866.4 (20065) GTAGTTTATGTTTATATAGTATCTCAATTTCCAACAAAAGTAAACAGTTATTTTAAAATTTCTGCGTAATCACATGTTACAAACTGTTTCTCTAAC
  SARS-CoV-2 NC_045512.2     (1) ------------------------------------------------------------------------------------------------
```

```
                                                                                                        Section 211
                        (20161) 20161        20174      20185      20196      20207      20218      20229      20240          20256
T4 NC_000866.4          (20161) TTGACGATTTTACGAAAGTATCTTTTGCATTGGCGAATCTGCCTCTTCGTAGGGCGAACAGCAAACTTAATAAATTCCACTCGACCAAATGGAGGG
SARS-CoV-2 NC_045512.2      (1) ------------------------------------------------------------------------------------------------
                                                                                                        Section 212
                        (20257) 20257 20262      20273      20284      20295      20306      20317      20328      20339       20352
T4 NC_000866.4          (20257) CTTTCTTCTGCTGGAATATCTAACACCAATTCCCACGTATCTGCAATAAGTGCTTTGAATTGCGTATTTTTCCTGACGTTATACGGAGTAGGTTTA
SARS-CoV-2 NC_045512.2      (1) ------------------------------------------------------------------------------------------------
                                                                                                        Section 213
                        (20353) 20353   20361      20372      20383      20394      20405      20416      20427                20448
T4 NC_000866.4          (20353) AATAAAACAATATGCATATTATCCTCGGCAATCCACTTCACATACTTTCTTGTCATCAATGAAAGCTTTAACTAATGCTTTATTAACTTCAGCATA
SARS-CoV-2 NC_045512.2      (1) ------------------------------------------------------------------------------------------------
                                                                                                        Section 214
                        (20449) 20449      20460      20471      20482      20493      20504      20515      20526              20544
T4 NC_000866.4          (20449) TTGAGTAGTAGCCCATTGAACGTCATCTTTCATCATTGTGATTTCTTTAGTAAACATGCTTTCATTCTTAAACCACCCCATAAAAACTACCTTTAC
SARS-CoV-2 NC_045512.2      (1) ------------------------------------------------------------------------------------------------
                                                                                                        Section 215
                        (20545) 20545         20559      20570      20581      20592      20603      20614      20625           20640
T4 NC_000866.4          (20545) CAATTCCATAACAATCTCCTCATTTAACCGACAAGACTACTATACCATAGTCTTGTCAGCTTGTAAACTAAAATTTTAATTCATTCGCCAAAGCAT
SARS-CoV-2 NC_045512.2      (1) ------------------------------------------------------------------------------------------------
                                                                                                        Section 216
                        (20641) 20641 20647      20658      20669      20680      20691      20702      20713      20724        20736
T4 NC_000866.4          (20641) CTAACTGAGCTCGAGTCGATTCATTTCTTTGATAGCGATTCTGCTCAGCCTGAATCTGTTGTGAACCTGCTACTTCGTTCACTTCAGTTGGAGTAG
SARS-CoV-2 NC_045512.2      (1) ------------------------------------------------------------------------------------------------
                                                                                                        Section 217
                        (20737) 20737    20746      20757      20768      20779      20790      20801      20812                20832
T4 NC_000866.4          (20737) AATCTTGTTCAATTTCTACCCATTTTTGATTTCCTTTTTGAACACCCATCAAAAACTTATTCCACTTATTCTTATCACCATATCGTGATTTGATTT
SARS-CoV-2 NC_045512.2      (1) ------------------------------------------------------------------------------------------------
```

```
                                                                                                  Section 218
                          (20833) 20833       20845      20856      20867      20878      20889      20900      20911           20928
       T4 NC_000866.4     (20833) GCTTAATGAGTTGTTGTTCAGCAGCTGCTAGCTCCTCGGTTTCAATGACCGCAAGCATAAAATCGGCTGTTGCTGGAAGACCGGCAGATTCTGCAA
SARS-CoV-2 NC_045512.2        (1) ------------------------------------------------------------------------------------------------
                                                                                                  Section 219
                          (20929) 20929          20944      20955      20966      20977      20988      20999      21010         21024
       T4 NC_000866.4     (20929) TATCGCTCATGTTAACATCGGAAGAGTCCCAAGCTTGTTTACCAACCTGTGCTGCAGTCCAAAGAACAGTTTCGGTTTCAACAGCCAGAGCACGTA
SARS-CoV-2 NC_045512.2        (1) ------------------------------------------------------------------------------------------------
                                                                                                  Section 220
                          (21025) 21025  21032      21043      21054      21065      21076      21087      21098      21109      21120
       T4 NC_000866.4     (21025) ATTCCTCTGCAATAGCTTTAACAGTTGTGTAACTATTTTCTGAATAAACTCTAATGCGGCAAGATTTACAAATACCTAGATAGTCGACAATAATGA
SARS-CoV-2 NC_045512.2        (1) ------------------------------------------------------------------------------------------------
                                                                                                  Section 221
                          (21121) 21121     21131      21142      21153      21164      21175      21186      21197              21216
       T4 NC_000866.4     (21121) TTGTTGGAACAAAATTCTTCTTGAGCTTCAATTCGTTTAAAAGTGATCGAAATGTATTAGCGTCTGCTCCACCAGTAGGATACTGTTTAACGATTA
SARS-CoV-2 NC_045512.2        (1) ------------------------------------------------------------------------------------------------
                                                                                                  Section 222
                          (21217) 21217        21230      21241      21252      21263      21274      21285      21296           21312
       T4 NC_000866.4     (21217) AACGACCAAGAGTAGATTTCTCACGCCATTTTTCCATTTTTCCTTTATACTCAGCGTAAGAAATATGCCCATCATCAATGTCATCAAGAGAAACAT
SARS-CoV-2 NC_045512.2        (1) ------------------------------------------------------------------------------------------------
                                                                                                  Section 223
                          (21313) 21313 21318      21329      21340      21351      21362      21373      21384      21395        21408
       T4 NC_000866.4     (21313) CAAGCATATTAGCGTCAATACGTTTAGCACAGACTTCTTCTGCCATTTCCATGGAAATGTAAAGAACATTATGTCCGAGCTGTAAATAATCTGCCG
SARS-CoV-2 NC_045512.2        (1) ------------------------------------------------------------------------------------------------
                                                                                                  Section 224
                          (21409) 21409   21417      21428      21439      21450      21461      21472      21483                21504
       T4 NC_000866.4     (21409) CTAATGAACACAATCCTAATGATTTACCAACGTTAACGCCAGCCATTAAAACGTTCAGTGTTCCAGTTTCAGCTCCGCCTTTCGTAATTTTGTTCA
SARS-CoV-2 NC_045512.2        (1) ------------------------------------------------------------------------------------------------
```

```
                                                                                                     Section 225
                         (21505) 21505      21516      21527      21538      21549      21560      21571      21582            21600
         T4 NC_000866.4  (21505) GAATTCTGAGTTTAAATGGAACCTTACGAGCTTTATTCATATAAGATAGCCAACGTGCTTCGTAGTCATCCATCCAATCATGACCAACGTAACTAT
SARS-CoV-2 NC_045512.2       (1) ------------------------------------------------------------------------------------------------
                                                                                                     Section 226
                         (21601) 21601         21615      21626      21637      21648      21659      21670      21681          21696
         T4 NC_000866.4  (21601) CAAATGAAATTGATAATGCCTGGCGCATGATGTCAGGAATAGCACCAACATCCGGCATTTTCTTATTTCGTTTTTCCGGAGGAAGCTCAGCATTAG
SARS-CoV-2 NC_045512.2       (1) ------------------------------------------------------------------------------------------------
                                                                                                     Section 227
                         (21697) 21697 21703      21714      21725      21736      21747      21758      21769      21780      21792
         T4 NC_000866.4  (21697) TTTGAATTTCGATTATTTTAGACGTAGCATTAAACATCGCCCTTTGCTGAACATATTTTTCTGTTTCTTTTACTAACCAGCTGTGGTCTTCCGGAG
SARS-CoV-2 NC_045512.2       (1) ------------------------------------------------------------------------------------------------
                                                                                                     Section 228
                         (21793) 21793    21802      21813      21824      21835      21846      21857      21868              21888
         T4 NC_000866.4  (21793) AATCAGCCAGTTTTGAAATAAGTGTTTTTACACCAGAATATTCTGTTTCAGTAAATGAACTATTTTCTAATGCAACATTTAACGCATTAATAGATG
SARS-CoV-2 NC_045512.2       (1) ------------------------------------------------------------------------------------------------
                                                                                                     Section 229
                         (21889) 21889       21901      21912      21923      21934      21945      21956      21967            21984
         T4 NC_000866.4  (21889) GAACGCTATGGTACTCATTAACATGAGATTTAATTAATTTGAATGTATTTTTAGCTGGACCACTTTCAAAATATTCTGAATCCATATATGGCCAAA
SARS-CoV-2 NC_045512.2       (1) ------------------------------------------------------------------------------------------------
                                                                                                     Section 230
                         (21985) 21985          22000      22011      22022      22033      22044      22055      22066          22080
         T4 NC_000866.4  (21985) CTTTTGAAAAATAAGCTTGATCAAATATGAGATGAGAAAGAATAATTTCTACCACACTTACTCCTTAAAAGAATTTAAATTTTTTCTTTGACCTTT
SARS-CoV-2 NC_045512.2       (1) ------------------------------------------------------------------------------------------------
                                                                                                     Section 231
                         (22081) 22081  22088      22099      22110      22121      22132      22143      22154      22165      22176
         T4 NC_000866.4  (22081) TATTAAATGCATCTTGCAGTTGCATTGTAATACATTTTTCTACATGAGGAGCTAACTCAGCTTTTCTTTCTTGGTCAAGAACAGCAAAGTCCATTA
SARS-CoV-2 NC_045512.2       (1) ------------------------------------------------------------------------------------------------
```

Section 232

```
                         22177     22187      22198      22209      22220      22231      22242      22253              22272
                (22177)
T4 NC_000866.4  (22177) CAACCTTTCCATCAACCCAATCCAGTTTAGTTACATACACTATATGCGTAGAACCATCTTCTAGTTTAATGACAATCTCCTGGATAACATTTTCCA
SARS-CoV-2 NC_045512.2  (1) ------------------------------------------------------------------------------------------------
```

Section 233

```
                         22273        22286      22297      22308      22319      22330      22341      22352          22368
                (22273)
T4 NC_000866.4  (22273) TAGCAGATTTAATTATCTTAAGAGACTCATTAAAAAGACGTTCTTTTCTTTCTTCTTCCCCCTCCGAAGAGGGGGATTCATCGATAATTTCTAGAT
SARS-CoV-2 NC_045512.2  (1) ------------------------------------------------------------------------------------------------
```

Section 234

```
                         22369 22374      22385      22396      22407      22418      22429      22440      22451      22464
                (22369)
T4 NC_000866.4  (22369) CTAAATCTAAATCATCTTTATTCATTAAATTCTTCCATATCACTTAGCTGTTCGAGGTCAGTTTCTAAATCAGCAGCTGATTTACTTTTACTTTCT
SARS-CoV-2 NC_045512.2  (1) ------------------------------------------------------------------------------------------------
```

Section 235

```
                         22465   22473      22484      22495      22506      22517      22528      22539              22560
                (22465)
T4 NC_000866.4  (22465) GGAGATTTAAATTTTTCAACCTTTGAGTTAATCAATTCATCAACTTCAGCTTCAACAATTTCATTACTATCAATAGCACCTAACTGATAAGCACGT
SARS-CoV-2 NC_045512.2  (1) ------------------------------------------------------------------------------------------------
```

Section 236

```
                         22561      22572      22583      22594      22605      22616      22627      22638          22656
                (22561)
T4 NC_000866.4  (22561) TTAATAGCATCTCGGAATGGTTGATGCTTAAATAAAGGACCCCAGAATGTAGTGCAGTTGGTATCTTTTGCACGCCAAGATTTTTCTTCGCGAATC
SARS-CoV-2 NC_045512.2  (1) ------------------------------------------------------------------------------------------------
```

Section 237

```
                         22657         22671      22682      22693      22704      22715      22726      22737          22752
                (22657)
T4 NC_000866.4  (22657) ATCTCGCCAGTTTCTTCGTCAAGAAATTCACGAGCATACCAGCCATTTTTAGGTTTTACCACGAATCCTAATTCTAGAGCCATATCTAACAATCCA
SARS-CoV-2 NC_045512.2  (1) ------------------------------------------------------------------------------------------------
```

Section 238

```
                         22753 22759      22770      22781      22792      22803      22814      22825      22836      22848
                (22753)
T4 NC_000866.4  (22753) GAATAAGGATCGATACCACCGTCAAATTTAACATCAATAAAGAATTTACTTTTTTCTTTAACGGTACGAGATTTTTCTACATTTAGAACAAATTGA
SARS-CoV-2 NC_045512.2  (1) ------------------------------------------------------------------------------------------------
```

```
                                                                                                              Section 239
                              (22849) 22849    22858      22869      22880      22891      22902      22913      22924           22944
           T4 NC_000866.4     (22849) TACCCCTGAAGATCAGAACCATCTTTAATCTGGCGTTTACCGATAATGAATACGGTATCAGCCGAATACATCGGTCCAGTACCACCTCCCATAACT
   SARS-CoV-2 NC_045512.2         (1) ------------------------------------------------------------------------------------------------
                                                                                                              Section 240
                              (22945) 22945       22957      22968      22979      22990      23001      23012      23023        23040
           T4 NC_000866.4     (22945) GTTTTACTAAACATTTCTTGTGTTTCGTATGTATGGTTAATAGCAATACATGGAATATTTTTAGTACTAAAATAGGGAGTTACGATACGAAATAAG
   SARS-CoV-2 NC_045512.2         (1) ------------------------------------------------------------------------------------------------
                                                                                                              Section 241
                              (23041) 23041          23056      23067      23078      23089      23100      23111      23122      23136
           T4 NC_000866.4     (23041) CTTTTCATTGTTTTAGCTCTAGTCATATCACTAACAACTTTTTCATTTAAAGCATCTTCAGTTTCTTTCTTAGAAGCTAAGTTACCAAGTGAATCG
   SARS-CoV-2 NC_045512.2         (1) ------------------------------------------------------------------------------------------------
                                                                                                              Section 242
                              (23137) 23137  23144      23155      23166      23177      23188      23199      23210      23221  23232
           T4 NC_000866.4     (23137) ATAAAAACGACTACCTTTTCGCCGCGTTCAATTGCATCCAATTGATTAACCATGTCAATACGTAATTGCTCAAGTGATTGAACCGGAGTATGAATT
   SARS-CoV-2 NC_045512.2         (1) ------------------------------------------------------------------------------------------------
                                                                                                              Section 243
                              (23233) 23233     23243      23254      23265      23276      23287      23298      23309          23328
           T4 NC_000866.4     (23233) ACTCGTTCTGGATCGACTCCCATAGACCGCAAATAAGCAGGAGTAATACCAAATTCACTATCATAAAACAAACATACTGCATCAGGATATTGACGC
   SARS-CoV-2 NC_045512.2         (1) ------------------------------------------------------------------------------------------------
                                                                                                              Section 244
                              (23329) 23329        23342      23353      23364      23375      23386      23397      23408          23424
           T4 NC_000866.4     (23329) ATGTAAGATGACACCATTGTTAATCCAAAGTTTGATTTAAATGATTTTGATGGACCTGCCAAAATTAACAGACCAGATTGCATACCACCAGTAATT
   SARS-CoV-2 NC_045512.2         (1) ------------------------------------------------------------------------------------------------
                                                                                                              Section 245
                              (23425) 23425 23430      23441      23452      23463      23474      23485      23496      23507    23520
           T4 NC_000866.4     (23425) TCACCAGAAAGTGCAATATTCATCATAGGAATTTTTGTTCGAACTACATCTTTTTCATTAAAGAATTTAGATGCTGTTAATTCTGCAGTCAATTTA
   SARS-CoV-2 NC_045512.2         (1) ------------------------------------------------------------------------------------------------
```

Section 246

```
                          (23521) 23521   23529      23540      23551      23562      23573      23584      23595                23616
T4 NC_000866.4            (23521) GAAGTAGAAGCTTTAATCAAACGAGATTTTAAATCAGACATATAATACCTTATAGTAGTGTTCTTGTTCCACGAAATACTTCGAGCATTCTTGCAT
SARS-CoV-2 NC_045512.2        (1) ------------------------------------------------------------------------------------------------
```

Section 247

```
                          (23617) 23617      23628      23639      23650      23661      23672      23683      23694            23712
T4 NC_000866.4            (23617) AAGATTTATTTGGAAATCCAGCTTCAATTGCTAATTTTTTAAGCTTAATAGCACCGGATTTTCCAGAGTTTTCCCATATTTCTTTAATCTTTTCAA
SARS-CoV-2 NC_045512.2        (1) ------------------------------------------------------------------------------------------------
```

Section 248

```
                          (23713) 23713         23727      23738      23749      23760      23771      23782      23793          23808
T4 NC_000866.4            (23713) TGTTATCCCACATTATTGGGTCACGTTTATGTGATGGTTTATTTCTATTATAACCATATGGATTATTAAGCAAAGCTTCTTTTCGTTTTGCTTTAG
SARS-CoV-2 NC_045512.2        (1) ------------------------------------------------------------------------------------------------
```

Section 249

```
                          (23809) 23809 23815      23826      23837      23848      23859      23870      23881      23892       23904
T4 NC_000866.4            (23809) TTTCTTCTGACTGCTTTTTGCCTAGCTGGGCTTTCCGGCAAACATCACTAAATCCAAGATGTTTTGGTTTTCCTTTTTGAGCTTTTGAAATTTTTT
SARS-CoV-2 NC_045512.2        (1) ------------------------------------------------------------------------------------------------
```

Section 250

```
                          (23905) 23905    23914      23925      23936      23947      23958      23969      23980               24000
T4 NC_000866.4            (23905) CTTTGGTTTCTGATGAAACTCTATAACCAATTCTTCTACCACCCTCTCCACCAATTTTTAAATTATAAGTCATAGGATCATTTACCACATCTATTG
SARS-CoV-2 NC_045512.2        (1) ------------------------------------------------------------------------------------------------
```

Section 251

```
                          (24001) 24001       24013      24024      24035      24046      24057      24068      24079            24096
T4 NC_000866.4            (24001) TTACTAATTCTCTTTCAGCATCACGGGCTGATTTAAAATCTTTATAAAACCCAAGAATGCTTAAATTAAAATTTTTCTTACCATACTTTTTCTTGG
SARS-CoV-2 NC_045512.2        (1) ------------------------------------------------------------------------------------------------
```

Section 252

```
                          (24097) 24097          24112      24123      24134      24145      24156      24167      24178         24192
T4 NC_000866.4            (24097) CTTGTGCTAATAAAGTTCCTGATCCCATATAACCATCATTTAAATCATCGGTTGAATGAGTTCCATAGTAAATTTTATTATTAACTAAATTAGTTA
SARS-CoV-2 NC_045512.2        (1) ------------------------------------------------------------------------------------------------
```

```
Section 253
                        (24193) 24193 24200 24211 24222 24233 24244 24255 24266 24277 24288
T4 NC_000866.4          (24193) TAACATAAGTATAATTATATTTCTTTTCTTTATGTCTTTTCATGTCATTAATCAACTGCTATTCGATGAACTTCTCTCTTTTCTAATCAATATAAC
SARS-CoV-2 NC_045512.2  (1)     ------------------------------------------------------------------------------------------------
Section 254
                        (24289) 24289 24299 24310 24321 24332 24343 24354 24365 24384
T4 NC_000866.4          (24289) AAAAGAAGTCCCAAAAAGCAAACAGACCTAAACCGATAATAAGCAATAAAGGTCCTAACATTTATTCCACCGGTTAAAGATAAATAACTTTCTAAT
SARS-CoV-2 NC_045512.2  (1)     ------------------------------------------------------------------------------------------------
Section 255
                        (24385) 24385 24398 24409 24420 24431 24442 24453 24464 24480
T4 NC_000866.4          (24385) AATAGTTCATAATTTTTATAAATCAATAGCTTTTTTGAACGCATCTTGCCATTCGGCTTTCTTTGCACGGGTTTATTTAAAATATCATGTTGAAT
SARS-CoV-2 NC_045512.2  (1)     ------------------------------------------------------------------------------------------------
Section 256
                        (24481) 24481 24486 24497 24508 24519 24530 24541 24552 24563 24576
T4 NC_000866.4          (24481) AGAAAGCATCTCTTTACGCAAAACATCACTGTGTTTTAACTCATTGACTCTATCAATGAGTTCAGCACGATTATTTACATAAAAACGAGCATCATT
SARS-CoV-2 NC_045512.2  (1)     ------------------------------------------------------------------------------------------------
Section 257
                        (24577) 24577 24585 24596 24607 24618 24629 24640 24651 24672
T4 NC_000866.4          (24577) AATAATTCGATGTTTGGTATCAAATTCTTCGTCAATTAGCATCACTGCATCAGATGCCATTGTTTCCCAGACGCGTAAGGTAATAAAGTTGTCATT
SARS-CoV-2 NC_045512.2  (1)     ------------------------------------------------------------------------------------------------
Section 258
                        (24673) 24673 24684 24695 24706 24717 24728 24739 24750 24768
T4 NC_000866.4          (24673) ATAATTCTTGTCACCAATAATTAATGCAGCAATAGCTTGACTATTCTTTTCAGATACCATGTTCATAGGAATTTTTCCAGTGAACACCGGAGCTTT
SARS-CoV-2 NC_045512.2  (1)     ------------------------------------------------------------------------------------------------
Section 259
                        (24769) 24769 24783 24794 24805 24816 24827 24838 24849 24864
T4 NC_000866.4          (24769) GGTCCAAGGATATTTAGGATTTTTAAACTGTTTTTCTCGTGCATTGCCAAAAAACTCAATATTTAAACCGGTGTCAAATAAGAATTCTACCATCTT
SARS-CoV-2 NC_045512.2  (1)     ------------------------------------------------------------------------------------------------
```

```
                                                                                                  Section 260
                           (24865) 24865 24871      24882      24893      24904      24915      24926      24937      24948  24960
           T4 NC_000866.4  (24865) GGATTCGCGTTGACCGGACCGAAATGAACCGCCATAAATAACATCCAAAGTTTTCTTGGTAGGCTTAGATAATTGAAAATCGTTCATATGAATTTT
   SARS-CoV-2 NC_045512.2      (1) ------------------------------------------------------------------------------------------------
                                                                                                  Section 261
                           (24961) 24961    24970      24981      24992      25003      25014      25025      25036              25056
           T4 NC_000866.4  (24961) ATATTGTTCAATAGGAAAATATTCAAATTCAATAACATTATCAACTTTCTTATGCGCAGCCTTAGCAATGTCTAAATTTATACCTTGGGAAATCAC
   SARS-CoV-2 NC_045512.2      (1) ------------------------------------------------------------------------------------------------
                                                                                                  Section 262
                           (25057) 25057       25069      25080      25091      25102      25113      25124      25135            25152
           T4 NC_000866.4  (25057) TTTAATTGGTGATTTAATTAATAGCTCTTCTTCAGTGTACAAATATGCCCATGGTCTATTTTTAACATTTGGCCAAGACTGCGAAAACGGCAAACG
   SARS-CoV-2 NC_045512.2      (1) ------------------------------------------------------------------------------------------------
                                                                                                  Section 263
                           (25153) 25153          25168      25179      25190      25201      25212      25223      25234         25248
           T4 NC_000866.4  (25153) TATATCTGTAAATAAATAATAAATTTTACTTTTGTATTTTGCCATAAATTTTTGCGCAGATAAAATTGCTAAATTAGGTTTACCGCCAAAAAAGTT
   SARS-CoV-2 NC_045512.2      (1) ------------------------------------------------------------------------------------------------
                                                                                                  Section 264
                           (25249) 25249  25256      25267      25278      25289      25300      25311      25322      25333  25344
           T4 NC_000866.4  (25249) AATAGAAGAATTAACAACTATCAAACGGTCATAATCATTAACATCTACTTCATCAAAAGATTTAGTGTAAACACCATTTTTAAGAGAAATAATGTC
   SARS-CoV-2 NC_045512.2      (1) ------------------------------------------------------------------------------------------------
                                                                                                  Section 265
                           (25345) 25345     25355      25366      25377      25388      25399      25410      25421              25440
           T4 NC_000866.4  (25345) GACATTAAGACCCATTTCAGAAATAACTTTAAAAAGATAAATAGTTTCAGAAGATGGAACAGTTTTAAAATTAATAACATTATTACCCATATTAAT
   SARS-CoV-2 NC_045512.2      (1) ------------------------------------------------------------------------------------------------
                                                                                                  Section 266
                           (25441) 25441        25454      25465      25476      25487      25498      25509      25520           25536
           T4 NC_000866.4  (25441) TATAGCAATTTTCATATTATTCCTTTTATGTTAAACGATTAAGCGTATTTTCCTACATAATCTTTTTTCGAAATATGTGTTTCGCCAGTTTTCCAC
   SARS-CoV-2 NC_045512.2      (1) ------------------------------------------------------------------------------------------------
```

Section 267

```
                         (25537) 25537 25542      25553      25564      25575      25586      25597      25608      25619        25632
T4 NC_000866.4           (25537) CAATGATCAACCAAATAAAAATGACGAGAATACACATGTAGGCTTCCAACATTCCATATAATGGAACCTGCTTTATACTGGCGAGTTGAATCACCT
SARS-CoV-2 NC_045512.2       (1) ------------------------------------------------------------------------------------------------
```

Section 268

```
                         (25633) 25633   25641      25652      25663      25674      25685      25696      25707                25728
T4 NC_000866.4           (25633) GCATTCAAATCAGATACTAATTTATCTAATACGTATTTTTGCCATGCATAATCATTACGGAATCCGAAGACCACGTCATTTGAGCGCATGTTAACA
SARS-CoV-2 NC_045512.2       (1) ------------------------------------------------------------------------------------------------
```

Section 269

```
                         (25729) 25729      25740      25751      25762      25773      25784      25795      25806              25824
T4 NC_000866.4           (25729) ACCGCATTGATTTTCTTGTCACGAATCAGGTATTGTACTGTATTCGTGCACATGAAATCTGACATACCATCTTTATTATAGTCAAACTGCATAGAT
SARS-CoV-2 NC_045512.2       (1) ------------------------------------------------------------------------------------------------
```

Section 270

```
                         (25825) 25825         25839      25850      25861      25872      25883      25894      25905          25920
T4 NC_000866.4           (25825) GGACGAGTATAAATCATGATACCACGTCGAGAATCAGGATTTTGACCAAGTTCAGCTAAACACATGTCATACTGAGCATAGTTATCTTCTGACCAG
SARS-CoV-2 NC_045512.2       (1) ------------------------------------------------------------------------------------------------
```

Section 271

```
                         (25921) 25921 25927      25938      25949      25960      25971      25982      25993      26004       26016
T4 NC_000866.4           (25921) ATAGCCCAACCATAATTCGAGTTAATTTCACCTTTAGAAGATGCTACTTGTTGCCAAATCTTCGGTGTTTCACCCGGAATATCTTTAACGAACAAG
SARS-CoV-2 NC_045512.2       (1) ------------------------------------------------------------------------------------------------
```

Section 272

```
                         (26017) 26017    26026      26037      26048      26059      26070      26081      26092               26112
T4 NC_000866.4           (26017) CTTTTAGATTTATACCATTCAAGTTCACGCTGAATGTATTCATCATTAAGAGCGCCAAAAATAAACGGTTCATCTGCTACAAATGATGCGCCAATA
SARS-CoV-2 NC_045512.2       (1) ------------------------------------------------------------------------------------------------
```

Section 273

```
                         (26113) 26113       26125      26136      26147      26158      26169      26180      26191            26208
T4 NC_000866.4           (26113) ATTTCAATAGTTTTAACACCTGTTTTATCAACTACGAAATCTTTTTCTTTTAATGCAAGCCCCAAATGAAGACGGATTTCTTCAACTGTCATAGAG
SARS-CoV-2 NC_045512.2       (1) ------------------------------------------------------------------------------------------------
```

```
                                                                                                                      Section 274
                         (26209) 26209          26224      26235      26246      26257      26268      26279      26290          26304
        T4 NC_000866.4   (26209) TCACTAATCATTTAAACCTCAATTGATACATTCATATTTAACTTGTAACAGTAATAAACTCCAACCTAAAATAATAGTTGGAATCATAAGAGGAAC
SARS-CoV-2 NC_045512.2       (1) ------------------------------------------------------------------------------------------------
                                                                                                                      Section 275
                         (26305) 26305  26312      26323      26334      26345      26356      26367      26378      26389   26400
        T4 NC_000866.4   (26305) CGTTACACTATAGTATATACTTATTATAATCATCAAGATTAAAAGCAATGCTGCTATAATTTTGCTTTTCATTCCTTCTCTCTGATGATAATTACC
SARS-CoV-2 NC_045512.2       (1) ------------------------------------------------------------------------------------------------
                                                                                                                      Section 276
                         (26401) 26401     26411      26422      26433      26444      26455      26466      26477          26496
        T4 NC_000866.4   (26401) TGATTTGGCTGCGCAGACTTTTTAGTTTCACCTGCAATTGACCAAATAAATGTAATAAACCAACCAATAATTGACCAGTTAAACAGTAAAGATGCG
SARS-CoV-2 NC_045512.2       (1) ------------------------------------------------------------------------------------------------
                                                                                                                      Section 277
                         (26497) 26497        26510      26521      26532      26543      26554      26565      26576          26592
        T4 NC_000866.4   (26497) AAAAAGATTCCTACTGTCGATTTTGACCCACGCATCAAGGCGATAAACCATGGAAGCATGTATATAATAATAGCCAACACGCCTGAAACTAAAACC
SARS-CoV-2 NC_045512.2       (1) ------------------------------------------------------------------------------------------------
                                                                                                                      Section 278
                         (26593) 26593 26598      26609      26620      26631      26642      26653      26664      26675    26688
        T4 NC_000866.4   (26593) ATAAAAATTGAACCTGCTACTAAAGTTTCCATGTTTTCCTCACTTAGGTCAAATTTTTTACACATGAATTATAAGAATTCACTACATACTCCATCG
SARS-CoV-2 NC_045512.2       (1) ------------------------------------------------------------------------------------------------
                                                                                                                      Section 279
                         (26689) 26689   26697      26708      26719      26730      26741      26752      26763             26784
        T4 NC_000866.4   (26689) GAGCGTTTTTACCTGTACGCCACTGGTAATTATTAGCCCAATTTGCCCAAAGCTCAGCGCAGTAGTTTTCAATTTTTTCTTCGCGTGTAATTACAT
SARS-CoV-2 NC_045512.2       (1) ------------------------------------------------------------------------------------------------
                                                                                                                      Section 280
                         (26785) 26785      26796      26807      26818      26829      26840      26851      26862          26880
        T4 NC_000866.4   (26785) CTGAATTACGATATGCTTGAGCAGATTCATCTGGACGAATAGCTTCGTCAAAATTCGCCTGCATTTGTTCTACTGTCTGTTTTGGAGCTTCTTTAT
SARS-CoV-2 NC_045512.2       (1) ------------------------------------------------------------------------------------------------
```

```
                                                                                                  Section 281
                       (26881) 26881         26895      26906      26917      26928      26939      26950      26961         26976
T4 NC_000866.4         (26881) AACACTTGACATTAGGATTATAAAATTTGCTTGAACAGTTTACAATTTTTCCTACATCAGACTGATTTACTACCGGTCCTTGAGCTACACAACCAG
SARS-CoV-2 NC_045512.2     (1) ------------------------------------------------------------------------------------------------
                                                                                                  Section 282
                       (26977) 26977 26983      26994      27005      27016      27027      27038      27049      27060       27072
T4 NC_000866.4         (26977) CAAGACCTAATGCAATAACCAAAATAGCGATTTTCATTTCATTCTCCAAATCCGTATCAGTAGTTGATAGTTGTATAGTACCATGGAAGAACAGTC
SARS-CoV-2 NC_045512.2     (1) ------------------------------------------------------------------------------------------------
                                                                                                  Section 283
                       (27073) 27073    27082      27093      27104      27115      27126      27137      27148               27168
T4 NC_000866.4         (27073) TTGTAAACAGTTTTGTGAAAAAATTTTTAGGGAATCCAAAGGGTCCAGAATCATCTCTTTTTCATAAGTATAGATTTATATTACTTGTATGAAAAA
SARS-CoV-2 NC_045512.2     (1) ------------------------------------------------------------------------------------------------
                                                                                                  Section 284
                       (27169) 27169       27181      27192      27203      27214      27225      27236      27247            27264
T4 NC_000866.4         (27169) GGGACCTGGAGGTCCTAGATTTATTCTATCAGCCAAACAGGAAGTCTAACGAAGCTTTTTCTTCATAGTCCATGCCAGCCGATTCACACATACCCG
SARS-CoV-2 NC_045512.2     (1) ------------------------------------------------------------------------------------------------
                                                                                                  Section 285
                       (27265) 27265          27280      27291      27302      27313      27324      27335      27346         27360
T4 NC_000866.4         (27265) CAAGCGGTTTAACAAACGATTTTTGGAACAAAGTTGAGTGGTCAATCCAAGATAGCACATCAGAACGAATTTCTTTTGGAAGTTCTGTACCCGATG
SARS-CoV-2 NC_045512.2     (1) ------------------------------------------------------------------------------------------------
                                                                                                  Section 286
                       (27361) 27361  27368      27379      27390      27401      27412      27423      27434      27445      27456
T4 NC_000866.4         (27361) GCCAAGCAATGCACTTGTCACCAAATGGATTTCCTTCACGTAATGGAAGAACCATTACTTTATTTCCATCCAAAATTGGAGCTACACCTAAACCGC
SARS-CoV-2 NC_045512.2     (1) ------------------------------------------------------------------------------------------------
                                                                                                  Section 287
                       (27457) 27457     27467      27478      27489      27500      27511      27522      27533               27552
T4 NC_000866.4         (27457) TAACAGCTCGACGATAAGTTAGCACACCACGAATATGGAACGGGCATTTAAATCCTGGCCAACCTTTATCATCATATTTCGCTATATCGTTCGCAG
SARS-CoV-2 NC_045512.2     (1) ------------------------------------------------------------------------------------------------
```

Section 288

	(27553)	27553 27566 27577 27588 27599 27610 27621 27632 27648
T4 NC_000866.4	(27553)	TTTTTACTTCAGCAATAACTTTATAGTCAAGTTGACGATATTCTTTCTCGAAGTTCTTGTAGTATTCTTGGACAGACTCTTCACCTTCCTGAAGAA
SARS-CoV-2 NC_045512.2	(1)	--

Section 289

	(27649)	27649 27654 27665 27676 27687 27698 27709 27720 27731 27744
T4 NC_000866.4	(27649)	TACGACGAATACTTTCTTCGAGAGCTTCTTGCACTGCTTTTGGTGTTGAACTCTGCTGAGTTTCCATACCCATGATTTTTAGATGCGGTTCAGCAA
SARS-CoV-2 NC_045512.2	(1)	--

Section 290

	(27745)	27745 27753 27764 27775 27786 27797 27808 27819 27840
T4 NC_000866.4	(27745)	ATCGCTTATCTTCCATATCATAAACGTTCAGAGCATAACGCTTTTTCGCTTTCCAAAATCCACCAACGCCCTTTGAACCAAGCGGAGGGCAAGAAA
SARS-CoV-2 NC_045512.2	(1)	--

Section 291

	(27841)	27841 27852 27863 27874 27885 27896 27907 27918 27936
T4 NC_000866.4	(27841)	TAGCTTCACGGTCCATATGCATCAGATGCTCGCGGTTATTCATATAATCACATAACTCACGATATGCAACATCAATCATAGGTTCCATCTTTTTCT
SARS-CoV-2 NC_045512.2	(1)	--

Section 292

	(27937)	27937 27951 27962 27973 27984 27995 28006 28017 28032
T4 NC_000866.4	(27937)	TACCGAACTGATTCATGAATTCAACCAAATCGTTCTGCTCTTTGAATCGGTCAAGACCAACTTTTTCAATAACTTTATCTACGCAAACATATACCG
SARS-CoV-2 NC_045512.2	(1)	--

Section 293

	(28033)	28033 28039 28050 28061 28072 28083 28094 28105 28116 28128
T4 NC_000866.4	(28033)	AATCAGTATCACCTGCTGCAATGAAATCTTCATCATTAGTTCCGCATACTTTATTCAGATATTCATTAATTTTACGAGCAATCCACTGAATACCGA
SARS-CoV-2 NC_045512.2	(1)	--

Section 294

	(28129)	28129 28138 28149 28160 28171 28182 28193 28204 28224
T4 NC_000866.4	(28129)	CTTGGCCGAAAATTGTGATAGCAGTAGCATTTCGCAAATCATAGTAACGGAAATGAATATTACCAAGAGCACCATAAAGACTGTTAATGAGAATTT
SARS-CoV-2 NC_045512.2	(1)	--

```
                                                                                                          Section 295
                           (28225) 28225       28237       28248       28259       28270       28281       28292       28303          28320
T4 NC_000866.4             (28225) TACGGTTCAGCTGATTTGTATTAGCAAGTGTAGCTGCTTTTTCACATTCTTCAATCAGACTATTGAGAACAGATTCGGTGTAATTCGATAGTTCAT
SARS-CoV-2 NC_045512.2         (1) ------------------------------------------------------------------------------------------------
                                                                                                          Section 296
                           (28321) 28321          28336       28347       28358       28369       28380       28391       28402       28416
T4 NC_000866.4             (28321) TTAAGAAATCATCACTGAACTTAACATATCGTTCAACTTCTGGTTTAGTTGAACAAGACCCTGCGCCTTTCATAATAATCTTTTTAATAGCTTCGG
SARS-CoV-2 NC_045512.2         (1) ------------------------------------------------------------------------------------------------
                                                                                                          Section 297
                           (28417) 28417  28424       28435       28446       28457       28468       28479       28490       28501 28512
T4 NC_000866.4             (28417) CATTCATTTCTTCAGCGAACATTTTCTTTTTCCAGTCTTTACGCTGGAAAAATACTTTAGCGATTTCCTTTGGAATGATACCTTCTTGATGTTTAT
SARS-CoV-2 NC_045512.2         (1) ------------------------------------------------------------------------------------------------
                                                                                                          Section 298
                           (28513) 28513     28523       28534       28545       28556       28567       28578       28589          28608
T4 NC_000866.4             (28513) CATACATCCATCCATTCGGAGAACAAGAATATTCATCACTCGGTTTAGGAGCTGTTCCTGCGATATATTCATGAATTGGATGAACTTTAAACTGAC
SARS-CoV-2 NC_045512.2         (1) ------------------------------------------------------------------------------------------------
                                                                                                          Section 299
                           (28609) 28609        28622       28633       28644       28655       28666       28677       28688         28704
T4 NC_000866.4             (28609) CACGAATAGTTTCAGGACTAATGTTAACCTGGCGAATAATGCTCGGATACAGAGACGTCAAGTCAAAACTCATAATGTATCGACGTGCAATTGGTT
SARS-CoV-2 NC_045512.2         (1) ------------------------------------------------------------------------------------------------
                                                                                                          Section 300
                           (28705) 28705 28710       28721       28732       28743       28754       28765       28776       28787     28800
T4 NC_000866.4             (28705) TAGGTTCAAACACAAATGCACCCGGAAAACTCTGTTTAACGTGCGAACCTTGTTGAGGAATAACCTTATGTTCACCTTTCAATGAGTTAAAAATAA
SARS-CoV-2 NC_045512.2         (1) ------------------------------------------------------------------------------------------------
                                                                                                          Section 301
                           (28801) 28801   28809       28820       28831       28842       28853       28864       28875            28896
T4 NC_000866.4             (28801) TAGCATCCCAAGTTTTAATAGGACTCATTACACCAGAAAAAGGCATTTTAGCGTAATAAGACATACTTAAAACTAGATCGATAAACCCACGAATTT
SARS-CoV-2 NC_045512.2         (1) ------------------------------------------------------------------------------------------------
```

Section 302

```
                        (28897) 28897      28908      28919      28930      28941      28952      28963      28974           28992
          T4 NC_000866.4 (28897) TATCGATTGCTTGAACTGATTCTACGTCAATGATGTTATAACTAATGTATCGTTGATGATTAGTCTCACGAAGTTTATTAATAGGACCGTCGTATG
   SARS-CoV-2 NC_045512.2 (1) ------------------------------------------------------------------------------------------------
```

Section 303

```
                        (28993) 28993         29007      29018      29029      29040      29051      29062      29073          29088
          T4 NC_000866.4 (28993) GTAATTTACCTTTTTTGGTTTCATGTTGAGCAACTGATTCCAAAGAGAATGACGGCAAATTAGTAAAAGCGAATTTCTTGTACAAATCTAAATAAT
   SARS-CoV-2 NC_045512.2 (1) ------------------------------------------------------------------------------------------------
```

Section 304

```
                        (29089) 29089 29095      29106      29117      29128      29139      29150      29161      29172      29184
          T4 NC_000866.4 (29089) CAAGAATAGATACGCCATCAATAGAATAAATTTCTTTGCTACCGTACATATTTTGAATTAGTTTAGATTTTACCCGACCGATTGGAGAGAAACGTT
   SARS-CoV-2 NC_045512.2 (1) ------------------------------------------------------------------------------------------------
```

Section 305

```
                        (29185) 29185    29194      29205      29216      29227      29238      29249      29260               29280
          T4 NC_000866.4 (29185) TCATACTACGTTCACCCAGAATCATTTTAACACGATTCATGATATACGGAACGTCAAACCCCTCAATATTCCAACCAGTAAAAATAGCAGGTCGTT
   SARS-CoV-2 NC_045512.2 (1) ------------------------------------------------------------------------------------------------
```

Section 306

```
                        (29281) 29281       29293      29304      29315      29326      29337      29348      29359          29376
          T4 NC_000866.4 (29281) TCTGTTCCCAAAGATTGATATATTCCATGAGCATATCACGCTCATTATCGAATGGCATATAAATTACTCGGTCAAGAATTTCTTGAGGAACTTCAT
   SARS-CoV-2 NC_045512.2 (1) ------------------------------------------------------------------------------------------------
```

Section 307

```
                        (29377) 29377          29392      29403      29414      29425      29436      29447      29458      29472
          T4 NC_000866.4 (29377) CACCACCTTCACAGTCAAGCTTAGCAGCTAACTTTGCATCCCATTTTGATACTGAACCGTACATTGAATTCAAAAGGTCGAAAACATAAAAACGAT
   SARS-CoV-2 NC_045512.2 (1) ------------------------------------------------------------------------------------------------
```

Section 308

```
                        (29473) 29473  29480      29491      29502      29513      29524      29535      29546      29557   29568
          T4 NC_000866.4 (29473) CGTCAATTGAATCGTAATGAGTGATAGCATCAATTTCATATTCTGCTTTCATTGGGTCAGGAAATTTATCACCAGTAACCTCAATGTCACAGTTAG
   SARS-CoV-2 NC_045512.2 (1) ------------------------------------------------------------------------------------------------
```

```
                                                                                                          Section 309
                          (29569) 29569    29579      29590      29601      29612      29623      29634      29645           29664
T4 NC_000866.4            (29569) CTACACGAACAAATTTTCGGTCATAAACAATTTCTGAACCATATGTATCACTTATATAAGCGAGTTTAAAATCGTTCATACCGAGAGCTTCGAGAC
SARS-CoV-2 NC_045512.2        (1) ------------------------------------------------------------------------------------------------
                                                                                                          Section 310
                          (29665) 29665        29678      29689      29700      29711      29722      29733      29744          29760
T4 NC_000866.4            (29665) CGATGTCTTCCATTCGCTTCATCCAATCTCGAGCATCTTTCATTGATGGAAATTTTTGAGGAGCGCAGTTTTTACCATAGATGTCTTTGTATTTTG
SARS-CoV-2 NC_045512.2        (1) ------------------------------------------------------------------------------------------------
                                                                                                          Section 311
                          (29761) 29761 29766      29777      29788      29799      29810      29821      29832      29843    29856
T4 NC_000866.4            (29761) ACTCTTCCTTACAATGCCTAAACATAGTTGGAAGATATTCTACTTCACGGGTACGTTCCTTTCCATTTTCATCAATATAACGTTCAACAATGTTAT
SARS-CoV-2 NC_045512.2        (1) ------------------------------------------------------------------------------------------------
                                                                                                          Section 312
                          (29857) 29857   29865      29876      29887      29898      29909      29920      29931           29952
T4 NC_000866.4            (29857) TTCCGACTGTTTCAATAGAGATATAAAATTCTTTCATAGATATTCCTTAGTTTATAGCCCGAGTTATTAGGCTCTTGATATATTATACTCCAAATA
SARS-CoV-2 NC_045512.2        (1) ------------------------------------------------------------------------------------------------
                                                                                                          Section 313
                          (29953) 29953      29964      29975      29986      29997      30008      30019      30030         30048
T4 NC_000866.4            (29953) AGGGGCCGAAGCCCCTTGCTTAATTACCAATCGTATATTTAGGAACGAGCTTCCATTCATGTTTTTGTTTAAAAGAAATAACTCGGAAGTTATTAG
SARS-CoV-2 NC_045512.2        (1) ------------------------------------------------------------------------------------------------
                                                                                                          Section 314
                          (30049) 30049         30063      30074      30085      30096      30107      30118      30129      30144
T4 NC_000866.4            (30049) TTAAATCTTTCATAAAAGTTCTTTGACCAGGAACGATTTCAATTAGTCCCCAATCTTCTAATAGCCATGCAATCGAATCACGACGAACTTCATCTT
SARS-CoV-2 NC_045512.2        (1) ------------------------------------------------------------------------------------------------
                                                                                                          Section 315
                          (30145) 30145 30151      30162      30173      30184      30195      30206      30217      30228    30240
T4 NC_000866.4            (30145) CTTCTGTCATTTCAACTTGACGACCATCCATACGAAGCATTTCTTTAAAATGAACGATATAGTATAGTCCTTTTTTCTGAAGAATATGACAGGACT
SARS-CoV-2 NC_045512.2        (1) ------------------------------------------------------------------------------------------------
```

```
                                                                                                                          Section 316
                           (30241) 30241   30250     30261     30272     30283     30294     30305     30316       30336
T4 NC_000866.4             (30241) GATATAGAACTTTATCTTTATTATTAGCAATTCCCATACGAGTCAAAGTTTCTTTTACTTTCAGAAAATCTTCAGGTTTTTTAAGAGTAATTTCAA
SARS-CoV-2 NC_045512.2         (1) ------------------------------------------------------------------------------------------------
                                                                                                                          Section 317
                           (30337) 30337     30349     30360     30371     30382     30393     30404     30415       30432
T4 NC_000866.4             (30337) TCATTTTACCATTCCAATGCTAGTTTTTTGAGTTGTTTCTGTTCTTTTACGTTCTTAGTCACTTCTTTCAAAAAATCATCCGTGACTAAACCTTTT
SARS-CoV-2 NC_045512.2         (1) ------------------------------------------------------------------------------------------------
                                                                                                                          Section 318
                           (30433) 30433          30448      30459      30470      30481      30492      30503      30514    30528
T4 NC_000866.4             (30433) AGTTCTTTTAATACTAAAGGAAGTTTTCCATTTTTAGTAAGAATTGATTTATAGTTAATTGCATCATTTGTATTAACTTGATACCGCTTAGCAAGT
SARS-CoV-2 NC_045512.2         (1) ------------------------------------------------------------------------------------------------
                                                                                                                          Section 319
                           (30529) 30529  30536      30547      30558      30569      30580      30591      30602      30613  30624
T4 NC_000866.4             (30529) AACTTAATAATCAATACTTCGGTGGAATCTTCAACCAGTTTTGCCCATTTACCATATCTTTTACCACGAGGAACTGCAGCCATTAGATAATTAAAA
SARS-CoV-2 NC_045512.2         (1) ------------------------------------------------------------------------------------------------
                                                                                                                          Section 320
                           (30625) 30625     30635      30646      30657      30668      30679      30690      30701      30720
T4 NC_000866.4             (30625) TGAGCTTCATCACTTAAGCCTGATCCAATTAAATTCATAGCATATACAGCTGGCATACACTCTGGAAATTGTGATAATGCATTTTCAACCATGAAT
SARS-CoV-2 NC_045512.2         (1) ------------------------------------------------------------------------------------------------
                                                                                                                          Section 321
                           (30721) 30721        30734      30745      30756      30767      30778      30789      30800    30816
T4 NC_000866.4             (30721) TTTGAATAATCTTTTTGAGCAATAGAGCATTTAGTTTTATTATTAATAGCTCCAATTATTTCAAAAAATTCATTTTCTGCTTTTTCTTTAAAAGAA
SARS-CoV-2 NC_045512.2         (1) ------------------------------------------------------------------------------------------------
                                                                                                                          Section 322
                           (30817) 30817 30822      30833      30844      30855      30866      30877      30888      30899  30912
T4 NC_000866.4             (30817) TCAGCAGCGGATTGGACAGCTGTCCAATCTTTTGAATACCAAGCAACTTGATGCTCGTTTAATTGAATATCATCTTTAAATAAGCTCATATCACTT
SARS-CoV-2 NC_045512.2         (1) ------------------------------------------------------------------------------------------------
```

Section 323

```
                                (30913) 30913 30921 30932 30943 30954 30965 30976 30987 31008
T4 NC_000866.4                  (30913) CCACTGCATTTCGCATGCTAATTGAATGAAAAGATAAGCTAAATGCAATTCAGTATTAGCTGCAATACCATGATACTGATTATTTTCGCCGACAAT
SARS-CoV-2 NC_045512.2              (1) ------------------------------------------------------------------------------------------------
```

Section 324

```
                                (31009) 31009 31020 31031 31042 31053 31064 31075 31086 31104
T4 NC_000866.4                  (31009) TTCGTACATACGAATAATACTTTGTGGAGTTACACGTGAATAGATTTCTTCGGCAAGTTTACCCACGAACCACGAATAATCAGCCGCATATTTTGG
SARS-CoV-2 NC_045512.2              (1) ------------------------------------------------------------------------------------------------
```

Section 325

```
                                (31105) 31105 31119 31130 31141 31152 31163 31174 31185 31200
T4 NC_000866.4                  (31105) TGCTAAAGCTCTGAGTTGTTTAACATCTTTATTTTTGAGAGACTCAAGAACATCATCAATAGCACCACGATCGTTAGTAACCAGTGATAAAATACC
SARS-CoV-2 NC_045512.2              (1) ------------------------------------------------------------------------------------------------
```

Section 326

```
                                (31201) 31201 31207 31218 31229 31240 31251 31262 31273 31284 31296
T4 NC_000866.4                  (31201) AGCATCCAAAACACCTTTAGACGAATAACTATCGAGCTCGCCAATAGTTTTACGAAAATCAGGAAAATTCTTTTTAACCAAAGCTGCTACAACTTT
SARS-CoV-2 NC_045512.2              (1) ------------------------------------------------------------------------------------------------
```

Section 327

```
                                (31297) 31297 31306 31317 31328 31339 31350 31361 31372 31392
T4 NC_000866.4                  (31297) CATATCAGCTATAGCAATTCCTTCATGCTTGCAGATTTCAGTCAATCGACGAATCATCTGCTTCATCATTTCAATTTTATCTTCATCAGTTGGTTG
SARS-CoV-2 NC_045512.2              (1) ------------------------------------------------------------------------------------------------
```

Section 328

```
                                (31393) 31393 31405 31416 31427 31438 31449 31460 31471 31488
T4 NC_000866.4                  (31393) ACCGAATGTAATAACTCGGCAGCGTGACTGAAGCGGTTTAATAATACCATCAATATTATTAGCAGTAATAATAATACTACAGTTTGAACTATAAGC
SARS-CoV-2 NC_045512.2              (1) ------------------------------------------------------------------------------------------------
```

Section 329

```
                                (31489) 31489 31504 31515 31526 31537 31548 31559 31570 31584
T4 NC_000866.4                  (31489) TTCCATAAAGGAACGAAGATGTCGCTGAGACTCTGCTAACCCTGAACGGTCAAATTCATCAATAACGATTACTTTTTGACGACCATCAAATGAAGC
SARS-CoV-2 NC_045512.2              (1) ------------------------------------------------------------------------------------------------
```

```
                                                                                                            Section 330
                           (31585) 31585 31592      31603      31614      31625      31636      31647      31658      31669 31680
           T4 NC_000866.4  (31585) GGCGCTGGCAAAATTAGTCAAAGGACCACGAACGAAATCAATTTTACAATCTGACCCATTCACAAACATCATATCAGCATTTACATCATGACATAA
SARS-CoV-2 NC_045512.2         (1) ------------------------------------------------------------------------------------------------
                                                                                                            Section 331
                           (31681) 31681     31691      31702      31713      31724      31735      31746      31757              31776
           T4 NC_000866.4  (31681) TGCTTTTGCTACAGTTGTTTTACCTGTTCCTGGAGAAGGAGAATGAAGAATAATATGTGGAATCTTACCTTTACTTGTAATAGATTTAAAGGTTTC
SARS-CoV-2 NC_045512.2         (1) ------------------------------------------------------------------------------------------------
                                                                                                            Section 332
                           (31777) 31777        31790      31801      31812      31823      31834      31845      31856           31872
           T4 NC_000866.4  (31777) TTTATCAAAAGCGGGAAGAATACATTCATCGATAGTAGATGGACGATATTTCTGTTCAAGAATGTGTTCTTTTTCATTTACAGTAATCATAATTTC
SARS-CoV-2 NC_045512.2         (1) ------------------------------------------------------------------------------------------------
                                                                                                            Section 333
                           (31873) 31873 31878      31889      31900      31911      31922      31933      31944      31955      31968
           T4 NC_000866.4  (31873) CTCATTCAAGTTTTAGTGTAAATTATAAAGGCCGAAGCCCTCTATTAAAAATCGTGGGTAGAATCAGCTTCAAGAGCTACCACATAATTCGCGTGT
SARS-CoV-2 NC_045512.2         (1) ------------------------------------------------------------------------------------------------
                                                                                                            Section 334
                           (31969) 31969   31977      31988      31999      32010      32021      32032      32043              32064
           T4 NC_000866.4  (31969) TCACCTTCAAATTTAGCAGCACCTTGTTTACCTTTTGCCCAAAGCAGAAGTTTATAATTTCCTGGTTGCATTTTCATATTTGCCATATTGATAATG
SARS-CoV-2 NC_045512.2         (1) ------------------------------------------------------------------------------------------------
                                                                                                            Section 335
                           (32065) 32065      32076      32087      32098      32109      32120      32131      32142              32160
           T4 NC_000866.4  (32065) AAATTAAATGTATTTTCACCATCATAATCACCAAGAGTCAAAGAATATTTAACACGGGTCAGAGCAGAATCTTCTACTTTATTAAAACCGTTAATT
SARS-CoV-2 NC_045512.2         (1) ------------------------------------------------------------------------------------------------
                                                                                                            Section 336
                           (32161) 32161         32175      32186      32197      32208      32219      32230      32241           32256
           T4 NC_000866.4  (32161) ACGATTTTACCTTCTTTTACCGTGATAGCAATTGTATCAATTTGCAGACCACGAGATACACGCAACAGCTGTTGAAGGTCTTCAGCTTTAATTTCA
SARS-CoV-2 NC_045512.2         (1) ------------------------------------------------------------------------------------------------
```

Section 337

```
                        (32257) 32257 32263 32274 32285 32296 32307 32318 32329 32340 32352
T4 NC_000866.4          (32257) GTAACAGCAGATGCTACCGGGAATGGAATTGGTTTATTAGGAGCAACTACTGTACTCGGATCGGCTGCTGGCCAAAAAATTGTTGAGCGGGCATCA
SARS-CoV-2 NC_045512.2      (1) ------------------------------------------------------------------------------------------------
```

Section 338

```
                        (32353) 32353 32362 32373 32384 32395 32406 32417 32428 32448
T4 NC_000866.4          (32353) GCAATTTTAATATTTCCATCTTCTGACTGGGAAATTTCTGCATCATCATTAACTAAAGACAGAATACCGAGAAAACCGTTCAAATCGTAAATTGCT
SARS-CoV-2 NC_045512.2      (1) ------------------------------------------------------------------------------------------------
```

Section 339

```
                        (32449) 32449 32461 32472 32483 32494 32505 32516 32527 32544
T4 NC_000866.4          (32449) ACATCAAAATCAATAACGTCAGAAATATTTGCTTCCGCATAAGTTGTACCATTAACTGCGCGAGTCATAATAAATTGACCGGATTTAAGCATAATA
SARS-CoV-2 NC_045512.2      (1) ------------------------------------------------------------------------------------------------
```

Section 340

```
                        (32545) 32545 32560 32571 32582 32593 32604 32615 32626 32640
T4 NC_000866.4          (32545) CCAGAGTTAATAGTAGCGAAATTTTTAAGCAGAGCAGTAGTATCTTTAGACAGTTTCATGTAATTTCCTTCAATTCAAATGAGATTTAATTTTATA
SARS-CoV-2 NC_045512.2      (1) ------------------------------------------------------------------------------------------------
```

Section 341

```
                        (32641) 32641 32648 32659 32670 32681 32692 32703 32714 32725 32736
T4 NC_000866.4          (32641) ACTAATTTAATAAAGCAATTAACGATTAAAATCAGCCGCAATTGTTTCCGCAACAATTTGAGCAGCAACAATTAGACGTTCATCTGCATTACCGCA
SARS-CoV-2 NC_045512.2      (1) ------------------------------------------------------------------------------------------------
```

Section 342

```
                        (32737) 32737 32747 32758 32769 32780 32791 32802 32813 32832
T4 NC_000866.4          (32737) ATAATCATCTTCAAGGCGTTCACCACATGAAGTCATAATAAATTTAGCACCGGCGTTTAGGGATTCTGTAGTATGTTTGCGCATTAGTTCAATCCA
SARS-CoV-2 NC_045512.2      (1) ------------------------------------------------------------------------------------------------
```

Section 343

```
                        (32833) 32833 32846 32857 32868 32879 32890 32901 32912 32928
T4 NC_000866.4          (32833) TTTATTACTTACTTCACGATCGATAGCTTCATAATACGCATGACGAGCAGGTGCAGATTTAATTTTGTTCTGAATAACTTCCATTGCGTTATCAGA
SARS-CoV-2 NC_045512.2      (1) ------------------------------------------------------------------------------------------------
```

```
                                                                                      Section 344
                          (32929) 32929 32934      32945      32956      32967      32978      32989      33000      33011        33024
         T4 NC_000866.4   (32929) AAGAGACAAAACCCATGCTCGACGAATTTTATTTTGGTTTTGTGGATTTGATTCAGAACGCACGTGTTTTGGCTGAATATCTTTTACATCAACAGT
SARS-CoV-2 NC_045512.2        (1) ------------------------------------------------------------------------------------------------
                                                                                      Section 345
                          (33025) 33025   33033      33044      33055      33066      33077      33088      33099                33120
         T4 NC_000866.4   (33025) ATAATTCACAGTAATTTTAGTCATAATACACCTTTAGTCATAATAATCAGTAACAGTCCAAGCTTCATTTCTATTGGACATTATTTTTGTATATTC
SARS-CoV-2 NC_045512.2        (1) ------------------------------------------------------------------------------------------------
                                                                                      Section 346
                          (33121) 33121      33132      33143      33154      33165      33176      33187      33198              33216
         T4 NC_000866.4   (33121) TGCTTTAAATGCATTCCTAAGCATAGATTCAGTAACTATATGCTCTTCATTAGAAAAATTATTTCTCAGAATATATCGTTTTATTTCAGGAATAGT
SARS-CoV-2 NC_045512.2        (1) ------------------------------------------------------------------------------------------------
                                                                                      Section 347
                          (33217) 33217         33231      33242      33253      33264      33275      33286      33297         33312
         T4 NC_000866.4   (33217) TAATAGATGCTGTCCAGTTGAATATTCCATGTTTTTCCTCCATAGAGATTATACTCTAATAAATTAAAGCATAATCTCTTATAAATTAAACCATTA
SARS-CoV-2 NC_045512.2        (1) ------------------------------------------------------------------------------------------------
                                                                                      Section 348
                          (33313) 33313 33319      33330      33341      33352      33363      33374      33385      33396       33408
         T4 NC_000866.4   (33313) CAGTAAATCGACCAACTTTCTTCATTTGAAGATGCTGACCATATTCTTGCGGGTCATGGTCTTTATGCGAAATTATAAAAACGTTAGTGTTTTTCA
SARS-CoV-2 NC_045512.2        (1) ------------------------------------------------------------------------------------------------
                                                                                      Section 349
                          (33409) 33409    33418      33429      33440      33451      33462      33473      33484               33504
         T4 NC_000866.4   (33409) TTGAATTTATAATATTAGCTACACCTTTAATACCTTCGGCATCAAATGACCCATCAAACACTTCATCAAGAATTAATGTACTAATACTAACACCAG
SARS-CoV-2 NC_045512.2        (1) ------------------------------------------------------------------------------------------------
                                                                                      Section 350
                          (33505) 33505       33517      33528      33539      33550      33561      33572      33583             33600
         T4 NC_000866.4   (33505) ATACGATAGAAGCAATATCACGCCAAGTAAATAAAAGAGCAATATCGATTCGTGCCTTTTCACCTTCACTAAATGAAGCATAACTAAAATCTTCAC
SARS-CoV-2 NC_045512.2        (1) ------------------------------------------------------------------------------------------------
```

```
                                                                                                                          Section 351
                              (33601) 33601          33616      33627      33638      33649      33660      33671      33682       33696
T4 NC_000866.4                (33601) GACCACGGGATTTAATTGTCTCATTAAATTCTTCATCTAATGTAAACACATAATCCGCTTCCATTATTTTAAGATAATGGTTAATCTGCTTATTAA
SARS-CoV-2 NC_045512.2            (1) ------------------------------------------------------------------------------------------------
                                                                                                                          Section 352
                              (33697) 33697  33704      33715      33726      33737      33748      33759      33770      33781      33792
T4 NC_000866.4                (33697) ATAATGGAATGTACTTTTTAATAATAGCACCTTTAATACCAGAATCTTTGAGCATATCAGTCAAAATTCCTCGGTGGTATTTTTCCATTACTAAAT
SARS-CoV-2 NC_045512.2            (1) ------------------------------------------------------------------------------------------------
                                                                                                                          Section 353
                              (33793) 33793     33803      33814      33825      33836      33847      33858      33869              33888
T4 NC_000866.4                (33793) TAGTTTTTGTCTTAACAATTTTATCAAGTTCTTCTTGAAGCAGTGCTATTTCATCAGCATGGTCAATAAACTCAGAAGATGCTTTTTCTATAGCCG
SARS-CoV-2 NC_045512.2            (1) ------------------------------------------------------------------------------------------------
                                                                                                                          Section 354
                              (33889) 33889        33902      33913      33924      33935      33946      33957      33968           33984
T4 NC_000866.4                (33889) CTTTAACTTTTTTAGCTTTATCTACTGCTGCGATCAGAGATTGCTTTTTATTGCGAATATCATTTGCCAACGACTGCTGGGTTTTAATATTATCTC
SARS-CoV-2 NC_045512.2            (1) ------------------------------------------------------------------------------------------------
                                                                                                                          Section 355
                              (33985) 33985 33990      34001      34012      34023      34034      34045      34056      34067        34080
T4 NC_000866.4                (33985) GGTATTCATCAACAAGAACTTTTAAATTATCACGATGTGTTGAAAGCTGTTCAAACGAATGTGTGCATTCAGAAACTTTATCTTTAATTTTAGAAA
SARS-CoV-2 NC_045512.2            (1) ------------------------------------------------------------------------------------------------
                                                                                                                          Section 356
                              (34081) 34081   34089      34100      34111      34122      34133      34144      34155                34176
T4 NC_000866.4                (34081) CAACTTTATCACCGGAACTCAATTGTGACAAACAGGTTGGACATAATCCACCTTCGTGATACATATTAATGACTTTATTATACGAGTCAATTTTTG
SARS-CoV-2 NC_045512.2            (1) ------------------------------------------------------------------------------------------------
                                                                                                                          Section 357
                              (34177) 34177      34188      34199      34210      34221      34232      34243      34254             34272
T4 NC_000866.4                (34177) ATTTAATTAAAAATGCTTCTTGACCGATTTTATTAAATGCATCAGTCGGGTCTTCGTCCAAAACAATATTAACTAATCTTTCGTTAGCTTCTTCTA
SARS-CoV-2 NC_045512.2            (1) ------------------------------------------------------------------------------------------------
```

Section 358

```
                         (34273) 34273         34287      34298      34309      34320      34331      34342      34353          34368
T4 NC_000866.4           (34273) TTTCCGATTTTAGCGTTCTAGCTTCTTTTGCCAAATCATCATACATATTTTGTAGACGAGTAAGGTTGTCACCCGTTAATTTTTTCTGGCGTTCAA
SARS-CoV-2 NC_045512.2       (1) ------------------------------------------------------------------------------------------------
```

Section 359

```
                         (34369) 34369 34375      34386      34397      34408      34419      34430      34441      34452       34464
T4 NC_000866.4           (34369) CATTATCATTATATATTTTAATTTGTTGGATAATACTATCTTTTTTAACATCAAGCACTTGGTTCTGCGAATTTAATTCACGTATTAGTGCTTTAT
SARS-CoV-2 NC_045512.2       (1) ------------------------------------------------------------------------------------------------
```

Section 360

```
                         (34465) 34465    34474      34485      34496      34507      34518      34529      34540                34560
T4 NC_000866.4           (34465) TAAGCTTATCCATTTCAGCTAATGTTCCTACCTCAAGCAGGTCTTCCACAAGCTTTCTTCGCGCAGGGGTCGACAAACCCATGAAAGGGGTATACC
SARS-CoV-2 NC_045512.2       (1) ------------------------------------------------------------------------------------------------
```

Section 361

```
                         (34561) 34561       34573      34584      34595      34606      34617      34628      34639            34656
T4 NC_000866.4           (34561) CTGCTGTACCAAGGACAACAATCTGCTTGAAACTGGCATATGACATTCCGATAAGCTGTTCAAATTCTGCTTGGAAATCTTTACTGCTGGCAGATT
SARS-CoV-2 NC_045512.2       (1) ------------------------------------------------------------------------------------------------
```

Section 362

```
                         (34657) 34657          34672      34683      34694      34705      34716      34727      34738        34752
T4 NC_000866.4           (34657) CATTAAGACGTGTACCGTTAACGGTGATTTCGAAAACGTTTGGTTTTTGTCCTCTTTTGATATAGTACTTTTTCTCATCATATTCCATCCACAGTT
SARS-CoV-2 NC_045512.2       (1) ------------------------------------------------------------------------------------------------
```

Section 363

```
                         (34753) 34753  34760      34771      34782      34793      34804      34815      34826      34837      34848
T4 NC_000866.4           (34753) CAACTAAAAGTTCTTTCTTATTTGTGCTGTTTATTAATTGACCTTTCTTTACATCGCGAAATGGCTTACCAAAAAGCCCAAATGTGATGGCTTCTA
SARS-CoV-2 NC_045512.2       (1) ------------------------------------------------------------------------------------------------
```

Section 364

```
                         (34849) 34849     34859      34870      34881      34892      34903      34914      34925              34944
T4 NC_000866.4           (34849) GCATAGTAGACTTACCACCGCCATTTCGTCCAGTAATAAGAGTTTTTTGAACCTTATCTAATTGAATGTCAATCCCATTTTGACCAACTGACATTA
SARS-CoV-2 NC_045512.2       (1) ------------------------------------------------------------------------------------------------
```

```
                                                                                                            Section 365
                          (34945) 34945        34958      34969      34980      34991      35002      35013      35024          35040
         T4 NC_000866.4   (34945) TATTTTTATATTTTACTCTATTAAGTTTAAAATTCTTCACAAAAGATTCCTTTTAATGTATCTTTTAGACCATTCTATCATATCATCATAATCTAA
SARS-CoV-2 NC_045512.2        (1) ------------------------------------------------------------------------------------------------
                                                                                                            Section 366
                          (35041) 35041 35046      35057      35068      35079      35090      35101      35112      35123      35136
         T4 NC_000866.4   (35041) AAAGTATTCATCAAATTCAGCCATGCAAACAACGCCTTGTGCTGTTTTTGATGTGATATAAATTATTCCAACATATCTAGAATCTTCTTCGGTATA
SARS-CoV-2 NC_045512.2        (1) ------------------------------------------------------------------------------------------------
                                                                                                            Section 367
                          (35137) 35137   35145      35156      35167      35178      35189      35200      35211                35232
         T4 NC_000866.4   (35137) ATCAATATTTGCTATGAATTCATCATTAATGTCAAATGTCGAAAACTTCACAGTATGCATCCTTAATACAAGATACAGCCATATCTCGTAATGATT
SARS-CoV-2 NC_045512.2        (1) ------------------------------------------------------------------------------------------------
                                                                                                            Section 368
                          (35233) 35233      35244      35255      35266      35277      35288      35299      35310            35328
         T4 NC_000866.4   (35233) TAGGTGTGTCATTATCTAAAATGTTGAAGTTAAAAGATACAGCCCAGTCAGTGCAGACAGTAACCTTTTTAATACAATTATCTTCAACCTCTAACG
SARS-CoV-2 NC_045512.2        (1) ------------------------------------------------------------------------------------------------
                                                                                                            Section 369
                          (35329) 35329         35343      35354      35365      35376      35387      35398      35409          35424
         T4 NC_000866.4   (35329) GTTCAAACCAGTATTCAATAATTTCTTTATGACCAATATCATCTTTTACTTCACATTTAAAATGCTGACTCATCATAACATTTTTAAATTCATCAA
SARS-CoV-2 NC_045512.2        (1) ------------------------------------------------------------------------------------------------
                                                                                                            Section 370
                          (35425) 35425 35431      35442      35453      35464      35475      35486      35497      35508       35520
         T4 NC_000866.4   (35425) AAGTCATTGTGTTGCCTCTACATATAGCTGATTTGCATATTGAATAAGTGCTTCACGGTCAGAATCAGTGATGTCTGGAATTGCATTAATATACTC
SARS-CoV-2 NC_045512.2        (1) ------------------------------------------------------------------------------------------------
                                                                                                            Section 371
                          (35521) 35521    35530      35541      35552      35563      35574      35585      35596               35616
         T4 NC_000866.4   (35521) TTCCATTAATGTCTGAAGCGATTGAACTTCAACTTCTTCACTGTCATCTGACTCGACAGAGTTATCAATCTTTGACACAACTCGTAATGAATGCAC
SARS-CoV-2 NC_045512.2        (1) ------------------------------------------------------------------------------------------------
```

```
                                                                                                                  Section 372
                          (35617) 35617       35629      35640      35651      35662      35673      35684      35695          35712
         T4 NC_000866.4   (35617) AACTTTTTCTAGTTCAGATTCGAACTTCGTCAGATTTTTGTCTACTTCAGTTACTATAACACGTACTGATAGATTTGTAAAATCTTTATAGTCAAT
  SARS-CoV-2 NC_045512.2      (1) ------------------------------------------------------------------------------------------------
                                                                                                                  Section 373
                          (35713) 35713          35728      35739      35750      35761      35772      35783      35794        35808
         T4 NC_000866.4   (35713) TTTTCCTTTAAATGGATAATGAATTCTACGATGCCAGGTAGTATTGTTTGGAATAAATTCCGTTCGTTCTGTTTCTGTATCAAACATCCAGAACCC
  SARS-CoV-2 NC_045512.2      (1) ------------------------------------------------------------------------------------------------
                                                                                                                  Section 374
                          (35809) 35809  35816      35827      35838      35849      35860      35871      35882      35893      35904
         T4 NC_000866.4   (35809) ACGAGGGTCATTCTCGTCACCTGCGGTTAGTGTCCATGGTGTCCCAATATATCTGACGTTTGCAGCCTCAGAAATAGTATGGAAGTGACCAGACCA
  SARS-CoV-2 NC_045512.2      (1) ------------------------------------------------------------------------------------------------
                                                                                                                  Section 375
                          (35905) 35905     35915      35926      35937      35948      35959      35970      35981            36000
         T4 NC_000866.4   (35905) CACTTCTTTATAAGTCTTAAGGAAATCGGGTTCAAGACCATGAGATTTCATTCCTTTATAAAAATAAAATCCATTCAGTTCCCAGTGACCAACACA
  SARS-CoV-2 NC_045512.2      (1) ------------------------------------------------------------------------------------------------
                                                                                                                  Section 376
                          (36001) 36001        36014      36025      36036      36047      36058      36069      36080          36096
         T4 NC_000866.4   (36001) AAAAGAAGCAGATGAAGTTTTGATATGCTCAAGAATTTCACCAGTATTTTCTTCGCACATCCAAGGAATCAAATCAATCAAACACCCGTCAAAATC
  SARS-CoV-2 NC_045512.2      (1) ------------------------------------------------------------------------------------------------
                                                                                                                  Section 377
                          (36097) 36097 36102      36113      36124      36135      36146      36157      36168      36179      36192
         T4 NC_000866.4   (36097) TACTGTAGTAGGCTTATCATACACTTTAACATTAGGATATTTAGCCAAAAGCTCAGTAGAAGCATTTGGATGCATTACATTTTTATAGTGGAGATC
  SARS-CoV-2 NC_045512.2      (1) ------------------------------------------------------------------------------------------------
                                                                                                                  Section 378
                          (36193) 36193   36201      36212      36223      36234      36245      36256      36267              36288
         T4 NC_000866.4   (36193) GTGATTTCCTACAATAGTGTGTAATGTAATTCCAGCATCATCAAGCGTTTGAACTATTTCACGGGCAAACTCCATAGTTTTATGTGTGATCGCTTT
  SARS-CoV-2 NC_045512.2      (1) ------------------------------------------------------------------------------------------------
```

```
                                                                                                                          Section 379
                        (36289) 36289      36300      36311      36322      36333      36344      36355      36366            36384
          T4 NC_000866.4 (36289) TCGCACATCAAAAATATCACCGTATTGAATCCAGGTAGTAATTCCATTTTTCTTAGAATATTCTATCGCTTGCTTAATTCCATCAATTTGAATACC
 SARS-CoV-2 NC_045512.2     (1) ------------------------------------------------------------------------------------------------
                                                                                                                          Section 380
                        (36385) 36385         36399      36410      36421      36432      36443      36454      36465          36480
          T4 NC_000866.4 (36385) GCGAATCCACTCATCATCAGCTTTAACGCCTAAATGCCAATCACCTAAATTTAAAATTTTCATATATCAAGAACCGTCATTGAAATGCAAAATAAA
 SARS-CoV-2 NC_045512.2     (1) ------------------------------------------------------------------------------------------------
                                                                                                                          Section 381
                        (36481) 36481 36487      36498      36509      36520      36531      36542      36553      36564       36576
          T4 NC_000866.4 (36481) ATTATTGAAATAAATCCATCTGGAGTGCTAAAGAACCCAATCCAACATGCTCTAGTGAATAGATAAAATGCAAGAAAAAGTATCACATATCCAAGA
 SARS-CoV-2 NC_045512.2     (1) ------------------------------------------------------------------------------------------------
                                                                                                                          Section 382
                        (36577) 36577    36586      36597      36608      36619      36630      36641      36652               36672
          T4 NC_000866.4 (36577) AATATCATTATATCAAACTCCGTATAAAGCTAAAGGGCCGAAGCCCTTTATTTTGTAATAATGTCAAACTGTTCTTTAAAGCAGAAGCTTGAATCT
 SARS-CoV-2 NC_045512.2     (1) ------------------------------------------------------------------------------------------------
                                                                                                                          Section 383
                        (36673) 36673       36685      36696      36707      36718      36729      36740      36751            36768
          T4 NC_000866.4 (36673) TGATGCTGATACAAAAATTCATATGCTTTTTCTCGCTCACGGTCATAAAGAGCTCGGTCAGATGACAGTTCTTTAATACGTTCAAATGTTGATTCC
 SARS-CoV-2 NC_045512.2     (1) ------------------------------------------------------------------------------------------------
                                                                                                                          Section 384
                        (36769) 36769          36784      36795      36806      36817      36828      36839      36850         36864
          T4 NC_000866.4 (36769) ATATCATTTTCATCAAACCAAATGATACCGCTATCATGCGAGGTCAAAGGAGTATTATCAACACGGAATTTTAAATTTTCGCCAGTAGATTTCCAA
 SARS-CoV-2 NC_045512.2     (1) ------------------------------------------------------------------------------------------------
                                                                                                                          Section 385
                        (36865) 36865  36872      36883      36894      36905      36916      36927      36938      36949      36960
          T4 NC_000866.4 (36865) AATACCGGAATTGTTCCACATGCACCAAGCTCGAGATGAGTATATTCGAGTGAGCGTTGTAAGTATTTCTGGTTAAGTTTACTCAACTGATATCCA
 SARS-CoV-2 NC_045512.2     (1) ------------------------------------------------------------------------------------------------
```

```
Section 386
                       (36961) 36961     36971       36982       36993       37004       37015       37026       37037         37056
        T4 NC_000866.4 (36961) AAGCCAGATTTACTCATTCGTTCAAGCATTTCACTATTAATATAACAATCTAGGATTTGTGCCGGTTGATTCGGCGCGAGATTCATTTTATCAATC
SARS-CoV-2 NC_045512.2     (1) ------------------------------------------------------------------------------------------------

Section 387
                       (37057) 37057        37070       37081       37092       37103       37114       37125       37136       37152
        T4 NC_000866.4 (37057) TCACGATTACCGTAATATTCATACGGAATACCTTTTTCCTTAATTGCAATAAAAGCAGGGGAACGTTCCAGACCTTCCATTACAGTGGATTTACCA
SARS-CoV-2 NC_045512.2     (1) ------------------------------------------------------------------------------------------------

Section 388
                       (37153) 37153 37158      37169       37180       37191       37202       37213       37224       37235   37248
        T4 NC_000866.4 (37153) GCAGGTTTTAAGAATTTTTCATGAAAATCAAACATCTGGTAAAAACCTTTCCATGTAGTCGTACGACCAATCCAACGGTTGATATTCATGTTAATT
SARS-CoV-2 NC_045512.2     (1) ------------------------------------------------------------------------------------------------

Section 389
                       (37249) 37249   37257       37268       37279       37290       37301       37312       37323           37344
        T4 NC_000866.4 (37249) TCAGAAACATCTTTCCAATAAGTTGACCGAACCTTCACAATATCCATAGGAGGCTGAAAATTATATACTGTCGGTGCTTCTTCAATATCATCAAAC
SARS-CoV-2 NC_045512.2     (1) ------------------------------------------------------------------------------------------------

Section 390
                       (37345) 37345      37356       37367       37378       37389       37400       37411       37422         37440
        T4 NC_000866.4 (37345) AGAGAAACAGTTTCTGGATACCATTCTTTCATCAGAACTTTATTAAAATCACCATTATCAGAATGGCTAAAAATAACATCAGCTCGACGAACAGTT
SARS-CoV-2 NC_045512.2     (1) ------------------------------------------------------------------------------------------------

Section 391
                       (37441) 37441         37455       37466       37477       37488       37499       37510       37521         37536
        T4 NC_000866.4 (37441) TCTTCTAATCCCAAATTTCGACGCAAAGAAAGAACAGAATGATCATGCTGATAAACTACAACACGAATAGAAGGTTTAATATTATCTAAAAGTTTT
SARS-CoV-2 NC_045512.2     (1) ------------------------------------------------------------------------------------------------

Section 392
                       (37537) 37537 37543      37554       37565       37576       37587       37598       37609       37620   37632
        T4 NC_000866.4 (37537) TTATAGTTATTAATCGTAGCTTCTTGAACGGAAGTAGCAGGAACAGAATTAATAATTAGAATATCACAATCATTTACTAGCTTAAGTGCTTTATCG
SARS-CoV-2 NC_045512.2     (1) ------------------------------------------------------------------------------------------------
```

Section 393

```
                          37633    37642      37653      37664      37675      37686      37697      37708          37728
                 (37633)
T4 NC_000866.4   (37633) TATTCTTTAGCTAAAATAACTGGAATTGAAAATGATTTGTGGTCATGAGAACTTGTACGAGTAAATGATTTATCTTTAGCATAAACCAAAGTTACT
SARS-CoV-2 NC_045512.2 (1) ------------------------------------------------------------------------------------------------
```

Section 394

```
                          37729       37741      37752      37763      37774      37785      37796      37807           37824
                 (37729)
T4 NC_000866.4   (37729) TCATGACCATTTTTAATAAACCAATCACGTTGCTCGAGTGAGAATTTTGTTACACCACAACCTTCAAGACCTCGAGCCATAAAAATGCAAATACGC
SARS-CoV-2 NC_045512.2 (1) ------------------------------------------------------------------------------------------------
```

Section 395

```
                          37825          37840      37851      37862      37873      37884      37895      37906        37920
                 (37825)
T4 NC_000866.4   (37825) ATAGTTTTCCTCTTTTCATTTAATAAATCATGTAAATAATATTTTATTTTCTATAAAACGCTACGAATAGGCCCAAACATATCCATAAGCAATTTT
SARS-CoV-2 NC_045512.2 (1) ------------------------------------------------------------------------------------------------
```

Section 396

```
                          37921  37928      37939      37950      37961      37972      37983      37994      38005    38016
                 (37921)
T4 NC_000866.4   (37921) GCTTTTTCCAGATATGCACTTTTTAATATTAGACATGCATCCTTTAACGGATACAGCTGCGTCTTTAATTCTAGGAAATACTCGTAATAATTTTCC
SARS-CoV-2 NC_045512.2 (1) ------------------------------------------------------------------------------------------------
```

Section 397

```
                          38017     38027      38038      38049      38060      38071      38082      38093           38112
                 (38017)
T4 NC_000866.4   (38017) AGTAGTATCATATTGAAATACTGGCACATTTCTTTTCTGAGTTATCCCACGTTTAGATTTAGACATTTTTTGTTTAGTTGCATTAGACATCCTAGA
SARS-CoV-2 NC_045512.2 (1) ------------------------------------------------------------------------------------------------
```

Section 398

```
                          38113        38126      38137      38148      38159      38170      38181      38192          38208
                 (38113)
T4 NC_000866.4   (38113) GGGCTTTCCTATGTTATCAGAATAATAATATTTCCATTGAAATCCTCCAGCGGTTTTCCTTTTACCATCTACACATTGTTTAATTGAAGTTGAACA
SARS-CoV-2 NC_045512.2 (1) ------------------------------------------------------------------------------------------------
```

Section 399

```
                          38209 38214      38225      38236      38247      38258      38269      38280      38291     38304
                 (38209)
T4 NC_000866.4   (38209) GCTATATGACATATCTTCTGCAGCATCTGTAATACATCTATATTTGCGAATAAAATTTCCATTTAAATCATATTGATAAATTGGTTTTCCAGCATT
SARS-CoV-2 NC_045512.2 (1) ------------------------------------------------------------------------------------------------
```

```
                                                                                                                  Section 400
                         (38305) 38305   38313      38324      38335      38346      38357      38368      38379                38400
T4 NC_000866.4           (38305) TCGTCTAGCGTTAGACATGTATTTTTTATTATCATCATCTTTCCAGAACTCTTTCATGCTTTCCGATATTGAAGAAGATACAGCTGTTTTAATCCA
SARS-CoV-2 NC_045512.2   (1)     ------------------------------------------------------------------------------------------------
                                                                                                                  Section 401
                         (38401) 38401      38412      38423      38434      38445      38456      38467      38478             38496
T4 NC_000866.4           (38401) TCCATACATTTTATTATTTAGTCTTGTTCCGTCAGAACTATAACACATCATACGAATAGCTAAAGCCAATTTAGGAAGTCTATAAATTTTAAATAA
SARS-CoV-2 NC_045512.2   (1)     ------------------------------------------------------------------------------------------------
                                                                                                                  Section 402
                         (38497) 38497         38511      38522      38533      38544      38555      38566      38577        38592
T4 NC_000866.4           (38497) TAATAAATGCGCGGTAAAATGTTCTTCTGGTGTCAAAAGAACTAAATTAGTTTTATCATCTGTACCACCCATACATCTAGGAATTATATGATGTGT
SARS-CoV-2 NC_045512.2   (1)     ------------------------------------------------------------------------------------------------
                                                                                                                  Section 403
                         (38593) 38593 38599      38610      38621      38632      38643      38654      38665      38676      38688
T4 NC_000866.4           (38593) TTCAGTATAGTATGTCAAAAGACTTTTATCATTGCCTCTGTTTAGTCCTTTTTCGATCAGTAAATTATATATGTTTAAATAATTCATTTTAGTTTA
SARS-CoV-2 NC_045512.2   (1)     ------------------------------------------------------------------------------------------------
                                                                                                                  Section 404
                         (38689) 38689    38698      38709      38720      38731      38742      38753      38764               38784
T4 NC_000866.4           (38689) TTTTACCAAAAAATTTATAAAGCAATATAGGAGCCGAAGCTCCTATCCACATAATACGCCATACAGAGGCTCGTTAGAACTTTTAAATTTTATGCG
SARS-CoV-2 NC_045512.2   (1)     ------------------------------------------------------------------------------------------------
                                                                                                                  Section 405
                         (38785) 38785       38797      38808      38819      38830      38841      38852      38863            38880
T4 NC_000866.4           (38785) CTTATATGTTATAGTTCCTTCTGCTTTAGCTTTATCATGAGCCTCTTTAAAGCGTCTCATCATTTCCTCTCTAGAGGAACGAATTTTATTATAATC
SARS-CoV-2 NC_045512.2   (1)     ------------------------------------------------------------------------------------------------
                                                                                                                  Section 406
                         (38881) 38881          38896      38907      38918      38929      38940      38951      38962         38976
T4 NC_000866.4           (38881) TATTTCAGAAGTCGGATGTTCATCTTTCACAGTTGCCACCATTTTTTGACTTGATAAGAATCAACCCACACTTTCATATTAGGGTCTGCTCTTACA
SARS-CoV-2 NC_045512.2   (1)     ------------------------------------------------------------------------------------------------
```

```
                                                                                                   Section 407
                     (38977) 38977  38984           38995           39006           39017           39028           39039           39050           39061      39072
        T4 NC_000866.4 (38977) ATAGGAGGATTAACTTCTTTGATTGAACTATCACGGTATTCTTTTTCAGAAATTTTCACGCAAAGATGACCATTCAAAGATTCAGTAAATCCTGCA
 SARS-CoV-2 NC_045512.2    (1) ------------------------------------------------------------------------------------------------
                                                                                                   Section 408
                     (39073) 39073     39083           39094           39105           39116           39127           39138           39149                39168
        T4 NC_000866.4 (39073) TTAATTTTAAGACGCTTGAATTTTACGTGTGTAAGCATCAATCATATCCTCAATCTGCGATCTAGTAGTCTTCCAAAGAATACTGATGAGTTCATC
 SARS-CoV-2 NC_045512.2    (1) ------------------------------------------------------------------------------------------------
                                                                                                   Section 409
                     (39169) 39169        39182           39193           39204           39215           39226           39237           39248           39264
        T4 NC_000866.4 (39169) GTTATATGGCTGTTTTAGAATATCCCGACTTTTCTTGATAGCATATTCGTATTGAGCAAAATTATTATTTTCAGCTGCGATCTGAGCGTGCTTATA
 SARS-CoV-2 NC_045512.2    (1) ------------------------------------------------------------------------------------------------
                                                                                                   Section 410
                     (39265) 39265 39270           39281           39292           39303           39314           39325           39336           39347      39360
        T4 NC_000866.4 (39265) AAGACGATTCAGTTCGCGTTTGTTTTTAGACAATAACTTATTTGCTTTTCTTTCTGCTTCAAGACGGTTCTTTTCTTCAATAGAAGAAATAAGCTT
 SARS-CoV-2 NC_045512.2    (1) ------------------------------------------------------------------------------------------------
                                                                                                   Section 411
                     (39361) 39361   39369           39380           39391           39402           39413           39424           39435                39456
        T4 NC_000866.4 (39361) TTCCACTTCATCATTAATTTCGGGTTTATCAGTCATATTATTTCTCTAATATAAAATAAAAATCATCATCTGTTAAATGATACCGATAGTTTAATT
 SARS-CoV-2 NC_045512.2    (1) ------------------------------------------------------------------------------------------------
                                                                                                   Section 412
                     (39457) 39457      39468           39479           39490           39501           39512           39523           39534                39552
        T4 NC_000866.4 (39457) CTACACCATTAGATTTAAAAGCGGTATCATACGGATTTTCTGGATCAATATCAATGTCAAGAGCTAAAACTTCCCTGAGATACATTTTAAGTAAAT
 SARS-CoV-2 NC_045512.2    (1) ------------------------------------------------------------------------------------------------
                                                                                                   Section 413
                     (39553) 39553         39567           39578           39589           39600           39611           39622           39633           39648
        T4 NC_000866.4 (39553) AGGGAATAGCTTCAACTTCAGGTATTTCTTCCAAGAATCCGGAGAGGTTAATCGTTAGCCTCATATAAAAAATCCAAACTAGGAGAATCATCTACA
 SARS-CoV-2 NC_045512.2    (1) ------------------------------------------------------------------------------------------------
```

```
                                                                                                                   Section 414
                              (39649) 39649 39655      39666      39677      39688      39699      39710      39721      39732      39744
T4 NC_000866.4                (39649) ACACTTTTCTTTTCAGCCCCCGGTGTTCTATAGGTTGATTCTTCGTAATGCGTCATTTTATCATAGATGTCTTGAATAAAAGTTTCATCTACTAAC
SARS-CoV-2 NC_045512.2            (1) ------------------------------------------------------------------------------------------------
                                                                                                                   Section 415
                              (39745) 39745    39754      39765      39776      39787      39798      39809      39820                39840
T4 NC_000866.4                (39745) GCAACCATATCGTCGTCACGGCTGTCATAGACATTGTGAACGAAGTAACTATATTTCTTTGCAACTTCCTTACGTTCTTTTTTAATACGTTGGACG
SARS-CoV-2 NC_045512.2            (1) ------------------------------------------------------------------------------------------------
                                                                                                                   Section 416
                              (39841) 39841       39853      39864      39875      39886      39897      39908      39919            39936
T4 NC_000866.4                (39841) AATGCATTAAAACAAGCTTGAGTTATATACGCATGTGGGTTTTTATATTTCGTTTCATCAAAATTGTGAAGCCCCTTAATAGAAGCTTCTATACCA
SARS-CoV-2 NC_045512.2            (1) ------------------------------------------------------------------------------------------------
                                                                                                                   Section 417
                              (39937) 39937          39952      39963      39974      39985      39996      40007      40018        40032
T4 NC_000866.4                (39937) TCTGCAATCATTTCTTGTTTCCAAGACTGGGTGTATCCTGAAAAGTTGAAACGTTTAGATAAGCCTTCTGCAATAAGCATAATGGCTAATCCGATA
SARS-CoV-2 NC_045512.2            (1) ------------------------------------------------------------------------------------------------
                                                                                                                   Section 418
                              (40033) 40033  40040      40051      40062      40073      40084      40095      40106      40117  40128
T4 NC_000866.4                (40033) GTATCATTCTGACGAACTACTTTATTTGGGTCTTTATTATTTGCTAATTCTGTTTTCCAATCAATAATAGCTTGTAAAAGCTCTTTATTGTTTACG
SARS-CoV-2 NC_045512.2            (1) ------------------------------------------------------------------------------------------------
                                                                                                                   Section 419
                              (40129) 40129     40139      40150      40161      40172      40183      40194      40205              40224
T4 NC_000866.4                (40129) TAATTATATTTAGGCTTAGTTTCTGACATTTTCACCTCTTAGCTCAATTCATAGATCTATTATATCATAATATTTGAAGACCTATCTTAAAGCATA
SARS-CoV-2 NC_045512.2            (1) ------------------------------------------------------------------------------------------------
                                                                                                                   Section 420
                              (40225) 40225        40238      40249      40260      40271      40282      40293      40304            40320
T4 NC_000866.4                (40225) GAGGATGAATCAATTTCAAGCACTTCATCAGATTAGCCGCTCCAAGAGCTGCATCTAGTGAATCAAATTGGTCAACATATTCAATTAATTCGCCGT
SARS-CoV-2 NC_045512.2            (1) ------------------------------------------------------------------------------------------------
```

```
                                                                                                               Section 421
                         (40321) 40321 40326      40337      40348      40359      40370      40381      40392      40403     40416
        T4 NC_000866.4   (40321) AATTAGCGTATAACCACCATTGGCTAAATTCATACTCAAGGATGAATCCATTTCCTTCAATTTGAGTTAAACCAATGCCATTTGTATTTACTTCAT
SARS-CoV-2 NC_045512.2       (1) ------------------------------------------------------------------------------------------------
                                                                                                               Section 422
                         (40417) 40417   40425      40436      40447      40458      40469      40480      40491                40512
        T4 NC_000866.4   (40417) ACCCAGCGAGACGTAAATCGTTAATAAGAGCTTCGTTCATAATTATACCTTAGTAATTTTCAGGTCTGCAAATTTTTTCTTGCGTTGATTTTTCAT
SARS-CoV-2 NC_045512.2       (1) ------------------------------------------------------------------------------------------------
                                                                                                               Section 423
                         (40513) 40513      40524      40535      40546      40557      40568      40579      40590             40608
        T4 NC_000866.4   (40513) GCGACGAATAGTTTTATCGGAAATTTCATGCTTTTGATAAGCTTTAGATTCTACACCAAAAGCTTTAACATCAAATTCTGACAAGATATATTGAAC
SARS-CoV-2 NC_045512.2       (1) ------------------------------------------------------------------------------------------------
                                                                                                               Section 424
                         (40609) 40609         40623      40634      40645      40656      40667      40678      40689          40704
        T4 NC_000866.4   (40609) CAACAATTCACGGACAGTATTGCGTCCAATCTTCTGATCATTCTGTTTCATCGTTTGATGAAGCTCTTTTTCCCATTTATCCAAAATTTGAGGAGT
SARS-CoV-2 NC_045512.2       (1) ------------------------------------------------------------------------------------------------
                                                                                                               Section 425
                         (40705) 40705 40711      40722      40733      40744      40755      40766      40777      40788     40800
        T4 NC_000866.4   (40705) TACAATATCGCCTTTTTCTAGCAAAGAAACTACTTTATCATATGCGTAAGAATTGATGGCGTTTTTAATTTGAATAGTCATACATTATCCTCAATT
SARS-CoV-2 NC_045512.2       (1) ------------------------------------------------------------------------------------------------
                                                                                                               Section 426
                         (40801) 40801    40810      40821      40832      40843      40854      40865      40876                40896
        T4 NC_000866.4   (40801) ACGTTAAAATTTTATTATCCAAAAAGGCCGAAGCCCTTAGGCTAAACTTTTTGGCACCCTTCCAGCCTTCGTACATCATTGCGACTGACAATGACA
SARS-CoV-2 NC_045512.2       (1) ------------------------------------------------------------------------------------------------
                                                                                                               Section 427
                         (40897) 40897       40909      40920      40931      40942      40953      40964      40975            40992
        T4 NC_000866.4   (40897) AAGCTCCTTCACATGCTGCATACTTATTATTCCAGAACCAATTTAGAAAAACTTCATCCTCAATACCGTTGTGTTTCATGTTCTGGAAAAATTTGG
SARS-CoV-2 NC_045512.2       (1) ------------------------------------------------------------------------------------------------
```

```
                                                                                                                      Section 428
                              (40993) 40993          41008      41019      41030      41041      41052      41063      41074         41088
T4 NC_000866.4                (40993) CGCGTTCTTCAGCAATTCGCTGCGATAATTTAGATTCAGGATTCATTTAAATTTCCCAATTGCCATTTTCATCAATAAATTTAATCCAGTCATTTA
SARS-CoV-2 NC_045512.2            (1) ------------------------------------------------------------------------------------------------
                                                                                                                      Section 429
                              (41089) 41089  41096      41107      41118      41129      41140      41151      41162      41173      41184
T4 NC_000866.4                (41089) CTGACCACTTGGTCGTATCGCCTTTTGGAGTAACATTTAAAGTGTATAGCCCTTGCTTAAAAAGCATGCGTTTGATATTCATATTTTCCTCAGCTG
SARS-CoV-2 NC_045512.2            (1) ------------------------------------------------------------------------------------------------
                                                                                                                      Section 430
                              (41185) 41185     41195      41206      41217      41228      41239      41250      41261              41280
T4 NC_000866.4                (41185) TAACGATAACACTCGTTTGATTTACGTTTAGCAACTCGTTGAGAAGTATTATAATCAAAATCATCATCAATGTAAACTGATTTTTTCAACTTTCTT
SARS-CoV-2 NC_045512.2            (1) ------------------------------------------------------------------------------------------------
                                                                                                                      Section 431
                              (41281) 41281        41294      41305      41316      41327      41338      41349      41360           41376
T4 NC_000866.4                (41281) ACTTCACCGCGTAATTGACGATTTAACTCATCTTTAACTTCTGAATCAATACCTTTCATTCTACGCCATTTATCTGAGCGAAAAATGTTTTCAACC
SARS-CoV-2 NC_045512.2            (1) ------------------------------------------------------------------------------------------------
                                                                                                                      Section 432
                              (41377) 41377 41382      41393      41404      41415      41426      41437      41448      41459        41472
T4 NC_000866.4                (41377) ATATCTTTATGACTTACCCCATCAGGAGCTTTACGCTTTCCGAAATAGTCATAATCACGCACTTTTAAGTCTTTACGACGATACGTTTTACCCATG
SARS-CoV-2 NC_045512.2            (1) ------------------------------------------------------------------------------------------------
                                                                                                                      Section 433
                              (41473) 41473   41481      41492      41503      41514      41525      41536      41547                41568
T4 NC_000866.4                (41473) GAGTTTAATTTCCTTAGCAACTGAACTAAATACAGCACGATCACAAATCATACGTTTATGTAACTTGAGTATAAGATAGTAAACATCAAAACCATT
SARS-CoV-2 NC_045512.2            (1) ------------------------------------------------------------------------------------------------
                                                                                                                      Section 434
                              (41569) 41569      41580      41591      41602      41613      41624      41635      41646             41664
T4 NC_000866.4                (41569) TACATAAGTAACACGAACAACATTACCATTCACGAGATAATACTGTCCCTTTTTAATCTCTTTATCAACCACAACCATATCAATTCCTCAAAGGTA
SARS-CoV-2 NC_045512.2            (1) ------------------------------------------------------------------------------------------------
```

```
                                                                                                    Section 435
                           (41665) 41665         41679       41690       41701       41712       41723       41734       41745      41760
          T4 NC_000866.4   (41665) ATTCATATGTTAATAATACCACGGTTTGAACTTGTTGTAAACAACTTTGTGAAAAATATTTTAGGGAATGATAAGAAAGGAACGATAGCTTAGAAT
SARS-CoV-2 NC_045512.2         (1) ------------------------------------------------------------------------------------------------
                                                                                                    Section 436
                           (41761) 41761 41767       41778       41789       41800       41811       41822       41833       41844  41856
          T4 NC_000866.4   (41761) GGTAATATACAGAATGTGAGAAAGAAAGGCCCAGAGGGCCCGTCTTAATCTTCTATGATATCTCTATCATATCCAAGTGAAATGAGAGTTTCTTTG
SARS-CoV-2 NC_045512.2         (1) ------------------------------------------------------------------------------------------------
                                                                                                    Section 437
                           (41857) 41857   41866       41877       41888       41899       41910       41921       41932            41952
          T4 NC_000866.4   (41857) AAGTGTTTAATGTTCTTTTGTCTAGAATCATTAATGAAAATGACTGGATAACGAATGTTAAGAGATGTGAATCCAGCGCGTTTAGCAAGAGATACA
SARS-CoV-2 NC_045512.2         (1) ------------------------------------------------------------------------------------------------
                                                                                                    Section 438
                           (41953) 41953      41965      41976       41987       41998       42009       42020       42031            42048
          T4 NC_000866.4   (41953) ATCAGCGGACGATCATACTCAATCTTACCATTATTTGTAAGAACTTTATAGAAAGTAAAAGGAGCATTGAGCTCCTTTAGAAGTTTTGTAACTGAT
SARS-CoV-2 NC_045512.2         (1) ------------------------------------------------------------------------------------------------
                                                                                                    Section 439
                           (42049) 42049          42064      42075       42086       42097       42108       42119       42130      42144
          T4 NC_000866.4   (42049) TGACATCCAGGACAACGACCTACTTCATCTGGAATTCCATAGACTTCAATCTTATTTTGTTTCACAACTATTCCTTACTAAGAGCAGCATTCAGCT
SARS-CoV-2 NC_045512.2         (1) ------------------------------------------------------------------------------------------------
                                                                                                    Section 440
                           (42145) 42145  42152      42163       42174       42185       42196       42207       42218       42229  42240
          T4 NC_000866.4   (42145) TGTTAGTAATTTTATCCAGACGCTCATTGAATTCACTAGAAGATAAGCCCTTTTCTGGAGAAATCAAACTAATCACGAAAAATATAGCAATAAAAG
SARS-CoV-2 NC_045512.2         (1) ------------------------------------------------------------------------------------------------
                                                                                                    Section 441
                           (42241) 42241     42251       42262       42273       42284       42295       42306       42317            42336
          T4 NC_000866.4   (42241) GAATAAGGAAAATAGCTCCAACTGCCATAAACAGAAAGAATGTTACAGTTGTAAGAAAATCAGCTAAACCTTTACGAAATTTATACATTTACATTT
SARS-CoV-2 NC_045512.2         (1) ------------------------------------------------------------------------------------------------
```

```
                                                                                                                                    Section 442
                         (42337) 42337        42350      42361      42372      42383      42394      42405      42416          42432
        T4 NC_000866.4   (42337) ACCTTTAATTGATTAACCAAGCATTGATAAGCACTAAACTATACTGCGAATAAAATTCTGGACCAAAATGAAAATCATATCATTTATAGTATCCAT
  SARS-CoV-2 NC_045512.2     (1) ------------------------------------------------------------------------------------------------
                                                                                                                                    Section 443
                         (42433) 42433 42438      42449      42460      42471      42482      42493      42504      42515          42528
        T4 NC_000866.4   (42433) AATGTAATTCAATTTAATCATGTTTCCACACCCCATCGGTATTTGACCAAAGTCGCTGATTATCTGATCCTCGCCACAGCTTTTTGGTCGGAAGAT
  SARS-CoV-2 NC_045512.2     (1) ------------------------------------------------------------------------------------------------
                                                                                                                                    Section 444
                         (42529) 42529   42537      42548      42559      42570      42581      42592      42603                42624
        T4 NC_000866.4   (42529) TTTTCTCATACTTCCCATCAATAATAACATCAACATATTTAAGCATTTCTAGTTGTTTAATATCTTCAAACTTATATCCTGTCCACAACCAGATAT
  SARS-CoV-2 NC_045512.2     (1) ------------------------------------------------------------------------------------------------
                                                                                                                                    Section 445
                         (42625) 42625      42636      42647      42658      42669      42680      42691      42702                42720
        T4 NC_000866.4   (42625) CTTTCTCCGGAAATCTTGCTTTAACCCAAGAAACTAAATTTGAAATCTCTTCTCGGTTCTGTGGATAAAGTGGGTCACCGCCGGTTAAGGTAAGGC
  SARS-CoV-2 NC_045512.2     (1) ------------------------------------------------------------------------------------------------
                                                                                                                                    Section 446
                         (42721) 42721         42735      42746      42757      42768      42779      42790      42801          42816
        T4 NC_000866.4   (42721) CTTGGATATACGATTTGCTTAAATGGGACGCAAGTTCTTTAACGGTATTCATAGTGAATAACTGTCCGTTACGAGCATTCCAAGTACTACGATTAT
  SARS-CoV-2 NC_045512.2     (1) ------------------------------------------------------------------------------------------------
                                                                                                                                    Section 447
                         (42817) 42817 42823      42834      42845      42856      42867      42878      42889      42900          42912
        T4 NC_000866.4   (42817) AACAACCTTCGCATTTATGCAAACATCCAGTGACGAAGAGAACGACCCTACATCCAGGGCCGTTAACGAAATCGCATGGATAAATTCTATCATAAT
  SARS-CoV-2 NC_045512.2     (1) ------------------------------------------------------------------------------------------------
                                                                                                                                    Section 448
                         (42913) 42913    42922      42933      42944      42955      42966      42977      42988                  43008
        T4 NC_000866.4   (42913) TCATCTTTATATTTCCAAGTATATCCCTTATGAATAGATTGAATTCCTTTGCAACATTTATAAACAGCAGAATGAAAAAATCCTGCATTTCTTATT
  SARS-CoV-2 NC_045512.2     (1) ------------------------------------------------------------------------------------------------
```

```
                                                                                                   Section 449
                          (43009) 43009       43021      43032      43043      43054      43065      43076      43087            43104
           T4 NC_000866.4 (43009) TCAGTTGCTCCAGTTAGTTCTATTGTATTTCCGTCTGGTGAATAGCCAATAACCGTTTTGGTGTTAAAGCGATTTCTAGAACTATTTTTGATATGC
SARS-CoV-2 NC_045512.2        (1) ------------------------------------------------------------------------------------------------
                                                                                                   Section 450
                          (43105) 43105          43120      43131      43142      43153      43164      43175      43186         43200
           T4 NC_000866.4 (43105) TCTTTATGATTAGTAGACAAAGTTCTTCCTGTTAGCCCATCAGATATCCTTTTTCCAAAATCATCAGGAAGAGTTACTTTAGACCTAGCTATGCTC
SARS-CoV-2 NC_045512.2        (1) ------------------------------------------------------------------------------------------------
                                                                                                   Section 451
                          (43201) 43201  43208      43219      43230      43241      43252      43263      43274      43285  43296
           T4 NC_000866.4 (43201) ATTAGCTTCTTAGTTTTATCGCTTCTTTTTGCGCCACGCTGATTTTCAAATTTCTTAGCTTTTGCATACGCGTATTGTGAAGAAGTAACTTTTCTA
SARS-CoV-2 NC_045512.2        (1) ------------------------------------------------------------------------------------------------
                                                                                                   Section 452
                          (43297) 43297     43307      43318      43329      43340      43351      43362      43373            43392
           T4 NC_000866.4 (43297) TGCTTACTATTACTCATTATCCAATATGCATCATACATCTTTCCACCATATATTTAGCCAATAAATGGTGAGCAGTATAGTGGGCCTTATAAGTT
SARS-CoV-2 NC_045512.2        (1) ------------------------------------------------------------------------------------------------
                                                                                                   Section 453
                          (43393) 43393        43406      43417      43428      43439      43450      43461      43472           43488
           T4 NC_000866.4 (43393) AAAAATACTAAGTTGTCTAAATCATCTGAACCACCCATACTTCTTGGAATTATATGATGAAGTTCATACCCGGCTTTAGTTTGACGGGGCTTAGAT
SARS-CoV-2 NC_045512.2        (1) ------------------------------------------------------------------------------------------------
                                                                                                   Section 454
                          (43489) 43489 43494      43505      43516      43527      43538      43549      43560      43571    43584
           T4 NC_000866.4 (43489) GGGTGTTCAGCAGAATTAACTAGATTTGAATAGATTCTGTCATAATTCATTGGTGCTTAACCCTATGCATGATTTCTTTATTTTTACCGAGATTAA
SARS-CoV-2 NC_045512.2        (1) ------------------------------------------------------------------------------------------------
                                                                                                   Section 455
                          (43585) 43585   43593      43604      43615      43626      43637      43648      43659                43680
           T4 NC_000866.4 (43585) ATCCGCGTTCGTTCGGATTTCCCAAATAACCACATGTTCTTCTTATGGTATTCATCTTTTTAGGATCAGTTTCTCCACAAATAGAACAAACAAATC
SARS-CoV-2 NC_045512.2        (1) ------------------------------------------------------------------------------------------------
```

```
                                                                                                       Section 456
                        (43681) 43681      43692      43703      43714      43725      43736      43747      43758          43776
T4 NC_000866.4          (43681) CGTTTTCAGTAGGAGTCATTTCATGGGTACTTCCACATGTAAAACATTTATCTACTGGCATATTAACACCAAAATAATCTAAATGCTGTGCAGCAT
SARS-CoV-2 NC_045512.2      (1) ------------------------------------------------------------------------------------------------
                                                                                                       Section 457
                        (43777) 43777         43791      43802      43813      43824      43835      43846      43857          43872
T4 NC_000866.4          (43777) AATCCCACACGGCCTCAAGACCTTTTAAGTTATTTTTCATATCAGGAAGTTCAACATAAGAAATGTGACCACCTGTCGCAATGAAATGATATGGCG
SARS-CoV-2 NC_045512.2      (1) ------------------------------------------------------------------------------------------------
                                                                                                       Section 458
                        (43873) 43873 43879      43890      43901      43912      43923      43934      43945      43956       43968
T4 NC_000866.4          (43873) CTTCACGAGAAATCTTTTCAAACGGAGTAATATTTTCTTCTACTGAAACATGGAAACTGTTAGTGTACCAGCCTTTATCGGTAACATCTTTTACGC
SARS-CoV-2 NC_045512.2      (1) ------------------------------------------------------------------------------------------------
                                                                                                       Section 459
                        (43969) 43969    43978      43989      44000      44011      44022      44033      44044               44064
T4 NC_000866.4          (43969) TTCCATATTTTTCTGTATCGAGTTTACAGAAGCGATAACACAGGTTTTCAGCAGGAGTCGAATATAAACTAAAAGCAAATCCGGTTCTTTCAGTCC
SARS-CoV-2 NC_045512.2      (1) ------------------------------------------------------------------------------------------------
                                                                                                       Section 460
                        (44065) 44065       44077      44088      44099      44110      44121      44132      44143            44160
T4 NC_000866.4          (44065) ACTGTTTAAGATGAGCATTCATTTTAGTTAAAATTTCTCGTCCAATATCACGACCGACAAGAATATTCAATTCGTGAATACCAATGTATCCTAAAG
SARS-CoV-2 NC_045512.2      (1) ------------------------------------------------------------------------------------------------
                                                                                                       Section 461
                        (44161) 44161          44176      44187      44198      44209      44220      44231      44242          44256
T4 NC_000866.4          (44161) ACACTGAACTTCTACCGTTTTTAAATAACTCAATTATGTCGTCATCAGGTTTAAGACGAACCCCGAATGCACCTTCTTGGTAAAGAATAGGAGCAA
SARS-CoV-2 NC_045512.2      (1) ------------------------------------------------------------------------------------------------
                                                                                                       Section 462
                        (44257) 44257  44264      44275      44286      44297      44308      44319      44330      44341      44352
T4 NC_000866.4          (44257) CAGTAGCTTTAACTCCTTTTAAGGAACTAATTCTACACATCAAAGCTTCAAAACATAAATCCATTCGTTCATTAAATAGCTCAACAAATTTCTGTT
SARS-CoV-2 NC_045512.2      (1) ------------------------------------------------------------------------------------------------
```

```
                                                                                                        Section 463
                       (44353) 44353     44363      44374      44385      44396      44407      44418      44429              44448
T4 NC_000866.4         (44353) CATTGAACTGTGTTCCAATATAAGAATCTAACGCGATGCGAGGAAGATTCAGTGTTACAACACCAAGATTATTGCGTCCATCAAGAATTTCATTGC
SARS-CoV-2 NC_045512.2     (1) ------------------------------------------------------------------------------------------------
                                                                                                        Section 464
                       (44449) 44449        44462      44473      44484      44495      44506      44517      44528           44544
T4 NC_000866.4         (44449) CAGTCGAATCTTTCCATACGCTCAAGAAACTACGGCAACCCATCGGAGAAACAGGAACAGATGAACCAGTGATAGCTTTATTGTTCTTAGCTGAAA
SARS-CoV-2 NC_045512.2     (1) ------------------------------------------------------------------------------------------------
                                                                                                        Section 465
                       (44545) 44545 44550      44561      44572      44583      44594      44605      44616      44627       44640
T4 NC_000866.4         (44545) TAATATCAGGATACATCCTTTTGCTTGCGCACTCTAGAGCAAGCTGTTTAATATCATAGTTCGGATCGTCTTTATAAAGATTAACACCTTCTTCAA
SARS-CoV-2 NC_045512.2     (1) ------------------------------------------------------------------------------------------------
                                                                                                        Section 466
                       (44641) 44641   44649      44660      44671      44682      44693      44704      44715                 44736
T4 NC_000866.4         (44641) CGAACATAACAAGCTTAGGGAAAATAGGAGTTATCCCATCACGACCAAGACCTTTAATACGATTTTTCAGAATTGCTTTCTGAATCATTCGTTCAG
SARS-CoV-2 NC_045512.2     (1) ------------------------------------------------------------------------------------------------
                                                                                                        Section 467
                       (44737) 44737      44748      44759      44770      44781      44792      44803      44814              44832
T4 NC_000866.4         (44737) TCCAGTCAGTTCCCGTACCAAATGTAATTGTTACAAAAGGAGTCTGTCCGTTTGAACTGAATAACGTGTTCACTTCATCAATGTGTTCAGTAGAGT
SARS-CoV-2 NC_045512.2     (1) ------------------------------------------------------------------------------------------------
                                                                                                        Section 468
                       (44833) 44833         44847      44858      44869      44880      44891      44902      44913           44928
T4 NC_000866.4         (44833) TCGCTACTTCTCTACCCGTTTATATGAAACAATGTAATTTGGATAATTGTCATCTAAACACAAATCTTTAATACGACTTGGGTGGAGTTTTAAATC
SARS-CoV-2 NC_045512.2     (1) ------------------------------------------------------------------------------------------------
                                                                                                        Section 469
                       (44929) 44929  44935      44946      44957      44968      44979      44990      45001      45012       45024
T4 NC_000866.4         (44929) TTTAGCAGCATCCACAAATGATTTATAGATATTGTCATTTATTTTAACATATTCTGGGACGGGATGAATTGTTATTTTTATATCTATAACATTTGG
SARS-CoV-2 NC_045512.2     (1) ------------------------------------------------------------------------------------------------
```

Section 470

```
                        (45025) 45025    45034      45045      45056      45067      45078      45089      45100               45120
T4 NC_000866.4          (45025) ATGATTACTAACCTGTTCTTCAGAGCATTTAAGAAATTTTGCAGCTGATTTAAAACTTCTAAATTTATTTCCGGATTTTAAAGCTATAGAAACAGT
SARS-CoV-2 NC_045512.2  (1)     ------------------------------------------------------------------------------------------------
```

Section 471

```
                        (45121) 45121       45133      45144      45155      45166      45177      45188      45199            45216
T4 NC_000866.4          (45121) CTTTTTAGCCGTTCTACTACCAATATTATTTTTAACATGAGCTTCACTTCTACTATTATTTTTATAATGCTCAATCAATTTCTCTCGTATCTTATC
SARS-CoV-2 NC_045512.2  (1)     ------------------------------------------------------------------------------------------------
```

Section 472

```
                        (45217) 45217          45232      45243      45254      45265      45276      45287      45298         45312
T4 NC_000866.4          (45217) TTTATGTTTTAAAGATAAGATTTTACCTTTATGGGCATTACTAAGTTTTTGCTTATGTTCTTCTGAATCCGGATATTTGTTAAATTTATATCCACC
SARS-CoV-2 NC_045512.2  (1)     ------------------------------------------------------------------------------------------------
```

Section 473

```
                        (45313) 45313  45320      45331      45342      45353      45364      45375      45386      45397      45408
T4 NC_000866.4          (45313) TATAGATTTATTAAGAATAAATTCGTTATTAAAATATTTCCTAATAAGCATTTCTTCATGTTTAAGGGCCGATTCATAAGAATCAAAAACTTGAAG
SARS-CoV-2 NC_045512.2  (1)     ------------------------------------------------------------------------------------------------
```

Section 474

```
                        (45409) 45409     45419      45430      45441      45452      45463      45474      45485              45504
T4 NC_000866.4          (45409) AATTATCCACTTAGCTTTATAATCTTTAAGCTTTTCTTTAACAAGCTTAGATGACGAATTGTATTCTTTCCAATTTGTATCTTTACCATATATAGT
SARS-CoV-2 NC_045512.2  (1)     ------------------------------------------------------------------------------------------------
```

Section 475

```
                        (45505) 45505        45518      45529      45540      45551      45562      45573      45584            45600
T4 NC_000866.4          (45505) TTTGAATTTTTTAAAACCTATATAAAAAGACTTATCAGGAAATCTTACCATATAGGTAAATGCTACAGAATTGGCAATTTTTAAGCTTTTTCTTAA
SARS-CoV-2 NC_045512.2  (1)     ------------------------------------------------------------------------------------------------
```

Section 476

```
                        (45601) 45601 45606      45617      45628      45639      45650      45661      45672      45683        45696
T4 NC_000866.4          (45601) TTTCCATTTCATCGTTTCACCTCGTATTCATATTTATACGAGGATAAACAGCTGCATGTCACCATGCAGTTCAGACTATATCTTCAACTCTTAGAG
SARS-CoV-2 NC_045512.2  (1)     ------------------------------------------------------------------------------------------------
```

```
                                                                                                          Section 477
                         (45697) 45697   45705      45716      45727      45738      45749      45760      45771                45792
          T4 NC_000866.4 (45697) TTGTCTGCCGTTTCGGGTCGCTTGACCCTACTCCCTTACATTCATCAGGGATAGTCGTTGGGCATTTACAGCTACTGCTGATTTAGCAACGGATTG
    SARS-CoV-2 NC_045512.2     (1) ------------------------------------------------------------------------------------------------
                                                                                                          Section 478
                         (45793) 45793      45804      45815      45826      45837      45848      45859      45870             45888
          T4 NC_000866.4 (45793) TCTCAGTGAGAGTTTCCCGTTTTAGGCAGATTTTACATGAGCTTGACTTACGTTAACTCATAAGCTTGGAATGCATCGTATACGTCTTTTTCTGTT
    SARS-CoV-2 NC_045512.2     (1) ------------------------------------------------------------------------------------------------
                                                                                                          Section 479
                         (45889) 45889         45903      45914      45925      45936      45947      45958      45969          45984
          T4 NC_000866.4 (45889) TTAGATTGAGCATAATTCAACGCATCAGCGATTTGCCATTTTTCTGCATCCTCAATATGTTTTGCATAGGTGCGTTTAACATAAGGAGAAAGTACT
    SARS-CoV-2 NC_045512.2     (1) ------------------------------------------------------------------------------------------------
                                                                                                          Section 480
                         (45985) 45985 45991      46002      46013      46024      46035      46046      46057      46068       46080
          T4 NC_000866.4 (45985) TTATCTACATTCGCAAAAGTCGTTCCGCCGTATTGGTGAGAAGCAACTTGCGCAGTAATTTGTGCCATAATTGCAGTAGCAACTCCAATTGATTTA
    SARS-CoV-2 NC_045512.2     (1) ------------------------------------------------------------------------------------------------
                                                                                                          Section 481
                         (46081) 46081    46090      46101      46112      46123      46134      46145      46156               46176
          T4 NC_000866.4 (46081) GGAGTTTCAATCTGCGCATTACCAAGCTTAAATCCGTTTTCAAGCATTCCTTTTAAATCTACTAAACAGCAATTAGTAAATGGAAGAGCAGGGGAA
    SARS-CoV-2 NC_045512.2     (1) ------------------------------------------------------------------------------------------------
                                                                                                          Section 482
                         (46177) 46177       46189      46200      46211      46222      46233      46244      46255            46272
          T4 NC_000866.4 (46177) TAATCAATATCATGCACGTGAATAATTCCGCTTTCATGCGCTTTCATAATAAAAGACGGGACCATATTTTTGGCAATGTGTTTAGACACAATACCA
    SARS-CoV-2 NC_045512.2     (1) ------------------------------------------------------------------------------------------------
                                                                                                          Section 483
                         (46273) 46273          46288      46299      46310      46321      46332      46343      46354         46368
          T4 NC_000866.4 (46273) GCCATAAGGTCCCGTTGAGTTGGAAAAACACGAGAATCTTTATTAGCATTCTCGTTTAAAAGGTCTTTATTAGTTTTATGAATTAATCCTTCAATT
    SARS-CoV-2 NC_045512.2     (1) ------------------------------------------------------------------------------------------------
```

```
Section 484
                             (46369) 46369  46376      46387      46398      46409      46420      46431      46442      46453      46464
T4 NC_000866.4               (46369) TCTTTTTCAATTGTCATTTTAAACTCTTTCTAAGCTGCTTCTTGAATGAAGCTATTAATTGTGTTTTGGTGTCAGATTCATTATATTCAAATCCTC
SARS-CoV-2 NC_045512.2           (1) ------------------------------------------------------------------------------------------------

Section 485
                             (46465) 46465     46475      46486      46497      46508      46519      46530      46541              46560
T4 NC_000866.4               (46465) TTTGAAGCATCTCGGCCATCATTTCCTCTTTTCCTAAACGAGAAAATTCCTTTGATTTATCTCCAACAAAGTTAGGGTGAATATTATTTTGGGTGT
SARS-CoV-2 NC_045512.2           (1) ------------------------------------------------------------------------------------------------

Section 486
                             (46561) 46561        46574      46585      46596      46607      46618      46629      46640           46656
T4 NC_000866.4               (46561) AATCGGATTTTAAATAAGTAAGTAAATTTTCTAACCATTCAAGATAATCAACACCTTGTCCCTTTAAGCCAGAACGATTAAATTTATGCTTCATTT
SARS-CoV-2 NC_045512.2           (1) ------------------------------------------------------------------------------------------------

Section 487
                             (46657) 46657 46662      46673      46684      46695      46706      46717      46728      46739      46752
T4 NC_000866.4               (46657) GACCTTCTGCAGCATTGCATAGATTACAAAGCAATCCACGCACCTTTCCTGCTTTTGGTCCATTTAATTCATGGTCATGGTCGAGGTGATTAGCTT
SARS-CoV-2 NC_045512.2           (1) ------------------------------------------------------------------------------------------------

Section 488
                             (46753) 46753   46761      46772      46783      46794      46805      46816      46827              46848
T4 NC_000866.4               (46753) GAACATCAGGATTTAGTTCTCGTTGGCAAATTAAGCATTTACCGTTTTGTGCATCATAAAATTTCTGTTTTTCTTCTTTGTATAATTTGCCAGTCA
SARS-CoV-2 NC_045512.2           (1) ------------------------------------------------------------------------------------------------

Section 489
                             (46849) 46849      46860      46871      46882      46893      46904      46915      46926              46944
T4 NC_000866.4               (46849) ATAACATAATAAACCCTTACCTTAAATAGATAAGGGTATTTATTATTTTCAAGTATTGTAAAACATTTGATGCAATCGCTTATATTGCTGAATCAT
SARS-CoV-2 NC_045512.2           (1) ------------------------------------------------------------------------------------------------

Section 490
                             (46945) 46945         46959      46970      46981      46992      47003      47014      47025           47040
T4 NC_000866.4               (46945) TCGGTCAGAAAAAGAAATTTGAGTTTCAAGCCATTCAATGTACTCTGCGGCAGCTTGCATTAAATTTCCTTCATAGCCATCGTTATTTTCTTGTGC
SARS-CoV-2 NC_045512.2           (1) ------------------------------------------------------------------------------------------------
```

Section 491

```
                        (47041) 47041 47047      47058      47069      47080      47091      47102      47113      47124      47136
T4 NC_000866.4          (47041) AGCTAATTTAGCTAATGCGTATGAAATACGTTCACCTTGAAAATCGGCTTTAGGCTTCTGAACAACTTGATTAGTTCGCTCTACAACTTCTTCAAT
SARS-CoV-2 NC_045512.2      (1) ------------------------------------------------------------------------------------------------
```

Section 492

```
                        (47137) 47137    47146      47157      47168      47179      47190      47201      47212               47232
T4 NC_000866.4          (47137) TTCGCCATTTTCTACTGATTCAGTATTCCACAAACACCAATACGTAATTGGCTTATCGTAGATGTTAATAATCTTTCCATCAGAAAGTTCAATTTC
SARS-CoV-2 NC_045512.2      (1) ------------------------------------------------------------------------------------------------
```

Section 493

```
                        (47233) 47233       47245      47256      47267      47278      47289      47300      47311            47328
T4 NC_000866.4          (47233) AATAATTCCAGTGTCAGGCTCTATATCATCTTCACATTCTTTTACAAGTTCACGAACTTTAAAGACAGTACCTGCACTAAGTTCCGGCCAGTAATT
SARS-CoV-2 NC_045512.2      (1) ------------------------------------------------------------------------------------------------
```

Section 494

```
                        (47329) 47329          47344      47355      47366      47377      47388      47399      47410         47424
T4 NC_000866.4          (47329) ACACAGCCCTGTATCAGCACGATTAATTCTAAACCATTTATCTACTGTAATCATGTCCCATCTCCATATCAATTAAGTCATTTATCGTTGGTTCAT
SARS-CoV-2 NC_045512.2      (1) ------------------------------------------------------------------------------------------------
```

Section 495

```
                        (47425) 47425  47432      47443      47454      47465      47476      47487      47498      47509      47520
T4 NC_000866.4          (47425) TATACACCGTTTCTTCATCAGTGTAAACCGGTTCTTCCGGCTCTGGCTCTACAGTTTCCCATCTAGCCGCCCACCAAGGTTTTATAGCGTAATCCA
SARS-CoV-2 NC_045512.2      (1) ------------------------------------------------------------------------------------------------
```

Section 496

```
                        (47521) 47521     47531      47542      47553      47564      47575      47586      47597              47616
T4 NC_000866.4          (47521) TTCTCGTACTGTCTGAGTTACTACACGTTCTCGAAGCTCAACCAGTTCAACAGTAGGAACTGCATAATACCAGTCAGAATGGTAAGAACCTGAGCG
SARS-CoV-2 NC_045512.2      (1) ------------------------------------------------------------------------------------------------
```

Section 497

```
                        (47617) 47617        47630      47641      47652      47663      47674      47685      47696           47712
T4 NC_000866.4          (47617) AGATTCATTTACAGCCACATGTACATTATGTTTTGGACTAAAATAAACACACTGACGATATTGGCATTTACCATCTTGCACCCAGTCTTCTATTTC
SARS-CoV-2 NC_045512.2      (1) ------------------------------------------------------------------------------------------------
```

```
                                                                                                                     Section 498
                            (47713) 47713 47718      47729      47740      47751      47762      47773      47784      47795      47808
T4 NC_000866.4              (47713) GATAGGTTTAAGATAGTCGGAAAAATCGAAATCATAGTTTTCCGAAAATGCATCATGGTCTTCAATGATTTCGCTCAGAACTGCATCAACATCTAA
SARS-CoV-2 NC_045512.2          (1) ------------------------------------------------------------------------------------------------
                                                                                                                     Section 499
                            (47809) 47809   47817      47828      47839      47850      47861      47872      47883                47904
T4 NC_000866.4              (47809) TTTATTTTCAATATTCATTTTTCACACCACCAACTGCTTACTTCAAAATCGGGTTCGCCATGTTCCCAGAACCAGGGGCCGATAGGAATCCACTTA
SARS-CoV-2 NC_045512.2          (1) ------------------------------------------------------------------------------------------------
                                                                                                                     Section 500
                            (47905) 47905      47916      47927      47938      47949      47960      47971      47982             48000
T4 NC_000866.4              (47905) CCTTCATCGGTGACGTCATATTCTTCGGATTTAAGCCCAACGTATCGCCATTCGACTTCATAAGCGGTACCACCGATTACAACTGTGTTCTCGCCA
SARS-CoV-2 NC_045512.2          (1) ------------------------------------------------------------------------------------------------
                                                                                                                     Section 501
                            (48001) 48001         48015      48026      48037      48048      48059      48070      48081          48096
T4 NC_000866.4              (48001) TACGGGTTAGACACAACAAAGTCTATACCTTCTGCTTTAGCTAACCAAATCATGTATTCATAGAGTTTATATTCCAGGTCGCCTTCTGTTCCATCG
SARS-CoV-2 NC_045512.2          (1) ------------------------------------------------------------------------------------------------
                                                                                                                     Section 502
                            (48097) 48097 48103      48114      48125      48136      48147      48158      48169      48180      48192
T4 NC_000866.4              (48097) CCTAGAAAAATAATTTGTTCATTTAATTCTATCATTTAAAGTATTCCCGCAATTGGTCAAATCCACCAATATGACTTCCATCAGGAGCAAATACCT
SARS-CoV-2 NC_045512.2          (1) ------------------------------------------------------------------------------------------------
                                                                                                                     Section 503
                            (48193) 48193    48202      48213      48224      48235      48246      48257      48268               48288
T4 NC_000866.4              (48193) GAGGCATTGTTAAGCCGATTTGAGTATCACGACCTAGTTTAGTCAGAAGCTCAGCAATTTTCTCATCATCAAAAACACCTTTTTCCGGCATAATGT
SARS-CoV-2 NC_045512.2          (1) ------------------------------------------------------------------------------------------------
                                                                                                                     Section 504
                            (48289) 48289       48301      48312      48323      48334      48345      48356      48367            48384
T4 NC_000866.4              (48289) TGATAAATTCAAACGGCTGTTTCTTCACAGTCAAAAGACGTTTTGCATTATCGCAATACACACATTTATGAATGTTGCTATCATAACCATATACTT
SARS-CoV-2 NC_045512.2          (1) ------------------------------------------------------------------------------------------------
```

```
                                                                                                                      Section 505
                          (48385) 48385          48400     48411      48422      48433      48444      48455      48466       48480
         T4 NC_000866.4   (48385) TAAACATATTATTCCTTAATTCCTAGTACTTGTTTAAAAGTCTCGTCGTAATCAAGACTTTGGCCCGTTTGTTCTTTATGCTTGTATATAATATCA
SARS-CoV-2 NC_045512.2        (1) ------------------------------------------------------------------------------------------------
                                                                                                                      Section 506
                          (48481) 48481  48488      48499      48510      48521      48532      48543      48554      48565   48576
         T4 NC_000866.4   (48481) CTTACTTCTGATAGCATATTTTTATATGAACGAGTTAAAGCAGATTTAAGCACGCTGTATCTATCAGGAAATTTACCGTGTTCATTATAATAAGCT
SARS-CoV-2 NC_045512.2        (1) ------------------------------------------------------------------------------------------------
                                                                                                                      Section 507
                          (48577) 48577    48587      48598      48609      48620      48631      48642      48653            48672
         T4 NC_000866.4   (48577) ATTGCTAATTCACGGACTGCCTTTTCAGCAGTCTCCATATATTCTTTACGCTTTGTCATTTTCTTCTCGGTCAAATCGGTGTTTACAATGGCGACA
SARS-CoV-2 NC_045512.2        (1) ------------------------------------------------------------------------------------------------
                                                                                                                      Section 508
                          (48673) 48673        48686      48697      48708      48719      48730      48741      48752       48768
         T4 NC_000866.4   (48673) TTTATAACGAAGATTATTAGTCTGCCAATGGACCAATTGCACCTGTTCAGTTCCACATTCAGGGCAATTAGGAACGTTTTTAGAAGCCCTTTCGCG
SARS-CoV-2 NC_045512.2        (1) ------------------------------------------------------------------------------------------------
                                                                                                                      Section 509
                          (48769) 48769 48774      48785      48796      48807      48818      48829      48840      48851    48864
         T4 NC_000866.4   (48769) ACGTTCAACCATGACCATTACAGAATCCCAATTAACAGGAGGGCTATAATCATCACAACCATAAATCTTTCCACGCATTTCCAGATCGTCTTCTTC
SARS-CoV-2 NC_045512.2        (1) ------------------------------------------------------------------------------------------------
                                                                                                                      Section 510
                          (48865) 48865   48873      48884      48895      48906      48917      48928      48939            48960
         T4 NC_000866.4   (48865) ACCAGCCATAATAATTTTAATTAGACTAGAATTACTTGAAGCTGCAATGTCTTCTAATAGACGCTTTTTCATTTCAATACCTCAATAGCATTACGT
SARS-CoV-2 NC_045512.2        (1) ------------------------------------------------------------------------------------------------
                                                                                                                      Section 511
                          (48961) 48961      48972      48983      48994      49005      49016      49027      49038            49056
         T4 NC_000866.4   (48961) AAACCATTTGCTTTGGCGGTTAAATCCTTAAGAACTTTAGTATGCTCTTCAATCTGTTTTTCAACTTCAATCAAACGGGTACTGAGATATTCGCGT
SARS-CoV-2 NC_045512.2        (1) ------------------------------------------------------------------------------------------------
```

```
                                                                                                   Section 512
                          (49057) 49057          49071      49082      49093      49104      49115      49126      49137          49152
         T4 NC_000866.4   (49057) TCTTCTTTCATAGTATCATTCCCATGATTGGGCTTTGGCGTAGGTTTAAATTTATTAGGGTCCTTTAACTTAATAACAACTTTAGTTTCAAACAGT
SARS-CoV-2 NC_045512.2        (1) ------------------------------------------------------------------------------------------------
                                                                                                   Section 513
                          (49153) 49153 49159      49170      49181      49192      49203      49214      49225      49236       49248
         T4 NC_000866.4   (49153) GAAAGACCATAATTCGTATTATCATCAAAGGATTCAACTACGTCCATTTTAGACAATAACGTACGAAGTTTATAAGTCAAAGAACGAATCGTTTTA
SARS-CoV-2 NC_045512.2        (1) ------------------------------------------------------------------------------------------------
                                                                                                   Section 514
                          (49249) 49249    49258      49269      49280      49291      49302      49313      49324               49344
         T4 NC_000866.4   (49249) TTATGTTTATTAGCTTCACATGGTTTCATATATGGAAGATTTTTATATATTTCATGCGGAGCACAAACAATAATTAACAATTCAAATGGTCCATTC
SARS-CoV-2 NC_045512.2        (1) ------------------------------------------------------------------------------------------------
                                                                                                   Section 515
                          (49345) 49345       49357      49368      49379      49390      49401      49412      49423            49440
         T4 NC_000866.4   (49345) TGAGATGATGGAATGAATTTAGCCGTAACCCAATCTTTTACATTTACATTAATAGTACGATCATCGCCATGACAAAACAGTCTACGAGAATGCTTA
SARS-CoV-2 NC_045512.2        (1) ------------------------------------------------------------------------------------------------
                                                                                                   Section 516
                          (49441) 49441          49456      49467      49478      49489      49500      49511      49522          49536
         T4 NC_000866.4   (49441) AAAAGCATATTCACATTATTATTAAATTCGTGTGAAAACTCACTGGCAAATTTTTCTTTACTATCTTTAGAAAACCATTCTTCTAAATAAGTTCTT
SARS-CoV-2 NC_045512.2        (1) ------------------------------------------------------------------------------------------------
                                                                                                   Section 517
                          (49537) 49537  49544      49555      49566      49577      49588      49599      49610      49621     49632
         T4 NC_000866.4   (49537) TTCACGTCGTTAATAATATTAAATGTAACAACATTATCATTTGATACATTAGTCTCAATGTAGCTTGGATACATAAATTTTTTAACATTATTAACA
SARS-CoV-2 NC_045512.2        (1) ------------------------------------------------------------------------------------------------
                                                                                                   Section 518
                          (49633) 49633     49643      49654      49665      49676      49687      49698      49709              49728
         T4 NC_000866.4   (49633) GCTATCACAGAAGATAGTAATCGGGAACGTTTGAACCACTCGAGCAAAGAACCAAGAGAATGTGAATACGCATTTTGTGTATCTTTACAAATATGG
SARS-CoV-2 NC_045512.2        (1) ------------------------------------------------------------------------------------------------
```

```
                                                                                                             Section 519
                           (49729) 49729        49742      49753      49764      49775      49786      49797      49808          49824
         T4 NC_000866.4    (49729) TTTTCCCATCCAATATAGTCTAAAAAATCACGAAGAATATTATTGATTACTTCTCTATGTCCTTTCTGATAATCACGATGTTTAGTAATAAGACTG
SARS-CoV-2 NC_045512.2         (1) ------------------------------------------------------------------------------------------------
                                                                                                             Section 520
                           (49825) 49825 49830      49841      49852      49863      49874      49885      49896      49907      49920
         T4 NC_000866.4    (49825) TCAAAATAATCAATATAATGTTTACGAGTTTTCATGTTCTTCTCACTTGGTTAATGATTTATACTCCGAGCCATCCTTGGCTTTAAATTTTACTTA
SARS-CoV-2 NC_045512.2         (1) ------------------------------------------------------------------------------------------------
                                                                                                             Section 521
                           (49921) 49921   49929      49940      49951      49962      49973      49984      49995               50016
         T4 NC_000866.4    (49921) ATTAACTGCAAAGCTTGTTCTAGACGATCAAGACGATTAACGGATTCTTCCCAAATCTTTTTAGCCTGCTCATATTCTTTCTGCGCTTTATTAGAA
SARS-CoV-2 NC_045512.2         (1) ------------------------------------------------------------------------------------------------
                                                                                                             Section 522
                           (50017) 50017      50028      50039      50050      50061      50072      50083      50094              50112
         T4 NC_000866.4    (50017) ATTTCTAGAACTTCTTTATAAGCTTTTTCTAGTGCAATAACTTCTGGACGAATTTCTATCGGTTCAGGTGAATCATCAAGACATTCCATTAGTTCC
SARS-CoV-2 NC_045512.2         (1) ------------------------------------------------------------------------------------------------
                                                                                                             Section 523
                           (50113) 50113         50127      50138      50149      50160      50171      50182      50193          50208
         T4 NC_000866.4    (50113) TCAAGGGTAGTTTCTTCTTTAGGAGTATTCACAATTTCATCACATTTTTGTTGGTAAATTTCTTTACTAGTAGGTGAATAAGCACATTTCACTTCA
SARS-CoV-2 NC_045512.2         (1) ------------------------------------------------------------------------------------------------
                                                                                                             Section 524
                           (50209) 50209 50215      50226      50237      50248      50259      50270      50281      50292       50304
         T4 NC_000866.4    (50209) CGAAGGCTAATTACGAGTTTATATCCTTCCCAACCGCATATATTCTTAAAAGGATATGTATGAATATTTTCTACTGTTTCCATACAAAGTAATGCG
SARS-CoV-2 NC_045512.2         (1) ------------------------------------------------------------------------------------------------
                                                                                                             Section 525
                           (50305) 50305    50314      50325      50336      50347      50358      50369      50380               50400
         T4 NC_000866.4    (50305) GTCTCTAATTGACGAGTTATAGTGCTAGCAATTGAGAAAAATTTATTAATATTCTTTTGGTTAGTTTCTGGTGTAAAAAATCCATAATTCACAAAA
SARS-CoV-2 NC_045512.2         (1) ------------------------------------------------------------------------------------------------
```

Section 526

(50401) 50401 50413 50424 50435 50446 50457 50468 50479 50496
T4 NC_000866.4 (50401) ATAGATACTTTATTTTTATCAAGTTCATATTCTTTTATGATAATCATATCAGAAGCCAAAGGATGAATTTGACGATAATCGCCATAACATAATTTA
SARS-CoV-2 NC_045512.2 (1) --

Section 527

(50497) 50497 50512 50523 50534 50545 50556 50567 50578 50592
T4 NC_000866.4 (50497) GAAGCTGATTTTAGAATTTGCTTCTTGAAAAGTCTGAAATTACTAATCCAACGACGAGTAAAAATATTCTCAGGGTCTTCTTTATTATTGAGATGA
SARS-CoV-2 NC_045512.2 (1) --

Section 528

(50593) 50593 50600 50611 50622 50633 50644 50655 50666 50677 50688
T4 NC_000866.4 (50593) TAAGAATTAACATCACCGAACCAATTATATCCTACTACATCTTTAGTTCGTTTAATTTCTTTCCCAAAATTACCAAGAATATCCCGATTAACCAAA
SARS-CoV-2 NC_045512.2 (1) --

Section 529

(50689) 50689 50699 50710 50721 50732 50743 50754 50765 50784
T4 NC_000866.4 (50689) TATGAAAGAAGACGAGACTCTTTAAAGGTTTTCTTGATAACATCTTTATCTACACGGCTAGAAATTTTACGAATTTCATGAATAAATTTAGTAGAA
SARS-CoV-2 NC_045512.2 (1) --

Section 530

(50785) 50785 50798 50809 50820 50831 50842 50853 50864 50880
T4 NC_000866.4 (50785) GAAACAATACTTGCATCAACACTATTGAGCCACTGATTGATAATAGAAAGAACGCTATTTTGGCCAAACATCGGTATAGCTTTATCATCAATAACG
SARS-CoV-2 NC_045512.2 (1) --

Section 531

(50881) 50881 50886 50897 50908 50919 50930 50941 50952 50963 50976
T4 NC_000866.4 (50881) CTATTGAATGATTTGATATATTCGTTACGAGTCATATTAATCTCCTCAGTAGAAAGTAAGAACATTATACCACATCCTTGTGGCAAAGTAAACTAG
SARS-CoV-2 NC_045512.2 (1) --

Section 532

(50977) 50977 50985 50996 51007 51018 51029 51040 51051 51072
T4 NC_000866.4 (50977) TTCAGTGCATTTAGTGCATTGTTCAGTTTAGAACGTTGCTTTGTCAGATTTTGAACCCTTGACTGAGCTTGTTCTAACGCCTTTTCAGCTTCTAGC
SARS-CoV-2 NC_045512.2 (1) --

```
                                                                                                            Section 533
                    (51073) 51073      51084      51095      51106      51117      51128      51139      51150           51168
T4 NC_000866.4      (51073) ACTTCATTGGTCGCTTTAACTAATTCAGCATCTACTGCCTTAAGAGACTTCTCAATCGCATCCGCGTGCCATTTTTCAACAGGTTTAAGACTTGGA
SARS-CoV-2 NC_045512.2  (1) ------------------------------------------------------------------------------------------------
                                                                                                            Section 534
                    (51169) 51169         51183      51194      51205      51216      51227      51238      51249      51264
T4 NC_000866.4      (51169) TTTTCAACAGGAAGAAAATTCACTTTATTGAATTTCCATGCATCTTTATTACCTGAGCTATACATCCAAAATTTAGGATCGTTTGATAAGTAATTA
SARS-CoV-2 NC_045512.2  (1) ------------------------------------------------------------------------------------------------
                                                                                                            Section 535
                    (51265) 51265 51271      51282      51293      51304      51315      51326      51337      51348   51360
T4 NC_000866.4      (51265) GATAAATTTAAGTTATCTTGCACTTCTTGCTTTTTCTCTTGTGTGACTTCGTCCTTTCTCAAAAATTCATATTTAAATGAAACGATCATATCAAGT
SARS-CoV-2 NC_045512.2  (1) ------------------------------------------------------------------------------------------------
                                                                                                            Section 536
                    (51361) 51361    51370      51381      51392      51403      51414      51425      51436           51456
T4 NC_000866.4      (51361) TCATATGATGTTGTATCTAACTTAAATCGTTCAAAACGAGGTAAAATCTTAGATTGAACAGCAGCAACAACATCCATATACTTAAATGCTTCTGTC
SARS-CoV-2 NC_045512.2  (1) ------------------------------------------------------------------------------------------------
                                                                                                            Section 537
                    (51457) 51457       51469      51480      51491      51502      51513      51524      51535          51552
T4 NC_000866.4      (51457) AACTGCGATTTAAGGCATTCGCAAATTGAAAGAGAATTTTTTGTATTAGGTTTAAAGCTAATTCGTGCAGTTCTATTATTTTCTTTTAACGGTCTT
SARS-CoV-2 NC_045512.2  (1) ------------------------------------------------------------------------------------------------
                                                                                                            Section 538
                    (51553) 51553          51568      51579      51590      51601      51612      51623      51634         51648
T4 NC_000866.4      (51553) ACTTCCATCTGAAGAGTATAACCATCAAATTTCAGATTCTTTAAGTTAATGTCAGAGCCTTTTAATCGACTAGCAGTAGACAAAATTTGCTTTAAT
SARS-CoV-2 NC_045512.2  (1) ------------------------------------------------------------------------------------------------
                                                                                                            Section 539
                    (51649) 51649  51656      51667      51678      51689      51700      51711      51722      51733   51744
T4 NC_000866.4      (51649) TGTCTACGGAACAGAGCAATAAATCTCCATTCAATACGATAATGATTTGGATTGAAAAATCCAGATGATAAATCGACCTTACTTTGACCAACGCCT
SARS-CoV-2 NC_045512.2  (1) ------------------------------------------------------------------------------------------------
```

Section 540

(51745) 51745 51755 51766 51777 51788 51799 51810 51821 51840
T4 NC_000866.4 (51745) TGAACAAATAGAGGATTTTTATAATCAATAGTTTTACAGAAATCGGTGAGCTGTTCACGAGCAGTCATTTGAGTAATATACCCTGCTTTACTAAAA
SARS-CoV-2 NC_045512.2 (1) --

Section 541

(51841) 51841 51854 51865 51876 51887 51898 51909 51920 51936
T4 NC_000866.4 (51841) TTACGCACCCATTCACTGCTATTCGTATACTTAAAGTGATGAATAACACGATTAACTTTATCAGGGTCTAAATTATTTTCACACAAAAATGTCATA
SARS-CoV-2 NC_045512.2 (1) --

Section 542

(51937) 51937 51942 51953 51964 51975 51986 51997 52008 52019 52032
T4 NC_000866.4 (51937) ACATCACGAGTATAGCTGGCATTACGAACCATATCTTCAATTTGAGAACGAGTTTTCATGGTGTTCCTTAAGATTTAAGTAAATCAACAATTTTAA
SARS-CoV-2 NC_045512.2 (1) --

Section 543

(52033) 52033 52041 52052 52063 52074 52085 52096 52107 52128
T4 NC_000866.4 (52033) TTAACTTTTCACGCTCAGATTTAGCTTTACTACTCAATCCAGATAGTCTGAAAATTTCATCATCATATTGTTGAATAGAAATATCAGCTCTTCAAT
SARS-CoV-2 NC_045512.2 (1) --

Section 544

(52129) 52129 52140 52151 52162 52173 52184 52195 52206 52224
T4 NC_000866.4 (52129) TTGCTTATTAAAATAATCAATTTGTTCAGAATGTTTTTCGTTACTACGAACTGGTACAGGTTTTGTAGGCAATTTAGTTGAACTGGATTCATTCGG
SARS-CoV-2 NC_045512.2 (1) --

Section 545

(52225) 52225 52239 52250 52261 52272 52283 52294 52305 52320
T4 NC_000866.4 (52225) GCGATAAATTAAAATGCAATTTGAACCAATCGGGCATTTAGCACCAGATGAATATTCTAACGTTCCAGCTTCTTTTAAAACTTCAAAAGCTAAACA
SARS-CoV-2 NC_045512.2 (1) --

Section 546

(52321) 52321 52327 52338 52349 52360 52371 52382 52393 52404 52416
T4 NC_000866.4 (52321) GAGATGATGCCCCATATTAACATAATCAGTAGAGCGAGCTCTGATGTAATAATCATCTCCACGAGTGCTAAATTTAAAGCATTTAAGGTCTTCTGT
SARS-CoV-2 NC_045512.2 (1) --

```
                                                                                              Section 547
                          (52417) 52417    52426      52437      52448      52459      52470      52481      52492                52512
          T4 NC_000866.4  (52417) ATTATTAGTTTTAAAAATCATTTGTTTATCTAGACCTTTAGCCAATCGAGCACCTAATGCTAATAATCGTCGTTGATTTTCCCACAAATCTGCGAT
SARS-CoV-2 NC_045512.2        (1) ------------------------------------------------------------------------------------------------
```

```
                                                                                              Section 548
                          (52513) 52513       52525      52536      52547      52558      52569      52580      52591            52608
          T4 NC_000866.4  (52513) CATTTGGTCAAATGATAATTTCGGCATTAGCCGACCATAAAGGTCATATCCTTTATTATAATTGCGAAGGATTTCACTCGCATTAATACGAGACAA
SARS-CoV-2 NC_045512.2        (1) ------------------------------------------------------------------------------------------------
```

```
                                                                                              Section 549
                          (52609) 52609          52624      52635      52646      52657      52668      52679      52690         52704
          T4 NC_000866.4  (52609) CGCTCCAATTTTATTAAGGGCTTTCATAATACGATTAGATAAACTCATTCCAGACCCGTTTGTTCGCTGTAAATGTTTTTCTAATCCAAATCCAAT
SARS-CoV-2 NC_045512.2        (1) ------------------------------------------------------------------------------------------------
```

```
                                                                                              Section 550
                          (52705) 52705  52712      52723      52734      52745      52756      52767      52778      52789     52800
          T4 NC_000866.4  (52705) ATCAACTTTAAATTTATCTAAAATTTCAGCATGAACATCTCTGTCAATAACATTCAAATCCAAAGTTGGGTTAAATCTATGAAAAAATTTATCTGG
SARS-CoV-2 NC_045512.2        (1) ------------------------------------------------------------------------------------------------
```

```
                                                                                              Section 551
                          (52801) 52801     52811      52822      52833      52844      52855      52866      52877            52896
          T4 NC_000866.4  (52801) CTCTCCACGACGTAAAACAGTCCATTCATTCATCATTTTTTTATTAACTAAAGATTTAATTACTGCGTTGACATTATTAATTACTACTGACATATT
SARS-CoV-2 NC_045512.2        (1) ------------------------------------------------------------------------------------------------
```

```
                                                                                              Section 552
                          (52897) 52897        52910      52921      52932      52943      52954      52965      52976            52992
          T4 NC_000866.4  (52897) TTCCTCACTCAATTTTACCAATTACGCGGAATAAGATTGAACAGACTATATAAGTACCACATATAGATTGAACTAATGCCATTCCAAAGAACCAAA
SARS-CoV-2 NC_045512.2        (1) ------------------------------------------------------------------------------------------------
```

```
                                                                                              Section 553
                          (52993) 52993 52998      53009      53020      53031      53042      53053      53064      53075      53088
          T4 NC_000866.4  (52993) CAATATTATCAAACCATGTCTGTTTTACGTCGAAAGGACGTAAACTTACAGTAATATTATCACCTTTTTCTATTGAAGAATACGTCTCTGGGGAAA
SARS-CoV-2 NC_045512.2        (1) ------------------------------------------------------------------------------------------------
```

```
Section 554
                         (53089) 53089 53097 53108 53119 53130 53141 53152 53163 53184
T4 NC_000866.4           (53089) TATATTCACTAAATCTATAACCGTCTTTGAGTTCATATACAGCAATAAACGATAAACTAGACCCCTTTCCTTGAGTTCCTGTAAGGGTATTAACTA
SARS-CoV-2 NC_045512.2       (1) ------------------------------------------------------------------------------------------------

Section 555
                         (53185) 53185 53196 53207 53218 53229 53240 53251 53262 53280
T4 NC_000866.4           (53185) CAGTAACATCATAATCTTTATAATGCATATAATCATTAATTGCGTAATAACCATATGCAATTACTATACATAAACAACATATCAATAAATTCAATC
SARS-CoV-2 NC_045512.2       (1) ------------------------------------------------------------------------------------------------

Section 556
                         (53281) 53281 53295 53306 53317 53328 53339 53350 53361 53376
T4 NC_000866.4           (53281) TTTTAATTATCAACTGTTTCATAATAATCTCAATTAAAAGGGCTTAGAACCATTATACCATCCTTGGTATAAAGCGGTTATGCGAGTACCGTATTT
SARS-CoV-2 NC_045512.2       (1) ------------------------------------------------------------------------------------------------

Section 557
                         (53377) 53377 53383 53394 53405 53416 53427 53438 53449 53460 53472
T4 NC_000866.4           (53377) AACCGTTCTTCAAACTTCCGAAGAGTGTTCTGGCGTTCAGCTCTTTGCTTTTTGTAAGTTTCAATACGCTCTGAAATGAGAGTGTATCGTTCATTT
SARS-CoV-2 NC_045512.2       (1) ------------------------------------------------------------------------------------------------

Section 558
                         (53473) 53473 53482 53493 53504 53515 53526 53537 53548 53568
T4 NC_000866.4           (53473) ACTGATTCTTTCATAAAATCAGGAATTTCTCGAGAAGCTTTAATCTCGTCAAATTTATCAATAACAGCTTGCTCTTCAGCAATTAAGTTATCATAC
SARS-CoV-2 NC_045512.2       (1) ------------------------------------------------------------------------------------------------

Section 559
                         (53569) 53569 53581 53592 53603 53614 53625 53636 53647 53664
T4 NC_000866.4           (53569) ATCAAAATATCTTTCTTGATGAACTCAATATCTTCTTGAGTTACACGAGATAATTTAGATGCTTTATCCTTTTTGTACTGTTCGTTAGTATCACGA
SARS-CoV-2 NC_045512.2       (1) ------------------------------------------------------------------------------------------------

Section 560
                         (53665) 53665 53680 53691 53702 53713 53724 53735 53746 53760
T4 NC_000866.4           (53665) GACCAGTGTAATGTACGATTTTTATTCGTATTCTTGTAAATTTCTACAATACCAATCTCATCGATAATAACGATCCAATTCCAACGGGATTTGTAA
SARS-CoV-2 NC_045512.2       (1) ------------------------------------------------------------------------------------------------
```

Section 561

```
                          (53761) 53761 53768      53779      53790      53801      53812      53823      53834      53845   53856
         T4 NC_000866.4   (53761) ATTTGTCCTCCATTAACAGTGATTTCACCTCCGATTGAGATGTCATTAAAGAACTTGCTTTGTGCTTCTGATTTAAATTTACCATCGTTGTAATTT
SARS-CoV-2 NC_045512.2        (1) ------------------------------------------------------------------------------------------------
```

Section 562

```
                          (53857) 53857     53867      53878      53889      53900      53911      53922      53933          53952
         T4 NC_000866.4   (53857) ACCAGGTTGAAAATATCTTTAGCGTTCATTTTGTGTTCCTCCGTAGTTGATAGTTGTATAGTACCACAGAGGAACGGTCTTGTAAACAACTAAAAG
SARS-CoV-2 NC_045512.2        (1) ------------------------------------------------------------------------------------------------
```

Section 563

```
                          (53953) 53953        53966      53977      53988      53999      54010      54021      54032          54048
         T4 NC_000866.4   (53953) AAACTTCTTTCACAATTTTTTCCACTGAACCAAGCGCTCACTGCTTTCTTAGTTTCAGGAGCAGTGTTATCCATAAACCATTCAAAGGCAGCCTTT
SARS-CoV-2 NC_045512.2        (1) ------------------------------------------------------------------------------------------------
```

Section 564

```
                          (54049) 54049 54054      54065      54076      54087      54098      54109      54120      54131     54144
         T4 NC_000866.4   (54049) TTATGATTCTGGAGGGCTTCTCGGGCTTTAATCTGCTCACGGTCTATTAACACTAACATATGAGCCTTTCTTGTCACCAGGGGCTTCTTATGATTT
SARS-CoV-2 NC_045512.2        (1) ------------------------------------------------------------------------------------------------
```

Section 565

```
                          (54145) 54145   54153      54164      54175      54186      54197      54208      54219          54240
         T4 NC_000866.4   (54145) TTTGAATACTCCCAATCATTTGTCCATCGCATCGTTGTTGCGAATTGAAATACAGCTTCTTTAATCTTAGTTTCGTAAATTTCACGAGCCTTTGAG
SARS-CoV-2 NC_045512.2        (1) ------------------------------------------------------------------------------------------------
```

Section 566

```
                          (54241) 54241      54252      54263      54274      54285      54296      54307      54318          54336
         T4 NC_000866.4   (54241) TATAACATCATTACCTCCATTTACCAGTTTAATTCTAGTCATCTTTTTGATGGCAGTCCATATAATCTATTTCTGAACTGCCTTTTTGTCTTAGAA
SARS-CoV-2 NC_045512.2        (1) ------------------------------------------------------------------------------------------------
```

Section 567

```
                          (54337) 54337         54351      54362      54373      54384      54395      54406      54417      54432
         T4 NC_000866.4   (54337) GGCCTCTTATGAATTTATTTCAGAAGAGTAACCCGTAGCGATTTCTTCCCAACCGTTTTTGTCGGTCATAATAAAGTCAGCAAGATAAAGTGCAGT
SARS-CoV-2 NC_045512.2        (1) ------------------------------------------------------------------------------------------------
```

```
                                                                                                         Section 568
                          (54433) 54433 54439      54450      54461      54472      54483      54494      54505      54516     54528
T4 NC_000866.4            (54433) ACGCAGTGAAACATTACGTAAACGGTTAACATTGACTTTCATCCATGATAATGCTTTATAAGTTTCTTCATCAGAAAGACCACGCTTTTGCATCAT
SARS-CoV-2 NC_045512.2        (1) ------------------------------------------------------------------------------------------------
                                                                                                         Section 569
                          (54529) 54529    54538      54549      54560      54571      54582      54593      54604               54624
T4 NC_000866.4            (54529) GTCGGTTGAAAGAATAACATCTTCAACCCTGACCATAATTTCTTCATTAGTGTGAACACCCAAATCCAAATAAACTGAGCGGGACACTAATGCTTG
SARS-CoV-2 NC_045512.2        (1) ------------------------------------------------------------------------------------------------
                                                                                                         Section 570
                          (54625) 54625       54637      54648      54659      54670      54681      54692      54703            54720
T4 NC_000866.4            (54625) TAAATGTGGAGCAAGTTTAGTACCACGGTCTAATTCGCGGTCAATGTCAACGTTTGTGATAAAAACAATTGTTCCTTTAAATTCGAGCTCACGCTC
SARS-CoV-2 NC_045512.2        (1) ------------------------------------------------------------------------------------------------
                                                                                                         Section 571
                          (54721) 54721          54736      54747      54758      54769      54780      54791      54802         54816
T4 NC_000866.4            (54721) AATGCCTTTTTCTTCTAAGTAAGAAGATGCAGTGCTCCAGCAGACTTTACGGGTCTCTCCAGTGTCCAGAGCAGCTTTCAGAAGATTAAGAATGTC
SARS-CoV-2 NC_045512.2        (1) ------------------------------------------------------------------------------------------------
                                                                                                         Section 572
                          (54817) 54817  54824      54835      54846      54857      54868      54879      54890      54901      54912
T4 NC_000866.4            (54817) CATATCAGAGAAAACATCCACATCATCAATCAAAAGGACAGAATTCTCTTCACGATTATTCCAAAGCTGTTCATAAAGACCGATACCAGAGATTTT
SARS-CoV-2 NC_045512.2        (1) ------------------------------------------------------------------------------------------------
                                                                                                         Section 573
                          (54913) 54913     54923      54934      54945      54956      54967      54978      54989              55008
T4 NC_000866.4            (54913) ACCGTTAATGCTTTTATACTCAATGTATCCAATATCATTTGCTTTATTCAAAGCTTTATCTAAAGAATATGTTTTACCAATACCCGCCGCACCAGA
SARS-CoV-2 NC_045512.2        (1) ------------------------------------------------------------------------------------------------
                                                                                                         Section 574
                          (55009) 55009        55022      55033      55044      55055      55066      55077      55088           55104
T4 NC_000866.4            (55009) GATAATTAATGAACGAATATTTCCGTTAATAATACCATTCGTCATCATTCCCATAACATTAAATCTTTTATTAATGCGGGTTTTCATATCTTCATA
SARS-CoV-2 NC_045512.2        (1) ------------------------------------------------------------------------------------------------
```

```
                                                                                                   Section 575
                    (55105) 55105 55110          55121          55132          55143          55154          55165          55176          55187      55200
         T4 NC_000866.4 (55105) TGATTCTTTAACTTCTTCAACTTTTACACCATCATATGAAATGTCTGATTTGTAAACCCAAACACCGCGACGTTTACCGTCAATTTCAACAAAAAC
   SARS-CoV-2 NC_045512.2     (1) ------------------------------------------------------------------------------------------------
                                                                                                   Section 576
                    (55201) 55201    55209          55220          55231          55242          55253          55264          55275                55296
         T4 NC_000866.4 (55201) TTTACCATCTCCTTGTGCATCTACCGGAGCATTATCAGGGAACCATTCACCTAAGAGCTCAAAAGTTCCAGAGATTTCTTTACCGAAGTACATACC
   SARS-CoV-2 NC_045512.2     (1) ------------------------------------------------------------------------------------------------
                                                                                                   Section 577
                    (55297) 55297          55308          55319          55330          55341          55352          55363          55374                55392
         T4 NC_000866.4 (55297) CTTATTGATAGTTACAGTTTTCATTTTATTCTCCAAATCCGTATCAGTTGATAGTTGTATAGTACCATAAAGCTTTATGCTTGTAAACCGTTTTGT
   SARS-CoV-2 NC_045512.2     (1) ------------------------------------------------------------------------------------------------
                                                                                                   Section 578
                    (55393) 55393               55407          55418          55429          55440          55451          55462          55473          55488
         T4 NC_000866.4 (55393) GAAAAAATTTTGAAATAAAAAAGGGAGCCCGAAGGCTCCCTATCATTTATAATAACTTCGATGGTTTTCAAGATAAACCCTCTCAAGGAAGTCATC
   SARS-CoV-2 NC_045512.2     (1) ------------------------------------------------------------------------------------------------
                                                                                                   Section 579
                    (55489) 55489 55495          55506          55517          55528          55539          55550          55561          55572      55584
         T4 NC_000866.4 (55489) CCAGAAACTCATGTCTACTTTTTGCTGCATACCGTTCTTAGAAGCTTCAGTAGATGCTGCTTCTACTTGATCGACCACATCTTCCAAAAACTCTTG
   SARS-CoV-2 NC_045512.2     (1) ------------------------------------------------------------------------------------------------
                                                                                                   Section 580
                    (55585) 55585    55594          55605          55616          55627          55638          55649          55660                55680
         T4 NC_000866.4 (55585) AACGGTTTTAAATGGATGCTTACCCAACTTCACGTCGAGAATAAATGGAGCATCTTGGAGTGGATAAACCAAGTCACCAGTTTTGTAAATTTCCAA
   SARS-CoV-2 NC_045512.2     (1) ------------------------------------------------------------------------------------------------
                                                                                                   Section 581
                    (55681) 55681          55693          55704          55715          55726          55737          55748          55759                55776
         T4 NC_000866.4 (55681) TAGTTGAAGTCCACCACGACAAGCATGGCTCAGAGCTTTCCAGTCAATGCCTTCATTGGCTTCGGCCTTACGAGCACGTTCGCCGTATTCAGCATC
   SARS-CoV-2 NC_045512.2     (1) ------------------------------------------------------------------------------------------------
```

```
                                                                                                   Section 582
                       (55777) 55777          55792      55803      55814      55825      55836      55847      55858          55872
T4 NC_000866.4         (55777) TAATTTGTTCAGTGACTGCTTAAGCTCAATAAGAGAAAGCGTTGTCTGATATTTACGACCCAACACTGTGTAAAACGTTTGTGGGCCTGTTTTCTC
SARS-CoV-2 NC_045512.2     (1) ------------------------------------------------------------------------------------------------
                                                                                                   Section 583
                       (55873) 55873  55880      55891      55902      55913      55924      55935      55946      55957    55968
T4 NC_000866.4         (55873) ATGATTATGGAACACCCATTCACAGAATTCGTTTTCTGGAAGACGATGCTTAATATCTTCAACTTTAGTACGACGCTGCTTAATGGAACCATCTTC
SARS-CoV-2 NC_045512.2     (1) ------------------------------------------------------------------------------------------------
                                                                                                   Section 584
                       (55969) 55969     55979      55990      56001      56012      56023      56034      56045              56064
T4 NC_000866.4         (55969) TTGGTAATCAACCCATTGCTCAGGGATTTGATTAACTACTTTCAATACATCGCGTAATGCAGCCAAACGAGAACCCTTGACGCCGTATTTAGAAGC
SARS-CoV-2 NC_045512.2     (1) ------------------------------------------------------------------------------------------------
                                                                                                   Section 585
                       (56065) 56065        56078      56089      56100      56111      56122      56133      56144            56160
T4 NC_000866.4         (56065) TTGCTTGCGGACATATCCTAAATATGATTTCATGTTAGTCGTATAAAAACGAGAACGGTTGTCTTGAATAAACTTCCAMACATCAGGCAAATCGGA
SARS-CoV-2 NC_045512.2     (1) ------------------------------------------------------------------------------------------------
                                                                                                   Section 586
                       (56161) 56161 56166      56177      56188      56199      56210      56221      56232      56243      56256
T4 NC_000866.4         (56161) TTTAACCACTAGTTCAGGTGGAGTGTGAAGCATATCCAATGCTACAGGTTCACCATCTGCTGCTAATTTAAAGAAATACTTAAGACTATACAATTC
SARS-CoV-2 NC_045512.2     (1) ------------------------------------------------------------------------------------------------
                                                                                                   Section 587
                       (56257) 56257   56265      56276      56287      56298      56309      56320      56331                56352
T4 NC_000866.4         (56257) GTGGTCAATATTATCTTTAGTGTTTTTAGATGATGTGTTGTTGGTATTTTTACTCATGTGCTCTTTGACGTTTCCAATAAGAATATCGCGAGCAGG
SARS-CoV-2 NC_045512.2     (1) ------------------------------------------------------------------------------------------------
                                                                                                   Section 588
                       (56353) 56353      56364      56375      56386      56397      56408      56419      56430            56448
T4 NC_000866.4         (56353) AGGAACAAAGATTTCTTTAAAATCTACATCAGATTCTGGAGTAGAAGTTCCATAAAGATGACTACCAAAATAAGACTTAACTACAGTTTTCATTAT
SARS-CoV-2 NC_045512.2     (1) ------------------------------------------------------------------------------------------------
```

```
                                                                                                                Section 589
                       (56449) 56449         56463      56474      56485      56496      56507      56518      56529          56544
T4 NC_000866.4         (56449) TAGACCTTTCATAATCTTCATTAAATTGTACAATCAATCGATGATAATTAGATTTTGGATATCCTAATTTACTTATATAAGTTCCGAATGACCCAC
SARS-CoV-2 NC_045512.2     (1) ------------------------------------------------------------------------------------------------
                                                                                                                Section 590
                       (56545) 56545 56551      56562      56573      56584      56595      56606      56617      56628       56640
T4 NC_000866.4         (56545) GTTTAGGTCTATTTAATTTAATCCATAACTTATAAAGTAAGTCATAGTCTTGCCAATGTTTACCAGTTCTTTGTCTAATTTTAGATGAATTTGATT
SARS-CoV-2 NC_045512.2     (1) ------------------------------------------------------------------------------------------------
                                                                                                                Section 591
                       (56641) 56641    56650      56661      56672      56683      56694      56705      56716               56736
T4 NC_000866.4         (56641) GTTTCTTTTTAGCTTCAAAATTATTCAAAGATTTTTTGACTGCAGCAGAGTGTTTTTCTTTAACTCCCGGTCGTTTAAAAGCAGCTTTCACCCCTT
SARS-CoV-2 NC_045512.2     (1) ------------------------------------------------------------------------------------------------
                                                                                                                Section 592
                       (56737) 56737       56749      56760      56771      56782      56793      56804      56815            56832
T4 NC_000866.4         (56737) TAGATATTTTTTCTTTAACTTCCGGTTTGTTTTGTGCTTCTAACTGGGATTCTGAAATTTTAGCCCTTATTTCAGGAAGACTTAATGTTATCGAAC
SARS-CoV-2 NC_045512.2     (1) ------------------------------------------------------------------------------------------------
                                                                                                                Section 593
                       (56833) 56833          56848      56859      56870      56881      56892      56903      56914          56928
T4 NC_000866.4         (56833) GTTTTTCTCTGTATTCATTAGACTTCCACATTTCTTTCATTGTATTAGAATGTATTTTTGAAAATTTTTCTCTAACTAACTGATATCCTCTTGAAG
SARS-CoV-2 NC_045512.2     (1) ------------------------------------------------------------------------------------------------
                                                                                                                Section 594
                       (56929) 56929  56936      56947      56958      56969      56980      56991      57002      57013      57024
T4 NC_000866.4         (56929) TTAATTTGAGATTTCTTCCTAAAGAATCTTCTCCAAAATTATAAAATGACCACCATGCATAAATTAATCCAGGCGAATTGTAATGAATTTTAGCTA
SARS-CoV-2 NC_045512.2     (1) ------------------------------------------------------------------------------------------------
                                                                                                                Section 595
                       (57025) 57025     57035      57046      57057      57068      57079      57090      57101               57120
T4 NC_000866.4         (57025) AAAGCCAATGGGCTATAAAATGCTCTCTAGCTGTTAATAAAACTAGATTATCAGAATCATCATTACCACCAATACAAGATGGAATAATATGATGCT
SARS-CoV-2 NC_045512.2     (1) ------------------------------------------------------------------------------------------------
```

```
                                                                                                              Section 596
                           (57121) 57121       57134     57145     57156     57167     57178     57189     57200          57216
T4 NC_000866.4             (57121) TTTCCGTATAAAAATTTAATTTAGATTTATCTAATTTTCTGAGTTTTCCTTTCTTAATTAAATTATTATATACTTTAGTATAATTCATTGGTTCTT
SARS-CoV-2 NC_045512.2         (1) ------------------------------------------------------------------------------------------------
                                                                                                              Section 597
                           (57217) 57217 57222      57233     57244     57255     57266     57277     57288     57299      57312
T4 NC_000866.4             (57217) CGTCTCATTTAATTTTGCTTTGCATTTATAACACATGTCTGTTCCACTTGAAATAAACATATTTCCTCACTTTGAAATCATAGTTGGAATAACAGA
SARS-CoV-2 NC_045512.2         (1) ------------------------------------------------------------------------------------------------
                                                                                                              Section 598
                           (57313) 57313   57321      57332     57343     57354     57365     57376     57387               57408
T4 NC_000866.4             (57313) ATCAAGATAAGTCTTTAGTGCAATAGCTTCCTCTTTCTTTAATGTAATGATATGTGATCGAAAATCATCAATTTGACGAATAGATACTACATCTCC
SARS-CoV-2 NC_045512.2         (1) ------------------------------------------------------------------------------------------------
                                                                                                              Section 599
                           (57409) 57409      57420     57431     57442     57453     57464     57475     57486               57504
T4 NC_000866.4             (57409) CTCTTCATAGCATTTTGAAACATTTAAAATAGTTTCATCATTCTGATTACAGGAGTTAGTAATAATAGCATTACATTTTTTAAACCATTTTAAATT
SARS-CoV-2 NC_045512.2         (1) ------------------------------------------------------------------------------------------------
                                                                                                              Section 600
                           (57505) 57505         57519     57530     57541     57552     57563     57574     57585          57600
T4 NC_000866.4             (57505) ATGTTTTCTTTTAGTAGAATCATAAAAATATTTAATGTTAGTTATAAAATTATCCCAATTATTAATTGATAAGCACATTGACTCGCTTTTAATATT
SARS-CoV-2 NC_045512.2         (1) ------------------------------------------------------------------------------------------------
                                                                                                              Section 601
                           (57601) 57601 57607      57618     57629     57640     57651     57662     57673     57684      57696
T4 NC_000866.4             (57601) AAATCCTGGGCATGAAGAATAAAAATGAATTTTATGCTCATCATTAATGCTTACAATTTTATCAGTGTAAGCATATTCAATTTGGGTTAAACGAAC
SARS-CoV-2 NC_045512.2         (1) ------------------------------------------------------------------------------------------------
                                                                                                              Section 602
                           (57697) 57697    57706      57717     57728     57739     57750     57761     57772               57792
T4 NC_000866.4             (57697) AATTTCGCTAGGCGTAAAATATAACATGTCATCTTCTTGCGTCAAACGATACATGTTATTTACTTTTTCTATAGCCAATTCACCAAAAAGTGGACT
SARS-CoV-2 NC_045512.2         (1) ------------------------------------------------------------------------------------------------
```

Section 603

```
                          (57793) 57793       57805      57816      57827      57838      57849      57860      57871            57888
T4 NC_000866.4            (57793) AACTTCTAATACTAGTCGTTTCGCCTCGCTCATCATTACATACTCCTCTGAATCATATTAATAATGTTATTCACCAGATTATAAGTAAACATTGGG
SARS-CoV-2 NC_045512.2        (1) ------------------------------------------------------------------------------------------------
```

Section 604

```
                          (57889) 57889          57904      57915      57926      57937      57948      57959      57970         57984
T4 NC_000866.4            (57889) TAATTATATTGAATCATCACATATATAACAAACAAAACTTTCATTCTCTTCTCCTTGGCAGTTGACAAGATTACTATACCATAATCTTGTCAACTT
SARS-CoV-2 NC_045512.2        (1) ------------------------------------------------------------------------------------------------
```

Section 605

```
                          (57985) 57985  57992      58003      58014      58025      58036      58047      58058      58069      58080
T4 NC_000866.4            (57985) GTAAACCATTAAATGACGTTTTCGATAAAATTTTGAAGCTTTGTATGAGCATCAACCATGATTTTCATTTCTTCCTTGGAAAAGCTGTGCCTCTTT
SARS-CoV-2 NC_045512.2        (1) ------------------------------------------------------------------------------------------------
```

Section 606

```
                          (58081) 58081     58091      58102      58113      58124      58135      58146      58157            58176
T4 NC_000866.4            (58081) CCCGAGAAGAACATTCATAGTCAGAAACTGCATTAGCATATTCTTCAATTAGCTTCATTAAAAACATCTGTTTTTCAGTTTTCATTATTCCACCTA
SARS-CoV-2 NC_045512.2        (1) ------------------------------------------------------------------------------------------------
```

Section 607

```
                          (58177) 58177        58190      58201      58212      58223      58234      58245      58256           58272
T4 NC_000866.4            (58177) ATCATTTCAAGATATTGAACTAACTTAGCTTTGGATTTATCCAAATCCCTTTTAGCTGCTTCTATACCGTCGTATGAATATCCTTCACAATGCTCA
SARS-CoV-2 NC_045512.2        (1) ------------------------------------------------------------------------------------------------
```

Section 608

```
                          (58273) 58273 58278      58289      58300      58311      58322      58333      58344      58355       58368
T4 NC_000866.4            (58273) ACTGCTAATTGATATGAATCTATTTCAATATCATGCGCTAATTTAATGATTTTTTCAAACTGTTCGCGTGTTAGCATACTTAAACTCTCGTATTAT
SARS-CoV-2 NC_045512.2        (1) ------------------------------------------------------------------------------------------------
```

Section 609

```
                          (58369) 58369   58377      58388      58399      58410      58421      58432      58443                58464
T4 NC_000866.4            (58369) GATCGATAATTTCATCAAGAAACATATCTAACGCTTCTACAGCATTATTAACTTTAGCTTCAAATTCTTCAATACCTGATAAGGCAAAGTTAATGC
SARS-CoV-2 NC_045512.2        (1) ------------------------------------------------------------------------------------------------
```

```
Section 610
                      (58465) 58465      58476      58487      58498      58509      58520      58531      58542           58560
T4 NC_000866.4        (58465) GTTCCTCATCTGCTTTACGAATTAAAGCTACCAATTCCTTAATTTTATCCGCTTGTTCGATACTAATCATTATTCCACCACATATGAAAGAGAGAA
SARS-CoV-2 NC_045512.2    (1) ------------------------------------------------------------------------------------------------
Section 611
                      (58561) 58561         58575      58586      58597      58608      58619      58630      58641          58656
T4 NC_000866.4        (58561) TATTGCACACGCCATGTGAGTTGCAACTTCATCACACATATTATAACGTTTCTTAAGAAGTTCTACAAGTTCTTCACTAGTAACTTCATCCATGTC
SARS-CoV-2 NC_045512.2    (1) ------------------------------------------------------------------------------------------------
Section 612
                      (58657) 58657 58663      58674      58685      58696      58707      58718      58729      58740      58752
T4 NC_000866.4        (58657) GACGAAAAAATCACCATTAATGATGACGTAGATATTTCCTTCTTGATTGAGTGCTTCAATTTTCATGATGTTCTCCTCTTTATCCGATGGTTGTAT
SARS-CoV-2 NC_045512.2    (1) ------------------------------------------------------------------------------------------------
Section 613
                      (58753) 58753    58762      58773      58784      58795      58806      58817      58828               58848
T4 NC_000866.4        (58753) AGTACCACAGCTCAAACGGAAAGTAAACCGGTAAAATGAAAAAAAGTCTCCCGAAGGAGACTAATGTTATTCGAGGGAAAGAAGATACTTACTCTG
SARS-CoV-2 NC_045512.2    (1) ------------------------------------------------------------------------------------------------
Section 614
                      (58849) 58849       58861      58872      58883      58894      58905      58916      58927           58944
T4 NC_000866.4        (58849) GTAAAACATTCCAGTAATATCATCTATCGTGCTTTGGATGGCTGGAGGCATTTCTTTATAAATGCTGTTAGATTGGTCTAGTATGCGATCAATCAT
SARS-CoV-2 NC_045512.2    (1) ------------------------------------------------------------------------------------------------
Section 615
                      (58945) 58945          58960      58971      58982      58993      59004      59015      59026          59040
T4 NC_000866.4        (58945) TTTAATTGTGTCGGTAGGAAGTTTACTGGCATCTGGAATTGAAGGCGTGTATTTTCGACCAGAATACCCCAAATATTGCTCACCAAATTTATCAAT
SARS-CoV-2 NC_045512.2    (1) ------------------------------------------------------------------------------------------------
Section 616
                      (59041) 59041  59048      59059      59070      59081      59092      59103      59114      59125    59136
T4 NC_000866.4        (59041) CAAATCTGGCAACTCGGAAAAAATAAAATCGTATGCTTTGTGTCTAGCATAACTTTTAGTTTCAAAATGTGCAGAATGAAAATAAGCTTGTGCAGC
SARS-CoV-2 NC_045512.2    (1) ------------------------------------------------------------------------------------------------
```

```
                                                                                                                          Section 617
                        (59137) 59137     59147      59158      59169      59180      59191      59202      59213            59232
        T4 NC_000866.4  (59137) CATTAATAAACCTAAGTATTCATCTGCCTTTGAAGGTTTTCCACTTTGTGAAAAGTCGCTGAATTTCATTCAGTCTCCAATTTAATGTTCATAATT
  SARS-CoV-2 NC_045512.2    (1) ------------------------------------------------------------------------------------------------
                                                                                                                          Section 618
                        (59233) 59233        59246      59257      59268      59279      59290      59301      59312          59328
        T4 NC_000866.4  (59233) CTAGCGTATGATTGTGCCATCTCCGCGCCTCGCTCTATACATTCAAAATCAGAAGAGCACGGGTCATTTTTATAGGTCGTTCTCATAAAACTATAG
  SARS-CoV-2 NC_045512.2    (1) ------------------------------------------------------------------------------------------------
                                                                                                                          Section 619
                        (59329) 59329 59334      59345      59356      59367      59378      59389      59400      59411      59424
        T4 NC_000866.4  (59329) AATTGTTCAGACGATTCTACGCTTTTATTTTCAAAAAGCATATAAACGTGCCTAATACCAGATTCCATAAATTTATCAAAATGAGGATCGACATTC
  SARS-CoV-2 NC_045512.2    (1) ------------------------------------------------------------------------------------------------
                                                                                                                          Section 620
                        (59425) 59425   59433      59444      59455      59466      59477      59488      59499            59520
        T4 NC_000866.4  (59425) GCTTCAATCGATGGAGATAAAACAAATGACAATCCTAGCATGGCAAAAAGTGCTGTTGCTTTTAAGGCCATAAAGGCCTCCTATCATTTTTGTCCT
  SARS-CoV-2 NC_045512.2    (1) ------------------------------------------------------------------------------------------------
                                                                                                                          Section 621
                        (59521) 59521      59532      59543      59554      59565      59576      59587      59598          59616
        T4 NC_000866.4  (59521) GTATTTACTTTGTGCCGATGCACGGCCTTAACTTTATCAAGGTATTTTTCAAAATTTCGCAATCTAGTATAGTCTGCCGGAGATTGGTTGAGTGAT
  SARS-CoV-2 NC_045512.2    (1) ------------------------------------------------------------------------------------------------
                                                                                                                          Section 622
                        (59617) 59617         59631      59642      59653      59664      59675      59686      59697        59712
        T4 NC_000866.4  (59617) ACTTCTCGACGCAAAGCTGAAATGATATTTCCAACTTCCCTACGAATTTCATCTAATTGAAGAACAGTAAGATTGCGAAGTTGCTTTTCAGTTAAT
  SARS-CoV-2 NC_045512.2    (1) ------------------------------------------------------------------------------------------------
                                                                                                                          Section 623
                        (59713) 59713 59719      59730      59741      59752      59763      59774      59785      59796    59808
        T4 NC_000866.4  (59713) TGTAGCATATATACCCCTTTAGTTAGATAAACCTATTTATAACTTTTGCACTAACCGAGCTTTTTAGTTAATTCATTCCAATGTTTTCTACACAAA
  SARS-CoV-2 NC_045512.2    (1) ------------------------------------------------------------------------------------------------
```

```
                                                                                                      Section 624
                        (59809) 59809    59818      59829      59840      59851      59862      59873      59884          59904
T4 NC_000866.4          (59809) GAAACATAAATTTCATCACCAATACAAATTTGATTACCTTCTTTAACTGGTGTTCCATCTTCCATTAATCGAGCTGTCATAATCGCTTTTTTACCA
SARS-CoV-2 NC_045512.2      (1) ------------------------------------------------------------------------------------------------
                                                                                                      Section 625
                        (59905) 59905       59917      59928      59939      59950      59961      59972      59983        60000
T4 NC_000866.4          (59905) CAATGACAAACTGCTTTTAGTTCAATAAGTTTATCTGCAATCGCTAAAAGTTCTTTAGAACCTTCAAATAATTTTCCAGCGAAATCAGTCCTTAGC
SARS-CoV-2 NC_045512.2      (1) ------------------------------------------------------------------------------------------------
                                                                                                      Section 626
                        (60001) 60001          60016      60027      60038      60049      60060      60071      60082        60096
T4 NC_000866.4          (60001) CCATAAGCCATAACAGGAACATTATATGTATCAACAATTCGGCTCAATTGATGCACCTGTTCAGTTTTTAAAAACTGAGCTTCATCTACAAATACG
SARS-CoV-2 NC_045512.2      (1) ------------------------------------------------------------------------------------------------
                                                                                                      Section 627
                        (60097) 60097  60104      60115      60126      60137      60148      60159      60170      60181  60192
T4 NC_000866.4          (60097) CAATGAATATCTTTTTGTGCTTCAGCCCATTTATAGAACTCGAAAATATCCATATCATCTGTAATAATATTCGCTTCCTGCTTAATTCCAATGCGA
SARS-CoV-2 NC_045512.2      (1) ------------------------------------------------------------------------------------------------
                                                                                                      Section 628
                        (60193) 60193     60203      60214      60225      60236      60247      60258      60269          60288
T4 NC_000866.4          (60193) GAAACGACTTCACAGACAGAATCGCGAGTATCAATAGCAGGCTTAAGAACTAATACACTCATTCCACGTTCTTTATAATTATGTGCAGCAATCAAA
SARS-CoV-2 NC_045512.2      (1) ------------------------------------------------------------------------------------------------
                                                                                                      Section 629
                        (60289) 60289        60302      60313      60324      60335      60346      60357      60368        60384
T4 NC_000866.4          (60289) AGAGAAGCAGATTTTCCAGCATTCATTGCTGCATAAGTAAAAATTAAACTCGCCATCTTAGTCCTTAGTTAAATTTTCTAAATATGTTTCTAAATC
SARS-CoV-2 NC_045512.2      (1) ------------------------------------------------------------------------------------------------
                                                                                                      Section 630
                        (60385) 60385 60390      60401      60412      60423      60434      60445      60456      60467    60480
T4 NC_000866.4          (60385) ATTTTCAGCTTTATCGATAGATTTTACTAATTCGTAATATGTTTCGGCATCTCCATATTCAGAAGATATTTCAAAAGACAAATCCTTTTCTAAACT
SARS-CoV-2 NC_045512.2      (1) ------------------------------------------------------------------------------------------------
```

```
                                                                                                                   Section 631
                          (60481) 60481   60489       60500       60511       60522       60533       60544       60555                 60576
           T4 NC_000866.4 (60481) AATAATTTCACCAACTAAAAATAATATTTCGTTCTTTTGTTCGCGAGTAATCATAAGGAATTTATATAATCAATGAGTTCTTGTTCTTTATTATCG
     SARS-CoV-2 NC_045512.2   (1) ------------------------------------------------------------------------------------------------
                                                                                                                   Section 632
                          (60577) 60577       60588       60599       60610       60621       60632       60643       60654             60672
           T4 NC_000866.4 (60577) AATTCTTTAGAAAGTTCTTCGTACTCGTTTGCGCTAAAAGGACCGCATTCATTACAAACTTTTTCCAATTCACTATTTTTATCCATAACTTCGTGG
     SARS-CoV-2 NC_045512.2   (1) ------------------------------------------------------------------------------------------------
                                                                                                                   Section 633
                          (60673) 60673         60687       60698       60709       60720       60731       60742       60753           60768
           T4 NC_000866.4 (60673) ATAAGAGAAAAGAGTGTGTCTTTTTGTTCTTTGCTTAAACTCATAACCATGTCACCTTTAAGCAGTATTCTTCTACATGCTGTTTACGACCTTTCT
     SARS-CoV-2 NC_045512.2   (1) ------------------------------------------------------------------------------------------------
                                                                                                                   Section 634
                          (60769) 60769 60775       60786       60797       60808       60819       60830       60841       60852       60864
           T4 NC_000866.4 (60769) TATCAATAAAGGTATATTCAACGAATGTTCCAATGTAGTCTTTATCTACATCATGTGGACTATTAATTGGACATTTAGTGCGACAAATGCGTTCCC
     SARS-CoV-2 NC_045512.2   (1) ------------------------------------------------------------------------------------------------
                                                                                                                   Section 635
                          (60865) 60865    60874       60885       60896       60907       60918       60929       60940                60960
           T4 NC_000866.4 (60865) ATTGACGAATAATTACTGCCTTATTCTTTGGGTCATATGGATGTGGATAATGTATATTCATAATAATGGTTCCCAATCAACAATCACAATTTCTAA
     SARS-CoV-2 NC_045512.2   (1) ------------------------------------------------------------------------------------------------
                                                                                                                   Section 636
                          (60961) 60961       60973       60984       60995       61006       61017       61028       61039             61056
           T4 NC_000866.4 (60961) TTTAGAGGAATATGTATCTAAAATCCCCTCAATAATATCCCAGTTCCCTTTACCTATGCCTGCACCAATCCTAGGCATATAGATTGTAGGTTTAAT
     SARS-CoV-2 NC_045512.2   (1) ------------------------------------------------------------------------------------------------
                                                                                                                   Section 637
                          (61057) 61057          61072       61083       61094       61105       61116       61127       61138          61152
           T4 NC_000866.4 (61057) CAGTTTATTTTCACCAAACTCATTTAATTCTAACATACAATTCATTAAAGCGGAATACTCAAAATTTGGCCCTGGTTGAAATTGAGTATAAAGATT
     SARS-CoV-2 NC_045512.2   (1) ------------------------------------------------------------------------------------------------
```

```
                                                                                                   Section 638
                          (61153) 61153  61160      61171      61182      61193      61204      61215      61226      61237     61248
         T4 NC_000866.4  (61153) GAAGCAGTAAGCTTTATGAGTCCTAAAGTATTTTTCATAGACTGAGTAAGAACCGAGTTTAGTTACATCACCCCATTCAGTCTGTAATTTATCAGC
    SARS-CoV-2 NC_045512.2     (1) ------------------------------------------------------------------------------------------------
                                                                                                   Section 639
                          (61249) 61249     61259      61270      61281      61292      61303      61314      61325              61344
         T4 NC_000866.4  (61249) TTCCAAAATTTTAGGGAAAGCTTTGGTTAATTGACCCGCTACGCCTGAACCCATAGTATGAAAACAATTACATCCATGTGCAATATTTTTACCTTC
    SARS-CoV-2 NC_045512.2     (1) ------------------------------------------------------------------------------------------------
                                                                                                   Section 640
                          (61345) 61345        61358      61369      61380      61391      61402      61413      61424           61440
         T4 NC_000866.4  (61345) AGCGAAAAGGGCGACAATATCGCCCTTGATATATTTTACAATCATCTAGTACTCAATCCTCGATTATAAGAATCTACCAAACGGTCAACCATTGAA
    SARS-CoV-2 NC_045512.2     (1) ------------------------------------------------------------------------------------------------
                                                                                                   Section 641
                          (61441) 61441 61446      61457      61468      61479      61490      61501      61512      61523       61536
         T4 NC_000866.4  (61441) TGACAAGCGGCTTTATCTTTCTCCTCCGCAACTGAACATTCTAAGGTATTCCACTTTTTAGCATATCGTTTTAACAATGTATCGTTTTTGTATCTG
    SARS-CoV-2 NC_045512.2     (1) ------------------------------------------------------------------------------------------------
                                                                                                   Section 642
                          (61537) 61537   61545      61556      61567      61578      61589      61600      61611               61632
         T4 NC_000866.4  (61537) CTTGATTTATCTCTTTCTCCGTCTTTATATGCATATATTAATTTCTGTGCAAATTCAGCTTGGCATGCCTTATTTTTCCCACAATAATCTGCCGCA
    SARS-CoV-2 NC_045512.2     (1) ------------------------------------------------------------------------------------------------
                                                                                                   Section 643
                          (61633) 61633      61644      61655      61666      61677      61688      61699      61710             61728
         T4 NC_000866.4  (61633) GTGCGGTTTACATATTCTCTAATTTCAGTATATGATGTATCTGCTGACGCAGAAGCAGAAAATGAAATTAATCCTATACATAAAACCAAAATTTTA
    SARS-CoV-2 NC_045512.2     (1) ------------------------------------------------------------------------------------------------
                                                                                                   Section 644
                          (61729) 61729         61743      61754      61765      61776      61787      61798      61809          61824
         T4 NC_000866.4  (61729) GTCATTTACTATTTCCAAAAGTTTATTATTTTTAAGGTAATTAGCCTTTTCTAGGACTTCAGAAGCATATTTAGAACCTGCTTTAACATTCCATCC
    SARS-CoV-2 NC_045512.2     (1) ------------------------------------------------------------------------------------------------
```

```
                                                                                                   Section 645
                          (61825) 61825 61831      61842      61853      61864      61875      61886      61897      61908 61920
         T4 NC_000866.4   (61825) CGAATTATAAGAGGATATTGCTTTTCTTATATCGCCCTTATGTATATTTAACCAATAAGAAAGTTCAATGTACGCCCAGGAAGCTGAATTGGATCG
   SARS-CoV-2 NC_045512.2     (1) ------------------------------------------------------------------------------------------------
                                                                                                   Section 646
                          (61921) 61921    61930      61941      61952      61963      61974      61985      61996           62016
         T4 NC_000866.4   (61921) TTTATTCAACATTCTTTTTATTTCAGCATCGGTCATATTATAACCAAGTTCCTTAACTCTTGCTCGCATAGTAGGCAAATAATTTTGGAACATTCC
   SARS-CoV-2 NC_045512.2     (1) ------------------------------------------------------------------------------------------------
                                                                                                   Section 647
                          (62017) 62017       62029      62040      62051      62062      62073      62084      62095           62112
         T4 NC_000866.4   (62017) GTAGGCGTGATGCTTTGGTTTAGATTTTAAATTAACTCCGCCAGAGCTTTCTTGCCATAAAATGGCAGCCATTATATGACCTAATCCGCTCTTGTG
   SARS-CoV-2 NC_045512.2     (1) ------------------------------------------------------------------------------------------------
                                                                                                   Section 648
                          (62113) 62113          62128      62139      62150      62161      62172      62183      62194         62208
         T4 NC_000866.4   (62113) GATATTTTTGTGTGTTTTATATTTTCCATCCTTAGAAAATTGTTCCCCGAATTGATACGCGTAACGCATGTTATCGAGTTGGACATTACTGAAAGT
   SARS-CoV-2 NC_045512.2     (1) ------------------------------------------------------------------------------------------------
                                                                                                   Section 649
                          (62209) 62209  62216      62227      62238      62249      62260      62271      62282      62293 62304
         T4 NC_000866.4   (62209) ATGCTCGGAGCTATGTGCCATCATTGAAATGGCCAATAGACCAGCGAGTAGTGCTTTTCTCATGCTTACCTCATTGAGTTTTAATTACTGCTTTAG
   SARS-CoV-2 NC_045512.2     (1) ------------------------------------------------------------------------------------------------
                                                                                                   Section 650
                          (62305) 62305     62315      62326      62337      62348      62359      62370      62381           62400
         T4 NC_000866.4   (62305) AAGCCTTTCCTGGTAAACGACGACTGTTGATAATTGCCATCCTACATTGAAGTGACGGGTCTTTGAACTTCTCGTTAGGTTTACAAACTGTAAATC
   SARS-CoV-2 NC_045512.2     (1) ------------------------------------------------------------------------------------------------
                                                                                                   Section 651
                          (62401) 62401        62414      62425      62436      62447      62458      62469      62480           62496
         T4 NC_000866.4   (62401) CAAGCCAAAGATTTCCATCTGTGATTTCTAAACGTCCAGGACGATATTCAACCCCATCAATAAAATCCTCGTCAATGTCAGGACGCGGAGGCATAC
   SARS-CoV-2 NC_045512.2     (1) ------------------------------------------------------------------------------------------------
```

```
                                                                                                    Section 652
                          (62497) 62497 62502        62513      62524      62535      62546      62557      62568      62579      62592
          T4 NC_000866.4  (62497) TCAGGAATTCATTAACTTCTAAAACATGGTCTTTTATTTTATGGAATAATTCAAAAACGTATGTCTCATCAATCTCCCGTTGAATTGCGCGATCAA
  SARS-CoV-2 NC_045512.2      (1) ------------------------------------------------------------------------------------------------
                                                                                                    Section 653
                          (62593) 62593   62601      62612      62623      62634      62645      62656      62667                62688
          T4 NC_000866.4  (62593) GAAGATGTTGAGAATATTTTAGATGAAACGATGAGACTCCTGCTGCTTTTGATGCCTCACGAATCTCATTGTTAATTTGACGAAACTCCGACTCAA
  SARS-CoV-2 NC_045512.2      (1) ------------------------------------------------------------------------------------------------
                                                                                                    Section 654
                          (62689) 62689      62700      62711      62722      62733      62744      62755      62766              62784
          T4 NC_000866.4  (62689) AGTGACGACGAAGCTTATTTCGACGGATAAAAACTTCTGTATTGATAGTCATGTTATTCTCCTCTTAACTGATAGAAAAATTATACCACAGTCAAG
  SARS-CoV-2 NC_045512.2      (1) ------------------------------------------------------------------------------------------------
                                                                                                    Section 655
                          (62785) 62785         62799      62810      62821      62832      62843      62854      62865          62880
          T4 NC_000866.4  (62785) AGGAAAAGTAAACAGTTATTCTTTAAATCTAATCAATTTATTCATAGACTTTGAAACTTCGGCACGAACCTCATGTAGATTTTTGAGCTGTTCAAG
  SARS-CoV-2 NC_045512.2      (1) ------------------------------------------------------------------------------------------------
                                                                                                    Section 656
                          (62881) 62881 62887      62898      62909      62920      62931      62942      62953      62964       62976
          T4 NC_000866.4  (62881) ACGCTGCGTATAGTAAGCAATTTCATCTTCTTCGAGACAGTCCTGCGAATCTTCTTTAAGATAACGTGCATAGTCCTGGAAAGCGTTACGGACTAC
  SARS-CoV-2 NC_045512.2      (1) ------------------------------------------------------------------------------------------------
                                                                                                    Section 657
                          (62977) 62977    62986      62997      63008      63019      63030      63041      63052                63072
          T4 NC_000866.4  (62977) TTCCTGGAAGTCATCAAGACTTTGAATTTTCTTAGGAGCAACAGATACACGACGAGGGGCAGTATAATACTCATAACCAAACCCTGCGCTTAATTG
  SARS-CoV-2 NC_045512.2      (1) ------------------------------------------------------------------------------------------------
                                                                                                    Section 658
                          (63073) 63073       63085      63096      63107      63118      63129      63140      63151            63168
          T4 NC_000866.4  (63073) AGCCATTAGTATTTTTCCTCTGGTTGGAACACTGCACGACAAGCCCACATACTGGCTTCTTTGAGTTTCGTTTTAGCAATAGTTAACTGATCGAGA
  SARS-CoV-2 NC_045512.2      (1) ------------------------------------------------------------------------------------------------
```

```
                                                                                                        Section 659
                          (63169) 63169          63184      63195      63206      63217      63228      63239      63250         63264
           T4 NC_000866.4 (63169) CTTTCAGCATAATTCTTCGCGAATTCACAGTCTTCGCAATTATCTAGTGCTTCCCAGAATTCATCATATAAAGCATCAAAGATAAGTCCTAAACGA
     SARS-CoV-2 NC_045512.2   (1) ------------------------------------------------------------------------------------------------
                                                                                                        Section 660
                          (63265) 63265  63272      63283      63294      63305      63316      63327      63338      63349    63360
           T4 NC_000866.4 (63265) ACTTCAGCGTCTTTAATAGCATTTACTTTACCGATTTTCTCTTCAGTATGTGGTTTATAACCCTTAATATCTTCAATCATATTTGACTTCCTCACC
     SARS-CoV-2 NC_045512.2   (1) ------------------------------------------------------------------------------------------------
                                                                                                        Section 661
                          (63361) 63361     63371      63382      63393      63404      63415      63426      63437              63456
           T4 NC_000866.4 (63361) AGTACATAATACGTATTCAACTAAACGAATAGGTTCATGAATGCCATAGCCTTGAACAGAAATTTCTGTCGTAGGATAAATTCCATCAATATCACC
     SARS-CoV-2 NC_045512.2   (1) ------------------------------------------------------------------------------------------------
                                                                                                        Section 662
                          (63457) 63457        63470      63481      63492      63503      63514      63525      63536          63552
           T4 NC_000866.4 (63457) CATATTCCACGCTTCATTAAATTGCTGTTCGCCTGAGTTACTAAACCACTCGCGAAAGCATTTAGCACATCTTCAGAACCTTCAATAATTATCTTT
     SARS-CoV-2 NC_045512.2   (1) ------------------------------------------------------------------------------------------------
                                                                                                        Section 663
                          (63553) 63553 63558      63569      63580      63591      63602      63613      63624      63635     63648
           T4 NC_000866.4 (63553) GCCATTACAAACTTTCAGTAAAGGTACGAGCGATAACGTCGCGCTGCTGTTCCGGAGTCAGAGAGTTAAAGCGAACTGCATAACCGGATACACGGA
     SARS-CoV-2 NC_045512.2   (1) ------------------------------------------------------------------------------------------------
                                                                                                        Section 664
                          (63649) 63649   63657      63668      63679      63690      63701      63712      63723              63744
           T4 NC_000866.4 (63649) TGGTCAGCTGCGGATATTTTTCCGGATGCTTAACTGCATCTTCCAGAGTTTCATGACGCAGAACGTTAACGTTCAGGTGTTGACCACCTTCAATTT
     SARS-CoV-2 NC_045512.2   (1) ------------------------------------------------------------------------------------------------
                                                                                                        Section 665
                          (63745) 63745      63756      63767      63778      63789      63800      63811      63822              63840
           T4 NC_000866.4 (63745) TAACTGTAGGTTGTGGCTCAATTTCAATTTCACGGGCATGCAAACCATAGAAAATTTCTGGGTCTACAAAAGAGTCCTCTTTAAAGGTTTTAGAGA
     SARS-CoV-2 NC_045512.2   (1) ------------------------------------------------------------------------------------------------
```

```
                                                                                                        Section 666
                      (63841) 63841         63855      63866      63877      63888      63899      63910      63921          63936
T4 NC_000866.4        (63841) CAATAATTCGTGCTTGAATACCATCTTCAAAATAAATAGTACCTTTATGTGTGCCTTCAAGAATTTGATATGCTTTCATATAAACCTCAATTAGAA
SARS-CoV-2 NC_045512.2    (1) ------------------------------------------------------------------------------------------------
                                                                                                        Section 667
                      (63937) 63937 63943      63954      63965      63976      63987      63998      64009      64020       64032
T4 NC_000866.4        (63937) AATAAATTTATCCAAGATTGTTCTTTAATTAAAAATGGCTCAGAATCATATGCCATTAAACTTTGCGTAATTAATCCTTTAAAAGGTCCATCAATA
SARS-CoV-2 NC_045512.2    (1) ------------------------------------------------------------------------------------------------
                                                                                                        Section 668
                      (64033) 64033    64042      64053      64064      64075      64086      64097      64108               64128
T4 NC_000866.4        (64033) AATTCCATGGTAAAATATGGAATTTTATTCATTAGCCGTGCATTAGGAGCAGTGCACAAAACTCTGCATCCTTTGAATACGCCTTTTTGTAATTTG
SARS-CoV-2 NC_045512.2    (1) ------------------------------------------------------------------------------------------------
                                                                                                        Section 669
                      (64129) 64129       64141      64152      64163      64174      64185      64196      64207            64224
T4 NC_000866.4        (64129) TATTGCTTGGGATAAAATTCGCTCAAAATGTTATTTTTTGCCAAAATTTCAAAATGATTCACCAATTTATTTTTAATAGTTTTTGGCGAAAAATAA
SARS-CoV-2 NC_045512.2    (1) ------------------------------------------------------------------------------------------------
                                                                                                        Section 670
                      (64225) 64225          64240      64251      64262      64273      64284      64295      64306         64320
T4 NC_000866.4        (64225) AGATATTCGAAAAGCTGAGTGTCTGTCATCATTGCATTCCGATTACGAAAAACTGTGGACGAGTAATACCACCAATGCAACATTTACTATTACAGC
SARS-CoV-2 NC_045512.2    (1) ------------------------------------------------------------------------------------------------
                                                                                                        Section 671
                      (64321) 64321  64328      64339      64350      64361      64372      64383      64394      64405       64416
T4 NC_000866.4        (64321) AGTAGTGTACGGTGTCAATATGGACACTATAAATCTTATCCATATCAGGAGATTTGACAGGCTCATCAATTATATACAAAATTCGCGAAAGCTTTA
SARS-CoV-2 NC_045512.2    (1) ------------------------------------------------------------------------------------------------
                                                                                                        Section 672
                      (64417) 64417     64427      64438      64449      64460      64471      64482      64493               64512
T4 NC_000866.4        (64417) AACCTCTGAACTTGCTTCCTTTATTACCAATAAAACTGCGTACAGAATCAGTAAATAAACGAAAACGTATATCATCATTAGAATAACGCGAAAATT
SARS-CoV-2 NC_045512.2    (1) ------------------------------------------------------------------------------------------------
```

```
                                                    Section 673
                        (64513) 64513 64526 64537 64548 64559 64570 64581 64592 64608
        T4 NC_000866.4  (64513) CCTTTTTGATGTTATTTGCAGAAATTTTAGCATAAGCTGAAGTATTAGAAAGAACAATAACTGTTCCGCCATCATACAACCAATTAGCAGCAAAGT
SARS-CoV-2 NC_045512.2      (1) ------------------------------------------------------------------------------------------------
                                                    Section 674
                        (64609) 64609 64614 64625 64636 64647 64658 64669 64680 64691 64704
        T4 NC_000866.4  (64609) TAGTCACAGCAATTGATTTACCGGATTGACGTCCACCATCTAGTCGAAGTGTACAATACTGTTTAAGTAAGTCTTCAAATGGCGGGATATATTCGT
SARS-CoV-2 NC_045512.2      (1) ------------------------------------------------------------------------------------------------
                                                    Section 675
                        (64705) 64705 64713 64724 64735 64746 64757 64768 64779 64800
        T4 NC_000866.4  (64705) TTTTACAAATTTCTTCTACTCTAGCATCAGAATGGTGTGTAAAAGCATTCATCAGGGATAGATAAGGACCAGTTAAAAATGTTCTCATTTCTTCTC
SARS-CoV-2 NC_045512.2      (1) ------------------------------------------------------------------------------------------------
                                                    Section 676
                        (64801) 64801 64812 64823 64834 64845 64856 64867 64878 64896
        T4 NC_000866.4  (64801) TATAAGCTCTATAAGTTTGGGCCATTCCGTGGCACATGAATTGTCCATTTCTGTATTTACCCATTACCGCGCTTGGGCTCGACCTTATTACAGGTT
SARS-CoV-2 NC_045512.2      (1) ------------------------------------------------------------------------------------------------
                                                    Section 677
                        (64897) 64897 64911 64922 64933 64944 64955 64966 64977 64992
        T4 NC_000866.4  (64897) GGCGGGAATCCCTCATATAATCATGAGGTCCAGGTTGTTCCCTTATGCATAAATCGCCTTACCGTAGTATTTGTACCAAGTAGGACGTTGTGCAAT
SARS-CoV-2 NC_045512.2      (1) ------------------------------------------------------------------------------------------------
                                                    Section 678
                        (64993) 64993 64999 65010 65021 65032 65043 65054 65065 65076 65088
        T4 NC_000866.4  (64993) TTTTTCATCTAAACGAGCTTGTGATATAGCAATAGAAGCTTCATGGGGAATATAATCACCACGGAATTCCTGAGGAATATCACTAATATCCTGGAC
SARS-CoV-2 NC_045512.2      (1) ------------------------------------------------------------------------------------------------
                                                    Section 679
                        (65089) 65089 65098 65109 65120 65131 65142 65153 65164 65184
        T4 NC_000866.4  (65089) TGTAGTATCCTTGATATTAAAACCACGTTTTAAACATTCAGCTATAAGCTCAATTTGACGTTTACGTAAGAACTCGAGCTTATCGTAAAAGAATGT
SARS-CoV-2 NC_045512.2      (1) ------------------------------------------------------------------------------------------------
```

```
                                                                                                       Section 680
                         (65185) 65185      65197      65208      65219      65230      65241      65252      65263           65280
T4 NC_000866.4           (65185) AACATGACCTGCGCCAAGGATAAAAGTAGGACTGATTTTAAAATCACGAACACGTTTACCGTTAGCAACATGCTTACGAACTGCACCAAAAACACG
SARS-CoV-2 NC_045512.2       (1) ------------------------------------------------------------------------------------------------
                                                                                                       Section 681
                         (65281) 65281          65296      65307      65318      65329      65340      65351      65362         65376
T4 NC_000866.4           (65281) CGGCAATTCACGATATTCAGCCATTAAGTGTTGGTCAGCCAATTCAGATACTAAAGTAAGGTTGATACGAGTCATTTTAGTGTTCTCCTGTAGTTG
SARS-CoV-2 NC_045512.2       (1) ------------------------------------------------------------------------------------------------
                                                                                                       Section 682
                         (65377) 65377  65384      65395      65406      65417      65428      65439      65450      65461      65472
T4 NC_000866.4           (65377) ATAGGTCTATAGTATCATACCTACAGGAGATGTAAACTGTTATTTATCTTTAATTGCTTTAGCTGCTTCGATAGCCGCTTGCTGAAGGTCATCCAT
SARS-CoV-2 NC_045512.2       (1) ------------------------------------------------------------------------------------------------
                                                                                                       Section 683
                         (65473) 65473     65483      65494      65505      65516      65527      65538      65549             65568
T4 NC_000866.4           (65473) AGACATGCCGAACTTAGAAGCAAAGTTATCAATTTTCTTTTCGACGGCATTCAAAGGCTTGGCCTGTTTACCTTCATTAGCGCCAGGAAGAGCGAG
SARS-CoV-2 NC_045512.2       (1) ------------------------------------------------------------------------------------------------
                                                                                                       Section 684
                         (65569) 65569        65582      65593      65604      65615      65626      65637      65648           65664
T4 NC_000866.4           (65569) AATACGCTCTCTGTCAATATAAAGGCTTACAAGTTTCTTACGGTCTTTATCGGGCAAATCATGAAATGAATGGGCCTTTTTATTTACAGCGGCTTC
SARS-CoV-2 NC_045512.2       (1) ------------------------------------------------------------------------------------------------
                                                                                                       Section 685
                         (65665) 65665 65670      65681      65692      65703      65714      65725      65736      65747        65760
T4 NC_000866.4           (65665) TAATTTACCTGCGCCCACTCGCGCTTCGGCAATAAATTCTTGATATGTTTTCATATGTTTCCTTTAAATGTAAATATTTTTATTATTCTATCCTAG
SARS-CoV-2 NC_045512.2       (1) ------------------------------------------------------------------------------------------------
                                                                                                       Section 686
                         (65761) 65761   65769      65780      65791      65802      65813      65824      65835                65856
T4 NC_000866.4           (65761) AATTGTGATAATATATTCACAATTCTAGGAGTTGTAAACTGCTTTTATTTAAGCGTCCCAAGTATAAGCTTTATTAAGAATTACCACGGGCTGCAT
SARS-CoV-2 NC_045512.2       (1) ------------------------------------------------------------------------------------------------
```

```
                                                                                                    Section 687
                            (65857) 65857      65868      65879      65890      65901      65912      65923      65934             65952
        T4 NC_000866.4      (65857) TAGCAACGGCGTAAGCGTACTGAATATTAGCGTCTTTAAACTTACCTTTAGAGGTATCTATTTCTGCCTTAAAGCCGCCTTTAGTCATAACATCGG
  SARS-CoV-2 NC_045512.2        (1) ------------------------------------------------------------------------------------------------
                                                                                                    Section 688
                            (65953) 65953         65967      65978      65989      66000      66011      66022      66033          66048
        T4 NC_000866.4      (65953) CAAATTCTTTACGGAAAGCCATTGCATCAAGACCTTTCCATGATGATTTATGCTTGGCAAATTCAAGACCTGCGAAGTTGACAGCTTTAGCCAATT
  SARS-CoV-2 NC_045512.2        (1) ------------------------------------------------------------------------------------------------
                                                                                                    Section 689
                            (66049) 66049 66055      66066      66077      66088      66099      66110      66121      66132       66144
        T4 NC_000866.4      (66049) TATTATCAATGACCCATTTACCGGCTTTAGGCACAAACTTCGGGCCTTTCTGTTTAGAGAACAGTTTTAAATTCCAGCGGCGAAGGTCTGCATCAG
  SARS-CoV-2 NC_045512.2        (1) ------------------------------------------------------------------------------------------------
                                                                                                    Section 690
                            (66145) 66145    66154      66165      66176      66187      66198      66209      66220               66240
        T4 NC_000866.4      (66145) CTTTAACAAATTCTGAGTCTAAGTTACTAGCAACAACATCTTCAATATGAGCAAATTTAAGTCCGTCAACTTCAATATTTAAATCGGTACGCCAGC
  SARS-CoV-2 NC_045512.2        (1) ------------------------------------------------------------------------------------------------
                                                                                                    Section 691
                            (66241) 66241       66253      66264      66275      66286      66297      66308      66319            66336
        T4 NC_000866.4      (66241) GAAGTCCTTCCCAAGCGAAGGCTTTAAAGTCGGAAGCCTTTGTAGCTAAGTACCGTTCAATAGGAGCTGTTTTAGGGTCAAAGCCGTTTCCTGATC
  SARS-CoV-2 NC_045512.2        (1) ------------------------------------------------------------------------------------------------
                                                                                                    Section 692
                            (66337) 66337          66352      66363      66374      66385      66396      66407      66418        66432
        T4 NC_000866.4      (66337) GGTAGGTCCACTCATCTTTGTTAATGCCTTTGGCCTTTACTACAGAAGCTTCGGCAATAAATTCTTGATATGTTTTCATATGTTTCCTTTAAATAT
  SARS-CoV-2 NC_045512.2        (1) ------------------------------------------------------------------------------------------------
                                                                                                    Section 693
                            (66433) 66433  66440      66451      66462      66473      66484      66495      66506      66517      66528
        T4 NC_000866.4      (66433) TTTAATTAGTAATTGTCTATTCAAGTAATTGTGAATATACTATCACAATTCCAAGAGAAAGTAAACAGCTTTATAGATTTTTATACGCGTCCCAAG
  SARS-CoV-2 NC_045512.2        (1) ------------------------------------------------------------------------------------------------
```

```
                                                                                                 Section 694
                        (66529) 66529     66539      66550      66561      66572      66583      66594      66605             66624
T4 NC_000866.4          (66529) TGCCAGTTCTAAACGTTGTAATGACTCGTTTTGCGCGATTAGGTGTTTGATTATACCATATACTTTTAGCTAAGTTAACTGCTGCTTCATCCCAGC
SARS-CoV-2 NC_045512.2      (1) ------------------------------------------------------------------------------------------------
                                                                                                 Section 695
                        (66625) 66625        66638      66649      66660      66671      66682      66693      66704           66720
T4 NC_000866.4          (66625) GTTTTTGTTGAAGCATACGTAAAGAGTTAGTAAATCCTGCCACACCGGTTTCTCCCATTTGGAAAACCATATTAATCAATGCACAGCGACGAACCG
SARS-CoV-2 NC_045512.2      (1) ------------------------------------------------------------------------------------------------
                                                                                                 Section 696
                        (66721) 66721 66726      66737      66748      66759      66770      66781      66792      66803       66816
T4 NC_000866.4          (66721) CATCAAGAGAATCATAAACCGGTTTTAATTTAGCATTTCTCAGAATTCCGCGAACAGCAGCATCAACATCCTGATTAAAGAGTTTTTCAGCCTCAT
SARS-CoV-2 NC_045512.2      (1) ------------------------------------------------------------------------------------------------
                                                                                                 Section 697
                        (66817) 66817   66825      66836      66847      66858      66869      66880      66891              66912
T4 NC_000866.4          (66817) CTTTTGTAATTACACCATTGCAATTACGCCCAATAGCTTTATCTAATTCAGATTTAGCAGCATTAAGTGATGGACTTTTTGTAAGCAAATGACCGA
SARS-CoV-2 NC_045512.2      (1) ------------------------------------------------------------------------------------------------
                                                                                                 Section 698
                        (66913) 66913      66924      66935      66946      66957      66968      66979      66990            67008
T4 NC_000866.4          (66913) TGCCAATAGTGTAATAGCCTTCTGTGTCTTTATAGATTTTAAGTCTAAGACGTTCATCTATACGTAACATTTCAAATATATTCATAATACCTCCTA
SARS-CoV-2 NC_045512.2      (1) ------------------------------------------------------------------------------------------------
                                                                                                 Section 699
                        (67009) 67009         67023      67034      67045      67056      67067      67078      67089          67104
T4 NC_000866.4          (67009) AGTATTTATAGAAGGTATTTATAAAATTAAAAGAGGTTGTTCATTATTCGGTAAAGTGAAGGACCCATCACATATTGCCACTGAGTACGAGGAATA
SARS-CoV-2 NC_045512.2      (1) ------------------------------------------------------------------------------------------------
                                                                                                 Section 700
                        (67105) 67105 67111      67122      67133      67144      67155      67166      67177      67188       67200
T4 NC_000866.4          (67105) AGAGCAAAAGCGTCCATCTCTGGAATCATAACGCCATCTTTATTTTCAAAATAAGACTCGCAACGGCAATTTCTGAACATCTCATGCTCTACTGGA
SARS-CoV-2 NC_045512.2      (1) ------------------------------------------------------------------------------------------------
```

Section 701

(67201) 67201 67210 67221 67232 67243 67254 67265 67276 67296
T4 NC_000866.4 (67201) ATCGTGTAATAAAATAACTGTAGGTCTTTATTACTAGAATATTTAAATACACCTAGGTCTTCTAGAAGGTCTGGATTATAATTGCTAAAACCAGTC
SARS-CoV-2 NC_045512.2 (1) --

Section 702

(67297) 67297 67309 67320 67331 67342 67353 67364 67375 67392
T4 NC_000866.4 (67297) TCTTCTAAACATTCTCTTCGTGCTGCATCTAATGCGCTTAAATCAGAATTTTCTACACGGCCCTTTGGAATATCCCAACGATGTGCCATCATTCCA
SARS-CoV-2 NC_045512.2 (1) --

Section 703

(67393) 67393 67408 67419 67430 67441 67452 67463 67474 67488
T4 NC_000866.4 (67393) GTCTTACGAGAACCAGTAACCCGACCCATAAATAAATCTTTATCTTCTGTCATAAAGATAATACCAGCTGATAATGTTTTCATTTTAATTTCCTGC
SARS-CoV-2 NC_045512.2 (1) --

Section 704

(67489) 67489 67496 67507 67518 67529 67540 67551 67562 67573 67584
T4 NC_000866.4 (67489) ATTCAGTGATAAAGTTATTTAAATTTTGAGCATATTTCTTTTCATCAAAAATCTTTTGCTGTCTGCGTAACCGCCATGGCATTTCAATGAACATAC
SARS-CoV-2 NC_045512.2 (1) --

Section 705

(67585) 67585 67595 67606 67617 67628 67639 67650 67661 67680
T4 NC_000866.4 (67585) GCCATATCCCTAGATAATACCGCTGCTGTAAAAATATTAACAAGTATAGTTAAAAGAATCCAATCGCCTATTCTGTCCATTGGATTTTTATAAAAA
SARS-CoV-2 NC_045512.2 (1) --

Section 706

(67681) 67681 67694 67705 67716 67727 67738 67749 67760 67776
T4 NC_000866.4 (67681) AGTAAAATACGAATGATGATATAGGAAGACTAATGATATACCACAGAAGAACCTTCTTATCTGTGAACCAATCAGCATTCGTTAACTTAGCGCGAC
SARS-CoV-2 NC_045512.2 (1) --

Section 707

(67777) 67777 67782 67793 67804 67815 67826 67837 67848 67859 67872
T4 NC_000866.4 (67777) CATTTTGAATACACACGAATTTATCATCTGTTACAGTAAATGGCTTAGCTGCTTGATATCCCATTCTAAACTCCCTAATTAATCGTTTCTTTGTAT
SARS-CoV-2 NC_045512.2 (1) --

```
                                                                                                   Section 708
                          (67873) 67873    67881      67892      67903      67914      67925      67936      67947        67968
           T4 NC_000866.4 (67873) CTTCGGAACAACCATTCCAATCAACTCTATCAACTGGAATGCCATCATCCCCATCATCTAAATCATACCAGCGAGTTTTTAAAATCATTTAATTTT
    SARS-CoV-2 NC_045512.2    (1) ------------------------------------------------------------------------------------------------
                                                                                                   Section 709
                          (67969) 67969      67980      67991      68002      68013      68024      68035      68046        68064
           T4 NC_000866.4 (67969) CCTGCAATCAATCACAAACTCTTTCATTGATTCATTTTCAATATAAGACATGTAGCTATTATATTCTTTTAATTGTATTTTGTAATCCTTTTTTCT
    SARS-CoV-2 NC_045512.2    (1) ------------------------------------------------------------------------------------------------
                                                                                                   Section 710
                          (68065) 68065         68079      68090      68101      68112      68123      68134      68145          68160
           T4 NC_000866.4 (68065) TTGCCAATTTATTTTAAAATTATCATAATGAAAATATAAAATGATACCAAAGAATGAAAACAATGAAATAATTTTAGTATAACAAAGCTCGCTCCA
    SARS-CoV-2 NC_045512.2    (1) ------------------------------------------------------------------------------------------------
                                                                                                   Section 711
                          (68161) 68161 68167      68178      68189      68200      68211      68222      68233      68244      68256
           T4 NC_000866.4 (68161) AACTTCTATTATATCTACTGTACCACTGATTTTTAAAATAAAACAGTCAATTAATAGTCCAATAAGACTACCTGTAAGAGCTGCAGCCAACGCCAC
    SARS-CoV-2 NC_045512.2    (1) ------------------------------------------------------------------------------------------------
                                                                                                   Section 712
                          (68257) 68257    68266      68277      68288      68299      68310      68321      68332          68352
           T4 NC_000866.4 (68257) AGCAAAAATTAAAAATGACTCAGAAAACGAATATTTGACTTTATTTAGCTTTGGCTTTTGCATCGTGATTCCTTAACAAATTTCATAATTTCATTA
    SARS-CoV-2 NC_045512.2    (1) ------------------------------------------------------------------------------------------------
                                                                                                   Section 713
                          (68353) 68353       68365      68376      68387      68398      68409      68420      68431          68448
           T4 NC_000866.4 (68353) AATTCATACTCAGCAAGTTTAAGCTGGTGTTCCTTTTTAATCTTTTTGCACTGGGCTTTCCAATCACGTACGCGTTTACGATAATGTCTTCCTTGA
    SARS-CoV-2 NC_045512.2    (1) ------------------------------------------------------------------------------------------------
                                                                                                   Section 714
                          (68449) 68449          68464      68475      68486      68497      68508      68519      68530        68544
           T4 NC_000866.4 (68449) TACCAGTATCCTATCCAATTTACGGGTACTAATAAAAATGGAACTACCAATGGAAGAGTTAGCATTAACATAATTATTACGCCAGAATCAATATCA
    SARS-CoV-2 NC_045512.2    (1) ------------------------------------------------------------------------------------------------
```

```
                                                                                                Section 715
                    (68545) 68545 68552      68563      68574      68585      68596      68607      68618      68629     68640
     T4 NC_000866.4 (68545) GTCATAACATCTAAAACACCTCCAATAATCAATAGAATTACAAATGATATAGCTATCACAGGACCTATTAATACATCAGTAGAAATTATCTGGCGC
SARS-CoV-2 NC_045512.2  (1) ------------------------------------------------------------------------------------------------
                                                                                                Section 716
                    (68641) 68641     68651      68662      68673      68684      68695      68706      68717              68736
     T4 NC_000866.4 (68641) TTTAATTCATACTTCAAAGGTTTACTTGGAAGGTATAGTGATGGCTTTGACATATTCTCTACATTCCTTAACAAATTTTTCTAGTAATAAATCACT
SARS-CoV-2 NC_045512.2  (1) ------------------------------------------------------------------------------------------------
                                                                                                Section 717
                    (68737) 68737        68750      68761      68772      68783      68794      68805      68816           68832
     T4 NC_000866.4 (68737) TTCAAAATTAGGATTTTCCACTAATTTATCAAAAAGATCATCAACAATATTCAAGATATTTCTTTTACTAAGAATACGTTTATTTTCATGCTTCGT
SARS-CoV-2 NC_045512.2  (1) ------------------------------------------------------------------------------------------------
                                                                                                Section 718
                    (68833) 68833 68838      68849      68860      68871      68882      68893      68904      68915     68928
     T4 NC_000866.4 (68833) TTCAGAATCAACTATAAGAGTAAAGAAATATTTCTTTCCCTGAAATTTTACCGTAGTATCAATATAAAATAAATTTGACTTTTGTAAATTACGTTT
SARS-CoV-2 NC_045512.2  (1) ------------------------------------------------------------------------------------------------
                                                                                                Section 719
                    (68929) 68929   68937      68948      68959      68970      68981      68992      69003              69024
     T4 NC_000866.4 (68929) AAACCATGCGTCACTTAAACTATAAACACCGAGATAATCAAAATCGTCGTTTAAATAACAAACTGACCATTCAGGAGAAATGAAATCAGTAAATTC
SARS-CoV-2 NC_045512.2  (1) ------------------------------------------------------------------------------------------------
                                                                                                Section 720
                    (69025) 69025      69036      69047      69058      69069      69080      69091      69102              69120
     T4 NC_000866.4 (69025) AACATCAAAATCACATGTCAATGAATGAATTGATTCAATACTGTTAATAAGTATTCCAGGACGTATTAAAGACTTTTTACCTCTGGAAAATCTTCC
SARS-CoV-2 NC_045512.2  (1) ------------------------------------------------------------------------------------------------
                                                                                                Section 721
                    (69121) 69121         69135      69146      69157      69168      69179      69190      69201          69216
     T4 NC_000866.4 (69121) AGAAAGACTTTCATCAGTTTCATATGAAGAACCCCAATAATAATTACGTCCTTTTGCCATATGTTTAAGAGCATTTAGTAATTGGTCTGGAACATC
SARS-CoV-2 NC_045512.2  (1) ------------------------------------------------------------------------------------------------
```

Section 722

```
                          (69217) 69217 69223 69234 69245 69256 69267 69278 69289 69300 69312
T4 NC_000866.4            (69217) AACGTGTCTTTGGAACTCTTCAAACATTGAATTGAAATCACTTTGCATTTTCATTCCTATTTACTCCAAGTAATAGGGGCCGAAGCCCCTTATCAT
SARS-CoV-2 NC_045512.2        (1) ------------------------------------------------------------------------------------------------
```

Section 723

```
                          (69313) 69313 69322 69333 69344 69355 69366 69377 69388 69408
T4 NC_000866.4            (69313) TATTTCAGAGAATTAATATATTCCTGAACATCGGCAGAGGTAGTTTCAACCCCAGAAATATTACCATTAAAGGTTTCAACTCGAGCAAGAGTATCT
SARS-CoV-2 NC_045512.2        (1) ------------------------------------------------------------------------------------------------
```

Section 724

```
                          (69409) 69409 69421 69432 69443 69454 69465 69476 69487 69504
T4 NC_000866.4            (69409) TCAATATCAACCTTAGTCAGTGCTGCAATTTCAACTACATCATCAGCAGTACTAATTCCAAGGGCATTTGCTGCACGAGTTTCACGGATATATTCC
SARS-CoV-2 NC_045512.2        (1) ------------------------------------------------------------------------------------------------
```

Section 725

```
                          (69505) 69505 69520 69531 69542 69553 69564 69575 69586 69600
T4 NC_000866.4            (69505) AATTTAACTGCAAGTTCTTGGCGAGCATCATCTAACTCAACTACTTTCTTGGCGATTTCAATTCGCATTTCAGCATAACCATCAGCTTTAGTAGTC
SARS-CoV-2 NC_045512.2        (1) ------------------------------------------------------------------------------------------------
```

Section 726

```
                          (69601) 69601 69608 69619 69630 69641 69652 69663 69674 69685 69696
T4 NC_000866.4            (69601) AGCTGTTCAGCTGTTCGACGATATAGCAAGCCGAGTTTAGCATGCATTGTTACATCTTGACCTTCGGAAAGAAGCTTGCGAATTTCACGCTCTTTT
SARS-CoV-2 NC_045512.2        (1) ------------------------------------------------------------------------------------------------
```

Section 727

```
                          (69697) 69697 69707 69718 69729 69740 69751 69762 69773 69792
T4 NC_000866.4            (69697) GATTCGGCCTGTTTATTCTTTTCAACAATAAGTTCACGAATACGTTTTTCTTCATTAATAGATTTAACAGAAGCAGTTTTTAGGTCTTTAATTTTA
SARS-CoV-2 NC_045512.2        (1) ------------------------------------------------------------------------------------------------
```

Section 728

```
                          (69793) 69793 69806 69817 69828 69839 69850 69861 69872 69888
T4 NC_000866.4            (69793) TCAAGTAGTTTTGCTGCTGCAGCAGTATACTGTTCTTCAACAGATAGATTTTTAGCCATTGCAGAACCAAGTTTAGTGCGAATAAACTCAACAATT
SARS-CoV-2 NC_045512.2        (1) ------------------------------------------------------------------------------------------------
```

```
                                                                                                   Section 729
                         (69889) 69889 69894     69905     69916     69927     69938     69949     69960     69971   69984
T4 NC_000866.4           (69889) TTCTTCAGTGTGTTCATAGTATTTCCTTAGGTTGGTATAATTAGATAATATAATATCACGTTTCTAATAGATTGTAAACTTATTCTTCGTCTAGCT
SARS-CoV-2 NC_045512.2       (1) ------------------------------------------------------------------------------------------------
                                                                                                   Section 730
                         (69985) 69985   69993     70004     70015     70026     70037     70048     70059           70080
T4 NC_000866.4           (69985) CGTCGATAAAGGCGTTGATGGCCTCGATAATGGCATCATTGATAGCCAATAAAATAAAATCATCGTCAGTACCTTTAGAAGATTCTAAAGCATTGA
SARS-CoV-2 NC_045512.2       (1) ------------------------------------------------------------------------------------------------
                                                                                                   Section 731
                         (70081) 70081     70092     70103     70114     70125     70136     70147     70158           70176
T4 NC_000866.4           (70081) TATATGCTTGGTTGACGAGTTCCCAAGCCTTTTTAAAATAAGGAGCTTCATCATCAGGACAAATATCCCGCACGCCTTCAAAGATACGTTTGGCAT
SARS-CoV-2 NC_045512.2       (1) ------------------------------------------------------------------------------------------------
                                                                                                   Section 732
                         (70177) 70177        70191     70202     70213     70224     70235     70246     70257        70272
T4 NC_000866.4           (70177) AATCTAACACCCATTGTACAGGCATGTTTTGAGAACGTTCGTTAAACTCTTTAAAGTCCTTAGATTCAAAAAGCTCTTCAGGATAATTATTTCTAT
SARS-CoV-2 NC_045512.2       (1) ------------------------------------------------------------------------------------------------
                                                                                                   Section 733
                         (70273) 70273 70279     70290     70301     70312     70323     70334     70345     70356   70368
T4 NC_000866.4           (70273) TACAAAAAGCTTTACTAAAGTTACGTTTCATAATGTTTTCCTCATTTGTATAGGCTCATAATATCTCAATCATSAGCCTATGTAAACTTATTTCAT
SARS-CoV-2 NC_045512.2       (1) ------------------------------------------------------------------------------------------------
                                                                                                   Section 734
                         (70369) 70369    70378     70389     70400     70411     70422     70433     70444            70464
T4 NC_000866.4           (70369) ATTATTGAAATATTCTTCTGCGATTTCGTCGTTATCATGGTAAACTTTAGAAGACAGTTTAACATAACTTTCAGCAGTGAACATGTTAATCACAAC
SARS-CoV-2 NC_045512.2       (1) ------------------------------------------------------------------------------------------------
                                                                                                   Section 735
                         (70465) 70465       70477     70488     70499     70510     70521     70532     70543         70560
T4 NC_000866.4           (70465) CTTTACAGTATACCACTGACCGTCTTCATTACCCATTACTGCGTAAGTTTCAAACATCGGATGGTCAGGACCGATAACTTTAATATCATTCACCGT
SARS-CoV-2 NC_045512.2       (1) ------------------------------------------------------------------------------------------------
```

Section 736

```
                       (70561) 70561          70576      70587      70598      70609      70620      70631      70642         70656
T4 NC_000866.4         (70561) ACGACCGAAATCTTCTGAAACACATTTCATAAAGAAGTTGAAAAGTTCACCGTAATTATCCATTTTATTCTCCAAGTTATTTTCTGTATCAGTAGT
SARS-CoV-2 NC_045512.2     (1) ------------------------------------------------------------------------------------------------
```

Section 737

```
                       (70657) 70657  70664      70675      70686      70697      70708      70719      70730      70741      70752
T4 NC_000866.4         (70657) TGATAGTTGTATAGTACCATGGAAGAACAAGGATGTAAACAGTTTTGTGAAAAAATTTTTAAAAAGTTTTAGGGAATTCTAGGGCGGAGAGGGGCA
SARS-CoV-2 NC_045512.2     (1) ------------------------------------------------------------------------------------------------
```

Section 738

```
                       (70753) 70753     70763      70774      70785      70796      70807      70818      70829              70848
T4 NC_000866.4         (70753) ATTAAAAGATAGGATAATATATTATAAAGGGTATAAACTAAATGATGCCTAGAGAGGTCTGGAAAGGCTTAGATACCAAAAAGCCCCAACCTTTCG
SARS-CoV-2 NC_045512.2     (1) ------------------------------------------------------------------------------------------------
```

Section 739

```
                       (70849) 70849        70862      70873      70884      70895      70906      70917      70928           70944
T4 NC_000866.4         (70849) GTCGGGGCTAACCGTTGCGGCAACCTTGTCGGGGTTCCACCTGCCAAGGCAAGTGTTTGTACGAAACGCCGGGATTCGAACCCGGTTATTAAGTAG
SARS-CoV-2 NC_045512.2     (1) ------------------------------------------------------------------------------------------------
```

Section 740

```
                       (70945) 70945 70950      70961      70972      70983      70994      71005      71016      71027        71040
T4 NC_000866.4         (70945) TTGACGCTACTCAATATTTTTAAAAGGCCATATCTCAACCATATCCGAACGTTCCGTCAAAAACGCTACTCGGCTTACGGCAAAGATATTTCCTCG
SARS-CoV-2 NC_045512.2     (1) ------------------------------------------------------------------------------------------------
```

Section 741

```
                       (71041) 71041   71049      71060      71071      71082      71093      71104      71115                71136
T4 NC_000866.4         (71041) AATCGATAATTTGGTGCGCCGTTTCTGCTGTGATGTAAGAGGGCATCAATAAACGCAAAGATTATTAACGCAATTCCTTACTCAGGGAACCATCAG
SARS-CoV-2 NC_045512.2     (1) ------------------------------------------------------------------------------------------------
```

Section 742

```
                       (71137) 71137      71148      71159      71170      71181      71192      71203      71214            71232
T4 NC_000866.4         (71137) TCCGACGACTTACCGGTAGCGACCCGGTTTCTCATTTGGTATCCCGCCCTGGGATCGAACCAGGACCGCAAACTTAGAAGGATCGTATGCTATCCA
SARS-CoV-2 NC_045512.2     (1) ------------------------------------------------------------------------------------------------
```

```
                                                                                                     Section 743
                          (71233) 71233         71247     71258      71269      71280      71291      71302      71313        71328
T4 NC_000866.4            (71233) TTACACCAGCGGGACGTAATTTAAAATTTCATTTTTCGACCTTTAAACCATCCTTCTGGAATTGGGTCAGTTTTCTTAATACGTTTAGAAACTTTT
SARS-CoV-2 NC_045512.2        (1) ------------------------------------------------------------------------------------------------
                                                                                                     Section 744
                          (71329) 71329 71335      71346      71357      71368      71379      71390      71401      71412     71424
T4 NC_000866.4            (71329) TCATCTAATGAATGAATCCACATCATACCGAATTGGGAATTCTTTTCACCTTTCTGGTGATTATTTTTGGCGTGAGATTCTTTCATTTTATTAATA
SARS-CoV-2 NC_045512.2        (1) ------------------------------------------------------------------------------------------------
                                                                                                     Section 745
                          (71425) 71425    71434      71445      71456      71467      71478      71489      71500               71520
T4 NC_000866.4            (71425) GTTTCAGGAGTATGATGCTTATTTAGAAATCTGCTATTATTTAAAAATTTTTCCCTGTATTCAGGAGTTGACCACAAACGTTTAAATACATTTGAA
SARS-CoV-2 NC_045512.2        (1) ------------------------------------------------------------------------------------------------
                                                                                                     Section 746
                          (71521) 71521       71533      71544      71555      71566      71577      71588      71599            71616
T4 NC_000866.4            (71521) CCAATTTTACGATATTTTTCTTGAAGTAAAATATCATTTTCAAAACGTGACTTAAACGATTTAGCTCCTTTTAAGCTAGCATCTTTCTTCTGGTTT
SARS-CoV-2 NC_045512.2        (1) ------------------------------------------------------------------------------------------------
                                                                                                     Section 747
                          (71617) 71617          71632      71643      71654      71665      71676      71687      71698          71712
T4 NC_000866.4            (71617) AGCATTCCAGGAATATTTACATGATCCCATCCACCTTCACCGCCAAGTTTTAAATTATACACATCTGGTCTATTTAAAAACTCTTCTGTGACAATA
SARS-CoV-2 NC_045512.2        (1) ------------------------------------------------------------------------------------------------
                                                                                                     Section 748
                          (71713) 71713  71720      71731      71742      71753      71764      71775      71786      71797     71808
T4 NC_000866.4            (71713) TTTTTCTCGGCTTCAAGCATAGATTCTTTATCGTCAAAATACTCTAATATTTCTTTAGAAAAATTTTCTATACCATATTTATCTTGGGCTCTTTTT
SARS-CoV-2 NC_045512.2        (1) ------------------------------------------------------------------------------------------------
                                                                                                     Section 749
                          (71809) 71809     71819      71830      71841      71852      71863      71874      71885              71904
T4 NC_000866.4            (71809) AATAATTTACCAGAACCCATATATCCATCATCTAAATTTTCGGTAGAATGCACACCAATATAAATTTTATTATTAATTTTATTTGTTATTTTATAA
SARS-CoV-2 NC_045512.2        (1) ------------------------------------------------------------------------------------------------
```

```
                                                                                                        Section 750
                         (71905) 71905        71918      71929      71940      71951      71962      71973      71984           72000
         T4 NC_000866.4  (71905) GTGTAATAGAACATAAATATCTCCTATTTCTAAGAGTATTTATGTTCTCAAAATATGACCCAGACCAGATTTGAACTGGTAACCTTTCCCTTATGA
  SARS-CoV-2 NC_045512.2     (1) ------------------------------------------------------------------------------------------------
                                                                                                        Section 751
                         (72001) 72001 72006      72017      72028      72039      72050      72061      72072      72083      72096
         T4 NC_000866.4  (72001) GGGGACTGCTGCTAACCATTGAGCTACAGGGCCTTGGTGCTGATTGACGGAATCGAACCGCCGACATCCTCATTACAAGTGAGGTGCTCTACCTAC
  SARS-CoV-2 NC_045512.2     (1) ------------------------------------------------------------------------------------------------
                                                                                                        Section 752
                         (72097) 72097   72105      72116      72127      72138      72149      72160      72171           72192
         T4 NC_000866.4  (72097) TGAGCTAAATCAGCAAAATTACGGAGGCGATAGGATTTGAACCTATGAGTCGCCGGAGCGACTGCCGGTTTTCAAGACCGGTGCATTAAACCACTC
  SARS-CoV-2 NC_045512.2     (1) ------------------------------------------------------------------------------------------------
                                                                                                        Section 753
                         (72193) 72193      72204      72215      72226      72237      72248      72259      72270           72288
         T4 NC_000866.4  (72193) TGCCACGCCTCCAGTCTCCATACAAGGATTTGAACCTTGGACCTCCTGATCCCAAATCAGGCGCTCTACCAAACTGAGCTACACGGAGTAAATTAA
  SARS-CoV-2 NC_045512.2     (1) ------------------------------------------------------------------------------------------------
                                                                                                        Section 754
                         (72289) 72289         72303      72314      72325      72336      72347      72358      72369      72384
         T4 NC_000866.4  (72289) ATTGGAGCGGATAATGAGAATCGAACTCACATCATCAGATTGGAAGTCTGAGGTAATACCATTATACGATATCCGCAAATTTGGTGCGAGAAGTGG
  SARS-CoV-2 NC_045512.2     (1) ------------------------------------------------------------------------------------------------
                                                                                                        Section 755
                         (72385) 72385 72391      72402      72413      72424      72435      72446      72457      72468      72480
         T4 NC_000866.4  (72385) GACTCGAACCCACAAGGAAATCATTCCGCAGCATTTTAAGTGCTGTGCCTTTACCAATTTGACCATTCTCGCGCTGGGAATAAAGGACTCGAACCT
  SARS-CoV-2 NC_045512.2     (1) ------------------------------------------------------------------------------------------------
                                                                                                        Section 756
                         (72481) 72481    72490      72501      72512      72523      72534      72545      72556           72576
         T4 NC_000866.4  (72481) TTGCATCTAGCAGTCAAAGTGCTATGCCTTACCAACTTGGCTAATTCCCAATTATTAACAAAGGCTCTCTAACAAGAACCCTTGATGATAGAGGGT
  SARS-CoV-2 NC_045512.2     (1) ------------------------------------------------------------------------------------------------
```

```
                                                                                                   Section 757
                         (72577) 72577       72589      72600      72611      72622      72633      72644      72655           72672
T4 NC_000866.4           (72577) ATTAATCAGTGCGGTATGAGTTAATAATAACAAATAATTCTTAAAGCATATTTACCATTTATGATGATACGTATTTACGATACATTCAAGACCCAA
SARS-CoV-2 NC_045512.2   (1)     ------------------------------------------------------------------------------------------------
                                                                                                   Section 758
                         (72673) 72673          72688      72699      72710      72721      72732      72743      72754         72768
T4 NC_000866.4           (72673) AGGATTCTTGAAAATATCATATTCAAGAGGACCTTTTTCTGTTTCAATAAAGAAATCAAAATTTACTGTATTAAATTTACGGTCTTCCTTTACTAA
SARS-CoV-2 NC_045512.2   (1)     ------------------------------------------------------------------------------------------------
                                                                                                   Section 759
                         (72769) 72769  72776      72787      72798      72809      72820      72831      72842      72853      72864
T4 NC_000866.4           (72769) TTTAACTTGAGAAGATGAACGATCAATGTAAACCTTTTCAACTTCAAAACACGTTAAAATGCCATAATCATCAATCAAGGCTTTAGCTGCTTCTTG
SARS-CoV-2 NC_045512.2   (1)     ------------------------------------------------------------------------------------------------
                                                                                                   Section 760
                         (72865) 72865     72875      72886      72897      72908      72919      72930      72941              72960
T4 NC_000866.4           (72865) ATCATATTTATATCCATTTTCAACGGATGATACTTTCGCATAAAGAATCATCATCAACCTCTATCAACAATAGCATGAGTATGGGCATTTACGATT
SARS-CoV-2 NC_045512.2   (1)     ------------------------------------------------------------------------------------------------
                                                                                                   Section 761
                         (72961) 72961        72974      72985      72996      73007      73018      73029      73040           73056
T4 NC_000866.4           (72961) TGCCACCAGTCGAAACGATTGGAACCATAATCTGGTTTATTTTCATTTTCTTTAATGATATCACGCAGTTTATCTTCTGTTTCAGCATACGCAATT
SARS-CoV-2 NC_045512.2   (1)     ------------------------------------------------------------------------------------------------
                                                                                                   Section 762
                         (73057) 73057 73062      73073      73084      73095      73106      73117      73128      73139      73152
T4 NC_000866.4           (73057) AAATCATCATATCCACCACAAGGATAATAATTATCACCTGCGAATAAAAGAAAATTTACCTTAGATGGATTTACGTAATAATGGTCTTTAGGATAT
SARS-CoV-2 NC_045512.2   (1)     ------------------------------------------------------------------------------------------------
                                                                                                   Section 763
                         (73153) 73153   73161      73172      73183      73194      73205      73216      73227                73248
T4 NC_000866.4           (73153) TTAGTTCCTCTCCAATCAGTTACTTCAACATAACGGTAAGAAAATCCATTTTTACTTTCAATCCAACTCCACGCTTCAAAAGGAGTATTAAAAACT
SARS-CoV-2 NC_045512.2   (1)     ------------------------------------------------------------------------------------------------
```

```
                                                                                                    Section 764
                         (73249) 73249      73260      73271      73282      73293      73304      73315      73326             73344
         T4 NC_000866.4  (73249) TTATCAGGTATTAAATTACCTTCAAAATGAGAAGGATTTGCATAATCCCCGGCATAAACATAATATTCGTTAATACTCATTTATTCACCTTTAGAA
SARS-CoV-2 NC_045512.2       (1) ------------------------------------------------------------------------------------------------
                                                                                                    Section 765
                         (73345) 73345         73359      73370      73381      73392      73403      73414      73425          73440
         T4 NC_000866.4  (73345) ATTTTATCCATAACGATAGCAATTAAACCAATTAAAAATGCTACTACAAGTGAAAACACATTTTCTGCTGTAGTTAATAATCCGCATATAAATCCA
SARS-CoV-2 NC_045512.2       (1) ------------------------------------------------------------------------------------------------
                                                                                                    Section 766
                         (73441) 73441 73447      73458      73469      73480      73491      73502      73513      73524       73536
         T4 NC_000866.4  (73441) ACAAACATTGAAAAACTAAAAGCAGAAGCAGAAATTGCAATAGCAACATTTCGAATTAATTCACAGCGTTTCATTTTATTCTCCTCAGTAGTTGAT
SARS-CoV-2 NC_045512.2       (1) ------------------------------------------------------------------------------------------------
                                                                                                    Section 767
                         (73537) 73537    73546      73557      73568      73579      73590      73601      73612              73632
         T4 NC_000866.4  (73537) AGGGTAATAGTATCACAGCTAAAACCCTATGTAAACAACTTTGTGAAATATTTATTACAAAAGATTTTTAGCAATAATCTTGAGATGTGCCGCAGA
SARS-CoV-2 NC_045512.2       (1) ------------------------------------------------------------------------------------------------
                                                                                                    Section 768
                         (73633) 73633       73645      73656      73667      73678      73689      73700      73711            73728
         T4 NC_000866.4  (73633) AATGTGTTTAGCTTTAAACAACGCAGTTTCTTCAGCAGGAGAGATAACGATTGTAGCACCATCCTTTTTAGCAGACCACCCATCACCTAGGTAAAC
SARS-CoV-2 NC_045512.2       (1) ------------------------------------------------------------------------------------------------
                                                                                                    Section 769
                         (73729) 73729          73744      73755      73766      73777      73788      73799      73810         73824
         T4 NC_000866.4  (73729) AGTACCTTTGATTTCTTCGCCATCAACCAGACTAATCATTGGTTTACCTTCTCGTCCTTTATTTGCTTTAATAACTTCAGAAGTAAGAGTAGCTTC
SARS-CoV-2 NC_045512.2       (1) ------------------------------------------------------------------------------------------------
                                                                                                    Section 770
                         (73825) 73825  73832      73843      73854      73865      73876      73887      73898      73909      73920
         T4 NC_000866.4  (73825) GGTAATGGTAGAAACCGGGGTAGTTGTAGAAGTAAATTCTTTAAATGTTTTCATTTTTATTTTCCTAATTAATTTTGATGAGGTAATAGTATCACT
SARS-CoV-2 NC_045512.2       (1) ------------------------------------------------------------------------------------------------
```

```
                                                                                                   Section 771
                          (73921) 73921     73931      73942      73953      73964      73975      73986      73997           74016
T4 NC_000866.4            (73921) ACCTCATCAGTATGTAAACAACTTTGTGAAATTATTTTAAATCATCTGCCCAATCGAGTTTAAGAGGCTCTTTGTATTCACGATCTAATACGACCG
SARS-CoV-2 NC_045512.2        (1) ------------------------------------------------------------------------------------------------
                                                                                                   Section 772
                          (74017) 74017        74030      74041      74052      74063      74074      74085      74096           74112
T4 NC_000866.4            (74017) GAATTTGTACATCACCGCTAAATGATAAGGGCCCAACATTATAAGACAATGTTATATGCGGTGTGTAATCATCAAAATCATGTGTAGCACCTAGTG
SARS-CoV-2 NC_045512.2        (1) ------------------------------------------------------------------------------------------------
                                                                                                   Section 773
                          (74113) 74113 74118      74129      74140      74151      74162      74173      74184      74195        74208
T4 NC_000866.4            (74113) CCCGCGCATACATGTGTCGACAGCGCAGATATTCAGAATCTAGCACAAGTACAAGAGTCGATCCATCTTGTGTTTTCCATACTTCTAAATGTCCAG
SARS-CoV-2 NC_045512.2        (1) ------------------------------------------------------------------------------------------------
                                                                                                   Section 774
                          (74209) 74209   74217      74228      74239      74250      74261      74272      74283                74304
T4 NC_000866.4            (74209) AAGAAGCTACTTCAAAACTTCCACTCGATGGAACATATGGAACATTTACTCTTGAATAACATATAGTCGAATGAATTTTTTCTCTAGGAACTGGAT
SARS-CoV-2 NC_045512.2        (1) ------------------------------------------------------------------------------------------------
                                                                                                   Section 775
                          (74305) 74305      74316      74327      74338      74349      74360      74371      74382           74400
T4 NC_000866.4            (74305) TAGGAACACGTAAAGAGCGCTGAAGTTCTTCCAGCGCATCAAGTGTTAATTCTGAAAACTTAGCTGCTACATAAAGACCCGTTGAAAAGTCTTTAA
SARS-CoV-2 NC_045512.2        (1) ------------------------------------------------------------------------------------------------
                                                                                                   Section 776
                          (74401) 74401         74415      74426      74437      74448      74459      74470      74481      74496
T4 NC_000866.4            (74401) ATTCCATCATTCTTCATCTTTTGCTTCATCTGCAGATTCAGCAGTAAGATTTTTGACAGCTTCAACGATTTCTTCAACTTTGATAGTATCGCCAGT
SARS-CoV-2 NC_045512.2        (1) ------------------------------------------------------------------------------------------------
                                                                                                   Section 777
                          (74497) 74497 74503      74514      74525      74536      74547      74558      74569      74580       74592
T4 NC_000866.4            (74497) GATACCTACTGCACGAGCAATTTCAGCCAAAGTTCCTTGCAGAATTTTGGATTCTTCCATCAGACGAGCAGCTTGATCCTGCGTATCAAGAATGCG
SARS-CoV-2 NC_045512.2        (1) ------------------------------------------------------------------------------------------------
```

```
                                                                                                   Section 778
                          (74593) 74593    74602      74613      74624      74635      74646      74657      74668          74688
        T4 NC_000866.4    (74593) AGATTTCAGAGTTACGATTTCAGCAGACAGTTTTTGTTCAACAGTTTGTTCAGACATTATAGTACCTTTAGTGTATTTTTAATTTTAGAAAAAAGT
  SARS-CoV-2 NC_045512.2      (1) ------------------------------------------------------------------------------------------------
                                                                                                   Section 779
                          (74689) 74689       74701      74712      74723      74734      74745      74756      74767           74784
        T4 NC_000866.4    (74689) TCTTCAAGAGAACCATCGTTTGTAATTACTAAATCGCCATCACGAATTGGCAATCCAGCTTCTGTAATATGTGTATCATTGGATTTTTGACCAGGA
  SARS-CoV-2 NC_045512.2      (1) ------------------------------------------------------------------------------------------------
                                                                                                   Section 780
                          (74785) 74785          74800      74811      74822      74833      74844      74855      74866         74880
        T4 NC_000866.4    (74785) CGAACTACATGAATTACTGTAGCACCCATCGCCCTAGCCGCATCCATTTCATGATCTTGACGGGTATCAGGAACGATATAATAATCATAACCTGAG
  SARS-CoV-2 NC_045512.2      (1) ------------------------------------------------------------------------------------------------
                                                                                                   Section 781
                          (74881) 74881  74888      74899      74910      74921      74932      74943      74954      74965    74976
        T4 NC_000866.4    (74881) TTAAATTTATCAAGATAATCTAAAGCAAATAATTTTACCCAGTACATGCGGTCGAAGTTATTAACAATCAAATCCGTACCTAGGGCTTGCATCAGA
  SARS-CoV-2 NC_045512.2      (1) ------------------------------------------------------------------------------------------------
                                                                                                   Section 782
                          (74977) 74977     74987      74998      75009      75020      75031      75042      75053          75072
        T4 NC_000866.4    (74977) CGACGGACTGACCATTGATCTTCAATATTATTTATAACGTCAGTAATCTTATTAAATGCTACGAAATTAACTGATTCTTTTCCTTCGTCATCAAAA
  SARS-CoV-2 NC_045512.2      (1) ------------------------------------------------------------------------------------------------
                                                                                                   Section 783
                          (75073) 75073        75086      75097      75108      75119      75130      75141      75152          75168
        T4 NC_000866.4    (75073) ACAAACACACCTTTAATTGGGCTTTTACCATTAAGATAACAAAATGCTTGTTCCATAATCGTGATTACTTCTAATTTAGTCAGATTTAAATTAGTC
  SARS-CoV-2 NC_045512.2      (1) ------------------------------------------------------------------------------------------------
                                                                                                   Section 784
                          (75169) 75169 75174      75185      75196      75207      75218      75229      75240      75251      75264
        T4 NC_000866.4    (75169) TCACGATCATAGTCAATTCCTTCAAACTCTTTACGAGTTAAGCAAGGATAGTCAGTGTTTGCTGCAAATACTCCCCATGCATAAGCCAATGCATCC
  SARS-CoV-2 NC_045512.2      (1) ------------------------------------------------------------------------------------------------
```

Section 785

```
                    (75265) 75265   75273      75284      75295      75306      75317      75328      75339                75360
       T4 NC_000866.4 (75265) TTAATAGGACCAGCAAGTTGGTATTTAACTGCAGAATAATTGCTCATGATAAAATCAGCAGTAGTATCTTTTCCACTACGCTTTACACCGCTTAAA
SARS-CoV-2 NC_045512.2     (1) ------------------------------------------------------------------------------------------------
```

Section 786

```
                    (75361) 75361      75372      75383      75394      75405      75416      75427      75438            75456
       T4 NC_000866.4 (75361) AAGATTAGTTTCATGTGTTTCTCCTCAAATTTAATTAAGATTATAACACACAAAACTGAAGCATTAAACTTCTGCTATAATTTTACCATCTTTTTC
SARS-CoV-2 NC_045512.2     (1) ------------------------------------------------------------------------------------------------
```

Section 787

```
                    (75457) 75457         75471      75482      75493      75504      75515      75526      75537          75552
       T4 NC_000866.4 (75457) TACTTGAAAATAGGTGTAAGGAATTGTTGCAGTACATACTAAAGCCGGGTCTGAATCTTCCGTGTAGCTAAATTCTACTTCAGATAGGTCAGAAAC
SARS-CoV-2 NC_045512.2     (1) ------------------------------------------------------------------------------------------------
```

Section 788

```
                    (75553) 75553 75559      75570      75581      75592      75603      75614      75625      75636      75648
       T4 NC_000866.4 (75553) CCAAGGCTTATAAAAATTTATTGACATCACGATTTCAGTTTTGCTATTATCTAAGATGTAAAGCGTAATGTACTCAGGACCTGTTTTTTGGGCAGT
SARS-CoV-2 NC_045512.2     (1) ------------------------------------------------------------------------------------------------
```

Section 789

```
                    (75649) 75649    75658      75669      75680      75691      75702      75713      75724            75744
       T4 NC_000866.4 (75649) ATTTTCACCTGTAAGATAGTTGCTAGTTCCTAGCATCCATTCATACATTCCTATCCACGACTTAAGTTCTTCATCAACTATAAATCTCACAATGAG
SARS-CoV-2 NC_045512.2     (1) ------------------------------------------------------------------------------------------------
```

Section 790

```
                    (75745) 75745       75757      75768      75779      75790      75801      75812      75823            75840
       T4 NC_000866.4 (75745) TGGATCATACTCAAATGTAACACCTGGACGTTGTGCTCGGCCCAGTCCAAACGGCCCAGTCACGGTATCAGTAACAGGTATTCTAATTCCTGGAAT
SARS-CoV-2 NC_045512.2     (1) ------------------------------------------------------------------------------------------------
```

Section 791

```
                    (75841) 75841          75856      75867      75878      75889      75900      75911      75922          75936
       T4 NC_000866.4 (75841) AGGAACTGACTGAGCATTTAAAGTAAAAGCAGATGTAGTATTACTATGTGGTATTGATACTACAAAGTTAGTTGTATTTGCTTGGTTAAAAATTTG
SARS-CoV-2 NC_045512.2     (1) ------------------------------------------------------------------------------------------------
```

```
                                                                                                    Section 792
                           (75937) 75937 75944      75955      75966      75977      75988      75999      76010      76021     76032
          T4 NC_000866.4   (75937) TTGCAGAGCTTGCGACATATATTCCTCATAATGCTTTATAACTGTTGGTGGTATAATGGGTCTAAGTCCCTTCCATTCAATTCCATTTAGAACAAA
 SARS-CoV-2 NC_045512.2        (1) ------------------------------------------------------------------------------------------------
                                                                                                    Section 793
                           (76033) 76033    76043      76054      76065      76076      76087      76098      76109               76128
          T4 NC_000866.4   (76033) CAACAGAAAAGAATGGAAGATAATAGAATTAGATATTTGACCAGACTTTGTTTGCAGAGAAACGTTTTCCTTTTGAAACGAACTGCTGAAGTGGCA
 SARS-CoV-2 NC_045512.2        (1) ------------------------------------------------------------------------------------------------
                                                                                                    Section 794
                           (76129) 76129        76142      76153      76164      76175      76186      76197      76208           76224
          T4 NC_000866.4   (76129) TCAACACAACGTTCGCCCAGTCTTTCGGGGCGATTTCAACAAGGCTACCCATAATATTACCAGGTATATATGCCTTAATCATTTGGTCTGCACCCC
 SARS-CoV-2 NC_045512.2        (1) ------------------------------------------------------------------------------------------------
                                                                                                    Section 795
                           (76225) 76225 76230      76241      76252      76263      76274      76285      76296      76307     76320
          T4 NC_000866.4   (76225) TAAATCCTTTCACTTGACTCCAATCAATTTTTAATTTCGTTTTATTAGTAATAGTAGGTGTATTTGCATATTGCTTTAAAAGCTCTTCTAGGAATT
 SARS-CoV-2 NC_045512.2        (1) ------------------------------------------------------------------------------------------------
                                                                                                    Section 796
                           (76321) 76321   76329      76340      76351      76362      76373      76384      76395                76416
          T4 NC_000866.4   (76321) GCTGACGAGCTTTAGGTGGAATATAGTGCAAGTTTAATCCGTACATTAAATTATGCTTACCTAAACCAAGGTAAATTATCAAAGGAAATTTATCCC
 SARS-CoV-2 NC_045512.2        (1) ------------------------------------------------------------------------------------------------
                                                                                                    Section 797
                           (76417) 76417      76428      76439      76450      76461      76472      76483      76494            76512
          T4 NC_000866.4   (76417) AGTAAGGAAGAGTTTCCTTGTGTTTAGCATCATAAGCAAAAGCATATATTCGTCCCGGCTGCGGGCGAACAACTTTATGTCCTTTTACTTGCTTAA
 SARS-CoV-2 NC_045512.2        (1) ------------------------------------------------------------------------------------------------
                                                                                                    Section 798
                           (76513) 76513         76527      76538      76549      76560      76571      76582      76593          76608
          T4 NC_000866.4   (76513) TAGTTTCAGCAAACCACTTTCTGGTTTTATTATTAATTGCTGCGCCTTCATTACGAATTTTATCACGCAATGTTTGTCTGAATGAATTTATCATAA
 SARS-CoV-2 NC_045512.2        (1) ------------------------------------------------------------------------------------------------
```

```
                                                                                                      Section 799
                          (76609) 76609 76615      76626      76637      76648      76659      76670      76681      76692      76704
T4 NC_000866.4            (76609) GCAGTTGTCTTTCTTGCTTATTGAGTTTATTCATTGGTTTTGATTCAAGTTTTTGAATCTTTTCAGCCGTTTTAATTCCTGAAGCATATTTTGACA
SARS-CoV-2 NC_045512.2        (1) ------------------------------------------------------------------------------------------------
                                                                                                      Section 800
                          (76705) 76705    76714      76725      76736      76747      76758      76769      76780               76800
T4 NC_000866.4            (76705) TTGCTGAAGTAAACGTAGAGTATTTGATTCCTCTTTCTTCAGCAAATTGCTTTCCTGTCATTCCTTTTGCTTTGGCCTTTTTATATTCAAGACCTA
SARS-CoV-2 NC_045512.2        (1) ------------------------------------------------------------------------------------------------
                                                                                                      Section 801
                          (76801) 76801       76813      76824      76835      76846      76857      76868      76879            76896
T4 NC_000866.4            (76801) TCTGAATCCATTTCTTTTCGTTTAATGATTGCTTAACCTTTGGAACTTGGGGAGTGCTTTCATTAATTATTTGAAAAATAGCCATTATGCCCCCTT
SARS-CoV-2 NC_045512.2        (1) ------------------------------------------------------------------------------------------------
                                                                                                      Section 802
                          (76897) 76897          76912      76923      76934      76945      76956      76967      76978         76992
T4 NC_000866.4            (76897) AAAGCCAAGAGCTCGTAATCCATCTTCTGTTAGAATTCTAAATTTATTCCACGCTTTTCAGCTAAAGATTGTGCTGCTTTCCATTTGTCAGTGTT
SARS-CoV-2 NC_045512.2        (1) ------------------------------------------------------------------------------------------------
                                                                                                      Section 803
                          (76993) 76993  77000      77011      77022      77033      77044      77055      77066      77077      77088
T4 NC_000866.4            (76993) CACAGACCAGGTATAAATTTCATTCATAAATCTTTTCTTCGCTGCGGTCGTTAGATGTGCTGGTTTAACTGGTGGTTGTGTTTCTTTTTTAGGTTT
SARS-CoV-2 NC_045512.2        (1) ------------------------------------------------------------------------------------------------
                                                                                                      Section 804
                          (77089) 77089     77099      77110      77121      77132      77143      77154      77165               77184
T4 NC_000866.4            (77089) TATTTCAATAAAAAATTCTTGTCCAGAAGAATCTTTCATCCAAATATCCATGAAGTATCTACGTTTTTCCCTTCTGCATTACAAAAATAAGGAAT
SARS-CoV-2 NC_045512.2        (1) ------------------------------------------------------------------------------------------------
                                                                                                      Section 805
                          (77185) 77185        77198      77209      77220      77231      77242      77253      77264            77280
T4 NC_000866.4            (77185) TACTGCTGTTTCACTACCCCATGCAATAATTTCTGGATTTTTATCTAACCATTCAAAAAAGAATTTTTCCCAATTTGATCTATACGTAATTTTTTT
SARS-CoV-2 NC_045512.2        (1) ------------------------------------------------------------------------------------------------
```

```
                                         Section 806
                    (77281) 77281 77286 77297 77308 77319 77330 77341 77352 77363 77376
T4 NC_000866.4      (77281) AGGGTCACCTCTATACTTTGATATATTTTTAGGAACCCATTTTCCAGAATATGCCATTGGATTCTCCTTATAAATAGATAATATATTTATAAACAG
SARS-CoV-2 NC_045512.2  (1) ------------------------------------------------------------------------------------------------
                                         Section 807
                    (77377) 77377 77385 77396 77407 77418 77429 77440 77451 77472
T4 NC_000866.4      (77377) GAGGGCCCATGCTCTTTACATTTTTTGATCCGATTGAATATGCGGCCAAAACGGTGAATAAAAACGCGCCGACTATTCCTATGACAGATATTTTTA
SARS-CoV-2 NC_045512.2  (1) ------------------------------------------------------------------------------------------------
                                         Section 808
                    (77473) 77473 77484 77495 77506 77517 77528 77539 77550 77568
T4 NC_000866.4      (77473) GAAACTATAAAGACTATTTTAAACGCGCTCTTGCGGGATACCGCTTACGTACTTATTATATTAAAGGTTCACCACGCCCGGAAGAATTAGCAAATG
SARS-CoV-2 NC_045512.2  (1) ------------------------------------------------------------------------------------------------
                                         Section 809
                    (77569) 77569 77583 77594 77605 77616 77627 77638 77649 77664
T4 NC_000866.4      (77569) CTATATATGGAAATCCACAGCTGTATTGGGTTTTATTGATGTGTAATGATAATTATGACCCGTATTATGGATGGATTACTTCGCAAGAAGCTGCTT
SARS-CoV-2 NC_045512.2  (1) ------------------------------------------------------------------------------------------------
                                         Section 810
                    (77665) 77665 77671 77682 77693 77704 77715 77726 77737 77748 77760
T4 NC_000866.4      (77665) ATCAAGCATCTATACAAAAATACAAAAACGTAGGTGGAGACCAAATAGTATATCATGTGAATGAGAACGGTGAAAAATTTTATAATTTAATATCAT
SARS-CoV-2 NC_045512.2  (1) ------------------------------------------------------------------------------------------------
                                         Section 811
                    (77761) 77761 77770 77781 77792 77803 77814 77825 77836 77856
T4 NC_000866.4      (77761) ACGATGATAATCCATATGTTTGGTATGATAAAGGCGATAAAGCTAGAAAATATCCTCAATATGAAGGAGCGCTTGCTGCGGTCGATACGTATGAAG
SARS-CoV-2 NC_045512.2  (1) ------------------------------------------------------------------------------------------------
                                         Section 812
                    (77857) 77857 77869 77880 77891 77902 77913 77924 77935 77952
T4 NC_000866.4      (77857) CTGCTGTTCTTGAAAATGAAAAACTTCGTCAAATAAAAATAATAGCAAAATCAGACATCAATTCATTTATGAACGACCTTATACGTATAATGGAGA
SARS-CoV-2 NC_045512.2  (1) ------------------------------------------------------------------------------------------------
```

```
                                                                                                              Section 813
                          (77953) 77953          77968      77979      77990      78001      78012      78023      78034        78048
T4 NC_000866.4            (77953) AATCTTATGGAAATGATAAGTAATAACCTTAATTGGTTTGTCGGTGTTGTTGAAGATAGAATGGACCCATTAAAATTAGGTCGTGTTCGTGTTCGT
SARS-CoV-2 NC_045512.2        (1) ------------------------------------------------------------------------------------------------
                                                                                                              Section 814
                          (78049) 78049  78056      78067      78078      78089      78100      78111      78122      78133  78144
T4 NC_000866.4            (78049) GTGGTTGGTCTGCATCCACCTCAAAGAGCACAAGGTGATGTAATGGGTATTCCAACTGAAAAATTACCATGGATGTCAGTTATTCAACCTATAACT
SARS-CoV-2 NC_045512.2        (1) ------------------------------------------------------------------------------------------------
                                                                                                              Section 815
                          (78145) 78145     78155      78166      78177      78188      78199      78210      78221              78240
T4 NC_000866.4            (78145) TCTGCAGCAATGTCTGGAATTGGAGGTTCTGTTACTGGACCAGTAGAAGGAACTAGAGTTTATGGTCATTTTTTAGACAAATGGAAAACTAATGGA
SARS-CoV-2 NC_045512.2        (1) ------------------------------------------------------------------------------------------------
                                                                                                              Section 816
                          (78241) 78241        78254      78265      78276      78287      78298      78309      78320           78336
T4 NC_000866.4            (78241) ATTGTCCTTGGCACGTATGGTGGAATAGTTCGCGAAAAACCGAATAGACTTGAAGGATTTTCTGACCCAACTGGGCAGTATCCTAGACGTTTAGGA
SARS-CoV-2 NC_045512.2        (1) ------------------------------------------------------------------------------------------------
                                                                                                              Section 817
                          (78337) 78337 78342      78353      78364      78375      78386      78397      78408      78419    78432
T4 NC_000866.4            (78337) AATGATACTAACGTACTAAACCAAGGTGGAGAAGTAGGATATGATTCGTCTTCTAACGTTATCCAAGATAGTAACTTAGACACCGCAATAAATCCC
SARS-CoV-2 NC_045512.2        (1) ------------------------------------------------------------------------------------------------
                                                                                                              Section 818
                          (78433) 78433   78441      78452      78463      78474      78485      78496      78507                78528
T4 NC_000866.4            (78433) GATGATAGACCGCTATCAGAGATTCCGACCGATGATAATCCAAATATGTCAATGGCTGAAATGCTTCGCCGTGATGAAGGATTAAGATTAAAAGTT
SARS-CoV-2 NC_045512.2        (1) ------------------------------------------------------------------------------------------------
                                                                                                              Section 819
                          (78529) 78529      78540      78551      78562      78573      78584      78595      78606              78624
T4 NC_000866.4            (78529) TATTGGGATACCGAAGGATATCCGACAATTGGTATTGGTCATCTTATCATGAAGCAGCCAGTTCGTGATATGGCTCAAATTAATAAAGTTTTATCA
SARS-CoV-2 NC_045512.2        (1) ------------------------------------------------------------------------------------------------
```

```
                                                                                                    Section 820
                          (78625) 78625        78639     78650     78661     78672     78683     78694     78705          78720
T4 NC_000866.4            (78625) AAACAAGTTGGTCGTGAAATTACAGGAAATCCAGGTTCTATTACAATGGAAGAGGCGACGACTTTATTTGAGCGTGATTTGGCTGATATGCAACGG
SARS-CoV-2 NC_045512.2        (1) ------------------------------------------------------------------------------------------------
                                                                                                    Section 821
                          (78721) 78721 78727      78738     78749     78760     78771     78782     78793     78804     78816
T4 NC_000866.4            (78721) GACATTAAATCACATTCTAAAGTAGGACCAGTCTGGCAAGCTGTCAACCGTTCTCGTCAAATGGCGTTAGAAAATATGGCATTTCAGATGGGTGTT
SARS-CoV-2 NC_045512.2        (1) ------------------------------------------------------------------------------------------------
                                                                                                    Section 822
                          (78817) 78817   78826      78837     78848     78859     78870     78881     78892               78912
T4 NC_000866.4            (78817) GGTGGTGTAGCTAAATTTAACACAATGTTAACTGCTATGTTAGCAGGAGATTGGGAAAAAGCGTATAAAGCCGGTCGTGATTCATTGTGGTATCAA
SARS-CoV-2 NC_045512.2        (1) ------------------------------------------------------------------------------------------------
                                                                                                    Section 823
                          (78913) 78913       78925      78936     78947     78958     78969     78980     78991          79008
T4 NC_000866.4            (78913) CAAACAAAAGGCCGTGCATCCCGTGTTACCATGATTATTCTTACGGGGAATTTGGAATCATATGGTGTTGAAGTGAAAACCCCAGCTAGGTCTCTA
SARS-CoV-2 NC_045512.2        (1) ------------------------------------------------------------------------------------------------
                                                                                                    Section 824
                          (79009) 79009          79024     79035     79046     79057     79068     79079     79090          79104
T4 NC_000866.4            (79009) TCAGCAATGGCTGCTACTGTAGCTAAATCTTCTGACCCTGCTGACCCTCCTATTCCAAATGACTCGAGAATTTTATTCAAAGAACCAGTTTCTTCA
SARS-CoV-2 NC_045512.2        (1) ------------------------------------------------------------------------------------------------
                                                                                                    Section 825
                          (79105) 79105 79112      79123     79134     79145     79156     79167     79178     79189     79200
T4 NC_000866.4            (79105) TATAAAGGTGAATATCCTTATGTGCATACAATGGAAACTGAAAGCGGACATATTCAGGAATTTGATGATACCCCTGGGCAAGAACGATATAGATTA
SARS-CoV-2 NC_045512.2        (1) ------------------------------------------------------------------------------------------------
                                                                                                    Section 826
                          (79201) 79201     79211      79222     79233     79244     79255     79266     79277               79296
T4 NC_000866.4            (79201) GTTCATCCAACTGGAACTTATGAAGAAGTATCACCATCAGGAAGAAGAACAAGAAAAACTGTTGATAATTTGTATGATATAACCAATGCTGATGGT
SARS-CoV-2 NC_045512.2        (1) ------------------------------------------------------------------------------------------------
```

```
                                                                                                  Section 827
                              79297        79310      79321      79332      79343      79354      79365      79376          79392
                     (79297)
T4 NC_000866.4       (79297)  AATTTTTTGGTAGCCGGTGATAAAAAGACTAACGTCGGTGGTTCAGAAATTTATTATAACATGGATAATCGTTTACATCAAATCGATGGAAGCAAT
SARS-CoV-2 NC_045512.2   (1)  ------------------------------------------------------------------------------------------------
                                                                                                  Section 828
                              79393 79398      79409      79420      79431      79442      79453      79464      79475        79488
                     (79393)
T4 NC_000866.4       (79393)  ACAATATTTGTACGTGGAGACGAAACGAAAACTGTTGAAGGTAATGGAACTATCCTAGTTAAAGGTAATGTTACTATTATAGTTGAAGGTAATGCT
SARS-CoV-2 NC_045512.2   (1)  ------------------------------------------------------------------------------------------------
                                                                                                  Section 829
                              79489   79497      79508      79519      79530      79541      79552      79563                79584
                     (79489)
T4 NC_000866.4       (79489)  GACATTACAGTTAAAGGAGATGCTACCACTTTAGTTGAAGGAAATCAAACTAACACAGTAAATGGAAATCTTTCTTGGAAAGTTGCCGGGACAGTT
SARS-CoV-2 NC_045512.2   (1)  ------------------------------------------------------------------------------------------------
                                                                                                  Section 830
                              79585      79596      79607      79618      79629      79640      79651      79662            79680
                     (79585)
T4 NC_000866.4       (79585)  GATTGGGATGTCGGTGGTGATTGGACAGAAAAAATGGCATCTATGAGTTCTATTTCATCTGGTCAATACACAATTGATGGATCGAGGATTGACATT
SARS-CoV-2 NC_045512.2   (1)  ------------------------------------------------------------------------------------------------
                                                                                                  Section 831
                              79681          79695      79706      79717      79728      79739      79750      79761          79776
                     (79681)
T4 NC_000866.4       (79681)  GGCTAATATACTTCCAATGAGCGCTGATTTAGGAGAATCCATGGAAGGTTCTTCTATCGACGTCACCTTTACCGCTCAATTAGAAACAGGTGAAAC
SARS-CoV-2 NC_045512.2   (1)  ------------------------------------------------------------------------------------------------
                                                                                                  Section 832
                              79777  79783      79794      79805      79816      79827      79838      79849      79860      79872
                     (79777)
T4 NC_000866.4       (79777)  GTTAGTATCTATAAATATAACTAGTTACGAAGAAACTCCTGGGGTTTTAGTAGAAGAAAATCGCTTATATGGAACATATGAATCTGTATTTGGTTT
SARS-CoV-2 NC_045512.2   (1)  ------------------------------------------------------------------------------------------------
                                                                                                  Section 833
                              79873    79882      79893      79904      79915      79926      79937      79948                79968
                     (79873)
T4 NC_000866.4       (79873)  CGGAAATGACGCGTTGAAATATCGTTTAGGCGATGAATTTAAAACTGCTGCTTCATGGGAAGAACTTCCTACTGATTCTGATACTCAGTTGTATTT
SARS-CoV-2 NC_045512.2   (1)  ------------------------------------------------------------------------------------------------
```

```
                                                                                                                  Section 834
                         (79969) 79969       79981      79992      80003      80014      80025      80036      80047           80064
         T4 NC_000866.4  (79969) GTGGAAAGCTCCTCAAAACCTCCAGAAGACATTCACTTACGAAGTAACATTAATATATGACTACCAAGAACAAAGTGAATCTGGGGGTTCTGGCAG
    SARS-CoV-2 NC_045512.2   (1) ------------------------------------------------------------------------------------------------
                                                                                                                  Section 835
                         (80065) 80065          80080      80091      80102      80113      80124      80135      80146           80160
         T4 NC_000866.4  (80065) TAATTCTAGGTCATCTTCTGATACTACTGAACCGACAGATCCTCCTGCTCCAGTAAGAAAAACTCTAGTTAAAAATTATACTAAAACTATAGTTGG
    SARS-CoV-2 NC_045512.2   (1) ------------------------------------------------------------------------------------------------
                                                                                                                  Section 836
                         (80161) 80161  80168      80179      80190      80201      80212      80223      80234      80245     80256
         T4 NC_000866.4  (80161) AAATTGGAGTCGTTGGGCTAATAAACTGAGAAAATATGCCTATGCAAGACCATAAATATTTTTATTTGTATTCAATAACTAATAAAACAACAGAAA
    SARS-CoV-2 NC_045512.2   (1) ------------------------------------------------------------------------------------------------
                                                                                                                  Section 837
                         (80257) 80257     80267      80278      80289      80300      80311      80322      80333               80352
         T4 NC_000866.4  (80257) AAATTTATGTAGGCGTCCACAAAACTTCAAATTTGGATGATGGGTATATGGGTTCTGGCGTTGCCATTAAAAATGCCATTAAAAAATATGGCATAG
    SARS-CoV-2 NC_045512.2   (1) ------------------------------------------------------------------------------------------------
                                                                                                                  Section 838
                         (80353) 80353        80366      80377      80388      80399      80410      80421      80432           80448
         T4 NC_000866.4  (80353) ATAATTTTTATAAGCATATTATAAAATTCTTTGAATCTGAAAAAGCTATGTATGACGCAGAGGCAGAAATAGTCACAGAGGAATTTGTTAAATCTA
    SARS-CoV-2 NC_045512.2   (1) ------------------------------------------------------------------------------------------------
                                                                                                                  Section 839
                         (80449) 80449 80454      80465      80476      80487      80498      80509      80520      80531       80544
         T4 NC_000866.4  (80449) AGAAAACTTATAATATGAAACTAGGCGGTATCGGTGGCTTCCCAAAACATAACACAGCGGGTGCTAAAAATGGATTTTACGGTAAATCTCATTCGC
    SARS-CoV-2 NC_045512.2   (1) ------------------------------------------------------------------------------------------------
                                                                                                                  Section 840
                         (80545) 80545   80553      80564      80575      80586      80597      80608      80619                 80640
         T4 NC_000866.4  (80545) GTGAAACTAGATTGAAAATTAGCATTAAATCGTCTAGAAAAAGAGGGCCTAGAGGGCTAGAGGTAAAACTCTGAAGATGTGTGGCGCCAATAACCC
    SARS-CoV-2 NC_045512.2   (1) ------------------------------------------------------------------------------------------------
```

```
                                                                                                     Section 841
                         (80641) 80641      80652      80663      80674      80685      80696      80707      80718            80736
        T4 NC_000866.4   (80641) AAGGTATGGCAAAATAGCCCCTAATGCTAAATCTGTTATTATCAACGGCGTTTTATATAAAAGTATTAAAATCGCAGCTAAAGCTCTTAATATAAA
SARS-CoV-2 NC_045512.2       (1) ------------------------------------------------------------------------------------------------
                                                                                                     Section 842
                         (80737) 80737         80751      80762      80773      80784      80795      80806      80817          80832
        T4 NC_000866.4   (80737) TTATAGTACCTTAAAGGGGCGAGTTAAAGCGGGGTATTATAAATGTCAGGATTAAGTTATGATAAGTGTGTTACTGCTGGCCATGAAGCGTGGCCT
SARS-CoV-2 NC_045512.2       (1) ------------------------------------------------------------------------------------------------
                                                                                                     Section 843
                         (80833) 80833 80839      80850      80861      80872      80883      80894      80905      80916       80928
        T4 NC_000866.4   (80833) CCAACAGTTGTGAATGCTACACAAAGTAAAGTATTCACTGGAGGAATTGCTGTTCTCGTAGCAGGCGATCCAATTACAGAACATACAGAAATTAAA
SARS-CoV-2 NC_045512.2       (1) ------------------------------------------------------------------------------------------------
                                                                                                     Section 844
                         (80929) 80929    80938      80949      80960      80971      80982      80993      81004               81024
        T4 NC_000866.4   (80929) AAGCCGTATGAAACACATGGCGGAGTGACACAACCTAGAACTTCTAAGGTATATGTCACTGGAAAGAAAGCTGTTCAAATGGCTGATCCAATATCA
SARS-CoV-2 NC_045512.2       (1) ------------------------------------------------------------------------------------------------
                                                                                                     Section 845
                         (81025) 81025       81037      81048      81059      81070      81081      81092      81103            81120
        T4 NC_000866.4   (81025) TGCGGTGATACTGTGGCTCAGGCATCATCTAAAGTATTCATTAAATAGGATTTAAAATGGCAAATACCCCTGTAAATTATCAATTAACAAGAACAG
SARS-CoV-2 NC_045512.2       (1) ------------------------------------------------------------------------------------------------
                                                                                                     Section 846
                         (81121) 81121          81136      81147      81158      81169      81180      81191      81202          81216
        T4 NC_000866.4   (81121) CAAATGCTATTCCCGAGATATTCGTCGGGGGTACATTTGCTGAAATAAAACAAAACCTCATTGAATGGCTTAATGGCCAAAATGAATTTTTGGATT
SARS-CoV-2 NC_045512.2       (1) ------------------------------------------------------------------------------------------------
                                                                                                     Section 847
                         (81217) 81217  81224      81235      81246      81257      81268      81279      81290      81301       81312
        T4 NC_000866.4   (81217) ATGATTTTGAAGGCTCAAGATTAAACGTTCTGTGTGACCTTTTAGCTTATAATACATTATACATTCAGCAGTTTGGTAATGCTGCTGTGTATGAAA
SARS-CoV-2 NC_045512.2       (1) ------------------------------------------------------------------------------------------------
```

```
                                                                                                             Section 848
                       (81313) 81313     81323     81334     81345     81356     81367     81378     81389     81408
T4 NC_000866.4         (81313) GCTTTATGCGTACTGCTAACTTACGAAGTTCAGTTGTTCAAGCTGCACAAGATAACGGATATTTACCTACTTCAAAATCCGCTGCGCAGACCGAAA
SARS-CoV-2 NC_045512.2     (1) ------------------------------------------------------------------------------------------------
                                                                                                             Section 849
                       (81409) 81409     81422     81433     81444     81455     81466     81477     81488     81504
T4 NC_000866.4         (81409) TTATGTTAACATGCACTGACGCATTGAATAGGAATTACATTACTATTCCTCGCGGAACTCGCTTTTTAGCATATGCAAAAGATACTTCTGTTAATC
SARS-CoV-2 NC_045512.2     (1) ------------------------------------------------------------------------------------------------
                                                                                                             Section 850
                       (81505) 81505 81510     81521     81532     81543     81554     81565     81576     81587     81600
T4 NC_000866.4         (81505) CATATAACTTCGTTTCTAGGGAAGACGTTATTGCTATTCGTGATAAAAATAACCAATATTTTCCGCGTTTAAAATTGGCCCAGGGACGTATAGTAA
SARS-CoV-2 NC_045512.2     (1) --------------------------------------------ATTAAAGGTTTATACCTTCCCAGGTAACAAACCAACCAAC-----TTTCGAT
                                                                                                             Section 851
                       (81601) 81601 81609     81620     81631     81642     81653     81664     81675     81696
T4 NC_000866.4         (81601) GAACTGAAATCATTTATGATAAATTAACACCTATTATCATTTATGATAAAAATATTGATAGAAACCAGGTTAAATTATAC--GTTGATGGAGCGGA
SARS-CoV-2 NC_045512.2    (48) CTCTTGTAGATCTGTTCTCTAAACGAACTTT--AAAATCTGTGTGGCTGTCACTCGGCTGCATGCTTAGTGCACTCACGCAGTATAATTAATAACT
                                                                                                             Section 852
                       (81697) 81697     81708     81719     81730     81741     81752     81763     81774     81792
T4 NC_000866.4         (81695) AT------GGATTAACTGGACGAGAAAGTCAATGGTTCATGCTGGTTCAACAT--CAACGATTTACTATATGCGTGAAACTATTGATGGAAACACT
SARS-CoV-2 NC_045512.2   (142) AATTACTGTCGTTGACAGGACACG--AGTAACTCGTCTAT-CTTCTGCAGGCTGCTTACGGTTTCGTCCGTGT-TGCAGCCGATCATCAGCACATC
                                                                                                             Section 853
                       (81793) 81793     81807     81818     81829     81840     81851     81862     81873     81888
T4 NC_000866.4         (81783) GAATTCTATTTTGGTGAAGGTGAAATTTCTGTTAATGCTTCTGAAGGAGCTTTGACCGCTAATTATATCGGAGGTCTTAAACCTACTCAGAACTCT
SARS-CoV-2 NC_045512.2   (234) TAGG----TTTC-GTCCGGGT---GTGACCGAAAGGTAAGATGGAG-AGCCTTGTCCC-TGGTTTCAACG-AGAAAACACACGTCCAACTCAGTTT
                                                                                                             Section 854
                       (81889) 81889 81895     81906     81917     81928     81939     81950     81961     81972     81984
T4 NC_000866.4         (81879) ACGATTGTTATTGAGTACATTAGTACTA--ATGGTGCTGACGCGAA-CGGAGCAGTCGGATTT-TCATACGCAGATACATTAACAAATATAACTGT
SARS-CoV-2 NC_045512.2   (319) GCCTGTTTTACAGGTTCGCGACGTGCTCGTACGTGGCTTTGGAGACTCCGTGGAGGAGGTCTTATCAGAGGCACGT-CAACATCTTAAAGATGGCA
```

```
Section 855
                         (81985) 81985    81994      82005      82016      82027      82038      82049      82060           82080
T4 NC_000866.4           (81971) CATCAATATTAATGAAAATCCAAACGATGATCCAGATTTTGTTGGGGCAGATGGAGGCGGTGATCCAGAAGATATTGAGCGTATTCGCGAATTGGG
SARS-CoV-2 NC_045512.2   (414)   CTTGTGGCTTAGTAGAAGTTGAAAAAGGCGTTTTGCCTCAACTTGAACAGCCCTATGTGTTCATC-AAACGTT------CGGATGCTCGAACTGCA

Section 856
                         (82081) 82081       82093      82104      82115      82126      82137      82148      82159           82176
T4 NC_000866.4           (82067) TACTATTAAACGCGAAA-CCCAACAACGATGCGTAACTGCGACTGACTATGATACATTCGT--TTCAGAGAGATTTGGTTCTATTATTCAAGCTGT
SARS-CoV-2 NC_045512.2   (503)   CCTCATGGTCATGTTATGGTTGAGCTGGTAGCAGAACT-CGAAGGCATTCAGTACGGTCGTAGTGGTGAGACACTTGGTGTCCTTGTCCCTCATGT

Section 857
                         (82177) 82177          82192      82203      82214      82225      82236      82247      82258         82272
T4 NC_000866.4           (82160) TCAGACTTTCACTGATTCTACTAAACCTGGGTATGCATTTATTGC-TGCTAAACCTAAATCAGGATTGTATTTAACTACCGTACAGCGTGAAGATA
SARS-CoV-2 NC_045512.2   (598)   -------------GGGCGAA--ATACCAGTG---GC--TTACCGCAAGGTTCTTCTTCGTAAGAACGGTAATAAA-----GGAGCTGGTGGCCATA

Section 858
                         (82273) 82273  82280      82291      82302      82313      82324      82335      82346      82357      82368
T4 NC_000866.4           (82255) TTAAAAAT-TATCTCAAAGACTATAATTTAGCTCCTATTACGCCATCAATTATTTCTCCTAATTATCTTTTTATTAAGACTAATTTA-AAAGTC--
SARS-CoV-2 NC_045512.2   (669)   GTTACGGCGCCGATCTAA-A--GTCATTTGACTTAGGCGACGAGCTTGGCACTGATCCTTATGAAGATTTTCAAGAAAACTGGAACACTAAACATA

Section 859
                         (82369) 82369     82379      82390      82401      82412      82423      82434      82445           82464
T4 NC_000866.4           (82347) ACATATGCTTTAAATAAACTGCAAGAATCCGAACAGTGGCTTGAAGGTCAAATAATTGATAAAATAGATCGCTATTATACCGAAGATGTAGAAATT
SARS-CoV-2 NC_045512.2   (762)   GCAGTGGTGTTAC---CCGTGAACTCATGCGTGAGCTTAACGGAGGGCATACACTCGCTATG-TCGATAACAACTT--CTGTGGCCCTG---ATG

Section 860
                         (82465) 82465        82478      82489      82500      82511      82522      82533      82544           82560
T4 NC_000866.4           (82443) TTTAACTCGTCTTTCGCTAAATCTAAGATGTTGA--CATATGTAGATGATGCAGATCATTCTGTCATTGGTTCATCAGCGACTATTCAAATGGTTC
SARS-CoV-2 NC_045512.2   (849)   GCTACCCT---CTTGAGTGCATTAAAGACCTTCTAGCACGTGCTGGTAAAGCTTCATGCACTTTGTCCGAACAACTGGACTTTATTGACACTAAGA

Section 861
                         (82561) 82561 82566      82577      82588      82599      82610      82621      82632      82643         82656
T4 NC_000866.4           (82537) GTGAAGTACAAAACTTCTATAAAACGCCTGAAGCGGGTATTAAATACAATAATCAAATAAAAGATCGTTCTATGGAATCTA-ATACGTTTTCATTT
SARS-CoV-2 NC_045512.2   (942)   GGGGTGTATACTGCTGCCGTGAACATGAGCATGAAATTGCTTGGTACACGGAACGTTCTGAAAAGAG--CTATGAATTGCAGACACCTTTTGAAAT
```

```
                                                                                                      Section 862
                           (82657) 82657   82665       82676       82687       82698       82709       82720       82731         82752
T4 NC_000866.4             (82632) AATTCTGGACGAAAGGTTGTAAATCCTGATACTGGTTTAGAAGAAGATGTATTATATGACGTTCGTATAGTATCAACAGACCGAGATTCTAAAGGA
SARS-CoV-2 NC_045512.2     (1036)  TAAATTGGCAAAGAAATTTGACACCTTC--AATGGG--GAATGTCCAAATTTTGTAT----TTCCCTTAAATTCCATAATCAAGACTATTCAACCA
                                                                                                      Section 863
                           (82753) 82753       82764       82775       82786       82797       82808       82819       82830     82848
T4 NC_000866.4             (82728) ATTG--GTAAAGTTATTATTGGTCCATTTGCTTCTGGCGATGTTACAGAAAATGAAAACATTCAGCCGTATACAGGCAACGATTTTAACAAATTAG
SARS-CoV-2 NC_045512.2     (1124)  AGGGTTGAAAAG---AAAAAGCTTGATGGCTTTATGGGTAGAATTC-GAT---CTGTCTATCCAGTTGCGT-CACCAATGAATGCAACCAAATGT
                                                                                                      Section 864
                           (82849) 82849         82863       82874       82885       82896       82907       82918       82929   82944
T4 NC_000866.4             (82822) CAAATTCTGATGGACGCGACAAATACTATGTTATC--GGTGAAATAAATTATCCAGCTGATGTGATTTATTGGAATATCGCTAAAATTAATTTAAC
SARS-CoV-2 NC_045512.2     (1212)  GCCTTTCAACT--------CTCATGAAGTGTGATCATTGTGGTG-AAACTTCATGGCAGACGGGC--------GATTTTGTTAAAGCCACTT----
                                                                                                      Section 865
                           (82945) 82945 82951       82962       82973       82984       82995       83006       83017       83028   83040
T4 NC_000866.4             (82916) ATCTGAAAAATTTGAAGTTCAGACCATTGAATTATATTCTGACCCAACCGATGATGTTATCTTTACTCGCGATGGTTCACTGATTGTATTTGAAAA
SARS-CoV-2 NC_045512.2     (1287)  ----GCGAATTTTGTGGCACTGAGAATTTGACTAAAGAAGGTGCCACTACTTGTGGTTACTTA----CCCCAAAATGCTGTTGTTAAAATTTA---
                                                                                                      Section 866
                           (83041) 83041    83050       83061       83072       83083       83094       83105       83116       83136
T4 NC_000866.4             (83012) TGACTTACGTCCACAATACTTAACTATCGATTTGGAGCCTATATCACA--ATGACAGTA--AAAGCACCTTCAGTCACTAGTCTCAGAATTTCCAA
SARS-CoV-2 NC_045512.2     (1372)  ---T--T-GTCCAGCATG------TCACAATTCAGAAGTAGGACCTGAGCATAGTCTTGCCGAATACCAT-------AATGAATCTGGCTTGAAAA
                                                                                                      Section 867
                           (83137) 83137       83149       83160       83171       83182       83193       83204       83215     83232
T4 NC_000866.4             (83104) GTTATCCGCAAATCAGGTGCAAGTACGCTGGGATGACGTTGGTGCTAATTTCTACTATTTTGTAGAAATCGCTGAGACAAAAACAAACTCGGGGGA
SARS-CoV-2 NC_045512.2     (1449)  CCATTCTTCGTAAG-GGTGGTCGCACTATT----GCCTTTGGAGGCTGTGTGTTCTCTTATGTTGGTTGCCATAACAAGTGTGCCTATTGGGTTCC
                                                                                                      Section 868
                           (83233) 83233          83248       83259       83270       83281       83292       83303       83314   83328
T4 NC_000866.4             (83200) AAATCTCCCGAGTAATCAATATCGTTGGATTAATTTAGGATATACAGCAAATAATAGTTTCTTTTTTGATGATG--CTGATCCATTAACAACATAC
SARS-CoV-2 NC_045512.2     (1540)  ACGTG---CTAGCGCTAACATAGGTTGTAACCATACAGGTGTTGTTGGAGAAGGTTCCGAAGGTCTTAATGACAACCTTCTTGAAATACTCCAAAA
```

Section 869

(83329) 83329 83336 83347 83358 83369 83380 83391 83402 83413 83424
T4 NC_000866.4 (83294) A-TT---ATTAGAGTAGCCACAGCTGCGCAAGATTTTGAGCAGTCTGATTGGATTTATACCGAAGAGTTTGAAACTTTTGCTACAAATGCTTATAC
SARS-CoV-2 NC_045512.2 (1633) AG-AGAAAGTCAAC--ATCAATATTGTTGGTGACTTTAAACTTAATGAAGAGAT---CGCCATTATTTTGGCATCTTTTTCTGCTTCCACAAGTGC

Section 870

(83425) 83425 83435 83446 83457 83468 83479 83490 83501 83520
T4 NC_000866.4 (83386) ATTTCAAAACATGATTGAAATGCAATTAGCCAATAAATTCATTCAGGAAAAATTTACTCTTAATAATTCTGATTATGTTAATTTTAATAATGATA-
SARS-CoV-2 NC_045512.2 (1723) TTTTGTGGAAACTGTGAAAGGT---TTGGATTATAAAG-CATTCAA--A---CAAATTGTTGAATCCTGTGGTAATTTTAAAGTTACAAAAGGAAA

Section 871

(83521) 83521 83534 83545 83556 83567 83578 83589 83600 83616
T4 NC_000866.4 (83481) --CTATAATGGCTGCATTGATGAATGAATCATTCCAATTCAGCCCATCGTATGTTGATGTTTC-ATCAATAAGTAATTTTATTATTGGTGAAAATG
SARS-CoV-2 NC_045512.2 (1810) AGCTAAAAAAGGTGCCTGGAATATTGGTGAACAGAAATCAATACTGAGTCCTCTTTATGCATTTGCATCAGAGGCTGCTCGTGTTGTACGATCAAT

Section 872

(83617) 83617 83622 83633 83644 83655 83666 83677 83688 83699 83712
T4 NC_000866.4 (83574) AGTATCATGAAATACAAGGTTCTATTCAGCAAGTATGTAAGGATATTAACCGAGTTTATTTGAT-GGAATCAGAAGGAATTCTATATCTTTT--TG
SARS-CoV-2 NC_045512.2 (1906) TTTCTCCCGCACT-CTTGAAACTGCTCAA-AATTCTGTGCGTGTTTTACAGAAGGCCGCTATAACAATACTAGATGGAATTTCACAGTATTCACTG

Section 873

(83713) 83713 83721 83732 83743 83754 83765 83776 83787 83808
T4 NC_000866.4 (83667) AGCGCTATCAACCTGTAGTTAAAGTATCCAATGATAAAGGACAAACCTGGAAAGCTGTAAAGCTCTTCAATGACCGTGTAGGATATCCTTTATCTA
SARS-CoV-2 NC_045512.2 (2000) AGACTCATTGA--TGCTATGATGTTCACATCTGATTTGGCTACTAACAATCTAGTTGTAATGGCCTACATTACAGGTGGTGTTGTTCAGTTGACTT

Section 874

(83809) 83809 83820 83831 83842 83853 83864 83875 83886 83904
T4 NC_000866.4 (83763) AGACAGTATATTACCAATCTGCGAACACAACATACGTTCTAGGATACGACAAGATTTTCTATGGCCGCAAATCTACTGATGTTAGATGGTCAGCCG
SARS-CoV-2 NC_045512.2 (2094) CG-CAGTGGCTAACTAACAT-CTTTGGCA-----CTGTTTATGAAAAAC-----TCAAACCCGTCCTTGATTGGCTTGAAG--AGAAGTTT-AAGG

Section 875

(83905) 83905 83919 83930 83941 83952 83963 83974 83985 84000
T4 NC_000866.4 (83859) ATGATGTCAGATTTAGTTCTCAGGATATAACATTTGCTAAACTTGGCG-ACCAATTACATCTAGGATTTGATGTAGAAATTTTTGCCACTTACGCG
SARS-CoV-2 NC_045512.2 (2175) AAGGTGTAGAGTTTCTTAGAGACGGTTGGGAAATTGTTAAATTTATCTCAACCTGTGCTTGTGAAATTGTCGGTGGACAAATT-GTCACCTGTGCA

```
                                                                                                          Section 876
                           (84001) 84001 84007           84018           84029           84040           84051           84062           84073           84084      84096
T4 NC_000866.4             (83954) ACTTTACCAGCGAAT-GTATACCG-CATTGCAGAAGCTATTACTTGCACCG-ATGATTACATTTACGTTGTCGCCAGAGACAAAGTTAGATACATA
SARS-CoV-2 NC_045512.2     (2270)  AAGGAAATTAAGGAGAGTGTTCAGACATTCTTTAAGCTTGTAAATAAATTTTTGGCTTTG-TGTG--CTGACTCTATCATTATTGGTGGAGCTAAA
                                                                                                          Section 877
                           (84097) 84097    84106            84117           84128           84139           84150           84161           84172                84192
T4 NC_000866.4             (84047) AAAAC-GAGTAATGCACTTA--TAGATTTTGATCCATTATCTCCAA---CATATTCGGAAAGACTTTTTGAACCTGATACCATGACTATAACCGGA
SARS-CoV-2 NC_045512.2     (2363)  CTTAAAGCCT--TGAATTTAGGTGAAACATTTGTCACGCACTCAAAGGGATTGTACAGAAAGTGTGTTAAATCCAGAGAAGAAA-CTGGCCTACTC
                                                                                                          Section 878
                           (84193) 84193       84205           84216           84227           84238           84249           84260           84271              84288
T4 NC_000866.4             (84137) AATCC---TAAAGCAGTATGCTATAAAATGGATTCTATCTGTGATAAAGTTTTTGCTCTTA----TTATT-GGTGAAGTTGAAACATTAAATGCTA
SARS-CoV-2 NC_045512.2     (2456)  ATGCCTCTAAAAGCCCCAAA--AGAAATTATCTTCTTAGAGGGAGAAACACTTCCCACAGAAGTGTTAACAGAGGAAGTTGTCTTGAAAACTGGTG
                                                                                                          Section 879
                           (84289) 84289          84304           84315           84326           84337           84348           84359           84370         84384
T4 NC_000866.4             (84225) ATCCTAGAACATCA-A--AAATAATTGATTCCGCTGATAAAGGAATATATGTTTTAAATCATGACGAAAAAACATGGAAAAGAGTTTTTGGTAATA
SARS-CoV-2 NC_045512.2     (2550)  ATTTACAACCATTAGAACAACCTACTAGTGAAGCTGTTGAAGCTCCATTGGTTGGTACACCAGTTTGTATTAACGGGCTTA----TGTTGCTCGAA
                                                                                                          Section 880
                           (84385) 84385  84392           84403           84414           84425           84436           84447           84458           84469  84480
T4 NC_000866.4             (84318) CCGAAGAAGAAAGAAGACGTATTCAACCC--GGATATGCGAATATGTCAACTGACGGTAAATTAGTTTCTCTGTCTTCGAGTAATTTTAAA----T
SARS-CoV-2 NC_045512.2     (2642)  ATCAAAGACACAGAAAA-GTACTGTGCCCTTGCACCTAATATGATGGTAACAAACAATACCTTCACA-CTCAAAGGCGGTGCACCAACAAAGGTTA
                                                                                                          Section 881
                           (84481) 84481     84491           84502           84513           84524           84535           84546           84557                84576
T4 NC_000866.4             (84408) TTTTAAGTGATAATGTTGTTAATGACCCTG-AAAC-TGCAGCAAA----ATATCAGTTAATTGGCGCTGTTA--AATATGAATTTCCTCGTGAATG
SARS-CoV-2 NC_045512.2     (2736)  CTTTTGGTGATGACACTGTGATAGAAG-TGCAAGGTTACAAGAGTGTGAATATCACTT--TTGAACTTGATGAAAGGATTGATAAAGTACTTAATG
                                                                                                          Section 882
                           (84577) 84577        84590           84601           84612           84623           84634           84645           84656             84672
T4 NC_000866.4             (84496) GTTAGCTGATAAGCATTATCATATGATGGCATT--TATAGCGGATGAAACATC---TGATTGGAGACTTTTACTCCTCAACCAATGAAATACTAC
SARS-CoV-2 NC_045512.2     (2829)  AGAAG-TGCTCTGCCTATACAGTTGAACTCGGTACAGAAGTAAATGAGTTCGCCTGTGTTGTGGCAGATGCTGTCATAAAAACTTTGCAACCAGTA
```

```
                                                                                                  Section 883
                          (84673) 84673 84678      84689      84700      84711      84722      84733      84744      84755     84768
T4 NC_000866.4            (84587) GCAGAACCAT-TCTTTAACTGGTCTAAAAAATCTAACACACGTTGTTGGATAAACAACTCTGATAGAGCTGTGGTAGTTTATGCTGATTTAAAATA
SARS-CoV-2 NC_045512.2    (2924)  TCTGAATTACTTACACCACTGGGCATTGATTTAGATGAGTGGAGTATGGCTACATACTACTTATT-TGATGAG------TCTGGTGAGTTTAAATT
                                                                                                  Section 884
                          (84769) 84769   84777      84788      84799      84810      84821      84832      84843                84864
T4 NC_000866.4            (84682) CACTAAAGTTATAGAAAATATTCCGGAAACATCACCAGATAGATTAGTTCATGAA--TACTGGG-ATGATGGT--GATTGCACTATAGT---AATG
SARS-CoV-2 NC_045512.2    (3013)  GGCTTCA--CATATGTATTGTTCTTTCTACCC-TCCAGATGAGGATGAAGAAGAAGGTGATTGTGAAGAAGAAGAGTTTGAGCCATCAACTCAATA
                                                                                                  Section 885
                          (84865) 84865      84876      84887      84898      84909      84920      84931      84942                84960
T4 NC_000866.4            (84770) CCAAATGTCAAATTCACTGGATTTAAAAAATACGCATCAGGAATGCTTTTCTATAAAGCCTCCGGTGAAATAATTTCTTACTATGATTTTAACTAT
SARS-CoV-2 NC_045512.2    (3106)  TGAGTATGGTACTGAAGATGATTACCAAGGTAAACCTTTGGAATTTGGTGCCA-----CTTCTGCTG------CTCTTCAACCTGAA---GAAGAG
                                                                                                  Section 886
                          (84961) 84961          84975      84986      84997      85008      85019      85030      85041          85056
T4 NC_000866.4            (84866) CGTGTGAGAGATACAGTAGAA-ATTATTTGGAAGCCAACTGAAGTATTTTTA-AAAGCATTTTTACAAACCAAGAGCATGAGACTCCTTGGTCAC
SARS-CoV-2 NC_045512.2    (3188)  CAAGAAGAAGATTGGTTAGATGATGATAGTCAA-CAAACTGTTGGTCAACAAGACGGCAG-TG-AGGACAATCAGACAA--CTACTATTCAAACAA
                                                                                                  Section 887
                          (85057) 85057 85063      85074      85085      85096      85107      85118      85129      85140     85152
T4 NC_000866.4            (84960) CAGAAGAAGAGCGTGGATTAGCTGACCCTGATTTAAGACCATTAATTGGCACAA-TGATGCCTGAT-TCTTATTTGTTACAGGATTCGAATTTTGA
SARS-CoV-2 NC_045512.2    (3279)  TTGTTGAGGTTCAACCTCAATTAGAGATGGAACTTACACCAGTTGTTCAGACTATTGAAGTGAATAGTTTTAGTGGTTATTTAAAACTTACTGA--
                                                                                                  Section 888
                          (85153) 85153    85162      85173      85184      85195      85206      85217      85228                85248
T4 NC_000866.4            (85054) GGCATTTTGCGAAGCATATATTCAGTATCTTTCTGATGGATATGGAACTCAATACAATAATTTACGAAATTTAATTCGTAACCAATATCCACGAGA
SARS-CoV-2 NC_045512.2    (3373)  --CAATGTATACATTAAAAATGCAGACATTGT-GGAAGAAGCTAAAAAGGTAAA-ACCAACAG-TGGTTGTTAATGC--AGCCAATGTTTACCTTA
                                                                                                  Section 889
                          (85249) 85249       85261      85272      85283      85294      85305      85316      85327          85344
T4 NC_000866.4            (85150) A-----GAGCACGCATGGAATATTTGTGGTCAGAGATATATAAAAGAAACATTTATTTAAATGCTGATAAACGCGATGCTGTTGCGAGATTCTTT
SARS-CoV-2 NC_045512.2    (3462)  AACATGGAGGAGGTGTTGCAGGAGCCTTAAATAAGGCTACTAACAATGC-CATGCAAGTTGAATCTGATGATTACATAGCTACTAATGGACCACTT
```

```
                                                                                                   Section 890
                            (85345) 85345          85360      85371      85382      85393      85404      85415      85426    85440
T4 NC_000866.4              (85241) GAATCACGTAGC-TATGATTTTTA-TTCTACTAAAGGAAT-TGAAGCATCATACAAGTTTCTTTTTAAAGTTCTTTATAA---TGAAGAAGTTGAA
SARS-CoV-2 NC_045512.2      (3557)  AAAGTGGGTGGTAGTTGTGTTTTAAGCGGACACAATCTTGCTAAACACTGTCTTCATGTTGTCGGCCCAAATGTTAACAAAGGTGAAGACATTCAA
                                                                                                   Section 891
                            (85441) 85441  85448      85459      85470      85481      85492      85503      85514      85525  85536
T4 NC_000866.4              (85331) ATT---GAAATTGAATCTGGGGCTGGTACTGAATATGATATAATCGTTCAATCTGATTCTTTGACTGAAGATTTAGTAGGACAAACGATTTATACG
SARS-CoV-2 NC_045512.2      (3653)  CTTCTTAAGAGTGCTTATGAAAATTTTAATCAGCACGAAGTTCTACTTGCACCATTATTATCAGCTGGTATTTTTG--GTGCTGACCCTATACAT-
                                                                                                   Section 892
                            (85537) 85537     85547      85558      85569      85580      85591      85602      85613         85632
T4 NC_000866.4              (85424) GCAACAGGAAGATGTAATGTTACTTA--TATAGAAAGAAG--CTATTCTAATGGTAAATTGCAATGGACCGTAA-CTATTCATAATCTTTTGGAC
SARS-CoV-2 NC_045512.2      (3746)  TCTTTAAGAGTTTGTGTAGATACTGTTCGCACAAATGTCTACTTAGCTGTCTTTGATAAAAATCTCTATGACAAACTTGTTTCAAGCTTTTTGGA-
                                                                                                   Section 893
                            (85633) 85633        85646      85657      85668      85679      85690      85701      85712      85728
T4 NC_000866.4              (85515) GATTAATTGCTGGTCAAGAAGTTAAAGCAGAAAGACTCCCTAGTTTTGAA--GGCGAAATTATTCGTGGGGTTAAAGGAAAGGATTTGCTTCAAAA
SARS-CoV-2 NC_045512.2      (3841)  -AATGAAGAGTGAAAAGCAAGTTGAACA--AAAGATCGCTGAGATTCCTAAAGAGGAAGTTAAGC-CATTTATAACTGAAAGTAAAC-CTTCAGTT
                                                                                                   Section 894
                            (85729) 85729 85734      85745      85756      85767      85778      85789      85800      85811    85824
T4 NC_000866.4              (85609) CAATATAGACTATATTAATAGAAGTAGATCATACTATGTAATGAAAATTAAATCCAATTTACCTTCTTCCCGCTGG----AAATCTGACGTTATTC
SARS-CoV-2 NC_045512.2      (3932)  GAACAGAGAAAACAAGATGATAAGAAAATCAAAGCTTGTGTTGAAGA-------AGTT--ACA-AC--AACTCTGGAAGAAACTAAGTTCCTCACA
                                                                                                   Section 895
                            (85825) 85825   85833      85844      85855      85866      85877      85888      85899         85920
T4 NC_000866.4              (85701) GTTTTGTTCATCCAGTAGGATTTGGATTTATAGCAATTACCCTTTTAACAAT-GTTTATTAATGTTGGTTTAACTCTTAAACATACAGAGACTATA
SARS-CoV-2 NC_045512.2      (4016)  GAAAACTTGTTACTTT-ATATTGACATTAATGGCAA-T-CTTCATCCAGATTCTGCCACTCTTGTTAGTGACATTGA----CAT-CACTTTCTTAA
                                                                                                   Section 896
                            (85921) 85921      85932      85943      85954      85965      85976      85987      85998         86016
T4 NC_000866.4              (85796) ATTAATAAATACAAAACTATAAATGG-GATTCTGGATTGCCTACTGAATATGCCGACAGAATAGCTAAATTAACTCCAACCGGTGAAATTGAGCA
SARS-CoV-2 NC_045512.2      (4104)  A------GAAAGATGCTCCATATATAGTGGGTGATGTTGTTCAAGAGGGTGTT----TAACT-GCTGTGGTTATACCTAC---TAAAAGGCTGG
```

```
                                                                                                    Section 897
                          (86017) 86017         86031      86042      86053      86064      86075      86086      86097       86112
T4 NC_000866.4            (85891) TGAT--TCAGTAACAGGCGAAGCAATTTATGAGCC---TGGCCCAATGGCTGGTGTAAAATATC--CTCTTCCTGATGACTATAATGCTGAAAATA
SARS-CoV-2 NC_045512.2    (4186)  TGGCACTACTGAAATGCTAGCGAAAGCTTTGAGAAAAGTG-CCAACAGACAATTATATAACCACTTACCCGGGTCAGGTTTAATGGTTACACTG
                                                                                                    Section 898
                          (86113) 86113 86119      86130      86141      86152      86163      86174      86185      86196       86208
T4 NC_000866.4            (85980) -ATAATTCAATATTTCAAGGTCA-ATTGCCGTCTGAACGACGTAAATTAATGAG--TCCTTTATTTG-ATGCATCTGGAACAACATTTGCGCAATT
SARS-CoV-2 NC_045512.2    (4281)  TAGAGGAGGCAAAGACAGTGCTTAAAAAGTGTAAAAGTGCCTTTTACATTCTACCATCTATTATCTCTAATGAGAAGCAAGAAATTCTTGGAACT-
                                                                                                    Section 899
                          (86209) 86209    86218      86229      86240      86251      86262      86273      86284             86304
T4 NC_000866.4            (86071) TAGAGATTTAGTTAATAAACGTCTAAAAGATAATATAGGAAATCCAAGAGACCCTGAAAATCCAACACAGGTTAAAATAGATGAATGATTCAAGTG
SARS-CoV-2 NC_045512.2    (4376)  ----------GTTTCTTGGAATTTGCGAGAAATGCTTGCACATGCAGAAGAAACACGCAAATTAATGCCTGTCTGTGTGGAA-ACTAAAGCCATAG
                                                                                                    Section 900
                          (86305) 86305       86317      86328      86339      86350      86361      86372      86383            86400
T4 NC_000866.4            (86167) TTATCTATCGTGCGATAGTTACTTCAAAATTTAGAACAGAAAAAATGTTGAATTTTTATAATTCAATTGGAAGTGGTCCGGATAAAAACACTATCT
SARS-CoV-2 NC_045512.2    (4461)  TT-TCAACTATAC-AGCGTAAATATAAGGGTATTAAAATACAAGAGGGTGTGG---------TTGATT----ATGGTGCT------AGATTTTACT
                                                                                                    Section 901
                          (86401) 86401          86416      86427      86438      86449      86460      86471      86482       86496
T4 NC_000866.4            (86263) TTATCACAT-TTGGAAGATCAGAACCGTGGTC-ATCAAATGAAAATGAGGTGGGCTTTGCCCCACCTTATCCAACCGATTCTGTATTAGGCGTAAC
SARS-CoV-2 NC_045512.2    (4536)  TTTACACCAG-TAAAACAACTGTAGCGTCACTTATCAACACACTTAACGATCTAAATGAAACT-----------C----TTGTTACAATGCCACTT
                                                                                                    Section 902
                          (86497) 86497  86504      86515      86526      86537      86548      86559      86570      86581  86592
T4 NC_000866.4            (86357) TGACATGTGGACGCATATGATGGGAACAGTAAAAGTTCTTCCATCAATGCTTGATGCTGTTATTCCTCG-CAGAGATTGGGGAGATACTAGATATC
SARS-CoV-2 NC_045512.2    (4616)  GGCTATGTA-ACACATGGCTTAAATTTGGAAGAAGCTGCTC-------GGTATATGAGATCTCTCAAAGTGCCAGCTACAGTTTCTGTTTCTTCAC
                                                                                                    Section 903
                          (86593) 86593     86603      86614      86625      86636      86647      86658      86669             86688
T4 NC_000866.4            (86452) CGGATCCATACACATTTAGAATTAACGATATTGTAG-TGTGTAACTCAG-CTCCT-TACAACGCT--ACTGAATCAGGCGCTGGTTGGTTAGTGTA
SARS-CoV-2 NC_045512.2    (4704)  CTGATGCTGTTACAGC--GTATAATGGTTATCTTACTTCTTCTTCTAAAACACCTGAAGAACATTTATTGAAACCATCTCAC-----TTGCTGGT
```

```
                                                                                                   Section 904
                        (86689) 86689        86702      86713      86724      86735      86746      86757      86768     86784
T4 NC_000866.4          (86543) TCGTTGTTTAGATGTTCCTGATACCGGAATGTGTTCAATAGCATCTTTAACTGATAAAGATGAATGCCTTAAGTTAGGTGGAAAATGGACTCCTTC
SARS-CoV-2 NC_045512.2  (4793)  TCCTATAAAGATTGGTCCT-ATTCTGGACAATCTACA----CAACTA-----GGTATAGA---ATTTCTTAAGAGAGGTGATAAAAGTGTATATTA
                                                                                                   Section 905
                        (86785) 86785 86790      86801      86812      86823      86834      86845      86856      86867     86880
T4 NC_000866.4          (86639) TGCTAGGTCAATGACTCCGCCTGAAGGTCGAGGAGATGCTGAAGGAACAATTGA-ACCCG---GAGACGGGTATGTGTGGGAATA------TCTAT
SARS-CoV-2 NC_045512.2  (4876)  CACTAG---TAATCCTACCACATTCCACCTAGATGGTGAAGTTATCACCTTTGACAATCTTAAGACACTTCTTTCTTTGAGAGAAGTGAGGACTAT
                                                                                                   Section 906
                        (86881) 86881   86889      86900      86911      86922      86933      86944      86955          86976
T4 NC_000866.4          (86725) TTGAGATTCCG-CCTGATGTATCTATAAATAGATGCACGAATGAATATATCGTGGTTCCTTGGCCTGAGGAATTAAAAGAAGACCCGACTAGATGG
SARS-CoV-2 NC_045512.2  (4969)  TA-AGGTGTTTACAACAGTAGACAACATTAACCTCCAC-ACGCAAGTTGTGGACATGTCAATGACATATGGACAACAGTTTGGTCCAACTTATTTG
                                                                                                   Section 907
                        (86977) 86977      86988      86999      87010      87021      87032      87043      87054          87072
T4 NC_000866.4          (86820) GGATATGAAGATAATCTCACTTGGCAACAAGATGATTTTGGATTAATTTACCGTGTTAAGGCAAATACTATCCGTTTTAAAGCATATTTAGATTCA
SARS-CoV-2 NC_045512.2  (5063)  GA---TGGAGCTGATGTTACTAAAATAAAACCTCA--------TAATTCACA-TGAAG-GTAAAACATTTTATGTTTTACCTAATGATGACACTCT
                                                                                                   Section 908
                        (87073) 87073         87087      87098      87109      87120      87131      87142      87153     87168
T4 NC_000866.4          (86916) GTTTATTTTCCTGAAGCTGCATTACCAGGAAATAAAGGATT-TAGACAAATATCAATAATCACGAATCCTC-TTGAAG-CTAAAGCTCATCCAAAT
SARS-CoV-2 NC_045512.2  (5146)  ACGT--GTTGAGGCTTTT-GAGTACTACCACACAACTGATCCTAGTTTTC--TGGGTAGGTACATGTCAGCATTAAATCACACTAAAAAGTGGAAA
                                                                                                   Section 909
                        (87169) 87169 87175      87186      87197      87208      87219      87230      87241      87252     87264
T4 NC_000866.4          (87009) GACCCAAACGTTAAAGCTGAAAAGGATTATTATGACC-CAGAAGATTTAATGAGGCATTCGGGTGAAATGATTTATATGGAAAATAGGCCACCTAT
SARS-CoV-2 NC_045512.2  (5237)  TACCCACAAGTTAATGGTTTAACTT-CTATTAAATGGGCAGA----TAACAACTGTTATCTTGCCACTGCATTGTT------AACACTCCAACAAA
                                                                                                   Section 910
                        (87265) 87265    87274      87285      87296      87307      87318      87329      87340          87360
T4 NC_000866.4          (87104) TATTATGGCAATGGATCAAACAGAAGAAATCAATATTCTGTTTACATTTTAAATTAAGGGAGCCCATGGGCTCCCTTTTTCTTTATAAATACTATA
SARS-CoV-2 NC_045512.2  (5322)  TAGAGTTGAAGT-------------TTAATCCACCTGC--TCTACAAGATG-CTTATTACAGAGCAAGGGCTGGTGAAGCTGC--TAACTTTTGTG
```

```
                                                                                      Section 911
                       (87361) 87361       87373      87384      87395      87406      87417      87428      87439           87456
T4 NC_000866.4         (87200) AACTCATAAGGAAACCGCTATGTTCATTCAAGAACCAAAGAAATTGATTGATACCGGCGAAATTGGTAACGCTTCTACTGGTGATATCTTATTCGA
SARS-CoV-2 NC_045512.2 (5400)  CACTTAT-CTTAGCCTACTGTAATAAGACAGTAGGTGAGTTAGGTGAT-GTTAGAGAAACAATGAGTTACTTGT---TTCAACATGCCAATTTAGA
                                                                                      Section 912
                       (87457) 87457          87472      87483      87494      87505      87516      87527      87538         87552
T4 NC_000866.4         (87296) CGGTGGTAATAAAATTAATAGTGATTTTAACGCAATTTATAATGCGTTTGGCGATCAGCGTAAAATGGCAGTAGCAAATGGCACTGGAGCAGATGG
SARS-CoV-2 NC_045512.2 (5491)  TTCTTGCAA-------AAGAGTC--TTGAACGTGGTGTGTAAAACTTGTGGACAACAGCAGA------CAACCCTTAAGGGTGTAGAAGCTGTT--
                                                                                      Section 913
                       (87553) 87553  87560      87571      87582      87593      87604      87615      87626      87637   87648
T4 NC_000866.4         (87392) TCAAATTATCCATGC-TACTGGATATTATCAAAAACACTCTATTACAGAGTACGCAACTCCAGTGAAAGTTGGCACTAGACATGATATTGA-TACC
SARS-CoV-2 NC_045512.2 (5570)  ----A-TGTACATGGGCACACTTTCTTATGAACAA---TTTAAGAAA-GGTGTTCAGATACCTTGTACGTG--TGGTAAACAAGCTACAAAATATC
                                                                                      Section 914
                       (87649) 87649     87659      87670      87681      87692      87703      87714      87725           87744
T4 NC_000866.4         (87486) TC-TACTGTAGGTGTTAAAGTTATCATTGAAAGAGGCGAACTCGGCGATTGTGT-TGAATTCATTAACTCTAATGGATCAATATCAGTTACTAATC
SARS-CoV-2 NC_045512.2 (5655)  TAGTACAACAGGAGTCACCTTTTGTTATGATGTCAGCACCACCTGC---TCAGTATGAACTTAAG------CATGGTACATTTAC-----------
                                                                                      Section 915
                       (87745) 87745        87758      87769      87780      87791      87802      87813      87824         87840
T4 NC_000866.4         (87580) CTTTGACAATTCAAGCTATTGATTCAATTAAAGGTGTTTCAGGTAATTTAGTAGTAACTAGCCCATATAGTAAAGTTACTTTACGCTGTATTTCAT
SARS-CoV-2 NC_045512.2 (5731)  TTGTGCTAG-TGAGTACACTGGT--AATTACCAG----TGTGGTCACT-ATAAACATATAACTTCTAAAGAAACTTTGTATTGCATAGACGGTGCT
                                                                                      Section 916
                       (87841) 87841 87846      87857      87868      87879      87890      87901      87912      87923      87936
T4 NC_000866.4         (87676) CTGAT-AATTCTACGTCGGTTTGGAATTATTCTATTGAAAGTATGTTTGGACAAAAGGAATCACCAGCTGAAGGTACATGGAATATTTCTACATCT
SARS-CoV-2 NC_045512.2 (5819)  TTACTTACAAAGTCCTCAGAATACAAAGGTCCTATT--ACGGATGTTTTCTACAAAGAAAACAGTTACACAACAACCATAAAACCAGT-TACTTAT
                                                                                      Section 917
                       (87937) 87937   87945      87956      87967      87978      87989      88000      88011                88032
T4 NC_000866.4         (87771) GGATCAGTTGACATTCCATTATTTCATCGTACTGAATACAATATGGCTAAATTGCTAGTTACGTGCCAATCGGTAGATGGAAGAAAAATTAAAACA
SARS-CoV-2 NC_045512.2 (5912)  AAATTGG------------------ATGGTGTTG-------TTTGT---A-----CAGAAATTGACCCTA----AGTTGGA---C-AATTATTATA
```

```
                                                                                                         Section 918
                          (88033) 88033      88044      88055      88066      88077      88088      88099      88110         88128
T4 NC_000866.4            (87867) GCAGAAATAAATATTCTTGTGGATA-CTGTTAATTCAG-AGGTAATTTCTTCTGAATATGCTGTCATGCGAGTTGGGAATGAAACCGAAGAAGACG
SARS-CoV-2 NC_045512.2    (5967)  AGAAAGACAATTCTTATTTCACAGAGCAACCAATTGATCTTGTACCAAACCAACCATATCC--AAACGCAAGCTTCGA--TAATTTTAAGTTTGT-
                                                                                                         Section 919
                          (88129) 88129            88143      88154      88165      88176      88187      88198      88209         88224
T4 NC_000866.4            (87961) AAATCGCTAATATTGCATTTAGTATTAAAGAAAATTATGTAACGGCGACTATAAGTTCTTCAACTGTCGGTATGAGAGC-AGCAGTTAAAGTTATC
SARS-CoV-2 NC_045512.2    (6058)  -ATGTGATAATATCAAATTTGCTGATGATTTAAACCAGTTAAC--TGGTTATAAGA-----AACCTGCTTCAAGAGAGCTTAAAGTTACATTTTTC
                                                                                                         Section 920
                          (88225) 88225 88231      88242      88253      88264      88275      88286      88297      88308         88320
T4 NC_000866.4            (88056) GCTACGCAGAAAATCGGGGTGGCTCAATAATGAAACAAAATATTAATATCGGTAATGTTGTAGATGATGGTA-CCGGTGACTACCTGCGTAAAGGT
SARS-CoV-2 NC_045512.2    (6146)  CCTGACTTAAATGGTGATGTGG-TGGCTATTGATTATAAACACT-AC-------ACACCCTCTTTAAGAAAGGAGCTAAATTGTTACATAAACC-
                                                                                                         Section 921
                          (88321) 88321   88330      88341      88352      88363      88374      88385      88396                  88416
T4 NC_000866.4            (88151) GGTATAAAAATAAATGAAAACTTTGATGAGCTTTATTATGAACTCGGTGATGGTGATGTTCCATATTCAGCCGGTGCCTGGAAAACTTATAATGCT
SARS-CoV-2 NC_045512.2    (6232)  --TATTGTTTGGCATGTTAACAATGC--AACTAA--T---AAAGCCACGT-----ATAAACCAAAT--ACCTGGTG-----------TATAC-GTT
                                                                                                         Section 922
                          (88417) 88417       88429      88440      88451      88462      88473      88484      88495             88512
T4 NC_000866.4            (88247) TCATCAGGACAAACATTAACAGCAGAATGGGGAAAATCATACGCTATTAATACATCTTCTGGAAGAGTGACTATAAATCTTCCAAAGGGTACAGTT
SARS-CoV-2 NC_045512.2    (6300)  GTCTTTGGAGCA-CAAAACCAGTTGAA------ACATCAAATTCGTTTGA----TGTACTGAA-----GTC---AGAGGACGCGCAGGGAATGGAT
                                                                                                         Section 923
                          (88513) 88513          88528      88539      88550      88561      88572      88583      88594         88608
T4 NC_000866.4            (88343) AATGATTACAACAAGGTAAT-TAGAGCTAGAGACGTATTTGCTACATGGAACGTCAACCCAGTTACACTAGTAGCTGCTTCCGGCGA-TACGATTA
SARS-CoV-2 NC_045512.2    (6377)  AATCTTGCCTGCGAAG--ATCTAAAACCAGTCTCTGAAGAAGTAG-TGGAAAATCCTACCA--TACAGAAAGACGTTCTT---GAGTGTAATGTGA
                                                                                                         Section 924
                          (88609) 88609 88616      88627      88638      88649      88660      88671      88682      88693      88704
T4 NC_000866.4            (88437) AAGGGTCTGCAGTACCAGTTGAAATTAATGTTCGATTCAGCGATTTAGAACTAGTGTATTGT-GCCCCAGGACGTTGGGAATATGTCAAAAATAAA
SARS-CoV-2 NC_045512.2    (6465)  AAACTACCGAAGTTGTAGGAGACATTATACTTAAAC-CAGCAAATAA----TAGTTTAAAATTACAGAAGAGGTTGG-----CCACACAGATCTA
```

```
                                                                                                    Section 925
                        (88705) 88705     88715          88726          88737          88748          88759          88770          88781        88800
T4 NC_000866.4          (88532) CAAATTGACAAAATTACCAGTTCAGACATTAGTAATGTAGCTCGCAAAGAATTTTTAGTTGAAGTTCAAGGACAAACAGACTTTTTAGATGTTTTC
SARS-CoV-2 NC_045512.2  (6551)  ATGGCTGCTTATGTAGACAATTCTAGTCTTACTA------------------TT----------------------AAGAAACCTAAT----------

                                                                                                    Section 926
                        (88801) 88801         88814          88825          88836          88847          88858          88869          88880       88896
T4 NC_000866.4          (88628) CGTGGAACTAGTTATAATGTAAATAACATCAGAGTAAAACATCGTGGTAACGAATTGTATTACGGCGATGTGTTTAGCGAAAACAGCGATTTTGGC
SARS-CoV-2 NC_045512.2  (6599)  ----GAATTATCTAG----------------A---------------------------GTATTAGGTT-----------TGAAAAC------CCTTGC

                                                                                                    Section 927
                        (88897) 88897 88902      88913          88924          88935          88946          88957          88968          88979     88992
T4 NC_000866.4          (88724) TCTCCAGGCGAAAATGAAGGAGAACTGGTTCCTCTTGATGGATTTAACATTCGATTAAGACAGCCTTGTAATATTGGTGACACTGTTCAAATTGAA
SARS-CoV-2 NC_045512.2  (6634)  TACTCATGGTTTAGCTGCTGTTAATAGTGTCC-CTTG--GGATAC-------------TATAGC----TAATTATGCTAAGCCT------------

                                                                                                    Section 928
                        (88993) 88993   89001          89012          89023          89034          89045          89056          89067          89088
T4 NC_000866.4          (88820) ACATTTATGGATGGTGTATCACAGTGGAGAAGTTCATATACAAGACGTCAATTAGATTGTTAGATTC-AAAATTAACGTCAAAACTTCTTTAGA
SARS-CoV-2 NC_045512.2  (6698)  -----------TTTCTTAACAAAGTTGTTA-------GTACAA--CTACTAA---CATAGTTACACGGT---------GTTTAAACCGTGTTT-GT

                                                                                                    Section 929
                        (89089) 89089      89100          89111          89122          89133          89144          89155          89166        89184
T4 NC_000866.4          (88915) AGGAAGCATTTACGTTACTGATTTATCAACAATGAAATCAATTCCATTTTCTGCTTTTGGATTAATTCCAGGAGAACCTATTAATCCTAACTCTCT
SARS-CoV-2 NC_045512.2  (6761)  ACTAATTATATGC----CTTATTTCTTTACTTTA---TTGCTACAATTGTGTACTTT-----TACTAGAAGTACA-------AATTCTAGAATTAA

                                                                                                    Section 930
                        (89185) 89185         89199          89210          89221          89232          89243          89254          89265        89280
T4 NC_000866.4          (89011) TGAGGTTCGTTTTAACGGGATTTTACAGGAATTGGCTGGCACAGTTGGAATGCCATTATTTCATTGTGTTGGTGCCGATTCAGACGATGAAGTAGA
SARS-CoV-2 NC_045512.2  (6838)  AGCATCTATGCCGACTACTATAGCAAAGAATACTGTTAAGAGTGTCGGTA---AATTTTGTCTAG----AGGCTTCATTTAATTATTTGAAGTCAC

                                                                                                    Section 931
                        (89281) 89281 89287      89298          89309          89320          89331          89342          89353          89364     89376
T4 NC_000866.4          (89107) ATGCTCTGTTTTAGGTGGAACTTGGGAACAA-TC-TCATACCGATTATTCAGTTGA--AACTGATGAAAACGGCATACCAGAAATTTTAC--ATTT
SARS-CoV-2 NC_045512.2  (6927)  CTAATTTTTCTAAACTGATAAATATTATAATTTGGTTTTACTATTAAGTGTTTGCCTAGGTTCTTTAATCTACTCAACCGCTGCTTTAGGTGTTT
```

```
                                    Section 932
                    89377 89386 89397 89408 89419 89430 89441 89452 89472
                  (89377)
T4 NC_000866.4    (89197) CGATAGCGTATTTGAGCATGGTGACATTATCAATATCACCTGGTT--TAATAATGATTTGGGTACATTATTAACAAAAGATGAGATTATTGATGAA
SARS-CoV-2 NC_045512.2 (7023) TAATGTCTAATTTAGGCATGC----CTTCTTACTGT--ACTGGTTACAGAGAAGGCTATTTGAACTCTACTAATGTCACTATTGCAACCTACTGTA
```

```
                                    Section 933
                    89473 89485 89496 89507 89518 89529 89540 89551 89568
                  (89473)
T4 NC_000866.4    (89291) ACTGATAATCTCTATGTATCGCAAGGACC--TGGAGTAGATATTTCTGGTGATGTAAATTTAACAGACTTCGATAAAATTGGTTGGCCAAATGTAG
SARS-CoV-2 NC_045512.2 (7113) CTGGTTCTATACCTTGTAGTGTTTGTCTTAGTGGTTTAGATTCTTTAGACACCTATCCTTCTTTAGAAACTATACAAATTA-----CCATTT----
```

```
                                    Section 934
                    89569 89584 89595 89606 89617 89628 89639 89650 89664
                  (89569)
T4 NC_000866.4    (89385) AAGCAGTTCAATCTTATCAACGCGCATTTAATGCTGTTTCA-AATATCT-TTGATACG-ATTTATCCTATTGGAACTATATATGAAAACGCTGTTA
SARS-CoV-2 NC_045512.2 (7200) CATCTTTTAAATGGGATTTAACTGCTTTTGGCTTAGTTGCAGAGTGGTTTTTGGCATATATTCTTTTCACTAGGTTTTTCTATGTACTTGGATTGG
```

```
                                    Section 935
                    89665 89672 89683 89694 89705 89716 89727 89738 89749 89760
                  (89665)
T4 NC_000866.4    (89478) ATCCAAATAACCCTGTTACATATATGGGATTCGGCTCATGGAAATTATTTGGGCAAGGAAAAGTTTTAGTTGGATGGAATGAAGATATTTCGGACC
SARS-CoV-2 NC_045512.2 (7296) CTGCA-ATCATGCAATTGTTTTTCAGCTATTTTGC-AGTACATTTTATTAGT---AATTCTTGGCTTATGTGGTT--AATAA--------------
```

```
                                    Section 936
                    89761 89771 89782 89793 89804 89815 89826 89837 89856
                  (89761)
T4 NC_000866.4    (89574) CTAACTTTGCTCTAAATAACAACGATTTAGATTCGGGTGGAAATCCTTCACATACCGCAGGTGGAACAGGTGGTTCTACTTCTGTTACATTGGAAA
SARS-CoV-2 NC_045512.2 (7371) TTAATCTTGTAC-AAATGGCCCCGATTTCAGCT---ATGGTTAGAATGTACAT----C---------------------TTCT-TTGCAT------
```

```
                                    Section 937
                    89857 89870 89881 89892 89903 89914 89925 89936 89952
                  (89857)
T4 NC_000866.4    (89670) ATGCTAATCTTCCTGCAACTGAAACAGATGAAGAAGTTCTAATAGTTGATGAAAATGGATCAGTCATTGTTGGTGGTGTCAATACGATCCAGATG
SARS-CoV-2 NC_045512.2 (7431) ---C--ATTTTATTATGTATGGAA-AAGTTATGTGCATGTTGTAGACGGTTGTAATTCATCAA-CTTGTATGATGTGT-TACAAACGTAATAGAGC
```

```
                                    Section 938
                    89953 89958 89969 89980 89991 90002 90013 90024 90035 90048
                  (89953)
T4 NC_000866.4    (89766) AATCCG-GTCCAATTTACACTAAATACC-GTGAAGCTAAAG-CATCTACTAACTCTACTCACACTCCGCCAACATCAATAACTAACATTCAACCAT
SARS-CoV-2 NC_045512.2 (7519) AACAAGAGTCGAATGTACAACTATTGTTAATGGTGTTAGAAGGTCCTTTTATGTCTATGCTAATGGAGGTAAAGGCTTTTGCAAAC--TACACAAT
```

Section 939

(90049) 90049 90057 90068 90079 90090 90101 90112 90123 90144

T4 NC_000866.4 (89859) -ATATTACAGTTTATCGTTGGATAAGGATTGCATAATG-AGTTTACTTAATAATAAAGC-GGGAGTTATTTCCCGCTTAGCCGATTTTCTTGGTTT

SARS-CoV-2 NC_045512.2 (7613) TGGAATTGTGTTAATTGTGATACATTCTGTGCTGGTAGTACATTTATTAGTGATGAAGTTGCGAGAGACTTGTCACTA--CAGTTTAAAAGACCAA

Section 940

(90145) 90145 90156 90167 90178 90189 90200 90211 90222 90240

T4 NC_000866.4 (89952) TAGACCTAAAACTGGCGACATTGATGTAATGAATCGTCAATCAGTCGGGTCAGTGACAATATCTCAATTAGCGAAAGGATTTT--ATGAACCAAAC

SARS-CoV-2 NC_045512.2 (7707) TAAATCCT--ACTGA--CCAGTCTTCT--TACATCGTTGAT-AGT-GTTACAGTGAAGAATGGT--TCCATCCATCTTTACTTTGATAAAGCTGGT

Section 941

(90241) 90241 90255 90266 90277 90288 90299 90310 90321 90336

T4 NC_000866.4 (90046) ATAGAATCAGCTATTAATGACGTTCATAATTTTTCTATAAAAGACGTTGGCACAATTATTACTAATAAAACTGGTGTTTC-TCCTGAGGGTGTTTC

SARS-CoV-2 NC_045512.2 (7793) CAAAAGACTT--ATGAAAGACATTCTCTCTCTCATTTTGTTA-ACTTAGACAACCTGAGAGCTAATAACACTAAAGGTTCAT--TGCCTATTAATG

Section 942

(90337) 90337 90343 90354 90365 90376 90387 90398 90409 90420 90432

T4 NC_000866.4 (90141) TCAAACTGATTATTGGGCATTTTCTGGAACTGTAACAGACGATTCTCTTCCTCCGGGTTCTCCTA-TTACGGTATTAGTATT-TGGTCTTCCAGTT

SARS-CoV-2 NC_045512.2 (7884) TTATAGTTTTTGATGGTA----AATCAAAATGTGAAGAATCATCTGCAAAATCAGCGTCTGTTTAC-TACAGT--CAGC-TTATGTGTCAACCTAT

Section 943

(90433) 90433 90442 90453 90464 90475 90486 90497 90508 90528

T4 NC_000866.4 (90235) TCAGCAACAACTGGAATGACGGCAATTGAGTTTGTTGCAAAAGTTCGCGTTGCACTACAAGAAGCTATTGCGTCATTTACTGCTATCAATTCATAT

SARS-CoV-2 NC_045512.2 (7972) ACTG---TTACTAGATCA--GGCATTAGTGTCTGATGTTGGTGATAGTGCGGAAGTT---GCAGTTAAAATGTTTGATGCTTACGTTAATACGTTT

Section 944

(90529) 90529 90541 90552 90563 90574 90585 90596 90607 90624

T4 NC_000866.4 (90331) AAAGACCATCCAACTGATGGTAGTAAATTAGAAGTTACTTATTTAGATAATCAAAA-ACATGTATTAAGCACATATTCTACATATGGAATAACTAT

SARS-CoV-2 NC_045512.2 (8060) -T----CATCAA-------CTTTTA-------ACGTACCAATGGAAAAACTCAAAACACTAGTTGCAACTGCAGAAGCTGAACTTGCAAAGAATGT

Section 945

(90625) 90625 90640 90651 90662 90673 90684 90695 90706 90720

T4 NC_000866.4 (90426) TTCCCAAGAAATTATATCTG--AGTCTAAGCCTGGCTATGGTACATGGAATTTATTGGGCGCACAAACTGTAACTTTAGATAATCAGCAGACTCCT

SARS-CoV-2 NC_045512.2 (8137) GTCCTTAGACAATGTCTTATCTACTTTTATTTCAGCAGCTCGGCAAGGGTTTGTTGATTCAGATGTA--GAAACTAAAGATGTTGTTGAATGTCTT

Section 946

```
                       (90721) 90721  90728      90739      90750      90761      90772      90783      90794      90805      90816
T4 NC_000866.4         (90520) ACAGTATTTTATCATTTTGAGAGAACAGCATGAGTAATAATACATATCAACACGTTT--CTAATGAATCTCGTTATGTAAAATTTGATCCTACCGA
SARS-CoV-2 NC_045512.2 (8231)  AAATTGTCACATCAATCTGACATAGAAG--TTACTGGCGATAGTTGTAATAACTATATGCTCACCTATAACAAAGTTGAAAACATGACAC-CCCGT
```

Section 947

```
                       (90817) 90817     90827      90838      90849      90860      90871      90882      90893              90912
T4 NC_000866.4         (90614) TACGAATTTTCCACCGGAGATTACTG-A-TGTTCACGCTGCTATAGCAGCCATTTCTCCTGCTGGAGTAAATGGAGTTCCTGATGCATCGTCAACA
SARS-CoV-2 NC_045512.2 (8324)  GAC---CTTGGTGCTTGTATTGACTGTAGTGCGCGTCATATTAATGC-GCAGGTAGCAAAAAGTCACAACATTGCTTTGATATGGAACGTTAAAGA
```

Section 948

```
                       (90913) 90913        90926      90937      90948      90959      90970      90981      90992          91008
T4 NC_000866.4         (90708) ACAAAGGGAATTCTATTTATTCCACTGAACAGGAAGT-TATAGATGGAACTAATAATACCAAAGCAGTTACACCAGCAACGTTGGCAACAAGATT
SARS-CoV-2 NC_045512.2 (8416)  TTTCATGTCATTGTCTGAACAACTACGAAACAAATACGTAGTGCTGCTAAAAAGAATAACTTACCTTTA---AGTTGACATGTGCAAC------
```

Section 949

```
                       (91009) 91009 91014      91025      91036      91047      91058      91069      91080      91091      91104
T4 NC_000866.4         (90803) ATCTTATCCAAATGCAACTGAAACTGTTTACGGATTAACAAGATATTCAACCAATGATGAAGCCATTGCCGGAGTTAATAATGAATCTTCTATAAC
SARS-CoV-2 NC_045512.2 (8503)  ----TA--------CTAGACAAGTTGTTAATGTTGTAACAACAAAGATAGCACTTAAGGGTGGTA---AAATTGTTAATAATTGG--TTGAAGCAG
```

Section 950

```
                       (91105) 91105   91113      91124      91135      91146      91157      91168      91179              91200
T4 NC_000866.4         (90899) TCCAGCTAAATTTACTGTCGCCCTTAATAATGCGTTTGAAACGCGAGTTTCAACTGAATCC--TCAAATG--GTGTTATTAAAATTTCATCTCTAC
SARS-CoV-2 NC_045512.2 (8582)  TT-AATTAAAGTTACACTTGTGTTCCTTTTTGTTGCTGCTATTTTCTATTTAATAACACCTGTTCATGTCATGTCTAAACATACTGACTTTTCAAG
```

Section 951

```
                       (91201) 91201      91212      91223      91234      91245      91256      91267      91278              91296
T4 NC_000866.4         (90991) CGCAAGCATTAGCTGGTGCAGATGATACTACTGCAATGACTCCATTAAAAACA-CAGCAGTTAGCTATTAAATTAATTGCGCAAATTGCTCCTTCT
SARS-CoV-2 NC_045512.2 (8677)  TGAAATCA-TAGGATACAAGGCTATTGATGGTGGTGTCACTCGTGACATAGCATCTACAGATACTTGTTTTGCTAACA----AACATGCTGATTTT
```

Section 952

```
                       (91297) 91297         91311      91322      91333      91344      91355      91366      91377          91392
T4 NC_000866.4         (91086) GAAACCACAGCTACCGAATCGGACCAAGGTGTTGTTCAATTAGCAACAGTAGCGCAGGTTCGTCAGGGAACTTTAAGAGAAGGCTATGCAATTTCT
SARS-CoV-2 NC_045512.2 (8768)  GACACATGGTTTAGCCAG------CGTGGTGGTAGTTA--TACTAA---TGACAAAGCTTGCCCATT---------GA--TTGCT--GCA----GT
```

```
                                                                                                        Section 953
                        (91393) 91393 91399      91410      91421      91432      91443      91454      91465      91476      91488
T4 NC_000866.4          (91182) CCTTATACGTTTATGAATTCATCTTCTACTGAAGAATATAAAGGCGTAATTAAATTAGGAACACAATCA-GAAGTTAACTCGAATAATGCTTCTGT
SARS-CoV-2 NC_045512.2  (8836)  CATAACAAG---A-GAAGT-GGGTTTTGTCGTGCCTGGTTTGCCTGGCACGATATTACGCACA-ACTAATG--GTGACTTTTTGCATTTCTTACCT
                                                                                                        Section 954
                        (91489) 91489    91498      91509      91520      91531      91542      91553      91564          91584
T4 NC_000866.4          (91277) TGCGGTTACTGGCGCAACTCTTAATGGTCGTGGTTCTACGACGTCAATGAGAGGCGTAGTTAAATTAACTACAACCGCCGGTTCACAGAGTGGAGG
SARS-CoV-2 NC_045512.2  (8924)  AGAGTTTTAG-TGCAGTTGGTAACATCTGTTACACACCATCAAAACTTATAG-AGTACACTGACT--TTGCAACATCAGCTT----GTGTTTGG
                                                                                                        Section 955
                        (91585) 91585       91597      91608      91619      91630      91641      91652      91663          91680
T4 NC_000866.4          (91373) CGATGCTTCATCAGCCTTAGCTTGGAATGCTGACGTTATCCAGCAAAGAGGTGGTCAAATTATCTATGGAACACTCC-GCATT-GAA--GACACA-
SARS-CoV-2 NC_045512.2  (9012)  C--TGCTGAATGTACAATTTTTAAAGATGCTTCTGGTAAGC--C--AG----TACCATATTGT-TATG--ATACCAATGTACTAGAAGGTTCTGTT
                                                                                                        Section 956
                        (91681) 91681          91696      91707      91718      91729      91740      91751      91762          91776
T4 NC_000866.4          (91464) -TTTACAATAGCTAATGGTGGAGCAAATATTACGGGTACCGTCAGAATGACTGGCGGTTATATTCAAGGTAACCGCATCGTAACACAAAATGAAAT
SARS-CoV-2 NC_045512.2  (9095)  GCTTATGAAAGTTTACGCCCTGACACACGTTATGTGCTCA---TGGATGGCT--CTATTATT----------CAATTTCCTAAC------------
                                                                                                        Section 957
                        (91777) 91777  91784      91795      91806      91817      91828      91839      91850      91861      91872
T4 NC_000866.4          (91559) TGATAGAACTATTCCTGTCGGAGCTATTATGATGTGGGCCGCTGATAGTCTTCCTAGTGATGCTTGGCGCTTCTGCCATGGTGGAACTGTTTCAGC
SARS-CoV-2 NC_045512.2  (9164)  ------ACCTACC----TTGAAGGTTCTGTTAGAGTGGTAACAACTTTTGATTCT---GAGTACTGTAGGCACGGCACTTGTGAAAGATCAGAAGC
                                                                                                        Section 958
                        (91873) 91873     91883      91894      91905      91916      91927      91938      91949          91968
T4 NC_000866.4          (91655) GTCAGATTGTCCATTATATGCTTCTAGAATTGGA---A-CAAGATATGGCGGAAACCCATCAAATCCTGGATTGCCTGACATGCGTGGTCTTTTG
SARS-CoV-2 NC_045512.2  (9247)  TGGTGTTTGTGTATCTACTAGTGGTAGATGGGTACTTAACA----ATGATTATTACAGATCTTTACCAGGA--GTTT--TCTG--TGGTGTAGAT-
                                                                                                        Section 959
                        (91969) 91969        91982      91993      92004      92015      92026      92037      92048          92064
T4 NC_000866.4          (91747) TTCGTGGTTCTGGTCGTGGTTCTCACTTAACAAATCCAAATGTTAATGGTAATGACCAAT--TTGGTAAACCTAGATTAGGTGTAGGTTGTACCGG
SARS-CoV-2 NC_045512.2  (9332)  ------------------GCTGTAAATTTACTTACTAATATGTTTA-------CACCACTAAT---TCAACCTA-TT--GGTGC----------TT
```

```
                                                                                                                    Section 960
                          (92065) 92065 92070     92081      92092      92103      92114      92125      92136      92147   92160
          T4 NC_000866.4  (91841) TGGATATGTTGGTGAAGTACAGATACAACAGATGTCTT-ATCATAAACATGCTGGTGGATTTGGTGAGCATGATGATCTGGGGGCATTCGGTAATA
    SARS-CoV-2 NC_045512.2 (9387) TGGACA-----TATCAG--CATCTATAGTAGCTGGTGGTATTGTAGCTATCGTAGTA-ACATGCCTTGCCTACT-----------ATTTTATGAGG
                                                                                                                    Section 961
                          (92161) 92161   92169      92180      92191      92202      92213      92224      92235           92256
          T4 NC_000866.4  (91936) CCCGTAGATCAAATTTTGTTGGTACACGTAAAGGACTTGACTGGGATAACCGTTCATACTTCACCAATGACGGATATGAAATTGACCCAGAATCAC
    SARS-CoV-2 NC_045512.2 (9464) TT--TAGAAGAGCTTTTGGTGAATACAGTCATGTAGTTGCCTTTAATA-CTTT--ACTATTC-CTTATGTCATTCACTGTACT----CTGTTTAAC
                                                                                                                    Section 962
                          (92257) 92257      92268      92279      92290      92301      92312      92323      92334      92352
          T4 NC_000866.4  (92032) AACGAAATTCCAAATATACATTAAATCGTCCTGAATTAATTGGAAATGAAACACGTCCATGGAACATTTCTTTAAACTACATAATTAAGGTAAAAG
    SARS-CoV-2 NC_045512.2 (9550) ACC--AGTT--TACTCATTCTTACCTGGTGTTTATTCTGTTAT-----TTACTTGTACTTG--ACATT--TT-ATCTTAC-TAATGATGTTTCTTT
                                                                                                                    Section 963
                          (92353) 92353         92367      92378      92389      92400      92411      92422      92433      92448
          T4 NC_000866.4  (92128) AATGACAGATATTGTACTGAATGACTT-ACCATTCGTTGACGGCCCTCCTGCAGAGGGCCAGAGCCGCATTTCCTGGATTAAAAACGGCGAAGAAA
    SARS-CoV-2 NC_045512.2 (9631) TTTAGCACATATTCAGTGGATGGTTATGTTCACACCTTTAGTACCTTTCTGGATAACAATTGCT-TATATCATTTGTATTTCCA----CAAAGCAT
                                                                                                                    Section 964
                          (92449) 92449 92455      92466      92477      92488      92499      92510      92521      92532   92544
          T4 NC_000866.4  (92223) TATTAGGAGCTGACACACAGTATGGAAGTGAAGGCTCAATGAATAGACC-TACGGTTTCTGTACTAAGAAATGTTGAAG----TTCTTGATAAAAA
    SARS-CoV-2 NC_045512.2 (9722) TTCTATTGGTT--C-TTTAGTAATTACCTAAAG---AGACGTGTAGTCTTTAATGGTGTTTCCTTTAGTACTTTTGAAGAAGCTGCGCTGTGCACC
                                                                                                                    Section 965
                          (92545) 92545    92554      92565      92576      92587      92598      92609      92620           92640
          T4 NC_000866.4  (92314) CATTGGAATACTTAAAACATCTTTAGAAACCGCAAATAGTGATATTAAAACAATTC----AGGGCATCTTAGATGTAT--CTGGTGATATTGAAGC
    SARS-CoV-2 NC_045512.2 (9812) TTTTTGTTAAATAAAGAAATGTATCTAAAGT-TGCGTAGTGATGTGCTATTACCTCTTACGCAATATAATAGATACTTAGCTCTTTATAATAAGTA
                                                                                                                    Section 966
                          (92641) 92641       92653      92664      92675      92686      92697      92708      92719           92736
          T4 NC_000866.4  (92404) TTTGGCCCAAATAGGTATCAA--TAAAAAGGATATTTCTGACCTCAAAACGCTAACCAGTGAACACACAGAAATATTAAATGGAACTAATAATACG
    SARS-CoV-2 NC_045512.2 (9907) CAAGTATTTTAGTGG-AGCAATGGATACAACTAGCTACAGAGA---AGCTGCTTGTT-GTCATCTCGCAAAGGCTCTCAATGACTTCAGTAACTCA
```

```
Section 967
                         92737          92752      92763      92774      92785      92796      92807      92818          92832
                 (92737)
T4 NC_000866.4   (92498) GTTGACAGTATTCTTGCCGATATTGGTCATTTAACGCCGAGGCCAACTCTGTATACAGAACGATCAGAAATGATTTACTGTGGATAAAGCGTGAA
SARS-CoV-2 NC_045512.2 (9998) GGTTCTGATGTTCTTTACCAACCACC-ACAAACCTCTATCACCTCAGCTGT-TTTGCAGAGTGGTTTAGA-AAAATGGCATTCCCATCTGGTAAA

Section 968
                         92833  92840      92851      92862      92873      92884      92895      92906      92917  92928
                 (92833)
T4 NC_000866.4   (92594) CTTGGACAATACACTGGTCAAGATATTAATGGTCTTCCTGTTGTAGGAAATCCTAGT-AGTGGAATGA-AGCATCGCATTATTAATAAT-ACTGAT
SARS-CoV-2 NC_045512.2 (10091) GTTGAGGGTTGTATGGTACAAGTAACTTGTGG---TACAACTACAC---TTAACGGTCTTTGGCTTGATGACGTAGTTTACTGTCCAAGACATGTG

Section 969
                         92929     92939      92950      92961      92972      92983      92994      93005          93024
                 (92929)
T4 NC_000866.4   (92687) GTCATCACTTCGCAGGGAATACGTTTAAGCGAATTAGAAACAAAATTTATTGAATCTGATGTAGGTTCTTTGACCATTG----AAGTTGGTAATCT
SARS-CoV-2 NC_045512.2 (10181) ATCTGCACCTCTGAAGACATGC-TTAACCCTAATTATGAAG---ATTTACTCATTCGTAAGTCTAATCATAATTTCTTGGTACAGGCTGGTAATGT

Section 970
                         93025        93038      93049      93060      93071      93082      93093      93104          93120
                 (93025)
T4 NC_000866.4   (92779) TCGTGAAGAGCTTGGACCGAAACCACCATCATTTTCACAGAACGTTTATAGTCGTTTAAATGAAATTGACACTAAACAGACAACAGTTGAGTCTGA
SARS-CoV-2 NC_045512.2 (10273) TCAACTCAGGGTT------ATTGGACATTCTATGCAAAATTGTGTACTTAAGCTTAAGGTTGATACAGCCAATCCTAAGACA-CC--TAAGTATAA

Section 971
                         93121 93126      93137      93148      93159      93170      93181      93192      93203     93216
                 (93121)
T4 NC_000866.4   (92875) CATTAGTGCTATTAAGACCTCAATAGGATATCCAGGAAATAATTCGATTATCACGAGTGTTAATACAAAC-------ACTGATAATATTGCATCTA
SARS-CoV-2 NC_045512.2 (10360) GTTTGTTCGCATTCAACCAGGACAGACTTTTCAGTG-----TTAGCTTGTTACAATGGTTCAC-CATCTGGTGTTTAC---CAATGTG----CTA

Section 972
                         93217   93225      93236      93247      93258      93269      93280      93291              93312
                 (93217)
T4 NC_000866.4   (92964) TTAATTTAGAGCTAAATCAAAGTGGAGGTATTAAACAGCGTTTAAC-CGTTATTGAAACTTCCATTGGTCAGATGATATTCCTTCGAGTATTAAA
SARS-CoV-2 NC_045512.2 (10443) TGAG----GCCCAATTTCACTATTAAGGGTTCATTC----------C--TTAATGGTTCATGTGGTAGTGTTGGTTTA--ACATAGATTATGACT

Section 973
                         93313      93324      93335      93346      93357      93368      93379      93390          93408
                 (93313)
T4 NC_000866.4   (93059) GGTCAAATCAAA---GATAATACAACTTCAATCGAATCTCTAAATGGAATCGTCGGTGAAAACACTTCATCTGGCTTAAGAGCGAATGTTTCATGG
SARS-CoV-2 NC_045512.2 (10521) GTGTC--TCTTTTTGTTACATGCACCATA--TGGAATTACCAACTGGAGTTCATGCTG-GCA---------CAGACTTAGAAG-GTAACTTTTATGG
```

Section 974
(93409) 93409 93423 93434 93445 93456 93467 93478 93489 93504
T4 NC_000866.4 (93152) TTAAACCAAATTGTTGGAACT--GATTCTAGCGGTGGACAACCTTCTCCTCCTGGGTCT-CTTTTAAACCGA--GTTTCTAC--AATTG-AAACTT
SARS-CoV-2 NC_045512.2 (10603) --------ACCTTTTG---TTGACAGGCAAACAGC--ACAAGCAGCTGGTACGGACACAACTATTACAGTTAATGTTTTAGCTTGGTTGTACGCTG

Section 975
(93505) 93505 93511 93522 93533 93544 93555 93566 93577 93588 93600
T4 NC_000866.4 (93240) CTGTTTCAGGCTTGAATAACGCTGTTCAAAACCTACAAGTAGAGATTGGTAATAACAGCGCAGGAATTAAAGGGCAAGT-TGTAGCGTTAAATACT
SARS-CoV-2 NC_045512.2 (10686) CTGTTATAAATGGAGACAGGTGGTTTCTCAATCGATTTACCACAACTCTTAATGACTTT--A--ACCTTGTGGCTATGAA-GTACAATTATGAACC

Section 976
(93601) 93601 93610 93621 93632 93643 93654 93665 93676 93696
T4 NC_000866.4 (93335) TTAGTAAATGGAACTAATCCAAACGGTTCAACTGTTGAAGAGCGCGGATTAACCAATTCAATAAAAGCTAACGAAACTAACATTGCATCAGTTACA
SARS-CoV-2 NC_045512.2 (10777) TCTAACACAAGA------CCATGTTGACATACTAGGACCTCTTTCTGCTCAA-----ACTGGAATTGCCGTTTTAGATATGTGTGCTTCATTAAAA

Section 977
(93697) 93697 93709 93720 93731 93742 93753 93764 93775 93792
T4 NC_000866.4 (93431) CAAGAAGTGAATACAGCTAAAGGCAATATATCTTCTTACAAGGTGATGTTCA-AGCTCTCCAAGAAGCCGGTTATATTCCTGAAGCTCCAAGAGA
SARS-CoV-2 NC_045512.2 (10862) GAATTA-------CTGCAAAATGGTATGAATGGACGT-ACCA--TATTGGGTAGTGCTTTATTAGAAGATGAATTTACACCTTTTGATGTT---GT

Section 978
(93793) 93793 93808 93819 93830 93841 93852 93863 93874 93888
T4 NC_000866.4 (93526) TGGGCAAGCTTACGTTCGTAAAGATGGCGAATGGGTATTCCTTTCTACCTTTTTATCACCAGCATA-ACATGGGGCCGCAAGGCCCCAAAGGATTT
SARS-CoV-2 NC_045512.2 (10945) TAGACAATGCTCAGGTGTTACTTT---CCAAAGTGCAGTGAAAA-GAACA----ATCAAGGGTACACACCACTGGTTGTTACTCACAATTTTGACT

Section 979
(93889) 93889 93896 93907 93918 93929 93940 93951 93962 93973 93984
T4 NC_000866.4 (93621) TAAATGTCAGGATATAATCCTCAGAATCAAAGGAACTCAAAGATGTCATT-CTAAGACGTTTAGGGGCTCCAATTATTAATGTTGAGTTAACACC
SARS-CoV-2 NC_045512.2 (11033) TCACTTTTAGT-TTTAGTCCAGAGTACTCAAT-GGTCTTTGTTCTTTTTTTGTATGAAATGCCTTTTTACCTTTTGCTATGG-GTATTATTGCT

Section 980
(93985) 93985 93995 94006 94017 94028 94039 94050 94061 94080
T4 NC_000866.4 (93716) CGATCAAATTTACGATTGTATCCAGCGTGCCCTAGAATTATA-CGGTGAATACCATTTTGATGGACTCAATAAAGGTTTTC-ATGTTTTTTATGTA
SARS-CoV-2 NC_045512.2 (11126) ATGTCTGCTTTTGCAATGATGTTTGTCAAACATAAGCATGCATTTCTCTGTTTGTTTTTGTTACCTTCTCTTGCCACTGTAGCTTATTTTAATATG

Section 981

(94081) 94081 94094 94105 94116 94127 94138 94149 94160 94176
T4 NC_000866.4 (93810) GGGGATGATGAAGAAAGG-----TACAAGACCGGAGTCTTCGATTTAAGAGGTTCTAACGTATTTGCAGTAACCCGCATTTTACGCACAAATATTG
SARS-CoV-2 NC_045512.2 (11222) GTCTATATGCCTGCTAGTTGGGTGATGCGTATTATGACATGGTTGGATATGGTTGATAC----T---AGTTTGTCTGGTTTTAAGCTAAAAGACTG

Section 982

(94177) 94177 94182 94193 94204 94215 94226 94237 94248 94259 94272
T4 NC_000866.4 (93901) GGTCAATAACATCTATGGATGGAAACGCTACATATCCGTGGTTTACTGA-CTTTCTTTTAGGAATGGCTGGTATTAATG-GCGGAATGGGAACGTC
SARS-CoV-2 NC_045512.2 (11311) TGT----------TATGTATGCATCAGCTGTA-----GTG--TTACTAATCCTTATGACAGCAAGAACTGTGTATGATGATGGTGCTAGGAGAGTG

Section 983

(94273) 94273 94281 94292 94303 94314 94325 94336 94347 94368
T4 NC_000866.4 (93995) TTGTAATAGATTTTATGGACCAAATGCCTTTGGAGCTGATTTAGGATATTTTACCCAGCTTACCAGTTATATGGGAATGATGCAAGATAT--GCTC
SARS-CoV-2 NC_045512.2 (11390) TGGACACTTAT-------G---AATGTCT-TGACACTCGTTTATAA-AGTTTATTATGGT----AATGCTTTAG--ATCAAGCCATTTCCATGTGG

Section 984

(94369) 94369 94380 94391 94402 94413 94424 94435 94446 94464
T4 NC_000866.4 (94089) TCTCCTATTCCAGACTTTTGGTTTAATTCAGCAAATGAACAGCTCAAAGTCATGGGAAACTTCCAAAAATATGATTTAATTATCGTAGAAAGCTGG
SARS-CoV-2 NC_045512.2 (11468) GCTCTTATAATCTCTGTTACTTCTAACTACTCAGGTGTAGTTACAACTGTCATGTTTTTGGCCAGAGGTATTGTTTTTATGTGTGTTGAGTATTGC

Section 985

(94465) 94465 94479 94490 94501 94512 94523 94534 94545 94560
T4 NC_000866.4 (94185) ACTAAATCATACATTGATACAAACAAAATGGTTGGAAATACAGTAGGATATGGAACAGTCGGTCCACAAGATAGCTGGT-CATTATCTGAA-CGAT
SARS-CoV-2 NC_045512.2 (11564) CCTATTTTCTTCATAACTGGTAATACACTTCAGTGTATAATGCTAGTTTATTGTTTCTTAGGCTATTTTTGTACTTGTTACTTTGGCCTCTTTTGT

Section 986

(94561) 94561 94567 94578 94589 94600 94611 94622 94633 94644 94656
T4 NC_000866.4 (94279) ATAATAACCCAGACCACAATTTAGTAGGTCGTGTTGTCGGCCAAGATCCGAATGTTAAACAGGGTGCTTATAATAATCGTTGGG-TGAAAGACTAT
SARS-CoV-2 NC_045512.2 (11660) TTACTCAACCG--CTACTTTAGACTGACTCTTGGTGTTTATGATTACTTAGTTTCTACACAGGAGTTTAGATATATGAATTCACAGGGACTACTCC

Section 987

(94657) 94657 94666 94677 94688 94699 94710 94721 94732 94752
T4 NC_000866.4 (94374) GCAACAGCTTTAGCTAAAGAATTGAACGGTCAAATTTTAGCACGCCACCAAGGTATGATGCTTCCGGGCGGTGTTAC--AATTGATGG-GCAGCGC
SARS-CoV-2 NC_045512.2 (11754) CACCCAAGAATAGCATAG---ATG--CCTTCAAAC-TCAACATTAAATTGTTGGGTGTTGGTGGCAAACCTTGTATCAAAGTAGCCACTGTACAGT

Section 988

(94753) 94753 94765 94776 94787 94798 94809 94820 94831 94848

T4 NC_000866.4 (94467) TTAATAGAAGAAGCCAGATTAGAAAAAGAAGCACT--GCGCGAAGAATTATACTTACTTGATCCTCCATTTGGAATTTTGGTAGGTTAATATGGCT

SARS-CoV-2 NC_045512.2 (11844) CTAAAA-----TGTCAG-ATGTAAAGTGCACATCAGTAGTCTTACTCTCAGTTTTGCAACA-ACTCAGAGTAGAATCATCATCTAAATTGTGGGCT

Section 989

(94849) 94849 94864 94875 94886 94897 94908 94919 94930 94944

T4 NC_000866.4 (94561) ACTTATGATAAAAATCTTTTTGCTAAATTGGAAAACCGCACAGGTTATTCT-CAGACCAATGAAACTGAAATATTAAATCCTTATGTAAATTTC--

SARS-CoV-2 NC_045512.2 (11933) CAATGTGTCCAGTTACACAATGACATTCTCTTAGCT---AAAGATACTACTGAAG-CCTTTGAAAAAATGGTTTCACTACTTTCTGTTTTGCTTTC

Section 990

(94945) 94945 94952 94963 94974 94985 94996 95007 95018 95029 95040

T4 NC_000866.4 (94654) -AATCATTATAA-AAACAGCCAAATATTAGCTGATGTATTAGTAGCTGAAAGCATTCAAATGCGAGGTGTAGAATGCTATTATGTTCCAAGAGAGT

SARS-CoV-2 NC_045512.2 (12025) CATGCAGGGTGCTGTAGACATAAA--CAAGCTTTGTGAAGAAATGCTGGA------CAACAGGGCAACCTTACAAGCTAT--AGCCTCA-GAGTTT

Section 991

(95041) 95041 95051 95062 95073 95084 95095 95106 95117 95136

T4 NC_000866.4 (94748) ATGTTTC--CCCTGATTTGATATTC-GGCGAAGACTTAAAAAATAAATTTACTAAAGCTTGGAAATTTGCTGCATATTTAAATTCATTTGAAGGAT

SARS-CoV-2 NC_045512.2 (12110) AGTTCCCTTCCATCATATGCAGCTTTTGCTACTGCTCAAGAAGCTTATGAGCAGGCTGTTGCTAAT--GGTG-ATTCTGAAGTTGTTCTTAAAAAG

Section 992

(95137) 95137 95150 95161 95172 95183 95194 95205 95216 95232

T4 NC_000866.4 (94841) ATGAAGGAGCTAAATCGTTCTTTAGTAACTTTGGTATGCAAGTACAAGACGAAG-TGACTTTATCTATTAACCCA---AATTTATTTAAGCATCAA

SARS-CoV-2 NC_045512.2 (12203) TTGAAGAAG-----TCTT---TG---AATGTGGCTA---------AATCTGAATTTGACCGT---GATGCAGCCATGCAACGTAAGTTGGAA--AA

Section 993

(95233) 95233 95238 95249 95260 95271 95282 95293 95304 95315 95328

T4 NC_000866.4 (94933) GTTAACGGAA-AAGAACCCAAGGAAGGTGATTTGATATATTTTCCTATGGATAACAGCTTATTTGAAATTAACTGGGTTGAACCATATGATCCATT

SARS-CoV-2 NC_045512.2 (12274) GATGGCTGATCAAGCTATGACCCAAATGTATAAACAGGCTAGATCTGAGGACAAGAGG----GCAAAAGTTACTAGTGCTATGCAGACAATGCTTT

Section 994

(95329) 95329 95337 95348 95359 95370 95381 95392 95403 95424

T4 NC_000866.4 (95028) TTATCAATTAG-GCCAAAAC---GCTATTCGTAAAATTACGGCAGGTAAATTCATTTATTCTGGAGAAGAAATTAATCCAGTTCTACAGAAAAATG

SARS-CoV-2 NC_045512.2 (12366) TCACTATGCTTAG---AAAGTTGGATAATGATGCACTCAACAA---CATTATCAACAATGCAAGAGATGGTTGTGTTCCC-TTGAACATAATACCT

```
                                                                                      Section 995
                           (95425) 95425      95436      95447      95458      95469      95480      95491      95502            95520
T4 NC_000866.4             (95120) AAGGAATTAACATTCCAGAATTTAG----TGAATTAGAATTAAATGCTGTTCGCAAT-CTTAACGGTATTCATGACATTAATATTGATCAGTATGC
SARS-CoV-2 NC_045512.2     (12455) CTTACAACAGCAGCCAAACTAATGGTTGTCATACCAGACTATAACACATATAAAAATACGTGTGATGGTACAACATTTACTTATGCATCAGCAT--
                                                                                      Section 996
                           (95521) 95521         95535      95546      95557      95568      95579      95590      95601         95616
T4 NC_000866.4             (95211) TGAAGTAGATCAAATTAATTCTGAAGCTAAAGAAT--AC--GTTGAA---CCTTATGTTGTTGTCAATAACAGAGGCA-------AATCTTTCGA-
SARS-CoV-2 NC_045512.2     (12549) TGTGGGAAATCCAACAGGTTGTAGATGCAGATAGTAAAATTGTTCAACTTAGTGAAATTAGTATGGACAATTCACCTAATTTAGCATGGCCTCTTA
                                                                                      Section 997
                           (95617) 95617 95623      95634      95645      95656      95667      95678      95689      95700      95712
T4 NC_000866.4             (95292) ATCTAGCCCATTTGACAATGATTTCATGGATTAATAAATATTATAAACTAATTAAAGCCCGGATTAGGAGAAGTCATGTTTGGTTAT---TTTTAT
SARS-CoV-2 NC_045512.2     (12645) TTGTAACAGCTTTAAGGGCCAATTCTGCTGTCAAATTACAGAATAATGAGCTTAGT-CCTGTTGCACTACGACAGATGTCTTGTGCTGCCGGTACT
                                                                                      Section 998
                           (95713) 95713    95722      95733      95744      95755      95766      95777      95788            95808
T4 NC_000866.4             (95385) AATTCGTCTTTTAGACGATATGCTACCTTGATGGGCGATTTGTTTTCAAATATCCAAATCAAACGTCAGTTAGAATCTGGT-GATAAGTTTATACG
SARS-CoV-2 NC_045512.2     (12740) ACACAAACTGCTTGCACTGATGACA-----ATGCGTTAGCTTACTACA-----ACACAACAAAGGAGGTAGGTTTGTACTTG-CACTGTTATCCG
                                                                                      Section 999
                           (95809) 95809       95821      95832      95843      95854      95865      95876      95887            95904
T4 NC_000866.4             (95480) TGTTCCTATTACGTATG--CATCAAAGGAACACTTTA---TGATGAAATTGAATAAATGGACATCAATAAATTCACAAGAAGATGTAGCTAAAGTT
SARS-CoV-2 NC_045512.2     (12825) ATTTACAGGA---TTTGAAATGGGCTAGATTCCCTAAGAGTGATGGAACTGGT--ACTATCTATACAGAA-CTGGAACCACCTTGTAGGTTTGTTA
                                                                                      Section 1000
                           (95905) 95905          95920      95931      95942      95953      95964      95975      95986         96000
T4 NC_000866.4             (95571) GAAA--CTATTCTACCTCGTATAA--ATTTACATTTAGTTGATTTTAGCTATAATGCTCCATTTAAAACAAACATTTTAAATCAGAATTTA-CTGC
SARS-CoV-2 NC_045512.2     (12915) CAGACACACCTAAAGGTCCTAAAGTGAAGTATTTATACTTTATTAAAGGATTAAACAACC--TAAATAGAGGTATGGTACTTGGTAGTTTAGCTGC
                                                                                      Section 1001
                           (96001) 96001  96008      96019      96030      96041      96052      96063      96074      96085      96096
T4 NC_000866.4             (95662) AAAA--AGGTGCAACTTCTGTAGTATCGCAGTATAATCCATCTCCTATTAAAATGATTTATGAATTGAGTATCTTTACTCGCTATGAAGA---TGA
SARS-CoV-2 NC_045512.2     (13009) CACAGTACGTCTACAAGCTGG--TAATGCAACAGAAGTGCCTGCCAATTCAA-------CTGTATTATCTTTCTGTGCTTTTGCTGTAGATGCTGC
```

Section 1002

	(96097)	96097 96107 96118 96129 96140 96151 96162 96173 96192
T4 NC_000866.4	(95753)	TATGTTTCAAATAGTTGAAC-AGATTCTTCCATATTTTCAACC--TCATTTTAATACAACTATGTACGAGCAGTTTGGAAATGATATTCCATTTAA
SARS-CoV-2 NC_045512.2	(13096)	TAAAGCTTACAAAGATTATCTAGCTAGTGGGGGACAACCAATCACTAATTGTGTTAAGATGTTGTGTACACACACTGGTACTGGTCAGGCAAT-AA

Section 1003

	(96193)	96193 96206 96217 96228 96239 96250 96261 96272 96288
T4 NC_000866.4	(95846)	AAGGGATAT--TAAAATTGTACTGATGTCTGCTGCTATAGACGAAGCTATAGATGGGGATAATTTA-TCTCGTCGTAGAATTGAATGGTCATTAAC
SARS-CoV-2 NC_045512.2	(13191)	CAGTTACACCGGAAGCCAATATGGATCAAGAATCCTTTGGTGGTGCATCGTGTTGTCTGTACTGCCGTTGCCACATAGATCATCCAAATCCTAAAG

Section 1004

	(96289)	96289 96294 96305 96316 96327 96338 96349 96360 96371 96384
T4 NC_000866.4	(95939)	A----TTTGAAGTAAATGGATGGATGTATCCTCCAGTAGATGATGCAGAAGGATTAATTCGTACTACTTATACAG--ATTTTCACGCCAATACAAG
SARS-CoV-2 NC_045512.2	(13287)	GATTTTGTGACTTAAAAGG---TAAGTATGTACAAATACCTA------------CAACTTGTGCTAATGACCCTGTGGGTTTTAC---ACTTAAAA

Section 1005

	(96385)	96385 96393 96404 96415 96426 96437 96448 96459 96480
T4 NC_000866.4	(96029)	AGATTTGCCTGACGGCGAAGGTGTTTTTGAATCTGTCGATAGCGAAGTTGTTCCTCGAGATATTGACCC---AGAAGACTGGGATGGAACAGTAAA
SARS-CoV-2 NC_045512.2	(13365)	ACA-CAGTCTGTACCGTCTGCGGTATGTGGAAAGGTTATGGCTGTAGTTGTG-ATCAACTCCGCGAACCCATGCTTCAGTCAGCTG--ATGCACAA

Section 1006

	(96481)	96481 96492 96503 96514 96525 96536 96547 96558 96576
T4 NC_000866.4	(96122)	ACAAACTTTCACTAGTAATGTAAATA-GACCAACACCGCCAGAACCTCCTGGCCCAAGAACATAGAGGTTATTATGGAAGGTCTTGATATAAACAA
SARS-CoV-2 NC_045512.2	(13457)	TCGTTTTTAAACGGGTTTGCGGTGTAAGTGCAGCCCGTCTTACACCGTGCGGCACAGGCACT-AGT---ACTGATGTCG----------TATACAG

Section 1007

	(96577)	96577 96591 96602 96613 96624 96635 96646 96657 96672
T4 NC_000866.4	(96217)	ACTTTTAGATATTTCTGACCTCCCCGGAATTGACGGGGAGGAAATCAAAGTGTATGAACCTCTGCAATTAGTAGAAG-TTAAAAGCAATCCACAAA
SARS-CoV-2 NC_045512.2	(13539)	GGCTTTTGACATCTACAATGATAAAGTAGCT-----GGTTTTGCTAAATTCCTAAAAAC-T----A-ATTGTTGTCGCTTCCAAGAAAAGGACGAA

Section 1008

	(96673)	96673 96679 96690 96701 96712 96723 96734 96745 96756 96768
T4 NC_000866.4	(96312)	ACCGTACTCCAGACTTAGAAGATGATTATGGAGTAGTTCGTCGAAATATGCATTTTCAGCAACAAATGCTAATGGACGCTGCCAAGATTTTTCTTG
SARS-CoV-2 NC_045512.2	(13624)	G-----ATGACAATTTAATTGATTCTTACTTTGTAGTTAAGAGACACACTT-TCTCTAACTACCAACATGAAGAAACAATTTATAATTTACTTAAG

```
                                                                                                      Section 1009
                       (96769) 96769    96778      96789      96800      96811      96822      96833      96844        96864
T4 NC_000866.4         (96408) AGACAGCAAAGAATGCTGATTCTCCTCGTCACA--TGGAAGTATTTGCAACTCTTATGGGCAAATGACTACGACGAACAGAGAAATACTGAAGCT
SARS-CoV-2 NC_045512.2 (13714) -GATTGTCCAG----CTGTTGCTAAACATGACTTCTTTAAGTTTAGAATAGAC-----GGTGACATG-GTACCAC--ATATATCACGTCAACGTCT
                                                                                                      Section 1010
                       (96865) 96865       96877      96888      96899      96910      96921      96932      96943        96960
T4 NC_000866.4         (96502) TCATAAAGATATGAAAG-ACATTACATCTGAGCAGGTTGGCACCAAAGGCGCTGTTCCTACAGGTCAAATGAATATTCAGAATGCGACAG-TATTC
SARS-CoV-2 NC_045512.2 (13797) TACTAAATACACAATGGCAGACCTCGTCTATGCTTTAAGGCATTTTGATGAAGGTAATTGTGACACATTAAAAGAAATACTTGTCACATACAATTG
                                                                                                      Section 1011
                       (96961) 96961          96976      96987      96998      97009      97020      97031      97042        97056
T4 NC_000866.4         (96596) ATGGGTTCACCAACAGAATTAATGGACGAAATTGGTGATGCTTACGAGGCTCAAGAAGCTCGTGAGAAGGTGATAAATGGAACAACCGATTAATGT
SARS-CoV-2 NC_045512.2 (13893) TTGTGATGATGATTATT-TCAATAAAAAGGACTGG-------TATGATTTTGTAGAAAACCCAGATATATT-A-----CGCGTATACGCCAACTTA
                                                                                                      Section 1012
                       (97057) 97057  97064      97075      97086      97097      97108      97119      97130      97141   97152
T4 NC_000866.4         (96692) ATTAAATGATTTC--CATCCGTTAAATGAAGCTGGAAAAATTTTAATAAAACACCCAAGCTTAGCGGAAAGAAAAGA-TGAAGATGGAATT-CATT
SARS-CoV-2 NC_045512.2 (13975) GGTGAACGTGTACGCCAAGCTTT-------GTTAAAAACAGTACAATT---CTGTGATGCCATGCGAAATGCTGGTATTGTTGGTGTACTGACATT
                                                                                                      Section 1013
                       (97153) 97153     97163      97174      97185      97196      97207      97218      97229        97248
T4 NC_000866.4         (96784) GGATAAAATCTCAGTG-GGATGGAAAATGGTATCCTGAAAAATTCAGTGATTACCTTCGT-CTACACAAAATAGTAAAAATTCCAAACAA--CTCT
SARS-CoV-2 NC_045512.2 (14061) AGATAA--TCAAGATCTCAATGGTAACTGGTATGAT------TTCGGTGATTTCATACAAACCACGCCAGGTAGTGGAGTTCCTGTTGTAGATTCT
                                                                                                      Section 1014
                       (97249) 97249       97262      97273      97284      97295      97306      97317      97328        97344
T4 NC_000866.4         (96876) GATAAGCCTGAATTATTTCAAACTTATAAAGATAAGAATAATAAAAGATCTCGGTATATGGGTCT--TCCTAACTTGAAACGAGCTAATATTAAAA
SARS-CoV-2 NC_045512.2 (14149) TATTATTCATTGTTAATGCCTA--TATTAACCT-----TGACCAGGGCTTTAACTGCA-GAGTCACATGTTGACACTGACTTAACAAAGCCTTACA
                                                                                                      Section 1015
                       (97345) 97345 97350      97361      97372      97383      97394      97405      97416      97427      97440
T4 NC_000866.4         (96970) CACAATGG-ACTCGTGAAATGGTTGAGGAATGGAAAAAATGCCGAGATGATATTGT--TTATTTTGCAGAAACATACTGTGCCATTA---CTCATA
SARS-CoV-2 NC_045512.2 (14237) TTAAGTGGGATTTGTTAAAATATGACTTCACGGAAGAGAGGTTAAAACTCTTTGACCGTTATTTT------AAATATTGGGATCAGACATACCACC
```

```
                                                                                                  Section 1016
                        (97441) 97441   97449      97460      97471      97482      97493      97504      97515           97536
T4 NC_000866.4          (97060) TTGACTATGGTGTCATAAAGGTTCAATTACGTGACTATCAGCGTGATATGCTCAAAATAATGTCATCTAAACGTATGACTGTTTGTAATCTATCGC
SARS-CoV-2 NC_045512.2  (14327) CAAATTGTGTTAACTGTTTGGATGACAGATGCATTCTGCATTGTGCAAACTTTAATGTTTATTCTCTACA-GTGTTCCCACCTACAAGTTTTGGA
                                                                                                  Section 1017
                        (97537) 97537      97548      97559      97570      97581      97592      97603      97614           97632
T4 NC_000866.4          (97156) GCCAGCTCGGTAAAACCACCGTAGTAGCTATTTTCCTTGCACACTTTGTATGTTTTAACAAAGATAAAGCTGTAGG----TA-TTCTTGCACACA-
SARS-CoV-2 NC_045512.2  (14422) CCACTAGTGAGAAAAATAT-----TTGTTGATGGTGTTCCA---TTTGTAG--TTTCAACTGGATACCACTTCAGAGAGCTAGGTGTTGTACATAA
                                                                                                  Section 1018
                        (97633) 97633          97647      97658      97669      97680      97691      97702      97713           97728
T4 NC_000866.4          (97246) -AAGGCTCAATGTCTGCGGAAGTTTTAGACCGTACTAAGCAAGCAATTGAACTGCTTCCTGACTTTTACAACCAGGAATTGTTGAATGGAATAAG
SARS-CoV-2 NC_045512.2  (14508) TCAGGATGTAAACTTAC-ATAGCTCTAGACT-TAGT-TTTAAGGAATTA--CTTGTGTAT-GCTGCT--GACCCT-GCTATGCACGCTGCTTCTGG
                                                                                                  Section 1019
                        (97729) 97729 97735      97746      97757      97768      97779      97790      97801      97812       97824
T4 NC_000866.4          (97341) GGTTCAATTGAACTAGATAATGGTTCTTCAATTGGCGCTTATGCTTCCTCTCCTGACGCAGTTCGTGGTAACTCGTTCGCAATGATTTACATTGAC
SARS-CoV-2 NC_045512.2  (14595) TAATCTATT--ACTAGATAAACGCACTACGTGCTTTTCAGTAGCTGCACTTACTAACAATGTTGCTTTCAAACTGTCAAACCCGGTAA-------
                                                                                                  Section 1020
                        (97825) 97825    97834      97845      97856      97867      97878      97889      97900            97920
T4 NC_000866.4          (97437) GAATGTGCGTTTATTCCAAACTTCCATGATTCCTGGCTTGCTATTCAACCAGTAATTTCATCTGGTCGTCGTTCGAAAATTATTATTACTACGACT
SARS-CoV-2 NC_045512.2  (14682) --------TTTTAACAAAGACTTCTATGACT--TTGCTGTGTCT--AAGGGTTTCTTTAA----------GGAAGGAAGTTCTGTTGAAT------
                                                                                                  Section 1021
                        (97921) 97921       97933      97944      97955      97966      97977      97988      97999           98016
T4 NC_000866.4          (97533) CCTAATGGATTAAATCATTTTTATGATATTTGGACTGCTGCTGTCGAAGGTAAATCTGGATTTGAACCATATACTGCTATTTGGAATTCAGTTAAA
SARS-CoV-2 NC_045512.2  (14750) --TAAAACACTTCTTC--TTTGCTCAGGATGGTAATGCTGCTATCAGCGATTATGACTACTATCGTTATAATCTACCAACAATGTGTGATATCAGA
                                                                                                  Section 1022
                        (98017) 98017          98032      98043      98054      98065      98076      98087      98098          98112
T4 NC_000866.4          (97629) GAACGTCTTTATAACGATGAAGATATTTTTGACGA--TGGATGGCAATGGAGCATACAAACCATTAATGGTTCTTCATTAGCTCAATTCCGTCAAG
SARS-CoV-2 NC_045512.2  (14842) CAACTACTATTTGTAGTTGAAGTTGTTGATAAGTACTTTGATTGTTACGATG--GTGGCTGTATTAATGCTAA----CCAAGTCA-T--CGTCAAC
```

```
                                                                                                    Section 1023
                           (98113) 98113  98120          98131          98142          98153          98164          98175          98186          98197      98208
T4 NC_000866.4             (97723) AACATACTGCAGCGTTTGAAGGGACTTCTGGTACATTAATTTCAGGAATGAAATTAGCTGTTATGGATTTTATTGAAGTAACACCAGATGATCATG
SARS-CoV-2 NC_045512.2     (14929) AACCT-----AGACAAATCAG------CTGGT-TTTCCATTTAATAAATGGGGTAAG--GCTA--GACTTTATTATGATTCAATGAG-TTATGAGG
                                                                                                    Section 1024
                           (98209) 98209        98219          98230          98241          98252          98263          98274          98285          98304
T4 NC_000866.4             (97819) GTTTTCACCAATTTAAAAAACCTGAACCAGATAGAAAATATATTGCAACTCTAGATTGCTCAGAAGGTCGTGGGCAAGATTACCACGCTTTGCATA
SARS-CoV-2 NC_045512.2     (15008) ------ATCAAGATGCA---CTTTTCGCATATACAAAACGTAATGT-------CATCCCTACTATA-----ACTCAAATGAATC---TTAAGTATG
                                                                                                    Section 1025
                           (98305) 98305           98318          98329          98340          98351          98362          98373          98384      98400
T4 NC_000866.4             (97915) TTATTGATGTTACTGATGATGTGTGGGAACAGGTTGGTGTTTTGCATTCAAACACTATTTCTCATTTAATTCTACCTGACATCGTTATGCGTTATT
SARS-CoV-2 NC_045512.2     (15080) CCATTAGTGCAAAGAATAGAGCTCGCACCGTAGCTGGTGTCTCTATCTGTAGTACTATGACCAATAGACAGTTTCAT--------CAAAAATTATT
                                                                                                    Section 1026
                           (98401) 98401 98406          98417          98428          98439          98450          98461          98472          98483      98496
T4 NC_000866.4             (98011) TAGTAGAATACAATGAATGCCCAGTTTATATTGAATTAAATAGTACTGGTGTGTCAGTTGCAAAATCGCTTTATATGGAT-TTAGAATACGAAGGT
SARS-CoV-2 NC_045512.2     (15168) -----GAAATCAATAGCCGCC-ACT--AGAG-GAGCTAC--TGTA-------GTAATTGGAACAAGCAAATTCTATGGTGGTTGGCACAACATGTT
                                                                                                    Section 1027
                           (98497) 98497    98505          98516          98527          98538          98549          98560          98571                98592
T4 NC_000866.4             (98106) GTTATCTGCGATTCATATACTGATTTAGGAATGAAACAAACTAAACGCACGAAAGCAGTAGGATGTTCCACGCTAAAAGACCTTATTGAAAAAGAT
SARS-CoV-2 NC_045512.2     (15246) AAAAACTGTT-----TATAGTGATGTAGAAAACCCTC-ACCTTATGGGT---------TGGGATTATCCTAAATGTGATAGAGCCATGCCTAACAT
                                                                                                    Section 1028
                           (98593) 98593         98604          98615          98626          98637          98648          98659          98670           98688
T4 NC_000866.4             (98202) AAGCTTATTATTCAT-CACCGAGCGACTATTCAAG--AATTTAGAACGTTTAGTGAAAAAGGCGTGTCTTGGGCGGCTGAAGAAGGTTATCATGAC
SARS-CoV-2 NC_045512.2     (15327) --GCTTAGAATTATGGCCTCACTTGTTCTTGCTCGCAAACATACAACGTGTTGT----A--GCTTGTC--ACACCGTTTCTATAGATTAGCTAATG
                                                                                                    Section 1029
                           (98689) 98689           98703          98714          98725          98736          98747          98758          98769      98784
T4 NC_000866.4             (98295) GATTTAGTAATGTCTTTAGTGATTTTTGGATGGTTATCAACGCAGTCAAAATTTATTGATTATGCGGATAAAGATGACATGCGATTAGCATCT---
SARS-CoV-2 NC_045512.2     (15413) AGTGTGCTCAAGTATTGAGTGAAATGGTCATGTGTGGCGGTTCACTATATGTTAAACCAGGTGGAACCTCATCAGGAGATGCCACAACTGCTTATG
```

```
                                                                                                        Section 1030
                            (98785) 98785 98791      98802      98813      98824      98835      98846      98857      98868  98880
T4 NC_000866.4              (98388) G---AAGTATTTTCAAAAGAGCTTCAGGATATGAGCGACGACTACGCGCCAGTTATATTTGTGGATT-CGGTTCATTCTGC--TGAGTATGTTC-C
SARS-CoV-2 NC_045512.2      (15509) CTAATAGTGTTTTTAACATTTGTCAAGCTG-TCACGGCCAATGTTAATGCACTTTATCTACTGATGGTAACAAAATTGCCGATAAGTATGTCCGC
                                                                                                        Section 1031
                            (98881) 98881    98890      98901      98912      98923      98934      98945      98956          98976
T4 NC_000866.4              (98477) AGT----ATCTCATGGTATGTCAATGGTATAAATATATTAAAGCATATTAAAGAGGA-TTAAAAATGACTTTAT----TATCTCCGGGCATTGAGC
SARS-CoV-2 NC_045512.2      (15604) AATTTACAACACAGACTTTATGAGTGTCTCTATAGAAATAGAGATGTTGACACAGACTTGTGAATGAGTTTTACGCATATTTGCGTAAACATTTC
                                                                                                        Section 1032
                            (98977) 98977       98989      99000      99011      99022      99033      99044      99055          99072
T4 NC_000866.4              (98564) TCAAAGAAACTACGGTTCAAAGCACC------GTAGTT-AATA--ACT-------CTACTGGTACAGCAGCTTTGGCCGGTAAATTCCAGTGGGT
SARS-CoV-2 NC_045512.2      (15700) TCAATGATGATACTCTCTGACGATGCTGTTGTGTGTTTCAATAGCACTTATGCATCTCAAGGTCTAGTGGCTAGCATAAAGAACTTTAAGTCAGTT
                                                                                                        Section 1033
                            (99073) 99073          99088      99099      99110      99121      99132      99143      99154      99168
T4 NC_000866.4              (98644) CCTGCTTTTCAGATTAAACAGGTTACAAATGAAGTAGATTTAGTTAATACTTTTGGTCAACCAACCGCTGAAACTGCTGACTATTTTATGTCTGCG
SARS-CoV-2 NC_045512.2      (15796) CTTTATTATCAAAACAATGTTTTTATGTCTGAAGCA-AA--ATGTTGGACTGAGACTGACCT---TACTAAAGGACCTCATGAATTTTGCTCTCAA
                                                                                                        Section 1034
                            (99169) 99169  99176      99187      99198      99209      99220      99231      99242      99253  99264
T4 NC_000866.4              (98740) -ATGAATTTCT--TGCAGTACGGAAATGACTTACGAGTAGTTCGTGCTGTTGATAGAGATACCGCTAAAAC-TCATCGCCAATTGCTGGTAATAT
SARS-CoV-2 NC_045512.2      (15886) CATACAATGCTAGTTAAACAGGGTGATGATT----ATGTGTACCTT--CCTTACCCAGATCCATCAAGAATCCTAGGGGCCGGCTGTTTTGTAGAT
                                                                                                        Section 1035
                            (99265) 99265     99275      99286      99297      99308      99319      99330      99341          99360
T4 NC_000866.4              (98832) TGATTACACAATTTCTACCCCAGGTAGTAACTATGCGGTTGGA--GATAAAATCACGGTC-AAATATGTTTCAGATGATATTGAAACTGAAGGTAA
SARS-CoV-2 NC_045512.2      (15976) -GATATC------GTAAAAACAGATGGTACACTTATGATTGAACGGTTCGTGTCTTTAGCTATAGATGCTTACCCACTTA------CTAAAC--A-
                                                                                                        Section 1036
                            (99361) 99361        99374      99385      99396      99407      99418      99429      99440          99456
T4 NC_000866.4              (98925) AATTACTGAAGTAGACGCAGATGGAAA-AATTAAGAAAATTAATATTCCTACTGGCAAAAT-TACGCTAAAGCGAAAGAAGTCGGTGAATATCCA
SARS-CoV-2 NC_045512.2      (16056) TCCTAATCAGGAGTATGCTGATGTCTTTCATTTGTACTTACAATACATAAGAAAGCTACATGATGAGTTAACAGGACACATGTTAGAC---ATGTA
```

```
                                                                                                       Section 1037
                    (99457) 99457 99462      99473      99484      99495      99506      99517      99528      99539    99552
T4 NC_000866.4      (99019) ACACTAGGTTCTAACTGGACTGCGGAAATTTCTTCATCTTCCTCTGGTTTAGCTGCAGTAATAACTCTTGGAAAAATTA--TTACTGATTCTGGTA
SARS-CoV-2 NC_045512.2 (16149) TTCTGTTATGCTTACTAAT-----GATAACACTTCAAGG---------------------------TATTGGGAACCTGAGTTTTATGAGGCTATGT
                                                                                                       Section 1038
                    (99553) 99553   99561      99572      99583      99594      99605      99616      99627         99648
T4 NC_000866.4      (99113) TTTTATTAGCTGAAATTGAAAATGCTGAAGCTGCTATGACAGCGGTTGACTTTCAAGCAAATCTTAAAAAATATGGAATTCCAGGAGTAGTAGCGC
SARS-CoV-2 NC_045512.2 (16214) ACACACCGCATACAGTCTTACAGGCTGTTGGGGCTTG--TGTTCTTTGCAATTCACAGACTTCATTAAGATGTGGTGCTTGCATACGTAG---ACC
                                                                                                       Section 1039
                    (99649) 99649      99660      99671      99682      99693      99704      99715      99726         99744
T4 NC_000866.4      (99209) TTTATCCAGGCGAATTAGGCGATAAAATTGAAATTGAAATCGTATCTAAAGCTGACTATGCAAAAGGAGCTTCTGCATTACTCCCAATTTATCCAG
SARS-CoV-2 NC_045512.2 (16305) ATTCTTATGTTGTAAATGCTGTTACGACCATG-------TC--ATATCAACATCAC---ATAAATTAGTCTTGTCTGTTAATCCGTATGTTT----
                                                                                                       Section 1040
                    (99745) 99745         99759      99770      99781      99792      99803      99814      99825          99840
T4 NC_000866.4      (99305) GTGGTGGTACTCGTGCATCTACTGCTAAAGCAGTGTTTGGATATGGACCGCAAACTGATTCACAGTACGCTATTATAGTTCGTCGTAATGATGCTA
SARS-CoV-2 NC_045512.2 (16385) ------GCAA----------TGCTCCAGGTTGTGATGTCACAGATGTGACTCAACTTTACTTAG--G-AGGTATGAGCTATTATTGTAA--ATCACA
                                                                                                       Section 1041
                    (99841) 99841 99847      99858      99869      99880      99891      99902      99913      99924    99936
T4 NC_000866.4      (99401) TTGTTCAAAGCGTTGTTCTTTCAACTAAGCGTGGTGAAAAAGATATTTACGATAGTAACATCTATATCGATGACTTTTTCGCAAAAGGTGGTTCAG
SARS-CoV-2 NC_045512.2 (16461) TA--AACCACCCATTAGTTTTCCATT--GTGTGCTAATGGACAAGTTTTTGGT--TTATATAAAAATACATGTGTTGGTAGCGATAA-TGTTACTG
                                                                                                       Section 1042
                    (99937) 99937    99946      99957      99968      99979      99990      100001     100012         100032
T4 NC_000866.4      (99497) AATATATTTTTGCAACTGCACAAAACTGGCCAGAAGGCTTCTCTGGAATTTTAACTCTGTCTGGTGGATTATCATCAAATGCTGAAGTAACAG-CA
SARS-CoV-2 NC_045512.2 (16550) ACTTTAA---TGCAATTG--CAACATGTGACTGGACAAAT-GCTGGTGATTACATTTTAGCTAACACCTG-TACTGAAAGACTCAAGCTTTTTGCA
                                                                                                       Section 1043
                   (100033) 100033      100045     100056     100067     100078     100089     100100     100111        100128
T4 NC_000866.4      (99592) GGAGATTTGATGGAAGCTTGGGACTTCTTTGCTGACCGTGAATCTGTTGACGTTCAACTGTTTATTGCAGGTTCTTGTGCCGGTGAATCTTTAGAA
SARS-CoV-2 NC_045512.2 (16639) GCAGAAACGCTCAAAGCTACTGA-------GGAGACATTTAAACTGTCTTATGGTATT------------GCTACTGTACGTGAAGTGCTGTCTGA
```

```
Section 1044
                            (100129) 100129         100144     100155     100166     100177     100188     100199     100210   100224
T4 NC_000866.4              (99688)  A-CAGCATCTACTGTCCAAAAACACGTCGTTTCAATTGGGATGCTCGCCAAGATTGCTTAGTATTG-TGCTCTCCTCCGCGTGAAACTGTAGTTG
SARS-CoV-2 NC_045512.2      (16716)  CAGAGAATTACATCTTTCATGGGAAGTTGGTAAACCTAGAC---CACCACTTAACCGA-AATTATGTCTTTACTGGTTATCGTGTAACTAAAAACA

Section 1045
                            (100225) 100225 100232     100243     100254     100265     100276     100287     100298     100309 100320
T4 NC_000866.4              (99782)  GAATTCCTGTAACCCGTGCTGTTGATAACCTAGTCAATTGGAGAACTGCGGCAGGTTCATACACTGATAATAACTTTAATATCAGTTCAACCTATG
SARS-CoV-2 NC_045512.2      (16808)  GTAAAGTACAAATAGGAGA-G--TACA--CCT------TTGAAAAAGGTGAC-TATGGTGATGCTGTTGTTTACCGAGGT---ACAACAACTTACA

Section 1046
                            (100321) 100321    100331     100342     100353     100364     100375     100386     100397        100416
T4 NC_000866.4              (99878)  CAGCAATTGATGGTAACCATAAGTATCAGTATGACAAATATAATGATGTGAATCGTTGGGTTCCATTAGCAGCTGATATTGCTGGTTTATGCGCAA
SARS-CoV-2 NC_045512.2      (16889)  AATTAAATGTTGGTGATTATTTTGTGCTG-ACATCACATACAGTAATGC--------------CATTA--AGTGCAC----CTACACTA-GTGCCA

Section 1047
                            (100417) 100417       100430     100441     100452     100463     100474     100485     100496     100512
T4 NC_000866.4              (99974)  GAACTGATAACGTATCTCAGACTTGGATGTCTCCAGCTGGTTATAAT-CGTGGTCAGATTCTTAACGTTATTAAACTTGCTATTGAAACTCGCCAG
SARS-CoV-2 NC_045512.2      (16963)  CAAGAGCAC---TATGTTAGAATT----------ACTGGCTTATACCCAACACTCAATATCTCA--G--ATG-AGTTTTCTAGCAATGTTGCAAAT

Section 1048
                            (100513) 100513100518    100529     100540     100551     100562     100573     100584     100595 100608
T4 NC_000866.4              (100069) GCTCAGCGCGACCGTTTATACC-AAGAAGCTATCAACCCGGTAACCGGTACAGGTGGTGATGGTTACGTATTGTATGGTGATAAAACAGCTACTTC
SARS-CoV-2 NC_045512.2      (17041) TATCAA----AAGGTTGGTATGCAA-AAGTATTCTACACTCC--AGGGACCACCTGGTACTGGTAAGAGTCATTTTGCTATTGGCCTAGCTCTCTA

Section 1049
                            (100609) 100609 100617     100628     100639     100650     100661     100672     100683        100704
T4 NC_000866.4              (100164) TGTTCCTTCTCCATTTGATCGTATTAACGTTCGTCGTCTGTTTAATATGTTGAAAACGAATATCGGACGTAGTTCAAAATATCGTTTGTTCGAATT
SARS-CoV-2 NC_045512.2      (17130) CTACCCTTCTGCTCG-CATAGTGTATAC-AGCTTGCTCTCATGCCGCTGTTGATGCACTATGTGAGAAGGCATT-AAAATAT--TTGCCTATAGAT

Section 1050
                            (100705) 100705     100716     100727     100738     100749     100760     100771     100782        100800
T4 NC_000866.4              (100260) AAA--CAACGCGTTTACTCGTTCATCATTCCGCACAGAAACTGCCCAGTACTTACAAGGGAATAAAGCTCTCGGTGGAATTTATGAATATCGTGTA
SARS-CoV-2 NC_045512.2      (17221) AAATGTAGTAGAATTATACCTGCACGTGCTCGTGTAGAGTGTTTTGATAAATTCAAAGTGAATTCAACATTAG--A-ACAGTAT--GTCTTTGTA
```

```
                                                                                    Section 1051
                             (100801) 100801        100815    100826     100837    100848     100859     100870     100881        100896
           T4 NC_000866.4 (100354) GTTTGCGATACAACAAATAACACTCCGTCAGTAATTGATAGAAATGAGTTTGTTGCAACATTCTACATCCAACCGGCTAGAAGCATTAACTACATT
 SARS-CoV-2 NC_045512.2  (17312) CTGTAA-ATGCATTGCCTGAGAC---GACAGCA---GATATAGTTGTCTTTGATGAA--ATTTCAATGGCCACAAATTATGAT--TTGAGTGTTGT
                                                                                    Section 1052
                             (100897) 100897 100903     100914     100925     100936     100947     100958     100969     100980  100992
           T4 NC_000866.4 (100450) ACCTT--AAACTTCGTAGCAACTGCTACTGGTGCAGATTTCGAT-GAGTTAACTGGTCT--TGCTGGTTAATACGGTGCATT-CTAAA-GGCCTGT
 SARS-CoV-2 NC_045512.2  (17397) CAATGCCAGATTACGTGCTAAGCACTATGTGTACA-TTGGCGACCCTGCTCAATTACCTGCACCACGC----ACATTGC-TAACTAAGGGCACACT
                                                                                    Section 1053
                             (100993) 100993   101002     101013     101024     101035     101046     101057     101068          101088
           T4 NC_000866.4 (100539) TTCGGCAGGCCATATAAATACACTATATCCTTAATTCTTTAATTCTATATGCCCTAGGTTAAACATAGGATATAAATACTACAGAGGCTAATATG
 SARS-CoV-2 NC_045512.2  (17487) AGAACCAGAATATTTCAATTCAGTGTGTAG--ACTTATG-AAAACTATAGGTCCAGACATGTTCCTCGGAACTTGTCGGCGTTGT--CCTGCTGAA
                                                                                    Section 1054
                             (101089) 101089      101101     101112     101123     101134     101145     101156     101167          101184
           T4 NC_000866.4 (100635) TTTGTAGATGATGTAACACGAGCGTTTGAATCTGGTGATTTGCTCGACCTAACTTATTCCAAGTAGAAATTTCTT-ATCTTGGACAAAATTTTAC
 SARS-CoV-2 NC_045512.2  (17578) ATTGTTGACACTGTGA---GTGC--T----TTGGTTTATGATAATAAGCTTAAAGCACATAAAGACAAATCAGCTCAATGCTTTAAAATGTTTTAT
                                                                                    Section 1055
                             (101185) 101185         101200     101211     101222     101233     101244     101255     101266     101280
           T4 NC_000866.4 (100730) GTTCCAATGTAAAGCTACTGCTCTACCAGCTGGTATTGTAGAAAAAATTCCAGTCGGATTTATGAACCGTAAAATTAACGTAGCAGGCGATCGTAC
 SARS-CoV-2 NC_045512.2  (17665) AAGGGTGT-TATCACGCATGATGTTTCATCTGCAATTAACAGGCCA---CAAATAGGCGTGGTAAGAGAATTCCTTA------CACGT--------
                                                                                    Section 1056
                             (101281) 101281 101288     101299     101310     101321     101332     101343     101354     101365 101376
           T4 NC_000866.4 (100826) ATTCGATGACTGGACTGTTACAGTAATGAACGATGAAGCTCATGATGCTCGTCAGAAGTTCGTTGATTGGCAAAGCATTGCTGCGGGGCAAGGAAA
 SARS-CoV-2 NC_045512.2  (17743) AACCC-TGCTTGGA--------GAAAAGCTGTCTTTATTTCACCTTATAATTCACAGAATGCTGTAGCCTCAAAGATTTT----GGGACTACCAAC
                                                                                    Section 1057
                             (101377) 101377    101387     101398     101409     101420     101431     101442     101453          101472
           T4 NC_000866.4 (100922) CGAAATTACTGGTGGAAAACCTG---CAGAGTATAAAAAGAGCGCTATCGTTCGTCAATATGCTCGTGACGCTAAAACA-GTAACAAAAGAAATTG
 SARS-CoV-2 NC_045512.2  (17826) TCAAACTGTTGATTCATCACAGGGCTCAGAATATGACTA--T-GTCATATTCACTCAACCACTGAAACAGCTCACTCTTGTAATGTAAACA----
```

Section 1058

(101473) 101473 101486 101497 101508 101519 101530 101541 101552 101568
T4 NC_000866.4 (101014) AAATTAAAGGTCTGTGGCCTACTAACGTGGGTGAACTTCAATTAGATTGGATTCAAACAATGAAATCCAAACTTTGAAGTAACTCTTGCTCTCG
SARS-CoV-2 NC_045512.2 (17915) -GATTTAATGTT-----GCTATTACCA-----GAGCAAAA---------GTAGGCATACTTTGCAT----AATGTCTGATAGAGACCTTTATGACA

Section 1059

(101569) 101569 101574 101585 101596 101607 101618 101629 101640 101651 101664
T4 NC_000866.4 (101110) ATTATTGGAATAAAATGAATGGGAGAAATCCCCATCCTGCTTAAAGCAGAGAAGTCCATTATAAATATAACTATAATTCCCATTTGGAGAATAC
SARS-CoV-2 NC_045512.2 (17987) AG--TTGCAATTTACAAGTCTTGA---AATTCCACGT--AGG---AATGTGGCAACTTTACAAGCTGAAAATGTAACAGGAC-----TCTTTAAAG

Section 1060

(101665) 101665 101673 101684 101695 101706 101717 101728 101739 101760
T4 NC_000866.4 (101206) AATGAAATTTAATGTATTAAGTTTGTTTGCTCCATGGGCTAAAATGGACGAACGAAATTTTAAAGACCAAGAAAAAGAAGATCTTGTTTCCATTAC
SARS-CoV-2 NC_045512.2 (18068) ATTGTAGT--AAGGTAATCACTGGGTT------ACATCCTACACAGGC------AC---CTACACACCTC---AGTGTTGACACTAAATTCAAAAC

Section 1061

(101761) 101761 101772 101783 101794 101805 101816 101827 101838 101856
T4 NC_000866.4 (101302) AGCCCCAAAGCTTGATGATGGAGCAAGAGAATTTGAAGTAAGCTCGAATG---AAGCTGCTTCTCCTTATAATGCTGCATTCCAAACAATTTTTGG
SARS-CoV-2 NC_045512.2 (18144) T-----GAAGGTTTATGT------------GTTGACAT--ACCTGGCATACCTAAGGACATGACCTATAGAAGACT-CATCTCTATGA--TGGGTT

Section 1062

(101857) 101857 101871 101882 101893 101904 101915 101926 101937 101952
T4 NC_000866.4 (101395) TTCATATGAACCAGGAATGAAAACTACTCGTGAGCTTATTGATACATATCGTAATCTCATGAATAACTATGAAGTAGATAATGCAGTTTCAGAAAT
SARS-CoV-2 NC_045512.2 (18218) TTAAAATGAATTA-----TCAAGTTAATGGTTACCCTA---A--CAT---GTT-TATCACCCGCGAAGAAGCTATAAGACATGTACGTGCATGGAT

Section 1063

(101953) 101953 101959 101970 101981 101992 102003 102014 102025 102036 102048
T4 NC_000866.4 (101491) CGTTTCAGATGCTATCGTCTATGAAGATGATACTGAAGTCGTAGCGTTAAATTTGGATAAATCTAAATTTAGCCCAAAAATTAAAAATATGATGTT
SARS-CoV-2 NC_045512.2 (18300) TGGCTTCGATGTCGAGGGGTGTCATGCTACTAGAGAAGCTGTTGGTACCAATTT-----------ACCTTTACAGCTAGGTTTTTCTACAGG-TGTT

Section 1064

(102049) 102049 102058 102069 102080 102091 102102 102113 102124 102144
T4 NC_000866.4 (101587) AGATGAATTTAGTGATGTAT-TAAATCATCTATCGTTTCAACGAAAAGGTTCTGATCATTTTAGACGTTGGTATGTTGATTCAAGAATTTTCTTTC
SARS-CoV-2 NC_045512.2 (18385) AACC----TAGTTGCTGTACCTACAGGTTATGTTGATACACCTAATAATA-CAGATTTTTCCAGAGTTAG---TGCTAAACCACCGCCTGGAGATC

```
                                                                                                Section 1065
                         (102145) 102145      102157     102168     102179     102190     102201     102212     102223          102240
        T4 NC_000866.4   (101682) ATAAAATCATTGATCCAAAACGTCCAAAAGAAGGCATAAAAGAATTACGTAGATTAGACCCTCGCCAAGTTCAGTATGTTCGTGAAATTATAACAG
SARS-CoV-2 NC_045512.2    (18473) A-------ATTTAAACACCTCATACCACTTAT-GTACAAAGGACTTCCTTGGAAT-GTAGTGCGTATAA---AGATTGTAC---AAATGTTAAGTG
                                                                                                Section 1066
                         (102241) 102241         102256     102267     102278     102289     102300     102311     102322         102336
        T4 NC_000866.4   (101778) AAACTGAAGCTGGCACAAAAATAGTTAAAGGTTACAAAGAATATTTTATATATGATACTGCCCATGAGTCATATGCATGTGATGGTAGAATGTATG
SARS-CoV-2 NC_045512.2    (18554) ACACAC------TTAA----------AAATCTCTCTGACAGAGTCGTATTTGT-------CTTATGGG-CACATGGCTTTGA-GTTGACATCTATG
                                                                                                Section 1067
                         (102337) 102337 102344     102355     102366     102377     102388     102399     102410     102421 102432
        T4 NC_000866.4   (101874) AAGC-TGGCACAAAAATAAAAATTCCTAAAGCTGCCGTCGTTTATGCCCATTCTGGATTAGTCGAT----TGTTGCGGTAAAAATATCATCGGGTA
SARS-CoV-2 NC_045512.2    (18625) AAGTATTTTGTGAAAATAGGAC--CTGAGCGCACCTGTTGTCTATGTGATAGACGTGCCACATGCTTTTCCACTGCTTCAGACACTTATGCC--TG
                                                                                                Section 1068
                         (102433) 102433    102443     102454     102465     102476     102487     102498     102509         102528
        T4 NC_000866.4   (101965) TTTGCATCGTGCTGTTAAACCTGCTAAC--CAATTAAAA--TTATTAG-AAGATGCTGTAGTCATTTATCGCA--TTACTCGTGCTCCTGACCGTC
SARS-CoV-2 NC_045512.2    (18717) TTGGCATCATTCTATTGGATTTGATTACGTCTATAATCCGTTTATGATTGATGTTCAACAATGGGGTTTTACAGG-TAACCTACAAAGCAACCATG
                                                                                                Section 1069
                         (102529) 102529       102542     102553     102564     102575     102586     102597     102608         102624
        T4 NC_000866.4   (102054) GTGTTTGGTATGTAGACACAGGTAATATGCCTGCTCGTAAAGCTGCTGAGCACATGCAACATGTTATGAACACGATGAAAAACCGTGTAGTATATG
SARS-CoV-2 NC_045512.2    (18812) ATCTGTATTGTCAAGTCCATGGTAATGCACATGTAGCTAGTTGTGATGCAATCATGACTAGG----TGTCTA-GCTGTCCACGAGTGCTTTGTTAA
                                                                                                Section 1070
                         (102625) 102625102630    102641     102652     102663     102674     102685     102696     102707 102720
        T4 NC_000866.4   (102150) ATGCATCAACAGGTAAA----ATAAAAAATCAACAGC-ATAATATGTCTATGACCGAAGACTATTG---GTTGCAGCGCCGTGATGGTAAAGCTGT
SARS-CoV-2 NC_045512.2    (18903) GCGTGTTGACTGGACTATTGAATATCCTATAATTGGTGATGAACTGAAGATTAATGCGGCTTGTAGAAAGGTTCAACACATGGTTGTTAAAGCTGC
                                                                                                Section 1071
                         (102721) 102721  102729     102740     102751     102762     102773     102784     102795          102816
        T4 NC_000866.4   (102238) GACAGAAGTTGATACTCTTCCTGGTGCTGATAATACTGGCAATATGGAAGATATTCGTTGGTTTAGACAAGCTC-TTTATATGGCA--TTACGTGT
SARS-CoV-2 NC_045512.2    (18999) ATTATTAGCAGACAAATTCCCAGTTCTTCACGACATTGGTAACCCTAAAGCTATTAAGTGTGTACCTCAAGCTGATGTAGAATGGAAGTTCTATGA
```

Section 1072

```
(102817)                    102817     102828     102839     102850     102861     102872     102883     102894           102912
T4 NC_000866.4 (102331)     TCCTCTTTCACGCATTCCGCAAGACCAACAAGGCGGTGTGATGTTTGATTCTGGAACTAGCATTACACGTGAT---GAATTAACGTTTGCTAAATT
SARS-CoV-2 NC_045512.2 (19095) TGCACAGCCTTG--T--------AGTGACAAAGC---------TTATAAAATAGAAGAATTATTCTA--TTCTTATGCCACACATTCTGACAAATT
```

Section 1073

```
(102913)                    102913        102927     102938     102949     102960     102971     102982     102993         103008
T4 NC_000866.4 (102424)     TATTCGTGAGTTACAGCACAAGTTT--GAAGAAGTTTTCCTAGATCCGCTTAAAACAAATCTTTTGCTTAAAGGTATAATCACAGAAGATGAGTGG
SARS-CoV-2 NC_045512.2 (19170) CACAGATG-GTGTATGCCTATTTTGGAATTGCAATGTCGATAGATATCCTGCTAA---TTCCATTGTTTGTAGATT---TGACACTA--------G
```

Section 1074

```
(103009)                    103009 103015     103026     103037     103048     103059     103070     103081     103092 103104
T4 NC_000866.4 (102518)     AATGATGAAATAAATAATATTAAGATAGAATTTCATCGGGATAGCTACTTTGCTGAGCTCAAAGAAGCAGAA-ATTTTGGAACGAAGAATTAATAT
SARS-CoV-2 NC_045512.2 (19251) AGTGCTATC-----TAACCTTAACTTGCCTGGTTGTGATGGTGGCAGTTTGTATGTAAATAAACATGCATTCCACACACCAGCTTTTGATAAAAGT
```

Section 1075

```
(103105)                    103105   103114     103125     103136     103147     103158     103169     103180          103200
T4 NC_000866.4 (102613)     GCTAACCATGGCAGAAC-CATTTATTGGTAAATATATTTCTCACAGAACTGCTATGAAAGACATTTTGCAGATGACTGATGA-AGAAATAGAACAA
SARS-CoV-2 NC_045512.2 (19342) GCTTTTGTTAATTTAAAACAATTACCATT-TTTCTATTACTCTGACAGT--CCATGTGAGTCTCATGGAAAACAAGTAGTGTCAGATATAGATT-A
```

Section 1076

```
(103201)                    103201      103213     103224     103235     103246     103257     103268     103279         103296
T4 NC_000866.4 (102707)     GAAGCCAAGCAAATTGAAGAAGAGTCTAAAGAGGCTCG-TTTCCAAGACCCCGACCAAGAACAAGAGGATTTTTAATGGAAGGTTTAATTGAAG--
SARS-CoV-2 NC_045512.2 (19434) TGTACCACTAAAGTCTGCTACGTGTATAACACGTTGCAATTTAGGTGGTGCTGTCTGTAGACATCATGCTAATGAGTACAGATTGTATCTCGATGC
```

Section 1077

```
(103297)                    103297         103312     103323     103334     103345     103356     103367     103378         103392
T4 NC_000866.4 (102800)     CTATTAAAT---CAAACGACCTCG---TAGCCG-CTCGTAAATTA----TTTGCTGAAGCCATGGCTGCAAGAACGATTGATTTAAT-TAAAGAAG
SARS-CoV-2 NC_045512.2 (19530) TTATAACATGATGATCTCAGCTGGCTTTAGCTTGTGGGTTTACAAACAATTTGAT--ACTTATAACCTCTGGAACACTTTTACAAGACTTCAGAGT
```

Section 1078

```
(103393)                    103393 103400     103411     103422     103433     103444     103455     103466     103477 103488
T4 NC_000866.4 (102884)     AAAAAATCGCTATCGCTCGCAATTTCTTAATCGAAGGTGAAGAACCTGAAGACGAGGATGAAGATGAAGATGACGAAGATAGTGATGATAAAGACG
SARS-CoV-2 NC_045512.2 (19624) TTAGAA--AATGTGGCTTTTAATGTTGTAAATAAGGGACACTTTGATGGACAACAGGGTGAAGTACCAGTT-TCTATCATTA--ATAACACTGTTT
```

Section 1079

```
                    (103489) 103489    103499      103510      103521      103532      103543      103554      103565         103584
      T4 NC_000866.4 (102980) ACAAAAAGACGAAGACTCTGACGAAGACGAGGATGATGAATAATGCTTCTGATCCCTGAAACTCATGAATTAGTTCTCGAGAATGTCGAAGCACT
SARS-CoV-2 NC_045512.2 (19715) ACACAAAAGTTGATGGTGTTGATGTAGAATTGTTTGAA-AATAAAACAACATTACCTGTTAATGTAGCATTTGAGCTTTGGGCTAAGCGCAACATT
```

Section 1080

```
                    (103585) 103585       103598      103609      103620      103631      103642      103653      103664        103680
      T4 NC_000866.4 (103076) TATTCCTGAAGCACAGGGTCGCTTTGACGAATTGTCTTCTGCTTTAAATAAAGACGA-TATAAATACAATTGTCGAGAATATGCTT-GATGATGAA
SARS-CoV-2 NC_045512.2 (19810) AAA-CCAGTACCAGAGG-TGAAAATACTCAATAATTTGGGTGTGGACATTGCTGCTAATACTGTGATCTGGGACTACAAAAGAGATGCTCCAGCAC
```

Section 1081

```
                    (103681) 103681103686      103697      103708      103719      103730      103741      103752      103763   103776
      T4 NC_000866.4 (103170) ACTGATTTAGCGGTTGCATTAGCTTCTATTAATGAAAATATGCCGTTAAATGAATTC---ATCGTTAAACATGTTTCTGCCCGTGGTGAAATTACT
SARS-CoV-2 NC_045512.2 (19904) ATATATCTA-CTATTGGTGTTTGTTCTATGACTGACAT--AGCCAAGAAACCAACTGAAACGATTTGTGCACCACTCACTGTCTTTTTGAT-GGT
```

Section 1082

```
                    (103777) 103777  103785      103796      103807      103818      103829      103840      103851          103872
      T4 NC_000866.4 (103263) CGCACTAAAGACC--GCAAAACGC-------GTGAACGAAATGCATTTCAAACCACTGGGCTGTCTAAAGCAAAACGTAGACAAATTGCTCGTAAA
SARS-CoV-2 NC_045512.2 (19996) AGAGTTGATGGTCAAGTAGACTTATTTAGAAATGCCCGTAATGGTGTTCTTATTACAGA---AGGTAGTGTTAAAGGTTTACAA---CCATCTGTA
```

Section 1083

```
                    (103873) 103873      103884      103895      103906      103917      103928      103939      103950          103968
      T4 NC_000866.4 (103350) GCTACCAAAACG-AAGATTGCCAATCCAGCAGGTCAATCTCGTGC-TCAGCGTAAGCGTAAAAAAGCTCTTAAACGCCGTAAA-GCATTAGGATTA
SARS-CoV-2 NC_045512.2 (20086) GGTCCCAAA---CAAGCTAGTCTT------AATGGAGTCACATTAATTGGAGAAGCCGTAAAAACACAGTTCAATTATTATAAGAAAGT-TGATGG
```

Section 1084

```
                    (103969) 103969        103983      103994      104005      104016      104027      104038      104049         104064
      T4 NC_000866.4 (103443) AGCTAATGAATGAACCCCAATTACTAATTGAAACTTGGGGTCAACCTGGCGAAATTATTGATGGCGTACCAATGCTTGAATCTCATGATGGAAAAG
SARS-CoV-2 NC_045512.2 (20172) TGTTGTCCAACAATTACCTGAAACTTACTTTACTCAGAGTAGAAATTTACAAGAATTT-A------AACCCAGGAGTCAAATGGAAATTG---ATT
```

Section 1085

```
                    (104065) 104065 104071      104082      104093      104104      104115      104126      104137      104148   104160
      T4 NC_000866.4 (103539) ACTTAGGTTTAAAACCGGGTTTATACATCGAAGGAATATTCATGCAAGCGGAAGTCGTCAATAGAAATAAACGTCTTTATCCAAAACGTATATTAG
SARS-CoV-2 NC_045512.2 (20258) TCTTAGAATTAGCTATG-GATGA--------------ATTCATTGAA-CGG---TATAAATTAGAAGGCTATGCCTTCGAACATATCGTTTA-TGG
```

```
Section 1086
(104161) 104161 104170 104181 104192 104203 104214 104225 104236 104256
T4 NC_000866.4 (103635) AAAAAGCGGTAAAAGACTATATTAATTGAGCAAGTTTTAACTAAACAAGCTCTCGGAGAATTAAATCATCCTCC-ACGCGCTAATGTTGACCCGATG
SARS-CoV-2 NC_045512.2 (20334) AGATTTTAGTCATAGTCAGT-TAGGTG-----GTTTACATCTA--------CTGATTGGACTAGCTAAACGTTTTAAGGAATCACCTTTTGAAT-TA

Section 1087
(104257) 104257 104269 104280 104291 104302 104313 104324 104335 104352
T4 NC_000866.4 (103730) CAAGCCGCTATCATTATAGAAGATATGTGGTGGAAAGGAAATGACGTATA-CGGA-CGAGCTCGTGTTATTGAAGGTGACCATGGTCCTGGAGATA
SARS-CoV-2 NC_045512.2 (20416) GAAGATTTTATTCCTATGGACAGTACAGT-TAAAAACTATTTCATA-ACAGATGCGCAAACAGGTTCATCTAAGTGTGTGTGTTCTGTTATTGATT

Section 1088
(104353) 104353 104368 104379 104390 104401 104412 104423 104434 104448
T4 NC_000866.4 (103824) AATTAGCAGCTAATATTCGTGCCGGATGGATTCCAGGAGTTTCTTCTCGTGGATTAGGTTCATTGACTGACACAAATGAAGGTTATCGTATCGTAA
SARS-CoV-2 NC_045512.2 (20510) TATTACTTGATGATTTTGTTGAAATAATAAAATCCCAAGATTTATCTG------TAGTTTCTAAGGTTG-------TCAAAGTGAC--TATTGACT

Section 1089
(104449) 104449 104456 104467 104478 104489 104500 104511 104522 104533 104544
T4 NC_000866.4 (103920) ACGAAGGATTCAAATTAACTGTAGGTGTTGATGCAGTATGGGGTCCAAGTGCTCCAGATGCATGGGTAACTCCTAAGGAAATTACCGAATCACAGA
SARS-CoV-2 NC_045512.2 (20591) ATACAGAAATTTCATT-----TATGCTTTGGTGTA--AAGATGGCCATGT-----AGAAACAT--TTTACCCAAAATTACAAT--CTAGTCA---A

Section 1090
(104545) 104545 104555 104566 104577 104588 104599 104610 104621 104640
T4 NC_000866.4 (104016) CGGCGGA-AGCCGATACAAG-TGCCGATGACGCCTATATGGCTCTCGCAGAGGCCATGAAAAAAGCGTTATAAATATTATTATCTAAACAACAGGA
SARS-CoV-2 NC_045512.2 (20668) GCGTGGCAACCGGGT----GTTGCT----ATGCCTA-AT--CTTTACAAAATGC---AAAGAATGCTATTAGAAAAGTGTGACCTTC---------

Section 1091
(104641) 104641 104654 104665 104676 104687 104698 104709 104720 104736
T4 NC_000866.4 (104110) CT-ACAAAATGCTTAAAGAACAACTGATTGCCGAAGCGCAGAAAATTGATGCTTCCGTTGCTCTTGATAGTATTTTCGAATCAGTTAATATTTCTC
SARS-CoV-2 NC_045512.2 (20741) --AAAATTATGGTGATAGTGCAACA--TTACCTAAAGGC---ATAATGATGAAT----GTCGC--AA-AATATACTCAACTGTGTCAATATTTAAA

Section 1092
(104737) 104737 104742 104753 104764 104775 104786 104797 104808 104819 104832
T4 NC_000866.4 (104205) CGGAAGCAAAAGAAACTTTCGGCACTGTATTCGAAGCTACCGTCAAGCAGCACGCCGTTA--AATTAGCTGAATCTCATATCGCTAAAATTGCTGA
SARS-CoV-2 NC_045512.2 (20823) CACATTAACATTAGCTGTACCCTATAATATGAGAGTTATACATTTTGGTGCTGGTTCTGATAAAGGAGTTGCACCAGGTACAGCTG---T-TTTAA
```

```
                                                                                                     Section 1093
                      (104833) 104833  104841      104852      104863      104874      104885      104896      104907         104928
     T4 NC_000866.4   (104299) AAAAGCAGAAGAAGAAGTAGAAAAAAATAAAGAAGAAGCCGAAGAAAAAGCTGAGAAGAAAATCGCTGAGCAAGCTTCTAAATTCATTGACCATCT
SARS-CoV-2 NC_045512.2 (20915) GACAGTGGTTGCCTACG--GGTACGCTGCTTGTCGATTCAGATCTTAATGACTT------TGTCTCTGATGCAGATTCAACTTTGATTGGTGATTG
                                                                                                     Section 1094
                      (104929) 104929      104940      104951      104962      104973      104984      104995      105006         105024
     T4 NC_000866.4   (104395) TGCAAAAGAATGGCTCGCTGAAAATA-------AATTAGCAGTTG-ATAAAGGCATCAAAGCCGAACTGTTTGAATCCATG-CTTGGTGGATTAAA
SARS-CoV-2 NC_045512.2 (21003) TGCAACTGTACATACAGCTAATAAATGGGATCTCATTATTAGTGATATGTACGACCCTAAGACTAAAAATG---TTACAAAGAAAATGACTCTAA
                                                                                                     Section 1095
                      (105025) 105025        105039      105050      105061      105072      105083      105094      105105       105120
     T4 NC_000866.4   (104482) AGAGCTCTTTGTTGAACACAACGTTGTTGTTCCAGAAGAATCAGTTGATGTTGTAGCTGAAATGGAAGAAGAGCTGCAAGAACATAAAGAAGAATC
SARS-CoV-2 NC_045512.2 (21096) AGAGGGTTTTTTCACTTACATTTGTGGGTTTATACAACAAAAGCTAGCTCTTGGAGGTTCCGTGGCTATAAAGATAACAGAACATTCTTG-GAATG
                                                                                                     Section 1096
                      (105121) 105121 105127     105138      105149      105160      105171      105182      105193      105204   105216
     T4 NC_000866.4   (104578) GCCTCGTCTGTTCGAAGAACTGAATATGCGCG-ACGCATATATCAATTATGTGCAGCGTGAAGTGGCATTGAGCGAAAGTACTAA-AGATCTGACT
SARS-CoV-2 NC_045512.2 (21191) CTGATCTTTATAAGCTCATGGGACACTTCGCAT-GGTGGACAGCCTTTGTTACTAATGTGAA-------TGCGTCATCATCTGAAGCATTTTTAAT
                                                                                                     Section 1097
                      (105217) 105217   105226      105237      105248      105259      105270      105281      105292           105312
     T4 NC_000866.4   (104672) GAGTCTCAAAAAGAAAAAGTCTCTGCTCTGGTCG--AAGGTATGGATTATTCAGATG-CATTCTCAAGTAAATTGAGT-GCAATCGTAGAAATGGT
SARS-CoV-2 NC_045512.2 (21279) TGG-------ATGTAATTATCTTGGCAAACCACGCGAACAAATAGATGGTT---ATGTCATGCATGC--AAATTACATAT--TTTGGAGGAA----
                                                                                                     Section 1098
                      (105313) 105313        105325      105336      105347      105358      105369      105380      105391       105408
     T4 NC_000866.4   (104764) GAAGAAATCTAATAAAGATG-AAAGC---ACTATTACTGAGA-GTATAAATACTCCTGATACTGAAGCAGCCGGACTGA-ATTTCGTCACTGAAGC
SARS-CoV-2 NC_045512.2 (21357) -TACAAATCCAATTCAGTTGTCTTCCTATTCTTTATTTGACATGAGTAAATTTCCCCTTAAATTAAGGG---GTACTGCTGTTATGTCTTTAAA--
                                                                                                     Section 1099
                      (105409) 105409          105424      105435      105446      105457      105468      105479      105490     105504
     T4 NC_000866.4   (104854) TGTAGAAGATAAAGCTGCACAGGGTGCAGAAGATATTGTAAGTGTATATGCGAAAGTCGCATCTCGTTTCTAATTTTAAAGGTTAACACAAATGAC
SARS-CoV-2 NC_045512.2 (21447) AGAAGGTCAAATCAATGATATG------------ATTTTATCTCTTCTTAGTAAAGGTAGA-----CTTATAATTAGAGA-----AAACAACAGAG
```

```
                                                                                                    Section 1100
                  (105505) 105505 105512     105523     105534     105545     105556     105567     105578     105589 105600
      T4 NC_000866.4 (104950) TATCAAAA-CTAAAGCTGAACTTTTGAACAAATGGAAGCCATTACTGGAAGGTGAAGGTTTACCGGAAATTGCTAATAGCAAACAAGCGATTATCG
SARS-CoV-2 NC_045512.2 (21521) TTGTTATTTCTAGTGATGTTCTTGTTAACAACT--AAACGAACAATGT------------T---TGTTTTTCTT-------------GTTTTATTG
                                                                                                    Section 1101
                  (105601) 105601    105611     105622     105633     105644     105655     105666     105677      105696
      T4 NC_000866.4 (105045) CTAAAATCTTTGAAAACCAGGAAAAAGATTTCCAGACAGCTCCGGAATATAAAGACGAAAAAATTGCTCAGGCATTCGGTTCTTTCTTAACAGAAG
SARS-CoV-2 NC_045512.2 (21587) C--CACTAGTCTCTAGTCAGTGT---GTTAATCTTACA--ACCAGAACTCAATTACCCC----CTGCATACAC-------TAATTCTTT-CACACG
                                                                                                    Section 1102
                  (105697) 105697      105710     105721     105732     105743     105754     105765     105776      105792
      T4 NC_000866.4 (105141) CTGAAATCGGTGGTGACCACGGTTACAATGCTACCAACATCGCTGCAGGTCAGACTTCTGGCGCAGTAACTCAGATTGGCCCAGCTGTTATGGGTA
SARS-CoV-2 NC_045512.2 (21664) T-------GGTGTTTATTACCCTGACAAAGTTTTCAG-ATCCT--CAGTTTTACATTC---------AACTCA----GGACTTGTTCT------TA
                                                                                                    Section 1103
                  (105793) 105793105798     105809     105820     105831     105842     105853     105864     105875 105888
      T4 NC_000866.4 (105237) TGGTACGTCGTG-CTATTCC----TAACCTGATTGCTTTCGATATTTGTGGTGTTCAGCCGAT---GAACAGCCCGACTGGCCAGGTATTCGCACT
SARS-CoV-2 NC_045512.2 (21731) --------CCTTTCTTTTCCAATGTTACTTGGTTCCATGCTATACATGTC-TCTGGGACCAATGGTACTAAGAG-GTTT-----GATAACCC-TGT
                                                                                                    Section 1104
                  (105889) 105889  105897     105908     105919     105930     105941     105952     105963      105984
      T4 NC_000866.4 (105325) GCGCGCAGTATATGGTAAAGACCCAGTGGCTGCCGGTGCTAAAGAAGCATTCCACCCAATGTATGGTC--CAGATGCAATGTTCTCTGGTCAGGGT
SARS-CoV-2 NC_045512.2 (21811) CCTACCATTTAATGAT------------------GGTGTTTATTTTGC-TTCCACTGAG---AAGTCTAACATAAT-AAGAGGCTGGATTTTTGGT
                                                                                                    Section 1105
                  (105985) 105985     105996     106007     106018     106029     106040     106051     106062      106080
      T4 NC_000866.4 (105419) GCTGCTAAGAAATTCCCAGCTCTGGCTGCTAGCACACAAACCACAGTAGGTGATATCTATACTCACTTCTTCCAGGAAACTGGTACTGTATATCTG
SARS-CoV-2 NC_045512.2 (21884) ACTACTT----------------------TAGATTCGAAGACCCAGT---CCCTACTTATTGTTAATAACGCTACTAATGTTGTTATTAAAGTCTG
                                                                                                    Section 1106
                  (106081) 106081        106095     106106     106117     106128     106139     106150     106161      106176
      T4 NC_000866.4 (105515) CAAGCTTCTGTTCAAGTAACAATCGATGCTGGTGCGACTGATGCTGCTAAATTAGATG-CTGAAATTAAGAAACAAATGGAAGCTGGTGCACTGGT
SARS-CoV-2 NC_045512.2 (21955) TGAATTTCAATTTT-GTAATGATCCAT------------TTTG--G--------GTGTTTATTACCACAAAACAACAAAAGTTGGA----TGGA
```

```
Section 1107
                    (106177) 106177 106183 106194 106205 106216 106227 106238 106249 106260 106272
T4 NC_000866.4      (105610) AG-AAATCGCTGAAGGTATGGCTACTTC-TATCGCTGAACTCCAGGAAGGTTTCAAT--GGTTCTACCGATAACCCATGGAATGAAATGGCTTCC
SARS-CoV-2 NC_045512.2 (22024) AAGTGAGTTCAGAGTTTAT-TCTAGTGCGAATAATTGCACTTT-TGAATATGTCTCTCAGCCTTTTCTTATGGACCTTGAAGGAAAACAGGGTAAT

Section 1108
                    (106273) 106273 106282 106293 106304 106315 106326 106337 106348 106368
T4 NC_000866.4      (105702) GTATCGATAAGCAAGTTATCGAAGCTAAATCTCGTCAGCTGAAAGCTGCTTACTCTATTGAATTAGCACAAGACCTCCGCGCTGTTCACGGTATGG
SARS-CoV-2 NC_045512.2 (22118) TT--C---AAAAATCTTAGGGAATTT--GTGTT-TAAGAATATTGATGGTTATTTTAAAATATATTCTAAGCACACGCC---TATTAA--TT-TAG

Section 1109
                    (106369) 106369 106381 106392 106403 106414 106425 106436 106447 106464
T4 NC_000866.4      (105798) ATGCTGATGCTGAACTGTCTGGTATTCTGGCTACAGAAATTATGCTGGAAATCAACCGTGAAGTTGTTGATTGGATTAACTACTCAGCTCAGGTTG
SARS-CoV-2 NC_045512.2 (22200) TGCGTGATC---TCCCTCAGGGTTTTTCGGCTTTAGAACCATTGGTAGATTTGCC---AATAGGTATTAACATCACTAGGTTTCAAACTTTACTTG

Section 1110
                    (106465) 106465 106480 106491 106502 106513 106524 106535 106546 106560
T4 NC_000866.4      (105894) GTAAATCTGGTATGACCCTGAC---TCCGGGTTC---TAAAGCTGGTGTATTTGACTTCCAGGACCCAATTGATATTCGTGGTGCTCGCTGGGCGG
SARS-CoV-2 NC_045512.2 (22290) CTTTACATAGAAGTTATTTGACTCCTGGTGATTCTTCTTCAGGTTGGACAGCTGG-TGCTGCA--GCTTATTATGTGGGTTATCTTCAACCTAGGA

Section 1111
                    (106561) 106561 106568 106579 106590 106601 106612 106623 106634 106645 106656
T4 NC_000866.4      (105984) GTGAATCCTTTAAAGCTCTGTTGTTCCAGATTGACAAAGAAGCAGTTGAAATTGCTCGTCAGACCGGTCGTGGTGAAGGTAACTTCATTATCGCTT
SARS-CoV-2 NC_045512.2 (22383) CTTTTCTATTAAAATAT---A---------ATGAAAATGGAACCATTACAGATGCT-GT-AGACTGTGC------------ACTTGACCCTCTCT-

Section 1112
                    (106657) 106657 106667 106678 106689 106700 106711 106722 106733 106752
T4 NC_000866.4      (106080) CCCGTAACGTAGTTAACGTTTTGG--CTTCAGTTGATACCGGCATTTCTTATGCTGCACAGGGTCTGGCTACCGGCTTTAGCACTGATACTACCAA
SARS-CoV-2 NC_045512.2 (22452) -CAGAAACAAAGTGTACGTTGAAATCCTTCACTGTAGAAA---AAGGA--ATCTATCAAACT---T-----CTAACTTTAGAG----TCCAACCAA

Section 1113
                    (106753) 106753 106766 106777 106788 106799 106810 106821 106832 106848
T4 NC_000866.4      (106174) GTCAGTATTTGCTGGTGTTCTGGGTGGTAAATACCGCGTATATATCGACCAGTATGCTAAACAGGATTATTTCACTGTAGGTTATAAAGGTCCGAA
SARS-CoV-2 NC_045512.2 (22530) --CAGAATCTATTGTTA-----------GATTTCCTAAT--ATTACAAACT-TGTGCCTTTTGG----------TGAAGTTTTAA--CGCCACC
```

```
Section 1114
(106849)                       106849 106854     106865     106876     106887     106898     106909     106920     106931      106944
T4 NC_000866.4 (106270)        CGAAATGGATGCTGGTATTTACTATGCTCCATATGTAGCTCTG--ACTCCGCTGC-GTGGTTCCGATCCGAAGAACTTCCAACCGGTAATGGGATT
SARS-CoV-2 NC_045512.2 (22598) AGATTTGCAT-CTGTT-TATGCTTGGAACAGGAAGAGAATCAGCAACTGTGTTGCTGATTATTCTGTCCTATATAATTCCGCATCATTTTCCACTT
```

```
Section 1115
(106945)                       106945  106953     106964     106975     106986     106997     107008     107019              107040
T4 NC_000866.4 (106363)        CAAAACTCGTTACGGTATCGGTATCAACCCATTTGCAGAATCCGCTGCTCAGGCTCCGGCTTCTCGCATCCAGAGCGGTATGCCTTCTATTCTGAA
SARS-CoV-2 NC_045512.2 (22692) TTA-AGT-GTTATGGAGTGTCT-CCTACTAAATTAAATGAT------CTCTGCTTTACTAATGTC-TATGCAGATTCATTTGTAATTAGAGGTGAT
```

```
Section 1116
(107041)                       107041     107052     107063     107074     107085     107096     107107     107118              107136
T4 NC_000866.4 (106459)        TAGCCTTGGTA-AAAACGCTTACTTTAGACGTGTATATGTTAAAGGTATCTAATCTCTAACGATAGAAACACAATTTTAGGGAACCTTCGGGTTCC
SARS-CoV-2 NC_045512.2 (22778) --GAAGTCAGACAAATCGCTCCAGGGCAAACTGG-AAAGATTGCTGATTATAATTATAAA---TTACCAGATGATTTTA--CAGGCTGCG------
```

```
Section 1117
(107137)                       107137        107151     107162     107173     107184     107195     107206     107217         107232
T4 NC_000866.4 (106554)        CTTTTTTCTATTTTATACGATAGCAATCAGGCATATCATCCGCATTTATCCAATTGCGAATAGTTTTAGGACTAACTTTAAAATGCTCCGCTGCGT
SARS-CoV-2 NC_045512.2 (22860) --TTATA--GCTTGGAATTCTAACAATCTTG-------------ATTCTAAGGTTGGTGGTAATTATA--ATTACCTGTATA----GATTGT----
```

```
Section 1118
(107233)                       107233 107239     107250     107261     107272     107283     107294     107305     107316     107328
T4 NC_000866.4 (106650)        AATCAGGATTATCAAATTTAACGCCCTTTATACATATTGGAATAAATTTTTTA--ATACCACCAAGTTTTTCAGAAATAGCTTTACGA--TGTGAA
SARS-CoV-2 NC_045512.2 (22929) -TT-AGGAAGTCTAATCTCAAACCTTTTGAGAGAGAT--------ATTTCAACTGAAATCTATCAGGCCGGTAGCACACCTTGTA--ATG-GTGTT
```

```
Section 1119
(107329)                       107329   107338     107349     107360     107371     107382     107393     107404              107424
T4 NC_000866.4 (106742)        ATCGATATAGGTTTGTTTTTTCGTGGATGAACATGTGTTTTATAAT--ATTCATTGCGCCCTTTAAC-TCGCTTCGCAATAGTTTCATCAGATTGC
SARS-CoV-2 NC_045512.2 (23012) GAAGGTTTTAATTGTTACTTTCCTTTACAATCATATGGTTTCCAACCCACTAATGGTGTTGGTTACCAACCATACAGAGTAGTAGTACTTTCTTT-
```

```
Section 1120
(107425)                       107425      107437     107448     107459     107470     107481     107492     107503              107520
T4 NC_000866.4 (106835)        TTAACGCCTGTTTTTGCCTTTGATATTTTTCGTTTAGCTTCCACAGTCATTCCTTCTTTTGTTCGTATTGATAACATATTACGATATGAAGGATCT
SARS-CoV-2 NC_045512.2 (23107) TGAACTTCTACATGCACCA--GCAACTGTTTGTGGACCTAA-AAAGTC--TACTAATTTGG------TTAAAAACA------AATGTG--------
```

Section 1121

(107521) 107521 107536 107547 107558 107569 107580 107591 107602 107616

T4 NC_000866.4 (106931) TGCAAATGAACTATAACTGGATTTCCTCTGCCACCAATAGCAGCATTATAGGTATCAGTTCTCATAACGAATTCCTCATTAACTAGTAAAGCTTCC

SARS-CoV-2 NC_045512.2 (23178) -TCAATTTCAACTTCAATGG--TTTAACAGGCACAGGTGTTCTTACTGAGTCTAACAAAAGTTTCTGCCTTTC--CAACAATTTG-GCAGAGA-C

Section 1122

(107617) 107617 107624 107635 107646 107657 107668 107679 107690 107701 107712

T4 NC_000866.4 (107027) ATTTTATACATCTCCTCAGATGAGGAGAAAGAATAAAGAATTTCTTTT-TTAAAGTTATGAATACCATATTTTTTGATGGATTTTTTGATGTTTAC

SARS-CoV-2 NC_045512.2 (23267) ATTGCTGACACTACTGATGCTGTCCGTGATCCACAGACACTTGAGATTCTTGACATTAC----ACCATGTTCTTTTGGTGGTGTCAGTGTTATAAC

Section 1123

(107713) 107713 107723 107734 107745 107756 107767 107778 107789 107808

T4 NC_000866.4 (107122) GCCAGAACCCATATAACCATCGTTTTCGTCAAGAGTAGCATGAGCTCCGATGTAAATTTTTCCATTGATGATATTAGTAATTTGATATATTAAATA

SARS-CoV-2 NC_045512.2 (23359) ACCAGGAAC---AAATACTTCTA--AC--CAG--GTTGC-TGTTC--TTT-----AT-----C-AGGATGTTAACTGCAC------------AGAA

Section 1124

(107809) 107809 107822 107833 107844 107855 107866 107877 107888 107904

T4 NC_000866.4 (107218) TTTCATTTTAAACATCACTCCGTTTGTATATGATTATAATATCA-TATTACTTTGGTCTTGTAAATAACTTTATAAATAGTATTATATTTCAACAA

SARS-CoV-2 NC_045512.2 (23420) GTCCCTGTT-------------GCTATTCATGCAGATCAACTTACTCCTACTTGG--CGTGTTTAT---TCTACAGGTCTAATGTTTTTCAA---

Section 1125

(107905) 107905 107910 107921 107932 107943 107954 107965 107976 107987 108000

T4 NC_000866.4 (107313) GGAAAATACAATGGCTAAAATCAACGAACTTCTGCGCGAATCAACCACAACGAATAGCAACTCAATCGGTC-GCCCAAATCTCGTTGCTTTGACTC

SARS-CoV-2 NC_045512.2 (23495) ---ACACGTGCAGGCTGTT--TAA------TAGGGGCTGAACA-TGTCAAC-------AACTCATATGA-GTGTGACATACCCATTGGTG---CAG

Section 1126

(108001) 108001 108009 108020 108031 108042 108053 108064 108075 108096

T4 NC_000866.4 (107408) GCGCTACCACTAAATTAATATATTCTGACATTGTAGCAACGCAAAGAACTAATCAACCTGTTGCTGCTTTTTATGGTATCAAATACCTTAACCCAG

SARS-CoV-2 NC_045512.2 (23568) GTATATGCGCTA-GTTATCAGACTCAGACTAATTCTCCTCGGCGGGCACGTAG-----TGTAGCTAGTCAATCCATC----ATTGCCTACACTATG

Section 1127

(108097) 108097 108108 108119 108130 108141 108152 108163 108174 108192

T4 NC_000866.4 (107504) ACAACGAATTTACATTTAAAACTGGTGCTACTTACGCTGGCGAAGCTGGATATGTAGACCGAGAACAAATCACAGAATTAACAGAAGAGTCTAAAT

SARS-CoV-2 NC_045512.2 (23654) TCACTTGGTGCAG-----AAAATTCAGTTGCTTACTCTAATAACTCTATTGCCATACCC-----ACAAATTTACTATTAG----TGTTACCACAG

```
                                                                                                             Section 1128
                     (108193) 108193        108207     108218     108229     108240     108251     108262     108273      108288
      T4 NC_000866.4 (107600) TAACTCTCAATAAAGGCGATTTATTCAAATATAATAATATCGTTTATAAAGTATTAGAAGATACTCCATTTGCTACTATCGAAGAAAGTGATTTAG
SARS-CoV-2 NC_045512.2 (23736) AAATTCT-ACCAGTGTCTATGACC-AAGACATCAGTAGATTGTACAATGTACATTTGTGGTGATTCAACT---GAATGCAGCAATCTTT-TGTTGC
                                                                                                             Section 1129
                     (108289) 108289 108295     108306     108317     108328     108339     108350     108361     108372  108384
      T4 NC_000866.4 (107696) AATTAGCTCTTCAGATTGCAATCGTTCTTTTAAAGGTTCGTCTATTTTCTGACGCAGCGTCAACAAGCAAATTTGAAAGCTCTGATAGTGAAATTG
SARS-CoV-2 NC_045512.2 (23826) AAT----ATGGCAGTTTTTGTAC--ACAATTAAACCGTGCTTTA---ACTGGAATAG-CTGTTGAACAAGACAAAAACACCCAAGAAGTTT--TTG
                                                                                                             Section 1130
                     (108385) 108385   108394     108405     108416     108427     108438     108449     108460         108480
      T4 NC_000866.4 (107792) CGGATGCTAGATTCCAGATTAATAAATGGCAAACTGCAGTTAAATCTCGTAAAC-TTAAAACTGGCATCACAGTTGAATTAGCGCAAGATTTAGAA
SARS-CoV-2 NC_045512.2 (23910) CACAAGTCAAA---CAAATTTACAAAACAC---CACCAATTAAAGATTTTGGTGGTTTTAATTTTT----CACAAATATTACC---AGATCCATCA
                                                                                                             Section 1131
                     (108481) 108481      108493     108504     108515     108526     108537     108548     108559      108576
      T4 NC_000866.4 (107887) GCAAATGGATTCGATGCTCCTAATTTCTTGGAAGATTTGCTTGCAACTGAAATGGCAGATGAAATCAATAAAGACATTCTGCAGTCTTTGATTACA
SARS-CoV-2 NC_045512.2 (23993) --AAACCAAGCAAGAGGTCAT----TTATTGAAGATCTACTTTTCAACAAAGTGACACTTGCA---------G--ATGCTGGCTTCAT--CAAACA
                                                                                                             Section 1132
                     (108577) 108577         108592     108603     108614     108625     108636     108647     108658     108672
      T4 NC_000866.4 (107983) GTGTCAAAACGCTATAAAGTTACAGGAATTACTGATAGTGGATTCATCGATTTGAGTTATGCATCTGCTCCTGAAGCTGGTCGTTCATTATACCGA
SARS-CoV-2 NC_045512.2 (24070) ATATGGTG-----ATTGCCTTGGTGATATTGCTGCTAGAGACCTCAT--TTGTGCACAAAAGTTTAACGGCCTTA----CTGTTTTGCCACCTTTG
                                                                                                             Section 1133
                     (108673) 108673 108680     108691     108702     108713     108724     108735     108746     108757 108768
      T4 NC_000866.4 (108079) ATGGTATGTGAAATGGTTTCGCATATCCAAAAAGAATCAACTTATACAGCAACGTTCTGTGTTGCTTCAGCTCGTGCCGCTGCGATTCTTGCTGCA
SARS-CoV-2 NC_045512.2 (24155) CTCACAGATGAAATGATT--G-----CTCAATACACT-------T-CTGCACTGTT---AGCGGGTACAATCACTTCTGGTTGGACCTTTGGTGCA
                                                                                                             Section 1134
                     (108769) 108769    108779     108790     108801     108812     108823     108834     108845         108864
      T4 NC_000866.4 (108175) TCAGGCTGGTTAAAACATAACCAGAAGATGA-CAAATATCTTTCACAA--AATGCCTACGGGTTCTTAGCTAATGGTTTACCGCTTTATTGCG-A
SARS-CoV-2 NC_045512.2 (24233) G-GTGCTGCATTA--CAAATACCATTTGCTATGCAAATGGCTTATAGGTTTAATGGTATTGGAGTTACACAGAATGTTCTCTATGAGAACCAAAAA
```

```
                                                                                                   Section 1135
                    (108865) 108865      108878     108889     108900     108911     108922     108933     108944       108960
   T4 NC_000866.4 (108267) T--ACTAACAGCCCATTAGATTATGTAATCGTTG--GCGTAGTAGAAAATATCGGTGAAAAAGAAATTG---TTGGATCAATTTTCTATGCTCCGT
SARS-CoV-2 NC_045512.2 (24326) TTGATTGCCAACCAATTTAATAGTGCTATTGGCAAAATTCAAGACTCACTTTC--TTCCACAGCAAGTGCACTTGGAAACTTCAAGATGT--GGT
                                                                                                   Section 1136
                    (108961) 108961108966      108977     108988     108999     109010     109021     109032     109043   109056
   T4 NC_000866.4 (108356) A-TACAGAAGGTCTCGACTTAGATGACCCTGAACATGTAGGTGCA-TTTAAAGTTGTTGTTGATCCAGAAAGCTTACAACCATCTATCGGTTTA--
SARS-CoV-2 NC_045512.2 (24418) CAACCAAAATGCACAAGCTTTAAACACGCTTGTTAAACAACTTAGCTCCAATTTTGGTGCAATTTCA---AGTGTTTTAAATGATATCCTTTCACG
                                                                                                   Section 1137
                    (109057) 109057  109065     109076     109087     109098     109109     109120     109131           109152
   T4 NC_000866.4 (108448) -TTAGTTAGATATGCTTTAT--CAGCAAATCCTTATACTGTAGCAAAAGATGAAAAAGAAGCAAGAATAATTGACGGTGGAGACATGGATAA-AAT
SARS-CoV-2 NC_045512.2 (24511) TCTTGACAAAGTTGAGGCTGAAGTGCAAATTGATAGGTTG-ATCACAGGCAGACTTCAAAGTTTGCAGACATATGTGACTCAACAA-----TTAAT
                                                                                                   Section 1138
                    (109153) 109153     109164     109175     109186     109197     109208     109219     109230       109248
   T4 NC_000866.4 (108540) GGCAGGTCGTTCAGATTTGTCTGTTTATTAGGTGTTAAGCTACCAAAAATTATCATTGATGAATAAAACAA-A--GG-GACCTTTCGGTCCCTTT
SARS-CoV-2 NC_045512.2 (24601) TAGAGCTGCAGAAATCA-GAGCTTCT-GCTAATCTTGCTGCTACTAAAATGTCAGAGTGTGTACTTGGACAATCAAAAAGAG-------TTGATTT
                                                                                                   Section 1139
                    (109249) 109249        109263     109274     109285     109296     109307     109318     109329       109344
   T4 NC_000866.4 (108632) TTATTTAACTTACCAACTCAATCCAAGCTGGACGAAGTACATCTTGTACCATTTTAACTAATTCCTTTTTAATCAAAGAAGGATTATCTGCTTGAG
SARS-CoV-2 NC_045512.2 (24688) TTGTGGAAAGGGC--TATCATCTTATGTCCT------TCCCTCAGTCAGCACCTCA--TGGTGTAGTCTTCTTGCATGT-GACTTATGTCCCTGCA
                                                                                                   Section 1140
                    (109345) 109345 109351     109362     109373     109384     109395     109406     109417     109428   109440
   T4 NC_000866.4 (108728) TTAGAGTAATACCTTCACGAGAAGTTTCTTCCAAAATATCTTGAACAGTTAGCCCCATCACCTTTCCAAAATC-CTTTGGACCAATTTCGCCAATT
SARS-CoV-2 NC_045512.2 (24773) CAAGAAAA-GAACTTCACAACT-GCTCCTGCCATT-TGTCATGATGGAAAAGCACACTTTCCTCGTGAAGGTGTCTTTGTTTCAAATGGCACA---
                                                                                                   Section 1141
                    (109441) 109441   109450     109461     109472     109483     109494     109505     109516           109536
   T4 NC_000866.4 (108823) TTAGAAATAACGTTATTTACGCGGTTCAGTGTAACGTAACAAGCTAAAATTCCCACCAATTTGTTATCAGCTTCTGATAGCTCAACTTTAGCTTTA
SARS-CoV-2 NC_045512.2 (24863) ---C-ACTG--GTTTGTAACACAAAGGAATTTTTATGAACCACAAATCATTACTACAGACAACACATTTGTGTCTGGTA-----ACTGTGATGTTG
```

```
                                                                                                        Section 1142
                 (109537) 109537      109549     109560     109571     109582     109593     109604     109615     109632
T4 NC_000866.4   (108919) ATAGGCTTATCAGACTTTTTCTTTTCACTAAATTTAGAGTTCTTGCATTTAATCGCTACACGATTTCCATTACG-AAGCCAAGAAGGATAACAAGG
SARS-CoV-2 NC_045512.2 (24948) TAA------TAGGAATTGTCAACAACAC--AGTTTATGATCCTTTGCAACCTGAATTAGAC----TCATTCAAGGAGG---AGTTAGATAAATA--
                                                                                                        Section 1143
                 (109633) 109633         109648     109659     109670     109681     109692     109703     109714     109728
T4 NC_000866.4   (109014) TTTCAATACATATCCTTCAGCAG-TAAATACTTCGCCTTTTGCTTCGGCATTCCAAACGCATTTATTTGCATCAACTAATCCAGCATGGTCTACTG
SARS-CoV-2 NC_045512.2 (25027) TTTTAAGAATCATACATCACCAGATGTTGATTTAGGTGACATCTCTGGCATTAATGCTTCAGTTGTAAACATTCAAAAAGAAATTGACCGCCTCAA
                                                                                                        Section 1144
                 (109729) 109729 109736     109747     109758     109769     109780     109791     109802     109813 109824
T4 NC_000866.4   (109109) TAAAATTATAATCTTGGACGACAGAATCTAAATCATTTGGCAATTTAATAAGCTCTTCAAATTTACCGCGACCTAAAAGTGGAGCCATTTTAAATT
SARS-CoV-2 NC_045512.2 (25123) TGAGGTTGCCAA---GAAT--TTAAATGAATCTCTCATCGATCTCCAAGAACTTGGAAAGTATGAGCAGTATATAAAA-TGG--CCATGGTACATT
                                                                                                        Section 1145
                 (109825) 109825    109835     109846     109857     109868     109879     109890     109901     109920
T4 NC_000866.4   (109205) TAAAT--GTATTACAGAATGATTCCATCATATAATCATCTACATAAGTCACATCACCGCTTTCTGTAGTAACAATAATGTCAAATACATAAAAATC
SARS-CoV-2 NC_045512.2 (25211) TGGCTAGGTTTTATAGCTGGCTT------GATTGCCATAGTAAT-GGTGACAATTATGCTTT--------GCTGTATGACCAGTTGCTGTAG----
                                                                                                        Section 1146
                 (109921) 109921       109934     109945     109956     109967     109978     109989     110000     110016
T4 NC_000866.4   (109299) TTTATCACAATAATCAACATTCTTCTGAATGCCAGGTCCAGCGAATTCGCCAAAGACTTGATAAGATACAACCGCTGAGGTTTCCATAATATCTTG
SARS-CoV-2 NC_045512.2 (25288) -TTGTCTCAAGGGCTGTTGTTCTTGTGGAT-CCT---GCTGCAAATTTGATGAAGA-----CGACT---------CTGAG---CCAGT-----GCTC
                                                                                                        Section 1147
                 (110017) 110017110022     110033     110044     110055     110066     110077     110088     110099     110112
T4 NC_000866.4   (109395) TACAGCTTTAATGGAATCAGCATAATTCTTCAAAATAATTTCATACCCAAAGA-AATCTTCAGCAGGAAGAATCGGTCCAGTGCGTTTAGCGCAAG
SARS-CoV-2 NC_045512.2 (25358) AAAGGAGTCAAAT--TACATTACACATAAACGAACTTATGGATTTGTTTATGAGAATCTTCAC-------AATTGGAACTGTAACTTTGAAGCAAG
                                                                                                        Section 1148
                 (110113) 110113  110121     110132     110143     110154     110165     110176     110187     110208
T4 NC_000866.4   (109490) TCACTTTATCACGCTCAATAATCAATGAGAAATTTGTGCCGTGAATCTTTTCACGAGCTACCCACTCCCCACCAGTCAATCCCAAGCTATAAAGTT
SARS-CoV-2 NC_045512.2 (25445) --GTGAAATCAAGGATGCTACTCCTTCAGAT-TTTGTTCGCGCTACTGCAACGATACCGATACAAGCCTCAC--TCCCTTTCGGATGGCTTATTGT
```

```
Section 1149
(110209)                     110209     110220     110231     110242     110253     110264     110275     110286     110304
T4 NC_000866.4 (109586)      TTTCAATAAATTTAGAGTTGTAATGATTTTCAAGACTGCTATACTTTTTAAACATAATTAATCCTCAAAATGTAATTTCTAACCAATCACCATCAC
SARS-CoV-2 NC_045512.2 (25536) TGGCGTTGCACTTC---TTGCTGTTTTCAGAGCGCTTCCAAAATCATAACCCTCAAAAAGAGATGGCAA--CTAGCACTCTCCAAGGGTGTTCAC

Section 1150
(110305)                     110305     110319     110330     110341     110352     110363     110374     110385     110400
T4 NC_000866.4 (109682)      GCTGATCACTATTGACTTTAAAGCTGAATCCTTCTTTTCTCAGCCAATCACCAATTTCTTCTGTAATCAATTTATCACGAGCAATACAATAATAAT
SARS-CoV-2 NC_045512.2 (25627) TTTGTTTGCAACTTGCTGTTGTTGTTTGTAACAGTTTACTCA------CACCTTTTGCTCGTT------GCTGCTGGCCTTGAAGCCCCTTTTCTC

Section 1151
(110401)                     110401 110407     110418     110429     110440     110451     110462     110473     110484 110496
T4 NC_000866.4 (109778)      TAAAATGTGTTTTACCTTGTTCAGCTGCTTTATTAGCAAGTTCTGAAAAATCTTTAATAAAACACTCTAGCTTAAACTGTTTA---CTTTTTAATG
SARS-CoV-2 NC_045512.2 (25711) TATC----TTTATGCTTTAGTCTACTTCTTGCAGAGTATAAACTTTGTAAGAATAATAATGAGGCTTTGGCT-TTGCTGGAAATGCCGTTCCAAAA

Section 1152
(110497)                     110497     110506     110517     110528     110539     110550     110561     110572     110592
T4 NC_000866.4 (109871)      CTTTTTCGCGTAATTGATTA-GCAAAAGATTCATTTTCATAAAGATCATACTGTT----CCATTTTTCACCTTTTTATTGATATGTCTTTTTTCTAT
SARS-CoV-2 NC_045512.2 (25802) ACCCAT----TACTTTATGATGCCAACTATTTTCTTTGCTGGCATACTAATTGTTACGACTATTGTATACCTTACAATAG-TGTAACTTCTTCAAT

Section 1153
(110593)                     110593     110605     110616     110627     110638     110649     110660     110671     110688
T4 NC_000866.4 (109962)      AGACAACTTTTTCTCGAGCC--CATAATACAGCC-ACTTCTTT---TGCCTGTAAGTTTAATTCACG-----AGCAATTTCAATGAATGACTTTCC
SARS-CoV-2 NC_045512.2 (25893) TGTC-ATTACTTCAGGTGATGGCACAACAAGTCCTATTTCTGAACATGACTACCAGATTGGTGGTTATACTGAAAAATGGGAATC--TGGAGTAAA

Section 1154
(110689)                     110689     110704     110715     110726     110737     110748     110759     110770     110784
T4 NC_000866.4 (110047)      AGACTCATGAAGAGTAAACACCACAACCTCAGTTCTCATAATCAATCTCATGTTATCGAGTTGGTGCCATTATATACATCATTTTCTGATTGTGTT
SARS-CoV-2 NC_045512.2 (25986) AGACTGTGTTGTATTACACAGT--TACTTCACTTCAGACTATT-ACCAGCTGTACTCAACTCA-----ATTGAGTACAGACACTGGTG-TTGAACA

Section 1155
(110785)                     110785 110792     110803     110814     110825     110836     110847     110858     110869 110880
T4 NC_000866.4 (110143)      TTGTGTGCT-TTCAAAATGAAGAAAGGGGCCGAAGCCCCTTATGATTATGGATAGGTATAGATGATACCAGTTTCTAAAGCAGTT--TTATGAA-T
SARS-CoV-2 NC_045512.2 (26073) TGTTACCTTCTTCATCTACAATAAAATTGTTGATGAGCCTGAAGAACATGTCCAAATTCACACAATCGACGGTTCATCCGGAGTTGTTAATCCAGT
```

```
                                                                                                          Section 1156
                          (110881) 110881    110891      110902      110913      110924      110935      110946      110957        110976
         T4 NC_000866.4   (110235) GATGTATCCATTACGCGATTCTTGGACATCAACTTCTGGATAGT--CTTTCATCATCTTCTGGAGAGTGTAACGATGCAGGTAATATTTACTATCT
SARS-CoV-2 NC_045512.2    (26169)  AATGGAACCAATTTATGATGAACCGACGACGACTACTAGCGTGCCTTTGTAAGCACAAGCTGATG--AGTACGAACTTATGTACTCATTCGTTTCG
                                                                                                          Section 1157
                          (110977) 110977      110990      111001      111012      111023      111034      111045      111056      111072
         T4 NC_000866.4   (110329) GGGTCGTCAGTTTTCCAAT-CTTTACCTTCTTCGGTCATTTTTTGGATTTCATCCATAACCCACCAACCGCACCAG--ATGTAAGCTGAGCTACGG
SARS-CoV-2 NC_045512.2    (26263)  GAAGAGACAGGTACGTTAATAGTTAATAGCGTACTTCTTTTTCTTGCTTTCGTGGTATTCTTGCTAGTTACACTAGCCATCCTTACTGCGCTTCGA
                                                                                                          Section 1158
                          (111073) 111073111078      111089      111100      111111      111122      111133      111144      111155  111168
         T4 NC_000866.4   (110422) TGTGGAAGAGGATGAACATAAGGTAATTCACCTTCTGGTTCTGGAGCA-ACTTTAGCCGTGACTGTTACATTACCAGTTTGGTAACGGTTACAGG
SARS-CoV-2 NC_045512.2    (26359)  TT-GTGTGCGTACTGCTGCAATATTGTTAACGTGAGTCTTGTAAAACCTTCTTTTTACGTTTACTCTCGTGTTAAAAATCTG--AATTCTTCTAGA
                                                                                                          Section 1159
                          (111169) 111169 111177      111188      111199      111210      111221      111232      111243            111264
         T4 NC_000866.4   (110517) GTTA-TAATCTGCAGCAGTAACAGTTGCAGTAACTTCAATAGTTTGACTTCCAACAGATGAGGTATCGACAGTATATACGTTAGTTGACCCTTCTA
SARS-CoV-2 NC_045512.2    (26452)  GTTCCTGATCTTCTGGTCTAAAC---GAACTAAATATTATATT------------AGTTTTTCTGTTTGGAACTTTAATTTTAG------CCATGG
                                                                                                          Section 1160
                          (111265) 111265     111276      111287      111298      111309      111320      111331      111342      111360
         T4 NC_000866.4   (110612) CAGGAGA---AGAATCTTTCTTCCATGAGTAAGTAATTTGTGCTTCTTCTGGAGCACCCGTAACATTAGCCGTAAATGTAGCCGAAGCATCTTGC-
SARS-CoV-2 NC_045512.2    (26527)  CAGATTCCAACGGTACTATTACCGTTGAAGAGCTTAAAAA-GCTCCTT-----GAACAATGGAACCTAGT--AATAGGTTCCTATTC--CTTACA
                                                                                                          Section 1161
                          (111361) 111361          111375      111386      111397      111408      111419      111430      111441    111456
         T4 NC_000866.4   (110704) TGAACATTAATAGAAGGAGGAGTCAATGTAACCTGTGGATTCATTGTCTTCTTATTAACCGTT-AATGATACTTCATTAGA-AGTAACGCTTAGTG
SARS-CoV-2 NC_045512.2    (26613)  TGGATTTGTCTTCTACAATTTGCCTATGCCAACAG--GAATAGGTTTTGTATATAATTAAGTTAATTTTCCTCTGGCTGTTATGGCCAGTAACTT
                                                                                                          Section 1162
                          (111457) 111457 111463      111474      111485      111496      111507      111518      111529      111540 111552
         T4 NC_000866.4   (110798) CATCATAATCTGTCGCGGTTACTTGGGCTACGCATTTAATTCTTTTTACTCCACTTGTAGTTGGAGTATAGCTAAATGTAGAGTTAGTTTCTCCAC
SARS-CoV-2 NC_045512.2    (26707)  TAGCTTGTTTTGTGCTTGCTGCT--GTTTACAGAATAAATTGGAT------CACCGGTGGAATTGCTATCGCAATGGCTTGTCTTGTAGGCT----
```

```
                                                                                                   Section 1163
                           (111553) 111553   111562     111573     111584     111595     111606     111617     111628         111648
           T4 NC_000866.4 (110894) CAACTTGTGAATCATCTACATACCACTGATACGTAGCAGATGCTCCATCAGGTTGAGAAGCTAAGGCAGCAGTAAATTGAACTGGGGTTCCAATCA
  SARS-CoV-2 NC_045512.2  (26791) --TGATGTGGCTCAGCTACTTCA-----------TTGCT-----TCTTTCAGACTG-----TTTGCGCGTACGCG-TTCCATGTGGTCAT----TCA
                                                                                                   Section 1164
                           (111649) 111649      111661     111672     111683     111694     111705     111716     111727       111744
           T4 NC_000866.4 (110990) CTCCAGCCGCAGGACTAGCAGGAGTTACGGCTAAGGTAGTCGTCTGTGTCTTATTTTTAACTGTGATAGTTGT-TGTCGCTTCAGCCGTTTCCGGG
  SARS-CoV-2 NC_045512.2  (26860) ATCCA---GAAA--CTAACATTCTTCTC----AACGTGCCACTCCATGGCACTATTCTGACCA-GACCGCTTCTAGAAAGTGAACTCGTAATCGGA
                                                                                                   Section 1165
                           (111745) 111745         111760     111771     111782     111793     111804     111815     111826     111840
           T4 NC_000866.4 (111085) CCT-----CCTTC----AGAAAGT-GTATTTGTTGCAACTACTTTAATAGTCTTTGACCGGCAGGTCCTTTTAGTACATAACTAAAAGTTGCTTC
  SARS-CoV-2 NC_045512.2  (26946) GCTGTGATCCTTCGTGGACATCTTCGTATTGCTGGACACCA-TCTAGGACGCTGT-GACATCAAGGACC--TGCCTAAAGAAATCACTGTTGCTAC
                                                                                                   Section 1166
                           (111841) 111841 111848     111859     111870     111881     111892     111903     111914     111925 111936
           T4 NC_000866.4 (111171) AGCTCCATC--TTGTGGAACATTATCTACGCTCCAAGCATA-TGTAATAGTTCCGCCTCCAGTTTGACCACTGGGTGTAGCAGTAA-ACTGCTTAG
  SARS-CoV-2 NC_045512.2  (27038) ATCACGAACGCTTTCTTATTACAAATTGGGAGCTTCGCAGCGTGTAGCAGGT--GACTCAGGTTTTGCTGCATACAGTCGCTACAGGATTGGCAAC
                                                                                                   Section 1167
                           (111937) 111937    111947     111958     111969     111980     111991     112002     112013         112032
           T4 NC_000866.4 (111263) TTTCATCAATAACCCCTGTAGGTGTTTTAGGAGTTATATCAACTGTAAAAGTCATAAGTTATCCTTATTTTAATGTTACGAAAGAAGAGTTGCGTG
  SARS-CoV-2 NC_045512.2  (27132) T---ATAAATTAAAC--ACAGACCATTCC--AG---TAGC-AGTGACAAT---ATTGCTTTGCTTGTACAGTAAGTGAC-AA--------CAGATG
                                                                                                   Section 1168
                           (112033) 112033       112046     112057     112068     112079     112090     112101     112112       112128
           T4 NC_000866.4 (111359) TTTCACGAATTAAAACTGATCCATCGCGATTAATGTAATAAATTAAGCTAA-ATAAAGTTTGGTGTGCTGACGCATGTTCAAAACTAGTTGGGTGA
  SARS-CoV-2 NC_045512.2  (27205) TTTCATCTC-----GTTGA-CTTTCA-GGTTACTAT----AGCAGAGATATTACTAATTATTATGAG-----GACTTTTAAAGTTTCCAT--TTGG
                                                                                                   Section 1169
                           (112129) 112129112134     112145     112156     112167     112178     112189     112200     112211    112224
           T4 NC_000866.4 (111454) GATTTCCAATCAGGAGTTTCAGCAATCCATTGATAAATCCACCAAG-GAACAGTACAGAATCCTAGATTTTTCCAATCAGTTGAAGATTCGGACT
  SARS-CoV-2 NC_045512.2  (27283) AATCTTGATT-ACATCATAAACCTCATAATTAAAAATTTATCTAAGTCACTAACTGAGAA---TAAA-TATTCTCAATTAGATGA--AGAGCAACC
```

Section 1170

```
                  (112225) 112225  112233     112244     112255     112266     112277     112288     112299       112320
       T4 NC_000866.4 (111549) AAAGTTTTCCGGAAGAGTAAATACAGACGGCTTTTCAGATTCAATAATCTCAGCT-AC--AGCCTGTTCGAATTTTTCTTCAACAAAAGGAGTATC
SARS-CoV-2 NC_045512.2 (27372) AATGGAGATT----GATTAAACGAACATGAAAATTATTCTTTTCTTGGCACTGATAACACTCGCTACTTGTGAGCTTTATCACTACCAAGAGTGTG
```

Section 1171

```
                  (112321) 112321     112332     112343     112354     112365     112376     112387     112398       112416
       T4 NC_000866.4 (111642) TTCAATCAAAA-CATCGGTATTTTCAGGAATTTTATCCGTTTCTACTACTTCA-ATTTTAATGTCAGATTTAATCG-------GAGAATCAATCAG
SARS-CoV-2 NC_045512.2 (27464) TTAGAGGTACAACAGTACTTTTAAAAGAACCTTGCTC---TTCTGGAACATACGAGGGCAATTCACCATTTCATCCTCTAGCTGATAACAAATTTG
```

Section 1172

```
                  (112417) 112417         112431     112442     112453     112464     112475     112486     112497     112512
       T4 NC_000866.4 (111729) AAGTG-CTGCTTCTGG-ATTGACTT--CTTCATC--GTCATATTTTAATCCCTCTGCGGCATCAGCAGCATC--AATTAAGTCTTTAATAGATAAC
SARS-CoV-2 NC_045512.2 (27557) CACTGACTTGCTTTAGCACTCAATTTGCTTTTGCTTGTCCTGACGGCGTAAAACACGTCTATCAGTTACGTGCCAGATCAGTTTCACCTAAACTGT
```

Section 1173

```
                  (112513) 112513 112519     112530     112541     112552     112563     112574     112585     112596 112608
       T4 NC_000866.4 (111817) CCATCAGTCTCTGGCATAGGTTCACTAGCGAGCTTCTGGAGGGCTTCTTCAA-TATCAACAACGATATTATCAAAAGATTTATTCTTTTTGACCTT
SARS-CoV-2 NC_045512.2 (27653) TCATCAGACA--AGAGGAAGTTCAAGAACTTTACTCTCCAATTTTTCTTATTGTTGCGGCAATAGTGTTTATAACACTTTGCTTCACACTCAAAAG
```

Section 1174

```
                  (112609) 112609   112618     112629     112640     112651     112662     112673     112684           112704
       T4 NC_000866.4 (111912) TATACCAAACTGTTCAGCATATTCAGCTAATTTAGCTTTAGCTTCTTTGTTATCATCAAGAGCCTTCAGCTCTGCAATATAATCTTTATCTATCAT
SARS-CoV-2 NC_045512.2 (27747) AAAGACAGAATGAT-TGAACTTTCAT-TAATTGACTTCTATTTGTGCTTTT----T----AGCCTT----TCTGCTA-------------------
```

Section 1175

```
                  (112705) 112705      112717     112728     112739     112750     112761     112772     112783       112800
       T4 NC_000866.4 (112008) AATATTTCCTCAGTATAAATATAGATATATTTATTACTCGGAAAATAGTATGTACCACTTTGTATATGAAACAACAAATC-TAATAAATGGTAAAA
SARS-CoV-2 NC_045512.2 (27810) -----TTCC------------TTGTTTTAATTA-TGCT-------TATTATCTTTGGTTCTCACTTGAACTGCAAGATCATAATGAAACTTGTCA
```

Section 1176

```
                  (112801) 112801         112816     112827     112838     112849     112860     112871     112882       112896
       T4 NC_000866.4 (112103) AGTATATAGGAAAGCACTCTACTGATGACTTGAATGATGGTTACCTTGGTTCCGGTAAG-GCAATTCAGCAGGCTATAAAGAAATATGGTGAAAAC
SARS-CoV-2 NC_045512.2 (27881) CGCCTAAACGAA--CATGAAATTTCTTGTTT---TCTTAGGAATCATCACAACTGTAGCTGCATTTCACCAAGAATG-------TAGTTTACAGTC
```

```
                                                                                                    Section 1177
                     (112897) 112897 112904     112915     112926     112937     112948     112959     112970     112981 112992
T4 NC_000866.4       (112198) AATTTCTCTAGAACAATACTAAAAGAGTTTAAAACTTCCGAAGAAGCGTACATGTATGAAGAAGAAATTATAACTCCTGAACTAATAAAAAGCAAA
SARS-CoV-2 NC_045512.2 (27965) ATGTACTCAACATCA--ACCATATGTAGTTGATGACCCGTGTCCTATTCACTTCTATTCTAAATGGTATATTAGAGTAGGAGCTAG-AAAATCAGC
                                                                                                    Section 1178
                     (112993) 112993    113003     113014     113025     113036     113047     113058     113069            113088
T4 NC_000866.4       (112294) AATTATTATAATATGAAACCTGGTGGAATTGGTGGAATTGTTATGACTACAGATGTTATAGCAAAGATGAAAGAATCTTCCGCTAAAAGATTTGAA
SARS-CoV-2 NC_045512.2 (28058) ACCTTTAATTGAATTGTGCGTGGATGAG---GCTGGTTCTAAATCACC-CATTCAGTACATCGATATCGGT--AATTATAC--------AGTTT--
                                                                                                    Section 1179
                     (113089) 113089       113102     113113     113124     113135     113146     113157     113168           113184
T4 NC_000866.4       (112390) AACTCACCGGGCACGGTATTAGGTAAAACTTGTTATACTAATGGAACTAAAAATATTTTTATTAA-ACCTGGAGAACTTGTTCCAGAAGGATTTGT
SARS-CoV-2 NC_045512.2 (28138) ----C-CTGTTTACCTTT-----TACAA----TTAATTGCCAGGAAC-----CTAAATTGGGTAGT-CTTGTAGTGCGTTGTTC----G---TTCT
                                                                                                    Section 1180
                     (113185) 113185113190    113201     113212     113223     113234     113245     113256     113267    113280
T4 NC_000866.4       (112485) AAAAGGGATGGTTCATCCTAATAGAAAGTCCAGAAAAGGATGTAAAGTCAAACCGACTACCACAGGAACTTTTTGGGTCAATAATGGCGCAATAAA
SARS-CoV-2 NC_045512.2 (28207) ATGAAG-ACTTTTTAGAGTATCATGACGTT-CGTGTTGTTTTAGATTTCATCTAAACGAACAAACTAAAATGTCTG----ATAATGGACCCCAAAA
                                                                                                    Section 1181
                     (113281) 113281  113289     113300     113311     113322     113333     113344     113355            113376
T4 NC_000866.4       (112581) TAAA-TTAATACAACCAGACGGTATTATTCCCGACGGATTTATTAAAGGTCGTCTCATGAAAAGAGATTCTAAAGGCAAATTTAGTAAGGCATAAT
SARS-CoV-2 NC_045512.2 (28297) TCAGCGAAATGCACCCCGCATTACGTTTGGTGGACCCTCAGATTCAAC-TGGCA----GTAACCAGA-----ATGGAGAACGCAGTGGGGCG----
                                                                                                    Section 1182
                     (113377) 113377     113388     113399     113410     113421     113432     113443     113454            113472
T4 NC_000866.4       (112676) TATGGATATTAAAGTACATTTTCACG-ACTTCAGTCATGTACGCATCGATT--GTGAAGAGAGCACGTTCCACGAATTAAGAGATTTCTT---TTC
SARS-CoV-2 NC_045512.2 (28379) ---CGATCAAAACAACGTCGGCCCCAAGGTTTACCCAATAATACTGCGTCTTGGTTCACCGCTCTCACTCAACATGGCAAGGAAGACCTTAAATTC
                                                                                                    Section 1183
                     (113473) 113473       113487     113498     113509     113520     113531     113542     113553           113568
T4 NC_000866.4       (112766) GTTTGAGGCCGATGGATATAGATTTAATCCTCGCTTCAGATATGGCAACTGGGATGGACGAATCCGTCTTTTAGATTATAATCGTCTTCTTCCATT
SARS-CoV-2 NC_045512.2 (28472) CCTCGAGGA-CAAGGCGTTCCAATTAACACCAATAGCAGTCCAGATGACCAAATTGGCTACTACCGA-----AG-AGCTACCAGACGAATTCG-TG
```

Section 1184

```
(113569)                    113569 113575 113586 113597 113608 113619 113630 113641 113652 113664
T4 NC_000866.4 (112862)     CGGCTTAGTCGGGCAAATTAAAAAATTCTGTGATA-ATTTTGGCTATAAAGCCTGG--ATTGACCCACAAATTAACGAAAAAGAAGAAT-TATCAA
SARS-CoV-2 NC_045512.2 (28560) GTGGTGA--CGGTAAAATGAAAGATCTCAGTCCAAGATGGTATTTCTACTACCTAGGAACTGGGCCAGAAGCTG--GACTTCCC--TATGGTGCTA
```

Section 1185

```
(113665)                    113665 113674 113685 113696 113707 113718 113729 113740 113760
T4 NC_000866.4 (112954)     GAAAAGATTTTGATGAATGGCTTTCTAAATTAGAAATCTATTCAGGAAATAAAAGAATTGAACCGCACTGGTATCAAAAAGATGCAGTGTTCGAAG
SARS-CoV-2 NC_045512.2 (28650) ACAAAGACGGCATCATATGGGTTGC-AACTGAGGGAGCCTTGAATACACCAAAAGATCACATTGGCACCCG-----CAATCCTGCTAAC----AAT
```

Section 1186

```
(113761)                    113761 113773 113784 113795 113806 113817 113828 113839 113856
T4 NC_000866.4 (113050)     GATTAGTTAATCGTCGTAGAA-TTCTTAATCTTCCAACATCTGCAGGTAAA-TCTTTAATTCAAGCTTTGCTTGCGCGATATTATTTGGAAAATTA
SARS-CoV-2 NC_045512.2 (28736) GCT---GCAATCGTGCTACAACTTCCTCAAGGAACAACATTGCCA---AAAGGCTTCTACGCAG--AAGG-------GAGCAGAGGCGGC------
```

Section 1187

```
(113857)                    113857 113872 113883 113894 113905 113916 113927 113938 113952
T4 NC_000866.4 (113144)     TGAAGGTAAAATTCTTATCATTGTTCCAACAACTGCTCTGACAACTCAGATGGCTGATGACTTCGTCGACTATCGTTTATTCAGCCATGCAATGAT
SARS-CoV-2 NC_045512.2 (28811) ---AGTCAA---GCCTCTTCTCGTTCC-----------TCATCACGTAG-TCGC--AACAGTTCAAGAA---------ATTCAACT---C---CAG
```

Section 1188

```
(113953)                    113953 113960 113971 113982 113993 114004 114015 114026 114037 114048
T4 NC_000866.4 (113240)     AAAGAAAATTGGTGGCGGAGCATCAAAAGATGATAAATATAAAAATGATGCACCAGTCGTTGTTGGTACATGGCAAACTGTAGTAAAACAACCGAA
SARS-CoV-2 NC_045512.2 (28872) GCAG-----CAGTAGGGGAACTTCTCCTG---------CTAGAATGGCTGGCAATGGCGGTGATGCTGCT---CTTGCTTTGCTGCTGCTT-----
```

Section 1189

```
(114049)                    114049 114059 114070 114081 114092 114103 114114 114125 114144
T4 NC_000866.4 (113336)     AGAATGGTTCTCACAGTTTGGAATGATGATGAATGATGAATGCCATCTTGCTACAGGAAAAAGTATTTCATCTATCATATCAGGTTTAAATAACTG
SARS-CoV-2 NC_045512.2 (28946) -GACAGATTGAACCAGCTTGAGAGCAAAATGTCTGGTAAAGGCC-------AACAACAACAAGG-------CCAAACTGTCA--CTAAGAAATCTG
```

Section 1190

```
(114145)                    114145 114158 114169 114180 114191 114202 114213 114224 114240
T4 NC_000866.4 (113432)     CATGTTCAAATTC-GGTTTGTCTGGTTCATTACGTGATG---GCAAAGC---CAATATCATGCAGTATGTTGGAATGTTTGGTGAAATATTTAAAC
SARS-CoV-2 NC_045512.2 (29025) CTGCTGAGGCTTCTAAGAAGCCTCGGCAAAACGTACTGCCACTAAAGCATACAATGTAACACAAGCTTTCGGCAGACGTGGTCCAGAACAAACCC
```

```
Section 1191
(114241) 114241 114246 114257 114268 114279 114290 114301 114312 114323 114336
T4 NC_000866.4 (113521) CAGTAACGACTTCTAAATTAATGGAAGATGGACAAGTAACTGAG-CTAAAAATTAATAGTATTTTTCTTCGCTATCCCGATGAG-TTCACTACTAA
SARS-CoV-2 NC_045512.2 (29121) AAGGAAATTTTGGGGACCAGGAACTAATCAGACAAGGAACTGATTACAAACATTGGCCGCAAATTGCACAATTTGCCCCCAGCGCTTCAGCGTTCT

Section 1192
(114337) 114337 114345 114356 114367 114378 114389 114400 114411 114432
T4 NC_000866.4 (113615) ATTAAAGGGAAAAACTTACCAAGAAGAAATAAAAATTATTACTGGGCTTAGTAAAAGAAATAAATGGATCGCTAAATTAGCTATTAAGCT-TGCGC
SARS-CoV-2 NC_045512.2 (29217) TCGGAATGTCGCGCATTGGCATG--GAAGTCACACCTTCGG--GAACGTGGTTGACCTACACA---G---GTGCCATCAAATTGGATGACAAAGAT

Section 1193
(114433) 114433 114444 114455 114466 114477 114488 114499 114510 114528
T4 NC_000866.4 (113710) AAAAAGATGAAAACGCTTTTGTCATGTTTAAACATGTATCGCATGGTAAAGCTATTTTCGATTTAATTAAAAATGAATACGATAAAGTTTATTACG
SARS-CoV-2 NC_045512.2 (29303) CCAAATTTCAAAGATCAAGTCATTTTGCTGAATAAGCATATTGACGCATACAAAA---CATTCCCACCAACAGAGCCT---AAAAAGGACAAAAAG

Section 1194
(114529) 114529 114543 114554 114565 114576 114587 114598 114609 114624
T4 NC_000866.4 (113806) TATCAGGGGAAGTTGATACCGAAACCCGCAATATAATGAAAACCTTAGCTGAAAATGGTAAAGGAATAATTATAGTAGCTAGTTATGGTGTATTTT
SARS-CoV-2 NC_045512.2 (29393) AAGAAGG--CTGATGAAACTCAAGCCTT-ACCGCAGAGACAGAAGAAACAGCAAACTGTGA--CTCTTCTTCCTGCTGCAGATTTGGATG--ATTT

Section 1195
(114625) 114625 114631 114642 114653 114664 114675 114686 114697 114708 114720
T4 NC_000866.4 (113902) CTACTGGTATTTCAGTTAAAAATCTGCATCACGTTGTTTTAGCGCACGGTGTTAAATCAA-AAATCATTGTATTACAAACAATCGGTCGTGTATTA
SARS-CoV-2 NC_045512.2 (29482) CTCCAAACAATTG---CAACAATC--CATGA-GCAGTGCTGACTCAAC----TCAGGCCTAAACTCAT-GCAGACCACACAAG-GCAGATGGGCTA

Section 1196
(114721) 114721 114730 114741 114752 114763 114774 114785 114796 114816
T4 NC_000866.4 (113997) CGTAAGCATGGTTCTAAGACAATAGC--AACAGTCTGGGACCTCATAGATAGCGCAGGCGTCAAGCCAAAATCTGCTAATACGAAAAGAAATATG
SARS-CoV-2 NC_045512.2 (29566) TATAAACGT--TT---------TCGCTTTTCCGTTTACGAT-ATATAGTCTACTCTTGTGCAGAATGAATTCTCGTAACTACATAGCACAAGTAGA

Section 1197
(114817) 114817 114829 114840 114851 114862 114873 114884 114895 114912
T4 NC_000866.4 (114091) TTCATTTGAACTATCTTTTAAAA--CACGGCATTGATCGTATTCAGCGCTACGCAGATGAAAAATTTAATTACGTAATGAAAACAGTTAATTTAAT
SARS-CoV-2 NC_045512.2 (29650) TGTAG-----TTAACTTTAATCTCACATAGCAATCTT--TAATCAGTGTGTAACATT---AGGGAGGACTTG-AAAGAGCCACCA---CATTTT--
```

```
                                                                                              Section 1198
                          (114913) 114913         114928     114939     114950     114961     114972     114983     114994       115008
        T4 NC_000866.4 (114185) AAGCTTCGGCCCTTTGGAGAAAAAGATGTTACTAGAATTTAAACAATTTCTTTATGAAGCTTCTATTGATGAA----TTTATGGGTAAAATTGCCT
SARS-CoV-2 NC_045512.2  (29730) CACCGAGGCCAC---GCGGAGTACGATCG-AGTGTACAGTGAACAATGCTAGGGAGAGCTGCCTATATGGAAGAGCCCTAATGTGTAAAATTAATT
                                                                                              Section 1199
                          (115009) 115009 115016     115027     115038     115049     115060     115071     115082     115093 115104
        T4 NC_000866.4 (114277) CTTGTC-AAACATTAGAAGGTTTAGAAGAACTTGAAGCTTATTATAA-GAAAAAGAGTCAAAGAAACTGAATTAAAAGATACTGATGACATCTCTGT
SARS-CoV-2 NC_045512.2  (29822) TTAGTAGTGCTATCCCCA--TGTGATTTTAATAGCTTCTTAGGAGAATGACAAAAAAAAAAAAAAAAAAAAAAAAAAAAAAAAA------------
                                                                                              Section 1200
                          (115105) 115105    115115     115126     115137     115148     115159     115170     115181       115200
        T4 NC_000866.4 (114371) GAGAGATGCTTTGGCAGGAAAAAGAGCTGAATTAGAAGATTCAGACGATGAAGTAGAAGAAAGCTTTTAAATTAAAAAAGGCCCAACCAAAAAGGA
SARS-CoV-2 NC_045512.2  (29904) ------------------------------------------------------------------------------------------------
                                                                                              Section 1201
                          (115201) 115201       115214     115225     115236     115247     115258     115269     115280       115296
        T4 NC_000866.4 (114467) AGGGCCAAAACTATAGACTAAAGGTCACACTATAGCAAAAGTTGTGTTTCATTTAATTGTTCTTCCGAACTTTCTGAAACTGGTAGTTCTTTAATG
SARS-CoV-2 NC_045512.2  (29904) ------------------------------------------------------------------------------------------------
                                                                                              Section 1202
                          (115297) 115297115302     115313     115324     115335     115346     115357     115368     115379 115392
        T4 NC_000866.4 (114563) TAATTATAGCAAGGCCCAGGATGTACAGGACCTTTGTCTGTTTCAACAACCAATGCAGAATCGATTGGAGTTTTACAGACAACACAAATCTTATCT
SARS-CoV-2 NC_045512.2  (29904) ------------------------------------------------------------------------------------------------
                                                                                              Section 1203
                          (115393) 115393 115401     115412     115423     115434     115445     115456     115467         115488
        T4 NC_000866.4 (114659) GACATGATTGTCTCCTCTGAATTATATCTATTTATACAACTCTCATATGCATATCAATGCCCATATCTTTAGAATAAAAATATTCATCAAGATATC
SARS-CoV-2 NC_045512.2  (29904) ------------------------------------------------------------------------------------------------
                                                                                              Section 1204
                          (115489) 115489    115500     115511     115522     115533     115544     115555     115566       115584
        T4 NC_000866.4 (114755) CGGCAAATTTTCCTTTAATATAAAGGACATCTTCACCACACGGGTGGTCGGCCAGGATACGAATATCCTGACGCTTAAGATTATGCTTTTTCATTA
SARS-CoV-2 NC_045512.2  (29904) ------------------------------------------------------------------------------------------------
```

```
Section 1205
                             (115585) 115585        115599   115610   115621   115632   115643   115654   115665   115680
            T4 NC_000866.4 (114851) AGAATTGAATTTCCGTTTCAAATTCTTCTTCATAATTAAAAGCATCATCAATGCTATATCTCATTATTTTCCAGCCTCAAATGCTCGCATGTCTTG
 SARS-CoV-2 NC_045512.2   (29904) ------------------------------------------------------------------------------------------------
Section 1206
                             (115681) 115681 115687   115698   115709   115720   115731   115742   115753   115764 115776
            T4 NC_000866.4 (114947) AATATGCTTAATAGCAAATCCACGTGATTTAATAGCATCAAGAGCTCCGCTACAGAAATCTAATAAAATCCCCCAATACTGCAACGAGGTATCAAC
 SARS-CoV-2 NC_045512.2   (29904) ------------------------------------------------------------------------------------------------
Section 1207
                             (115777) 115777   115786   115797   115808   115819   115830   115841   115852        115872
            T4 NC_000866.4 (115043) CTTTAAAACATCCTTATCCGCTGATAGAACTGTCTTCATTTCTGATTTCTCGTAACGATCCATACTAAATTCATCACCATCTCCTCGTCCCGAGTA
 SARS-CoV-2 NC_045512.2   (29904) ------------------------------------------------------------------------------------------------
Section 1208
                             (115873) 115873      115885   115896   115907   115918   115929   115940   115951        115968
            T4 NC_000866.4 (115139) GTAGTCTAATCTAGCTTTAAGAGCAACTTTTTTCTGTGCTTCAATTCTAAGCATTTCCTTTTTAATACTTGAATGCTTATTAAGCCATTTACTATA
 SARS-CoV-2 NC_045512.2   (29904) ------------------------------------------------------------------------------------------------
Section 1209
                             (115969) 115969         115984   115995   116006   116017   116028   116039   116050   116064
            T4 NC_000866.4 (115235) TAACATCACATTATTAGCTGCTTCATACTGTAATTTAGTCGAATCTATAAACACATCTTTCTTCAATTCTTCTTGAAGATCTTCTAATCTCATATT
 SARS-CoV-2 NC_045512.2   (29904) ------------------------------------------------------------------------------------------------
Section 1210
                             (116065) 116065 116072   116083   116094   116105   116116   116127   116138   116149 116160
            T4 NC_000866.4 (115331) GTTCTCTATTCAATTGTTATTGGTTGTTATTGGATGGACTTAGATTCATTATACCACGTTTTAACGTGAAGCATTATACTCTATTACTGGAAGCCA
 SARS-CoV-2 NC_045512.2   (29904) ------------------------------------------------------------------------------------------------
Section 1211
                             (116161) 116161   116171   116182   116193   116204   116215   116226   116237        116256
            T4 NC_000866.4 (115427) GCTGCAGTTTTATCTGCTCAATATCATCAGGATTATCGATGACCGAAAAGCGTATTTCTACTATCAGAGTATAATCGTCATAAACGGGTATCACAT
 SARS-CoV-2 NC_045512.2   (29904) ------------------------------------------------------------------------------------------------
```

```
                                                                                                                  Section 1212
                              (116257) 116257       116270     116281     116292     116303     116314     116325     116336          116352
             T4 NC_000866.4 (115523) TAACTGCTAATTTATCAATACGTGGCTCATAGTTTCTTACTGCGCTTTCGATATTGCGTTCAACCGTGTCAGCAGTAAGAGGAGTCATATTTTCAA
     SARS-CoV-2 NC_045512.2  (29904) ------------------------------------------------------------------------------------------------
                                                                                                                  Section 1213
                              (116353) 116353116358       116369     116380     116391     116402     116413     116424     116435     116448
             T4 NC_000866.4 (115619) AAAGCTGGTCTGATAAATCACATCCAAATTCAGGGTCAAACGGTCTTGAACCTTTTCTTGTTGTAATAATTCCCAAAAGACTGTTTTTAATTGACC
     SARS-CoV-2 NC_045512.2  (29904) ------------------------------------------------------------------------------------------------
                                                                                                                  Section 1214
                              (116449) 116449   116457     116468     116479     116490     116501     116512     116523               116544
             T4 NC_000866.4 (115715) TTAATCCAAGCGATCTGGAAACGTCTTTGTTCCAATCCATTTTCATTTCCGGGTCAATATCAGAATAAAGCTTATTAATATTTGCCATTATAGTAA
     SARS-CoV-2 NC_045512.2  (29904) ------------------------------------------------------------------------------------------------
                                                                                                                  Section 1215
                              (116545) 116545     116556     116567     116578     116589     116600     116611     116622               116640
             T4 NC_000866.4 (115811) CTCAAAGAACTCTTTGAGGCCTCTTATTACGTGAGCATGGGTTTTTCCACACTCTGGACACTTAATTGGAACAGCCAAATAAACGGTAGGCTTTAA
     SARS-CoV-2 NC_045512.2  (29904) ------------------------------------------------------------------------------------------------
                                                                                                                  Section 1216
                              (116641) 116641         116655     116666     116677     116688     116699     116710     116721          116736
             T4 NC_000866.4 (115907) AAGCATATCTTTTATAGCTACAATATCTGACTCTGTGATGATAGAATATAAATCTTCTAGTTCCTTTTCATTTAAGTCTTCAACTGGAATGCTTTC
     SARS-CoV-2 NC_045512.2  (29904) ------------------------------------------------------------------------------------------------
                                                                                                                  Section 1217
                              (116737) 116737 116743     116754     116765     116776     116787     116798     116809     116820     116832
             T4 NC_000866.4 (116003) CCCGTTAGCATGAATCGTTTCTATACATGATACTATCATGTGGGCTATATTTTTATCATCAAAAATTTTAGGGTATCGGAATTTAATTTTAATGTC
     SARS-CoV-2 NC_045512.2  (29904) ------------------------------------------------------------------------------------------------
                                                                                                                  Section 1218
                              (116833) 116833   116842     116853     116864     116875     116886     116897     116908               116928
             T4 NC_000866.4 (116099) ACCTAGTGTATACCAGAGGTCTTCTGGTGCATCTATTTGTGTATGTAATAGATTTATATGGGTTGGTATTTCAGTTCCACAGGTGCACTTCCAGGA
     SARS-CoV-2 NC_045512.2  (29904) ------------------------------------------------------------------------------------------------
```

```
                                                                                                   Section 1219
                     (116929) 116929      116941     116952     116963     116974     116985     116996     117007          117024
   T4 NC_000866.4 (116195) GTTTTCGTGATTAACTTCACCGAGAGAATGTGCCCATAAATGAATCAACAATAGTTCTGATTCTTGGCGGTTTAAATCTTTTGCATTTGTGCAGTC
SARS-CoV-2 NC_045512.2 (29904) ------------------------------------------------------------------------------------------------
                                                                                                   Section 1220
                     (117025) 117025         117040     117051     117062     117073     117084     117095     117106        117120
   T4 NC_000866.4 (116291) TTTGATTAGCTTTTTAACAATTACTTCTACGGAACCATTATTTTTGGCAGTAATAAGTTCTAGATATTCTTTAAGCGTGAATGCGCGACAATTGAT
SARS-CoV-2 NC_045512.2 (29904) ------------------------------------------------------------------------------------------------
                                                                                                   Section 1221
                     (117121) 117121 117128     117139     117150     117161     117172     117183     117194     117205 117216
   T4 NC_000866.4 (116387) TATTTTAGAACCAACTCTCACATCAAATTTGTATTCATACATATTTAGCTCCTTTATTTATCATATTTATAAATAGAATAAAAGGAGCATCTATGG
SARS-CoV-2 NC_045512.2 (29904) ------------------------------------------------------------------------------------------------
                                                                                                   Section 1222
                     (117217) 117217    117227     117238     117249     117260     117271     117282     117293            117312
   T4 NC_000866.4 (116483) CAAACATTATTCGTTGTAAATTACCAGATGGTGTTCATCGTTTTAAACCATTTACGGTAGAAGATTATCGAGATTTTTTGTTAGTTCGAAACGATA
SARS-CoV-2 NC_045512.2 (29904) ------------------------------------------------------------------------------------------------
                                                                                                   Section 1223
                     (117313) 117313       117326     117337     117348     117359     117370     117381     117392          117408
   T4 NC_000866.4 (116579) TAGAACATCGGTCACCACAAGAACAAAAGCAAATAATTACTGATTTAATTGATGATTATTTTGGAGACTATCCGAAGACTTGGCAACCATTTATAT
SARS-CoV-2 NC_045512.2 (29904) ------------------------------------------------------------------------------------------------
                                                                                                   Section 1224
                     (117409) 117409117414     117425     117436     117447     117458     117469     117480     117491       117504
   T4 NC_000866.4 (116675) TTTTGCAGGTATTTGTAGGGTCAATAGGTAAAACTAAAAGTACGGTCACATTTATATGTCCAAAATGTAAAAAAGAAAAGACAGTTCCATTTGAAA
SARS-CoV-2 NC_045512.2 (29904) ------------------------------------------------------------------------------------------------
                                                                                                   Section 1225
                     (117505) 117505  117513     117524     117535     117546     117557     117568     117579             117600
   T4 NC_000866.4 (116771) TATATCAAAAAGAATTAAAGGACCTTGTTTTTGATGTAGCTAATGTTAAAATTAAATTAAAGTTTCCTTCTGAGTTTTATGAAAATAAAGCAAAGA
SARS-CoV-2 NC_045512.2 (29904) ------------------------------------------------------------------------------------------------
```

Section 1226

```
                          (117601) 117601     117612     117623     117634     117645     117656     117667     117678            117696
         T4 NC_000866.4 (116867) TGATTACTGAAAATATTCATTCTGTTCAAGTAGATGAAATATGGTATGATTGGAAGGAAATTAGCGAGTCCAGTCAAATAGAACTAGTTGACGCCA
SARS-CoV-2 NC_045512.2  (29904) ------------------------------------------------------------------------------------------------
```

Section 1227

```
                          (117697) 117697        117711     117722     117733     117744     117755     117766     117777         117792
         T4 NC_000866.4 (116963) TCGAGATAGAAACATTAGAAAAAATTCTCGATGCAATGAATCCTATTAATTTAACTCTACACATGTCATGCTGTAATAAGTACATTAAAAAATACA
SARS-CoV-2 NC_045512.2  (29904) ------------------------------------------------------------------------------------------------
```

Section 1228

```
                          (117793) 117793 117799     117810     117821     117832     117843     117854     117865     117876      117888
         T4 NC_000866.4 (117059) CTGATATAGTAGACGTGTTTAAGCTATTAGTTAACCCAGATGAGATATTTACTTTTTATCAAATTAATCACACACTCGTAAAAAGTAATTATAGCT
SARS-CoV-2 NC_045512.2  (29904) ------------------------------------------------------------------------------------------------
```

Section 1229

```
                          (117889) 117889   117898     117909     117920     117931     117942     117953     117964              117984
         T4 NC_000866.4 (117155) TAAATTCAATAAGTAAAATGATTCCTGCCGAGCGCGGATTCGTATTAAAACTGATTGAGAAGGATAAACAATAATGAGTATGTTGCAACGCCCCGG
SARS-CoV-2 NC_045512.2  (29904) ------------------------------------------------------------------------------------------------
```

Section 1230

```
                          (117985) 117985      117997     118008     118019     118030     118041     118052     118063            118080
         T4 NC_000866.4 (117251) ATATCCAAATCTCAGCGTTAAATTATTTGATAGCTACGACGCTTGGAGTAATAATAGATTTGTTGAATTAGCTGCTACTATTACCACATTAACTAT
SARS-CoV-2 NC_045512.2  (29904) ------------------------------------------------------------------------------------------------
```

Section 1231

```
                          (118081) 118081         118096     118107     118118     118129     118140     118151     118162         118176
         T4 NC_000866.4 (117347) GCGGGATTCTCTTTATGGCCGAAATGAAGGAATGCTGCAGTTTTATGATTCTAAAAACATCCATACAAAAATGGATGGAAATGAAATAATTCAGAT
SARS-CoV-2 NC_045512.2  (29904) ------------------------------------------------------------------------------------------------
```

Section 1232

```
                          (118177) 118177 118184     118195     118206     118217     118228     118239     118250     118261      118272
         T4 NC_000866.4 (117443) TTCTGTAGCTAATGCAAATGATATTAATAATGTTAAAACACGAATTTATGGATGTAAGCATTTTTCCGTGTCAGTAGATTCAAAAGGTGATAACAT
SARS-CoV-2 NC_045512.2  (29904) ------------------------------------------------------------------------------------------------
```

```
                                                                                            Section 1233
                           (118273) 118273    118283     118294     118305     118316     118327     118338     118349          118368
           T4 NC_000866.4 (117539) CATTGCTATTGAATTGGGAACTATTCATTCTATAGAAAATCTTAAATTTGGTAGACCATTTTTCCCTGATGCAGGTGAATCTATAAAAGAAATGCT
SARS-CoV-2 NC_045512.2   (29904) ------------------------------------------------------------------------------------------------
                                                                                            Section 1234
                           (118369) 118369       118382     118393     118404     118415     118426     118437     118448          118464
           T4 NC_000866.4 (117635) TGGTGTCATTTATCAGGATCGCACATTATTAACTCCAGCAATAAATGCTATAAATGCTTATGTTCCTGATATTCCATGGACTAGCACATTTGAAAA
SARS-CoV-2 NC_045512.2   (29904) ------------------------------------------------------------------------------------------------
                                                                                            Section 1235
                           (118465) 118465118470     118481     118492     118503     118514     118525     118536     118547   118560
           T4 NC_000866.4 (117731) CTATTTGTCATATGTAAGAGAAGTTGCTCTAGCTGTAGGAAGCGACAAATTTGTATTTGTATGGCAAGACATCATGGGCGTTAACATGATGGACTA
SARS-CoV-2 NC_045512.2   (29904) ------------------------------------------------------------------------------------------------
                                                                                            Section 1236
                           (118561) 118561  118569     118580     118591     118602     118613     118624     118635          118656
           T4 NC_000866.4 (117827) TGATATGATGATAAATCAAGAACCATATCCAATGATTGTCGGTGAGCCATCTTTAATAGGTCAATTCATCCAAGAATTAAAATATCCATTAGCATA
SARS-CoV-2 NC_045512.2   (29904) ------------------------------------------------------------------------------------------------
                                                                                            Section 1237
                           (118657) 118657     118668     118679     118690     118701     118712     118723     118734          118752
           T4 NC_000866.4 (117923) TGATTTCGTTTGGTTGACTAAATCGAATCCTCACAAACGTGACCCAATGAAAAACGCTACTATCTATGCGCATTCATTTTTAGATTCTTCAATACC
SARS-CoV-2 NC_045512.2   (29904) ------------------------------------------------------------------------------------------------
                                                                                            Section 1238
                           (118753) 118753        118767     118778     118789     118800     118811     118822     118833     118848
           T4 NC_000866.4 (118019) AATGATTACTACAGGAAAGGGTGAAAACTCTATTGTGGTGTCAAGGTCAGGTGCTTATTCTGAAATGACTTATAGGAATGGATATGAAGAAGCTAT
SARS-CoV-2 NC_045512.2   (29904) ------------------------------------------------------------------------------------------------
                                                                                            Section 1239
                           (118849) 118849 118855     118866     118877     118888     118899     118910     118921     118932 118944
           T4 NC_000866.4 (118115) TCGTCTTCAAACTATGGCACAATATGACGGCTATGCTAAATGTTCTACTATCGGTAATTTTAACTTGACTCCTGGTGTTAAAATTATTTTTAATGA
SARS-CoV-2 NC_045512.2   (29904) ------------------------------------------------------------------------------------------------
```

```
                                                                                                  Section 1240
                        (118945) 118945   118954     118965     118976     118987     118998     119009     119020          119040
       T4 NC_000866.4 (118211) TAGTAAAAACCAATTTAAAACAGAATTTTACGTTGATGAAGTTATCCATGAATTATCCAATAATAATTCAGTAACTCATCTATATATGTTCACTAA
SARS-CoV-2 NC_045512.2  (29904) ------------------------------------------------------------------------------------------------
                                                                                                  Section 1241
                        (119041) 119041      119053     119064     119075     119086     119097     119108     119119          119136
       T4 NC_000866.4 (118307) TGCAACGAAACTGGAAACAATAGACCCAGTTAAGGTTAAAAATGAATTTAAATCTGATACTACCACTGAAGAAAGTAGTTCTTCCAATAAGCAATA
SARS-CoV-2 NC_045512.2  (29904) ------------------------------------------------------------------------------------------------
                                                                                                  Section 1242
                        (119137) 119137         119152     119163     119174     119185     119196     119207     119218     119232
       T4 NC_000866.4 (118403) AAGAAGTTTCTATTCCTAAAATGGGTCTTAAACATTATAACATTTTAAAAGATGTTAAAGGTCCTGATGAAAATTTAAAACTTCTCATTGATTCTA
SARS-CoV-2 NC_045512.2  (29904) ------------------------------------------------------------------------------------------------
                                                                                                  Section 1243
                        (119233) 119233 119240     119251     119262     119273     119284     119295     119306     119317 119328
       T4 NC_000866.4 (118499) TTTGTCCGAATTTATCACCGGCAGAAGTTGATTTCGTTTCTATTCATTTATTGGAATTTAATGGAAAGATTAAATCTCGTAAAGAAATAGATGGTT
SARS-CoV-2 NC_045512.2  (29904) ------------------------------------------------------------------------------------------------
                                                                                                  Section 1244
                        (119329) 119329   119339     119350     119361     119372     119383     119394     119405          119424
       T4 NC_000866.4 (118595) ATACTTATGACATTAATGATGTTTATGTATGCCAAAGATTGGAATTTCAATACCAAGGAAATACATTTTATTTTAGACCTCCTGGAAAATTTGAAC
SARS-CoV-2 NC_045512.2  (29904) ------------------------------------------------------------------------------------------------
                                                                                                  Section 1245
                        (119425) 119425       119438     119449     119460     119471     119482     119493     119504          119520
       T4 NC_000866.4 (118691) AATTTTTAACGGTGAGCGATATGTTATCTAAATGCTTACTTAGGGTCAACGATGAAGTTAAAGAAATTAATTTTCTTGAGATGCCAGCATTCGTTT
SARS-CoV-2 NC_045512.2  (29904) ------------------------------------------------------------------------------------------------
                                                                                                  Section 1246
                        (119521) 119521119526     119537     119548     119559     119570     119581     119592     119603 119616
       T4 NC_000866.4 (118787) TAAAATGGGCAAATGATATTTTTACAACTTTAGCAATTCCTGGCCCTAATGGTCCAATAACTGGAATTGGCAATATTATTGGATTATTTGAATGAA
SARS-CoV-2 NC_045512.2  (29904) ------------------------------------------------------------------------------------------------
```

```
                                                                                               Section 1247
                        (119617) 119617 119625 119636 119647 119658 119669 119680 119691 119712
      T4 NC_000866.4 (118883) AAAGCCACAAGAAATGCAAACGATGCGTAGAAAAGTTATTTCAGATAATAAACCAACACAGGAAGCGGCTAAATCCGCTTCTAATACTTTATCTGG
SARS-CoV-2 NC_045512.2  (29904) ------------------------------------------------------------------------------------------------
                                                                                               Section 1248
                        (119713) 119713 119724 119735 119746 119757 119768 119779 119790 119808
      T4 NC_000866.4 (118979) GCTTAATGACATATCTACGAAATTGGATGATGCTCAAGCTGCTTCTGAATTAATAGCTCAAACTGTCGAAGAAAAATCGAATGAAATAATTGGAGC
SARS-CoV-2 NC_045512.2  (29904) ------------------------------------------------------------------------------------------------
                                                                                               Section 1249
                        (119809) 119809 119823 119834 119845 119856 119867 119878 119889 119904
      T4 NC_000866.4 (119075) AATTGACAATGTAGAAAGCGCAGTGAGTGATACATCTGCCGGTTCTGAGTTAATTGCTGAAACTGTCGAAATTGGCAACAATATTAATAAAGAAAT
SARS-CoV-2 NC_045512.2  (29904) ------------------------------------------------------------------------------------------------
                                                                                               Section 1250
                        (119905) 119905 119911 119922 119933 119944 119955 119966 119977 119988 120000
      T4 NC_000866.4 (119171) CGGTGAATCGCTCGGAAGCAAATTAGATAAATTAACAAGTTTACTAGAGCAAAAAATCCAGACAGCTGGAATTCAACAGACTGGAACTAGTTTAGC
SARS-CoV-2 NC_045512.2  (29904) ------------------------------------------------------------------------------------------------
                                                                                               Section 1251
                        (120001) 120001 120010 120021 120032 120043 120054 120065 120076 120096
      T4 NC_000866.4 (119267) TACGGTTGAAAGCGCTATTCCTGTTAAAGTCGTTGAGGATGATACTGCTGAATCTGTGGGTCCTTTATTACCAGCTCCTGAAGCAGTTAATAATGA
SARS-CoV-2 NC_045512.2  (29904) ------------------------------------------------------------------------------------------------
                                                                                               Section 1252
                        (120097) 120097 120109 120120 120131 120142 120153 120164 120175 120192
      T4 NC_000866.4 (119363) TCCTGACGCTGATTTTTTCCCTACCCCTCAGCCAGTTGAGCCAAAGCAAGAATCACCAGAAGAAAAACAGAAAAAAGAAGCATTTAACTTAAAATT
SARS-CoV-2 NC_045512.2  (29904) ------------------------------------------------------------------------------------------------
                                                                                               Section 1253
                        (120193) 120193 120208 120219 120230 120241 120252 120263 120274 120288
      T4 NC_000866.4 (119459) ATCTCAAGCTTTAGATAAATTAACGAAGACTGTTGATTTTGGATTTAAAAAATCCATTTCAATTACTGATAAAATATCAAGCATGCTATTTAAGTA
SARS-CoV-2 NC_045512.2  (29904) ------------------------------------------------------------------------------------------------
```

```
                                                                                                    Section 1254
                          (120289) 120289 120296     120307     120318     120329     120340     120351     120362     120373  120384
           T4 NC_000866.4 (119555) CACCGTCAGTGCTGCTATTGAAGCTGCTAAAATGACTGCAATGATATTGGCTGTTGTTGTTGGAATAGACCTTTTGATGATTCACTTTAAATACTG
    SARS-CoV-2 NC_045512.2 (29904) ------------------------------------------------------------------------------------------------
                                                                                                    Section 1255
                          (120385) 120385    120395     120406     120417     120428     120439     120450     120461            120480
           T4 NC_000866.4 (119651) GTCAGATAAATTTTCAAAAGCCTGGGATTTGTTTAGTACAGACTTTACCAAATTCTCTAGCGAAACCGGAACTTGGGGTCCTTTATTACAGAGCAT
    SARS-CoV-2 NC_045512.2 (29904) ------------------------------------------------------------------------------------------------
                                                                                                    Section 1256
                          (120481) 120481       120494     120505     120516     120527     120538     120549     120560          120576
           T4 NC_000866.4 (119747) CTTTGATTCTATTGATAAAATTAAACAACTTTGGGAAGCGGGAGATTGGGGTGGATTGACAGTAGCTATTGTTGAAGGGCTTGGAAAGGTTCTTTT
    SARS-CoV-2 NC_045512.2 (29904) ------------------------------------------------------------------------------------------------
                                                                                                    Section 1257
                          (120577) 120577120582     120593     120604     120615     120626     120637     120648     120659  120672
           T4 NC_000866.4 (119843) TAATTTAGGTGAACTTATTCAATTAGGTATGGCTAAATTATCTGCAGCAATTCTTCGAGTCATTCCTGGTATGAAGGATACTGCTGATGAAGTAGA
    SARS-CoV-2 NC_045512.2 (29904) ------------------------------------------------------------------------------------------------
                                                                                                    Section 1258
                          (120673) 120673  120681     120692     120703     120714     120725     120736     120747            120768
           T4 NC_000866.4 (119939) AGGAAGAGCATTAGAAAATTTCCAAAATTCTACTGGAGCATCTCTCAATAAAGAAGACCAAGAAAAAGTAGCAAATTATCAAGATAAACGAATGAA
    SARS-CoV-2 NC_045512.2 (29904) ------------------------------------------------------------------------------------------------
                                                                                                    Section 1259
                          (120769) 120769     120780     120791     120802     120813     120824     120835     120846            120864
           T4 NC_000866.4 (120035) TGGAGACCTTGGCCCAATAGCAGAAGGACTAGACAAAATCTCTAACTGGAAAACTCGTGCATCTAACTGGATTCGTGGTGTAGATAATAAAGAAGC
    SARS-CoV-2 NC_045512.2 (29904) ------------------------------------------------------------------------------------------------
                                                                                                    Section 1260
                          (120865) 120865        120879     120890     120901     120912     120923     120934     120945          120960
           T4 NC_000866.4 (120131) GCTGACTACCGACGAAGAGCGTGCGGCAGAAGAAGAAAAATTAAAGCAACTTTCACCGGAAGAAAGAAAAAATGCTTTAATGAAGGCTAATGAAGC
    SARS-CoV-2 NC_045512.2 (29904) ------------------------------------------------------------------------------------------------
```

```
                                                                                                                         Section 1261
                          (120961) 120961 120967     120978     120989     121000     121011     121022     121033     121044      121056
          T4 NC_000866.4 (120227) TCGTGCTGCGATGATTCGTTTTGAAAAATATGCCGATTCAGCTGATATGAGTAAAGACTCAACGGTTAAATCAGTTGAAGCTGCCTATGAAGACCT
  SARS-CoV-2 NC_045512.2  (29904) ------------------------------------------------------------------------------------------------
                                                                                                                         Section 1262
                          (121057) 121057   121066     121077     121088     121099     121110     121121     121132                 121152
          T4 NC_000866.4 (120323) TAAAAAACGGATGGATGACCCGGATTTAAATAATTCACCGGCAGTTAAAAAAGAACTTGCTGCTAGATTTTCTAAAATTGATGCTACTTATCAAGA
  SARS-CoV-2 NC_045512.2  (29904) ------------------------------------------------------------------------------------------------
                                                                                                                         Section 1263
                          (121153) 121153      121165     121176     121187     121198     121209     121220     121231           121248
          T4 NC_000866.4 (120419) GCTCAAGAAAAATCAGCCTAATGCCAAACCTGAAACTTCTGCTAAATCACCAGAAGCGAAACAAGTCCAGGTGATTGAAAAGAACAAAGCACAGCA
  SARS-CoV-2 NC_045512.2  (29904) ------------------------------------------------------------------------------------------------
                                                                                                                         Section 1264
                          (121249) 121249         121264     121275     121286     121297     121308     121319     121330      121344
          T4 NC_000866.4 (120515) AGCTCCTGTTCAACAAGCATCTCCTTCGATCAATAATACTAATAATGTTATTAAGAAAAATACTGTCGTTCATAATATGACACCTGTAACGAGCAC
  SARS-CoV-2 NC_045512.2  (29904) ------------------------------------------------------------------------------------------------
                                                                                                                         Section 1265
                          (121345) 121345 121352     121363     121374     121385     121396     121407     121418     121429     121440
          T4 NC_000866.4 (120611) GACTGCTCCTGGTGTATTTGATGCGACTGGAGTTAATTAAGGAATAATATGGCAATTGTTAAAGAAATAACTGCTGATTTAATTAAAAAGTCCGGT
  SARS-CoV-2 NC_045512.2  (29904) ------------------------------------------------------------------------------------------------
                                                                                                                         Section 1266
                          (121441) 121441    121451     121462     121473     121484     121495     121506     121517                 121536
          T4 NC_000866.4 (120707) GAGAAAATTTCAGCCGGACAGAGTACTAAATCAGAAGTAGGAACTAAAACATACACAGCCCAGTTTCCAACTGGGCGTGCTAGTGGTAATGACACT
  SARS-CoV-2 NC_045512.2  (29904) ------------------------------------------------------------------------------------------------
                                                                                                                         Section 1267
                          (121537) 121537       121550     121561     121572     121583     121594     121605     121616           121632
          T4 NC_000866.4 (120803) ACAGAGGACTTCCAGGTAACAGATCTATATAAGAATGGATTATTATTTACTGCATACAATATGTCATCTAGGGATTCTGGAAGTCTTAGATCGATG
  SARS-CoV-2 NC_045512.2  (29904) ------------------------------------------------------------------------------------------------
```

```
                                                                                             Section 1268
                     (121633) 121633121638      121649      121660      121671      121682      121693      121704      121715     121728
       T4 NC_000866.4 (120899) AGATCTAACTACTCTTCTTCATCTTCGAGTATTTTACGTACAGCTAGAAACACTATTAGTAGTACAGTATCAAAACTATCAAATGGATTAATATCA
SARS-CoV-2 NC_045512.2  (29904) ------------------------------------------------------------------------------------------------
                                                                                             Section 1269
                     (121729) 121729   121737      121748      121759      121770      121781      121792      121803               121824
       T4 NC_000866.4 (120995) AATAATAATTCAGGAACAATAAGTAAATCTCCTATCGCAAACATTCTTTTACCGAGATCTAAATCTGATGTTGATACATCATCACATAGATTTAAT
SARS-CoV-2 NC_045512.2  (29904) ------------------------------------------------------------------------------------------------
                                                                                             Section 1270
                     (121825) 121825      121836      121847      121858      121869      121880      121891      121902             121920
       T4 NC_000866.4 (121091) GATGTTCAAGAAAGCCTTATCAGTAGAGGCGGAGGTACTGCTACTGGTGTGCTAAGTAATATTGCTTCAACCGCAGTATTTGGGGCACTGGAAAGT
SARS-CoV-2 NC_045512.2  (29904) ------------------------------------------------------------------------------------------------
                                                                                             Section 1271
                     (121921) 121921         121935      121946      121957      121968      121979      121990      122001     122016
       T4 NC_000866.4 (121187) ATAACACAAGGTATAATGGCTGATAATAATGAACAGATTTATACGACAGCCAGAAGTATGTATGGTGGTGCTGAAAATAGAACTAAAGTGTTTACA
SARS-CoV-2 NC_045512.2  (29904) ------------------------------------------------------------------------------------------------
                                                                                             Section 1272
                     (122017) 122017 122023      122034      122045      122056      122067      122078      122089      122100 122112
       T4 NC_000866.4 (121283) TGGGATTTGACTCCACGTTCAACAGAAGATTTAATGGCTATTATTAATATCTATCAATATTTTAACTATTTTTCTTATGGTGAAACGGGTAAATCT
SARS-CoV-2 NC_045512.2  (29904) ------------------------------------------------------------------------------------------------
                                                                                             Section 1273
                     (122113) 122113   122122      122133      122144      122155      122166      122177      122188              122208
       T4 NC_000866.4 (121379) CAATATGCTGCTGAAATAAAGGGGTATTTAGATGATTGGTATCGTTCTACGTTAATTGAACCTTTATCTCCGGAAGACGCAGCTAAAAATAAAACA
SARS-CoV-2 NC_045512.2  (29904) ------------------------------------------------------------------------------------------------
                                                                                             Section 1274
                     (122209) 122209      122221      122232      122243      122254      122265      122276      122287             122304
       T4 NC_000866.4 (121475) CTATTTGAGAAAATGACATCGAGTTTAACTAACGTTCTAGTAGTTTCAAACCCGACAGTTTGGATGGTGAAAAACTTTGGCGCAACATCTAAGTTT
SARS-CoV-2 NC_045512.2  (29904) ------------------------------------------------------------------------------------------------
```

```
                                                                                                  Section 1275
                        (122305) 122305         122320     122331     122342     122353     122364     122375     122386     122400
         T4 NC_000866.4 (121571) GATGGAAAAACGGAAATATTTGGTCCATGTCAAATACAGAGCATTAGATTTGATAAAACACCTAATGGTAACTTTAACGGATTAGCTATTGCTCCA
  SARS-CoV-2 NC_045512.2 (29904) ------------------------------------------------------------------------------------------------
                                                                                                  Section 1276
                        (122401) 122401 122408     122419     122430     122441     122452     122463     122474     122485 122496
         T4 NC_000866.4 (121667) AACCTCCCTAGTACATTTACTCTCGAGATTACTATGAGAGAAATTATCACGTTAAACCGTGCTTCTTTATATGCGGGGACTTTTTAATGTATTCTT
  SARS-CoV-2 NC_045512.2 (29904) ------------------------------------------------------------------------------------------------
                                                                                                  Section 1277
                        (122497) 122497    122507     122518     122529     122540     122551     122562     122573         122592
         T4 NC_000866.4 (121763) TAGAGGAATTTAATAATCAAGCAATAAACGCAGATTTCCAACGTAATAATATGTTTAGCTGCGTTTTTGCGACAACTCCATCAACTAAAAGCTCTT
  SARS-CoV-2 NC_045512.2 (29904) ------------------------------------------------------------------------------------------------
                                                                                                  Section 1278
                        (122593) 122593       122606     122617     122628     122639     122650     122661     122672          122688
         T4 NC_000866.4 (121859) CGTTGATAAGTTCAATTAGCAACTTTTCTTATAATAACTTGGGCCTAAATTCAGATTGGTTAGGATTAACTCAAGGTGATATTAATCAGGGAATTA
  SARS-CoV-2 NC_045512.2 (29904) ------------------------------------------------------------------------------------------------
                                                                                                  Section 1279
                        (122689) 122689122694     122705     122716     122727     122738     122749     122760     122771     122784
         T4 NC_000866.4 (121955) CCACGCTAATTACAGCTGGCACACAAAAACTGATAAGAAAATCAGGAGTCAGTAAATATCTTATTGGTGCCATGAGTCAACGTACAGTTCAAAGTT
  SARS-CoV-2 NC_045512.2 (29904) ------------------------------------------------------------------------------------------------
                                                                                                  Section 1280
                        (122785) 122785 122793     122804     122815     122826     122837     122848     122859               122880
         T4 NC_000866.4 (122051) TATTAGGCTCATTTACAGTTGGTACATATTTAATTGACTTCTTTAACATGGCATATAACTCATCTGGATTGATGATATACTCTGTAAAAATGCCAG
  SARS-CoV-2 NC_045512.2 (29904) ------------------------------------------------------------------------------------------------
                                                                                                  Section 1281
                        (122881) 122881     122892     122903     122914     122925     122936     122947     122958         122976
         T4 NC_000866.4 (122147) AGAATAGATTATCCTATGAAACTGACTGGAACTATAATTCTCCTAATATTCGTATAACCGGAAGAGAATTAGACCCTTTGGTTATTTCATTTAGAA
  SARS-CoV-2 NC_045512.2 (29904) ------------------------------------------------------------------------------------------------
```

```
                                                                                                   Section 1282
                         (122977) 122977        122991     123002     123013     123024     123035     123046     123057      123072
         T4 NC_000866.4 (122243) TGGATTCAGAAGCTTGTAACTATCGTGCAATGCAAGACTGGGTTAACTCCGTTCAAGACCCAGTAACTGGACTGCGTGCTTTGCCACAAGATGTCG
SARS-CoV-2 NC_045512.2   (29904) ------------------------------------------------------------------------------------------------
                                                                                                   Section 1283
                         (123073) 123073 123079     123090     123101     123112     123123     123134     123145     123156 123168
         T4 NC_000866.4 (122339) AGGCAGATATTCAGGTTAATCTTCATTCTCGCAATGGATTACCTCATACTGCGGTGATGTTCACGATGCATTCAATATCAGTGAGCGCTCCTGAGT
SARS-CoV-2 NC_045512.2   (29904) ------------------------------------------------------------------------------------------------
                                                                                                   Section 1284
                         (123169) 123169   123178     123189     123200     123211     123222     123233     123244           123264
         T4 NC_000866.4 (122435) TATCATATGATGGAGATAACCAAATAACTACATTTGATGTTACTTTTGCGTACAGAGTGATGCAGGCTGGAGCAGTTGATAGGCAACGTGCGCTTG
SARS-CoV-2 NC_045512.2   (29904) ------------------------------------------------------------------------------------------------
                                                                                                   Section 1285
                         (123265) 123265      123277     123288     123299     123310     123321     123332     123343          123360
         T4 NC_000866.4 (122531) AATGGCTTGAATCTGCTGCTATAAATGGTATTCAAAGCGTTCTCGGAAATAGTGGAGGTGTTACTGGACTATCTAATTCGCTTTCACGACTTAGTA
SARS-CoV-2 NC_045512.2   (29904) ------------------------------------------------------------------------------------------------
                                                                                                   Section 1286
                         (123361) 123361         123376     123387     123398     123409     123420     123431     123442     123456
         T4 NC_000866.4 (122627) GATTAGGGGGAACTGCAGGAAGCATTTCAAACATTAATACTATGACAGGAATTGTCAATTCGCAGAGTAAAATATTAGGAGCAATATAACAATGGG
SARS-CoV-2 NC_045512.2   (29904) ------------------------------------------------------------------------------------------------
                                                                                                   Section 1287
                         (123457) 123457 123464     123475     123486     123497     123508     123519     123530     123541 123552
         T4 NC_000866.4 (122723) GACCGAAAGGTCCATATTTTTATTTACGGAATGAAATGAAAGCAGCAACTGAAGCAACTAAACTGTCTTCAATATAAACTTCAATTTTTACAGGAG
SARS-CoV-2 NC_045512.2   (29904) ------------------------------------------------------------------------------------------------
                                                                                                   Section 1288
                         (123553) 123553    123563     123574     123585     123596     123607     123618     123629          123648
         T4 NC_000866.4 (122819) CTTCTGACTCAAATTTACCTGTTACTACACCCTGAAAAATACTTTCAGTCTGTTCTGGCTTTGAAAAATTTTCAGAAGGAAAAATTCCGAACTTTT
SARS-CoV-2 NC_045512.2   (29904) ------------------------------------------------------------------------------------------------
```

```
                                                                                                   Section 1289
              (123649) 123649      123662     123673     123684     123695     123706     123717     123728     123744
T4 NC_000866.4 (122915) TATCTGTTCCAAAAATTTTGATAAATTCATCGTAAACCGCTTCGTTAAAAGCATTATCAGCAGGAATAACGCCTTCAACTACAAGTTCTTGACCTA
SARS-CoV-2 NC_045512.2 (29904) ------------------------------------------------------------------------------------------------
```

```
                                                                                                   Section 1290
              (123745) 123745123750     123761     123772     123783     123794     123805     123816     123827   123840
T4 NC_000866.4 (123011) AAAAGCGTAAAGAAGATTTCATTTTGTGTTCCTCATGTTATGTTAGTAAGACTACTATAACACAACACGAGGGACTTGTAAACTACATTTTGAACT
SARS-CoV-2 NC_045512.2 (29904) ------------------------------------------------------------------------------------------------
```

```
                                                                                                   Section 1291
              (123841) 123841  123849     123860     123871     123882     123893     123904     123915          123936
T4 NC_000866.4 (123107) TTTTAGTACGCGTAATAGGCATGCGTCGATTTTATACTGTTTCATTGTTTGAAGAGCAGTATCAAAAACAGCATTAATGACACCAATTGGATTTCC
SARS-CoV-2 NC_045512.2 (29904) ------------------------------------------------------------------------------------------------
```

```
                                                                                                   Section 1292
              (123937) 123937     123948     123959     123970     123981     123992     124003     124014          124032
T4 NC_000866.4 (123203) ACCCAAGTTTTTAAGCTTAACTAATGATAGTTTCTCATTAACACCTATCATTACGATATGCATCATTTTATCGCCTGGTTTAACGTTTTATTTGTA
SARS-CoV-2 NC_045512.2 (29904) ------------------------------------------------------------------------------------------------
```

```
                                                                                                   Section 1293
              (124033) 124033        124047     124058     124069     124080     124091     124102     124113     124128
T4 NC_000866.4 (123299) TCACCGCCAGAGGTGTATGTACAAAATCTAAATCCAGGAAGTACACTTTCTTCACCGGCTTGAATAGCAAAAATTTGTGGTATTTTATGCTTAGGA
SARS-CoV-2 NC_045512.2 (29904) ------------------------------------------------------------------------------------------------
```

```
                                                                                                   Section 1294
              (124129) 124129 124135     124146     124157     124168     124179     124190     124201     124212  124224
T4 NC_000866.4 (123395) TTTAATGTAACAACCGGCGCCGAAGCGCCGTCAAATAATTCTGTAATAAGTTCCATGATTTATCCTTGAACGAACTTGTAAGGCATGTTTGCAATA
SARS-CoV-2 NC_045512.2 (29904) ------------------------------------------------------------------------------------------------
```

```
                                                                                                   Section 1295
              (124225) 124225  124234     124245     124256     124267     124278     124289     124300          124320
T4 NC_000866.4 (123491) TCTATGCAAGACGCAATAATTCCAAGAGATGATTCTACTTTCTGTTTAACATTTGATGAAACAAATGATGCAAAGTCAACTCTATCTTCTATATCA
SARS-CoV-2 NC_045512.2 (29904) ------------------------------------------------------------------------------------------------
```

Section 1296

```
(124321)                     124321 124333 124344 124355 124366 124377 124388 124399 124416
T4 NC_000866.4 (123587)      CCTGTCATTGTAACCAATTCACCAGTTTCCATTAAATGGTCTCCGTCATAGATTACCGATTCGGTGAGTTCTTCTGTGGTCATAACTTCAGCTTGA
SARS-CoV-2 NC_045512.2 (29904) ------------------------------------------------------------------------------------------------
```

Section 1297

```
(124417)                     124417 124432 124443 124454 124465 124476 124487 124498 124512
T4 NC_000866.4 (123683)      ATAAGCTTGTTGTTAGTTTTAACTGTTCCATCATTGTAAGATGCATCGGTTATTTTATTAACTTTGACCATTAATCCACGTGGCAAAATGACTTCC
SARS-CoV-2 NC_045512.2 (29904) ------------------------------------------------------------------------------------------------
```

Section 1298

```
(124513)                     124513 124520 124531 124542 124553 124564 124575 124586 124597 124608
T4 NC_000866.4 (123779)      ATTTCATTTGAAGGCGCTAAACTTCCACCGGGTAAAACAACATTGACCTTATCAGCCCCAGTAATAACCCATCCAATTCCAACTAAATTATCGCTA
SARS-CoV-2 NC_045512.2 (29904) ------------------------------------------------------------------------------------------------
```

Section 1299

```
(124609)                     124609 124619 124630 124641 124652 124663 124674 124685 124704
T4 NC_000866.4 (123875)      GAATTTACAAGTCCTTCATCAGTTTTATCAATAGAAACGCTTAAACGCTTTTCGTCTGGTAAAACACCTATAGATGAATCAGTCATCCAAGTACCA
SARS-CoV-2 NC_045512.2 (29904) ------------------------------------------------------------------------------------------------
```

Section 1300

```
(124705)                     124705 124718 124729 124740 124751 124762 124773 124784 124800
T4 NC_000866.4 (123971)      AAAATATTTGGATATAATGATGTTGACACAAAGTTTCTAAAATAAAAAACTCGATTTTTTACCATTGCTTCGTATATTGAAGGTAACATTCGTTGT
SARS-CoV-2 NC_045512.2 (29904) ------------------------------------------------------------------------------------------------
```

Section 1301

```
(124801)                     124801 124806 124817 124828 124839 124850 124861 124872 124883 124896
T4 NC_000866.4 (124067)      GAACGATACAAAGTAATACCTTTTGGTAATCGTTCACCATTTTTAAAGGCTGAATCTAAATTATCAATAGCTTTTTCTATGTCAGATGCTGTCAAA
SARS-CoV-2 NC_045512.2 (29904) ------------------------------------------------------------------------------------------------
```

Section 1302

```
(124897)                     124897 124905 124916 124927 124938 124949 124960 124971 124992
T4 NC_000866.4 (124163)      ATACTTGTACGCTCATCTGGATTATATAATCCCAAAAGAGCATTATTTATGTCTACATATCCTGAACCTACGTATTCACGAATTCCGCGTTTTTGC
SARS-CoV-2 NC_045512.2 (29904) ------------------------------------------------------------------------------------------------
```

```
Section 1303
(124993)                   124993     125004     125015     125026     125037     125048     125059     125070     125088
T4 NC_000866.4 (124259)    GCTGGTGTGTATTTAGATGAATCTTTATTTTCGACTATAGGATGTAATGACCATCCAGCGGTAAGAGCATATCCACGTAATTCTCTTCGAATAATC
SARS-CoV-2 NC_045512.2 (29904) ------------------------------------------------------------------------------------------------

Section 1304
(125089)                   125089     125103     125114     125125     125136     125147     125158     125169     125184
T4 NC_000866.4 (124355)    TTTGTTTTTATTGCATTCCAAGAATTTTGTACTAACTCATTTGCAGCATCTTGGTTTAAATGCGAATGTTTGTTAATATTTCTTTCAAACCAAGCA
SARS-CoV-2 NC_045512.2 (29904) ------------------------------------------------------------------------------------------------

Section 1305
(125185)                   125185 125191     125202     125213     125224     125235     125246     125257     125268     125280
T4 NC_000866.4 (124451)    CCCTTATATTTTTCTAAAGTATCATCGACGATAGAAGCAATAGTTTCTAAGGTTTTTATTGAAGTAATAGATTCTTTTCGTAATCTTTCTAAAGCT
SARS-CoV-2 NC_045512.2 (29904) ------------------------------------------------------------------------------------------------

Section 1306
(125281)                   125281     125290     125301     125312     125323     125334     125345     125356     125376
T4 NC_000866.4 (124547)    TTATTTTTTATTTCTGCGTTAATAATGGATTCTTTATCTTCAGGAATTATAGAAGCTTCTCTGAATGCCATCCCAGCTGTAACACTGCTAAGAGCA
SARS-CoV-2 NC_045512.2 (29904) ------------------------------------------------------------------------------------------------

Section 1307
(125377)                   125377     125389     125400     125411     125422     125433     125444     125455     125472
T4 NC_000866.4 (124643)    TTCTCTAGTGCAAATCCTGAAGTAGAAATTACCGTTAATTCATTAGAATCAGAAATTAAAGGCGCGTCCGCTGGTTTATTTAATTCGGCCGCTGAA
SARS-CoV-2 NC_045512.2 (29904) ------------------------------------------------------------------------------------------------

Section 1308
(125473)                   125473     125488     125499     125510     125521     125532     125543     125554     125568
T4 NC_000866.4 (124739)    GCCTCAAATTTTTGAAACATTGGTGTTTCAAATCTAGAAGATTCCAATGACTGACTTTGCGCAATTGCTCTACGGGAAATTTTAACTTTAACGATT
SARS-CoV-2 NC_045512.2 (29904) ------------------------------------------------------------------------------------------------

Section 1309
(125569)                   125569 125576     125587     125598     125609     125620     125631     125642     125653 125664
T4 NC_000866.4 (124835)    ACAGCTTGGTCAGAACGTTTATCATTTTCTTGCGCAATAGATGCTGCAATTGCCTCATTTTTAGTTACTTGAGCCCCAGTATCTTTATTGATATAA
SARS-CoV-2 NC_045512.2 (29904) ------------------------------------------------------------------------------------------------
```

Section 1310
```
(125665) 125665    125675    125686     125697     125708     125719     125730     125741           125760
T4 NC_000866.4 (124931) ACATCACCGACCTTCGATTCAACTTTAGTAAAGAGCTCGGTACTAATTTCCGGAACTCCTGGAATGTCTTCTAGTGATACATTTTTGCGATGTATA
SARS-CoV-2 NC_045512.2 (29904) ------------------------------------------------------------------------------------------------
```

Section 1311
```
(125761) 125761    125774     125785     125796     125807     125818     125829     125840          125856
T4 NC_000866.4 (125027) AGAATATATGCATACTTTTTATCGTAATCCCAGAGTTCCTTAAGAAGGACGTATCTACCACCTGAACGACTACGGATAAGTCTATCAGCAATAACT
SARS-CoV-2 NC_045512.2 (29904) ------------------------------------------------------------------------------------------------
```

Section 1312
```
(125857) 125857125862    125873     125884     125895     125906     125917     125928     125939    125952
T4 NC_000866.4 (125123) TGAATTTGTCGAGCTTGGCCAGCAGTTTTAGACTTAAGAATACGGAGCATACAGGCATCAATTTTATACTGGCGCATTGTTTGCATTGCAACAGTA
SARS-CoV-2 NC_045512.2 (29904) ------------------------------------------------------------------------------------------------
```

Section 1313
```
(125953) 125953  125961     125972     125983     125994     126005     126016     126027           126048
T4 NC_000866.4 (125219) AAAACTGAATTGATATAATTAATTGGGCTTGGACCAAGACCTTTTAATTTAGCAATTGAACCTTTAGCAGTTAATGTAAAAGGAACAATATGCATC
SARS-CoV-2 NC_045512.2 (29904) ------------------------------------------------------------------------------------------------
```

Section 1314
```
(126049) 126049     126060     126071     126082     126093     126104     126115     126126           126144
T4 NC_000866.4 (125315) ATTTTATCGCCCATCTTTAAATCACGATTAGTATCACCTCCAGATGTATAGGTACATAAACGAAAGCCTGGTTGTTCAATTGCATCATCAACATGA
SARS-CoV-2 NC_045512.2 (29904) ------------------------------------------------------------------------------------------------
```

Section 1315
```
(126145) 126145        126159     126170     126181     126192     126203     126214     126225         126240
T4 NC_000866.4 (125411) ACTGAAAAAATTTGCGGTATTTTCTTCTTTGGATATAAGTTTGTAATTGGAAGAGTAGTATCTTCGTCAAATAATTCTGTAATAAGTTCCATCATA
SARS-CoV-2 NC_045512.2 (29904) ------------------------------------------------------------------------------------------------
```

Section 1316
```
(126241) 126241 126247     126258     126269     126280     126291     126302     126313     126324  126336
T4 NC_000866.4 (125507) TCCTCTCTAGTGTTTATTCTATTCTATTTATAAAATTAAAGGCCCGAAGGCCTTTAATAATCTATTGGTAAGAGAGTACGATATATTTCAAACTTT
SARS-CoV-2 NC_045512.2 (29904) ------------------------------------------------------------------------------------------------
```

```
                                                                                                      Section 1317
                   (126337) 126337   126346     126357     126368     126379     126390     126401     126412         126432
  T4 NC_000866.4 (125603) GGACCTTTTTCATAAGCATCAAATGTTTCTGTGAATTTATTATAAGCATATGCATCTATAAATTCAATCATGATTTGTGATACAGAAGTAGAAACA
SARS-CoV-2 NC_045512.2  (29904) ------------------------------------------------------------------------------------------------
                                                                                                      Section 1318
                   (126433) 126433      126445     126456     126467     126478     126489     126500     126511         126528
  T4 NC_000866.4 (125699) TCTCCACCTTCTTTTTGAGCCACGACAATTGTTTCTAAGTAAGCTTTCATAGACCAGTTACCTCATGAAAATCACCAAATACATCTTCGAATGTAT
SARS-CoV-2 NC_045512.2  (29904) ------------------------------------------------------------------------------------------------
                                                                                                      Section 1319
                   (126529) 126529         126544     126555     126566     126577     126588     126599     126610       126624
  T4 NC_000866.4 (125795) TAGCTTTAGTTTTATCTTCACGTAAACGAATCGCAATCGGAAGAAATAATTTAACGTAATCAGTGCGGCCATCAGATTTTAACCAACCGTTGCATT
SARS-CoV-2 NC_045512.2  (29904) ------------------------------------------------------------------------------------------------
                                                                                                      Section 1320
                   (126625) 126625 126632     126643     126654     126665     126676     126687     126698     126709 126720
  T4 NC_000866.4 (125891) CGCACTCTAGAATTTTTCCAATATAATAATTTTGGTTTTCCATAATGCGAGTACGGTCAAGTTCATGCGATTTTACACCGGCTTTATCTTTTAAGC
SARS-CoV-2 NC_045512.2  (29904) ------------------------------------------------------------------------------------------------
                                                                                                      Section 1321
                   (126721) 126721    126731     126742     126753     126764     126775     126786     126797         126816
  T4 NC_000866.4 (125987) CTGAACCAGCATTTACCTTAATTTTTCCACACTCTGACTCAAGAATAAATCCACCCGCTTTAGTAGGGTCTTTACGGTGAGGATAAATTCCTACAA
SARS-CoV-2 NC_045512.2  (29904) ------------------------------------------------------------------------------------------------
                                                                                                      Section 1322
                   (126817) 126817       126830     126841     126852     126863     126874     126885     126896         126912
  T4 NC_000866.4 (126083) TTTTTAAATCAACATCAATTACTTCTTTAAATTTATAAAGATTTTTTGAACGAGCATTTTCCCATAATCCATCGATATTTTTGAGAATAATACCTT
SARS-CoV-2 NC_045512.2  (29904) ------------------------------------------------------------------------------------------------
                                                                                                      Section 1323
                   (126913) 126913126918     126929     126940     126951     126962     126973     126984     126995     127008
  T4 NC_000866.4 (126179) CAAGACCTTGGTCAATATACTTTTTATAAATTACCTTAGCTTCATCTAGGTTATTTACTACCTGGTTTTCAATTAAAATTACTTTATCATATCCAG
SARS-CoV-2 NC_045512.2  (29904) ------------------------------------------------------------------------------------------------
```

```
                                                                                                        Section 1324
                       (127009) 127009  127017     127028     127039     127050     127061     127072     127083     127104
T4 NC_000866.4         (126275) ATGTCATTTGTTCTAGTTTAGAAAAACGTACATCATATTTCAAACGAAATGCAGGAAGACTGTATATTTCTACCAACGGGACATAATCCCAGACCT
SARS-CoV-2 NC_045512.2  (29904) ------------------------------------------------------------------------------------------------
                                                                                                        Section 1325
                       (127105) 127105     127116     127127     127138     127149     127160     127171     127182     127200
T4 NC_000866.4         (126371) GAAACTTCATGCATTGTGCTTCTTTTTCAGAAATGGTTCCCTTTAAAGATTTATTGGCGATTCCATTAGAAGCAGTACGTGATTCAGCTACTTCGG
SARS-CoV-2 NC_045512.2  (29904) ------------------------------------------------------------------------------------------------
                                                                                                        Section 1326
                       (127201) 127201        127215     127226     127237     127248     127259     127270     127281     127296
T4 NC_000866.4         (126467) CGAATTCTTTAGCTTTACTGTTTTCAGGATAAGCATCAAAAAGAAAATCTAGGCCTTCTGGCTCCTTTTTAACTTGCTCATGGTATACCAATTCGC
SARS-CoV-2 NC_045512.2  (29904) ------------------------------------------------------------------------------------------------
                                                                                                        Section 1327
                       (127297) 127297 127303     127314     127325     127336     127347     127358     127369     127380 127392
T4 NC_000866.4         (126563) CATCAATCAACACACCTTCTGGATGAATCTGGCGGGCTTCAGCGGTCATTTTAATTAACTCTTCCTTAAGAAGATCTAATCCTAGATATTCATTAC
SARS-CoV-2 NC_045512.2  (29904) ------------------------------------------------------------------------------------------------
                                                                                                        Section 1328
                       (127393) 127393   127402     127413     127424     127435     127446     127457     127468     127488
T4 NC_000866.4         (126659) CAGCTCGTGATAAAAGACGAACATCATCTAATTCATCACCTCTAACTTCAGCAAAACACCGAGCTCCATCAGCTTTTAACTGAGCAAAGGCTGGAA
SARS-CoV-2 NC_045512.2  (29904) ------------------------------------------------------------------------------------------------
                                                                                                        Section 1329
                       (127489) 127489      127501     127512     127523     127534     127545     127556     127567     127584
T4 NC_000866.4         (126755) ATTTGATATTCTTATTAATGCCTTTTTCATCATAAGAACTTGCGAGCATTTGAGGTTGTTCAGGAATTAAACCTGGCCAAACTTTGTTTGCAATAG
SARS-CoV-2 NC_045512.2  (29904) ------------------------------------------------------------------------------------------------
                                                                                                        Section 1330
                       (127585) 127585         127600     127611     127622     127633     127644     127655     127666     127680
T4 NC_000866.4         (126851) ATACTGAAGCACCACATTCAAGGTCTCGCATCATCACTCGACGCAAAACTTCAACATCATCTTTTTTACCATCGGTGATATATCCAGTTAATTCCT
SARS-CoV-2 NC_045512.2  (29904) ------------------------------------------------------------------------------------------------
```

```
                                                                                                            Section 1331
                   (127681) 127681 127688     127699     127710     127721     127732     127743     127754     127765 127776
T4 NC_000866.4     (126947) CAATTGCTGCATTTCCAGTCAATTTCCGAGTAGCTAATGTGAATTCAATGAAGTCAAGCATATCGGTAAGAGTCAACATTCCAAAACTCTGGGTAG
SARS-CoV-2 NC_045512.2 (29904) ------------------------------------------------------------------------------------------------
                                                                                                            Section 1332
                   (127777) 127777    127787     127798     127809     127820     127831     127842     127853          127872
T4 NC_000866.4     (127043) CAATACCAGGTTTAGGCCATTTCTTGATATAATACTGTAACCCACGAGAATAAGTCAGACGATATACTCGTTTAAGCAATTCATTATCTTTATTCT
SARS-CoV-2 NC_045512.2 (29904) ------------------------------------------------------------------------------------------------
                                                                                                            Section 1333
                   (127873) 127873       127886     127897     127908     127919     127930     127941     127952          127968
T4 NC_000866.4     (127139) TTTCAAGAATTGCTTGCTTCTGTTTAGTTGAACCAATAGATGCTATTTCGTTCAGAATTTTAAGAATCATTGTTCATCCTTTAGAGTTTGGTTTAC
SARS-CoV-2 NC_045512.2 (29904) ------------------------------------------------------------------------------------------------
                                                                                                            Section 1334
                   (127969) 127969127974    127985     127996     128007     128018     128029     128040     128051       128064
T4 NC_000866.4     (127235) AGCTCTATTATAAATCAATTCATCATTAAGCTCAGTCAAAGACCTGTGGTACGTGGTTCTAACTTTATTTCCTTGCATCCAGTGCTTGATATAAAT
SARS-CoV-2 NC_045512.2 (29904) ------------------------------------------------------------------------------------------------
                                                                                                            Section 1335
                   (128065) 128065  128073     128084     128095     128106     128117     128128     128139               128160
T4 NC_000866.4     (127331) GAAACCTTGCTCTACACATTTTTTAAAAATTCGTTCGTCTTTTTGAGCTCGGAATTCTGGATTGCATCTAAAAAATTGATTTACGTGACCGTAATC
SARS-CoV-2 NC_045512.2 (29904) ------------------------------------------------------------------------------------------------
                                                                                                            Section 1336
                   (128161) 128161     128172     128183     128194     128205     128216     128227     128238          128256
T4 NC_000866.4     (127427) ACGTGTAGTATTACCTTCATTTTCATAAATAGTGTGAACAACAAACATTAGAATGCTCCTTGGAAAATATTATCACCACAAGTAGGTCTATTATAC
SARS-CoV-2 NC_045512.2 (29904) ------------------------------------------------------------------------------------------------
                                                                                                            Section 1337
                   (128257) 128257        128271     128282     128293     128304     128315     128326     128337          128352
T4 NC_000866.4     (127523) AAATACTCTATACCGCCGGGCTTAATATAGTTCCATGTCTGAAACGGATGCGTCTGATATGGATGATATGGATTATAAGGATTAAATCCAGGAGTT
SARS-CoV-2 NC_045512.2 (29904) ------------------------------------------------------------------------------------------------
```

```
                                                                                                     Section 1338
                          (128353) 128353 128359    128370    128381    128392    128403    128414    128425    128436   128448
        T4 NC_000866.4 (127619) CTCCAGGTAGTGTTCCAAGGAAAAGAATCGTTTTTAATCATCTTTTCAATTACATCTTTTATAGCTTTTTCACTATCAAAACTTTCTTTATTTTCC
SARS-CoV-2 NC_045512.2  (29904) ------------------------------------------------------------------------------------------------
                                                                                                     Section 1339
                          (128449) 128449   128458    128469    128480    128491    128502    128513    128524              128544
        T4 NC_000866.4 (127715) TTTGGTGAAAAAAGCTTATTCTCTACATCGTTCCATGTATAAACTCGCTGAGCAGTTTTTGGAATATTGTCACGCTCTCCTCGAGCCATCCAATAC
SARS-CoV-2 NC_045512.2  (29904) ------------------------------------------------------------------------------------------------
                                                                                                     Section 1340
                          (128545) 128545      128557    128568    128579    128590    128601    128612    128623           128640
        T4 NC_000866.4 (127811) ACAGGAACACGTAATATTTCACTCGCGTGATCGCAGTGGTGAGCGAGATCGTCAATATAACAAATTACGTTATATTTCTCTTTTGCTTTTTTGAAC
SARS-CoV-2 NC_045512.2  (29904) ------------------------------------------------------------------------------------------------
                                                                                                     Section 1341
                          (128641) 128641         128656    128667    128678    128689    128700    128711    128722        128736
        T4 NC_000866.4 (127907) AACTCTTCTTTTGAAGAATCATGACTACACATCAGTACTTCTGAGAAAGCACCAGGAAAAAGAGCATTCAAATTAAATTGACGATTTAATAGAGCG
SARS-CoV-2 NC_045512.2  (29904) ------------------------------------------------------------------------------------------------
                                                                                                     Section 1342
                          (128737) 128737 128744    128755    128766    128777    128788    128799    128810    128821   128832
        T4 NC_000866.4 (128003) TCAATAGAATCACCCAATGCTGTAACAGCAACAAAATTATAATCTTCTTTTAATTTGTTAATTACACACAGAGCATCTTTATATGGAGACAAGTAA
SARS-CoV-2 NC_045512.2  (29904) ------------------------------------------------------------------------------------------------
                                                                                                     Section 1343
                          (128833) 128833    128843    128854    128865    128876    128887    128898    128909              128928
        T4 NC_000866.4 (128099) CGAATAAAATCCGAACGATTGTATTTTTCAATTAACTTGACGCCAAGTTCTTCATCACAGTTAAAGAGTTTACCAGGAGAAATAAATTTCTCATCT
SARS-CoV-2 NC_045512.2  (29904) ------------------------------------------------------------------------------------------------
                                                                                                     Section 1344
                          (128929) 128929       128942    128953    128964    128975    128986    128997    129008           129024
        T4 NC_000866.4 (128195) TGGATCATTTTTAAAATATGTTCTAACGGAAGATTATATTTCTGAGCAAAATAAGGAAGGCCTGATTGCCAGCTTAAACATACTCCATCAATATCA
SARS-CoV-2 NC_045512.2  (29904) ------------------------------------------------------------------------------------------------
```

```
                                                                                          Section 1345
                    (129025) 129025129030    129041    129052    129063    129074    129085    129096    129107  129120
T4 NC_000866.4      (128291) GTTAAAATAGTAGGCTTCATAGAGAGTCTCTTAATAGGTTTAACACATCAATAAATTCAGCTTCGGTTAGTATTGTATCATCTTTTGTTAGACCAC
SARS-CoV-2 NC_045512.2 (29904) ------------------------------------------------------------------------------------------------
                                                                                          Section 1346
                    (129121) 129121  129129    129140    129151    129162    129173    129184    129195          129216
T4 NC_000866.4      (128387) TAGCAATGCTGTGCTTCAAAACTTTTCCTTTCGAGGCTTGTAATGCATCACGAAAGCCCTTGTTTTGAATCGCTGCTTCAAAATATGCATTTGTGT
SARS-CoV-2 NC_045512.2 (29904) ------------------------------------------------------------------------------------------------
                                                                                          Section 1347
                    (129217) 129217    129228    129239    129250    129261    129272    129283    129294          129312
T4 NC_000866.4      (128483) ATAATTCTTTCCACGCCGGGGAGTATCTTGAAAACGGAACTCCAAGCCAAAAGAGGGTCCCACGGTCCTGAGCTCTAGCATAAGACCTTCCAGCTT
SARS-CoV-2 NC_045512.2 (29904) ------------------------------------------------------------------------------------------------
                                                                                          Section 1348
                    (129313) 129313        129327    129338    129349    129360    129371    129382    129393      129408
T4 NC_000866.4      (128579) GTTGGGCGGCAAGCCCGGATAACCCAAATATACGACGTTGTTGTTCAACATTTTTCACCTTACACCCTTGGAGGAATCCTTCAAGACCTCCAAATT
SARS-CoV-2 NC_045512.2 (29904) ------------------------------------------------------------------------------------------------
                                                                                          Section 1349
                    (129409) 129409 129415    129426    129437    129448    129459    129470    129481    129492  129504
T4 NC_000866.4      (128675) GAATACCATCCATAACGAAAGGCCATTGGGCGAAATTACTTAATGCACATGATGGCCACCTAAAATTGCTTCTAATCTCTAACTCAGACATTTTCA
SARS-CoV-2 NC_045512.2 (29904) ------------------------------------------------------------------------------------------------
                                                                                          Section 1350
                    (129505) 129505  129514    129525    129536    129547    129558    129569    129580          129600
T4 NC_000866.4      (128771) ATGCTTATAATTTCAACATCAGCCCAATGACCATAGCAAGGAAGACGAAATTCAACTGGCCAGTTAGGGTCCCTTTCTAATATAATAGACTCAACT
SARS-CoV-2 NC_045512.2 (29904) ------------------------------------------------------------------------------------------------
                                                                                          Section 1351
                    (129601) 129601     129613    129624    129635    129646    129657    129668    129679          129696
T4 NC_000866.4      (128867) TGTGGTTCTTCGTGTTCATCAGTATACGGATTTTTATCTGTTACTTTATATGTGACCTTAATGTACTGAATTCCAAAAATCTTATTAATTATATTC
SARS-CoV-2 NC_045512.2 (29904) ------------------------------------------------------------------------------------------------
```

```
                                                                                            Section 1352
                  (129697) 129697                  129712    129723    129734    129745    129756    129767    129778      129792
      T4 NC_000866.4 (128963) ATACTAATTCCTTTAATCCGTAGATAGGAGATAATTCATCACCCATACGAAGGTCTTCATTTCCATCTACCCAGGAAACAATATAAGCCTCTTTTA
SARS-CoV-2 NC_045512.2 (29904) ------------------------------------------------------------------------------------------------
                                                                                            Section 1353
                  (129793) 129793 129800    129811    129822    129833    129844    129855    129866    129877 129888
      T4 NC_000866.4 (129059) TTTGAATACCACTCCATTTAAATGGAGGTAGAACCTTAGAAATTAATCCAGGTATACCAACTCCCTTTAATTCAACAGTTTGACCTAAAAAGAATT
SARS-CoV-2 NC_045512.2 (29904) ------------------------------------------------------------------------------------------------
                                                                                            Section 1354
                  (129889) 129889   129899    129910    129921    129932    129943    129954    129965        129984
      T4 NC_000866.4 (129155) TCATTAGAACCTCATCTGAAAACCGTGCGATTTAACATTACCGCCGCCAATATCAGAAATGTTAATTTCACGCGCAATTGAAGGGTCAATGTCAAT
SARS-CoV-2 NC_045512.2 (29904) ------------------------------------------------------------------------------------------------
                                                                                            Section 1355
                  (129985) 129985      129998    130009    130020    130031    130042    130053    130064        130080
      T4 NC_000866.4 (129251) TTCACGATTGAGTTTAGTGATAGCTAAAGTATCACGCCCATTTACAGTACGGAACTCTAAAGGACAAACCACATCAACGTAGTTTTTAATCGATTC
SARS-CoV-2 NC_045512.2 (29904) ------------------------------------------------------------------------------------------------
                                                                                            Section 1356
                  (130081) 130081130086    130097    130108    130119    130130    130141    130152    130163    130176
      T4 NC_000866.4 (129347) GCTAGTAAGTTGCAAATGTGCAGGAAGGTCTTTAGGTGCCTTTGAGAAAACCACTTCACAGAAATTTTTGCGGGTATCAAAATAAGTAGTCATAAA
SARS-CoV-2 NC_045512.2 (29904) ------------------------------------------------------------------------------------------------
                                                                                            Section 1357
                  (130177) 130177 130185    130196    130207    130218    130229    130240    130251            130272
      T4 NC_000866.4 (129443) CATAATATTTTCCTCAGTAAGGGGCTGAAGCCCCTCATTTTATTTTAAATATCAAATTCATTAAGAACTACATCAAAGATTGCTTCAAGATGCTCA
SARS-CoV-2 NC_045512.2 (29904) ------------------------------------------------------------------------------------------------
                                                                                            Section 1358
                  (130273) 130273    130284    130295    130306    130317    130328    130339    130350        130368
      T4 NC_000866.4 (129539) GGTTTAGCTCTGTTACTCAGAATATGACGAATCCAAGTTTTAACTAAGAGTTTACGATTAGCACCATTCCAGCAAGGATGAGTCCCTAAATCGCGT
SARS-CoV-2 NC_045512.2 (29904) ------------------------------------------------------------------------------------------------
```

```
                                                                                                   Section 1359
                     (130369) 130369        130383     130394     130405     130416     130427     130438     130449         130464
     T4 NC_000866.4 (129635) TGGCGGAAATCATCATCCAGAGCGATTTTGAAGTTGGAACCTTTCATCGTGATTGAAACCGTGATACCGTTTTCAAATCGCATATAAACGTAGTTA
SARS-CoV-2 NC_045512.2 (29904) ------------------------------------------------------------------------------------------------
                                                                                                   Section 1360
                     (130465) 130465 130471     130482     130493     130504     130515     130526     130537     130548 130560
     T4 NC_000866.4 (129731) GGAGTCATATACTGTTCAATTTCGCATACTGATCCATTTTGATGTTTCCAGAGGCAAATAGTATCAATAGAACCTGCAATACCATTAGAAACATAT
SARS-CoV-2 NC_045512.2 (29904) ------------------------------------------------------------------------------------------------
                                                                                                   Section 1361
                     (130561) 130561   130570     130581     130592     130603     130614     130625     130636         130656
     T4 NC_000866.4 (129827) TTACGTTCAAAGTTAATGTAGTTCATTTTTATTCTCCGAGATGTTTAATTGCGGTACAGGTATATAATATCATATCCTGTACCAAAGTAAACAATT
SARS-CoV-2 NC_045512.2 (29904) ------------------------------------------------------------------------------------------------
                                                                                                   Section 1362
                     (130657) 130657      130669     130680     130691     130702     130713     130724     130735         130752
     T4 NC_000866.4 (129923) ATTTTACTACTTTCCAATGCTGCATGTCAAGTTTACCAACTTTTTTCATCTTCTCAATTAAGCGTTCTGCACGTTGGCGAGCTGTAACATAATGCC
SARS-CoV-2 NC_045512.2 (29904) ------------------------------------------------------------------------------------------------
                                                                                                   Section 1363
                     (130753) 130753         130768     130779     130790     130801     130812     130823     130834     130848
     T4 NC_000866.4 (130019) ATTCGCCTAATTCATTTTGTTCAATTTTTCCAACGATTACTGTATTCAATTCATAAATCCAACCAGTAAAGAAATTATGAACTTGAATTGTAAAGG
SARS-CoV-2 NC_045512.2 (29904) ------------------------------------------------------------------------------------------------
                                                                                                   Section 1364
                     (130849) 130849 130856     130867     130878     130889     130900     130911     130922     130933 130944
     T4 NC_000866.4 (130115) TGAAATCTGTTCCCATACCTTCTGTTGTTTCTACTTCAATAATATCACCTTCAACTGCCATTAAGAACCACATAGTTTCATCATATTTACCATTGA
SARS-CoV-2 NC_045512.2 (29904) ------------------------------------------------------------------------------------------------
                                                                                                   Section 1365
                     (130945) 130945    130955     130966     130977     130988     130999     131010     131021         131040
     T4 NC_000866.4 (130211) AGCATTTAGTTTTAACTGCAGCGTTCAGATTAATCGTTTTCATTTTATTCTCCTTTGTTTGTGTAAGATAATACTATCACAAAGGAACTATACTGT
SARS-CoV-2 NC_045512.2 (29904) ------------------------------------------------------------------------------------------------
```

Section 1366
(131041) 131041 131054 131065 131076 131087 131098 131109 131120 131136
T4 NC_000866.4 (130307) AAACAACTTTGTGCAATCTTTGGAAAATAAAAAAGGACTCCCGAAGGAGTCCTCAACTTATGCTTTCTGCTTACCAAAACGAGAAGCATCATCTCG
SARS-CoV-2 NC_045512.2 (29904) --

Section 1367
(131137) 131137 131142 131153 131164 131175 131186 131197 131208 131219 131232
T4 NC_000866.4 (130403) AAGAACCGCACGTGCTCGGCGCATGATCTTCTCAACAGTTTGATTGATACGAGAGTTCGACCCACGCTTGTAGCCAGCGCGTTTAGAATCACCAAC
SARS-CoV-2 NC_045512.2 (29904) --

Section 1368
(131233) 131233 131241 131252 131263 131274 131285 131296 131307 131328
T4 NC_000866.4 (130499) TTTCTTTTCAACTGCTTTCTTTGCTTTAGCTTGTTTTGCCATTATAAATTCTCTTTTAAATGAAAATGCAGGACTTATTGGCATTGCCTGCGCAAG
SARS-CoV-2 NC_045512.2 (29904) --

Section 1369
(131329) 131329 131340 131351 131362 131373 131384 131395 131406 131424
T4 NC_000866.4 (130595) CCCTCAAGGGGAACATAGGTTTTTGGATATTTAACGACCAGGATAACCATAAACCCGTCATCATTCACATTCAAGAGGTACACCGTAAAACTGTCG
SARS-CoV-2 NC_045512.2 (29904) --

Section 1370
(131425) 131425 131439 131450 131461 131472 131483 131494 131505 131520
T4 NC_000866.4 (130691) GGGTCTTAAAACTATAATGATTCGCAAATCATTAATCAGACAGTTCGACGGCTCCTCGATTTAGCTCACACTAAGGCAGTGAATCTCCAATAAATT
SARS-CoV-2 NC_045512.2 (29904) --

Section 1371
(131521) 131521 131527 131538 131549 131560 131571 131582 131593 131604 131616
T4 NC_000866.4 (130787) ACTTCAGTGTTACCACAAAGTGACGAACTGCTTTTCGTGCAGCAGAAGCCAGAGGCTTAGCATATTTAAGTTCATCTTTTTCCTGAAGCTCAGCAG
SARS-CoV-2 NC_045512.2 (29904) --

Section 1372
(131617) 131617 131626 131637 131648 131659 131670 131681 131692 131712
T4 NC_000866.4 (130883) CTAATGCAGTTTGAGCAGGATTCAGATGTTTGAAATAACGCAGGATTTCAAGAGCTTCGGCTTCAACATCAATAGATGCGCCATAGTTTTCGTGAC
SARS-CoV-2 NC_045512.2 (29904) --

```
                                                                                                                    Section 1373
                         (131713) 131713      131725     131736     131747     131758     131769     131780     131791          131808
        T4 NC_000866.4 (130979) CATTATTCCATGCGTTTCGTTGCAGTTCAAGAGCGTGTTGTAATTGTTTAATCATTTAAAAATTCTCGTTAGAGATTAAAACTCGGTAGTCACGTT
 SARS-CoV-2 NC_045512.2  (29904) ------------------------------------------------------------------------------------------------
                                                                                                                    Section 1374
                         (131809) 131809         131824     131835     131846     131857     131868     131879     131890        131904
        T4 NC_000866.4 (131075) CTTCTGAATTTCATCTTCTTTCGACAGATCTCTCAGTTGTAGACTACCACATAGAATTGTTCGGTTAACTTATTATTCCGACACCCAATTCATATT
 SARS-CoV-2 NC_045512.2  (29904) ------------------------------------------------------------------------------------------------
                                                                                                                    Section 1375
                         (131905) 131905 131912     131923     131934     131945     131956     131967     131978     131989 132000
        T4 NC_000866.4 (131171) ATTATTTATATCACTTATAAAGACACGGAATAGCTTTATAGTGACAGGTAACGAATTTTTGTTTAATTTCTTTTGGCTGCTTAAGACCCAGAGCTA
 SARS-CoV-2 NC_045512.2  (29904) ------------------------------------------------------------------------------------------------
                                                                                                                    Section 1376
                         (132001) 132001    132011     132022     132033     132044     132055     132066     132077           132096
        T4 NC_000866.4 (131267) CAAAAGGATGCGGAACATTTCGAATTTGACCAACTGGAAGAGAAGTCAAATCACCAACTTCGCAGAAACCTTCAGGAACATCAGGACCGACAGAGT
 SARS-CoV-2 NC_045512.2  (29904) ------------------------------------------------------------------------------------------------
                                                                                                                    Section 1377
                         (132097) 132097       132110     132121     132132     132143     132154     132165     132176         132192
        T4 NC_000866.4 (131363) GAACTACACACAGTTCAGGAACTTCACCTTGAACACGTTTACCGATAATAAGTCCTGATTCTGTAACTTCTTCATCACCGGCTTGTGCAGGTTCAG
 SARS-CoV-2 NC_045512.2  (29904) ------------------------------------------------------------------------------------------------
                                                                                                                    Section 1378
                         (132193) 132193132198     132209     132220     132231     132242     132253     132264     132275    132288
        T4 NC_000866.4 (131459) AAACTAAAATAACATATTCACCGACAGCACGAATTGGTAGCTGTTGTACTTCAGACATCGTTTTTCCTTTTTGTTAACAGATGAATTAATAATAAC
 SARS-CoV-2 NC_045512.2  (29904) ------------------------------------------------------------------------------------------------
                                                                                                                    Section 1379
                         (132289) 132289  132297     132308     132319     132330     132341     132352     132363               132384
        T4 NC_000866.4 (131555) AAATAGTTCTTAAAGCATTTATTTACCAATAAATTGAAGCAAATGCTCAACTTTCATACCATTAACGGAAATCAATTTGTCAATAGAAAAACCTCG
 SARS-CoV-2 NC_045512.2  (29904) ------------------------------------------------------------------------------------------------
```

Section 1380

(132385) 132385 132396 132407 132418 132429 132440 132451 132462 132480
T4 NC_000866.4 (131651) CCACGCACCAAGCTCAACATCAAATACTGGAATCATGTCAGTAGATTCTTTCCGAGTAGATTCAGTCAATTTGCCAGTTTGCATGGTTGGCATAAA
SARS-CoV-2 NC_045512.2 (29904) --

Section 1381

(132481) 132481 132495 132506 132517 132528 132539 132550 132561 132576
T4 NC_000866.4 (131747) GTCTGCATCACGAGTACCTTTCATAGTACGAATAGTACCATCAGACTTTTCAAAAACTACGTTTGAAACACCCATGGACAATTTAGTTTTCAAAAT
SARS-CoV-2 NC_045512.2 (29904) --

Section 1382

(132577) 132577 132583 132594 132605 132616 132627 132638 132649 132660 132672
T4 NC_000866.4 (131843) TTCACGAATTGCTACTTTCTGCTCAGTTGTCAGTTTCATTTATTTACCTATTACAGTTTTAATATGAGTTGTTCCACGTTCTTTAAGGGTGGAAAG
SARS-CoV-2 NC_045512.2 (29904) --

Section 1383

(132673) 132673 132682 132693 132704 132715 132726 132737 132748 132768
T4 NC_000866.4 (131939) TAATTTTTGGCATTTTTCTAAATCAGATTTCCAACTATATGGTCTATCAATACAAACCCAATTTGTCTTATAATACTGTTTCCATTTAGAGAAAAA
SARS-CoV-2 NC_045512.2 (29904) --

Section 1384

(132769) 132769 132781 132792 132803 132814 132825 132836 132847 132864
T4 NC_000866.4 (132035) ATATTTCTTATACTCTACAGCAAATGAGATGTTCTCGTTAGAATAAGAACTAATTGCTGTGAGTTTTACCAAACGAAATTTCATTATTCACCACAG
SARS-CoV-2 NC_045512.2 (29904) --

Section 1385

(132865) 132865 132880 132891 132902 132913 132924 132935 132946 132960
T4 NC_000866.4 (132131) AATTCGTTGATATTTTCCCAGTTTAACTTATTCAAGTTTTTCTTAGGAACATTAAACACTTCAATACCTGCATTTCGCAGAATATCATCCCAACCG
SARS-CoV-2 NC_045512.2 (29904) --

Section 1386

(132961) 132961 132968 132979 132990 133001 133012 133023 133034 133045 133056
T4 NC_000866.4 (132227) GGTTTATTTTTGTCGTATGTTTCACAATAAACCAGCTTTTTAATACCAGATTGAGCTATCGCTTTTGCGCAATCTGGACAAGGAGAAAGTGTTACA
SARS-CoV-2 NC_045512.2 (29904) --

```
                                                                                                    Section 1387
                          (133057) 133057    133067      133078      133089      133100      133111      133122      133133        133152
        T4 NC_000866.4 (132323) TACATAGTAGCACCTTCAATAGAAGAACCATTTCGTGCAGCAAACAAAATTGCATTTAGTTCAGCATGAATTTCATTTTTAGATGACCATTCCGAG
SARS-CoV-2 NC_045512.2  (29904) ------------------------------------------------------------------------------------------------
                                                                                                    Section 1388
                          (133153) 133153       133166      133177      133188      133199      133210      133221      133232        133248
        T4 NC_000866.4 (132419) TGAGCACTACGATGTTCTTTCGCCAAAACAAAACGATCAGTTGAACCAAATGATACGCATTCAGGCTTATGACCTTGAATGATAGCATGTTTAGGC
SARS-CoV-2 NC_045512.2  (29904) ------------------------------------------------------------------------------------------------
                                                                                                    Section 1389
                          (133249) 133249133254    133265      133276      133287      133298      133309      133320      133331     133344
        T4 NC_000866.4 (132515) TTATTCAACAACCATCCTTGCTCAGCAGCATAATCACAACAGTTCACACCCCCTGCGGGTGAACCATTATACCCAGTAGAAATAATACGTCCATTC
SARS-CoV-2 NC_045512.2  (29904) ------------------------------------------------------------------------------------------------
                                                                                                    Section 1390
                          (133345) 133345 133353      133364      133375      133386      133397      133408      133419             133440
        T4 NC_000866.4 (132611) TTTTCAATTACTGCTCCTACCTTCCAGGAGCAACATTTTGATTCCTGCGATACTAAATATGCAATTTGAAGTACTGTACTCGCTTTCATTTCATAA
SARS-CoV-2 NC_045512.2  (29904) ------------------------------------------------------------------------------------------------
                                                                                                    Section 1391
                          (133441) 133441     133452      133463      133474      133485      133496      133507      133518        133536
        T4 NC_000866.4 (132707) TCACCAGATAAGCAGATTTAGCAGTTTCAACACGATAAATTTCGTGACGAAGTTTAGTTATACTTTTAATAACAGAACTAATTATATTCTGCCCAT
SARS-CoV-2 NC_045512.2  (29904) ------------------------------------------------------------------------------------------------
                                                                                                    Section 1392
                          (133537) 133537        133551      133562      133573      133584      133595      133606      133617      133632
        T4 NC_000866.4 (132803) CTTTAAAGCGGTTTTTCTTATCAATAAAAACTGCGCCAGTCATCTTTTTGTGAAGCTCAACTGGATACTTCGTCACAATAATAGCATCATACACAG
SARS-CoV-2 NC_045512.2  (29904) ------------------------------------------------------------------------------------------------
                                                                                                    Section 1393
                          (133633) 133633 133639     133650      133661      133672      133683      133694      133705      133716  133728
        T4 NC_000866.4 (132899) AAGGATGAATACTATTCACCAGAGTATCATTCATTAAAGTTATTCTAATGAACTGTGCTGTTTCAGAATCAAGCGCTCTATGATCGCCAGTATCAT
SARS-CoV-2 NC_045512.2  (29904) ------------------------------------------------------------------------------------------------
```

Section 1394

```
                            (133729) 133729  133738     133749     133760     133771     133782     133793     133804            133824
T4 NC_000866.4              (132995) TTTCAAGACAATTATCAATTATATCAGTTAAATTCATCATAGTACGCCATACACCCTTTGTGCTTCAACTAATCCATCAAAATCCAGTTTAAGATG
SARS-CoV-2 NC_045512.2      (29904)  ------------------------------------------------------------------------------------------------
```

Section 1395

```
                            (133825) 133825      133837     133848     133859     133870     133881     133892     133903           133920
T4 NC_000866.4              (133091) CGATATTTGATCGCCATCACCTGGATTCACAATTACTAATACTGAACGAGGAGTTTCGGTAATAACACGAACCGATGTTTCAGGAAATCGTTCAGA
SARS-CoV-2 NC_045512.2      (29904)  ------------------------------------------------------------------------------------------------
```

Section 1396

```
                            (133921) 133921         133936     133947     133958     133969     133980     133991     134002        134016
T4 NC_000866.4              (133187) AACCTTATTTACTAATTCCTGCGCAAATAGTTTAACTTTTTCTTGGAATTCCTTTAACAGTAATCGGTTTTTCACTTAGCAACATTTTGTTTTCCT
SARS-CoV-2 NC_045512.2      (29904)  ------------------------------------------------------------------------------------------------
```

Section 1397

```
                            (134017) 134017 134024     134035     134046     134057     134068     134079     134090     134101 134112
T4 NC_000866.4              (133283) CATTTGTTTTGGTAGAGCTATAATATCACAACTCTACCGTAAAGTAAACCATTAAATCGCTTTGAATTCCGCAGTTTGAGATTCAAAGCGAATATC
SARS-CoV-2 NC_045512.2      (29904)  ------------------------------------------------------------------------------------------------
```

Section 1398

```
                            (134113) 134113    134123     134134     134145     134156     134167     134178     134189            134208
T4 NC_000866.4              (133379) GCCTTTGATAACAAGCTCAGCATCAAGACCAAATACGACAATGATATGCGCGGAACCTGGATACAGTGTAATGGCAATAGAATCCACCTGGTCTGG
SARS-CoV-2 NC_045512.2      (29904)  ------------------------------------------------------------------------------------------------
```

Section 1399

```
                            (134209) 134209       134222     134233     134244     134255     134266     134277     134288           134304
T4 NC_000866.4              (133475) AAGCAAAGTGTTCAATACATGAGTCACTTGAGCATGGATTCGAAGCTCAGCTGCGTTATCAAGTTTTTCAAACATATTATTAGCGATAATTTGGCT
SARS-CoV-2 NC_045512.2      (29904)  ------------------------------------------------------------------------------------------------
```

Section 1400

```
                            (134305) 134305134310     134321     134332     134343     134354     134365     134376     134387     134400
T4 NC_000866.4              (133571) AAACACTACTTCTACGATTTTAGAGTAAGTCGGAAACATATTTACCTCACATAATTTTCTTCAAGCCAATCAATAACATCCAACGCATTATCAAAA
SARS-CoV-2 NC_045512.2      (29904)  ------------------------------------------------------------------------------------------------
```

```
                                                                                              Section 1401
                   (134401) 134401 134409     134420     134431     134442     134453     134464     134475          134496
       T4 NC_000866.4 (133667) GTTGAACCATCTACTCTGTCTTCTGTTTCATAATCAAGAACATCTAGGCCTACTCTTCCGTCAACAATAGGCCATAGACAAAATAGATATTTCTTT
 SARS-CoV-2 NC_045512.2 (29904) ------------------------------------------------------------------------------------------------
                                                                                              Section 1402
                   (134497) 134497     134508     134519     134530     134541     134552     134563     134574          134592
       T4 NC_000866.4 (133763) TCTTTTTCAATTTTATCACAAAGACGATAAATCTTTTCTAGGTTATTCATAAGTTTTCCATGGTAAAGGCAGTTTAGTTTTCTTTACTACTAGTTC
 SARS-CoV-2 NC_045512.2 (29904) ------------------------------------------------------------------------------------------------
                                                                                              Section 1403
                   (134593) 134593        134607     134618     134629     134640     134651     134662     134673         134688
       T4 NC_000866.4 (133859) AACATCGGGATTCTTTTCTCTTAATTTAAGACATTCCTCCCATGCTCTATTTTCACTAGTAAATACACAAAATTGCCCATTACTAGTACCAACTAA
 SARS-CoV-2 NC_045512.2 (29904) ------------------------------------------------------------------------------------------------
                                                                                              Section 1404
                   (134689) 134689 134695     134706     134717     134728     134739     134750     134761     134772 134784
       T4 NC_000866.4 (133955) ACCGCTATTTACAATAACAATAGCCCAAGTTTCATGGTGCCAAGCCATTAAAAATCTCCCGAAGCGACTTGCCAGCATTCAACACCGATACGACGC
 SARS-CoV-2 NC_045512.2 (29904) ------------------------------------------------------------------------------------------------
                                                                                              Section 1405
                   (134785) 134785  134794     134805     134816     134827     134838     134849     134860           134880
       T4 NC_000866.4 (134051) CACATTTCAACTACTTGAGTTCGGTCATCAATAGCTAATTTCACGTCAAAATGCGGTGCAATGTGTTTCCAGAAAATTTCTTCTTTAACTACATCG
 SARS-CoV-2 NC_045512.2 (29904) ------------------------------------------------------------------------------------------------
                                                                                              Section 1406
                   (134881) 134881      134893     134904     134915     134926     134937     134948     134959          134976
       T4 NC_000866.4 (134147) TCTTTACGGGTATCGCCTTGTTCGCGCTGACATTGCATAACTAATGGAACGCCAGCAATGTCCTCAACCCATTTACGGGTCATACGATAATATTTC
 SARS-CoV-2 NC_045512.2 (29904) ------------------------------------------------------------------------------------------------
                                                                                              Section 1407
                   (134977) 134977         134992     135003     135014     135025     135036     135047     135058     135072
       T4 NC_000866.4 (134243) GTTGGGTCTTCTTTAGTTCCACTTTCACGACCTGAAACGACTACGATTTGATAACCCATAAGAGCATACATCTTAGACAGTTCAACAACCATAGGA
 SARS-CoV-2 NC_045512.2 (29904) ------------------------------------------------------------------------------------------------
```

```
                                                                                                   Section 1408
                      (135073) 135073 135080     135091     135102     135113     135124     135135     135146     135157 135168
       T4 NC_000866.4 (134339) TTGATAACATCGGTATCGCATTTTTCAAGGTCATAAGGACCACGACCATTCATTTTAGCTAGTGTACCATCAACATCAAAAATAACTGCTTTTGGT
SARS-CoV-2 NC_045512.2  (29904) ------------------------------------------------------------------------------------------------
                                                                                                   Section 1409
                      (135169) 135169    135179     135190     135201     135212     135223     135234     135245          135264
       T4 NC_000866.4 (134435) TTACCAGGAGTCCCATTATATACTGGAAGACCGAGATACTCTCGCATGCTTTTATACATTGAACGTAAAACATCAATTGGTACTGCTTTAGTTCCG
SARS-CoV-2 NC_045512.2  (29904) ------------------------------------------------------------------------------------------------
                                                                                                   Section 1410
                      (135265) 135265       135278     135289     135300     135311     135322     135333     135344         135360
       T4 NC_000866.4 (134531) CGTTTTGAGTTACGTTTAACCAATTCAGTCCAAGGAACATCAAACACTTTATGTTCAACTTTCCAGCCGTATTCTTTGGCAAAAGTTTCCCATGCT
SARS-CoV-2 NC_045512.2  (29904) ------------------------------------------------------------------------------------------------
                                                                                                   Section 1411
                      (135361) 135361135366     135377     135388     135399     135410     135421     135432     135443   135456
       T4 NC_000866.4 (134627) AGGCGACGTTCAGGATTCAGGTTAGTATCTGAAATGATTACTCCCTTAACAGAATCGCCACCGTACAGAATACTTTTAGCTGTATCAAACTGCATA
SARS-CoV-2 NC_045512.2  (29904) ------------------------------------------------------------------------------------------------
                                                                                                   Section 1412
                      (135457) 135457  135465     135476     135487     135498     135509     135520     135531          135552
       T4 NC_000866.4 (134723) CCAGTTACGATACCTTCTTTCTTTTTGGTATACTTGTACTCATCGCGTTCTTCATGCGCCATAATAGATTGGCGATAGTCATCACGATTGATATTA
SARS-CoV-2 NC_045512.2  (29904) ------------------------------------------------------------------------------------------------
                                                                                                   Section 1413
                      (135553) 135553     135564     135575     135586     135597     135608     135619     135630          135648
       T4 NC_000866.4 (134819) TAAAACCCGGGATTCTTAGCAATAAATTCACGAGCCCAAGTACTCTTACCAGAACCAGGACAGCCAATAGTCAAAATAATCTTTTTCATTTATTTT
SARS-CoV-2 NC_045512.2  (29904) ------------------------------------------------------------------------------------------------
                                                                                                   Section 1414
                      (135649) 135649        135663     135674     135685     135696     135707     135718     135729         135744
       T4 NC_000866.4 (134915) TTCTCAACTAATGATTGAATATAATCATGTAGGTCTTTAGATGCTTTACCCCACTTATTTTGATATTCATTTTTGAGATTAGCACGTGATTGAGCT
SARS-CoV-2 NC_045512.2  (29904) ------------------------------------------------------------------------------------------------
```

```
                                                                                                          Section 1415
                         (135745) 135745 135751     135762     135773     135784     135795     135806     135817     135828   135840
       T4 NC_000866.4 (135011) AATAAAACATCATTAGTTGGAGGTAAAGATTCTAACCGCTGAATCTGGCGTCCATAAATCATTGCAGCCATCTCGGATTCATAAATCAATCCTTTG
SARS-CoV-2 NC_045512.2  (29904) ------------------------------------------------------------------------------------------------
                                                                                                          Section 1416
                         (135841) 135841   135850     135861     135872     135883     135894     135905     135916         135936
       T4 NC_000866.4 (135107) AGATGTTCAAATTGATGCCATGAAATCATTTACATTTATCCTCTTTTAGCTCTTGACGATAATAACATRTCATAGTTTTCTGGTCATGTACATATC
SARS-CoV-2 NC_045512.2  (29904) ------------------------------------------------------------------------------------------------
                                                                                                          Section 1417
                         (135937) 135937      135949     135960     135971     135982     135993     136004     136015         136032
       T4 NC_000866.4 (135203) GTTTTACATCATTAAGCCAAATACGAAATTCCTGAGAATCTTCAAATGGCATACCGACCCAGGCTTTACCATCAATAACTTTAACTTGCCAAGATA
SARS-CoV-2 NC_045512.2  (29904) ------------------------------------------------------------------------------------------------
                                                                                                          Section 1418
                         (136033) 136033         136048     136059     136070     136081     136092     136103     136114   136128
       T4 NC_000866.4 (135299) GTTTAGCTTCATCATATGACTTTATTTGCACAGGCCAATTAGGATGAACTGTTTCTTTCTTTACTTCTAGAGGCTTTGTCGAACAACCAACTAGAA
SARS-CoV-2 NC_045512.2  (29904) ------------------------------------------------------------------------------------------------
                                                                                                          Section 1419
                         (136129) 136129 136136     136147     136158     136169     136180     136191     136202     136213 136224
       T4 NC_000866.4 (135395) GACCAATAGATAATATTACTGCTGATAGTTTAATCATTTAGAAAGGTCCTGGATGTCTTCTGCGAACTTGTTGAAGGAGTTGTTGATTTGTTTTTC
SARS-CoV-2 NC_045512.2  (29904) ------------------------------------------------------------------------------------------------
                                                                                                          Section 1420
                         (136225) 136225    136235     136246     136257     136268     136279     136290     136301         136320
       T4 NC_000866.4 (135491) AACCAATCCTGGCTTATGAGCCACCACATCCGCCTTCTTTGCATCTTTGCGCAGTTTTTCATTTTCACGCTCAATAGCAGCAATTGCCTCACGATT
SARS-CoV-2 NC_045512.2  (29904) ------------------------------------------------------------------------------------------------
                                                                                                          Section 1421
                         (136321) 136321       136334     136345     136356     136367     136378     136389     136400         136416
       T4 NC_000866.4 (135587) TTTATTATTCATCGCATCAATATAATTATACTGAATTCGCAAATTATTTAATGCTAAGGCGTTTTCATTGGCCGTTTTTGTAATTTCAGTAACTGA
SARS-CoV-2 NC_045512.2  (29904) ------------------------------------------------------------------------------------------------
```

```
                                                                                                    Section 1422
                         (136417) 136417136422      136433     136444     136455     136466     136477     136488     136499   136512
         T4 NC_000866.4 (135683) TGTTTCTAATCTTTCTACCTTATGTTTTAAAATAATAGATGTTCCGCCAAATGCTATTACAAGTAATAGCAATCCAGCTGTAAAATTACTTACATG
SARS-CoV-2 NC_045512.2   (29904) ------------------------------------------------------------------------------------------------
                                                                                                    Section 1423
                         (136513) 136513  136521     136532     136543     136554     136565     136576     136587               136608
         T4 NC_000866.4 (135779) CATAAAGTTTTAATAACCTCTACAATATCGTCTTGAGAAAGACCGTTAATTAAAATATGATGTTCAGCTGGAGATTTAGAAATTTTAAAGCATGCC
SARS-CoV-2 NC_045512.2   (29904) ------------------------------------------------------------------------------------------------
                                                                                                    Section 1424
                         (136609) 136609     136620     136631     136642     136653     136664     136675     136686               136704
         T4 NC_000866.4 (135875) TCAACATCTTCTGCCATATCCGATGCACTACGATTTGGATTACTAATACCAAGGCGATGTTTTCCCGTCAAAGGATTAACGATGATATAGCATTTG
SARS-CoV-2 NC_045512.2   (29904) ------------------------------------------------------------------------------------------------
                                                                                                    Section 1425
                         (136705) 136705        136719     136730     136741     136752     136763     136774     136785           136800
         T4 NC_000866.4 (135971) CAGCTATTGATATGAACATTGGGTTGAGTCTGATTAATGAACACTTCACAATCATACTTAGCAAGTTGATTTTCTAAAAAGACTTTCATCTCTTCA
SARS-CoV-2 NC_045512.2   (29904) ------------------------------------------------------------------------------------------------
                                                                                                    Section 1426
                         (136801) 136801 136807     136818     136829     136840     136851     136862     136873     136884   136896
         T4 NC_000866.4 (136067) ACCGCATCAGGAAGCATATCACGGGCTTGCTCAAGACGACGATTTCGATATTCTTTAATGGTCGTTTTCCGCTTGACTTGCTTAGCTAAATCTTTC
SARS-CoV-2 NC_045512.2   (29904) ------------------------------------------------------------------------------------------------
                                                                                                    Section 1427
                         (136897) 136897   136906     136917     136928     136939     136950     136961     136972               136992
         T4 NC_000866.4 (136163) TTAAGACCAGTGATATATCCAACTCGACGATTTCCTTTGAATACAGAAATCCCATCTGTAGTATCACCGTATGCTTCAACGACCATTTCAGTAGTA
SARS-CoV-2 NC_045512.2   (29904) ------------------------------------------------------------------------------------------------
                                                                                                    Section 1428
                         (136993) 136993      137005     137016     137027     137038     137049     137060     137071               137088
         T4 NC_000866.4 (136259) ATAAGTTGTAAATCCATCATAAAGTCCTCATGTTATGTCAGTAAGACTACTATAACACAACACGAGGGACTTGTAAACAACTTAGTATCCTTCTGG
SARS-CoV-2 NC_045512.2   (29904) ------------------------------------------------------------------------------------------------
```

Section 1429

```
(137089)                   137089         137104     137115     137126     137137     137148     137159     137170       137184
T4 NC_000866.4 (136355)    GATAAATTTTTTATAATTTTTCAAAAAATTCTGTTCGATTTCACACATGACCTTTTCTTGACTATCGTACCCCTGGTATAAGCTCATGATGATACC
SARS-CoV-2 NC_045512.2 (29904) ------------------------------------------------------------------------------------------------
```

Section 1430

```
(137185)                   137185 137192     137203     137214     137225     137236     137247     137258     137269 137280
T4 NC_000866.4 (136451)    GAACAGGTGATCCATTCCAGCACCTTTAGCAACACCTTGTGCTTCCATTGCATAAGTCTTTCTATCCTTACCGCAATGCTTATTATGACAGTCAAG
SARS-CoV-2 NC_045512.2 (29904) ------------------------------------------------------------------------------------------------
```

Section 1431

```
(137281)                   137281    137291     137302     137313     137324     137335     137346     137357             137376
T4 NC_000866.4 (136547)    AACTAAAAACAGAGCTCGGTCTAAGTACTTCAGATAAGTCGTTTCAAATGCTTCAATTTTTCTGTATGAATATTCATCGTCAGCATACATTGCTTT
SARS-CoV-2 NC_045512.2 (29904) ------------------------------------------------------------------------------------------------
```

Section 1432

```
(137377)                   137377       137390     137401     137412     137423     137434     137445     137456          137472
T4 NC_000866.4 (136643)    AAGATCATCTGATGCACCATCAATAATAGTCTTAAACAATTTTTCTGGATTATCTAATGAACTTTTTGTACTATGAAGAGACACGTACCAGTCAGA
SARS-CoV-2 NC_045512.2 (29904) ------------------------------------------------------------------------------------------------
```

Section 1433

```
(137473)                   137473137478     137489     137500     137511     137522     137533     137544     137555   137568
T4 NC_000866.4 (136739)    CTTAATTTTAAAATGAGAACCATCTTTCATCACAGCAACATAGCCTTCGATGTTTTCTGCATTTTTAGCTTCTTCTATCCATTTAGGGCTATCGAT
SARS-CoV-2 NC_045512.2 (29904) ------------------------------------------------------------------------------------------------
```

Section 1434

```
(137569)                   137569  137577     137588     137599     137610     137621     137632     137643             137664
T4 NC_000866.4 (136835)    TTCGTATCGTTCAACTAGATACGGACGAAGAGTAGCATCTTTATAAATATCATCGTATGAAATGTATTCACCCGTTTCGTTTTCACGAACATTCAG
SARS-CoV-2 NC_045512.2 (29904) ------------------------------------------------------------------------------------------------
```

Section 1435

```
(137665)                   137665     137676     137687     137698     137709     137720     137731     137742             137760
T4 NC_000866.4 (136931)    TAAAATAATTTTCATCTCTTGATAAGCAAGAACGATTCTATTCGTCGGGGCAACGAATTCGAAGTTAGCAGTAAATCCATCTTCAGCTAATTCTTT
SARS-CoV-2 NC_045512.2 (29904) ------------------------------------------------------------------------------------------------
```

```
                                                                                                         Section 1436
                      (137761) 137761          137775     137786     137797     137808     137819     137830     137841       137856
      T4 NC_000866.4 (137027) AAGTCTATCACGCAACCGATGGTGATTAATATTCATCAAAATTCCATTAGCCATTAAAGCCTGCTCAGATTTGATTGAACCCTTTGATTTGAACAG
SARS-CoV-2 NC_045512.2  (29904) ------------------------------------------------------------------------------------------------
                                                                                                         Section 1437
                      (137857) 137857 137863     137874     137885     137896     137907     137918     137929     137940  137952
      T4 NC_000866.4 (137123) AATTTCATCACCGTCTAAATAAGTTGATACCAAAGACCCGTCTTCTTTTGTTAGAATATAATCAACATCGTTTAAATCGATATTCATCGTGAACGG
SARS-CoV-2 NC_045512.2  (29904) ------------------------------------------------------------------------------------------------
                                                                                                         Section 1438
                      (137953) 137953   137962     137973     137984     137995     138006     138017     138028          138048
      T4 NC_000866.4 (137219) ATTTTCATTCAAGTTAAAAAACTTTTCCATAGGACGAGAAGCAATTCTTACTGGTTTTTCTCCATCCATTTCAAACATAATTCCACGACATTCTAG
SARS-CoV-2 NC_045512.2  (29904) ------------------------------------------------------------------------------------------------
                                                                                                         Section 1439
                      (138049) 138049      138061     138072     138083     138094     138105     138116     138127          138144
      T4 NC_000866.4 (137315) TGCATCTGGAAGTAACCAATCAGAATAAGATGCATAATTATATGAGAAAATTCTGTAAGTTCTTCCAGATGCACTTACATCATCTGAGTAAAAAAA
SARS-CoV-2 NC_045512.2  (29904) ------------------------------------------------------------------------------------------------
                                                                                                         Section 1440
                      (138145) 138145         138160     138171     138182     138193     138204     138215     138226       138240
      T4 NC_000866.4 (137411) CTTACGCTGCGAATCCTTACATAGTTCCATTAAATTGTTAAAAAGTTCTTGCATTGTGTATCCTCTTTTGTGTTTTGAATATAGTACCACACTCCA
SARS-CoV-2 NC_045512.2  (29904) ------------------------------------------------------------------------------------------------
                                                                                                         Section 1441
                      (138241) 138241 138248     138259     138270     138281     138292     138303     138314     138325 138336
      T4 NC_000866.4 (137507) TGTGGAAGCATCATTTTTTCTTGTGTTGAATATTCCAAGGCGGGTTAAACAGTTTAATGAATAGAGGCTCCTCTAAGTCAATCGTTGCGATTGTCA
SARS-CoV-2 NC_045512.2  (29904) ------------------------------------------------------------------------------------------------
                                                                                                         Section 1442
                      (138337) 138337    138347     138358     138369     138380     138391     138402     138413          138432
      T4 NC_000866.4 (137603) TTGTACCTAACTCATTTGTCATAGAAAGATTAAAACATTGGCGGGCGTAAAATTCAACTTTGCTTCCTTCCTTTAGCGCAGAATGAATTAATGCAG
SARS-CoV-2 NC_045512.2  (29904) ------------------------------------------------------------------------------------------------
```

```
                                                                                              Section 1443
                         (138433) 138433      138446    138457    138468    138479    138490    138501    138512       138528
       T4 NC_000866.4 (137699) ATTTAGTAGAATCAGACGTTTTGTCTTTGCGGTTAATAGCAGTTCTATAATAGTTTATTCTTTTACGTAAATTTTTAGTTTTTCCAATATAAACAA
SARS-CoV-2 NC_045512.2  (29904) ------------------------------------------------------------------------------------------------
                                                                                              Section 1444
                         (138529) 138529138534    138545    138556    138567    138578    138589    138600    138611   138624
       T4 NC_000866.4 (137795) GCTCATCATTTATAGCAATAGCATAAATTACGTTATACTTGTTTGGAATAGATAATTGTTTTATACTTCCATTGTCGTCTAATTCTAGCTCAGTAT
SARS-CoV-2 NC_045512.2  (29904) ------------------------------------------------------------------------------------------------
                                                                                              Section 1445
                         (138625) 138625  138633    138644    138655    138666    138677    138688    138699             138720
       T4 NC_000866.4 (137891) ATTTAATAAATGAATATTCTGTTGCAATTTCTTTCATAATAAAATGGGCCTTGCGGCCCACTCCTTAAAAGTATTTTTTAAAACTCATCATAACTT
SARS-CoV-2 NC_045512.2  (29904) ------------------------------------------------------------------------------------------------
                                                                                              Section 1446
                         (138721) 138721     138732    138743    138754    138765    138776    138787    138798          138816
       T4 NC_000866.4 (137987) TATCATCAACATCATTATCAATCTGTGCAACAAGGTAAGATGACAGTTCTACTTCTTGCGGCGCGGATTGAACATTATCAGAATTAAGATATTCAC
SARS-CoV-2 NC_045512.2  (29904) ------------------------------------------------------------------------------------------------
                                                                                              Section 1447
                         (138817) 138817        138831    138842    138853    138864    138875    138886    138897       138912
       T4 NC_000866.4 (138083) GAATCCAAGGATATGGATGTTTAACCGGAGCATCGGTAATTGGGCATGGAAGACCGCACTGTTTCATACGAGATACAGTTAAGTAATCAATAAAGC
SARS-CoV-2 NC_045512.2  (29904) ------------------------------------------------------------------------------------------------
                                                                                              Section 1448
                         (138913) 138913 138919    138930    138941    138952    138963    138974    138985    138996   139008
       T4 NC_000866.4 (138179) TCCACATGCTATTTGTATTTAATCCAGGAACATCACCATCTTTAAATAAATGAACTGCCCAGTCTTTTTCCTGGCGGTTAACTTCCATGAAAATAT
SARS-CoV-2 NC_045512.2  (29904) ------------------------------------------------------------------------------------------------
                                                                                              Section 1449
                         (139009) 139009  139018    139029    139040    139051    139062    139073    139084             139104
       T4 NC_000866.4 (138275) CAACTGCTTCTTGTTCACACTCTTGGGCAATTTTAACCCATTCATCACCATCAGTACCGGATTGAAGTTGACGAATAATATATTGGGTGCCTTTAA
SARS-CoV-2 NC_045512.2  (29904) ------------------------------------------------------------------------------------------------
```

Section 1450

```
                              139105     139117     139128     139139     139150     139161     139172     139183     139200
                     (139105)
T4 NC_000866.4       (138371) GGTGAAGCTGTTCATCACGTGCAATGAACTTCATAATCTTGGCATTACCTTCCATGATTTCCATGTTCTTATGGAAGTTAAAGGTACCGTATGTTC
SARS-CoV-2 NC_045512.2 (29904) ------------------------------------------------------------------------------------------------
```

Section 1451

```
                              139201     139216     139227     139238     139249     139260     139271     139282     139296
                     (139201)
T4 NC_000866.4       (138467) GATTGAGCTCGCTAATTCTCAACCCGTTCTCTTATGAACTGCTGCATGTTACCATGCAGTCCAGACTATATCACAATCCCGGAGGGATTCTCCCCA
SARS-CoV-2 NC_045512.2 (29904) ------------------------------------------------------------------------------------------------
```

Section 1452

```
                              139297 139304 139315 139326 139337 139348 139359 139370 139381 139392
                     (139297)
T4 NC_000866.4       (138563) TTTCGAGTCGCTTGACCCTACGTCCGAAGACTAGTCGTTGAACCTTGTTTTGCAATTGTCGCCGTGCCATCGTTTATATGTTGATGGTGATAAATC
SARS-CoV-2 NC_045512.2 (29904) ------------------------------------------------------------------------------------------------
```

Section 1453

```
                              139393 139403 139414 139425 139436 139447 139458 139469 139488
                     (139393)
T4 NC_000866.4       (138659) TTTCTTATCACAATATGGACAATTCAATTGAATTCTATTTGGATTTAATTTACATCTGTCATTATGTTTTAGATAAAATCCAGGACCTTTACCAAT
SARS-CoV-2 NC_045512.2 (29904) ------------------------------------------------------------------------------------------------
```

Section 1454

```
                              139489 139502 139513 139524 139535 139546 139557 139568 139584
                     (139489)
T4 NC_000866.4       (138755) GTGACCACAGAAATCACATGTGATTTCTTTTTGTGCAGGATGGGTCCCGTTCTTAACCATTTCTAATGTTTTTGCTGATGTTCTTTTCTTATGTTC
SARS-CoV-2 NC_045512.2 (29904) ------------------------------------------------------------------------------------------------
```

Section 1455

```
                              139585139590 139601 139612 139623 139634 139645 139656 139667 139680
                     (139585)
T4 NC_000866.4       (138851) TTCCATCAGAATCTTTAGGTATAGGTCCGTTAGCATCTATCCAAATTTTTCTATAGTTCAATTGTGCATCCTCTTAAAAGTATAATCATATTTATA
SARS-CoV-2 NC_045512.2 (29904) ------------------------------------------------------------------------------------------------
```

Section 1456

```
                              139681 139689 139700 139711 139722 139733 139744 139755 139776
                     (139681)
T4 NC_000866.4       (138947) TTATACTAATTAAAGGTGCAAGCAAAACCTTGGCTGCTAGTTTTCCATAAAGGACTTTCCAGCAATTAAAGGAGTTTTCGATAAACGTTACCGTTT
SARS-CoV-2 NC_045512.2 (29904) ------------------------------------------------------------------------------------------------
```

```
                                                                                                    Section 1457
                       (139777) 139777     139788     139799     139810     139821     139832     139843     139854         139872
        T4 NC_000866.4 (139043) AAAGGCGCATTTTACGCAAAAGATACATAAAAACGAATAGCTTCTAAGGCATTAATAACGTGCAAACAGAGGTAAAGAGATTTCATCAGATCTCGT
SARS-CoV-2 NC_045512.2  (29904) ------------------------------------------------------------------------------------------------
                                                                                                    Section 1458
                       (139873) 139873        139887     139898     139909     139920     139931     139942     139953         139968
        T4 NC_000866.4 (139139) TTAGCTCTTTGCTCAACGTCTTTATCAGCTAGAATTAGACCTTGGTCTTTATAATATTCAACCATGTCTTTAGCATTTTCCCATTCACGGGTTTTA
SARS-CoV-2 NC_045512.2  (29904) ------------------------------------------------------------------------------------------------
                                                                                                    Section 1459
                       (139969) 139969 139975     139986     139997     140008     140019     140030     140041     140052  140064
        T4 NC_000866.4 (139235) ACCAAGACATCATCGTAATATCGCCCAATGGACTCAGCACGTTTCATAATAGCTTCATCTAATACAATCTCATCAAACACCTTCGATGGATCAGTA
SARS-CoV-2 NC_045512.2  (29904) ------------------------------------------------------------------------------------------------
                                                                                                    Section 1460
                       (140065) 140065   140074     140085     140096     140107     140118     140129     140140              140160
        T4 NC_000866.4 (139331) TAAAGATTTCGCATGATATGAGTATATGAACGACTGTGAATAGTTTCACTAAAAGTCCATGTAGCAACCCATGTATCAAGGCTTGGGTCTGAAATT
SARS-CoV-2 NC_045512.2  (29904) ------------------------------------------------------------------------------------------------
                                                                                                    Section 1461
                       (140161) 140161      140173     140184     140195     140206     140217     140228     140239         140256
        T4 NC_000866.4 (139427) AATGACATAAGTACAGCAGATGGCGCACGACCCTGAATGCTATCCAAAAGTGATTGATACTTCAGGTTGTTAGTAAAAATATTTTGCTGATACTGA
SARS-CoV-2 NC_045512.2  (29904) ------------------------------------------------------------------------------------------------
                                                                                                    Section 1462
                       (140257) 140257          140272     140283     140294     140305     140316     140327     140338       140352
        T4 NC_000866.4 (139523) GGAAGCTTATTAAATTGCGCAGCATCCATCATTAAGTTTACTTCTTCAGGACGCCAAAAAAAACTGATCTGCCGCTCAATGAGTTCTTCAAATACT
SARS-CoV-2 NC_045512.2  (29904) ------------------------------------------------------------------------------------------------
                                                                                                    Section 1463
                       (140353) 140353 140360     140371     140382     140393     140404     140415     140426     140437  140448
        T4 NC_000866.4 (139619) CGATGTCGTTGAATATCATATCGAGCTAAACCTAATCCAGAACCAAAGAACATCGGTTCATTCAAAACATCAACTGGATTTGTATTAAAAACTGTA
SARS-CoV-2 NC_045512.2  (29904) ------------------------------------------------------------------------------------------------
```

```
                                                Section 1464
                       (140449) 140449    140459    140470    140481    140492    140503    140514    140525    140544
          T4 NC_000866.4 (139715) CTCATTTAGAATCCTTAAATTTACATTTATCATAATGCCATCTTAAAGCATTGCCTTTATTAACTTTCTTACCACAGTGCGGGCAAGGTGGATACT
   SARS-CoV-2 NC_045512.2  (29904) ------------------------------------------------------------------------------------------------

                                                Section 1465
                       (140545) 140545    140558    140569    140580    140591    140602    140613    140624    140640
          T4 NC_000866.4 (139811) CTGCTCCTTGTTTAGTTCTCAAAGAAATCAAATCACGGGTTTCTTTAGTATGAGGAACATCTCTTGTCGGAGAAATTGTACCATACATCGGATTAA
   SARS-CoV-2 NC_045512.2  (29904) ------------------------------------------------------------------------------------------------

                                                Section 1466
                       (140641) 140641 140646    140657    140668    140679    140690    140701    140712    140723    140736
          T4 NC_000866.4 (139907) GCACGCCGACTTTAGCAGCAGCTATATTCTTTTTAGCTTCTTCAGTTTTAGGCTTTCTCATCTTTTTCTTAGGTTTCTTCGCTTTTAGCCCTTCCT
   SARS-CoV-2 NC_045512.2  (29904) ------------------------------------------------------------------------------------------------

                                                Section 1467
                       (140737) 140737    140745    140756    140767    140778    140789    140800    140811    140832
          T4 NC_000866.4 (140003) TTGGTTGCCGCCGATATTTTGTGTTTGGTTTCTTCTGTTAAACGCAGACCTGTTTTCGCTTTTTGACATTTTCAGTCTCGTTTCTTCAGAAATGAC
   SARS-CoV-2 NC_045512.2  (29904) ------------------------------------------------------------------------------------------------

                                                Section 1468
                       (140833) 140833    140844    140855    140866    140877    140888    140899    140910    140928
          T4 NC_000866.4 (140099) CGTACCTTTGCGAAATTGCGAATTTAACTTCTTTGCATGGGCATATATTTTAGAATGAACTTTATAATGACGTTTCTTAGTTCCTTTCATATTGCA
   SARS-CoV-2 NC_045512.2  (29904) ------------------------------------------------------------------------------------------------

                                                Section 1469
                       (140929) 140929    140943    140954    140965    140976    140987    140998    141009    141024
          T4 NC_000866.4 (140195) CATCATGAAAAATGCGAATATAACAGATTTTACCGGATAAATCTTTGATAAAATAGCATGCGCTATAAAATGCTCTCTAGCTGTTAATTCAACTAA
   SARS-CoV-2 NC_045512.2  (29904) ------------------------------------------------------------------------------------------------

                                                Section 1470
                       (141025) 141025 141031    141042    141053    141064    141075    141086    141097    141108    141120
          T4 NC_000866.4 (140291) ATTTTCTTTATCATCAGAACCTCCCATGCATCTAGGGATTATATGATGTGTCTCTTTATATTCGGATAAAGGTTCCCGAGCCTGAGCTCGGGAAAT
   SARS-CoV-2 NC_045512.2  (29904) ------------------------------------------------------------------------------------------------
```

```
                                                                                                        Section 1471
                           (141121) 141121   141130     141141     141152     141163     141174     141185     141196          141216
           T4 NC_000866.4 (140387) TAGGTCGTTATAGATTTTTTGATAATTCATTACAATTTACACGCTGCACAATCATCGGCTTTAGGAGTTTCTATTTCATAATCATCAGTACCAGAA
      SARS-CoV-2 NC_045512.2 (29904) ------------------------------------------------------------------------------------------------
                                                                                                        Section 1472
                           (141217) 141217      141229     141240     141251     141262     141273     141284     141295          141312
           T4 NC_000866.4 (140483) CCATCACGGGTATTATGATAATAGAAATTTTTAATGCCATAATACCATCCGTATAGCATGTCATCAATCATTATTGACATTGGAACCTTTCCTTTT
      SARS-CoV-2 NC_045512.2 (29904) ------------------------------------------------------------------------------------------------
                                                                                                        Section 1473
                           (141313) 141313         141328     141339     141350     141361     141372     141383     141394     141408
           T4 NC_000866.4 (140579) GGAAAAATCTGCGGGTCATAATATGTATTCGCTGAAGCTGATTGACATACCCATTTCAGCATAATAGCTACCTGCGTAAGATAAGGTTTATTACCT
      SARS-CoV-2 NC_045512.2 (29904) ------------------------------------------------------------------------------------------------
                                                                                                        Section 1474
                           (141409) 141409 141416     141427     141438     141449     141460     141471     141482     141493 141504
           T4 NC_000866.4 (140675) TTCTTAGCTAATTTCCATGTATAATCATAGAGGTCTATGTTATGCTCAATATTGGGCACGACTTGATTAAAGGAACCCTCTTTTGATTCTTTAACA
      SARS-CoV-2 NC_045512.2 (29904) ------------------------------------------------------------------------------------------------
                                                                                                        Section 1475
                           (141505) 141505    141515     141526     141537     141548     141559     141570     141581          141600
           T4 NC_000866.4 (140771) CTTACCGGTCCACGTGGAGGCTCGTAGCCGTTTGTACTGTTAGAAACTTGGGAAGATGACTCACATGGCATAAGTGCTGATAATGTGCTATTACGG
      SARS-CoV-2 NC_045512.2 (29904) ------------------------------------------------------------------------------------------------
                                                                                                        Section 1476
                           (141601) 141601       141614     141625     141636     141647     141658     141669     141680          141696
           T4 NC_000866.4 (140867) ATGCCAAATAGCTTAAGGTCTTCCCGCAGCGCCGACCAGTCACAAACGTATTTTGGAGCTGCGATTTGGTCAATCTTTTTATTGTACCAGTCGATA
      SARS-CoV-2 NC_045512.2 (29904) ------------------------------------------------------------------------------------------------
                                                                                                        Section 1477
                           (141697) 141697141702     141713     141724     141735     141746     141757     141768     141779    141792
           T4 NC_000866.4 (140963) GGTAATTCGCCTCGAGACCAACGAGTGTCTGAATAATATTCCGAAGGTCCTTTTTCTTTGGCGAGCTTAATGGATGCTTTAATGAGTCCATACTGT
      SARS-CoV-2 NC_045512.2 (29904) ------------------------------------------------------------------------------------------------
```

```
                                                                                                        Section 1478
                   (141793) 141793  141801      141812      141823      141834      141845      141856      141867         141888
 T4 NC_000866.4    (141059) AATCTCTCAAATAGTTCATGTGTTAAATCGTTAGCATCTTCATAAGAAGCAAAGTTACTTGCCAGCCAAGCTGCATAGTTGGTAACACCTACACCG
 SARS-CoV-2 NC_045512.2 (29904) ------------------------------------------------------------------------------------------------
                                                                                                        Section 1479
                   (141889) 141889     141900      141911      141922      141933      141944      141955      141966         141984
 T4 NC_000866.4    (141155) AGGTTACGACGCTTTTTAGCTTTTTCTGCTTCAGGAACCGGATATCCTTGGTAATCCAACAGATTATCAAGAGCACGAACTTGAACTTCTGCCAAT
 SARS-CoV-2 NC_045512.2 (29904) ------------------------------------------------------------------------------------------------
                                                                                                        Section 1480
                   (141985) 141985        141999      142010      142021      142032      142043      142054      142065         142080
 T4 NC_000866.4    (141251) TCATTAATTTTATCTTGGTCTTGCCAGTCAAAATTATCTAATACGAATGCAGAGAGAGTACACAATCCAATTTCAGCATCAGGGCTATTCACATCA
 SARS-CoV-2 NC_045512.2 (29904) ------------------------------------------------------------------------------------------------
                                                                                                        Section 1481
                   (142081) 142081 142087     142098      142109      142120      142131      142142      142153      142164  142176
 T4 NC_000866.4    (141347) TTTGTTGGAATAGCAATTTCACAGCACAAGTTACTCTGACGAATAGGTGCCTTTTCACGAATAAACGGAGTATAGTTATTCGTATTATCAATGAAC
 SARS-CoV-2 NC_045512.2 (29904) ------------------------------------------------------------------------------------------------
                                                                                                        Section 1482
                   (142177) 142177   142186      142197      142208      142219      142230      142241      142252         142272
 T4 NC_000866.4    (141443) TGCACATAAATCCTTGCTGTTCCTGAACGTTCAGTCATGAGCAATTCAAATAGTTCACGGGCTTTAATACGCTTTTTACGAATATTAGGGTCTTTT
 SARS-CoV-2 NC_045512.2 (29904) ------------------------------------------------------------------------------------------------
                                                                                                        Section 1483
                   (142273) 142273      142285      142296      142307      142318      142329      142340      142351         142368
 T4 NC_000866.4    (141539) TCTGCTGCTTCGTATAATTCACGGAAACGGTCTTGGTCTTTAAAATAAGAATAATAAAGCTCGCCACCCATTTCATGCGGACTGAACAAAGTAATG
 SARS-CoV-2 NC_045512.2 (29904) ------------------------------------------------------------------------------------------------
                                                                                                        Section 1484
                   (142369) 142369         142384      142395      142406      142417      142428      142439      142450    142464
 T4 NC_000866.4    (141635) TAATCGTTTTTTCCAAAACGTTCCATCATCAAATCATTCAGTTGAACACCATAATCCATATGACGAATGCGGTTTTCTTCTACGCCTTTGTTATTT
 SARS-CoV-2 NC_045512.2 (29904) ------------------------------------------------------------------------------------------------
```

```
                                                                                                                        Section 1485
                        (142465) 142465 142472 142483 142494 142505 142516 142527 142538 142549 142560
      T4 NC_000866.4 (141731) TTCAAAACGAGAAGATTTTCAACTTCCAAATGCCAAATAGGATAATAAGCAGTAGCAGCGCCGCCACGAATTCCACCCTGTGAACATGATTTAACT
SARS-CoV-2 NC_045512.2  (29904) ------------------------------------------------------------------------------------------------
                                                                                                                        Section 1486
                        (142561) 142561 142571 142582 142593 142604 142615 142626 142637 142656
      T4 NC_000866.4 (141827) GCAGTCTGAAAATGTTTCCAAAAAGGAATAACACCAGTATGGCGTACTTCACCCATGCCAATCTTAGAACCTTCGGCACGAATCATACCAACGTTA
SARS-CoV-2 NC_045512.2  (29904) ------------------------------------------------------------------------------------------------
                                                                                                                        Section 1487
                        (142657) 142657 142670 142681 142692 142703 142714 142725 142736 142752
      T4 NC_000866.4 (141923) ATACCAATTCCAGCGCGTTTAGAGATATATTCAACAATTGAAGCAGAAGCCTTATTGATAGACTTCAATGAATCTCCTGCCTCAATAACAACACAT
SARS-CoV-2 NC_045512.2  (29904) ------------------------------------------------------------------------------------------------
                                                                                                                        Section 1488
                        (142753) 142753142758 142769 142780 142791 142802 142813 142824 142835 142848
      T4 NC_000866.4 (142019) GAACTAAACTGTCGAGTCGGAGTACGACAACCAGCCATAATAGGAGTTGGCAATGAAATCTGTCGAGTTGATACTGCTTCATAAAAACGGATAACA
SARS-CoV-2 NC_045512.2  (29904) ------------------------------------------------------------------------------------------------
                                                                                                                        Section 1489
                        (142849) 142849 142857 142868 142879 142890 142901 142912 142923 142944
      T4 NC_000866.4 (142115) TGTTTTAATCTATCAACAGGTTCATCTTGATGCAGTGCCATTCCAATAGTCATAAATGCAAACTGTGGAGTTTCATAAATTTGACCAGTGGTTTTA
SARS-CoV-2 NC_045512.2  (29904) ------------------------------------------------------------------------------------------------
                                                                                                                        Section 1490
                        (142945) 142945 142956 142967 142978 142989 143000 143011 143022 143040
      T4 NC_000866.4 (142211) TCTTTAACTAGATATTTTTCTTTTAATTGCATTGCCCCGGAATAAGTAAATTCCATATCCCGTTCGTGCTTAATTTTTGATTCTAAAAATGTAATT
SARS-CoV-2 NC_045512.2  (29904) ------------------------------------------------------------------------------------------------
                                                                                                                        Section 1491
                        (143041) 143041 143055 143066 143077 143088 143099 143110 143121 143136
      T4 NC_000866.4 (142307) TCTTCTGCTGAATATTTTGACAATAATTCAGGGTCGTATTTACCTGCATTTACACAATAAGAAATATGGTCAATAAATGAACGTGGTTCATACTGC
SARS-CoV-2 NC_045512.2  (29904) ------------------------------------------------------------------------------------------------
```

```
                                                                                                                   Section 1492
                         (143137) 143137 143143      143154      143165      143176      143187      143198      143209      143220    143232
           T4 NC_000866.4 (142403) CCATAAACATGCTTACGAAGAGCAAACATTAAACAGCGTGCAGCTACATATTGATAATCAGGTTCTTCAACCGAAATAGAATTCGCAGCAGCCTTA
      SARS-CoV-2 NC_045512.2 (29904) ------------------------------------------------------------------------------------------------
                                                                                                                   Section 1493
                         (143233) 143233   143242      143253      143264      143275      143286      143297      143308              143328
           T4 NC_000866.4 (142499) ATGACAATAGTCTGAATATCATCAGTTGTCATTCCATCACGGAGATAAGATTTAATATTTTCATATAATTCATAAGGATCTACTGATGTTCCTTCA
      SARS-CoV-2 NC_045512.2 (29904) ------------------------------------------------------------------------------------------------
                                                                                                                   Section 1494
                         (143329) 143329      143341      143352      143363      143374      143385      143396      143407          143424
           T4 NC_000866.4 (142595) GCTGCCCAAGATAAAACTTTAATAATTTTTTGTGGGTCAAAGCTCTGAGAAACACCACTACTTTTGATAACATTAATTAATTGCATAAGTCCTCAA
      SARS-CoV-2 NC_045512.2 (29904) ------------------------------------------------------------------------------------------------
                                                                                                                   Section 1495
                         (143425) 143425         143440      143451      143462      143473      143484      143495      143506     143520
           T4 NC_000866.4 (142691) CTTGAAAATCGTCTTTAAACAATCGGTTAACTATATGAGCTATTATATCACCATGACACGGCTTTGGTTTACATGTGCATCCTAGCCTCATTCCAC
      SARS-CoV-2 NC_045512.2 (29904) ------------------------------------------------------------------------------------------------
                                                                                                                   Section 1496
                         (143521) 143521 143528      143539      143550      143561      143572      143583      143594      143605 143616
           T4 NC_000866.4 (142787) GTAAAGGCTCTAAATGTGCTTTAGTTATTTCTCCGGATTTAATTCGACGTATAAAATCTTTTTTGAATAATTCAATGGCAGCCTCCCGGCTGCCAG
      SARS-CoV-2 NC_045512.2 (29904) ------------------------------------------------------------------------------------------------
                                                                                                                   Section 1497
                         (143617) 143617    143627      143638      143649      143660      143671      143682      143693             143712
           T4 NC_000866.4 (142883) CATCTTTACCGACGTAATTTCCCCAAAATGTACCGCGGTGAATATTAACATCAAAGTCGGATTTATATTTATTCACTACCCGGCATAGACGGCCCA
      SARS-CoV-2 NC_045512.2 (29904) ------------------------------------------------------------------------------------------------
                                                                                                                   Section 1498
                         (143713) 143713       143726      143737      143748      143759      143770      143781      143792          143808
           T4 NC_000866.4 (142979) CGCTGGAATAATTCGTCATATTGTTTTTCCGTTAAAACAGTAATATCGTAGTAACAGTCAGAAGAAGTTTTAACTGTGGAAATTTTATTATCAAAA
      SARS-CoV-2 NC_045512.2 (29904) ------------------------------------------------------------------------------------------------
```

```
                                                                                                Section 1499
                          (143809) 143809 143814    143825     143836     143847     143858     143869     143880     143891   143904
         T4 NC_000866.4   (143075) TACTCACGAGTCATTTTATGAGTATAGTATTTTTTACCATAAATGGTAATAGGCTGTTCTGGTCCTGGAACTTCTAACTCGCTTGGGTTAGGAAGT
SARS-CoV-2 NC_045512.2     (29904) ------------------------------------------------------------------------------------------------
                                                                                                Section 1500
                          (143905) 143905  143913     143924     143935     143946     143957     143968     143979         144000
         T4 NC_000866.4   (143171) GTAAAAAGAACTACACCAGAAGTATCTTTAAATCGTAAAATCATATATCCTCGCAATAATAAAATTACACCGCCATCTTTCCTTTAATAGGAGGGT
SARS-CoV-2 NC_045512.2     (29904) ------------------------------------------------------------------------------------------------
                                                                                                Section 1501
                          (144001) 144001     144012     144023     144034     144045     144056     144067     144078       144096
         T4 NC_000866.4   (143267) GTGATACATAGTTGTTAAGAACGAAATCTTTAGGCCTAAGTTTAAGAACATATTTTAATTGTTCTTTAGTAGAAAGATATCGGAATTTATAAGGTA
SARS-CoV-2 NC_045512.2     (29904) ------------------------------------------------------------------------------------------------
                                                                                                Section 1502
                          (144097) 144097        144111     144122     144133     144144     144155     144166     144177     144192
         T4 NC_000866.4   (143363) GACCACTTATTACCAGCTCACAAAGCTCTTTAGGTTCACGCCTCAAAATTTCTTTACATTGTTCTACGTGATTCATATAGATATGAGTATTACCAC
SARS-CoV-2 NC_045512.2     (29904) ------------------------------------------------------------------------------------------------
                                                                                                Section 1503
                          (144193) 144193 144199    144210     144221     144232     144243     144254     144265     144276   144288
         T4 NC_000866.4   (143459) CAGAAAATATCAAATCCCCTGGAATAAGATTACACATCTTAGCTACAATATGAACTAACGTAGCATATGACGCAATATTAAACGGTAGCATTATGT
SARS-CoV-2 NC_045512.2     (29904) ------------------------------------------------------------------------------------------------
                                                                                                Section 1504
                          (144289) 144289   144298     144309     144320     144331     144342     144353     144364         144384
         T4 NC_000866.4   (143555) TCAGATAAGGTCGTTAATCTTACCCCGGAATTATATCCAGCTGCATGTCACCATGCAGAGCAGACTATATCTCCAACTTGTTAAAGCAAGTTGTCT
SARS-CoV-2 NC_045512.2     (29904) ------------------------------------------------------------------------------------------------
                                                                                                Section 1505
                          (144385) 144385      144397     144408     144419     144430     144441     144452     144463       144480
         T4 NC_000866.4   (143651) ATCGTTTCGAGTCACTTGACCCTACTCCCCAAAGGGATAGTCGTTAGGCATTTATGTAGAACCAATTCCATTTATCAGATTTTACACGATAAGTAA
SARS-CoV-2 NC_045512.2     (29904) ------------------------------------------------------------------------------------------------
```

```
                                                                                                  Section 1506
                       (144481) 144481         144496     144507     144518     144529     144540     144551     144562   144576
         T4 NC_000866.4 (143747) CTAATCCAGACGAAATTTTAAAATGTCTAGCTGCATCTGCTGCACAATCAAAAATAACCCCATCACATGAAATCTTTTTAATATTACTAGGCTTTT
  SARS-CoV-2 NC_045512.2 (29904) ------------------------------------------------------------------------------------------------
                                                                                                  Section 1507
                       (144577) 144577 144584     144595     144606     144617     144628     144639     144650     144661 144672
         T4 NC_000866.4 (143843) TACCTTTCATCTTTTCTGATATTTTAGATTTAGTTATGTCTGAATGCTTATGATTAAAGAATGAATTATTTTCACCTGAACGATTTCTGCATTTAC
  SARS-CoV-2 NC_045512.2 (29904) ------------------------------------------------------------------------------------------------
                                                                                                  Section 1508
                       (144673) 144673   144683     144694     144705     144716     144727     144738     144749         144768
         T4 NC_000866.4 (143939) TACAAGTATAAGCAGAAGTTTGTATGCGAACACCGCACTTACAAAACTTATGGGTTTCTGGATTCCAACGCCCGTTTTTACTTCCGGGTTTACTGT
  SARS-CoV-2 NC_045512.2 (29904) ------------------------------------------------------------------------------------------------
                                                                                                  Section 1509
                       (144769) 144769       144782     144793     144804     144815     144826     144837     144848       144864
         T4 NC_000866.4 (144035) AAAGAGCTTTCCGACCATCAGGTCCAAGTTTAAGCATCTTAGCTTTAACAGTTTCAGAACGTTTCTTAATAATTTCTTCTTTTAATGGATGCGTAG
  SARS-CoV-2 NC_045512.2 (29904) ------------------------------------------------------------------------------------------------
                                                                                                  Section 1510
                       (144865) 144865144870     144881     144892     144903     144914     144925     144936     144947   144960
         T4 NC_000866.4 (144131) AACATGTATCACCAAACGTTGCATCAGCAATATTGTATCCATTAATTTTAGAATTAAGCTCTTTAATCCAAAAATTTTCTCGTTCAATAATCAAAT
  SARS-CoV-2 NC_045512.2 (29904) ------------------------------------------------------------------------------------------------
                                                                                                  Section 1511
                       (144961) 144961 144969     144980     144991     145002     145013     145024     145035           145056
         T4 NC_000866.4 (144227) CTTTCTCATATGGAATTTCTTCCAAAATAGAACATTCAAACACATTACCATGTTTGTTAAAAGACCTCTGAAGTTTTATAGAAGAATGGCATCCTT
  SARS-CoV-2 NC_045512.2 (29904) ------------------------------------------------------------------------------------------------
                                                                                                  Section 1512
                       (145057) 145057     145068     145079     145090     145101     145112     145123     145134         145152
         T4 NC_000866.4 (144323) TTTCTAAATCTTTAAAATGCCTCTTCCATCTCTTTTCAAAATCTTTAGCACTTCCTACATATACTTTATTGTTTAAAGTATTTTTAATCTGATAAA
  SARS-CoV-2 NC_045512.2 (29904) ------------------------------------------------------------------------------------------------
```

```
                                                                                                Section 1513
                 (145153) 145153        145167     145178     145189     145200     145211     145222     145233    145248
    T4 NC_000866.4 (144419) TTCCGCTTTTCATAAATACCTCTTTAAATATAGAAGTATTTATTAAAGGGCAAGTCCTACAATTTAGCACGGGATTGTCTACTAGAGAGGTTCCCC
SARS-CoV-2 NC_045512.2 (29904) ------------------------------------------------------------------------------------------------
                                                                                                Section 1514
                 (145249) 145249 145255     145266     145277     145288     145299     145310     145321     145332 145344
    T4 NC_000866.4 (144515) GTTTAGATAGATTACAAGTATAAGTCACCTTATACTCAGGCCTCAATTAACCCAAGAAAACATCTACTGAGCGTTGATACCACTGCAAATCCAAAT
SARS-CoV-2 NC_045512.2 (29904) ------------------------------------------------------------------------------------------------
                                                                                                Section 1515
                 (145345) 145345 145354     145365     145376     145387     145398     145409     145420         145440
    T4 NC_000866.4 (144611) AGCCATTACGCACATTAAACTGATAGAACATATGACAAGGCGGTAATGCCATATATTTAAGTTCAGCTGGATTCCATGCAGAAACAATTTGACGCC
SARS-CoV-2 NC_045512.2 (29904) ------------------------------------------------------------------------------------------------
                                                                                                Section 1516
                 (145441) 145441      145453     145464     145475     145486     145497     145508     145519    145536
    T4 NC_000866.4 (144707) TATCATTTGGCAGTTTTTTAATACGATCAATAACTTCTATAATTTGGTCTACACCACCAAAATCACGCCACTGTTTTCCATAAATTGGACCAAGTT
SARS-CoV-2 NC_045512.2 (29904) ------------------------------------------------------------------------------------------------
                                                                                                Section 1517
                 (145537) 145537         145552     145563     145574     145585     145596     145607     145618    145632
    T4 NC_000866.4 (144803) CACCGCTATGGTATCCTAAATCTTTTGCTTGATTTTCGTAATTTTCATCCCAGACTGTTTTGCCTTGGATTAACGAATCGTGTTGAATTAATCGTA
SARS-CoV-2 NC_045512.2 (29904) ------------------------------------------------------------------------------------------------
                                                                                                Section 1518
                 (145633) 145633  145640     145651     145662     145673     145684     145695     145706     145717 145728
    T4 NC_000866.4 (144899) AATCATTGACATTTGTGCTTCCTGATAAAAACCATATTAGCTCAGCAATGCAAGCTTTCCAGGCGAGCTTCTTAGTTGTTACCGCAGGAAAACCTT
SARS-CoV-2 NC_045512.2 (29904) ------------------------------------------------------------------------------------------------
                                                                                                Section 1519
                 (145729) 145729    145739     145750     145761     145772     145783     145794     145805         145824
    T4 NC_000866.4 (144995) TAGTTAAATCCCAGCGTAATTTAGATCCGAACAGAGCAATTGTTCCTGTGCCTGTACGATCATCGGTTTCATAACCATTTTCAAAAATGTCTTTAA
SARS-CoV-2 NC_045512.2 (29904) ------------------------------------------------------------------------------------------------
```

Section 1520

```
                            (145825) 145825       145838     145849     145860     145871     145882     145893     145904         145920
        T4 NC_000866.4 (145091) TTAAATCTTGGTATTGTTTCATTTATATACTGATTCCGTAAGGGTTGTTACTTCATCTATTTTATACCAATGCGTTTCAACCATTTCACGCTTGCT
SARS-CoV-2 NC_045512.2  (29904) ------------------------------------------------------------------------------------------------
```

Section 1521

```
                            (145921) 145921145926      145937     145948     145959     145970     145981     145992     146003   146016
        T4 NC_000866.4 (145187) TATATCATCAAGAAAACTTGCGTCTAATTGAACTGTTGAATTAACACGATGCCTTTTAACGATGCGAGAAACAACTACTTCATCTGCATAAGGTAA
SARS-CoV-2 NC_045512.2  (29904) ------------------------------------------------------------------------------------------------
```

Section 1522

```
                            (146017) 146017  146025     146036     146047     146058     146069     146080     146091             146112
        T4 NC_000866.4 (145283) TGCAGCATATAACAGAGCAGGCCCGCCAATTACACTTACTTTAGAATTCTGATCAAGCATAGTTTCGAATGGTGCATTAGGGCTTGACACTTGAAT
SARS-CoV-2 NC_045512.2  (29904) ------------------------------------------------------------------------------------------------
```

Section 1523

```
                            (146113) 146113     146124     146135     146146     146157     146168     146179     146190         146208
        T4 NC_000866.4 (145379) TTCGCCGCCAGAAATGTAAGTTATATATTGCTCCCAAGTAATATAGAAATGTGCTAAATCGCCGTCTTTAGTTACAGGATAATCACGCGCAAGGTC
SARS-CoV-2 NC_045512.2  (29904) ------------------------------------------------------------------------------------------------
```

Section 1524

```
                            (146209) 146209        146223     146234     146245     146256     146267     146278     146289     146304
        T4 NC_000866.4 (145475) ACACACCACAATATGGCTACGACCAGGAAGTAATGTAGGCAATGACTGGAACGTTTTAGCACCCATAATCATAATTGTGCCTTCAGTACGAGCTTT
SARS-CoV-2 NC_045512.2  (29904) ------------------------------------------------------------------------------------------------
```

Section 1525

```
                            (146305) 146305 146311     146322     146333     146344     146355     146366     146377     146388  146400
        T4 NC_000866.4 (145571) AAAATTCTGGAGGTCCTTTTTAACTCGTCCCCATGGTAAACCATCACCTAAACCGAATGCTAATTCATTAAAGCCGTCGACCGTTTTAGTTGGAGA
SARS-CoV-2 NC_045512.2  (29904) ------------------------------------------------------------------------------------------------
```

Section 1526

```
                            (146401) 146401   146410     146421     146432     146443     146454     146465     146476             146496
        T4 NC_000866.4 (145667) ATAACGGAATACCAATTTAATCATTACGTAAATCCTATTTTAATTGAAAACGAATGCTTACTTGGATAATTTCAATGACATACATAATATTTTCCT
SARS-CoV-2 NC_045512.2  (29904) ------------------------------------------------------------------------------------------------
```

```
                                                                                           Section 1527
                          (146497) 146497          146509         146520         146531         146542         146553         146564         146575       146592
T4 NC_000866.4 (145763) CAAACAGACTTTTTCACAATTTTCCAATCAGCTTTAAACTGCTCGACGTCAGAATGGTAAATCCAAAATCCTGCGCTTTCTCCGTCTTCATAAAGA
SARS-CoV-2 NC_045512.2 (29904) ------------------------------------------------------------------------------------------------
                                                                                           Section 1528
                          (146593) 146593              146608         146619         146630         146641         146652         146663         146674       146688
T4 NC_000866.4 (145859) GGACATCCATCGCATTCATCTTCCCATCCCATATCACGTAAAAGATGTTCAGCTTTTTCAACAAGTTCAGAATCTTTACCGATGATATTAAAATAC
SARS-CoV-2 NC_045512.2 (29904) ------------------------------------------------------------------------------------------------
                                                                                           Section 1529
                          (146689) 146689  146696         146707         146718         146729         146740         146751         146762         146773  146784
T4 NC_000866.4 (145955) CATTTACCTCTAACTTCTGAATCTTTGATGCTCTGGCGTTGTAATCTCATTTTATTCTCCTTAGCAAGCTTTAATCAAAAGATATAAACAGACCAA
SARS-CoV-2 NC_045512.2 (29904) ------------------------------------------------------------------------------------------------
                                                                                           Section 1530
                          (146785) 146785    146795         146806         146817         146828         146839         146850         146861            146880
T4 NC_000866.4 (146051) CATAACTGCTGCCATAATATAAGGTGCGAACATTTTCTTTTCTCCATTAGTTTTGATAGGGTAATAGTATCACACTACTACCCTGATGTAAACTAC
SARS-CoV-2 NC_045512.2 (29904) ------------------------------------------------------------------------------------------------
                                                                                           Section 1531
                          (146881) 146881          146894         146905         146916         146927         146938         146949         146960       146976
T4 NC_000866.4 (146147) TTTTTGAAAGTTTTTCGCAAAAGTTCAATGATTTCATCTACATTGTTTTCGTCAACAATGCAGTGAATTTTTGTTACGCCAGAAACCTTGTCTTTG
SARS-CoV-2 NC_045512.2 (29904) ------------------------------------------------------------------------------------------------
                                                                                           Section 1532
                          (146977) 146977146982         146993         147004         147015         147026         147037         147048         147059  147072
T4 NC_000866.4 (146243) ACTTCATCTTCTTCAGAAGTCGGTTCTTTATATTCGCGGAAACAATAAAACTCTTCTTCACTAAGTTCAAAATAATCATCACCCATACCATCATCA
SARS-CoV-2 NC_045512.2 (29904) ------------------------------------------------------------------------------------------------
                                                                                           Section 1533
                          (147073) 147073  147081         147092         147103         147114         147125         147136         147147            147168
T4 NC_000866.4 (146339) TTATAGATTTCACCATTAGCACAAATGATTTCGGTTACATAATCAAAGCCATCTAAACTTGATATTGATTTAACTTCAAACCAACCGCCATTTTCT
SARS-CoV-2 NC_045512.2 (29904) ------------------------------------------------------------------------------------------------
```

```
                                                                                                         Section 1534
                            (147169) 147169     147180     147191     147202     147213     147224     147235     147246           147264
               T4 NC_000866.4 (146435) TGAATGATGCCGACCATACTAGCATTTGATGAACTAATATCAATGAAAGATTTAATACGATGTGGATTTAACTCGTATTTTTTGCCGATTTCCATT
      SARS-CoV-2 NC_045512.2  (29904) ------------------------------------------------------------------------------------------------
                                                                                                         Section 1535
                            (147265) 147265        147279     147290     147301     147312     147323     147334     147345      147360
               T4 NC_000866.4 (146531) TTGATTTTCCTCATTTTAATAGGGGCTTGATAGCCCCTTGATAATTATTGTTCAATCAGTCCCATGTAAAATTCTGCGTCTTCAGAATCCATGCCA
      SARS-CoV-2 NC_045512.2  (29904) ------------------------------------------------------------------------------------------------
                                                                                                         Section 1536
                            (147361) 147361 147367     147378     147389     147400     147411     147422     147433     147444  147456
               T4 NC_000866.4 (146627) TCACAATATTCATTAGCCATAAAGCGGGTGAGGTCTTCAAGAGGACCTTCAATAACGATTTGAATACTCCAAAACTTAGAATCTTGCACGCTTGTG
      SARS-CoV-2 NC_045512.2  (29904) ------------------------------------------------------------------------------------------------
                                                                                                         Section 1537
                            (147457) 147457   147466     147477     147488     147499     147510     147521     147532           147552
               T4 NC_000866.4 (146723) ATACTAAGTTCAGGATAACGATTACGAATAATCTCTTCAATATATTCAAAATCAACGATGTCAATATCAACTTTAGCCATATTATTTTCCTCTTTA
      SARS-CoV-2 NC_045512.2  (29904) ------------------------------------------------------------------------------------------------
                                                                                                         Section 1538
                            (147553) 147553      147565     147576     147587     147598     147609     147620     147631           147648
               T4 NC_000866.4 (146819) ATTATTAGCAGTATTGCCGATAGTTGTATAGTACCATAAAGCTTTATGCTTGTAAACCGTTTTGTGAAAAAATTTTTAAAATAAAAAAGGGGACCT
      SARS-CoV-2 NC_045512.2  (29904) ------------------------------------------------------------------------------------------------
                                                                                                         Section 1539
                            (147649) 147649         147664     147675     147686     147697     147708     147719     147730      147744
               T4 NC_000866.4 (146915) CTAGGGTCCCCAATTAATTAGTAATATAATCTATTAAAGGTCATTCAAAAGGTCATCCAGGTCCGTGTCATCAGCACTAGATGAACTACCAGAGCT
      SARS-CoV-2 NC_045512.2  (29904) ------------------------------------------------------------------------------------------------
                                                                                                         Section 1540
                            (147745) 147745 147752     147763     147774     147785     147796     147807     147818     147829 147840
               T4 NC_000866.4 (147011) TGAGCTCATAAAATCATCTTCAGTTTTTGTATTGAAGTCATCAACATTGAATGCATCCAAATCATCAGCCACTTTATCAGCTTTCTTAGCAGCAGT
      SARS-CoV-2 NC_045512.2  (29904) ------------------------------------------------------------------------------------------------
```

Section 1541

(147841) 147841 147851 147862 147873 147884 147895 147906 147917 147936
T4 NC_000866.4 (147107) TGCAGCAGCACCGCCCATCACAGCAGTTCCCATAACTTGACCGAATTTAGTATTAAGTTCTTCAAACGATTTGAATTTATCTTTAGAAGTCATTTC
SARS-CoV-2 NC_045512.2 (29904) --

Section 1542

(147937) 147937 147950 147961 147972 147983 147994 148005 148016 148032
T4 NC_000866.4 (147203) AGAAAGGTCAACCATTTGTTCGAACAGTTCTTTCTGGAAAGATTCATCGTCAATGTTTGGAATCGCAGATTGATTCAGGAATTTAGATTCATCGTA
SARS-CoV-2 NC_045512.2 (29904) --

Section 1543

(148033) 148033 148038 148049 148060 148071 148082 148093 148104 148115 148128
T4 NC_000866.4 (147299) GTTACTAAATCCAGAAACTTGTTTAACTTTCAGTACAAAGTTAGCACCTTCCCACGGACAAGTTACATCAACTGGAGTTTCACCCATTTCAACATC
SARS-CoV-2 NC_045512.2 (29904) --

Section 1544

(148129) 148129 148137 148148 148159 148170 148181 148192 148203 148224
T4 NC_000866.4 (147395) AACCGCAATCATTGCATTGATTTTATCCCAGATTTTCTTACCAAAGCGGTATTTAAATACTTTACCTTCGTTTTCTGGAGCAGCTGGGTCTTTTAC
SARS-CoV-2 NC_045512.2 (29904) --

Section 1545

(148225) 148225 148236 148247 148258 148269 148280 148291 148302 148320
T4 NC_000866.4 (147491) TACAAGAATGTTAGCCCAGTAAGAAGTTTTACGTTTAACAAGACTGTACTCTTTATTGTCAGTGTTGTATAGATCATTTTTACTGATGTATTGACA
SARS-CoV-2 NC_045512.2 (29904) --

Section 1546

(148321) 148321 148335 148346 148357 148368 148379 148390 148401 148416
T4 NC_000866.4 (147587) TACTGGGCAAGAATCGTAATCACCATGGGTAGATGAACATGTTTCAATATACCATTTACCATTTTTCTTGAAACCGTGATTTACAAGAATTGCGAA
SARS-CoV-2 NC_045512.2 (29904) --

Section 1547

(148417) 148417 148423 148434 148445 148456 148467 148478 148489 148500 148512
T4 NC_000866.4 (147683) TGGTGCTTGTTCATCATTTTTAGACGGAAGAAAACGAATTACTGCTTGACCGTTACCCGCATTATCGAGTTTCAGTTTCCACTCGCCTTTATCTTC
SARS-CoV-2 NC_045512.2 (29904) --

Section 1548

```
                              (148513) 148513  148522     148533     148544     148555     148566     148577     148588          148608
          T4 NC_000866.4 (147779) AGAAGAAAAACCTTTATTGCCATTCAGTTTAGCCATTTGTGCAGCGAGTTCAGCAGTAGATTTACGTTTAAACATTTTTATTTCCTTTTTAATTTA
  SARS-CoV-2 NC_045512.2   (29904) ------------------------------------------------------------------------------------------------
```

Section 1549

```
                              (148609) 148609      148621     148632     148643     148654     148665     148676     148687          148704
          T4 NC_000866.4 (147875) ATTTAATTAACAGTTGGTGCTATGACACTTTACCTCATAGCTGGCATAATTCGCAATACTCTGGGTCTTCGAGAGGTATCCAACCTGAGTTGAAAT
  SARS-CoV-2 NC_045512.2   (29904) ------------------------------------------------------------------------------------------------
```

Section 1550

```
                              (148705) 148705         148720     148731     148742     148753     148764     148775     148786        148800
          T4 NC_000866.4 (147971) ACTTTACCATCGATTTAGCAGTTGTATCAGTTATATTTATATTACCTTTAACTCTTCGCCATCCAGGAGTTTTACCGTACAGATTAGAGGATAATA
  SARS-CoV-2 NC_045512.2   (29904) ------------------------------------------------------------------------------------------------
```

Section 1551

```
                              (148801) 148801 148808     148819     148830     148841     148852     148863     148874     148885 148896
          T4 NC_000866.4 (148067) ATAACACATAATTCTCGTAAGCAATATGAGATAATTTCCAAGACTCTATATTAGCTCGTGATGTTTTCCAAGGTCTAAAATCGTCACGGTTCATAT
  SARS-CoV-2 NC_045512.2   (29904) ------------------------------------------------------------------------------------------------
```

Section 1552

```
                              (148897) 148897    148907     148918     148929     148940     148951     148962     148973          148992
          T4 NC_000866.4 (148163) AATTAGCCAATCTCATATGCTCTCTAACTTCCGGGTCTTTGGCTGGATGAGTTTCACCACTCACACCAAATCCACCACCAGCATATACCAGATTAA
  SARS-CoV-2 NC_045512.2   (29904) ------------------------------------------------------------------------------------------------
```

Section 1553

```
                              (148993) 148993       149006     149017     149028     149039     149050     149061     149072          149088
          T4 NC_000866.4 (148259) AATAGTCTGGATTATCTCTGGCATTTACTTCAAGTTGGTATTTTCGTTCTGCTTCAATAACATCCAAGTCATCATCAATTTGAATTATTTTAACGC
  SARS-CoV-2 NC_045512.2   (29904) ------------------------------------------------------------------------------------------------
```

Section 1554

```
                              (149089) 149089149094      149105     149116     149127     149138     149149     149160     149171       149184
          T4 NC_000866.4 (148355) TCGGTTTTTGAAGCATTAGCGCATTCAAAAATCTTTTTTGTTTACATGAGCTCCAGTATTCCTTTCCGGAAGAGTCATATATTATTCCGTTCTCAA
  SARS-CoV-2 NC_045512.2   (29904) ------------------------------------------------------------------------------------------------
```

Section 1555

```
                        (149185) 149185 149193 149204 149215 149226 149237 149248 149259 149280
T4 NC_000866.4          (148451) ATGAGCAATTTAATTTACTACCGATATAGTAGTATGGCGGAGTCTTATTTTTGACACGGTCTTCAAATGTAAACCAATATACTATATTCATATCAA
SARS-CoV-2 NC_045512.2   (29904) ------------------------------------------------------------------------------------------------
```

Section 1556

```
                        (149281) 149281 149292 149303 149314 149325 149336 149347 149358 149376
T4 NC_000866.4          (148547) TACTTGCAAGATTTCACAGTTTCAATGAAAACATTTTTAGCTTTCTGTGAATCAATATTTAAAATTTTTCTATAAGCCTTTAACTTTATAGAATAA
SARS-CoV-2 NC_045512.2   (29904) ------------------------------------------------------------------------------------------------
```

Section 1557

```
                        (149377) 149377 149391 149402 149413 149424 149435 149446 149457 149472
T4 NC_000866.4          (148643) TTATTCCAGACTAAATTATCAGTCTGTTCATCATGTTTATCAATTATATTTAAAAACGAATCAAGCAAGATAAACGTCTCAAACGAAATTATGTTC
SARS-CoV-2 NC_045512.2   (29904) ------------------------------------------------------------------------------------------------
```

Section 1558

```
                        (149473) 149473 149479 149490 149501 149512 149523 149534 149545 149556 149568
T4 NC_000866.4          (148739) GATTGCAGAAGTTTAAAAATATAACTTGATTGAACTTTTGGATTATACTCAAAAATTTCTTTAAAAGCAGAAACTTCAACTTTTTTACTAAAATAA
SARS-CoV-2 NC_045512.2   (29904) ------------------------------------------------------------------------------------------------
```

Section 1559

```
                        (149569) 149569 149578 149589 149600 149611 149622 149633 149644 149664
T4 NC_000866.4          (148835) TAAATGTTGCGAATATCTTCTTCAAACTTAAATTTAATTTGCTTTAAGCGTCCGATATATTCACGATAAAACACAAGTGCATCAGCGTCAGAGATG
SARS-CoV-2 NC_045512.2   (29904) ------------------------------------------------------------------------------------------------
```

Section 1560

```
                        (149665) 149665 149677 149688 149699 149710 149721 149732 149743 149760
T4 NC_000866.4          (148931) TCACCAATCCAAGCATCTTGGTTAGCAACCAAATTGCTTATAAAGATTAAAGCAAGTTCCTTTAATTTATATTTTTCTGATAACTTCTGGAAAAAA
SARS-CoV-2 NC_045512.2   (29904) ------------------------------------------------------------------------------------------------
```

Section 1561

```
                        (149761) 149761 149776 149787 149798 149809 149820 149831 149842 149856
T4 NC_000866.4          (149027) TACTTATCCCTTCGCTTTTGATAAGCGGCATCAGACACCCGCATGCACCAATTATACTTAATTACATCATACTTTCCATTCATATGTTGTTTTATC
SARS-CoV-2 NC_045512.2   (29904) ------------------------------------------------------------------------------------------------
```

Section 1562

```
(149857) 149857 149864 149875 149886 149897 149908 149919 149930 149941 149952
T4 NC_000866.4 (149123) ATTAAGTATAATTTATAAACTGATTTACCATCAATGTATCTTTCACCACCAGCAGGCATGCGGAGTTTAATCATAGTAGAAAATCTAATGTATTAG
SARS-CoV-2 NC_045512.2 (29904) ------------------------------------------------------------------------------------------------
```

Section 1563

```
(149953) 149953 149963 149974 149985 149996 150007 150018 150029 150048
T4 NC_000866.4 (149219) TTTTTTCACAACGAACAACAGAAGGACGTAAAAGATTTTCGTCAATAGCTTCTGACTGAATTTTTTCAATTATACCCGAAGGAATAAATTTAGCAA
SARS-CoV-2 NC_045512.2 (29904) ------------------------------------------------------------------------------------------------
```

Section 1564

```
(150049) 150049 150062 150073 150084 150095 150106 150117 150128 150144
T4 NC_000866.4 (149315) ATTGAGTTTCAGGAATAGAATTTTCTTCTAAGAATGCTGTTGTAGCTTCAAGATAACTCATTCCAAACTCTTCTACCATTTTTTCAATAATAAATC
SARS-CoV-2 NC_045512.2 (29904) ------------------------------------------------------------------------------------------------
```

Section 1565

```
(150145) 150145 150150 150161 150172 150183 150194 150205 150216 150227 150240
T4 NC_000866.4 (149411) CATTTTCTTGGCGGTCAAGAAGCTTTGCTATTTCATCCTTTTCTTTCTTAATTGAAAGTTCTTTTTCTGAAAGACCGGTCTCATCGACCGGACGAA
SARS-CoV-2 NC_045512.2 (29904) ------------------------------------------------------------------------------------------------
```

Section 1566

```
(150241) 150241 150249 150260 150271 150282 150293 150304 150315 150336
T4 NC_000866.4 (149507) TATCATTTAGAGAAAACTGTGTCATAAAGTTCAACTACCTCTTCAGTTTCAGCTTCAAACACATCACGGTTATCTTTATGATACAAAGCTAATAGA
SARS-CoV-2 NC_045512.2 (29904) ------------------------------------------------------------------------------------------------
```

Section 1567

```
(150337) 150337 150348 150359 150370 150381 150392 150403 150414 150432
T4 NC_000866.4 (149603) CGATTAAACATCTTACCATCAACGCCAAGTTCATCTTTAGCACGAATTCGAATATCTTTAATCAGTTCATTATAACCGGAAATTTTCAGTTTATGA
SARS-CoV-2 NC_045512.2 (29904) ------------------------------------------------------------------------------------------------
```

Section 1568

```
(150433) 150433 150447 150458 150469 150480 150491 150502 150513 150528
T4 NC_000866.4 (149699) TCAGATGCTTCTTTAATAAATTTAGCCAAGTCTTCGCCATGGATAGCTTCATCAAATTCAACCATTTCTTTTTTAGCCATTATTCACCTCAAAATT
SARS-CoV-2 NC_045512.2 (29904) ------------------------------------------------------------------------------------------------
```

```
                                                                                                  Section 1569
                    (150529) 150529 150535     150546     150557     150568     150579     150590     150601     150612 150624
T4 NC_000866.4      (149795) CATTAATGCTATTAGTTAATTTAGAAAGACCCGCTTTTACAAAATATGAATAAATTTTGCCACGCGGTGGTAATTTATATGAATTATAGTAATTCA
SARS-CoV-2 NC_045512.2 (29904) ------------------------------------------------------------------------------------------------
                                                                                                  Section 1570
                    (150625) 150625   150634     150645     150656     150667     150678     150689     150700          150720
T4 NC_000866.4      (149891) CAATGTTTGAAGCAATATTATCAGGAATATAATCAAAATCAATTAGAACTAAATTTTCTTTATAACGATTATATTCAGATTCAGTGAGAAGCACCT
SARS-CoV-2 NC_045512.2 (29904) ------------------------------------------------------------------------------------------------
                                                                                                  Section 1571
                    (150721) 150721      150733     150744     150755     150766     150777     150788     150799          150816
T4 NC_000866.4      (149987) TAGCTTGCTCACGGTCATTAGCAATAGCTTCAACGATTGAAGTTTTCATTGAAGGAGTTCGTTCACCTTCAACTCTGGTAAACCAAAAGTCAGATC
SARS-CoV-2 NC_045512.2 (29904) ------------------------------------------------------------------------------------------------
                                                                                                  Section 1572
                    (150817) 150817         150832     150843     150854     150865     150876     150887     150898        150912
T4 NC_000866.4      (150083) GTACTTTAACTGAAGCAACGTTATCCTTTTTGTCGCCTTTAAGGATTTTAGTCATACAGTCAATTTCAGCAGAACCGCTTTTAATTTTAACCCATT
SARS-CoV-2 NC_045512.2 (29904) ------------------------------------------------------------------------------------------------
                                                                                                  Section 1573
                    (150913) 150913 150920     150931     150942     150953     150964     150975     150986     150997 151008
T4 NC_000866.4      (150179) TCTTATGCATCGGAGACCATTGCTTAACATTTGGATATTTGTGAAGCTGAGTAAAGTCACCATCTGACGAAATGATTAAAATCTTATGTCCTTCTA
SARS-CoV-2 NC_045512.2 (29904) ------------------------------------------------------------------------------------------------
                                                                                                  Section 1574
                    (151009) 151009    151019     151030     151041     151052     151063     151074     151085          151104
T4 NC_000866.4      (150275) AAGAGAACTTTTTAACAAGAACAGCAATGTGGTCATCTGCTTCATACTTATCAATATCCATAACAATGTATGGCATATAAGCTTTCAATTCATCTA
SARS-CoV-2 NC_045512.2 (29904) ------------------------------------------------------------------------------------------------
                                                                                                  Section 1575
                    (151105) 151105       151118     151129     151140     151151     151162     151173     151184          151200
T4 NC_000866.4      (150371) TAACTTTATGGCTGGATTCAAAATAACCTTCCCAGTCCCAAGTAGATTCTTCTCGTGCTTTTCCACGGTTTTTCTTATAATAATAAGCGAAATCAC
SARS-CoV-2 NC_045512.2 (29904) ------------------------------------------------------------------------------------------------
```

```
                                                                                                  Section 1576
                    (151201) 151201151206     151217     151228     151239     151250     151261     151272     151283   151296
          T4 NC_000866.4 (150467) GACGCCAATATCCAGATTTCGCGTTATCAATACACAGTACAATTTTAGTGTATCCAAGCGTTTTTGCTTTTTTGACATTAAACTTAATTGAGTTCA
SARS-CoV-2 NC_045512.2  (29904) ------------------------------------------------------------------------------------------------
                                                                                                  Section 1577
                    (151297) 151297 151305     151316     151327     151338     151349     151360     151371          151392
          T4 NC_000866.4 (150563) ATATCAAATGACGAACCATTGATAAATTAATTTTTTTCTTTATCTGGGAAGTTTACCAAAGCAGTTGAAAGCGCAATTTGACTAAAGTCAATTAAGC
SARS-CoV-2 NC_045512.2  (29904) ------------------------------------------------------------------------------------------------
                                                                                                  Section 1578
                    (151393) 151393     151404     151415     151426     151437     151448     151459     151470          151488
          T4 NC_000866.4 (150659) AGATTCCTTCTTTGTAATCTTCATCCAACATCATTTCTAAATCCATATGAACCTCGTTCAATTAGTGAGATTTCTATTATATACCATCCAAATCTT
SARS-CoV-2 NC_045512.2  (29904) ------------------------------------------------------------------------------------------------
                                                                                                  Section 1579
                    (151489) 151489        151503     151514     151525     151536     151547     151558     151569     151584
          T4 NC_000866.4 (150755) AAAGTAAACAAGTATAAATACTTATTATTGAAAACACAATAGGAGCCCGGGAGAATGGCCGAGATTAAAAGAGAATTCAGAGCAGAAGATGGTCTG
SARS-CoV-2 NC_045512.2  (29904) ------------------------------------------------------------------------------------------------
                                                                                                  Section 1580
                    (151585) 151585 151591     151602     151613     151624     151635     151646     151657     151668   151680
          T4 NC_000866.4 (150851) GACGCAGGTGGTGATAAAATAATCAACGTAGCTTTAGCTGATCGTACCGTAGGAACTGACGGTGTTAACGTTGATTACTTAATTCAAGAAAACACA
SARS-CoV-2 NC_045512.2  (29904) ------------------------------------------------------------------------------------------------
                                                                                                  Section 1581
                    (151681) 151681   151690     151701     151712     151723     151734     151745     151756          151776
          T4 NC_000866.4 (150947) GTTCAACAGTATGATCCAACTCGTGGATATTTAAAAGATTTTGTAATCATTTATGATAACCGCTTTTGGGCTGCTATAAATGATATTCCAAAACCA
SARS-CoV-2 NC_045512.2  (29904) ------------------------------------------------------------------------------------------------
                                                                                                  Section 1582
                    (151777) 151777      151789     151800     151811     151822     151833     151844     151855        151872
          T4 NC_000866.4 (151043) GCAGGAGCTTTTAATAGCGGACGCTGGAGAGCATTACGTACCGATGCTAACTGGATTACGGTTTCATCTGGTTCATATCAATTAAAATCTGGTGAA
SARS-CoV-2 NC_045512.2  (29904) ------------------------------------------------------------------------------------------------
```

Section 1583

```
(151873) 151873 151888 151899 151910 151921 151932 151943 151954 151968
T4 NC_000866.4 (151139) GCAATTTCGGTTAACACCGCAGCTGGAAATGACATCACGTTTACTTTACCATCTTCTCCAATTGATGGTGATACTATCGTTCTCCAAGATATTGGA
SARS-CoV-2 NC_045512.2 (29904) ------------------------------------------------------------------------------------------------
```

Section 1584

```
(151969) 151969 151976 151987 151998 152009 152020 152031 152042 152053 152064
T4 NC_000866.4 (151235) GGAAAACCTGGAGTTAACCAAGTTTTAATTGTAGCTCCAGTACAAAGTATTGTAAACTTTAGAGGTGAACAGGTACGTTCAGTACTAATGACTCAT
SARS-CoV-2 NC_045512.2 (29904) ------------------------------------------------------------------------------------------------
```

Section 1585

```
(152065) 152065 152075 152086 152097 152108 152119 152130 152141 152160
T4 NC_000866.4 (151331) CCAAAGTCACAGCTAGTTTTAATTTTTAGTAATCGTCTGTGGCAAATGTATGTTGCTGATTATAGTAGAGAAGCTATAGTTGTAACACCAGCGAAT
SARS-CoV-2 NC_045512.2 (29904) ------------------------------------------------------------------------------------------------
```

Section 1586

```
(152161) 152161 152174 152185 152196 152207 152218 152229 152240 152256
T4 NC_000866.4 (151427) ACTTATCAAGCGCAATCCAACGATTTTATCGTACGTAGATTTACTTCTGCTGCACCAATTAATGTCAAACTTCCAAGATTTGCTAATCATGGCGAT
SARS-CoV-2 NC_045512.2 (29904) ------------------------------------------------------------------------------------------------
```

Section 1587

```
(152257) 152257 152262 152273 152284 152295 152306 152317 152328 152339 152352
T4 NC_000866.4 (151523) ATTATTAATTTCGTCGATTTAGATAAACTAAATCCGCTTTATCATACAATTGTTACTACATACGATGAAACGACTTCAGTACAAGAAGTTGGAACT
SARS-CoV-2 NC_045512.2 (29904) ------------------------------------------------------------------------------------------------
```

Section 1588

```
(152353) 152353 152361 152372 152383 152394 152405 152416 152427 152448
T4 NC_000866.4 (151619) CATTCCATTGAAGGCCGTACATCGATTGACGGTTTCTTGATGTTTGATGATAATGAGAAATTATGGAGACTGTTTGACGGGGATAGTAAAGCGCGT
SARS-CoV-2 NC_045512.2 (29904) ------------------------------------------------------------------------------------------------
```

Section 1589

```
(152449) 152449 152460 152471 152482 152493 152504 152515 152526 152544
T4 NC_000866.4 (151715) TTACGTATCATAACGACTAATTCAAACATTCGTCCAAATGAAGAAGTTATGGTATTTGGTGCGAATAACGGAACAACTCAAACAATTGAGCTTAAG
SARS-CoV-2 NC_045512.2 (29904) ------------------------------------------------------------------------------------------------
```

```
                                                                                                   Section 1590
                 (152545) 152545       152559     152570     152581     152592     152603     152614     152625        152640
T4 NC_000866.4   (151811) CTTCCAACTAATATTTCTGTTGGTGATACTGTTAAAATTTCCATGAATTACATGAGAAAAGGACAAACAGTTAAAATCAAAGCTGCTGATGAAGAT
SARS-CoV-2 NC_045512.2 (29904) ------------------------------------------------------------------------------------------------
                                                                                                   Section 1591
                 (152641) 152641 152647    152658     152669     152680     152691     152702     152713     152724      152736
T4 NC_000866.4   (151907) AAAATTGCTTCTTCAGTTCAATTGCTGCAATTCCCAAAACGCTCAGAATATCCACCTGAAGCTGAATGGGTTACAGTTCAAGAATTAGTTTTTAAC
SARS-CoV-2 NC_045512.2 (29904) ------------------------------------------------------------------------------------------------
                                                                                                   Section 1592
                 (152737) 152737   152746     152757     152768     152779     152790     152801     152812              152832
T4 NC_000866.4   (152003) GATGAAACTAATTATGTTCCAGTTTTGGAGCTTGCTTACATAGAAGATTCTGATGGAAAATATTGGGTTGTACAGCAAAACGTTCCAACTGTAGAA
SARS-CoV-2 NC_045512.2 (29904) ------------------------------------------------------------------------------------------------
                                                                                                   Section 1593
                 (152833) 152833      152845     152856     152867     152878     152889     152900     152911           152928
T4 NC_000866.4   (152099) AGAGTAGATTCTTTAAATGATTCTACTAGAGCAAGATTAGGCGTAATTGCTTTAGCTACACAAGCTCAAGCTAATGTCGATTTAGAAAATTCTCCA
SARS-CoV-2 NC_045512.2 (29904) ------------------------------------------------------------------------------------------------
                                                                                                   Section 1594
                 (152929) 152929         152944     152955     152966     152977     152988     152999     153010      153024
T4 NC_000866.4   (152195) CAAAAAGAATTAGCAATTACTCCAGAAACGTTAGCTAATCGTACTGCTACAGAAACTCGCAGAGGTATTGCAAGAATAGCAACTACTGCTCAAGTG
SARS-CoV-2 NC_045512.2 (29904) ------------------------------------------------------------------------------------------------
                                                                                                   Section 1595
                 (153025) 153025 153032     153043     153054     153065     153076     153087     153098     153109 153120
T4 NC_000866.4   (152291) AATCAGAACACCACATTCTCTTTTGCTGATGATATTATCATCACTCCTAAAAAGCTGAATGAAAGAACTGCTACAGAAACTCGTAGAGGTGTCGCA
SARS-CoV-2 NC_045512.2 (29904) ------------------------------------------------------------------------------------------------
                                                                                                   Section 1596
                 (153121) 153121    153131     153142     153153     153164     153175     153186     153197           153216
T4 NC_000866.4   (152387) GAAATTGCTACGCAGCAAGAAACTAATGCAGGAACCGATGATACTACAATCATCACTCCTAAAAAGCTTCAAGCTCGTCAAGGTTCTGAATCATTA
SARS-CoV-2 NC_045512.2 (29904) ------------------------------------------------------------------------------------------------
```

```
                                                                                          Section 1597
                      (153217) 153217       153230     153241     153252     153263     153274     153285     153296         153312
     T4 NC_000866.4 (152483) TCTGGTATTGTAACCTTTGTATCTACTGCAGGTGCTACTCCAGCTTCTAGCCGTGAATTAAATGGTACGAATGTTTATAATAAAAACACTGATAAT
SARS-CoV-2 NC_045512.2 (29904) ------------------------------------------------------------------------------------------------
                                                                                          Section 1598
                      (153313) 153313153318       153329     153340     153351     153362     153373     153384     153395   153408
     T4 NC_000866.4 (152579) TTAGTTGTTTCACCTAAAGCTTTGGATCAGTATAAAGCTACTCCAACACAGCAAGGTGCAGTAATTTTAGCAGTTGAAAGTGAAGTAATTGCTGGA
SARS-CoV-2 NC_045512.2 (29904) ------------------------------------------------------------------------------------------------
                                                                                          Section 1599
                      (153409) 153409  153417     153428     153439     153450     153461     153472     153483           153504
     T4 NC_000866.4 (152675) CAAAGTCAGCAAGGATGGGCAAATGCTGTTGTAACGCCAGAAACGTTACATAAAAAGACATCAACTGATGGAAGAATTGGTTTAATTGAAATTGCT
SARS-CoV-2 NC_045512.2 (29904) ------------------------------------------------------------------------------------------------
                                                                                          Section 1600
                      (153505) 153505     153516     153527     153538     153549     153560     153571     153582           153600
     T4 NC_000866.4 (152771) ACGCAAAGTGAAGTTAATACAGGAACTGATTATACTCGTGCAGTCACTCCTAAAACTTTAAATGACCGTAGAGCAACTGAAAGTTTAAGTGGTATA
SARS-CoV-2 NC_045512.2 (29904) ------------------------------------------------------------------------------------------------
                                                                                          Section 1601
                      (153601) 153601          153615     153626     153637     153648     153659     153670     153681     153696
     T4 NC_000866.4 (152867) GCTGAAATTGCTACACAAGTTGAATTCGACGCAGGCGTCGACGATACTCGTATCTCTACACCATTAAAAATTAAAACCAGATTTAATAGTACTGAT
SARS-CoV-2 NC_045512.2 (29904) ------------------------------------------------------------------------------------------------
                                                                                          Section 1602
                      (153697) 153697 153703     153714     153725     153736     153747     153758     153769     153780   153792
     T4 NC_000866.4 (152963) CGTACTTCTGTTGTTGCTCTATCTGGATTAGTTGAATCAGGAACTCTCTGGGACCATTATACACTTAATATTCTTGAAGCAAATGAGACACAACGT
SARS-CoV-2 NC_045512.2 (29904) ------------------------------------------------------------------------------------------------
                                                                                          Section 1603
                      (153793) 153793   153802     153813     153824     153835     153846     153857     153868           153888
     T4 NC_000866.4 (153059) GGTACACTTCGTGTAGCTACGCAGGTCGAAGCTGCTGCGGGAACATTAGATAATGTTTTAATAACTCCTAAAAAGCTTTTAGGTACTAAATCTACT
SARS-CoV-2 NC_045512.2 (29904) ------------------------------------------------------------------------------------------------
```

```
Section 1604
(153889) 153889 153901 153912 153923 153934 153945 153956 153967 153984
T4 NC_000866.4 (153155) GAAGCGCAAGAGGGTGTTATTAAAGTTGCAACTCAGTCTGAAACTGTGACTGGAACGTCAGCAAATACTGCTGTATCTCCAAAAAATTTAAAATGG
SARS-CoV-2 NC_045512.2 (29904) ------------------------------------------------------------------------------------------------
Section 1605
(153985) 153985 154000 154011 154022 154033 154044 154055 154066 154080
T4 NC_000866.4 (153251) ATTGCGCAGAGTGAACCTACTTGGGCAGCTACTACTGCAATAAGAGGTTTTGTTAAAACTTCATCTGGTTCAATTACATTCGTTGGTAATGATACA
SARS-CoV-2 NC_045512.2 (29904) ------------------------------------------------------------------------------------------------
Section 1606
(154081) 154081 154088 154099 154110 154121 154132 154143 154154 154165 154176
T4 NC_000866.4 (153347) GTCGGTTCTACCCAAGATTTAGAACTGTATGAGAAAAATAGCTATGCGGTATCACCATATGAATTAAACCGTGTATTAGCAAATTATTTGCCACTA
SARS-CoV-2 NC_045512.2 (29904) ------------------------------------------------------------------------------------------------
Section 1607
(154177) 154177 154187 154198 154209 154220 154231 154242 154253 154272
T4 NC_000866.4 (153443) AAAGCAAAAGCTGCTGATACAAATTTATTGGATGGTCTAGATTCATCTCAGTTCATTCGTAGGGATATTGCACAGACGGTTAATGGTTCACTAACC
SARS-CoV-2 NC_045512.2 (29904) ------------------------------------------------------------------------------------------------
Section 1608
(154273) 154273 154286 154297 154308 154319 154330 154341 154352 154368
T4 NC_000866.4 (153539) TTAACCCAACAAACGAATCTGAGTGCCCCTCTTGTATCATCTAGTACTGGTGAATTTGGTGGTTCATTGGCCGCTAATAGAACATTTACCATCCGT
SARS-CoV-2 NC_045512.2 (29904) ------------------------------------------------------------------------------------------------
Section 1609
(154369) 154369154374 154385 154396 154407 154418 154429 154440 154451 154464
T4 NC_000866.4 (153635) AATACAGGAGCCCCGACTAGTATCGTTTTCGAAAAAGGTCCTGCATCCGGGGCAAATCCTGCACAGTCAATGAGTATTCGTGTATGGGGTAACCAA
SARS-CoV-2 NC_045512.2 (29904) ------------------------------------------------------------------------------------------------
Section 1610
(154465) 154465 154473 154484 154495 154506 154517 154528 154539 154560
T4 NC_000866.4 (153731) TTTGGCGGCGGTAGTGATACGACCCGTTCGACAGTGTTTGAAGTTGGCGATGACACATCTCATCACTTTTATTCTCAACGTAATAAAGACGGTAAT
SARS-CoV-2 NC_045512.2 (29904) ------------------------------------------------------------------------------------------------
```

```
                                                                                                   Section 1611
                    (154561) 154561     154572     154583     154594     154605     154616     154627     154638           154656
     T4 NC_000866.4 (153827) ATAGCGTTTAACATTAATGGTACTGTAATGCCAATAAACATTAATGCTTCCGGTTTGATGAATGTGAATGGCACTGCAACATTCGGTCGTTCAGTT
SARS-CoV-2 NC_045512.2 (29904) ------------------------------------------------------------------------------------------------
                                                                                                   Section 1612
                    (154657) 154657        154671     154682     154693     154704     154715     154726     154737          154752
     T4 NC_000866.4 (153923) ACAGCCAATGGTGAATTCATCAGCAAGTCTGCAAATGCTTTTAGAGCAATAAACGGTGATTACGGATTCTTTATTCGTAATGATGCCTCTAATACC
SARS-CoV-2 NC_045512.2 (29904) ------------------------------------------------------------------------------------------------
                                                                                                   Section 1613
                    (154753) 154753 154759     154770     154781     154792     154803     154814     154825     154836      154848
     T4 NC_000866.4 (154019) TATTTTTTGCTCACTGCAGCCGGTGATCAGACTGGTGGTTTTAATGGATTACGCCCATTATTAATTAATAATCAATCCGGTCAGATTACAATTGGT
SARS-CoV-2 NC_045512.2 (29904) ------------------------------------------------------------------------------------------------
                                                                                                   Section 1614
                    (154849) 154849   154858     154869     154880     154891     154902     154913     154924               154944
     T4 NC_000866.4 (154115) GAAGGCTTAATCATTGCCAAAGGTGTTACTATAAATTCAGGCGGTTTAACTGTTAACTCGAGAATTCGTTCTCAGGGTACTAAAACATCTGATTTA
SARS-CoV-2 NC_045512.2 (29904) ------------------------------------------------------------------------------------------------
                                                                                                   Section 1615
                    (154945) 154945      154957     154968     154979     154990     155001     155012     155023           155040
     T4 NC_000866.4 (154211) TATACCCGTGCGCCAACATCTGATACTGTAGGATTCTGGTCAATCGATATTAATGATTCAGCCACTTATAACCAGTTCCCGGGTTATTTTAAAATG
SARS-CoV-2 NC_045512.2 (29904) ------------------------------------------------------------------------------------------------
                                                                                                   Section 1616
                    (155041) 155041         155056     155067     155078     155089     155100     155111     155122          155136
     T4 NC_000866.4 (154307) GTTGAAAAAACTAATGAAGTGACTGGGCTTCCATACTTAGAACGTGGCGAAGAAGTTAAATCTCCTGGTACACTGACTCAGTTTGGTAACACACTT
SARS-CoV-2 NC_045512.2 (29904) ------------------------------------------------------------------------------------------------
                                                                                                   Section 1617
                    (155137) 155137 155144     155155     155166     155177     155188     155199     155210     155221     155232
     T4 NC_000866.4 (154403) GATTCGCTTTACCAAGATTGGATTACTTATCCAACGACGCCAGAAGCGCGTACCACTCGCTGGACACGTACATGGCAGAAAACCAAAAACTCTTGG
SARS-CoV-2 NC_045512.2 (29904) ------------------------------------------------------------------------------------------------
```

```
                                                                                                    Section 1618
                 (155233) 155233    155243     155254     155265     155276     155287     155298     155309           155328
      T4 NC_000866.4 (154499) TCAAGTTTTGTTCAGGTATTTGACGGAGGTAACCCTCCTCAACCATCTGATATCGGTGCTTTACCATCTGATAATGCTACAATGGGGAATCTTACT
 SARS-CoV-2 NC_045512.2 (29904) ------------------------------------------------------------------------------------------------
                                                                                                    Section 1619
                 (155329) 155329       155342     155353     155364     155375     155386     155397     155408          155424
      T4 NC_000866.4 (154595) ATTCGTGATTTCTTGCGAATTGGTAATGTTCGCATTGTTCCTGACCCAGTGAATAAAACGGTTAAATTTGAATGGGTTGAATAAGAGGTATTATGG
 SARS-CoV-2 NC_045512.2 (29904) ------------------------------------------------------------------------------------------------
                                                                                                    Section 1620
                 (155425) 155425155430     155441     155452     155463     155474     155485     155496     155507      155520
      T4 NC_000866.4 (154691) AAAAATTTATGGCCGAGTTTGGACAAGGATATGTCCAAACGCCATTTTTATCGGAAAGTAATTCAGTAAGATATAAAATAAGTATAGCGGGTTCTT
 SARS-CoV-2 NC_045512.2 (29904) ------------------------------------------------------------------------------------------------
                                                                                                    Section 1621
                 (155521) 155521  155529     155540     155551     155562     155573     155584     155595               155616
      T4 NC_000866.4 (154787) GCCCGCTTTTCTACAGCAGGACCATCATATGTTAAATTTCAGGATAATCCTGTAGGAAGTCAAACATTTAGCGCAGGCCTCCATTTAAGAGTTTTG
 SARS-CoV-2 NC_045512.2 (29904) ------------------------------------------------------------------------------------------------
                                                                                                    Section 1622
                 (155617) 155617     155628     155639     155650     155661     155672     155683     155694           155712
      T4 NC_000866.4 (154883) ACCCTTCCACCGGAGCATTAGTTGATAGTAAGTCATATGCCTTTTCGACTTCAAATGATACTACATCAGCTGCTTTTGTTAGTTTCATGAATTCTT
 SARS-CoV-2 NC_045512.2 (29904) ------------------------------------------------------------------------------------------------
                                                                                                    Section 1623
                 (155713) 155713         155727     155738     155749     155760     155771     155782     155793       155808
      T4 NC_000866.4 (154979) TGACGAATAATCGAATTGTTGCTATATTAACTAGTGGAAAGGTTAATTTTCCTCCTGAAGTAGTATCTTGGTTAAGAACCGCCGGAACGTCTGCCT
 SARS-CoV-2 NC_045512.2 (29904) ------------------------------------------------------------------------------------------------
                                                                                                    Section 1624
                 (155809) 155809 155815     155826     155837     155848     155859     155870     155881     155892  155904
      T4 NC_000866.4 (155075) TTCCATCTGATTCTATATTGTCAAGATTTGACGTATCATATGCTGCTTTTTATACTTCTTCTAAAAGAGCTATCGCATTAGAGCATGTTAAACTGA
 SARS-CoV-2 NC_045512.2 (29904) ------------------------------------------------------------------------------------------------
```

```
                                                                                                   Section 1625
                      (155905) 155905   155914         155925         155936         155947         155958         155969         155980           156000
          T4 NC_000866.4 (155171) GTAATAGAAAAAGCACAGATGATTATCAAACTATTTTAGATGTTGTATTTGACAGTTTAGAAGATGTAGGGGCTACCGGGTTTCCAAGAGGAACGT
SARS-CoV-2 NC_045512.2  (29904) ------------------------------------------------------------------------------------------------
                                                                                                   Section 1626
                      (156001) 156001          156013         156024         156035         156046         156057         156068         156079           156096
          T4 NC_000866.4 (155267) ATGAAAGTGTTGAGCAATTCATGTCGGCAGTTGGTGGAACTAATGACGAAATTGCGAGATTGCCAACTTCAGCTGCTATAAGTAAATTATCTGATT
SARS-CoV-2 NC_045512.2  (29904) ------------------------------------------------------------------------------------------------
                                                                                                   Section 1627
                      (156097) 156097              156112         156123         156134         156145         156156         156167         156178     156192
          T4 NC_000866.4 (155363) ATAATTTAATTCCTGGAGATGTTCTTTATCTTAAAGCTCAGTTATATGCTGATGCTGATTTACTTGCTCTTGGAACTACAAATATATCTATCCGTT
SARS-CoV-2 NC_045512.2  (29904) ------------------------------------------------------------------------------------------------
                                                                                                   Section 1628
                      (156193) 156193  156200         156211         156222         156233         156244         156255         156266         156277  156288
          T4 NC_000866.4 (155459) TTTATAATGCATCTAACGGATATATTTCTTCAACACAAGCTGAATTTACTGGGCAAGCTGGGTCATGGGAATTAAAGGAAGATTATGTAGTTGTTC
SARS-CoV-2 NC_045512.2  (29904) ------------------------------------------------------------------------------------------------
                                                                                                   Section 1629
                      (156289) 156289    156299         156310         156321         156332         156343         156354         156365            156384
          T4 NC_000866.4 (155555) CAGAAAACGCAGTAGGATTTACGATATACGCACAGAGAACTGCACAAGCTGGCCAAGGTGGCATGAGAAATTTAAGCTTTTCTGAAGTATCAAGAA
SARS-CoV-2 NC_045512.2  (29904) ------------------------------------------------------------------------------------------------
                                                                                                   Section 1630
                      (156385) 156385           156398         156409         156420         156431         156442         156453         156464           156480
          T4 NC_000866.4 (155651) ATGGCGGCATTTCGAAACCTGCTGAATTTGGCGTCAATGGTATTCGTGTTAATTATATCTGCGAATCCGCTTCACCCCCGGATATAATGGTACTTC
SARS-CoV-2 NC_045512.2  (29904) ------------------------------------------------------------------------------------------------
                                                                                                   Section 1631
                      (156481) 156481156486         156497         156508         156519         156530         156541         156552         156563     156576
          T4 NC_000866.4 (155747) CTACGCAAGCATCGTCTAAAACTGGTAAAGTGTTTGGGCAAGAATTTAGAGAAGTTTAAATTGAGGGACCCTTCGGGTTCCCTTTTTCTTTATAAA
SARS-CoV-2 NC_045512.2  (29904) ------------------------------------------------------------------------------------------------
```

Section 1632

```
                          (156577) 156577  156585  156596  156607  156618  156629  156640  156651  156672
T4 NC_000866.4            (155843) TACTATTCAAATAAAGGGGCATACAATGGCTGATTTAAAAGTAGGTTCAACAACTGGAGGCTCTGTCATTTGGCATCAAGGAAATTTTCCATTGAA
SARS-CoV-2 NC_045512.2    (29904)  ------------------------------------------------------------------------------------------------
```

Section 1633

```
                          (156673) 156673  156684  156695  156706  156717  156728  156739  156750  156768
T4 NC_000866.4            (155939) TCCAGCCGGTGACGATGTACTCTATAAATCATTTAAAATATATTCAGAATATAACAAACCACAAGCTGCTGATAACGATTTCGTTTCTAAAGCTAA
SARS-CoV-2 NC_045512.2    (29904)  ------------------------------------------------------------------------------------------------
```

Section 1634

```
                          (156769) 156769  156783  156794  156805  156816  156827  156838  156849  156864
T4 NC_000866.4            (156035) TGGTGGTACTTATGCATCAAAGGTAACATTTAACGCTGGCATTCAAGTCCCATATGCTCCAAACATCATGAGCCCATGCGGGATTTATGGGGGTAA
SARS-CoV-2 NC_045512.2    (29904)  ------------------------------------------------------------------------------------------------
```

Section 1635

```
                          (156865) 156865  156871  156882  156893  156904  156915  156926  156937  156948  156960
T4 NC_000866.4            (156131) CGGTGATGGTGCTACTTTTGATAAAGCAAATATCGATATTGTTTCATGGTATGGCGTAGGATTTAAATCGTCATTTGGTTCAACAGGCCGAACTGT
SARS-CoV-2 NC_045512.2    (29904)  ------------------------------------------------------------------------------------------------
```

Section 1636

```
                          (156961) 156961  156970  156981  156992  157003  157014  157025  157036  157056
T4 NC_000866.4            (156227) TGTAATTAATACACGCAATGGTGATATTAACACAAAAGGTGTTGTGTCGGCAGCTGGTCAAGTAAGAAGTGGTGCGGCTGCTCCTATAGCAGCGAA
SARS-CoV-2 NC_045512.2    (29904)  ------------------------------------------------------------------------------------------------
```

Section 1637

```
                          (157057) 157057  157069  157080  157091  157102  157113  157124  157135  157152
T4 NC_000866.4            (156323) TGACCTTACTAGAAAGGACTATGTTGATGGAGCAATAAATACTGTTACTGCAAATGCAAACTCTAGGGTGCTACGGTCTGGTGACACCATGACAGG
SARS-CoV-2 NC_045512.2    (29904)  ------------------------------------------------------------------------------------------------
```

Section 1638

```
                          (157153) 157153  157168  157179  157190  157201  157212  157223  157234  157248
T4 NC_000866.4            (156419) TAATTTAACAGCGCCAAACTTTTTCTCGCAGAATCCTGCATCTCAACCCTCACACGTTCCACGATTTGACCAAATCGTAATTAAGGATTCTGTTCA
SARS-CoV-2 NC_045512.2    (29904)  ------------------------------------------------------------------------------------------------
```

Section 1639

```
                          (157249) 157249 157256 157267 157278 157289 157300 157311 157322 157333 157344
        T4 NC_000866.4 (156515) AGATTTCGGCTATTATTAAGAGGACTTATGGCTACTTTAAAACAAATACAATTTAAAAGAAGCAAAATCGCAGGAACACGTCCTGCTGCTTCAGTA
SARS-CoV-2 NC_045512.2  (29904) ------------------------------------------------------------------------------------------------
```

Section 1640

```
                          (157345) 157345 157355 157366 157377 157388 157399 157410 157421 157440
        T4 NC_000866.4 (156611) TTAGCCGAAGGTGAATTGGCTATAAACTTAAAAGATAGAACAATTTTTACTAAAGATGATTCAGGAAATATCATCGATCTAGGTTTTGCTAAAGGC
SARS-CoV-2 NC_045512.2  (29904) ------------------------------------------------------------------------------------------------
```

Section 1641

```
                          (157441) 157441 157454 157465 157476 157487 157498 157509 157520 157536
        T4 NC_000866.4 (156707) GGGCAAGTTGATGGCAACGTTACTATTAACGGACTTTTGAGATTAAATGGCGATTATGTACAAACAGGTGGAATGACTGTAAACGGACCCATTGGT
SARS-CoV-2 NC_045512.2  (29904) ------------------------------------------------------------------------------------------------
```

Section 1642

```
                          (157537) 157537157542 157553 157564 157575 157586 157597 157608 157619 157632
        T4 NC_000866.4 (156803) TCTACTGATGGCGTCACTGGAAAAATTTTCAGATCTACACAGGGTTCATTTTATGCAAGAGCAACAAACGATACTTCAAATGCCCATTTATGGTTT
SARS-CoV-2 NC_045512.2  (29904) ------------------------------------------------------------------------------------------------
```

Section 1643

```
                          (157633) 157633 157641 157652 157663 157674 157685 157696 157707 157728
        T4 NC_000866.4 (156899) GAAAATGCCGATGGCACTGAACGTGGCGTTATATATGCTCGCCCTCAAACTACAACTGACGGTGAAATACGCCTTAGGGTTAGACAAGGAACAGGA
SARS-CoV-2 NC_045512.2  (29904) ------------------------------------------------------------------------------------------------
```

Section 1644

```
                          (157729) 157729 157740 157751 157762 157773 157784 157795 157806 157824
        T4 NC_000866.4 (156995) AGCACTGCCAACAGTGAATTCTATTTCCGCTCTATAAATGGAGGCGAATTTCAGGCTAACCGTATTTTAGCATCAGATTCGTTAGTAACAAAACGC
SARS-CoV-2 NC_045512.2  (29904) ------------------------------------------------------------------------------------------------
```

Section 1645

```
                          (157825) 157825 157839 157850 157861 157872 157883 157894 157905 157920
        T4 NC_000866.4 (157091) ATTGCGGTTGATACCGTTATTCATGATGCCAAAGCATTTGGACAATATGATTCTCACTCTTTGGTTAATTATGTTTATCCTGGAACCGGTGAAACA
SARS-CoV-2 NC_045512.2  (29904) ------------------------------------------------------------------------------------------------
```

Section 1646

```
                          (157921) 157921 157927     157938     157949     157960     157971     157982     157993     158004     158016
         T4 NC_000866.4 (157187) AATGGTGTAAACTATCTTCGTAAAGTTCGCGCTAAGTCCGGTGGTACAATTTATCATGAAATTGTTACTGCACAAACAGGCCTGGCTGATGAAGTT
SARS-CoV-2 NC_045512.2   (29904) ------------------------------------------------------------------------------------------------
```

Section 1647

```
                          (158017) 158017   158026     158037     158048     158059     158070     158081     158092               158112
         T4 NC_000866.4 (157283) TCTTGGTGGTCTGGTGATACACCAGTATTTAAACTATACGGTATTCGTGACGATGGCAGAATGATTATCCGTAATAGCCTTGCATTAGGTACATTC
SARS-CoV-2 NC_045512.2   (29904) ------------------------------------------------------------------------------------------------
```

Section 1648

```
                          (158113) 158113      158125     158136     158147     158158     158169     158180     158191           158208
         T4 NC_000866.4 (157379) ACTACAAATTTCCCGTCTAGTGATTATGGCAACGTCGGTGTAATGGGCGATAAGTATCTTGTTCTCGGCGACACTGTAACTGGCTTGTCATACAAA
SARS-CoV-2 NC_045512.2   (29904) ------------------------------------------------------------------------------------------------
```

Section 1649

```
                          (158209) 158209         158224     158235     158246     158257     158268     158279     158290        158304
         T4 NC_000866.4 (157475) AAAACTGGTGTATTTGATCTAGTTGGCGGTGGATATTCTGTTGCTTCTATTACTCCTGACAGTTTCCGTAGTACTCGTAAAGGTATATTTGGTCGT
SARS-CoV-2 NC_045512.2   (29904) ------------------------------------------------------------------------------------------------
```

Section 1650

```
                          (158305) 158305 158312     158323     158334     158345     158356     158367     158378     158389   158400
         T4 NC_000866.4 (157571) TCTGAGGACCAAGGCGCAACTTGGATAATGCCTGGTACAAATGCTGCTCTCTTGTCTGTTCAAACACAAGCTGATAATAACAATGCTGGAGACGGA
SARS-CoV-2 NC_045512.2   (29904) ------------------------------------------------------------------------------------------------
```

Section 1651

```
                          (158401) 158401    158411     158422     158433     158444     158455     158466     158477               158496
         T4 NC_000866.4 (157667) CAAACCCATATCGGGTACAATGCTGGCGGTAAAATGAACCACTATTTCCGTGGTACAGGTCAGATGAATATCAATACCCAACAAGGTATGGAAATT
SARS-CoV-2 NC_045512.2   (29904) ------------------------------------------------------------------------------------------------
```

Section 1652

```
                          (158497) 158497       158510     158521     158532     158543     158554     158565     158576           158592
         T4 NC_000866.4 (157763) AACCCGGGTATTTTGAAATTGGTAACTGGCTCTAATAATGTACAATTTTACGCTGACGGAACTATTTCTTCCATTCAACCTATTAAATTAGATAAC
SARS-CoV-2 NC_045512.2   (29904) ------------------------------------------------------------------------------------------------
```

Section 1653

```
                          (158593) 158593 158598 158609 158620 158631 158642 158653 158664 158675 158688
T4 NC_000866.4            (157859) GAGATATTTTTAACTAAATCTAATAATACTGCGGGTCTTAAATTTGGAGCTCCTAGCCAAGTTGATGGCACAAGGACTATCCAATGGAACGGTGGT
SARS-CoV-2 NC_045512.2    (29904)  ------------------------------------------------------------------------------------------------
```

Section 1654

```
                          (158689) 158689 158697 158708 158719 158730 158741 158752 158763 158784
T4 NC_000866.4            (157955) ACTCGCGAAGGACAGAATAAAAACTATGTGATTATTAAAGCATGGGGTAACTCATTTAATGCCACTGGTGATAGATCTCGCGAAACGGTTTTCCAA
SARS-CoV-2 NC_045512.2    (29904)  ------------------------------------------------------------------------------------------------
```

Section 1655

```
                          (158785) 158785 158796 158807 158818 158829 158840 158851 158862 158880
T4 NC_000866.4            (158051) GTATCAGATAGTCAAGGATATTATTTTTATGCTCATCGTAAAGCTCCAACCGGCGACGAAACTATTGGACGTATTGAAGCTCAATTTGCTGGGGAT
SARS-CoV-2 NC_045512.2    (29904)  ------------------------------------------------------------------------------------------------
```

Section 1656

```
                          (158881) 158881 158895 158906 158917 158928 158939 158950 158961 158976
T4 NC_000866.4            (158147) GTTTATGCTAAAGGTATTATTGCCAACGGAAATTTTAGAGTTGTTGGGTCAAGCGCTTTAGCCGGCAATGTTACTATGTCTAACGGTTTGTTTGTC
SARS-CoV-2 NC_045512.2    (29904)  ------------------------------------------------------------------------------------------------
```

Section 1657

```
                          (158977) 158977 158983 158994 159005 159016 159027 159038 159049 159060 159072
T4 NC_000866.4            (158243) CAAGGTGGTTCTTCTATTACTGGACAAGTTAAAATTGGCGGAACAGCAAACGCACTGAGAATTTGGAACGCTGAATATGGTGCTATTTTCCGTCGT
SARS-CoV-2 NC_045512.2    (29904)  ------------------------------------------------------------------------------------------------
```

Section 1658

```
                          (159073) 159073 159082 159093 159104 159115 159126 159137 159148 159168
T4 NC_000866.4            (158339) TCGGAAAGTAACTTTTATATTATTCCAACCAATCAAAATGAAGGAGAAAGTGGAGACATTCACAGCTCTTTGAGACCTGTGAGAATAGGATTAAAC
SARS-CoV-2 NC_045512.2    (29904)  ------------------------------------------------------------------------------------------------
```

Section 1659

```
                          (159169) 159169 159181 159192 159203 159214 159225 159236 159247 159264
T4 NC_000866.4            (158435) GATGGCATGGTTGGGTTAGGAAGAGATTCTTTTATAGTAGATCAAAATAATGCTTTAACTACGATAAACAGTAACTCTCGCATTAATGCCAACTTT
SARS-CoV-2 NC_045512.2    (29904)  ------------------------------------------------------------------------------------------------
```

```
                                                                                                    Section 1660
                 (159265) 159265         159280     159291     159302     159313     159324     159335     159346     159360
T4 NC_000866.4   (158531) AGAATGCAATTGGGGCAGTCGGCATACATTGATGCAGAATGTACTGATGCTGTTCGCCCGGCGGGTGCAGGTTCATTTGCTTCCCAGAATAATGAA
SARS-CoV-2 NC_045512.2 (29904) ------------------------------------------------------------------------------------------------
                                                                                                    Section 1661
                 (159361) 159361 159368     159379     159390     159401     159412     159423     159434     159445 159456
T4 NC_000866.4   (158627) GACGTCCGTGCGCCGTTCTATATGAATATTGATAGAACTGATGCTAGTGCATATGTTCCTATTTTGAAACAACGTTATGTTCAAGGCAATGGCTGC
SARS-CoV-2 NC_045512.2 (29904) ------------------------------------------------------------------------------------------------
                                                                                                    Section 1662
                 (159457) 159457    159467     159478     159489     159500     159511     159522     159533          159552
T4 NC_000866.4   (158723) TATTCATTAGGGACTTTAATTAATAATGGTAATTTCCGAGTTCATTACCATGGCGGCGGAGATAACGGTTCTACAGGTCCACAGACTGCTGATTTT
SARS-CoV-2 NC_045512.2 (29904) ------------------------------------------------------------------------------------------------
                                                                                                    Section 1663
                 (159553) 159553       159566     159577     159588     159599     159610     159621     159632          159648
T4 NC_000866.4   (158819) GGATGGGAATTTATTAAAAACGGTGATTTTATTTCACCTCGCGATTTAATAGCAGGCAAAGTCAGATTTGATAGAACTGGTAATATCACTGGTGGT
SARS-CoV-2 NC_045512.2 (29904) ------------------------------------------------------------------------------------------------
                                                                                                    Section 1664
                 (159649) 159649159654      159665     159676     159687     159698     159709     159720     159731   159744
T4 NC_000866.4   (158915) TCTGGTAATTTTGCTAACTTAAACAGTACAATTGAATCACTTAAAACTGATATCATGTCGAGTTACCCAATTGGTGCTCCGATTCCTTGGCCGAGT
SARS-CoV-2 NC_045512.2 (29904) ------------------------------------------------------------------------------------------------
                                                                                                    Section 1665
                 (159745) 159745  159753     159764     159775     159786     159797     159808     159819          159840
T4 NC_000866.4   (159011) GATTCAGTTCCTGCTGGATTTGCTTTGATGGAAGGTCAGACCTTTGATAAGTCCGCATATCCAAAGTTAGCTGTTGCATATCCTAGCGGTGTTATT
SARS-CoV-2 NC_045512.2 (29904) ------------------------------------------------------------------------------------------------
                                                                                                    Section 1666
                 (159841) 159841     159852     159863     159874     159885     159896     159907     159918          159936
T4 NC_000866.4   (159107) CCAGATATGCGCGGGCAAACTATCAAGGGTAAACCAAGTGGTCGTGCTGTTTTGAGCGCTGAGGCAGATGGTGTTAAGGCTCATAGCCATAGTGCA
SARS-CoV-2 NC_045512.2 (29904) ------------------------------------------------------------------------------------------------
```

```
                                                                                                  Section 1667
                     (159937) 159937        159951     159962     159973     159984     159995     160006     160017       160032
     T4 NC_000866.4 (159203) TCGGCTTCAAGTACTGACTTAGGTACTAAAACCACATCAAGCTTTGACTATGGTACGAAGGGAACTAACAGTACGGGTGGACACACTCACTCTGGT
SARS-CoV-2 NC_045512.2 (29904) ------------------------------------------------------------------------------------------------
                                                                                                  Section 1668
                     (160033) 160033 160039     160050     160061     160072     160083     160094     160105     160116 160128
     T4 NC_000866.4 (159299) AGTGGTTCTACTAGCACAAATGGTGAGCACAGCCACTACATCGAGGCATGGAATGGTACTGGTGTAGGTGGTAATAAGATGTCATCATATGCCATA
SARS-CoV-2 NC_045512.2 (29904) ------------------------------------------------------------------------------------------------
                                                                                                  Section 1669
                     (160129) 160129   160138     160149     160160     160171     160182     160193     160204             160224
     T4 NC_000866.4 (159395) TCATACAGGGCGGGTGGGAGTAACACTAATGCAGCAGGGAACCACAGTCACACTTTCTCTTTTGGGACTAGCAGTGCTGGCGACCATTCCCACTCT
SARS-CoV-2 NC_045512.2 (29904) ------------------------------------------------------------------------------------------------
                                                                                                  Section 1670
                     (160225) 160225      160237     160248     160259     160270     160281     160292     160303           160320
     T4 NC_000866.4 (159491) GTAGGTATTGGTGCTCATACCCACACGGTAGCAATTGGATCACATGGTCATACTATCACTGTAAATAGTACAGGTAATACAGAAAACACGGTTAAA
SARS-CoV-2 NC_045512.2 (29904) ------------------------------------------------------------------------------------------------
                                                                                                  Section 1671
                     (160321) 160321         160336     160347     160358     160369     160380     160391     160402       160416
     T4 NC_000866.4 (159587) AACATTGCTTTTAACTATATCGTTCGTTTAGCATAAGGAGAGGGGCTTCGGCCCTTCTAAATATGAAAATATATCATTATTATTTTGACACTAAAG
SARS-CoV-2 NC_045512.2 (29904) ------------------------------------------------------------------------------------------------
                                                                                                  Section 1672
                     (160417) 160417 160424     160435     160446     160457     160468     160479     160490     160501 160512
     T4 NC_000866.4 (159683) AATTTTACAAAGAAGAAAATTACAAACCGGTTAAAGGCCTCGGTCTTCCTGCTCATTCAACAATTAAAAAACCTTTAGAACCTAAAGAAGGATACG
SARS-CoV-2 NC_045512.2 (29904) ------------------------------------------------------------------------------------------------
                                                                                                  Section 1673
                     (160513) 160513    160523     160534     160545     160556     160567     160578     160589             160608
     T4 NC_000866.4 (159779) CGGTTGTATTTGATGAACGTACTCAGGATTGGATTTATGAAGAAGACCATCGCGGAAAACGCGCATGGACTTTTAATAAAGAAGAAATTTTTATAA
SARS-CoV-2 NC_045512.2 (29904) ------------------------------------------------------------------------------------------------
```

```
                                                                                                    Section 1674
(160609)                     160609       160622     160633     160644     160655     160666     160677     160688          160704
T4 NC_000866.4 (159875)      GTGACATTGGAAGCCCGGTTGGTATAACTTTCGATGAGCCCGGCGAATTTGATATATGGACTGATGACGGTTGGAAAGAAGACGAAACATATAAGC
SARS-CoV-2 NC_045512.2 (29904) ------------------------------------------------------------------------------------------------

                                                                                                    Section 1675
(160705)                     160705160710    160721     160732     160743     160754     160765     160776     160787     160800
T4 NC_000866.4 (159971)      GAGTTTTAATTCGTAATAGAAAAATTGAAGAATTATATAAAGAGTTCCAAGTTTTAAATAATATGATTGAAGCTTCTGTCGCCAATAAAAAGGAAA
SARS-CoV-2 NC_045512.2 (29904) ------------------------------------------------------------------------------------------------

                                                                                                    Section 1676
(160801)                     160801  160809     160820     160831     160842     160853     160864     160875               160896
T4 NC_000866.4 (160067)      AATTCTATTATAAAAACCTTAAGCGGTTCTTTGCTCTTTTAGAAAAGCATGAGCATTTAGGTGGTGAATTCCCTTCATGGCCTGAAAAAGAACAGA
SARS-CoV-2 NC_045512.2 (29904) ------------------------------------------------------------------------------------------------

                                                                                                    Section 1677
(160897)                     160897     160908     160919     160930     160941     160952     160963     160974          160992
T4 NC_000866.4 (160163)      AGCCTTGGTATAAGCGTTTATTCAAGCATTACGTATAAATATCTTAAAAGGAGGGTCTATGGCAGCACCTAGAATATCATTTTCGCCCTCTGATAT
SARS-CoV-2 NC_045512.2 (29904) ------------------------------------------------------------------------------------------------

                                                                                                    Section 1678
(160993)                     160993        161007     161018     161029     161040     161051     161062     161073          161088
T4 NC_000866.4 (160259)      TCTATTTGGTGTTCTAGATCGCTTGTTCAAAGATAACGCTACCGGGAAGGTTCTTGCTTCCCGGGTAGCTGTCGTAATTCTTTTGTTTATAATGGC
SARS-CoV-2 NC_045512.2 (29904) ------------------------------------------------------------------------------------------------

                                                                                                    Section 1679
(161089)                     161089 161095     161106     161117     161128     161139     161150     161161     161172     161184
T4 NC_000866.4 (160355)      GATTGTTTGGTATAGGGGAGATAGTTTCTTTGAGTACTATAAGCAATCAAAGTATGAAACATACAGTGAAATTATTGAAAAGGAAAGAACTGCACG
SARS-CoV-2 NC_045512.2 (29904) ------------------------------------------------------------------------------------------------

                                                                                                    Section 1680
(161185)                     161185   161194     161205     161216     161227     161238     161249     161260               161280
T4 NC_000866.4 (160451)      CTTTGAATCTGTCGCCCTGGAACAACTCCAGATAGTTCATATATCATCTGAGGCAGACTTTAGTGCGGTGTATTCTTTCCGCCCTAAAAACTTAAA
SARS-CoV-2 NC_045512.2 (29904) ------------------------------------------------------------------------------------------------
```

```
                                                                                                                    Section 1681
                      (161281) 161281      161293    161304    161315    161326    161337    161348    161359        161376
        T4 NC_000866.4 (160547) CTATTTTGTTGATATTATAGCATACGAAGGAAAATTACCTTCAACAATAAGTGAAAAATCACTTGGAGGATATCCTGTTGATAAAACTATGGATGA
SARS-CoV-2 NC_045512.2  (29904) ------------------------------------------------------------------------------------------------
                                                                                                                    Section 1682
                      (161377) 161377         161392    161403    161414    161425    161436    161447    161458       161472
        T4 NC_000866.4 (160643) ATATACAGTTCATTTAAATGGACGTCATTATTATTCCAACTCAAAATTTGCTTTTTTACCAACTAAAAAGCCTACTCCCGAAATAAACTACATGTA
SARS-CoV-2 NC_045512.2  (29904) ------------------------------------------------------------------------------------------------
                                                                                                                    Section 1683
                      (161473) 161473 161480     161491    161502    161513    161524    161535    161546    161557 161568
        T4 NC_000866.4 (160739) CAGTTGTCCATATTTTAATTTGGATAATATCTATGCTGGAACGATAACCATGTACTGGTATAGAAATGATCATATAAGTAATGACCGCCTTGAATC
SARS-CoV-2 NC_045512.2  (29904) ------------------------------------------------------------------------------------------------
                                                                                                                    Section 1684
                      (161569) 161569    161579     161590    161601    161612    161623    161634    161645          161664
        T4 NC_000866.4 (160835) AATATGTGCTCAGGCGGCCAGAATATTAGGAAGGGCTAAATAATTATTTGTTCGTATACATCTCTAGATATCGATATACACCCTCAAAACCCTCGT
SARS-CoV-2 NC_045512.2  (29904) ------------------------------------------------------------------------------------------------
                                                                                                                    Section 1685
                      (161665) 161665       161678     161689    161700    161711    161722    161733    161744         161760
        T4 NC_000866.4 (160931) TGAATTCGTCGATGAGGGTTTTCTTATCTTCTTGAGTTAATTCAGAAACAATTTTACGGAATGAATTTTGATTTAACTTTCTACCTTCATGCGTTA
SARS-CoV-2 NC_045512.2  (29904) ------------------------------------------------------------------------------------------------
                                                                                                                    Section 1686
                      (161761) 161761161766     161777    161788    161799    161810    161821    161832    161843      161856
        T4 NC_000866.4 (161027) CTCCAATCTCATTTAGAAATGCAATAAAATTAGCACGATTCTCAACAATATCTTCTCTGGAAAATTTAATCAAAATAGATGCAACAGTAATAATTT
SARS-CoV-2 NC_045512.2  (29904) ------------------------------------------------------------------------------------------------
                                                                                                                    Section 1687
                      (161857) 161857  161865     161876    161887    161898    161909    161920    161931            161952
        T4 NC_000866.4 (161123) CACGAACTGTATCAATGTTTTTATTCATTAACTATACCACTCAATTAGTTGACTTTGTTATAATATCATCAGACGCTTGATTTGTAAACTGGTCTG
SARS-CoV-2 NC_045512.2  (29904) ------------------------------------------------------------------------------------------------
```

```
                                                                                                        Section 1688
                           (161953) 161953     161964     161975     161986     161997     162008     162019     162030          162048
          T4 NC_000866.4 (161219) TGTAATTTTCTTCAAAAATTTTTTCTACGAATTCCTTGAACGATTCACGTTCCTGAGCTACATTATGCTCGATTACCTTTTCAAGATTATGACTCA
   SARS-CoV-2 NC_045512.2  (29904) ------------------------------------------------------------------------------------------------
                                                                                                        Section 1689
                           (162049) 162049        162063     162074     162085     162096     162107     162118     162129          162144
          T4 NC_000866.4 (161315) TTCGAAATAATCTTCAATTTCATAATCATGGACATAAATCATTATAGTTTTTAATACATCATCAATATTTTTTCCTGGAGCTGGAATTACGTAAAA
   SARS-CoV-2 NC_045512.2  (29904) ------------------------------------------------------------------------------------------------
                                                                                                        Section 1690
                           (162145) 162145 162151     162162     162173     162184     162195     162206     162217     162228    162240
          T4 NC_000866.4 (161411) ATACCCTGCTTTTGAGAGGTCTTTATAAGTTCCAATCAAGAAATCATTATTCTCAAGATGTAACTCTTCAACTAATTCATTGACAATTGAATGGTA
   SARS-CoV-2 NC_045512.2  (29904) ------------------------------------------------------------------------------------------------
                                                                                                        Section 1691
                           (162241) 162241   162250     162261     162272     162283     162294     162305     162316               162336
          T4 NC_000866.4 (161507) TAGGTTTGGCAGAAACTTATATAGCTTTTCTAGAATATCAATTTTGAATGTATATTGAACCACGGACTGAGAATCAATAATCATAGACCTTCCCCT
   SARS-CoV-2 NC_045512.2  (29904) ------------------------------------------------------------------------------------------------
                                                                                                        Section 1692
                           (162337) 162337      162349     162360     162371     162382     162393     162404     162415          162432
          T4 NC_000866.4 (161603) TATGTTTCTGTTTGCGATTAGATTCTTTAAACGCTTTCTTCTTATCCTTATGAACAGAAGCTTTATTAAAATTATGCTTTGCGACTAAATTGTTCA
   SARS-CoV-2 NC_045512.2  (29904) ------------------------------------------------------------------------------------------------
                                                                                                        Section 1693
                           (162433) 162433         162448     162459     162470     162481     162492     162503     162514          162528
          T4 NC_000866.4 (161699) TAGTGCTGAATTACCTCTCTTAAACATTTGCATGTGAATGAAAACTTTTTAGCTACACCACATTCAAATATATGTTCTCTTAAATCGCGTGTATCG
   SARS-CoV-2 NC_045512.2  (29904) ------------------------------------------------------------------------------------------------
                                                                                                        Section 1694
                           (162529) 162529 162536     162547     162558     162569     162580     162591     162602     162613 162624
          T4 NC_000866.4 (161795) GTATATCCCATCTCAACAATAAAATGCCGTATTAGATTTTTATCTTTATCGTTTAGAGAATTAAAATAATCAGATTTTGAATTAATTTCCCTGGCC
   SARS-CoV-2 NC_045512.2  (29904) ------------------------------------------------------------------------------------------------
```

```
                                          Section 1695
                    (162625) 162625    162635     162646     162657     162668     162679     162690     162701          162720
     T4 NC_000866.4 (161891) AAATTGAATCACCTTCAGTTGACGTTTTAACTCTTTTATCATCTCTTCGTTCATCGCAATATAAAGATCGCGTAGAGCAGGTTTTAGCATTCCATT
SARS-CoV-2 NC_045512.2 (29904) ------------------------------------------------------------------------------------------------
                                          Section 1696
                    (162721) 162721       162734     162745     162756     162767     162778     162789     162800       162816
     T4 NC_000866.4 (161987) TACTGGAGAACTAAATGGACATACATAATCTTTTCCTACGAGCTTTTTAGTGAATTCCATATCACAGAACTGAAATCCCGGCTCATTGGTATAAAT
SARS-CoV-2 NC_045512.2 (29904) ------------------------------------------------------------------------------------------------
                                          Section 1697
                    (162817) 162817162822     162833     162844     162855     162866     162877     162888     162899   162912
     T4 NC_000866.4 (162083) TCCCCAATTAGTTGACATCATTTTATTGGCATATTCCAGTGCCTGGATTTGATTCATAATTCCATCAATTTGAAACTTTTTAATATTCATTAGTAA
SARS-CoV-2 NC_045512.2 (29904) ------------------------------------------------------------------------------------------------
                                          Section 1698
                    (162913) 162913  162921     162932     162943     162954     162965     162976     162987            163008
     T4 NC_000866.4 (162179) AGGTCCTCAGAGTAAAGTTCTTTTTCACTACCACGTTCAATACTTACTTGTCCAGCGTAAGTTGCAATAATCATTGCTTCTTCACGTGTCCAATAA
SARS-CoV-2 NC_045512.2 (29904) ------------------------------------------------------------------------------------------------
                                          Section 1699
                    (163009) 163009     163020     163031     163042     163053     163064     163075     163086          163104
     T4 NC_000866.4 (162275) TTACTATATTGGTCAATAAACCCTTGGTCATCATCACAAACTTGCTGAGTAACTAATTGAGGTTTAACTACATCTAAAACTTCTGCCATATCTTTA
SARS-CoV-2 NC_045512.2 (29904) ------------------------------------------------------------------------------------------------
                                          Section 1700
                    (163105) 163105        163119     163130     163141     163152     163163     163174     163185      163200
     T4 NC_000866.4 (162371) GAATAATGACGAGCACCTGGAATAATAAGAGTTCGTCCATCTTTTAATTTAAAACGGTTGGCTGCGCAAACAATTCGTCGTTGATACTTTTGGTTT
SARS-CoV-2 NC_045512.2 (29904) ------------------------------------------------------------------------------------------------
                                          Section 1701
                    (163201) 163201 163207     163218     163229     163240     163251     163262     163273     163284   163296
     T4 NC_000866.4 (162467) TCATCCCAGTACGCAGTCTGCCAACAGATTTCAGGAACTTCTTTCAGAATATCTTCTTCTGTGCATTTATAACCATGCGCTTTAAATTTTTCAACA
SARS-CoV-2 NC_045512.2 (29904) ------------------------------------------------------------------------------------------------
```

```
                                                                                                Section 1702
                         (163297) 163297  163306    163317    163328    163339    163350    163361    163372       163392
        T4 NC_000866.4 (162563) AGACTTTCTGGAGTTTCACGAGATAAAGGAACATTCAGCATTTTTAAACGATTGATAAATGGGTTCATTTAAACCATCCTTTAATACGCTGCCACA
SARS-CoV-2 NC_045512.2  (29904) ------------------------------------------------------------------------------------------------
                                                                                                Section 1703
                         (163393) 163393     163405    163416    163427    163438    163449    163460    163471        163488
        T4 NC_000866.4 (162659) AAGTTTTCTGTTGAGCTTTGTTGACGCCAATTGAGCGAATAACCGGTTGAGATTCCTGGAATTCTTTATAATCAGCAAGGTAAATTTCGTAAGCTG
SARS-CoV-2 NC_045512.2  (29904) ------------------------------------------------------------------------------------------------
                                                                                                Section 1704
                         (163489) 163489        163504    163515    163526    163537    163548    163559    163570   163584
        T4 NC_000866.4 (162755) CATCCGTAAATGAACTTATCGCTGCCATAAAATTATTGCGAATACCTACTGGAGCATCTTTACTTTCACGAATGATCATGTATTTACCAGTCTTAA
SARS-CoV-2 NC_045512.2  (29904) ------------------------------------------------------------------------------------------------
                                                                                                Section 1705
                         (163585) 163585 163592    163603    163614    163625    163636    163647    163658    163669 163680
        T4 NC_000866.4 (162851) TCTTTACGATAGTTCCAAGATAAGCTCCATGGTACCAAATATCCCAACCCTCTTGAGTAGGTTCTGCGCAACGACGAAGTTCATTGACAATTTCTA
SARS-CoV-2 NC_045512.2  (29904) ------------------------------------------------------------------------------------------------
                                                                                                Section 1706
                         (163681) 163681   163691    163702    163713    163724    163735    163746    163757       163776
        T4 NC_000866.4 (162947) ACTTGTTTATTATTTATTCCTCACAGTTCAGATGCTACAGTGATTACAGCTTCAATGTTTTCTGCCGAGCGTTTAATGTCAAGATACACATTACCG
SARS-CoV-2 NC_045512.2  (29904) ------------------------------------------------------------------------------------------------
                                                                                                Section 1707
                         (163777) 163777      163790    163801    163812    163823    163834    163845    163856       163872
        T4 NC_000866.4 (163043) TTTTTAGCGATTTTACATGACATTCCGATGTCAGTAAATTTCTGAATATGATGTTCCATCATTTTGTATCCAAAAATTCGCATATTTCCATTGTTA
SARS-CoV-2 NC_045512.2  (29904) ------------------------------------------------------------------------------------------------
                                                                                                Section 1708
                         (163873) 163873163878    163889    163900    163911    163922    163933    163944    163955  163968
        T4 NC_000866.4 (163139) TTAATTTCAAAATTACGAATTCCGTTAGTGCGTTTTTCTAAAATAGCAAGATAATTACTACGATAAATTTCAACCTTTTTAAGAACAAATCCATTT
SARS-CoV-2 NC_045512.2  (29904) ------------------------------------------------------------------------------------------------
```

```
                                                                                                    Section 1709
                         (163969) 163969 163977     163988     163999     164010     164021     164032     164043               164064
          T4 NC_000866.4 (163235) TTATCTAAAAGTTTTAACATGAGGTCTTTATCTTCTTCCATATCGGAAGTAATCTCGCGAGCTTTACGAGTTGCTCGTTTTTTCAGCAGTTCCGGA
SARS-CoV-2 NC_045512.2    (29904) ------------------------------------------------------------------------------------------------
                                                                                                    Section 1710
                         (164065) 164065     164076     164087     164098     164109     164120     164131     164142           164160
          T4 NC_000866.4 (163331) GCATTTTCCTGTGCATATAAAGTTGCTGCATTTGAAATAATATCCTGAGCTTCACCAGTAATGATTAATCCATCACCAGATTTCTCCACCAGGCCT
SARS-CoV-2 NC_045512.2    (29904) ------------------------------------------------------------------------------------------------
                                                                                                    Section 1711
                         (164161) 164161        164175     164186     164197     164208     164219     164230     164241         164256
          T4 NC_000866.4 (163427) TTTTTAATCAATACCCCAATATTACTATTAACTACTGCGTTACCTAAATCTGGATGCACCTCACGAACTTCTGCAGCTGTAATGAAATCTTTCTTA
SARS-CoV-2 NC_045512.2    (29904) ------------------------------------------------------------------------------------------------
                                                                                                    Section 1712
                         (164257) 164257 164263     164274     164285     164296     164307     164318     164329     164340  164352
          T4 NC_000866.4 (163523) GCAATGGTAATTAAAATCGTAGCAGTTTTTTCATTCAGAACATCGTTAGAAGCTTTGATGATGTAAGTTACTTTAGACATTTTCTAATCTCCGTAA
SARS-CoV-2 NC_045512.2    (29904) ------------------------------------------------------------------------------------------------
                                                                                                    Section 1713
                         (164353) 164353   164362     164373     164384     164395     164406     164417     164428             164448
          T4 NC_000866.4 (163619) TTCTGTATCAGTAGTTGATAGTTGTATAGTACCACAGTATGCTTTGGTTGTAAACCGTTTTGTGAAAAAATTTTAAAATAAAAAAGGGAGAGCCT
SARS-CoV-2 NC_045512.2    (29904) ------------------------------------------------------------------------------------------------
                                                                                                    Section 1714
                         (164449) 164449      164461     164472     164483     164494     164505     164516     164527           164544
          T4 NC_000866.4 (163715) CGGCTCTCCCTAAAATTACTGCATGACTGTGATAACTGTCATGATAACACGTTGAATTCCGAACGCAAGAAGACCTCCTGCTACGGCTGGAACAAC
SARS-CoV-2 NC_045512.2    (29904) ------------------------------------------------------------------------------------------------
                                                                                                    Section 1715
                         (164545) 164545         164560     164571     164582     164593     164604     164615     164626        164640
          T4 NC_000866.4 (163811) GCCTAAACCCGCCAGTAAAATGCTACCAGATACTAATGCAGCGCTTGTAATACCAATGAATGGACTCATTTGATTTCCTCTAAATCTTTGGTGTAT
SARS-CoV-2 NC_045512.2    (29904) ------------------------------------------------------------------------------------------------
```

```
                                                                                                 Section 1716
                  (164641) 164641 164648     164659     164670     164681     164692     164703     164714     164725 164736
T4 NC_000866.4    (163907) TCAGTAACTACATCAGTAGTTTTCCAATATTCGTTTTCTTCTTTTTTAGCTTTAGCTTCTTCAGCAAGTTTCTTTGCTTCATCGGAAGTCATATGA
SARS-CoV-2 NC_045512.2 (29904) ------------------------------------------------------------------------------------------------
                                                                                                 Section 1717
                  (164737) 164737    164747     164758     164769     164780     164791     164802     164813          164832
T4 NC_000866.4    (164003) AAAATATTCATTCCAACTAGTTTATCAACATAAGAAGAATACATATCGATTTTCGAAAGTTCTTCGGTCAGTTCTTTACGAGTTTTACCCTGTACA
SARS-CoV-2 NC_045512.2 (29904) ------------------------------------------------------------------------------------------------
                                                                                                 Section 1718
                  (164833) 164833       164846     164857     164868     164879     164890     164901     164912          164928
T4 NC_000866.4    (164099) ACAATTTCACCTGAAATTACTTTCTTAATGAAATGTGCTTTGGCAAAGGCTAAACGAAAAGCTGACTCAGTTTCTTTAATTTTGTTATCAATTCGT
SARS-CoV-2 NC_045512.2 (29904) ------------------------------------------------------------------------------------------------
                                                                                                 Section 1719
                  (164929) 164929164934     164945     164956     164967     164978     164989     165000     165011   165024
T4 NC_000866.4    (164195) TTTTGGACATAAGTTTTACGAACTTCAACAAAGTCTTTAATTAAATCAACTACGTTATCGTAAACTTGCAGCTTTCCTTTCTCATTAATAACCGTA
SARS-CoV-2 NC_045512.2 (29904) ------------------------------------------------------------------------------------------------
                                                                                                 Section 1720
                  (165025) 165025  165033     165044     165055     165066     165077     165088     165099          165120
T4 NC_000866.4    (164291) ATATTCTGGGAACGACGCTCAATCAGTCCGAAGTCTTTCATAATTTTTGCATGGCGTTCTTCTTCGTTATCGCTCAAAGAATATTCTTTGCGGAAT
SARS-CoV-2 NC_045512.2 (29904) ------------------------------------------------------------------------------------------------
                                                                                                 Section 1721
                  (165121) 165121     165132     165143     165154     165165     165176     165187     165198          165216
T4 NC_000866.4    (164387) TTAACTTTGAAGCCAAAACCATGCTCACCACAAGCATCATCCCATGTAATGAAGCCTTTATTTTCAAGTGGGTCTAAGATTTTACTCACATAAGTT
SARS-CoV-2 NC_045512.2 (29904) ------------------------------------------------------------------------------------------------
                                                                                                 Section 1722
                  (165217) 165217        165231     165242     165253     165264     165275     165286     165297     165312
T4 NC_000866.4    (164483) TCACGATCATACTTATACGGAATCTCAGTGATATGCATTTGAGTTCGTGAAGTAAACTTATATGTTCCACGAATTTCATATTGCCCATCAATTTCA
SARS-CoV-2 NC_045512.2 (29904) ------------------------------------------------------------------------------------------------
```

```
                                                                                                              Section 1723
                         (165313) 165313 165319    165330    165341    165352    165363    165374    165385    165396  165408
         T4 NC_000866.4 (164579) ACGACTTCACCACGAAATTCTGGGAATTCTACTTTCGGTTTAGTTACTTTCTTTCCTTGAAGAGCTTGCAGTACAGCTTTCTTGACAGAAGAAACA
SARS-CoV-2 NC_045512.2  (29904) ------------------------------------------------------------------------------------------------
                                                                                                              Section 1724
                         (165409) 165409   165418    165429    165440    165451    165462    165473    165484        165504
         T4 NC_000866.4 (164675) CTATGAGGAAGAATGTAAGTTGCATAACCAGTTGCAATACCGGAAACGCCATTAAGAAGAACAGTAGGAATAATAGGCAAATAGAAAGCAGGCGGA
SARS-CoV-2 NC_045512.2  (29904) ------------------------------------------------------------------------------------------------
                                                                                                              Section 1725
                         (165505) 165505      165517    165528    165539    165550    165561    165572    165583       165600
         T4 NC_000866.4 (164771) ATGTGTTCTTTATCTTGATGTACCGGAGCATATTCAGTATCTTTATATACGTTATAGAAATTTTTACTTACACGAGCAAAAATATAACGACTTGCC
SARS-CoV-2 NC_045512.2  (29904) ------------------------------------------------------------------------------------------------
                                                                                                              Section 1726
                         (165601) 165601         165616    165627    165638    165649    165660    165671    165682    165696
         T4 NC_000866.4 (164867) GCTGCCTTTTGGACAGTACGAGAACCAAAGTTTCCTTGACCATCTAACAGAGGAAAGTTATTATTCCAAGTATTAGCCATCAAAGCACCTGCGTCT
SARS-CoV-2 NC_045512.2  (29904) ------------------------------------------------------------------------------------------------
                                                                                                              Section 1727
                         (165697) 165697 165704    165715    165726    165737    165748    165759    165770    165781  165792
         T4 NC_000866.4 (164963) TGTGCAGAGTTTTCACCATGATGATATCCAAGGTCCGCTACACCACCTGCAATAGAAGCGAGTTTGTGAAACTTATCTTTATTTCCTCGTGCCAAA
SARS-CoV-2 NC_045512.2  (29904) ------------------------------------------------------------------------------------------------
                                                                                                              Section 1728
                         (165793) 165793    165803    165814    165825    165836    165847    165858    165869        165888
         T4 NC_000866.4 (165059) TCAAGAGCTCGAGCAATAACAAATCGTTGAACTGGCTTAAATCCATCAATCATATTTGGGATAGCACGATTTTCAACCGTGTACATAGCATAAGCC
SARS-CoV-2 NC_045512.2  (29904) ------------------------------------------------------------------------------------------------
                                                                                                              Section 1729
                         (165889) 165889       165902    165913    165924    165935    165946    165957    165968       165984
         T4 NC_000866.4 (165155) AATGCTTCATTATCAATGATACTTTTTAAATCGCGATTATTCAGTTGCATAAATTTACCATACTAGTGAATGTAGTGCCATAATAACATCAGAAAT
SARS-CoV-2 NC_045512.2  (29904) ------------------------------------------------------------------------------------------------
```

Section 1730
```
(165985)                     165985 165990 166001 166012 166023 166034 166045 166056 166067 166080
T4 NC_000866.4 (165251)      GAAAAGCACGACTTGAATTAATCCGAACATTACTCCGTAATATAGTGCTACCAATAAAGCAGCAAGGGCTAATGAATAGCCCAAGATTTTCTTAAT
SARS-CoV-2 NC_045512.2 (29904) ------------------------------------------------------------------------------------------------
```

Section 1731
```
(166081)                     166081 166089 166100 166111 166122 166133 166144 166155 166176
T4 NC_000866.4 (165347)      CATTAGATAACAACACAAATGTTAAATATGCACACATACCCTGGGCTAAAGCTTGTGAAAACACACTGCTAGCATCGATACAGATAGTTAAAACAC
SARS-CoV-2 NC_045512.2 (29904) ------------------------------------------------------------------------------------------------
```

Section 1732
```
(166177)                     166177 166188 166199 166210 166221 166232 166243 166254 166272
T4 NC_000866.4 (165443)      ATGCTACTATCCAACAAATAAATGAAATAACTCCTAATAATTTTGCAATATTCATATTTTCCTCACTGGCGTCCGAAGACGCCTTTAGTTTTAAGA
SARS-CoV-2 NC_045512.2 (29904) ------------------------------------------------------------------------------------------------
```

Section 1733
```
(166273)                     166273 166287 166298 166309 166320 166331 166342 166353 166368
T4 NC_000866.4 (165539)      TTGTTACGATAGAACTGCATCACGTGTTCGTTATGGAAATTACTCATTAATATGCCTGTAAAACAAATTTAAAGTTATCAGCCAACATACGGTTCA
SARS-CoV-2 NC_045512.2 (29904) ------------------------------------------------------------------------------------------------
```

Section 1734
```
(166369)                     166369 166375 166386 166397 166408 166419 166430 166441 166452 166464
T4 NC_000866.4 (165635)      TTTCTTCGAGTGTTTGATACTCAGAATGATGATTACGAGTAAACGCCAAAGCTAGCTGACCTTTTCCAAATCCCGTCGTTAGAGGTTTCATCTTAG
SARS-CoV-2 NC_045512.2 (29904) ------------------------------------------------------------------------------------------------
```

Section 1735
```
(166465)                     166465 166474 166485 166496 166507 166518 166529 166540 166560
T4 NC_000866.4 (165731)      AAGCAGGCAGATAAAACACTGTGTATGGAACATTGTTATTTGCAATAGTACGCGCAAGTTGAGACCGGCGTTGACGAATATGACTTAAAACCGCAC
SARS-CoV-2 NC_045512.2 (29904) ------------------------------------------------------------------------------------------------
```

Section 1736
```
(166561)                     166561 166573 166584 166595 166606 166617 166628 166639 166656
T4 NC_000866.4 (165827)      TGAATCCTTGCTTAGAACGCTGATTACCTACATAAAATCGTGCAGATACGCATGGATTACTAAATGGACCACCGAGTTTACTCACTAAAAAGTAAA
SARS-CoV-2 NC_045512.2 (29904) ------------------------------------------------------------------------------------------------
```

```
                                                                                                 Section 1737
                         (166657) 166657         166672     166683     166694     166705     166716     166727     166738     166752
      T4 NC_000866.4 (165923) ATCCAGGTTTAGATAAAATATCTTTATGCGGAGTTCCTAAAAACCATTCACCACCCTTGATTGTACCAATAACAGTAGCGCCTGCATCATTCAGAT
SARS-CoV-2 NC_045512.2 (29904) ------------------------------------------------------------------------------------------------
                                                                                                 Section 1738
                         (166753) 166753 166760     166771     166782     166793     166804     166815     166826     166837 166848
      T4 NC_000866.4 (166019) CAGTAACAGTCATATATTTCATATTAATTTCCTCTAAATTATTTTCTACTCCAAGGCCGCATGAATACACACGGCCATTAAATTACTCGTCGCAGT
SARS-CoV-2 NC_045512.2 (29904) ------------------------------------------------------------------------------------------------
                                                                                                 Section 1739
                         (166849) 166849    166859     166870     166881     166892     166903     166914     166925         166944
      T4 NC_000866.4 (166115) CGACGCTCAATTCCCAAAACTCTTCTACAGTATAAGTTTCAGTATCATTTTCAATACAGAAACGTTCATTACTATTATTTGCTAAAGTAGCATTAA
SARS-CoV-2 NC_045512.2 (29904) ------------------------------------------------------------------------------------------------
                                                                                                 Section 1740
                         (166945) 166945       166958     166969     166980     166991     167002     167013     167024         167040
      T4 NC_000866.4 (166211) CTGTCATTTTTTCGCTAGTGCTCTTAAGAGGTGAAATACGAATTAACTGATCACCGTTATCTAAACAAAAAATTTCACCAACTTTTACATCTTTAA
SARS-CoV-2 NC_045512.2 (29904) ------------------------------------------------------------------------------------------------
                                                                                                 Section 1741
                         (167041) 167041167046     167057     167068     167079     167090     167101     167112     167123 167136
      T4 NC_000866.4 (166307) AACTTTTCATAATTCACCTCAAGGAGTATAAAATCCAAATGCAGTTGTTGACCATCCCATCCAATATGGAAAATTTACACCAATGTAAAACATAAG
SARS-CoV-2 NC_045512.2 (29904) ------------------------------------------------------------------------------------------------
                                                                                                 Section 1742
                         (167137) 167137  167145     167156     167167     167178     167189     167200     167211         167232
      T4 NC_000866.4 (166403) AATATAAAACCAACCGCTCAGCAAATTCATCATTTTACACCATTCCAAATTGTTTCAACCACGGATTTTAAACCATTTTGATGAATATCCATTCCT
SARS-CoV-2 NC_045512.2 (29904) ------------------------------------------------------------------------------------------------
                                                                                                 Section 1743
                         (167233) 167233     167244     167255     167266     167277     167288     167299     167310         167328
      T4 NC_000866.4 (166499) ACTACCGCCATCAAATAAATTCCAACTACAACTGAACCTAAGGCAAAAATCAGCATGAAAATGAATAAAGCCGGAAAAATATTATCGAAAAACCAT
SARS-CoV-2 NC_045512.2 (29904) ------------------------------------------------------------------------------------------------
```

```
                                                                                                  Section 1744
                 (167329) 167329       167343     167354     167365     167376     167387     167398     167409         167424
T4 NC_000866.4   (166595) TCAATAAATGTAAAAGCACTGCGTTTACGTTCATATTTTCCTCACATAAATCCAAAGTAAACGTTTAATACATCAATCATTAAAACGATTGGGAAT
SARS-CoV-2 NC_045512.2 (29904) ------------------------------------------------------------------------------------------------
                                                                                                  Section 1745
                 (167425) 167425 167431     167442     167453     167464     167475     167486     167497     167508      167520
T4 NC_000866.4   (166691) ATACTCAAAACTATTAGTATTATAACTACATTCCATATAGCTTTAATAATCTTTTTCATTTTCTGTTCCTCCATAGTTGATAGGGTAATAGTACCA
SARS-CoV-2 NC_045512.2 (29904) ------------------------------------------------------------------------------------------------
                                                                                                  Section 1746
                 (167521) 167521   167530     167541     167552     167563     167574     167585     167596                167616
T4 NC_000866.4   (166787) CGGAAGAACAGTCTTGTAAACAACTTTTTTAAAAATATTCGTAATAAATGTGAATACCAACTACTACCGCTGAAACCTGTGCAACCCACCACGCAC
SARS-CoV-2 NC_045512.2 (29904) ------------------------------------------------------------------------------------------------
                                                                                                  Section 1747
                 (167617) 167617      167629     167640     167651     167662     167673     167684     167695                167712
T4 NC_000866.4   (166883) AAGCAATAAGTACAGAATTCAAAATTTTCATAATAACCTCATTACAAAAGTAAATGTTAAACAAATTACTGGAATACTAATTAACCAAACAAAACA
SARS-CoV-2 NC_045512.2 (29904) ------------------------------------------------------------------------------------------------
                                                                                                  Section 1748
                 (167713) 167713         167728     167739     167750     167761     167772     167783     167794         167808
T4 NC_000866.4   (166979) CCACCATAATGAACTCATAGTTCAATCTCAGCGATTTTCATTTTATTCTCCAAATCCGTATCAGTAGTTGATAGTTGTATAGTACCACGGTCCTTG
SARS-CoV-2 NC_045512.2 (29904) ------------------------------------------------------------------------------------------------
                                                                                                  Section 1749
                 (167809) 167809 167816     167827     167838     167849     167860     167871     167882     167893     167904
T4 NC_000866.4   (167075) TGGTATGTAAACTGTTTTGTGAAATTTTTTAAATGGAAAGATACCATCCGTTGTAGTTGCTTTTTCTTACAACTTTACGAAGGTCTTCTCTGTCAC
SARS-CoV-2 NC_045512.2 (29904) ------------------------------------------------------------------------------------------------
                                                                                                  Section 1750
                 (167905) 167905    167915     167926     167937     167948     167959     167970     167981                168000
T4 NC_000866.4   (167171) CGATGAACTTCGGAGTGTACTGGATGACACCTGGATGAATTTCTTTAGTGTTGAATATAATTATACAGTCAGCGACTTGATGATTTAGAATGGGCC
SARS-CoV-2 NC_045512.2 (29904) ------------------------------------------------------------------------------------------------
```

```
                                                                                                      Section 1751
                        (168001) 168001      168014    168025    168036    168047    168058    168069    168080         168096
       T4 NC_000866.4 (167267) CTAGATTTATTCCAGAACCATATGGATACTCTCCGCTGCATCCCGTTGTTACCGAAATCCAACGTGAGTCAGTTTGATGTGTCTTAACTTCTACAC
SARS-CoV-2 NC_045512.2  (29904) ------------------------------------------------------------------------------------------------
                                                                                                      Section 1752
                        (168097) 168097168102    168113    168124    168135    168146    168157    168168    168179   168192
       T4 NC_000866.4 (167363) GAAGCCCGCAGTATTTTGGATGAGCCAATACATCCCATGCATATGTGTACGGATCATCGACATCCTCTTGGCCTTTATTGACATATCCACTCAACC
SARS-CoV-2 NC_045512.2  (29904) ------------------------------------------------------------------------------------------------
                                                                                                      Section 1753
                        (168193) 168193  168201    168212    168223    168234    168245    168256    168267          168288
       T4 NC_000866.4 (167459) AATCTGCCACAAAAAACTCTGCGTACACAGCGATACGGCATCTTTCGATAACTTCTGCCTTATCTTGATTTGGGTTTTGTTTTAAAGAGTATCTTG
SARS-CoV-2 NC_045512.2  (29904) ------------------------------------------------------------------------------------------------
                                                                                                      Section 1754
                        (168289) 168289     168300    168311    168322    168333    168344    168355    168366         168384
       T4 NC_000866.4 (167555) CAGTATCAGCAATTTTGACCTTCATTTCACAGGTCAAGTCACTGTTCGATAGGGTAAATGTCGGAATCTGAAATAGTCTCTGTAACCCAGGATTCG
SARS-CoV-2 NC_045512.2  (29904) ------------------------------------------------------------------------------------------------
                                                                                                      Section 1755
                        (168385) 168385        168399    168410    168421    168432    168443    168454    168465      168480
       T4 NC_000866.4 (167651) TTTTCTGCATTTAAACTTTCCTTTATGTCGGAATCACCGATATTCATATAAATCATAATTTCTCTTAAAACAAAAGGCCGAAGCCCTTTATTTTAC
SARS-CoV-2 NC_045512.2  (29904) ------------------------------------------------------------------------------------------------
                                                                                                      Section 1756
                        (168481) 168481 168487    168498    168509    168520    168531    168542    168553    168564   168576
       T4 NC_000866.4 (167747) TTGAATTGTGCAATTCTTTTCTCTAGACATTCAGCATAAGATTTCATTGAGATGAACTGCGAAAGTAGCAGTTCTTGCTCAACTGCGCTAACTGTT
SARS-CoV-2 NC_045512.2  (29904) ------------------------------------------------------------------------------------------------
                                                                                                      Section 1757
                        (168577) 168577   168586    168597    168608    168619    168630    168641    168652          168672
       T4 NC_000866.4 (167843) AGAAACTTTGCGCTTTCTAAAAATTTGCTCAGTGCATTAATTTTGAGCATTAATTGATCGTATTCTTCTTTTACTCGTGCTTGATAAGCTAACATA
SARS-CoV-2 NC_045512.2  (29904) ------------------------------------------------------------------------------------------------
```

Section 1758

(168673) 168673 168685 168696 168707 168718 168729 168740 168751 168768
T4 NC_000866.4 (167939) ATTTTCCTTAGTTAAGGGCCGAAGCCTTATTTAAATTGTTCAGTAACGTCTTCAACTACTTCATATTGGCAGGTACGCATTTTAGCATCGTTGTAA
SARS-CoV-2 NC_045512.2 (29904) --

Section 1759

(168769) 168769 168784 168795 168806 168817 168828 168839 168850 168864
T4 NC_000866.4 (168035) TCAATCGGAATTGATACTACATCGCGAGGATGAACTTTAACTTTTACAACTCGGCTGGTTGAACTACCAAAGTGACGAATATAAGATTTAGAACAC
SARS-CoV-2 NC_045512.2 (29904) --

Section 1760

(168865) 168865 168872 168883 168894 168905 168916 168927 168938 168949 168960
T4 NC_000866.4 (168131) ACATGCAAACCACGAGAACAAGTTTGTGTATCATCGTCATTCACACGAGTACGTGGCATTTTAACTACTTTACCCGGACTGTTATCAAAGGTGTTT
SARS-CoV-2 NC_045512.2 (29904) --

Section 1761

(168961) 168961 168971 168982 168993 169004 169015 169026 169037 169056
T4 NC_000866.4 (168227) GAGTGACAGTCAAAGTAATTGCTGCGAACTACTTTCCAAGCATAGAAGTAACCATCTTCTGTAATTTCAATATCGTTTGCTACCAAGAAATCAAAG
SARS-CoV-2 NC_045512.2 (29904) --

Section 1762

(169057) 169057 169070 169081 169092 169103 169114 169125 169136 169152
T4 NC_000866.4 (168323) AGTCGAGATACCGCTTTTTGGCTTGGGTTTTCCAACAGATTTTCCAAGAACGGAAAATAAAATTCAAAGTTTTCGCCTTTTTCCATCGAGTCAAGA
SARS-CoV-2 NC_045512.2 (29904) --

Section 1763

(169153) 169153169158 169169 169180 169191 169202 169213 169224 169235 169248
T4 NC_000866.4 (168419) ATACGATCAACCAAACCAGACCGCAATTCAATATTTTGATAGAACAAGCTTCCACCTTCAATTCGAACATCGCCGGAAATATATTTTTCAACAGCA
SARS-CoV-2 NC_045512.2 (29904) --

Section 1764

(169249) 169249 169257 169268 169279 169290 169301 169312 169323 169344
T4 NC_000866.4 (168515) CGACGAACATTAATTTTTTGTGCCGCTTCTTCCAACTTATCCGCTACAAGCAGATTAAGAATTTCCTGGAAGTTTGAATGAGTATTAGGAGTTGCG
SARS-CoV-2 NC_045512.2 (29904) --

```
                                                                                                                     Section 1765
                        (169345) 169345     169356     169367     169378     169389     169400     169411     169422           169440
           T4 NC_000866.4 (168611) TTATAAGTTACGCCATCAACAGTAATTGAAATGAATTTTTTAGATGCATTCCAAATAATGTCAGATTTAGCAACTGGAGCAATAACTGCATCGCTA
SARS-CoV-2 NC_045512.2  (29904) ------------------------------------------------------------------------------------------------
                                                                                                                     Section 1766
                        (169441) 169441        169455     169466     169477     169488     169499     169510     169521         169536
           T4 NC_000866.4 (168707) TTAACTTTAACTGTAATATCACCGCTAATAGTAACTTTAGGGCGTTTAGCTTCTTCAGCATTTTTCAAAACACGACGGATTGTGTCAACCGATACA
SARS-CoV-2 NC_045512.2  (29904) ------------------------------------------------------------------------------------------------
                                                                                                                     Section 1767
                        (169537) 169537 169543     169554     169565     169576     169587     169598     169609     169620  169632
           T4 NC_000866.4 (168803) CCTTGCCAATCAGCCAATTCCTGTTGGGTGTAATTACCACTTGAATACAGTTTAACAATTTCAGCTTGTTCGTTTTTGGTCAGGCATTTAATATTG
SARS-CoV-2 NC_045512.2  (29904) ------------------------------------------------------------------------------------------------
                                                                                                                     Section 1768
                        (169633) 169633 169637
           T4 NC_000866.4 (168899) TACAT
SARS-CoV-2 NC_045512.2  (29904) -----
```
