# Supplementary material for: Ubiquitous Micro-Modular Homologies among Genomes from Viruses to Bacteria to Human Mitochondrial DNA: Platforms for Recombination during Evolution?
Source: Viruses. 2022 Apr 24;14(5):885. doi: 10.3390/v14050885 (PMC9147251; doi:10.3390/v14050885)
Supplement: Supplementary file 1 [file viruses-14-00885-s001.zip › Revised Table S1 Accession Numbers.pdf]

Genomes	**NCBI Nucleotide Accession Number https://www.ncbi.nlm.nih.gov/nuccore**
Human adenovirus type 2	J01917.1
Human adenovirus type 5	AC_000008.1
Human adenovirus type 12	X73487
Autographa californica Nuclear Polyhedrosis Virus	NC_001623.1
Hepatitis B Virus	NC_003977.2
HIV-1 HXB2	K03455.1
Escherichia virus lambda	NC_001416.1
Escherichia virus Mu	NC_000929.1
Escherichia virus T4	NC_000866.4
Acidianus rod-shaped virus 1	NC_009965.1
Nitrosopumilus spindle-shaped virus isolate NSV1	NC_048199.1
Sulfolobus turreted icosahedral virus	NC_005892
Alternaria brassicicola endornavirus isolate 1	NC_026136.1
Carrot mottle mimic umbravirus (CMoMV) dsRNA1	NC_001726.1
Fig badnavirus 1	NC_017830.1
Candidatus Carsonella ruddii strain *BT* chromosome	CP024798.1
Escherichia coli K-12	NC_000913.3
Homo sapiens mitochondrion	NC_012920.1
SARS-CoV-2	NC_045512.2
SARS-CoV-2 Spike region original: 21,563-25,384nt	NC_045512.2
SARS-CoV-2 Spike region delta: 21,561-25,376nt*	MZ437368.1

Table S1 - NCBI Nucleotide Accession Numbers of nucleotide sequences analyzed in this article.
