# Supplementary material for: Ubiquitous Micro-Modular Homologies among Genomes from Viruses to Bacteria to Human Mitochondrial DNA: Platforms for Recombination during Evolution?
Source: Viruses. 2022 Apr 24;14(5):885. doi: 10.3390/v14050885 (PMC9147251; doi:10.3390/v14050885)
Supplement: Supplementary file 1 [file viruses-14-00885-s001.zip › Revised Table S2 Nucleotide Sequence Comparisons to the SARS-CoV-2 Nucleotide Sequence.pdf]

Genomes	N-mer	Count	% of total	∑
Sulfolobus turreted icosahedral virus	3	463	60.1	770 positions
	4	201	26.1	
	5	72	9.4	
	6	24	3.1	
	7	10	1.3	
Alternaria brassicicola betaendornavirus	3	230	65.9	349 positions
	4	77	22.1	
	5	29	8.3	
	6	7	2.0	
	7	3	0.9	
	8	2	0.6	
	9	0	0	
	10	1	0.3	
Fig badnavirus 1	3	222	55.9	397 positions
	4	99	24.9	
	5	55	13.9	
	6	15	3.8	
	7	4	1.0	
	8	1	0.3	
	9	0	0	
	10	1	0.3	
Autographa californica Nuclear Polyhedrosis Virus	3	383	70.3	545 positions
	4	112	20.6	
	5	33	6.1	
	6	10	1.8	
	7	4	0.7	
	8	2	0.4	
	9	1	0.2	
Escherichia virus lambda	3	375	70.1	535 positions
	4	108	20.2	
	5	35	6.5	
	6	11	2.1	
	7	1	0.2	
	8	5	0.9	
Human Adenovirus type 12	3	565	61.2	923 positions
	4	245	26.5	
	5	73	7.9	
	6	28	3.0	
	7	4	0.4	
	8	6	0.6	
	9	2	0.2	
Hepatitis B Virus	3	56	77.8	72 positions
	4	13	18.1	
	5	3	4.2	

Genomes	N-mer	Count	% of total	∑
Human Immunodeficiency Virus	3	160	66.1	242 positions
	4	45	18.6	
	5	23	9.5	
	6	13	5.4	
	7	1	0.4	
Candidatus Carsonella Proteobacterium BT	3	930	54.2	1714 positions
	4	456	26.6	
	5	216	12.6	
	6	68	4.0	
	7	28	1.6	
	8	13	0.8	
	9	2	0.1	
	10	2	0.1	
Escherichia Coli K12	3	947	59.2	1599 positions
	4	412	25.8	
	5	161	10.1	
	6	49	3.1	
	7	23	1.4	
	8	6	0.4	
	9	0	0	
	10	0	0	
	11	1	0.06	
***Homo sapiens* mitochondrial DNA**	3	490	55.2	887 positions
	4	243	27.4	
	5	96	10.8	
	6	31	3.5	
	7	15	1.7	
	8	11	1.2	
	9	0	0	
	10	0	0	
	11	0	0	
	12	0	0	
	13	1	0.1	

Table S2 – Nucleotide sequence comparisons of lengths of identical sequence stretches between the genomes of organisms as indicated and SARS-CoV-2 RNA.
