# Supplementary figures and images for: Ubiquitous Micro-Modular Homologies among Genomes from Viruses to Bacteria to Human Mitochondrial DNA: Platforms for Recombination during Evolution?
Source: Viruses. 2022 Apr 24;14(5):885. doi: 10.3390/v14050885 (PMC9147251; doi:10.3390/v14050885)

**E. Coli 1–200,00nt**

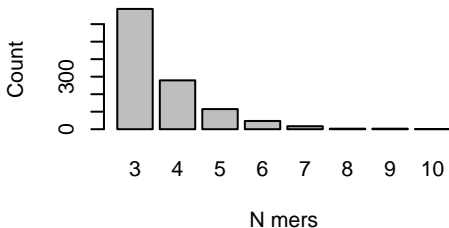

**E. Coli 200,001–400,00nt**

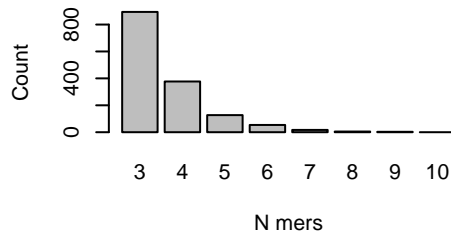

**E. Coli 400,001–600,00nt**

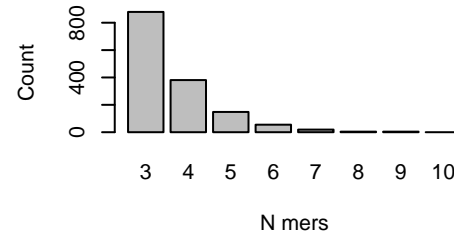

**E. Coli 600,001–800,00nt**

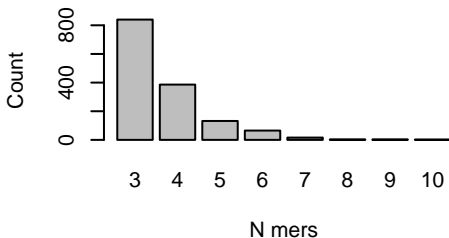

**E. Coli 800,001–1,000,000nt**

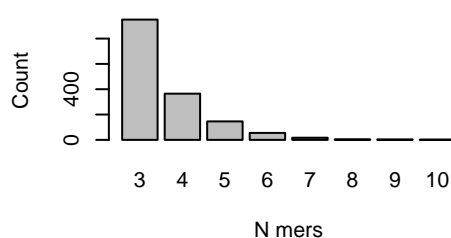

**E. Coli 1,000,001–1,200,000nt**

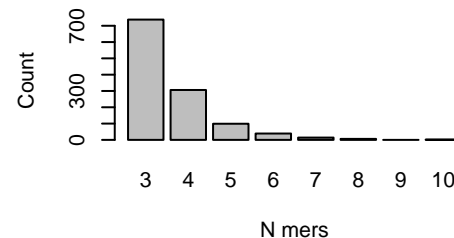

**E. Coli 1,200,001–1,400,000nt**

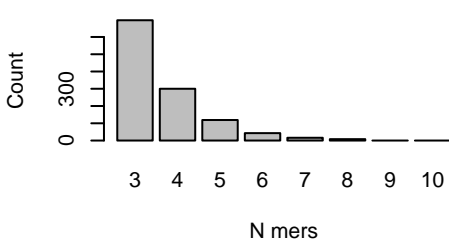

**E. Coli 1,400,001–1,600,000nt**

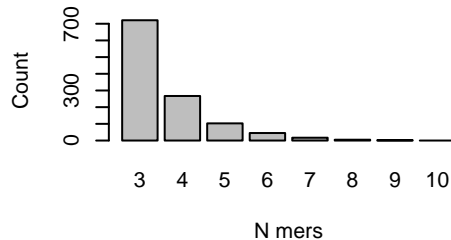

**E. Coli 1,600,001–1,800,000nt**

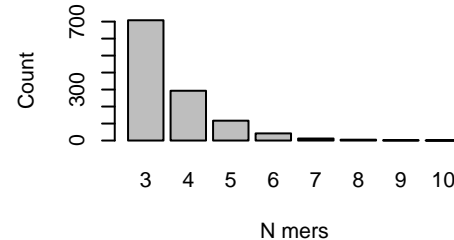

Supplement: Supplementary file 1 [file viruses-14-00885-s001.zip › Fig. S6A, E.coli1-1800000.pdf]

**E. Coli 3,600,001–3,800,000nt**

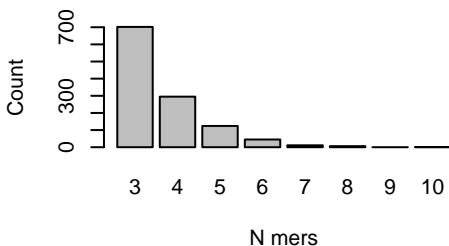

**E. Coli 3,800,001–4,000,000nt**

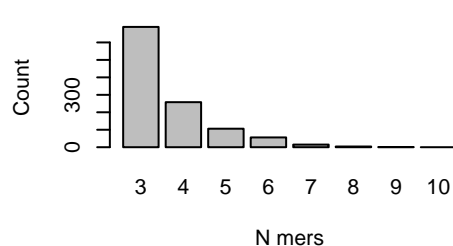

**E. Coli 4,000,001–4,200,000nt**

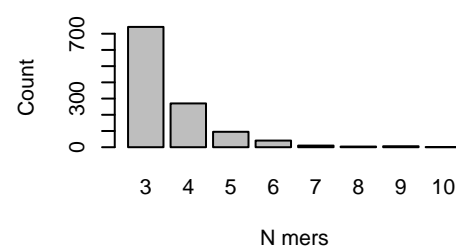

**E. Coli 4,200,001–4,400,000nt**

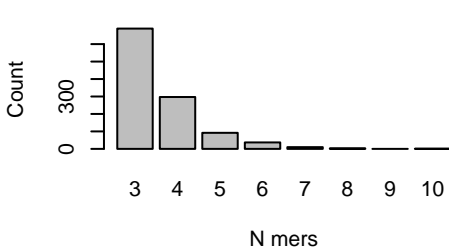

**E. Coli 4,400,001–4,600,000nt**

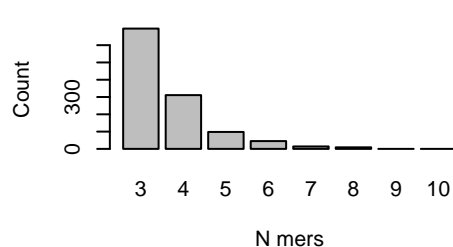

**E. Coli 4,600,000–4,641,652nt**

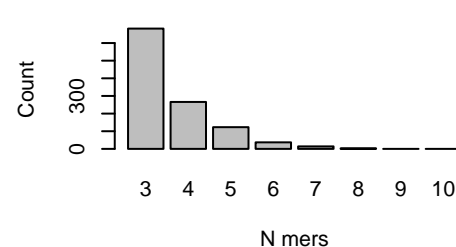

Supplement: Supplementary file 1 [file viruses-14-00885-s001.zip › Fig. S6B, E.coli, 3600001-4641652.pdf]
